# The human transketolase-like proteins TKTL1 and TKTL2 are *bona fide* transketolases

**DOI:** 10.1186/s12900-018-0099-y

**Published:** 2019-01-15

**Authors:** Gaurang P. Deshpande, Hugh-George Patterton, M. Faadiel Essop

**Affiliations:** 1 Cardio-Metabolic Research Group (CMRG), Department of Physiological Sciences, Stellenbosch University, Room 2005, Mike De Vries Building, Merriman Avenue, Stellenbosch, 7600 South Africa; 2 Centre for Bioinformatics and Computational Biology, Stellenbosch University, Stellenbosch, 7600 South Africa

**Keywords:** Transketolase, Transketolase-like 1, Transketolase-like 2, Thiamine, Pentose phosphate pathway, Glycolysis

## Abstract

**Background:**

Three transketolase genes have been identified in the human genome to date: transketolase (TKT), transketolase-like 1 (TKTL1) and transketolase-like 2 (TKTL2). Altered TKT functionality is strongly implicated in the development of diabetes and various cancers, thus offering possible therapeutic utility. It will be of great value to know whether TKTL1 and TKTL2 are, similarly, potential therapeutic targets. However, it remains unclear whether TKTL1 and TKTL2 are functional transketolases.

**Results:**

Homology modelling of TKTL1 and TKTL2 using TKT as template, revealed that both TKTL1 and TKTL2 could assume a folded structure like TKT. TKTL1/2 presented a cleft of suitable dimensions between the homodimer surfaces that could accommodate the co-factor-substrate. An appropriate cavity and a hydrophobic nodule were also present in TKTL1/2, into which the diphosphate group fitted, and that was implicated in aminopyrimidine and thiazole ring binding in TKT, respectively. The presence of several identical residues at structurally equivalent positions in TKTL1/2 and TKT identified a network of interactions between the protein and co-factor-substrate, suggesting the functional fidelity of TKTL1/2 as transketolases.

**Conclusions:**

Our data support the hypothesis that TKTL1 and TKTL2 are functional transketolases and represent novel therapeutic targets for diabetes and cancer.

**Electronic supplementary material:**

The online version of this article (10.1186/s12900-018-0099-y) contains supplementary material, which is available to authorized users.

## Background

Three transketolase genes have been identified in the human genome to date. Transketolase (EC 2.2.1.1; TKT) is a crucial enzyme that links the pentose phosphate pathway (PPP), a non-oxidative glucose pathway (NOGP), to glycolysis. The TKT gene is located on chromosome 3 at position 3p21.1 and is responsible for generating sugar phosphates for intracellular nucleotide metabolism as well as for the production of nicotinamide adenine dinucleotide phosphate (NADPH), a reducing agent and anti-oxidant [1]. TKT is a homodimer and known thiamine diphosphate (TDP)-dependent enzyme that possesses two active sites located at the monomer contact surfaces [2, 3]. In addition to TKT, transketolase-like 1 (TKTL1) and transketolase-like 2 (TKTL2) loci are found on the X chromosome at Xq28 and on chromosome 4 at 4q32.2, respectively [1].

An altered function of transketolase is linked to various pathophysiologic complications, suggesting TKT as a potential therapeutic target. TKT is the best studied transketolase, and previous studies reported an altered activity in patients suffering from diabetes and various cancers [3–5]. For example, hyperglycemic individuals display lowered TKT activity that may be ameliorated by thiamine treatment, thus offering potential as a novel treatment for type 2 diabetes [6]. TKT pathways are also intricately involved in cancer progression and metastasis. For example, PPP activation satisfies the high demand by cancer cells for nucleotides (to ensure proliferation) by the terminal conversion of glucose to ribose and conversion to lactate. Moreover, TKT inhibition by oxythiamine (a thiamine antagonist) substantially decreased pancreatic cancer cell growth [7]. Taken together, these studies strongly implicate perturbed TKT function in the development of diabetes and various cancers [8].

What about TKTL1 and TKTL2? There is limited information regarding the role of TKTL2; more is known about TKTL1. TKTL1, previously postulated to be a pseudogene, encodes a transketolase like-protein that is also linked to cancer [9]. For example, TKTL1 mutations are associated with cancers, making it a promising target for anti-cancer treatments [6, 10]. TKTL1 silencing in colon cancer cells attenuated cell proliferation, and significantly decreased TKT activity, suggesting an interplay between the two transketolases [11]. However, some researchers found that this relationship was experimentally variable [12].

Human TKT and TKTL1 differ in both primary structure and in amino acid composition [13], and several groups have raised doubt whether TKTL1 indeed was an actual transketolase [14, 15]. There are no experimental data confirming the enzymatic activity of TKTL1 compared to TKT. TKTL1 has 38 amino acids less in the active site compared to TKT, and this may affect its enzymatic activity. TKTL1 also lacks two vital histidine residues that are otherwise conserved in transketolases, and are required for catalytic processes [15]. There is also a substitution mutation (W124S) in the TKTL1 amino acid sequence. Moreover, the functional identity of TKTL1 and TKTL2 as possible transketolases was based only on an analysis of the one-dimensional sequence alignment of these proteins with the structure of human TKT [13]. A significant limitation of such an approach is that it could miss the identification of alternative residues and possible interactions that may be involved in co-factor and substrate binding.

In light of conflicting findings and uncertainties, the current study investigated the possible function of TKTL1 and TKTL2 as putative transketolases. We employed homology modelling and structural analysis of TKTL1 and TKTL2 using the 0.97 Å crystal structure of TKT in complex with a TDP co-factor-substrate as the template [13].

## Materials and methods

### Modelling

**Fig. 1:**
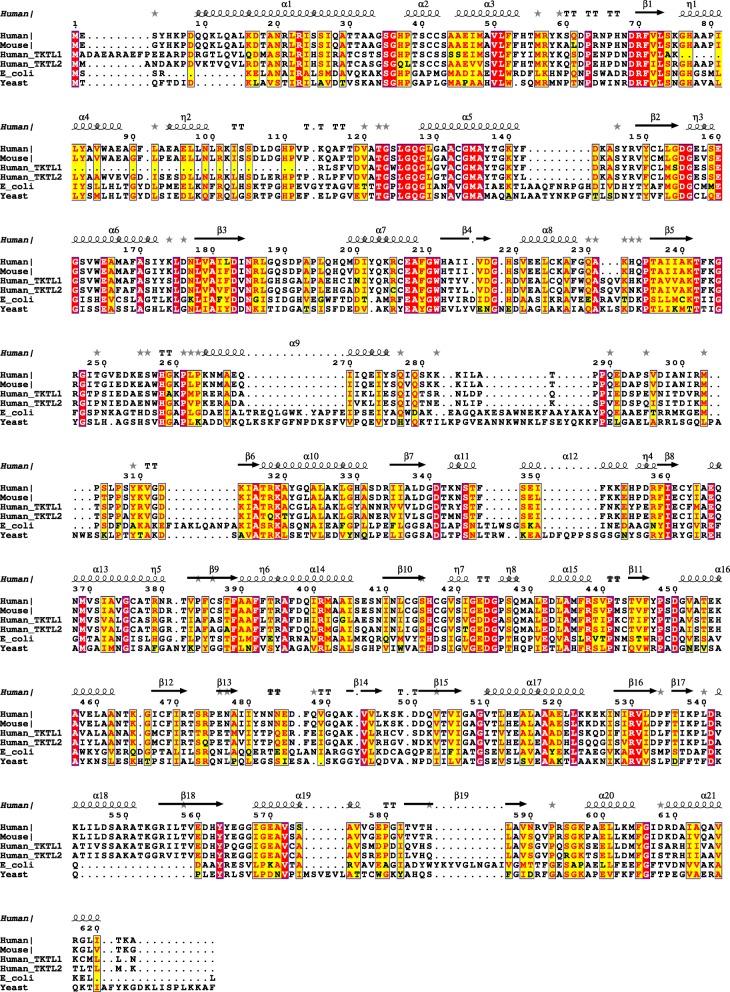
The multiple alignment of TKT, TKTL1 and TKTL2. The multiple alignment of human (P29401.3), mouse (P40142.1), *Escherichia coli* (P27302.5) and *Saccharomyces cerevisiae* (P23254.4) transketolase (TKT) sequences as well as human transketolase-like protein 1 (TKTL1) (P51854.2) and 2 (TKTL2) (Q9H0I9.1) sequences. The secondary structures visible in the human TKT crystal structure (PDB accession 4KXW) is shown aligned above the human TKT sequence. The α-helices, 3_10_-helices and β-strands are indicated. Identical and conserved residues at aligned sequence positions are identified by red and yellow blocks, respectively. The alignment was calculated with T-coffee [33] and the alignment figure generated with ESPript [34]

To assess the relationship between TKT and transketolase-like proteins, we performed a multiple alignment of several transketolase enzyme sequences that included the putative transketolase family members TKTL1 and TKTL2. The multiple alignment revealed a high degree of conservation among the mammalian enzymes (Fig. 1). Sequence regions present in yeast and *Escherichia coli* TKT, that appear to have been lost in the mammalian group enzymes during evolution, map to unstructured regions between α5-β2 and α9-β6 and within the α9 and α12 helices (Fig. 1). These deletions, however, do not abolish the functionality of the mammalian TKT [16]. Within the mammalian group of transketolase enzymes, TKTL1 contains an additional deletion from G76 to P113. This incorporates α4 and 3_10_-helix 5 (η5), including H77, that is involved in stabilizing TDP in the active site of TKT. However, as we show below, this deletion does not affect the overall structure of the modeled TKTL1 enzyme and the absence of an H77 homolog may be compensated for by alternative stabilizing interactions to TDP. TKTL2 does not contain this deletion and is 64% identical and 92% conserved compared to TKT in this region (Fig. 1). TKTL1 is 60% identical and 81% conserved relative to TKT.

We performed a PHI-BLAST search and identified human TKT (UniProtKB P29401) as an appropriate template for modelling (Fig. 1). A co-crystal structure of this protein in complex with ketose D-xylulose-5-phosphate (X5P) is available at a resolution of 0.97 Å (PDB 4KXW) [17]. We generated multiple homology models of TKTL1 and TKTL2 using this crystal structure and the Robetta [18] and SwissProt servers [19]. Although all models were energy minimized, we did not proceed with molecular dynamics simulations, thinking that an additional predictive refinement would contribute little to our primary structural analysis.

### Model verification

We verified the TKTL1 and TKTL2 model qualities using MolProbity [20] and Verify3D [21] available on the SAVE server (services.mbi.ucla.edu/SAVES). The Verify3D method calculates a 3D-1D score for the presence of a given residue within its environment in the calculated model and reports the average score in a sliding 21-residue window. For the TKTL1 model, 96% of the residues have a 3D-1D score > 0.2, where Verify3D defines a score of > 0.2 for more than 80% of the residues as acceptable. The Ramachandran plot showed that 98.7% (585/593) of all residues were in allowed regions, 99.8% of all rotamers were favored and no bad rotamers were present in the model. In the TKTL2 model, 93% of residues had a 3D-1D score > 0.2, 99.4% (625/626) of residues had ϕ and ψ angles within the allowed regions, no bad rotamers were present and 98.2% of the rotamers were favored. By comparison, in the crystal structure of TKT [17], 92% of residues had a 3D-1D score > 0.2, 99.7% of residues exhibited favored ϕ and ψ angles, 94% were favored rotamers and 3% bad rotamers were present, emphasizing the quality of our proposed models.

### Fitting the TDP substrate ligand in the TKTL1 and TKTL2 models

**Fig. 2:**
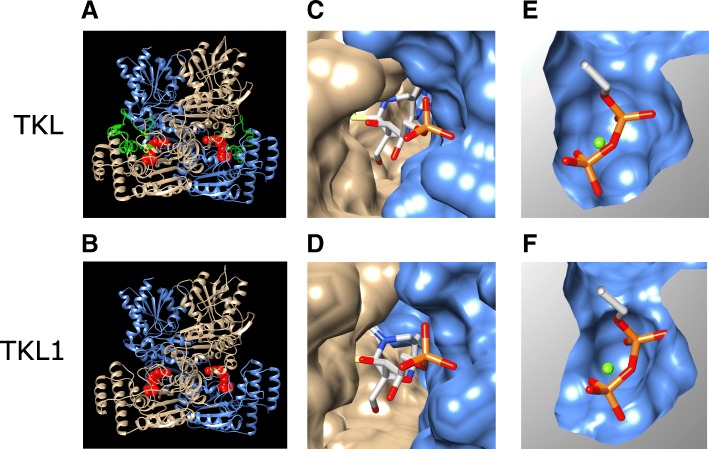
Homology of TKTL1. Homology model of TKTL1 based on the TKT co-crystal structure with a TDP-D-xylulose-5-phosphate (X5P) adduct. **a** Ribbon representation of the homodimer (tan and blue sub-units) of TKT with the TDP-X5P adduct ligand shown as red spheres. The η1, α4 and η2 helices (green), as well as H77 and H110 (yellow) (absent in TKTL1) are shown. **b** Homology model of TKTL1 shown as a homodimer (tan and blue sub-units) and fitted to the coordinates of TKT to show the location of the substrate adduct, indicated by the red spheres. **c** The ligand present in the substrate cleft that is formed between the surfaces of the two units in the TKT homodimer is shown. **d** A similar substrate cleft is visible between the two units of the homodimer in the homology model of TKTL1. **e** A di-phosphate binding cavity in visible on the surface within the substrate cleft of each monomer in the TKT homodimer. **f** A di-phosphate binding cavity is also visible within the substrate cleft of the TKTL1 homology model

To allow analysis of the substrate binding to the modeled TKTL1 and TKTL2, chain A of human transketolase, crystallized as a covalent complex with its physiological substrate X5P (PDB accession: 4KXW), was aligned to chain A of the TKTL1 or TKTL2 model using the Matchmaker tool in Chimera [22, 23]. This superposition gave a RMSD (α-carbon) of 0.4 and 0.3 Å for the TKTL1 and TKTL2 models, respectively, and placed the X5P ligand in the same relative position in the model compared to its position in the TKT crystal structure (Fig. 2a and b, Figure S1A). The region G76 to P113 of TKT, absent in TKTL1, is shown by the green section and the H77 and H110 residues (where H77 was proposed to be involved in binding to TDP) are shown in yellow (Fig. 2a). Note that the peptide chain in TKTL1 proceeds smoothly as an unstructured coil from A82 to D89, bypassing the α4 and η5 secondary structures observed between S74 and D119 in TKT (Fig. 2a). The absence of this section of the structure appears to have negligible impact on the global structure of the modeled TKTL1, based on the close structural co-alignment (α-carbon RMSD = 0.4 Å) of the minimized model (Fig. 2a and b, Figure S1A).

## Results

### Homology models of TKTL1 and TKTL2 show substrate clefts

The binding of the TDP substrate and substrate adduct in the active site of TKT has been pursued by a number of previous studies [13, 16, 17]. For the current study, TDP fits in a channel formed at the interface surfaces of the homodimer subunits (Fig. 2c). When viewing the position of the aligned TDP ligand in the structure of the modeled TKTL1, the interface surface between the homodimer subunits similarly provides a crevice into which the TDP fits (Fig. 2d; Figure S1B shows a similar crevice in TKTL2). TKTL1 provides a surface architecture, distribution of polar/hydrophobic regions and spatial placement of residue sidechains very similar to that found in the TKT crystal structure. This allows for the stabilization of the bound TDP in a similar orientation relative to key surface elements, and the two identified active site residues.

### Identical and similar hydrogen-bonds stabilize the TDP diphosphate in TKTL1/2

**Fig. 3:**
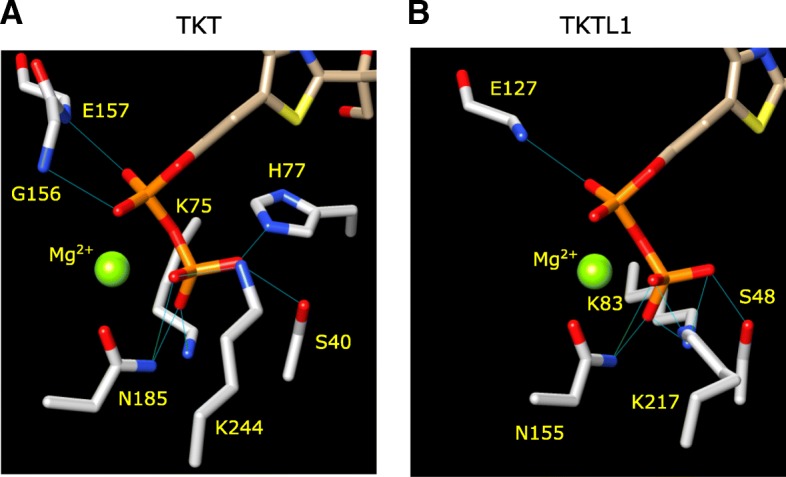
Binding of TDP to enzyme. Binding of the thiamine diphosphate (TDP) group to the enzyme. The residues involved in hydrogen bonding to the di-phosphate group of TDP are shown for (**a**) TKT and (**b**) TKTL1. The equivalent of TKT H77 is not present in TKTL1

For TKT the TDP diphosphate group is inserted into a cavity on the surface and this is coordinated with an Mg^2+^ cation (Fig. 2e). Several hydrogen-bonds are made to the phosphate oxygen atoms by residue side-chains that protrude into the cavity volume. This includes hydrogen-bonds from the G156 and E157 backbone amide groups, the K75 and K244 ε-NH_2_, the N185 NH_2_ and the S40 hydroxyl and H77 τ-NH groups (Fig. 3a). Hydrogen-bonds were identified in Chimera using the criteria of Mills and Dean. [24] When analyzing the TKTL1 model, the diphosphate group of TDP is similarly inserted into a surface cavity (Fig. 2f; the cavity in TKTL2 is shown in Figure S1C) and a network of possible hydrogen-bond interactions can be observed in the binding to the TDP diphosphate (Fig. 3b). A similar series of hydrogen bonds involving the equivalent residues are visible in TKTL2 (Figure S2A). Importantly, TKTL1 lacks the equivalent of H77 and there is no alternative residue in a suitable position that can substitute for H77. However, five groups are involved in hydrogen-bonding to the TDP diphosphate in TKTL1. These include the E127 backbone amide, the K83 and K217 ε-NH_2_, the N155 NH_2_ and the S48 hydroxyl groups (Fig. 3b). It is likely that this extensive set of interactions, combined with the coordinate binding to the localized Mg2^+^, is sufficient to retain the TDP diphosphate tail stably bound within the cavity. TKTL2 contains H78 at the equivalent position of TKT H77, and the former residue makes a similar hydrogen-bond to the β-phosphate hydroxyl group (Figure S2A; refer Additional files 1 and 2 for TKTL1 and TKTL2 in covalent complex X-ray diffraction data).

### Interactions with the aminopyrimidine and thiazole rings

**Fig. 4:**
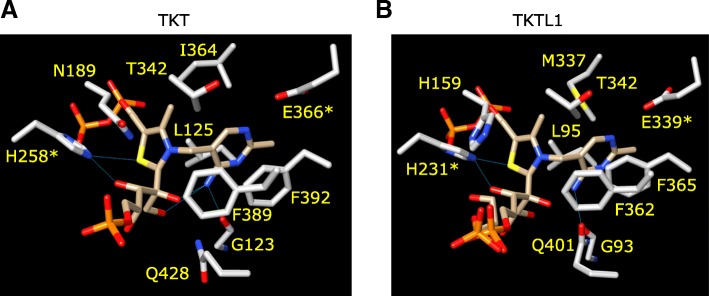
Binding of thiamine backbone to the enzyme. The residues involved in hydrogen bonds, hydrophobic interactions and stacking interactions in (**a**) TKT and (**b**) TKTL1 are shown. The residues indicated by asterisks have been implicated in catalysis

Numerous residues that are contributed by both subunits of the homodimer are involved in interactions with the aminopyrimidine and thiazole rings of TDP. N189, H258, Q428, G125 and E366 were previously identified to be involved in a hydrogen-bond network with the TDP ring structures [13, 16, 17] (Fig. 4a). The T342, I364, L125, F389 and F392 side-chains were proposed to be involved in non-polar and hydrophobic interactions with the TDP rings [13, 16, 17] (Fig. 4a). A similar array of potential interacting residues are found in the TKTL1 (Fig. 4b) and TKTL2 models (Figure S2B). There are many identical residues present at similar positions relative to the TDP ligand in the TKTL1 model, e.g. the equivalent TKT-TKTL1 residue pairs H258-H231, Q428-Q421, E366-E369, G123-G93, F389-F362, F392-F365, T342-T342 and L125-L95. Two residues that may be involved in binding in TKT are absent in TKTL1, but may be substituted by residues in comparable positions that can participate in a similar interaction. For instance, I364 of TKT is absent in TKTL1, but may be substituted by M337. N185 is similarly absent in TKTL1, but H158 is observed at a similar position, where the τ-NH group of the imidazole ring may be involved in a hydrogen-bond to the thiazole sulphur (Fig. 4b). In the case of TKTL2 the residues H262, Q432, E370, G124, F393, F396, T346, L126, I368 and N186 matches the corresponding residues in TKT (Figure S2B).

### Binding of the substrate in TKTL1/2

**Fig. 5:**
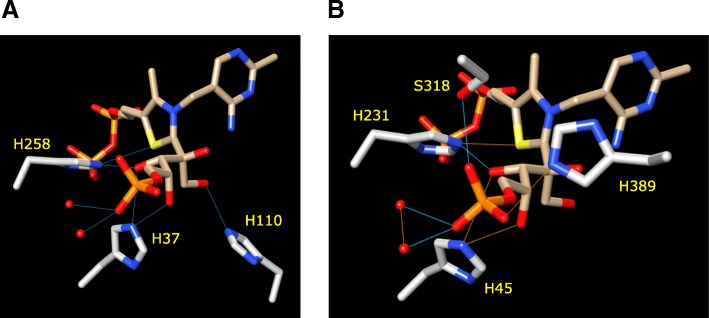
Binding of X5P to TKT. Binding of D-xylulose-5-phosphate (X5P) in the active site of TKT. Residues and water molecules involved in hydrogen bonding to the xylulose adduct are shown for (**a**) TKT and (**b**) TKTL1

**Fig. 6:**
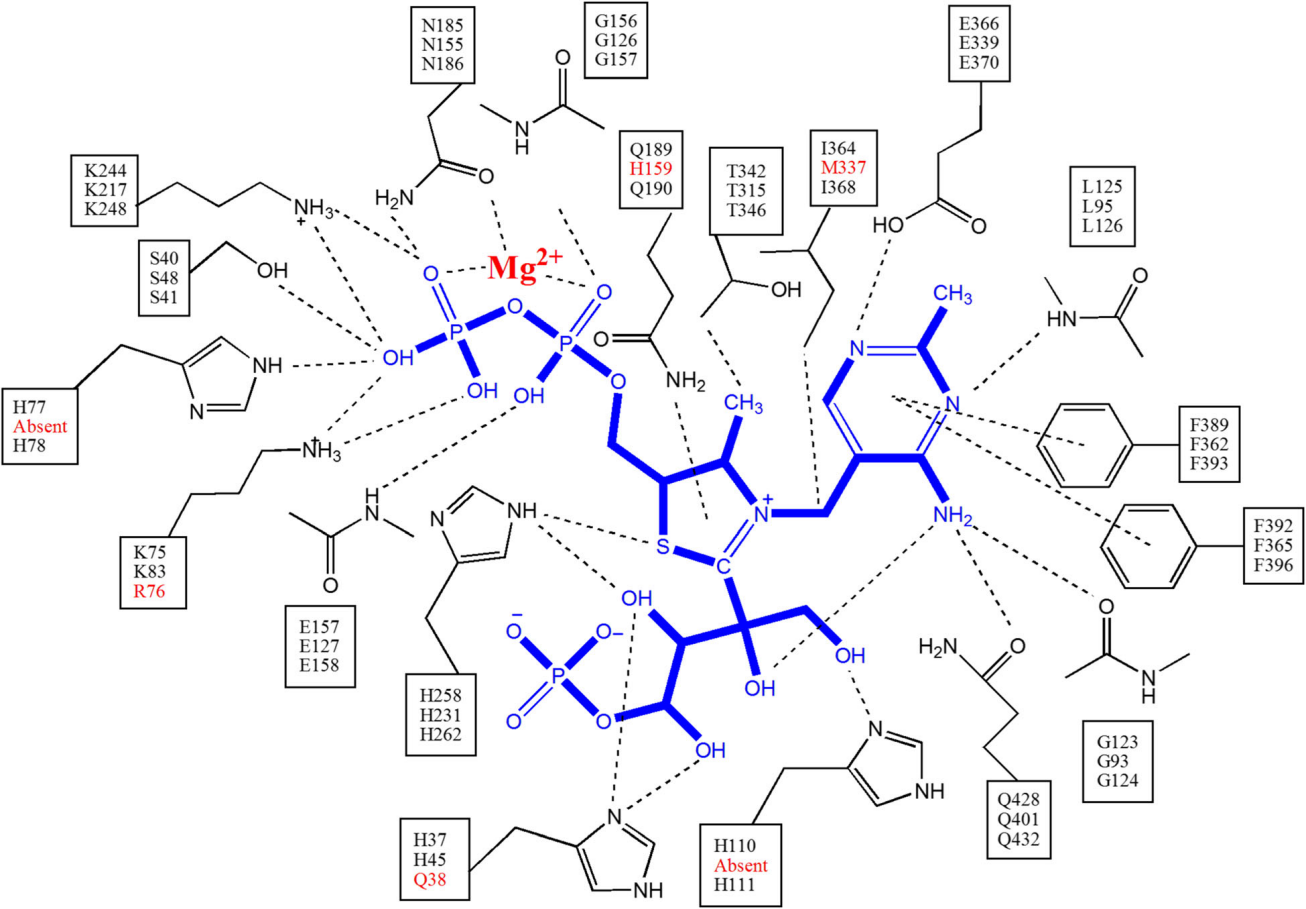
Interaction between TDP and TKT. Interactions between thiamine diphosphate (TDP) co-factor and the xylulose adduct, and residue sidechains and peptide backbone in the active site of TKT. Residues and backbone groups involved in hydrogen bonding, hydrophobic interactions and stacking interactions are indicated, based on the analysis of Tittmann and co-workers [6]. The equivalent residues in TKTL1 and TKTL2, based on the multiple alignment shown in Fig. 1, are indicated for each residue (order TKT, TKTL1 and TKTL2, top to bottom, in each square). Residues that are absent or not identical in the alignment are indicated

The co-crystal structure of TKT and the TDP co-factor reveals a reaction intermediate, a covalent adduct between X5P and TDP [17]. The sugar adduct is stabilized in the active site by a series of hydrogen bonds between H37, H258 and H110, and the hydroxyl groups on C1, C3 and C4 of the X5P (Fig. 5a). TKTL1 lacks H110 and there does not seem to be another residue in a suitable position to make a hydrogen-bond to C1 of X5P. However, an extensive network of hydrogen-bonds is still associated with the sugar adduct in the active site. Both H389 and S318 make hydrogen-bonds to the X5P phosphate group, while H45 and H231 make hydrogen-bonds to the C3 and C4 hydroxyl groups of X5P (Fig. 5b). In the case of TKTL2, Q38 substitutes for TKT H37, and is seen to make a similar hydrogen-bond to X5P C3 (Figure S2C). The residues equivalent to TKT H258 and H110 are present as H262 and H111 in TKTL2, and make hydrogen-bonds to C1 and the thiazole sulphur (Figure S2C). The residues involved in interactions with the co-factor in TKT, TKTL1 and TKTL2 are summarized in Fig. 6.

## Discussion

We have constructed homology models for TKTL1 and TKTL2 using the crystal structure of human TKT in complex with a reaction intermediate of the TDP co-factor as a structural template. This allows a more rigorous assessment of the possible enzyme-co-factor and enzyme-substrate interactions compared to a simple alignment of sequences. When employing superposition of models with the TKT crystal structure to position the co-factor adduct in the models, we have shown that a similar co-factor and substrate crevice is formed at the interface between the two sub-units of the homodimer in the TKTL1 and TKTL2 models (Fig. 2c and d and Figure S1B). This location of the co-factor adduct further allowed the required insertion of the TDP diphosphate tail into a cavity, where numerous conserved and new interactions were possible that anchored TDP to the protein (Fig. 2e and f and Figure S1B, Fig. 5 and Figure S2A). The environment and spatial distribution of residues similarly allowed mostly conserved interactions in TKTL1 and TKTL2 with the aminopyrimidine and thiazole rings (Fig. 4 and Figure S2B). The residues that may be involved in interactions with the TDP co-factor and X5PA intermediate in TKT [13] and the equivalent residues identified here in the TKTL1 and TKTL2 models are summarized in Fig. 6.

**Fig. 7:**
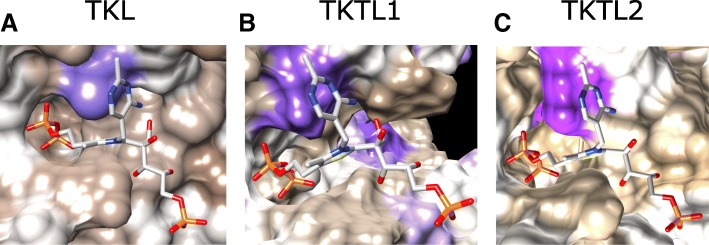
Hydrophobic interactions with aminopyrimidine and thiazole rings. Association of thiamine diphosphate (TDP) with a hydrophobic nodule. The aminopyrimidine and thiazole rings (linked by a methylene bridge) is stabilized in a V-conformation by binding of the di-phosphate in a cavity, and association of a hydrophobic nodule formed by (**a**) L125 in TKT, (**b**) L95 in TKTL1, and (**c**) L126 in TKTL2, with ring structures. The molecular surface is shown colored according to the Kyte-Doolittle hydrophobic value in the range − 4.5 (purple) to 4.5 (tan)

A hydrophobic knob observed in TKT was also formed by L95 and L125 in TKTL1 and TKTL2, respectively (Fig. 7). This protrusion is associated with the methylene group connecting the aminopyrimidine and thiazole rings of TDP and stabilized the co-factor in the active site. Numerous hydrogen-bonds to the X5P hydroxyl groups were also conserved in TKTL1 and TKTL2 and are expected to stabilize the reaction intermediate. Taken together, the sum of these interactions to the diphosphate tail, aminopyrimidine and thiazole rings, and X5P adduct, demonstrates a largely conserved pattern of interactions between the modeled TKTL1 and TKTL2 and the co-factor adduct, when compared to the TKT crystal structure (Fig. 6). This strongly suggests that TKTL1 and TKTL2 can stably associate with the TDP-X5P intermediate. Of note, the catalytic residue E366, shown to be essential for catalysis [25], is conserved in TKTL1 and TKTL2, while H110 is conserved in TKTL2. Thus, apart from a close structural equivalence, residues crucial for catalytic function is also conserved in TKTL2. It therefore seems very likely that TKTL2 can act as a transketolase.

However, will the absence of H110 in TKTL1 abolish its transketolase activity? It appears that alternative active residue patterns are possible in transketolases, suggesting some structural and functional degeneracy. For example, yeast transketolase utilizes H481 and H103 to bind to a water molecule, through which the thiazole C2 proton is indirectly abstracted. Mutation of the H481 residue abolished enzyme activity [26]. Intriguingly, in human TKT a glutamine residue (Q428) is present at the position equivalent to yeast H481 (Fig. 1). Mutation of Q428 resulted in a 3- to 4-fold reduction, but not a loss of TKT activity [27]. However, the mutation of H110 in human TKT (equivalent to yeast H103) resulted in a 50-fold reduction in activity, effectively abolishing enzyme function [27]. Thus, for human TKT the proposal is made that H110 and H77 pair functioned like the yeast H481 and H103 pair, where H110 and H481, respectively, acted as ultimate acceptors of the thiazole C2 proton.

Because of its value in biocatalysis, there has been a number of mutational and directed evolution studies of transketolases [28]. Although several interesting mutations were identified that resulted in relaxed substrate specificity and enhanced activity [28], there was no clear identification of a residue that could act as alternative proton acceptor to H110 or H481. However, the possible mechanistic impact of the absence of H110 and H77 in TKTL1 on possible catalytic activity is far from resolved in the literature. For example, Titmann and colleagues reported that deletion of a 38 residue region in TKT mimicked the observed deletion found in TKTL1 (containing H110 and H77) and abolished transketolase activity [29]. However, the recombinant deletion protein was not properly folded and it did not dimerize efficiently. Since dimerization is a requirement for the formation of a functional active site, where both sub-units contribute essential residues, it is not clear that this study provides any insights into TKTL1 functionality. A related study also reported the absence of transketolase activity for a recombinant human transketolase (38 residues deleted). However, other than noting that the protein was recovered from inclusion bodies in the bacterial hosts, this study did not assess folding and dimerization of the overexpressed protein [14].

Transketolase catalyzes the transfer of a 2-carbon ketose from a donor such as X5P to an acceptor such as ribose-5-P to produce sedoheptulose-7-P and glyceraldehyde-3-P in a classic ping-pong-bi-bi two-substrate reaction. An alternative reaction is the one-substrate reaction, where the ketose derived from the X5P donor dissociates from the active site to produce glycolaldehyde, which then acts as acceptor for another ketose to produce erythrose [30]. Some reported that the two-substrate reaction was decreased by two-fold in a H103A mutant yeast protein [31]. Of note, the one-substrate X5P reaction was enhanced by ~ two-fold to a level similar to the two-substrate reaction in the H103A mutant protein [31]. However, in yeast the H481 proton sink is still present in the H103 mutant. This is unlike the human enzyme where the structural homolog of H103 is H110, the functional equivalent of yeast H481.

Studies that expressed recombinant TKTL1 (versus TKT deletion mutants to mimic TKTL1) or that assayed endogenous TKTL1, reported transketolase activity [6, 32]. Here the recombinant TKTL1 protein exhibited both classic two-substrate activity, utilizing X5P and ribose-5-phosphate, as well as one-substrate specificity, utilizing only X5P [6]. Furthermore, others demonstrated that siRNA knockdown of TKTL1 in cultured human leukemia cells caused a significant decrease in TKT activity that was measured by glyceraldehyde-3-P production in the two-substrate X5P and ribose-5-P reaction [32]. No direct data on possible non-specific knockdown of TKT was provided, but the authors concluded that TKTL1 possesses TKT activity [32].

In light of these two studies demonstrating TKT activity for recombinant [6] and endogenous [32] TKTL1, and that the latter apparently lacks the equivalent of H110, the question naturally arises which residue acts as terminal acceptor to the thiazole C2 proton? An evaluation of the distribution of histidine residues in the region of the thiazole C2 and aminopyrimidine amino groups, shows that H231 in TKTL1 is structurally equivalent to H258 in TKT (Fig. 4a). The τ-nitrogen of H231 (like H258) is also within hydrogen-bond distance of the thiazole sulphur. It is therefore likely that H231 may act as acceptor to the C2 proton to activate the thiazole ring. For TKT the H110 residue is the preferred acceptor and may in addition stabilize the co-factor-substrate intermediate (Fig. 5a). It is thus possible that the absence of H110 in TKTL1 weakens the interaction with the ketose reaction intermediate, allowing for the dissociation of glycolaldehyde and enhancing the one-substrate reaction.

## Conclusions

Together these results support our hypothesis that TKTL1 and TKTL2 are functional transketolases and thus opens up the possibility that altered activity/function may be implicated in diseases such as diabetes and cancer. Additional physiological and biochemical studies should therefore be pursued to assess how modulation of such enzymes may be exploited as putative therapeutic interventions for diabetes and cancer.

## Additional files

Additional file 1: TKTL1_model: TKTL1 in covalent complex. X-ray diffraction data. (PDF 2100 kb)

Additional file 2: TKTL2_model: TKTL2 in covalent complex. X-ray diffraction data. (PDF 2185 kb)

## Abbreviations

NADPH: Nicotinamide adenine dinucleotide phosphate
NOGP: Non-oxidative glucose pathway
PPP: Pentose phosphate pathway
TDP: Thiamine diphosphate
TKT: Transketolase
TKTL1: Transketolase-like 1
TKTL2: Transketolase-like 2
X5P: D-xylulose-5-phosphate

## References

[1] Coy JF, Dubel S, Kioschis P, Thomas K, Micklem G, Delius H, Poustka A (1996). Molecular cloning of tissue-specific transcripts of a transketolase-related gene: implications for the evolution of new vertebrate genes. Genomics.

[2] Lindqvist Y, Schneider G, Ermler U, Sundstrom M (1992). Three-dimensional structure of transketolase, a thiamine diphosphate dependent enzyme, at 2.5 a resolution. EMBO J.

[3] Zhao J, Zhong CJ (2009). A review on research progress of transketolase. Neurosci Bull.

[4] Butterworth RF, Gaudreau C, Vincelette J, Bourgault AM, Lamothe F, Nutini AM (1991). Thiamine deficiency and Wernicke's encephalopathy in AIDS. Metab Brain Dis.

[5] Gibson GE, Sheu KF, Blass JP, Baker A, Carlson KC, Harding B, Perrino P (1988). Reduced activities of thiamine-dependent enzymes in the brains and peripheral tissues of patients with Alzheimer's disease. Arch Neurol.

[6] Coy JF, Dressler D, Wilde J, Schubert P (2005). Mutations in the transketolase-like gene TKTL1: clinical implications for neurodegenerative diseases, diabetes and cancer. Clin Lab.

[7] Rais B, Comin B, Puigjaner J, Brandes JL, Creppy E, Saboureau D, Ennamany R, Lee WN, Boros LG, Cascante M (1999). Oxythiamine and dehydroepiandrosterone induce a G1 phase cycle arrest in Ehrlich's tumor cells through inhibition of the pentose cycle. FEBS Lett.

[8] Jayachandran A, Lo PH, Chueh AC, Prithviraj P, Molania R, Davalos-Salas M, Anaka M, Walkiewicz M, Cebon J, Behren A (2016). Transketolase-like 1 ectopic expression is associated with DNA hypomethylation and induces the Warburg effect in melanoma cells. BMC Cancer.

[9] Foldi M, Stickeler E, Bau L, Kretz O, Watermann D, Gitsch G, Kayser G, Zur Hausen A, Coy JF (2007). Transketolase protein TKTL1 overexpression: a potential biomarker and therapeutic target in breast cancer. Oncol Rep.

[10] Langbein S, Zerilli M, Zur Hausen A, Staiger W, Rensch-Boschert K, Lukan N, Popa J, Ternullo MP, Steidler A, Weiss C (2006). Expression of transketolase TKTL1 predicts colon and urothelial cancer patient survival: Warburg effect reinterpreted. Br J Cancer.

[11] Hu LH, Yang JH, Zhang DT, Zhang S, Wang L, Cai PC, Zheng JF, Huang JS (2007). The TKTL1 gene influences total transketolase activity and cell proliferation in human colon cancer LoVo cells. Anti-Cancer Drugs.

[12] Zhang M, Chai YD, Brumbaugh J, Liu X, Rabii R, Feng S, Misuno K, Messadi D, Hu S (2014). Oral cancer cells may rewire alternative metabolic pathways to survive from siRNA silencing of metabolic enzymes. BMC Cancer.

[13] Mitschke L, Parthier C, Schroder-Tittmann K, Coy J, Ludtke S, Tittmann K (2010). The crystal structure of human transketolase and new insights into its mode of action. J Biol Chem.

[14] Meshalkina LE, Drutsa VL, Koroleva ON, Solovjeva ON, Kochetov GA (2013). Is transketolase-like protein, TKTL1, transketolase?. Biochim Biophys Acta.

[15] Maslova AO, Meshalkina LE, Kochetov GA (2012). Computer modeling of transketolase-like protein, TKTL1, a marker of certain tumor tissues. Biochemistry (Mosc).

[16] Nilsson U, Meshalkina L, Lindqvist Y, Schneider G (1997). Examination of substrate binding in thiamin diphosphate-dependent transketolase by protein crystallography and site-directed mutagenesis. J Biol Chem.

[17] Ludtke S, Neumann P, Erixon KM, Leeper F, Kluger R, Ficner R, Tittmann K (2013). Sub-angstrom-resolution crystallography reveals physical distortions that enhance reactivity of a covalent enzymatic intermediate. Nat Chem.

[18] Kim DE, Chivian D, Baker D (2004). Protein structure prediction and analysis using the Robetta server. Nucleic Acids Res.

[19] Benkert P, Kunzli M, Schwede T (2009). QMEAN server for protein model quality estimation. Nucleic Acids Res.

[20] Chen VB, Arendall WB, Headd JJ, Keedy DA, Immormino RM, Kapral GJ, Murray LW, Richardson JS, Richardson DC (2010). MolProbity: all-atom structure validation for macromolecular crystallography. Acta Crystallogr D Biol Crystallogr.

[21] Luthy R, Bowie JU, Eisenberg D (1992). Assessment of protein models with three-dimensional profiles. Nature.

[22] Pettersen EF, Goddard TD, Huang CC, Couch GS, Greenblatt DM, Meng EC, Ferrin TE (2004). UCSF chimera--a visualization system for exploratory research and analysis. J Comput Chem.

[23] Meng EC, Pettersen EF, Couch GS, Huang CC, Ferrin TE (2006). Tools for integrated sequence-structure analysis with UCSF chimera. BMC Bioinformatics.

[24] Mills JE, Dean PM (1996). Three-dimensional hydrogen-bond geometry and probability information from a crystal survey. J Comput Aided Mol Des.

[25] Di Tommaso P, Moretti S, Xenarios I, Orobitg M, Montanyola A, Chang JM, Taly JF, Notredame C (2011). T-coffee: a web server for the multiple sequence alignment of protein and RNA sequences using structural information and homology extension. Nucleic Acids Res.

[26] Robert X, Gouet P (2014). Deciphering key features in protein structures with the new ENDscript server. Nucleic Acids Res.

[27] Singleton CK, Wang JJ, Shan L, Martin PR (1996). Conserved residues are functionally distinct within transketolases of different species. Biochemistry.

[28] Ranoux A, Hanefeld U (2013). Improving Transketolase. Top Catalyst.

[29] Lyskov S, Gray JJ (2008). The RosettaDock server for local protein-protein docking. Nucleic Acids Res.

[30] Bykova IA, Solovjeva ON, Meshalkina LE, Kovina MV, Kochetov GA (2001). One-substrate transketolase-catalyzed reaction. Biochem Biophys Res Commun.

[31] Selivanov VA, Kovina MV, Kochevova NV, Meshalkina LE, Kochetov GA (2004). Kinetic study of the H103A mutant yeast transketolase. FEBS Lett.

[32] Diaz-Moralli S, Aguilar E, Marin S, Coy JF, Dewerchin M, Antoniewicz MR, Meca-Cortes O, Notebaert L, Ghesquiere B, Eelen G, et al. A key role for transketolase-like 1 in tumor metabolic reprogramming. Oncotarget. 2016. 10.18632/oncotarget.10429 PMC5239521 27391434

[33] Xu ZP, Wawrousek EF, Piatigorsky J (2002). Transketolase haploinsufficiency reduces adipose tissue and female fertility in mice. Mol Cell Biol.

[34] Boyle L, Wamelink MM, Salomons GS, Roos B, Pop A, Dauber A, Hwa V, Andrew M, Douglas J, Feingold M (2016). Mutations in TKT are the cause of a syndrome including short stature, developmental delay, and congenital heart defects. Am J Hum Genet.

