# Supplementary material for: The human transketolase-like proteins TKTL1 and TKTL2 are bona fide transketolases
Source: BMC Struct Biol. 2019 Jan 15;19:2. doi: 10.1186/s12900-018-0099-y (PMC6334435; doi:10.1186/s12900-018-0099-y)
Supplement: Supplementary file 1 — TKTL1_model: TKTL1 in covalent complex. X-ray diffraction data. (PDF 2100 kb) [file 12900_2018_99_MOESM1_ESM.pdf]

```
HEADER    TRANSFERASE                             28-MAY-13   XXXX
TITLE     HUMAN TRANSKETOLASE IN COVALENT COMPLEX WITH DONOR KETOSE D-
XYLULOSE-
TITLE    2 5-PHOSPHATE, CRYSTAL 2
KEYWDS    THIAMIN DIPHOSPHATE, ENZYME CATALYSIS, PENTOSE PHOSPHATE
PATHWAY,
KEYWDS   2 TRANSFERASE
EXPDTA    X-RAY DIFFRACTION
AUTHOR    P.NEUMANN, S.LUEDTKE, R.FICNER, K.TITTMANN
JRNL        AUTH   S.LUDTKE, P.NEUMANN, K.M.ERIXON, F.LEEPER, R.KLUGER,
JRNL        AUTH 2 R.FICNER, K.TITTMANN
JRNL        TITL   SUB-ANGSTROM-RESOLUTION CRYSTALLOGRAPHY REVEALS
PHYSICAL
JRNL        TITL 2 DISTORTIONS THAT ENHANCE REACTIVITY OF A COVALENT
ENZYMATIC
JRNL        TITL 3 INTERMEDIATE.
JRNL        REF    NAT CHEM                      V.   5   762 2013
JRNL        REFN
JRNL        PMID   23965678
JRNL        DOI    10.1038/NCHEM.1728
SEQRES   1 A  637  MET GLU SER TYR HIS LYS PRO ASP GLN GLN LYS LEU GLN
SEQRES   2 A  637  ALA LEU LYS ASP THR ALA ASN ARG LEU ARG ILE SER SER
SEQRES   3 A  637  ILE GLN ALA THR THR ALA ALA GLY SER GLY HIS PRO THR
SEQRES   4 A  637  SER CYS CYS SER ALA ALA GLU ILE MET ALA VAL LEU PHE
SEQRES   5 A  637  PHE HIS THR MET ARG TYR LYS SER GLN ASP PRO ARG ASN
SEQRES   6 A  637  PRO HIS ASN ASP ARG PHE VAL LEU SER LYS GLY HIS ALA
SEQRES   7 A  637  ALA PRO ILE LEU TYR ALA VAL TRP ALA GLU ALA GLY PHE
SEQRES   8 A  637  LEU ALA GLU ALA GLU LEU LEU ASN LEU ARG LYS ILE SER
SEQRES   9 A  637  SER ASP LEU ASP GLY HIS PRO VAL PRO LYS GLN ALA PHE
SEQRES  10 A  637  THR ASP VAL ALA THR GLY SER LEU GLY GLN GLY LEU GLY
SEQRES  11 A  637  ALA ALA CYS GLY MET ALA TYR THR GLY LYS TYR PHE ASP
SEQRES  12 A  637  LYS ALA SER TYR ARG VAL TYR CYS LEU LEU GLY ASP GLY
SEQRES  13 A  637  GLU LEU SER GLU GLY SER VAL TRP GLU ALA MET ALA PHE
SEQRES  14 A  637  ALA SER ILE TYR LYS LEU ASP ASN LEU VAL ALA ILE LEU
SEQRES  15 A  637  ASP ILE ASN ARG LEU GLY GLN SER ASP PRO ALA PRO LEU
SEQRES  16 A  637  GLN HIS GLN MET ASP ILE TYR GLN LYS ARG CYS GLU ALA
SEQRES  17 A  637  PHE GLY TRP HIS ALA ILE ILE VAL ASP GLY HIS SER VAL
SEQRES  18 A  637  GLU GLU LEU CYS LYS ALA PHE GLY GLN ALA LYS HIS GLN
SEQRES  19 A  637  PRO THR ALA ILE ILE ALA LYS THR PHE LYS GLY ARG GLY
SEQRES  20 A  637  ILE THR GLY VAL GLU ASP LYS GLU SER TRP HIS GLY LYS
SEQRES  21 A  637  PRO LEU PRO LYS ASN MET ALA GLU GLN ILE ILE GLN GLU
SEQRES  22 A  637  ILE TYR SER GLN ILE GLN SER LYS LYS LYS ILE LEU ALA
SEQRES  23 A  637  THR PRO PRO GLN GLU ASP ALA PRO SER VAL ASP ILE ALA
SEQRES  24 A  637  ASN ILE ARG MET PRO SER LEU PRO SER TYR LYS VAL GLY
SEQRES  25 A  637  ASP LYS ILE ALA THR ARG LYS ALA TYR GLY GLN ALA LEU
SEQRES  26 A  637  ALA LYS LEU GLY HIS ALA SER ASP ARG ILE ILE ALA LEU
SEQRES  27 A  637  ASP GLY ASP THR LYS ASN SER THR PHE SER GLU ILE PHE
SEQRES  28 A  637  LYS LYS GLU HIS PRO ASP ARG PHE ILE GLU CYS TYR ILE
SEQRES  29 A  637  ALA GLU GLN ASN MET VAL SER ILE ALA VAL GLY CYS ALA
SEQRES  30 A  637  THR ARG ASN ARG THR VAL PRO PHE CYS SER THR PHE ALA
SEQRES  31 A  637  ALA PHE PHE THR ARG ALA PHE ASP GLN ILE ARG MET ALA
SEQRES  32 A  637  ALA ILE SER GLU SER ASN ILE ASN LEU CYS GLY SER HIS
SEQRES  33 A  637  CYS GLY VAL SER ILE GLY GLU ASP GLY PRO SER GLN MET
SEQRES  34 A  637  ALA LEU GLU ASP LEU ALA MET PHE ARG SER VAL PRO THR
SEQRES  35 A  637  SER THR VAL PHE TYR PRO SER ASP GLY VAL ALA THR GLU
SEQRES  36 A  637  LYS ALA VAL GLU LEU ALA ALA ASN THR LYS GLY ILE CYS
SEQRES  37 A  637  PHE ILE ARG THR SER ARG PRO GLU ASN ALA ILE ILE TYR
SEQRES  38 A  637  ASN ASN ASN GLU ASP PHE GLN VAL GLY GLN ALA LYS VAL
```

```
SEQRES  39 A  637  VAL LEU LYS SER LYS ASP ASP GLN VAL THR VAL ILE GLY
SEQRES  40 A  637  ALA GLY VAL THR LEU HIS GLU ALA LEU ALA ALA ALA GLU
SEQRES  41 A  637  LEU LEU LYS LYS GLU LYS ILE ASN ILE ARG VAL LEU ASP
SEQRES  42 A  637  PRO PHE THR ILE LYS PRO LEU ASP ARG LYS LEU ILE LEU
SEQRES  43 A  637  ASP SER ALA ARG ALA THR LYS GLY ARG ILE LEU THR VAL
SEQRES  44 A  637  GLU ASP HIS TYR TYR GLU GLY GLY ILE GLY GLU ALA VAL
SEQRES  45 A  637  SER SER ALA VAL VAL GLY GLU PRO GLY ILE THR VAL THR
SEQRES  46 A  637  HIS LEU ALA VAL ASN ARG VAL PRO ARG SER GLY LYS PRO
SEQRES  47 A  637  ALA GLU LEU LEU LYS MET PHE GLY ILE ASP ARG ASP ALA
SEQRES  48 A  637  ILE ALA GLN ALA VAL ARG GLY LEU ILE THR LYS ALA LEU
SEQRES  49 A  637  VAL PRO ARG GLY SER LEU GLU HIS HIS HIS HIS HIS HIS
HETNAM      MG MAGNESIUM ION
HETNAM     EDO 1,2-ETHANEDIOL
HETNAM      NA SODIUM ION
HETNAM     TDP THIAMIN DIPHOSPHATE
HETNAM     DX5 D-XYLITOL-5-PHOSPHATE
HETSYN     EDO ETHYLENE GLYCOL
HETSYN     DX5 D-HYLITOL-5-PHOSPHATE
FORMUL   2   MG    MG 2+
FORMUL   3  EDO    12(C2 H6 O2)
FORMUL  14   NA    NA 1+
FORMUL  16  TDP    C12 H18 N4 O7 P2 S
FORMUL  17  DX5    2(C5 H13 O8 P)
FORMUL  19  HOH   *963(H2 O)
LINK         C2  TDP A1015                 C2 ADX5 A1016     1555   1555
1.54
LINK         OD1 ASP A 155                MG    MG A1001     1555   1555
2.03
LINK        MG    MG A1001                 O13 TDP A1015     1555   1555
2.06
LINK         O   LEU A 187                MG    MG A1001     1555   1555
2.06
LINK        MG    MG A1001                 O21 TDP A1015     1555   1555
2.07
LINK         OD1 ASN A 185                MG    MG A1001     1555   1555
2.11
LINK        MG    MG A1001                 O   HOH A7001     1555   1555
2.22
LINK         O   ALA A 461                NA    NA A1013     1555   1555
2.25
LINK        NA    NA A1013                 O   HOH A7081     1555   1555
2.25
LINK         O   THR A 464                NA    NA A1013     1555   1555
2.33
LINK         OD1 ASN A 411                NA    NA A1013     1555   1555
2.35
LINK        NA    NA A1013                 O   HOH A7070     1555   1555
2.41
CISPEP   1 LYS A  538    PRO A  539          1        -3.69
SITE     1 AC1  5 ASP A 155  ASN A 185  LEU A 187  TDP A1015
SITE     2 AC1  5 HOH A7001
SITE     1 AC2  8 PRO A  63  ASN A  68  ASP A  69  ARG A  70
SITE     2 AC2  8 PHE A  71  ARG A 379  HOH A7186  HOH A7847
SITE     1 AC3  7 TYR A 564  ALA A 588  ASN A 590  HOH A7051
SITE     2 AC3  7 HOH A7055  HOH A7126  HOH A7247
SITE     1 AC4  8 PHE A  71  VAL A  72  LEU A  73  LEU A  82
SITE     2 AC4  8 PHE A 117  HOH A7040  HOH A7056  HOH A7068
SITE     1 AC5 13 ASP A 155  GLY A 156  LEU A 158  SER A 159
```

```
SITE     2 AC5 13 TRP A 164  PRO A 194  LEU A 195  TYR A 202
SITE     3 AC5 13 ARG A 205  EDO A1012  HOH A7467  HOH A7960
SITE     4 AC5 13 HOH A7961
SITE     1 AC6  7 LEU A  92  LEU A 107  GLN A 115  HOH A7110
SITE     2 AC6  7 HOH A7124  HOH A7174  HOH A7920
SITE     1 AC7  4 GLN A  10  ASN A 590  ARG A 591  HOH A7260
SITE     1 AC8  6 TYR A 137  TYR A 141  TYR A 173  HOH A7409
SITE     2 AC8  6 HOH A7610  HOH A7914
SITE     1 AC9  8 ALA A  33  ARG A 101  GLU A 423  LYS A 597
SITE     2 AC9  8 HOH A7103  HOH A7586  HOH A7742  HOH A7934
SITE     1 BC1 10 ARG A  21  SER A  25  ALA A  84  GLU A  88
SITE     2 BC1 10 GLU A  94  LEU A  97  LYS A 283  HOH A7177
SITE     3 BC1 10 HOH A7472  HOH A7672
SITE     1 BC2  7 PHE A 142  ASP A 333  ARG A 358  HOH A7406
SITE     2 BC2  7 HOH A7539  HOH A7700  HOH A7894
SITE     1 BC3  9 LEU A 158  SER A 159  TRP A 164  GLU A 165
SITE     2 BC3  9 ALA A 168  PHE A 209  EDO A1005  EDO A1014
SITE     3 BC3  9 HOH A7961
SITE     1 BC4  6 ASN A 411  ALA A 461  THR A 464  CYS A 468
SITE     2 BC4  6 HOH A7070  HOH A7081
SITE     1 BC5  6 SER A 159  GLU A 165  ALA A 168  EDO A1012
SITE     2 BC5  6 HOH A7467  HOH A7715
SITE     1 BC6 26 SER A  40  LYS A  75  HIS A  77  GLY A 123
SITE     2 BC6 26 SER A 124  LEU A 125  GLY A 154  ASP A 155
SITE     3 BC6 26 GLY A 156  GLU A 157  GLU A 160  ASN A 185
SITE     4 BC6 26 LEU A 187  GLY A 188  GLN A 189  LYS A 244
SITE     5 BC6 26 HIS A 258  ASP A 341  ILE A 364  GLU A 366
SITE     6 BC6 26 PHE A 392  ARG A 395  GLN A 428   MG A1001
SITE     7 BC6 26 DX5 A1016  HOH A7001
SITE     1 BC7 19 HIS A  37  HIS A  77  HIS A 110  GLN A 189
SITE     2 BC7 19 HIS A 258  SER A 345  PHE A 389  HIS A 416
SITE     3 BC7 19 ASP A 424  GLN A 428  ARG A 474  TDP A1015
SITE     4 BC7 19 DX5 A1017  HOH A7053  HOH A7095  HOH A7108
SITE     5 BC7 19 HOH A7246  HOH A7263  HOH A7299
SITE     1 BC8 14 HIS A  37  GLN A 189  HIS A 258  ARG A 318
SITE     2 BC8 14 SER A 345  HIS A 416  ASP A 424  ARG A 474
SITE     3 BC8 14 DX5 A1016  HOH A7108  HOH A7246  HOH A7263
SITE     4 BC8 14 HOH A7272  HOH A7299
CRYST1  113.756   86.017   72.994  90.00 125.46  90.00 C 1 2 1       4
ORIGX1      1.000000  0.000000  0.000000        0.00000
ORIGX2      0.000000  1.000000  0.000000        0.00000
ORIGX3      0.000000  0.000000  1.000000        0.00000
SCALE1      0.008791  0.000000  0.006261        0.00000
SCALE2      0.000000  0.011626  0.000000        0.00000
SCALE3      0.000000  0.000000  0.016819        0.00000
MODEL        1
HETATM    1 MG   MG  A1001      -8.737  -1.113  15.346  1.00  4.15
Mg
ANISOU    1 MG   MG  A1001      632    472    472    -12    -10     33
Mg
HETATM    2  N1' TDP A1015      -6.862   9.553   8.850  1.00  5.75
N
ANISOU    2  N1' TDP A1015      909    538    738      5    -74     23
N
HETATM    3  C2' TDP A1015      -5.581   9.664   9.301  1.00  5.58
C
ANISOU    3  C2' TDP A1015      832    524    766     89    -22    -18
C
```

```
HETATM    4  C2A TDP A1015      -4.497   9.698   8.295  1.00  6.48
C
ANISOU    4  C2A TDP A1015      919    743    800     13     73    -49
C
HETATM    5  N3' TDP A1015      -5.288   9.772  10.576  1.00  5.24
N
ANISOU    5  N3' TDP A1015      737    594    661     73    -52    -68
N
HETATM    6  C4' TDP A1015      -6.316   9.849  11.502  1.00  5.34
C
ANISOU    6  C4' TDP A1015      833    524    671     91    -92     34
C
HETATM    7  N4' TDP A1015      -6.013  10.129  12.738  1.00  6.32
N
ANISOU    7  N4' TDP A1015      865    886    651     80   -111      0
N
HETATM    8  C5' TDP A1015      -7.678   9.646  11.074  1.00  5.31
C
ANISOU    8  C5' TDP A1015      800    527    690     21     -4     52
C
HETATM    9  C6' TDP A1015      -7.909   9.531   9.748  1.00  5.48
C
ANISOU    9  C6' TDP A1015      881    498    705      7     27     38
C
HETATM   10  C35 TDP A1015      -8.849   9.737  12.025  1.00  5.81
C
ANISOU   10  C35 TDP A1015      817    671    718     85    -10    116
C
HETATM   11  N3  TDP A1015      -8.863   8.649  13.036  1.00  5.49
N
ANISOU   11  N3  TDP A1015      738    669    679    -46    -68     13
N
HETATM   12  C2  TDP A1015      -8.355   8.763  14.320  1.00  6.54
C
ANISOU   12  C2  TDP A1015      968    716    802     62    -54     -4
C
HETATM   13  S1  TDP A1015      -8.234   7.200  15.036  1.00  6.08
S
ANISOU   13  S1  TDP A1015      977    665    669    -72    -68     25
S
HETATM   14  C5  TDP A1015      -9.000   6.438  13.685  1.00  4.69
C
ANISOU   14  C5  TDP A1015      583    583    617     27      6    -46
C
HETATM   15  C4  TDP A1015      -9.277   7.349  12.698  1.00  5.07
C
ANISOU   15  C4  TDP A1015      679    580    665    -28    -23    -11
C
HETATM   16  C4A TDP A1015      -9.974   7.111  11.413  1.00  6.57
C
ANISOU   16  C4A TDP A1015     1025    678    794    -66   -263      5
C
HETATM   17  C5A TDP A1015      -9.347   4.983  13.702  1.00  5.27
C
ANISOU   17  C5A TDP A1015      702    565    736     32    -12     29
C
HETATM   18  C5B TDP A1015      -8.109   4.126  13.877  1.00  5.40
C
```

```
ANISOU   18  C5B TDP A1015       777    447    828      -6    -84    -15
C
HETATM   19  O5G TDP A1015       -8.540   2.749  13.942  1.00  4.98
O
ANISOU   19  O5G TDP A1015       678    567    648     -18    -13     22
O
HETATM   20  P1  TDP A1015       -7.437   1.640  14.162  1.00  4.37
P
ANISOU   20  P1  TDP A1015       652    530    480       3    -34     13
P
HETATM   21  O11 TDP A1015       -6.992   1.835  15.687  1.00  4.67
O
ANISOU   21  O11 TDP A1015       676    578    520     -37    -16    -47
O
HETATM   22  O12 TDP A1015       -6.236   1.879  13.318  1.00  4.89
O
ANISOU   22  O12 TDP A1015       688    628    543      -3     56     49
O
HETATM   23  O13 TDP A1015       -8.141   0.342  14.015  1.00  4.59
O
ANISOU   23  O13 TDP A1015       702    545    497     -27    -10     31
O
HETATM   24  P2  TDP A1015       -7.686   1.386  17.073  1.00  4.67
P
ANISOU   24  P2  TDP A1015       752    517    505     -36    -18    -13
P
HETATM   25  O21 TDP A1015       -8.797   0.420  16.734  1.00  4.67
O
ANISOU   25  O21 TDP A1015       741    506    529     -50    -20     13
O
HETATM   26  O22 TDP A1015       -6.604   0.681  17.829  1.00  5.74
O
ANISOU   26  O22 TDP A1015       929    581    672       8   -193     58
O
HETATM   27  O23 TDP A1015       -8.180   2.655  17.693  1.00  5.73
O
ANISOU   27  O23 TDP A1015       855    648    673    -109    110    -92
O
HETATM   28  C1 ADX5 A1016       -7.461   9.833  16.404  0.76  5.96
C
ANISOU   28  C1 ADX5 A1016       795    809    659      29   -137    -50
C
HETATM   29  C2 ADX5 A1016       -8.313  10.045  15.173  0.76  5.25
C
ANISOU   29  C2 ADX5 A1016       785    479    733     -70   -100    -91
C
HETATM   30  C3 ADX5 A1016       -9.843  10.331  15.596  0.76  6.24
C
ANISOU   30  C3 ADX5 A1016       763    662    946    -105   -130    -74
C
HETATM   31  C4 ADX5 A1016      -10.094  11.696  16.217  0.76  5.84
C
ANISOU   31  C4 ADX5 A1016       776    751    693      45   -125    -71
C
HETATM   32  C5 ADX5 A1016      -11.618  11.938  16.316  0.76  8.12
C
ANISOU   32  C5 ADX5 A1016      1055    714   1315      73    164   -166
C
```

```
HETATM   33  O1 ADX5 A1016      -6.075   9.760  15.987  0.76  7.77
O
ANISOU   33  O1 ADX5 A1016      744   1298    909    149    -85   -308
O
HETATM   34  O2 ADX5 A1016      -7.914  11.143  14.412  0.76  6.23
O
ANISOU   34  O2 ADX5 A1016     1114    566    686    -43     17     44
O
HETATM   35  O3 ADX5 A1016     -10.311   9.338  16.438  0.76  7.72
O
ANISOU   35  O3 ADX5 A1016     1005    603   1325    -45    238     28
O
HETATM   36  O4 ADX5 A1016      -9.493  11.773  17.506  0.76  6.89
O
ANISOU   36  O4 ADX5 A1016      897    895    824     22   -127    -40
O
HETATM   37  O5 ADX5 A1016     -11.827  13.339  16.621  0.76  7.56
O
ANISOU   37  O5 ADX5 A1016      962    909   1002     90     55   -116
O
HETATM   38  P  ADX5 A1016     -13.254  13.788  17.212  0.76  7.30
P
ANISOU   38  P  ADX5 A1016     1027    858    889    101    -40    -83
P
HETATM   39  O1PADX5 A1016     -13.213  15.317  17.176  0.76  8.51
O
ANISOU   39  O1PADX5 A1016     1157    842   1236     15     51     16
O
HETATM   40  O2PADX5 A1016     -14.329  13.233  16.339  0.76  8.88
O
ANISOU   40  O2PADX5 A1016     1032   1294   1049    117    -99   -283
O
HETATM   41  O3PADX5 A1016     -13.372  13.253  18.617  0.76  8.82
O
ANISOU   41  O3PADX5 A1016     1514    979    860   -146     67     40
O
HETATM   42  C3 BDX5 A1017     -11.582  10.867  16.531  0.24 46.37
C
ANISOU   42  C3 BDX5 A1017    13940   1236   2443  -1122  -1222    753
C
HETATM   43  C4 BDX5 A1017     -11.323  12.188  17.218  0.24 23.15
C
ANISOU   43  C4 BDX5 A1017     2446   1768   4583   1622  -1990   -617
C
HETATM   44  C5 BDX5 A1017     -12.512  12.667  18.007  0.24 21.75
C
ANISOU   44  C5 BDX5 A1017     2263    984   5017    821  -1926  -1703
C
HETATM   45  O3 BDX5 A1017     -11.734   9.905  17.486  0.24 26.32
O
ANISOU   45  O3 BDX5 A1017     6197   1208   2596  -1737  -2980    829
O
HETATM   46  O4 BDX5 A1017     -10.185  12.075  18.070  0.24 17.60
O
ANISOU   46  O4 BDX5 A1017     1421   2006   3261   -514   -657   1691
O
HETATM   47  O5 BDX5 A1017     -13.819  12.773  17.553  0.24  9.90
O
```

```
ANISOU   47  O5 BDX5 A1017      1689    861   1212   -159   -437      20
O
HETATM   48  P  BDX5 A1017      -14.584  14.204  17.629  0.24  8.44
P
ANISOU   48  P  BDX5 A1017      1207    948   1053     39   -155     142
P
HETATM   49  O1PBDX5 A1017      -15.628  13.948  16.581  0.24  9.47
O
ANISOU   49  O1PBDX5 A1017      1020   1090   1489   -140   -610      28
O
HETATM   50  O2PBDX5 A1017      -15.129  14.270  19.028  0.24 16.45
O
ANISOU   50  O2PBDX5 A1017      2618   2692    941  -1758    727    -434
O
HETATM   51  O3PBDX5 A1017      -13.692  15.369  17.297  0.24 14.94
O
ANISOU   51  O3PBDX5 A1017      2826   2158    694  -1368     -2     124
O
ENDMDL
CONECT    1   23   25
CONECT   12   13   11   29
CONECT    3    5    4    2
CONECT    4    3
CONECT   10    8   11
CONECT   15   16   11   14
CONECT    6    5    8    7
CONECT   16   15
CONECT   14   13   17   15
CONECT    8    9    6   10
CONECT   17   14   18
CONECT   18   19   17
CONECT    9    8    2
CONECT    2    9    3
CONECT   11   12   10   15
CONECT    5    6    3
CONECT    7    6
CONECT   21   24   20
CONECT   22   20
CONECT   23   20
CONECT   23    1
CONECT   25   24
CONECT   25    1
CONECT   26   24
CONECT   27   24
CONECT   19   20   18
CONECT   20   21   23   19   22
CONECT   24   21   25   27   26
CONECT   13   14   12
CONECT   28   29   33
CONECT   29   28   30   12   34
CONECT   30   29   35   31
CONECT   31   30   36   32
CONECT   32   37   31
CONECT   33   28
CONECT   39   38
CONECT   34   29
CONECT   40   38
CONECT   35   30
```

```
CONECT   41   38
CONECT   36   31
CONECT   37   38   32
CONECT   38   41   37   40   39
CONECT   42   45   43
CONECT   43   46   44   42
CONECT   44   47   43
CONECT   49   48
CONECT   50   48
CONECT   45   42
CONECT   51   48
CONECT   46   43
CONECT   47   48   44
CONECT   48   49   51   47   50
MODEL        2
HETATM    1 MG   MG  A1001       8.737  -1.113 -15.346  1.00  4.15
Mg
ANISOU    1 MG   MG  A1001      632    472    472    -12    -10     33
Mg
HETATM    2  N1' TDP A1015       6.862   9.553  -8.850  1.00  5.75
N
ANISOU    2  N1' TDP A1015      909    538    738      5    -74     23
N
HETATM    3  C2' TDP A1015       5.581   9.664  -9.301  1.00  5.58
C
ANISOU    3  C2' TDP A1015      832    524    766     89    -22    -18
C
HETATM    4  C2A TDP A1015       4.497   9.698  -8.295  1.00  6.48
C
ANISOU    4  C2A TDP A1015      919    743    800     13     73    -49
C
HETATM    5  N3' TDP A1015       5.288   9.772 -10.576  1.00  5.24
N
ANISOU    5  N3' TDP A1015      737    594    661     73    -52    -68
N
HETATM    6  C4' TDP A1015       6.316   9.849 -11.502  1.00  5.34
C
ANISOU    6  C4' TDP A1015      833    524    671     91    -92     34
C
HETATM    7  N4' TDP A1015       6.013  10.129 -12.738  1.00  6.32
N
ANISOU    7  N4' TDP A1015      865    886    651     80   -111      0
N
HETATM    8  C5' TDP A1015       7.678   9.646 -11.074  1.00  5.31
C
ANISOU    8  C5' TDP A1015      800    527    690     21     -4     52
C
HETATM    9  C6' TDP A1015       7.909   9.531  -9.748  1.00  5.48
C
ANISOU    9  C6' TDP A1015      881    498    705      7     27     38
C
HETATM   10  C35 TDP A1015       8.849   9.737 -12.025  1.00  5.81
C
ANISOU   10  C35 TDP A1015      817    671    718     85    -10    116
C
HETATM   11  N3  TDP A1015       8.863   8.649 -13.036  1.00  5.49
N
```

```
ANISOU   11  N3  TDP A1015      738    669    679    -46    -68     13
N
HETATM   12  C2  TDP A1015       8.355   8.763 -14.320  1.00  6.54
C
ANISOU   12  C2  TDP A1015      968    716    802     62    -54     -4
C
HETATM   13  S1  TDP A1015       8.234   7.200 -15.036  1.00  6.08
S
ANISOU   13  S1  TDP A1015      977    665    669    -72    -68     25
S
HETATM   14  C5  TDP A1015       9.000   6.438 -13.685  1.00  4.69
C
ANISOU   14  C5  TDP A1015      583    583    617     27      6    -46
C
HETATM   15  C4  TDP A1015       9.277   7.349 -12.698  1.00  5.07
C
ANISOU   15  C4  TDP A1015      679    580    665    -28    -23    -11
C
HETATM   16  C4A TDP A1015       9.974   7.111 -11.413  1.00  6.57
C
ANISOU   16  C4A TDP A1015     1025    678    794    -66   -263      5
C
HETATM   17  C5A TDP A1015       9.347   4.983 -13.702  1.00  5.27
C
ANISOU   17  C5A TDP A1015      702    565    736     32    -12     29
C
HETATM   18  C5B TDP A1015       8.109   4.126 -13.877  1.00  5.40
C
ANISOU   18  C5B TDP A1015      777    447    828     -6    -84    -15
C
HETATM   19  O5G TDP A1015       8.540   2.749 -13.942  1.00  4.98
O
ANISOU   19  O5G TDP A1015      678    567    648    -18    -13     22
O
HETATM   20  P1  TDP A1015       7.437   1.640 -14.162  1.00  4.37
P
ANISOU   20  P1  TDP A1015      652    530    480      3    -34     13
P
HETATM   21  O11 TDP A1015       6.992   1.835 -15.687  1.00  4.67
O
ANISOU   21  O11 TDP A1015      676    578    520    -37    -16    -47
O
HETATM   22  O12 TDP A1015       6.236   1.879 -13.318  1.00  4.89
O
ANISOU   22  O12 TDP A1015      688    628    543     -3     56     49
O
HETATM   23  O13 TDP A1015       8.141   0.342 -14.015  1.00  4.59
O
ANISOU   23  O13 TDP A1015      702    545    497    -27    -10     31
O
HETATM   24  P2  TDP A1015       7.686   1.386 -17.073  1.00  4.67
P
ANISOU   24  P2  TDP A1015      752    517    505    -36    -18    -13
P
HETATM   25  O21 TDP A1015       8.797   0.420 -16.734  1.00  4.67
O
ANISOU   25  O21 TDP A1015      741    506    529    -50    -20     13
O
```

```
HETATM   26  O22 TDP A1015       6.604   0.681 -17.829  1.00  5.74
O
ANISOU   26  O22 TDP A1015      929    581    672      8   -193     58
O
HETATM   27  O23 TDP A1015       8.180   2.655 -17.693  1.00  5.73
O
ANISOU   27  O23 TDP A1015      855    648    673   -109    110    -92
O
HETATM   28  C1 ADX5 A1016       7.461   9.833 -16.404  0.76  5.96
C
ANISOU   28  C1 ADX5 A1016      795    809    659     29   -137    -50
C
HETATM   29  C2 ADX5 A1016       8.313  10.045 -15.173  0.76  5.25
C
ANISOU   29  C2 ADX5 A1016      785    479    733    -70   -100    -91
C
HETATM   30  C3 ADX5 A1016       9.843  10.331 -15.596  0.76  6.24
C
ANISOU   30  C3 ADX5 A1016      763    662    946   -105   -130    -74
C
HETATM   31  C4 ADX5 A1016      10.094  11.696 -16.217  0.76  5.84
C
ANISOU   31  C4 ADX5 A1016      776    751    693     45   -125    -71
C
HETATM   32  C5 ADX5 A1016      11.618  11.938 -16.316  0.76  8.12
C
ANISOU   32  C5 ADX5 A1016     1055    714   1315     73    164   -166
C
HETATM   33  O1 ADX5 A1016       6.075   9.760 -15.987  0.76  7.77
O
ANISOU   33  O1 ADX5 A1016      744   1298    909    149    -85   -308
O
HETATM   34  O2 ADX5 A1016       7.914  11.143 -14.412  0.76  6.23
O
ANISOU   34  O2 ADX5 A1016     1114    566    686    -43     17     44
O
HETATM   35  O3 ADX5 A1016      10.311   9.338 -16.438  0.76  7.72
O
ANISOU   35  O3 ADX5 A1016     1005    603   1325    -45    238     28
O
HETATM   36  O4 ADX5 A1016       9.493  11.773 -17.506  0.76  6.89
O
ANISOU   36  O4 ADX5 A1016      897    895    824     22   -127    -40
O
HETATM   37  O5 ADX5 A1016      11.827  13.339 -16.621  0.76  7.56
O
ANISOU   37  O5 ADX5 A1016      962    909   1002     90     55   -116
O
HETATM   38  P  ADX5 A1016      13.254  13.788 -17.212  0.76  7.30
P
ANISOU   38  P  ADX5 A1016     1027    858    889    101    -40    -83
P
HETATM   39  O1PADX5 A1016      13.213  15.317 -17.176  0.76  8.51
O
ANISOU   39  O1PADX5 A1016     1157    842   1236     15     51     16
O
HETATM   40  O2PADX5 A1016      14.329  13.233 -16.339  0.76  8.88
O
```

```
ANISOU   40  O2PADX5 A1016     1032   1294   1049    117    -99   -283
O
HETATM   41  O3PADX5 A1016      13.372  13.253 -18.617  0.76  8.82
O
ANISOU   41  O3PADX5 A1016     1514    979    860   -146     67     40
O
HETATM   42  C3 BDX5 A1017      11.582  10.867 -16.531  0.24 46.37
C
ANISOU   42  C3 BDX5 A1017    13940   1236   2443  -1122  -1222    753
C
HETATM   43  C4 BDX5 A1017      11.323  12.188 -17.218  0.24 23.15
C
ANISOU   43  C4 BDX5 A1017     2446   1768   4583   1622  -1990   -617
C
HETATM   44  C5 BDX5 A1017      12.512  12.667 -18.007  0.24 21.75
C
ANISOU   44  C5 BDX5 A1017     2263    984   5017    821  -1926  -1703
C
HETATM   45  O3 BDX5 A1017      11.734   9.905 -17.486  0.24 26.32
O
ANISOU   45  O3 BDX5 A1017     6197   1208   2596  -1737  -2980    829
O
HETATM   46  O4 BDX5 A1017      10.185  12.075 -18.070  0.24 17.60
O
ANISOU   46  O4 BDX5 A1017     1421   2006   3261   -514   -657   1691
O
HETATM   47  O5 BDX5 A1017      13.819  12.773 -17.553  0.24  9.90
O
ANISOU   47  O5 BDX5 A1017     1689    861   1212   -159   -437     20
O
HETATM   48  P  BDX5 A1017      14.584  14.204 -17.629  0.24  8.44
P
ANISOU   48  P  BDX5 A1017     1207    948   1053     39   -155    142
P
HETATM   49  O1PBDX5 A1017      15.628  13.948 -16.581  0.24  9.47
O
ANISOU   49  O1PBDX5 A1017     1020   1090   1489   -140   -610     28
O
HETATM   50  O2PBDX5 A1017      15.129  14.270 -19.028  0.24 16.45
O
ANISOU   50  O2PBDX5 A1017     2618   2692    941  -1758    727   -434
O
HETATM   51  O3PBDX5 A1017      13.692  15.369 -17.297  0.24 14.94
O
ANISOU   51  O3PBDX5 A1017     2826   2158    694  -1368     -2    124
O
ENDMDL
CONECT   12   11   29   13
CONECT    3    4    2    5
CONECT    4    3
CONECT   10    8   11
CONECT   15   16   14   11
CONECT    6    8    7    5
CONECT   16   15
CONECT   14   15   13   17
CONECT    8    6   10    9
CONECT   17   18   14
CONECT   18   19   17
```

```
CONECT    9    8    2
CONECT    2    3    9
CONECT   11   15   12   10
CONECT    5    3    6
CONECT    7    6
CONECT   21   24   20
CONECT   22   20
CONECT   23   20
CONECT   25   24
CONECT   26   24
CONECT   27   24
CONECT   19   18   20
CONECT   20   19   21   22   23
CONECT   24   27   26   21   25
CONECT   13   12   14
CONECT   28   33   29
CONECT   29   30   34   28   12
CONECT   30   31   35   29
CONECT   31   30   32   36
CONECT   32   31   37
CONECT   33   28
CONECT   39   38
CONECT   34   29
CONECT   40   38
CONECT   35   30
CONECT   41   38
CONECT   36   31
CONECT   37   32   38
CONECT   38   41   37   40   39
CONECT   42   45   43
CONECT   43   42   44   46
CONECT   44   47   43
CONECT   49   48
CONECT   50   48
CONECT   45   42
CONECT   51   48
CONECT   46   43
CONECT   47   44   48
CONECT   48   47   49   50   51
MODEL        3
ATOM      1  N   ALA A   1      39.043 -11.179   8.396  1.00400.00
N
ATOM      2  CA  ALA A   1      38.022 -10.244   8.859  1.00400.00
C
ATOM      3  C   ALA A   1      36.884 -10.983   9.553  1.00400.00
C
ATOM      4  O   ALA A   1      36.314 -11.923   8.998  1.00400.00
O
ATOM      5  CB  ALA A   1      37.484  -9.427   7.693  1.00600.00
C
ATOM      6 1H   ALA A   1      39.380 -10.888   7.489  1.00480.00
H
ATOM      7 2H   ALA A   1      39.810 -11.193   9.052  1.00480.00
H
ATOM      8 3H   ALA A   1      38.645 -12.104   8.325  1.00480.00
H
ATOM      9  HA  ALA A   1      38.480  -9.571   9.585  1.00480.00
H
```

```
ATOM     10 1HB  ALA A   1      36.741  -8.718   8.057  1.00720.00
H
ATOM     11 2HB  ALA A   1      38.302  -8.884   7.222  1.00720.00
H
ATOM     12 3HB  ALA A   1      37.023 -10.093   6.965  1.00720.00
H
ATOM     13  N   ASP A   2      36.559 -10.552  10.767  1.00400.00
N
ATOM     14  CA  ASP A   2      35.487 -11.167  11.537  1.00400.00
C
ATOM     15  C   ASP A   2      34.172 -10.427  11.344  1.00400.00
C
ATOM     16  O   ASP A   2      34.159  -9.220  11.094  1.00400.00
O
ATOM     17  CB  ASP A   2      35.847 -11.201  13.022  1.00600.00
C
ATOM     18  CG  ASP A   2      37.017 -12.127  13.321  1.00600.00
C
ATOM     19  OD1 ASP A   2      37.286 -12.991  12.521  1.00600.00
O
ATOM     20  OD2 ASP A   2      37.632 -11.961  14.347  1.00600.00
O
ATOM     21  H   ASP A   2      37.067  -9.776  11.168  1.00480.00
H
ATOM     22  HA  ASP A   2      35.355 -12.192  11.189  1.00480.00
H
ATOM     23 1HB  ASP A   2      36.103 -10.195  13.358  1.00720.00
H
ATOM     24 2HB  ASP A   2      34.983 -11.529  13.601  1.00720.00
H
ATOM     25  N   ALA A   3      33.067 -11.157  11.455  1.00400.00
N
ATOM     26  CA  ALA A   3      31.743 -10.563  11.326  1.00400.00
C
ATOM     27  C   ALA A   3      31.447  -9.663  12.520  1.00400.00
C
ATOM     28  O   ALA A   3      31.841  -9.962  13.648  1.00400.00
O
ATOM     29  CB  ALA A   3      30.684 -11.648  11.207  1.00600.00
C
ATOM     30  H   ALA A   3      33.147 -12.146  11.646  1.00480.00
H
ATOM     31  HA  ALA A   3      31.730  -9.949  10.426  1.00480.00
H
ATOM     32 1HB  ALA A   3      29.704 -11.189  11.092  1.00720.00
H
ATOM     33 2HB  ALA A   3      30.898 -12.271  10.338  1.00720.00
H
ATOM     34 3HB  ALA A   3      30.693 -12.265  12.105  1.00720.00
H
ATOM     35  N   GLU A   4      30.736  -8.569  12.268  1.00400.00
N
ATOM     36  CA  GLU A   4      30.382  -7.637  13.330  1.00400.00
C
ATOM     37  C   GLU A   4      29.118  -8.127  14.030  1.00400.00
C
ATOM     38  O   GLU A   4      28.018  -8.061  13.476  1.00400.00
O
```

ATOM 39 CB GLU A 4 30.188 -6.237 12.757 1.00600.00
C
ATOM 40 CG GLU A 4 29.930 -5.162 13.792 1.00600.00
C
ATOM 41 CD GLU A 4 29.741 -3.809 13.174 1.00600.00
C
ATOM 42 OE1 GLU A 4 29.788 -3.720 11.968 1.00600.00
O
ATOM 43 OE2 GLU A 4 29.556 -2.861 13.899 1.00600.00
O
ATOM 44 H GLU A 4 30.440 -8.378 11.323 1.00480.00
H
ATOM 45 HA GLU A 4 31.191 -7.610 14.059 1.00480.00
H
ATOM 46 1HB GLU A 4 31.077 -5.951 12.193 1.00720.00
H
ATOM 47 2HB GLU A 4 29.349 -6.243 12.063 1.00720.00
H
ATOM 48 1HG GLU A 4 29.043 -5.426 14.357 1.00720.00
H
ATOM 49 2HG GLU A 4 30.772 -5.127 14.482 1.00720.00
H
ATOM 50 N ALA A 5 29.285 -8.630 15.251 1.00400.00
N
ATOM 51 CA ALA A 5 28.199 -9.273 15.990 1.00400.00
C
ATOM 52 C ALA A 5 27.207 -8.310 16.630 1.00400.00
C
ATOM 53 O ALA A 5 27.172 -8.155 17.850 1.00400.00
O
ATOM 54 CB ALA A 5 28.788 -10.168 17.070 1.00600.00
C
ATOM 55 H ALA A 5 30.208 -8.611 15.662 1.00480.00
H
ATOM 56 HA ALA A 5 27.647 -9.894 15.284 1.00480.00
H
ATOM 57 1HB ALA A 5 27.987 -10.693 17.590 1.00720.00
H
ATOM 58 2HB ALA A 5 29.459 -10.896 16.612 1.00720.00
H
ATOM 59 3HB ALA A 5 29.345 -9.561 17.782 1.00720.00
H
ATOM 60 N ARG A 6 26.366 -7.708 15.798 1.00400.00
N
ATOM 61 CA ARG A 6 25.282 -6.857 16.272 1.00400.00
C
ATOM 62 C ARG A 6 24.000 -7.667 16.286 1.00400.00
C
ATOM 63 O ARG A 6 23.002 -7.280 16.894 1.00400.00
O
ATOM 64 CB ARG A 6 25.134 -5.641 15.400 1.00600.00
C
ATOM 65 CG ARG A 6 26.358 -4.773 15.424 1.00600.00
C
ATOM 66 CD ARG A 6 26.591 -4.197 16.768 1.00600.00
C
ATOM 67 NE ARG A 6 27.741 -3.321 16.765 1.00600.00
N

ATOM 68 CZ ARG A 6 28.987 -3.681 17.130 1.00600.00
C
ATOM 69 NH1 ARG A 6 29.222 -4.901 17.567 1.00600.00
N
ATOM 70 NH2 ARG A 6 29.976 -2.807 17.048 1.00600.00
N
ATOM 71 H ARG A 6 26.496 -7.859 14.806 1.00480.00
H
ATOM 72 HA ARG A 6 25.504 -6.532 17.289 1.00480.00
H
ATOM 73 1HB ARG A 6 24.947 -5.940 14.369 1.00720.00
H
ATOM 74 2HB ARG A 6 24.285 -5.043 15.734 1.00720.00
H
ATOM 75 1HG ARG A 6 27.219 -5.382 15.165 1.00720.00
H
ATOM 76 2HG ARG A 6 26.253 -3.961 14.703 1.00720.00
H
ATOM 77 1HD ARG A 6 25.724 -3.622 17.080 1.00720.00
H
ATOM 78 2HD ARG A 6 26.773 -4.999 17.482 1.00720.00
H
ATOM 79 HE ARG A 6 27.593 -2.380 16.416 1.00720.00
H
ATOM 80 1HH1 ARG A 6 28.464 -5.567 17.631 1.00720.00
H
ATOM 81 2HH1 ARG A 6 30.156 -5.167 17.842 1.00720.00
H
ATOM 82 1HH2 ARG A 6 29.793 -1.875 16.703 1.00720.00
H
ATOM 83 2HH2 ARG A 6 30.911 -3.074 17.318 1.00720.00
H
ATOM 84 N ALA A 7 24.058 -8.820 15.619 1.00400.00
N
ATOM 85 CA ALA A 7 22.980 -9.786 15.557 1.00400.00
C
ATOM 86 C ALA A 7 23.013 -10.726 16.762 1.00400.00
C
ATOM 87 O ALA A 7 22.171 -11.617 16.880 1.00400.00
O
ATOM 88 CB ALA A 7 23.089 -10.584 14.269 1.00600.00
C
ATOM 89 H ALA A 7 24.904 -9.055 15.123 1.00480.00
H
ATOM 90 HA ALA A 7 22.034 -9.248 15.572 1.00480.00
H
ATOM 91 1HB ALA A 7 22.274 -11.301 14.209 1.00720.00
H
ATOM 92 2HB ALA A 7 23.042 -9.906 13.416 1.00720.00
H
ATOM 93 3HB ALA A 7 24.038 -11.118 14.254 1.00720.00
H
ATOM 94 N GLU A 8 23.990 -10.539 17.650 1.00400.00
N
ATOM 95 CA GLU A 8 24.097 -11.369 18.832 1.00400.00
C
ATOM 96 C GLU A 8 23.267 -10.788 19.960 1.00400.00
C

```
ATOM     97  O   GLU A   8      23.503  -9.668  20.416  1.00400.00
O
ATOM     98  CB  GLU A   8      25.556 -11.497  19.265  1.00600.00
C
ATOM     99  CG  GLU A   8      25.772 -12.395  20.475  1.00600.00
C
ATOM    100  CD  GLU A   8      27.219 -12.512  20.865  1.00600.00
C
ATOM    101  OE1 GLU A   8      28.045 -11.941  20.194  1.00600.00
O
ATOM    102  OE2 GLU A   8      27.499 -13.174  21.836  1.00600.00
O
ATOM    103  H   GLU A   8      24.672  -9.810  17.515  1.00480.00
H
ATOM    104  HA  GLU A   8      23.712 -12.362  18.599  1.00480.00
H
ATOM    105 1HB  GLU A   8      26.145 -11.899  18.442  1.00720.00
H
ATOM    106 2HB  GLU A   8      25.954 -10.511  19.505  1.00720.00
H
ATOM    107 1HG  GLU A   8      25.211 -11.993  21.317  1.00720.00
H
ATOM    108 2HG  GLU A   8      25.379 -13.387  20.253  1.00720.00
H
ATOM    109  N   PHE A   9      22.294 -11.567  20.409  1.00400.00
N
ATOM    110  CA  PHE A   9      21.405 -11.165  21.487  1.00400.00
C
ATOM    111  C   PHE A   9      21.463 -12.163  22.642  1.00251.85
C
ATOM    112  O   PHE A   9      21.828 -13.321  22.436  1.00258.82
O
ATOM    113  CB  PHE A   9      19.977 -10.984  20.947  1.00600.00
C
ATOM    114  CG  PHE A   9      19.860  -9.811  20.011  1.00600.00
C
ATOM    115  CD1 PHE A   9      20.008  -9.964  18.644  1.00600.00
C
ATOM    116  CD2 PHE A   9      19.620  -8.539  20.512  1.00600.00
C
ATOM    117  CE1 PHE A   9      19.925  -8.874  17.800  1.00600.00
C
ATOM    118  CE2 PHE A   9      19.532  -7.448  19.667  1.00600.00
C
ATOM    119  CZ  PHE A   9      19.686  -7.617  18.307  1.00600.00
C
ATOM    120  H   PHE A   9      22.161 -12.472  19.984  1.00480.00
H
ATOM    121  HA  PHE A   9      21.746 -10.196  21.850  1.00480.00
H
ATOM    122 1HB  PHE A   9      19.682 -11.881  20.403  1.00720.00
H
ATOM    123 2HB  PHE A   9      19.270 -10.849  21.763  1.00720.00
H
ATOM    124  HD1 PHE A   9      20.199 -10.957  18.239  1.00720.00
H
ATOM    125  HD2 PHE A   9      19.505  -8.403  21.587  1.00720.00
H
```

```
ATOM    126  HE1 PHE A   9      20.051  -9.010  16.727  1.00720.00
H
ATOM    127  HE2 PHE A   9      19.345  -6.455  20.075  1.00720.00
H
ATOM    128  HZ  PHE A   9      19.621  -6.759  17.640  1.00720.00
H
ATOM    129  N   PRO A  10      21.127 -11.719  23.861  1.00154.22
N
ATOM    130  CA  PRO A  10      21.115 -12.451  25.124  1.00 62.05
C
ATOM    131  C   PRO A  10      20.010 -13.498  25.186  1.00 26.21
C
ATOM    132  O   PRO A  10      19.051 -13.464  24.413  1.00 52.11
O
ATOM    133  CB  PRO A  10      20.921 -11.350  26.176  1.00 93.07
C
ATOM    134  CG  PRO A  10      20.260 -10.237  25.448  1.00 93.07
C
ATOM    135  CD  PRO A  10      20.888 -10.275  24.084  1.00 93.07
C
ATOM    136  HA  PRO A  10      22.094 -12.933  25.257  1.00 74.46
H
ATOM    137 1HB  PRO A  10      20.309 -11.731  27.008  1.00111.69
H
ATOM    138 2HB  PRO A  10      21.893 -11.062  26.601  1.00111.69
H
ATOM    139 1HG  PRO A  10      19.172 -10.392  25.419  1.00111.69
H
ATOM    140 2HG  PRO A  10      20.434  -9.285  25.969  1.00111.69
H
ATOM    141 1HD  PRO A  10      20.184  -9.863  23.354  1.00111.69
H
ATOM    142 2HD  PRO A  10      21.844  -9.730  24.082  1.00111.69
H
ATOM    143  N   GLU A  11      20.179 -14.432  26.103  1.00 40.37
N
ATOM    144  CA  GLU A  11      19.275 -15.551  26.295  1.00 69.18
C
ATOM    145  C   GLU A  11      17.836 -15.182  26.611  1.00 48.13
C
ATOM    146  O   GLU A  11      17.552 -14.319  27.446  1.00116.32
O
ATOM    147  CB  GLU A  11      19.798 -16.449  27.415  1.00103.77
C
ATOM    148  CG  GLU A  11      18.967 -17.702  27.658  1.00103.77
C
ATOM    149  CD  GLU A  11      19.546 -18.587  28.727  1.00103.77
C
ATOM    150  OE1 GLU A  11      20.569 -18.241  29.266  1.00103.77
O
ATOM    151  OE2 GLU A  11      18.966 -19.610  29.002  1.00103.77
O
ATOM    152  H   GLU A  11      20.991 -14.369  26.700  1.00 48.44
H
ATOM    153  HA  GLU A  11      19.269 -16.130  25.371  1.00 83.02
H
ATOM    154 1HB  GLU A  11      20.816 -16.763  27.184  1.00124.52
H
```

```
ATOM    155 2HB  GLU A  11      19.835 -15.885  28.347  1.00124.52
H
ATOM    156 1HG  GLU A  11      17.962 -17.407  27.958  1.00124.52
H
ATOM    157 2HG  GLU A  11      18.891 -18.263  26.728  1.00124.52
H
ATOM    158  N   GLU A  12      16.943 -15.853  25.910  1.00 20.24
N
ATOM    159  CA  GLU A  12      15.493 -15.800  26.075  1.00 18.63
C
ATOM    160  C   GLU A  12      14.940 -16.059  27.469  1.00 21.58
C
ATOM    161  O   GLU A  12      14.252 -17.059  27.622  1.00 73.51
O
ATOM    162  CB  GLU A  12      14.806 -16.826  25.175  1.00 27.95
C
ATOM    163  CG  GLU A  12      14.912 -16.604  23.681  1.00 27.95
C
ATOM    164  CD  GLU A  12      14.132 -17.651  22.923  1.00 27.95
C
ATOM    165  OE1 GLU A  12      13.594 -18.537  23.557  1.00 27.95
O
ATOM    166  OE2 GLU A  12      14.063 -17.566  21.721  1.00 27.95
O
ATOM    167  H   GLU A  12      17.298 -16.498  25.218  1.00 24.29
H
ATOM    168  HA  GLU A  12      15.173 -14.808  25.776  1.00 22.36
H
ATOM    169 1HB  GLU A  12      15.218 -17.813  25.384  1.00 33.53
H
ATOM    170 2HB  GLU A  12      13.745 -16.860  25.421  1.00 33.53
H
ATOM    171 1HG  GLU A  12      14.539 -15.613  23.428  1.00 33.53
H
ATOM    172 2HG  GLU A  12      15.960 -16.654  23.388  1.00 33.53
H
ATOM    173  N   ALA A  13      15.191 -15.226  28.463  1.00 16.10
N
ATOM    174  CA  ALA A  13      14.646 -15.501  29.802  1.00 12.78
C
ATOM    175  C   ALA A  13      13.136 -15.755  29.758  1.00 13.22
C
ATOM    176  O   ALA A  13      12.357 -14.812  29.741  1.00 49.69
O
ATOM    177  CB  ALA A  13      14.948 -14.341  30.734  1.00 19.17
C
ATOM    178  H   ALA A  13      15.775 -14.417  28.296  1.00 19.32
H
ATOM    179  HA  ALA A  13      15.129 -16.402  30.185  1.00 15.34
H
ATOM    180 1HB  ALA A  13      14.569 -14.563  31.730  1.00 23.00
H
ATOM    181 2HB  ALA A  13      16.027 -14.186  30.782  1.00 23.00
H
ATOM    182 3HB  ALA A  13      14.471 -13.437  30.357  1.00 23.00
H
ATOM    183  N   ARG A  14      12.721 -17.022  29.772  1.00 16.84
N
```

```
ATOM    184  CA  ARG A  14      11.303 -17.356  29.685  1.00 19.95
C
ATOM    185  C   ARG A  14      10.631 -17.221  31.057  1.00 15.18
C
ATOM    186  O   ARG A  14      10.964 -17.970  31.978  1.00 22.07
O
ATOM    187  CB  ARG A  14      11.124 -18.757  29.112  1.00 29.92
C
ATOM    188  CG  ARG A  14      11.598 -18.856  27.663  1.00 29.92
C
ATOM    189  CD  ARG A  14      11.390 -20.180  27.032  1.00 29.92
C
ATOM    190  NE  ARG A  14      11.922 -20.195  25.672  1.00 29.92
N
ATOM    191  CZ  ARG A  14      11.846 -21.227  24.813  1.00 29.92
C
ATOM    192  NH1 ARG A  14      11.252 -22.349  25.160  1.00 29.92
N
ATOM    193  NH2 ARG A  14      12.380 -21.093  23.612  1.00 29.92
N
ATOM    194  H   ARG A  14      13.404 -17.765  29.810  1.00 20.21
H
ATOM    195  HA  ARG A  14      10.846 -16.682  28.972  1.00 23.94
H
ATOM    196 1HB  ARG A  14      11.688 -19.476  29.705  1.00 35.91
H
ATOM    197 2HB  ARG A  14      10.074 -19.044  29.148  1.00 35.91
H
ATOM    198 1HG  ARG A  14      11.089 -18.104  27.061  1.00 35.91
H
ATOM    199 2HG  ARG A  14      12.666 -18.679  27.636  1.00 35.91
H
ATOM    200 1HD  ARG A  14      11.902 -20.949  27.610  1.00 35.91
H
ATOM    201 2HD  ARG A  14      10.324 -20.404  26.988  1.00 35.91
H
ATOM    202  HE  ARG A  14      12.402 -19.364  25.334  1.00 35.91
H
ATOM    203 1HH1 ARG A  14      10.848 -22.442  26.081  1.00 35.91
H
ATOM    204 2HH1 ARG A  14      11.203 -23.116  24.506  1.00 35.91
H
ATOM    205 1HH2 ARG A  14      12.835 -20.214  23.365  1.00 35.91
H
ATOM    206 2HH2 ARG A  14      12.339 -21.849  22.947  1.00 35.91
H
ATOM    207  N   PRO A  15       9.705 -16.255  31.219  1.00  9.75
N
ATOM    208  CA  PRO A  15       8.979 -15.917  32.436  1.00  6.87
C
ATOM    209  C   PRO A  15       8.187 -17.091  32.983  1.00  5.32
C
ATOM    210  O   PRO A  15       7.594 -17.866  32.231  1.00  8.19
O
ATOM    211  CB  PRO A  15       8.024 -14.796  31.987  1.00 10.30
C
ATOM    212  CG  PRO A  15       8.663 -14.207  30.789  1.00 10.30
C
```

```
ATOM    213  CD  PRO A  15       9.274 -15.386  30.095  1.00 10.30
C
ATOM    214  HA  PRO A  15       9.690 -15.546  33.188  1.00  8.24
H
ATOM    215 1HB  PRO A  15       7.035 -15.220  31.771  1.00 12.37
H
ATOM    216 2HB  PRO A  15       7.891 -14.062  32.793  1.00 12.37
H
ATOM    217 1HG  PRO A  15       7.917 -13.688  30.173  1.00 12.37
H
ATOM    218 2HG  PRO A  15       9.410 -13.458  31.088  1.00 12.37
H
ATOM    219 1HD  PRO A  15       8.531 -15.908  29.474  1.00 12.37
H
ATOM    220 2HD  PRO A  15      10.116 -15.021  29.504  1.00 12.37
H
ATOM    221  N   ASP A  16       8.142 -17.188  34.305  1.00  4.02
N
ATOM    222  CA  ASP A  16       7.354 -18.222  34.945  1.00  3.79
C
ATOM    223  C   ASP A  16       5.965 -17.675  35.210  1.00  3.19
C
ATOM    224  O   ASP A  16       5.710 -16.504  34.934  1.00  3.53
O
ATOM    225  CB  ASP A  16       8.007 -18.671  36.250  1.00  5.69
C
ATOM    226  CG  ASP A  16       8.006 -17.568  37.295  1.00  5.69
C
ATOM    227  OD1 ASP A  16       7.340 -16.581  37.073  1.00  5.69
O
ATOM    228  OD2 ASP A  16       8.659 -17.718  38.299  1.00  5.69
O
ATOM    229  H   ASP A  16       8.659 -16.527  34.866  1.00  4.82
H
ATOM    230  HA  ASP A  16       7.272 -19.078  34.273  1.00  4.55
H
ATOM    231 1HB  ASP A  16       7.483 -19.538  36.649  1.00  6.82
H
ATOM    232 2HB  ASP A  16       9.039 -18.969  36.057  1.00  6.82
H
ATOM    233  N   ARG A  17       5.096 -18.495  35.790  1.00  3.35
N
ATOM    234  CA  ARG A  17       3.715 -18.107  36.065  1.00  3.39
C
ATOM    235  C   ARG A  17       3.596 -16.815  36.873  1.00  2.55
C
ATOM    236  O   ARG A  17       2.742 -15.980  36.577  1.00  2.53
O
ATOM    237  CB  ARG A  17       3.002 -19.223  36.813  1.00  5.08
C
ATOM    238  CG  ARG A  17       1.554 -18.941  37.190  1.00  5.08
C
ATOM    239  CD  ARG A  17       0.666 -18.881  35.997  1.00  5.08
C
ATOM    240  NE  ARG A  17      -0.706 -18.566  36.368  1.00  5.08
N
ATOM    241  CZ  ARG A  17      -1.193 -17.316  36.493  1.00  5.08
C
```

```
ATOM    242  NH1 ARG A  17      -0.427 -16.285  36.245  1.00  5.08
N
ATOM    243  NH2 ARG A  17      -2.440 -17.098  36.866  1.00  5.08
N
ATOM    244  H   ARG A  17       5.389 -19.434  36.016  1.00  4.02
H
ATOM    245  HA  ARG A  17       3.210 -17.959  35.109  1.00  4.07
H
ATOM    246 1HB  ARG A  17       3.010 -20.127  36.205  1.00  6.10
H
ATOM    247 2HB  ARG A  17       3.542 -19.446  37.733  1.00  6.10
H
ATOM    248 1HG  ARG A  17       1.191 -19.733  37.845  1.00  6.10
H
ATOM    249 2HG  ARG A  17       1.494 -17.985  37.710  1.00  6.10
H
ATOM    250 1HD  ARG A  17       1.021 -18.109  35.314  1.00  6.10
H
ATOM    251 2HD  ARG A  17       0.670 -19.844  35.489  1.00  6.10
H
ATOM    252  HE  ARG A  17      -1.330 -19.337  36.569  1.00  6.10
H
ATOM    253 1HH1 ARG A  17       0.532 -16.417  35.959  1.00  6.10
H
ATOM    254 2HH1 ARG A  17      -0.813 -15.355  36.355  1.00  6.10
H
ATOM    255 1HH2 ARG A  17      -3.058 -17.873  37.065  1.00  6.10
H
ATOM    256 2HH2 ARG A  17      -2.760 -16.141  36.956  1.00  6.10
H
ATOM    257  N   GLY A  18       4.436 -16.672  37.899  1.00  2.41
N
ATOM    258  CA  GLY A  18       4.414 -15.500  38.769  1.00  2.35
C
ATOM    259  C   GLY A  18       4.759 -14.226  38.020  1.00  2.22
C
ATOM    260  O   GLY A  18       4.068 -13.215  38.155  1.00  2.65
O
ATOM    261  H   GLY A  18       5.118 -17.394  38.082  1.00  2.89
H
ATOM    262 1HA  GLY A  18       3.425 -15.403  39.218  1.00  2.82
H
ATOM    263 2HA  GLY A  18       5.120 -15.645  39.586  1.00  2.82
H
ATOM    264  N   THR A  19       5.818 -14.283  37.217  1.00  2.65
N
ATOM    265  CA  THR A  19       6.239 -13.133  36.436  1.00  3.24
C
ATOM    266  C   THR A  19       5.249 -12.817  35.323  1.00  1.92
C
ATOM    267  O   THR A  19       5.053 -11.648  34.987  1.00  1.57
O
ATOM    268  CB  THR A  19       7.640 -13.387  35.867  1.00  4.86
C
ATOM    269  OG1 THR A  19       7.624 -14.613  35.136  1.00  4.86
O
ATOM    270  CG2 THR A  19       8.675 -13.469  36.975  1.00  4.86
C
```

```
ATOM    271  H   THR A  19       6.351 -15.143  37.156  1.00  3.18
H
ATOM    272  HA  THR A  19       6.290 -12.268  37.096  1.00  3.89
H
ATOM    273  HB  THR A  19       7.903 -12.583  35.187  1.00  5.83
H
ATOM    274  HG1 THR A  19       7.364 -15.328  35.733  1.00  5.83
H
ATOM    275 1HG2 THR A  19       9.659 -13.654  36.542  1.00  5.83
H
ATOM    276 2HG2 THR A  19       8.693 -12.530  37.527  1.00  5.83
H
ATOM    277 3HG2 THR A  19       8.419 -14.284  37.652  1.00  5.83
H
ATOM    278  N   LEU A  20       4.565 -13.843  34.814  1.00  1.95
N
ATOM    279  CA  LEU A  20       3.511 -13.642  33.834  1.00  1.70
C
ATOM    280  C   LEU A  20       2.300 -12.990  34.489  1.00  1.35
C
ATOM    281  O   LEU A  20       1.618 -12.182  33.860  1.00  1.28
O
ATOM    282  CB  LEU A  20       3.122 -14.974  33.192  1.00  2.55
C
ATOM    283  CG  LEU A  20       4.180 -15.592  32.271  1.00  2.55
C
ATOM    284  CD1 LEU A  20       3.753 -17.001  31.892  1.00  2.55
C
ATOM    285  CD2 LEU A  20       4.333 -14.722  31.031  1.00  2.55
C
ATOM    286  H   LEU A  20       4.794 -14.787  35.097  1.00  2.34
H
ATOM    287  HA  LEU A  20       3.882 -12.976  33.056  1.00  2.04
H
ATOM    288 1HB  LEU A  20       2.909 -15.692  33.981  1.00  3.06
H
ATOM    289 2HB  LEU A  20       2.215 -14.828  32.606  1.00  3.06
H
ATOM    290  HG  LEU A  20       5.133 -15.651  32.790  1.00  3.06
H
ATOM    291 1HD1 LEU A  20       4.505 -17.445  31.240  1.00  3.06
H
ATOM    292 2HD1 LEU A  20       3.655 -17.604  32.793  1.00  3.06
H
ATOM    293 3HD1 LEU A  20       2.797 -16.966  31.371  1.00  3.06
H
ATOM    294 1HD2 LEU A  20       5.086 -15.157  30.373  1.00  3.06
H
ATOM    295 2HD2 LEU A  20       3.380 -14.666  30.505  1.00  3.06
H
ATOM    296 3HD2 LEU A  20       4.644 -13.719  31.326  1.00  3.06
H
ATOM    297  N   GLN A  21       2.041 -13.325  35.757  1.00  1.36
N
ATOM    298  CA  GLN A  21       0.939 -12.702  36.469  1.00  1.31
C
ATOM    299  C   GLN A  21       1.198 -11.245  36.742  1.00  1.29
C
```

```
ATOM    300  O   GLN A  21       0.294 -10.430  36.584  1.00  1.18
O
ATOM    301  CB  GLN A  21       0.658 -13.386  37.799  1.00  1.97
C
ATOM    302  CG  GLN A  21      -0.558 -12.806  38.500  1.00  1.97
C
ATOM    303  CD  GLN A  21      -1.828 -12.956  37.675  1.00  1.97
C
ATOM    304  OE1 GLN A  21      -2.142 -14.043  37.169  1.00  1.97
O
ATOM    305  NE2 GLN A  21      -2.568 -11.859  37.529  1.00  1.97
N
ATOM    306  H   GLN A  21       2.599 -14.026  36.226  1.00  1.63
H
ATOM    307  HA  GLN A  21       0.045 -12.773  35.849  1.00  1.57
H
ATOM    308 1HB  GLN A  21       0.517 -14.451  37.664  1.00  2.36
H
ATOM    309 2HB  GLN A  21       1.514 -13.255  38.460  1.00  2.36
H
ATOM    310 1HG  GLN A  21      -0.701 -13.327  39.447  1.00  2.36
H
ATOM    311 2HG  GLN A  21      -0.391 -11.744  38.679  1.00  2.36
H
ATOM    312 1HE2 GLN A  21      -3.414 -11.895  36.995  1.00  2.36
H
ATOM    313 2HE2 GLN A  21      -2.288 -10.986  37.945  1.00  2.36
H
ATOM    314  N   VAL A  22       2.433 -10.911  37.126  1.00  1.50
N
ATOM    315  CA  VAL A  22       2.771  -9.522  37.399  1.00  1.49
C
ATOM    316  C   VAL A  22       2.677  -8.738  36.107  1.00  1.42
C
ATOM    317  O   VAL A  22       2.188  -7.615  36.091  1.00  1.39
O
ATOM    318  CB  VAL A  22       4.173  -9.373  38.000  1.00  2.23
C
ATOM    319  CG1 VAL A  22       4.497  -7.895  38.119  1.00  2.23
C
ATOM    320  CG2 VAL A  22       4.237 -10.045  39.358  1.00  2.23
C
ATOM    321  H   VAL A  22       3.133 -11.633  37.258  1.00  1.80
H
ATOM    322  HA  VAL A  22       2.048  -9.119  38.109  1.00  1.79
H
ATOM    323  HB  VAL A  22       4.905  -9.825  37.331  1.00  2.68
H
ATOM    324 1HG1 VAL A  22       5.498  -7.772  38.532  1.00  2.68
H
ATOM    325 2HG1 VAL A  22       4.449  -7.438  37.132  1.00  2.68
H
ATOM    326 3HG1 VAL A  22       3.773  -7.417  38.778  1.00  2.68
H
ATOM    327 1HG2 VAL A  22       5.237  -9.930  39.774  1.00  2.68
H
ATOM    328 2HG2 VAL A  22       3.511  -9.584  40.020  1.00  2.68
H
```

```
ATOM    329 3HG2 VAL A  22       4.007 -11.103  39.254  1.00  2.68
H
ATOM    330  N   LEU A  23       3.141  -9.348  35.028  1.00  1.46
N
ATOM    331  CA  LEU A  23       3.057  -8.789  33.697  1.00  1.29
C
ATOM    332  C   LEU A  23       1.602  -8.470  33.325  1.00  0.97
C
ATOM    333  O   LEU A  23       1.309  -7.381  32.823  1.00  0.96
O
ATOM    334  CB  LEU A  23       3.706  -9.792  32.763  1.00  1.94
C
ATOM    335  CG  LEU A  23       3.776  -9.474  31.300  1.00  1.94
C
ATOM    336  CD1 LEU A  23       4.519  -8.171  31.116  1.00  1.94
C
ATOM    337  CD2 LEU A  23       4.517 -10.631  30.647  1.00  1.94
C
ATOM    338  H   LEU A  23       3.581 -10.255  35.120  1.00  1.75
H
ATOM    339  HA  LEU A  23       3.630  -7.863  33.673  1.00  1.55
H
ATOM    340 1HB  LEU A  23       4.721  -9.962  33.107  1.00  2.32
H
ATOM    341 2HB  LEU A  23       3.170 -10.731  32.848  1.00  2.32
H
ATOM    342  HG  LEU A  23       2.778  -9.372  30.874  1.00  2.32
H
ATOM    343 1HD1 LEU A  23       4.592  -7.952  30.057  1.00  2.32
H
ATOM    344 2HD1 LEU A  23       3.975  -7.371  31.617  1.00  2.32
H
ATOM    345 3HD1 LEU A  23       5.513  -8.247  31.544  1.00  2.32
H
ATOM    346 1HD2 LEU A  23       4.617 -10.458  29.584  1.00  2.32
H
ATOM    347 2HD2 LEU A  23       5.507 -10.725  31.090  1.00  2.32
H
ATOM    348 3HD2 LEU A  23       3.961 -11.553  30.813  1.00  2.32
H
ATOM    349  N   GLN A  24       0.694  -9.408  33.604  1.00  0.90
N
ATOM    350  CA  GLN A  24      -0.726  -9.184  33.379  1.00  0.91
C
ATOM    351  C   GLN A  24      -1.252  -8.084  34.310  1.00  0.86
C
ATOM    352  O   GLN A  24      -2.090  -7.278  33.901  1.00  0.80
O
ATOM    353  CB  GLN A  24      -1.511 -10.477  33.582  1.00  1.36
C
ATOM    354  CG  GLN A  24      -2.980 -10.368  33.217  1.00  1.36
C
ATOM    355  CD  GLN A  24      -3.189 -10.068  31.742  1.00  1.36
C
ATOM    356  OE1 GLN A  24      -2.636 -10.748  30.874  1.00  1.36
O
ATOM    357  NE2 GLN A  24      -3.992  -9.049  31.453  1.00  1.36
N
```

```
ATOM    358  H   GLN A  24       0.994 -10.301  33.973  1.00  1.08
H
ATOM    359  HA  GLN A  24      -0.865  -8.851  32.352  1.00  1.09
H
ATOM    360 1HB  GLN A  24      -1.068 -11.270  32.979  1.00  1.64
H
ATOM    361 2HB  GLN A  24      -1.445 -10.783  34.626  1.00  1.64
H
ATOM    362 1HG  GLN A  24      -3.470 -11.314  33.447  1.00  1.64
H
ATOM    363 2HG  GLN A  24      -3.429  -9.563  33.800  1.00  1.64
H
ATOM    364 1HE2 GLN A  24      -4.169  -8.798  30.495  1.00  1.64
H
ATOM    365 2HE2 GLN A  24      -4.419  -8.529  32.192  1.00  1.64
H
ATOM    366  N   ASP A  25      -0.740  -8.036  35.546  1.00  1.04
N
ATOM    367  CA  ASP A  25      -1.113  -6.992  36.490  1.00  1.41
C
ATOM    368  C   ASP A  25      -0.687  -5.624  35.964  1.00  1.15
C
ATOM    369  O   ASP A  25      -1.421  -4.652  36.128  1.00  1.22
O
ATOM    370  CB  ASP A  25      -0.488  -7.229  37.875  1.00  2.11
C
ATOM    371  CG  ASP A  25      -1.086  -8.393  38.672  1.00  2.11
C
ATOM    372  OD1 ASP A  25      -2.124  -8.907  38.323  1.00  2.11
O
ATOM    373  OD2 ASP A  25      -0.507  -8.729  39.674  1.00  2.11
O
ATOM    374  H   ASP A  25      -0.085  -8.743  35.842  1.00  1.25
H
ATOM    375  HA  ASP A  25      -2.198  -6.994  36.597  1.00  1.69
H
ATOM    376 1HB  ASP A  25       0.576  -7.411  37.769  1.00  2.54
H
ATOM    377 2HB  ASP A  25      -0.590  -6.321  38.469  1.00  2.54
H
ATOM    378  N   MET A  26       0.482  -5.555  35.308  1.00  1.10
N
ATOM    379  CA  MET A  26       0.952  -4.296  34.745  1.00  1.22
C
ATOM    380  C   MET A  26       0.038  -3.858  33.614  1.00  0.90
C
ATOM    381  O   MET A  26      -0.267  -2.673  33.501  1.00  0.96
O
ATOM    382  CB  MET A  26       2.385  -4.408  34.212  1.00  1.83
C
ATOM    383  CG  MET A  26       3.475  -4.674  35.243  1.00  1.83
C
ATOM    384  SD  MET A  26       3.562  -3.433  36.538  1.00  1.83
S
ATOM    385  CE  MET A  26       2.678  -4.312  37.822  1.00  1.83
C
ATOM    386  H   MET A  26       1.058  -6.381  35.224  1.00  1.32
H
```

```
ATOM    387  HA  MET A  26       0.920  -3.531  35.518  1.00  1.46
H
ATOM    388 1HB  MET A  26       2.433  -5.207  33.474  1.00  2.20
H
ATOM    389 2HB  MET A  26       2.640  -3.481  33.699  1.00  2.20
H
ATOM    390 1HG  MET A  26       3.325  -5.642  35.706  1.00  2.20
H
ATOM    391 2HG  MET A  26       4.436  -4.691  34.733  1.00  2.20
H
ATOM    392 1HE  MET A  26       2.628  -3.695  38.719  1.00  2.20
H
ATOM    393 2HE  MET A  26       1.667  -4.538  37.480  1.00  2.20
H
ATOM    394 3HE  MET A  26       3.198  -5.241  38.052  1.00  2.20
H
ATOM    395  N   ALA A  27      -0.432  -4.819  32.809  1.00  0.73
N
ATOM    396  CA  ALA A  27      -1.371  -4.518  31.732  1.00  0.67
C
ATOM    397  C   ALA A  27      -2.651  -3.933  32.304  1.00  0.68
C
ATOM    398  O   ALA A  27      -3.187  -2.959  31.774  1.00  0.70
O
ATOM    399  CB  ALA A  27      -1.671  -5.770  30.920  1.00  1.01
C
ATOM    400  H   ALA A  27      -0.109  -5.773  32.930  1.00  0.88
H
ATOM    401  HA  ALA A  27      -0.921  -3.769  31.083  1.00  0.80
H
ATOM    402 1HB  ALA A  27      -2.358  -5.523  30.110  1.00  1.21
H
ATOM    403 2HB  ALA A  27      -0.744  -6.161  30.503  1.00  1.21
H
ATOM    404 3HB  ALA A  27      -2.125  -6.524  31.559  1.00  1.21
H
ATOM    405  N   SER A  28      -3.122  -4.524  33.404  1.00  0.71
N
ATOM    406  CA  SER A  28      -4.313  -4.046  34.085  1.00  0.78
C
ATOM    407  C   SER A  28      -4.110  -2.640  34.631  1.00  0.88
C
ATOM    408  O   SER A  28      -5.005  -1.800  34.518  1.00  0.96
O
ATOM    409  CB  SER A  28      -4.692  -5.023  35.181  1.00  1.17
C
ATOM    410  OG  SER A  28      -5.096  -6.262  34.644  1.00  1.17
O
ATOM    411  H   SER A  28      -2.646  -5.340  33.770  1.00  0.85
H
ATOM    412  HA  SER A  28      -5.122  -3.997  33.369  1.00  0.94
H
ATOM    413 1HB  SER A  28      -3.836  -5.175  35.836  1.00  1.40
H
ATOM    414 2HB  SER A  28      -5.492  -4.606  35.788  1.00  1.40
H
ATOM    415  HG  SER A  28      -5.158  -6.863  35.392  1.00  1.40
H
```

ATOM 416 N ARG A 29 -2.926 -2.372 35.179 1.00 0.93
N
ATOM 417 CA ARG A 29 -2.606 -1.044 35.673 1.00 1.09
C
ATOM 418 C ARG A 29 -2.584 -0.032 34.535 1.00 0.88
C
ATOM 419 O ARG A 29 -3.054 1.090 34.707 1.00 0.97
O
ATOM 420 CB ARG A 29 -1.272 -1.045 36.392 1.00 1.64
C
ATOM 421 CG ARG A 29 -1.285 -1.755 37.734 1.00 1.64
C
ATOM 422 CD ARG A 29 0.073 -1.834 38.321 1.00 1.64
C
ATOM 423 NE ARG A 29 0.611 -0.521 38.635 1.00 1.64
N
ATOM 424 CZ ARG A 29 0.371 0.152 39.777 1.00 1.64
C
ATOM 425 NH1 ARG A 29 -0.402 -0.376 40.702 1.00 1.64
N
ATOM 426 NH2 ARG A 29 0.912 1.343 39.967 1.00 1.64
N
ATOM 427 H ARG A 29 -2.239 -3.109 35.273 1.00 1.12
H
ATOM 428 HA ARG A 29 -3.377 -0.746 36.384 1.00 1.31
H
ATOM 429 1HB ARG A 29 -0.520 -1.527 35.771 1.00 1.96
H
ATOM 430 2HB ARG A 29 -0.949 -0.018 36.564 1.00 1.96
H
ATOM 431 1HG ARG A 29 -1.921 -1.203 38.428 1.00 1.96
H
ATOM 432 2HG ARG A 29 -1.672 -2.765 37.617 1.00 1.96
H
ATOM 433 1HD ARG A 29 0.047 -2.423 39.237 1.00 1.96
H
ATOM 434 2HD ARG A 29 0.738 -2.305 37.602 1.00 1.96
H
ATOM 435 HE ARG A 29 1.213 -0.083 37.950 1.00 1.96
H
ATOM 436 1HH1 ARG A 29 -0.813 -1.287 40.556 1.00 1.96
H
ATOM 437 2HH1 ARG A 29 -0.580 0.129 41.558 1.00 1.96
H
ATOM 438 1HH2 ARG A 29 1.503 1.748 39.255 1.00 1.96
H
ATOM 439 2HH2 ARG A 29 0.730 1.848 40.822 1.00 1.96
H
ATOM 440 N LEU A 30 -2.073 -0.437 33.364 1.00 0.73
N
ATOM 441 CA LEU A 30 -2.069 0.441 32.199 1.00 0.67
C
ATOM 442 C LEU A 30 -3.480 0.789 31.761 1.00 0.67
C
ATOM 443 O LEU A 30 -3.744 1.934 31.389 1.00 0.68
O
ATOM 444 CB LEU A 30 -1.343 -0.210 31.009 1.00 1.01
C

ATOM 445 CG LEU A 30 0.176 -0.372 31.120 1.00 1.01
C
ATOM 446 CD1 LEU A 30 0.672 -1.187 29.933 1.00 1.01
C
ATOM 447 CD2 LEU A 30 0.827 1.000 31.148 1.00 1.01
C
ATOM 448 H LEU A 30 -1.668 -1.359 33.285 1.00 0.88
H
ATOM 449 HA LEU A 30 -1.552 1.363 32.465 1.00 0.80
H
ATOM 450 1HB LEU A 30 -1.761 -1.199 30.848 1.00 1.21
H
ATOM 451 2HB LEU A 30 -1.541 0.388 30.120 1.00 1.21
H
ATOM 452 HG LEU A 30 0.425 -0.910 32.032 1.00 1.21
H
ATOM 453 1HD1 LEU A 30 1.753 -1.316 30.006 1.00 1.21
H
ATOM 454 2HD1 LEU A 30 0.189 -2.163 29.938 1.00 1.21
H
ATOM 455 3HD1 LEU A 30 0.430 -0.667 29.008 1.00 1.21
H
ATOM 456 1HD2 LEU A 30 1.909 0.887 31.226 1.00 1.21
H
ATOM 457 2HD2 LEU A 30 0.582 1.538 30.231 1.00 1.21
H
ATOM 458 3HD2 LEU A 30 0.457 1.561 32.007 1.00 1.21
H
ATOM 459 N ARG A 31 -4.392 -0.190 31.819 1.00 0.73
N
ATOM 460 CA ARG A 31 -5.771 0.072 31.428 1.00 0.83
C
ATOM 461 C ARG A 31 -6.418 1.045 32.394 1.00 0.85
C
ATOM 462 O ARG A 31 -7.116 1.964 31.966 1.00 0.89
O
ATOM 463 CB ARG A 31 -6.582 -1.210 31.357 1.00 1.24
C
ATOM 464 CG ARG A 31 -6.170 -2.118 30.214 1.00 1.24
C
ATOM 465 CD ARG A 31 -7.151 -3.193 29.937 1.00 1.24
C
ATOM 466 NE ARG A 31 -7.382 -4.016 31.102 1.00 1.24
N
ATOM 467 CZ ARG A 31 -6.611 -5.045 31.498 1.00 1.24
C
ATOM 468 NH1 ARG A 31 -5.529 -5.390 30.827 1.00 1.24
N
ATOM 469 NH2 ARG A 31 -6.944 -5.689 32.604 1.00 1.24
N
ATOM 470 H ARG A 31 -4.118 -1.124 32.103 1.00 0.88
H
ATOM 471 HA ARG A 31 -5.769 0.523 30.436 1.00 1.00
H
ATOM 472 1HB ARG A 31 -6.472 -1.768 32.286 1.00 1.49
H
ATOM 473 2HB ARG A 31 -7.638 -0.973 31.235 1.00 1.49
H

```
ATOM    474 1HG  ARG A  31      -6.047  -1.523  29.312  1.00  1.49
H
ATOM    475 2HG  ARG A  31      -5.223  -2.593  30.463  1.00  1.49
H
ATOM    476 1HD  ARG A  31      -8.098  -2.745  29.642  1.00  1.49
H
ATOM    477 2HD  ARG A  31      -6.782  -3.831  29.134  1.00  1.49
H
ATOM    478  HE  ARG A  31      -8.188  -3.797  31.670  1.00  1.49
H
ATOM    479 1HH1 ARG A  31      -5.243  -4.893  29.989  1.00  1.49
H
ATOM    480 2HH1 ARG A  31      -4.962  -6.160  31.153  1.00  1.49
H
ATOM    481 1HH2 ARG A  31      -7.767  -5.408  33.118  1.00  1.49
H
ATOM    482 2HH2 ARG A  31      -6.349  -6.416  32.970  1.00  1.49
H
ATOM    483  N   ILE A  32      -6.148   0.874  33.687  1.00  0.88
N
ATOM    484  CA  ILE A  32      -6.663   1.782  34.700  1.00  1.00
C
ATOM    485  C   ILE A  32      -6.165   3.191  34.505  1.00  0.98
C
ATOM    486  O   ILE A  32      -6.946   4.140  34.571  1.00  1.09
O
ATOM    487  CB  ILE A  32      -6.273   1.323  36.103  1.00  1.50
C
ATOM    488  CG1 ILE A  32      -7.014   0.044  36.456  1.00  1.50
C
ATOM    489  CG2 ILE A  32      -6.543   2.428  37.102  1.00  1.50
C
ATOM    490  CD1 ILE A  32      -6.480  -0.634  37.689  1.00  1.50
C
ATOM    491  H   ILE A  32      -5.585   0.083  33.980  1.00  1.06
H
ATOM    492  HA  ILE A  32      -7.750   1.793  34.629  1.00  1.20
H
ATOM    493  HB  ILE A  32      -5.210   1.085  36.121  1.00  1.80
H
ATOM    494 1HG1 ILE A  32      -8.067   0.274  36.614  1.00  1.80
H
ATOM    495 2HG1 ILE A  32      -6.939  -0.642  35.617  1.00  1.80
H
ATOM    496 1HG2 ILE A  32      -6.248   2.098  38.098  1.00  1.80
H
ATOM    497 2HG2 ILE A  32      -5.969   3.313  36.831  1.00  1.80
H
ATOM    498 3HG2 ILE A  32      -7.602   2.672  37.098  1.00  1.80
H
ATOM    499 1HD1 ILE A  32      -7.052  -1.540  37.888  1.00  1.80
H
ATOM    500 2HD1 ILE A  32      -5.432  -0.892  37.537  1.00  1.80
H
ATOM    501 3HD1 ILE A  32      -6.566   0.041  38.540  1.00  1.80
H
ATOM    502  N   HIS A  33      -4.864   3.325  34.270  1.00  0.90
N
```

ATOM 503 CA HIS A 33 -4.254 4.626 34.094 1.00 0.89
C
ATOM 504 C HIS A 33 -4.796 5.317 32.857 1.00 0.88
C
ATOM 505 O HIS A 33 -5.042 6.521 32.883 1.00 1.04
O
ATOM 506 CB HIS A 33 -2.739 4.471 33.979 1.00 1.33
C
ATOM 507 CG HIS A 33 -2.105 4.000 35.249 1.00 1.33
C
ATOM 508 ND1 HIS A 33 -0.797 3.569 35.312 1.00 1.33
N
ATOM 509 CD2 HIS A 33 -2.605 3.875 36.500 1.00 1.33
C
ATOM 510 CE1 HIS A 33 -0.520 3.198 36.551 1.00 1.33
C
ATOM 511 NE2 HIS A 33 -1.599 3.375 37.290 1.00 1.33
N
ATOM 512 H HIS A 33 -4.275 2.503 34.237 1.00 1.08
H
ATOM 513 HA HIS A 33 -4.471 5.255 34.955 1.00 1.07
H
ATOM 514 1HB HIS A 33 -2.501 3.755 33.192 1.00 1.60
H
ATOM 515 2HB HIS A 33 -2.291 5.427 33.706 1.00 1.60
H
ATOM 516 HD1 HIS A 33 -0.136 3.574 34.563 1.00 1.60
H
ATOM 517 HD2 HIS A 33 -3.585 4.084 36.929 1.00 1.60
H
ATOM 518 HE1 HIS A 33 0.471 2.825 36.808 1.00 1.60
H
ATOM 519 N SER A 34 -5.016 4.547 31.788 1.00 0.77
N
ATOM 520 CA SER A 34 -5.568 5.084 30.557 1.00 0.77
C
ATOM 521 C SER A 34 -6.969 5.629 30.792 1.00 0.87
C
ATOM 522 O SER A 34 -7.302 6.725 30.335 1.00 1.00
O
ATOM 523 CB SER A 34 -5.611 4.000 29.499 1.00 1.16
C
ATOM 524 OG SER A 34 -4.316 3.575 29.167 1.00 1.16
O
ATOM 525 H SER A 34 -4.772 3.565 31.815 1.00 0.92
H
ATOM 526 HA SER A 34 -4.930 5.899 30.212 1.00 0.92
H
ATOM 527 1HB SER A 34 -6.192 3.153 29.861 1.00 1.39
H
ATOM 528 2HB SER A 34 -6.107 4.383 28.609 1.00 1.39
H
ATOM 529 HG SER A 34 -3.983 3.116 29.948 1.00 1.39
H
ATOM 530 N ILE A 35 -7.775 4.878 31.543 1.00 0.85
N
ATOM 531 CA ILE A 35 -9.127 5.299 31.866 1.00 0.92
C

ATOM 532 C ILE A 35 -9.126 6.560 32.714 1.00 1.12
C
ATOM 533 O ILE A 35 -9.869 7.495 32.423 1.00 1.26
O
ATOM 534 CB ILE A 35 -9.892 4.191 32.604 1.00 1.38
C
ATOM 535 CG1 ILE A 35 -10.141 3.011 31.671 1.00 1.38
C
ATOM 536 CG2 ILE A 35 -11.216 4.730 33.117 1.00 1.38
C
ATOM 537 CD1 ILE A 35 -10.616 1.781 32.399 1.00 1.38
C
ATOM 538 H ILE A 35 -7.453 3.978 31.876 1.00 1.02
H
ATOM 539 HA ILE A 35 -9.652 5.516 30.938 1.00 1.10
H
ATOM 540 HB ILE A 35 -9.298 3.831 33.442 1.00 1.66
H
ATOM 541 1HG1 ILE A 35 -10.898 3.290 30.939 1.00 1.66
H
ATOM 542 2HG1 ILE A 35 -9.225 2.769 31.137 1.00 1.66
H
ATOM 543 1HG2 ILE A 35 -11.750 3.941 33.644 1.00 1.66
H
ATOM 544 2HG2 ILE A 35 -11.028 5.560 33.797 1.00 1.66
H
ATOM 545 3HG2 ILE A 35 -11.817 5.079 32.277 1.00 1.66
H
ATOM 546 1HD1 ILE A 35 -10.785 0.975 31.684 1.00 1.66
H
ATOM 547 2HD1 ILE A 35 -9.863 1.473 33.123 1.00 1.66
H
ATOM 548 3HD1 ILE A 35 -11.547 2.008 32.917 1.00 1.66
H
ATOM 549 N ARG A 36 -8.291 6.587 33.755 1.00 1.24
N
ATOM 550 CA ARG A 36 -8.206 7.743 34.639 1.00 1.64
C
ATOM 551 C ARG A 36 -7.752 8.999 33.912 1.00 1.46
C
ATOM 552 O ARG A 36 -8.294 10.081 34.145 1.00 1.77
O
ATOM 553 CB ARG A 36 -7.234 7.480 35.785 1.00 2.46
C
ATOM 554 CG ARG A 36 -7.706 6.488 36.830 1.00 2.46
C
ATOM 555 CD ARG A 36 -6.689 6.263 37.890 1.00 2.46
C
ATOM 556 NE ARG A 36 -7.161 5.330 38.905 1.00 2.46
N
ATOM 557 CZ ARG A 36 -6.396 4.800 39.880 1.00 2.46
C
ATOM 558 NH1 ARG A 36 -5.125 5.124 39.967 1.00 2.46
N
ATOM 559 NH2 ARG A 36 -6.920 3.950 40.748 1.00 2.46
N
ATOM 560 H ARG A 36 -7.709 5.783 33.948 1.00 1.49
H

```
ATOM    561  HA  ARG A  36      -9.196   7.926  35.058  1.00  1.97
H
ATOM    562 1HB  ARG A  36      -6.295   7.104  35.381  1.00  2.95
H
ATOM    563 2HB  ARG A  36      -7.016   8.416  36.298  1.00  2.95
H
ATOM    564 1HG  ARG A  36      -8.620   6.856  37.299  1.00  2.95
H
ATOM    565 2HG  ARG A  36      -7.904   5.534  36.347  1.00  2.95
H
ATOM    566 1HD  ARG A  36      -5.784   5.853  37.444  1.00  2.95
H
ATOM    567 2HD  ARG A  36      -6.458   7.209  38.377  1.00  2.95
H
ATOM    568  HE  ARG A  36      -8.133   5.054  38.873  1.00  2.95
H
ATOM    569 1HH1 ARG A  36      -4.721   5.771  39.306  1.00  2.95
H
ATOM    570 2HH1 ARG A  36      -4.552   4.723  40.696  1.00  2.95
H
ATOM    571 1HH2 ARG A  36      -7.896   3.691  40.685  1.00  2.95
H
ATOM    572 2HH2 ARG A  36      -6.349   3.547  41.476  1.00  2.95
H
ATOM    573  N   ALA A  37      -6.757   8.849  33.037  1.00  1.30
N
ATOM    574  CA  ALA A  37      -6.210   9.964  32.279  1.00  1.45
C
ATOM    575  C   ALA A  37      -7.235  10.561  31.335  1.00  1.57
C
ATOM    576  O   ALA A  37      -7.398  11.782  31.288  1.00  1.82
O
ATOM    577  CB  ALA A  37      -4.991   9.509  31.495  1.00  2.17
C
ATOM    578  H   ALA A  37      -6.344   7.935  32.902  1.00  1.56
H
ATOM    579  HA  ALA A  37      -5.912  10.738  32.986  1.00  1.74
H
ATOM    580 1HB  ALA A  37      -4.569  10.355  30.953  1.00  2.61
H
ATOM    581 2HB  ALA A  37      -4.245   9.109  32.182  1.00  2.61
H
ATOM    582 3HB  ALA A  37      -5.281   8.734  30.786  1.00  2.61
H
ATOM    583  N   THR A  38      -7.930   9.699  30.591  1.00  1.43
N
ATOM    584  CA  THR A  38      -8.932  10.149  29.636  1.00  1.44
C
ATOM    585  C   THR A  38     -10.185  10.678  30.313  1.00  1.48
C
ATOM    586  O   THR A  38     -10.825  11.604  29.813  1.00  1.61
O
ATOM    587  CB  THR A  38      -9.309   8.990  28.715  1.00  2.16
C
ATOM    588  OG1 THR A  38      -9.740   7.898  29.532  1.00  2.16
O
ATOM    589  CG2 THR A  38      -8.133   8.567  27.845  1.00  2.16
C
```

```
ATOM    590  H   THR A  38      -7.748   8.705  30.666  1.00  1.72
H
ATOM    591  HA  THR A  38      -8.503  10.951  29.036  1.00  1.73
H
ATOM    592  HB  THR A  38     -10.136   9.291  28.077  1.00  2.59
H
ATOM    593  HG1 THR A  38      -8.989   7.568  30.040  1.00  2.59
H
ATOM    594 1HG2 THR A  38      -8.432   7.737  27.206  1.00  2.59
H
ATOM    595 2HG2 THR A  38      -7.821   9.406  27.227  1.00  2.59
H
ATOM    596 3HG2 THR A  38      -7.301   8.258  28.475  1.00  2.59
H
ATOM    597  N   CYS A  39     -10.508  10.126  31.479  1.00  1.42
N
ATOM    598  CA  CYS A  39     -11.645  10.591  32.257  1.00  1.48
C
ATOM    599  C   CYS A  39     -11.385  11.996  32.761  1.00  1.77
C
ATOM    600  O   CYS A  39     -12.215  12.891  32.591  1.00  1.88
O
ATOM    601  CB  CYS A  39     -11.920   9.662  33.438  1.00  2.22
C
ATOM    602  SG  CYS A  39     -13.350  10.144  34.429  1.00  2.22
S
ATOM    603  H   CYS A  39      -9.975   9.346  31.837  1.00  1.70
H
ATOM    604  HA  CYS A  39     -12.526  10.605  31.615  1.00  1.78
H
ATOM    605 1HB  CYS A  39     -12.092   8.650  33.069  1.00  2.66
H
ATOM    606 2HB  CYS A  39     -11.048   9.629  34.090  1.00  2.66
H
ATOM    607  HG  CYS A  39     -14.231  10.088  33.436  1.00  2.66
H
ATOM    608  N   SER A  40     -10.219  12.185  33.386  1.00  1.93
N
ATOM    609  CA  SER A  40      -9.813  13.484  33.902  1.00  2.38
C
ATOM    610  C   SER A  40      -9.720  14.511  32.780  1.00  2.65
C
ATOM    611  O   SER A  40     -10.152  15.654  32.935  1.00  3.30
O
ATOM    612  CB  SER A  40      -8.469  13.372  34.597  1.00  3.57
C
ATOM    613  OG  SER A  40      -8.556  12.559  35.735  1.00  3.57
O
ATOM    614  H   SER A  40      -9.587  11.405  33.512  1.00  2.32
H
ATOM    615  HA  SER A  40     -10.560  13.824  34.620  1.00  2.86
H
ATOM    616 1HB  SER A  40      -7.733  12.958  33.908  1.00  4.28
H
ATOM    617 2HB  SER A  40      -8.126  14.365  34.883  1.00  4.28
H
ATOM    618  HG  SER A  40      -8.700  11.666  35.406  1.00  4.28
H
```

```
ATOM    619  N   THR A  41      -9.169  14.084  31.643  1.00  2.36
N
ATOM    620  CA  THR A  41      -9.041  14.908  30.451  1.00  2.90
C
ATOM    621  C   THR A  41     -10.388  15.259  29.831  1.00  3.01
C
ATOM    622  O   THR A  41     -10.552  16.353  29.291  1.00  6.29
O
ATOM    623  CB  THR A  41      -8.178  14.203  29.388  1.00  4.35
C
ATOM    624  OG1 THR A  41      -6.864  13.967  29.911  1.00  4.35
O
ATOM    625  CG2 THR A  41      -8.079  15.060  28.139  1.00  4.35
C
ATOM    626  H   THR A  41      -8.803  13.142  31.593  1.00  2.83
H
ATOM    627  HA  THR A  41      -8.549  15.838  30.731  1.00  3.48
H
ATOM    628  HB  THR A  41      -8.631  13.247  29.130  1.00  5.22
H
ATOM    629  HG1 THR A  41      -6.913  13.297  30.602  1.00  5.22
H
ATOM    630 1HG2 THR A  41      -7.470  14.549  27.394  1.00  5.22
H
ATOM    631 2HG2 THR A  41      -9.077  15.232  27.738  1.00  5.22
H
ATOM    632 3HG2 THR A  41      -7.621  16.016  28.390  1.00  5.22
H
ATOM    633  N   SER A  42     -11.333  14.310  29.885  1.00  2.66
N
ATOM    634  CA  SER A  42     -12.656  14.411  29.264  1.00  2.56
C
ATOM    635  C   SER A  42     -12.515  14.285  27.752  1.00  2.40
C
ATOM    636  O   SER A  42     -13.241  14.924  26.988  1.00  2.65
O
ATOM    637  CB  SER A  42     -13.372  15.706  29.634  1.00  3.84
C
ATOM    638  OG  SER A  42     -13.576  15.801  31.018  1.00  3.84
O
ATOM    639  H   SER A  42     -11.117  13.442  30.351  1.00  3.19
H
ATOM    640  HA  SER A  42     -13.266  13.578  29.617  1.00  3.07
H
ATOM    641 1HB  SER A  42     -12.816  16.572  29.282  1.00  4.61
H
ATOM    642 2HB  SER A  42     -14.338  15.728  29.131  1.00  4.61
H
ATOM    643  HG  SER A  42     -12.705  15.741  31.421  1.00  4.61
H
ATOM    644  N   SER A  43     -11.556  13.456  27.338  1.00  2.14
N
ATOM    645  CA  SER A  43     -11.266  13.187  25.932  1.00  2.17
C
ATOM    646  C   SER A  43     -10.316  12.005  25.822  1.00  2.61
C
ATOM    647  O   SER A  43     -10.075  11.307  26.798  1.00 12.74
O
```

ATOM 648 CB SER A 43 -10.662 14.401 25.251 1.00 3.25
C
ATOM 649 OG SER A 43 -10.547 14.179 23.871 1.00 3.25
O
ATOM 650 H SER A 43 -11.026 12.954 28.043 1.00 2.57
H
ATOM 651 HA SER A 43 -12.196 12.929 25.426 1.00 2.60
H
ATOM 652 1HB SER A 43 -11.288 15.274 25.430 1.00 3.91
H
ATOM 653 2HB SER A 43 -9.680 14.609 25.672 1.00 3.91
H
ATOM 654 HG SER A 43 -10.192 14.992 23.501 1.00 3.91
H
ATOM 655 N GLY A 44 -9.751 11.789 24.649 1.00 3.64
N
ATOM 656 CA GLY A 44 -8.812 10.686 24.477 1.00 2.32
C
ATOM 657 C GLY A 44 -9.488 9.418 23.981 1.00 1.82
C
ATOM 658 O GLY A 44 -10.648 9.437 23.575 1.00 2.57
O
ATOM 659 H GLY A 44 -9.974 12.406 23.880 1.00 4.37
H
ATOM 660 1HA GLY A 44 -8.037 10.983 23.770 1.00 2.78
H
ATOM 661 2HA GLY A 44 -8.312 10.488 25.423 1.00 2.78
H
ATOM 662 N HIS A 45 -8.751 8.312 24.000 1.00 1.38
N
ATOM 663 CA HIS A 45 -9.278 7.077 23.450 1.00 1.26
C
ATOM 664 C HIS A 45 -8.916 5.791 24.224 1.00 0.96
C
ATOM 665 O HIS A 45 -8.118 4.976 23.742 1.00 1.00
O
ATOM 666 CB HIS A 45 -8.761 6.974 22.040 1.00 1.89
C
ATOM 667 CG HIS A 45 -9.423 5.920 21.291 1.00 1.89
C
ATOM 668 ND1 HIS A 45 -9.082 5.608 20.005 1.00 1.89
N
ATOM 669 CD2 HIS A 45 -10.414 5.082 21.644 1.00 1.89
C
ATOM 670 CE1 HIS A 45 -9.834 4.618 19.593 1.00 1.89
C
ATOM 671 NE2 HIS A 45 -10.663 4.279 20.564 1.00 1.89
N
ATOM 672 H HIS A 45 -7.811 8.337 24.369 1.00 1.66
H
ATOM 673 HA HIS A 45 -10.364 7.139 23.391 1.00 1.51
H
ATOM 674 1HB HIS A 45 -8.914 7.922 21.521 1.00 2.27
H
ATOM 675 2HB HIS A 45 -7.690 6.772 22.053 1.00 2.27
H
ATOM 676 HD1 HIS A 45 -8.308 5.980 19.490 1.00 2.27
H

```
ATOM    677  HD2 HIS A  45      -10.987   4.967  22.564  1.00  2.27
H
ATOM    678  HE1 HIS A  45       -9.687   4.211  18.599  1.00  2.27
H
ATOM    679  N   PRO A  46       -9.553   5.591  25.389  1.00  0.99
N
ATOM    680  CA  PRO A  46       -9.406   4.514  26.376  1.00  1.14
C
ATOM    681  C   PRO A  46       -9.397   3.114  25.793  1.00  0.80
C
ATOM    682  O   PRO A  46       -8.676   2.239  26.278  1.00  0.94
O
ATOM    683  CB  PRO A  46      -10.685   4.628  27.214  1.00  1.71
C
ATOM    684  CG  PRO A  46      -11.060   6.042  27.116  1.00  1.71
C
ATOM    685  CD  PRO A  46      -10.766   6.387  25.685  1.00  1.71
C
ATOM    686  HA  PRO A  46       -8.510   4.692  26.988  1.00  1.37
H
ATOM    687 1HB  PRO A  46      -11.460   3.953  26.819  1.00  2.05
H
ATOM    688 2HB  PRO A  46      -10.485   4.314  28.249  1.00  2.05
H
ATOM    689 1HG  PRO A  46      -12.119   6.163  27.381  1.00  2.05
H
ATOM    690 2HG  PRO A  46      -10.479   6.633  27.821  1.00  2.05
H
ATOM    691 1HD  PRO A  46      -11.593   6.078  25.029  1.00  2.05
H
ATOM    692 2HD  PRO A  46      -10.563   7.460  25.612  1.00  2.05
H
ATOM    693  N   THR A  47      -10.235   2.901  24.776  1.00  0.78
N
ATOM    694  CA  THR A  47      -10.414   1.575  24.209  1.00  0.74
C
ATOM    695  C   THR A  47       -9.296   1.186  23.260  1.00  0.79
C
ATOM    696  O   THR A  47       -9.090  -0.006  23.009  1.00  0.91
O
ATOM    697  CB  THR A  47      -11.760   1.469  23.469  1.00  1.11
C
ATOM    698  OG1 THR A  47      -11.776   2.367  22.363  1.00  1.11
O
ATOM    699  CG2 THR A  47      -12.902   1.817  24.404  1.00  1.11
C
ATOM    700  H   THR A  47      -10.781   3.669  24.414  1.00  0.94
H
ATOM    701  HA  THR A  47      -10.421   0.858  25.028  1.00  0.89
H
ATOM    702  HB  THR A  47      -11.892   0.458  23.101  1.00  1.33
H
ATOM    703  HG1 THR A  47      -11.978   3.250  22.682  1.00  1.33
H
ATOM    704 1HG2 THR A  47      -13.847   1.737  23.867  1.00  1.33
H
ATOM    705 2HG2 THR A  47      -12.905   1.129  25.248  1.00  1.33
H
```

```
ATOM    706 3HG2 THR A  47     -12.777   2.837  24.767  1.00  1.33
H
ATOM    707  N   SER A  48      -8.564   2.178  22.735  1.00  0.91
N
ATOM    708  CA  SER A  48      -7.433   1.863  21.871  1.00  1.26
C
ATOM    709  C   SER A  48      -6.264   1.537  22.727  1.00  1.85
C
ATOM    710  O   SER A  48      -5.390   0.765  22.337  1.00  6.41
O
ATOM    711  CB  SER A  48      -7.041   3.009  20.993  1.00  1.89
C
ATOM    712  OG  SER A  48      -6.564   4.088  21.754  1.00  1.89
O
ATOM    713  H   SER A  48      -8.751   3.145  22.963  1.00  1.09
H
ATOM    714  HA  SER A  48      -7.677   0.995  21.258  1.00  1.51
H
ATOM    715 1HB  SER A  48      -6.265   2.681  20.309  1.00  2.27
H
ATOM    716 2HB  SER A  48      -7.881   3.304  20.396  1.00  2.27
H
ATOM    717  HG  SER A  48      -7.280   4.331  22.351  1.00  2.27
H
ATOM    718  N   CYS A  49      -6.244   2.131  23.911  1.00  0.96
N
ATOM    719  CA  CYS A  49      -5.206   1.822  24.865  1.00  0.79
C
ATOM    720  C   CYS A  49      -5.424   0.411  25.389  1.00  0.76
C
ATOM    721  O   CYS A  49      -4.503  -0.407  25.404  1.00  1.46
O
ATOM    722  CB  CYS A  49      -5.220   2.820  26.018  1.00  1.19
C
ATOM    723  SG  CYS A  49      -4.713   4.492  25.554  1.00  1.19
S
ATOM    724  H   CYS A  49      -6.959   2.815  24.145  1.00  1.15
H
ATOM    725  HA  CYS A  49      -4.239   1.870  24.365  1.00  0.95
H
ATOM    726 1HB  CYS A  49      -6.222   2.878  26.440  1.00  1.42
H
ATOM    727 2HB  CYS A  49      -4.552   2.472  26.805  1.00  1.42
H
ATOM    728  HG  CYS A  49      -5.663   4.677  24.644  1.00  1.42
H
ATOM    729  N   SER A  50      -6.667   0.134  25.792  1.00  0.53
N
ATOM    730  CA  SER A  50      -7.060  -1.148  26.357  1.00  0.51
C
ATOM    731  C   SER A  50      -6.737  -2.342  25.469  1.00  0.45
C
ATOM    732  O   SER A  50      -6.188  -3.339  25.948  1.00  0.48
O
ATOM    733  CB  SER A  50      -8.548  -1.128  26.641  1.00  0.77
C
ATOM    734  OG  SER A  50      -8.858  -0.185  27.632  1.00  0.77
O
```

ATOM	735	H	SER	A	50	-7.373	0.858	25.743	1.00	0.64	H
ATOM	736	HA	SER	A	50	-6.527	-1.275	27.296	1.00	0.61	H
ATOM	737	1HB	SER	A	50	-9.090	-0.893	25.727	1.00	0.92	H
ATOM	738	2HB	SER	A	50	-8.867	-2.117	26.965	1.00	0.92	H
ATOM	739	HG	SER	A	50	-8.662	0.677	27.248	1.00	0.92	H
ATOM	740	N	SER	A	51	-7.016	-2.231	24.165	1.00	0.43	N
ATOM	741	CA	SER	A	51	-6.777	-3.344	23.244	1.00	0.47	C
ATOM	742	C	SER	A	51	-5.313	-3.795	23.126	1.00	0.50	C
ATOM	743	O	SER	A	51	-5.060	-4.932	22.711	1.00	1.27	O
ATOM	744	CB	SER	A	51	-7.337	-3.002	21.861	1.00	0.70	C
ATOM	745	OG	SER	A	51	-6.679	-1.927	21.236	1.00	0.70	O
ATOM	746	H	SER	A	51	-7.460	-1.388	23.810	1.00	0.52	H
ATOM	747	HA	SER	A	51	-7.346	-4.197	23.616	1.00	0.56	H
ATOM	748	1HB	SER	A	51	-7.281	-3.882	21.221	1.00	0.85	H
ATOM	749	2HB	SER	A	51	-8.389	-2.757	21.974	1.00	0.85	H
ATOM	750	HG	SER	A	51	-5.870	-2.268	20.838	1.00	0.85	H
ATOM	751	N	SER	A	52	-4.353	-2.952	23.531	1.00	0.43	N
ATOM	752	CA	SER	A	52	-2.949	-3.346	23.447	1.00	0.44	C
ATOM	753	C	SER	A	52	-2.216	-3.218	24.780	1.00	0.45	C
ATOM	754	O	SER	A	52	-0.990	-3.093	24.795	1.00	0.57	O
ATOM	755	CB	SER	A	52	-2.230	-2.528	22.389	1.00	0.66	C
ATOM	756	OG	SER	A	52	-2.298	-1.151	22.655	1.00	0.66	O
ATOM	757	H	SER	A	52	-4.580	-2.037	23.906	1.00	0.52	H
ATOM	758	HA	SER	A	52	-2.910	-4.393	23.147	1.00	0.53	H
ATOM	759	1HB	SER	A	52	-1.188	-2.839	22.348	1.00	0.79	H
ATOM	760	2HB	SER	A	52	-2.670	-2.734	21.414	1.00	0.79	H
ATOM	761	HG	SER	A	52	-2.036	-1.049	23.573	1.00	0.79	H
ATOM	762	N	SER	A	53	-2.953	-3.253	25.897	1.00	0.43	N
ATOM	763	CA	SER	A	53	-2.315	-3.122	27.210	1.00	0.47	C

```
ATOM    764  C   SER A  53      -1.341  -4.263  27.521  1.00  0.54
C
ATOM    765  O   SER A  53      -0.305  -4.040  28.155  1.00  0.82
O
ATOM    766  CB  SER A  53      -3.384  -3.061  28.284  1.00  0.70
C
ATOM    767  OG  SER A  53      -4.092  -4.271  28.364  1.00  0.70
O
ATOM    768  H   SER A  53      -3.963  -3.356  25.846  1.00  0.52
H
ATOM    769  HA  SER A  53      -1.756  -2.188  27.222  1.00  0.56
H
ATOM    770 1HB  SER A  53      -2.922  -2.838  29.245  1.00  0.85
H
ATOM    771 2HB  SER A  53      -4.073  -2.250  28.056  1.00  0.85
H
ATOM    772  HG  SER A  53      -4.425  -4.447  27.478  1.00  0.85
H
ATOM    773  N   GLU A  54      -1.644  -5.467  27.034  1.00  0.46
N
ATOM    774  CA  GLU A  54      -0.744  -6.596  27.218  1.00  0.46
C
ATOM    775  C   GLU A  54       0.485  -6.463  26.346  1.00  0.50
C
ATOM    776  O   GLU A  54       1.589  -6.766  26.789  1.00  0.80
O
ATOM    777  CB  GLU A  54      -1.442  -7.926  26.938  1.00  0.69
C
ATOM    778  CG  GLU A  54      -2.454  -8.364  27.990  1.00  0.69
C
ATOM    779  CD  GLU A  54      -3.823  -7.773  27.843  1.00  0.69
C
ATOM    780  OE1 GLU A  54      -4.072  -7.093  26.877  1.00  0.69
O
ATOM    781  OE2 GLU A  54      -4.632  -8.014  28.713  1.00  0.69
O
ATOM    782  H   GLU A  54      -2.509  -5.601  26.530  1.00  0.55
H
ATOM    783  HA  GLU A  54      -0.414  -6.604  28.256  1.00  0.55
H
ATOM    784 1HB  GLU A  54      -1.970  -7.853  25.990  1.00  0.83
H
ATOM    785 2HB  GLU A  54      -0.700  -8.717  26.836  1.00  0.83
H
ATOM    786 1HG  GLU A  54      -2.539  -9.447  27.953  1.00  0.83
H
ATOM    787 2HG  GLU A  54      -2.062  -8.100  28.971  1.00  0.83
H
ATOM    788  N   ILE A  55       0.295  -5.991  25.115  1.00  0.36
N
ATOM    789  CA  ILE A  55       1.398  -5.809  24.183  1.00  0.34
C
ATOM    790  C   ILE A  55       2.405  -4.825  24.737  1.00  0.37
C
ATOM    791  O   ILE A  55       3.612  -5.075  24.698  1.00  0.41
O
ATOM    792  CB  ILE A  55       0.895  -5.308  22.820  1.00  0.51
C
```

ATOM 793 CG1 ILE A 55 0.072 -6.399 22.131 1.00 0.51
C
ATOM 794 CG2 ILE A 55 2.078 -4.895 21.956 1.00 0.51
C
ATOM 795 CD1 ILE A 55 -0.704 -5.904 20.931 1.00 0.51
C
ATOM 796 H ILE A 55 -0.641 -5.759 24.815 1.00 0.43
H
ATOM 797 HA ILE A 55 1.900 -6.763 24.039 1.00 0.41
H
ATOM 798 HB ILE A 55 0.242 -4.451 22.968 1.00 0.61
H
ATOM 799 1HG1 ILE A 55 0.739 -7.195 21.805 1.00 0.61
H
ATOM 800 2HG1 ILE A 55 -0.636 -6.817 22.848 1.00 0.61
H
ATOM 801 1HG2 ILE A 55 1.714 -4.530 20.997 1.00 0.61
H
ATOM 802 2HG2 ILE A 55 2.636 -4.105 22.457 1.00 0.61
H
ATOM 803 3HG2 ILE A 55 2.729 -5.754 21.796 1.00 0.61
H
ATOM 804 1HD1 ILE A 55 -1.263 -6.730 20.493 1.00 0.61
H
ATOM 805 2HD1 ILE A 55 -1.396 -5.123 21.242 1.00 0.61
H
ATOM 806 3HD1 ILE A 55 -0.013 -5.501 20.193 1.00 0.61
H
ATOM 807 N MET A 56 1.898 -3.711 25.262 1.00 0.39
N
ATOM 808 CA MET A 56 2.754 -2.694 25.834 1.00 0.45
C
ATOM 809 C MET A 56 3.458 -3.206 27.077 1.00 0.51
C
ATOM 810 O MET A 56 4.648 -2.964 27.249 1.00 0.72
O
ATOM 811 CB MET A 56 1.936 -1.450 26.168 1.00 0.68
C
ATOM 812 CG MET A 56 1.441 -0.673 24.958 1.00 0.68
C
ATOM 813 SD MET A 56 0.686 0.903 25.412 1.00 0.68
S
ATOM 814 CE MET A 56 -0.858 0.356 26.141 1.00 0.68
C
ATOM 815 H MET A 56 0.898 -3.560 25.247 1.00 0.47
H
ATOM 816 HA MET A 56 3.517 -2.431 25.103 1.00 0.54
H
ATOM 817 1HB MET A 56 1.066 -1.735 26.758 1.00 0.81
H
ATOM 818 2HB MET A 56 2.536 -0.773 26.777 1.00 0.81
H
ATOM 819 1HG MET A 56 2.275 -0.474 24.287 1.00 0.81
H
ATOM 820 2HG MET A 56 0.704 -1.266 24.418 1.00 0.81
H
ATOM 821 1HE MET A 56 -1.432 1.223 26.469 1.00 0.81
H

ATOM 822 2HE MET A 56 -1.433 -0.203 25.403 1.00 0.81
H
ATOM 823 3HE MET A 56 -0.648 -0.283 26.999 1.00 0.81
H
ATOM 824 N SER A 57 2.746 -3.948 27.928 1.00 0.47
N
ATOM 825 CA SER A 57 3.364 -4.485 29.132 1.00 0.54
C
ATOM 826 C SER A 57 4.521 -5.415 28.801 1.00 0.54
C
ATOM 827 O SER A 57 5.593 -5.323 29.398 1.00 0.85
O
ATOM 828 CB SER A 57 2.339 -5.244 29.940 1.00 0.81
C
ATOM 829 OG SER A 57 1.339 -4.385 30.394 1.00 0.81
O
ATOM 830 H SER A 57 1.760 -4.121 27.769 1.00 0.56
H
ATOM 831 HA SER A 57 3.747 -3.654 29.725 1.00 0.65
H
ATOM 832 1HB SER A 57 1.899 -6.035 29.333 1.00 0.97
H
ATOM 833 2HB SER A 57 2.826 -5.715 30.791 1.00 0.97
H
ATOM 834 HG SER A 57 0.821 -4.153 29.615 1.00 0.97
H
ATOM 835 N VAL A 58 4.309 -6.298 27.832 1.00 0.47
N
ATOM 836 CA VAL A 58 5.332 -7.248 27.432 1.00 0.45
C
ATOM 837 C VAL A 58 6.558 -6.574 26.866 1.00 0.38
C
ATOM 838 O VAL A 58 7.683 -6.873 27.281 1.00 0.45
O
ATOM 839 CB VAL A 58 4.759 -8.228 26.400 1.00 0.68
C
ATOM 840 CG1 VAL A 58 5.868 -9.075 25.817 1.00 0.68
C
ATOM 841 CG2 VAL A 58 3.737 -9.119 27.074 1.00 0.68
C
ATOM 842 H VAL A 58 3.408 -6.332 27.375 1.00 0.56
H
ATOM 843 HA VAL A 58 5.634 -7.818 28.309 1.00 0.54
H
ATOM 844 HB VAL A 58 4.290 -7.668 25.589 1.00 0.81
H
ATOM 845 1HG1 VAL A 58 5.449 -9.766 25.087 1.00 0.81
H
ATOM 846 2HG1 VAL A 58 6.597 -8.431 25.331 1.00 0.81
H
ATOM 847 3HG1 VAL A 58 6.352 -9.639 26.614 1.00 0.81
H
ATOM 848 1HG2 VAL A 58 3.319 -9.810 26.352 1.00 0.81
H
ATOM 849 2HG2 VAL A 58 4.226 -9.676 27.862 1.00 0.81
H
ATOM 850 3HG2 VAL A 58 2.939 -8.517 27.500 1.00 0.81
H

```
ATOM    851  N   LEU A  59       6.349  -5.649  25.936  1.00  0.42
N
ATOM    852  CA  LEU A  59       7.474  -4.962  25.343  1.00  0.49
C
ATOM    853  C   LEU A  59       8.213  -4.075  26.330  1.00  0.41
C
ATOM    854  O   LEU A  59       9.441  -4.063  26.363  1.00  0.51
O
ATOM    855  CB  LEU A  59       7.010  -4.130  24.146  1.00  0.73
C
ATOM    856  CG  LEU A  59       6.579  -4.948  22.924  1.00  0.73
C
ATOM    857  CD1 LEU A  59       5.988  -4.030  21.865  1.00  0.73
C
ATOM    858  CD2 LEU A  59       7.793  -5.685  22.391  1.00  0.73
C
ATOM    859  H   LEU A  59       5.409  -5.431  25.624  1.00  0.50
H
ATOM    860  HA  LEU A  59       8.163  -5.710  24.980  1.00  0.59
H
ATOM    861 1HB  LEU A  59       6.162  -3.518  24.452  1.00  0.88
H
ATOM    862 2HB  LEU A  59       7.821  -3.469  23.839  1.00  0.88
H
ATOM    863  HG  LEU A  59       5.812  -5.667  23.212  1.00  0.88
H
ATOM    864 1HD1 LEU A  59       5.683  -4.620  21.000  1.00  0.88
H
ATOM    865 2HD1 LEU A  59       5.120  -3.513  22.276  1.00  0.88
H
ATOM    866 3HD1 LEU A  59       6.735  -3.298  21.561  1.00  0.88
H
ATOM    867 1HD2 LEU A  59       7.509  -6.276  21.522  1.00  0.88
H
ATOM    868 2HD2 LEU A  59       8.558  -4.964  22.104  1.00  0.88
H
ATOM    869 3HD2 LEU A  59       8.185  -6.343  23.163  1.00  0.88
H
ATOM    870  N   PHE A  60       7.485  -3.364  27.176  1.00  0.44
N
ATOM    871  CA  PHE A  60       8.142  -2.464  28.106  1.00  0.57
C
ATOM    872  C   PHE A  60       8.760  -3.127  29.329  1.00  0.62
C
ATOM    873  O   PHE A  60       9.656  -2.542  29.943  1.00  1.17
O
ATOM    874  CB  PHE A  60       7.212  -1.328  28.523  1.00  0.85
C
ATOM    875  CG  PHE A  60       6.991  -0.328  27.417  1.00  0.85
C
ATOM    876  CD1 PHE A  60       5.719  -0.043  26.939  1.00  0.85
C
ATOM    877  CD2 PHE A  60       8.073   0.320  26.840  1.00  0.85
C
ATOM    878  CE1 PHE A  60       5.534   0.874  25.922  1.00  0.85
C
ATOM    879  CE2 PHE A  60       7.892   1.236  25.823  1.00  0.85
C
```

```
ATOM    880  CZ  PHE A  60       6.620   1.515  25.369  1.00  0.85
C
ATOM    881  H   PHE A  60       6.476  -3.400  27.155  1.00  0.53
H
ATOM    882  HA  PHE A  60       8.961  -1.992  27.562  1.00  0.68
H
ATOM    883 1HB  PHE A  60       6.246  -1.732  28.820  1.00  1.03
H
ATOM    884 2HB  PHE A  60       7.630  -0.804  29.381  1.00  1.03
H
ATOM    885  HD1 PHE A  60       4.857  -0.544  27.379  1.00  1.03
H
ATOM    886  HD2 PHE A  60       9.076   0.102  27.200  1.00  1.03
H
ATOM    887  HE1 PHE A  60       4.530   1.093  25.561  1.00  1.03
H
ATOM    888  HE2 PHE A  60       8.751   1.742  25.384  1.00  1.03
H
ATOM    889  HZ  PHE A  60       6.477   2.242  24.575  1.00  1.03
H
ATOM    890  N   PHE A  61       8.308  -4.329  29.695  1.00  0.55
N
ATOM    891  CA  PHE A  61       8.865  -4.955  30.887  1.00  0.67
C
ATOM    892  C   PHE A  61       9.743  -6.186  30.666  1.00  0.99
C
ATOM    893  O   PHE A  61      10.623  -6.446  31.488  1.00  1.94
O
ATOM    894  CB  PHE A  61       7.765  -5.262  31.885  1.00  1.01
C
ATOM    895  CG  PHE A  61       7.098  -4.016  32.386  1.00  1.01
C
ATOM    896  CD1 PHE A  61       5.760  -3.757  32.140  1.00  1.01
C
ATOM    897  CD2 PHE A  61       7.833  -3.082  33.093  1.00  1.01
C
ATOM    898  CE1 PHE A  61       5.172  -2.595  32.597  1.00  1.01
C
ATOM    899  CE2 PHE A  61       7.250  -1.922  33.556  1.00  1.01
C
ATOM    900  CZ  PHE A  61       5.917  -1.678  33.307  1.00  1.01
C
ATOM    901  H   PHE A  61       7.560  -4.788  29.192  1.00  0.66
H
ATOM    902  HA  PHE A  61       9.499  -4.210  31.368  1.00  0.80
H
ATOM    903 1HB  PHE A  61       7.011  -5.901  31.428  1.00  1.21
H
ATOM    904 2HB  PHE A  61       8.187  -5.794  32.733  1.00  1.21
H
ATOM    905  HD1 PHE A  61       5.169  -4.480  31.584  1.00  1.21
H
ATOM    906  HD2 PHE A  61       8.888  -3.277  33.285  1.00  1.21
H
ATOM    907  HE1 PHE A  61       4.118  -2.403  32.397  1.00  1.21
H
ATOM    908  HE2 PHE A  61       7.843  -1.198  34.115  1.00  1.21
H
```

ATOM 909 HZ PHE A 61 5.452 -0.761 33.670 1.00 1.21
H
ATOM 910 N TYR A 62 9.541 -6.949 29.589 1.00 0.78
N
ATOM 911 CA TYR A 62 10.426 -8.098 29.374 1.00 1.53
C
ATOM 912 C TYR A 62 11.180 -8.130 28.056 1.00 1.26
C
ATOM 913 O TYR A 62 12.277 -8.688 28.003 1.00 2.87
O
ATOM 914 CB TYR A 62 9.662 -9.409 29.546 1.00 2.29
C
ATOM 915 CG TYR A 62 9.300 -9.659 30.985 1.00 2.29
C
ATOM 916 CD1 TYR A 62 8.016 -9.456 31.437 1.00 2.29
C
ATOM 917 CD2 TYR A 62 10.288 -10.080 31.861 1.00 2.29
C
ATOM 918 CE1 TYR A 62 7.718 -9.672 32.768 1.00 2.29
C
ATOM 919 CE2 TYR A 62 9.991 -10.292 33.189 1.00 2.29
C
ATOM 920 CZ TYR A 62 8.711 -10.086 33.643 1.00 2.29
C
ATOM 921 OH TYR A 62 8.420 -10.285 34.969 1.00 2.29
O
ATOM 922 H TYR A 62 8.804 -6.754 28.926 1.00 0.94
H
ATOM 923 HA TYR A 62 11.185 -8.073 30.156 1.00 1.84
H
ATOM 924 1HB TYR A 62 8.744 -9.387 28.957 1.00 2.75
H
ATOM 925 2HB TYR A 62 10.268 -10.243 29.193 1.00 2.75
H
ATOM 926 HD1 TYR A 62 7.248 -9.122 30.743 1.00 2.75
H
ATOM 927 HD2 TYR A 62 11.304 -10.235 31.499 1.00 2.75
H
ATOM 928 HE1 TYR A 62 6.709 -9.508 33.133 1.00 2.75
H
ATOM 929 HE2 TYR A 62 10.769 -10.617 33.879 1.00 2.75
H
ATOM 930 HH TYR A 62 9.227 -10.506 35.441 1.00 2.75
H
ATOM 931 N ILE A 63 10.648 -7.527 27.003 1.00 0.46
N
ATOM 932 CA ILE A 63 11.331 -7.639 25.717 1.00 0.40
C
ATOM 933 C ILE A 63 12.323 -6.531 25.392 1.00 0.55
C
ATOM 934 O ILE A 63 13.469 -6.814 25.041 1.00 1.23
O
ATOM 935 CB ILE A 63 10.308 -7.704 24.602 1.00 0.60
C
ATOM 936 CG1 ILE A 63 9.390 -8.898 24.851 1.00 0.60
C
ATOM 937 CG2 ILE A 63 10.999 -7.780 23.253 1.00 0.60
C

```
ATOM    938  CD1 ILE A  63      10.118 -10.224  24.922  1.00  0.60
C
ATOM    939  H   ILE A  63       9.751  -7.062  27.069  1.00  0.55
H
ATOM    940  HA  ILE A  63      11.879  -8.580  25.718  1.00  0.48
H
ATOM    941  HB  ILE A  63       9.704  -6.810  24.631  1.00  0.72
H
ATOM    942 1HG1 ILE A  63       8.869  -8.747  25.795  1.00  0.72
H
ATOM    943 2HG1 ILE A  63       8.647  -8.944  24.060  1.00  0.72
H
ATOM    944 1HG2 ILE A  63      10.254  -7.800  22.463  1.00  0.72
H
ATOM    945 2HG2 ILE A  63      11.634  -6.904  23.122  1.00  0.72
H
ATOM    946 3HG2 ILE A  63      11.610  -8.681  23.206  1.00  0.72
H
ATOM    947 1HD1 ILE A  63       9.402 -11.024  25.108  1.00  0.72
H
ATOM    948 2HD1 ILE A  63      10.632 -10.409  23.979  1.00  0.72
H
ATOM    949 3HD1 ILE A  63      10.846 -10.195  25.732  1.00  0.72
H
ATOM    950  N   MET A  64      11.892  -5.280  25.487  1.00  0.54
N
ATOM    951  CA  MET A  64      12.756  -4.157  25.145  1.00  0.76
C
ATOM    952  C   MET A  64      13.774  -3.838  26.221  1.00  0.82
C
ATOM    953  O   MET A  64      13.586  -4.153  27.399  1.00  2.41
O
ATOM    954  CB  MET A  64      11.933  -2.906  24.874  1.00  1.14
C
ATOM    955  CG  MET A  64      11.045  -2.956  23.647  1.00  1.14
C
ATOM    956  SD  MET A  64      10.110  -1.433  23.464  1.00  1.14
S
ATOM    957  CE  MET A  64      11.475  -0.318  23.149  1.00  1.14
C
ATOM    958  H   MET A  64      10.946  -5.087  25.784  1.00  0.65
H
ATOM    959  HA  MET A  64      13.307  -4.417  24.241  1.00  0.91
H
ATOM    960 1HB  MET A  64      11.308  -2.683  25.736  1.00  1.37
H
ATOM    961 2HB  MET A  64      12.609  -2.060  24.743  1.00  1.37
H
ATOM    962 1HG  MET A  64      11.658  -3.095  22.760  1.00  1.37
H
ATOM    963 2HG  MET A  64      10.346  -3.788  23.723  1.00  1.37
H
ATOM    964 1HE  MET A  64      11.103   0.697  23.018  1.00  1.37
H
ATOM    965 2HE  MET A  64      12.165  -0.340  23.995  1.00  1.37
H
ATOM    966 3HE  MET A  64      12.000  -0.632  22.246  1.00  1.37
H
```

ATOM	967	N	ARG	A	65	14.850	-3.185	25.804	1.00	0.69	N
ATOM	968	CA	ARG	A	65	15.888	-2.742	26.714	1.00	0.68	C
ATOM	969	C	ARG	A	65	16.124	-1.262	26.490	1.00	0.68	C
ATOM	970	O	ARG	A	65	16.321	-0.815	25.359	1.00	0.72	O
ATOM	971	CB	ARG	A	65	17.170	-3.516	26.508	1.00	1.02	C
ATOM	972	CG	ARG	A	65	17.074	-4.998	26.795	1.00	1.02	C
ATOM	973	CD	ARG	A	65	16.881	-5.247	28.242	1.00	1.02	C
ATOM	974	NE	ARG	A	65	16.835	-6.665	28.545	1.00	1.02	N
ATOM	975	CZ	ARG	A	65	15.727	-7.432	28.494	1.00	1.02	C
ATOM	976	NH1	ARG	A	65	14.564	-6.924	28.146	1.00	1.02	N
ATOM	977	NH2	ARG	A	65	15.805	-8.715	28.794	1.00	1.02	N
ATOM	978	H	ARG	A	65	14.960	-2.995	24.816	1.00	0.83	H
ATOM	979	HA	ARG	A	65	15.548	-2.895	27.737	1.00	0.82	H
ATOM	980	1HB	ARG	A	65	17.509	-3.396	25.479	1.00	1.22	H
ATOM	981	2HB	ARG	A	65	17.939	-3.114	27.157	1.00	1.22	H
ATOM	982	1HG	ARG	A	65	16.229	-5.425	26.254	1.00	1.22	H
ATOM	983	2HG	ARG	A	65	17.993	-5.492	26.480	1.00	1.22	H
ATOM	984	1HD	ARG	A	65	17.701	-4.803	28.803	1.00	1.22	H
ATOM	985	2HD	ARG	A	65	15.939	-4.802	28.562	1.00	1.22	H
ATOM	986	HE	ARG	A	65	17.701	-7.111	28.817	1.00	1.22	H
ATOM	987	1HH1	ARG	A	65	14.465	-5.942	27.915	1.00	1.22	H
ATOM	988	2HH1	ARG	A	65	13.754	-7.529	28.107	1.00	1.22	H
ATOM	989	1HH2	ARG	A	65	16.692	-9.120	29.063	1.00	1.22	H
ATOM	990	2HH2	ARG	A	65	14.976	-9.288	28.756	1.00	1.22	H
ATOM	991	N	TYR	A	66	16.043	-0.502	27.570	1.00	0.77	N
ATOM	992	CA	TYR	A	66	16.134	0.945	27.490	1.00	0.78	C
ATOM	993	C	TYR	A	66	16.344	1.582	28.858	1.00	0.92	C
ATOM	994	O	TYR	A	66	15.885	1.059	29.874	1.00	1.27	O
ATOM	995	CB	TYR	A	66	14.854	1.499	26.854	1.00	1.17	C

```
ATOM    996  CG  TYR A  66      13.604   1.124  27.622  1.00  1.17
C
ATOM    997  CD1 TYR A  66      13.144   1.951  28.638  1.00  1.17
C
ATOM    998  CD2 TYR A  66      12.919  -0.047  27.316  1.00  1.17
C
ATOM    999  CE1 TYR A  66      12.008   1.606  29.345  1.00  1.17
C
ATOM   1000  CE2 TYR A  66      11.786  -0.392  28.024  1.00  1.17
C
ATOM   1001  CZ  TYR A  66      11.331   0.432  29.034  1.00  1.17
C
ATOM   1002  OH  TYR A  66      10.204   0.094  29.744  1.00  1.17
O
ATOM   1003  H   TYR A  66      15.928  -0.953  28.469  1.00  0.92
H
ATOM   1004  HA  TYR A  66      16.984   1.205  26.862  1.00  0.94
H
ATOM   1005 1HB  TYR A  66      14.909   2.586  26.805  1.00  1.40
H
ATOM   1006 2HB  TYR A  66      14.753   1.128  25.834  1.00  1.40
H
ATOM   1007  HD1 TYR A  66      13.680   2.869  28.879  1.00  1.40
H
ATOM   1008  HD2 TYR A  66      13.279  -0.696  26.519  1.00  1.40
H
ATOM   1009  HE1 TYR A  66      11.646   2.254  30.143  1.00  1.40
H
ATOM   1010  HE2 TYR A  66      11.252  -1.313  27.786  1.00  1.40
H
ATOM   1011  HH  TYR A  66      10.073  -0.861  29.711  1.00  1.40
H
ATOM   1012  N   LYS A  67      17.011   2.731  28.877  1.00  0.84
N
ATOM   1013  CA  LYS A  67      17.220   3.465  30.114  1.00  1.31
C
ATOM   1014  C   LYS A  67      15.934   4.155  30.520  1.00  2.73
C
ATOM   1015  O   LYS A  67      15.505   5.102  29.869  1.00 19.37
O
ATOM   1016  CB  LYS A  67      18.334   4.496  29.965  1.00  1.97
C
ATOM   1017  CG  LYS A  67      18.599   5.292  31.235  1.00  1.97
C
ATOM   1018  CD  LYS A  67      19.214   4.406  32.307  1.00  1.97
C
ATOM   1019  CE  LYS A  67      19.573   5.201  33.553  1.00  1.97
C
ATOM   1020  NZ  LYS A  67      20.172   4.338  34.607  1.00  1.97
N
ATOM   1021  H   LYS A  67      17.383   3.102  28.014  1.00  1.01
H
ATOM   1022  HA  LYS A  67      17.491   2.760  30.902  1.00  1.57
H
ATOM   1023 1HB  LYS A  67      19.260   3.996  29.679  1.00  2.36
H
ATOM   1024 2HB  LYS A  67      18.081   5.198  29.170  1.00  2.36
H
```

```
ATOM   1025 1HG  LYS A  67      19.282   6.114  31.017  1.00  2.36
H
ATOM   1026 2HG  LYS A  67      17.665   5.707  31.609  1.00  2.36
H
ATOM   1027 1HD  LYS A  67      18.509   3.621  32.579  1.00  2.36
H
ATOM   1028 2HD  LYS A  67      20.119   3.940  31.915  1.00  2.36
H
ATOM   1029 1HE  LYS A  67      20.284   5.982  33.288  1.00  2.36
H
ATOM   1030 2HE  LYS A  67      18.672   5.665  33.953  1.00  2.36
H
ATOM   1031 1HZ  LYS A  67      20.394   4.900  35.416  1.00  2.36
H
ATOM   1032 2HZ  LYS A  67      19.513   3.618  34.869  1.00  2.36
H
ATOM   1033 3HZ  LYS A  67      21.015   3.911  34.253  1.00  2.36
H
ATOM   1034  N   GLN A  68      15.335   3.698  31.609  1.00  3.72
N
ATOM   1035  CA  GLN A  68      14.054   4.210  32.093  1.00  3.01
C
ATOM   1036  C   GLN A  68      13.995   5.728  32.218  1.00  5.44
C
ATOM   1037  O   GLN A  68      13.027   6.358  31.791  1.00 11.09
O
ATOM   1038  CB  GLN A  68      13.759   3.619  33.468  1.00  4.51
C
ATOM   1039  CG  GLN A  68      13.458   2.140  33.484  1.00  4.51
C
ATOM   1040  CD  GLN A  68      13.320   1.630  34.903  1.00  4.51
C
ATOM   1041  OE1 GLN A  68      12.762   2.310  35.770  1.00  4.51
O
ATOM   1042  NE2 GLN A  68      13.843   0.438  35.150  1.00  4.51
N
ATOM   1043  H   GLN A  68      15.766   2.933  32.109  1.00  4.46
H
ATOM   1044  HA  GLN A  68      13.277   3.899  31.394  1.00  3.61
H
ATOM   1045 1HB  GLN A  68      14.612   3.790  34.125  1.00  5.42
H
ATOM   1046 2HB  GLN A  68      12.905   4.136  33.906  1.00  5.42
H
ATOM   1047 1HG  GLN A  68      12.521   1.959  32.958  1.00  5.42
H
ATOM   1048 2HG  GLN A  68      14.272   1.600  33.000  1.00  5.42
H
ATOM   1049 1HE2 GLN A  68      13.795   0.032  36.067  1.00  5.42
H
ATOM   1050 2HE2 GLN A  68      14.286  -0.087  34.423  1.00  5.42
H
ATOM   1051  N   SER A  69      15.032   6.310  32.817  1.00  7.31
N
ATOM   1052  CA  SER A  69      15.084   7.747  33.058  1.00 12.36
C
ATOM   1053  C   SER A  69      15.616   8.555  31.878  1.00  9.75
C
```

```
ATOM   1054  O   SER A  69      15.765   9.773  31.983  1.00 18.84
O
ATOM   1055  CB  SER A  69      15.938   8.036  34.277  1.00 18.54
C
ATOM   1056  OG  SER A  69      17.277   7.699  34.048  1.00 18.54
O
ATOM   1057  H   SER A  69      15.800   5.737  33.132  1.00  8.77
H
ATOM   1058  HA  SER A  69      14.069   8.089  33.264  1.00 14.83
H
ATOM   1059 1HB  SER A  69      15.863   9.093  34.530  1.00 22.25
H
ATOM   1060 2HB  SER A  69      15.557   7.470  35.126  1.00 22.25
H
ATOM   1061  HG  SER A  69      17.568   8.260  33.325  1.00 22.25
H
ATOM   1062  N   ASP A  70      15.893   7.902  30.757  1.00  3.33
N
ATOM   1063  CA  ASP A  70      16.393   8.613  29.597  1.00  2.44
C
ATOM   1064  C   ASP A  70      15.923   7.959  28.306  1.00  2.00
C
ATOM   1065  O   ASP A  70      16.723   7.340  27.605  1.00  1.95
O
ATOM   1066  CB  ASP A  70      17.923   8.660  29.618  1.00  3.66
C
ATOM   1067  CG  ASP A  70      18.506   9.552  28.531  1.00  3.66
C
ATOM   1068  OD1 ASP A  70      17.751  10.265  27.914  1.00  3.66
O
ATOM   1069  OD2 ASP A  70      19.695   9.492  28.309  1.00  3.66
O
ATOM   1070  H   ASP A  70      15.753   6.905  30.680  1.00  4.00
H
ATOM   1071  HA  ASP A  70      16.016   9.636  29.626  1.00  2.93
H
ATOM   1072 1HB  ASP A  70      18.262   9.023  30.588  1.00  4.39
H
ATOM   1073 2HB  ASP A  70      18.318   7.653  29.488  1.00  4.39
H
ATOM   1074  N   PRO A  71      14.622   8.081  27.980  1.00  1.88
N
ATOM   1075  CA  PRO A  71      13.917   7.572  26.801  1.00  1.49
C
ATOM   1076  C   PRO A  71      14.627   7.909  25.494  1.00  1.67
C
ATOM   1077  O   PRO A  71      14.609   7.118  24.553  1.00  3.87
O
ATOM   1078  CB  PRO A  71      12.568   8.301  26.852  1.00  2.23
C
ATOM   1079  CG  PRO A  71      12.352   8.562  28.293  1.00  2.23
C
ATOM   1080  CD  PRO A  71      13.719   8.927  28.797  1.00  2.23
C
ATOM   1081  HA  PRO A  71      13.784   6.486  26.902  1.00  1.79
H
ATOM   1082 1HB  PRO A  71      12.612   9.222  26.254  1.00  2.68
H
```

```
ATOM   1083 2HB  PRO A  71      11.783   7.668  26.412  1.00  2.68
H
ATOM   1084 1HG  PRO A  71      11.616   9.368  28.430  1.00  2.68
H
ATOM   1085 2HG  PRO A  71      11.944   7.667  28.785  1.00  2.68
H
ATOM   1086 1HD  PRO A  71      13.933   9.993  28.622  1.00  2.68
H
ATOM   1087 2HD  PRO A  71      13.767   8.669  29.856  1.00  2.68
H
ATOM   1088  N   GLU A  72      15.265   9.078  25.456  1.00  2.05
N
ATOM   1089  CA  GLU A  72      15.951   9.583  24.276  1.00  3.01
C
ATOM   1090  C   GLU A  72      17.390   9.085  24.100  1.00  2.30
C
ATOM   1091  O   GLU A  72      18.058   9.481  23.144  1.00  2.88
O
ATOM   1092  CB  GLU A  72      15.957  11.110  24.312  1.00  4.51
C
ATOM   1093  CG  GLU A  72      14.574  11.743  24.219  1.00  4.51
C
ATOM   1094  CD  GLU A  72      14.613  13.246  24.281  1.00  4.51
C
ATOM   1095  OE1 GLU A  72      15.688  13.791  24.356  1.00  4.51
O
ATOM   1096  OE2 GLU A  72      13.566  13.850  24.264  1.00  4.51
O
ATOM   1097  H   GLU A  72      15.254   9.662  26.281  1.00  2.46
H
ATOM   1098  HA  GLU A  72      15.383   9.266  23.401  1.00  3.61
H
ATOM   1099 1HB  GLU A  72      16.420  11.449  25.240  1.00  5.42
H
ATOM   1100 2HB  GLU A  72      16.557  11.493  23.487  1.00  5.42
H
ATOM   1101 1HG  GLU A  72      14.111  11.442  23.278  1.00  5.42
H
ATOM   1102 2HG  GLU A  72      13.958  11.366  25.033  1.00  5.42
H
ATOM   1103  N   ASN A  73      17.878   8.248  25.013  1.00  1.59
N
ATOM   1104  CA  ASN A  73      19.251   7.767  24.919  1.00  1.38
C
ATOM   1105  C   ASN A  73      19.451   6.947  23.637  1.00  1.24
C
ATOM   1106  O   ASN A  73      18.667   6.038  23.370  1.00  1.40
O
ATOM   1107  CB  ASN A  73      19.609   6.926  26.124  1.00  2.07
C
ATOM   1108  CG  ASN A  73      21.077   6.667  26.241  1.00  2.07
C
ATOM   1109  OD1 ASN A  73      21.689   5.919  25.466  1.00  2.07
O
ATOM   1110  ND2 ASN A  73      21.668   7.310  27.218  1.00  2.07
N
ATOM   1111  H   ASN A  73      17.313   7.943  25.794  1.00  1.91
H
```

ATOM 1112 HA ASN A 73 19.907 8.632 24.906 1.00 1.66
H
ATOM 1113 1HB ASN A 73 19.263 7.410 27.032 1.00 2.48
H
ATOM 1114 2HB ASN A 73 19.101 5.968 26.059 1.00 2.48
H
ATOM 1115 1HD2 ASN A 73 22.652 7.202 27.368 1.00 2.48
H
ATOM 1116 2HD2 ASN A 73 21.127 7.920 27.805 1.00 2.48
H
ATOM 1117 N PRO A 74 20.492 7.244 22.839 1.00 1.12
N
ATOM 1118 CA PRO A 74 20.906 6.594 21.591 1.00 0.96
C
ATOM 1119 C PRO A 74 21.033 5.069 21.687 1.00 1.14
C
ATOM 1120 O PRO A 74 20.854 4.370 20.686 1.00 1.56
O
ATOM 1121 CB PRO A 74 22.263 7.236 21.305 1.00 1.44
C
ATOM 1122 CG PRO A 74 22.146 8.605 21.873 1.00 1.44
C
ATOM 1123 CD PRO A 74 21.318 8.441 23.112 1.00 1.44
C
ATOM 1124 HA PRO A 74 20.191 6.862 20.804 1.00 1.15
H
ATOM 1125 1HB PRO A 74 23.066 6.647 21.771 1.00 1.73
H
ATOM 1126 2HB PRO A 74 22.456 7.240 20.220 1.00 1.73
H
ATOM 1127 1HG PRO A 74 23.148 9.010 22.087 1.00 1.73
H
ATOM 1128 2HG PRO A 74 21.680 9.280 21.141 1.00 1.73
H
ATOM 1129 1HD PRO A 74 21.960 8.272 23.990 1.00 1.73
H
ATOM 1130 2HD PRO A 74 20.680 9.329 23.230 1.00 1.73
H
ATOM 1131 N ASP A 75 21.329 4.545 22.871 1.00 1.51
N
ATOM 1132 CA ASP A 75 21.466 3.105 23.026 1.00 2.16
C
ATOM 1133 C ASP A 75 20.159 2.378 23.301 1.00 2.66
C
ATOM 1134 O ASP A 75 20.149 1.153 23.377 1.00 6.17
O
ATOM 1135 CB ASP A 75 22.449 2.794 24.143 1.00 3.24
C
ATOM 1136 CG ASP A 75 23.867 3.129 23.752 1.00 3.24
C
ATOM 1137 OD1 ASP A 75 24.216 2.850 22.630 1.00 3.24
O
ATOM 1138 OD2 ASP A 75 24.586 3.664 24.562 1.00 3.24
O
ATOM 1139 H ASP A 75 21.463 5.140 23.682 1.00 1.81
H
ATOM 1140 HA ASP A 75 21.873 2.706 22.098 1.00 2.59
H

ATOM 1141 1HB ASP A 75 22.184 3.364 25.034 1.00 3.89
H
ATOM 1142 2HB ASP A 75 22.395 1.735 24.398 1.00 3.89
H
ATOM 1143 N ASN A 76 19.061 3.109 23.435 1.00 1.39
N
ATOM 1144 CA ASN A 76 17.782 2.484 23.726 1.00 1.46
C
ATOM 1145 C ASN A 76 17.139 1.826 22.529 1.00 1.63
C
ATOM 1146 O ASN A 76 17.220 2.341 21.410 1.00 2.75
O
ATOM 1147 CB ASN A 76 16.794 3.495 24.268 1.00 2.19
C
ATOM 1148 CG ASN A 76 17.150 4.032 25.606 1.00 2.19
C
ATOM 1149 OD1 ASN A 76 17.954 3.447 26.340 1.00 2.19
O
ATOM 1150 ND2 ASN A 76 16.549 5.138 25.954 1.00 2.19
N
ATOM 1151 H ASN A 76 19.095 4.116 23.352 1.00 1.67
H
ATOM 1152 HA ASN A 76 17.943 1.706 24.473 1.00 1.75
H
ATOM 1153 1HB ASN A 76 16.718 4.331 23.571 1.00 2.63
H
ATOM 1154 2HB ASN A 76 15.807 3.036 24.329 1.00 2.63
H
ATOM 1155 1HD2 ASN A 76 16.737 5.559 26.845 1.00 2.63
H
ATOM 1156 2HD2 ASN A 76 15.899 5.579 25.330 1.00 2.63
H
ATOM 1157 N ASP A 77 16.425 0.731 22.793 1.00 1.06
N
ATOM 1158 CA ASP A 77 15.579 0.113 21.788 1.00 0.87
C
ATOM 1159 C ASP A 77 14.483 1.118 21.472 1.00 0.86
C
ATOM 1160 O ASP A 77 14.072 1.888 22.343 1.00 1.93
O
ATOM 1161 CB ASP A 77 14.974 -1.202 22.292 1.00 1.30
C
ATOM 1162 CG ASP A 77 15.965 -2.352 22.359 1.00 1.30
C
ATOM 1163 OD1 ASP A 77 16.871 -2.389 21.562 1.00 1.30
O
ATOM 1164 OD2 ASP A 77 15.821 -3.178 23.226 1.00 1.30
O
ATOM 1165 H ASP A 77 16.440 0.335 23.725 1.00 1.27
H
ATOM 1166 HA ASP A 77 16.158 -0.075 20.884 1.00 1.04
H
ATOM 1167 1HB ASP A 77 14.562 -1.047 23.289 1.00 1.57
H
ATOM 1168 2HB ASP A 77 14.150 -1.494 21.641 1.00 1.57
H
ATOM 1169 N ARG A 78 14.028 1.140 20.234 1.00 0.66
N

```
ATOM   1170  CA  ARG A  78      13.023   2.104  19.831  1.00  0.73
C
ATOM   1171  C   ARG A  78      11.631   1.522  19.819  1.00  0.96
C
ATOM   1172  O   ARG A  78      11.427   0.393  19.382  1.00  3.00
O
ATOM   1173  CB  ARG A  78      13.334   2.657  18.454  1.00  1.09
C
ATOM   1174  CG  ARG A  78      12.300   3.625  17.891  1.00  1.09
C
ATOM   1175  CD  ARG A  78      12.305   4.938  18.586  1.00  1.09
C
ATOM   1176  NE  ARG A  78      11.310   5.835  18.025  1.00  1.09
N
ATOM   1177  CZ  ARG A  78      11.542   6.679  17.000  1.00  1.09
C
ATOM   1178  NH1 ARG A  78      12.739   6.746  16.465  1.00  1.09
N
ATOM   1179  NH2 ARG A  78      10.566   7.440  16.536  1.00  1.09
N
ATOM   1180  H   ARG A  78      14.374   0.466  19.565  1.00  0.79
H
ATOM   1181  HA  ARG A  78      13.040   2.931  20.541  1.00  0.88
H
ATOM   1182 1HB  ARG A  78      14.295   3.167  18.476  1.00  1.31
H
ATOM   1183 2HB  ARG A  78      13.417   1.831  17.754  1.00  1.31
H
ATOM   1184 1HG  ARG A  78      12.501   3.806  16.838  1.00  1.31
H
ATOM   1185 2HG  ARG A  78      11.305   3.194  17.984  1.00  1.31
H
ATOM   1186 1HD  ARG A  78      12.077   4.792  19.642  1.00  1.31
H
ATOM   1187 2HD  ARG A  78      13.283   5.405  18.485  1.00  1.31
H
ATOM   1188  HE  ARG A  78      10.377   5.806  18.413  1.00  1.31
H
ATOM   1189 1HH1 ARG A  78      13.486   6.166  16.821  1.00  1.31
H
ATOM   1190 2HH1 ARG A  78      12.918   7.377  15.697  1.00  1.31
H
ATOM   1191 1HH2 ARG A  78       9.644   7.381  16.945  1.00  1.31
H
ATOM   1192 2HH2 ARG A  78      10.742   8.070  15.766  1.00  1.31
H
ATOM   1193  N   PHE A  79      10.674   2.303  20.295  1.00  0.87
N
ATOM   1194  CA  PHE A  79       9.280   1.918  20.225  1.00  1.11
C
ATOM   1195  C   PHE A  79       8.492   3.028  19.581  1.00  1.19
C
ATOM   1196  O   PHE A  79       8.393   4.132  20.116  1.00  3.28
O
ATOM   1197  CB  PHE A  79       8.672   1.622  21.587  1.00  1.67
C
ATOM   1198  CG  PHE A  79       7.234   1.199  21.493  1.00  1.67
C
```

```
ATOM   1199  CD1 PHE A  79       6.890  -0.050  21.000  1.00  1.67
C
ATOM   1200  CD2 PHE A  79       6.219   2.051  21.898  1.00  1.67
C
ATOM   1201  CE1 PHE A  79       5.566  -0.438  20.916  1.00  1.67
C
ATOM   1202  CE2 PHE A  79       4.898   1.663  21.824  1.00  1.67
C
ATOM   1203  CZ  PHE A  79       4.571   0.419  21.332  1.00  1.67
C
ATOM   1204  H   PHE A  79      10.916   3.204  20.682  1.00  1.04
H
ATOM   1205  HA  PHE A  79       9.191   1.026  19.608  1.00  1.33
H
ATOM   1206 1HB  PHE A  79       9.223   0.822  22.065  1.00  2.00
H
ATOM   1207 2HB  PHE A  79       8.736   2.501  22.226  1.00  2.00
H
ATOM   1208  HD1 PHE A  79       7.678  -0.727  20.678  1.00  2.00
H
ATOM   1209  HD2 PHE A  79       6.476   3.036  22.288  1.00  2.00
H
ATOM   1210  HE1 PHE A  79       5.310  -1.422  20.526  1.00  2.00
H
ATOM   1211  HE2 PHE A  79       4.112   2.338  22.155  1.00  2.00
H
ATOM   1212  HZ  PHE A  79       3.528   0.115  21.272  1.00  2.00
H
ATOM   1213  N   VAL A  80       7.930   2.726  18.428  1.00  0.64
N
ATOM   1214  CA  VAL A  80       7.128   3.678  17.711  1.00  0.60
C
ATOM   1215  C   VAL A  80       5.686   3.279  17.805  1.00  0.68
C
ATOM   1216  O   VAL A  80       5.246   2.378  17.091  1.00  1.41
O
ATOM   1217  CB  VAL A  80       7.509   3.729  16.217  1.00  0.90
C
ATOM   1218  CG1 VAL A  80       6.638   4.754  15.509  1.00  0.90
C
ATOM   1219  CG2 VAL A  80       8.979   4.057  16.057  1.00  0.90
C
ATOM   1220  H   VAL A  80       8.059   1.804  18.042  1.00  0.77
H
ATOM   1221  HA  VAL A  80       7.257   4.665  18.154  1.00  0.72
H
ATOM   1222  HB  VAL A  80       7.306   2.758  15.764  1.00  1.08
H
ATOM   1223 1HG1 VAL A  80       6.894   4.777  14.451  1.00  1.08
H
ATOM   1224 2HG1 VAL A  80       5.589   4.484  15.625  1.00  1.08
H
ATOM   1225 3HG1 VAL A  80       6.810   5.739  15.943  1.00  1.08
H
ATOM   1226 1HG2 VAL A  80       9.233   4.080  14.997  1.00  1.08
H
ATOM   1227 2HG2 VAL A  80       9.179   5.028  16.501  1.00  1.08
H
```

```
ATOM   1228 3HG2 VAL A  80       9.576   3.294  16.557  1.00  1.08
H
ATOM   1229  N   LEU A  81       4.926   3.934  18.664  1.00  0.65
N
ATOM   1230  CA  LEU A  81       3.522   3.623  18.632  1.00  0.84
C
ATOM   1231  C   LEU A  81       2.995   4.399  17.456  1.00  1.10
C
ATOM   1232  O   LEU A  81       2.885   5.624  17.531  1.00  4.30
O
ATOM   1233  CB  LEU A  81       2.769   4.036  19.898  1.00  1.26
C
ATOM   1234  CG  LEU A  81       1.294   3.608  19.909  1.00  1.26
C
ATOM   1235  CD1 LEU A  81       1.247   2.114  19.863  1.00  1.26
C
ATOM   1236  CD2 LEU A  81       0.562   4.098  21.145  1.00  1.26
C
ATOM   1237  H   LEU A  81       5.301   4.644  19.278  1.00  0.78
H
ATOM   1238  HA  LEU A  81       3.379   2.557  18.452  1.00  1.01
H
ATOM   1239 1HB  LEU A  81       3.261   3.632  20.774  1.00  1.51
H
ATOM   1240 2HB  LEU A  81       2.791   5.119  19.948  1.00  1.51
H
ATOM   1241  HG  LEU A  81       0.807   3.985  19.018  1.00  1.51
H
ATOM   1242 1HD1 LEU A  81       0.211   1.780  19.842  1.00  1.51
H
ATOM   1243 2HD1 LEU A  81       1.760   1.772  18.971  1.00  1.51
H
ATOM   1244 3HD1 LEU A  81       1.742   1.713  20.747  1.00  1.51
H
ATOM   1245 1HD2 LEU A  81      -0.472   3.753  21.110  1.00  1.51
H
ATOM   1246 2HD2 LEU A  81       1.045   3.706  22.039  1.00  1.51
H
ATOM   1247 3HD2 LEU A  81       0.569   5.184  21.170  1.00  1.51
H
ATOM   1248  N   ALA A  82       2.699   3.697  16.370  1.00  0.82
N
ATOM   1249  CA  ALA A  82       2.166   4.328  15.178  1.00  0.65
C
ATOM   1250  C   ALA A  82       0.811   4.893  15.546  1.00  0.71
C
ATOM   1251  O   ALA A  82       0.431   5.986  15.132  1.00  1.02
O
ATOM   1252  CB  ALA A  82       2.093   3.340  14.033  1.00  0.98
C
ATOM   1253  H   ALA A  82       2.811   2.692  16.390  1.00  0.98
H
ATOM   1254  HA  ALA A  82       2.817   5.154  14.896  1.00  0.78
H
ATOM   1255 1HB  ALA A  82       1.697   3.834  13.149  1.00  1.17
H
ATOM   1256 2HB  ALA A  82       3.090   2.960  13.818  1.00  1.17
H
```

```
ATOM   1257 3HB  ALA A  82       1.442   2.515  14.313  1.00  1.17
H
ATOM   1258  N   LYS A  83       0.103   4.146  16.379  1.00  1.01
N
ATOM   1259  CA  LYS A  83      -1.158   4.557  16.976  1.00  2.11
C
ATOM   1260  C   LYS A  83      -1.053   5.666  18.084  1.00  3.92
C
ATOM   1261  O   LYS A  83      -1.564   5.460  19.170  1.00 48.75
O
ATOM   1262  CB  LYS A  83      -1.807   3.289  17.557  1.00  3.17
C
ATOM   1263  CG  LYS A  83      -3.145   3.452  18.254  1.00  3.17
C
ATOM   1264  CD  LYS A  83      -3.705   2.090  18.686  1.00  3.17
C
ATOM   1265  CE  LYS A  83      -2.838   1.445  19.769  1.00  3.17
C
ATOM   1266  NZ  LYS A  83      -3.497   0.268  20.422  1.00  3.17
N
ATOM   1267  H   LYS A  83       0.474   3.230  16.615  1.00  1.21
H
ATOM   1268  HA  LYS A  83      -1.793   4.947  16.179  1.00  2.53
H
ATOM   1269 1HB  LYS A  83      -1.950   2.567  16.751  1.00  3.80
H
ATOM   1270 2HB  LYS A  83      -1.124   2.834  18.272  1.00  3.80
H
ATOM   1271 1HG  LYS A  83      -3.027   4.082  19.136  1.00  3.80
H
ATOM   1272 2HG  LYS A  83      -3.854   3.929  17.578  1.00  3.80
H
ATOM   1273 1HD  LYS A  83      -4.717   2.213  19.063  1.00  3.80
H
ATOM   1274 2HD  LYS A  83      -3.738   1.424  17.823  1.00  3.80
H
ATOM   1275 1HE  LYS A  83      -1.906   1.112  19.313  1.00  3.80
H
ATOM   1276 2HE  LYS A  83      -2.609   2.187  20.534  1.00  3.80
H
ATOM   1277 1HZ  LYS A  83      -2.886  -0.122  21.126  1.00  3.80
H
ATOM   1278 2HZ  LYS A  83      -4.349   0.553  20.886  1.00  3.80
H
ATOM   1279 3HZ  LYS A  83      -3.721  -0.466  19.762  1.00  3.80
H
ATOM   1280  N   ARG A  84      -0.449   6.828  17.806  1.00 14.95
N
ATOM   1281  CA  ARG A  84      -0.396   8.010  18.730  1.00 10.65
C
ATOM   1282  C   ARG A  84       0.166   7.877  20.200  1.00 13.63
C
ATOM   1283  O   ARG A  84      -0.599   7.624  21.127  1.00 51.84
O
ATOM   1284  CB  ARG A  84      -1.821   8.546  18.855  1.00 15.98
C
ATOM   1285  CG  ARG A  84      -2.432   9.024  17.547  1.00 15.98
C
```

```
ATOM   1286  CD  ARG A  84      -3.849   9.452  17.697  1.00 15.98
C
ATOM   1287  NE  ARG A  84      -3.996  10.635  18.533  1.00 15.98
N
ATOM   1288  CZ  ARG A  84      -3.919  11.913  18.103  1.00 15.98
C
ATOM   1289  NH1 ARG A  84      -3.705  12.192  16.832  1.00 15.98
N
ATOM   1290  NH2 ARG A  84      -4.063  12.898  18.975  1.00 15.98
N
ATOM   1291  H   ARG A  84      -0.041   6.922  16.885  1.00 17.94
H
ATOM   1292  HA  ARG A  84       0.205   8.766  18.226  1.00 12.78
H
ATOM   1293 1HB  ARG A  84      -2.473   7.778  19.266  1.00 19.17
H
ATOM   1294 2HB  ARG A  84      -1.831   9.388  19.547  1.00 19.17
H
ATOM   1295 1HG  ARG A  84      -1.857   9.866  17.190  1.00 19.17
H
ATOM   1296 2HG  ARG A  84      -2.391   8.221  16.810  1.00 19.17
H
ATOM   1297 1HD  ARG A  84      -4.260   9.679  16.714  1.00 19.17
H
ATOM   1298 2HD  ARG A  84      -4.424   8.644  18.149  1.00 19.17
H
ATOM   1299  HE  ARG A  84      -4.163  10.487  19.520  1.00 19.17
H
ATOM   1300 1HH1 ARG A  84      -3.588  11.457  16.143  1.00 19.17
H
ATOM   1301 2HH1 ARG A  84      -3.639  13.157  16.532  1.00 19.17
H
ATOM   1302 1HH2 ARG A  84      -4.228  12.689  19.950  1.00 19.17
H
ATOM   1303 2HH2 ARG A  84      -4.012  13.859  18.668  1.00 19.17
H
ATOM   1304  N   LEU A  85       1.462   8.167  20.434  1.00 13.51
N
ATOM   1305  CA  LEU A  85       2.050   8.096  21.808  1.00 15.42
C
ATOM   1306  C   LEU A  85       2.930   9.299  22.191  1.00  8.44
C
ATOM   1307  O   LEU A  85       3.675   9.822  21.367  1.00 18.80
O
ATOM   1308  CB  LEU A  85       2.892   6.826  21.982  1.00 23.13
C
ATOM   1309  CG  LEU A  85       3.592   6.628  23.338  1.00 23.13
C
ATOM   1310  CD1 LEU A  85       2.543   6.436  24.424  1.00 23.13
C
ATOM   1311  CD2 LEU A  85       4.492   5.401  23.269  1.00 23.13
C
ATOM   1312  H   LEU A  85       2.060   8.404  19.655  1.00 16.21
H
ATOM   1313  HA  LEU A  85       1.225   8.052  22.519  1.00 18.50
H
ATOM   1314 1HB  LEU A  85       2.246   5.979  21.859  1.00 27.76
H
```

```
ATOM   1315 2HB  LEU A  85       3.646   6.797  21.201  1.00 27.76
H
ATOM   1316  HG  LEU A  85       4.189   7.504  23.578  1.00 27.76
H
ATOM   1317 1HD1 LEU A  85       3.036   6.295  25.386  1.00 27.76
H
ATOM   1318 2HD1 LEU A  85       1.898   7.311  24.475  1.00 27.76
H
ATOM   1319 3HD1 LEU A  85       1.941   5.557  24.193  1.00 27.76
H
ATOM   1320 1HD2 LEU A  85       4.984   5.257  24.232  1.00 27.76
H
ATOM   1321 2HD2 LEU A  85       3.889   4.524  23.036  1.00 27.76
H
ATOM   1322 3HD2 LEU A  85       5.245   5.535  22.497  1.00 27.76
H
ATOM   1323  N   SER A  86       2.805   9.743  23.447  1.00  9.29
N
ATOM   1324  CA  SER A  86       3.550  10.877  24.006  1.00 10.18
C
ATOM   1325  C   SER A  86       5.084  10.754  23.993  1.00  8.41
C
ATOM   1326  O   SER A  86       5.773  11.650  23.513  1.00 19.70
O
ATOM   1327  CB  SER A  86       3.104  11.106  25.437  1.00 15.27
C
ATOM   1328  OG  SER A  86       3.806  12.170  26.017  1.00 15.27
O
ATOM   1329  H   SER A  86       2.155   9.262  24.054  1.00 11.15
H
ATOM   1330  HA  SER A  86       3.286  11.759  23.421  1.00 12.22
H
ATOM   1331 1HB  SER A  86       2.036  11.319  25.454  1.00 18.32
H
ATOM   1332 2HB  SER A  86       3.266  10.200  26.020  1.00 18.32
H
ATOM   1333  HG  SER A  86       4.730  11.911  26.013  1.00 18.32
H
ATOM   1334  N   PHE A  87       5.632   9.652  24.496  1.00  9.59
N
ATOM   1335  CA  PHE A  87       7.092   9.521  24.620  1.00  9.56
C
ATOM   1336  C   PHE A  87       7.770   8.962  23.371  1.00  4.70
C
ATOM   1337  O   PHE A  87       8.475   7.957  23.436  1.00 11.68
O
ATOM   1338  CB  PHE A  87       7.401   8.614  25.803  1.00 14.34
C
ATOM   1339  CG  PHE A  87       6.922   9.188  27.099  1.00 14.34
C
ATOM   1340  CD1 PHE A  87       5.691   8.811  27.615  1.00 14.34
C
ATOM   1341  CD2 PHE A  87       7.688  10.104  27.801  1.00 14.34
C
ATOM   1342  CE1 PHE A  87       5.235   9.341  28.807  1.00 14.34
C
ATOM   1343  CE2 PHE A  87       7.235  10.635  28.992  1.00 14.34
C
```

```
ATOM   1344  CZ  PHE A  87       6.006  10.253  29.495  1.00 14.34
C
ATOM   1345  H   PHE A  87       5.038   8.914  24.845  1.00 11.51
H
ATOM   1346  HA  PHE A  87       7.507  10.511  24.816  1.00 11.47
H
ATOM   1347 1HB  PHE A  87       6.928   7.644  25.660  1.00 17.21
H
ATOM   1348 2HB  PHE A  87       8.476   8.452  25.875  1.00 17.21
H
ATOM   1349  HD1 PHE A  87       5.082   8.091  27.068  1.00 17.21
H
ATOM   1350  HD2 PHE A  87       8.657  10.407  27.401  1.00 17.21
H
ATOM   1351  HE1 PHE A  87       4.266   9.038  29.201  1.00 17.21
H
ATOM   1352  HE2 PHE A  87       7.845  11.357  29.536  1.00 17.21
H
ATOM   1353  HZ  PHE A  87       5.648  10.672  30.434  1.00 17.21
H
ATOM   1354  N   VAL A  88       7.557   9.623  22.237  1.00  8.32
N
ATOM   1355  CA  VAL A  88       8.093   9.171  20.955  1.00  4.68
C
ATOM   1356  C   VAL A  88       8.822  10.303  20.228  1.00  3.11
C
ATOM   1357  O   VAL A  88       8.385  11.452  20.250  1.00  6.16
O
ATOM   1358  CB  VAL A  88       6.962   8.588  20.064  1.00  7.02
C
ATOM   1359  CG1 VAL A  88       7.499   8.152  18.712  1.00  7.02
C
ATOM   1360  CG2 VAL A  88       6.337   7.391  20.747  1.00  7.02
C
ATOM   1361  H   VAL A  88       6.997  10.465  22.281  1.00  9.98
H
ATOM   1362  HA  VAL A  88       8.811   8.373  21.147  1.00  5.62
H
ATOM   1363  HB  VAL A  88       6.204   9.355  19.894  1.00  8.42
H
ATOM   1364 1HG1 VAL A  88       6.684   7.750  18.110  1.00  8.42
H
ATOM   1365 2HG1 VAL A  88       7.929   9.005  18.210  1.00  8.42
H
ATOM   1366 3HG1 VAL A  88       8.258   7.383  18.854  1.00  8.42
H
ATOM   1367 1HG2 VAL A  88       5.543   6.991  20.118  1.00  8.42
H
ATOM   1368 2HG2 VAL A  88       7.096   6.624  20.904  1.00  8.42
H
ATOM   1369 3HG2 VAL A  88       5.919   7.690  21.706  1.00  8.42
H
ATOM   1370  N   ASP A  89       9.962   9.969  19.621  1.00  1.62
N
ATOM   1371  CA  ASP A  89      10.790  10.940  18.903  1.00  1.71
C
ATOM   1372  C   ASP A  89      10.046  11.620  17.736  1.00  1.23
C
```

```
ATOM   1373  O   ASP A  89      10.180  12.828  17.534  1.00  2.49
O
ATOM   1374  CB  ASP A  89      12.062  10.252  18.383  1.00  2.56
C
ATOM   1375  CG  ASP A  89      13.047   9.879  19.497  1.00  2.56
C
ATOM   1376  OD1 ASP A  89      12.934  10.425  20.568  1.00  2.56
O
ATOM   1377  OD2 ASP A  89      13.882   9.033  19.268  1.00  2.56
O
ATOM   1378  H   ASP A  89      10.265   9.007  19.658  1.00  1.94
H
ATOM   1379  HA  ASP A  89      11.089  11.716  19.607  1.00  2.05
H
ATOM   1380 1HB  ASP A  89      11.793   9.351  17.842  1.00  3.08
H
ATOM   1381 2HB  ASP A  89      12.568  10.915  17.681  1.00  3.08
H
ATOM   1382  N   VAL A  90       9.263  10.851  16.981  1.00  0.62
N
ATOM   1383  CA  VAL A  90       8.483  11.401  15.872  1.00  0.43
C
ATOM   1384  C   VAL A  90       6.982  11.298  16.132  1.00  0.42
C
ATOM   1385  O   VAL A  90       6.472  10.224  16.449  1.00  0.47
O
ATOM   1386  CB  VAL A  90       8.832  10.681  14.550  1.00  0.65
C
ATOM   1387  CG1 VAL A  90       7.977  11.218  13.409  1.00  0.65
C
ATOM   1388  CG2 VAL A  90      10.310  10.884  14.233  1.00  0.65
C
ATOM   1389  H   VAL A  90       9.207   9.866  17.178  1.00  0.74
H
ATOM   1390  HA  VAL A  90       8.742  12.453  15.765  1.00  0.52
H
ATOM   1391  HB  VAL A  90       8.616   9.619  14.655  1.00  0.77
H
ATOM   1392 1HG1 VAL A  90       8.229  10.697  12.488  1.00  0.77
H
ATOM   1393 2HG1 VAL A  90       6.922  11.062  13.635  1.00  0.77
H
ATOM   1394 3HG1 VAL A  90       8.168  12.278  13.278  1.00  0.77
H
ATOM   1395 1HG2 VAL A  90      10.557  10.371  13.304  1.00  0.77
H
ATOM   1396 2HG2 VAL A  90      10.513  11.950  14.122  1.00  0.77
H
ATOM   1397 3HG2 VAL A  90      10.917  10.482  15.042  1.00  0.77
H
ATOM   1398  N   ALA A  91       6.286  12.423  16.000  1.00  0.47
N
ATOM   1399  CA  ALA A  91       4.846  12.474  16.202  1.00  0.57
C
ATOM   1400  C   ALA A  91       4.127  11.521  15.257  1.00  0.57
C
ATOM   1401  O   ALA A  91       4.395  11.502  14.055  1.00  0.76
O
```

```
ATOM   1402  CB  ALA A  91       4.342  13.897  15.997  1.00  0.85
C
ATOM   1403  H   ALA A  91       6.773  13.271  15.751  1.00  0.56
H
ATOM   1404  HA  ALA A  91       4.634  12.161  17.225  1.00  0.68
H
ATOM   1405 1HB  ALA A  91       3.267  13.933  16.174  1.00  1.03
H
ATOM   1406 2HB  ALA A  91       4.846  14.566  16.695  1.00  1.03
H
ATOM   1407 3HB  ALA A  91       4.552  14.213  14.976  1.00  1.03
H
ATOM   1408  N   THR A  92       3.193  10.747  15.802  1.00  0.56
N
ATOM   1409  CA  THR A  92       2.412   9.809  15.003  1.00  0.65
C
ATOM   1410  C   THR A  92       0.928  10.068  15.191  1.00  0.80
C
ATOM   1411  O   THR A  92       0.144   9.143  15.394  1.00  2.75
O
ATOM   1412  CB  THR A  92       2.717   8.341  15.360  1.00  0.98
C
ATOM   1413  OG1 THR A  92       2.474   8.129  16.755  1.00  0.98
O
ATOM   1414  CG2 THR A  92       4.157   7.972  15.031  1.00  0.98
C
ATOM   1415  H   THR A  92       3.024  10.807  16.797  1.00  0.67
H
ATOM   1416  HA  THR A  92       2.650   9.961  13.951  1.00  0.78
H
ATOM   1417  HB  THR A  92       2.054   7.699  14.786  1.00  1.17
H
ATOM   1418  HG1 THR A  92       2.596   7.196  16.959  1.00  1.17
H
ATOM   1419 1HG2 THR A  92       4.331   6.927  15.287  1.00  1.17
H
ATOM   1420 2HG2 THR A  92       4.336   8.121  13.967  1.00  1.17
H
ATOM   1421 3HG2 THR A  92       4.833   8.601  15.604  1.00  1.17
H
ATOM   1422  N   GLY A  93       0.553  11.341  15.133  1.00  0.92
N
ATOM   1423  CA  GLY A  93      -0.837  11.745  15.256  1.00  0.92
C
ATOM   1424  C   GLY A  93      -1.647  11.234  14.073  1.00  0.88
C
ATOM   1425  O   GLY A  93      -2.827  10.896  14.215  1.00  1.98
O
ATOM   1426  H   GLY A  93       1.256  12.049  14.982  1.00  1.10
H
ATOM   1427 1HA  GLY A  93      -1.246  11.378  16.187  1.00  1.10
H
ATOM   1428 2HA  GLY A  93      -0.890  12.830  15.295  1.00  1.10
H
ATOM   1429  N   TRP A  94      -0.995  11.215  12.905  1.00  1.25
N
ATOM   1430  CA  TRP A  94      -1.573  10.734  11.664  1.00  1.21
C
```

```
ATOM   1431  C   TRP A  94      -1.268   9.269  11.468  1.00  1.27
C
ATOM   1432  O   TRP A  94      -0.103   8.850  11.470  1.00  3.12
O
ATOM   1433  CB  TRP A  94      -1.017  11.487  10.482  1.00  1.81
C
ATOM   1434  CG  TRP A  94      -1.405  12.906  10.441  1.00  1.81
C
ATOM   1435  CD1 TRP A  94      -1.467  13.742  11.501  1.00  1.81
C
ATOM   1436  CD2 TRP A  94      -1.776  13.690   9.291  1.00  1.81
C
ATOM   1437  NE1 TRP A  94      -1.849  14.980  11.094  1.00  1.81
N
ATOM   1438  CE2 TRP A  94      -2.030  14.976   9.755  1.00  1.81
C
ATOM   1439  CE3 TRP A  94      -1.907  13.414   7.929  1.00  1.81
C
ATOM   1440  CZ2 TRP A  94      -2.393  15.993   8.922  1.00  1.81
C
ATOM   1441  CZ3 TRP A  94      -2.274  14.449   7.083  1.00  1.81
C
ATOM   1442  CH2 TRP A  94      -2.497  15.704   7.577  1.00  1.81
C
ATOM   1443  H   TRP A  94      -0.038  11.535  12.880  1.00  1.50
H
ATOM   1444  HA  TRP A  94      -2.654  10.867  11.703  1.00  1.45
H
ATOM   1445 1HB  TRP A  94       0.062  11.447  10.529  1.00  2.18
H
ATOM   1446 2HB  TRP A  94      -1.331  11.013   9.557  1.00  2.18
H
ATOM   1447  HD1 TRP A  94      -1.245  13.467  12.526  1.00  2.18
H
ATOM   1448  HE1 TRP A  94      -1.988  15.783  11.682  1.00  2.18
H
ATOM   1449  HE3 TRP A  94      -1.717  12.415   7.542  1.00  2.18
H
ATOM   1450  HZ2 TRP A  94      -2.583  16.995   9.295  1.00  2.18
H
ATOM   1451  HZ3 TRP A  94      -2.371  14.245   6.016  1.00  2.18
H
ATOM   1452  HH2 TRP A  94      -2.764  16.503   6.904  1.00  2.18
H
ATOM   1453  N   LEU A  95      -2.318   8.505  11.257  1.00  1.48
N
ATOM   1454  CA  LEU A  95      -2.184   7.079  11.111  1.00  1.40
C
ATOM   1455  C   LEU A  95      -1.476   6.702   9.820  1.00  0.83
C
ATOM   1456  O   LEU A  95      -1.644   7.347   8.783  1.00  1.23
O
ATOM   1457  CB  LEU A  95      -3.576   6.429  11.149  1.00  2.10
C
ATOM   1458  CG  LEU A  95      -4.346   6.571  12.471  1.00  2.10
C
ATOM   1459  CD1 LEU A  95      -5.721   5.933  12.316  1.00  2.10
C
```

ATOM 1460 CD2 LEU A 95 -3.558 5.920 13.603 1.00 2.10
C
ATOM 1461 H LEU A 95 -3.239 8.922 11.239 1.00 1.78
H
ATOM 1462 HA LEU A 95 -1.597 6.702 11.948 1.00 1.68
H
ATOM 1463 1HB LEU A 95 -4.187 6.879 10.368 1.00 2.52
H
ATOM 1464 2HB LEU A 95 -3.484 5.370 10.935 1.00 2.52
H
ATOM 1465 HG LEU A 95 -4.489 7.628 12.695 1.00 2.52
H
ATOM 1466 1HD1 LEU A 95 -6.280 6.043 13.245 1.00 2.52
H
ATOM 1467 2HD1 LEU A 95 -6.262 6.423 11.510 1.00 2.52
H
ATOM 1468 3HD1 LEU A 95 -5.607 4.875 12.085 1.00 2.52
H
ATOM 1469 1HD2 LEU A 95 -4.108 6.031 14.538 1.00 2.52
H
ATOM 1470 2HD2 LEU A 95 -3.421 4.862 13.388 1.00 2.52
H
ATOM 1471 3HD2 LEU A 95 -2.585 6.401 13.694 1.00 2.52
H
ATOM 1472 N GLY A 96 -0.674 5.645 9.908 1.00 0.42
N
ATOM 1473 CA GLY A 96 0.034 5.075 8.773 1.00 0.30
C
ATOM 1474 C GLY A 96 1.457 5.586 8.598 1.00 0.83
C
ATOM 1475 O GLY A 96 2.164 5.143 7.689 1.00 6.62
O
ATOM 1476 H GLY A 96 -0.569 5.194 10.810 1.00 0.50
H
ATOM 1477 1HA GLY A 96 0.058 3.994 8.888 1.00 0.36
H
ATOM 1478 2HA GLY A 96 -0.533 5.276 7.864 1.00 0.36
H
ATOM 1479 N GLN A 97 1.886 6.532 9.433 1.00 0.53
N
ATOM 1480 CA GLN A 97 3.215 7.104 9.244 1.00 0.38
C
ATOM 1481 C GLN A 97 4.329 6.475 10.103 1.00 0.30
C
ATOM 1482 O GLN A 97 5.507 6.511 9.728 1.00 0.57
O
ATOM 1483 CB GLN A 97 3.123 8.595 9.540 1.00 0.57
C
ATOM 1484 CG GLN A 97 2.173 9.317 8.604 1.00 0.57
C
ATOM 1485 CD GLN A 97 2.106 10.786 8.875 1.00 0.57
C
ATOM 1486 OE1 GLN A 97 2.298 11.203 10.018 1.00 0.57
O
ATOM 1487 NE2 GLN A 97 1.835 11.579 7.847 1.00 0.57
N
ATOM 1488 H GLN A 97 1.281 6.906 10.167 1.00 0.64
H

```
ATOM   1489  HA  GLN A  97       3.491   6.974   8.197  1.00  0.46
H
ATOM   1490 1HB  GLN A  97       2.780   8.748  10.564  1.00  0.68
H
ATOM   1491 2HB  GLN A  97       4.108   9.052   9.446  1.00  0.68
H
ATOM   1492 1HG  GLN A  97       2.477   9.165   7.579  1.00  0.68
H
ATOM   1493 2HG  GLN A  97       1.174   8.906   8.752  1.00  0.68
H
ATOM   1494 1HE2 GLN A  97       1.782  12.570   7.979  1.00  0.68
H
ATOM   1495 2HE2 GLN A  97       1.692  11.188   6.937  1.00  0.68
H
ATOM   1496  N   GLY A  98       3.944   5.910  11.246  1.00  0.32
N
ATOM   1497  CA  GLY A  98       4.881   5.361  12.223  1.00  0.33
C
ATOM   1498  C   GLY A  98       5.803   4.263  11.709  1.00  0.28
C
ATOM   1499  O   GLY A  98       6.988   4.264  12.035  1.00  0.33
O
ATOM   1500  H   GLY A  98       2.959   5.890  11.468  1.00  0.38
H
ATOM   1501 1HA  GLY A  98       5.490   6.175  12.614  1.00  0.40
H
ATOM   1502 2HA  GLY A  98       4.316   4.977  13.071  1.00  0.40
H
ATOM   1503  N   LEU A  99       5.269   3.323  10.929  1.00  0.23
N
ATOM   1504  CA  LEU A  99       6.075   2.209  10.433  1.00  0.20
C
ATOM   1505  C   LEU A  99       7.215   2.689   9.541  1.00  0.15
C
ATOM   1506  O   LEU A  99       8.320   2.156   9.619  1.00  0.17
O
ATOM   1507  CB  LEU A  99       5.206   1.194   9.684  1.00  0.30
C
ATOM   1508  CG  LEU A  99       5.953  -0.059   9.192  1.00  0.30
C
ATOM   1509  CD1 LEU A  99       6.570  -0.779  10.387  1.00  0.30
C
ATOM   1510  CD2 LEU A  99       4.980  -0.971   8.461  1.00  0.30
C
ATOM   1511  H   LEU A  99       4.289   3.373  10.688  1.00  0.28
H
ATOM   1512  HA  LEU A  99       6.514   1.703  11.290  1.00  0.24
H
ATOM   1513 1HB  LEU A  99       4.403   0.867  10.343  1.00  0.36
H
ATOM   1514 2HB  LEU A  99       4.763   1.682   8.816  1.00  0.36
H
ATOM   1515  HG  LEU A  99       6.759   0.232   8.523  1.00  0.36
H
ATOM   1516 1HD1 LEU A  99       7.105  -1.663  10.040  1.00  0.36
H
ATOM   1517 2HD1 LEU A  99       7.266  -0.111  10.894  1.00  0.36
H
```

```
ATOM   1518 3HD1 LEU A  99       5.783  -1.079  11.078  1.00  0.36
H
ATOM   1519 1HD2 LEU A  99       5.507  -1.860   8.114  1.00  0.36
H
ATOM   1520 2HD2 LEU A  99       4.178  -1.268   9.139  1.00  0.36
H
ATOM   1521 3HD2 LEU A  99       4.554  -0.444   7.607  1.00  0.36
H
ATOM   1522  N   GLY A 100       6.945   3.675   8.687  1.00  0.20
N
ATOM   1523  CA  GLY A 100       7.973   4.236   7.816  1.00  0.13
C
ATOM   1524  C   GLY A 100       9.098   4.852   8.642  1.00  0.12
C
ATOM   1525  O   GLY A 100      10.279   4.662   8.338  1.00  0.11
O
ATOM   1526  H   GLY A 100       6.010   4.060   8.651  1.00  0.24
H
ATOM   1527 1HA  GLY A 100       8.372   3.456   7.168  1.00  0.16
H
ATOM   1528 2HA  GLY A 100       7.524   4.990   7.173  1.00  0.16
H
ATOM   1529  N   VAL A 101       8.719   5.566   9.702  1.00  0.14
N
ATOM   1530  CA  VAL A 101       9.684   6.152  10.622  1.00  0.16
C
ATOM   1531  C   VAL A 101      10.510   5.064  11.296  1.00  0.15
C
ATOM   1532  O   VAL A 101      11.734   5.180  11.399  1.00  0.15
O
ATOM   1533  CB  VAL A 101       8.964   6.996  11.677  1.00  0.24
C
ATOM   1534  CG1 VAL A 101       9.938   7.431  12.755  1.00  0.24
C
ATOM   1535  CG2 VAL A 101       8.330   8.196  10.998  1.00  0.24
C
ATOM   1536  H   VAL A 101       7.727   5.714   9.872  1.00  0.17
H
ATOM   1537  HA  VAL A 101      10.354   6.798  10.059  1.00  0.19
H
ATOM   1538  HB  VAL A 101       8.192   6.394  12.153  1.00  0.29
H
ATOM   1539 1HG1 VAL A 101       9.409   8.017  13.503  1.00  0.29
H
ATOM   1540 2HG1 VAL A 101      10.376   6.551  13.227  1.00  0.29
H
ATOM   1541 3HG1 VAL A 101      10.726   8.036  12.309  1.00  0.29
H
ATOM   1542 1HG2 VAL A 101       7.799   8.791  11.737  1.00  0.29
H
ATOM   1543 2HG2 VAL A 101       9.105   8.801  10.527  1.00  0.29
H
ATOM   1544 3HG2 VAL A 101       7.626   7.854  10.239  1.00  0.29
H
ATOM   1545  N   ALA A 102       9.827   4.007  11.737  1.00  0.17
N
ATOM   1546  CA  ALA A 102      10.460   2.859  12.364  1.00  0.19
C
```

ATOM 1547 C ALA A 102 11.461 2.210 11.418 1.00 0.16
C
ATOM 1548 O ALA A 102 12.538 1.803 11.849 1.00 0.18
O
ATOM 1549 CB ALA A 102 9.403 1.851 12.792 1.00 0.29
C
ATOM 1550 H ALA A 102 8.822 3.999 11.638 1.00 0.20
H
ATOM 1551 HA ALA A 102 11.001 3.205 13.244 1.00 0.23
H
ATOM 1552 1HB ALA A 102 9.880 1.005 13.281 1.00 0.34
H
ATOM 1553 2HB ALA A 102 8.709 2.326 13.485 1.00 0.34
H
ATOM 1554 3HB ALA A 102 8.859 1.499 11.920 1.00 0.34
H
ATOM 1555 N CYS A 103 11.112 2.134 10.130 1.00 0.13
N
ATOM 1556 CA CYS A 103 11.999 1.583 9.119 1.00 0.12
C
ATOM 1557 C CYS A 103 13.264 2.419 8.986 1.00 0.10
C
ATOM 1558 O CYS A 103 14.355 1.865 8.877 1.00 0.11
O
ATOM 1559 CB CYS A 103 11.301 1.506 7.765 1.00 0.18
C
ATOM 1560 SG CYS A 103 9.995 0.264 7.661 1.00 0.18
S
ATOM 1561 H CYS A 103 10.196 2.452 9.846 1.00 0.16
H
ATOM 1562 HA CYS A 103 12.280 0.574 9.419 1.00 0.14
H
ATOM 1563 1HB CYS A 103 10.867 2.471 7.518 1.00 0.22
H
ATOM 1564 2HB CYS A 103 12.040 1.278 7.004 1.00 0.22
H
ATOM 1565 HG CYS A 103 9.650 0.535 6.407 1.00 0.22
H
ATOM 1566 N GLY A 104 13.124 3.748 9.016 1.00 0.10
N
ATOM 1567 CA GLY A 104 14.285 4.637 8.965 1.00 0.10
C
ATOM 1568 C GLY A 104 15.204 4.403 10.162 1.00 0.15
C
ATOM 1569 O GLY A 104 16.429 4.345 10.015 1.00 0.22
O
ATOM 1570 H GLY A 104 12.193 4.149 9.063 1.00 0.12
H
ATOM 1571 1HA GLY A 104 14.837 4.462 8.043 1.00 0.12
H
ATOM 1572 2HA GLY A 104 13.950 5.674 8.951 1.00 0.12
H
ATOM 1573 N MET A 105 14.597 4.249 11.338 1.00 0.18
N
ATOM 1574 CA MET A 105 15.327 3.988 12.569 1.00 0.28
C
ATOM 1575 C MET A 105 16.069 2.656 12.516 1.00 0.20
C

```
ATOM   1576  O   MET A 105      17.240   2.580  12.897  1.00  0.23
O
ATOM   1577  CB  MET A 105      14.362   4.001  13.744  1.00  0.42
C
ATOM   1578  CG  MET A 105      15.029   3.920  15.097  1.00  0.42
C
ATOM   1579  SD  MET A 105      15.975   5.394  15.499  1.00  0.42
S
ATOM   1580  CE  MET A 105      16.284   5.098  17.234  1.00  0.42
C
ATOM   1581  H   MET A 105      13.588   4.335  11.383  1.00  0.22
H
ATOM   1582  HA  MET A 105      16.066   4.776  12.705  1.00  0.34
H
ATOM   1583 1HB  MET A 105      13.768   4.914  13.716  1.00  0.50
H
ATOM   1584 2HB  MET A 105      13.672   3.161  13.662  1.00  0.50
H
ATOM   1585 1HG  MET A 105      14.284   3.766  15.867  1.00  0.50
H
ATOM   1586 2HG  MET A 105      15.705   3.068  15.104  1.00  0.50
H
ATOM   1587 1HE  MET A 105      16.858   5.927  17.646  1.00  0.50
H
ATOM   1588 2HE  MET A 105      15.336   5.016  17.765  1.00  0.50
H
ATOM   1589 3HE  MET A 105      16.846   4.172  17.353  1.00  0.50
H
ATOM   1590  N   ALA A 106      15.370   1.614  12.058  1.00  0.20
N
ATOM   1591  CA  ALA A 106      15.929   0.276  11.942  1.00  0.22
C
ATOM   1592  C   ALA A 106      17.045   0.247  10.914  1.00  0.20
C
ATOM   1593  O   ALA A 106      18.075  -0.390  11.139  1.00  0.22
O
ATOM   1594  CB  ALA A 106      14.838  -0.714  11.575  1.00  0.33
C
ATOM   1595  H   ALA A 106      14.407   1.750  11.787  1.00  0.24
H
ATOM   1596  HA  ALA A 106      16.353  -0.004  12.906  1.00  0.26
H
ATOM   1597 1HB  ALA A 106      15.258  -1.717  11.510  1.00  0.40
H
ATOM   1598 2HB  ALA A 106      14.060  -0.696  12.338  1.00  0.40
H
ATOM   1599 3HB  ALA A 106      14.408  -0.436  10.617  1.00  0.40
H
ATOM   1600  N   TYR A 107      16.840   0.951   9.800  1.00  0.17
N
ATOM   1601  CA  TYR A 107      17.823   1.050   8.736  1.00  0.15
C
ATOM   1602  C   TYR A 107      19.111   1.633   9.287  1.00  0.14
C
ATOM   1603  O   TYR A 107      20.193   1.119   9.023  1.00  0.13
O
ATOM   1604  CB  TYR A 107      17.314   1.932   7.594  1.00  0.22
C
```

ATOM 1605 CG TYR A 107 18.245 1.955 6.408 1.00 0.22
C
ATOM 1606 CD1 TYR A 107 17.936 1.197 5.289 1.00 0.22
C
ATOM 1607 CD2 TYR A 107 19.428 2.681 6.450 1.00 0.22
C
ATOM 1608 CE1 TYR A 107 18.796 1.183 4.213 1.00 0.22
C
ATOM 1609 CE2 TYR A 107 20.288 2.649 5.370 1.00 0.22
C
ATOM 1610 CZ TYR A 107 19.975 1.906 4.256 1.00 0.22
C
ATOM 1611 OH TYR A 107 20.835 1.874 3.184 1.00 0.22
O
ATOM 1612 H TYR A 107 15.963 1.430 9.672 1.00 0.20
H
ATOM 1613 HA TYR A 107 18.039 0.053 8.356 1.00 0.18
H
ATOM 1614 1HB TYR A 107 16.342 1.572 7.258 1.00 0.27
H
ATOM 1615 2HB TYR A 107 17.183 2.955 7.947 1.00 0.27
H
ATOM 1616 HD1 TYR A 107 17.014 0.617 5.259 1.00 0.27
H
ATOM 1617 HD2 TYR A 107 19.680 3.266 7.334 1.00 0.27
H
ATOM 1618 HE1 TYR A 107 18.557 0.600 3.335 1.00 0.27
H
ATOM 1619 HE2 TYR A 107 21.219 3.207 5.396 1.00 0.27
H
ATOM 1620 HH TYR A 107 20.468 1.312 2.497 1.00 0.27
H
ATOM 1621 N THR A 108 18.989 2.716 10.053 1.00 0.20
N
ATOM 1622 CA THR A 108 20.145 3.365 10.646 1.00 0.28
C
ATOM 1623 C THR A 108 20.851 2.439 11.641 1.00 0.28
C
ATOM 1624 O THR A 108 22.072 2.267 11.591 1.00 0.39
O
ATOM 1625 CB THR A 108 19.715 4.670 11.347 1.00 0.42
C
ATOM 1626 OG1 THR A 108 19.127 5.554 10.385 1.00 0.42
O
ATOM 1627 CG2 THR A 108 20.905 5.362 11.984 1.00 0.42
C
ATOM 1628 H THR A 108 18.072 3.116 10.215 1.00 0.24
H
ATOM 1629 HA THR A 108 20.849 3.608 9.853 1.00 0.34
H
ATOM 1630 HB THR A 108 18.979 4.442 12.116 1.00 0.50
H
ATOM 1631 HG1 THR A 108 18.302 5.172 10.066 1.00 0.50
H
ATOM 1632 1HG2 THR A 108 20.570 6.278 12.465 1.00 0.50
H
ATOM 1633 2HG2 THR A 108 21.359 4.706 12.726 1.00 0.50
H

```
ATOM   1634 3HG2 THR A 108      21.637   5.613  11.223  1.00  0.50
H
ATOM   1635  N   GLY A 109      20.077   1.797  12.512  1.00  0.25
N
ATOM   1636  CA  GLY A 109      20.645   0.878  13.490  1.00  0.31
C
ATOM   1637  C   GLY A 109      21.392  -0.274  12.835  1.00  0.33
C
ATOM   1638  O   GLY A 109      22.403  -0.742  13.354  1.00  0.50
O
ATOM   1639  H   GLY A 109      19.078   1.964  12.521  1.00  0.30
H
ATOM   1640 1HA  GLY A 109      21.320   1.425  14.148  1.00  0.37
H
ATOM   1641 2HA  GLY A 109      19.847   0.480  14.114  1.00  0.37
H
ATOM   1642  N   LYS A 110      20.902  -0.735  11.689  1.00  0.28
N
ATOM   1643  CA  LYS A 110      21.520  -1.840  10.974  1.00  0.31
C
ATOM   1644  C   LYS A 110      22.686  -1.446  10.062  1.00  0.25
C
ATOM   1645  O   LYS A 110      23.706  -2.135  10.024  1.00  0.36
O
ATOM   1646  CB  LYS A 110      20.462  -2.556  10.135  1.00  0.46
C
ATOM   1647  CG  LYS A 110      20.981  -3.763   9.373  1.00  0.46
C
ATOM   1648  CD  LYS A 110      19.860  -4.499   8.657  1.00  0.46
C
ATOM   1649  CE  LYS A 110      20.410  -5.671   7.866  1.00  0.46
C
ATOM   1650  NZ  LYS A 110      20.968  -6.717   8.772  1.00  0.46
N
ATOM   1651  H   LYS A 110      20.051  -0.337  11.313  1.00  0.34
H
ATOM   1652  HA  LYS A 110      21.907  -2.542  11.713  1.00  0.37
H
ATOM   1653 1HB  LYS A 110      19.652  -2.893  10.783  1.00  0.56
H
ATOM   1654 2HB  LYS A 110      20.034  -1.858   9.413  1.00  0.56
H
ATOM   1655 1HG  LYS A 110      21.715  -3.437   8.635  1.00  0.56
H
ATOM   1656 2HG  LYS A 110      21.467  -4.447  10.066  1.00  0.56
H
ATOM   1657 1HD  LYS A 110      19.144  -4.873   9.392  1.00  0.56
H
ATOM   1658 2HD  LYS A 110      19.340  -3.820   7.984  1.00  0.56
H
ATOM   1659 1HE  LYS A 110      19.615  -6.109   7.267  1.00  0.56
H
ATOM   1660 2HE  LYS A 110      21.200  -5.321   7.201  1.00  0.56
H
ATOM   1661 1HZ  LYS A 110      21.323  -7.489   8.228  1.00  0.56
H
ATOM   1662 2HZ  LYS A 110      21.712  -6.327   9.332  1.00  0.56
H
```

```
ATOM   1663 3HZ  LYS A 110      20.223  -7.041   9.376  1.00  0.56
H
ATOM   1664  N   TYR A 111      22.517  -0.366   9.303  1.00  0.19
N
ATOM   1665  CA  TYR A 111      23.487   0.018   8.284  1.00  0.17
C
ATOM   1666  C   TYR A 111      24.383   1.217   8.593  1.00  0.21
C
ATOM   1667  O   TYR A 111      25.487   1.304   8.055  1.00  0.55
O
ATOM   1668  CB  TYR A 111      22.736   0.330   6.986  1.00  0.26
C
ATOM   1669  CG  TYR A 111      22.009  -0.840   6.353  1.00  0.26
C
ATOM   1670  CD1 TYR A 111      20.633  -0.972   6.483  1.00  0.26
C
ATOM   1671  CD2 TYR A 111      22.721  -1.771   5.618  1.00  0.26
C
ATOM   1672  CE1 TYR A 111      19.973  -2.021   5.877  1.00  0.26
C
ATOM   1673  CE2 TYR A 111      22.063  -2.822   5.013  1.00  0.26
C
ATOM   1674  CZ  TYR A 111      20.695  -2.948   5.137  1.00  0.26
C
ATOM   1675  OH  TYR A 111      20.056  -3.997   4.521  1.00  0.26
O
ATOM   1676  H   TYR A 111      21.677   0.179   9.393  1.00  0.23
H
ATOM   1677  HA  TYR A 111      24.141  -0.837   8.113  1.00  0.20
H
ATOM   1678 1HB  TYR A 111      22.004   1.113   7.182  1.00  0.31
H
ATOM   1679 2HB  TYR A 111      23.439   0.725   6.253  1.00  0.31
H
ATOM   1680  HD1 TYR A 111      20.070  -0.242   7.056  1.00  0.31
H
ATOM   1681  HD2 TYR A 111      23.800  -1.669   5.510  1.00  0.31
H
ATOM   1682  HE1 TYR A 111      18.892  -2.118   5.976  1.00  0.31
H
ATOM   1683  HE2 TYR A 111      22.624  -3.552   4.431  1.00  0.31
H
ATOM   1684  HH  TYR A 111      19.130  -4.019   4.786  1.00  0.31
H
ATOM   1685  N   PHE A 112      23.924   2.151   9.417  1.00  0.15
N
ATOM   1686  CA  PHE A 112      24.712   3.350   9.671  1.00  0.20
C
ATOM   1687  C   PHE A 112      25.410   3.283  11.010  1.00  0.33
C
ATOM   1688  O   PHE A 112      26.640   3.283  11.086  1.00  0.63
O
ATOM   1689  CB  PHE A 112      23.823   4.589   9.628  1.00  0.30
C
ATOM   1690  CG  PHE A 112      23.295   4.943   8.265  1.00  0.30
C
ATOM   1691  CD1 PHE A 112      24.073   4.756   7.135  1.00  0.30
C
```

```
ATOM   1692  CD2 PHE A 112      22.022   5.480   8.110  1.00  0.30
C
ATOM   1693  CE1 PHE A 112      23.592   5.089   5.886  1.00  0.30
C
ATOM   1694  CE2 PHE A 112      21.541   5.816   6.862  1.00  0.30
C
ATOM   1695  CZ  PHE A 112      22.330   5.620   5.746  1.00  0.30
C
ATOM   1696  H   PHE A 112      23.032   2.054   9.879  1.00  0.18
H
ATOM   1697  HA  PHE A 112      25.471   3.439   8.893  1.00  0.24
H
ATOM   1698 1HB  PHE A 112      22.975   4.424  10.280  1.00  0.36
H
ATOM   1699 2HB  PHE A 112      24.372   5.447  10.014  1.00  0.36
H
ATOM   1700  HD1 PHE A 112      25.075   4.339   7.240  1.00  0.36
H
ATOM   1701  HD2 PHE A 112      21.400   5.638   8.990  1.00  0.36
H
ATOM   1702  HE1 PHE A 112      24.216   4.933   5.012  1.00  0.36
H
ATOM   1703  HE2 PHE A 112      20.540   6.236   6.756  1.00  0.36
H
ATOM   1704  HZ  PHE A 112      21.955   5.882   4.757  1.00  0.36
H
ATOM   1705  N   ASP A 113      24.624   3.249  12.070  1.00  0.55
N
ATOM   1706  CA  ASP A 113      25.182   3.159  13.398  1.00  0.87
C
ATOM   1707  C   ASP A 113      25.673   1.750  13.662  1.00  0.56
C
ATOM   1708  O   ASP A 113      26.705   1.572  14.309  1.00  0.63
O
ATOM   1709  CB  ASP A 113      24.175   3.655  14.429  1.00  1.30
C
ATOM   1710  CG  ASP A 113      24.048   5.173  14.307  1.00  1.30
C
ATOM   1711  OD1 ASP A 113      24.877   5.741  13.637  1.00  1.30
O
ATOM   1712  OD2 ASP A 113      23.169   5.759  14.891  1.00  1.30
O
ATOM   1713  H   ASP A 113      23.624   3.246  11.943  1.00  0.66
H
ATOM   1714  HA  ASP A 113      26.044   3.822  13.446  1.00  1.04
H
ATOM   1715 1HB  ASP A 113      23.202   3.196  14.251  1.00  1.57
H
ATOM   1716 2HB  ASP A 113      24.496   3.390  15.435  1.00  1.57
H
ATOM   1717  N   ARG A 114      24.958   0.757  13.123  1.00  0.65
N
ATOM   1718  CA  ARG A 114      25.395  -0.639  13.226  1.00  0.70
C
ATOM   1719  C   ARG A 114      25.505  -1.039  14.689  1.00  0.60
C
ATOM   1720  O   ARG A 114      26.560  -1.479  15.150  1.00  0.75
O
```

ATOM 1721 CB ARG A 114 26.720 -0.827 12.504 1.00 1.05
C
ATOM 1722 CG ARG A 114 26.667 -0.482 11.023 1.00 1.05
C
ATOM 1723 CD ARG A 114 27.979 -0.659 10.364 1.00 1.05
C
ATOM 1724 NE ARG A 114 28.375 -2.052 10.329 1.00 1.05
N
ATOM 1725 CZ ARG A 114 27.957 -2.953 9.424 1.00 1.05
C
ATOM 1726 NH1 ARG A 114 27.127 -2.602 8.465 1.00 1.05
N
ATOM 1727 NH2 ARG A 114 28.395 -4.195 9.519 1.00 1.05
N
ATOM 1728 H ARG A 114 24.097 0.987 12.628 1.00 0.78
H
ATOM 1729 HA ARG A 114 24.655 -1.276 12.742 1.00 0.84
H
ATOM 1730 1HB ARG A 114 27.497 -0.219 12.963 1.00 1.26
H
ATOM 1731 2HB ARG A 114 27.034 -1.868 12.586 1.00 1.26
H
ATOM 1732 1HG ARG A 114 25.948 -1.132 10.526 1.00 1.26
H
ATOM 1733 2HG ARG A 114 26.362 0.557 10.900 1.00 1.26
H
ATOM 1734 1HD ARG A 114 27.926 -0.293 9.340 1.00 1.26
H
ATOM 1735 2HD ARG A 114 28.739 -0.102 10.911 1.00 1.26
H
ATOM 1736 HE ARG A 114 29.012 -2.386 11.048 1.00 1.26
H
ATOM 1737 1HH1 ARG A 114 26.797 -1.648 8.407 1.00 1.26
H
ATOM 1738 2HH1 ARG A 114 26.817 -3.286 7.791 1.00 1.26
H
ATOM 1739 1HH2 ARG A 114 29.030 -4.430 10.278 1.00 1.26
H
ATOM 1740 2HH2 ARG A 114 28.097 -4.893 8.856 1.00 1.26
H
ATOM 1741 N ALA A 115 24.407 -0.835 15.407 1.00 0.57
N
ATOM 1742 CA ALA A 115 24.307 -1.070 16.841 1.00 0.76
C
ATOM 1743 C ALA A 115 23.253 -2.129 17.125 1.00 1.17
C
ATOM 1744 O ALA A 115 22.338 -2.326 16.328 1.00 6.21
O
ATOM 1745 CB ALA A 115 23.978 0.230 17.558 1.00 1.14
C
ATOM 1746 H ALA A 115 23.587 -0.502 14.915 1.00 0.68
H
ATOM 1747 HA ALA A 115 25.266 -1.442 17.201 1.00 0.91
H
ATOM 1748 1HB ALA A 115 23.919 0.055 18.631 1.00 1.37
H
ATOM 1749 2HB ALA A 115 24.758 0.962 17.352 1.00 1.37
H

```
ATOM   1750 3HB  ALA A 115      23.023   0.609  17.200  1.00  1.37
H
ATOM   1751  N   SER A 116      23.362  -2.807  18.264  1.00  1.57
N
ATOM   1752  CA  SER A 116      22.436  -3.882  18.610  1.00  1.77
C
ATOM   1753  C   SER A 116      21.103  -3.442  19.228  1.00  2.23
C
ATOM   1754  O   SER A 116      20.589  -4.125  20.111  1.00 13.57
O
ATOM   1755  CB  SER A 116      23.104  -4.860  19.557  1.00  2.66
C
ATOM   1756  OG  SER A 116      23.434  -4.239  20.766  1.00  2.66
O
ATOM   1757  H   SER A 116      24.123  -2.611  18.901  1.00  1.88
H
ATOM   1758  HA  SER A 116      22.204  -4.419  17.690  1.00  2.12
H
ATOM   1759 1HB  SER A 116      22.433  -5.697  19.746  1.00  3.19
H
ATOM   1760 2HB  SER A 116      24.004  -5.260  19.094  1.00  3.19
H
ATOM   1761  HG  SER A 116      24.117  -3.598  20.545  1.00  3.19
H
ATOM   1762  N   TYR A 117      20.538  -2.314  18.799  1.00  2.43
N
ATOM   1763  CA  TYR A 117      19.225  -1.973  19.317  1.00  1.87
C
ATOM   1764  C   TYR A 117      18.198  -2.413  18.281  1.00  0.77
C
ATOM   1765  O   TYR A 117      18.509  -2.516  17.090  1.00  1.79
O
ATOM   1766  CB  TYR A 117      19.076  -0.478  19.627  1.00  2.81
C
ATOM   1767  CG  TYR A 117      19.045   0.435  18.424  1.00  2.81
C
ATOM   1768  CD1 TYR A 117      17.834   0.672  17.790  1.00  2.81
C
ATOM   1769  CD2 TYR A 117      20.199   1.050  17.969  1.00  2.81
C
ATOM   1770  CE1 TYR A 117      17.777   1.512  16.700  1.00  2.81
C
ATOM   1771  CE2 TYR A 117      20.140   1.897  16.876  1.00  2.81
C
ATOM   1772  CZ  TYR A 117      18.933   2.129  16.243  1.00  2.81
C
ATOM   1773  OH  TYR A 117      18.874   2.972  15.156  1.00  2.81
O
ATOM   1774  H   TYR A 117      20.977  -1.734  18.101  1.00  2.92
H
ATOM   1775  HA  TYR A 117      19.041  -2.536  20.232  1.00  2.24
H
ATOM   1776 1HB  TYR A 117      18.159  -0.320  20.194  1.00  3.37
H
ATOM   1777 2HB  TYR A 117      19.902  -0.164  20.263  1.00  3.37
H
ATOM   1778  HD1 TYR A 117      16.926   0.190  18.155  1.00  3.37
H
```

ATOM 1779 HD2 TYR A 117 21.149 0.869 18.472 1.00 3.37
H
ATOM 1780 HE1 TYR A 117 16.826 1.693 16.205 1.00 3.37
H
ATOM 1781 HE2 TYR A 117 21.047 2.384 16.516 1.00 3.37
H
ATOM 1782 HH TYR A 117 18.021 2.882 14.720 1.00 3.37
H
ATOM 1783 N ARG A 118 16.985 -2.665 18.743 1.00 0.66
N
ATOM 1784 CA ARG A 118 15.905 -3.113 17.879 1.00 0.73
C
ATOM 1785 C ARG A 118 14.809 -2.076 17.839 1.00 0.68
C
ATOM 1786 O ARG A 118 14.738 -1.211 18.712 1.00 1.63
O
ATOM 1787 CB ARG A 118 15.348 -4.458 18.343 1.00 1.09
C
ATOM 1788 CG ARG A 118 16.321 -5.629 18.225 1.00 1.09
C
ATOM 1789 CD ARG A 118 15.703 -6.925 18.620 1.00 1.09
C
ATOM 1790 NE ARG A 118 15.427 -6.991 20.047 1.00 1.09
N
ATOM 1791 CZ ARG A 118 14.725 -7.974 20.645 1.00 1.09
C
ATOM 1792 NH1 ARG A 118 14.229 -8.958 19.930 1.00 1.09
N
ATOM 1793 NH2 ARG A 118 14.534 -7.944 21.953 1.00 1.09
N
ATOM 1794 H ARG A 118 16.816 -2.548 19.737 1.00 0.79
H
ATOM 1795 HA ARG A 118 16.296 -3.237 16.869 1.00 0.88
H
ATOM 1796 1HB ARG A 118 15.048 -4.386 19.388 1.00 1.31
H
ATOM 1797 2HB ARG A 118 14.459 -4.706 17.763 1.00 1.31
H
ATOM 1798 1HG ARG A 118 16.659 -5.719 17.194 1.00 1.31
H
ATOM 1799 2HG ARG A 118 17.179 -5.450 18.873 1.00 1.31
H
ATOM 1800 1HD ARG A 118 14.761 -7.054 18.085 1.00 1.31
H
ATOM 1801 2HD ARG A 118 16.379 -7.740 18.366 1.00 1.31
H
ATOM 1802 HE ARG A 118 15.793 -6.251 20.633 1.00 1.31
H
ATOM 1803 1HH1 ARG A 118 14.374 -8.981 18.930 1.00 1.31
H
ATOM 1804 2HH1 ARG A 118 13.701 -9.694 20.380 1.00 1.31
H
ATOM 1805 1HH2 ARG A 118 14.915 -7.186 22.505 1.00 1.31
H
ATOM 1806 2HH2 ARG A 118 14.009 -8.678 22.405 1.00 1.31
H
ATOM 1807 N VAL A 119 13.980 -2.142 16.806 1.00 0.40
N

ATOM 1808 CA VAL A 119 12.901 -1.191 16.641 1.00 0.37
C
ATOM 1809 C VAL A 119 11.561 -1.901 16.589 1.00 0.39
C
ATOM 1810 O VAL A 119 11.341 -2.763 15.741 1.00 0.57
O
ATOM 1811 CB VAL A 119 13.087 -0.374 15.352 1.00 0.55
C
ATOM 1812 CG1 VAL A 119 11.945 0.617 15.207 1.00 0.55
C
ATOM 1813 CG2 VAL A 119 14.430 0.328 15.370 1.00 0.55
C
ATOM 1814 H VAL A 119 14.099 -2.878 16.123 1.00 0.48
H
ATOM 1815 HA VAL A 119 12.904 -0.509 17.491 1.00 0.44
H
ATOM 1816 HB VAL A 119 13.045 -1.045 14.494 1.00 0.67
H
ATOM 1817 1HG1 VAL A 119 12.069 1.182 14.284 1.00 0.67
H
ATOM 1818 2HG1 VAL A 119 10.998 0.081 15.180 1.00 0.67
H
ATOM 1819 3HG1 VAL A 119 11.949 1.302 16.053 1.00 0.67
H
ATOM 1820 1HG2 VAL A 119 14.551 0.895 14.452 1.00 0.67
H
ATOM 1821 2HG2 VAL A 119 14.483 1.005 16.219 1.00 0.67
H
ATOM 1822 3HG2 VAL A 119 15.228 -0.410 15.448 1.00 0.67
H
ATOM 1823 N PHE A 120 10.673 -1.528 17.493 1.00 0.37
N
ATOM 1824 CA PHE A 120 9.340 -2.089 17.542 1.00 0.37
C
ATOM 1825 C PHE A 120 8.346 -1.022 17.117 1.00 0.52
C
ATOM 1826 O PHE A 120 8.517 0.145 17.459 1.00 1.43
O
ATOM 1827 CB PHE A 120 9.039 -2.570 18.962 1.00 0.55
C
ATOM 1828 CG PHE A 120 9.888 -3.732 19.387 1.00 0.55
C
ATOM 1829 CD1 PHE A 120 11.159 -3.525 19.907 1.00 0.55
C
ATOM 1830 CD2 PHE A 120 9.422 -5.032 19.276 1.00 0.55
C
ATOM 1831 CE1 PHE A 120 11.945 -4.593 20.298 1.00 0.55
C
ATOM 1832 CE2 PHE A 120 10.203 -6.102 19.667 1.00 0.55
C
ATOM 1833 CZ PHE A 120 11.467 -5.880 20.175 1.00 0.55
C
ATOM 1834 H PHE A 120 10.928 -0.826 18.166 1.00 0.44
H
ATOM 1835 HA PHE A 120 9.284 -2.929 16.856 1.00 0.44
H
ATOM 1836 1HB PHE A 120 9.210 -1.755 19.661 1.00 0.67
H

```
ATOM   1837 2HB  PHE A 120       7.992  -2.862  19.040  1.00  0.67
H
ATOM   1838  HD1 PHE A 120      11.534  -2.505  20.002  1.00  0.67
H
ATOM   1839  HD2 PHE A 120       8.427  -5.208  18.872  1.00  0.67
H
ATOM   1840  HE1 PHE A 120      12.941  -4.416  20.702  1.00  0.67
H
ATOM   1841  HE2 PHE A 120       9.825  -7.119  19.572  1.00  0.67
H
ATOM   1842  HZ  PHE A 120      12.084  -6.722  20.483  1.00  0.67
H
ATOM   1843  N   CYS A 121       7.310  -1.419  16.390  1.00  0.31
N
ATOM   1844  CA  CYS A 121       6.305  -0.465  15.942  1.00  0.32
C
ATOM   1845  C   CYS A 121       4.911  -1.044  16.062  1.00  0.26
C
ATOM   1846  O   CYS A 121       4.597  -2.044  15.424  1.00  0.45
O
ATOM   1847  CB  CYS A 121       6.552  -0.058  14.494  1.00  0.48
C
ATOM   1848  SG  CYS A 121       5.369   1.155  13.868  1.00  0.48
S
ATOM   1849  H   CYS A 121       7.240  -2.391  16.121  1.00  0.37
H
ATOM   1850  HA  CYS A 121       6.373   0.420  16.567  1.00  0.38
H
ATOM   1851 1HB  CYS A 121       7.552   0.364  14.404  1.00  0.58
H
ATOM   1852 2HB  CYS A 121       6.507  -0.938  13.853  1.00  0.58
H
ATOM   1853  HG  CYS A 121       4.287   0.412  14.075  1.00  0.58
H
ATOM   1854  N   LEU A 122       4.082  -0.410  16.880  1.00  0.25
N
ATOM   1855  CA  LEU A 122       2.730  -0.901  17.123  1.00  0.23
C
ATOM   1856  C   LEU A 122       1.688  -0.020  16.448  1.00  0.28
C
ATOM   1857  O   LEU A 122       1.619   1.191  16.671  1.00  0.55
O
ATOM   1858  CB  LEU A 122       2.496  -1.026  18.639  1.00  0.35
C
ATOM   1859  CG  LEU A 122       1.073  -1.379  19.119  1.00  0.35
C
ATOM   1860  CD1 LEU A 122       0.658  -2.741  18.610  1.00  0.35
C
ATOM   1861  CD2 LEU A 122       1.056  -1.389  20.643  1.00  0.35
C
ATOM   1862  H   LEU A 122       4.402   0.441  17.329  1.00  0.30
H
ATOM   1863  HA  LEU A 122       2.649  -1.898  16.695  1.00  0.28
H
ATOM   1864 1HB  LEU A 122       3.159  -1.803  19.015  1.00  0.41
H
ATOM   1865 2HB  LEU A 122       2.787  -0.090  19.108  1.00  0.41
H
```

```
ATOM   1866  HG  LEU A 122       0.366  -0.640  18.741  1.00  0.41
H
ATOM   1867 1HD1 LEU A 122      -0.347  -2.970  18.962  1.00  0.41
H
ATOM   1868 2HD1 LEU A 122       0.670  -2.743  17.522  1.00  0.41
H
ATOM   1869 3HD1 LEU A 122       1.353  -3.483  18.989  1.00  0.41
H
ATOM   1870 1HD2 LEU A 122       0.054  -1.633  20.990  1.00  0.41
H
ATOM   1871 2HD2 LEU A 122       1.758  -2.139  21.007  1.00  0.41
H
ATOM   1872 3HD2 LEU A 122       1.342  -0.415  21.028  1.00  0.41
H
ATOM   1873  N   MET A 123       0.908  -0.656  15.587  1.00  0.25
N
ATOM   1874  CA  MET A 123      -0.095   0.014  14.780  1.00  0.31
C
ATOM   1875  C   MET A 123      -1.449  -0.662  14.877  1.00  0.24
C
ATOM   1876  O   MET A 123      -1.538  -1.793  15.348  1.00  0.33
O
ATOM   1877  CB  MET A 123       0.379   0.014  13.342  1.00  0.46
C
ATOM   1878  CG  MET A 123       0.536  -1.380  12.761  1.00  0.46
C
ATOM   1879  SD  MET A 123       1.029  -1.328  11.044  1.00  0.46
S
ATOM   1880  CE  MET A 123      -0.517  -0.700  10.425  1.00  0.46
C
ATOM   1881  H   MET A 123       1.031  -1.657  15.479  1.00  0.30
H
ATOM   1882  HA  MET A 123      -0.204   1.039  15.132  1.00  0.37
H
ATOM   1883 1HB  MET A 123      -0.326   0.557  12.718  1.00  0.56
H
ATOM   1884 2HB  MET A 123       1.343   0.518  13.270  1.00  0.56
H
ATOM   1885 1HG  MET A 123       1.289  -1.932  13.323  1.00  0.56
H
ATOM   1886 2HG  MET A 123      -0.410  -1.917  12.834  1.00  0.56
H
ATOM   1887 1HE  MET A 123      -0.457  -0.577   9.350  1.00  0.56
H
ATOM   1888 2HE  MET A 123      -1.317  -1.400  10.671  1.00  0.56
H
ATOM   1889 3HE  MET A 123      -0.725   0.262  10.890  1.00  0.56
H
ATOM   1890  N   SER A 124      -2.499   0.021  14.416  1.00  0.26
N
ATOM   1891  CA  SER A 124      -3.832  -0.588  14.437  1.00  0.33
C
ATOM   1892  C   SER A 124      -4.191  -1.159  13.079  1.00  0.33
C
ATOM   1893  O   SER A 124      -3.597  -0.792  12.067  1.00  0.56
O
ATOM   1894  CB  SER A 124      -4.896   0.413  14.850  1.00  0.49
C
```

ATOM 1895 OG SER A 124 -5.044 1.434 13.901 1.00 0.49
O
ATOM 1896 H SER A 124 -2.357 0.958 14.042 1.00 0.31
H
ATOM 1897 HA SER A 124 -3.829 -1.407 15.154 1.00 0.40
H
ATOM 1898 1HB SER A 124 -5.846 -0.105 14.978 1.00 0.59
H
ATOM 1899 2HB SER A 124 -4.633 0.847 15.813 1.00 0.59
H
ATOM 1900 HG SER A 124 -4.277 2.005 14.014 1.00 0.59
H
ATOM 1901 N ASP A 125 -5.169 -2.050 13.035 1.00 0.51
N
ATOM 1902 CA ASP A 125 -5.576 -2.549 11.731 1.00 0.45
C
ATOM 1903 C ASP A 125 -6.355 -1.524 10.924 1.00 0.54
C
ATOM 1904 O ASP A 125 -6.324 -1.569 9.696 1.00 1.43
O
ATOM 1905 CB ASP A 125 -6.341 -3.849 11.814 1.00 0.68
C
ATOM 1906 CG ASP A 125 -5.447 -5.019 12.104 1.00 0.68
C
ATOM 1907 OD1 ASP A 125 -4.248 -4.857 12.129 1.00 0.68
O
ATOM 1908 OD2 ASP A 125 -5.977 -6.089 12.237 1.00 0.68
O
ATOM 1909 H ASP A 125 -5.630 -2.362 13.880 1.00 0.61
H
ATOM 1910 HA ASP A 125 -4.666 -2.771 11.172 1.00 0.54
H
ATOM 1911 1HB ASP A 125 -7.073 -3.788 12.616 1.00 0.81
H
ATOM 1912 2HB ASP A 125 -6.877 -4.031 10.882 1.00 0.81
H
ATOM 1913 N GLY A 126 -7.021 -0.574 11.583 1.00 0.35
N
ATOM 1914 CA GLY A 126 -7.670 0.497 10.835 1.00 0.40
C
ATOM 1915 C GLY A 126 -6.591 1.269 10.121 1.00 0.36
C
ATOM 1916 O GLY A 126 -6.692 1.554 8.926 1.00 0.41
O
ATOM 1917 H GLY A 126 -7.056 -0.580 12.593 1.00 0.42
H
ATOM 1918 1HA GLY A 126 -8.352 0.088 10.099 1.00 0.48
H
ATOM 1919 2HA GLY A 126 -8.232 1.147 11.505 1.00 0.48
H
ATOM 1920 N GLU A 127 -5.531 1.559 10.861 1.00 0.37
N
ATOM 1921 CA GLU A 127 -4.345 2.207 10.351 1.00 0.35
C
ATOM 1922 C GLU A 127 -3.716 1.490 9.151 1.00 0.30
C
ATOM 1923 O GLU A 127 -3.233 2.150 8.228 1.00 0.36
O

ATOM 1924 CB GLU A 127 -3.367 2.346 11.500 1.00 0.52
C
ATOM 1925 CG GLU A 127 -2.054 2.945 11.172 1.00 0.52
C
ATOM 1926 CD GLU A 127 -1.298 3.270 12.414 1.00 0.52
C
ATOM 1927 OE1 GLU A 127 -1.517 2.651 13.434 1.00 0.52
O
ATOM 1928 OE2 GLU A 127 -0.524 4.192 12.348 1.00 0.52
O
ATOM 1929 H GLU A 127 -5.528 1.319 11.851 1.00 0.44
H
ATOM 1930 HA GLU A 127 -4.627 3.208 10.023 1.00 0.42
H
ATOM 1931 1HB GLU A 127 -3.819 2.975 12.265 1.00 0.63
H
ATOM 1932 2HB GLU A 127 -3.173 1.387 11.952 1.00 0.63
H
ATOM 1933 1HG GLU A 127 -1.477 2.239 10.577 1.00 0.63
H
ATOM 1934 2HG GLU A 127 -2.220 3.831 10.577 1.00 0.63
H
ATOM 1935 N SER A 128 -3.740 0.146 9.148 1.00 0.48
N
ATOM 1936 CA SER A 128 -3.130 -0.632 8.060 1.00 0.45
C
ATOM 1937 C SER A 128 -3.758 -0.385 6.690 1.00 0.43
C
ATOM 1938 O SER A 128 -3.154 -0.730 5.679 1.00 0.62
O
ATOM 1939 CB SER A 128 -3.197 -2.132 8.326 1.00 0.68
C
ATOM 1940 OG SER A 128 -4.489 -2.646 8.146 1.00 0.68
O
ATOM 1941 H SER A 128 -4.132 -0.343 9.944 1.00 0.58
H
ATOM 1942 HA SER A 128 -2.083 -0.346 7.993 1.00 0.54
H
ATOM 1943 1HB SER A 128 -2.506 -2.645 7.657 1.00 0.81
H
ATOM 1944 2HB SER A 128 -2.869 -2.332 9.344 1.00 0.81
H
ATOM 1945 HG SER A 128 -5.059 -2.197 8.773 1.00 0.81
H
ATOM 1946 N SER A 129 -4.958 0.207 6.634 1.00 0.41
N
ATOM 1947 CA SER A 129 -5.576 0.495 5.346 1.00 0.43
C
ATOM 1948 C SER A 129 -4.872 1.634 4.590 1.00 0.42
C
ATOM 1949 O SER A 129 -5.109 1.815 3.396 1.00 0.56
O
ATOM 1950 CB SER A 129 -7.046 0.814 5.536 1.00 0.65
C
ATOM 1951 OG SER A 129 -7.224 1.990 6.270 1.00 0.65
O
ATOM 1952 H SER A 129 -5.448 0.484 7.478 1.00 0.49
H

```
ATOM   1953  HA  SER A 129      -5.506  -0.403   4.733  1.00  0.52
H
ATOM   1954 1HB  SER A 129      -7.523   0.914   4.562  1.00  0.77
H
ATOM   1955 2HB  SER A 129      -7.528  -0.014   6.053  1.00  0.77
H
ATOM   1956  HG  SER A 129      -6.838   2.706   5.755  1.00  0.77
H
ATOM   1957  N   GLU A 130      -3.998   2.384   5.273  1.00  0.36
N
ATOM   1958  CA  GLU A 130      -3.248   3.445   4.615  1.00  0.42
C
ATOM   1959  C   GLU A 130      -2.102   2.878   3.791  1.00  0.38
C
ATOM   1960  O   GLU A 130      -1.314   2.066   4.278  1.00  0.68
O
ATOM   1961  CB  GLU A 130      -2.694   4.432   5.646  1.00  0.63
C
ATOM   1962  CG  GLU A 130      -3.747   5.261   6.359  1.00  0.63
C
ATOM   1963  CD  GLU A 130      -4.399   6.252   5.433  1.00  0.63
C
ATOM   1964  OE1 GLU A 130      -3.984   6.348   4.305  1.00  0.63
O
ATOM   1965  OE2 GLU A 130      -5.281   6.957   5.870  1.00  0.63
O
ATOM   1966  H   GLU A 130      -3.834   2.217   6.258  1.00  0.43
H
ATOM   1967  HA  GLU A 130      -3.919   3.978   3.942  1.00  0.50
H
ATOM   1968 1HB  GLU A 130      -2.133   3.885   6.404  1.00  0.76
H
ATOM   1969 2HB  GLU A 130      -2.002   5.118   5.159  1.00  0.76
H
ATOM   1970 1HG  GLU A 130      -4.511   4.597   6.764  1.00  0.76
H
ATOM   1971 2HG  GLU A 130      -3.282   5.792   7.189  1.00  0.76
H
ATOM   1972  N   GLY A 131      -1.992   3.353   2.550  1.00  0.26
N
ATOM   1973  CA  GLY A 131      -0.985   2.909   1.591  1.00  0.32
C
ATOM   1974  C   GLY A 131       0.450   3.004   2.086  1.00  0.28
C
ATOM   1975  O   GLY A 131       1.267   2.130   1.790  1.00  0.48
O
ATOM   1976  H   GLY A 131      -2.670   4.039   2.226  1.00  0.31
H
ATOM   1977 1HA  GLY A 131      -1.197   1.889   1.299  1.00  0.38
H
ATOM   1978 2HA  GLY A 131      -1.087   3.511   0.692  1.00  0.38
H
ATOM   1979  N   SER A 132       0.743   4.058   2.846  1.00  0.36
N
ATOM   1980  CA  SER A 132       2.073   4.300   3.393  1.00  0.51
C
ATOM   1981  C   SER A 132       2.592   3.157   4.261  1.00  0.34
C
```

```
ATOM   1982  O   SER A 132       3.805   2.966   4.370  1.00  0.74
O
ATOM   1983  CB  SER A 132       2.048   5.585   4.190  1.00  0.77
C
ATOM   1984  OG  SER A 132       1.190   5.468   5.292  1.00  0.77
O
ATOM   1985  H   SER A 132       0.014   4.729   3.045  1.00  0.43
H
ATOM   1986  HA  SER A 132       2.763   4.427   2.556  1.00  0.61
H
ATOM   1987 1HB  SER A 132       3.053   5.836   4.529  1.00  0.92
H
ATOM   1988 2HB  SER A 132       1.706   6.394   3.548  1.00  0.92
H
ATOM   1989  HG  SER A 132       1.712   5.126   6.026  1.00  0.92
H
ATOM   1990  N   VAL A 133       1.679   2.382   4.856  1.00  0.25
N
ATOM   1991  CA  VAL A 133       2.063   1.240   5.666  1.00  0.29
C
ATOM   1992  C   VAL A 133       2.682   0.181   4.795  1.00  0.26
C
ATOM   1993  O   VAL A 133       3.739  -0.360   5.113  1.00  0.44
O
ATOM   1994  CB  VAL A 133       0.845   0.630   6.377  1.00  0.43
C
ATOM   1995  CG1 VAL A 133       1.249  -0.673   7.050  1.00  0.43
C
ATOM   1996  CG2 VAL A 133       0.291   1.623   7.385  1.00  0.43
C
ATOM   1997  H   VAL A 133       0.691   2.564   4.729  1.00  0.30
H
ATOM   1998  HA  VAL A 133       2.792   1.563   6.411  1.00  0.35
H
ATOM   1999  HB  VAL A 133       0.079   0.394   5.637  1.00  0.52
H
ATOM   2000 1HG1 VAL A 133       0.382  -1.119   7.532  1.00  0.52
H
ATOM   2001 2HG1 VAL A 133       1.636  -1.364   6.300  1.00  0.52
H
ATOM   2002 3HG1 VAL A 133       2.020  -0.476   7.794  1.00  0.52
H
ATOM   2003 1HG2 VAL A 133      -0.577   1.195   7.881  1.00  0.52
H
ATOM   2004 2HG2 VAL A 133       1.057   1.854   8.126  1.00  0.52
H
ATOM   2005 3HG2 VAL A 133      -0.001   2.538   6.869  1.00  0.52
H
ATOM   2006  N   TRP A 134       2.002  -0.108   3.696  1.00  0.42
N
ATOM   2007  CA  TRP A 134       2.421  -1.108   2.739  1.00  0.33
C
ATOM   2008  C   TRP A 134       3.723  -0.707   2.053  1.00  0.34
C
ATOM   2009  O   TRP A 134       4.555  -1.568   1.759  1.00  0.56
O
ATOM   2010  CB  TRP A 134       1.278  -1.319   1.750  1.00  0.49
C
```

ATOM 2011 CG TRP A 134 0.114 -2.005 2.412 1.00 0.49
C
ATOM 2012 CD1 TRP A 134 0.195 -3.031 3.304 1.00 0.49
C
ATOM 2013 CD2 TRP A 134 -1.298 -1.690 2.299 1.00 0.49
C
ATOM 2014 NE1 TRP A 134 -1.059 -3.395 3.728 1.00 0.49
N
ATOM 2015 CE2 TRP A 134 -1.984 -2.583 3.125 1.00 0.49
C
ATOM 2016 CE3 TRP A 134 -2.021 -0.738 1.582 1.00 0.49
C
ATOM 2017 CZ2 TRP A 134 -3.367 -2.560 3.244 1.00 0.49
C
ATOM 2018 CZ3 TRP A 134 -3.403 -0.708 1.705 1.00 0.49
C
ATOM 2019 CH2 TRP A 134 -4.061 -1.596 2.511 1.00 0.49
C
ATOM 2020 H TRP A 134 1.142 0.392 3.517 1.00 0.50
H
ATOM 2021 HA TRP A 134 2.589 -2.044 3.271 1.00 0.40
H
ATOM 2022 1HB TRP A 134 0.944 -0.361 1.352 1.00 0.59
H
ATOM 2023 2HB TRP A 134 1.616 -1.930 0.913 1.00 0.59
H
ATOM 2024 HD1 TRP A 134 1.122 -3.498 3.625 1.00 0.59
H
ATOM 2025 HE1 TRP A 134 -1.267 -4.132 4.384 1.00 0.59
H
ATOM 2026 HE3 TRP A 134 -1.508 -0.034 0.935 1.00 0.59
H
ATOM 2027 HZ2 TRP A 134 -3.904 -3.255 3.888 1.00 0.59
H
ATOM 2028 HZ3 TRP A 134 -3.952 0.043 1.140 1.00 0.59
H
ATOM 2029 HH2 TRP A 134 -5.146 -1.545 2.584 1.00 0.59
H
ATOM 2030 N GLU A 135 3.919 0.602 1.844 1.00 0.23
N
ATOM 2031 CA GLU A 135 5.175 1.087 1.276 1.00 0.21
C
ATOM 2032 C GLU A 135 6.325 0.760 2.221 1.00 0.19
C
ATOM 2033 O GLU A 135 7.372 0.267 1.792 1.00 0.21
O
ATOM 2034 CB GLU A 135 5.144 2.603 1.053 1.00 0.32
C
ATOM 2035 CG GLU A 135 4.207 3.100 -0.030 1.00 0.32
C
ATOM 2036 CD GLU A 135 4.228 4.604 -0.190 1.00 0.32
C
ATOM 2037 OE1 GLU A 135 4.999 5.256 0.483 1.00 0.32
O
ATOM 2038 OE2 GLU A 135 3.467 5.094 -0.993 1.00 0.32
O
ATOM 2039 H GLU A 135 3.181 1.261 2.067 1.00 0.28
H

```
ATOM   2040  HA  GLU A 135       5.350   0.585   0.325  1.00  0.25
H
ATOM   2041 1HB  GLU A 135       4.880   3.106   1.983  1.00  0.38
H
ATOM   2042 2HB  GLU A 135       6.144   2.931   0.776  1.00  0.38
H
ATOM   2043 1HG  GLU A 135       4.489   2.642  -0.976  1.00  0.38
H
ATOM   2044 2HG  GLU A 135       3.195   2.779   0.209  1.00  0.38
H
ATOM   2045  N   ALA A 136       6.107   1.023   3.514  1.00  0.17
N
ATOM   2046  CA  ALA A 136       7.090   0.740   4.547  1.00  0.14
C
ATOM   2047  C   ALA A 136       7.348  -0.757   4.678  1.00  0.16
C
ATOM   2048  O   ALA A 136       8.491  -1.173   4.877  1.00  0.17
O
ATOM   2049  CB  ALA A 136       6.628   1.319   5.869  1.00  0.21
C
ATOM   2050  H   ALA A 136       5.233   1.457   3.791  1.00  0.20
H
ATOM   2051  HA  ALA A 136       8.028   1.217   4.263  1.00  0.17
H
ATOM   2052 1HB  ALA A 136       7.382   1.134   6.632  1.00  0.25
H
ATOM   2053 2HB  ALA A 136       6.479   2.393   5.759  1.00  0.25
H
ATOM   2054 3HB  ALA A 136       5.691   0.853   6.164  1.00  0.25
H
ATOM   2055  N   MET A 137       6.296  -1.569   4.549  1.00  0.19
N
ATOM   2056  CA  MET A 137       6.466  -3.012   4.616  1.00  0.31
C
ATOM   2057  C   MET A 137       7.359  -3.512   3.493  1.00  0.24
C
ATOM   2058  O   MET A 137       8.247  -4.340   3.730  1.00  0.32
O
ATOM   2059  CB  MET A 137       5.115  -3.720   4.596  1.00  0.46
C
ATOM   2060  CG  MET A 137       4.312  -3.524   5.870  1.00  0.46
C
ATOM   2061  SD  MET A 137       2.677  -4.259   5.792  1.00  0.46
S
ATOM   2062  CE  MET A 137       3.132  -5.985   5.839  1.00  0.46
C
ATOM   2063  H   MET A 137       5.369  -1.183   4.422  1.00  0.23
H
ATOM   2064  HA  MET A 137       6.957  -3.248   5.551  1.00  0.37
H
ATOM   2065 1HB  MET A 137       4.519  -3.352   3.761  1.00  0.56
H
ATOM   2066 2HB  MET A 137       5.263  -4.790   4.450  1.00  0.56
H
ATOM   2067 1HG  MET A 137       4.848  -3.975   6.704  1.00  0.56
H
ATOM   2068 2HG  MET A 137       4.202  -2.464   6.075  1.00  0.56
H
```

```
ATOM   2069 1HE  MET A 137       2.231  -6.595   5.801  1.00  0.56
H
ATOM   2070 2HE  MET A 137       3.768  -6.217   4.983  1.00  0.56
H
ATOM   2071 3HE  MET A 137       3.675  -6.193   6.762  1.00  0.56
H
ATOM   2072  N   ALA A 138       7.142  -2.997   2.286  1.00  0.25
N
ATOM   2073  CA  ALA A 138       7.961  -3.361   1.144  1.00  0.27
C
ATOM   2074  C   ALA A 138       9.420  -2.940   1.354  1.00  0.27
C
ATOM   2075  O   ALA A 138      10.329  -3.720   1.063  1.00  0.45
O
ATOM   2076  CB  ALA A 138       7.404  -2.727  -0.118  1.00  0.41
C
ATOM   2077  H   ALA A 138       6.379  -2.344   2.151  1.00  0.30
H
ATOM   2078  HA  ALA A 138       7.936  -4.446   1.040  1.00  0.32
H
ATOM   2079 1HB  ALA A 138       8.008  -3.025  -0.975  1.00  0.49
H
ATOM   2080 2HB  ALA A 138       6.375  -3.055  -0.265  1.00  0.49
H
ATOM   2081 3HB  ALA A 138       7.426  -1.642  -0.021  1.00  0.49
H
ATOM   2082  N   PHE A 139       9.624  -1.718   1.867  1.00  0.19
N
ATOM   2083  CA  PHE A 139      10.955  -1.163   2.138  1.00  0.17
C
ATOM   2084  C   PHE A 139      11.756  -2.049   3.072  1.00  0.17
C
ATOM   2085  O   PHE A 139      12.910  -2.376   2.790  1.00  0.19
O
ATOM   2086  CB  PHE A 139      10.821   0.221   2.796  1.00  0.26
C
ATOM   2087  CG  PHE A 139      12.117   0.909   3.183  1.00  0.26
C
ATOM   2088  CD1 PHE A 139      12.757   1.777   2.306  1.00  0.26
C
ATOM   2089  CD2 PHE A 139      12.698   0.698   4.427  1.00  0.26
C
ATOM   2090  CE1 PHE A 139      13.924   2.424   2.670  1.00  0.26
C
ATOM   2091  CE2 PHE A 139      13.871   1.339   4.789  1.00  0.26
C
ATOM   2092  CZ  PHE A 139      14.484   2.205   3.911  1.00  0.26
C
ATOM   2093  H   PHE A 139       8.822  -1.130   2.059  1.00  0.23
H
ATOM   2094  HA  PHE A 139      11.493  -1.068   1.195  1.00  0.20
H
ATOM   2095 1HB  PHE A 139      10.284   0.880   2.114  1.00  0.31
H
ATOM   2096 2HB  PHE A 139      10.213   0.133   3.693  1.00  0.31
H
ATOM   2097  HD1 PHE A 139      12.319   1.955   1.323  1.00  0.31
H
```

ATOM 2098 HD2 PHE A 139 12.211 0.019 5.128 1.00 0.31
H
ATOM 2099 HE1 PHE A 139 14.404 3.107 1.970 1.00 0.31
H
ATOM 2100 HE2 PHE A 139 14.309 1.162 5.770 1.00 0.31
H
ATOM 2101 HZ PHE A 139 15.405 2.712 4.195 1.00 0.31
H
ATOM 2102 N ALA A 140 11.140 -2.409 4.194 1.00 0.18
N
ATOM 2103 CA ALA A 140 11.786 -3.216 5.210 1.00 0.20
C
ATOM 2104 C ALA A 140 12.216 -4.570 4.671 1.00 0.23
C
ATOM 2105 O ALA A 140 13.296 -5.063 4.999 1.00 0.25
O
ATOM 2106 CB ALA A 140 10.852 -3.380 6.383 1.00 0.30
C
ATOM 2107 H ALA A 140 10.191 -2.094 4.358 1.00 0.22
H
ATOM 2108 HA ALA A 140 12.680 -2.689 5.534 1.00 0.24
H
ATOM 2109 1HB ALA A 140 11.346 -3.947 7.168 1.00 0.36
H
ATOM 2110 2HB ALA A 140 10.578 -2.399 6.768 1.00 0.36
H
ATOM 2111 3HB ALA A 140 9.959 -3.906 6.056 1.00 0.36
H
ATOM 2112 N SER A 141 11.379 -5.188 3.839 1.00 0.26
N
ATOM 2113 CA SER A 141 11.770 -6.458 3.247 1.00 0.47
C
ATOM 2114 C SER A 141 12.893 -6.282 2.232 1.00 0.42
C
ATOM 2115 O SER A 141 13.835 -7.074 2.216 1.00 0.88
O
ATOM 2116 CB SER A 141 10.586 -7.116 2.606 1.00 0.70
C
ATOM 2117 OG SER A 141 9.662 -7.492 3.580 1.00 0.70
O
ATOM 2118 H SER A 141 10.472 -4.787 3.618 1.00 0.31
H
ATOM 2119 HA SER A 141 12.131 -7.108 4.044 1.00 0.56
H
ATOM 2120 1HB SER A 141 10.118 -6.424 1.905 1.00 0.85
H
ATOM 2121 2HB SER A 141 10.906 -7.991 2.043 1.00 0.85
H
ATOM 2122 HG SER A 141 8.888 -7.787 3.099 1.00 0.85
H
ATOM 2123 N TYR A 142 12.820 -5.226 1.414 1.00 0.40
N
ATOM 2124 CA TYR A 142 13.864 -4.940 0.425 1.00 0.40
C
ATOM 2125 C TYR A 142 15.225 -4.791 1.100 1.00 0.33
C
ATOM 2126 O TYR A 142 16.228 -5.309 0.608 1.00 0.42
O

```
ATOM   2127  CB  TYR A 142      13.543  -3.678  -0.387  1.00  0.60
C
ATOM   2128  CG  TYR A 142      14.561  -3.382  -1.483  1.00  0.60
C
ATOM   2129  CD1 TYR A 142      14.474  -4.013  -2.719  1.00  0.60
C
ATOM   2130  CD2 TYR A 142      15.589  -2.489  -1.251  1.00  0.60
C
ATOM   2131  CE1 TYR A 142      15.408  -3.738  -3.706  1.00  0.60
C
ATOM   2132  CE2 TYR A 142      16.523  -2.209  -2.233  1.00  0.60
C
ATOM   2133  CZ  TYR A 142      16.436  -2.829  -3.460  1.00  0.60
C
ATOM   2134  OH  TYR A 142      17.360  -2.552  -4.451  1.00  0.60
O
ATOM   2135  H   TYR A 142      12.019  -4.609   1.459  1.00  0.48
H
ATOM   2136  HA  TYR A 142      13.928  -5.783  -0.262  1.00  0.48
H
ATOM   2137 1HB  TYR A 142      12.561  -3.785  -0.852  1.00  0.72
H
ATOM   2138 2HB  TYR A 142      13.499  -2.817   0.278  1.00  0.72
H
ATOM   2139  HD1 TYR A 142      13.669  -4.723  -2.912  1.00  0.72
H
ATOM   2140  HD2 TYR A 142      15.657  -2.005  -0.286  1.00  0.72
H
ATOM   2141  HE1 TYR A 142      15.337  -4.233  -4.674  1.00  0.72
H
ATOM   2142  HE2 TYR A 142      17.327  -1.500  -2.038  1.00  0.72
H
ATOM   2143  HH  TYR A 142      17.658  -1.632  -4.385  1.00  0.72
H
ATOM   2144  N   TYR A 143      15.260  -4.083   2.233  1.00  0.28
N
ATOM   2145  CA  TYR A 143      16.506  -3.899   2.968  1.00  0.26
C
ATOM   2146  C   TYR A 143      16.691  -4.857   4.143  1.00  0.28
C
ATOM   2147  O   TYR A 143      17.519  -4.591   5.018  1.00  0.35
O
ATOM   2148  CB  TYR A 143      16.611  -2.467   3.479  1.00  0.39
C
ATOM   2149  CG  TYR A 143      16.826  -1.462   2.383  1.00  0.39
C
ATOM   2150  CD1 TYR A 143      15.826  -0.572   2.032  1.00  0.39
C
ATOM   2151  CD2 TYR A 143      18.043  -1.441   1.719  1.00  0.39
C
ATOM   2152  CE1 TYR A 143      16.051   0.344   1.022  1.00  0.39
C
ATOM   2153  CE2 TYR A 143      18.267  -0.522   0.716  1.00  0.39
C
ATOM   2154  CZ  TYR A 143      17.274   0.369   0.371  1.00  0.39
C
ATOM   2155  OH  TYR A 143      17.490   1.281  -0.628  1.00  0.39
O
```

ATOM 2156 H TYR A 143 14.411 -3.654 2.581 1.00 0.34
H
ATOM 2157 HA TYR A 143 17.330 -4.074 2.277 1.00 0.31
H
ATOM 2158 1HB TYR A 143 15.697 -2.202 4.013 1.00 0.47
H
ATOM 2159 2HB TYR A 143 17.439 -2.388 4.182 1.00 0.47
H
ATOM 2160 HD1 TYR A 143 14.866 -0.594 2.549 1.00 0.47
H
ATOM 2161 HD2 TYR A 143 18.826 -2.147 1.996 1.00 0.47
H
ATOM 2162 HE1 TYR A 143 15.274 1.049 0.734 1.00 0.47
H
ATOM 2163 HE2 TYR A 143 19.225 -0.500 0.196 1.00 0.47
H
ATOM 2164 HH TYR A 143 16.655 1.702 -0.863 1.00 0.47
H
ATOM 2165 N SER A 144 15.960 -5.981 4.143 1.00 0.35
N
ATOM 2166 CA SER A 144 16.061 -7.026 5.166 1.00 0.43
C
ATOM 2167 C SER A 144 16.242 -6.497 6.590 1.00 0.33
C
ATOM 2168 O SER A 144 17.171 -6.909 7.287 1.00 0.40
O
ATOM 2169 CB SER A 144 17.218 -7.958 4.841 1.00 0.65
C
ATOM 2170 OG SER A 144 17.049 -8.594 3.601 1.00 0.65
O
ATOM 2171 H SER A 144 15.301 -6.148 3.395 1.00 0.42
H
ATOM 2172 HA SER A 144 15.137 -7.606 5.143 1.00 0.52
H
ATOM 2173 1HB SER A 144 18.146 -7.386 4.833 1.00 0.77
H
ATOM 2174 2HB SER A 144 17.308 -8.709 5.626 1.00 0.77
H
ATOM 2175 HG SER A 144 17.858 -9.093 3.452 1.00 0.77
H
ATOM 2176 N LEU A 145 15.361 -5.594 7.027 1.00 0.24
N
ATOM 2177 CA LEU A 145 15.462 -5.014 8.370 1.00 0.21
C
ATOM 2178 C LEU A 145 15.088 -6.019 9.469 1.00 0.30
C
ATOM 2179 O LEU A 145 14.015 -5.939 10.066 1.00 0.37
O
ATOM 2180 CB LEU A 145 14.590 -3.754 8.471 1.00 0.32
C
ATOM 2181 CG LEU A 145 14.962 -2.630 7.487 1.00 0.32
C
ATOM 2182 CD1 LEU A 145 14.021 -1.450 7.682 1.00 0.32
C
ATOM 2183 CD2 LEU A 145 16.412 -2.223 7.705 1.00 0.32
C
ATOM 2184 H LEU A 145 14.615 -5.297 6.411 1.00 0.29
H

```
ATOM   2185  HA  LEU A 145      16.497  -4.716   8.529  1.00  0.25
H
ATOM   2186 1HB  LEU A 145      13.553  -4.026   8.288  1.00  0.38
H
ATOM   2187 2HB  LEU A 145      14.670  -3.352   9.481  1.00  0.38
H
ATOM   2188  HG  LEU A 145      14.839  -2.991   6.466  1.00  0.38
H
ATOM   2189 1HD1 LEU A 145      14.278  -0.663   6.974  1.00  0.38
H
ATOM   2190 2HD1 LEU A 145      12.994  -1.766   7.515  1.00  0.38
H
ATOM   2191 3HD1 LEU A 145      14.120  -1.070   8.694  1.00  0.38
H
ATOM   2192 1HD2 LEU A 145      16.677  -1.434   7.001  1.00  0.38
H
ATOM   2193 2HD2 LEU A 145      16.539  -1.859   8.723  1.00  0.38
H
ATOM   2194 3HD2 LEU A 145      17.059  -3.085   7.545  1.00  0.38
H
ATOM   2195  N   ASP A 146      15.998  -6.952   9.738  1.00  0.42
N
ATOM   2196  CA  ASP A 146      15.795  -8.022  10.710  1.00  0.84
C
ATOM   2197  C   ASP A 146      15.685  -7.563  12.164  1.00  0.84
C
ATOM   2198  O   ASP A 146      15.242  -8.333  13.016  1.00  3.54
O
ATOM   2199  CB  ASP A 146      16.924  -9.052  10.580  1.00  1.26
C
ATOM   2200  CG  ASP A 146      18.315  -8.455  10.786  1.00  1.26
C
ATOM   2201  OD1 ASP A 146      18.607  -7.470  10.150  1.00  1.26
O
ATOM   2202  OD2 ASP A 146      19.075  -8.984  11.561  1.00  1.26
O
ATOM   2203  H   ASP A 146      16.854  -6.934   9.202  1.00  0.50
H
ATOM   2204  HA  ASP A 146      14.861  -8.524  10.456  1.00  1.01
H
ATOM   2205 1HB  ASP A 146      16.776  -9.850  11.307  1.00  1.51
H
ATOM   2206 2HB  ASP A 146      16.889  -9.503   9.589  1.00  1.51
H
ATOM   2207  N   ASN A 147      16.046  -6.311  12.460  1.00  0.46
N
ATOM   2208  CA  ASN A 147      15.855  -5.810  13.815  1.00  0.67
C
ATOM   2209  C   ASN A 147      14.556  -5.007  13.927  1.00  0.59
C
ATOM   2210  O   ASN A 147      14.334  -4.340  14.936  1.00  1.45
O
ATOM   2211  CB  ASN A 147      17.028  -4.960  14.272  1.00  1.01
C
ATOM   2212  CG  ASN A 147      17.164  -3.680  13.503  1.00  1.01
C
ATOM   2213  OD1 ASN A 147      16.834  -3.621  12.314  1.00  1.01
O
```

```
ATOM   2214  ND2 ASN A 147      17.629  -2.648  14.165  1.00  1.01
N
ATOM   2215  H   ASN A 147      16.437  -5.709  11.751  1.00  0.55
H
ATOM   2216  HA  ASN A 147      15.767  -6.662  14.490  1.00  0.80
H
ATOM   2217 1HB  ASN A 147      16.915  -4.722  15.328  1.00  1.21
H
ATOM   2218 2HB  ASN A 147      17.952  -5.531  14.164  1.00  1.21
H
ATOM   2219 1HD2 ASN A 147      17.737  -1.768  13.700  1.00  1.21
H
ATOM   2220 2HD2 ASN A 147      17.877  -2.731  15.134  1.00  1.21
H
ATOM   2221  N   LEU A 148      13.693  -5.089  12.907  1.00  0.29
N
ATOM   2222  CA  LEU A 148      12.423  -4.382  12.914  1.00  0.27
C
ATOM   2223  C   LEU A 148      11.268  -5.344  13.166  1.00  0.30
C
ATOM   2224  O   LEU A 148      11.113  -6.338  12.450  1.00  0.41
O
ATOM   2225  CB  LEU A 148      12.213  -3.659  11.575  1.00  0.41
C
ATOM   2226  CG  LEU A 148      10.895  -2.891  11.407  1.00  0.41
C
ATOM   2227  CD1 LEU A 148      10.852  -1.729  12.388  1.00  0.41
C
ATOM   2228  CD2 LEU A 148      10.794  -2.393   9.977  1.00  0.41
C
ATOM   2229  H   LEU A 148      13.915  -5.636  12.085  1.00  0.35
H
ATOM   2230  HA  LEU A 148      12.440  -3.643  13.713  1.00  0.32
H
ATOM   2231 1HB  LEU A 148      13.028  -2.953  11.433  1.00  0.49
H
ATOM   2232 2HB  LEU A 148      12.259  -4.396  10.776  1.00  0.49
H
ATOM   2233  HG  LEU A 148      10.056  -3.551  11.625  1.00  0.49
H
ATOM   2234 1HD1 LEU A 148       9.914  -1.188  12.269  1.00  0.49
H
ATOM   2235 2HD1 LEU A 148      10.924  -2.110  13.405  1.00  0.49
H
ATOM   2236 3HD1 LEU A 148      11.686  -1.055  12.192  1.00  0.49
H
ATOM   2237 1HD2 LEU A 148       9.859  -1.848   9.846  1.00  0.49
H
ATOM   2238 2HD2 LEU A 148      11.633  -1.730   9.761  1.00  0.49
H
ATOM   2239 3HD2 LEU A 148      10.819  -3.244   9.298  1.00  0.49
H
ATOM   2240  N   VAL A 149      10.473  -5.037  14.186  1.00  0.29
N
ATOM   2241  CA  VAL A 149       9.313  -5.832  14.550  1.00  0.37
C
ATOM   2242  C   VAL A 149       8.055  -4.979  14.554  1.00  0.29
C
```

ATOM 2243 O VAL A 149 7.946 -4.037 15.337 1.00 0.39
O
ATOM 2244 CB VAL A 149 9.482 -6.448 15.950 1.00 0.55
C
ATOM 2245 CG1 VAL A 149 8.243 -7.259 16.309 1.00 0.55
C
ATOM 2246 CG2 VAL A 149 10.739 -7.307 16.007 1.00 0.55
C
ATOM 2247 H VAL A 149 10.684 -4.212 14.732 1.00 0.35
H
ATOM 2248 HA VAL A 149 9.193 -6.633 13.819 1.00 0.44
H
ATOM 2249 HB VAL A 149 9.567 -5.642 16.672 1.00 0.67
H
ATOM 2250 1HG1 VAL A 149 8.354 -7.671 17.312 1.00 0.67
H
ATOM 2251 2HG1 VAL A 149 7.364 -6.615 16.278 1.00 0.67
H
ATOM 2252 3HG1 VAL A 149 8.122 -8.074 15.595 1.00 0.67
H
ATOM 2253 1HG2 VAL A 149 10.849 -7.719 17.009 1.00 0.67
H
ATOM 2254 2HG2 VAL A 149 10.664 -8.120 15.289 1.00 0.67
H
ATOM 2255 3HG2 VAL A 149 11.609 -6.695 15.771 1.00 0.67
H
ATOM 2256 N ALA A 150 7.106 -5.319 13.698 1.00 0.26
N
ATOM 2257 CA ALA A 150 5.845 -4.600 13.669 1.00 0.29
C
ATOM 2258 C ALA A 150 4.821 -5.415 14.418 1.00 0.31
C
ATOM 2259 O ALA A 150 4.806 -6.640 14.323 1.00 0.34
O
ATOM 2260 CB ALA A 150 5.382 -4.333 12.250 1.00 0.43
C
ATOM 2261 H ALA A 150 7.250 -6.105 13.083 1.00 0.31
H
ATOM 2262 HA ALA A 150 5.976 -3.652 14.185 1.00 0.35
H
ATOM 2263 1HB ALA A 150 4.437 -3.792 12.274 1.00 0.52
H
ATOM 2264 2HB ALA A 150 6.130 -3.736 11.729 1.00 0.52
H
ATOM 2265 3HB ALA A 150 5.244 -5.280 11.730 1.00 0.52
H
ATOM 2266 N ILE A 151 3.974 -4.734 15.168 1.00 0.32
N
ATOM 2267 CA ILE A 151 2.947 -5.396 15.933 1.00 0.28
C
ATOM 2268 C ILE A 151 1.611 -4.864 15.453 1.00 0.29
C
ATOM 2269 O ILE A 151 1.406 -3.654 15.353 1.00 0.28
O
ATOM 2270 CB ILE A 151 3.106 -5.154 17.436 1.00 0.42
C
ATOM 2271 CG1 ILE A 151 4.506 -5.543 17.898 1.00 0.42
C

ATOM 2272 CG2 ILE A 151 2.087 -6.013 18.174 1.00 0.42
C
ATOM 2273 CD1 ILE A 151 5.490 -4.394 17.914 1.00 0.42
C
ATOM 2274 H ILE A 151 4.052 -3.732 15.210 1.00 0.38
H
ATOM 2275 HA ILE A 151 2.985 -6.464 15.739 1.00 0.34
H
ATOM 2276 HB ILE A 151 2.957 -4.101 17.663 1.00 0.50
H
ATOM 2277 1HG1 ILE A 151 4.437 -5.937 18.905 1.00 0.50
H
ATOM 2278 2HG1 ILE A 151 4.894 -6.325 17.245 1.00 0.50
H
ATOM 2279 1HG2 ILE A 151 2.191 -5.858 19.244 1.00 0.50
H
ATOM 2280 2HG2 ILE A 151 1.081 -5.738 17.862 1.00 0.50
H
ATOM 2281 3HG2 ILE A 151 2.262 -7.064 17.942 1.00 0.50
H
ATOM 2282 1HD1 ILE A 151 6.456 -4.751 18.263 1.00 0.50
H
ATOM 2283 2HD1 ILE A 151 5.596 -3.988 16.908 1.00 0.50
H
ATOM 2284 3HD1 ILE A 151 5.128 -3.616 18.583 1.00 0.50
H
ATOM 2285 N PHE A 152 0.719 -5.777 15.116 1.00 0.32
N
ATOM 2286 CA PHE A 152 -0.562 -5.405 14.558 1.00 0.33
C
ATOM 2287 C PHE A 152 -1.683 -5.596 15.558 1.00 0.34
C
ATOM 2288 O PHE A 152 -2.037 -6.718 15.928 1.00 0.40
O
ATOM 2289 CB PHE A 152 -0.811 -6.231 13.301 1.00 0.49
C
ATOM 2290 CG PHE A 152 0.206 -5.945 12.235 1.00 0.49
C
ATOM 2291 CD1 PHE A 152 1.428 -6.607 12.240 1.00 0.49
C
ATOM 2292 CD2 PHE A 152 -0.042 -5.018 11.235 1.00 0.49
C
ATOM 2293 CE1 PHE A 152 2.378 -6.342 11.273 1.00 0.49
C
ATOM 2294 CE2 PHE A 152 0.907 -4.756 10.263 1.00 0.49
C
ATOM 2295 CZ PHE A 152 2.118 -5.418 10.285 1.00 0.49
C
ATOM 2296 H PHE A 152 0.942 -6.754 15.239 1.00 0.38
H
ATOM 2297 HA PHE A 152 -0.528 -4.352 14.280 1.00 0.40
H
ATOM 2298 1HB PHE A 152 -0.778 -7.287 13.536 1.00 0.59
H
ATOM 2299 2HB PHE A 152 -1.800 -6.008 12.903 1.00 0.59
H
ATOM 2300 HD1 PHE A 152 1.635 -7.337 13.023 1.00 0.59
H

ATOM 2301 HD2 PHE A 152 -0.998 -4.493 11.222 1.00 0.59
H
ATOM 2302 HE1 PHE A 152 3.332 -6.862 11.292 1.00 0.59
H
ATOM 2303 HE2 PHE A 152 0.701 -4.025 9.483 1.00 0.59
H
ATOM 2304 HZ PHE A 152 2.868 -5.210 9.522 1.00 0.59
H
ATOM 2305 N ASP A 153 -2.243 -4.481 16.008 1.00 0.32
N
ATOM 2306 CA ASP A 153 -3.322 -4.506 16.974 1.00 0.34
C
ATOM 2307 C ASP A 153 -4.636 -4.866 16.294 1.00 0.36
C
ATOM 2308 O ASP A 153 -5.427 -3.988 15.939 1.00 0.44
O
ATOM 2309 CB ASP A 153 -3.417 -3.133 17.680 1.00 0.51
C
ATOM 2310 CG ASP A 153 -4.477 -3.089 18.753 1.00 0.51
C
ATOM 2311 OD1 ASP A 153 -5.262 -3.993 18.763 1.00 0.51
O
ATOM 2312 OD2 ASP A 153 -4.525 -2.173 19.544 1.00 0.51
O
ATOM 2313 H ASP A 153 -1.919 -3.586 15.675 1.00 0.38
H
ATOM 2314 HA ASP A 153 -3.103 -5.268 17.722 1.00 0.41
H
ATOM 2315 1HB ASP A 153 -2.454 -2.890 18.128 1.00 0.61
H
ATOM 2316 2HB ASP A 153 -3.637 -2.361 16.947 1.00 0.61
H
ATOM 2317 N VAL A 154 -4.880 -6.178 16.136 1.00 0.42
N
ATOM 2318 CA VAL A 154 -6.104 -6.654 15.488 1.00 0.48
C
ATOM 2319 C VAL A 154 -7.266 -6.581 16.454 1.00 0.57
C
ATOM 2320 O VAL A 154 -7.710 -7.607 16.973 1.00 0.71
O
ATOM 2321 CB VAL A 154 -5.968 -8.126 15.023 1.00 0.72
C
ATOM 2322 CG1 VAL A 154 -7.233 -8.555 14.289 1.00 0.72
C
ATOM 2323 CG2 VAL A 154 -4.717 -8.327 14.174 1.00 0.72
C
ATOM 2324 H VAL A 154 -4.189 -6.850 16.458 1.00 0.50
H
ATOM 2325 HA VAL A 154 -6.319 -6.018 14.632 1.00 0.58
H
ATOM 2326 HB VAL A 154 -5.896 -8.753 15.898 1.00 0.86
H
ATOM 2327 1HG1 VAL A 154 -7.151 -9.601 14.001 1.00 0.86
H
ATOM 2328 2HG1 VAL A 154 -8.095 -8.425 14.942 1.00 0.86
H
ATOM 2329 3HG1 VAL A 154 -7.359 -7.942 13.396 1.00 0.86
H

```
ATOM   2330 1HG2 VAL A 154      -4.644  -9.376  13.892  1.00  0.86
H
ATOM   2331 2HG2 VAL A 154      -4.762  -7.721  13.278  1.00  0.86
H
ATOM   2332 3HG2 VAL A 154      -3.839  -8.047  14.754  1.00  0.86
H
ATOM   2333  N   ASN A 155      -7.776  -5.373  16.668  1.00  0.60
N
ATOM   2334  CA  ASN A 155      -8.865  -5.136  17.606  1.00  0.85
C
ATOM   2335  C   ASN A 155     -10.247  -5.062  16.969  1.00  1.13
C
ATOM   2336  O   ASN A 155     -11.193  -4.582  17.599  1.00  3.11
O
ATOM   2337  CB  ASN A 155      -8.561  -3.894  18.424  1.00  1.27
C
ATOM   2338  CG  ASN A 155      -8.474  -2.636  17.621  1.00  1.27
C
ATOM   2339  OD1 ASN A 155      -8.894  -2.588  16.459  1.00  1.27
O
ATOM   2340  ND2 ASN A 155      -7.914  -1.612  18.220  1.00  1.27
N
ATOM   2341  H   ASN A 155      -7.344  -4.591  16.191  1.00  0.72
H
ATOM   2342  HA  ASN A 155      -8.893  -5.976  18.296  1.00  1.02
H
ATOM   2343 1HB  ASN A 155      -9.334  -3.761  19.173  1.00  1.53
H
ATOM   2344 2HB  ASN A 155      -7.624  -4.029  18.956  1.00  1.53
H
ATOM   2345 1HD2 ASN A 155      -7.815  -0.743  17.735  1.00  1.53
H
ATOM   2346 2HD2 ASN A 155      -7.570  -1.703  19.162  1.00  1.53
H
ATOM   2347  N   ARG A 156     -10.371  -5.576  15.738  1.00  0.68
N
ATOM   2348  CA  ARG A 156     -11.634  -5.680  14.989  1.00  0.75
C
ATOM   2349  C   ARG A 156     -12.247  -4.393  14.438  1.00  1.00
C
ATOM   2350  O   ARG A 156     -12.685  -4.382  13.286  1.00  2.97
O
ATOM   2351  CB  ARG A 156     -12.718  -6.301  15.843  1.00  1.12
C
ATOM   2352  CG  ARG A 156     -13.968  -6.640  15.063  1.00  1.12
C
ATOM   2353  CD  ARG A 156     -15.024  -7.236  15.908  1.00  1.12
C
ATOM   2354  NE  ARG A 156     -16.102  -7.747  15.090  1.00  1.12
N
ATOM   2355  CZ  ARG A 156     -17.280  -8.202  15.541  1.00  1.12
C
ATOM   2356  NH1 ARG A 156     -17.554  -8.192  16.827  1.00  1.12
N
ATOM   2357  NH2 ARG A 156     -18.147  -8.658  14.654  1.00  1.12
N
ATOM   2358  H   ARG A 156      -9.543  -5.933  15.287  1.00  0.82
H
```

```
ATOM   2359  HA  ARG A 156      -11.451  -6.345  14.147  1.00  0.90
H
ATOM   2360 1HB  ARG A 156      -12.346  -7.200  16.317  1.00  1.35
H
ATOM   2361 2HB  ARG A 156      -13.024  -5.617  16.632  1.00  1.35
H
ATOM   2362 1HG  ARG A 156      -14.368  -5.737  14.604  1.00  1.35
H
ATOM   2363 2HG  ARG A 156      -13.717  -7.360  14.288  1.00  1.35
H
ATOM   2364 1HD  ARG A 156      -14.621  -8.057  16.498  1.00  1.35
H
ATOM   2365 2HD  ARG A 156      -15.431  -6.478  16.576  1.00  1.35
H
ATOM   2366  HE  ARG A 156      -15.955  -7.799  14.088  1.00  1.35
H
ATOM   2367 1HH1 ARG A 156      -16.870  -7.839  17.491  1.00  1.35
H
ATOM   2368 2HH1 ARG A 156      -18.441  -8.537  17.162  1.00  1.35
H
ATOM   2369 1HH2 ARG A 156      -17.884  -8.651  13.672  1.00  1.35
H
ATOM   2370 2HH2 ARG A 156      -19.045  -9.011  14.947  1.00  1.35
H
ATOM   2371  N   LEU A 157      -12.367  -3.366  15.276  1.00  1.24
N
ATOM   2372  CA  LEU A 157      -13.063  -2.138  14.921  1.00  1.20
C
ATOM   2373  C   LEU A 157      -12.164  -0.916  14.660  1.00  1.83
C
ATOM   2374  O   LEU A 157      -11.070  -0.794  15.211  1.00  4.59
O
ATOM   2375  CB  LEU A 157      -14.015  -1.808  16.069  1.00  1.80
C
ATOM   2376  CG  LEU A 157      -14.990  -2.926  16.469  1.00  1.80
C
ATOM   2377  CD1 LEU A 157      -15.790  -2.482  17.678  1.00  1.80
C
ATOM   2378  CD2 LEU A 157      -15.898  -3.265  15.305  1.00  1.80
C
ATOM   2379  H   LEU A 157      -11.991  -3.460  16.207  1.00  1.49
H
ATOM   2380  HA  LEU A 157      -13.638  -2.330  14.022  1.00  1.44
H
ATOM   2381 1HB  LEU A 157      -13.419  -1.569  16.944  1.00  2.16
H
ATOM   2382 2HB  LEU A 157      -14.603  -0.930  15.800  1.00  2.16
H
ATOM   2383  HG  LEU A 157      -14.427  -3.816  16.754  1.00  2.16
H
ATOM   2384 1HD1 LEU A 157      -16.473  -3.279  17.975  1.00  2.16
H
ATOM   2385 2HD1 LEU A 157      -15.112  -2.263  18.502  1.00  2.16
H
ATOM   2386 3HD1 LEU A 157      -16.360  -1.589  17.429  1.00  2.16
H
ATOM   2387 1HD2 LEU A 157      -16.584  -4.061  15.596  1.00  2.16
H
```

```
ATOM   2388 2HD2 LEU A 157     -16.467  -2.383  15.013  1.00  2.16
H
ATOM   2389 3HD2 LEU A 157     -15.291  -3.600  14.466  1.00  2.16
H
ATOM   2390  N   GLY A 158     -12.660  -0.012  13.810  1.00  0.90
N
ATOM   2391  CA  GLY A 158     -12.000   1.253  13.476  1.00  1.13
C
ATOM   2392  C   GLY A 158     -12.736   2.414  14.150  1.00  1.21
C
ATOM   2393  O   GLY A 158     -13.187   2.279  15.288  1.00  2.84
O
ATOM   2394  H   GLY A 158     -13.553  -0.194  13.380  1.00  1.08
H
ATOM   2395 1HA  GLY A 158     -10.961   1.227  13.805  1.00  1.36
H
ATOM   2396 2HA  GLY A 158     -11.999   1.393  12.396  1.00  1.36
H
ATOM   2397  N   HIS A 159     -12.852   3.555  13.458  1.00  1.13
N
ATOM   2398  CA  HIS A 159     -13.557   4.712  14.026  1.00  1.51
C
ATOM   2399  C   HIS A 159     -15.059   4.675  13.842  1.00  1.76
C
ATOM   2400  O   HIS A 159     -15.806   4.669  14.822  1.00  2.21
O
ATOM   2401  CB  HIS A 159     -13.009   6.027  13.448  1.00  2.27
C
ATOM   2402  CG  HIS A 159     -13.658   7.303  13.955  1.00  2.27
C
ATOM   2403  ND1 HIS A 159     -13.471   7.787  15.236  1.00  2.27
N
ATOM   2404  CD2 HIS A 159     -14.462   8.201  13.334  1.00  2.27
C
ATOM   2405  CE1 HIS A 159     -14.126   8.927  15.374  1.00  2.27
C
ATOM   2406  NE2 HIS A 159     -14.725   9.206  14.234  1.00  2.27
N
ATOM   2407  H   HIS A 159     -12.459   3.633  12.522  1.00  1.36
H
ATOM   2408  HA  HIS A 159     -13.397   4.728  15.083  1.00  1.81
H
ATOM   2409 1HB  HIS A 159     -11.943   6.089  13.665  1.00  2.72
H
ATOM   2410 2HB  HIS A 159     -13.111   6.006  12.369  1.00  2.72
H
ATOM   2411  HD2 HIS A 159     -14.814   8.145  12.308  1.00  2.72
H
ATOM   2412  HE1 HIS A 159     -14.152   9.541  16.274  1.00  2.72
H
ATOM   2413  HE2 HIS A 159     -15.277  10.038  14.061  1.00  2.72
H
ATOM   2414  N   SER A 160     -15.504   4.690  12.591  1.00  1.78
N
ATOM   2415  CA  SER A 160     -16.926   4.734  12.284  1.00  1.98
C
ATOM   2416  C   SER A 160     -17.574   3.356  12.255  1.00  1.83
C
```

ATOM 2417 O SER A 160 -18.797 3.241 12.308 1.00 7.20
O
ATOM 2418 CB SER A 160 -17.151 5.419 10.944 1.00 2.97
C
ATOM 2419 OG SER A 160 -16.668 4.643 9.882 1.00 2.97
O
ATOM 2420 H SER A 160 -14.843 4.670 11.826 1.00 2.14
H
ATOM 2421 HA SER A 160 -17.419 5.321 13.059 1.00 2.38
H
ATOM 2422 1HB SER A 160 -18.215 5.597 10.807 1.00 3.56
H
ATOM 2423 2HB SER A 160 -16.654 6.388 10.945 1.00 3.56
H
ATOM 2424 HG SER A 160 -15.715 4.587 9.999 1.00 3.56
H
ATOM 2425 N GLY A 161 -16.774 2.308 12.177 1.00 2.28
N
ATOM 2426 CA GLY A 161 -17.336 0.968 12.120 1.00 1.95
C
ATOM 2427 C GLY A 161 -16.249 -0.080 12.169 1.00 1.66
C
ATOM 2428 O GLY A 161 -15.122 0.214 12.554 1.00 2.86
O
ATOM 2429 H GLY A 161 -15.773 2.433 12.141 1.00 2.74
H
ATOM 2430 1HA GLY A 161 -18.023 0.825 12.955 1.00 2.34
H
ATOM 2431 2HA GLY A 161 -17.915 0.854 11.205 1.00 2.34
H
ATOM 2432 N ALA A 162 -16.611 -1.299 11.798 1.00 1.68
N
ATOM 2433 CA ALA A 162 -15.702 -2.432 11.785 1.00 1.60
C
ATOM 2434 C ALA A 162 -14.671 -2.329 10.674 1.00 1.43
C
ATOM 2435 O ALA A 162 -14.911 -1.712 9.639 1.00 1.96
O
ATOM 2436 CB ALA A 162 -16.491 -3.718 11.644 1.00 2.40
C
ATOM 2437 H ALA A 162 -17.569 -1.449 11.510 1.00 2.02
H
ATOM 2438 HA ALA A 162 -15.170 -2.442 12.727 1.00 1.92
H
ATOM 2439 1HB ALA A 162 -15.813 -4.563 11.672 1.00 2.88
H
ATOM 2440 2HB ALA A 162 -17.204 -3.798 12.466 1.00 2.88
H
ATOM 2441 3HB ALA A 162 -17.029 -3.715 10.699 1.00 2.88
H
ATOM 2442 N LEU A 163 -13.528 -2.952 10.895 1.00 1.24
N
ATOM 2443 CA LEU A 163 -12.445 -2.949 9.931 1.00 1.40
C
ATOM 2444 C LEU A 163 -12.718 -3.875 8.755 1.00 0.80
C
ATOM 2445 O LEU A 163 -13.415 -4.875 8.917 1.00 1.59
O

```
ATOM   2446  CB  LEU A 163     -11.181  -3.342  10.661  1.00  2.10
C
ATOM   2447  CG  LEU A 163     -10.853  -2.330  11.724  1.00  2.10
C
ATOM   2448  CD1 LEU A 163      -9.623  -2.746  12.482  1.00  2.10
C
ATOM   2449  CD2 LEU A 163     -10.693  -0.999  11.041  1.00  2.10
C
ATOM   2450  H   LEU A 163     -13.387  -3.434  11.771  1.00  1.49
H
ATOM   2451  HA  LEU A 163     -12.339  -1.933   9.565  1.00  1.68
H
ATOM   2452 1HB  LEU A 163     -11.328  -4.307  11.142  1.00  2.52
H
ATOM   2453 2HB  LEU A 163     -10.338  -3.411   9.981  1.00  2.52
H
ATOM   2454  HG  LEU A 163     -11.671  -2.271  12.431  1.00  2.52
H
ATOM   2455 1HD1 LEU A 163      -9.396  -2.007  13.250  1.00  2.52
H
ATOM   2456 2HD1 LEU A 163      -9.789  -3.716  12.949  1.00  2.52
H
ATOM   2457 3HD1 LEU A 163      -8.799  -2.814  11.782  1.00  2.52
H
ATOM   2458 1HD2 LEU A 163     -10.457  -0.231  11.775  1.00  2.52
H
ATOM   2459 2HD2 LEU A 163      -9.890  -1.081  10.317  1.00  2.52
H
ATOM   2460 3HD2 LEU A 163     -11.618  -0.739  10.526  1.00  2.52
H
ATOM   2461  N   PRO A 164     -12.148  -3.576   7.570  1.00  0.79
N
ATOM   2462  CA  PRO A 164     -12.307  -4.257   6.280  1.00  1.45
C
ATOM   2463  C   PRO A 164     -12.247  -5.782   6.355  1.00  1.34
C
ATOM   2464  O   PRO A 164     -12.887  -6.463   5.554  1.00  2.69
O
ATOM   2465  CB  PRO A 164     -11.110  -3.755   5.464  1.00  2.17
C
ATOM   2466  CG  PRO A 164     -10.805  -2.426   6.029  1.00  2.17
C
ATOM   2467  CD  PRO A 164     -11.034  -2.599   7.502  1.00  2.17
C
ATOM   2468  HA  PRO A 164     -13.250  -3.932   5.823  1.00  1.74
H
ATOM   2469 1HB  PRO A 164     -10.270  -4.459   5.554  1.00  2.61
H
ATOM   2470 2HB  PRO A 164     -11.378  -3.709   4.398  1.00  2.61
H
ATOM   2471 1HG  PRO A 164      -9.770  -2.139   5.792  1.00  2.61
H
ATOM   2472 2HG  PRO A 164     -11.454  -1.670   5.577  1.00  2.61
H
ATOM   2473 1HD  PRO A 164     -10.130  -3.011   7.971  1.00  2.61
H
ATOM   2474 2HD  PRO A 164     -11.320  -1.633   7.937  1.00  2.61
H
```

ATOM 2475 N ALA A 165 -11.499 -6.332 7.312 1.00 0.86
N
ATOM 2476 CA ALA A 165 -11.447 -7.777 7.435 1.00 0.97
C
ATOM 2477 C ALA A 165 -11.420 -8.214 8.898 1.00 1.11
C
ATOM 2478 O ALA A 165 -10.852 -9.253 9.208 1.00 3.82
O
ATOM 2479 CB ALA A 165 -10.228 -8.319 6.702 1.00 1.46
C
ATOM 2480 H ALA A 165 -10.971 -5.752 7.948 1.00 1.03
H
ATOM 2481 HA ALA A 165 -12.347 -8.191 6.980 1.00 1.16
H
ATOM 2482 1HB ALA A 165 -10.214 -9.404 6.766 1.00 1.75
H
ATOM 2483 2HB ALA A 165 -10.274 -8.019 5.655 1.00 1.75
H
ATOM 2484 3HB ALA A 165 -9.324 -7.914 7.155 1.00 1.75
H
ATOM 2485 N GLU A 166 -12.037 -7.405 9.766 1.00 0.78
N
ATOM 2486 CA GLU A 166 -12.232 -7.607 11.217 1.00 1.34
C
ATOM 2487 C GLU A 166 -11.234 -8.528 11.948 1.00 0.64
C
ATOM 2488 O GLU A 166 -10.214 -8.060 12.448 1.00 0.72
O
ATOM 2489 CB GLU A 166 -13.651 -8.123 11.465 1.00 2.01
C
ATOM 2490 CG GLU A 166 -14.735 -7.108 11.146 1.00 2.01
C
ATOM 2491 CD GLU A 166 -16.132 -7.592 11.454 1.00 2.01
C
ATOM 2492 OE1 GLU A 166 -16.284 -8.369 12.368 1.00 2.01
O
ATOM 2493 OE2 GLU A 166 -17.046 -7.186 10.777 1.00 2.01
O
ATOM 2494 H GLU A 166 -12.461 -6.566 9.389 1.00 0.94
H
ATOM 2495 HA GLU A 166 -12.157 -6.623 11.684 1.00 1.61
H
ATOM 2496 1HB GLU A 166 -13.831 -8.990 10.832 1.00 2.41
H
ATOM 2497 2HB GLU A 166 -13.756 -8.444 12.499 1.00 2.41
H
ATOM 2498 1HG GLU A 166 -14.538 -6.204 11.723 1.00 2.41
H
ATOM 2499 2HG GLU A 166 -14.672 -6.846 10.090 1.00 2.41
H
ATOM 2500 N HIS A 167 -11.514 -9.834 11.956 1.00 0.74
N
ATOM 2501 CA HIS A 167 -10.666 -10.828 12.626 1.00 0.75
C
ATOM 2502 C HIS A 167 -10.144 -11.888 11.700 1.00 0.84
C
ATOM 2503 O HIS A 167 -9.803 -12.984 12.146 1.00 1.11
O

```
ATOM   2504  CB  HIS A 167     -11.377 -11.561  13.761  1.00  1.12
C
ATOM   2505  CG  HIS A 167     -11.701 -10.759  14.970  1.00  1.12
C
ATOM   2506  ND1 HIS A 167     -10.724 -10.296  15.827  1.00  1.12
N
ATOM   2507  CD2 HIS A 167     -12.883 -10.381  15.502  1.00  1.12
C
ATOM   2508  CE1 HIS A 167     -11.294  -9.663  16.832  1.00  1.12
C
ATOM   2509  NE2 HIS A 167     -12.603  -9.706  16.661  1.00  1.12
N
ATOM   2510  H   HIS A 167     -12.354 -10.162  11.504  1.00  0.89
H
ATOM   2511  HA  HIS A 167      -9.802 -10.322  13.059  1.00  0.90
H
ATOM   2512 1HB  HIS A 167     -12.313 -11.973  13.382  1.00  1.35
H
ATOM   2513 2HB  HIS A 167     -10.762 -12.405  14.074  1.00  1.35
H
ATOM   2514  HD1 HIS A 167      -9.754 -10.204  15.601  1.00  1.35
H
ATOM   2515  HD2 HIS A 167     -13.917 -10.532  15.191  1.00  1.35
H
ATOM   2516  HE1 HIS A 167     -10.692  -9.220  17.621  1.00  1.35
H
ATOM   2517  N   CYS A 168     -10.081 -11.588  10.413  1.00  0.92
N
ATOM   2518  CA  CYS A 168      -9.582 -12.541   9.432  1.00  1.06
C
ATOM   2519  C   CYS A 168      -8.068 -12.590   9.472  1.00  0.83
C
ATOM   2520  O   CYS A 168      -7.394 -12.118   8.555  1.00  1.03
O
ATOM   2521  CB  CYS A 168     -10.037 -12.175   8.032  1.00  1.59
C
ATOM   2522  SG  CYS A 168     -11.829 -12.233   7.784  1.00  1.59
S
ATOM   2523  H   CYS A 168     -10.377 -10.682  10.100  1.00  1.10
H
ATOM   2524  HA  CYS A 168      -9.970 -13.530   9.676  1.00  1.27
H
ATOM   2525 1HB  CYS A 168      -9.692 -11.170   7.793  1.00  1.91
H
ATOM   2526 2HB  CYS A 168      -9.578 -12.857   7.316  1.00  1.91
H
ATOM   2527  HG  CYS A 168     -12.135 -11.347   8.725  1.00  1.91
H
ATOM   2528  N   ILE A 169      -7.565 -13.192  10.543  1.00  0.75
N
ATOM   2529  CA  ILE A 169      -6.158 -13.334  10.839  1.00  0.95
C
ATOM   2530  C   ILE A 169      -5.372 -13.929   9.702  1.00  0.91
C
ATOM   2531  O   ILE A 169      -4.248 -13.509   9.448  1.00  1.70
O
ATOM   2532  CB  ILE A 169      -5.970 -14.201  12.073  1.00  1.42
C
```

```
ATOM   2533  CG1 ILE A 169      -6.543 -13.510  13.319  1.00  1.42
C
ATOM   2534  CG2 ILE A 169      -4.510 -14.500  12.234  1.00  1.42
C
ATOM   2535  CD1 ILE A 169      -5.873 -12.199  13.657  1.00  1.42
C
ATOM   2536  H   ILE A 169      -8.226 -13.555  11.214  1.00  0.90
H
ATOM   2537  HA  ILE A 169      -5.751 -12.350  11.050  1.00  1.14
H
ATOM   2538  HB  ILE A 169      -6.513 -15.136  11.945  1.00  1.71
H
ATOM   2539 1HG1 ILE A 169      -7.603 -13.323  13.165  1.00  1.71
H
ATOM   2540 2HG1 ILE A 169      -6.434 -14.177  14.174  1.00  1.71
H
ATOM   2541 1HG2 ILE A 169      -4.396 -15.137  13.099  1.00  1.71
H
ATOM   2542 2HG2 ILE A 169      -4.139 -15.015  11.349  1.00  1.71
H
ATOM   2543 3HG2 ILE A 169      -3.954 -13.574  12.377  1.00  1.71
H
ATOM   2544 1HD1 ILE A 169      -6.333 -11.774  14.548  1.00  1.71
H
ATOM   2545 2HD1 ILE A 169      -4.815 -12.373  13.842  1.00  1.71
H
ATOM   2546 3HD1 ILE A 169      -5.989 -11.507  12.824  1.00  1.71
H
ATOM   2547  N   ASN A 170      -5.957 -14.912   9.028  1.00  0.76
N
ATOM   2548  CA  ASN A 170      -5.306 -15.575   7.913  1.00  0.95
C
ATOM   2549  C   ASN A 170      -5.053 -14.624   6.747  1.00  0.93
C
ATOM   2550  O   ASN A 170      -4.077 -14.795   6.017  1.00  0.98
O
ATOM   2551  CB  ASN A 170      -6.129 -16.760   7.462  1.00  1.42
C
ATOM   2552  CG  ASN A 170      -6.059 -17.904   8.434  1.00  1.42
C
ATOM   2553  OD1 ASN A 170      -5.125 -17.999   9.238  1.00  1.42
O
ATOM   2554  ND2 ASN A 170      -7.034 -18.773   8.378  1.00  1.42
N
ATOM   2555  H   ASN A 170      -6.882 -15.212   9.299  1.00  0.91
H
ATOM   2556  HA  ASN A 170      -4.334 -15.938   8.250  1.00  1.14
H
ATOM   2557 1HB  ASN A 170      -7.170 -16.461   7.343  1.00  1.71
H
ATOM   2558 2HB  ASN A 170      -5.772 -17.103   6.489  1.00  1.71
H
ATOM   2559 1HD2 ASN A 170      -7.043 -19.555   9.003  1.00  1.71
H
ATOM   2560 2HD2 ASN A 170      -7.771 -18.657   7.713  1.00  1.71
H
ATOM   2561  N   ILE A 171      -5.932 -13.633   6.558  1.00  0.91
N
```

ATOM 2562 CA ILE A 171 -5.740 -12.653 5.499 1.00 0.90
C
ATOM 2563 C ILE A 171 -4.642 -11.700 5.889 1.00 0.70
C
ATOM 2564 O ILE A 171 -3.781 -11.370 5.073 1.00 0.71
O
ATOM 2565 CB ILE A 171 -7.018 -11.855 5.174 1.00 1.35
C
ATOM 2566 CG1 ILE A 171 -8.069 -12.774 4.547 1.00 1.35
C
ATOM 2567 CG2 ILE A 171 -6.687 -10.692 4.251 1.00 1.35
C
ATOM 2568 CD1 ILE A 171 -9.428 -12.129 4.394 1.00 1.35
C
ATOM 2569 H ILE A 171 -6.715 -13.516 7.183 1.00 1.09
H
ATOM 2570 HA ILE A 171 -5.431 -13.175 4.594 1.00 1.08
H
ATOM 2571 HB ILE A 171 -7.443 -11.465 6.098 1.00 1.62
H
ATOM 2572 1HG1 ILE A 171 -7.724 -13.090 3.563 1.00 1.62
H
ATOM 2573 2HG1 ILE A 171 -8.180 -13.661 5.171 1.00 1.62
H
ATOM 2574 1HG2 ILE A 171 -7.594 -10.126 4.040 1.00 1.62
H
ATOM 2575 2HG2 ILE A 171 -5.958 -10.041 4.734 1.00 1.62
H
ATOM 2576 3HG2 ILE A 171 -6.271 -11.073 3.318 1.00 1.62
H
ATOM 2577 1HD1 ILE A 171 -10.120 -12.841 3.944 1.00 1.62
H
ATOM 2578 2HD1 ILE A 171 -9.806 -11.822 5.365 1.00 1.62
H
ATOM 2579 3HD1 ILE A 171 -9.342 -11.254 3.751 1.00 1.62
H
ATOM 2580 N TYR A 172 -4.674 -11.265 7.150 1.00 0.59
N
ATOM 2581 CA TYR A 172 -3.661 -10.351 7.650 1.00 0.47
C
ATOM 2582 C TYR A 172 -2.293 -11.019 7.551 1.00 0.45
C
ATOM 2583 O TYR A 172 -1.308 -10.388 7.155 1.00 0.40
O
ATOM 2584 CB TYR A 172 -3.968 -9.972 9.107 1.00 0.70
C
ATOM 2585 CG TYR A 172 -5.226 -9.142 9.284 1.00 0.70
C
ATOM 2586 CD1 TYR A 172 -6.152 -9.478 10.265 1.00 0.70
C
ATOM 2587 CD2 TYR A 172 -5.460 -8.049 8.464 1.00 0.70
C
ATOM 2588 CE1 TYR A 172 -7.299 -8.725 10.427 1.00 0.70
C
ATOM 2589 CE2 TYR A 172 -6.606 -7.295 8.627 1.00 0.70
C
ATOM 2590 CZ TYR A 172 -7.522 -7.627 9.604 1.00 0.70
C

```
ATOM   2591  OH  TYR A 172      -8.655  -6.865   9.766  1.00  0.70
O
ATOM   2592  H   TYR A 172      -5.425 -11.571   7.761  1.00  0.71
H
ATOM   2593  HA  TYR A 172      -3.651  -9.454   7.032  1.00  0.56
H
ATOM   2594 1HB  TYR A 172      -4.073 -10.879   9.704  1.00  0.85
H
ATOM   2595 2HB  TYR A 172      -3.131  -9.408   9.518  1.00  0.85
H
ATOM   2596  HD1 TYR A 172      -5.972 -10.332  10.912  1.00  0.85
H
ATOM   2597  HD2 TYR A 172      -4.738  -7.783   7.692  1.00  0.85
H
ATOM   2598  HE1 TYR A 172      -8.021  -8.988  11.200  1.00  0.85
H
ATOM   2599  HE2 TYR A 172      -6.786  -6.433   7.984  1.00  0.85
H
ATOM   2600  HH  TYR A 172      -9.106  -7.137  10.571  1.00  0.85
H
ATOM   2601  N   GLN A 173      -2.268 -12.314   7.866  1.00  0.53
N
ATOM   2602  CA  GLN A 173      -1.087 -13.138   7.773  1.00  0.55
C
ATOM   2603  C   GLN A 173      -0.558 -13.230   6.362  1.00  0.53
C
ATOM   2604  O   GLN A 173       0.623 -12.978   6.121  1.00  0.47
O
ATOM   2605  CB  GLN A 173      -1.384 -14.548   8.291  1.00  0.83
C
ATOM   2606  CG  GLN A 173      -0.203 -15.494   8.206  1.00  0.83
C
ATOM   2607  CD  GLN A 173      -0.532 -16.900   8.658  1.00  0.83
C
ATOM   2608  OE1 GLN A 173      -1.544 -17.145   9.323  1.00  0.83
O
ATOM   2609  NE2 GLN A 173       0.329 -17.844   8.291  1.00  0.83
N
ATOM   2610  H   GLN A 173      -3.112 -12.751   8.205  1.00  0.64
H
ATOM   2611  HA  GLN A 173      -0.327 -12.681   8.386  1.00  0.66
H
ATOM   2612 1HB  GLN A 173      -1.700 -14.493   9.333  1.00  0.99
H
ATOM   2613 2HB  GLN A 173      -2.203 -14.984   7.724  1.00  0.99
H
ATOM   2614 1HG  GLN A 173       0.128 -15.542   7.169  1.00  0.99
H
ATOM   2615 2HG  GLN A 173       0.601 -15.122   8.835  1.00  0.99
H
ATOM   2616 1HE2 GLN A 173       0.175 -18.797   8.553  1.00  0.99
H
ATOM   2617 2HE2 GLN A 173       1.139 -17.598   7.750  1.00  0.99
H
ATOM   2618  N   ARG A 174      -1.447 -13.581   5.435  1.00  0.65
N
ATOM   2619  CA  ARG A 174      -1.102 -13.748   4.037  1.00  0.75
C
```

```
ATOM   2620  C   ARG A 174      -0.495 -12.480   3.459  1.00  0.64
C
ATOM   2621  O   ARG A 174       0.491 -12.559   2.730  1.00  0.71
O
ATOM   2622  CB  ARG A 174      -2.339 -14.142   3.240  1.00  1.12
C
ATOM   2623  CG  ARG A 174      -2.112 -14.467   1.771  1.00  1.12
C
ATOM   2624  CD  ARG A 174      -1.325 -15.711   1.588  1.00  1.12
C
ATOM   2625  NE  ARG A 174      -1.235 -16.105   0.185  1.00  1.12
N
ATOM   2626  CZ  ARG A 174      -0.496 -17.136  -0.272  1.00  1.12
C
ATOM   2627  NH1 ARG A 174       0.212 -17.852   0.576  1.00  1.12
N
ATOM   2628  NH2 ARG A 174      -0.474 -17.442  -1.560  1.00  1.12
N
ATOM   2629  H   ARG A 174      -2.400 -13.777   5.710  1.00  0.78
H
ATOM   2630  HA  ARG A 174      -0.368 -14.551   3.957  1.00  0.90
H
ATOM   2631 1HB  ARG A 174      -2.793 -15.020   3.698  1.00  1.35
H
ATOM   2632 2HB  ARG A 174      -3.072 -13.336   3.289  1.00  1.35
H
ATOM   2633 1HG  ARG A 174      -3.070 -14.581   1.265  1.00  1.35
H
ATOM   2634 2HG  ARG A 174      -1.550 -13.660   1.312  1.00  1.35
H
ATOM   2635 1HD  ARG A 174      -0.313 -15.559   1.963  1.00  1.35
H
ATOM   2636 2HD  ARG A 174      -1.797 -16.524   2.140  1.00  1.35
H
ATOM   2637  HE  ARG A 174      -1.770 -15.575  -0.492  1.00  1.35
H
ATOM   2638 1HH1 ARG A 174       0.201 -17.625   1.562  1.00  1.35
H
ATOM   2639 2HH1 ARG A 174       0.768 -18.625   0.243  1.00  1.35
H
ATOM   2640 1HH2 ARG A 174      -1.017 -16.913  -2.237  1.00  1.35
H
ATOM   2641 2HH2 ARG A 174       0.083 -18.220  -1.882  1.00  1.35
H
ATOM   2642  N   ARG A 175      -1.070 -11.316   3.787  1.00  0.56
N
ATOM   2643  CA  ARG A 175      -0.546 -10.050   3.277  1.00  0.56
C
ATOM   2644  C   ARG A 175       0.835  -9.740   3.802  1.00  0.54
C
ATOM   2645  O   ARG A 175       1.708  -9.338   3.031  1.00  0.68
O
ATOM   2646  CB  ARG A 175      -1.481  -8.894   3.615  1.00  0.84
C
ATOM   2647  CG  ARG A 175      -2.789  -8.891   2.842  1.00  0.84
C
ATOM   2648  CD  ARG A 175      -3.668  -7.757   3.197  1.00  0.84
C
```

```
ATOM   2649  NE  ARG A 175      -4.920  -7.812   2.463  1.00  0.84
N
ATOM   2650  CZ  ARG A 175      -5.886  -6.875   2.527  1.00  0.84
C
ATOM   2651  NH1 ARG A 175      -5.720  -5.808   3.280  1.00  0.84
N
ATOM   2652  NH2 ARG A 175      -7.002  -7.026   1.836  1.00  0.84
N
ATOM   2653  H   ARG A 175      -1.893 -11.309   4.379  1.00  0.67
H
ATOM   2654  HA  ARG A 175      -0.463 -10.115   2.199  1.00  0.67
H
ATOM   2655 1HB  ARG A 175      -1.728  -8.926   4.676  1.00  1.01
H
ATOM   2656 2HB  ARG A 175      -0.979  -7.947   3.423  1.00  1.01
H
ATOM   2657 1HG  ARG A 175      -2.571  -8.813   1.777  1.00  1.01
H
ATOM   2658 2HG  ARG A 175      -3.330  -9.819   3.034  1.00  1.01
H
ATOM   2659 1HD  ARG A 175      -3.892  -7.787   4.263  1.00  1.01
H
ATOM   2660 2HD  ARG A 175      -3.170  -6.818   2.955  1.00  1.01
H
ATOM   2661  HE  ARG A 175      -5.080  -8.628   1.878  1.00  1.01
H
ATOM   2662 1HH1 ARG A 175      -4.868  -5.693   3.811  1.00  1.01
H
ATOM   2663 2HH1 ARG A 175      -6.445  -5.107   3.329  1.00  1.01
H
ATOM   2664 1HH2 ARG A 175      -7.133  -7.844   1.260  1.00  1.01
H
ATOM   2665 2HH2 ARG A 175      -7.724  -6.323   1.887  1.00  1.01
H
ATOM   2666  N   CYS A 176       1.055  -9.954   5.093  1.00  0.47
N
ATOM   2667  CA  CYS A 176       2.363  -9.678   5.654  1.00  0.47
C
ATOM   2668  C   CYS A 176       3.427 -10.600   5.062  1.00  0.38
C
ATOM   2669  O   CYS A 176       4.520 -10.144   4.716  1.00  0.38
O
ATOM   2670  CB  CYS A 176       2.315  -9.809   7.168  1.00  0.70
C
ATOM   2671  SG  CYS A 176       1.372  -8.504   7.996  1.00  0.70
S
ATOM   2672  H   CYS A 176       0.310 -10.281   5.700  1.00  0.56
H
ATOM   2673  HA  CYS A 176       2.629  -8.655   5.408  1.00  0.56
H
ATOM   2674 1HB  CYS A 176       1.857 -10.763   7.427  1.00  0.85
H
ATOM   2675 2HB  CYS A 176       3.327  -9.804   7.570  1.00  0.85
H
ATOM   2676  HG  CYS A 176       1.433  -9.041   9.210  1.00  0.85
H
ATOM   2677  N   GLU A 177       3.082 -11.879   4.898  1.00  0.39
N
```

```
ATOM   2678  CA  GLU A 177       3.981 -12.848   4.287  1.00  0.39
C
ATOM   2679  C   GLU A 177       4.256 -12.489   2.833  1.00  0.34
C
ATOM   2680  O   GLU A 177       5.390 -12.586   2.365  1.00  0.40
O
ATOM   2681  CB  GLU A 177       3.377 -14.250   4.375  1.00  0.58
C
ATOM   2682  CG  GLU A 177       3.331 -14.828   5.781  1.00  0.58
C
ATOM   2683  CD  GLU A 177       2.605 -16.143   5.857  1.00  0.58
C
ATOM   2684  OE1 GLU A 177       1.983 -16.519   4.894  1.00  0.58
O
ATOM   2685  OE2 GLU A 177       2.674 -16.776   6.886  1.00  0.58
O
ATOM   2686  H   GLU A 177       2.175 -12.196   5.214  1.00  0.47
H
ATOM   2687  HA  GLU A 177       4.926 -12.836   4.830  1.00  0.47
H
ATOM   2688 1HB  GLU A 177       2.358 -14.231   3.988  1.00  0.70
H
ATOM   2689 2HB  GLU A 177       3.952 -14.934   3.751  1.00  0.70
H
ATOM   2690 1HG  GLU A 177       4.352 -14.970   6.134  1.00  0.70
H
ATOM   2691 2HG  GLU A 177       2.847 -14.111   6.441  1.00  0.70
H
ATOM   2692  N   ALA A 178       3.220 -12.038   2.130  1.00  0.35
N
ATOM   2693  CA  ALA A 178       3.325 -11.636   0.737  1.00  0.43
C
ATOM   2694  C   ALA A 178       4.280 -10.456   0.551  1.00  0.57
C
ATOM   2695  O   ALA A 178       4.982 -10.378  -0.461  1.00  1.30
O
ATOM   2696  CB  ALA A 178       1.962 -11.295   0.209  1.00  0.65
C
ATOM   2697  H   ALA A 178       2.308 -11.996   2.561  1.00  0.42
H
ATOM   2698  HA  ALA A 178       3.723 -12.479   0.173  1.00  0.52
H
ATOM   2699 1HB  ALA A 178       2.057 -11.041  -0.839  1.00  0.77
H
ATOM   2700 2HB  ALA A 178       1.302 -12.156   0.317  1.00  0.77
H
ATOM   2701 3HB  ALA A 178       1.551 -10.451   0.760  1.00  0.77
H
ATOM   2702  N   PHE A 179       4.353  -9.568   1.549  1.00  0.92
N
ATOM   2703  CA  PHE A 179       5.308  -8.469   1.489  1.00  2.30
C
ATOM   2704  C   PHE A 179       6.686  -8.824   2.041  1.00  0.44
C
ATOM   2705  O   PHE A 179       7.522  -7.932   2.159  1.00  0.73
O
ATOM   2706  CB  PHE A 179       4.822  -7.218   2.239  1.00  3.45
C
```

ATOM 2707 CG PHE A 179 3.734 -6.430 1.568 1.00 3.45
C
ATOM 2708 CD1 PHE A 179 2.449 -6.410 2.075 1.00 3.45
C
ATOM 2709 CD2 PHE A 179 4.006 -5.689 0.425 1.00 3.45
C
ATOM 2710 CE1 PHE A 179 1.462 -5.672 1.457 1.00 3.45
C
ATOM 2711 CE2 PHE A 179 3.017 -4.950 -0.198 1.00 3.45
C
ATOM 2712 CZ PHE A 179 1.740 -4.943 0.320 1.00 3.45
C
ATOM 2713 H PHE A 179 3.721 -9.642 2.339 1.00 1.10
H
ATOM 2714 HA PHE A 179 5.433 -8.194 0.444 1.00 2.76
H
ATOM 2715 1HB PHE A 179 4.459 -7.516 3.222 1.00 4.14
H
ATOM 2716 2HB PHE A 179 5.667 -6.550 2.399 1.00 4.14
H
ATOM 2717 HD1 PHE A 179 2.221 -6.978 2.975 1.00 4.14
H
ATOM 2718 HD2 PHE A 179 5.018 -5.693 0.020 1.00 4.14
H
ATOM 2719 HE1 PHE A 179 0.456 -5.667 1.875 1.00 4.14
H
ATOM 2720 HE2 PHE A 179 3.245 -4.375 -1.095 1.00 4.14
H
ATOM 2721 HZ PHE A 179 0.957 -4.362 -0.166 1.00 4.14
H
ATOM 2722 N GLY A 180 6.949 -10.109 2.326 1.00 0.49
N
ATOM 2723 CA GLY A 180 8.263 -10.544 2.799 1.00 0.44
C
ATOM 2724 C GLY A 180 8.481 -10.508 4.317 1.00 0.43
C
ATOM 2725 O GLY A 180 9.628 -10.489 4.761 1.00 0.78
O
ATOM 2726 H GLY A 180 6.243 -10.819 2.216 1.00 0.59
H
ATOM 2727 1HA GLY A 180 8.435 -11.562 2.446 1.00 0.53
H
ATOM 2728 2HA GLY A 180 9.025 -9.931 2.319 1.00 0.53
H
ATOM 2729 N TRP A 181 7.413 -10.491 5.116 1.00 0.30
N
ATOM 2730 CA TRP A 181 7.576 -10.440 6.573 1.00 0.35
C
ATOM 2731 C TRP A 181 7.372 -11.786 7.274 1.00 0.48
C
ATOM 2732 O TRP A 181 6.491 -12.574 6.920 1.00 0.65
O
ATOM 2733 CB TRP A 181 6.594 -9.423 7.172 1.00 0.52
C
ATOM 2734 CG TRP A 181 6.955 -8.001 6.883 1.00 0.52
C
ATOM 2735 CD1 TRP A 181 7.102 -7.422 5.664 1.00 0.52
C

```
ATOM   2736  CD2 TRP A 181       7.199  -6.957   7.852  1.00  0.52
C
ATOM   2737  NE1 TRP A 181       7.453  -6.105   5.809  1.00  0.52
N
ATOM   2738  CE2 TRP A 181       7.511  -5.802   7.141  1.00  0.52
C
ATOM   2739  CE3 TRP A 181       7.185  -6.915   9.241  1.00  0.52
C
ATOM   2740  CZ2 TRP A 181       7.815  -4.608   7.779  1.00  0.52
C
ATOM   2741  CZ3 TRP A 181       7.498  -5.725   9.882  1.00  0.52
C
ATOM   2742  CH2 TRP A 181       7.804  -4.600   9.170  1.00  0.52
C
ATOM   2743  H   TRP A 181       6.475 -10.505   4.732  1.00  0.36
H
ATOM   2744  HA  TRP A 181       8.590 -10.101   6.786  1.00  0.42
H
ATOM   2745 1HB  TRP A 181       5.590  -9.608   6.801  1.00  0.63
H
ATOM   2746 2HB  TRP A 181       6.570  -9.549   8.253  1.00  0.63
H
ATOM   2747  HD1 TRP A 181       6.979  -7.933   4.715  1.00  0.63
H
ATOM   2748  HE1 TRP A 181       7.637  -5.466   5.050  1.00  0.63
H
ATOM   2749  HE3 TRP A 181       6.944  -7.808   9.802  1.00  0.63
H
ATOM   2750  HZ2 TRP A 181       8.063  -3.701   7.233  1.00  0.63
H
ATOM   2751  HZ3 TRP A 181       7.493  -5.712  10.970  1.00  0.63
H
ATOM   2752  HH2 TRP A 181       8.043  -3.680   9.705  1.00  0.63
H
ATOM   2753  N   ASN A 182       8.182 -12.021   8.309  1.00  0.52
N
ATOM   2754  CA  ASN A 182       8.087 -13.225   9.129  1.00  0.64
C
ATOM   2755  C   ASN A 182       6.919 -13.072  10.069  1.00  0.43
C
ATOM   2756  O   ASN A 182       7.058 -12.606  11.198  1.00  0.38
O
ATOM   2757  CB  ASN A 182       9.380 -13.503   9.879  1.00  0.96
C
ATOM   2758  CG  ASN A 182      10.463 -14.071   8.999  1.00  0.96
C
ATOM   2759  OD1 ASN A 182      10.264 -15.127   8.378  1.00  0.96
O
ATOM   2760  ND2 ASN A 182      11.592 -13.417   8.922  1.00  0.96
N
ATOM   2761  H   ASN A 182       8.869 -11.324   8.553  1.00  0.62
H
ATOM   2762  HA  ASN A 182       7.874 -14.077   8.483  1.00  0.77
H
ATOM   2763 1HB  ASN A 182       9.745 -12.578  10.329  1.00  1.15
H
ATOM   2764 2HB  ASN A 182       9.186 -14.205  10.689  1.00  1.15
H
```

```
ATOM   2765 1HD2 ASN A 182      12.327 -13.767   8.344  1.00  1.15
H
ATOM   2766 2HD2 ASN A 182      11.729 -12.561   9.424  1.00  1.15
H
ATOM   2767  N   THR A 183       5.760 -13.441   9.563  1.00  0.59
N
ATOM   2768  CA  THR A 183       4.512 -13.287  10.269  1.00  0.71
C
ATOM   2769  C   THR A 183       4.220 -14.351  11.293  1.00  0.95
C
ATOM   2770  O   THR A 183       4.361 -15.546  11.032  1.00  1.50
O
ATOM   2771  CB  THR A 183       3.367 -13.262   9.282  1.00  1.06
C
ATOM   2772  OG1 THR A 183       3.573 -12.193   8.362  1.00  1.06
O
ATOM   2773  CG2 THR A 183       2.073 -13.035  10.027  1.00  1.06
C
ATOM   2774  H   THR A 183       5.754 -13.799   8.614  1.00  0.71
H
ATOM   2775  HA  THR A 183       4.542 -12.337  10.793  1.00  0.85
H
ATOM   2776  HB  THR A 183       3.321 -14.205   8.737  1.00  1.28
H
ATOM   2777  HG1 THR A 183       4.396 -12.338   7.885  1.00  1.28
H
ATOM   2778 1HG2 THR A 183       1.268 -13.005   9.316  1.00  1.28
H
ATOM   2779 2HG2 THR A 183       1.899 -13.848  10.730  1.00  1.28
H
ATOM   2780 3HG2 THR A 183       2.124 -12.090  10.565  1.00  1.28
H
ATOM   2781  N   TYR A 184       3.785 -13.900  12.461  1.00  0.96
N
ATOM   2782  CA  TYR A 184       3.377 -14.800  13.515  1.00  1.34
C
ATOM   2783  C   TYR A 184       2.018 -14.383  14.053  1.00  0.99
C
ATOM   2784  O   TYR A 184       1.705 -13.199  14.151  1.00  2.29
O
ATOM   2785  CB  TYR A 184       4.416 -14.831  14.625  1.00  2.01
C
ATOM   2786  CG  TYR A 184       5.757 -15.325  14.142  1.00  2.01
C
ATOM   2787  CD1 TYR A 184       6.726 -14.415  13.758  1.00  2.01
C
ATOM   2788  CD2 TYR A 184       6.010 -16.684  14.057  1.00  2.01
C
ATOM   2789  CE1 TYR A 184       7.949 -14.858  13.295  1.00  2.01
C
ATOM   2790  CE2 TYR A 184       7.231 -17.130  13.593  1.00  2.01
C
ATOM   2791  CZ  TYR A 184       8.199 -16.222  13.212  1.00  2.01
C
ATOM   2792  OH  TYR A 184       9.416 -16.668  12.752  1.00  2.01
O
ATOM   2793  H   TYR A 184       3.716 -12.901  12.599  1.00  1.15
H
```

```
ATOM   2794  HA  TYR A 184       3.278 -15.803  13.101  1.00  1.61
H
ATOM   2795 1HB  TYR A 184       4.546 -13.828  15.031  1.00  2.41
H
ATOM   2796 2HB  TYR A 184       4.079 -15.480  15.432  1.00  2.41
H
ATOM   2797  HD1 TYR A 184       6.518 -13.348  13.821  1.00  2.41
H
ATOM   2798  HD2 TYR A 184       5.244 -17.401  14.354  1.00  2.41
H
ATOM   2799  HE1 TYR A 184       8.711 -14.140  12.994  1.00  2.41
H
ATOM   2800  HE2 TYR A 184       7.431 -18.199  13.525  1.00  2.41
H
ATOM   2801  HH  TYR A 184       9.487 -17.613  12.908  1.00  2.41
H
ATOM   2802  N   VAL A 185       1.210 -15.374  14.391  1.00  1.12
N
ATOM   2803  CA  VAL A 185      -0.123 -15.139  14.920  1.00  1.35
C
ATOM   2804  C   VAL A 185      -0.224 -15.461  16.389  1.00  1.37
C
ATOM   2805  O   VAL A 185      -0.013 -16.604  16.793  1.00  3.40
O
ATOM   2806  CB  VAL A 185      -1.148 -15.985  14.178  1.00  2.03
C
ATOM   2807  CG1 VAL A 185      -2.506 -15.770  14.814  1.00  2.03
C
ATOM   2808  CG2 VAL A 185      -1.155 -15.595  12.704  1.00  2.03
C
ATOM   2809  H   VAL A 185       1.530 -16.326  14.281  1.00  1.34
H
ATOM   2810  HA  VAL A 185      -0.369 -14.088  14.777  1.00  1.62
H
ATOM   2811  HB  VAL A 185      -0.895 -17.041  14.281  1.00  2.43
H
ATOM   2812 1HG1 VAL A 185      -3.246 -16.384  14.302  1.00  2.43
H
ATOM   2813 2HG1 VAL A 185      -2.465 -16.057  15.864  1.00  2.43
H
ATOM   2814 3HG1 VAL A 185      -2.784 -14.719  14.736  1.00  2.43
H
ATOM   2815 1HG2 VAL A 185      -1.889 -16.200  12.171  1.00  2.43
H
ATOM   2816 2HG2 VAL A 185      -1.413 -14.541  12.608  1.00  2.43
H
ATOM   2817 3HG2 VAL A 185      -0.167 -15.767  12.277  1.00  2.43
H
ATOM   2818  N   VAL A 186      -0.559 -14.458  17.188  1.00  0.63
N
ATOM   2819  CA  VAL A 186      -0.717 -14.659  18.620  1.00  0.91
C
ATOM   2820  C   VAL A 186      -1.996 -14.042  19.142  1.00  0.92
C
ATOM   2821  O   VAL A 186      -2.581 -13.168  18.505  1.00  1.50
O
ATOM   2822  CB  VAL A 186       0.465 -14.047  19.405  1.00  1.36
C
```

ATOM 2823 CG1 VAL A 186 1.777 -14.701 19.015 1.00 1.36
C
ATOM 2824 CG2 VAL A 186 0.519 -12.550 19.139 1.00 1.36
C
ATOM 2825 H VAL A 186 -0.715 -13.540 16.791 1.00 0.76
H
ATOM 2826 HA VAL A 186 -0.745 -15.732 18.815 1.00 1.09
H
ATOM 2827 HB VAL A 186 0.315 -14.226 20.470 1.00 1.64
H
ATOM 2828 1HG1 VAL A 186 2.580 -14.256 19.600 1.00 1.64
H
ATOM 2829 2HG1 VAL A 186 1.727 -15.769 19.221 1.00 1.64
H
ATOM 2830 3HG1 VAL A 186 1.970 -14.541 17.955 1.00 1.64
H
ATOM 2831 1HG2 VAL A 186 1.340 -12.112 19.703 1.00 1.64
H
ATOM 2832 2HG2 VAL A 186 0.675 -12.375 18.075 1.00 1.64
H
ATOM 2833 3HG2 VAL A 186 -0.420 -12.092 19.449 1.00 1.64
H
ATOM 2834 N ASP A 187 -2.412 -14.495 20.316 1.00 0.73
N
ATOM 2835 CA ASP A 187 -3.535 -13.885 20.993 1.00 0.74
C
ATOM 2836 C ASP A 187 -2.963 -12.711 21.750 1.00 0.67
C
ATOM 2837 O ASP A 187 -2.138 -12.888 22.645 1.00 0.69
O
ATOM 2838 CB ASP A 187 -4.251 -14.869 21.924 1.00 1.11
C
ATOM 2839 CG ASP A 187 -5.523 -14.304 22.576 1.00 1.11
C
ATOM 2840 OD1 ASP A 187 -5.598 -13.127 22.851 1.00 1.11
O
ATOM 2841 OD2 ASP A 187 -6.434 -15.077 22.770 1.00 1.11
O
ATOM 2842 H ASP A 187 -1.910 -15.248 20.765 1.00 0.88
H
ATOM 2843 HA ASP A 187 -4.248 -13.514 20.255 1.00 0.89
H
ATOM 2844 1HB ASP A 187 -4.521 -15.762 21.360 1.00 1.33
H
ATOM 2845 2HB ASP A 187 -3.566 -15.181 22.713 1.00 1.33
H
ATOM 2846 N GLY A 188 -3.377 -11.509 21.375 1.00 0.63
N
ATOM 2847 CA GLY A 188 -2.858 -10.269 21.946 1.00 0.58
C
ATOM 2848 C GLY A 188 -3.141 -10.092 23.434 1.00 0.67
C
ATOM 2849 O GLY A 188 -2.540 -9.234 24.077 1.00 0.79
O
ATOM 2850 H GLY A 188 -4.088 -11.448 20.664 1.00 0.76
H
ATOM 2851 1HA GLY A 188 -1.783 -10.228 21.780 1.00 0.70
H

```
ATOM   2852 2HA  GLY A 188      -3.285  -9.427  21.402  1.00  0.70
H
ATOM   2853  N   ARG A 189      -4.053 -10.890  23.986  1.00  0.71
N
ATOM   2854  CA  ARG A 189      -4.382 -10.819  25.400  1.00  0.84
C
ATOM   2855  C   ARG A 189      -3.485 -11.747  26.223  1.00  0.92
C
ATOM   2856  O   ARG A 189      -3.471 -11.681  27.454  1.00  1.04
O
ATOM   2857  CB  ARG A 189      -5.824 -11.224  25.608  1.00  1.26
C
ATOM   2858  CG  ARG A 189      -6.846 -10.340  24.920  1.00  1.26
C
ATOM   2859  CD  ARG A 189      -8.186 -10.958  25.005  1.00  1.26
C
ATOM   2860  NE  ARG A 189      -8.187 -12.246  24.327  1.00  1.26
N
ATOM   2861  CZ  ARG A 189      -9.215 -13.114  24.307  1.00  1.26
C
ATOM   2862  NH1 ARG A 189     -10.350 -12.818  24.905  1.00  1.26
N
ATOM   2863  NH2 ARG A 189      -9.070 -14.270  23.685  1.00  1.26
N
ATOM   2864  H   ARG A 189      -4.542 -11.579  23.421  1.00  0.85
H
ATOM   2865  HA  ARG A 189      -4.242  -9.793  25.741  1.00  1.01
H
ATOM   2866 1HB  ARG A 189      -5.971 -12.240  25.244  1.00  1.51
H
ATOM   2867 2HB  ARG A 189      -6.051 -11.221  26.674  1.00  1.51
H
ATOM   2868 1HG  ARG A 189      -6.874  -9.364  25.403  1.00  1.51
H
ATOM   2869 2HG  ARG A 189      -6.581 -10.222  23.868  1.00  1.51
H
ATOM   2870 1HD  ARG A 189      -8.455 -11.119  26.048  1.00  1.51
H
ATOM   2871 2HD  ARG A 189      -8.923 -10.316  24.528  1.00  1.51
H
ATOM   2872  HE  ARG A 189      -7.331 -12.526  23.850  1.00  1.51
H
ATOM   2873 1HH1 ARG A 189     -10.457 -11.933  25.380  1.00  1.51
H
ATOM   2874 2HH1 ARG A 189     -11.117 -13.475  24.887  1.00  1.51
H
ATOM   2875 1HH2 ARG A 189      -8.183 -14.497  23.242  1.00  1.51
H
ATOM   2876 2HH2 ARG A 189      -9.829 -14.934  23.662  1.00  1.51
H
ATOM   2877  N   ASP A 190      -2.777 -12.647  25.544  1.00  0.96
N
ATOM   2878  CA  ASP A 190      -1.924 -13.624  26.198  1.00  1.09
C
ATOM   2879  C   ASP A 190      -0.486 -13.147  26.281  1.00  0.99
C
ATOM   2880  O   ASP A 190       0.263 -13.257  25.309  1.00  0.95
O
```

ATOM 2881 CB ASP A 190 -2.018 -14.958 25.457 1.00 1.64
C
ATOM 2882 CG ASP A 190 -1.197 -16.070 26.080 1.00 1.64
C
ATOM 2883 OD1 ASP A 190 -0.278 -15.779 26.809 1.00 1.64
O
ATOM 2884 OD2 ASP A 190 -1.493 -17.211 25.818 1.00 1.64
O
ATOM 2885 H ASP A 190 -2.803 -12.659 24.534 1.00 1.15
H
ATOM 2886 HA ASP A 190 -2.289 -13.771 27.216 1.00 1.31
H
ATOM 2887 1HB ASP A 190 -3.060 -15.276 25.424 1.00 1.96
H
ATOM 2888 2HB ASP A 190 -1.690 -14.821 24.427 1.00 1.96
H
ATOM 2889 N VAL A 191 -0.105 -12.637 27.454 1.00 1.00
N
ATOM 2890 CA VAL A 191 1.238 -12.113 27.686 1.00 0.95
C
ATOM 2891 C VAL A 191 2.351 -13.157 27.569 1.00 1.04
C
ATOM 2892 O VAL A 191 3.508 -12.792 27.378 1.00 1.00
O
ATOM 2893 CB VAL A 191 1.327 -11.445 29.077 1.00 1.42
C
ATOM 2894 CG1 VAL A 191 0.375 -10.268 29.166 1.00 1.42
C
ATOM 2895 CG2 VAL A 191 1.016 -12.456 30.174 1.00 1.42
C
ATOM 2896 H VAL A 191 -0.779 -12.597 28.207 1.00 1.20
H
ATOM 2897 HA VAL A 191 1.424 -11.342 26.938 1.00 1.14
H
ATOM 2898 HB VAL A 191 2.330 -11.058 29.208 1.00 1.71
H
ATOM 2899 1HG1 VAL A 191 0.469 -9.796 30.143 1.00 1.71
H
ATOM 2900 2HG1 VAL A 191 0.622 -9.546 28.389 1.00 1.71
H
ATOM 2901 3HG1 VAL A 191 -0.650 -10.616 29.029 1.00 1.71
H
ATOM 2902 1HG2 VAL A 191 1.098 -11.974 31.147 1.00 1.71
H
ATOM 2903 2HG2 VAL A 191 0.002 -12.835 30.042 1.00 1.71
H
ATOM 2904 3HG2 VAL A 191 1.721 -13.284 30.120 1.00 1.71
H
ATOM 2905 N GLU A 192 2.019 -14.445 27.684 1.00 1.34
N
ATOM 2906 CA GLU A 192 3.034 -15.481 27.561 1.00 1.70
C
ATOM 2907 C GLU A 192 3.345 -15.670 26.100 1.00 1.60
C
ATOM 2908 O GLU A 192 4.511 -15.719 25.706 1.00 1.74
O
ATOM 2909 CB GLU A 192 2.581 -16.803 28.176 1.00 2.55
C

ATOM 2910 CG GLU A 192 3.642 -17.894 28.113 1.00 2.55
C
ATOM 2911 CD GLU A 192 3.232 -19.180 28.777 1.00 2.55
C
ATOM 2912 OE1 GLU A 192 2.171 -19.230 29.350 1.00 2.55
O
ATOM 2913 OE2 GLU A 192 3.987 -20.122 28.703 1.00 2.55
O
ATOM 2914 H GLU A 192 1.053 -14.723 27.814 1.00 1.61
H
ATOM 2915 HA GLU A 192 3.943 -15.151 28.066 1.00 2.04
H
ATOM 2916 1HB GLU A 192 2.310 -16.648 29.219 1.00 3.06
H
ATOM 2917 2HB GLU A 192 1.695 -17.166 27.656 1.00 3.06
H
ATOM 2918 1HG GLU A 192 3.870 -18.099 27.067 1.00 3.06
H
ATOM 2919 2HG GLU A 192 4.552 -17.525 28.586 1.00 3.06
H
ATOM 2920 N ALA A 193 2.282 -15.759 25.301 1.00 1.51
N
ATOM 2921 CA ALA A 193 2.402 -15.890 23.856 1.00 1.61
C
ATOM 2922 C ALA A 193 3.151 -14.699 23.291 1.00 1.26
C
ATOM 2923 O ALA A 193 4.010 -14.863 22.423 1.00 1.39
O
ATOM 2924 CB ALA A 193 1.031 -16.001 23.205 1.00 2.42
C
ATOM 2925 H ALA A 193 1.359 -15.728 25.723 1.00 1.81
H
ATOM 2926 HA ALA A 193 2.977 -16.791 23.639 1.00 1.93
H
ATOM 2927 1HB ALA A 193 1.147 -16.114 22.127 1.00 2.90
H
ATOM 2928 2HB ALA A 193 0.508 -16.870 23.606 1.00 2.90
H
ATOM 2929 3HB ALA A 193 0.452 -15.103 23.415 1.00 2.90
H
ATOM 2930 N LEU A 194 2.831 -13.506 23.801 1.00 0.98
N
ATOM 2931 CA LEU A 194 3.509 -12.293 23.384 1.00 0.86
C
ATOM 2932 C LEU A 194 4.983 -12.311 23.756 1.00 0.91
C
ATOM 2933 O LEU A 194 5.832 -11.994 22.926 1.00 1.09
O
ATOM 2934 CB LEU A 194 2.836 -11.082 24.015 1.00 1.29
C
ATOM 2935 CG LEU A 194 1.455 -10.710 23.471 1.00 1.29
C
ATOM 2936 CD1 LEU A 194 0.844 -9.648 24.366 1.00 1.29
C
ATOM 2937 CD2 LEU A 194 1.606 -10.189 22.047 1.00 1.29
C
ATOM 2938 H LEU A 194 2.090 -13.438 24.489 1.00 1.18
H

```
ATOM   2939  HA  LEU A 194       3.420 -12.209  22.305  1.00  1.03
H
ATOM   2940 1HB  LEU A 194       2.716 -11.286  25.076  1.00  1.55
H
ATOM   2941 2HB  LEU A 194       3.487 -10.215  23.901  1.00  1.55
H
ATOM   2942  HG  LEU A 194       0.804 -11.584  23.473  1.00  1.55
H
ATOM   2943 1HD1 LEU A 194      -0.136  -9.371  23.984  1.00  1.55
H
ATOM   2944 2HD1 LEU A 194       0.741 -10.036  25.378  1.00  1.55
H
ATOM   2945 3HD1 LEU A 194       1.489  -8.773  24.379  1.00  1.55
H
ATOM   2946 1HD2 LEU A 194       0.626  -9.918  21.654  1.00  1.55
H
ATOM   2947 2HD2 LEU A 194       2.253  -9.311  22.048  1.00  1.55
H
ATOM   2948 3HD2 LEU A 194       2.048 -10.965  21.424  1.00  1.55
H
ATOM   2949  N   CYS A 195       5.313 -12.733  24.974  1.00  1.00
N
ATOM   2950  CA  CYS A 195       6.717 -12.796  25.341  1.00  1.17
C
ATOM   2951  C   CYS A 195       7.471 -13.706  24.395  1.00  1.42
C
ATOM   2952  O   CYS A 195       8.538 -13.338  23.909  1.00  1.81
O
ATOM   2953  CB  CYS A 195       6.896 -13.296  26.774  1.00  1.75
C
ATOM   2954  SG  CYS A 195       6.394 -12.112  28.039  1.00  1.75
S
ATOM   2955  H   CYS A 195       4.605 -12.977  25.658  1.00  1.20
H
ATOM   2956  HA  CYS A 195       7.140 -11.794  25.270  1.00  1.40
H
ATOM   2957 1HB  CYS A 195       6.307 -14.202  26.912  1.00  2.11
H
ATOM   2958 2HB  CYS A 195       7.941 -13.555  26.946  1.00  2.11
H
ATOM   2959  HG  CYS A 195       6.287 -12.997  29.024  1.00  2.11
H
ATOM   2960  N   GLN A 196       6.907 -14.881  24.124  1.00  1.52
N
ATOM   2961  CA  GLN A 196       7.532 -15.860  23.252  1.00  1.87
C
ATOM   2962  C   GLN A 196       7.738 -15.353  21.824  1.00  1.70
C
ATOM   2963  O   GLN A 196       8.835 -15.489  21.270  1.00  2.26
O
ATOM   2964  CB  GLN A 196       6.680 -17.129  23.223  1.00  2.81
C
ATOM   2965  CG  GLN A 196       6.679 -17.913  24.524  1.00  2.81
C
ATOM   2966  CD  GLN A 196       5.712 -19.083  24.489  1.00  2.81
C
ATOM   2967  OE1 GLN A 196       5.002 -19.288  23.501  1.00  2.81
O
```

```
ATOM   2968  NE2 GLN A 196       5.676 -19.854  25.569  1.00  2.81
N
ATOM   2969  H   GLN A 196       6.023 -15.116  24.557  1.00  1.82
H
ATOM   2970  HA  GLN A 196       8.512 -16.105  23.663  1.00  2.24
H
ATOM   2971 1HB  GLN A 196       5.649 -16.868  22.988  1.00  3.37
H
ATOM   2972 2HB  GLN A 196       7.038 -17.791  22.433  1.00  3.37
H
ATOM   2973 1HG  GLN A 196       7.680 -18.306  24.697  1.00  3.37
H
ATOM   2974 2HG  GLN A 196       6.393 -17.253  25.341  1.00  3.37
H
ATOM   2975 1HE2 GLN A 196       5.054 -20.639  25.607  1.00  3.37
H
ATOM   2976 2HE2 GLN A 196       6.265 -19.651  26.352  1.00  3.37
H
ATOM   2977  N   VAL A 197       6.691 -14.759  21.231  1.00  1.31
N
ATOM   2978  CA  VAL A 197       6.787 -14.291  19.850  1.00  1.27
C
ATOM   2979  C   VAL A 197       7.786 -13.162  19.694  1.00  1.40
C
ATOM   2980  O   VAL A 197       8.392 -13.028  18.631  1.00  2.91
O
ATOM   2981  CB  VAL A 197       5.405 -13.831  19.302  1.00  1.91
C
ATOM   2982  CG1 VAL A 197       4.977 -12.507  19.895  1.00  1.91
C
ATOM   2983  CG2 VAL A 197       5.460 -13.702  17.793  1.00  1.91
C
ATOM   2984  H   VAL A 197       5.814 -14.662  21.726  1.00  1.57
H
ATOM   2985  HA  VAL A 197       7.121 -15.129  19.241  1.00  1.52
H
ATOM   2986  HB  VAL A 197       4.661 -14.575  19.581  1.00  2.29
H
ATOM   2987 1HG1 VAL A 197       3.997 -12.234  19.503  1.00  2.29
H
ATOM   2988 2HG1 VAL A 197       4.917 -12.616  20.963  1.00  2.29
H
ATOM   2989 3HG1 VAL A 197       5.696 -11.730  19.639  1.00  2.29
H
ATOM   2990 1HG2 VAL A 197       4.481 -13.398  17.422  1.00  2.29
H
ATOM   2991 2HG2 VAL A 197       6.202 -12.953  17.518  1.00  2.29
H
ATOM   2992 3HG2 VAL A 197       5.728 -14.661  17.358  1.00  2.29
H
ATOM   2993  N   PHE A 198       7.992 -12.374  20.747  1.00  0.89
N
ATOM   2994  CA  PHE A 198       8.981 -11.321  20.672  1.00  0.80
C
ATOM   2995  C   PHE A 198      10.388 -11.855  20.963  1.00  1.17
C
ATOM   2996  O   PHE A 198      11.352 -11.430  20.324  1.00  5.14
O
```

```
ATOM   2997  CB  PHE A 198       8.646 -10.200  21.634  1.00  1.20
C
ATOM   2998  CG  PHE A 198       7.428  -9.400  21.279  1.00  1.20
C
ATOM   2999  CD1 PHE A 198       6.425  -9.213  22.211  1.00  1.20
C
ATOM   3000  CD2 PHE A 198       7.270  -8.848  20.022  1.00  1.20
C
ATOM   3001  CE1 PHE A 198       5.284  -8.499  21.912  1.00  1.20
C
ATOM   3002  CE2 PHE A 198       6.132  -8.130  19.715  1.00  1.20
C
ATOM   3003  CZ  PHE A 198       5.136  -7.958  20.659  1.00  1.20
C
ATOM   3004  H   PHE A 198       7.444 -12.484  21.593  1.00  1.07
H
ATOM   3005  HA  PHE A 198       8.974 -10.915  19.660  1.00  0.96
H
ATOM   3006 1HB  PHE A 198       8.489 -10.625  22.623  1.00  1.44
H
ATOM   3007 2HB  PHE A 198       9.491  -9.526  21.693  1.00  1.44
H
ATOM   3008  HD1 PHE A 198       6.549  -9.651  23.198  1.00  1.44
H
ATOM   3009  HD2 PHE A 198       8.051  -8.987  19.274  1.00  1.44
H
ATOM   3010  HE1 PHE A 198       4.506  -8.369  22.662  1.00  1.44
H
ATOM   3011  HE2 PHE A 198       6.021  -7.704  18.721  1.00  1.44
H
ATOM   3012  HZ  PHE A 198       4.237  -7.395  20.415  1.00  1.44
H
ATOM   3013  N   TRP A 199      10.504 -12.810  21.902  1.00  2.02
N
ATOM   3014  CA  TRP A 199      11.800 -13.404  22.252  1.00  3.14
C
ATOM   3015  C   TRP A 199      12.485 -14.106  21.096  1.00  2.89
C
ATOM   3016  O   TRP A 199      13.711 -14.065  20.987  1.00  5.79
O
ATOM   3017  CB  TRP A 199      11.683 -14.462  23.372  1.00  4.71
C
ATOM   3018  CG  TRP A 199      11.664 -13.977  24.797  1.00  4.71
C
ATOM   3019  CD1 TRP A 199      10.711 -14.228  25.739  1.00  4.71
C
ATOM   3020  CD2 TRP A 199      12.665 -13.164  25.454  1.00  4.71
C
ATOM   3021  NE1 TRP A 199      11.048 -13.633  26.927  1.00  4.71
N
ATOM   3022  CE2 TRP A 199      12.240 -12.982  26.774  1.00  4.71
C
ATOM   3023  CE3 TRP A 199      13.870 -12.588  25.037  1.00  4.71
C
ATOM   3024  CZ2 TRP A 199      12.978 -12.252  27.689  1.00  4.71
C
ATOM   3025  CZ3 TRP A 199      14.613 -11.859  25.957  1.00  4.71
C
```

```
ATOM   3026  CH2 TRP A 199      14.177 -11.698  27.248  1.00  4.71
C
ATOM   3027  H   TRP A 199       9.687 -13.121  22.407  1.00  2.42
H
ATOM   3028  HA  TRP A 199      12.453 -12.604  22.600  1.00  3.77
H
ATOM   3029 1HB  TRP A 199      10.764 -15.027  23.214  1.00  5.65
H
ATOM   3030 2HB  TRP A 199      12.501 -15.168  23.273  1.00  5.65
H
ATOM   3031  HD1 TRP A 199       9.816 -14.822  25.572  1.00  5.65
H
ATOM   3032  HE1 TRP A 199      10.511 -13.674  27.780  1.00  5.65
H
ATOM   3033  HE3 TRP A 199      14.221 -12.716  24.014  1.00  5.65
H
ATOM   3034  HZ2 TRP A 199      12.649 -12.113  28.718  1.00  5.65
H
ATOM   3035  HZ3 TRP A 199      15.553 -11.417  25.628  1.00  5.65
H
ATOM   3036  HH2 TRP A 199      14.785 -11.124  27.942  1.00  5.65
H
ATOM   3037  N   GLN A 200      11.708 -14.748  20.220  1.00  3.22
N
ATOM   3038  CA  GLN A 200      12.304 -15.482  19.103  1.00  4.07
C
ATOM   3039  C   GLN A 200      13.024 -14.575  18.114  1.00  4.54
C
ATOM   3040  O   GLN A 200      13.810 -15.057  17.303  1.00 14.68
O
ATOM   3041  CB  GLN A 200      11.263 -16.280  18.346  1.00  6.11
C
ATOM   3042  CG  GLN A 200      10.368 -15.415  17.515  1.00  6.11
C
ATOM   3043  CD  GLN A 200       9.262 -16.183  16.861  1.00  6.11
C
ATOM   3044  OE1 GLN A 200       9.419 -17.312  16.384  1.00  6.11
O
ATOM   3045  NE2 GLN A 200       8.109 -15.548  16.839  1.00  6.11
N
ATOM   3046  H   GLN A 200      10.702 -14.771  20.359  1.00  3.86
H
ATOM   3047  HA  GLN A 200      13.037 -16.178  19.509  1.00  4.88
H
ATOM   3048 1HB  GLN A 200      11.753 -17.000  17.691  1.00  7.33
H
ATOM   3049 2HB  GLN A 200      10.644 -16.837  19.049  1.00  7.33
H
ATOM   3050 1HG  GLN A 200       9.933 -14.663  18.161  1.00  7.33
H
ATOM   3051 2HG  GLN A 200      10.960 -14.939  16.733  1.00  7.33
H
ATOM   3052 1HE2 GLN A 200       7.304 -15.975  16.426  1.00  7.33
H
ATOM   3053 2HE2 GLN A 200       8.051 -14.633  17.250  1.00  7.33
H
ATOM   3054  N   ALA A 201      12.780 -13.259  18.181  1.00  7.77
N
```

ATOM 3055 CA ALA A 201 13.442 -12.304 17.313 1.00 12.81
C
ATOM 3056 C ALA A 201 14.837 -12.010 17.809 1.00 30.15
C
ATOM 3057 O ALA A 201 15.567 -11.256 17.172 1.00 69.43
O
ATOM 3058 CB ALA A 201 12.633 -11.021 17.219 1.00 19.21
C
ATOM 3059 H ALA A 201 12.131 -12.884 18.863 1.00 9.32
H
ATOM 3060 HA ALA A 201 13.522 -12.747 16.320 1.00 15.37
H
ATOM 3061 1HB ALA A 201 13.132 -10.326 16.544 1.00 23.06
H
ATOM 3062 2HB ALA A 201 11.638 -11.247 16.835 1.00 23.06
H
ATOM 3063 3HB ALA A 201 12.546 -10.570 18.206 1.00 23.06
H
ATOM 3064 N SER A 202 15.220 -12.589 18.960 1.00 50.10
N
ATOM 3065 CA SER A 202 16.596 -12.515 19.387 1.00 75.07
C
ATOM 3066 C SER A 202 17.377 -13.275 18.340 1.00 57.22
C
ATOM 3067 O SER A 202 18.518 -12.948 18.019 1.00 81.96
O
ATOM 3068 CB SER A 202 16.793 -13.148 20.752 1.00112.60
C
ATOM 3069 OG SER A 202 16.562 -14.532 20.700 1.00112.60
O
ATOM 3070 H SER A 202 14.578 -13.134 19.516 1.00 60.12
H
ATOM 3071 HA SER A 202 16.927 -11.475 19.402 1.00 90.08
H
ATOM 3072 1HB SER A 202 17.810 -12.962 21.094 1.00135.13
H
ATOM 3073 2HB SER A 202 16.116 -12.691 21.472 1.00135.13
H
ATOM 3074 HG SER A 202 15.605 -14.637 20.659 1.00135.13
H
ATOM 3075 N GLN A 203 16.708 -14.255 17.716 1.00 79.06
N
ATOM 3076 CA GLN A 203 17.277 -14.988 16.631 1.00118.23
C
ATOM 3077 C GLN A 203 16.840 -14.255 15.384 1.00108.97
C
ATOM 3078 O GLN A 203 15.981 -14.727 14.636 1.00126.99
O
ATOM 3079 CB GLN A 203 16.790 -16.432 16.634 1.00177.34
C
ATOM 3080 CG GLN A 203 17.045 -17.137 17.954 1.00177.34
C
ATOM 3081 CD GLN A 203 18.509 -17.201 18.316 1.00177.34
C
ATOM 3082 OE1 GLN A 203 19.316 -17.851 17.646 1.00177.34
O
ATOM 3083 NE2 GLN A 203 18.861 -16.506 19.394 1.00177.34
N

```
ATOM   3084  H   GLN A 203      15.767 -14.503  17.985  1.00 94.87
H
ATOM   3085  HA  GLN A 203      18.365 -14.962  16.695  1.00141.88
H
ATOM   3086 1HB  GLN A 203      15.721 -16.463  16.430  1.00212.81
H
ATOM   3087 2HB  GLN A 203      17.293 -16.991  15.846  1.00212.81
H
ATOM   3088 1HG  GLN A 203      16.523 -16.599  18.744  1.00212.81
H
ATOM   3089 2HG  GLN A 203      16.667 -18.157  17.887  1.00212.81
H
ATOM   3090 1HE2 GLN A 203      19.816 -16.496  19.695  1.00212.81
H
ATOM   3091 2HE2 GLN A 203      18.169 -15.983  19.901  1.00212.81
H
ATOM   3092  N   VAL A 204      17.426 -13.066  15.197  1.00131.29
N
ATOM   3093  CA  VAL A 204      17.174 -12.178  14.067  1.00160.30
C
ATOM   3094  C   VAL A 204      17.403 -12.902  12.775  1.00 71.78
C
ATOM   3095  O   VAL A 204      18.481 -12.814  12.188  1.00136.33
O
ATOM   3096  CB  VAL A 204      18.054 -10.920  14.128  1.00240.45
C
ATOM   3097  CG1 VAL A 204      17.720 -10.084  15.345  1.00240.45
C
ATOM   3098  CG2 VAL A 204      19.508 -11.334  14.173  1.00240.45
C
ATOM   3099  H   VAL A 204      18.103 -12.782  15.896  1.00157.55
H
ATOM   3100  HA  VAL A 204      16.133 -11.859  14.106  1.00192.36
H
ATOM   3101  HB  VAL A 204      17.865 -10.311  13.246  1.00288.54
H
ATOM   3102 1HG1 VAL A 204      18.349  -9.194  15.353  1.00288.54
H
ATOM   3103 2HG1 VAL A 204      16.672  -9.785  15.305  1.00288.54
H
ATOM   3104 3HG1 VAL A 204      17.898 -10.665  16.249  1.00288.54
H
ATOM   3105 1HG2 VAL A 204      20.132 -10.442  14.203  1.00288.54
H
ATOM   3106 2HG2 VAL A 204      19.686 -11.935  15.066  1.00288.54
H
ATOM   3107 3HG2 VAL A 204      19.751 -11.919  13.287  1.00288.54
H
ATOM   3108  N   LYS A 205      16.395 -13.674  12.385  1.00 27.07
N
ATOM   3109  CA  LYS A 205      16.427 -14.515  11.228  1.00 39.13
C
ATOM   3110  C   LYS A 205      16.892 -13.818   9.976  1.00 29.28
C
ATOM   3111  O   LYS A 205      18.060 -13.928   9.588  1.00 90.14
O
ATOM   3112  CB  LYS A 205      15.030 -15.105  11.000  1.00 58.70
C
```

ATOM 3113 CG LYS A 205 14.892 -16.039 9.802 1.00 58.70
C
ATOM 3114 CD LYS A 205 13.459 -16.518 9.681 1.00 58.70
C
ATOM 3115 CE LYS A 205 13.226 -17.302 8.401 1.00 58.70
C
ATOM 3116 NZ LYS A 205 11.787 -17.644 8.231 1.00 58.70
N
ATOM 3117 H LYS A 205 15.570 -13.692 12.967 1.00 32.48
H
ATOM 3118 HA LYS A 205 17.121 -15.334 11.423 1.00 46.96
H
ATOM 3119 1HB LYS A 205 14.747 -15.678 11.882 1.00 70.43
H
ATOM 3120 2HB LYS A 205 14.296 -14.306 10.895 1.00 70.43
H
ATOM 3121 1HG LYS A 205 15.174 -15.537 8.878 1.00 70.43
H
ATOM 3122 2HG LYS A 205 15.544 -16.902 9.936 1.00 70.43
H
ATOM 3123 1HD LYS A 205 13.219 -17.158 10.534 1.00 70.43
H
ATOM 3124 2HD LYS A 205 12.790 -15.662 9.699 1.00 70.43
H
ATOM 3125 1HE LYS A 205 13.550 -16.705 7.551 1.00 70.43
H
ATOM 3126 2HE LYS A 205 13.809 -18.223 8.428 1.00 70.43
H
ATOM 3127 1HZ LYS A 205 11.659 -18.158 7.372 1.00 70.43
H
ATOM 3128 2HZ LYS A 205 11.478 -18.206 9.011 1.00 70.43
H
ATOM 3129 3HZ LYS A 205 11.245 -16.784 8.199 1.00 70.43
H
ATOM 3130 N HIS A 206 16.003 -13.157 9.291 1.00 23.25
N
ATOM 3131 CA HIS A 206 16.439 -12.546 8.068 1.00 25.60
C
ATOM 3132 C HIS A 206 15.571 -11.393 7.685 1.00 10.78
C
ATOM 3133 O HIS A 206 16.065 -10.339 7.310 1.00 47.16
O
ATOM 3134 CB HIS A 206 16.517 -13.583 6.947 1.00 38.40
C
ATOM 3135 CG HIS A 206 17.075 -13.040 5.675 1.00 38.40
C
ATOM 3136 ND1 HIS A 206 18.371 -12.582 5.568 1.00 38.40
N
ATOM 3137 CD2 HIS A 206 16.524 -12.901 4.452 1.00 38.40
C
ATOM 3138 CE1 HIS A 206 18.587 -12.170 4.330 1.00 38.40
C
ATOM 3139 NE2 HIS A 206 17.481 -12.352 3.633 1.00 38.40
N
ATOM 3140 H HIS A 206 15.053 -13.062 9.613 1.00 27.90
H
ATOM 3141 HA HIS A 206 17.445 -12.147 8.213 1.00 30.72
H

```
ATOM   3142 1HB  HIS A 206      17.144 -14.417   7.261  1.00 46.08
H
ATOM   3143 2HB  HIS A 206      15.523 -13.978   6.743  1.00 46.08
H
ATOM   3144  HD1 HIS A 206      19.079 -12.645   6.273  1.00 46.08
H
ATOM   3145  HD2 HIS A 206      15.536 -13.138   4.061  1.00 46.08
H
ATOM   3146  HE1 HIS A 206      19.558 -11.767   4.042  1.00 46.08
H
ATOM   3147  N   LYS A 207      14.271 -11.593   7.759  1.00  2.38
N
ATOM   3148  CA  LYS A 207      13.334 -10.547   7.401  1.00  7.66
C
ATOM   3149  C   LYS A 207      12.607 -10.025   8.628  1.00  5.02
C
ATOM   3150  O   LYS A 207      12.517 -10.734   9.632  1.00 27.10
O
ATOM   3151  CB  LYS A 207      12.336 -11.059   6.356  1.00 11.49
C
ATOM   3152  CG  LYS A 207      12.947 -11.498   5.036  1.00 11.49
C
ATOM   3153  CD  LYS A 207      13.580 -10.311   4.316  1.00 11.49
C
ATOM   3154  CE  LYS A 207      14.044 -10.684   2.916  1.00 11.49
C
ATOM   3155  NZ  LYS A 207      14.768  -9.569   2.253  1.00 11.49
N
ATOM   3156  H   LYS A 207      13.918 -12.486   8.065  1.00  2.86
H
ATOM   3157  HA  LYS A 207      13.904  -9.735   6.959  1.00  9.19
H
ATOM   3158 1HB  LYS A 207      11.798 -11.915   6.760  1.00 13.79
H
ATOM   3159 2HB  LYS A 207      11.596 -10.298   6.133  1.00 13.79
H
ATOM   3160 1HG  LYS A 207      13.709 -12.256   5.218  1.00 13.79
H
ATOM   3161 2HG  LYS A 207      12.173 -11.928   4.401  1.00 13.79
H
ATOM   3162 1HD  LYS A 207      12.850  -9.503   4.242  1.00 13.79
H
ATOM   3163 2HD  LYS A 207      14.436  -9.953   4.888  1.00 13.79
H
ATOM   3164 1HE  LYS A 207      14.702 -11.544   2.969  1.00 13.79
H
ATOM   3165 2HE  LYS A 207      13.178 -10.948   2.309  1.00 13.79
H
ATOM   3166 1HZ  LYS A 207      15.062  -9.854   1.330  1.00 13.79
H
ATOM   3167 2HZ  LYS A 207      14.159  -8.765   2.178  1.00 13.79
H
ATOM   3168 3HZ  LYS A 207      15.579  -9.316   2.801  1.00 13.79
H
ATOM   3169  N   PRO A 208      12.052  -8.807   8.555  1.00  1.18
N
ATOM   3170  CA  PRO A 208      11.294  -8.115   9.585  1.00  0.48
C
```

ATOM 3171 C PRO A 208 10.151 -9.002 10.070 1.00 0.47
C
ATOM 3172 O PRO A 208 9.601 -9.787 9.299 1.00 1.87
O
ATOM 3173 CB PRO A 208 10.779 -6.874 8.865 1.00 0.72
C
ATOM 3174 CG PRO A 208 11.764 -6.616 7.794 1.00 0.72
C
ATOM 3175 CD PRO A 208 12.212 -7.966 7.345 1.00 0.72
C
ATOM 3176 HA PRO A 208 11.964 -7.847 10.415 1.00 0.58
H
ATOM 3177 1HB PRO A 208 9.789 -7.080 8.455 1.00 0.86
H
ATOM 3178 2HB PRO A 208 10.674 -6.038 9.573 1.00 0.86
H
ATOM 3179 1HG PRO A 208 11.273 -6.074 6.978 1.00 0.86
H
ATOM 3180 2HG PRO A 208 12.579 -5.979 8.151 1.00 0.86
H
ATOM 3181 1HD PRO A 208 11.561 -8.316 6.533 1.00 0.86
H
ATOM 3182 2HD PRO A 208 13.264 -7.903 7.041 1.00 0.86
H
ATOM 3183 N THR A 209 9.814 -8.869 11.353 1.00 0.48
N
ATOM 3184 CA THR A 209 8.802 -9.705 12.001 1.00 0.71
C
ATOM 3185 C THR A 209 7.445 -9.023 12.081 1.00 0.31
C
ATOM 3186 O THR A 209 7.360 -7.840 12.401 1.00 0.30
O
ATOM 3187 CB THR A 209 9.256 -10.100 13.422 1.00 1.06
C
ATOM 3188 OG1 THR A 209 10.464 -10.871 13.346 1.00 1.06
O
ATOM 3189 CG2 THR A 209 8.186 -10.912 14.125 1.00 1.06
C
ATOM 3190 H THR A 209 10.288 -8.157 11.898 1.00 0.58
H
ATOM 3191 HA THR A 209 8.685 -10.616 11.415 1.00 0.85
H
ATOM 3192 HB THR A 209 9.450 -9.197 13.996 1.00 1.28
H
ATOM 3193 HG1 THR A 209 11.141 -10.362 12.894 1.00 1.28
H
ATOM 3194 1HG2 THR A 209 8.528 -11.177 15.126 1.00 1.28
H
ATOM 3195 2HG2 THR A 209 7.271 -10.328 14.201 1.00 1.28
H
ATOM 3196 3HG2 THR A 209 7.995 -11.817 13.555 1.00 1.28
H
ATOM 3197 N ALA A 210 6.387 -9.768 11.762 1.00 0.35
N
ATOM 3198 CA ALA A 210 5.024 -9.234 11.775 1.00 0.39
C
ATOM 3199 C ALA A 210 4.163 -9.922 12.837 1.00 0.47
C

ATOM   3200 O   ALA A 210   3.557 -10.968 12.597 1.00 0.86
O
ATOM   3201 CB ALA A 210   4.387 -9.388 10.401 1.00 0.58
C
ATOM   3202 H   ALA A 210   6.536 -10.738 11.516 1.00 0.42
H
ATOM   3203 HA ALA A 210   5.076 -8.175 12.024 1.00 0.47
H
ATOM   3204 1HB ALA A 210   3.385 -8.963 10.416 1.00 0.70
H
ATOM   3205 2HB ALA A 210   4.988 -8.863   9.661 1.00 0.70
H
ATOM   3206 3HB ALA A 210   4.327 -10.436 10.133 1.00 0.70
H
ATOM   3207 N   VAL A 211   4.104 -9.331 14.024 1.00 0.52
N
ATOM   3208 CA VAL A 211   3.341 -9.924 15.108 1.00 0.64
C
ATOM   3209 C   VAL A 211   1.878 -9.544 15.021 1.00 0.57
C
ATOM   3210 O   VAL A 211   1.455 -8.512 15.541 1.00 1.01
O
ATOM   3211 CB VAL A 211   3.914 -9.493 16.470 1.00 0.96
C
ATOM   3212 CG1 VAL A 211   3.105 -10.109 17.604 1.00 0.96
C
ATOM   3213 CG2 VAL A 211   5.366 -9.930 16.546 1.00 0.96
C
ATOM   3214 H   VAL A 211   4.582 -8.457 14.177 1.00 0.62
H
ATOM   3215 HA VAL A 211   3.420 -11.010 15.030 1.00 0.77
H
ATOM   3216 HB VAL A 211   3.848 -8.409 16.566 1.00 1.15
H
ATOM   3217 1HG1 VAL A 211   3.519 -9.792 18.561 1.00 1.15
H
ATOM   3218 2HG1 VAL A 211   2.068 -9.781 17.531 1.00 1.15
H
ATOM   3219 3HG1 VAL A 211   3.149 -11.195 17.533 1.00 1.15
H
ATOM   3220 1HG2 VAL A 211   5.792 -9.631 17.498 1.00 1.15
H
ATOM   3221 2HG2 VAL A 211   5.423 -11.013 16.449 1.00 1.15
H
ATOM   3222 3HG2 VAL A 211   5.927 -9.463 15.737 1.00 1.15
H
ATOM   3223 N   VAL A 212   1.108 -10.416 14.388 1.00 0.61
N
ATOM   3224 CA VAL A 212   -0.329 -10.230 14.256 1.00 0.49
C
ATOM   3225 C   VAL A 212   -0.988 -10.638 15.561 1.00 0.54
C
ATOM   3226 O   VAL A 212   -0.994 -11.817 15.910 1.00 1.26
O
ATOM   3227 CB VAL A 212   -0.868 -11.070 13.076 1.00 0.73
C
ATOM   3228 CG1 VAL A 212   -2.376 -10.937 12.961 1.00 0.73
C

```
ATOM   3229  CG2 VAL A 212      -0.198 -10.607  11.789  1.00  0.73
C
ATOM   3230  H   VAL A 212       1.555 -11.234  13.984  1.00  0.73
H
ATOM   3231  HA  VAL A 212      -0.536  -9.183  14.069  1.00  0.59
H
ATOM   3232  HB  VAL A 212      -0.640 -12.121  13.253  1.00  0.88
H
ATOM   3233 1HG1 VAL A 212      -2.733 -11.546  12.130  1.00  0.88
H
ATOM   3234 2HG1 VAL A 212      -2.841 -11.276  13.886  1.00  0.88
H
ATOM   3235 3HG1 VAL A 212      -2.635  -9.894  12.784  1.00  0.88
H
ATOM   3236 1HG2 VAL A 212      -0.560 -11.205  10.954  1.00  0.88
H
ATOM   3237 2HG2 VAL A 212      -0.432  -9.557  11.614  1.00  0.88
H
ATOM   3238 3HG2 VAL A 212       0.882 -10.727  11.880  1.00  0.88
H
ATOM   3239  N   ALA A 213      -1.531  -9.663  16.291  1.00  0.45
N
ATOM   3240  CA  ALA A 213      -2.097  -9.952  17.603  1.00  0.54
C
ATOM   3241  C   ALA A 213      -3.607  -9.772  17.641  1.00  0.45
C
ATOM   3242  O   ALA A 213      -4.126  -8.676  17.436  1.00  0.49
O
ATOM   3243  CB  ALA A 213      -1.441  -9.059  18.643  1.00  0.81
C
ATOM   3244  H   ALA A 213      -1.534  -8.707  15.956  1.00  0.54
H
ATOM   3245  HA  ALA A 213      -1.876 -10.988  17.842  1.00  0.65
H
ATOM   3246 1HB  ALA A 213      -1.833  -9.297  19.627  1.00  0.97
H
ATOM   3247 2HB  ALA A 213      -0.364  -9.221  18.631  1.00  0.97
H
ATOM   3248 3HB  ALA A 213      -1.653  -8.016  18.410  1.00  0.97
H
ATOM   3249  N   LYS A 214      -4.313 -10.861  17.937  1.00  0.51
N
ATOM   3250  CA  LYS A 214      -5.767 -10.824  18.006  1.00  0.52
C
ATOM   3251  C   LYS A 214      -6.210 -10.288  19.352  1.00  0.47
C
ATOM   3252  O   LYS A 214      -5.822 -10.805  20.394  1.00  0.60
O
ATOM   3253  CB  LYS A 214      -6.359 -12.211  17.762  1.00  0.78
C
ATOM   3254  CG  LYS A 214      -7.884 -12.244  17.720  1.00  0.78
C
ATOM   3255  CD  LYS A 214      -8.394 -13.647  17.421  1.00  0.78
C
ATOM   3256  CE  LYS A 214      -9.915 -13.706  17.420  1.00  0.78
C
ATOM   3257  NZ  LYS A 214     -10.411 -15.088  17.158  1.00  0.78
N
```

```
ATOM   3258  H   LYS A 214      -3.827 -11.730  18.115  1.00  0.61
H
ATOM   3259  HA  LYS A 214      -6.142 -10.153  17.241  1.00  0.62
H
ATOM   3260 1HB  LYS A 214      -5.988 -12.603  16.814  1.00  0.94
H
ATOM   3261 2HB  LYS A 214      -6.030 -12.891  18.548  1.00  0.94
H
ATOM   3262 1HG  LYS A 214      -8.284 -11.916  18.680  1.00  0.94
H
ATOM   3263 2HG  LYS A 214      -8.241 -11.565  16.947  1.00  0.94
H
ATOM   3264 1HD  LYS A 214      -8.028 -13.967  16.445  1.00  0.94
H
ATOM   3265 2HD  LYS A 214      -8.016 -14.338  18.175  1.00  0.94
H
ATOM   3266 1HE  LYS A 214     -10.288 -13.374  18.388  1.00  0.94
H
ATOM   3267 2HE  LYS A 214     -10.301 -13.041  16.648  1.00  0.94
H
ATOM   3268 1HZ  LYS A 214     -11.421 -15.091  17.165  1.00  0.94
H
ATOM   3269 2HZ  LYS A 214     -10.080 -15.398  16.255  1.00  0.94
H
ATOM   3270 3HZ  LYS A 214     -10.068 -15.711  17.875  1.00  0.94
H
ATOM   3271  N   THR A 215      -7.015  -9.241  19.336  1.00  0.59
N
ATOM   3272  CA  THR A 215      -7.470  -8.613  20.569  1.00  0.72
C
ATOM   3273  C   THR A 215      -8.827  -7.955  20.392  1.00  0.47
C
ATOM   3274  O   THR A 215      -9.505  -8.176  19.392  1.00  0.61
O
ATOM   3275  CB  THR A 215      -6.419  -7.589  21.045  1.00  1.08
C
ATOM   3276  OG1 THR A 215      -6.744  -7.096  22.354  1.00  1.08
O
ATOM   3277  CG2 THR A 215      -6.335  -6.449  20.074  1.00  1.08
C
ATOM   3278  H   THR A 215      -7.302  -8.842  18.450  1.00  0.71
H
ATOM   3279  HA  THR A 215      -7.569  -9.385  21.332  1.00  0.86
H
ATOM   3280  HB  THR A 215      -5.446  -8.076  21.094  1.00  1.30
H
ATOM   3281  HG1 THR A 215      -6.154  -6.352  22.560  1.00  1.30
H
ATOM   3282 1HG2 THR A 215      -5.577  -5.746  20.414  1.00  1.30
H
ATOM   3283 2HG2 THR A 215      -6.061  -6.829  19.091  1.00  1.30
H
ATOM   3284 3HG2 THR A 215      -7.297  -5.946  20.015  1.00  1.30
H
ATOM   3285  N   PHE A 216      -9.246  -7.178  21.381  1.00  0.50
N
ATOM   3286  CA  PHE A 216     -10.546  -6.529  21.292  1.00  0.72
C
```

```
ATOM   3287  C   PHE A 216     -10.526  -5.087  21.740  1.00  0.76
C
ATOM   3288  O   PHE A 216     -10.005  -4.766  22.808  1.00  1.75
O
ATOM   3289  CB  PHE A 216     -11.597  -7.289  22.110  1.00  1.08
C
ATOM   3290  CG  PHE A 216     -11.842  -8.684  21.621  1.00  1.08
C
ATOM   3291  CD1 PHE A 216     -11.154  -9.762  22.161  1.00  1.08
C
ATOM   3292  CD2 PHE A 216     -12.751  -8.919  20.601  1.00  1.08
C
ATOM   3293  CE1 PHE A 216     -11.369 -11.043  21.688  1.00  1.08
C
ATOM   3294  CE2 PHE A 216     -12.971 -10.198  20.131  1.00  1.08
C
ATOM   3295  CZ  PHE A 216     -12.278 -11.261  20.674  1.00  1.08
C
ATOM   3296  H   PHE A 216      -8.635  -7.034  22.171  1.00  0.60
H
ATOM   3297  HA  PHE A 216     -10.861  -6.546  20.248  1.00  0.86
H
ATOM   3298 1HB  PHE A 216     -11.279  -7.344  23.151  1.00  1.30
H
ATOM   3299 2HB  PHE A 216     -12.542  -6.748  22.085  1.00  1.30
H
ATOM   3300  HD1 PHE A 216     -10.434  -9.588  22.960  1.00  1.30
H
ATOM   3301  HD2 PHE A 216     -13.294  -8.079  20.166  1.00  1.30
H
ATOM   3302  HE1 PHE A 216     -10.822 -11.882  22.117  1.00  1.30
H
ATOM   3303  HE2 PHE A 216     -13.689 -10.367  19.328  1.00  1.30
H
ATOM   3304  HZ  PHE A 216     -12.450 -12.270  20.302  1.00  1.30
H
ATOM   3305  N   LYS A 217     -11.136  -4.228  20.931  1.00  0.80
N
ATOM   3306  CA  LYS A 217     -11.291  -2.827  21.273  1.00  0.71
C
ATOM   3307  C   LYS A 217     -11.977  -2.697  22.622  1.00  0.80
C
ATOM   3308  O   LYS A 217     -13.060  -3.237  22.818  1.00  1.42
O
ATOM   3309  CB  LYS A 217     -12.104  -2.127  20.189  1.00  1.06
C
ATOM   3310  CG  LYS A 217     -12.273  -0.636  20.386  1.00  1.06
C
ATOM   3311  CD  LYS A 217     -12.989  -0.010  19.205  1.00  1.06
C
ATOM   3312  CE  LYS A 217     -13.145   1.476  19.406  1.00  1.06
C
ATOM   3313  NZ  LYS A 217     -11.834   2.056  19.742  1.00  1.06
N
ATOM   3314  H   LYS A 217     -11.484  -4.545  20.029  1.00  0.96
H
ATOM   3315  HA  LYS A 217     -10.310  -2.363  21.340  1.00  0.85
H
```

```
ATOM   3316 1HB  LYS A 217     -11.627  -2.284  19.222  1.00  1.28
H
ATOM   3317 2HB  LYS A 217     -13.099  -2.571  20.138  1.00  1.28
H
ATOM   3318 1HG  LYS A 217     -12.854  -0.452  21.289  1.00  1.28
H
ATOM   3319 2HG  LYS A 217     -11.294  -0.169  20.500  1.00  1.28
H
ATOM   3320 1HD  LYS A 217     -12.416  -0.187  18.294  1.00  1.28
H
ATOM   3321 2HD  LYS A 217     -13.976  -0.458  19.092  1.00  1.28
H
ATOM   3322 1HE  LYS A 217     -13.506   1.924  18.481  1.00  1.28
H
ATOM   3323 2HE  LYS A 217     -13.853   1.689  20.205  1.00  1.28
H
ATOM   3324 1HZ  LYS A 217     -11.874   3.054  19.872  1.00  1.28
H
ATOM   3325 2HZ  LYS A 217     -11.518   1.656  20.612  1.00  1.28
H
ATOM   3326 3HZ  LYS A 217     -11.173   1.850  19.007  1.00  1.28
H
ATOM   3327  N   GLY A 218     -11.362  -1.985  23.562  1.00  0.66
N
ATOM   3328  CA  GLY A 218     -11.969  -1.848  24.882  1.00  0.93
C
ATOM   3329  C   GLY A 218     -11.743  -3.064  25.782  1.00  0.93
C
ATOM   3330  O   GLY A 218     -12.457  -3.231  26.772  1.00  1.16
O
ATOM   3331  H   GLY A 218     -10.482  -1.521  23.367  1.00  0.79
H
ATOM   3332 1HA  GLY A 218     -11.567  -0.962  25.371  1.00  1.12
H
ATOM   3333 2HA  GLY A 218     -13.038  -1.679  24.766  1.00  1.12
H
ATOM   3334  N   ARG A 219     -10.754  -3.895  25.436  1.00  0.81
N
ATOM   3335  CA  ARG A 219     -10.405  -5.101  26.186  1.00  0.84
C
ATOM   3336  C   ARG A 219     -10.349  -4.899  27.692  1.00  1.04
C
ATOM   3337  O   ARG A 219      -9.496  -4.171  28.199  1.00  1.51
O
ATOM   3338  CB  ARG A 219      -9.043  -5.619  25.752  1.00  1.26
C
ATOM   3339  CG  ARG A 219      -8.618  -6.914  26.415  1.00  1.26
C
ATOM   3340  CD  ARG A 219      -7.976  -6.649  27.729  1.00  1.26
C
ATOM   3341  NE  ARG A 219      -7.462  -7.851  28.350  1.00  1.26
N
ATOM   3342  CZ  ARG A 219      -8.206  -8.750  29.022  1.00  1.26
C
ATOM   3343  NH1 ARG A 219      -9.512  -8.603  29.109  1.00  1.26
N
ATOM   3344  NH2 ARG A 219      -7.618  -9.788  29.593  1.00  1.26
N
```

ATOM	3345	H	ARG	A	219	-10.230	-3.710	24.591	1.00	0.97	H
ATOM	3346	HA	ARG	A	219	-11.150	-5.861	25.971	1.00	1.01	H
ATOM	3347	1HB	ARG	A	219	-9.037	-5.796	24.682	1.00	1.51	H
ATOM	3348	2HB	ARG	A	219	-8.281	-4.871	25.967	1.00	1.51	H
ATOM	3349	1HG	ARG	A	219	-9.492	-7.547	26.577	1.00	1.51	H
ATOM	3350	2HG	ARG	A	219	-7.902	-7.434	25.778	1.00	1.51	H
ATOM	3351	1HD	ARG	A	219	-7.143	-5.962	27.584	1.00	1.51	H
ATOM	3352	2HD	ARG	A	219	-8.696	-6.200	28.409	1.00	1.51	H
ATOM	3353	HE	ARG	A	219	-6.457	-7.991	28.330	1.00	1.51	H
ATOM	3354	1HH1	ARG	A	219	-9.963	-7.811	28.668	1.00	1.51	H
ATOM	3355	2HH1	ARG	A	219	-10.066	-9.281	29.613	1.00	1.51	H
ATOM	3356	1HH2	ARG	A	219	-6.613	-9.895	29.521	1.00	1.51	H
ATOM	3357	2HH2	ARG	A	219	-8.167	-10.467	30.097	1.00	1.51	H
ATOM	3358	N	GLY	A	220	-11.240	-5.585	28.405	1.00	0.88	N
ATOM	3359	CA	GLY	A	220	-11.279	-5.542	29.862	1.00	0.90	C
ATOM	3360	C	GLY	A	220	-12.296	-4.561	30.431	1.00	0.92	C
ATOM	3361	O	GLY	A	220	-12.508	-4.531	31.645	1.00	1.02	O
ATOM	3362	H	GLY	A	220	-11.911	-6.168	27.913	1.00	1.06	H
ATOM	3363	1HA	GLY	A	220	-11.499	-6.541	30.239	1.00	1.08	H
ATOM	3364	2HA	GLY	A	220	-10.289	-5.284	30.235	1.00	1.08	H
ATOM	3365	N	THR	A	221	-12.930	-3.767	29.573	1.00	0.91	N
ATOM	3366	CA	THR	A	221	-13.917	-2.806	30.041	1.00	0.96	C
ATOM	3367	C	THR	A	221	-15.366	-3.313	29.839	1.00	1.07	C
ATOM	3368	O	THR	A	221	-15.807	-3.488	28.699	1.00	1.17	O
ATOM	3369	CB	THR	A	221	-13.749	-1.466	29.320	1.00	1.44	C
ATOM	3370	OG1	THR	A	221	-12.444	-0.930	29.577	1.00	1.44	O
ATOM	3371	CG2	THR	A	221	-14.782	-0.508	29.844	1.00	1.44	C
ATOM	3372	H	THR	A	221	-12.723	-3.809	28.585	1.00	1.09	H
ATOM	3373	HA	THR	A	221	-13.730	-2.623	31.093	1.00	1.15	H

```
ATOM   3374  HB  THR A 221     -13.875  -1.602  28.247  1.00  1.73
H
ATOM   3375  HG1 THR A 221     -11.780  -1.510  29.197  1.00  1.73
H
ATOM   3376 1HG2 THR A 221     -14.677   0.457  29.349  1.00  1.73
H
ATOM   3377 2HG2 THR A 221     -15.776  -0.910  29.661  1.00  1.73
H
ATOM   3378 3HG2 THR A 221     -14.632  -0.392  30.912  1.00  1.73
H
ATOM   3379  N   PRO A 222     -16.098  -3.583  30.935  1.00  1.45
N
ATOM   3380  CA  PRO A 222     -17.494  -4.028  31.016  1.00  1.59
C
ATOM   3381  C   PRO A 222     -18.407  -3.303  30.034  1.00  1.66
C
ATOM   3382  O   PRO A 222     -18.537  -2.077  30.090  1.00  1.65
O
ATOM   3383  CB  PRO A 222     -17.883  -3.681  32.456  1.00  2.39
C
ATOM   3384  CG  PRO A 222     -16.613  -3.777  33.210  1.00  2.39
C
ATOM   3385  CD  PRO A 222     -15.588  -3.218  32.275  1.00  2.39
C
ATOM   3386  HA  PRO A 222     -17.533  -5.115  30.849  1.00  1.91
H
ATOM   3387 1HB  PRO A 222     -18.327  -2.676  32.496  1.00  2.86
H
ATOM   3388 2HB  PRO A 222     -18.647  -4.385  32.818  1.00  2.86
H
ATOM   3389 1HG  PRO A 222     -16.683  -3.201  34.143  1.00  2.86
H
ATOM   3390 2HG  PRO A 222     -16.410  -4.821  33.490  1.00  2.86
H
ATOM   3391 1HD  PRO A 222     -15.514  -2.127  32.372  1.00  2.86
H
ATOM   3392 2HD  PRO A 222     -14.630  -3.717  32.470  1.00  2.86
H
ATOM   3393  N   SER A 223     -19.062  -4.074  29.159  1.00  1.80
N
ATOM   3394  CA  SER A 223     -20.023  -3.577  28.159  1.00  1.92
C
ATOM   3395  C   SER A 223     -19.420  -2.704  27.043  1.00  2.11
C
ATOM   3396  O   SER A 223     -20.160  -2.188  26.203  1.00  4.83
O
ATOM   3397  CB  SER A 223     -21.136  -2.791  28.839  1.00  2.88
C
ATOM   3398  OG  SER A 223     -21.808  -3.577  29.785  1.00  2.88
O
ATOM   3399  H   SER A 223     -18.887  -5.070  29.188  1.00  2.16
H
ATOM   3400  HA  SER A 223     -20.476  -4.447  27.682  1.00  2.30
H
ATOM   3401 1HB  SER A 223     -20.736  -1.899  29.314  1.00  3.46
H
ATOM   3402 2HB  SER A 223     -21.845  -2.457  28.082  1.00  3.46
H
```

ATOM 3403 HG SER A 223 -22.206 -4.300 29.294 1.00 3.46
H
ATOM 3404 N ILE A 224 -18.099 -2.525 27.035 1.00 1.29
N
ATOM 3405 CA ILE A 224 -17.432 -1.698 26.039 1.00 1.21
C
ATOM 3406 C ILE A 224 -16.607 -2.574 25.109 1.00 1.15
C
ATOM 3407 O ILE A 224 -16.569 -2.338 23.898 1.00 1.23
O
ATOM 3408 CB ILE A 224 -16.522 -0.655 26.700 1.00 1.81
C
ATOM 3409 CG1 ILE A 224 -17.321 0.225 27.669 1.00 1.81
C
ATOM 3410 CG2 ILE A 224 -15.860 0.201 25.640 1.00 1.81
C
ATOM 3411 CD1 ILE A 224 -18.440 0.985 27.033 1.00 1.81
C
ATOM 3412 H ILE A 224 -17.520 -2.969 27.733 1.00 1.55
H
ATOM 3413 HA ILE A 224 -18.185 -1.180 25.447 1.00 1.45
H
ATOM 3414 HB ILE A 224 -15.756 -1.167 27.275 1.00 2.18
H
ATOM 3415 1HG1 ILE A 224 -17.738 -0.406 28.451 1.00 2.18
H
ATOM 3416 2HG1 ILE A 224 -16.647 0.945 28.132 1.00 2.18
H
ATOM 3417 1HG2 ILE A 224 -15.206 0.927 26.121 1.00 2.18
H
ATOM 3418 2HG2 ILE A 224 -15.275 -0.430 24.973 1.00 2.18
H
ATOM 3419 3HG2 ILE A 224 -16.624 0.724 25.068 1.00 2.18
H
ATOM 3420 1HD1 ILE A 224 -18.953 1.582 27.789 1.00 2.18
H
ATOM 3421 2HD1 ILE A 224 -18.030 1.643 26.270 1.00 2.18
H
ATOM 3422 3HD1 ILE A 224 -19.146 0.292 26.578 1.00 2.18
H
ATOM 3423 N GLU A 225 -15.935 -3.569 25.697 1.00 1.09
N
ATOM 3424 CA GLU A 225 -15.119 -4.519 24.952 1.00 1.01
C
ATOM 3425 C GLU A 225 -15.871 -5.111 23.767 1.00 0.96
C
ATOM 3426 O GLU A 225 -16.952 -5.682 23.929 1.00 1.06
O
ATOM 3427 CB GLU A 225 -14.644 -5.657 25.861 1.00 1.52
C
ATOM 3428 CG GLU A 225 -13.749 -6.676 25.158 1.00 1.52
C
ATOM 3429 CD GLU A 225 -13.292 -7.801 26.055 1.00 1.52
C
ATOM 3430 OE1 GLU A 225 -12.725 -7.544 27.093 1.00 1.52
O
ATOM 3431 OE2 GLU A 225 -13.519 -8.933 25.693 1.00 1.52
O

```
ATOM   3432  H   GLU A 225     -15.981  -3.663  26.703  1.00  1.31
H
ATOM   3433  HA  GLU A 225     -14.248  -3.995  24.580  1.00  1.21
H
ATOM   3434 1HB  GLU A 225     -14.091  -5.245  26.704  1.00  1.82
H
ATOM   3435 2HB  GLU A 225     -15.507  -6.187  26.263  1.00  1.82
H
ATOM   3436 1HG  GLU A 225     -14.304  -7.104  24.324  1.00  1.82
H
ATOM   3437 2HG  GLU A 225     -12.883  -6.161  24.748  1.00  1.82
H
ATOM   3438  N   ASP A 226     -15.265  -4.996  22.583  1.00  0.85
N
ATOM   3439  CA  ASP A 226     -15.827  -5.490  21.328  1.00  0.86
C
ATOM   3440  C   ASP A 226     -17.141  -4.818  20.912  1.00  1.44
C
ATOM   3441  O   ASP A 226     -17.906  -5.390  20.135  1.00  4.62
O
ATOM   3442  CB  ASP A 226     -16.045  -7.010  21.422  1.00  1.29
C
ATOM   3443  CG  ASP A 226     -16.264  -7.692  20.078  1.00  1.29
C
ATOM   3444  OD1 ASP A 226     -15.848  -7.169  19.073  1.00  1.29
O
ATOM   3445  OD2 ASP A 226     -16.859  -8.743  20.066  1.00  1.29
O
ATOM   3446  H   ASP A 226     -14.380  -4.511  22.551  1.00  1.02
H
ATOM   3447  HA  ASP A 226     -15.096  -5.302  20.541  1.00  1.03
H
ATOM   3448 1HB  ASP A 226     -15.184  -7.468  21.909  1.00  1.55
H
ATOM   3449 2HB  ASP A 226     -16.915  -7.212  22.047  1.00  1.55
H
ATOM   3450  N   ALA A 227     -17.376  -3.588  21.371  1.00  1.21
N
ATOM   3451  CA  ALA A 227     -18.579  -2.858  20.986  1.00  1.31
C
ATOM   3452  C   ALA A 227     -18.223  -1.619  20.172  1.00  1.25
C
ATOM   3453  O   ALA A 227     -17.176  -1.002  20.382  1.00  1.23
O
ATOM   3454  CB  ALA A 227     -19.381  -2.467  22.219  1.00  1.97
C
ATOM   3455  H   ALA A 227     -16.736  -3.157  22.025  1.00  1.45
H
ATOM   3456  HA  ALA A 227     -19.188  -3.509  20.359  1.00  1.57
H
ATOM   3457 1HB  ALA A 227     -20.288  -1.945  21.914  1.00  2.36
H
ATOM   3458 2HB  ALA A 227     -19.648  -3.364  22.777  1.00  2.36
H
ATOM   3459 3HB  ALA A 227     -18.783  -1.814  22.851  1.00  2.36
H
ATOM   3460  N   GLU A 228     -19.101  -1.264  19.239  1.00  1.37
N
```

```
ATOM   3461  CA  GLU A 228     -18.906  -0.098  18.385  1.00  1.37
C
ATOM   3462  C   GLU A 228     -19.412   1.177  19.049  1.00  1.55
C
ATOM   3463  O   GLU A 228     -20.110   1.122  20.061  1.00  2.14
O
ATOM   3464  CB  GLU A 228     -19.613  -0.292  17.037  1.00  2.06
C
ATOM   3465  CG  GLU A 228     -19.041  -1.400  16.165  1.00  2.06
C
ATOM   3466  CD  GLU A 228     -19.757  -1.546  14.849  1.00  2.06
C
ATOM   3467  OE1 GLU A 228     -20.816  -0.986  14.709  1.00  2.06
O
ATOM   3468  OE2 GLU A 228     -19.240  -2.211  13.981  1.00  2.06
O
ATOM   3469  H   GLU A 228     -19.936  -1.820  19.120  1.00  1.64
H
ATOM   3470  HA  GLU A 228     -17.837   0.018  18.201  1.00  1.64
H
ATOM   3471 1HB  GLU A 228     -20.666  -0.518  17.211  1.00  2.47
H
ATOM   3472 2HB  GLU A 228     -19.570   0.634  16.464  1.00  2.47
H
ATOM   3473 1HG  GLU A 228     -17.994  -1.176  15.969  1.00  2.47
H
ATOM   3474 2HG  GLU A 228     -19.089  -2.342  16.710  1.00  2.47
H
ATOM   3475  N   SER A 229     -19.041   2.316  18.457  1.00  1.64
N
ATOM   3476  CA  SER A 229     -19.405   3.668  18.900  1.00  2.01
C
ATOM   3477  C   SER A 229     -18.704   4.087  20.189  1.00  2.16
C
ATOM   3478  O   SER A 229     -19.258   4.845  20.984  1.00  5.71
O
ATOM   3479  CB  SER A 229     -20.910   3.796  19.080  1.00  3.01
C
ATOM   3480  OG  SER A 229     -21.585   3.534  17.879  1.00  3.01
O
ATOM   3481  H   SER A 229     -18.465   2.241  17.630  1.00  1.97
H
ATOM   3482  HA  SER A 229     -19.103   4.367  18.119  1.00  2.41
H
ATOM   3483 1HB  SER A 229     -21.263   3.124  19.858  1.00  3.62
H
ATOM   3484 2HB  SER A 229     -21.142   4.808  19.407  1.00  3.62
H
ATOM   3485  HG  SER A 229     -22.519   3.641  18.073  1.00  3.62
H
ATOM   3486  N   TRP A 230     -17.477   3.612  20.377  1.00  1.62
N
ATOM   3487  CA  TRP A 230     -16.686   3.991  21.542  1.00  2.60
C
ATOM   3488  C   TRP A 230     -15.350   4.620  21.150  1.00  1.65
C
ATOM   3489  O   TRP A 230     -14.547   4.977  22.017  1.00  1.77
O
```

ATOM	3490	CB	TRP	A	230	-16.494	2.791	22.460	1.00	3.90
C										
ATOM	3491	CG	TRP	A	230	-17.808	2.366	23.030	1.00	3.90
C										
ATOM	3492	CD1	TRP	A	230	-18.500	1.226	22.765	1.00	3.90
C										
ATOM	3493	CD2	TRP	A	230	-18.624	3.125	23.949	1.00	3.90
C										
ATOM	3494	NE1	TRP	A	230	-19.688	1.222	23.453	1.00	3.90
N										
ATOM	3495	CE2	TRP	A	230	-19.782	2.379	24.179	1.00	3.90
C										
ATOM	3496	CE3	TRP	A	230	-18.471	4.364	24.578	1.00	3.90
C										
ATOM	3497	CZ2	TRP	A	230	-20.788	2.827	25.020	1.00	3.90
C										
ATOM	3498	CZ3	TRP	A	230	-19.476	4.815	25.423	1.00	3.90
C										
ATOM	3499	CH2	TRP	A	230	-20.605	4.066	25.637	1.00	3.90
C										
ATOM	3500	H	TRP	A	230	-17.083	2.976	19.701	1.00	1.94
H										
ATOM	3501	HA	TRP	A	230	-17.248	4.737	22.100	1.00	3.12
H										
ATOM	3502	1HB	TRP	A	230	-16.061	1.958	21.907	1.00	4.68
H										
ATOM	3503	2HB	TRP	A	230	-15.819	3.045	23.276	1.00	4.68
H										
ATOM	3504	HD1	TRP	A	230	-18.163	0.438	22.099	1.00	4.68
H										
ATOM	3505	HE1	TRP	A	230	-20.382	0.489	23.423	1.00	4.68
H										
ATOM	3506	HE3	TRP	A	230	-17.576	4.961	24.411	1.00	4.68
H										
ATOM	3507	HZ2	TRP	A	230	-21.692	2.247	25.203	1.00	4.68
H										
ATOM	3508	HZ3	TRP	A	230	-19.346	5.781	25.912	1.00	4.68
H										
ATOM	3509	HH2	TRP	A	230	-21.376	4.449	26.306	1.00	4.68
H										
ATOM	3510	N	HIS	A	231	-15.108	4.742	19.842	1.00	2.50
N										
ATOM	3511	CA	HIS	A	231	-13.867	5.324	19.365	1.00	3.81
C										
ATOM	3512	C	HIS	A	231	-13.818	6.786	19.759	1.00	2.91
C										
ATOM	3513	O	HIS	A	231	-14.772	7.534	19.538	1.00	3.84
O										
ATOM	3514	CB	HIS	A	231	-13.738	5.163	17.849	1.00	5.71
C										
ATOM	3515	CG	HIS	A	231	-12.376	5.468	17.292	1.00	5.71
C										
ATOM	3516	ND1	HIS	A	231	-11.885	6.749	17.169	1.00	5.71
N										
ATOM	3517	CD2	HIS	A	231	-11.432	4.651	16.774	1.00	5.71
C										
ATOM	3518	CE1	HIS	A	231	-10.673	6.697	16.639	1.00	5.71
C										

ATOM 3519 NE2 HIS A 231 -10.383 5.438 16.379 1.00 5.71
N
ATOM 3520 H HIS A 231 -15.791 4.437 19.166 1.00 3.00
H
ATOM 3521 HA HIS A 231 -13.028 4.824 19.834 1.00 4.57
H
ATOM 3522 1HB HIS A 231 -13.994 4.140 17.569 1.00 6.86
H
ATOM 3523 2HB HIS A 231 -14.455 5.820 17.355 1.00 6.86
H
ATOM 3524 HD1 HIS A 231 -12.499 7.468 16.842 1.00 6.86
H
ATOM 3525 HD2 HIS A 231 -11.385 3.570 16.638 1.00 6.86
H
ATOM 3526 HE1 HIS A 231 -10.106 7.611 16.466 1.00 6.86
H
ATOM 3527 N ALA A 232 -12.699 7.178 20.353 1.00 2.33
N
ATOM 3528 CA ALA A 232 -12.454 8.517 20.876 1.00 2.79
C
ATOM 3529 C ALA A 232 -13.440 8.912 21.987 1.00 2.30
C
ATOM 3530 O ALA A 232 -13.640 10.102 22.237 1.00 3.03
O
ATOM 3531 CB ALA A 232 -12.515 9.535 19.746 1.00 4.19
C
ATOM 3532 H ALA A 232 -11.969 6.495 20.449 1.00 2.80
H
ATOM 3533 HA ALA A 232 -11.451 8.529 21.302 1.00 3.35
H
ATOM 3534 1HB ALA A 232 -12.287 10.525 20.140 1.00 5.02
H
ATOM 3535 2HB ALA A 232 -11.783 9.270 18.983 1.00 5.02
H
ATOM 3536 3HB ALA A 232 -13.509 9.546 19.306 1.00 5.02
H
ATOM 3537 N LYS A 233 -14.063 7.929 22.648 1.00 1.67
N
ATOM 3538 CA LYS A 233 -14.996 8.227 23.727 1.00 1.81
C
ATOM 3539 C LYS A 233 -14.416 7.876 25.105 1.00 1.64
C
ATOM 3540 O LYS A 233 -14.151 6.704 25.368 1.00 2.61
O
ATOM 3541 CB LYS A 233 -16.301 7.453 23.536 1.00 2.71
C
ATOM 3542 CG LYS A 233 -17.005 7.706 22.214 1.00 2.71
C
ATOM 3543 CD LYS A 233 -17.473 9.144 22.102 1.00 2.71
C
ATOM 3544 CE LYS A 233 -18.261 9.365 20.820 1.00 2.71
C
ATOM 3545 NZ LYS A 233 -18.698 10.781 20.674 1.00 2.71
N
ATOM 3546 H LYS A 233 -13.907 6.959 22.406 1.00 2.00
H
ATOM 3547 HA LYS A 233 -15.225 9.286 23.681 1.00 2.17
H

```
ATOM   3548 1HB  LYS A 233     -16.099   6.385  23.606  1.00  3.26
H
ATOM   3549 2HB  LYS A 233     -16.994   7.709  24.337  1.00  3.26
H
ATOM   3550 1HG  LYS A 233     -16.324   7.491  21.394  1.00  3.26
H
ATOM   3551 2HG  LYS A 233     -17.867   7.045  22.130  1.00  3.26
H
ATOM   3552 1HD  LYS A 233     -18.105   9.391  22.955  1.00  3.26
H
ATOM   3553 2HD  LYS A 233     -16.609   9.809  22.105  1.00  3.26
H
ATOM   3554 1HE  LYS A 233     -17.640   9.097  19.966  1.00  3.26
H
ATOM   3555 2HE  LYS A 233     -19.142   8.723  20.827  1.00  3.26
H
ATOM   3556 1HZ  LYS A 233     -19.218  10.887  19.815  1.00  3.26
H
ATOM   3557 2HZ  LYS A 233     -19.285  11.034  21.456  1.00  3.26
H
ATOM   3558 3HZ  LYS A 233     -17.886  11.381  20.654  1.00  3.26
H
ATOM   3559  N   PRO A 234     -14.220   8.862  25.993  1.00  1.36
N
ATOM   3560  CA  PRO A 234     -13.768   8.740  27.371  1.00  1.31
C
ATOM   3561  C   PRO A 234     -14.908   8.199  28.223  1.00  1.04
C
ATOM   3562  O   PRO A 234     -16.078   8.393  27.889  1.00  1.24
O
ATOM   3563  CB  PRO A 234     -13.394  10.170  27.756  1.00  1.97
C
ATOM   3564  CG  PRO A 234     -14.294  11.018  26.924  1.00  1.97
C
ATOM   3565  CD  PRO A 234     -14.451  10.272  25.625  1.00  1.97
C
ATOM   3566  HA  PRO A 234     -12.895   8.080  27.415  1.00  1.57
H
ATOM   3567 1HB  PRO A 234     -13.543  10.321  28.835  1.00  2.36
H
ATOM   3568 2HB  PRO A 234     -12.329  10.348  27.551  1.00  2.36
H
ATOM   3569 1HG  PRO A 234     -15.253  11.167  27.442  1.00  2.36
H
ATOM   3570 2HG  PRO A 234     -13.858  12.013  26.780  1.00  2.36
H
ATOM   3571 1HD  PRO A 234     -15.480  10.413  25.266  1.00  2.36
H
ATOM   3572 2HD  PRO A 234     -13.705  10.601  24.886  1.00  2.36
H
ATOM   3573  N   MET A 235     -14.583   7.530  29.320  1.00  1.17
N
ATOM   3574  CA  MET A 235     -15.634   7.016  30.185  1.00  1.13
C
ATOM   3575  C   MET A 235     -16.127   8.125  31.121  1.00  1.51
C
ATOM   3576  O   MET A 235     -15.366   9.038  31.442  1.00  3.09
O
```

ATOM 3577 CB MET A 235 -15.123 5.826 30.999 1.00 1.69
C
ATOM 3578 CG MET A 235 -14.708 4.611 30.174 1.00 1.69
C
ATOM 3579 SD MET A 235 -16.095 3.831 29.313 1.00 1.69
S
ATOM 3580 CE MET A 235 -15.895 4.426 27.634 1.00 1.69
C
ATOM 3581 H MET A 235 -13.612 7.385 29.559 1.00 1.40
H
ATOM 3582 HA MET A 235 -16.451 6.680 29.556 1.00 1.36
H
ATOM 3583 1HB MET A 235 -14.260 6.134 31.587 1.00 2.03
H
ATOM 3584 2HB MET A 235 -15.897 5.505 31.696 1.00 2.03
H
ATOM 3585 1HG MET A 235 -13.970 4.915 29.431 1.00 2.03
H
ATOM 3586 2HG MET A 235 -14.246 3.871 30.826 1.00 2.03
H
ATOM 3587 1HE MET A 235 -16.684 4.013 27.006 1.00 2.03
H
ATOM 3588 2HE MET A 235 -15.954 5.514 27.623 1.00 2.03
H
ATOM 3589 3HE MET A 235 -14.924 4.111 27.249 1.00 2.03
H
ATOM 3590 N PRO A 236 -17.399 8.072 31.537 1.00 1.14
N
ATOM 3591 CA PRO A 236 -18.048 8.936 32.519 1.00 1.43
C
ATOM 3592 C PRO A 236 -17.360 8.762 33.857 1.00 1.68
C
ATOM 3593 O PRO A 236 -16.835 7.691 34.126 1.00 2.50
O
ATOM 3594 CB PRO A 236 -19.499 8.451 32.533 1.00 2.15
C
ATOM 3595 CG PRO A 236 -19.716 7.873 31.173 1.00 2.15
C
ATOM 3596 CD PRO A 236 -18.388 7.307 30.753 1.00 2.15
C
ATOM 3597 HA PRO A 236 -17.983 9.980 32.180 1.00 1.72
H
ATOM 3598 1HB PRO A 236 -19.646 7.719 33.339 1.00 2.57
H
ATOM 3599 2HB PRO A 236 -20.172 9.296 32.745 1.00 2.57
H
ATOM 3600 1HG PRO A 236 -20.504 7.107 31.213 1.00 2.57
H
ATOM 3601 2HG PRO A 236 -20.070 8.655 30.487 1.00 2.57
H
ATOM 3602 1HD PRO A 236 -18.319 6.236 30.989 1.00 2.57
H
ATOM 3603 2HD PRO A 236 -18.250 7.500 29.679 1.00 2.57
H
ATOM 3604 N ARG A 237 -17.365 9.796 34.690 1.00 2.24
N
ATOM 3605 CA ARG A 237 -16.722 9.741 36.008 1.00 3.01
C

```
ATOM   3606  C   ARG A 237     -17.105   8.501  36.821  1.00  2.46
C
ATOM   3607  O   ARG A 237     -16.237   7.720  37.217  1.00  2.86
O
ATOM   3608  CB  ARG A 237     -17.059  10.999  36.797  1.00  4.51
C
ATOM   3609  CG  ARG A 237     -16.468  11.070  38.196  1.00  4.51
C
ATOM   3610  CD  ARG A 237     -16.823  12.349  38.868  1.00  4.51
C
ATOM   3611  NE  ARG A 237     -18.255  12.480  39.061  1.00  4.51
N
ATOM   3612  CZ  ARG A 237     -18.905  13.631  39.307  1.00  4.51
C
ATOM   3613  NH1 ARG A 237     -18.251  14.769  39.401  1.00  4.51
N
ATOM   3614  NH2 ARG A 237     -20.216  13.591  39.449  1.00  4.51
N
ATOM   3615  H   ARG A 237     -17.820  10.652  34.405  1.00  2.69
H
ATOM   3616  HA  ARG A 237     -15.645   9.722  35.856  1.00  3.61
H
ATOM   3617 1HB  ARG A 237     -16.709  11.872  36.250  1.00  5.42
H
ATOM   3618 2HB  ARG A 237     -18.142  11.089  36.894  1.00  5.42
H
ATOM   3619 1HG  ARG A 237     -16.854  10.246  38.797  1.00  5.42
H
ATOM   3620 2HG  ARG A 237     -15.381  11.001  38.138  1.00  5.42
H
ATOM   3621 1HD  ARG A 237     -16.343  12.389  39.845  1.00  5.42
H
ATOM   3622 2HD  ARG A 237     -16.483  13.186  38.261  1.00  5.42
H
ATOM   3623  HE  ARG A 237     -18.827  11.645  39.003  1.00  5.42
H
ATOM   3624 1HH1 ARG A 237     -17.247  14.783  39.289  1.00  5.42
H
ATOM   3625 2HH1 ARG A 237     -18.753  15.625  39.585  1.00  5.42
H
ATOM   3626 1HH2 ARG A 237     -20.689  12.694  39.365  1.00  5.42
H
ATOM   3627 2HH2 ARG A 237     -20.738  14.434  39.633  1.00  5.42
H
ATOM   3628  N   GLU A 238     -18.401   8.335  37.071  1.00  2.05
N
ATOM   3629  CA  GLU A 238     -18.905   7.204  37.840  1.00  2.04
C
ATOM   3630  C   GLU A 238     -18.684   5.875  37.137  1.00  1.96
C
ATOM   3631  O   GLU A 238     -18.421   4.862  37.790  1.00  1.98
O
ATOM   3632  CB  GLU A 238     -20.390   7.395  38.135  1.00  3.06
C
ATOM   3633  CG  GLU A 238     -20.680   8.536  39.096  1.00  3.06
C
ATOM   3634  CD  GLU A 238     -20.637   9.885  38.443  1.00  3.06
C
```

```
ATOM   3635  OE1 GLU A 238     -20.587   9.953  37.239  1.00  3.06
O
ATOM   3636  OE2 GLU A 238     -20.619  10.859  39.161  1.00  3.06
O
ATOM   3637  H   GLU A 238     -19.060   9.015  36.719  1.00  2.46
H
ATOM   3638  HA  GLU A 238     -18.367   7.174  38.789  1.00  2.45
H
ATOM   3639 1HB  GLU A 238     -20.928   7.591  37.206  1.00  3.67
H
ATOM   3640 2HB  GLU A 238     -20.799   6.482  38.565  1.00  3.67
H
ATOM   3641 1HG  GLU A 238     -21.670   8.388  39.527  1.00  3.67
H
ATOM   3642 2HG  GLU A 238     -19.952   8.506  39.907  1.00  3.67
H
ATOM   3643  N   ARG A 239     -18.794   5.882  35.811  1.00  2.15
N
ATOM   3644  CA  ARG A 239     -18.600   4.672  35.036  1.00  2.27
C
ATOM   3645  C   ARG A 239     -17.160   4.215  35.141  1.00  1.99
C
ATOM   3646  O   ARG A 239     -16.900   3.034  35.366  1.00  2.16
O
ATOM   3647  CB  ARG A 239     -18.960   4.896  33.580  1.00  3.41
C
ATOM   3648  CG  ARG A 239     -18.843   3.658  32.718  1.00  3.41
C
ATOM   3649  CD  ARG A 239     -19.812   2.619  33.153  1.00  3.41
C
ATOM   3650  NE  ARG A 239     -19.701   1.384  32.388  1.00  3.41
N
ATOM   3651  CZ  ARG A 239     -20.444   0.283  32.626  1.00  3.41
C
ATOM   3652  NH1 ARG A 239     -21.341   0.303  33.587  1.00  3.41
N
ATOM   3653  NH2 ARG A 239     -20.286  -0.815  31.908  1.00  3.41
N
ATOM   3654  H   ARG A 239     -19.016   6.744  35.332  1.00  2.58
H
ATOM   3655  HA  ARG A 239     -19.246   3.891  35.440  1.00  2.72
H
ATOM   3656 1HB  ARG A 239     -19.983   5.262  33.506  1.00  4.09
H
ATOM   3657 2HB  ARG A 239     -18.306   5.658  33.160  1.00  4.09
H
ATOM   3658 1HG  ARG A 239     -19.044   3.911  31.677  1.00  4.09
H
ATOM   3659 2HG  ARG A 239     -17.841   3.259  32.811  1.00  4.09
H
ATOM   3660 1HD  ARG A 239     -19.637   2.383  34.202  1.00  4.09
H
ATOM   3661 2HD  ARG A 239     -20.826   3.001  33.035  1.00  4.09
H
ATOM   3662  HE  ARG A 239     -19.019   1.344  31.642  1.00  4.09
H
ATOM   3663 1HH1 ARG A 239     -21.472   1.137  34.142  1.00  4.09
H
```

ATOM   3664 2HH1 ARG A 239   -21.902 -0.518 33.768 1.00 4.09
H
ATOM   3665 1HH2 ARG A 239   -19.594 -0.863 31.168 1.00 4.09
H
ATOM   3666 2HH2 ARG A 239   -20.854 -1.627 32.101 1.00 4.09
H
ATOM   3667 N   ALA A 240   -16.238   5.163 35.008 1.00 1.73
N
ATOM   3668 CA ALA A 240   -14.815   4.918 35.106 1.00 1.54
C
ATOM   3669 C   ALA A 240   -14.463   4.362 36.468 1.00 1.61
C
ATOM   3670 O   ALA A 240   -13.695   3.409 36.561 1.00 1.52
O
ATOM   3671 CB ALA A 240   -14.045   6.201 34.849 1.00 2.31
C
ATOM   3672 H   ALA A 240   -16.536   6.104 34.818 1.00 2.08
H
ATOM   3673 HA ALA A 240   -14.540   4.176 34.356 1.00 1.85
H
ATOM   3674 1HB ALA A 240   -12.977   6.007 34.912 1.00 2.77
H
ATOM   3675 2HB ALA A 240   -14.289   6.580 33.857 1.00 2.77
H
ATOM   3676 3HB ALA A 240   -14.319   6.944 35.597 1.00 2.77
H
ATOM   3677 N   ASP A 241   -15.043   4.934 37.524 1.00 1.80
N
ATOM   3678 CA ASP A 241   -14.796   4.437 38.870 1.00 1.88
C
ATOM   3679 C   ASP A 241   -15.196   2.974 39.004 1.00 1.85
C
ATOM   3680 O   ASP A 241   -14.428   2.171 39.533 1.00 1.80
O
ATOM   3681 CB ASP A 241   -15.544   5.279 39.905 1.00 2.82
C
ATOM   3682 CG ASP A 241   -14.934   6.660 40.107 1.00 2.82
C
ATOM   3683 OD1 ASP A 241   -13.820   6.865 39.686 1.00 2.82
O
ATOM   3684 OD2 ASP A 241   -15.583   7.493 40.693 1.00 2.82
O
ATOM   3685 H   ASP A 241   -15.650   5.737 37.399 1.00 2.16
H
ATOM   3686 HA ASP A 241   -13.727   4.516 39.073 1.00 2.26
H
ATOM   3687 1HB ASP A 241   -16.582   5.400 39.594 1.00 3.38
H
ATOM   3688 2HB ASP A 241   -15.549   4.758 40.863 1.00 3.38
H
ATOM   3689 N   ALA A 242   -16.388   2.629 38.510 1.00 1.95
N
ATOM   3690 CA ALA A 242   -16.868   1.253 38.572 1.00 1.99
C
ATOM   3691 C   ALA A 242   -15.960   0.304 37.797 1.00 1.77
C
ATOM   3692 O   ALA A 242   -15.607 -0.767 38.297 1.00 1.81
O

ATOM 3693 CB ALA A 242 -18.285 1.174 38.026 1.00 2.98
C
ATOM 3694 H ALA A 242 -16.984 3.341 38.099 1.00 2.34
H
ATOM 3695 HA ALA A 242 -16.870 0.942 39.616 1.00 2.39
H
ATOM 3696 1HB ALA A 242 -18.649 0.150 38.103 1.00 3.58
H
ATOM 3697 2HB ALA A 242 -18.934 1.834 38.604 1.00 3.58
H
ATOM 3698 3HB ALA A 242 -18.292 1.485 36.983 1.00 3.58
H
ATOM 3699 N ILE A 243 -15.574 0.717 36.587 1.00 1.62
N
ATOM 3700 CA ILE A 243 -14.719 -0.075 35.712 1.00 1.45
C
ATOM 3701 C ILE A 243 -13.376 -0.346 36.344 1.00 1.45
C
ATOM 3702 O ILE A 243 -12.921 -1.487 36.377 1.00 1.51
O
ATOM 3703 CB ILE A 243 -14.481 0.655 34.385 1.00 2.17
C
ATOM 3704 CG1 ILE A 243 -15.762 0.743 33.567 1.00 2.17
C
ATOM 3705 CG2 ILE A 243 -13.415 -0.084 33.595 1.00 2.17
C
ATOM 3706 CD1 ILE A 243 -15.651 1.743 32.443 1.00 2.17
C
ATOM 3707 H ILE A 243 -15.907 1.609 36.244 1.00 1.94
H
ATOM 3708 HA ILE A 243 -15.208 -1.029 35.515 1.00 1.74
H
ATOM 3709 HB ILE A 243 -14.147 1.673 34.581 1.00 2.61
H
ATOM 3710 1HG1 ILE A 243 -15.970 -0.232 33.134 1.00 2.61
H
ATOM 3711 2HG1 ILE A 243 -16.598 1.013 34.207 1.00 2.61
H
ATOM 3712 1HG2 ILE A 243 -13.237 0.435 32.654 1.00 2.61
H
ATOM 3713 2HG2 ILE A 243 -12.494 -0.120 34.171 1.00 2.61
H
ATOM 3714 3HG2 ILE A 243 -13.752 -1.100 33.388 1.00 2.61
H
ATOM 3715 1HD1 ILE A 243 -16.579 1.755 31.871 1.00 2.61
H
ATOM 3716 2HD1 ILE A 243 -15.466 2.733 32.858 1.00 2.61
H
ATOM 3717 3HD1 ILE A 243 -14.827 1.474 31.788 1.00 2.61
H
ATOM 3718 N ILE A 244 -12.749 0.715 36.838 1.00 1.49
N
ATOM 3719 CA ILE A 244 -11.454 0.632 37.479 1.00 1.51
C
ATOM 3720 C ILE A 244 -11.468 -0.259 38.691 1.00 1.57
C
ATOM 3721 O ILE A 244 -10.552 -1.054 38.858 1.00 1.68
O

```
ATOM   3722  CB  ILE A 244     -10.945   2.021  37.842  1.00  2.27
C
ATOM   3723  CG1 ILE A 244     -10.596   2.772  36.553  1.00  2.27
C
ATOM   3724  CG2 ILE A 244      -9.760   1.922  38.787  1.00  2.27
C
ATOM   3725  CD1 ILE A 244     -10.361   4.242  36.762  1.00  2.27
C
ATOM   3726  H   ILE A 244     -13.184   1.624  36.764  1.00  1.79
H
ATOM   3727  HA  ILE A 244     -10.754   0.205  36.762  1.00  1.81
H
ATOM   3728  HB  ILE A 244     -11.742   2.581  38.330  1.00  2.72
H
ATOM   3729 1HG1 ILE A 244      -9.694   2.338  36.125  1.00  2.72
H
ATOM   3730 2HG1 ILE A 244     -11.408   2.650  35.835  1.00  2.72
H
ATOM   3731 1HG2 ILE A 244      -9.413   2.922  39.042  1.00  2.72
H
ATOM   3732 2HG2 ILE A 244     -10.063   1.402  39.695  1.00  2.72
H
ATOM   3733 3HG2 ILE A 244      -8.956   1.368  38.308  1.00  2.72
H
ATOM   3734 1HD1 ILE A 244     -10.111   4.712  35.812  1.00  2.72
H
ATOM   3735 2HD1 ILE A 244     -11.263   4.700  37.170  1.00  2.72
H
ATOM   3736 3HD1 ILE A 244      -9.539   4.376  37.461  1.00  2.72
H
ATOM   3737  N   LYS A 245     -12.498  -0.152  39.532  1.00  1.58
N
ATOM   3738  CA  LYS A 245     -12.590  -1.030  40.691  1.00  1.65
C
ATOM   3739  C   LYS A 245     -12.638  -2.493  40.258  1.00  1.66
C
ATOM   3740  O   LYS A 245     -11.985  -3.346  40.866  1.00  1.69
O
ATOM   3741  CB  LYS A 245     -13.821  -0.684  41.526  1.00  2.47
C
ATOM   3742  CG  LYS A 245     -13.719   0.633  42.284  1.00  2.47
C
ATOM   3743  CD  LYS A 245     -15.002   0.929  43.046  1.00  2.47
C
ATOM   3744  CE  LYS A 245     -14.918   2.256  43.786  1.00  2.47
C
ATOM   3745  NZ  LYS A 245     -16.176   2.559  44.524  1.00  2.47
N
ATOM   3746  H   LYS A 245     -13.221   0.541  39.376  1.00  1.90
H
ATOM   3747  HA  LYS A 245     -11.699  -0.889  41.306  1.00  1.98
H
ATOM   3748 1HB  LYS A 245     -14.696  -0.627  40.877  1.00  2.97
H
ATOM   3749 2HB  LYS A 245     -14.002  -1.475  42.253  1.00  2.97
H
ATOM   3750 1HG  LYS A 245     -12.896   0.571  42.998  1.00  2.97
H
```

```
ATOM   3751 2HG  LYS A 245     -13.509   1.447  41.596  1.00  2.97
H
ATOM   3752 1HD  LYS A 245     -15.838   0.965  42.347  1.00  2.97
H
ATOM   3753 2HD  LYS A 245     -15.188   0.133  43.767  1.00  2.97
H
ATOM   3754 1HE  LYS A 245     -14.092   2.218  44.496  1.00  2.97
H
ATOM   3755 2HE  LYS A 245     -14.728   3.054  43.069  1.00  2.97
H
ATOM   3756 1HZ  LYS A 245     -16.082   3.445  45.000  1.00  2.97
H
ATOM   3757 2HZ  LYS A 245     -16.944   2.609  43.869  1.00  2.97
H
ATOM   3758 3HZ  LYS A 245     -16.355   1.830  45.200  1.00  2.97
H
ATOM   3759  N   LEU A 246     -13.385  -2.773  39.187  1.00  1.72
N
ATOM   3760  CA  LEU A 246     -13.460  -4.125  38.659  1.00  1.86
C
ATOM   3761  C   LEU A 246     -12.113  -4.582  38.097  1.00  1.73
C
ATOM   3762  O   LEU A 246     -11.723  -5.735  38.291  1.00  1.91
O
ATOM   3763  CB  LEU A 246     -14.546  -4.198  37.582  1.00  2.79
C
ATOM   3764  CG  LEU A 246     -15.981  -4.045  38.104  1.00  2.79
C
ATOM   3765  CD1 LEU A 246     -16.942  -3.933  36.931  1.00  2.79
C
ATOM   3766  CD2 LEU A 246     -16.324  -5.243  38.975  1.00  2.79
C
ATOM   3767  H   LEU A 246     -13.928  -2.041  38.742  1.00  2.06
H
ATOM   3768  HA  LEU A 246     -13.735  -4.795  39.472  1.00  2.23
H
ATOM   3769 1HB  LEU A 246     -14.375  -3.413  36.850  1.00  3.35
H
ATOM   3770 2HB  LEU A 246     -14.476  -5.161  37.078  1.00  3.35
H
ATOM   3771  HG  LEU A 246     -16.062  -3.135  38.697  1.00  3.35
H
ATOM   3772 1HD1 LEU A 246     -17.960  -3.819  37.304  1.00  3.35
H
ATOM   3773 2HD1 LEU A 246     -16.677  -3.065  36.330  1.00  3.35
H
ATOM   3774 3HD1 LEU A 246     -16.877  -4.833  36.321  1.00  3.35
H
ATOM   3775 1HD2 LEU A 246     -17.341  -5.139  39.354  1.00  3.35
H
ATOM   3776 2HD2 LEU A 246     -16.249  -6.156  38.383  1.00  3.35
H
ATOM   3777 3HD2 LEU A 246     -15.628  -5.295  39.812  1.00  3.35
H
ATOM   3778  N   ILE A 247     -11.374  -3.674  37.455  1.00  1.48
N
ATOM   3779  CA  ILE A 247     -10.052  -4.018  36.943  1.00  1.32
C
```

ATOM   3780 C   ILE A 247   -9.103 -4.304 38.099 1.00 1.47
C
ATOM   3781 O   ILE A 247   -8.361 -5.285 38.070 1.00 1.66
O
ATOM   3782 CB ILE A 247   -9.443 -2.909 36.075 1.00 1.98
C
ATOM   3783 CG1 ILE A 247   -10.236 -2.717 34.785 1.00 1.98
C
ATOM   3784 CG2 ILE A 247   -8.019 -3.310 35.728 1.00 1.98
C
ATOM   3785 CD1 ILE A 247   -9.846 -1.464 34.034 1.00 1.98
C
ATOM   3786 H   ILE A 247   -11.743 -2.744 37.296 1.00 1.78
H
ATOM   3787 HA ILE A 247   -10.137 -4.920 36.339 1.00 1.58
H
ATOM   3788 HB ILE A 247   -9.442 -1.963 36.615 1.00 2.38
H
ATOM   3789 1HG1 ILE A 247   -10.075 -3.576 34.136 1.00 2.38
H
ATOM   3790 2HG1 ILE A 247   -11.298 -2.664 35.022 1.00 2.38
H
ATOM   3791 1HG2 ILE A 247   -7.562 -2.543 35.108 1.00 2.38
H
ATOM   3792 2HG2 ILE A 247   -7.441 -3.430 36.644 1.00 2.38
H
ATOM   3793 3HG2 ILE A 247   -8.031 -4.252 35.182 1.00 2.38
H
ATOM   3794 1HD1 ILE A 247   -10.443 -1.383 33.126 1.00 2.38
H
ATOM   3795 2HD1 ILE A 247   -10.022 -0.593 34.665 1.00 2.38
H
ATOM   3796 3HD1 ILE A 247   -8.791 -1.513 33.770 1.00 2.38
H
ATOM   3797 N   GLU A 248   -9.143 -3.448 39.123 1.00 1.60
N
ATOM   3798 CA GLU A 248   -8.302 -3.586 40.304 1.00 1.98
C
ATOM   3799 C   GLU A 248   -8.514 -4.916 41.005 1.00 2.17
C
ATOM   3800 O   GLU A 248   -7.555 -5.514 41.490 1.00 2.46
O
ATOM   3801 CB GLU A 248   -8.558 -2.446 41.296 1.00 2.97
C
ATOM   3802 CG GLU A 248   -8.025 -1.083 40.871 1.00 2.97
C
ATOM   3803 CD GLU A 248   -8.376   0.014 41.841 1.00 2.97
C
ATOM   3804 OE1 GLU A 248   -9.177 -0.226 42.713 1.00 2.97
O
ATOM   3805 OE2 GLU A 248   -7.836   1.089 41.717 1.00 2.97
O
ATOM   3806 H   GLU A 248   -9.771 -2.662 39.077 1.00 1.92
H
ATOM   3807 HA GLU A 248   -7.264 -3.538 39.988 1.00 2.38
H
ATOM   3808 1HB GLU A 248   -9.630 -2.343 41.458 1.00 3.56
H

ATOM   3809 2HB GLU A 248   -8.106 -2.694 42.256 1.00 3.56
H
ATOM   3810 1HG GLU A 248   -6.940 -1.142 40.790 1.00 3.56
H
ATOM   3811 2HG GLU A 248   -8.418 -0.833 39.889 1.00 3.56
H
ATOM   3812 N   SER A 249   -9.754 -5.421 41.010 1.00 2.18
N
ATOM   3813 CA SER A 249   -10.042 -6.711 41.638 1.00 2.44
C
ATOM   3814 C   SER A 249   -9.326 -7.881 40.955 1.00 2.91
C
ATOM   3815 O   SER A 249   -9.214 -8.961 41.537 1.00 4.84
O
ATOM   3816 CB SER A 249   -11.533 -7.010 41.622 1.00 3.66
C
ATOM   3817 OG SER A 249   -11.988 -7.329 40.331 1.00 3.66
O
ATOM   3818 H   SER A 249   -10.517 -4.879 40.619 1.00 2.62
H
ATOM   3819 HA SER A 249   -9.705 -6.667 42.675 1.00 2.93
H
ATOM   3820 1HB SER A 249   -11.743 -7.838 42.299 1.00 4.39
H
ATOM   3821 2HB SER A 249   -12.077 -6.143 41.994 1.00 4.39
H
ATOM   3822 HG SER A 249   -11.803 -6.568 39.777 1.00 4.39
H
ATOM   3823 N   GLN A 250   -8.858 -7.681 39.721 1.00 2.21
N
ATOM   3824 CA GLN A 250   -8.178 -8.723 38.978 1.00 2.78
C
ATOM   3825 C   GLN A 250   -6.656 -8.610 39.099 1.00 3.61
C
ATOM   3826 O   GLN A 250   -5.926 -9.450 38.569 1.00 4.66
O
ATOM   3827 CB GLN A 250   -8.606 -8.657 37.515 1.00 4.17
C
ATOM   3828 CG GLN A 250   -10.104 -8.813 37.338 1.00 4.17
C
ATOM   3829 CD GLN A 250   -10.617 -10.135 37.872 1.00 4.17
C
ATOM   3830 OE1 GLN A 250   -10.224 -11.206 37.398 1.00 4.17
O
ATOM   3831 NE2 GLN A 250   -11.486 -10.070 38.876 1.00 4.17
N
ATOM   3832 H   GLN A 250   -8.959 -6.784 39.269 1.00 2.65
H
ATOM   3833 HA GLN A 250   -8.478 -9.687 39.386 1.00 3.34
H
ATOM   3834 1HB GLN A 250   -8.308 -7.699 37.087 1.00 5.00
H
ATOM   3835 2HB GLN A 250   -8.109 -9.443 36.949 1.00 5.00
H
ATOM   3836 1HG GLN A 250   -10.609 -8.011 37.876 1.00 5.00
H
ATOM   3837 2HG GLN A 250   -10.343 -8.758 36.276 1.00 5.00
H

```
ATOM   3838 1HE2 GLN A 250     -11.855 -10.910  39.274  1.00  5.00
H
ATOM   3839 2HE2 GLN A 250     -11.765  -9.179  39.243  1.00  5.00
H
ATOM   3840  N   ILE A 251      -6.179  -7.572  39.795  1.00  4.15
N
ATOM   3841  CA  ILE A 251      -4.751  -7.371  39.998  1.00  6.44
C
ATOM   3842  C   ILE A 251      -4.295  -8.091  41.253  1.00  3.42
C
ATOM   3843  O   ILE A 251      -4.794  -7.826  42.347  1.00  3.11
O
ATOM   3844  CB  ILE A 251      -4.406  -5.867  40.087  1.00  9.66
C
ATOM   3845  CG1 ILE A 251      -4.750  -5.180  38.762  1.00  9.66
C
ATOM   3846  CG2 ILE A 251      -2.949  -5.659  40.449  1.00  9.66
C
ATOM   3847  CD1 ILE A 251      -4.617  -3.675  38.796  1.00  9.66
C
ATOM   3848  H   ILE A 251      -6.806  -6.906  40.222  1.00  4.98
H
ATOM   3849  HA  ILE A 251      -4.217  -7.793  39.149  1.00  7.73
H
ATOM   3850  HB  ILE A 251      -5.027  -5.409  40.855  1.00 11.59
H
ATOM   3851 1HG1 ILE A 251      -4.092  -5.568  37.987  1.00 11.59
H
ATOM   3852 2HG1 ILE A 251      -5.778  -5.426  38.496  1.00 11.59
H
ATOM   3853 1HG2 ILE A 251      -2.740  -4.591  40.522  1.00 11.59
H
ATOM   3854 2HG2 ILE A 251      -2.741  -6.134  41.407  1.00 11.59
H
ATOM   3855 3HG2 ILE A 251      -2.320  -6.098  39.683  1.00 11.59
H
ATOM   3856 1HD1 ILE A 251      -4.879  -3.265  37.821  1.00 11.59
H
ATOM   3857 2HD1 ILE A 251      -5.280  -3.263  39.554  1.00 11.59
H
ATOM   3858 3HD1 ILE A 251      -3.588  -3.409  39.035  1.00 11.59
H
ATOM   3859  N   GLN A 252      -3.349  -9.009  41.086  1.00  5.35
N
ATOM   3860  CA  GLN A 252      -2.861  -9.817  42.198  1.00  6.48
C
ATOM   3861  C   GLN A 252      -1.825  -9.119  43.077  1.00  3.40
C
ATOM   3862  O   GLN A 252      -1.828  -9.305  44.293  1.00 11.21
O
ATOM   3863  CB  GLN A 252      -2.279 -11.123  41.667  1.00  9.72
C
ATOM   3864  CG  GLN A 252      -3.312 -12.029  41.021  1.00  9.72
C
ATOM   3865  CD  GLN A 252      -4.396 -12.453  41.993  1.00  9.72
C
ATOM   3866  OE1 GLN A 252      -4.110 -12.900  43.107  1.00  9.72
O
```

ATOM 3867 NE2 GLN A 252 -5.650 -12.316 41.576 1.00 9.72
N
ATOM 3868 H GLN A 252 -2.977 -9.163 40.153 1.00 6.42
H
ATOM 3869 HA GLN A 252 -3.715 -10.059 42.832 1.00 7.78
H
ATOM 3870 1HB GLN A 252 -1.512 -10.904 40.923 1.00 11.66
H
ATOM 3871 2HB GLN A 252 -1.803 -11.672 42.479 1.00 11.66
H
ATOM 3872 1HG GLN A 252 -3.781 -11.497 40.194 1.00 11.66
H
ATOM 3873 2HG GLN A 252 -2.814 -12.926 40.654 1.00 11.66
H
ATOM 3874 1HE2 GLN A 252 -6.408 -12.577 42.174 1.00 11.66
H
ATOM 3875 2HE2 GLN A 252 -5.837 -11.947 40.665 1.00 11.66
H
ATOM 3876 N THR A 253 -0.961 -8.298 42.492 1.00 6.24
N
ATOM 3877 CA THR A 253 0.029 -7.615 43.321 1.00 7.13
C
ATOM 3878 C THR A 253 0.299 -6.193 42.901 1.00 8.14
C
ATOM 3879 O THR A 253 0.077 -5.802 41.757 1.00 18.88
O
ATOM 3880 CB THR A 253 1.382 -8.340 43.326 1.00 10.70
C
ATOM 3881 OG1 THR A 253 2.241 -7.712 44.289 1.00 10.70
O
ATOM 3882 CG2 THR A 253 2.034 -8.244 41.958 1.00 10.70
C
ATOM 3883 H THR A 253 -0.962 -8.180 41.485 1.00 7.49
H
ATOM 3884 HA THR A 253 -0.345 -7.590 44.344 1.00 8.56
H
ATOM 3885 HB THR A 253 1.243 -9.387 43.593 1.00 12.83
H
ATOM 3886 HG1 THR A 253 1.917 -7.901 45.174 1.00 12.83
H
ATOM 3887 1HG2 THR A 253 2.999 -8.750 41.983 1.00 12.83
H
ATOM 3888 2HG2 THR A 253 1.395 -8.717 41.215 1.00 12.83
H
ATOM 3889 3HG2 THR A 253 2.183 -7.196 41.696 1.00 12.83
H
ATOM 3890 N SER A 254 0.792 -5.420 43.863 1.00 4.32
N
ATOM 3891 CA SER A 254 1.162 -4.031 43.653 1.00 5.02
C
ATOM 3892 C SER A 254 2.623 -3.897 43.223 1.00 3.26
C
ATOM 3893 O SER A 254 3.073 -2.803 42.882 1.00 6.89
O
ATOM 3894 CB SER A 254 0.929 -3.231 44.920 1.00 7.53
C
ATOM 3895 OG SER A 254 1.801 -3.638 45.939 1.00 7.53
O

ATOM 3896 H SER A 254 0.928 -5.822 44.779 1.00 5.18
H
ATOM 3897 HA SER A 254 0.535 -3.623 42.860 1.00 6.02
H
ATOM 3898 1HB SER A 254 1.077 -2.171 44.712 1.00 9.04
H
ATOM 3899 2HB SER A 254 -0.101 -3.360 45.246 1.00 9.04
H
ATOM 3900 HG SER A 254 2.688 -3.502 45.598 1.00 9.04
H
ATOM 3901 N ARG A 255 3.361 -5.009 43.252 1.00 2.87
N
ATOM 3902 CA ARG A 255 4.760 -5.008 42.850 1.00 2.41
C
ATOM 3903 C ARG A 255 4.909 -4.664 41.381 1.00 2.20
C
ATOM 3904 O ARG A 255 4.275 -5.276 40.524 1.00 2.94
O
ATOM 3905 CB ARG A 255 5.388 -6.367 43.105 1.00 3.62
C
ATOM 3906 CG ARG A 255 6.874 -6.454 42.803 1.00 3.62
C
ATOM 3907 CD ARG A 255 7.421 -7.790 43.153 1.00 3.62
C
ATOM 3908 NE ARG A 255 8.843 -7.890 42.859 1.00 3.62
N
ATOM 3909 CZ ARG A 255 9.602 -8.974 43.114 1.00 3.62
C
ATOM 3910 NH1 ARG A 255 9.067 -10.040 43.667 1.00 3.62
N
ATOM 3911 NH2 ARG A 255 10.888 -8.966 42.808 1.00 3.62
N
ATOM 3912 H ARG A 255 2.950 -5.885 43.556 1.00 3.44
H
ATOM 3913 HA ARG A 255 5.290 -4.260 43.440 1.00 2.89
H
ATOM 3914 1HB ARG A 255 5.247 -6.642 44.150 1.00 4.34
H
ATOM 3915 2HB ARG A 255 4.885 -7.120 42.499 1.00 4.34
H
ATOM 3916 1HG ARG A 255 7.043 -6.279 41.740 1.00 4.34
H
ATOM 3917 2HG ARG A 255 7.410 -5.701 43.384 1.00 4.34
H
ATOM 3918 1HD ARG A 255 7.279 -7.974 44.217 1.00 4.34
H
ATOM 3919 2HD ARG A 255 6.901 -8.555 42.579 1.00 4.34
H
ATOM 3920 HE ARG A 255 9.292 -7.091 42.434 1.00 4.34
H
ATOM 3921 1HH1 ARG A 255 8.084 -10.046 43.903 1.00 4.34
H
ATOM 3922 2HH1 ARG A 255 9.637 -10.850 43.858 1.00 4.34
H
ATOM 3923 1HH2 ARG A 255 11.301 -8.146 42.382 1.00 4.34
H
ATOM 3924 2HH2 ARG A 255 11.457 -9.776 42.998 1.00 4.34
H

ATOM 3925 N ASN A 256 5.763 -3.693 41.096 1.00 2.04
N
ATOM 3926 CA ASN A 256 5.991 -3.261 39.727 1.00 2.13
C
ATOM 3927 C ASN A 256 7.226 -3.912 39.128 1.00 2.43
C
ATOM 3928 O ASN A 256 8.165 -4.265 39.842 1.00 4.85
O
ATOM 3929 CB ASN A 256 6.105 -1.751 39.669 1.00 3.19
C
ATOM 3930 CG ASN A 256 4.819 -1.056 40.026 1.00 3.19
C
ATOM 3931 OD1 ASN A 256 3.734 -1.426 39.561 1.00 3.19
O
ATOM 3932 ND2 ASN A 256 4.922 -0.047 40.853 1.00 3.19
N
ATOM 3933 H ASN A 256 6.260 -3.233 41.845 1.00 2.45
H
ATOM 3934 HA ASN A 256 5.139 -3.569 39.123 1.00 2.56
H
ATOM 3935 1HB ASN A 256 6.885 -1.419 40.354 1.00 3.83
H
ATOM 3936 2HB ASN A 256 6.402 -1.447 38.664 1.00 3.83
H
ATOM 3937 1HD2 ASN A 256 4.105 0.458 41.133 1.00 3.83
H
ATOM 3938 2HD2 ASN A 256 5.818 0.221 41.206 1.00 3.83
H
ATOM 3939 N LEU A 257 7.215 -4.064 37.811 1.00 1.67
N
ATOM 3940 CA LEU A 257 8.340 -4.620 37.078 1.00 1.66
C
ATOM 3941 C LEU A 257 9.390 -3.550 36.813 1.00 1.67
C
ATOM 3942 O LEU A 257 9.073 -2.363 36.735 1.00 1.81
O
ATOM 3943 CB LEU A 257 7.845 -5.255 35.781 1.00 2.49
C
ATOM 3944 CG LEU A 257 6.918 -6.451 35.977 1.00 2.49
C
ATOM 3945 CD1 LEU A 257 6.384 -6.938 34.641 1.00 2.49
C
ATOM 3946 CD2 LEU A 257 7.702 -7.554 36.665 1.00 2.49
C
ATOM 3947 H LEU A 257 6.398 -3.766 37.294 1.00 2.00
H
ATOM 3948 HA LEU A 257 8.797 -5.399 37.687 1.00 1.99
H
ATOM 3949 1HB LEU A 257 7.277 -4.507 35.241 1.00 2.99
H
ATOM 3950 2HB LEU A 257 8.695 -5.567 35.176 1.00 2.99
H
ATOM 3951 HG LEU A 257 6.073 -6.153 36.593 1.00 2.99
H
ATOM 3952 1HD1 LEU A 257 5.725 -7.791 34.803 1.00 2.99
H
ATOM 3953 2HD1 LEU A 257 5.830 -6.141 34.151 1.00 2.99
H

```
ATOM   3954 3HD1 LEU A 257       7.218  -7.238  34.012  1.00  2.99
H
ATOM   3955 1HD2 LEU A 257       7.059  -8.419  36.822  1.00  2.99
H
ATOM   3956 2HD2 LEU A 257       8.546  -7.841  36.039  1.00  2.99
H
ATOM   3957 3HD2 LEU A 257       8.069  -7.193  37.626  1.00  2.99
H
ATOM   3958  N   ASP A 258      10.644  -3.979  36.711  1.00  1.71
N
ATOM   3959  CA  ASP A 258      11.783  -3.083  36.532  1.00  1.89
C
ATOM   3960  C   ASP A 258      12.486  -3.304  35.177  1.00  1.79
C
ATOM   3961  O   ASP A 258      13.258  -4.255  35.044  1.00  2.42
O
ATOM   3962  CB  ASP A 258      12.767  -3.312  37.691  1.00  2.83
C
ATOM   3963  CG  ASP A 258      13.972  -2.385  37.709  1.00  2.83
C
ATOM   3964  OD1 ASP A 258      14.122  -1.610  36.799  1.00  2.83
O
ATOM   3965  OD2 ASP A 258      14.734  -2.462  38.642  1.00  2.83
O
ATOM   3966  H   ASP A 258      10.819  -4.971  36.775  1.00  2.05
H
ATOM   3967  HA  ASP A 258      11.428  -2.056  36.586  1.00  2.27
H
ATOM   3968 1HB  ASP A 258      12.236  -3.197  38.636  1.00  3.40
H
ATOM   3969 2HB  ASP A 258      13.129  -4.340  37.651  1.00  3.40
H
ATOM   3970  N   PRO A 259      12.205  -2.468  34.161  1.00  1.56
N
ATOM   3971  CA  PRO A 259      12.777  -2.480  32.815  1.00  1.53
C
ATOM   3972  C   PRO A 259      14.298  -2.430  32.848  1.00  1.65
C
ATOM   3973  O   PRO A 259      14.897  -1.586  33.518  1.00  2.06
O
ATOM   3974  CB  PRO A 259      12.188  -1.230  32.173  1.00  2.29
C
ATOM   3975  CG  PRO A 259      10.876  -1.056  32.850  1.00  2.29
C
ATOM   3976  CD  PRO A 259      11.111  -1.486  34.271  1.00  2.29
C
ATOM   3977  HA  PRO A 259      12.436  -3.385  32.290  1.00  1.84
H
ATOM   3978 1HB  PRO A 259      12.865  -0.376  32.317  1.00  2.75
H
ATOM   3979 2HB  PRO A 259      12.090  -1.383  31.089  1.00  2.75
H
ATOM   3980 1HG  PRO A 259      10.549  -0.009  32.776  1.00  2.75
H
ATOM   3981 2HG  PRO A 259      10.115  -1.660  32.340  1.00  2.75
H
ATOM   3982 1HD  PRO A 259      11.415  -0.636  34.896  1.00  2.75
H
```

```
ATOM   3983 2HD  PRO A 259      10.207  -1.971  34.657  1.00  2.75
H
ATOM   3984  N   GLN A 260      14.921  -3.314  32.085  1.00  1.50
N
ATOM   3985  CA  GLN A 260      16.368  -3.384  32.061  1.00  1.66
C
ATOM   3986  C   GLN A 260      16.945  -2.519  30.943  1.00  1.41
C
ATOM   3987  O   GLN A 260      16.259  -2.272  29.951  1.00  1.18
O
ATOM   3988  CB  GLN A 260      16.801  -4.843  31.918  1.00  2.49
C
ATOM   3989  CG  GLN A 260      16.331  -5.704  33.074  1.00  2.49
C
ATOM   3990  CD  GLN A 260      16.942  -5.266  34.391  1.00  2.49
C
ATOM   3991  OE1 GLN A 260      18.166  -5.288  34.555  1.00  2.49
O
ATOM   3992  NE2 GLN A 260      16.100  -4.856  35.333  1.00  2.49
N
ATOM   3993  H   GLN A 260      14.379  -3.966  31.536  1.00  1.80
H
ATOM   3994  HA  GLN A 260      16.729  -3.014  33.017  1.00  1.99
H
ATOM   3995 1HB  GLN A 260      16.382  -5.260  31.005  1.00  2.99
H
ATOM   3996 2HB  GLN A 260      17.886  -4.915  31.850  1.00  2.99
H
ATOM   3997 1HG  GLN A 260      15.246  -5.629  33.156  1.00  2.99
H
ATOM   3998 2HG  GLN A 260      16.622  -6.738  32.887  1.00  2.99
H
ATOM   3999 1HE2 GLN A 260      16.451  -4.550  36.221  1.00  2.99
H
ATOM   4000 2HE2 GLN A 260      15.113  -4.839  35.161  1.00  2.99
H
ATOM   4001  N   PRO A 261      18.182  -2.030  31.126  1.00  1.65
N
ATOM   4002  CA  PRO A 261      18.984  -1.199  30.235  1.00  1.55
C
ATOM   4003  C   PRO A 261      19.569  -2.007  29.077  1.00  1.72
C
ATOM   4004  O   PRO A 261      19.736  -3.222  29.185  1.00  2.67
O
ATOM   4005  CB  PRO A 261      20.080  -0.648  31.154  1.00  2.33
C
ATOM   4006  CG  PRO A 261      20.232  -1.676  32.212  1.00  2.33
C
ATOM   4007  CD  PRO A 261      18.835  -2.174  32.445  1.00  2.33
C
ATOM   4008  HA  PRO A 261      18.344  -0.387  29.873  1.00  1.86
H
ATOM   4009 1HB  PRO A 261      21.007  -0.491  30.583  1.00  2.79
H
ATOM   4010 2HB  PRO A 261      19.779   0.332  31.553  1.00  2.79
H
ATOM   4011 1HG  PRO A 261      20.913  -2.471  31.875  1.00  2.79
H
```

```
ATOM   4012 2HG  PRO A 261      20.681  -1.234  33.114  1.00  2.79
H
ATOM   4013 1HD  PRO A 261      18.869  -3.227  32.756  1.00  2.79
H
ATOM   4014 2HD  PRO A 261      18.322  -1.546  33.190  1.00  2.79
H
ATOM   4015  N   PRO A 262      19.872  -1.338  27.962  1.00  1.51
N
ATOM   4016  CA  PRO A 262      20.421  -1.830  26.708  1.00  1.56
C
ATOM   4017  C   PRO A 262      21.935  -1.969  26.729  1.00  1.37
C
ATOM   4018  O   PRO A 262      22.600  -1.559  27.682  1.00  1.77
O
ATOM   4019  CB  PRO A 262      20.000  -0.749  25.727  1.00  2.34
C
ATOM   4020  CG  PRO A 262      20.091   0.505  26.527  1.00  2.34
C
ATOM   4021  CD  PRO A 262      19.652   0.118  27.907  1.00  2.34
C
ATOM   4022  HA  PRO A 262      19.953  -2.792  26.457  1.00  1.87
H
ATOM   4023 1HB  PRO A 262      20.671  -0.751  24.856  1.00  2.81
H
ATOM   4024 2HB  PRO A 262      18.986  -0.949  25.353  1.00  2.81
H
ATOM   4025 1HG  PRO A 262      21.121   0.893  26.505  1.00  2.81
H
ATOM   4026 2HG  PRO A 262      19.453   1.284  26.086  1.00  2.81
H
ATOM   4027 1HD  PRO A 262      20.269   0.628  28.659  1.00  2.81
H
ATOM   4028 2HD  PRO A 262      18.595   0.340  28.031  1.00  2.81
H
ATOM   4029  N   ILE A 263      22.472  -2.525  25.647  1.00  1.70
N
ATOM   4030  CA  ILE A 263      23.909  -2.662  25.473  1.00  1.87
C
ATOM   4031  C   ILE A 263      24.462  -1.388  24.854  1.00  1.44
C
ATOM   4032  O   ILE A 263      24.056  -0.991  23.761  1.00  2.46
O
ATOM   4033  CB  ILE A 263      24.252  -3.865  24.579  1.00  2.81
C
ATOM   4034  CG1 ILE A 263      23.753  -5.159  25.229  1.00  2.81
C
ATOM   4035  CG2 ILE A 263      25.752  -3.921  24.330  1.00  2.81
C
ATOM   4036  CD1 ILE A 263      23.820  -6.361  24.314  1.00  2.81
C
ATOM   4037  H   ILE A 263      21.861  -2.851  24.911  1.00  2.04
H
ATOM   4038  HA  ILE A 263      24.371  -2.804  26.449  1.00  2.24
H
ATOM   4039  HB  ILE A 263      23.736  -3.763  23.624  1.00  3.37
H
ATOM   4040 1HG1 ILE A 263      24.355  -5.367  26.114  1.00  3.37
H
```

ATOM 4041 2HG1 ILE A 263 22.718 -5.024 25.543 1.00 3.37
H
ATOM 4042 1HG2 ILE A 263 25.982 -4.769 23.685 1.00 3.37
H
ATOM 4043 2HG2 ILE A 263 26.075 -3.000 23.846 1.00 3.37
H
ATOM 4044 3HG2 ILE A 263 26.275 -4.035 25.279 1.00 3.37
H
ATOM 4045 1HD1 ILE A 263 23.451 -7.242 24.840 1.00 3.37
H
ATOM 4046 2HD1 ILE A 263 23.203 -6.181 23.432 1.00 3.37
H
ATOM 4047 3HD1 ILE A 263 24.851 -6.529 24.007 1.00 3.37
H
ATOM 4048 N GLU A 264 25.381 -0.746 25.565 1.00 1.51
N
ATOM 4049 CA GLU A 264 25.960 0.524 25.137 1.00 1.91
C
ATOM 4050 C GLU A 264 26.900 0.415 23.938 1.00 2.04
C
ATOM 4051 O GLU A 264 28.120 0.474 24.101 1.00 2.98
O
ATOM 4052 CB GLU A 264 26.708 1.164 26.306 1.00 2.86
C
ATOM 4053 CG GLU A 264 25.818 1.560 27.476 1.00 2.86
C
ATOM 4054 CD GLU A 264 26.585 2.177 28.613 1.00 2.86
C
ATOM 4055 OE1 GLU A 264 27.790 2.218 28.540 1.00 2.86
O
ATOM 4056 OE2 GLU A 264 25.966 2.606 29.559 1.00 2.86
O
ATOM 4057 H GLU A 264 25.677 -1.141 26.446 1.00 1.81
H
ATOM 4058 HA GLU A 264 25.143 1.176 24.860 1.00 2.29
H
ATOM 4059 1HB GLU A 264 27.465 0.474 26.677 1.00 3.44
H
ATOM 4060 2HB GLU A 264 27.222 2.061 25.960 1.00 3.44
H
ATOM 4061 1HG GLU A 264 25.074 2.274 27.124 1.00 3.44
H
ATOM 4062 2HG GLU A 264 25.292 0.676 27.836 1.00 3.44
H
ATOM 4063 N ASP A 265 26.344 0.278 22.741 1.00 1.83
N
ATOM 4064 CA ASP A 265 27.190 0.159 21.559 1.00 2.98
C
ATOM 4065 C ASP A 265 26.872 1.136 20.424 1.00 1.44
C
ATOM 4066 O ASP A 265 27.408 0.980 19.326 1.00 2.42
O
ATOM 4067 CB ASP A 265 27.122 -1.260 21.028 1.00 4.47
C
ATOM 4068 CG ASP A 265 25.730 -1.614 20.584 1.00 4.47
C
ATOM 4069 OD1 ASP A 265 24.851 -0.804 20.746 1.00 4.47
O

ATOM 4070 OD2 ASP A 265 25.543 -2.686 20.068 1.00 4.47
O
ATOM 4071 H ASP A 265 25.335 0.224 22.673 1.00 2.20
H
ATOM 4072 HA ASP A 265 28.217 0.348 21.869 1.00 3.58
H
ATOM 4073 1HB ASP A 265 27.802 -1.371 20.183 1.00 5.36
H
ATOM 4074 2HB ASP A 265 27.437 -1.959 21.802 1.00 5.36
H
ATOM 4075 N SER A 266 26.032 2.145 20.662 1.00 1.03
N
ATOM 4076 CA SER A 266 25.791 3.108 19.596 1.00 0.97
C
ATOM 4077 C SER A 266 27.036 3.978 19.495 1.00 1.24
C
ATOM 4078 O SER A 266 27.599 4.356 20.523 1.00 3.11
O
ATOM 4079 CB SER A 266 24.588 3.981 19.887 1.00 1.46
C
ATOM 4080 OG SER A 266 23.430 3.209 20.018 1.00 1.46
O
ATOM 4081 H SER A 266 25.570 2.267 21.556 1.00 1.24
H
ATOM 4082 HA SER A 266 25.633 2.566 18.667 1.00 1.16
H
ATOM 4083 1HB SER A 266 24.758 4.549 20.802 1.00 1.75
H
ATOM 4084 2HB SER A 266 24.460 4.697 19.076 1.00 1.75
H
ATOM 4085 HG SER A 266 23.492 2.835 20.907 1.00 1.75
H
ATOM 4086 N PRO A 267 27.475 4.319 18.287 1.00 0.64
N
ATOM 4087 CA PRO A 267 28.637 5.130 17.971 1.00 0.65
C
ATOM 4088 C PRO A 267 28.387 6.590 18.293 1.00 0.65
C
ATOM 4089 O PRO A 267 27.250 7.061 18.247 1.00 0.72
O
ATOM 4090 CB PRO A 267 28.826 4.911 16.470 1.00 0.98
C
ATOM 4091 CG PRO A 267 27.462 4.601 15.965 1.00 0.98
C
ATOM 4092 CD PRO A 267 26.808 3.820 17.074 1.00 0.98
C
ATOM 4093 HA PRO A 267 29.503 4.753 18.536 1.00 0.78
H
ATOM 4094 1HB PRO A 267 29.254 5.814 16.009 1.00 1.17
H
ATOM 4095 2HB PRO A 267 29.540 4.093 16.297 1.00 1.17
H
ATOM 4096 1HG PRO A 267 26.923 5.530 15.722 1.00 1.17
H
ATOM 4097 2HG PRO A 267 27.535 4.025 15.031 1.00 1.17
H
ATOM 4098 1HD PRO A 267 25.734 4.048 17.113 1.00 1.17
H

```
ATOM   4099 2HD  PRO A 267      26.998   2.743  16.953  1.00  1.17
H
ATOM   4100  N   GLU A 268      29.456   7.307  18.607  1.00  0.81
N
ATOM   4101  CA  GLU A 268      29.344   8.725  18.897  1.00  0.81
C
ATOM   4102  C   GLU A 268      29.126   9.499  17.610  1.00  0.59
C
ATOM   4103  O   GLU A 268      29.826   9.283  16.619  1.00  0.58
O
ATOM   4104  CB  GLU A 268      30.593   9.232  19.618  1.00  1.22
C
ATOM   4105  CG  GLU A 268      30.516  10.686  20.060  1.00  1.22
C
ATOM   4106  CD  GLU A 268      31.736  11.128  20.818  1.00  1.22
C
ATOM   4107  OE1 GLU A 268      32.641  10.341  20.960  1.00  1.22
O
ATOM   4108  OE2 GLU A 268      31.762  12.251  21.265  1.00  1.22
O
ATOM   4109  H   GLU A 268      30.363   6.863  18.638  1.00  0.97
H
ATOM   4110  HA  GLU A 268      28.478   8.882  19.542  1.00  0.97
H
ATOM   4111 1HB  GLU A 268      30.776   8.622  20.502  1.00  1.46
H
ATOM   4112 2HB  GLU A 268      31.459   9.126  18.963  1.00  1.46
H
ATOM   4113 1HG  GLU A 268      30.401  11.319  19.180  1.00  1.46
H
ATOM   4114 2HG  GLU A 268      29.636  10.819  20.688  1.00  1.46
H
ATOM   4115  N   VAL A 269      28.156  10.402  17.631  1.00  0.53
N
ATOM   4116  CA  VAL A 269      27.829  11.213  16.467  1.00  0.39
C
ATOM   4117  C   VAL A 269      28.187  12.671  16.719  1.00  0.45
C
ATOM   4118  O   VAL A 269      27.821  13.239  17.749  1.00  0.51
O
ATOM   4119  CB  VAL A 269      26.328  11.082  16.123  1.00  0.58
C
ATOM   4120  CG1 VAL A 269      25.979  11.970  14.941  1.00  0.58
C
ATOM   4121  CG2 VAL A 269      26.002   9.627  15.804  1.00  0.58
C
ATOM   4122  H   VAL A 269      27.622  10.526  18.480  1.00  0.64
H
ATOM   4123  HA  VAL A 269      28.411  10.853  15.618  1.00  0.47
H
ATOM   4124  HB  VAL A 269      25.733  11.413  16.975  1.00  0.70
H
ATOM   4125 1HG1 VAL A 269      24.917  11.874  14.716  1.00  0.70
H
ATOM   4126 2HG1 VAL A 269      26.205  13.007  15.187  1.00  0.70
H
ATOM   4127 3HG1 VAL A 269      26.562  11.666  14.073  1.00  0.70
H
```

```
ATOM   4128 1HG2 VAL A 269      24.943   9.535  15.570  1.00  0.70
H
ATOM   4129 2HG2 VAL A 269      26.593   9.302  14.948  1.00  0.70
H
ATOM   4130 3HG2 VAL A 269      26.238   9.002  16.666  1.00  0.70
H
ATOM   4131  N   ASN A 270      28.907  13.270  15.777  1.00  0.46
N
ATOM   4132  CA  ASN A 270      29.327  14.658  15.906  1.00  0.52
C
ATOM   4133  C   ASN A 270      28.160  15.611  15.722  1.00  0.52
C
ATOM   4134  O   ASN A 270      27.241  15.345  14.950  1.00  0.47
O
ATOM   4135  CB  ASN A 270      30.426  14.973  14.911  1.00  0.78
C
ATOM   4136  CG  ASN A 270      31.711  14.267  15.236  1.00  0.78
C
ATOM   4137  OD1 ASN A 270      32.059  14.096  16.410  1.00  0.78
O
ATOM   4138  ND2 ASN A 270      32.422  13.850  14.221  1.00  0.78
N
ATOM   4139  H   ASN A 270      29.173  12.747  14.955  1.00  0.55
H
ATOM   4140  HA  ASN A 270      29.715  14.810  16.914  1.00  0.62
H
ATOM   4141 1HB  ASN A 270      30.107  14.678  13.910  1.00  0.94
H
ATOM   4142 2HB  ASN A 270      30.609  16.047  14.894  1.00  0.94
H
ATOM   4143 1HD2 ASN A 270      33.287  13.372  14.379  1.00  0.94
H
ATOM   4144 2HD2 ASN A 270      32.102  14.009  13.287  1.00  0.94
H
ATOM   4145  N   ILE A 271      28.200  16.719  16.453  1.00  0.62
N
ATOM   4146  CA  ILE A 271      27.167  17.747  16.397  1.00  0.68
C
ATOM   4147  C   ILE A 271      27.712  19.059  15.835  1.00  0.78
C
ATOM   4148  O   ILE A 271      27.175  20.131  16.119  1.00  0.92
O
ATOM   4149  CB  ILE A 271      26.574  17.995  17.794  1.00  1.02
C
ATOM   4150  CG1 ILE A 271      27.681  18.441  18.754  1.00  1.02
C
ATOM   4151  CG2 ILE A 271      25.897  16.732  18.308  1.00  1.02
C
ATOM   4152  CD1 ILE A 271      27.162  18.944  20.081  1.00  1.02
C
ATOM   4153  H   ILE A 271      28.984  16.857  17.077  1.00  0.74
H
ATOM   4154  HA  ILE A 271      26.369  17.401  15.742  1.00  0.82
H
ATOM   4155  HB  ILE A 271      25.842  18.799  17.740  1.00  1.22
H
ATOM   4156 1HG1 ILE A 271      28.349  17.601  18.940  1.00  1.22
H
```

```
ATOM   4157 2HG1 ILE A 271      28.256  19.241  18.287  1.00  1.22
H
ATOM   4158 1HG2 ILE A 271      25.475  16.921  19.294  1.00  1.22
H
ATOM   4159 2HG2 ILE A 271      25.102  16.441  17.622  1.00  1.22
H
ATOM   4160 3HG2 ILE A 271      26.629  15.926  18.375  1.00  1.22
H
ATOM   4161 1HD1 ILE A 271      28.001  19.243  20.710  1.00  1.22
H
ATOM   4162 2HD1 ILE A 271      26.508  19.801  19.916  1.00  1.22
H
ATOM   4163 3HD1 ILE A 271      26.602  18.152  20.577  1.00  1.22
H
ATOM   4164  N   THR A 272      28.802  18.975  15.073  1.00  0.84
N
ATOM   4165  CA  THR A 272      29.476  20.163  14.556  1.00  0.94
C
ATOM   4166  C   THR A 272      28.872  20.692  13.255  1.00  0.68
C
ATOM   4167  O   THR A 272      28.081  20.018  12.594  1.00  0.88
O
ATOM   4168  CB  THR A 272      30.969  19.862  14.330  1.00  1.41
C
ATOM   4169  OG1 THR A 272      31.110  18.861  13.314  1.00  1.41
O
ATOM   4170  CG2 THR A 272      31.596  19.351  15.618  1.00  1.41
C
ATOM   4171  H   THR A 272      29.187  18.067  14.856  1.00  1.01
H
ATOM   4172  HA  THR A 272      29.395  20.951  15.305  1.00  1.13
H
ATOM   4173  HB  THR A 272      31.482  20.769  14.011  1.00  1.69
H
ATOM   4174  HG1 THR A 272      32.043  18.729  13.124  1.00  1.69
H
ATOM   4175 1HG2 THR A 272      32.652  19.139  15.449  1.00  1.69
H
ATOM   4176 2HG2 THR A 272      31.498  20.109  16.396  1.00  1.69
H
ATOM   4177 3HG2 THR A 272      31.089  18.440  15.934  1.00  1.69
H
ATOM   4178  N   ASP A 273      29.267  21.911  12.889  1.00  0.69
N
ATOM   4179  CA  ASP A 273      28.794  22.529  11.655  1.00  1.30
C
ATOM   4180  C   ASP A 273      29.463  21.895  10.444  1.00  1.64
C
ATOM   4181  O   ASP A 273      30.679  21.692  10.431  1.00  4.21
O
ATOM   4182  CB  ASP A 273      29.068  24.034  11.666  1.00  1.95
C
ATOM   4183  CG  ASP A 273      28.210  24.792  12.671  1.00  1.95
C
ATOM   4184  OD1 ASP A 273      27.244  24.241  13.141  1.00  1.95
O
ATOM   4185  OD2 ASP A 273      28.535  25.919  12.960  1.00  1.95
O
```

```
ATOM   4186  H   ASP A 273      29.913  22.417  13.476  1.00  0.83
H
ATOM   4187  HA  ASP A 273      27.718  22.369  11.576  1.00  1.56
H
ATOM   4188 1HB  ASP A 273      30.117  24.210  11.901  1.00  2.34
H
ATOM   4189 2HB  ASP A 273      28.882  24.443  10.672  1.00  2.34
H
ATOM   4190  N   VAL A 274      28.674  21.602   9.417  1.00  0.61
N
ATOM   4191  CA  VAL A 274      29.210  20.982   8.218  1.00  0.55
C
ATOM   4192  C   VAL A 274      29.567  22.037   7.194  1.00  0.57
C
ATOM   4193  O   VAL A 274      28.729  22.848   6.802  1.00  1.47
O
ATOM   4194  CB  VAL A 274      28.199  20.005   7.589  1.00  0.83
C
ATOM   4195  CG1 VAL A 274      28.793  19.398   6.328  1.00  0.83
C
ATOM   4196  CG2 VAL A 274      27.832  18.927   8.582  1.00  0.83
C
ATOM   4197  H   VAL A 274      27.684  21.808   9.473  1.00  0.73
H
ATOM   4198  HA  VAL A 274      30.112  20.430   8.484  1.00  0.66
H
ATOM   4199  HB  VAL A 274      27.303  20.554   7.300  1.00  0.99
H
ATOM   4200 1HG1 VAL A 274      28.070  18.719   5.874  1.00  0.99
H
ATOM   4201 2HG1 VAL A 274      29.038  20.193   5.625  1.00  0.99
H
ATOM   4202 3HG1 VAL A 274      29.698  18.847   6.581  1.00  0.99
H
ATOM   4203 1HG2 VAL A 274      27.112  18.247   8.132  1.00  0.99
H
ATOM   4204 2HG2 VAL A 274      28.728  18.375   8.865  1.00  0.99
H
ATOM   4205 3HG2 VAL A 274      27.393  19.385   9.470  1.00  0.99
H
ATOM   4206  N   ARG A 275      30.812  22.045   6.762  1.00  0.64
N
ATOM   4207  CA  ARG A 275      31.226  23.006   5.759  1.00  1.00
C
ATOM   4208  C   ARG A 275      31.792  22.278   4.568  1.00  0.81
C
ATOM   4209  O   ARG A 275      32.477  21.270   4.729  1.00  1.16
O
ATOM   4210  CB  ARG A 275      32.267  23.963   6.317  1.00  1.50
C
ATOM   4211  CG  ARG A 275      31.785  24.863   7.444  1.00  1.50
C
ATOM   4212  CD  ARG A 275      32.861  25.778   7.905  1.00  1.50
C
ATOM   4213  NE  ARG A 275      32.425  26.635   8.995  1.00  1.50
N
ATOM   4214  CZ  ARG A 275      33.190  27.579   9.577  1.00  1.50
C
```

ATOM 4215 NH1 ARG A 275 34.422 27.773 9.164 1.00 1.50
N
ATOM 4216 NH2 ARG A 275 32.701 28.308 10.566 1.00 1.50
N
ATOM 4217 H ARG A 275 31.477 21.372 7.120 1.00 0.77
H
ATOM 4218 HA ARG A 275 30.357 23.581 5.437 1.00 1.20
H
ATOM 4219 1HB ARG A 275 33.116 23.394 6.694 1.00 1.80
H
ATOM 4220 2HB ARG A 275 32.636 24.606 5.517 1.00 1.80
H
ATOM 4221 1HG ARG A 275 30.946 25.467 7.096 1.00 1.80
H
ATOM 4222 2HG ARG A 275 31.468 24.254 8.290 1.00 1.80
H
ATOM 4223 1HD ARG A 275 33.710 25.190 8.254 1.00 1.80
H
ATOM 4224 2HD ARG A 275 33.177 26.413 7.077 1.00 1.80
H
ATOM 4225 HE ARG A 275 31.482 26.515 9.340 1.00 1.80
H
ATOM 4226 1HH1 ARG A 275 34.797 27.216 8.410 1.00 1.80
H
ATOM 4227 2HH1 ARG A 275 34.996 28.480 9.601 1.00 1.80
H
ATOM 4228 1HH2 ARG A 275 31.754 28.158 10.884 1.00 1.80
H
ATOM 4229 2HH2 ARG A 275 33.275 29.015 11.003 1.00 1.80
H
ATOM 4230 N MET A 276 31.524 22.783 3.371 1.00 1.06
N
ATOM 4231 CA MET A 276 32.080 22.146 2.193 1.00 1.28
C
ATOM 4232 C MET A 276 33.573 22.403 2.190 1.00 1.21
C
ATOM 4233 O MET A 276 34.032 23.451 2.646 1.00 1.73
O
ATOM 4234 CB MET A 276 31.394 22.644 0.923 1.00 1.92
C
ATOM 4235 CG MET A 276 29.946 22.178 0.793 1.00 1.92
C
ATOM 4236 SD MET A 276 29.156 22.731 -0.732 1.00 1.92
S
ATOM 4237 CE MET A 276 27.549 21.952 -0.581 1.00 1.92
C
ATOM 4238 H MET A 276 30.948 23.608 3.281 1.00 1.27
H
ATOM 4239 HA MET A 276 31.928 21.070 2.267 1.00 1.54
H
ATOM 4240 1HB MET A 276 31.401 23.733 0.904 1.00 2.30
H
ATOM 4241 2HB MET A 276 31.943 22.295 0.047 1.00 2.30
H
ATOM 4242 1HG MET A 276 29.913 21.089 0.821 1.00 2.30
H
ATOM 4243 2HG MET A 276 29.368 22.557 1.634 1.00 2.30
H

```
ATOM   4244 1HE  MET A 276      26.940  22.207  -1.448  1.00  2.30
H
ATOM   4245 2HE  MET A 276      27.673  20.871  -0.529  1.00  2.30
H
ATOM   4246 3HE  MET A 276      27.055  22.306   0.323  1.00  2.30
H
ATOM   4247  N   THR A 277      34.338  21.442   1.686  1.00  2.23
N
ATOM   4248  CA  THR A 277      35.793  21.537   1.743  1.00  4.18
C
ATOM   4249  C   THR A 277      36.390  22.562   0.785  1.00  3.57
C
ATOM   4250  O   THR A 277      37.561  22.919   0.915  1.00  5.63
O
ATOM   4251  CB  THR A 277      36.433  20.165   1.492  1.00  6.27
C
ATOM   4252  OG1 THR A 277      36.059  19.686   0.194  1.00  6.27
O
ATOM   4253  CG2 THR A 277      35.967  19.184   2.559  1.00  6.27
C
ATOM   4254  H   THR A 277      33.910  20.616   1.293  1.00  2.68
H
ATOM   4255  HA  THR A 277      36.065  21.842   2.754  1.00  5.02
H
ATOM   4256  HB  THR A 277      37.518  20.257   1.538  1.00  7.52
H
ATOM   4257  HG1 THR A 277      36.348  20.314  -0.473  1.00  7.52
H
ATOM   4258 1HG2 THR A 277      36.426  18.214   2.393  1.00  7.52
H
ATOM   4259 2HG2 THR A 277      36.257  19.554   3.542  1.00  7.52
H
ATOM   4260 3HG2 THR A 277      34.883  19.085   2.515  1.00  7.52
H
ATOM   4261  N   SER A 278      35.595  23.056  -0.151  1.00  2.54
N
ATOM   4262  CA  SER A 278      36.063  24.084  -1.059  1.00  4.28
C
ATOM   4263  C   SER A 278      34.889  24.954  -1.479  1.00  3.61
C
ATOM   4264  O   SER A 278      33.737  24.571  -1.267  1.00  5.30
O
ATOM   4265  CB  SER A 278      36.707  23.443  -2.280  1.00  6.42
C
ATOM   4266  OG  SER A 278      35.750  22.776  -3.055  1.00  6.42
O
ATOM   4267  H   SER A 278      34.646  22.725  -0.233  1.00  3.05
H
ATOM   4268  HA  SER A 278      36.798  24.700  -0.539  1.00  5.14
H
ATOM   4269 1HB  SER A 278      37.202  24.198  -2.888  1.00  7.70
H
ATOM   4270 2HB  SER A 278      37.468  22.736  -1.955  1.00  7.70
H
ATOM   4271  HG  SER A 278      35.164  23.461  -3.390  1.00  7.70
H
ATOM   4272  N   PRO A 279      35.158  26.138  -2.029  1.00  5.45
N
```

```
ATOM   4273  CA  PRO A 279      34.217  27.054  -2.640  1.00  2.23
C
ATOM   4274  C   PRO A 279      33.816  26.456  -3.983  1.00  2.24
C
ATOM   4275  O   PRO A 279      34.529  25.589  -4.494  1.00  4.90
O
ATOM   4276  CB  PRO A 279      34.995  28.370  -2.752  1.00  3.34
C
ATOM   4277  CG  PRO A 279      36.421  27.952  -2.781  1.00  3.34
C
ATOM   4278  CD  PRO A 279      36.471  26.785  -1.825  1.00  3.34
C
ATOM   4279  HA  PRO A 279      33.354  27.158  -1.970  1.00  2.68
H
ATOM   4280 1HB  PRO A 279      34.693  28.911  -3.661  1.00  4.01
H
ATOM   4281 2HB  PRO A 279      34.758  29.020  -1.897  1.00  4.01
H
ATOM   4282 1HG  PRO A 279      36.718  27.678  -3.804  1.00  4.01
H
ATOM   4283 2HG  PRO A 279      37.070  28.784  -2.470  1.00  4.01
H
ATOM   4284 1HD  PRO A 279      37.296  26.118  -2.102  1.00  4.01
H
ATOM   4285 2HD  PRO A 279      36.560  27.135  -0.787  1.00  4.01
H
ATOM   4286  N   PRO A 280      32.688  26.882  -4.555  1.00  1.38
N
ATOM   4287  CA  PRO A 280      32.127  26.456  -5.828  1.00  1.47
C
ATOM   4288  C   PRO A 280      33.162  26.577  -6.936  1.00  1.80
C
ATOM   4289  O   PRO A 280      33.942  27.525  -6.981  1.00  6.39
O
ATOM   4290  CB  PRO A 280      30.952  27.422  -6.033  1.00  2.21
C
ATOM   4291  CG  PRO A 280      30.544  27.793  -4.653  1.00  2.21
C
ATOM   4292  CD  PRO A 280      31.833  27.880  -3.888  1.00  2.21
C
ATOM   4293  HA  PRO A 280      31.773  25.418  -5.739  1.00  1.76
H
ATOM   4294 1HB  PRO A 280      31.265  28.283  -6.640  1.00  2.65
H
ATOM   4295 2HB  PRO A 280      30.145  26.917  -6.580  1.00  2.65
H
ATOM   4296 1HG  PRO A 280      29.994  28.743  -4.652  1.00  2.65
H
ATOM   4297 2HG  PRO A 280      29.863  27.031  -4.244  1.00  2.65
H
ATOM   4298 1HD  PRO A 280      32.281  28.879  -3.976  1.00  2.65
H
ATOM   4299 2HD  PRO A 280      31.647  27.601  -2.843  1.00  2.65
H
ATOM   4300  N   ASP A 281      33.165  25.613  -7.837  1.00  1.93
N
ATOM   4301  CA  ASP A 281      34.121  25.601  -8.935  1.00  1.67
C
```

```
ATOM   4302  C   ASP A 281      33.460  25.903 -10.264  1.00  2.32
C
ATOM   4303  O   ASP A 281      33.706  25.214 -11.254  1.00  5.84
O
ATOM   4304  CB  ASP A 281      34.882  24.269  -8.994  1.00  2.50
C
ATOM   4305  CG  ASP A 281      34.010  23.037  -9.184  1.00  2.50
C
ATOM   4306  OD1 ASP A 281      32.824  23.117  -8.967  1.00  2.50
O
ATOM   4307  OD2 ASP A 281      34.544  22.010  -9.526  1.00  2.50
O
ATOM   4308  H   ASP A 281      32.493  24.863  -7.758  1.00  2.32
H
ATOM   4309  HA  ASP A 281      34.857  26.384  -8.750  1.00  2.00
H
ATOM   4310 1HB  ASP A 281      35.601  24.308  -9.813  1.00  3.01
H
ATOM   4311 2HB  ASP A 281      35.453  24.146  -8.073  1.00  3.01
H
ATOM   4312  N   TYR A 282      32.594  26.914 -10.282  1.00  1.35
N
ATOM   4313  CA  TYR A 282      31.897  27.257 -11.507  1.00  1.54
C
ATOM   4314  C   TYR A 282      32.384  28.589 -12.035  1.00  1.67
C
ATOM   4315  O   TYR A 282      32.740  29.482 -11.267  1.00  2.26
O
ATOM   4316  CB  TYR A 282      30.402  27.339 -11.268  1.00  2.31
C
ATOM   4317  CG  TYR A 282      29.855  26.078 -10.685  1.00  2.31
C
ATOM   4318  CD1 TYR A 282      29.656  26.052  -9.328  1.00  2.31
C
ATOM   4319  CD2 TYR A 282      29.589  24.958 -11.460  1.00  2.31
C
ATOM   4320  CE1 TYR A 282      29.189  24.921  -8.726  1.00  2.31
C
ATOM   4321  CE2 TYR A 282      29.120  23.806 -10.849  1.00  2.31
C
ATOM   4322  CZ  TYR A 282      28.922  23.787  -9.481  1.00  2.31
C
ATOM   4323  OH  TYR A 282      28.465  22.650  -8.852  1.00  2.31
O
ATOM   4324  H   TYR A 282      32.425  27.448  -9.444  1.00  1.62
H
ATOM   4325  HA  TYR A 282      32.104  26.498 -12.261  1.00  1.85
H
ATOM   4326 1HB  TYR A 282      30.174  28.164 -10.593  1.00  2.77
H
ATOM   4327 2HB  TYR A 282      29.899  27.525 -12.211  1.00  2.77
H
ATOM   4328  HD1 TYR A 282      29.875  26.936  -8.732  1.00  2.77
H
ATOM   4329  HD2 TYR A 282      29.757  24.980 -12.537  1.00  2.77
H
ATOM   4330  HE1 TYR A 282      29.037  24.923  -7.657  1.00  2.77
H
```

```
ATOM   4331  HE2 TYR A 282      28.914  22.916 -11.443  1.00  2.77
H
ATOM   4332  HH  TYR A 282      28.704  22.686  -7.917  1.00  2.77
H
ATOM   4333  N   ARG A 283      32.388  28.724 -13.349  1.00  1.68
N
ATOM   4334  CA  ARG A 283      32.800  29.961 -13.983  1.00  1.84
C
ATOM   4335  C   ARG A 283      31.593  30.734 -14.461  1.00  1.86
C
ATOM   4336  O   ARG A 283      30.686  30.164 -15.068  1.00  1.82
O
ATOM   4337  CB  ARG A 283      33.726  29.684 -15.153  1.00  2.76
C
ATOM   4338  CG  ARG A 283      34.246  30.908 -15.883  1.00  2.76
C
ATOM   4339  CD  ARG A 283      35.142  30.520 -17.001  1.00  2.76
C
ATOM   4340  NE  ARG A 283      34.433  29.752 -18.015  1.00  2.76
N
ATOM   4341  CZ  ARG A 283      34.984  29.228 -19.122  1.00  2.76
C
ATOM   4342  NH1 ARG A 283      36.262  29.397 -19.388  1.00  2.76
N
ATOM   4343  NH2 ARG A 283      34.208  28.538 -19.937  1.00  2.76
N
ATOM   4344  H   ARG A 283      32.092  27.950 -13.930  1.00  2.02
H
ATOM   4345  HA  ARG A 283      33.336  30.567 -13.253  1.00  2.21
H
ATOM   4346 1HB  ARG A 283      34.589  29.119 -14.805  1.00  3.31
H
ATOM   4347 2HB  ARG A 283      33.211  29.069 -15.884  1.00  3.31
H
ATOM   4348 1HG  ARG A 283      33.409  31.471 -16.295  1.00  3.31
H
ATOM   4349 2HG  ARG A 283      34.806  31.538 -15.191  1.00  3.31
H
ATOM   4350 1HD  ARG A 283      35.546  31.416 -17.472  1.00  3.31
H
ATOM   4351 2HD  ARG A 283      35.959  29.909 -16.619  1.00  3.31
H
ATOM   4352  HE  ARG A 283      33.442  29.574 -17.880  1.00  3.31
H
ATOM   4353 1HH1 ARG A 283      36.843  29.926 -18.755  1.00  3.31
H
ATOM   4354 2HH1 ARG A 283      36.661  28.995 -20.224  1.00  3.31
H
ATOM   4355 1HH2 ARG A 283      33.228  28.422 -19.693  1.00  3.31
H
ATOM   4356 2HH2 ARG A 283      34.584  28.126 -20.776  1.00  3.31
H
ATOM   4357  N   VAL A 284      31.593  32.034 -14.185  1.00  1.93
N
ATOM   4358  CA  VAL A 284      30.490  32.893 -14.567  1.00  1.98
C
ATOM   4359  C   VAL A 284      30.343  32.898 -16.076  1.00  2.13
C
```

ATOM 4360 O VAL A 284 31.310 33.124 -16.807 1.00 2.37
O
ATOM 4361 CB VAL A 284 30.716 34.320 -14.030 1.00 2.97
C
ATOM 4362 CG1 VAL A 284 29.643 35.268 -14.540 1.00 2.97
C
ATOM 4363 CG2 VAL A 284 30.705 34.285 -12.507 1.00 2.97
C
ATOM 4364 H VAL A 284 32.376 32.432 -13.687 1.00 2.32
H
ATOM 4365 HA VAL A 284 29.574 32.505 -14.127 1.00 2.38
H
ATOM 4366 HB VAL A 284 31.679 34.688 -14.385 1.00 3.56
H
ATOM 4367 1HG1 VAL A 284 29.831 36.271 -14.153 1.00 3.56
H
ATOM 4368 2HG1 VAL A 284 29.662 35.291 -15.629 1.00 3.56
H
ATOM 4369 3HG1 VAL A 284 28.666 34.931 -14.200 1.00 3.56
H
ATOM 4370 1HG2 VAL A 284 30.878 35.287 -12.117 1.00 3.56
H
ATOM 4371 2HG2 VAL A 284 29.736 33.922 -12.160 1.00 3.56
H
ATOM 4372 3HG2 VAL A 284 31.490 33.617 -12.152 1.00 3.56
H
ATOM 4373 N GLY A 285 29.128 32.620 -16.532 1.00 2.02
N
ATOM 4374 CA GLY A 285 28.827 32.526 -17.950 1.00 2.13
C
ATOM 4375 C GLY A 285 28.587 31.082 -18.410 1.00 2.03
C
ATOM 4376 O GLY A 285 27.938 30.862 -19.433 1.00 2.07
O
ATOM 4377 H GLY A 285 28.378 32.488 -15.867 1.00 2.42
H
ATOM 4378 1HA GLY A 285 27.947 33.127 -18.165 1.00 2.56
H
ATOM 4379 2HA GLY A 285 29.649 32.956 -18.521 1.00 2.56
H
ATOM 4380 N ASP A 286 29.109 30.101 -17.667 1.00 1.93
N
ATOM 4381 CA ASP A 286 28.908 28.697 -18.026 1.00 1.89
C
ATOM 4382 C ASP A 286 27.468 28.280 -17.778 1.00 1.77
C
ATOM 4383 O ASP A 286 26.831 28.775 -16.857 1.00 1.70
O
ATOM 4384 CB ASP A 286 29.834 27.774 -17.232 1.00 2.83
C
ATOM 4385 CG ASP A 286 31.304 27.887 -17.616 1.00 2.83
C
ATOM 4386 OD1 ASP A 286 31.622 28.569 -18.563 1.00 2.83
O
ATOM 4387 OD2 ASP A 286 32.100 27.260 -16.962 1.00 2.83
O
ATOM 4388 H ASP A 286 29.643 30.315 -16.831 1.00 2.32
H

```
ATOM   4389  HA  ASP A 286      29.122  28.575 -19.088  1.00  2.27
H
ATOM   4390 1HB  ASP A 286      29.738  27.998 -16.170  1.00  3.40
H
ATOM   4391 2HB  ASP A 286      29.519  26.739 -17.372  1.00  3.40
H
ATOM   4392  N   LYS A 287      26.940  27.366 -18.588  1.00  1.81
N
ATOM   4393  CA  LYS A 287      25.565  26.939 -18.358  1.00  1.74
C
ATOM   4394  C   LYS A 287      25.483  25.583 -17.674  1.00  1.68
C
ATOM   4395  O   LYS A 287      26.194  24.642 -18.027  1.00  2.66
O
ATOM   4396  CB  LYS A 287      24.771  26.911 -19.657  1.00  2.61
C
ATOM   4397  CG  LYS A 287      24.540  28.278 -20.278  1.00  2.61
C
ATOM   4398  CD  LYS A 287      23.678  28.168 -21.523  1.00  2.61
C
ATOM   4399  CE  LYS A 287      23.435  29.527 -22.160  1.00  2.61
C
ATOM   4400  NZ  LYS A 287      22.607  29.418 -23.392  1.00  2.61
N
ATOM   4401  H   LYS A 287      27.482  26.973 -19.344  1.00  2.17
H
ATOM   4402  HA  LYS A 287      25.100  27.666 -17.703  1.00  2.09
H
ATOM   4403 1HB  LYS A 287      25.292  26.294 -20.390  1.00  3.13
H
ATOM   4404 2HB  LYS A 287      23.797  26.457 -19.481  1.00  3.13
H
ATOM   4405 1HG  LYS A 287      24.040  28.924 -19.557  1.00  3.13
H
ATOM   4406 2HG  LYS A 287      25.496  28.728 -20.544  1.00  3.13
H
ATOM   4407 1HD  LYS A 287      24.169  27.518 -22.248  1.00  3.13
H
ATOM   4408 2HD  LYS A 287      22.717  27.725 -21.260  1.00  3.13
H
ATOM   4409 1HE  LYS A 287      22.923  30.171 -21.446  1.00  3.13
H
ATOM   4410 2HE  LYS A 287      24.393  29.979 -22.417  1.00  3.13
H
ATOM   4411 1HZ  LYS A 287      22.466  30.337 -23.787  1.00  3.13
H
ATOM   4412 2HZ  LYS A 287      23.080  28.831 -24.065  1.00  3.13
H
ATOM   4413 3HZ  LYS A 287      21.712  29.010 -23.160  1.00  3.13
H
ATOM   4414  N   ILE A 288      24.597  25.506 -16.689  1.00  1.48
N
ATOM   4415  CA  ILE A 288      24.369  24.298 -15.910  1.00  1.44
C
ATOM   4416  C   ILE A 288      23.018  24.335 -15.205  1.00  1.17
C
ATOM   4417  O   ILE A 288      22.577  25.384 -14.737  1.00  1.18
O
```

```
ATOM   4418  CB  ILE A 288      25.496  24.098 -14.894  1.00  2.16
C
ATOM   4419  CG1 ILE A 288      25.306  22.773 -14.137  1.00  2.16
C
ATOM   4420  CG2 ILE A 288      25.562  25.286 -13.968  1.00  2.16
C
ATOM   4421  CD1 ILE A 288      26.511  22.341 -13.338  1.00  2.16
C
ATOM   4422  H   ILE A 288      24.062  26.335 -16.466  1.00  1.78
H
ATOM   4423  HA  ILE A 288      24.372  23.447 -16.589  1.00  1.73
H
ATOM   4424  HB  ILE A 288      26.443  24.022 -15.428  1.00  2.59
H
ATOM   4425 1HG1 ILE A 288      24.467  22.875 -13.453  1.00  2.59
H
ATOM   4426 2HG1 ILE A 288      25.073  21.987 -14.856  1.00  2.59
H
ATOM   4427 1HG2 ILE A 288      26.386  25.146 -13.272  1.00  2.59
H
ATOM   4428 2HG2 ILE A 288      25.727  26.192 -14.548  1.00  2.59
H
ATOM   4429 3HG2 ILE A 288      24.625  25.372 -13.418  1.00  2.59
H
ATOM   4430 1HD1 ILE A 288      26.295  21.398 -12.834  1.00  2.59
H
ATOM   4431 2HD1 ILE A 288      27.364  22.209 -14.005  1.00  2.59
H
ATOM   4432 3HD1 ILE A 288      26.745  23.102 -12.596  1.00  2.59
H
ATOM   4433  N   ALA A 289      22.341  23.196 -15.174  1.00  1.06
N
ATOM   4434  CA  ALA A 289      21.047  23.107 -14.513  1.00  0.96
C
ATOM   4435  C   ALA A 289      21.239  23.112 -13.012  1.00  0.83
C
ATOM   4436  O   ALA A 289      22.216  22.550 -12.513  1.00  0.78
O
ATOM   4437  CB  ALA A 289      20.307  21.851 -14.948  1.00  1.44
C
ATOM   4438  H   ALA A 289      22.739  22.370 -15.596  1.00  1.27
H
ATOM   4439  HA  ALA A 289      20.459  23.984 -14.784  1.00  1.15
H
ATOM   4440 1HB  ALA A 289      19.337  21.812 -14.453  1.00  1.73
H
ATOM   4441 2HB  ALA A 289      20.162  21.871 -16.028  1.00  1.73
H
ATOM   4442 3HB  ALA A 289      20.890  20.974 -14.676  1.00  1.73
H
ATOM   4443  N   THR A 290      20.297  23.713 -12.286  1.00  0.82
N
ATOM   4444  CA  THR A 290      20.414  23.743 -10.835  1.00  0.76
C
ATOM   4445  C   THR A 290      20.234  22.344 -10.253  1.00  0.65
C
ATOM   4446  O   THR A 290      20.806  22.041  -9.207  1.00  0.60
O
```

ATOM 4447 CB THR A 290 19.411 24.727 -10.202 1.00 1.14
C
ATOM 4448 OG1 THR A 290 18.080 24.367 -10.575 1.00 1.14
O
ATOM 4449 CG2 THR A 290 19.693 26.155 -10.640 1.00 1.14
C
ATOM 4450 H THR A 290 19.510 24.158 -12.740 1.00 0.98
H
ATOM 4451 HA THR A 290 21.418 24.083 -10.579 1.00 0.91
H
ATOM 4452 HB THR A 290 19.498 24.668 -9.118 1.00 1.37
H
ATOM 4453 HG1 THR A 290 17.851 24.751 -11.431 1.00 1.37
H
ATOM 4454 1HG2 THR A 290 18.976 26.828 -10.171 1.00 1.37
H
ATOM 4455 2HG2 THR A 290 20.703 26.435 -10.341 1.00 1.37
H
ATOM 4456 3HG2 THR A 290 19.603 26.227 -11.724 1.00 1.37
H
ATOM 4457 N ARG A 291 19.510 21.467 -10.961 1.00 0.64
N
ATOM 4458 CA ARG A 291 19.420 20.068 -10.539 1.00 0.57
C
ATOM 4459 C ARG A 291 20.790 19.405 -10.533 1.00 0.58
C
ATOM 4460 O ARG A 291 21.138 18.689 -9.590 1.00 0.55
O
ATOM 4461 CB ARG A 291 18.568 19.258 -11.482 1.00 0.85
C
ATOM 4462 CG ARG A 291 17.091 19.506 -11.481 1.00 0.85
C
ATOM 4463 CD ARG A 291 16.495 18.681 -12.552 1.00 0.85
C
ATOM 4464 NE ARG A 291 17.012 19.072 -13.849 1.00 0.85
N
ATOM 4465 CZ ARG A 291 17.271 18.240 -14.881 1.00 0.85
C
ATOM 4466 NH1 ARG A 291 17.090 16.939 -14.784 1.00 0.85
N
ATOM 4467 NH2 ARG A 291 17.729 18.743 -16.012 1.00 0.85
N
ATOM 4468 H ARG A 291 18.993 21.768 -11.784 1.00 0.77
H
ATOM 4469 HA ARG A 291 18.997 20.028 -9.534 1.00 0.68
H
ATOM 4470 1HB ARG A 291 18.917 19.412 -12.502 1.00 1.03
H
ATOM 4471 2HB ARG A 291 18.699 18.202 -11.253 1.00 1.03
H
ATOM 4472 1HG ARG A 291 16.679 19.195 -10.523 1.00 1.03
H
ATOM 4473 2HG ARG A 291 16.874 20.554 -11.644 1.00 1.03
H
ATOM 4474 1HD ARG A 291 16.744 17.644 -12.381 1.00 1.03
H
ATOM 4475 2HD ARG A 291 15.414 18.804 -12.562 1.00 1.03
H

ATOM	4476	HE	ARG	A	291	17.189	20.060	-13.994	1.00	1.03	H
ATOM	4477	1HH1	ARG	A	291	16.776	16.515	-13.921	1.00	1.03	H
ATOM	4478	2HH1	ARG	A	291	17.300	16.345	-15.573	1.00	1.03	H
ATOM	4479	1HH2	ARG	A	291	17.852	19.745	-16.110	1.00	1.03	H
ATOM	4480	2HH2	ARG	A	291	17.933	18.132	-16.789	1.00	1.03	H
ATOM	4481	N	LYS	A	292	21.559	19.647	-11.602	1.00	0.66	N
ATOM	4482	CA	LYS	A	292	22.888	19.070	-11.747	1.00	0.69	C
ATOM	4483	C	LYS	A	292	23.800	19.586	-10.671	1.00	0.67	C
ATOM	4484	O	LYS	A	292	24.550	18.821	-10.061	1.00	0.66	O
ATOM	4485	CB	LYS	A	292	23.488	19.403	-13.117	1.00	1.03	C
ATOM	4486	CG	LYS	A	292	22.838	18.694	-14.287	1.00	1.03	C
ATOM	4487	CD	LYS	A	292	23.481	19.071	-15.614	1.00	1.03	C
ATOM	4488	CE	LYS	A	292	22.827	18.311	-16.762	1.00	1.03	C
ATOM	4489	NZ	LYS	A	292	23.459	18.609	-18.079	1.00	1.03	N
ATOM	4490	H	LYS	A	292	21.208	20.246	-12.335	1.00	0.79	H
ATOM	4491	HA	LYS	A	292	22.818	17.987	-11.640	1.00	0.83	H
ATOM	4492	1HB	LYS	A	292	23.415	20.475	-13.296	1.00	1.24	H
ATOM	4493	2HB	LYS	A	292	24.548	19.144	-13.121	1.00	1.24	H
ATOM	4494	1HG	LYS	A	292	22.938	17.618	-14.149	1.00	1.24	H
ATOM	4495	2HG	LYS	A	292	21.778	18.942	-14.320	1.00	1.24	H
ATOM	4496	1HD	LYS	A	292	23.368	20.142	-15.787	1.00	1.24	H
ATOM	4497	2HD	LYS	A	292	24.544	18.832	-15.588	1.00	1.24	H
ATOM	4498	1HE	LYS	A	292	22.911	17.243	-16.568	1.00	1.24	H
ATOM	4499	2HE	LYS	A	292	21.771	18.577	-16.811	1.00	1.24	H
ATOM	4500	1HZ	LYS	A	292	22.995	18.076	-18.801	1.00	1.24	H
ATOM	4501	2HZ	LYS	A	292	23.385	19.593	-18.306	1.00	1.24	H
ATOM	4502	3HZ	LYS	A	292	24.435	18.352	-18.051	1.00	1.24	H
ATOM	4503	N	ALA	A	293	23.703	20.890	-10.426	1.00	0.69	N
ATOM	4504	CA	ALA	A	293	24.490	21.550	-9.409	1.00	0.70	C

ATOM	4505	C	ALA	A	293	24.208	20.969	-8.040	1.00	0.66	C
ATOM	4506	O	ALA	A	293	25.135	20.777	-7.257	1.00	0.71	O
ATOM	4507	CB	ALA	A	293	24.204	23.029	-9.422	1.00	1.05	C
ATOM	4508	H	ALA	A	293	23.069	21.451	-10.986	1.00	0.83	H
ATOM	4509	HA	ALA	A	293	25.544	21.389	-9.637	1.00	0.84	H
ATOM	4510	1HB	ALA	A	293	24.821	23.525	-8.674	1.00	1.26	H
ATOM	4511	2HB	ALA	A	293	24.432	23.430	-10.409	1.00	1.26	H
ATOM	4512	3HB	ALA	A	293	23.152	23.198	-9.198	1.00	1.26	H
ATOM	4513	N	CYS	A	294	22.937	20.680	-7.759	1.00	0.60	N
ATOM	4514	CA	CYS	A	294	22.542	20.084	-6.492	1.00	0.63	C
ATOM	4515	C	CYS	A	294	23.184	18.727	-6.297	1.00	0.57	C
ATOM	4516	O	CYS	A	294	23.719	18.447	-5.228	1.00	0.63	O
ATOM	4517	CB	CYS	A	294	21.034	19.942	-6.386	1.00	0.95	C
ATOM	4518	SG	CYS	A	294	20.517	19.263	-4.799	1.00	0.95	S
ATOM	4519	H	CYS	A	294	22.215	20.898	-8.433	1.00	0.72	H
ATOM	4520	HA	CYS	A	294	22.875	20.740	-5.688	1.00	0.76	H
ATOM	4521	1HB	CYS	A	294	20.563	20.916	-6.514	1.00	1.13	H
ATOM	4522	2HB	CYS	A	294	20.664	19.288	-7.176	1.00	1.13	H
ATOM	4523	HG	CYS	A	294	21.107	18.084	-4.957	1.00	1.13	H
ATOM	4524	N	GLY	A	295	23.113	17.872	-7.320	1.00	0.53	N
ATOM	4525	CA	GLY	A	295	23.730	16.551	-7.234	1.00	0.51	C
ATOM	4526	C	GLY	A	295	25.225	16.660	-6.931	1.00	0.56	C
ATOM	4527	O	GLY	A	295	25.743	15.943	-6.066	1.00	0.55	O
ATOM	4528	H	GLY	A	295	22.626	18.144	-8.168	1.00	0.64	H
ATOM	4529	1HA	GLY	A	295	23.236	15.965	-6.460	1.00	0.61	H
ATOM	4530	2HA	GLY	A	295	23.585	16.028	-8.178	1.00	0.61	H
ATOM	4531	N	LEU	A	296	25.902	17.576	-7.629	1.00	0.64	N
ATOM	4532	CA	LEU	A	296	27.322	17.815	-7.417	1.00	0.73	C
ATOM	4533	C	LEU	A	296	27.591	18.325	-6.007	1.00	0.72	C

```
ATOM   4534  O   LEU A 296      28.538  17.881  -5.355  1.00  0.74
O
ATOM   4535  CB  LEU A 296      27.823  18.845  -8.436  1.00  1.09
C
ATOM   4536  CG  LEU A 296      27.874  18.381  -9.896  1.00  1.09
C
ATOM   4537  CD1 LEU A 296      28.156  19.578 -10.793  1.00  1.09
C
ATOM   4538  CD2 LEU A 296      28.959  17.326 -10.046  1.00  1.09
C
ATOM   4539  H   LEU A 296      25.419  18.116  -8.338  1.00  0.77
H
ATOM   4540  HA  LEU A 296      27.859  16.878  -7.558  1.00  0.88
H
ATOM   4541 1HB  LEU A 296      27.177  19.720  -8.393  1.00  1.31
H
ATOM   4542 2HB  LEU A 296      28.830  19.151  -8.153  1.00  1.31
H
ATOM   4543  HG  LEU A 296      26.912  17.957 -10.181  1.00  1.31
H
ATOM   4544 1HD1 LEU A 296      28.188  19.253 -11.833  1.00  1.31
H
ATOM   4545 2HD1 LEU A 296      27.365  20.319 -10.669  1.00  1.31
H
ATOM   4546 3HD1 LEU A 296      29.113  20.020 -10.520  1.00  1.31
H
ATOM   4547 1HD2 LEU A 296      29.000  16.990 -11.082  1.00  1.31
H
ATOM   4548 2HD2 LEU A 296      29.922  17.754  -9.765  1.00  1.31
H
ATOM   4549 3HD2 LEU A 296      28.737  16.480  -9.399  1.00  1.31
H
ATOM   4550  N   ALA A 297      26.726  19.231  -5.542  1.00  0.72
N
ATOM   4551  CA  ALA A 297      26.811  19.835  -4.220  1.00  0.74
C
ATOM   4552  C   ALA A 297      26.722  18.806  -3.125  1.00  0.54
C
ATOM   4553  O   ALA A 297      27.522  18.824  -2.191  1.00  0.53
O
ATOM   4554  CB  ALA A 297      25.689  20.841  -4.030  1.00  1.11
C
ATOM   4555  H   ALA A 297      25.983  19.549  -6.146  1.00  0.86
H
ATOM   4556  HA  ALA A 297      27.773  20.341  -4.142  1.00  0.89
H
ATOM   4557 1HB  ALA A 297      25.777  21.309  -3.051  1.00  1.33
H
ATOM   4558 2HB  ALA A 297      25.735  21.600  -4.803  1.00  1.33
H
ATOM   4559 3HB  ALA A 297      24.732  20.331  -4.098  1.00  1.33
H
ATOM   4560  N   LEU A 298      25.738  17.910  -3.243  1.00  0.50
N
ATOM   4561  CA  LEU A 298      25.525  16.877  -2.245  1.00  0.46
C
ATOM   4562  C   LEU A 298      26.699  15.936  -2.154  1.00  0.42
C
```

ATOM 4563 O LEU A 298 27.096 15.562 -1.052 1.00 0.38
O
ATOM 4564 CB LEU A 298 24.261 16.068 -2.564 1.00 0.69
C
ATOM 4565 CG LEU A 298 22.933 16.809 -2.382 1.00 0.69
C
ATOM 4566 CD1 LEU A 298 21.791 15.940 -2.899 1.00 0.69
C
ATOM 4567 CD2 LEU A 298 22.750 17.133 -0.913 1.00 0.69
C
ATOM 4568 H LEU A 298 25.112 17.963 -4.034 1.00 0.60
H
ATOM 4569 HA LEU A 298 25.396 17.358 -1.277 1.00 0.55
H
ATOM 4570 1HB LEU A 298 24.314 15.738 -3.600 1.00 0.83
H
ATOM 4571 2HB LEU A 298 24.240 15.187 -1.923 1.00 0.83
H
ATOM 4572 HG LEU A 298 22.946 17.733 -2.958 1.00 0.83
H
ATOM 4573 1HD1 LEU A 298 20.846 16.470 -2.773 1.00 0.83
H
ATOM 4574 2HD1 LEU A 298 21.948 15.725 -3.955 1.00 0.83
H
ATOM 4575 3HD1 LEU A 298 21.761 15.007 -2.338 1.00 0.83
H
ATOM 4576 1HD2 LEU A 298 21.809 17.666 -0.775 1.00 0.83
H
ATOM 4577 2HD2 LEU A 298 22.732 16.208 -0.335 1.00 0.83
H
ATOM 4578 3HD2 LEU A 298 23.575 17.755 -0.572 1.00 0.83
H
ATOM 4579 N ALA A 299 27.270 15.565 -3.301 1.00 0.53
N
ATOM 4580 CA ALA A 299 28.427 14.680 -3.302 1.00 0.60
C
ATOM 4581 C ALA A 299 29.606 15.336 -2.601 1.00 0.65
C
ATOM 4582 O ALA A 299 30.288 14.694 -1.797 1.00 0.64
O
ATOM 4583 CB ALA A 299 28.795 14.300 -4.714 1.00 0.90
C
ATOM 4584 H ALA A 299 26.888 15.880 -4.189 1.00 0.64
H
ATOM 4585 HA ALA A 299 28.170 13.778 -2.752 1.00 0.72
H
ATOM 4586 1HB ALA A 299 29.647 13.620 -4.698 1.00 1.08
H
ATOM 4587 2HB ALA A 299 27.943 13.808 -5.179 1.00 1.08
H
ATOM 4588 3HB ALA A 299 29.055 15.194 -5.281 1.00 1.08
H
ATOM 4589 N LYS A 300 29.828 16.622 -2.885 1.00 0.74
N
ATOM 4590 CA LYS A 300 30.908 17.357 -2.245 1.00 0.80
C
ATOM 4591 C LYS A 300 30.689 17.437 -0.741 1.00 0.68
C

ATOM 4592 O LYS A 300 31.624 17.265 0.044 1.00 0.83
O
ATOM 4593 CB LYS A 300 31.014 18.763 -2.824 1.00 1.20
C
ATOM 4594 CG LYS A 300 31.530 18.830 -4.254 1.00 1.20
C
ATOM 4595 CD LYS A 300 31.564 20.271 -4.737 1.00 1.20
C
ATOM 4596 CE LYS A 300 32.023 20.387 -6.183 1.00 1.20
C
ATOM 4597 NZ LYS A 300 32.057 21.807 -6.610 1.00 1.20
N
ATOM 4598 H LYS A 300 29.252 17.098 -3.571 1.00 0.89
H
ATOM 4599 HA LYS A 300 31.846 16.828 -2.422 1.00 0.96
H
ATOM 4600 1HB LYS A 300 30.033 19.240 -2.803 1.00 1.44
H
ATOM 4601 2HB LYS A 300 31.680 19.363 -2.203 1.00 1.44
H
ATOM 4602 1HG LYS A 300 32.539 18.419 -4.296 1.00 1.44
H
ATOM 4603 2HG LYS A 300 30.890 18.244 -4.909 1.00 1.44
H
ATOM 4604 1HD LYS A 300 30.566 20.705 -4.652 1.00 1.44
H
ATOM 4605 2HD LYS A 300 32.242 20.846 -4.108 1.00 1.44
H
ATOM 4606 1HE LYS A 300 33.018 19.958 -6.290 1.00 1.44
H
ATOM 4607 2HE LYS A 300 31.333 19.841 -6.827 1.00 1.44
H
ATOM 4608 1HZ LYS A 300 32.349 21.903 -7.573 1.00 1.44
H
ATOM 4609 2HZ LYS A 300 31.133 22.203 -6.514 1.00 1.44
H
ATOM 4610 3HZ LYS A 300 32.696 22.316 -6.021 1.00 1.44
H
ATOM 4611 N LEU A 301 29.440 17.680 -0.350 1.00 0.55
N
ATOM 4612 CA LEU A 301 29.066 17.766 1.046 1.00 0.59
C
ATOM 4613 C LEU A 301 29.328 16.423 1.745 1.00 0.68
C
ATOM 4614 O LEU A 301 29.768 16.391 2.897 1.00 1.10
O
ATOM 4615 CB LEU A 301 27.587 18.127 1.153 1.00 0.89
C
ATOM 4616 CG LEU A 301 27.100 18.510 2.538 1.00 0.89
C
ATOM 4617 CD1 LEU A 301 27.811 19.784 2.973 1.00 0.89
C
ATOM 4618 CD2 LEU A 301 25.605 18.675 2.511 1.00 0.89
C
ATOM 4619 H LEU A 301 28.723 17.833 -1.046 1.00 0.66
H
ATOM 4620 HA LEU A 301 29.665 18.537 1.528 1.00 0.71
H

```
ATOM   4621 1HB  LEU A 301      27.386  18.963   0.488  1.00  1.06
H
ATOM   4622 2HB  LEU A 301      26.995  17.276   0.821  1.00  1.06
H
ATOM   4623  HG  LEU A 301      27.364  17.735   3.248  1.00  1.06
H
ATOM   4624 1HD1 LEU A 301      27.472  20.067   3.968  1.00  1.06
H
ATOM   4625 2HD1 LEU A 301      28.887  19.616   2.987  1.00  1.06
H
ATOM   4626 3HD1 LEU A 301      27.576  20.585   2.273  1.00  1.06
H
ATOM   4627 1HD2 LEU A 301      25.258  18.941   3.506  1.00  1.06
H
ATOM   4628 2HD2 LEU A 301      25.342  19.460   1.811  1.00  1.06
H
ATOM   4629 3HD2 LEU A 301      25.141  17.738   2.201  1.00  1.06
H
ATOM   4630  N   GLY A 302      29.069  15.321   1.023  1.00  0.58
N
ATOM   4631  CA  GLY A 302      29.306  13.966   1.511  1.00  0.70
C
ATOM   4632  C   GLY A 302      30.768  13.752   1.866  1.00  0.86
C
ATOM   4633  O   GLY A 302      31.081  13.111   2.868  1.00  2.79
O
ATOM   4634  H   GLY A 302      28.674  15.426   0.100  1.00  0.70
H
ATOM   4635 1HA  GLY A 302      28.686  13.779   2.385  1.00  0.84
H
ATOM   4636 2HA  GLY A 302      29.010  13.249   0.746  1.00  0.84
H
ATOM   4637  N   TYR A 303      31.663  14.301   1.045  1.00  1.04
N
ATOM   4638  CA  TYR A 303      33.095  14.257   1.323  1.00  0.75
C
ATOM   4639  C   TYR A 303      33.410  14.975   2.632  1.00  1.10
C
ATOM   4640  O   TYR A 303      34.192  14.488   3.451  1.00  4.36
O
ATOM   4641  CB  TYR A 303      33.888  14.868   0.162  1.00  1.12
C
ATOM   4642  CG  TYR A 303      35.386  14.877   0.365  1.00  1.12
C
ATOM   4643  CD1 TYR A 303      36.117  13.699   0.285  1.00  1.12
C
ATOM   4644  CD2 TYR A 303      36.036  16.073   0.616  1.00  1.12
C
ATOM   4645  CE1 TYR A 303      37.486  13.720   0.463  1.00  1.12
C
ATOM   4646  CE2 TYR A 303      37.406  16.095   0.792  1.00  1.12
C
ATOM   4647  CZ  TYR A 303      38.130  14.925   0.717  1.00  1.12
C
ATOM   4648  OH  TYR A 303      39.494  14.947   0.893  1.00  1.12
O
ATOM   4649  H   TYR A 303      31.333  14.760   0.202  1.00  1.25
H
```

```
ATOM   4650  HA  TYR A 303      33.395  13.216   1.441  1.00  0.90
H
ATOM   4651 1HB  TYR A 303      33.672  14.325  -0.757  1.00  1.35
H
ATOM   4652 2HB  TYR A 303      33.570  15.896   0.008  1.00  1.35
H
ATOM   4653  HD1 TYR A 303      35.615  12.755   0.085  1.00  1.35
H
ATOM   4654  HD2 TYR A 303      35.462  16.997   0.672  1.00  1.35
H
ATOM   4655  HE1 TYR A 303      38.058  12.795   0.401  1.00  1.35
H
ATOM   4656  HE2 TYR A 303      37.914  17.039   0.989  1.00  1.35
H
ATOM   4657  HH  TYR A 303      39.842  14.056   0.802  1.00  1.35
H
ATOM   4658  N   ALA A 304      32.812  16.151   2.808  1.00  1.14
N
ATOM   4659  CA  ALA A 304      33.012  16.958   4.006  1.00  1.07
C
ATOM   4660  C   ALA A 304      32.553  16.278   5.309  1.00  0.79
C
ATOM   4661  O   ALA A 304      33.234  16.395   6.330  1.00  0.87
O
ATOM   4662  CB  ALA A 304      32.285  18.280   3.851  1.00  1.60
C
ATOM   4663  H   ALA A 304      32.206  16.505   2.073  1.00  1.37
H
ATOM   4664  HA  ALA A 304      34.081  17.154   4.096  1.00  1.28
H
ATOM   4665 1HB  ALA A 304      32.481  18.893   4.728  1.00  1.93
H
ATOM   4666 2HB  ALA A 304      32.643  18.792   2.958  1.00  1.93
H
ATOM   4667 3HB  ALA A 304      31.216  18.107   3.763  1.00  1.93
H
ATOM   4668  N   ASN A 305      31.416  15.572   5.298  1.00  0.56
N
ATOM   4669  CA  ASN A 305      30.974  14.979   6.562  1.00  0.40
C
ATOM   4670  C   ASN A 305      30.161  13.695   6.412  1.00  0.29
C
ATOM   4671  O   ASN A 305      29.202  13.624   5.643  1.00  0.34
O
ATOM   4672  CB  ASN A 305      30.193  16.008   7.352  1.00  0.60
C
ATOM   4673  CG  ASN A 305      29.949  15.609   8.780  1.00  0.60
C
ATOM   4674  OD1 ASN A 305      29.128  14.748   9.116  1.00  0.60
O
ATOM   4675  ND2 ASN A 305      30.693  16.233   9.658  1.00  0.60
N
ATOM   4676  H   ASN A 305      30.866  15.492   4.450  1.00  0.67
H
ATOM   4677  HA  ASN A 305      31.864  14.718   7.136  1.00  0.48
H
ATOM   4678 1HB  ASN A 305      30.736  16.953   7.344  1.00  0.72
H
```

```
ATOM   4679 2HB  ASN A 305      29.231  16.183   6.868  1.00  0.72
H
ATOM   4680 1HD2 ASN A 305      30.606  16.020  10.632  1.00  0.72
H
ATOM   4681 2HD2 ASN A 305      31.355  16.917   9.355  1.00  0.72
H
ATOM   4682  N   ASN A 306      30.557  12.692   7.196  1.00  0.35
N
ATOM   4683  CA  ASN A 306      29.984  11.346   7.207  1.00  0.31
C
ATOM   4684  C   ASN A 306      28.498  11.245   7.567  1.00  0.36
C
ATOM   4685  O   ASN A 306      27.870  10.220   7.288  1.00  0.60
O
ATOM   4686  CB  ASN A 306      30.782  10.485   8.162  1.00  0.46
C
ATOM   4687  CG  ASN A 306      32.131  10.113   7.615  1.00  0.46
C
ATOM   4688  OD1 ASN A 306      32.351  10.124   6.399  1.00  0.46
O
ATOM   4689  ND2 ASN A 306      33.043   9.781   8.493  1.00  0.46
N
ATOM   4690  H   ASN A 306      31.340  12.866   7.812  1.00  0.42
H
ATOM   4691  HA  ASN A 306      30.091  10.936   6.202  1.00  0.37
H
ATOM   4692 1HB  ASN A 306      30.924  11.025   9.099  1.00  0.56
H
ATOM   4693 2HB  ASN A 306      30.226   9.579   8.392  1.00  0.56
H
ATOM   4694 1HD2 ASN A 306      33.960   9.523   8.188  1.00  0.56
H
ATOM   4695 2HD2 ASN A 306      32.823   9.785   9.468  1.00  0.56
H
ATOM   4696  N   ARG A 307      27.925  12.291   8.166  1.00  0.34
N
ATOM   4697  CA  ARG A 307      26.507  12.273   8.505  1.00  0.48
C
ATOM   4698  C   ARG A 307      25.605  12.515   7.304  1.00  0.44
C
ATOM   4699  O   ARG A 307      24.396  12.333   7.403  1.00  0.57
O
ATOM   4700  CB  ARG A 307      26.186  13.304   9.567  1.00  0.72
C
ATOM   4701  CG  ARG A 307      26.762  13.016  10.939  1.00  0.72
C
ATOM   4702  CD  ARG A 307      26.546  14.157  11.857  1.00  0.72
C
ATOM   4703  NE  ARG A 307      27.346  15.298  11.461  1.00  0.72
N
ATOM   4704  CZ  ARG A 307      27.223  16.540  11.971  1.00  0.72
C
ATOM   4705  NH1 ARG A 307      26.314  16.803  12.881  1.00  0.72
N
ATOM   4706  NH2 ARG A 307      28.021  17.500  11.558  1.00  0.72
N
ATOM   4707  H   ARG A 307      28.464  13.119   8.392  1.00  0.41
H
```

```
ATOM   4708  HA  ARG A 307      26.273  11.288   8.911  1.00  0.58
H
ATOM   4709 1HB  ARG A 307      26.559  14.277   9.253  1.00  0.86
H
ATOM   4710 2HB  ARG A 307      25.106  13.389   9.677  1.00  0.86
H
ATOM   4711 1HG  ARG A 307      26.281  12.135  11.364  1.00  0.86
H
ATOM   4712 2HG  ARG A 307      27.836  12.840  10.856  1.00  0.86
H
ATOM   4713 1HD  ARG A 307      25.497  14.450  11.841  1.00  0.86
H
ATOM   4714 2HD  ARG A 307      26.832  13.874  12.868  1.00  0.86
H
ATOM   4715  HE  ARG A 307      28.058  15.142  10.753  1.00  0.86
H
ATOM   4716 1HH1 ARG A 307      25.702  16.069  13.213  1.00  0.86
H
ATOM   4717 2HH1 ARG A 307      26.234  17.738  13.255  1.00  0.86
H
ATOM   4718 1HH2 ARG A 307      28.723  17.310  10.857  1.00  0.86
H
ATOM   4719 2HH2 ARG A 307      27.938  18.430  11.948  1.00  0.86
H
ATOM   4720  N   VAL A 308      26.167  12.934   6.174  1.00  0.36
N
ATOM   4721  CA  VAL A 308      25.349  13.204   4.998  1.00  0.39
C
ATOM   4722  C   VAL A 308      24.820  11.931   4.344  1.00  0.29
C
ATOM   4723  O   VAL A 308      25.589  11.115   3.835  1.00  0.44
O
ATOM   4724  CB  VAL A 308      26.177  13.995   3.972  1.00  0.58
C
ATOM   4725  CG1 VAL A 308      25.391  14.218   2.685  1.00  0.58
C
ATOM   4726  CG2 VAL A 308      26.585  15.320   4.588  1.00  0.58
C
ATOM   4727  H   VAL A 308      27.170  13.076   6.109  1.00  0.43
H
ATOM   4728  HA  VAL A 308      24.499  13.814   5.307  1.00  0.47
H
ATOM   4729  HB  VAL A 308      27.068  13.420   3.721  1.00  0.70
H
ATOM   4730 1HG1 VAL A 308      26.006  14.769   1.973  1.00  0.70
H
ATOM   4731 2HG1 VAL A 308      25.115  13.255   2.257  1.00  0.70
H
ATOM   4732 3HG1 VAL A 308      24.494  14.787   2.902  1.00  0.70
H
ATOM   4733 1HG2 VAL A 308      27.196  15.863   3.875  1.00  0.70
H
ATOM   4734 2HG2 VAL A 308      25.696  15.901   4.834  1.00  0.70
H
ATOM   4735 3HG2 VAL A 308      27.163  15.140   5.495  1.00  0.70
H
ATOM   4736  N   VAL A 309      23.496  11.795   4.338  1.00  0.20
N
```

```
ATOM   4737  CA  VAL A 309      22.784  10.670   3.724  1.00  0.38
C
ATOM   4738  C   VAL A 309      21.780  11.175   2.704  1.00  0.30
C
ATOM   4739  O   VAL A 309      21.068  12.141   2.966  1.00  0.67
O
ATOM   4740  CB  VAL A 309      22.066   9.824   4.800  1.00  0.57
C
ATOM   4741  CG1 VAL A 309      21.205   8.738   4.164  1.00  0.57
C
ATOM   4742  CG2 VAL A 309      23.116   9.174   5.689  1.00  0.57
C
ATOM   4743  H   VAL A 309      22.943  12.508   4.796  1.00  0.24
H
ATOM   4744  HA  VAL A 309      23.510  10.038   3.214  1.00  0.46
H
ATOM   4745  HB  VAL A 309      21.417  10.469   5.393  1.00  0.68
H
ATOM   4746 1HG1 VAL A 309      20.714   8.163   4.949  1.00  0.68
H
ATOM   4747 2HG1 VAL A 309      20.449   9.195   3.527  1.00  0.68
H
ATOM   4748 3HG1 VAL A 309      21.829   8.076   3.570  1.00  0.68
H
ATOM   4749 1HG2 VAL A 309      22.623   8.584   6.461  1.00  0.68
H
ATOM   4750 2HG2 VAL A 309      23.752   8.526   5.086  1.00  0.68
H
ATOM   4751 3HG2 VAL A 309      23.726   9.944   6.158  1.00  0.68
H
ATOM   4752  N   VAL A 310      21.739  10.542   1.536  1.00  0.16
N
ATOM   4753  CA  VAL A 310      20.835  10.989   0.480  1.00  0.17
C
ATOM   4754  C   VAL A 310      19.836   9.894   0.105  1.00  0.15
C
ATOM   4755  O   VAL A 310      20.207   8.731  -0.025  1.00  0.17
O
ATOM   4756  CB  VAL A 310      21.635  11.452  -0.760  1.00  0.26
C
ATOM   4757  CG1 VAL A 310      20.692  11.873  -1.876  1.00  0.26
C
ATOM   4758  CG2 VAL A 310      22.539  12.621  -0.384  1.00  0.26
C
ATOM   4759  H   VAL A 310      22.336   9.735   1.391  1.00  0.19
H
ATOM   4760  HA  VAL A 310      20.280  11.846   0.854  1.00  0.20
H
ATOM   4761  HB  VAL A 310      22.236  10.623  -1.125  1.00  0.31
H
ATOM   4762 1HG1 VAL A 310      21.273  12.184  -2.743  1.00  0.31
H
ATOM   4763 2HG1 VAL A 310      20.051  11.038  -2.151  1.00  0.31
H
ATOM   4764 3HG1 VAL A 310      20.076  12.705  -1.537  1.00  0.31
H
ATOM   4765 1HG2 VAL A 310      23.104  12.936  -1.260  1.00  0.31
H
```

ATOM 4766 2HG2 VAL A 310 21.937 13.451 -0.030 1.00 0.31
H
ATOM 4767 3HG2 VAL A 310 23.231 12.313 0.399 1.00 0.31
H
ATOM 4768 N LEU A 311 18.569 10.280 -0.034 1.00 0.13
N
ATOM 4769 CA LEU A 311 17.491 9.344 -0.346 1.00 0.11
C
ATOM 4770 C LEU A 311 16.818 9.748 -1.651 1.00 0.12
C
ATOM 4771 O LEU A 311 16.724 10.938 -1.944 1.00 0.12
O
ATOM 4772 CB LEU A 311 16.431 9.363 0.770 1.00 0.17
C
ATOM 4773 CG LEU A 311 16.886 8.969 2.181 1.00 0.17
C
ATOM 4774 CD1 LEU A 311 17.460 10.192 2.891 1.00 0.17
C
ATOM 4775 CD2 LEU A 311 15.698 8.408 2.946 1.00 0.17
C
ATOM 4776 H LEU A 311 18.353 11.262 0.077 1.00 0.16
H
ATOM 4777 HA LEU A 311 17.905 8.345 -0.451 1.00 0.13
H
ATOM 4778 1HB LEU A 311 16.014 10.365 0.832 1.00 0.20
H
ATOM 4779 2HB LEU A 311 15.628 8.683 0.488 1.00 0.20
H
ATOM 4780 HG LEU A 311 17.673 8.222 2.121 1.00 0.20
H
ATOM 4781 1HD1 LEU A 311 17.787 9.910 3.893 1.00 0.20
H
ATOM 4782 2HD1 LEU A 311 18.309 10.582 2.333 1.00 0.20
H
ATOM 4783 3HD1 LEU A 311 16.692 10.961 2.964 1.00 0.20
H
ATOM 4784 1HD2 LEU A 311 16.013 8.125 3.950 1.00 0.20
H
ATOM 4785 2HD2 LEU A 311 14.915 9.164 3.011 1.00 0.20
H
ATOM 4786 3HD2 LEU A 311 15.312 7.531 2.426 1.00 0.20
H
ATOM 4787 N ASP A 312 16.337 8.781 -2.431 1.00 0.14
N
ATOM 4788 CA ASP A 312 15.671 9.168 -3.678 1.00 0.17
C
ATOM 4789 C ASP A 312 14.649 8.153 -4.191 1.00 0.27
C
ATOM 4790 O ASP A 312 14.934 6.960 -4.323 1.00 0.75
O
ATOM 4791 CB ASP A 312 16.701 9.417 -4.763 1.00 0.26
C
ATOM 4792 CG ASP A 312 16.135 10.115 -5.979 1.00 0.26
C
ATOM 4793 OD1 ASP A 312 15.247 9.568 -6.591 1.00 0.26
O
ATOM 4794 OD2 ASP A 312 16.612 11.190 -6.287 1.00 0.26
O

```
ATOM   4795  H   ASP A 312      16.454   7.810  -2.164  1.00  0.17
H
ATOM   4796  HA  ASP A 312      15.140  10.101  -3.493  1.00  0.20
H
ATOM   4797 1HB  ASP A 312      17.528  10.006  -4.364  1.00  0.31
H
ATOM   4798 2HB  ASP A 312      17.098   8.459  -5.087  1.00  0.31
H
ATOM   4799  N   GLY A 313      13.457   8.655  -4.514  1.00  0.32
N
ATOM   4800  CA  GLY A 313      12.348   7.833  -4.993  1.00  0.36
C
ATOM   4801  C   GLY A 313      12.390   7.477  -6.490  1.00  0.41
C
ATOM   4802  O   GLY A 313      11.497   7.860  -7.247  1.00  0.61
O
ATOM   4803  H   GLY A 313      13.307   9.649  -4.407  1.00  0.38
H
ATOM   4804 1HA  GLY A 313      12.341   6.914  -4.411  1.00  0.43
H
ATOM   4805 2HA  GLY A 313      11.411   8.343  -4.772  1.00  0.43
H
ATOM   4806  N   ASP A 314      13.413   6.728  -6.897  1.00  0.38
N
ATOM   4807  CA  ASP A 314      13.593   6.283  -8.288  1.00  0.49
C
ATOM   4808  C   ASP A 314      13.675   7.403  -9.328  1.00  0.57
C
ATOM   4809  O   ASP A 314      13.245   7.213 -10.464  1.00  1.07
O
ATOM   4810  CB  ASP A 314      12.448   5.335  -8.698  1.00  0.73
C
ATOM   4811  CG  ASP A 314      12.742   4.556  -9.976  1.00  0.73
C
ATOM   4812  OD1 ASP A 314      13.895   4.368 -10.273  1.00  0.73
O
ATOM   4813  OD2 ASP A 314      11.823   4.161 -10.658  1.00  0.73
O
ATOM   4814  H   ASP A 314      14.096   6.464  -6.198  1.00  0.46
H
ATOM   4815  HA  ASP A 314      14.526   5.723  -8.334  1.00  0.59
H
ATOM   4816 1HB  ASP A 314      12.264   4.624  -7.902  1.00  0.88
H
ATOM   4817 2HB  ASP A 314      11.533   5.908  -8.838  1.00  0.88
H
ATOM   4818  N   THR A 315      14.249   8.550  -8.961  1.00  0.38
N
ATOM   4819  CA  THR A 315      14.441   9.656  -9.891  1.00  0.41
C
ATOM   4820  C   THR A 315      15.911  10.086  -9.912  1.00  0.34
C
ATOM   4821  O   THR A 315      16.214  11.208 -10.306  1.00  0.44
O
ATOM   4822  CB  THR A 315      13.559  10.862  -9.522  1.00  0.61
C
ATOM   4823  OG1 THR A 315      13.877  11.287  -8.195  1.00  0.61
O
```

```
ATOM   4824  CG2 THR A 315      12.079  10.513  -9.601  1.00  0.61
C
ATOM   4825  H   THR A 315      14.570   8.681  -8.010  1.00  0.46
H
ATOM   4826  HA  THR A 315      14.165   9.329 -10.888  1.00  0.49
H
ATOM   4827  HB  THR A 315      13.763  11.679 -10.211  1.00  0.74
H
ATOM   4828  HG1 THR A 315      14.227  10.546  -7.688  1.00  0.74
H
ATOM   4829 1HG2 THR A 315      11.485  11.389  -9.337  1.00  0.74
H
ATOM   4830 2HG2 THR A 315      11.832  10.200 -10.615  1.00  0.74
H
ATOM   4831 3HG2 THR A 315      11.856   9.705  -8.908  1.00  0.74
H
ATOM   4832  N   ARG A 316      16.803   9.189  -9.469  1.00  0.32
N
ATOM   4833  CA  ARG A 316      18.249   9.438  -9.300  1.00  0.38
C
ATOM   4834  C   ARG A 316      19.008  10.081 -10.452  1.00  0.35
C
ATOM   4835  O   ARG A 316      19.971  10.820 -10.231  1.00  0.42
O
ATOM   4836  CB  ARG A 316      18.958   8.123  -9.023  1.00  0.57
C
ATOM   4837  CG  ARG A 316      18.728   7.547  -7.656  1.00  0.57
C
ATOM   4838  CD  ARG A 316      19.438   6.253  -7.471  1.00  0.57
C
ATOM   4839  NE  ARG A 316      18.778   5.152  -8.142  1.00  0.57
N
ATOM   4840  CZ  ARG A 316      19.108   3.859  -7.997  1.00  0.57
C
ATOM   4841  NH1 ARG A 316      20.095   3.496  -7.210  1.00  0.57
N
ATOM   4842  NH2 ARG A 316      18.396   2.965  -8.649  1.00  0.57
N
ATOM   4843  H   ARG A 316      16.461   8.279  -9.196  1.00  0.38
H
ATOM   4844  HA  ARG A 316      18.362  10.081  -8.428  1.00  0.46
H
ATOM   4845 1HB  ARG A 316      18.649   7.379  -9.753  1.00  0.68
H
ATOM   4846 2HB  ARG A 316      20.031   8.258  -9.132  1.00  0.68
H
ATOM   4847 1HG  ARG A 316      19.101   8.257  -6.922  1.00  0.68
H
ATOM   4848 2HG  ARG A 316      17.661   7.385  -7.501  1.00  0.68
H
ATOM   4849 1HD  ARG A 316      20.449   6.336  -7.868  1.00  0.68
H
ATOM   4850 2HD  ARG A 316      19.484   6.008  -6.421  1.00  0.68
H
ATOM   4851  HE  ARG A 316      17.991   5.342  -8.747  1.00  0.68
H
ATOM   4852 1HH1 ARG A 316      20.627   4.191  -6.706  1.00  0.68
H
```

```
ATOM   4853 2HH1 ARG A 316      20.285   2.510  -7.072  1.00  0.68
H
ATOM   4854 1HH2 ARG A 316      17.641   3.299  -9.236  1.00  0.68
H
ATOM   4855 2HH2 ARG A 316      18.602   1.981  -8.556  1.00  0.68
H
ATOM   4856  N   TYR A 317      18.600   9.800 -11.681  1.00  0.57
N
ATOM   4857  CA  TYR A 317      19.299  10.345 -12.839  1.00  0.83
C
ATOM   4858  C   TYR A 317      18.623  11.591 -13.372  1.00  0.62
C
ATOM   4859  O   TYR A 317      19.103  12.204 -14.326  1.00  0.68
O
ATOM   4860  CB  TYR A 317      19.379   9.292 -13.941  1.00  1.24
C
ATOM   4861  CG  TYR A 317      20.276   8.125 -13.609  1.00  1.24
C
ATOM   4862  CD1 TYR A 317      19.765   7.047 -12.915  1.00  1.24
C
ATOM   4863  CD2 TYR A 317      21.599   8.124 -14.020  1.00  1.24
C
ATOM   4864  CE1 TYR A 317      20.574   5.970 -12.617  1.00  1.24
C
ATOM   4865  CE2 TYR A 317      22.407   7.041 -13.733  1.00  1.24
C
ATOM   4866  CZ  TYR A 317      21.899   5.968 -13.030  1.00  1.24
C
ATOM   4867  OH  TYR A 317      22.703   4.890 -12.737  1.00  1.24
O
ATOM   4868  H   TYR A 317      17.807   9.193 -11.820  1.00  0.68
H
ATOM   4869  HA  TYR A 317      20.310  10.616 -12.539  1.00  1.00
H
ATOM   4870 1HB  TYR A 317      18.381   8.906 -14.150  1.00  1.49
H
ATOM   4871 2HB  TYR A 317      19.749   9.753 -14.857  1.00  1.49
H
ATOM   4872  HD1 TYR A 317      18.724   7.056 -12.604  1.00  1.49
H
ATOM   4873  HD2 TYR A 317      21.999   8.973 -14.575  1.00  1.49
H
ATOM   4874  HE1 TYR A 317      20.173   5.121 -12.065  1.00  1.49
H
ATOM   4875  HE2 TYR A 317      23.448   7.036 -14.057  1.00  1.49
H
ATOM   4876  HH  TYR A 317      23.625   5.146 -12.824  1.00  1.49
H
ATOM   4877  N   SER A 318      17.505  11.956 -12.762  1.00  0.57
N
ATOM   4878  CA  SER A 318      16.750  13.126 -13.151  1.00  0.59
C
ATOM   4879  C   SER A 318      16.979  14.239 -12.131  1.00  0.49
C
ATOM   4880  O   SER A 318      17.004  15.421 -12.469  1.00  0.54
O
ATOM   4881  CB  SER A 318      15.290  12.744 -13.271  1.00  0.89
C
```

```
ATOM   4882  OG  SER A 318      15.112  11.816 -14.309  1.00  0.89
O
ATOM   4883  H   SER A 318      17.156  11.423 -11.983  1.00  0.68
H
ATOM   4884  HA  SER A 318      17.106  13.468 -14.124  1.00  0.71
H
ATOM   4885 1HB  SER A 318      14.947  12.313 -12.331  1.00  1.06
H
ATOM   4886 2HB  SER A 318      14.689  13.624 -13.468  1.00  1.06
H
ATOM   4887  HG  SER A 318      15.412  12.255 -15.110  1.00  1.06
H
ATOM   4888  N   THR A 319      17.193  13.841 -10.879  1.00  0.41
N
ATOM   4889  CA  THR A 319      17.513  14.751  -9.783  1.00  0.38
C
ATOM   4890  C   THR A 319      19.014  14.892  -9.636  1.00  0.36
C
ATOM   4891  O   THR A 319      19.502  15.775  -8.930  1.00  0.37
O
ATOM   4892  CB  THR A 319      16.980  14.212  -8.448  1.00  0.57
C
ATOM   4893  OG1 THR A 319      17.639  12.961  -8.169  1.00  0.57
O
ATOM   4894  CG2 THR A 319      15.475  14.007  -8.510  1.00  0.57
C
ATOM   4895  H   THR A 319      17.113  12.858 -10.665  1.00  0.49
H
ATOM   4896  HA  THR A 319      17.084  15.732  -9.991  1.00  0.46
H
ATOM   4897  HB  THR A 319      17.208  14.918  -7.649  1.00  0.68
H
ATOM   4898  HG1 THR A 319      17.114  12.440  -7.546  1.00  0.68
H
ATOM   4899 1HG2 THR A 319      15.124  13.616  -7.555  1.00  0.68
H
ATOM   4900 2HG2 THR A 319      14.986  14.958  -8.715  1.00  0.68
H
ATOM   4901 3HG2 THR A 319      15.236  13.299  -9.300  1.00  0.68
H
ATOM   4902  N   PHE A 320      19.736  13.984 -10.291  1.00  0.38
N
ATOM   4903  CA  PHE A 320      21.181  13.850 -10.218  1.00  0.39
C
ATOM   4904  C   PHE A 320      21.693  13.537  -8.822  1.00  0.37
C
ATOM   4905  O   PHE A 320      22.879  13.719  -8.539  1.00  0.41
O
ATOM   4906  CB  PHE A 320      21.871  15.073 -10.800  1.00  0.58
C
ATOM   4907  CG  PHE A 320      21.527  15.251 -12.239  1.00  0.58
C
ATOM   4908  CD1 PHE A 320      20.650  16.235 -12.640  1.00  0.58
C
ATOM   4909  CD2 PHE A 320      22.074  14.413 -13.196  1.00  0.58
C
ATOM   4910  CE1 PHE A 320      20.328  16.385 -13.968  1.00  0.58
C
```

```
ATOM   4911  CE2 PHE A 320      21.749  14.560 -14.529  1.00  0.58
C
ATOM   4912  CZ  PHE A 320      20.873  15.549 -14.916  1.00  0.58
C
ATOM   4913  H   PHE A 320      19.254  13.310 -10.865  1.00  0.46
H
ATOM   4914  HA  PHE A 320      21.459  13.009 -10.854  1.00  0.47
H
ATOM   4915 1HB  PHE A 320      21.578  15.970 -10.259  1.00  0.70
H
ATOM   4916 2HB  PHE A 320      22.948  14.964 -10.715  1.00  0.70
H
ATOM   4917  HD1 PHE A 320      20.217  16.893 -11.891  1.00  0.70
H
ATOM   4918  HD2 PHE A 320      22.765  13.629 -12.886  1.00  0.70
H
ATOM   4919  HE1 PHE A 320      19.644  17.170 -14.270  1.00  0.70
H
ATOM   4920  HE2 PHE A 320      22.185  13.894 -15.274  1.00  0.70
H
ATOM   4921  HZ  PHE A 320      20.615  15.669 -15.967  1.00  0.70
H
ATOM   4922  N   SER A 321      20.828  12.937  -7.984  1.00  0.33
N
ATOM   4923  CA  SER A 321      21.250  12.446  -6.678  1.00  0.33
C
ATOM   4924  C   SER A 321      22.192  11.253  -6.865  1.00  0.40
C
ATOM   4925  O   SER A 321      22.991  10.937  -5.980  1.00  0.62
O
ATOM   4926  CB  SER A 321      20.046  12.071  -5.850  1.00  0.49
C
ATOM   4927  OG  SER A 321      19.402  10.952  -6.385  1.00  0.49
O
ATOM   4928  H   SER A 321      19.846  12.835  -8.235  1.00  0.40
H
ATOM   4929  HA  SER A 321      21.794  13.239  -6.163  1.00  0.40
H
ATOM   4930 1HB  SER A 321      20.341  11.875  -4.822  1.00  0.59
H
ATOM   4931 2HB  SER A 321      19.354  12.912  -5.837  1.00  0.59
H
ATOM   4932  HG  SER A 321      18.464  11.084  -6.205  1.00  0.59
H
ATOM   4933  N   GLU A 322      22.126  10.642  -8.056  1.00  0.38
N
ATOM   4934  CA  GLU A 322      23.042   9.606  -8.496  1.00  0.56
C
ATOM   4935  C   GLU A 322      24.508   9.992  -8.341  1.00  0.60
C
ATOM   4936  O   GLU A 322      25.345   9.118  -8.144  1.00  0.72
O
ATOM   4937  CB  GLU A 322      22.786   9.237  -9.948  1.00  0.84
C
ATOM   4938  CG  GLU A 322      23.741   8.186 -10.472  1.00  0.84
C
ATOM   4939  CD  GLU A 322      23.643   6.874  -9.748  1.00  0.84
C
```

ATOM	4940	OE1	GLU	A	322	22.706	6.674	-9.014	1.00	0.84	O
ATOM	4941	OE2	GLU	A	322	24.518	6.061	-9.929	1.00	0.84	O
ATOM	4942	H	GLU	A	322	21.395	10.908	-8.707	1.00	0.46	H
ATOM	4943	HA	GLU	A	322	22.863	8.721	-7.887	1.00	0.67	H
ATOM	4944	1HB	GLU	A	322	21.773	8.859	-10.065	1.00	1.01	H
ATOM	4945	2HB	GLU	A	322	22.881	10.124	-10.574	1.00	1.01	H
ATOM	4946	1HG	GLU	A	322	23.547	8.029	-11.528	1.00	1.01	H
ATOM	4947	2HG	GLU	A	322	24.755	8.569	-10.375	1.00	1.01	H
ATOM	4948	N	ILE	A	323	24.831	11.284	-8.470	1.00	0.56	N
ATOM	4949	CA	ILE	A	323	26.208	11.731	-8.332	1.00	0.64	C
ATOM	4950	C	ILE	A	323	26.705	11.372	-6.927	1.00	0.63	C
ATOM	4951	O	ILE	A	323	27.801	10.824	-6.767	1.00	0.95	O
ATOM	4952	CB	ILE	A	323	26.306	13.249	-8.589	1.00	0.96	C
ATOM	4953	CG1	ILE	A	323	25.991	13.547	-10.059	1.00	0.96	C
ATOM	4954	CG2	ILE	A	323	27.698	13.747	-8.253	1.00	0.96	C
ATOM	4955	CD1	ILE	A	323	25.780	15.016	-10.354	1.00	0.96	C
ATOM	4956	H	ILE	A	323	24.113	11.981	-8.636	1.00	0.67	H
ATOM	4957	HA	ILE	A	323	26.825	11.211	-9.063	1.00	0.77	H
ATOM	4958	HB	ILE	A	323	25.573	13.770	-7.976	1.00	1.15	H
ATOM	4959	1HG1	ILE	A	323	26.812	13.189	-10.677	1.00	1.15	H
ATOM	4960	2HG1	ILE	A	323	25.086	13.009	-10.345	1.00	1.15	H
ATOM	4961	1HG2	ILE	A	323	27.752	14.818	-8.432	1.00	1.15	H
ATOM	4962	2HG2	ILE	A	323	27.910	13.540	-7.212	1.00	1.15	H
ATOM	4963	3HG2	ILE	A	323	28.430	13.236	-8.879	1.00	1.15	H
ATOM	4964	1HD1	ILE	A	323	25.562	15.148	-11.414	1.00	1.15	H
ATOM	4965	2HD1	ILE	A	323	24.945	15.389	-9.763	1.00	1.15	H
ATOM	4966	3HD1	ILE	A	323	26.681	15.568	-10.097	1.00	1.15	H
ATOM	4967	N	PHE	A	324	25.877	11.666	-5.921	1.00	0.43	N
ATOM	4968	CA	PHE	A	324	26.163	11.292	-4.543	1.00	0.39	C

```
ATOM   4969  C   PHE A 324      26.234   9.779  -4.412  1.00  0.42
C
ATOM   4970  O   PHE A 324      27.144   9.260  -3.776  1.00  0.48
O
ATOM   4971  CB  PHE A 324      25.108  11.796  -3.571  1.00  0.58
C
ATOM   4972  CG  PHE A 324      25.419  11.404  -2.154  1.00  0.58
C
ATOM   4973  CD1 PHE A 324      26.123  12.249  -1.315  1.00  0.58
C
ATOM   4974  CD2 PHE A 324      25.032  10.168  -1.664  1.00  0.58
C
ATOM   4975  CE1 PHE A 324      26.415  11.881  -0.016  1.00  0.58
C
ATOM   4976  CE2 PHE A 324      25.323   9.797  -0.371  1.00  0.58
C
ATOM   4977  CZ  PHE A 324      26.011  10.655   0.458  1.00  0.58
C
ATOM   4978  H   PHE A 324      25.009  12.142  -6.123  1.00  0.52
H
ATOM   4979  HA  PHE A 324      27.130  11.708  -4.259  1.00  0.47
H
ATOM   4980 1HB  PHE A 324      25.046  12.883  -3.622  1.00  0.70
H
ATOM   4981 2HB  PHE A 324      24.132  11.394  -3.834  1.00  0.70
H
ATOM   4982  HD1 PHE A 324      26.440  13.217  -1.688  1.00  0.70
H
ATOM   4983  HD2 PHE A 324      24.494   9.486  -2.317  1.00  0.70
H
ATOM   4984  HE1 PHE A 324      26.963  12.561   0.634  1.00  0.70
H
ATOM   4985  HE2 PHE A 324      25.010   8.823  -0.009  1.00  0.70
H
ATOM   4986  HZ  PHE A 324      26.241  10.360   1.481  1.00  0.70
H
ATOM   4987  N   ASN A 325      25.249   9.081  -4.994  1.00  0.40
N
ATOM   4988  CA  ASN A 325      25.194   7.619  -4.949  1.00  0.43
C
ATOM   4989  C   ASN A 325      26.486   6.990  -5.454  1.00  0.53
C
ATOM   4990  O   ASN A 325      26.998   6.055  -4.847  1.00  0.62
O
ATOM   4991  CB  ASN A 325      24.003   7.100  -5.739  1.00  0.65
C
ATOM   4992  CG  ASN A 325      23.832   5.606  -5.633  1.00  0.65
C
ATOM   4993  OD1 ASN A 325      24.111   5.008  -4.587  1.00  0.65
O
ATOM   4994  ND2 ASN A 325      23.365   4.991  -6.692  1.00  0.65
N
ATOM   4995  H   ASN A 325      24.516   9.584  -5.480  1.00  0.48
H
ATOM   4996  HA  ASN A 325      25.075   7.312  -3.908  1.00  0.52
H
ATOM   4997 1HB  ASN A 325      23.091   7.589  -5.401  1.00  0.77
H
```

```
ATOM   4998 2HB  ASN A 325      24.134   7.350  -6.790  1.00  0.77
H
ATOM   4999 1HD2 ASN A 325      23.229   4.000  -6.677  1.00  0.77
H
ATOM   5000 2HD2 ASN A 325      23.142   5.513  -7.518  1.00  0.77
H
ATOM   5001  N   LYS A 326      27.015   7.501  -6.565  1.00  0.57
N
ATOM   5002  CA  LYS A 326      28.267   6.997  -7.112  1.00  0.83
C
ATOM   5003  C   LYS A 326      29.445   7.231  -6.176  1.00  0.88
C
ATOM   5004  O   LYS A 326      30.277   6.341  -5.986  1.00  1.00
O
ATOM   5005  CB  LYS A 326      28.576   7.663  -8.454  1.00  1.24
C
ATOM   5006  CG  LYS A 326      27.696   7.247  -9.622  1.00  1.24
C
ATOM   5007  CD  LYS A 326      28.105   7.994 -10.884  1.00  1.24
C
ATOM   5008  CE  LYS A 326      27.269   7.587 -12.088  1.00  1.24
C
ATOM   5009  NZ  LYS A 326      27.675   8.330 -13.316  1.00  1.24
N
ATOM   5010  H   LYS A 326      26.537   8.248  -7.045  1.00  0.68
H
ATOM   5011  HA  LYS A 326      28.168   5.922  -7.264  1.00  1.00
H
ATOM   5012 1HB  LYS A 326      28.485   8.744  -8.347  1.00  1.49
H
ATOM   5013 2HB  LYS A 326      29.610   7.452  -8.730  1.00  1.49
H
ATOM   5014 1HG  LYS A 326      27.796   6.175  -9.793  1.00  1.49
H
ATOM   5015 2HG  LYS A 326      26.654   7.464  -9.398  1.00  1.49
H
ATOM   5016 1HD  LYS A 326      27.991   9.066 -10.723  1.00  1.49
H
ATOM   5017 2HD  LYS A 326      29.153   7.788 -11.102  1.00  1.49
H
ATOM   5018 1HE  LYS A 326      27.390   6.520 -12.265  1.00  1.49
H
ATOM   5019 2HE  LYS A 326      26.219   7.792 -11.884  1.00  1.49
H
ATOM   5020 1HZ  LYS A 326      27.101   8.036 -14.093  1.00  1.49
H
ATOM   5021 2HZ  LYS A 326      27.554   9.322 -13.166  1.00  1.49
H
ATOM   5022 3HZ  LYS A 326      28.645   8.138 -13.521  1.00  1.49
H
ATOM   5023  N   GLU A 327      29.515   8.425  -5.588  1.00  0.93
N
ATOM   5024  CA  GLU A 327      30.623   8.763  -4.704  1.00  1.25
C
ATOM   5025  C   GLU A 327      30.544   8.088  -3.337  1.00  1.34
C
ATOM   5026  O   GLU A 327      31.568   7.671  -2.794  1.00  2.71
O
```

```
ATOM   5027  CB  GLU A 327      30.682  10.279  -4.519  1.00  1.88
C
ATOM   5028  CG  GLU A 327      31.059  11.055  -5.772  1.00  1.88
C
ATOM   5029  CD  GLU A 327      32.440  10.732  -6.271  1.00  1.88
C
ATOM   5030  OE1 GLU A 327      33.345  10.695  -5.474  1.00  1.88
O
ATOM   5031  OE2 GLU A 327      32.592  10.526  -7.452  1.00  1.88
O
ATOM   5032  H   GLU A 327      28.810   9.129  -5.785  1.00  1.12
H
ATOM   5033  HA  GLU A 327      31.546   8.436  -5.183  1.00  1.50
H
ATOM   5034 1HB  GLU A 327      29.713  10.643  -4.177  1.00  2.25
H
ATOM   5035 2HB  GLU A 327      31.415  10.520  -3.751  1.00  2.25
H
ATOM   5036 1HG  GLU A 327      30.341  10.829  -6.558  1.00  2.25
H
ATOM   5037 2HG  GLU A 327      30.999  12.121  -5.559  1.00  2.25
H
ATOM   5038  N   TYR A 328      29.330   7.957  -2.793  1.00  0.62
N
ATOM   5039  CA  TYR A 328      29.124   7.359  -1.472  1.00  0.70
C
ATOM   5040  C   TYR A 328      27.894   6.457  -1.405  1.00  0.67
C
ATOM   5041  O   TYR A 328      26.947   6.779  -0.685  1.00  0.97
O
ATOM   5042  CB  TYR A 328      28.976   8.467  -0.421  1.00  1.05
C
ATOM   5043  CG  TYR A 328      30.165   9.380  -0.401  1.00  1.05
C
ATOM   5044  CD1 TYR A 328      30.099  10.625  -1.013  1.00  1.05
C
ATOM   5045  CD2 TYR A 328      31.339   8.944   0.186  1.00  1.05
C
ATOM   5046  CE1 TYR A 328      31.223  11.423  -1.047  1.00  1.05
C
ATOM   5047  CE2 TYR A 328      32.458   9.744   0.151  1.00  1.05
C
ATOM   5048  CZ  TYR A 328      32.402  10.970  -0.469  1.00  1.05
C
ATOM   5049  OH  TYR A 328      33.531  11.741  -0.529  1.00  1.05
O
ATOM   5050  H   TYR A 328      28.528   8.304  -3.288  1.00  0.74
H
ATOM   5051  HA  TYR A 328      29.997   6.755  -1.227  1.00  0.84
H
ATOM   5052 1HB  TYR A 328      28.092   9.061  -0.640  1.00  1.26
H
ATOM   5053 2HB  TYR A 328      28.857   8.030   0.570  1.00  1.26
H
ATOM   5054  HD1 TYR A 328      29.172  10.960  -1.480  1.00  1.26
H
ATOM   5055  HD2 TYR A 328      31.383   7.962   0.658  1.00  1.26
H
```

ATOM	5056	HE1	TYR	A	328	31.189	12.394	-1.539	1.00	1.26	H
ATOM	5057	HE2	TYR	A	328	33.389	9.398	0.599	1.00	1.26	H
ATOM	5058	HH	TYR	A	328	33.437	12.381	-1.239	1.00	1.26	H
ATOM	5059	N	PRO	A	329	27.893	5.330	-2.140	1.00	0.66	N
ATOM	5060	CA	PRO	A	329	26.849	4.305	-2.245	1.00	0.66	C
ATOM	5061	C	PRO	A	329	26.292	3.882	-0.896	1.00	0.65	C
ATOM	5062	O	PRO	A	329	25.084	3.706	-0.730	1.00	0.93	O
ATOM	5063	CB	PRO	A	329	27.591	3.107	-2.851	1.00	0.99	C
ATOM	5064	CG	PRO	A	329	28.672	3.712	-3.654	1.00	0.99	C
ATOM	5065	CD	PRO	A	329	29.138	4.859	-2.797	1.00	0.99	C
ATOM	5066	HA	PRO	A	329	26.047	4.661	-2.909	1.00	0.79	H
ATOM	5067	1HB	PRO	A	329	27.971	2.450	-2.055	1.00	1.19	H
ATOM	5068	2HB	PRO	A	329	26.899	2.506	-3.460	1.00	1.19	H
ATOM	5069	1HG	PRO	A	329	29.465	2.974	-3.838	1.00	1.19	H
ATOM	5070	2HG	PRO	A	329	28.296	4.027	-4.637	1.00	1.19	H
ATOM	5071	1HD	PRO	A	329	29.868	4.522	-2.045	1.00	1.19	H
ATOM	5072	2HD	PRO	A	329	29.556	5.616	-3.462	1.00	1.19	H
ATOM	5073	N	GLU	A	330	27.191	3.716	0.072	1.00	0.46	N
ATOM	5074	CA	GLU	A	330	26.836	3.235	1.402	1.00	0.58	C
ATOM	5075	C	GLU	A	330	25.995	4.217	2.217	1.00	0.25	C
ATOM	5076	O	GLU	A	330	25.430	3.839	3.242	1.00	0.68	O
ATOM	5077	CB	GLU	A	330	28.109	2.904	2.175	1.00	0.87	C
ATOM	5078	CG	GLU	A	330	28.905	4.130	2.573	1.00	0.87	C
ATOM	5079	CD	GLU	A	330	29.797	4.652	1.492	1.00	0.87	C
ATOM	5080	OE1	GLU	A	330	29.848	4.061	0.441	1.00	0.87	O
ATOM	5081	OE2	GLU	A	330	30.403	5.676	1.712	1.00	0.87	O
ATOM	5082	H	GLU	A	330	28.162	3.900	-0.130	1.00	0.55	H
ATOM	5083	HA	GLU	A	330	26.255	2.319	1.283	1.00	0.70	H
ATOM	5084	1HB	GLU	A	330	27.857	2.355	3.082	1.00	1.04	H

```
ATOM   5085 2HB  GLU A 330      28.751   2.264   1.568  1.00  1.04
H
ATOM   5086 1HG  GLU A 330      28.216   4.910   2.864  1.00  1.04
H
ATOM   5087 2HG  GLU A 330      29.511   3.880   3.442  1.00  1.04
H
ATOM   5088  N   ARG A 331      25.907   5.469   1.767  1.00  0.44
N
ATOM   5089  CA  ARG A 331      25.107   6.484   2.427  1.00  0.30
C
ATOM   5090  C   ARG A 331      23.947   6.915   1.552  1.00  0.27
C
ATOM   5091  O   ARG A 331      23.384   7.991   1.756  1.00  0.50
O
ATOM   5092  CB  ARG A 331      25.963   7.685   2.799  1.00  0.45
C
ATOM   5093  CG  ARG A 331      26.948   7.427   3.928  1.00  0.45
C
ATOM   5094  CD  ARG A 331      27.973   8.494   4.069  1.00  0.45
C
ATOM   5095  NE  ARG A 331      28.768   8.294   5.273  1.00  0.45
N
ATOM   5096  CZ  ARG A 331      29.822   7.456   5.402  1.00  0.45
C
ATOM   5097  NH1 ARG A 331      30.269   6.735   4.395  1.00  0.45
N
ATOM   5098  NH2 ARG A 331      30.421   7.351   6.573  1.00  0.45
N
ATOM   5099  H   ARG A 331      26.397   5.742   0.927  1.00  0.53
H
ATOM   5100  HA  ARG A 331      24.702   6.062   3.344  1.00  0.36
H
ATOM   5101 1HB  ARG A 331      26.537   8.006   1.931  1.00  0.54
H
ATOM   5102 2HB  ARG A 331      25.324   8.516   3.098  1.00  0.54
H
ATOM   5103 1HG  ARG A 331      26.399   7.369   4.869  1.00  0.54
H
ATOM   5104 2HG  ARG A 331      27.463   6.487   3.764  1.00  0.54
H
ATOM   5105 1HD  ARG A 331      28.642   8.480   3.210  1.00  0.54
H
ATOM   5106 2HD  ARG A 331      27.487   9.466   4.139  1.00  0.54
H
ATOM   5107  HE  ARG A 331      28.490   8.824   6.092  1.00  0.54
H
ATOM   5108 1HH1 ARG A 331      29.847   6.781   3.475  1.00  0.54
H
ATOM   5109 2HH1 ARG A 331      31.058   6.120   4.529  1.00  0.54
H
ATOM   5110 1HH2 ARG A 331      30.089   7.892   7.357  1.00  0.54
H
ATOM   5111 2HH2 ARG A 331      31.209   6.730   6.681  1.00  0.54
H
ATOM   5112  N   PHE A 332      23.598   6.089   0.569  1.00  0.27
N
ATOM   5113  CA  PHE A 332      22.513   6.437  -0.317  1.00  0.26
C
```

```
ATOM   5114  C   PHE A 332      21.381   5.409  -0.228  1.00  0.23
C
ATOM   5115  O   PHE A 332      21.623   4.203  -0.310  1.00  0.28
O
ATOM   5116  CB  PHE A 332      23.012   6.532  -1.741  1.00  0.39
C
ATOM   5117  CG  PHE A 332      21.930   6.990  -2.641  1.00  0.39
C
ATOM   5118  CD1 PHE A 332      21.804   8.332  -2.947  1.00  0.39
C
ATOM   5119  CD2 PHE A 332      21.010   6.092  -3.141  1.00  0.39
C
ATOM   5120  CE1 PHE A 332      20.785   8.768  -3.751  1.00  0.39
C
ATOM   5121  CE2 PHE A 332      19.982   6.529  -3.931  1.00  0.39
C
ATOM   5122  CZ  PHE A 332      19.878   7.867  -4.236  1.00  0.39
C
ATOM   5123  H   PHE A 332      24.088   5.216   0.411  1.00  0.32
H
ATOM   5124  HA  PHE A 332      22.125   7.407  -0.020  1.00  0.31
H
ATOM   5125 1HB  PHE A 332      23.841   7.236  -1.805  1.00  0.47
H
ATOM   5126 2HB  PHE A 332      23.364   5.560  -2.084  1.00  0.47
H
ATOM   5127  HD1 PHE A 332      22.519   9.042  -2.545  1.00  0.47
H
ATOM   5128  HD2 PHE A 332      21.099   5.035  -2.888  1.00  0.47
H
ATOM   5129  HE1 PHE A 332      20.692   9.824  -3.996  1.00  0.47
H
ATOM   5130  HE2 PHE A 332      19.251   5.821  -4.318  1.00  0.47
H
ATOM   5131  HZ  PHE A 332      19.074   8.213  -4.866  1.00  0.47
H
ATOM   5132  N   ILE A 333      20.148   5.889  -0.046  1.00  0.18
N
ATOM   5133  CA  ILE A 333      18.998   5.002   0.103  1.00  0.16
C
ATOM   5134  C   ILE A 333      18.025   5.060  -1.078  1.00  0.18
C
ATOM   5135  O   ILE A 333      17.395   6.090  -1.337  1.00  0.21
O
ATOM   5136  CB  ILE A 333      18.217   5.334   1.391  1.00  0.24
C
ATOM   5137  CG1 ILE A 333      19.124   5.199   2.617  1.00  0.24
C
ATOM   5138  CG2 ILE A 333      17.015   4.407   1.527  1.00  0.24
C
ATOM   5139  CD1 ILE A 333      18.494   5.691   3.901  1.00  0.24
C
ATOM   5140  H   ILE A 333      20.013   6.888  -0.004  1.00  0.22
H
ATOM   5141  HA  ILE A 333      19.366   3.981   0.184  1.00  0.19
H
ATOM   5142  HB  ILE A 333      17.870   6.361   1.345  1.00  0.29
H
```

ATOM 5143 1HG1 ILE A 333 19.394 4.153 2.739 1.00 0.29
H
ATOM 5144 2HG1 ILE A 333 20.037 5.769 2.447 1.00 0.29
H
ATOM 5145 1HG2 ILE A 333 16.464 4.656 2.434 1.00 0.29
H
ATOM 5146 2HG2 ILE A 333 16.365 4.524 0.663 1.00 0.29
H
ATOM 5147 3HG2 ILE A 333 17.360 3.374 1.585 1.00 0.29
H
ATOM 5148 1HD1 ILE A 333 19.198 5.562 4.724 1.00 0.29
H
ATOM 5149 2HD1 ILE A 333 18.240 6.746 3.802 1.00 0.29
H
ATOM 5150 3HD1 ILE A 333 17.590 5.117 4.105 1.00 0.29
H
ATOM 5151 N GLU A 334 17.885 3.922 -1.761 1.00 0.24
N
ATOM 5152 CA GLU A 334 16.927 3.766 -2.853 1.00 0.29
C
ATOM 5153 C GLU A 334 15.556 3.624 -2.229 1.00 0.31
C
ATOM 5154 O GLU A 334 15.339 2.678 -1.469 1.00 0.65
O
ATOM 5155 CB GLU A 334 17.200 2.485 -3.663 1.00 0.43
C
ATOM 5156 CG GLU A 334 18.515 2.411 -4.409 1.00 0.43
C
ATOM 5157 CD GLU A 334 18.718 1.056 -5.061 1.00 0.43
C
ATOM 5158 OE1 GLU A 334 18.116 0.109 -4.598 1.00 0.43
O
ATOM 5159 OE2 GLU A 334 19.458 0.972 -6.019 1.00 0.43
O
ATOM 5160 H GLU A 334 18.453 3.127 -1.499 1.00 0.29
H
ATOM 5161 HA GLU A 334 16.947 4.645 -3.496 1.00 0.35
H
ATOM 5162 1HB GLU A 334 17.154 1.620 -3.003 1.00 0.52
H
ATOM 5163 2HB GLU A 334 16.409 2.362 -4.402 1.00 0.52
H
ATOM 5164 1HG GLU A 334 18.519 3.185 -5.170 1.00 0.52
H
ATOM 5165 2HG GLU A 334 19.332 2.609 -3.716 1.00 0.52
H
ATOM 5166 N CYS A 335 14.631 4.527 -2.529 1.00 0.20
N
ATOM 5167 CA CYS A 335 13.314 4.421 -1.913 1.00 0.17
C
ATOM 5168 C CYS A 335 12.232 4.007 -2.897 1.00 0.39
C
ATOM 5169 O CYS A 335 11.117 3.685 -2.483 1.00 1.81
O
ATOM 5170 CB CYS A 335 12.926 5.753 -1.277 1.00 0.26
C
ATOM 5171 SG CYS A 335 14.029 6.272 0.057 1.00 0.26
S

ATOM 5172 H CYS A 335 14.835 5.294 -3.160 1.00 0.24
H
ATOM 5173 HA CYS A 335 13.365 3.670 -1.124 1.00 0.20
H
ATOM 5174 1HB CYS A 335 12.929 6.533 -2.040 1.00 0.31
H
ATOM 5175 2HB CYS A 335 11.915 5.688 -0.878 1.00 0.31
H
ATOM 5176 HG CYS A 335 13.779 5.237 0.853 1.00 0.31
H
ATOM 5177 N PHE A 336 12.581 3.979 -4.187 1.00 0.22
N
ATOM 5178 CA PHE A 336 11.670 3.587 -5.260 1.00 0.20
C
ATOM 5179 C PHE A 336 10.494 4.553 -5.357 1.00 0.19
C
ATOM 5180 O PHE A 336 10.477 5.580 -4.680 1.00 0.30
O
ATOM 5181 CB PHE A 336 11.213 2.142 -5.083 1.00 0.30
C
ATOM 5182 CG PHE A 336 12.362 1.194 -5.222 1.00 0.30
C
ATOM 5183 CD1 PHE A 336 12.998 0.685 -4.101 1.00 0.30
C
ATOM 5184 CD2 PHE A 336 12.826 0.827 -6.475 1.00 0.30
C
ATOM 5185 CE1 PHE A 336 14.067 -0.173 -4.229 1.00 0.30
C
ATOM 5186 CE2 PHE A 336 13.896 -0.032 -6.604 1.00 0.30
C
ATOM 5187 CZ PHE A 336 14.517 -0.534 -5.481 1.00 0.30
C
ATOM 5188 H PHE A 336 13.518 4.249 -4.443 1.00 0.26
H
ATOM 5189 HA PHE A 336 12.220 3.630 -6.197 1.00 0.24
H
ATOM 5190 1HB PHE A 336 10.758 1.994 -4.107 1.00 0.36
H
ATOM 5191 2HB PHE A 336 10.470 1.887 -5.836 1.00 0.36
H
ATOM 5192 HD1 PHE A 336 12.645 0.972 -3.109 1.00 0.36
H
ATOM 5193 HD2 PHE A 336 12.338 1.229 -7.364 1.00 0.36
H
ATOM 5194 HE1 PHE A 336 14.555 -0.561 -3.338 1.00 0.36
H
ATOM 5195 HE2 PHE A 336 14.253 -0.311 -7.593 1.00 0.36
H
ATOM 5196 HZ PHE A 336 15.363 -1.210 -5.587 1.00 0.36
H
ATOM 5197 N MET A 337 9.503 4.251 -6.188 1.00 0.22
N
ATOM 5198 CA MET A 337 8.363 5.161 -6.291 1.00 0.24
C
ATOM 5199 C MET A 337 7.463 5.009 -5.065 1.00 0.29
C
ATOM 5200 O MET A 337 6.469 4.284 -5.082 1.00 0.62
O

ATOM 5201 CB MET A 337 7.580 4.916 -7.587 1.00 0.36
C
ATOM 5202 CG MET A 337 8.338 5.243 -8.872 1.00 0.36
C
ATOM 5203 SD MET A 337 8.685 7.006 -9.046 1.00 0.36
S
ATOM 5204 CE MET A 337 9.513 7.072 -10.635 1.00 0.36
C
ATOM 5205 H MET A 337 9.529 3.407 -6.744 1.00 0.26
H
ATOM 5206 HA MET A 337 8.736 6.185 -6.303 1.00 0.29
H
ATOM 5207 1HB MET A 337 7.280 3.874 -7.647 1.00 0.43
H
ATOM 5208 2HB MET A 337 6.669 5.516 -7.577 1.00 0.43
H
ATOM 5209 1HG MET A 337 9.284 4.702 -8.885 1.00 0.43
H
ATOM 5210 2HG MET A 337 7.750 4.920 -9.732 1.00 0.43
H
ATOM 5211 1HE MET A 337 9.782 8.103 -10.863 1.00 0.43
H
ATOM 5212 2HE MET A 337 10.414 6.458 -10.605 1.00 0.43
H
ATOM 5213 3HE MET A 337 8.844 6.692 -11.408 1.00 0.43
H
ATOM 5214 N ALA A 338 7.875 5.654 -3.983 1.00 0.22
N
ATOM 5215 CA ALA A 338 7.190 5.648 -2.701 1.00 0.21
C
ATOM 5216 C ALA A 338 7.528 6.940 -1.977 1.00 0.23
C
ATOM 5217 O ALA A 338 8.333 6.928 -1.045 1.00 0.34
O
ATOM 5218 CB ALA A 338 7.596 4.429 -1.885 1.00 0.32
C
ATOM 5219 H ALA A 338 8.725 6.193 -4.072 1.00 0.26
H
ATOM 5220 HA ALA A 338 6.114 5.624 -2.878 1.00 0.25
H
ATOM 5221 1HB ALA A 338 7.072 4.440 -0.931 1.00 0.38
H
ATOM 5222 2HB ALA A 338 7.336 3.524 -2.432 1.00 0.38
H
ATOM 5223 3HB ALA A 338 8.671 4.451 -1.709 1.00 0.38
H
ATOM 5224 N GLU A 339 6.942 8.049 -2.429 1.00 0.36
N
ATOM 5225 CA GLU A 339 7.296 9.376 -1.926 1.00 0.50
C
ATOM 5226 C GLU A 339 7.016 9.543 -0.446 1.00 0.31
C
ATOM 5227 O GLU A 339 7.809 10.147 0.283 1.00 0.29
O
ATOM 5228 CB GLU A 339 6.491 10.441 -2.674 1.00 0.75
C
ATOM 5229 CG GLU A 339 6.784 10.533 -4.159 1.00 0.75
C

```
ATOM   5230  CD  GLU A 339       8.213  10.817  -4.451  1.00  0.75
C
ATOM   5231  OE1 GLU A 339       8.946  11.076  -3.530  1.00  0.75
O
ATOM   5232  OE2 GLU A 339       8.580  10.785  -5.602  1.00  0.75
O
ATOM   5233  H   GLU A 339       6.258   7.970  -3.172  1.00  0.43
H
ATOM   5234  HA  GLU A 339       8.362   9.536  -2.095  1.00  0.60
H
ATOM   5235 1HB  GLU A 339       5.428  10.236  -2.559  1.00  0.90
H
ATOM   5236 2HB  GLU A 339       6.686  11.419  -2.235  1.00  0.90
H
ATOM   5237 1HG  GLU A 339       6.507   9.591  -4.630  1.00  0.90
H
ATOM   5238 2HG  GLU A 339       6.166  11.318  -4.591  1.00  0.90
H
ATOM   5239  N   GLN A 340       5.885   8.999  -0.014  1.00  0.32
N
ATOM   5240  CA  GLN A 340       5.458   9.069   1.367  1.00  0.35
C
ATOM   5241  C   GLN A 340       6.439   8.361   2.271  1.00  0.20
C
ATOM   5242  O   GLN A 340       6.938   8.944   3.241  1.00  0.24
O
ATOM   5243  CB  GLN A 340       4.065   8.471   1.482  1.00  0.52
C
ATOM   5244  CG  GLN A 340       3.007   9.346   0.851  1.00  0.52
C
ATOM   5245  CD  GLN A 340       1.614   8.769   0.934  1.00  0.52
C
ATOM   5246  OE1 GLN A 340       1.404   7.597   1.262  1.00  0.52
O
ATOM   5247  NE2 GLN A 340       0.641   9.616   0.630  1.00  0.52
N
ATOM   5248  H   GLN A 340       5.292   8.518  -0.675  1.00  0.38
H
ATOM   5249  HA  GLN A 340       5.416  10.114   1.669  1.00  0.42
H
ATOM   5250 1HB  GLN A 340       4.044   7.516   0.960  1.00  0.63
H
ATOM   5251 2HB  GLN A 340       3.816   8.273   2.515  1.00  0.63
H
ATOM   5252 1HG  GLN A 340       3.000  10.307   1.363  1.00  0.63
H
ATOM   5253 2HG  GLN A 340       3.252   9.486  -0.200  1.00  0.63
H
ATOM   5254 1HE2 GLN A 340      -0.314   9.321   0.660  1.00  0.63
H
ATOM   5255 2HE2 GLN A 340       0.875  10.554   0.361  1.00  0.63
H
ATOM   5256  N   ASN A 341       6.746   7.120   1.907  1.00  0.15
N
ATOM   5257  CA  ASN A 341       7.732   6.326   2.608  1.00  0.13
C
ATOM   5258  C   ASN A 341       9.084   7.014   2.666  1.00  0.09
C
```

```
ATOM   5259  O   ASN A 341       9.678   7.073   3.739  1.00  0.14
O
ATOM   5260  CB  ASN A 341       7.861   4.970   1.969  1.00  0.20
C
ATOM   5261  CG  ASN A 341       8.806   4.069   2.689  1.00  0.20
C
ATOM   5262  OD1 ASN A 341       8.612   3.725   3.861  1.00  0.20
O
ATOM   5263  ND2 ASN A 341       9.844   3.677   2.004  1.00  0.20
N
ATOM   5264  H   ASN A 341       6.252   6.689   1.130  1.00  0.18
H
ATOM   5265  HA  ASN A 341       7.393   6.190   3.636  1.00  0.16
H
ATOM   5266 1HB  ASN A 341       6.882   4.503   1.955  1.00  0.23
H
ATOM   5267 2HB  ASN A 341       8.190   5.081   0.936  1.00  0.23
H
ATOM   5268 1HD2 ASN A 341      10.518   3.073   2.425  1.00  0.23
H
ATOM   5269 2HD2 ASN A 341       9.961   3.981   1.059  1.00  0.23
H
ATOM   5270  N   MET A 342       9.559   7.542   1.533  1.00  0.11
N
ATOM   5271  CA  MET A 342      10.855   8.221   1.475  1.00  0.12
C
ATOM   5272  C   MET A 342      11.000   9.272   2.561  1.00  0.11
C
ATOM   5273  O   MET A 342      12.005   9.291   3.277  1.00  0.12
O
ATOM   5274  CB  MET A 342      11.057   8.880   0.109  1.00  0.18
C
ATOM   5275  CG  MET A 342      12.374   9.641  -0.028  1.00  0.18
C
ATOM   5276  SD  MET A 342      12.513  10.532  -1.592  1.00  0.18
S
ATOM   5277  CE  MET A 342      11.333  11.854  -1.331  1.00  0.18
C
ATOM   5278  H   MET A 342       9.022   7.457   0.679  1.00  0.13
H
ATOM   5279  HA  MET A 342      11.638   7.480   1.626  1.00  0.14
H
ATOM   5280 1HB  MET A 342      11.025   8.119  -0.670  1.00  0.22
H
ATOM   5281 2HB  MET A 342      10.245   9.581  -0.085  1.00  0.22
H
ATOM   5282 1HG  MET A 342      12.460  10.361   0.785  1.00  0.22
H
ATOM   5283 2HG  MET A 342      13.208   8.946   0.046  1.00  0.22
H
ATOM   5284 1HE  MET A 342      11.304  12.495  -2.211  1.00  0.22
H
ATOM   5285 2HE  MET A 342      10.342  11.428  -1.160  1.00  0.22
H
ATOM   5286 3HE  MET A 342      11.629  12.443  -0.463  1.00  0.22
H
ATOM   5287  N   VAL A 343       9.996  10.142   2.681  1.00  0.10
N
```

```
ATOM   5288  CA  VAL A 343      10.039  11.184   3.692  1.00  0.12
C
ATOM   5289  C   VAL A 343      10.046  10.593   5.098  1.00  0.10
C
ATOM   5290  O   VAL A 343      10.813  11.053   5.942  1.00  0.12
O
ATOM   5291  CB  VAL A 343       8.859  12.161   3.537  1.00  0.18
C
ATOM   5292  CG1 VAL A 343       8.824  13.123   4.721  1.00  0.18
C
ATOM   5293  CG2 VAL A 343       9.012  12.930   2.232  1.00  0.18
C
ATOM   5294  H   VAL A 343       9.201  10.086   2.048  1.00  0.12
H
ATOM   5295  HA  VAL A 343      10.959  11.752   3.556  1.00  0.14
H
ATOM   5296  HB  VAL A 343       7.923  11.603   3.530  1.00  0.22
H
ATOM   5297 1HG1 VAL A 343       7.984  13.808   4.609  1.00  0.22
H
ATOM   5298 2HG1 VAL A 343       8.711  12.558   5.647  1.00  0.22
H
ATOM   5299 3HG1 VAL A 343       9.754  13.691   4.755  1.00  0.22
H
ATOM   5300 1HG2 VAL A 343       8.178  13.622   2.117  1.00  0.22
H
ATOM   5301 2HG2 VAL A 343       9.948  13.490   2.247  1.00  0.22
H
ATOM   5302 3HG2 VAL A 343       9.021  12.231   1.395  1.00  0.22
H
ATOM   5303  N   SER A 344       9.206   9.578   5.354  1.00  0.11
N
ATOM   5304  CA  SER A 344       9.170   8.972   6.687  1.00  0.18
C
ATOM   5305  C   SER A 344      10.474   8.277   7.060  1.00  0.13
C
ATOM   5306  O   SER A 344      10.865   8.292   8.226  1.00  0.24
O
ATOM   5307  CB  SER A 344       8.066   7.951   6.768  1.00  0.27
C
ATOM   5308  OG  SER A 344       8.376   6.837   5.987  1.00  0.27
O
ATOM   5309  H   SER A 344       8.580   9.236   4.630  1.00  0.13
H
ATOM   5310  HA  SER A 344       8.983   9.757   7.413  1.00  0.22
H
ATOM   5311 1HB  SER A 344       7.907   7.652   7.803  1.00  0.32
H
ATOM   5312 2HB  SER A 344       7.147   8.391   6.406  1.00  0.32
H
ATOM   5313  HG  SER A 344       8.696   7.182   5.151  1.00  0.32
H
ATOM   5314  N   VAL A 345      11.166   7.715   6.067  1.00  0.14
N
ATOM   5315  CA  VAL A 345      12.454   7.080   6.304  1.00  0.12
C
ATOM   5316  C   VAL A 345      13.460   8.131   6.701  1.00  0.13
C
```

```
ATOM   5317  O   VAL A 345      14.223   7.929   7.642  1.00  0.14
O
ATOM   5318  CB  VAL A 345      12.936   6.294   5.065  1.00  0.18
C
ATOM   5319  CG1 VAL A 345      14.373   5.821   5.254  1.00  0.18
C
ATOM   5320  CG2 VAL A 345      12.036   5.080   4.866  1.00  0.18
C
ATOM   5321  H   VAL A 345      10.786   7.709   5.132  1.00  0.17
H
ATOM   5322  HA  VAL A 345      12.346   6.374   7.128  1.00  0.14
H
ATOM   5323  HB  VAL A 345      12.900   6.941   4.188  1.00  0.22
H
ATOM   5324 1HG1 VAL A 345      14.695   5.272   4.370  1.00  0.22
H
ATOM   5325 2HG1 VAL A 345      15.023   6.682   5.403  1.00  0.22
H
ATOM   5326 3HG1 VAL A 345      14.428   5.170   6.122  1.00  0.22
H
ATOM   5327 1HG2 VAL A 345      12.365   4.523   3.990  1.00  0.22
H
ATOM   5328 2HG2 VAL A 345      12.094   4.440   5.745  1.00  0.22
H
ATOM   5329 3HG2 VAL A 345      11.008   5.401   4.723  1.00  0.22
H
ATOM   5330  N   ALA A 346      13.443   9.263   5.994  1.00  0.12
N
ATOM   5331  CA  ALA A 346      14.317  10.374   6.326  1.00  0.12
C
ATOM   5332  C   ALA A 346      14.032  10.870   7.748  1.00  0.12
C
ATOM   5333  O   ALA A 346      14.970  11.131   8.505  1.00  0.14
O
ATOM   5334  CB  ALA A 346      14.143  11.497   5.313  1.00  0.18
C
ATOM   5335  H   ALA A 346      12.814   9.353   5.203  1.00  0.14
H
ATOM   5336  HA  ALA A 346      15.347  10.020   6.291  1.00  0.14
H
ATOM   5337 1HB  ALA A 346      14.819  12.315   5.555  1.00  0.22
H
ATOM   5338 2HB  ALA A 346      14.371  11.123   4.316  1.00  0.22
H
ATOM   5339 3HB  ALA A 346      13.117  11.857   5.338  1.00  0.22
H
ATOM   5340  N   LEU A 347      12.740  10.961   8.118  1.00  0.12
N
ATOM   5341  CA  LEU A 347      12.342  11.395   9.461  1.00  0.13
C
ATOM   5342  C   LEU A 347      12.901  10.468  10.528  1.00  0.14
C
ATOM   5343  O   LEU A 347      13.399  10.928  11.559  1.00  0.16
O
ATOM   5344  CB  LEU A 347      10.800  11.419   9.600  1.00  0.20
C
ATOM   5345  CG  LEU A 347      10.047  12.503   8.820  1.00  0.20
C
```

```
ATOM   5346  CD1 LEU A 347       8.540  12.268   8.910  1.00  0.20
C
ATOM   5347  CD2 LEU A 347      10.396  13.845   9.403  1.00  0.20
C
ATOM   5348  H   LEU A 347      12.017  10.750   7.444  1.00  0.14
H
ATOM   5349  HA  LEU A 347      12.731  12.398   9.632  1.00  0.16
H
ATOM   5350 1HB  LEU A 347      10.409  10.457   9.280  1.00  0.23
H
ATOM   5351 2HB  LEU A 347      10.555  11.548  10.655  1.00  0.23
H
ATOM   5352  HG  LEU A 347      10.342  12.475   7.775  1.00  0.23
H
ATOM   5353 1HD1 LEU A 347       8.019  13.047   8.354  1.00  0.23
H
ATOM   5354 2HD1 LEU A 347       8.288  11.298   8.492  1.00  0.23
H
ATOM   5355 3HD1 LEU A 347       8.232  12.300   9.954  1.00  0.23
H
ATOM   5356 1HD2 LEU A 347       9.879  14.628   8.849  1.00  0.23
H
ATOM   5357 2HD2 LEU A 347      10.092  13.878  10.449  1.00  0.23
H
ATOM   5358 3HD2 LEU A 347      11.469  14.001   9.337  1.00  0.23
H
ATOM   5359  N   GLY A 348      12.839   9.163  10.265  1.00  0.13
N
ATOM   5360  CA  GLY A 348      13.373   8.168  11.180  1.00  0.14
C
ATOM   5361  C   GLY A 348      14.885   8.268  11.289  1.00  0.15
C
ATOM   5362  O   GLY A 348      15.436   8.265  12.389  1.00  0.20
O
ATOM   5363  H   GLY A 348      12.389   8.849   9.414  1.00  0.16
H
ATOM   5364 1HA  GLY A 348      12.925   8.300  12.164  1.00  0.17
H
ATOM   5365 2HA  GLY A 348      13.098   7.174  10.831  1.00  0.17
H
ATOM   5366  N   CYS A 349      15.553   8.392  10.145  1.00  0.14
N
ATOM   5367  CA  CYS A 349      17.005   8.483  10.097  1.00  0.18
C
ATOM   5368  C   CYS A 349      17.546   9.684  10.877  1.00  0.32
C
ATOM   5369  O   CYS A 349      18.558   9.574  11.570  1.00  1.03
O
ATOM   5370  CB  CYS A 349      17.469   8.556   8.642  1.00  0.27
C
ATOM   5371  SG  CYS A 349      17.263   7.015   7.715  1.00  0.27
S
ATOM   5372  H   CYS A 349      15.044   8.394   9.272  1.00  0.17
H
ATOM   5373  HA  CYS A 349      17.416   7.575  10.539  1.00  0.22
H
ATOM   5374 1HB  CYS A 349      16.915   9.338   8.123  1.00  0.32
H
```

```
ATOM   5375 2HB  CYS A 349      18.519   8.820   8.609  1.00  0.32
H
ATOM   5376  HG  CYS A 349      17.728   7.484   6.562  1.00  0.32
H
ATOM   5377  N   ALA A 350      16.862  10.824  10.793  1.00  0.17
N
ATOM   5378  CA  ALA A 350      17.301  12.011  11.516  1.00  0.26
C
ATOM   5379  C   ALA A 350      16.899  12.013  12.997  1.00  0.20
C
ATOM   5380  O   ALA A 350      17.205  12.978  13.708  1.00  0.22
O
ATOM   5381  CB  ALA A 350      16.746  13.252  10.851  1.00  0.39
C
ATOM   5382  H   ALA A 350      16.046  10.879  10.197  1.00  0.20
H
ATOM   5383  HA  ALA A 350      18.389  12.045  11.467  1.00  0.31
H
ATOM   5384 1HB  ALA A 350      17.114  14.138  11.368  1.00  0.47
H
ATOM   5385 2HB  ALA A 350      17.068  13.279   9.810  1.00  0.47
H
ATOM   5386 3HB  ALA A 350      15.659  13.229  10.897  1.00  0.47
H
ATOM   5387  N   SER A 351      16.215  10.963  13.477  1.00  0.20
N
ATOM   5388  CA  SER A 351      15.818  10.969  14.872  1.00  0.29
C
ATOM   5389  C   SER A 351      17.061  10.843  15.718  1.00  0.24
C
ATOM   5390  O   SER A 351      18.051  10.232  15.307  1.00  0.39
O
ATOM   5391  CB  SER A 351      14.842   9.845  15.167  1.00  0.43
C
ATOM   5392  OG  SER A 351      15.449   8.592  15.022  1.00  0.43
O
ATOM   5393  H   SER A 351      15.973  10.166  12.906  1.00  0.24
H
ATOM   5394  HA  SER A 351      15.337  11.920  15.100  1.00  0.35
H
ATOM   5395 1HB  SER A 351      14.465   9.954  16.183  1.00  0.52
H
ATOM   5396 2HB  SER A 351      13.991   9.920  14.492  1.00  0.52
H
ATOM   5397  HG  SER A 351      15.645   8.498  14.083  1.00  0.52
H
ATOM   5398  N   ARG A 352      17.006  11.445  16.903  1.00  0.37
N
ATOM   5399  CA  ARG A 352      18.126  11.508  17.842  1.00  0.53
C
ATOM   5400  C   ARG A 352      19.399  12.184  17.261  1.00  0.36
C
ATOM   5401  O   ARG A 352      20.453  12.145  17.895  1.00  0.64
O
ATOM   5402  CB  ARG A 352      18.454  10.104  18.368  1.00  0.80
C
ATOM   5403  CG  ARG A 352      17.281   9.437  19.096  1.00  0.80
C
```

ATOM 5404 CD ARG A 352 17.610 8.109 19.690 1.00 0.80
C
ATOM 5405 NE ARG A 352 16.424 7.486 20.281 1.00 0.80
N
ATOM 5406 CZ ARG A 352 16.366 6.242 20.820 1.00 0.80
C
ATOM 5407 NH1 ARG A 352 17.431 5.466 20.865 1.00 0.80
N
ATOM 5408 NH2 ARG A 352 15.217 5.797 21.299 1.00 0.80
N
ATOM 5409 H ARG A 352 16.143 11.900 17.166 1.00 0.44
H
ATOM 5410 HA ARG A 352 17.803 12.101 18.698 1.00 0.64
H
ATOM 5411 1HB ARG A 352 18.750 9.455 17.546 1.00 0.95
H
ATOM 5412 2HB ARG A 352 19.291 10.158 19.063 1.00 0.95
H
ATOM 5413 1HG ARG A 352 16.947 10.090 19.904 1.00 0.95
H
ATOM 5414 2HG ARG A 352 16.457 9.293 18.395 1.00 0.95
H
ATOM 5415 1HD ARG A 352 17.996 7.453 18.916 1.00 0.95
H
ATOM 5416 2HD ARG A 352 18.359 8.234 20.473 1.00 0.95
H
ATOM 5417 HE ARG A 352 15.562 8.029 20.253 1.00 0.95
H
ATOM 5418 1HH1 ARG A 352 18.313 5.802 20.514 1.00 0.95
H
ATOM 5419 2HH1 ARG A 352 17.365 4.527 21.251 1.00 0.95
H
ATOM 5420 1HH2 ARG A 352 14.398 6.388 21.269 1.00 0.95
H
ATOM 5421 2HH2 ARG A 352 15.161 4.872 21.699 1.00 0.95
H
ATOM 5422 N GLY A 353 19.312 12.804 16.065 1.00 0.28
N
ATOM 5423 CA GLY A 353 20.461 13.467 15.463 1.00 0.31
C
ATOM 5424 C GLY A 353 21.439 12.482 14.829 1.00 0.28
C
ATOM 5425 O GLY A 353 22.590 12.838 14.577 1.00 0.37
O
ATOM 5426 H GLY A 353 18.443 12.834 15.546 1.00 0.34
H
ATOM 5427 1HA GLY A 353 20.113 14.171 14.708 1.00 0.37
H
ATOM 5428 2HA GLY A 353 20.977 14.052 16.224 1.00 0.37
H
ATOM 5429 N ARG A 354 20.993 11.247 14.580 1.00 0.22
N
ATOM 5430 CA ARG A 354 21.877 10.215 14.043 1.00 0.28
C
ATOM 5431 C ARG A 354 22.482 10.573 12.679 1.00 0.29
C
ATOM 5432 O ARG A 354 23.657 10.295 12.431 1.00 1.54
O

```
ATOM   5433  CB  ARG A 354      21.125   8.899  13.942  1.00  0.42
C
ATOM   5434  CG  ARG A 354      20.783   8.265  15.286  1.00  0.42
C
ATOM   5435  CD  ARG A 354      20.075   6.969  15.124  1.00  0.42
C
ATOM   5436  NE  ARG A 354      19.575   6.454  16.382  1.00  0.42
N
ATOM   5437  CZ  ARG A 354      20.272   5.729  17.283  1.00  0.42
C
ATOM   5438  NH1 ARG A 354      21.529   5.399  17.090  1.00  0.42
N
ATOM   5439  NH2 ARG A 354      19.672   5.341  18.387  1.00  0.42
N
ATOM   5440  H   ARG A 354      20.032  11.000  14.795  1.00  0.26
H
ATOM   5441  HA  ARG A 354      22.697  10.078  14.749  1.00  0.34
H
ATOM   5442 1HB  ARG A 354      20.193   9.048  13.402  1.00  0.50
H
ATOM   5443 2HB  ARG A 354      21.721   8.179  13.381  1.00  0.50
H
ATOM   5444 1HG  ARG A 354      21.702   8.082  15.843  1.00  0.50
H
ATOM   5445 2HG  ARG A 354      20.143   8.939  15.855  1.00  0.50
H
ATOM   5446 1HD  ARG A 354      19.229   7.100  14.450  1.00  0.50
H
ATOM   5447 2HD  ARG A 354      20.760   6.234  14.709  1.00  0.50
H
ATOM   5448  HE  ARG A 354      18.614   6.664  16.606  1.00  0.50
H
ATOM   5449 1HH1 ARG A 354      22.015   5.668  16.244  1.00  0.50
H
ATOM   5450 2HH1 ARG A 354      22.016   4.854  17.786  1.00  0.50
H
ATOM   5451 1HH2 ARG A 354      18.704   5.577  18.541  1.00  0.50
H
ATOM   5452 2HH2 ARG A 354      20.179   4.811  19.082  1.00  0.50
H
ATOM   5453  N   THR A 355      21.704  11.199  11.799  1.00  0.58
N
ATOM   5454  CA  THR A 355      22.253  11.595  10.506  1.00  0.53
C
ATOM   5455  C   THR A 355      21.553  12.807   9.911  1.00  0.28
C
ATOM   5456  O   THR A 355      20.531  13.268  10.422  1.00  0.45
O
ATOM   5457  CB  THR A 355      22.187  10.448   9.492  1.00  0.80
C
ATOM   5458  OG1 THR A 355      22.960  10.797   8.348  1.00  0.80
O
ATOM   5459  CG2 THR A 355      20.770  10.211   9.044  1.00  0.80
C
ATOM   5460  H   THR A 355      20.741  11.403  12.026  1.00  0.70
H
ATOM   5461  HA  THR A 355      23.301  11.858  10.649  1.00  0.64
H
```

```
ATOM   5462  HB  THR A 355      22.591   9.539   9.939  1.00  0.95
H
ATOM   5463  HG1 THR A 355      23.860  10.997   8.612  1.00  0.95
H
ATOM   5464 1HG2 THR A 355      20.751   9.400   8.317  1.00  0.95
H
ATOM   5465 2HG2 THR A 355      20.169   9.941   9.903  1.00  0.95
H
ATOM   5466 3HG2 THR A 355      20.372  11.116   8.588  1.00  0.95
H
ATOM   5467  N   ILE A 356      22.120  13.312   8.818  1.00  0.25
N
ATOM   5468  CA  ILE A 356      21.581  14.466   8.105  1.00  0.21
C
ATOM   5469  C   ILE A 356      21.021  13.988   6.770  1.00  0.16
C
ATOM   5470  O   ILE A 356      21.774  13.712   5.836  1.00  0.17
O
ATOM   5471  CB  ILE A 356      22.673  15.523   7.853  1.00  0.32
C
ATOM   5472  CG1 ILE A 356      23.310  15.968   9.170  1.00  0.32
C
ATOM   5473  CG2 ILE A 356      22.057  16.725   7.157  1.00  0.32
C
ATOM   5474  CD1 ILE A 356      24.531  16.840   8.979  1.00  0.32
C
ATOM   5475  H   ILE A 356      22.954  12.871   8.458  1.00  0.30
H
ATOM   5476  HA  ILE A 356      20.777  14.909   8.690  1.00  0.25
H
ATOM   5477  HB  ILE A 356      23.457  15.099   7.227  1.00  0.38
H
ATOM   5478 1HG1 ILE A 356      22.576  16.525   9.752  1.00  0.38
H
ATOM   5479 2HG1 ILE A 356      23.603  15.088   9.741  1.00  0.38
H
ATOM   5480 1HG2 ILE A 356      22.826  17.472   6.967  1.00  0.38
H
ATOM   5481 2HG2 ILE A 356      21.612  16.408   6.218  1.00  0.38
H
ATOM   5482 3HG2 ILE A 356      21.283  17.154   7.794  1.00  0.38
H
ATOM   5483 1HD1 ILE A 356      24.934  17.121   9.953  1.00  0.38
H
ATOM   5484 2HD1 ILE A 356      25.287  16.290   8.417  1.00  0.38
H
ATOM   5485 3HD1 ILE A 356      24.253  17.739   8.430  1.00  0.38
H
ATOM   5486  N   ALA A 357      19.699  13.885   6.687  1.00  0.16
N
ATOM   5487  CA  ALA A 357      19.057  13.332   5.499  1.00  0.14
C
ATOM   5488  C   ALA A 357      18.684  14.377   4.453  1.00  0.16
C
ATOM   5489  O   ALA A 357      18.090  15.407   4.770  1.00  0.20
O
ATOM   5490  CB  ALA A 357      17.808  12.569   5.916  1.00  0.21
C
```

ATOM 5491 H ALA A 357 19.130 14.160 7.474 1.00 0.19
H
ATOM 5492 HA ALA A 357 19.758 12.637 5.038 1.00 0.17
H
ATOM 5493 1HB ALA A 357 17.348 12.116 5.040 1.00 0.25
H
ATOM 5494 2HB ALA A 357 18.080 11.790 6.628 1.00 0.25
H
ATOM 5495 3HB ALA A 357 17.102 13.254 6.381 1.00 0.25
H
ATOM 5496 N PHE A 358 19.003 14.050 3.198 1.00 0.19
N
ATOM 5497 CA PHE A 358 18.682 14.836 2.012 1.00 0.19
C
ATOM 5498 C PHE A 358 17.862 13.979 1.054 1.00 0.16
C
ATOM 5499 O PHE A 358 18.422 13.176 0.313 1.00 0.20
O
ATOM 5500 CB PHE A 358 19.951 15.276 1.296 1.00 0.29
C
ATOM 5501 CG PHE A 358 20.819 16.198 2.087 1.00 0.29
C
ATOM 5502 CD1 PHE A 358 21.765 15.692 2.961 1.00 0.29
C
ATOM 5503 CD2 PHE A 358 20.702 17.573 1.949 1.00 0.29
C
ATOM 5504 CE1 PHE A 358 22.568 16.543 3.686 1.00 0.29
C
ATOM 5505 CE2 PHE A 358 21.507 18.426 2.672 1.00 0.29
C
ATOM 5506 CZ PHE A 358 22.437 17.904 3.544 1.00 0.29
C
ATOM 5507 H PHE A 358 19.524 13.197 3.057 1.00 0.23
H
ATOM 5508 HA PHE A 358 18.104 15.709 2.304 1.00 0.23
H
ATOM 5509 1HB PHE A 358 20.535 14.396 1.051 1.00 0.34
H
ATOM 5510 2HB PHE A 358 19.693 15.769 0.359 1.00 0.34
H
ATOM 5511 HD1 PHE A 358 21.869 14.613 3.074 1.00 0.34
H
ATOM 5512 HD2 PHE A 358 19.962 17.977 1.258 1.00 0.34
H
ATOM 5513 HE1 PHE A 358 23.308 16.137 4.375 1.00 0.34
H
ATOM 5514 HE2 PHE A 358 21.408 19.506 2.560 1.00 0.34
H
ATOM 5515 HZ PHE A 358 23.071 18.568 4.121 1.00 0.34
H
ATOM 5516 N ALA A 359 16.547 14.127 1.085 1.00 0.17
N
ATOM 5517 CA ALA A 359 15.685 13.334 0.218 1.00 0.17
C
ATOM 5518 C ALA A 359 15.398 14.099 -1.065 1.00 0.20
C
ATOM 5519 O ALA A 359 15.139 15.298 -1.016 1.00 0.32
O

ATOM 5520 CB ALA A 359 14.393 12.981 0.938 1.00 0.26
C
ATOM 5521 H ALA A 359 16.143 14.819 1.702 1.00 0.20
H
ATOM 5522 HA ALA A 359 16.208 12.417 -0.043 1.00 0.20
H
ATOM 5523 1HB ALA A 359 13.768 12.374 0.286 1.00 0.31
H
ATOM 5524 2HB ALA A 359 14.623 12.421 1.844 1.00 0.31
H
ATOM 5525 3HB ALA A 359 13.862 13.894 1.202 1.00 0.31
H
ATOM 5526 N SER A 360 15.426 13.416 -2.208 1.00 0.25
N
ATOM 5527 CA SER A 360 15.161 14.105 -3.461 1.00 0.33
C
ATOM 5528 C SER A 360 14.227 13.372 -4.413 1.00 0.33
C
ATOM 5529 O SER A 360 14.228 12.147 -4.518 1.00 0.98
O
ATOM 5530 CB SER A 360 16.484 14.385 -4.160 1.00 0.49
C
ATOM 5531 OG SER A 360 17.190 13.200 -4.440 1.00 0.49
O
ATOM 5532 H SER A 360 15.658 12.434 -2.205 1.00 0.30
H
ATOM 5533 HA SER A 360 14.697 15.057 -3.217 1.00 0.40
H
ATOM 5534 1HB SER A 360 16.296 14.927 -5.086 1.00 0.59
H
ATOM 5535 2HB SER A 360 17.091 15.028 -3.525 1.00 0.59
H
ATOM 5536 HG SER A 360 16.694 12.693 -5.095 1.00 0.59
H
ATOM 5537 N THR A 361 13.410 14.174 -5.084 1.00 0.23
N
ATOM 5538 CA THR A 361 12.454 13.751 -6.104 1.00 0.23
C
ATOM 5539 C THR A 361 12.000 14.967 -6.890 1.00 0.29
C
ATOM 5540 O THR A 361 12.577 16.040 -6.743 1.00 0.57
O
ATOM 5541 CB THR A 361 11.261 12.993 -5.512 1.00 0.35
C
ATOM 5542 OG1 THR A 361 10.488 12.441 -6.589 1.00 0.35
O
ATOM 5543 CG2 THR A 361 10.410 13.900 -4.658 1.00 0.35
C
ATOM 5544 H THR A 361 13.486 15.168 -4.883 1.00 0.28
H
ATOM 5545 HA THR A 361 12.960 13.079 -6.795 1.00 0.28
H
ATOM 5546 HB THR A 361 11.629 12.172 -4.897 1.00 0.41
H
ATOM 5547 HG1 THR A 361 9.870 11.792 -6.234 1.00 0.41
H
ATOM 5548 1HG2 THR A 361 9.573 13.342 -4.246 1.00 0.41
H

ATOM 5549 2HG2 THR A 361 11.020 14.292 -3.846 1.00 0.41
H
ATOM 5550 3HG2 THR A 361 10.035 14.720 -5.251 1.00 0.41
H
ATOM 5551 N PHE A 362 10.991 14.803 -7.737 1.00 0.30
N
ATOM 5552 CA PHE A 362 10.460 15.965 -8.433 1.00 0.46
C
ATOM 5553 C PHE A 362 9.659 16.754 -7.405 1.00 0.54
C
ATOM 5554 O PHE A 362 8.934 16.168 -6.601 1.00 0.78
O
ATOM 5555 CB PHE A 362 9.526 15.566 -9.569 1.00 0.69
C
ATOM 5556 CG PHE A 362 10.202 14.846 -10.694 1.00 0.69
C
ATOM 5557 CD1 PHE A 362 11.509 14.409 -10.565 1.00 0.69
C
ATOM 5558 CD2 PHE A 362 9.533 14.594 -11.878 1.00 0.69
C
ATOM 5559 CE1 PHE A 362 12.133 13.732 -11.588 1.00 0.69
C
ATOM 5560 CE2 PHE A 362 10.157 13.914 -12.904 1.00 0.69
C
ATOM 5561 CZ PHE A 362 11.458 13.482 -12.759 1.00 0.69
C
ATOM 5562 H PHE A 362 10.577 13.886 -7.828 1.00 0.36
H
ATOM 5563 HA PHE A 362 11.277 16.578 -8.809 1.00 0.55
H
ATOM 5564 1HB PHE A 362 8.737 14.931 -9.184 1.00 0.83
H
ATOM 5565 2HB PHE A 362 9.055 16.459 -9.978 1.00 0.83
H
ATOM 5566 HD1 PHE A 362 12.049 14.602 -9.641 1.00 0.83
H
ATOM 5567 HD2 PHE A 362 8.502 14.931 -11.994 1.00 0.83
H
ATOM 5568 HE1 PHE A 362 13.160 13.396 -11.465 1.00 0.83
H
ATOM 5569 HE2 PHE A 362 9.626 13.714 -13.829 1.00 0.83
H
ATOM 5570 HZ PHE A 362 11.948 12.945 -13.570 1.00 0.83
H
ATOM 5571 N ALA A 363 9.747 18.074 -7.445 1.00 0.51
N
ATOM 5572 CA ALA A 363 9.025 18.890 -6.473 1.00 0.56
C
ATOM 5573 C ALA A 363 7.530 18.690 -6.583 1.00 0.46
C
ATOM 5574 O ALA A 363 6.808 18.852 -5.601 1.00 0.46
O
ATOM 5575 CB ALA A 363 9.346 20.345 -6.644 1.00 0.84
C
ATOM 5576 H ALA A 363 10.334 18.510 -8.141 1.00 0.61
H
ATOM 5577 HA ALA A 363 9.332 18.577 -5.480 1.00 0.67
H

```
ATOM   5578 1HB  ALA A 363       8.812  20.929  -5.895  1.00  1.01
H
ATOM   5579 2HB  ALA A 363      10.412  20.508  -6.533  1.00  1.01
H
ATOM   5580 3HB  ALA A 363       9.042  20.657  -7.634  1.00  1.01
H
ATOM   5581  N   ALA A 364       7.058  18.345  -7.778  1.00  0.41
N
ATOM   5582  CA  ALA A 364       5.658  18.055  -7.968  1.00  0.36
C
ATOM   5583  C   ALA A 364       5.294  16.826  -7.159  1.00  0.33
C
ATOM   5584  O   ALA A 364       4.301  16.818  -6.431  1.00  0.50
O
ATOM   5585  CB  ALA A 364       5.352  17.842  -9.446  1.00  0.54
C
ATOM   5586  H   ALA A 364       7.688  18.260  -8.562  1.00  0.49
H
ATOM   5587  HA  ALA A 364       5.078  18.897  -7.593  1.00  0.43
H
ATOM   5588 1HB  ALA A 364       4.290  17.634  -9.574  1.00  0.65
H
ATOM   5589 2HB  ALA A 364       5.615  18.739 -10.005  1.00  0.65
H
ATOM   5590 3HB  ALA A 364       5.932  16.999  -9.820  1.00  0.65
H
ATOM   5591  N   PHE A 365       6.141  15.803  -7.246  1.00  0.28
N
ATOM   5592  CA  PHE A 365       5.903  14.535  -6.581  1.00  0.41
C
ATOM   5593  C   PHE A 365       5.910  14.669  -5.064  1.00  0.26
C
ATOM   5594  O   PHE A 365       5.209  13.925  -4.382  1.00  0.48
O
ATOM   5595  CB  PHE A 365       6.923  13.507  -7.048  1.00  0.61
C
ATOM   5596  CG  PHE A 365       6.735  13.137  -8.492  1.00  0.61
C
ATOM   5597  CD1 PHE A 365       5.627  13.594  -9.200  1.00  0.61
C
ATOM   5598  CD2 PHE A 365       7.662  12.340  -9.151  1.00  0.61
C
ATOM   5599  CE1 PHE A 365       5.455  13.271 -10.533  1.00  0.61
C
ATOM   5600  CE2 PHE A 365       7.490  12.014 -10.483  1.00  0.61
C
ATOM   5601  CZ  PHE A 365       6.388  12.482 -11.175  1.00  0.61
C
ATOM   5602  H   PHE A 365       6.960  15.893  -7.828  1.00  0.34
H
ATOM   5603  HA  PHE A 365       4.915  14.180  -6.876  1.00  0.49
H
ATOM   5604 1HB  PHE A 365       7.931  13.894  -6.920  1.00  0.74
H
ATOM   5605 2HB  PHE A 365       6.837  12.602  -6.449  1.00  0.74
H
ATOM   5606  HD1 PHE A 365       4.892  14.219  -8.694  1.00  0.74
H
```

ATOM 5607 HD2 PHE A 365 8.534 11.975 -8.605 1.00 0.74
H
ATOM 5608 HE1 PHE A 365 4.584 13.639 -11.075 1.00 0.74
H
ATOM 5609 HE2 PHE A 365 8.226 11.389 -10.989 1.00 0.74
H
ATOM 5610 HZ PHE A 365 6.255 12.225 -12.224 1.00 0.74
H
ATOM 5611 N LEU A 366 6.644 15.655 -4.536 1.00 0.29
N
ATOM 5612 CA LEU A 366 6.655 15.910 -3.092 1.00 0.33
C
ATOM 5613 C LEU A 366 5.278 16.198 -2.495 1.00 0.34
C
ATOM 5614 O LEU A 366 5.077 15.981 -1.301 1.00 0.41
O
ATOM 5615 CB LEU A 366 7.579 17.083 -2.751 1.00 0.49
C
ATOM 5616 CG LEU A 366 9.064 16.804 -2.910 1.00 0.49
C
ATOM 5617 CD1 LEU A 366 9.863 18.079 -2.688 1.00 0.49
C
ATOM 5618 CD2 LEU A 366 9.444 15.733 -1.897 1.00 0.49
C
ATOM 5619 H LEU A 366 7.231 16.214 -5.149 1.00 0.35
H
ATOM 5620 HA LEU A 366 7.048 15.018 -2.603 1.00 0.40
H
ATOM 5621 1HB LEU A 366 7.327 17.927 -3.389 1.00 0.59
H
ATOM 5622 2HB LEU A 366 7.405 17.373 -1.715 1.00 0.59
H
ATOM 5623 HG LEU A 366 9.268 16.450 -3.920 1.00 0.59
H
ATOM 5624 1HD1 LEU A 366 10.925 17.868 -2.804 1.00 0.59
H
ATOM 5625 2HD1 LEU A 366 9.562 18.830 -3.416 1.00 0.59
H
ATOM 5626 3HD1 LEU A 366 9.675 18.456 -1.683 1.00 0.59
H
ATOM 5627 1HD2 LEU A 366 10.505 15.502 -1.990 1.00 0.59
H
ATOM 5628 2HD2 LEU A 366 9.237 16.094 -0.890 1.00 0.59
H
ATOM 5629 3HD2 LEU A 366 8.860 14.833 -2.087 1.00 0.59
H
ATOM 5630 N THR A 367 4.311 16.634 -3.310 1.00 0.37
N
ATOM 5631 CA THR A 367 2.972 16.908 -2.797 1.00 0.57
C
ATOM 5632 C THR A 367 2.284 15.626 -2.321 1.00 0.60
C
ATOM 5633 O THR A 367 1.418 15.664 -1.447 1.00 0.82
O
ATOM 5634 CB THR A 367 2.116 17.578 -3.868 1.00 0.85
C
ATOM 5635 OG1 THR A 367 2.036 16.715 -4.998 1.00 0.85
O

```
ATOM   5636  CG2 THR A 367       2.715  18.916  -4.285  1.00  0.85
C
ATOM   5637  H   THR A 367       4.492  16.783  -4.297  1.00  0.44
H
ATOM   5638  HA  THR A 367       3.057  17.585  -1.947  1.00  0.68
H
ATOM   5639  HB  THR A 367       1.118  17.725  -3.481  1.00  1.03
H
ATOM   5640  HG1 THR A 367       2.919  16.591  -5.364  1.00  1.03
H
ATOM   5641 1HG2 THR A 367       2.088  19.373  -5.050  1.00  1.03
H
ATOM   5642 2HG2 THR A 367       2.770  19.576  -3.419  1.00  1.03
H
ATOM   5643 3HG2 THR A 367       3.717  18.757  -4.683  1.00  1.03
H
ATOM   5644  N   ARG A 368       2.722  14.486  -2.850  1.00  0.69
N
ATOM   5645  CA  ARG A 368       2.238  13.175  -2.447  1.00  1.20
C
ATOM   5646  C   ARG A 368       2.573  12.873  -0.992  1.00  1.05
C
ATOM   5647  O   ARG A 368       1.843  12.147  -0.314  1.00  1.94
O
ATOM   5648  CB  ARG A 368       2.827  12.118  -3.374  1.00  1.80
C
ATOM   5649  CG  ARG A 368       2.515  10.676  -3.044  1.00  1.80
C
ATOM   5650  CD  ARG A 368       2.782   9.792  -4.213  1.00  1.80
C
ATOM   5651  NE  ARG A 368       1.731   9.929  -5.211  1.00  1.80
N
ATOM   5652  CZ  ARG A 368       1.725   9.377  -6.441  1.00  1.80
C
ATOM   5653  NH1 ARG A 368       2.724   8.663  -6.897  1.00  1.80
N
ATOM   5654  NH2 ARG A 368       0.684   9.524  -7.232  1.00  1.80
N
ATOM   5655  H   ARG A 368       3.445  14.509  -3.555  1.00  0.83
H
ATOM   5656  HA  ARG A 368       1.155  13.161  -2.558  1.00  1.44
H
ATOM   5657 1HB  ARG A 368       2.492  12.302  -4.393  1.00  2.16
H
ATOM   5658 2HB  ARG A 368       3.913  12.203  -3.368  1.00  2.16
H
ATOM   5659 1HG  ARG A 368       3.149  10.349  -2.222  1.00  2.16
H
ATOM   5660 2HG  ARG A 368       1.468  10.579  -2.757  1.00  2.16
H
ATOM   5661 1HD  ARG A 368       3.728  10.071  -4.676  1.00  2.16
H
ATOM   5662 2HD  ARG A 368       2.824   8.751  -3.897  1.00  2.16
H
ATOM   5663  HE  ARG A 368       0.906  10.448  -4.931  1.00  2.16
H
ATOM   5664 1HH1 ARG A 368       3.554   8.494  -6.343  1.00  2.16
H
```

```
ATOM   5665 2HH1 ARG A 368       2.628   8.242  -7.815  1.00  2.16
H
ATOM   5666 1HH2 ARG A 368      -0.120  10.052  -6.925  1.00  2.16
H
ATOM   5667 2HH2 ARG A 368       0.706   9.063  -8.132  1.00  2.16
H
ATOM   5668  N   ALA A 369       3.697  13.408  -0.520  1.00  0.49
N
ATOM   5669  CA  ALA A 369       4.146  13.186   0.837  1.00  0.54
C
ATOM   5670  C   ALA A 369       3.900  14.393   1.725  1.00  0.62
C
ATOM   5671  O   ALA A 369       4.532  14.505   2.774  1.00  0.95
O
ATOM   5672  CB  ALA A 369       5.620  12.846   0.840  1.00  0.81
C
ATOM   5673  H   ALA A 369       4.254  14.021  -1.101  1.00  0.59
H
ATOM   5674  HA  ALA A 369       3.584  12.349   1.247  1.00  0.65
H
ATOM   5675 1HB  ALA A 369       5.947  12.648   1.860  1.00  0.97
H
ATOM   5676 2HB  ALA A 369       5.788  11.964   0.226  1.00  0.97
H
ATOM   5677 3HB  ALA A 369       6.186  13.683   0.433  1.00  0.97
H
ATOM   5678  N   PHE A 370       2.997  15.291   1.319  1.00  0.63
N
ATOM   5679  CA  PHE A 370       2.731  16.497   2.090  1.00  0.95
C
ATOM   5680  C   PHE A 370       2.361  16.188   3.531  1.00  0.68
C
ATOM   5681  O   PHE A 370       2.779  16.898   4.443  1.00  0.75
O
ATOM   5682  CB  PHE A 370       1.615  17.307   1.485  1.00  1.42
C
ATOM   5683  CG  PHE A 370       1.438  18.599   2.205  1.00  1.42
C
ATOM   5684  CD1 PHE A 370       2.054  19.745   1.740  1.00  1.42
C
ATOM   5685  CD2 PHE A 370       0.696  18.670   3.368  1.00  1.42
C
ATOM   5686  CE1 PHE A 370       1.891  20.940   2.406  1.00  1.42
C
ATOM   5687  CE2 PHE A 370       0.530  19.865   4.031  1.00  1.42
C
ATOM   5688  CZ  PHE A 370       1.123  21.000   3.536  1.00  1.42
C
ATOM   5689  H   PHE A 370       2.487  15.166   0.451  1.00  0.76
H
ATOM   5690  HA  PHE A 370       3.636  17.105   2.095  1.00  1.14
H
ATOM   5691 1HB  PHE A 370       1.830  17.519   0.439  1.00  1.71
H
ATOM   5692 2HB  PHE A 370       0.679  16.752   1.530  1.00  1.71
H
ATOM   5693  HD1 PHE A 370       2.659  19.698   0.835  1.00  1.71
H
```

```
ATOM   5694  HD2 PHE A 370       0.229  17.764   3.753  1.00  1.71
H
ATOM   5695  HE1 PHE A 370       2.365  21.842   2.033  1.00  1.71
H
ATOM   5696  HE2 PHE A 370      -0.072  19.915   4.937  1.00  1.71
H
ATOM   5697  HZ  PHE A 370       0.995  21.950   4.038  1.00  1.71
H
ATOM   5698  N   ASP A 371       1.547  15.150   3.727  1.00  0.85
N
ATOM   5699  CA  ASP A 371       1.131  14.712   5.050  1.00  0.98
C
ATOM   5700  C   ASP A 371       2.330  14.373   5.944  1.00  0.98
C
ATOM   5701  O   ASP A 371       2.349  14.722   7.125  1.00  2.70
O
ATOM   5702  CB  ASP A 371       0.206  13.491   4.917  1.00  1.47
C
ATOM   5703  CG  ASP A 371       0.815  12.312   4.161  1.00  1.47
C
ATOM   5704  OD1 ASP A 371       1.859  12.471   3.568  1.00  1.47
O
ATOM   5705  OD2 ASP A 371       0.257  11.242   4.216  1.00  1.47
O
ATOM   5706  H   ASP A 371       1.218  14.634   2.923  1.00  1.02
H
ATOM   5707  HA  ASP A 371       0.573  15.520   5.522  1.00  1.18
H
ATOM   5708 1HB  ASP A 371      -0.059  13.140   5.910  1.00  1.76
H
ATOM   5709 2HB  ASP A 371      -0.717  13.788   4.418  1.00  1.76
H
ATOM   5710  N   HIS A 372       3.327  13.707   5.356  1.00  0.55
N
ATOM   5711  CA  HIS A 372       4.551  13.320   6.046  1.00  0.68
C
ATOM   5712  C   HIS A 372       5.444  14.512   6.285  1.00  0.87
C
ATOM   5713  O   HIS A 372       6.125  14.593   7.306  1.00  1.45
O
ATOM   5714  CB  HIS A 372       5.318  12.261   5.260  1.00  1.02
C
ATOM   5715  CG  HIS A 372       4.679  10.923   5.311  1.00  1.02
C
ATOM   5716  ND1 HIS A 372       3.634  10.566   4.495  1.00  1.02
N
ATOM   5717  CD2 HIS A 372       4.930   9.857   6.095  1.00  1.02
C
ATOM   5718  CE1 HIS A 372       3.267   9.329   4.779  1.00  1.02
C
ATOM   5719  NE2 HIS A 372       4.048   8.872   5.736  1.00  1.02
N
ATOM   5720  H   HIS A 372       3.198  13.440   4.388  1.00  0.66
H
ATOM   5721  HA  HIS A 372       4.297  12.892   7.011  1.00  0.82
H
ATOM   5722 1HB  HIS A 372       5.404  12.564   4.217  1.00  1.22
H
```

```
ATOM   5723 2HB  HIS A 372       6.324  12.168   5.663  1.00  1.22
H
ATOM   5724  HD1 HIS A 372       3.071  11.204   3.963  1.00  1.22
H
ATOM   5725  HD2 HIS A 372       5.653   9.683   6.889  1.00  1.22
H
ATOM   5726  HE1 HIS A 372       2.446   8.858   4.237  1.00  1.22
H
ATOM   5727  N   ILE A 373       5.433  15.451   5.356  1.00  0.78
N
ATOM   5728  CA  ILE A 373       6.175  16.679   5.530  1.00  0.85
C
ATOM   5729  C   ILE A 373       5.599  17.460   6.701  1.00  0.67
C
ATOM   5730  O   ILE A 373       6.341  17.973   7.544  1.00  0.77
O
ATOM   5731  CB  ILE A 373       6.146  17.518   4.244  1.00  1.27
C
ATOM   5732  CG1 ILE A 373       6.956  16.806   3.147  1.00  1.27
C
ATOM   5733  CG2 ILE A 373       6.670  18.911   4.523  1.00  1.27
C
ATOM   5734  CD1 ILE A 373       6.766  17.381   1.759  1.00  1.27
C
ATOM   5735  H   ILE A 373       4.895  15.312   4.511  1.00  0.94
H
ATOM   5736  HA  ILE A 373       7.211  16.433   5.757  1.00  1.02
H
ATOM   5737  HB  ILE A 373       5.120  17.592   3.885  1.00  1.53
H
ATOM   5738 1HG1 ILE A 373       8.015  16.868   3.400  1.00  1.53
H
ATOM   5739 2HG1 ILE A 373       6.673  15.755   3.124  1.00  1.53
H
ATOM   5740 1HG2 ILE A 373       6.630  19.505   3.610  1.00  1.53
H
ATOM   5741 2HG2 ILE A 373       6.055  19.381   5.287  1.00  1.53
H
ATOM   5742 3HG2 ILE A 373       7.699  18.851   4.873  1.00  1.53
H
ATOM   5743 1HD1 ILE A 373       7.374  16.823   1.048  1.00  1.53
H
ATOM   5744 2HD1 ILE A 373       5.717  17.306   1.474  1.00  1.53
H
ATOM   5745 3HD1 ILE A 373       7.068  18.427   1.753  1.00  1.53
H
ATOM   5746  N   ARG A 374       4.271  17.536   6.743  1.00  0.53
N
ATOM   5747  CA  ARG A 374       3.575  18.253   7.783  1.00  0.56
C
ATOM   5748  C   ARG A 374       3.811  17.622   9.154  1.00  0.60
C
ATOM   5749  O   ARG A 374       4.096  18.343  10.114  1.00  0.60
O
ATOM   5750  CB  ARG A 374       2.092  18.301   7.492  1.00  0.84
C
ATOM   5751  CG  ARG A 374       1.348  19.228   8.417  1.00  0.84
C
```

ATOM 5752 CD ARG A 374 -0.090 19.343 8.102 1.00 0.84
C
ATOM 5753 NE ARG A 374 -0.640 20.468 8.823 1.00 0.84
N
ATOM 5754 CZ ARG A 374 -1.894 20.597 9.270 1.00 0.84
C
ATOM 5755 NH1 ARG A 374 -2.770 19.640 9.098 1.00 0.84
N
ATOM 5756 NH2 ARG A 374 -2.237 21.700 9.898 1.00 0.84
N
ATOM 5757 H ARG A 374 3.726 17.102 6.013 1.00 0.64
H
ATOM 5758 HA ARG A 374 3.951 19.274 7.810 1.00 0.67
H
ATOM 5759 1HB ARG A 374 1.927 18.637 6.470 1.00 1.01
H
ATOM 5760 2HB ARG A 374 1.659 17.305 7.589 1.00 1.01
H
ATOM 5761 1HG ARG A 374 1.441 18.864 9.439 1.00 1.01
H
ATOM 5762 2HG ARG A 374 1.786 20.224 8.349 1.00 1.01
H
ATOM 5763 1HD ARG A 374 -0.231 19.512 7.036 1.00 1.01
H
ATOM 5764 2HD ARG A 374 -0.617 18.440 8.409 1.00 1.01
H
ATOM 5765 HE ARG A 374 -0.022 21.258 8.960 1.00 1.01
H
ATOM 5766 1HH1 ARG A 374 -2.503 18.796 8.614 1.00 1.01
H
ATOM 5767 2HH1 ARG A 374 -3.709 19.753 9.454 1.00 1.01
H
ATOM 5768 1HH2 ARG A 374 -1.555 22.433 10.035 1.00 1.01
H
ATOM 5769 2HH2 ARG A 374 -3.173 21.785 10.263 1.00 1.01
H
ATOM 5770 N ILE A 375 3.729 16.280 9.247 1.00 0.73
N
ATOM 5771 CA ILE A 375 3.981 15.622 10.530 1.00 0.87
C
ATOM 5772 C ILE A 375 5.450 15.760 10.897 1.00 0.53
C
ATOM 5773 O ILE A 375 5.776 15.791 12.079 1.00 0.58
O
ATOM 5774 CB ILE A 375 3.611 14.116 10.535 1.00 1.30
C
ATOM 5775 CG1 ILE A 375 3.446 13.599 11.970 1.00 1.30
C
ATOM 5776 CG2 ILE A 375 4.707 13.295 9.884 1.00 1.30
C
ATOM 5777 CD1 ILE A 375 2.285 14.199 12.722 1.00 1.30
C
ATOM 5778 H ILE A 375 3.471 15.723 8.441 1.00 0.88
H
ATOM 5779 HA ILE A 375 3.388 16.117 11.294 1.00 1.04
H
ATOM 5780 HB ILE A 375 2.671 13.962 10.006 1.00 1.57
H

```
ATOM   5781 1HG1 ILE A 375       3.306  12.520  11.936  1.00  1.57
H
ATOM   5782 2HG1 ILE A 375       4.359  13.807  12.529  1.00  1.57
H
ATOM   5783 1HG2 ILE A 375       4.440  12.242   9.897  1.00  1.57
H
ATOM   5784 2HG2 ILE A 375       4.826  13.622   8.868  1.00  1.57
H
ATOM   5785 3HG2 ILE A 375       5.641  13.429  10.428  1.00  1.57
H
ATOM   5786 1HD1 ILE A 375       2.241  13.772  13.723  1.00  1.57
H
ATOM   5787 2HD1 ILE A 375       2.424  15.273  12.793  1.00  1.57
H
ATOM   5788 3HD1 ILE A 375       1.360  13.983  12.193  1.00  1.57
H
ATOM   5789  N   GLY A 376       6.331  15.873   9.893  1.00  0.42
N
ATOM   5790  CA  GLY A 376       7.752  16.083  10.127  1.00  0.35
C
ATOM   5791  C   GLY A 376       7.967  17.394  10.861  1.00  0.34
C
ATOM   5792  O   GLY A 376       8.779  17.472  11.789  1.00  0.36
O
ATOM   5793  H   GLY A 376       6.019  15.791   8.934  1.00  0.50
H
ATOM   5794 1HA  GLY A 376       8.154  15.257  10.713  1.00  0.42
H
ATOM   5795 2HA  GLY A 376       8.281  16.100   9.175  1.00  0.42
H
ATOM   5796  N   GLY A 377       7.222  18.423  10.450  1.00  0.38
N
ATOM   5797  CA  GLY A 377       7.249  19.707  11.138  1.00  0.42
C
ATOM   5798  C   GLY A 377       6.727  19.544  12.565  1.00  0.44
C
ATOM   5799  O   GLY A 377       7.335  20.032  13.518  1.00  0.48
O
ATOM   5800  H   GLY A 377       6.623  18.305   9.640  1.00  0.46
H
ATOM   5801 1HA  GLY A 377       8.268  20.094  11.155  1.00  0.50
H
ATOM   5802 2HA  GLY A 377       6.635  20.426  10.596  1.00  0.50
H
ATOM   5803  N   LEU A 378       5.626  18.804  12.714  1.00  0.45
N
ATOM   5804  CA  LEU A 378       5.024  18.556  14.022  1.00  0.46
C
ATOM   5805  C   LEU A 378       5.954  17.725  14.924  1.00  0.55
C
ATOM   5806  O   LEU A 378       5.935  17.865  16.149  1.00  0.90
O
ATOM   5807  CB  LEU A 378       3.674  17.848  13.831  1.00  0.69
C
ATOM   5808  CG  LEU A 378       2.603  18.706  13.131  1.00  0.69
C
ATOM   5809  CD1 LEU A 378       1.331  17.900  12.891  1.00  0.69
C
```

```
ATOM   5810  CD2 LEU A 378       2.329  19.918  13.978  1.00  0.69
C
ATOM   5811  H   LEU A 378       5.167  18.435  11.889  1.00  0.54
H
ATOM   5812  HA  LEU A 378       4.848  19.515  14.506  1.00  0.55
H
ATOM   5813 1HB  LEU A 378       3.827  16.950  13.242  1.00  0.83
H
ATOM   5814 2HB  LEU A 378       3.286  17.562  14.809  1.00  0.83
H
ATOM   5815  HG  LEU A 378       2.975  19.032  12.162  1.00  0.83
H
ATOM   5816 1HD1 LEU A 378       0.595  18.530  12.393  1.00  0.83
H
ATOM   5817 2HD1 LEU A 378       1.555  17.045  12.259  1.00  0.83
H
ATOM   5818 3HD1 LEU A 378       0.930  17.557  13.844  1.00  0.83
H
ATOM   5819 1HD2 LEU A 378       1.584  20.545  13.487  1.00  0.83
H
ATOM   5820 2HD2 LEU A 378       1.956  19.601  14.952  1.00  0.83
H
ATOM   5821 3HD2 LEU A 378       3.251  20.481  14.108  1.00  0.83
H
ATOM   5822  N   ALA A 379       6.788  16.889  14.305  1.00  0.49
N
ATOM   5823  CA  ALA A 379       7.771  16.067  14.992  1.00  0.48
C
ATOM   5824  C   ALA A 379       9.065  16.816  15.303  1.00  0.46
C
ATOM   5825  O   ALA A 379       9.979  16.235  15.892  1.00  0.52
O
ATOM   5826  CB  ALA A 379       8.101  14.860  14.141  1.00  0.72
C
ATOM   5827  H   ALA A 379       6.726  16.799  13.303  1.00  0.59
H
ATOM   5828  HA  ALA A 379       7.336  15.740  15.936  1.00  0.58
H
ATOM   5829 1HB  ALA A 379       8.816  14.230  14.667  1.00  0.86
H
ATOM   5830 2HB  ALA A 379       7.190  14.296  13.940  1.00  0.86
H
ATOM   5831 3HB  ALA A 379       8.536  15.190  13.199  1.00  0.86
H
ATOM   5832  N   GLU A 380       9.157  18.090  14.898  1.00  0.46
N
ATOM   5833  CA  GLU A 380      10.354  18.901  15.088  1.00  0.47
C
ATOM   5834  C   GLU A 380      11.588  18.240  14.480  1.00  0.41
C
ATOM   5835  O   GLU A 380      12.665  18.244  15.081  1.00  0.43
O
ATOM   5836  CB  GLU A 380      10.582  19.174  16.578  1.00  0.70
C
ATOM   5837  CG  GLU A 380       9.449  19.940  17.248  1.00  0.70
C
ATOM   5838  CD  GLU A 380       9.738  20.279  18.684  1.00  0.70
C
```

```
ATOM   5839  OE1 GLU A 380      10.714  19.794  19.204  1.00  0.70
O
ATOM   5840  OE2 GLU A 380       8.984  21.026  19.261  1.00  0.70
O
ATOM   5841  H   GLU A 380       8.382  18.540  14.435  1.00  0.55
H
ATOM   5842  HA  GLU A 380      10.203  19.857  14.585  1.00  0.56
H
ATOM   5843 1HB  GLU A 380      10.714  18.234  17.113  1.00  0.85
H
ATOM   5844 2HB  GLU A 380      11.496  19.753  16.704  1.00  0.85
H
ATOM   5845 1HG  GLU A 380       9.273  20.864  16.698  1.00  0.85
H
ATOM   5846 2HG  GLU A 380       8.540  19.342  17.198  1.00  0.85
H
ATOM   5847  N   SER A 381      11.427  17.672  13.283  1.00  0.40
N
ATOM   5848  CA  SER A 381      12.525  17.005  12.603  1.00  0.37
C
ATOM   5849  C   SER A 381      13.422  17.993  11.874  1.00  0.34
C
ATOM   5850  O   SER A 381      13.089  19.170  11.734  1.00  0.56
O
ATOM   5851  CB  SER A 381      11.993  16.001  11.617  1.00  0.55
C
ATOM   5852  OG  SER A 381      11.353  16.654  10.559  1.00  0.55
O
ATOM   5853  H   SER A 381      10.520  17.695  12.834  1.00  0.48
H
ATOM   5854  HA  SER A 381      13.126  16.483  13.348  1.00  0.44
H
ATOM   5855 1HB  SER A 381      12.811  15.390  11.235  1.00  0.67
H
ATOM   5856 2HB  SER A 381      11.291  15.336  12.118  1.00  0.67
H
ATOM   5857  HG  SER A 381      10.639  17.158  10.960  1.00  0.67
H
ATOM   5858  N   ASN A 382      14.556  17.495  11.398  1.00  0.31
N
ATOM   5859  CA  ASN A 382      15.515  18.319  10.679  1.00  0.36
C
ATOM   5860  C   ASN A 382      15.854  17.646   9.355  1.00  0.35
C
ATOM   5861  O   ASN A 382      16.945  17.099   9.188  1.00  1.23
O
ATOM   5862  CB  ASN A 382      16.758  18.538  11.514  1.00  0.54
C
ATOM   5863  CG  ASN A 382      16.458  19.192  12.836  1.00  0.54
C
ATOM   5864  OD1 ASN A 382      15.999  20.336  12.916  1.00  0.54
O
ATOM   5865  ND2 ASN A 382      16.715  18.469  13.897  1.00  0.54
N
ATOM   5866  H   ASN A 382      14.765  16.519  11.546  1.00  0.37
H
ATOM   5867  HA  ASN A 382      15.060  19.282  10.461  1.00  0.43
H
```

ATOM 5868 1HB ASN A 382 17.247 17.580 11.698 1.00 0.65
H
ATOM 5869 2HB ASN A 382 17.461 19.164 10.963 1.00 0.65
H
ATOM 5870 1HD2 ASN A 382 16.539 18.840 14.809 1.00 0.65
H
ATOM 5871 2HD2 ASN A 382 17.086 17.546 13.796 1.00 0.65
H
ATOM 5872 N ILE A 383 14.893 17.662 8.434 1.00 0.40
N
ATOM 5873 CA ILE A 383 15.031 16.995 7.140 1.00 0.42
C
ATOM 5874 C ILE A 383 15.229 17.962 5.990 1.00 0.30
C
ATOM 5875 O ILE A 383 14.531 18.974 5.896 1.00 0.48
O
ATOM 5876 CB ILE A 383 13.795 16.132 6.835 1.00 0.63
C
ATOM 5877 CG1 ILE A 383 13.568 15.160 7.970 1.00 0.63
C
ATOM 5878 CG2 ILE A 383 13.976 15.368 5.529 1.00 0.63
C
ATOM 5879 CD1 ILE A 383 14.738 14.258 8.204 1.00 0.63
C
ATOM 5880 H ILE A 383 14.031 18.147 8.640 1.00 0.48
H
ATOM 5881 HA ILE A 383 15.903 16.343 7.184 1.00 0.50
H
ATOM 5882 HB ILE A 383 12.916 16.769 6.763 1.00 0.76
H
ATOM 5883 1HG1 ILE A 383 13.360 15.713 8.885 1.00 0.76
H
ATOM 5884 2HG1 ILE A 383 12.703 14.543 7.734 1.00 0.76
H
ATOM 5885 1HG2 ILE A 383 13.090 14.765 5.336 1.00 0.76
H
ATOM 5886 2HG2 ILE A 383 14.118 16.069 4.712 1.00 0.76
H
ATOM 5887 3HG2 ILE A 383 14.848 14.718 5.604 1.00 0.76
H
ATOM 5888 1HD1 ILE A 383 14.506 13.580 9.024 1.00 0.76
H
ATOM 5889 2HD1 ILE A 383 14.940 13.682 7.302 1.00 0.76
H
ATOM 5890 3HD1 ILE A 383 15.613 14.850 8.458 1.00 0.76
H
ATOM 5891 N ASN A 384 16.176 17.638 5.118 1.00 0.24
N
ATOM 5892 CA ASN A 384 16.417 18.423 3.930 1.00 0.30
C
ATOM 5893 C ASN A 384 15.757 17.715 2.750 1.00 0.29
C
ATOM 5894 O ASN A 384 15.911 16.506 2.587 1.00 0.34
O
ATOM 5895 CB ASN A 384 17.904 18.589 3.719 1.00 0.45
C
ATOM 5896 CG ASN A 384 18.555 19.332 4.851 1.00 0.45
C

ATOM 5897 OD1 ASN A 384 18.148 20.442 5.200 1.00 0.45
O
ATOM 5898 ND2 ASN A 384 19.553 18.735 5.448 1.00 0.45
N
ATOM 5899 H ASN A 384 16.737 16.807 5.257 1.00 0.29
H
ATOM 5900 HA ASN A 384 15.952 19.402 4.043 1.00 0.36
H
ATOM 5901 1HB ASN A 384 18.369 17.609 3.628 1.00 0.54
H
ATOM 5902 2HB ASN A 384 18.086 19.129 2.790 1.00 0.54
H
ATOM 5903 1HD2 ASN A 384 20.016 19.183 6.214 1.00 0.54
H
ATOM 5904 2HD2 ASN A 384 19.850 17.831 5.140 1.00 0.54
H
ATOM 5905 N ILE A 385 14.999 18.453 1.955 1.00 0.28
N
ATOM 5906 CA ILE A 385 14.340 17.872 0.799 1.00 0.35
C
ATOM 5907 C ILE A 385 14.557 18.719 -0.451 1.00 0.38
C
ATOM 5908 O ILE A 385 14.352 19.931 -0.439 1.00 0.97
O
ATOM 5909 CB ILE A 385 12.842 17.660 1.055 1.00 0.52
C
ATOM 5910 CG1 ILE A 385 12.609 16.700 2.232 1.00 0.52
C
ATOM 5911 CG2 ILE A 385 12.186 17.121 -0.191 1.00 0.52
C
ATOM 5912 CD1 ILE A 385 11.162 16.559 2.648 1.00 0.52
C
ATOM 5913 H ILE A 385 14.884 19.435 2.149 1.00 0.34
H
ATOM 5914 HA ILE A 385 14.780 16.896 0.622 1.00 0.42
H
ATOM 5915 HB ILE A 385 12.399 18.608 1.329 1.00 0.63
H
ATOM 5916 1HG1 ILE A 385 12.986 15.715 1.962 1.00 0.63
H
ATOM 5917 2HG1 ILE A 385 13.161 17.056 3.097 1.00 0.63
H
ATOM 5918 1HG2 ILE A 385 11.123 16.992 -0.003 1.00 0.63
H
ATOM 5919 2HG2 ILE A 385 12.329 17.824 -1.010 1.00 0.63
H
ATOM 5920 3HG2 ILE A 385 12.630 16.161 -0.452 1.00 0.63
H
ATOM 5921 1HD1 ILE A 385 11.091 15.864 3.484 1.00 0.63
H
ATOM 5922 2HD1 ILE A 385 10.777 17.530 2.954 1.00 0.63
H
ATOM 5923 3HD1 ILE A 385 10.577 16.182 1.812 1.00 0.63
H
ATOM 5924 N ILE A 386 14.982 18.072 -1.524 1.00 0.31
N
ATOM 5925 CA ILE A 386 15.247 18.757 -2.774 1.00 0.40
C

ATOM	5926	C	ILE	A	386	14.232	18.348	-3.823	1.00	0.40	C
ATOM	5927	O	ILE	A	386	14.054	17.159	-4.095	1.00	0.42	O
ATOM	5928	CB	ILE	A	386	16.658	18.456	-3.289	1.00	0.60	C
ATOM	5929	CG1	ILE	A	386	17.706	18.880	-2.252	1.00	0.60	C
ATOM	5930	CG2	ILE	A	386	16.871	19.185	-4.607	1.00	0.60	C
ATOM	5931	CD1	ILE	A	386	18.093	17.773	-1.289	1.00	0.60	C
ATOM	5932	H	ILE	A	386	15.120	17.074	-1.469	1.00	0.37	H
ATOM	5933	HA	ILE	A	386	15.159	19.829	-2.611	1.00	0.48	H
ATOM	5934	HB	ILE	A	386	16.769	17.384	-3.447	1.00	0.72	H
ATOM	5935	1HG1	ILE	A	386	18.602	19.218	-2.768	1.00	0.72	H
ATOM	5936	2HG1	ILE	A	386	17.311	19.713	-1.670	1.00	0.72	H
ATOM	5937	1HG2	ILE	A	386	17.864	18.965	-4.988	1.00	0.72	H
ATOM	5938	2HG2	ILE	A	386	16.127	18.853	-5.331	1.00	0.72	H
ATOM	5939	3HG2	ILE	A	386	16.770	20.258	-4.450	1.00	0.72	H
ATOM	5940	1HD1	ILE	A	386	18.834	18.150	-0.587	1.00	0.72	H
ATOM	5941	2HD1	ILE	A	386	17.211	17.437	-0.743	1.00	0.72	H
ATOM	5942	3HD1	ILE	A	386	18.512	16.938	-1.847	1.00	0.72	H
ATOM	5943	N	GLY	A	387	13.561	19.336	-4.394	1.00	0.43	N
ATOM	5944	CA	GLY	A	387	12.557	19.081	-5.408	1.00	0.42	C
ATOM	5945	C	GLY	A	387	13.041	19.565	-6.770	1.00	0.48	C
ATOM	5946	O	GLY	A	387	13.441	20.718	-6.920	1.00	0.76	O
ATOM	5947	H	GLY	A	387	13.777	20.289	-4.130	1.00	0.52	H
ATOM	5948	1HA	GLY	A	387	12.333	18.018	-5.444	1.00	0.50	H
ATOM	5949	2HA	GLY	A	387	11.638	19.591	-5.133	1.00	0.50	H
ATOM	5950	N	SER	A	388	12.994	18.676	-7.756	1.00	0.39	N
ATOM	5951	CA	SER	A	388	13.446	18.974	-9.109	1.00	0.40	C
ATOM	5952	C	SER	A	388	12.300	19.208	-10.076	1.00	0.44	C
ATOM	5953	O	SER	A	388	11.139	18.986	-9.740	1.00	0.47	O
ATOM	5954	CB	SER	A	388	14.303	17.835	-9.614	1.00	0.60	C

```
ATOM   5955  OG  SER A 388      13.535  16.701  -9.886  1.00  0.60
O
ATOM   5956  H   SER A 388      12.655  17.747  -7.547  1.00  0.47
H
ATOM   5957  HA  SER A 388      14.040  19.881  -9.082  1.00  0.48
H
ATOM   5958 1HB  SER A 388      14.820  18.139 -10.513  1.00  0.72
H
ATOM   5959 2HB  SER A 388      15.055  17.597  -8.864  1.00  0.72
H
ATOM   5960  HG  SER A 388      13.228  16.809 -10.793  1.00  0.72
H
ATOM   5961  N   HIS A 389      12.630  19.674 -11.284  1.00  0.46
N
ATOM   5962  CA  HIS A 389      11.627  19.872 -12.330  1.00  0.48
C
ATOM   5963  C   HIS A 389      10.538  20.849 -11.937  1.00  0.49
C
ATOM   5964  O   HIS A 389       9.346  20.576 -12.116  1.00  0.55
O
ATOM   5965  CB  HIS A 389      11.012  18.522 -12.721  1.00  0.72
C
ATOM   5966  CG  HIS A 389      12.009  17.633 -13.368  1.00  0.72
C
ATOM   5967  ND1 HIS A 389      12.992  16.980 -12.662  1.00  0.72
N
ATOM   5968  CD2 HIS A 389      12.176  17.284 -14.661  1.00  0.72
C
ATOM   5969  CE1 HIS A 389      13.733  16.279 -13.499  1.00  0.72
C
ATOM   5970  NE2 HIS A 389      13.257  16.443 -14.719  1.00  0.72
N
ATOM   5971  H   HIS A 389      13.600  19.868 -11.488  1.00  0.55
H
ATOM   5972  HA  HIS A 389      12.115  20.277 -13.215  1.00  0.58
H
ATOM   5973 1HB  HIS A 389      10.611  18.012 -11.846  1.00  0.86
H
ATOM   5974 2HB  HIS A 389      10.186  18.675 -13.413  1.00  0.86
H
ATOM   5975  HD2 HIS A 389      11.565  17.617 -15.501  1.00  0.86
H
ATOM   5976  HE1 HIS A 389      14.589  15.669 -13.224  1.00  0.86
H
ATOM   5977  HE2 HIS A 389      13.624  16.022 -15.561  1.00  0.86
H
ATOM   5978  N   CYS A 390      10.931  21.992 -11.420  1.00  0.52
N
ATOM   5979  CA  CYS A 390       9.915  22.917 -11.015  1.00  0.77
C
ATOM   5980  C   CYS A 390       9.543  23.884 -12.116  1.00  0.74
C
ATOM   5981  O   CYS A 390      10.402  24.361 -12.859  1.00  1.30
O
ATOM   5982  CB  CYS A 390      10.401  23.660  -9.815  1.00  1.16
C
ATOM   5983  SG  CYS A 390      10.761  22.569  -8.445  1.00  1.16
S
```

```
ATOM   5984  H   CYS A 390      11.914  22.207 -11.265  1.00  0.62
H
ATOM   5985  HA  CYS A 390       9.030  22.351 -10.745  1.00  0.92
H
ATOM   5986 1HB  CYS A 390      11.301  24.224 -10.056  1.00  1.39
H
ATOM   5987 2HB  CYS A 390       9.629  24.356  -9.495  1.00  1.39
H
ATOM   5988  HG  CYS A 390      11.708  21.875  -9.064  1.00  1.39
H
ATOM   5989  N   GLY A 391       8.252  24.163 -12.221  1.00  0.77
N
ATOM   5990  CA  GLY A 391       7.760  25.109 -13.205  1.00  0.86
C
ATOM   5991  C   GLY A 391       7.461  24.532 -14.592  1.00  0.96
C
ATOM   5992  O   GLY A 391       7.816  23.397 -14.910  1.00  0.92
O
ATOM   5993  H   GLY A 391       7.594  23.756 -11.571  1.00  0.92
H
ATOM   5994 1HA  GLY A 391       6.875  25.597 -12.817  1.00  1.03
H
ATOM   5995 2HA  GLY A 391       8.515  25.880 -13.307  1.00  1.03
H
ATOM   5996  N   VAL A 392       6.851  25.373 -15.442  1.00  1.14
N
ATOM   5997  CA  VAL A 392       6.510  25.011 -16.813  1.00  1.26
C
ATOM   5998  C   VAL A 392       7.772  24.906 -17.647  1.00  1.30
C
ATOM   5999  O   VAL A 392       7.783  24.226 -18.674  1.00  1.43
O
ATOM   6000  CB  VAL A 392       5.544  26.031 -17.463  1.00  1.89
C
ATOM   6001  CG1 VAL A 392       4.290  26.160 -16.614  1.00  1.89
C
ATOM   6002  CG2 VAL A 392       6.228  27.374 -17.670  1.00  1.89
C
ATOM   6003  H   VAL A 392       6.603  26.297 -15.110  1.00  1.37
H
ATOM   6004  HA  VAL A 392       6.028  24.040 -16.800  1.00  1.51
H
ATOM   6005  HB  VAL A 392       5.231  25.644 -18.433  1.00  2.27
H
ATOM   6006 1HG1 VAL A 392       3.592  26.848 -17.085  1.00  2.27
H
ATOM   6007 2HG1 VAL A 392       3.821  25.181 -16.510  1.00  2.27
H
ATOM   6008 3HG1 VAL A 392       4.560  26.537 -15.628  1.00  2.27
H
ATOM   6009 1HG2 VAL A 392       5.538  28.068 -18.142  1.00  2.27
H
ATOM   6010 2HG2 VAL A 392       6.539  27.776 -16.710  1.00  2.27
H
ATOM   6011 3HG2 VAL A 392       7.102  27.247 -18.307  1.00  2.27
H
ATOM   6012  N   SER A 393       8.853  25.556 -17.183  1.00  1.64
N
```

ATOM   6013 CA SER A 393   10.165 25.499 -17.821 1.00 1.58
C
ATOM   6014 C   SER A 393   10.689 24.081 -18.135 1.00 1.43
C
ATOM   6015 O   SER A 393   11.612 23.949 -18.942 1.00 1.57
O
ATOM   6016 CB SER A 393   11.184 26.216 -16.956 1.00 2.37
C
ATOM   6017 OG SER A 393   11.394 25.536 -15.750 1.00 2.37
O
ATOM   6018 H   SER A 393   8.762 26.138 -16.349 1.00 1.97
H
ATOM   6019 HA SER A 393   10.092 26.039 -18.767 1.00 1.90
H
ATOM   6020 1HB SER A 393   12.125 26.301 -17.498 1.00 2.84
H
ATOM   6021 2HB SER A 393   10.834 27.226 -16.748 1.00 2.84
H
ATOM   6022 HG SER A 393   10.570 25.608 -15.258 1.00 2.84
H
ATOM   6023 N   VAL A 394   10.103 23.022 -17.542 1.00 1.18
N
ATOM   6024 CA VAL A 394   10.538 21.657 -17.845 1.00 1.06
C
ATOM   6025 C   VAL A 394   10.236 21.239 -19.292 1.00 1.12
C
ATOM   6026 O   VAL A 394   10.859 20.311 -19.811 1.00 1.14
O
ATOM   6027 CB VAL A 394   9.867 20.644 -16.907 1.00 1.59
C
ATOM   6028 CG1 VAL A 394   10.198 20.980 -15.468 1.00 1.59
C
ATOM   6029 CG2 VAL A 394   8.379 20.637 -17.147 1.00 1.59
C
ATOM   6030 H   VAL A 394   9.362 23.155 -16.857 1.00 1.42
H
ATOM   6031 HA VAL A 394   11.607 21.613 -17.687 1.00 1.27
H
ATOM   6032 HB VAL A 394   10.267 19.648 -17.106 1.00 1.91
H
ATOM   6033 1HG1 VAL A 394   9.727 20.248 -14.813 1.00 1.91
H
ATOM   6034 2HG1 VAL A 394   11.274 20.958 -15.324 1.00 1.91
H
ATOM   6035 3HG1 VAL A 394   9.820 21.973 -15.230 1.00 1.91
H
ATOM   6036 1HG2 VAL A 394   7.908 19.913 -16.482 1.00 1.91
H
ATOM   6037 2HG2 VAL A 394   7.985 21.631 -16.947 1.00 1.91
H
ATOM   6038 3HG2 VAL A 394   8.171 20.363 -18.181 1.00 1.91
H
ATOM   6039 N   GLY A 395   9.293 21.920 -19.949 1.00 1.23
N
ATOM   6040 CA GLY A 395   8.984 21.621 -21.342 1.00 1.37
C
ATOM   6041 C   GLY A 395   7.903 20.554 -21.554 1.00 1.29
C

ATOM   6042 O   GLY A 395   6.927 20.465 -20.800 1.00 1.27
O
ATOM   6043 H   GLY A 395   8.797 22.671 -19.486 1.00 1.48
H
ATOM   6044 1HA GLY A 395   8.682 22.540 -21.840 1.00 1.64
H
ATOM   6045 2HA GLY A 395   9.897 21.299 -21.840 1.00 1.64
H
ATOM   6046 N   ASP A 396   8.117 19.740 -22.600 1.00 1.45
N
ATOM   6047 CA ASP A 396   7.193 18.705 -23.101 1.00 1.49
C
ATOM   6048 C   ASP A 396   6.735 17.598 -22.153 1.00 1.81
C
ATOM   6049 O   ASP A 396   5.952 16.741 -22.562 1.00 5.71
O
ATOM   6050 CB ASP A 396   7.818 18.005 -24.310 1.00 2.23
C
ATOM   6051 CG ASP A 396   7.842 18.849 -25.563 1.00 2.23
C
ATOM   6052 OD1 ASP A 396   7.074 19.774 -25.647 1.00 2.23
O
ATOM   6053 OD2 ASP A 396   8.630 18.561 -26.431 1.00 2.23
O
ATOM   6054 H   ASP A 396   8.973 19.879 -23.119 1.00 1.74
H
ATOM   6055 HA ASP A 396   6.298 19.220 -23.438 1.00 1.79
H
ATOM   6056 1HB ASP A 396   8.842 17.720 -24.068 1.00 2.68
H
ATOM   6057 2HB ASP A 396   7.267 17.087 -24.521 1.00 2.68
H
ATOM   6058 N   ASP A 397   7.179 17.610 -20.907 1.00 1.53
N
ATOM   6059 CA ASP A 397   6.732 16.630 -19.942 1.00 1.33
C
ATOM   6060 C   ASP A 397   5.250 16.830 -19.628 1.00 1.17
C
ATOM   6061 O   ASP A 397   4.526 15.871 -19.359 1.00 1.25
O
ATOM   6062 CB ASP A 397   7.546 16.765 -18.668 1.00 2.00
C
ATOM   6063 CG ASP A 397   8.993 16.353 -18.860 1.00 2.00
C
ATOM   6064 OD1 ASP A 397   9.303 15.711 -19.834 1.00 2.00
O
ATOM   6065 OD2 ASP A 397   9.787 16.697 -18.018 1.00 2.00
O
ATOM   6066 H   ASP A 397   7.837 18.308 -20.606 1.00 1.84
H
ATOM   6067 HA ASP A 397   6.870 15.633 -20.361 1.00 1.60
H
ATOM   6068 1HB ASP A 397   7.517 17.798 -18.321 1.00 2.39
H
ATOM   6069 2HB ASP A 397   7.104 16.143 -17.889 1.00 2.39
H
ATOM   6070 N   GLY A 398   4.811 18.090 -19.661 1.00 1.13
N

```
ATOM   6071  CA  GLY A 398       3.424  18.429 -19.367  1.00  1.05
C
ATOM   6072  C   GLY A 398       3.226  18.711 -17.879  1.00  0.99
C
ATOM   6073  O   GLY A 398       4.109  18.433 -17.066  1.00  1.31
O
ATOM   6074  H   GLY A 398       5.456  18.834 -19.914  1.00  1.36
H
ATOM   6075 1HA  GLY A 398       3.133  19.304 -19.948  1.00  1.26
H
ATOM   6076 2HA  GLY A 398       2.776  17.610 -19.675  1.00  1.26
H
ATOM   6077  N   ALA A 399       2.043  19.235 -17.540  1.00  0.82
N
ATOM   6078  CA  ALA A 399       1.668  19.641 -16.178  1.00  0.83
C
ATOM   6079  C   ALA A 399       1.923  18.590 -15.114  1.00  0.95
C
ATOM   6080  O   ALA A 399       2.240  18.927 -13.973  1.00  0.89
O
ATOM   6081  CB  ALA A 399       0.197  19.998 -16.139  1.00  1.24
C
ATOM   6082  H   ALA A 399       1.371  19.398 -18.279  1.00  0.98
H
ATOM   6083  HA  ALA A 399       2.255  20.520 -15.922  1.00  1.00
H
ATOM   6084 1HB  ALA A 399      -0.062  20.341 -15.138  1.00  1.49
H
ATOM   6085 2HB  ALA A 399      -0.009  20.788 -16.854  1.00  1.49
H
ATOM   6086 3HB  ALA A 399      -0.397  19.122 -16.388  1.00  1.49
H
ATOM   6087  N   SER A 400       1.804  17.320 -15.482  1.00  1.29
N
ATOM   6088  CA  SER A 400       1.994  16.234 -14.543  1.00  1.75
C
ATOM   6089  C   SER A 400       3.360  16.233 -13.853  1.00  1.78
C
ATOM   6090  O   SER A 400       3.464  15.762 -12.724  1.00  2.89
O
ATOM   6091  CB  SER A 400       1.813  14.918 -15.263  1.00  2.62
C
ATOM   6092  OG  SER A 400       2.825  14.722 -16.213  1.00  2.62
O
ATOM   6093  H   SER A 400       1.553  17.101 -16.435  1.00  1.55
H
ATOM   6094  HA  SER A 400       1.225  16.321 -13.777  1.00  2.10
H
ATOM   6095 1HB  SER A 400       1.830  14.104 -14.541  1.00  3.15
H
ATOM   6096 2HB  SER A 400       0.841  14.902 -15.754  1.00  3.15
H
ATOM   6097  HG  SER A 400       2.738  15.435 -16.850  1.00  3.15
H
ATOM   6098  N   GLN A 401       4.390  16.776 -14.498  1.00  1.14
N
ATOM   6099  CA  GLN A 401       5.708  16.817 -13.878  1.00  0.85
C
```

```
ATOM   6100  C   GLN A 401       6.181  18.244 -13.636  1.00  0.99
C
ATOM   6101  O   GLN A 401       7.378  18.477 -13.480  1.00  1.70
O
ATOM   6102  CB  GLN A 401       6.737  16.064 -14.724  1.00  1.27
C
ATOM   6103  CG  GLN A 401       6.464  14.574 -14.849  1.00  1.27
C
ATOM   6104  CD  GLN A 401       7.527  13.850 -15.655  1.00  1.27
C
ATOM   6105  OE1 GLN A 401       7.435  12.638 -15.887  1.00  1.27
O
ATOM   6106  NE2 GLN A 401       8.547  14.581 -16.084  1.00  1.27
N
ATOM   6107  H   GLN A 401       4.263  17.176 -15.420  1.00  1.37
H
ATOM   6108  HA  GLN A 401       5.645  16.323 -12.908  1.00  1.02
H
ATOM   6109 1HB  GLN A 401       6.759  16.489 -15.727  1.00  1.53
H
ATOM   6110 2HB  GLN A 401       7.729  16.192 -14.291  1.00  1.53
H
ATOM   6111 1HG  GLN A 401       6.436  14.138 -13.850  1.00  1.53
H
ATOM   6112 2HG  GLN A 401       5.503  14.432 -15.344  1.00  1.53
H
ATOM   6113 1HE2 GLN A 401       9.278  14.159 -16.623  1.00  1.53
H
ATOM   6114 2HE2 GLN A 401       8.588  15.559 -15.879  1.00  1.53
H
ATOM   6115  N   MET A 402       5.245  19.192 -13.604  1.00  0.69
N
ATOM   6116  CA  MET A 402       5.562  20.599 -13.379  1.00  0.66
C
ATOM   6117  C   MET A 402       5.180  21.024 -11.987  1.00  0.70
C
ATOM   6118  O   MET A 402       3.998  21.048 -11.660  1.00  1.17
O
ATOM   6119  CB  MET A 402       4.809  21.477 -14.379  1.00  0.99
C
ATOM   6120  CG  MET A 402       5.234  21.302 -15.815  1.00  0.99
C
ATOM   6121  SD  MET A 402       4.208  22.214 -16.974  1.00  0.99
S
ATOM   6122  CE  MET A 402       5.007  21.874 -18.536  1.00  0.99
C
ATOM   6123  H   MET A 402       4.271  18.946 -13.731  1.00  0.83
H
ATOM   6124  HA  MET A 402       6.636  20.743 -13.499  1.00  0.79
H
ATOM   6125 1HB  MET A 402       3.744  21.257 -14.320  1.00  1.19
H
ATOM   6126 2HB  MET A 402       4.942  22.526 -14.116  1.00  1.19
H
ATOM   6127 1HG  MET A 402       6.243  21.683 -15.899  1.00  1.19
H
ATOM   6128 2HG  MET A 402       5.237  20.251 -16.087  1.00  1.19
H
```

ATOM   6129 1HE MET A 402   4.465 22.376 -19.338 1.00 1.19
H
ATOM   6130 2HE MET A 402   6.033 22.241 -18.507 1.00 1.19
H
ATOM   6131 3HE MET A 402   5.013 20.802 -18.719 1.00 1.19
H
ATOM   6132 N   ALA A 403   6.153 21.402 -11.172 1.00 0.65
N
ATOM   6133 CA ALA A 403   5.788 21.866 -9.844 1.00 0.72
C
ATOM   6134 C   ALA A 403   5.384 23.325 -9.891 1.00 1.06
C
ATOM   6135 O   ALA A 403   6.192 24.189 -10.222 1.00 2.33
O
ATOM   6136 CB ALA A 403   6.921 21.685 -8.880 1.00 1.08
C
ATOM   6137 H   ALA A 403   7.128 21.348 -11.463 1.00 0.78
H
ATOM   6138 HA ALA A 403   4.938 21.281 -9.501 1.00 0.86
H
ATOM   6139 1HB ALA A 403   6.609 22.012 -7.889 1.00 1.30
H
ATOM   6140 2HB ALA A 403   7.196 20.637 -8.848 1.00 1.30
H
ATOM   6141 3HB ALA A 403   7.776 22.273 -9.195 1.00 1.30
H
ATOM   6142 N   LEU A 404   4.122 23.593 -9.594 1.00 1.22
N
ATOM   6143 CA LEU A 404   3.612 24.957 -9.590 1.00 1.06
C
ATOM   6144 C   LEU A 404   2.858 25.239 -8.300 1.00 0.96
C
ATOM   6145 O   LEU A 404   2.246 26.295 -8.148 1.00 1.60
O
ATOM   6146 CB LEU A 404   2.695 25.174 -10.804 1.00 1.59
C
ATOM   6147 CG LEU A 404   3.356 25.049 -12.188 1.00 1.59
C
ATOM   6148 CD1 LEU A 404   2.308 25.067 -13.266 1.00 1.59
C
ATOM   6149 CD2 LEU A 404   4.294 26.209 -12.406 1.00 1.59
C
ATOM   6150 H   LEU A 404   3.499 22.837 -9.356 1.00 1.46
H
ATOM   6151 HA LEU A 404   4.455 25.644 -9.655 1.00 1.27
H
ATOM   6152 1HB LEU A 404   1.888 24.446 -10.761 1.00 1.91
H
ATOM   6153 2HB LEU A 404   2.259 26.171 -10.736 1.00 1.91
H
ATOM   6154 HG LEU A 404   3.901 24.108 -12.252 1.00 1.91
H
ATOM   6155 1HD1 LEU A 404   2.793 24.980 -14.236 1.00 1.91
H
ATOM   6156 2HD1 LEU A 404   1.618 24.235 -13.126 1.00 1.91
H
ATOM   6157 3HD1 LEU A 404   1.765 26.006 -13.220 1.00 1.91
H

ATOM	6158	1HD2	LEU	A	404	4.759	26.121	-13.387	1.00	1.91	H
ATOM	6159	2HD2	LEU	A	404	3.736	27.143	-12.352	1.00	1.91	H
ATOM	6160	3HD2	LEU	A	404	5.066	26.202	-11.636	1.00	1.91	H
ATOM	6161	N	GLU	A	405	2.896	24.283	-7.370	1.00	0.79	N
ATOM	6162	CA	GLU	A	405	2.198	24.407	-6.098	1.00	0.96	C
ATOM	6163	C	GLU	A	405	3.142	24.226	-4.909	1.00	0.58	C
ATOM	6164	O	GLU	A	405	2.739	24.404	-3.759	1.00	0.93	O
ATOM	6165	CB	GLU	A	405	1.075	23.355	-6.053	1.00	1.44	C
ATOM	6166	CG	GLU	A	405	0.108	23.523	-7.214	1.00	1.44	C
ATOM	6167	CD	GLU	A	405	-1.059	22.586	-7.284	1.00	1.44	C
ATOM	6168	OE1	GLU	A	405	-1.934	22.653	-6.455	1.00	1.44	O
ATOM	6169	OE2	GLU	A	405	-1.093	21.820	-8.213	1.00	1.44	O
ATOM	6170	H	GLU	A	405	3.417	23.439	-7.547	1.00	0.95	H
ATOM	6171	HA	GLU	A	405	1.758	25.403	-6.038	1.00	1.15	H
ATOM	6172	1HB	GLU	A	405	1.503	22.352	-6.096	1.00	1.73	H
ATOM	6173	2HB	GLU	A	405	0.519	23.443	-5.120	1.00	1.73	H
ATOM	6174	1HG	GLU	A	405	-0.265	24.532	-7.172	1.00	1.73	H
ATOM	6175	2HG	GLU	A	405	0.671	23.425	-8.139	1.00	1.73	H
ATOM	6176	N	ASP	A	406	4.392	23.864	-5.198	1.00	0.75	N
ATOM	6177	CA	ASP	A	406	5.350	23.503	-4.166	1.00	0.80	C
ATOM	6178	C	ASP	A	406	5.805	24.624	-3.253	1.00	0.72	C
ATOM	6179	O	ASP	A	406	6.141	24.359	-2.105	1.00	1.16	O
ATOM	6180	CB	ASP	A	406	6.582	22.893	-4.829	1.00	1.20	C
ATOM	6181	CG	ASP	A	406	7.236	23.851	-5.812	1.00	1.20	C
ATOM	6182	OD1	ASP	A	406	6.529	24.382	-6.635	1.00	1.20	O
ATOM	6183	OD2	ASP	A	406	8.426	24.050	-5.731	1.00	1.20	O
ATOM	6184	H	ASP	A	406	4.680	23.791	-6.161	1.00	0.90	H
ATOM	6185	HA	ASP	A	406	4.886	22.739	-3.542	1.00	0.96	H
ATOM	6186	1HB	ASP	A	406	7.311	22.621	-4.069	1.00	1.44	H

```
ATOM   6187 2HB  ASP A 406       6.302  21.981  -5.356  1.00  1.44
H
ATOM   6188  N   ILE A 407       5.778  25.870  -3.702  1.00  1.10
N
ATOM   6189  CA  ILE A 407       6.230  26.929  -2.818  1.00  1.41
C
ATOM   6190  C   ILE A 407       5.158  27.211  -1.818  1.00  1.18
C
ATOM   6191  O   ILE A 407       5.447  27.431  -0.646  1.00  2.34
O
ATOM   6192  CB  ILE A 407       6.628  28.201  -3.570  1.00  2.11
C
ATOM   6193  CG1 ILE A 407       7.805  27.876  -4.488  1.00  2.11
C
ATOM   6194  CG2 ILE A 407       6.972  29.310  -2.593  1.00  2.11
C
ATOM   6195  CD1 ILE A 407       8.988  27.298  -3.748  1.00  2.11
C
ATOM   6196  H   ILE A 407       5.457  26.098  -4.634  1.00  1.32
H
ATOM   6197  HA  ILE A 407       7.109  26.579  -2.279  1.00  1.69
H
ATOM   6198  HB  ILE A 407       5.804  28.531  -4.198  1.00  2.54
H
ATOM   6199 1HG1 ILE A 407       7.487  27.155  -5.241  1.00  2.54
H
ATOM   6200 2HG1 ILE A 407       8.124  28.787  -4.993  1.00  2.54
H
ATOM   6201 1HG2 ILE A 407       7.250  30.208  -3.146  1.00  2.54
H
ATOM   6202 2HG2 ILE A 407       6.106  29.523  -1.969  1.00  2.54
H
ATOM   6203 3HG2 ILE A 407       7.806  28.998  -1.964  1.00  2.54
H
ATOM   6204 1HD1 ILE A 407       9.795  27.092  -4.449  1.00  2.54
H
ATOM   6205 2HD1 ILE A 407       9.328  28.012  -3.002  1.00  2.54
H
ATOM   6206 3HD1 ILE A 407       8.691  26.374  -3.256  1.00  2.54
H
ATOM   6207  N   ALA A 408       3.913  27.182  -2.271  1.00  1.00
N
ATOM   6208  CA  ALA A 408       2.799  27.362  -1.371  1.00  0.88
C
ATOM   6209  C   ALA A 408       2.810  26.283  -0.312  1.00  0.86
C
ATOM   6210  O   ALA A 408       2.648  26.569   0.874  1.00  1.63
O
ATOM   6211  CB  ALA A 408       1.502  27.323  -2.129  1.00  1.32
C
ATOM   6212  H   ALA A 408       3.739  27.017  -3.253  1.00  1.20
H
ATOM   6213  HA  ALA A 408       2.905  28.328  -0.878  1.00  1.06
H
ATOM   6214 1HB  ALA A 408       0.670  27.472  -1.441  1.00  1.58
H
ATOM   6215 2HB  ALA A 408       1.495  28.109  -2.884  1.00  1.58
H
```

```
ATOM   6216 3HB  ALA A 408       1.402  26.355  -2.615  1.00  1.58
H
ATOM   6217  N   MET A 409       3.031  25.040  -0.744  1.00  0.65
N
ATOM   6218  CA  MET A 409       3.051  23.922   0.179  1.00  0.80
C
ATOM   6219  C   MET A 409       4.162  24.008   1.193  1.00  0.71
C
ATOM   6220  O   MET A 409       3.936  23.755   2.375  1.00  1.12
O
ATOM   6221  CB  MET A 409       3.167  22.619  -0.602  1.00  1.20
C
ATOM   6222  CG  MET A 409       1.935  22.329  -1.417  1.00  1.20
C
ATOM   6223  SD  MET A 409       0.484  22.184  -0.370  1.00  1.20
S
ATOM   6224  CE  MET A 409      -0.083  23.874  -0.442  1.00  1.20
C
ATOM   6225  H   MET A 409       3.154  24.862  -1.735  1.00  0.78
H
ATOM   6226  HA  MET A 409       2.110  23.918   0.726  1.00  0.96
H
ATOM   6227 1HB  MET A 409       4.015  22.675  -1.284  1.00  1.44
H
ATOM   6228 2HB  MET A 409       3.345  21.788   0.075  1.00  1.44
H
ATOM   6229 1HG  MET A 409       1.757  23.125  -2.131  1.00  1.44
H
ATOM   6230 2HG  MET A 409       2.072  21.396  -1.961  1.00  1.44
H
ATOM   6231 1HE  MET A 409      -0.976  23.982   0.169  1.00  1.44
H
ATOM   6232 2HE  MET A 409       0.692  24.532  -0.054  1.00  1.44
H
ATOM   6233 3HE  MET A 409      -0.307  24.143  -1.475  1.00  1.44
H
ATOM   6234  N   PHE A 410       5.353  24.396   0.756  1.00  0.57
N
ATOM   6235  CA  PHE A 410       6.475  24.472   1.670  1.00  0.57
C
ATOM   6236  C   PHE A 410       6.295  25.633   2.642  1.00  0.54
C
ATOM   6237  O   PHE A 410       6.610  25.507   3.824  1.00  0.74
O
ATOM   6238  CB  PHE A 410       7.765  24.668   0.883  1.00  0.85
C
ATOM   6239  CG  PHE A 410       8.057  23.530  -0.041  1.00  0.85
C
ATOM   6240  CD1 PHE A 410       7.392  22.319   0.095  1.00  0.85
C
ATOM   6241  CD2 PHE A 410       8.968  23.683  -1.076  1.00  0.85
C
ATOM   6242  CE1 PHE A 410       7.624  21.287  -0.791  1.00  0.85
C
ATOM   6243  CE2 PHE A 410       9.205  22.649  -1.960  1.00  0.85
C
ATOM   6244  CZ  PHE A 410       8.529  21.453  -1.818  1.00  0.85
C
```

```
ATOM   6245  H   PHE A 410       5.500  24.596  -0.225  1.00  0.68
H
ATOM   6246  HA  PHE A 410       6.531  23.543   2.239  1.00  0.68
H
ATOM   6247 1HB  PHE A 410       7.694  25.575   0.288  1.00  1.03
H
ATOM   6248 2HB  PHE A 410       8.604  24.778   1.567  1.00  1.03
H
ATOM   6249  HD1 PHE A 410       6.669  22.194   0.903  1.00  1.03
H
ATOM   6250  HD2 PHE A 410       9.488  24.633  -1.195  1.00  1.03
H
ATOM   6251  HE1 PHE A 410       7.092  20.343  -0.682  1.00  1.03
H
ATOM   6252  HE2 PHE A 410       9.918  22.778  -2.775  1.00  1.03
H
ATOM   6253  HZ  PHE A 410       8.710  20.644  -2.519  1.00  1.03
H
ATOM   6254  N   ARG A 411       5.742  26.753   2.162  1.00  0.68
N
ATOM   6255  CA  ARG A 411       5.529  27.908   3.028  1.00  1.01
C
ATOM   6256  C   ARG A 411       4.585  27.626   4.169  1.00  1.10
C
ATOM   6257  O   ARG A 411       4.785  28.125   5.277  1.00  2.41
O
ATOM   6258  CB  ARG A 411       4.973  29.098   2.273  1.00  1.52
C
ATOM   6259  CG  ARG A 411       5.925  29.855   1.376  1.00  1.52
C
ATOM   6260  CD  ARG A 411       5.189  30.900   0.649  1.00  1.52
C
ATOM   6261  NE  ARG A 411       4.600  31.870   1.556  1.00  1.52
N
ATOM   6262  CZ  ARG A 411       3.486  32.566   1.289  1.00  1.52
C
ATOM   6263  NH1 ARG A 411       2.860  32.383   0.162  1.00  1.52
N
ATOM   6264  NH2 ARG A 411       3.019  33.412   2.179  1.00  1.52
N
ATOM   6265  H   ARG A 411       5.494  26.825   1.184  1.00  0.82
H
ATOM   6266  HA  ARG A 411       6.493  28.194   3.446  1.00  1.21
H
ATOM   6267 1HB  ARG A 411       4.138  28.772   1.654  1.00  1.82
H
ATOM   6268 2HB  ARG A 411       4.584  29.820   2.988  1.00  1.82
H
ATOM   6269 1HG  ARG A 411       6.711  30.323   1.970  1.00  1.82
H
ATOM   6270 2HG  ARG A 411       6.365  29.183   0.646  1.00  1.82
H
ATOM   6271 1HD  ARG A 411       5.863  31.427  -0.027  1.00  1.82
H
ATOM   6272 2HD  ARG A 411       4.388  30.439   0.078  1.00  1.82
H
ATOM   6273  HE  ARG A 411       5.022  32.023   2.464  1.00  1.82
H
```

```
ATOM   6274 1HH1 ARG A 411       3.218  31.714  -0.505  1.00  1.82
H
ATOM   6275 2HH1 ARG A 411       2.004  32.889  -0.033  1.00  1.82
H
ATOM   6276 1HH2 ARG A 411       3.516  33.522   3.048  1.00  1.82
H
ATOM   6277 2HH2 ARG A 411       2.158  33.909   2.000  1.00  1.82
H
ATOM   6278  N   THR A 412       3.569  26.795   3.940  1.00  1.11
N
ATOM   6279  CA  THR A 412       2.636  26.497   5.023  1.00  1.34
C
ATOM   6280  C   THR A 412       3.216  25.600   6.118  1.00  1.56
C
ATOM   6281  O   THR A 412       2.574  25.418   7.148  1.00  3.63
O
ATOM   6282  CB  THR A 412       1.352  25.832   4.511  1.00  2.01
C
ATOM   6283  OG1 THR A 412       1.650  24.577   3.895  1.00  2.01
O
ATOM   6284  CG2 THR A 412       0.644  26.722   3.535  1.00  2.01
C
ATOM   6285  H   THR A 412       3.414  26.412   3.012  1.00  1.33
H
ATOM   6286  HA  THR A 412       2.354  27.441   5.490  1.00  1.61
H
ATOM   6287  HB  THR A 412       0.690  25.660   5.348  1.00  2.41
H
ATOM   6288  HG1 THR A 412       1.628  23.891   4.573  1.00  2.41
H
ATOM   6289 1HG2 THR A 412      -0.270  26.228   3.209  1.00  2.41
H
ATOM   6290 2HG2 THR A 412       0.401  27.657   4.029  1.00  2.41
H
ATOM   6291 3HG2 THR A 412       1.279  26.919   2.681  1.00  2.41
H
ATOM   6292  N   ILE A 413       4.417  25.048   5.916  1.00  0.89
N
ATOM   6293  CA  ILE A 413       5.033  24.188   6.916  1.00  1.09
C
ATOM   6294  C   ILE A 413       5.792  25.026   7.960  1.00  1.28
C
ATOM   6295  O   ILE A 413       6.656  25.830   7.597  1.00  1.63
O
ATOM   6296  CB  ILE A 413       6.004  23.189   6.238  1.00  1.64
C
ATOM   6297  CG1 ILE A 413       5.261  22.347   5.208  1.00  1.64
C
ATOM   6298  CG2 ILE A 413       6.670  22.292   7.272  1.00  1.64
C
ATOM   6299  CD1 ILE A 413       4.163  21.510   5.809  1.00  1.64
C
ATOM   6300  H   ILE A 413       4.935  25.220   5.065  1.00  1.07
H
ATOM   6301  HA  ILE A 413       4.251  23.611   7.395  1.00  1.31
H
ATOM   6302  HB  ILE A 413       6.763  23.745   5.696  1.00  1.96
H
```

```
ATOM   6303 1HG1 ILE A 413       4.819  23.010   4.469  1.00  1.96
H
ATOM   6304 2HG1 ILE A 413       5.965  21.691   4.700  1.00  1.96
H
ATOM   6305 1HG2 ILE A 413       7.355  21.606   6.773  1.00  1.96
H
ATOM   6306 2HG2 ILE A 413       7.224  22.905   7.980  1.00  1.96
H
ATOM   6307 3HG2 ILE A 413       5.908  21.722   7.804  1.00  1.96
H
ATOM   6308 1HD1 ILE A 413       3.663  20.940   5.026  1.00  1.96
H
ATOM   6309 2HD1 ILE A 413       4.594  20.826   6.537  1.00  1.96
H
ATOM   6310 3HD1 ILE A 413       3.446  22.158   6.303  1.00  1.96
H
ATOM   6311  N   PRO A 414       5.448  24.892   9.250  1.00  1.54
N
ATOM   6312  CA  PRO A 414       6.098  25.516  10.384  1.00  3.43
C
ATOM   6313  C   PRO A 414       7.541  25.070  10.388  1.00 12.38
C
ATOM   6314  O   PRO A 414       7.839  23.911  10.099  1.00 86.38
O
ATOM   6315  CB  PRO A 414       5.331  24.986  11.595  1.00  5.15
C
ATOM   6316  CG  PRO A 414       3.989  24.643  11.054  1.00  5.15
C
ATOM   6317  CD  PRO A 414       4.254  24.129   9.669  1.00  5.15
C
ATOM   6318  HA  PRO A 414       6.019  26.610  10.301  1.00  4.12
H
ATOM   6319 1HB  PRO A 414       5.853  24.116  12.021  1.00  6.17
H
ATOM   6320 2HB  PRO A 414       5.289  25.753  12.380  1.00  6.17
H
ATOM   6321 1HG  PRO A 414       3.490  23.902  11.697  1.00  6.17
H
ATOM   6322 2HG  PRO A 414       3.358  25.540  11.048  1.00  6.17
H
ATOM   6323 1HD  PRO A 414       4.477  23.052   9.683  1.00  6.17
H
ATOM   6324 2HD  PRO A 414       3.393  24.360   9.035  1.00  6.17
H
ATOM   6325  N   LYS A 415       8.436  25.972  10.729  1.00  5.12
N
ATOM   6326  CA  LYS A 415       9.867  25.692  10.746  1.00  2.56
C
ATOM   6327  C   LYS A 415      10.424  25.308   9.369  1.00  1.76
C
ATOM   6328  O   LYS A 415      11.438  24.615   9.289  1.00  5.39
O
ATOM   6329  CB  LYS A 415      10.191  24.583  11.756  1.00  3.84
C
ATOM   6330  CG  LYS A 415       9.655  24.830  13.160  1.00  3.84
C
ATOM   6331  CD  LYS A 415      10.279  26.069  13.780  1.00  3.84
C
```

```
ATOM   6332  CE  LYS A 415       9.781  26.288  15.201  1.00  3.84
C
ATOM   6333  NZ  LYS A 415      10.320  27.544  15.790  1.00  3.84
N
ATOM   6334  H   LYS A 415       8.120  26.900  10.976  1.00  6.14
H
ATOM   6335  HA  LYS A 415      10.384  26.600  11.063  1.00  3.07
H
ATOM   6336 1HB  LYS A 415       9.801  23.628  11.407  1.00  4.61
H
ATOM   6337 2HB  LYS A 415      11.273  24.477  11.836  1.00  4.61
H
ATOM   6338 1HG  LYS A 415       8.573  24.957  13.125  1.00  4.61
H
ATOM   6339 2HG  LYS A 415       9.879  23.970  13.789  1.00  4.61
H
ATOM   6340 1HD  LYS A 415      11.364  25.961  13.797  1.00  4.61
H
ATOM   6341 2HD  LYS A 415      10.029  26.943  13.179  1.00  4.61
H
ATOM   6342 1HE  LYS A 415       8.694  26.338  15.196  1.00  4.61
H
ATOM   6343 2HE  LYS A 415      10.091  25.447  15.821  1.00  4.61
H
ATOM   6344 1HZ  LYS A 415       9.968  27.652  16.731  1.00  4.61
H
ATOM   6345 2HZ  LYS A 415      11.329  27.501  15.811  1.00  4.61
H
ATOM   6346 3HZ  LYS A 415      10.028  28.331  15.229  1.00  4.61
H
ATOM   6347  N   CYS A 416       9.785  25.777   8.293  1.00  1.60
N
ATOM   6348  CA  CYS A 416      10.284  25.514   6.954  1.00  1.01
C
ATOM   6349  C   CYS A 416      11.128  26.644   6.368  1.00  0.74
C
ATOM   6350  O   CYS A 416      10.751  27.814   6.434  1.00  1.18
O
ATOM   6351  CB  CYS A 416       9.143  25.244   5.998  1.00  1.52
C
ATOM   6352  SG  CYS A 416       9.665  24.944   4.302  1.00  1.52
S
ATOM   6353  H   CYS A 416       8.921  26.298   8.393  1.00  1.92
H
ATOM   6354  HA  CYS A 416      10.890  24.622   7.012  1.00  1.21
H
ATOM   6355 1HB  CYS A 416       8.598  24.370   6.344  1.00  1.82
H
ATOM   6356 2HB  CYS A 416       8.457  26.087   5.991  1.00  1.82
H
ATOM   6357  HG  CYS A 416      10.153  26.161   4.095  1.00  1.82
H
ATOM   6358  N   THR A 417      12.269  26.276   5.791  1.00  0.55
N
ATOM   6359  CA  THR A 417      13.134  27.219   5.088  1.00  0.53
C
ATOM   6360  C   THR A 417      13.092  26.884   3.606  1.00  0.60
C
```

```
ATOM   6361  O   THR A 417      13.363  25.749   3.218  1.00  0.89
O
ATOM   6362  CB  THR A 417      14.595  27.173   5.592  1.00  0.80
C
ATOM   6363  OG1 THR A 417      14.642  27.532   6.980  1.00  0.80
O
ATOM   6364  CG2 THR A 417      15.471  28.128   4.788  1.00  0.80
C
ATOM   6365  H   THR A 417      12.516  25.298   5.806  1.00  0.66
H
ATOM   6366  HA  THR A 417      12.747  28.226   5.228  1.00  0.64
H
ATOM   6367  HB  THR A 417      14.980  26.160   5.480  1.00  0.95
H
ATOM   6368  HG1 THR A 417      14.125  26.903   7.490  1.00  0.95
H
ATOM   6369 1HG2 THR A 417      16.496  28.076   5.154  1.00  0.95
H
ATOM   6370 2HG2 THR A 417      15.447  27.846   3.735  1.00  0.95
H
ATOM   6371 3HG2 THR A 417      15.097  29.145   4.900  1.00  0.95
H
ATOM   6372  N   ILE A 418      12.744  27.860   2.781  1.00  0.64
N
ATOM   6373  CA  ILE A 418      12.654  27.624   1.350  1.00  0.56
C
ATOM   6374  C   ILE A 418      13.759  28.305   0.602  1.00  0.68
C
ATOM   6375  O   ILE A 418      13.824  29.533   0.569  1.00  0.83
O
ATOM   6376  CB  ILE A 418      11.348  28.134   0.748  1.00  0.84
C
ATOM   6377  CG1 ILE A 418      10.149  27.483   1.387  1.00  0.84
C
ATOM   6378  CG2 ILE A 418      11.345  27.851  -0.743  1.00  0.84
C
ATOM   6379  CD1 ILE A 418       8.876  28.126   0.931  1.00  0.84
C
ATOM   6380  H   ILE A 418      12.550  28.775   3.154  1.00  0.77
H
ATOM   6381  HA  ILE A 418      12.727  26.553   1.167  1.00  0.67
H
ATOM   6382  HB  ILE A 418      11.266  29.205   0.913  1.00  1.01
H
ATOM   6383 1HG1 ILE A 418      10.126  26.425   1.131  1.00  1.01
H
ATOM   6384 2HG1 ILE A 418      10.217  27.578   2.470  1.00  1.01
H
ATOM   6385 1HG2 ILE A 418      10.420  28.227  -1.176  1.00  1.01
H
ATOM   6386 2HG2 ILE A 418      12.193  28.345  -1.213  1.00  1.01
H
ATOM   6387 3HG2 ILE A 418      11.414  26.775  -0.910  1.00  1.01
H
ATOM   6388 1HD1 ILE A 418       8.035  27.639   1.413  1.00  1.01
H
ATOM   6389 2HD1 ILE A 418       8.886  29.183   1.198  1.00  1.01
H
```

```
ATOM   6390 3HD1 ILE A 418       8.784  28.026  -0.149  1.00  1.01
H
ATOM   6391  N   PHE A 419      14.587  27.504  -0.041  1.00  0.76
N
ATOM   6392  CA  PHE A 419      15.655  27.997  -0.877  1.00  1.19
C
ATOM   6393  C   PHE A 419      15.223  27.830  -2.316  1.00  2.24
C
ATOM   6394  O   PHE A 419      14.684  26.790  -2.688  1.00  8.95
O
ATOM   6395  CB  PHE A 419      16.907  27.122  -0.737  1.00  1.78
C
ATOM   6396  CG  PHE A 419      17.588  27.103   0.596  1.00  1.78
C
ATOM   6397  CD1 PHE A 419      17.221  26.172   1.558  1.00  1.78
C
ATOM   6398  CD2 PHE A 419      18.620  27.983   0.877  1.00  1.78
C
ATOM   6399  CE1 PHE A 419      17.873  26.126   2.773  1.00  1.78
C
ATOM   6400  CE2 PHE A 419      19.273  27.935   2.089  1.00  1.78
C
ATOM   6401  CZ  PHE A 419      18.899  27.005   3.036  1.00  1.78
C
ATOM   6402  H   PHE A 419      14.468  26.507   0.044  1.00  0.91
H
ATOM   6403  HA  PHE A 419      15.858  29.045  -0.661  1.00  1.43
H
ATOM   6404 1HB  PHE A 419      16.639  26.097  -0.982  1.00  2.14
H
ATOM   6405 2HB  PHE A 419      17.639  27.441  -1.476  1.00  2.14
H
ATOM   6406  HD1 PHE A 419      16.416  25.470   1.343  1.00  2.14
H
ATOM   6407  HD2 PHE A 419      18.917  28.711   0.122  1.00  2.14
H
ATOM   6408  HE1 PHE A 419      17.582  25.392   3.522  1.00  2.14
H
ATOM   6409  HE2 PHE A 419      20.085  28.629   2.298  1.00  2.14
H
ATOM   6410  HZ  PHE A 419      19.415  26.962   3.993  1.00  2.14
H
ATOM   6411  N   TYR A 420      15.530  28.805  -3.141  1.00  1.01
N
ATOM   6412  CA  TYR A 420      15.263  28.688  -4.561  1.00  0.93
C
ATOM   6413  C   TYR A 420      16.425  29.294  -5.321  1.00  1.06
C
ATOM   6414  O   TYR A 420      16.380  30.468  -5.690  1.00  2.09
O
ATOM   6415  CB  TYR A 420      13.950  29.367  -4.944  1.00  1.40
C
ATOM   6416  CG  TYR A 420      13.563  29.137  -6.392  1.00  1.40
C
ATOM   6417  CD1 TYR A 420      14.364  28.351  -7.207  1.00  1.40
C
ATOM   6418  CD2 TYR A 420      12.417  29.714  -6.912  1.00  1.40
C
```

ATOM 6419 CE1 TYR A 420 14.027 28.149 -8.529 1.00 1.40
C
ATOM 6420 CE2 TYR A 420 12.080 29.517 -8.235 1.00 1.40
C
ATOM 6421 CZ TYR A 420 12.879 28.739 -9.043 1.00 1.40
C
ATOM 6422 OH TYR A 420 12.534 28.545 -10.361 1.00 1.40
O
ATOM 6423 H TYR A 420 15.955 29.650 -2.787 1.00 1.21
H
ATOM 6424 HA TYR A 420 15.187 27.634 -4.824 1.00 1.12
H
ATOM 6425 1HB TYR A 420 13.146 28.989 -4.311 1.00 1.67
H
ATOM 6426 2HB TYR A 420 14.028 30.441 -4.775 1.00 1.67
H
ATOM 6427 HD1 TYR A 420 15.266 27.891 -6.808 1.00 1.67
H
ATOM 6428 HD2 TYR A 420 11.783 30.333 -6.284 1.00 1.67
H
ATOM 6429 HE1 TYR A 420 14.662 27.532 -9.162 1.00 1.67
H
ATOM 6430 HE2 TYR A 420 11.179 29.979 -8.642 1.00 1.67
H
ATOM 6431 HH TYR A 420 11.817 29.142 -10.593 1.00 1.67
H
ATOM 6432 N PRO A 421 17.481 28.493 -5.539 1.00 1.25
N
ATOM 6433 CA PRO A 421 18.713 28.785 -6.252 1.00 1.38
C
ATOM 6434 C PRO A 421 18.454 29.265 -7.660 1.00 1.05
C
ATOM 6435 O PRO A 421 17.441 28.936 -8.279 1.00 2.55
O
ATOM 6436 CB PRO A 421 19.455 27.451 -6.260 1.00 2.07
C
ATOM 6437 CG PRO A 421 18.963 26.739 -5.058 1.00 2.07
C
ATOM 6438 CD PRO A 421 17.529 27.139 -4.941 1.00 2.07
C
ATOM 6439 HA PRO A 421 19.281 29.541 -5.689 1.00 1.66
H
ATOM 6440 1HB PRO A 421 19.244 26.907 -7.192 1.00 2.48
H
ATOM 6441 2HB PRO A 421 20.538 27.632 -6.233 1.00 2.48
H
ATOM 6442 1HG PRO A 421 19.089 25.653 -5.182 1.00 2.48
H
ATOM 6443 2HG PRO A 421 19.555 27.032 -4.181 1.00 2.48
H
ATOM 6444 1HD PRO A 421 16.890 26.451 -5.500 1.00 2.48
H
ATOM 6445 2HD PRO A 421 17.272 27.165 -3.879 1.00 2.48
H
ATOM 6446 N THR A 422 19.391 30.047 -8.159 1.00 1.45
N
ATOM 6447 CA THR A 422 19.301 30.630 -9.483 1.00 1.26
C

```
ATOM   6448  C   THR A 422      20.304  29.977 -10.393  1.00  1.35
C
ATOM   6449  O   THR A 422      19.987  29.604 -11.521  1.00  2.18
O
ATOM   6450  CB  THR A 422      19.557  32.128  -9.425  1.00  1.89
C
ATOM   6451  OG1 THR A 422      18.581  32.725  -8.573  1.00  1.89
O
ATOM   6452  CG2 THR A 422      19.487  32.745 -10.806  1.00  1.89
C
ATOM   6453  H   THR A 422      20.217  30.229  -7.600  1.00  1.74
H
ATOM   6454  HA  THR A 422      18.305  30.453  -9.884  1.00  1.51
H
ATOM   6455  HB  THR A 422      20.545  32.305  -9.001  1.00  2.27
H
ATOM   6456  HG1 THR A 422      18.995  33.438  -8.082  1.00  2.27
H
ATOM   6457 1HG2 THR A 422      19.669  33.815 -10.733  1.00  2.27
H
ATOM   6458 2HG2 THR A 422      20.244  32.289 -11.442  1.00  2.27
H
ATOM   6459 3HG2 THR A 422      18.499  32.572 -11.232  1.00  2.27
H
ATOM   6460  N   ASP A 423      21.521  29.823  -9.889  1.00  1.16
N
ATOM   6461  CA  ASP A 423      22.570  29.165 -10.642  1.00  1.15
C
ATOM   6462  C   ASP A 423      23.249  28.130  -9.759  1.00  1.06
C
ATOM   6463  O   ASP A 423      22.826  27.905  -8.623  1.00  1.04
O
ATOM   6464  CB  ASP A 423      23.613  30.160 -11.148  1.00  1.72
C
ATOM   6465  CG  ASP A 423      24.388  30.821 -10.028  1.00  1.72
C
ATOM   6466  OD1 ASP A 423      24.205  30.425  -8.893  1.00  1.72
O
ATOM   6467  OD2 ASP A 423      25.165  31.706 -10.316  1.00  1.72
O
ATOM   6468  H   ASP A 423      21.715  30.145  -8.953  1.00  1.39
H
ATOM   6469  HA  ASP A 423      22.127  28.652 -11.498  1.00  1.38
H
ATOM   6470 1HB  ASP A 423      24.312  29.653 -11.811  1.00  2.07
H
ATOM   6471 2HB  ASP A 423      23.121  30.939 -11.728  1.00  2.07
H
ATOM   6472  N   ALA A 424      24.305  27.518 -10.282  1.00  1.08
N
ATOM   6473  CA  ALA A 424      25.035  26.477  -9.574  1.00  1.09
C
ATOM   6474  C   ALA A 424      25.731  26.948  -8.311  1.00  1.02
C
ATOM   6475  O   ALA A 424      25.790  26.200  -7.334  1.00  1.05
O
ATOM   6476  CB  ALA A 424      26.054  25.868 -10.488  1.00  1.64
C
```

```
ATOM   6477  H   ALA A 424      24.600  27.763 -11.216  1.00  1.30
H
ATOM   6478  HA  ALA A 424      24.316  25.718  -9.283  1.00  1.31
H
ATOM   6479 1HB  ALA A 424      26.556  25.056  -9.969  1.00  1.96
H
ATOM   6480 2HB  ALA A 424      25.541  25.477 -11.355  1.00  1.96
H
ATOM   6481 3HB  ALA A 424      26.782  26.619 -10.791  1.00  1.96
H
ATOM   6482  N   VAL A 425      26.273  28.162  -8.329  1.00  0.98
N
ATOM   6483  CA  VAL A 425      26.962  28.698  -7.162  1.00  0.99
C
ATOM   6484  C   VAL A 425      25.980  28.859  -6.011  1.00  0.94
C
ATOM   6485  O   VAL A 425      26.224  28.372  -4.903  1.00  0.95
O
ATOM   6486  CB  VAL A 425      27.637  30.046  -7.513  1.00  1.48
C
ATOM   6487  CG1 VAL A 425      28.182  30.731  -6.269  1.00  1.48
C
ATOM   6488  CG2 VAL A 425      28.769  29.790  -8.500  1.00  1.48
C
ATOM   6489  H   VAL A 425      26.200  28.732  -9.161  1.00  1.18
H
ATOM   6490  HA  VAL A 425      27.738  27.992  -6.862  1.00  1.19
H
ATOM   6491  HB  VAL A 425      26.898  30.709  -7.963  1.00  1.78
H
ATOM   6492 1HG1 VAL A 425      28.644  31.678  -6.546  1.00  1.78
H
ATOM   6493 2HG1 VAL A 425      27.366  30.915  -5.572  1.00  1.78
H
ATOM   6494 3HG1 VAL A 425      28.924  30.092  -5.798  1.00  1.78
H
ATOM   6495 1HG2 VAL A 425      29.242  30.734  -8.764  1.00  1.78
H
ATOM   6496 2HG2 VAL A 425      29.507  29.131  -8.042  1.00  1.78
H
ATOM   6497 3HG2 VAL A 425      28.370  29.320  -9.398  1.00  1.78
H
ATOM   6498  N   SER A 426      24.844  29.494  -6.299  1.00  0.91
N
ATOM   6499  CA  SER A 426      23.795  29.668  -5.312  1.00  0.88
C
ATOM   6500  C   SER A 426      23.224  28.326  -4.852  1.00  0.84
C
ATOM   6501  O   SER A 426      22.854  28.182  -3.687  1.00  1.05
O
ATOM   6502  CB  SER A 426      22.697  30.540  -5.907  1.00  1.32
C
ATOM   6503  OG  SER A 426      22.119  29.906  -7.016  1.00  1.32
O
ATOM   6504  H   SER A 426      24.707  29.873  -7.230  1.00  1.09
H
ATOM   6505  HA  SER A 426      24.217  30.176  -4.445  1.00  1.06
H
```

```
ATOM   6506 1HB  SER A 426      21.935  30.736  -5.153  1.00  1.58
H
ATOM   6507 2HB  SER A 426      23.116  31.499  -6.210  1.00  1.58
H
ATOM   6508  HG  SER A 426      22.832  29.860  -7.673  1.00  1.58
H
ATOM   6509  N   THR A 427      23.219  27.328  -5.747  1.00  0.78
N
ATOM   6510  CA  THR A 427      22.735  25.997  -5.410  1.00  0.74
C
ATOM   6511  C   THR A 427      23.637  25.305  -4.402  1.00  0.70
C
ATOM   6512  O   THR A 427      23.139  24.728  -3.434  1.00  0.80
O
ATOM   6513  CB  THR A 427      22.612  25.108  -6.658  1.00  1.11
C
ATOM   6514  OG1 THR A 427      21.695  25.696  -7.586  1.00  1.11
O
ATOM   6515  CG2 THR A 427      22.094  23.741  -6.253  1.00  1.11
C
ATOM   6516  H   THR A 427      23.510  27.506  -6.700  1.00  0.94
H
ATOM   6517  HA  THR A 427      21.749  26.093  -4.960  1.00  0.89
H
ATOM   6518  HB  THR A 427      23.584  25.002  -7.135  1.00  1.33
H
ATOM   6519  HG1 THR A 427      22.030  26.555  -7.865  1.00  1.33
H
ATOM   6520 1HG2 THR A 427      22.000  23.115  -7.139  1.00  1.33
H
ATOM   6521 2HG2 THR A 427      22.788  23.281  -5.551  1.00  1.33
H
ATOM   6522 3HG2 THR A 427      21.118  23.848  -5.780  1.00  1.33
H
ATOM   6523  N   GLU A 428      24.960  25.366  -4.609  1.00  0.77
N
ATOM   6524  CA  GLU A 428      25.855  24.717  -3.656  1.00  0.76
C
ATOM   6525  C   GLU A 428      25.785  25.371  -2.298  1.00  0.80
C
ATOM   6526  O   GLU A 428      25.828  24.683  -1.276  1.00  0.89
O
ATOM   6527  CB  GLU A 428      27.319  24.724  -4.100  1.00  1.14
C
ATOM   6528  CG  GLU A 428      27.641  23.835  -5.283  1.00  1.14
C
ATOM   6529  CD  GLU A 428      29.110  23.599  -5.439  1.00  1.14
C
ATOM   6530  OE1 GLU A 428      29.873  24.138  -4.677  1.00  1.14
O
ATOM   6531  OE2 GLU A 428      29.473  22.878  -6.341  1.00  1.14
O
ATOM   6532  H   GLU A 428      25.329  25.835  -5.427  1.00  0.92
H
ATOM   6533  HA  GLU A 428      25.539  23.681  -3.543  1.00  0.91
H
ATOM   6534 1HB  GLU A 428      27.610  25.741  -4.363  1.00  1.37
H
```

```
ATOM   6535 2HB  GLU A 428      27.952  24.410  -3.270  1.00  1.37
H
ATOM   6536 1HG  GLU A 428      27.156  22.879  -5.161  1.00  1.37
H
ATOM   6537 2HG  GLU A 428      27.251  24.298  -6.188  1.00  1.37
H
ATOM   6538  N   HIS A 429      25.646  26.694  -2.278  1.00  0.87
N
ATOM   6539  CA  HIS A 429      25.543  27.384  -1.010  1.00  1.06
C
ATOM   6540  C   HIS A 429      24.229  27.065  -0.315  1.00  1.16
C
ATOM   6541  O   HIS A 429      24.204  26.935   0.908  1.00  1.46
O
ATOM   6542  CB  HIS A 429      25.736  28.887  -1.188  1.00  1.59
C
ATOM   6543  CG  HIS A 429      27.166  29.267  -1.442  1.00  1.59
C
ATOM   6544  ND1 HIS A 429      28.134  29.208  -0.461  1.00  1.59
N
ATOM   6545  CD2 HIS A 429      27.791  29.710  -2.557  1.00  1.59
C
ATOM   6546  CE1 HIS A 429      29.289  29.608  -0.965  1.00  1.59
C
ATOM   6547  NE2 HIS A 429      29.107  29.919  -2.232  1.00  1.59
N
ATOM   6548  H   HIS A 429      25.636  27.227  -3.142  1.00  1.04
H
ATOM   6549  HA  HIS A 429      26.341  27.034  -0.355  1.00  1.27
H
ATOM   6550 1HB  HIS A 429      25.129  29.238  -2.023  1.00  1.91
H
ATOM   6551 2HB  HIS A 429      25.397  29.403  -0.292  1.00  1.91
H
ATOM   6552  HD1 HIS A 429      27.966  29.089   0.517  1.00  1.91
H
ATOM   6553  HD2 HIS A 429      27.446  29.917  -3.566  1.00  1.91
H
ATOM   6554  HE1 HIS A 429      30.185  29.637  -0.345  1.00  1.91
H
ATOM   6555  N   ALA A 430      23.153  26.884  -1.090  1.00  1.12
N
ATOM   6556  CA  ALA A 430      21.862  26.498  -0.530  1.00  1.50
C
ATOM   6557  C   ALA A 430      21.958  25.147   0.160  1.00  0.86
C
ATOM   6558  O   ALA A 430      21.427  24.974   1.256  1.00  0.82
O
ATOM   6559  CB  ALA A 430      20.800  26.452  -1.618  1.00  2.25
C
ATOM   6560  H   ALA A 430      23.216  27.038  -2.088  1.00  1.34
H
ATOM   6561  HA  ALA A 430      21.579  27.241   0.216  1.00  1.80
H
ATOM   6562 1HB  ALA A 430      19.840  26.181  -1.180  1.00  2.70
H
ATOM   6563 2HB  ALA A 430      20.721  27.430  -2.092  1.00  2.70
H
```

```
ATOM   6564 3HB  ALA A 430      21.075  25.710  -2.365  1.00  2.70
H
ATOM   6565  N   VAL A 431      22.651  24.199  -0.477  1.00  0.70
N
ATOM   6566  CA  VAL A 431      22.843  22.869   0.090  1.00  0.61
C
ATOM   6567  C   VAL A 431      23.630  22.916   1.398  1.00  0.53
C
ATOM   6568  O   VAL A 431      23.254  22.260   2.372  1.00  0.63
O
ATOM   6569  CB  VAL A 431      23.544  21.954  -0.934  1.00  0.92
C
ATOM   6570  CG1 VAL A 431      23.942  20.650  -0.279  1.00  0.92
C
ATOM   6571  CG2 VAL A 431      22.596  21.677  -2.095  1.00  0.92
C
ATOM   6572  H   VAL A 431      23.041  24.402  -1.391  1.00  0.84
H
ATOM   6573  HA  VAL A 431      21.861  22.446   0.299  1.00  0.73
H
ATOM   6574  HB  VAL A 431      24.448  22.441  -1.297  1.00  1.10
H
ATOM   6575 1HG1 VAL A 431      24.443  20.012  -1.006  1.00  1.10
H
ATOM   6576 2HG1 VAL A 431      24.618  20.866   0.544  1.00  1.10
H
ATOM   6577 3HG1 VAL A 431      23.054  20.144   0.099  1.00  1.10
H
ATOM   6578 1HG2 VAL A 431      23.086  21.033  -2.823  1.00  1.10
H
ATOM   6579 2HG2 VAL A 431      21.700  21.184  -1.722  1.00  1.10
H
ATOM   6580 3HG2 VAL A 431      22.319  22.616  -2.572  1.00  1.10
H
ATOM   6581  N   ALA A 432      24.715  23.694   1.419  1.00  0.56
N
ATOM   6582  CA  ALA A 432      25.524  23.842   2.625  1.00  0.61
C
ATOM   6583  C   ALA A 432      24.720  24.478   3.767  1.00  0.62
C
ATOM   6584  O   ALA A 432      24.774  24.014   4.910  1.00  0.83
O
ATOM   6585  CB  ALA A 432      26.756  24.679   2.323  1.00  0.92
C
ATOM   6586  H   ALA A 432      24.992  24.189   0.578  1.00  0.67
H
ATOM   6587  HA  ALA A 432      25.838  22.848   2.947  1.00  0.73
H
ATOM   6588 1HB  ALA A 432      27.370  24.760   3.220  1.00  1.10
H
ATOM   6589 2HB  ALA A 432      27.335  24.204   1.531  1.00  1.10
H
ATOM   6590 3HB  ALA A 432      26.450  25.673   2.001  1.00  1.10
H
ATOM   6591  N   LEU A 433      23.971  25.535   3.450  1.00  0.61
N
ATOM   6592  CA  LEU A 433      23.150  26.212   4.444  1.00  0.64
C
```

ATOM 6593 C LEU A 433 22.042 25.313 4.957 1.00 0.65
C
ATOM 6594 O LEU A 433 21.752 25.312 6.154 1.00 1.02
O
ATOM 6595 CB LEU A 433 22.557 27.491 3.844 1.00 0.96
C
ATOM 6596 CG LEU A 433 23.558 28.625 3.581 1.00 0.96
C
ATOM 6597 CD1 LEU A 433 22.883 29.727 2.789 1.00 0.96
C
ATOM 6598 CD2 LEU A 433 24.063 29.162 4.910 1.00 0.96
C
ATOM 6599 H LEU A 433 23.974 25.889 2.503 1.00 0.73
H
ATOM 6600 HA LEU A 433 23.787 26.487 5.284 1.00 0.77
H
ATOM 6601 1HB LEU A 433 22.084 27.243 2.896 1.00 1.15
H
ATOM 6602 2HB LEU A 433 21.795 27.873 4.522 1.00 1.15
H
ATOM 6603 HG LEU A 433 24.398 28.247 2.998 1.00 1.15
H
ATOM 6604 1HD1 LEU A 433 23.598 30.527 2.599 1.00 1.15
H
ATOM 6605 2HD1 LEU A 433 22.532 29.320 1.843 1.00 1.15
H
ATOM 6606 3HD1 LEU A 433 22.040 30.121 3.351 1.00 1.15
H
ATOM 6607 1HD2 LEU A 433 24.777 29.966 4.729 1.00 1.15
H
ATOM 6608 2HD2 LEU A 433 23.224 29.545 5.491 1.00 1.15
H
ATOM 6609 3HD2 LEU A 433 24.552 28.360 5.464 1.00 1.15
H
ATOM 6610 N ALA A 434 21.450 24.519 4.062 1.00 0.50
N
ATOM 6611 CA ALA A 434 20.403 23.578 4.432 1.00 0.50
C
ATOM 6612 C ALA A 434 20.940 22.542 5.405 1.00 0.43
C
ATOM 6613 O ALA A 434 20.292 22.230 6.403 1.00 0.48
O
ATOM 6614 CB ALA A 434 19.840 22.893 3.195 1.00 0.75
C
ATOM 6615 H ALA A 434 21.711 24.578 3.088 1.00 0.60
H
ATOM 6616 HA ALA A 434 19.606 24.130 4.929 1.00 0.60
H
ATOM 6617 1HB ALA A 434 19.044 22.208 3.488 1.00 0.90
H
ATOM 6618 2HB ALA A 434 19.440 23.645 2.514 1.00 0.90
H
ATOM 6619 3HB ALA A 434 20.632 22.338 2.696 1.00 0.90
H
ATOM 6620 N ALA A 435 22.153 22.047 5.147 1.00 0.40
N
ATOM 6621 CA ALA A 435 22.794 21.063 6.015 1.00 0.42
C

```
ATOM   6622  C    ALA A 435      22.894  21.549   7.454  1.00  0.51
C
ATOM   6623  O    ALA A 435      22.708  20.771   8.392  1.00  1.87
O
ATOM   6624  CB   ALA A 435      24.185  20.739   5.497  1.00  0.63
C
ATOM   6625  H    ALA A 435      22.636  22.331   4.303  1.00  0.48
H
ATOM   6626  HA   ALA A 435      22.187  20.158   6.002  1.00  0.50
H
ATOM   6627 1HB   ALA A 435      24.643  19.979   6.128  1.00  0.76
H
ATOM   6628 2HB   ALA A 435      24.110  20.375   4.477  1.00  0.76
H
ATOM   6629 3HB   ALA A 435      24.799  21.637   5.512  1.00  0.76
H
ATOM   6630  N    ASN A 436      23.182  22.839   7.629  1.00  0.65
N
ATOM   6631  CA   ASN A 436      23.317  23.404   8.964  1.00  0.78
C
ATOM   6632  C    ASN A 436      22.068  24.120   9.480  1.00  1.32
C
ATOM   6633  O    ASN A 436      22.125  24.791  10.512  1.00  7.58
O
ATOM   6634  CB   ASN A 436      24.486  24.351   8.979  1.00  1.17
C
ATOM   6635  CG   ASN A 436      25.770  23.623   8.768  1.00  1.17
C
ATOM   6636  OD1 ASN A 436      26.051  22.619   9.433  1.00  1.17
O
ATOM   6637  ND2 ASN A 436      26.548  24.104   7.836  1.00  1.17
N
ATOM   6638  H    ASN A 436      23.356  23.425   6.815  1.00  0.78
H
ATOM   6639  HA   ASN A 436      23.524  22.587   9.657  1.00  0.94
H
ATOM   6640 1HB   ASN A 436      24.365  25.096   8.191  1.00  1.40
H
ATOM   6641 2HB   ASN A 436      24.525  24.877   9.932  1.00  1.40
H
ATOM   6642 1HD2 ASN A 436      27.414  23.653   7.620  1.00  1.40
H
ATOM   6643 2HD2 ASN A 436      26.269  24.913   7.319  1.00  1.40
H
ATOM   6644  N    ALA A 437      20.945  23.984   8.785  1.00  0.88
N
ATOM   6645  CA   ALA A 437      19.725  24.646   9.208  1.00  0.68
C
ATOM   6646  C    ALA A 437      18.792  23.642   9.861  1.00  0.70
C
ATOM   6647  O    ALA A 437      18.709  22.488   9.441  1.00  1.82
O
ATOM   6648  CB   ALA A 437      19.053  25.321   8.024  1.00  1.02
C
ATOM   6649  H    ALA A 437      20.912  23.410   7.952  1.00  1.06
H
ATOM   6650  HA   ALA A 437      19.980  25.401   9.951  1.00  0.82
H
```

```
ATOM   6651 1HB  ALA A 437      18.146  25.826   8.356  1.00  1.22
H
ATOM   6652 2HB  ALA A 437      19.736  26.050   7.589  1.00  1.22
H
ATOM   6653 3HB  ALA A 437      18.803  24.570   7.279  1.00  1.22
H
ATOM   6654  N   LYS A 438      18.100  24.080  10.904  1.00  0.61
N
ATOM   6655  CA  LYS A 438      17.161  23.220  11.610  1.00  0.87
C
ATOM   6656  C   LYS A 438      15.773  23.329  10.997  1.00  3.30
C
ATOM   6657  O   LYS A 438      15.435  24.345  10.397  1.00 28.68
O
ATOM   6658  CB  LYS A 438      17.117  23.580  13.094  1.00  1.30
C
ATOM   6659  CG  LYS A 438      18.419  23.318  13.836  1.00  1.30
C
ATOM   6660  CD  LYS A 438      18.294  23.670  15.310  1.00  1.30
C
ATOM   6661  CE  LYS A 438      19.595  23.410  16.054  1.00  1.30
C
ATOM   6662  NZ  LYS A 438      19.490  23.765  17.495  1.00  1.30
N
ATOM   6663  H   LYS A 438      18.220  25.035  11.210  1.00  0.73
H
ATOM   6664  HA  LYS A 438      17.492  22.186  11.512  1.00  1.04
H
ATOM   6665 1HB  LYS A 438      16.872  24.636  13.206  1.00  1.57
H
ATOM   6666 2HB  LYS A 438      16.331  23.006  13.584  1.00  1.57
H
ATOM   6667 1HG  LYS A 438      18.683  22.263  13.744  1.00  1.57
H
ATOM   6668 2HG  LYS A 438      19.217  23.914  13.394  1.00  1.57
H
ATOM   6669 1HD  LYS A 438      18.031  24.723  15.411  1.00  1.57
H
ATOM   6670 2HD  LYS A 438      17.503  23.071  15.761  1.00  1.57
H
ATOM   6671 1HE  LYS A 438      19.851  22.355  15.968  1.00  1.57
H
ATOM   6672 2HE  LYS A 438      20.391  24.003  15.603  1.00  1.57
H
ATOM   6673 1HZ  LYS A 438      20.370  23.579  17.954  1.00  1.57
H
ATOM   6674 2HZ  LYS A 438      19.264  24.747  17.585  1.00  1.57
H
ATOM   6675 3HZ  LYS A 438      18.763  23.213  17.927  1.00  1.57
H
ATOM   6676  N   GLY A 439      14.971  22.288  11.154  1.00  2.56
N
ATOM   6677  CA  GLY A 439      13.617  22.293  10.615  1.00  0.83
C
ATOM   6678  C   GLY A 439      13.547  21.646   9.237  1.00  1.23
C
ATOM   6679  O   GLY A 439      14.408  20.852   8.865  1.00  4.96
O
```

ATOM 6680 H GLY A 439 15.306 21.481 11.666 1.00 3.07
H
ATOM 6681 1HA GLY A 439 12.955 21.762 11.301 1.00 1.00
H
ATOM 6682 2HA GLY A 439 13.254 23.318 10.556 1.00 1.00
H
ATOM 6683 N MET A 440 12.507 21.982 8.485 1.00 0.70
N
ATOM 6684 CA MET A 440 12.308 21.400 7.165 1.00 0.77
C
ATOM 6685 C MET A 440 12.890 22.325 6.121 1.00 0.91
C
ATOM 6686 O MET A 440 12.393 23.426 5.921 1.00 2.52
O
ATOM 6687 CB MET A 440 10.819 21.163 6.899 1.00 1.16
C
ATOM 6688 CG MET A 440 10.134 20.239 7.898 1.00 1.16
C
ATOM 6689 SD MET A 440 10.805 18.563 7.873 1.00 1.16
S
ATOM 6690 CE MET A 440 10.218 17.970 6.286 1.00 1.16
C
ATOM 6691 H MET A 440 11.856 22.676 8.831 1.00 0.84
H
ATOM 6692 HA MET A 440 12.839 20.450 7.111 1.00 0.92
H
ATOM 6693 1HB MET A 440 10.291 22.114 6.903 1.00 1.39
H
ATOM 6694 2HB MET A 440 10.698 20.722 5.911 1.00 1.39
H
ATOM 6695 1HG MET A 440 10.250 20.642 8.904 1.00 1.39
H
ATOM 6696 2HG MET A 440 9.069 20.187 7.674 1.00 1.39
H
ATOM 6697 1HE MET A 440 10.559 16.945 6.132 1.00 1.39
H
ATOM 6698 2HE MET A 440 9.128 17.996 6.271 1.00 1.39
H
ATOM 6699 3HE MET A 440 10.609 18.607 5.492 1.00 1.39
H
ATOM 6700 N CYS A 441 13.946 21.899 5.456 1.00 0.35
N
ATOM 6701 CA CYS A 441 14.549 22.777 4.472 1.00 0.45
C
ATOM 6702 C CYS A 441 14.300 22.255 3.088 1.00 0.50
C
ATOM 6703 O CYS A 441 14.592 21.100 2.796 1.00 1.66
O
ATOM 6704 CB CYS A 441 16.040 22.913 4.704 1.00 0.68
C
ATOM 6705 SG CYS A 441 16.461 23.622 6.309 1.00 0.68
S
ATOM 6706 H CYS A 441 14.320 20.973 5.624 1.00 0.42
H
ATOM 6707 HA CYS A 441 14.099 23.765 4.557 1.00 0.54
H
ATOM 6708 1HB CYS A 441 16.502 21.935 4.631 1.00 0.81
H

```
ATOM   6709 2HB  CYS A 441      16.476  23.542   3.930  1.00  0.81
H
ATOM   6710  HG  CYS A 441      15.954  22.631   7.037  1.00  0.81
H
ATOM   6711  N   PHE A 442      13.752  23.107   2.241  1.00  0.46
N
ATOM   6712  CA  PHE A 442      13.466  22.722   0.880  1.00  0.50
C
ATOM   6713  C   PHE A 442      14.274  23.494  -0.125  1.00  0.53
C
ATOM   6714  O   PHE A 442      14.388  24.713  -0.035  1.00  0.85
O
ATOM   6715  CB  PHE A 442      11.991  22.869   0.569  1.00  0.75
C
ATOM   6716  CG  PHE A 442      11.133  21.875   1.280  1.00  0.75
C
ATOM   6717  CD1 PHE A 442      10.659  22.125   2.552  1.00  0.75
C
ATOM   6718  CD2 PHE A 442      10.792  20.688   0.663  1.00  0.75
C
ATOM   6719  CE1 PHE A 442       9.855  21.208   3.196  1.00  0.75
C
ATOM   6720  CE2 PHE A 442       9.987  19.770   1.299  1.00  0.75
C
ATOM   6721  CZ  PHE A 442       9.523  20.032   2.567  1.00  0.75
C
ATOM   6722  H   PHE A 442      13.539  24.043   2.550  1.00  0.55
H
ATOM   6723  HA  PHE A 442      13.715  21.675   0.780  1.00  0.60
H
ATOM   6724 1HB  PHE A 442      11.657  23.868   0.847  1.00  0.90
H
ATOM   6725 2HB  PHE A 442      11.831  22.754  -0.501  1.00  0.90
H
ATOM   6726  HD1 PHE A 442      10.931  23.058   3.038  1.00  0.90
H
ATOM   6727  HD2 PHE A 442      11.162  20.484  -0.343  1.00  0.90
H
ATOM   6728  HE1 PHE A 442       9.485  21.416   4.198  1.00  0.90
H
ATOM   6729  HE2 PHE A 442       9.722  18.838   0.804  1.00  0.90
H
ATOM   6730  HZ  PHE A 442       8.897  19.302   3.064  1.00  0.90
H
ATOM   6731  N   ILE A 443      14.815  22.776  -1.098  1.00  0.39
N
ATOM   6732  CA  ILE A 443      15.586  23.387  -2.164  1.00  0.50
C
ATOM   6733  C   ILE A 443      14.902  23.148  -3.495  1.00  0.48
C
ATOM   6734  O   ILE A 443      14.724  22.007  -3.922  1.00  0.76
O
ATOM   6735  CB  ILE A 443      17.030  22.851  -2.205  1.00  0.75
C
ATOM   6736  CG1 ILE A 443      17.740  23.146  -0.878  1.00  0.75
C
ATOM   6737  CG2 ILE A 443      17.786  23.477  -3.367  1.00  0.75
C
```

```
ATOM   6738  CD1 ILE A 443      19.092  22.487  -0.754  1.00  0.75
C
ATOM   6739  H   ILE A 443      14.684  21.772  -1.088  1.00  0.47
H
ATOM   6740  HA  ILE A 443      15.628  24.461  -1.994  1.00  0.60
H
ATOM   6741  HB  ILE A 443      17.008  21.771  -2.333  1.00  0.90
H
ATOM   6742 1HG1 ILE A 443      17.874  24.222  -0.777  1.00  0.90
H
ATOM   6743 2HG1 ILE A 443      17.118  22.797  -0.054  1.00  0.90
H
ATOM   6744 1HG2 ILE A 443      18.802  23.083  -3.395  1.00  0.90
H
ATOM   6745 2HG2 ILE A 443      17.278  23.240  -4.301  1.00  0.90
H
ATOM   6746 3HG2 ILE A 443      17.820  24.558  -3.236  1.00  0.90
H
ATOM   6747 1HD1 ILE A 443      19.533  22.741   0.209  1.00  0.90
H
ATOM   6748 2HD1 ILE A 443      18.978  21.405  -0.828  1.00  0.90
H
ATOM   6749 3HD1 ILE A 443      19.742  22.838  -1.553  1.00  0.90
H
ATOM   6750  N   ARG A 444      14.497  24.234  -4.135  1.00  0.76
N
ATOM   6751  CA  ARG A 444      13.830  24.166  -5.420  1.00  0.99
C
ATOM   6752  C   ARG A 444      14.805  24.219  -6.594  1.00  1.18
C
ATOM   6753  O   ARG A 444      15.454  25.239  -6.821  1.00  2.77
O
ATOM   6754  CB  ARG A 444      12.822  25.296  -5.527  1.00  1.48
C
ATOM   6755  CG  ARG A 444      12.137  25.338  -6.859  1.00  1.48
C
ATOM   6756  CD  ARG A 444      11.017  26.297  -6.942  1.00  1.48
C
ATOM   6757  NE  ARG A 444      10.639  26.467  -8.329  1.00  1.48
N
ATOM   6758  CZ  ARG A 444       9.544  27.089  -8.797  1.00  1.48
C
ATOM   6759  NH1 ARG A 444       8.670  27.614  -7.974  1.00  1.48
N
ATOM   6760  NH2 ARG A 444       9.371  27.156 -10.106  1.00  1.48
N
ATOM   6761  H   ARG A 444      14.654  25.140  -3.714  1.00  0.91
H
ATOM   6762  HA  ARG A 444      13.287  23.222  -5.472  1.00  1.19
H
ATOM   6763 1HB  ARG A 444      12.057  25.182  -4.760  1.00  1.78
H
ATOM   6764 2HB  ARG A 444      13.317  26.253  -5.364  1.00  1.78
H
ATOM   6765 1HG  ARG A 444      12.859  25.581  -7.637  1.00  1.78
H
ATOM   6766 2HG  ARG A 444      11.736  24.353  -7.039  1.00  1.78
H
```

ATOM 6767 1HD ARG A 444 10.158 25.904 -6.398 1.00 1.78
H
ATOM 6768 2HD ARG A 444 11.304 27.257 -6.530 1.00 1.78
H
ATOM 6769 HE ARG A 444 11.276 26.088 -9.018 1.00 1.78
H
ATOM 6770 1HH1 ARG A 444 8.814 27.546 -6.977 1.00 1.78
H
ATOM 6771 2HH1 ARG A 444 7.850 28.075 -8.338 1.00 1.78
H
ATOM 6772 1HH2 ARG A 444 10.053 26.744 -10.726 1.00 1.78
H
ATOM 6773 2HH2 ARG A 444 8.551 27.602 -10.494 1.00 1.78
H
ATOM 6774 N THR A 445 14.891 23.130 -7.358 1.00 1.07
N
ATOM 6775 CA THR A 445 15.808 23.078 -8.495 1.00 1.00
C
ATOM 6776 C THR A 445 15.065 23.011 -9.832 1.00 0.87
C
ATOM 6777 O THR A 445 13.869 22.703 -9.890 1.00 0.90
O
ATOM 6778 CB THR A 445 16.791 21.896 -8.366 1.00 1.50
C
ATOM 6779 OG1 THR A 445 16.072 20.660 -8.357 1.00 1.50
O
ATOM 6780 CG2 THR A 445 17.607 22.014 -7.091 1.00 1.50
C
ATOM 6781 H THR A 445 14.330 22.319 -7.142 1.00 1.28
H
ATOM 6782 HA THR A 445 16.400 23.993 -8.496 1.00 1.20
H
ATOM 6783 HB THR A 445 17.466 21.902 -9.218 1.00 1.80
H
ATOM 6784 HG1 THR A 445 15.410 20.686 -7.660 1.00 1.80
H
ATOM 6785 1HG2 THR A 445 18.299 21.176 -7.023 1.00 1.80
H
ATOM 6786 2HG2 THR A 445 18.168 22.949 -7.103 1.00 1.80
H
ATOM 6787 3HG2 THR A 445 16.938 22.003 -6.230 1.00 1.80
H
ATOM 6788 N THR A 446 15.800 23.335 -10.896 1.00 0.78
N
ATOM 6789 CA THR A 446 15.228 23.490 -12.225 1.00 0.74
C
ATOM 6790 C THR A 446 15.846 22.615 -13.314 1.00 0.73
C
ATOM 6791 O THR A 446 17.005 22.186 -13.246 1.00 0.76
O
ATOM 6792 CB THR A 446 15.363 24.963 -12.679 1.00 1.11
C
ATOM 6793 OG1 THR A 446 16.757 25.302 -12.784 1.00 1.11
O
ATOM 6794 CG2 THR A 446 14.694 25.907 -11.689 1.00 1.11
C
ATOM 6795 H THR A 446 16.781 23.517 -10.756 1.00 0.94
H

ATOM 6796 HA THR A 446 14.168 23.243 -12.164 1.00 0.89
H
ATOM 6797 HB THR A 446 14.889 25.084 -13.650 1.00 1.33
H
ATOM 6798 HG1 THR A 446 16.846 26.168 -13.193 1.00 1.33
H
ATOM 6799 1HG2 THR A 446 14.802 26.934 -12.037 1.00 1.33
H
ATOM 6800 2HG2 THR A 446 13.634 25.660 -11.611 1.00 1.33
H
ATOM 6801 3HG2 THR A 446 15.162 25.806 -10.712 1.00 1.33
H
ATOM 6802 N ARG A 447 15.031 22.397 -14.346 1.00 0.80
N
ATOM 6803 CA ARG A 447 15.341 21.591 -15.526 1.00 0.85
C
ATOM 6804 C ARG A 447 16.433 22.146 -16.455 1.00 1.08
C
ATOM 6805 O ARG A 447 17.470 21.501 -16.630 1.00 1.29
O
ATOM 6806 CB ARG A 447 14.057 21.366 -16.329 1.00 1.27
C
ATOM 6807 CG ARG A 447 14.178 20.587 -17.638 1.00 1.27
C
ATOM 6808 CD ARG A 447 14.538 19.176 -17.408 1.00 1.27
C
ATOM 6809 NE ARG A 447 14.446 18.382 -18.622 1.00 1.27
N
ATOM 6810 CZ ARG A 447 13.334 17.705 -18.969 1.00 1.27
C
ATOM 6811 NH1 ARG A 447 12.296 17.756 -18.171 1.00 1.27
N
ATOM 6812 NH2 ARG A 447 13.283 16.998 -20.083 1.00 1.27
N
ATOM 6813 H ARG A 447 14.110 22.811 -14.302 1.00 0.96
H
ATOM 6814 HA ARG A 447 15.661 20.621 -15.180 1.00 1.02
H
ATOM 6815 1HB ARG A 447 13.363 20.805 -15.707 1.00 1.53
H
ATOM 6816 2HB ARG A 447 13.574 22.311 -16.559 1.00 1.53
H
ATOM 6817 1HG ARG A 447 13.239 20.603 -18.185 1.00 1.53
H
ATOM 6818 2HG ARG A 447 14.958 21.036 -18.253 1.00 1.53
H
ATOM 6819 1HD ARG A 447 15.557 19.131 -17.057 1.00 1.53
H
ATOM 6820 2HD ARG A 447 13.873 18.742 -16.663 1.00 1.53
H
ATOM 6821 HE ARG A 447 15.245 18.343 -19.240 1.00 1.53
H
ATOM 6822 1HH1 ARG A 447 12.342 18.299 -17.322 1.00 1.53
H
ATOM 6823 2HH1 ARG A 447 11.444 17.252 -18.388 1.00 1.53
H
ATOM 6824 1HH2 ARG A 447 14.085 16.960 -20.696 1.00 1.53
H

```
ATOM   6825 2HH2 ARG A 447      12.438  16.498 -20.322  1.00  1.53
H
ATOM   6826  N   PRO A 448      16.209  23.312 -17.082  1.00  1.27
N
ATOM   6827  CA  PRO A 448      17.069  23.891 -18.096  1.00  1.44
C
ATOM   6828  C   PRO A 448      18.408  24.321 -17.542  1.00  1.51
C
ATOM   6829  O   PRO A 448      18.522  24.703 -16.375  1.00  1.54
O
ATOM   6830  CB  PRO A 448      16.293  25.129 -18.532  1.00  2.16
C
ATOM   6831  CG  PRO A 448      15.645  25.604 -17.271  1.00  2.16
C
ATOM   6832  CD  PRO A 448      15.444  24.382 -16.412  1.00  2.16
C
ATOM   6833  HA  PRO A 448      17.192  23.178 -18.924  1.00  1.73
H
ATOM   6834 1HB  PRO A 448      16.977  25.871 -18.966  1.00  2.59
H
ATOM   6835 2HB  PRO A 448      15.564  24.864 -19.312  1.00  2.59
H
ATOM   6836 1HG  PRO A 448      16.286  26.354 -16.784  1.00  2.59
H
ATOM   6837 2HG  PRO A 448      14.695  26.107 -17.503  1.00  2.59
H
ATOM   6838 1HD  PRO A 448      15.883  24.577 -15.423  1.00  2.59
H
ATOM   6839 2HD  PRO A 448      14.374  24.138 -16.355  1.00  2.59
H
ATOM   6840  N   GLU A 449      19.419  24.298 -18.400  1.00  1.60
N
ATOM   6841  CA  GLU A 449      20.730  24.767 -18.007  1.00  1.68
C
ATOM   6842  C   GLU A 449      20.748  26.272 -18.106  1.00  1.83
C
ATOM   6843  O   GLU A 449      20.318  26.836 -19.114  1.00  2.08
O
ATOM   6844  CB  GLU A 449      21.827  24.162 -18.885  1.00  2.52
C
ATOM   6845  CG  GLU A 449      22.013  22.660 -18.737  1.00  2.52
C
ATOM   6846  CD  GLU A 449      23.129  22.135 -19.594  1.00  2.52
C
ATOM   6847  OE1 GLU A 449      23.572  22.855 -20.457  1.00  2.52
O
ATOM   6848  OE2 GLU A 449      23.546  21.019 -19.387  1.00  2.52
O
ATOM   6849  H   GLU A 449      19.268  23.961 -19.340  1.00  1.92
H
ATOM   6850  HA  GLU A 449      20.904  24.489 -16.973  1.00  2.02
H
ATOM   6851 1HB  GLU A 449      21.606  24.367 -19.933  1.00  3.02
H
ATOM   6852 2HB  GLU A 449      22.779  24.637 -18.655  1.00  3.02
H
ATOM   6853 1HG  GLU A 449      22.226  22.423 -17.698  1.00  3.02
H
```

ATOM 6854 2HG GLU A 449 21.085 22.161 -19.013 1.00 3.02
H
ATOM 6855 N THR A 450 21.244 26.928 -17.066 1.00 1.72
N
ATOM 6856 CA THR A 450 21.254 28.376 -17.063 1.00 1.69
C
ATOM 6857 C THR A 450 22.637 28.897 -16.730 1.00 1.59
C
ATOM 6858 O THR A 450 23.470 28.174 -16.189 1.00 1.60
O
ATOM 6859 CB THR A 450 20.231 28.920 -16.063 1.00 2.54
C
ATOM 6860 OG1 THR A 450 20.620 28.559 -14.726 1.00 2.54
O
ATOM 6861 CG2 THR A 450 18.836 28.372 -16.343 1.00 2.54
C
ATOM 6862 H THR A 450 21.605 26.423 -16.265 1.00 2.06
H
ATOM 6863 HA THR A 450 20.989 28.732 -18.059 1.00 2.03
H
ATOM 6864 HB THR A 450 20.197 29.998 -16.159 1.00 3.04
H
ATOM 6865 HG1 THR A 450 21.087 29.293 -14.307 1.00 3.04
H
ATOM 6866 1HG2 THR A 450 18.134 28.792 -15.624 1.00 3.04
H
ATOM 6867 2HG2 THR A 450 18.531 28.649 -17.352 1.00 3.04
H
ATOM 6868 3HG2 THR A 450 18.840 27.288 -16.250 1.00 3.04
H
ATOM 6869 N MET A 451 22.891 30.148 -17.069 1.00 1.64
N
ATOM 6870 CA MET A 451 24.220 30.715 -16.894 1.00 1.67
C
ATOM 6871 C MET A 451 24.616 30.943 -15.440 1.00 1.72
C
ATOM 6872 O MET A 451 23.806 31.348 -14.611 1.00 1.84
O
ATOM 6873 CB MET A 451 24.299 32.030 -17.662 1.00 2.50
C
ATOM 6874 CG MET A 451 24.249 31.864 -19.173 1.00 2.50
C
ATOM 6875 SD MET A 451 24.212 33.434 -20.061 1.00 2.50
S
ATOM 6876 CE MET A 451 25.873 34.038 -19.795 1.00 2.50
C
ATOM 6877 H MET A 451 22.160 30.711 -17.483 1.00 1.97
H
ATOM 6878 HA MET A 451 24.933 30.022 -17.328 1.00 2.00
H
ATOM 6879 1HB MET A 451 23.474 32.677 -17.367 1.00 3.01
H
ATOM 6880 2HB MET A 451 25.227 32.546 -17.410 1.00 3.01
H
ATOM 6881 1HG MET A 451 25.125 31.304 -19.502 1.00 3.01
H
ATOM 6882 2HG MET A 451 23.361 31.295 -19.445 1.00 3.01
H

```
ATOM   6883 1HE  MET A 451      25.993  35.001 -20.291  1.00  3.01
H
ATOM   6884 2HE  MET A 451      26.048  34.155 -18.725  1.00  3.01
H
ATOM   6885 3HE  MET A 451      26.590  33.326 -20.206  1.00  3.01
H
ATOM   6886  N   VAL A 452      25.888  30.729 -15.135  1.00  1.72
N
ATOM   6887  CA  VAL A 452      26.377  31.042 -13.805  1.00  1.71
C
ATOM   6888  C   VAL A 452      26.482  32.546 -13.723  1.00  1.74
C
ATOM   6889  O   VAL A 452      27.074  33.174 -14.598  1.00  1.80
O
ATOM   6890  CB  VAL A 452      27.731  30.371 -13.529  1.00  2.56
C
ATOM   6891  CG1 VAL A 452      28.280  30.815 -12.179  1.00  2.56
C
ATOM   6892  CG2 VAL A 452      27.554  28.861 -13.546  1.00  2.56
C
ATOM   6893  H   VAL A 452      26.515  30.346 -15.829  1.00  2.06
H
ATOM   6894  HA  VAL A 452      25.658  30.697 -13.068  1.00  2.05
H
ATOM   6895  HB  VAL A 452      28.439  30.668 -14.300  1.00  3.08
H
ATOM   6896 1HG1 VAL A 452      29.243  30.336 -12.001  1.00  3.08
H
ATOM   6897 2HG1 VAL A 452      28.408  31.897 -12.173  1.00  3.08
H
ATOM   6898 3HG1 VAL A 452      27.582  30.530 -11.391  1.00  3.08
H
ATOM   6899 1HG2 VAL A 452      28.513  28.384 -13.370  1.00  3.08
H
ATOM   6900 2HG2 VAL A 452      26.850  28.570 -12.767  1.00  3.08
H
ATOM   6901 3HG2 VAL A 452      27.170  28.547 -14.513  1.00  3.08
H
ATOM   6902  N   ILE A 453      25.879  33.132 -12.699  1.00  1.73
N
ATOM   6903  CA  ILE A 453      25.872  34.585 -12.585  1.00  1.78
C
ATOM   6904  C   ILE A 453      26.553  35.064 -11.317  1.00  1.94
C
ATOM   6905  O   ILE A 453      26.944  36.230 -11.228  1.00  2.15
O
ATOM   6906  CB  ILE A 453      24.425  35.127 -12.656  1.00  2.67
C
ATOM   6907  CG1 ILE A 453      23.594  34.572 -11.500  1.00  2.67
C
ATOM   6908  CG2 ILE A 453      23.783  34.793 -13.993  1.00  2.67
C
ATOM   6909  CD1 ILE A 453      22.235  35.210 -11.383  1.00  2.67
C
ATOM   6910  H   ILE A 453      25.426  32.568 -11.989  1.00  2.08
H
ATOM   6911  HA  ILE A 453      26.420  34.996 -13.431  1.00  2.14
H
```

```
ATOM   6912  HB  ILE A 453      24.450  36.210 -12.541  1.00  3.20
H
ATOM   6913 1HG1 ILE A 453      23.468  33.498 -11.628  1.00  3.20
H
ATOM   6914 2HG1 ILE A 453      24.127  34.746 -10.567  1.00  3.20
H
ATOM   6915 1HG2 ILE A 453      22.774  35.203 -14.025  1.00  3.20
H
ATOM   6916 2HG2 ILE A 453      24.375  35.225 -14.799  1.00  3.20
H
ATOM   6917 3HG2 ILE A 453      23.738  33.714 -14.115  1.00  3.20
H
ATOM   6918 1HD1 ILE A 453      21.703  34.779 -10.535  1.00  3.20
H
ATOM   6919 2HD1 ILE A 453      22.351  36.280 -11.234  1.00  3.20
H
ATOM   6920 3HD1 ILE A 453      21.669  35.029 -12.297  1.00  3.20
H
ATOM   6921  N   TYR A 454      26.738  34.169 -10.353  1.00  2.00
N
ATOM   6922  CA  TYR A 454      27.453  34.540  -9.152  1.00  2.33
C
ATOM   6923  C   TYR A 454      28.889  34.075  -9.175  1.00  2.26
C
ATOM   6924  O   TYR A 454      29.204  33.000  -9.684  1.00  2.23
O
ATOM   6925  CB  TYR A 454      26.789  33.965  -7.913  1.00  3.50
C
ATOM   6926  CG  TYR A 454      25.458  34.570  -7.588  1.00  3.50
C
ATOM   6927  CD1 TYR A 454      24.293  33.872  -7.856  1.00  3.50
C
ATOM   6928  CD2 TYR A 454      25.404  35.824  -7.012  1.00  3.50
C
ATOM   6929  CE1 TYR A 454      23.075  34.420  -7.536  1.00  3.50
C
ATOM   6930  CE2 TYR A 454      24.187  36.375  -6.696  1.00  3.50
C
ATOM   6931  CZ  TYR A 454      23.032  35.673  -6.950  1.00  3.50
C
ATOM   6932  OH  TYR A 454      21.822  36.220  -6.627  1.00  3.50
O
ATOM   6933  H   TYR A 454      26.364  33.228 -10.437  1.00  2.40
H
ATOM   6934  HA  TYR A 454      27.460  35.627  -9.075  1.00  2.80
H
ATOM   6935 1HB  TYR A 454      26.645  32.893  -8.051  1.00  4.19
H
ATOM   6936 2HB  TYR A 454      27.442  34.099  -7.052  1.00  4.19
H
ATOM   6937  HD1 TYR A 454      24.345  32.888  -8.307  1.00  4.19
H
ATOM   6938  HD2 TYR A 454      26.324  36.372  -6.806  1.00  4.19
H
ATOM   6939  HE1 TYR A 454      22.155  33.871  -7.738  1.00  4.19
H
ATOM   6940  HE2 TYR A 454      24.138  37.364  -6.240  1.00  4.19
H
```

```
ATOM   6941  HH  TYR A 454      21.120  35.620  -6.904  1.00  4.19
H
ATOM   6942  N   THR A 455      29.750  34.880  -8.581  1.00  2.33
N
ATOM   6943  CA  THR A 455      31.137  34.517  -8.399  1.00  2.25
C
ATOM   6944  C   THR A 455      31.149  33.553  -7.213  1.00  1.95
C
ATOM   6945  O   THR A 455      30.437  33.800  -6.245  1.00  2.07
O
ATOM   6946  CB  THR A 455      32.014  35.762  -8.116  1.00  3.38
C
ATOM   6947  OG1 THR A 455      31.923  36.668  -9.224  1.00  3.38
O
ATOM   6948  CG2 THR A 455      33.474  35.388  -7.908  1.00  3.38
C
ATOM   6949  H   THR A 455      29.425  35.763  -8.212  1.00  2.80
H
ATOM   6950  HA  THR A 455      31.502  34.021  -9.295  1.00  2.70
H
ATOM   6951  HB  THR A 455      31.643  36.264  -7.222  1.00  4.05
H
ATOM   6952  HG1 THR A 455      32.465  37.442  -9.051  1.00  4.05
H
ATOM   6953 1HG2 THR A 455      34.056  36.289  -7.713  1.00  4.05
H
ATOM   6954 2HG2 THR A 455      33.565  34.712  -7.063  1.00  4.05
H
ATOM   6955 3HG2 THR A 455      33.855  34.899  -8.804  1.00  4.05
H
ATOM   6956  N   PRO A 456      31.893  32.441  -7.264  1.00  1.82
N
ATOM   6957  CA  PRO A 456      32.037  31.412  -6.224  1.00  1.79
C
ATOM   6958  C   PRO A 456      32.367  31.938  -4.825  1.00  2.02
C
ATOM   6959  O   PRO A 456      32.074  31.278  -3.829  1.00  2.66
O
ATOM   6960  CB  PRO A 456      33.164  30.556  -6.768  1.00  2.69
C
ATOM   6961  CG  PRO A 456      32.995  30.627  -8.250  1.00  2.69
C
ATOM   6962  CD  PRO A 456      32.515  32.021  -8.532  1.00  2.69
C
ATOM   6963  HA  PRO A 456      31.110  30.825  -6.189  1.00  2.15
H
ATOM   6964 1HB  PRO A 456      34.133  30.944  -6.430  1.00  3.22
H
ATOM   6965 2HB  PRO A 456      33.063  29.545  -6.363  1.00  3.22
H
ATOM   6966 1HG  PRO A 456      33.951  30.403  -8.748  1.00  3.22
H
ATOM   6967 2HG  PRO A 456      32.279  29.862  -8.586  1.00  3.22
H
ATOM   6968 1HD  PRO A 456      33.362  32.674  -8.782  1.00  3.22
H
ATOM   6969 2HD  PRO A 456      31.765  31.996  -9.335  1.00  3.22
H
```

```
ATOM   6970  N   GLN A 457      32.965  33.122  -4.743  1.00  1.82
N
ATOM   6971  CA  GLN A 457      33.314  33.733  -3.471  1.00  1.96
C
ATOM   6972  C   GLN A 457      32.146  34.513  -2.847  1.00  1.94
C
ATOM   6973  O   GLN A 457      32.267  35.023  -1.731  1.00  2.08
O
ATOM   6974  CB  GLN A 457      34.508  34.668  -3.660  1.00  2.94
C
ATOM   6975  CG  GLN A 457      35.765  33.978  -4.162  1.00  2.94
C
ATOM   6976  CD  GLN A 457      36.264  32.913  -3.205  1.00  2.94
C
ATOM   6977  OE1 GLN A 457      36.424  33.159  -2.007  1.00  2.94
O
ATOM   6978  NE2 GLN A 457      36.518  31.720  -3.733  1.00  2.94
N
ATOM   6979  H   GLN A 457      33.194  33.616  -5.589  1.00  2.18
H
ATOM   6980  HA  GLN A 457      33.596  32.940  -2.777  1.00  2.35
H
ATOM   6981 1HB  GLN A 457      34.248  35.451  -4.373  1.00  3.53
H
ATOM   6982 2HB  GLN A 457      34.746  35.153  -2.714  1.00  3.53
H
ATOM   6983 1HG  GLN A 457      35.550  33.503  -5.118  1.00  3.53
H
ATOM   6984 2HG  GLN A 457      36.551  34.723  -4.283  1.00  3.53
H
ATOM   6985 1HE2 GLN A 457      36.852  30.978  -3.151  1.00  3.53
H
ATOM   6986 2HE2 GLN A 457      36.376  31.565  -4.710  1.00  3.53
H
ATOM   6987  N   GLU A 458      31.029  34.619  -3.570  1.00  1.85
N
ATOM   6988  CA  GLU A 458      29.844  35.321  -3.092  1.00  1.86
C
ATOM   6989  C   GLU A 458      29.293  34.677  -1.825  1.00  1.72
C
ATOM   6990  O   GLU A 458      29.106  33.461  -1.763  1.00  1.80
O
ATOM   6991  CB  GLU A 458      28.764  35.351  -4.176  1.00  2.79
C
ATOM   6992  CG  GLU A 458      27.531  36.165  -3.812  1.00  2.79
C
ATOM   6993  CD  GLU A 458      27.815  37.643  -3.748  1.00  2.79
C
ATOM   6994  OE1 GLU A 458      28.692  38.089  -4.451  1.00  2.79
O
ATOM   6995  OE2 GLU A 458      27.153  38.332  -3.005  1.00  2.79
O
ATOM   6996  H   GLU A 458      30.975  34.194  -4.481  1.00  2.22
H
ATOM   6997  HA  GLU A 458      30.126  36.348  -2.854  1.00  2.23
H
ATOM   6998 1HB  GLU A 458      29.181  35.774  -5.089  1.00  3.35
H
```

```
ATOM   6999 2HB  GLU A 458      28.447  34.334  -4.403  1.00  3.35
H
ATOM   7000 1HG  GLU A 458      26.754  35.983  -4.551  1.00  3.35
H
ATOM   7001 2HG  GLU A 458      27.162  35.825  -2.847  1.00  3.35
H
ATOM   7002  N   ARG A 459      29.039  35.497  -0.813  1.00  1.81
N
ATOM   7003  CA  ARG A 459      28.521  35.006   0.457  1.00  1.77
C
ATOM   7004  C   ARG A 459      27.000  34.954   0.480  1.00  1.69
C
ATOM   7005  O   ARG A 459      26.334  35.980   0.340  1.00  2.74
O
ATOM   7006  CB  ARG A 459      28.985  35.884   1.605  1.00  2.66
C
ATOM   7007  CG  ARG A 459      28.509  35.426   2.974  1.00  2.66
C
ATOM   7008  CD  ARG A 459      28.865  36.400   4.036  1.00  2.66
C
ATOM   7009  NE  ARG A 459      28.413  35.955   5.343  1.00  2.66
N
ATOM   7010  CZ  ARG A 459      27.171  36.168   5.825  1.00  2.66
C
ATOM   7011  NH1 ARG A 459      26.277  36.797   5.101  1.00  2.66
N
ATOM   7012  NH2 ARG A 459      26.829  35.745   7.026  1.00  2.66
N
ATOM   7013  H   ARG A 459      29.213  36.487  -0.927  1.00  2.17
H
ATOM   7014  HA  ARG A 459      28.905  33.997   0.616  1.00  2.12
H
ATOM   7015 1HB  ARG A 459      30.074  35.912   1.625  1.00  3.19
H
ATOM   7016 2HB  ARG A 459      28.632  36.903   1.452  1.00  3.19
H
ATOM   7017 1HG  ARG A 459      27.424  35.313   2.964  1.00  3.19
H
ATOM   7018 2HG  ARG A 459      28.970  34.469   3.219  1.00  3.19
H
ATOM   7019 1HD  ARG A 459      29.947  36.522   4.073  1.00  3.19
H
ATOM   7020 2HD  ARG A 459      28.398  37.360   3.822  1.00  3.19
H
ATOM   7021  HE  ARG A 459      29.072  35.465   5.932  1.00  3.19
H
ATOM   7022 1HH1 ARG A 459      26.509  37.130   4.176  1.00  3.19
H
ATOM   7023 2HH1 ARG A 459      25.344  36.930   5.482  1.00  3.19
H
ATOM   7024 1HH2 ARG A 459      27.493  35.255   7.608  1.00  3.19
H
ATOM   7025 2HH2 ARG A 459      25.884  35.920   7.350  1.00  3.19
H
ATOM   7026  N   PHE A 460      26.460  33.758   0.685  1.00  1.27
N
ATOM   7027  CA  PHE A 460      25.016  33.559   0.757  1.00  1.35
C
```

```
ATOM   7028  C   PHE A 460      24.558  33.318   2.182  1.00  1.25
C
ATOM   7029  O   PHE A 460      25.288  32.754   2.996  1.00  1.21
O
ATOM   7030  CB  PHE A 460      24.602  32.378  -0.104  1.00  2.03
C
ATOM   7031  CG  PHE A 460      24.746  32.625  -1.569  1.00  2.03
C
ATOM   7032  CD1 PHE A 460      25.973  32.488  -2.180  1.00  2.03
C
ATOM   7033  CD2 PHE A 460      23.654  32.976  -2.341  1.00  2.03
C
ATOM   7034  CE1 PHE A 460      26.116  32.691  -3.533  1.00  2.03
C
ATOM   7035  CE2 PHE A 460      23.791  33.182  -3.697  1.00  2.03
C
ATOM   7036  CZ  PHE A 460      25.027  33.033  -4.288  1.00  2.03
C
ATOM   7037  H   PHE A 460      27.069  32.958   0.789  1.00  1.52
H
ATOM   7038  HA  PHE A 460      24.521  34.456   0.384  1.00  1.62
H
ATOM   7039 1HB  PHE A 460      25.208  31.514   0.158  1.00  2.43
H
ATOM   7040 2HB  PHE A 460      23.563  32.126   0.100  1.00  2.43
H
ATOM   7041  HD1 PHE A 460      26.838  32.211  -1.577  1.00  2.43
H
ATOM   7042  HD2 PHE A 460      22.681  33.087  -1.865  1.00  2.43
H
ATOM   7043  HE1 PHE A 460      27.094  32.575  -4.000  1.00  2.43
H
ATOM   7044  HE2 PHE A 460      22.926  33.456  -4.301  1.00  2.43
H
ATOM   7045  HZ  PHE A 460      25.142  33.186  -5.352  1.00  2.43
H
ATOM   7046  N   GLU A 461      23.339  33.748   2.473  1.00  1.35
N
ATOM   7047  CA  GLU A 461      22.763  33.591   3.798  1.00  1.31
C
ATOM   7048  C   GLU A 461      21.249  33.492   3.734  1.00  1.18
C
ATOM   7049  O   GLU A 461      20.605  34.160   2.924  1.00  1.24
O
ATOM   7050  CB  GLU A 461      23.182  34.747   4.712  1.00  1.97
C
ATOM   7051  CG  GLU A 461      22.666  34.625   6.141  1.00  1.97
C
ATOM   7052  CD  GLU A 461      23.189  35.687   7.073  1.00  1.97
C
ATOM   7053  OE1 GLU A 461      24.042  36.448   6.679  1.00  1.97
O
ATOM   7054  OE2 GLU A 461      22.743  35.728   8.196  1.00  1.97
O
ATOM   7055  H   GLU A 461      22.793  34.202   1.754  1.00  1.62
H
ATOM   7056  HA  GLU A 461      23.143  32.663   4.227  1.00  1.57
H
```

ATOM 7057 1HB GLU A 461 24.271 34.799 4.751 1.00 2.36
H
ATOM 7058 2HB GLU A 461 22.821 35.689 4.301 1.00 2.36
H
ATOM 7059 1HG GLU A 461 21.578 34.688 6.127 1.00 2.36
H
ATOM 7060 2HG GLU A 461 22.938 33.645 6.530 1.00 2.36
H
ATOM 7061 N ILE A 462 20.697 32.636 4.583 1.00 1.09
N
ATOM 7062 CA ILE A 462 19.263 32.422 4.656 1.00 1.05
C
ATOM 7063 C ILE A 462 18.575 33.711 5.083 1.00 1.25
C
ATOM 7064 O ILE A 462 18.935 34.297 6.104 1.00 1.39
O
ATOM 7065 CB ILE A 462 18.944 31.299 5.658 1.00 1.58
C
ATOM 7066 CG1 ILE A 462 19.539 29.979 5.174 1.00 1.58
C
ATOM 7067 CG2 ILE A 462 17.447 31.168 5.847 1.00 1.58
C
ATOM 7068 CD1 ILE A 462 19.490 28.880 6.210 1.00 1.58
C
ATOM 7069 H ILE A 462 21.297 32.119 5.212 1.00 1.31
H
ATOM 7070 HA ILE A 462 18.898 32.140 3.671 1.00 1.26
H
ATOM 7071 HB ILE A 462 19.406 31.531 6.617 1.00 1.89
H
ATOM 7072 1HG1 ILE A 462 18.994 29.652 4.291 1.00 1.89
H
ATOM 7073 2HG1 ILE A 462 20.579 30.141 4.896 1.00 1.89
H
ATOM 7074 1HG2 ILE A 462 17.238 30.378 6.567 1.00 1.89
H
ATOM 7075 2HG2 ILE A 462 17.048 32.110 6.217 1.00 1.89
H
ATOM 7076 3HG2 ILE A 462 16.978 30.924 4.894 1.00 1.89
H
ATOM 7077 1HD1 ILE A 462 19.930 27.971 5.804 1.00 1.89
H
ATOM 7078 2HD1 ILE A 462 20.049 29.189 7.093 1.00 1.89
H
ATOM 7079 3HD1 ILE A 462 18.454 28.688 6.486 1.00 1.89
H
ATOM 7080 N GLY A 463 17.597 34.168 4.298 1.00 1.32
N
ATOM 7081 CA GLY A 463 16.924 35.423 4.599 1.00 1.61
C
ATOM 7082 C GLY A 463 17.617 36.637 3.975 1.00 1.80
C
ATOM 7083 O GLY A 463 17.205 37.769 4.224 1.00 2.10
O
ATOM 7084 H GLY A 463 17.288 33.657 3.479 1.00 1.58
H
ATOM 7085 1HA GLY A 463 15.896 35.370 4.241 1.00 1.93
H

ATOM 7086 2HA GLY A 463 16.873 35.551 5.679 1.00 1.93
H
ATOM 7087 N GLN A 464 18.664 36.412 3.172 1.00 1.71
N
ATOM 7088 CA GLN A 464 19.359 37.515 2.511 1.00 1.87
C
ATOM 7089 C GLN A 464 19.214 37.457 0.987 1.00 2.01
C
ATOM 7090 O GLN A 464 19.643 36.493 0.349 1.00 3.25
O
ATOM 7091 CB GLN A 464 20.844 37.506 2.878 1.00 2.81
C
ATOM 7092 CG GLN A 464 21.654 38.584 2.185 1.00 2.81
C
ATOM 7093 CD GLN A 464 21.314 39.975 2.688 1.00 2.81
C
ATOM 7094 OE1 GLN A 464 21.520 40.288 3.866 1.00 2.81
O
ATOM 7095 NE2 GLN A 464 20.789 40.815 1.804 1.00 2.81
N
ATOM 7096 H GLN A 464 19.003 35.472 3.015 1.00 2.05
H
ATOM 7097 HA GLN A 464 18.923 38.453 2.853 1.00 2.24
H
ATOM 7098 1HB GLN A 464 20.954 37.637 3.955 1.00 3.37
H
ATOM 7099 2HB GLN A 464 21.279 36.542 2.619 1.00 3.37
H
ATOM 7100 1HG GLN A 464 22.713 38.404 2.367 1.00 3.37
H
ATOM 7101 2HG GLN A 464 21.445 38.550 1.116 1.00 3.37
H
ATOM 7102 1HE2 GLN A 464 20.540 41.744 2.082 1.00 3.37
H
ATOM 7103 2HE2 GLN A 464 20.629 40.518 0.861 1.00 3.37
H
ATOM 7104 N ALA A 465 18.615 38.502 0.416 1.00 1.67
N
ATOM 7105 CA ALA A 465 18.428 38.625 -1.029 1.00 1.60
C
ATOM 7106 C ALA A 465 19.607 39.351 -1.654 1.00 1.55
C
ATOM 7107 O ALA A 465 20.359 40.048 -0.970 1.00 1.58
O
ATOM 7108 CB ALA A 465 17.137 39.364 -1.350 1.00 2.40
C
ATOM 7109 H ALA A 465 18.282 39.259 1.006 1.00 2.00
H
ATOM 7110 HA ALA A 465 18.381 37.623 -1.454 1.00 1.92
H
ATOM 7111 1HB ALA A 465 17.015 39.427 -2.431 1.00 2.88
H
ATOM 7112 2HB ALA A 465 16.295 38.822 -0.921 1.00 2.88
H
ATOM 7113 3HB ALA A 465 17.179 40.367 -0.930 1.00 2.88
H
ATOM 7114 N LYS A 466 19.777 39.187 -2.960 1.00 1.57
N

ATOM 7115 CA LYS A 466 20.870 39.842 -3.649 1.00 1.69
C
ATOM 7116 C LYS A 466 20.411 40.689 -4.824 1.00 2.08
C
ATOM 7117 O LYS A 466 19.464 40.357 -5.537 1.00 2.48
O
ATOM 7118 CB LYS A 466 21.880 38.795 -4.099 1.00 2.54
C
ATOM 7119 CG LYS A 466 22.561 38.115 -2.923 1.00 2.54
C
ATOM 7120 CD LYS A 466 23.602 37.110 -3.350 1.00 2.54
C
ATOM 7121 CE LYS A 466 24.265 36.516 -2.131 1.00 2.54
C
ATOM 7122 NZ LYS A 466 25.017 37.552 -1.376 1.00 2.54
N
ATOM 7123 H LYS A 466 19.144 38.600 -3.478 1.00 1.88
H
ATOM 7124 HA LYS A 466 21.367 40.508 -2.943 1.00 2.03
H
ATOM 7125 1HB LYS A 466 21.381 38.029 -4.696 1.00 3.04
H
ATOM 7126 2HB LYS A 466 22.647 39.257 -4.720 1.00 3.04
H
ATOM 7127 1HG LYS A 466 23.038 38.871 -2.298 1.00 3.04
H
ATOM 7128 2HG LYS A 466 21.814 37.598 -2.323 1.00 3.04
H
ATOM 7129 1HD LYS A 466 23.134 36.316 -3.931 1.00 3.04
H
ATOM 7130 2HD LYS A 466 24.360 37.599 -3.964 1.00 3.04
H
ATOM 7131 1HE LYS A 466 23.506 36.085 -1.480 1.00 3.04
H
ATOM 7132 2HE LYS A 466 24.952 35.731 -2.437 1.00 3.04
H
ATOM 7133 1HZ LYS A 466 25.451 37.113 -0.578 1.00 3.04
H
ATOM 7134 2HZ LYS A 466 25.737 37.937 -1.974 1.00 3.04
H
ATOM 7135 3HZ LYS A 466 24.396 38.285 -1.070 1.00 3.04
H
ATOM 7136 N VAL A 467 21.114 41.793 -5.031 1.00 2.95
N
ATOM 7137 CA VAL A 467 20.816 42.679 -6.139 1.00 3.13
C
ATOM 7138 C VAL A 467 21.700 42.303 -7.309 1.00 3.13
C
ATOM 7139 O VAL A 467 22.917 42.474 -7.251 1.00 3.60
O
ATOM 7140 CB VAL A 467 21.076 44.144 -5.761 1.00 4.70
C
ATOM 7141 CG1 VAL A 467 20.764 45.024 -6.952 1.00 4.70
C
ATOM 7142 CG2 VAL A 467 20.242 44.536 -4.549 1.00 4.70
C
ATOM 7143 H VAL A 467 21.877 42.019 -4.409 1.00 3.54
H

```
ATOM   7144  HA  VAL A 467      19.772  42.559  -6.424  1.00  3.76
H
ATOM   7145  HB  VAL A 467      22.133  44.270  -5.526  1.00  5.63
H
ATOM   7146 1HG1 VAL A 467      20.968  46.063  -6.697  1.00  5.63
H
ATOM   7147 2HG1 VAL A 467      21.389  44.729  -7.796  1.00  5.63
H
ATOM   7148 3HG1 VAL A 467      19.714  44.912  -7.220  1.00  5.63
H
ATOM   7149 1HG2 VAL A 467      20.446  45.575  -4.291  1.00  5.63
H
ATOM   7150 2HG2 VAL A 467      19.186  44.424  -4.776  1.00  5.63
H
ATOM   7151 3HG2 VAL A 467      20.501  43.896  -3.706  1.00  5.63
H
ATOM   7152  N   LEU A 468      21.093  41.777  -8.362  1.00  2.91
N
ATOM   7153  CA  LEU A 468      21.842  41.345  -9.534  1.00  3.06
C
ATOM   7154  C   LEU A 468      22.131  42.466 -10.491  1.00  3.20
C
ATOM   7155  O   LEU A 468      23.233  42.561 -11.033  1.00  3.51
O
ATOM   7156  CB  LEU A 468      21.078  40.263 -10.275  1.00  4.59
C
ATOM   7157  CG  LEU A 468      20.956  38.960  -9.518  1.00  4.59
C
ATOM   7158  CD1 LEU A 468      20.082  38.016 -10.302  1.00  4.59
C
ATOM   7159  CD2 LEU A 468      22.346  38.392  -9.322  1.00  4.59
C
ATOM   7160  H   LEU A 468      20.089  41.682  -8.360  1.00  3.49
H
ATOM   7161  HA  LEU A 468      22.792  40.933  -9.197  1.00  3.67
H
ATOM   7162 1HB  LEU A 468      20.074  40.624 -10.485  1.00  5.51
H
ATOM   7163 2HB  LEU A 468      21.581  40.059 -11.219  1.00  5.51
H
ATOM   7164  HG  LEU A 468      20.489  39.129  -8.547  1.00  5.51
H
ATOM   7165 1HD1 LEU A 468      19.992  37.071  -9.767  1.00  5.51
H
ATOM   7166 2HD1 LEU A 468      19.093  38.456 -10.427  1.00  5.51
H
ATOM   7167 3HD1 LEU A 468      20.526  37.837 -11.281  1.00  5.51
H
ATOM   7168 1HD2 LEU A 468      22.273  37.451  -8.789  1.00  5.51
H
ATOM   7169 2HD2 LEU A 468      22.812  38.225 -10.293  1.00  5.51
H
ATOM   7170 3HD2 LEU A 468      22.949  39.095  -8.747  1.00  5.51
H
ATOM   7171  N   ARG A 469      21.141  43.311 -10.714  1.00  3.20
N
ATOM   7172  CA  ARG A 469      21.335  44.424 -11.626  1.00  3.58
C
```

ATOM 7173 C ARG A 469 20.783 45.683 -11.033 1.00 3.70
C
ATOM 7174 O ARG A 469 19.664 45.700 -10.536 1.00 4.27
O
ATOM 7175 CB ARG A 469 20.663 44.174 -12.967 1.00 5.37
C
ATOM 7176 CG ARG A 469 21.221 43.011 -13.767 1.00 5.37
C
ATOM 7177 CD ARG A 469 22.585 43.307 -14.275 1.00 5.37
C
ATOM 7178 NE ARG A 469 23.104 42.231 -15.101 1.00 5.37
N
ATOM 7179 CZ ARG A 469 23.771 41.152 -14.644 1.00 5.37
C
ATOM 7180 NH1 ARG A 469 24.011 40.995 -13.358 1.00 5.37
N
ATOM 7181 NH2 ARG A 469 24.194 40.238 -15.501 1.00 5.37
N
ATOM 7182 H ARG A 469 20.252 43.168 -10.243 1.00 3.84
H
ATOM 7183 HA ARG A 469 22.403 44.560 -11.793 1.00 4.30
H
ATOM 7184 1HB ARG A 469 19.604 43.997 -12.812 1.00 6.44
H
ATOM 7185 2HB ARG A 469 20.754 45.066 -13.587 1.00 6.44
H
ATOM 7186 1HG ARG A 469 21.271 42.121 -13.142 1.00 6.44
H
ATOM 7187 2HG ARG A 469 20.575 42.819 -14.623 1.00 6.44
H
ATOM 7188 1HD ARG A 469 22.558 44.216 -14.876 1.00 6.44
H
ATOM 7189 2HD ARG A 469 23.264 43.450 -13.435 1.00 6.44
H
ATOM 7190 HE ARG A 469 22.951 42.297 -16.099 1.00 6.44
H
ATOM 7191 1HH1 ARG A 469 23.698 41.682 -12.681 1.00 6.44
H
ATOM 7192 2HH1 ARG A 469 24.515 40.181 -13.038 1.00 6.44
H
ATOM 7193 1HH2 ARG A 469 24.015 40.352 -16.489 1.00 6.44
H
ATOM 7194 2HH2 ARG A 469 24.695 39.427 -15.169 1.00 6.44
H
ATOM 7195 N HIS A 470 21.563 46.750 -11.104 1.00 3.62
N
ATOM 7196 CA HIS A 470 21.126 48.018 -10.555 1.00 3.75
C
ATOM 7197 C HIS A 470 21.753 49.221 -11.234 1.00 3.73
C
ATOM 7198 O HIS A 470 22.795 49.121 -11.882 1.00 4.17
O
ATOM 7199 CB HIS A 470 21.434 48.056 -9.064 1.00 5.62
C
ATOM 7200 CG HIS A 470 22.885 47.894 -8.755 1.00 5.62
C
ATOM 7201 ND1 HIS A 470 23.493 46.657 -8.696 1.00 5.62
N

```
ATOM   7202  CD2 HIS A 470      23.850  48.802  -8.487  1.00  5.62
C
ATOM   7203  CE1 HIS A 470      24.771  46.811  -8.403  1.00  5.62
C
ATOM   7204  NE2 HIS A 470      25.014  48.104  -8.271  1.00  5.62
N
ATOM   7205  H   HIS A 470      22.474  46.681 -11.535  1.00  4.34
H
ATOM   7206  HA  HIS A 470      20.048  48.099 -10.677  1.00  4.50
H
ATOM   7207 1HB  HIS A 470      21.097  49.003  -8.643  1.00  6.75
H
ATOM   7208 2HB  HIS A 470      20.887  47.264  -8.566  1.00  6.75
H
ATOM   7209  HD2 HIS A 470      23.728  49.885  -8.447  1.00  6.75
H
ATOM   7210  HE1 HIS A 470      25.500  46.011  -8.290  1.00  6.75
H
ATOM   7211  HE2 HIS A 470      25.908  48.517  -8.049  1.00  6.75
H
ATOM   7212  N   CYS A 471      21.122  50.367 -11.021  1.00  4.45
N
ATOM   7213  CA  CYS A 471      21.526  51.642 -11.590  1.00  4.41
C
ATOM   7214  C   CYS A 471      20.735  52.784 -10.963  1.00  4.44
C
ATOM   7215  O   CYS A 471      19.586  52.616 -10.557  1.00  4.83
O
ATOM   7216  CB  CYS A 471      21.302  51.663 -13.106  1.00  6.62
C
ATOM   7217  SG  CYS A 471      21.912  53.156 -13.926  1.00  6.62
S
ATOM   7218  H   CYS A 471      20.267  50.329 -10.484  1.00  5.34
H
ATOM   7219  HA  CYS A 471      22.586  51.795 -11.389  1.00  5.29
H
ATOM   7220 1HB  CYS A 471      21.798  50.804 -13.559  1.00  7.94
H
ATOM   7221 2HB  CYS A 471      20.238  51.574 -13.319  1.00  7.94
H
ATOM   7222  HG  CYS A 471      21.517  52.813 -15.148  1.00  7.94
H
ATOM   7223  N   VAL A 472      21.374  53.943 -10.846  1.00  4.42
N
ATOM   7224  CA  VAL A 472      20.743  55.141 -10.288  1.00  4.63
C
ATOM   7225  C   VAL A 472      19.380  55.487 -10.916  1.00  4.72
C
ATOM   7226  O   VAL A 472      18.529  56.084 -10.256  1.00  5.60
O
ATOM   7227  CB  VAL A 472      21.696  56.348 -10.431  1.00  6.95
C
ATOM   7228  CG1 VAL A 472      21.867  56.736 -11.896  1.00  6.95
C
ATOM   7229  CG2 VAL A 472      21.144  57.520  -9.636  1.00  6.95
C
ATOM   7230  H   VAL A 472      22.326  54.007 -11.176  1.00  5.30
H
```

```
ATOM   7231  HA  VAL A 472      20.586  54.965  -9.222  1.00  5.56
H
ATOM   7232  HB  VAL A 472      22.678  56.070 -10.047  1.00  8.33
H
ATOM   7233 1HG1 VAL A 472      22.558  57.577 -11.967  1.00  8.33
H
ATOM   7234 2HG1 VAL A 472      22.269  55.890 -12.452  1.00  8.33
H
ATOM   7235 3HG1 VAL A 472      20.907  57.027 -12.317  1.00  8.33
H
ATOM   7236 1HG2 VAL A 472      21.822  58.369  -9.721  1.00  8.33
H
ATOM   7237 2HG2 VAL A 472      20.165  57.798 -10.028  1.00  8.33
H
ATOM   7238 3HG2 VAL A 472      21.048  57.236  -8.588  1.00  8.33
H
ATOM   7239  N   SER A 473      19.177  55.119 -12.184  1.00  5.30
N
ATOM   7240  CA  SER A 473      17.946  55.425 -12.894  1.00  5.12
C
ATOM   7241  C   SER A 473      16.969  54.250 -13.021  1.00  5.66
C
ATOM   7242  O   SER A 473      16.111  54.267 -13.900  1.00 22.75
O
ATOM   7243  CB  SER A 473      18.284  55.949 -14.276  1.00  7.68
C
ATOM   7244  OG  SER A 473      18.996  54.998 -15.017  1.00  7.68
O
ATOM   7245  H   SER A 473      19.908  54.626 -12.678  1.00  6.36
H
ATOM   7246  HA  SER A 473      17.438  56.220 -12.347  1.00  6.14
H
ATOM   7247 1HB  SER A 473      17.364  56.208 -14.801  1.00  9.22
H
ATOM   7248 2HB  SER A 473      18.875  56.859 -14.185  1.00  9.22
H
ATOM   7249  HG  SER A 473      18.459  54.201 -15.010  1.00  9.22
H
ATOM   7250  N   ASP A 474      17.081  53.239 -12.162  1.00  6.85
N
ATOM   7251  CA  ASP A 474      16.138  52.117 -12.211  1.00  4.42
C
ATOM   7252  C   ASP A 474      14.720  52.589 -11.913  1.00  5.16
C
ATOM   7253  O   ASP A 474      14.495  53.308 -10.942  1.00 26.00
O
ATOM   7254  CB  ASP A 474      16.528  51.053 -11.198  1.00  6.63
C
ATOM   7255  CG  ASP A 474      17.805  50.348 -11.559  1.00  6.63
C
ATOM   7256  OD1 ASP A 474      18.137  50.299 -12.718  1.00  6.63
O
ATOM   7257  OD2 ASP A 474      18.477  49.910 -10.653  1.00  6.63
O
ATOM   7258  H   ASP A 474      17.822  53.232 -11.473  1.00  8.22
H
ATOM   7259  HA  ASP A 474      16.157  51.688 -13.211  1.00  5.30
H
```

ATOM 7260 1HB ASP A 474 16.648 51.514 -10.217 1.00 7.96
H
ATOM 7261 2HB ASP A 474 15.732 50.314 -11.119 1.00 7.96
H
ATOM 7262 N LYS A 475 13.760 52.176 -12.728 1.00 7.24
N
ATOM 7263 CA LYS A 475 12.382 52.608 -12.525 1.00 4.62
C
ATOM 7264 C LYS A 475 11.419 51.519 -12.073 1.00 4.40
C
ATOM 7265 O LYS A 475 10.213 51.754 -12.015 1.00 16.54
O
ATOM 7266 CB LYS A 475 11.852 53.283 -13.778 1.00 6.93
C
ATOM 7267 CG LYS A 475 12.607 54.548 -14.149 1.00 6.93
C
ATOM 7268 CD LYS A 475 12.478 55.565 -13.022 1.00 6.93
C
ATOM 7269 CE LYS A 475 13.075 56.916 -13.378 1.00 6.93
C
ATOM 7270 NZ LYS A 475 13.039 57.849 -12.214 1.00 6.93
N
ATOM 7271 H LYS A 475 13.995 51.576 -13.508 1.00 8.69
H
ATOM 7272 HA LYS A 475 12.389 53.363 -11.738 1.00 5.54
H
ATOM 7273 1HB LYS A 475 11.916 52.593 -14.620 1.00 8.32
H
ATOM 7274 2HB LYS A 475 10.804 53.543 -13.638 1.00 8.32
H
ATOM 7275 1HG LYS A 475 13.659 54.317 -14.314 1.00 8.32
H
ATOM 7276 2HG LYS A 475 12.194 54.969 -15.064 1.00 8.32
H
ATOM 7277 1HD LYS A 475 11.423 55.704 -12.779 1.00 8.32
H
ATOM 7278 2HD LYS A 475 12.984 55.185 -12.134 1.00 8.32
H
ATOM 7279 1HE LYS A 475 14.106 56.792 -13.705 1.00 8.32
H
ATOM 7280 2HE LYS A 475 12.496 57.350 -14.194 1.00 8.32
H
ATOM 7281 1HZ LYS A 475 13.422 58.744 -12.480 1.00 8.32
H
ATOM 7282 2HZ LYS A 475 12.082 57.966 -11.900 1.00 8.32
H
ATOM 7283 3HZ LYS A 475 13.583 57.460 -11.459 1.00 8.32
H
ATOM 7284 N VAL A 476 11.935 50.342 -11.746 1.00 6.48
N
ATOM 7285 CA VAL A 476 11.097 49.272 -11.207 1.00 4.20
C
ATOM 7286 C VAL A 476 11.943 48.110 -10.725 1.00 3.67
C
ATOM 7287 O VAL A 476 12.902 47.710 -11.388 1.00 3.93
O
ATOM 7288 CB VAL A 476 10.073 48.771 -12.245 1.00 6.30
C

```
ATOM   7289  CG1 VAL A 476      10.783  48.193 -13.430  1.00  6.30
C
ATOM   7290  CG2 VAL A 476       9.174  47.719 -11.626  1.00  6.30
C
ATOM   7291  H   VAL A 476      12.927  50.189 -11.842  1.00  7.78
H
ATOM   7292  HA  VAL A 476      10.543  49.669 -10.356  1.00  5.04
H
ATOM   7293  HB  VAL A 476       9.466  49.607 -12.590  1.00  7.56
H
ATOM   7294 1HG1 VAL A 476      10.045  47.858 -14.152  1.00  7.56
H
ATOM   7295 2HG1 VAL A 476      11.412  48.961 -13.878  1.00  7.56
H
ATOM   7296 3HG1 VAL A 476      11.399  47.351 -13.117  1.00  7.56
H
ATOM   7297 1HG2 VAL A 476       8.452  47.394 -12.369  1.00  7.56
H
ATOM   7298 2HG2 VAL A 476       9.766  46.866 -11.300  1.00  7.56
H
ATOM   7299 3HG2 VAL A 476       8.650  48.140 -10.773  1.00  7.56
H
ATOM   7300  N   THR A 477      11.566  47.570  -9.570  1.00  3.45
N
ATOM   7301  CA  THR A 477      12.215  46.415  -8.988  1.00  3.21
C
ATOM   7302  C   THR A 477      11.574  45.156  -9.491  1.00  3.03
C
ATOM   7303  O   THR A 477      10.422  44.885  -9.181  1.00  3.31
O
ATOM   7304  CB  THR A 477      12.120  46.422  -7.448  1.00  4.81
C
ATOM   7305  OG1 THR A 477      12.805  47.559  -6.905  1.00  4.81
O
ATOM   7306  CG2 THR A 477      12.702  45.149  -6.880  1.00  4.81
C
ATOM   7307  H   THR A 477      10.776  47.966  -9.082  1.00  4.14
H
ATOM   7308  HA  THR A 477      13.254  46.398  -9.287  1.00  3.85
H
ATOM   7309  HB  THR A 477      11.070  46.486  -7.160  1.00  5.78
H
ATOM   7310  HG1 THR A 477      12.383  48.376  -7.193  1.00  5.78
H
ATOM   7311 1HG2 THR A 477      12.615  45.158  -5.795  1.00  5.78
H
ATOM   7312 2HG2 THR A 477      12.164  44.291  -7.282  1.00  5.78
H
ATOM   7313 3HG2 THR A 477      13.750  45.086  -7.163  1.00  5.78
H
ATOM   7314  N   VAL A 478      12.327  44.373 -10.239  1.00  2.90
N
ATOM   7315  CA  VAL A 478      11.822  43.120 -10.758  1.00  2.80
C
ATOM   7316  C   VAL A 478      12.428  41.957  -9.982  1.00  2.78
C
ATOM   7317  O   VAL A 478      13.638  41.732 -10.012  1.00  3.00
O
```

```
ATOM   7318  CB  VAL A 478      12.115  42.990 -12.264  1.00  4.20
C
ATOM   7319  CG1 VAL A 478      11.592  41.654 -12.782  1.00  4.20
C
ATOM   7320  CG2 VAL A 478      11.455  44.150 -13.004  1.00  4.20
C
ATOM   7321  H   VAL A 478      13.269  44.651 -10.451  1.00  3.48
H
ATOM   7322  HA  VAL A 478      10.746  43.111 -10.628  1.00  3.36
H
ATOM   7323  HB  VAL A 478      13.193  43.013 -12.427  1.00  5.04
H
ATOM   7324 1HG1 VAL A 478      11.809  41.565 -13.846  1.00  5.04
H
ATOM   7325 2HG1 VAL A 478      12.077  40.840 -12.243  1.00  5.04
H
ATOM   7326 3HG1 VAL A 478      10.515  41.601 -12.626  1.00  5.04
H
ATOM   7327 1HG2 VAL A 478      11.662  44.068 -14.070  1.00  5.04
H
ATOM   7328 2HG2 VAL A 478      10.379  44.121 -12.841  1.00  5.04
H
ATOM   7329 3HG2 VAL A 478      11.852  45.094 -12.629  1.00  5.04
H
ATOM   7330  N   ILE A 479      11.565  41.236  -9.285  1.00  2.55
N
ATOM   7331  CA  ILE A 479      11.955  40.108  -8.469  1.00  2.40
C
ATOM   7332  C   ILE A 479      11.721  38.809  -9.205  1.00  2.27
C
ATOM   7333  O   ILE A 479      10.612  38.534  -9.659  1.00  2.29
O
ATOM   7334  CB  ILE A 479      11.175  40.092  -7.142  1.00  3.60
C
ATOM   7335  CG1 ILE A 479      11.477  41.351  -6.321  1.00  3.60
C
ATOM   7336  CG2 ILE A 479      11.521  38.847  -6.363  1.00  3.60
C
ATOM   7337  CD1 ILE A 479      10.588  41.516  -5.109  1.00  3.60
C
ATOM   7338  H   ILE A 479      10.589  41.480  -9.338  1.00  3.06
H
ATOM   7339  HA  ILE A 479      13.017  40.189  -8.245  1.00  2.88
H
ATOM   7340  HB  ILE A 479      10.107  40.095  -7.352  1.00  4.32
H
ATOM   7341 1HG1 ILE A 479      12.508  41.317  -5.980  1.00  4.32
H
ATOM   7342 2HG1 ILE A 479      11.353  42.227  -6.958  1.00  4.32
H
ATOM   7343 1HG2 ILE A 479      10.954  38.837  -5.433  1.00  4.32
H
ATOM   7344 2HG2 ILE A 479      11.271  37.965  -6.951  1.00  4.32
H
ATOM   7345 3HG2 ILE A 479      12.587  38.846  -6.141  1.00  4.32
H
ATOM   7346 1HD1 ILE A 479      10.858  42.429  -4.580  1.00  4.32
H
```

```
ATOM   7347 2HD1 ILE A 479       9.547  41.576  -5.429  1.00  4.32
H
ATOM   7348 3HD1 ILE A 479      10.715  40.662  -4.447  1.00  4.32
H
ATOM   7349  N   GLY A 480      12.765  38.005  -9.327  1.00  2.16
N
ATOM   7350  CA  GLY A 480      12.625  36.724 -10.002  1.00  2.07
C
ATOM   7351  C   GLY A 480      13.340  35.627  -9.238  1.00  2.14
C
ATOM   7352  O   GLY A 480      13.981  35.881  -8.217  1.00  2.42
O
ATOM   7353  H   GLY A 480      13.659  38.286  -8.936  1.00  2.59
H
ATOM   7354 1HA  GLY A 480      11.569  36.474 -10.102  1.00  2.48
H
ATOM   7355 2HA  GLY A 480      13.033  36.796 -11.010  1.00  2.48
H
ATOM   7356  N   ALA A 481      13.235  34.405  -9.737  1.00  1.93
N
ATOM   7357  CA  ALA A 481      13.912  33.271  -9.120  1.00  1.90
C
ATOM   7358  C   ALA A 481      14.052  32.143 -10.145  1.00  1.71
C
ATOM   7359  O   ALA A 481      13.140  31.903 -10.944  1.00  2.15
O
ATOM   7360  CB  ALA A 481      13.164  32.830  -7.887  1.00  2.85
C
ATOM   7361  H   ALA A 481      12.683  34.259 -10.569  1.00  2.32
H
ATOM   7362  HA  ALA A 481      14.909  33.583  -8.813  1.00  2.28
H
ATOM   7363 1HB  ALA A 481      13.701  32.007  -7.425  1.00  3.42
H
ATOM   7364 2HB  ALA A 481      13.101  33.660  -7.181  1.00  3.42
H
ATOM   7365 3HB  ALA A 481      12.159  32.513  -8.161  1.00  3.42
H
ATOM   7366  N   GLY A 482      15.193  31.457 -10.114  1.00  1.83
N
ATOM   7367  CA  GLY A 482      15.463  30.386 -11.064  1.00  1.58
C
ATOM   7368  C   GLY A 482      15.435  30.930 -12.481  1.00  1.47
C
ATOM   7369  O   GLY A 482      16.024  31.976 -12.769  1.00  1.43
O
ATOM   7370  H   GLY A 482      15.894  31.693  -9.424  1.00  2.20
H
ATOM   7371 1HA  GLY A 482      16.437  29.943 -10.855  1.00  1.90
H
ATOM   7372 2HA  GLY A 482      14.721  29.601 -10.959  1.00  1.90
H
ATOM   7373  N   ILE A 483      14.706  30.235 -13.353  1.00  1.54
N
ATOM   7374  CA  ILE A 483      14.578  30.627 -14.753  1.00  1.62
C
ATOM   7375  C   ILE A 483      14.000  32.029 -14.932  1.00  1.82
C
```

```
ATOM   7376  O   ILE A 483      14.285  32.681 -15.935  1.00  2.07
O
ATOM   7377  CB  ILE A 483      13.715  29.617 -15.538  1.00  2.43
C
ATOM   7378  CG1 ILE A 483      13.884  29.863 -17.039  1.00  2.43
C
ATOM   7379  CG2 ILE A 483      12.246  29.721 -15.146  1.00  2.43
C
ATOM   7380  CD1 ILE A 483      15.290  29.621 -17.536  1.00  2.43
C
ATOM   7381  H   ILE A 483      14.239  29.395 -13.038  1.00  1.85
H
ATOM   7382  HA  ILE A 483      15.571  30.617 -15.195  1.00  1.94
H
ATOM   7383  HB  ILE A 483      14.069  28.608 -15.327  1.00  2.92
H
ATOM   7384 1HG1 ILE A 483      13.208  29.206 -17.583  1.00  2.92
H
ATOM   7385 2HG1 ILE A 483      13.613  30.895 -17.264  1.00  2.92
H
ATOM   7386 1HG2 ILE A 483      11.668  28.989 -15.708  1.00  2.92
H
ATOM   7387 2HG2 ILE A 483      12.139  29.526 -14.080  1.00  2.92
H
ATOM   7388 3HG2 ILE A 483      11.875  30.720 -15.372  1.00  2.92
H
ATOM   7389 1HD1 ILE A 483      15.335  29.813 -18.608  1.00  2.92
H
ATOM   7390 2HD1 ILE A 483      15.979  30.289 -17.019  1.00  2.92
H
ATOM   7391 3HD1 ILE A 483      15.571  28.588 -17.341  1.00  2.92
H
ATOM   7392  N   THR A 484      13.202  32.500 -13.966  1.00  1.85
N
ATOM   7393  CA  THR A 484      12.604  33.817 -14.089  1.00  2.10
C
ATOM   7394  C   THR A 484      13.585  34.928 -13.726  1.00  2.06
C
ATOM   7395  O   THR A 484      13.347  36.087 -14.068  1.00  2.16
O
ATOM   7396  CB  THR A 484      11.301  33.911 -13.280  1.00  3.15
C
ATOM   7397  OG1 THR A 484      11.534  33.573 -11.907  1.00  3.15
O
ATOM   7398  CG2 THR A 484      10.252  32.981 -13.872  1.00  3.15
C
ATOM   7399  H   THR A 484      13.016  31.955 -13.132  1.00  2.22
H
ATOM   7400  HA  THR A 484      12.329  33.962 -15.134  1.00  2.52
H
ATOM   7401  HB  THR A 484      10.927  34.931 -13.329  1.00  3.78
H
ATOM   7402  HG1 THR A 484      11.909  32.688 -11.853  1.00  3.78
H
ATOM   7403 1HG2 THR A 484       9.328  33.071 -13.307  1.00  3.78
H
ATOM   7404 2HG2 THR A 484      10.068  33.258 -14.911  1.00  3.78
H
```

```
ATOM   7405 3HG2 THR A 484      10.607  31.954 -13.828  1.00  3.78
H
ATOM   7406  N   VAL A 485      14.713  34.579 -13.089  1.00  1.84
N
ATOM   7407  CA  VAL A 485      15.779  35.558 -12.909  1.00  1.74
C
ATOM   7408  C   VAL A 485      16.364  35.853 -14.235  1.00  1.91
C
ATOM   7409  O   VAL A 485      16.581  37.009 -14.585  1.00  2.47
O
ATOM   7410  CB  VAL A 485      16.952  35.074 -12.049  1.00  2.61
C
ATOM   7411  CG1 VAL A 485      18.072  36.077 -12.158  1.00  2.61
C
ATOM   7412  CG2 VAL A 485      16.552  34.961 -10.615  1.00  2.61
C
ATOM   7413  H   VAL A 485      14.874  33.628 -12.790  1.00  2.21
H
ATOM   7414  HA  VAL A 485      15.364  36.473 -12.483  1.00  2.09
H
ATOM   7415  HB  VAL A 485      17.310  34.115 -12.420  1.00  3.13
H
ATOM   7416 1HG1 VAL A 485      18.916  35.740 -11.560  1.00  3.13
H
ATOM   7417 2HG1 VAL A 485      18.376  36.164 -13.199  1.00  3.13
H
ATOM   7418 3HG1 VAL A 485      17.729  37.045 -11.797  1.00  3.13
H
ATOM   7419 1HG2 VAL A 485      17.399  34.629 -10.016  1.00  3.13
H
ATOM   7420 2HG2 VAL A 485      16.206  35.928 -10.250  1.00  3.13
H
ATOM   7421 3HG2 VAL A 485      15.758  34.238 -10.555  1.00  3.13
H
ATOM   7422  N   TYR A 486      16.634  34.784 -14.969  1.00  1.80
N
ATOM   7423  CA  TYR A 486      17.261  34.912 -16.265  1.00  1.98
C
ATOM   7424  C   TYR A 486      16.368  35.608 -17.246  1.00  2.02
C
ATOM   7425  O   TYR A 486      16.849  36.394 -18.059  1.00  2.33
O
ATOM   7426  CB  TYR A 486      17.626  33.555 -16.780  1.00  2.97
C
ATOM   7427  CG  TYR A 486      18.672  32.952 -15.918  1.00  2.97
C
ATOM   7428  CD1 TYR A 486      18.266  32.080 -14.939  1.00  2.97
C
ATOM   7429  CD2 TYR A 486      20.008  33.266 -16.091  1.00  2.97
C
ATOM   7430  CE1 TYR A 486      19.189  31.500 -14.122  1.00  2.97
C
ATOM   7431  CE2 TYR A 486      20.943  32.676 -15.273  1.00  2.97
C
ATOM   7432  CZ  TYR A 486      20.529  31.795 -14.297  1.00  2.97
C
ATOM   7433  OH  TYR A 486      21.440  31.145 -13.519  1.00  2.97
O
```

```
ATOM   7434  H   TYR A 486      16.420  33.862 -14.588  1.00  2.16
H
ATOM   7435  HA  TYR A 486      18.166  35.508 -16.157  1.00  2.38
H
ATOM   7436 1HB  TYR A 486      16.754  32.900 -16.783  1.00  3.56
H
ATOM   7437 2HB  TYR A 486      18.008  33.623 -17.797  1.00  3.56
H
ATOM   7438  HD1 TYR A 486      17.212  31.850 -14.825  1.00  3.56
H
ATOM   7439  HD2 TYR A 486      20.317  33.959 -16.872  1.00  3.56
H
ATOM   7440  HE1 TYR A 486      18.872  30.796 -13.355  1.00  3.56
H
ATOM   7441  HE2 TYR A 486      22.002  32.895 -15.407  1.00  3.56
H
ATOM   7442  HH  TYR A 486      22.320  31.317 -13.859  1.00  3.56
H
ATOM   7443  N   GLU A 487      15.064  35.366 -17.146  1.00  1.88
N
ATOM   7444  CA  GLU A 487      14.148  36.058 -18.023  1.00  2.06
C
ATOM   7445  C   GLU A 487      14.118  37.539 -17.658  1.00  2.22
C
ATOM   7446  O   GLU A 487      14.065  38.384 -18.548  1.00  2.40
O
ATOM   7447  CB  GLU A 487      12.748  35.448 -17.942  1.00  3.09
C
ATOM   7448  CG  GLU A 487      12.636  34.037 -18.520  1.00  3.09
C
ATOM   7449  CD  GLU A 487      13.030  33.924 -19.976  1.00  3.09
C
ATOM   7450  OE1 GLU A 487      12.561  34.687 -20.789  1.00  3.09
O
ATOM   7451  OE2 GLU A 487      13.814  33.053 -20.274  1.00  3.09
O
ATOM   7452  H   GLU A 487      14.713  34.680 -16.487  1.00  2.26
H
ATOM   7453  HA  GLU A 487      14.508  35.966 -19.048  1.00  2.47
H
ATOM   7454 1HB  GLU A 487      12.438  35.400 -16.899  1.00  3.71
H
ATOM   7455 2HB  GLU A 487      12.040  36.089 -18.456  1.00  3.71
H
ATOM   7456 1HG  GLU A 487      13.274  33.373 -17.946  1.00  3.71
H
ATOM   7457 2HG  GLU A 487      11.610  33.693 -18.401  1.00  3.71
H
ATOM   7458  N   ALA A 488      14.202  37.857 -16.360  1.00  2.21
N
ATOM   7459  CA  ALA A 488      14.257  39.249 -15.918  1.00  2.43
C
ATOM   7460  C   ALA A 488      15.542  39.925 -16.397  1.00  2.66
C
ATOM   7461  O   ALA A 488      15.513  41.089 -16.795  1.00  2.99
O
ATOM   7462  CB  ALA A 488      14.154  39.336 -14.402  1.00  3.65
C
```

ATOM 7463 H ALA A 488 14.204 37.129 -15.655 1.00 2.65
H
ATOM 7464 HA ALA A 488 13.415 39.779 -16.359 1.00 2.92
H
ATOM 7465 1HB ALA A 488 14.169 40.382 -14.095 1.00 4.37
H
ATOM 7466 2HB ALA A 488 13.221 38.875 -14.076 1.00 4.37
H
ATOM 7467 3HB ALA A 488 14.992 38.814 -13.946 1.00 4.37
H
ATOM 7468 N LEU A 489 16.661 39.195 -16.362 1.00 2.58
N
ATOM 7469 CA LEU A 489 17.938 39.720 -16.828 1.00 2.93
C
ATOM 7470 C LEU A 489 17.891 39.999 -18.330 1.00 3.21
C
ATOM 7471 O LEU A 489 18.371 41.037 -18.792 1.00 3.70
O
ATOM 7472 CB LEU A 489 19.063 38.714 -16.520 1.00 4.40
C
ATOM 7473 CG LEU A 489 19.427 38.518 -15.035 1.00 4.40
C
ATOM 7474 CD1 LEU A 489 20.392 37.343 -14.898 1.00 4.40
C
ATOM 7475 CD2 LEU A 489 20.057 39.789 -14.498 1.00 4.40
C
ATOM 7476 H LEU A 489 16.628 38.256 -15.992 1.00 3.10
H
ATOM 7477 HA LEU A 489 18.142 40.654 -16.306 1.00 3.52
H
ATOM 7478 1HB LEU A 489 18.773 37.742 -16.915 1.00 5.27
H
ATOM 7479 2HB LEU A 489 19.965 39.036 -17.038 1.00 5.27
H
ATOM 7480 HG LEU A 489 18.532 38.294 -14.462 1.00 5.27
H
ATOM 7481 1HD1 LEU A 489 20.645 37.201 -13.848 1.00 5.27
H
ATOM 7482 2HD1 LEU A 489 19.923 36.439 -15.282 1.00 5.27
H
ATOM 7483 3HD1 LEU A 489 21.298 37.550 -15.466 1.00 5.27
H
ATOM 7484 1HD2 LEU A 489 20.312 39.651 -13.446 1.00 5.27
H
ATOM 7485 2HD2 LEU A 489 20.960 40.014 -15.065 1.00 5.27
H
ATOM 7486 3HD2 LEU A 489 19.351 40.613 -14.596 1.00 5.27
H
ATOM 7487 N ALA A 490 17.277 39.082 -19.080 1.00 2.99
N
ATOM 7488 CA ALA A 490 17.109 39.235 -20.518 1.00 3.20
C
ATOM 7489 C ALA A 490 16.205 40.418 -20.816 1.00 3.41
C
ATOM 7490 O ALA A 490 16.469 41.189 -21.745 1.00 3.74
O
ATOM 7491 CB ALA A 490 16.539 37.963 -21.119 1.00 4.80
C

ATOM 7492 H ALA A 490 16.928 38.238 -18.646 1.00 3.59
H
ATOM 7493 HA ALA A 490 18.086 39.433 -20.958 1.00 3.84
H
ATOM 7494 1HB ALA A 490 16.436 38.083 -22.197 1.00 5.76
H
ATOM 7495 2HB ALA A 490 17.211 37.130 -20.910 1.00 5.76
H
ATOM 7496 3HB ALA A 490 15.565 37.758 -20.681 1.00 5.76
H
ATOM 7497 N ALA A 491 15.149 40.561 -20.013 1.00 3.26
N
ATOM 7498 CA ALA A 491 14.222 41.664 -20.142 1.00 3.46
C
ATOM 7499 C ALA A 491 14.947 42.974 -19.943 1.00 3.81
C
ATOM 7500 O ALA A 491 14.753 43.898 -20.725 1.00 4.22
O
ATOM 7501 CB ALA A 491 13.088 41.529 -19.138 1.00 5.19
C
ATOM 7502 H ALA A 491 14.973 39.874 -19.294 1.00 3.91
H
ATOM 7503 HA ALA A 491 13.811 41.652 -21.150 1.00 4.15
H
ATOM 7504 1HB ALA A 491 12.392 42.358 -19.258 1.00 6.23
H
ATOM 7505 2HB ALA A 491 12.565 40.588 -19.307 1.00 6.23
H
ATOM 7506 3HB ALA A 491 13.490 41.542 -18.128 1.00 6.23
H
ATOM 7507 N ALA A 492 15.809 43.035 -18.919 1.00 3.76
N
ATOM 7508 CA ALA A 492 16.593 44.228 -18.619 1.00 4.16
C
ATOM 7509 C ALA A 492 17.426 44.654 -19.812 1.00 4.11
C
ATOM 7510 O ALA A 492 17.472 45.840 -20.143 1.00 4.21
O
ATOM 7511 CB ALA A 492 17.512 43.979 -17.428 1.00 6.24
C
ATOM 7512 H ALA A 492 15.904 42.235 -18.307 1.00 4.51
H
ATOM 7513 HA ALA A 492 15.903 45.035 -18.377 1.00 4.99
H
ATOM 7514 1HB ALA A 492 18.070 44.888 -17.205 1.00 7.49
H
ATOM 7515 2HB ALA A 492 16.926 43.692 -16.559 1.00 7.49
H
ATOM 7516 3HB ALA A 492 18.210 43.180 -17.667 1.00 7.49
H
ATOM 7517 N ASP A 493 18.078 43.686 -20.458 1.00 4.13
N
ATOM 7518 CA ASP A 493 18.892 43.977 -21.628 1.00 4.24
C
ATOM 7519 C ASP A 493 18.064 44.600 -22.751 1.00 4.25
C
ATOM 7520 O ASP A 493 18.458 45.615 -23.333 1.00 4.52
O

ATOM 7521 CB ASP A 493 19.581 42.705 -22.126 1.00 6.36
C
ATOM 7522 CG ASP A 493 20.701 42.238 -21.202 1.00 6.36
C
ATOM 7523 OD1 ASP A 493 21.124 43.005 -20.369 1.00 6.36
O
ATOM 7524 OD2 ASP A 493 21.132 41.119 -21.348 1.00 6.36
O
ATOM 7525 H ASP A 493 18.022 42.732 -20.114 1.00 4.96
H
ATOM 7526 HA ASP A 493 19.662 44.692 -21.339 1.00 5.09
H
ATOM 7527 1HB ASP A 493 18.849 41.903 -22.217 1.00 7.63
H
ATOM 7528 2HB ASP A 493 19.998 42.883 -23.117 1.00 7.63
H
ATOM 7529 N GLU A 494 16.896 44.025 -23.023 1.00 4.13
N
ATOM 7530 CA GLU A 494 16.022 44.562 -24.060 1.00 4.19
C
ATOM 7531 C GLU A 494 15.415 45.906 -23.676 1.00 4.34
C
ATOM 7532 O GLU A 494 15.218 46.769 -24.529 1.00 5.03
O
ATOM 7533 CB GLU A 494 14.925 43.565 -24.373 1.00 6.29
C
ATOM 7534 CG GLU A 494 15.407 42.326 -25.106 1.00 6.29
C
ATOM 7535 CD GLU A 494 15.963 42.665 -26.461 1.00 6.29
C
ATOM 7536 OE1 GLU A 494 15.299 43.355 -27.195 1.00 6.29
O
ATOM 7537 OE2 GLU A 494 17.058 42.251 -26.765 1.00 6.29
O
ATOM 7538 H GLU A 494 16.619 43.186 -22.520 1.00 4.96
H
ATOM 7539 HA GLU A 494 16.618 44.710 -24.961 1.00 5.03
H
ATOM 7540 1HB GLU A 494 14.480 43.243 -23.435 1.00 7.54
H
ATOM 7541 2HB GLU A 494 14.146 44.040 -24.968 1.00 7.54
H
ATOM 7542 1HG GLU A 494 16.183 41.840 -24.513 1.00 7.54
H
ATOM 7543 2HG GLU A 494 14.577 41.630 -25.216 1.00 7.54
H
ATOM 7544 N LEU A 495 15.135 46.090 -22.394 1.00 4.22
N
ATOM 7545 CA LEU A 495 14.616 47.349 -21.889 1.00 4.38
C
ATOM 7546 C LEU A 495 15.634 48.474 -22.012 1.00 4.48
C
ATOM 7547 O LEU A 495 15.265 49.599 -22.352 1.00 4.80
O
ATOM 7548 CB LEU A 495 14.172 47.162 -20.435 1.00 6.57
C
ATOM 7549 CG LEU A 495 12.895 46.334 -20.258 1.00 6.57
C

ATOM 7550 CD1 LEU A 495 12.707 45.985 -18.805 1.00 6.57
C
ATOM 7551 CD2 LEU A 495 11.711 47.150 -20.745 1.00 6.57
C
ATOM 7552 H LEU A 495 15.283 45.338 -21.738 1.00 5.06
H
ATOM 7553 HA LEU A 495 13.742 47.618 -22.481 1.00 5.26
H
ATOM 7554 1HB LEU A 495 14.968 46.656 -19.893 1.00 7.88
H
ATOM 7555 2HB LEU A 495 14.012 48.132 -19.975 1.00 7.88
H
ATOM 7556 HG LEU A 495 12.969 45.417 -20.833 1.00 7.88
H
ATOM 7557 1HD1 LEU A 495 11.796 45.399 -18.686 1.00 7.88
H
ATOM 7558 2HD1 LEU A 495 13.560 45.404 -18.456 1.00 7.88
H
ATOM 7559 3HD1 LEU A 495 12.627 46.903 -18.229 1.00 7.88
H
ATOM 7560 1HD2 LEU A 495 10.795 46.572 -20.620 1.00 7.88
H
ATOM 7561 2HD2 LEU A 495 11.637 48.066 -20.163 1.00 7.88
H
ATOM 7562 3HD2 LEU A 495 11.846 47.397 -21.797 1.00 7.88
H
ATOM 7563 N SER A 496 16.924 48.163 -21.811 1.00 4.51
N
ATOM 7564 CA SER A 496 17.979 49.172 -21.929 1.00 4.85
C
ATOM 7565 C SER A 496 18.076 49.722 -23.351 1.00 5.21
C
ATOM 7566 O SER A 496 18.502 50.858 -23.555 1.00 5.13
O
ATOM 7567 CB SER A 496 19.330 48.601 -21.534 1.00 7.27
C
ATOM 7568 OG SER A 496 19.822 47.712 -22.503 1.00 7.27
O
ATOM 7569 H SER A 496 17.176 47.225 -21.523 1.00 5.41
H
ATOM 7570 HA SER A 496 17.742 49.998 -21.258 1.00 5.82
H
ATOM 7571 1HB SER A 496 20.040 49.415 -21.392 1.00 8.73
H
ATOM 7572 2HB SER A 496 19.236 48.083 -20.581 1.00 8.73
H
ATOM 7573 HG SER A 496 19.169 47.011 -22.601 1.00 8.73
H
ATOM 7574 N LYS A 497 17.618 48.944 -24.335 1.00 6.34
N
ATOM 7575 CA LYS A 497 17.599 49.383 -25.727 1.00 6.67
C
ATOM 7576 C LYS A 497 16.601 50.532 -25.939 1.00 6.58
C
ATOM 7577 O LYS A 497 16.685 51.259 -26.931 1.00 6.95
O
ATOM 7578 CB LYS A 497 17.270 48.203 -26.639 1.00 10.00
C

ATOM 7579 CG LYS A 497 18.370 47.148 -26.689 1.00 10.00
C
ATOM 7580 CD LYS A 497 17.986 45.955 -27.554 1.00 10.00
C
ATOM 7581 CE LYS A 497 19.135 44.956 -27.639 1.00 10.00
C
ATOM 7582 NZ LYS A 497 18.774 43.738 -28.419 1.00 10.00
N
ATOM 7583 H LYS A 497 17.285 48.015 -24.120 1.00 7.61
H
ATOM 7584 HA LYS A 497 18.593 49.754 -25.985 1.00 8.00
H
ATOM 7585 1HB LYS A 497 16.347 47.727 -26.323 1.00 12.01
H
ATOM 7586 2HB LYS A 497 17.115 48.565 -27.655 1.00 12.01
H
ATOM 7587 1HG LYS A 497 19.281 47.595 -27.089 1.00 12.01
H
ATOM 7588 2HG LYS A 497 18.576 46.796 -25.679 1.00 12.01
H
ATOM 7589 1HD LYS A 497 17.112 45.458 -27.131 1.00 12.01
H
ATOM 7590 2HD LYS A 497 17.738 46.296 -28.559 1.00 12.01
H
ATOM 7591 1HE LYS A 497 19.982 45.441 -28.121 1.00 12.01
H
ATOM 7592 2HE LYS A 497 19.429 44.656 -26.632 1.00 12.01
H
ATOM 7593 1HZ LYS A 497 19.567 43.114 -28.454 1.00 12.01
H
ATOM 7594 2HZ LYS A 497 18.002 43.264 -27.963 1.00 12.01
H
ATOM 7595 3HZ LYS A 497 18.506 43.998 -29.356 1.00 12.01
H
ATOM 7596 N GLN A 498 15.662 50.692 -25.003 1.00 6.83
N
ATOM 7597 CA GLN A 498 14.678 51.757 -25.031 1.00 7.23
C
ATOM 7598 C GLN A 498 14.945 52.770 -23.917 1.00 6.54
C
ATOM 7599 O GLN A 498 14.053 53.538 -23.553 1.00 7.22
O
ATOM 7600 CB GLN A 498 13.274 51.179 -24.876 1.00 10.85
C
ATOM 7601 CG GLN A 498 12.886 50.210 -25.975 1.00 10.85
C
ATOM 7602 CD GLN A 498 12.849 50.868 -27.340 1.00 10.85
C
ATOM 7603 OE1 GLN A 498 12.252 51.935 -27.511 1.00 10.85
O
ATOM 7604 NE2 GLN A 498 13.486 50.238 -28.319 1.00 10.85
N
ATOM 7605 H GLN A 498 15.624 50.067 -24.214 1.00 8.20
H
ATOM 7606 HA GLN A 498 14.748 52.276 -25.987 1.00 8.68
H
ATOM 7607 1HB GLN A 498 13.199 50.658 -23.922 1.00 13.01
H

```
ATOM   7608 2HB  GLN A 498      12.545  51.990 -24.866  1.00 13.01
H
ATOM   7609 1HG  GLN A 498      13.614  49.400 -26.005  1.00 13.01
H
ATOM   7610 2HG  GLN A 498      11.893  49.812 -25.760  1.00 13.01
H
ATOM   7611 1HE2 GLN A 498      13.496  50.626 -29.241  1.00 13.01
H
ATOM   7612 2HE2 GLN A 498      13.957  49.375 -28.135  1.00 13.01
H
ATOM   7613  N   ASP A 499      16.166  52.744 -23.361  1.00  5.78
N
ATOM   7614  CA  ASP A 499      16.574  53.614 -22.257  1.00  5.87
C
ATOM   7615  C   ASP A 499      15.775  53.351 -20.982  1.00  5.48
C
ATOM   7616  O   ASP A 499      15.549  54.265 -20.187  1.00  6.08
O
ATOM   7617  CB  ASP A 499      16.455  55.091 -22.650  1.00  8.80
C
ATOM   7618  CG  ASP A 499      17.391  55.476 -23.787  1.00  8.80
C
ATOM   7619  OD1 ASP A 499      18.499  54.995 -23.809  1.00  8.80
O
ATOM   7620  OD2 ASP A 499      16.990  56.249 -24.622  1.00  8.80
O
ATOM   7621  H   ASP A 499      16.863  52.098 -23.701  1.00  6.94
H
ATOM   7622  HA  ASP A 499      17.623  53.409 -22.039  1.00  7.04
H
ATOM   7623 1HB  ASP A 499      15.434  55.329 -22.942  1.00 10.57
H
ATOM   7624 2HB  ASP A 499      16.694  55.710 -21.785  1.00 10.57
H
ATOM   7625  N   ILE A 500      15.359  52.101 -20.784  1.00  4.98
N
ATOM   7626  CA  ILE A 500      14.618  51.712 -19.594  1.00  4.78
C
ATOM   7627  C   ILE A 500      15.512  50.890 -18.690  1.00  4.81
C
ATOM   7628  O   ILE A 500      16.114  49.908 -19.122  1.00  4.90
O
ATOM   7629  CB  ILE A 500      13.360  50.899 -19.948  1.00  7.17
C
ATOM   7630  CG1 ILE A 500      12.411  51.728 -20.808  1.00  7.17
C
ATOM   7631  CG2 ILE A 500      12.676  50.427 -18.672  1.00  7.17
C
ATOM   7632  CD1 ILE A 500      11.284  50.916 -21.403  1.00  7.17
C
ATOM   7633  H   ILE A 500      15.557  51.383 -21.468  1.00  5.98
H
ATOM   7634  HA  ILE A 500      14.314  52.609 -19.058  1.00  5.74
H
ATOM   7635  HB  ILE A 500      13.644  50.039 -20.543  1.00  8.60
H
ATOM   7636 1HG1 ILE A 500      11.981  52.524 -20.200  1.00  8.60
H
```

ATOM 7637 2HG1 ILE A 500 12.972 52.186 -21.622 1.00 8.60
H
ATOM 7638 1HG2 ILE A 500 11.794 49.843 -18.927 1.00 8.60
H
ATOM 7639 2HG2 ILE A 500 13.363 49.811 -18.095 1.00 8.60
H
ATOM 7640 3HG2 ILE A 500 12.379 51.291 -18.079 1.00 8.60
H
ATOM 7641 1HD1 ILE A 500 10.646 51.566 -22.003 1.00 8.60
H
ATOM 7642 2HD1 ILE A 500 11.697 50.129 -22.035 1.00 8.60
H
ATOM 7643 3HD1 ILE A 500 10.695 50.470 -20.603 1.00 8.60
H
ATOM 7644 N PHE A 501 15.615 51.291 -17.437 1.00 5.31
N
ATOM 7645 CA PHE A 501 16.475 50.570 -16.522 1.00 5.57
C
ATOM 7646 C PHE A 501 15.660 49.913 -15.433 1.00 4.44
C
ATOM 7647 O PHE A 501 14.709 50.505 -14.921 1.00 8.24
O
ATOM 7648 CB PHE A 501 17.499 51.534 -15.941 1.00 8.36
C
ATOM 7649 CG PHE A 501 18.388 52.089 -17.013 1.00 8.36
C
ATOM 7650 CD1 PHE A 501 18.040 53.258 -17.675 1.00 8.36
C
ATOM 7651 CD2 PHE A 501 19.552 51.437 -17.382 1.00 8.36
C
ATOM 7652 CE1 PHE A 501 18.839 53.765 -18.681 1.00 8.36
C
ATOM 7653 CE2 PHE A 501 20.353 51.942 -18.389 1.00 8.36
C
ATOM 7654 CZ PHE A 501 19.995 53.107 -19.039 1.00 8.36
C
ATOM 7655 H PHE A 501 15.101 52.101 -17.120 1.00 6.37
H
ATOM 7656 HA PHE A 501 17.002 49.791 -17.074 1.00 6.68
H
ATOM 7657 1HB PHE A 501 16.994 52.361 -15.445 1.00 10.03
H
ATOM 7658 2HB PHE A 501 18.119 51.025 -15.203 1.00 10.03
H
ATOM 7659 HD1 PHE A 501 17.122 53.776 -17.396 1.00 10.03
H
ATOM 7660 HD2 PHE A 501 19.832 50.515 -16.872 1.00 10.03
H
ATOM 7661 HE1 PHE A 501 18.552 54.684 -19.194 1.00 10.03
H
ATOM 7662 HE2 PHE A 501 21.267 51.420 -18.672 1.00 10.03
H
ATOM 7663 HZ PHE A 501 20.625 53.504 -19.833 1.00 10.03
H
ATOM 7664 N ILE A 502 16.012 48.673 -15.100 1.00 4.77
N
ATOM 7665 CA ILE A 502 15.295 47.983 -14.044 1.00 3.98
C

```
ATOM   7666  C   ILE A 502      16.263  47.386 -13.039  1.00  4.21
C
ATOM   7667  O   ILE A 502      17.431  47.137 -13.339  1.00  5.30
O
ATOM   7668  CB  ILE A 502      14.381  46.854 -14.578  1.00  5.97
C
ATOM   7669  CG1 ILE A 502      15.178  45.722 -15.220  1.00  5.97
C
ATOM   7670  CG2 ILE A 502      13.441  47.424 -15.621  1.00  5.97
C
ATOM   7671  CD1 ILE A 502      14.319  44.512 -15.542  1.00  5.97
C
ATOM   7672  H   ILE A 502      16.788  48.223 -15.564  1.00  5.72
H
ATOM   7673  HA  ILE A 502      14.670  48.703 -13.519  1.00  4.78
H
ATOM   7674  HB  ILE A 502      13.800  46.440 -13.756  1.00  7.16
H
ATOM   7675 1HG1 ILE A 502      15.632  46.081 -16.142  1.00  7.16
H
ATOM   7676 2HG1 ILE A 502      15.974  45.411 -14.543  1.00  7.16
H
ATOM   7677 1HG2 ILE A 502      12.780  46.636 -15.976  1.00  7.16
H
ATOM   7678 2HG2 ILE A 502      12.855  48.226 -15.189  1.00  7.16
H
ATOM   7679 3HG2 ILE A 502      14.018  47.816 -16.457  1.00  7.16
H
ATOM   7680 1HD1 ILE A 502      14.925  43.732 -15.997  1.00  7.16
H
ATOM   7681 2HD1 ILE A 502      13.877  44.132 -14.622  1.00  7.16
H
ATOM   7682 3HD1 ILE A 502      13.527  44.800 -16.230  1.00  7.16
H
ATOM   7683  N   ARG A 503      15.743  47.143 -11.854  1.00  4.06
N
ATOM   7684  CA  ARG A 503      16.478  46.556 -10.756  1.00  4.32
C
ATOM   7685  C   ARG A 503      16.173  45.067 -10.710  1.00  4.74
C
ATOM   7686  O   ARG A 503      15.015  44.684 -10.624  1.00  7.59
O
ATOM   7687  CB  ARG A 503      16.062  47.251  -9.472  1.00  6.48
C
ATOM   7688  CG  ARG A 503      16.654  46.776  -8.166  1.00  6.48
C
ATOM   7689  CD  ARG A 503      18.057  47.168  -8.037  1.00  6.48
C
ATOM   7690  NE  ARG A 503      18.204  48.592  -8.244  1.00  6.48
N
ATOM   7691  CZ  ARG A 503      18.110  49.550  -7.310  1.00  6.48
C
ATOM   7692  NH1 ARG A 503      17.894  49.256  -6.046  1.00  6.48
N
ATOM   7693  NH2 ARG A 503      18.243  50.796  -7.713  1.00  6.48
N
ATOM   7694  H   ARG A 503      14.772  47.374 -11.699  1.00  4.87
H
```

```
ATOM   7695  HA  ARG A 503      17.542  46.705 -10.922  1.00  5.18
H
ATOM   7696 1HB  ARG A 503      16.332  48.303  -9.556  1.00  7.78
H
ATOM   7697 2HB  ARG A 503      14.986  47.220  -9.372  1.00  7.78
H
ATOM   7698 1HG  ARG A 503      16.113  47.256  -7.363  1.00  7.78
H
ATOM   7699 2HG  ARG A 503      16.572  45.694  -8.082  1.00  7.78
H
ATOM   7700 1HD  ARG A 503      18.411  46.930  -7.034  1.00  7.78
H
ATOM   7701 2HD  ARG A 503      18.659  46.649  -8.767  1.00  7.78
H
ATOM   7702  HE  ARG A 503      18.354  48.910  -9.194  1.00  7.78
H
ATOM   7703 1HH1 ARG A 503      17.791  48.291  -5.765  1.00  7.78
H
ATOM   7704 2HH1 ARG A 503      17.827  49.995  -5.362  1.00  7.78
H
ATOM   7705 1HH2 ARG A 503      18.398  50.957  -8.703  1.00  7.78
H
ATOM   7706 2HH2 ARG A 503      18.180  51.560  -7.057  1.00  7.78
H
ATOM   7707  N   VAL A 504      17.187  44.216 -10.798  1.00  3.36
N
ATOM   7708  CA  VAL A 504      16.915  42.778 -10.781  1.00  3.12
C
ATOM   7709  C   VAL A 504      17.292  42.179  -9.440  1.00  2.88
C
ATOM   7710  O   VAL A 504      18.452  42.250  -9.031  1.00  2.82
O
ATOM   7711  CB  VAL A 504      17.654  42.043 -11.922  1.00  4.68
C
ATOM   7712  CG1 VAL A 504      17.373  40.547 -11.839  1.00  4.68
C
ATOM   7713  CG2 VAL A 504      17.184  42.588 -13.267  1.00  4.68
C
ATOM   7714  H   VAL A 504      18.133  44.566 -10.857  1.00  4.03
H
ATOM   7715  HA  VAL A 504      15.846  42.628 -10.932  1.00  3.74
H
ATOM   7716  HB  VAL A 504      18.727  42.189 -11.812  1.00  5.62
H
ATOM   7717 1HG1 VAL A 504      17.900  40.030 -12.640  1.00  5.62
H
ATOM   7718 2HG1 VAL A 504      17.713  40.165 -10.878  1.00  5.62
H
ATOM   7719 3HG1 VAL A 504      16.302  40.373 -11.940  1.00  5.62
H
ATOM   7720 1HG2 VAL A 504      17.705  42.072 -14.072  1.00  5.62
H
ATOM   7721 2HG2 VAL A 504      16.111  42.427 -13.368  1.00  5.62
H
ATOM   7722 3HG2 VAL A 504      17.397  43.655 -13.322  1.00  5.62
H
ATOM   7723  N   ILE A 505      16.287  41.618  -8.759  1.00  2.80
N
```

ATOM 7724 CA ILE A 505 16.446 41.029 -7.435 1.00 2.62
C
ATOM 7725 C ILE A 505 16.331 39.513 -7.402 1.00 2.39
C
ATOM 7726 O ILE A 505 15.388 38.923 -7.936 1.00 2.56
O
ATOM 7727 CB ILE A 505 15.406 41.610 -6.463 1.00 3.93
C
ATOM 7728 CG1 ILE A 505 15.539 43.127 -6.414 1.00 3.93
C
ATOM 7729 CG2 ILE A 505 15.557 41.002 -5.071 1.00 3.93
C
ATOM 7730 CD1 ILE A 505 16.890 43.604 -5.952 1.00 3.93
C
ATOM 7731 H ILE A 505 15.369 41.605 -9.182 1.00 3.36
H
ATOM 7732 HA ILE A 505 17.436 41.295 -7.067 1.00 3.14
H
ATOM 7733 HB ILE A 505 14.412 41.391 -6.843 1.00 4.72
H
ATOM 7734 1HG1 ILE A 505 15.350 43.534 -7.406 1.00 4.72
H
ATOM 7735 2HG1 ILE A 505 14.790 43.525 -5.730 1.00 4.72
H
ATOM 7736 1HG2 ILE A 505 14.801 41.422 -4.409 1.00 4.72
H
ATOM 7737 2HG2 ILE A 505 15.426 39.923 -5.127 1.00 4.72
H
ATOM 7738 3HG2 ILE A 505 16.549 41.228 -4.681 1.00 4.72
H
ATOM 7739 1HD1 ILE A 505 16.905 44.694 -5.938 1.00 4.72
H
ATOM 7740 2HD1 ILE A 505 17.085 43.226 -4.949 1.00 4.72
H
ATOM 7741 3HD1 ILE A 505 17.655 43.239 -6.633 1.00 4.72
H
ATOM 7742 N ASP A 506 17.305 38.897 -6.745 1.00 2.11
N
ATOM 7743 CA ASP A 506 17.338 37.472 -6.499 1.00 2.09
C
ATOM 7744 C ASP A 506 17.077 37.214 -5.035 1.00 1.80
C
ATOM 7745 O ASP A 506 17.922 37.485 -4.190 1.00 1.53
O
ATOM 7746 CB ASP A 506 18.666 36.874 -6.871 1.00 3.13
C
ATOM 7747 CG ASP A 506 18.665 35.379 -6.661 1.00 3.13
C
ATOM 7748 OD1 ASP A 506 17.734 34.862 -6.087 1.00 3.13
O
ATOM 7749 OD2 ASP A 506 19.621 34.760 -7.060 1.00 3.13
O
ATOM 7750 H ASP A 506 18.054 39.453 -6.361 1.00 2.53
H
ATOM 7751 HA ASP A 506 16.553 36.991 -7.085 1.00 2.51
H
ATOM 7752 1HB ASP A 506 18.879 37.083 -7.915 1.00 3.76
H

ATOM 7753 2HB ASP A 506 19.457 37.324 -6.269 1.00 3.76
H
ATOM 7754 N LEU A 507 15.911 36.700 -4.709 1.00 1.95
N
ATOM 7755 CA LEU A 507 15.574 36.505 -3.310 1.00 1.80
C
ATOM 7756 C LEU A 507 16.403 35.453 -2.573 1.00 1.53
C
ATOM 7757 O LEU A 507 16.467 35.491 -1.344 1.00 1.47
O
ATOM 7758 CB LEU A 507 14.114 36.113 -3.200 1.00 2.70
C
ATOM 7759 CG LEU A 507 13.129 37.186 -3.629 1.00 2.70
C
ATOM 7760 CD1 LEU A 507 11.735 36.601 -3.561 1.00 2.70
C
ATOM 7761 CD2 LEU A 507 13.289 38.415 -2.746 1.00 2.70
C
ATOM 7762 H LEU A 507 15.244 36.463 -5.431 1.00 2.34
H
ATOM 7763 HA LEU A 507 15.712 37.455 -2.804 1.00 2.16
H
ATOM 7764 1HB LEU A 507 13.940 35.234 -3.818 1.00 3.24
H
ATOM 7765 2HB LEU A 507 13.898 35.856 -2.164 1.00 3.24
H
ATOM 7766 HG LEU A 507 13.326 37.466 -4.662 1.00 3.24
H
ATOM 7767 1HD1 LEU A 507 11.007 37.346 -3.874 1.00 3.24
H
ATOM 7768 2HD1 LEU A 507 11.678 35.736 -4.221 1.00 3.24
H
ATOM 7769 3HD1 LEU A 507 11.520 36.289 -2.545 1.00 3.24
H
ATOM 7770 1HD2 LEU A 507 12.582 39.183 -3.060 1.00 3.24
H
ATOM 7771 2HD2 LEU A 507 13.099 38.147 -1.709 1.00 3.24
H
ATOM 7772 3HD2 LEU A 507 14.305 38.799 -2.838 1.00 3.24
H
ATOM 7773 N PHE A 508 17.047 34.537 -3.308 1.00 1.60
N
ATOM 7774 CA PHE A 508 17.748 33.360 -2.763 1.00 1.46
C
ATOM 7775 C PHE A 508 16.830 32.471 -1.899 1.00 1.09
C
ATOM 7776 O PHE A 508 16.537 31.346 -2.301 1.00 1.02
O
ATOM 7777 CB PHE A 508 19.028 33.759 -2.024 1.00 2.19
C
ATOM 7778 CG PHE A 508 19.793 32.577 -1.494 1.00 2.19
C
ATOM 7779 CD1 PHE A 508 20.270 31.603 -2.362 1.00 2.19
C
ATOM 7780 CD2 PHE A 508 20.058 32.446 -0.141 1.00 2.19
C
ATOM 7781 CE1 PHE A 508 20.980 30.517 -1.886 1.00 2.19
C

```
ATOM   7782  CE2 PHE A 508      20.768  31.364   0.337  1.00  2.19
C
ATOM   7783  CZ  PHE A 508      21.230  30.397  -0.537  1.00  2.19
C
ATOM   7784  H   PHE A 508      17.026  34.624  -4.321  1.00  1.92
H
ATOM   7785  HA  PHE A 508      18.069  32.753  -3.610  1.00  1.75
H
ATOM   7786 1HB  PHE A 508      19.680  34.315  -2.698  1.00  2.63
H
ATOM   7787 2HB  PHE A 508      18.784  34.412  -1.187  1.00  2.63
H
ATOM   7788  HD1 PHE A 508      20.073  31.700  -3.430  1.00  2.63
H
ATOM   7789  HD2 PHE A 508      19.696  33.210   0.548  1.00  2.63
H
ATOM   7790  HE1 PHE A 508      21.343  29.760  -2.578  1.00  2.63
H
ATOM   7791  HE2 PHE A 508      20.963  31.275   1.403  1.00  2.63
H
ATOM   7792  HZ  PHE A 508      21.789  29.542  -0.164  1.00  2.63
H
ATOM   7793  N   THR A 509      16.348  32.959  -0.755  1.00  1.06
N
ATOM   7794  CA  THR A 509      15.353  32.195  -0.021  1.00  1.03
C
ATOM   7795  C   THR A 509      14.006  32.890  -0.165  1.00  1.06
C
ATOM   7796  O   THR A 509      13.917  34.115  -0.248  1.00  1.15
O
ATOM   7797  CB  THR A 509      15.695  32.013   1.487  1.00  1.54
C
ATOM   7798  OG1 THR A 509      15.797  33.290   2.141  1.00  1.54
O
ATOM   7799  CG2 THR A 509      16.991  31.247   1.654  1.00  1.54
C
ATOM   7800  H   THR A 509      16.636  33.872  -0.435  1.00  1.27
H
ATOM   7801  HA  THR A 509      15.269  31.206  -0.469  1.00  1.24
H
ATOM   7802  HB  THR A 509      14.897  31.446   1.965  1.00  1.85
H
ATOM   7803  HG1 THR A 509      14.918  33.602   2.399  1.00  1.85
H
ATOM   7804 1HG2 THR A 509      17.200  31.111   2.714  1.00  1.85
H
ATOM   7805 2HG2 THR A 509      16.897  30.273   1.177  1.00  1.85
H
ATOM   7806 3HG2 THR A 509      17.801  31.801   1.193  1.00  1.85
H
ATOM   7807  N   ILE A 510      12.961  32.091  -0.181  1.00  1.02
N
ATOM   7808  CA  ILE A 510      11.597  32.570  -0.288  1.00  1.07
C
ATOM   7809  C   ILE A 510      11.062  32.681   1.108  1.00  1.07
C
ATOM   7810  O   ILE A 510      10.299  33.591   1.435  1.00  1.20
O
```

```
ATOM   7811  CB  ILE A 510      10.737  31.628  -1.132  1.00  1.60
C
ATOM   7812  CG1 ILE A 510      11.271  31.611  -2.561  1.00  1.60
C
ATOM   7813  CG2 ILE A 510       9.284  32.077  -1.105  1.00  1.60
C
ATOM   7814  CD1 ILE A 510      10.667  30.531  -3.411  1.00  1.60
C
ATOM   7815  H   ILE A 510      13.128  31.101  -0.097  1.00  1.22
H
ATOM   7816  HA  ILE A 510      11.596  33.557  -0.746  1.00  1.28
H
ATOM   7817  HB  ILE A 510      10.809  30.619  -0.741  1.00  1.93
H
ATOM   7818 1HG1 ILE A 510      11.067  32.575  -3.026  1.00  1.93
H
ATOM   7819 2HG1 ILE A 510      12.351  31.465  -2.538  1.00  1.93
H
ATOM   7820 1HG2 ILE A 510       8.684  31.401  -1.709  1.00  1.93
H
ATOM   7821 2HG2 ILE A 510       8.920  32.069  -0.079  1.00  1.93
H
ATOM   7822 3HG2 ILE A 510       9.210  33.088  -1.509  1.00  1.93
H
ATOM   7823 1HD1 ILE A 510      11.089  30.582  -4.409  1.00  1.93
H
ATOM   7824 2HD1 ILE A 510      10.887  29.559  -2.975  1.00  1.93
H
ATOM   7825 3HD1 ILE A 510       9.589  30.670  -3.467  1.00  1.93
H
ATOM   7826  N   LYS A 511      11.466  31.724   1.929  1.00  1.02
N
ATOM   7827  CA  LYS A 511      11.093  31.709   3.322  1.00  1.03
C
ATOM   7828  C   LYS A 511      12.336  31.405   4.152  1.00  0.90
C
ATOM   7829  O   LYS A 511      12.883  30.311   4.071  1.00  0.74
O
ATOM   7830  CB  LYS A 511      10.009  30.669   3.583  1.00  1.54
C
ATOM   7831  CG  LYS A 511       9.500  30.661   5.011  1.00  1.54
C
ATOM   7832  CD  LYS A 511       8.419  29.614   5.218  1.00  1.54
C
ATOM   7833  CE  LYS A 511       7.909  29.654   6.651  1.00  1.54
C
ATOM   7834  NZ  LYS A 511       6.961  28.555   6.940  1.00  1.54
N
ATOM   7835  H   LYS A 511      12.085  30.997   1.578  1.00  1.22
H
ATOM   7836  HA  LYS A 511      10.709  32.689   3.599  1.00  1.24
H
ATOM   7837 1HB  LYS A 511       9.162  30.849   2.921  1.00  1.85
H
ATOM   7838 2HB  LYS A 511      10.397  29.675   3.360  1.00  1.85
H
ATOM   7839 1HG  LYS A 511      10.328  30.451   5.690  1.00  1.85
H
```

```
ATOM   7840 2HG  LYS A 511       9.095  31.642   5.257  1.00  1.85
H
ATOM   7841 1HD  LYS A 511       7.587  29.811   4.542  1.00  1.85
H
ATOM   7842 2HD  LYS A 511       8.815  28.622   5.006  1.00  1.85
H
ATOM   7843 1HE  LYS A 511       8.751  29.588   7.338  1.00  1.85
H
ATOM   7844 2HE  LYS A 511       7.399  30.605   6.811  1.00  1.85
H
ATOM   7845 1HZ  LYS A 511       6.639  28.627   7.895  1.00  1.85
H
ATOM   7846 2HZ  LYS A 511       6.167  28.593   6.315  1.00  1.85
H
ATOM   7847 3HZ  LYS A 511       7.426  27.666   6.815  1.00  1.85
H
ATOM   7848  N   PRO A 512      12.806  32.359   4.946  1.00  1.02
N
ATOM   7849  CA  PRO A 512      12.352  33.722   5.176  1.00  1.22
C
ATOM   7850  C   PRO A 512      12.620  34.624   3.978  1.00  1.33
C
ATOM   7851  O   PRO A 512      13.535  34.391   3.179  1.00  1.24
O
ATOM   7852  CB  PRO A 512      13.161  34.158   6.405  1.00  1.83
C
ATOM   7853  CG  PRO A 512      14.388  33.316   6.368  1.00  1.83
C
ATOM   7854  CD  PRO A 512      13.920  31.997   5.828  1.00  1.83
C
ATOM   7855  HA  PRO A 512      11.278  33.708   5.413  1.00  1.46
H
ATOM   7856 1HB  PRO A 512      13.383  35.234   6.347  1.00  2.20
H
ATOM   7857 2HB  PRO A 512      12.568  34.002   7.318  1.00  2.20
H
ATOM   7858 1HG  PRO A 512      15.149  33.782   5.733  1.00  2.20
H
ATOM   7859 2HG  PRO A 512      14.821  33.233   7.376  1.00  2.20
H
ATOM   7860 1HD  PRO A 512      14.710  31.511   5.239  1.00  2.20
H
ATOM   7861 2HD  PRO A 512      13.561  31.347   6.640  1.00  2.20
H
ATOM   7862  N   LEU A 513      11.799  35.659   3.869  1.00  1.57
N
ATOM   7863  CA  LEU A 513      11.871  36.601   2.770  1.00  1.73
C
ATOM   7864  C   LEU A 513      12.562  37.893   3.182  1.00  1.78
C
ATOM   7865  O   LEU A 513      12.147  38.545   4.143  1.00  2.21
O
ATOM   7866  CB  LEU A 513      10.456  36.920   2.289  1.00  2.59
C
ATOM   7867  CG  LEU A 513      10.369  37.885   1.123  1.00  2.59
C
ATOM   7868  CD1 LEU A 513      10.979  37.212  -0.082  1.00  2.59
C
```

ATOM 7869 CD2 LEU A 513 8.923 38.250 0.869 1.00 2.59
C
ATOM 7870 H LEU A 513 11.084 35.789 4.571 1.00 1.88
H
ATOM 7871 HA LEU A 513 12.437 36.145 1.958 1.00 2.08
H
ATOM 7872 1HB LEU A 513 9.970 35.993 1.992 1.00 3.11
H
ATOM 7873 2HB LEU A 513 9.895 37.349 3.118 1.00 3.11
H
ATOM 7874 HG LEU A 513 10.940 38.786 1.342 1.00 3.11
H
ATOM 7875 1HD1 LEU A 513 10.934 37.883 -0.937 1.00 3.11
H
ATOM 7876 2HD1 LEU A 513 12.020 36.960 0.129 1.00 3.11
H
ATOM 7877 3HD1 LEU A 513 10.426 36.301 -0.308 1.00 3.11
H
ATOM 7878 1HD2 LEU A 513 8.868 38.936 0.024 1.00 3.11
H
ATOM 7879 2HD2 LEU A 513 8.355 37.348 0.643 1.00 3.11
H
ATOM 7880 3HD2 LEU A 513 8.512 38.727 1.756 1.00 3.11
H
ATOM 7881 N ASP A 514 13.609 38.271 2.455 1.00 1.68
N
ATOM 7882 CA ASP A 514 14.341 39.503 2.742 1.00 1.80
C
ATOM 7883 C ASP A 514 13.591 40.783 2.399 1.00 2.05
C
ATOM 7884 O ASP A 514 13.934 41.463 1.428 1.00 2.21
O
ATOM 7885 CB ASP A 514 15.650 39.517 1.994 1.00 2.70
C
ATOM 7886 CG ASP A 514 16.477 40.736 2.337 1.00 2.70
C
ATOM 7887 OD1 ASP A 514 15.974 41.599 3.020 1.00 2.70
O
ATOM 7888 OD2 ASP A 514 17.604 40.804 1.906 1.00 2.70
O
ATOM 7889 H ASP A 514 13.907 37.692 1.682 1.00 2.02
H
ATOM 7890 HA ASP A 514 14.557 39.520 3.811 1.00 2.16
H
ATOM 7891 1HB ASP A 514 16.219 38.617 2.211 1.00 3.24
H
ATOM 7892 2HB ASP A 514 15.444 39.527 0.927 1.00 3.24
H
ATOM 7893 N VAL A 515 12.616 41.133 3.231 1.00 2.25
N
ATOM 7894 CA VAL A 515 11.821 42.337 3.044 1.00 2.66
C
ATOM 7895 C VAL A 515 12.628 43.630 3.155 1.00 2.52
C
ATOM 7896 O VAL A 515 12.236 44.646 2.585 1.00 2.49
O
ATOM 7897 CB VAL A 515 10.654 42.366 4.050 1.00 3.99
C

```
ATOM   7898  CG1 VAL A 515      11.161  42.637   5.458  1.00   3.99
C
ATOM   7899  CG2 VAL A 515       9.660  43.437   3.628  1.00   3.99
C
ATOM   7900  H   VAL A 515      12.409  40.511   4.002  1.00   2.70
H
ATOM   7901  HA  VAL A 515      11.390  42.296   2.046  1.00   3.19
H
ATOM   7902  HB  VAL A 515      10.166  41.391   4.057  1.00   4.79
H
ATOM   7903 1HG1 VAL A 515      10.319  42.638   6.152  1.00   4.79
H
ATOM   7904 2HG1 VAL A 515      11.867  41.858   5.747  1.00   4.79
H
ATOM   7905 3HG1 VAL A 515      11.654  43.607   5.492  1.00   4.79
H
ATOM   7906 1HG2 VAL A 515       8.824  43.453   4.326  1.00   4.79
H
ATOM   7907 2HG2 VAL A 515      10.154  44.407   3.633  1.00   4.79
H
ATOM   7908 3HG2 VAL A 515       9.293  43.220   2.626  1.00   4.79
H
ATOM   7909  N   ALA A 516      13.745  43.607   3.886  1.00   2.82
N
ATOM   7910  CA  ALA A 516      14.567  44.804   4.045  1.00   2.93
C
ATOM   7911  C   ALA A 516      15.131  45.250   2.701  1.00   2.92
C
ATOM   7912  O   ALA A 516      15.023  46.426   2.336  1.00   3.05
O
ATOM   7913  CB  ALA A 516      15.692  44.543   5.031  1.00   4.40
C
ATOM   7914  H   ALA A 516      14.033  42.751   4.337  1.00   3.38
H
ATOM   7915  HA  ALA A 516      13.934  45.604   4.427  1.00   3.52
H
ATOM   7916 1HB  ALA A 516      16.284  45.449   5.157  1.00   5.27
H
ATOM   7917 2HB  ALA A 516      15.270  44.247   5.992  1.00   5.27
H
ATOM   7918 3HB  ALA A 516      16.327  43.743   4.653  1.00   5.27
H
ATOM   7919  N   THR A 517      15.714  44.298   1.961  1.00   2.76
N
ATOM   7920  CA  THR A 517      16.232  44.582   0.628  1.00   2.73
C
ATOM   7921  C   THR A 517      15.107  44.897  -0.336  1.00   2.79
C
ATOM   7922  O   THR A 517      15.236  45.821  -1.136  1.00   2.97
O
ATOM   7923  CB  THR A 517      17.080  43.424   0.050  1.00   4.09
C
ATOM   7924  OG1 THR A 517      18.243  43.210   0.865  1.00   4.09
O
ATOM   7925  CG2 THR A 517      17.511  43.741  -1.378  1.00   4.09
C
ATOM   7926  H   THR A 517      15.794  43.356   2.331  1.00   3.31
H
```

ATOM 7927 HA THR A 517 16.873 45.461 0.690 1.00 3.28
H
ATOM 7928 HB THR A 517 16.487 42.510 0.039 1.00 4.91
H
ATOM 7929 HG1 THR A 517 18.075 42.481 1.471 1.00 4.91
H
ATOM 7930 1HG2 THR A 517 18.101 42.912 -1.771 1.00 4.91
H
ATOM 7931 2HG2 THR A 517 16.629 43.887 -2.001 1.00 4.91
H
ATOM 7932 3HG2 THR A 517 18.114 44.648 -1.383 1.00 4.91
H
ATOM 7933 N ILE A 518 14.004 44.137 -0.262 1.00 2.67
N
ATOM 7934 CA ILE A 518 12.871 44.366 -1.161 1.00 2.72
C
ATOM 7935 C ILE A 518 12.329 45.780 -1.056 1.00 2.95
C
ATOM 7936 O ILE A 518 12.141 46.443 -2.075 1.00 3.14
O
ATOM 7937 CB ILE A 518 11.713 43.390 -0.880 1.00 4.08
C
ATOM 7938 CG1 ILE A 518 12.102 41.966 -1.265 1.00 4.08
C
ATOM 7939 CG2 ILE A 518 10.464 43.821 -1.636 1.00 4.08
C
ATOM 7940 CD1 ILE A 518 11.130 40.935 -0.750 1.00 4.08
C
ATOM 7941 H ILE A 518 13.953 43.378 0.411 1.00 3.20
H
ATOM 7942 HA ILE A 518 13.210 44.208 -2.184 1.00 3.26
H
ATOM 7943 HB ILE A 518 11.498 43.390 0.186 1.00 4.90
H
ATOM 7944 1HG1 ILE A 518 12.143 41.890 -2.350 1.00 4.90
H
ATOM 7945 2HG1 ILE A 518 13.091 41.742 -0.869 1.00 4.90
H
ATOM 7946 1HG2 ILE A 518 9.649 43.133 -1.415 1.00 4.90
H
ATOM 7947 2HG2 ILE A 518 10.180 44.827 -1.329 1.00 4.90
H
ATOM 7948 3HG2 ILE A 518 10.665 43.812 -2.707 1.00 4.90
H
ATOM 7949 1HD1 ILE A 518 11.459 39.947 -1.061 1.00 4.90
H
ATOM 7950 2HD1 ILE A 518 11.091 40.980 0.337 1.00 4.90
H
ATOM 7951 3HD1 ILE A 518 10.140 41.133 -1.157 1.00 4.90
H
ATOM 7952 N VAL A 519 12.076 46.233 0.168 1.00 2.96
N
ATOM 7953 CA VAL A 519 11.567 47.576 0.405 1.00 3.16
C
ATOM 7954 C VAL A 519 12.559 48.656 0.028 1.00 3.39
C
ATOM 7955 O VAL A 519 12.175 49.648 -0.590 1.00 3.53
O

```
ATOM   7956  CB  VAL A 519      11.161  47.752   1.872  1.00  4.74
C
ATOM   7957  CG1 VAL A 519      10.852  49.210   2.151  1.00  4.74
C
ATOM   7958  CG2 VAL A 519       9.927  46.910   2.148  1.00  4.74
C
ATOM   7959  H   VAL A 519      12.234  45.632   0.965  1.00  3.55
H
ATOM   7960  HA  VAL A 519      10.674  47.707  -0.206  1.00  3.79
H
ATOM   7961  HB  VAL A 519      11.981  47.443   2.519  1.00  5.69
H
ATOM   7962 1HG1 VAL A 519      10.563  49.326   3.196  1.00  5.69
H
ATOM   7963 2HG1 VAL A 519      11.737  49.815   1.951  1.00  5.69
H
ATOM   7964 3HG1 VAL A 519      10.036  49.537   1.511  1.00  5.69
H
ATOM   7965 1HG2 VAL A 519       9.632  47.029   3.190  1.00  5.69
H
ATOM   7966 2HG2 VAL A 519       9.113  47.237   1.502  1.00  5.69
H
ATOM   7967 3HG2 VAL A 519      10.148  45.864   1.950  1.00  5.69
H
ATOM   7968  N   SER A 520      13.829  48.478   0.401  1.00  3.47
N
ATOM   7969  CA  SER A 520      14.853  49.459   0.064  1.00  3.75
C
ATOM   7970  C   SER A 520      14.933  49.642  -1.447  1.00  3.89
C
ATOM   7971  O   SER A 520      14.989  50.769  -1.948  1.00  4.47
O
ATOM   7972  CB  SER A 520      16.199  49.003   0.597  1.00  5.62
C
ATOM   7973  OG  SER A 520      16.197  48.950   1.998  1.00  5.62
O
ATOM   7974  H   SER A 520      14.103  47.658   0.931  1.00  4.16
H
ATOM   7975  HA  SER A 520      14.588  50.415   0.519  1.00  4.50
H
ATOM   7976 1HB  SER A 520      16.439  48.021   0.194  1.00  6.75
H
ATOM   7977 2HB  SER A 520      16.971  49.694   0.261  1.00  6.75
H
ATOM   7978  HG  SER A 520      15.644  48.194   2.230  1.00  6.75
H
ATOM   7979  N   SER A 521      14.914  48.514  -2.157  1.00  3.60
N
ATOM   7980  CA  SER A 521      14.944  48.468  -3.605  1.00  3.77
C
ATOM   7981  C   SER A 521      13.719  49.146  -4.197  1.00  3.88
C
ATOM   7982  O   SER A 521      13.834  49.966  -5.112  1.00  4.75
O
ATOM   7983  CB  SER A 521      15.011  47.022  -4.047  1.00  5.66
C
ATOM   7984  OG  SER A 521      15.052  46.914  -5.435  1.00  5.66
O
```

```
ATOM   7985  H   SER A 521      14.886  47.630  -1.668  1.00  4.32
H
ATOM   7986  HA  SER A 521      15.834  48.992  -3.954  1.00  4.52
H
ATOM   7987 1HB  SER A 521      15.899  46.557  -3.619  1.00  6.79
H
ATOM   7988 2HB  SER A 521      14.148  46.483  -3.664  1.00  6.79
H
ATOM   7989  HG  SER A 521      14.196  47.204  -5.772  1.00  6.79
H
ATOM   7990  N   ALA A 522      12.550  48.802  -3.656  1.00  3.42
N
ATOM   7991  CA  ALA A 522      11.289  49.362  -4.102  1.00  3.60
C
ATOM   7992  C   ALA A 522      11.305  50.868  -4.028  1.00  4.09
C
ATOM   7993  O   ALA A 522      11.014  51.536  -5.014  1.00  4.71
O
ATOM   7994  CB  ALA A 522      10.148  48.824  -3.263  1.00  5.40
C
ATOM   7995  H   ALA A 522      12.524  48.111  -2.920  1.00  4.10
H
ATOM   7996  HA  ALA A 522      11.137  49.072  -5.142  1.00  4.32
H
ATOM   7997 1HB  ALA A 522       9.204  49.229  -3.628  1.00  6.48
H
ATOM   7998 2HB  ALA A 522      10.136  47.740  -3.341  1.00  6.48
H
ATOM   7999 3HB  ALA A 522      10.288  49.111  -2.224  1.00  6.48
H
ATOM   8000  N   LYS A 523      11.705  51.407  -2.885  1.00  4.27
N
ATOM   8001  CA  LYS A 523      11.744  52.848  -2.703  1.00  5.33
C
ATOM   8002  C   LYS A 523      12.773  53.525  -3.613  1.00  5.05
C
ATOM   8003  O   LYS A 523      12.555  54.651  -4.061  1.00  6.94
O
ATOM   8004  CB  LYS A 523      11.966  53.147  -1.227  1.00  8.00
C
ATOM   8005  CG  LYS A 523      10.728  52.791  -0.406  1.00  8.00
C
ATOM   8006  CD  LYS A 523      10.884  53.050   1.081  1.00  8.00
C
ATOM   8007  CE  LYS A 523       9.582  52.713   1.806  1.00  8.00
C
ATOM   8008  NZ  LYS A 523       9.680  52.898   3.279  1.00  8.00
N
ATOM   8009  H   LYS A 523      11.952  50.807  -2.110  1.00  5.12
H
ATOM   8010  HA  LYS A 523      10.765  53.247  -2.972  1.00  6.40
H
ATOM   8011 1HB  LYS A 523      12.812  52.572  -0.851  1.00  9.59
H
ATOM   8012 2HB  LYS A 523      12.188  54.206  -1.087  1.00  9.59
H
ATOM   8013 1HG  LYS A 523       9.880  53.372  -0.772  1.00  9.59
H
```

ATOM 8014 2HG LYS A 523 10.501 51.735 -0.550 1.00 9.59
H
ATOM 8015 1HD LYS A 523 11.689 52.433 1.481 1.00 9.59
H
ATOM 8016 2HD LYS A 523 11.128 54.098 1.250 1.00 9.59
H
ATOM 8017 1HE LYS A 523 8.789 53.355 1.425 1.00 9.59
H
ATOM 8018 2HE LYS A 523 9.318 51.676 1.600 1.00 9.59
H
ATOM 8019 1HZ LYS A 523 8.791 52.658 3.698 1.00 9.59
H
ATOM 8020 2HZ LYS A 523 10.400 52.294 3.648 1.00 9.59
H
ATOM 8021 3HZ LYS A 523 9.906 53.859 3.488 1.00 9.59
H
ATOM 8022 N ALA A 524 13.859 52.821 -3.945 1.00 4.49
N
ATOM 8023 CA ALA A 524 14.842 53.338 -4.897 1.00 4.58
C
ATOM 8024 C ALA A 524 14.308 53.310 -6.343 1.00 5.45
C
ATOM 8025 O ALA A 524 14.834 54.001 -7.216 1.00 11.92
O
ATOM 8026 CB ALA A 524 16.128 52.531 -4.807 1.00 6.87
C
ATOM 8027 H ALA A 524 14.030 51.919 -3.515 1.00 5.39
H
ATOM 8028 HA ALA A 524 15.052 54.375 -4.636 1.00 5.50
H
ATOM 8029 1HB ALA A 524 16.863 52.943 -5.497 1.00 8.24
H
ATOM 8030 2HB ALA A 524 16.515 52.580 -3.789 1.00 8.24
H
ATOM 8031 3HB ALA A 524 15.924 51.494 -5.068 1.00 8.24
H
ATOM 8032 N THR A 525 13.252 52.526 -6.584 1.00 4.05
N
ATOM 8033 CA THR A 525 12.638 52.376 -7.896 1.00 4.01
C
ATOM 8034 C THR A 525 11.204 52.912 -7.910 1.00 4.07
C
ATOM 8035 O THR A 525 10.295 52.270 -8.440 1.00 4.43
O
ATOM 8036 CB THR A 525 12.645 50.908 -8.323 1.00 6.01
C
ATOM 8037 OG1 THR A 525 11.976 50.118 -7.335 1.00 6.01
O
ATOM 8038 CG2 THR A 525 14.068 50.407 -8.488 1.00 6.01
C
ATOM 8039 H THR A 525 12.859 51.972 -5.836 1.00 4.86
H
ATOM 8040 HA THR A 525 13.219 52.949 -8.618 1.00 4.81
H
ATOM 8041 HB THR A 525 12.116 50.816 -9.261 1.00 7.22
H
ATOM 8042 HG1 THR A 525 12.363 50.308 -6.473 1.00 7.22
H

```
ATOM   8043 1HG2 THR A 525      14.053  49.363  -8.799  1.00  7.22
H
ATOM   8044 2HG2 THR A 525      14.572  51.005  -9.243  1.00  7.22
H
ATOM   8045 3HG2 THR A 525      14.599  50.495  -7.542  1.00  7.22
H
ATOM   8046  N   GLU A 526      11.025  54.088  -7.301  1.00  4.80
N
ATOM   8047  CA  GLU A 526       9.748  54.807  -7.180  1.00  4.86
C
ATOM   8048  C   GLU A 526       8.577  53.986  -6.634  1.00  4.85
C
ATOM   8049  O   GLU A 526       7.427  54.234  -6.996  1.00  5.22
O
ATOM   8050  CB  GLU A 526       9.325  55.384  -8.544  1.00  7.29
C
ATOM   8051  CG  GLU A 526      10.291  56.395  -9.158  1.00  7.29
C
ATOM   8052  CD  GLU A 526       9.751  57.071 -10.404  1.00  7.29
C
ATOM   8053  OE1 GLU A 526       8.612  56.848 -10.749  1.00  7.29
O
ATOM   8054  OE2 GLU A 526      10.489  57.805 -11.024  1.00  7.29
O
ATOM   8055  H   GLU A 526      11.839  54.527  -6.895  1.00  5.76
H
ATOM   8056  HA  GLU A 526       9.907  55.641  -6.497  1.00  5.83
H
ATOM   8057 1HB  GLU A 526       9.206  54.568  -9.257  1.00  8.75
H
ATOM   8058 2HB  GLU A 526       8.355  55.872  -8.443  1.00  8.75
H
ATOM   8059 1HG  GLU A 526      10.516  57.159  -8.415  1.00  8.75
H
ATOM   8060 2HG  GLU A 526      11.221  55.884  -9.404  1.00  8.75
H
ATOM   8061  N   GLY A 527       8.861  53.026  -5.756  1.00  4.65
N
ATOM   8062  CA  GLY A 527       7.825  52.216  -5.126  1.00  4.61
C
ATOM   8063  C   GLY A 527       7.269  51.112  -6.027  1.00  4.44
C
ATOM   8064  O   GLY A 527       6.258  50.496  -5.701  1.00  6.22
O
ATOM   8065  H   GLY A 527       9.820  52.848  -5.499  1.00  5.58
H
ATOM   8066 1HA  GLY A 527       8.230  51.768  -4.218  1.00  5.53
H
ATOM   8067 2HA  GLY A 527       7.012  52.867  -4.812  1.00  5.53
H
ATOM   8068  N   ARG A 528       7.898  50.851  -7.168  1.00  4.78
N
ATOM   8069  CA  ARG A 528       7.345  49.840  -8.060  1.00  4.09
C
ATOM   8070  C   ARG A 528       8.044  48.492  -7.960  1.00  4.14
C
ATOM   8071  O   ARG A 528       9.256  48.398  -8.139  1.00  4.91
O
```

```
ATOM   8072  CB  ARG A 528       7.407  50.320  -9.506  1.00  6.13
C
ATOM   8073  CG  ARG A 528       6.524  51.510  -9.851  1.00  6.13
C
ATOM   8074  CD  ARG A 528       6.635  51.850 -11.297  1.00  6.13
C
ATOM   8075  NE  ARG A 528       5.674  52.867 -11.717  1.00  6.13
N
ATOM   8076  CZ  ARG A 528       5.893  54.190 -11.613  1.00  6.13
C
ATOM   8077  NH1 ARG A 528       7.033  54.593 -11.092  1.00  6.13
N
ATOM   8078  NH2 ARG A 528       4.985  55.065 -12.030  1.00  6.13
N
ATOM   8079  H   ARG A 528       8.733  51.355  -7.441  1.00  5.74
H
ATOM   8080  HA  ARG A 528       6.295  49.697  -7.799  1.00  4.91
H
ATOM   8081 1HB  ARG A 528       8.433  50.593  -9.752  1.00  7.36
H
ATOM   8082 2HB  ARG A 528       7.115  49.506 -10.167  1.00  7.36
H
ATOM   8083 1HG  ARG A 528       5.484  51.273  -9.629  1.00  7.36
H
ATOM   8084 2HG  ARG A 528       6.834  52.380  -9.268  1.00  7.36
H
ATOM   8085 1HD  ARG A 528       7.636  52.235 -11.492  1.00  7.36
H
ATOM   8086 2HD  ARG A 528       6.478  50.958 -11.889  1.00  7.36
H
ATOM   8087  HE  ARG A 528       4.798  52.562 -12.125  1.00  7.36
H
ATOM   8088 1HH1 ARG A 528       7.708  53.911 -10.782  1.00  7.36
H
ATOM   8089 2HH1 ARG A 528       7.258  55.577 -10.993  1.00  7.36
H
ATOM   8090 1HH2 ARG A 528       4.102  54.757 -12.434  1.00  7.36
H
ATOM   8091 2HH2 ARG A 528       5.169  56.054 -11.946  1.00  7.36
H
ATOM   8092  N   ILE A 529       7.254  47.445  -7.708  1.00  3.59
N
ATOM   8093  CA  ILE A 529       7.745  46.071  -7.655  1.00  3.49
C
ATOM   8094  C   ILE A 529       6.988  45.165  -8.622  1.00  3.34
C
ATOM   8095  O   ILE A 529       5.762  45.162  -8.632  1.00  3.46
O
ATOM   8096  CB  ILE A 529       7.598  45.448  -6.248  1.00  5.24
C
ATOM   8097  CG1 ILE A 529       8.396  46.206  -5.194  1.00  5.24
C
ATOM   8098  CG2 ILE A 529       8.021  43.986  -6.268  1.00  5.24
C
ATOM   8099  CD1 ILE A 529       8.109  45.709  -3.792  1.00  5.24
C
ATOM   8100  H   ILE A 529       6.269  47.603  -7.534  1.00  4.31
H
```

```
ATOM   8101  HA  ILE A 529       8.796  46.072  -7.929  1.00  4.19
H
ATOM   8102  HB  ILE A 529       6.555  45.510  -5.956  1.00  6.28
H
ATOM   8103 1HG1 ILE A 529       9.461  46.090  -5.396  1.00  6.28
H
ATOM   8104 2HG1 ILE A 529       8.149  47.266  -5.243  1.00  6.28
H
ATOM   8105 1HG2 ILE A 529       7.883  43.556  -5.276  1.00  6.28
H
ATOM   8106 2HG2 ILE A 529       7.414  43.435  -6.983  1.00  6.28
H
ATOM   8107 3HG2 ILE A 529       9.071  43.914  -6.551  1.00  6.28
H
ATOM   8108 1HD1 ILE A 529       8.696  46.270  -3.068  1.00  6.28
H
ATOM   8109 2HD1 ILE A 529       7.051  45.836  -3.572  1.00  6.28
H
ATOM   8110 3HD1 ILE A 529       8.368  44.653  -3.723  1.00  6.28
H
ATOM   8111  N   ILE A 530       7.721  44.397  -9.415  1.00  3.13
N
ATOM   8112  CA  ILE A 530       7.154  43.373 -10.285  1.00  3.01
C
ATOM   8113  C   ILE A 530       7.747  42.037  -9.910  1.00  2.68
C
ATOM   8114  O   ILE A 530       8.950  41.844 -10.033  1.00  2.52
O
ATOM   8115  CB  ILE A 530       7.426  43.611 -11.782  1.00  4.51
C
ATOM   8116  CG1 ILE A 530       6.805  44.917 -12.261  1.00  4.51
C
ATOM   8117  CG2 ILE A 530       6.911  42.427 -12.595  1.00  4.51
C
ATOM   8118  CD1 ILE A 530       7.221  45.271 -13.666  1.00  4.51
C
ATOM   8119  H   ILE A 530       8.720  44.491  -9.380  1.00  3.76
H
ATOM   8120  HA  ILE A 530       6.079  43.334 -10.131  1.00  3.61
H
ATOM   8121  HB  ILE A 530       8.499  43.699 -11.935  1.00  5.42
H
ATOM   8122 1HG1 ILE A 530       5.722  44.828 -12.240  1.00  5.42
H
ATOM   8123 2HG1 ILE A 530       7.097  45.724 -11.593  1.00  5.42
H
ATOM   8124 1HG2 ILE A 530       7.128  42.589 -13.651  1.00  5.42
H
ATOM   8125 2HG2 ILE A 530       7.402  41.516 -12.260  1.00  5.42
H
ATOM   8126 3HG2 ILE A 530       5.836  42.332 -12.455  1.00  5.42
H
ATOM   8127 1HD1 ILE A 530       6.750  46.206 -13.965  1.00  5.42
H
ATOM   8128 2HD1 ILE A 530       8.304  45.382 -13.704  1.00  5.42
H
ATOM   8129 3HD1 ILE A 530       6.914  44.479 -14.346  1.00  5.42
H
```

ATOM 8130 N THR A 531 6.921 41.111 -9.465 1.00 2.60
N
ATOM 8131 CA THR A 531 7.440 39.806 -9.115 1.00 2.32
C
ATOM 8132 C THR A 531 6.982 38.790 -10.139 1.00 2.16
C
ATOM 8133 O THR A 531 5.787 38.644 -10.379 1.00 2.23
O
ATOM 8134 CB THR A 531 6.998 39.379 -7.713 1.00 3.48
C
ATOM 8135 OG1 THR A 531 7.483 40.320 -6.746 1.00 3.48
O
ATOM 8136 CG2 THR A 531 7.562 38.008 -7.400 1.00 3.48
C
ATOM 8137 H THR A 531 5.936 41.312 -9.379 1.00 3.12
H
ATOM 8138 HA THR A 531 8.529 39.841 -9.135 1.00 2.78
H
ATOM 8139 HB THR A 531 5.910 39.347 -7.664 1.00 4.18
H
ATOM 8140 HG1 THR A 531 7.212 40.044 -5.868 1.00 4.18
H
ATOM 8141 1HG2 THR A 531 7.245 37.716 -6.404 1.00 4.18
H
ATOM 8142 2HG2 THR A 531 7.194 37.285 -8.127 1.00 4.18
H
ATOM 8143 3HG2 THR A 531 8.650 38.043 -7.442 1.00 4.18
H
ATOM 8144 N VAL A 532 7.938 38.078 -10.717 1.00 2.04
N
ATOM 8145 CA VAL A 532 7.642 37.070 -11.722 1.00 1.95
C
ATOM 8146 C VAL A 532 8.132 35.712 -11.266 1.00 1.89
C
ATOM 8147 O VAL A 532 9.252 35.576 -10.774 1.00 2.07
O
ATOM 8148 CB VAL A 532 8.301 37.451 -13.066 1.00 2.92
C
ATOM 8149 CG1 VAL A 532 9.798 37.643 -12.872 1.00 2.92
C
ATOM 8150 CG2 VAL A 532 8.039 36.357 -14.100 1.00 2.92
C
ATOM 8151 H VAL A 532 8.899 38.249 -10.451 1.00 2.45
H
ATOM 8152 HA VAL A 532 6.566 37.028 -11.861 1.00 2.34
H
ATOM 8153 HB VAL A 532 7.886 38.396 -13.416 1.00 3.51
H
ATOM 8154 1HG1 VAL A 532 10.260 37.921 -13.817 1.00 3.51
H
ATOM 8155 2HG1 VAL A 532 9.970 38.433 -12.142 1.00 3.51
H
ATOM 8156 3HG1 VAL A 532 10.240 36.718 -12.511 1.00 3.51
H
ATOM 8157 1HG2 VAL A 532 8.500 36.635 -15.047 1.00 3.51
H
ATOM 8158 2HG2 VAL A 532 8.469 35.419 -13.752 1.00 3.51
H

ATOM	8159	3HG2	VAL	A	532	6.967	36.233	-14.242	1.00	3.51	H
ATOM	8160	N	GLU	A	533	7.293	34.699	-11.437	1.00	1.81	N
ATOM	8161	CA	GLU	A	533	7.682	33.367	-11.010	1.00	2.21	C
ATOM	8162	C	GLU	A	533	7.026	32.256	-11.799	1.00	1.78	C
ATOM	8163	O	GLU	A	533	5.868	32.346	-12.204	1.00	3.80	O
ATOM	8164	CB	GLU	A	533	7.327	33.197	-9.548	1.00	3.31	C
ATOM	8165	CG	GLU	A	533	5.832	33.237	-9.299	1.00	3.31	C
ATOM	8166	CD	GLU	A	533	5.513	33.232	-7.851	1.00	3.31	C
ATOM	8167	OE1	GLU	A	533	5.976	32.362	-7.152	1.00	3.31	O
ATOM	8168	OE2	GLU	A	533	4.815	34.122	-7.425	1.00	3.31	O
ATOM	8169	H	GLU	A	533	6.379	34.870	-11.836	1.00	2.17	H
ATOM	8170	HA	GLU	A	533	8.763	33.275	-11.128	1.00	2.65	H
ATOM	8171	1HB	GLU	A	533	7.710	32.244	-9.182	1.00	3.98	H
ATOM	8172	2HB	GLU	A	533	7.793	33.990	-8.962	1.00	3.98	H
ATOM	8173	1HG	GLU	A	533	5.418	34.139	-9.751	1.00	3.98	H
ATOM	8174	2HG	GLU	A	533	5.366	32.377	-9.777	1.00	3.98	H
ATOM	8175	N	ASP	A	534	7.756	31.160	-11.947	1.00	1.71	N
ATOM	8176	CA	ASP	A	534	7.244	30.000	-12.651	1.00	1.48	C
ATOM	8177	C	ASP	A	534	6.522	29.111	-11.636	1.00	2.65	C
ATOM	8178	O	ASP	A	534	7.022	28.071	-11.207	1.00	11.70	O
ATOM	8179	CB	ASP	A	534	8.411	29.301	-13.359	1.00	2.22	C
ATOM	8180	CG	ASP	A	534	7.990	28.212	-14.305	1.00	2.22	C
ATOM	8181	OD1	ASP	A	534	6.822	27.908	-14.337	1.00	2.22	O
ATOM	8182	OD2	ASP	A	534	8.832	27.677	-14.996	1.00	2.22	O
ATOM	8183	H	ASP	A	534	8.696	31.139	-11.579	1.00	2.05	H
ATOM	8184	HA	ASP	A	534	6.520	30.324	-13.396	1.00	1.78	H
ATOM	8185	1HB	ASP	A	534	8.983	30.039	-13.921	1.00	2.66	H
ATOM	8186	2HB	ASP	A	534	9.081	28.871	-12.614	1.00	2.66	H
ATOM	8187	N	HIS	A	535	5.340	29.565	-11.240	1.00	1.36	N

ATOM 8188 CA HIS A 535 4.556 28.954 -10.171 1.00 1.64
C
ATOM 8189 C HIS A 535 3.147 29.510 -10.234 1.00 1.68
C
ATOM 8190 O HIS A 535 2.945 30.575 -10.812 1.00 2.45
O
ATOM 8191 CB HIS A 535 5.205 29.310 -8.821 1.00 2.46
C
ATOM 8192 CG HIS A 535 4.737 28.535 -7.623 1.00 2.46
C
ATOM 8193 ND1 HIS A 535 3.625 28.889 -6.886 1.00 2.46
N
ATOM 8194 CD2 HIS A 535 5.246 27.433 -7.030 1.00 2.46
C
ATOM 8195 CE1 HIS A 535 3.475 28.038 -5.887 1.00 2.46
C
ATOM 8196 NE2 HIS A 535 4.449 27.145 -5.951 1.00 2.46
N
ATOM 8197 H HIS A 535 5.007 30.419 -11.676 1.00 1.63
H
ATOM 8198 HA HIS A 535 4.519 27.874 -10.272 1.00 1.97
H
ATOM 8199 1HB HIS A 535 6.282 29.186 -8.900 1.00 2.95
H
ATOM 8200 2HB HIS A 535 5.024 30.365 -8.614 1.00 2.95
H
ATOM 8201 HD1 HIS A 535 2.876 29.467 -7.217 1.00 2.95
H
ATOM 8202 HD2 HIS A 535 6.110 26.805 -7.250 1.00 2.95
H
ATOM 8203 HE1 HIS A 535 2.649 28.152 -5.185 1.00 2.95
H
ATOM 8204 N TYR A 536 2.168 28.789 -9.693 1.00 1.40
N
ATOM 8205 CA TYR A 536 0.822 29.336 -9.665 1.00 1.84
C
ATOM 8206 C TYR A 536 0.810 30.542 -8.715 1.00 2.29
C
ATOM 8207 O TYR A 536 1.505 30.533 -7.698 1.00 4.96
O
ATOM 8208 CB TYR A 536 -0.212 28.315 -9.180 1.00 2.76
C
ATOM 8209 CG TYR A 536 -0.499 27.168 -10.120 1.00 2.76
C
ATOM 8210 CD1 TYR A 536 -0.770 25.929 -9.595 1.00 2.76
C
ATOM 8211 CD2 TYR A 536 -0.486 27.339 -11.485 1.00 2.76
C
ATOM 8212 CE1 TYR A 536 -1.032 24.854 -10.416 1.00 2.76
C
ATOM 8213 CE2 TYR A 536 -0.754 26.273 -12.316 1.00 2.76
C
ATOM 8214 CZ TYR A 536 -1.024 25.030 -11.789 1.00 2.76
C
ATOM 8215 OH TYR A 536 -1.279 23.962 -12.617 1.00 2.76
O
ATOM 8216 H TYR A 536 2.353 27.895 -9.258 1.00 1.68
H

ATOM   8217 HA TYR A 536   0.564 29.651 -10.671 1.00 2.21
H
ATOM   8218 1HB TYR A 536   0.128 27.891 -8.233 1.00 3.31
H
ATOM   8219 2HB TYR A 536   -1.152 28.827 -8.983 1.00 3.31
H
ATOM   8220 HD1 TYR A 536   -0.778 25.813 -8.518 1.00 3.31
H
ATOM   8221 HD2 TYR A 536   -0.262 28.309 -11.902 1.00 3.31
H
ATOM   8222 HE1 TYR A 536   -1.245 23.874 -9.988 1.00 3.31
H
ATOM   8223 HE2 TYR A 536   -0.739 26.412 -13.390 1.00 3.31
H
ATOM   8224 HH TYR A 536   -1.404 23.172 -12.084 1.00 3.31
H
ATOM   8225 N   PRO A 537   0.012 31.571 -9.009 1.00 1.89
N
ATOM   8226 CA PRO A 537   -0.183 32.815 -8.278 1.00 1.93
C
ATOM   8227 C   PRO A 537   -0.504 32.630 -6.805 1.00 6.32
C
ATOM   8228 O   PRO A 537   -0.207 33.499 -6.006 1.00 43.14
O
ATOM   8229 CB PRO A 537   -1.390 33.443 -8.981 1.00 2.90
C
ATOM   8230 CG PRO A 537   -1.317 32.954 -10.376 1.00 2.90
C
ATOM   8231 CD PRO A 537   -0.735 31.578 -10.273 1.00 2.90
C
ATOM   8232 HA PRO A 537   0.710 33.447 -8.398 1.00 2.32
H
ATOM   8233 1HB PRO A 537   -2.313 33.140 -8.476 1.00 3.47
H
ATOM   8234 2HB PRO A 537   -1.333 34.540 -8.912 1.00 3.47
H
ATOM   8235 1HG PRO A 537   -2.329 32.936 -10.809 1.00 3.47
H
ATOM   8236 2HG PRO A 537   -0.731 33.635 -11.001 1.00 3.47
H
ATOM   8237 1HD PRO A 537   -1.514 30.801 -10.278 1.00 3.47
H
ATOM   8238 2HD PRO A 537   -0.021 31.466 -11.095 1.00 3.47
H
ATOM   8239 N   GLN A 538   -1.137 31.526 -6.447 1.00 2.91
N
ATOM   8240 CA GLN A 538   -1.601 31.339 -5.085 1.00 1.75
C
ATOM   8241 C   GLN A 538   -0.512 30.733 -4.190 1.00 2.11
C
ATOM   8242 O   GLN A 538   -0.008 29.646 -4.461 1.00 4.06
O
ATOM   8243 CB GLN A 538   -2.827 30.428 -5.114 1.00 2.62
C
ATOM   8244 CG GLN A 538   -3.910 30.840 -6.127 1.00 2.62
C
ATOM   8245 CD GLN A 538   -4.575 32.189 -5.911 1.00 2.62
C

```
ATOM   8246  OE1 GLN A 538      -5.149  32.496  -4.864  1.00  2.62
O
ATOM   8247  NE2 GLN A 538      -4.519  33.023  -6.940  1.00  2.62
N
ATOM   8248  H   GLN A 538      -1.337  30.817  -7.136  1.00  3.49
H
ATOM   8249  HA  GLN A 538      -1.881  32.310  -4.676  1.00  2.10
H
ATOM   8250 1HB  GLN A 538      -2.518  29.409  -5.348  1.00  3.15
H
ATOM   8251 2HB  GLN A 538      -3.279  30.411  -4.124  1.00  3.15
H
ATOM   8252 1HG  GLN A 538      -3.453  30.857  -7.116  1.00  3.15
H
ATOM   8253 2HG  GLN A 538      -4.697  30.096  -6.108  1.00  3.15
H
ATOM   8254 1HE2 GLN A 538      -4.942  33.928  -6.878  1.00  3.15
H
ATOM   8255 2HE2 GLN A 538      -4.062  32.746  -7.784  1.00  3.15
H
ATOM   8256  N   GLY A 539      -0.134  31.449  -3.138  1.00  1.63
N
ATOM   8257  CA  GLY A 539       0.861  30.958  -2.193  1.00  1.65
C
ATOM   8258  C   GLY A 539       2.318  31.037  -2.680  1.00  1.65
C
ATOM   8259  O   GLY A 539       3.215  30.529  -2.006  1.00  2.04
O
ATOM   8260  H   GLY A 539      -0.576  32.340  -2.964  1.00  1.96
H
ATOM   8261 1HA  GLY A 539       0.766  31.517  -1.266  1.00  1.98
H
ATOM   8262 2HA  GLY A 539       0.622  29.929  -1.942  1.00  1.98
H
ATOM   8263  N   GLY A 540       2.574  31.703  -3.798  1.00  1.55
N
ATOM   8264  CA  GLY A 540       3.935  31.772  -4.321  1.00  1.55
C
ATOM   8265  C   GLY A 540       4.737  32.963  -3.804  1.00  1.75
C
ATOM   8266  O   GLY A 540       4.420  33.557  -2.770  1.00  2.29
O
ATOM   8267  H   GLY A 540       1.823  32.142  -4.313  1.00  1.86
H
ATOM   8268 1HA  GLY A 540       4.458  30.849  -4.070  1.00  1.86
H
ATOM   8269 2HA  GLY A 540       3.893  31.816  -5.410  1.00  1.86
H
ATOM   8270  N   ILE A 541       5.788  33.287  -4.545  1.00  1.88
N
ATOM   8271  CA  ILE A 541       6.707  34.362  -4.216  1.00  1.95
C
ATOM   8272  C   ILE A 541       6.022  35.712  -4.260  1.00  2.18
C
ATOM   8273  O   ILE A 541       6.238  36.540  -3.380  1.00  2.52
O
ATOM   8274  CB  ILE A 541       7.896  34.397  -5.179  1.00  2.92
C
```

ATOM 8275 CG1 ILE A 541 8.762 33.155 -5.017 1.00 2.92
C
ATOM 8276 CG2 ILE A 541 8.718 35.629 -4.881 1.00 2.92
C
ATOM 8277 CD1 ILE A 541 9.784 32.996 -6.118 1.00 2.92
C
ATOM 8278 H ILE A 541 5.955 32.754 -5.389 1.00 2.26
H
ATOM 8279 HA ILE A 541 7.082 34.200 -3.206 1.00 2.34
H
ATOM 8280 HB ILE A 541 7.541 34.431 -6.208 1.00 3.51
H
ATOM 8281 1HG1 ILE A 541 9.280 33.207 -4.062 1.00 3.51
H
ATOM 8282 2HG1 ILE A 541 8.121 32.273 -5.013 1.00 3.51
H
ATOM 8283 1HG2 ILE A 541 9.567 35.676 -5.563 1.00 3.51
H
ATOM 8284 2HG2 ILE A 541 8.104 36.516 -5.000 1.00 3.51
H
ATOM 8285 3HG2 ILE A 541 9.076 35.578 -3.853 1.00 3.51
H
ATOM 8286 1HD1 ILE A 541 10.368 32.093 -5.949 1.00 3.51
H
ATOM 8287 2HD1 ILE A 541 9.273 32.924 -7.079 1.00 3.51
H
ATOM 8288 3HD1 ILE A 541 10.448 33.861 -6.126 1.00 3.51
H
ATOM 8289 N GLY A 542 5.216 35.940 -5.297 1.00 2.16
N
ATOM 8290 CA GLY A 542 4.508 37.204 -5.461 1.00 2.40
C
ATOM 8291 C GLY A 542 3.634 37.494 -4.259 1.00 2.37
C
ATOM 8292 O GLY A 542 3.563 38.631 -3.794 1.00 2.70
O
ATOM 8293 H GLY A 542 5.084 35.214 -5.992 1.00 2.59
H
ATOM 8294 1HA GLY A 542 5.224 38.012 -5.596 1.00 2.88
H
ATOM 8295 2HA GLY A 542 3.893 37.162 -6.360 1.00 2.88
H
ATOM 8296 N GLU A 543 2.983 36.455 -3.760 1.00 2.12
N
ATOM 8297 CA GLU A 543 2.107 36.561 -2.617 1.00 2.50
C
ATOM 8298 C GLU A 543 2.898 36.834 -1.351 1.00 2.27
C
ATOM 8299 O GLU A 543 2.488 37.649 -0.524 1.00 2.34
O
ATOM 8300 CB GLU A 543 1.355 35.272 -2.475 1.00 3.75
C
ATOM 8301 CG GLU A 543 0.433 34.963 -3.621 1.00 3.75
C
ATOM 8302 CD GLU A 543 -0.789 35.809 -3.710 1.00 3.75
C
ATOM 8303 OE1 GLU A 543 -1.603 35.747 -2.816 1.00 3.75
O

ATOM 8304 OE2 GLU A 543 -0.919 36.517 -4.680 1.00 3.75
O
ATOM 8305 H GLU A 543 3.093 35.553 -4.199 1.00 2.54
H
ATOM 8306 HA GLU A 543 1.409 37.381 -2.782 1.00 3.00
H
ATOM 8307 1HB GLU A 543 2.043 34.439 -2.335 1.00 4.50
H
ATOM 8308 2HB GLU A 543 0.739 35.353 -1.594 1.00 4.50
H
ATOM 8309 1HG GLU A 543 0.998 35.088 -4.543 1.00 4.50
H
ATOM 8310 2HG GLU A 543 0.139 33.929 -3.557 1.00 4.50
H
ATOM 8311 N ALA A 544 4.057 36.175 -1.225 1.00 2.48
N
ATOM 8312 CA ALA A 544 4.939 36.389 -0.089 1.00 2.57
C
ATOM 8313 C ALA A 544 5.404 37.838 -0.050 1.00 2.61
C
ATOM 8314 O ALA A 544 5.400 38.461 1.014 1.00 2.66
O
ATOM 8315 CB ALA A 544 6.132 35.443 -0.156 1.00 3.85
C
ATOM 8316 H ALA A 544 4.323 35.488 -1.921 1.00 2.98
H
ATOM 8317 HA ALA A 544 4.376 36.188 0.824 1.00 3.08
H
ATOM 8318 1HB ALA A 544 6.770 35.599 0.713 1.00 4.63
H
ATOM 8319 2HB ALA A 544 5.776 34.412 -0.164 1.00 4.63
H
ATOM 8320 3HB ALA A 544 6.701 35.635 -1.064 1.00 4.63
H
ATOM 8321 N VAL A 545 5.781 38.375 -1.216 1.00 2.67
N
ATOM 8322 CA VAL A 545 6.226 39.757 -1.323 1.00 2.89
C
ATOM 8323 C VAL A 545 5.107 40.723 -0.997 1.00 3.31
C
ATOM 8324 O VAL A 545 5.326 41.664 -0.238 1.00 3.52
O
ATOM 8325 CB VAL A 545 6.780 40.065 -2.734 1.00 4.33
C
ATOM 8326 CG1 VAL A 545 7.040 41.563 -2.877 1.00 4.33
C
ATOM 8327 CG2 VAL A 545 8.078 39.290 -2.959 1.00 4.33
C
ATOM 8328 H VAL A 545 5.773 37.800 -2.048 1.00 3.20
H
ATOM 8329 HA VAL A 545 7.030 39.915 -0.606 1.00 3.47
H
ATOM 8330 HB VAL A 545 6.041 39.777 -3.483 1.00 5.20
H
ATOM 8331 1HG1 VAL A 545 7.421 41.774 -3.877 1.00 5.20
H
ATOM 8332 2HG1 VAL A 545 6.110 42.109 -2.722 1.00 5.20
H

ATOM 8333 3HG1 VAL A 545 7.774 41.878 -2.135 1.00 5.20
H
ATOM 8334 1HG2 VAL A 545 8.463 39.505 -3.956 1.00 5.20
H
ATOM 8335 2HG2 VAL A 545 8.814 39.592 -2.214 1.00 5.20
H
ATOM 8336 3HG2 VAL A 545 7.889 38.223 -2.866 1.00 5.20
H
ATOM 8337 N CYS A 546 3.912 40.484 -1.549 1.00 3.58
N
ATOM 8338 CA CYS A 546 2.768 41.347 -1.289 1.00 4.06
C
ATOM 8339 C CYS A 546 2.456 41.418 0.191 1.00 4.54
C
ATOM 8340 O CYS A 546 2.255 42.506 0.729 1.00 6.46
O
ATOM 8341 CB CYS A 546 1.520 40.849 -2.025 1.00 6.09
C
ATOM 8342 SG CYS A 546 1.564 41.053 -3.819 1.00 6.09
S
ATOM 8343 H CYS A 546 3.794 39.699 -2.179 1.00 4.30
H
ATOM 8344 HA CYS A 546 3.003 42.350 -1.645 1.00 4.87
H
ATOM 8345 1HB CYS A 546 1.379 39.789 -1.814 1.00 7.31
H
ATOM 8346 2HB CYS A 546 0.643 41.375 -1.649 1.00 7.31
H
ATOM 8347 HG CYS A 546 0.413 40.432 -4.059 1.00 7.31
H
ATOM 8348 N ALA A 547 2.424 40.265 0.852 1.00 3.57
N
ATOM 8349 CA ALA A 547 2.154 40.215 2.279 1.00 4.02
C
ATOM 8350 C ALA A 547 3.225 40.962 3.065 1.00 4.23
C
ATOM 8351 O ALA A 547 2.916 41.679 4.018 1.00 5.59
O
ATOM 8352 CB ALA A 547 2.070 38.770 2.742 1.00 6.03
C
ATOM 8353 H ALA A 547 2.583 39.397 0.357 1.00 4.28
H
ATOM 8354 HA ALA A 547 1.198 40.705 2.461 1.00 4.82
H
ATOM 8355 1HB ALA A 547 1.841 38.741 3.807 1.00 7.24
H
ATOM 8356 2HB ALA A 547 1.284 38.255 2.189 1.00 7.24
H
ATOM 8357 3HB ALA A 547 3.022 38.274 2.561 1.00 7.24
H
ATOM 8358 N ALA A 548 4.484 40.796 2.658 1.00 3.39
N
ATOM 8359 CA ALA A 548 5.604 41.447 3.321 1.00 3.39
C
ATOM 8360 C ALA A 548 5.573 42.973 3.216 1.00 3.75
C
ATOM 8361 O ALA A 548 5.883 43.659 4.193 1.00 4.12
O

```
ATOM   8362  CB  ALA A 548       6.907  40.929   2.738  1.00  5.08
C
ATOM   8363  H   ALA A 548       4.686  40.177   1.883  1.00  4.07
H
ATOM   8364  HA  ALA A 548       5.559  41.185   4.377  1.00  4.07
H
ATOM   8365 1HB  ALA A 548       7.745  41.379   3.268  1.00  6.10
H
ATOM   8366 2HB  ALA A 548       6.946  39.846   2.852  1.00  6.10
H
ATOM   8367 3HB  ALA A 548       6.963  41.186   1.683  1.00  6.10
H
ATOM   8368  N   VAL A 549       5.212  43.513   2.048  1.00  3.64
N
ATOM   8369  CA  VAL A 549       5.252  44.967   1.884  1.00  3.78
C
ATOM   8370  C   VAL A 549       3.894  45.652   2.022  1.00  4.28
C
ATOM   8371  O   VAL A 549       3.837  46.884   2.028  1.00  6.95
O
ATOM   8372  CB  VAL A 549       5.834  45.357   0.511  1.00  5.67
C
ATOM   8373  CG1 VAL A 549       7.223  44.760   0.335  1.00  5.67
C
ATOM   8374  CG2 VAL A 549       4.890  44.934  -0.591  1.00  5.67
C
ATOM   8375  H   VAL A 549       4.965  42.918   1.266  1.00  4.37
H
ATOM   8376  HA  VAL A 549       5.911  45.370   2.654  1.00  4.54
H
ATOM   8377  HB  VAL A 549       5.950  46.440   0.479  1.00  6.80
H
ATOM   8378 1HG1 VAL A 549       7.635  45.070  -0.626  1.00  6.80
H
ATOM   8379 2HG1 VAL A 549       7.872  45.107   1.138  1.00  6.80
H
ATOM   8380 3HG1 VAL A 549       7.157  43.672   0.368  1.00  6.80
H
ATOM   8381 1HG2 VAL A 549       5.307  45.241  -1.547  1.00  6.80
H
ATOM   8382 2HG2 VAL A 549       4.763  43.857  -0.579  1.00  6.80
H
ATOM   8383 3HG2 VAL A 549       3.921  45.412  -0.447  1.00  6.80
H
ATOM   8384  N   SER A 550       2.809  44.877   2.113  1.00  3.27
N
ATOM   8385  CA  SER A 550       1.489  45.477   2.245  1.00  4.07
C
ATOM   8386  C   SER A 550       1.442  46.413   3.429  1.00  6.51
C
ATOM   8387  O   SER A 550       1.935  46.083   4.502  1.00 31.85
O
ATOM   8388  CB  SER A 550       0.421  44.416   2.409  1.00  6.11
C
ATOM   8389  OG  SER A 550      -0.831  45.003   2.637  1.00  6.11
O
ATOM   8390  H   SER A 550       2.884  43.870   2.074  1.00  3.92
H
```

ATOM	8391	HA	SER	A	550	1.277	46.052	1.342	1.00	4.88	H
ATOM	8392	1HB	SER	A	550	0.374	43.802	1.512	1.00	7.33	H
ATOM	8393	2HB	SER	A	550	0.680	43.762	3.242	1.00	7.33	H
ATOM	8394	HG	SER	A	550	-1.439	44.272	2.773	1.00	7.33	H
ATOM	8395	N	MET	A	551	0.824	47.572	3.209	1.00	4.64	N
ATOM	8396	CA	MET	A	551	0.639	48.668	4.172	1.00	3.92	C
ATOM	8397	C	MET	A	551	1.763	49.714	4.108	1.00	3.45	C
ATOM	8398	O	MET	A	551	1.613	50.796	4.676	1.00	4.93	O
ATOM	8399	CB	MET	A	551	0.544	48.176	5.631	1.00	5.88	C
ATOM	8400	CG	MET	A	551	-0.634	47.267	5.953	1.00	5.88	C
ATOM	8401	SD	MET	A	551	-0.663	46.749	7.680	1.00	5.88	S
ATOM	8402	CE	MET	A	551	-1.181	48.269	8.467	1.00	5.88	C
ATOM	8403	H	MET	A	551	0.447	47.714	2.282	1.00	5.57	H
ATOM	8404	HA	MET	A	551	-0.291	49.175	3.923	1.00	4.70	H
ATOM	8405	1HB	MET	A	551	1.466	47.701	5.952	1.00	7.06	H
ATOM	8406	2HB	MET	A	551	0.423	49.051	6.271	1.00	7.06	H
ATOM	8407	1HG	MET	A	551	-1.561	47.771	5.729	1.00	7.06	H
ATOM	8408	2HG	MET	A	551	-0.586	46.370	5.336	1.00	7.06	H
ATOM	8409	1HE	MET	A	551	-1.246	48.116	9.545	1.00	7.06	H
ATOM	8410	2HE	MET	A	551	-0.454	49.054	8.256	1.00	7.06	H
ATOM	8411	3HE	MET	A	551	-2.157	48.564	8.083	1.00	7.06	H
ATOM	8412	N	ASP	A	552	2.874	49.409	3.425	1.00	3.31	N
ATOM	8413	CA	ASP	A	552	4.003	50.343	3.343	1.00	4.01	C
ATOM	8414	C	ASP	A	552	3.797	51.367	2.204	1.00	4.98	C
ATOM	8415	O	ASP	A	552	4.001	51.021	1.037	1.00	4.78	O
ATOM	8416	CB	ASP	A	552	5.320	49.572	3.148	1.00	6.01	C
ATOM	8417	CG	ASP	A	552	6.575	50.454	3.166	1.00	6.01	C
ATOM	8418	OD1	ASP	A	552	6.457	51.647	3.037	1.00	6.01	O
ATOM	8419	OD2	ASP	A	552	7.646	49.918	3.329	1.00	6.01	O

```
ATOM   8420  H   ASP A 552       2.967  48.513   2.960  1.00  3.97
H
ATOM   8421  HA  ASP A 552       4.080  50.858   4.293  1.00  4.81
H
ATOM   8422 1HB  ASP A 552       5.417  48.822   3.935  1.00  7.22
H
ATOM   8423 2HB  ASP A 552       5.286  49.038   2.198  1.00  7.22
H
ATOM   8424  N   PRO A 553       3.327  52.591   2.549  1.00  6.91
N
ATOM   8425  CA  PRO A 553       3.035  53.722   1.651  1.00  8.85
C
ATOM   8426  C   PRO A 553       3.894  53.790   0.395  1.00  6.16
C
ATOM   8427  O   PRO A 553       5.120  53.678   0.441  1.00  6.27
O
ATOM   8428  CB  PRO A 553       3.324  54.962   2.506  1.00 13.27
C
ATOM   8429  CG  PRO A 553       3.170  54.507   3.897  1.00 13.27
C
ATOM   8430  CD  PRO A 553       3.717  53.120   3.879  1.00 13.27
C
ATOM   8431  HA  PRO A 553       1.970  53.690   1.378  1.00 10.62
H
ATOM   8432 1HB  PRO A 553       4.340  55.334   2.292  1.00 15.93
H
ATOM   8433 2HB  PRO A 553       2.628  55.771   2.244  1.00 15.93
H
ATOM   8434 1HG  PRO A 553       3.717  55.172   4.583  1.00 15.93
H
ATOM   8435 2HG  PRO A 553       2.111  54.543   4.194  1.00 15.93
H
ATOM   8436 1HD  PRO A 553       4.812  53.135   3.981  1.00 15.93
H
ATOM   8437 2HD  PRO A 553       3.238  52.555   4.687  1.00 15.93
H
ATOM   8438  N   ASP A 554       3.204  54.034  -0.714  1.00  6.12
N
ATOM   8439  CA  ASP A 554       3.732  54.175  -2.071  1.00  5.05
C
ATOM   8440  C   ASP A 554       4.296  52.903  -2.722  1.00  4.69
C
ATOM   8441  O   ASP A 554       4.639  52.945  -3.902  1.00  5.07
O
ATOM   8442  CB  ASP A 554       4.814  55.259  -2.104  1.00  7.57
C
ATOM   8443  CG  ASP A 554       4.280  56.633  -1.723  1.00  7.57
C
ATOM   8444  OD1 ASP A 554       3.160  56.930  -2.067  1.00  7.57
O
ATOM   8445  OD2 ASP A 554       4.994  57.372  -1.088  1.00  7.57
O
ATOM   8446  H   ASP A 554       2.204  54.118  -0.611  1.00  7.34
H
ATOM   8447  HA  ASP A 554       2.911  54.517  -2.699  1.00  6.06
H
ATOM   8448 1HB  ASP A 554       5.635  55.003  -1.435  1.00  9.09
H
```

ATOM	8449	2HB	ASP	A	554	5.227	55.321	-3.110	1.00	9.09	H
ATOM	8450	N	ILE	A	555	4.335	51.765	-2.020	1.00	4.44	N
ATOM	8451	CA	ILE	A	555	4.802	50.561	-2.692	1.00	4.14	C
ATOM	8452	C	ILE	A	555	3.652	49.800	-3.332	1.00	3.46	C
ATOM	8453	O	ILE	A	555	2.677	49.434	-2.671	1.00	3.65	O
ATOM	8454	CB	ILE	A	555	5.592	49.619	-1.768	1.00	6.21	C
ATOM	8455	CG1	ILE	A	555	6.862	50.313	-1.273	1.00	6.21	C
ATOM	8456	CG2	ILE	A	555	5.934	48.331	-2.499	1.00	6.21	C
ATOM	8457	CD1	ILE	A	555	7.605	49.522	-0.227	1.00	6.21	C
ATOM	8458	H	ILE	A	555	4.066	51.715	-1.043	1.00	5.33	H
ATOM	8459	HA	ILE	A	555	5.479	50.863	-3.486	1.00	4.97	H
ATOM	8460	HB	ILE	A	555	4.989	49.386	-0.890	1.00	7.45	H
ATOM	8461	1HG1	ILE	A	555	7.530	50.485	-2.118	1.00	7.45	H
ATOM	8462	2HG1	ILE	A	555	6.595	51.279	-0.844	1.00	7.45	H
ATOM	8463	1HG2	ILE	A	555	6.485	47.669	-1.832	1.00	7.45	H
ATOM	8464	2HG2	ILE	A	555	5.017	47.838	-2.820	1.00	7.45	H
ATOM	8465	3HG2	ILE	A	555	6.547	48.559	-3.372	1.00	7.45	H
ATOM	8466	1HD1	ILE	A	555	8.492	50.074	0.083	1.00	7.45	H
ATOM	8467	2HD1	ILE	A	555	6.958	49.360	0.633	1.00	7.45	H
ATOM	8468	3HD1	ILE	A	555	7.902	48.560	-0.640	1.00	7.45	H
ATOM	8469	N	GLN	A	556	3.787	49.574	-4.632	1.00	3.34	N
ATOM	8470	CA	GLN	A	556	2.817	48.845	-5.439	1.00	2.71	C
ATOM	8471	C	GLN	A	556	3.450	47.580	-6.012	1.00	3.16	C
ATOM	8472	O	GLN	A	556	4.522	47.644	-6.615	1.00	3.55	O
ATOM	8473	CB	GLN	A	556	2.298	49.738	-6.575	1.00	4.06	C
ATOM	8474	CG	GLN	A	556	1.464	50.925	-6.127	1.00	4.06	C
ATOM	8475	CD	GLN	A	556	0.946	51.732	-7.305	1.00	4.06	C
ATOM	8476	OE1	GLN	A	556	1.732	52.285	-8.079	1.00	4.06	O
ATOM	8477	NE2	GLN	A	556	-0.374	51.800	-7.458	1.00	4.06	N

```
ATOM   8478  H   GLN A 556       4.621  49.925  -5.079  1.00  4.01
H
ATOM   8479  HA  GLN A 556       1.982  48.553  -4.803  1.00  3.25
H
ATOM   8480 1HB  GLN A 556       3.152  50.135  -7.125  1.00  4.88
H
ATOM   8481 2HB  GLN A 556       1.713  49.157  -7.279  1.00  4.88
H
ATOM   8482 1HG  GLN A 556       0.613  50.573  -5.551  1.00  4.88
H
ATOM   8483 2HG  GLN A 556       2.085  51.579  -5.515  1.00  4.88
H
ATOM   8484 1HE2 GLN A 556      -0.760  52.319  -8.222  1.00  4.88
H
ATOM   8485 2HE2 GLN A 556      -0.991  51.334  -6.819  1.00  4.88
H
ATOM   8486  N   VAL A 557       2.780  46.437  -5.834  1.00  3.37
N
ATOM   8487  CA  VAL A 557       3.315  45.168  -6.323  1.00  3.57
C
ATOM   8488  C   VAL A 557       2.469  44.547  -7.435  1.00  3.55
C
ATOM   8489  O   VAL A 557       1.292  44.240  -7.249  1.00  3.64
O
ATOM   8490  CB  VAL A 557       3.438  44.136  -5.185  1.00  5.35
C
ATOM   8491  CG1 VAL A 557       3.990  42.825  -5.736  1.00  5.35
C
ATOM   8492  CG2 VAL A 557       4.334  44.665  -4.089  1.00  5.35
C
ATOM   8493  H   VAL A 557       1.899  46.447  -5.341  1.00  4.04
H
ATOM   8494  HA  VAL A 557       4.313  45.354  -6.716  1.00  4.28
H
ATOM   8495  HB  VAL A 557       2.447  43.933  -4.780  1.00  6.43
H
ATOM   8496 1HG1 VAL A 557       4.058  42.093  -4.932  1.00  6.43
H
ATOM   8497 2HG1 VAL A 557       3.327  42.448  -6.514  1.00  6.43
H
ATOM   8498 3HG1 VAL A 557       4.981  42.996  -6.155  1.00  6.43
H
ATOM   8499 1HG2 VAL A 557       4.400  43.924  -3.293  1.00  6.43
H
ATOM   8500 2HG2 VAL A 557       5.325  44.853  -4.488  1.00  6.43
H
ATOM   8501 3HG2 VAL A 557       3.919  45.591  -3.691  1.00  6.43
H
ATOM   8502  N   HIS A 558       3.110  44.333  -8.572  1.00  3.55
N
ATOM   8503  CA  HIS A 558       2.544  43.697  -9.753  1.00  3.44
C
ATOM   8504  C   HIS A 558       3.087  42.282  -9.818  1.00  3.15
C
ATOM   8505  O   HIS A 558       4.215  42.046  -9.389  1.00  3.29
O
ATOM   8506  CB  HIS A 558       2.927  44.471 -11.017  1.00  5.16
C
```

```
ATOM   8507  CG  HIS A 558       2.410  43.863 -12.282  1.00  5.16
C
ATOM   8508  ND1 HIS A 558       1.074  43.876 -12.624  1.00  5.16
N
ATOM   8509  CD2 HIS A 558       3.050  43.226 -13.291  1.00  5.16
C
ATOM   8510  CE1 HIS A 558       0.916  43.268 -13.787  1.00  5.16
C
ATOM   8511  NE2 HIS A 558       2.099  42.868 -14.212  1.00  5.16
N
ATOM   8512  H   HIS A 558       4.070  44.626  -8.625  1.00  4.26
H
ATOM   8513  HA  HIS A 558       1.458  43.651  -9.678  1.00  4.13
H
ATOM   8514 1HB  HIS A 558       2.545  45.490 -10.946  1.00  6.19
H
ATOM   8515 2HB  HIS A 558       4.011  44.535 -11.083  1.00  6.19
H
ATOM   8516  HD1 HIS A 558       0.320  44.169 -12.037  1.00  6.19
H
ATOM   8517  HD2 HIS A 558       4.095  42.979 -13.468  1.00  6.19
H
ATOM   8518  HE1 HIS A 558      -0.073  43.171 -14.233  1.00  6.19
H
ATOM   8519  N   SER A 559       2.290  41.330 -10.293  1.00  2.94
N
ATOM   8520  CA  SER A 559       2.773  39.956 -10.310  1.00  2.67
C
ATOM   8521  C   SER A 559       2.480  39.181 -11.593  1.00  2.68
C
ATOM   8522  O   SER A 559       1.385  39.251 -12.153  1.00  2.91
O
ATOM   8523  CB  SER A 559       2.192  39.199  -9.137  1.00  4.00
C
ATOM   8524  OG  SER A 559       2.590  37.857  -9.166  1.00  4.00
O
ATOM   8525  H   SER A 559       1.368  41.554 -10.639  1.00  3.53
H
ATOM   8526  HA  SER A 559       3.853  39.987 -10.186  1.00  3.20
H
ATOM   8527 1HB  SER A 559       2.525  39.661  -8.209  1.00  4.81
H
ATOM   8528 2HB  SER A 559       1.104  39.261  -9.162  1.00  4.81
H
ATOM   8529  HG  SER A 559       3.546  37.867  -9.271  1.00  4.81
H
ATOM   8530  N   LEU A 560       3.493  38.429 -12.029  1.00  2.51
N
ATOM   8531  CA  LEU A 560       3.435  37.565 -13.200  1.00  2.48
C
ATOM   8532  C   LEU A 560       3.591  36.110 -12.754  1.00  2.20
C
ATOM   8533  O   LEU A 560       4.604  35.741 -12.161  1.00  2.01
O
ATOM   8534  CB  LEU A 560       4.554  37.921 -14.190  1.00  3.72
C
ATOM   8535  CG  LEU A 560       4.574  39.357 -14.729  1.00  3.72
C
```

ATOM 8536 CD1 LEU A 560 5.812 39.544 -15.602 1.00 3.72
C
ATOM 8537 CD2 LEU A 560 3.305 39.620 -15.523 1.00 3.72
C
ATOM 8538 H LEU A 560 4.353 38.450 -11.504 1.00 3.01
H
ATOM 8539 HA LEU A 560 2.467 37.685 -13.687 1.00 2.98
H
ATOM 8540 1HB LEU A 560 5.506 37.754 -13.697 1.00 4.46
H
ATOM 8541 2HB LEU A 560 4.489 37.247 -15.044 1.00 4.46
H
ATOM 8542 HG LEU A 560 4.635 40.061 -13.899 1.00 4.46
H
ATOM 8543 1HD1 LEU A 560 5.838 40.564 -15.983 1.00 4.46
H
ATOM 8544 2HD1 LEU A 560 6.707 39.355 -15.010 1.00 4.46
H
ATOM 8545 3HD1 LEU A 560 5.775 38.846 -16.437 1.00 4.46
H
ATOM 8546 1HD2 LEU A 560 3.319 40.640 -15.906 1.00 4.46
H
ATOM 8547 2HD2 LEU A 560 3.248 38.920 -16.357 1.00 4.46
H
ATOM 8548 3HD2 LEU A 560 2.438 39.486 -14.877 1.00 4.46
H
ATOM 8549 N ALA A 561 2.591 35.288 -13.038 1.00 2.46
N
ATOM 8550 CA ALA A 561 2.619 33.893 -12.607 1.00 2.28
C
ATOM 8551 C ALA A 561 1.744 33.033 -13.524 1.00 2.19
C
ATOM 8552 O ALA A 561 1.045 33.562 -14.388 1.00 2.29
O
ATOM 8553 CB ALA A 561 2.181 33.809 -11.158 1.00 3.42
C
ATOM 8554 H ALA A 561 1.789 35.636 -13.544 1.00 2.95
H
ATOM 8555 HA ALA A 561 3.642 33.527 -12.688 1.00 2.74
H
ATOM 8556 1HB ALA A 561 2.213 32.781 -10.814 1.00 4.10
H
ATOM 8557 2HB ALA A 561 2.848 34.415 -10.543 1.00 4.10
H
ATOM 8558 3HB ALA A 561 1.173 34.192 -11.068 1.00 4.10
H
ATOM 8559 N VAL A 562 1.814 31.707 -13.362 1.00 2.03
N
ATOM 8560 CA VAL A 562 1.088 30.792 -14.253 1.00 1.89
C
ATOM 8561 C VAL A 562 -0.399 30.741 -13.903 1.00 1.90
C
ATOM 8562 O VAL A 562 -0.784 30.243 -12.853 1.00 3.02
O
ATOM 8563 CB VAL A 562 1.697 29.373 -14.168 1.00 2.83
C
ATOM 8564 CG1 VAL A 562 0.914 28.413 -15.052 1.00 2.83
C

```
ATOM   8565  CG2 VAL A 562       3.162  29.421 -14.583  1.00  2.83
C
ATOM   8566  H   VAL A 562       2.372  31.326 -12.609  1.00  2.44
H
ATOM   8567  HA  VAL A 562       1.192  31.153 -15.276  1.00  2.27
H
ATOM   8568  HB  VAL A 562       1.620  29.013 -13.143  1.00  3.40
H
ATOM   8569 1HG1 VAL A 562       1.340  27.414 -14.974  1.00  3.40
H
ATOM   8570 2HG1 VAL A 562      -0.126  28.390 -14.727  1.00  3.40
H
ATOM   8571 3HG1 VAL A 562       0.963  28.747 -16.089  1.00  3.40
H
ATOM   8572 1HG2 VAL A 562       3.593  28.423 -14.510  1.00  3.40
H
ATOM   8573 2HG2 VAL A 562       3.240  29.775 -15.610  1.00  3.40
H
ATOM   8574 3HG2 VAL A 562       3.705  30.099 -13.925  1.00  3.40
H
ATOM   8575  N   SER A 563      -1.240  31.243 -14.797  1.00  1.64
N
ATOM   8576  CA  SER A 563      -2.681  31.359 -14.544  1.00  1.77
C
ATOM   8577  C   SER A 563      -3.472  30.051 -14.385  1.00  1.57
C
ATOM   8578  O   SER A 563      -4.556  30.062 -13.801  1.00  1.66
O
ATOM   8579  CB  SER A 563      -3.316  32.155 -15.666  1.00  2.66
C
ATOM   8580  OG  SER A 563      -3.287  31.432 -16.868  1.00  2.66
O
ATOM   8581  H   SER A 563      -0.869  31.601 -15.667  1.00  1.97
H
ATOM   8582  HA  SER A 563      -2.798  31.926 -13.618  1.00  2.12
H
ATOM   8583 1HB  SER A 563      -4.348  32.393 -15.406  1.00  3.19
H
ATOM   8584 2HB  SER A 563      -2.784  33.096 -15.793  1.00  3.19
H
ATOM   8585  HG  SER A 563      -3.793  30.631 -16.706  1.00  3.19
H
ATOM   8586  N   GLY A 564      -2.958  28.934 -14.890  1.00  1.42
N
ATOM   8587  CA  GLY A 564      -3.708  27.681 -14.796  1.00  1.38
C
ATOM   8588  C   GLY A 564      -2.922  26.494 -15.324  1.00  1.21
C
ATOM   8589  O   GLY A 564      -1.707  26.572 -15.480  1.00  2.02
O
ATOM   8590  H   GLY A 564      -2.061  28.951 -15.352  1.00  1.70
H
ATOM   8591 1HA  GLY A 564      -3.979  27.503 -13.755  1.00  1.66
H
ATOM   8592 2HA  GLY A 564      -4.639  27.779 -15.353  1.00  1.66
H
ATOM   8593  N   VAL A 565      -3.624  25.394 -15.605  1.00  1.14
N
```

ATOM   8594 CA VAL A 565   -2.983 24.160 -16.064 1.00 0.99
C
ATOM   8595 C   VAL A 565   -2.167 24.358 -17.340 1.00 1.15
C
ATOM   8596 O   VAL A 565   -2.696 24.844 -18.341 1.00 1.46
O
ATOM   8597 CB VAL A 565   -4.039 23.075 -16.343 1.00 1.48
C
ATOM   8598 CG1 VAL A 565   -3.372 21.861 -16.973 1.00 1.48
C
ATOM   8599 CG2 VAL A 565   -4.733 22.695 -15.047 1.00 1.48
C
ATOM   8600 H   VAL A 565   -4.626 25.406 -15.475 1.00 1.37
H
ATOM   8601 HA VAL A 565   -2.347 23.808 -15.257 1.00 1.19
H
ATOM   8602 HB VAL A 565   -4.773 23.456 -17.053 1.00 1.78
H
ATOM   8603 1HG1 VAL A 565   -4.122 21.097 -17.176 1.00 1.78
H
ATOM   8604 2HG1 VAL A 565   -2.892 22.152 -17.907 1.00 1.78
H
ATOM   8605 3HG1 VAL A 565   -2.622 21.465 -16.292 1.00 1.78
H
ATOM   8606 1HG2 VAL A 565   -5.484 21.931 -15.247 1.00 1.78
H
ATOM   8607 2HG2 VAL A 565   -3.999 22.309 -14.340 1.00 1.78
H
ATOM   8608 3HG2 VAL A 565   -5.215 23.575 -14.629 1.00 1.78
H
ATOM   8609 N   PRO A 566   -0.880 23.989 -17.321 1.00 1.34
N
ATOM   8610 CA PRO A 566   0.064 24.076 -18.417 1.00 1.33
C
ATOM   8611 C   PRO A 566   0.046 22.872 -19.352 1.00 1.25
C
ATOM   8612 O   PRO A 566   -0.301 21.756 -18.964 1.00 1.79
O
ATOM   8613 CB PRO A 566   1.399 24.229 -17.710 1.00 2.00
C
ATOM   8614 CG PRO A 566   1.230 23.482 -16.449 1.00 2.00
C
ATOM   8615 CD PRO A 566   -0.188 23.747 -16.038 1.00 2.00
C
ATOM   8616 HA PRO A 566   -0.173 24.983 -18.977 1.00 1.60
H
ATOM   8617 1HB PRO A 566   2.208 23.832 -18.339 1.00 2.39
H
ATOM   8618 2HB PRO A 566   1.613 25.295 -17.546 1.00 2.39
H
ATOM   8619 1HG PRO A 566   1.428 22.423 -16.629 1.00 2.39
H
ATOM   8620 2HG PRO A 566   1.967 23.814 -15.713 1.00 2.39
H
ATOM   8621 1HD PRO A 566   -0.598 22.854 -15.545 1.00 2.39
H
ATOM   8622 2HD PRO A 566   -0.245 24.635 -15.393 1.00 2.39
H

ATOM   8623 N   GLN A 567   0.448 23.123 -20.586 1.00 1.42
N
ATOM   8624 CA GLN A 567   0.523 22.116 -21.637 1.00 1.38
C
ATOM   8625 C   GLN A 567   1.971 21.795 -22.004 1.00 1.39
C
ATOM   8626 O   GLN A 567   2.902 22.384 -21.457 1.00 1.56
O
ATOM   8627 CB GLN A 567   -0.231 22.603 -22.876 1.00 2.07
C
ATOM   8628 CG GLN A 567   -1.690 22.932 -22.616 1.00 2.07
C
ATOM   8629 CD GLN A 567   -2.494 21.717 -22.200 1.00 2.07
C
ATOM   8630 OE1 GLN A 567   -2.469 20.680 -22.869 1.00 2.07
O
ATOM   8631 NE2 GLN A 567   -3.214 21.837 -21.091 1.00 2.07
N
ATOM   8632 H   GLN A 567   0.691 24.077 -20.812 1.00 1.70
H
ATOM   8633 HA GLN A 567   0.054 21.201 -21.276 1.00 1.66
H
ATOM   8634 1HB GLN A 567   0.253 23.499 -23.267 1.00 2.48
H
ATOM   8635 2HB GLN A 567   -0.192 21.843 -23.655 1.00 2.48
H
ATOM   8636 1HG GLN A 567   -1.750 23.671 -21.817 1.00 2.48
H
ATOM   8637 2HG GLN A 567   -2.129 23.335 -23.529 1.00 2.48
H
ATOM   8638 1HE2 GLN A 567   -3.765 21.067 -20.766 1.00 2.48
H
ATOM   8639 2HE2 GLN A 567   -3.205 22.695 -20.579 1.00 2.48
H
ATOM   8640 N   SER A 568   2.147 20.835 -22.915 1.00 1.33
N
ATOM   8641 CA SER A 568   3.466 20.481 -23.440 1.00 1.39
C
ATOM   8642 C   SER A 568   3.953 21.589 -24.382 1.00 1.45
C
ATOM   8643 O   SER A 568   3.179 22.471 -24.758 1.00 1.40
O
ATOM   8644 CB SER A 568   3.409 19.155 -24.176 1.00 2.08
C
ATOM   8645 OG SER A 568   2.659 19.268 -25.354 1.00 2.08
O
ATOM   8646 H   SER A 568   1.335 20.363 -23.287 1.00 1.60
H
ATOM   8647 HA SER A 568   4.167 20.400 -22.608 1.00 1.67
H
ATOM   8648 1HB SER A 568   4.418 18.825 -24.418 1.00 2.50
H
ATOM   8649 2HB SER A 568   2.965 18.400 -23.529 1.00 2.50
H
ATOM   8650 HG SER A 568   3.113 19.921 -25.894 1.00 2.50
H
ATOM   8651 N   GLY A 569   5.226 21.552 -24.762 1.00 1.60
N

ATOM 8652 CA GLY A 569 5.806 22.591 -25.611 1.00 1.67
C
ATOM 8653 C GLY A 569 7.239 22.881 -25.178 1.00 1.67
C
ATOM 8654 O GLY A 569 7.704 22.353 -24.168 1.00 1.52
O
ATOM 8655 H GLY A 569 5.826 20.786 -24.482 1.00 1.92
H
ATOM 8656 1HA GLY A 569 5.792 22.264 -26.651 1.00 2.00
H
ATOM 8657 2HA GLY A 569 5.207 23.498 -25.545 1.00 2.00
H
ATOM 8658 N LYS A 570 7.946 23.705 -25.947 1.00 1.92
N
ATOM 8659 CA LYS A 570 9.323 24.047 -25.610 1.00 1.94
C
ATOM 8660 C LYS A 570 9.347 24.866 -24.342 1.00 1.86
C
ATOM 8661 O LYS A 570 8.448 25.670 -24.120 1.00 2.03
O
ATOM 8662 CB LYS A 570 10.013 24.846 -26.718 1.00 2.91
C
ATOM 8663 CG LYS A 570 11.508 25.064 -26.492 1.00 2.91
C
ATOM 8664 CD LYS A 570 12.144 25.891 -27.596 1.00 2.91
C
ATOM 8665 CE LYS A 570 13.654 25.983 -27.401 1.00 2.91
C
ATOM 8666 NZ LYS A 570 14.288 26.934 -28.353 1.00 2.91
N
ATOM 8667 H LYS A 570 7.521 24.120 -26.772 1.00 2.30
H
ATOM 8668 HA LYS A 570 9.882 23.128 -25.433 1.00 2.33
H
ATOM 8669 1HB LYS A 570 9.880 24.339 -27.675 1.00 3.49
H
ATOM 8670 2HB LYS A 570 9.550 25.823 -26.797 1.00 3.49
H
ATOM 8671 1HG LYS A 570 11.669 25.584 -25.551 1.00 3.49
H
ATOM 8672 2HG LYS A 570 12.010 24.098 -26.442 1.00 3.49
H
ATOM 8673 1HD LYS A 570 11.936 25.433 -28.563 1.00 3.49
H
ATOM 8674 2HD LYS A 570 11.725 26.899 -27.592 1.00 3.49
H
ATOM 8675 1HE LYS A 570 13.874 26.300 -26.383 1.00 3.49
H
ATOM 8676 2HE LYS A 570 14.083 24.993 -27.560 1.00 3.49
H
ATOM 8677 1HZ LYS A 570 15.284 26.952 -28.197 1.00 3.49
H
ATOM 8678 2HZ LYS A 570 14.100 26.653 -29.305 1.00 3.49
H
ATOM 8679 3HZ LYS A 570 13.910 27.859 -28.183 1.00 3.49
H
ATOM 8680 N SER A 571 10.383 24.677 -23.534 1.00 1.74
N

```
ATOM   8681  CA   SER A 571       10.577  25.428 -22.298  1.00  1.71
C
ATOM   8682  C    SER A 571       10.303  26.921 -22.510  1.00  1.88
C
ATOM   8683  O    SER A 571        9.512  27.525 -21.782  1.00  1.91
O
ATOM   8684  CB   SER A 571       12.011  25.228 -21.833  1.00  2.56
C
ATOM   8685  OG   SER A 571       12.279  25.914 -20.646  1.00  2.56
O
ATOM   8686  H    SER A 571       11.073  23.982 -23.787  1.00  2.09
H
ATOM   8687  HA   SER A 571        9.887  25.046 -21.543  1.00  2.05
H
ATOM   8688 1HB   SER A 571       12.205  24.164 -21.692  1.00  3.08
H
ATOM   8689 2HB   SER A 571       12.686  25.578 -22.612  1.00  3.08
H
ATOM   8690  HG   SER A 571       12.062  25.293 -19.943  1.00  3.08
H
ATOM   8691  N    GLU A 572       10.946  27.491 -23.532  1.00  2.03
N
ATOM   8692  CA   GLU A 572       10.796  28.894 -23.902  1.00  2.25
C
ATOM   8693  C    GLU A 572        9.373  29.237 -24.310  1.00  2.32
C
ATOM   8694  O    GLU A 572        8.839  30.274 -23.908  1.00  2.39
O
ATOM   8695  CB   GLU A 572       11.727  29.221 -25.067  1.00  3.38
C
ATOM   8696  CG   GLU A 572       13.208  29.223 -24.731  1.00  3.38
C
ATOM   8697  CD   GLU A 572       14.067  29.392 -25.954  1.00  3.38
C
ATOM   8698  OE1 GLU A 572       13.538  29.322 -27.042  1.00  3.38
O
ATOM   8699  OE2 GLU A 572       15.252  29.573 -25.808  1.00  3.38
O
ATOM   8700  H    GLU A 572       11.579  26.922 -24.073  1.00  2.44
H
ATOM   8701  HA   GLU A 572       11.066  29.508 -23.042  1.00  2.70
H
ATOM   8702 1HB   GLU A 572       11.570  28.502 -25.870  1.00  4.05
H
ATOM   8703 2HB   GLU A 572       11.478  30.207 -25.460  1.00  4.05
H
ATOM   8704 1HG   GLU A 572       13.414  30.039 -24.039  1.00  4.05
H
ATOM   8705 2HG   GLU A 572       13.462  28.287 -24.237  1.00  4.05
H
ATOM   8706  N    GLU A 573        8.762  28.356 -25.105  1.00  2.31
N
ATOM   8707  CA   GLU A 573        7.403  28.556 -25.578  1.00  2.31
C
ATOM   8708  C    GLU A 573        6.425  28.611 -24.433  1.00  2.20
C
ATOM   8709  O    GLU A 573        5.512  29.430 -24.444  1.00  2.34
O
```

```
ATOM   8710  CB  GLU A 573       6.986  27.437 -26.540  1.00  3.46
C
ATOM   8711  CG  GLU A 573       7.655  27.474 -27.905  1.00  3.46
C
ATOM   8712  CD  GLU A 573       7.312  26.278 -28.757  1.00  3.46
C
ATOM   8713  OE1 GLU A 573       6.952  25.255 -28.216  1.00  3.46
O
ATOM   8714  OE2 GLU A 573       7.409  26.390 -29.956  1.00  3.46
O
ATOM   8715  H   GLU A 573       9.259  27.526 -25.392  1.00  2.77
H
ATOM   8716  HA  GLU A 573       7.359  29.508 -26.110  1.00  2.77
H
ATOM   8717 1HB  GLU A 573       7.204  26.470 -26.090  1.00  4.16
H
ATOM   8718 2HB  GLU A 573       5.908  27.484 -26.701  1.00  4.16
H
ATOM   8719 1HG  GLU A 573       7.332  28.375 -28.424  1.00  4.16
H
ATOM   8720 2HG  GLU A 573       8.734  27.533 -27.775  1.00  4.16
H
ATOM   8721  N   LEU A 574       6.623  27.747 -23.445  1.00  1.99
N
ATOM   8722  CA  LEU A 574       5.741  27.688 -22.299  1.00  1.83
C
ATOM   8723  C   LEU A 574       5.900  28.925 -21.426  1.00  1.91
C
ATOM   8724  O   LEU A 574       4.912  29.459 -20.923  1.00  1.95
O
ATOM   8725  CB  LEU A 574       6.042  26.424 -21.502  1.00  2.75
C
ATOM   8726  CG  LEU A 574       5.733  25.123 -22.253  1.00  2.75
C
ATOM   8727  CD1 LEU A 574       6.187  23.947 -21.418  1.00  2.75
C
ATOM   8728  CD2 LEU A 574       4.258  25.040 -22.571  1.00  2.75
C
ATOM   8729  H   LEU A 574       7.393  27.096 -23.507  1.00  2.39
H
ATOM   8730  HA  LEU A 574       4.713  27.644 -22.655  1.00  2.20
H
ATOM   8731 1HB  LEU A 574       7.097  26.417 -21.236  1.00  3.29
H
ATOM   8732 2HB  LEU A 574       5.451  26.433 -20.588  1.00  3.29
H
ATOM   8733  HG  LEU A 574       6.294  25.108 -23.186  1.00  3.29
H
ATOM   8734 1HD1 LEU A 574       5.990  23.019 -21.955  1.00  3.29
H
ATOM   8735 2HD1 LEU A 574       7.254  24.037 -21.222  1.00  3.29
H
ATOM   8736 3HD1 LEU A 574       5.643  23.945 -20.476  1.00  3.29
H
ATOM   8737 1HD2 LEU A 574       4.059  24.122 -23.118  1.00  3.29
H
ATOM   8738 2HD2 LEU A 574       3.689  25.032 -21.649  1.00  3.29
H
```

ATOM   8739 3HD2 LEU A 574   3.967 25.894 -23.179 1.00 3.29
H
ATOM   8740 N   LEU A 575   7.132 29.425 -21.296 1.00 2.00
N
ATOM   8741 CA LEU A 575   7.354 30.644 -20.527 1.00 2.14
C
ATOM   8742 C   LEU A 575   6.584 31.800 -21.178 1.00 2.43
C
ATOM   8743 O   LEU A 575   5.977 32.627 -20.492 1.00 2.62
O
ATOM   8744 CB LEU A 575   8.855 30.975 -20.468 1.00 3.21
C
ATOM   8745 CG LEU A 575   9.752 30.022 -19.657 1.00 3.21
C
ATOM   8746 CD1 LEU A 575   11.207 30.390 -19.892 1.00 3.21
C
ATOM   8747 CD2 LEU A 575   9.411 30.114 -18.184 1.00 3.21
C
ATOM   8748 H   LEU A 575   7.927 28.943 -21.704 1.00 2.40
H
ATOM   8749 HA LEU A 575   6.977 30.496 -19.515 1.00 2.57
H
ATOM   8750 1HB LEU A 575   9.244 31.004 -21.483 1.00 3.85
H
ATOM   8751 2HB LEU A 575   8.967 31.966 -20.032 1.00 3.85
H
ATOM   8752 HG LEU A 575   9.600 29.000 -19.993 1.00 3.85
H
ATOM   8753 1HD1 LEU A 575   11.850 29.713 -19.331 1.00 3.85
H
ATOM   8754 2HD1 LEU A 575   11.438 30.309 -20.954 1.00 3.85
H
ATOM   8755 3HD1 LEU A 575   11.383 31.412 -19.560 1.00 3.85
H
ATOM   8756 1HD2 LEU A 575   10.050 29.435 -17.619 1.00 3.85
H
ATOM   8757 2HD2 LEU A 575   9.566 31.135 -17.834 1.00 3.85
H
ATOM   8758 3HD2 LEU A 575   8.370 29.833 -18.043 1.00 3.85
H
ATOM   8759 N   ASP A 576   6.583 31.831 -22.519 1.00 2.59
N
ATOM   8760 CA ASP A 576   5.814 32.829 -23.253 1.00 2.95
C
ATOM   8761 C   ASP A 576   4.311 32.633 -23.033 1.00 2.75
C
ATOM   8762 O   ASP A 576   3.594 33.576 -22.698 1.00 2.89
O
ATOM   8763 CB ASP A 576   6.102 32.761 -24.761 1.00 4.43
C
ATOM   8764 CG ASP A 576   7.479 33.263 -25.190 1.00 4.43
C
ATOM   8765 OD1 ASP A 576   8.134 33.938 -24.433 1.00 4.43
O
ATOM   8766 OD2 ASP A 576   7.856 32.973 -26.301 1.00 4.43
O
ATOM   8767 H   ASP A 576   7.133 31.154 -23.036 1.00 3.11
H

```
ATOM   8768  HA  ASP A 576       6.088  33.817 -22.886  1.00  3.54
H
ATOM   8769 1HB  ASP A 576       6.001  31.731 -25.099  1.00  5.31
H
ATOM   8770 2HB  ASP A 576       5.349  33.344 -25.292  1.00  5.31
H
ATOM   8771  N   MET A 577       3.847  31.392 -23.197  1.00  2.51
N
ATOM   8772  CA  MET A 577       2.433  31.054 -23.056  1.00  2.47
C
ATOM   8773  C   MET A 577       1.834  31.420 -21.718  1.00  2.41
C
ATOM   8774  O   MET A 577       0.673  31.826 -21.656  1.00  2.48
O
ATOM   8775  CB  MET A 577       2.217  29.559 -23.289  1.00  3.71
C
ATOM   8776  CG  MET A 577       2.366  29.101 -24.729  1.00  3.71
C
ATOM   8777  SD  MET A 577       2.307  27.307 -24.893  1.00  3.71
S
ATOM   8778  CE  MET A 577       0.604  26.970 -24.456  1.00  3.71
C
ATOM   8779  H   MET A 577       4.487  30.657 -23.462  1.00  3.01
H
ATOM   8780  HA  MET A 577       1.881  31.605 -23.816  1.00  2.96
H
ATOM   8781 1HB  MET A 577       2.926  28.994 -22.686  1.00  4.45
H
ATOM   8782 2HB  MET A 577       1.216  29.282 -22.956  1.00  4.45
H
ATOM   8783 1HG  MET A 577       1.553  29.522 -25.320  1.00  4.45
H
ATOM   8784 2HG  MET A 577       3.301  29.461 -25.149  1.00  4.45
H
ATOM   8785 1HE  MET A 577       0.420  25.897 -24.513  1.00  4.45
H
ATOM   8786 2HE  MET A 577       0.414  27.319 -23.440  1.00  4.45
H
ATOM   8787 3HE  MET A 577      -0.059  27.490 -25.148  1.00  4.45
H
ATOM   8788  N   TYR A 578       2.606  31.293 -20.642  1.00  2.27
N
ATOM   8789  CA  TYR A 578       2.034  31.562 -19.336  1.00  2.19
C
ATOM   8790  C   TYR A 578       2.436  32.907 -18.745  1.00  2.33
C
ATOM   8791  O   TYR A 578       2.269  33.127 -17.547  1.00  2.24
O
ATOM   8792  CB  TYR A 578       2.363  30.395 -18.412  1.00  3.29
C
ATOM   8793  CG  TYR A 578       1.803  29.149 -19.047  1.00  3.29
C
ATOM   8794  CD1 TYR A 578       2.641  28.124 -19.438  1.00  3.29
C
ATOM   8795  CD2 TYR A 578       0.447  29.076 -19.325  1.00  3.29
C
ATOM   8796  CE1 TYR A 578       2.135  27.037 -20.108  1.00  3.29
C
```

ATOM 8797 CE2 TYR A 578 -0.060 27.987 -20.002 1.00 3.29
C
ATOM 8798 CZ TYR A 578 0.782 26.976 -20.403 1.00 3.29
C
ATOM 8799 OH TYR A 578 0.276 25.909 -21.110 1.00 3.29
O
ATOM 8800 H TYR A 578 3.556 30.953 -20.726 1.00 2.72
H
ATOM 8801 HA TYR A 578 0.950 31.584 -19.449 1.00 2.63
H
ATOM 8802 1HB TYR A 578 3.442 30.284 -18.292 1.00 3.94
H
ATOM 8803 2HB TYR A 578 1.910 30.534 -17.432 1.00 3.94
H
ATOM 8804 HD1 TYR A 578 3.708 28.185 -19.231 1.00 3.94
H
ATOM 8805 HD2 TYR A 578 -0.214 29.891 -19.031 1.00 3.94
H
ATOM 8806 HE1 TYR A 578 2.801 26.243 -20.417 1.00 3.94
H
ATOM 8807 HE2 TYR A 578 -1.124 27.938 -20.239 1.00 3.94
H
ATOM 8808 HH TYR A 578 -0.648 26.077 -21.317 1.00 3.94
H
ATOM 8809 N GLY A 579 2.923 33.829 -19.586 1.00 2.63
N
ATOM 8810 CA GLY A 579 3.154 35.199 -19.131 1.00 2.94
C
ATOM 8811 C GLY A 579 4.358 35.422 -18.219 1.00 2.61
C
ATOM 8812 O GLY A 579 4.372 36.391 -17.459 1.00 2.59
O
ATOM 8813 H GLY A 579 3.087 33.609 -20.563 1.00 3.16
H
ATOM 8814 1HA GLY A 579 3.235 35.843 -20.000 1.00 3.53
H
ATOM 8815 2HA GLY A 579 2.261 35.534 -18.606 1.00 3.53
H
ATOM 8816 N ILE A 580 5.342 34.528 -18.248 1.00 2.45
N
ATOM 8817 CA ILE A 580 6.477 34.648 -17.337 1.00 2.16
C
ATOM 8818 C ILE A 580 7.846 34.724 -18.022 1.00 2.12
C
ATOM 8819 O ILE A 580 8.873 34.521 -17.374 1.00 3.89
O
ATOM 8820 CB ILE A 580 6.435 33.493 -16.322 1.00 3.24
C
ATOM 8821 CG1 ILE A 580 6.464 32.163 -17.062 1.00 3.24
C
ATOM 8822 CG2 ILE A 580 5.208 33.586 -15.421 1.00 3.24
C
ATOM 8823 CD1 ILE A 580 6.622 30.988 -16.143 1.00 3.24
C
ATOM 8824 H ILE A 580 5.323 33.755 -18.902 1.00 2.94
H
ATOM 8825 HA ILE A 580 6.349 35.572 -16.774 1.00 2.59
H

```
ATOM   8826  HB  ILE A 580       7.329  33.536 -15.703  1.00  3.89
H
ATOM   8827 1HG1 ILE A 580       5.538  32.044 -17.621  1.00  3.89
H
ATOM   8828 2HG1 ILE A 580       7.293  32.166 -17.769  1.00  3.89
H
ATOM   8829 1HG2 ILE A 580       5.220  32.763 -14.707  1.00  3.89
H
ATOM   8830 2HG2 ILE A 580       5.224  34.533 -14.883  1.00  3.89
H
ATOM   8831 3HG2 ILE A 580       4.303  33.527 -16.024  1.00  3.89
H
ATOM   8832 1HD1 ILE A 580       6.635  30.069 -16.724  1.00  3.89
H
ATOM   8833 2HD1 ILE A 580       7.557  31.083 -15.591  1.00  3.89
H
ATOM   8834 3HD1 ILE A 580       5.788  30.960 -15.443  1.00  3.89
H
ATOM   8835  N   SER A 581       7.863  35.014 -19.323  1.00  3.42
N
ATOM   8836  CA  SER A 581       9.127  35.161 -20.042  1.00  3.06
C
ATOM   8837  C   SER A 581       9.547  36.615 -20.098  1.00  3.03
C
ATOM   8838  O   SER A 581       8.747  37.504 -19.801  1.00  3.80
O
ATOM   8839  CB  SER A 581       9.028  34.631 -21.450  1.00  4.59
C
ATOM   8840  OG  SER A 581       8.213  35.445 -22.241  1.00  4.59
O
ATOM   8841  H   SER A 581       6.995  35.154 -19.819  1.00  4.10
H
ATOM   8842  HA  SER A 581       9.897  34.601 -19.511  1.00  3.67
H
ATOM   8843 1HB  SER A 581      10.024  34.572 -21.888  1.00  5.51
H
ATOM   8844 2HB  SER A 581       8.625  33.626 -21.433  1.00  5.51
H
ATOM   8845  HG  SER A 581       8.101  34.962 -23.068  1.00  5.51
H
ATOM   8846  N   ALA A 582      10.785  36.842 -20.534  1.00  2.67
N
ATOM   8847  CA  ALA A 582      11.381  38.168 -20.661  1.00  2.75
C
ATOM   8848  C   ALA A 582      10.478  39.149 -21.379  1.00  3.07
C
ATOM   8849  O   ALA A 582      10.347  40.286 -20.942  1.00  3.23
O
ATOM   8850  CB  ALA A 582      12.700  38.073 -21.403  1.00  4.12
C
ATOM   8851  H   ALA A 582      11.365  36.040 -20.750  1.00  3.20
H
ATOM   8852  HA  ALA A 582      11.563  38.551 -19.658  1.00  3.30
H
ATOM   8853 1HB  ALA A 582      13.159  39.060 -21.463  1.00  4.95
H
ATOM   8854 2HB  ALA A 582      13.365  37.394 -20.870  1.00  4.95
H
```

```
ATOM   8855 3HB  ALA A 582      12.527  37.693 -22.409  1.00  4.95
H
ATOM   8856  N   ARG A 583       9.842  38.714 -22.465  1.00  3.20
N
ATOM   8857  CA  ARG A 583       8.966  39.583 -23.240  1.00  3.66
C
ATOM   8858  C   ARG A 583       7.773  40.075 -22.436  1.00  3.53
C
ATOM   8859  O   ARG A 583       7.314  41.200 -22.632  1.00  4.43
O
ATOM   8860  CB  ARG A 583       8.479  38.856 -24.478  1.00  5.49
C
ATOM   8861  CG  ARG A 583       9.545  38.632 -25.538  1.00  5.49
C
ATOM   8862  CD  ARG A 583       9.029  37.845 -26.688  1.00  5.49
C
ATOM   8863  NE  ARG A 583      10.042  37.660 -27.717  1.00  5.49
N
ATOM   8864  CZ  ARG A 583       9.927  36.820 -28.765  1.00  5.49
C
ATOM   8865  NH1 ARG A 583       8.844  36.089 -28.910  1.00  5.49
N
ATOM   8866  NH2 ARG A 583      10.905  36.731 -29.650  1.00  5.49
N
ATOM   8867  H   ARG A 583       9.979  37.759 -22.769  1.00  3.84
H
ATOM   8868  HA  ARG A 583       9.539  40.448 -23.561  1.00  4.39
H
ATOM   8869 1HB  ARG A 583       8.081  37.883 -24.197  1.00  6.59
H
ATOM   8870 2HB  ARG A 583       7.667  39.421 -24.938  1.00  6.59
H
ATOM   8871 1HG  ARG A 583       9.894  39.594 -25.913  1.00  6.59
H
ATOM   8872 2HG  ARG A 583      10.384  38.088 -25.100  1.00  6.59
H
ATOM   8873 1HD  ARG A 583       8.710  36.862 -26.343  1.00  6.59
H
ATOM   8874 2HD  ARG A 583       8.182  38.366 -27.134  1.00  6.59
H
ATOM   8875  HE  ARG A 583      10.891  38.205 -27.643  1.00  6.59
H
ATOM   8876 1HH1 ARG A 583       8.096  36.151 -28.232  1.00  6.59
H
ATOM   8877 2HH1 ARG A 583       8.760  35.459 -29.694  1.00  6.59
H
ATOM   8878 1HH2 ARG A 583      11.739  37.294 -29.539  1.00  6.59
H
ATOM   8879 2HH2 ARG A 583      10.821  36.103 -30.434  1.00  6.59
H
ATOM   8880  N   HIS A 584       7.278  39.244 -21.530  1.00  2.98
N
ATOM   8881  CA  HIS A 584       6.139  39.612 -20.718  1.00  2.98
C
ATOM   8882  C   HIS A 584       6.581  40.496 -19.581  1.00  3.04
C
ATOM   8883  O   HIS A 584       5.839  41.386 -19.163  1.00  3.13
O
```

```
ATOM   8884  CB  HIS A 584       5.440  38.367 -20.212  1.00  4.47
C
ATOM   8885  CG  HIS A 584       4.765  37.644 -21.322  1.00  4.47
C
ATOM   8886  ND1 HIS A 584       3.642  38.139 -21.951  1.00  4.47
N
ATOM   8887  CD2 HIS A 584       5.051  36.472 -21.932  1.00  4.47
C
ATOM   8888  CE1 HIS A 584       3.263  37.298 -22.895  1.00  4.47
C
ATOM   8889  NE2 HIS A 584       4.099  36.280 -22.904  1.00  4.47
N
ATOM   8890  H   HIS A 584       7.706  38.341 -21.378  1.00  3.58
H
ATOM   8891  HA  HIS A 584       5.426  40.176 -21.319  1.00  3.58
H
ATOM   8892 1HB  HIS A 584       6.161  37.697 -19.742  1.00  5.36
H
ATOM   8893 2HB  HIS A 584       4.694  38.634 -19.463  1.00  5.36
H
ATOM   8894  HD2 HIS A 584       5.876  35.803 -21.695  1.00  5.36
H
ATOM   8895  HE1 HIS A 584       2.403  37.424 -23.555  1.00  5.36
H
ATOM   8896  HE2 HIS A 584       4.053  35.481 -23.522  1.00  5.36
H
ATOM   8897  N   ILE A 585       7.807  40.272 -19.112  1.00  3.04
N
ATOM   8898  CA  ILE A 585       8.398  41.103 -18.080  1.00  3.14
C
ATOM   8899  C   ILE A 585       8.585  42.509 -18.635  1.00  3.39
C
ATOM   8900  O   ILE A 585       8.246  43.483 -17.968  1.00  3.49
O
ATOM   8901  CB  ILE A 585       9.730  40.513 -17.591  1.00  4.71
C
ATOM   8902  CG1 ILE A 585       9.454  39.191 -16.866  1.00  4.71
C
ATOM   8903  CG2 ILE A 585      10.429  41.499 -16.662  1.00  4.71
C
ATOM   8904  CD1 ILE A 585      10.689  38.373 -16.599  1.00  4.71
C
ATOM   8905  H   ILE A 585       8.340  39.494 -19.479  1.00  3.65
H
ATOM   8906  HA  ILE A 585       7.716  41.157 -17.234  1.00  3.77
H
ATOM   8907  HB  ILE A 585      10.371  40.296 -18.442  1.00  5.65
H
ATOM   8908 1HG1 ILE A 585       8.970  39.405 -15.913  1.00  5.65
H
ATOM   8909 2HG1 ILE A 585       8.770  38.594 -17.469  1.00  5.65
H
ATOM   8910 1HG2 ILE A 585      11.370  41.070 -16.321  1.00  5.65
H
ATOM   8911 2HG2 ILE A 585      10.626  42.426 -17.199  1.00  5.65
H
ATOM   8912 3HG2 ILE A 585       9.791  41.705 -15.804  1.00  5.65
H
```

ATOM 8913 1HD1 ILE A 585 10.411 37.452 -16.085 1.00 5.65
H
ATOM 8914 2HD1 ILE A 585 11.174 38.127 -17.544 1.00 5.65
H
ATOM 8915 3HD1 ILE A 585 11.373 38.943 -15.975 1.00 5.65
H
ATOM 8916 N ILE A 586 9.089 42.598 -19.873 1.00 3.54
N
ATOM 8917 CA ILE A 586 9.266 43.864 -20.585 1.00 3.90
C
ATOM 8918 C ILE A 586 7.968 44.638 -20.678 1.00 4.02
C
ATOM 8919 O ILE A 586 7.929 45.824 -20.348 1.00 4.20
O
ATOM 8920 CB ILE A 586 9.799 43.605 -22.011 1.00 5.85
C
ATOM 8921 CG1 ILE A 586 11.236 43.104 -21.949 1.00 5.85
C
ATOM 8922 CG2 ILE A 586 9.671 44.836 -22.890 1.00 5.85
C
ATOM 8923 CD1 ILE A 586 11.731 42.528 -23.248 1.00 5.85
C
ATOM 8924 H ILE A 586 9.367 41.749 -20.342 1.00 4.25
H
ATOM 8925 HA ILE A 586 9.990 44.469 -20.042 1.00 4.68
H
ATOM 8926 HB ILE A 586 9.212 42.806 -22.458 1.00 7.02
H
ATOM 8927 1HG1 ILE A 586 11.887 43.927 -21.676 1.00 7.02
H
ATOM 8928 2HG1 ILE A 586 11.312 42.345 -21.180 1.00 7.02
H
ATOM 8929 1HG2 ILE A 586 10.031 44.601 -23.892 1.00 7.02
H
ATOM 8930 2HG2 ILE A 586 8.627 45.139 -22.944 1.00 7.02
H
ATOM 8931 3HG2 ILE A 586 10.261 45.647 -22.481 1.00 7.02
H
ATOM 8932 1HD1 ILE A 586 12.756 42.186 -23.123 1.00 7.02
H
ATOM 8933 2HD1 ILE A 586 11.110 41.686 -23.537 1.00 7.02
H
ATOM 8934 3HD1 ILE A 586 11.691 43.290 -24.025 1.00 7.02
H
ATOM 8935 N VAL A 587 6.904 43.956 -21.112 1.00 3.93
N
ATOM 8936 CA VAL A 587 5.591 44.573 -21.202 1.00 4.00
C
ATOM 8937 C VAL A 587 5.104 45.060 -19.850 1.00 3.96
C
ATOM 8938 O VAL A 587 4.603 46.179 -19.748 1.00 4.13
O
ATOM 8939 CB VAL A 587 4.566 43.592 -21.790 1.00 6.00
C
ATOM 8940 CG1 VAL A 587 3.163 44.165 -21.657 1.00 6.00
C
ATOM 8941 CG2 VAL A 587 4.885 43.350 -23.256 1.00 6.00
C

```
ATOM   8942  H   VAL A 587       7.011  42.988 -21.401  1.00  4.72
H
ATOM   8943  HA  VAL A 587       5.663  45.431 -21.870  1.00  4.80
H
ATOM   8944  HB  VAL A 587       4.604  42.652 -21.239  1.00  7.20
H
ATOM   8945 1HG1 VAL A 587       2.442  43.461 -22.072  1.00  7.20
H
ATOM   8946 2HG1 VAL A 587       2.937  44.335 -20.605  1.00  7.20
H
ATOM   8947 3HG1 VAL A 587       3.102  45.109 -22.198  1.00  7.20
H
ATOM   8948 1HG2 VAL A 587       4.163  42.651 -23.676  1.00  7.20
H
ATOM   8949 2HG2 VAL A 587       4.834  44.295 -23.798  1.00  7.20
H
ATOM   8950 3HG2 VAL A 587       5.887  42.938 -23.351  1.00  7.20
H
ATOM   8951  N   ALA A 588       5.252  44.220 -18.820  1.00  3.78
N
ATOM   8952  CA  ALA A 588       4.839  44.570 -17.466  1.00  3.79
C
ATOM   8953  C   ALA A 588       5.545  45.830 -16.990  1.00  4.00
C
ATOM   8954  O   ALA A 588       4.904  46.711 -16.419  1.00  4.24
O
ATOM   8955  CB  ALA A 588       5.123  43.421 -16.513  1.00  5.69
C
ATOM   8956  H   ALA A 588       5.651  43.304 -18.975  1.00  4.54
H
ATOM   8957  HA  ALA A 588       3.768  44.767 -17.478  1.00  4.55
H
ATOM   8958 1HB  ALA A 588       4.787  43.690 -15.513  1.00  6.82
H
ATOM   8959 2HB  ALA A 588       4.590  42.531 -16.848  1.00  6.82
H
ATOM   8960 3HB  ALA A 588       6.192  43.217 -16.493  1.00  6.82
H
ATOM   8961  N   VAL A 589       6.854  45.920 -17.250  1.00  3.98
N
ATOM   8962  CA  VAL A 589       7.631  47.099 -16.891  1.00  4.28
C
ATOM   8963  C   VAL A 589       7.089  48.337 -17.561  1.00  4.47
C
ATOM   8964  O   VAL A 589       6.879  49.351 -16.899  1.00  4.63
O
ATOM   8965  CB  VAL A 589       9.115  46.922 -17.273  1.00  6.42
C
ATOM   8966  CG1 VAL A 589       9.846  48.250 -17.131  1.00  6.42
C
ATOM   8967  CG2 VAL A 589       9.754  45.877 -16.364  1.00  6.42
C
ATOM   8968  H   VAL A 589       7.325  45.147 -17.699  1.00  4.78
H
ATOM   8969  HA  VAL A 589       7.569  47.238 -15.812  1.00  5.14
H
ATOM   8970  HB  VAL A 589       9.191  46.605 -18.312  1.00  7.70
H
```

```
ATOM   8971 1HG1 VAL A 589      10.893  48.122 -17.405  1.00  7.70
H
ATOM   8972 2HG1 VAL A 589       9.388  48.988 -17.789  1.00  7.70
H
ATOM   8973 3HG1 VAL A 589       9.781  48.597 -16.102  1.00  7.70
H
ATOM   8974 1HG2 VAL A 589      10.800  45.748 -16.632  1.00  7.70
H
ATOM   8975 2HG2 VAL A 589       9.684  46.202 -15.329  1.00  7.70
H
ATOM   8976 3HG2 VAL A 589       9.236  44.929 -16.470  1.00  7.70
H
ATOM   8977  N   LYS A 590       6.847  48.244 -18.868  1.00  4.50
N
ATOM   8978  CA  LYS A 590       6.319  49.368 -19.624  1.00  4.77
C
ATOM   8979  C   LYS A 590       4.955  49.803 -19.106  1.00  4.98
C
ATOM   8980  O   LYS A 590       4.690  51.000 -19.001  1.00  5.31
O
ATOM   8981  CB  LYS A 590       6.226  49.006 -21.102  1.00  7.15
C
ATOM   8982  CG  LYS A 590       7.567  48.888 -21.815  1.00  7.15
C
ATOM   8983  CD  LYS A 590       7.366  48.495 -23.271  1.00  7.15
C
ATOM   8984  CE  LYS A 590       8.684  48.401 -24.022  1.00  7.15
C
ATOM   8985  NZ  LYS A 590       8.475  47.985 -25.439  1.00  7.15
N
ATOM   8986  H   LYS A 590       7.051  47.379 -19.354  1.00  5.40
H
ATOM   8987  HA  LYS A 590       7.001  50.210 -19.514  1.00  5.72
H
ATOM   8988 1HB  LYS A 590       5.709  48.053 -21.211  1.00  8.59
H
ATOM   8989 2HB  LYS A 590       5.636  49.759 -21.625  1.00  8.59
H
ATOM   8990 1HG  LYS A 590       8.088  49.845 -21.773  1.00  8.59
H
ATOM   8991 2HG  LYS A 590       8.183  48.138 -21.321  1.00  8.59
H
ATOM   8992 1HD  LYS A 590       6.862  47.529 -23.318  1.00  8.59
H
ATOM   8993 2HD  LYS A 590       6.735  49.238 -23.760  1.00  8.59
H
ATOM   8994 1HE  LYS A 590       9.176  49.371 -24.008  1.00  8.59
H
ATOM   8995 2HE  LYS A 590       9.329  47.674 -23.531  1.00  8.59
H
ATOM   8996 1HZ  LYS A 590       9.366  47.932 -25.912  1.00  8.59
H
ATOM   8997 2HZ  LYS A 590       8.028  47.079 -25.462  1.00  8.59
H
ATOM   8998 3HZ  LYS A 590       7.887  48.662 -25.905  1.00  8.59
H
ATOM   8999  N   CYS A 591       4.107  48.834 -18.759  1.00  4.87
N
```

```
ATOM   9000  CA  CYS A 591       2.790  49.132 -18.220  1.00  5.08
C
ATOM   9001  C   CYS A 591       2.908  49.895 -16.916  1.00  5.22
C
ATOM   9002  O   CYS A 591       2.213  50.889 -16.714  1.00  5.73
O
ATOM   9003  CB  CYS A 591       1.993  47.848 -17.988  1.00  7.62
C
ATOM   9004  SG  CYS A 591       1.468  47.018 -19.507  1.00  7.62
S
ATOM   9005  H   CYS A 591       4.372  47.867 -18.888  1.00  5.84
H
ATOM   9006  HA  CYS A 591       2.252  49.748 -18.939  1.00  6.10
H
ATOM   9007 1HB  CYS A 591       2.594  47.145 -17.412  1.00  9.14
H
ATOM   9008 2HB  CYS A 591       1.103  48.074 -17.403  1.00  9.14
H
ATOM   9009  HG  CYS A 591       0.880  45.988 -18.905  1.00  9.14
H
ATOM   9010  N   MET A 592       3.805  49.445 -16.039  1.00  5.01
N
ATOM   9011  CA  MET A 592       4.031  50.115 -14.768  1.00  5.18
C
ATOM   9012  C   MET A 592       4.543  51.542 -14.990  1.00  5.08
C
ATOM   9013  O   MET A 592       4.091  52.475 -14.325  1.00  5.27
O
ATOM   9014  CB  MET A 592       5.014  49.296 -13.926  1.00  7.77
C
ATOM   9015  CG  MET A 592       4.466  47.969 -13.407  1.00  7.77
C
ATOM   9016  SD  MET A 592       3.099  48.151 -12.244  1.00  7.77
S
ATOM   9017  CE  MET A 592       3.970  48.717 -10.782  1.00  7.77
C
ATOM   9018  H   MET A 592       4.335  48.609 -16.250  1.00  6.01
H
ATOM   9019  HA  MET A 592       3.082  50.180 -14.236  1.00  6.22
H
ATOM   9020 1HB  MET A 592       5.911  49.087 -14.508  1.00  9.32
H
ATOM   9021 2HB  MET A 592       5.311  49.878 -13.061  1.00  9.32
H
ATOM   9022 1HG  MET A 592       4.118  47.367 -14.243  1.00  9.32
H
ATOM   9023 2HG  MET A 592       5.265  47.425 -12.906  1.00  9.32
H
ATOM   9024 1HE  MET A 592       3.258  48.875  -9.972  1.00  9.32
H
ATOM   9025 2HE  MET A 592       4.702  47.966 -10.482  1.00  9.32
H
ATOM   9026 3HE  MET A 592       4.481  49.654 -11.004  1.00  9.32
H
ATOM   9027  N   LEU A 593       5.428  51.722 -15.976  1.00  4.91
N
ATOM   9028  CA  LEU A 593       5.954  53.044 -16.317  1.00  5.00
C
```

```
ATOM   9029  C   LEU A 593       4.855  53.987 -16.800  1.00  5.34
C
ATOM   9030  O   LEU A 593       4.887  55.187 -16.517  1.00  7.31
O
ATOM   9031  CB  LEU A 593       7.020  52.924 -17.415  1.00  7.50
C
ATOM   9032  CG  LEU A 593       8.333  52.248 -17.012  1.00  7.50
C
ATOM   9033  CD1 LEU A 593       9.194  52.050 -18.250  1.00  7.50
C
ATOM   9034  CD2 LEU A 593       9.043  53.104 -15.988  1.00  7.50
C
ATOM   9035  H   LEU A 593       5.773  50.920 -16.486  1.00  5.89
H
ATOM   9036  HA  LEU A 593       6.409  53.474 -15.426  1.00  6.00
H
ATOM   9037 1HB  LEU A 593       6.603  52.361 -18.246  1.00  9.00
H
ATOM   9038 2HB  LEU A 593       7.261  53.925 -17.771  1.00  9.00
H
ATOM   9039  HG  LEU A 593       8.130  51.274 -16.579  1.00  9.00
H
ATOM   9040 1HD1 LEU A 593      10.125  51.563 -17.964  1.00  9.00
H
ATOM   9041 2HD1 LEU A 593       8.666  51.427 -18.969  1.00  9.00
H
ATOM   9042 3HD1 LEU A 593       9.413  53.017 -18.700  1.00  9.00
H
ATOM   9043 1HD2 LEU A 593       9.972  52.615 -15.704  1.00  9.00
H
ATOM   9044 2HD2 LEU A 593       9.259  54.083 -16.415  1.00  9.00
H
ATOM   9045 3HD2 LEU A 593       8.409  53.222 -15.108  1.00  9.00
H
ATOM   9046  N   LEU A 594       3.878  53.438 -17.513  1.00  4.44
N
ATOM   9047  CA  LEU A 594       2.760  54.211 -18.029  1.00  4.71
C
ATOM   9048  C   LEU A 594       1.601  54.374 -17.040  1.00  5.26
C
ATOM   9049  O   LEU A 594       0.596  55.005 -17.377  1.00  6.06
O
ATOM   9050  CB  LEU A 594       2.240  53.548 -19.307  1.00  7.06
C
ATOM   9051  CG  LEU A 594       3.226  53.518 -20.481  1.00  7.06
C
ATOM   9052  CD1 LEU A 594       2.622  52.720 -21.626  1.00  7.06
C
ATOM   9053  CD2 LEU A 594       3.537  54.943 -20.908  1.00  7.06
C
ATOM   9054  H   LEU A 594       3.927  52.452 -17.740  1.00  5.33
H
ATOM   9055  HA  LEU A 594       3.129  55.204 -18.281  1.00  5.65
H
ATOM   9056 1HB  LEU A 594       1.965  52.520 -19.076  1.00  8.48
H
ATOM   9057 2HB  LEU A 594       1.346  54.079 -19.636  1.00  8.48
H
```

```
ATOM   9058  HG  LEU A 594       4.148  53.028 -20.174  1.00  8.48
H
ATOM   9059 1HD1 LEU A 594       3.323  52.691 -22.460  1.00  8.48
H
ATOM   9060 2HD1 LEU A 594       2.414  51.703 -21.291  1.00  8.48
H
ATOM   9061 3HD1 LEU A 594       1.695  53.192 -21.948  1.00  8.48
H
ATOM   9062 1HD2 LEU A 594       4.241  54.927 -21.741  1.00  8.48
H
ATOM   9063 2HD2 LEU A 594       2.617  55.438 -21.221  1.00  8.48
H
ATOM   9064 3HD2 LEU A 594       3.975  55.486 -20.071  1.00  8.48
H
ATOM   9065  N   ASN A 595       1.714  53.797 -15.837  1.00  5.52
N
ATOM   9066  CA  ASN A 595       0.628  53.877 -14.869  1.00  6.67
C
ATOM   9067  C   ASN A 595       0.900  54.818 -13.721  1.00 46.72
C
ATOM   9068  O   ASN A 595       2.017  54.883 -13.185  1.00103.98
O
ATOM   9069  OXT ASN A 595      -0.096  55.178 -13.097  1.00  0.00
O
ATOM   9070  CB  ASN A 595       0.274  52.506 -14.319  1.00 10.00
C
ATOM   9071  CG  ASN A 595      -0.389  51.611 -15.329  1.00 10.00
C
ATOM   9072  OD1 ASN A 595      -1.113  52.080 -16.214  1.00 10.00
O
ATOM   9073  ND2 ASN A 595      -0.173  50.326 -15.202  1.00 10.00
N
ATOM   9074  H   ASN A 595       2.553  53.302 -15.566  1.00  6.62
H
ATOM   9075  HA  ASN A 595      -0.253  54.262 -15.385  1.00  8.00
H
ATOM   9076 1HB  ASN A 595       1.181  52.016 -13.963  1.00 12.01
H
ATOM   9077 2HB  ASN A 595      -0.393  52.619 -13.465  1.00 12.01
H
ATOM   9078 1HD2 ASN A 595      -0.594  49.683 -15.841  1.00 12.01
H
ATOM   9079 2HD2 ASN A 595       0.414  49.989 -14.466  1.00 12.01
H
TER    9080      ASN A 595
ATOM   9081  N   ALA B   1     -38.989 -12.094  -7.957  1.00400.00
N
ATOM   9082  CA  ALA B   1     -37.984 -11.153  -8.446  1.00400.00
C
ATOM   9083  C   ALA B   1     -36.833 -11.892  -9.119  1.00400.00
C
ATOM   9084  O   ALA B   1     -36.247 -12.806  -8.538  1.00400.00
O
ATOM   9085  CB  ALA B   1     -37.461 -10.297  -7.303  1.00600.00
C
ATOM   9086 1H   ALA B   1     -39.333 -11.784  -7.059  1.00480.00
H
```

ATOM 9087 2H ALA B 1 -39.757 -12.139 -8.613 1.00480.00
H
ATOM 9088 3H ALA B 1 -38.575 -13.011 -7.861 1.00480.00
H
ATOM 9089 HA ALA B 1 -38.454 -10.510 -9.189 1.00480.00
H
ATOM 9090 1HB ALA B 1 -36.730 -9.585 -7.686 1.00720.00
H
ATOM 9091 2HB ALA B 1 -38.290 -9.755 -6.845 1.00720.00
H
ATOM 9092 3HB ALA B 1 -36.990 -10.934 -6.556 1.00720.00
H
ATOM 9093 N ASP B 2 -36.517 -11.488 -10.344 1.00400.00
N
ATOM 9094 CA ASP B 2 -35.434 -12.104 -11.098 1.00400.00
C
ATOM 9095 C ASP B 2 -34.131 -11.336 -10.926 1.00400.00
C
ATOM 9096 O ASP B 2 -34.140 -10.123 -10.709 1.00400.00
O
ATOM 9097 CB ASP B 2 -35.792 -12.184 -12.582 1.00600.00
C
ATOM 9098 CG ASP B 2 -36.945 -13.139 -12.856 1.00600.00
C
ATOM 9099 OD1 ASP B 2 -37.200 -13.985 -12.032 1.00600.00
O
ATOM 9100 OD2 ASP B 2 -37.563 -13.012 -13.885 1.00600.00
O
ATOM 9101 H ASP B 2 -37.037 -10.732 -10.767 1.00480.00
H
ATOM 9102 HA ASP B 2 -35.283 -13.117 -10.724 1.00480.00
H
ATOM 9103 1HB ASP B 2 -36.066 -11.193 -12.945 1.00720.00
H
ATOM 9104 2HB ASP B 2 -34.923 -12.514 -13.152 1.00720.00
H
ATOM 9105 N ALA B 3 -33.013 -12.050 -11.017 1.00400.00
N
ATOM 9106 CA ALA B 3 -31.702 -11.429 -10.905 1.00400.00
C
ATOM 9107 C ALA B 3 -31.421 -10.556 -12.122 1.00400.00
C
ATOM 9108 O ALA B 3 -31.809 -10.893 -13.242 1.00400.00
O
ATOM 9109 CB ALA B 3 -30.623 -12.491 -10.756 1.00600.00
C
ATOM 9110 H ALA B 3 -33.076 -13.045 -11.181 1.00480.00
H
ATOM 9111 HA ALA B 3 -31.698 -10.791 -10.021 1.00480.00
H
ATOM 9112 1HB ALA B 3 -29.650 -12.012 -10.655 1.00720.00
H
ATOM 9113 2HB ALA B 3 -30.826 -13.093 -9.871 1.00720.00
H
ATOM 9114 3HB ALA B 3 -30.620 -13.131 -11.637 1.00720.00
H
ATOM 9115 N GLU B 4 -30.729 -9.443 -11.901 1.00400.00
N

```
ATOM   9116  CA  GLU B   4      -30.392  -8.534 -12.988  1.00400.00
C
ATOM   9117  C   GLU B   4      -29.118  -9.021 -13.673  1.00400.00
C
ATOM   9118  O   GLU B   4      -28.021  -8.921 -13.122  1.00400.00
O
ATOM   9119  CB  GLU B   4      -30.224  -7.116 -12.452  1.00600.00
C
ATOM   9120  CG  GLU B   4      -29.984  -6.065 -13.516  1.00600.00
C
ATOM   9121  CD  GLU B   4      -29.819  -4.694 -12.934  1.00600.00
C
ATOM   9122  OE1 GLU B   4      -29.866  -4.573 -11.731  1.00600.00
O
ATOM   9123  OE2 GLU B   4      -29.652  -3.762 -13.686  1.00600.00
O
ATOM   9124  H   GLU B   4      -30.438  -9.221 -10.961  1.00480.00
H
ATOM   9125  HA  GLU B   4      -31.202  -8.540 -13.717  1.00480.00
H
ATOM   9126 1HB  GLU B   4      -31.119  -6.831 -11.897  1.00720.00
H
ATOM   9127 2HB  GLU B   4      -29.384  -7.088 -11.758  1.00720.00
H
ATOM   9128 1HG  GLU B   4      -29.093  -6.329 -14.075  1.00720.00
H
ATOM   9129 2HG  GLU B   4      -30.827  -6.064 -14.206  1.00720.00
H
ATOM   9130  N   ALA B   5      -29.277  -9.558 -14.882  1.00400.00
N
ATOM   9131  CA  ALA B   5      -28.179 -10.203 -15.601  1.00400.00
C
ATOM   9132  C   ALA B   5      -27.205  -9.239 -16.269  1.00400.00
C
ATOM   9133  O   ALA B   5      -27.170  -9.117 -17.492  1.00400.00
O
ATOM   9134  CB  ALA B   5      -28.753 -11.135 -16.657  1.00600.00
C
ATOM   9135  H   ALA B   5      -30.200  -9.565 -15.292  1.00480.00
H
ATOM   9136  HA  ALA B   5      -27.616 -10.794 -14.880  1.00480.00
H
ATOM   9137 1HB  ALA B   5      -27.942 -11.661 -17.163  1.00720.00
H
ATOM   9138 2HB  ALA B   5      -29.412 -11.862 -16.180  1.00720.00
H
ATOM   9139 3HB  ALA B   5      -29.320 -10.557 -17.386  1.00720.00
H
ATOM   9140  N   ARG B   6      -26.374  -8.602 -15.453  1.00400.00
N
ATOM   9141  CA  ARG B   6      -25.306  -7.743 -15.949  1.00400.00
C
ATOM   9142  C   ARG B   6      -24.007  -8.530 -15.942  1.00400.00
C
ATOM   9143  O   ARG B   6      -23.017  -8.141 -16.561  1.00400.00
O
ATOM   9144  CB  ARG B   6      -25.179  -6.501 -15.111  1.00600.00
C
```

```
ATOM   9145  CG  ARG B   6      -26.419  -5.656 -15.158  1.00600.00
C
ATOM   9146  CD  ARG B   6      -26.662  -5.121 -16.518  1.00600.00
C
ATOM   9147  NE  ARG B   6      -27.828  -4.268 -16.537  1.00600.00
N
ATOM   9148  CZ  ARG B   6      -29.068  -4.662 -16.892  1.00600.00
C
ATOM   9149  NH1 ARG B   6      -29.279  -5.896 -17.296  1.00600.00
N
ATOM   9150  NH2 ARG B   6      -30.072  -3.805 -16.832  1.00600.00
N
ATOM   9151  H   ARG B   6      -26.502  -8.726 -14.456  1.00480.00
H
ATOM   9152  HA  ARG B   6      -25.533  -7.450 -16.975  1.00480.00
H
ATOM   9153 1HB  ARG B   6      -24.988  -6.769 -14.073  1.00720.00
H
ATOM   9154 2HB  ARG B   6      -24.342  -5.898 -15.462  1.00720.00
H
ATOM   9155 1HG  ARG B   6      -27.268  -6.274 -14.883  1.00720.00
H
ATOM   9156 2HG  ARG B   6      -26.329  -4.824 -14.460  1.00720.00
H
ATOM   9157 1HD  ARG B   6      -25.805  -4.538 -16.845  1.00720.00
H
ATOM   9158 2HD  ARG B   6      -26.829  -5.945 -17.211  1.00720.00
H
ATOM   9159  HE  ARG B   6      -27.699  -3.316 -16.212  1.00720.00
H
ATOM   9160 1HH1 ARG B   6      -28.510  -6.550 -17.344  1.00720.00
H
ATOM   9161 2HH1 ARG B   6      -30.208  -6.188 -17.563  1.00720.00
H
ATOM   9162 1HH2 ARG B   6      -29.908  -2.860 -16.513  1.00720.00
H
ATOM   9163 2HH2 ARG B   6      -31.002  -4.096 -17.094  1.00720.00
H
ATOM   9164  N   ALA B   7      -24.046  -9.666 -15.243  1.00400.00
N
ATOM   9165  CA  ALA B   7      -22.952 -10.611 -15.156  1.00400.00
C
ATOM   9166  C   ALA B   7      -22.970 -11.585 -16.334  1.00400.00
C
ATOM   9167  O   ALA B   7      -22.115 -12.465 -16.427  1.00400.00
O
ATOM   9168  CB  ALA B   7      -23.044 -11.373 -13.845  1.00600.00
C
ATOM   9169  H   ALA B   7      -24.887  -9.903 -14.741  1.00480.00
H
ATOM   9170  HA  ALA B   7      -22.015 -10.056 -15.189  1.00480.00
H
ATOM   9171 1HB  ALA B   7      -22.216 -12.073 -13.767  1.00720.00
H
ATOM   9172 2HB  ALA B   7      -23.007 -10.671 -13.012  1.00720.00
H
ATOM   9173 3HB  ALA B   7      -23.983 -11.923 -13.814  1.00720.00
H
```

```
ATOM   9174  N   GLU B   8      -23.950 -11.437 -17.227  1.00400.00
N
ATOM   9175  CA  GLU B   8      -24.045 -12.299 -18.386  1.00400.00
C
ATOM   9176  C   GLU B   8      -23.223 -11.736 -19.530  1.00400.00
C
ATOM   9177  O   GLU B   8      -23.480 -10.635 -20.019  1.00400.00
O
ATOM   9178  CB  GLU B   8      -25.501 -12.463 -18.813  1.00600.00
C
ATOM   9179  CG  GLU B   8      -25.703 -13.395 -19.999  1.00600.00
C
ATOM   9180  CD  GLU B   8      -27.148 -13.544 -20.386  1.00600.00
C
ATOM   9181  OE1 GLU B   8      -27.983 -12.966 -19.731  1.00600.00
O
ATOM   9182  OE2 GLU B   8      -27.418 -14.236 -21.338  1.00600.00
O
ATOM   9183  H   GLU B   8      -24.641 -10.713 -17.113  1.00480.00
H
ATOM   9184  HA  GLU B   8      -23.644 -13.280 -18.126  1.00480.00
H
ATOM   9185 1HB  GLU B   8      -26.084 -12.852 -17.979  1.00720.00
H
ATOM   9186 2HB  GLU B   8      -25.914 -11.489 -19.078  1.00720.00
H
ATOM   9187 1HG  GLU B   8      -25.147 -13.006 -20.852  1.00720.00
H
ATOM   9188 2HG  GLU B   8      -25.296 -14.374 -19.751  1.00720.00
H
ATOM   9189  N   PHE B   9      -22.236 -12.508 -19.953  1.00400.00
N
ATOM   9190  CA  PHE B   9      -21.352 -12.124 -21.039  1.00400.00
C
ATOM   9191  C   PHE B   9      -21.395 -13.157 -22.164  1.00251.90
C
ATOM   9192  O   PHE B   9      -21.743 -14.315 -21.923  1.00258.80
O
ATOM   9193  CB  PHE B   9      -19.929 -11.903 -20.504  1.00600.00
C
ATOM   9194  CG  PHE B   9      -19.832 -10.701 -19.604  1.00600.00
C
ATOM   9195  CD1 PHE B   9      -19.983 -10.814 -18.233  1.00600.00
C
ATOM   9196  CD2 PHE B   9      -19.609  -9.442 -20.141  1.00600.00
C
ATOM   9197  CE1 PHE B   9      -19.919  -9.698 -17.421  1.00600.00
C
ATOM   9198  CE2 PHE B   9      -19.540  -8.324 -19.331  1.00600.00
C
ATOM   9199  CZ  PHE B   9      -19.697  -8.453 -17.966  1.00600.00
C
ATOM   9200  H   PHE B   9      -22.088 -13.399 -19.499  1.00480.00
H
ATOM   9201  HA  PHE B   9      -21.710 -11.172 -21.432  1.00480.00
H
ATOM   9202 1HB  PHE B   9      -19.620 -12.778 -19.933  1.00720.00
H
```

```
ATOM   9203 2HB  PHE B   9      -19.223 -11.782 -21.323  1.00720.00
H
ATOM   9204  HD1 PHE B   9      -20.162 -11.797 -17.798  1.00720.00
H
ATOM   9205  HD2 PHE B   9      -19.492  -9.336 -21.221  1.00720.00
H
ATOM   9206  HE1 PHE B   9      -20.046  -9.803 -16.345  1.00720.00
H
ATOM   9207  HE2 PHE B   9      -19.366  -7.341 -19.767  1.00720.00
H
ATOM   9208  HZ  PHE B   9      -19.646  -7.574 -17.325  1.00720.00
H
ATOM   9209  N   PRO B  10      -21.068 -12.744 -23.394  1.00154.19
N
ATOM   9210  CA  PRO B  10      -21.050 -13.515 -24.633  1.00 62.07
C
ATOM   9211  C   PRO B  10      -19.923 -14.539 -24.681  1.00 26.21
C
ATOM   9212  O   PRO B  10      -18.972 -14.484 -23.898  1.00 52.12
O
ATOM   9213  CB  PRO B  10      -20.890 -12.441 -25.719  1.00 93.11
C
ATOM   9214  CG  PRO B  10      -20.240 -11.298 -25.031  1.00 93.11
C
ATOM   9215  CD  PRO B  10      -20.853 -11.305 -23.661  1.00 93.11
C
ATOM   9216  HA  PRO B  10      -22.019 -14.022 -24.743  1.00 74.48
H
ATOM   9217 1HB  PRO B  10      -20.285 -12.832 -26.549  1.00111.73
H
ATOM   9218 2HB  PRO B  10      -21.875 -12.182 -26.135  1.00111.73
H
ATOM   9219 1HG  PRO B  10      -19.149 -11.434 -25.012  1.00111.73
H
ATOM   9220 2HG  PRO B  10      -20.435 -10.362 -25.579  1.00111.73
H
ATOM   9221 1HD  PRO B  10      -20.147 -10.864 -22.949  1.00111.73
H
ATOM   9222 2HD  PRO B  10      -21.815 -10.774 -23.663  1.00111.73
H
ATOM   9223  N   GLU B  11      -20.068 -15.475 -25.602  1.00 40.35
N
ATOM   9224  CA  GLU B  11      -19.144 -16.578 -25.820  1.00 69.20
C
ATOM   9225  C   GLU B  11      -17.705 -16.156 -26.049  1.00 48.11
C
ATOM   9226  O   GLU B  11      -17.407 -15.189 -26.757  1.00116.31
O
ATOM   9227  CB  GLU B  11      -19.611 -17.413 -27.014  1.00103.80
C
ATOM   9228  CG  GLU B  11      -18.757 -18.640 -27.308  1.00103.80
C
ATOM   9229  CD  GLU B  11      -19.302 -19.470 -28.436  1.00103.80
C
ATOM   9230  OE1 GLU B  11      -20.315 -19.103 -28.981  1.00103.80
O
ATOM   9231  OE2 GLU B  11      -18.706 -20.473 -28.752  1.00103.80
O
```

```
ATOM   9232  H   GLU B  11     -20.884 -15.426 -26.196  1.00 48.42
H
ATOM   9233  HA  GLU B  11     -19.166 -17.209 -24.930  1.00 83.04
H
ATOM   9234 1HB  GLU B  11     -20.633 -17.753 -26.840  1.00124.56
H
ATOM   9235 2HB  GLU B  11     -19.623 -16.792 -27.910  1.00124.56
H
ATOM   9236 1HG  GLU B  11     -17.750 -18.318 -27.571  1.00124.56
H
ATOM   9237 2HG  GLU B  11     -18.691 -19.252 -26.409  1.00124.56
H
ATOM   9238  N   GLU B  12     -16.809 -16.930 -25.469  1.00 20.25
N
ATOM   9239  CA  GLU B  12     -15.379 -16.703 -25.573  1.00 18.63
C
ATOM   9240  C   GLU B  12     -14.795 -17.092 -26.912  1.00 21.57
C
ATOM   9241  O   GLU B  12     -14.119 -18.115 -26.998  1.00 73.52
O
ATOM   9242  CB  GLU B  12     -14.631 -17.511 -24.519  1.00 27.95
C
ATOM   9243  CG  GLU B  12     -14.922 -17.174 -23.071  1.00 27.95
C
ATOM   9244  CD  GLU B  12     -14.123 -18.059 -22.158  1.00 27.95
C
ATOM   9245  OE1 GLU B  12     -13.344 -18.832 -22.669  1.00 27.95
O
ATOM   9246  OE2 GLU B  12     -14.281 -17.972 -20.963  1.00 27.95
O
ATOM   9247  H   GLU B  12     -17.134 -17.708 -24.915  1.00 24.30
H
ATOM   9248  HA  GLU B  12     -15.193 -15.647 -25.415  1.00 22.36
H
ATOM   9249 1HB  GLU B  12     -14.852 -18.570 -24.657  1.00 33.53
H
ATOM   9250 2HB  GLU B  12     -13.559 -17.383 -24.671  1.00 33.53
H
ATOM   9251 1HG  GLU B  12     -14.667 -16.132 -22.880  1.00 33.53
H
ATOM   9252 2HG  GLU B  12     -15.985 -17.308 -22.874  1.00 33.53
H
ATOM   9253  N   ALA B  13     -15.025 -16.297 -27.943  1.00 16.10
N
ATOM   9254  CA  ALA B  13     -14.488 -16.622 -29.262  1.00 12.78
C
ATOM   9255  C   ALA B  13     -12.978 -16.831 -29.218  1.00 13.21
C
ATOM   9256  O   ALA B  13     -12.217 -15.876 -29.316  1.00 49.68
O
ATOM   9257  CB  ALA B  13     -14.830 -15.521 -30.250  1.00 19.17
C
ATOM   9258  H   ALA B  13     -15.600 -15.474 -27.803  1.00 19.32
H
ATOM   9259  HA  ALA B  13     -14.947 -17.556 -29.594  1.00 15.34
H
ATOM   9260 1HB  ALA B  13     -14.450 -15.785 -31.236  1.00 23.00
H
```

```
ATOM   9261 2HB  ALA B  13      -15.912 -15.399 -30.298  1.00 23.00
H
ATOM   9262 3HB  ALA B  13      -14.374 -14.587 -29.924  1.00 23.00
H
ATOM   9263  N   ARG B  14      -12.549 -18.083 -29.138  1.00 16.84
N
ATOM   9264  CA  ARG B  14      -11.128 -18.404 -29.083  1.00 19.95
C
ATOM   9265  C   ARG B  14      -10.468 -18.239 -30.459  1.00 15.17
C
ATOM   9266  O   ARG B  14      -10.825 -18.954 -31.398  1.00 22.07
O
ATOM   9267  CB  ARG B  14      -10.913 -19.822 -28.583  1.00 29.92
C
ATOM   9268  CG  ARG B  14      -11.477 -20.115 -27.195  1.00 29.92
C
ATOM   9269  CD  ARG B  14      -10.838 -19.279 -26.140  1.00 29.92
C
ATOM   9270  NE  ARG B  14      -11.346 -19.589 -24.809  1.00 29.92
N
ATOM   9271  CZ  ARG B  14      -10.834 -20.531 -23.995  1.00 29.92
C
ATOM   9272  NH1 ARG B  14       -9.809 -21.259 -24.383  1.00 29.92
N
ATOM   9273  NH2 ARG B  14      -11.371 -20.717 -22.803  1.00 29.92
N
ATOM   9274  H   ARG B  14      -13.236 -18.815 -29.039  1.00 20.21
H
ATOM   9275  HA  ARG B  14      -10.668 -17.745 -28.356  1.00 23.94
H
ATOM   9276 1HB  ARG B  14      -11.372 -20.525 -29.278  1.00 35.91
H
ATOM   9277 2HB  ARG B  14       -9.845 -20.039 -28.556  1.00 35.91
H
ATOM   9278 1HG  ARG B  14      -12.548 -19.925 -27.189  1.00 35.91
H
ATOM   9279 2HG  ARG B  14      -11.296 -21.162 -26.949  1.00 35.91
H
ATOM   9280 1HD  ARG B  14       -9.762 -19.450 -26.144  1.00 35.91
H
ATOM   9281 2HD  ARG B  14      -11.039 -18.226 -26.342  1.00 35.91
H
ATOM   9282  HE  ARG B  14      -12.139 -19.061 -24.451  1.00 35.91
H
ATOM   9283 1HH1 ARG B  14       -9.404 -21.114 -25.296  1.00 35.91
H
ATOM   9284 2HH1 ARG B  14       -9.431 -21.964 -23.768  1.00 35.91
H
ATOM   9285 1HH2 ARG B  14      -12.159 -20.141 -22.518  1.00 35.91
H
ATOM   9286 2HH2 ARG B  14      -11.001 -21.418 -22.180  1.00 35.91
H
ATOM   9287  N   PRO B  15       -9.509 -17.306 -30.592  1.00  9.75
N
ATOM   9288  CA  PRO B  15       -8.742 -16.965 -31.787  1.00  6.87
C
ATOM   9289  C   PRO B  15       -7.997 -18.153 -32.366  1.00  5.32
C
```

```
ATOM   9290  O   PRO B  15      -7.408 -18.942 -31.626  1.00  8.19
O
ATOM   9291  CB  PRO B  15      -7.733 -15.930 -31.271  1.00 10.30
C
ATOM   9292  CG  PRO B  15      -8.379 -15.318 -30.090  1.00 10.30
C
ATOM   9293  CD  PRO B  15      -9.101 -16.458 -29.445  1.00 10.30
C
ATOM   9294  HA  PRO B  15      -9.416 -16.524 -32.537  1.00  8.24
H
ATOM   9295 1HB  PRO B  15      -6.802 -16.445 -31.005  1.00 12.37
H
ATOM   9296 2HB  PRO B  15      -7.481 -15.204 -32.048  1.00 12.37
H
ATOM   9297 1HG  PRO B  15      -7.621 -14.867 -29.433  1.00 12.37
H
ATOM   9298 2HG  PRO B  15      -9.056 -14.509 -30.403  1.00 12.37
H
ATOM   9299 1HD  PRO B  15      -8.433 -17.019 -28.774  1.00 12.37
H
ATOM   9300 2HD  PRO B  15      -9.966 -16.050 -28.914  1.00 12.37
H
ATOM   9301  N   ASP B  16      -7.964 -18.249 -33.690  1.00  4.03
N
ATOM   9302  CA  ASP B  16      -7.178 -19.297 -34.317  1.00  3.78
C
ATOM   9303  C   ASP B  16      -5.793 -18.740 -34.609  1.00  3.19
C
ATOM   9304  O   ASP B  16      -5.534 -17.574 -34.324  1.00  3.52
O
ATOM   9305  CB  ASP B  16      -7.859 -19.819 -35.591  1.00  5.67
C
ATOM   9306  CG  ASP B  16      -7.944 -18.799 -36.715  1.00  5.67
C
ATOM   9307  OD1 ASP B  16      -7.260 -17.803 -36.649  1.00  5.67
O
ATOM   9308  OD2 ASP B  16      -8.697 -19.023 -37.633  1.00  5.67
O
ATOM   9309  H   ASP B  16      -8.476 -17.590 -34.259  1.00  4.84
H
ATOM   9310  HA  ASP B  16      -7.074 -20.128 -33.619  1.00  4.54
H
ATOM   9311 1HB  ASP B  16      -7.323 -20.695 -35.956  1.00  6.80
H
ATOM   9312 2HB  ASP B  16      -8.872 -20.143 -35.344  1.00  6.80
H
ATOM   9313  N   ARG B  17      -4.925 -19.557 -35.195  1.00  3.35
N
ATOM   9314  CA  ARG B  17      -3.545 -19.161 -35.478  1.00  3.39
C
ATOM   9315  C   ARG B  17      -3.428 -17.877 -36.296  1.00  2.55
C
ATOM   9316  O   ARG B  17      -2.596 -17.024 -35.989  1.00  2.53
O
ATOM   9317  CB  ARG B  17      -2.829 -20.282 -36.219  1.00  5.08
C
ATOM   9318  CG  ARG B  17      -1.391 -19.989 -36.627  1.00  5.08
C
```

ATOM   9319 CD ARG B 17   -0.472 -19.897 -35.458 1.00 5.08
C
ATOM   9320 NE ARG B 17   0.887 -19.577 -35.877 1.00 5.08
N
ATOM   9321 CZ ARG B 17   1.364 -18.327 -36.034 1.00 5.08
C
ATOM   9322 NH1 ARG B 17   0.605 -17.293 -35.773 1.00 5.08
N
ATOM   9323 NH2 ARG B 17   2.596 -18.115 -36.456 1.00 5.08
N
ATOM   9324 H   ARG B 17   -5.217 -20.496 -35.426 1.00 4.02
H
ATOM   9325 HA ARG B 17   -3.040 -19.004 -34.525 1.00 4.07
H
ATOM   9326 1HB ARG B 17   -2.815 -21.174 -35.596 1.00 6.10
H
ATOM   9327 2HB ARG B 17   -3.381 -20.527 -37.126 1.00 6.10
H
ATOM   9328 1HG ARG B 17   -1.033 -20.790 -37.275 1.00 6.10
H
ATOM   9329 2HG ARG B 17   -1.352 -19.043 -37.166 1.00 6.10
H
ATOM   9330 1HD ARG B 17   -0.816 -19.113 -34.783 1.00 6.10
H
ATOM   9331 2HD ARG B 17   -0.453 -20.850 -34.930 1.00 6.10
H
ATOM   9332 HE ARG B 17   1.506 -20.348 -36.092 1.00 6.10
H
ATOM   9333 1HH1 ARG B 17   -0.342 -17.424 -35.448 1.00 6.10
H
ATOM   9334 2HH1 ARG B 17   0.983 -16.363 -35.908 1.00 6.10
H
ATOM   9335 1HH2 ARG B 17   3.206 -18.892 -36.669 1.00 6.10
H
ATOM   9336 2HH2 ARG B 17   2.910 -17.158 -36.572 1.00 6.10
H
ATOM   9337 N   GLY B 18   -4.246 -17.754 -37.342 1.00 2.40
N
ATOM   9338 CA GLY B 18   -4.215 -16.583 -38.214 1.00 2.35
C
ATOM   9339 C   GLY B 18   -4.653 -15.333 -37.474 1.00 2.22
C
ATOM   9340 O   GLY B 18   -4.052 -14.269 -37.629 1.00 2.65
O
ATOM   9341 H   GLY B 18   -4.912 -18.489 -37.539 1.00 2.88
H
ATOM   9342 1HA GLY B 18   -3.205 -16.447 -38.603 1.00 2.82
H
ATOM   9343 2HA GLY B 18   -4.869 -16.751 -39.069 1.00 2.82
H
ATOM   9344 N   THR B 19   -5.688 -15.489 -36.656 1.00 2.65
N
ATOM   9345 CA THR B 19   -6.252 -14.427 -35.847 1.00 3.23
C
ATOM   9346 C   THR B 19   -5.201 -13.921 -34.862 1.00 1.92
C
ATOM   9347 O   THR B 19   -5.021 -12.711 -34.690 1.00 1.58
O

```
ATOM   9348  CB  THR B  19      -7.486 -14.971 -35.103  1.00  4.84
C
ATOM   9349  OG1 THR B  19      -8.446 -15.438 -36.059  1.00  4.84
O
ATOM   9350  CG2 THR B  19      -8.130 -13.911 -34.267  1.00  4.84
C
ATOM   9351  H   THR B  19      -6.127 -16.402 -36.603  1.00  3.18
H
ATOM   9352  HA  THR B  19      -6.551 -13.603 -36.496  1.00  3.88
H
ATOM   9353  HB  THR B  19      -7.185 -15.794 -34.461  1.00  5.81
H
ATOM   9354  HG1 THR B  19      -8.088 -16.216 -36.503  1.00  5.81
H
ATOM   9355 1HG2 THR B  19      -8.999 -14.325 -33.757  1.00  5.81
H
ATOM   9356 2HG2 THR B  19      -7.419 -13.534 -33.532  1.00  5.81
H
ATOM   9357 3HG2 THR B  19      -8.446 -13.107 -34.924  1.00  5.81
H
ATOM   9358  N   LEU B  20      -4.496 -14.871 -34.249  1.00  1.96
N
ATOM   9359  CA  LEU B  20      -3.418 -14.597 -33.318  1.00  1.70
C
ATOM   9360  C   LEU B  20      -2.232 -13.947 -34.019  1.00  1.35
C
ATOM   9361  O   LEU B  20      -1.568 -13.095 -33.434  1.00  1.28
O
ATOM   9362  CB  LEU B  20      -2.982 -15.900 -32.649  1.00  2.55
C
ATOM   9363  CG  LEU B  20      -4.005 -16.518 -31.693  1.00  2.55
C
ATOM   9364  CD1 LEU B  20      -3.537 -17.904 -31.288  1.00  2.55
C
ATOM   9365  CD2 LEU B  20      -4.160 -15.623 -30.474  1.00  2.55
C
ATOM   9366  H   LEU B  20      -4.730 -15.838 -34.430  1.00  2.35
H
ATOM   9367  HA  LEU B  20      -3.786 -13.915 -32.554  1.00  2.04
H
ATOM   9368 1HB  LEU B  20      -2.766 -16.634 -33.423  1.00  3.06
H
ATOM   9369 2HB  LEU B  20      -2.068 -15.713 -32.085  1.00  3.06
H
ATOM   9370  HG  LEU B  20      -4.964 -16.614 -32.195  1.00  3.06
H
ATOM   9371 1HD1 LEU B  20      -4.267 -18.352 -30.612  1.00  3.06
H
ATOM   9372 2HD1 LEU B  20      -3.440 -18.528 -32.177  1.00  3.06
H
ATOM   9373 3HD1 LEU B  20      -2.574 -17.834 -30.787  1.00  3.06
H
ATOM   9374 1HD2 LEU B  20      -4.890 -16.061 -29.792  1.00  3.06
H
ATOM   9375 2HD2 LEU B  20      -3.201 -15.532 -29.966  1.00  3.06
H
ATOM   9376 3HD2 LEU B  20      -4.502 -14.637 -30.788  1.00  3.06
H
```

```
ATOM   9377  N   GLN B  21      -1.971 -14.330 -35.271  1.00  1.36
N
ATOM   9378  CA  GLN B  21      -0.883 -13.714 -36.012  1.00  1.30
C
ATOM   9379  C   GLN B  21      -1.154 -12.264 -36.315  1.00  1.28
C
ATOM   9380  O   GLN B  21      -0.253 -11.438 -36.186  1.00  1.18
O
ATOM   9381  CB  GLN B  21      -0.622 -14.426 -37.332  1.00  1.95
C
ATOM   9382  CG  GLN B  21       0.572 -13.852 -38.079  1.00  1.95
C
ATOM   9383  CD  GLN B  21       1.867 -13.987 -37.296  1.00  1.95
C
ATOM   9384  OE1 GLN B  21       2.218 -15.079 -36.825  1.00  1.95
O
ATOM   9385  NE2 GLN B  21       2.588 -12.877 -37.145  1.00  1.95
N
ATOM   9386  H   GLN B  21      -2.513 -15.064 -35.706  1.00  1.63
H
ATOM   9387  HA  GLN B  21       0.020 -13.767 -35.404  1.00  1.56
H
ATOM   9388 1HB  GLN B  21      -0.460 -15.485 -37.168  1.00  2.34
H
ATOM   9389 2HB  GLN B  21      -1.495 -14.326 -37.976  1.00  2.34
H
ATOM   9390 1HG  GLN B  21       0.685 -14.383 -39.023  1.00  2.34
H
ATOM   9391 2HG  GLN B  21       0.396 -12.792 -38.265  1.00  2.34
H
ATOM   9392 1HE2 GLN B  21       3.449 -12.907 -36.634  1.00  2.34
H
ATOM   9393 2HE2 GLN B  21       2.279 -12.002 -37.535  1.00  2.34
H
ATOM   9394  N   VAL B  22      -2.392 -11.941 -36.694  1.00  1.50
N
ATOM   9395  CA  VAL B  22      -2.724 -10.558 -36.997  1.00  1.49
C
ATOM   9396  C   VAL B  22      -2.655  -9.756 -35.715  1.00  1.42
C
ATOM   9397  O   VAL B  22      -2.195  -8.620 -35.713  1.00  1.39
O
ATOM   9398  CB  VAL B  22      -4.111 -10.405 -37.629  1.00  2.23
C
ATOM   9399  CG1 VAL B  22      -4.405  -8.922 -37.792  1.00  2.23
C
ATOM   9400  CG2 VAL B  22      -4.160 -11.119 -38.965  1.00  2.23
C
ATOM   9401  H   VAL B  22      -3.093 -12.666 -36.803  1.00  1.80
H
ATOM   9402  HA  VAL B  22      -1.986 -10.165 -37.697  1.00  1.79
H
ATOM   9403  HB  VAL B  22      -4.864 -10.825 -36.962  1.00  2.68
H
ATOM   9404 1HG1 VAL B  22      -5.393  -8.790 -38.230  1.00  2.68
H
ATOM   9405 2HG1 VAL B  22      -4.368  -8.437 -36.818  1.00  2.68
H
```

```
ATOM   9406 3HG1 VAL B  22      -3.659  -8.474 -38.448  1.00  2.68
H
ATOM   9407 1HG2 VAL B  22      -5.150 -10.999 -39.405  1.00  2.68
H
ATOM   9408 2HG2 VAL B  22      -3.413 -10.692 -39.626  1.00  2.68
H
ATOM   9409 3HG2 VAL B  22      -3.953 -12.177 -38.823  1.00  2.68
H
ATOM   9410  N   LEU B  23      -3.111 -10.361 -34.628  1.00  1.46
N
ATOM   9411  CA  LEU B  23      -3.049  -9.781 -33.304  1.00  1.29
C
ATOM   9412  C   LEU B  23      -1.593  -9.413 -32.944  1.00  0.97
C
ATOM   9413  O   LEU B  23      -1.331  -8.307 -32.454  1.00  0.96
O
ATOM   9414  CB  LEU B  23      -3.674 -10.796 -32.354  1.00  1.94
C
ATOM   9415  CG  LEU B  23      -3.784 -10.450 -30.900  1.00  1.94
C
ATOM   9416  CD1 LEU B  23      -4.598  -9.187 -30.765  1.00  1.94
C
ATOM   9417  CD2 LEU B  23      -4.475 -11.621 -30.222  1.00  1.94
C
ATOM   9418  H   LEU B  23      -3.528 -11.282 -34.711  1.00  1.75
H
ATOM   9419  HA  LEU B  23      -3.648  -8.871 -33.293  1.00  1.55
H
ATOM   9420 1HB  LEU B  23      -4.675 -11.023 -32.711  1.00  2.32
H
ATOM   9421 2HB  LEU B  23      -3.094 -11.708 -32.402  1.00  2.32
H
ATOM   9422  HG  LEU B  23      -2.800 -10.282 -30.466  1.00  2.32
H
ATOM   9423 1HD1 LEU B  23      -4.695  -8.934 -29.718  1.00  2.32
H
ATOM   9424 2HD1 LEU B  23      -4.093  -8.385 -31.291  1.00  2.32
H
ATOM   9425 3HD1 LEU B  23      -5.577  -9.330 -31.200  1.00  2.32
H
ATOM   9426 1HD2 LEU B  23      -4.599 -11.421 -29.165  1.00  2.32
H
ATOM   9427 2HD2 LEU B  23      -5.455 -11.777 -30.675  1.00  2.32
H
ATOM   9428 3HD2 LEU B  23      -3.871 -12.518 -30.352  1.00  2.32
H
ATOM   9429  N   GLN B  24      -0.658 -10.327 -33.233  1.00  0.90
N
ATOM   9430  CA  GLN B  24       0.769 -10.074 -33.051  1.00  0.91
C
ATOM   9431  C   GLN B  24       1.249  -8.968 -33.987  1.00  0.85
C
ATOM   9432  O   GLN B  24       2.049  -8.122 -33.584  1.00  0.80
O
ATOM   9433  CB  GLN B  24       1.586 -11.343 -33.305  1.00  1.36
C
ATOM   9434  CG  GLN B  24       1.424 -12.422 -32.256  1.00  1.36
C
```

ATOM 9435 CD GLN B 24 2.154 -13.698 -32.626 1.00 1.36
C
ATOM 9436 OE1 GLN B 24 2.355 -13.994 -33.807 1.00 1.36
O
ATOM 9437 NE2 GLN B 24 2.559 -14.463 -31.619 1.00 1.36
N
ATOM 9438 H GLN B 24 -0.945 -11.230 -33.587 1.00 1.08
H
ATOM 9439 HA GLN B 24 0.936 -9.747 -32.024 1.00 1.09
H
ATOM 9440 1HB GLN B 24 1.306 -11.770 -34.267 1.00 1.64
H
ATOM 9441 2HB GLN B 24 2.644 -11.087 -33.359 1.00 1.64
H
ATOM 9442 1HG GLN B 24 1.827 -12.060 -31.310 1.00 1.64
H
ATOM 9443 2HG GLN B 24 0.370 -12.651 -32.148 1.00 1.64
H
ATOM 9444 1HE2 GLN B 24 3.046 -15.317 -31.804 1.00 1.64
H
ATOM 9445 2HE2 GLN B 24 2.377 -14.186 -30.676 1.00 1.64
H
ATOM 9446 N ASP B 25 0.746 -8.969 -35.226 1.00 1.04
N
ATOM 9447 CA ASP B 25 1.093 -7.945 -36.201 1.00 1.41
C
ATOM 9448 C ASP B 25 0.648 -6.567 -35.716 1.00 1.15
C
ATOM 9449 O ASP B 25 1.363 -5.586 -35.917 1.00 1.22
O
ATOM 9450 CB ASP B 25 0.459 -8.240 -37.569 1.00 2.11
C
ATOM 9451 CG ASP B 25 1.074 -9.422 -38.327 1.00 2.11
C
ATOM 9452 OD1 ASP B 25 2.119 -9.909 -37.959 1.00 2.11
O
ATOM 9453 OD2 ASP B 25 0.502 -9.800 -39.319 1.00 2.11
O
ATOM 9454 H ASP B 25 0.119 -9.708 -35.506 1.00 1.25
H
ATOM 9455 HA ASP B 25 2.176 -7.931 -36.318 1.00 1.69
H
ATOM 9456 1HB ASP B 25 -0.601 -8.435 -37.445 1.00 2.54
H
ATOM 9457 2HB ASP B 25 0.541 -7.352 -38.196 1.00 2.54
H
ATOM 9458 N MET B 26 -0.519 -6.498 -35.062 1.00 1.10
N
ATOM 9459 CA MET B 26 -1.010 -5.237 -34.531 1.00 1.22
C
ATOM 9460 C MET B 26 -0.134 -4.769 -33.381 1.00 0.90
C
ATOM 9461 O MET B 26 0.143 -3.577 -33.276 1.00 0.96
O
ATOM 9462 CB MET B 26 -2.463 -5.358 -34.071 1.00 1.83
C
ATOM 9463 CG MET B 26 -3.486 -5.608 -35.172 1.00 1.83
C

ATOM 9464 SD MET B 26 -3.515 -4.308 -36.417 1.00 1.83
S
ATOM 9465 CE MET B 26 -2.502 -5.062 -37.687 1.00 1.83
C
ATOM 9466 H MET B 26 -1.079 -7.332 -34.952 1.00 1.32
H
ATOM 9467 HA MET B 26 -0.955 -4.485 -35.317 1.00 1.46
H
ATOM 9468 1HB MET B 26 -2.549 -6.174 -33.356 1.00 2.20
H
ATOM 9469 2HB MET B 26 -2.748 -4.442 -33.555 1.00 2.20
H
ATOM 9470 1HG MET B 26 -3.278 -6.550 -35.669 1.00 2.20
H
ATOM 9471 2HG MET B 26 -4.477 -5.671 -34.726 1.00 2.20
H
ATOM 9472 1HE MET B 26 -2.420 -4.384 -38.537 1.00 2.20
H
ATOM 9473 2HE MET B 26 -1.507 -5.264 -37.287 1.00 2.20
H
ATOM 9474 3HE MET B 26 -2.958 -5.997 -38.012 1.00 2.20
H
ATOM 9475 N ALA B 27 0.340 -5.706 -32.554 1.00 0.73
N
ATOM 9476 CA ALA B 27 1.256 -5.358 -31.473 1.00 0.67
C
ATOM 9477 C ALA B 27 2.541 -4.769 -32.052 1.00 0.68
C
ATOM 9478 O ALA B 27 3.060 -3.775 -31.540 1.00 0.69
O
ATOM 9479 CB ALA B 27 1.559 -6.579 -30.618 1.00 1.01
C
ATOM 9480 H ALA B 27 0.046 -6.670 -32.665 1.00 0.88
H
ATOM 9481 HA ALA B 27 0.783 -4.598 -30.853 1.00 0.80
H
ATOM 9482 1HB ALA B 27 2.230 -6.297 -29.806 1.00 1.21
H
ATOM 9483 2HB ALA B 27 0.632 -6.970 -30.201 1.00 1.21
H
ATOM 9484 3HB ALA B 27 2.034 -7.346 -31.225 1.00 1.21
H
ATOM 9485 N SER B 28 3.030 -5.377 -33.135 1.00 0.71
N
ATOM 9486 CA SER B 28 4.218 -4.899 -33.823 1.00 0.79
C
ATOM 9487 C SER B 28 3.987 -3.515 -34.413 1.00 0.88
C
ATOM 9488 O SER B 28 4.857 -2.648 -34.317 1.00 0.96
O
ATOM 9489 CB SER B 28 4.625 -5.900 -34.888 1.00 1.19
C
ATOM 9490 OG SER B 28 5.049 -7.115 -34.315 1.00 1.19
O
ATOM 9491 H SER B 28 2.570 -6.209 -33.487 1.00 0.85
H
ATOM 9492 HA SER B 28 5.021 -4.810 -33.103 1.00 0.95
H

```
ATOM   9493 1HB  SER B  28       3.778  -6.087 -35.544  1.00  1.42
H
ATOM   9494 2HB  SER B  28       5.422  -5.484 -35.502  1.00  1.42
H
ATOM   9495  HG  SER B  28       5.122  -7.738 -35.045  1.00  1.42
H
ATOM   9496  N   ARG B  29       2.801  -3.301 -34.986  1.00  0.94
N
ATOM   9497  CA  ARG B  29       2.430  -2.001 -35.514  1.00  1.09
C
ATOM   9498  C   ARG B  29       2.424  -0.943 -34.410  1.00  0.88
C
ATOM   9499  O   ARG B  29       2.888   0.178 -34.625  1.00  0.97
O
ATOM   9500  CB  ARG B  29       1.069  -2.050 -36.197  1.00  1.64
C
ATOM   9501  CG  ARG B  29       0.674  -0.715 -36.787  1.00  1.64
C
ATOM   9502  CD  ARG B  29      -0.582  -0.720 -37.580  1.00  1.64
C
ATOM   9503  NE  ARG B  29      -0.889   0.641 -38.002  1.00  1.64
N
ATOM   9504  CZ  ARG B  29      -1.918   1.038 -38.772  1.00  1.64
C
ATOM   9505  NH1 ARG B  29      -2.796   0.189 -39.257  1.00  1.64
N
ATOM   9506  NH2 ARG B  29      -2.042   2.326 -39.042  1.00  1.64
N
ATOM   9507  H   ARG B  29       2.143  -4.064 -35.068  1.00  1.13
H
ATOM   9508  HA  ARG B  29       3.169  -1.710 -36.260  1.00  1.31
H
ATOM   9509 1HB  ARG B  29       1.082  -2.788 -36.998  1.00  1.96
H
ATOM   9510 2HB  ARG B  29       0.302  -2.344 -35.483  1.00  1.96
H
ATOM   9511 1HG  ARG B  29       0.529  -0.025 -35.963  1.00  1.96
H
ATOM   9512 2HG  ARG B  29       1.479  -0.349 -37.426  1.00  1.96
H
ATOM   9513 1HD  ARG B  29      -0.464  -1.340 -38.468  1.00  1.96
H
ATOM   9514 2HD  ARG B  29      -1.408  -1.091 -36.974  1.00  1.96
H
ATOM   9515  HE  ARG B  29      -0.268   1.372 -37.673  1.00  1.96
H
ATOM   9516 1HH1 ARG B  29      -2.733  -0.805 -39.068  1.00  1.96
H
ATOM   9517 2HH1 ARG B  29      -3.555   0.530 -39.827  1.00  1.96
H
ATOM   9518 1HH2 ARG B  29      -1.367   2.982 -38.668  1.00  1.96
H
ATOM   9519 2HH2 ARG B  29      -2.804   2.651 -39.616  1.00  1.96
H
ATOM   9520  N   LEU B  30       1.907  -1.306 -33.228  1.00  0.73
N
ATOM   9521  CA  LEU B  30       1.891  -0.402 -32.084  1.00  0.67
C
```

```
ATOM   9522  C   LEU B  30       3.300  -0.015 -31.657  1.00  0.67
C
ATOM   9523  O   LEU B  30       3.545   1.147 -31.323  1.00  0.68
O
ATOM   9524  CB  LEU B  30       1.176  -1.050 -30.882  1.00  1.01
C
ATOM   9525  CG  LEU B  30      -0.339  -1.244 -30.997  1.00  1.01
C
ATOM   9526  CD1 LEU B  30      -0.828  -2.072 -29.811  1.00  1.01
C
ATOM   9527  CD2 LEU B  30      -1.019   0.114 -31.026  1.00  1.01
C
ATOM   9528  H   LEU B  30       1.507  -2.229 -33.124  1.00  0.88
H
ATOM   9529  HA  LEU B  30       1.356   0.503 -32.367  1.00  0.80
H
ATOM   9530 1HB  LEU B  30       1.616  -2.026 -30.701  1.00  1.21
H
ATOM   9531 2HB  LEU B  30       1.358  -0.430 -30.004  1.00  1.21
H
ATOM   9532  HG  LEU B  30      -0.576  -1.783 -31.910  1.00  1.21
H
ATOM   9533 1HD1 LEU B  30      -1.906  -2.217 -29.890  1.00  1.21
H
ATOM   9534 2HD1 LEU B  30      -0.330  -3.040 -29.813  1.00  1.21
H
ATOM   9535 3HD1 LEU B  30      -0.600  -1.549 -28.884  1.00  1.21
H
ATOM   9536 1HD2 LEU B  30      -2.097  -0.020 -31.108  1.00  1.21
H
ATOM   9537 2HD2 LEU B  30      -0.789   0.656 -30.108  1.00  1.21
H
ATOM   9538 3HD2 LEU B  30      -0.658   0.683 -31.883  1.00  1.21
H
ATOM   9539  N   ARG B  31       4.230  -0.979 -31.684  1.00  0.73
N
ATOM   9540  CA  ARG B  31       5.607  -0.680 -31.307  1.00  0.83
C
ATOM   9541  C   ARG B  31       6.233   0.279 -32.302  1.00  0.85
C
ATOM   9542  O   ARG B  31       6.925   1.214 -31.903  1.00  0.89
O
ATOM   9543  CB  ARG B  31       6.445  -1.944 -31.207  1.00  1.24
C
ATOM   9544  CG  ARG B  31       6.058  -2.834 -30.041  1.00  1.24
C
ATOM   9545  CD  ARG B  31       7.064  -3.881 -29.739  1.00  1.24
C
ATOM   9546  NE  ARG B  31       7.313  -4.732 -30.883  1.00  1.24
N
ATOM   9547  CZ  ARG B  31       6.565  -5.788 -31.247  1.00  1.24
C
ATOM   9548  NH1 ARG B  31       5.489  -6.136 -30.567  1.00  1.24
N
ATOM   9549  NH2 ARG B  31       6.908  -6.460 -32.331  1.00  1.24
N
ATOM   9550  H   ARG B  31       3.972  -1.927 -31.936  1.00  0.88
H
```

```
ATOM   9551  HA  ARG B  31       5.600  -0.203 -30.327  1.00  1.00
H
ATOM   9552 1HB  ARG B  31       6.345  -2.527 -32.121  1.00  1.49
H
ATOM   9553 2HB  ARG B  31       7.497  -1.682 -31.094  1.00  1.49
H
ATOM   9554 1HG  ARG B  31       5.924  -2.221 -29.152  1.00  1.49
H
ATOM   9555 2HG  ARG B  31       5.122  -3.335 -30.276  1.00  1.49
H
ATOM   9556 1HD  ARG B  31       8.002  -3.405 -29.458  1.00  1.49
H
ATOM   9557 2HD  ARG B  31       6.710  -4.504 -28.918  1.00  1.49
H
ATOM   9558  HE  ARG B  31       8.115  -4.512 -31.456  1.00  1.49
H
ATOM   9559 1HH1 ARG B  31       5.192  -5.620 -29.746  1.00  1.49
H
ATOM   9560 2HH1 ARG B  31       4.941  -6.928 -30.870  1.00  1.49
H
ATOM   9561 1HH2 ARG B  31       7.725  -6.183 -32.858  1.00  1.49
H
ATOM   9562 2HH2 ARG B  31       6.326  -7.212 -32.670  1.00  1.49
H
ATOM   9563  N   ILE B  32       5.949   0.075 -33.588  1.00  0.88
N
ATOM   9564  CA  ILE B  32       6.446   0.962 -34.627  1.00  1.00
C
ATOM   9565  C   ILE B  32       5.939   2.375 -34.466  1.00  0.98
C
ATOM   9566  O   ILE B  32       6.723   3.321 -34.547  1.00  1.08
O
ATOM   9567  CB  ILE B  32       6.055   0.461 -36.017  1.00  1.50
C
ATOM   9568  CG1 ILE B  32       6.812  -0.819 -36.336  1.00  1.50
C
ATOM   9569  CG2 ILE B  32       6.314   1.539 -37.048  1.00  1.50
C
ATOM   9570  CD1 ILE B  32       6.285  -1.538 -37.550  1.00  1.50
C
ATOM   9571  H   ILE B  32       5.391  -0.729 -33.855  1.00  1.06
H
ATOM   9572  HA  ILE B  32       7.533   0.984 -34.566  1.00  1.20
H
ATOM   9573  HB  ILE B  32       4.996   0.212 -36.025  1.00  1.80
H
ATOM   9574 1HG1 ILE B  32       7.861  -0.581 -36.502  1.00  1.80
H
ATOM   9575 2HG1 ILE B  32       6.744  -1.483 -35.479  1.00  1.80
H
ATOM   9576 1HG2 ILE B  32       6.022   1.179 -38.033  1.00  1.80
H
ATOM   9577 2HG2 ILE B  32       5.733   2.426 -36.800  1.00  1.80
H
ATOM   9578 3HG2 ILE B  32       7.372   1.793 -37.051  1.00  1.80
H
ATOM   9579 1HD1 ILE B  32       6.867  -2.442 -37.723  1.00  1.80
H
```

```
ATOM   9580 2HD1 ILE B  32       5.239  -1.803 -37.390  1.00  1.80
H
ATOM   9581 3HD1 ILE B  32       6.364  -0.886 -38.419  1.00  1.80
H
ATOM   9582  N   HIS B  33       4.632   2.525 -34.235  1.00  0.90
N
ATOM   9583  CA  HIS B  33       4.071   3.856 -34.072  1.00  0.89
C
ATOM   9584  C   HIS B  33       4.603   4.539 -32.838  1.00  0.88
C
ATOM   9585  O   HIS B  33       4.851   5.742 -32.869  1.00  1.04
O
ATOM   9586  CB  HIS B  33       2.549   3.843 -34.004  1.00  1.33
C
ATOM   9587  CG  HIS B  33       1.892   3.577 -35.312  1.00  1.33
C
ATOM   9588  ND1 HIS B  33       2.129   4.340 -36.435  1.00  1.33
N
ATOM   9589  CD2 HIS B  33       0.979   2.653 -35.667  1.00  1.33
C
ATOM   9590  CE1 HIS B  33       1.401   3.879 -37.436  1.00  1.33
C
ATOM   9591  NE2 HIS B  33       0.691   2.855 -36.997  1.00  1.33
N
ATOM   9592  H   HIS B  33       4.028   1.715 -34.190  1.00  1.08
H
ATOM   9593  HA  HIS B  33       4.357   4.467 -34.927  1.00  1.07
H
ATOM   9594 1HB  HIS B  33       2.223   3.079 -33.298  1.00  1.60
H
ATOM   9595 2HB  HIS B  33       2.194   4.804 -33.635  1.00  1.60
H
ATOM   9596  HD1 HIS B  33       2.586   5.230 -36.445  1.00  1.60
H
ATOM   9597  HD2 HIS B  33       0.487   1.857 -35.111  1.00  1.60
H
ATOM   9598  HE1 HIS B  33       1.449   4.347 -38.418  1.00  1.60
H
ATOM   9599  N   SER B  34       4.801   3.778 -31.761  1.00  0.77
N
ATOM   9600  CA  SER B  34       5.344   4.338 -30.536  1.00  0.77
C
ATOM   9601  C   SER B  34       6.735   4.906 -30.789  1.00  0.87
C
ATOM   9602  O   SER B  34       7.050   6.015 -30.355  1.00  1.00
O
ATOM   9603  CB  SER B  34       5.409   3.265 -29.468  1.00  1.16
C
ATOM   9604  OG  SER B  34       4.124   2.819 -29.131  1.00  1.16
O
ATOM   9605  H   SER B  34       4.554   2.798 -31.777  1.00  0.92
H
ATOM   9606  HA  SER B  34       4.693   5.144 -30.197  1.00  0.92
H
ATOM   9607 1HB  SER B  34       6.007   2.426 -29.823  1.00  1.39
H
ATOM   9608 2HB  SER B  34       5.901   3.666 -28.584  1.00  1.39
H
```

```
ATOM   9609  HG  SER B  34       3.797   2.350 -29.906  1.00  1.39
H
ATOM   9610  N   ILE B  35       7.548   4.156 -31.534  1.00  0.85
N
ATOM   9611  CA  ILE B  35       8.891   4.594 -31.875  1.00  0.92
C
ATOM   9612  C   ILE B  35       8.864   5.833 -32.755  1.00  1.12
C
ATOM   9613  O   ILE B  35       9.589   6.790 -32.487  1.00  1.26
O
ATOM   9614  CB  ILE B  35       9.672   3.482 -32.588  1.00  1.38
C
ATOM   9615  CG1 ILE B  35       9.943   2.328 -31.628  1.00  1.38
C
ATOM   9616  CG2 ILE B  35      10.985   4.030 -33.117  1.00  1.38
C
ATOM   9617  CD1 ILE B  35      10.437   1.089 -32.327  1.00  1.38
C
ATOM   9618  H   ILE B  35       7.240   3.243 -31.849  1.00  1.02
H
ATOM   9619  HA  ILE B  35       9.416   4.843 -30.954  1.00  1.10
H
ATOM   9620  HB  ILE B  35       9.081   3.091 -33.415  1.00  1.66
H
ATOM   9621 1HG1 ILE B  35      10.696   2.638 -30.904  1.00  1.66
H
ATOM   9622 2HG1 ILE B  35       9.031   2.084 -31.087  1.00  1.66
H
ATOM   9623 1HG2 ILE B  35      11.530   3.238 -33.628  1.00  1.66
H
ATOM   9624 2HG2 ILE B  35      10.781   4.840 -33.817  1.00  1.66
H
ATOM   9625 3HG2 ILE B  35      11.583   4.408 -32.288  1.00  1.66
H
ATOM   9626 1HD1 ILE B  35      10.619   0.304 -31.593  1.00  1.66
H
ATOM   9627 2HD1 ILE B  35       9.687   0.753 -33.042  1.00  1.66
H
ATOM   9628 3HD1 ILE B  35      11.363   1.320 -32.852  1.00  1.66
H
ATOM   9629  N   ARG B  36       8.030   5.818 -33.796  1.00  1.24
N
ATOM   9630  CA  ARG B  36       7.926   6.949 -34.712  1.00  1.64
C
ATOM   9631  C   ARG B  36       7.451   8.217 -34.022  1.00  1.46
C
ATOM   9632  O   ARG B  36       7.972   9.302 -34.289  1.00  1.76
O
ATOM   9633  CB  ARG B  36       6.959   6.639 -35.850  1.00  2.46
C
ATOM   9634  CG  ARG B  36       7.450   5.628 -36.868  1.00  2.46
C
ATOM   9635  CD  ARG B  36       6.437   5.353 -37.921  1.00  2.46
C
ATOM   9636  NE  ARG B  36       6.930   4.409 -38.914  1.00  2.46
N
ATOM   9637  CZ  ARG B  36       6.176   3.833 -39.873  1.00  2.46
C
```

```
ATOM   9638  NH1 ARG B  36       4.896   4.119 -39.962  1.00  2.46
N
ATOM   9639  NH2 ARG B  36       6.722   2.977 -40.722  1.00  2.46
N
ATOM   9640  H   ARG B  36       7.462   4.999 -33.969  1.00  1.49
H
ATOM   9641  HA  ARG B  36       8.913   7.136 -35.136  1.00  1.97
H
ATOM   9642 1HB  ARG B  36       6.026   6.256 -35.437  1.00  2.95
H
ATOM   9643 2HB  ARG B  36       6.725   7.558 -36.388  1.00  2.95
H
ATOM   9644 1HG  ARG B  36       8.355   6.000 -37.348  1.00  2.95
H
ATOM   9645 2HG  ARG B  36       7.666   4.691 -36.360  1.00  2.95
H
ATOM   9646 1HD  ARG B  36       5.543   4.932 -37.464  1.00  2.95
H
ATOM   9647 2HD  ARG B  36       6.181   6.281 -38.431  1.00  2.95
H
ATOM   9648  HE  ARG B  36       7.910   4.160 -38.880  1.00  2.95
H
ATOM   9649 1HH1 ARG B  36       4.476   4.771 -39.315  1.00  2.95
H
ATOM   9650 2HH1 ARG B  36       4.333   3.685 -40.680  1.00  2.95
H
ATOM   9651 1HH2 ARG B  36       7.704   2.747 -40.657  1.00  2.95
H
ATOM   9652 2HH2 ARG B  36       6.158   2.540 -41.438  1.00  2.95
H
ATOM   9653  N   ALA B  37       6.459   8.076 -33.142  1.00  1.30
N
ATOM   9654  CA  ALA B  37       5.891   9.202 -32.418  1.00  1.45
C
ATOM   9655  C   ALA B  37       6.903   9.840 -31.487  1.00  1.57
C
ATOM   9656  O   ALA B  37       7.044  11.063 -31.472  1.00  1.82
O
ATOM   9657  CB  ALA B  37       4.676   8.748 -31.624  1.00  2.17
C
ATOM   9658  H   ALA B  37       6.067   7.161 -32.979  1.00  1.56
H
ATOM   9659  HA  ALA B  37       5.583   9.952 -33.146  1.00  1.74
H
ATOM   9660 1HB  ALA B  37       4.238   9.602 -31.108  1.00  2.61
H
ATOM   9661 2HB  ALA B  37       3.940   8.317 -32.303  1.00  2.61
H
ATOM   9662 3HB  ALA B  37       4.977   7.997 -30.895  1.00  2.61
H
ATOM   9663  N   THR B  38       7.610   9.010 -30.720  1.00  1.43
N
ATOM   9664  CA  THR B  38       8.603   9.504 -29.777  1.00  1.44
C
ATOM   9665  C   THR B  38       9.847  10.036 -30.468  1.00  1.48
C
ATOM   9666  O   THR B  38      10.470  10.987 -29.994  1.00  1.61
O
```

```
ATOM   9667  CB  THR B  38       8.998   8.378 -28.825  1.00  2.16
C
ATOM   9668  OG1 THR B  38       9.447   7.269 -29.611  1.00  2.16
O
ATOM   9669  CG2 THR B  38       7.829   7.960 -27.942  1.00  2.16
C
ATOM   9670  H   THR B  38       7.446   8.010 -30.767  1.00  1.72
H
ATOM   9671  HA  THR B  38       8.159  10.315 -29.200  1.00  1.73
H
ATOM   9672  HB  THR B  38       9.822   8.710 -28.196  1.00  2.59
H
ATOM   9673  HG1 THR B  38       8.701   6.909 -30.102  1.00  2.59
H
ATOM   9674 1HG2 THR B  38       8.142   7.153 -27.280  1.00  2.59
H
ATOM   9675 2HG2 THR B  38       7.503   8.812 -27.347  1.00  2.59
H
ATOM   9676 3HG2 THR B  38       7.003   7.620 -28.563  1.00  2.59
H
ATOM   9677  N   CYS B  39      10.181   9.457 -31.618  1.00  1.42
N
ATOM   9678  CA  CYS B  39      11.310   9.921 -32.408  1.00  1.48
C
ATOM   9679  C   CYS B  39      11.025  11.306 -32.951  1.00  1.76
C
ATOM   9680  O   CYS B  39      11.839  12.221 -32.806  1.00  1.88
O
ATOM   9681  CB  CYS B  39      11.602   8.964 -33.562  1.00  2.22
C
ATOM   9682  SG  CYS B  39      13.025   9.444 -34.566  1.00  2.22
S
ATOM   9683  H   CYS B  39       9.662   8.658 -31.954  1.00  1.70
H
ATOM   9684  HA  CYS B  39      12.190   9.969 -31.767  1.00  1.78
H
ATOM   9685 1HB  CYS B  39      11.793   7.967 -33.166  1.00  2.66
H
ATOM   9686 2HB  CYS B  39      10.733   8.899 -34.215  1.00  2.66
H
ATOM   9687  HG  CYS B  39      13.906   9.432 -33.570  1.00  2.66
H
ATOM   9688  N   SER B  40       9.856  11.457 -33.581  1.00  1.93
N
ATOM   9689  CA  SER B  40       9.427  12.734 -34.132  1.00  2.38
C
ATOM   9690  C   SER B  40       9.315  13.790 -33.039  1.00  2.65
C
ATOM   9691  O   SER B  40       9.727  14.937 -33.226  1.00  3.30
O
ATOM   9692  CB  SER B  40       8.086  12.578 -34.824  1.00  3.57
C
ATOM   9693  OG  SER B  40       8.188  11.736 -35.940  1.00  3.57
O
ATOM   9694  H   SER B  40       9.239  10.663 -33.686  1.00  2.32
H
ATOM   9695  HA  SER B  40      10.168  13.067 -34.860  1.00  2.86
H
```

```
ATOM   9696 1HB  SER B  40       7.357  12.171 -34.125  1.00  4.28
H
ATOM   9697 2HB  SER B  40       7.726  13.557 -35.138  1.00  4.28
H
ATOM   9698  HG  SER B  40       8.347  10.855 -35.586  1.00  4.28
H
ATOM   9699  N   THR B  41       8.771  13.384 -31.892  1.00  2.37
N
ATOM   9700  CA  THR B  41       8.627  14.238 -30.723  1.00  2.90
C
ATOM   9701  C   THR B  41       9.967  14.632 -30.113  1.00  3.01
C
ATOM   9702  O   THR B  41      10.110  15.742 -29.602  1.00  6.30
O
ATOM   9703  CB  THR B  41       7.777  13.546 -29.640  1.00  4.35
C
ATOM   9704  OG1 THR B  41       6.468  13.273 -30.155  1.00  4.35
O
ATOM   9705  CG2 THR B  41       7.664  14.436 -28.415  1.00  4.35
C
ATOM   9706  H   THR B  41       8.421  12.438 -31.815  1.00  2.84
H
ATOM   9707  HA  THR B  41       8.117  15.152 -31.028  1.00  3.48
H
ATOM   9708  HB  THR B  41       8.248  12.607 -29.356  1.00  5.22
H
ATOM   9709  HG1 THR B  41       6.529  12.586 -30.828  1.00  5.22
H
ATOM   9710 1HG2 THR B  41       7.063  13.934 -27.656  1.00  5.22
H
ATOM   9711 2HG2 THR B  41       8.658  14.637 -28.019  1.00  5.22
H
ATOM   9712 3HG2 THR B  41       7.187  15.376 -28.691  1.00  5.22
H
ATOM   9713  N   SER B  42      10.928  13.699 -30.141  1.00  2.66
N
ATOM   9714  CA  SER B  42      12.249  13.842 -29.525  1.00  2.56
C
ATOM   9715  C   SER B  42      12.111  13.754 -28.010  1.00  2.40
C
ATOM   9716  O   SER B  42      12.825  14.427 -27.264  1.00  2.65
O
ATOM   9717  CB  SER B  42      12.941  15.140 -29.930  1.00  3.84
C
ATOM   9718  OG  SER B  42      13.143  15.201 -31.316  1.00  3.84
O
ATOM   9719  H   SER B  42      10.729  12.814 -30.584  1.00  3.19
H
ATOM   9720  HA  SER B  42      12.875  13.012 -29.855  1.00  3.07
H
ATOM   9721 1HB  SER B  42      12.370  16.004 -29.601  1.00  4.61
H
ATOM   9722 2HB  SER B  42      13.906  15.193 -29.428  1.00  4.61
H
ATOM   9723  HG  SER B  42      12.274  15.114 -31.717  1.00  4.61
H
ATOM   9724  N   SER B  43      11.168  12.918 -27.573  1.00  2.14
N
```

```
ATOM   9725  CA  SER B  43      10.886  12.679 -26.161  1.00  2.17
C
ATOM   9726  C   SER B  43       9.958  11.482 -26.017  1.00  2.61
C
ATOM   9727  O   SER B  43       9.729  10.754 -26.973  1.00 12.73
O
ATOM   9728  CB  SER B  43      10.262  13.899 -25.508  1.00  3.25
C
ATOM   9729  OG  SER B  43      10.155  13.710 -24.123  1.00  3.25
O
ATOM   9730  H   SER B  43      10.647  12.388 -28.265  1.00  2.57
H
ATOM   9731  HA  SER B  43      11.823  12.451 -25.650  1.00  2.60
H
ATOM   9732 1HB  SER B  43      10.871  14.780 -25.713  1.00  3.91
H
ATOM   9733 2HB  SER B  43       9.275  14.079 -25.932  1.00  3.91
H
ATOM   9734  HG  SER B  43       9.786  14.526 -23.774  1.00  3.91
H
ATOM   9735  N   GLY B  44       9.399  11.291 -24.836  1.00  3.64
N
ATOM   9736  CA  GLY B  44       8.479  10.176 -24.632  1.00  2.32
C
ATOM   9737  C   GLY B  44       9.175   8.933 -24.102  1.00  1.81
C
ATOM   9738  O   GLY B  44      10.336   8.981 -23.697  1.00  2.57
O
ATOM   9739  H   GLY B  44       9.613  11.932 -24.085  1.00  4.37
H
ATOM   9740 1HA  GLY B  44       7.699  10.480 -23.933  1.00  2.78
H
ATOM   9741 2HA  GLY B  44       7.981   9.943 -25.571  1.00  2.78
H
ATOM   9742  N   HIS B  45       8.454   7.815 -24.087  1.00  1.38
N
ATOM   9743  CA  HIS B  45       8.997   6.603 -23.507  1.00  1.26
C
ATOM   9744  C   HIS B  45       8.655   5.290 -24.246  1.00  0.96
C
ATOM   9745  O   HIS B  45       7.870   4.477 -23.738  1.00  1.00
O
ATOM   9746  CB  HIS B  45       8.481   6.523 -22.097  1.00  1.89
C
ATOM   9747  CG  HIS B  45       9.161   5.493 -21.332  1.00  1.89
C
ATOM   9748  ND1 HIS B  45       8.814   5.180 -20.048  1.00  1.89
N
ATOM   9749  CD2 HIS B  45      10.179   4.681 -21.668  1.00  1.89
C
ATOM   9750  CE1 HIS B  45       9.589   4.215 -19.621  1.00  1.89
C
ATOM   9751  NE2 HIS B  45      10.438   3.894 -20.579  1.00  1.89
N
ATOM   9752  H   HIS B  45       7.513   7.817 -24.456  1.00  1.66
H
ATOM   9753  HA  HIS B  45      10.082   6.681 -23.448  1.00  1.51
H
```

```
ATOM   9754 1HB  HIS B  45       8.621   7.481 -21.595  1.00  2.27
H
ATOM   9755 2HB  HIS B  45       7.414   6.304 -22.105  1.00  2.27
H
ATOM   9756  HD1 HIS B  45       8.014   5.520 -19.552  1.00  2.27
H
ATOM   9757  HD2 HIS B  45      10.766   4.576 -22.580  1.00  2.27
H
ATOM   9758  HE1 HIS B  45       9.444   3.811 -18.625  1.00  2.27
H
ATOM   9759  N   PRO B  46       9.294   5.069 -25.405  1.00  0.99
N
ATOM   9760  CA  PRO B  46       9.165   3.963 -26.362  1.00  1.13
C
ATOM   9761  C   PRO B  46       9.187   2.582 -25.742  1.00  0.80
C
ATOM   9762  O   PRO B  46       8.489   1.677 -26.206  1.00  0.95
O
ATOM   9763  CB  PRO B  46      10.439   4.079 -27.206  1.00  1.69
C
ATOM   9764  CG  PRO B  46      10.790   5.499 -27.147  1.00  1.69
C
ATOM   9765  CD  PRO B  46      10.493   5.878 -25.725  1.00  1.69
C
ATOM   9766  HA  PRO B  46       8.265   4.108 -26.973  1.00  1.36
H
ATOM   9767 1HB  PRO B  46      11.226   3.428 -26.798  1.00  2.03
H
ATOM   9768 2HB  PRO B  46      10.241   3.735 -28.233  1.00  2.03
H
ATOM   9769 1HG  PRO B  46      11.846   5.631 -27.418  1.00  2.03
H
ATOM   9770 2HG  PRO B  46      10.196   6.062 -27.865  1.00  2.03
H
ATOM   9771 1HD  PRO B  46      11.326   5.601 -25.063  1.00  2.03
H
ATOM   9772 2HD  PRO B  46      10.272   6.949 -25.681  1.00  2.03
H
ATOM   9773  N   THR B  47      10.024   2.414 -24.715  1.00  0.78
N
ATOM   9774  CA  THR B  47      10.231   1.107 -24.118  1.00  0.74
C
ATOM   9775  C   THR B  47       9.125   0.715 -23.156  1.00  0.79
C
ATOM   9776  O   THR B  47       8.953  -0.474 -22.870  1.00  0.91
O
ATOM   9777  CB  THR B  47      11.581   1.041 -23.383  1.00  1.11
C
ATOM   9778  OG1 THR B  47      11.586   1.965 -22.296  1.00  1.11
O
ATOM   9779  CG2 THR B  47      12.715   1.390 -24.328  1.00  1.11
C
ATOM   9780  H   THR B  47      10.550   3.203 -24.369  1.00  0.94
H
ATOM   9781  HA  THR B  47      10.251   0.370 -24.919  1.00  0.89
H
ATOM   9782  HB  THR B  47      11.735   0.043 -22.991  1.00  1.33
H
```

```
ATOM   9783  HG1 THR B  47      11.755   2.846 -22.636  1.00  1.33
H
ATOM   9784 1HG2 THR B  47      13.663   1.338 -23.793  1.00  1.33
H
ATOM   9785 2HG2 THR B  47      12.727   0.683 -25.157  1.00  1.33
H
ATOM   9786 3HG2 THR B  47      12.572   2.398 -24.714  1.00  1.33
H
ATOM   9787  N   SER B  48       8.360   1.697 -22.660  1.00  0.91
N
ATOM   9788  CA  SER B  48       7.239   1.362 -21.790  1.00  1.26
C
ATOM   9789  C   SER B  48       6.079   0.993 -22.641  1.00  1.85
C
ATOM   9790  O   SER B  48       5.246   0.175 -22.255  1.00  6.41
O
ATOM   9791  CB  SER B  48       6.813   2.503 -20.919  1.00  1.89
C
ATOM   9792  OG  SER B  48       6.319   3.566 -21.690  1.00  1.89
O
ATOM   9793  H   SER B  48       8.516   2.664 -22.913  1.00  1.09
H
ATOM   9794  HA  SER B  48       7.507   0.505 -21.169  1.00  1.51
H
ATOM   9795 1HB  SER B  48       6.034   2.158 -20.240  1.00  2.27
H
ATOM   9796 2HB  SER B  48       7.640   2.820 -20.313  1.00  2.27
H
ATOM   9797  HG  SER B  48       7.028   3.816 -22.290  1.00  2.27
H
ATOM   9798  N   CYS B  49       6.039   1.583 -23.827  1.00  0.96
N
ATOM   9799  CA  CYS B  49       5.014   1.232 -24.779  1.00  0.79
C
ATOM   9800  C   CYS B  49       5.267  -0.186 -25.267  1.00  0.77
C
ATOM   9801  O   CYS B  49       4.367  -1.028 -25.255  1.00  1.46
O
ATOM   9802  CB  CYS B  49       5.014   2.203 -25.954  1.00  1.19
C
ATOM   9803  SG  CYS B  49       4.470   3.874 -25.532  1.00  1.19
S
ATOM   9804  H   CYS B  49       6.724   2.297 -24.060  1.00  1.15
H
ATOM   9805  HA  CYS B  49       4.042   1.271 -24.286  1.00  0.95
H
ATOM   9806 1HB  CYS B  49       6.018   2.272 -26.372  1.00  1.42
H
ATOM   9807 2HB  CYS B  49       4.359   1.823 -26.739  1.00  1.42
H
ATOM   9808  HG  CYS B  49       5.411   4.099 -24.622  1.00  1.42
H
ATOM   9809  N   SER B  50       6.517  -0.444 -25.666  1.00  0.53
N
ATOM   9810  CA  SER B  50       6.937  -1.733 -26.197  1.00  0.51
C
ATOM   9811  C   SER B  50       6.636  -2.912 -25.280  1.00  0.45
C
```

ATOM 9812 O SER B 50 6.105 -3.928 -25.735 1.00 0.48
O
ATOM 9813 CB SER B 50 8.426 -1.690 -26.476 1.00 0.77
C
ATOM 9814 OG SER B 50 8.721 -0.767 -27.489 1.00 0.77
O
ATOM 9815 H SER B 50 7.204 0.297 -25.641 1.00 0.64
H
ATOM 9816 HA SER B 50 6.412 -1.895 -27.135 1.00 0.61
H
ATOM 9817 1HB SER B 50 8.960 -1.420 -25.566 1.00 0.92
H
ATOM 9818 2HB SER B 50 8.765 -2.680 -26.772 1.00 0.92
H
ATOM 9819 HG SER B 50 8.506 0.101 -27.129 1.00 0.92
H
ATOM 9820 N SER B 51 6.912 -2.769 -23.978 1.00 0.43
N
ATOM 9821 CA SER B 51 6.686 -3.871 -23.040 1.00 0.47
C
ATOM 9822 C SER B 51 5.223 -4.326 -22.909 1.00 0.50
C
ATOM 9823 O SER B 51 4.974 -5.458 -22.477 1.00 1.27
O
ATOM 9824 CB SER B 51 7.244 -3.503 -21.666 1.00 0.70
C
ATOM 9825 OG SER B 51 6.566 -2.435 -21.052 1.00 0.70
O
ATOM 9826 H SER B 51 7.340 -1.913 -23.639 1.00 0.52
H
ATOM 9827 HA SER B 51 7.256 -4.726 -23.402 1.00 0.56
H
ATOM 9828 1HB SER B 51 7.208 -4.376 -21.016 1.00 0.85
H
ATOM 9829 2HB SER B 51 8.290 -3.237 -21.785 1.00 0.85
H
ATOM 9830 HG SER B 51 5.744 -2.784 -20.686 1.00 0.85
H
ATOM 9831 N SER B 52 4.263 -3.479 -23.310 1.00 0.43
N
ATOM 9832 CA SER B 52 2.851 -3.836 -23.213 1.00 0.44
C
ATOM 9833 C SER B 52 2.138 -3.853 -24.563 1.00 0.45
C
ATOM 9834 O SER B 52 0.908 -3.787 -24.599 1.00 0.57
O
ATOM 9835 CB SER B 52 2.128 -2.870 -22.302 1.00 0.66
C
ATOM 9836 OG SER B 52 2.213 -1.556 -22.784 1.00 0.66
O
ATOM 9837 H SER B 52 4.500 -2.573 -23.695 1.00 0.52
H
ATOM 9838 HA SER B 52 2.781 -4.835 -22.783 1.00 0.53
H
ATOM 9839 1HB SER B 52 1.083 -3.166 -22.224 1.00 0.79
H
ATOM 9840 2HB SER B 52 2.559 -2.921 -21.303 1.00 0.79
H

ATOM 9841 HG SER B 52 3.151 -1.351 -22.832 1.00 0.79
H
ATOM 9842 N SER B 53 2.890 -3.904 -25.673 1.00 0.42
N
ATOM 9843 CA SER B 53 2.250 -3.832 -26.991 1.00 0.47
C
ATOM 9844 C SER B 53 1.281 -4.984 -27.260 1.00 0.54
C
ATOM 9845 O SER B 53 0.249 -4.790 -27.904 1.00 0.83
O
ATOM 9846 CB SER B 53 3.316 -3.807 -28.074 1.00 0.70
C
ATOM 9847 OG SER B 53 3.993 -5.037 -28.200 1.00 0.70
O
ATOM 9848 H SER B 53 3.901 -3.971 -25.615 1.00 0.50
H
ATOM 9849 HA SER B 53 1.686 -2.902 -27.043 1.00 0.56
H
ATOM 9850 1HB SER B 53 2.865 -3.535 -29.027 1.00 0.85
H
ATOM 9851 2HB SER B 53 4.036 -3.029 -27.825 1.00 0.85
H
ATOM 9852 HG SER B 53 3.951 -5.499 -27.355 1.00 0.85
H
ATOM 9853 N GLU B 54 1.585 -6.161 -26.723 1.00 0.46
N
ATOM 9854 CA GLU B 54 0.713 -7.316 -26.863 1.00 0.46
C
ATOM 9855 C GLU B 54 -0.523 -7.177 -26.013 1.00 0.50
C
ATOM 9856 O GLU B 54 -1.618 -7.515 -26.453 1.00 0.80
O
ATOM 9857 CB GLU B 54 1.455 -8.588 -26.502 1.00 0.69
C
ATOM 9858 CG GLU B 54 2.510 -9.002 -27.507 1.00 0.69
C
ATOM 9859 CD GLU B 54 3.785 -8.226 -27.414 1.00 0.69
C
ATOM 9860 OE1 GLU B 54 3.846 -7.256 -26.689 1.00 0.69
O
ATOM 9861 OE2 GLU B 54 4.712 -8.621 -28.083 1.00 0.69
O
ATOM 9862 H GLU B 54 2.446 -6.264 -26.206 1.00 0.55
H
ATOM 9863 HA GLU B 54 0.400 -7.386 -27.905 1.00 0.55
H
ATOM 9864 1HB GLU B 54 1.952 -8.448 -25.544 1.00 0.83
H
ATOM 9865 2HB GLU B 54 0.750 -9.411 -26.391 1.00 0.83
H
ATOM 9866 1HG GLU B 54 2.732 -10.056 -27.363 1.00 0.83
H
ATOM 9867 2HG GLU B 54 2.096 -8.885 -28.507 1.00 0.83
H
ATOM 9868 N ILE B 55 -0.346 -6.657 -24.801 1.00 0.36
N
ATOM 9869 CA ILE B 55 -1.451 -6.461 -23.876 1.00 0.35
C

```
ATOM   9870  C   ILE B  55      -2.474  -5.519 -24.466  1.00  0.37
C
ATOM   9871  O   ILE B  55      -3.676  -5.791 -24.424  1.00  0.41
O
ATOM   9872  CB  ILE B  55      -0.954  -5.889 -22.538  1.00  0.52
C
ATOM   9873  CG1 ILE B  55      -0.103  -6.927 -21.802  1.00  0.52
C
ATOM   9874  CG2 ILE B  55      -2.143  -5.461 -21.690  1.00  0.52
C
ATOM   9875  CD1 ILE B  55       0.665  -6.353 -20.632  1.00  0.52
C
ATOM   9876  H   ILE B  55       0.585  -6.398 -24.507  1.00  0.43
H
ATOM   9877  HA  ILE B  55      -1.933  -7.418 -23.691  1.00  0.42
H
ATOM   9878  HB  ILE B  55      -0.320  -5.024 -22.726  1.00  0.63
H
ATOM   9879 1HG1 ILE B  55      -0.751  -7.721 -21.434  1.00  0.63
H
ATOM   9880 2HG1 ILE B  55       0.611  -7.363 -22.502  1.00  0.63
H
ATOM   9881 1HG2 ILE B  55      -1.785  -5.044 -20.750  1.00  0.63
H
ATOM   9882 2HG2 ILE B  55      -2.719  -4.707 -22.224  1.00  0.63
H
ATOM   9883 3HG2 ILE B  55      -2.776  -6.326 -21.487  1.00  0.63
H
ATOM   9884 1HD1 ILE B  55       1.246  -7.143 -20.156  1.00  0.63
H
ATOM   9885 2HD1 ILE B  55       1.337  -5.572 -20.985  1.00  0.63
H
ATOM   9886 3HD1 ILE B  55      -0.033  -5.932 -19.911  1.00  0.63
H
ATOM   9887  N   MET B  56      -1.986  -4.413 -25.024  1.00  0.39
N
ATOM   9888  CA  MET B  56      -2.853  -3.425 -25.627  1.00  0.45
C
ATOM   9889  C   MET B  56      -3.541  -3.977 -26.858  1.00  0.51
C
ATOM   9890  O   MET B  56      -4.738  -3.772 -27.037  1.00  0.72
O
ATOM   9891  CB  MET B  56      -2.049  -2.180 -25.990  1.00  0.68
C
ATOM   9892  CG  MET B  56      -1.566  -1.367 -24.799  1.00  0.68
C
ATOM   9893  SD  MET B  56      -0.825   0.202 -25.294  1.00  0.68
S
ATOM   9894  CE  MET B  56       0.729  -0.356 -25.991  1.00  0.68
C
ATOM   9895  H   MET B  56      -0.986  -4.248 -25.012  1.00  0.47
H
ATOM   9896  HA  MET B  56      -3.624  -3.153 -24.906  1.00  0.54
H
ATOM   9897 1HB  MET B  56      -1.174  -2.471 -26.569  1.00  0.81
H
ATOM   9898 2HB  MET B  56      -2.655  -1.526 -26.618  1.00  0.81
H
```

```
ATOM   9899 1HG  MET B  56       -2.404  -1.159 -24.135  1.00  0.81
H
ATOM   9900 2HG  MET B  56       -0.823  -1.938 -24.243  1.00  0.81
H
ATOM   9901 1HE  MET B  56        1.301   0.505 -26.340  1.00  0.81
H
ATOM   9902 2HE  MET B  56        1.301  -0.887 -25.229  1.00  0.81
H
ATOM   9903 3HE  MET B  56        0.531  -1.024 -26.830  1.00  0.81
H
ATOM   9904  N   SER B  57       -2.804  -4.711 -27.690  1.00  0.47
N
ATOM   9905  CA  SER B  57       -3.385  -5.292 -28.888  1.00  0.54
C
ATOM   9906  C   SER B  57       -4.533  -6.244 -28.536  1.00  0.54
C
ATOM   9907  O   SER B  57       -5.613  -6.172 -29.125  1.00  0.84
O
ATOM   9908  CB  SER B  57       -2.303  -6.007 -29.672  1.00  0.81
C
ATOM   9909  OG  SER B  57       -2.809  -6.550 -30.854  1.00  0.81
O
ATOM   9910  H   SER B  57       -1.815  -4.850 -27.517  1.00  0.56
H
ATOM   9911  HA  SER B  57       -3.785  -4.484 -29.503  1.00  0.65
H
ATOM   9912 1HB  SER B  57       -1.499  -5.309 -29.904  1.00  0.97
H
ATOM   9913 2HB  SER B  57       -1.878  -6.802 -29.059  1.00  0.97
H
ATOM   9914  HG  SER B  57       -2.098  -7.073 -31.238  1.00  0.97
H
ATOM   9915  N   VAL B  58       -4.306  -7.121 -27.561  1.00  0.47
N
ATOM   9916  CA  VAL B  58       -5.319  -8.074 -27.128  1.00  0.45
C
ATOM   9917  C   VAL B  58       -6.562  -7.409 -26.571  1.00  0.37
C
ATOM   9918  O   VAL B  58       -7.682  -7.744 -26.974  1.00  0.45
O
ATOM   9919  CB  VAL B  58       -4.729  -9.016 -26.068  1.00  0.68
C
ATOM   9920  CG1 VAL B  58       -5.824  -9.858 -25.450  1.00  0.68
C
ATOM   9921  CG2 VAL B  58       -3.702  -9.918 -26.725  1.00  0.68
C
ATOM   9922  H   VAL B  58       -3.398  -7.142 -27.114  1.00  0.56
H
ATOM   9923  HA  VAL B  58       -5.618  -8.673 -27.984  1.00  0.54
H
ATOM   9924  HB  VAL B  58       -4.260  -8.427 -25.280  1.00  0.81
H
ATOM   9925 1HG1 VAL B  58       -5.392 -10.520 -24.701  1.00  0.81
H
ATOM   9926 2HG1 VAL B  58       -6.557  -9.206 -24.977  1.00  0.81
H
ATOM   9927 3HG1 VAL B  58       -6.308 -10.451 -26.224  1.00  0.81
H
```

ATOM 9928 1HG2 VAL B 58 -3.272 -10.581 -25.985 1.00 0.81
H
ATOM 9929 2HG2 VAL B 58 -4.189 -10.503 -27.492 1.00 0.81
H
ATOM 9930 3HG2 VAL B 58 -2.914 -9.319 -27.174 1.00 0.81
H
ATOM 9931 N LEU B 59 -6.374 -6.457 -25.663 1.00 0.42
N
ATOM 9932 CA LEU B 59 -7.516 -5.785 -25.084 1.00 0.49
C
ATOM 9933 C LEU B 59 -8.277 -4.936 -26.088 1.00 0.41
C
ATOM 9934 O LEU B 59 -9.504 -4.954 -26.121 1.00 0.51
O
ATOM 9935 CB LEU B 59 -7.074 -4.914 -23.904 1.00 0.73
C
ATOM 9936 CG LEU B 59 -6.627 -5.694 -22.662 1.00 0.73
C
ATOM 9937 CD1 LEU B 59 -6.055 -4.738 -21.628 1.00 0.73
C
ATOM 9938 CD2 LEU B 59 -7.826 -6.441 -22.111 1.00 0.73
C
ATOM 9939 H LEU B 59 -5.440 -6.208 -25.358 1.00 0.50
H
ATOM 9940 HA LEU B 59 -8.187 -6.541 -24.704 1.00 0.59
H
ATOM 9941 1HB LEU B 59 -6.240 -4.291 -24.223 1.00 0.88
H
ATOM 9942 2HB LEU B 59 -7.901 -4.265 -23.614 1.00 0.88
H
ATOM 9943 HG LEU B 59 -5.845 -6.405 -22.933 1.00 0.88
H
ATOM 9944 1HD1 LEU B 59 -5.740 -5.302 -20.749 1.00 0.88
H
ATOM 9945 2HD1 LEU B 59 -5.198 -4.216 -22.050 1.00 0.88
H
ATOM 9946 3HD1 LEU B 59 -6.818 -4.015 -21.342 1.00 0.88
H
ATOM 9947 1HD2 LEU B 59 -7.529 -7.003 -21.228 1.00 0.88
H
ATOM 9948 2HD2 LEU B 59 -8.605 -5.728 -21.843 1.00 0.88
H
ATOM 9949 3HD2 LEU B 59 -8.204 -7.125 -22.868 1.00 0.88
H
ATOM 9950 N PHE B 60 -7.565 -4.225 -26.947 1.00 0.44
N
ATOM 9951 CA PHE B 60 -8.241 -3.361 -27.896 1.00 0.57
C
ATOM 9952 C PHE B 60 -8.842 -4.067 -29.103 1.00 0.62
C
ATOM 9953 O PHE B 60 -9.753 -3.519 -29.728 1.00 1.17
O
ATOM 9954 CB PHE B 60 -7.341 -2.209 -28.330 1.00 0.85
C
ATOM 9955 CG PHE B 60 -7.158 -1.183 -27.242 1.00 0.85
C
ATOM 9956 CD1 PHE B 60 -5.903 -0.873 -26.736 1.00 0.85
C

ATOM 9957 CD2 PHE B 60 -8.262 -0.533 -26.708 1.00 0.85
C
ATOM 9958 CE1 PHE B 60 -5.757 0.069 -25.735 1.00 0.85
C
ATOM 9959 CE2 PHE B 60 -8.120 0.407 -25.709 1.00 0.85
C
ATOM 9960 CZ PHE B 60 -6.866 0.710 -25.227 1.00 0.85
C
ATOM 9961 H PHE B 60 -6.555 -4.237 -26.924 1.00 0.53
H
ATOM 9962 HA PHE B 60 -9.074 -2.900 -27.362 1.00 0.68
H
ATOM 9963 1HB PHE B 60 -6.361 -2.591 -28.612 1.00 1.03
H
ATOM 9964 2HB PHE B 60 -7.768 -1.714 -29.202 1.00 1.03
H
ATOM 9965 HD1 PHE B 60 -5.025 -1.374 -27.140 1.00 1.03
H
ATOM 9966 HD2 PHE B 60 -9.253 -0.770 -27.091 1.00 1.03
H
ATOM 9967 HE1 PHE B 60 -4.765 0.307 -25.351 1.00 1.03
H
ATOM 9968 HE2 PHE B 60 -8.997 0.912 -25.304 1.00 1.03
H
ATOM 9969 HZ PHE B 60 -6.752 1.457 -24.445 1.00 1.03
H
ATOM 9970 N PHE B 61 -8.362 -5.267 -29.442 1.00 0.55
N
ATOM 9971 CA PHE B 61 -8.911 -5.931 -30.614 1.00 0.67
C
ATOM 9972 C PHE B 61 -9.783 -7.156 -30.350 1.00 0.99
C
ATOM 9973 O PHE B 61 -10.667 -7.441 -31.159 1.00 1.94
O
ATOM 9974 CB PHE B 61 -7.809 -6.266 -31.603 1.00 1.01
C
ATOM 9975 CG PHE B 61 -7.154 -5.031 -32.145 1.00 1.01
C
ATOM 9976 CD1 PHE B 61 -5.811 -4.764 -31.927 1.00 1.01
C
ATOM 9977 CD2 PHE B 61 -7.902 -4.112 -32.857 1.00 1.01
C
ATOM 9978 CE1 PHE B 61 -5.235 -3.610 -32.418 1.00 1.01
C
ATOM 9979 CE2 PHE B 61 -7.331 -2.961 -33.353 1.00 1.01
C
ATOM 9980 CZ PHE B 61 -5.994 -2.710 -33.134 1.00 1.01
C
ATOM 9981 H PHE B 61 -7.597 -5.693 -28.935 1.00 0.66
H
ATOM 9982 HA PHE B 61 -9.550 -5.206 -31.120 1.00 0.80
H
ATOM 9983 1HB PHE B 61 -7.050 -6.882 -31.124 1.00 1.21
H
ATOM 9984 2HB PHE B 61 -8.225 -6.831 -32.435 1.00 1.21
H
ATOM 9985 HD1 PHE B 61 -5.208 -5.475 -31.366 1.00 1.21
H

```
ATOM   9986  HD2 PHE B  61       -8.960  -4.315 -33.025  1.00  1.21
H
ATOM   9987  HE1 PHE B  61       -4.179  -3.412 -32.240  1.00  1.21
H
ATOM   9988  HE2 PHE B  61       -7.935  -2.249 -33.915  1.00  1.21
H
ATOM   9989  HZ  PHE B  61       -5.538  -1.799 -33.523  1.00  1.21
H
ATOM   9990  N   TYR B  62       -9.574  -7.887 -29.252  1.00  0.78
N
ATOM   9991  CA  TYR B  62      -10.457  -9.034 -29.010  1.00  1.53
C
ATOM   9992  C   TYR B  62      -11.193  -9.059 -27.686  1.00  1.26
C
ATOM   9993  O   TYR B  62      -12.278  -9.638 -27.611  1.00  2.88
O
ATOM   9994  CB  TYR B  62       -9.703 -10.341 -29.197  1.00  2.29
C
ATOM   9995  CG  TYR B  62       -9.350 -10.507 -30.640  1.00  2.29
C
ATOM   9996  CD1 TYR B  62      -10.374 -10.688 -31.552  1.00  2.29
C
ATOM   9997  CD2 TYR B  62       -8.044 -10.456 -31.059  1.00  2.29
C
ATOM   9998  CE1 TYR B  62      -10.091 -10.799 -32.891  1.00  2.29
C
ATOM   9999  CE2 TYR B  62       -7.757 -10.574 -32.401  1.00  2.29
C
ATOM  10000  CZ  TYR B  62       -8.777 -10.735 -33.310  1.00  2.29
C
ATOM  10001  OH  TYR B  62       -8.497 -10.836 -34.645  1.00  2.29
O
ATOM  10002  H   TYR B  62       -8.837  -7.672 -28.594  1.00  0.94
H
ATOM  10003  HA  TYR B  62      -11.229  -9.011 -29.778  1.00  1.84
H
ATOM  10004 1HB  TYR B  62       -8.784 -10.342 -28.609  1.00  2.75
H
ATOM  10005 2HB  TYR B  62      -10.315 -11.186 -28.886  1.00  2.75
H
ATOM  10006  HD1 TYR B  62      -11.407 -10.725 -31.209  1.00  2.75
H
ATOM  10007  HD2 TYR B  62       -7.249 -10.315 -30.329  1.00  2.75
H
ATOM  10008  HE1 TYR B  62      -10.893 -10.930 -33.616  1.00  2.75
H
ATOM  10009  HE2 TYR B  62       -6.728 -10.530 -32.750  1.00  2.75
H
ATOM  10010  HH  TYR B  62       -7.546 -10.888 -34.769  1.00  2.75
H
ATOM  10011  N   ILE B  63      -10.658  -8.435 -26.649  1.00  0.46
N
ATOM  10012  CA  ILE B  63      -11.331  -8.533 -25.360  1.00  0.40
C
ATOM  10013  C   ILE B  63      -12.346  -7.438 -25.064  1.00  0.55
C
ATOM  10014  O   ILE B  63      -13.484  -7.735 -24.699  1.00  1.23
O
```

```
ATOM  10015  CB  ILE B  63     -10.302  -8.549 -24.249  1.00  0.60
C
ATOM  10016  CG1 ILE B  63      -9.361  -9.733 -24.470  1.00  0.60
C
ATOM  10017  CG2 ILE B  63     -10.983  -8.598 -22.895  1.00  0.60
C
ATOM  10018  CD1 ILE B  63     -10.063 -11.075 -24.504  1.00  0.60
C
ATOM  10019  H   ILE B  63      -9.769  -7.955 -26.731  1.00  0.55
H
ATOM  10020  HA  ILE B  63     -11.858  -9.485 -25.335  1.00  0.48
H
ATOM  10021  HB  ILE B  63      -9.713  -7.647 -24.306  1.00  0.72
H
ATOM  10022 1HG1 ILE B  63      -8.846  -9.597 -25.420  1.00  0.72
H
ATOM  10023 2HG1 ILE B  63      -8.615  -9.744 -23.680  1.00  0.72
H
ATOM  10024 1HG2 ILE B  63     -10.232  -8.583 -22.110  1.00  0.72
H
ATOM  10025 2HG2 ILE B  63     -11.632  -7.730 -22.786  1.00  0.72
H
ATOM  10026 3HG2 ILE B  63     -11.578  -9.507 -22.818  1.00  0.72
H
ATOM  10027 1HD1 ILE B  63      -9.331 -11.867 -24.670  1.00  0.72
H
ATOM  10028 2HD1 ILE B  63     -10.571 -11.246 -23.554  1.00  0.72
H
ATOM  10029 3HD1 ILE B  63     -10.793 -11.085 -25.312  1.00  0.72
H
ATOM  10030  N   MET B  64     -11.944  -6.183 -25.206  1.00  0.54
N
ATOM  10031  CA  MET B  64     -12.829  -5.067 -24.897  1.00  0.76
C
ATOM  10032  C   MET B  64     -13.857  -4.798 -25.979  1.00  0.82
C
ATOM  10033  O   MET B  64     -13.665  -5.141 -27.147  1.00  2.40
O
ATOM  10034  CB  MET B  64     -12.025  -3.796 -24.671  1.00  1.14
C
ATOM  10035  CG  MET B  64     -11.132  -3.797 -23.447  1.00  1.14
C
ATOM  10036  SD  MET B  64     -10.217  -2.256 -23.315  1.00  1.14
S
ATOM  10037  CE  MET B  64     -11.597  -1.150 -23.039  1.00  1.14
C
ATOM  10038  H   MET B  64     -11.004  -5.979 -25.513  1.00  0.65
H
ATOM  10039  HA  MET B  64     -13.372  -5.306 -23.982  1.00  0.91
H
ATOM  10040 1HB  MET B  64     -11.407  -3.594 -25.542  1.00  1.37
H
ATOM  10041 2HB  MET B  64     -12.712  -2.956 -24.566  1.00  1.37
H
ATOM  10042 1HG  MET B  64     -11.739  -3.919 -22.552  1.00  1.37
H
ATOM  10043 2HG  MET B  64     -10.422  -4.621 -23.503  1.00  1.37
H
```

ATOM 10044 1HE MET B 64   -11.237 -0.128 -22.940 1.00 1.37
H
ATOM 10045 2HE MET B 64   -12.285 -1.209 -23.883 1.00 1.37
H
ATOM 10046 3HE MET B 64   -12.119 -1.442 -22.126 1.00 1.37
H
ATOM 10047 N   ARG B 65   -14.943 -4.153 -25.577 1.00 0.70
N
ATOM 10048 CA ARG B 65   -15.991 -3.752 -26.494 1.00 0.68
C
ATOM 10049 C   ARG B 65   -16.247 -2.270 -26.308 1.00 0.68
C
ATOM 10050 O   ARG B 65   -16.455 -1.797 -25.190 1.00 0.72
O
ATOM 10051 CB ARG B 65   -17.262 -4.535 -26.261 1.00 1.02
C
ATOM 10052 CG ARG B 65   -17.144 -6.022 -26.508 1.00 1.02
C
ATOM 10053 CD ARG B 65   -16.956 -6.311 -27.948 1.00 1.02
C
ATOM 10054 NE ARG B 65   -16.887 -7.737 -28.206 1.00 1.02
N
ATOM 10055 CZ ARG B 65   -15.764 -8.480 -28.139 1.00 1.02
C
ATOM 10056 NH1 ARG B 65   -14.608 -7.938 -27.822 1.00 1.02
N
ATOM 10057 NH2 ARG B 65   -15.820 -9.774 -28.393 1.00 1.02
N
ATOM 10058 H   ARG B 65   -15.056 -3.939 -24.593 1.00 0.84
H
ATOM 10059 HA ARG B 65   -15.653 -3.928 -27.514 1.00 0.82
H
ATOM 10060 1HB ARG B 65   -17.597 -4.391 -25.234 1.00 1.22
H
ATOM 10061 2HB ARG B 65   -18.039 -4.162 -26.919 1.00 1.22
H
ATOM 10062 1HG ARG B 65   -16.292 -6.422 -25.960 1.00 1.22
H
ATOM 10063 2HG ARG B 65   -18.055 -6.520 -26.174 1.00 1.22
H
ATOM 10064 1HD ARG B 65   -17.792 -5.900 -28.514 1.00 1.22
H
ATOM 10065 2HD ARG B 65   -16.028 -5.856 -28.291 1.00 1.22
H
ATOM 10066 HE ARG B 65   -17.747 -8.208 -28.454 1.00 1.22
H
ATOM 10067 1HH1 ARG B 65   -14.525 -6.947 -27.625 1.00 1.22
H
ATOM 10068 2HH1 ARG B 65   -13.785 -8.525 -27.768 1.00 1.22
H
ATOM 10069 1HH2 ARG B 65   -16.700 -10.206 -28.638 1.00 1.22
H
ATOM 10070 2HH2 ARG B 65   -14.978 -10.328 -28.342 1.00 1.22
H
ATOM 10071 N   TYR B 66   -16.172 -1.539 -27.407 1.00 0.76
N
ATOM 10072 CA TYR B 66   -16.284 -0.093 -27.365 1.00 0.78
C

```
ATOM  10073  C   TYR B  66     -16.504   0.485 -28.755  1.00  0.92
C
ATOM  10074  O   TYR B  66     -16.037  -0.071 -29.749  1.00  1.27
O
ATOM  10075  CB  TYR B  66     -15.014   0.501 -26.748  1.00  1.17
C
ATOM  10076  CG  TYR B  66     -13.760   0.132 -27.512  1.00  1.17
C
ATOM  10077  CD1 TYR B  66     -13.318   0.943 -28.547  1.00  1.17
C
ATOM  10078  CD2 TYR B  66     -13.056  -1.020 -27.181  1.00  1.17
C
ATOM  10079  CE1 TYR B  66     -12.178   0.603 -29.249  1.00  1.17
C
ATOM  10080  CE2 TYR B  66     -11.919  -1.361 -27.885  1.00  1.17
C
ATOM  10081  CZ  TYR B  66     -11.481  -0.553 -28.914  1.00  1.17
C
ATOM  10082  OH  TYR B  66     -10.348  -0.888 -29.618  1.00  1.17
O
ATOM  10083  H   TYR B  66     -16.047  -2.012 -28.292  1.00  0.91
H
ATOM  10084  HA  TYR B  66     -17.140   0.172 -26.748  1.00  0.94
H
ATOM  10085 1HB  TYR B  66     -15.089   1.589 -26.726  1.00  1.40
H
ATOM  10086 2HB  TYR B  66     -14.904   0.158 -25.721  1.00  1.40
H
ATOM  10087  HD1 TYR B  66     -13.871   1.846 -28.807  1.00  1.40
H
ATOM  10088  HD2 TYR B  66     -13.403  -1.657 -26.369  1.00  1.40
H
ATOM  10089  HE1 TYR B  66     -11.828   1.240 -30.062  1.00  1.40
H
ATOM  10090  HE2 TYR B  66     -11.368  -2.266 -27.628  1.00  1.40
H
ATOM  10091  HH  TYR B  66     -10.196  -1.837 -29.553  1.00  1.40
H
ATOM  10092  N   LYS B  67     -17.192   1.614 -28.824  1.00  0.83
N
ATOM  10093  CA  LYS B  67     -17.418   2.257 -30.102  1.00  1.31
C
ATOM  10094  C   LYS B  67     -16.170   3.026 -30.504  1.00  2.73
C
ATOM  10095  O   LYS B  67     -15.801   4.005 -29.867  1.00 19.36
O
ATOM  10096  CB  LYS B  67     -18.648   3.154 -30.026  1.00  1.97
C
ATOM  10097  CG  LYS B  67     -19.930   2.356 -29.810  1.00  1.97
C
ATOM  10098  CD  LYS B  67     -21.166   3.235 -29.784  1.00  1.97
C
ATOM  10099  CE  LYS B  67     -22.419   2.394 -29.566  1.00  1.97
C
ATOM  10100  NZ  LYS B  67     -23.655   3.220 -29.558  1.00  1.97
N
ATOM  10101  H   LYS B  67     -17.569   2.026 -27.980  1.00  1.00
H
```

```
ATOM  10102  HA  LYS B  67     -17.597   1.488 -30.855  1.00  1.57
H
ATOM  10103 1HB  LYS B  67     -18.544   3.860 -29.202  1.00  2.36
H
ATOM  10104 2HB  LYS B  67     -18.752   3.726 -30.947  1.00  2.36
H
ATOM  10105 1HG  LYS B  67     -20.037   1.623 -30.611  1.00  2.36
H
ATOM  10106 2HG  LYS B  67     -19.862   1.824 -28.863  1.00  2.36
H
ATOM  10107 1HD  LYS B  67     -21.082   3.965 -28.978  1.00  2.36
H
ATOM  10108 2HD  LYS B  67     -21.257   3.771 -30.729  1.00  2.36
H
ATOM  10109 1HE  LYS B  67     -22.494   1.654 -30.363  1.00  2.36
H
ATOM  10110 2HE  LYS B  67     -22.338   1.874 -28.612  1.00  2.36
H
ATOM  10111 1HZ  LYS B  67     -24.463   2.623 -29.412  1.00  2.36
H
ATOM  10112 2HZ  LYS B  67     -23.604   3.899 -28.813  1.00  2.36
H
ATOM  10113 3HZ  LYS B  67     -23.750   3.697 -30.443  1.00  2.36
H
ATOM  10114  N   GLN B  68     -15.536   2.573 -31.578  1.00  3.73
N
ATOM  10115  CA  GLN B  68     -14.264   3.109 -32.064  1.00  3.00
C
ATOM  10116  C   GLN B  68     -14.243   4.620 -32.234  1.00  5.45
C
ATOM  10117  O   GLN B  68     -13.293   5.288 -31.823  1.00 11.10
O
ATOM  10118  CB  GLN B  68     -13.938   2.485 -33.418  1.00  4.50
C
ATOM  10119  CG  GLN B  68     -13.595   1.013 -33.384  1.00  4.50
C
ATOM  10120  CD  GLN B  68     -13.436   0.461 -34.785  1.00  4.50
C
ATOM  10121  OE1 GLN B  68     -12.870   1.117 -35.665  1.00  4.50
O
ATOM  10122  NE2 GLN B  68     -13.950  -0.741 -35.003  1.00  4.50
N
ATOM  10123  H   GLN B  68     -15.934   1.782 -32.065  1.00  4.48
H
ATOM  10124  HA  GLN B  68     -13.486   2.840 -31.348  1.00  3.60
H
ATOM  10125 1HB  GLN B  68     -14.788   2.611 -34.088  1.00  5.40
H
ATOM  10126 2HB  GLN B  68     -13.094   3.011 -33.863  1.00  5.40
H
ATOM  10127 1HG  GLN B  68     -12.656   0.876 -32.849  1.00  5.40
H
ATOM  10128 2HG  GLN B  68     -14.397   0.467 -32.887  1.00  5.40
H
ATOM  10129 1HE2 GLN B  68     -13.888  -1.175 -35.907  1.00  5.40
H
ATOM  10130 2HE2 GLN B  68     -14.400  -1.246 -34.266  1.00  5.40
H
```

```
ATOM  10131  N   SER B  69     -15.294   5.154 -32.847  1.00  7.31
N
ATOM  10132  CA  SER B  69     -15.391   6.579 -33.135  1.00 12.36
C
ATOM  10133  C   SER B  69     -15.928   7.411 -31.974  1.00  9.75
C
ATOM  10134  O   SER B  69     -16.093   8.623 -32.110  1.00 18.85
O
ATOM  10135  CB  SER B  69     -16.272   6.795 -34.349  1.00 18.54
C
ATOM  10136  OG  SER B  69     -17.592   6.404 -34.092  1.00 18.54
O
ATOM  10137  H   SER B  69     -16.045   4.549 -33.146  1.00  8.77
H
ATOM  10138  HA  SER B  69     -14.388   6.941 -33.369  1.00 14.83
H
ATOM  10139 1HB  SER B  69     -16.248   7.847 -34.632  1.00 22.25
H
ATOM  10140 2HB  SER B  69     -15.878   6.222 -35.189  1.00 22.25
H
ATOM  10141  HG  SER B  69     -17.889   6.944 -33.357  1.00 22.25
H
ATOM  10142  N   ASP B  70     -16.186   6.784 -30.832  1.00  3.33
N
ATOM  10143  CA  ASP B  70     -16.688   7.518 -29.687  1.00  2.44
C
ATOM  10144  C   ASP B  70     -16.202   6.906 -28.382  1.00  2.00
C
ATOM  10145  O   ASP B  70     -16.987   6.290 -27.660  1.00  1.95
O
ATOM  10146  CB  ASP B  70     -18.219   7.542 -29.695  1.00  3.66
C
ATOM  10147  CG  ASP B  70     -18.804   8.454 -28.623  1.00  3.66
C
ATOM  10148  OD1 ASP B  70     -18.050   9.175 -28.012  1.00  3.66
O
ATOM  10149  OD2 ASP B  70     -19.993   8.400 -28.404  1.00  3.66
O
ATOM  10150  H   ASP B  70     -16.035   5.791 -30.730  1.00  4.00
H
ATOM  10151  HA  ASP B  70     -16.326   8.546 -29.746  1.00  2.93
H
ATOM  10152 1HB  ASP B  70     -18.572   7.881 -30.670  1.00  4.39
H
ATOM  10153 2HB  ASP B  70     -18.600   6.533 -29.542  1.00  4.39
H
ATOM  10154  N   PRO B  71     -14.905   7.070 -28.063  1.00  1.88
N
ATOM  10155  CA  PRO B  71     -14.183   6.610 -26.877  1.00  1.49
C
ATOM  10156  C   PRO B  71     -14.893   6.950 -25.575  1.00  1.67
C
ATOM  10157  O   PRO B  71     -14.842   6.184 -24.616  1.00  3.87
O
ATOM  10158  CB  PRO B  71     -12.852   7.366 -26.952  1.00  2.23
C
ATOM  10159  CG  PRO B  71     -12.649   7.601 -28.399  1.00  2.23
C
```

ATOM 10160 CD PRO B 71 -14.028 7.921 -28.903 1.00 2.23
C
ATOM 10161 HA PRO B 71 -14.021 5.520 -26.955 1.00 1.79
H
ATOM 10162 1HB PRO B 71 -12.918 8.301 -26.376 1.00 2.68
H
ATOM 10163 2HB PRO B 71 -12.049 6.766 -26.501 1.00 2.68
H
ATOM 10164 1HG PRO B 71 -11.934 8.420 -28.558 1.00 2.68
H
ATOM 10165 2HG PRO B 71 -12.222 6.703 -28.873 1.00 2.68
H
ATOM 10166 1HD PRO B 71 -14.268 8.985 -28.747 1.00 2.68
H
ATOM 10167 2HD PRO B 71 -14.076 7.641 -29.956 1.00 2.68
H
ATOM 10168 N GLU B 72 -15.571 8.098 -25.555 1.00 2.06
N
ATOM 10169 CA GLU B 72 -16.250 8.587 -24.365 1.00 3.00
C
ATOM 10170 C GLU B 72 -17.698 8.117 -24.218 1.00 2.29
C
ATOM 10171 O GLU B 72 -18.397 8.573 -23.311 1.00 2.89
O
ATOM 10172 CB GLU B 72 -16.222 10.113 -24.363 1.00 4.50
C
ATOM 10173 CG GLU B 72 -14.829 10.720 -24.269 1.00 4.50
C
ATOM 10174 CD GLU B 72 -14.847 12.223 -24.299 1.00 4.50
C
ATOM 10175 OE1 GLU B 72 -15.917 12.781 -24.353 1.00 4.50
O
ATOM 10176 OE2 GLU B 72 -13.793 12.814 -24.283 1.00 4.50
O
ATOM 10177 H GLU B 72 -15.593 8.669 -26.388 1.00 2.47
H
ATOM 10178 HA GLU B 72 -15.695 8.233 -23.494 1.00 3.60
H
ATOM 10179 1HB GLU B 72 -16.688 10.485 -25.277 1.00 5.40
H
ATOM 10180 2HB GLU B 72 -16.807 10.487 -23.522 1.00 5.40
H
ATOM 10181 1HG GLU B 72 -14.361 10.390 -23.340 1.00 5.40
H
ATOM 10182 2HG GLU B 72 -14.226 10.351 -25.098 1.00 5.40
H
ATOM 10183 N ASN B 73 -18.153 7.218 -25.086 1.00 1.58
N
ATOM 10184 CA ASN B 73 -19.525 6.739 -24.999 1.00 1.37
C
ATOM 10185 C ASN B 73 -19.727 5.957 -23.695 1.00 1.23
C
ATOM 10186 O ASN B 73 -18.930 5.071 -23.391 1.00 1.41
O
ATOM 10187 CB ASN B 73 -19.870 5.863 -26.183 1.00 2.06
C
ATOM 10188 CG ASN B 73 -21.337 5.610 -26.304 1.00 2.06
C

```
ATOM  10189  OD1 ASN B  73      -21.947   4.856 -25.535  1.00  2.06
O
ATOM  10190  ND2 ASN B  73      -21.930   6.266 -27.270  1.00  2.06
N
ATOM  10191  H   ASN B  73      -17.565   6.868 -25.831  1.00  1.90
H
ATOM  10192  HA  ASN B  73      -20.184   7.602 -25.016  1.00  1.64
H
ATOM  10193 1HB  ASN B  73      -19.512   6.317 -27.101  1.00  2.47
H
ATOM  10194 2HB  ASN B  73      -19.366   4.904 -26.082  1.00  2.47
H
ATOM  10195 1HD2 ASN B  73      -22.914   6.168 -27.416  1.00  2.47
H
ATOM  10196 2HD2 ASN B  73      -21.390   6.883 -27.853  1.00  2.47
H
ATOM  10197  N   PRO B  74      -20.779   6.262 -22.918  1.00  1.12
N
ATOM  10198  CA  PRO B  74      -21.196   5.640 -21.658  1.00  0.96
C
ATOM  10199  C   PRO B  74      -21.318   4.113 -21.715  1.00  1.14
C
ATOM  10200  O   PRO B  74      -21.186   3.442 -20.691  1.00  1.56
O
ATOM  10201  CB  PRO B  74      -22.554   6.288 -21.387  1.00  1.44
C
ATOM  10202  CG  PRO B  74      -22.443   7.641 -21.995  1.00  1.44
C
ATOM  10203  CD  PRO B  74      -21.609   7.448 -23.225  1.00  1.44
C
ATOM  10204  HA  PRO B  74      -20.480   5.929 -20.876  1.00  1.15
H
ATOM  10205 1HB  PRO B  74      -23.355   5.684 -21.836  1.00  1.73
H
ATOM  10206 2HB  PRO B  74      -22.746   6.323 -20.304  1.00  1.73
H
ATOM  10207 1HG  PRO B  74      -23.444   8.034 -22.224  1.00  1.73
H
ATOM  10208 2HG  PRO B  74      -21.982   8.340 -21.281  1.00  1.73
H
ATOM  10209 1HD  PRO B  74      -22.246   7.255 -24.100  1.00  1.73
H
ATOM  10210 2HD  PRO B  74      -20.974   8.335 -23.364  1.00  1.73
H
ATOM  10211  N   ASP B  75      -21.566   3.556 -22.895  1.00  1.51
N
ATOM  10212  CA  ASP B  75      -21.706   2.115 -23.008  1.00  2.16
C
ATOM  10213  C   ASP B  75      -20.383   1.389 -23.240  1.00  2.66
C
ATOM  10214  O   ASP B  75      -20.358   0.162 -23.269  1.00  6.17
O
ATOM  10215  CB  ASP B  75      -22.675   1.789 -24.140  1.00  3.24
C
ATOM  10216  CG  ASP B  75      -24.100   2.185 -23.803  1.00  3.24
C
ATOM  10217  OD1 ASP B  75      -24.554   1.809 -22.749  1.00  3.24
O
```

ATOM 10218 OD2 ASP B 75 -24.720 2.858 -24.593 1.00 3.24
O
ATOM 10219 H ASP B 75 -21.668 4.125 -23.728 1.00 1.81
H
ATOM 10220 HA ASP B 75 -22.133 1.743 -22.078 1.00 2.59
H
ATOM 10221 1HB ASP B 75 -22.368 2.312 -25.045 1.00 3.89
H
ATOM 10222 2HB ASP B 75 -22.647 0.719 -24.351 1.00 3.89
H
ATOM 10223 N ASN B 76 -19.289 2.129 -23.390 1.00 1.38
N
ATOM 10224 CA ASN B 76 -17.992 1.518 -23.653 1.00 1.46
C
ATOM 10225 C ASN B 76 -17.332 0.904 -22.436 1.00 1.62
C
ATOM 10226 O ASN B 76 -17.423 1.447 -21.330 1.00 2.75
O
ATOM 10227 CB ASN B 76 -17.018 2.532 -24.222 1.00 2.19
C
ATOM 10228 CG ASN B 76 -17.371 3.024 -25.583 1.00 2.19
C
ATOM 10229 OD1 ASN B 76 -18.159 2.407 -26.308 1.00 2.19
O
ATOM 10230 ND2 ASN B 76 -16.782 4.128 -25.957 1.00 2.19
N
ATOM 10231 H ASN B 76 -19.338 3.138 -23.340 1.00 1.66
H
ATOM 10232 HA ASN B 76 -18.135 0.716 -24.379 1.00 1.75
H
ATOM 10233 1HB ASN B 76 -16.961 3.390 -23.551 1.00 2.63
H
ATOM 10234 2HB ASN B 76 -16.024 2.088 -24.263 1.00 2.63
H
ATOM 10235 1HD2 ASN B 76 -16.969 4.519 -26.863 1.00 2.63
H
ATOM 10236 2HD2 ASN B 76 -16.144 4.597 -25.341 1.00 2.63
H
ATOM 10237 N ASP B 77 -16.594 -0.183 -22.674 1.00 1.05
N
ATOM 10238 CA ASP B 77 -15.733 -0.764 -21.655 1.00 0.87
C
ATOM 10239 C ASP B 77 -14.645 0.259 -21.378 1.00 0.86
C
ATOM 10240 O ASP B 77 -14.237 0.994 -22.280 1.00 1.93
O
ATOM 10241 CB ASP B 77 -15.114 -2.085 -22.126 1.00 1.30
C
ATOM 10242 CG ASP B 77 -16.089 -3.249 -22.160 1.00 1.30
C
ATOM 10243 OD1 ASP B 77 -16.986 -3.284 -21.354 1.00 1.30
O
ATOM 10244 OD2 ASP B 77 -15.939 -4.091 -23.010 1.00 1.30
O
ATOM 10245 H ASP B 77 -16.602 -0.600 -23.597 1.00 1.26
H
ATOM 10246 HA ASP B 77 -16.303 -0.931 -20.742 1.00 1.04
H

```
ATOM  10247 1HB  ASP B  77      -14.706  -1.950 -23.128  1.00  1.57
H
ATOM  10248 2HB  ASP B  77      -14.284  -2.347 -21.469  1.00  1.57
H
ATOM  10249  N   ARG B  78      -14.200   0.344 -20.139  1.00  0.66
N
ATOM  10250  CA  ARG B  78      -13.200   1.334 -19.791  1.00  0.73
C
ATOM  10251  C   ARG B  78      -11.802   0.767 -19.764  1.00  0.96
C
ATOM  10252  O   ARG B  78      -11.589  -0.361 -19.325  1.00  3.00
O
ATOM  10253  CB  ARG B  78      -13.487   1.945 -18.442  1.00  1.09
C
ATOM  10254  CG  ARG B  78      -12.551   3.079 -18.056  1.00  1.09
C
ATOM  10255  CD  ARG B  78      -12.926   3.636 -16.751  1.00  1.09
C
ATOM  10256  NE  ARG B  78      -12.119   4.782 -16.364  1.00  1.09
N
ATOM  10257  CZ  ARG B  78      -12.431   6.066 -16.617  1.00  1.09
C
ATOM  10258  NH1 ARG B  78      -13.529   6.349 -17.283  1.00  1.09
N
ATOM  10259  NH2 ARG B  78      -11.640   7.024 -16.167  1.00  1.09
N
ATOM  10260  H   ARG B  78      -14.541  -0.297 -19.437  1.00  0.79
H
ATOM  10261  HA  ARG B  78      -13.232   2.128 -20.538  1.00  0.88
H
ATOM  10262 1HB  ARG B  78      -14.506   2.327 -18.421  1.00  1.31
H
ATOM  10263 2HB  ARG B  78      -13.405   1.177 -17.679  1.00  1.31
H
ATOM  10264 1HG  ARG B  78      -11.530   2.708 -17.977  1.00  1.31
H
ATOM  10265 2HG  ARG B  78      -12.599   3.872 -18.802  1.00  1.31
H
ATOM  10266 1HD  ARG B  78      -13.962   3.953 -16.795  1.00  1.31
H
ATOM  10267 2HD  ARG B  78      -12.816   2.870 -15.986  1.00  1.31
H
ATOM  10268  HE  ARG B  78      -11.285   4.606 -15.830  1.00  1.31
H
ATOM  10269 1HH1 ARG B  78      -14.121   5.602 -17.613  1.00  1.31
H
ATOM  10270 2HH1 ARG B  78      -13.803   7.310 -17.460  1.00  1.31
H
ATOM  10271 1HH2 ARG B  78      -10.807   6.789 -15.648  1.00  1.31
H
ATOM  10272 2HH2 ARG B  78      -11.877   7.990 -16.329  1.00  1.31
H
ATOM  10273  N   PHE B  79      -10.847   1.565 -20.214  1.00  0.87
N
ATOM  10274  CA  PHE B  79       -9.449   1.195 -20.129  1.00  1.11
C
ATOM  10275  C   PHE B  79       -8.669   2.330 -19.521  1.00  1.19
C
```

```
ATOM  10276  O   PHE B  79      -8.574   3.416 -20.095  1.00  3.29
O
ATOM  10277  CB  PHE B  79      -8.836   0.861 -21.480  1.00  1.67
C
ATOM  10278  CG  PHE B  79      -7.389   0.467 -21.373  1.00  1.67
C
ATOM  10279  CD1 PHE B  79      -7.019  -0.767 -20.862  1.00  1.67
C
ATOM  10280  CD2 PHE B  79      -6.392   1.337 -21.781  1.00  1.67
C
ATOM  10281  CE1 PHE B  79      -5.686  -1.122 -20.767  1.00  1.67
C
ATOM  10282  CE2 PHE B  79      -5.062   0.984 -21.696  1.00  1.67
C
ATOM  10283  CZ  PHE B  79      -4.709  -0.247 -21.187  1.00  1.67
C
ATOM  10284  H   PHE B  79     -11.095   2.467 -20.596  1.00  1.04
H
ATOM  10285  HA  PHE B  79      -9.355   0.325 -19.484  1.00  1.33
H
ATOM  10286 1HB  PHE B  79      -9.373   0.035 -21.929  1.00  2.00
H
ATOM  10287 2HB  PHE B  79      -8.915   1.716 -22.149  1.00  2.00
H
ATOM  10288  HD1 PHE B  79      -7.791  -1.460 -20.537  1.00  2.00
H
ATOM  10289  HD2 PHE B  79      -6.672   2.311 -22.183  1.00  2.00
H
ATOM  10290  HE1 PHE B  79      -5.407  -2.095 -20.363  1.00  2.00
H
ATOM  10291  HE2 PHE B  79      -4.291   1.674 -22.032  1.00  2.00
H
ATOM  10292  HZ  PHE B  79      -3.659  -0.524 -21.119  1.00  2.00
H
ATOM  10293  N   VAL B  80      -8.102   2.066 -18.360  1.00  0.63
N
ATOM  10294  CA  VAL B  80      -7.306   3.044 -17.673  1.00  0.60
C
ATOM  10295  C   VAL B  80      -5.864   2.681 -17.771  1.00  0.68
C
ATOM  10296  O   VAL B  80      -5.404   1.781 -17.071  1.00  1.41
O
ATOM  10297  CB  VAL B  80      -7.654   3.116 -16.182  1.00  0.90
C
ATOM  10298  CG1 VAL B  80      -6.788   4.168 -15.511  1.00  0.90
C
ATOM  10299  CG2 VAL B  80      -9.114   3.421 -16.008  1.00  0.90
C
ATOM  10300  H   VAL B  80      -8.225   1.156 -17.947  1.00  0.76
H
ATOM  10301  HA  VAL B  80      -7.463   4.020 -18.133  1.00  0.72
H
ATOM  10302  HB  VAL B  80      -7.428   2.158 -15.714  1.00  1.08
H
ATOM  10303 1HG1 VAL B  80      -7.023   4.208 -14.450  1.00  1.08
H
ATOM  10304 2HG1 VAL B  80      -5.737   3.912 -15.641  1.00  1.08
H
```

```
ATOM  10305 3HG1 VAL B  80      -6.984   5.141 -15.962  1.00  1.08
H
ATOM  10306 1HG2 VAL B  80      -9.352   3.464 -14.946  1.00  1.08
H
ATOM  10307 2HG2 VAL B  80      -9.335   4.383 -16.472  1.00  1.08
H
ATOM  10308 3HG2 VAL B  80      -9.708   2.641 -16.484  1.00  1.08
H
ATOM  10309  N   LEU B  81      -5.124   3.378 -18.604  1.00  0.65
N
ATOM  10310  CA  LEU B  81      -3.712   3.117 -18.586  1.00  0.84
C
ATOM  10311  C   LEU B  81      -3.173   3.912 -17.431  1.00  1.10
C
ATOM  10312  O   LEU B  81      -3.037   5.136 -17.516  1.00  4.31
O
ATOM  10313  CB  LEU B  81      -3.025   3.533 -19.869  1.00  1.26
C
ATOM  10314  CG  LEU B  81      -1.531   3.220 -19.955  1.00  1.26
C
ATOM  10315  CD1 LEU B  81      -1.340   1.731 -19.841  1.00  1.26
C
ATOM  10316  CD2 LEU B  81      -0.967   3.709 -21.278  1.00  1.26
C
ATOM  10317  H   LEU B  81      -5.524   4.089 -19.199  1.00  0.78
H
ATOM  10318  HA  LEU B  81      -3.535   2.057 -18.405  1.00  1.01
H
ATOM  10319 1HB  LEU B  81      -3.531   3.100 -20.724  1.00  1.51
H
ATOM  10320 2HB  LEU B  81      -3.113   4.611 -19.915  1.00  1.51
H
ATOM  10321  HG  LEU B  81      -1.010   3.699 -19.132  1.00  1.51
H
ATOM  10322 1HD1 LEU B  81      -0.277   1.493 -19.893  1.00  1.51
H
ATOM  10323 2HD1 LEU B  81      -1.743   1.388 -18.892  1.00  1.51
H
ATOM  10324 3HD1 LEU B  81      -1.863   1.239 -20.659  1.00  1.51
H
ATOM  10325 1HD2 LEU B  81       0.097   3.474 -21.329  1.00  1.51
H
ATOM  10326 2HD2 LEU B  81      -1.486   3.216 -22.099  1.00  1.51
H
ATOM  10327 3HD2 LEU B  81      -1.100   4.783 -21.361  1.00  1.51
H
ATOM  10328  N   ALA B  82      -2.868   3.221 -16.343  1.00  0.82
N
ATOM  10329  CA  ALA B  82      -2.333   3.863 -15.159  1.00  0.65
C
ATOM  10330  C   ALA B  82      -0.970   4.440 -15.508  1.00  0.71
C
ATOM  10331  O   ALA B  82      -0.550   5.460 -14.965  1.00  1.02
O
ATOM  10332  CB  ALA B  82      -2.264   2.880 -14.009  1.00  0.98
C
ATOM  10333  H   ALA B  82      -2.986   2.217 -16.350  1.00  0.98
H
```

ATOM 10334 HA ALA B 82 -2.990 4.687 -14.883 1.00 0.78
H
ATOM 10335 1HB ALA B 82 -1.878 3.380 -13.126 1.00 1.17
H
ATOM 10336 2HB ALA B 82 -3.263 2.498 -13.800 1.00 1.17
H
ATOM 10337 3HB ALA B 82 -1.609 2.057 -14.280 1.00 1.17
H
ATOM 10338 N LYS B 83 -0.319 3.804 -16.480 1.00 1.01
N
ATOM 10339 CA LYS B 83 0.937 4.272 -17.036 1.00 2.10
C
ATOM 10340 C LYS B 83 0.905 5.389 -18.088 1.00 3.92
C
ATOM 10341 O LYS B 83 1.831 5.436 -18.876 1.00 48.76
O
ATOM 10342 CB LYS B 83 1.685 3.115 -17.666 1.00 3.15
C
ATOM 10343 CG LYS B 83 2.148 2.117 -16.665 1.00 3.15
C
ATOM 10344 CD LYS B 83 3.159 2.777 -15.759 1.00 3.15
C
ATOM 10345 CE LYS B 83 4.493 2.985 -16.463 1.00 3.15
C
ATOM 10346 NZ LYS B 83 5.565 3.340 -15.498 1.00 3.15
N
ATOM 10347 H LYS B 83 -0.714 2.932 -16.820 1.00 1.21
H
ATOM 10348 HA LYS B 83 1.532 4.643 -16.202 1.00 2.52
H
ATOM 10349 1HB LYS B 83 1.045 2.604 -18.382 1.00 3.78
H
ATOM 10350 2HB LYS B 83 2.557 3.487 -18.202 1.00 3.78
H
ATOM 10351 1HG LYS B 83 1.303 1.780 -16.072 1.00 3.78
H
ATOM 10352 2HG LYS B 83 2.599 1.260 -17.164 1.00 3.78
H
ATOM 10353 1HD LYS B 83 2.772 3.750 -15.459 1.00 3.78
H
ATOM 10354 2HD LYS B 83 3.292 2.183 -14.867 1.00 3.78
H
ATOM 10355 1HE LYS B 83 4.772 2.068 -16.979 1.00 3.78
H
ATOM 10356 2HE LYS B 83 4.397 3.786 -17.197 1.00 3.78
H
ATOM 10357 1HZ LYS B 83 6.445 3.460 -15.981 1.00 3.78
H
ATOM 10358 2HZ LYS B 83 5.326 4.194 -15.015 1.00 3.78
H
ATOM 10359 3HZ LYS B 83 5.635 2.579 -14.833 1.00 3.78
H
ATOM 10360 N ARG B 84 -0.111 6.259 -18.134 1.00 14.95
N
ATOM 10361 CA ARG B 84 -0.111 7.481 -19.004 1.00 10.64
C
ATOM 10362 C ARG B 84 -0.645 7.340 -20.435 1.00 13.63
C

```
ATOM  10363  O   ARG B  84       0.136   7.190 -21.372  1.00 51.83
O
ATOM  10364  CB  ARG B  84       1.276   8.106 -19.145  1.00 15.96
C
ATOM  10365  CG  ARG B  84       1.859   8.664 -17.876  1.00 15.96
C
ATOM  10366  CD  ARG B  84       3.181   9.284 -18.109  1.00 15.96
C
ATOM  10367  NE  ARG B  84       3.683   9.917 -16.910  1.00 15.96
N
ATOM  10368  CZ  ARG B  84       4.865  10.538 -16.788  1.00 15.96
C
ATOM  10369  NH1 ARG B  84       5.714  10.606 -17.790  1.00 15.96
N
ATOM  10370  NH2 ARG B  84       5.142  11.088 -15.627  1.00 15.96
N
ATOM  10371  H   ARG B  84      -0.909   6.111 -17.528  1.00 17.94
H
ATOM  10372  HA  ARG B  84      -0.739   8.219 -18.503  1.00 12.77
H
ATOM  10373 1HB  ARG B  84       1.995   7.456 -19.629  1.00 19.15
H
ATOM  10374 2HB  ARG B  84       1.176   8.967 -19.805  1.00 19.15
H
ATOM  10375 1HG  ARG B  84       1.176   9.414 -17.498  1.00 19.15
H
ATOM  10376 2HG  ARG B  84       1.975   7.873 -17.136  1.00 19.15
H
ATOM  10377 1HD  ARG B  84       3.895   8.520 -18.418  1.00 19.15
H
ATOM  10378 2HD  ARG B  84       3.100  10.041 -18.887  1.00 19.15
H
ATOM  10379  HE  ARG B  84       3.094   9.923 -16.087  1.00 19.15
H
ATOM  10380 1HH1 ARG B  84       5.484  10.182 -18.677  1.00 19.15
H
ATOM  10381 2HH1 ARG B  84       6.595  11.086 -17.667  1.00 19.15
H
ATOM  10382 1HH2 ARG B  84       4.451  11.020 -14.888  1.00 19.15
H
ATOM  10383 2HH2 ARG B  84       6.016  11.580 -15.498  1.00 19.15
H
ATOM  10384  N   LEU B  85      -1.956   7.454 -20.605  1.00 13.51
N
ATOM  10385  CA  LEU B  85      -2.640   7.356 -21.913  1.00 15.43
C
ATOM  10386  C   LEU B  85      -3.312   8.648 -22.362  1.00  8.42
C
ATOM  10387  O   LEU B  85      -3.897   9.354 -21.549  1.00 18.81
O
ATOM  10388  CB  LEU B  85      -3.709   6.254 -21.862  1.00 23.14
C
ATOM  10389  CG  LEU B  85      -4.641   6.091 -23.062  1.00 23.14
C
ATOM  10390  CD1 LEU B  85      -3.850   5.616 -24.272  1.00 23.14
C
ATOM  10391  CD2 LEU B  85      -5.730   5.090 -22.706  1.00 23.14
C
```

ATOM 10392 H   LEU B 85   -2.527   7.599 -19.784 1.00 16.21
H
ATOM 10393 HA LEU B 85   -1.897   7.085 -22.662 1.00 18.52
H
ATOM 10394 1HB LEU B 85   -3.185   5.314 -21.790 1.00 27.77
H
ATOM 10395 2HB LEU B 85   -4.319   6.388 -20.968 1.00 27.77
H
ATOM 10396 HG LEU B 85   -5.096   7.045 -23.296 1.00 27.77
H
ATOM 10397 1HD1 LEU B 85   -4.520   5.504 -25.124 1.00 27.77
H
ATOM 10398 2HD1 LEU B 85   -3.076   6.342 -24.514 1.00 27.77
H
ATOM 10399 3HD1 LEU B 85   -3.387   4.656 -24.046 1.00 27.77
H
ATOM 10400 1HD2 LEU B 85   -6.406   4.971 -23.554 1.00 27.77
H
ATOM 10401 2HD2 LEU B 85   -5.272   4.130 -22.470 1.00 27.77
H
ATOM 10402 3HD2 LEU B 85   -6.290   5.447 -21.844 1.00 27.77
H
ATOM 10403 N   SER B 86   -3.207   8.967 -23.651 1.00 9.29
N
ATOM 10404 CA SER B 86   -3.874 10.137 -24.228 1.00 10.18
C
ATOM 10405 C   SER B 86   -5.413 10.040 -24.194 1.00 8.41
C
ATOM 10406 O   SER B 86   -6.094 10.945 -23.718 1.00 19.70
O
ATOM 10407 CB SER B 86   -3.422 10.327 -25.663 1.00 15.27
C
ATOM 10408 OG SER B 86   -4.054 11.431 -26.248 1.00 15.27
O
ATOM 10409 H   SER B 86   -2.672   8.364 -24.261 1.00 11.15
H
ATOM 10410 HA SER B 86   -3.580 11.014 -23.650 1.00 12.22
H
ATOM 10411 1HB SER B 86   -2.341 10.469 -25.686 1.00 18.32
H
ATOM 10412 2HB SER B 86   -3.645   9.429 -26.238 1.00 18.32
H
ATOM 10413 HG SER B 86   -4.994 11.235 -26.237 1.00 18.32
H
ATOM 10414 N   PHE B 87   -5.962   8.929 -24.670 1.00 9.58
N
ATOM 10415 CA PHE B 87   -7.412   8.751 -24.778 1.00 9.55
C
ATOM 10416 C   PHE B 87   -8.067   8.211 -23.509 1.00 4.70
C
ATOM 10417 O   PHE B 87   -8.713   7.165 -23.535 1.00 11.68
O
ATOM 10418 CB PHE B 87   -7.705   7.797 -25.930 1.00 14.33
C
ATOM 10419 CG PHE B 87   -7.261   8.341 -27.251 1.00 14.33
C
ATOM 10420 CD1 PHE B 87   -6.021   7.998 -27.766 1.00 14.33
C

```
ATOM  10421  CD2 PHE B  87      -8.073   9.196 -27.979  1.00 14.33
C
ATOM  10422  CE1 PHE B  87      -5.599   8.500 -28.981  1.00 14.33
C
ATOM  10423  CE2 PHE B  87      -7.655   9.698 -29.196  1.00 14.33
C
ATOM  10424  CZ  PHE B  87      -6.415   9.350 -29.696  1.00 14.33
C
ATOM  10425  H   PHE B  87      -5.359   8.196 -25.013  1.00 11.50
H
ATOM  10426  HA  PHE B  87      -7.857   9.722 -25.004  1.00 11.46
H
ATOM  10427 1HB  PHE B  87      -7.199   6.848 -25.760  1.00 17.19
H
ATOM  10428 2HB  PHE B  87      -8.775   7.598 -25.982  1.00 17.19
H
ATOM  10429  HD1 PHE B  87      -5.375   7.327 -27.198  1.00 17.19
H
ATOM  10430  HD2 PHE B  87      -9.051   9.471 -27.581  1.00 17.19
H
ATOM  10431  HE1 PHE B  87      -4.620   8.224 -29.375  1.00 17.19
H
ATOM  10432  HE2 PHE B  87      -8.301  10.369 -29.760  1.00 17.19
H
ATOM  10433  HZ  PHE B  87      -6.084   9.746 -30.655  1.00 17.19
H
ATOM  10434  N   VAL B  88      -7.916   8.933 -22.404  1.00  8.32
N
ATOM  10435  CA  VAL B  88      -8.468   8.476 -21.130  1.00  4.68
C
ATOM  10436  C   VAL B  88      -9.211   9.615 -20.447  1.00  3.12
C
ATOM  10437  O   VAL B  88      -8.825  10.779 -20.563  1.00  6.15
O
ATOM  10438  CB  VAL B  88      -7.341   7.963 -20.209  1.00  7.02
C
ATOM  10439  CG1 VAL B  88      -6.457   9.128 -19.811  1.00  7.02
C
ATOM  10440  CG2 VAL B  88      -7.907   7.243 -18.989  1.00  7.02
C
ATOM  10441  H   VAL B  88      -7.399   9.802 -22.463  1.00  9.98
H
ATOM  10442  HA  VAL B  88      -9.168   7.661 -21.321  1.00  5.62
H
ATOM  10443  HB  VAL B  88      -6.721   7.271 -20.775  1.00  8.42
H
ATOM  10444 1HG1 VAL B  88      -5.633   8.770 -19.192  1.00  8.42
H
ATOM  10445 2HG1 VAL B  88      -6.065   9.604 -20.705  1.00  8.42
H
ATOM  10446 3HG1 VAL B  88      -7.045   9.852 -19.246  1.00  8.42
H
ATOM  10447 1HG2 VAL B  88      -7.086   6.880 -18.371  1.00  8.42
H
ATOM  10448 2HG2 VAL B  88      -8.516   7.926 -18.405  1.00  8.42
H
ATOM  10449 3HG2 VAL B  88      -8.517   6.400 -19.314  1.00  8.42
H
```

ATOM 10450 N   ASP B 89   -10.290   9.277 -19.752 1.00 1.62
N
ATOM 10451 CA ASP B 89   -11.113 10.272 -19.076 1.00 1.71
C
ATOM 10452 C   ASP B 89   -10.378 10.971 -17.922 1.00 1.23
C
ATOM 10453 O   ASP B 89   -10.554 12.169 -17.711 1.00 2.49
O
ATOM 10454 CB ASP B 89   -12.388   9.620 -18.549 1.00 2.56
C
ATOM 10455 CG ASP B 89   -13.396   9.214 -19.622 1.00 2.56
C
ATOM 10456 OD1 ASP B 89   -13.321   9.710 -20.720 1.00 2.56
O
ATOM 10457 OD2 ASP B 89   -14.217   8.373 -19.321 1.00 2.56
O
ATOM 10458 H   ASP B 89   -10.552   8.303 -19.698 1.00 1.94
H
ATOM 10459 HA ASP B 89   -11.392 11.034 -19.804 1.00 2.05
H
ATOM 10460 1HB ASP B 89   -12.113   8.724 -18.010 1.00 3.08
H
ATOM 10461 2HB ASP B 89   -12.876 10.293 -17.844 1.00 3.08
H
ATOM 10462 N   VAL B 90   -9.555 10.227 -17.182 1.00 0.62
N
ATOM 10463 CA VAL B 90   -8.801 10.807 -16.074 1.00 0.43
C
ATOM 10464 C   VAL B 90   -7.302 10.721 -16.316 1.00 0.42
C
ATOM 10465 O   VAL B 90   -6.774   9.647 -16.600 1.00 0.47
O
ATOM 10466 CB VAL B 90   -9.151 10.111 -14.741 1.00 0.65
C
ATOM 10467 CG1 VAL B 90   -8.312 10.685 -13.607 1.00 0.65
C
ATOM 10468 CG2 VAL B 90   -10.632 10.302 -14.436 1.00 0.65
C
ATOM 10469 H   VAL B 90   -9.451   9.247 -17.388 1.00 0.74
H
ATOM 10470 HA VAL B 90   -9.074 11.857 -15.990 1.00 0.52
H
ATOM 10471 HB VAL B 90   -8.920   9.049 -14.821 1.00 0.77
H
ATOM 10472 1HG1 VAL B 90   -8.564 10.183 -12.676 1.00 0.77
H
ATOM 10473 2HG1 VAL B 90   -7.254 10.540 -13.823 1.00 0.77
H
ATOM 10474 3HG1 VAL B 90   -8.519 11.746 -13.504 1.00 0.77
H
ATOM 10475 1HG2 VAL B 90   -10.879   9.805 -13.498 1.00 0.77
H
ATOM 10476 2HG2 VAL B 90   -10.851 11.366 -14.351 1.00 0.77
H
ATOM 10477 3HG2 VAL B 90   -11.231   9.873 -15.240 1.00 0.77
H
ATOM 10478 N   ALA B 91   -6.628 11.863 -16.210 1.00 0.47
N

```
ATOM  10479  CA  ALA B  91      -5.188  11.944 -16.407  1.00  0.57
C
ATOM  10480  C   ALA B  91      -4.448  11.024 -15.444  1.00  0.57
C
ATOM  10481  O   ALA B  91      -4.721  11.015 -14.244  1.00  0.77
O
ATOM  10482  CB  ALA B  91      -4.719  13.381 -16.223  1.00  0.85
C
ATOM  10483  H   ALA B  91      -7.133  12.706 -15.985  1.00  0.56
H
ATOM  10484  HA  ALA B  91      -4.964  11.619 -17.423  1.00  0.68
H
ATOM  10485 1HB  ALA B  91      -3.644  13.439 -16.396  1.00  1.03
H
ATOM  10486 2HB  ALA B  91      -5.235  14.026 -16.933  1.00  1.03
H
ATOM  10487 3HB  ALA B  91      -4.940  13.708 -15.208  1.00  1.03
H
ATOM  10488  N   THR B  92      -3.493  10.267 -15.979  1.00  0.55
N
ATOM  10489  CA  THR B  92      -2.688   9.351 -15.177  1.00  0.64
C
ATOM  10490  C   THR B  92      -1.203   9.629 -15.356  1.00  0.80
C
ATOM  10491  O   THR B  92      -0.407   8.707 -15.525  1.00  2.75
O
ATOM  10492  CB  THR B  92      -2.974   7.876 -15.535  1.00  0.96
C
ATOM  10493  OG1 THR B  92      -2.751   7.673 -16.936  1.00  0.96
O
ATOM  10494  CG2 THR B  92      -4.403   7.481 -15.184  1.00  0.96
C
ATOM  10495  H   THR B  92      -3.324  10.323 -16.973  1.00  0.66
H
ATOM  10496  HA  THR B  92      -2.933   9.503 -14.126  1.00  0.77
H
ATOM  10497  HB  THR B  92      -2.290   7.241 -14.975  1.00  1.15
H
ATOM  10498  HG1 THR B  92      -2.998   6.773 -17.169  1.00  1.15
H
ATOM  10499 1HG2 THR B  92      -4.563   6.435 -15.444  1.00  1.15
H
ATOM  10500 2HG2 THR B  92      -4.569   7.621 -14.117  1.00  1.15
H
ATOM  10501 3HG2 THR B  92      -5.099   8.101 -15.744  1.00  1.15
H
ATOM  10502  N   GLY B  93      -0.835  10.908 -15.322  1.00  0.92
N
ATOM  10503  CA  GLY B  93       0.562  11.321 -15.426  1.00  0.92
C
ATOM  10504  C   GLY B  93       1.341  10.865 -14.198  1.00  0.88
C
ATOM  10505  O   GLY B  93       2.507  10.466 -14.287  1.00  1.98
O
ATOM  10506  H   GLY B  93      -1.545  11.617 -15.202  1.00  1.10
H
ATOM  10507 1HA  GLY B  93       1.009  10.924 -16.327  1.00  1.10
H
```

```
ATOM  10508 2HA  GLY B  93       0.608  12.404 -15.507  1.00  1.10
H
ATOM  10509  N   TRP B  94       0.677  10.940 -13.043  1.00  1.26
N
ATOM  10510  CA  TRP B  94       1.254  10.514 -11.781  1.00  1.21
C
ATOM  10511  C   TRP B  94       0.981   9.046 -11.567  1.00  1.27
C
ATOM  10512  O   TRP B  94      -0.162   8.596 -11.657  1.00  3.12
O
ATOM  10513  CB  TRP B  94       0.667  11.290 -10.626  1.00  1.81
C
ATOM  10514  CG  TRP B  94       1.014  12.725 -10.639  1.00  1.81
C
ATOM  10515  CD1 TRP B  94       1.084  13.506 -11.740  1.00  1.81
C
ATOM  10516  CD2 TRP B  94       1.330  13.581  -9.523  1.00  1.81
C
ATOM  10517  NE1 TRP B  94       1.416  14.775 -11.390  1.00  1.81
N
ATOM  10518  CE2 TRP B  94       1.563  14.849 -10.050  1.00  1.81
C
ATOM  10519  CE3 TRP B  94       1.432  13.383  -8.143  1.00  1.81
C
ATOM  10520  CZ2 TRP B  94       1.878  15.923  -9.266  1.00  1.81
C
ATOM  10521  CZ3 TRP B  94       1.751  14.475  -7.348  1.00  1.81
C
ATOM  10522  CH2 TRP B  94       1.958  15.707  -7.908  1.00  1.81
C
ATOM  10523  H   TRP B  94      -0.268  11.294 -13.040  1.00  1.51
H
ATOM  10524  HA  TRP B  94       2.333  10.670 -11.811  1.00  1.45
H
ATOM  10525 1HB  TRP B  94      -0.409  11.219 -10.675  1.00  2.18
H
ATOM  10526 2HB  TRP B  94       0.991  10.858  -9.680  1.00  2.18
H
ATOM  10527  HD1 TRP B  94       0.901  13.173 -12.756  1.00  2.18
H
ATOM  10528  HE1 TRP B  94       1.549  15.548 -12.015  1.00  2.18
H
ATOM  10529  HE3 TRP B  94       1.258  12.401  -7.706  1.00  2.18
H
ATOM  10530  HZ2 TRP B  94       2.055  16.915  -9.682  1.00  2.18
H
ATOM  10531  HZ3 TRP B  94       1.828  14.332  -6.270  1.00  2.18
H
ATOM  10532  HH2 TRP B  94       2.194  16.552  -7.276  1.00  2.18
H
ATOM  10533  N   LEU B  95       2.028   8.307 -11.259  1.00  1.48
N
ATOM  10534  CA  LEU B  95       1.920   6.872 -11.080  1.00  1.40
C
ATOM  10535  C   LEU B  95       1.175   6.477  -9.814  1.00  0.82
C
ATOM  10536  O   LEU B  95       1.304   7.121  -8.769  1.00  1.23
O
```

ATOM 10537 CB LEU B 95   3.326   6.263 -11.043 1.00 2.10
C
ATOM 10538 CG LEU B 95   4.140   6.398 -12.330 1.00 2.10
C
ATOM 10539 CD1 LEU B 95   5.524   5.799 -12.107 1.00 2.10
C
ATOM 10540 CD2 LEU B 95   3.403   5.707 -13.467 1.00 2.10
C
ATOM 10541 H   LEU B 95   2.934   8.745 -11.176 1.00 1.78
H
ATOM 10542 HA LEU B 95   1.382   6.464 -11.937 1.00 1.68
H
ATOM 10543 1HB LEU B 95   3.891   6.748 -10.249 1.00 2.52
H
ATOM 10544 2HB LEU B 95   3.254   5.206 -10.806 1.00 2.52
H
ATOM 10545 HG LEU B 95   4.266   7.453 -12.573 1.00 2.52
H
ATOM 10546 1HD1 LEU B 95   6.118   5.902 -13.015 1.00 2.52
H
ATOM 10547 2HD1 LEU B 95   6.018   6.324 -11.289 1.00 2.52
H
ATOM 10548 3HD1 LEU B 95   5.426   4.744 -11.854 1.00 2.52
H
ATOM 10549 1HD2 LEU B 95   3.979   5.809 -14.388 1.00 2.52
H
ATOM 10550 2HD2 LEU B 95   3.279   4.651 -13.232 1.00 2.52
H
ATOM 10551 3HD2 LEU B 95   2.424   6.166 -13.600 1.00 2.52
H
ATOM 10552 N   GLY B 96   0.414   5.384 -9.914 1.00 0.42
N
ATOM 10553 CA GLY B 96   -0.311   4.786 -8.792 1.00 0.29
C
ATOM 10554 C   GLY B 96   -1.726   5.316 -8.584 1.00 0.83
C
ATOM 10555 O   GLY B 96   -2.365   5.002 -7.578 1.00 6.62
O
ATOM 10556 H   GLY B 96   0.343   4.931 -10.817 1.00 0.50
H
ATOM 10557 1HA GLY B 96   -0.357   3.711 -8.950 1.00 0.35
H
ATOM 10558 2HA GLY B 96   0.264   4.939 -7.881 1.00 0.35
H
ATOM 10559 N   GLN B 97   -2.219   6.126 -9.509 1.00 0.53
N
ATOM 10560 CA GLN B 97   -3.530   6.737 -9.328 1.00 0.38
C
ATOM 10561 C   GLN B 97   -4.636   6.075 -10.153 1.00 0.30
C
ATOM 10562 O   GLN B 97   -5.805   6.074 -9.754 1.00 0.57
O
ATOM 10563 CB GLN B 97   -3.440   8.210 -9.701 1.00 0.57
C
ATOM 10564 CG GLN B 97   -2.480   8.988 -8.828 1.00 0.57
C
ATOM 10565 CD GLN B 97   -2.468 10.444 -9.169 1.00 0.57
C

ATOM 10566 OE1 GLN B 97 -2.661 10.794 -10.334 1.00 0.57
O
ATOM 10567 NE2 GLN B 97 -2.247 11.297 -8.177 1.00 0.57
N
ATOM 10568 H GLN B 97 -1.674 6.346 -10.330 1.00 0.64
H
ATOM 10569 HA GLN B 97 -3.803 6.657 -8.276 1.00 0.46
H
ATOM 10570 1HB GLN B 97 -3.113 8.308 -10.737 1.00 0.68
H
ATOM 10571 2HB GLN B 97 -4.423 8.670 -9.618 1.00 0.68
H
ATOM 10572 1HG GLN B 97 -2.742 8.872 -7.786 1.00 0.68
H
ATOM 10573 2HG GLN B 97 -1.476 8.599 -8.998 1.00 0.68
H
ATOM 10574 1HE2 GLN B 97 -2.233 12.283 -8.356 1.00 0.68
H
ATOM 10575 2HE2 GLN B 97 -2.102 10.958 -7.247 1.00 0.68
H
ATOM 10576 N GLY B 98 -4.250 5.532 -11.307 1.00 0.32
N
ATOM 10577 CA GLY B 98 -5.176 4.943 -12.264 1.00 0.33
C
ATOM 10578 C GLY B 98 -6.058 3.832 -11.712 1.00 0.28
C
ATOM 10579 O GLY B 98 -7.244 3.788 -12.029 1.00 0.33
O
ATOM 10580 H GLY B 98 -3.270 5.555 -11.549 1.00 0.38
H
ATOM 10581 1HA GLY B 98 -5.813 5.731 -12.665 1.00 0.40
H
ATOM 10582 2HA GLY B 98 -4.607 4.555 -13.108 1.00 0.40
H
ATOM 10583 N LEU B 99 -5.491 2.934 -10.906 1.00 0.23
N
ATOM 10584 CA LEU B 99 -6.263 1.814 -10.368 1.00 0.20
C
ATOM 10585 C LEU B 99 -7.412 2.293 -9.488 1.00 0.15
C
ATOM 10586 O LEU B 99 -8.506 1.734 -9.546 1.00 0.17
O
ATOM 10587 CB LEU B 99 -5.360 0.851 -9.589 1.00 0.30
C
ATOM 10588 CG LEU B 99 -6.068 -0.405 -9.050 1.00 0.30
C
ATOM 10589 CD1 LEU B 99 -6.666 -1.187 -10.215 1.00 0.30
C
ATOM 10590 CD2 LEU B 99 -5.069 -1.262 -8.285 1.00 0.30
C
ATOM 10591 H LEU B 99 -4.512 3.018 -10.674 1.00 0.28
H
ATOM 10592 HA LEU B 99 -6.691 1.265 -11.205 1.00 0.24
H
ATOM 10593 1HB LEU B 99 -4.552 0.524 -10.242 1.00 0.36
H
ATOM 10594 2HB LEU B 99 -4.927 1.381 -8.742 1.00 0.36
H

```
ATOM  10595  HG  LEU B  99      -6.883  -0.113  -8.391  1.00  0.36
H
ATOM  10596 1HD1 LEU B  99      -7.175  -2.071  -9.836  1.00  0.36
H
ATOM  10597 2HD1 LEU B  99      -7.380  -0.557 -10.746  1.00  0.36
H
ATOM  10598 3HD1 LEU B  99      -5.872  -1.489 -10.896  1.00  0.36
H
ATOM  10599 1HD2 LEU B  99      -5.570  -2.151  -7.903  1.00  0.36
H
ATOM  10600 2HD2 LEU B  99      -4.259  -1.560  -8.952  1.00  0.36
H
ATOM  10601 3HD2 LEU B  99      -4.659  -0.689  -7.453  1.00  0.36
H
ATOM  10602  N   GLY B 100      -7.162   3.310  -8.662  1.00  0.20
N
ATOM  10603  CA  GLY B 100      -8.201   3.871  -7.804  1.00  0.13
C
ATOM  10604  C   GLY B 100      -9.340   4.446  -8.643  1.00  0.12
C
ATOM  10605  O   GLY B 100     -10.518   4.245  -8.331  1.00  0.11
O
ATOM  10606  H   GLY B 100      -6.238   3.716  -8.640  1.00  0.24
H
ATOM  10607 1HA  GLY B 100      -8.586   3.100  -7.138  1.00  0.16
H
ATOM  10608 2HA  GLY B 100      -7.768   4.649  -7.179  1.00  0.16
H
ATOM  10609  N   VAL B 101      -8.977   5.137  -9.722  1.00  0.14
N
ATOM  10610  CA  VAL B 101      -9.955   5.686 -10.652  1.00  0.16
C
ATOM  10611  C   VAL B 101     -10.761   4.569 -11.302  1.00  0.15
C
ATOM  10612  O   VAL B 101     -11.988   4.662 -11.412  1.00  0.15
O
ATOM  10613  CB  VAL B 101      -9.250   6.522 -11.724  1.00  0.24
C
ATOM  10614  CG1 VAL B 101     -10.234   6.920 -12.807  1.00  0.24
C
ATOM  10615  CG2 VAL B 101      -8.636   7.746 -11.067  1.00  0.24
C
ATOM  10616  H   VAL B 101      -7.987   5.294  -9.899  1.00  0.17
H
ATOM  10617  HA  VAL B 101     -10.635   6.331 -10.101  1.00  0.19
H
ATOM  10618  HB  VAL B 101      -8.469   5.925 -12.191  1.00  0.29
H
ATOM  10619 1HG1 VAL B 101      -9.716   7.501 -13.566  1.00  0.29
H
ATOM  10620 2HG1 VAL B 101     -10.657   6.024 -13.260  1.00  0.29
H
ATOM  10621 3HG1 VAL B 101     -11.030   7.519 -12.369  1.00  0.29
H
ATOM  10622 1HG2 VAL B 101      -8.117   8.336 -11.820  1.00  0.29
H
ATOM  10623 2HG2 VAL B 101      -9.420   8.345 -10.607  1.00  0.29
H
```

```
ATOM  10624 3HG2 VAL B 101      -7.925   7.430 -10.304  1.00  0.29
H
ATOM  10625  N   ALA B 102     -10.059   3.513 -11.715  1.00  0.17
N
ATOM  10626  CA  ALA B 102     -10.668   2.337 -12.313  1.00  0.18
C
ATOM  10627  C   ALA B 102     -11.665   1.704 -11.354  1.00  0.16
C
ATOM  10628  O   ALA B 102     -12.740   1.284 -11.775  1.00  0.18
O
ATOM  10629  CB  ALA B 102      -9.594   1.333 -12.705  1.00  0.27
C
ATOM  10630  H   ALA B 102      -9.054   3.525 -11.614  1.00  0.20
H
ATOM  10631  HA  ALA B 102     -11.210   2.649 -13.207  1.00  0.22
H
ATOM  10632 1HB  ALA B 102     -10.055   0.465 -13.171  1.00  0.32
H
ATOM  10633 2HB  ALA B 102      -8.902   1.797 -13.408  1.00  0.32
H
ATOM  10634 3HB  ALA B 102      -9.049   1.016 -11.820  1.00  0.32
H
ATOM  10635  N   CYS B 103     -11.311   1.660 -10.064  1.00  0.13
N
ATOM  10636  CA  CYS B 103     -12.192   1.126  -9.038  1.00  0.11
C
ATOM  10637  C   CYS B 103     -13.466   1.951  -8.923  1.00  0.10
C
ATOM  10638  O   CYS B 103     -14.547   1.387  -8.782  1.00  0.11
O
ATOM  10639  CB  CYS B 103     -11.492   1.092  -7.681  1.00  0.17
C
ATOM  10640  SG  CYS B 103     -10.171  -0.131  -7.546  1.00  0.17
S
ATOM  10641  H   CYS B 103     -10.396   1.991  -9.790  1.00  0.16
H
ATOM  10642  HA  CYS B 103     -12.461   0.107  -9.312  1.00  0.13
H
ATOM  10643 1HB  CYS B 103     -11.069   2.069  -7.460  1.00  0.20
H
ATOM  10644 2HB  CYS B 103     -12.228   0.875  -6.914  1.00  0.20
H
ATOM  10645  HG  CYS B 103      -9.833   0.168  -6.296  1.00  0.20
H
ATOM  10646  N   GLY B 104     -13.344   3.281  -9.005  1.00  0.10
N
ATOM  10647  CA  GLY B 104     -14.515   4.157  -8.967  1.00  0.10
C
ATOM  10648  C   GLY B 104     -15.468   3.851 -10.120  1.00  0.15
C
ATOM  10649  O   GLY B 104     -16.682   3.745  -9.924  1.00  0.22
O
ATOM  10650  H   GLY B 104     -12.418   3.693  -9.079  1.00  0.12
H
ATOM  10651 1HA  GLY B 104     -15.037   4.023  -8.023  1.00  0.12
H
ATOM  10652 2HA  GLY B 104     -14.197   5.197  -9.017  1.00  0.12
H
```

ATOM 10653 N   MET B 105   -14.913   3.695 -11.317 1.00 0.18
N
ATOM 10654 CA MET B 105   -15.710   3.340 -12.479 1.00 0.28
C
ATOM 10655 C   MET B 105   -16.309   1.958 -12.433 1.00 0.19
C
ATOM 10656 O   MET B 105   -17.454   1.778 -12.848 1.00 0.23
O
ATOM 10657 CB MET B 105   -14.889   3.537 -13.716 1.00 0.42
C
ATOM 10658 CG MET B 105   -14.806   4.992 -14.066 1.00 0.42
C
ATOM 10659 SD MET B 105   -16.439   5.588 -14.524 1.00 0.42
S
ATOM 10660 CE MET B 105   -16.640   4.609 -16.011 1.00 0.42
C
ATOM 10661 H   MET B 105   -13.911   3.828 -11.419 1.00 0.22
H
ATOM 10662 HA MET B 105   -16.543   4.039 -12.529 1.00 0.34
H
ATOM 10663 1HB MET B 105   -13.881   3.159 -13.561 1.00 0.50
H
ATOM 10664 2HB MET B 105   -15.331   2.997 -14.554 1.00 0.50
H
ATOM 10665 1HG MET B 105   -14.447   5.561 -13.210 1.00 0.50
H
ATOM 10666 2HG MET B 105   -14.139   5.154 -14.887 1.00 0.50
H
ATOM 10667 1HE MET B 105   -17.604   4.809 -16.459 1.00 0.50
H
ATOM 10668 2HE MET B 105   -15.855   4.863 -16.721 1.00 0.50
H
ATOM 10669 3HE MET B 105   -16.575   3.550 -15.760 1.00 0.50
H
ATOM 10670 N   ALA B 106   -15.555   0.989 -11.922 1.00 0.20
N
ATOM 10671 CA ALA B 106   -16.055 -0.366 -11.786 1.00 0.22
C
ATOM 10672 C   ALA B 106   -17.185 -0.394 -10.770 1.00 0.20
C
ATOM 10673 O   ALA B 106   -18.201 -1.055 -10.988 1.00 0.22
O
ATOM 10674 CB ALA B 106   -14.932 -1.304 -11.383 1.00 0.33
C
ATOM 10675 H   ALA B 106   -14.610   1.189 -11.627 1.00 0.24
H
ATOM 10676 HA ALA B 106   -16.457 -0.685 -12.748 1.00 0.26
H
ATOM 10677 1HB ALA B 106   -15.316 -2.320 -11.295 1.00 0.40
H
ATOM 10678 2HB ALA B 106   -14.148 -1.275 -12.139 1.00 0.40
H
ATOM 10679 3HB ALA B 106   -14.524 -0.985 -10.429 1.00 0.40
H
ATOM 10680 N   TYR B 107   -17.008   0.351 -9.676 1.00 0.17
N
ATOM 10681 CA TYR B 107   -18.000   0.463 -8.622 1.00 0.15
C

ATOM 10682 C   TYR B 107   -19.292   1.023 -9.192 1.00 0.14
C
ATOM 10683 O   TYR B 107   -20.369   0.507 -8.918 1.00 0.12
O
ATOM 10684 CB TYR B 107   -17.502   1.371 -7.494 1.00 0.22
C
ATOM 10685 CG TYR B 107   -18.424   1.397 -6.299 1.00 0.22
C
ATOM 10686 CD1 TYR B 107   -18.098   0.650 -5.176 1.00 0.22
C
ATOM 10687 CD2 TYR B 107   -19.608   2.118 -6.333 1.00 0.22
C
ATOM 10688 CE1 TYR B 107   -18.944   0.640 -4.089 1.00 0.22
C
ATOM 10689 CE2 TYR B 107   -20.456   2.092 -5.242 1.00 0.22
C
ATOM 10690 CZ TYR B 107   -20.126   1.359 -4.125 1.00 0.22
C
ATOM 10691 OH TYR B 107   -20.973   1.333 -3.042 1.00 0.22
O
ATOM 10692 H   TYR B 107   -16.141   0.851 -9.554 1.00 0.20
H
ATOM 10693 HA TYR B 107   -18.210 -0.528 -8.224 1.00 0.18
H
ATOM 10694 1HB TYR B 107   -16.521   1.033 -7.161 1.00 0.27
H
ATOM 10695 2HB TYR B 107   -17.391   2.390 -7.862 1.00 0.27
H
ATOM 10696 HD1 TYR B 107   -17.173   0.074 -5.153 1.00 0.27
H
ATOM 10697 HD2 TYR B 107   -19.874   2.696 -7.218 1.00 0.27
H
ATOM 10698 HE1 TYR B 107   -18.692   0.063 -3.208 1.00 0.27
H
ATOM 10699 HE2 TYR B 107   -21.389   2.648 -5.261 1.00 0.27
H
ATOM 10700 HH TYR B 107   -20.594   0.780 -2.354 1.00 0.27
H
ATOM 10701 N   THR B 108   -19.179   2.085 -9.989 1.00 0.20
N
ATOM 10702 CA THR B 108   -20.342   2.707 -10.601 1.00 0.28
C
ATOM 10703 C   THR B 108   -21.039   1.749 -11.573 1.00 0.28
C
ATOM 10704 O   THR B 108   -22.259   1.568 -11.520 1.00 0.39
O
ATOM 10705 CB THR B 108   -19.924   3.995 -11.337 1.00 0.42
C
ATOM 10706 OG1 THR B 108   -19.349   4.911 -10.398 1.00 0.42
O
ATOM 10707 CG2 THR B 108   -21.121   4.654 -11.997 1.00 0.42
C
ATOM 10708 H   THR B 108   -18.266   2.489 -10.160 1.00 0.24
H
ATOM 10709 HA THR B 108   -21.049   2.964 -9.816 1.00 0.34
H
ATOM 10710 HB THR B 108   -19.183   3.754 -12.097 1.00 0.50
H

```
ATOM  10711  HG1 THR B 108     -18.522   4.548 -10.064  1.00  0.50
H
ATOM  10712 1HG2 THR B 108     -20.796   5.560 -12.503  1.00  0.50
H
ATOM  10713 2HG2 THR B 108     -21.564   3.971 -12.721  1.00  0.50
H
ATOM  10714 3HG2 THR B 108     -21.858   4.915 -11.245  1.00  0.50
H
ATOM  10715  N   GLY B 109     -20.259   1.092 -12.427  1.00  0.25
N
ATOM  10716  CA  GLY B 109     -20.814   0.144 -13.382  1.00  0.31
C
ATOM  10717  C   GLY B 109     -21.536  -1.006 -12.696  1.00  0.34
C
ATOM  10718  O   GLY B 109     -22.538  -1.505 -13.201  1.00  0.50
O
ATOM  10719  H   GLY B 109     -19.263   1.266 -12.438  1.00  0.30
H
ATOM  10720 1HA  GLY B 109     -21.502   0.665 -14.049  1.00  0.37
H
ATOM  10721 2HA  GLY B 109     -20.011  -0.253 -14.002  1.00  0.37
H
ATOM  10722  N   LYS B 110     -21.037  -1.426 -11.539  1.00  0.28
N
ATOM  10723  CA  LYS B 110     -21.638  -2.521 -10.792  1.00  0.31
C
ATOM  10724  C   LYS B 110     -22.811  -2.122  -9.892  1.00  0.25
C
ATOM  10725  O   LYS B 110     -23.819  -2.827  -9.837  1.00  0.36
O
ATOM  10726  CB  LYS B 110     -20.569  -3.196  -9.934  1.00  0.46
C
ATOM  10727  CG  LYS B 110     -21.075  -4.385  -9.137  1.00  0.46
C
ATOM  10728  CD  LYS B 110     -19.947  -5.087  -8.399  1.00  0.46
C
ATOM  10729  CE  LYS B 110     -20.488  -6.238  -7.568  1.00  0.46
C
ATOM  10730  NZ  LYS B 110     -21.043  -7.314  -8.438  1.00  0.46
N
ATOM  10731  H   LYS B 110     -20.193  -1.004 -11.174  1.00  0.34
H
ATOM  10732  HA  LYS B 110     -22.013  -3.249 -11.513  1.00  0.37
H
ATOM  10733 1HB  LYS B 110     -19.755  -3.541 -10.572  1.00  0.56
H
ATOM  10734 2HB  LYS B 110     -20.151  -2.472  -9.234  1.00  0.56
H
ATOM  10735 1HG  LYS B 110     -21.815  -4.048  -8.412  1.00  0.56
H
ATOM  10736 2HG  LYS B 110     -21.551  -5.097  -9.811  1.00  0.56
H
ATOM  10737 1HD  LYS B 110     -19.229  -5.480  -9.120  1.00  0.56
H
ATOM  10738 2HD  LYS B 110     -19.432  -4.382  -7.749  1.00  0.56
H
ATOM  10739 1HE  LYS B 110     -19.687  -6.652  -6.959  1.00  0.56
H
```

ATOM 10740 2HE LYS B 110   -21.276 -5.872 -6.910 1.00 0.56
H
ATOM 10741 1HZ LYS B 110   -21.391 -8.071 -7.869 1.00 0.56
H
ATOM 10742 2HZ LYS B 110   -21.793 -6.946 -9.006 1.00 0.56
H
ATOM 10743 3HZ LYS B 110   -20.299 -7.651 -9.035 1.00 0.56
H
ATOM 10744 N   TYR B 111   -22.659 -1.023 -9.159 1.00 0.19
N
ATOM 10745 CA TYR B 111   -23.635 -0.628 -8.150 1.00 0.17
C
ATOM 10746 C   TYR B 111   -24.550   0.548 -8.489 1.00 0.21
C
ATOM 10747 O   TYR B 111   -25.655   0.633 -7.951 1.00 0.55
O
ATOM 10748 CB TYR B 111   -22.890 -0.270 -6.861 1.00 0.26
C
ATOM 10749 CG TYR B 111   -22.148 -1.411 -6.193 1.00 0.26
C
ATOM 10750 CD1 TYR B 111   -20.772 -1.532 -6.328 1.00 0.26
C
ATOM 10751 CD2 TYR B 111   -22.843 -2.323 -5.421 1.00 0.26
C
ATOM 10752 CE1 TYR B 111   -20.094 -2.552 -5.691 1.00 0.26
C
ATOM 10753 CE2 TYR B 111   -22.168 -3.345 -4.785 1.00 0.26
C
ATOM 10754 CZ TYR B 111   -20.801 -3.460 -4.914 1.00 0.26
C
ATOM 10755 OH TYR B 111   -20.142 -4.478 -4.267 1.00 0.26
O
ATOM 10756 H   TYR B 111   -21.828 -0.467 -9.262 1.00 0.23
H
ATOM 10757 HA TYR B 111   -24.275 -1.488 -7.958 1.00 0.20
H
ATOM 10758 1HB TYR B 111   -22.170   0.520 -7.075 1.00 0.31
H
ATOM 10759 2HB TYR B 111   -23.600   0.133 -6.139 1.00 0.31
H
ATOM 10760 HD1 TYR B 111   -20.220 -0.816 -6.930 1.00 0.31
H
ATOM 10761 HD2 TYR B 111   -23.923 -2.230 -5.309 1.00 0.31
H
ATOM 10762 HE1 TYR B 111   -19.014 -2.639 -5.794 1.00 0.31
H
ATOM 10763 HE2 TYR B 111   -22.718 -4.061 -4.173 1.00 0.31
H
ATOM 10764 HH TYR B 111   -19.214 -4.486 -4.526 1.00 0.31
H
ATOM 10765 N   PHE B 112   -24.108   1.468 -9.338 1.00 0.15
N
ATOM 10766 CA PHE B 112   -24.917   2.647 -9.616 1.00 0.20
C
ATOM 10767 C   PHE B 112   -25.623   2.533 -10.949 1.00 0.33
C
ATOM 10768 O   PHE B 112   -26.853   2.508 -11.015 1.00 0.63
O

ATOM 10769 CB PHE B 112 -24.049 3.903 -9.610 1.00 0.30
C
ATOM 10770 CG PHE B 112 -23.515 4.299 -8.260 1.00 0.30
C
ATOM 10771 CD1 PHE B 112 -22.247 4.850 -8.129 1.00 0.30
C
ATOM 10772 CD2 PHE B 112 -24.277 4.121 -7.116 1.00 0.30
C
ATOM 10773 CE1 PHE B 112 -21.756 5.218 -6.892 1.00 0.30
C
ATOM 10774 CE2 PHE B 112 -23.787 4.486 -5.879 1.00 0.30
C
ATOM 10775 CZ PHE B 112 -22.529 5.036 -5.765 1.00 0.30
C
ATOM 10776 H PHE B 112 -23.216 1.375 -9.801 1.00 0.18
H
ATOM 10777 HA PHE B 112 -25.672 2.743 -8.836 1.00 0.24
H
ATOM 10778 1HB PHE B 112 -23.201 3.738 -10.266 1.00 0.36
H
ATOM 10779 2HB PHE B 112 -24.615 4.741 -10.012 1.00 0.36
H
ATOM 10780 HD1 PHE B 112 -21.635 4.997 -9.018 1.00 0.36
H
ATOM 10781 HD2 PHE B 112 -25.273 3.688 -7.202 1.00 0.36
H
ATOM 10782 HE1 PHE B 112 -20.759 5.650 -6.807 1.00 0.36
H
ATOM 10783 HE2 PHE B 112 -24.397 4.338 -4.994 1.00 0.36
H
ATOM 10784 HZ PHE B 112 -22.146 5.322 -4.787 1.00 0.36
H
ATOM 10785 N ASP B 113 -24.845 2.489 -12.013 1.00 0.55
N
ATOM 10786 CA ASP B 113 -25.416 2.354 -13.331 1.00 0.87
C
ATOM 10787 C ASP B 113 -25.861 0.925 -13.568 1.00 0.55
C
ATOM 10788 O ASP B 113 -26.886 0.703 -14.214 1.00 0.63
O
ATOM 10789 CB ASP B 113 -24.448 2.888 -14.376 1.00 1.30
C
ATOM 10790 CG ASP B 113 -24.406 4.412 -14.258 1.00 1.30
C
ATOM 10791 OD1 ASP B 113 -25.258 4.930 -13.578 1.00 1.30
O
ATOM 10792 OD2 ASP B 113 -23.572 5.050 -14.853 1.00 1.30
O
ATOM 10793 H ASP B 113 -23.843 2.508 -11.895 1.00 0.66
H
ATOM 10794 HA ASP B 113 -26.304 2.983 -13.377 1.00 1.04
H
ATOM 10795 1HB ASP B 113 -23.450 2.483 -14.204 1.00 1.57
H
ATOM 10796 2HB ASP B 113 -24.763 2.599 -15.375 1.00 1.57
H
ATOM 10797 N ARG B 114 -25.120 -0.037 -13.006 1.00 0.65
N

ATOM 10798 CA ARG B 114   -25.528 -1.445 -13.073 1.00 0.70
C
ATOM 10799 C   ARG B 114   -25.630 -1.888 -14.524 1.00 0.60
C
ATOM 10800 O   ARG B 114   -26.677 -2.361 -14.972 1.00 0.75
O
ATOM 10801 CB ARG B 114   -26.847 -1.642 -12.345 1.00 1.05
C
ATOM 10802 CG ARG B 114   -26.799 -1.258 -10.874 1.00 1.05
C
ATOM 10803 CD ARG B 114   -28.109 -1.443 -10.209 1.00 1.05
C
ATOM 10804 NE ARG B 114   -28.479 -2.842 -10.138 1.00 1.05
N
ATOM 10805 CZ ARG B 114   -28.043 -3.711 -9.211 1.00 1.05
C
ATOM 10806 NH1 ARG B 114   -27.219 -3.322 -8.264 1.00 1.05
N
ATOM 10807 NH2 ARG B 114   -28.459 -4.962 -9.275 1.00 1.05
N
ATOM 10808 H   ARG B 114   -24.266   0.225 -12.517 1.00 0.78
H
ATOM 10809 HA ARG B 114   -24.773 -2.054 -12.574 1.00 0.84
H
ATOM 10810 1HB ARG B 114   -27.638 -1.063 -12.818 1.00 1.26
H
ATOM 10811 2HB ARG B 114   -27.138 -2.692 -12.399 1.00 1.26
H
ATOM 10812 1HG ARG B 114   -26.070 -1.882 -10.360 1.00 1.26
H
ATOM 10813 2HG ARG B 114   -26.514 -0.212 -10.778 1.00 1.26
H
ATOM 10814 1HD ARG B 114   -28.062 -1.050 -9.194 1.00 1.26
H
ATOM 10815 2HD ARG B 114   -28.879 -0.915 -10.769 1.00 1.26
H
ATOM 10816 HE ARG B 114   -29.111 -3.205 -10.847 1.00 1.26
H
ATOM 10817 1HH1 ARG B 114   -26.907 -2.360 -8.231 1.00 1.26
H
ATOM 10818 2HH1 ARG B 114   -26.896 -3.983 -7.574 1.00 1.26
H
ATOM 10819 1HH2 ARG B 114   -29.091 -5.228 -10.025 1.00 1.26
H
ATOM 10820 2HH2 ARG B 114   -28.148 -5.639 -8.594 1.00 1.26
H
ATOM 10821 N   ALA B 115   -24.537 -1.684 -15.250 1.00 0.57
N
ATOM 10822 CA ALA B 115   -24.433 -1.958 -16.676 1.00 0.76
C
ATOM 10823 C   ALA B 115   -23.359 -3.004 -16.932 1.00 1.17
C
ATOM 10824 O   ALA B 115   -22.441 -3.163 -16.129 1.00 6.21
O
ATOM 10825 CB ALA B 115   -24.128 -0.672 -17.426 1.00 1.14
C
ATOM 10826 H   ALA B 115   -23.724 -1.323 -14.766 1.00 0.68
H

ATOM 10827 HA ALA B 115 -25.386 -2.357 -17.024 1.00 0.91
H
ATOM 10828 1HB ALA B 115 -24.066 -0.873 -18.494 1.00 1.37
H
ATOM 10829 2HB ALA B 115 -24.922 0.050 -17.237 1.00 1.37
H
ATOM 10830 3HB ALA B 115 -23.182 -0.266 -17.077 1.00 1.37
H
ATOM 10831 N SER B 116 -23.456 -3.714 -18.051 1.00 1.57
N
ATOM 10832 CA SER B 116 -22.509 -4.779 -18.369 1.00 1.76
C
ATOM 10833 C SER B 116 -21.186 -4.330 -18.997 1.00 2.22
C
ATOM 10834 O SER B 116 -20.656 -5.027 -19.862 1.00 13.57
O
ATOM 10835 CB SER B 116 -23.156 -5.796 -19.289 1.00 2.64
C
ATOM 10836 OG SER B 116 -23.496 -5.215 -20.516 1.00 2.64
O
ATOM 10837 H SER B 116 -24.220 -3.551 -18.694 1.00 1.88
H
ATOM 10838 HA SER B 116 -22.269 -5.289 -17.434 1.00 2.11
H
ATOM 10839 1HB SER B 116 -22.468 -6.624 -19.454 1.00 3.17
H
ATOM 10840 2HB SER B 116 -24.049 -6.201 -18.816 1.00 3.17
H
ATOM 10841 HG SER B 116 -24.192 -4.582 -20.313 1.00 3.17
H
ATOM 10842 N TYR B 117 -20.641 -3.180 -18.597 1.00 2.42
N
ATOM 10843 CA TYR B 117 -19.335 -2.827 -19.121 1.00 1.88
C
ATOM 10844 C TYR B 117 -18.301 -3.216 -18.073 1.00 0.77
C
ATOM 10845 O TYR B 117 -18.612 -3.295 -16.880 1.00 1.80
O
ATOM 10846 CB TYR B 117 -19.217 -1.338 -19.476 1.00 2.82
C
ATOM 10847 CG TYR B 117 -19.231 -0.376 -18.310 1.00 2.82
C
ATOM 10848 CD1 TYR B 117 -18.039 -0.069 -17.664 1.00 2.82
C
ATOM 10849 CD2 TYR B 117 -20.414 0.217 -17.899 1.00 2.82
C
ATOM 10850 CE1 TYR B 117 -18.031 0.823 -16.610 1.00 2.82
C
ATOM 10851 CE2 TYR B 117 -20.406 1.111 -16.844 1.00 2.82
C
ATOM 10852 CZ TYR B 117 -19.220 1.415 -16.202 1.00 2.82
C
ATOM 10853 OH TYR B 117 -19.213 2.304 -15.153 1.00 2.82
O
ATOM 10854 H TYR B 117 -21.093 -2.591 -17.915 1.00 2.90
H
ATOM 10855 HA TYR B 117 -19.137 -3.412 -20.020 1.00 2.26
H

```
ATOM  10856 1HB  TYR B 117     -18.295  -1.178 -20.033  1.00  3.38
H
ATOM  10857 2HB  TYR B 117     -20.038  -1.068 -20.139  1.00  3.38
H
ATOM  10858  HD1 TYR B 117     -17.108  -0.533 -17.994  1.00  3.38
H
ATOM  10859  HD2 TYR B 117     -21.347  -0.019 -18.411  1.00  3.38
H
ATOM  10860  HE1 TYR B 117     -17.097   1.063 -16.104  1.00  3.38
H
ATOM  10861  HE2 TYR B 117     -21.336   1.580 -16.522  1.00  3.38
H
ATOM  10862  HH  TYR B 117     -18.362   2.268 -14.704  1.00  3.38
H
ATOM  10863  N   ARG B 118     -17.083  -3.457 -18.529  1.00  0.66
N
ATOM  10864  CA  ARG B 118     -15.996  -3.859 -17.654  1.00  0.74
C
ATOM  10865  C   ARG B 118     -14.919  -2.799 -17.645  1.00  0.68
C
ATOM  10866  O   ARG B 118     -14.863  -1.965 -18.548  1.00  1.63
O
ATOM  10867  CB  ARG B 118     -15.412  -5.204 -18.081  1.00  1.11
C
ATOM  10868  CG  ARG B 118     -16.362  -6.391 -17.934  1.00  1.11
C
ATOM  10869  CD  ARG B 118     -15.718  -7.684 -18.293  1.00  1.11
C
ATOM  10870  NE  ARG B 118     -15.440  -7.785 -19.718  1.00  1.11
N
ATOM  10871  CZ  ARG B 118     -14.723  -8.773 -20.290  1.00  1.11
C
ATOM  10872  NH1 ARG B 118     -14.216  -9.732 -19.549  1.00  1.11
N
ATOM  10873  NH2 ARG B 118     -14.532  -8.775 -21.598  1.00  1.11
N
ATOM  10874  H   ARG B 118     -16.917  -3.364 -19.525  1.00  0.79
H
ATOM  10875  HA  ARG B 118     -16.382  -3.962 -16.640  1.00  0.89
H
ATOM  10876 1HB  ARG B 118     -15.112  -5.154 -19.127  1.00  1.33
H
ATOM  10877 2HB  ARG B 118     -14.520  -5.421 -17.493  1.00  1.33
H
ATOM  10878 1HG  ARG B 118     -16.702  -6.461 -16.903  1.00  1.33
H
ATOM  10879 2HG  ARG B 118     -17.222  -6.246 -18.589  1.00  1.33
H
ATOM  10880 1HD  ARG B 118     -14.777  -7.782 -17.756  1.00  1.33
H
ATOM  10881 2HD  ARG B 118     -16.379  -8.507 -18.018  1.00  1.33
H
ATOM  10882  HE  ARG B 118     -15.814  -7.064 -20.324  1.00  1.33
H
ATOM  10883 1HH1 ARG B 118     -14.363  -9.730 -18.549  1.00  1.33
H
ATOM  10884 2HH1 ARG B 118     -13.677 -10.472 -19.978  1.00  1.33
H
```

```
ATOM  10885 1HH2 ARG B 118     -14.921  -8.036 -22.169  1.00  1.33
H
ATOM  10886 2HH2 ARG B 118     -13.996  -9.513 -22.030  1.00  1.33
H
ATOM  10887  N   VAL B 119     -14.093  -2.814 -16.609  1.00  0.40
N
ATOM  10888  CA  VAL B 119     -13.023  -1.843 -16.482  1.00  0.37
C
ATOM  10889  C   VAL B 119     -11.674  -2.537 -16.405  1.00  0.39
C
ATOM  10890  O   VAL B 119     -11.444  -3.365 -15.527  1.00  0.56
O
ATOM  10891  CB  VAL B 119     -13.216  -0.967 -15.228  1.00  0.55
C
ATOM  10892  CG1 VAL B 119     -12.069   0.024 -15.124  1.00  0.55
C
ATOM  10893  CG2 VAL B 119     -14.553  -0.244 -15.287  1.00  0.55
C
ATOM  10894  H   VAL B 119     -14.200  -3.526 -15.899  1.00  0.48
H
ATOM  10895  HA  VAL B 119     -13.033  -1.198 -17.361  1.00  0.44
H
ATOM  10896  HB  VAL B 119     -13.184  -1.600 -14.341  1.00  0.67
H
ATOM  10897 1HG1 VAL B 119     -12.196   0.630 -14.228  1.00  0.67
H
ATOM  10898 2HG1 VAL B 119     -11.125  -0.517 -15.066  1.00  0.67
H
ATOM  10899 3HG1 VAL B 119     -12.065   0.670 -16.001  1.00  0.67
H
ATOM  10900 1HG2 VAL B 119     -14.672   0.364 -14.392  1.00  0.67
H
ATOM  10901 2HG2 VAL B 119     -14.590   0.395 -16.164  1.00  0.67
H
ATOM  10902 3HG2 VAL B 119     -15.361  -0.974 -15.338  1.00  0.67
H
ATOM  10903  N   PHE B 120     -10.791  -2.186 -17.324  1.00  0.37
N
ATOM  10904  CA  PHE B 120      -9.450  -2.728 -17.352  1.00  0.37
C
ATOM  10905  C   PHE B 120      -8.475  -1.633 -16.955  1.00  0.52
C
ATOM  10906  O   PHE B 120      -8.675  -0.477 -17.318  1.00  1.43
O
ATOM  10907  CB  PHE B 120      -9.135  -3.242 -18.757  1.00  0.55
C
ATOM  10908  CG  PHE B 120      -9.962  -4.431 -19.155  1.00  0.55
C
ATOM  10909  CD1 PHE B 120     -11.234  -4.259 -19.684  1.00  0.55
C
ATOM  10910  CD2 PHE B 120      -9.473  -5.718 -19.008  1.00  0.55
C
ATOM  10911  CE1 PHE B 120     -12.000  -5.351 -20.049  1.00  0.55
C
ATOM  10912  CE2 PHE B 120     -10.234  -6.811 -19.371  1.00  0.55
C
ATOM  10913  CZ  PHE B 120     -11.500  -6.626 -19.891  1.00  0.55
C
```

```
ATOM  10914  H   PHE B 120     -11.058  -1.512 -18.025  1.00  0.44
H
ATOM  10915  HA  PHE B 120      -9.384  -3.547 -16.643  1.00  0.44
H
ATOM  10916 1HB  PHE B 120      -9.319  -2.450 -19.480  1.00  0.67
H
ATOM  10917 2HB  PHE B 120      -8.083  -3.517 -18.826  1.00  0.67
H
ATOM  10918  HD1 PHE B 120     -11.625  -3.249 -19.808  1.00  0.67
H
ATOM  10919  HD2 PHE B 120      -8.476  -5.866 -18.596  1.00  0.67
H
ATOM  10920  HE1 PHE B 120     -12.998  -5.202 -20.461  1.00  0.67
H
ATOM  10921  HE2 PHE B 120      -9.839  -7.819 -19.248  1.00  0.67
H
ATOM  10922  HZ  PHE B 120     -12.101  -7.487 -20.177  1.00  0.67
H
ATOM  10923  N   CYS B 121      -7.426  -1.993 -16.227  1.00  0.32
N
ATOM  10924  CA  CYS B 121      -6.438  -1.008 -15.802  1.00  0.32
C
ATOM  10925  C   CYS B 121      -5.029  -1.558 -15.913  1.00  0.26
C
ATOM  10926  O   CYS B 121      -4.690  -2.533 -15.251  1.00  0.45
O
ATOM  10927  CB  CYS B 121      -6.690  -0.575 -14.363  1.00  0.48
C
ATOM  10928  SG  CYS B 121      -5.531   0.678 -13.769  1.00  0.48
S
ATOM  10929  H   CYS B 121      -7.332  -2.958 -15.939  1.00  0.38
H
ATOM  10930  HA  CYS B 121      -6.528  -0.140 -16.447  1.00  0.38
H
ATOM  10931 1HB  CYS B 121      -7.699  -0.173 -14.278  1.00  0.58
H
ATOM  10932 2HB  CYS B 121      -6.624  -1.439 -13.703  1.00  0.58
H
ATOM  10933  HG  CYS B 121      -4.434  -0.045 -13.970  1.00  0.58
H
ATOM  10934  N   LEU B 122      -4.214  -0.922 -16.749  1.00  0.25
N
ATOM  10935  CA  LEU B 122      -2.849  -1.382 -16.984  1.00  0.23
C
ATOM  10936  C   LEU B 122      -1.825  -0.479 -16.314  1.00  0.28
C
ATOM  10937  O   LEU B 122      -1.760   0.725 -16.574  1.00  0.55
O
ATOM  10938  CB  LEU B 122      -2.596  -1.494 -18.495  1.00  0.35
C
ATOM  10939  CG  LEU B 122      -1.164  -1.839 -18.948  1.00  0.35
C
ATOM  10940  CD1 LEU B 122      -0.759  -3.204 -18.433  1.00  0.35
C
ATOM  10941  CD2 LEU B 122      -1.116  -1.846 -20.470  1.00  0.35
C
ATOM  10942  H   LEU B 122      -4.556  -0.093 -17.219  1.00  0.30
H
```

```
ATOM  10943  HA  LEU B 122      -2.747  -2.378 -16.555  1.00  0.28
H
ATOM  10944 1HB  LEU B 122      -3.255  -2.264 -18.894  1.00  0.41
H
ATOM  10945 2HB  LEU B 122      -2.870  -0.548 -18.956  1.00  0.41
H
ATOM  10946  HG  LEU B 122      -0.466  -1.098 -18.556  1.00  0.41
H
ATOM  10947 1HD1 LEU B 122       0.253  -3.432 -18.765  1.00  0.41
H
ATOM  10948 2HD1 LEU B 122      -0.793  -3.206 -17.345  1.00  0.41
H
ATOM  10949 3HD1 LEU B 122      -1.446  -3.948 -18.824  1.00  0.41
H
ATOM  10950 1HD2 LEU B 122      -0.106  -2.084 -20.794  1.00  0.41
H
ATOM  10951 2HD2 LEU B 122      -1.807  -2.596 -20.853  1.00  0.41
H
ATOM  10952 3HD2 LEU B 122      -1.397  -0.870 -20.857  1.00  0.41
H
ATOM  10953  N   MET B 123      -1.048  -1.089 -15.426  1.00  0.25
N
ATOM  10954  CA  MET B 123      -0.050  -0.398 -14.626  1.00  0.31
C
ATOM  10955  C   MET B 123       1.310  -1.074 -14.713  1.00  0.24
C
ATOM  10956  O   MET B 123       1.397  -2.205 -15.184  1.00  0.33
O
ATOM  10957  CB  MET B 123      -0.525  -0.377 -13.187  1.00  0.46
C
ATOM  10958  CG  MET B 123      -0.666  -1.755 -12.572  1.00  0.46
C
ATOM  10959  SD  MET B 123      -1.154  -1.658 -10.853  1.00  0.46
S
ATOM  10960  CE  MET B 123       0.380  -0.982 -10.254  1.00  0.46
C
ATOM  10961  H   MET B 123      -1.171  -2.086 -15.292  1.00  0.30
H
ATOM  10962  HA  MET B 123       0.047   0.621 -14.990  1.00  0.37
H
ATOM  10963 1HB  MET B 123       0.174   0.186 -12.573  1.00  0.56
H
ATOM  10964 2HB  MET B 123      -1.494   0.118 -13.124  1.00  0.56
H
ATOM  10965 1HG  MET B 123      -1.415  -2.329 -13.116  1.00  0.56
H
ATOM  10966 2HG  MET B 123       0.285  -2.285 -12.633  1.00  0.56
H
ATOM  10967 1HE  MET B 123       0.319  -0.827  -9.182  1.00  0.56
H
ATOM  10968 2HE  MET B 123       1.194  -1.672 -10.479  1.00  0.56
H
ATOM  10969 3HE  MET B 123       0.570  -0.028 -10.746  1.00  0.56
H
ATOM  10970  N   SER B 124       2.366  -0.398 -14.248  1.00  0.26
N
ATOM  10971  CA  SER B 124       3.688  -1.045 -14.253  1.00  0.33
C
```

```
ATOM  10972  C   SER B 124       4.057  -1.513 -12.877  1.00  0.33
C
ATOM  10973  O   SER B 124       3.461  -1.094 -11.886  1.00  0.56
O
ATOM  10974  CB  SER B 124       4.802  -0.143 -14.745  1.00  0.49
C
ATOM  10975  OG  SER B 124       5.075   0.905 -13.846  1.00  0.49
O
ATOM  10976  H   SER B 124       2.231   0.542 -13.877  1.00  0.31
H
ATOM  10977  HA  SER B 124       3.644  -1.918 -14.903  1.00  0.40
H
ATOM  10978 1HB  SER B 124       5.705  -0.733 -14.901  1.00  0.59
H
ATOM  10979 2HB  SER B 124       4.519   0.262 -15.713  1.00  0.59
H
ATOM  10980  HG  SER B 124       4.215   1.223 -13.564  1.00  0.59
H
ATOM  10981  N   ASP B 125       5.049  -2.376 -12.794  1.00  0.51
N
ATOM  10982  CA  ASP B 125       5.472  -2.808 -11.480  1.00  0.45
C
ATOM  10983  C   ASP B 125       6.232  -1.733 -10.720  1.00  0.54
C
ATOM  10984  O   ASP B 125       6.205  -1.727  -9.492  1.00  1.43
O
ATOM  10985  CB  ASP B 125       6.261  -4.091 -11.532  1.00  0.68
C
ATOM  10986  CG  ASP B 125       5.379  -5.273 -11.797  1.00  0.68
C
ATOM  10987  OD1 ASP B 125       4.177  -5.129 -11.787  1.00  0.68
O
ATOM  10988  OD2 ASP B 125       5.919  -6.332 -11.963  1.00  0.68
O
ATOM  10989  H   ASP B 125       5.510  -2.720 -13.627  1.00  0.61
H
ATOM  10990  HA  ASP B 125       4.572  -3.028 -10.906  1.00  0.54
H
ATOM  10991 1HB  ASP B 125       6.993  -4.035 -12.334  1.00  0.81
H
ATOM  10992 2HB  ASP B 125       6.797  -4.243 -10.595  1.00  0.81
H
ATOM  10993  N   GLY B 126       6.877  -0.796 -11.419  1.00  0.36
N
ATOM  10994  CA  GLY B 126       7.508   0.315 -10.714  1.00  0.40
C
ATOM  10995  C   GLY B 126       6.420   1.094 -10.025  1.00  0.36
C
ATOM  10996  O   GLY B 126       6.523   1.438  -8.846  1.00  0.41
O
ATOM  10997  H   GLY B 126       6.909  -0.840 -12.428  1.00  0.43
H
ATOM  10998 1HA  GLY B 126       8.199  -0.054  -9.966  1.00  0.48
H
ATOM  10999 2HA  GLY B 126       8.056   0.949 -11.410  1.00  0.48
H
ATOM  11000  N   GLU B 127       5.349   1.325 -10.768  1.00  0.37
N
```

```
ATOM  11001  CA  GLU B 127       4.158   1.979 -10.278  1.00  0.35
C
ATOM  11002  C   GLU B 127       3.543   1.301  -9.048  1.00  0.30
C
ATOM  11003  O   GLU B 127       3.059   1.989  -8.146  1.00  0.36
O
ATOM  11004  CB  GLU B 127       3.166   2.057 -11.416  1.00  0.52
C
ATOM  11005  CG  GLU B 127       1.868   2.684 -11.091  1.00  0.52
C
ATOM  11006  CD  GLU B 127       1.078   2.927 -12.322  1.00  0.52
C
ATOM  11007  OE1 GLU B 127       1.275   2.244 -13.303  1.00  0.52
O
ATOM  11008  OE2 GLU B 127       0.296   3.844 -12.292  1.00  0.52
O
ATOM  11009  H   GLU B 127       5.351   1.026 -11.741  1.00  0.44
H
ATOM  11010  HA  GLU B 127       4.428   2.996  -9.992  1.00  0.42
H
ATOM  11011 1HB  GLU B 127       3.608   2.627 -12.232  1.00  0.63
H
ATOM  11012 2HB  GLU B 127       2.949   1.070 -11.793  1.00  0.63
H
ATOM  11013 1HG  GLU B 127       1.307   2.024 -10.432  1.00  0.63
H
ATOM  11014 2HG  GLU B 127       2.052   3.611 -10.566  1.00  0.63
H
ATOM  11015  N   SER B 128       3.585  -0.043  -8.997  1.00  0.48
N
ATOM  11016  CA  SER B 128       2.992  -0.796  -7.879  1.00  0.45
C
ATOM  11017  C   SER B 128       3.629  -0.501  -6.522  1.00  0.43
C
ATOM  11018  O   SER B 128       3.055  -0.854  -5.490  1.00  0.62
O
ATOM  11019  CB  SER B 128       3.076  -2.301  -8.100  1.00  0.68
C
ATOM  11020  OG  SER B 128       4.378  -2.790  -7.917  1.00  0.68
O
ATOM  11021  H   SER B 128       3.978  -0.554  -9.779  1.00  0.58
H
ATOM  11022  HA  SER B 128       1.941  -0.522  -7.813  1.00  0.54
H
ATOM  11023 1HB  SER B 128       2.399  -2.804  -7.410  1.00  0.81
H
ATOM  11024 2HB  SER B 128       2.742  -2.537  -9.108  1.00  0.81
H
ATOM  11025  HG  SER B 128       4.940  -2.337  -8.548  1.00  0.81
H
ATOM  11026  N   SER B 129       4.809   0.129  -6.498  1.00  0.41
N
ATOM  11027  CA  SER B 129       5.435   0.475  -5.230  1.00  0.43
C
ATOM  11028  C   SER B 129       4.708   1.614  -4.496  1.00  0.42
C
ATOM  11029  O   SER B 129       4.935   1.814  -3.303  1.00  0.57
O
```

ATOM 11030 CB SER B 129   6.891   0.830 -5.455 1.00 0.65
C
ATOM 11031 OG SER B 129   7.020   1.985 -6.229 1.00 0.65
O
ATOM 11032 H   SER B 129   5.278   0.402 -7.355 1.00 0.49
H
ATOM 11033 HA SER B 129   5.401 -0.406 -4.590 1.00 0.52
H
ATOM 11034 1HB SER B 129   7.380   0.979 -4.493 1.00 0.77
H
ATOM 11035 2HB SER B 129   7.391   0.000 -5.952 1.00 0.77
H
ATOM 11036 HG SER B 129   6.610   2.702 -5.737 1.00 0.77
H
ATOM 11037 N   GLU B 130   3.824   2.344 -5.190 1.00 0.36
N
ATOM 11038 CA GLU B 130   3.051   3.391 -4.528 1.00 0.42
C
ATOM 11039 C   GLU B 130   1.907   2.798 -3.724 1.00 0.38
C
ATOM 11040 O   GLU B 130   1.146   1.969 -4.224 1.00 0.68
O
ATOM 11041 CB GLU B 130   2.499   4.396 -5.538 1.00 0.63
C
ATOM 11042 CG GLU B 130   3.541   5.300 -6.173 1.00 0.63
C
ATOM 11043 CD GLU B 130   4.098   6.284 -5.173 1.00 0.63
C
ATOM 11044 OE1 GLU B 130   3.766   6.175 -4.019 1.00 0.63
O
ATOM 11045 OE2 GLU B 130   4.791   7.194 -5.572 1.00 0.63
O
ATOM 11046 H   GLU B 130   3.663   2.165 -6.174 1.00 0.43
H
ATOM 11047 HA GLU B 130   3.709   3.920 -3.838 1.00 0.50
H
ATOM 11048 1HB GLU B 130   1.995   3.859 -6.342 1.00 0.76
H
ATOM 11049 2HB GLU B 130   1.759   5.031 -5.052 1.00 0.76
H
ATOM 11050 1HG GLU B 130   4.355   4.689 -6.561 1.00 0.76
H
ATOM 11051 2HG GLU B 130   3.090   5.839 -7.006 1.00 0.76
H
ATOM 11052 N   GLY B 131   1.776   3.256 -2.479 1.00 0.26
N
ATOM 11053 CA GLY B 131   0.779   2.754 -1.536 1.00 0.32
C
ATOM 11054 C   GLY B 131   -0.661   2.878 -2.010 1.00 0.28
C
ATOM 11055 O   GLY B 131   -1.490   2.024 -1.691 1.00 0.48
O
ATOM 11056 H   GLY B 131   2.423   3.964 -2.141 1.00 0.31
H
ATOM 11057 1HA GLY B 131   0.996   1.709 -1.312 1.00 0.38
H
ATOM 11058 2HA GLY B 131   0.892   3.299 -0.603 1.00 0.38
H

ATOM 11059 N SER B 132 -0.944 3.922 -2.789 1.00 0.36
N
ATOM 11060 CA SER B 132 -2.274 4.163 -3.335 1.00 0.51
C
ATOM 11061 C SER B 132 -2.779 3.005 -4.194 1.00 0.34
C
ATOM 11062 O SER B 132 -3.990 2.796 -4.303 1.00 0.74
O
ATOM 11063 CB SER B 132 -2.245 5.446 -4.130 1.00 0.77
C
ATOM 11064 OG SER B 132 -1.370 5.335 -5.219 1.00 0.77
O
ATOM 11065 H SER B 132 -0.209 4.580 -3.005 1.00 0.43
H
ATOM 11066 HA SER B 132 -2.965 4.291 -2.500 1.00 0.61
H
ATOM 11067 1HB SER B 132 -3.247 5.690 -4.485 1.00 0.92
H
ATOM 11068 2HB SER B 132 -1.918 6.259 -3.484 1.00 0.92
H
ATOM 11069 HG SER B 132 -1.873 4.965 -5.949 1.00 0.92
H
ATOM 11070 N VAL B 133 -1.852 2.234 -4.772 1.00 0.25
N
ATOM 11071 CA VAL B 133 -2.211 1.068 -5.553 1.00 0.29
C
ATOM 11072 C VAL B 133 -2.829 0.025 -4.665 1.00 0.26
C
ATOM 11073 O VAL B 133 -3.878 -0.525 -4.985 1.00 0.44
O
ATOM 11074 CB VAL B 133 -0.976 0.453 -6.233 1.00 0.43
C
ATOM 11075 CG1 VAL B 133 -1.353 -0.880 -6.866 1.00 0.43
C
ATOM 11076 CG2 VAL B 133 -0.434 1.420 -7.272 1.00 0.43
C
ATOM 11077 H VAL B 133 -0.868 2.436 -4.648 1.00 0.30
H
ATOM 11078 HA VAL B 133 -2.934 1.361 -6.315 1.00 0.35
H
ATOM 11079 HB VAL B 133 -0.213 0.255 -5.482 1.00 0.52
H
ATOM 11080 1HG1 VAL B 133 -0.474 -1.326 -7.326 1.00 0.52
H
ATOM 11081 2HG1 VAL B 133 -1.733 -1.552 -6.096 1.00 0.52
H
ATOM 11082 3HG1 VAL B 133 -2.122 -0.721 -7.622 1.00 0.52
H
ATOM 11083 1HG2 VAL B 133 0.445 0.992 -7.749 1.00 0.52
H
ATOM 11084 2HG2 VAL B 133 -1.199 1.611 -8.025 1.00 0.52
H
ATOM 11085 3HG2 VAL B 133 -0.161 2.357 -6.787 1.00 0.52
H
ATOM 11086 N TRP B 134 -2.168 -0.239 -3.543 1.00 0.42
N
ATOM 11087 CA TRP B 134 -2.627 -1.250 -2.614 1.00 0.33
C

ATOM 11088 C TRP B 134 -3.907 -0.826 -1.911 1.00 0.34
C
ATOM 11089 O TRP B 134 -4.725 -1.679 -1.563 1.00 0.56
O
ATOM 11090 CB TRP B 134 -1.525 -1.568 -1.617 1.00 0.49
C
ATOM 11091 CG TRP B 134 -0.374 -2.255 -2.271 1.00 0.49
C
ATOM 11092 CD1 TRP B 134 0.893 -1.777 -2.417 1.00 0.49
C
ATOM 11093 CD2 TRP B 134 -0.394 -3.545 -2.922 1.00 0.49
C
ATOM 11094 NE1 TRP B 134 1.663 -2.684 -3.105 1.00 0.49
N
ATOM 11095 CE2 TRP B 134 0.887 -3.770 -3.426 1.00 0.49
C
ATOM 11096 CE3 TRP B 134 -1.386 -4.509 -3.123 1.00 0.49
C
ATOM 11097 CZ2 TRP B 134 1.207 -4.923 -4.124 1.00 0.49
C
ATOM 11098 CZ3 TRP B 134 -1.069 -5.664 -3.822 1.00 0.49
C
ATOM 11099 CH2 TRP B 134 0.193 -5.863 -4.312 1.00 0.49
C
ATOM 11100 H TRP B 134 -1.315 0.263 -3.339 1.00 0.50
H
ATOM 11101 HA TRP B 134 -2.835 -2.157 -3.178 1.00 0.40
H
ATOM 11102 1HB TRP B 134 -1.163 -0.647 -1.163 1.00 0.59
H
ATOM 11103 2HB TRP B 134 -1.910 -2.209 -0.827 1.00 0.59
H
ATOM 11104 HD1 TRP B 134 1.244 -0.815 -2.046 1.00 0.59
H
ATOM 11105 HE1 TRP B 134 2.637 -2.564 -3.348 1.00 0.59
H
ATOM 11106 HE3 TRP B 134 -2.392 -4.352 -2.740 1.00 0.59
H
ATOM 11107 HZ2 TRP B 134 2.205 -5.100 -4.522 1.00 0.59
H
ATOM 11108 HZ3 TRP B 134 -1.850 -6.409 -3.975 1.00 0.59
H
ATOM 11109 HH2 TRP B 134 0.408 -6.779 -4.861 1.00 0.59
H
ATOM 11110 N GLU B 135 -4.111 0.486 -1.743 1.00 0.23
N
ATOM 11111 CA GLU B 135 -5.369 0.962 -1.177 1.00 0.21
C
ATOM 11112 C GLU B 135 -6.511 0.587 -2.117 1.00 0.19
C
ATOM 11113 O GLU B 135 -7.550 0.089 -1.674 1.00 0.21
O
ATOM 11114 CB GLU B 135 -5.368 2.483 -0.976 1.00 0.32
C
ATOM 11115 CG GLU B 135 -4.442 3.021 0.100 1.00 0.32
C
ATOM 11116 CD GLU B 135 -4.513 4.529 0.243 1.00 0.32
C

ATOM 11117 OE1 GLU B 135   -5.270   5.148 -0.476 1.00 0.32
O
ATOM 11118 OE2 GLU B 135   -3.809   5.051   1.077 1.00 0.32
O
ATOM 11119 H   GLU B 135   -3.387   1.150 -1.996 1.00 0.28
H
ATOM 11120 HA GLU B 135   -5.538   0.474 -0.218 1.00 0.25
H
ATOM 11121 1HB GLU B 135   -5.114   2.975 -1.915 1.00 0.38
H
ATOM 11122 2HB GLU B 135   -6.375   2.794 -0.705 1.00 0.38
H
ATOM 11123 1HG GLU B 135   -4.706   2.563   1.052 1.00 0.38
H
ATOM 11124 2HG GLU B 135   -3.420   2.731 -0.141 1.00 0.38
H
ATOM 11125 N   ALA B 136   -6.293   0.808 -3.417 1.00 0.17
N
ATOM 11126 CA ALA B 136   -7.267   0.468 -4.446 1.00 0.14
C
ATOM 11127 C   ALA B 136   -7.478 -1.045 -4.540 1.00 0.16
C
ATOM 11128 O   ALA B 136   -8.608 -1.499 -4.732 1.00 0.17
O
ATOM 11129 CB ALA B 136   -6.819   1.027 -5.781 1.00 0.21
C
ATOM 11130 H   ALA B 136   -5.426   1.252 -3.706 1.00 0.20
H
ATOM 11131 HA ALA B 136   -8.218   0.922 -4.176 1.00 0.17
H
ATOM 11132 1HB ALA B 136   -7.565   0.798 -6.539 1.00 0.25
H
ATOM 11133 2HB ALA B 136   -6.704   2.108 -5.698 1.00 0.25
H
ATOM 11134 3HB ALA B 136   -5.867   0.584 -6.061 1.00 0.25
H
ATOM 11135 N   MET B 137   -6.398 -1.818 -4.383 1.00 0.19
N
ATOM 11136 CA MET B 137   -6.481 -3.275 -4.407 1.00 0.31
C
ATOM 11137 C   MET B 137   -7.414 -3.760 -3.297 1.00 0.24
C
ATOM 11138 O   MET B 137   -8.299 -4.590 -3.538 1.00 0.31
O
ATOM 11139 CB MET B 137   -5.080 -3.887 -4.237 1.00 0.46
C
ATOM 11140 CG MET B 137   -4.134 -3.711 -5.428 1.00 0.46
C
ATOM 11141 SD MET B 137   -4.544 -4.715 -6.863 1.00 0.46
S
ATOM 11142 CE MET B 137   -4.001 -6.326 -6.301 1.00 0.46
C
ATOM 11143 H   MET B 137   -5.490 -1.386 -4.264 1.00 0.23
H
ATOM 11144 HA MET B 137   -6.897 -3.588 -5.360 1.00 0.37
H
ATOM 11145 1HB MET B 137   -4.599 -3.448 -3.366 1.00 0.56
H

ATOM 11146 2HB MET B 137   -5.176 -4.956 -4.049 1.00 0.56
H
ATOM 11147 1HG MET B 137   -4.154 -2.674 -5.742 1.00 0.56
H
ATOM 11148 2HG MET B 137   -3.119 -3.948 -5.129 1.00 0.56
H
ATOM 11149 1HE MET B 137   -4.191 -7.065 -7.078 1.00 0.56
H
ATOM 11150 2HE MET B 137   -2.933 -6.294 -6.084 1.00 0.56
H
ATOM 11151 3HE MET B 137   -4.547 -6.599 -5.398 1.00 0.56
H
ATOM 11152 N   ALA B 138   -7.219 -3.223 -2.094 1.00 0.25
N
ATOM 11153 CA ALA B 138   -8.045 -3.569 -0.950 1.00 0.27
C
ATOM 11154 C   ALA B 138   -9.508 -3.163 -1.164 1.00 0.27
C
ATOM 11155 O   ALA B 138   -10.409 -3.945 -0.855 1.00 0.45
O
ATOM 11156 CB ALA B 138   -7.491 -2.906   0.301 1.00 0.41
C
ATOM 11157 H   ALA B 138   -6.462 -2.563 -1.963 1.00 0.30
H
ATOM 11158 HA ALA B 138   -8.012 -4.651 -0.825 1.00 0.32
H
ATOM 11159 1HB ALA B 138   -8.094 -3.192   1.163 1.00 0.49
H
ATOM 11160 2HB ALA B 138   -6.461 -3.227   0.456 1.00 0.49
H
ATOM 11161 3HB ALA B 138   -7.518 -1.824   0.182 1.00 0.49
H
ATOM 11162 N   PHE B 139   -9.729 -1.955 -1.701 1.00 0.19
N
ATOM 11163 CA PHE B 139   -11.071 -1.426 -1.973 1.00 0.17
C
ATOM 11164 C   PHE B 139   -11.869 -2.345 -2.878 1.00 0.17
C
ATOM 11165 O   PHE B 139   -13.016 -2.681 -2.580 1.00 0.19
O
ATOM 11166 CB PHE B 139   -10.967 -0.056 -2.660 1.00 0.26
C
ATOM 11167 CG PHE B 139   -12.284   0.590 -3.043 1.00 0.26
C
ATOM 11168 CD1 PHE B 139   -12.950   1.432 -2.162 1.00 0.26
C
ATOM 11169 CD2 PHE B 139   -12.858   0.362 -4.287 1.00 0.26
C
ATOM 11170 CE1 PHE B 139   -14.141   2.038 -2.521 1.00 0.26
C
ATOM 11171 CE2 PHE B 139   -14.054   0.961 -4.645 1.00 0.26
C
ATOM 11172 CZ PHE B 139   -14.697   1.802 -3.761 1.00 0.26
C
ATOM 11173 H   PHE B 139   -8.937 -1.359 -1.912 1.00 0.23
H
ATOM 11174 HA PHE B 139   -11.602 -1.320 -1.026 1.00 0.20
H

```
ATOM  11175 1HB  PHE B 139     -10.437   0.630  -1.998  1.00  0.31
H
ATOM  11176 2HB  PHE B 139     -10.367  -0.152  -3.562  1.00  0.31
H
ATOM  11177  HD1 PHE B 139     -12.516   1.623  -1.181  1.00  0.31
H
ATOM  11178  HD2 PHE B 139     -12.350  -0.299  -4.990  1.00  0.31
H
ATOM  11179  HE1 PHE B 139     -14.644   2.701  -1.818  1.00  0.31
H
ATOM  11180  HE2 PHE B 139     -14.489   0.771  -5.626  1.00  0.31
H
ATOM  11181  HZ  PHE B 139     -15.636   2.276  -4.041  1.00  0.31
H
ATOM  11182  N   ALA B 140     -11.260  -2.722  -3.998  1.00  0.18
N
ATOM  11183  CA  ALA B 140     -11.910  -3.558  -4.987  1.00  0.20
C
ATOM  11184  C   ALA B 140     -12.303  -4.914  -4.422  1.00  0.23
C
ATOM  11185  O   ALA B 140     -13.375  -5.432  -4.731  1.00  0.25
O
ATOM  11186  CB  ALA B 140     -10.999  -3.724  -6.171  1.00  0.30
C
ATOM  11187  H   ALA B 140     -10.317  -2.400  -4.179  1.00  0.22
H
ATOM  11188  HA  ALA B 140     -12.819  -3.053  -5.303  1.00  0.24
H
ATOM  11189 1HB  ALA B 140     -11.496  -4.312  -6.937  1.00  0.36
H
ATOM  11190 2HB  ALA B 140     -10.748  -2.745  -6.578  1.00  0.36
H
ATOM  11191 3HB  ALA B 140     -10.091  -4.228  -5.850  1.00  0.36
H
ATOM  11192  N   SER B 141     -11.446  -5.505  -3.592  1.00  0.26
N
ATOM  11193  CA  SER B 141     -11.811  -6.772  -2.978  1.00  0.47
C
ATOM  11194  C   SER B 141     -12.928  -6.598  -1.962  1.00  0.42
C
ATOM  11195  O   SER B 141     -13.856  -7.407  -1.916  1.00  0.88
O
ATOM  11196  CB  SER B 141     -10.618  -7.402  -2.328  1.00  0.70
C
ATOM  11197  OG  SER B 141      -9.689  -7.779  -3.296  1.00  0.70
O
ATOM  11198  H   SER B 141     -10.543  -5.084  -3.386  1.00  0.31
H
ATOM  11199  HA  SER B 141     -12.166  -7.441  -3.763  1.00  0.56
H
ATOM  11200 1HB  SER B 141     -10.161  -6.692  -1.639  1.00  0.85
H
ATOM  11201 2HB  SER B 141     -10.925  -8.272  -1.751  1.00  0.85
H
ATOM  11202  HG  SER B 141      -8.907  -8.042  -2.809  1.00  0.85
H
ATOM  11203  N   TYR B 142     -12.865  -5.521  -1.173  1.00  0.40
N
```

ATOM 11204 CA TYR B 142 -13.898 -5.240 -0.181 1.00 0.40
C
ATOM 11205 C TYR B 142 -15.269 -5.112 -0.842 1.00 0.33
C
ATOM 11206 O TYR B 142 -16.262 -5.628 -0.327 1.00 0.42
O
ATOM 11207 CB TYR B 142 -13.579 -3.974 0.612 1.00 0.60
C
ATOM 11208 CG TYR B 142 -14.567 -3.734 1.733 1.00 0.60
C
ATOM 11209 CD1 TYR B 142 -14.407 -4.381 2.951 1.00 0.60
C
ATOM 11210 CD2 TYR B 142 -15.641 -2.888 1.542 1.00 0.60
C
ATOM 11211 CE1 TYR B 142 -15.321 -4.174 3.967 1.00 0.60
C
ATOM 11212 CE2 TYR B 142 -16.554 -2.675 2.555 1.00 0.60
C
ATOM 11213 CZ TYR B 142 -16.396 -3.315 3.765 1.00 0.60
C
ATOM 11214 OH TYR B 142 -17.302 -3.105 4.781 1.00 0.60
O
ATOM 11215 H TYR B 142 -12.078 -4.890 -1.243 1.00 0.48
H
ATOM 11216 HA TYR B 142 -13.945 -6.078 0.515 1.00 0.48
H
ATOM 11217 1HB TYR B 142 -12.579 -4.050 1.043 1.00 0.72
H
ATOM 11218 2HB TYR B 142 -13.591 -3.109 -0.050 1.00 0.72
H
ATOM 11219 HD1 TYR B 142 -13.564 -5.055 3.104 1.00 0.72
H
ATOM 11220 HD2 TYR B 142 -15.763 -2.394 0.586 1.00 0.72
H
ATOM 11221 HE1 TYR B 142 -15.195 -4.683 4.923 1.00 0.72
H
ATOM 11222 HE2 TYR B 142 -17.399 -2.004 2.397 1.00 0.72
H
ATOM 11223 HH TYR B 142 -17.792 -2.296 4.610 1.00 0.72
H
ATOM 11224 N TYR B 143 -15.321 -4.426 -1.985 1.00 0.28
N
ATOM 11225 CA TYR B 143 -16.576 -4.270 -2.710 1.00 0.26
C
ATOM 11226 C TYR B 143 -16.755 -5.248 -3.875 1.00 0.28
C
ATOM 11227 O TYR B 143 -17.584 -5.002 -4.756 1.00 0.35
O
ATOM 11228 CB TYR B 143 -16.712 -2.838 -3.217 1.00 0.39
C
ATOM 11229 CG TYR B 143 -16.940 -1.847 -2.106 1.00 0.39
C
ATOM 11230 CD1 TYR B 143 -15.962 -0.935 -1.745 1.00 0.39
C
ATOM 11231 CD2 TYR B 143 -18.153 -1.867 -1.434 1.00 0.39
C
ATOM 11232 CE1 TYR B 143 -16.206 -0.038 -0.717 1.00 0.39
C

ATOM 11233 CE2 TYR B 143   -18.395 -0.972 -0.413 1.00 0.39
C
ATOM 11234 CZ TYR B 143   -17.427 -0.060 -0.055 1.00 0.39
C
ATOM 11235 OH TYR B 143   -17.672   0.825   0.968 1.00 0.39
O
ATOM 11236 H   TYR B 143   -14.481 -3.995 -2.352 1.00 0.34
H
ATOM 11237 HA TYR B 143   -17.391 -4.456 -2.010 1.00 0.31
H
ATOM 11238 1HB TYR B 143   -15.806 -2.550 -3.752 1.00 0.47
H
ATOM 11239 2HB TYR B 143   -17.545 -2.770 -3.915 1.00 0.47
H
ATOM 11240 HD1 TYR B 143   -15.005 -0.923 -2.267 1.00 0.47
H
ATOM 11241 HD2 TYR B 143   -18.919 -2.589 -1.717 1.00 0.47
H
ATOM 11242 HE1 TYR B 143   -15.442   0.681 -0.425 1.00 0.47
H
ATOM 11243 HE2 TYR B 143   -19.350 -0.988   0.112 1.00 0.47
H
ATOM 11244 HH TYR B 143   -16.871   1.321   1.167 1.00 0.47
H
ATOM 11245 N   SER B 144   -16.020 -6.371 -3.852 1.00 0.35
N
ATOM 11246 CA SER B 144   -16.119 -7.439 -4.855 1.00 0.43
C
ATOM 11247 C   SER B 144   -16.294 -6.947 -6.290 1.00 0.33
C
ATOM 11248 O   SER B 144   -17.218 -7.386 -6.980 1.00 0.40
O
ATOM 11249 CB SER B 144   -17.284 -8.357 -4.524 1.00 0.65
C
ATOM 11250 OG SER B 144   -17.136 -8.972 -3.271 1.00 0.65
O
ATOM 11251 H   SER B 144   -15.360 -6.521 -3.102 1.00 0.42
H
ATOM 11252 HA SER B 144   -15.196 -8.020 -4.814 1.00 0.52
H
ATOM 11253 1HB SER B 144   -18.206 -7.776 -4.536 1.00 0.77
H
ATOM 11254 2HB SER B 144   -17.372 -9.120 -5.296 1.00 0.77
H
ATOM 11255 HG SER B 144   -17.969 -9.422 -3.104 1.00 0.77
H
ATOM 11256 N   LEU B 145   -15.421 -6.050 -6.750 1.00 0.24
N
ATOM 11257 CA LEU B 145   -15.532 -5.509 -8.106 1.00 0.21
C
ATOM 11258 C   LEU B 145   -15.143 -6.534 -9.183 1.00 0.30
C
ATOM 11259 O   LEU B 145   -14.072 -6.452 -9.784 1.00 0.37
O
ATOM 11260 CB LEU B 145   -14.685 -4.234 -8.235 1.00 0.32
C
ATOM 11261 CG LEU B 145   -15.085 -3.101 -7.272 1.00 0.32
C

ATOM 11262 CD1 LEU B 145 -14.177 -1.899 -7.484 1.00 0.32
C
ATOM 11263 CD2 LEU B 145 -16.545 -2.739 -7.497 1.00 0.32
C
ATOM 11264 H LEU B 145 -14.678 -5.729 -6.141 1.00 0.29
H
ATOM 11265 HA LEU B 145 -16.573 -5.233 -8.272 1.00 0.25
H
ATOM 11266 1HB LEU B 145 -13.643 -4.479 -8.045 1.00 0.38
H
ATOM 11267 2HB LEU B 145 -14.772 -3.854 -9.253 1.00 0.38
H
ATOM 11268 HG LEU B 145 -14.954 -3.440 -6.245 1.00 0.38
H
ATOM 11269 1HD1 LEU B 145 -14.456 -1.108 -6.790 1.00 0.38
H
ATOM 11270 2HD1 LEU B 145 -13.142 -2.184 -7.311 1.00 0.38
H
ATOM 11271 3HD1 LEU B 145 -14.285 -1.538 -8.502 1.00 0.38
H
ATOM 11272 1HD2 LEU B 145 -16.834 -1.944 -6.808 1.00 0.38
H
ATOM 11273 2HD2 LEU B 145 -16.682 -2.396 -8.523 1.00 0.38
H
ATOM 11274 3HD2 LEU B 145 -17.169 -3.615 -7.322 1.00 0.38
H
ATOM 11275 N ASP B 146 -16.040 -7.488 -9.429 1.00 0.42
N
ATOM 11276 CA ASP B 146 -15.826 -8.579 -10.378 1.00 0.84
C
ATOM 11277 C ASP B 146 -15.704 -8.155 -11.838 1.00 0.84
C
ATOM 11278 O ASP B 146 -15.238 -8.939 -12.664 1.00 3.54
O
ATOM 11279 CB ASP B 146 -16.962 -9.603 -10.247 1.00 1.26
C
ATOM 11280 CG ASP B 146 -18.346 -9.006 -10.483 1.00 1.26
C
ATOM 11281 OD1 ASP B 146 -18.682 -8.076 -9.789 1.00 1.26
O
ATOM 11282 OD2 ASP B 146 -19.057 -9.478 -11.337 1.00 1.26
O
ATOM 11283 H ASP B 146 -16.894 -7.470 -8.891 1.00 0.50
H
ATOM 11284 HA ASP B 146 -14.897 -9.078 -10.102 1.00 1.01
H
ATOM 11285 1HB ASP B 146 -16.803 -10.414 -10.957 1.00 1.51
H
ATOM 11286 2HB ASP B 146 -16.945 -10.036 -9.252 1.00 1.51
H
ATOM 11287 N ASN B 147 -16.085 -6.919 -12.169 1.00 0.45
N
ATOM 11288 CA ASN B 147 -15.896 -6.450 -13.534 1.00 0.67
C
ATOM 11289 C ASN B 147 -14.610 -5.631 -13.664 1.00 0.59
C
ATOM 11290 O ASN B 147 -14.397 -4.986 -14.690 1.00 1.45
O

```
ATOM  11291  CB  ASN B 147     -17.080  -5.632 -14.015  1.00  1.01
C
ATOM  11292  CG  ASN B 147     -17.238  -4.336 -13.279  1.00  1.01
C
ATOM  11293  OD1 ASN B 147     -16.903  -4.240 -12.093  1.00  1.01
O
ATOM  11294  ND2 ASN B 147     -17.729  -3.331 -13.962  1.00  1.01
N
ATOM  11295  H   ASN B 147     -16.492  -6.305 -11.477  1.00  0.54
H
ATOM  11296  HA  ASN B 147     -15.792  -7.318 -14.186  1.00  0.80
H
ATOM  11297 1HB  ASN B 147     -16.969  -5.416 -15.076  1.00  1.21
H
ATOM  11298 2HB  ASN B 147     -17.995  -6.213 -13.895  1.00  1.21
H
ATOM  11299 1HD2 ASN B 147     -17.853  -2.442 -13.520  1.00  1.21
H
ATOM  11300 2HD2 ASN B 147     -17.979  -3.444 -14.929  1.00  1.21
H
ATOM  11301  N   LEU B 148     -13.748  -5.675 -12.641  1.00  0.29
N
ATOM  11302  CA  LEU B 148     -12.490  -4.947 -12.666  1.00  0.27
C
ATOM  11303  C   LEU B 148     -11.320  -5.896 -12.892  1.00  0.30
C
ATOM  11304  O   LEU B 148     -11.149  -6.870 -12.153  1.00  0.41
O
ATOM  11305  CB  LEU B 148     -12.294  -4.187 -11.346  1.00  0.41
C
ATOM  11306  CG  LEU B 148     -10.993  -3.390 -11.196  1.00  0.41
C
ATOM  11307  CD1 LEU B 148     -10.970  -2.254 -12.207  1.00  0.41
C
ATOM  11308  CD2 LEU B 148     -10.908  -2.851  -9.780  1.00  0.41
C
ATOM  11309  H   LEU B 148     -13.962  -6.204 -11.807  1.00  0.35
H
ATOM  11310  HA  LEU B 148     -12.519  -4.230 -13.484  1.00  0.32
H
ATOM  11311 1HB  LEU B 148     -13.123  -3.496 -11.220  1.00  0.49
H
ATOM  11312 2HB  LEU B 148     -12.326  -4.907 -10.530  1.00  0.49
H
ATOM  11313  HG  LEU B 148     -10.139  -4.038 -11.393  1.00  0.49
H
ATOM  11314 1HD1 LEU B 148     -10.043  -1.691 -12.099  1.00  0.49
H
ATOM  11315 2HD1 LEU B 148     -11.031  -2.662 -13.213  1.00  0.49
H
ATOM  11316 3HD1 LEU B 148     -11.817  -1.592 -12.031  1.00  0.49
H
ATOM  11317 1HD2 LEU B 148      -9.984  -2.285  -9.660  1.00  0.49
H
ATOM  11318 2HD2 LEU B 148     -11.761  -2.201  -9.585  1.00  0.49
H
ATOM  11319 3HD2 LEU B 148     -10.918  -3.684  -9.080  1.00  0.49
H
```

```
ATOM  11320  N   VAL B 149     -10.529  -5.601 -13.921  1.00  0.29
N
ATOM  11321  CA  VAL B 149      -9.354  -6.387 -14.263  1.00  0.37
C
ATOM  11322  C   VAL B 149      -8.112  -5.513 -14.292  1.00  0.29
C
ATOM  11323  O   VAL B 149      -8.019  -4.590 -15.101  1.00  0.39
O
ATOM  11324  CB  VAL B 149      -9.515  -7.044 -15.645  1.00  0.55
C
ATOM  11325  CG1 VAL B 149      -8.263  -7.844 -15.983  1.00  0.55
C
ATOM  11326  CG2 VAL B 149     -10.757  -7.926 -15.678  1.00  0.55
C
ATOM  11327  H   VAL B 149     -10.753  -4.794 -14.486  1.00  0.35
H
ATOM  11328  HA  VAL B 149      -9.221  -7.165 -13.511  1.00  0.44
H
ATOM  11329  HB  VAL B 149      -9.615  -6.260 -16.389  1.00  0.67
H
ATOM  11330 1HG1 VAL B 149      -8.368  -8.285 -16.974  1.00  0.67
H
ATOM  11331 2HG1 VAL B 149      -7.395  -7.184 -15.970  1.00  0.67
H
ATOM  11332 3HG1 VAL B 149      -8.127  -8.635 -15.246  1.00  0.67
H
ATOM  11333 1HG2 VAL B 149     -10.861  -8.367 -16.668  1.00  0.67
H
ATOM  11334 2HG2 VAL B 149     -10.669  -8.717 -14.937  1.00  0.67
H
ATOM  11335 3HG2 VAL B 149     -11.638  -7.322 -15.458  1.00  0.67
H
ATOM  11336  N   ALA B 150      -7.156  -5.810 -13.429  1.00  0.26
N
ATOM  11337  CA  ALA B 150      -5.909  -5.069 -13.423  1.00  0.29
C
ATOM  11338  C   ALA B 150      -4.869  -5.883 -14.149  1.00  0.31
C
ATOM  11339  O   ALA B 150      -4.828  -7.105 -14.015  1.00  0.34
O
ATOM  11340  CB  ALA B 150      -5.448  -4.748 -12.014  1.00  0.43
C
ATOM  11341  H   ALA B 150      -7.284  -6.581 -12.790  1.00  0.31
H
ATOM  11342  HA  ALA B 150      -6.057  -4.137 -13.968  1.00  0.35
H
ATOM  11343 1HB  ALA B 150      -4.514  -4.187 -12.054  1.00  0.52
H
ATOM  11344 2HB  ALA B 150      -6.208  -4.149 -11.510  1.00  0.52
H
ATOM  11345 3HB  ALA B 150      -5.291  -5.673 -11.463  1.00  0.52
H
ATOM  11346  N   ILE B 151      -4.038  -5.208 -14.920  1.00  0.32
N
ATOM  11347  CA  ILE B 151      -2.995  -5.866 -15.668  1.00  0.28
C
ATOM  11348  C   ILE B 151      -1.671  -5.304 -15.197  1.00  0.29
C
```

```
ATOM  11349  O   ILE B 151      -1.487  -4.087 -15.129  1.00  0.28
O
ATOM  11350  CB  ILE B 151      -3.153  -5.658 -17.177  1.00  0.42
C
ATOM  11351  CG1 ILE B 151      -4.544  -6.084 -17.637  1.00  0.42
C
ATOM  11352  CG2 ILE B 151      -2.110  -6.504 -17.896  1.00  0.42
C
ATOM  11353  CD1 ILE B 151      -5.551  -4.956 -17.678  1.00  0.42
C
ATOM  11354  H   ILE B 151      -4.137  -4.209 -14.992  1.00  0.38
H
ATOM  11355  HA  ILE B 151      -3.016  -6.933 -15.455  1.00  0.34
H
ATOM  11356  HB  ILE B 151      -3.027  -4.606 -17.424  1.00  0.50
H
ATOM  11357 1HG1 ILE B 151      -4.464  -6.498 -18.635  1.00  0.50
H
ATOM  11358 2HG1 ILE B 151      -4.918  -6.860 -16.969  1.00  0.50
H
ATOM  11359 1HG2 ILE B 151      -2.213  -6.369 -18.971  1.00  0.50
H
ATOM  11360 2HG2 ILE B 151      -1.112  -6.197 -17.587  1.00  0.50
H
ATOM  11361 3HG2 ILE B 151      -2.260  -7.555 -17.648  1.00  0.50
H
ATOM  11362 1HD1 ILE B 151      -6.509  -5.339 -18.022  1.00  0.50
H
ATOM  11363 2HD1 ILE B 151      -5.666  -4.531 -16.681  1.00  0.50
H
ATOM  11364 3HD1 ILE B 151      -5.202  -4.185 -18.363  1.00  0.50
H
ATOM  11365  N   PHE B 152      -0.767  -6.193 -14.832  1.00  0.32
N
ATOM  11366  CA  PHE B 152       0.506  -5.788 -14.279  1.00  0.33
C
ATOM  11367  C   PHE B 152       1.632  -5.993 -15.270  1.00  0.34
C
ATOM  11368  O   PHE B 152       2.008  -7.120 -15.599  1.00  0.40
O
ATOM  11369  CB  PHE B 152       0.761  -6.578 -13.001  1.00  0.49
C
ATOM  11370  CG  PHE B 152      -0.268  -6.283 -11.948  1.00  0.49
C
ATOM  11371  CD1 PHE B 152      -1.478  -6.964 -11.946  1.00  0.49
C
ATOM  11372  CD2 PHE B 152      -0.041  -5.329 -10.969  1.00  0.49
C
ATOM  11373  CE1 PHE B 152      -2.438  -6.692 -10.993  1.00  0.49
C
ATOM  11374  CE2 PHE B 152      -1.002  -5.059 -10.011  1.00  0.49
C
ATOM  11375  CZ  PHE B 152      -2.201  -5.742 -10.025  1.00  0.49
C
ATOM  11376  H   PHE B 152      -0.975  -7.176 -14.930  1.00  0.38
H
ATOM  11377  HA  PHE B 152       0.457  -4.728 -14.030  1.00  0.40
H
```

```
ATOM  11378 1HB  PHE B 152       0.746  -7.640 -13.211  1.00  0.59
H
ATOM  11379 2HB  PHE B 152       1.744  -6.329 -12.603  1.00  0.59
H
ATOM  11380  HD1 PHE B 152      -1.668  -7.716 -12.713  1.00  0.59
H
ATOM  11381  HD2 PHE B 152       0.906  -4.788 -10.963  1.00  0.59
H
ATOM  11382  HE1 PHE B 152      -3.384  -7.227 -11.006  1.00  0.59
H
ATOM  11383  HE2 PHE B 152      -0.813  -4.306  -9.247  1.00  0.59
H
ATOM  11384  HZ  PHE B 152      -2.960  -5.528  -9.273  1.00  0.59
H
ATOM  11385  N   ASP B 153       2.170  -4.886 -15.762  1.00  0.32
N
ATOM  11386  CA  ASP B 153       3.251  -4.936 -16.722  1.00  0.34
C
ATOM  11387  C   ASP B 153       4.573  -5.240 -16.032  1.00  0.36
C
ATOM  11388  O   ASP B 153       5.342  -4.332 -15.704  1.00  0.44
O
ATOM  11389  CB  ASP B 153       3.316  -3.609 -17.502  1.00  0.51
C
ATOM  11390  CG  ASP B 153       4.395  -3.602 -18.558  1.00  0.51
C
ATOM  11391  OD1 ASP B 153       5.270  -4.411 -18.431  1.00  0.51
O
ATOM  11392  OD2 ASP B 153       4.351  -2.820 -19.479  1.00  0.51
O
ATOM  11393  H   ASP B 153       1.832  -3.983 -15.463  1.00  0.38
H
ATOM  11394  HA  ASP B 153       3.048  -5.740 -17.430  1.00  0.41
H
ATOM  11395 1HB  ASP B 153       2.356  -3.421 -17.981  1.00  0.61
H
ATOM  11396 2HB  ASP B 153       3.501  -2.787 -16.813  1.00  0.61
H
ATOM  11397  N   VAL B 154       4.847  -6.539 -15.838  1.00  0.42
N
ATOM  11398  CA  VAL B 154       6.080  -6.976 -15.183  1.00  0.48
C
ATOM  11399  C   VAL B 154       7.240  -6.902 -16.151  1.00  0.57
C
ATOM  11400  O   VAL B 154       7.706  -7.931 -16.641  1.00  0.71
O
ATOM  11401  CB  VAL B 154       5.977  -8.441 -14.687  1.00  0.72
C
ATOM  11402  CG1 VAL B 154       7.253  -8.827 -13.953  1.00  0.72
C
ATOM  11403  CG2 VAL B 154       4.734  -8.655 -13.832  1.00  0.72
C
ATOM  11404  H   VAL B 154       4.169  -7.233 -16.140  1.00  0.50
H
ATOM  11405  HA  VAL B 154       6.284  -6.315 -14.340  1.00  0.58
H
ATOM  11406  HB  VAL B 154       5.915  -9.084 -15.552  1.00  0.86
H
```

```
ATOM  11407 1HG1 VAL B 154       7.195  -9.870 -13.645  1.00  0.86
H
ATOM  11408 2HG1 VAL B 154       8.109  -8.691 -14.613  1.00  0.86
H
ATOM  11409 3HG1 VAL B 154       7.368  -8.194 -13.073  1.00  0.86
H
ATOM  11410 1HG2 VAL B 154       4.686  -9.701 -13.532  1.00  0.86
H
ATOM  11411 2HG2 VAL B 154       4.767  -8.033 -12.946  1.00  0.86
H
ATOM  11412 3HG2 VAL B 154       3.847  -8.404 -14.413  1.00  0.86
H
ATOM  11413  N   ASN B 155       7.728  -5.692 -16.398  1.00  0.60
N
ATOM  11414  CA  ASN B 155       8.817  -5.463 -17.338  1.00  0.85
C
ATOM  11415  C   ASN B 155      10.195  -5.345 -16.703  1.00  1.13
C
ATOM  11416  O   ASN B 155      11.129  -4.856 -17.343  1.00  3.11
O
ATOM  11417  CB  ASN B 155       8.498  -4.254 -18.198  1.00  1.27
C
ATOM  11418  CG  ASN B 155       8.378  -2.975 -17.437  1.00  1.27
C
ATOM  11419  OD1 ASN B 155       8.796  -2.876 -16.277  1.00  1.27
O
ATOM  11420  ND2 ASN B 155       7.790  -1.988 -18.069  1.00  1.27
N
ATOM  11421  H   ASN B 155       7.280  -4.905 -15.944  1.00  0.72
H
ATOM  11422  HA  ASN B 155       8.862  -6.326 -18.002  1.00  1.02
H
ATOM  11423 1HB  ASN B 155       9.281  -4.128 -18.937  1.00  1.53
H
ATOM  11424 2HB  ASN B 155       7.576  -4.425 -18.742  1.00  1.53
H
ATOM  11425 1HD2 ASN B 155       7.667  -1.106 -17.615  1.00  1.53
H
ATOM  11426 2HD2 ASN B 155       7.449  -2.120 -19.007  1.00  1.53
H
ATOM  11427  N   ARG B 156      10.330  -5.826 -15.457  1.00  0.68
N
ATOM  11428  CA  ARG B 156      11.597  -5.871 -14.705  1.00  0.75
C
ATOM  11429  C   ARG B 156      12.172  -4.555 -14.187  1.00  1.00
C
ATOM  11430  O   ARG B 156      12.599  -4.497 -13.034  1.00  2.97
O
ATOM  11431  CB  ARG B 156      12.707  -6.485 -15.533  1.00  1.12
C
ATOM  11432  CG  ARG B 156      13.988  -6.674 -14.754  1.00  1.12
C
ATOM  11433  CD  ARG B 156      15.079  -7.229 -15.582  1.00  1.12
C
ATOM  11434  NE  ARG B 156      16.266  -7.454 -14.781  1.00  1.12
N
ATOM  11435  CZ  ARG B 156      17.440  -7.911 -15.237  1.00  1.12
C
```

```
ATOM  11436  NH1 ARG B 156      17.605  -8.178 -16.513  1.00  1.12
N
ATOM  11437  NH2 ARG B 156      18.417  -8.085 -14.364  1.00  1.12
N
ATOM  11438  H   ARG B 156       9.513  -6.196 -14.998  1.00  0.82
H
ATOM  11439  HA  ARG B 156      11.430  -6.517 -13.842  1.00  0.90
H
ATOM  11440 1HB  ARG B 156      12.390  -7.446 -15.920  1.00  1.35
H
ATOM  11441 2HB  ARG B 156      12.954  -5.852 -16.382  1.00  1.35
H
ATOM  11442 1HG  ARG B 156      14.316  -5.718 -14.353  1.00  1.35
H
ATOM  11443 2HG  ARG B 156      13.809  -7.368 -13.933  1.00  1.35
H
ATOM  11444 1HD  ARG B 156      14.774  -8.179 -16.016  1.00  1.35
H
ATOM  11445 2HD  ARG B 156      15.330  -6.528 -16.377  1.00  1.35
H
ATOM  11446  HE  ARG B 156      16.210  -7.265 -13.786  1.00  1.35
H
ATOM  11447 1HH1 ARG B 156      16.843  -8.028 -17.169  1.00  1.35
H
ATOM  11448 2HH1 ARG B 156      18.491  -8.523 -16.852  1.00  1.35
H
ATOM  11449 1HH2 ARG B 156      18.235  -7.870 -13.385  1.00  1.35
H
ATOM  11450 2HH2 ARG B 156      19.317  -8.429 -14.660  1.00  1.35
H
ATOM  11451  N   LEU B 157      12.277  -3.552 -15.057  1.00  1.24
N
ATOM  11452  CA  LEU B 157      12.944  -2.299 -14.735  1.00  1.20
C
ATOM  11453  C   LEU B 157      12.021  -1.094 -14.495  1.00  1.83
C
ATOM  11454  O   LEU B 157      10.922  -1.007 -15.043  1.00  4.59
O
ATOM  11455  CB  LEU B 157      13.883  -1.969 -15.894  1.00  1.80
C
ATOM  11456  CG  LEU B 157      14.895  -3.061 -16.264  1.00  1.80
C
ATOM  11457  CD1 LEU B 157      15.679  -2.625 -17.486  1.00  1.80
C
ATOM  11458  CD2 LEU B 157      15.816  -3.324 -15.091  1.00  1.80
C
ATOM  11459  H   LEU B 157      11.910  -3.684 -15.987  1.00  1.49
H
ATOM  11460  HA  LEU B 157      13.528  -2.457 -13.835  1.00  1.44
H
ATOM  11461 1HB  LEU B 157      13.278  -1.777 -16.774  1.00  2.16
H
ATOM  11462 2HB  LEU B 157      14.438  -1.063 -15.651  1.00  2.16
H
ATOM  11463  HG  LEU B 157      14.367  -3.980 -16.519  1.00  2.16
H
ATOM  11464 1HD1 LEU B 157      16.391  -3.404 -17.758  1.00  2.16
H
```

```
ATOM  11465 2HD1 LEU B 157      14.994  -2.458 -18.316  1.00  2.16
H
ATOM  11466 3HD1 LEU B 157      16.214  -1.704 -17.265  1.00  2.16
H
ATOM  11467 1HD2 LEU B 157      16.533  -4.101 -15.357  1.00  2.16
H
ATOM  11468 2HD2 LEU B 157      16.349  -2.411 -14.829  1.00  2.16
H
ATOM  11469 3HD2 LEU B 157      15.223  -3.655 -14.241  1.00  2.16
H
ATOM  11470  N   GLY B 158      12.506  -0.162 -13.672  1.00  0.90
N
ATOM  11471  CA  GLY B 158      11.823   1.096 -13.364  1.00  1.13
C
ATOM  11472  C   GLY B 158      12.525   2.252 -14.080  1.00  1.21
C
ATOM  11473  O   GLY B 158      12.962   2.097 -15.221  1.00  2.84
O
ATOM  11474  H   GLY B 158      13.406  -0.315 -13.243  1.00  1.08
H
ATOM  11475 1HA  GLY B 158      10.782   1.040 -13.678  1.00  1.36
H
ATOM  11476 2HA  GLY B 158      11.833   1.267 -12.289  1.00  1.36
H
ATOM  11477  N   HIS B 159      12.630   3.411 -13.416  1.00  1.13
N
ATOM  11478  CA  HIS B 159      13.293   4.568 -14.026  1.00  1.52
C
ATOM  11479  C   HIS B 159      14.798   4.568 -13.872  1.00  1.76
C
ATOM  11480  O   HIS B 159      15.527   4.553 -14.866  1.00  2.22
O
ATOM  11481  CB  HIS B 159      12.726   5.882 -13.463  1.00  2.28
C
ATOM  11482  CG  HIS B 159      13.333   7.161 -14.014  1.00  2.28
C
ATOM  11483  ND1 HIS B 159      13.105   7.612 -15.300  1.00  2.28
N
ATOM  11484  CD2 HIS B 159      14.129   8.090 -13.432  1.00  2.28
C
ATOM  11485  CE1 HIS B 159      13.730   8.763 -15.478  1.00  2.28
C
ATOM  11486  NE2 HIS B 159      14.349   9.082 -14.359  1.00  2.28
N
ATOM  11487  H   HIS B 159      12.252   3.502 -12.476  1.00  1.36
H
ATOM  11488  HA  HIS B 159      13.110   4.558 -15.078  1.00  1.82
H
ATOM  11489 1HB  HIS B 159      11.654   5.915 -13.657  1.00  2.74
H
ATOM  11490 2HB  HIS B 159      12.855   5.889 -12.388  1.00  2.74
H
ATOM  11491  HD2 HIS B 159      14.506   8.062 -12.414  1.00  2.74
H
ATOM  11492  HE1 HIS B 159      13.722   9.358 -16.392  1.00  2.74
H
ATOM  11493  HE2 HIS B 159      14.887   9.928 -14.216  1.00  2.74
H
```

ATOM 11494 N SER B 160 15.269 4.622 -12.634 1.00 1.78
N
ATOM 11495 CA SER B 160 16.696 4.709 -12.375 1.00 1.98
C
ATOM 11496 C SER B 160 17.371 3.352 -12.242 1.00 1.83
C
ATOM 11497 O SER B 160 18.596 3.266 -12.237 1.00 7.20
O
ATOM 11498 CB SER B 160 16.944 5.520 -11.123 1.00 2.97
C
ATOM 11499 OG SER B 160 16.519 4.849 -9.969 1.00 2.97
O
ATOM 11500 H SER B 160 14.630 4.606 -11.852 1.00 2.14
H
ATOM 11501 HA SER B 160 17.157 5.230 -13.214 1.00 2.38
H
ATOM 11502 1HB SER B 160 18.007 5.731 -11.042 1.00 3.56
H
ATOM 11503 2HB SER B 160 16.426 6.475 -11.204 1.00 3.56
H
ATOM 11504 HG SER B 160 15.566 4.733 -10.062 1.00 3.56
H
ATOM 11505 N GLY B 161 16.590 2.288 -12.139 1.00 2.27
N
ATOM 11506 CA GLY B 161 17.174 0.963 -11.992 1.00 1.95
C
ATOM 11507 C GLY B 161 16.109 -0.108 -12.028 1.00 1.66
C
ATOM 11508 O GLY B 161 14.981 0.153 -12.436 1.00 2.87
O
ATOM 11509 H GLY B 161 15.586 2.390 -12.151 1.00 2.72
H
ATOM 11510 1HA GLY B 161 17.896 0.793 -12.791 1.00 2.34
H
ATOM 11511 2HA GLY B 161 17.721 0.908 -11.051 1.00 2.34
H
ATOM 11512 N ALA B 162 16.490 -1.310 -11.620 1.00 1.68
N
ATOM 11513 CA ALA B 162 15.597 -2.457 -11.590 1.00 1.61
C
ATOM 11514 C ALA B 162 14.566 -2.345 -10.483 1.00 1.43
C
ATOM 11515 O ALA B 162 14.796 -1.698 -9.462 1.00 1.96
O
ATOM 11516 CB ALA B 162 16.400 -3.732 -11.422 1.00 2.42
C
ATOM 11517 H ALA B 162 17.446 -1.435 -11.315 1.00 2.02
H
ATOM 11518 HA ALA B 162 15.066 -2.490 -12.531 1.00 1.93
H
ATOM 11519 1HB ALA B 162 15.730 -4.589 -11.433 1.00 2.90
H
ATOM 11520 2HB ALA B 162 17.116 -3.821 -12.240 1.00 2.90
H
ATOM 11521 3HB ALA B 162 16.935 -3.703 -10.475 1.00 2.90
H
ATOM 11522 N LEU B 163 13.435 -2.995 -10.688 1.00 1.24
N

ATOM 11523 CA LEU B 163   12.366 -2.987 -9.715 1.00 1.40
C
ATOM 11524 C   LEU B 163   12.686 -3.883 -8.532 1.00 0.80
C
ATOM 11525 O   LEU B 163   13.393 -4.877 -8.691 1.00 1.59
O
ATOM 11526 CB LEU B 163   11.101 -3.422 -10.407 1.00 2.10
C
ATOM 11527 CG LEU B 163   10.738 -2.450 -11.497 1.00 2.10
C
ATOM 11528 CD1 LEU B 163   9.509 -2.914 -12.225 1.00 2.10
C
ATOM 11529 CD2 LEU B 163   10.564 -1.102 -10.852 1.00 2.10
C
ATOM 11530 H   LEU B 163   13.298 -3.500 -11.553 1.00 1.49
H
ATOM 11531 HA LEU B 163   12.246 -1.965 -9.374 1.00 1.68
H
ATOM 11532 1HB LEU B 163   11.249 -4.404 -10.852 1.00 2.52
H
ATOM 11533 2HB LEU B 163   10.276 -3.471 -9.700 1.00 2.52
H
ATOM 11534 HG LEU B 163   11.546 -2.397 -12.217 1.00 2.52
H
ATOM 11535 1HD1 LEU B 163   9.258 -2.204 -13.012 1.00 2.52
H
ATOM 11536 2HD1 LEU B 163   9.689 -3.895 -12.664 1.00 2.52
H
ATOM 11537 3HD1 LEU B 163   8.693 -2.978 -11.514 1.00 2.52
H
ATOM 11538 1HD2 LEU B 163   10.304 -0.361 -11.607 1.00 2.52
H
ATOM 11539 2HD2 LEU B 163   9.773 -1.176 -10.114 1.00 2.52
H
ATOM 11540 3HD2 LEU B 163   11.490 -0.810 -10.358 1.00 2.52
H
ATOM 11541 N   PRO B 164   12.159 -3.561 -7.338 1.00 0.79
N
ATOM 11542 CA PRO B 164   12.372 -4.222 -6.051 1.00 1.45
C
ATOM 11543 C   PRO B 164   12.265 -5.746 -6.117 1.00 1.34
C
ATOM 11544 O   PRO B 164   12.992 -6.446 -5.415 1.00 2.68
O
ATOM 11545 CB PRO B 164   11.237 -3.667 -5.186 1.00 2.17
C
ATOM 11546 CG PRO B 164   10.985 -2.320 -5.738 1.00 2.17
C
ATOM 11547 CD PRO B 164   11.130 -2.501 -7.222 1.00 2.17
C
ATOM 11548 HA PRO B 164   13.349 -3.924 -5.655 1.00 1.74
H
ATOM 11549 1HB PRO B 164   10.357 -4.324 -5.242 1.00 2.61
H
ATOM 11550 2HB PRO B 164   11.552 -3.639 -4.131 1.00 2.61
H
ATOM 11551 1HG PRO B 164   9.982 -1.969 -5.451 1.00 2.61
H

ATOM 11552 2HG PRO B 164   11.703 -1.607 -5.321 1.00 2.61
H
ATOM 11553 1HD PRO B 164   10.180 -2.834 -7.662 1.00 2.61
H
ATOM 11554 2HD PRO B 164   11.479 -1.557 -7.657 1.00 2.61
H
ATOM 11555 N   ALA B 165   11.349 -6.256 -6.938 1.00 0.85
N
ATOM 11556 CA ALA B 165   11.181 -7.699 -7.073 1.00 0.97
C
ATOM 11557 C   ALA B 165   11.290 -8.176 -8.522 1.00 1.11
C
ATOM 11558 O   ALA B 165   10.709 -9.206 -8.841 1.00 3.82
O
ATOM 11559 CB ALA B 165   9.832 -8.112 -6.492 1.00 1.46
C
ATOM 11560 H   ALA B 165   10.765 -5.637 -7.479 1.00 1.02
H
ATOM 11561 HA ALA B 165   11.973 -8.189 -6.508 1.00 1.16
H
ATOM 11562 1HB ALA B 165   9.717 -9.192 -6.564 1.00 1.75
H
ATOM 11563 2HB ALA B 165   9.788 -7.814 -5.444 1.00 1.75
H
ATOM 11564 3HB ALA B 165   9.029 -7.624 -7.039 1.00 1.75
H
ATOM 11565 N   GLU B 166   12.036 -7.450 -9.368 1.00 0.78
N
ATOM 11566 CA GLU B 166   12.213 -7.728 -10.815 1.00 1.34
C
ATOM 11567 C   GLU B 166   11.205 -8.716 -11.432 1.00 0.64
C
ATOM 11568 O   GLU B 166   10.057 -8.340 -11.694 1.00 0.72
O
ATOM 11569 CB GLU B 166   13.629 -8.235 -11.088 1.00 2.01
C
ATOM 11570 CG GLU B 166   14.725 -7.203 -10.931 1.00 2.01
C
ATOM 11571 CD GLU B 166   16.089 -7.782 -11.198 1.00 2.01
C
ATOM 11572 OE1 GLU B 166   16.416 -8.781 -10.607 1.00 2.01
O
ATOM 11573 OE2 GLU B 166   16.786 -7.246 -12.030 1.00 2.01
O
ATOM 11574 H   GLU B 166   12.508 -6.630 -9.005 1.00 0.94
H
ATOM 11575 HA GLU B 166   12.094 -6.784 -11.344 1.00 1.61
H
ATOM 11576 1HB GLU B 166   13.865 -9.064 -10.421 1.00 2.41
H
ATOM 11577 2HB GLU B 166   13.699 -8.597 -12.113 1.00 2.41
H
ATOM 11578 1HG GLU B 166   14.536 -6.396 -11.636 1.00 2.41
H
ATOM 11579 2HG GLU B 166   14.689 -6.798 -9.921 1.00 2.41
H
ATOM 11580 N   HIS B 167   11.642 -9.953 -11.703 1.00 0.74
N

ATOM 11581 CA HIS B 167   10.794 -10.989 -12.302 1.00 0.75
C
ATOM 11582 C   HIS B 167   10.255 -12.008 -11.312 1.00 0.84
C
ATOM 11583 O   HIS B 167   9.929 -13.130 -11.700 1.00 1.11
O
ATOM 11584 CB HIS B 167   11.530 -11.765 -13.399 1.00 1.12
C
ATOM 11585 CG HIS B 167   11.845 -10.993 -14.635 1.00 1.12
C
ATOM 11586 ND1 HIS B 167   10.869 -10.595 -15.520 1.00 1.12
N
ATOM 11587 CD2 HIS B 167   13.023 -10.582 -15.160 1.00 1.12
C
ATOM 11588 CE1 HIS B 167   11.430 -9.969 -16.537 1.00 1.12
C
ATOM 11589 NE2 HIS B 167   12.736 -9.952 -16.342 1.00 1.12
N
ATOM 11590 H   HIS B 167   12.591 -10.207 -11.471 1.00 0.89
H
ATOM 11591 HA HIS B 167   9.932 -10.508 -12.765 1.00 0.90
H
ATOM 11592 1HB HIS B 167   12.466 -12.156 -12.999 1.00 1.35
H
ATOM 11593 2HB HIS B 167   10.926 -12.622 -13.695 1.00 1.35
H
ATOM 11594 HD1 HIS B 167   9.888 -10.553 -15.323 1.00 1.35
H
ATOM 11595 HD2 HIS B 167   14.058 -10.677 -14.831 1.00 1.35
H
ATOM 11596 HE1 HIS B 167   10.824 -9.576 -17.349 1.00 1.35
H
ATOM 11597 N   CYS B 168   10.163 -11.647 -10.038 1.00 0.92
N
ATOM 11598 CA CYS B 168   9.671 -12.565 -9.012 1.00 1.06
C
ATOM 11599 C   CYS B 168   8.157 -12.670 -9.042 1.00 0.83
C
ATOM 11600 O   CYS B 168   7.469 -12.202 -8.132 1.00 1.03
O
ATOM 11601 CB CYS B 168   10.113 -12.119 -7.632 1.00 1.59
C
ATOM 11602 SG CYS B 168   11.906 -12.127 -7.387 1.00 1.59
S
ATOM 11603 H   CYS B 168   10.437 -10.719 -9.761 1.00 1.10
H
ATOM 11604 HA CYS B 168   10.089 -13.554 -9.205 1.00 1.27
H
ATOM 11605 1HB CYS B 168   9.751 -11.109 -7.446 1.00 1.91
H
ATOM 11606 2HB CYS B 168   9.668 -12.771 -6.884 1.00 1.91
H
ATOM 11607 HG CYS B 168   12.180 -11.249 -8.346 1.00 1.91
H
ATOM 11608 N   ILE B 169   7.670 -13.317 -10.095 1.00 0.75
N
ATOM 11609 CA ILE B 169   6.268 -13.528 -10.382 1.00 0.95
C

ATOM 11610 C   ILE B 169   5.495 -14.099 -9.220 1.00 0.91
C
ATOM 11611 O   ILE B 169   4.362 -13.692 -8.978 1.00 1.70
O
ATOM 11612 CB ILE B 169   6.119 -14.455 -11.575 1.00 1.42
C
ATOM 11613 CG1 ILE B 169   6.662 -13.791 -12.850 1.00 1.42
C
ATOM 11614 CG2 ILE B 169   4.677 -14.838 -11.723 1.00 1.42
C
ATOM 11615 CD1 ILE B 169   5.935 -12.526 -13.243 1.00 1.42
C
ATOM 11616 H   ILE B 169   8.343 -13.670 -10.761 1.00 0.90
H
ATOM 11617 HA ILE B 169   5.824 -12.576 -10.644 1.00 1.14
H
ATOM 11618 HB ILE B 169   6.709 -15.356 -11.406 1.00 1.71
H
ATOM 11619 1HG1 ILE B 169   7.712 -13.551 -12.703 1.00 1.71
H
ATOM 11620 2HG1 ILE B 169   6.586 -14.499 -13.677 1.00 1.71
H
ATOM 11621 1HG2 ILE B 169   4.599 -15.519 -12.560 1.00 1.71
H
ATOM 11622 2HG2 ILE B 169   4.332 -15.332 -10.815 1.00 1.71
H
ATOM 11623 3HG2 ILE B 169   4.073 -13.949 -11.907 1.00 1.71
H
ATOM 11624 1HD1 ILE B 169   6.377 -12.120 -14.153 1.00 1.71
H
ATOM 11625 2HD1 ILE B 169   4.884 -12.755 -13.421 1.00 1.71
H
ATOM 11626 3HD1 ILE B 169   6.018 -11.796 -12.441 1.00 1.71
H
ATOM 11627 N   ASN B 170   6.104 -15.042 -8.511 1.00 0.75
N
ATOM 11628 CA ASN B 170   5.467 -15.680 -7.376 1.00 0.95
C
ATOM 11629 C   ASN B 170   5.187 -14.693 -6.251 1.00 0.93
C
ATOM 11630 O   ASN B 170   4.202 -14.848 -5.536 1.00 0.98
O
ATOM 11631 CB ASN B 170   6.318 -16.826 -6.876 1.00 1.42
C
ATOM 11632 CG ASN B 170   6.282 -18.006 -7.805 1.00 1.42
C
ATOM 11633 OD1 ASN B 170   5.354 -18.156 -8.607 1.00 1.42
O
ATOM 11634 ND2 ASN B 170   7.280 -18.849 -7.713 1.00 1.42
N
ATOM 11635 H   ASN B 170   7.037 -15.329 -8.772 1.00 0.90
H
ATOM 11636 HA ASN B 170   4.506 -16.080 -7.701 1.00 1.14
H
ATOM 11637 1HB ASN B 170   7.352 -16.495 -6.765 1.00 1.71
H
ATOM 11638 2HB ASN B 170   5.967 -17.141 -5.894 1.00 1.71
H

ATOM 11639 1HD2 ASN B 170 7.311 -19.653 -8.307 1.00 1.71
H
ATOM 11640 2HD2 ASN B 170 8.010 -18.688 -7.051 1.00 1.71
H
ATOM 11641 N ILE B 171 6.050 -13.688 -6.082 1.00 0.91
N
ATOM 11642 CA ILE B 171 5.833 -12.682 -5.055 1.00 0.90
C
ATOM 11643 C ILE B 171 4.708 -11.767 -5.466 1.00 0.70
C
ATOM 11644 O ILE B 171 3.843 -11.438 -4.654 1.00 0.71
O
ATOM 11645 CB ILE B 171 7.096 -11.854 -4.757 1.00 1.35
C
ATOM 11646 CG1 ILE B 171 8.158 -12.738 -4.101 1.00 1.35
C
ATOM 11647 CG2 ILE B 171 6.748 -10.668 -3.871 1.00 1.35
C
ATOM 11648 CD1 ILE B 171 9.511 -12.073 -3.968 1.00 1.35
C
ATOM 11649 H ILE B 171 6.842 -13.580 -6.700 1.00 1.09
H
ATOM 11650 HA ILE B 171 5.540 -13.185 -4.135 1.00 1.08
H
ATOM 11651 HB ILE B 171 7.515 -11.486 -5.693 1.00 1.62
H
ATOM 11652 1HG1 ILE B 171 7.818 -13.024 -3.106 1.00 1.62
H
ATOM 11653 2HG1 ILE B 171 8.280 -13.643 -4.694 1.00 1.62
H
ATOM 11654 1HG2 ILE B 171 7.648 -10.084 -3.678 1.00 1.62
H
ATOM 11655 2HG2 ILE B 171 6.011 -10.043 -4.372 1.00 1.62
H
ATOM 11656 3HG2 ILE B 171 6.339 -11.026 -2.925 1.00 1.62
H
ATOM 11657 1HD1 ILE B 171 10.210 -12.763 -3.495 1.00 1.62
H
ATOM 11658 2HD1 ILE B 171 9.886 -11.794 -4.948 1.00 1.62
H
ATOM 11659 3HD1 ILE B 171 9.415 -11.179 -3.353 1.00 1.62
H
ATOM 11660 N TYR B 172 4.720 -11.368 -6.740 1.00 0.59
N
ATOM 11661 CA TYR B 172 3.677 -10.496 -7.259 1.00 0.48
C
ATOM 11662 C TYR B 172 2.331 -11.195 -7.122 1.00 0.45
C
ATOM 11663 O TYR B 172 1.333 -10.575 -6.742 1.00 0.40
O
ATOM 11664 CB TYR B 172 3.936 -10.165 -8.737 1.00 0.72
C
ATOM 11665 CG TYR B 172 5.170 -9.324 -9.001 1.00 0.72
C
ATOM 11666 CD1 TYR B 172 6.067 -9.724 -9.980 1.00 0.72
C
ATOM 11667 CD2 TYR B 172 5.416 -8.168 -8.277 1.00 0.72
C

ATOM 11668 CE1 TYR B 172   7.197 -8.983 -10.240 1.00 0.72
C
ATOM 11669 CE2 TYR B 172   6.549 -7.419 -8.543 1.00 0.72
C
ATOM 11670 CZ TYR B 172   7.438 -7.824 -9.522 1.00 0.72
C
ATOM 11671 OH TYR B 172   8.562 -7.075 -9.794 1.00 0.72
O
ATOM 11672 H   TYR B 172   5.476 -11.671 -7.347 1.00 0.71
H
ATOM 11673 HA TYR B 172   3.651 -9.578 -6.672 1.00 0.58
H
ATOM 11674 1HB TYR B 172   4.037 -11.094 -9.301 1.00 0.86
H
ATOM 11675 2HB TYR B 172   3.076 -9.634 -9.144 1.00 0.86
H
ATOM 11676 HD1 TYR B 172   5.876 -10.624 -10.551 1.00 0.86
H
ATOM 11677 HD2 TYR B 172   4.716 -7.844 -7.505 1.00 0.86
H
ATOM 11678 HE1 TYR B 172   7.891 -9.306 -11.014 1.00 0.86
H
ATOM 11679 HE2 TYR B 172   6.741 -6.506 -7.980 1.00 0.86
H
ATOM 11680 HH TYR B 172   9.053 -7.483 -10.518 1.00 0.86
H
ATOM 11681 N   GLN B 173   2.338 -12.503 -7.394 1.00 0.53
N
ATOM 11682 CA GLN B 173   1.175 -13.350 -7.269 1.00 0.55
C
ATOM 11683 C   GLN B 173   0.647 -13.402 -5.855 1.00 0.53
C
ATOM 11684 O   GLN B 173   -0.535 -13.142 -5.625 1.00 0.47
O
ATOM 11685 CB GLN B 173   1.498 -14.769 -7.737 1.00 0.83
C
ATOM 11686 CG GLN B 173   0.335 -15.734 -7.617 1.00 0.83
C
ATOM 11687 CD GLN B 173   0.693 -17.147 -8.023 1.00 0.83
C
ATOM 11688 OE1 GLN B 173   1.668 -17.383 -8.743 1.00 0.83
O
ATOM 11689 NE2 GLN B 173   -0.101 -18.104 -7.556 1.00 0.83
N
ATOM 11690 H   GLN B 173   3.190 -12.931 -7.723 1.00 0.64
H
ATOM 11691 HA GLN B 173   0.404 -12.932 -7.895 1.00 0.66
H
ATOM 11692 1HB GLN B 173   1.810 -14.745 -8.781 1.00 0.99
H
ATOM 11693 2HB GLN B 173   2.326 -15.170 -7.158 1.00 0.99
H
ATOM 11694 1HG GLN B 173   0.008 -15.754 -6.578 1.00 0.99
H
ATOM 11695 2HG GLN B 173   -0.478 -15.397 -8.255 1.00 0.99
H
ATOM 11696 1HE2 GLN B 173   0.077 -19.062 -7.782 1.00 0.99
H

```
ATOM  11697 2HE2 GLN B 173      -0.883 -17.864  -6.971  1.00  0.99
H
ATOM  11698  N   ARG B 174       1.533 -13.724  -4.911  1.00  0.65
N
ATOM  11699  CA  ARG B 174       1.166 -13.817  -3.508  1.00  0.75
C
ATOM  11700  C   ARG B 174       0.547 -12.544  -2.990  1.00  0.64
C
ATOM  11701  O   ARG B 174      -0.435 -12.605  -2.253  1.00  0.71
O
ATOM  11702  CB  ARG B 174       2.381 -14.150  -2.654  1.00  1.12
C
ATOM  11703  CG  ARG B 174       2.888 -15.577  -2.766  1.00  1.12
C
ATOM  11704  CD  ARG B 174       4.155 -15.763  -2.011  1.00  1.12
C
ATOM  11705  NE  ARG B 174       4.657 -17.124  -2.117  1.00  1.12
N
ATOM  11706  CZ  ARG B 174       5.849 -17.539  -1.644  1.00  1.12
C
ATOM  11707  NH1 ARG B 174       6.647 -16.692  -1.034  1.00  1.12
N
ATOM  11708  NH2 ARG B 174       6.215 -18.800  -1.792  1.00  1.12
N
ATOM  11709  H   ARG B 174       2.487 -13.936  -5.172  1.00  0.78
H
ATOM  11710  HA  ARG B 174       0.439 -14.622  -3.399  1.00  0.90
H
ATOM  11711 1HB  ARG B 174       3.204 -13.490  -2.924  1.00  1.35
H
ATOM  11712 2HB  ARG B 174       2.146 -13.967  -1.604  1.00  1.35
H
ATOM  11713 1HG  ARG B 174       2.146 -16.248  -2.335  1.00  1.35
H
ATOM  11714 2HG  ARG B 174       3.048 -15.847  -3.805  1.00  1.35
H
ATOM  11715 1HD  ARG B 174       4.913 -15.088  -2.405  1.00  1.35
H
ATOM  11716 2HD  ARG B 174       3.986 -15.543  -0.957  1.00  1.35
H
ATOM  11717  HE  ARG B 174       4.072 -17.806  -2.580  1.00  1.35
H
ATOM  11718 1HH1 ARG B 174       6.368 -15.728  -0.917  1.00  1.35
H
ATOM  11719 2HH1 ARG B 174       7.538 -17.006  -0.678  1.00  1.35
H
ATOM  11720 1HH2 ARG B 174       5.601 -19.453  -2.260  1.00  1.35
H
ATOM  11721 2HH2 ARG B 174       7.106 -19.112  -1.437  1.00  1.35
H
ATOM  11722  N   ARG B 175       1.107 -11.394  -3.367  1.00  0.56
N
ATOM  11723  CA  ARG B 175       0.580 -10.123  -2.892  1.00  0.56
C
ATOM  11724  C   ARG B 175      -0.801  -9.838  -3.442  1.00  0.54
C
ATOM  11725  O   ARG B 175      -1.685  -9.422  -2.698  1.00  0.68
O
```

```
ATOM  11726  CB  ARG B 175       1.524  -8.990  -3.252  1.00  0.84
C
ATOM  11727  CG  ARG B 175       2.834  -8.992  -2.491  1.00  0.84
C
ATOM  11728  CD  ARG B 175       3.742  -7.903  -2.913  1.00  0.84
C
ATOM  11729  NE  ARG B 175       5.015  -7.981  -2.216  1.00  0.84
N
ATOM  11730  CZ  ARG B 175       6.055  -7.150  -2.419  1.00  0.84
C
ATOM  11731  NH1 ARG B 175       5.959  -6.171  -3.292  1.00  0.84
N
ATOM  11732  NH2 ARG B 175       7.177  -7.322  -1.741  1.00  0.84
N
ATOM  11733  H   ARG B 175       1.925 -11.400  -3.966  1.00  0.67
H
ATOM  11734  HA  ARG B 175       0.496 -10.163  -1.811  1.00  0.67
H
ATOM  11735 1HB  ARG B 175       1.749  -9.017  -4.318  1.00  1.01
H
ATOM  11736 2HB  ARG B 175       1.038  -8.045  -3.035  1.00  1.01
H
ATOM  11737 1HG  ARG B 175       2.629  -8.859  -1.429  1.00  1.01
H
ATOM  11738 2HG  ARG B 175       3.346  -9.943  -2.644  1.00  1.01
H
ATOM  11739 1HD  ARG B 175       3.933  -7.978  -3.983  1.00  1.01
H
ATOM  11740 2HD  ARG B 175       3.287  -6.939  -2.689  1.00  1.01
H
ATOM  11741  HE  ARG B 175       5.128  -8.731  -1.540  1.00  1.01
H
ATOM  11742 1HH1 ARG B 175       5.103  -6.039  -3.812  1.00  1.01
H
ATOM  11743 2HH1 ARG B 175       6.742  -5.552  -3.444  1.00  1.01
H
ATOM  11744 1HH2 ARG B 175       7.253  -8.077  -1.072  1.00  1.01
H
ATOM  11745 2HH2 ARG B 175       7.958  -6.702  -1.894  1.00  1.01
H
ATOM  11746  N   CYS B 176      -1.017 -10.099  -4.722  1.00  0.47
N
ATOM  11747  CA  CYS B 176      -2.334  -9.852  -5.282  1.00  0.47
C
ATOM  11748  C   CYS B 176      -3.383 -10.781  -4.658  1.00  0.38
C
ATOM  11749  O   CYS B 176      -4.490 -10.340  -4.338  1.00  0.38
O
ATOM  11750  CB  CYS B 176      -2.290 -10.017  -6.792  1.00  0.70
C
ATOM  11751  SG  CYS B 176      -1.370  -8.715  -7.650  1.00  0.70
S
ATOM  11752  H   CYS B 176      -0.270 -10.437  -5.321  1.00  0.56
H
ATOM  11753  HA  CYS B 176      -2.612  -8.821  -5.061  1.00  0.56
H
ATOM  11754 1HB  CYS B 176      -1.818 -10.968  -7.031  1.00  0.85
H
```

```
ATOM  11755 2HB  CYS B 176      -3.303 -10.036  -7.192  1.00  0.85
H
ATOM  11756  HG  CYS B 176      -1.418  -9.282  -8.851  1.00  0.85
H
ATOM  11757  N   GLU B 177      -3.007 -12.047  -4.437  1.00  0.40
N
ATOM  11758  CA  GLU B 177      -3.878 -13.018  -3.781  1.00  0.39
C
ATOM  11759  C   GLU B 177      -4.178 -12.601  -2.351  1.00  0.35
C
ATOM  11760  O   GLU B 177      -5.312 -12.711  -1.884  1.00  0.40
O
ATOM  11761  CB  GLU B 177      -3.223 -14.400  -3.764  1.00  0.58
C
ATOM  11762  CG  GLU B 177      -3.121 -15.090  -5.113  1.00  0.58
C
ATOM  11763  CD  GLU B 177      -2.345 -16.377  -5.036  1.00  0.58
C
ATOM  11764  OE1 GLU B 177      -1.739 -16.631  -4.020  1.00  0.58
O
ATOM  11765  OE2 GLU B 177      -2.367 -17.114  -5.996  1.00  0.58
O
ATOM  11766  H   GLU B 177      -2.091 -12.352  -4.739  1.00  0.48
H
ATOM  11767  HA  GLU B 177      -4.819 -13.073  -4.331  1.00  0.47
H
ATOM  11768 1HB  GLU B 177      -2.215 -14.314  -3.361  1.00  0.70
H
ATOM  11769 2HB  GLU B 177      -3.786 -15.057  -3.100  1.00  0.70
H
ATOM  11770 1HG  GLU B 177      -4.125 -15.305  -5.476  1.00  0.70
H
ATOM  11771 2HG  GLU B 177      -2.643 -14.419  -5.823  1.00  0.70
H
ATOM  11772  N   ALA B 178      -3.156 -12.098  -1.665  1.00  0.35
N
ATOM  11773  CA  ALA B 178      -3.254 -11.637  -0.289  1.00  0.43
C
ATOM  11774  C   ALA B 178      -4.263 -10.512  -0.126  1.00  0.57
C
ATOM  11775  O   ALA B 178      -4.968 -10.433   0.883  1.00  1.31
O
ATOM  11776  CB  ALA B 178      -1.906 -11.179   0.178  1.00  0.65
C
ATOM  11777  H   ALA B 178      -2.246 -12.052  -2.103  1.00  0.42
H
ATOM  11778  HA  ALA B 178      -3.582 -12.476   0.321  1.00  0.52
H
ATOM  11779 1HB  ALA B 178      -1.997 -10.886   1.214  1.00  0.77
H
ATOM  11780 2HB  ALA B 178      -1.186 -11.990   0.089  1.00  0.77
H
ATOM  11781 3HB  ALA B 178      -1.572 -10.332  -0.418  1.00  0.77
H
ATOM  11782  N   PHE B 179      -4.356  -9.658  -1.140  1.00  0.92
N
ATOM  11783  CA  PHE B 179      -5.338  -8.590  -1.142  1.00  2.30
C
```

```
ATOM  11784  C   PHE B 179      -6.698  -8.985  -1.697  1.00  0.44
C
ATOM  11785  O   PHE B 179      -7.548  -8.113  -1.854  1.00  0.73
O
ATOM  11786  CB  PHE B 179      -4.806  -7.365  -1.879  1.00  3.45
C
ATOM  11787  CG  PHE B 179      -3.852  -6.550  -1.063  1.00  3.45
C
ATOM  11788  CD1 PHE B 179      -2.588  -7.015  -0.763  1.00  3.45
C
ATOM  11789  CD2 PHE B 179      -4.218  -5.291  -0.612  1.00  3.45
C
ATOM  11790  CE1 PHE B 179      -1.711  -6.258  -0.027  1.00  3.45
C
ATOM  11791  CE2 PHE B 179      -3.340  -4.525   0.122  1.00  3.45
C
ATOM  11792  CZ  PHE B 179      -2.083  -5.010   0.416  1.00  3.45
C
ATOM  11793  H   PHE B 179      -3.712  -9.740  -1.920  1.00  1.10
H
ATOM  11794  HA  PHE B 179      -5.491  -8.289  -0.109  1.00  2.76
H
ATOM  11795 1HB  PHE B 179      -4.295  -7.681  -2.787  1.00  4.14
H
ATOM  11796 2HB  PHE B 179      -5.636  -6.725  -2.173  1.00  4.14
H
ATOM  11797  HD1 PHE B 179      -2.289  -7.997  -1.105  1.00  4.14
H
ATOM  11798  HD2 PHE B 179      -5.211  -4.907  -0.848  1.00  4.14
H
ATOM  11799  HE1 PHE B 179      -0.725  -6.655   0.196  1.00  4.14
H
ATOM  11800  HE2 PHE B 179      -3.639  -3.537   0.466  1.00  4.14
H
ATOM  11801  HZ  PHE B 179      -1.384  -4.409   0.995  1.00  4.14
H
ATOM  11802  N   GLY B 180      -6.933 -10.284  -1.929  1.00  0.49
N
ATOM  11803  CA  GLY B 180      -8.232 -10.767  -2.386  1.00  0.44
C
ATOM  11804  C   GLY B 180      -8.453 -10.780  -3.900  1.00  0.43
C
ATOM  11805  O   GLY B 180      -9.599 -10.807  -4.340  1.00  0.78
O
ATOM  11806  H   GLY B 180      -6.211 -10.973  -1.789  1.00  0.59
H
ATOM  11807 1HA  GLY B 180      -8.376 -11.779  -2.004  1.00  0.53
H
ATOM  11808 2HA  GLY B 180      -9.009 -10.162  -1.923  1.00  0.53
H
ATOM  11809  N   TRP B 181      -7.388 -10.750  -4.703  1.00  0.30
N
ATOM  11810  CA  TRP B 181      -7.568 -10.745  -6.158  1.00  0.35
C
ATOM  11811  C   TRP B 181      -7.319 -12.098  -6.826  1.00  0.48
C
ATOM  11812  O   TRP B 181      -6.418 -12.853  -6.451  1.00  0.65
O
```

ATOM 11813 CB TRP B 181   -6.644 -9.702 -6.794 1.00 0.52
C
ATOM 11814 CG TRP B 181   -7.079 -8.293 -6.549 1.00 0.52
C
ATOM 11815 CD1 TRP B 181   -7.311 -7.699 -5.350 1.00 0.52
C
ATOM 11816 CD2 TRP B 181   -7.312 -7.276 -7.547 1.00 0.52
C
ATOM 11817 NE1 TRP B 181   -7.700 -6.398 -5.535 1.00 0.52
N
ATOM 11818 CE2 TRP B 181   -7.699 -6.121 -6.874 1.00 0.52
C
ATOM 11819 CE3 TRP B 181   -7.229 -7.257 -8.934 1.00 0.52
C
ATOM 11820 CZ2 TRP B 181   -8.005 -4.947 -7.548 1.00 0.52
C
ATOM 11821 CZ3 TRP B 181   -7.546 -6.091 -9.610 1.00 0.52
C
ATOM 11822 CH2 TRP B 181   -7.923 -4.962 -8.936 1.00 0.52
C
ATOM 11823 H   TRP B 181   -6.449 -10.726 -4.327 1.00 0.36
H
ATOM 11824 HA TRP B 181   -8.597 -10.454 -6.368 1.00 0.42
H
ATOM 11825 1HB TRP B 181   -5.632 -9.822 -6.416 1.00 0.63
H
ATOM 11826 2HB TRP B 181   -6.612 -9.862 -7.871 1.00 0.63
H
ATOM 11827 HD1 TRP B 181   -7.218 -8.187 -4.385 1.00 0.63
H
ATOM 11828 HE1 TRP B 181   -7.943 -5.750 -4.800 1.00 0.63
H
ATOM 11829 HE3 TRP B 181   -6.928 -8.149 -9.468 1.00 0.63
H
ATOM 11830 HZ2 TRP B 181   -8.303 -4.039 -7.029 1.00 0.63
H
ATOM 11831 HZ3 TRP B 181   -7.487 -6.096 -10.695 1.00 0.63
H
ATOM 11832 HH2 TRP B 181   -8.162 -4.060 -9.500 1.00 0.63
H
ATOM 11833 N   ASN B 182   -8.118 -12.378 -7.857 1.00 0.52
N
ATOM 11834 CA ASN B 182   -7.994 -13.600 -8.644 1.00 0.64
C
ATOM 11835 C   ASN B 182   -6.829 -13.454 -9.587 1.00 0.43
C
ATOM 11836 O   ASN B 182   -6.976 -13.024 -10.729 1.00 0.38
O
ATOM 11837 CB ASN B 182   -9.283 -13.922 -9.379 1.00 0.96
C
ATOM 11838 CG ASN B 182   -10.341 -14.485 -8.468 1.00 0.96
C
ATOM 11839 OD1 ASN B 182   -10.107 -15.523 -7.834 1.00 0.96
O
ATOM 11840 ND2 ASN B 182   -11.478 -13.848 -8.376 1.00 0.96
N
ATOM 11841 H   ASN B 182   -8.820 -11.704 -8.121 1.00 0.62
H

```
ATOM  11842  HA  ASN B 182      -7.766 -14.429  -7.973  1.00  0.77
H
ATOM  11843 1HB  ASN B 182      -9.669 -13.018  -9.852  1.00  1.15
H
ATOM  11844 2HB  ASN B 182      -9.082 -14.644 -10.170  1.00  1.15
H
ATOM  11845 1HD2 ASN B 182     -12.194 -14.200  -7.776  1.00  1.15
H
ATOM  11846 2HD2 ASN B 182     -11.645 -13.004  -8.891  1.00  1.15
H
ATOM  11847  N   THR B 183      -5.665 -13.788  -9.069  1.00  0.59
N
ATOM  11848  CA  THR B 183      -4.419 -13.637  -9.776  1.00  0.71
C
ATOM  11849  C   THR B 183      -4.113 -14.723 -10.774  1.00  0.95
C
ATOM  11850  O   THR B 183      -4.235 -15.914 -10.483  1.00  1.50
O
ATOM  11851  CB  THR B 183      -3.273 -13.576  -8.788  1.00  1.06
C
ATOM  11852  OG1 THR B 183      -3.490 -12.487  -7.895  1.00  1.06
O
ATOM  11853  CG2 THR B 183      -1.980 -13.358  -9.538  1.00  1.06
C
ATOM  11854  H   THR B 183      -5.656 -14.116  -8.109  1.00  0.71
H
ATOM  11855  HA  THR B 183      -4.457 -12.700 -10.322  1.00  0.85
H
ATOM  11856  HB  THR B 183      -3.220 -14.505  -8.218  1.00  1.28
H
ATOM  11857  HG1 THR B 183      -4.315 -12.624  -7.421  1.00  1.28
H
ATOM  11858 1HG2 THR B 183      -1.176 -13.302  -8.827  1.00  1.28
H
ATOM  11859 2HG2 THR B 183      -1.799 -14.186 -10.220  1.00  1.28
H
ATOM  11860 3HG2 THR B 183      -2.038 -12.427 -10.100  1.00  1.28
H
ATOM  11861  N   TYR B 184      -3.684 -14.295 -11.953  1.00  0.95
N
ATOM  11862  CA  TYR B 184      -3.262 -15.215 -12.984  1.00  1.34
C
ATOM  11863  C   TYR B 184      -1.911 -14.789 -13.534  1.00  0.99
C
ATOM  11864  O   TYR B 184      -1.621 -13.602 -13.666  1.00  2.29
O
ATOM  11865  CB  TYR B 184      -4.300 -15.294 -14.092  1.00  2.01
C
ATOM  11866  CG  TYR B 184      -5.633 -15.798 -13.597  1.00  2.01
C
ATOM  11867  CD1 TYR B 184      -6.617 -14.896 -13.236  1.00  2.01
C
ATOM  11868  CD2 TYR B 184      -5.862 -17.159 -13.477  1.00  2.01
C
ATOM  11869  CE1 TYR B 184      -7.833 -15.348 -12.762  1.00  2.01
C
ATOM  11870  CE2 TYR B 184      -7.076 -17.613 -13.002  1.00  2.01
C
```

ATOM 11871 CZ TYR B 184   -8.059 -16.713 -12.644 1.00 2.01
C
ATOM 11872 OH TYR B 184   -9.269 -17.168 -12.173 1.00 2.01
O
ATOM 11873 H   TYR B 184   -3.630 -13.299 -12.118 1.00 1.14
H
ATOM 11874 HA TYR B 184   -3.144 -16.206 -12.545 1.00 1.61
H
ATOM 11875 1HB TYR B 184   -4.447 -14.304 -14.524 1.00 2.41
H
ATOM 11876 2HB TYR B 184   -3.953 -15.957 -14.883 1.00 2.41
H
ATOM 11877 HD1 TYR B 184   -6.427 -13.827 -13.326 1.00 2.41
H
ATOM 11878 HD2 TYR B 184   -5.083 -17.870 -13.756 1.00 2.41
H
ATOM 11879 HE1 TYR B 184   -8.607 -14.635 -12.479 1.00 2.41
H
ATOM 11880 HE2 TYR B 184   -7.257 -18.684 -12.906 1.00 2.41
H
ATOM 11881 HH TYR B 184   -9.325 -18.116 -12.310 1.00 2.41
H
ATOM 11882 N   VAL B 185   -1.083 -15.772 -13.843 1.00 1.13
N
ATOM 11883 CA VAL B 185   0.244 -15.527 -14.379 1.00 1.35
C
ATOM 11884 C   VAL B 185   0.352 -15.889 -15.837 1.00 1.37
C
ATOM 11885 O   VAL B 185   0.162 -17.048 -16.208 1.00 3.40
O
ATOM 11886 CB VAL B 185   1.288 -16.327 -13.613 1.00 2.03
C
ATOM 11887 CG1 VAL B 185   2.642 -16.101 -14.257 1.00 2.03
C
ATOM 11888 CG2 VAL B 185   1.290 -15.893 -12.151 1.00 2.03
C
ATOM 11889 H   VAL B 185   -1.383 -16.727 -13.705 1.00 1.36
H
ATOM 11890 HA VAL B 185   0.469 -14.466 -14.268 1.00 1.62
H
ATOM 11891 HB VAL B 185   1.059 -17.390 -13.685 1.00 2.43
H
ATOM 11892 1HG1 VAL B 185   3.392 -16.686 -13.729 1.00 2.43
H
ATOM 11893 2HG1 VAL B 185   2.607 -16.419 -15.299 1.00 2.43
H
ATOM 11894 3HG1 VAL B 185   2.898 -15.043 -14.209 1.00 2.43
H
ATOM 11895 1HG2 VAL B 185   2.037 -16.467 -11.602 1.00 2.43
H
ATOM 11896 2HG2 VAL B 185   1.527 -14.831 -12.088 1.00 2.43
H
ATOM 11897 3HG2 VAL B 185   0.307 -16.073 -11.716 1.00 2.43
H
ATOM 11898 N   VAL B 186   0.668 -14.904 -16.666 1.00 0.63
N
ATOM 11899 CA VAL B 186   0.828 -15.144 -18.091 1.00 0.91
C

ATOM 11900 C   VAL B 186   2.096 -14.519 -18.633 1.00 0.93
C
ATOM 11901 O   VAL B 186   2.666 -13.617 -18.020 1.00 1.50
O
ATOM 11902 CB VAL B 186   -0.363 -14.575 -18.893 1.00 1.36
C
ATOM 11903 CG1 VAL B 186   -1.664 -15.240 -18.484 1.00 1.36
C
ATOM 11904 CG2 VAL B 186   -0.443 -13.073 -18.671 1.00 1.36
C
ATOM 11905 H   VAL B 186   0.807 -13.973 -16.297 1.00 0.76
H
ATOM 11906 HA VAL B 186   0.876 -16.221 -18.256 1.00 1.09
H
ATOM 11907 HB VAL B 186   -0.210 -14.782 -19.952 1.00 1.64
H
ATOM 11908 1HG1 VAL B 186   -2.473 -14.826 -19.081 1.00 1.64
H
ATOM 11909 2HG1 VAL B 186   -1.595 -16.314 -18.658 1.00 1.64
H
ATOM 11910 3HG1 VAL B 186   -1.859 -15.053 -17.430 1.00 1.64
H
ATOM 11911 1HG2 VAL B 186   -1.271 -12.665 -19.246 1.00 1.64
H
ATOM 11912 2HG2 VAL B 186   -0.602 -12.870 -17.612 1.00 1.64
H
ATOM 11913 3HG2 VAL B 186   0.488 -12.607 -18.994 1.00 1.64
H
ATOM 11914 N   ASP B 187   2.520 -14.997 -19.792 1.00 0.73
N
ATOM 11915 CA ASP B 187   3.633 -14.389 -20.487 1.00 0.74
C
ATOM 11916 C   ASP B 187   3.040 -13.245 -21.275 1.00 0.67
C
ATOM 11917 O   ASP B 187   2.220 -13.460 -22.165 1.00 0.69
O
ATOM 11918 CB ASP B 187   4.364 -15.387 -21.392 1.00 1.11
C
ATOM 11919 CG ASP B 187   5.624 -14.820 -22.063 1.00 1.11
C
ATOM 11920 OD1 ASP B 187   5.682 -13.648 -22.365 1.00 1.11
O
ATOM 11921 OD2 ASP B 187   6.545 -15.585 -22.244 1.00 1.11
O
ATOM 11922 H   ASP B 187   2.032 -15.772 -20.220 1.00 0.88
H
ATOM 11923 HA ASP B 187   4.340 -13.987 -19.761 1.00 0.89
H
ATOM 11924 1HB ASP B 187   4.649 -16.259 -20.805 1.00 1.33
H
ATOM 11925 2HB ASP B 187   3.681 -15.731 -22.171 1.00 1.33
H
ATOM 11926 N   GLY B 188   3.434 -12.027 -20.931 1.00 0.63
N
ATOM 11927 CA GLY B 188   2.895 -10.813 -21.535 1.00 0.58
C
ATOM 11928 C   GLY B 188   3.174 -10.671 -23.029 1.00 0.68
C

```
ATOM  11929  O   GLY B 188       2.556  -9.843 -23.692  1.00  0.79
O
ATOM  11930  H   GLY B 188       4.145 -11.934 -20.222  1.00  0.76
H
ATOM  11931 1HA  GLY B 188       1.818 -10.785 -21.370  1.00  0.70
H
ATOM  11932 2HA  GLY B 188       3.308  -9.949 -21.014  1.00  0.70
H
ATOM  11933  N   ARG B 189       4.101 -11.467 -23.559  1.00  0.71
N
ATOM  11934  CA  ARG B 189       4.428 -11.429 -24.975  1.00  0.84
C
ATOM  11935  C   ARG B 189       3.544 -12.392 -25.773  1.00  0.92
C
ATOM  11936  O   ARG B 189       3.524 -12.356 -27.004  1.00  1.04
O
ATOM  11937  CB  ARG B 189       5.875 -11.819 -25.176  1.00  1.26
C
ATOM  11938  CG  ARG B 189       6.887 -10.905 -24.513  1.00  1.26
C
ATOM  11939  CD  ARG B 189       8.234 -11.511 -24.589  1.00  1.26
C
ATOM  11940  NE  ARG B 189       8.253 -12.781 -23.878  1.00  1.26
N
ATOM  11941  CZ  ARG B 189       9.290 -13.640 -23.844  1.00  1.26
C
ATOM  11942  NH1 ARG B 189      10.418 -13.348 -24.457  1.00  1.26
N
ATOM  11943  NH2 ARG B 189       9.160 -14.781 -23.192  1.00  1.26
N
ATOM  11944  H   ARG B 189       4.603 -12.130 -22.975  1.00  0.85
H
ATOM  11945  HA  ARG B 189       4.272 -10.415 -25.345  1.00  1.01
H
ATOM  11946 1HB  ARG B 189       6.035 -12.823 -24.786  1.00  1.51
H
ATOM  11947 2HB  ARG B 189       6.101 -11.841 -26.241  1.00  1.51
H
ATOM  11948 1HG  ARG B 189       6.904  -9.941 -25.020  1.00  1.51
H
ATOM  11949 2HG  ARG B 189       6.626 -10.765 -23.463  1.00  1.51
H
ATOM  11950 1HD  ARG B 189       8.503 -11.694 -25.628  1.00  1.51
H
ATOM  11951 2HD  ARG B 189       8.967 -10.848 -24.131  1.00  1.51
H
ATOM  11952  HE  ARG B 189       7.403 -13.056 -23.389  1.00  1.51
H
ATOM  11953 1HH1 ARG B 189      10.513 -12.475 -24.956  1.00  1.51
H
ATOM  11954 2HH1 ARG B 189      11.190 -13.998 -24.428  1.00  1.51
H
ATOM  11955 1HH2 ARG B 189       8.278 -15.004 -22.737  1.00  1.51
H
ATOM  11956 2HH2 ARG B 189       9.924 -15.437 -23.158  1.00  1.51
H
ATOM  11957  N   ASP B 190       2.857 -13.289 -25.068  1.00  0.96
N
```

```
ATOM  11958  CA  ASP B 190       2.018 -14.296 -25.695  1.00  1.09
C
ATOM  11959  C   ASP B 190       0.573 -13.844 -25.782  1.00  0.99
C
ATOM  11960  O   ASP B 190      -0.170 -13.939 -24.803  1.00  0.95
O
ATOM  11961  CB  ASP B 190       2.136 -15.609 -24.921  1.00  1.64
C
ATOM  11962  CG  ASP B 190       1.332 -16.750 -25.515  1.00  1.64
C
ATOM  11963  OD1 ASP B 190       0.409 -16.490 -26.252  1.00  1.64
O
ATOM  11964  OD2 ASP B 190       1.646 -17.879 -25.223  1.00  1.64
O
ATOM  11965  H   ASP B 190       2.887 -13.276 -24.059  1.00  1.15
H
ATOM  11966  HA  ASP B 190       2.382 -14.462 -26.709  1.00  1.31
H
ATOM  11967 1HB  ASP B 190       3.183 -15.909 -24.883  1.00  1.96
H
ATOM  11968 2HB  ASP B 190       1.807 -15.452 -23.893  1.00  1.96
H
ATOM  11969  N   VAL B 191       0.178 -13.369 -26.965  1.00  1.00
N
ATOM  11970  CA  VAL B 191      -1.174 -12.868 -27.193  1.00  0.95
C
ATOM  11971  C   VAL B 191      -2.268 -13.931 -27.058  1.00  1.04
C
ATOM  11972  O   VAL B 191      -3.433 -13.583 -26.880  1.00  1.00
O
ATOM  11973  CB  VAL B 191      -1.288 -12.224 -28.589  1.00  1.42
C
ATOM  11974  CG1 VAL B 191      -0.338 -11.044 -28.716  1.00  1.42
C
ATOM  11975  CG2 VAL B 191      -1.009 -13.260 -29.666  1.00  1.42
C
ATOM  11976  H   VAL B 191       0.845 -13.339 -27.724  1.00  1.20
H
ATOM  11977  HA  VAL B 191      -1.368 -12.091 -26.454  1.00  1.14
H
ATOM  11978  HB  VAL B 191      -2.294 -11.837 -28.705  1.00  1.71
H
ATOM  11979 1HG1 VAL B 191      -0.454 -10.589 -29.700  1.00  1.71
H
ATOM  11980 2HG1 VAL B 191      -0.570 -10.310 -27.947  1.00  1.71
H
ATOM  11981 3HG1 VAL B 191       0.690 -11.388 -28.595  1.00  1.71
H
ATOM  11982 1HG2 VAL B 191      -1.118 -12.797 -30.646  1.00  1.71
H
ATOM  11983 2HG2 VAL B 191       0.006 -13.641 -29.552  1.00  1.71
H
ATOM  11984 3HG2 VAL B 191      -1.716 -14.080 -29.575  1.00  1.71
H
ATOM  11985  N   GLU B 192      -1.913 -15.215 -27.148  1.00  1.34
N
ATOM  11986  CA  GLU B 192      -2.908 -16.267 -27.003  1.00  1.70
C
```

ATOM 11987 C   GLU B 192   -3.215 -16.428 -25.538 1.00 1.61
C
ATOM 11988 O   GLU B 192   -4.379 -16.492 -25.142 1.00 1.74
O
ATOM 11989 CB GLU B 192   -2.429 -17.596 -27.583 1.00 2.55
C
ATOM 11990 CG GLU B 192   -3.473 -18.702 -27.497 1.00 2.55
C
ATOM 11991 CD GLU B 192   -3.040 -20.001 -28.124 1.00 2.55
C
ATOM 11992 OE1 GLU B 192   -1.977 -20.048 -28.694 1.00 2.55
O
ATOM 11993 OE2 GLU B 192   -3.779 -20.951 -28.022 1.00 2.55
O
ATOM 11994 H   GLU B 192   -0.941 -15.478 -27.268 1.00 1.61
H
ATOM 11995 HA GLU B 192   -3.821 -15.967 -27.515 1.00 2.04
H
ATOM 11996 1HB GLU B 192   -2.155 -17.462 -28.629 1.00 3.06
H
ATOM 11997 2HB GLU B 192   -1.539 -17.931 -27.051 1.00 3.06
H
ATOM 11998 1HG GLU B 192   -3.702 -18.883 -26.447 1.00 3.06
H
ATOM 11999 2HG GLU B 192   -4.385 -18.361 -27.984 1.00 3.06
H
ATOM 12000 N   ALA B 193   -2.149 -16.472 -24.738 1.00 1.51
N
ATOM 12001 CA ALA B 193   -2.264 -16.567 -23.290 1.00 1.62
C
ATOM 12002 C   ALA B 193   -3.037 -15.375 -22.760 1.00 1.26
C
ATOM 12003 O   ALA B 193   -3.893 -15.530 -21.889 1.00 1.39
O
ATOM 12004 CB ALA B 193   -0.891 -16.633 -22.638 1.00 2.43
C
ATOM 12005 H   ALA B 193   -1.227 -16.434 -25.163 1.00 1.81
H
ATOM 12006 HA ALA B 193   -2.822 -17.472 -23.048 1.00 1.94
H
ATOM 12007 1HB ALA B 193   -1.006 -16.718 -21.558 1.00 2.92
H
ATOM 12008 2HB ALA B 193   -0.352 -17.501 -23.015 1.00 2.92
H
ATOM 12009 3HB ALA B 193   -0.331 -15.730 -22.873 1.00 2.92
H
ATOM 12010 N   LEU B 194   -2.739 -14.190 -23.302 1.00 0.98
N
ATOM 12011 CA LEU B 194   -3.441 -12.980 -22.920 1.00 0.86
C
ATOM 12012 C   LEU B 194   -4.915 -13.037 -23.294 1.00 0.91
C
ATOM 12013 O   LEU B 194   -5.770 -12.711 -22.475 1.00 1.09
O
ATOM 12014 CB LEU B 194   -2.792 -11.770 -23.581 1.00 1.29
C
ATOM 12015 CG LEU B 194   -1.422 -11.354 -23.044 1.00 1.29
C

```
ATOM  12016  CD1 LEU B 194      -0.832 -10.299 -23.963  1.00  1.29
C
ATOM  12017  CD2 LEU B 194      -1.585 -10.803 -21.633  1.00  1.29
C
ATOM  12018  H   LEU B 194      -1.998 -14.126 -23.992  1.00  1.18
H
ATOM  12019  HA  LEU B 194      -3.357 -12.865 -21.844  1.00  1.03
H
ATOM  12020 1HB  LEU B 194      -2.668 -11.997 -24.636  1.00  1.55
H
ATOM  12021 2HB  LEU B 194      -3.462 -10.915 -23.487  1.00  1.55
H
ATOM  12022  HG  LEU B 194      -0.751 -12.212 -23.027  1.00  1.55
H
ATOM  12023 1HD1 LEU B 194       0.141  -9.992 -23.584  1.00  1.55
H
ATOM  12024 2HD1 LEU B 194      -0.719 -10.707 -24.964  1.00  1.55
H
ATOM  12025 3HD1 LEU B 194      -1.495  -9.438 -23.995  1.00  1.55
H
ATOM  12026 1HD2 LEU B 194      -0.611 -10.502 -21.245  1.00  1.55
H
ATOM  12027 2HD2 LEU B 194      -2.251  -9.941 -21.654  1.00  1.55
H
ATOM  12028 3HD2 LEU B 194      -2.010 -11.574 -20.992  1.00  1.55
H
ATOM  12029  N   CYS B 195      -5.234 -13.502 -24.500  1.00  1.00
N
ATOM  12030  CA  CYS B 195      -6.638 -13.603 -24.862  1.00  1.17
C
ATOM  12031  C   CYS B 195      -7.375 -14.494 -23.888  1.00  1.42
C
ATOM  12032  O   CYS B 195      -8.446 -14.127 -23.408  1.00  1.80
O
ATOM  12033  CB  CYS B 195      -6.821 -14.157 -26.277  1.00  1.75
C
ATOM  12034  SG  CYS B 195      -6.354 -13.020 -27.595  1.00  1.75
S
ATOM  12035  H   CYS B 195      -4.523 -13.755 -25.175  1.00  1.20
H
ATOM  12036  HA  CYS B 195      -7.078 -12.607 -24.823  1.00  1.40
H
ATOM  12037 1HB  CYS B 195      -6.223 -15.063 -26.389  1.00  2.11
H
ATOM  12038 2HB  CYS B 195      -7.863 -14.435 -26.426  1.00  2.11
H
ATOM  12039  HG  CYS B 195      -7.298 -12.121 -27.341  1.00  2.11
H
ATOM  12040  N   GLN B 196      -6.793 -15.652 -23.585  1.00  1.52
N
ATOM  12041  CA  GLN B 196      -7.407 -16.614 -22.686  1.00  1.87
C
ATOM  12042  C   GLN B 196      -7.613 -16.074 -21.272  1.00  1.70
C
ATOM  12043  O   GLN B 196      -8.705 -16.213 -20.710  1.00  2.26
O
ATOM  12044  CB  GLN B 196      -6.545 -17.877 -22.628  1.00  2.81
C
```

ATOM 12045 CG GLN B 196 -6.542 -18.693 -23.912 1.00 2.81
C
ATOM 12046 CD GLN B 196 -5.578 -19.863 -23.846 1.00 2.81
C
ATOM 12047 OE1 GLN B 196 -4.936 -20.098 -22.819 1.00 2.81
O
ATOM 12048 NE2 GLN B 196 -5.465 -20.603 -24.944 1.00 2.81
N
ATOM 12049 H GLN B 196 -5.906 -15.888 -24.013 1.00 1.82
H
ATOM 12050 HA GLN B 196 -8.385 -16.878 -23.089 1.00 2.24
H
ATOM 12051 1HB GLN B 196 -5.515 -17.603 -22.404 1.00 3.37
H
ATOM 12052 2HB GLN B 196 -6.896 -18.521 -21.822 1.00 3.37
H
ATOM 12053 1HG GLN B 196 -7.544 -19.086 -24.078 1.00 3.37
H
ATOM 12054 2HG GLN B 196 -6.251 -18.052 -24.743 1.00 3.37
H
ATOM 12055 1HE2 GLN B 196 -4.841 -21.385 -24.962 1.00 3.37
H
ATOM 12056 2HE2 GLN B 196 -5.997 -20.377 -25.760 1.00 3.37
H
ATOM 12057 N VAL B 197 -6.576 -15.445 -20.699 1.00 1.31
N
ATOM 12058 CA VAL B 197 -6.677 -14.943 -19.331 1.00 1.27
C
ATOM 12059 C VAL B 197 -7.696 -13.828 -19.204 1.00 1.40
C
ATOM 12060 O VAL B 197 -8.303 -13.676 -18.144 1.00 2.91
O
ATOM 12061 CB VAL B 197 -5.303 -14.443 -18.798 1.00 1.91
C
ATOM 12062 CG1 VAL B 197 -4.902 -13.126 -19.425 1.00 1.91
C
ATOM 12063 CG2 VAL B 197 -5.358 -14.276 -17.292 1.00 1.91
C
ATOM 12064 H VAL B 197 -5.701 -15.345 -21.200 1.00 1.57
H
ATOM 12065 HA VAL B 197 -6.996 -15.770 -18.698 1.00 1.52
H
ATOM 12066 HB VAL B 197 -4.545 -15.179 -19.059 1.00 2.29
H
ATOM 12067 1HG1 VAL B 197 -3.927 -12.824 -19.044 1.00 2.29
H
ATOM 12068 2HG1 VAL B 197 -4.844 -13.261 -20.491 1.00 2.29
H
ATOM 12069 3HG1 VAL B 197 -5.635 -12.357 -19.186 1.00 2.29
H
ATOM 12070 1HG2 VAL B 197 -4.385 -13.944 -16.931 1.00 2.29
H
ATOM 12071 2HG2 VAL B 197 -6.113 -13.535 -17.033 1.00 2.29
H
ATOM 12072 3HG2 VAL B 197 -5.608 -15.229 -16.832 1.00 2.29
H
ATOM 12073 N PHE B 198 -7.914 -13.073 -20.277 1.00 0.89
N

```
ATOM  12074  CA  PHE B 198      -8.919 -12.034 -20.231  1.00  0.80
C
ATOM  12075  C   PHE B 198     -10.318 -12.599 -20.510  1.00  1.17
C
ATOM  12076  O   PHE B 198     -11.290 -12.173 -19.885  1.00  5.15
O
ATOM  12077  CB  PHE B 198      -8.600 -10.936 -21.220  1.00  1.20
C
ATOM  12078  CG  PHE B 198      -7.395 -10.105 -20.886  1.00  1.20
C
ATOM  12079  CD1 PHE B 198      -6.396  -9.923 -21.825  1.00  1.20
C
ATOM  12080  CD2 PHE B 198      -7.245  -9.519 -19.644  1.00  1.20
C
ATOM  12081  CE1 PHE B 198      -5.269  -9.181 -21.545  1.00  1.20
C
ATOM  12082  CE2 PHE B 198      -6.120  -8.772 -19.358  1.00  1.20
C
ATOM  12083  CZ  PHE B 198      -5.128  -8.606 -20.307  1.00  1.20
C
ATOM  12084  H   PHE B 198      -7.364 -13.197 -21.120  1.00  1.07
H
ATOM  12085  HA  PHE B 198      -8.921 -11.602 -19.228  1.00  0.96
H
ATOM  12086 1HB  PHE B 198      -8.434 -11.385 -22.197  1.00  1.44
H
ATOM  12087 2HB  PHE B 198      -9.456 -10.278 -21.299  1.00  1.44
H
ATOM  12088  HD1 PHE B 198      -6.514 -10.386 -22.802  1.00  1.44
H
ATOM  12089  HD2 PHE B 198      -8.022  -9.653 -18.890  1.00  1.44
H
ATOM  12090  HE1 PHE B 198      -4.493  -9.055 -22.300  1.00  1.44
H
ATOM  12091  HE2 PHE B 198      -6.014  -8.320 -18.374  1.00  1.44
H
ATOM  12092  HZ  PHE B 198      -4.239  -8.021 -20.079  1.00  1.44
H
ATOM  12093  N   TRP B 199     -10.416 -13.583 -21.421  1.00  2.02
N
ATOM  12094  CA  TRP B 199     -11.702 -14.206 -21.760  1.00  3.13
C
ATOM  12095  C   TRP B 199     -12.379 -14.881 -20.581  1.00  2.88
C
ATOM  12096  O   TRP B 199     -13.604 -14.847 -20.472  1.00  5.79
O
ATOM  12097  CB  TRP B 199     -11.568 -15.301 -22.842  1.00  4.70
C
ATOM  12098  CG  TRP B 199     -11.535 -14.876 -24.287  1.00  4.70
C
ATOM  12099  CD1 TRP B 199     -10.570 -15.167 -25.204  1.00  4.70
C
ATOM  12100  CD2 TRP B 199     -12.527 -14.094 -24.990  1.00  4.70
C
ATOM  12101  NE1 TRP B 199     -10.892 -14.624 -26.421  1.00  4.70
N
ATOM  12102  CE2 TRP B 199     -12.085 -13.967 -26.311  1.00  4.70
C
```

```
ATOM  12103  CE3 TRP B 199     -13.738 -13.502 -24.613  1.00  4.70
C
ATOM  12104  CZ2 TRP B 199     -12.813 -13.278 -27.264  1.00  4.70
C
ATOM  12105  CZ3 TRP B 199     -14.469 -12.813 -25.572  1.00  4.70
C
ATOM  12106  CH2 TRP B 199     -14.018 -12.706 -26.863  1.00  4.70
C
ATOM  12107  H   TRP B 199      -9.593 -13.895 -21.916  1.00  2.42
H
ATOM  12108  HA  TRP B 199     -12.366 -13.427 -22.133  1.00  3.76
H
ATOM  12109 1HB  TRP B 199     -10.653 -15.863 -22.651  1.00  5.63
H
ATOM  12110 2HB  TRP B 199     -12.389 -16.000 -22.730  1.00  5.63
H
ATOM  12111  HD1 TRP B 199      -9.677 -15.752 -25.001  1.00  5.63
H
ATOM  12112  HE1 TRP B 199     -10.343 -14.698 -27.265  1.00  5.63
H
ATOM  12113  HE3 TRP B 199     -14.102 -13.586 -23.589  1.00  5.63
H
ATOM  12114  HZ2 TRP B 199     -12.471 -13.182 -28.295  1.00  5.63
H
ATOM  12115  HZ3 TRP B 199     -15.413 -12.357 -25.273  1.00  5.63
H
ATOM  12116  HH2 TRP B 199     -14.617 -12.162 -27.590  1.00  5.63
H
ATOM  12117  N   GLN B 200     -11.593 -15.490 -19.688  1.00  3.22
N
ATOM  12118  CA  GLN B 200     -12.177 -16.203 -18.551  1.00  4.07
C
ATOM  12119  C   GLN B 200     -12.914 -15.285 -17.586  1.00  4.54
C
ATOM  12120  O   GLN B 200     -13.692 -15.760 -16.764  1.00 14.68
O
ATOM  12121  CB  GLN B 200     -11.124 -16.962 -17.772  1.00  6.11
C
ATOM  12122  CG  GLN B 200     -10.243 -16.060 -16.966  1.00  6.11
C
ATOM  12123  CD  GLN B 200      -9.124 -16.791 -16.291  1.00  6.11
C
ATOM  12124  OE1 GLN B 200      -9.262 -17.908 -15.786  1.00  6.11
O
ATOM  12125  NE2 GLN B 200      -7.982 -16.135 -16.287  1.00  6.11
N
ATOM  12126  H   GLN B 200     -10.588 -15.505 -19.829  1.00  3.86
H
ATOM  12127  HA  GLN B 200     -12.899 -16.922 -18.938  1.00  4.88
H
ATOM  12128 1HB  GLN B 200     -11.602 -17.671 -17.097  1.00  7.33
H
ATOM  12129 2HB  GLN B 200     -10.496 -17.530 -18.460  1.00  7.33
H
ATOM  12130 1HG  GLN B 200      -9.820 -15.320 -17.634  1.00  7.33
H
ATOM  12131 2HG  GLN B 200     -10.842 -15.572 -16.198  1.00  7.33
H
```

```
ATOM  12132 1HE2 GLN B 200      -7.170 -16.537 -15.861  1.00  7.33
H
ATOM  12133 2HE2 GLN B 200      -7.939 -15.231 -16.721  1.00  7.33
H
ATOM  12134  N   ALA B 201     -12.694 -13.967 -17.688  1.00  7.76
N
ATOM  12135  CA  ALA B 201     -13.375 -13.005 -16.847  1.00 12.80
C
ATOM  12136  C   ALA B 201     -14.776 -12.749 -17.350  1.00 30.16
C
ATOM  12137  O   ALA B 201     -15.518 -11.988 -16.734  1.00 69.42
O
ATOM  12138  CB  ALA B 201     -12.589 -11.704 -16.785  1.00 19.20
C
ATOM  12139  H   ALA B 201     -12.051 -13.599 -18.380  1.00  9.31
H
ATOM  12140  HA  ALA B 201     -13.447 -13.422 -15.842  1.00 15.36
H
ATOM  12141 1HB  ALA B 201     -13.102 -11.001 -16.129  1.00 23.04
H
ATOM  12142 2HB  ALA B 201     -11.591 -11.901 -16.396  1.00 23.04
H
ATOM  12143 3HB  ALA B 201     -12.511 -11.279 -17.784  1.00 23.04
H
ATOM  12144  N   SER B 202     -15.148 -13.365 -18.484  1.00 50.10
N
ATOM  12145  CA  SER B 202     -16.526 -13.322 -18.913  1.00 75.08
C
ATOM  12146  C   SER B 202     -17.296 -14.066 -17.846  1.00 57.21
C
ATOM  12147  O   SER B 202     -18.443 -13.747 -17.537  1.00 81.96
O
ATOM  12148  CB  SER B 202     -16.717 -13.998 -20.259  1.00112.62
C
ATOM  12149  OG  SER B 202     -16.491 -15.381 -20.162  1.00112.62
O
ATOM  12150  H   SER B 202     -14.499 -13.916 -19.025  1.00 60.12
H
ATOM  12151  HA  SER B 202     -16.872 -12.288 -18.956  1.00 90.10
H
ATOM  12152 1HB  SER B 202     -17.732 -13.821 -20.611  1.00135.14
H
ATOM  12153 2HB  SER B 202     -16.037 -13.566 -20.989  1.00135.14
H
ATOM  12154  HG  SER B 202     -15.534 -15.492 -20.149  1.00135.14
H
ATOM  12155  N   GLN B 203     -16.615 -15.019 -17.198  1.00 79.09
N
ATOM  12156  CA  GLN B 203     -17.178 -15.733 -16.098  1.00118.25
C
ATOM  12157  C   GLN B 203     -16.755 -14.967 -14.866  1.00108.93
C
ATOM  12158  O   GLN B 203     -15.891 -15.411 -14.108  1.00127.02
O
ATOM  12159  CB  GLN B 203     -16.672 -17.171 -16.071  1.00177.38
C
ATOM  12160  CG  GLN B 203     -16.930 -17.905 -17.373  1.00177.38
C
```

ATOM 12161 CD GLN B 203   -18.400 -17.998 -17.714 1.00177.38
C
ATOM 12162 OE1 GLN B 203   -19.185 -18.649 -17.017 1.00177.38
O
ATOM 12163 NE2 GLN B 203   -18.781 -17.327 -18.796 1.00177.38
N
ATOM 12164 H   GLN B 203   -15.668 -15.259 -17.457 1.00 94.91
H
ATOM 12165 HA GLN B 203   -18.265 -15.723 -16.166 1.00141.90
H
ATOM 12166 1HB GLN B 203   -15.600 -17.183 -15.877 1.00212.85
H
ATOM 12167 2HB GLN B 203   -17.161 -17.719 -15.266 1.00212.85
H
ATOM 12168 1HG GLN B 203   -16.428 -17.374 -18.181 1.00212.85
H
ATOM 12169 2HG GLN B 203   -16.536 -18.918 -17.292 1.00212.85
H
ATOM 12170 1HE2 GLN B 203   -19.741 -17.336 -19.081 1.00212.85
H
ATOM 12171 2HE2 GLN B 203   -18.106 -16.802 -19.323 1.00212.85
H
ATOM 12172 N   VAL B 204   -17.354 -13.779 -14.711 1.00131.30
N
ATOM 12173 CA VAL B 204   -17.114 -12.858 -13.602 1.00160.29
C
ATOM 12174 C   VAL B 204   -17.332 -13.542 -12.283 1.00 71.78
C
ATOM 12175 O   VAL B 204   -18.402 -13.426 -11.687 1.00136.31
O
ATOM 12176 CB VAL B 204   -18.008 -11.613 -13.701 1.00240.44
C
ATOM 12177 CG1 VAL B 204   -17.678 -10.809 -14.940 1.00240.44
C
ATOM 12178 CG2 VAL B 204   -19.458 -12.040 -13.738 1.00240.44
C
ATOM 12179 H   VAL B 204   -18.034 -13.519 -15.417 1.00157.56
H
ATOM 12180 HA VAL B 204   -16.075 -12.529 -13.653 1.00192.35
H
ATOM 12181 HB VAL B 204   -17.827 -10.978 -12.837 1.00288.52
H
ATOM 12182 1HG1 VAL B 204   -18.315 -9.926 -14.976 1.00288.52
H
ATOM 12183 2HG1 VAL B 204   -16.633 -10.500 -14.904 1.00288.52
H
ATOM 12184 3HG1 VAL B 204   -17.848 -11.418 -15.827 1.00288.52
H
ATOM 12185 1HG2 VAL B 204   -20.090 -11.155 -13.795 1.00288.52
H
ATOM 12186 2HG2 VAL B 204   -19.631 -12.668 -14.614 1.00288.52
H
ATOM 12187 3HG2 VAL B 204   -19.700 -12.601 -12.836 1.00288.52
H
ATOM 12188 N   LYS B 205   -16.328 -14.309 -11.888 1.00 27.05
N
ATOM 12189 CA LYS B 205   -16.309 -15.101 -10.698 1.00 39.12
C

```
ATOM  12190  C   LYS B 205      -16.797 -14.382  -9.451  1.00 29.29
C
ATOM  12191  O   LYS B 205      -17.982 -14.443  -9.100  1.00 90.15
O
ATOM  12192  CB  LYS B 205      -14.878 -15.632 -10.490  1.00 58.68
C
ATOM  12193  CG  LYS B 205      -14.668 -16.546  -9.287  1.00 58.68
C
ATOM  12194  CD  LYS B 205      -13.223 -16.992  -9.197  1.00 58.68
C
ATOM  12195  CE  LYS B 205      -12.956 -17.767  -7.918  1.00 58.68
C
ATOM  12196  NZ  LYS B 205      -11.510 -18.075  -7.760  1.00 58.68
N
ATOM  12197  H   LYS B 205      -15.519 -14.353 -12.490  1.00 32.46
H
ATOM  12198  HA  LYS B 205      -16.965 -15.955 -10.855  1.00 46.94
H
ATOM  12199 1HB  LYS B 205      -14.594 -16.208 -11.373  1.00 70.42
H
ATOM  12200 2HB  LYS B 205      -14.176 -14.803 -10.421  1.00 70.42
H
ATOM  12201 1HG  LYS B 205      -14.915 -16.033  -8.363  1.00 70.42
H
ATOM  12202 2HG  LYS B 205      -15.308 -17.423  -9.379  1.00 70.42
H
ATOM  12203 1HD  LYS B 205      -12.991 -17.633 -10.050  1.00 70.42
H
ATOM  12204 2HD  LYS B 205      -12.569 -16.125  -9.234  1.00 70.42
H
ATOM  12205 1HE  LYS B 205      -13.289 -17.178  -7.064  1.00 70.42
H
ATOM  12206 2HE  LYS B 205      -13.517 -18.702  -7.939  1.00 70.42
H
ATOM  12207 1HZ  LYS B 205      -11.361 -18.586  -6.901  1.00 70.42
H
ATOM  12208 2HZ  LYS B 205      -11.192 -18.628  -8.541  1.00 70.42
H
ATOM  12209 3HZ  LYS B 205      -10.990 -17.202  -7.729  1.00 70.42
H
ATOM  12210  N   HIS B 206      -15.891 -13.778  -8.736  1.00 23.25
N
ATOM  12211  CA  HIS B 206      -16.228 -13.159  -7.481  1.00 25.59
C
ATOM  12212  C   HIS B 206      -15.456 -11.900  -7.299  1.00 10.78
C
ATOM  12213  O   HIS B 206      -16.018 -10.818  -7.211  1.00 47.15
O
ATOM  12214  CB  HIS B 206      -15.978 -14.094  -6.282  1.00 38.38
C
ATOM  12215  CG  HIS B 206      -16.903 -15.271  -6.208  1.00 38.38
C
ATOM  12216  ND1 HIS B 206      -18.258 -15.133  -5.991  1.00 38.38
N
ATOM  12217  CD2 HIS B 206      -16.671 -16.600  -6.307  1.00 38.38
C
ATOM  12218  CE1 HIS B 206      -18.820 -16.330  -5.971  1.00 38.38
C
```

```
ATOM  12219  NE2 HIS B 206     -17.878 -17.235  -6.160  1.00 38.38
N
ATOM  12220  H   HIS B 206     -14.939 -13.735  -9.060  1.00 27.90
H
ATOM  12221  HA  HIS B 206     -17.283 -12.895  -7.482  1.00 30.71
H
ATOM  12222 1HB  HIS B 206     -14.957 -14.475  -6.323  1.00 46.06
H
ATOM  12223 2HB  HIS B 206     -16.077 -13.528  -5.356  1.00 46.06
H
ATOM  12224  HD1 HIS B 206     -18.777 -14.283  -6.082  1.00 46.06
H
ATOM  12225  HD2 HIS B 206     -15.766 -17.185  -6.470  1.00 46.06
H
ATOM  12226  HE1 HIS B 206     -19.892 -16.437  -5.814  1.00 46.06
H
ATOM  12227  N   LYS B 207     -14.158 -12.059  -7.158  1.00  2.38
N
ATOM  12228  CA  LYS B 207     -13.273 -10.948  -6.908  1.00  7.66
C
ATOM  12229  C   LYS B 207     -12.581 -10.470  -8.181  1.00  5.02
C
ATOM  12230  O   LYS B 207     -12.493 -11.224  -9.153  1.00 27.12
O
ATOM  12231  CB  LYS B 207     -12.252 -11.366  -5.852  1.00 11.49
C
ATOM  12232  CG  LYS B 207     -12.874 -11.797  -4.535  1.00 11.49
C
ATOM  12233  CD  LYS B 207     -13.607 -10.636  -3.882  1.00 11.49
C
ATOM  12234  CE  LYS B 207     -14.152 -11.025  -2.516  1.00 11.49
C
ATOM  12235  NZ  LYS B 207     -14.897  -9.907  -1.880  1.00 11.49
N
ATOM  12236  H   LYS B 207     -13.767 -12.985  -7.230  1.00  2.86
H
ATOM  12237  HA  LYS B 207     -13.873 -10.136  -6.510  1.00  9.19
H
ATOM  12238 1HB  LYS B 207     -11.659 -12.201  -6.225  1.00 13.79
H
ATOM  12239 2HB  LYS B 207     -11.566 -10.557  -5.638  1.00 13.79
H
ATOM  12240 1HG  LYS B 207     -13.575 -12.614  -4.708  1.00 13.79
H
ATOM  12241 2HG  LYS B 207     -12.091 -12.148  -3.863  1.00 13.79
H
ATOM  12242 1HD  LYS B 207     -12.924  -9.793  -3.763  1.00 13.79
H
ATOM  12243 2HD  LYS B 207     -14.436 -10.325  -4.516  1.00 13.79
H
ATOM  12244 1HE  LYS B 207     -14.821 -11.876  -2.629  1.00 13.79
H
ATOM  12245 2HE  LYS B 207     -13.325 -11.312  -1.867  1.00 13.79
H
ATOM  12246 1HZ  LYS B 207     -15.245 -10.199  -0.978  1.00 13.79
H
ATOM  12247 2HZ  LYS B 207     -14.280  -9.112  -1.760  1.00 13.79
H
```

```
ATOM  12248 3HZ  LYS B 207     -15.672  -9.633  -2.467  1.00 13.79
H
ATOM  12249  N   PRO B 208     -12.051  -9.239  -8.177  1.00  1.17
N
ATOM  12250  CA  PRO B 208     -11.323  -8.577  -9.249  1.00  0.48
C
ATOM  12251  C   PRO B 208     -10.157  -9.451  -9.705  1.00  0.47
C
ATOM  12252  O   PRO B 208      -9.592 -10.202  -8.907  1.00  1.87
O
ATOM  12253  CB  PRO B 208     -10.841  -7.286  -8.605  1.00  0.72
C
ATOM  12254  CG  PRO B 208     -11.809  -7.008  -7.523  1.00  0.72
C
ATOM  12255  CD  PRO B 208     -12.202  -8.347  -7.002  1.00  0.72
C
ATOM  12256  HA  PRO B 208     -12.010  -8.374 -10.085  1.00  0.58
H
ATOM  12257 1HB  PRO B 208      -9.839  -7.440  -8.209  1.00  0.86
H
ATOM  12258 2HB  PRO B 208     -10.779  -6.483  -9.353  1.00  0.86
H
ATOM  12259 1HG  PRO B 208     -11.314  -6.417  -6.744  1.00  0.86
H
ATOM  12260 2HG  PRO B 208     -12.650  -6.411  -7.887  1.00  0.86
H
ATOM  12261 1HD  PRO B 208     -11.514  -8.637  -6.197  1.00  0.86
H
ATOM  12262 2HD  PRO B 208     -13.246  -8.304  -6.666  1.00  0.86
H
ATOM  12263  N   THR B 209      -9.816  -9.349 -10.988  1.00  0.48
N
ATOM  12264  CA  THR B 209      -8.782 -10.181 -11.611  1.00  0.71
C
ATOM  12265  C   THR B 209      -7.438  -9.474 -11.704  1.00  0.31
C
ATOM  12266  O   THR B 209      -7.374  -8.297 -12.053  1.00  0.30
O
ATOM  12267  CB  THR B 209      -9.222 -10.622 -13.022  1.00  1.06
C
ATOM  12268  OG1 THR B 209     -10.414 -11.416 -12.930  1.00  1.06
O
ATOM  12269  CG2 THR B 209      -8.134 -11.431 -13.701  1.00  1.06
C
ATOM  12270  H   THR B 209     -10.304  -8.665 -11.556  1.00  0.58
H
ATOM  12271  HA  THR B 209      -8.649 -11.074 -11.000  1.00  0.85
H
ATOM  12272  HB  THR B 209      -9.433  -9.739 -13.621  1.00  1.28
H
ATOM  12273  HG1 THR B 209     -11.104 -10.909 -12.494  1.00  1.28
H
ATOM  12274 1HG2 THR B 209      -8.468 -11.729 -14.694  1.00  1.28
H
ATOM  12275 2HG2 THR B 209      -7.230 -10.830 -13.789  1.00  1.28
H
ATOM  12276 3HG2 THR B 209      -7.926 -12.316 -13.106  1.00  1.28
H
```

```
ATOM  12277  N   ALA B 210      -6.367 -10.192 -11.366  1.00  0.35
N
ATOM  12278  CA  ALA B 210      -5.014  -9.635 -11.390  1.00  0.39
C
ATOM  12279  C   ALA B 210      -4.136 -10.336 -12.434  1.00  0.47
C
ATOM  12280  O   ALA B 210      -3.513 -11.364 -12.164  1.00  0.86
O
ATOM  12281  CB  ALA B 210      -4.379  -9.738 -10.010  1.00  0.58
C
ATOM  12282  H   ALA B 210      -6.498 -11.158 -11.096  1.00  0.42
H
ATOM  12283  HA  ALA B 210      -5.084  -8.584 -11.669  1.00  0.47
H
ATOM  12284 1HB  ALA B 210      -3.384  -9.295 -10.033  1.00  0.70
H
ATOM  12285 2HB  ALA B 210      -4.992  -9.205  -9.287  1.00  0.70
H
ATOM  12286 3HB  ALA B 210      -4.299 -10.777  -9.713  1.00  0.70
H
ATOM  12287  N   VAL B 211      -4.084  -9.776 -13.636  1.00  0.52
N
ATOM  12288  CA  VAL B 211      -3.309 -10.385 -14.702  1.00  0.64
C
ATOM  12289  C   VAL B 211      -1.852  -9.979 -14.629  1.00  0.57
C
ATOM  12290  O   VAL B 211      -1.447  -8.955 -15.177  1.00  1.01
O
ATOM  12291  CB  VAL B 211      -3.888 -10.003 -16.076  1.00  0.96
C
ATOM  12292  CG1 VAL B 211      -3.066 -10.635 -17.192  1.00  0.96
C
ATOM  12293  CG2 VAL B 211      -5.332 -10.469 -16.142  1.00  0.96
C
ATOM  12294  H   VAL B 211      -4.577  -8.914 -13.813  1.00  0.62
H
ATOM  12295  HA  VAL B 211      -3.370 -11.469 -14.595  1.00  0.77
H
ATOM  12296  HB  VAL B 211      -3.844  -8.921 -16.203  1.00  1.15
H
ATOM  12297 1HG1 VAL B 211      -3.486 -10.352 -18.158  1.00  1.15
H
ATOM  12298 2HG1 VAL B 211      -2.037 -10.285 -17.128  1.00  1.15
H
ATOM  12299 3HG1 VAL B 211      -3.090 -11.720 -17.091  1.00  1.15
H
ATOM  12300 1HG2 VAL B 211      -5.762 -10.206 -17.102  1.00  1.15
H
ATOM  12301 2HG2 VAL B 211      -5.368 -11.551 -16.015  1.00  1.15
H
ATOM  12302 3HG2 VAL B 211      -5.903  -9.991 -15.346  1.00  1.15
H
ATOM  12303  N   VAL B 212      -1.068 -10.822 -13.974  1.00  0.61
N
ATOM  12304  CA  VAL B 212       0.369 -10.614 -13.851  1.00  0.49
C
ATOM  12305  C   VAL B 212       1.029 -11.039 -15.149  1.00  0.54
C
```

```
ATOM  12306  O   VAL B 212       1.054 -12.226 -15.471  1.00  1.26
O
ATOM  12307  CB  VAL B 212       0.925 -11.423 -12.657  1.00  0.73
C
ATOM  12308  CG1 VAL B 212       2.432 -11.268 -12.551  1.00  0.73
C
ATOM  12309  CG2 VAL B 212       0.254 -10.945 -11.377  1.00  0.73
C
ATOM  12310  H   VAL B 212      -1.501 -11.635 -13.547  1.00  0.73
H
ATOM  12311  HA  VAL B 212       0.562  -9.560 -13.685  1.00  0.59
H
ATOM  12312  HB  VAL B 212       0.709 -12.480 -12.812  1.00  0.88
H
ATOM  12313 1HG1 VAL B 212       2.800 -11.856 -11.710  1.00  0.88
H
ATOM  12314 2HG1 VAL B 212       2.898 -11.619 -13.471  1.00  0.88
H
ATOM  12315 3HG1 VAL B 212       2.678 -10.218 -12.394  1.00  0.88
H
ATOM  12316 1HG2 VAL B 212       0.628 -11.521 -10.531  1.00  0.88
H
ATOM  12317 2HG2 VAL B 212       0.475  -9.888 -11.222  1.00  0.88
H
ATOM  12318 3HG2 VAL B 212      -0.825 -11.081 -11.460  1.00  0.88
H
ATOM  12319  N   ALA B 213       1.556 -10.072 -15.901  1.00  0.45
N
ATOM  12320  CA  ALA B 213       2.124 -10.380 -17.208  1.00  0.54
C
ATOM  12321  C   ALA B 213       3.631 -10.181 -17.253  1.00  0.45
C
ATOM  12322  O   ALA B 213       4.134  -9.072 -17.079  1.00  0.49
O
ATOM  12323  CB  ALA B 213       1.454  -9.519 -18.266  1.00  0.81
C
ATOM  12324  H   ALA B 213       1.544  -9.109 -15.587  1.00  0.54
H
ATOM  12325  HA  ALA B 213       1.917 -11.426 -17.426  1.00  0.65
H
ATOM  12326 1HB  ALA B 213       1.848  -9.772 -19.246  1.00  0.97
H
ATOM  12327 2HB  ALA B 213       0.379  -9.696 -18.250  1.00  0.97
H
ATOM  12328 3HB  ALA B 213       1.651  -8.469 -18.056  1.00  0.97
H
ATOM  12329  N   LYS B 214       4.353 -11.266 -17.519  1.00  0.51
N
ATOM  12330  CA  LYS B 214       5.807 -11.211 -17.591  1.00  0.52
C
ATOM  12331  C   LYS B 214       6.241 -10.697 -18.950  1.00  0.47
C
ATOM  12332  O   LYS B 214       5.862 -11.247 -19.979  1.00  0.60
O
ATOM  12333  CB  LYS B 214       6.417 -12.585 -17.322  1.00  0.78
C
ATOM  12334  CG  LYS B 214       7.943 -12.598 -17.280  1.00  0.78
C
```

```
ATOM  12335  CD  LYS B 214       8.471 -13.988 -16.955  1.00  0.78
C
ATOM  12336  CE  LYS B 214       9.994 -14.028 -16.951  1.00  0.78
C
ATOM  12337  NZ  LYS B 214      10.506 -15.397 -16.663  1.00  0.78
N
ATOM  12338  H   LYS B 214       3.879 -12.147 -17.673  1.00  0.61
H
ATOM  12339  HA  LYS B 214       6.175 -10.520 -16.841  1.00  0.62
H
ATOM  12340 1HB  LYS B 214       6.051 -12.964 -16.367  1.00  0.94
H
ATOM  12341 2HB  LYS B 214       6.098 -13.284 -18.095  1.00  0.94
H
ATOM  12342 1HG  LYS B 214       8.337 -12.285 -18.248  1.00  0.94
H
ATOM  12343 2HG  LYS B 214       8.292 -11.899 -16.521  1.00  0.94
H
ATOM  12344 1HD  LYS B 214       8.109 -14.293 -15.972  1.00  0.94
H
ATOM  12345 2HD  LYS B 214       8.102 -14.698 -17.694  1.00  0.94
H
ATOM  12346 1HE  LYS B 214      10.362 -13.710 -17.926  1.00  0.94
H
ATOM  12347 2HE  LYS B 214      10.371 -13.342 -16.193  1.00  0.94
H
ATOM  12348 1HZ  LYS B 214      11.517 -15.388 -16.669  1.00  0.94
H
ATOM  12349 2HZ  LYS B 214      10.179 -15.695 -15.754  1.00  0.94
H
ATOM  12350 3HZ  LYS B 214      10.172 -16.039 -17.368  1.00  0.94
H
ATOM  12351  N   THR B 215       7.026  -9.635 -18.960  1.00  0.59
N
ATOM  12352  CA  THR B 215       7.468  -9.030 -20.209  1.00  0.72
C
ATOM  12353  C   THR B 215       8.815  -8.344 -20.050  1.00  0.47
C
ATOM  12354  O   THR B 215       9.499  -8.531 -19.047  1.00  0.61
O
ATOM  12355  CB  THR B 215       6.396  -8.038 -20.711  1.00  1.08
C
ATOM  12356  OG1 THR B 215       6.709  -7.572 -22.033  1.00  1.08
O
ATOM  12357  CG2 THR B 215       6.291  -6.875 -19.770  1.00  1.08
C
ATOM  12358  H   THR B 215       7.307  -9.210 -18.084  1.00  0.71
H
ATOM  12359  HA  THR B 215       7.579  -9.819 -20.953  1.00  0.86
H
ATOM  12360  HB  THR B 215       5.432  -8.546 -20.745  1.00  1.30
H
ATOM  12361  HG1 THR B 215       6.100  -6.850 -22.260  1.00  1.30
H
ATOM  12362 1HG2 THR B 215       5.519  -6.193 -20.125  1.00  1.30
H
ATOM  12363 2HG2 THR B 215       6.026  -7.235 -18.776  1.00  1.30
H
```

ATOM 12364 3HG2 THR B 215   7.242 -6.354 -19.726 1.00 1.30
H
ATOM 12365 N   PHE B 216   9.219 -7.585 -21.059 1.00 0.50
N
ATOM 12366 CA PHE B 216   10.510 -6.915 -20.992 1.00 0.72
C
ATOM 12367 C   PHE B 216   10.468 -5.484 -21.472 1.00 0.76
C
ATOM 12368 O   PHE B 216   9.939 -5.196 -22.546 1.00 1.75
O
ATOM 12369 CB PHE B 216   11.565 -7.677 -21.802 1.00 1.08
C
ATOM 12370 CG PHE B 216   11.832 -9.061 -21.289 1.00 1.08
C
ATOM 12371 CD1 PHE B 216   11.149 -10.156 -21.801 1.00 1.08
C
ATOM 12372 CD2 PHE B 216   12.758 -9.267 -20.279 1.00 1.08
C
ATOM 12373 CE1 PHE B 216   11.387 -11.426 -21.310 1.00 1.08
C
ATOM 12374 CE2 PHE B 216   12.999 -10.535 -19.790 1.00 1.08
C
ATOM 12375 CZ PHE B 216   12.311 -11.615 -20.304 1.00 1.08
C
ATOM 12376 H   PHE B 216   8.602 -7.469 -21.850 1.00 0.60
H
ATOM 12377 HA PHE B 216   10.831 -6.905 -19.948 1.00 0.86
H
ATOM 12378 1HB PHE B 216   11.241 -7.755 -22.839 1.00 1.30
H
ATOM 12379 2HB PHE B 216   12.502 -7.123 -21.793 1.00 1.30
H
ATOM 12380 HD1 PHE B 216   10.418 -10.003 -22.593 1.00 1.30
H
ATOM 12381 HD2 PHE B 216   13.296 -8.413 -19.867 1.00 1.30
H
ATOM 12382 HE1 PHE B 216   10.844 -12.277 -21.717 1.00 1.30
H
ATOM 12383 HE2 PHE B 216   13.729 -10.681 -18.995 1.00 1.30
H
ATOM 12384 HZ PHE B 216   12.500 -12.614 -19.918 1.00 1.30
H
ATOM 12385 N   LYS B 217   11.066 -4.596 -20.687 1.00 0.80
N
ATOM 12386 CA LYS B 217   11.196 -3.202 -21.068 1.00 0.71
C
ATOM 12387 C   LYS B 217   11.880 -3.094 -22.420 1.00 0.80
C
ATOM 12388 O   LYS B 217   12.972 -3.621 -22.601 1.00 1.42
O
ATOM 12389 CB LYS B 217   11.995 -2.457 -20.004 1.00 1.06
C
ATOM 12390 CG LYS B 217   12.137 -0.970 -20.244 1.00 1.06
C
ATOM 12391 CD LYS B 217   12.845 -0.297 -19.085 1.00 1.06
C
ATOM 12392 CE LYS B 217   12.979   1.182 -19.332 1.00 1.06
C

```
ATOM  12393  NZ  LYS B 217      11.659   1.730 -19.682  1.00  1.06
N
ATOM  12394  H   LYS B 217      11.421  -4.882 -19.779  1.00  0.96
H
ATOM  12395  HA  LYS B 217      10.205  -2.758 -21.147  1.00  0.85
H
ATOM  12396 1HB  LYS B 217      11.521  -2.594 -19.032  1.00  1.28
H
ATOM  12397 2HB  LYS B 217      12.998  -2.881 -19.941  1.00  1.28
H
ATOM  12398 1HG  LYS B 217      12.712  -0.801 -21.155  1.00  1.28
H
ATOM  12399 2HG  LYS B 217      11.150  -0.524 -20.369  1.00  1.28
H
ATOM  12400 1HD  LYS B 217      12.276  -0.454 -18.168  1.00  1.28
H
ATOM  12401 2HD  LYS B 217      13.838  -0.725 -18.960  1.00  1.28
H
ATOM  12402 1HE  LYS B 217      13.335   1.665 -18.424  1.00  1.28
H
ATOM  12403 2HE  LYS B 217      13.681   1.380 -20.142  1.00  1.28
H
ATOM  12404 1HZ  LYS B 217      11.684   2.724 -19.845  1.00  1.28
H
ATOM  12405 2HZ  LYS B 217      11.346   1.298 -20.537  1.00  1.28
H
ATOM  12406 3HZ  LYS B 217      11.005   1.538 -18.938  1.00  1.28
H
ATOM  12407  N   GLY B 218      11.253  -2.418 -23.378  1.00  0.66
N
ATOM  12408  CA  GLY B 218      11.859  -2.301 -24.701  1.00  0.93
C
ATOM  12409  C   GLY B 218      11.656  -3.545 -25.568  1.00  0.93
C
ATOM  12410  O   GLY B 218      12.371  -3.723 -26.556  1.00  1.16
O
ATOM  12411  H   GLY B 218      10.366  -1.964 -23.193  1.00  0.79
H
ATOM  12412 1HA  GLY B 218      11.439  -1.436 -25.212  1.00  1.12
H
ATOM  12413 2HA  GLY B 218      12.925  -2.110 -24.589  1.00  1.12
H
ATOM  12414  N   ARG B 219      10.688  -4.386 -25.195  1.00  0.81
N
ATOM  12415  CA  ARG B 219      10.364  -5.618 -25.911  1.00  0.84
C
ATOM  12416  C   ARG B 219      10.293  -5.461 -27.422  1.00  1.04
C
ATOM  12417  O   ARG B 219       9.424  -4.764 -27.945  1.00  1.51
O
ATOM  12418  CB  ARG B 219       9.020  -6.152 -25.449  1.00  1.26
C
ATOM  12419  CG  ARG B 219       8.621  -7.471 -26.068  1.00  1.26
C
ATOM  12420  CD  ARG B 219       7.968  -7.250 -27.380  1.00  1.26
C
ATOM  12421  NE  ARG B 219       7.463  -8.474 -27.956  1.00  1.26
N
```

```
ATOM  12422  CZ  ARG B 219       8.203  -9.386 -28.614  1.00  1.26
C
ATOM  12423  NH1 ARG B 219       9.504  -9.227 -28.737  1.00  1.26
N
ATOM  12424  NH2 ARG B 219       7.610 -10.445 -29.134  1.00  1.26
N
ATOM  12425  H   ARG B 219      10.163  -4.190 -24.354  1.00  0.97
H
ATOM  12426  HA  ARG B 219      11.127  -6.355 -25.680  1.00  1.01
H
ATOM  12427 1HB  ARG B 219       9.027  -6.301 -24.375  1.00  1.51
H
ATOM  12428 2HB  ARG B 219       8.238  -5.428 -25.677  1.00  1.51
H
ATOM  12429 1HG  ARG B 219       9.506  -8.090 -26.220  1.00  1.51
H
ATOM  12430 2HG  ARG B 219       7.918  -7.986 -25.412  1.00  1.51
H
ATOM  12431 1HD  ARG B 219       7.127  -6.568 -27.251  1.00  1.51
H
ATOM  12432 2HD  ARG B 219       8.676  -6.818 -28.081  1.00  1.51
H
ATOM  12433  HE  ARG B 219       6.462  -8.625 -27.901  1.00  1.51
H
ATOM  12434 1HH1 ARG B 219       9.956  -8.417 -28.334  1.00  1.51
H
ATOM  12435 2HH1 ARG B 219      10.053  -9.914 -29.233  1.00  1.51
H
ATOM  12436 1HH2 ARG B 219       6.608 -10.557 -29.034  1.00  1.51
H
ATOM  12437 2HH2 ARG B 219       8.150 -11.137 -29.631  1.00  1.51
H
ATOM  12438  N   GLY B 220      11.197  -6.147 -28.120  1.00  0.88
N
ATOM  12439  CA  GLY B 220      11.228  -6.144 -29.579  1.00  0.90
C
ATOM  12440  C   GLY B 220      12.228  -5.163 -30.177  1.00  0.92
C
ATOM  12441  O   GLY B 220      12.438  -5.162 -31.390  1.00  1.02
O
ATOM  12442  H   GLY B 220      11.880  -6.704 -27.615  1.00  1.06
H
ATOM  12443 1HA  GLY B 220      11.463  -7.149 -29.929  1.00  1.08
H
ATOM  12444 2HA  GLY B 220      10.234  -5.912 -29.955  1.00  1.08
H
ATOM  12445  N   THR B 221      12.848  -4.333 -29.340  1.00  0.91
N
ATOM  12446  CA  THR B 221      13.819  -3.370 -29.834  1.00  0.96
C
ATOM  12447  C   THR B 221      15.276  -3.845 -29.619  1.00  1.07
C
ATOM  12448  O   THR B 221      15.720  -3.981 -28.475  1.00  1.17
O
ATOM  12449  CB  THR B 221      13.627  -2.013 -29.150  1.00  1.44
C
ATOM  12450  OG1 THR B 221      12.315  -1.506 -29.422  1.00  1.44
O
```

ATOM 12451 CG2 THR B 221   14.645 -1.053 -29.700 1.00 1.44
C
ATOM 12452 H   THR B 221   12.642 -4.353 -28.351 1.00 1.09
H
ATOM 12453 HA THR B 221   13.629 -3.219 -30.891 1.00 1.15
H
ATOM 12454 HB THR B 221   13.755 -2.117 -28.074 1.00 1.73
H
ATOM 12455 HG1 THR B 221   11.659 -2.086 -29.024 1.00 1.73
H
ATOM 12456 1HG2 THR B 221   14.524 -0.077 -29.231 1.00 1.73
H
ATOM 12457 2HG2 THR B 221   15.646 -1.433 -29.505 1.00 1.73
H
ATOM 12458 3HG2 THR B 221   14.495 -0.968 -30.771 1.00 1.73
H
ATOM 12459 N   PRO B 222   16.014 -4.131 -30.707 1.00 1.45
N
ATOM 12460 CA PRO B 222   17.417 -4.554 -30.776 1.00 1.59
C
ATOM 12461 C   PRO B 222   18.316 -3.786 -29.815 1.00 1.66
C
ATOM 12462 O   PRO B 222   18.424 -2.560 -29.903 1.00 1.65
O
ATOM 12463 CB PRO B 222   17.801 -4.238 -32.225 1.00 2.39
C
ATOM 12464 CG PRO B 222   16.532 -4.377 -32.977 1.00 2.39
C
ATOM 12465 CD PRO B 222   15.497 -3.811 -32.057 1.00 2.39
C
ATOM 12466 HA PRO B 222   17.475 -5.635 -30.581 1.00 1.91
H
ATOM 12467 1HB PRO B 222   18.226 -3.227 -32.292 1.00 2.86
H
ATOM 12468 2HB PRO B 222   18.577 -4.938 -32.568 1.00 2.86
H
ATOM 12469 1HG PRO B 222   16.593 -3.824 -33.925 1.00 2.86
H
ATOM 12470 2HG PRO B 222   16.348 -5.432 -33.228 1.00 2.86
H
ATOM 12471 1HD PRO B 222   15.404 -2.726 -32.183 1.00 2.86
H
ATOM 12472 2HD PRO B 222   14.548 -4.332 -32.238 1.00 2.86
H
ATOM 12473 N   SER B 223   18.985 -4.522 -28.919 1.00 1.80
N
ATOM 12474 CA SER B 223   19.936 -3.981 -27.933 1.00 1.91
C
ATOM 12475 C   SER B 223   19.318 -3.089 -26.841 1.00 2.12
C
ATOM 12476 O   SER B 223   20.048 -2.538 -26.016 1.00 4.82
O
ATOM 12477 CB SER B 223   21.035 -3.194 -28.634 1.00 2.86
C
ATOM 12478 OG SER B 223   21.722 -3.993 -29.559 1.00 2.86
O
ATOM 12479 H   SER B 223   18.828 -5.521 -28.922 1.00 2.16
H

ATOM 12480 HA SER B 223   20.405 -4.829 -27.433 1.00 2.29
H
ATOM 12481 1HB SER B 223   20.619 -2.322 -29.134 1.00 3.44
H
ATOM 12482 2HB SER B 223   21.738 -2.826 -27.887 1.00 3.44
H
ATOM 12483 HG SER B 223   22.131 -4.696 -29.048 1.00 3.44
H
ATOM 12484 N   ILE B 224   17.994 -2.932 -26.838 1.00 1.29
N
ATOM 12485 CA ILE B 224   17.312 -2.090 -25.863 1.00 1.21
C
ATOM 12486 C   ILE B 224   16.503 -2.956 -24.910 1.00 1.15
C
ATOM 12487 O   ILE B 224   16.459 -2.689 -23.707 1.00 1.24
O
ATOM 12488 CB ILE B 224   16.385 -1.080 -26.551 1.00 1.81
C
ATOM 12489 CG1 ILE B 224   17.169 -0.214 -27.543 1.00 1.81
C
ATOM 12490 CG2 ILE B 224   15.711 -0.207 -25.512 1.00 1.81
C
ATOM 12491 CD1 ILE B 224   18.276   0.582 -26.929 1.00 1.81
C
ATOM 12492 H   ILE B 224   17.422 -3.405 -27.523 1.00 1.55
H
ATOM 12493 HA ILE B 224   18.057 -1.545 -25.285 1.00 1.45
H
ATOM 12494 HB ILE B 224   15.628 -1.620 -27.111 1.00 2.18
H
ATOM 12495 1HG1 ILE B 224   17.595 -0.857 -28.309 1.00 2.18
H
ATOM 12496 2HG1 ILE B 224   16.481   0.482 -28.024 1.00 2.18
H
ATOM 12497 1HG2 ILE B 224   15.044   0.496 -26.010 1.00 2.18
H
ATOM 12498 2HG2 ILE B 224   15.138 -0.831 -24.827 1.00 2.18
H
ATOM 12499 3HG2 ILE B 224   16.468   0.343 -24.955 1.00 2.18
H
ATOM 12500 1HD1 ILE B 224   18.778   1.166 -27.700 1.00 2.18
H
ATOM 12501 2HD1 ILE B 224   17.854   1.252 -26.182 1.00 2.18
H
ATOM 12502 3HD1 ILE B 224   18.993 -0.088 -26.457 1.00 2.18
H
ATOM 12503 N   GLU B 225   15.849 -3.978 -25.473 1.00 1.09
N
ATOM 12504 CA GLU B 225   15.051 -4.922 -24.703 1.00 1.00
C
ATOM 12505 C   GLU B 225   15.813 -5.471 -23.503 1.00 0.96
C
ATOM 12506 O   GLU B 225   16.905 -6.023 -23.649 1.00 1.06
O
ATOM 12507 CB GLU B 225   14.597 -6.093 -25.581 1.00 1.50
C
ATOM 12508 CG GLU B 225   13.727 -7.112 -24.850 1.00 1.50
C

```
ATOM  12509  CD  GLU B 225      13.288  -8.267 -25.717  1.00  1.50
C
ATOM  12510  OE1 GLU B 225      12.720  -8.045 -26.764  1.00  1.50
O
ATOM  12511  OE2 GLU B 225      13.530  -9.385 -25.326  1.00  1.50
O
ATOM  12512  H   GLU B 225      15.898  -4.097 -26.476  1.00  1.31
H
ATOM  12513  HA  GLU B 225      14.171  -4.406 -24.345  1.00  1.20
H
ATOM  12514 1HB  GLU B 225      14.033  -5.713 -26.433  1.00  1.80
H
ATOM  12515 2HB  GLU B 225      15.471  -6.615 -25.972  1.00  1.80
H
ATOM  12516 1HG  GLU B 225      14.294  -7.509 -24.009  1.00  1.80
H
ATOM  12517 2HG  GLU B 225      12.853  -6.604 -24.448  1.00  1.80
H
ATOM  12518  N   ASP B 226      15.204  -5.337 -22.322  1.00  0.85
N
ATOM  12519  CA  ASP B 226      15.775  -5.790 -21.055  1.00  0.86
C
ATOM  12520  C   ASP B 226      17.074  -5.080 -20.655  1.00  1.44
C
ATOM  12521  O   ASP B 226      17.849  -5.615 -19.862  1.00  4.63
O
ATOM  12522  CB  ASP B 226      16.024  -7.308 -21.119  1.00  1.29
C
ATOM  12523  CG  ASP B 226      16.245  -7.967 -19.762  1.00  1.29
C
ATOM  12524  OD1 ASP B 226      15.785  -7.455 -18.771  1.00  1.29
O
ATOM  12525  OD2 ASP B 226      16.888  -8.989 -19.728  1.00  1.29
O
ATOM  12526  H   ASP B 226      14.311  -4.868 -22.302  1.00  1.02
H
ATOM  12527  HA  ASP B 226      15.039  -5.600 -20.273  1.00  1.03
H
ATOM  12528 1HB  ASP B 226      15.177  -7.792 -21.605  1.00  1.55
H
ATOM  12529 2HB  ASP B 226      16.903  -7.505 -21.733  1.00  1.55
H
ATOM  12530  N   ALA B 227      17.289  -3.858 -21.150  1.00  1.22
N
ATOM  12531  CA  ALA B 227      18.477  -3.095 -20.787  1.00  1.31
C
ATOM  12532  C   ALA B 227      18.101  -1.841 -20.007  1.00  1.25
C
ATOM  12533  O   ALA B 227      17.051  -1.240 -20.241  1.00  1.23
O
ATOM  12534  CB  ALA B 227      19.270  -2.722 -22.032  1.00  1.97
C
ATOM  12535  H   ALA B 227      16.641  -3.459 -21.817  1.00  1.46
H
ATOM  12536  HA  ALA B 227      19.100  -3.717 -20.145  1.00  1.57
H
ATOM  12537 1HB  ALA B 227      20.168  -2.175 -21.743  1.00  2.36
H
```

```
ATOM  12538 2HB  ALA B 227      19.555  -3.628 -22.567  1.00  2.36
H
ATOM  12539 3HB  ALA B 227      18.660  -2.097 -22.680  1.00  2.36
H
ATOM  12540  N   GLU B 228      18.970  -1.453 -19.080  1.00  1.37
N
ATOM  12541  CA  GLU B 228      18.759  -0.272 -18.252  1.00  1.37
C
ATOM  12542  C   GLU B 228      19.240   0.994 -18.944  1.00  1.55
C
ATOM  12543  O   GLU B 228      19.937   0.929 -19.956  1.00  2.14
O
ATOM  12544  CB  GLU B 228      19.478  -0.417 -16.904  1.00  2.06
C
ATOM  12545  CG  GLU B 228      18.933  -1.510 -16.000  1.00  2.06
C
ATOM  12546  CD  GLU B 228      19.661  -1.604 -14.685  1.00  2.06
C
ATOM  12547  OE1 GLU B 228      20.715  -1.027 -14.571  1.00  2.06
O
ATOM  12548  OE2 GLU B 228      19.160  -2.246 -13.792  1.00  2.06
O
ATOM  12549  H   GLU B 228      19.811  -1.997 -18.941  1.00  1.64
H
ATOM  12550  HA  GLU B 228      17.689  -0.170 -18.065  1.00  1.64
H
ATOM  12551 1HB  GLU B 228      20.533  -0.629 -17.080  1.00  2.47
H
ATOM  12552 2HB  GLU B 228      19.423   0.523 -16.356  1.00  2.47
H
ATOM  12553 1HG  GLU B 228      17.885  -1.297 -15.802  1.00  2.47
H
ATOM  12554 2HG  GLU B 228      18.994  -2.466 -16.516  1.00  2.47
H
ATOM  12555  N   SER B 229      18.849   2.138 -18.377  1.00  1.65
N
ATOM  12556  CA  SER B 229      19.203   3.479 -18.848  1.00  2.01
C
ATOM  12557  C   SER B 229      18.511   3.856 -20.153  1.00  2.16
C
ATOM  12558  O   SER B 229      19.072   4.596 -20.962  1.00  5.71
O
ATOM  12559  CB  SER B 229      20.710   3.616 -19.009  1.00  3.01
C
ATOM  12560  OG  SER B 229      21.368   3.386 -17.793  1.00  3.01
O
ATOM  12561  H   SER B 229      18.272   2.073 -17.550  1.00  1.98
H
ATOM  12562  HA  SER B 229      18.884   4.194 -18.087  1.00  2.41
H
ATOM  12563 1HB  SER B 229      21.080   2.929 -19.766  1.00  3.62
H
ATOM  12564 2HB  SER B 229      20.941   4.621 -19.356  1.00  3.62
H
ATOM  12565  HG  SER B 229      22.306   3.463 -17.980  1.00  3.62
H
ATOM  12566  N   TRP B 230      17.285   3.368 -20.340  1.00  1.62
N
```

ATOM 12567 CA TRP B 230   16.490   3.710 -21.515 1.00 2.60
C
ATOM 12568 C   TRP B 230   15.144   4.330 -21.147 1.00 1.65
C
ATOM 12569 O   TRP B 230   14.347   4.668 -22.030 1.00 1.76
O
ATOM 12570 CB TRP B 230   16.321   2.486 -22.404 1.00 3.90
C
ATOM 12571 CG TRP B 230   17.639   2.095 -22.981 1.00 3.90
C
ATOM 12572 CD1 TRP B 230   18.383   0.993 -22.701 1.00 3.90
C
ATOM 12573 CD2 TRP B 230   18.405   2.866 -23.932 1.00 3.90
C
ATOM 12574 NE1 TRP B 230   19.558   1.022 -23.410 1.00 3.90
N
ATOM 12575 CE2 TRP B 230   19.589   2.164 -24.167 1.00 3.90
C
ATOM 12576 CE3 TRP B 230   18.185   4.083 -24.588 1.00 3.90
C
ATOM 12577 CZ2 TRP B 230   20.559   2.637 -25.036 1.00 3.90
C
ATOM 12578 CZ3 TRP B 230   19.154   4.555 -25.462 1.00 3.90
C
ATOM 12579 CH2 TRP B 230   20.312   3.850 -25.680 1.00 3.90
C
ATOM 12580 H   TRP B 230   16.894   2.748 -19.647 1.00 1.94
H
ATOM 12581 HA TRP B 230   17.044   4.447 -22.092 1.00 3.12
H
ATOM 12582 1HB TRP B 230   15.924   1.651 -21.827 1.00 4.68
H
ATOM 12583 2HB TRP B 230   15.625   2.702 -23.215 1.00 4.68
H
ATOM 12584 HD1 TRP B 230   18.093   0.207 -22.008 1.00 4.68
H
ATOM 12585 HE1 TRP B 230   20.283   0.320 -23.376 1.00 4.68
H
ATOM 12586 HE3 TRP B 230   17.269   4.647 -24.417 1.00 4.68
H
ATOM 12587 HZ2 TRP B 230   21.485   2.091 -25.223 1.00 4.68
H
ATOM 12588 HZ3 TRP B 230   18.975   5.503 -25.970 1.00 4.68
H
ATOM 12589 HH2 TRP B 230   21.053   4.250 -26.372 1.00 4.68
H
ATOM 12590 N   HIS B 231   14.889   4.467 -19.844 1.00 2.50
N
ATOM 12591 CA HIS B 231   13.637   5.034 -19.385 1.00 3.81
C
ATOM 12592 C   HIS B 231   13.554   6.488 -19.802 1.00 2.91
C
ATOM 12593 O   HIS B 231   14.480   7.269 -19.581 1.00 3.83
O
ATOM 12594 CB HIS B 231   13.496   4.892 -17.868 1.00 5.71
C
ATOM 12595 CG HIS B 231   12.122   5.175 -17.330 1.00 5.71
C

ATOM 12596 ND1 HIS B 231   11.602   6.447 -17.231 1.00 5.71
N
ATOM 12597 CD2 HIS B 231   11.192   4.345 -16.810 1.00 5.71
C
ATOM 12598 CE1 HIS B 231   10.386   6.375 -16.714 1.00 5.71
C
ATOM 12599 NE2 HIS B 231   10.121   5.114 -16.437 1.00 5.71
N
ATOM 12600 H   HIS B 231   15.572   4.182 -19.158 1.00 3.00
H
ATOM 12601 HA HIS B 231   12.810   4.508 -19.853 1.00 4.57
H
ATOM 12602 1HB HIS B 231   13.771   3.878 -17.573 1.00 6.86
H
ATOM 12603 2HB HIS B 231   14.195   5.570 -17.377 1.00 6.86
H
ATOM 12604 HD1 HIS B 231   12.194   7.187 -16.912 1.00 6.86
H
ATOM 12605 HD2 HIS B 231   11.167   3.266 -16.658 1.00 6.86
H
ATOM 12606 HE1 HIS B 231   9.797   7.279 -16.562 1.00 6.86
H
ATOM 12607 N   ALA B 232   12.436   6.824 -20.424 1.00 2.33
N
ATOM 12608 CA ALA B 232   12.133   8.142 -20.954 1.00 2.79
C
ATOM 12609 C   ALA B 232   13.119   8.563 -22.046 1.00 2.30
C
ATOM 12610 O   ALA B 232   13.340   9.759 -22.247 1.00 3.03
O
ATOM 12611 CB ALA B 232   12.122   9.168 -19.832 1.00 4.19
C
ATOM 12612 H   ALA B 232   11.741   6.107 -20.527 1.00 2.80
H
ATOM 12613 HA ALA B 232   11.140   8.103 -21.401 1.00 3.35
H
ATOM 12614 1HB ALA B 232   11.855 10.144 -20.237 1.00 5.02
H
ATOM 12615 2HB ALA B 232   11.389   8.873 -19.082 1.00 5.02
H
ATOM 12616 3HB ALA B 232   13.106   9.229 -19.370 1.00 5.02
H
ATOM 12617 N   LYS B 233   13.687   7.600 -22.778 1.00 1.67
N
ATOM 12618 CA LYS B 233   14.588   7.965 -23.856 1.00 1.81
C
ATOM 12619 C   LYS B 233   14.068   7.494 -25.216 1.00 1.64
C
ATOM 12620 O   LYS B 233   13.861   6.296 -25.412 1.00 2.61
O
ATOM 12621 CB LYS B 233   15.974   7.380 -23.627 1.00 2.71
C
ATOM 12622 CG LYS B 233   16.690   7.926 -22.408 1.00 2.71
C
ATOM 12623 CD LYS B 233   18.090   7.359 -22.317 1.00 2.71
C
ATOM 12624 CE LYS B 233   18.813   7.844 -21.072 1.00 2.71
C

ATOM 12625 NZ LYS B 233   20.154   7.216 -20.947 1.00 2.71
N
ATOM 12626 H   LYS B 233   13.524   6.621 -22.575 1.00 2.00
H
ATOM 12627 HA LYS B 233   14.683   9.043 -23.849 1.00 2.17
H
ATOM 12628 1HB LYS B 233   15.896   6.298 -23.511 1.00 3.26
H
ATOM 12629 2HB LYS B 233   16.600   7.573 -24.499 1.00 3.26
H
ATOM 12630 1HG LYS B 233   16.748   9.013 -22.471 1.00 3.26
H
ATOM 12631 2HG LYS B 233   16.139   7.658 -21.508 1.00 3.26
H
ATOM 12632 1HD LYS B 233   18.041   6.273 -22.312 1.00 3.26
H
ATOM 12633 2HD LYS B 233   18.660   7.669 -23.192 1.00 3.26
H
ATOM 12634 1HE LYS B 233   18.932   8.925 -21.121 1.00 3.26
H
ATOM 12635 2HE LYS B 233   18.222   7.593 -20.192 1.00 3.26
H
ATOM 12636 1HZ LYS B 233   20.612   7.546 -20.109 1.00 3.26
H
ATOM 12637 2HZ LYS B 233   20.023   6.210 -20.893 1.00 3.26
H
ATOM 12638 3HZ LYS B 233   20.716   7.444 -21.753 1.00 3.26
H
ATOM 12639 N   PRO B 234   13.870   8.419 -26.167 1.00 1.35
N
ATOM 12640 CA PRO B 234   13.448   8.218 -27.542 1.00 1.31
C
ATOM 12641 C   PRO B 234   14.609   7.673 -28.349 1.00 1.04
C
ATOM 12642 O   PRO B 234   15.770   7.900 -28.002 1.00 1.23
O
ATOM 12643 CB PRO B 234   13.049   9.617 -28.006 1.00 1.97
C
ATOM 12644 CG PRO B 234   13.924 10.523 -27.214 1.00 1.97
C
ATOM 12645 CD PRO B 234   14.076   9.853 -25.878 1.00 1.97
C
ATOM 12646 HA PRO B 234   12.593   7.537 -27.569 1.00 1.57
H
ATOM 12647 1HB PRO B 234   13.205   9.714 -29.091 1.00 2.36
H
ATOM 12648 2HB PRO B 234   11.977   9.786 -27.819 1.00 2.36
H
ATOM 12649 1HG PRO B 234   14.887 10.658 -27.728 1.00 2.36
H
ATOM 12650 2HG PRO B 234   13.471 11.517 -27.128 1.00 2.36
H
ATOM 12651 1HD PRO B 234   15.099 10.026 -25.512 1.00 2.36
H
ATOM 12652 2HD PRO B 234   13.315 10.211 -25.167 1.00 2.36
H
ATOM 12653 N   MET B 235   14.310   6.964 -29.426 1.00 1.18
N

ATOM 12654 CA MET B 235 15.379 6.451 -30.262 1.00 1.13
C
ATOM 12655 C MET B 235 15.848 7.549 -31.221 1.00 1.51
C
ATOM 12656 O MET B 235 15.067 8.439 -31.562 1.00 3.09
O
ATOM 12657 CB MET B 235 14.904 5.227 -31.047 1.00 1.69
C
ATOM 12658 CG MET B 235 14.503 4.030 -30.190 1.00 1.69
C
ATOM 12659 SD MET B 235 15.895 3.302 -29.292 1.00 1.69
S
ATOM 12660 CE MET B 235 15.661 3.943 -27.633 1.00 1.69
C
ATOM 12661 H MET B 235 13.346 6.792 -29.671 1.00 1.42
H
ATOM 12662 HA MET B 235 16.201 6.155 -29.618 1.00 1.36
H
ATOM 12663 1HB MET B 235 14.042 5.501 -31.654 1.00 2.03
H
ATOM 12664 2HB MET B 235 15.693 4.902 -31.725 1.00 2.03
H
ATOM 12665 1HG MET B 235 13.752 4.341 -29.465 1.00 2.03
H
ATOM 12666 2HG MET B 235 14.062 3.261 -30.824 1.00 2.03
H
ATOM 12667 1HE MET B 235 16.452 3.565 -26.984 1.00 2.03
H
ATOM 12668 2HE MET B 235 15.699 5.032 -27.655 1.00 2.03
H
ATOM 12669 3HE MET B 235 14.693 3.620 -27.250 1.00 2.03
H
ATOM 12670 N PRO B 236 17.120 7.517 -31.634 1.00 1.14
N
ATOM 12671 CA PRO B 236 17.749 8.372 -32.635 1.00 1.43
C
ATOM 12672 C PRO B 236 17.064 8.150 -33.967 1.00 1.69
C
ATOM 12673 O PRO B 236 16.561 7.062 -34.208 1.00 2.50
O
ATOM 12674 CB PRO B 236 19.211 7.921 -32.639 1.00 2.15
C
ATOM 12675 CG PRO B 236 19.440 7.383 -31.265 1.00 2.15
C
ATOM 12676 CD PRO B 236 18.126 6.798 -30.829 1.00 2.15
C
ATOM 12677 HA PRO B 236 17.659 9.423 -32.321 1.00 1.72
H
ATOM 12678 1HB PRO B 236 19.374 7.172 -33.426 1.00 2.57
H
ATOM 12679 2HB PRO B 236 19.864 8.775 -32.872 1.00 2.57
H
ATOM 12680 1HG PRO B 236 20.246 6.633 -31.285 1.00 2.57
H
ATOM 12681 2HG PRO B 236 19.778 8.190 -30.599 1.00 2.57
H
ATOM 12682 1HD PRO B 236 18.082 5.719 -31.036 1.00 2.57
H

```
ATOM  12683 2HD  PRO B 236      17.983   7.015 -29.762  1.00  2.57
H
ATOM  12684  N   ARG B 237      17.045   9.162 -34.826  1.00  2.25
N
ATOM  12685  CA  ARG B 237      16.402   9.060 -36.141  1.00  3.01
C
ATOM  12686  C   ARG B 237      16.807   7.805 -36.920  1.00  2.46
C
ATOM  12687  O   ARG B 237      15.952   6.999 -37.296  1.00  2.86
O
ATOM  12688  CB  ARG B 237      16.715  10.301 -36.964  1.00  4.51
C
ATOM  12689  CG  ARG B 237      16.121  10.323 -38.364  1.00  4.51
C
ATOM  12690  CD  ARG B 237      16.453  11.588 -39.072  1.00  4.51
C
ATOM  12691  NE  ARG B 237      17.882  11.740 -39.271  1.00  4.51
N
ATOM  12692  CZ  ARG B 237      18.512  12.895 -39.550  1.00  4.51
C
ATOM  12693  NH1 ARG B 237      17.837  14.017 -39.675  1.00  4.51
N
ATOM  12694  NH2 ARG B 237      19.823  12.875 -39.691  1.00  4.51
N
ATOM  12695  H   ARG B 237      17.483  10.034 -34.564  1.00  2.70
H
ATOM  12696  HA  ARG B 237      15.326   9.025 -35.987  1.00  3.61
H
ATOM  12697 1HB  ARG B 237      16.349  11.184 -36.441  1.00  5.42
H
ATOM  12698 2HB  ARG B 237      17.796  10.409 -37.065  1.00  5.42
H
ATOM  12699 1HG  ARG B 237      16.522   9.490 -38.943  1.00  5.42
H
ATOM  12700 2HG  ARG B 237      15.036  10.236 -38.302  1.00  5.42
H
ATOM  12701 1HD  ARG B 237      15.971  11.594 -40.049  1.00  5.42
H
ATOM  12702 2HD  ARG B 237      16.098  12.436 -38.488  1.00  5.42
H
ATOM  12703  HE  ARG B 237      18.470  10.918 -39.188  1.00  5.42
H
ATOM  12704 1HH1 ARG B 237      16.834  14.018 -39.563  1.00  5.42
H
ATOM  12705 2HH1 ARG B 237      18.322  14.877 -39.884  1.00  5.42
H
ATOM  12706 1HH2 ARG B 237      20.311  11.989 -39.583  1.00  5.42
H
ATOM  12707 2HH2 ARG B 237      20.329  13.722 -39.900  1.00  5.42
H
ATOM  12708  N   GLU B 238      18.106   7.656 -37.165  1.00  2.05
N
ATOM  12709  CA  GLU B 238      18.630   6.512 -37.903  1.00  2.04
C
ATOM  12710  C   GLU B 238      18.431   5.199 -37.165  1.00  1.96
C
ATOM  12711  O   GLU B 238      18.187   4.165 -37.790  1.00  1.98
O
```

ATOM 12712 CB GLU B 238 20.111 6.723 -38.204 1.00 3.06
C
ATOM 12713 CG GLU B 238 20.380 7.841 -39.196 1.00 3.06
C
ATOM 12714 CD GLU B 238 20.312 9.207 -38.580 1.00 3.06
C
ATOM 12715 OE1 GLU B 238 20.263 9.307 -37.379 1.00 3.06
O
ATOM 12716 OE2 GLU B 238 20.275 10.159 -39.325 1.00 3.06
O
ATOM 12717 H GLU B 238 18.753 8.355 -36.831 1.00 2.46
H
ATOM 12718 HA GLU B 238 18.092 6.447 -38.851 1.00 2.45
H
ATOM 12719 1HB GLU B 238 20.645 6.953 -37.281 1.00 3.67
H
ATOM 12720 2HB GLU B 238 20.535 5.804 -38.609 1.00 3.67
H
ATOM 12721 1HG GLU B 238 21.372 7.699 -39.623 1.00 3.67
H
ATOM 12722 2HG GLU B 238 19.654 7.775 -40.005 1.00 3.67
H
ATOM 12723 N ARG B 239 18.543 5.244 -35.839 1.00 2.14
N
ATOM 12724 CA ARG B 239 18.371 4.052 -35.032 1.00 2.27
C
ATOM 12725 C ARG B 239 16.938 3.566 -35.124 1.00 1.99
C
ATOM 12726 O ARG B 239 16.699 2.376 -35.317 1.00 2.16
O
ATOM 12727 CB ARG B 239 18.727 4.321 -33.584 1.00 3.41
C
ATOM 12728 CG ARG B 239 18.634 3.107 -32.688 1.00 3.41
C
ATOM 12729 CD ARG B 239 19.620 2.072 -33.095 1.00 3.41
C
ATOM 12730 NE ARG B 239 19.531 0.857 -32.297 1.00 3.41
N
ATOM 12731 CZ ARG B 239 20.294 -0.236 -32.504 1.00 3.41
C
ATOM 12732 NH1 ARG B 239 21.190 -0.228 -33.466 1.00 3.41
N
ATOM 12733 NH2 ARG B 239 20.155 -1.317 -31.756 1.00 3.41
N
ATOM 12734 H ARG B 239 18.750 6.121 -35.385 1.00 2.57
H
ATOM 12735 HA ARG B 239 19.029 3.272 -35.415 1.00 2.72
H
ATOM 12736 1HB ARG B 239 19.744 4.708 -33.521 1.00 4.09
H
ATOM 12737 2HB ARG B 239 18.060 5.083 -33.183 1.00 4.09
H
ATOM 12738 1HG ARG B 239 18.831 3.391 -31.654 1.00 4.09
H
ATOM 12739 2HG ARG B 239 17.638 2.687 -32.768 1.00 4.09
H
ATOM 12740 1HD ARG B 239 19.449 1.805 -34.137 1.00 4.09
H

```
ATOM  12741 2HD  ARG B 239      20.626   2.475 -32.987  1.00  4.09
H
ATOM  12742  HE  ARG B 239      18.850   0.825 -31.549  1.00  4.09
H
ATOM  12743 1HH1 ARG B 239      21.306   0.594 -34.044  1.00  4.09
H
ATOM  12744 2HH1 ARG B 239      21.765  -1.043 -33.626  1.00  4.09
H
ATOM  12745 1HH2 ARG B 239      19.464  -1.357 -31.015  1.00  4.09
H
ATOM  12746 2HH2 ARG B 239      20.737  -2.124 -31.928  1.00  4.09
H
ATOM  12747  N   ALA B 240      16.000   4.502 -35.016  1.00  1.73
N
ATOM  12748  CA  ALA B 240      14.581   4.230 -35.107  1.00  1.55
C
ATOM  12749  C   ALA B 240      14.240   3.630 -36.453  1.00  1.60
C
ATOM  12750  O   ALA B 240      13.487   2.663 -36.519  1.00  1.52
O
ATOM  12751  CB  ALA B 240      13.789   5.507 -34.885  1.00  2.33
C
ATOM  12752  H   ALA B 240      16.283   5.453 -34.852  1.00  2.08
H
ATOM  12753  HA  ALA B 240      14.319   3.505 -34.337  1.00  1.86
H
ATOM  12754 1HB  ALA B 240      12.724   5.293 -34.942  1.00  2.79
H
ATOM  12755 2HB  ALA B 240      14.027   5.918 -33.904  1.00  2.79
H
ATOM  12756 3HB  ALA B 240      14.051   6.232 -35.653  1.00  2.79
H
ATOM  12757  N   ASP B 241      14.809   4.184 -37.526  1.00  1.80
N
ATOM  12758  CA  ASP B 241      14.569   3.647 -38.856  1.00  1.88
C
ATOM  12759  C   ASP B 241      14.996   2.188 -38.952  1.00  1.85
C
ATOM  12760  O   ASP B 241      14.243   1.357 -39.457  1.00  1.80
O
ATOM  12761  CB  ASP B 241      15.303   4.472 -39.914  1.00  2.82
C
ATOM  12762  CG  ASP B 241      14.668   5.838 -40.153  1.00  2.82
C
ATOM  12763  OD1 ASP B 241      13.550   6.035 -39.737  1.00  2.82
O
ATOM  12764  OD2 ASP B 241      15.302   6.666 -40.762  1.00  2.82
O
ATOM  12765  H   ASP B 241      15.402   5.001 -37.422  1.00  2.16
H
ATOM  12766  HA  ASP B 241      13.499   3.700 -39.061  1.00  2.26
H
ATOM  12767 1HB  ASP B 241      16.339   4.621 -39.607  1.00  3.38
H
ATOM  12768 2HB  ASP B 241      15.316   3.926 -40.857  1.00  3.38
H
ATOM  12769  N   ALA B 242      16.194   1.878 -38.449  1.00  1.95
N
```

ATOM 12770 CA ALA B 242   16.699   0.509 -38.474 1.00 1.99
C
ATOM 12771 C   ALA B 242   15.808 -0.435 -37.673 1.00 1.77
C
ATOM 12772 O   ALA B 242   15.475 -1.524 -38.146 1.00 1.81
O
ATOM 12773 CB ALA B 242   18.117   0.470 -37.927 1.00 2.98
C
ATOM 12774 H   ALA B 242   16.778   2.611 -38.058 1.00 2.34
H
ATOM 12775 HA ALA B 242   16.706   0.170 -39.509 1.00 2.39
H
ATOM 12776 1HB ALA B 242   18.499 -0.549 -37.976 1.00 3.58
H
ATOM 12777 2HB ALA B 242   18.754   1.126 -38.523 1.00 3.58
H
ATOM 12778 3HB ALA B 242   18.119   0.809 -36.892 1.00 3.58
H
ATOM 12779 N   ILE B 243   15.414   0.003 -36.475 1.00 1.63
N
ATOM 12780 CA ILE B 243   14.573 -0.781 -35.580 1.00 1.45
C
ATOM 12781 C   ILE B 243   13.235 -1.091 -36.202 1.00 1.45
C
ATOM 12782 O   ILE B 243   12.801 -2.241 -36.204 1.00 1.51
O
ATOM 12783 CB ILE B 243   14.326 -0.021 -34.273 1.00 2.17
C
ATOM 12784 CG1 ILE B 243   15.606   0.110 -33.460 1.00 2.17
C
ATOM 12785 CG2 ILE B 243   13.274 -0.756 -33.461 1.00 2.17
C
ATOM 12786 CD1 ILE B 243   15.481   1.136 -32.362 1.00 2.17
C
ATOM 12787 H   ILE B 243   15.730   0.910 -36.157 1.00 1.96
H
ATOM 12788 HA ILE B 243   15.080 -1.720 -35.359 1.00 1.74
H
ATOM 12789 HB ILE B 243   13.974   0.986 -34.494 1.00 2.61
H
ATOM 12790 1HG1 ILE B 243   15.832 -0.850 -33.004 1.00 2.61
H
ATOM 12791 2HG1 ILE B 243   16.436   0.376 -34.110 1.00 2.61
H
ATOM 12792 1HG2 ILE B 243   13.087 -0.214 -32.534 1.00 2.61
H
ATOM 12793 2HG2 ILE B 243   12.351 -0.821 -34.035 1.00 2.61
H
ATOM 12794 3HG2 ILE B 243   13.627 -1.759 -33.228 1.00 2.61
H
ATOM 12795 1HD1 ILE B 243   16.411   1.179 -31.795 1.00 2.61
H
ATOM 12796 2HD1 ILE B 243   15.280   2.113 -32.804 1.00 2.61
H
ATOM 12797 3HD1 ILE B 243   14.664   0.872 -31.700 1.00 2.61
H
ATOM 12798 N   ILE B 244   12.589 -0.056 -36.723 1.00 1.50
N

ATOM 12799 CA ILE B 244 11.295 -0.181 -37.359 1.00 1.50
C
ATOM 12800 C ILE B 244 11.325 -1.103 -38.548 1.00 1.57
C
ATOM 12801 O ILE B 244 10.425 -1.919 -38.694 1.00 1.68
O
ATOM 12802 CB ILE B 244 10.757 1.188 -37.757 1.00 2.25
C
ATOM 12803 CG1 ILE B 244 10.396 1.967 -36.490 1.00 2.25
C
ATOM 12804 CG2 ILE B 244 9.569 1.038 -38.693 1.00 2.25
C
ATOM 12805 CD1 ILE B 244 10.133 3.425 -36.741 1.00 2.25
C
ATOM 12806 H ILE B 244 13.007 0.862 -36.674 1.00 1.80
H
ATOM 12807 HA ILE B 244 10.603 -0.604 -36.631 1.00 1.80
H
ATOM 12808 HB ILE B 244 11.541 1.749 -38.265 1.00 2.70
H
ATOM 12809 1HG1 ILE B 244 9.503 1.528 -36.048 1.00 2.70
H
ATOM 12810 2HG1 ILE B 244 11.211 1.881 -35.771 1.00 2.70
H
ATOM 12811 1HG2 ILE B 244 9.201 2.024 -38.974 1.00 2.70
H
ATOM 12812 2HG2 ILE B 244 9.878 0.498 -39.587 1.00 2.70
H
ATOM 12813 3HG2 ILE B 244 8.779 0.483 -38.194 1.00 2.70
H
ATOM 12814 1HD1 ILE B 244 9.875 3.919 -35.805 1.00 2.70
H
ATOM 12815 2HD1 ILE B 244 11.025 3.888 -37.164 1.00 2.70
H
ATOM 12816 3HD1 ILE B 244 9.307 3.522 -37.442 1.00 2.70
H
ATOM 12817 N LYS B 245 12.354 -0.999 -39.391 1.00 1.58
N
ATOM 12818 CA LYS B 245 12.463 -1.906 -40.527 1.00 1.65
C
ATOM 12819 C LYS B 245 12.536 -3.357 -40.055 1.00 1.66
C
ATOM 12820 O LYS B 245 11.898 -4.237 -40.639 1.00 1.69
O
ATOM 12821 CB LYS B 245 13.688 -1.561 -41.370 1.00 2.47
C
ATOM 12822 CG LYS B 245 13.564 -0.268 -42.165 1.00 2.47
C
ATOM 12823 CD LYS B 245 14.842 0.031 -42.933 1.00 2.47
C
ATOM 12824 CE LYS B 245 14.735 1.336 -43.708 1.00 2.47
C
ATOM 12825 NZ LYS B 245 15.989 1.642 -44.453 1.00 2.47
N
ATOM 12826 H LYS B 245 13.064 -0.290 -39.254 1.00 1.90
H
ATOM 12827 HA LYS B 245 11.570 -1.798 -41.146 1.00 1.98
H

```
ATOM  12828 1HB  LYS B 245      14.562  -1.472 -40.722  1.00  2.97
H
ATOM  12829 2HB  LYS B 245      13.884  -2.369 -42.075  1.00  2.97
H
ATOM  12830 1HG  LYS B 245      12.743  -0.365 -42.877  1.00  2.97
H
ATOM  12831 2HG  LYS B 245      13.338   0.561 -41.499  1.00  2.97
H
ATOM  12832 1HD  LYS B 245      15.677   0.101 -42.234  1.00  2.97
H
ATOM  12833 2HD  LYS B 245      15.043  -0.781 -43.632  1.00  2.97
H
ATOM  12834 1HE  LYS B 245      13.912   1.265 -44.418  1.00  2.97
H
ATOM  12835 2HE  LYS B 245      14.529   2.150 -43.014  1.00  2.97
H
ATOM  12836 1HZ  LYS B 245      15.880   2.513 -44.953  1.00  2.97
H
ATOM  12837 2HZ  LYS B 245      16.756   1.723 -43.799  1.00  2.97
H
ATOM  12838 3HZ  LYS B 245      16.181   0.899 -45.109  1.00  2.97
H
ATOM  12839  N   LEU B 246      13.287  -3.594 -38.977  1.00  1.72
N
ATOM  12840  CA  LEU B 246      13.385  -4.929 -38.412  1.00  1.85
C
ATOM  12841  C   LEU B 246      12.046  -5.394 -37.838  1.00  1.73
C
ATOM  12842  O   LEU B 246      11.676  -6.558 -38.001  1.00  1.92
O
ATOM  12843  CB  LEU B 246      14.473  -4.954 -37.333  1.00  2.78
C
ATOM  12844  CG  LEU B 246      15.905  -4.791 -37.860  1.00  2.78
C
ATOM  12845  CD1 LEU B 246      16.862  -4.631 -36.691  1.00  2.78
C
ATOM  12846  CD2 LEU B 246      16.268  -6.006 -38.698  1.00  2.78
C
ATOM  12847  H   LEU B 246      13.819  -2.841 -38.551  1.00  2.06
H
ATOM  12848  HA  LEU B 246      13.673  -5.616 -39.206  1.00  2.22
H
ATOM  12849 1HB  LEU B 246      14.287  -4.152 -36.623  1.00  3.33
H
ATOM  12850 2HB  LEU B 246      14.419  -5.904 -36.802  1.00  3.33
H
ATOM  12851  HG  LEU B 246      15.969  -3.897 -38.478  1.00  3.33
H
ATOM  12852 1HD1 LEU B 246      17.879  -4.509 -37.067  1.00  3.33
H
ATOM  12853 2HD1 LEU B 246      16.582  -3.750 -36.114  1.00  3.33
H
ATOM  12854 3HD1 LEU B 246      16.814  -5.514 -36.056  1.00  3.33
H
ATOM  12855 1HD2 LEU B 246      17.282  -5.894 -39.080  1.00  3.33
H
ATOM  12856 2HD2 LEU B 246      16.208  -6.903 -38.082  1.00  3.33
H
```

ATOM 12857 3HD2 LEU B 246 15.573 -6.093 -39.533 1.00 3.33
H
ATOM 12858 N ILE B 247 11.292 -4.482 -37.221 1.00 1.48
N
ATOM 12859 CA ILE B 247 9.975 -4.833 -36.701 1.00 1.32
C
ATOM 12860 C ILE B 247 9.032 -5.170 -37.847 1.00 1.47
C
ATOM 12861 O ILE B 247 8.309 -6.164 -37.790 1.00 1.66
O
ATOM 12862 CB ILE B 247 9.345 -3.707 -35.868 1.00 1.98
C
ATOM 12863 CG1 ILE B 247 10.132 -3.465 -34.583 1.00 1.98
C
ATOM 12864 CG2 ILE B 247 7.925 -4.117 -35.514 1.00 1.98
C
ATOM 12865 CD1 ILE B 247 9.720 -2.196 -33.871 1.00 1.98
C
ATOM 12866 H ILE B 247 11.645 -3.542 -37.087 1.00 1.78
H
ATOM 12867 HA ILE B 247 10.074 -5.714 -36.068 1.00 1.58
H
ATOM 12868 HB ILE B 247 9.333 -2.779 -36.436 1.00 2.38
H
ATOM 12869 1HG1 ILE B 247 9.981 -4.307 -33.908 1.00 2.38
H
ATOM 12870 2HG1 ILE B 247 11.193 -3.403 -34.817 1.00 2.38
H
ATOM 12871 1HG2 ILE B 247 7.456 -3.338 -34.919 1.00 2.38
H
ATOM 12872 2HG2 ILE B 247 7.353 -4.273 -36.428 1.00 2.38
H
ATOM 12873 3HG2 ILE B 247 7.948 -5.043 -34.940 1.00 2.38
H
ATOM 12874 1HD1 ILE B 247 10.313 -2.079 -32.964 1.00 2.38
H
ATOM 12875 2HD1 ILE B 247 9.885 -1.341 -34.527 1.00 2.38
H
ATOM 12876 3HD1 ILE B 247 8.664 -2.253 -33.608 1.00 2.38
H
ATOM 12877 N GLU B 248 9.057 -4.342 -38.894 1.00 1.60
N
ATOM 12878 CA GLU B 248 8.218 -4.530 -40.070 1.00 1.98
C
ATOM 12879 C GLU B 248 8.455 -5.872 -40.735 1.00 2.17
C
ATOM 12880 O GLU B 248 7.508 -6.502 -41.203 1.00 2.46
O
ATOM 12881 CB GLU B 248 8.450 -3.410 -41.091 1.00 2.97
C
ATOM 12882 CG GLU B 248 7.892 -2.049 -40.700 1.00 2.97
C
ATOM 12883 CD GLU B 248 8.219 -0.970 -41.700 1.00 2.97
C
ATOM 12884 OE1 GLU B 248 9.022 -1.215 -42.567 1.00 2.97
O
ATOM 12885 OE2 GLU B 248 7.659 0.098 -41.601 1.00 2.97
O

ATOM 12886 H   GLU B 248   9.670 -3.544 -38.871 1.00 1.92
H
ATOM 12887 HA GLU B 248   7.179 -4.492 -39.755 1.00 2.38
H
ATOM 12888 1HB GLU B 248   9.521 -3.290 -41.258 1.00 3.56
H
ATOM 12889 2HB GLU B 248   8.003 -3.691 -42.045 1.00 3.56
H
ATOM 12890 1HG GLU B 248   6.808 -2.127 -40.616 1.00 3.56
H
ATOM 12891 2HG GLU B 248   8.280 -1.766 -39.726 1.00 3.56
H
ATOM 12892 N   SER B 249   9.705 -6.354 -40.727 1.00 2.18
N
ATOM 12893 CA SER B 249   10.016 -7.656 -41.321 1.00 2.44
C
ATOM 12894 C   SER B 249   9.324 -8.820 -40.605 1.00 2.91
C
ATOM 12895 O   SER B 249   9.230 -9.917 -41.157 1.00 4.85
O
ATOM 12896 CB SER B 249   11.513 -7.925 -41.298 1.00 3.66
C
ATOM 12897 OG SER B 249   11.975 -8.202 -40.000 1.00 3.66
O
ATOM 12898 H   SER B 249   10.458 -5.788 -40.351 1.00 2.62
H
ATOM 12899 HA SER B 249   9.678 -7.646 -42.357 1.00 2.93
H
ATOM 12900 1HB SER B 249   11.737 -8.768 -41.952 1.00 4.39
H
ATOM 12901 2HB SER B 249   12.040 -7.058 -41.694 1.00 4.39
H
ATOM 12902 HG SER B 249   11.774 -7.430 -39.465 1.00 4.39
H
ATOM 12903 N   GLN B 250   8.852 -8.595 -39.376 1.00 2.21
N
ATOM 12904 CA GLN B 250   8.193 -9.630 -38.605 1.00 2.78
C
ATOM 12905 C   GLN B 250   6.668 -9.551 -38.729 1.00 3.61
C
ATOM 12906 O   GLN B 250   5.955 -10.389 -38.175 1.00 4.66
O
ATOM 12907 CB GLN B 250   8.618 -9.516 -37.144 1.00 4.17
C
ATOM 12908 CG GLN B 250   10.119 -9.639 -36.962 1.00 4.17
C
ATOM 12909 CD GLN B 250   10.657 -10.966 -37.459 1.00 4.17
C
ATOM 12910 OE1 GLN B 250   10.282 -12.030 -36.958 1.00 4.17
O
ATOM 12911 NE2 GLN B 250   11.529 -10.912 -38.460 1.00 4.17
N
ATOM 12912 H   GLN B 250   8.935 -7.685 -38.949 1.00 2.65
H
ATOM 12913 HA GLN B 250   8.512 -10.600 -38.986 1.00 3.34
H
ATOM 12914 1HB GLN B 250   8.302 -8.554 -36.743 1.00 5.00
H

```
ATOM  12915 2HB  GLN B 250       8.135 -10.297 -36.556  1.00  5.00
H
ATOM  12916 1HG  GLN B 250      10.609  -8.843 -37.522  1.00  5.00
H
ATOM  12917 2HG  GLN B 250      10.356  -9.550 -35.903  1.00  5.00
H
ATOM  12918 1HE2 GLN B 250      11.914 -11.756 -38.835  1.00  5.00
H
ATOM  12919 2HE2 GLN B 250      11.793 -10.026 -38.850  1.00  5.00
H
ATOM  12920  N   ILE B 251       6.172  -8.544 -39.453  1.00  4.14
N
ATOM  12921  CA  ILE B 251       4.741  -8.376 -39.662  1.00  6.44
C
ATOM  12922  C   ILE B 251       4.298  -9.133 -40.899  1.00  3.42
C
ATOM  12923  O   ILE B 251       4.793  -8.886 -41.999  1.00  3.11
O
ATOM  12924  CB  ILE B 251       4.370  -6.883 -39.786  1.00  9.66
C
ATOM  12925  CG1 ILE B 251       4.701  -6.163 -38.476  1.00  9.66
C
ATOM  12926  CG2 ILE B 251       2.911  -6.711 -40.159  1.00  9.66
C
ATOM  12927  CD1 ILE B 251       4.542  -4.661 -38.541  1.00  9.66
C
ATOM  12928  H   ILE B 251       6.786  -7.879 -39.900  1.00  4.97
H
ATOM  12929  HA  ILE B 251       4.214  -8.786 -38.802  1.00  7.73
H
ATOM  12930  HB  ILE B 251       4.984  -6.431 -40.562  1.00 11.59
H
ATOM  12931 1HG1 ILE B 251       4.050  -6.545 -37.693  1.00 11.59
H
ATOM  12932 2HG1 ILE B 251       5.731  -6.384 -38.203  1.00 11.59
H
ATOM  12933 1HG2 ILE B 251       2.682  -5.650 -40.255  1.00 11.59
H
ATOM  12934 2HG2 ILE B 251       2.715  -7.212 -41.106  1.00 11.59
H
ATOM  12935 3HG2 ILE B 251       2.288  -7.144 -39.384  1.00 11.59
H
ATOM  12936 1HD1 ILE B 251       4.797  -4.226 -37.574  1.00 11.59
H
ATOM  12937 2HD1 ILE B 251       5.198  -4.253 -39.307  1.00 11.59
H
ATOM  12938 3HD1 ILE B 251       3.509  -4.418 -38.787  1.00 11.59
H
ATOM  12939  N   GLN B 252       3.367 -10.063 -40.711  1.00  5.35
N
ATOM  12940  CA  GLN B 252       2.896 -10.904 -41.806  1.00  6.48
C
ATOM  12941  C   GLN B 252       1.850 -10.247 -42.702  1.00  3.40
C
ATOM  12942  O   GLN B 252       1.861 -10.460 -43.915  1.00 11.21
O
ATOM  12943  CB  GLN B 252       2.337 -12.210 -41.248  1.00  9.72
C
```

```
ATOM  12944  CG  GLN B 252       3.387 -13.084 -40.587  1.00  9.72
C
ATOM  12945  CD  GLN B 252       4.478 -13.506 -41.552  1.00  9.72
C
ATOM  12946  OE1 GLN B 252       4.198 -13.976 -42.659  1.00  9.72
O
ATOM  12947  NE2 GLN B 252       5.729 -13.339 -41.140  1.00  9.72
N
ATOM  12948  H   GLN B 252       2.995 -10.201 -39.774  1.00  6.42
H
ATOM  12949  HA  GLN B 252       3.755 -11.145 -42.431  1.00  7.78
H
ATOM  12950 1HB  GLN B 252       1.567 -11.989 -40.508  1.00 11.66
H
ATOM  12951 2HB  GLN B 252       1.870 -12.782 -42.049  1.00 11.66
H
ATOM  12952 1HG  GLN B 252       3.848 -12.527 -39.771  1.00 11.66
H
ATOM  12953 2HG  GLN B 252       2.906 -13.983 -40.202  1.00 11.66
H
ATOM  12954 1HE2 GLN B 252       6.492 -13.599 -41.735  1.00 11.66
H
ATOM  12955 2HE2 GLN B 252       5.912 -12.953 -40.236  1.00 11.66
H
ATOM  12956  N   THR B 253       0.969  -9.428 -42.141  1.00  6.24
N
ATOM  12957  CA  THR B 253      -0.026  -8.781 -42.990  1.00  7.13
C
ATOM  12958  C   THR B 253      -0.327  -7.358 -42.596  1.00  8.14
C
ATOM  12959  O   THR B 253      -0.119  -6.944 -41.457  1.00 18.87
O
ATOM  12960  CB  THR B 253      -1.363  -9.532 -42.997  1.00 10.70
C
ATOM  12961  OG1 THR B 253      -2.226  -8.936 -43.977  1.00 10.70
O
ATOM  12962  CG2 THR B 253      -2.029  -9.430 -41.639  1.00 10.70
C
ATOM  12963  H   THR B 253       0.963  -9.288 -41.137  1.00  7.49
H
ATOM  12964  HA  THR B 253       0.358  -8.763 -44.010  1.00  8.56
H
ATOM  12965  HB  THR B 253      -1.199 -10.581 -43.248  1.00 12.83
H
ATOM  12966  HG1 THR B 253      -1.890  -9.133 -44.856  1.00 12.83
H
ATOM  12967 1HG2 THR B 253      -2.982  -9.959 -41.664  1.00 12.83
H
ATOM  12968 2HG2 THR B 253      -1.386  -9.880 -40.884  1.00 12.83
H
ATOM  12969 3HG2 THR B 253      -2.200  -8.384 -41.392  1.00 12.83
H
ATOM  12970  N   SER B 254      -0.829  -6.608 -43.572  1.00  4.32
N
ATOM  12971  CA  SER B 254      -1.226  -5.224 -43.382  1.00  5.01
C
ATOM  12972  C   SER B 254      -2.693  -5.114 -42.968  1.00  3.25
C
```

ATOM 12973 O   SER B 254   -3.173 -4.021 -42.662 1.00 6.89
O
ATOM 12974 CB SER B 254   -0.994 -4.433 -44.655 1.00 7.51
C
ATOM 12975 OG SER B 254   -1.845 -4.870 -45.679 1.00 7.51
O
ATOM 12976 H   SER B 254   -0.953 -7.027 -44.483 1.00 5.18
H
ATOM 12977 HA SER B 254   -0.614 -4.796 -42.586 1.00 6.01
H
ATOM 12978 1HB SER B 254   -1.166 -3.375 -44.462 1.00 9.02
H
ATOM 12979 2HB SER B 254   0.043 -4.545 -44.967 1.00 9.02
H
ATOM 12980 HG SER B 254   -2.738 -4.749 -45.351 1.00 9.02
H
ATOM 12981 N   ARG B 255   -3.402 -6.245 -42.968 1.00 2.87
N
ATOM 12982 CA ARG B 255   -4.803 -6.269 -42.574 1.00 2.41
C
ATOM 12983 C   ARG B 255   -4.967 -5.870 -41.120 1.00 2.20
C
ATOM 12984 O   ARG B 255   -4.317 -6.424 -40.235 1.00 2.94
O
ATOM 12985 CB ARG B 255   -5.392 -7.654 -42.777 1.00 3.62
C
ATOM 12986 CG ARG B 255   -6.876 -7.767 -42.480 1.00 3.62
C
ATOM 12987 CD ARG B 255   -7.389 -9.127 -42.784 1.00 3.62
C
ATOM 12988 NE ARG B 255   -8.808 -9.251 -42.496 1.00 3.62
N
ATOM 12989 CZ ARG B 255   -9.544 -10.356 -42.725 1.00 3.62
C
ATOM 12990 NH1 ARG B 255   -8.981 -11.424 -43.248 1.00 3.62
N
ATOM 12991 NH2 ARG B 255   -10.830 -10.368 -42.426 1.00 3.62
N
ATOM 12992 H   ARG B 255   -2.968 -7.117 -43.245 1.00 3.44
H
ATOM 12993 HA ARG B 255   -5.351 -5.559 -43.194 1.00 2.89
H
ATOM 12994 1HB ARG B 255   -5.236 -7.965 -43.808 1.00 4.34
H
ATOM 12995 2HB ARG B 255   -4.872 -8.367 -42.138 1.00 4.34
H
ATOM 12996 1HG ARG B 255   -7.055 -7.560 -41.424 1.00 4.34
H
ATOM 12997 2HG ARG B 255   -7.427 -7.049 -43.089 1.00 4.34
H
ATOM 12998 1HD ARG B 255   -7.234 -9.345 -43.840 1.00 4.34
H
ATOM 12999 2HD ARG B 255   -6.852 -9.860 -42.181 1.00 4.34
H
ATOM 13000 HE ARG B 255   -9.277 -8.450 -42.092 1.00 4.34
H
ATOM 13001 1HH1 ARG B 255   -7.998 -11.416 -43.478 1.00 4.34
H

```
ATOM  13002 2HH1 ARG B 255      -9.534 -12.252 -43.420  1.00  4.34
H
ATOM  13003 1HH2 ARG B 255     -11.262  -9.547 -42.025  1.00  4.34
H
ATOM  13004 2HH2 ARG B 255     -11.382 -11.195 -42.598  1.00  4.34
H
ATOM  13005  N   ASN B 256      -5.852  -4.917 -40.882  1.00  2.04
N
ATOM  13006  CA  ASN B 256      -6.101  -4.420 -39.543  1.00  2.13
C
ATOM  13007  C   ASN B 256      -7.306  -5.087 -38.909  1.00  2.43
C
ATOM  13008  O   ASN B 256      -8.226  -5.526 -39.600  1.00  4.86
O
ATOM  13009  CB  ASN B 256      -6.290  -2.922 -39.578  1.00  3.19
C
ATOM  13010  CG  ASN B 256      -5.041  -2.196 -39.979  1.00  3.19
C
ATOM  13011  OD1 ASN B 256      -3.978  -2.348 -39.362  1.00  3.19
O
ATOM  13012  ND2 ASN B 256      -5.147  -1.408 -41.020  1.00  3.19
N
ATOM  13013  H   ASN B 256      -6.359  -4.511 -41.657  1.00  2.45
H
ATOM  13014  HA  ASN B 256      -5.237  -4.653 -38.920  1.00  2.56
H
ATOM  13015 1HB  ASN B 256      -7.084  -2.672 -40.282  1.00  3.83
H
ATOM  13016 2HB  ASN B 256      -6.602  -2.570 -38.594  1.00  3.83
H
ATOM  13017 1HD2 ASN B 256      -4.351  -0.899 -41.348  1.00  3.83
H
ATOM  13018 2HD2 ASN B 256      -6.024  -1.318 -41.490  1.00  3.83
H
ATOM  13019  N   LEU B 257      -7.295  -5.149 -37.588  1.00  1.67
N
ATOM  13020  CA  LEU B 257      -8.397  -5.713 -36.832  1.00  1.66
C
ATOM  13021  C   LEU B 257      -9.474  -4.670 -36.587  1.00  1.67
C
ATOM  13022  O   LEU B 257      -9.192  -3.472 -36.548  1.00  1.81
O
ATOM  13023  CB  LEU B 257      -7.868  -6.291 -35.534  1.00  2.49
C
ATOM  13024  CG  LEU B 257      -6.899  -7.441 -35.740  1.00  2.49
C
ATOM  13025  CD1 LEU B 257      -6.369  -7.931 -34.404  1.00  2.49
C
ATOM  13026  CD2 LEU B 257      -7.640  -8.527 -36.492  1.00  2.49
C
ATOM  13027  H   LEU B 257      -6.498  -4.782 -37.088  1.00  2.00
H
ATOM  13028  HA  LEU B 257      -8.835  -6.522 -37.414  1.00  1.99
H
ATOM  13029 1HB  LEU B 257      -7.326  -5.512 -35.014  1.00  2.99
H
ATOM  13030 2HB  LEU B 257      -8.697  -6.632 -34.915  1.00  2.99
H
```

```
ATOM  13031  HG  LEU B 257      -6.049  -7.104 -36.330  1.00  2.99
H
ATOM  13032 1HD1 LEU B 257      -5.678  -8.759 -34.566  1.00  2.99
H
ATOM  13033 2HD1 LEU B 257      -5.850  -7.120 -33.895  1.00  2.99
H
ATOM  13034 3HD1 LEU B 257      -7.203  -8.265 -33.794  1.00  2.99
H
ATOM  13035 1HD2 LEU B 257      -6.981  -9.377 -36.667  1.00  2.99
H
ATOM  13036 2HD2 LEU B 257      -8.499  -8.847 -35.905  1.00  2.99
H
ATOM  13037 3HD2 LEU B 257      -7.987  -8.133 -37.448  1.00  2.99
H
ATOM  13038  N   ASP B 258     -10.712  -5.132 -36.458  1.00  1.71
N
ATOM  13039  CA  ASP B 258     -11.873  -4.261 -36.301  1.00  1.90
C
ATOM  13040  C   ASP B 258     -12.573  -4.469 -34.945  1.00  1.79
C
ATOM  13041  O   ASP B 258     -13.329  -5.432 -34.792  1.00  2.42
O
ATOM  13042  CB  ASP B 258     -12.848  -4.537 -37.456  1.00  2.85
C
ATOM  13043  CG  ASP B 258     -14.072  -3.636 -37.496  1.00  2.85
C
ATOM  13044  OD1 ASP B 258     -14.237  -2.838 -36.608  1.00  2.85
O
ATOM  13045  OD2 ASP B 258     -14.836  -3.758 -38.424  1.00  2.85
O
ATOM  13046  H   ASP B 258     -10.858  -6.131 -36.487  1.00  2.05
H
ATOM  13047  HA  ASP B 258     -11.541  -3.227 -36.376  1.00  2.28
H
ATOM  13048 1HB  ASP B 258     -12.317  -4.431 -38.402  1.00  3.42
H
ATOM  13049 2HB  ASP B 258     -13.189  -5.572 -37.395  1.00  3.42
H
ATOM  13050  N   PRO B 259     -12.306  -3.609 -33.946  1.00  1.56
N
ATOM  13051  CA  PRO B 259     -12.881  -3.602 -32.602  1.00  1.53
C
ATOM  13052  C   PRO B 259     -14.401  -3.573 -32.639  1.00  1.64
C
ATOM  13053  O   PRO B 259     -15.011  -2.754 -33.331  1.00  2.06
O
ATOM  13054  CB  PRO B 259     -12.310  -2.331 -31.984  1.00  2.29
C
ATOM  13055  CG  PRO B 259     -10.996  -2.156 -32.660  1.00  2.29
C
ATOM  13056  CD  PRO B 259     -11.218  -2.620 -34.071  1.00  2.29
C
ATOM  13057  HA  PRO B 259     -12.528  -4.490 -32.057  1.00  1.84
H
ATOM  13058 1HB  PRO B 259     -12.996  -1.489 -32.149  1.00  2.75
H
ATOM  13059 2HB  PRO B 259     -12.214  -2.459 -30.896  1.00  2.75
H
```

ATOM 13060 1HG PRO B 259   -10.683 -1.103 -32.609 1.00 2.75
H
ATOM 13061 2HG PRO B 259   -10.230 -2.738 -32.132 1.00 2.75
H
ATOM 13062 1HD PRO B 259   -11.523 -1.789 -34.719 1.00 2.75
H
ATOM 13063 2HD PRO B 259   -10.308 -3.109 -34.440 1.00 2.75
H
ATOM 13064 N   GLN B 260   -15.013 -4.446 -31.855 1.00 1.50
N
ATOM 13065 CA GLN B 260   -16.459 -4.537 -31.831 1.00 1.66
C
ATOM 13066 C   GLN B 260   -17.048 -3.653 -30.735 1.00 1.41
C
ATOM 13067 O   GLN B 260   -16.367 -3.376 -29.747 1.00 1.18
O
ATOM 13068 CB GLN B 260   -16.872 -5.997 -31.651 1.00 2.49
C
ATOM 13069 CG GLN B 260   -16.387 -6.882 -32.782 1.00 2.49
C
ATOM 13070 CD GLN B 260   -17.002 -6.488 -34.112 1.00 2.49
C
ATOM 13071 OE1 GLN B 260   -18.225 -6.531 -34.278 1.00 2.49
O
ATOM 13072 NE2 GLN B 260   -16.163 -6.089 -35.063 1.00 2.49
N
ATOM 13073 H   GLN B 260   -14.463 -5.077 -31.289 1.00 1.80
H
ATOM 13074 HA GLN B 260   -16.824 -4.195 -32.796 1.00 1.99
H
ATOM 13075 1HB GLN B 260   -16.451 -6.384 -30.725 1.00 2.99
H
ATOM 13076 2HB GLN B 260   -17.956 -6.081 -31.584 1.00 2.99
H
ATOM 13077 1HG GLN B 260   -15.304 -6.794 -32.864 1.00 2.99
H
ATOM 13078 2HG GLN B 260   -16.664 -7.915 -32.569 1.00 2.99
H
ATOM 13079 1HE2 GLN B 260   -16.516 -5.811 -35.958 1.00 2.99
H
ATOM 13080 2HE2 GLN B 260   -15.178 -6.053 -34.888 1.00 2.99
H
ATOM 13081 N   PRO B 261   -18.290 -3.181 -30.932 1.00 1.65
N
ATOM 13082 CA PRO B 261   -19.102 -2.338 -30.063 1.00 1.55
C
ATOM 13083 C   PRO B 261   -19.677 -3.126 -28.885 1.00 1.71
C
ATOM 13084 O   PRO B 261   -19.822 -4.346 -28.963 1.00 2.67
O
ATOM 13085 CB PRO B 261   -20.203 -1.819 -30.994 1.00 2.33
C
ATOM 13086 CG PRO B 261   -20.341 -2.873 -32.030 1.00 2.33
C
ATOM 13087 CD PRO B 261   -18.937 -3.362 -32.249 1.00 2.33
C
ATOM 13088 HA PRO B 261   -18.470 -1.512 -29.717 1.00 1.86
H

ATOM 13089 1HB PRO B 261   -21.132 -1.659 -30.429 1.00 2.79
H
ATOM 13090 2HB PRO B 261   -19.911 -0.846 -31.416 1.00 2.79
H
ATOM 13091 1HG PRO B 261   -21.015 -3.667 -31.676 1.00 2.79
H
ATOM 13092 2HG PRO B 261   -20.793 -2.455 -32.941 1.00 2.79
H
ATOM 13093 1HD PRO B 261   -18.960 -4.422 -32.534 1.00 2.79
H
ATOM 13094 2HD PRO B 261   -18.431 -2.746 -33.006 1.00 2.79
H
ATOM 13095 N   PRO B 262   -19.994 -2.432 -27.790 1.00 1.51
N
ATOM 13096 CA PRO B 262   -20.537 -2.899 -26.524 1.00 1.56
C
ATOM 13097 C   PRO B 262   -22.047 -3.073 -26.545 1.00 1.37
C
ATOM 13098 O   PRO B 262   -22.718 -2.702 -27.510 1.00 1.77
O
ATOM 13099 CB PRO B 262   -20.146 -1.780 -25.574 1.00 2.34
C
ATOM 13100 CG PRO B 262   -20.259 -0.552 -26.413 1.00 2.34
C
ATOM 13101 CD PRO B 262   -19.802 -0.972 -27.777 1.00 2.34
C
ATOM 13102 HA PRO B 262   -20.049 -3.842 -26.242 1.00 1.87
H
ATOM 13103 1HB PRO B 262   -20.822 -1.772 -24.707 1.00 2.81
H
ATOM 13104 2HB PRO B 262   -19.129 -1.946 -25.190 1.00 2.81
H
ATOM 13105 1HG PRO B 262   -21.297 -0.187 -26.408 1.00 2.81
H
ATOM 13106 2HG PRO B 262   -19.640   0.253 -25.992 1.00 2.81
H
ATOM 13107 1HD PRO B 262   -20.424 -0.496 -28.548 1.00 2.81
H
ATOM 13108 2HD PRO B 262   -18.749 -0.732 -27.898 1.00 2.81
H
ATOM 13109 N   ILE B 263   -22.575 -3.611 -25.450 1.00 1.70
N
ATOM 13110 CA ILE B 263   -24.010 -3.770 -25.281 1.00 1.87
C
ATOM 13111 C   ILE B 263   -24.581 -2.497 -24.677 1.00 1.44
C
ATOM 13112 O   ILE B 263   -24.192 -2.091 -23.581 1.00 2.47
O
ATOM 13113 CB ILE B 263   -24.337 -4.969 -24.376 1.00 2.81
C
ATOM 13114 CG1 ILE B 263   -23.811 -6.261 -25.007 1.00 2.81
C
ATOM 13115 CG2 ILE B 263   -25.838 -5.051 -24.137 1.00 2.81
C
ATOM 13116 CD1 ILE B 263   -23.863 -7.454 -24.079 1.00 2.81
C
ATOM 13117 H   ILE B 263   -21.961 -3.906 -24.705 1.00 2.04
H

```
ATOM  13118  HA  ILE B 263     -24.468  -3.929 -26.257  1.00  2.24
H
ATOM  13119  HB  ILE B 263     -23.831  -4.847 -23.418  1.00  3.37
H
ATOM  13120 1HG1 ILE B 263     -24.401  -6.489 -25.894  1.00  3.37
H
ATOM  13121 2HG1 ILE B 263     -22.775  -6.111 -25.314  1.00  3.37
H
ATOM  13122 1HG2 ILE B 263     -26.057  -5.895 -23.485  1.00  3.37
H
ATOM  13123 2HG2 ILE B 263     -26.183  -4.131 -23.666  1.00  3.37
H
ATOM  13124 3HG2 ILE B 263     -26.352  -5.185 -25.090  1.00  3.37
H
ATOM  13125 1HD1 ILE B 263     -23.475  -8.333 -24.593  1.00  3.37
H
ATOM  13126 2HD1 ILE B 263     -23.258  -7.254 -23.194  1.00  3.37
H
ATOM  13127 3HD1 ILE B 263     -24.895  -7.636 -23.779  1.00  3.37
H
ATOM  13128  N   GLU B 264     -25.490  -1.861 -25.405  1.00  1.51
N
ATOM  13129  CA  GLU B 264     -26.081  -0.593 -24.987  1.00  1.91
C
ATOM  13130  C   GLU B 264     -27.033  -0.699 -23.798  1.00  2.04
C
ATOM  13131  O   GLU B 264     -28.252  -0.657 -23.973  1.00  2.98
O
ATOM  13132  CB  GLU B 264     -26.811   0.048 -26.167  1.00  2.86
C
ATOM  13133  CG  GLU B 264     -25.899   0.480 -27.309  1.00  2.86
C
ATOM  13134  CD  GLU B 264     -26.652   1.096 -28.458  1.00  2.86
C
ATOM  13135  OE1 GLU B 264     -27.851   0.955 -28.497  1.00  2.86
O
ATOM  13136  OE2 GLU B 264     -26.033   1.721 -29.290  1.00  2.86
O
ATOM  13137  H   GLU B 264     -25.771  -2.261 -26.289  1.00  1.81
H
ATOM  13138  HA  GLU B 264     -25.268   0.062 -24.700  1.00  2.29
H
ATOM  13139 1HB  GLU B 264     -27.543  -0.653 -26.568  1.00  3.44
H
ATOM  13140 2HB  GLU B 264     -27.354   0.928 -25.822  1.00  3.44
H
ATOM  13141 1HG  GLU B 264     -25.181   1.206 -26.927  1.00  3.44
H
ATOM  13142 2HG  GLU B 264     -25.345  -0.387 -27.666  1.00  3.44
H
ATOM  13143  N   ASP B 265     -26.483  -0.813 -22.595  1.00  1.82
N
ATOM  13144  CA  ASP B 265     -27.332  -0.918 -21.415  1.00  2.99
C
ATOM  13145  C   ASP B 265     -27.020   0.087 -20.302  1.00  1.45
C
ATOM  13146  O   ASP B 265     -27.566  -0.036 -19.205  1.00  2.42
O
```

```
ATOM  13147  CB  ASP B 265     -27.245  -2.322 -20.851  1.00  4.49
C
ATOM  13148  CG  ASP B 265     -25.848  -2.637 -20.394  1.00  4.49
C
ATOM  13149  OD1 ASP B 265     -24.988  -1.810 -20.563  1.00  4.49
O
ATOM  13150  OD2 ASP B 265     -25.640  -3.697 -19.859  1.00  4.49
O
ATOM  13151  H   ASP B 265     -25.475  -0.856 -22.523  1.00  2.18
H
ATOM  13152  HA  ASP B 265     -28.361  -0.746 -21.729  1.00  3.59
H
ATOM  13153 1HB  ASP B 265     -27.928  -2.425 -20.008  1.00  5.38
H
ATOM  13154 2HB  ASP B 265     -27.543  -3.045 -21.611  1.00  5.38
H
ATOM  13155  N   SER B 266     -26.171   1.083 -20.560  1.00  1.04
N
ATOM  13156  CA  SER B 266     -25.932   2.079 -19.527  1.00  0.97
C
ATOM  13157  C   SER B 266     -27.170   2.980 -19.480  1.00  1.24
C
ATOM  13158  O   SER B 266     -27.683   3.365 -20.533  1.00  3.12
O
ATOM  13159  CB  SER B 266     -24.669   2.885 -19.814  1.00  1.46
C
ATOM  13160  OG  SER B 266     -24.468   3.885 -18.849  1.00  1.46
O
ATOM  13161  H   SER B 266     -25.709   1.177 -21.459  1.00  1.25
H
ATOM  13162  HA  SER B 266     -25.810   1.566 -18.576  1.00  1.16
H
ATOM  13163 1HB  SER B 266     -23.808   2.216 -19.838  1.00  1.75
H
ATOM  13164 2HB  SER B 266     -24.753   3.343 -20.798  1.00  1.75
H
ATOM  13165  HG  SER B 266     -25.273   4.413 -18.847  1.00  1.75
H
ATOM  13166  N   PRO B 267     -27.678   3.309 -18.290  1.00  0.64
N
ATOM  13167  CA  PRO B 267     -28.861   4.114 -18.027  1.00  0.65
C
ATOM  13168  C   PRO B 267     -28.628   5.577 -18.350  1.00  0.65
C
ATOM  13169  O   PRO B 267     -27.500   6.069 -18.290  1.00  0.72
O
ATOM  13170  CB  PRO B 267     -29.107   3.905 -16.531  1.00  0.98
C
ATOM  13171  CG  PRO B 267     -27.767   3.584 -15.970  1.00  0.98
C
ATOM  13172  CD  PRO B 267     -27.086   2.785 -17.048  1.00  0.98
C
ATOM  13173  HA  PRO B 267     -29.700   3.719 -18.618  1.00  0.78
H
ATOM  13174 1HB  PRO B 267     -29.543   4.814 -16.090  1.00  1.17
H
ATOM  13175 2HB  PRO B 267     -29.836   3.095 -16.380  1.00  1.17
H
```

```
ATOM  13176 1HG  PRO B 267     -27.227   4.509 -15.720  1.00  1.17
H
ATOM  13177 2HG  PRO B 267     -27.878   3.019 -15.034  1.00  1.17
H
ATOM  13178 1HD  PRO B 267     -26.006   2.981 -17.034  1.00  1.17
H
ATOM  13179 2HD  PRO B 267     -27.313   1.714 -16.942  1.00  1.17
H
ATOM  13180  N   GLU B 268     -29.706   6.272 -18.689  1.00  0.81
N
ATOM  13181  CA  GLU B 268     -29.625   7.687 -19.000  1.00  0.81
C
ATOM  13182  C   GLU B 268     -29.427   8.494 -17.729  1.00  0.59
C
ATOM  13183  O   GLU B 268     -30.124   8.285 -16.734  1.00  0.59
O
ATOM  13184  CB  GLU B 268     -30.883   8.154 -19.731  1.00  1.22
C
ATOM  13185  CG  GLU B 268     -30.839   9.601 -20.198  1.00  1.22
C
ATOM  13186  CD  GLU B 268     -32.067  10.002 -20.969  1.00  1.22
C
ATOM  13187  OE1 GLU B 268     -32.953   9.191 -21.097  1.00  1.22
O
ATOM  13188  OE2 GLU B 268     -32.117  11.115 -21.438  1.00  1.22
O
ATOM  13189  H   GLU B 268     -30.603   5.808 -18.728  1.00  0.97
H
ATOM  13190  HA  GLU B 268     -28.763   7.853 -19.647  1.00  0.97
H
ATOM  13191 1HB  GLU B 268     -31.048   7.525 -20.607  1.00  1.46
H
ATOM  13192 2HB  GLU B 268     -31.748   8.038 -19.078  1.00  1.46
H
ATOM  13193 1HG  GLU B 268     -30.743  10.251 -19.329  1.00  1.46
H
ATOM  13194 2HG  GLU B 268     -29.960   9.745 -20.825  1.00  1.46
H
ATOM  13195  N   VAL B 269     -28.478   9.418 -17.772  1.00  0.53
N
ATOM  13196  CA  VAL B 269     -28.169  10.265 -16.630  1.00  0.39
C
ATOM  13197  C   VAL B 269     -28.554  11.709 -16.922  1.00  0.45
C
ATOM  13198  O   VAL B 269     -28.199  12.255 -17.967  1.00  0.51
O
ATOM  13199  CB  VAL B 269     -26.666  10.170 -16.281  1.00  0.58
C
ATOM  13200  CG1 VAL B 269     -26.335  11.097 -15.122  1.00  0.58
C
ATOM  13201  CG2 VAL B 269     -26.312   8.732 -15.925  1.00  0.58
C
ATOM  13202  H   VAL B 269     -27.948   9.535 -18.624  1.00  0.64
H
ATOM  13203  HA  VAL B 269     -28.745   9.917 -15.773  1.00  0.47
H
ATOM  13204  HB  VAL B 269     -26.077  10.491 -17.140  1.00  0.70
H
```

ATOM 13205 1HG1 VAL B 269   -25.271 11.026 -14.894 1.00 0.70
H
ATOM 13206 2HG1 VAL B 269   -26.579 12.123 -15.394 1.00 0.70
H
ATOM 13207 3HG1 VAL B 269   -26.912 10.803 -14.246 1.00 0.70
H
ATOM 13208 1HG2 VAL B 269   -25.251   8.665 -15.688 1.00 0.70
H
ATOM 13209 2HG2 VAL B 269   -26.897   8.416 -15.060 1.00 0.70
H
ATOM 13210 3HG2 VAL B 269   -26.535   8.079 -16.770 1.00 0.70
H
ATOM 13211 N   ASN B 270   -29.286 12.320 -15.998 1.00 0.45
N
ATOM 13212 CA ASN B 270   -29.732 13.697 -16.165 1.00 0.52
C
ATOM 13213 C   ASN B 270   -28.583 14.677 -16.009 1.00 0.52
C
ATOM 13214 O   ASN B 270   -27.659 14.447 -15.230 1.00 0.47
O
ATOM 13215 CB ASN B 270   -30.837 14.019 -15.180 1.00 0.78
C
ATOM 13216 CG ASN B 270   -32.108 13.281 -15.484 1.00 0.78
C
ATOM 13217 OD1 ASN B 270   -32.453 13.070 -16.652 1.00 0.78
O
ATOM 13218 ND2 ASN B 270   -32.812 12.879 -14.457 1.00 0.78
N
ATOM 13219 H   ASN B 270   -29.543 11.816 -15.160 1.00 0.54
H
ATOM 13220 HA ASN B 270   -30.122 13.812 -17.178 1.00 0.62
H
ATOM 13221 1HB ASN B 270   -30.513 13.760 -14.170 1.00 0.94
H
ATOM 13222 2HB ASN B 270   -31.040 15.090 -15.193 1.00 0.94
H
ATOM 13223 1HD2 ASN B 270   -33.668 12.381 -14.600 1.00 0.94
H
ATOM 13224 2HD2 ASN B 270   -32.495 13.069 -13.528 1.00 0.94
H
ATOM 13225 N   ILE B 271   -28.642 15.762 -16.770 1.00 0.62
N
ATOM 13226 CA ILE B 271   -27.628 16.810 -16.742 1.00 0.68
C
ATOM 13227 C   ILE B 271   -28.197 18.128 -16.215 1.00 0.78
C
ATOM 13228 O   ILE B 271   -27.678 19.200 -16.528 1.00 0.92
O
ATOM 13229 CB ILE B 271   -27.040 17.031 -18.146 1.00 1.02
C
ATOM 13230 CG1 ILE B 271   -28.154 17.430 -19.117 1.00 1.02
C
ATOM 13231 CG2 ILE B 271   -26.341 15.767 -18.625 1.00 1.02
C
ATOM 13232 CD1 ILE B 271   -27.645 17.906 -20.458 1.00 1.02
C
ATOM 13233 H   ILE B 271   -29.429 15.870 -17.396 1.00 0.74
H

ATOM 13234 HA ILE B 271   -26.824 16.496 -16.078 1.00 0.82
H
ATOM 13235 HB ILE B 271   -26.323 17.849 -18.114 1.00 1.22
H
ATOM 13236 1HG1 ILE B 271   -28.808 16.574 -19.280 1.00 1.22
H
ATOM 13237 2HG1 ILE B 271   -28.745 18.231 -18.670 1.00 1.22
H
ATOM 13238 1HG2 ILE B 271   -25.922 15.936 -19.617 1.00 1.22
H
ATOM 13239 2HG2 ILE B 271   -25.540 15.509 -17.932 1.00 1.22
H
ATOM 13240 3HG2 ILE B 271   -27.057 14.946 -18.670 1.00 1.22
H
ATOM 13241 1HD1 ILE B 271   -28.488 18.173 -21.094 1.00 1.22
H
ATOM 13242 2HD1 ILE B 271   -27.006 18.779 -20.315 1.00 1.22
H
ATOM 13243 3HD1 ILE B 271   -27.071 17.111 -20.931 1.00 1.22
H
ATOM 13244 N   THR B 272   -29.284 18.045 -15.450 1.00 0.84
N
ATOM 13245 CA THR B 272   -29.980 19.234 -14.965 1.00 0.94
C
ATOM 13246 C   THR B 272   -29.387 19.809 -13.680 1.00 0.68
C
ATOM 13247 O   THR B 272   -28.584 19.166 -13.000 1.00 0.88
O
ATOM 13248 CB THR B 272   -31.468 18.913 -14.731 1.00 1.41
C
ATOM 13249 OG1 THR B 272   -31.591 17.938 -13.688 1.00 1.41
O
ATOM 13250 CG2 THR B 272   -32.085 18.357 -16.006 1.00 1.41
C
ATOM 13251 H   THR B 272   -29.653 17.137 -15.210 1.00 1.01
H
ATOM 13252 HA THR B 272   -29.911 20.003 -15.735 1.00 1.13
H
ATOM 13253 HB THR B 272   -31.996 19.820 -14.438 1.00 1.69
H
ATOM 13254 HG1 THR B 272   -32.522 17.796 -13.495 1.00 1.69
H
ATOM 13255 1HG2 THR B 272   -33.137 18.131 -15.831 1.00 1.69
H
ATOM 13256 2HG2 THR B 272   -31.999 19.095 -16.804 1.00 1.69
H
ATOM 13257 3HG2 THR B 272   -31.563 17.446 -16.296 1.00 1.69
H
ATOM 13258 N   ASP B 273   -29.801 21.029 -13.346 1.00 0.70
N
ATOM 13259 CA ASP B 273   -29.341 21.691 -12.131 1.00 1.31
C
ATOM 13260 C   ASP B 273   -30.000 21.077 -10.903 1.00 1.64
C
ATOM 13261 O   ASP B 273   -31.211 20.854 -10.885 1.00 4.22
O
ATOM 13262 CB ASP B 273   -29.640 23.189 -12.182 1.00 1.97
C

ATOM 13263 CG ASP B 273 -28.797 23.934 -13.207 1.00 1.97
C
ATOM 13264 OD1 ASP B 273 -27.821 23.388 -13.663 1.00 1.97
O
ATOM 13265 OD2 ASP B 273 -29.142 25.046 -13.527 1.00 1.97
O
ATOM 13266 H ASP B 273 -30.456 21.510 -13.948 1.00 0.84
H
ATOM 13267 HA ASP B 273 -28.262 21.552 -12.046 1.00 1.57
H
ATOM 13268 1HB ASP B 273 -30.694 23.340 -12.422 1.00 2.36
H
ATOM 13269 2HB ASP B 273 -29.463 23.628 -11.200 1.00 2.36
H
ATOM 13270 N VAL B 274 -29.206 20.826 -9.868 1.00 0.61
N
ATOM 13271 CA VAL B 274 -29.735 20.230 -8.653 1.00 0.55
C
ATOM 13272 C VAL B 274 -30.107 21.305 -7.657 1.00 0.57
C
ATOM 13273 O VAL B 274 -29.283 22.139 -7.288 1.00 1.47
O
ATOM 13274 CB VAL B 274 -28.709 19.285 -7.998 1.00 0.83
C
ATOM 13275 CG1 VAL B 274 -29.294 18.702 -6.722 1.00 0.83
C
ATOM 13276 CG2 VAL B 274 -28.329 18.187 -8.964 1.00 0.83
C
ATOM 13277 H VAL B 274 -28.220 21.048 -9.928 1.00 0.73
H
ATOM 13278 HA VAL B 274 -30.628 19.658 -8.905 1.00 0.66
H
ATOM 13279 HB VAL B 274 -27.820 19.854 -7.725 1.00 0.99
H
ATOM 13280 1HG1 VAL B 274 -28.562 18.047 -6.250 1.00 0.99
H
ATOM 13281 2HG1 VAL B 274 -29.551 19.512 -6.040 1.00 0.99
H
ATOM 13282 3HG1 VAL B 274 -30.191 18.132 -6.960 1.00 0.99
H
ATOM 13283 1HG2 VAL B 274 -27.599 17.528 -8.495 1.00 0.99
H
ATOM 13284 2HG2 VAL B 274 -29.216 17.615 -9.231 1.00 0.99
H
ATOM 13285 3HG2 VAL B 274 -27.896 18.628 -9.862 1.00 0.99
H
ATOM 13286 N ARG B 275 -31.352 21.304 -7.225 1.00 0.64
N
ATOM 13287 CA ARG B 275 -31.782 22.285 -6.247 1.00 1.00
C
ATOM 13288 C ARG B 275 -32.335 21.578 -5.037 1.00 0.81
C
ATOM 13289 O ARG B 275 -33.005 20.556 -5.172 1.00 1.16
O
ATOM 13290 CB ARG B 275 -32.840 23.209 -6.829 1.00 1.50
C
ATOM 13291 CG ARG B 275 -32.374 24.087 -7.980 1.00 1.50
C

```
ATOM  13292  CD  ARG B 275     -33.466  24.970  -8.465  1.00  1.50
C
ATOM  13293  NE  ARG B 275     -33.043  25.805  -9.579  1.00  1.50
N
ATOM  13294  CZ  ARG B 275     -33.825  26.718 -10.187  1.00  1.50
C
ATOM  13295  NH1 ARG B 275     -35.061  26.903  -9.779  1.00  1.50
N
ATOM  13296  NH2 ARG B 275     -33.349  27.428 -11.195  1.00  1.50
N
ATOM  13297  H   ARG B 275     -32.005  20.612  -7.565  1.00  0.77
H
ATOM  13298  HA  ARG B 275     -30.923  22.882  -5.941  1.00  1.20
H
ATOM  13299 1HB  ARG B 275     -33.680  22.616  -7.191  1.00  1.80
H
ATOM  13300 2HB  ARG B 275     -33.218  23.867  -6.047  1.00  1.80
H
ATOM  13301 1HG  ARG B 275     -31.545  24.714  -7.650  1.00  1.80
H
ATOM  13302 2HG  ARG B 275     -32.046  23.460  -8.811  1.00  1.80
H
ATOM  13303 1HD  ARG B 275     -34.305  24.358  -8.797  1.00  1.80
H
ATOM  13304 2HD  ARG B 275     -33.791  25.621  -7.655  1.00  1.80
H
ATOM  13305  HE  ARG B 275     -32.099  25.690  -9.921  1.00  1.80
H
ATOM  13306 1HH1 ARG B 275     -35.425  26.359  -9.009  1.00  1.80
H
ATOM  13307 2HH1 ARG B 275     -35.647  27.587 -10.235  1.00  1.80
H
ATOM  13308 1HH2 ARG B 275     -32.399  27.286 -11.510  1.00  1.80
H
ATOM  13309 2HH2 ARG B 275     -33.935  28.112 -11.651  1.00  1.80
H
ATOM  13310  N   MET B 276     -32.076  22.119  -3.855  1.00  1.07
N
ATOM  13311  CA  MET B 276     -32.621  21.504  -2.660  1.00  1.28
C
ATOM  13312  C   MET B 276     -34.118  21.735  -2.663  1.00  1.22
C
ATOM  13313  O   MET B 276     -34.595  22.763  -3.146  1.00  1.73
O
ATOM  13314  CB  MET B 276     -31.944  22.044  -1.404  1.00  1.92
C
ATOM  13315  CG  MET B 276     -30.488  21.608  -1.262  1.00  1.92
C
ATOM  13316  SD  MET B 276     -29.707  22.212   0.249  1.00  1.92
S
ATOM  13317  CE  MET B 276     -28.086  21.458   0.119  1.00  1.92
C
ATOM  13318  H   MET B 276     -31.513  22.956  -3.786  1.00  1.28
H
ATOM  13319  HA  MET B 276     -32.451  20.428  -2.707  1.00  1.54
H
ATOM  13320 1HB  MET B 276     -31.969  23.133  -1.413  1.00  2.30
H
```

```
ATOM  13321 2HB  MET B 276     -32.486  21.709  -0.519  1.00  2.30
H
ATOM  13322 1HG  MET B 276     -30.436  20.519  -1.262  1.00  2.30
H
ATOM  13323 2HG  MET B 276     -29.916  21.974  -2.113  1.00  2.30
H
ATOM  13324 1HE  MET B 276     -27.484  21.746   0.979  1.00  2.30
H
ATOM  13325 2HE  MET B 276     -28.192  20.374   0.095  1.00  2.30
H
ATOM  13326 3HE  MET B 276     -27.598  21.796  -0.795  1.00  2.30
H
ATOM  13327  N   THR B 277     -34.867  20.774  -2.135  1.00  2.23
N
ATOM  13328  CA  THR B 277     -36.324  20.843  -2.193  1.00  4.18
C
ATOM  13329  C   THR B 277     -36.937  21.881  -1.260  1.00  3.57
C
ATOM  13330  O   THR B 277     -38.115  22.214  -1.395  1.00  5.63
O
ATOM  13331  CB  THR B 277     -36.939  19.467  -1.909  1.00  6.27
C
ATOM  13332  OG1 THR B 277     -36.558  19.027  -0.600  1.00  6.27
O
ATOM  13333  CG2 THR B 277     -36.455  18.468  -2.951  1.00  6.27
C
ATOM  13334  H   THR B 277     -34.424  19.966  -1.721  1.00  2.68
H
ATOM  13335  HA  THR B 277     -36.600  21.119  -3.212  1.00  5.02
H
ATOM  13336  HB  THR B 277     -38.026  19.539  -1.957  1.00  7.52
H
ATOM  13337  HG1 THR B 277     -36.859  19.665   0.052  1.00  7.52
H
ATOM  13338 1HG2 THR B 277     -36.898  17.493  -2.761  1.00  7.52
H
ATOM  13339 2HG2 THR B 277     -36.751  18.809  -3.944  1.00  7.52
H
ATOM  13340 3HG2 THR B 277     -35.371  18.389  -2.904  1.00  7.52
H
ATOM  13341  N   SER B 278     -36.149  22.412  -0.338  1.00  2.54
N
ATOM  13342  CA  SER B 278     -36.635  23.452   0.547  1.00  4.28
C
ATOM  13343  C   SER B 278     -35.477  24.360   0.936  1.00  3.61
C
ATOM  13344  O   SER B 278     -34.319  23.998   0.729  1.00  5.31
O
ATOM  13345  CB  SER B 278     -37.253  22.829   1.791  1.00  6.42
C
ATOM  13346  OG  SER B 278     -36.276  22.202   2.574  1.00  6.42
O
ATOM  13347  H   SER B 278     -35.194  22.101  -0.249  1.00  3.05
H
ATOM  13348  HA  SER B 278     -37.387  24.038   0.017  1.00  5.14
H
ATOM  13349 1HB  SER B 278     -37.761  23.587   2.382  1.00  7.70
H
```

ATOM 13350 2HB SER B 278 -38.002 22.097 1.489 1.00 7.70
H
ATOM 13351 HG SER B 278 -35.702 22.908 2.887 1.00 7.70
H
ATOM 13352 N PRO B 279 -35.771 25.552 1.456 1.00 5.45
N
ATOM 13353 CA PRO B 279 -34.851 26.507 2.038 1.00 2.24
C
ATOM 13354 C PRO B 279 -34.435 25.957 3.397 1.00 2.24
C
ATOM 13355 O PRO B 279 -35.129 25.090 3.934 1.00 4.90
O
ATOM 13356 CB PRO B 279 -35.658 27.807 2.115 1.00 3.36
C
ATOM 13357 CG PRO B 279 -37.073 27.358 2.162 1.00 3.36
C
ATOM 13358 CD PRO B 279 -37.101 26.161 1.245 1.00 3.36
C
ATOM 13359 HA PRO B 279 -33.991 26.610 1.365 1.00 2.69
H
ATOM 13360 1HB PRO B 279 -35.364 28.382 3.005 1.00 4.03
H
ATOM 13361 2HB PRO B 279 -35.440 28.436 1.239 1.00 4.03
H
ATOM 13362 1HG PRO B 279 -37.361 27.108 3.195 1.00 4.03
H
ATOM 13363 2HG PRO B 279 -37.743 28.165 1.829 1.00 4.03
H
ATOM 13364 1HD PRO B 279 -37.907 25.483 1.547 1.00 4.03
H
ATOM 13365 2HD PRO B 279 -37.207 26.476 0.196 1.00 4.03
H
ATOM 13366 N PRO B 280 -33.314 26.422 3.955 1.00 1.38
N
ATOM 13367 CA PRO B 280 -32.740 26.043 5.236 1.00 1.47
C
ATOM 13368 C PRO B 280 -33.776 26.166 6.341 1.00 1.80
C
ATOM 13369 O PRO B 280 -34.586 27.091 6.360 1.00 6.39
O
ATOM 13370 CB PRO B 280 -31.587 27.041 5.413 1.00 2.21
C
ATOM 13371 CG PRO B 280 -31.192 27.387 4.022 1.00 2.21
C
ATOM 13372 CD PRO B 280 -32.488 27.425 3.261 1.00 2.21
C
ATOM 13373 HA PRO B 280 -32.363 25.011 5.171 1.00 1.76
H
ATOM 13374 1HB PRO B 280 -31.917 27.908 5.999 1.00 2.65
H
ATOM 13375 2HB PRO B 280 -30.767 26.567 5.968 1.00 2.65
H
ATOM 13376 1HG PRO B 280 -30.667 28.350 3.995 1.00 2.65
H
ATOM 13377 2HG PRO B 280 -30.495 26.632 3.628 1.00 2.65
H
ATOM 13378 1HD PRO B 280 -32.957 28.414 3.327 1.00 2.65
H

ATOM 13379 2HD PRO B 280 -32.299 27.127 2.221 1.00 2.65
H
ATOM 13380 N ASP B 281 -33.743 25.233 7.271 1.00 1.93
N
ATOM 13381 CA ASP B 281 -34.700 25.205 8.367 1.00 1.67
C
ATOM 13382 C ASP B 281 -34.054 25.571 9.686 1.00 2.32
C
ATOM 13383 O ASP B 281 -34.277 24.902 10.695 1.00 5.84
O
ATOM 13384 CB ASP B 281 -35.366 23.831 8.463 1.00 2.50
C
ATOM 13385 CG ASP B 281 -34.383 22.700 8.712 1.00 2.50
C
ATOM 13386 OD1 ASP B 281 -33.200 22.931 8.628 1.00 2.50
O
ATOM 13387 OD2 ASP B 281 -34.821 21.601 8.954 1.00 2.50
O
ATOM 13388 H ASP B 281 -33.042 24.507 7.212 1.00 2.32
H
ATOM 13389 HA ASP B 281 -35.478 25.940 8.160 1.00 2.00
H
ATOM 13390 1HB ASP B 281 -36.096 23.841 9.272 1.00 3.01
H
ATOM 13391 2HB ASP B 281 -35.907 23.627 7.538 1.00 3.01
H
ATOM 13392 N TYR B 282 -33.228 26.612 9.677 1.00 1.35
N
ATOM 13393 CA TYR B 282 -32.538 27.004 10.891 1.00 1.54
C
ATOM 13394 C TYR B 282 -33.047 28.338 11.386 1.00 1.67
C
ATOM 13395 O TYR B 282 -33.415 29.206 10.596 1.00 2.26
O
ATOM 13396 CB TYR B 282 -31.045 27.107 10.649 1.00 2.31
C
ATOM 13397 CG TYR B 282 -30.472 25.839 10.105 1.00 2.31
C
ATOM 13398 CD1 TYR B 282 -30.275 25.771 8.748 1.00 2.31
C
ATOM 13399 CD2 TYR B 282 -30.183 24.750 10.915 1.00 2.31
C
ATOM 13400 CE1 TYR B 282 -29.784 24.629 8.183 1.00 2.31
C
ATOM 13401 CE2 TYR B 282 -29.690 23.589 10.343 1.00 2.31
C
ATOM 13402 CZ TYR B 282 -29.494 23.527 8.977 1.00 2.31
C
ATOM 13403 OH TYR B 282 -29.013 22.378 8.388 1.00 2.31
O
ATOM 13404 H TYR B 282 -33.079 27.133 8.826 1.00 1.62
H
ATOM 13405 HA TYR B 282 -32.729 26.261 11.665 1.00 1.85
H
ATOM 13406 1HB TYR B 282 -30.832 27.913 9.949 1.00 2.77
H
ATOM 13407 2HB TYR B 282 -30.545 27.331 11.584 1.00 2.77
H

ATOM 13408 HD1 TYR B 282   -30.511 26.630   8.122 1.00 2.77
H
ATOM 13409 HD2 TYR B 282   -30.351 24.805 11.991 1.00 2.77
H
ATOM 13410 HE1 TYR B 282   -29.632 24.597   7.114 1.00 2.77
H
ATOM 13411 HE2 TYR B 282   -29.466 22.724 10.968 1.00 2.77
H
ATOM 13412 HH TYR B 282   -29.257 22.374   7.453 1.00 2.77
H
ATOM 13413 N   ARG B 283   -33.057 28.503 12.696 1.00 1.68
N
ATOM 13414 CA ARG B 283   -33.490 29.747 13.301 1.00 1.84
C
ATOM 13415 C   ARG B 283   -32.294 30.552 13.756 1.00 1.86
C
ATOM 13416 O   ARG B 283   -31.378 30.013 14.378 1.00 1.82
O
ATOM 13417 CB ARG B 283   -34.407 29.482 14.480 1.00 2.76
C
ATOM 13418 CG ARG B 283   -34.947 30.715 15.180 1.00 2.76
C
ATOM 13419 CD ARG B 283   -35.836 30.339 16.309 1.00 2.76
C
ATOM 13420 NE ARG B 283   -35.113 29.610 17.341 1.00 2.76
N
ATOM 13421 CZ ARG B 283   -35.654 29.103 18.461 1.00 2.76
C
ATOM 13422 NH1 ARG B 283   -36.935 29.255 18.723 1.00 2.76
N
ATOM 13423 NH2 ARG B 283   -34.866 28.447 19.292 1.00 2.76
N
ATOM 13424 H   ARG B 283   -32.750 27.747 13.295 1.00 2.02
H
ATOM 13425 HA ARG B 283   -34.038 30.326 12.558 1.00 2.21
H
ATOM 13426 1HB ARG B 283   -35.261 28.893 14.149 1.00 3.31
H
ATOM 13427 2HB ARG B 283   -33.879 28.895 15.226 1.00 3.31
H
ATOM 13428 1HG ARG B 283   -34.118 31.303 15.578 1.00 3.31
H
ATOM 13429 2HG ARG B 283   -35.519 31.319 14.475 1.00 3.31
H
ATOM 13430 1HD ARG B 283   -36.255 31.239 16.758 1.00 3.31
H
ATOM 13431 2HD ARG B 283   -36.641 29.705 15.945 1.00 3.31
H
ATOM 13432 HE ARG B 283   -34.119 29.445 17.210 1.00 3.31
H
ATOM 13433 1HH1 ARG B 283   -37.525 29.759 18.077 1.00 3.31
H
ATOM 13434 2HH1 ARG B 283   -37.327 28.867 19.569 1.00 3.31
H
ATOM 13435 1HH2 ARG B 283   -33.883 28.343 19.052 1.00 3.31
H
ATOM 13436 2HH2 ARG B 283   -35.235 28.050 20.142 1.00 3.31
H

ATOM 13437 N   VAL B 284   -32.316 31.843 13.445 1.00 1.93
N
ATOM 13438 CA VAL B 284   -31.226 32.730 13.803 1.00 1.99
C
ATOM 13439 C   VAL B 284   -31.080 32.778 15.310 1.00 2.13
C
ATOM 13440 O   VAL B 284   -32.050 33.006 16.035 1.00 2.37
O
ATOM 13441 CB VAL B 284   -31.477 34.139 13.227 1.00 2.98
C
ATOM 13442 CG1 VAL B 284   -30.418 35.119 13.710 1.00 2.98
C
ATOM 13443 CG2 VAL B 284   -31.464 34.061 11.705 1.00 2.98
C
ATOM 13444 H   VAL B 284   -33.105 32.215 12.937 1.00 2.32
H
ATOM 13445 HA VAL B 284   -30.305 32.345 13.374 1.00 2.39
H
ATOM 13446 HB VAL B 284   -32.445 34.500 13.572 1.00 3.58
H
ATOM 13447 1HG1 VAL B 284   -30.623 36.106 13.298 1.00 3.58
H
ATOM 13448 2HG1 VAL B 284   -30.438 35.169 14.799 1.00 3.58
H
ATOM 13449 3HG1 VAL B 284   -29.436 34.788 13.379 1.00 3.58
H
ATOM 13450 1HG2 VAL B 284   -31.655 35.051 11.289 1.00 3.58
H
ATOM 13451 2HG2 VAL B 284   -30.492 33.706 11.368 1.00 3.58
H
ATOM 13452 3HG2 VAL B 284   -32.240 33.372 11.369 1.00 3.58
H
ATOM 13453 N   GLY B 285   -29.860 32.533 15.774 1.00 2.02
N
ATOM 13454 CA GLY B 285   -29.559 32.482 17.194 1.00 2.13
C
ATOM 13455 C   GLY B 285   -29.293 31.056 17.693 1.00 2.03
C
ATOM 13456 O   GLY B 285   -28.639 30.874 18.720 1.00 2.07
O
ATOM 13457 H   GLY B 285   -29.108 32.396 15.113 1.00 2.42
H
ATOM 13458 1HA GLY B 285   -28.691 33.104 17.393 1.00 2.56
H
ATOM 13459 2HA GLY B 285   -30.389 32.913 17.753 1.00 2.56
H
ATOM 13460 N   ASP B 286   -29.798 30.047 16.976 1.00 1.94
N
ATOM 13461 CA ASP B 286   -29.572 28.655 17.373 1.00 1.89
C
ATOM 13462 C   ASP B 286   -28.125 28.257 17.136 1.00 1.77
C
ATOM 13463 O   ASP B 286   -27.496 28.740 16.202 1.00 1.70
O
ATOM 13464 CB ASP B 286   -30.480 27.695 16.604 1.00 2.83
C
ATOM 13465 CG ASP B 286   -31.951 27.792 16.986 1.00 2.83
C

ATOM 13466 OD1 ASP B 286 -32.283 28.492 17.916 1.00 2.83
O
ATOM 13467 OD2 ASP B 286 -32.737 27.134 16.349 1.00 2.83
O
ATOM 13468 H ASP B 286 -30.335 30.227 16.136 1.00 2.33
H
ATOM 13469 HA ASP B 286 -29.783 28.559 18.438 1.00 2.27
H
ATOM 13470 1HB ASP B 286 -30.388 27.894 15.536 1.00 3.40
H
ATOM 13471 2HB ASP B 286 -30.147 26.671 16.772 1.00 3.40
H
ATOM 13472 N LYS B 287 -27.580 27.376 17.969 1.00 1.81
N
ATOM 13473 CA LYS B 287 -26.197 26.967 17.751 1.00 1.74
C
ATOM 13474 C LYS B 287 -26.090 25.596 17.103 1.00 1.68
C
ATOM 13475 O LYS B 287 -26.785 24.652 17.480 1.00 2.66
O
ATOM 13476 CB LYS B 287 -25.403 26.988 19.050 1.00 2.61
C
ATOM 13477 CG LYS B 287 -25.197 28.375 19.635 1.00 2.61
C
ATOM 13478 CD LYS B 287 -24.333 28.313 20.881 1.00 2.61
C
ATOM 13479 CE LYS B 287 -24.115 29.693 21.482 1.00 2.61
C
ATOM 13480 NZ LYS B 287 -23.285 29.631 22.718 1.00 2.61
N
ATOM 13481 H LYS B 287 -28.115 26.994 18.737 1.00 2.17
H
ATOM 13482 HA LYS B 287 -25.745 27.685 17.077 1.00 2.09
H
ATOM 13483 1HB LYS B 287 -25.914 26.381 19.799 1.00 3.13
H
ATOM 13484 2HB LYS B 287 -24.422 26.546 18.885 1.00 3.13
H
ATOM 13485 1HG LYS B 287 -24.709 29.010 18.896 1.00 3.13
H
ATOM 13486 2HG LYS B 287 -26.161 28.815 19.889 1.00 3.13
H
ATOM 13487 1HD LYS B 287 -24.813 27.673 21.624 1.00 3.13
H
ATOM 13488 2HD LYS B 287 -23.365 27.880 20.630 1.00 3.13
H
ATOM 13489 1HE LYS B 287 -23.615 30.327 20.753 1.00 3.13
H
ATOM 13490 2HE LYS B 287 -25.081 30.135 21.728 1.00 3.13
H
ATOM 13491 1HZ LYS B 287 -23.161 30.562 23.088 1.00 3.13
H
ATOM 13492 2HZ LYS B 287 -23.748 29.053 23.406 1.00 3.13
H
ATOM 13493 3HZ LYS B 287 -22.383 29.234 22.497 1.00 3.13
H
ATOM 13494 N ILE B 288 -25.203 25.508 16.121 1.00 1.48
N

```
ATOM  13495  CA  ILE B 288     -24.952  24.286  15.372  1.00  1.44
C
ATOM  13496  C   ILE B 288     -23.602  24.327  14.667  1.00  1.17
C
ATOM  13497  O   ILE B 288     -23.179  25.371  14.173  1.00  1.18
O
ATOM  13498  CB  ILE B 288     -26.075  24.041  14.360  1.00  2.16
C
ATOM  13499  CG1 ILE B 288     -25.861  22.700  13.637  1.00  2.16
C
ATOM  13500  CG2 ILE B 288     -26.160  25.205  13.404  1.00  2.16
C
ATOM  13501  CD1 ILE B 288     -27.057  22.228  12.848  1.00  2.16
C
ATOM  13502  H   ILE B 288     -24.683  26.341  15.877  1.00  1.78
H
ATOM  13503  HA  ILE B 288     -24.942  23.452  16.073  1.00  1.73
H
ATOM  13504  HB  ILE B 288     -27.021  23.962  14.896  1.00  2.59
H
ATOM  13505 1HG1 ILE B 288     -25.022  22.799  12.953  1.00  2.59
H
ATOM  13506 2HG1 ILE B 288     -25.615  21.937  14.376  1.00  2.59
H
ATOM  13507 1HG2 ILE B 288     -26.980  25.034  12.711  1.00  2.59
H
ATOM  13508 2HG2 ILE B 288     -26.342  26.121  13.962  1.00  2.59
H
ATOM  13509 3HG2 ILE B 288     -25.223  25.293  12.854  1.00  2.59
H
ATOM  13510 1HD1 ILE B 288     -26.824  21.277  12.369  1.00  2.59
H
ATOM  13511 2HD1 ILE B 288     -27.908  22.099  13.518  1.00  2.59
H
ATOM  13512 3HD1 ILE B 288     -27.302  22.966  12.087  1.00  2.59
H
ATOM  13513  N   ALA B 289     -22.904  23.200  14.666  1.00  1.06
N
ATOM  13514  CA  ALA B 289     -21.609  23.115  14.008  1.00  0.96
C
ATOM  13515  C   ALA B 289     -21.800  23.070  12.506  1.00  0.83
C
ATOM  13516  O   ALA B 289     -22.764  22.473  12.024  1.00  0.78
O
ATOM  13517  CB  ALA B 289     -20.844  21.888  14.481  1.00  1.44
C
ATOM  13518  H   ALA B 289     -23.289  22.379  15.110  1.00  1.27
H
ATOM  13519  HA  ALA B 289     -21.038  24.012  14.251  1.00  1.15
H
ATOM  13520 1HB  ALA B 289     -19.873  21.852  13.988  1.00  1.73
H
ATOM  13521 2HB  ALA B 289     -20.700  21.945  15.560  1.00  1.73
H
ATOM  13522 3HB  ALA B 289     -21.408  20.992  14.239  1.00  1.73
H
ATOM  13523  N   THR B 290     -20.870  23.669  11.761  1.00  0.81
N
```

ATOM 13524 CA THR B 290 -20.988 23.651 10.309 1.00 0.76
C
ATOM 13525 C THR B 290 -20.776 22.241 9.766 1.00 0.65
C
ATOM 13526 O THR B 290 -21.333 21.900 8.724 1.00 0.60
O
ATOM 13527 CB THR B 290 -20.015 24.644 9.646 1.00 1.14
C
ATOM 13528 OG1 THR B 290 -18.674 24.332 10.031 1.00 1.14
O
ATOM 13529 CG2 THR B 290 -20.338 26.076 10.040 1.00 1.14
C
ATOM 13530 H THR B 290 -20.092 24.144 12.201 1.00 0.97
H
ATOM 13531 HA THR B 290 -22.000 23.960 10.045 1.00 0.91
H
ATOM 13532 HB THR B 290 -20.097 24.547 8.564 1.00 1.37
H
ATOM 13533 HG1 THR B 290 -18.473 24.720 10.892 1.00 1.37
H
ATOM 13534 1HG2 THR B 290 -19.640 26.754 9.549 1.00 1.37
H
ATOM 13535 2HG2 THR B 290 -21.354 26.316 9.731 1.00 1.37
H
ATOM 13536 3HG2 THR B 290 -20.251 26.184 11.120 1.00 1.37
H
ATOM 13537 N ARG B 291 -20.035 21.398 10.501 1.00 0.64
N
ATOM 13538 CA ARG B 291 -19.920 19.990 10.116 1.00 0.57
C
ATOM 13539 C ARG B 291 -21.282 19.308 10.120 1.00 0.58
C
ATOM 13540 O ARG B 291 -21.621 18.574 9.187 1.00 0.55
O
ATOM 13541 CB ARG B 291 -19.063 19.214 11.086 1.00 0.85
C
ATOM 13542 CG ARG B 291 -17.588 19.476 11.090 1.00 0.85
C
ATOM 13543 CD ARG B 291 -16.994 18.685 12.185 1.00 0.85
C
ATOM 13544 NE ARG B 291 -17.526 19.099 13.467 1.00 0.85
N
ATOM 13545 CZ ARG B 291 -17.779 18.290 14.519 1.00 0.85
C
ATOM 13546 NH1 ARG B 291 -17.580 16.989 14.457 1.00 0.85
N
ATOM 13547 NH2 ARG B 291 -18.248 18.813 15.638 1.00 0.85
N
ATOM 13548 H ARG B 291 -19.530 21.729 11.319 1.00 0.77
H
ATOM 13549 HA ARG B 291 -19.492 19.930 9.116 1.00 0.68
H
ATOM 13550 1HB ARG B 291 -19.421 19.390 12.099 1.00 1.03
H
ATOM 13551 2HB ARG B 291 -19.181 18.151 10.883 1.00 1.03
H
ATOM 13552 1HG ARG B 291 -17.163 19.144 10.145 1.00 1.03
H

```
ATOM  13553 2HG  ARG B 291     -17.382  20.531  11.228  1.00  1.03
H
ATOM  13554 1HD  ARG B 291     -17.229  17.642  12.034  1.00  1.03
H
ATOM  13555 2HD  ARG B 291     -15.913  18.820  12.202  1.00  1.03
H
ATOM  13556  HE  ARG B 291     -17.718  20.086  13.586  1.00  1.03
H
ATOM  13557 1HH1 ARG B 291     -17.255  16.548  13.606  1.00  1.03
H
ATOM  13558 2HH1 ARG B 291     -17.786  16.412  15.259  1.00  1.03
H
ATOM  13559 1HH2 ARG B 291     -18.382  19.815  15.713  1.00  1.03
H
ATOM  13560 2HH2 ARG B 291     -18.448  18.218  16.427  1.00  1.03
H
ATOM  13561  N   LYS B 292     -22.054  19.560  11.185  1.00  0.66
N
ATOM  13562  CA  LYS B 292     -23.377  18.973  11.341  1.00  0.69
C
ATOM  13563  C   LYS B 292     -24.287  19.450  10.245  1.00  0.68
C
ATOM  13564  O   LYS B 292     -25.040  18.663   9.666  1.00  0.66
O
ATOM  13565  CB  LYS B 292     -23.988  19.338  12.698  1.00  1.03
C
ATOM  13566  CG  LYS B 292     -23.337  18.670  13.891  1.00  1.03
C
ATOM  13567  CD  LYS B 292     -23.990  19.079  15.203  1.00  1.03
C
ATOM  13568  CE  LYS B 292     -23.340  18.354  16.375  1.00  1.03
C
ATOM  13569  NZ  LYS B 292     -23.980  18.686  17.680  1.00  1.03
N
ATOM  13570  H   LYS B 292     -21.708  20.176  11.908  1.00  0.79
H
ATOM  13571  HA  LYS B 292     -23.295  17.888  11.265  1.00  0.83
H
ATOM  13572 1HB  LYS B 292     -23.929  20.415  12.848  1.00  1.24
H
ATOM  13573 2HB  LYS B 292     -25.044  19.067  12.704  1.00  1.24
H
ATOM  13574 1HG  LYS B 292     -23.426  17.589  13.784  1.00  1.24
H
ATOM  13575 2HG  LYS B 292     -22.280  18.930  13.922  1.00  1.24
H
ATOM  13576 1HD  LYS B 292     -23.885  20.155  15.347  1.00  1.24
H
ATOM  13577 2HD  LYS B 292     -25.051  18.834  15.177  1.00  1.24
H
ATOM  13578 1HE  LYS B 292     -23.418  17.281  16.210  1.00  1.24
H
ATOM  13579 2HE  LYS B 292     -22.285  18.625  16.423  1.00  1.24
H
ATOM  13580 1HZ  LYS B 292     -23.518  18.176  18.418  1.00  1.24
H
ATOM  13581 2HZ  LYS B 292     -23.910  19.676  17.879  1.00  1.24
H
```

ATOM 13582 3HZ LYS B 292 -24.954 18.424 17.654 1.00 1.24
H
ATOM 13583 N ALA B 293 -24.182 20.743 9.950 1.00 0.70
N
ATOM 13584 CA ALA B 293 -24.966 21.365 8.909 1.00 0.71
C
ATOM 13585 C ALA B 293 -24.686 20.728 7.556 1.00 0.66
C
ATOM 13586 O ALA B 293 -25.627 20.485 6.804 1.00 0.72
O
ATOM 13587 CB ALA B 293 -24.681 22.843 8.871 1.00 1.06
C
ATOM 13588 H ALA B 293 -23.550 21.323 10.489 1.00 0.84
H
ATOM 13589 HA ALA B 293 -26.020 21.214 9.140 1.00 0.85
H
ATOM 13590 1HB ALA B 293 -25.296 23.313 8.104 1.00 1.28
H
ATOM 13591 2HB ALA B 293 -24.912 23.278 9.844 1.00 1.28
H
ATOM 13592 3HB ALA B 293 -23.630 23.008 8.646 1.00 1.28
H
ATOM 13593 N CYS B 294 -23.411 20.441 7.244 1.00 0.60
N
ATOM 13594 CA CYS B 294 -23.093 19.762 5.983 1.00 0.63
C
ATOM 13595 C CYS B 294 -23.692 18.390 5.899 1.00 0.57
C
ATOM 13596 O CYS B 294 -24.211 18.021 4.851 1.00 0.63
O
ATOM 13597 CB CYS B 294 -21.596 19.616 5.733 1.00 0.95
C
ATOM 13598 SG CYS B 294 -20.772 21.127 5.247 1.00 0.95
S
ATOM 13599 H CYS B 294 -22.663 20.698 7.876 1.00 0.72
H
ATOM 13600 HA CYS B 294 -23.503 20.361 5.169 1.00 0.76
H
ATOM 13601 1HB CYS B 294 -21.113 19.249 6.639 1.00 1.13
H
ATOM 13602 2HB CYS B 294 -21.429 18.876 4.951 1.00 1.13
H
ATOM 13603 HG CYS B 294 -21.456 21.303 4.123 1.00 1.13
H
ATOM 13604 N GLY B 295 -23.629 17.625 6.985 1.00 0.52
N
ATOM 13605 CA GLY B 295 -24.219 16.294 6.972 1.00 0.51
C
ATOM 13606 C GLY B 295 -25.706 16.371 6.640 1.00 0.56
C
ATOM 13607 O GLY B 295 -26.205 15.615 5.797 1.00 0.55
O
ATOM 13608 H GLY B 295 -23.161 17.964 7.821 1.00 0.62
H
ATOM 13609 1HA GLY B 295 -23.705 15.670 6.239 1.00 0.61
H
ATOM 13610 2HA GLY B 295 -24.082 15.831 7.946 1.00 0.61
H

ATOM 13611 N   LEU B 296   -26.398 17.309   7.289 1.00 0.64
N
ATOM 13612 CA LEU B 296   -27.815 17.529   7.058 1.00 0.73
C
ATOM 13613 C   LEU B 296   -28.078 17.989   5.630 1.00 0.72
C
ATOM 13614 O   LEU B 296   -29.010 17.509   4.985 1.00 0.74
O
ATOM 13615 CB LEU B 296   -28.332 18.588   8.039 1.00 1.09
C
ATOM 13616 CG LEU B 296   -28.387 18.168   9.512 1.00 1.09
C
ATOM 13617 CD1 LEU B 296   -28.686 19.390 10.371 1.00 1.09
C
ATOM 13618 CD2 LEU B 296   -29.465 17.109   9.687 1.00 1.09
C
ATOM 13619 H   LEU B 296   -25.924 17.882   7.980 1.00 0.77
H
ATOM 13620 HA LEU B 296   -28.345 16.594   7.226 1.00 0.88
H
ATOM 13621 1HB LEU B 296   -27.692 19.466   7.972 1.00 1.31
H
ATOM 13622 2HB LEU B 296   -29.339 18.875   7.741 1.00 1.31
H
ATOM 13623 HG LEU B 296   -27.423 17.763   9.816 1.00 1.31
H
ATOM 13624 1HD1 LEU B 296   -28.722 19.097 11.420 1.00 1.31
H
ATOM 13625 2HD1 LEU B 296   -27.902 20.134 10.228 1.00 1.31
H
ATOM 13626 3HD1 LEU B 296   -29.645 19.814 10.079 1.00 1.31
H
ATOM 13627 1HD2 LEU B 296   -29.509 16.805 10.734 1.00 1.31
H
ATOM 13628 2HD2 LEU B 296   -30.429 17.518   9.388 1.00 1.31
H
ATOM 13629 3HD2 LEU B 296   -29.230 16.244   9.068 1.00 1.31
H
ATOM 13630 N   ALA B 297   -27.221 18.893   5.143 1.00 0.71
N
ATOM 13631 CA ALA B 297   -27.303 19.453   3.802 1.00 0.74
C
ATOM 13632 C   ALA B 297   -27.198 18.389   2.741 1.00 0.54
C
ATOM 13633 O   ALA B 297   -27.994 18.373   1.802 1.00 0.53
O
ATOM 13634 CB ALA B 297   -26.187 20.462   3.588 1.00 1.11
C
ATOM 13635 H   ALA B 297   -26.488 19.238   5.742 1.00 0.85
H
ATOM 13636 HA ALA B 297   -28.269 19.948   3.702 1.00 0.89
H
ATOM 13637 1HB ALA B 297   -26.273 20.898   2.593 1.00 1.33
H
ATOM 13638 2HB ALA B 297   -26.248 21.247   4.335 1.00 1.33
H
ATOM 13639 3HB ALA B 297   -25.227 19.962   3.679 1.00 1.33
H

ATOM 13640 N LEU B 298 -26.211 17.506 2.896 1.00 0.50
N
ATOM 13641 CA LEU B 298 -25.976 16.437 1.942 1.00 0.46
C
ATOM 13642 C LEU B 298 -27.144 15.496 1.854 1.00 0.42
C
ATOM 13643 O LEU B 298 -27.530 15.103 0.755 1.00 0.38
O
ATOM 13644 CB LEU B 298 -24.742 15.615 2.335 1.00 0.69
C
ATOM 13645 CG LEU B 298 -23.386 16.299 2.183 1.00 0.69
C
ATOM 13646 CD1 LEU B 298 -22.312 15.403 2.791 1.00 0.69
C
ATOM 13647 CD2 LEU B 298 -23.125 16.556 0.712 1.00 0.69
C
ATOM 13648 H LEU B 298 -25.593 17.591 3.689 1.00 0.60
H
ATOM 13649 HA LEU B 298 -25.812 16.880 0.960 1.00 0.55
H
ATOM 13650 1HB LEU B 298 -24.844 15.322 3.379 1.00 0.83
H
ATOM 13651 2HB LEU B 298 -24.722 14.710 1.727 1.00 0.83
H
ATOM 13652 HG LEU B 298 -23.386 17.246 2.719 1.00 0.83
H
ATOM 13653 1HD1 LEU B 298 -21.340 15.881 2.695 1.00 0.83
H
ATOM 13654 2HD1 LEU B 298 -22.532 15.241 3.847 1.00 0.83
H
ATOM 13655 3HD1 LEU B 298 -22.299 14.447 2.271 1.00 0.83
H
ATOM 13656 1HD2 LEU B 298 -22.158 17.045 0.599 1.00 0.83
H
ATOM 13657 2HD2 LEU B 298 -23.120 15.610 0.172 1.00 0.83
H
ATOM 13658 3HD2 LEU B 298 -23.907 17.199 0.311 1.00 0.83
H
ATOM 13659 N ALA B 299 -27.717 15.141 3.003 1.00 0.53
N
ATOM 13660 CA ALA B 299 -28.859 14.242 3.015 1.00 0.60
C
ATOM 13661 C ALA B 299 -30.042 14.865 2.299 1.00 0.65
C
ATOM 13662 O ALA B 299 -30.716 14.194 1.512 1.00 0.64
O
ATOM 13663 CB ALA B 299 -29.229 13.883 4.431 1.00 0.90
C
ATOM 13664 H ALA B 299 -27.344 15.478 3.887 1.00 0.64
H
ATOM 13665 HA ALA B 299 -28.588 13.333 2.483 1.00 0.72
H
ATOM 13666 1HB ALA B 299 -30.070 13.190 4.422 1.00 1.08
H
ATOM 13667 2HB ALA B 299 -28.372 13.411 4.907 1.00 1.08
H
ATOM 13668 3HB ALA B 299 -29.505 14.783 4.980 1.00 1.08
H

```
ATOM  13669  N   LYS B 300     -30.280  16.154   2.550  1.00  0.74
N
ATOM  13670  CA  LYS B 300     -31.372  16.854   1.893  1.00  0.81
C
ATOM  13671  C   LYS B 300     -31.150  16.905   0.391  1.00  0.68
C
ATOM  13672  O   LYS B 300     -32.082  16.700  -0.391  1.00  0.83
O
ATOM  13673  CB  LYS B 300     -31.499  18.267   2.448  1.00  1.22
C
ATOM  13674  CG  LYS B 300     -32.006  18.325   3.878  1.00  1.22
C
ATOM  13675  CD  LYS B 300     -32.073  19.756   4.375  1.00  1.22
C
ATOM  13676  CE  LYS B 300     -32.478  19.815   5.837  1.00  1.22
C
ATOM  13677  NZ  LYS B 300     -32.570  21.213   6.317  1.00  1.22
N
ATOM  13678  H   LYS B 300     -29.709  16.655   3.221  1.00  0.89
H
ATOM  13679  HA  LYS B 300     -32.299  16.314   2.081  1.00  0.97
H
ATOM  13680 1HB  LYS B 300     -30.528  18.762   2.417  1.00  1.46
H
ATOM  13681 2HB  LYS B 300     -32.183  18.844   1.826  1.00  1.46
H
ATOM  13682 1HG  LYS B 300     -33.007  17.892   3.922  1.00  1.46
H
ATOM  13683 2HG  LYS B 300     -31.356  17.745   4.528  1.00  1.46
H
ATOM  13684 1HD  LYS B 300     -31.095  20.228   4.260  1.00  1.46
H
ATOM  13685 2HD  LYS B 300     -32.798  20.315   3.783  1.00  1.46
H
ATOM  13686 1HE  LYS B 300     -33.443  19.328   5.971  1.00  1.46
H
ATOM  13687 2HE  LYS B 300     -31.732  19.288   6.434  1.00  1.46
H
ATOM  13688 1HZ  LYS B 300     -32.827  21.225   7.294  1.00  1.46
H
ATOM  13689 2HZ  LYS B 300     -31.675  21.671   6.200  1.00  1.46
H
ATOM  13690 3HZ  LYS B 300     -33.267  21.709   5.782  1.00  1.46
H
ATOM  13691  N   LEU B 301     -29.905  17.158  -0.005  1.00  0.55
N
ATOM  13692  CA  LEU B 301     -29.532  17.216  -1.403  1.00  0.59
C
ATOM  13693  C   LEU B 301     -29.769  15.849  -2.065  1.00  0.68
C
ATOM  13694  O   LEU B 301     -30.208  15.777  -3.215  1.00  1.10
O
ATOM  13695  CB  LEU B 301     -28.063  17.603  -1.524  1.00  0.89
C
ATOM  13696  CG  LEU B 301     -27.592  17.962  -2.921  1.00  0.89
C
ATOM  13697  CD1 LEU B 301     -28.327  19.214  -3.378  1.00  0.89
C
```

ATOM 13698 CD2 LEU B 301   -26.099 18.150 -2.910 1.00 0.89
C
ATOM 13699 H   LEU B 301   -29.191 17.339   0.687 1.00 0.66
H
ATOM 13700 HA LEU B 301   -30.148 17.962 -1.903 1.00 0.71
H
ATOM 13701 1HB LEU B 301   -27.875 18.458 -0.879 1.00 1.06
H
ATOM 13702 2HB LEU B 301   -27.452 16.771 -1.175 1.00 1.06
H
ATOM 13703 HG LEU B 301   -27.850 17.168 -3.611 1.00 1.06
H
ATOM 13704 1HD1 LEU B 301   -28.001 19.479 -4.381 1.00 1.06
H
ATOM 13705 2HD1 LEU B 301   -29.401 19.028 -3.379 1.00 1.06
H
ATOM 13706 3HD1 LEU B 301   -28.100 20.034 -2.699 1.00 1.06
H
ATOM 13707 1HD2 LEU B 301   -25.763 18.398 -3.913 1.00 1.06
H
ATOM 13708 2HD2 LEU B 301   -25.842 18.954 -2.230 1.00 1.06
H
ATOM 13709 3HD2 LEU B 301   -25.619 17.228 -2.582 1.00 1.06
H
ATOM 13710 N   GLY B 302   -29.489 14.772 -1.314 1.00 0.58
N
ATOM 13711 CA GLY B 302   -29.699 13.398 -1.764 1.00 0.70
C
ATOM 13712 C   GLY B 302   -31.158 13.149 -2.111 1.00 0.86
C
ATOM 13713 O   GLY B 302   -31.459 12.475 -3.094 1.00 2.79
O
ATOM 13714 H   GLY B 302   -29.093 14.910 -0.394 1.00 0.70
H
ATOM 13715 1HA GLY B 302   -29.076 13.201 -2.634 1.00 0.84
H
ATOM 13716 2HA GLY B 302   -29.388 12.710 -0.981 1.00 0.84
H
ATOM 13717 N   TYR B 303   -32.062 13.703 -1.304 1.00 1.04
N
ATOM 13718 CA TYR B 303   -33.495 13.626 -1.579 1.00 0.75
C
ATOM 13719 C   TYR B 303   -33.821 14.304 -2.907 1.00 1.10
C
ATOM 13720 O   TYR B 303   -34.595 13.781 -3.712 1.00 4.35
O
ATOM 13721 CB TYR B 303   -34.298 14.253 -0.434 1.00 1.12
C
ATOM 13722 CG TYR B 303   -35.797 14.231 -0.638 1.00 1.12
C
ATOM 13723 CD1 TYR B 303   -36.509 13.045 -0.524 1.00 1.12
C
ATOM 13724 CD2 TYR B 303   -36.465 15.409 -0.921 1.00 1.12
C
ATOM 13725 CE1 TYR B 303   -37.879 13.040 -0.701 1.00 1.12
C
ATOM 13726 CE2 TYR B 303   -37.835 15.405 -1.097 1.00 1.12
C

ATOM 13727 CZ TYR B 303   -38.542 14.227 -0.989 1.00 1.12
C
ATOM 13728 OH TYR B 303   -39.906 14.223 -1.165 1.00 1.12
O
ATOM 13729 H   TYR B 303   -31.742 14.192 -0.474 1.00 1.25
H
ATOM 13730 HA TYR B 303   -33.775 12.578 -1.671 1.00 0.90
H
ATOM 13731 1HB TYR B 303   -34.073 13.735   0.498 1.00 1.35
H
ATOM 13732 2HB TYR B 303   -33.998 15.289 -0.305 1.00 1.35
H
ATOM 13733 HD1 TYR B 303   -35.992 12.115 -0.297 1.00 1.35
H
ATOM 13734 HD2 TYR B 303   -35.907 16.341 -1.003 1.00 1.35
H
ATOM 13735 HE1 TYR B 303   -38.436 12.107 -0.614 1.00 1.35
H
ATOM 13736 HE2 TYR B 303   -38.359 16.335 -1.320 1.00 1.35
H
ATOM 13737 HH TYR B 303   -40.240 13.329 -1.050 1.00 1.35
H
ATOM 13738 N   ALA B 304   -33.245 15.486 -3.114 1.00 1.14
N
ATOM 13739 CA ALA B 304   -33.460 16.257 -4.332 1.00 1.07
C
ATOM 13740 C   ALA B 304   -32.988 15.550 -5.616 1.00 0.79
C
ATOM 13741 O   ALA B 304   -33.672 15.626 -6.640 1.00 0.87
O
ATOM 13742 CB ALA B 304   -32.754 17.596 -4.213 1.00 1.60
C
ATOM 13743 H   ALA B 304   -32.647 15.871 -2.389 1.00 1.37
H
ATOM 13744 HA ALA B 304   -34.532 16.431 -4.427 1.00 1.28
H
ATOM 13745 1HB ALA B 304   -32.962 18.181 -5.106 1.00 1.93
H
ATOM 13746 2HB ALA B 304   -33.121 18.125 -3.334 1.00 1.93
H
ATOM 13747 3HB ALA B 304   -31.682 17.444 -4.122 1.00 1.93
H
ATOM 13748 N   ASN B 305   -31.840 14.864 -5.586 1.00 0.56
N
ATOM 13749 CA ASN B 305   -31.388 14.245 -6.834 1.00 0.40
C
ATOM 13750 C   ASN B 305   -30.551 12.980 -6.648 1.00 0.29
C
ATOM 13751 O   ASN B 305   -29.587 12.950 -5.882 1.00 0.34
O
ATOM 13752 CB ASN B 305   -30.626 15.264 -7.654 1.00 0.60
C
ATOM 13753 CG ASN B 305   -30.378 14.829 -9.071 1.00 0.60
C
ATOM 13754 OD1 ASN B 305   -29.542 13.972 -9.383 1.00 0.60
O
ATOM 13755 ND2 ASN B 305   -31.132 15.415 -9.966 1.00 0.60
N

ATOM 13756 H ASN B 305 -31.286 14.817 -4.738 1.00 0.67
H
ATOM 13757 HA ASN B 305 -32.273 13.950 -7.399 1.00 0.48
H
ATOM 13758 1HB ASN B 305 -31.188 16.199 -7.672 1.00 0.72
H
ATOM 13759 2HB ASN B 305 -29.668 15.470 -7.178 1.00 0.72
H
ATOM 13760 1HD2 ASN B 305 -31.043 15.177 -10.933 1.00 0.72
H
ATOM 13761 2HD2 ASN B 305 -31.805 16.097 -9.680 1.00 0.72
H
ATOM 13762 N ASN B 306 -30.932 11.946 -7.402 1.00 0.35
N
ATOM 13763 CA ASN B 306 -30.334 10.612 -7.375 1.00 0.30
C
ATOM 13764 C ASN B 306 -28.848 10.526 -7.735 1.00 0.36
C
ATOM 13765 O ASN B 306 -28.201 9.521 -7.429 1.00 0.60
O
ATOM 13766 CB ASN B 306 -31.119 9.710 -8.304 1.00 0.45
C
ATOM 13767 CG ASN B 306 -32.461 9.330 -7.745 1.00 0.45
C
ATOM 13768 OD1 ASN B 306 -32.678 9.370 -6.530 1.00 0.45
O
ATOM 13769 ND2 ASN B 306 -33.367 8.957 -8.612 1.00 0.45
N
ATOM 13770 H ASN B 306 -31.720 12.089 -8.018 1.00 0.42
H
ATOM 13771 HA ASN B 306 -30.433 10.229 -6.358 1.00 0.36
H
ATOM 13772 1HB ASN B 306 -31.272 10.220 -9.256 1.00 0.54
H
ATOM 13773 2HB ASN B 306 -30.547 8.808 -8.510 1.00 0.54
H
ATOM 13774 1HD2 ASN B 306 -34.279 8.691 -8.298 1.00 0.54
H
ATOM 13775 2HD2 ASN B 306 -33.149 8.937 -9.587 1.00 0.54
H
ATOM 13776 N ARG B 307 -28.295 11.565 -8.364 1.00 0.34
N
ATOM 13777 CA ARG B 307 -26.879 11.561 -8.706 1.00 0.49
C
ATOM 13778 C ARG B 307 -25.979 11.852 -7.516 1.00 0.44
C
ATOM 13779 O ARG B 307 -24.767 11.686 -7.614 1.00 0.57
O
ATOM 13780 CB ARG B 307 -26.577 12.567 -9.798 1.00 0.73
C
ATOM 13781 CG ARG B 307 -27.151 12.232 -11.161 1.00 0.73
C
ATOM 13782 CD ARG B 307 -26.954 13.350 -12.109 1.00 0.73
C
ATOM 13783 NE ARG B 307 -27.772 14.490 -11.743 1.00 0.73
N
ATOM 13784 CZ ARG B 307 -27.669 15.719 -12.286 1.00 0.73
C

ATOM 13785 NH1 ARG B 307 -26.762 15.973 -13.201 1.00 0.73
N
ATOM 13786 NH2 ARG B 307 -28.484 16.677 -11.898 1.00 0.73
N
ATOM 13787 H ARG B 307 -28.848 12.376 -8.614 1.00 0.41
H
ATOM 13788 HA ARG B 307 -26.630 10.570 -9.085 1.00 0.59
H
ATOM 13789 1HB ARG B 307 -26.965 13.543 -9.510 1.00 0.88
H
ATOM 13790 2HB ARG B 307 -25.498 12.665 -9.913 1.00 0.88
H
ATOM 13791 1HG ARG B 307 -26.654 11.347 -11.562 1.00 0.88
H
ATOM 13792 2HG ARG B 307 -28.220 12.041 -11.070 1.00 0.88
H
ATOM 13793 1HD ARG B 307 -25.910 13.661 -12.102 1.00 0.88
H
ATOM 13794 2HD ARG B 307 -27.234 13.035 -13.111 1.00 0.88
H
ATOM 13795 HE ARG B 307 -28.482 14.341 -11.033 1.00 0.88
H
ATOM 13796 1HH1 ARG B 307 -26.138 15.242 -13.514 1.00 0.88
H
ATOM 13797 2HH1 ARG B 307 -26.697 16.900 -13.599 1.00 0.88
H
ATOM 13798 1HH2 ARG B 307 -29.183 16.493 -11.195 1.00 0.88
H
ATOM 13799 2HH2 ARG B 307 -28.414 17.597 -12.313 1.00 0.88
H
ATOM 13800 N VAL B 308 -26.541 12.295 -6.395 1.00 0.36
N
ATOM 13801 CA VAL B 308 -25.713 12.617 -5.240 1.00 0.39
C
ATOM 13802 C VAL B 308 -25.173 11.371 -4.540 1.00 0.29
C
ATOM 13803 O VAL B 308 -25.935 10.565 -4.007 1.00 0.44
O
ATOM 13804 CB VAL B 308 -26.527 13.449 -4.237 1.00 0.58
C
ATOM 13805 CG1 VAL B 308 -25.714 13.731 -2.977 1.00 0.58
C
ATOM 13806 CG2 VAL B 308 -26.955 14.743 -4.905 1.00 0.58
C
ATOM 13807 H VAL B 308 -27.546 12.422 -6.325 1.00 0.43
H
ATOM 13808 HA VAL B 308 -24.867 13.215 -5.583 1.00 0.47
H
ATOM 13809 HB VAL B 308 -27.409 12.882 -3.943 1.00 0.70
H
ATOM 13810 1HG1 VAL B 308 -26.315 14.311 -2.278 1.00 0.70
H
ATOM 13811 2HG1 VAL B 308 -25.425 12.789 -2.513 1.00 0.70
H
ATOM 13812 3HG1 VAL B 308 -24.822 14.293 -3.241 1.00 0.70
H
ATOM 13813 1HG2 VAL B 308 -27.553 15.315 -4.204 1.00 0.70
H

```
ATOM  13814 2HG2 VAL B 308      -26.075  15.317  -5.195  1.00  0.70
H
ATOM  13815 3HG2 VAL B 308      -27.550  14.518  -5.790  1.00  0.70
H
ATOM  13816  N   VAL B 309      -23.848  11.250  -4.529  1.00  0.20
N
ATOM  13817  CA  VAL B 309      -23.119  10.157  -3.878  1.00  0.38
C
ATOM  13818  C   VAL B 309      -22.126  10.712  -2.873  1.00  0.30
C
ATOM  13819  O   VAL B 309      -21.440  11.689  -3.160  1.00  0.67
O
ATOM  13820  CB  VAL B 309      -22.385   9.289  -4.929  1.00  0.57
C
ATOM  13821  CG1 VAL B 309      -21.503   8.240  -4.262  1.00  0.57
C
ATOM  13822  CG2 VAL B 309      -23.423   8.591  -5.795  1.00  0.57
C
ATOM  13823  H   VAL B 309      -23.306  11.957  -5.008  1.00  0.24
H
ATOM  13824  HA  VAL B 309      -23.835   9.528  -3.351  1.00  0.46
H
ATOM  13825  HB  VAL B 309      -21.750   9.928  -5.543  1.00  0.68
H
ATOM  13826 1HG1 VAL B 309      -21.002   7.649  -5.029  1.00  0.68
H
ATOM  13827 2HG1 VAL B 309      -20.755   8.730  -3.639  1.00  0.68
H
ATOM  13828 3HG1 VAL B 309      -22.113   7.583  -3.647  1.00  0.68
H
ATOM  13829 1HG2 VAL B 309      -22.920   7.987  -6.548  1.00  0.68
H
ATOM  13830 2HG2 VAL B 309      -24.046   7.950  -5.170  1.00  0.68
H
ATOM  13831 3HG2 VAL B 309      -24.048   9.333  -6.288  1.00  0.68
H
ATOM  13832  N   VAL B 310      -22.071  10.111  -1.689  1.00  0.16
N
ATOM  13833  CA  VAL B 310      -21.176  10.602  -0.644  1.00  0.17
C
ATOM  13834  C   VAL B 310      -20.159   9.537  -0.241  1.00  0.15
C
ATOM  13835  O   VAL B 310      -20.508   8.371  -0.079  1.00  0.17
O
ATOM  13836  CB  VAL B 310      -21.985  11.080   0.583  1.00  0.26
C
ATOM  13837  CG1 VAL B 310      -21.052  11.534   1.694  1.00  0.26
C
ATOM  13838  CG2 VAL B 310      -22.896  12.234   0.178  1.00  0.26
C
ATOM  13839  H   VAL B 310      -22.649   9.295  -1.524  1.00  0.19
H
ATOM  13840  HA  VAL B 310      -20.637  11.458  -1.040  1.00  0.20
H
ATOM  13841  HB  VAL B 310      -22.581  10.254   0.961  1.00  0.31
H
ATOM  13842 1HG1 VAL B 310      -21.641  11.859   2.551  1.00  0.31
H
```

```
ATOM  13843 2HG1 VAL B 310      -20.406  10.709   1.992  1.00  0.31
H
ATOM  13844 3HG1 VAL B 310      -20.441  12.364   1.340  1.00  0.31
H
ATOM  13845 1HG2 VAL B 310      -23.468  12.564   1.043  1.00  0.31
H
ATOM  13846 2HG2 VAL B 310      -22.296  13.061  -0.190  1.00  0.31
H
ATOM  13847 3HG2 VAL B 310      -23.581  11.906  -0.604  1.00  0.31
H
ATOM  13848  N   LEU B 311      -18.898   9.949  -0.112  1.00  0.13
N
ATOM  13849  CA  LEU B 311      -17.807   9.041   0.226  1.00  0.12
C
ATOM  13850  C   LEU B 311      -17.144   9.492   1.521  1.00  0.12
C
ATOM  13851  O   LEU B 311      -17.069  10.691   1.779  1.00  0.12
O
ATOM  13852  CB  LEU B 311      -16.745   9.042  -0.888  1.00  0.18
C
ATOM  13853  CG  LEU B 311      -17.193   8.601  -2.288  1.00  0.18
C
ATOM  13854  CD1 LEU B 311      -17.781   9.794  -3.035  1.00  0.18
C
ATOM  13855  CD2 LEU B 311      -15.997   8.031  -3.032  1.00  0.18
C
ATOM  13856  H   LEU B 311      -18.699  10.930  -0.253  1.00  0.16
H
ATOM  13857  HA  LEU B 311      -18.208   8.039   0.359  1.00  0.14
H
ATOM  13858 1HB  LEU B 311      -16.343  10.049  -0.979  1.00  0.22
H
ATOM  13859 2HB  LEU B 311      -15.934   8.381  -0.585  1.00  0.22
H
ATOM  13860  HG  LEU B 311      -17.972   7.847  -2.206  1.00  0.22
H
ATOM  13861 1HD1 LEU B 311      -18.102   9.478  -4.027  1.00  0.22
H
ATOM  13862 2HD1 LEU B 311      -18.635  10.190  -2.490  1.00  0.22
H
ATOM  13863 3HD1 LEU B 311      -17.022  10.569  -3.130  1.00  0.22
H
ATOM  13864 1HD2 LEU B 311      -16.308   7.713  -4.028  1.00  0.22
H
ATOM  13865 2HD2 LEU B 311      -15.223   8.794  -3.119  1.00  0.22
H
ATOM  13866 3HD2 LEU B 311      -15.602   7.174  -2.486  1.00  0.22
H
ATOM  13867  N   ASP B 312      -16.649   8.555   2.330  1.00  0.14
N
ATOM  13868  CA  ASP B 312      -15.993   8.985   3.566  1.00  0.17
C
ATOM  13869  C   ASP B 312      -14.952   8.002   4.103  1.00  0.26
C
ATOM  13870  O   ASP B 312      -15.216   6.809   4.268  1.00  0.75
O
ATOM  13871  CB  ASP B 312      -17.032   9.242   4.645  1.00  0.26
C
```

ATOM 13872 CG ASP B 312   -16.475   9.982   5.843 1.00 0.26
C
ATOM 13873 OD1 ASP B 312   -15.578   9.465   6.473 1.00 0.26
O
ATOM 13874 OD2 ASP B 312   -16.967 11.059   6.118 1.00 0.26
O
ATOM 13875 H   ASP B 312   -16.752   7.575   2.088 1.00 0.17
H
ATOM 13876 HA ASP B 312   -15.481   9.924   3.359 1.00 0.20
H
ATOM 13877 1HB ASP B 312   -17.866   9.808   4.231 1.00 0.31
H
ATOM 13878 2HB ASP B 312   -17.415   8.286   4.991 1.00 0.31
H
ATOM 13879 N   GLY B 313   -13.768   8.533   4.407 1.00 0.32
N
ATOM 13880 CA GLY B 313   -12.643   7.742   4.902 1.00 0.36
C
ATOM 13881 C   GLY B 313   -12.665   7.435   6.411 1.00 0.41
C
ATOM 13882 O   GLY B 313   -11.774   7.861   7.145 1.00 0.61
O
ATOM 13883 H   GLY B 313   -13.635   9.526   4.271 1.00 0.38
H
ATOM 13884 1HA GLY B 313   -12.629   6.805   4.350 1.00 0.43
H
ATOM 13885 2HA GLY B 313   -11.716   8.258   4.656 1.00 0.43
H
ATOM 13886 N   ASP B 314   -13.669   6.681   6.854 1.00 0.38
N
ATOM 13887 CA ASP B 314   -13.816   6.276   8.264 1.00 0.49
C
ATOM 13888 C   ASP B 314   -13.925   7.426   9.271 1.00 0.57
C
ATOM 13889 O   ASP B 314   -13.511   7.271 10.417 1.00 1.07
O
ATOM 13890 CB ASP B 314   -12.634   5.384   8.699 1.00 0.73
C
ATOM 13891 CG ASP B 314   -12.906   4.616   9.992 1.00 0.73
C
ATOM 13892 OD1 ASP B 314   -14.051   4.377 10.278 1.00 0.73
O
ATOM 13893 OD2 ASP B 314   -11.980   4.281 10.697 1.00 0.73
O
ATOM 13894 H   ASP B 314   -14.354   6.380   6.173 1.00 0.46
H
ATOM 13895 HA ASP B 314   -14.730   5.687   8.340 1.00 0.59
H
ATOM 13896 1HB ASP B 314   -12.404   4.670   7.915 1.00 0.88
H
ATOM 13897 2HB ASP B 314   -11.749   6.002   8.846 1.00 0.88
H
ATOM 13898 N   THR B 315   -14.492   8.563   8.863 1.00 0.38
N
ATOM 13899 CA THR B 315   -14.696   9.700   9.758 1.00 0.41
C
ATOM 13900 C   THR B 315   -16.167 10.102   9.746 1.00 0.34
C

ATOM 13901 O   THR B 315   -16.511 11.221 10.113 1.00 0.44
O
ATOM 13902 CB THR B 315   -13.837 10.903   9.336 1.00 0.61
C
ATOM 13903 OG1 THR B 315   -14.180 11.270   7.998 1.00 0.61
O
ATOM 13904 CG2 THR B 315   -12.352 10.577   9.409 1.00 0.61
C
ATOM 13905 H   THR B 315   -14.805   8.665   7.906 1.00 0.46
H
ATOM 13906 HA THR B 315   -14.421   9.415 10.767 1.00 0.49
H
ATOM 13907 HB THR B 315   -14.047 11.742   9.996 1.00 0.74
H
ATOM 13908 HG1 THR B 315   -14.540 10.508   7.528 1.00 0.74
H
ATOM 13909 1HG2 THR B 315   -11.774 11.449   9.103 1.00 0.74
H
ATOM 13910 2HG2 THR B 315   -12.087 10.307 10.431 1.00 0.74
H
ATOM 13911 3HG2 THR B 315   -12.127   9.746   8.744 1.00 0.74
H
ATOM 13912 N   ARG B 316   -17.009   9.175   9.299 1.00 0.32
N
ATOM 13913 CA ARG B 316   -18.441   9.343   9.053 1.00 0.38
C
ATOM 13914 C   ARG B 316   -19.274   9.965 10.199 1.00 0.35
C
ATOM 13915 O   ARG B 316   -20.253 10.678   9.952 1.00 0.42
O
ATOM 13916 CB ARG B 316   -18.979   7.969   8.661 1.00 0.57
C
ATOM 13917 CG ARG B 316   -20.389   7.937   8.155 1.00 0.57
C
ATOM 13918 CD ARG B 316   -20.751   6.635   7.543 1.00 0.57
C
ATOM 13919 NE ARG B 316   -20.776   5.540   8.487 1.00 0.57
N
ATOM 13920 CZ ARG B 316   -19.887   4.530   8.517 1.00 0.57
C
ATOM 13921 NH1 ARG B 316   -18.905   4.491   7.643 1.00 0.57
N
ATOM 13922 NH2 ARG B 316   -20.003   3.569   9.414 1.00 0.57
N
ATOM 13923 H   ARG B 316   -16.625   8.272   9.067 1.00 0.38
H
ATOM 13924 HA ARG B 316   -18.541   9.996   8.187 1.00 0.46
H
ATOM 13925 1HB ARG B 316   -18.345   7.542   7.886 1.00 0.68
H
ATOM 13926 2HB ARG B 316   -18.934   7.305   9.523 1.00 0.68
H
ATOM 13927 1HG ARG B 316   -21.052   8.094   8.994 1.00 0.68
H
ATOM 13928 2HG ARG B 316   -20.535   8.720   7.413 1.00 0.68
H
ATOM 13929 1HD ARG B 316   -21.732   6.710   7.079 1.00 0.68
H

```
ATOM  13930 2HD  ARG B 316     -20.006   6.392   6.790  1.00  0.68
H
ATOM  13931  HE  ARG B 316     -21.527   5.546   9.169  1.00  0.68
H
ATOM  13932 1HH1 ARG B 316     -18.811   5.219   6.951  1.00  0.68
H
ATOM  13933 2HH1 ARG B 316     -18.250   3.715   7.655  1.00  0.68
H
ATOM  13934 1HH2 ARG B 316     -20.757   3.592  10.087  1.00  0.68
H
ATOM  13935 2HH2 ARG B 316     -19.333   2.814   9.425  1.00  0.68
H
ATOM  13936  N   TYR B 317     -18.897   9.707  11.449  1.00  0.57
N
ATOM  13937  CA  TYR B 317     -19.626  10.265  12.589  1.00  0.83
C
ATOM  13938  C   TYR B 317     -18.995  11.553  13.115  1.00  0.63
C
ATOM  13939  O   TYR B 317     -19.509  12.167  14.050  1.00  0.68
O
ATOM  13940  CB  TYR B 317     -19.691   9.241  13.715  1.00  1.24
C
ATOM  13941  CG  TYR B 317     -20.541   8.037  13.403  1.00  1.24
C
ATOM  13942  CD1 TYR B 317     -19.979   6.962  12.744  1.00  1.24
C
ATOM  13943  CD2 TYR B 317     -21.867   7.992  13.802  1.00  1.24
C
ATOM  13944  CE1 TYR B 317     -20.739   5.846  12.475  1.00  1.24
C
ATOM  13945  CE2 TYR B 317     -22.628   6.870  13.538  1.00  1.24
C
ATOM  13946  CZ  TYR B 317     -22.064   5.798  12.877  1.00  1.24
C
ATOM  13947  OH  TYR B 317     -22.815   4.675  12.612  1.00  1.24
O
ATOM  13948  H   TYR B 317     -18.096   9.114  11.616  1.00  0.68
H
ATOM  13949  HA  TYR B 317     -20.641  10.500  12.268  1.00  1.00
H
ATOM  13950 1HB  TYR B 317     -18.685   8.896  13.951  1.00  1.49
H
ATOM  13951 2HB  TYR B 317     -20.092   9.714  14.611  1.00  1.49
H
ATOM  13952  HD1 TYR B 317     -18.936   7.003  12.440  1.00  1.49
H
ATOM  13953  HD2 TYR B 317     -22.308   8.839  14.329  1.00  1.49
H
ATOM  13954  HE1 TYR B 317     -20.299   5.002  11.952  1.00  1.49
H
ATOM  13955  HE2 TYR B 317     -23.670   6.829  13.855  1.00  1.49
H
ATOM  13956  HH  TYR B 317     -23.743   4.870  12.756  1.00  1.49
H
ATOM  13957  N   SER B 318     -17.875  11.946  12.522  1.00  0.57
N
ATOM  13958  CA  SER B 318     -17.159  13.147  12.904  1.00  0.60
C
```

```
ATOM  13959  C   SER B 318      -17.403  14.241  11.867  1.00  0.50
C
ATOM  13960  O   SER B 318      -17.449  15.425  12.185  1.00  0.54
O
ATOM  13961  CB  SER B 318      -15.688  12.820  13.055  1.00  0.90
C
ATOM  13962  OG  SER B 318      -15.496  11.916  14.112  1.00  0.90
O
ATOM  13963  H   SER B 318      -17.497  11.411  11.757  1.00  0.68
H
ATOM  13964  HA  SER B 318      -17.541  13.493  13.867  1.00  0.72
H
ATOM  13965 1HB  SER B 318      -15.313  12.386  12.128  1.00  1.08
H
ATOM  13966 2HB  SER B 318      -15.125  13.726  13.247  1.00  1.08
H
ATOM  13967  HG  SER B 318      -15.816  12.362  14.900  1.00  1.08
H
ATOM  13968  N   THR B 319      -17.597  13.818  10.620  1.00  0.41
N
ATOM  13969  CA  THR B 319      -17.931  14.699   9.507  1.00  0.38
C
ATOM  13970  C   THR B 319      -19.435  14.817   9.359  1.00  0.36
C
ATOM  13971  O   THR B 319      -19.935  15.671   8.627  1.00  0.37
O
ATOM  13972  CB  THR B 319      -17.393  14.142   8.181  1.00  0.57
C
ATOM  13973  OG1 THR B 319      -18.032  12.873   7.934  1.00  0.57
O
ATOM  13974  CG2 THR B 319      -15.884  13.964   8.238  1.00  0.57
C
ATOM  13975  H   THR B 319      -17.495  12.832  10.423  1.00  0.49
H
ATOM  13976  HA  THR B 319      -17.515  15.690   9.693  1.00  0.46
H
ATOM  13977  HB  THR B 319      -17.638  14.825   7.369  1.00  0.68
H
ATOM  13978  HG1 THR B 319      -17.496  12.342   7.329  1.00  0.68
H
ATOM  13979 1HG2 THR B 319      -15.531  13.560   7.290  1.00  0.68
H
ATOM  13980 2HG2 THR B 319      -15.411  14.928   8.420  1.00  0.68
H
ATOM  13981 3HG2 THR B 319      -15.626  13.278   9.042  1.00  0.68
H
ATOM  13982  N   PHE B 320      -20.144  13.920  10.042  1.00  0.38
N
ATOM  13983  CA  PHE B 320      -21.587  13.766   9.972  1.00  0.39
C
ATOM  13984  C   PHE B 320      -22.095  13.403   8.589  1.00  0.37
C
ATOM  13985  O   PHE B 320      -23.283  13.559   8.303  1.00  0.40
O
ATOM  13986  CB  PHE B 320      -22.296  14.994  10.521  1.00  0.58
C
ATOM  13987  CG  PHE B 320      -21.972  15.201  11.961  1.00  0.58
C
```

ATOM 13988 CD1 PHE B 320 -21.113 16.203 12.356 1.00 0.58
C
ATOM 13989 CD2 PHE B 320 -22.518 14.371 12.924 1.00 0.58
C
ATOM 13990 CE1 PHE B 320 -20.809 16.377 13.687 1.00 0.58
C
ATOM 13991 CE2 PHE B 320 -22.213 14.540 14.258 1.00 0.58
C
ATOM 13992 CZ PHE B 320 -21.355 15.547 14.640 1.00 0.58
C
ATOM 13993 H PHE B 320 -19.654 13.270 10.636 1.00 0.46
H
ATOM 13994 HA PHE B 320 -21.854 12.942 10.633 1.00 0.47
H
ATOM 13995 1HB PHE B 320 -22.005 15.884 9.967 1.00 0.70
H
ATOM 13996 2HB PHE B 320 -23.373 14.874 10.425 1.00 0.70
H
ATOM 13997 HD1 PHE B 320 -20.678 16.856 11.604 1.00 0.70
H
ATOM 13998 HD2 PHE B 320 -23.195 13.573 12.616 1.00 0.70
H
ATOM 13999 HE1 PHE B 320 -20.141 17.174 13.986 1.00 0.70
H
ATOM 14000 HE2 PHE B 320 -22.648 13.880 15.007 1.00 0.70
H
ATOM 14001 HZ PHE B 320 -21.112 15.687 15.692 1.00 0.70
H
ATOM 14002 N SER B 321 -21.225 12.784 7.771 1.00 0.33
N
ATOM 14003 CA SER B 321 -21.650 12.240 6.489 1.00 0.33
C
ATOM 14004 C SER B 321 -22.580 11.042 6.734 1.00 0.40
C
ATOM 14005 O SER B 321 -23.364 10.670 5.857 1.00 0.62
O
ATOM 14006 CB SER B 321 -20.447 11.859 5.660 1.00 0.49
C
ATOM 14007 OG SER B 321 -19.764 10.783 6.226 1.00 0.49
O
ATOM 14008 H SER B 321 -20.240 12.703 8.020 1.00 0.40
H
ATOM 14009 HA SER B 321 -22.211 13.006 5.951 1.00 0.40
H
ATOM 14010 1HB SER B 321 -20.753 11.612 4.645 1.00 0.59
H
ATOM 14011 2HB SER B 321 -19.778 12.718 5.598 1.00 0.59
H
ATOM 14012 HG SER B 321 -18.831 10.934 6.032 1.00 0.59
H
ATOM 14013 N GLU B 322 -22.527 10.500 7.966 1.00 0.39
N
ATOM 14014 CA GLU B 322 -23.447 9.484 8.465 1.00 0.56
C
ATOM 14015 C GLU B 322 -24.898 9.816 8.222 1.00 0.60
C
ATOM 14016 O GLU B 322 -25.704 8.911 8.033 1.00 0.72
O

ATOM 14017 CB GLU B 322   -23.310   9.275   9.972 1.00 0.84
C
ATOM 14018 CG GLU B 322   -24.277   8.215 10.475 1.00 0.84
C
ATOM 14019 CD GLU B 322   -23.962   6.860   9.917 1.00 0.84
C
ATOM 14020 OE1 GLU B 322   -22.811   6.513   9.918 1.00 0.84
O
ATOM 14021 OE2 GLU B 322   -24.851   6.186   9.456 1.00 0.84
O
ATOM 14022 H   GLU B 322   -21.799 10.803   8.599 1.00 0.47
H
ATOM 14023 HA GLU B 322   -23.226   8.546   7.956 1.00 0.67
H
ATOM 14024 1HB GLU B 322   -22.299   8.985 10.242 1.00 1.01
H
ATOM 14025 2HB GLU B 322   -23.534 10.206 10.492 1.00 1.01
H
ATOM 14026 1HG GLU B 322   -24.230   8.177 11.561 1.00 1.01
H
ATOM 14027 2HG GLU B 322   -25.289   8.498 10.191 1.00 1.01
H
ATOM 14028 N   ILE B 323   -25.248 11.100   8.290 1.00 0.56
N
ATOM 14029 CA ILE B 323   -26.626 11.514   8.117 1.00 0.65
C
ATOM 14030 C   ILE B 323   -27.091 11.097   6.721 1.00 0.63
C
ATOM 14031 O   ILE B 323   -28.172 10.522   6.564 1.00 0.95
O
ATOM 14032 CB ILE B 323   -26.751 13.033   8.336 1.00 0.98
C
ATOM 14033 CG1 ILE B 323   -26.457 13.363   9.806 1.00 0.98
C
ATOM 14034 CG2 ILE B 323   -28.148 13.501   7.981 1.00 0.98
C
ATOM 14035 CD1 ILE B 323   -26.265 14.838 10.079 1.00 0.98
C
ATOM 14036 H   ILE B 323   -24.546 11.817   8.443 1.00 0.67
H
ATOM 14037 HA ILE B 323   -27.243 11.004   8.854 1.00 0.78
H
ATOM 14038 HB ILE B 323   -26.019 13.555   7.720 1.00 1.17
H
ATOM 14039 1HG1 ILE B 323   -27.285 13.007 10.418 1.00 1.17
H
ATOM 14040 2HG1 ILE B 323   -25.553 12.838 10.113 1.00 1.17
H
ATOM 14041 1HG2 ILE B 323   -28.222 14.575   8.138 1.00 1.17
H
ATOM 14042 2HG2 ILE B 323   -28.347 13.270   6.943 1.00 1.17
H
ATOM 14043 3HG2 ILE B 323   -28.876 12.989   8.611 1.00 1.17
H
ATOM 14044 1HD1 ILE B 323   -26.065 14.989 11.140 1.00 1.17
H
ATOM 14045 2HD1 ILE B 323   -25.425 15.209   9.496 1.00 1.17
H

```
ATOM  14046 3HD1 ILE B 323     -27.167  15.377   9.800  1.00  1.17
H
ATOM  14047  N   PHE B 324     -26.252  11.371   5.720  1.00  0.43
N
ATOM  14048  CA  PHE B 324     -26.510  10.943   4.353  1.00  0.40
C
ATOM  14049  C   PHE B 324     -26.552   9.425   4.277  1.00  0.42
C
ATOM  14050  O   PHE B 324     -27.445   8.867   3.648  1.00  0.48
O
ATOM  14051  CB  PHE B 324     -25.451  11.437   3.382  1.00  0.60
C
ATOM  14052  CG  PHE B 324     -25.742  11.001   1.975  1.00  0.60
C
ATOM  14053  CD1 PHE B 324     -26.463  11.809   1.113  1.00  0.60
C
ATOM  14054  CD2 PHE B 324     -25.323   9.763   1.521  1.00  0.60
C
ATOM  14055  CE1 PHE B 324     -26.741  11.402  -0.178  1.00  0.60
C
ATOM  14056  CE2 PHE B 324     -25.601   9.354   0.238  1.00  0.60
C
ATOM  14057  CZ  PHE B 324     -26.307  10.173  -0.617  1.00  0.60
C
ATOM  14058  H   PHE B 324     -25.395  11.870   5.918  1.00  0.52
H
ATOM  14059  HA  PHE B 324     -27.480  11.332   4.042  1.00  0.48
H
ATOM  14060 1HB  PHE B 324     -25.404  12.525   3.404  1.00  0.72
H
ATOM  14061 2HB  PHE B 324     -24.473  11.055   3.665  1.00  0.72
H
ATOM  14062  HD1 PHE B 324     -26.806  12.778   1.461  1.00  0.72
H
ATOM  14063  HD2 PHE B 324     -24.770   9.110   2.194  1.00  0.72
H
ATOM  14064  HE1 PHE B 324     -27.302  12.052  -0.847  1.00  0.72
H
ATOM  14065  HE2 PHE B 324     -25.265   8.379  -0.097  1.00  0.72
H
ATOM  14066  HZ  PHE B 324     -26.526   9.846  -1.632  1.00  0.72
H
ATOM  14067  N   ASN B 325     -25.568   8.763   4.901  1.00  0.40
N
ATOM  14068  CA  ASN B 325     -25.498   7.299   4.922  1.00  0.43
C
ATOM  14069  C   ASN B 325     -26.791   6.675   5.414  1.00  0.53
C
ATOM  14070  O   ASN B 325     -27.285   5.722   4.818  1.00  0.62
O
ATOM  14071  CB  ASN B 325     -24.347   6.817   5.786  1.00  0.65
C
ATOM  14072  CG  ASN B 325     -24.220   5.314   5.781  1.00  0.65
C
ATOM  14073  OD1 ASN B 325     -24.048   4.695   4.726  1.00  0.65
O
ATOM  14074  ND2 ASN B 325     -24.309   4.712   6.941  1.00  0.65
N
```

```
ATOM  14075  H   ASN B 325     -24.849   9.295   5.379  1.00  0.48
H
ATOM  14076  HA  ASN B 325     -25.338   6.949   3.902  1.00  0.52
H
ATOM  14077 1HB  ASN B 325     -23.413   7.256   5.435  1.00  0.77
H
ATOM  14078 2HB  ASN B 325     -24.499   7.143   6.813  1.00  0.77
H
ATOM  14079 1HD2 ASN B 325     -24.235   3.716   6.994  1.00  0.77
H
ATOM  14080 2HD2 ASN B 325     -24.457   5.242   7.781  1.00  0.77
H
ATOM  14081  N   LYS B 326     -27.338   7.204   6.508  1.00  0.57
N
ATOM  14082  CA  LYS B 326     -28.587   6.696   7.055  1.00  0.84
C
ATOM  14083  C   LYS B 326     -29.753   6.878   6.100  1.00  0.88
C
ATOM  14084  O   LYS B 326     -30.564   5.966   5.925  1.00  1.00
O
ATOM  14085  CB  LYS B 326     -28.939   7.403   8.367  1.00  1.26
C
ATOM  14086  CG  LYS B 326     -28.081   7.053   9.569  1.00  1.26
C
ATOM  14087  CD  LYS B 326     -28.547   7.827  10.796  1.00  1.26
C
ATOM  14088  CE  LYS B 326     -27.739   7.475  12.036  1.00  1.26
C
ATOM  14089  NZ  LYS B 326     -28.200   8.243  13.229  1.00  1.26
N
ATOM  14090  H   LYS B 326     -26.873   7.967   6.974  1.00  0.68
H
ATOM  14091  HA  LYS B 326     -28.472   5.629   7.245  1.00  1.01
H
ATOM  14092 1HB  LYS B 326     -28.867   8.481   8.221  1.00  1.51
H
ATOM  14093 2HB  LYS B 326     -29.974   7.181   8.627  1.00  1.51
H
ATOM  14094 1HG  LYS B 326     -28.146   5.983   9.771  1.00  1.51
H
ATOM  14095 2HG  LYS B 326     -27.045   7.304   9.363  1.00  1.51
H
ATOM  14096 1HD  LYS B 326     -28.452   8.897  10.605  1.00  1.51
H
ATOM  14097 2HD  LYS B 326     -29.596   7.603  10.986  1.00  1.51
H
ATOM  14098 1HE  LYS B 326     -27.843   6.410  12.240  1.00  1.51
H
ATOM  14099 2HE  LYS B 326     -26.689   7.698  11.858  1.00  1.51
H
ATOM  14100 1HZ  LYS B 326     -27.644   7.985  14.032  1.00  1.51
H
ATOM  14101 2HZ  LYS B 326     -28.096   9.233  13.053  1.00  1.51
H
ATOM  14102 3HZ  LYS B 326     -29.170   8.034  13.410  1.00  1.51
H
ATOM  14103  N   GLU B 327     -29.838   8.053   5.478  1.00  0.93
N
```

ATOM 14104 CA GLU B 327 -30.943 8.344 4.578 1.00 1.25
C
ATOM 14105 C GLU B 327 -30.842 7.634 3.232 1.00 1.33
C
ATOM 14106 O GLU B 327 -31.854 7.180 2.696 1.00 2.71
O
ATOM 14107 CB GLU B 327 -31.030 9.853 4.351 1.00 1.88
C
ATOM 14108 CG GLU B 327 -31.433 10.655 5.579 1.00 1.88
C
ATOM 14109 CD GLU B 327 -32.810 10.318 6.078 1.00 1.88
C
ATOM 14110 OE1 GLU B 327 -33.709 10.241 5.275 1.00 1.88
O
ATOM 14111 OE2 GLU B 327 -32.967 10.141 7.263 1.00 1.88
O
ATOM 14112 H GLU B 327 -29.149 8.776 5.661 1.00 1.12
H
ATOM 14113 HA GLU B 327 -31.864 8.014 5.060 1.00 1.50
H
ATOM 14114 1HB GLU B 327 -30.066 10.226 4.007 1.00 2.25
H
ATOM 14115 2HB GLU B 327 -31.761 10.058 3.571 1.00 2.25
H
ATOM 14116 1HG GLU B 327 -30.716 10.464 6.377 1.00 2.25
H
ATOM 14117 2HG GLU B 327 -31.392 11.716 5.339 1.00 2.25
H
ATOM 14118 N TYR B 328 -29.622 7.510 2.700 1.00 0.63
N
ATOM 14119 CA TYR B 328 -29.397 6.880 1.398 1.00 0.70
C
ATOM 14120 C TYR B 328 -28.152 5.997 1.360 1.00 0.67
C
ATOM 14121 O TYR B 328 -27.208 6.316 0.638 1.00 0.97
O
ATOM 14122 CB TYR B 328 -29.263 7.960 0.316 1.00 1.05
C
ATOM 14123 CG TYR B 328 -30.470 8.847 0.264 1.00 1.05
C
ATOM 14124 CD1 TYR B 328 -30.436 10.110 0.838 1.00 1.05
C
ATOM 14125 CD2 TYR B 328 -31.632 8.368 -0.317 1.00 1.05
C
ATOM 14126 CE1 TYR B 328 -31.578 10.883 0.844 1.00 1.05
C
ATOM 14127 CE2 TYR B 328 -32.768 9.143 -0.312 1.00 1.05
C
ATOM 14128 CZ TYR B 328 -32.744 10.387 0.273 1.00 1.05
C
ATOM 14129 OH TYR B 328 -33.889 11.134 0.306 1.00 1.05
O
ATOM 14130 H TYR B 328 -28.830 7.887 3.190 1.00 0.76
H
ATOM 14131 HA TYR B 328 -30.259 6.252 1.166 1.00 0.84
H
ATOM 14132 1HB TYR B 328 -28.392 8.578 0.522 1.00 1.26
H

ATOM 14133 2HB TYR B 328   -29.129   7.498 -0.660 1.00 1.26
H
ATOM 14134 HD1 TYR B 328   -29.520 10.479   1.301 1.00 1.26
H
ATOM 14135 HD2 TYR B 328   -31.650   7.372 -0.760 1.00 1.26
H
ATOM 14136 HE1 TYR B 328   -31.569 11.868   1.308 1.00 1.26
H
ATOM 14137 HE2 TYR B 328   -33.689   8.764 -0.754 1.00 1.26
H
ATOM 14138 HH TYR B 328   -33.814 11.797   0.995 1.00 1.26
H
ATOM 14139 N   PRO B 329   -28.135   4.890   2.127 1.00 0.66
N
ATOM 14140 CA PRO B 329   -27.074   3.886   2.262 1.00 0.66
C
ATOM 14141 C   PRO B 329   -26.508   3.436   0.926 1.00 0.65
C
ATOM 14142 O   PRO B 329   -25.298   3.277   0.765 1.00 0.93
O
ATOM 14143 CB PRO B 329   -27.798   2.694   2.901 1.00 0.99
C
ATOM 14144 CG PRO B 329   -28.889   3.303   3.684 1.00 0.99
C
ATOM 14145 CD PRO B 329   -29.374   4.417   2.794 1.00 0.99
C
ATOM 14146 HA PRO B 329   -26.280   4.274   2.917 1.00 0.79
H
ATOM 14147 1HB PRO B 329   -28.164   2.009   2.122 1.00 1.19
H
ATOM 14148 2HB PRO B 329   -27.097   2.121   3.526 1.00 1.19
H
ATOM 14149 1HG PRO B 329   -29.671   2.556   3.887 1.00 1.19
H
ATOM 14150 2HG PRO B 329   -28.521   3.651   4.658 1.00 1.19
H
ATOM 14151 1HD PRO B 329   -30.095   4.049   2.050 1.00 1.19
H
ATOM 14152 2HD PRO B 329   -29.806   5.186   3.437 1.00 1.19
H
ATOM 14153 N   GLU B 330   -27.403   3.227 -0.039 1.00 0.46
N
ATOM 14154 CA GLU B 330   -27.038   2.718 -1.355 1.00 0.57
C
ATOM 14155 C   GLU B 330   -26.212   3.694 -2.193 1.00 0.25
C
ATOM 14156 O   GLU B 330   -25.642   3.300 -3.209 1.00 0.68
O
ATOM 14157 CB GLU B 330   -28.304   2.346 -2.120 1.00 0.85
C
ATOM 14158 CG GLU B 330   -29.120   3.548 -2.550 1.00 0.85
C
ATOM 14159 CD GLU B 330   -30.025   4.078 -1.484 1.00 0.85
C
ATOM 14160 OE1 GLU B 330   -30.066   3.511 -0.418 1.00 0.85
O
ATOM 14161 OE2 GLU B 330   -30.649   5.084 -1.730 1.00 0.85
O

ATOM 14162 H   GLU B 330   -28.379   3.399   0.158 1.00 0.55
H
ATOM 14163 HA GLU B 330   -26.443   1.816 -1.212 1.00 0.68
H
ATOM 14164 1HB GLU B 330   -28.039   1.779 -3.014 1.00 1.03
H
ATOM 14165 2HB GLU B 330   -28.935   1.710 -1.500 1.00 1.03
H
ATOM 14166 1HG GLU B 330   -28.444   4.333 -2.857 1.00 1.03
H
ATOM 14167 2HG GLU B 330   -29.719   3.267 -3.415 1.00 1.03
H
ATOM 14168 N   ARG B 331   -26.144   4.958 -1.775 1.00 0.44
N
ATOM 14169 CA ARG B 331   -25.360   5.969 -2.458 1.00 0.30
C
ATOM 14170 C   ARG B 331   -24.208   6.445 -1.593 1.00 0.27
C
ATOM 14171 O   ARG B 331   -23.654   7.518 -1.837 1.00 0.50
O
ATOM 14172 CB ARG B 331   -26.238   7.144 -2.863 1.00 0.45
C
ATOM 14173 CG ARG B 331   -27.216   6.840 -3.987 1.00 0.45
C
ATOM 14174 CD ARG B 331   -28.259   7.885 -4.156 1.00 0.45
C
ATOM 14175 NE ARG B 331   -29.050   7.639 -5.355 1.00 0.45
N
ATOM 14176 CZ ARG B 331   -30.089   6.781 -5.461 1.00 0.45
C
ATOM 14177 NH1 ARG B 331   -30.525   6.080 -4.436 1.00 0.45
N
ATOM 14178 NH2 ARG B 331   -30.685   6.634 -6.630 1.00 0.45
N
ATOM 14179 H   ARG B 331   -26.640   5.242 -0.941 1.00 0.53
H
ATOM 14180 HA ARG B 331   -24.946   5.530 -3.363 1.00 0.36
H
ATOM 14181 1HB ARG B 331   -26.819   7.476 -2.004 1.00 0.54
H
ATOM 14182 2HB ARG B 331   -25.614   7.979 -3.183 1.00 0.54
H
ATOM 14183 1HG ARG B 331   -26.663   6.770 -4.925 1.00 0.54
H
ATOM 14184 2HG ARG B 331   -27.713   5.896 -3.800 1.00 0.54
H
ATOM 14185 1HD ARG B 331   -28.929   7.880 -3.296 1.00 0.54
H
ATOM 14186 2HD ARG B 331   -27.791   8.864 -4.250 1.00 0.54
H
ATOM 14187 HE ARG B 331   -28.779   8.151 -6.188 1.00 0.54
H
ATOM 14188 1HH1 ARG B 331   -30.106   6.157 -3.517 1.00 0.54
H
ATOM 14189 2HH1 ARG B 331   -31.304   5.449 -4.554 1.00 0.54
H
ATOM 14190 1HH2 ARG B 331   -30.361   7.158 -7.429 1.00 0.54
H

ATOM 14191 2HH2 ARG B 331   -31.462   5.996 -6.722 1.00 0.54
H
ATOM 14192 N   PHE B 332   -23.857   5.664 -0.573 1.00 0.27
N
ATOM 14193 CA PHE B 332   -22.772   6.054   0.302 1.00 0.26
C
ATOM 14194 C   PHE B 332   -21.628   5.043   0.221 1.00 0.23
C
ATOM 14195 O   PHE B 332   -21.848   3.836   0.331 1.00 0.28
O
ATOM 14196 CB PHE B 332   -23.259   6.168   1.735 1.00 0.39
C
ATOM 14197 CG PHE B 332   -22.188   6.651   2.652 1.00 0.39
C
ATOM 14198 CD1 PHE B 332   -22.048   8.005   2.904 1.00 0.39
C
ATOM 14199 CD2 PHE B 332   -21.304   5.765   3.240 1.00 0.39
C
ATOM 14200 CE1 PHE B 332   -21.056   8.463   3.735 1.00 0.39
C
ATOM 14201 CE2 PHE B 332   -20.305   6.221   4.066 1.00 0.39
C
ATOM 14202 CZ PHE B 332   -20.188   7.573   4.313 1.00 0.39
C
ATOM 14203 H   PHE B 332   -24.340   4.795 -0.382 1.00 0.32
H
ATOM 14204 HA PHE B 332   -22.403   7.025 -0.018 1.00 0.31
H
ATOM 14205 1HB PHE B 332   -24.099   6.860   1.787 1.00 0.47
H
ATOM 14206 2HB PHE B 332   -23.605   5.198   2.089 1.00 0.47
H
ATOM 14207 HD1 PHE B 332   -22.734   8.707   2.440 1.00 0.47
H
ATOM 14208 HD2 PHE B 332   -21.404   4.697   3.038 1.00 0.47
H
ATOM 14209 HE1 PHE B 332   -20.957   9.528   3.934 1.00 0.47
H
ATOM 14210 HE2 PHE B 332   -19.612   5.519   4.528 1.00 0.47
H
ATOM 14211 HZ PHE B 332   -19.408   7.935   4.973 1.00 0.47
H
ATOM 14212 N   ILE B 333   -20.408   5.540   0.014 1.00 0.18
N
ATOM 14213 CA ILE B 333   -19.247   4.671 -0.119 1.00 0.16
C
ATOM 14214 C   ILE B 333   -18.275   4.824   1.047 1.00 0.18
C
ATOM 14215 O   ILE B 333   -17.651   5.873   1.218 1.00 0.21
O
ATOM 14216 CB ILE B 333   -18.482   4.962 -1.427 1.00 0.24
C
ATOM 14217 CG1 ILE B 333   -19.397   4.773 -2.639 1.00 0.24
C
ATOM 14218 CG2 ILE B 333   -17.273   4.042 -1.536 1.00 0.24
C
ATOM 14219 CD1 ILE B 333   -18.776   5.223 -3.944 1.00 0.24
C

```
ATOM  14220  H   ILE B 333     -20.292   6.540  -0.053  1.00  0.22
H
ATOM  14221  HA  ILE B 333     -19.590   3.638  -0.142  1.00  0.19
H
ATOM  14222  HB  ILE B 333     -18.146   5.993  -1.423  1.00  0.29
H
ATOM  14223 1HG1 ILE B 333     -19.658   3.721  -2.723  1.00  0.29
H
ATOM  14224 2HG1 ILE B 333     -20.314   5.342  -2.486  1.00  0.29
H
ATOM  14225 1HG2 ILE B 333     -16.730   4.262  -2.454  1.00  0.29
H
ATOM  14226 2HG2 ILE B 333     -16.618   4.200  -0.681  1.00  0.29
H
ATOM  14227 3HG2 ILE B 333     -17.606   3.004  -1.551  1.00  0.29
H
ATOM  14228 1HD1 ILE B 333     -19.482   5.061  -4.758  1.00  0.29
H
ATOM  14229 2HD1 ILE B 333     -18.531   6.285  -3.883  1.00  0.29
H
ATOM  14230 3HD1 ILE B 333     -17.868   4.652  -4.131  1.00  0.29
H
ATOM  14231  N   GLU B 334     -18.132   3.760   1.824  1.00  0.24
N
ATOM  14232  CA  GLU B 334     -17.175   3.750   2.916  1.00  0.29
C
ATOM  14233  C   GLU B 334     -15.808   3.445   2.325  1.00  0.31
C
ATOM  14234  O   GLU B 334     -15.631   2.417   1.668  1.00  0.65
O
ATOM  14235  CB  GLU B 334     -17.578   2.708   3.961  1.00  0.43
C
ATOM  14236  CG  GLU B 334     -16.697   2.639   5.197  1.00  0.43
C
ATOM  14237  CD  GLU B 334     -17.207   1.624   6.183  1.00  0.43
C
ATOM  14238  OE1 GLU B 334     -17.237   0.465   5.849  1.00  0.43
O
ATOM  14239  OE2 GLU B 334     -17.611   2.011   7.256  1.00  0.43
O
ATOM  14240  H   GLU B 334     -18.686   2.934   1.644  1.00  0.29
H
ATOM  14241  HA  GLU B 334     -17.145   4.736   3.381  1.00  0.35
H
ATOM  14242 1HB  GLU B 334     -18.596   2.910   4.293  1.00  0.52
H
ATOM  14243 2HB  GLU B 334     -17.577   1.719   3.503  1.00  0.52
H
ATOM  14244 1HG  GLU B 334     -15.684   2.369   4.901  1.00  0.52
H
ATOM  14245 2HG  GLU B 334     -16.664   3.620   5.670  1.00  0.52
H
ATOM  14246  N   CYS B 335     -14.847   4.339   2.537  1.00  0.20
N
ATOM  14247  CA  CYS B 335     -13.535   4.152   1.931  1.00  0.17
C
ATOM  14248  C   CYS B 335     -12.451   3.792   2.933  1.00  0.39
C
```

ATOM 14249 O   CYS B 335   -11.329   3.482   2.529 1.00 1.80
O
ATOM 14250 CB CYS B 335   -13.110   5.420   1.192 1.00 0.26
C
ATOM 14251 SG CYS B 335   -14.191   5.871 -0.183 1.00 0.26
S
ATOM 14252 H   CYS B 335   -15.029   5.162   3.098 1.00 0.24
H
ATOM 14253 HA CYS B 335   -13.608   3.343   1.205 1.00 0.20
H
ATOM 14254 1HB CYS B 335   -13.085   6.257   1.890 1.00 0.31
H
ATOM 14255 2HB CYS B 335   -12.101   5.290   0.799 1.00 0.31
H
ATOM 14256 HG CYS B 335   -15.264   6.112   0.563 1.00 0.31
H
ATOM 14257 N   PHE B 336   -12.795   3.817   4.224 1.00 0.22
N
ATOM 14258 CA PHE B 336   -11.868   3.490   5.311 1.00 0.20
C
ATOM 14259 C   PHE B 336   -10.726   4.498   5.371 1.00 0.19
C
ATOM 14260 O   PHE B 336   -10.741   5.503   4.660 1.00 0.30
O
ATOM 14261 CB PHE B 336   -11.329   2.064   5.171 1.00 0.30
C
ATOM 14262 CG PHE B 336   -12.390   1.020   5.325 1.00 0.30
C
ATOM 14263 CD1 PHE B 336   -12.947   0.403   4.213 1.00 0.30
C
ATOM 14264 CD2 PHE B 336   -12.840   0.658   6.583 1.00 0.30
C
ATOM 14265 CE1 PHE B 336   -13.923 -0.560   4.360 1.00 0.30
C
ATOM 14266 CE2 PHE B 336   -13.817 -0.302   6.729 1.00 0.30
C
ATOM 14267 CZ PHE B 336   -14.355 -0.914   5.619 1.00 0.30
C
ATOM 14268 H   PHE B 336   -13.737   4.076   4.472 1.00 0.26
H
ATOM 14269 HA PHE B 336   -12.417   3.540   6.249 1.00 0.24
H
ATOM 14270 1HB PHE B 336   -10.852   1.925   4.204 1.00 0.36
H
ATOM 14271 2HB PHE B 336   -10.572   1.880   5.930 1.00 0.36
H
ATOM 14272 HD1 PHE B 336   -12.602   0.684   3.218 1.00 0.36
H
ATOM 14273 HD2 PHE B 336   -12.413   1.141   7.463 1.00 0.36
H
ATOM 14274 HE1 PHE B 336   -14.350 -1.038   3.480 1.00 0.36
H
ATOM 14275 HE2 PHE B 336   -14.163 -0.577   7.722 1.00 0.36
H
ATOM 14276 HZ PHE B 336   -15.128 -1.672   5.742 1.00 0.36
H
ATOM 14277 N   MET B 337   -9.717   4.246   6.201 1.00 0.22
N

ATOM 14278 CA MET B 337 -8.593 5.178 6.255 1.00 0.24
C
ATOM 14279 C MET B 337 -7.711 4.998 5.024 1.00 0.29
C
ATOM 14280 O MET B 337 -6.718 4.270 5.046 1.00 0.62
O
ATOM 14281 CB MET B 337 -7.777 5.000 7.538 1.00 0.36
C
ATOM 14282 CG MET B 337 -8.531 5.308 8.823 1.00 0.36
C
ATOM 14283 SD MET B 337 -9.082 7.027 8.900 1.00 0.36
S
ATOM 14284 CE MET B 337 -7.527 7.910 9.028 1.00 0.36
C
ATOM 14285 H MET B 337 -9.719 3.424 6.787 1.00 0.26
H
ATOM 14286 HA MET B 337 -8.983 6.196 6.233 1.00 0.29
H
ATOM 14287 1HB MET B 337 -7.420 3.976 7.609 1.00 0.43
H
ATOM 14288 2HB MET B 337 -6.902 5.650 7.504 1.00 0.43
H
ATOM 14289 1HG MET B 337 -9.404 4.661 8.901 1.00 0.43
H
ATOM 14290 2HG MET B 337 -7.886 5.113 9.679 1.00 0.43
H
ATOM 14291 1HE MET B 337 -7.721 8.982 9.078 1.00 0.43
H
ATOM 14292 2HE MET B 337 -6.999 7.595 9.926 1.00 0.43
H
ATOM 14293 3HE MET B 337 -6.914 7.694 8.151 1.00 0.43
H
ATOM 14294 N ALA B 338 -8.134 5.621 3.937 1.00 0.22
N
ATOM 14295 CA ALA B 338 -7.458 5.585 2.652 1.00 0.21
C
ATOM 14296 C ALA B 338 -7.795 6.859 1.897 1.00 0.23
C
ATOM 14297 O ALA B 338 -8.607 6.822 0.972 1.00 0.35
O
ATOM 14298 CB ALA B 338 -7.870 4.346 1.872 1.00 0.32
C
ATOM 14299 H ALA B 338 -8.984 6.160 4.022 1.00 0.26
H
ATOM 14300 HA ALA B 338 -6.380 5.563 2.824 1.00 0.25
H
ATOM 14301 1HB ALA B 338 -7.351 4.327 0.915 1.00 0.38
H
ATOM 14302 2HB ALA B 338 -7.608 3.454 2.443 1.00 0.38
H
ATOM 14303 3HB ALA B 338 -8.945 4.363 1.702 1.00 0.38
H
ATOM 14304 N GLU B 339 -7.205 7.982 2.313 1.00 0.36
N
ATOM 14305 CA GLU B 339 -7.565 9.291 1.766 1.00 0.50
C
ATOM 14306 C GLU B 339 -7.287 9.416 0.280 1.00 0.31
C

```
ATOM  14307  O   GLU B 339      -8.106   9.966  -0.464  1.00  0.29
O
ATOM  14308  CB  GLU B 339      -6.775  10.384   2.485  1.00  0.75
C
ATOM  14309  CG  GLU B 339      -7.095  10.520   3.960  1.00  0.75
C
ATOM  14310  CD  GLU B 339      -8.532  10.811   4.213  1.00  0.75
C
ATOM  14311  OE1 GLU B 339      -9.237  11.078   3.274  1.00  0.75
O
ATOM  14312  OE2 GLU B 339      -8.932  10.769   5.353  1.00  0.75
O
ATOM  14313  H   GLU B 339      -6.518   7.927   3.053  1.00  0.43
H
ATOM  14314  HA  GLU B 339      -8.632   9.449   1.928  1.00  0.60
H
ATOM  14315 1HB  GLU B 339      -5.709  10.182   2.394  1.00  0.90
H
ATOM  14316 2HB  GLU B 339      -6.968  11.346   2.010  1.00  0.90
H
ATOM  14317 1HG  GLU B 339      -6.830   9.592   4.466  1.00  0.90
H
ATOM  14318 2HG  GLU B 339      -6.487  11.319   4.381  1.00  0.90
H
ATOM  14319  N   GLN B 340      -6.136   8.894  -0.145  1.00  0.32
N
ATOM  14320  CA  GLN B 340      -5.749   8.911  -1.549  1.00  0.34
C
ATOM  14321  C   GLN B 340      -6.750   8.173  -2.398  1.00  0.20
C
ATOM  14322  O   GLN B 340      -7.273   8.720  -3.375  1.00  0.24
O
ATOM  14323  CB  GLN B 340      -4.366   8.287  -1.731  1.00  0.51
C
ATOM  14324  CG  GLN B 340      -3.191   9.135  -1.276  1.00  0.51
C
ATOM  14325  CD  GLN B 340      -1.879   8.377  -1.409  1.00  0.51
C
ATOM  14326  OE1 GLN B 340      -1.698   7.351  -0.750  1.00  0.51
O
ATOM  14327  NE2 GLN B 340      -0.968   8.845  -2.257  1.00  0.51
N
ATOM  14328  H   GLN B 340      -5.514   8.464   0.525  1.00  0.38
H
ATOM  14329  HA  GLN B 340      -5.715   9.944  -1.888  1.00  0.41
H
ATOM  14330 1HB  GLN B 340      -4.326   7.368  -1.147  1.00  0.61
H
ATOM  14331 2HB  GLN B 340      -4.222   8.000  -2.765  1.00  0.61
H
ATOM  14332 1HG  GLN B 340      -3.138  10.038  -1.887  1.00  0.61
H
ATOM  14333 2HG  GLN B 340      -3.330   9.396  -0.228  1.00  0.61
H
ATOM  14334 1HE2 GLN B 340      -0.098   8.359  -2.368  1.00  0.61
H
ATOM  14335 2HE2 GLN B 340      -1.131   9.679  -2.790  1.00  0.61
H
```

ATOM 14336 N ASN B 341 -7.035 6.939 -1.988 1.00 0.15
N
ATOM 14337 CA ASN B 341 -8.017 6.107 -2.649 1.00 0.13
C
ATOM 14338 C ASN B 341 -9.375 6.773 -2.728 1.00 0.09
C
ATOM 14339 O ASN B 341 -9.970 6.788 -3.803 1.00 0.14
O
ATOM 14340 CB ASN B 341 -8.130 4.777 -1.953 1.00 0.20
C
ATOM 14341 CG ASN B 341 -9.076 3.837 -2.626 1.00 0.20
C
ATOM 14342 OD1 ASN B 341 -8.881 3.432 -3.777 1.00 0.20
O
ATOM 14343 ND2 ASN B 341 -10.116 3.484 -1.922 1.00 0.20
N
ATOM 14344 H ASN B 341 -6.524 6.540 -1.203 1.00 0.18
H
ATOM 14345 HA ASN B 341 -7.682 5.932 -3.672 1.00 0.16
H
ATOM 14346 1HB ASN B 341 -7.147 4.319 -1.928 1.00 0.23
H
ATOM 14347 2HB ASN B 341 -8.451 4.929 -0.924 1.00 0.23
H
ATOM 14348 1HD2 ASN B 341 -10.792 2.860 -2.310 1.00 0.23
H
ATOM 14349 2HD2 ASN B 341 -10.235 3.838 -0.994 1.00 0.23
H
ATOM 14350 N MET B 342 -9.859 7.327 -1.612 1.00 0.11
N
ATOM 14351 CA MET B 342 -11.166 7.981 -1.578 1.00 0.12
C
ATOM 14352 C MET B 342 -11.320 9.004 -2.689 1.00 0.11
C
ATOM 14353 O MET B 342 -12.326 8.996 -3.406 1.00 0.12
O
ATOM 14354 CB MET B 342 -11.399 8.656 -0.227 1.00 0.18
C
ATOM 14355 CG MET B 342 -12.737 9.384 -0.118 1.00 0.18
C
ATOM 14356 SD MET B 342 -12.930 10.277 1.437 1.00 0.18
S
ATOM 14357 CE MET B 342 -11.767 11.618 1.203 1.00 0.18
C
ATOM 14358 H MET B 342 -9.319 7.279 -0.756 1.00 0.13
H
ATOM 14359 HA MET B 342 -11.934 7.220 -1.724 1.00 0.14
H
ATOM 14360 1HB MET B 342 -11.353 7.911 0.565 1.00 0.22
H
ATOM 14361 2HB MET B 342 -10.607 9.382 -0.040 1.00 0.22
H
ATOM 14362 1HG MET B 342 -12.829 10.097 -0.937 1.00 0.22
H
ATOM 14363 2HG MET B 342 -13.550 8.664 -0.203 1.00 0.22
H
ATOM 14364 1HE MET B 342 -11.772 12.260 2.083 1.00 0.22
H

```
ATOM  14365 2HE  MET B 342     -10.766  11.210   1.058  1.00  0.22
H
ATOM  14366 3HE  MET B 342     -12.051  12.201   0.326  1.00  0.22
H
ATOM  14367  N   VAL B 343     -10.326   9.883  -2.827  1.00  0.10
N
ATOM  14368  CA  VAL B 343     -10.375  10.896  -3.867  1.00  0.12
C
ATOM  14369  C   VAL B 343     -10.381  10.269  -5.256  1.00  0.10
C
ATOM  14370  O   VAL B 343     -11.155  10.696  -6.110  1.00  0.12
O
ATOM  14371  CB  VAL B 343      -9.200  11.882  -3.740  1.00  0.18
C
ATOM  14372  CG1 VAL B 343      -9.166  12.810  -4.951  1.00  0.18
C
ATOM  14373  CG2 VAL B 343      -9.358  12.689  -2.459  1.00  0.18
C
ATOM  14374  H   VAL B 343      -9.532   9.850  -2.193  1.00  0.12
H
ATOM  14375  HA  VAL B 343     -11.299  11.463  -3.744  1.00  0.14
H
ATOM  14376  HB  VAL B 343      -8.261  11.328  -3.715  1.00  0.22
H
ATOM  14377 1HG1 VAL B 343      -8.327  13.500  -4.856  1.00  0.22
H
ATOM  14378 2HG1 VAL B 343      -9.048  12.218  -5.859  1.00  0.22
H
ATOM  14379 3HG1 VAL B 343     -10.096  13.374  -5.002  1.00  0.22
H
ATOM  14380 1HG2 VAL B 343      -8.528  13.387  -2.363  1.00  0.22
H
ATOM  14381 2HG2 VAL B 343     -10.295  13.244  -2.493  1.00  0.22
H
ATOM  14382 3HG2 VAL B 343      -9.366  12.014  -1.602  1.00  0.22
H
ATOM  14383  N   SER B 344      -9.531   9.257  -5.484  1.00  0.11
N
ATOM  14384  CA  SER B 344      -9.484   8.615  -6.799  1.00  0.18
C
ATOM  14385  C   SER B 344     -10.776   7.890  -7.151  1.00  0.13
C
ATOM  14386  O   SER B 344     -11.169   7.867  -8.317  1.00  0.24
O
ATOM  14387  CB  SER B 344      -8.365   7.610  -6.850  1.00  0.27
C
ATOM  14388  OG  SER B 344      -8.660   6.512  -6.040  1.00  0.27
O
ATOM  14389  H   SER B 344      -8.900   8.943  -4.751  1.00  0.13
H
ATOM  14390  HA  SER B 344      -9.310   9.381  -7.548  1.00  0.22
H
ATOM  14391 1HB  SER B 344      -8.196   7.287  -7.876  1.00  0.32
H
ATOM  14392 2HB  SER B 344      -7.455   8.074  -6.493  1.00  0.32
H
ATOM  14393  HG  SER B 344      -8.992   6.874  -5.216  1.00  0.32
H
```

ATOM 14394 N VAL B 345 -11.459 7.343 -6.143 1.00 0.14
N
ATOM 14395 CA VAL B 345 -12.737 6.681 -6.360 1.00 0.12
C
ATOM 14396 C VAL B 345 -13.758 7.705 -6.786 1.00 0.13
C
ATOM 14397 O VAL B 345 -14.517 7.463 -7.721 1.00 0.14
O
ATOM 14398 CB VAL B 345 -13.208 5.922 -5.098 1.00 0.18
C
ATOM 14399 CG1 VAL B 345 -14.636 5.424 -5.274 1.00 0.18
C
ATOM 14400 CG2 VAL B 345 -12.294 4.724 -4.866 1.00 0.18
C
ATOM 14401 H VAL B 345 -11.077 7.370 -5.208 1.00 0.17
H
ATOM 14402 HA VAL B 345 -12.618 5.955 -7.165 1.00 0.14
H
ATOM 14403 HB VAL B 345 -13.180 6.593 -4.239 1.00 0.22
H
ATOM 14404 1HG1 VAL B 345 -14.949 4.894 -4.375 1.00 0.22
H
ATOM 14405 2HG1 VAL B 345 -15.299 6.271 -5.446 1.00 0.22
H
ATOM 14406 3HG1 VAL B 345 -14.682 4.749 -6.124 1.00 0.22
H
ATOM 14407 1HG2 VAL B 345 -12.618 4.187 -3.976 1.00 0.22
H
ATOM 14408 2HG2 VAL B 345 -12.345 4.059 -5.729 1.00 0.22
H
ATOM 14409 3HG2 VAL B 345 -11.269 5.059 -4.733 1.00 0.22
H
ATOM 14410 N ALA B 346 -13.760 8.856 -6.111 1.00 0.12
N
ATOM 14411 CA ALA B 346 -14.653 9.944 -6.473 1.00 0.12
C
ATOM 14412 C ALA B 346 -14.374 10.408 -7.907 1.00 0.12
C
ATOM 14413 O ALA B 346 -15.315 10.631 -8.672 1.00 0.15
O
ATOM 14414 CB ALA B 346 -14.500 11.096 -5.489 1.00 0.18
C
ATOM 14415 H ALA B 346 -13.132 8.979 -5.325 1.00 0.14
H
ATOM 14416 HA ALA B 346 -15.677 9.574 -6.431 1.00 0.14
H
ATOM 14417 1HB ALA B 346 -15.190 11.896 -5.753 1.00 0.22
H
ATOM 14418 2HB ALA B 346 -14.723 10.742 -4.482 1.00 0.22
H
ATOM 14419 3HB ALA B 346 -13.480 11.473 -5.522 1.00 0.22
H
ATOM 14420 N LEU B 347 -13.084 10.513 -8.278 1.00 0.12
N
ATOM 14421 CA LEU B 347 -12.692 10.919 -9.630 1.00 0.13
C
ATOM 14422 C LEU B 347 -13.233 9.956 -10.671 1.00 0.14
C

```
ATOM  14423  O   LEU B 347     -13.738  10.377 -11.713  1.00  0.16
O
ATOM  14424  CB  LEU B 347     -11.150  10.966  -9.769  1.00  0.20
C
ATOM  14425  CG  LEU B 347     -10.418  12.083  -9.017  1.00  0.20
C
ATOM  14426  CD1 LEU B 347      -8.908  11.871  -9.096  1.00  0.20
C
ATOM  14427  CD2 LEU B 347     -10.787  13.403  -9.639  1.00  0.20
C
ATOM  14428  H   LEU B 347     -12.358  10.333  -7.597  1.00  0.14
H
ATOM  14429  HA  LEU B 347     -13.098  11.911  -9.829  1.00  0.16
H
ATOM  14430 1HB  LEU B 347     -10.742  10.019  -9.422  1.00  0.23
H
ATOM  14431 2HB  LEU B 347     -10.905  11.070 -10.825  1.00  0.23
H
ATOM  14432  HG  LEU B 347     -10.716  12.079  -7.972  1.00  0.23
H
ATOM  14433 1HD1 LEU B 347      -8.401  12.675  -8.560  1.00  0.23
H
ATOM  14434 2HD1 LEU B 347      -8.641  10.919  -8.649  1.00  0.23
H
ATOM  14435 3HD1 LEU B 347      -8.595  11.880 -10.139  1.00  0.23
H
ATOM  14436 1HD2 LEU B 347     -10.286  14.209  -9.104  1.00  0.23
H
ATOM  14437 2HD2 LEU B 347     -10.480  13.410 -10.684  1.00  0.23
H
ATOM  14438 3HD2 LEU B 347     -11.863  13.541  -9.581  1.00  0.23
H
ATOM  14439  N   GLY B 348     -13.150   8.660 -10.369  1.00  0.13
N
ATOM  14440  CA  GLY B 348     -13.672   7.633 -11.252  1.00  0.14
C
ATOM  14441  C   GLY B 348     -15.184   7.721 -11.358  1.00  0.15
C
ATOM  14442  O   GLY B 348     -15.735   7.703 -12.454  1.00  0.20
O
ATOM  14443  H   GLY B 348     -12.694   8.378  -9.511  1.00  0.16
H
ATOM  14444 1HA  GLY B 348     -13.228   7.741 -12.240  1.00  0.17
H
ATOM  14445 2HA  GLY B 348     -13.388   6.652 -10.874  1.00  0.17
H
ATOM  14446  N   CYS B 349     -15.854   7.853 -10.220  1.00  0.14
N
ATOM  14447  CA  CYS B 349     -17.307   7.928 -10.185  1.00  0.18
C
ATOM  14448  C   CYS B 349     -17.864   9.094 -11.010  1.00  0.32
C
ATOM  14449  O   CYS B 349     -18.872   8.945 -11.702  1.00  1.02
O
ATOM  14450  CB  CYS B 349     -17.778   8.043  -8.734  1.00  0.27
C
ATOM  14451  SG  CYS B 349     -17.549   6.538  -7.755  1.00  0.27
S
```

```
ATOM  14452  H   CYS B 349     -15.350   7.873  -9.346  1.00  0.17
H
ATOM  14453  HA  CYS B 349     -17.703   6.999 -10.597  1.00  0.22
H
ATOM  14454 1HB  CYS B 349     -17.241   8.852  -8.240  1.00  0.32
H
ATOM  14455 2HB  CYS B 349     -18.833   8.288  -8.713  1.00  0.32
H
ATOM  14456  HG  CYS B 349     -18.029   7.036  -6.620  1.00  0.32
H
ATOM  14457  N   ALA B 350     -17.197  10.247 -10.966  1.00  0.17
N
ATOM  14458  CA  ALA B 350     -17.648  11.409 -11.730  1.00  0.26
C
ATOM  14459  C   ALA B 350     -17.262  11.362 -13.219  1.00  0.20
C
ATOM  14460  O   ALA B 350     -17.609  12.282 -13.967  1.00  0.22
O
ATOM  14461  CB  ALA B 350     -17.091  12.676 -11.114  1.00  0.39
C
ATOM  14462  H   ALA B 350     -16.382  10.335 -10.370  1.00  0.20
H
ATOM  14463  HA  ALA B 350     -18.737  11.440 -11.671  1.00  0.31
H
ATOM  14464 1HB  ALA B 350     -17.468  13.541 -11.659  1.00  0.47
H
ATOM  14465 2HB  ALA B 350     -17.403  12.738 -10.072  1.00  0.47
H
ATOM  14466 3HB  ALA B 350     -16.005  12.656 -11.169  1.00  0.47
H
ATOM  14467  N   SER B 351     -16.538  10.320 -13.653  1.00  0.20
N
ATOM  14468  CA  SER B 351     -16.140  10.222 -15.048  1.00  0.29
C
ATOM  14469  C   SER B 351     -17.383  10.067 -15.901  1.00  0.24
C
ATOM  14470  O   SER B 351     -18.355   9.425 -15.493  1.00  0.39
O
ATOM  14471  CB  SER B 351     -15.181   9.067 -15.270  1.00  0.43
C
ATOM  14472  OG  SER B 351     -14.828   8.959 -16.625  1.00  0.43
O
ATOM  14473  H   SER B 351     -16.268   9.571 -13.032  1.00  0.24
H
ATOM  14474  HA  SER B 351     -15.639  11.149 -15.335  1.00  0.35
H
ATOM  14475 1HB  SER B 351     -14.286   9.214 -14.665  1.00  0.52
H
ATOM  14476 2HB  SER B 351     -15.651   8.145 -14.941  1.00  0.52
H
ATOM  14477  HG  SER B 351     -15.653   8.909 -17.109  1.00  0.52
H
ATOM  14478  N   ARG B 352     -17.359  10.701 -17.074  1.00  0.37
N
ATOM  14479  CA  ARG B 352     -18.484  10.736 -18.013  1.00  0.53
C
ATOM  14480  C   ARG B 352     -19.765  11.385 -17.437  1.00  0.36
C
```

ATOM 14481 O   ARG B 352   -20.821 11.314 -18.067 1.00 0.63
O
ATOM 14482 CB ARG B 352   -18.799   9.317 -18.523 1.00 0.80
C
ATOM 14483 CG ARG B 352   -17.631   8.643 -19.252 1.00 0.80
C
ATOM 14484 CD ARG B 352   -17.948   7.288 -19.792 1.00 0.80
C
ATOM 14485 NE ARG B 352   -16.755   6.667 -20.371 1.00 0.80
N
ATOM 14486 CZ ARG B 352   -16.671   5.398 -20.844 1.00 0.80
C
ATOM 14487 NH1 ARG B 352   -17.714   4.591 -20.825 1.00 0.80
N
ATOM 14488 NH2 ARG B 352   -15.522   4.959 -21.325 1.00 0.80
N
ATOM 14489 H   ARG B 352   -16.513 11.193 -17.329 1.00 0.44
H
ATOM 14490 HA ARG B 352   -18.174 11.327 -18.875 1.00 0.64
H
ATOM 14491 1HB ARG B 352   -19.090   8.677 -17.695 1.00 0.95
H
ATOM 14492 2HB ARG B 352   -19.639   9.359 -19.216 1.00 0.95
H
ATOM 14493 1HG ARG B 352   -17.327   9.273 -20.090 1.00 0.95
H
ATOM 14494 2HG ARG B 352   -16.792   8.534 -18.569 1.00 0.95
H
ATOM 14495 1HD ARG B 352   -18.315   6.651 -18.992 1.00 0.95
H
ATOM 14496 2HD ARG B 352   -18.703   7.373 -20.573 1.00 0.95
H
ATOM 14497 HE ARG B 352   -15.909   7.236 -20.388 1.00 0.95
H
ATOM 14498 1HH1 ARG B 352   -18.597   4.920 -20.471 1.00 0.95
H
ATOM 14499 2HH1 ARG B 352   -17.630   3.636 -21.166 1.00 0.95
H
ATOM 14500 1HH2 ARG B 352   -14.718   5.571 -21.349 1.00 0.95
H
ATOM 14501 2HH2 ARG B 352   -15.449   4.015 -21.676 1.00 0.95
H
ATOM 14502 N   GLY B 353   -19.681 12.021 -16.255 1.00 0.28
N
ATOM 14503 CA GLY B 353   -20.834 12.682 -15.663 1.00 0.31
C
ATOM 14504 C   GLY B 353   -21.806 11.706 -15.015 1.00 0.28
C
ATOM 14505 O   GLY B 353   -22.964 12.056 -14.783 1.00 0.37
O
ATOM 14506 H   GLY B 353   -18.809 12.069 -15.742 1.00 0.34
H
ATOM 14507 1HA GLY B 353   -20.491 13.401 -14.919 1.00 0.37
H
ATOM 14508 2HA GLY B 353   -21.353 13.252 -16.433 1.00 0.37
H
ATOM 14509 N   ARG B 354   -21.352 10.483 -14.731 1.00 0.22
N

```
ATOM  14510  CA  ARG B 354     -22.242   9.470 -14.171  1.00  0.28
C
ATOM  14511  C   ARG B 354     -22.838   9.850 -12.815  1.00  0.29
C
ATOM  14512  O   ARG B 354     -24.007   9.563 -12.553  1.00  1.54
O
ATOM  14513  CB  ARG B 354     -21.506   8.157 -14.045  1.00  0.42
C
ATOM  14514  CG  ARG B 354     -21.187   7.508 -15.376  1.00  0.42
C
ATOM  14515  CD  ARG B 354     -20.518   6.205 -15.201  1.00  0.42
C
ATOM  14516  NE  ARG B 354     -20.061   5.667 -16.457  1.00  0.42
N
ATOM  14517  CZ  ARG B 354     -20.797   4.970 -17.343  1.00  0.42
C
ATOM  14518  NH1 ARG B 354     -22.069   4.706 -17.127  1.00  0.42
N
ATOM  14519  NH2 ARG B 354     -20.207   4.555 -18.442  1.00  0.42
N
ATOM  14520  H   ARG B 354     -20.388  10.234 -14.932  1.00  0.26
H
ATOM  14521  HA  ARG B 354     -23.064   9.326 -14.872  1.00  0.34
H
ATOM  14522 1HB  ARG B 354     -20.569   8.307 -13.513  1.00  0.50
H
ATOM  14523 2HB  ARG B 354     -22.107   7.452 -13.467  1.00  0.50
H
ATOM  14524 1HG  ARG B 354     -22.113   7.343 -15.929  1.00  0.50
H
ATOM  14525 2HG  ARG B 354     -20.530   8.159 -15.952  1.00  0.50
H
ATOM  14526 1HD  ARG B 354     -19.654   6.326 -14.547  1.00  0.50
H
ATOM  14527 2HD  ARG B 354     -21.208   5.492 -14.759  1.00  0.50
H
ATOM  14528  HE  ARG B 354     -19.100   5.842 -16.698  1.00  0.50
H
ATOM  14529 1HH1 ARG B 354     -22.516   5.010 -16.271  1.00  0.50
H
ATOM  14530 2HH1 ARG B 354     -22.629   4.204 -17.806  1.00  0.50
H
ATOM  14531 1HH2 ARG B 354     -19.230   4.754 -18.589  1.00  0.50
H
ATOM  14532 2HH2 ARG B 354     -20.723   4.034 -19.134  1.00  0.50
H
ATOM  14533  N   THR B 355     -22.062  10.507 -11.957  1.00  0.58
N
ATOM  14534  CA  THR B 355     -22.610  10.923 -10.672  1.00  0.53
C
ATOM  14535  C   THR B 355     -21.929  12.161 -10.110  1.00  0.28
C
ATOM  14536  O   THR B 355     -20.912  12.624 -10.627  1.00  0.45
O
ATOM  14537  CB  THR B 355     -22.519   9.801  -9.634  1.00  0.80
C
ATOM  14538  OG1 THR B 355     -23.289  10.162  -8.493  1.00  0.80
O
```

ATOM 14539 CG2 THR B 355   -21.096   9.601 -9.190 1.00 0.80
C
ATOM 14540 H   THR B 355   -21.104 10.720 -12.195 1.00 0.70
H
ATOM 14541 HA THR B 355   -23.663 11.163 -10.815 1.00 0.64
H
ATOM 14542 HB THR B 355   -22.908   8.875 -10.057 1.00 0.95
H
ATOM 14543 HG1 THR B 355   -24.193 10.351 -8.755 1.00 0.95
H
ATOM 14544 1HG2 THR B 355   -21.058   8.807 -8.445 1.00 0.95
H
ATOM 14545 2HG2 THR B 355   -20.495   9.321 -10.049 1.00 0.95
H
ATOM 14546 3HG2 THR B 355   -20.711 10.524 -8.759 1.00 0.95
H
ATOM 14547 N   ILE B 356   -22.515 12.683 -9.035 1.00 0.25
N
ATOM 14548 CA ILE B 356   -22.014 13.863 -8.338 1.00 0.21
C
ATOM 14549 C   ILE B 356   -21.434 13.427 -6.999 1.00 0.16
C
ATOM 14550 O   ILE B 356   -22.176 13.158 -6.054 1.00 0.17
O
ATOM 14551 CB ILE B 356   -23.146 14.882 -8.098 1.00 0.32
C
ATOM 14552 CG1 ILE B 356   -23.791 15.292 -9.424 1.00 0.32
C
ATOM 14553 CG2 ILE B 356   -22.592 16.104 -7.388 1.00 0.32
C
ATOM 14554 CD1 ILE B 356   -25.043 16.120 -9.249 1.00 0.32
C
ATOM 14555 H   ILE B 356   -23.343 12.234 -8.673 1.00 0.30
H
ATOM 14556 HA ILE B 356   -21.228 14.327 -8.932 1.00 0.25
H
ATOM 14557 HB ILE B 356   -23.920 14.425 -7.483 1.00 0.38
H
ATOM 14558 1HG1 ILE B 356   -23.072 15.871 -10.005 1.00 0.38
H
ATOM 14559 2HG1 ILE B 356   -24.045 14.398 -9.989 1.00 0.38
H
ATOM 14560 1HG2 ILE B 356   -23.395 16.818 -7.208 1.00 0.38
H
ATOM 14561 2HG2 ILE B 356   -22.151 15.800 -6.443 1.00 0.38
H
ATOM 14562 3HG2 ILE B 356   -21.827 16.569 -8.012 1.00 0.38
H
ATOM 14563 1HD1 ILE B 356   -25.449 16.377 -10.228 1.00 0.38
H
ATOM 14564 2HD1 ILE B 356   -25.783 15.547 -8.688 1.00 0.38
H
ATOM 14565 3HD1 ILE B 356   -24.803 17.032 -8.705 1.00 0.38
H
ATOM 14566 N   ALA B 357   -20.111 13.358 -6.919 1.00 0.16
N
ATOM 14567 CA ALA B 357   -19.459 12.844 -5.719 1.00 0.14
C

ATOM 14568 C ALA B 357 -19.087 13.921 -4.704 1.00 0.16
C
ATOM 14569 O ALA B 357 -18.488 14.938 -5.050 1.00 0.20
O
ATOM 14570 CB ALA B 357 -18.206 12.078 -6.121 1.00 0.21
C
ATOM 14571 H ALA B 357 -19.549 13.626 -7.714 1.00 0.19
H
ATOM 14572 HA ALA B 357 -20.152 12.157 -5.235 1.00 0.17
H
ATOM 14573 1HB ALA B 357 -17.737 11.653 -5.236 1.00 0.25
H
ATOM 14574 2HB ALA B 357 -18.476 11.277 -6.810 1.00 0.25
H
ATOM 14575 3HB ALA B 357 -17.508 12.755 -6.610 1.00 0.25
H
ATOM 14576 N PHE B 358 -19.407 13.633 -3.439 1.00 0.20
N
ATOM 14577 CA PHE B 358 -19.071 14.447 -2.272 1.00 0.19
C
ATOM 14578 C PHE B 358 -18.242 13.619 -1.304 1.00 0.16
C
ATOM 14579 O PHE B 358 -18.789 12.824 -0.545 1.00 0.20
O
ATOM 14580 CB PHE B 358 -20.323 14.913 -1.537 1.00 0.29
C
ATOM 14581 CG PHE B 358 -21.183 15.870 -2.291 1.00 0.29
C
ATOM 14582 CD1 PHE B 358 -22.142 15.416 -3.181 1.00 0.29
C
ATOM 14583 CD2 PHE B 358 -21.047 17.236 -2.087 1.00 0.29
C
ATOM 14584 CE1 PHE B 358 -22.938 16.315 -3.859 1.00 0.29
C
ATOM 14585 CE2 PHE B 358 -21.842 18.135 -2.764 1.00 0.29
C
ATOM 14586 CZ PHE B 358 -22.784 17.668 -3.652 1.00 0.29
C
ATOM 14587 H PHE B 358 -19.933 12.788 -3.273 1.00 0.24
H
ATOM 14588 HA PHE B 358 -18.493 15.309 -2.592 1.00 0.23
H
ATOM 14589 1HB PHE B 358 -20.926 14.045 -1.294 1.00 0.34
H
ATOM 14590 2HB PHE B 358 -20.036 15.384 -0.597 1.00 0.34
H
ATOM 14591 HD1 PHE B 358 -22.262 14.345 -3.341 1.00 0.34
H
ATOM 14592 HD2 PHE B 358 -20.297 17.596 -1.381 1.00 0.34
H
ATOM 14593 HE1 PHE B 358 -23.689 15.953 -4.559 1.00 0.34
H
ATOM 14594 HE2 PHE B 358 -21.727 19.205 -2.600 1.00 0.34
H
ATOM 14595 HZ PHE B 358 -23.411 18.369 -4.189 1.00 0.34
H
ATOM 14596 N ALA B 359 -16.930 13.794 -1.339 1.00 0.17
N

```
ATOM  14597  CA  ALA B 359     -16.052  13.042  -0.454  1.00  0.17
C
ATOM  14598  C   ALA B 359     -15.777  13.852   0.805  1.00  0.20
C
ATOM  14599  O   ALA B 359     -15.515  15.050   0.716  1.00  0.32
O
ATOM  14600  CB  ALA B 359     -14.757  12.693  -1.171  1.00  0.26
C
ATOM  14601  H   ALA B 359     -16.541  14.477  -1.973  1.00  0.20
H
ATOM  14602  HA  ALA B 359     -16.558  12.123  -0.164  1.00  0.20
H
ATOM  14603 1HB  ALA B 359     -14.118  12.117  -0.504  1.00  0.31
H
ATOM  14604 2HB  ALA B 359     -14.981  12.102  -2.059  1.00  0.31
H
ATOM  14605 3HB  ALA B 359     -14.245  13.608  -1.465  1.00  0.31
H
ATOM  14606  N   SER B 360     -15.819  13.205   1.969  1.00  0.25
N
ATOM  14607  CA  SER B 360     -15.571  13.925   3.208  1.00  0.33
C
ATOM  14608  C   SER B 360     -14.621  13.234   4.174  1.00  0.33
C
ATOM  14609  O   SER B 360     -14.594  12.011   4.303  1.00  0.98
O
ATOM  14610  CB  SER B 360     -16.900  14.189   3.902  1.00  0.49
C
ATOM  14611  OG  SER B 360     -17.581  12.995   4.208  1.00  0.49
O
ATOM  14612  H   SER B 360     -16.050  12.223   1.992  1.00  0.30
H
ATOM  14613  HA  SER B 360     -15.129  14.884   2.945  1.00  0.40
H
ATOM  14614 1HB  SER B 360     -16.726  14.754   4.817  1.00  0.59
H
ATOM  14615 2HB  SER B 360     -17.522  14.806   3.255  1.00  0.59
H
ATOM  14616  HG  SER B 360     -17.080  12.519   4.881  1.00  0.59
H
ATOM  14617  N   THR B 361     -13.822  14.067   4.826  1.00  0.23
N
ATOM  14618  CA  THR B 361     -12.851  13.682   5.847  1.00  0.23
C
ATOM  14619  C   THR B 361     -12.413  14.919   6.605  1.00  0.29
C
ATOM  14620  O   THR B 361     -13.003  15.981   6.432  1.00  0.57
O
ATOM  14621  CB  THR B 361     -11.649  12.937   5.258  1.00  0.35
C
ATOM  14622  OG1 THR B 361     -10.856  12.417   6.334  1.00  0.35
O
ATOM  14623  CG2 THR B 361     -10.823  13.849   4.383  1.00  0.35
C
ATOM  14624  H   THR B 361     -13.923  15.055   4.609  1.00  0.28
H
ATOM  14625  HA  THR B 361     -13.339  13.014   6.554  1.00  0.28
H
```

ATOM 14626 HB THR B 361   -12.006 12.101   4.657 1.00 0.41
H
ATOM 14627 HG1 THR B 361   -10.229 11.776   5.982 1.00 0.41
H
ATOM 14628 1HG2 THR B 361   -9.978 13.302   3.973 1.00 0.41
H
ATOM 14629 2HG2 THR B 361   -11.446 14.218   3.571 1.00 0.41
H
ATOM 14630 3HG2 THR B 361   -10.460 14.683   4.963 1.00 0.41
H
ATOM 14631 N   PHE B 362   -11.402 14.787   7.457 1.00 0.30
N
ATOM 14632 CA PHE B 362   -10.884 15.975   8.117 1.00 0.46
C
ATOM 14633 C   PHE B 362   -10.120 16.770   7.069 1.00 0.54
C
ATOM 14634 O   PHE B 362   -9.390 16.197   6.260 1.00 0.78
O
ATOM 14635 CB PHE B 362   -9.923 15.633   9.250 1.00 0.69
C
ATOM 14636 CG PHE B 362   -10.557 14.981 10.438 1.00 0.69
C
ATOM 14637 CD1 PHE B 362   -11.795 14.368 10.338 1.00 0.69
C
ATOM 14638 CD2 PHE B 362   -9.912 14.975 11.663 1.00 0.69
C
ATOM 14639 CE1 PHE B 362   -12.372 13.758 11.433 1.00 0.69
C
ATOM 14640 CE2 PHE B 362   -10.489 14.367 12.760 1.00 0.69
C
ATOM 14641 CZ PHE B 362   -11.718 13.756 12.644 1.00 0.69
C
ATOM 14642 H   PHE B 362   -10.976 13.877   7.569 1.00 0.36
H
ATOM 14643 HA PHE B 362   -11.711 16.577   8.494 1.00 0.55
H
ATOM 14644 1HB PHE B 362   -9.146 14.976   8.876 1.00 0.83
H
ATOM 14645 2HB PHE B 362   -9.437 16.545   9.591 1.00 0.83
H
ATOM 14646 HD1 PHE B 362   -12.316 14.366   9.382 1.00 0.83
H
ATOM 14647 HD2 PHE B 362   -8.937 15.454 11.756 1.00 0.83
H
ATOM 14648 HE1 PHE B 362   -13.345 13.281 11.339 1.00 0.83
H
ATOM 14649 HE2 PHE B 362   -9.972 14.368 13.719 1.00 0.83
H
ATOM 14650 HZ PHE B 362   -12.172 13.276 13.510 1.00 0.83
H
ATOM 14651 N   ALA B 363   -10.241 18.086   7.109 1.00 0.51
N
ATOM 14652 CA ALA B 363   -9.561 18.943   6.149 1.00 0.56
C
ATOM 14653 C   ALA B 363   -8.052 18.754   6.232 1.00 0.46
C
ATOM 14654 O   ALA B 363   -7.347 18.906   5.235 1.00 0.47
O

```
ATOM  14655  CB  ALA B 363      -9.936  20.391   6.377  1.00  0.84
C
ATOM  14656  H   ALA B 363     -10.835  18.500   7.809  1.00  0.61
H
ATOM  14657  HA  ALA B 363      -9.876  18.652   5.153  1.00  0.67
H
ATOM  14658 1HB  ALA B 363      -9.435  21.019   5.643  1.00  1.01
H
ATOM  14659 2HB  ALA B 363     -11.010  20.517   6.281  1.00  1.01
H
ATOM  14660 3HB  ALA B 363      -9.641  20.688   7.376  1.00  1.01
H
ATOM  14661  N   ALA B 364      -7.560  18.416   7.423  1.00  0.41
N
ATOM  14662  CA  ALA B 364      -6.156  18.132   7.614  1.00  0.35
C
ATOM  14663  C   ALA B 364      -5.780  16.895   6.819  1.00  0.33
C
ATOM  14664  O   ALA B 364      -4.802  16.898   6.071  1.00  0.50
O
ATOM  14665  CB  ALA B 364      -5.855  17.932   9.094  1.00  0.52
C
ATOM  14666  H   ALA B 364      -8.183  18.338   8.213  1.00  0.49
H
ATOM  14667  HA  ALA B 364      -5.577  18.974   7.233  1.00  0.42
H
ATOM  14668 1HB  ALA B 364      -4.795  17.728   9.228  1.00  0.63
H
ATOM  14669 2HB  ALA B 364      -6.124  18.834   9.644  1.00  0.63
H
ATOM  14670 3HB  ALA B 364      -6.435  17.093   9.473  1.00  0.63
H
ATOM  14671  N   PHE B 365      -6.601  15.852   6.942  1.00  0.28
N
ATOM  14672  CA  PHE B 365      -6.351  14.573   6.292  1.00  0.42
C
ATOM  14673  C   PHE B 365      -6.331  14.678   4.780  1.00  0.26
C
ATOM  14674  O   PHE B 365      -5.599  13.941   4.123  1.00  0.49
O
ATOM  14675  CB  PHE B 365      -7.383  13.544   6.727  1.00  0.63
C
ATOM  14676  CG  PHE B 365      -7.210  13.095   8.144  1.00  0.63
C
ATOM  14677  CD1 PHE B 365      -6.056  13.415   8.850  1.00  0.63
C
ATOM  14678  CD2 PHE B 365      -8.196  12.349   8.775  1.00  0.63
C
ATOM  14679  CE1 PHE B 365      -5.895  13.003  10.157  1.00  0.63
C
ATOM  14680  CE2 PHE B 365      -8.037  11.936  10.082  1.00  0.63
C
ATOM  14681  CZ  PHE B 365      -6.885  12.263  10.771  1.00  0.63
C
ATOM  14682  H   PHE B 365      -7.409  15.933   7.543  1.00  0.34
H
ATOM  14683  HA  PHE B 365      -5.370  14.219   6.613  1.00  0.50
H
```

```
ATOM  14684 1HB  PHE B 365      -8.386  13.953   6.617  1.00  0.76
H
ATOM  14685 2HB  PHE B 365      -7.316  12.669   6.085  1.00  0.76
H
ATOM  14686  HD1 PHE B 365      -5.275  13.998   8.362  1.00  0.76
H
ATOM  14687  HD2 PHE B 365      -9.103  12.092   8.227  1.00  0.76
H
ATOM  14688  HE1 PHE B 365      -4.986  13.260  10.702  1.00  0.76
H
ATOM  14689  HE2 PHE B 365      -8.817  11.353  10.570  1.00  0.76
H
ATOM  14690  HZ  PHE B 365      -6.760  11.936  11.803  1.00  0.76
H
ATOM  14691  N   LEU B 366      -7.085  15.630   4.225  1.00  0.29
N
ATOM  14692  CA  LEU B 366      -7.098  15.850   2.779  1.00  0.33
C
ATOM  14693  C   LEU B 366      -5.734  16.137   2.177  1.00  0.34
C
ATOM  14694  O   LEU B 366      -5.527  15.888   0.990  1.00  0.41
O
ATOM  14695  CB  LEU B 366      -8.010  17.016   2.424  1.00  0.49
C
ATOM  14696  CG  LEU B 366      -9.488  16.759   2.609  1.00  0.49
C
ATOM  14697  CD1 LEU B 366     -10.245  18.047   2.393  1.00  0.49
C
ATOM  14698  CD2 LEU B 366      -9.916  15.697   1.606  1.00  0.49
C
ATOM  14699  H   LEU B 366      -7.690  16.186   4.821  1.00  0.35
H
ATOM  14700  HA  LEU B 366      -7.491  14.949   2.309  1.00  0.40
H
ATOM  14701 1HB  LEU B 366      -7.741  17.872   3.039  1.00  0.59
H
ATOM  14702 2HB  LEU B 366      -7.848  17.280   1.380  1.00  0.59
H
ATOM  14703  HG  LEU B 366      -9.681  16.415   3.625  1.00  0.59
H
ATOM  14704 1HD1 LEU B 366     -11.311  17.868   2.529  1.00  0.59
H
ATOM  14705 2HD1 LEU B 366      -9.910  18.796   3.108  1.00  0.59
H
ATOM  14706 3HD1 LEU B 366     -10.062  18.407   1.383  1.00  0.59
H
ATOM  14707 1HD2 LEU B 366     -10.980  15.496   1.720  1.00  0.59
H
ATOM  14708 2HD2 LEU B 366      -9.718  16.052   0.594  1.00  0.59
H
ATOM  14709 3HD2 LEU B 366      -9.352  14.781   1.785  1.00  0.59
H
ATOM  14710  N   THR B 367      -4.783  16.621   2.976  1.00  0.37
N
ATOM  14711  CA  THR B 367      -3.470  16.922   2.438  1.00  0.57
C
ATOM  14712  C   THR B 367      -2.734  15.651   2.011  1.00  0.60
C
```

ATOM 14713 O   THR B 367   -1.867 15.688   1.139 1.00 0.82
O
ATOM 14714 CB THR B 367   -2.638 17.683   3.456 1.00 0.85
C
ATOM 14715 OG1 THR B 367   -2.457 16.871   4.613 1.00 0.85
O
ATOM 14716 CG2 THR B 367   -3.315 18.984   3.868 1.00 0.85
C
ATOM 14717 H   THR B 367   -4.962 16.793   3.959 1.00 0.44
H
ATOM 14718 HA THR B 367   -3.592 17.543   1.558 1.00 0.68
H
ATOM 14719 HB THR B 367   -1.682 17.898   3.011 1.00 1.03
H
ATOM 14720 HG1 THR B 367   -3.312 16.738   5.039 1.00 1.03
H
ATOM 14721 1HG2 THR B 367   -2.690 19.504   4.594 1.00 1.03
H
ATOM 14722 2HG2 THR B 367   -3.457 19.619   2.998 1.00 1.03
H
ATOM 14723 3HG2 THR B 367   -4.284 18.764   4.317 1.00 1.03
H
ATOM 14724 N   ARG B 368   -3.129 14.514   2.580 1.00 0.69
N
ATOM 14725 CA ARG B 368   -2.601 13.211   2.210 1.00 1.20
C
ATOM 14726 C   ARG B 368   -2.931 12.851   0.767 1.00 1.05
C
ATOM 14727 O   ARG B 368   -2.187 12.116   0.118 1.00 1.94
O
ATOM 14728 CB ARG B 368   -3.145 12.152   3.151 1.00 1.80
C
ATOM 14729 CG ARG B 368   -2.755 10.725   2.833 1.00 1.80
C
ATOM 14730 CD ARG B 368   -3.080   9.821   3.963 1.00 1.80
C
ATOM 14731 NE ARG B 368   -2.139   9.997   5.058 1.00 1.80
N
ATOM 14732 CZ ARG B 368   -2.314   9.547   6.315 1.00 1.80
C
ATOM 14733 NH1 ARG B 368   -3.421   8.934   6.655 1.00 1.80
N
ATOM 14734 NH2 ARG B 368   -1.366   9.703   7.218 1.00 1.80
N
ATOM 14735 H   ARG B 368   -3.851 14.533   3.287 1.00 0.83
H
ATOM 14736 HA ARG B 368   -1.516 13.239   2.315 1.00 1.44
H
ATOM 14737 1HB ARG B 368   -2.814 12.364   4.167 1.00 2.16
H
ATOM 14738 2HB ARG B 368   -4.233 12.191   3.146 1.00 2.16
H
ATOM 14739 1HG ARG B 368   -3.298 10.384   1.951 1.00 2.16
H
ATOM 14740 2HG ARG B 368   -1.681 10.671   2.644 1.00 2.16
H
ATOM 14741 1HD ARG B 368   -4.080 10.041   4.334 1.00 2.16
H

```
ATOM  14742 2HD  ARG B 368      -3.033   8.784   3.632  1.00  2.16
H
ATOM  14743  HE  ARG B 368      -1.263  10.462   4.845  1.00  2.16
H
ATOM  14744 1HH1 ARG B 368      -4.158   8.789   5.974  1.00  2.16
H
ATOM  14745 2HH1 ARG B 368      -3.536   8.586   7.596  1.00  2.16
H
ATOM  14746 1HH2 ARG B 368      -0.500  10.162   6.970  1.00  2.16
H
ATOM  14747 2HH2 ARG B 368      -1.503   9.313   8.142  1.00  2.16
H
ATOM  14748  N   ALA B 369      -4.072  13.337   0.283  1.00  0.50
N
ATOM  14749  CA  ALA B 369      -4.519  13.058  -1.065  1.00  0.54
C
ATOM  14750  C   ALA B 369      -4.303  14.238  -1.992  1.00  0.62
C
ATOM  14751  O   ALA B 369      -4.919  14.290  -3.054  1.00  0.95
O
ATOM  14752  CB  ALA B 369      -5.986  12.687  -1.051  1.00  0.81
C
ATOM  14753  H   ALA B 369      -4.643  13.954   0.844  1.00  0.60
H
ATOM  14754  HA  ALA B 369      -3.941  12.217  -1.447  1.00  0.65
H
ATOM  14755 1HB  ALA B 369      -6.311  12.448  -2.063  1.00  0.97
H
ATOM  14756 2HB  ALA B 369      -6.132  11.823  -0.408  1.00  0.97
H
ATOM  14757 3HB  ALA B 369      -6.569  13.525  -0.670  1.00  0.97
H
ATOM  14758  N   PHE B 370      -3.438  15.179  -1.609  1.00  0.63
N
ATOM  14759  CA  PHE B 370      -3.212  16.365  -2.417  1.00  0.95
C
ATOM  14760  C   PHE B 370      -2.777  16.035  -3.835  1.00  0.68
C
ATOM  14761  O   PHE B 370      -3.191  16.706  -4.778  1.00  0.75
O
ATOM  14762  CB  PHE B 370      -2.230  17.288  -1.733  1.00  1.42
C
ATOM  14763  CG  PHE B 370      -1.999  18.541  -2.496  1.00  1.42
C
ATOM  14764  CD1 PHE B 370      -3.022  19.115  -3.240  1.00  1.42
C
ATOM  14765  CD2 PHE B 370      -0.775  19.172  -2.446  1.00  1.42
C
ATOM  14766  CE1 PHE B 370      -2.806  20.292  -3.929  1.00  1.42
C
ATOM  14767  CE2 PHE B 370      -0.562  20.341  -3.133  1.00  1.42
C
ATOM  14768  CZ  PHE B 370      -1.574  20.902  -3.868  1.00  1.42
C
ATOM  14769  H   PHE B 370      -2.932  15.104  -0.734  1.00  0.76
H
ATOM  14770  HA  PHE B 370      -4.157  16.905  -2.484  1.00  1.14
H
```

```
ATOM  14771 1HB  PHE B 370      -2.597  17.547  -0.741  1.00  1.71
H
ATOM  14772 2HB  PHE B 370      -1.274  16.781  -1.609  1.00  1.71
H
ATOM  14773  HD1 PHE B 370      -3.998  18.629  -3.276  1.00  1.71
H
ATOM  14774  HD2 PHE B 370       0.027  18.730  -1.853  1.00  1.71
H
ATOM  14775  HE1 PHE B 370      -3.607  20.741  -4.511  1.00  1.71
H
ATOM  14776  HE2 PHE B 370       0.406  20.828  -3.096  1.00  1.71
H
ATOM  14777  HZ  PHE B 370      -1.391  21.830  -4.395  1.00  1.71
H
ATOM  14778  N   ASP B 371      -1.949  15.005  -3.978  1.00  0.85
N
ATOM  14779  CA  ASP B 371      -1.516  14.502  -5.277  1.00  0.97
C
ATOM  14780  C   ASP B 371      -2.702  14.140  -6.190  1.00  0.99
C
ATOM  14781  O   ASP B 371      -2.712  14.478  -7.375  1.00  2.70
O
ATOM  14782  CB  ASP B 371      -0.600  13.282  -5.063  1.00  1.46
C
ATOM  14783  CG  ASP B 371      -1.214  12.151  -4.216  1.00  1.46
C
ATOM  14784  OD1 ASP B 371      -2.300  12.311  -3.708  1.00  1.46
O
ATOM  14785  OD2 ASP B 371      -0.604  11.113  -4.118  1.00  1.46
O
ATOM  14786  H   ASP B 371      -1.623  14.532  -3.147  1.00  1.02
H
ATOM  14787  HA  ASP B 371      -0.938  15.285  -5.771  1.00  1.16
H
ATOM  14788 1HB  ASP B 371      -0.340  12.867  -6.034  1.00  1.75
H
ATOM  14789 2HB  ASP B 371       0.327  13.606  -4.593  1.00  1.75
H
ATOM  14790  N   HIS B 372      -3.690  13.450  -5.617  1.00  0.54
N
ATOM  14791  CA  HIS B 372      -4.893  13.023  -6.322  1.00  0.68
C
ATOM  14792  C   HIS B 372      -5.837  14.167  -6.574  1.00  0.87
C
ATOM  14793  O   HIS B 372      -6.526  14.205  -7.591  1.00  1.45
O
ATOM  14794  CB  HIS B 372      -5.641  11.947  -5.537  1.00  1.02
C
ATOM  14795  CG  HIS B 372      -4.981  10.621  -5.568  1.00  1.02
C
ATOM  14796  ND1 HIS B 372      -3.925  10.295  -4.752  1.00  1.02
N
ATOM  14797  CD2 HIS B 372      -5.225   9.532  -6.325  1.00  1.02
C
ATOM  14798  CE1 HIS B 372      -3.544   9.057  -5.012  1.00  1.02
C
ATOM  14799  NE2 HIS B 372      -4.328   8.567  -5.951  1.00  1.02
N
```

```
ATOM  14800  H   HIS B 372      -3.576  13.201  -4.643  1.00  0.65
H
ATOM  14801  HA  HIS B 372      -4.614  12.599  -7.283  1.00  0.82
H
ATOM  14802 1HB  HIS B 372      -5.742  12.257  -4.496  1.00  1.22
H
ATOM  14803 2HB  HIS B 372      -6.642  11.832  -5.946  1.00  1.22
H
ATOM  14804  HD1 HIS B 372      -3.407  10.945  -4.189  1.00  1.22
H
ATOM  14805  HD2 HIS B 372      -5.956   9.329  -7.107  1.00  1.22
H
ATOM  14806  HE1 HIS B 372      -2.714   8.607  -4.467  1.00  1.22
H
ATOM  14807  N   ILE B 373      -5.874  15.105  -5.656  1.00  0.78
N
ATOM  14808  CA  ILE B 373      -6.725  16.251  -5.825  1.00  0.85
C
ATOM  14809  C   ILE B 373      -6.164  17.079  -6.996  1.00  0.67
C
ATOM  14810  O   ILE B 373      -6.908  17.544  -7.865  1.00  0.77
O
ATOM  14811  CB  ILE B 373      -6.804  17.036  -4.515  1.00  1.27
C
ATOM  14812  CG1 ILE B 373      -7.501  16.201  -3.436  1.00  1.27
C
ATOM  14813  CG2 ILE B 373      -7.621  18.261  -4.754  1.00  1.27
C
ATOM  14814  CD1 ILE B 373      -7.364  16.767  -2.040  1.00  1.27
C
ATOM  14815  H   ILE B 373      -5.316  15.016  -4.817  1.00  0.94
H
ATOM  14816  HA  ILE B 373      -7.727  15.915  -6.087  1.00  1.02
H
ATOM  14817  HB  ILE B 373      -5.805  17.304  -4.173  1.00  1.53
H
ATOM  14818 1HG1 ILE B 373      -8.561  16.136  -3.676  1.00  1.53
H
ATOM  14819 2HG1 ILE B 373      -7.091  15.193  -3.443  1.00  1.53
H
ATOM  14820 1HG2 ILE B 373      -7.688  18.844  -3.838  1.00  1.53
H
ATOM  14821 2HG2 ILE B 373      -7.138  18.834  -5.534  1.00  1.53
H
ATOM  14822 3HG2 ILE B 373      -8.620  17.974  -5.076  1.00  1.53
H
ATOM  14823 1HD1 ILE B 373      -7.889  16.124  -1.333  1.00  1.53
H
ATOM  14824 2HD1 ILE B 373      -6.310  16.817  -1.768  1.00  1.53
H
ATOM  14825 3HD1 ILE B 373      -7.794  17.764  -2.012  1.00  1.53
H
ATOM  14826  N   ARG B 374      -4.841  17.224  -7.010  1.00  0.53
N
ATOM  14827  CA  ARG B 374      -4.116  17.906  -8.061  1.00  0.55
C
ATOM  14828  C   ARG B 374      -4.319  17.265  -9.440  1.00  0.60
C
```

ATOM 14829 O   ARG B 374   -4.616 17.975 -10.407 1.00 0.60
O
ATOM 14830 CB ARG B 374   -2.652 17.927 -7.715 1.00 0.83
C
ATOM 14831 CG ARG B 374   -1.813 18.651 -8.711 1.00 0.83
C
ATOM 14832 CD ARG B 374   -0.430 18.806 -8.241 1.00 0.83
C
ATOM 14833 NE ARG B 374   0.306 19.582 -9.201 1.00 0.83
N
ATOM 14834 CZ ARG B 374   1.596 19.922 -9.113 1.00 0.83
C
ATOM 14835 NH1 ARG B 374   2.332 19.536 -8.095 1.00 0.83
N
ATOM 14836 NH2 ARG B 374   2.095 20.653 -10.082 1.00 0.83
N
ATOM 14837 H   ARG B 374   -4.301 16.843 -6.249 1.00 0.64
H
ATOM 14838 HA ARG B 374   -4.471 18.935 -8.105 1.00 0.66
H
ATOM 14839 1HB ARG B 374   -2.511 18.406 -6.747 1.00 0.99
H
ATOM 14840 2HB ARG B 374   -2.273 16.909 -7.636 1.00 0.99
H
ATOM 14841 1HG ARG B 374   -1.794 18.093 -9.647 1.00 0.99
H
ATOM 14842 2HG ARG B 374   -2.230 19.643 -8.886 1.00 0.99
H
ATOM 14843 1HD ARG B 374   -0.417 19.333 -7.287 1.00 0.99
H
ATOM 14844 2HD ARG B 374   0.044 17.835 -8.138 1.00 0.99
H
ATOM 14845 HE ARG B 374   -0.205 19.922 -10.004 1.00 0.99
H
ATOM 14846 1HH1 ARG B 374   1.926 18.973 -7.361 1.00 0.99
H
ATOM 14847 2HH1 ARG B 374   3.302 19.804 -8.047 1.00 0.99
H
ATOM 14848 1HH2 ARG B 374   1.505 20.929 -10.854 1.00 0.99
H
ATOM 14849 2HH2 ARG B 374   3.066 20.922 -10.095 1.00 0.99
H
ATOM 14850 N   ILE B 375   -4.183 15.928 -9.531 1.00 0.73
N
ATOM 14851 CA ILE B 375   -4.405 15.254 -10.812 1.00 0.87
C
ATOM 14852 C   ILE B 375   -5.873 15.357 -11.195 1.00 0.53
C
ATOM 14853 O   ILE B 375   -6.190 15.363 -12.378 1.00 0.58
O
ATOM 14854 CB ILE B 375   -4.002 13.753 -10.805 1.00 1.30
C
ATOM 14855 CG1 ILE B 375   -3.815 13.227 -12.234 1.00 1.30
C
ATOM 14856 CG2 ILE B 375   -5.087 12.915 -10.161 1.00 1.30
C
ATOM 14857 CD1 ILE B 375   -2.657 13.836 -12.980 1.00 1.30
C

ATOM 14858 H ILE B 375 -3.909 15.384 -8.720 1.00 0.88
H
ATOM 14859 HA ILE B 375 -3.815 15.758 -11.575 1.00 1.04
H
ATOM 14860 HB ILE B 375 -3.065 13.625 -10.265 1.00 1.57
H
ATOM 14861 1HG1 ILE B 375 -3.658 12.150 -12.187 1.00 1.57
H
ATOM 14862 2HG1 ILE B 375 -4.725 13.413 -12.803 1.00 1.57
H
ATOM 14863 1HG2 ILE B 375 -4.797 11.867 -10.165 1.00 1.57
H
ATOM 14864 2HG2 ILE B 375 -5.226 13.242 -9.147 1.00 1.57
H
ATOM 14865 3HG2 ILE B 375 -6.018 13.024 -10.716 1.00 1.57
H
ATOM 14866 1HD1 ILE B 375 -2.597 13.399 -13.977 1.00 1.57
H
ATOM 14867 2HD1 ILE B 375 -2.814 14.905 -13.064 1.00 1.57
H
ATOM 14868 3HD1 ILE B 375 -1.733 13.639 -12.441 1.00 1.57
H
ATOM 14869 N GLY B 376 -6.765 15.462 -10.198 1.00 0.42
N
ATOM 14870 CA GLY B 376 -8.187 15.644 -10.446 1.00 0.35
C
ATOM 14871 C GLY B 376 -8.423 16.936 -11.208 1.00 0.34
C
ATOM 14872 O GLY B 376 -9.230 16.980 -12.141 1.00 0.36
O
ATOM 14873 H GLY B 376 -6.459 15.398 -9.236 1.00 0.50
H
ATOM 14874 1HA GLY B 376 -8.572 14.799 -11.016 1.00 0.42
H
ATOM 14875 2HA GLY B 376 -8.721 15.672 -9.498 1.00 0.42
H
ATOM 14876 N GLY B 377 -7.699 17.989 -10.817 1.00 0.38
N
ATOM 14877 CA GLY B 377 -7.748 19.256 -11.533 1.00 0.42
C
ATOM 14878 C GLY B 377 -7.222 19.069 -12.957 1.00 0.44
C
ATOM 14879 O GLY B 377 -7.838 19.520 -13.922 1.00 0.48
O
ATOM 14880 H GLY B 377 -7.104 17.899 -10.000 1.00 0.46
H
ATOM 14881 1HA GLY B 377 -8.773 19.625 -11.560 1.00 0.50
H
ATOM 14882 2HA GLY B 377 -7.147 19.998 -11.010 1.00 0.50
H
ATOM 14883 N LEU B 378 -6.107 18.345 -13.085 1.00 0.45
N
ATOM 14884 CA LEU B 378 -5.501 18.072 -14.386 1.00 0.46
C
ATOM 14885 C LEU B 378 -6.414 17.202 -15.269 1.00 0.55
C
ATOM 14886 O LEU B 378 -6.395 17.311 -16.497 1.00 0.90
O

```
ATOM  14887  CB  LEU B 378      -4.137  17.394 -14.171  1.00  0.69
C
ATOM  14888  CG  LEU B 378      -3.083  18.296 -13.501  1.00  0.69
C
ATOM  14889  CD1 LEU B 378      -1.797  17.525 -13.224  1.00  0.69
C
ATOM  14890  CD2 LEU B 378      -2.823  19.471 -14.400  1.00  0.69
C
ATOM  14891  H   LEU B 378      -5.646  18.006 -12.248  1.00  0.54
H
ATOM  14892  HA  LEU B 378      -5.340  19.021 -14.895  1.00  0.55
H
ATOM  14893 1HB  LEU B 378      -4.272  16.513 -13.554  1.00  0.83
H
ATOM  14894 2HB  LEU B 378      -3.744  17.082 -15.140  1.00  0.83
H
ATOM  14895  HG  LEU B 378      -3.468  18.654 -12.548  1.00  0.83
H
ATOM  14896 1HD1 LEU B 378      -1.074  18.188 -12.750  1.00  0.83
H
ATOM  14897 2HD1 LEU B 378      -2.007  16.693 -12.555  1.00  0.83
H
ATOM  14898 3HD1 LEU B 378      -1.386  17.149 -14.159  1.00  0.83
H
ATOM  14899 1HD2 LEU B 378      -2.092  20.133 -13.934  1.00  0.83
H
ATOM  14900 2HD2 LEU B 378      -2.440  19.117 -15.356  1.00  0.83
H
ATOM  14901 3HD2 LEU B 378      -3.754  20.011 -14.559  1.00  0.83
H
ATOM  14902  N   ALA B 379      -7.235  16.368 -14.631  1.00  0.48
N
ATOM  14903  CA  ALA B 379      -8.203  15.512 -15.300  1.00  0.48
C
ATOM  14904  C   ALA B 379      -9.512  16.230 -15.628  1.00  0.46
C
ATOM  14905  O   ALA B 379     -10.416  15.616 -16.198  1.00  0.52
O
ATOM  14906  CB  ALA B 379      -8.509  14.318 -14.423  1.00  0.72
C
ATOM  14907  H   ALA B 379      -7.175  16.305 -13.627  1.00  0.58
H
ATOM  14908  HA  ALA B 379      -7.761  15.173 -16.237  1.00  0.58
H
ATOM  14909 1HB  ALA B 379      -9.211  13.662 -14.937  1.00  0.86
H
ATOM  14910 2HB  ALA B 379      -7.588  13.775 -14.211  1.00  0.86
H
ATOM  14911 3HB  ALA B 379      -8.952  14.659 -13.489  1.00  0.86
H
ATOM  14912  N   GLU B 380      -9.626  17.513 -15.257  1.00  0.46
N
ATOM  14913  CA  GLU B 380     -10.839  18.295 -15.469  1.00  0.47
C
ATOM  14914  C   GLU B 380     -12.058  17.630 -14.835  1.00  0.41
C
ATOM  14915  O   GLU B 380     -13.136  17.594 -15.433  1.00  0.43
O
```

ATOM 14916 CB GLU B 380 -11.076 18.520 -16.964 1.00 0.70
C
ATOM 14917 CG GLU B 380 -9.960 19.286 -17.662 1.00 0.70
C
ATOM 14918 CD GLU B 380 -10.260 19.576 -19.106 1.00 0.70
C
ATOM 14919 OE1 GLU B 380 -11.230 19.060 -19.608 1.00 0.70
O
ATOM 14920 OE2 GLU B 380 -9.520 20.318 -19.709 1.00 0.70
O
ATOM 14921 H GLU B 380 -8.858 17.990 -14.809 1.00 0.55
H
ATOM 14922 HA GLU B 380 -10.704 19.268 -14.994 1.00 0.56
H
ATOM 14923 1HB GLU B 380 -11.194 17.562 -17.471 1.00 0.85
H
ATOM 14924 2HB GLU B 380 -12.002 19.078 -17.106 1.00 0.85
H
ATOM 14925 1HG GLU B 380 -9.799 20.229 -17.140 1.00 0.85
H
ATOM 14926 2HG GLU B 380 -9.040 18.706 -17.598 1.00 0.85
H
ATOM 14927 N SER B 381 -11.883 17.106 -13.622 1.00 0.40
N
ATOM 14928 CA SER B 381 -12.962 16.439 -12.909 1.00 0.37
C
ATOM 14929 C SER B 381 -13.889 17.428 -12.225 1.00 0.34
C
ATOM 14930 O SER B 381 -13.588 18.618 -12.123 1.00 0.56
O
ATOM 14931 CB SER B 381 -12.400 15.502 -11.874 1.00 0.55
C
ATOM 14932 OG SER B 381 -11.783 16.227 -10.848 1.00 0.55
O
ATOM 14933 H SER B 381 -10.973 17.161 -13.178 1.00 0.48
H
ATOM 14934 HA SER B 381 -13.545 15.863 -13.629 1.00 0.44
H
ATOM 14935 1HB SER B 381 -13.197 14.883 -11.463 1.00 0.67
H
ATOM 14936 2HB SER B 381 -11.674 14.837 -12.340 1.00 0.67
H
ATOM 14937 HG SER B 381 -11.083 16.731 -11.273 1.00 0.67
H
ATOM 14938 N ASN B 382 -15.013 16.918 -11.736 1.00 0.31
N
ATOM 14939 CA ASN B 382 -15.994 17.738 -11.046 1.00 0.36
C
ATOM 14940 C ASN B 382 -16.321 17.096 -9.703 1.00 0.35
C
ATOM 14941 O ASN B 382 -17.400 16.531 -9.518 1.00 1.23
O
ATOM 14942 CB ASN B 382 -17.240 17.904 -11.891 1.00 0.54
C
ATOM 14943 CG ASN B 382 -16.950 18.532 -13.228 1.00 0.54
C
ATOM 14944 OD1 ASN B 382 -16.518 19.685 -13.334 1.00 0.54
O

ATOM 14945 ND2 ASN B 382   -17.182 17.776 -14.270 1.00 0.54
N
ATOM 14946 H   ASN B 382   -15.196 15.933 -11.853 1.00 0.37
H
ATOM 14947 HA ASN B 382   -15.565 18.717 -10.854 1.00 0.43
H
ATOM 14948 1HB ASN B 382   -17.703 16.930 -12.052 1.00 0.65
H
ATOM 14949 2HB ASN B 382   -17.961 18.525 -11.358 1.00 0.65
H
ATOM 14950 1HD2 ASN B 382   -17.011 18.128 -15.191 1.00 0.65
H
ATOM 14951 2HD2 ASN B 382   -17.530 16.847 -14.148 1.00 0.65
H
ATOM 14952 N   ILE B 383   -15.359 17.159 -8.784 1.00 0.40
N
ATOM 14953 CA ILE B 383   -15.471 16.531 -7.469 1.00 0.42
C
ATOM 14954 C   ILE B 383   -15.695 17.521 -6.348 1.00 0.30
C
ATOM 14955 O   ILE B 383   -15.042 18.563 -6.294 1.00 0.48
O
ATOM 14956 CB ILE B 383   -14.207 15.714 -7.146 1.00 0.63
C
ATOM 14957 CG1 ILE B 383   -13.981 14.700 -8.245 1.00 0.63
C
ATOM 14958 CG2 ILE B 383   -14.326 15.017 -5.795 1.00 0.63
C
ATOM 14959 CD1 ILE B 383   -15.138 13.769 -8.424 1.00 0.63
C
ATOM 14960 H   ILE B 383   -14.509 17.657 -9.009 1.00 0.48
H
ATOM 14961 HA ILE B 383   -16.321 15.849 -7.490 1.00 0.50
H
ATOM 14962 HB ILE B 383   -13.345 16.377 -7.134 1.00 0.76
H
ATOM 14963 1HG1 ILE B 383   -13.796 15.218 -9.185 1.00 0.76
H
ATOM 14964 2HG1 ILE B 383   -13.101 14.107 -7.999 1.00 0.76
H
ATOM 14965 1HG2 ILE B 383   -13.416 14.451 -5.600 1.00 0.76
H
ATOM 14966 2HG2 ILE B 383   -14.460 15.759 -5.010 1.00 0.76
H
ATOM 14967 3HG2 ILE B 383   -15.180 14.341 -5.806 1.00 0.76
H
ATOM 14968 1HD1 ILE B 383   -14.908 13.063 -9.221 1.00 0.76
H
ATOM 14969 2HD1 ILE B 383   -15.318 13.227 -7.497 1.00 0.76
H
ATOM 14970 3HD1 ILE B 383   -16.029 14.336 -8.689 1.00 0.76
H
ATOM 14971 N   ASN B 384   -16.619 17.184 -5.457 1.00 0.24
N
ATOM 14972 CA ASN B 384   -16.882 17.994 -4.291 1.00 0.30
C
ATOM 14973 C   ASN B 384   -16.173 17.358 -3.094 1.00 0.29
C

ATOM 14974 O   ASN B 384   -16.271 16.150 -2.881 1.00 0.34
O
ATOM 14975 CB ASN B 384   -18.372 18.090 -4.062 1.00 0.45
C
ATOM 14976 CG ASN B 384   -19.089 18.740 -5.212 1.00 0.45
C
ATOM 14977 OD1 ASN B 384   -18.812 19.883 -5.577 1.00 0.45
O
ATOM 14978 ND2 ASN B 384   -20.004 18.022 -5.805 1.00 0.45
N
ATOM 14979 H   ASN B 384   -17.147 16.326 -5.565 1.00 0.29
H
ATOM 14980 HA ASN B 384   -16.466 18.990 -4.439 1.00 0.36
H
ATOM 14981 1HB ASN B 384   -18.783 17.094 -3.911 1.00 0.54
H
ATOM 14982 2HB ASN B 384   -18.565 18.665 -3.157 1.00 0.54
H
ATOM 14983 1HD2 ASN B 384   -20.509 18.400 -6.581 1.00 0.54
H
ATOM 14984 2HD2 ASN B 384   -20.197 17.094 -5.484 1.00 0.54
H
ATOM 14985 N   ILE B 385   -15.456 18.168 -2.331 1.00 0.28
N
ATOM 14986 CA ILE B 385   -14.739 17.703 -1.156 1.00 0.35
C
ATOM 14987 C   ILE B 385   -15.084 18.540   0.065 1.00 0.38
C
ATOM 14988 O   ILE B 385   -15.031 19.764   0.015 1.00 0.96
O
ATOM 14989 CB ILE B 385   -13.214 17.730 -1.375 1.00 0.52
C
ATOM 14990 CG1 ILE B 385   -12.804 16.806 -2.528 1.00 0.52
C
ATOM 14991 CG2 ILE B 385   -12.513 17.329 -0.105 1.00 0.52
C
ATOM 14992 CD1 ILE B 385   -11.340 16.910 -2.890 1.00 0.52
C
ATOM 14993 H   ILE B 385   -15.395 19.142 -2.578 1.00 0.34
H
ATOM 14994 HA ILE B 385   -15.034 16.675 -0.960 1.00 0.42
H
ATOM 14995 HB ILE B 385   -12.908 18.739 -1.649 1.00 0.63
H
ATOM 14996 1HG1 ILE B 385   -13.021 15.775 -2.253 1.00 0.63
H
ATOM 14997 2HG1 ILE B 385   -13.386 17.056 -3.414 1.00 0.63
H
ATOM 14998 1HG2 ILE B 385   -11.437 17.368 -0.266 1.00 0.63
H
ATOM 14999 2HG2 ILE B 385   -12.785 18.014   0.695 1.00 0.63
H
ATOM 15000 3HG2 ILE B 385   -12.805 16.315   0.168 1.00 0.63
H
ATOM 15001 1HD1 ILE B 385   -11.120 16.231 -3.715 1.00 0.63
H
ATOM 15002 2HD1 ILE B 385   -11.112 17.932 -3.191 1.00 0.63
H

ATOM 15003 3HD1 ILE B 385 -10.731 16.641 -2.028 1.00 0.63
H
ATOM 15004 N ILE B 386 -15.440 17.878 1.155 1.00 0.31
N
ATOM 15005 CA ILE B 386 -15.769 18.574 2.386 1.00 0.39
C
ATOM 15006 C ILE B 386 -14.755 18.201 3.460 1.00 0.40
C
ATOM 15007 O ILE B 386 -14.574 17.022 3.771 1.00 0.42
O
ATOM 15008 CB ILE B 386 -17.200 18.246 2.854 1.00 0.58
C
ATOM 15009 CG1 ILE B 386 -18.218 18.650 1.774 1.00 0.58
C
ATOM 15010 CG2 ILE B 386 -17.493 18.979 4.154 1.00 0.58
C
ATOM 15011 CD1 ILE B 386 -19.626 18.184 2.053 1.00 0.58
C
ATOM 15012 H ILE B 386 -15.474 16.869 1.130 1.00 0.37
H
ATOM 15013 HA ILE B 386 -15.704 19.647 2.212 1.00 0.47
H
ATOM 15014 HB ILE B 386 -17.296 17.173 3.013 1.00 0.70
H
ATOM 15015 1HG1 ILE B 386 -18.233 19.731 1.687 1.00 0.70
H
ATOM 15016 2HG1 ILE B 386 -17.906 18.231 0.819 1.00 0.70
H
ATOM 15017 1HG2 ILE B 386 -18.502 18.742 4.488 1.00 0.70
H
ATOM 15018 2HG2 ILE B 386 -16.777 18.671 4.915 1.00 0.70
H
ATOM 15019 3HG2 ILE B 386 -17.408 20.054 3.992 1.00 0.70
H
ATOM 15020 1HD1 ILE B 386 -20.285 18.507 1.246 1.00 0.70
H
ATOM 15021 2HD1 ILE B 386 -19.639 17.096 2.120 1.00 0.70
H
ATOM 15022 3HD1 ILE B 386 -19.971 18.609 2.994 1.00 0.70
H
ATOM 15023 N GLY B 387 -14.080 19.209 3.997 1.00 0.43
N
ATOM 15024 CA GLY B 387 -13.067 18.984 5.015 1.00 0.42
C
ATOM 15025 C GLY B 387 -13.543 19.492 6.370 1.00 0.48
C
ATOM 15026 O GLY B 387 -13.951 20.643 6.499 1.00 0.76
O
ATOM 15027 H GLY B 387 -14.295 20.152 3.705 1.00 0.52
H
ATOM 15028 1HA GLY B 387 -12.834 17.924 5.072 1.00 0.50
H
ATOM 15029 2HA GLY B 387 -12.153 19.499 4.727 1.00 0.50
H
ATOM 15030 N SER B 388 -13.474 18.624 7.375 1.00 0.39
N
ATOM 15031 CA SER B 388 -13.910 18.944 8.730 1.00 0.40
C

```
ATOM  15032  C   SER B 388     -12.767  19.249   9.676  1.00  0.44
C
ATOM  15033  O   SER B 388     -11.601  19.048   9.345  1.00  0.47
O
ATOM  15034  CB  SER B 388     -14.727  17.796   9.279  1.00  0.60
C
ATOM  15035  OG  SER B 388     -13.926  16.687   9.565  1.00  0.60
O
ATOM  15036  H   SER B 388     -13.128  17.696   7.182  1.00  0.47
H
ATOM  15037  HA  SER B 388     -14.523  19.831   8.689  1.00  0.48
H
ATOM  15038 1HB  SER B 388     -15.239  18.109  10.179  1.00  0.72
H
ATOM  15039 2HB  SER B 388     -15.486  17.520   8.548  1.00  0.72
H
ATOM  15040  HG  SER B 388     -13.602  16.829  10.460  1.00  0.72
H
ATOM  15041  N   HIS B 389     -13.104  19.748  10.863  1.00  0.46
N
ATOM  15042  CA  HIS B 389     -12.103  20.028  11.889  1.00  0.48
C
ATOM  15043  C   HIS B 389     -11.059  21.042  11.449  1.00  0.49
C
ATOM  15044  O   HIS B 389      -9.856  20.841  11.645  1.00  0.55
O
ATOM  15045  CB  HIS B 389     -11.434  18.715  12.320  1.00  0.72
C
ATOM  15046  CG  HIS B 389     -12.403  17.811  12.993  1.00  0.72
C
ATOM  15047  ND1 HIS B 389     -13.362  17.103  12.306  1.00  0.72
N
ATOM  15048  CD2 HIS B 389     -12.561  17.497  14.296  1.00  0.72
C
ATOM  15049  CE1 HIS B 389     -14.083  16.405  13.164  1.00  0.72
C
ATOM  15050  NE2 HIS B 389     -13.614  16.622  14.379  1.00  0.72
N
ATOM  15051  H   HIS B 389     -14.079  19.918  11.067  1.00  0.55
H
ATOM  15052  HA  HIS B 389     -12.601  20.442  12.765  1.00  0.58
H
ATOM  15053 1HB  HIS B 389     -11.015  18.193  11.462  1.00  0.86
H
ATOM  15054 2HB  HIS B 389     -10.616  18.918  13.008  1.00  0.86
H
ATOM  15055  HD2 HIS B 389     -11.963  17.875  15.124  1.00  0.86
H
ATOM  15056  HE1 HIS B 389     -14.919  15.758  12.906  1.00  0.86
H
ATOM  15057  HE2 HIS B 389     -13.969  16.215  15.232  1.00  0.86
H
ATOM  15058  N   CYS B 390     -11.527  22.139  10.877  1.00  0.52
N
ATOM  15059  CA  CYS B 390     -10.622  23.173  10.445  1.00  0.77
C
ATOM  15060  C   CYS B 390     -10.186  24.075  11.587  1.00  0.74
C
```

```
ATOM  15061  O   CYS B 390     -11.013  24.524  12.382  1.00  1.30
O
ATOM  15062  CB  CYS B 390     -11.292  24.015   9.366  1.00  1.16
C
ATOM  15063  SG  CYS B 390     -11.633  23.122   7.831  1.00  1.16
S
ATOM  15064  H   CYS B 390     -12.523  22.264  10.715  1.00  0.62
H
ATOM  15065  HA  CYS B 390      -9.742  22.701  10.030  1.00  0.92
H
ATOM  15066 1HB  CYS B 390     -12.241  24.391   9.749  1.00  1.39
H
ATOM  15067 2HB  CYS B 390     -10.673  24.877   9.137  1.00  1.39
H
ATOM  15068  HG  CYS B 390     -12.291  22.114   8.393  1.00  1.39
H
ATOM  15069  N   GLY B 391      -8.890  24.353  11.655  1.00  0.77
N
ATOM  15070  CA  GLY B 391      -8.365  25.291  12.636  1.00  0.86
C
ATOM  15071  C   GLY B 391      -8.063  24.756  14.039  1.00  0.96
C
ATOM  15072  O   GLY B 391      -8.396  23.622  14.388  1.00  0.92
O
ATOM  15073  H   GLY B 391      -8.255  23.956  10.976  1.00  0.92
H
ATOM  15074 1HA  GLY B 391      -7.477  25.755  12.230  1.00  1.03
H
ATOM  15075 2HA  GLY B 391      -9.097  26.083  12.728  1.00  1.03
H
ATOM  15076  N   VAL B 392      -7.464  25.633  14.865  1.00  1.14
N
ATOM  15077  CA  VAL B 392      -7.121  25.321  16.249  1.00  1.26
C
ATOM  15078  C   VAL B 392      -8.380  25.229  17.089  1.00  1.30
C
ATOM  15079  O   VAL B 392      -8.377  24.580  18.136  1.00  1.43
O
ATOM  15080  CB  VAL B 392      -6.172  26.379  16.868  1.00  1.89
C
ATOM  15081  CG1 VAL B 392      -4.923  26.500  16.009  1.00  1.89
C
ATOM  15082  CG2 VAL B 392      -6.878  27.714  17.040  1.00  1.89
C
ATOM  15083  H   VAL B 392      -7.237  26.550  14.505  1.00  1.37
H
ATOM  15084  HA  VAL B 392      -6.623  24.361  16.272  1.00  1.51
H
ATOM  15085  HB  VAL B 392      -5.846  26.023  17.845  1.00  2.27
H
ATOM  15086 1HG1 VAL B 392      -4.233  27.214  16.459  1.00  2.27
H
ATOM  15087 2HG1 VAL B 392      -4.441  25.528  15.929  1.00  2.27
H
ATOM  15088 3HG1 VAL B 392      -5.202  26.849  15.015  1.00  2.27
H
ATOM  15089 1HG2 VAL B 392      -6.198  28.432  17.491  1.00  2.27
H
```

```
ATOM  15090 2HG2 VAL B 392      -7.203  28.085  16.074  1.00  2.27
H
ATOM  15091 3HG2 VAL B 392      -7.746  27.587  17.688  1.00  2.27
H
ATOM  15092  N   SER B 393      -9.469  25.849  16.606  1.00  1.64
N
ATOM  15093  CA  SER B 393     -10.780  25.794  17.248  1.00  1.58
C
ATOM  15094  C   SER B 393     -11.282  24.377  17.602  1.00  1.43
C
ATOM  15095  O   SER B 393     -12.203  24.253  18.414  1.00  1.57
O
ATOM  15096  CB  SER B 393     -11.807  26.471  16.361  1.00  2.37
C
ATOM  15097  OG  SER B 393     -12.007  25.752  15.174  1.00  2.37
O
ATOM  15098  H   SER B 393      -9.389  26.405  15.755  1.00  1.97
H
ATOM  15099  HA  SER B 393     -10.715  26.362  18.177  1.00  1.90
H
ATOM  15100 1HB  SER B 393     -12.751  26.561  16.898  1.00  2.84
H
ATOM  15101 2HB  SER B 393     -11.468  27.478  16.123  1.00  2.84
H
ATOM  15102  HG  SER B 393     -11.189  25.834  14.675  1.00  2.84
H
ATOM  15103  N   VAL B 394     -10.678  23.312  17.040  1.00  1.18
N
ATOM  15104  CA  VAL B 394     -11.087  21.951  17.384  1.00  1.06
C
ATOM  15105  C   VAL B 394     -10.761  21.577  18.840  1.00  1.13
C
ATOM  15106  O   VAL B 394     -11.356  20.650  19.389  1.00  1.14
O
ATOM  15107  CB  VAL B 394     -10.410  20.926  16.464  1.00  1.59
C
ATOM  15108  CG1 VAL B 394     -10.759  21.224  15.021  1.00  1.59
C
ATOM  15109  CG2 VAL B 394      -8.918  20.945  16.687  1.00  1.59
C
ATOM  15110  H   VAL B 394      -9.940  23.436  16.351  1.00  1.42
H
ATOM  15111  HA  VAL B 394     -12.158  21.889  17.244  1.00  1.27
H
ATOM  15112  HB  VAL B 394     -10.794  19.931  16.690  1.00  1.91
H
ATOM  15113 1HG1 VAL B 394     -10.285  20.485  14.379  1.00  1.91
H
ATOM  15114 2HG1 VAL B 394     -11.837  21.185  14.888  1.00  1.91
H
ATOM  15115 3HG1 VAL B 394     -10.398  22.217  14.756  1.00  1.91
H
ATOM  15116 1HG2 VAL B 394      -8.445  20.212  16.036  1.00  1.91
H
ATOM  15117 2HG2 VAL B 394      -8.538  21.940  16.459  1.00  1.91
H
ATOM  15118 3HG2 VAL B 394      -8.697  20.701  17.726  1.00  1.91
H
```

ATOM 15119 N   GLY B 395   -9.824 22.297 19.464 1.00 1.24
N
ATOM 15120 CA GLY B 395   -9.482 22.048 20.859 1.00 1.37
C
ATOM 15121 C   GLY B 395   -8.413 20.971 21.081 1.00 1.29
C
ATOM 15122 O   GLY B 395   -7.482 20.810 20.285 1.00 1.27
O
ATOM 15123 H   GLY B 395   -9.353 23.046 18.974 1.00 1.49
H
ATOM 15124 1HA GLY B 395   -9.152 22.979 21.313 1.00 1.64
H
ATOM 15125 2HA GLY B 395   -10.387 21.760 21.392 1.00 1.64
H
ATOM 15126 N   ASP B 396   -8.590 20.241 22.193 1.00 1.45
N
ATOM 15127 CA ASP B 396   -7.702 19.190 22.727 1.00 1.49
C
ATOM 15128 C   ASP B 396   -7.188 18.088 21.799 1.00 1.81
C
ATOM 15129 O   ASP B 396   -6.326 17.312 22.211 1.00 5.72
O
ATOM 15130 CB ASP B 396   -8.397 18.498 23.897 1.00 2.23
C
ATOM 15131 CG ASP B 396   -8.436 19.357 25.143 1.00 2.23
C
ATOM 15132 OD1 ASP B 396   -7.670 20.286 25.223 1.00 2.23
O
ATOM 15133 OD2 ASP B 396   -9.232 19.078 26.006 1.00 2.23
O
ATOM 15134 H   ASP B 396   -9.406 20.462 22.744 1.00 1.74
H
ATOM 15135 HA ASP B 396   -6.828 19.692 23.124 1.00 1.79
H
ATOM 15136 1HB ASP B 396   -9.418 18.244 23.616 1.00 2.68
H
ATOM 15137 2HB ASP B 396   -7.879 17.567 24.132 1.00 2.68
H
ATOM 15138 N   ASP B 397   -7.679 18.002 20.572 1.00 1.53
N
ATOM 15139 CA ASP B 397   -7.196 17.002 19.640 1.00 1.33
C
ATOM 15140 C   ASP B 397   -5.718 17.237 19.299 1.00 1.18
C
ATOM 15141 O   ASP B 397   -4.971 16.287 19.053 1.00 1.25
O
ATOM 15142 CB ASP B 397   -8.028 17.042 18.368 1.00 2.00
C
ATOM 15143 CG ASP B 397   -9.451 16.554 18.575 1.00 2.00
C
ATOM 15144 OD1 ASP B 397   -9.723 15.938 19.578 1.00 2.00
O
ATOM 15145 OD2 ASP B 397   -10.263 16.814 17.722 1.00 2.00
O
ATOM 15146 H   ASP B 397   -8.401 18.637 20.267 1.00 1.84
H
ATOM 15147 HA ASP B 397   -7.293 16.019 20.100 1.00 1.60
H

ATOM 15148 1HB ASP B 397 -8.057 18.060 17.979 1.00 2.39
H
ATOM 15149 2HB ASP B 397 -7.555 16.415 17.615 1.00 2.39
H
ATOM 15150 N GLY B 398 -5.309 18.507 19.287 1.00 1.13
N
ATOM 15151 CA GLY B 398 -3.928 18.872 18.983 1.00 1.06
C
ATOM 15152 C GLY B 398 -3.723 19.118 17.491 1.00 0.99
C
ATOM 15153 O GLY B 398 -4.595 18.808 16.679 1.00 1.31
O
ATOM 15154 H GLY B 398 -5.973 19.242 19.514 1.00 1.36
H
ATOM 15155 1HA GLY B 398 -3.661 19.769 19.540 1.00 1.27
H
ATOM 15156 2HA GLY B 398 -3.262 18.079 19.318 1.00 1.27
H
ATOM 15157 N ALA B 399 -2.543 19.649 17.148 1.00 0.82
N
ATOM 15158 CA ALA B 399 -2.159 20.029 15.780 1.00 0.83
C
ATOM 15159 C ALA B 399 -2.396 18.957 14.737 1.00 0.95
C
ATOM 15160 O ALA B 399 -2.725 19.266 13.592 1.00 0.89
O
ATOM 15161 CB ALA B 399 -0.691 20.395 15.742 1.00 1.24
C
ATOM 15162 H ALA B 399 -1.881 19.839 17.888 1.00 0.98
H
ATOM 15163 HA ALA B 399 -2.751 20.897 15.500 1.00 1.00
H
ATOM 15164 1HB ALA B 399 -0.428 20.717 14.735 1.00 1.49
H
ATOM 15165 2HB ALA B 399 -0.494 21.200 16.441 1.00 1.49
H
ATOM 15166 3HB ALA B 399 -0.093 19.527 16.014 1.00 1.49
H
ATOM 15167 N SER B 400 -2.250 17.696 15.124 1.00 1.29
N
ATOM 15168 CA SER B 400 -2.418 16.601 14.192 1.00 1.74
C
ATOM 15169 C SER B 400 -3.796 16.565 13.538 1.00 1.78
C
ATOM 15170 O SER B 400 -3.923 16.062 12.425 1.00 2.89
O
ATOM 15171 CB SER B 400 -2.196 15.278 14.908 1.00 2.61
C
ATOM 15172 OG SER B 400 -3.229 15.012 15.822 1.00 2.61
O
ATOM 15173 H SER B 400 -1.993 17.494 16.080 1.00 1.55
H
ATOM 15174 HA SER B 400 -1.668 16.709 13.408 1.00 2.09
H
ATOM 15175 1HB SER B 400 -2.142 14.475 14.179 1.00 3.13
H
ATOM 15176 2HB SER B 400 -1.242 15.309 15.434 1.00 3.13
H

ATOM 15177 HG SER B 400 -3.256 15.768 16.417 1.00 3.13
H
ATOM 15178 N GLN B 401 -4.817 17.095 14.214 1.00 1.15
N
ATOM 15179 CA GLN B 401 -6.169 17.066 13.676 1.00 0.85
C
ATOM 15180 C GLN B 401 -6.649 18.464 13.286 1.00 0.99
C
ATOM 15181 O GLN B 401 -7.844 18.669 13.075 1.00 1.71
O
ATOM 15182 CB GLN B 401 -7.126 16.489 14.719 1.00 1.27
C
ATOM 15183 CG GLN B 401 -6.708 15.132 15.264 1.00 1.27
C
ATOM 15184 CD GLN B 401 -6.586 14.048 14.225 1.00 1.27
C
ATOM 15185 OE1 GLN B 401 -7.537 13.701 13.523 1.00 1.27
O
ATOM 15186 NE2 GLN B 401 -5.381 13.503 14.123 1.00 1.27
N
ATOM 15187 H GLN B 401 -4.664 17.530 15.115 1.00 1.38
H
ATOM 15188 HA GLN B 401 -6.180 16.437 12.787 1.00 1.02
H
ATOM 15189 1HB GLN B 401 -7.205 17.182 15.556 1.00 1.53
H
ATOM 15190 2HB GLN B 401 -8.120 16.387 14.283 1.00 1.53
H
ATOM 15191 1HG GLN B 401 -5.741 15.241 15.751 1.00 1.53
H
ATOM 15192 2HG GLN B 401 -7.452 14.812 15.993 1.00 1.53
H
ATOM 15193 1HE2 GLN B 401 -5.209 12.778 13.455 1.00 1.53
H
ATOM 15194 2HE2 GLN B 401 -4.642 13.838 14.713 1.00 1.53
H
ATOM 15195 N MET B 402 -5.726 19.425 13.213 1.00 0.69
N
ATOM 15196 CA MET B 402 -6.064 20.816 12.922 1.00 0.67
C
ATOM 15197 C MET B 402 -5.693 21.212 11.520 1.00 0.70
C
ATOM 15198 O MET B 402 -4.512 21.224 11.181 1.00 1.17
O
ATOM 15199 CB MET B 402 -5.332 21.741 13.894 1.00 1.01
C
ATOM 15200 CG MET B 402 -5.765 21.615 15.334 1.00 1.01
C
ATOM 15201 SD MET B 402 -4.757 22.581 16.462 1.00 1.01
S
ATOM 15202 CE MET B 402 -5.549 22.279 18.035 1.00 1.01
C
ATOM 15203 H MET B 402 -4.750 19.208 13.368 1.00 0.83
H
ATOM 15204 HA MET B 402 -7.141 20.945 13.036 1.00 0.80
H
ATOM 15205 1HB MET B 402 -4.264 21.535 13.851 1.00 1.21
H

```
ATOM  15206 2HB  MET B 402      -5.479  22.778  13.591  1.00  1.21
H
ATOM  15207 1HG  MET B 402      -6.782  21.978  15.401  1.00  1.21
H
ATOM  15208 2HG  MET B 402      -5.752  20.574  15.643  1.00  1.21
H
ATOM  15209 1HE  MET B 402      -5.014  22.814  18.820  1.00  1.21
H
ATOM  15210 2HE  MET B 402      -6.581  22.628  17.996  1.00  1.21
H
ATOM  15211 3HE  MET B 402      -5.538  21.212  18.252  1.00  1.21
H
ATOM  15212  N   ALA B 403      -6.677  21.576  10.716  1.00  0.65
N
ATOM  15213  CA  ALA B 403      -6.344  22.005   9.372  1.00  0.72
C
ATOM  15214  C   ALA B 403      -6.006  23.474   9.361  1.00  1.05
C
ATOM  15215  O   ALA B 403      -6.852  24.322   9.636  1.00  2.34
O
ATOM  15216  CB  ALA B 403      -7.474  21.738   8.433  1.00  1.08
C
ATOM  15217  H   ALA B 403      -7.647  21.535  11.025  1.00  0.78
H
ATOM  15218  HA  ALA B 403      -5.471  21.445   9.044  1.00  0.86
H
ATOM  15219 1HB  ALA B 403      -7.187  22.035   7.427  1.00  1.30
H
ATOM  15220 2HB  ALA B 403      -7.702  20.680   8.451  1.00  1.30
H
ATOM  15221 3HB  ALA B 403      -8.347  22.299   8.734  1.00  1.30
H
ATOM  15222  N   LEU B 404      -4.758  23.774   9.071  1.00  1.21
N
ATOM  15223  CA  LEU B 404      -4.302  25.146   9.029  1.00  1.06
C
ATOM  15224  C   LEU B 404      -3.545  25.413   7.738  1.00  0.96
C
ATOM  15225  O   LEU B 404      -2.965  26.482   7.560  1.00  1.60
O
ATOM  15226  CB  LEU B 404      -3.399  25.428  10.231  1.00  1.59
C
ATOM  15227  CG  LEU B 404      -4.001  25.331  11.636  1.00  1.59
C
ATOM  15228  CD1 LEU B 404      -2.888  25.412  12.659  1.00  1.59
C
ATOM  15229  CD2 LEU B 404      -4.956  26.474  11.858  1.00  1.59
C
ATOM  15230  H   LEU B 404      -4.106  23.030   8.868  1.00  1.45
H
ATOM  15231  HA  LEU B 404      -5.169  25.806   9.068  1.00  1.27
H
ATOM  15232 1HB  LEU B 404      -2.582  24.713  10.195  1.00  1.91
H
ATOM  15233 2HB  LEU B 404      -2.989  26.430  10.126  1.00  1.91
H
ATOM  15234  HG  LEU B 404      -4.521  24.381  11.756  1.00  1.91
H
```

```
ATOM  15235 1HD1 LEU B 404      -3.317  25.352  13.658  1.00  1.91
H
ATOM  15236 2HD1 LEU B 404      -2.191  24.588  12.511  1.00  1.91
H
ATOM  15237 3HD1 LEU B 404      -2.361  26.358  12.548  1.00  1.91
H
ATOM  15238 1HD2 LEU B 404      -5.378  26.408  12.860  1.00  1.91
H
ATOM  15239 2HD2 LEU B 404      -4.424  27.418  11.750  1.00  1.91
H
ATOM  15240 3HD2 LEU B 404      -5.758  26.423  11.122  1.00  1.91
H
ATOM  15241  N   GLU B 405      -3.528  24.431   6.839  1.00  0.79
N
ATOM  15242  CA  GLU B 405      -2.790  24.564   5.592  1.00  0.96
C
ATOM  15243  C   GLU B 405      -3.663  24.301   4.353  1.00  0.58
C
ATOM  15244  O   GLU B 405      -3.198  24.441   3.219  1.00  0.93
O
ATOM  15245  CB  GLU B 405      -1.591  23.604   5.649  1.00  1.44
C
ATOM  15246  CG  GLU B 405      -0.710  23.890   6.864  1.00  1.44
C
ATOM  15247  CD  GLU B 405       0.532  23.076   7.013  1.00  1.44
C
ATOM  15248  OE1 GLU B 405       1.242  22.916   6.059  1.00  1.44
O
ATOM  15249  OE2 GLU B 405       0.791  22.633   8.110  1.00  1.44
O
ATOM  15250  H   GLU B 405      -4.025  23.572   7.018  1.00  0.95
H
ATOM  15251  HA  GLU B 405      -2.414  25.585   5.525  1.00  1.15
H
ATOM  15252 1HB  GLU B 405      -1.939  22.571   5.704  1.00  1.73
H
ATOM  15253 2HB  GLU B 405      -0.985  23.707   4.748  1.00  1.73
H
ATOM  15254 1HG  GLU B 405      -0.428  24.936   6.827  1.00  1.73
H
ATOM  15255 2HG  GLU B 405      -1.312  23.746   7.758  1.00  1.73
H
ATOM  15256  N   ASP B 406      -4.913  23.894   4.573  1.00  0.75
N
ATOM  15257  CA  ASP B 406      -5.763  23.481   3.466  1.00  0.80
C
ATOM  15258  C   ASP B 406      -6.363  24.610   2.621  1.00  0.72
C
ATOM  15259  O   ASP B 406      -6.798  24.348   1.500  1.00  1.16
O
ATOM  15260  CB  ASP B 406      -6.885  22.610   3.990  1.00  1.20
C
ATOM  15261  CG  ASP B 406      -7.750  23.377   4.945  1.00  1.20
C
ATOM  15262  OD1 ASP B 406      -7.244  23.953   5.877  1.00  1.20
O
ATOM  15263  OD2 ASP B 406      -8.931  23.409   4.722  1.00  1.20
O
```

```
ATOM  15264  H   ASP B 406      -5.270  23.833   5.514  1.00  0.90
H
ATOM  15265  HA  ASP B 406      -5.154  22.867   2.805  1.00  0.96
H
ATOM  15266 1HB  ASP B 406      -7.497  22.252   3.161  1.00  1.44
H
ATOM  15267 2HB  ASP B 406      -6.472  21.739   4.500  1.00  1.44
H
ATOM  15268  N   ILE B 407      -6.372  25.860   3.103  1.00  1.10
N
ATOM  15269  CA  ILE B 407      -6.855  26.937   2.239  1.00  1.41
C
ATOM  15270  C   ILE B 407      -5.813  27.155   1.190  1.00  1.18
C
ATOM  15271  O   ILE B 407      -6.132  27.287   0.013  1.00  2.35
O
ATOM  15272  CB  ILE B 407      -7.131  28.279   2.953  1.00  2.11
C
ATOM  15273  CG1 ILE B 407      -8.308  28.155   3.911  1.00  2.11
C
ATOM  15274  CG2 ILE B 407      -7.403  29.358   1.921  1.00  2.11
C
ATOM  15275  CD1 ILE B 407      -8.480  29.351   4.821  1.00  2.11
C
ATOM  15276  H   ILE B 407      -6.018  26.081   4.025  1.00  1.32
H
ATOM  15277  HA  ILE B 407      -7.775  26.617   1.752  1.00  1.69
H
ATOM  15278  HB  ILE B 407      -6.264  28.572   3.535  1.00  2.54
H
ATOM  15279 1HG1 ILE B 407      -9.221  28.027   3.333  1.00  2.54
H
ATOM  15280 2HG1 ILE B 407      -8.165  27.270   4.529  1.00  2.54
H
ATOM  15281 1HG2 ILE B 407      -7.582  30.307   2.426  1.00  2.54
H
ATOM  15282 2HG2 ILE B 407      -6.540  29.455   1.264  1.00  2.54
H
ATOM  15283 3HG2 ILE B 407      -8.280  29.088   1.333  1.00  2.54
H
ATOM  15284 1HD1 ILE B 407      -9.336  29.193   5.475  1.00  2.54
H
ATOM  15285 2HD1 ILE B 407      -7.585  29.492   5.424  1.00  2.54
H
ATOM  15286 3HD1 ILE B 407      -8.645  30.236   4.215  1.00  2.54
H
ATOM  15287  N   ALA B 408      -4.559  27.191   1.624  1.00  1.00
N
ATOM  15288  CA  ALA B 408      -3.450  27.356   0.711  1.00  0.87
C
ATOM  15289  C   ALA B 408      -3.427  26.228  -0.294  1.00  0.86
C
ATOM  15290  O   ALA B 408      -3.286  26.468  -1.492  1.00  1.63
O
ATOM  15291  CB  ALA B 408      -2.149  27.395   1.470  1.00  1.30
C
ATOM  15292  H   ALA B 408      -4.371  27.091   2.611  1.00  1.20
H
```

ATOM 15293 HA ALA B 408 -3.584 28.293 0.172 1.00 1.04
H
ATOM 15294 1HB ALA B 408 -1.322 27.534 0.774 1.00 1.57
H
ATOM 15295 2HB ALA B 408 -2.165 28.217 2.184 1.00 1.57
H
ATOM 15296 3HB ALA B 408 -2.017 26.456 2.004 1.00 1.57
H
ATOM 15297 N MET B 409 -3.591 25.000 0.199 1.00 0.64
N
ATOM 15298 CA MET B 409 -3.581 23.832 -0.659 1.00 0.81
C
ATOM 15299 C MET B 409 -4.649 23.892 -1.723 1.00 0.71
C
ATOM 15300 O MET B 409 -4.364 23.698 -2.904 1.00 1.12
O
ATOM 15301 CB MET B 409 -3.722 22.574 0.173 1.00 1.22
C
ATOM 15302 CG MET B 409 -3.658 21.315 -0.652 1.00 1.22
C
ATOM 15303 SD MET B 409 -3.793 19.824 0.327 1.00 1.22
S
ATOM 15304 CE MET B 409 -5.526 19.799 0.723 1.00 1.22
C
ATOM 15305 H MET B 409 -3.692 24.866 1.199 1.00 0.77
H
ATOM 15306 HA MET B 409 -2.633 23.795 -1.183 1.00 0.97
H
ATOM 15307 1HB MET B 409 -2.933 22.535 0.923 1.00 1.46
H
ATOM 15308 2HB MET B 409 -4.678 22.584 0.696 1.00 1.46
H
ATOM 15309 1HG MET B 409 -4.459 21.319 -1.390 1.00 1.46
H
ATOM 15310 2HG MET B 409 -2.707 21.292 -1.179 1.00 1.46
H
ATOM 15311 1HE MET B 409 -5.748 18.928 1.337 1.00 1.46
H
ATOM 15312 2HE MET B 409 -5.789 20.706 1.269 1.00 1.46
H
ATOM 15313 3HE MET B 409 -6.101 19.749 -0.201 1.00 1.46
H
ATOM 15314 N PHE B 410 -5.882 24.164 -1.323 1.00 0.57
N
ATOM 15315 CA PHE B 410 -6.932 24.238 -2.310 1.00 0.57
C
ATOM 15316 C PHE B 410 -6.848 25.463 -3.223 1.00 0.54
C
ATOM 15317 O PHE B 410 -7.212 25.368 -4.394 1.00 0.74
O
ATOM 15318 CB PHE B 410 -8.302 24.037 -1.679 1.00 0.85
C
ATOM 15319 CG PHE B 410 -8.637 22.577 -1.463 1.00 0.85
C
ATOM 15320 CD1 PHE B 410 -8.384 21.937 -0.260 1.00 0.85
C
ATOM 15321 CD2 PHE B 410 -9.239 21.840 -2.474 1.00 0.85
C

```
ATOM  15322  CE1 PHE B 410      -8.725  20.610  -0.078  1.00  0.85
C
ATOM  15323  CE2 PHE B 410      -9.579  20.514  -2.294  1.00  0.85
C
ATOM  15324  CZ  PHE B 410      -9.322  19.898  -1.091  1.00  0.85
C
ATOM  15325  H   PHE B 410      -6.096  24.307  -0.344  1.00  0.68
H
ATOM  15326  HA  PHE B 410      -6.801  23.380  -2.951  1.00  0.68
H
ATOM  15327 1HB  PHE B 410      -8.339  24.543  -0.716  1.00  1.03
H
ATOM  15328 2HB  PHE B 410      -9.069  24.476  -2.314  1.00  1.03
H
ATOM  15329  HD1 PHE B 410      -7.918  22.491   0.552  1.00  1.03
H
ATOM  15330  HD2 PHE B 410      -9.449  22.324  -3.426  1.00  1.03
H
ATOM  15331  HE1 PHE B 410      -8.522  20.126   0.877  1.00  1.03
H
ATOM  15332  HE2 PHE B 410     -10.052  19.957  -3.102  1.00  1.03
H
ATOM  15333  HZ  PHE B 410      -9.590  18.853  -0.943  1.00  1.03
H
ATOM  15334  N   ARG B 411      -6.309  26.592  -2.738  1.00  0.68
N
ATOM  15335  CA  ARG B 411      -6.148  27.756  -3.615  1.00  1.01
C
ATOM  15336  C   ARG B 411      -5.224  27.468  -4.775  1.00  1.10
C
ATOM  15337  O   ARG B 411      -5.456  27.932  -5.892  1.00  2.40
O
ATOM  15338  CB  ARG B 411      -5.572  28.978  -2.904  1.00  1.52
C
ATOM  15339  CG  ARG B 411      -6.476  29.769  -1.985  1.00  1.52
C
ATOM  15340  CD  ARG B 411      -5.700  30.837  -1.300  1.00  1.52
C
ATOM  15341  NE  ARG B 411      -5.177  31.848  -2.220  1.00  1.52
N
ATOM  15342  CZ  ARG B 411      -4.267  32.790  -1.890  1.00  1.52
C
ATOM  15343  NH1 ARG B 411      -3.790  32.835  -0.676  1.00  1.52
N
ATOM  15344  NH2 ARG B 411      -3.867  33.656  -2.806  1.00  1.52
N
ATOM  15345  H   ARG B 411      -6.029  26.660  -1.770  1.00  0.82
H
ATOM  15346  HA  ARG B 411      -7.126  28.022  -4.012  1.00  1.21
H
ATOM  15347 1HB  ARG B 411      -4.707  28.673  -2.317  1.00  1.82
H
ATOM  15348 2HB  ARG B 411      -5.218  29.682  -3.655  1.00  1.82
H
ATOM  15349 1HG  ARG B 411      -7.269  30.239  -2.566  1.00  1.82
H
ATOM  15350 2HG  ARG B 411      -6.914  29.120  -1.232  1.00  1.82
H
```

ATOM 15351 1HD ARG B 411   -6.328 31.334 -0.561 1.00 1.82
H
ATOM 15352 2HD ARG B 411   -4.855 30.371 -0.804 1.00 1.82
H
ATOM 15353 HE ARG B 411   -5.496 31.848 -3.181 1.00 1.82
H
ATOM 15354 1HH1 ARG B 411   -4.096 32.149   0.003 1.00 1.82
H
ATOM 15355 2HH1 ARG B 411   -3.108 33.536 -0.424 1.00 1.82
H
ATOM 15356 1HH2 ARG B 411   -4.260 33.585 -3.735 1.00 1.82
H
ATOM 15357 2HH2 ARG B 411   -3.172 34.369 -2.599 1.00 1.82
H
ATOM 15358 N   THR B 412   -4.183 26.670 -4.541 1.00 1.11
N
ATOM 15359 CA THR B 412   -3.246 26.362 -5.617 1.00 1.34
C
ATOM 15360 C   THR B 412   -3.795 25.424 -6.689 1.00 1.55
C
ATOM 15361 O   THR B 412   -3.115 25.176 -7.678 1.00 3.63
O
ATOM 15362 CB THR B 412   -1.955 25.743 -5.084 1.00 2.01
C
ATOM 15363 OG1 THR B 412   -2.230 24.502 -4.428 1.00 2.01
O
ATOM 15364 CG2 THR B 412   -1.275 26.675 -4.132 1.00 2.01
C
ATOM 15365 H   THR B 412   -4.011 26.315 -3.605 1.00 1.33
H
ATOM 15366 HA THR B 412   -2.985 27.299 -6.108 1.00 1.61
H
ATOM 15367 HB THR B 412   -1.285 25.564 -5.909 1.00 2.41
H
ATOM 15368 HG1 THR B 412   -2.251 23.799 -5.089 1.00 2.41
H
ATOM 15369 1HG2 THR B 412   -0.356 26.209 -3.790 1.00 2.41
H
ATOM 15370 2HG2 THR B 412   -1.050 27.600 -4.653 1.00 2.41
H
ATOM 15371 3HG2 THR B 412   -1.919 26.883 -3.288 1.00 2.41
H
ATOM 15372 N   ILE B 413   -4.999 24.895 -6.506 1.00 0.89
N
ATOM 15373 CA ILE B 413   -5.572 23.992 -7.484 1.00 1.09
C
ATOM 15374 C   ILE B 413   -6.370 24.757 -8.542 1.00 1.27
C
ATOM 15375 O   ILE B 413   -7.285 25.512 -8.192 1.00 1.63
O
ATOM 15376 CB ILE B 413   -6.481 23.000 -6.781 1.00 1.64
C
ATOM 15377 CG1 ILE B 413   -5.669 22.211 -5.783 1.00 1.64
C
ATOM 15378 CG2 ILE B 413   -7.151 22.065 -7.780 1.00 1.64
C
ATOM 15379 CD1 ILE B 413   -6.547 21.431 -4.878 1.00 1.64
C

```
ATOM  15380  H   ILE B 413      -5.551  25.109  -5.687  1.00  1.07
H
ATOM  15381  HA  ILE B 413      -4.764  23.438  -7.952  1.00  1.31
H
ATOM  15382  HB  ILE B 413      -7.239  23.541  -6.222  1.00  1.96
H
ATOM  15383 1HG1 ILE B 413      -5.012  21.522  -6.314  1.00  1.96
H
ATOM  15384 2HG1 ILE B 413      -5.053  22.885  -5.188  1.00  1.96
H
ATOM  15385 1HG2 ILE B 413      -7.795  21.366  -7.250  1.00  1.96
H
ATOM  15386 2HG2 ILE B 413      -7.746  22.648  -8.477  1.00  1.96
H
ATOM  15387 3HG2 ILE B 413      -6.387  21.512  -8.327  1.00  1.96
H
ATOM  15388 1HD1 ILE B 413      -5.946  20.856  -4.172  1.00  1.96
H
ATOM  15389 2HD1 ILE B 413      -7.203  22.105  -4.341  1.00  1.96
H
ATOM  15390 3HD1 ILE B 413      -7.148  20.774  -5.489  1.00  1.96
H
ATOM  15391  N   PRO B 414      -6.021  24.619  -9.828  1.00  1.54
N
ATOM  15392  CA  PRO B 414      -6.707  25.190 -10.965  1.00  3.43
C
ATOM  15393  C   PRO B 414      -8.129  24.679 -10.951  1.00 12.38
C
ATOM  15394  O   PRO B 414      -8.378  23.512 -10.645  1.00 86.39
O
ATOM  15395  CB  PRO B 414      -5.919  24.684 -12.171  1.00  5.15
C
ATOM  15396  CG  PRO B 414      -4.553  24.439 -11.636  1.00  5.15
C
ATOM  15397  CD  PRO B 414      -4.777  23.936 -10.238  1.00  5.15
C
ATOM  15398  HA  PRO B 414      -6.681  26.287 -10.898  1.00  4.12
H
ATOM  15399 1HB  PRO B 414      -6.390  23.775 -12.574  1.00  6.17
H
ATOM  15400 2HB  PRO B 414      -5.932  25.437 -12.972  1.00  6.17
H
ATOM  15401 1HG  PRO B 414      -4.021  23.711 -12.267  1.00  6.17
H
ATOM  15402 2HG  PRO B 414      -3.970  25.370 -11.660  1.00  6.17
H
ATOM  15403 1HD  PRO B 414      -4.919  22.846 -10.228  1.00  6.17
H
ATOM  15404 2HD  PRO B 414      -3.934  24.245  -9.612  1.00  6.17
H
ATOM  15405  N   LYS B 415      -9.059  25.541 -11.297  1.00  5.12
N
ATOM  15406  CA  LYS B 415     -10.481  25.221 -11.300  1.00  2.56
C
ATOM  15407  C   LYS B 415     -11.026  24.846  -9.916  1.00  1.76
C
ATOM  15408  O   LYS B 415     -12.034  24.146  -9.827  1.00  5.39
O
```

ATOM 15409 CB LYS B 415 -10.776 24.084 -12.288 1.00 3.84
C
ATOM 15410 CG LYS B 415 -10.256 24.323 -13.699 1.00 3.84
C
ATOM 15411 CD LYS B 415 -10.921 25.532 -14.336 1.00 3.84
C
ATOM 15412 CE LYS B 415 -10.438 25.739 -15.764 1.00 3.84
C
ATOM 15413 NZ LYS B 415 -11.020 26.966 -16.373 1.00 3.84
N
ATOM 15414 H LYS B 415 -8.777 26.475 -11.560 1.00 6.14
H
ATOM 15415 HA LYS B 415 -11.023 26.107 -11.633 1.00 3.07
H
ATOM 15416 1HB LYS B 415 -10.352 23.149 -11.925 1.00 4.61
H
ATOM 15417 2HB LYS B 415 -11.855 23.943 -12.358 1.00 4.61
H
ATOM 15418 1HG LYS B 415 -9.178 24.483 -13.674 1.00 4.61
H
ATOM 15419 2HG LYS B 415 -10.458 23.446 -14.312 1.00 4.61
H
ATOM 15420 1HD LYS B 415 -12.002 25.391 -14.345 1.00 4.61
H
ATOM 15421 2HD LYS B 415 -10.693 26.423 -13.752 1.00 4.61
H
ATOM 15422 1HE LYS B 415 -9.353 25.825 -15.765 1.00 4.61
H
ATOM 15423 2HE LYS B 415 -10.722 24.878 -16.367 1.00 4.61
H
ATOM 15424 1HZ LYS B 415 -10.675 27.068 -17.318 1.00 4.61
H
ATOM 15425 2HZ LYS B 415 -12.027 26.891 -16.388 1.00 4.61
H
ATOM 15426 3HZ LYS B 415 -10.751 27.773 -15.828 1.00 4.61
H
ATOM 15427 N CYS B 416 -10.393 25.334 -8.843 1.00 1.60
N
ATOM 15428 CA CYS B 416 -10.907 25.080 -7.504 1.00 1.01
C
ATOM 15429 C CYS B 416 -11.752 26.227 -6.949 1.00 0.74
C
ATOM 15430 O CYS B 416 -11.376 27.395 -7.048 1.00 1.18
O
ATOM 15431 CB CYS B 416 -9.783 24.808 -6.519 1.00 1.52
C
ATOM 15432 SG CYS B 416 -10.357 24.497 -4.834 1.00 1.52
S
ATOM 15433 H CYS B 416 -9.534 25.861 -8.943 1.00 1.92
H
ATOM 15434 HA CYS B 416 -11.522 24.193 -7.560 1.00 1.21
H
ATOM 15435 1HB CYS B 416 -9.217 23.941 -6.851 1.00 1.82
H
ATOM 15436 2HB CYS B 416 -9.105 25.659 -6.490 1.00 1.82
H
ATOM 15437 HG CYS B 416 -10.933 23.330 -5.095 1.00 1.82
H

ATOM 15438 N THR B 417 -12.893 25.875 -6.365 1.00 0.54
N
ATOM 15439 CA THR B 417 -13.762 26.837 -5.695 1.00 0.54
C
ATOM 15440 C THR B 417 -13.738 26.541 -4.204 1.00 0.61
C
ATOM 15441 O THR B 417 -14.051 25.427 -3.788 1.00 0.89
O
ATOM 15442 CB THR B 417 -15.218 26.780 -6.212 1.00 0.81
C
ATOM 15443 OG1 THR B 417 -15.250 27.104 -7.608 1.00 0.81
O
ATOM 15444 CG2 THR B 417 -16.102 27.753 -5.440 1.00 0.81
C
ATOM 15445 H THR B 417 -13.136 24.895 -6.350 1.00 0.65
H
ATOM 15446 HA THR B 417 -13.372 27.838 -5.857 1.00 0.65
H
ATOM 15447 HB THR B 417 -15.605 25.770 -6.080 1.00 0.97
H
ATOM 15448 HG1 THR B 417 -14.720 26.469 -8.096 1.00 0.97
H
ATOM 15449 1HG2 THR B 417 -17.124 27.692 -5.814 1.00 0.97
H
ATOM 15450 2HG2 THR B 417 -16.088 27.497 -4.381 1.00 0.97
H
ATOM 15451 3HG2 THR B 417 -15.728 28.767 -5.572 1.00 0.97
H
ATOM 15452 N ILE B 418 -13.362 27.528 -3.403 1.00 0.64
N
ATOM 15453 CA ILE B 418 -13.278 27.321 -1.968 1.00 0.56
C
ATOM 15454 C ILE B 418 -14.388 28.028 -1.241 1.00 0.68
C
ATOM 15455 O ILE B 418 -14.469 29.255 -1.262 1.00 0.83
O
ATOM 15456 CB ILE B 418 -11.961 27.823 -1.376 1.00 0.84
C
ATOM 15457 CG1 ILE B 418 -10.767 27.135 -2.003 1.00 0.84
C
ATOM 15458 CG2 ILE B 418 -11.963 27.559 0.122 1.00 0.84
C
ATOM 15459 CD1 ILE B 418 -9.475 27.775 -1.578 1.00 0.84
C
ATOM 15460 H ILE B 418 -13.142 28.429 -3.799 1.00 0.77
H
ATOM 15461 HA ILE B 418 -13.363 26.255 -1.765 1.00 0.67
H
ATOM 15462 HB ILE B 418 -11.859 28.890 -1.560 1.00 1.01
H
ATOM 15463 1HG1 ILE B 418 -10.754 26.086 -1.711 1.00 1.01
H
ATOM 15464 2HG1 ILE B 418 -10.843 27.189 -3.089 1.00 1.01
H
ATOM 15465 1HG2 ILE B 418 -11.031 27.922 0.555 1.00 1.01
H
ATOM 15466 2HG2 ILE B 418 -12.803 28.075 0.584 1.00 1.01
H

ATOM 15467 3HG2 ILE B 418 -12.054 26.488 0.303 1.00 1.01
H
ATOM 15468 1HD1 ILE B 418 -8.649 27.255 -2.048 1.00 1.01
H
ATOM 15469 2HD1 ILE B 418 -9.468 28.822 -1.883 1.00 1.01
H
ATOM 15470 3HD1 ILE B 418 -9.375 27.711 -0.495 1.00 1.01
H
ATOM 15471 N PHE B 419 -15.208 27.245 -0.564 1.00 0.76
N
ATOM 15472 CA PHE B 419 -16.289 27.745 0.251 1.00 1.19
C
ATOM 15473 C PHE B 419 -15.867 27.621 1.698 1.00 2.24
C
ATOM 15474 O PHE B 419 -15.326 26.594 2.104 1.00 8.94
O
ATOM 15475 CB PHE B 419 -17.520 26.842 0.130 1.00 1.78
C
ATOM 15476 CG PHE B 419 -18.181 26.748 -1.209 1.00 1.78
C
ATOM 15477 CD1 PHE B 419 -17.761 25.798 -2.130 1.00 1.78
C
ATOM 15478 CD2 PHE B 419 -19.245 27.572 -1.538 1.00 1.78
C
ATOM 15479 CE1 PHE B 419 -18.389 25.677 -3.351 1.00 1.78
C
ATOM 15480 CE2 PHE B 419 -19.875 27.450 -2.759 1.00 1.78
C
ATOM 15481 CZ PHE B 419 -19.446 26.501 -3.664 1.00 1.78
C
ATOM 15482 H PHE B 419 -15.067 26.252 -0.608 1.00 0.91
H
ATOM 15483 HA PHE B 419 -16.508 28.784 0.004 1.00 1.43
H
ATOM 15484 1HB PHE B 419 -17.235 25.836 0.424 1.00 2.14
H
ATOM 15485 2HB PHE B 419 -18.271 27.179 0.843 1.00 2.14
H
ATOM 15486 HD1 PHE B 419 -16.929 25.140 -1.874 1.00 2.14
H
ATOM 15487 HD2 PHE B 419 -19.587 28.315 -0.818 1.00 2.14
H
ATOM 15488 HE1 PHE B 419 -18.053 24.926 -4.065 1.00 2.14
H
ATOM 15489 HE2 PHE B 419 -20.714 28.100 -3.007 1.00 2.14
H
ATOM 15490 HZ PHE B 419 -19.945 26.399 -4.625 1.00 2.14
H
ATOM 15491 N TYR B 420 -16.181 28.617 2.493 1.00 1.00
N
ATOM 15492 CA TYR B 420 -15.917 28.534 3.913 1.00 0.93
C
ATOM 15493 C TYR B 420 -17.089 29.132 4.664 1.00 1.07
C
ATOM 15494 O TYR B 420 -17.065 30.314 5.009 1.00 2.09
O
ATOM 15495 CB TYR B 420 -14.618 29.243 4.277 1.00 1.40
C

ATOM 15496 CG TYR B 420   -14.213 29.032   5.720 1.00 1.40
C
ATOM 15497 CD1 TYR B 420   -14.941 28.169   6.524 1.00 1.40
C
ATOM 15498 CD2 TYR B 420   -13.121 29.700   6.241 1.00 1.40
C
ATOM 15499 CE1 TYR B 420   -14.579 27.972   7.840 1.00 1.40
C
ATOM 15500 CE2 TYR B 420   -12.764 29.516   7.563 1.00 1.40
C
ATOM 15501 CZ TYR B 420   -13.486 28.652   8.358 1.00 1.40
C
ATOM 15502 OH TYR B 420   -13.118 28.459   9.669 1.00 1.40
O
ATOM 15503 H   TYR B 420   -16.607 29.451   2.117 1.00 1.20
H
ATOM 15504 HA TYR B 420   -15.820 27.486   4.197 1.00 1.12
H
ATOM 15505 1HB TYR B 420   -13.813 28.879   3.638 1.00 1.67
H
ATOM 15506 2HB TYR B 420   -14.721 30.314   4.102 1.00 1.67
H
ATOM 15507 HD1 TYR B 420   -15.801 27.639   6.118 1.00 1.67
H
ATOM 15508 HD2 TYR B 420   -12.547 30.380   5.616 1.00 1.67
H
ATOM 15509 HE1 TYR B 420   -15.153 27.289   8.466 1.00 1.67
H
ATOM 15510 HE2 TYR B 420   -11.906 30.048   7.974 1.00 1.67
H
ATOM 15511 HH TYR B 420   -13.643 27.748 10.045 1.00 1.67
H
ATOM 15512 N   PRO B 421   -18.131 28.319   4.899 1.00 1.26
N
ATOM 15513 CA PRO B 421   -19.369 28.607   5.603 1.00 1.39
C
ATOM 15514 C   PRO B 421   -19.121 29.129   6.998 1.00 1.05
C
ATOM 15515 O   PRO B 421   -18.101 28.837   7.625 1.00 2.54
O
ATOM 15516 CB PRO B 421   -20.087 27.260   5.647 1.00 2.08
C
ATOM 15517 CG PRO B 421   -19.576 26.522   4.469 1.00 2.08
C
ATOM 15518 CD PRO B 421   -18.147 26.944   4.348 1.00 2.08
C
ATOM 15519 HA PRO B 421   -19.950 29.336   5.019 1.00 1.67
H
ATOM 15520 1HB PRO B 421   -19.870 26.747   6.596 1.00 2.50
H
ATOM 15521 2HB PRO B 421   -21.173 27.419   5.610 1.00 2.50
H
ATOM 15522 1HG PRO B 421   -19.685 25.439   4.625 1.00 2.50
H
ATOM 15523 2HG PRO B 421   -20.167 26.779   3.580 1.00 2.50
H
ATOM 15524 1HD PRO B 421   -17.501 26.289   4.938 1.00 2.50
H

```
ATOM  15525 2HD  PRO B 421     -17.880  26.939   3.290  1.00  2.50
H
ATOM  15526  N   THR B 422     -20.075  29.905   7.476  1.00  1.44
N
ATOM  15527  CA  THR B 422     -19.999  30.525   8.783  1.00  1.26
C
ATOM  15528  C   THR B 422     -20.994  29.881   9.709  1.00  1.36
C
ATOM  15529  O   THR B 422     -20.671  29.544  10.847  1.00  2.18
O
ATOM  15530  CB  THR B 422     -20.278  32.017   8.682  1.00  1.89
C
ATOM  15531  OG1 THR B 422     -19.308  32.604   7.817  1.00  1.89
O
ATOM  15532  CG2 THR B 422     -20.224  32.675  10.044  1.00  1.89
C
ATOM  15533  H   THR B 422     -20.903  30.056   6.911  1.00  1.73
H
ATOM  15534  HA  THR B 422     -19.000  30.376   9.191  1.00  1.51
H
ATOM  15535  HB  THR B 422     -21.267  32.166   8.248  1.00  2.27
H
ATOM  15536  HG1 THR B 422     -19.733  33.292   7.299  1.00  2.27
H
ATOM  15537 1HG2 THR B 422     -20.423  33.740   9.938  1.00  2.27
H
ATOM  15538 2HG2 THR B 422     -20.976  32.226  10.690  1.00  2.27
H
ATOM  15539 3HG2 THR B 422     -19.236  32.530  10.478  1.00  2.27
H
ATOM  15540  N   ASP B 423     -22.207  29.697   9.209  1.00  1.16
N
ATOM  15541  CA  ASP B 423     -23.247  29.041   9.980  1.00  1.15
C
ATOM  15542  C   ASP B 423     -23.907  27.974   9.123  1.00  1.06
C
ATOM  15543  O   ASP B 423     -23.485  27.737   7.990  1.00  1.04
O
ATOM  15544  CB  ASP B 423     -24.307  30.030  10.459  1.00  1.72
C
ATOM  15545  CG  ASP B 423     -25.093  30.646   9.322  1.00  1.72
C
ATOM  15546  OD1 ASP B 423     -24.902  30.224   8.198  1.00  1.72
O
ATOM  15547  OD2 ASP B 423     -25.886  31.525   9.587  1.00  1.72
O
ATOM  15548  H   ASP B 423     -22.407  29.991   8.265  1.00  1.39
H
ATOM  15549  HA  ASP B 423     -22.795  28.557  10.847  1.00  1.38
H
ATOM  15550 1HB  ASP B 423     -24.997  29.529  11.135  1.00  2.07
H
ATOM  15551 2HB  ASP B 423     -23.828  30.833  11.018  1.00  2.07
H
ATOM  15552  N   ALA B 424     -24.948  27.350   9.661  1.00  1.08
N
ATOM  15553  CA  ALA B 424     -25.656  26.276   8.977  1.00  1.09
C
```

ATOM 15554 C ALA B 424 -26.372 26.704 7.709 1.00 1.02
C
ATOM 15555 O ALA B 424 -26.439 25.926 6.757 1.00 1.06
O
ATOM 15556 CB ALA B 424 -26.652 25.657 9.909 1.00 1.64
C
ATOM 15557 H ALA B 424 -25.246 27.608 10.591 1.00 1.30
H
ATOM 15558 HA ALA B 424 -24.918 25.531 8.698 1.00 1.31
H
ATOM 15559 1HB ALA B 424 -27.135 24.823 9.409 1.00 1.96
H
ATOM 15560 2HB ALA B 424 -26.124 25.296 10.781 1.00 1.96
H
ATOM 15561 3HB ALA B 424 -27.399 26.392 10.203 1.00 1.96
H
ATOM 15562 N VAL B 425 -26.918 27.916 7.691 1.00 0.98
N
ATOM 15563 CA VAL B 425 -27.623 28.407 6.514 1.00 0.99
C
ATOM 15564 C VAL B 425 -26.647 28.560 5.357 1.00 0.95
C
ATOM 15565 O VAL B 425 -26.880 28.035 4.263 1.00 0.95
O
ATOM 15566 CB VAL B 425 -28.324 29.750 6.834 1.00 1.48
C
ATOM 15567 CG1 VAL B 425 -28.889 30.388 5.574 1.00 1.48
C
ATOM 15568 CG2 VAL B 425 -29.445 29.495 7.832 1.00 1.48
C
ATOM 15569 H VAL B 425 -26.839 28.514 8.502 1.00 1.18
H
ATOM 15570 HA VAL B 425 -28.385 27.679 6.234 1.00 1.19
H
ATOM 15571 HB VAL B 425 -27.597 30.440 7.261 1.00 1.78
H
ATOM 15572 1HG1 VAL B 425 -29.370 31.334 5.828 1.00 1.78
H
ATOM 15573 2HG1 VAL B 425 -28.082 30.572 4.868 1.00 1.78
H
ATOM 15574 3HG1 VAL B 425 -29.620 29.722 5.124 1.00 1.78
H
ATOM 15575 1HG2 VAL B 425 -29.937 30.434 8.075 1.00 1.78
H
ATOM 15576 2HG2 VAL B 425 -30.171 28.809 7.396 1.00 1.78
H
ATOM 15577 3HG2 VAL B 425 -29.030 29.057 8.740 1.00 1.78
H
ATOM 15578 N SER B 426 -25.527 29.233 5.623 1.00 0.91
N
ATOM 15579 CA SER B 426 -24.479 29.406 4.630 1.00 0.88
C
ATOM 15580 C SER B 426 -23.889 28.063 4.204 1.00 0.84
C
ATOM 15581 O SER B 426 -23.515 27.891 3.043 1.00 1.05
O
ATOM 15582 CB SER B 426 -23.396 30.306 5.207 1.00 1.32
C

ATOM 15583 OG SER B 426 -22.808 29.703 6.328 1.00 1.32
O
ATOM 15584 H SER B 426 -25.398 29.643 6.542 1.00 1.09
H
ATOM 15585 HA SER B 426 -24.911 29.886 3.751 1.00 1.06
H
ATOM 15586 1HB SER B 426 -22.638 30.499 4.450 1.00 1.58
H
ATOM 15587 2HB SER B 426 -23.829 31.265 5.492 1.00 1.58
H
ATOM 15588 HG SER B 426 -23.518 29.659 6.987 1.00 1.58
H
ATOM 15589 N THR B 427 -23.874 27.097 5.127 1.00 0.78
N
ATOM 15590 CA THR B 427 -23.382 25.762 4.843 1.00 0.74
C
ATOM 15591 C THR B 427 -24.260 25.011 3.856 1.00 0.70
C
ATOM 15592 O THR B 427 -23.744 24.414 2.908 1.00 0.80
O
ATOM 15593 CB THR B 427 -23.267 24.940 6.125 1.00 1.11
C
ATOM 15594 OG1 THR B 427 -22.346 25.560 7.027 1.00 1.11
O
ATOM 15595 CG2 THR B 427 -22.770 23.572 5.775 1.00 1.11
C
ATOM 15596 H THR B 427 -24.167 27.303 6.074 1.00 0.94
H
ATOM 15597 HA THR B 427 -22.391 25.851 4.402 1.00 0.89
H
ATOM 15598 HB THR B 427 -24.238 24.859 6.607 1.00 1.33
H
ATOM 15599 HG1 THR B 427 -22.668 26.438 7.260 1.00 1.33
H
ATOM 15600 1HG2 THR B 427 -22.672 22.976 6.679 1.00 1.33
H
ATOM 15601 2HG2 THR B 427 -23.470 23.091 5.093 1.00 1.33
H
ATOM 15602 3HG2 THR B 427 -21.797 23.664 5.294 1.00 1.33
H
ATOM 15603 N GLU B 428 -25.585 25.042 4.056 1.00 0.77
N
ATOM 15604 CA GLU B 428 -26.460 24.337 3.123 1.00 0.75
C
ATOM 15605 C GLU B 428 -26.399 24.956 1.745 1.00 0.80
C
ATOM 15606 O GLU B 428 -26.425 24.237 0.744 1.00 0.89
O
ATOM 15607 CB GLU B 428 -27.930 24.309 3.568 1.00 1.12
C
ATOM 15608 CG GLU B 428 -28.230 23.440 4.777 1.00 1.12
C
ATOM 15609 CD GLU B 428 -29.699 23.170 4.951 1.00 1.12
C
ATOM 15610 OE1 GLU B 428 -30.479 23.662 4.174 1.00 1.12
O
ATOM 15611 OE2 GLU B 428 -30.042 22.474 5.883 1.00 1.12
O

ATOM 15612 H   GLU B 428   -25.970 25.527   4.857 1.00 0.92
H
ATOM 15613 HA GLU B 428   -26.112 23.308   3.042 1.00 0.90
H
ATOM 15614 1HB GLU B 428   -28.252 25.323   3.805 1.00 1.35
H
ATOM 15615 2HB GLU B 428   -28.550 23.955   2.745 1.00 1.35
H
ATOM 15616 1HG GLU B 428   -27.723 22.493   4.676 1.00 1.35
H
ATOM 15617 2HG GLU B 428   -27.846 23.934   5.668 1.00 1.35
H
ATOM 15618 N   HIS B 429   -26.286 26.281   1.688 1.00 0.87
N
ATOM 15619 CA HIS B 429   -26.191 26.939   0.401 1.00 1.06
C
ATOM 15620 C   HIS B 429   -24.870 26.625 -0.285 1.00 1.17
C
ATOM 15621 O   HIS B 429   -24.843 26.462 -1.503 1.00 1.46
O
ATOM 15622 CB HIS B 429   -26.408 28.441   0.539 1.00 1.59
C
ATOM 15623 CG HIS B 429   -27.845 28.806   0.780 1.00 1.59
C
ATOM 15624 ND1 HIS B 429   -28.809 28.706 -0.202 1.00 1.59
N
ATOM 15625 CD2 HIS B 429   -28.481 29.270   1.881 1.00 1.59
C
ATOM 15626 CE1 HIS B 429   -29.973 29.100   0.287 1.00 1.59
C
ATOM 15627 NE2 HIS B 429   -29.798 29.450   1.546 1.00 1.59
N
ATOM 15628 H   HIS B 429   -26.289 26.838   2.536 1.00 1.04
H
ATOM 15629 HA HIS B 429   -26.982 26.558 -0.245 1.00 1.27
H
ATOM 15630 1HB HIS B 429   -25.811 28.824   1.367 1.00 1.91
H
ATOM 15631 2HB HIS B 429   -26.075 28.941 -0.369 1.00 1.91
H
ATOM 15632 HD1 HIS B 429   -28.636 28.561 -1.176 1.00 1.91
H
ATOM 15633 HD2 HIS B 429   -28.142 29.511   2.885 1.00 1.91
H
ATOM 15634 HE1 HIS B 429   -30.866 29.099 -0.336 1.00 1.91
H
ATOM 15635 N   ALA B 430   -23.791 26.488   0.494 1.00 1.12
N
ATOM 15636 CA ALA B 430   -22.493 26.115 -0.058 1.00 1.50
C
ATOM 15637 C   ALA B 430   -22.559 24.741 -0.710 1.00 0.86
C
ATOM 15638 O   ALA B 430   -22.021 24.550 -1.798 1.00 0.82
O
ATOM 15639 CB ALA B 430   -21.428 26.123   1.030 1.00 2.25
C
ATOM 15640 H   ALA B 430   -23.856 26.668   1.487 1.00 1.34
H

```
ATOM  15641  HA  ALA B 430     -22.226  26.841  -0.824  1.00  1.80
H
ATOM  15642 1HB  ALA B 430     -20.464  25.863   0.597  1.00  2.70
H
ATOM  15643 2HB  ALA B 430     -21.371  27.117   1.474  1.00  2.70
H
ATOM  15644 3HB  ALA B 430     -21.687  25.398   1.799  1.00  2.70
H
ATOM  15645  N   VAL B 431     -23.234  23.795  -0.050  1.00  0.70
N
ATOM  15646  CA  VAL B 431     -23.387  22.445  -0.584  1.00  0.61
C
ATOM  15647  C   VAL B 431     -24.182  22.444  -1.890  1.00  0.53
C
ATOM  15648  O   VAL B 431     -23.796  21.769  -2.847  1.00  0.63
O
ATOM  15649  CB  VAL B 431     -24.056  21.528   0.460  1.00  0.92
C
ATOM  15650  CG1 VAL B 431     -24.406  20.194  -0.163  1.00  0.92
C
ATOM  15651  CG2 VAL B 431     -23.102  21.317   1.629  1.00  0.92
C
ATOM  15652  H   VAL B 431     -23.634  24.012   0.857  1.00  0.84
H
ATOM  15653  HA  VAL B 431     -22.394  22.047  -0.790  1.00  0.73
H
ATOM  15654  HB  VAL B 431     -24.978  21.991   0.811  1.00  1.10
H
ATOM  15655 1HG1 VAL B 431     -24.886  19.556   0.580  1.00  1.10
H
ATOM  15656 2HG1 VAL B 431     -25.089  20.364  -0.992  1.00  1.10
H
ATOM  15657 3HG1 VAL B 431     -23.500  19.709  -0.528  1.00  1.10
H
ATOM  15658 1HG2 VAL B 431     -23.569  20.674   2.371  1.00  1.10
H
ATOM  15659 2HG2 VAL B 431     -22.187  20.847   1.269  1.00  1.10
H
ATOM  15660 3HG2 VAL B 431     -22.861  22.277   2.082  1.00  1.10
H
ATOM  15661  N   ALA B 432     -25.282  23.199  -1.928  1.00  0.57
N
ATOM  15662  CA  ALA B 432     -26.097  23.301  -3.136  1.00  0.61
C
ATOM  15663  C   ALA B 432     -25.305  23.923  -4.296  1.00  0.62
C
ATOM  15664  O   ALA B 432     -25.352  23.428  -5.427  1.00  0.83
O
ATOM  15665  CB  ALA B 432     -27.343  24.123  -2.853  1.00  0.92
C
ATOM  15666  H   ALA B 432     -25.567  23.710  -1.099  1.00  0.68
H
ATOM  15667  HA  ALA B 432     -26.392  22.294  -3.432  1.00  0.73
H
ATOM  15668 1HB  ALA B 432     -27.959  24.170  -3.751  1.00  1.10
H
ATOM  15669 2HB  ALA B 432     -27.911  23.657  -2.048  1.00  1.10
H
```

```
ATOM  15670 3HB  ALA B 432      -27.054  25.130  -2.558  1.00  1.10
H
ATOM  15671  N   LEU B 433      -24.575  25.001  -4.008  1.00  0.61
N
ATOM  15672  CA  LEU B 433      -23.768  25.666  -5.021  1.00  0.64
C
ATOM  15673  C   LEU B 433      -22.642  24.772  -5.509  1.00  0.65
C
ATOM  15674  O   LEU B 433      -22.351  24.746  -6.705  1.00  1.02
O
ATOM  15675  CB  LEU B 433      -23.197  26.972  -4.458  1.00  0.96
C
ATOM  15676  CG  LEU B 433      -24.221  28.092  -4.224  1.00  0.96
C
ATOM  15677  CD1 LEU B 433      -23.566  29.228  -3.466  1.00  0.96
C
ATOM  15678  CD2 LEU B 433      -24.739  28.581  -5.567  1.00  0.96
C
ATOM  15679  H   LEU B 433      -24.583  25.379  -3.070  1.00  0.73
H
ATOM  15680  HA  LEU B 433      -24.409  25.905  -5.868  1.00  0.77
H
ATOM  15681 1HB  LEU B 433      -22.719  26.759  -3.505  1.00  1.15
H
ATOM  15682 2HB  LEU B 433      -22.444  27.349  -5.147  1.00  1.15
H
ATOM  15683  HG  LEU B 433      -25.051  27.714  -3.629  1.00  1.15
H
ATOM  15684 1HD1 LEU B 433      -24.295  30.019  -3.296  1.00  1.15
H
ATOM  15685 2HD1 LEU B 433      -23.206  28.853  -2.511  1.00  1.15
H
ATOM  15686 3HD1 LEU B 433      -22.732  29.622  -4.041  1.00  1.15
H
ATOM  15687 1HD2 LEU B 433      -25.470  29.376  -5.406  1.00  1.15
H
ATOM  15688 2HD2 LEU B 433      -23.910  28.964  -6.161  1.00  1.15
H
ATOM  15689 3HD2 LEU B 433      -25.215  27.755  -6.096  1.00  1.15
H
ATOM  15690  N   ALA B 434      -22.038  24.013  -4.591  1.00  0.50
N
ATOM  15691  CA  ALA B 434      -20.977  23.078  -4.935  1.00  0.50
C
ATOM  15692  C   ALA B 434      -21.495  22.010  -5.884  1.00  0.43
C
ATOM  15693  O   ALA B 434      -20.842  21.687  -6.874  1.00  0.48
O
ATOM  15694  CB  ALA B 434      -20.409  22.435  -3.678  1.00  0.75
C
ATOM  15695  H   ALA B 434      -22.303  24.095  -3.620  1.00  0.60
H
ATOM  15696  HA  ALA B 434      -20.186  23.630  -5.442  1.00  0.60
H
ATOM  15697 1HB  ALA B 434      -19.602  21.755  -3.949  1.00  0.90
H
ATOM  15698 2HB  ALA B 434      -20.023  23.209  -3.015  1.00  0.90
H
```

ATOM 15699 3HB ALA B 434   -21.193 21.880 -3.166 1.00 0.90
H
ATOM 15700 N   ALA B 435   -22.702 21.502 -5.615 1.00 0.40
N
ATOM 15701 CA ALA B 435   -23.327 20.487 -6.459 1.00 0.42
C
ATOM 15702 C   ALA B 435   -23.426 20.933 -7.910 1.00 0.51
C
ATOM 15703 O   ALA B 435   -23.223 20.134 -8.827 1.00 1.86
O
ATOM 15704 CB ALA B 435   -24.720 20.162 -5.944 1.00 0.63
C
ATOM 15705 H   ALA B 435   -23.189 21.798 -4.778 1.00 0.48
H
ATOM 15706 HA ALA B 435   -22.710 19.590 -6.418 1.00 0.50
H
ATOM 15707 1HB ALA B 435   -25.163 19.378 -6.558 1.00 0.76
H
ATOM 15708 2HB ALA B 435   -24.653 19.827 -4.914 1.00 0.76
H
ATOM 15709 3HB ALA B 435   -25.343 21.051 -5.990 1.00 0.76
H
ATOM 15710 N   ASN B 436   -23.738 22.211 -8.119 1.00 0.65
N
ATOM 15711 CA ASN B 436   -23.883 22.737 -9.469 1.00 0.78
C
ATOM 15712 C   ASN B 436   -22.649 23.463 -10.004 1.00 1.32
C
ATOM 15713 O   ASN B 436   -22.719 24.102 -11.055 1.00 7.58
O
ATOM 15714 CB ASN B 436   -25.070 23.662 -9.508 1.00 1.17
C
ATOM 15715 CG ASN B 436   -26.340 22.916 -9.277 1.00 1.17
C
ATOM 15716 OD1 ASN B 436   -26.601 21.888 -9.913 1.00 1.17
O
ATOM 15717 ND2 ASN B 436   -27.125 23.408 -8.358 1.00 1.17
N
ATOM 15718 H   ASN B 436   -23.924 22.815 -7.322 1.00 0.78
H
ATOM 15719 HA ASN B 436   -24.074 21.898 -10.139 1.00 0.94
H
ATOM 15720 1HB ASN B 436   -24.962 24.431 -8.741 1.00 1.40
H
ATOM 15721 2HB ASN B 436   -25.118 24.161 -10.475 1.00 1.40
H
ATOM 15722 1HD2 ASN B 436   -27.983 22.946 -8.130 1.00 1.40
H
ATOM 15723 2HD2 ASN B 436   -26.862 24.236 -7.865 1.00 1.40
H
ATOM 15724 N   ALA B 437   -21.524 23.368 -9.305 1.00 0.88
N
ATOM 15725 CA ALA B 437   -20.320 24.044 -9.755 1.00 0.67
C
ATOM 15726 C   ALA B 437   -19.366 23.038 -10.374 1.00 0.70
C
ATOM 15727 O   ALA B 437   -19.256 21.903 -9.913 1.00 1.82
O

ATOM 15728 CB ALA B 437 -19.663 24.777 -8.598 1.00 1.01
C
ATOM 15729 H ALA B 437 -21.478 22.822 -8.457 1.00 1.06
H
ATOM 15730 HA ALA B 437 -20.592 24.766 -10.524 1.00 0.80
H
ATOM 15731 1HB ALA B 437 -18.768 25.290 -8.949 1.00 1.21
H
ATOM 15732 2HB ALA B 437 -20.362 25.505 -8.188 1.00 1.21
H
ATOM 15733 3HB ALA B 437 -19.394 24.061 -7.826 1.00 1.21
H
ATOM 15734 N LYS B 438 -18.690 23.454 -11.435 1.00 0.61
N
ATOM 15735 CA LYS B 438 -17.736 22.592 -12.115 1.00 0.87
C
ATOM 15736 C LYS B 438 -16.353 22.747 -11.507 1.00 3.31
C
ATOM 15737 O LYS B 438 -16.034 23.787 -10.935 1.00 28.68
O
ATOM 15738 CB LYS B 438 -17.698 22.909 -13.608 1.00 1.30
C
ATOM 15739 CG LYS B 438 -18.994 22.599 -14.344 1.00 1.30
C
ATOM 15740 CD LYS B 438 -18.875 22.910 -15.827 1.00 1.30
C
ATOM 15741 CE LYS B 438 -20.170 22.604 -16.565 1.00 1.30
C
ATOM 15742 NZ LYS B 438 -20.069 22.918 -18.015 1.00 1.30
N
ATOM 15743 H LYS B 438 -18.833 24.395 -11.773 1.00 0.73
H
ATOM 15744 HA LYS B 438 -18.046 21.555 -11.986 1.00 1.04
H
ATOM 15745 1HB LYS B 438 -17.474 23.966 -13.753 1.00 1.57
H
ATOM 15746 2HB LYS B 438 -16.900 22.336 -14.082 1.00 1.57
H
ATOM 15747 1HG LYS B 438 -19.238 21.544 -14.221 1.00 1.57
H
ATOM 15748 2HG LYS B 438 -19.804 23.193 -13.920 1.00 1.57
H
ATOM 15749 1HD LYS B 438 -18.631 23.966 -15.959 1.00 1.57
H
ATOM 15750 2HD LYS B 438 -18.072 22.313 -16.260 1.00 1.57
H
ATOM 15751 1HE LYS B 438 -20.406 21.547 -16.448 1.00 1.57
H
ATOM 15752 2HE LYS B 438 -20.977 23.194 -16.131 1.00 1.57
H
ATOM 15753 1HZ LYS B 438 -20.946 22.703 -18.469 1.00 1.57
H
ATOM 15754 2HZ LYS B 438 -19.862 23.901 -18.133 1.00 1.57
H
ATOM 15755 3HZ LYS B 438 -19.331 22.367 -18.430 1.00 1.57
H
ATOM 15756 N GLY B 439 -15.531 21.720 -11.632 1.00 2.57
N

ATOM 15757 CA GLY B 439 -14.180 21.774 -11.092 1.00 0.83
C
ATOM 15758 C GLY B 439 -14.098 21.162 -9.701 1.00 1.23
C
ATOM 15759 O GLY B 439 -14.931 20.344 -9.316 1.00 4.96
O
ATOM 15760 H GLY B 439 -15.848 20.890 -12.119 1.00 3.08
H
ATOM 15761 1HA GLY B 439 -13.504 21.245 -11.764 1.00 1.00
H
ATOM 15762 2HA GLY B 439 -13.846 22.809 -11.057 1.00 1.00
H
ATOM 15763 N MET B 440 -13.079 21.553 -8.953 1.00 0.71
N
ATOM 15764 CA MET B 440 -12.859 20.995 -7.629 1.00 0.77
C
ATOM 15765 C MET B 440 -13.473 21.907 -6.584 1.00 0.91
C
ATOM 15766 O MET B 440 -12.985 23.007 -6.354 1.00 2.52
O
ATOM 15767 CB MET B 440 -11.360 20.804 -7.392 1.00 1.16
C
ATOM 15768 CG MET B 440 -11.005 20.146 -6.071 1.00 1.16
C
ATOM 15769 SD MET B 440 -11.639 18.460 -5.982 1.00 1.16
S
ATOM 15770 CE MET B 440 -10.642 17.609 -7.206 1.00 1.16
C
ATOM 15771 H MET B 440 -12.455 22.266 -9.307 1.00 0.85
H
ATOM 15772 HA MET B 440 -13.355 20.027 -7.567 1.00 0.92
H
ATOM 15773 1HB MET B 440 -10.944 20.192 -8.191 1.00 1.39
H
ATOM 15774 2HB MET B 440 -10.860 21.772 -7.429 1.00 1.39
H
ATOM 15775 1HG MET B 440 -9.922 20.117 -5.953 1.00 1.39
H
ATOM 15776 2HG MET B 440 -11.427 20.720 -5.247 1.00 1.39
H
ATOM 15777 1HE MET B 440 -10.932 16.558 -7.246 1.00 1.39
H
ATOM 15778 2HE MET B 440 -10.800 18.066 -8.183 1.00 1.39
H
ATOM 15779 3HE MET B 440 -9.588 17.683 -6.941 1.00 1.39
H
ATOM 15780 N CYS B 441 -14.559 21.472 -5.967 1.00 0.35
N
ATOM 15781 CA CYS B 441 -15.206 22.313 -4.973 1.00 0.45
C
ATOM 15782 C CYS B 441 -14.799 21.876 -3.594 1.00 0.50
C
ATOM 15783 O CYS B 441 -14.815 20.690 -3.286 1.00 1.66
O
ATOM 15784 CB CYS B 441 -16.712 22.242 -5.096 1.00 0.68
C
ATOM 15785 SG CYS B 441 -17.338 22.835 -6.678 1.00 0.68
S

```
ATOM  15786  H   CYS B 441     -14.921  20.547  -6.168  1.00  0.42
H
ATOM  15787  HA  CYS B 441     -14.891  23.345  -5.124  1.00  0.54
H
ATOM  15788 1HB  CYS B 441     -17.029  21.213  -4.963  1.00  0.81
H
ATOM  15789 2HB  CYS B 441     -17.171  22.833  -4.305  1.00  0.81
H
ATOM  15790  HG  CYS B 441     -16.821  21.852  -7.409  1.00  0.81
H
ATOM  15791  N   PHE B 442     -14.467  22.835  -2.754  1.00  0.46
N
ATOM  15792  CA  PHE B 442     -14.015  22.542  -1.415  1.00  0.50
C
ATOM  15793  C   PHE B 442     -14.772  23.315  -0.358  1.00  0.53
C
ATOM  15794  O   PHE B 442     -14.813  24.536  -0.397  1.00  0.85
O
ATOM  15795  CB  PHE B 442     -12.535  22.834  -1.303  1.00  0.75
C
ATOM  15796  CG  PHE B 442     -11.995  22.574   0.059  1.00  0.75
C
ATOM  15797  CD1 PHE B 442     -12.240  21.367   0.688  1.00  0.75
C
ATOM  15798  CD2 PHE B 442     -11.228  23.523   0.705  1.00  0.75
C
ATOM  15799  CE1 PHE B 442     -11.742  21.116   1.944  1.00  0.75
C
ATOM  15800  CE2 PHE B 442     -10.710  23.263   1.952  1.00  0.75
C
ATOM  15801  CZ  PHE B 442     -10.973  22.059   2.577  1.00  0.75
C
ATOM  15802  H   PHE B 442     -14.486  23.792  -3.068  1.00  0.55
H
ATOM  15803  HA  PHE B 442     -14.163  21.483  -1.235  1.00  0.60
H
ATOM  15804 1HB  PHE B 442     -11.993  22.214  -2.013  1.00  0.90
H
ATOM  15805 2HB  PHE B 442     -12.342  23.874  -1.559  1.00  0.90
H
ATOM  15806  HD1 PHE B 442     -12.838  20.617   0.178  1.00  0.90
H
ATOM  15807  HD2 PHE B 442     -11.024  24.473   0.213  1.00  0.90
H
ATOM  15808  HE1 PHE B 442     -11.955  20.169   2.431  1.00  0.90
H
ATOM  15809  HE2 PHE B 442     -10.097  24.011   2.454  1.00  0.90
H
ATOM  15810  HZ  PHE B 442     -10.569  21.859   3.569  1.00  0.90
H
ATOM  15811  N   ILE B 443     -15.384  22.595   0.576  1.00  0.39
N
ATOM  15812  CA  ILE B 443     -16.121  23.220   1.667  1.00  0.49
C
ATOM  15813  C   ILE B 443     -15.408  23.002   3.000  1.00  0.48
C
ATOM  15814  O   ILE B 443     -15.165  21.865   3.403  1.00  0.76
O
```

```
ATOM  15815  CB  ILE B 443     -17.558  22.676   1.746  1.00  0.73
C
ATOM  15816  CG1 ILE B 443     -18.309  22.974   0.441  1.00  0.73
C
ATOM  15817  CG2 ILE B 443     -18.287  23.282   2.934  1.00  0.73
C
ATOM  15818  CD1 ILE B 443     -19.648  22.286   0.337  1.00  0.73
C
ATOM  15819  H   ILE B 443     -15.321  21.586   0.535  1.00  0.47
H
ATOM  15820  HA  ILE B 443     -16.172  24.290   1.482  1.00  0.59
H
ATOM  15821  HB  ILE B 443     -17.521  21.598   1.864  1.00  0.88
H
ATOM  15822 1HG1 ILE B 443     -18.473  24.046   0.364  1.00  0.88
H
ATOM  15823 2HG1 ILE B 443     -17.696  22.657  -0.402  1.00  0.88
H
ATOM  15824 1HG2 ILE B 443     -19.297  22.876   2.988  1.00  0.88
H
ATOM  15825 2HG2 ILE B 443     -17.751  23.041   3.851  1.00  0.88
H
ATOM  15826 3HG2 ILE B 443     -18.335  24.363   2.814  1.00  0.88
H
ATOM  15827 1HD1 ILE B 443     -20.118  22.547  -0.611  1.00  0.88
H
ATOM  15828 2HD1 ILE B 443     -19.509  21.206   0.389  1.00  0.88
H
ATOM  15829 3HD1 ILE B 443     -20.287  22.608   1.159  1.00  0.88
H
ATOM  15830  N   ARG B 444     -15.079  24.100   3.684  1.00  0.76
N
ATOM  15831  CA  ARG B 444     -14.392  24.012   4.972  1.00  0.99
C
ATOM  15832  C   ARG B 444     -15.379  24.071   6.120  1.00  1.18
C
ATOM  15833  O   ARG B 444     -16.084  25.065   6.285  1.00  2.77
O
ATOM  15834  CB  ARG B 444     -13.408  25.155   5.146  1.00  1.48
C
ATOM  15835  CG  ARG B 444     -12.340  25.197   4.098  1.00  1.48
C
ATOM  15836  CD  ARG B 444     -11.289  26.208   4.344  1.00  1.48
C
ATOM  15837  NE  ARG B 444     -10.284  25.733   5.290  1.00  1.48
N
ATOM  15838  CZ  ARG B 444     -10.080  26.182   6.540  1.00  1.48
C
ATOM  15839  NH1 ARG B 444     -10.839  27.126   7.022  1.00  1.48
N
ATOM  15840  NH2 ARG B 444      -9.107  25.676   7.277  1.00  1.48
N
ATOM  15841  H   ARG B 444     -15.287  25.009   3.291  1.00  0.91
H
ATOM  15842  HA  ARG B 444     -13.852  23.066   5.019  1.00  1.19
H
ATOM  15843 1HB  ARG B 444     -13.939  26.100   5.097  1.00  1.78
H
```

```
ATOM  15844 2HB  ARG B 444     -12.927  25.090   6.121  1.00  1.78
H
ATOM  15845 1HG  ARG B 444     -11.855  24.226   4.110  1.00  1.78
H
ATOM  15846 2HG  ARG B 444     -12.783  25.376   3.118  1.00  1.78
H
ATOM  15847 1HD  ARG B 444     -10.790  26.441   3.405  1.00  1.78
H
ATOM  15848 2HD  ARG B 444     -11.740  27.112   4.750  1.00  1.78
H
ATOM  15849  HE  ARG B 444      -9.684  24.982   4.979  1.00  1.78
H
ATOM  15850 1HH1 ARG B 444     -11.580  27.505   6.453  1.00  1.78
H
ATOM  15851 2HH1 ARG B 444     -10.690  27.468   7.959  1.00  1.78
H
ATOM  15852 1HH2 ARG B 444      -8.504  24.956   6.893  1.00  1.78
H
ATOM  15853 2HH2 ARG B 444      -8.949  26.015   8.214  1.00  1.78
H
ATOM  15854  N   THR B 445     -15.413  23.026   6.941  1.00  1.07
N
ATOM  15855  CA  THR B 445     -16.341  22.998   8.061  1.00  0.99
C
ATOM  15856  C   THR B 445     -15.617  23.011   9.397  1.00  0.87
C
ATOM  15857  O   THR B 445     -14.417  22.727   9.478  1.00  0.90
O
ATOM  15858  CB  THR B 445     -17.262  21.780   7.982  1.00  1.48
C
ATOM  15859  OG1 THR B 445     -16.504  20.581   8.098  1.00  1.48
O
ATOM  15860  CG2 THR B 445     -17.961  21.763   6.659  1.00  1.48
C
ATOM  15861  H   THR B 445     -14.810  22.234   6.776  1.00  1.28
H
ATOM  15862  HA  THR B 445     -16.965  23.891   8.014  1.00  1.19
H
ATOM  15863  HB  THR B 445     -17.996  21.820   8.782  1.00  1.78
H
ATOM  15864  HG1 THR B 445     -15.869  20.541   7.376  1.00  1.78
H
ATOM  15865 1HG2 THR B 445     -18.599  20.885   6.620  1.00  1.78
H
ATOM  15866 2HG2 THR B 445     -18.560  22.666   6.547  1.00  1.78
H
ATOM  15867 3HG2 THR B 445     -17.225  21.713   5.858  1.00  1.78
H
ATOM  15868  N   THR B 446     -16.371  23.360  10.437  1.00  0.77
N
ATOM  15869  CA  THR B 446     -15.823  23.570  11.766  1.00  0.74
C
ATOM  15870  C   THR B 446     -16.403  22.675  12.854  1.00  0.73
C
ATOM  15871  O   THR B 446     -17.544  22.202  12.786  1.00  0.76
O
ATOM  15872  CB  THR B 446     -16.051  25.036  12.204  1.00  1.11
C
```

ATOM 15873 OG1 THR B 446   -17.463 25.298 12.278 1.00 1.11
O
ATOM 15874 CG2 THR B 446   -15.413 26.004 11.222 1.00 1.11
C
ATOM 15875 H   THR B 446   -17.352 23.520 10.276 1.00 0.92
H
ATOM 15876 HA THR B 446   -14.750 23.386 11.721 1.00 0.89
H
ATOM 15877 HB THR B 446   -15.611 25.191 13.187 1.00 1.33
H
ATOM 15878 HG1 THR B 446   -17.610 26.146 12.708 1.00 1.33
H
ATOM 15879 1HG2 THR B 446   -15.587 27.027 11.556 1.00 1.33
H
ATOM 15880 2HG2 THR B 446   -14.341 25.815 11.170 1.00 1.33
H
ATOM 15881 3HG2 THR B 446   -15.853 25.868 10.234 1.00 1.33
H
ATOM 15882 N   ARG B 447   -15.581 22.494 13.886 1.00 0.80
N
ATOM 15883 CA ARG B 447   -15.872 21.680 15.057 1.00 0.85
C
ATOM 15884 C   ARG B 447   -16.930 22.245 16.025 1.00 1.08
C
ATOM 15885 O   ARG B 447   -17.933 21.570 16.273 1.00 1.29
O
ATOM 15886 CB ARG B 447   -14.557 21.398 15.796 1.00 1.27
C
ATOM 15887 CG ARG B 447   -14.628 20.654 17.129 1.00 1.27
C
ATOM 15888 CD ARG B 447   -15.054 19.252 16.982 1.00 1.27
C
ATOM 15889 NE ARG B 447   -14.944 18.516 18.232 1.00 1.27
N
ATOM 15890 CZ ARG B 447   -13.836 17.840 18.597 1.00 1.27
C
ATOM 15891 NH1 ARG B 447   -12.805 17.833 17.788 1.00 1.27
N
ATOM 15892 NH2 ARG B 447   -13.783 17.189 19.746 1.00 1.27
N
ATOM 15893 H   ARG B 447   -14.678 22.944 13.846 1.00 0.96
H
ATOM 15894 HA ARG B 447   -16.230 20.726 14.706 1.00 1.02
H
ATOM 15895 1HB ARG B 447   -13.953 20.763 15.150 1.00 1.53
H
ATOM 15896 2HB ARG B 447   -13.983 22.307 15.941 1.00 1.53
H
ATOM 15897 1HG ARG B 447   -13.654 20.644 17.612 1.00 1.53
H
ATOM 15898 2HG ARG B 447   -15.343 21.157 17.781 1.00 1.53
H
ATOM 15899 1HD ARG B 447   -16.088 19.237 16.675 1.00 1.53
H
ATOM 15900 2HD ARG B 447   -14.439 18.754 16.234 1.00 1.53
H
ATOM 15901 HE ARG B 447   -15.734 18.519 18.865 1.00 1.53
H

ATOM 15902 1HH1 ARG B 447   -12.850 18.334 16.915 1.00 1.53
H
ATOM 15903 2HH1 ARG B 447   -11.958 17.330 18.026 1.00 1.53
H
ATOM 15904 1HH2 ARG B 447   -14.577 17.196 20.369 1.00 1.53
H
ATOM 15905 2HH2 ARG B 447   -12.943 16.689 20.000 1.00 1.53
H
ATOM 15906 N   PRO B 448   -16.772 23.470 16.566 1.00 1.27
N
ATOM 15907 CA PRO B 448   -17.646 24.070 17.561 1.00 1.44
C
ATOM 15908 C   PRO B 448   -18.988 24.472 16.991 1.00 1.51
C
ATOM 15909 O   PRO B 448   -19.102 24.819 15.816 1.00 1.54
O
ATOM 15910 CB PRO B 448   -16.866 25.303 18.029 1.00 2.16
C
ATOM 15911 CG PRO B 448   -16.019 25.677 16.867 1.00 2.16
C
ATOM 15912 CD PRO B 448   -15.637 24.370 16.240 1.00 2.16
C
ATOM 15913 HA PRO B 448   -17.782 23.360 18.391 1.00 1.73
H
ATOM 15914 1HB PRO B 448   -17.565 26.101 18.320 1.00 2.59
H
ATOM 15915 2HB PRO B 448   -16.274 25.055 18.922 1.00 2.59
H
ATOM 15916 1HG PRO B 448   -16.583 26.325 16.181 1.00 2.59
H
ATOM 15917 2HG PRO B 448   -15.147 26.255 17.205 1.00 2.59
H
ATOM 15918 1HD PRO B 448   -15.534 24.525 15.165 1.00 2.59
H
ATOM 15919 2HD PRO B 448   -14.709 24.011 16.704 1.00 2.59
H
ATOM 15920 N   GLU B 449   -20.000 24.448 17.849 1.00 1.59
N
ATOM 15921 CA GLU B 449   -21.321 24.880 17.449 1.00 1.68
C
ATOM 15922 C   GLU B 449   -21.369 26.386 17.511 1.00 1.84
C
ATOM 15923 O   GLU B 449   -20.949 26.983 18.506 1.00 2.08
O
ATOM 15924 CB GLU B 449   -22.402 24.278 18.348 1.00 2.52
C
ATOM 15925 CG GLU B 449   -22.558 22.767 18.241 1.00 2.52
C
ATOM 15926 CD GLU B 449   -23.654 22.243 19.123 1.00 2.52
C
ATOM 15927 OE1 GLU B 449   -24.093 22.969 19.981 1.00 2.52
O
ATOM 15928 OE2 GLU B 449   -24.063 21.120 18.936 1.00 2.52
O
ATOM 15929 H   GLU B 449   -19.844 24.137 18.798 1.00 1.91
H
ATOM 15930 HA GLU B 449   -21.492 24.575 16.423 1.00 2.02
H

ATOM 15931 1HB GLU B 449 -22.181 24.516 19.388 1.00 3.02
H
ATOM 15932 2HB GLU B 449 -23.364 24.725 18.108 1.00 3.02
H
ATOM 15933 1HG GLU B 449 -22.775 22.498 17.211 1.00 3.02
H
ATOM 15934 2HG GLU B 449 -21.617 22.296 18.521 1.00 3.02
H
ATOM 15935 N THR B 450 -21.878 27.005 16.457 1.00 1.72
N
ATOM 15936 CA THR B 450 -21.916 28.452 16.418 1.00 1.69
C
ATOM 15937 C THR B 450 -23.308 28.936 16.071 1.00 1.60
C
ATOM 15938 O THR B 450 -24.127 28.184 15.550 1.00 1.60
O
ATOM 15939 CB THR B 450 -20.906 28.992 15.402 1.00 2.54
C
ATOM 15940 OG1 THR B 450 -21.289 28.587 14.076 1.00 2.54
O
ATOM 15941 CG2 THR B 450 -19.500 28.481 15.695 1.00 2.54
C
ATOM 15942 H THR B 450 -22.227 26.473 15.668 1.00 2.06
H
ATOM 15943 HA THR B 450 -21.657 28.838 17.403 1.00 2.03
H
ATOM 15944 HB THR B 450 -20.896 30.072 15.470 1.00 3.04
H
ATOM 15945 HG1 THR B 450 -21.770 29.301 13.638 1.00 3.04
H
ATOM 15946 1HG2 THR B 450 -18.806 28.897 14.963 1.00 3.04
H
ATOM 15947 2HG2 THR B 450 -19.200 28.790 16.696 1.00 3.04
H
ATOM 15948 3HG2 THR B 450 -19.482 27.395 15.630 1.00 3.04
H
ATOM 15949 N MET B 451 -23.585 30.192 16.377 1.00 1.65
N
ATOM 15950 CA MET B 451 -24.923 30.729 16.187 1.00 1.67
C
ATOM 15951 C MET B 451 -25.322 30.910 14.728 1.00 1.72
C
ATOM 15952 O MET B 451 -24.518 31.306 13.888 1.00 1.83
O
ATOM 15953 CB MET B 451 -25.028 32.062 16.919 1.00 2.50
C
ATOM 15954 CG MET B 451 -24.975 31.937 18.435 1.00 2.50
C
ATOM 15955 SD MET B 451 -24.966 33.531 19.280 1.00 2.50
S
ATOM 15956 CE MET B 451 -26.636 34.100 18.998 1.00 2.50
C
ATOM 15957 H MET B 451 -22.866 30.777 16.777 1.00 1.98
H
ATOM 15958 HA MET B 451 -25.625 30.035 16.639 1.00 2.00
H
ATOM 15959 1HB MET B 451 -24.213 32.716 16.607 1.00 3.01
H

ATOM 15960 2HB MET B 451   -25.964 32.554 16.655 1.00 3.01
H
ATOM 15961 1HG MET B 451   -25.841 31.372 18.779 1.00 3.01
H
ATOM 15962 2HG MET B 451   -24.077 31.391 18.721 1.00 3.01
H
ATOM 15963 1HE MET B 451   -26.774 35.074 19.469 1.00 3.01
H
ATOM 15964 2HE MET B 451   -26.814 34.185 17.926 1.00 3.01
H
ATOM 15965 3HE MET B 451   -27.341 33.386 19.428 1.00 3.01
H
ATOM 15966 N   VAL B 452   -26.589 30.665 14.428 1.00 1.72
N
ATOM 15967 CA VAL B 452   -27.082 30.932 13.091 1.00 1.71
C
ATOM 15968 C   VAL B 452   -27.214 32.431 12.968 1.00 1.74
C
ATOM 15969 O   VAL B 452   -27.817 33.073 13.825 1.00 1.80
O
ATOM 15970 CB VAL B 452   -28.425 30.229 12.833 1.00 2.56
C
ATOM 15971 CG1 VAL B 452   -28.980 30.627 11.472 1.00 2.56
C
ATOM 15972 CG2 VAL B 452   -28.220 28.725 12.890 1.00 2.56
C
ATOM 15973 H   VAL B 452   -27.210 30.289 15.132 1.00 2.06
H
ATOM 15974 HA VAL B 452   -26.357 30.579 12.362 1.00 2.05
H
ATOM 15975 HB VAL B 452   -29.137 30.534 13.596 1.00 3.08
H
ATOM 15976 1HG1 VAL B 452   -29.935 30.126 11.307 1.00 3.08
H
ATOM 15977 2HG1 VAL B 452   -29.129 31.707 11.437 1.00 3.08
H
ATOM 15978 3HG1 VAL B 452   -28.278 30.333 10.691 1.00 3.08
H
ATOM 15979 1HG2 VAL B 452   -29.169 28.226 12.727 1.00 3.08
H
ATOM 15980 2HG2 VAL B 452   -27.511 28.425 12.119 1.00 3.08
H
ATOM 15981 3HG2 VAL B 452   -27.830 28.444 13.865 1.00 3.08
H
ATOM 15982 N   ILE B 453   -26.622 33.000 11.929 1.00 1.74
N
ATOM 15983 CA ILE B 453   -26.640 34.449 11.774 1.00 1.78
C
ATOM 15984 C   ILE B 453   -27.328 34.883 10.495 1.00 1.95
C
ATOM 15985 O   ILE B 453   -27.738 36.037 10.373 1.00 2.15
O
ATOM 15986 CB ILE B 453   -25.203 35.018 11.831 1.00 2.67
C
ATOM 15987 CG1 ILE B 453   -24.361 34.448 10.690 1.00 2.67
C
ATOM 15988 CG2 ILE B 453   -24.556 34.732 13.177 1.00 2.67
C

```
ATOM  15989  CD1 ILE B 453     -23.012  35.105  10.557  1.00  2.67
C
ATOM  15990  H   ILE B 453     -26.158  32.424  11.234  1.00  2.09
H
ATOM  15991  HA  ILE B 453     -27.195  34.873  12.609  1.00  2.14
H
ATOM  15992  HB  ILE B 453     -25.246  36.097  11.687  1.00  3.20
H
ATOM  15993 1HG1 ILE B 453     -24.216  33.379  10.847  1.00  3.20
H
ATOM  15994 2HG1 ILE B 453     -24.896  34.586   9.753  1.00  3.20
H
ATOM  15995 1HG2 ILE B 453     -23.554  35.160  13.198  1.00  3.20
H
ATOM  15996 2HG2 ILE B 453     -25.155  35.175  13.971  1.00  3.20
H
ATOM  15997 3HG2 ILE B 453     -24.491  33.657  13.328  1.00  3.20
H
ATOM  15998 1HD1 ILE B 453     -22.473  34.661   9.721  1.00  3.20
H
ATOM  15999 2HD1 ILE B 453     -23.147  36.169  10.379  1.00  3.20
H
ATOM  16000 3HD1 ILE B 453     -22.445  34.960  11.475  1.00  3.20
H
ATOM  16001  N   TYR B 454     -27.495  33.958   9.555  1.00  2.00
N
ATOM  16002  CA  TYR B 454     -28.216  34.286   8.344  1.00  2.33
C
ATOM  16003  C   TYR B 454     -29.645  33.794   8.378  1.00  2.27
C
ATOM  16004  O   TYR B 454     -29.940  32.726   8.914  1.00  2.23
O
ATOM  16005  CB  TYR B 454     -27.541  33.691   7.120  1.00  3.50
C
ATOM  16006  CG  TYR B 454     -26.218  34.306   6.779  1.00  3.50
C
ATOM  16007  CD1 TYR B 454     -25.045  33.634   7.068  1.00  3.50
C
ATOM  16008  CD2 TYR B 454     -26.181  35.545   6.169  1.00  3.50
C
ATOM  16009  CE1 TYR B 454     -23.833  34.188   6.734  1.00  3.50
C
ATOM  16010  CE2 TYR B 454     -24.971  36.103   5.837  1.00  3.50
C
ATOM  16011  CZ  TYR B 454     -23.806  35.425   6.112  1.00  3.50
C
ATOM  16012  OH  TYR B 454     -22.603  35.979   5.775  1.00  3.50
O
ATOM  16013  H   TYR B 454     -27.108  33.026   9.664  1.00  2.40
H
ATOM  16014  HA  TYR B 454     -28.244  35.369   8.239  1.00  2.80
H
ATOM  16015 1HB  TYR B 454     -27.382  32.625   7.283  1.00  4.19
H
ATOM  16016 2HB  TYR B 454     -28.195  33.793   6.255  1.00  4.19
H
ATOM  16017  HD1 TYR B 454     -25.083  32.663   7.546  1.00  4.19
H
```

```
ATOM  16018  HD2 TYR B 454     -27.108  36.074   5.946  1.00   4.19
H
ATOM  16019  HE1 TYR B 454     -22.906  33.658   6.952  1.00   4.19
H
ATOM  16020  HE2 TYR B 454     -24.935  37.078   5.352  1.00   4.19
H
ATOM  16021  HH  TYR B 454     -21.892  35.393   6.062  1.00   4.19
H
ATOM  16022  N   THR B 455     -30.522  34.568   7.762  1.00   2.33
N
ATOM  16023  CA  THR B 455     -31.904  34.178   7.590  1.00   2.26
C
ATOM  16024  C   THR B 455     -31.897  33.179   6.432  1.00   1.95
C
ATOM  16025  O   THR B 455     -31.182  33.406   5.462  1.00   2.07
O
ATOM  16026  CB  THR B 455     -32.799  35.402   7.273  1.00   3.39
C
ATOM  16027  OG1 THR B 455     -32.724  36.339   8.355  1.00   3.39
O
ATOM  16028  CG2 THR B 455     -34.250  34.994   7.074  1.00   3.39
C
ATOM  16029  H   THR B 455     -30.210  35.447   7.376  1.00   2.80
H
ATOM  16030  HA  THR B 455     -32.260  33.703   8.501  1.00   2.71
H
ATOM  16031  HB  THR B 455     -32.433  35.882   6.363  1.00   4.07
H
ATOM  16032  HG1 THR B 455     -33.283  37.095   8.160  1.00   4.07
H
ATOM  16033 1HG2 THR B 455     -34.847  35.878   6.852  1.00   4.07
H
ATOM  16034 2HG2 THR B 455     -34.327  34.293   6.249  1.00   4.07
H
ATOM  16035 3HG2 THR B 455     -34.623  34.525   7.982  1.00   4.07
H
ATOM  16036  N   PRO B 456     -32.627  32.061   6.512  1.00   1.83
N
ATOM  16037  CA  PRO B 456     -32.748  30.999   5.503  1.00   1.80
C
ATOM  16038  C   PRO B 456     -33.093  31.479   4.088  1.00   2.03
C
ATOM  16039  O   PRO B 456     -32.788  30.796   3.111  1.00   2.66
O
ATOM  16040  CB  PRO B 456     -33.854  30.133   6.071  1.00   2.70
C
ATOM  16041  CG  PRO B 456     -33.691  30.249   7.550  1.00   2.70
C
ATOM  16042  CD  PRO B 456     -33.240  31.660   7.793  1.00   2.70
C
ATOM  16043  HA  PRO B 456     -31.810  30.433   5.482  1.00   2.16
H
ATOM  16044 1HB  PRO B 456     -34.834  30.485   5.717  1.00   3.24
H
ATOM  16045 2HB  PRO B 456     -33.728  29.112   5.695  1.00   3.24
H
ATOM  16046 1HG  PRO B 456     -34.648  30.027   8.048  1.00   3.24
H
```

ATOM 16047 2HG PRO B 456 -32.968 29.509 7.912 1.00 3.24
H
ATOM 16048 1HD PRO B 456 -34.096 32.305 8.030 1.00 3.24
H
ATOM 16049 2HD PRO B 456 -32.483 31.672 8.593 1.00 3.24
H
ATOM 16050 N GLN B 457 -33.707 32.651 3.976 1.00 1.82
N
ATOM 16051 CA GLN B 457 -34.066 33.219 2.685 1.00 1.96
C
ATOM 16052 C GLN B 457 -32.911 34.005 2.044 1.00 1.94
C
ATOM 16053 O GLN B 457 -33.040 34.484 0.915 1.00 2.09
O
ATOM 16054 CB GLN B 457 -35.277 34.137 2.848 1.00 2.94
C
ATOM 16055 CG GLN B 457 -36.522 33.438 3.367 1.00 2.94
C
ATOM 16056 CD GLN B 457 -37.000 32.338 2.441 1.00 2.94
C
ATOM 16057 OE1 GLN B 457 -37.164 32.548 1.236 1.00 2.94
O
ATOM 16058 NE2 GLN B 457 -37.231 31.156 3.000 1.00 2.94
N
ATOM 16059 H GLN B 457 -33.945 33.164 4.807 1.00 2.18
H
ATOM 16060 HA GLN B 457 -34.332 32.403 2.014 1.00 2.35
H
ATOM 16061 1HB GLN B 457 -35.031 34.944 3.539 1.00 3.53
H
ATOM 16062 2HB GLN B 457 -35.521 34.591 1.888 1.00 3.53
H
ATOM 16063 1HG GLN B 457 -36.303 32.996 4.337 1.00 3.53
H
ATOM 16064 2HG GLN B 457 -37.322 34.174 3.463 1.00 3.53
H
ATOM 16065 1HE2 GLN B 457 -37.551 30.391 2.439 1.00 3.53
H
ATOM 16066 2HE2 GLN B 457 -37.087 31.030 3.982 1.00 3.53
H
ATOM 16067 N GLU B 458 -31.795 34.148 2.764 1.00 1.85
N
ATOM 16068 CA GLU B 458 -30.624 34.858 2.267 1.00 1.87
C
ATOM 16069 C GLU B 458 -30.059 34.186 1.019 1.00 1.71
C
ATOM 16070 O GLU B 458 -29.852 32.974 0.992 1.00 1.80
O
ATOM 16071 CB GLU B 458 -29.544 34.937 3.350 1.00 2.81
C
ATOM 16072 CG GLU B 458 -28.328 35.763 2.964 1.00 2.81
C
ATOM 16073 CD GLU B 458 -28.638 37.235 2.863 1.00 2.81
C
ATOM 16074 OE1 GLU B 458 -29.522 37.682 3.554 1.00 2.81
O
ATOM 16075 OE2 GLU B 458 -27.989 37.917 2.102 1.00 2.81
O

ATOM 16076 H GLU B 458 -31.736 33.748 3.687 1.00 2.22
H
ATOM 16077 HA GLU B 458 -30.923 35.872 2.001 1.00 2.24
H
ATOM 16078 1HB GLU B 458 -29.970 35.378 4.252 1.00 3.37
H
ATOM 16079 2HB GLU B 458 -29.209 33.933 3.605 1.00 3.37
H
ATOM 16080 1HG GLU B 458 -27.547 35.614 3.707 1.00 3.37
H
ATOM 16081 2HG GLU B 458 -27.953 35.406 2.008 1.00 3.37
H
ATOM 16082 N ARG B 459 -29.817 34.984 -0.015 1.00 1.81
N
ATOM 16083 CA ARG B 459 -29.290 34.466 -1.272 1.00 1.78
C
ATOM 16084 C ARG B 459 -27.769 34.444 -1.296 1.00 1.69
C
ATOM 16085 O ARG B 459 -27.121 35.485 -1.184 1.00 2.74
O
ATOM 16086 CB ARG B 459 -29.773 35.302 -2.444 1.00 2.67
C
ATOM 16087 CG ARG B 459 -29.287 34.815 -3.800 1.00 2.67
C
ATOM 16088 CD ARG B 459 -29.661 35.752 -4.889 1.00 2.67
C
ATOM 16089 NE ARG B 459 -29.202 35.279 -6.184 1.00 2.67
N
ATOM 16090 CZ ARG B 459 -27.966 35.502 -6.673 1.00 2.67
C
ATOM 16091 NH1 ARG B 459 -27.080 36.166 -5.969 1.00 2.67
N
ATOM 16092 NH2 ARG B 459 -27.617 35.051 -7.864 1.00 2.67
N
ATOM 16093 H ARG B 459 -30.007 35.973 0.071 1.00 2.17
H
ATOM 16094 HA ARG B 459 -29.653 33.446 -1.401 1.00 2.14
H
ATOM 16095 1HB ARG B 459 -30.862 35.306 -2.464 1.00 3.20
H
ATOM 16096 2HB ARG B 459 -29.441 36.332 -2.321 1.00 3.20
H
ATOM 16097 1HG ARG B 459 -28.201 34.720 -3.788 1.00 3.20
H
ATOM 16098 2HG ARG B 459 -29.733 33.843 -4.018 1.00 3.20
H
ATOM 16099 1HD ARG B 459 -30.745 35.855 -4.927 1.00 3.20
H
ATOM 16100 2HD ARG B 459 -29.210 36.725 -4.701 1.00 3.20
H
ATOM 16101 HE ARG B 459 -29.853 34.761 -6.758 1.00 3.20
H
ATOM 16102 1HH1 ARG B 459 -27.316 36.519 -5.054 1.00 3.20
H
ATOM 16103 2HH1 ARG B 459 -26.150 36.306 -6.357 1.00 3.20
H
ATOM 16104 1HH2 ARG B 459 -28.274 34.534 -8.430 1.00 3.20
H

```
ATOM  16105 2HH2 ARG B 459     -26.676  35.233  -8.195  1.00  3.20
H
ATOM  16106  N   PHE B 460     -27.206  33.253  -1.467  1.00  1.27
N
ATOM  16107  CA  PHE B 460     -25.759  33.078  -1.536  1.00  1.35
C
ATOM  16108  C   PHE B 460     -25.298  32.805  -2.954  1.00  1.25
C
ATOM  16109  O   PHE B 460     -26.018  32.203  -3.751  1.00  1.21
O
ATOM  16110  CB  PHE B 460     -25.324  31.929  -0.644  1.00  2.03
C
ATOM  16111  CG  PHE B 460     -25.478  32.216   0.813  1.00  2.03
C
ATOM  16112  CD1 PHE B 460     -26.704  32.066   1.426  1.00  2.03
C
ATOM  16113  CD2 PHE B 460     -24.399  32.620   1.577  1.00  2.03
C
ATOM  16114  CE1 PHE B 460     -26.856  32.307   2.771  1.00  2.03
C
ATOM  16115  CE2 PHE B 460     -24.545  32.864   2.925  1.00  2.03
C
ATOM  16116  CZ  PHE B 460     -25.779  32.702   3.517  1.00  2.03
C
ATOM  16117  H   PHE B 460     -27.801  32.440  -1.551  1.00  1.52
H
ATOM  16118  HA  PHE B 460     -25.281  33.994  -1.189  1.00  1.62
H
ATOM  16119 1HB  PHE B 460     -25.914  31.046  -0.881  1.00  2.43
H
ATOM  16120 2HB  PHE B 460     -24.281  31.692  -0.837  1.00  2.43
H
ATOM  16121  HD1 PHE B 460     -27.558  31.749   0.828  1.00  2.43
H
ATOM  16122  HD2 PHE B 460     -23.428  32.742   1.099  1.00  2.43
H
ATOM  16123  HE1 PHE B 460     -27.832  32.180   3.239  1.00  2.43
H
ATOM  16124  HE2 PHE B 460     -23.689  33.180   3.522  1.00  2.43
H
ATOM  16125  HZ  PHE B 460     -25.901  32.884   4.575  1.00  2.43
H
ATOM  16126  N   GLU B 461     -24.087  33.249  -3.258  1.00  1.35
N
ATOM  16127  CA  GLU B 461     -23.509  33.063  -4.578  1.00  1.31
C
ATOM  16128  C   GLU B 461     -21.994  32.996  -4.513  1.00  1.19
C
ATOM  16129  O   GLU B 461     -21.362  33.703  -3.727  1.00  1.24
O
ATOM  16130  CB  GLU B 461     -23.951  34.183  -5.525  1.00  1.97
C
ATOM  16131  CG  GLU B 461     -23.433  34.029  -6.952  1.00  1.97
C
ATOM  16132  CD  GLU B 461     -23.976  35.052  -7.915  1.00  1.97
C
ATOM  16133  OE1 GLU B 461     -24.841  35.810  -7.541  1.00  1.97
O
```

ATOM 16134 OE2 GLU B 461 -23.534 35.066 -9.039 1.00 1.97
O
ATOM 16135 H GLU B 461 -23.549 33.732 -2.553 1.00 1.62
H
ATOM 16136 HA GLU B 461 -23.872 32.115 -4.979 1.00 1.57
H
ATOM 16137 1HB GLU B 461 -25.040 34.213 -5.566 1.00 2.36
H
ATOM 16138 2HB GLU B 461 -23.608 35.144 -5.143 1.00 2.36
H
ATOM 16139 1HG GLU B 461 -22.346 34.114 -6.940 1.00 2.36
H
ATOM 16140 2HG GLU B 461 -23.686 33.032 -7.311 1.00 2.36
H
ATOM 16141 N ILE B 462 -21.425 32.123 -5.334 1.00 1.09
N
ATOM 16142 CA ILE B 462 -19.986 31.936 -5.401 1.00 1.05
C
ATOM 16143 C ILE B 462 -19.323 33.225 -5.865 1.00 1.24
C
ATOM 16144 O ILE B 462 -19.694 33.777 -6.903 1.00 1.39
O
ATOM 16145 CB ILE B 462 -19.647 30.789 -6.369 1.00 1.58
C
ATOM 16146 CG1 ILE B 462 -20.211 29.474 -5.837 1.00 1.58
C
ATOM 16147 CG2 ILE B 462 -18.149 30.686 -6.568 1.00 1.58
C
ATOM 16148 CD1 ILE B 462 -20.143 28.341 -6.835 1.00 1.58
C
ATOM 16149 H ILE B 462 -22.015 31.573 -5.943 1.00 1.31
H
ATOM 16150 HA ILE B 462 -19.616 31.690 -4.409 1.00 1.26
H
ATOM 16151 HB ILE B 462 -20.122 30.979 -7.331 1.00 1.89
H
ATOM 16152 1HG1 ILE B 462 -19.656 29.188 -4.948 1.00 1.89
H
ATOM 16153 2HG1 ILE B 462 -21.253 29.623 -5.561 1.00 1.89
H
ATOM 16154 1HG2 ILE B 462 -17.929 29.878 -7.265 1.00 1.89
H
ATOM 16155 2HG2 ILE B 462 -17.774 31.624 -6.973 1.00 1.89
H
ATOM 16156 3HG2 ILE B 462 -17.666 30.483 -5.613 1.00 1.89
H
ATOM 16157 1HD1 ILE B 462 -20.563 27.437 -6.397 1.00 1.89
H
ATOM 16158 2HD1 ILE B 462 -20.714 28.609 -7.726 1.00 1.89
H
ATOM 16159 3HD1 ILE B 462 -19.104 28.161 -7.111 1.00 1.89
H
ATOM 16160 N GLY B 463 -18.354 33.722 -5.094 1.00 1.32
N
ATOM 16161 CA GLY B 463 -17.704 34.979 -5.428 1.00 1.61
C
ATOM 16162 C GLY B 463 -18.418 36.198 -4.837 1.00 1.80
C

ATOM 16163 O GLY B 463 -18.025 37.331 -5.115 1.00 2.10
O
ATOM 16164 H GLY B 463 -18.035 33.237 -4.261 1.00 1.58
H
ATOM 16165 1HA GLY B 463 -16.674 34.955 -5.069 1.00 1.93
H
ATOM 16166 2HA GLY B 463 -17.654 35.079 -6.512 1.00 1.93
H
ATOM 16167 N GLN B 464 -19.461 35.976 -4.030 1.00 1.70
N
ATOM 16168 CA GLN B 464 -20.177 37.083 -3.398 1.00 1.87
C
ATOM 16169 C GLN B 464 -20.032 37.070 -1.873 1.00 2.02
C
ATOM 16170 O GLN B 464 -20.446 36.118 -1.210 1.00 3.25
O
ATOM 16171 CB GLN B 464 -21.661 37.037 -3.766 1.00 2.81
C
ATOM 16172 CG GLN B 464 -22.491 38.120 -3.101 1.00 2.81
C
ATOM 16173 CD GLN B 464 -22.177 39.503 -3.641 1.00 2.81
C
ATOM 16174 OE1 GLN B 464 -22.387 39.779 -4.825 1.00 2.81
O
ATOM 16175 NE2 GLN B 464 -21.668 40.374 -2.779 1.00 2.81
N
ATOM 16176 H GLN B 464 -19.784 35.034 -3.849 1.00 2.04
H
ATOM 16177 HA GLN B 464 -19.758 38.021 -3.765 1.00 2.24
H
ATOM 16178 1HB GLN B 464 -21.774 37.137 -4.845 1.00 3.37
H
ATOM 16179 2HB GLN B 464 -22.079 36.074 -3.479 1.00 3.37
H
ATOM 16180 1HG GLN B 464 -23.547 37.914 -3.277 1.00 3.37
H
ATOM 16181 2HG GLN B 464 -22.282 38.117 -2.031 1.00 3.37
H
ATOM 16182 1HE2 GLN B 464 -21.436 41.300 -3.081 1.00 3.37
H
ATOM 16183 2HE2 GLN B 464 -21.502 40.106 -1.828 1.00 3.37
H
ATOM 16184 N ALA B 465 -19.449 38.140 -1.331 1.00 1.66
N
ATOM 16185 CA ALA B 465 -19.265 38.304 0.110 1.00 1.60
C
ATOM 16186 C ALA B 465 -20.457 39.026 0.716 1.00 1.55
C
ATOM 16187 O ALA B 465 -21.221 39.691 0.013 1.00 1.58
O
ATOM 16188 CB ALA B 465 -17.989 39.073 0.412 1.00 2.40
C
ATOM 16189 H ALA B 465 -19.129 38.886 -1.940 1.00 1.99
H
ATOM 16190 HA ALA B 465 -19.202 37.315 0.562 1.00 1.92
H
ATOM 16191 1HB ALA B 465 -17.867 39.168 1.491 1.00 2.88
H

```
ATOM  16192 2HB  ALA B 465      -17.137  38.535  -0.002  1.00  2.88
H
ATOM  16193 3HB  ALA B 465      -18.047  40.064  -0.036  1.00  2.88
H
ATOM  16194  N   LYS B 466      -20.624  38.895   2.026  1.00  1.57
N
ATOM  16195  CA  LYS B 466      -21.728  39.550   2.697  1.00  1.69
C
ATOM  16196  C   LYS B 466      -21.285  40.436   3.850  1.00  2.08
C
ATOM  16197  O   LYS B 466      -20.332  40.141   4.571  1.00  2.49
O
ATOM  16198  CB  LYS B 466      -22.721  38.499   3.176  1.00  2.54
C
ATOM  16199  CG  LYS B 466      -23.391  37.779   2.017  1.00  2.54
C
ATOM  16200  CD  LYS B 466      -24.418  36.770   2.469  1.00  2.54
C
ATOM  16201  CE  LYS B 466      -25.071  36.136   1.267  1.00  2.54
C
ATOM  16202  NZ  LYS B 466      -25.842  37.139   0.486  1.00  2.54
N
ATOM  16203  H   LYS B 466      -19.981  38.333   2.560  1.00  1.88
H
ATOM  16204  HA  LYS B 466      -22.235  40.189   1.974  1.00  2.03
H
ATOM  16205 1HB  LYS B 466      -22.209  37.757   3.791  1.00  3.04
H
ATOM  16206 2HB  LYS B 466      -23.494  38.965   3.786  1.00  3.04
H
ATOM  16207 1HG  LYS B 466      -23.879  38.512   1.373  1.00  3.04
H
ATOM  16208 2HG  LYS B 466      -22.636  37.258   1.431  1.00  3.04
H
ATOM  16209 1HD  LYS B 466      -23.937  35.996   3.070  1.00  3.04
H
ATOM  16210 2HD  LYS B 466      -25.181  37.263   3.071  1.00  3.04
H
ATOM  16211 1HE  LYS B 466      -24.306  35.700   0.625  1.00  3.04
H
ATOM  16212 2HE  LYS B 466      -25.747  35.347   1.593  1.00  3.04
H
ATOM  16213 1HZ  LYS B 466      -26.269  36.673  -0.300  1.00  3.04
H
ATOM  16214 2HZ  LYS B 466      -26.566  37.529   1.074  1.00  3.04
H
ATOM  16215 3HZ  LYS B 466      -25.233  37.875   0.161  1.00  3.04
H
ATOM  16216  N   VAL B 467      -22.007  41.532   4.028  1.00  2.95
N
ATOM  16217  CA  VAL B 467      -21.727  42.454   5.110  1.00  3.13
C
ATOM  16218  C   VAL B 467      -22.604  42.093   6.291  1.00  3.13
C
ATOM  16219  O   VAL B 467      -23.824  42.240   6.227  1.00  3.60
O
ATOM  16220  CB  VAL B 467      -22.013  43.902   4.692  1.00  4.70
C
```

ATOM 16221 CG1 VAL B 467   -21.718 44.820   5.860 1.00 4.70
C
ATOM 16222 CG2 VAL B 467   -21.187 44.277   3.470 1.00 4.70
C
ATOM 16223 H   VAL B 467   -22.774 41.727   3.398 1.00 3.54
H
ATOM 16224 HA VAL B 467   -20.680 42.360   5.399 1.00 3.76
H
ATOM 16225 HB VAL B 467   -23.071 44.003   4.456 1.00 5.63
H
ATOM 16226 1HG1 VAL B 467   -21.940 45.848   5.576 1.00 5.63
H
ATOM 16227 2HG1 VAL B 467   -22.336 44.536   6.712 1.00 5.63
H
ATOM 16228 3HG1 VAL B 467   -20.667 44.735   6.129 1.00 5.63
H
ATOM 16229 1HG2 VAL B 467   -21.410 45.304   3.184 1.00 5.63
H
ATOM 16230 2HG2 VAL B 467   -20.129 44.190   3.700 1.00 5.63
H
ATOM 16231 3HG2 VAL B 467   -21.434 43.610   2.645 1.00 5.63
H
ATOM 16232 N   LEU B 468   -21.989 41.606   7.357 1.00 2.91
N
ATOM 16233 CA LEU B 468   -22.729 41.193   8.540 1.00 3.06
C
ATOM 16234 C   LEU B 468   -23.038 42.334   9.466 1.00 3.19
C
ATOM 16235 O   LEU B 468   -24.143 42.424 10.007 1.00 3.52
O
ATOM 16236 CB LEU B 468   -21.946 40.145   9.308 1.00 4.59
C
ATOM 16237 CG LEU B 468   -21.800 38.825   8.586 1.00 4.59
C
ATOM 16238 CD1 LEU B 468   -20.908 37.918   9.394 1.00 4.59
C
ATOM 16239 CD2 LEU B 468   -23.179 38.227   8.405 1.00 4.59
C
ATOM 16240 H   LEU B 468   -20.982 41.531   7.357 1.00 3.49
H
ATOM 16241 HA LEU B 468   -23.671 40.755   8.214 1.00 3.67
H
ATOM 16242 1HB LEU B 468   -20.949 40.530   9.510 1.00 5.51
H
ATOM 16243 2HB LEU B 468   -22.445 39.957 10.259 1.00 5.51
H
ATOM 16244 HG LEU B 468   -21.336 38.977   7.611 1.00 5.51
H
ATOM 16245 1HD1 LEU B 468   -20.802 36.961   8.883 1.00 5.51
H
ATOM 16246 2HD1 LEU B 468   -19.928 38.380   9.507 1.00 5.51
H
ATOM 16247 3HD1 LEU B 468   -21.349 37.756 10.376 1.00 5.51
H
ATOM 16248 1HD2 LEU B 468   -23.090 37.275   7.897 1.00 5.51
H
ATOM 16249 2HD2 LEU B 468   -23.642 38.077   9.381 1.00 5.51
H

ATOM 16250 3HD2 LEU B 468   -23.795 38.903   7.813 1.00 5.51
H
ATOM 16251 N   ARG B 469   -22.063 43.202   9.667 1.00 3.19
N
ATOM 16252 CA ARG B 469   -22.277 44.336 10.548 1.00 3.57
C
ATOM 16253 C   ARG B 469   -21.748 45.588   9.921 1.00 3.70
C
ATOM 16254 O   ARG B 469   -20.628 45.612   9.423 1.00 4.27
O
ATOM 16255 CB ARG B 469   -21.602 44.135 11.896 1.00 5.35
C
ATOM 16256 CG ARG B 469   -22.139 42.984 12.726 1.00 5.35
C
ATOM 16257 CD ARG B 469   -23.508 43.268 13.227 1.00 5.35
C
ATOM 16258 NE ARG B 469   -24.008 42.206 14.082 1.00 5.35
N
ATOM 16259 CZ ARG B 469   -24.656 41.103 13.654 1.00 5.35
C
ATOM 16260 NH1 ARG B 469   -24.892 40.907 12.373 1.00 5.35
N
ATOM 16261 NH2 ARG B 469   -25.062 40.206 14.536 1.00 5.35
N
ATOM 16262 H   ARG B 469   -21.173 43.062   9.200 1.00 3.83
H
ATOM 16263 HA ARG B 469   -23.348 44.457 10.712 1.00 4.28
H
ATOM 16264 1HB ARG B 469   -20.539 43.972 11.745 1.00 6.43
H
ATOM 16265 2HB ARG B 469   -21.709 45.041 12.491 1.00 6.43
H
ATOM 16266 1HG ARG B 469   -22.172 42.075 12.126 1.00 6.43
H
ATOM 16267 2HG ARG B 469   -21.489 42.826 13.587 1.00 6.43
H
ATOM 16268 1HD ARG B 469   -23.497 44.193 13.803 1.00 6.43
H
ATOM 16269 2HD ARG B 469   -24.189 43.376 12.383 1.00 6.43
H
ATOM 16270 HE ARG B 469   -23.857 42.301 15.077 1.00 6.43
H
ATOM 16271 1HH1 ARG B 469   -24.591 41.581 11.677 1.00 6.43
H
ATOM 16272 2HH1 ARG B 469   -25.382 40.076 12.075 1.00 6.43
H
ATOM 16273 1HH2 ARG B 469   -24.885 40.349 15.520 1.00 6.43
H
ATOM 16274 2HH2 ARG B 469   -25.548 39.377 14.225 1.00 6.43
H
ATOM 16275 N   HIS B 470   -22.546 46.643   9.964 1.00 3.63
N
ATOM 16276 CA HIS B 470   -22.132 47.902   9.381 1.00 3.75
C
ATOM 16277 C   HIS B 470   -22.779 49.113 10.027 1.00 3.73
C
ATOM 16278 O   HIS B 470   -23.820 49.012 10.678 1.00 4.17
O

ATOM 16279 CB HIS B 470   -22.441 47.895   7.889 1.00 5.62
C
ATOM 16280 CG HIS B 470   -23.889 47.699   7.584 1.00 5.62
C
ATOM 16281 ND1 HIS B 470   -24.475 46.450   7.560 1.00 5.62
N
ATOM 16282 CD2 HIS B 470   -24.869 48.582   7.293 1.00 5.62
C
ATOM 16283 CE1 HIS B 470   -25.755 46.574   7.263 1.00 5.62
C
ATOM 16284 NE2 HIS B 470   -26.021 47.859   7.097 1.00 5.62
N
ATOM 16285 H   HIS B 470   -23.457 46.569 10.396 1.00 4.36
H
ATOM 16286 HA HIS B 470   -21.056 48.005   9.499 1.00 4.50
H
ATOM 16287 1HB HIS B 470   -22.120 48.837   7.443 1.00 6.75
H
ATOM 16288 2HB HIS B 470   -21.879 47.100   7.411 1.00 6.75
H
ATOM 16289 HD2 HIS B 470   -24.767 49.665   7.224 1.00 6.75
H
ATOM 16290 HE1 HIS B 470   -26.471 45.757   7.173 1.00 6.75
H
ATOM 16291 HE2 HIS B 470   -26.922 48.250   6.863 1.00 6.75
H
ATOM 16292 N   CYS B 471   -22.170 50.264   9.782 1.00 4.46
N
ATOM 16293 CA CYS B 471   -22.596 51.547 10.317 1.00 4.42
C
ATOM 16294 C   CYS B 471   -21.827 52.684   9.659 1.00 4.44
C
ATOM 16295 O   CYS B 471   -20.674 52.527   9.257 1.00 4.83
O
ATOM 16296 CB CYS B 471   -22.374 51.612 11.831 1.00 6.63
C
ATOM 16297 SG CYS B 471   -23.011 53.115 12.611 1.00 6.63
S
ATOM 16298 H   CYS B 471   -21.314 50.227   9.246 1.00 5.35
H
ATOM 16299 HA CYS B 471   -23.660 51.674 10.111 1.00 5.30
H
ATOM 16300 1HB CYS B 471   -22.854 50.757 12.308 1.00 7.96
H
ATOM 16301 2HB CYS B 471   -21.308 51.547 12.046 1.00 7.96
H
ATOM 16302 HG CYS B 471   -22.610 52.813 13.842 1.00 7.96
H
ATOM 16303 N   VAL B 472   -22.486 53.828   9.510 1.00 4.42
N
ATOM 16304 CA VAL B 472   -21.876 55.022   8.920 1.00 4.62
C
ATOM 16305 C   VAL B 472   -20.519 55.410   9.539 1.00 4.72
C
ATOM 16306 O   VAL B 472   -19.679 56.003   8.863 1.00 5.60
O
ATOM 16307 CB VAL B 472   -22.851 56.214   9.031 1.00 6.93
C

```
ATOM  16308  CG1 VAL B 472     -23.029  56.639  10.485  1.00  6.93
C
ATOM  16309  CG2 VAL B 472     -22.320  57.374   8.204  1.00  6.93
C
ATOM  16310  H   VAL B 472     -23.440  53.885   9.840  1.00  5.30
H
ATOM  16311  HA  VAL B 472     -21.716  54.820   7.860  1.00  5.54
H
ATOM  16312  HB  VAL B 472     -23.827  55.910   8.655  1.00  8.32
H
ATOM  16313 1HG1 VAL B 472     -23.735  57.470  10.533  1.00  8.32
H
ATOM  16314 2HG1 VAL B 472     -23.416  55.802  11.064  1.00  8.32
H
ATOM  16315 3HG1 VAL B 472     -22.075  56.959  10.897  1.00  8.32
H
ATOM  16316 1HG2 VAL B 472     -23.014  58.213   8.266  1.00  8.32
H
ATOM  16317 2HG2 VAL B 472     -21.346  57.681   8.588  1.00  8.32
H
ATOM  16318 3HG2 VAL B 472     -22.218  57.064   7.165  1.00  8.32
H
ATOM  16319  N   SER B 473     -20.311  55.079  10.816  1.00  5.29
N
ATOM  16320  CA  SER B 473     -19.086  55.426  11.518  1.00  5.13
C
ATOM  16321  C   SER B 473     -18.087  54.273  11.676  1.00  5.65
C
ATOM  16322  O   SER B 473     -17.230  54.329  12.553  1.00 22.74
O
ATOM  16323  CB  SER B 473     -19.432  55.981  12.885  1.00  7.70
C
ATOM  16324  OG  SER B 473     -20.128  55.038  13.651  1.00  7.70
O
ATOM  16325  H   SER B 473     -21.033  54.588  11.323  1.00  6.35
H
ATOM  16326  HA  SER B 473     -18.591  56.215  10.948  1.00  6.16
H
ATOM  16327 1HB  SER B 473     -18.517  56.270  13.401  1.00  9.23
H
ATOM  16328 2HB  SER B 473     -20.039  56.878  12.769  1.00  9.23
H
ATOM  16329  HG  SER B 473     -19.578  54.252  13.666  1.00  9.23
H
ATOM  16330  N   ASP B 474     -18.181  53.236  10.845  1.00  6.86
N
ATOM  16331  CA  ASP B 474     -17.218  52.134  10.923  1.00  4.41
C
ATOM  16332  C   ASP B 474     -15.810  52.623  10.613  1.00  5.14
C
ATOM  16333  O   ASP B 474     -15.597  53.318   9.622  1.00 25.99
O
ATOM  16334  CB  ASP B 474     -17.590  51.036   9.940  1.00  6.62
C
ATOM  16335  CG  ASP B 474     -18.855  50.317  10.319  1.00  6.62
C
ATOM  16336  OD1 ASP B 474     -19.186  50.294  11.481  1.00  6.62
O
```

```
ATOM  16337  OD2 ASP B 474     -19.517  49.844   9.426  1.00  6.62
O
ATOM  16338  H   ASP B 474     -18.923  53.197  10.156  1.00  8.23
H
ATOM  16339  HA  ASP B 474     -17.231  51.732  11.934  1.00  5.29
H
ATOM  16340 1HB  ASP B 474     -17.717  51.468   8.947  1.00  7.94
H
ATOM  16341 2HB  ASP B 474     -16.780  50.309   9.882  1.00  7.94
H
ATOM  16342  N   LYS B 475     -14.841  52.248  11.439  1.00  7.23
N
ATOM  16343  CA  LYS B 475     -13.471  52.699  11.223  1.00  4.62
C
ATOM  16344  C   LYS B 475     -12.488  51.615  10.801  1.00  4.40
C
ATOM  16345  O   LYS B 475     -11.287  51.870  10.735  1.00 16.54
O
ATOM  16346  CB  LYS B 475     -12.955  53.416  12.457  1.00  6.93
C
ATOM  16347  CG  LYS B 475     -13.732  54.677  12.794  1.00  6.93
C
ATOM  16348  CD  LYS B 475     -13.621  55.666  11.640  1.00  6.93
C
ATOM  16349  CE  LYS B 475     -14.242  57.016  11.961  1.00  6.93
C
ATOM  16350  NZ  LYS B 475     -14.222  57.917  10.772  1.00  6.93
N
ATOM  16351  H   LYS B 475     -15.066  51.666  12.234  1.00  8.68
H
ATOM  16352  HA  LYS B 475     -13.492  53.432  10.415  1.00  5.54
H
ATOM  16353 1HB  LYS B 475     -13.006  52.748  13.317  1.00  8.32
H
ATOM  16354 2HB  LYS B 475     -11.910  53.692  12.310  1.00  8.32
H
ATOM  16355 1HG  LYS B 475     -14.779  54.433  12.966  1.00  8.32
H
ATOM  16356 2HG  LYS B 475     -13.326  55.131  13.698  1.00  8.32
H
ATOM  16357 1HD  LYS B 475     -12.569  55.818  11.394  1.00  8.32
H
ATOM  16358 2HD  LYS B 475     -14.120  55.254  10.763  1.00  8.32
H
ATOM  16359 1HE  LYS B 475     -15.270  56.882  12.292  1.00  8.32
H
ATOM  16360 2HE  LYS B 475     -13.671  57.482  12.763  1.00  8.32
H
ATOM  16361 1HZ  LYS B 475     -14.621  58.812  11.013  1.00  8.32
H
ATOM  16362 2HZ  LYS B 475     -13.267  58.043  10.455  1.00  8.32
H
ATOM  16363 3HZ  LYS B 475     -14.758  57.498  10.028  1.00  8.32
H
ATOM  16364  N   VAL B 476     -12.984  50.421  10.506  1.00  6.49
N
ATOM  16365  CA  VAL B 476     -12.127  49.352   9.995  1.00  4.21
C
```

```
ATOM  16366  C   VAL B 476     -12.953  48.161   9.545  1.00  3.67
C
ATOM  16367  O   VAL B 476     -13.904  47.764  10.219  1.00  3.93
O
ATOM  16368  CB  VAL B 476     -11.094  48.898  11.047  1.00  6.31
C
ATOM  16369  CG1 VAL B 476     -11.795  48.339  12.247  1.00  6.31
C
ATOM  16370  CG2 VAL B 476     -10.176  47.845  10.455  1.00  6.31
C
ATOM  16371  H   VAL B 476     -13.974  50.252  10.606  1.00  7.79
H
ATOM  16372  HA  VAL B 476     -11.580  49.734   9.133  1.00  5.05
H
ATOM  16373  HB  VAL B 476     -10.504  49.753  11.369  1.00  7.58
H
ATOM  16374 1HG1 VAL B 476     -11.050  48.036  12.977  1.00  7.58
H
ATOM  16375 2HG1 VAL B 476     -12.437  49.107  12.674  1.00  7.58
H
ATOM  16376 3HG1 VAL B 476     -12.394  47.477  11.957  1.00  7.58
H
ATOM  16377 1HG2 VAL B 476      -9.449  47.553  11.208  1.00  7.58
H
ATOM  16378 2HG2 VAL B 476     -10.754  46.972  10.155  1.00  7.58
H
ATOM  16379 3HG2 VAL B 476      -9.660  48.251   9.593  1.00  7.58
H
ATOM  16380  N   THR B 477     -12.566  47.598   8.406  1.00  3.45
N
ATOM  16381  CA  THR B 477     -13.193  46.415   7.855  1.00  3.22
C
ATOM  16382  C   THR B 477     -12.530  45.183   8.391  1.00  3.03
C
ATOM  16383  O   THR B 477     -11.374  44.925   8.088  1.00  3.32
O
ATOM  16384  CB  THR B 477     -13.099  46.383   6.315  1.00  4.83
C
ATOM  16385  OG1 THR B 477     -13.805  47.493   5.742  1.00  4.83
O
ATOM  16386  CG2 THR B 477     -13.659  45.084   5.783  1.00  4.83
C
ATOM  16387  H   THR B 477     -11.783  47.994   7.907  1.00  4.14
H
ATOM  16388  HA  THR B 477     -14.232  46.388   8.155  1.00  3.86
H
ATOM  16389  HB  THR B 477     -12.052  46.458   6.026  1.00  5.80
H
ATOM  16390  HG1 THR B 477     -13.397  48.324   6.008  1.00  5.80
H
ATOM  16391 1HG2 THR B 477     -13.572  45.066   4.697  1.00  5.80
H
ATOM  16392 2HG2 THR B 477     -13.105  44.247   6.207  1.00  5.80
H
ATOM  16393 3HG2 THR B 477     -14.705  45.011   6.068  1.00  5.80
H
ATOM  16394  N   VAL B 478     -13.270  44.406   9.159  1.00  2.91
N
```

ATOM 16395 CA VAL B 478 -12.743 43.177 9.711 1.00 2.80
C
ATOM 16396 C VAL B 478 -13.326 41.984 8.967 1.00 2.78
C
ATOM 16397 O VAL B 478 -14.532 41.737 9.004 1.00 3.00
O
ATOM 16398 CB VAL B 478 -13.033 43.082 11.220 1.00 4.20
C
ATOM 16399 CG1 VAL B 478 -12.486 41.770 11.773 1.00 4.20
C
ATOM 16400 CG2 VAL B 478 -12.395 44.272 11.930 1.00 4.20
C
ATOM 16401 H VAL B 478 -14.217 44.674 9.364 1.00 3.49
H
ATOM 16402 HA VAL B 478 -11.666 43.184 9.582 1.00 3.36
H
ATOM 16403 HB VAL B 478 -14.110 43.089 11.383 1.00 5.04
H
ATOM 16404 1HG1 VAL B 478 -12.701 41.703 12.839 1.00 5.04
H
ATOM 16405 2HG1 VAL B 478 -12.956 40.933 11.256 1.00 5.04
H
ATOM 16406 3HG1 VAL B 478 -11.407 41.732 11.617 1.00 5.04
H
ATOM 16407 1HG2 VAL B 478 -12.600 44.214 12.998 1.00 5.04
H
ATOM 16408 2HG2 VAL B 478 -11.319 44.258 11.768 1.00 5.04
H
ATOM 16409 3HG2 VAL B 478 -12.808 45.199 11.531 1.00 5.04
H
ATOM 16410 N ILE B 479 -12.452 41.261 8.287 1.00 2.55
N
ATOM 16411 CA ILE B 479 -12.822 40.103 7.502 1.00 2.41
C
ATOM 16412 C ILE B 479 -12.564 38.830 8.275 1.00 2.27
C
ATOM 16413 O ILE B 479 -11.450 38.587 8.734 1.00 2.28
O
ATOM 16414 CB ILE B 479 -12.045 40.062 6.174 1.00 3.62
C
ATOM 16415 CG1 ILE B 479 -12.368 41.294 5.319 1.00 3.62
C
ATOM 16416 CG2 ILE B 479 -12.374 38.790 5.430 1.00 3.62
C
ATOM 16417 CD1 ILE B 479 -11.484 41.438 4.102 1.00 3.62
C
ATOM 16418 H ILE B 479 -11.480 41.522 8.333 1.00 3.06
H
ATOM 16419 HA ILE B 479 -13.887 40.158 7.280 1.00 2.89
H
ATOM 16420 HB ILE B 479 -10.976 40.088 6.382 1.00 4.34
H
ATOM 16421 1HG1 ILE B 479 -13.400 41.233 4.981 1.00 4.34
H
ATOM 16422 2HG1 ILE B 479 -12.257 42.188 5.931 1.00 4.34
H
ATOM 16423 1HG2 ILE B 479 -11.810 38.762 4.500 1.00 4.34
H

ATOM 16424 2HG2 ILE B 479 -12.110 37.928 6.042 1.00 4.34
H
ATOM 16425 3HG2 ILE B 479 -13.441 38.767 5.212 1.00 4.34
H
ATOM 16426 1HD1 ILE B 479 -11.770 42.331 3.548 1.00 4.34
H
ATOM 16427 2HD1 ILE B 479 -10.444 41.523 4.417 1.00 4.34
H
ATOM 16428 3HD1 ILE B 479 -11.599 40.563 3.464 1.00 4.34
H
ATOM 16429 N GLY B 480 -13.595 38.014 8.424 1.00 2.17
N
ATOM 16430 CA GLY B 480 -13.432 36.754 9.134 1.00 2.06
C
ATOM 16431 C GLY B 480 -14.127 35.626 8.400 1.00 2.14
C
ATOM 16432 O GLY B 480 -14.774 35.841 7.374 1.00 2.42
O
ATOM 16433 H GLY B 480 -14.494 38.269 8.027 1.00 2.60
H
ATOM 16434 1HA GLY B 480 -12.371 36.526 9.239 1.00 2.47
H
ATOM 16435 2HA GLY B 480 -13.840 36.848 10.140 1.00 2.47
H
ATOM 16436 N ALA B 481 -14.002 34.420 8.930 1.00 1.93
N
ATOM 16437 CA ALA B 481 -14.660 33.262 8.341 1.00 1.90
C
ATOM 16438 C ALA B 481 -14.773 32.155 9.392 1.00 1.71
C
ATOM 16439 O ALA B 481 -13.858 31.961 10.201 1.00 2.14
O
ATOM 16440 CB ALA B 481 -13.910 32.811 7.114 1.00 2.85
C
ATOM 16441 H ALA B 481 -13.447 34.304 9.766 1.00 2.32
H
ATOM 16442 HA ALA B 481 -15.664 33.548 8.035 1.00 2.28
H
ATOM 16443 1HB ALA B 481 -14.436 31.968 6.673 1.00 3.42
H
ATOM 16444 2HB ALA B 481 -13.868 33.627 6.391 1.00 3.42
H
ATOM 16445 3HB ALA B 481 -12.899 32.518 7.388 1.00 3.42
H
ATOM 16446 N GLY B 482 -15.894 31.438 9.376 1.00 1.84
N
ATOM 16447 CA GLY B 482 -16.135 30.386 10.352 1.00 1.59
C
ATOM 16448 C GLY B 482 -16.127 30.969 11.757 1.00 1.47
C
ATOM 16449 O GLY B 482 -16.743 32.008 12.015 1.00 1.43
O
ATOM 16450 H GLY B 482 -16.600 31.636 8.678 1.00 2.21
H
ATOM 16451 1HA GLY B 482 -17.093 29.907 10.152 1.00 1.91
H
ATOM 16452 2HA GLY B 482 -15.366 29.621 10.268 1.00 1.91
H

ATOM 16453 N ILE B 483 -15.387 30.315 12.651 1.00 1.54
N
ATOM 16454 CA ILE B 483 -15.272 30.746 14.040 1.00 1.63
C
ATOM 16455 C ILE B 483 -14.725 32.164 14.184 1.00 1.82
C
ATOM 16456 O ILE B 483 -15.025 32.837 15.171 1.00 2.07
O
ATOM 16457 CB ILE B 483 -14.391 29.773 14.851 1.00 2.44
C
ATOM 16458 CG1 ILE B 483 -14.569 30.049 16.346 1.00 2.44
C
ATOM 16459 CG2 ILE B 483 -12.923 29.900 14.459 1.00 2.44
C
ATOM 16460 CD1 ILE B 483 -15.970 29.782 16.846 1.00 2.44
C
ATOM 16461 H ILE B 483 -14.899 29.479 12.360 1.00 1.85
H
ATOM 16462 HA ILE B 483 -16.267 30.727 14.479 1.00 1.96
H
ATOM 16463 HB ILE B 483 -14.722 28.752 14.664 1.00 2.93
H
ATOM 16464 1HG1 ILE B 483 -13.878 29.421 16.907 1.00 2.93
H
ATOM 16465 2HG1 ILE B 483 -14.325 31.092 16.549 1.00 2.93
H
ATOM 16466 1HG2 ILE B 483 -12.330 29.195 15.039 1.00 2.93
H
ATOM 16467 2HG2 ILE B 483 -12.809 29.683 13.399 1.00 2.93
H
ATOM 16468 3HG2 ILE B 483 -12.575 30.912 14.664 1.00 2.93
H
ATOM 16469 1HD1 ILE B 483 -16.022 29.999 17.913 1.00 2.93
H
ATOM 16470 2HD1 ILE B 483 -16.674 30.421 16.312 1.00 2.93
H
ATOM 16471 3HD1 ILE B 483 -16.225 28.738 16.674 1.00 2.93
H
ATOM 16472 N THR B 484 -13.934 32.626 13.208 1.00 1.86
N
ATOM 16473 CA THR B 484 -13.362 33.958 13.297 1.00 2.10
C
ATOM 16474 C THR B 484 -14.362 35.039 12.903 1.00 2.07
C
ATOM 16475 O THR B 484 -14.145 36.211 13.213 1.00 2.16
O
ATOM 16476 CB THR B 484 -12.058 34.055 12.487 1.00 3.15
C
ATOM 16477 OG1 THR B 484 -12.282 33.678 11.122 1.00 3.15
O
ATOM 16478 CG2 THR B 484 -10.993 33.161 13.104 1.00 3.15
C
ATOM 16479 H THR B 484 -13.735 32.064 12.389 1.00 2.23
H
ATOM 16480 HA THR B 484 -13.091 34.135 14.337 1.00 2.52
H
ATOM 16481 HB THR B 484 -11.705 35.083 12.511 1.00 3.78
H

ATOM 16482 HG1 THR B 484   -12.630 32.779 11.089 1.00 3.78
H
ATOM 16483 1HG2 THR B 484   -10.070 33.254 12.539 1.00 3.78
H
ATOM 16484 2HG2 THR B 484   -10.817 33.467 14.136 1.00 3.78
H
ATOM 16485 3HG2 THR B 484   -11.329 32.126 13.087 1.00 3.78
H
ATOM 16486 N   VAL B 485   -15.484 34.652 12.276 1.00 1.84
N
ATOM 16487 CA VAL B 485   -16.566 35.607 12.070 1.00 1.74
C
ATOM 16488 C   VAL B 485   -17.155 35.929 13.387 1.00 1.92
C
ATOM 16489 O   VAL B 485   -17.392 37.088 13.706 1.00 2.47
O
ATOM 16490 CB VAL B 485   -17.731 35.079 11.224 1.00 2.61
C
ATOM 16491 CG1 VAL B 485   -18.867 36.066 11.307 1.00 2.61
C
ATOM 16492 CG2 VAL B 485   -17.330 34.931   9.795 1.00 2.61
C
ATOM 16493 H   VAL B 485   -15.628 33.691 12.004 1.00 2.21
H
ATOM 16494 HA VAL B 485   -16.167 36.517 11.619 1.00 2.09
H
ATOM 16495 HB VAL B 485   -18.073 34.126 11.623 1.00 3.13
H
ATOM 16496 1HG1 VAL B 485   -19.707 35.698 10.720 1.00 3.13
H
ATOM 16497 2HG1 VAL B 485   -19.173 36.178 12.345 1.00 3.13
H
ATOM 16498 3HG1 VAL B 485   -18.542 37.030 10.917 1.00 3.13
H
ATOM 16499 1HG2 VAL B 485   -18.171 34.568   9.206 1.00 3.13
H
ATOM 16500 2HG2 VAL B 485   -16.999 35.892   9.403 1.00 3.13
H
ATOM 16501 3HG2 VAL B 485   -16.524 34.220   9.757 1.00 3.13
H
ATOM 16502 N   TYR B 486   -17.407 34.874 14.150 1.00 1.80
N
ATOM 16503 CA TYR B 486   -18.035 35.026 15.443 1.00 1.98
C
ATOM 16504 C   TYR B 486   -17.155 35.763 16.403 1.00 2.02
C
ATOM 16505 O   TYR B 486   -17.651 36.563 17.195 1.00 2.33
O
ATOM 16506 CB TYR B 486   -18.376 33.676 15.992 1.00 2.97
C
ATOM 16507 CG TYR B 486   -19.411 33.034 15.147 1.00 2.97
C
ATOM 16508 CD1 TYR B 486   -18.991 32.147 14.189 1.00 2.97
C
ATOM 16509 CD2 TYR B 486   -20.752 33.329 15.313 1.00 2.97
C
ATOM 16510 CE1 TYR B 486   -19.905 31.531 13.387 1.00 2.97
C

ATOM 16511 CE2 TYR B 486 -21.678 32.705 14.512 1.00 2.97
C
ATOM 16512 CZ TYR B 486 -21.250 31.808 13.557 1.00 2.97
C
ATOM 16513 OH TYR B 486 -22.150 31.123 12.797 1.00 2.97
O
ATOM 16514 H TYR B 486 -17.177 33.946 13.791 1.00 2.16
H
ATOM 16515 HA TYR B 486 -18.951 35.602 15.319 1.00 2.38
H
ATOM 16516 1HB TYR B 486 -17.492 33.038 16.012 1.00 3.56
H
ATOM 16517 2HB TYR B 486 -18.759 33.764 17.008 1.00 3.56
H
ATOM 16518 HD1 TYR B 486 -17.933 31.932 14.078 1.00 3.56
H
ATOM 16519 HD2 TYR B 486 -21.072 34.036 16.078 1.00 3.56
H
ATOM 16520 HE1 TYR B 486 -19.576 30.814 12.637 1.00 3.56
H
ATOM 16521 HE2 TYR B 486 -22.739 32.908 14.642 1.00 3.56
H
ATOM 16522 HH TYR B 486 -23.033 31.288 13.134 1.00 3.56
H
ATOM 16523 N GLU B 487 -15.847 35.544 16.309 1.00 1.89
N
ATOM 16524 CA GLU B 487 -14.944 36.275 17.166 1.00 2.06
C
ATOM 16525 C GLU B 487 -14.940 37.746 16.762 1.00 2.23
C
ATOM 16526 O GLU B 487 -14.903 38.615 17.629 1.00 2.40
O
ATOM 16527 CB GLU B 487 -13.533 35.689 17.102 1.00 3.09
C
ATOM 16528 CG GLU B 487 -13.395 34.296 17.717 1.00 3.09
C
ATOM 16529 CD GLU B 487 -13.788 34.214 19.176 1.00 3.09
C
ATOM 16530 OE1 GLU B 487 -13.333 35.007 19.967 1.00 3.09
O
ATOM 16531 OE2 GLU B 487 -14.555 33.338 19.498 1.00 3.09
O
ATOM 16532 H GLU B 487 -15.484 34.845 15.669 1.00 2.27
H
ATOM 16533 HA GLU B 487 -15.302 36.204 18.193 1.00 2.47
H
ATOM 16534 1HB GLU B 487 -13.222 35.619 16.060 1.00 3.71
H
ATOM 16535 2HB GLU B 487 -12.838 36.356 17.598 1.00 3.71
H
ATOM 16536 1HG GLU B 487 -14.022 33.605 17.160 1.00 3.71
H
ATOM 16537 2HG GLU B 487 -12.364 33.967 17.606 1.00 3.71
H
ATOM 16538 N ALA B 488 -15.030 38.028 15.456 1.00 2.21
N
ATOM 16539 CA ALA B 488 -15.110 39.406 14.976 1.00 2.43
C

ATOM 16540 C   ALA B 488   -16.407 40.072 15.437 1.00 2.66
C
ATOM 16541 O   ALA B 488   -16.398 41.247 15.803 1.00 2.99
O
ATOM 16542 CB ALA B 488   -15.008 39.454 13.459 1.00 3.65
C
ATOM 16543 H   ALA B 488   -15.018 37.282 14.772 1.00 2.65
H
ATOM 16544 HA ALA B 488   -14.277 39.963 15.404 1.00 2.92
H
ATOM 16545 1HB ALA B 488   -15.041 40.490 13.124 1.00 4.37
H
ATOM 16546 2HB ALA B 488   -14.066 39.001 13.146 1.00 4.37
H
ATOM 16547 3HB ALA B 488   -15.837 38.905 13.018 1.00 4.37
H
ATOM 16548 N   LEU B 489   -17.512 39.321 15.422 1.00 2.58
N
ATOM 16549 CA LEU B 489   -18.799 39.836 15.874 1.00 2.94
C
ATOM 16550 C   LEU B 489   -18.757 40.156 17.369 1.00 3.21
C
ATOM 16551 O   LEU B 489   -19.256 41.198 17.802 1.00 3.71
O
ATOM 16552 CB LEU B 489   -19.906 38.801 15.593 1.00 4.41
C
ATOM 16553 CG LEU B 489   -20.265 38.558 14.115 1.00 4.41
C
ATOM 16554 CD1 LEU B 489   -21.209 37.363 14.011 1.00 4.41
C
ATOM 16555 CD2 LEU B 489   -20.918 39.802 13.543 1.00 4.41
C
ATOM 16556 H   LEU B 489   -17.462 38.373 15.078 1.00 3.10
H
ATOM 16557 HA LEU B 489   -19.020 40.752 15.328 1.00 3.53
H
ATOM 16558 1HB LEU B 489   -19.599 37.846 16.015 1.00 5.29
H
ATOM 16559 2HB LEU B 489   -20.814 39.121 16.104 1.00 5.29
H
ATOM 16560 HG LEU B 489   -19.365 38.335 13.548 1.00 5.29
H
ATOM 16561 1HD1 LEU B 489   -21.459 37.188 12.964 1.00 5.29
H
ATOM 16562 2HD1 LEU B 489   -20.723 36.478 14.419 1.00 5.29
H
ATOM 16563 3HD1 LEU B 489   -22.119 37.569 14.572 1.00 5.29
H
ATOM 16564 1HD2 LEU B 489   -21.170 39.631 12.497 1.00 5.29
H
ATOM 16565 2HD2 LEU B 489   -21.825 40.027 14.104 1.00 5.29
H
ATOM 16566 3HD2 LEU B 489   -20.227 40.642 13.619 1.00 5.29
H
ATOM 16567 N   ALA B 490   -18.127 39.270 18.143 1.00 2.98
N
ATOM 16568 CA ALA B 490   -17.963 39.465 19.576 1.00 3.19
C

ATOM 16569 C ALA B 490 -17.080 40.673 19.842 1.00 3.41
C
ATOM 16570 O ALA B 490 -17.357 41.464 20.749 1.00 3.75
O
ATOM 16571 CB ALA B 490 -17.370 38.220 20.212 1.00 4.79
C
ATOM 16572 H ALA B 490 -17.764 38.420 17.732 1.00 3.58
H
ATOM 16573 HA ALA B 490 -18.942 39.658 20.011 1.00 3.83
H
ATOM 16574 1HB ALA B 490 -17.269 38.371 21.286 1.00 5.74
H
ATOM 16575 2HB ALA B 490 -18.027 37.370 20.024 1.00 5.74
H
ATOM 16576 3HB ALA B 490 -16.393 38.021 19.778 1.00 5.74
H
ATOM 16577 N ALA B 491 -16.027 40.812 19.035 1.00 3.26
N
ATOM 16578 CA ALA B 491 -15.118 41.935 19.133 1.00 3.46
C
ATOM 16579 C ALA B 491 -15.867 43.226 18.900 1.00 3.82
C
ATOM 16580 O ALA B 491 -15.689 44.173 19.656 1.00 4.22
O
ATOM 16581 CB ALA B 491 -13.982 41.792 18.134 1.00 5.19
C
ATOM 16582 H ALA B 491 -15.838 40.109 18.335 1.00 3.91
H
ATOM 16583 HA ALA B 491 -14.708 41.956 20.142 1.00 4.15
H
ATOM 16584 1HB ALA B 491 -13.302 42.637 18.231 1.00 6.23
H
ATOM 16585 2HB ALA B 491 -13.443 40.866 18.328 1.00 6.23
H
ATOM 16586 3HB ALA B 491 -14.384 41.771 17.124 1.00 6.23
H
ATOM 16587 N ALA B 492 -16.729 43.244 17.875 1.00 3.77
N
ATOM 16588 CA ALA B 492 -17.534 44.413 17.543 1.00 4.16
C
ATOM 16589 C ALA B 492 -18.375 44.857 18.724 1.00 4.11
C
ATOM 16590 O ALA B 492 -18.442 46.050 19.023 1.00 4.21
O
ATOM 16591 CB ALA B 492 -18.448 44.117 16.359 1.00 6.24
C
ATOM 16592 H ALA B 492 -16.810 42.426 17.285 1.00 4.52
H
ATOM 16593 HA ALA B 492 -16.859 45.226 17.279 1.00 4.99
H
ATOM 16594 1HB ALA B 492 -19.021 45.009 16.111 1.00 7.49
H
ATOM 16595 2HB ALA B 492 -17.857 43.816 15.498 1.00 7.49
H
ATOM 16596 3HB ALA B 492 -19.131 43.312 16.619 1.00 7.49
H
ATOM 16597 N ASP B 493 -19.010 43.895 19.396 1.00 4.13
N

ATOM 16598 CA ASP B 493 -19.830 44.203 20.558 1.00 4.25
C
ATOM 16599 C ASP B 493 -19.014 44.871 21.664 1.00 4.25
C
ATOM 16600 O ASP B 493 -19.425 45.894 22.217 1.00 4.52
O
ATOM 16601 CB ASP B 493 -20.496 42.933 21.089 1.00 6.38
C
ATOM 16602 CG ASP B 493 -21.608 42.422 20.180 1.00 6.38
C
ATOM 16603 OD1 ASP B 493 -22.043 43.158 19.326 1.00 6.38
O
ATOM 16604 OD2 ASP B 493 -22.018 41.299 20.356 1.00 6.38
O
ATOM 16605 H ASP B 493 -18.937 42.933 19.078 1.00 4.96
H
ATOM 16606 HA ASP B 493 -20.613 44.896 20.250 1.00 5.10
H
ATOM 16607 1HB ASP B 493 -19.749 42.148 21.203 1.00 7.65
H
ATOM 16608 2HB ASP B 493 -20.916 43.130 22.076 1.00 7.65
H
ATOM 16609 N GLU B 494 -17.835 44.325 21.951 1.00 4.13
N
ATOM 16610 CA GLU B 494 -16.971 44.905 22.973 1.00 4.18
C
ATOM 16611 C GLU B 494 -16.388 46.248 22.552 1.00 4.34
C
ATOM 16612 O GLU B 494 -16.206 47.138 23.382 1.00 5.03
O
ATOM 16613 CB GLU B 494 -15.856 43.937 23.312 1.00 6.27
C
ATOM 16614 CG GLU B 494 -16.317 42.710 24.078 1.00 6.27
C
ATOM 16615 CD GLU B 494 -16.879 43.074 25.424 1.00 6.27
C
ATOM 16616 OE1 GLU B 494 -16.228 43.797 26.139 1.00 6.27
O
ATOM 16617 OE2 GLU B 494 -17.966 42.651 25.739 1.00 6.27
O
ATOM 16618 H GLU B 494 -17.544 43.477 21.471 1.00 4.96
H
ATOM 16619 HA GLU B 494 -17.569 45.067 23.869 1.00 5.02
H
ATOM 16620 1HB GLU B 494 -15.405 43.597 22.383 1.00 7.52
H
ATOM 16621 2HB GLU B 494 -15.087 44.441 23.894 1.00 7.52
H
ATOM 16622 1HG GLU B 494 -17.083 42.194 23.499 1.00 7.52
H
ATOM 16623 2HG GLU B 494 -15.475 42.032 24.208 1.00 7.52
H
ATOM 16624 N LEU B 495 -16.111 46.403 21.265 1.00 4.23
N
ATOM 16625 CA LEU B 495 -15.614 47.657 20.726 1.00 4.38
C
ATOM 16626 C LEU B 495 -16.653 48.766 20.819 1.00 4.48
C

ATOM 16627 O LEU B 495 -16.304 49.906 21.129 1.00 4.80
O
ATOM 16628 CB LEU B 495 -15.167 47.438 19.278 1.00 6.57
C
ATOM 16629 CG LEU B 495 -13.876 46.629 19.123 1.00 6.57
C
ATOM 16630 CD1 LEU B 495 -13.681 46.243 17.680 1.00 6.57
C
ATOM 16631 CD2 LEU B 495 -12.705 47.478 19.586 1.00 6.57
C
ATOM 16632 H LEU B 495 -16.245 45.631 20.630 1.00 5.08
H
ATOM 16633 HA LEU B 495 -14.745 47.957 21.311 1.00 5.26
H
ATOM 16634 1HB LEU B 495 -15.954 46.903 18.751 1.00 7.88
H
ATOM 16635 2HB LEU B 495 -15.023 48.397 18.792 1.00 7.88
H
ATOM 16636 HG LEU B 495 -13.933 45.728 19.722 1.00 7.88
H
ATOM 16637 1HD1 LEU B 495 -12.759 45.671 17.577 1.00 7.88
H
ATOM 16638 2HD1 LEU B 495 -14.524 45.637 17.348 1.00 7.88
H
ATOM 16639 3HD1 LEU B 495 -13.617 47.146 17.079 1.00 7.88
H
ATOM 16640 1HD2 LEU B 495 -11.781 46.914 19.478 1.00 7.88
H
ATOM 16641 2HD2 LEU B 495 -12.649 48.380 18.981 1.00 7.88
H
ATOM 16642 3HD2 LEU B 495 -12.846 47.752 20.633 1.00 7.88
H
ATOM 16643 N SER B 496 -17.937 48.427 20.626 1.00 4.51
N
ATOM 16644 CA SER B 496 -19.010 49.420 20.718 1.00 4.84
C
ATOM 16645 C SER B 496 -19.116 50.006 22.125 1.00 5.22
C
ATOM 16646 O SER B 496 -19.563 51.140 22.298 1.00 5.12
O
ATOM 16647 CB SER B 496 -20.350 48.815 20.340 1.00 7.26
C
ATOM 16648 OG SER B 496 -20.825 47.944 21.331 1.00 7.26
O
ATOM 16649 H SER B 496 -18.171 47.478 20.364 1.00 5.41
H
ATOM 16650 HA SER B 496 -18.786 50.232 20.024 1.00 5.81
H
ATOM 16651 1HB SER B 496 -21.074 49.613 20.176 1.00 8.71
H
ATOM 16652 2HB SER B 496 -20.245 48.274 19.400 1.00 8.71
H
ATOM 16653 HG SER B 496 -20.161 47.258 21.447 1.00 8.71
H
ATOM 16654 N LYS B 497 -18.645 49.264 23.129 1.00 6.35
N
ATOM 16655 CA LYS B 497 -18.635 49.742 24.508 1.00 6.68
C

ATOM 16656 C LYS B 497 -17.657 50.913 24.690 1.00 6.58
C
ATOM 16657 O LYS B 497 -17.754 51.665 25.661 1.00 6.95
O
ATOM 16658 CB LYS B 497 -18.284 48.592 25.453 1.00 10.02
C
ATOM 16659 CG LYS B 497 -19.366 47.520 25.531 1.00 10.02
C
ATOM 16660 CD LYS B 497 -18.961 46.357 26.429 1.00 10.02
C
ATOM 16661 CE LYS B 497 -20.091 45.340 26.540 1.00 10.02
C
ATOM 16662 NZ LYS B 497 -19.709 44.151 27.353 1.00 10.02
N
ATOM 16663 H LYS B 497 -18.295 48.335 22.939 1.00 7.62
H
ATOM 16664 HA LYS B 497 -19.635 50.100 24.757 1.00 8.02
H
ATOM 16665 1HB LYS B 497 -17.353 48.125 25.148 1.00 12.02
H
ATOM 16666 2HB LYS B 497 -18.137 48.985 26.459 1.00 12.02
H
ATOM 16667 1HG LYS B 497 -20.284 47.961 25.919 1.00 12.02
H
ATOM 16668 2HG LYS B 497 -19.565 47.137 24.530 1.00 12.02
H
ATOM 16669 1HD LYS B 497 -18.077 45.864 26.018 1.00 12.02
H
ATOM 16670 2HD LYS B 497 -18.719 46.729 27.424 1.00 12.02
H
ATOM 16671 1HE LYS B 497 -20.947 45.824 27.009 1.00 12.02
H
ATOM 16672 2HE LYS B 497 -20.379 45.008 25.543 1.00 12.02
H
ATOM 16673 1HZ LYS B 497 -20.490 43.514 27.406 1.00 12.02
H
ATOM 16674 2HZ LYS B 497 -18.928 43.679 26.909 1.00 12.02
H
ATOM 16675 3HZ LYS B 497 -19.446 44.440 28.283 1.00 12.02
H
ATOM 16676 N GLN B 498 -16.720 51.064 23.749 1.00 6.83
N
ATOM 16677 CA GLN B 498 -15.755 52.147 23.748 1.00 7.22
C
ATOM 16678 C GLN B 498 -16.040 53.124 22.607 1.00 6.55
C
ATOM 16679 O GLN B 498 -15.163 53.898 22.222 1.00 7.22
O
ATOM 16680 CB GLN B 498 -14.341 51.590 23.609 1.00 10.83
C
ATOM 16681 CG GLN B 498 -13.936 50.657 24.733 1.00 10.83
C
ATOM 16682 CD GLN B 498 -13.911 51.353 26.080 1.00 10.83
C
ATOM 16683 OE1 GLN B 498 -13.334 52.435 26.222 1.00 10.83
O
ATOM 16684 NE2 GLN B 498 -14.537 50.738 27.076 1.00 10.83
N

```
ATOM  16685  H   GLN B 498      -16.671  50.418  22.977  1.00  8.20
H
ATOM  16686  HA  GLN B 498      -15.835  52.690  24.689  1.00  8.66
H
ATOM  16687 1HB  GLN B 498      -14.257  51.044  22.669  1.00 13.00
H
ATOM  16688 2HB  GLN B 498      -13.627  52.413  23.577  1.00 13.00
H
ATOM  16689 1HG  GLN B 498      -14.650  49.836  24.785  1.00 13.00
H
ATOM  16690 2HG  GLN B 498      -12.937  50.271  24.529  1.00 13.00
H
ATOM  16691 1HE2 GLN B 498      -14.556  51.151  27.987  1.00 13.00
H
ATOM  16692 2HE2 GLN B 498      -14.993  49.862  26.916  1.00 13.00
H
ATOM  16693  N   ASP B 499      -17.260  53.063  22.052  1.00  5.77
N
ATOM  16694  CA  ASP B 499      -17.684  53.893  20.927  1.00  5.87
C
ATOM  16695  C   ASP B 499      -16.880  53.611  19.658  1.00  5.48
C
ATOM  16696  O   ASP B 499      -16.669  54.507  18.839  1.00  6.09
O
ATOM  16697  CB  ASP B 499      -17.591  55.383  21.278  1.00  8.80
C
ATOM  16698  CG  ASP B 499      -18.534  55.781  22.404  1.00  8.80
C
ATOM  16699  OD1 ASP B 499      -19.634  55.282  22.440  1.00  8.80
O
ATOM  16700  OD2 ASP B 499      -18.146  56.584  23.219  1.00  8.80
O
ATOM  16701  H   ASP B 499      -17.946  52.412  22.410  1.00  6.92
H
ATOM  16702  HA  ASP B 499      -18.729  53.666  20.714  1.00  7.04
H
ATOM  16703 1HB  ASP B 499      -16.575  55.647  21.563  1.00 10.57
H
ATOM  16704 2HB  ASP B 499      -17.841  55.973  20.397  1.00 10.57
H
ATOM  16705  N   ILE B 500      -16.441  52.363  19.493  1.00  4.98
N
ATOM  16706  CA  ILE B 500      -15.693  51.955  18.315  1.00  4.78
C
ATOM  16707  C   ILE B 500      -16.572  51.094  17.433  1.00  4.81
C
ATOM  16708  O   ILE B 500      -17.158  50.114  17.892  1.00  4.90
O
ATOM  16709  CB  ILE B 500      -14.422  51.175  18.689  1.00  7.17
C
ATOM  16710  CG1 ILE B 500      -13.487  52.044  19.527  1.00  7.17
C
ATOM  16711  CG2 ILE B 500      -13.729  50.681  17.427  1.00  7.17
C
ATOM  16712  CD1 ILE B 500      -12.346  51.268  20.143  1.00  7.17
C
ATOM  16713  H   ILE B 500      -16.627  51.661  20.197  1.00  5.98
H
```

```
ATOM  16714  HA  ILE B 500     -15.406  52.842  17.754  1.00  5.74
H
ATOM  16715  HB  ILE B 500     -14.691  50.326  19.308  1.00  8.60
H
ATOM  16716 1HG1 ILE B 500     -13.072  52.831  18.897  1.00  8.60
H
ATOM  16717 2HG1 ILE B 500     -14.057  52.513  20.328  1.00  8.60
H
ATOM  16718 1HG2 ILE B 500     -12.836  50.120  17.697  1.00  8.60
H
ATOM  16719 2HG2 ILE B 500     -14.405  50.037  16.867  1.00  8.60
H
ATOM  16720 3HG2 ILE B 500     -13.448  51.534  16.810  1.00  8.60
H
ATOM  16721 1HD1 ILE B 500     -11.720  51.945  20.726  1.00  8.60
H
ATOM  16722 2HD1 ILE B 500     -12.745  50.491  20.796  1.00  8.60
H
ATOM  16723 3HD1 ILE B 500     -11.749  50.810  19.356  1.00  8.60
H
ATOM  16724  N   PHE B 501     -16.682  51.458  16.170  1.00  5.31
N
ATOM  16725  CA  PHE B 501     -17.529  50.697  15.275  1.00  5.57
C
ATOM  16726  C   PHE B 501     -16.703  50.026  14.204  1.00  4.44
C
ATOM  16727  O   PHE B 501     -15.762  50.621  13.675  1.00  8.24
O
ATOM  16728  CB  PHE B 501     -18.570  51.627  14.668  1.00  8.36
C
ATOM  16729  CG  PHE B 501     -19.468  52.195  15.726  1.00  8.36
C
ATOM  16730  CD1 PHE B 501     -19.142  53.388  16.355  1.00  8.36
C
ATOM  16731  CD2 PHE B 501     -20.622  51.533  16.113  1.00  8.36
C
ATOM  16732  CE1 PHE B 501     -19.949  53.908  17.348  1.00  8.36
C
ATOM  16733  CE2 PHE B 501     -21.432  52.050  17.105  1.00  8.36
C
ATOM  16734  CZ  PHE B 501     -21.094  53.239  17.723  1.00  8.36
C
ATOM  16735  H   PHE B 501     -16.182  52.268  15.831  1.00  6.37
H
ATOM  16736  HA  PHE B 501     -18.043  49.925  15.847  1.00  6.68
H
ATOM  16737 1HB  PHE B 501     -18.079  52.449  14.150  1.00 10.03
H
ATOM  16738 2HB  PHE B 501     -19.181  51.088  13.945  1.00 10.03
H
ATOM  16739  HD1 PHE B 501     -18.233  53.914  16.062  1.00 10.03
H
ATOM  16740  HD2 PHE B 501     -20.884  50.593  15.628  1.00 10.03
H
ATOM  16741  HE1 PHE B 501     -19.680  54.845  17.834  1.00 10.03
H
ATOM  16742  HE2 PHE B 501     -22.337  51.520  17.403  1.00 10.03
H
```

```
ATOM  16743  HZ  PHE B 501     -21.731  53.648  18.506  1.00 10.03
H
ATOM  16744  N   ILE B 502     -17.032  48.770  13.905  1.00  4.77
N
ATOM  16745  CA  ILE B 502     -16.304  48.065  12.867  1.00  3.98
C
ATOM  16746  C   ILE B 502     -17.260  47.424  11.879  1.00  4.21
C
ATOM  16747  O   ILE B 502     -18.425  47.163  12.186  1.00  5.29
O
ATOM  16748  CB  ILE B 502     -15.369  46.969  13.432  1.00  5.97
C
ATOM  16749  CG1 ILE B 502     -16.146  45.840  14.105  1.00  5.97
C
ATOM  16750  CG2 ILE B 502     -14.440  47.583  14.458  1.00  5.97
C
ATOM  16751  CD1 ILE B 502     -15.265  44.655  14.459  1.00  5.97
C
ATOM  16752  H   ILE B 502     -17.800  48.320  14.381  1.00  5.72
H
ATOM  16753  HA  ILE B 502     -15.691  48.782  12.324  1.00  4.78
H
ATOM  16754  HB  ILE B 502     -14.782  46.542  12.620  1.00  7.16
H
ATOM  16755 1HG1 ILE B 502     -16.606  46.216  15.017  1.00  7.16
H
ATOM  16756 2HG1 ILE B 502     -16.936  45.497  13.437  1.00  7.16
H
ATOM  16757 1HG2 ILE B 502     -13.764  46.818  14.834  1.00  7.16
H
ATOM  16758 2HG2 ILE B 502     -13.867  48.383  14.003  1.00  7.16
H
ATOM  16759 3HG2 ILE B 502     -15.023  47.987  15.284  1.00  7.16
H
ATOM  16760 1HD1 ILE B 502     -15.857  43.876  14.935  1.00  7.16
H
ATOM  16761 2HD1 ILE B 502     -14.816  44.258  13.549  1.00  7.16
H
ATOM  16762 3HD1 ILE B 502     -14.479  44.976  15.139  1.00  7.16
H
ATOM  16763  N   ARG B 503     -16.736  47.159  10.700  1.00  4.06
N
ATOM  16764  CA  ARG B 503     -17.459  46.528   9.620  1.00  4.31
C
ATOM  16765  C   ARG B 503     -17.128  45.046   9.614  1.00  4.73
C
ATOM  16766  O   ARG B 503     -15.963  44.680   9.537  1.00  7.59
O
ATOM  16767  CB  ARG B 503     -17.056  47.197   8.317  1.00  6.46
C
ATOM  16768  CG  ARG B 503     -17.638  46.677   7.025  1.00  6.46
C
ATOM  16769  CD  ARG B 503     -19.048  47.039   6.886  1.00  6.46
C
ATOM  16770  NE  ARG B 503     -19.220  48.465   7.053  1.00  6.46
N
ATOM  16771  CZ  ARG B 503     -19.144  49.399   6.094  1.00  6.46
C
```

```
ATOM  16772  NH1 ARG B 503     -18.923  49.076   4.840  1.00  6.46
N
ATOM  16773  NH2 ARG B 503     -19.300  50.653   6.463  1.00  6.46
N
ATOM  16774  H   ARG B 503     -15.769  47.402  10.539  1.00  4.87
H
ATOM  16775  HA  ARG B 503     -18.527  46.664   9.781  1.00  5.17
H
ATOM  16776 1HB  ARG B 503     -17.344  48.245   8.373  1.00  7.76
H
ATOM  16777 2HB  ARG B 503     -15.979  47.182   8.218  1.00  7.76
H
ATOM  16778 1HG  ARG B 503     -17.106  47.145   6.209  1.00  7.76
H
ATOM  16779 2HG  ARG B 503     -17.537  45.594   6.968  1.00  7.76
H
ATOM  16780 1HD  ARG B 503     -19.398  46.768   5.890  1.00  7.76
H
ATOM  16781 2HD  ARG B 503     -19.641  46.530   7.630  1.00  7.76
H
ATOM  16782  HE  ARG B 503     -19.377  48.807   7.995  1.00  7.76
H
ATOM  16783 1HH1 ARG B 503     -18.802  48.106   4.584  1.00  7.76
H
ATOM  16784 2HH1 ARG B 503     -18.869  49.796   4.134  1.00  7.76
H
ATOM  16785 1HH2 ARG B 503     -19.457  50.838   7.449  1.00  7.76
H
ATOM  16786 2HH2 ARG B 503     -19.251  51.401   5.787  1.00  7.76
H
ATOM  16787  N   VAL B 504     -18.126  44.179   9.726  1.00  3.36
N
ATOM  16788  CA  VAL B 504     -17.828  42.745   9.746  1.00  3.12
C
ATOM  16789  C   VAL B 504     -18.195  42.104   8.423  1.00  2.88
C
ATOM  16790  O   VAL B 504     -19.356  42.143   8.012  1.00  2.82
O
ATOM  16791  CB  VAL B 504     -18.555  42.029  10.906  1.00  4.68
C
ATOM  16792  CG1 VAL B 504     -18.248  40.536  10.866  1.00  4.68
C
ATOM  16793  CG2 VAL B 504     -18.094  42.619  12.237  1.00  4.68
C
ATOM  16794  H   VAL B 504     -19.079  44.513   9.774  1.00  4.03
H
ATOM  16795  HA  VAL B 504     -16.758  42.618   9.901  1.00  3.74
H
ATOM  16796  HB  VAL B 504     -19.630  42.154  10.794  1.00  5.62
H
ATOM  16797 1HG1 VAL B 504     -18.765  40.032  11.681  1.00  5.62
H
ATOM  16798 2HG1 VAL B 504     -18.581  40.123   9.914  1.00  5.62
H
ATOM  16799 3HG1 VAL B 504     -17.174  40.385  10.971  1.00  5.62
H
ATOM  16800 1HG2 VAL B 504     -18.606  42.116  13.056  1.00  5.62
H
```

```
ATOM  16801 2HG2 VAL B 504     -17.018  42.479  12.342  1.00  5.62
H
ATOM  16802 3HG2 VAL B 504     -18.326  43.684  12.263  1.00  5.62
H
ATOM  16803  N   ILE B 505     -17.182  41.541   7.757  1.00  2.80
N
ATOM  16804  CA  ILE B 505     -17.330  40.915   6.449  1.00  2.62
C
ATOM  16805  C   ILE B 505     -17.186  39.399   6.458  1.00  2.40
C
ATOM  16806  O   ILE B 505     -16.231  38.844   7.006  1.00  2.56
O
ATOM  16807  CB  ILE B 505     -16.301  41.488   5.461  1.00  3.93
C
ATOM  16808  CG1 ILE B 505     -16.462  43.001   5.371  1.00  3.93
C
ATOM  16809  CG2 ILE B 505     -16.440  40.839   4.086  1.00  3.93
C
ATOM  16810  CD1 ILE B 505     -17.820  43.441   4.897  1.00  3.93
C
ATOM  16811  H   ILE B 505     -16.263  41.557   8.179  1.00  3.36
H
ATOM  16812  HA  ILE B 505     -18.325  41.151   6.075  1.00  3.14
H
ATOM  16813  HB  ILE B 505     -15.303  41.297   5.846  1.00  4.72
H
ATOM  16814 1HG1 ILE B 505     -16.279  43.437   6.353  1.00  4.72
H
ATOM  16815 2HG1 ILE B 505     -15.719  43.394   4.677  1.00  4.72
H
ATOM  16816 1HG2 ILE B 505     -15.693  41.255   3.411  1.00  4.72
H
ATOM  16817 2HG2 ILE B 505     -16.291  39.765   4.171  1.00  4.72
H
ATOM  16818 3HG2 ILE B 505     -17.437  41.037   3.690  1.00  4.72
H
ATOM  16819 1HD1 ILE B 505     -17.855  44.530   4.854  1.00  4.72
H
ATOM  16820 2HD1 ILE B 505     -18.008  43.032   3.903  1.00  4.72
H
ATOM  16821 3HD1 ILE B 505     -18.580  43.080   5.588  1.00  4.72
H
ATOM  16822  N   ASP B 506     -18.147  38.747   5.819  1.00  2.11
N
ATOM  16823  CA  ASP B 506     -18.150  37.314   5.610  1.00  2.09
C
ATOM  16824  C   ASP B 506     -17.888  37.022   4.152  1.00  1.80
C
ATOM  16825  O   ASP B 506     -18.744  37.254   3.303  1.00  1.53
O
ATOM  16826  CB  ASP B 506     -19.462  36.695   6.000  1.00  3.13
C
ATOM  16827  CG  ASP B 506     -19.425  35.195   5.823  1.00  3.13
C
ATOM  16828  OD1 ASP B 506     -18.477  34.686   5.270  1.00  3.13
O
ATOM  16829  OD2 ASP B 506     -20.372  34.564   6.227  1.00  3.13
O
```

ATOM 16830 H   ASP B 506   -18.909 39.276   5.420 1.00 2.53
H
ATOM 16831 HA ASP B 506   -17.352 36.866   6.204 1.00 2.51
H
ATOM 16832 1HB ASP B 506   -19.675 36.921   7.041 1.00 3.76
H
ATOM 16833 2HB ASP B 506   -20.266 37.112   5.393 1.00 3.76
H
ATOM 16834 N   LEU B 507   -16.714 36.526   3.836 1.00 1.95
N
ATOM 16835 CA LEU B 507   -16.380 36.301   2.441 1.00 1.80
C
ATOM 16836 C   LEU B 507   -17.210 35.226   1.737 1.00 1.53
C
ATOM 16837 O   LEU B 507   -17.316 35.248   0.509 1.00 1.47
O
ATOM 16838 CB LEU B 507   -14.918 35.918   2.338 1.00 2.70
C
ATOM 16839 CG LEU B 507   -13.946 37.017   2.733 1.00 2.70
C
ATOM 16840 CD1 LEU B 507   -12.544 36.450   2.684 1.00 2.70
C
ATOM 16841 CD2 LEU B 507   -14.123 38.215   1.811 1.00 2.70
C
ATOM 16842 H   LEU B 507   -16.040 36.323   4.561 1.00 2.34
H
ATOM 16843 HA LEU B 507   -16.526 37.238   1.912 1.00 2.16
H
ATOM 16844 1HB LEU B 507   -14.733 35.060   2.981 1.00 3.24
H
ATOM 16845 2HB LEU B 507   -14.702 35.633   1.310 1.00 3.24
H
ATOM 16846 HG LEU B 507   -14.146 37.327   3.756 1.00 3.24
H
ATOM 16847 1HD1 LEU B 507   -11.827 37.215   2.974 1.00 3.24
H
ATOM 16848 2HD1 LEU B 507   -12.476 35.606   3.371 1.00 3.24
H
ATOM 16849 3HD1 LEU B 507   -12.323 36.108   1.679 1.00 3.24
H
ATOM 16850 1HD2 LEU B 507   -13.426 39.002   2.100 1.00 3.24
H
ATOM 16851 2HD2 LEU B 507   -13.930 37.917   0.783 1.00 3.24
H
ATOM 16852 3HD2 LEU B 507   -15.144 38.587   1.892 1.00 3.24
H
ATOM 16853 N   PHE B 508   -17.777 34.282   2.498 1.00 1.60
N
ATOM 16854 CA PHE B 508   -18.493 33.125   1.946 1.00 1.47
C
ATOM 16855 C   PHE B 508   -17.524 32.225   1.169 1.00 1.09
C
ATOM 16856 O   PHE B 508   -17.153 31.161   1.661 1.00 1.02
O
ATOM 16857 CB PHE B 508   -19.737 33.512   1.143 1.00 2.21
C
ATOM 16858 CG PHE B 508   -20.495 32.306   0.656 1.00 2.21
C

```
ATOM  16859  CD1 PHE B 508     -20.973  31.369   1.564  1.00  2.21
C
ATOM  16860  CD2 PHE B 508     -20.752  32.111  -0.691  1.00  2.21
C
ATOM  16861  CE1 PHE B 508     -21.673  30.257   1.136  1.00  2.21
C
ATOM  16862  CE2 PHE B 508     -21.450  31.001  -1.122  1.00  2.21
C
ATOM  16863  CZ  PHE B 508     -21.911  30.072  -0.206  1.00  2.21
C
ATOM  16864  H   PHE B 508     -17.717  34.358   3.510  1.00  1.92
H
ATOM  16865  HA  PHE B 508     -18.861  32.536   2.788  1.00  1.76
H
ATOM  16866 1HB  PHE B 508     -20.403  34.112   1.761  1.00  2.65
H
ATOM  16867 2HB  PHE B 508     -19.458  34.111   0.278  1.00  2.65
H
ATOM  16868  HD1 PHE B 508     -20.784  31.519   2.628  1.00  2.65
H
ATOM  16869  HD2 PHE B 508     -20.388  32.843  -1.413  1.00  2.65
H
ATOM  16870  HE1 PHE B 508     -22.036  29.530   1.861  1.00  2.65
H
ATOM  16871  HE2 PHE B 508     -21.636  30.861  -2.184  1.00  2.65
H
ATOM  16872  HZ  PHE B 508     -22.461  29.196  -0.542  1.00  2.65
H
ATOM  16873  N   THR B 509     -17.110  32.640  -0.031  1.00  1.07
N
ATOM  16874  CA  THR B 509     -16.082  31.886  -0.730  1.00  1.03
C
ATOM  16875  C   THR B 509     -14.748  32.611  -0.586  1.00  1.06
C
ATOM  16876  O   THR B 509     -14.682  33.838  -0.532  1.00  1.15
O
ATOM  16877  CB  THR B 509     -16.398  31.663  -2.239  1.00  1.54
C
ATOM  16878  OG1 THR B 509     -16.533  32.920  -2.922  1.00  1.54
O
ATOM  16879  CG2 THR B 509     -17.667  30.849  -2.404  1.00  1.54
C
ATOM  16880  H   THR B 509     -17.466  33.509  -0.408  1.00  1.28
H
ATOM  16881  HA  THR B 509     -15.985  30.910  -0.259  1.00  1.24
H
ATOM  16882  HB  THR B 509     -15.577  31.111  -2.694  1.00  1.85
H
ATOM  16883  HG1 THR B 509     -15.660  33.247  -3.185  1.00  1.85
H
ATOM  16884 1HG2 THR B 509     -17.860  30.685  -3.463  1.00  1.85
H
ATOM  16885 2HG2 THR B 509     -17.547  29.889  -1.905  1.00  1.85
H
ATOM  16886 3HG2 THR B 509     -18.499  31.388  -1.965  1.00  1.85
H
ATOM  16887  N   ILE B 510     -13.689  31.828  -0.540  1.00  1.02
N
```

ATOM 16888 CA ILE B 510 -12.330 32.321 -0.436 1.00 1.07
C
ATOM 16889 C ILE B 510 -11.800 32.426 -1.838 1.00 1.07
C
ATOM 16890 O ILE B 510 -11.063 33.349 -2.185 1.00 1.20
O
ATOM 16891 CB ILE B 510 -11.451 31.386 0.407 1.00 1.60
C
ATOM 16892 CG1 ILE B 510 -11.970 31.347 1.843 1.00 1.60
C
ATOM 16893 CG2 ILE B 510 -10.003 31.854 0.372 1.00 1.60
C
ATOM 16894 CD1 ILE B 510 -11.329 30.272 2.680 1.00 1.60
C
ATOM 16895 H ILE B 510 -13.843 30.835 -0.598 1.00 1.22
H
ATOM 16896 HA ILE B 510 -12.335 33.313 0.015 1.00 1.28
H
ATOM 16897 HB ILE B 510 -11.511 30.377 0.010 1.00 1.93
H
ATOM 16898 1HG1 ILE B 510 -11.785 32.311 2.312 1.00 1.93
H
ATOM 16899 2HG1 ILE B 510 -13.047 31.176 1.830 1.00 1.93
H
ATOM 16900 1HG2 ILE B 510 -9.392 31.181 0.969 1.00 1.93
H
ATOM 16901 2HG2 ILE B 510 -9.646 31.855 -0.656 1.00 1.93
H
ATOM 16902 3HG2 ILE B 510 -9.939 32.862 0.782 1.00 1.93
H
ATOM 16903 1HD1 ILE B 510 -11.738 30.303 3.687 1.00 1.93
H
ATOM 16904 2HD1 ILE B 510 -11.525 29.296 2.242 1.00 1.93
H
ATOM 16905 3HD1 ILE B 510 -10.255 30.442 2.719 1.00 1.93
H
ATOM 16906 N LYS B 511 -12.182 31.442 -2.641 1.00 1.02
N
ATOM 16907 CA LYS B 511 -11.810 31.401 -4.034 1.00 1.02
C
ATOM 16908 C LYS B 511 -13.045 31.052 -4.857 1.00 0.90
C
ATOM 16909 O LYS B 511 -13.568 29.948 -4.752 1.00 0.74
O
ATOM 16910 CB LYS B 511 -10.710 30.373 -4.267 1.00 1.53
C
ATOM 16911 CG LYS B 511 -10.205 30.331 -5.695 1.00 1.53
C
ATOM 16912 CD LYS B 511 -9.110 29.296 -5.872 1.00 1.53
C
ATOM 16913 CE LYS B 511 -8.613 29.290 -7.308 1.00 1.53
C
ATOM 16914 NZ LYS B 511 -7.645 28.199 -7.558 1.00 1.53
N
ATOM 16915 H LYS B 511 -12.781 30.710 -2.273 1.00 1.22
H
ATOM 16916 HA LYS B 511 -11.444 32.380 -4.334 1.00 1.22
H

ATOM 16917 1HB LYS B 511 -9.863 30.591 -3.616 1.00 1.84
H
ATOM 16918 2HB LYS B 511 -11.076 29.380 -4.010 1.00 1.84
H
ATOM 16919 1HG LYS B 511 -11.031 30.087 -6.364 1.00 1.84
H
ATOM 16920 2HG LYS B 511 -9.816 31.310 -5.972 1.00 1.84
H
ATOM 16921 1HD LYS B 511 -8.276 29.531 -5.209 1.00 1.84
H
ATOM 16922 2HD LYS B 511 -9.488 28.306 -5.617 1.00 1.84
H
ATOM 16923 1HE LYS B 511 -9.458 29.177 -7.985 1.00 1.84
H
ATOM 16924 2HE LYS B 511 -8.125 30.242 -7.512 1.00 1.84
H
ATOM 16925 1HZ LYS B 511 -7.331 28.238 -8.517 1.00 1.84
H
ATOM 16926 2HZ LYS B 511 -6.850 28.283 -6.941 1.00 1.84
H
ATOM 16927 3HZ LYS B 511 -8.091 27.307 -7.390 1.00 1.84
H
ATOM 16928 N PRO B 512 -13.531 31.975 -5.681 1.00 1.03
N
ATOM 16929 CA PRO B 512 -13.101 33.338 -5.952 1.00 1.22
C
ATOM 16930 C PRO B 512 -13.385 34.271 -4.782 1.00 1.32
C
ATOM 16931 O PRO B 512 -14.294 34.045 -3.975 1.00 1.24
O
ATOM 16932 CB PRO B 512 -13.917 33.723 -7.193 1.00 1.83
C
ATOM 16933 CG PRO B 512 -15.129 32.863 -7.130 1.00 1.83
C
ATOM 16934 CD PRO B 512 -14.640 31.569 -6.550 1.00 1.83
C
ATOM 16935 HA PRO B 512 -12.026 33.335 -6.188 1.00 1.46
H
ATOM 16936 1HB PRO B 512 -14.156 34.797 -7.168 1.00 2.20
H
ATOM 16937 2HB PRO B 512 -13.321 33.551 -8.102 1.00 2.20
H
ATOM 16938 1HG PRO B 512 -15.899 33.335 -6.509 1.00 2.20
H
ATOM 16939 2HG PRO B 512 -15.561 32.741 -8.134 1.00 2.20
H
ATOM 16940 1HD PRO B 512 -15.421 31.090 -5.945 1.00 2.20
H
ATOM 16941 2HD PRO B 512 -14.271 30.900 -7.342 1.00 2.20
H
ATOM 16942 N LEU B 513 -12.582 35.323 -4.705 1.00 1.57
N
ATOM 16943 CA LEU B 513 -12.671 36.295 -3.633 1.00 1.73
C
ATOM 16944 C LEU B 513 -13.384 37.563 -4.080 1.00 1.78
C
ATOM 16945 O LEU B 513 -12.983 38.195 -5.059 1.00 2.21
O

```
ATOM  16946  CB  LEU B 513     -11.261  36.651  -3.165  1.00  2.59
C
ATOM  16947  CG  LEU B 513     -11.188  37.650  -2.026  1.00  2.59
C
ATOM  16948  CD1 LEU B 513     -11.784  36.999  -0.800  1.00  2.59
C
ATOM  16949  CD2 LEU B 513      -9.750  38.049  -1.786  1.00  2.59
C
ATOM  16950  H   LEU B 513     -11.871  35.446  -5.411  1.00  1.88
H
ATOM  16951  HA  LEU B 513     -13.226  35.852  -2.807  1.00  2.08
H
ATOM  16952 1HB  LEU B 513     -10.759  35.742  -2.842  1.00  3.11
H
ATOM  16953 2HB  LEU B 513     -10.709  37.067  -4.007  1.00  3.11
H
ATOM  16954  HG  LEU B 513     -11.777  38.534  -2.268  1.00  3.11
H
ATOM  16955 1HD1 LEU B 513     -11.747  37.695   0.035  1.00  3.11
H
ATOM  16956 2HD1 LEU B 513     -12.818  36.724  -1.001  1.00  3.11
H
ATOM  16957 3HD1 LEU B 513     -11.213  36.106  -0.552  1.00  3.11
H
ATOM  16958 1HD2 LEU B 513      -9.704  38.758  -0.963  1.00  3.11
H
ATOM  16959 2HD2 LEU B 513      -9.164  37.164  -1.538  1.00  3.11
H
ATOM  16960 3HD2 LEU B 513      -9.349  38.507  -2.688  1.00  3.11
H
ATOM  16961  N   ASP B 514     -14.439  37.942  -3.362  1.00  1.68
N
ATOM  16962  CA  ASP B 514     -15.193  39.151  -3.683  1.00  1.81
C
ATOM  16963  C   ASP B 514     -14.466  40.455  -3.376  1.00  2.05
C
ATOM  16964  O   ASP B 514     -14.822  41.154  -2.424  1.00  2.21
O
ATOM  16965  CB  ASP B 514     -16.502  39.164  -2.933  1.00  2.71
C
ATOM  16966  CG  ASP B 514     -17.350  40.358  -3.308  1.00  2.71
C
ATOM  16967  OD1 ASP B 514     -16.862  41.211  -4.014  1.00  2.71
O
ATOM  16968  OD2 ASP B 514     -18.476  40.417  -2.878  1.00  2.71
O
ATOM  16969  H   ASP B 514     -14.726  37.378  -2.573  1.00  2.02
H
ATOM  16970  HA  ASP B 514     -15.410  39.134  -4.752  1.00  2.17
H
ATOM  16971 1HB  ASP B 514     -17.055  38.247  -3.125  1.00  3.26
H
ATOM  16972 2HB  ASP B 514     -16.294  39.205  -1.866  1.00  3.26
H
ATOM  16973  N   VAL B 515     -13.500  40.799  -4.220  1.00  2.25
N
ATOM  16974  CA  VAL B 515     -12.727  42.023  -4.069  1.00  2.67
C
```

ATOM 16975 C VAL B 515 -13.558 43.297 -4.211 1.00 2.52
C
ATOM 16976 O VAL B 515 -13.184 44.334 -3.670 1.00 2.48
O
ATOM 16977 CB VAL B 515 -11.563 42.045 -5.079 1.00 4.00
C
ATOM 16978 CG1 VAL B 515 -12.081 42.264 -6.493 1.00 4.00
C
ATOM 16979 CG2 VAL B 515 -10.592 43.150 -4.693 1.00 4.00
C
ATOM 16980 H VAL B 515 -13.280 40.159 -4.972 1.00 2.70
H
ATOM 16981 HA VAL B 515 -12.291 42.016 -3.070 1.00 3.20
H
ATOM 16982 HB VAL B 515 -11.055 41.081 -5.057 1.00 4.81
H
ATOM 16983 1HG1 VAL B 515 -11.241 42.262 -7.189 1.00 4.81
H
ATOM 16984 2HG1 VAL B 515 -12.771 41.463 -6.755 1.00 4.81
H
ATOM 16985 3HG1 VAL B 515 -12.594 43.222 -6.554 1.00 4.81
H
ATOM 16986 1HG2 VAL B 515 -9.759 43.162 -5.395 1.00 4.81
H
ATOM 16987 2HG2 VAL B 515 -11.108 44.108 -4.726 1.00 4.81
H
ATOM 16988 3HG2 VAL B 515 -10.216 42.972 -3.686 1.00 4.81
H
ATOM 16989 N ALA B 516 -14.675 43.234 -4.940 1.00 2.82
N
ATOM 16990 CA ALA B 516 -15.517 44.412 -5.129 1.00 2.93
C
ATOM 16991 C ALA B 516 -16.087 44.883 -3.796 1.00 2.92
C
ATOM 16992 O ALA B 516 -16.000 46.071 -3.464 1.00 3.05
O
ATOM 16993 CB ALA B 516 -16.639 44.104 -6.106 1.00 4.40
C
ATOM 16994 H ALA B 516 -14.947 42.360 -5.368 1.00 3.38
H
ATOM 16995 HA ALA B 516 -14.900 45.213 -5.535 1.00 3.52
H
ATOM 16996 1HB ALA B 516 -17.247 44.995 -6.255 1.00 5.27
H
ATOM 16997 2HB ALA B 516 -16.214 43.791 -7.060 1.00 5.27
H
ATOM 16998 3HB ALA B 516 -17.260 43.303 -5.706 1.00 5.27
H
ATOM 16999 N THR B 517 -16.654 43.943 -3.031 1.00 2.76
N
ATOM 17000 CA THR B 517 -17.177 44.253 -1.705 1.00 2.73
C
ATOM 17001 C THR B 517 -16.057 44.614 -0.753 1.00 2.80
C
ATOM 17002 O THR B 517 -16.202 45.557 0.023 1.00 2.97
O
ATOM 17003 CB THR B 517 -18.003 43.096 -1.095 1.00 4.09
C

```
ATOM  17004  OG1 THR B 517     -19.162  42.839  -1.903  1.00  4.09
O
ATOM  17005  CG2 THR B 517     -18.440  43.444   0.324  1.00  4.09
C
ATOM  17006  H   THR B 517     -16.718  42.989  -3.376  1.00  3.31
H
ATOM  17007  HA  THR B 517     -17.833  45.119  -1.791  1.00  3.28
H
ATOM  17008  HB  THR B 517     -17.394  42.194  -1.060  1.00  4.91
H
ATOM  17009  HG1 THR B 517     -18.982  42.096  -2.487  1.00  4.91
H
ATOM  17010 1HG2 THR B 517     -19.014  42.617   0.740  1.00  4.91
H
ATOM  17011 2HG2 THR B 517     -17.560  43.623   0.942  1.00  4.91
H
ATOM  17012 3HG2 THR B 517     -19.059  44.340   0.304  1.00  4.91
H
ATOM  17013  N   ILE B 518     -14.941  43.872  -0.806  1.00  2.67
N
ATOM  17014  CA  ILE B 518     -13.811  44.146   0.085  1.00  2.72
C
ATOM  17015  C   ILE B 518     -13.294  45.566  -0.060  1.00  2.95
C
ATOM  17016  O   ILE B 518     -13.117  46.259   0.940  1.00  3.15
O
ATOM  17017  CB  ILE B 518     -12.637  43.183  -0.173  1.00  4.08
C
ATOM  17018  CG1 ILE B 518     -13.000  41.763   0.250  1.00  4.08
C
ATOM  17019  CG2 ILE B 518     -11.393  43.655   0.568  1.00  4.08
C
ATOM  17020  CD1 ILE B 518     -12.012  40.735  -0.238  1.00  4.08
C
ATOM  17021  H   ILE B 518     -14.877  43.096  -1.458  1.00  3.20
H
ATOM  17022  HA  ILE B 518     -14.145  44.010   1.112  1.00  3.26
H
ATOM  17023  HB  ILE B 518     -12.424  43.158  -1.240  1.00  4.90
H
ATOM  17024 1HG1 ILE B 518     -13.038  41.715   1.338  1.00  4.90
H
ATOM  17025 2HG1 ILE B 518     -13.986  41.512  -0.136  1.00  4.90
H
ATOM  17026 1HG2 ILE B 518     -10.568  42.976   0.362  1.00  4.90
H
ATOM  17027 2HG2 ILE B 518     -11.128  44.657   0.234  1.00  4.90
H
ATOM  17028 3HG2 ILE B 518     -11.592  43.672   1.639  1.00  4.90
H
ATOM  17029 1HD1 ILE B 518     -12.323  39.750   0.100  1.00  4.90
H
ATOM  17030 2HD1 ILE B 518     -11.975  40.752  -1.327  1.00  4.90
H
ATOM  17031 3HD1 ILE B 518     -11.024  40.961   0.161  1.00  4.90
H
ATOM  17032  N   VAL B 519     -13.052  45.989  -1.297  1.00  2.96
N
```

ATOM 17033 CA VAL B 519 -12.569 47.333 -1.574 1.00 3.15
C
ATOM 17034 C VAL B 519 -13.577 48.406 -1.221 1.00 3.39
C
ATOM 17035 O VAL B 519 -13.208 49.420 -0.630 1.00 3.52
O
ATOM 17036 CB VAL B 519 -12.178 47.472 -3.050 1.00 4.72
C
ATOM 17037 CG1 VAL B 519 -11.898 48.927 -3.378 1.00 4.72
C
ATOM 17038 CG2 VAL B 519 -10.931 46.647 -3.312 1.00 4.72
C
ATOM 17039 H VAL B 519 -13.199 45.362 -2.075 1.00 3.55
H
ATOM 17040 HA VAL B 519 -11.673 47.497 -0.975 1.00 3.78
H
ATOM 17041 HB VAL B 519 -12.998 47.129 -3.681 1.00 5.67
H
ATOM 17042 1HG1 VAL B 519 -11.620 49.015 -4.428 1.00 5.67
H
ATOM 17043 2HG1 VAL B 519 -12.793 49.521 -3.188 1.00 5.67
H
ATOM 17044 3HG1 VAL B 519 -11.083 49.289 -2.756 1.00 5.67
H
ATOM 17045 1HG2 VAL B 519 -10.646 46.739 -4.360 1.00 5.67
H
ATOM 17046 2HG2 VAL B 519 -10.117 47.009 -2.684 1.00 5.67
H
ATOM 17047 3HG2 VAL B 519 -11.130 45.603 -3.080 1.00 5.67
H
ATOM 17048 N SER B 520 -14.845 48.197 -1.587 1.00 3.47
N
ATOM 17049 CA SER B 520 -15.884 49.169 -1.275 1.00 3.75
C
ATOM 17050 C SER B 520 -15.967 49.390 0.232 1.00 3.89
C
ATOM 17051 O SER B 520 -16.043 50.529 0.703 1.00 4.46
O
ATOM 17052 CB SER B 520 -17.222 48.677 -1.794 1.00 5.62
C
ATOM 17053 OG SER B 520 -17.220 48.588 -3.192 1.00 5.62
O
ATOM 17054 H SER B 520 -15.106 47.359 -2.095 1.00 4.16
H
ATOM 17055 HA SER B 520 -15.636 50.117 -1.753 1.00 4.50
H
ATOM 17056 1HB SER B 520 -17.446 47.700 -1.365 1.00 6.75
H
ATOM 17057 2HB SER B 520 -18.006 49.362 -1.473 1.00 6.75
H
ATOM 17058 HG SER B 520 -16.655 47.837 -3.406 1.00 6.75
H
ATOM 17059 N SER B 521 -15.928 48.282 0.971 1.00 3.60
N
ATOM 17060 CA SER B 521 -15.957 48.274 2.421 1.00 3.77
C
ATOM 17061 C SER B 521 -14.747 48.990 2.994 1.00 3.88
C

ATOM	17062	O	SER	B	521	-14.876	49.832	3.886	1.00	4.75	O
ATOM	17063	CB	SER	B	521	-15.998	46.840	2.901	1.00	5.66	C
ATOM	17064	OG	SER	B	521	-16.038	46.767	4.291	1.00	5.66	O
ATOM	17065	H	SER	B	521	-15.885	47.386	0.507	1.00	4.32	H
ATOM	17066	HA	SER	B	521	-16.858	48.791	2.754	1.00	4.52	H
ATOM	17067	1HB	SER	B	521	-16.878	46.347	2.485	1.00	6.79	H
ATOM	17068	2HB	SER	B	521	-15.126	46.305	2.531	1.00	6.79	H
ATOM	17069	HG	SER	B	521	-15.189	47.082	4.621	1.00	6.79	H
ATOM	17070	N	ALA	B	522	-13.571	48.653	2.463	1.00	3.42	N
ATOM	17071	CA	ALA	B	522	-12.321	49.247	2.894	1.00	3.60	C
ATOM	17072	C	ALA	B	522	-12.362	50.750	2.776	1.00	4.10	C
ATOM	17073	O	ALA	B	522	-12.082	51.451	3.742	1.00	4.72	O
ATOM	17074	CB	ALA	B	522	-11.170	48.703	2.074	1.00	5.40	C
ATOM	17075	H	ALA	B	522	-13.532	47.942	1.746	1.00	4.10	H
ATOM	17076	HA	ALA	B	522	-12.167	48.989	3.942	1.00	4.32	H
ATOM	17077	1HB	ALA	B	522	-10.233	49.135	2.428	1.00	6.48	H
ATOM	17078	2HB	ALA	B	522	-11.140	47.623	2.184	1.00	6.48	H
ATOM	17079	3HB	ALA	B	522	-11.312	48.956	1.026	1.00	6.48	H
ATOM	17080	N	LYS	B	523	-12.773	51.249	1.619	1.00	4.26	N
ATOM	17081	CA	LYS	B	523	-12.839	52.682	1.395	1.00	5.32	C
ATOM	17082	C	LYS	B	523	-13.879	53.366	2.290	1.00	5.06	C
ATOM	17083	O	LYS	B	523	-13.678	54.507	2.707	1.00	6.94	O
ATOM	17084	CB	LYS	B	523	-13.075	52.935	-0.088	1.00	7.98	C
ATOM	17085	CG	LYS	B	523	-11.836	52.579	-0.907	1.00	7.98	C
ATOM	17086	CD	LYS	B	523	-12.010	52.790	-2.399	1.00	7.98	C
ATOM	17087	CE	LYS	B	523	-10.708	52.456	-3.127	1.00	7.98	C
ATOM	17088	NZ	LYS	B	523	-10.828	52.595	-4.604	1.00	7.98	N
ATOM	17089	H	LYS	B	523	-13.011	50.622	0.862	1.00	5.11	H
ATOM	17090	HA	LYS	B	523	-11.867	53.107	1.647	1.00	6.38	H

ATOM 17091 1HB LYS B 523   -13.913 52.334 -0.442 1.00 9.58
H
ATOM 17092 2HB LYS B 523   -13.317 53.985 -0.257 1.00 9.58
H
ATOM 17093 1HG LYS B 523   -10.997 53.187 -0.566 1.00 9.58
H
ATOM 17094 2HG LYS B 523   -11.588 51.531 -0.734 1.00 9.58
H
ATOM 17095 1HD LYS B 523   -12.807 52.147 -2.773 1.00 9.58
H
ATOM 17096 2HD LYS B 523   -12.275 53.829 -2.596 1.00 9.58
H
ATOM 17097 1HE LYS B 523   -9.925 53.126 -2.775 1.00 9.58
H
ATOM 17098 2HE LYS B 523   -10.422 51.432 -2.893 1.00 9.58
H
ATOM 17099 1HZ LYS B 523   -9.940 52.361 -5.028 1.00 9.58
H
ATOM 17100 2HZ LYS B 523   -11.541 51.965 -4.945 1.00 9.58
H
ATOM 17101 3HZ LYS B 523   -11.076 53.545 -4.838 1.00 9.58
H
ATOM 17102 N   ALA B 524   -14.953 52.653   2.641 1.00 4.49
N
ATOM 17103 CA ALA B 524   -15.943 53.179   3.580 1.00 4.58
C
ATOM 17104 C   ALA B 524   -15.408 53.199   5.026 1.00 5.45
C
ATOM 17105 O   ALA B 524   -15.946 53.905   5.881 1.00 11.94
O
ATOM 17106 CB ALA B 524   -17.214 52.347   3.513 1.00 6.87
C
ATOM 17107 H   ALA B 524   -15.109 51.737   2.235 1.00 5.39
H
ATOM 17108 HA ALA B 524   -16.171 54.205   3.291 1.00 5.50
H
ATOM 17109 1HB ALA B 524   -17.957 52.765   4.193 1.00 8.24
H
ATOM 17110 2HB ALA B 524   -17.604 52.363   2.495 1.00 8.24
H
ATOM 17111 3HB ALA B 524   -16.992 51.323   3.801 1.00 8.24
H
ATOM 17112 N   THR B 525   -14.339 52.441   5.286 1.00 4.05
N
ATOM 17113 CA THR B 525   -13.722 52.338   6.602 1.00 4.01
C
ATOM 17114 C   THR B 525   -12.298 52.899   6.601 1.00 4.07
C
ATOM 17115 O   THR B 525   -11.378 52.288   7.148 1.00 4.43
O
ATOM 17116 CB THR B 525   -13.703 50.882   7.069 1.00 6.01
C
ATOM 17117 OG1 THR B 525   -13.020 50.077   6.102 1.00 6.01
O
ATOM 17118 CG2 THR B 525   -15.117 50.360   7.248 1.00 6.01
C
ATOM 17119 H   THR B 525   -13.936 51.873   4.554 1.00 4.86
H

ATOM 17120 HA THR B 525 -14.313 52.920 7.309 1.00 4.81
H
ATOM 17121 HB THR B 525 -13.173 50.824 8.009 1.00 7.22
H
ATOM 17122 HG1 THR B 525 -13.411 50.236 5.236 1.00 7.22
H
ATOM 17123 1HG2 THR B 525 -15.084 49.325 7.587 1.00 7.22
H
ATOM 17124 2HG2 THR B 525 -15.631 50.970 7.987 1.00 7.22
H
ATOM 17125 3HG2 THR B 525 -15.649 50.413 6.300 1.00 7.22
H
ATOM 17126 N GLU B 526 -12.139 54.060 5.960 1.00 4.80
N
ATOM 17127 CA GLU B 526 -10.876 54.799 5.820 1.00 4.86
C
ATOM 17128 C GLU B 526 -9.690 53.986 5.297 1.00 4.85
C
ATOM 17129 O GLU B 526 -8.545 54.265 5.653 1.00 5.22
O
ATOM 17130 CB GLU B 526 -10.464 55.420 7.168 1.00 7.29
C
ATOM 17131 CG GLU B 526 -11.449 56.430 7.754 1.00 7.29
C
ATOM 17132 CD GLU B 526 -10.921 57.148 8.981 1.00 7.29
C
ATOM 17133 OE1 GLU B 526 -9.779 56.955 9.333 1.00 7.29
O
ATOM 17134 OE2 GLU B 526 -11.672 57.886 9.581 1.00 7.29
O
ATOM 17135 H GLU B 526 -12.961 54.474 5.541 1.00 5.76
H
ATOM 17136 HA GLU B 526 -11.049 55.612 5.114 1.00 5.83
H
ATOM 17137 1HB GLU B 526 -10.331 54.626 7.902 1.00 8.75
H
ATOM 17138 2HB GLU B 526 -9.503 55.923 7.055 1.00 8.75
H
ATOM 17139 1HG GLU B 526 -11.687 57.170 6.991 1.00 8.75
H
ATOM 17140 2HG GLU B 526 -12.370 55.909 8.013 1.00 8.75
H
ATOM 17141 N GLY B 527 -9.954 52.999 4.443 1.00 4.65
N
ATOM 17142 CA GLY B 527 -8.902 52.191 3.837 1.00 4.61
C
ATOM 17143 C GLY B 527 -8.329 51.121 4.768 1.00 4.44
C
ATOM 17144 O GLY B 527 -7.309 50.513 4.459 1.00 6.22
O
ATOM 17145 H GLY B 527 -10.909 52.795 4.190 1.00 5.58
H
ATOM 17146 1HA GLY B 527 -9.295 51.712 2.940 1.00 5.53
H
ATOM 17147 2HA GLY B 527 -8.096 52.848 3.509 1.00 5.53
H
ATOM 17148 N ARG B 528 -8.956 50.878 5.913 1.00 4.78
N

ATOM 17149 CA ARG B 528   -8.385 49.901   6.831 1.00 4.09
C
ATOM 17150 C   ARG B 528   -9.060 48.537   6.769 1.00 4.14
C
ATOM 17151 O   ARG B 528   -10.271 48.429   6.952 1.00 4.90
O
ATOM 17152 CB ARG B 528   -8.456 50.419   8.263 1.00 6.13
C
ATOM 17153 CG ARG B 528   -7.593 51.634   8.576 1.00 6.13
C
ATOM 17154 CD ARG B 528   -7.711 52.010 10.013 1.00 6.13
C
ATOM 17155 NE ARG B 528   -6.769 53.055 10.405 1.00 6.13
N
ATOM 17156 CZ ARG B 528   -7.011 54.371 10.266 1.00 6.13
C
ATOM 17157 NH1 ARG B 528   -8.157 54.739   9.734 1.00 6.13
N
ATOM 17158 NH2 ARG B 528   -6.119 55.273 10.658 1.00 6.13
N
ATOM 17159 H   ARG B 528   -9.799 51.374   6.173 1.00 5.74
H
ATOM 17160 HA ARG B 528   -7.333 49.770   6.575 1.00 4.91
H
ATOM 17161 1HB ARG B 528   -9.486 50.680   8.503 1.00 7.36
H
ATOM 17162 2HB ARG B 528   -8.151 49.628   8.946 1.00 7.36
H
ATOM 17163 1HG ARG B 528   -6.549 51.409   8.361 1.00 7.36
H
ATOM 17164 2HG ARG B 528   -7.919 52.481   7.972 1.00 7.36
H
ATOM 17165 1HD ARG B 528   -8.719 52.382 10.198 1.00 7.36
H
ATOM 17166 2HD ARG B 528   -7.538 51.137 10.629 1.00 7.36
H
ATOM 17167 HE ARG B 528   -5.887 52.777 10.820 1.00 7.36
H
ATOM 17168 1HH1 ARG B 528   -8.822 54.037   9.443 1.00 7.36
H
ATOM 17169 2HH1 ARG B 528   -8.401 55.715   9.609 1.00 7.36
H
ATOM 17170 1HH2 ARG B 528   -5.230 54.991 11.070 1.00 7.36
H
ATOM 17171 2HH2 ARG B 528   -6.321 56.254 10.548 1.00 7.36
H
ATOM 17172 N   ILE B 529   -8.252 47.498   6.546 1.00 3.59
N
ATOM 17173 CA ILE B 529   -8.720 46.116   6.535 1.00 3.49
C
ATOM 17174 C   ILE B 529   -7.945 45.250   7.524 1.00 3.34
C
ATOM 17175 O   ILE B 529   -6.720 45.269   7.533 1.00 3.46
O
ATOM 17176 CB ILE B 529   -8.571 45.451   5.147 1.00 5.24
C
ATOM 17177 CG1 ILE B 529   -9.388 46.160   4.075 1.00 5.24
C

ATOM 17178 CG2 ILE B 529 -8.969 43.982 5.214 1.00 5.24
C
ATOM 17179 CD1 ILE B 529 -9.105 45.615 2.690 1.00 5.24
C
ATOM 17180 H ILE B 529 -7.271 47.667 6.366 1.00 4.31
H
ATOM 17181 HA ILE B 529 -9.770 46.106 6.813 1.00 4.19
H
ATOM 17182 HB ILE B 529 -7.530 45.521 4.847 1.00 6.28
H
ATOM 17183 1HG1 ILE B 529 -10.449 46.040 4.290 1.00 6.28
H
ATOM 17184 2HG1 ILE B 529 -9.153 47.224 4.083 1.00 6.28
H
ATOM 17185 1HG2 ILE B 529 -8.828 43.524 4.235 1.00 6.28
H
ATOM 17186 2HG2 ILE B 529 -8.348 43.463 5.943 1.00 6.28
H
ATOM 17187 3HG2 ILE B 529 -10.017 43.900 5.505 1.00 6.28
H
ATOM 17188 1HD1 ILE B 529 -9.703 46.142 1.951 1.00 6.28
H
ATOM 17189 2HD1 ILE B 529 -8.050 45.745 2.457 1.00 6.28
H
ATOM 17190 3HD1 ILE B 529 -9.352 44.555 2.662 1.00 6.28
H
ATOM 17191 N ILE B 530 -8.665 44.491 8.340 1.00 3.13
N
ATOM 17192 CA ILE B 530 -8.079 43.501 9.237 1.00 3.00
C
ATOM 17193 C ILE B 530 -8.650 42.146 8.897 1.00 2.67
C
ATOM 17194 O ILE B 530 -9.849 41.935 9.024 1.00 2.52
O
ATOM 17195 CB ILE B 530 -8.355 43.773 10.725 1.00 4.50
C
ATOM 17196 CG1 ILE B 530 -7.759 45.102 11.171 1.00 4.50
C
ATOM 17197 CG2 ILE B 530 -7.820 42.622 11.570 1.00 4.50
C
ATOM 17198 CD1 ILE B 530 -8.182 45.486 12.564 1.00 4.50
C
ATOM 17199 H ILE B 530 -9.665 44.567 8.304 1.00 3.76
H
ATOM 17200 HA ILE B 530 -7.004 43.476 9.083 1.00 3.60
H
ATOM 17201 HB ILE B 530 -9.430 43.846 10.877 1.00 5.40
H
ATOM 17202 1HG1 ILE B 530 -6.674 45.032 11.152 1.00 5.40
H
ATOM 17203 2HG1 ILE B 530 -8.065 45.886 10.480 1.00 5.40
H
ATOM 17204 1HG2 ILE B 530 -8.040 42.806 12.621 1.00 5.40
H
ATOM 17205 2HG2 ILE B 530 -8.294 41.691 11.259 1.00 5.40
H
ATOM 17206 3HG2 ILE B 530 -6.743 42.540 11.432 1.00 5.40
H

```
ATOM  17207 1HD1 ILE B 530      -7.728  46.438  12.838  1.00  5.40
H
ATOM  17208 2HD1 ILE B 530      -9.268  45.579  12.599  1.00  5.40
H
ATOM  17209 3HD1 ILE B 530      -7.861  44.718  13.265  1.00  5.40
H
ATOM  17210  N   THR B 531      -7.808  41.222   8.477  1.00  2.60
N
ATOM  17211  CA  THR B 531      -8.305  39.900   8.164  1.00  2.31
C
ATOM  17212  C   THR B 531      -7.830  38.919   9.213  1.00  2.16
C
ATOM  17213  O   THR B 531      -6.632  38.811   9.465  1.00  2.22
O
ATOM  17214  CB  THR B 531      -7.856  39.444   6.773  1.00  3.46
C
ATOM  17215  OG1 THR B 531      -8.358  40.351   5.783  1.00  3.46
O
ATOM  17216  CG2 THR B 531      -8.397  38.056   6.496  1.00  3.46
C
ATOM  17217  H   THR B 531      -6.825  41.438   8.386  1.00  3.12
H
ATOM  17218  HA  THR B 531      -9.395  39.917   8.183  1.00  2.77
H
ATOM  17219  HB  THR B 531      -6.767  39.429   6.724  1.00  4.16
H
ATOM  17220  HG1 THR B 531      -8.084  40.055   4.912  1.00  4.16
H
ATOM  17221 1HG2 THR B 531      -8.074  37.744   5.508  1.00  4.16
H
ATOM  17222 2HG2 THR B 531      -8.015  37.358   7.241  1.00  4.16
H
ATOM  17223 3HG2 THR B 531      -9.485  38.073   6.537  1.00  4.16
H
ATOM  17224  N   VAL B 532      -8.771  38.202   9.808  1.00  2.03
N
ATOM  17225  CA  VAL B 532      -8.457  37.228  10.838  1.00  1.95
C
ATOM  17226  C   VAL B 532      -8.920  35.848  10.422  1.00  1.89
C
ATOM  17227  O   VAL B 532     -10.038  35.677   9.936  1.00  2.07
O
ATOM  17228  CB  VAL B 532      -9.119  37.634  12.173  1.00  2.92
C
ATOM  17229  CG1 VAL B 532     -10.620  37.796  11.975  1.00  2.92
C
ATOM  17230  CG2 VAL B 532      -8.840  36.574  13.235  1.00  2.92
C
ATOM  17231  H   VAL B 532      -9.733  38.342   9.532  1.00  2.44
H
ATOM  17232  HA  VAL B 532      -7.379  37.209  10.977  1.00  2.34
H
ATOM  17233  HB  VAL B 532      -8.719  38.594  12.495  1.00  3.51
H
ATOM  17234 1HG1 VAL B 532     -11.085  38.093  12.912  1.00  3.51
H
ATOM  17235 2HG1 VAL B 532     -10.805  38.562  11.223  1.00  3.51
H
```

ATOM 17236 3HG1 VAL B 532   -11.047 36.853 11.641 1.00 3.51
H
ATOM 17237 1HG2 VAL B 532   -9.304 36.871 14.175 1.00 3.51
H
ATOM 17238 2HG2 VAL B 532   -9.254 35.619 12.915 1.00 3.51
H
ATOM 17239 3HG2 VAL B 532   -7.766 36.471 13.381 1.00 3.51
H
ATOM 17240 N   GLU B 533   -8.063 34.854 10.620 1.00 1.81
N
ATOM 17241 CA GLU B 533   -8.430 33.505 10.236 1.00 2.21
C
ATOM 17242 C   GLU B 533   -7.750 32.429 11.053 1.00 1.78
C
ATOM 17243 O   GLU B 533   -6.591 32.551 11.447 1.00 3.79
O
ATOM 17244 CB GLU B 533   -8.082 33.297   8.776 1.00 3.31
C
ATOM 17245 CG GLU B 533   -6.588 33.353   8.518 1.00 3.31
C
ATOM 17246 CD GLU B 533   -6.277 33.318   7.069 1.00 3.31
C
ATOM 17247 OE1 GLU B 533   -6.739 32.435   6.390 1.00 3.31
O
ATOM 17248 OE2 GLU B 533   -5.583 34.202   6.622 1.00 3.31
O
ATOM 17249 H   GLU B 533   -7.151 35.054 11.011 1.00 2.17
H
ATOM 17250 HA GLU B 533   -9.508 33.396 10.361 1.00 2.65
H
ATOM 17251 1HB GLU B 533   -8.453 32.329   8.441 1.00 3.98
H
ATOM 17252 2HB GLU B 533   -8.562 34.065   8.171 1.00 3.98
H
ATOM 17253 1HG GLU B 533   -6.186 34.270   8.947 1.00 3.98
H
ATOM 17254 2HG GLU B 533   -6.108 32.509   9.012 1.00 3.98
H
ATOM 17255 N   ASP B 534   -8.459 31.327 11.240 1.00 1.71
N
ATOM 17256 CA ASP B 534   -7.927 30.196 11.975 1.00 1.47
C
ATOM 17257 C   ASP B 534   -7.201 29.280 10.987 1.00 2.65
C
ATOM 17258 O   ASP B 534   -7.682 28.206 10.628 1.00 11.70
O
ATOM 17259 CB ASP B 534   -9.084 29.508 12.709 1.00 2.21
C
ATOM 17260 CG ASP B 534   -8.651 28.443 13.676 1.00 2.21
C
ATOM 17261 OD1 ASP B 534   -7.481 28.149 13.711 1.00 2.21
O
ATOM 17262 OD2 ASP B 534   -9.487 27.913 14.378 1.00 2.21
O
ATOM 17263 H   ASP B 534   -9.402 31.279 10.880 1.00 2.05
H
ATOM 17264 HA ASP B 534   -7.204 30.553 12.706 1.00 1.76
H

```
ATOM  17265 1HB  ASP B 534      -9.654  30.257  13.259  1.00  2.65
H
ATOM  17266 2HB  ASP B 534      -9.758  29.060  11.980  1.00  2.65
H
ATOM  17267  N   HIS B 535      -6.026  29.741  10.563  1.00  1.36
N
ATOM  17268  CA  HIS B 535      -5.226  29.111   9.514  1.00  1.64
C
ATOM  17269  C   HIS B 535      -3.829  29.716   9.529  1.00  1.68
C
ATOM  17270  O   HIS B 535      -3.648  30.801  10.079  1.00  2.45
O
ATOM  17271  CB  HIS B 535      -5.910  29.382   8.159  1.00  2.46
C
ATOM  17272  CG  HIS B 535      -5.421  28.588   6.986  1.00  2.46
C
ATOM  17273  ND1 HIS B 535      -4.335  28.966   6.223  1.00  2.46
N
ATOM  17274  CD2 HIS B 535      -5.892  27.446   6.435  1.00  2.46
C
ATOM  17275  CE1 HIS B 535      -4.162  28.090   5.249  1.00  2.46
C
ATOM  17276  NE2 HIS B 535      -5.097  27.159   5.354  1.00  2.46
N
ATOM  17277  H   HIS B 535      -5.715  30.621  10.958  1.00  1.63
H
ATOM  17278  HA  HIS B 535      -5.151  28.039   9.662  1.00  1.97
H
ATOM  17279 1HB  HIS B 535      -6.980  29.198   8.260  1.00  2.95
H
ATOM  17280 2HB  HIS B 535      -5.794  30.436   7.911  1.00  2.95
H
ATOM  17281  HD1 HIS B 535      -3.616  29.600   6.514  1.00  2.95
H
ATOM  17282  HD2 HIS B 535      -6.728  26.793   6.684  1.00  2.95
H
ATOM  17283  HE1 HIS B 535      -3.349  28.215   4.534  1.00  2.95
H
ATOM  17284  N   TYR B 536      -2.837  29.017   8.974  1.00  1.40
N
ATOM  17285  CA  TYR B 536      -1.505  29.608   8.878  1.00  1.84
C
ATOM  17286  C   TYR B 536      -1.541  30.796   7.924  1.00  2.29
C
ATOM  17287  O   TYR B 536      -2.253  30.756   6.921  1.00  4.96
O
ATOM  17288  CB  TYR B 536      -0.450  28.604   8.376  1.00  2.76
C
ATOM  17289  CG  TYR B 536      -0.029  27.641   9.438  1.00  2.76
C
ATOM  17290  CD1 TYR B 536      -0.336  26.307   9.368  1.00  2.76
C
ATOM  17291  CD2 TYR B 536       0.650  28.126  10.515  1.00  2.76
C
ATOM  17292  CE1 TYR B 536       0.014  25.452  10.388  1.00  2.76
C
ATOM  17293  CE2 TYR B 536       0.992  27.294  11.537  1.00  2.76
C
```

ATOM 17294 CZ TYR B 536   0.667 25.960 11.490 1.00 2.76
C
ATOM 17295 OH TYR B 536   0.978 25.140 12.543 1.00 2.76
O
ATOM 17296 H   TYR B 536   -3.008 28.105   8.572 1.00 1.68
H
ATOM 17297 HA TYR B 536   -1.220 29.940   9.870 1.00 2.21
H
ATOM 17298 1HB TYR B 536   -0.852 28.035   7.535 1.00 3.31
H
ATOM 17299 2HB TYR B 536   0.433 29.136   8.025 1.00 3.31
H
ATOM 17300 HD1 TYR B 536   -0.874 25.936   8.512 1.00 3.31
H
ATOM 17301 HD2 TYR B 536   0.902 29.182 10.559 1.00 3.31
H
ATOM 17302 HE1 TYR B 536   -0.247 24.397 10.334 1.00 3.31
H
ATOM 17303 HE2 TYR B 536   1.510 27.706 12.386 1.00 3.31
H
ATOM 17304 HH TYR B 536   0.926 25.654 13.356 1.00 3.31
H
ATOM 17305 N   PRO B 537   -0.761 31.850   8.199 1.00 1.89
N
ATOM 17306 CA PRO B 537   -0.595 33.085   7.441 1.00 1.93
C
ATOM 17307 C   PRO B 537   -0.252 32.851   5.978 1.00 6.32
C
ATOM 17308 O   PRO B 537   -0.559 33.676   5.126 1.00 43.14
O
ATOM 17309 CB PRO B 537   0.594 33.763   8.132 1.00 2.90
C
ATOM 17310 CG PRO B 537   0.548 33.287   9.534 1.00 2.90
C
ATOM 17311 CD PRO B 537   0.012 31.889   9.452 1.00 2.90
C
ATOM 17312 HA PRO B 537   -1.506 33.695   7.534 1.00 2.32
H
ATOM 17313 1HB PRO B 537   1.526 33.492   7.623 1.00 3.47
H
ATOM 17314 2HB PRO B 537   0.495 34.856   8.055 1.00 3.47
H
ATOM 17315 1HG PRO B 537   1.564 33.308   9.957 1.00 3.47
H
ATOM 17316 2HG PRO B 537   -0.054 33.955 10.156 1.00 3.47
H
ATOM 17317 1HD PRO B 537   0.820 31.144   9.440 1.00 3.47
H
ATOM 17318 2HD PRO B 537   -0.673 31.755 10.295 1.00 3.47
H
ATOM 17319 N   GLN B 538   0.413 31.743   5.695 1.00 2.91
N
ATOM 17320 CA GLN B 538   0.911 31.483   4.367 1.00 1.74
C
ATOM 17321 C   GLN B 538   -0.163 30.838   3.486 1.00 2.11
C
ATOM 17322 O   GLN B 538   -0.614 29.724   3.745 1.00 4.06
O

ATOM 17323 CB GLN B 538 2.135 30.567 4.475 1.00 2.61
C
ATOM 17324 CG GLN B 538 3.195 31.009 5.505 1.00 2.61
C
ATOM 17325 CD GLN B 538 3.896 32.344 5.282 1.00 2.61
C
ATOM 17326 OE1 GLN B 538 4.506 32.640 4.250 1.00 2.61
O
ATOM 17327 NE2 GLN B 538 3.827 33.187 6.303 1.00 2.61
N
ATOM 17328 H GLN B 538 0.616 31.080 6.427 1.00 3.49
H
ATOM 17329 HA GLN B 538 1.202 32.429 3.913 1.00 2.09
H
ATOM 17330 1HB GLN B 538 1.810 29.560 4.743 1.00 3.13
H
ATOM 17331 2HB GLN B 538 2.618 30.503 3.501 1.00 3.13
H
ATOM 17332 1HG GLN B 538 2.706 31.062 6.477 1.00 3.13
H
ATOM 17333 2HG GLN B 538 3.971 30.254 5.540 1.00 3.13
H
ATOM 17334 1HE2 GLN B 538 4.268 34.085 6.246 1.00 3.13
H
ATOM 17335 2HE2 GLN B 538 3.340 32.924 7.136 1.00 3.13
H
ATOM 17336 N GLY B 539 -0.578 31.554 2.453 1.00 1.64
N
ATOM 17337 CA GLY B 539 -1.555 31.054 1.488 1.00 1.65
C
ATOM 17338 C GLY B 539 -3.013 31.095 1.963 1.00 1.65
C
ATOM 17339 O GLY B 539 -3.893 30.568 1.284 1.00 2.03
O
ATOM 17340 H GLY B 539 -0.175 32.471 2.322 1.00 1.97
H
ATOM 17341 1HA GLY B 539 -1.467 31.638 0.572 1.00 1.98
H
ATOM 17342 2HA GLY B 539 -1.290 30.035 1.219 1.00 1.98
H
ATOM 17343 N GLY B 540 -3.291 31.749 3.082 1.00 1.55
N
ATOM 17344 CA GLY B 540 -4.659 31.793 3.590 1.00 1.54
C
ATOM 17345 C GLY B 540 -5.474 32.962 3.040 1.00 1.75
C
ATOM 17346 O GLY B 540 -5.152 33.535 1.995 1.00 2.29
O
ATOM 17347 H GLY B 540 -2.554 32.198 3.606 1.00 1.86
H
ATOM 17348 1HA GLY B 540 -5.160 30.856 3.348 1.00 1.85
H
ATOM 17349 2HA GLY B 540 -4.631 31.857 4.679 1.00 1.85
H
ATOM 17350 N ILE B 541 -6.538 33.289 3.763 1.00 1.88
N
ATOM 17351 CA ILE B 541 -7.464 34.349 3.400 1.00 1.95
C

ATOM 17352 C ILE B 541 -6.799 35.710 3.423 1.00 2.18
C
ATOM 17353 O ILE B 541 -7.026 36.517 2.527 1.00 2.51
O
ATOM 17354 CB ILE B 541 -8.668 34.388 4.341 1.00 2.92
C
ATOM 17355 CG1 ILE B 541 -9.515 33.137 4.179 1.00 2.92
C
ATOM 17356 CG2 ILE B 541 -9.496 35.608 4.011 1.00 2.92
C
ATOM 17357 CD1 ILE B 541 -10.555 32.970 5.262 1.00 2.92
C
ATOM 17358 H ILE B 541 -6.708 32.774 4.617 1.00 2.26
H
ATOM 17359 HA ILE B 541 -7.822 34.161 2.388 1.00 2.34
H
ATOM 17360 HB ILE B 541 -8.332 34.439 5.376 1.00 3.51
H
ATOM 17361 1HG1 ILE B 541 -10.018 33.172 3.214 1.00 3.51
H
ATOM 17362 2HG1 ILE B 541 -8.860 32.270 4.192 1.00 3.51
H
ATOM 17363 1HG2 ILE B 541 -10.358 35.656 4.677 1.00 3.51
H
ATOM 17364 2HG2 ILE B 541 -8.894 36.501 4.129 1.00 3.51
H
ATOM 17365 3HG2 ILE B 541 -9.836 35.539 2.978 1.00 3.51
H
ATOM 17366 1HD1 ILE B 541 -11.121 32.056 5.088 1.00 3.51
H
ATOM 17367 2HD1 ILE B 541 -10.061 32.912 6.232 1.00 3.51
H
ATOM 17368 3HD1 ILE B 541 -11.232 33.823 5.250 1.00 3.51
H
ATOM 17369 N GLY B 542 -6.002 35.973 4.460 1.00 2.16
N
ATOM 17370 CA GLY B 542 -5.319 37.255 4.601 1.00 2.40
C
ATOM 17371 C GLY B 542 -4.461 37.540 3.388 1.00 2.37
C
ATOM 17372 O GLY B 542 -4.444 38.660 2.879 1.00 2.70
O
ATOM 17373 H GLY B 542 -5.860 35.262 5.170 1.00 2.59
H
ATOM 17374 1HA GLY B 542 -6.051 38.051 4.726 1.00 2.88
H
ATOM 17375 2HA GLY B 542 -4.699 37.241 5.497 1.00 2.88
H
ATOM 17376 N GLU B 543 -3.760 36.516 2.929 1.00 2.12
N
ATOM 17377 CA GLU B 543 -2.920 36.609 1.761 1.00 2.50
C
ATOM 17378 C GLU B 543 -3.738 36.821 0.498 1.00 2.27
C
ATOM 17379 O GLU B 543 -3.375 37.648 -0.338 1.00 2.34
O
ATOM 17380 CB GLU B 543 -2.062 35.377 1.675 1.00 3.75
C

```
ATOM  17381  CG  GLU B 543      -1.094  35.378   0.531  1.00  3.75
C
ATOM  17382  CD  GLU B 543      -0.095  34.310   0.722  1.00  3.75
C
ATOM  17383  OE1 GLU B 543       0.354  34.173   1.831  1.00  3.75
O
ATOM  17384  OE2 GLU B 543       0.206  33.587  -0.192  1.00  3.75
O
ATOM  17385  H   GLU B 543      -3.819  35.631   3.413  1.00  2.54
H
ATOM  17386  HA  GLU B 543      -2.263  37.470   1.882  1.00  3.00
H
ATOM  17387 1HB  GLU B 543      -1.492  35.268   2.598  1.00  4.50
H
ATOM  17388 2HB  GLU B 543      -2.700  34.497   1.579  1.00  4.50
H
ATOM  17389 1HG  GLU B 543      -1.631  35.209  -0.400  1.00  4.50
H
ATOM  17390 2HG  GLU B 543      -0.596  36.344   0.471  1.00  4.50
H
ATOM  17391  N   ALA B 544      -4.849  36.088   0.370  1.00  2.49
N
ATOM  17392  CA  ALA B 544      -5.744  36.262  -0.768  1.00  2.56
C
ATOM  17393  C   ALA B 544      -6.233  37.709  -0.848  1.00  2.61
C
ATOM  17394  O   ALA B 544      -6.242  38.300  -1.931  1.00  2.66
O
ATOM  17395  CB  ALA B 544      -6.928  35.308  -0.662  1.00  3.84
C
ATOM  17396  H   ALA B 544      -5.081  35.392   1.069  1.00  2.99
H
ATOM  17397  HA  ALA B 544      -5.186  36.041  -1.677  1.00  3.07
H
ATOM  17398 1HB  ALA B 544      -7.575  35.432  -1.531  1.00  4.61
H
ATOM  17399 2HB  ALA B 544      -6.565  34.280  -0.627  1.00  4.61
H
ATOM  17400 3HB  ALA B 544      -7.493  35.522   0.242  1.00  4.61
H
ATOM  17401  N   VAL B 545      -6.617  38.275   0.303  1.00  2.67
N
ATOM  17402  CA  VAL B 545      -7.088  39.653   0.370  1.00  2.89
C
ATOM  17403  C   VAL B 545      -5.989  40.634   0.022  1.00  3.32
C
ATOM  17404  O   VAL B 545      -6.225  41.550  -0.765  1.00  3.52
O
ATOM  17405  CB  VAL B 545      -7.656  39.988   1.769  1.00  4.33
C
ATOM  17406  CG1 VAL B 545      -7.944  41.485   1.870  1.00  4.33
C
ATOM  17407  CG2 VAL B 545      -8.938  39.194   2.010  1.00  4.33
C
ATOM  17408  H   VAL B 545      -6.597  37.727   1.151  1.00  3.20
H
ATOM  17409  HA  VAL B 545      -7.892  39.775  -0.354  1.00  3.47
H
```

```
ATOM  17410  HB  VAL B 545      -6.915  39.734   2.528  1.00  5.20
H
ATOM  17411 1HG1 VAL B 545      -8.333  41.715   2.862  1.00  5.20
H
ATOM  17412 2HG1 VAL B 545      -7.023  42.044   1.704  1.00  5.20
H
ATOM  17413 3HG1 VAL B 545      -8.680  41.764   1.117  1.00  5.20
H
ATOM  17414 1HG2 VAL B 545      -9.332  39.428   2.998  1.00  5.20
H
ATOM  17415 2HG2 VAL B 545      -9.676  39.461   1.254  1.00  5.20
H
ATOM  17416 3HG2 VAL B 545      -8.728  38.129   1.947  1.00  5.20
H
ATOM  17417  N   CYS B 546      -4.791  40.439   0.588  1.00  3.58
N
ATOM  17418  CA  CYS B 546      -3.665  41.322   0.311  1.00  4.06
C
ATOM  17419  C   CYS B 546      -3.361  41.373  -1.172  1.00  4.54
C
ATOM  17420  O   CYS B 546      -3.179  42.453  -1.728  1.00  6.45
O
ATOM  17421  CB  CYS B 546      -2.404  40.861   1.049  1.00  6.09
C
ATOM  17422  SG  CYS B 546      -2.448  41.087   2.841  1.00  6.09
S
ATOM  17423  H   CYS B 546      -4.659  39.674   1.236  1.00  4.30
H
ATOM  17424  HA  CYS B 546      -3.921  42.326   0.650  1.00  4.87
H
ATOM  17425 1HB  CYS B 546      -2.239  39.802   0.851  1.00  7.31
H
ATOM  17426 2HB  CYS B 546      -1.541  41.404   0.664  1.00  7.31
H
ATOM  17427  HG  CYS B 546      -1.262  40.539   3.082  1.00  7.31
H
ATOM  17428  N   ALA B 547      -3.318  40.210  -1.811  1.00  3.56
N
ATOM  17429  CA  ALA B 547      -3.056  40.133  -3.236  1.00  4.02
C
ATOM  17430  C   ALA B 547      -4.132  40.839  -4.038  1.00  4.23
C
ATOM  17431  O   ALA B 547      -3.835  41.542  -5.004  1.00  5.60
O
ATOM  17432  CB  ALA B 547      -2.968  38.693  -3.660  1.00  6.03
C
ATOM  17433  H   ALA B 547      -3.461  39.350  -1.296  1.00  4.27
H
ATOM  17434  HA  ALA B 547      -2.106  40.627  -3.433  1.00  4.82
H
ATOM  17435 1HB  ALA B 547      -2.743  38.636  -4.724  1.00  7.24
H
ATOM  17436 2HB  ALA B 547      -2.177  38.204  -3.094  1.00  7.24
H
ATOM  17437 3HB  ALA B 547      -3.916  38.193  -3.463  1.00  7.24
H
ATOM  17438  N   ALA B 548      -5.387  40.647  -3.635  1.00  3.39
N
```

ATOM 17439 CA ALA B 548   -6.516 41.255 -4.315 1.00 3.39
C
ATOM 17440 C   ALA B 548   -6.509 42.781 -4.250 1.00 3.75
C
ATOM 17441 O   ALA B 548   -6.833 43.438 -5.242 1.00 4.13
O
ATOM 17442 CB ALA B 548   -7.810 40.729 -3.721 1.00 5.08
C
ATOM 17443 H   ALA B 548   -5.576 40.039 -2.848 1.00 4.07
H
ATOM 17444 HA ALA B 548   -6.466 40.966 -5.365 1.00 4.07
H
ATOM 17445 1HB ALA B 548   -8.656 41.147 -4.262 1.00 6.10
H
ATOM 17446 2HB ALA B 548   -7.828 39.642 -3.806 1.00 6.10
H
ATOM 17447 3HB ALA B 548   -7.873 41.010 -2.671 1.00 6.10
H
ATOM 17448 N   VAL B 549   -6.147 43.354 -3.098 1.00 3.64
N
ATOM 17449 CA VAL B 549   -6.201 44.811 -2.971 1.00 3.78
C
ATOM 17450 C   VAL B 549   -4.848 45.503 -3.117 1.00 4.28
C
ATOM 17451 O   VAL B 549   -4.800 46.735 -3.138 1.00 6.94
O
ATOM 17452 CB VAL B 549   -6.793 45.231 -1.611 1.00 5.67
C
ATOM 17453 CG1 VAL B 549   -8.180 44.628 -1.429 1.00 5.67
C
ATOM 17454 CG2 VAL B 549   -5.852 44.840 -0.494 1.00 5.67
C
ATOM 17455 H   VAL B 549   -5.889 42.782 -2.303 1.00 4.37
H
ATOM 17456 HA VAL B 549   -6.859 45.189 -3.753 1.00 4.54
H
ATOM 17457 HB VAL B 549   -6.917 46.314 -1.605 1.00 6.80
H
ATOM 17458 1HG1 VAL B 549   -8.599 44.960 -0.477 1.00 6.80
H
ATOM 17459 2HG1 VAL B 549   -8.826 44.954 -2.243 1.00 6.80
H
ATOM 17460 3HG1 VAL B 549   -8.107 43.541 -1.434 1.00 6.80
H
ATOM 17461 1HG2 VAL B 549   -6.277 45.167   0.452 1.00 6.80
H
ATOM 17462 2HG2 VAL B 549   -5.718 43.764 -0.480 1.00 6.80
H
ATOM 17463 3HG2 VAL B 549   -4.886 45.320 -0.645 1.00 6.80
H
ATOM 17464 N   SER B 550   -3.758 44.736 -3.197 1.00 3.27
N
ATOM 17465 CA SER B 550   -2.442 45.343 -3.338 1.00 4.07
C
ATOM 17466 C   SER B 550   -2.403 46.257 -4.537 1.00 6.51
C
ATOM 17467 O   SER B 550   -2.895 45.903 -5.604 1.00 31.83
O

```
ATOM  17468  CB  SER B 550      -1.365  44.289  -3.487  1.00  6.11
C
ATOM  17469  OG  SER B 550      -0.118  44.884  -3.725  1.00  6.11
O
ATOM  17470  H   SER B 550      -3.825  43.728  -3.146  1.00  3.92
H
ATOM  17471  HA  SER B 550      -2.235  45.935  -2.446  1.00  4.88
H
ATOM  17472 1HB  SER B 550      -1.310  43.690  -2.580  1.00  7.33
H
ATOM  17473 2HB  SER B 550      -1.619  43.620  -4.307  1.00  7.33
H
ATOM  17474  HG  SER B 550       0.496  44.156  -3.851  1.00  7.33
H
ATOM  17475  N   MET B 551      -1.797  47.426  -4.338  1.00  4.63
N
ATOM  17476  CA  MET B 551      -1.625  48.499  -5.325  1.00  3.92
C
ATOM  17477  C   MET B 551      -2.756  49.529  -5.272  1.00  3.44
C
ATOM  17478  O   MET B 551      -2.637  50.581  -5.898  1.00  4.93
O
ATOM  17479  CB  MET B 551      -1.538  47.980  -6.776  1.00  5.88
C
ATOM  17480  CG  MET B 551      -0.358  47.072  -7.091  1.00  5.88
C
ATOM  17481  SD  MET B 551      -0.342  46.518  -8.806  1.00  5.88
S
ATOM  17482  CE  MET B 551       0.158  48.022  -9.637  1.00  5.88
C
ATOM  17483  H   MET B 551      -1.420  47.589  -3.414  1.00  5.56
H
ATOM  17484  HA  MET B 551      -0.699  49.021  -5.092  1.00  4.70
H
ATOM  17485 1HB  MET B 551      -2.460  47.497  -7.083  1.00  7.06
H
ATOM  17486 2HB  MET B 551      -1.425  48.846  -7.430  1.00  7.06
H
ATOM  17487 1HG  MET B 551       0.567  47.590  -6.889  1.00  7.06
H
ATOM  17488 2HG  MET B 551      -0.391  46.189  -6.455  1.00  7.06
H
ATOM  17489 1HE  MET B 551       0.213  47.841 -10.711  1.00  7.06
H
ATOM  17490 2HE  MET B 551      -0.571  48.808  -9.438  1.00  7.06
H
ATOM  17491 3HE  MET B 551       1.137  48.331  -9.272  1.00  7.06
H
ATOM  17492  N   ASP B 552      -3.847  49.237  -4.547  1.00  3.32
N
ATOM  17493  CA  ASP B 552      -5.006  50.134  -4.518  1.00  4.00
C
ATOM  17494  C   ASP B 552      -4.928  51.284  -3.471  1.00  4.97
C
ATOM  17495  O   ASP B 552      -5.280  51.066  -2.308  1.00  4.78
O
ATOM  17496  CB  ASP B 552      -6.288  49.316  -4.278  1.00  6.00
C
```

```
ATOM  17497  CG  ASP B 552      -7.588  50.131  -4.349  1.00  6.00
C
ATOM  17498  OD1 ASP B 552      -7.537  51.335  -4.273  1.00  6.00
O
ATOM  17499  OD2 ASP B 552      -8.626  49.532  -4.499  1.00  6.00
O
ATOM  17500  H   ASP B 552      -3.908  48.368  -4.029  1.00  3.98
H
ATOM  17501  HA  ASP B 552      -5.097  50.553  -5.515  1.00  4.80
H
ATOM  17502 1HB  ASP B 552      -6.345  48.513  -5.015  1.00  7.20
H
ATOM  17503 2HB  ASP B 552      -6.232  48.846  -3.297  1.00  7.20
H
ATOM  17504  N   PRO B 553      -4.378  52.453  -3.886  1.00  6.92
N
ATOM  17505  CA  PRO B 553      -4.386  53.756  -3.153  1.00  8.84
C
ATOM  17506  C   PRO B 553      -5.081  53.863  -1.798  1.00  6.16
C
ATOM  17507  O   PRO B 553      -6.299  54.026  -1.768  1.00  6.28
O
ATOM  17508  CB  PRO B 553      -5.063  54.738  -4.109  1.00 13.26
C
ATOM  17509  CG  PRO B 553      -4.755  54.230  -5.442  1.00 13.26
C
ATOM  17510  CD  PRO B 553      -4.801  52.763  -5.289  1.00 13.26
C
ATOM  17511  HA  PRO B 553      -3.333  54.059  -3.028  1.00 10.61
H
ATOM  17512 1HB  PRO B 553      -6.143  54.767  -3.909  1.00 15.91
H
ATOM  17513 2HB  PRO B 553      -4.677  55.754  -3.939  1.00 15.91
H
ATOM  17514 1HG  PRO B 553      -5.487  54.605  -6.172  1.00 15.91
H
ATOM  17515 2HG  PRO B 553      -3.766  54.589  -5.767  1.00 15.91
H
ATOM  17516 1HD  PRO B 553      -5.830  52.429  -5.465  1.00 15.91
H
ATOM  17517 2HD  PRO B 553      -4.107  52.318  -6.008  1.00 15.91
H
ATOM  17518  N   ASP B 554      -4.322  53.742  -0.707  1.00  6.12
N
ATOM  17519  CA  ASP B 554      -4.789  53.981   0.675  1.00  5.05
C
ATOM  17520  C   ASP B 554      -5.252  52.745   1.414  1.00  4.69
C
ATOM  17521  O   ASP B 554      -5.385  52.788   2.639  1.00  5.06
O
ATOM  17522  CB  ASP B 554      -5.905  55.041   0.755  1.00  7.57
C
ATOM  17523  CG  ASP B 554      -5.471  56.404   0.227  1.00  7.57
C
ATOM  17524  OD1 ASP B 554      -4.316  56.732   0.365  1.00  7.57
O
ATOM  17525  OD2 ASP B 554      -6.298  57.105  -0.306  1.00  7.57
O
```

```
ATOM  17526  H   ASP B 554      -3.343  53.539  -0.842  1.00  7.34
H
ATOM  17527  HA  ASP B 554      -3.942  54.382   1.232  1.00  6.06
H
ATOM  17528 1HB  ASP B 554      -6.817  54.714   0.264  1.00  9.09
H
ATOM  17529 2HB  ASP B 554      -6.162  55.173   1.807  1.00  9.09
H
ATOM  17530  N   ILE B 555      -5.507  51.648   0.711  1.00  4.44
N
ATOM  17531  CA  ILE B 555      -5.912  50.460   1.441  1.00  4.14
C
ATOM  17532  C   ILE B 555      -4.720  49.778   2.095  1.00  3.46
C
ATOM  17533  O   ILE B 555      -3.730  49.444   1.443  1.00  3.65
O
ATOM  17534  CB  ILE B 555      -6.668  49.447   0.562  1.00  6.21
C
ATOM  17535  CG1 ILE B 555      -7.982  50.053   0.068  1.00  6.21
C
ATOM  17536  CG2 ILE B 555      -6.926  48.169   1.343  1.00  6.21
C
ATOM  17537  CD1 ILE B 555      -8.692  49.191  -0.946  1.00  6.21
C
ATOM  17538  H   ILE B 555      -5.412  51.620  -0.298  1.00  5.33
H
ATOM  17539  HA  ILE B 555      -6.594  50.770   2.227  1.00  4.97
H
ATOM  17540  HB  ILE B 555      -6.066  49.216  -0.318  1.00  7.45
H
ATOM  17541 1HG1 ILE B 555      -8.646  50.210   0.917  1.00  7.45
H
ATOM  17542 2HG1 ILE B 555      -7.776  51.020  -0.391  1.00  7.45
H
ATOM  17543 1HG2 ILE B 555      -7.452  47.457   0.707  1.00  7.45
H
ATOM  17544 2HG2 ILE B 555      -5.979  47.738   1.662  1.00  7.45
H
ATOM  17545 3HG2 ILE B 555      -7.536  48.393   2.217  1.00  7.45
H
ATOM  17546 1HD1 ILE B 555      -9.612  49.682  -1.257  1.00  7.45
H
ATOM  17547 2HD1 ILE B 555      -8.047  49.042  -1.811  1.00  7.45
H
ATOM  17548 3HD1 ILE B 555      -8.927  48.225  -0.501  1.00  7.45
H
ATOM  17549  N   GLN B 556      -4.845  49.578   3.401  1.00  3.34
N
ATOM  17550  CA  GLN B 556      -3.849  48.911   4.226  1.00  2.72
C
ATOM  17551  C   GLN B 556      -4.448  47.653   4.846  1.00  3.16
C
ATOM  17552  O   GLN B 556      -5.516  47.714   5.456  1.00  3.56
O
ATOM  17553  CB  GLN B 556      -3.347  49.859   5.326  1.00  4.08
C
ATOM  17554  CG  GLN B 556      -2.546  51.051   4.829  1.00  4.08
C
```

ATOM 17555 CD GLN B 556   -2.046 51.916   5.974 1.00 4.08
C
ATOM 17556 OE1 GLN B 556   -2.846 52.482   6.724 1.00 4.08
O
ATOM 17557 NE2 GLN B 556   -0.729 52.018   6.126 1.00 4.08
N
ATOM 17558 H   GLN B 556   -5.691 49.904   3.839 1.00 4.01
H
ATOM 17559 HA GLN B 556   -3.009 48.616   3.597 1.00 3.26
H
ATOM 17560 1HB GLN B 556   -4.208 50.256   5.863 1.00 4.90
H
ATOM 17561 2HB GLN B 556   -2.746 49.321   6.049 1.00 4.90
H
ATOM 17562 1HG GLN B 556   -1.686 50.699   4.264 1.00 4.90
H
ATOM 17563 2HG GLN B 556   -3.184 51.664   4.193 1.00 4.90
H
ATOM 17564 1HE2 GLN B 556   -0.356 52.576   6.870 1.00 4.90
H
ATOM 17565 2HE2 GLN B 556   -0.099 51.541   5.509 1.00 4.90
H
ATOM 17566 N   VAL B 557   -3.760 46.518   4.696 1.00 3.38
N
ATOM 17567 CA VAL B 557   -4.270 45.255   5.225 1.00 3.57
C
ATOM 17568 C   VAL B 557   -3.413 44.681   6.352 1.00 3.54
C
ATOM 17569 O   VAL B 557   -2.230 44.391   6.174 1.00 3.65
O
ATOM 17570 CB VAL B 557   -4.377 44.189   4.117 1.00 5.35
C
ATOM 17571 CG1 VAL B 557   -4.904 42.884   4.708 1.00 5.35
C
ATOM 17572 CG2 VAL B 557   -5.285 44.670   3.008 1.00 5.35
C
ATOM 17573 H   VAL B 557   -2.881 46.529   4.195 1.00 4.06
H
ATOM 17574 HA VAL B 557   -5.271 45.436   5.613 1.00 4.28
H
ATOM 17575 HB VAL B 557   -3.384 43.992   3.714 1.00 6.43
H
ATOM 17576 1HG1 VAL B 557   -4.962 42.128   3.925 1.00 6.43
H
ATOM 17577 2HG1 VAL B 557   -4.232 42.542   5.493 1.00 6.43
H
ATOM 17578 3HG1 VAL B 557   -5.897 43.050   5.125 1.00 6.43
H
ATOM 17579 1HG2 VAL B 557   -5.341 43.907   2.234 1.00 6.43
H
ATOM 17580 2HG2 VAL B 557   -6.279 44.853   3.405 1.00 6.43
H
ATOM 17581 3HG2 VAL B 557   -4.888 45.592   2.583 1.00 6.43
H
ATOM 17582 N   HIS B 558   -4.050 44.486   7.494 1.00 3.54
N
ATOM 17583 CA HIS B 558   -3.475 43.894   8.692 1.00 3.44
C

```
ATOM  17584  C   HIS B 558      -3.995  42.471   8.794  1.00  3.15
C
ATOM  17585  O   HIS B 558      -5.117  42.203   8.372  1.00  3.29
O
ATOM  17586  CB  HIS B 558      -3.874  44.694   9.933  1.00  5.16
C
ATOM  17587  CG  HIS B 558      -3.347  44.130  11.216  1.00  5.16
C
ATOM  17588  ND1 HIS B 558      -2.012  44.176  11.557  1.00  5.16
N
ATOM  17589  CD2 HIS B 558      -3.975  43.508  12.242  1.00  5.16
C
ATOM  17590  CE1 HIS B 558      -1.842  43.604  12.736  1.00  5.16
C
ATOM  17591  NE2 HIS B 558      -3.018  43.193  13.173  1.00  5.16
N
ATOM  17592  H   HIS B 558      -5.016  44.765   7.539  1.00  4.25
H
ATOM  17593  HA  HIS B 558      -2.389  43.864   8.619  1.00  4.13
H
ATOM  17594 1HB  HIS B 558      -3.510  45.717   9.837  1.00  6.19
H
ATOM  17595 2HB  HIS B 558      -4.959  44.739   9.998  1.00  6.19
H
ATOM  17596  HD1 HIS B 558      -1.262  44.468  10.962  1.00  6.19
H
ATOM  17597  HD2 HIS B 558      -5.015  43.247  12.426  1.00  6.19
H
ATOM  17598  HE1 HIS B 558      -0.851  43.538  13.184  1.00  6.19
H
ATOM  17599  N   SER B 559      -3.181  41.545   9.295  1.00  2.95
N
ATOM  17600  CA  SER B 559      -3.639  40.164   9.348  1.00  2.67
C
ATOM  17601  C   SER B 559      -3.332  39.431  10.651  1.00  2.68
C
ATOM  17602  O   SER B 559      -2.237  39.534  11.209  1.00  2.91
O
ATOM  17603  CB  SER B 559      -3.043  39.387   8.195  1.00  4.00
C
ATOM  17604  OG  SER B 559      -3.418  38.040   8.259  1.00  4.00
O
ATOM  17605  H   SER B 559      -2.263  41.796   9.634  1.00  3.54
H
ATOM  17606  HA  SER B 559      -4.718  40.173   9.224  1.00  3.20
H
ATOM  17607 1HB  SER B 559      -3.384  39.819   7.255  1.00  4.81
H
ATOM  17608 2HB  SER B 559      -1.957  39.469   8.220  1.00  4.81
H
ATOM  17609  HG  SER B 559      -4.374  38.035   8.366  1.00  4.81
H
ATOM  17610  N   LEU B 560      -4.331  38.673  11.109  1.00  2.51
N
ATOM  17611  CA  LEU B 560      -4.260  37.843  12.304  1.00  2.48
C
ATOM  17612  C   LEU B 560      -4.391  36.374  11.899  1.00  2.20
C
```

```
ATOM  17613  O   LEU B 560      -5.395  35.974  11.309  1.00  2.01
O
ATOM  17614  CB  LEU B 560      -5.384  38.206  13.284  1.00  3.72
C
ATOM  17615  CG  LEU B 560      -5.429  39.657  13.785  1.00  3.72
C
ATOM  17616  CD1 LEU B 560      -6.670  39.847  14.651  1.00  3.72
C
ATOM  17617  CD2 LEU B 560      -4.165  39.962  14.570  1.00  3.72
C
ATOM  17618  H   LEU B 560      -5.193  38.666  10.586  1.00  3.01
H
ATOM  17619  HA  LEU B 560      -3.293  37.992  12.786  1.00  2.98
H
ATOM  17620 1HB  LEU B 560      -6.332  38.009  12.795  1.00  4.46
H
ATOM  17621 2HB  LEU B 560      -5.308  37.559  14.156  1.00  4.46
H
ATOM  17622  HG  LEU B 560      -5.501  40.336  12.935  1.00  4.46
H
ATOM  17623 1HD1 LEU B 560      -6.713  40.876  15.004  1.00  4.46
H
ATOM  17624 2HD1 LEU B 560      -7.561  39.627  14.065  1.00  4.46
H
ATOM  17625 3HD1 LEU B 560      -6.621  39.172  15.505  1.00  4.46
H
ATOM  17626 1HD2 LEU B 560      -4.195  40.992  14.925  1.00  4.46
H
ATOM  17627 2HD2 LEU B 560      -4.095  39.286  15.423  1.00  4.46
H
ATOM  17628 3HD2 LEU B 560      -3.295  39.825  13.928  1.00  4.46
H
ATOM  17629  N   ALA B 561      -3.383  35.574  12.212  1.00  2.46
N
ATOM  17630  CA  ALA B 561      -3.394  34.167  11.819  1.00  2.27
C
ATOM  17631  C   ALA B 561      -2.504  33.345  12.754  1.00  2.19
C
ATOM  17632  O   ALA B 561      -1.804  33.907  13.597  1.00  2.28
O
ATOM  17633  CB  ALA B 561      -2.961  34.051  10.372  1.00  3.41
C
ATOM  17634  H   ALA B 561      -2.589  35.945  12.713  1.00  2.95
H
ATOM  17635  HA  ALA B 561      -4.411  33.790  11.916  1.00  2.72
H
ATOM  17636 1HB  ALA B 561      -2.979  33.014  10.057  1.00  4.09
H
ATOM  17637 2HB  ALA B 561      -3.639  34.631   9.745  1.00  4.09
H
ATOM  17638 3HB  ALA B 561      -1.959  34.446  10.268  1.00  4.09
H
ATOM  17639  N   VAL B 562      -2.562  32.014  12.633  1.00  2.03
N
ATOM  17640  CA  VAL B 562      -1.816  31.132  13.540  1.00  1.89
C
ATOM  17641  C   VAL B 562      -0.340  31.092  13.165  1.00  1.89
C
```

```
ATOM  17642  O   VAL B 562       0.025  30.566  12.126  1.00  3.02
O
ATOM  17643  CB  VAL B 562      -2.405  29.703  13.502  1.00  2.83
C
ATOM  17644  CG1 VAL B 562      -1.597  28.775  14.403  1.00  2.83
C
ATOM  17645  CG2 VAL B 562      -3.867  29.744  13.933  1.00  2.83
C
ATOM  17646  H   VAL B 562      -3.122  31.606  11.895  1.00  2.44
H
ATOM  17647  HA  VAL B 562      -1.910  31.521  14.555  1.00  2.27
H
ATOM  17648  HB  VAL B 562      -2.335  29.315  12.485  1.00  3.40
H
ATOM  17649 1HG1 VAL B 562      -2.013  27.769  14.357  1.00  3.40
H
ATOM  17650 2HG1 VAL B 562      -0.560  28.755  14.067  1.00  3.40
H
ATOM  17651 3HG1 VAL B 562      -1.639  29.137  15.429  1.00  3.40
H
ATOM  17652 1HG2 VAL B 562      -4.287  28.739  13.894  1.00  3.40
H
ATOM  17653 2HG2 VAL B 562      -3.936  30.126  14.951  1.00  3.40
H
ATOM  17654 3HG2 VAL B 562      -4.426  30.397  13.263  1.00  3.40
H
ATOM  17655  N   SER B 563       0.510  31.644  14.023  1.00  1.63
N
ATOM  17656  CA  SER B 563       1.944  31.773  13.741  1.00  1.76
C
ATOM  17657  C   SER B 563       2.790  30.488  13.710  1.00  1.57
C
ATOM  17658  O   SER B 563       3.879  30.494  13.135  1.00  1.66
O
ATOM  17659  CB  SER B 563       2.559  32.703  14.767  1.00  2.64
C
ATOM  17660  OG  SER B 563       2.567  32.108  16.036  1.00  2.64
O
ATOM  17661  H   SER B 563       0.149  32.030  14.885  1.00  1.96
H
ATOM  17662  HA  SER B 563       2.035  32.244  12.761  1.00  2.11
H
ATOM  17663 1HB  SER B 563       3.579  32.950  14.471  1.00  3.17
H
ATOM  17664 2HB  SER B 563       1.994  33.633  14.801  1.00  3.17
H
ATOM  17665  HG  SER B 563       3.111  31.320  15.954  1.00  3.17
H
ATOM  17666  N   GLY B 564       2.320  29.398  14.312  1.00  1.42
N
ATOM  17667  CA  GLY B 564       3.148  28.186  14.355  1.00  1.38
C
ATOM  17668  C   GLY B 564       2.375  26.942  14.777  1.00  1.21
C
ATOM  17669  O   GLY B 564       1.151  26.891  14.660  1.00  2.02
O
ATOM  17670  H   GLY B 564       1.415  29.410  14.756  1.00  1.70
H
```

```
ATOM  17671 1HA  GLY B 564       3.590  28.020  13.372  1.00  1.66
H
ATOM  17672 2HA  GLY B 564       3.976  28.346  15.045  1.00  1.66
H
ATOM  17673  N   VAL B 565       3.092  25.908  15.207  1.00  1.14
N
ATOM  17674  CA  VAL B 565       2.455  24.655  15.617  1.00  0.99
C
ATOM  17675  C   VAL B 565       1.589  24.849  16.858  1.00  1.15
C
ATOM  17676  O   VAL B 565       2.074  25.367  17.863  1.00  1.46
O
ATOM  17677  CB  VAL B 565       3.511  23.580  15.927  1.00  1.48
C
ATOM  17678  CG1 VAL B 565       2.832  22.346  16.499  1.00  1.48
C
ATOM  17679  CG2 VAL B 565       4.269  23.229  14.656  1.00  1.48
C
ATOM  17680  H   VAL B 565       4.096  25.995  15.270  1.00  1.37
H
ATOM  17681  HA  VAL B 565       1.852  24.303  14.786  1.00  1.19
H
ATOM  17682  HB  VAL B 565       4.208  23.957  16.677  1.00  1.78
H
ATOM  17683 1HG1 VAL B 565       3.581  21.585  16.722  1.00  1.78
H
ATOM  17684 2HG1 VAL B 565       2.305  22.611  17.414  1.00  1.78
H
ATOM  17685 3HG1 VAL B 565       2.121  21.957  15.775  1.00  1.78
H
ATOM  17686 1HG2 VAL B 565       5.019  22.471  14.878  1.00  1.78
H
ATOM  17687 2HG2 VAL B 565       3.573  22.845  13.911  1.00  1.78
H
ATOM  17688 3HG2 VAL B 565       4.759  24.122  14.271  1.00  1.78
H
ATOM  17689  N   PRO B 566       0.306  24.464  16.806  1.00  1.34
N
ATOM  17690  CA  PRO B 566      -0.664  24.558  17.878  1.00  1.33
C
ATOM  17691  C   PRO B 566      -0.622  23.384  18.851  1.00  1.25
C
ATOM  17692  O   PRO B 566      -0.246  22.266  18.500  1.00  1.79
O
ATOM  17693  CB  PRO B 566      -1.989  24.638  17.138  1.00  2.00
C
ATOM  17694  CG  PRO B 566      -1.766  23.858  15.905  1.00  2.00
C
ATOM  17695  CD  PRO B 566      -0.346  24.152  15.518  1.00  2.00
C
ATOM  17696  HA  PRO B 566      -0.472  25.490  18.413  1.00  1.60
H
ATOM  17697 1HB  PRO B 566      -2.795  24.230  17.765  1.00  2.39
H
ATOM  17698 2HB  PRO B 566      -2.239  25.690  16.937  1.00  2.39
H
ATOM  17699 1HG  PRO B 566      -1.940  22.801  16.115  1.00  2.39
H
```

```
ATOM  17700 2HG  PRO B 566      -2.493  24.147  15.139  1.00  2.39
H
ATOM  17701 1HD  PRO B 566       0.110  23.261  15.065  1.00  2.39
H
ATOM  17702 2HD  PRO B 566      -0.310  25.019  14.848  1.00  2.39
H
ATOM  17703  N   GLN B 567      -1.034  23.662  20.076  1.00  1.42
N
ATOM  17704  CA  GLN B 567      -1.086  22.686  21.156  1.00  1.38
C
ATOM  17705  C   GLN B 567      -2.526  22.344  21.532  1.00  1.39
C
ATOM  17706  O   GLN B 567      -3.469  22.902  20.971  1.00  1.56
O
ATOM  17707  CB  GLN B 567      -0.343  23.226  22.380  1.00  2.07
C
ATOM  17708  CG  GLN B 567       1.108  23.581  22.110  1.00  2.07
C
ATOM  17709  CD  GLN B 567       1.942  22.373  21.731  1.00  2.07
C
ATOM  17710  OE1 GLN B 567       1.942  21.356  22.431  1.00  2.07
O
ATOM  17711  NE2 GLN B 567       2.658  22.478  20.618  1.00  2.07
N
ATOM  17712  H   GLN B 567      -1.303  24.615  20.273  1.00  1.70
H
ATOM  17713  HA  GLN B 567      -0.597  21.772  20.821  1.00  1.66
H
ATOM  17714 1HB  GLN B 567      -0.847  24.121  22.745  1.00  2.48
H
ATOM  17715 2HB  GLN B 567      -0.365  22.487  23.180  1.00  2.48
H
ATOM  17716 1HG  GLN B 567       1.151  24.297  21.289  1.00  2.48
H
ATOM  17717 2HG  GLN B 567       1.536  24.022  23.011  1.00  2.48
H
ATOM  17718 1HE2 GLN B 567       3.228  21.712  20.316  1.00  2.48
H
ATOM  17719 2HE2 GLN B 567       2.628  23.321  20.080  1.00  2.48
H
ATOM  17720  N   SER B 568      -2.682  21.406  22.468  1.00  1.33
N
ATOM  17721  CA  SER B 568      -3.994  21.036  23.005  1.00  1.39
C
ATOM  17722  C   SER B 568      -4.505  22.155  23.920  1.00  1.45
C
ATOM  17723  O   SER B 568      -3.750  23.065  24.271  1.00  1.40
O
ATOM  17724  CB  SER B 568      -3.904  19.730  23.772  1.00  2.08
C
ATOM  17725  OG  SER B 568      -3.155  19.889  24.945  1.00  2.08
O
ATOM  17726  H   SER B 568      -1.859  20.960  22.851  1.00  1.60
H
ATOM  17727  HA  SER B 568      -4.694  20.918  22.177  1.00  1.67
H
ATOM  17728 1HB  SER B 568      -4.905  19.382  24.024  1.00  2.50
H
```

```
ATOM  17729 2HB  SER B 568      -3.444  18.970  23.143  1.00  2.50
H
ATOM  17730  HG  SER B 568      -3.623  20.542  25.471  1.00  2.50
H
ATOM  17731  N   GLY B 569      -5.778  22.098  24.305  1.00  1.60
N
ATOM  17732  CA  GLY B 569      -6.375  23.148  25.128  1.00  1.67
C
ATOM  17733  C   GLY B 569      -7.809  23.410  24.678  1.00  1.67
C
ATOM  17734  O   GLY B 569      -8.261  22.852  23.679  1.00  1.52
O
ATOM  17735  H   GLY B 569      -6.363  21.314  24.047  1.00  1.92
H
ATOM  17736 1HA  GLY B 569      -6.365  22.844  26.174  1.00  2.00
H
ATOM  17737 2HA  GLY B 569      -5.788  24.061  25.046  1.00  2.00
H
ATOM  17738  N   LYS B 570      -8.530  24.245  25.423  1.00  1.92
N
ATOM  17739  CA  LYS B 570      -9.911  24.560  25.072  1.00  1.94
C
ATOM  17740  C   LYS B 570      -9.946  25.345  23.784  1.00  1.86
C
ATOM  17741  O   LYS B 570      -9.059  26.157  23.540  1.00  2.03
O
ATOM  17742  CB  LYS B 570     -10.614  25.376  26.159  1.00  2.91
C
ATOM  17743  CG  LYS B 570     -12.112  25.565  25.925  1.00  2.91
C
ATOM  17744  CD  LYS B 570     -12.764  26.411  27.007  1.00  2.91
C
ATOM  17745  CE  LYS B 570     -14.274  26.472  26.807  1.00  2.91
C
ATOM  17746  NZ  LYS B 570     -14.926  27.436  27.735  1.00  2.91
N
ATOM  17747  H   LYS B 570      -8.113  24.688  26.238  1.00  2.30
H
ATOM  17748  HA  LYS B 570     -10.457  23.628  24.919  1.00  2.33
H
ATOM  17749 1HB  LYS B 570     -10.474  24.898  27.128  1.00  3.49
H
ATOM  17750 2HB  LYS B 570     -10.168  26.364  26.210  1.00  3.49
H
ATOM  17751 1HG  LYS B 570     -12.280  26.057  24.971  1.00  3.49
H
ATOM  17752 2HG  LYS B 570     -12.598  24.590  25.900  1.00  3.49
H
ATOM  17753 1HD  LYS B 570     -12.550  25.979  27.986  1.00  3.49
H
ATOM  17754 2HD  LYS B 570     -12.360  27.423  26.978  1.00  3.49
H
ATOM  17755 1HE  LYS B 570     -14.497  26.761  25.781  1.00  3.49
H
ATOM  17756 2HE  LYS B 570     -14.687  25.481  26.989  1.00  3.49
H
ATOM  17757 1HZ  LYS B 570     -15.923  27.435  27.575  1.00  3.49
H
```

```
ATOM  17758 2HZ  LYS B 570     -14.736  27.181  28.693  1.00  3.49
H
ATOM  17759 3HZ  LYS B 570     -14.562  28.363  27.543  1.00  3.49
H
ATOM  17760  N   SER B 571     -10.979  25.117  22.980  1.00  1.74
N
ATOM  17761  CA  SER B 571     -11.186  25.831  21.726  1.00  1.71
C
ATOM  17762  C   SER B 571     -10.938  27.334  21.898  1.00  1.88
C
ATOM  17763  O   SER B 571     -10.154  27.931  21.156  1.00  1.91
O
ATOM  17764  CB  SER B 571     -12.618  25.597  21.270  1.00  2.56
C
ATOM  17765  OG  SER B 571     -12.899  26.249  20.066  1.00  2.56
O
ATOM  17766  H   SER B 571     -11.657  24.418  23.253  1.00  2.09
H
ATOM  17767  HA  SER B 571     -10.492  25.443  20.980  1.00  2.05
H
ATOM  17768 1HB  SER B 571     -12.795  24.527  21.154  1.00  3.08
H
ATOM  17769 2HB  SER B 571     -13.299  25.955  22.040  1.00  3.08
H
ATOM  17770  HG  SER B 571     -12.671  25.616  19.378  1.00  3.08
H
ATOM  17771  N   GLU B 572     -11.591  27.920  22.904  1.00  2.03
N
ATOM  17772  CA  GLU B 572     -11.466  29.335  23.235  1.00  2.25
C
ATOM  17773  C   GLU B 572     -10.049  29.712  23.636  1.00  2.32
C
ATOM  17774  O   GLU B 572      -9.533  30.747  23.207  1.00  2.39
O
ATOM  17775  CB  GLU B 572     -12.403  29.677  24.392  1.00  3.38
C
ATOM  17776  CG  GLU B 572     -13.884  29.645  24.058  1.00  3.38
C
ATOM  17777  CD  GLU B 572     -14.746  29.834  25.275  1.00  3.38
C
ATOM  17778  OE1 GLU B 572     -14.216  29.805  26.364  1.00  3.38
O
ATOM  17779  OE2 GLU B 572     -15.935  29.987  25.124  1.00  3.38
O
ATOM  17780  H   GLU B 572     -12.217  27.356  23.458  1.00  2.44
H
ATOM  17781  HA  GLU B 572     -11.746  29.921  22.360  1.00  2.70
H
ATOM  17782 1HB  GLU B 572     -12.234  28.982  25.213  1.00  4.05
H
ATOM  17783 2HB  GLU B 572     -12.170  30.677  24.760  1.00  4.05
H
ATOM  17784 1HG  GLU B 572     -14.103  30.436  23.342  1.00  4.05
H
ATOM  17785 2HG  GLU B 572     -14.122  28.690  23.590  1.00  4.05
H
ATOM  17786  N   GLU B 573      -9.425  28.862  24.453  1.00  2.31
N
```

```
ATOM  17787  CA  GLU B 573      -8.069  29.095  24.923  1.00  2.32
C
ATOM  17788  C   GLU B 573      -7.089  29.138  23.777  1.00  2.21
C
ATOM  17789  O   GLU B 573      -6.185  29.966  23.774  1.00  2.34
O
ATOM  17790  CB  GLU B 573      -7.636  28.009  25.914  1.00  3.48
C
ATOM  17791  CG  GLU B 573      -8.307  28.072  27.276  1.00  3.48
C
ATOM  17792  CD  GLU B 573      -7.944  26.906  28.161  1.00  3.48
C
ATOM  17793  OE1 GLU B 573      -7.567  25.875  27.648  1.00  3.48
O
ATOM  17794  OE2 GLU B 573      -8.044  27.050  29.357  1.00  3.48
O
ATOM  17795  H   GLU B 573      -9.909  28.031  24.761  1.00  2.77
H
ATOM  17796  HA  GLU B 573      -8.041  30.062  25.429  1.00  2.78
H
ATOM  17797 1HB  GLU B 573      -7.839  27.028  25.489  1.00  4.18
H
ATOM  17798 2HB  GLU B 573      -6.560  28.077  26.074  1.00  4.18
H
ATOM  17799 1HG  GLU B 573      -8.000  28.993  27.771  1.00  4.18
H
ATOM  17800 2HG  GLU B 573      -9.387  28.109  27.144  1.00  4.18
H
ATOM  17801  N   LEU B 574      -7.277  28.253  22.804  1.00  2.00
N
ATOM  17802  CA  LEU B 574      -6.381  28.184  21.669  1.00  1.84
C
ATOM  17803  C   LEU B 574      -6.569  29.389  20.759  1.00  1.91
C
ATOM  17804  O   LEU B 574      -5.594  29.928  20.237  1.00  1.95
O
ATOM  17805  CB  LEU B 574      -6.634  26.889  20.906  1.00  2.76
C
ATOM  17806  CG  LEU B 574      -6.306  25.618  21.697  1.00  2.76
C
ATOM  17807  CD1 LEU B 574      -6.696  24.409  20.879  1.00  2.76
C
ATOM  17808  CD2 LEU B 574      -4.838  25.592  22.064  1.00  2.76
C
ATOM  17809  H   LEU B 574      -8.039  27.595  22.873  1.00  2.40
H
ATOM  17810  HA  LEU B 574      -5.356  28.183  22.036  1.00  2.21
H
ATOM  17811 1HB  LEU B 574      -7.684  26.846  20.622  1.00  3.31
H
ATOM  17812 2HB  LEU B 574      -6.028  26.888  20.001  1.00  3.31
H
ATOM  17813  HG  LEU B 574      -6.894  25.607  22.613  1.00  3.31
H
ATOM  17814 1HD1 LEU B 574      -6.488  23.500  21.444  1.00  3.31
H
ATOM  17815 2HD1 LEU B 574      -7.758  24.460  20.649  1.00  3.31
H
```

```
ATOM  17816 3HD1 LEU B 574      -6.124  24.402  19.954  1.00  3.31
H
ATOM  17817 1HD2 LEU B 574      -4.628  24.694  22.639  1.00  3.31
H
ATOM  17818 2HD2 LEU B 574      -4.238  25.582  21.163  1.00  3.31
H
ATOM  17819 3HD2 LEU B 574      -4.597  26.469  22.660  1.00  3.31
H
ATOM  17820  N   LEU B 575      -7.811  29.859  20.616  1.00  2.00
N
ATOM  17821  CA  LEU B 575      -8.056  31.052  19.813  1.00  2.14
C
ATOM  17822  C   LEU B 575      -7.309  32.239  20.433  1.00  2.44
C
ATOM  17823  O   LEU B 575      -6.717  33.059  19.725  1.00  2.62
O
ATOM  17824  CB  LEU B 575      -9.564  31.353  19.746  1.00  3.21
C
ATOM  17825  CG  LEU B 575     -10.443  30.361  18.963  1.00  3.21
C
ATOM  17826  CD1 LEU B 575     -11.907  30.709  19.189  1.00  3.21
C
ATOM  17827  CD2 LEU B 575     -10.105  30.419  17.488  1.00  3.21
C
ATOM  17828  H   LEU B 575      -8.595  29.373  21.039  1.00  2.40
H
ATOM  17829  HA  LEU B 575      -7.677  30.883  18.806  1.00  2.57
H
ATOM  17830 1HB  LEU B 575      -9.953  31.403  20.762  1.00  3.85
H
ATOM  17831 2HB  LEU B 575      -9.696  32.329  19.283  1.00  3.85
H
ATOM  17832  HG  LEU B 575     -10.273  29.353  19.327  1.00  3.85
H
ATOM  17833 1HD1 LEU B 575     -12.536  30.004  18.647  1.00  3.85
H
ATOM  17834 2HD1 LEU B 575     -12.135  30.654  20.253  1.00  3.85
H
ATOM  17835 3HD1 LEU B 575     -12.100  31.718  18.829  1.00  3.85
H
ATOM  17836 1HD2 LEU B 575     -10.732  29.713  16.942  1.00  3.85
H
ATOM  17837 2HD2 LEU B 575     -10.279  31.427  17.111  1.00  3.85
H
ATOM  17838 3HD2 LEU B 575      -9.059  30.152  17.355  1.00  3.85
H
ATOM  17839  N   ASP B 576      -7.308  32.306  21.773  1.00  2.60
N
ATOM  17840  CA  ASP B 576      -6.557  33.336  22.481  1.00  2.95
C
ATOM  17841  C   ASP B 576      -5.052  33.162  22.267  1.00  2.75
C
ATOM  17842  O   ASP B 576      -4.352  34.109  21.905  1.00  2.89
O
ATOM  17843  CB  ASP B 576      -6.846  33.301  23.990  1.00  4.43
C
ATOM  17844  CG  ASP B 576      -8.231  33.789  24.406  1.00  4.43
C
```

ATOM 17845 OD1 ASP B 576 -8.898 34.434 23.630 1.00 4.43
O
ATOM 17846 OD2 ASP B 576 -8.604 33.523 25.524 1.00 4.43
O
ATOM 17847 H ASP B 576 -7.846 31.632 22.309 1.00 3.12
H
ATOM 17848 HA ASP B 576 -6.850 34.310 22.089 1.00 3.54
H
ATOM 17849 1HB ASP B 576 -6.726 32.282 24.354 1.00 5.31
H
ATOM 17850 2HB ASP B 576 -6.104 33.910 24.506 1.00 5.31
H
ATOM 17851 N MET B 577 -4.562 31.935 22.465 1.00 2.51
N
ATOM 17852 CA MET B 577 -3.142 31.624 22.332 1.00 2.47
C
ATOM 17853 C MET B 577 -2.551 31.963 20.983 1.00 2.40
C
ATOM 17854 O MET B 577 -1.396 32.386 20.909 1.00 2.48
O
ATOM 17855 CB MET B 577 -2.892 30.143 22.612 1.00 3.71
C
ATOM 17856 CG MET B 577 -3.029 29.728 24.066 1.00 3.71
C
ATOM 17857 SD MET B 577 -2.923 27.942 24.288 1.00 3.71
S
ATOM 17858 CE MET B 577 -1.214 27.635 23.855 1.00 3.71
C
ATOM 17859 H MET B 577 -5.188 31.195 22.752 1.00 3.01
H
ATOM 17860 HA MET B 577 -2.602 32.210 23.076 1.00 2.96
H
ATOM 17861 1HB MET B 577 -3.590 29.544 22.029 1.00 4.45
H
ATOM 17862 2HB MET B 577 -1.886 29.879 22.285 1.00 4.45
H
ATOM 17863 1HG MET B 577 -2.227 30.189 24.642 1.00 4.45
H
ATOM 17864 2HG MET B 577 -3.972 30.077 24.476 1.00 4.45
H
ATOM 17865 1HE MET B 577 -1.002 26.570 23.947 1.00 4.45
H
ATOM 17866 2HE MET B 577 -1.035 27.955 22.828 1.00 4.45
H
ATOM 17867 3HE MET B 577 -0.562 28.194 24.528 1.00 4.45
H
ATOM 17868 N TYR B 578 -3.321 31.793 19.913 1.00 2.27
N
ATOM 17869 CA TYR B 578 -2.754 32.034 18.601 1.00 2.19
C
ATOM 17870 C TYR B 578 -3.179 33.356 17.973 1.00 2.33
C
ATOM 17871 O TYR B 578 -3.016 33.545 16.769 1.00 2.25
O
ATOM 17872 CB TYR B 578 -3.066 30.839 17.708 1.00 3.29
C
ATOM 17873 CG TYR B 578 -2.490 29.617 18.379 1.00 3.29
C

```
ATOM  17874  CD1 TYR B 578      -3.315  28.586  18.780  1.00  3.29
C
ATOM  17875  CD2 TYR B 578      -1.137  29.576  18.677  1.00  3.29
C
ATOM  17876  CE1 TYR B 578      -2.798  27.522  19.479  1.00  3.29
C
ATOM  17877  CE2 TYR B 578      -0.620  28.513  19.385  1.00  3.29
C
ATOM  17878  CZ  TYR B 578      -1.448  27.494  19.796  1.00  3.29
C
ATOM  17879  OH  TYR B 578      -0.932  26.453  20.533  1.00  3.29
O
ATOM  17880  H   TYR B 578      -4.266  31.439  20.007  1.00  2.72
H
ATOM  17881  HA  TYR B 578      -1.671  32.077  18.712  1.00  2.63
H
ATOM  17882 1HB  TYR B 578      -4.142  30.709  17.589  1.00  3.94
H
ATOM  17883 2HB  TYR B 578      -2.612  30.955  16.725  1.00  3.94
H
ATOM  17884  HD1 TYR B 578      -4.379  28.622  18.557  1.00  3.94
H
ATOM  17885  HD2 TYR B 578      -0.488  30.398  18.375  1.00  3.94
H
ATOM  17886  HE1 TYR B 578      -3.455  26.722  19.797  1.00  3.94
H
ATOM  17887  HE2 TYR B 578       0.441  28.489  19.637  1.00  3.94
H
ATOM  17888  HH  TYR B 578      -0.013  26.643  20.745  1.00  3.94
H
ATOM  17889  N   GLY B 579      -3.682  34.291  18.787  1.00  2.63
N
ATOM  17890  CA  GLY B 579      -3.938  35.645  18.295  1.00  2.94
C
ATOM  17891  C   GLY B 579      -5.145  35.820  17.378  1.00  2.61
C
ATOM  17892  O   GLY B 579      -5.176  36.767  16.590  1.00  2.59
O
ATOM  17893  H   GLY B 579      -3.844  34.096  19.770  1.00  3.16
H
ATOM  17894 1HA  GLY B 579      -4.030  36.310  19.146  1.00  3.53
H
ATOM  17895 2HA  GLY B 579      -3.051  35.981  17.761  1.00  3.53
H
ATOM  17896  N   ILE B 580      -6.112  34.909  17.431  1.00  2.45
N
ATOM  17897  CA  ILE B 580      -7.249  34.983  16.519  1.00  2.17
C
ATOM  17898  C   ILE B 580      -8.619  35.054  17.202  1.00  2.12
C
ATOM  17899  O   ILE B 580      -9.643  34.814  16.560  1.00  3.89
O
ATOM  17900  CB  ILE B 580      -7.185  33.799  15.539  1.00  3.25
C
ATOM  17901  CG1 ILE B 580      -7.191  32.493  16.318  1.00  3.25
C
ATOM  17902  CG2 ILE B 580      -5.961  33.888  14.634  1.00  3.25
C
```

ATOM 17903 CD1 ILE B 580   -7.325 31.287 15.435 1.00 3.25
C
ATOM 17904 H   ILE B 580   -6.079 34.156 18.108 1.00 2.94
H
ATOM 17905 HA ILE B 580   -7.139 35.892 15.929 1.00 2.60
H
ATOM 17906 HB ILE B 580   -8.080 33.808 14.918 1.00 3.91
H
ATOM 17907 1HG1 ILE B 580   -6.262 32.407 16.880 1.00 3.91
H
ATOM 17908 2HG1 ILE B 580   -8.019 32.501 17.025 1.00 3.91
H
ATOM 17909 1HG2 ILE B 580   -5.959 33.045 13.945 1.00 3.91
H
ATOM 17910 2HG2 ILE B 580   -5.995 34.818 14.068 1.00 3.91
H
ATOM 17911 3HG2 ILE B 580   -5.055 33.863 15.237 1.00 3.91
H
ATOM 17912 1HD1 ILE B 580   -7.321 30.386 16.044 1.00 3.91
H
ATOM 17913 2HD1 ILE B 580   -8.262 31.347 14.881 1.00 3.91
H
ATOM 17914 3HD1 ILE B 580   -6.491 31.255 14.736 1.00 3.91
H
ATOM 17915 N   SER B 581   -8.642 35.380 18.494 1.00 3.42
N
ATOM 17916 CA SER B 581   -9.909 35.523 19.207 1.00 3.06
C
ATOM 17917 C   SER B 581   -10.355 36.971 19.226 1.00 3.03
C
ATOM 17918 O   SER B 581   -9.570 37.865 18.904 1.00 3.80
O
ATOM 17919 CB SER B 581   -9.802 35.032 20.629 1.00 4.59
C
ATOM 17920 OG SER B 581   -9.001 35.881 21.400 1.00 4.59
O
ATOM 17921 H   SER B 581   -7.777 35.548 18.986 1.00 4.10
H
ATOM 17922 HA SER B 581   -10.668 34.936 18.692 1.00 3.67
H
ATOM 17923 1HB SER B 581   -10.796 34.968 21.070 1.00 5.51
H
ATOM 17924 2HB SER B 581   -9.382 34.034 20.639 1.00 5.51
H
ATOM 17925 HG SER B 581   -8.883 35.423 22.239 1.00 5.51
H
ATOM 17926 N   ALA B 582   -11.597 37.187 19.655 1.00 2.66
N
ATOM 17927 CA ALA B 582   -12.216 38.505 19.746 1.00 2.75
C
ATOM 17928 C   ALA B 582   -11.332 39.521 20.437 1.00 3.07
C
ATOM 17929 O   ALA B 582   -11.220 40.648 19.970 1.00 3.23
O
ATOM 17930 CB ALA B 582   -13.534 38.407 20.491 1.00 4.12
C
ATOM 17931 H   ALA B 582   -12.162 36.381 19.893 1.00 3.19
H

```
ATOM  17932  HA  ALA B 582     -12.405  38.857  18.733  1.00  3.30
H
ATOM  17933 1HB  ALA B 582     -14.009  39.386  20.524  1.00  4.95
H
ATOM  17934 2HB  ALA B 582     -14.187  37.702  19.977  1.00  4.95
H
ATOM  17935 3HB  ALA B 582     -13.354  38.056  21.507  1.00  4.95
H
ATOM  17936  N   ARG B 583     -10.688  39.126  21.534  1.00  3.20
N
ATOM  17937  CA  ARG B 583      -9.828  40.032  22.285  1.00  3.66
C
ATOM  17938  C   ARG B 583      -8.643  40.524  21.469  1.00  3.53
C
ATOM  17939  O   ARG B 583      -8.205  41.662  21.633  1.00  4.43
O
ATOM  17940  CB  ARG B 583      -9.328  39.348  23.543  1.00  5.49
C
ATOM  17941  CG  ARG B 583     -10.392  39.133  24.610  1.00  5.49
C
ATOM  17942  CD  ARG B 583      -9.860  38.388  25.780  1.00  5.49
C
ATOM  17943  NE  ARG B 583     -10.870  38.212  26.813  1.00  5.49
N
ATOM  17944  CZ  ARG B 583     -10.740  37.404  27.885  1.00  5.49
C
ATOM  17945  NH1 ARG B 583      -9.643  36.697  28.049  1.00  5.49
N
ATOM  17946  NH2 ARG B 583     -11.716  37.321  28.771  1.00  5.49
N
ATOM  17947  H   ARG B 583     -10.808  38.179  21.865  1.00  3.84
H
ATOM  17948  HA  ARG B 583     -10.417  40.895  22.583  1.00  4.39
H
ATOM  17949 1HB  ARG B 583      -8.913  38.374  23.289  1.00  6.59
H
ATOM  17950 2HB  ARG B 583      -8.527  39.940  23.987  1.00  6.59
H
ATOM  17951 1HG  ARG B 583     -10.757  40.099  24.957  1.00  6.59
H
ATOM  17952 2HG  ARG B 583     -11.218  38.562  24.186  1.00  6.59
H
ATOM  17953 1HD  ARG B 583      -9.523  37.401  25.461  1.00  6.59
H
ATOM  17954 2HD  ARG B 583      -9.023  38.935  26.211  1.00  6.59
H
ATOM  17955  HE  ARG B 583     -11.729  38.738  26.725  1.00  6.59
H
ATOM  17956 1HH1 ARG B 583      -8.898  36.755  27.370  1.00  6.59
H
ATOM  17957 2HH1 ARG B 583      -9.548  36.091  28.851  1.00  6.59
H
ATOM  17958 1HH2 ARG B 583     -12.561  37.863  28.645  1.00  6.59
H
ATOM  17959 2HH2 ARG B 583     -11.620  36.715  29.572  1.00  6.59
H
ATOM  17960  N   HIS B 584      -8.133  39.677  20.586  1.00  2.98
N
```

```
ATOM  17961  CA  HIS B 584      -7.001  40.044  19.763  1.00  2.98
C
ATOM  17962  C   HIS B 584      -7.458  40.889  18.602  1.00  3.05
C
ATOM  17963  O   HIS B 584      -6.732  41.779  18.160  1.00  3.13
O
ATOM  17964  CB  HIS B 584      -6.281  38.798  19.291  1.00  4.47
C
ATOM  17965  CG  HIS B 584      -5.592  38.115  20.420  1.00  4.47
C
ATOM  17966  ND1 HIS B 584      -4.479  38.648  21.036  1.00  4.47
N
ATOM  17967  CD2 HIS B 584      -5.858  36.956  21.062  1.00  4.47
C
ATOM  17968  CE1 HIS B 584      -4.086  37.839  22.001  1.00  4.47
C
ATOM  17969  NE2 HIS B 584      -4.905  36.807  22.038  1.00  4.47
N
ATOM  17970  H   HIS B 584      -8.546  38.763  20.458  1.00  3.58
H
ATOM  17971  HA  HIS B 584      -6.298  40.635  20.349  1.00  3.58
H
ATOM  17972 1HB  HIS B 584      -6.989  38.102  18.840  1.00  5.36
H
ATOM  17973 2HB  HIS B 584      -5.539  39.057  18.535  1.00  5.36
H
ATOM  17974  HD2 HIS B 584      -6.672  36.267  20.843  1.00  5.36
H
ATOM  17975  HE1 HIS B 584      -3.230  37.998  22.658  1.00  5.36
H
ATOM  17976  HE2 HIS B 584      -4.845  36.027  22.678  1.00  5.36
H
ATOM  17977  N   ILE B 585      -8.679  40.631  18.140  1.00  3.04
N
ATOM  17978  CA  ILE B 585      -9.286  41.422  17.086  1.00  3.15
C
ATOM  17979  C   ILE B 585      -9.498  42.839  17.604  1.00  3.39
C
ATOM  17980  O   ILE B 585      -9.175  43.800  16.911  1.00  3.50
O
ATOM  17981  CB  ILE B 585     -10.605  40.796  16.613  1.00  4.72
C
ATOM  17982  CG1 ILE B 585     -10.306  39.460  15.925  1.00  4.72
C
ATOM  17983  CG2 ILE B 585     -11.323  41.743  15.659  1.00  4.72
C
ATOM  17984  CD1 ILE B 585     -11.526  38.612  15.680  1.00  4.72
C
ATOM  17985  H   ILE B 585      -9.200  39.853  18.528  1.00  3.65
H
ATOM  17986  HA  ILE B 585      -8.604  41.465  16.239  1.00  3.78
H
ATOM  17987  HB  ILE B 585     -11.244  40.590  17.471  1.00  5.67
H
ATOM  17988 1HG1 ILE B 585      -9.826  39.657  14.967  1.00  5.67
H
ATOM  17989 2HG1 ILE B 585      -9.612  38.891  16.544  1.00  5.67
H
```

ATOM 17990 1HG2 ILE B 585   -12.255 41.289 15.329 1.00 5.67
H
ATOM 17991 2HG2 ILE B 585   -11.537 42.681 16.170 1.00 5.67
H
ATOM 17992 3HG2 ILE B 585   -10.687 41.938 14.794 1.00 5.67
H
ATOM 17993 1HD1 ILE B 585   -11.232 37.684 15.191 1.00 5.67
H
ATOM 17994 2HD1 ILE B 585   -12.007 38.384 16.630 1.00 5.67
H
ATOM 17995 3HD1 ILE B 585   -12.220 39.154 15.041 1.00 5.67
H
ATOM 17996 N   ILE B 586   -10.003 42.952 18.838 1.00 3.54
N
ATOM 17997 CA ILE B 586   -10.203 44.233 19.516 1.00 3.90
C
ATOM 17998 C   ILE B 586   -8.919 45.032 19.587 1.00 4.02
C
ATOM 17999 O   ILE B 586   -8.901 46.211 19.225 1.00 4.20
O
ATOM 18000 CB ILE B 586   -10.732 44.004 20.948 1.00 5.85
C
ATOM 18001 CG1 ILE B 586   -12.160 43.475 20.901 1.00 5.85
C
ATOM 18002 CG2 ILE B 586   -10.626 45.260 21.795 1.00 5.85
C
ATOM 18003 CD1 ILE B 586   -12.645 42.927 22.215 1.00 5.85
C
ATOM 18004 H   ILE B 586   -10.267 42.111 19.330 1.00 4.25
H
ATOM 18005 HA ILE B 586   -10.938 44.811 18.957 1.00 4.68
H
ATOM 18006 HB ILE B 586   -10.131 43.227 21.417 1.00 7.02
H
ATOM 18007 1HG1 ILE B 586   -12.825 44.279 20.605 1.00 7.02
H
ATOM 18008 2HG1 ILE B 586   -12.222 42.695 20.153 1.00 7.02
H
ATOM 18009 1HG2 ILE B 586   -10.982 45.046 22.802 1.00 7.02
H
ATOM 18010 2HG2 ILE B 586   -9.588 45.584 21.840 1.00 7.02
H
ATOM 18011 3HG2 ILE B 586   -11.231 46.049 21.362 1.00 7.02
H
ATOM 18012 1HD1 ILE B 586   -13.663 42.564 22.099 1.00 7.02
H
ATOM 18013 2HD1 ILE B 586   -12.009 42.104 22.527 1.00 7.02
H
ATOM 18014 3HD1 ILE B 586   -12.619 43.710 22.970 1.00 7.02
H
ATOM 18015 N   VAL B 587   -7.844 44.382 20.040 1.00 3.92
N
ATOM 18016 CA VAL B 587   -6.542 45.024 20.113 1.00 4.00
C
ATOM 18017 C   VAL B 587   -6.063 45.483 18.748 1.00 3.96
C
ATOM 18018 O   VAL B 587   -5.582 46.607 18.616 1.00 4.13
O

ATOM 18019 CB VAL B 587   -5.499 44.079 20.727 1.00 6.00
C
ATOM 18020 CG1 VAL B 587   -4.106 44.672 20.579 1.00 6.00
C
ATOM 18021 CG2 VAL B 587   -5.815 43.871 22.199 1.00 6.00
C
ATOM 18022 H   VAL B 587   -7.932 43.421 20.355 1.00 4.70
H
ATOM 18023 HA VAL B 587   -6.629 45.899 20.758 1.00 4.80
H
ATOM 18024 HB VAL B 587   -5.520 43.124 20.202 1.00 7.20
H
ATOM 18025 1HG1 VAL B 587   -3.373 43.993 21.013 1.00 7.20
H
ATOM 18026 2HG1 VAL B 587   -3.883 44.818 19.522 1.00 7.20
H
ATOM 18027 3HG1 VAL B 587   -4.063 45.631 21.095 1.00 7.20
H
ATOM 18028 1HG2 VAL B 587   -5.080 43.196 22.638 1.00 7.20
H
ATOM 18029 2HG2 VAL B 587   -5.780 44.830 22.715 1.00 7.20
H
ATOM 18030 3HG2 VAL B 587   -6.809 43.443 22.306 1.00 7.20
H
ATOM 18031 N   ALA B 588   -6.195 44.613 17.741 1.00 3.78
N
ATOM 18032 CA ALA B 588   -5.788 44.934 16.379 1.00 3.79
C
ATOM 18033 C   ALA B 588   -6.515 46.167 15.869 1.00 4.00
C
ATOM 18034 O   ALA B 588   -5.891 47.045 15.274 1.00 4.24
O
ATOM 18035 CB ALA B 588   -6.051 43.755 15.457 1.00 5.69
C
ATOM 18036 H   ALA B 588   -6.579 43.694 17.921 1.00 4.54
H
ATOM 18037 HA ALA B 588   -4.720 45.150 16.385 1.00 4.55
H
ATOM 18038 1HB ALA B 588   -5.719 44.002 14.451 1.00 6.82
H
ATOM 18039 2HB ALA B 588   -5.503 42.883 15.816 1.00 6.82
H
ATOM 18040 3HB ALA B 588   -7.115 43.531 15.442 1.00 6.82
H
ATOM 18041 N   VAL B 589   -7.826 46.241 16.125 1.00 3.99
N
ATOM 18042 CA VAL B 589   -8.624 47.396 15.736 1.00 4.27
C
ATOM 18043 C   VAL B 589   -8.105 48.660 16.373 1.00 4.48
C
ATOM 18044 O   VAL B 589   -7.913 49.660 15.682 1.00 4.63
O
ATOM 18045 CB VAL B 589   -10.106 47.203 16.123 1.00 6.40
C
ATOM 18046 CG1 VAL B 589   -10.860 48.514 15.944 1.00 6.40
C
ATOM 18047 CG2 VAL B 589   -10.725 46.122 15.243 1.00 6.40
C

```
ATOM  18048  H   VAL B 589      -8.284  45.472  16.597  1.00  4.79
H
ATOM  18049  HA  VAL B 589      -8.564  47.506  14.654  1.00  5.12
H
ATOM  18050  HB  VAL B 589     -10.175  46.913  17.170  1.00  7.69
H
ATOM  18051 1HG1 VAL B 589     -11.904  48.374  16.222  1.00  7.69
H
ATOM  18052 2HG1 VAL B 589     -10.414  49.276  16.582  1.00  7.69
H
ATOM  18053 3HG1 VAL B 589     -10.801  48.834  14.908  1.00  7.69
H
ATOM  18054 1HG2 VAL B 589     -11.769  45.983  15.514  1.00  7.69
H
ATOM  18055 2HG2 VAL B 589     -10.662  46.421  14.200  1.00  7.69
H
ATOM  18056 3HG2 VAL B 589     -10.191  45.187  15.375  1.00  7.69
H
ATOM  18057  N   LYS B 590      -7.861  48.609  17.681  1.00  4.50
N
ATOM  18058  CA  LYS B 590      -7.353  49.761  18.406  1.00  4.78
C
ATOM  18059  C   LYS B 590      -5.998  50.206  17.877  1.00  4.98
C
ATOM  18060  O   LYS B 590      -5.754  51.404  17.739  1.00  5.32
O
ATOM  18061  CB  LYS B 590      -7.255  49.441  19.894  1.00  7.17
C
ATOM  18062  CG  LYS B 590      -8.594  49.318  20.609  1.00  7.17
C
ATOM  18063  CD  LYS B 590      -8.386  48.969  22.076  1.00  7.17
C
ATOM  18064  CE  LYS B 590      -9.702  48.871  22.830  1.00  7.17
C
ATOM  18065  NZ  LYS B 590      -9.485  48.497  24.257  1.00  7.17
N
ATOM  18066  H   LYS B 590      -8.050  47.753  18.190  1.00  5.40
H
ATOM  18067  HA  LYS B 590      -8.050  50.587  18.273  1.00  5.74
H
ATOM  18068 1HB  LYS B 590      -6.720  48.500  20.028  1.00  8.60
H
ATOM  18069 2HB  LYS B 590      -6.678  50.218  20.396  1.00  8.60
H
ATOM  18070 1HG  LYS B 590      -9.131  50.266  20.543  1.00  8.60
H
ATOM  18071 2HG  LYS B 590      -9.195  48.544  20.137  1.00  8.60
H
ATOM  18072 1HD  LYS B 590      -7.865  48.013  22.149  1.00  8.60
H
ATOM  18073 2HD  LYS B 590      -7.768  49.734  22.544  1.00  8.60
H
ATOM  18074 1HE  LYS B 590     -10.211  49.833  22.790  1.00  8.60
H
ATOM  18075 2HE  LYS B 590     -10.334  48.120  22.358  1.00  8.60
H
ATOM  18076 1HZ  LYS B 590     -10.375  48.440  24.731  1.00  8.60
H
```

```
ATOM  18077 2HZ  LYS B 590      -9.022  47.600  24.304  1.00  8.60
H
ATOM  18078 3HZ  LYS B 590      -8.910  49.197  24.705  1.00  8.60
H
ATOM  18079  N   CYS B 591      -5.132  49.244  17.556  1.00  4.87
N
ATOM  18080  CA  CYS B 591      -3.822  49.550  17.007  1.00  5.09
C
ATOM  18081  C   CYS B 591      -3.951  50.276  15.684  1.00  5.22
C
ATOM  18082  O   CYS B 591      -3.275  51.276  15.453  1.00  5.73
O
ATOM  18083  CB  CYS B 591      -3.002  48.275  16.811  1.00  7.63
C
ATOM  18084  SG  CYS B 591      -2.461  47.496  18.351  1.00  7.63
S
ATOM  18085  H   CYS B 591      -5.381  48.276  17.710  1.00  5.84
H
ATOM  18086  HA  CYS B 591      -3.294  50.195  17.709  1.00  6.11
H
ATOM  18087 1HB  CYS B 591      -3.590  47.546  16.254  1.00  9.16
H
ATOM  18088 2HB  CYS B 591      -2.114  48.501  16.219  1.00  9.16
H
ATOM  18089  HG  CYS B 591      -1.855  46.460  17.778  1.00  9.16
H
ATOM  18090  N   MET B 592      -4.841  49.786  14.820  1.00  5.00
N
ATOM  18091  CA  MET B 592      -5.079  50.417  13.530  1.00  5.17
C
ATOM  18092  C   MET B 592      -5.616  51.840  13.714  1.00  5.07
C
ATOM  18093  O   MET B 592      -5.181  52.762  13.025  1.00  5.28
O
ATOM  18094  CB  MET B 592      -6.050  49.559  12.713  1.00  7.75
C
ATOM  18095  CG  MET B 592      -5.478  48.227  12.231  1.00  7.75
C
ATOM  18096  SD  MET B 592      -4.117  48.399  11.060  1.00  7.75
S
ATOM  18097  CE  MET B 592      -5.002  48.905   9.584  1.00  7.75
C
ATOM  18098  H   MET B 592      -5.355  48.946  15.055  1.00  6.00
H
ATOM  18099  HA  MET B 592      -4.132  50.483  12.996  1.00  6.20
H
ATOM  18100 1HB  MET B 592      -6.941  49.349  13.302  1.00  9.31
H
ATOM  18101 2HB  MET B 592      -6.357  50.110  11.833  1.00  9.31
H
ATOM  18102 1HG  MET B 592      -5.120  47.654  13.083  1.00  9.31
H
ATOM  18103 2HG  MET B 592      -6.269  47.655  11.747  1.00  9.31
H
ATOM  18104 1HE  MET B 592      -4.294  49.053   8.768  1.00  9.31
H
ATOM  18105 2HE  MET B 592      -5.721  48.133   9.308  1.00  9.31
H
```

ATOM 18106 3HE MET B 592 -5.528 49.839 9.780 1.00 9.31
H
ATOM 18107 N LEU B 593 -6.504 52.032 14.695 1.00 4.91
N
ATOM 18108 CA LEU B 593 -7.053 53.353 15.001 1.00 5.00
C
ATOM 18109 C LEU B 593 -5.973 54.328 15.457 1.00 5.34
C
ATOM 18110 O LEU B 593 -6.025 55.518 15.143 1.00 7.30
O
ATOM 18111 CB LEU B 593 -8.118 53.243 16.101 1.00 7.50
C
ATOM 18112 CG LEU B 593 -9.418 52.533 15.719 1.00 7.50
C
ATOM 18113 CD1 LEU B 593 -10.277 52.354 16.961 1.00 7.50
C
ATOM 18114 CD2 LEU B 593 -10.144 53.348 14.670 1.00 7.50
C
ATOM 18115 H LEU B 593 -6.835 51.237 15.227 1.00 5.89
H
ATOM 18116 HA LEU B 593 -7.517 53.750 14.098 1.00 6.00
H
ATOM 18117 1HB LEU B 593 -7.691 52.710 16.947 1.00 9.00
H
ATOM 18118 2HB LEU B 593 -8.377 54.250 16.430 1.00 9.00
H
ATOM 18119 HG LEU B 593 -9.198 51.552 15.310 1.00 9.00
H
ATOM 18120 1HD1 LEU B 593 -11.198 51.843 16.689 1.00 9.00
H
ATOM 18121 2HD1 LEU B 593 -9.737 51.759 17.696 1.00 9.00
H
ATOM 18122 3HD1 LEU B 593 -10.512 53.329 17.385 1.00 9.00
H
ATOM 18123 1HD2 LEU B 593 -11.065 52.837 14.402 1.00 9.00
H
ATOM 18124 2HD2 LEU B 593 -10.376 54.335 15.072 1.00 9.00
H
ATOM 18125 3HD2 LEU B 593 -9.512 53.454 13.789 1.00 9.00
H
ATOM 18126 N LEU B 594 -4.985 53.815 16.184 1.00 4.44
N
ATOM 18127 CA LEU B 594 -3.881 54.622 16.680 1.00 4.71
C
ATOM 18128 C LEU B 594 -2.725 54.779 15.686 1.00 5.26
C
ATOM 18129 O LEU B 594 -1.732 55.435 16.006 1.00 6.06
O
ATOM 18130 CB LEU B 594 -3.349 54.003 17.974 1.00 7.06
C
ATOM 18131 CG LEU B 594 -4.335 53.987 19.149 1.00 7.06
C
ATOM 18132 CD1 LEU B 594 -3.716 53.230 20.315 1.00 7.06
C
ATOM 18133 CD2 LEU B 594 -4.671 55.417 19.539 1.00 7.06
C
ATOM 18134 H LEU B 594 -5.016 52.834 16.438 1.00 5.33
H

```
ATOM  18135  HA  LEU B 594      -4.267  55.614  16.904  1.00  5.65
H
ATOM  18136 1HB  LEU B 594      -3.057  52.974  17.772  1.00  8.48
H
ATOM  18137 2HB  LEU B 594      -2.464  54.557  18.288  1.00  8.48
H
ATOM  18138  HG  LEU B 594      -5.248  53.472  18.857  1.00  8.48
H
ATOM  18139 1HD1 LEU B 594      -4.418  53.212  21.150  1.00  8.48
H
ATOM  18140 2HD1 LEU B 594      -3.491  52.210  20.008  1.00  8.48
H
ATOM  18141 3HD1 LEU B 594      -2.798  53.728  20.624  1.00  8.48
H
ATOM  18142 1HD2 LEU B 594      -5.375  55.412  20.371  1.00  8.48
H
ATOM  18143 2HD2 LEU B 594      -3.760  55.936  19.836  1.00  8.48
H
ATOM  18144 3HD2 LEU B 594      -5.119  55.930  18.687  1.00  8.48
H
ATOM  18145  N   ASN B 595      -2.827  54.166  14.499  1.00  5.52
N
ATOM  18146  CA  ASN B 595      -1.742  54.240  13.530  1.00  6.67
C
ATOM  18147  C   ASN B 595      -2.031  55.144  12.356  1.00 46.72
C
ATOM  18148  O   ASN B 595      -3.149  55.174  11.818  1.00104.00
O
ATOM  18149  OXT ASN B 595      -1.041  55.505  11.722  1.00  0.00
O
ATOM  18150  CB  ASN B 595      -1.364  52.860  13.017  1.00 10.00
C
ATOM  18151  CG  ASN B 595      -0.686  52.006  14.050  1.00 10.00
C
ATOM  18152  OD1 ASN B 595       0.030  52.512  14.922  1.00 10.00
O
ATOM  18153  ND2 ASN B 595      -0.879  50.714  13.958  1.00 10.00
N
ATOM  18154  H   ASN B 595      -3.658  53.651  14.242  1.00  6.62
H
ATOM  18155  HA  ASN B 595      -0.869  54.655  14.034  1.00  8.00
H
ATOM  18156 1HB  ASN B 595      -2.262  52.345  12.674  1.00 12.01
H
ATOM  18157 2HB  ASN B 595      -0.699  52.963  12.158  1.00 12.01
H
ATOM  18158 1HD2 ASN B 595      -0.446  50.096  14.615  1.00 12.01
H
ATOM  18159 2HD2 ASN B 595      -1.459  50.347  13.232  1.00 12.01
H
ENDMDL
END
```
