# Supplementary material for: The human transketolase-like proteins TKTL1 and TKTL2 are bona fide transketolases
Source: BMC Struct Biol. 2019 Jan 15;19:2. doi: 10.1186/s12900-018-0099-y (PMC6334435; doi:10.1186/s12900-018-0099-y)
Supplement: Supplementary file 2 — TKTL2_model: TKTL2 in covalent complex. X-ray diffraction data. (PDF 2185 kb) [file 12900_2018_99_MOESM2_ESM.pdf]

```
HEADER    TRANSFERASE                             28-MAY-13   XXXX
TITLE     HUMAN TRANSKETOLASE IN COVALENT COMPLEX WITH DONOR KETOSE D-
XYLULOSE-
TITLE    2 5-PHOSPHATE, CRYSTAL 2
KEYWDS    THIAMIN DIPHOSPHATE, ENZYME CATALYSIS, PENTOSE PHOSPHATE
PATHWAY,
KEYWDS   2 TRANSFERASE
EXPDTA    X-RAY DIFFRACTION
AUTHOR    P.NEUMANN, S.LUEDTKE, R.FICNER, K.TITTMANN
JRNL        AUTH   S.LUDTKE, P.NEUMANN, K.M.ERIXON, F.LEEPER, R.KLUGER,
JRNL        AUTH 2 R.FICNER, K.TITTMANN
JRNL        TITL   SUB-ANGSTROM-RESOLUTION CRYSTALLOGRAPHY REVEALS
PHYSICAL
JRNL        TITL 2 DISTORTIONS THAT ENHANCE REACTIVITY OF A COVALENT
ENZYMATIC
JRNL        TITL 3 INTERMEDIATE.
JRNL        REF    NAT CHEM                      V.   5   762 2013
JRNL        REFN
JRNL        PMID   23965678
JRNL        DOI    10.1038/NCHEM.1728
SEQRES   1 A  637  MET GLU SER TYR HIS LYS PRO ASP GLN GLN LYS LEU GLN
SEQRES   2 A  637  ALA LEU LYS ASP THR ALA ASN ARG LEU ARG ILE SER SER
SEQRES   3 A  637  ILE GLN ALA THR THR ALA ALA GLY SER GLY HIS PRO THR
SEQRES   4 A  637  SER CYS CYS SER ALA ALA GLU ILE MET ALA VAL LEU PHE
SEQRES   5 A  637  PHE HIS THR MET ARG TYR LYS SER GLN ASP PRO ARG ASN
SEQRES   6 A  637  PRO HIS ASN ASP ARG PHE VAL LEU SER LYS GLY HIS ALA
SEQRES   7 A  637  ALA PRO ILE LEU TYR ALA VAL TRP ALA GLU ALA GLY PHE
SEQRES   8 A  637  LEU ALA GLU ALA GLU LEU LEU ASN LEU ARG LYS ILE SER
SEQRES   9 A  637  SER ASP LEU ASP GLY HIS PRO VAL PRO LYS GLN ALA PHE
SEQRES  10 A  637  THR ASP VAL ALA THR GLY SER LEU GLY GLN GLY LEU GLY
SEQRES  11 A  637  ALA ALA CYS GLY MET ALA TYR THR GLY LYS TYR PHE ASP
SEQRES  12 A  637  LYS ALA SER TYR ARG VAL TYR CYS LEU LEU GLY ASP GLY
SEQRES  13 A  637  GLU LEU SER GLU GLY SER VAL TRP GLU ALA MET ALA PHE
SEQRES  14 A  637  ALA SER ILE TYR LYS LEU ASP ASN LEU VAL ALA ILE LEU
SEQRES  15 A  637  ASP ILE ASN ARG LEU GLY GLN SER ASP PRO ALA PRO LEU
SEQRES  16 A  637  GLN HIS GLN MET ASP ILE TYR GLN LYS ARG CYS GLU ALA
SEQRES  17 A  637  PHE GLY TRP HIS ALA ILE ILE VAL ASP GLY HIS SER VAL
SEQRES  18 A  637  GLU GLU LEU CYS LYS ALA PHE GLY GLN ALA LYS HIS GLN
SEQRES  19 A  637  PRO THR ALA ILE ILE ALA LYS THR PHE LYS GLY ARG GLY
SEQRES  20 A  637  ILE THR GLY VAL GLU ASP LYS GLU SER TRP HIS GLY LYS
SEQRES  21 A  637  PRO LEU PRO LYS ASN MET ALA GLU GLN ILE ILE GLN GLU
SEQRES  22 A  637  ILE TYR SER GLN ILE GLN SER LYS LYS LYS ILE LEU ALA
SEQRES  23 A  637  THR PRO PRO GLN GLU ASP ALA PRO SER VAL ASP ILE ALA
SEQRES  24 A  637  ASN ILE ARG MET PRO SER LEU PRO SER TYR LYS VAL GLY
SEQRES  25 A  637  ASP LYS ILE ALA THR ARG LYS ALA TYR GLY GLN ALA LEU
SEQRES  26 A  637  ALA LYS LEU GLY HIS ALA SER ASP ARG ILE ILE ALA LEU
SEQRES  27 A  637  ASP GLY ASP THR LYS ASN SER THR PHE SER GLU ILE PHE
SEQRES  28 A  637  LYS LYS GLU HIS PRO ASP ARG PHE ILE GLU CYS TYR ILE
SEQRES  29 A  637  ALA GLU GLN ASN MET VAL SER ILE ALA VAL GLY CYS ALA
SEQRES  30 A  637  THR ARG ASN ARG THR VAL PRO PHE CYS SER THR PHE ALA
SEQRES  31 A  637  ALA PHE PHE THR ARG ALA PHE ASP GLN ILE ARG MET ALA
SEQRES  32 A  637  ALA ILE SER GLU SER ASN ILE ASN LEU CYS GLY SER HIS
SEQRES  33 A  637  CYS GLY VAL SER ILE GLY GLU ASP GLY PRO SER GLN MET
SEQRES  34 A  637  ALA LEU GLU ASP LEU ALA MET PHE ARG SER VAL PRO THR
SEQRES  35 A  637  SER THR VAL PHE TYR PRO SER ASP GLY VAL ALA THR GLU
SEQRES  36 A  637  LYS ALA VAL GLU LEU ALA ALA ASN THR LYS GLY ILE CYS
SEQRES  37 A  637  PHE ILE ARG THR SER ARG PRO GLU ASN ALA ILE ILE TYR
SEQRES  38 A  637  ASN ASN ASN GLU ASP PHE GLN VAL GLY GLN ALA LYS VAL
```

```
SEQRES  39 A  637  VAL LEU LYS SER LYS ASP ASP GLN VAL THR VAL ILE GLY
SEQRES  40 A  637  ALA GLY VAL THR LEU HIS GLU ALA LEU ALA ALA ALA GLU
SEQRES  41 A  637  LEU LEU LYS LYS GLU LYS ILE ASN ILE ARG VAL LEU ASP
SEQRES  42 A  637  PRO PHE THR ILE LYS PRO LEU ASP ARG LYS LEU ILE LEU
SEQRES  43 A  637  ASP SER ALA ARG ALA THR LYS GLY ARG ILE LEU THR VAL
SEQRES  44 A  637  GLU ASP HIS TYR TYR GLU GLY GLY ILE GLY GLU ALA VAL
SEQRES  45 A  637  SER SER ALA VAL VAL GLY GLU PRO GLY ILE THR VAL THR
SEQRES  46 A  637  HIS LEU ALA VAL ASN ARG VAL PRO ARG SER GLY LYS PRO
SEQRES  47 A  637  ALA GLU LEU LEU LYS MET PHE GLY ILE ASP ARG ASP ALA
SEQRES  48 A  637  ILE ALA GLN ALA VAL ARG GLY LEU ILE THR LYS ALA LEU
SEQRES  49 A  637  VAL PRO ARG GLY SER LEU GLU HIS HIS HIS HIS HIS HIS
HETNAM      MG MAGNESIUM ION
HETNAM     EDO 1,2-ETHANEDIOL
HETNAM      NA SODIUM ION
HETNAM     TDP THIAMIN DIPHOSPHATE
HETNAM     DX5 D-XYLITOL-5-PHOSPHATE
HETSYN     EDO ETHYLENE GLYCOL
HETSYN     DX5 D-HYLITOL-5-PHOSPHATE
FORMUL   2   MG    MG 2+
FORMUL   3  EDO    12(C2 H6 O2)
FORMUL  14   NA    NA 1+
FORMUL  16  TDP    C12 H18 N4 O7 P2 S
FORMUL  17  DX5    2(C5 H13 O8 P)
FORMUL  19  HOH   *963(H2 O)
LINK         C2  TDP A1015                 C2 ADX5 A1016     1555   1555
1.54
LINK         OD1 ASP A 155                MG    MG A1001     1555   1555
2.03
LINK        MG    MG A1001                 O13 TDP A1015     1555   1555
2.06
LINK         O   LEU A 187                MG    MG A1001     1555   1555
2.06
LINK        MG    MG A1001                 O21 TDP A1015     1555   1555
2.07
LINK         OD1 ASN A 185                MG    MG A1001     1555   1555
2.11
LINK        MG    MG A1001                 O   HOH A7001     1555   1555
2.22
LINK         O   ALA A 461                NA    NA A1013     1555   1555
2.25
LINK        NA    NA A1013                 O   HOH A7081     1555   1555
2.25
LINK         O   THR A 464                NA    NA A1013     1555   1555
2.33
LINK         OD1 ASN A 411                NA    NA A1013     1555   1555
2.35
LINK        NA    NA A1013                 O   HOH A7070     1555   1555
2.41
CISPEP   1 LYS A  538    PRO A  539          1        -3.69
SITE     1 AC1  5 ASP A 155  ASN A 185  LEU A 187  TDP A1015
SITE     2 AC1  5 HOH A7001
SITE     1 AC2  8 PRO A  63  ASN A  68  ASP A  69  ARG A  70
SITE     2 AC2  8 PHE A  71  ARG A 379  HOH A7186  HOH A7847
SITE     1 AC3  7 TYR A 564  ALA A 588  ASN A 590  HOH A7051
SITE     2 AC3  7 HOH A7055  HOH A7126  HOH A7247
SITE     1 AC4  8 PHE A  71  VAL A  72  LEU A  73  LEU A  82
SITE     2 AC4  8 PHE A 117  HOH A7040  HOH A7056  HOH A7068
SITE     1 AC5 13 ASP A 155  GLY A 156  LEU A 158  SER A 159
```

```
SITE     2 AC5 13 TRP A 164  PRO A 194  LEU A 195  TYR A 202
SITE     3 AC5 13 ARG A 205  EDO A1012  HOH A7467  HOH A7960
SITE     4 AC5 13 HOH A7961
SITE     1 AC6  7 LEU A  92  LEU A 107  GLN A 115  HOH A7110
SITE     2 AC6  7 HOH A7124  HOH A7174  HOH A7920
SITE     1 AC7  4 GLN A  10  ASN A 590  ARG A 591  HOH A7260
SITE     1 AC8  6 TYR A 137  TYR A 141  TYR A 173  HOH A7409
SITE     2 AC8  6 HOH A7610  HOH A7914
SITE     1 AC9  8 ALA A  33  ARG A 101  GLU A 423  LYS A 597
SITE     2 AC9  8 HOH A7103  HOH A7586  HOH A7742  HOH A7934
SITE     1 BC1 10 ARG A  21  SER A  25  ALA A  84  GLU A  88
SITE     2 BC1 10 GLU A  94  LEU A  97  LYS A 283  HOH A7177
SITE     3 BC1 10 HOH A7472  HOH A7672
SITE     1 BC2  7 PHE A 142  ASP A 333  ARG A 358  HOH A7406
SITE     2 BC2  7 HOH A7539  HOH A7700  HOH A7894
SITE     1 BC3  9 LEU A 158  SER A 159  TRP A 164  GLU A 165
SITE     2 BC3  9 ALA A 168  PHE A 209  EDO A1005  EDO A1014
SITE     3 BC3  9 HOH A7961
SITE     1 BC4  6 ASN A 411  ALA A 461  THR A 464  CYS A 468
SITE     2 BC4  6 HOH A7070  HOH A7081
SITE     1 BC5  6 SER A 159  GLU A 165  ALA A 168  EDO A1012
SITE     2 BC5  6 HOH A7467  HOH A7715
SITE     1 BC6 26 SER A  40  LYS A  75  HIS A  77  GLY A 123
SITE     2 BC6 26 SER A 124  LEU A 125  GLY A 154  ASP A 155
SITE     3 BC6 26 GLY A 156  GLU A 157  GLU A 160  ASN A 185
SITE     4 BC6 26 LEU A 187  GLY A 188  GLN A 189  LYS A 244
SITE     5 BC6 26 HIS A 258  ASP A 341  ILE A 364  GLU A 366
SITE     6 BC6 26 PHE A 392  ARG A 395  GLN A 428   MG A1001
SITE     7 BC6 26 DX5 A1016  HOH A7001
SITE     1 BC7 19 HIS A  37  HIS A  77  HIS A 110  GLN A 189
SITE     2 BC7 19 HIS A 258  SER A 345  PHE A 389  HIS A 416
SITE     3 BC7 19 ASP A 424  GLN A 428  ARG A 474  TDP A1015
SITE     4 BC7 19 DX5 A1017  HOH A7053  HOH A7095  HOH A7108
SITE     5 BC7 19 HOH A7246  HOH A7263  HOH A7299
SITE     1 BC8 14 HIS A  37  GLN A 189  HIS A 258  ARG A 318
SITE     2 BC8 14 SER A 345  HIS A 416  ASP A 424  ARG A 474
SITE     3 BC8 14 DX5 A1016  HOH A7108  HOH A7246  HOH A7263
SITE     4 BC8 14 HOH A7272  HOH A7299
CRYST1  113.756   86.017   72.994  90.00 125.46  90.00 C 1 2 1        4
ORIGX1      1.000000  0.000000  0.000000        0.00000
ORIGX2      0.000000  1.000000  0.000000        0.00000
ORIGX3      0.000000  0.000000  1.000000        0.00000
SCALE1      0.008791  0.000000  0.006261        0.00000
SCALE2      0.000000  0.011626  0.000000        0.00000
SCALE3      0.000000  0.000000  0.016819        0.00000
MODEL        1
HETATM    1 MG   MG  A1001      -8.737  -1.113  15.346  1.00  4.15
Mg
ANISOU    1 MG   MG  A1001      632    472    472    -12    -10     33
Mg
HETATM    2  N1' TDP A1015      -6.862   9.553   8.850  1.00  5.75
N
ANISOU    2  N1' TDP A1015      909    538    738      5    -74     23
N
HETATM    3  C2' TDP A1015      -5.581   9.664   9.301  1.00  5.58
C
ANISOU    3  C2' TDP A1015      832    524    766     89    -22    -18
C
```

```
HETATM    4  C2A TDP A1015      -4.497   9.698   8.295  1.00  6.48
C
ANISOU    4  C2A TDP A1015     919    743    800     13     73    -49
C
HETATM    5  N3' TDP A1015      -5.288   9.772  10.576  1.00  5.24
N
ANISOU    5  N3' TDP A1015     737    594    661     73    -52    -68
N
HETATM    6  C4' TDP A1015      -6.316   9.849  11.502  1.00  5.34
C
ANISOU    6  C4' TDP A1015     833    524    671     91    -92     34
C
HETATM    7  N4' TDP A1015      -6.013  10.129  12.738  1.00  6.32
N
ANISOU    7  N4' TDP A1015     865    886    651     80   -111      0
N
HETATM    8  C5' TDP A1015      -7.678   9.646  11.074  1.00  5.31
C
ANISOU    8  C5' TDP A1015     800    527    690     21     -4     52
C
HETATM    9  C6' TDP A1015      -7.909   9.531   9.748  1.00  5.48
C
ANISOU    9  C6' TDP A1015     881    498    705      7     27     38
C
HETATM   10  C35 TDP A1015      -8.849   9.737  12.025  1.00  5.81
C
ANISOU   10  C35 TDP A1015     817    671    718     85    -10    116
C
HETATM   11  N3  TDP A1015      -8.863   8.649  13.036  1.00  5.49
N
ANISOU   11  N3  TDP A1015     738    669    679    -46    -68     13
N
HETATM   12  C2  TDP A1015      -8.355   8.763  14.320  1.00  6.54
C
ANISOU   12  C2  TDP A1015     968    716    802     62    -54     -4
C
HETATM   13  S1  TDP A1015      -8.234   7.200  15.036  1.00  6.08
S
ANISOU   13  S1  TDP A1015     977    665    669    -72    -68     25
S
HETATM   14  C5  TDP A1015      -9.000   6.438  13.685  1.00  4.69
C
ANISOU   14  C5  TDP A1015     583    583    617     27      6    -46
C
HETATM   15  C4  TDP A1015      -9.277   7.349  12.698  1.00  5.07
C
ANISOU   15  C4  TDP A1015     679    580    665    -28    -23    -11
C
HETATM   16  C4A TDP A1015      -9.974   7.111  11.413  1.00  6.57
C
ANISOU   16  C4A TDP A1015    1025    678    794    -66   -263      5
C
HETATM   17  C5A TDP A1015      -9.347   4.983  13.702  1.00  5.27
C
ANISOU   17  C5A TDP A1015     702    565    736     32    -12     29
C
HETATM   18  C5B TDP A1015      -8.109   4.126  13.877  1.00  5.40
C
```

```
ANISOU   18  C5B TDP A1015       777    447    828      -6    -84    -15
C
HETATM   19  O5G TDP A1015       -8.540   2.749  13.942  1.00  4.98
O
ANISOU   19  O5G TDP A1015       678    567    648     -18    -13     22
O
HETATM   20  P1  TDP A1015       -7.437   1.640  14.162  1.00  4.37
P
ANISOU   20  P1  TDP A1015       652    530    480       3    -34     13
P
HETATM   21  O11 TDP A1015       -6.992   1.835  15.687  1.00  4.67
O
ANISOU   21  O11 TDP A1015       676    578    520     -37    -16    -47
O
HETATM   22  O12 TDP A1015       -6.236   1.879  13.318  1.00  4.89
O
ANISOU   22  O12 TDP A1015       688    628    543      -3     56     49
O
HETATM   23  O13 TDP A1015       -8.141   0.342  14.015  1.00  4.59
O
ANISOU   23  O13 TDP A1015       702    545    497     -27    -10     31
O
HETATM   24  P2  TDP A1015       -7.686   1.386  17.073  1.00  4.67
P
ANISOU   24  P2  TDP A1015       752    517    505     -36    -18    -13
P
HETATM   25  O21 TDP A1015       -8.797   0.420  16.734  1.00  4.67
O
ANISOU   25  O21 TDP A1015       741    506    529     -50    -20     13
O
HETATM   26  O22 TDP A1015       -6.604   0.681  17.829  1.00  5.74
O
ANISOU   26  O22 TDP A1015       929    581    672       8   -193     58
O
HETATM   27  O23 TDP A1015       -8.180   2.655  17.693  1.00  5.73
O
ANISOU   27  O23 TDP A1015       855    648    673    -109    110    -92
O
HETATM   28  C1 ADX5 A1016       -7.461   9.833  16.404  0.76  5.96
C
ANISOU   28  C1 ADX5 A1016       795    809    659      29   -137    -50
C
HETATM   29  C2 ADX5 A1016       -8.313  10.045  15.173  0.76  5.25
C
ANISOU   29  C2 ADX5 A1016       785    479    733     -70   -100    -91
C
HETATM   30  C3 ADX5 A1016       -9.843  10.331  15.596  0.76  6.24
C
ANISOU   30  C3 ADX5 A1016       763    662    946    -105   -130    -74
C
HETATM   31  C4 ADX5 A1016      -10.094  11.696  16.217  0.76  5.84
C
ANISOU   31  C4 ADX5 A1016       776    751    693      45   -125    -71
C
HETATM   32  C5 ADX5 A1016      -11.618  11.938  16.316  0.76  8.12
C
ANISOU   32  C5 ADX5 A1016      1055    714   1315      73    164   -166
C
```

```
HETATM   33  O1 ADX5 A1016      -6.075   9.760  15.987  0.76  7.77
O
ANISOU   33  O1 ADX5 A1016      744   1298    909    149    -85   -308
O
HETATM   34  O2 ADX5 A1016      -7.914  11.143  14.412  0.76  6.23
O
ANISOU   34  O2 ADX5 A1016     1114    566    686    -43     17     44
O
HETATM   35  O3 ADX5 A1016     -10.311   9.338  16.438  0.76  7.72
O
ANISOU   35  O3 ADX5 A1016     1005    603   1325    -45    238     28
O
HETATM   36  O4 ADX5 A1016      -9.493  11.773  17.506  0.76  6.89
O
ANISOU   36  O4 ADX5 A1016      897    895    824     22   -127    -40
O
HETATM   37  O5 ADX5 A1016     -11.827  13.339  16.621  0.76  7.56
O
ANISOU   37  O5 ADX5 A1016      962    909   1002     90     55   -116
O
HETATM   38  P  ADX5 A1016     -13.254  13.788  17.212  0.76  7.30
P
ANISOU   38  P  ADX5 A1016     1027    858    889    101    -40    -83
P
HETATM   39  O1PADX5 A1016     -13.213  15.317  17.176  0.76  8.51
O
ANISOU   39  O1PADX5 A1016     1157    842   1236     15     51     16
O
HETATM   40  O2PADX5 A1016     -14.329  13.233  16.339  0.76  8.88
O
ANISOU   40  O2PADX5 A1016     1032   1294   1049    117    -99   -283
O
HETATM   41  O3PADX5 A1016     -13.372  13.253  18.617  0.76  8.82
O
ANISOU   41  O3PADX5 A1016     1514    979    860   -146     67     40
O
HETATM   42  C3 BDX5 A1017     -11.582  10.867  16.531  0.24 46.37
C
ANISOU   42  C3 BDX5 A1017    13940   1236   2443  -1122  -1222    753
C
HETATM   43  C4 BDX5 A1017     -11.323  12.188  17.218  0.24 23.15
C
ANISOU   43  C4 BDX5 A1017     2446   1768   4583   1622  -1990   -617
C
HETATM   44  C5 BDX5 A1017     -12.512  12.667  18.007  0.24 21.75
C
ANISOU   44  C5 BDX5 A1017     2263    984   5017    821  -1926  -1703
C
HETATM   45  O3 BDX5 A1017     -11.734   9.905  17.486  0.24 26.32
O
ANISOU   45  O3 BDX5 A1017     6197   1208   2596  -1737  -2980    829
O
HETATM   46  O4 BDX5 A1017     -10.185  12.075  18.070  0.24 17.60
O
ANISOU   46  O4 BDX5 A1017     1421   2006   3261   -514   -657   1691
O
HETATM   47  O5 BDX5 A1017     -13.819  12.773  17.553  0.24  9.90
O
```

```
ANISOU   47  O5 BDX5 A1017      1689    861   1212   -159   -437      20
O
HETATM   48  P  BDX5 A1017     -14.584  14.204  17.629  0.24  8.44
P
ANISOU   48  P  BDX5 A1017      1207    948   1053     39   -155     142
P
HETATM   49  O1PBDX5 A1017     -15.628  13.948  16.581  0.24  9.47
O
ANISOU   49  O1PBDX5 A1017      1020   1090   1489   -140   -610      28
O
HETATM   50  O2PBDX5 A1017     -15.129  14.270  19.028  0.24 16.45
O
ANISOU   50  O2PBDX5 A1017      2618   2692    941  -1758    727    -434
O
HETATM   51  O3PBDX5 A1017     -13.692  15.369  17.297  0.24 14.94
O
ANISOU   51  O3PBDX5 A1017      2826   2158    694  -1368     -2     124
O
ENDMDL
CONECT    1   23   25
CONECT   12   11   13   29
CONECT    3    2    5    4
CONECT    4    3
CONECT   10   11    8
CONECT   15   11   16   14
CONECT    6    7    5    8
CONECT   16   15
CONECT   14   15   13   17
CONECT    8   10    6    9
CONECT   17   14   18
CONECT   18   17   19
CONECT    9    2    8
CONECT    2    3    9
CONECT   11   10   15   12
CONECT    5    6    3
CONECT    7    6
CONECT   21   20   24
CONECT   22   20
CONECT   23   20
CONECT   23    1
CONECT   25   24
CONECT   25    1
CONECT   26   24
CONECT   27   24
CONECT   19   20   18
CONECT   20   21   22   23   19
CONECT   24   21   27   25   26
CONECT   13   12   14
CONECT   28   33   29
CONECT   29   12   28   34   30
CONECT   30   29   31   35
CONECT   31   36   32   30
CONECT   32   31   37
CONECT   33   28
CONECT   39   38
CONECT   34   29
CONECT   40   38
CONECT   35   30
```

```
CONECT   41   38
CONECT   36   31
CONECT   37   38   32
CONECT   38   40   37   39   41
CONECT   42   43   45
CONECT   43   42   46   44
CONECT   44   43   47
CONECT   49   48
CONECT   50   48
CONECT   45   42
CONECT   51   48
CONECT   46   43
CONECT   47   48   44
CONECT   48   51   50   47   49
MODEL        2
HETATM    1 MG   MG  A1001       8.737  -1.113 -15.346  1.00  4.15
Mg
ANISOU    1 MG   MG  A1001      632    472    472    -12    -10     33
Mg
HETATM    2  N1' TDP A1015       6.862   9.553  -8.850  1.00  5.75
N
ANISOU    2  N1' TDP A1015      909    538    738      5    -74     23
N
HETATM    3  C2' TDP A1015       5.581   9.664  -9.301  1.00  5.58
C
ANISOU    3  C2' TDP A1015      832    524    766     89    -22    -18
C
HETATM    4  C2A TDP A1015       4.497   9.698  -8.295  1.00  6.48
C
ANISOU    4  C2A TDP A1015      919    743    800     13     73    -49
C
HETATM    5  N3' TDP A1015       5.288   9.772 -10.576  1.00  5.24
N
ANISOU    5  N3' TDP A1015      737    594    661     73    -52    -68
N
HETATM    6  C4' TDP A1015       6.316   9.849 -11.502  1.00  5.34
C
ANISOU    6  C4' TDP A1015      833    524    671     91    -92     34
C
HETATM    7  N4' TDP A1015       6.013  10.129 -12.738  1.00  6.32
N
ANISOU    7  N4' TDP A1015      865    886    651     80   -111      0
N
HETATM    8  C5' TDP A1015       7.678   9.646 -11.074  1.00  5.31
C
ANISOU    8  C5' TDP A1015      800    527    690     21     -4     52
C
HETATM    9  C6' TDP A1015       7.909   9.531  -9.748  1.00  5.48
C
ANISOU    9  C6' TDP A1015      881    498    705      7     27     38
C
HETATM   10  C35 TDP A1015       8.849   9.737 -12.025  1.00  5.81
C
ANISOU   10  C35 TDP A1015      817    671    718     85    -10    116
C
HETATM   11  N3  TDP A1015       8.863   8.649 -13.036  1.00  5.49
N
```

```
ANISOU   11  N3  TDP A1015      738    669    679    -46    -68     13
N
HETATM   12  C2  TDP A1015       8.355   8.763 -14.320  1.00  6.54
C
ANISOU   12  C2  TDP A1015      968    716    802     62    -54     -4
C
HETATM   13  S1  TDP A1015       8.234   7.200 -15.036  1.00  6.08
S
ANISOU   13  S1  TDP A1015      977    665    669    -72    -68     25
S
HETATM   14  C5  TDP A1015       9.000   6.438 -13.685  1.00  4.69
C
ANISOU   14  C5  TDP A1015      583    583    617     27      6    -46
C
HETATM   15  C4  TDP A1015       9.277   7.349 -12.698  1.00  5.07
C
ANISOU   15  C4  TDP A1015      679    580    665    -28    -23    -11
C
HETATM   16  C4A TDP A1015       9.974   7.111 -11.413  1.00  6.57
C
ANISOU   16  C4A TDP A1015     1025    678    794    -66   -263      5
C
HETATM   17  C5A TDP A1015       9.347   4.983 -13.702  1.00  5.27
C
ANISOU   17  C5A TDP A1015      702    565    736     32    -12     29
C
HETATM   18  C5B TDP A1015       8.109   4.126 -13.877  1.00  5.40
C
ANISOU   18  C5B TDP A1015      777    447    828     -6    -84    -15
C
HETATM   19  O5G TDP A1015       8.540   2.749 -13.942  1.00  4.98
O
ANISOU   19  O5G TDP A1015      678    567    648    -18    -13     22
O
HETATM   20  P1  TDP A1015       7.437   1.640 -14.162  1.00  4.37
P
ANISOU   20  P1  TDP A1015      652    530    480      3    -34     13
P
HETATM   21  O11 TDP A1015       6.992   1.835 -15.687  1.00  4.67
O
ANISOU   21  O11 TDP A1015      676    578    520    -37    -16    -47
O
HETATM   22  O12 TDP A1015       6.236   1.879 -13.318  1.00  4.89
O
ANISOU   22  O12 TDP A1015      688    628    543     -3     56     49
O
HETATM   23  O13 TDP A1015       8.141   0.342 -14.015  1.00  4.59
O
ANISOU   23  O13 TDP A1015      702    545    497    -27    -10     31
O
HETATM   24  P2  TDP A1015       7.686   1.386 -17.073  1.00  4.67
P
ANISOU   24  P2  TDP A1015      752    517    505    -36    -18    -13
P
HETATM   25  O21 TDP A1015       8.797   0.420 -16.734  1.00  4.67
O
ANISOU   25  O21 TDP A1015      741    506    529    -50    -20     13
O
```

```
HETATM   26  O22 TDP A1015       6.604   0.681 -17.829  1.00  5.74
O
ANISOU   26  O22 TDP A1015      929    581    672      8   -193     58
O
HETATM   27  O23 TDP A1015       8.180   2.655 -17.693  1.00  5.73
O
ANISOU   27  O23 TDP A1015      855    648    673   -109    110    -92
O
HETATM   28  C1 ADX5 A1016       7.461   9.833 -16.404  0.76  5.96
C
ANISOU   28  C1 ADX5 A1016      795    809    659     29   -137    -50
C
HETATM   29  C2 ADX5 A1016       8.313  10.045 -15.173  0.76  5.25
C
ANISOU   29  C2 ADX5 A1016      785    479    733    -70   -100    -91
C
HETATM   30  C3 ADX5 A1016       9.843  10.331 -15.596  0.76  6.24
C
ANISOU   30  C3 ADX5 A1016      763    662    946   -105   -130    -74
C
HETATM   31  C4 ADX5 A1016      10.094  11.696 -16.217  0.76  5.84
C
ANISOU   31  C4 ADX5 A1016      776    751    693     45   -125    -71
C
HETATM   32  C5 ADX5 A1016      11.618  11.938 -16.316  0.76  8.12
C
ANISOU   32  C5 ADX5 A1016     1055    714   1315     73    164   -166
C
HETATM   33  O1 ADX5 A1016       6.075   9.760 -15.987  0.76  7.77
O
ANISOU   33  O1 ADX5 A1016      744   1298    909    149    -85   -308
O
HETATM   34  O2 ADX5 A1016       7.914  11.143 -14.412  0.76  6.23
O
ANISOU   34  O2 ADX5 A1016     1114    566    686    -43     17     44
O
HETATM   35  O3 ADX5 A1016      10.311   9.338 -16.438  0.76  7.72
O
ANISOU   35  O3 ADX5 A1016     1005    603   1325    -45    238     28
O
HETATM   36  O4 ADX5 A1016       9.493  11.773 -17.506  0.76  6.89
O
ANISOU   36  O4 ADX5 A1016      897    895    824     22   -127    -40
O
HETATM   37  O5 ADX5 A1016      11.827  13.339 -16.621  0.76  7.56
O
ANISOU   37  O5 ADX5 A1016      962    909   1002     90     55   -116
O
HETATM   38  P  ADX5 A1016      13.254  13.788 -17.212  0.76  7.30
P
ANISOU   38  P  ADX5 A1016     1027    858    889    101    -40    -83
P
HETATM   39  O1PADX5 A1016      13.213  15.317 -17.176  0.76  8.51
O
ANISOU   39  O1PADX5 A1016     1157    842   1236     15     51     16
O
HETATM   40  O2PADX5 A1016      14.329  13.233 -16.339  0.76  8.88
O
```

```
ANISOU   40  O2PADX5 A1016     1032   1294   1049    117    -99   -283
O
HETATM   41  O3PADX5 A1016      13.372  13.253 -18.617  0.76  8.82
O
ANISOU   41  O3PADX5 A1016     1514    979    860   -146     67     40
O
HETATM   42  C3 BDX5 A1017      11.582  10.867 -16.531  0.24 46.37
C
ANISOU   42  C3 BDX5 A1017    13940   1236   2443  -1122  -1222    753
C
HETATM   43  C4 BDX5 A1017      11.323  12.188 -17.218  0.24 23.15
C
ANISOU   43  C4 BDX5 A1017     2446   1768   4583   1622  -1990   -617
C
HETATM   44  C5 BDX5 A1017      12.512  12.667 -18.007  0.24 21.75
C
ANISOU   44  C5 BDX5 A1017     2263    984   5017    821  -1926  -1703
C
HETATM   45  O3 BDX5 A1017      11.734   9.905 -17.486  0.24 26.32
O
ANISOU   45  O3 BDX5 A1017     6197   1208   2596  -1737  -2980    829
O
HETATM   46  O4 BDX5 A1017      10.185  12.075 -18.070  0.24 17.60
O
ANISOU   46  O4 BDX5 A1017     1421   2006   3261   -514   -657   1691
O
HETATM   47  O5 BDX5 A1017      13.819  12.773 -17.553  0.24  9.90
O
ANISOU   47  O5 BDX5 A1017     1689    861   1212   -159   -437     20
O
HETATM   48  P  BDX5 A1017      14.584  14.204 -17.629  0.24  8.44
P
ANISOU   48  P  BDX5 A1017     1207    948   1053     39   -155    142
P
HETATM   49  O1PBDX5 A1017      15.628  13.948 -16.581  0.24  9.47
O
ANISOU   49  O1PBDX5 A1017     1020   1090   1489   -140   -610     28
O
HETATM   50  O2PBDX5 A1017      15.129  14.270 -19.028  0.24 16.45
O
ANISOU   50  O2PBDX5 A1017     2618   2692    941  -1758    727   -434
O
HETATM   51  O3PBDX5 A1017      13.692  15.369 -17.297  0.24 14.94
O
ANISOU   51  O3PBDX5 A1017     2826   2158    694  -1368     -2    124
O
ENDMDL
CONECT   12   13   11   29
CONECT    3    5    4    2
CONECT    4    3
CONECT   10   11    8
CONECT   15   14   16   11
CONECT    6    5    7    8
CONECT   16   15
CONECT   14   15   17   13
CONECT    8   10    9    6
CONECT   17   14   18
CONECT   18   17   19
```

```
CONECT    9    2    8
CONECT    2    3    9
CONECT   11   10   15   12
CONECT    5    3    6
CONECT    7    6
CONECT   21   24   20
CONECT   22   20
CONECT   23   20
CONECT   25   24
CONECT   26   24
CONECT   27   24
CONECT   19   20   18
CONECT   20   23   22   19   21
CONECT   24   26   27   25   21
CONECT   13   14   12
CONECT   28   33   29
CONECT   29   28   30   34   12
CONECT   30   31   35   29
CONECT   31   30   36   32
CONECT   32   31   37
CONECT   33   28
CONECT   39   38
CONECT   34   29
CONECT   40   38
CONECT   35   30
CONECT   41   38
CONECT   36   31
CONECT   37   38   32
CONECT   38   40   37   39   41
CONECT   42   45   43
CONECT   43   42   44   46
CONECT   44   43   47
CONECT   49   48
CONECT   50   48
CONECT   45   42
CONECT   51   48
CONECT   46   43
CONECT   47   48   44
CONECT   48   50   47   49   51
MODEL        3
ATOM      1  N   MET A   1      21.311  -6.290  29.993  1.00221.78
N
ATOM      2  CA  MET A   1      20.405  -7.409  29.728  1.00178.30
C
ATOM      3  C   MET A   1      21.055  -8.498  28.861  1.00 88.57
C
ATOM      4  O   MET A   1      21.805  -8.193  27.934  1.00142.36
O
ATOM      5  CB  MET A   1      19.132  -6.893  29.075  1.00267.45
C
ATOM      6  CG  MET A   1      18.088  -7.960  28.792  1.00267.45
C
ATOM      7  SD  MET A   1      17.456  -8.727  30.293  1.00267.45
S
ATOM      8  CE  MET A   1      16.560 -10.102  29.590  1.00267.45
C
ATOM      9 1H   MET A   1      20.861  -5.418  29.720  1.00266.14
H
```

```
ATOM     10 2H   MET A   1      21.532  -6.257  30.978  1.00266.14
H
ATOM     11 3H   MET A   1      22.163  -6.407  29.462  1.00266.14
H
ATOM     12  HA  MET A   1      20.143  -7.866  30.683  1.00213.96
H
ATOM     13 1HB  MET A   1      18.679  -6.136  29.710  1.00320.94
H
ATOM     14 2HB  MET A   1      19.387  -6.415  28.133  1.00320.94
H
ATOM     15 1HG  MET A   1      17.251  -7.513  28.257  1.00320.94
H
ATOM     16 2HG  MET A   1      18.516  -8.738  28.160  1.00320.94
H
ATOM     17 1HE  MET A   1      16.104 -10.689  30.388  1.00320.94
H
ATOM     18 2HE  MET A   1      15.783  -9.721  28.927  1.00320.94
H
ATOM     19 3HE  MET A   1      17.243 -10.730  29.019  1.00320.94
H
ATOM     20  N   MET A   2      20.784  -9.769  29.196  1.00 34.53
N
ATOM     21  CA  MET A   2      21.309 -10.930  28.480  1.00 18.92
C
ATOM     22  C   MET A   2      20.760 -11.072  27.063  1.00  9.21
C
ATOM     23  O   MET A   2      19.704 -10.536  26.725  1.00  9.08
O
ATOM     24  CB  MET A   2      20.992 -12.207  29.257  1.00 28.38
C
ATOM     25  CG  MET A   2      19.506 -12.531  29.334  1.00 28.38
C
ATOM     26  SD  MET A   2      19.149 -14.030  30.266  1.00 28.38
S
ATOM     27  CE  MET A   2      19.424 -13.458  31.939  1.00 28.38
C
ATOM     28  H   MET A   2      20.168  -9.933  29.980  1.00 41.44
H
ATOM     29  HA  MET A   2      22.391 -10.822  28.403  1.00 22.70
H
ATOM     30 1HB  MET A   2      21.499 -13.054  28.796  1.00 34.06
H
ATOM     31 2HB  MET A   2      21.369 -12.115  30.278  1.00 34.06
H
ATOM     32 1HG  MET A   2      18.995 -11.702  29.819  1.00 34.06
H
ATOM     33 2HG  MET A   2      19.097 -12.641  28.330  1.00 34.06
H
ATOM     34 1HE  MET A   2      19.242 -14.275  32.637  1.00 34.06
H
ATOM     35 2HE  MET A   2      20.455 -13.115  32.042  1.00 34.06
H
ATOM     36 3HE  MET A   2      18.744 -12.635  32.158  1.00 34.06
H
ATOM     37  N   ALA A   3      21.481 -11.832  26.244  1.00  7.90
N
ATOM     38  CA  ALA A   3      21.070 -12.120  24.876  1.00  7.72
C
```

```
ATOM     39  C   ALA A   3      20.217 -13.393  24.792  1.00  8.07
C
ATOM     40  O   ALA A   3      19.867 -13.835  23.699  1.00 16.05
O
ATOM     41  CB  ALA A   3      22.293 -12.258  23.984  1.00 11.58
C
ATOM     42  H   ALA A   3      22.346 -12.229  26.583  1.00  9.48
H
ATOM     43  HA  ALA A   3      20.463 -11.286  24.522  1.00  9.26
H
ATOM     44 1HB  ALA A   3      21.976 -12.443  22.958  1.00 13.90
H
ATOM     45 2HB  ALA A   3      22.877 -11.339  24.024  1.00 13.90
H
ATOM     46 3HB  ALA A   3      22.904 -13.091  24.330  1.00 13.90
H
ATOM     47  N   ASN A   4      19.904 -13.991  25.939  1.00  6.92
N
ATOM     48  CA  ASN A   4      19.138 -15.223  25.965  1.00  9.82
C
ATOM     49  C   ASN A   4      17.645 -15.013  26.206  1.00  7.13
C
ATOM     50  O   ASN A   4      17.219 -14.053  26.856  1.00  6.89
O
ATOM     51  CB  ASN A   4      19.704 -16.151  27.021  1.00 14.73
C
ATOM     52  CG  ASN A   4      21.097 -16.610  26.696  1.00 14.73
C
ATOM     53  OD1 ASN A   4      21.415 -16.901  25.537  1.00 14.73
O
ATOM     54  ND2 ASN A   4      21.934 -16.685  27.698  1.00 14.73
N
ATOM     55  H   ASN A   4      20.206 -13.596  26.813  1.00  8.30
H
ATOM     56  HA  ASN A   4      19.239 -15.707  24.993  1.00 11.78
H
ATOM     57 1HB  ASN A   4      19.718 -15.642  27.985  1.00 17.68
H
ATOM     58 2HB  ASN A   4      19.060 -17.025  27.122  1.00 17.68
H
ATOM     59 1HD2 ASN A   4      22.875 -16.987  27.542  1.00 17.68
H
ATOM     60 2HD2 ASN A   4      21.634 -16.442  28.620  1.00 17.68
H
ATOM     61  N   ASP A   5      16.854 -15.946  25.688  1.00  7.53
N
ATOM     62  CA  ASP A   5      15.414 -15.937  25.879  1.00  8.13
C
ATOM     63  C   ASP A   5      15.067 -16.240  27.316  1.00  5.50
C
ATOM     64  O   ASP A   5      14.933 -17.404  27.695  1.00  8.85
O
ATOM     65  CB  ASP A   5      14.760 -16.976  24.959  1.00 12.20
C
ATOM     66  CG  ASP A   5      13.246 -17.074  25.095  1.00 12.20
C
ATOM     67  OD1 ASP A   5      12.733 -16.675  26.113  1.00 12.20
O
```

```
ATOM     68  OD2 ASP A   5      12.616 -17.542  24.174  1.00 12.20
O
ATOM     69  H   ASP A   5      17.271 -16.696  25.153  1.00  9.04
H
ATOM     70  HA  ASP A   5      15.033 -14.946  25.631  1.00  9.76
H
ATOM     71 1HB  ASP A   5      14.997 -16.734  23.923  1.00 14.63
H
ATOM     72 2HB  ASP A   5      15.188 -17.956  25.168  1.00 14.63
H
ATOM     73  N   ALA A   6      14.872 -15.193  28.109  1.00  4.73
N
ATOM     74  CA  ALA A   6      14.573 -15.368  29.527  1.00  3.32
C
ATOM     75  C   ALA A   6      13.114 -15.757  29.772  1.00  4.21
C
ATOM     76  O   ALA A   6      12.322 -14.949  30.258  1.00  9.80
O
ATOM     77  CB  ALA A   6      14.904 -14.088  30.284  1.00  4.98
C
ATOM     78  H   ALA A   6      15.014 -14.269  27.722  1.00  5.68
H
ATOM     79  HA  ALA A   6      15.200 -16.173  29.905  1.00  3.98
H
ATOM     80 1HB  ALA A   6      14.717 -14.236  31.347  1.00  5.98
H
ATOM     81 2HB  ALA A   6      15.955 -13.840  30.132  1.00  5.98
H
ATOM     82 3HB  ALA A   6      14.284 -13.275  29.918  1.00  5.98
H
ATOM     83  N   LYS A   7      12.796 -17.016  29.444  1.00  2.99
N
ATOM     84  CA  LYS A   7      11.489 -17.642  29.651  1.00  3.80
C
ATOM     85  C   LYS A   7      10.847 -17.235  30.978  1.00  3.39
C
ATOM     86  O   LYS A   7      11.321 -17.652  32.036  1.00  4.85
O
ATOM     87  CB  LYS A   7      11.619 -19.161  29.609  1.00  5.70
C
ATOM     88  CG  LYS A   7      10.290 -19.893  29.739  1.00  5.70
C
ATOM     89  CD  LYS A   7      10.472 -21.398  29.625  1.00  5.70
C
ATOM     90  CE  LYS A   7       9.143 -22.128  29.748  1.00  5.70
C
ATOM     91  NZ  LYS A   7       9.309 -23.603  29.636  1.00  5.70
N
ATOM     92  H   LYS A   7      13.526 -17.570  29.020  1.00  3.59
H
ATOM     93  HA  LYS A   7      10.842 -17.352  28.830  1.00  4.56
H
ATOM     94 1HB  LYS A   7      12.078 -19.461  28.669  1.00  6.84
H
ATOM     95 2HB  LYS A   7      12.271 -19.497  30.417  1.00  6.84
H
ATOM     96 1HG  LYS A   7       9.837 -19.661  30.704  1.00  6.84
H
```

```
ATOM     97 2HG  LYS A   7       9.615 -19.559  28.953  1.00  6.84
H
ATOM     98 1HD  LYS A   7      10.921 -21.638  28.659  1.00  6.84
H
ATOM     99 2HD  LYS A   7      11.140 -21.745  30.413  1.00  6.84
H
ATOM    100 1HE  LYS A   7       8.696 -21.896  30.714  1.00  6.84
H
ATOM    101 2HE  LYS A   7       8.472 -21.788  28.960  1.00  6.84
H
ATOM    102 1HZ  LYS A   7       8.407 -24.051  29.724  1.00  6.84
H
ATOM    103 2HZ  LYS A   7       9.712 -23.831  28.737  1.00  6.84
H
ATOM    104 3HZ  LYS A   7       9.920 -23.931  30.370  1.00  6.84
H
ATOM    105  N   PRO A   8       9.780 -16.428  30.948  1.00  3.55
N
ATOM    106  CA  PRO A   8       9.014 -15.939  32.084  1.00  4.66
C
ATOM    107  C   PRO A   8       8.460 -17.074  32.931  1.00  2.38
C
ATOM    108  O   PRO A   8       7.962 -18.070  32.403  1.00  4.15
O
ATOM    109  CB  PRO A   8       7.881 -15.140  31.423  1.00  6.99
C
ATOM    110  CG  PRO A   8       8.449 -14.694  30.121  1.00  6.99
C
ATOM    111  CD  PRO A   8       9.313 -15.838  29.677  1.00  6.99
C
ATOM    112  HA  PRO A   8       9.654 -15.282  32.693  1.00  5.59
H
ATOM    113 1HB  PRO A   8       6.997 -15.783  31.298  1.00  8.39
H
ATOM    114 2HB  PRO A   8       7.578 -14.304  32.064  1.00  8.39
H
ATOM    115 1HG  PRO A   8       7.638 -14.474  29.411  1.00  8.39
H
ATOM    116 2HG  PRO A   8       9.017 -13.762  30.255  1.00  8.39
H
ATOM    117 1HD  PRO A   8       8.726 -16.575  29.110  1.00  8.39
H
ATOM    118 2HD  PRO A   8      10.163 -15.454  29.096  1.00  8.39
H
ATOM    119  N   ASP A   9       8.507 -16.908  34.247  1.00  3.05
N
ATOM    120  CA  ASP A   9       7.911 -17.897  35.130  1.00  3.91
C
ATOM    121  C   ASP A   9       6.494 -17.453  35.453  1.00  3.22
C
ATOM    122  O   ASP A   9       6.058 -16.409  34.972  1.00  4.52
O
ATOM    123  CB  ASP A   9       8.756 -18.100  36.397  1.00  5.87
C
ATOM    124  CG  ASP A   9       8.831 -16.885  37.307  1.00  5.87
C
ATOM    125  OD1 ASP A   9       8.048 -15.982  37.130  1.00  5.87
O
```

```
ATOM    126  OD2 ASP A   9       9.675 -16.868  38.171  1.00  5.87
O
ATOM    127  H   ASP A   9       8.948 -16.085  34.634  1.00  3.66
H
ATOM    128  HA  ASP A   9       7.857 -18.851  34.605  1.00  4.69
H
ATOM    129 1HB  ASP A   9       8.356 -18.936  36.970  1.00  7.04
H
ATOM    130 2HB  ASP A   9       9.773 -18.370  36.106  1.00  7.04
H
ATOM    131  N   VAL A  10       5.793 -18.221  36.280  1.00  3.38
N
ATOM    132  CA  VAL A  10       4.398 -17.934  36.608  1.00  3.22
C
ATOM    133  C   VAL A  10       4.198 -16.556  37.217  1.00  1.85
C
ATOM    134  O   VAL A  10       3.272 -15.838  36.834  1.00  1.61
O
ATOM    135  CB  VAL A  10       3.855 -18.991  37.584  1.00  4.83
C
ATOM    136  CG1 VAL A  10       2.483 -18.573  38.093  1.00  4.83
C
ATOM    137  CG2 VAL A  10       3.781 -20.336  36.880  1.00  4.83
C
ATOM    138  H   VAL A  10       6.224 -19.050  36.664  1.00  4.06
H
ATOM    139  HA  VAL A  10       3.818 -17.988  35.686  1.00  3.86
H
ATOM    140  HB  VAL A  10       4.520 -19.064  38.445  1.00  5.80
H
ATOM    141 1HG1 VAL A  10       2.110 -19.322  38.792  1.00  5.80
H
ATOM    142 2HG1 VAL A  10       2.559 -17.611  38.600  1.00  5.80
H
ATOM    143 3HG1 VAL A  10       1.794 -18.487  37.253  1.00  5.80
H
ATOM    144 1HG2 VAL A  10       3.404 -21.089  37.573  1.00  5.80
H
ATOM    145 2HG2 VAL A  10       3.110 -20.263  36.024  1.00  5.80
H
ATOM    146 3HG2 VAL A  10       4.774 -20.625  36.538  1.00  5.80
H
ATOM    147  N   LYS A  11       5.050 -16.209  38.177  1.00  1.73
N
ATOM    148  CA  LYS A  11       4.980 -14.921  38.849  1.00  1.50
C
ATOM    149  C   LYS A  11       5.184 -13.787  37.865  1.00  1.15
C
ATOM    150  O   LYS A  11       4.404 -12.838  37.846  1.00  1.94
O
ATOM    151  CB  LYS A  11       6.014 -14.852  39.969  1.00  2.25
C
ATOM    152  CG  LYS A  11       6.007 -13.548  40.756  1.00  2.25
C
ATOM    153  CD  LYS A  11       7.006 -13.597  41.906  1.00  2.25
C
ATOM    154  CE  LYS A  11       6.941 -12.332  42.749  1.00  2.25
C
```

```
ATOM    155  NZ  LYS A  11       7.463 -11.154  42.013  1.00  2.25
N
ATOM    156  H   LYS A  11       5.780 -16.855  38.443  1.00  2.08
H
ATOM    157  HA  LYS A  11       3.986 -14.811  39.284  1.00  1.80
H
ATOM    158 1HB  LYS A  11       5.841 -15.666  40.673  1.00  2.70
H
ATOM    159 2HB  LYS A  11       7.012 -14.987  39.552  1.00  2.70
H
ATOM    160 1HG  LYS A  11       6.270 -12.721  40.094  1.00  2.70
H
ATOM    161 2HG  LYS A  11       5.010 -13.368  41.157  1.00  2.70
H
ATOM    162 1HD  LYS A  11       6.789 -14.457  42.540  1.00  2.70
H
ATOM    163 2HD  LYS A  11       8.014 -13.706  41.507  1.00  2.70
H
ATOM    164 1HE  LYS A  11       5.905 -12.144  43.025  1.00  2.70
H
ATOM    165 2HE  LYS A  11       7.527 -12.471  43.657  1.00  2.70
H
ATOM    166 1HZ  LYS A  11       7.396 -10.329  42.597  1.00  2.70
H
ATOM    167 2HZ  LYS A  11       8.430 -11.307  41.762  1.00  2.70
H
ATOM    168 3HZ  LYS A  11       6.919 -11.010  41.176  1.00  2.70
H
ATOM    169  N   THR A  12       6.232 -13.906  37.050  1.00  1.14
N
ATOM    170  CA  THR A  12       6.575 -12.936  36.020  1.00  2.63
C
ATOM    171  C   THR A  12       5.388 -12.651  35.120  1.00  1.55
C
ATOM    172  O   THR A  12       5.062 -11.492  34.858  1.00  1.47
O
ATOM    173  CB  THR A  12       7.743 -13.461  35.171  1.00  3.94
C
ATOM    174  OG1 THR A  12       8.887 -13.668  36.008  1.00  3.94
O
ATOM    175  CG2 THR A  12       8.087 -12.482  34.084  1.00  3.94
C
ATOM    176  H   THR A  12       6.832 -14.719  37.147  1.00  1.37
H
ATOM    177  HA  THR A  12       6.874 -12.005  36.500  1.00  3.16
H
ATOM    178  HB  THR A  12       7.463 -14.409  34.717  1.00  4.73
H
ATOM    179  HG1 THR A  12       8.692 -14.384  36.625  1.00  4.73
H
ATOM    180 1HG2 THR A  12       8.916 -12.868  33.491  1.00  4.73
H
ATOM    181 2HG2 THR A  12       7.221 -12.326  33.441  1.00  4.73
H
ATOM    182 3HG2 THR A  12       8.373 -11.543  34.546  1.00  4.73
H
ATOM    183  N   VAL A  13       4.741 -13.722  34.670  1.00  1.60
N
```

```
ATOM    184  CA  VAL A  13       3.560 -13.635  33.834  1.00  1.55
C
ATOM    185  C   VAL A  13       2.414 -12.924  34.527  1.00  1.18
C
ATOM    186  O   VAL A  13       1.758 -12.080  33.922  1.00  1.09
O
ATOM    187  CB  VAL A  13       3.120 -15.038  33.419  1.00  2.33
C
ATOM    188  CG1 VAL A  13       1.761 -14.981  32.751  1.00  2.33
C
ATOM    189  CG2 VAL A  13       4.155 -15.614  32.468  1.00  2.33
C
ATOM    190  H   VAL A  13       5.089 -14.642  34.913  1.00  1.92
H
ATOM    191  HA  VAL A  13       3.819 -13.077  32.935  1.00  1.86
H
ATOM    192  HB  VAL A  13       3.035 -15.669  34.303  1.00  2.79
H
ATOM    193 1HG1 VAL A  13       1.455 -15.987  32.465  1.00  2.79
H
ATOM    194 2HG1 VAL A  13       1.031 -14.564  33.443  1.00  2.79
H
ATOM    195 3HG1 VAL A  13       1.821 -14.356  31.865  1.00  2.79
H
ATOM    196 1HG2 VAL A  13       3.856 -16.619  32.174  1.00  2.79
H
ATOM    197 2HG2 VAL A  13       4.224 -14.981  31.583  1.00  2.79
H
ATOM    198 3HG2 VAL A  13       5.124 -15.653  32.956  1.00  2.79
H
ATOM    199  N   GLN A  14       2.164 -13.263  35.791  1.00  1.17
N
ATOM    200  CA  GLN A  14       1.089 -12.616  36.522  1.00  1.09
C
ATOM    201  C   GLN A  14       1.332 -11.135  36.755  1.00  0.94
C
ATOM    202  O   GLN A  14       0.403 -10.345  36.628  1.00  0.88
O
ATOM    203  CB  GLN A  14       0.857 -13.288  37.868  1.00  1.64
C
ATOM    204  CG  GLN A  14      -0.308 -12.686  38.644  1.00  1.64
C
ATOM    205  CD  GLN A  14      -1.636 -12.849  37.924  1.00  1.64
C
ATOM    206  OE1 GLN A  14      -1.985 -13.956  37.502  1.00  1.64
O
ATOM    207  NE2 GLN A  14      -2.387 -11.760  37.779  1.00  1.64
N
ATOM    208  H   GLN A  14       2.712 -13.985  36.245  1.00  1.40
H
ATOM    209  HA  GLN A  14       0.176 -12.710  35.933  1.00  1.31
H
ATOM    210 1HB  GLN A  14       0.657 -14.350  37.719  1.00  1.96
H
ATOM    211 2HB  GLN A  14       1.755 -13.204  38.480  1.00  1.96
H
ATOM    212 1HG  GLN A  14      -0.380 -13.183  39.610  1.00  1.96
H
```

```
ATOM    213 2HG  GLN A  14      -0.125 -11.620  38.785  1.00  1.96
H
ATOM    214 1HE2 GLN A  14      -3.270 -11.820  37.314  1.00  1.96
H
ATOM    215 2HE2 GLN A  14      -2.079 -10.870  38.132  1.00  1.96
H
ATOM    216  N   VAL A  15       2.573 -10.756  37.073  1.00  0.97
N
ATOM    217  CA  VAL A  15       2.894  -9.352  37.322  1.00  0.94
C
ATOM    218  C   VAL A  15       2.782  -8.576  36.018  1.00  0.87
C
ATOM    219  O   VAL A  15       2.358  -7.422  36.000  1.00  1.04
O
ATOM    220  CB  VAL A  15       4.295  -9.184  37.926  1.00  1.41
C
ATOM    221  CG1 VAL A  15       4.603  -7.704  38.038  1.00  1.41
C
ATOM    222  CG2 VAL A  15       4.363  -9.846  39.292  1.00  1.41
C
ATOM    223  H   VAL A  15       3.299 -11.452  37.169  1.00  1.16
H
ATOM    224  HA  VAL A  15       2.169  -8.951  38.031  1.00  1.13
H
ATOM    225  HB  VAL A  15       5.031  -9.632  37.259  1.00  1.69
H
ATOM    226 1HG1 VAL A  15       5.602  -7.566  38.451  1.00  1.69
H
ATOM    227 2HG1 VAL A  15       4.547  -7.252  37.051  1.00  1.69
H
ATOM    228 3HG1 VAL A  15       3.874  -7.229  38.696  1.00  1.69
H
ATOM    229 1HG2 VAL A  15       5.361  -9.716  39.708  1.00  1.69
H
ATOM    230 2HG2 VAL A  15       3.632  -9.384  39.950  1.00  1.69
H
ATOM    231 3HG2 VAL A  15       4.144 -10.907  39.199  1.00  1.69
H
ATOM    232  N   LEU A  16       3.181  -9.218  34.936  1.00  0.92
N
ATOM    233  CA  LEU A  16       3.054  -8.703  33.595  1.00  0.87
C
ATOM    234  C   LEU A  16       1.574  -8.434  33.253  1.00  0.83
C
ATOM    235  O   LEU A  16       1.236  -7.380  32.710  1.00  1.01
O
ATOM    236  CB  LEU A  16       3.743  -9.707  32.688  1.00  1.30
C
ATOM    237  CG  LEU A  16       3.760  -9.461  31.217  1.00  1.30
C
ATOM    238  CD1 LEU A  16       4.386  -8.117  30.948  1.00  1.30
C
ATOM    239  CD2 LEU A  16       4.581 -10.588  30.606  1.00  1.30
C
ATOM    240  H   LEU A  16       3.589 -10.141  35.032  1.00  1.10
H
ATOM    241  HA  LEU A  16       3.594  -7.757  33.536  1.00  1.04
H
```

```
ATOM    242 1HB  LEU A  16       4.782  -9.782  33.006  1.00  1.57
H
ATOM    243 2HB  LEU A  16       3.282 -10.676  32.838  1.00  1.57
H
ATOM    244  HG  LEU A  16       2.751  -9.459  30.816  1.00  1.57
H
ATOM    245 1HD1 LEU A  16       4.409  -7.955  29.879  1.00  1.57
H
ATOM    246 2HD1 LEU A  16       3.787  -7.345  31.424  1.00  1.57
H
ATOM    247 3HD1 LEU A  16       5.391  -8.078  31.346  1.00  1.57
H
ATOM    248 1HD2 LEU A  16       4.642 -10.465  29.531  1.00  1.57
H
ATOM    249 2HD2 LEU A  16       5.584 -10.581  31.024  1.00  1.57
H
ATOM    250 3HD2 LEU A  16       4.105 -11.543  30.830  1.00  1.57
H
ATOM    251  N   ARG A  17       0.698  -9.380  33.612  1.00  0.71
N
ATOM    252  CA  ARG A  17      -0.742  -9.202  33.443  1.00  0.77
C
ATOM    253  C   ARG A  17      -1.266  -8.089  34.358  1.00  0.72
C
ATOM    254  O   ARG A  17      -2.120  -7.302  33.947  1.00  0.81
O
ATOM    255  CB  ARG A  17      -1.475 -10.496  33.754  1.00  1.16
C
ATOM    256  CG  ARG A  17      -1.284 -11.598  32.732  1.00  1.16
C
ATOM    257  CD  ARG A  17      -1.914 -12.869  33.164  1.00  1.16
C
ATOM    258  NE  ARG A  17      -1.733 -13.922  32.178  1.00  1.16
N
ATOM    259  CZ  ARG A  17      -1.936 -15.233  32.411  1.00  1.16
C
ATOM    260  NH1 ARG A  17      -2.326 -15.634  33.601  1.00  1.16
N
ATOM    261  NH2 ARG A  17      -1.738 -16.115  31.445  1.00  1.16
N
ATOM    262  H   ARG A  17       1.036 -10.247  34.007  1.00  0.85
H
ATOM    263  HA  ARG A  17      -0.940  -8.926  32.407  1.00  0.92
H
ATOM    264 1HB  ARG A  17      -1.147 -10.883  34.716  1.00  1.39
H
ATOM    265 2HB  ARG A  17      -2.545 -10.300  33.828  1.00  1.39
H
ATOM    266 1HG  ARG A  17      -1.731 -11.298  31.783  1.00  1.39
H
ATOM    267 2HG  ARG A  17      -0.222 -11.776  32.590  1.00  1.39
H
ATOM    268 1HD  ARG A  17      -1.466 -13.198  34.101  1.00  1.39
H
ATOM    269 2HD  ARG A  17      -2.984 -12.716  33.307  1.00  1.39
H
ATOM    270  HE  ARG A  17      -1.433 -13.651  31.251  1.00  1.39
H
```

```
ATOM    271 1HH1 ARG A  17      -2.472 -14.960  34.340  1.00  1.39
H
ATOM    272 2HH1 ARG A  17      -2.473 -16.617  33.778  1.00  1.39
H
ATOM    273 1HH2 ARG A  17      -1.433 -15.809  30.529  1.00  1.39
H
ATOM    274 2HH2 ARG A  17      -1.883 -17.099  31.620  1.00  1.39
H
ATOM    275  N   ASP A  18      -0.728  -8.010  35.580  1.00  0.66
N
ATOM    276  CA  ASP A  18      -1.091  -6.966  36.529  1.00  0.67
C
ATOM    277  C   ASP A  18      -0.713  -5.603  35.980  1.00  0.61
C
ATOM    278  O   ASP A  18      -1.462  -4.642  36.145  1.00  0.62
O
ATOM    279  CB  ASP A  18      -0.402  -7.174  37.879  1.00  1.01
C
ATOM    280  CG  ASP A  18      -0.942  -8.345  38.685  1.00  1.01
C
ATOM    281  OD1 ASP A  18      -1.984  -8.868  38.362  1.00  1.01
O
ATOM    282  OD2 ASP A  18      -0.316  -8.687  39.657  1.00  1.01
O
ATOM    283  H   ASP A  18      -0.056  -8.705  35.869  1.00  0.79
H
ATOM    284  HA  ASP A  18      -2.171  -6.990  36.679  1.00  0.80
H
ATOM    285 1HB  ASP A  18       0.662  -7.334  37.722  1.00  1.21
H
ATOM    286 2HB  ASP A  18      -0.499  -6.268  38.476  1.00  1.21
H
ATOM    287  N   THR A  19       0.443  -5.534  35.317  1.00  0.62
N
ATOM    288  CA  THR A  19       0.923  -4.314  34.696  1.00  0.69
C
ATOM    289  C   THR A  19      -0.046  -3.869  33.621  1.00  0.59
C
ATOM    290  O   THR A  19      -0.412  -2.697  33.575  1.00  0.61
O
ATOM    291  CB  THR A  19       2.325  -4.511  34.092  1.00  1.03
C
ATOM    292  OG1 THR A  19       3.247  -4.860  35.133  1.00  1.03
O
ATOM    293  CG2 THR A  19       2.790  -3.236  33.406  1.00  1.03
C
ATOM    294  H   THR A  19       1.021  -6.358  35.252  1.00  0.74
H
ATOM    295  HA  THR A  19       0.975  -3.532  35.454  1.00  0.83
H
ATOM    296  HB  THR A  19       2.297  -5.315  33.361  1.00  1.24
H
ATOM    297  HG1 THR A  19       2.972  -5.691  35.537  1.00  1.24
H
ATOM    298 1HG2 THR A  19       3.778  -3.398  32.987  1.00  1.24
H
ATOM    299 2HG2 THR A  19       2.093  -2.977  32.609  1.00  1.24
H
```

```
ATOM    300 3HG2 THR A  19       2.830  -2.425  34.132  1.00  1.24
H
ATOM    301  N   ALA A  20      -0.485  -4.814  32.784  1.00  0.59
N
ATOM    302  CA  ALA A  20      -1.466  -4.527  31.742  1.00  0.56
C
ATOM    303  C   ALA A  20      -2.752  -3.986  32.347  1.00  0.58
C
ATOM    304  O   ALA A  20      -3.341  -3.042  31.817  1.00  0.60
O
ATOM    305  CB  ALA A  20      -1.752  -5.781  30.927  1.00  0.84
C
ATOM    306  H   ALA A  20      -0.114  -5.755  32.862  1.00  0.71
H
ATOM    307  HA  ALA A  20      -1.056  -3.761  31.087  1.00  0.67
H
ATOM    308 1HB  ALA A  20      -2.467  -5.549  30.139  1.00  1.01
H
ATOM    309 2HB  ALA A  20      -0.826  -6.142  30.482  1.00  1.01
H
ATOM    310 3HB  ALA A  20      -2.166  -6.552  31.573  1.00  1.01
H
ATOM    311  N   ASN A  21      -3.167  -4.570  33.472  1.00  0.62
N
ATOM    312  CA  ASN A  21      -4.364  -4.134  34.170  1.00  0.68
C
ATOM    313  C   ASN A  21      -4.201  -2.712  34.691  1.00  0.70
C
ATOM    314  O   ASN A  21      -5.119  -1.898  34.572  1.00  0.80
O
ATOM    315  CB  ASN A  21      -4.682  -5.091  35.303  1.00  1.02
C
ATOM    316  CG  ASN A  21      -5.201  -6.433  34.848  1.00  1.02
C
ATOM    317  OD1 ASN A  21      -5.783  -6.586  33.765  1.00  1.02
O
ATOM    318  ND2 ASN A  21      -4.992  -7.429  35.670  1.00  1.02
N
ATOM    319  H   ASN A  21      -2.653  -5.360  33.840  1.00  0.74
H
ATOM    320  HA  ASN A  21      -5.186  -4.122  33.469  1.00  0.82
H
ATOM    321 1HB  ASN A  21      -3.785  -5.258  35.893  1.00  1.22
H
ATOM    322 2HB  ASN A  21      -5.421  -4.637  35.962  1.00  1.22
H
ATOM    323 1HD2 ASN A  21      -5.306  -8.349  35.434  1.00  1.22
H
ATOM    324 2HD2 ASN A  21      -4.517  -7.273  36.536  1.00  1.22
H
ATOM    325  N   ARG A  22      -3.023  -2.411  35.233  1.00  0.64
N
ATOM    326  CA  ARG A  22      -2.720  -1.081  35.725  1.00  0.68
C
ATOM    327  C   ARG A  22      -2.702  -0.065  34.593  1.00  0.74
C
ATOM    328  O   ARG A  22      -3.189   1.050  34.764  1.00  0.93
O
```

ATOM 329 CB ARG A 22 -1.387 -1.082 36.458 1.00 1.02
C
ATOM 330 CG ARG A 22 -1.418 -1.766 37.820 1.00 1.02
C
ATOM 331 CD ARG A 22 -0.065 -1.864 38.432 1.00 1.02
C
ATOM 332 NE ARG A 22 0.543 -0.553 38.585 1.00 1.02
N
ATOM 333 CZ ARG A 22 0.281 0.320 39.574 1.00 1.02
C
ATOM 334 NH1 ARG A 22 -0.586 0.020 40.519 1.00 1.02
N
ATOM 335 NH2 ARG A 22 0.897 1.488 39.581 1.00 1.02
N
ATOM 336 H ARG A 22 -2.320 -3.133 35.324 1.00 0.77
H
ATOM 337 HA ARG A 22 -3.497 -0.792 36.433 1.00 0.82
H
ATOM 338 1HB ARG A 22 -0.635 -1.585 35.854 1.00 1.22
H
ATOM 339 2HB ARG A 22 -1.052 -0.056 36.611 1.00 1.22
H
ATOM 340 1HG ARG A 22 -2.053 -1.192 38.496 1.00 1.22
H
ATOM 341 2HG ARG A 22 -1.820 -2.773 37.715 1.00 1.22
H
ATOM 342 1HD ARG A 22 -0.139 -2.322 39.417 1.00 1.22
H
ATOM 343 2HD ARG A 22 0.582 -2.467 37.796 1.00 1.22
H
ATOM 344 HE ARG A 22 1.242 -0.281 37.899 1.00 1.22
H
ATOM 345 1HH1 ARG A 22 -1.056 -0.874 40.508 1.00 1.22
H
ATOM 346 2HH1 ARG A 22 -0.781 0.684 41.255 1.00 1.22
H
ATOM 347 1HH2 ARG A 22 1.538 1.715 38.826 1.00 1.22
H
ATOM 348 2HH2 ARG A 22 0.712 2.161 40.310 1.00 1.22
H
ATOM 349 N LEU A 23 -2.174 -0.463 33.433 1.00 0.67
N
ATOM 350 CA LEU A 23 -2.152 0.407 32.268 1.00 0.67
C
ATOM 351 C LEU A 23 -3.569 0.736 31.812 1.00 0.69
C
ATOM 352 O LEU A 23 -3.852 1.882 31.451 1.00 0.72
O
ATOM 353 CB LEU A 23 -1.396 -0.260 31.119 1.00 1.01
C
ATOM 354 CG LEU A 23 0.119 -0.432 31.277 1.00 1.01
C
ATOM 355 CD1 LEU A 23 0.632 -1.264 30.115 1.00 1.01
C
ATOM 356 CD2 LEU A 23 0.793 0.923 31.309 1.00 1.01
C
ATOM 357 H LEU A 23 -1.764 -1.383 33.360 1.00 0.80
H

```
ATOM    358  HA  LEU A  23      -1.648   1.335  32.536  1.00  0.80
H
ATOM    359 1HB  LEU A  23      -1.819  -1.249  30.956  1.00  1.21
H
ATOM    360 2HB  LEU A  23      -1.560   0.327  30.227  1.00  1.21
H
ATOM    361  HG  LEU A  23       0.338  -0.963  32.201  1.00  1.21
H
ATOM    362 1HD1 LEU A  23       1.708  -1.407  30.214  1.00  1.21
H
ATOM    363 2HD1 LEU A  23       0.134  -2.230  30.117  1.00  1.21
H
ATOM    364 3HD1 LEU A  23       0.419  -0.748  29.181  1.00  1.21
H
ATOM    365 1HD2 LEU A  23       1.869   0.792  31.416  1.00  1.21
H
ATOM    366 2HD2 LEU A  23       0.583   1.456  30.381  1.00  1.21
H
ATOM    367 3HD2 LEU A  23       0.411   1.496  32.154  1.00  1.21
H
ATOM    368  N   ARG A  24      -4.456  -0.270  31.853  1.00  0.69
N
ATOM    369  CA  ARG A  24      -5.864  -0.086  31.516  1.00  0.68
C
ATOM    370  C   ARG A  24      -6.487   0.955  32.437  1.00  0.68
C
ATOM    371  O   ARG A  24      -7.138   1.888  31.968  1.00  0.63
O
ATOM    372  CB  ARG A  24      -6.634  -1.403  31.632  1.00  1.02
C
ATOM    373  CG  ARG A  24      -8.088  -1.341  31.182  1.00  1.02
C
ATOM    374  CD  ARG A  24      -8.788  -2.649  31.342  1.00  1.02
C
ATOM    375  NE  ARG A  24      -8.239  -3.698  30.510  1.00  1.02
N
ATOM    376  CZ  ARG A  24      -7.485  -4.711  30.976  1.00  1.02
C
ATOM    377  NH1 ARG A  24      -7.200  -4.750  32.253  1.00  1.02
N
ATOM    378  NH2 ARG A  24      -7.041  -5.652  30.161  1.00  1.02
N
ATOM    379  H   ARG A  24      -4.141  -1.196  32.118  1.00  0.83
H
ATOM    380  HA  ARG A  24      -5.933   0.268  30.488  1.00  0.82
H
ATOM    381 1HB  ARG A  24      -6.136  -2.168  31.036  1.00  1.22
H
ATOM    382 2HB  ARG A  24      -6.631  -1.742  32.665  1.00  1.22
H
ATOM    383 1HG  ARG A  24      -8.621  -0.590  31.765  1.00  1.22
H
ATOM    384 2HG  ARG A  24      -8.117  -1.069  30.130  1.00  1.22
H
ATOM    385 1HD  ARG A  24      -8.702  -2.974  32.375  1.00  1.22
H
ATOM    386 2HD  ARG A  24      -9.840  -2.529  31.086  1.00  1.22
H
```

```
ATOM    387  HE  ARG A  24      -8.491  -3.703  29.531  1.00  1.22
H
ATOM    388 1HH1 ARG A  24      -7.545  -4.027  32.867  1.00  1.22
H
ATOM    389 2HH1 ARG A  24      -6.631  -5.498  32.634  1.00  1.22
H
ATOM    390 1HH2 ARG A  24      -7.264  -5.614  29.176  1.00  1.22
H
ATOM    391 2HH2 ARG A  24      -6.473  -6.406  30.517  1.00  1.22
H
ATOM    392  N   ILE A  25      -6.252   0.799  33.742  1.00  0.76
N
ATOM    393  CA  ILE A  25      -6.758   1.725  34.749  1.00  0.80
C
ATOM    394  C   ILE A  25      -6.250   3.133  34.551  1.00  0.82
C
ATOM    395  O   ILE A  25      -7.024   4.085  34.645  1.00  0.85
O
ATOM    396  CB  ILE A  25      -6.376   1.272  36.157  1.00  1.20
C
ATOM    397  CG1 ILE A  25      -7.135   0.003  36.513  1.00  1.20
C
ATOM    398  CG2 ILE A  25      -6.644   2.383  37.151  1.00  1.20
C
ATOM    399  CD1 ILE A  25      -6.622  -0.671  37.759  1.00  1.20
C
ATOM    400  H   ILE A  25      -5.717  -0.006  34.052  1.00  0.91
H
ATOM    401  HA  ILE A  25      -7.844   1.744  34.679  1.00  0.96
H
ATOM    402  HB  ILE A  25      -5.316   1.024  36.182  1.00  1.44
H
ATOM    403 1HG1 ILE A  25      -8.187   0.244  36.655  1.00  1.44
H
ATOM    404 2HG1 ILE A  25      -7.053  -0.689  35.681  1.00  1.44
H
ATOM    405 1HG2 ILE A  25      -6.357   2.055  38.150  1.00  1.44
H
ATOM    406 2HG2 ILE A  25      -6.063   3.263  36.879  1.00  1.44
H
ATOM    407 3HG2 ILE A  25      -7.703   2.634  37.141  1.00  1.44
H
ATOM    408 1HD1 ILE A  25      -7.204  -1.571  37.955  1.00  1.44
H
ATOM    409 2HD1 ILE A  25      -5.574  -0.939  37.621  1.00  1.44
H
ATOM    410 3HD1 ILE A  25      -6.713   0.010  38.603  1.00  1.44
H
ATOM    411  N   HIS A  26      -4.952   3.265  34.279  1.00  0.86
N
ATOM    412  CA  HIS A  26      -4.357   4.570  34.060  1.00  0.96
C
ATOM    413  C   HIS A  26      -4.989   5.258  32.868  1.00  0.97
C
ATOM    414  O   HIS A  26      -5.278   6.454  32.920  1.00  1.12
O
ATOM    415  CB  HIS A  26      -2.854   4.462  33.791  1.00  1.44
C
```

ATOM	416	CG	HIS	A	26	-1.991	4.097	34.959	1.00	1.44	C
ATOM	417	ND1	HIS	A	26	-2.128	4.676	36.203	1.00	1.44	N
ATOM	418	CD2	HIS	A	26	-0.932	3.256	35.049	1.00	1.44	C
ATOM	419	CE1	HIS	A	26	-1.197	4.194	37.012	1.00	1.44	C
ATOM	420	NE2	HIS	A	26	-0.456	3.338	36.333	1.00	1.44	N
ATOM	421	H	HIS	A	26	-4.361	2.446	34.237	1.00	1.03	H
ATOM	422	HA	HIS	A	26	-4.513	5.200	34.936	1.00	1.15	H
ATOM	423	1HB	HIS	A	26	-2.691	3.710	33.019	1.00	1.73	H
ATOM	424	2HB	HIS	A	26	-2.498	5.409	33.391	1.00	1.73	H
ATOM	425	HD2	HIS	A	26	-0.521	2.643	34.248	1.00	1.73	H
ATOM	426	HE1	HIS	A	26	-1.056	4.468	38.057	1.00	1.73	H
ATOM	427	HE2	HIS	A	26	0.346	2.825	36.680	1.00	1.73	H
ATOM	428	N	SER	A	27	-5.230	4.490	31.804	1.00	0.89	N
ATOM	429	CA	SER	A	27	-5.849	5.019	30.605	1.00	0.92	C
ATOM	430	C	SER	A	27	-7.255	5.515	30.905	1.00	1.23	C
ATOM	431	O	SER	A	27	-7.641	6.587	30.440	1.00	1.91	O
ATOM	432	CB	SER	A	27	-5.891	3.953	29.528	1.00	1.38	C
ATOM	433	OG	SER	A	27	-4.597	3.591	29.132	1.00	1.38	O
ATOM	434	H	SER	A	27	-4.952	3.516	31.813	1.00	1.07	H
ATOM	435	HA	SER	A	27	-5.254	5.860	30.248	1.00	1.10	H
ATOM	436	1HB	SER	A	27	-6.420	3.075	29.897	1.00	1.66	H
ATOM	437	2HB	SER	A	27	-6.443	4.329	28.669	1.00	1.66	H
ATOM	438	HG	SER	A	27	-4.207	3.148	29.893	1.00	1.66	H
ATOM	439	N	ILE	A	28	-8.000	4.744	31.708	1.00	0.90	N
ATOM	440	CA	ILE	A	28	-9.367	5.099	32.085	1.00	0.96	C
ATOM	441	C	ILE	A	28	-9.422	6.376	32.875	1.00	0.98	C
ATOM	442	O	ILE	A	28	-10.194	7.278	32.550	1.00	1.08	O
ATOM	443	CB	ILE	A	28	-10.024	4.007	32.955	1.00	1.44	C
ATOM	444	CG1	ILE	A	28	-10.251	2.734	32.165	1.00	1.44	C

```
ATOM    445  CG2 ILE A  28     -11.352   4.516  33.493  1.00  1.44
C
ATOM    446  CD1 ILE A  28     -10.608   1.561  33.019  1.00  1.44
C
ATOM    447  H   ILE A  28      -7.621   3.865  32.042  1.00  1.08
H
ATOM    448  HA  ILE A  28      -9.957   5.235  31.183  1.00  1.15
H
ATOM    449  HB  ILE A  28      -9.370   3.761  33.790  1.00  1.73
H
ATOM    450 1HG1 ILE A  28     -11.063   2.896  31.468  1.00  1.73
H
ATOM    451 2HG1 ILE A  28      -9.355   2.495  31.602  1.00  1.73
H
ATOM    452 1HG2 ILE A  28     -11.811   3.750  34.116  1.00  1.73
H
ATOM    453 2HG2 ILE A  28     -11.186   5.414  34.086  1.00  1.73
H
ATOM    454 3HG2 ILE A  28     -12.016   4.750  32.660  1.00  1.73
H
ATOM    455 1HD1 ILE A  28     -10.764   0.687  32.386  1.00  1.73
H
ATOM    456 2HD1 ILE A  28      -9.797   1.364  33.720  1.00  1.73
H
ATOM    457 3HD1 ILE A  28     -11.520   1.778  33.570  1.00  1.73
H
ATOM    458  N   ARG A  29      -8.611   6.441  33.927  1.00  1.00
N
ATOM    459  CA  ARG A  29      -8.605   7.582  34.818  1.00  1.09
C
ATOM    460  C   ARG A  29      -8.156   8.848  34.125  1.00  1.17
C
ATOM    461  O   ARG A  29      -8.754   9.904  34.324  1.00  1.31
O
ATOM    462  CB  ARG A  29      -7.698   7.314  36.011  1.00  1.64
C
ATOM    463  CG  ARG A  29      -8.217   6.287  37.008  1.00  1.64
C
ATOM    464  CD  ARG A  29      -7.242   6.066  38.109  1.00  1.64
C
ATOM    465  NE  ARG A  29      -7.732   5.121  39.104  1.00  1.64
N
ATOM    466  CZ  ARG A  29      -6.986   4.614  40.108  1.00  1.64
C
ATOM    467  NH1 ARG A  29      -5.728   4.974  40.235  1.00  1.64
N
ATOM    468  NH2 ARG A  29      -7.515   3.757  40.966  1.00  1.64
N
ATOM    469  H   ARG A  29      -7.997   5.663  34.126  1.00  1.20
H
ATOM    470  HA  ARG A  29      -9.620   7.733  35.186  1.00  1.31
H
ATOM    471 1HB  ARG A  29      -6.728   6.966  35.658  1.00  1.96
H
ATOM    472 2HB  ARG A  29      -7.534   8.242  36.556  1.00  1.96
H
ATOM    473 1HG  ARG A  29      -9.154   6.639  37.442  1.00  1.96
H
```

ATOM	474	2HG	ARG	A	29	-8.386	5.337	36.500	1.00	1.96	H
ATOM	475	1HD	ARG	A	29	-6.314	5.673	37.697	1.00	1.96	H
ATOM	476	2HD	ARG	A	29	-7.042	7.012	38.610	1.00	1.96	H
ATOM	477	HE	ARG	A	29	-8.695	4.822	39.037	1.00	1.96	H
ATOM	478	1HH1	ARG	A	29	-5.318	5.626	39.581	1.00	1.96	H
ATOM	479	2HH1	ARG	A	29	-5.168	4.594	40.985	1.00	1.96	H
ATOM	480	1HH2	ARG	A	29	-8.480	3.472	40.873	1.00	1.96	H
ATOM	481	2HH2	ARG	A	29	-6.956	3.372	41.713	1.00	1.96	H
ATOM	482	N	ALA	A	30	-7.115	8.743	33.305	1.00	1.12	N
ATOM	483	CA	ALA	A	30	-6.600	9.889	32.584	1.00	1.24	C
ATOM	484	C	ALA	A	30	-7.617	10.397	31.565	1.00	1.34	C
ATOM	485	O	ALA	A	30	-7.847	11.603	31.468	1.00	1.47	O
ATOM	486	CB	ALA	A	30	-5.295	9.517	31.911	1.00	1.86	C
ATOM	487	H	ALA	A	30	-6.649	7.853	33.184	1.00	1.34	H
ATOM	488	HA	ALA	A	30	-6.414	10.688	33.301	1.00	1.49	H
ATOM	489	1HB	ALA	A	30	-4.889	10.388	31.405	1.00	2.23	H
ATOM	490	2HB	ALA	A	30	-4.584	9.178	32.666	1.00	2.23	H
ATOM	491	3HB	ALA	A	30	-5.466	8.718	31.191	1.00	2.23	H
ATOM	492	N	THR	A	31	-8.233	9.466	30.827	1.00	1.42	N
ATOM	493	CA	THR	A	31	-9.240	9.776	29.814	1.00	2.04	C
ATOM	494	C	THR	A	31	-10.452	10.462	30.437	1.00	1.57	C
ATOM	495	O	THR	A	31	-10.951	11.468	29.922	1.00	2.16	O
ATOM	496	CB	THR	A	31	-9.682	8.475	29.103	1.00	3.06	C
ATOM	497	OG1	THR	A	31	-8.559	7.883	28.437	1.00	3.06	O
ATOM	498	CG2	THR	A	31	-10.774	8.745	28.099	1.00	3.06	C
ATOM	499	H	THR	A	31	-7.990	8.491	30.949	1.00	1.70	H
ATOM	500	HA	THR	A	31	-8.802	10.452	29.080	1.00	2.45	H
ATOM	501	HB	THR	A	31	-10.056	7.773	29.848	1.00	3.67	H
ATOM	502	HG1	THR	A	31	-7.950	7.537	29.096	1.00	3.67	H

```
ATOM    503 1HG2 THR A  31      -11.072   7.814  27.621  1.00  3.67
H
ATOM    504 2HG2 THR A  31      -11.627   9.180  28.614  1.00  3.67
H
ATOM    505 3HG2 THR A  31      -10.412   9.436  27.342  1.00  3.67
H
ATOM    506  N   CYS A  32      -10.915   9.903  31.554  1.00  1.11
N
ATOM    507  CA  CYS A  32      -12.039  10.426  32.316  1.00  1.20
C
ATOM    508  C   CYS A  32      -11.758  11.823  32.835  1.00  1.28
C
ATOM    509  O   CYS A  32      -12.596  12.717  32.708  1.00  2.03
O
ATOM    510  CB  CYS A  32      -12.360   9.514  33.496  1.00  1.80
C
ATOM    511  SG  CYS A  32      -13.758  10.080  34.487  1.00  1.80
S
ATOM    512  H   CYS A  32      -10.471   9.065  31.904  1.00  1.33
H
ATOM    513  HA  CYS A  32      -12.910  10.467  31.662  1.00  1.44
H
ATOM    514 1HB  CYS A  32      -12.588   8.513  33.128  1.00  2.16
H
ATOM    515 2HB  CYS A  32      -11.490   9.435  34.148  1.00  2.16
H
ATOM    516  HG  CYS A  32      -14.626  10.132  33.480  1.00  2.16
H
ATOM    517  N   ALA A  33      -10.585  12.002  33.445  1.00  1.26
N
ATOM    518  CA  ALA A  33      -10.174  13.296  33.975  1.00  1.51
C
ATOM    519  C   ALA A  33      -10.136  14.347  32.873  1.00  1.61
C
ATOM    520  O   ALA A  33      -10.568  15.484  33.074  1.00  1.95
O
ATOM    521  CB  ALA A  33       -8.807  13.182  34.641  1.00  2.27
C
ATOM    522  H   ALA A  33       -9.948  11.223  33.549  1.00  1.51
H
ATOM    523  HA  ALA A  33      -10.908  13.610  34.717  1.00  1.81
H
ATOM    524 1HB  ALA A  33       -8.521  14.150  35.053  1.00  2.72
H
ATOM    525 2HB  ALA A  33       -8.853  12.445  35.441  1.00  2.72
H
ATOM    526 3HB  ALA A  33       -8.068  12.870  33.904  1.00  2.72
H
ATOM    527  N   SER A  34       -9.633  13.957  31.702  1.00  1.47
N
ATOM    528  CA  SER A  34       -9.562  14.836  30.548  1.00  2.07
C
ATOM    529  C   SER A  34      -10.944  15.146  29.986  1.00  1.72
C
ATOM    530  O   SER A  34      -11.217  16.271  29.563  1.00  2.09
O
ATOM    531  CB  SER A  34       -8.741  14.177  29.465  1.00  3.10
C
```

ATOM	532	OG	SER	A	34	-7.421	13.980	29.880	1.00	3.10
O										
ATOM	533	H	SER	A	34	-9.263	13.020	31.607	1.00	1.76
H										
ATOM	534	HA	SER	A	34	-9.089	15.772	30.850	1.00	2.48
H										
ATOM	535	1HB	SER	A	34	-9.188	13.219	29.205	1.00	3.73
H										
ATOM	536	2HB	SER	A	34	-8.754	14.799	28.570	1.00	3.73
H										
ATOM	537	HG	SER	A	34	-7.018	14.846	29.972	1.00	3.73
H										
ATOM	538	N	GLY	A	35	-11.817	14.137	29.987	1.00	1.68
N										
ATOM	539	CA	GLY	A	35	-13.141	14.263	29.406	1.00	1.64
C										
ATOM	540	C	GLY	A	35	-13.096	13.933	27.914	1.00	1.15
C										
ATOM	541	O	GLY	A	35	-13.989	14.324	27.161	1.00	0.94
O										
ATOM	542	H	GLY	A	35	-11.544	13.238	30.360	1.00	2.02
H										
ATOM	543	1HA	GLY	A	35	-13.831	13.590	29.916	1.00	1.97
H										
ATOM	544	2HA	GLY	A	35	-13.511	15.278	29.551	1.00	1.97
H										
ATOM	545	N	SER	A	36	-12.027	13.249	27.487	1.00	2.30
N										
ATOM	546	CA	SER	A	36	-11.843	12.885	26.080	1.00	2.39
C										
ATOM	547	C	SER	A	36	-10.677	11.916	25.922	1.00	3.34
C										
ATOM	548	O	SER	A	36	-9.848	11.796	26.819	1.00	11.86
O										
ATOM	549	CB	SER	A	36	-11.591	14.128	25.249	1.00	3.58
C										
ATOM	550	OG	SER	A	36	-10.361	14.708	25.579	1.00	3.58
O										
ATOM	551	H	SER	A	36	-11.348	12.924	28.166	1.00	2.76
H										
ATOM	552	HA	SER	A	36	-12.750	12.396	25.725	1.00	2.87
H										
ATOM	553	1HB	SER	A	36	-11.604	13.874	24.189	1.00	4.30
H										
ATOM	554	2HB	SER	A	36	-12.388	14.849	25.416	1.00	4.30
H										
ATOM	555	HG	SER	A	36	-10.441	15.643	25.354	1.00	4.30
H										
ATOM	556	N	GLY	A	37	-10.587	11.257	24.767	1.00	1.40
N										
ATOM	557	CA	GLY	A	37	-9.486	10.328	24.497	1.00	1.13
C										
ATOM	558	C	GLY	A	37	-9.952	8.901	24.196	1.00	1.15
C										
ATOM	559	O	GLY	A	37	-11.000	8.459	24.664	1.00	3.78
O										
ATOM	560	H	GLY	A	37	-11.296	11.399	24.063	1.00	1.68
H										

```
ATOM    561 1HA  GLY A  37      -8.903  10.702  23.656  1.00  1.36
H
ATOM    562 2HA  GLY A  37      -8.819  10.309  25.356  1.00  1.36
H
ATOM    563  N   GLN A  38      -9.144   8.169  23.421  1.00  1.41
N
ATOM    564  CA  GLN A  38      -9.482   6.801  23.004  1.00  1.37
C
ATOM    565  C   GLN A  38      -9.126   5.691  23.984  1.00  1.61
C
ATOM    566  O   GLN A  38      -8.284   4.841  23.689  1.00  5.39
O
ATOM    567  CB  GLN A  38      -8.845   6.468  21.650  1.00  2.06
C
ATOM    568  CG  GLN A  38      -9.418   7.259  20.498  1.00  2.06
C
ATOM    569  CD  GLN A  38      -8.841   6.906  19.160  1.00  2.06
C
ATOM    570  OE1 GLN A  38      -8.300   5.815  18.942  1.00  2.06
O
ATOM    571  NE2 GLN A  38      -8.957   7.845  18.226  1.00  2.06
N
ATOM    572  H   GLN A  38      -8.282   8.579  23.089  1.00  1.69
H
ATOM    573  HA  GLN A  38     -10.563   6.766  22.868  1.00  1.64
H
ATOM    574 1HB  GLN A  38      -7.771   6.647  21.693  1.00  2.47
H
ATOM    575 2HB  GLN A  38      -8.999   5.414  21.435  1.00  2.47
H
ATOM    576 1HG  GLN A  38     -10.472   7.032  20.440  1.00  2.47
H
ATOM    577 2HG  GLN A  38      -9.270   8.323  20.678  1.00  2.47
H
ATOM    578 1HE2 GLN A  38      -8.597   7.685  17.306  1.00  2.47
H
ATOM    579 2HE2 GLN A  38      -9.407   8.712  18.441  1.00  2.47
H
ATOM    580  N   LEU A  39      -9.808   5.689  25.116  1.00  0.43
N
ATOM    581  CA  LEU A  39      -9.674   4.642  26.121  1.00  0.40
C
ATOM    582  C   LEU A  39      -9.649   3.214  25.587  1.00  0.36
C
ATOM    583  O   LEU A  39      -8.798   2.417  25.982  1.00  0.36
O
ATOM    584  CB  LEU A  39     -10.805   4.750  27.115  1.00  0.60
C
ATOM    585  CG  LEU A  39     -10.859   3.593  28.086  1.00  0.60
C
ATOM    586  CD1 LEU A  39      -9.545   3.514  28.818  1.00  0.60
C
ATOM    587  CD2 LEU A  39     -12.018   3.817  29.028  1.00  0.60
C
ATOM    588  H   LEU A  39     -10.464   6.449  25.265  1.00  0.52
H
ATOM    589  HA  LEU A  39      -8.737   4.816  26.651  1.00  0.48
H
```

```
ATOM    590 1HB  LEU A  39      -10.686   5.666  27.689  1.00  0.72
H
ATOM    591 2HB  LEU A  39      -11.754   4.793  26.579  1.00  0.72
H
ATOM    592  HG  LEU A  39      -11.004   2.657  27.545  1.00  0.72
H
ATOM    593 1HD1 LEU A  39       -9.567   2.681  29.514  1.00  0.72
H
ATOM    594 2HD1 LEU A  39       -8.739   3.363  28.102  1.00  0.72
H
ATOM    595 3HD1 LEU A  39       -9.380   4.443  29.353  1.00  0.72
H
ATOM    596 1HD2 LEU A  39      -12.084   2.993  29.734  1.00  0.72
H
ATOM    597 2HD2 LEU A  39      -11.875   4.752  29.570  1.00  0.72
H
ATOM    598 3HD2 LEU A  39      -12.935   3.867  28.446  1.00  0.72
H
ATOM    599  N   THR A  40      -10.597   2.890  24.713  1.00  0.36
N
ATOM    600  CA  THR A  40      -10.726   1.539  24.179  1.00  0.39
C
ATOM    601  C   THR A  40       -9.564   1.134  23.267  1.00  0.42
C
ATOM    602  O   THR A  40       -9.289  -0.061  23.100  1.00  0.56
O
ATOM    603  CB  THR A  40      -12.049   1.403  23.405  1.00  0.58
C
ATOM    604  OG1 THR A  40      -12.073   2.342  22.322  1.00  0.58
O
ATOM    605  CG2 THR A  40      -13.226   1.670  24.325  1.00  0.58
C
ATOM    606  H   THR A  40      -11.260   3.595  24.421  1.00  0.43
H
ATOM    607  HA  THR A  40      -10.745   0.848  25.020  1.00  0.47
H
ATOM    608  HB  THR A  40      -12.131   0.397  23.002  1.00  0.70
H
ATOM    609  HG1 THR A  40      -12.112   3.234  22.677  1.00  0.70
H
ATOM    610 1HG2 THR A  40      -14.155   1.569  23.766  1.00  0.70
H
ATOM    611 2HG2 THR A  40      -13.216   0.953  25.146  1.00  0.70
H
ATOM    612 3HG2 THR A  40      -13.153   2.680  24.726  1.00  0.70
H
ATOM    613  N   SER A  41       -8.886   2.128  22.677  1.00  0.40
N
ATOM    614  CA  SER A  41       -7.743   1.871  21.813  1.00  0.54
C
ATOM    615  C   SER A  41       -6.561   1.506  22.680  1.00  1.01
C
ATOM    616  O   SER A  41       -5.755   0.646  22.327  1.00  3.82
O
ATOM    617  CB  SER A  41       -7.418   3.085  20.959  1.00  0.81
C
ATOM    618  OG  SER A  41       -8.456   3.358  20.055  1.00  0.81
O
```

```
ATOM    619  H   SER A  41      -9.132   3.091  22.857  1.00  0.48
H
ATOM    620  HA  SER A  41      -7.971   1.026  21.161  1.00  0.65
H
ATOM    621 1HB  SER A  41      -7.255   3.947  21.602  1.00  0.97
H
ATOM    622 2HB  SER A  41      -6.493   2.911  20.411  1.00  0.97
H
ATOM    623  HG  SER A  41      -8.296   4.247  19.717  1.00  0.97
H
ATOM    624  N   CYS A  42      -6.481   2.158  23.838  1.00  0.44
N
ATOM    625  CA  CYS A  42      -5.450   1.854  24.813  1.00  0.44
C
ATOM    626  C   CYS A  42      -5.641   0.440  25.356  1.00  0.60
C
ATOM    627  O   CYS A  42      -4.696  -0.348  25.399  1.00  0.98
O
ATOM    628  CB  CYS A  42      -5.495   2.853  25.966  1.00  0.66
C
ATOM    629  SG  CYS A  42      -4.982   4.527  25.523  1.00  0.66
S
ATOM    630  H   CYS A  42      -7.156   2.890  24.040  1.00  0.53
H
ATOM    631  HA  CYS A  42      -4.477   1.918  24.327  1.00  0.53
H
ATOM    632 1HB  CYS A  42      -6.505   2.910  26.363  1.00  0.79
H
ATOM    633 2HB  CYS A  42      -4.855   2.503  26.774  1.00  0.79
H
ATOM    634  HG  CYS A  42      -5.931   4.724  24.615  1.00  0.79
H
ATOM    635  N   CYS A  43      -6.880   0.121  25.742  1.00  0.40
N
ATOM    636  CA  CYS A  43      -7.225  -1.175  26.321  1.00  0.45
C
ATOM    637  C   CYS A  43      -6.832  -2.367  25.462  1.00  0.41
C
ATOM    638  O   CYS A  43      -6.234  -3.320  25.965  1.00  0.44
O
ATOM    639  CB  CYS A  43      -8.723  -1.240  26.574  1.00  0.68
C
ATOM    640  SG  CYS A  43      -9.278  -0.184  27.917  1.00  0.68
S
ATOM    641  H   CYS A  43      -7.607   0.822  25.667  1.00  0.48
H
ATOM    642  HA  CYS A  43      -6.711  -1.261  27.278  1.00  0.54
H
ATOM    643 1HB  CYS A  43      -9.250  -0.935  25.669  1.00  0.81
H
ATOM    644 2HB  CYS A  43      -9.019  -2.262  26.796  1.00  0.81
H
ATOM    645  HG  CYS A  43      -8.436   0.812  27.672  1.00  0.81
H
ATOM    646  N   SER A  44      -7.105  -2.307  24.160  1.00  0.46
N
ATOM    647  CA  SER A  44      -6.760  -3.431  23.290  1.00  0.52
C
```

ATOM	648	C	SER	A	44	-5.248	-3.665	23.104	1.00	0.56	C
ATOM	649	O	SER	A	44	-4.855	-4.692	22.553	1.00	0.78	O
ATOM	650	CB	SER	A	44	-7.434	-3.267	21.942	1.00	0.78	C
ATOM	651	OG	SER	A	44	-6.973	-2.143	21.240	1.00	0.78	O
ATOM	652	H	SER	A	44	-7.598	-1.504	23.777	1.00	0.55	H
ATOM	653	HA	SER	A	44	-7.174	-4.328	23.746	1.00	0.62	H
ATOM	654	1HB	SER	A	44	-7.272	-4.163	21.343	1.00	0.94	H
ATOM	655	2HB	SER	A	44	-8.500	-3.179	22.110	1.00	0.94	H
ATOM	656	HG	SER	A	44	-6.104	-2.372	20.894	1.00	0.94	H
ATOM	657	N	ALA	A	45	-4.403	-2.743	23.566	1.00	0.64	N
ATOM	658	CA	ALA	A	45	-2.962	-2.918	23.447	1.00	0.73	C
ATOM	659	C	ALA	A	45	-2.275	-3.088	24.807	1.00	0.50	C
ATOM	660	O	ALA	A	45	-1.049	-3.125	24.860	1.00	6.33	O
ATOM	661	CB	ALA	A	45	-2.357	-1.729	22.725	1.00	1.09	C
ATOM	662	H	ALA	A	45	-4.740	-1.906	24.022	1.00	0.77	H
ATOM	663	HA	ALA	A	45	-2.778	-3.820	22.864	1.00	0.88	H
ATOM	664	1HB	ALA	A	45	-1.285	-1.883	22.612	1.00	1.31	H
ATOM	665	2HB	ALA	A	45	-2.817	-1.631	21.742	1.00	1.31	H
ATOM	666	3HB	ALA	A	45	-2.536	-0.824	23.304	1.00	1.31	H
ATOM	667	N	ALA	A	46	-3.050	-3.171	25.894	1.00	1.48	N
ATOM	668	CA	ALA	A	46	-2.484	-3.221	27.251	1.00	0.36	C
ATOM	669	C	ALA	A	46	-1.427	-4.300	27.454	1.00	0.39	C
ATOM	670	O	ALA	A	46	-0.356	-4.021	28.002	1.00	1.59	O
ATOM	671	CB	ALA	A	46	-3.606	-3.419	28.258	1.00	0.54	C
ATOM	672	H	ALA	A	46	-4.061	-3.177	25.804	1.00	1.78	H
ATOM	673	HA	ALA	A	46	-2.002	-2.272	27.438	1.00	0.43	H
ATOM	674	1HB	ALA	A	46	-3.196	-3.405	29.267	1.00	0.65	H
ATOM	675	2HB	ALA	A	46	-4.334	-2.615	28.150	1.00	0.65	H
ATOM	676	3HB	ALA	A	46	-4.093	-4.376	28.076	1.00	0.65	H

```
ATOM    677  N   GLU A  47      -1.720  -5.521  27.025  1.00  0.76
N
ATOM    678  CA  GLU A  47      -0.778  -6.617  27.186  1.00  0.58
C
ATOM    679  C   GLU A  47       0.450  -6.442  26.313  1.00  0.40
C
ATOM    680  O   GLU A  47       1.559  -6.738  26.750  1.00  0.32
O
ATOM    681  CB  GLU A  47      -1.467  -7.945  26.903  1.00  0.87
C
ATOM    682  CG  GLU A  47      -2.496  -8.322  27.959  1.00  0.87
C
ATOM    683  CD  GLU A  47      -3.816  -7.643  27.759  1.00  0.87
C
ATOM    684  OE1 GLU A  47      -4.027  -7.122  26.691  1.00  0.87
O
ATOM    685  OE2 GLU A  47      -4.615  -7.639  28.667  1.00  0.87
O
ATOM    686  H   GLU A  47      -2.612  -5.695  26.583  1.00  0.91
H
ATOM    687  HA  GLU A  47      -0.447  -6.629  28.225  1.00  0.70
H
ATOM    688 1HB  GLU A  47      -1.981  -7.887  25.945  1.00  1.04
H
ATOM    689 2HB  GLU A  47      -0.731  -8.743  26.833  1.00  1.04
H
ATOM    690 1HG  GLU A  47      -2.646  -9.399  27.941  1.00  1.04
H
ATOM    691 2HG  GLU A  47      -2.103  -8.058  28.941  1.00  1.04
H
ATOM    692  N   VAL A  48       0.259  -5.942  25.093  1.00  0.44
N
ATOM    693  CA  VAL A  48       1.370  -5.703  24.178  1.00  0.35
C
ATOM    694  C   VAL A  48       2.364  -4.715  24.762  1.00  0.28
C
ATOM    695  O   VAL A  48       3.574  -4.951  24.733  1.00  0.23
O
ATOM    696  CB  VAL A  48       0.860  -5.162  22.833  1.00  0.52
C
ATOM    697  CG1 VAL A  48       2.037  -4.708  21.984  1.00  0.52
C
ATOM    698  CG2 VAL A  48       0.060  -6.243  22.119  1.00  0.52
C
ATOM    699  H   VAL A  48      -0.680  -5.719  24.790  1.00  0.53
H
ATOM    700  HA  VAL A  48       1.883  -6.648  24.002  1.00  0.42
H
ATOM    701  HB  VAL A  48       0.227  -4.293  23.011  1.00  0.63
H
ATOM    702 1HG1 VAL A  48       1.668  -4.310  21.041  1.00  0.63
H
ATOM    703 2HG1 VAL A  48       2.589  -3.931  22.512  1.00  0.63
H
ATOM    704 3HG1 VAL A  48       2.695  -5.555  21.791  1.00  0.63
H
ATOM    705 1HG2 VAL A  48      -0.310  -5.856  21.170  1.00  0.63
H
```

```
ATOM    706 2HG2 VAL A  48       0.698  -7.107  21.936  1.00  0.63
H
ATOM    707 3HG2 VAL A  48      -0.785  -6.540  22.741  1.00  0.63
H
ATOM    708  N   VAL A  49       1.836  -3.615  25.294  1.00  0.35
N
ATOM    709  CA  VAL A  49       2.643  -2.576  25.912  1.00  0.28
C
ATOM    710  C   VAL A  49       3.368  -3.116  27.133  1.00  0.32
C
ATOM    711  O   VAL A  49       4.561  -2.877  27.307  1.00  0.36
O
ATOM    712  CB  VAL A  49       1.753  -1.374  26.302  1.00  0.42
C
ATOM    713  CG1 VAL A  49       2.538  -0.368  27.130  1.00  0.42
C
ATOM    714  CG2 VAL A  49       1.226  -0.709  25.039  1.00  0.42
C
ATOM    715  H   VAL A  49       0.833  -3.490  25.264  1.00  0.42
H
ATOM    716  HA  VAL A  49       3.383  -2.238  25.187  1.00  0.34
H
ATOM    717  HB  VAL A  49       0.919  -1.728  26.909  1.00  0.50
H
ATOM    718 1HG1 VAL A  49       1.889   0.465  27.400  1.00  0.50
H
ATOM    719 2HG1 VAL A  49       2.907  -0.850  28.035  1.00  0.50
H
ATOM    720 3HG1 VAL A  49       3.377   0.004  26.546  1.00  0.50
H
ATOM    721 1HG2 VAL A  49       0.589   0.130  25.310  1.00  0.50
H
ATOM    722 2HG2 VAL A  49       2.065  -0.350  24.442  1.00  0.50
H
ATOM    723 3HG2 VAL A  49       0.652  -1.428  24.458  1.00  0.50
H
ATOM    724  N   SER A  50       2.653  -3.860  27.973  1.00  0.34
N
ATOM    725  CA  SER A  50       3.251  -4.435  29.166  1.00  0.49
C
ATOM    726  C   SER A  50       4.438  -5.330  28.834  1.00  0.48
C
ATOM    727  O   SER A  50       5.490  -5.237  29.467  1.00  0.68
O
ATOM    728  CB  SER A  50       2.216  -5.246  29.903  1.00  0.73
C
ATOM    729  OG  SER A  50       1.179  -4.430  30.344  1.00  0.73
O
ATOM    730  H   SER A  50       1.667  -4.022  27.801  1.00  0.41
H
ATOM    731  HA  SER A  50       3.597  -3.622  29.806  1.00  0.59
H
ATOM    732 1HB  SER A  50       1.822  -6.028  29.254  1.00  0.88
H
ATOM    733 2HB  SER A  50       2.679  -5.729  30.758  1.00  0.88
H
ATOM    734  HG  SER A  50       0.705  -4.158  29.549  1.00  0.88
H
```

ATOM 735 N VAL A 51 4.273 -6.184 27.825 1.00 0.43
N
ATOM 736 CA VAL A 51 5.324 -7.109 27.429 1.00 0.49
C
ATOM 737 C VAL A 51 6.530 -6.407 26.862 1.00 0.40
C
ATOM 738 O VAL A 51 7.659 -6.675 27.284 1.00 0.51
O
ATOM 739 CB VAL A 51 4.795 -8.112 26.385 1.00 0.73
C
ATOM 740 CG1 VAL A 51 5.941 -8.937 25.827 1.00 0.73
C
ATOM 741 CG2 VAL A 51 3.784 -9.035 27.033 1.00 0.73
C
ATOM 742 H VAL A 51 3.385 -6.220 27.340 1.00 0.52
H
ATOM 743 HA VAL A 51 5.644 -7.666 28.307 1.00 0.59
H
ATOM 744 HB VAL A 51 4.330 -7.567 25.563 1.00 0.88
H
ATOM 745 1HG1 VAL A 51 5.552 -9.640 25.093 1.00 0.88
H
ATOM 746 2HG1 VAL A 51 6.670 -8.279 25.352 1.00 0.88
H
ATOM 747 3HG1 VAL A 51 6.419 -9.484 26.637 1.00 0.88
H
ATOM 748 1HG2 VAL A 51 3.408 -9.738 26.297 1.00 0.88
H
ATOM 749 2HG2 VAL A 51 4.269 -9.579 27.833 1.00 0.88
H
ATOM 750 3HG2 VAL A 51 2.957 -8.460 27.440 1.00 0.88
H
ATOM 751 N LEU A 52 6.302 -5.504 25.917 1.00 0.30
N
ATOM 752 CA LEU A 52 7.417 -4.820 25.303 1.00 0.27
C
ATOM 753 C LEU A 52 8.168 -3.925 26.265 1.00 0.23
C
ATOM 754 O LEU A 52 9.395 -3.934 26.294 1.00 0.23
O
ATOM 755 CB LEU A 52 6.934 -4.001 24.104 1.00 0.41
C
ATOM 756 CG LEU A 52 6.490 -4.838 22.903 1.00 0.41
C
ATOM 757 CD1 LEU A 52 5.896 -3.939 21.830 1.00 0.41
C
ATOM 758 CD2 LEU A 52 7.694 -5.595 22.386 1.00 0.41
C
ATOM 759 H LEU A 52 5.358 -5.304 25.606 1.00 0.36
H
ATOM 760 HA LEU A 52 8.103 -5.572 24.936 1.00 0.32
H
ATOM 761 1HB LEU A 52 6.089 -3.387 24.415 1.00 0.49
H
ATOM 762 2HB LEU A 52 7.739 -3.343 23.778 1.00 0.49
H
ATOM 763 HG LEU A 52 5.720 -5.546 23.212 1.00 0.49
H

ATOM 764 1HD1 LEU A 52 5.583 -4.545 20.981 1.00 0.49
H
ATOM 765 2HD1 LEU A 52 5.034 -3.410 22.237 1.00 0.49
H
ATOM 766 3HD1 LEU A 52 6.645 -3.218 21.505 1.00 0.49
H
ATOM 767 1HD2 LEU A 52 7.402 -6.202 21.532 1.00 0.49
H
ATOM 768 2HD2 LEU A 52 8.464 -4.887 22.082 1.00 0.49
H
ATOM 769 3HD2 LEU A 52 8.082 -6.238 23.173 1.00 0.49
H
ATOM 770 N PHE A 53 7.460 -3.189 27.102 1.00 0.23
N
ATOM 771 CA PHE A 53 8.150 -2.295 28.011 1.00 0.22
C
ATOM 772 C PHE A 53 8.757 -2.964 29.239 1.00 0.26
C
ATOM 773 O PHE A 53 9.716 -2.437 29.804 1.00 0.25
O
ATOM 774 CB PHE A 53 7.239 -1.128 28.402 1.00 0.33
C
ATOM 775 CG PHE A 53 7.126 -0.079 27.322 1.00 0.33
C
ATOM 776 CD1 PHE A 53 6.178 -0.168 26.311 1.00 0.33
C
ATOM 777 CD2 PHE A 53 7.972 1.020 27.325 1.00 0.33
C
ATOM 778 CE1 PHE A 53 6.083 0.804 25.333 1.00 0.33
C
ATOM 779 CE2 PHE A 53 7.881 1.994 26.351 1.00 0.33
C
ATOM 780 CZ PHE A 53 6.935 1.886 25.354 1.00 0.33
C
ATOM 781 H PHE A 53 6.449 -3.205 27.090 1.00 0.28
H
ATOM 782 HA PHE A 53 8.979 -1.854 27.455 1.00 0.26
H
ATOM 783 1HB PHE A 53 6.240 -1.500 28.625 1.00 0.40
H
ATOM 784 2HB PHE A 53 7.621 -0.650 29.304 1.00 0.40
H
ATOM 785 HD1 PHE A 53 5.501 -1.017 26.287 1.00 0.40
H
ATOM 786 HD2 PHE A 53 8.719 1.113 28.112 1.00 0.40
H
ATOM 787 HE1 PHE A 53 5.333 0.714 24.547 1.00 0.40
H
ATOM 788 HE2 PHE A 53 8.557 2.848 26.370 1.00 0.40
H
ATOM 789 HZ PHE A 53 6.862 2.654 24.587 1.00 0.40
H
ATOM 790 N PHE A 54 8.225 -4.105 29.673 1.00 0.33
N
ATOM 791 CA PHE A 54 8.772 -4.688 30.888 1.00 0.43
C
ATOM 792 C PHE A 54 9.515 -6.013 30.764 1.00 0.95
C

ATOM 793 O PHE A 54 10.154 -6.433 31.729 1.00 3.13
O
ATOM 794 CB PHE A 54 7.692 -4.714 31.939 1.00 0.65
C
ATOM 795 CG PHE A 54 7.275 -3.309 32.248 1.00 0.65
C
ATOM 796 CD1 PHE A 54 6.147 -2.755 31.664 1.00 0.65
C
ATOM 797 CD2 PHE A 54 8.039 -2.524 33.094 1.00 0.65
C
ATOM 798 CE1 PHE A 54 5.795 -1.444 31.911 1.00 0.65
C
ATOM 799 CE2 PHE A 54 7.689 -1.219 33.350 1.00 0.65
C
ATOM 800 CZ PHE A 54 6.571 -0.679 32.750 1.00 0.65
C
ATOM 801 H PHE A 54 7.431 -4.539 29.220 1.00 0.40
H
ATOM 802 HA PHE A 54 9.507 -3.978 31.268 1.00 0.52
H
ATOM 803 1HB PHE A 54 6.827 -5.278 31.599 1.00 0.77
H
ATOM 804 2HB PHE A 54 8.075 -5.173 32.844 1.00 0.77
H
ATOM 805 HD1 PHE A 54 5.542 -3.360 30.992 1.00 0.77
H
ATOM 806 HD2 PHE A 54 8.932 -2.949 33.554 1.00 0.77
H
ATOM 807 HE1 PHE A 54 4.911 -1.016 31.439 1.00 0.77
H
ATOM 808 HE2 PHE A 54 8.300 -0.609 34.014 1.00 0.77
H
ATOM 809 HZ PHE A 54 6.305 0.355 32.942 1.00 0.77
H
ATOM 810 N HIS A 55 9.511 -6.641 29.587 1.00 0.68
N
ATOM 811 CA HIS A 55 10.282 -7.876 29.419 1.00 1.58
C
ATOM 812 C HIS A 55 11.026 -8.008 28.098 1.00 0.59
C
ATOM 813 O HIS A 55 12.093 -8.620 28.068 1.00 2.15
O
ATOM 814 CB HIS A 55 9.480 -9.141 29.747 1.00 2.37
C
ATOM 815 CG HIS A 55 9.191 -9.236 31.207 1.00 2.37
C
ATOM 816 ND1 HIS A 55 10.195 -9.374 32.143 1.00 2.37
N
ATOM 817 CD2 HIS A 55 8.035 -9.202 31.898 1.00 2.37
C
ATOM 818 CE1 HIS A 55 9.663 -9.402 33.349 1.00 2.37
C
ATOM 819 NE2 HIS A 55 8.354 -9.301 33.227 1.00 2.37
N
ATOM 820 H HIS A 55 8.959 -6.299 28.813 1.00 0.82
H
ATOM 821 HA HIS A 55 11.069 -7.848 30.171 1.00 1.90
H

ATOM 822 1HB HIS A 55 8.534 -9.132 29.204 1.00 2.84
H
ATOM 823 2HB HIS A 55 10.037 -10.025 29.440 1.00 2.84
H
ATOM 824 HD1 HIS A 55 11.166 -9.202 31.976 1.00 2.84
H
ATOM 825 HD2 HIS A 55 7.003 -9.109 31.594 1.00 2.84
H
ATOM 826 HE1 HIS A 55 10.293 -9.502 34.232 1.00 2.84
H
ATOM 827 N THR A 56 10.505 -7.449 27.012 1.00 0.70
N
ATOM 828 CA THR A 56 11.219 -7.585 25.745 1.00 0.57
C
ATOM 829 C THR A 56 12.242 -6.484 25.459 1.00 0.33
C
ATOM 830 O THR A 56 13.405 -6.782 25.184 1.00 0.54
O
ATOM 831 CB THR A 56 10.247 -7.622 24.567 1.00 0.85
C
ATOM 832 OG1 THR A 56 9.378 -8.753 24.692 1.00 0.85
O
ATOM 833 CG2 THR A 56 11.012 -7.693 23.260 1.00 0.85
C
ATOM 834 H THR A 56 9.619 -6.963 27.053 1.00 0.84
H
ATOM 835 HA THR A 56 11.756 -8.534 25.765 1.00 0.68
H
ATOM 836 HB THR A 56 9.653 -6.717 24.573 1.00 1.03
H
ATOM 837 HG1 THR A 56 9.902 -9.559 24.681 1.00 1.03
H
ATOM 838 1HG2 THR A 56 10.312 -7.699 22.428 1.00 1.03
H
ATOM 839 2HG2 THR A 56 11.665 -6.824 23.173 1.00 1.03
H
ATOM 840 3HG2 THR A 56 11.614 -8.600 23.238 1.00 1.03
H
ATOM 841 N MET A 57 11.816 -5.223 25.494 1.00 0.32
N
ATOM 842 CA MET A 57 12.705 -4.111 25.154 1.00 0.34
C
ATOM 843 C MET A 57 13.744 -3.775 26.210 1.00 0.42
C
ATOM 844 O MET A 57 13.501 -3.889 27.412 1.00 0.71
O
ATOM 845 CB MET A 57 11.901 -2.848 24.890 1.00 0.51
C
ATOM 846 CG MET A 57 11.028 -2.867 23.657 1.00 0.51
C
ATOM 847 SD MET A 57 10.081 -1.346 23.514 1.00 0.51
S
ATOM 848 CE MET A 57 11.440 -0.204 23.280 1.00 0.51
C
ATOM 849 H MET A 57 10.859 -5.013 25.738 1.00 0.38
H
ATOM 850 HA MET A 57 13.243 -4.380 24.246 1.00 0.41
H

```
ATOM    851 1HB  MET A  57      11.278  -2.622  25.751  1.00  0.61
H
ATOM    852 2HB  MET A  57      12.592  -2.014  24.772  1.00  0.61
H
ATOM    853 1HG  MET A  57      11.657  -2.967  22.777  1.00  0.61
H
ATOM    854 2HG  MET A  57      10.338  -3.709  23.695  1.00  0.61
H
ATOM    855 1HE  MET A  57      11.057   0.811  23.182  1.00  0.61
H
ATOM    856 2HE  MET A  57      12.108  -0.254  24.142  1.00  0.61
H
ATOM    857 3HE  MET A  57      11.990  -0.474  22.379  1.00  0.61
H
ATOM    858  N   LYS A  58      14.890  -3.303  25.731  1.00  0.37
N
ATOM    859  CA  LYS A  58      15.976  -2.827  26.572  1.00  0.54
C
ATOM    860  C   LYS A  58      16.072  -1.322  26.398  1.00  0.43
C
ATOM    861  O   LYS A  58      16.030  -0.837  25.271  1.00  0.55
O
ATOM    862  CB  LYS A  58      17.294  -3.450  26.160  1.00  0.81
C
ATOM    863  CG  LYS A  58      17.352  -4.956  26.234  1.00  0.81
C
ATOM    864  CD  LYS A  58      18.675  -5.451  25.677  1.00  0.81
C
ATOM    865  CE  LYS A  58      18.813  -5.083  24.206  1.00  0.81
C
ATOM    866  NZ  LYS A  58      17.757  -5.711  23.369  1.00  0.81
N
ATOM    867  H   LYS A  58      15.016  -3.269  24.728  1.00  0.44
H
ATOM    868  HA  LYS A  58      15.758  -3.052  27.615  1.00  0.65
H
ATOM    869 1HB  LYS A  58      17.531  -3.155  25.137  1.00  0.97
H
ATOM    870 2HB  LYS A  58      18.078  -3.065  26.805  1.00  0.97
H
ATOM    871 1HG  LYS A  58      17.252  -5.278  27.271  1.00  0.97
H
ATOM    872 2HG  LYS A  58      16.536  -5.384  25.653  1.00  0.97
H
ATOM    873 1HD  LYS A  58      19.500  -5.007  26.237  1.00  0.97
H
ATOM    874 2HD  LYS A  58      18.731  -6.535  25.777  1.00  0.97
H
ATOM    875 1HE  LYS A  58      18.750  -4.001  24.099  1.00  0.97
H
ATOM    876 2HE  LYS A  58      19.788  -5.414  23.847  1.00  0.97
H
ATOM    877 1HZ  LYS A  58      17.888  -5.438  22.406  1.00  0.97
H
ATOM    878 2HZ  LYS A  58      17.808  -6.716  23.447  1.00  0.97
H
ATOM    879 3HZ  LYS A  58      16.848  -5.393  23.682  1.00  0.97
H
```

```
ATOM    880  N   TYR A  59      16.221  -0.591  27.489  1.00  0.40
N
ATOM    881  CA  TYR A  59      16.297   0.867  27.419  1.00  0.33
C
ATOM    882  C   TYR A  59      16.486   1.474  28.798  1.00  0.35
C
ATOM    883  O   TYR A  59      16.001   0.943  29.797  1.00  0.44
O
ATOM    884  CB  TYR A  59      15.025   1.461  26.790  1.00  0.49
C
ATOM    885  CG  TYR A  59      13.767   1.108  27.546  1.00  0.49
C
ATOM    886  CD1 TYR A  59      13.356   1.904  28.606  1.00  0.49
C
ATOM    887  CD2 TYR A  59      13.026  -0.009  27.183  1.00  0.49
C
ATOM    888  CE1 TYR A  59      12.214   1.576  29.304  1.00  0.49
C
ATOM    889  CE2 TYR A  59      11.887  -0.337  27.881  1.00  0.49
C
ATOM    890  CZ  TYR A  59      11.487   0.451  28.936  1.00  0.49
C
ATOM    891  OH  TYR A  59      10.363   0.121  29.635  1.00  0.49
O
ATOM    892  H   TYR A  59      16.284  -1.055  28.383  1.00  0.48
H
ATOM    893  HA  TYR A  59      17.157   1.137  26.809  1.00  0.40
H
ATOM    894 1HB  TYR A  59      15.109   2.549  26.775  1.00  0.59
H
ATOM    895 2HB  TYR A  59      14.910   1.137  25.760  1.00  0.59
H
ATOM    896  HD1 TYR A  59      13.937   2.782  28.887  1.00  0.59
H
ATOM    897  HD2 TYR A  59      13.348  -0.629  26.349  1.00  0.59
H
ATOM    898  HE1 TYR A  59      11.888   2.195  30.140  1.00  0.59
H
ATOM    899  HE2 TYR A  59      11.306  -1.217  27.604  1.00  0.59
H
ATOM    900  HH  TYR A  59      10.204  -0.825  29.564  1.00  0.59
H
ATOM    901  N   LYS A  60      17.170   2.608  28.844  1.00  0.43
N
ATOM    902  CA  LYS A  60      17.412   3.282  30.102  1.00  0.90
C
ATOM    903  C   LYS A  60      16.172   4.047  30.540  1.00  1.97
C
ATOM    904  O   LYS A  60      15.743   4.996  29.886  1.00 15.60
O
ATOM    905  CB  LYS A  60      18.633   4.191  29.974  1.00  1.35
C
ATOM    906  CG  LYS A  60      19.917   3.396  29.740  1.00  1.35
C
ATOM    907  CD  LYS A  60      21.156   4.272  29.681  1.00  1.35
C
ATOM    908  CE  LYS A  60      22.404   3.419  29.458  1.00  1.35
C
```

```
ATOM    909  NZ  LYS A  60      23.650   4.232  29.456  1.00  1.35
N
ATOM    910  H   LYS A  60      17.553   2.992  27.991  1.00  0.52
H
ATOM    911  HA  LYS A  60      17.624   2.529  30.863  1.00  1.08
H
ATOM    912 1HB  LYS A  60      18.499   4.880  29.140  1.00  1.62
H
ATOM    913 2HB  LYS A  60      18.756   4.781  30.881  1.00  1.62
H
ATOM    914 1HG  LYS A  60      20.040   2.669  30.544  1.00  1.62
H
ATOM    915 2HG  LYS A  60      19.830   2.855  28.799  1.00  1.62
H
ATOM    916 1HD  LYS A  60      21.061   4.989  28.864  1.00  1.62
H
ATOM    917 2HD  LYS A  60      21.263   4.823  30.616  1.00  1.62
H
ATOM    918 1HE  LYS A  60      22.472   2.675  30.250  1.00  1.62
H
ATOM    919 2HE  LYS A  60      22.318   2.906  28.500  1.00  1.62
H
ATOM    920 1HZ  LYS A  60      24.457   3.634  29.306  1.00  1.62
H
ATOM    921 2HZ  LYS A  60      23.603   4.917  28.715  1.00  1.62
H
ATOM    922 3HZ  LYS A  60      23.747   4.702  30.343  1.00  1.62
H
ATOM    923  N   GLN A  61      15.624   3.627  31.673  1.00  2.29
N
ATOM    924  CA  GLN A  61      14.382   4.145  32.250  1.00  1.17
C
ATOM    925  C   GLN A  61      14.327   5.658  32.342  1.00  1.62
C
ATOM    926  O   GLN A  61      13.357   6.283  31.916  1.00  3.58
O
ATOM    927  CB  GLN A  61      14.236   3.597  33.667  1.00  1.75
C
ATOM    928  CG  GLN A  61      13.982   2.113  33.772  1.00  1.75
C
ATOM    929  CD  GLN A  61      14.100   1.662  35.213  1.00  1.75
C
ATOM    930  OE1 GLN A  61      13.864   2.445  36.136  1.00  1.75
O
ATOM    931  NE2 GLN A  61      14.492   0.414  35.427  1.00  1.75
N
ATOM    932  H   GLN A  61      16.076   2.862  32.154  1.00  2.75
H
ATOM    933  HA  GLN A  61      13.550   3.807  31.633  1.00  1.40
H
ATOM    934 1HB  GLN A  61      15.140   3.816  34.234  1.00  2.11
H
ATOM    935 2HB  GLN A  61      13.410   4.108  34.165  1.00  2.11
H
ATOM    936 1HG  GLN A  61      12.974   1.896  33.422  1.00  2.11
H
ATOM    937 2HG  GLN A  61      14.718   1.574  33.176  1.00  2.11
H
```

```
ATOM    938 1HE2 GLN A  61      14.599   0.087  36.364  1.00  2.11
H
ATOM    939 2HE2 GLN A  61      14.688  -0.207  34.665  1.00  2.11
H
ATOM    940  N   THR A  62      15.384   6.241  32.903  1.00  0.88
N
ATOM    941  CA  THR A  62      15.447   7.676  33.127  1.00  0.94
C
ATOM    942  C   THR A  62      16.255   8.407  32.069  1.00  0.99
C
ATOM    943  O   THR A  62      16.711   9.529  32.296  1.00  4.48
O
ATOM    944  CB  THR A  62      16.028   7.981  34.518  1.00  1.41
C
ATOM    945  OG1 THR A  62      17.339   7.410  34.631  1.00  1.41
O
ATOM    946  CG2 THR A  62      15.123   7.402  35.596  1.00  1.41
C
ATOM    947  H   THR A  62      16.156   5.668  33.212  1.00  1.06
H
ATOM    948  HA  THR A  62      14.432   8.068  33.094  1.00  1.13
H
ATOM    949  HB  THR A  62      16.100   9.061  34.651  1.00  1.69
H
ATOM    950  HG1 THR A  62      17.907   7.781  33.952  1.00  1.69
H
ATOM    951 1HG2 THR A  62      15.537   7.626  36.578  1.00  1.69
H
ATOM    952 2HG2 THR A  62      14.130   7.844  35.511  1.00  1.69
H
ATOM    953 3HG2 THR A  62      15.051   6.323  35.470  1.00  1.69
H
ATOM    954  N   ASP A  63      16.413   7.791  30.907  1.00  0.74
N
ATOM    955  CA  ASP A  63      17.114   8.422  29.809  1.00  0.76
C
ATOM    956  C   ASP A  63      16.666   7.801  28.498  1.00  0.55
C
ATOM    957  O   ASP A  63      17.455   7.156  27.808  1.00  0.63
O
ATOM    958  CB  ASP A  63      18.625   8.284  29.968  1.00  1.14
C
ATOM    959  CG  ASP A  63      19.379   9.106  28.937  1.00  1.14
C
ATOM    960  OD1 ASP A  63      18.738   9.698  28.100  1.00  1.14
O
ATOM    961  OD2 ASP A  63      20.584   9.133  28.988  1.00  1.14
O
ATOM    962  H   ASP A  63      16.036   6.865  30.752  1.00  0.89
H
ATOM    963  HA  ASP A  63      16.880   9.484  29.803  1.00  0.91
H
ATOM    964 1HB  ASP A  63      18.920   8.614  30.964  1.00  1.37
H
ATOM    965 2HB  ASP A  63      18.911   7.238  29.867  1.00  1.37
H
ATOM    966  N   PRO A  64      15.402   8.029  28.121  1.00  0.47
N
```

```
ATOM    967  CA  PRO A  64      14.699   7.526  26.947  1.00  0.37
C
ATOM    968  C   PRO A  64      15.236   8.081  25.644  1.00  0.88
C
ATOM    969  O   PRO A  64      14.957   7.541  24.572  1.00  3.30
O
ATOM    970  CB  PRO A  64      13.288   8.002  27.159  1.00  0.55
C
ATOM    971  CG  PRO A  64      13.411   9.229  27.984  1.00  0.55
C
ATOM    972  CD  PRO A  64      14.555   8.957  28.897  1.00  0.55
C
ATOM    973  HA  PRO A  64      14.743   6.427  26.947  1.00  0.44
H
ATOM    974 1HB  PRO A  64      12.798   8.168  26.193  1.00  0.67
H
ATOM    975 2HB  PRO A  64      12.745   7.205  27.672  1.00  0.67
H
ATOM    976 1HG  PRO A  64      13.597  10.094  27.335  1.00  0.67
H
ATOM    977 2HG  PRO A  64      12.477   9.427  28.525  1.00  0.67
H
ATOM    978 1HD  PRO A  64      15.085   9.897  29.105  1.00  0.67
H
ATOM    979 2HD  PRO A  64      14.192   8.475  29.815  1.00  0.67
H
ATOM    980  N   GLU A  65      16.015   9.149  25.739  1.00  0.72
N
ATOM    981  CA  GLU A  65      16.638   9.768  24.593  1.00  1.12
C
ATOM    982  C   GLU A  65      17.997   9.142  24.284  1.00  1.10
C
ATOM    983  O   GLU A  65      18.632   9.510  23.295  1.00  1.56
O
ATOM    984  CB  GLU A  65      16.819  11.268  24.839  1.00  1.68
C
ATOM    985  CG  GLU A  65      15.526  12.057  24.990  1.00  1.68
C
ATOM    986  CD  GLU A  65      15.766  13.522  25.240  1.00  1.68
C
ATOM    987  OE1 GLU A  65      16.906  13.915  25.309  1.00  1.68
O
ATOM    988  OE2 GLU A  65      14.809  14.249  25.363  1.00  1.68
O
ATOM    989  H   GLU A  65      16.191   9.556  26.645  1.00  0.86
H
ATOM    990  HA  GLU A  65      15.992   9.626  23.727  1.00  1.34
H
ATOM    991 1HB  GLU A  65      17.405  11.415  25.747  1.00  2.02
H
ATOM    992 2HB  GLU A  65      17.380  11.706  24.014  1.00  2.02
H
ATOM    993 1HG  GLU A  65      14.933  11.945  24.083  1.00  2.02
H
ATOM    994 2HG  GLU A  65      14.955  11.641  25.818  1.00  2.02
H
ATOM    995  N   HIS A  66      18.458   8.217  25.135  1.00  0.85
N
```

```
ATOM    996  CA  HIS A  66      19.783   7.649  24.953  1.00  0.86
C
ATOM    997  C   HIS A  66      19.880   6.838  23.649  1.00  0.64
C
ATOM    998  O   HIS A  66      19.022   5.995  23.378  1.00  0.67
O
ATOM    999  CB  HIS A  66      20.149   6.742  26.128  1.00  1.29
C
ATOM   1000  CG  HIS A  66      21.608   6.475  26.203  1.00  1.29
C
ATOM   1001  ND1 HIS A  66      22.266   5.622  25.343  1.00  1.29
N
ATOM   1002  CD2 HIS A  66      22.550   6.988  27.025  1.00  1.29
C
ATOM   1003  CE1 HIS A  66      23.556   5.626  25.639  1.00  1.29
C
ATOM   1004  NE2 HIS A  66      23.752   6.446  26.654  1.00  1.29
N
ATOM   1005  H   HIS A  66      17.919   7.919  25.937  1.00  1.02
H
ATOM   1006  HA  HIS A  66      20.501   8.458  24.943  1.00  1.03
H
ATOM   1007 1HB  HIS A  66      19.837   7.202  27.062  1.00  1.55
H
ATOM   1008 2HB  HIS A  66      19.630   5.790  26.038  1.00  1.55
H
ATOM   1009  HD2 HIS A  66      22.385   7.709  27.826  1.00  1.55
H
ATOM   1010  HE1 HIS A  66      24.336   5.066  25.139  1.00  1.55
H
ATOM   1011  HE2 HIS A  66      24.643   6.649  27.087  1.00  1.55
H
ATOM   1012  N   PRO A  67      20.938   7.069  22.850  1.00  0.73
N
ATOM   1013  CA  PRO A  67      21.280   6.446  21.570  1.00  0.67
C
ATOM   1014  C   PRO A  67      21.278   4.923  21.562  1.00  0.53
C
ATOM   1015  O   PRO A  67      21.010   4.331  20.517  1.00  0.55
O
ATOM   1016  CB  PRO A  67      22.686   6.987  21.296  1.00  1.01
C
ATOM   1017  CG  PRO A  67      22.679   8.342  21.904  1.00  1.01
C
ATOM   1018  CD  PRO A  67      21.833   8.217  23.130  1.00  1.01
C
ATOM   1019  HA  PRO A  67      20.585   6.821  20.809  1.00  0.80
H
ATOM   1020 1HB  PRO A  67      23.440   6.324  21.744  1.00  1.21
H
ATOM   1021 2HB  PRO A  67      22.879   7.008  20.213  1.00  1.21
H
ATOM   1022 1HG  PRO A  67      23.709   8.656  22.136  1.00  1.21
H
ATOM   1023 2HG  PRO A  67      22.279   9.075  21.189  1.00  1.21
H
ATOM   1024 1HD  PRO A  67      22.459   8.017  24.009  1.00  1.21
H
```

```
ATOM   1025 2HD  PRO A  67      21.244   9.139  23.244  1.00  1.21
H
ATOM   1026  N   ASP A  68      21.561   4.287  22.698  1.00  0.75
N
ATOM   1027  CA  ASP A  68      21.601   2.827  22.757  1.00  0.99
C
ATOM   1028  C   ASP A  68      20.289   2.142  23.101  1.00  1.13
C
ATOM   1029  O   ASP A  68      20.245   0.916  23.174  1.00  2.63
O
ATOM   1030  CB  ASP A  68      22.646   2.388  23.767  1.00  1.48
C
ATOM   1031  CG  ASP A  68      24.037   2.653  23.265  1.00  1.48
C
ATOM   1032  OD1 ASP A  68      24.277   2.329  22.133  1.00  1.48
O
ATOM   1033  OD2 ASP A  68      24.848   3.174  23.988  1.00  1.48
O
ATOM   1034  H   ASP A  68      21.770   4.814  23.540  1.00  0.90
H
ATOM   1035  HA  ASP A  68      21.910   2.467  21.776  1.00  1.19
H
ATOM   1036 1HB  ASP A  68      22.499   2.919  24.708  1.00  1.78
H
ATOM   1037 2HB  ASP A  68      22.541   1.322  23.968  1.00  1.78
H
ATOM   1038  N   ASN A  69      19.223   2.903  23.297  1.00  0.46
N
ATOM   1039  CA  ASN A  69      17.946   2.307  23.669  1.00  0.34
C
ATOM   1040  C   ASN A  69      17.218   1.613  22.521  1.00  0.47
C
ATOM   1041  O   ASN A  69      17.329   1.997  21.359  1.00  0.79
O
ATOM   1042  CB  ASN A  69      17.027   3.363  24.252  1.00  0.51
C
ATOM   1043  CG  ASN A  69      17.479   3.900  25.576  1.00  0.51
C
ATOM   1044  OD1 ASN A  69      18.238   3.264  26.312  1.00  0.51
O
ATOM   1045  ND2 ASN A  69      17.006   5.070  25.907  1.00  0.51
N
ATOM   1046  H   ASN A  69      19.283   3.912  23.216  1.00  0.55
H
ATOM   1047  HA  ASN A  69      18.135   1.549  24.431  1.00  0.41
H
ATOM   1048 1HB  ASN A  69      16.952   4.196  23.551  1.00  0.61
H
ATOM   1049 2HB  ASN A  69      16.027   2.948  24.366  1.00  0.61
H
ATOM   1050 1HD2 ASN A  69      17.261   5.491  26.781  1.00  0.61
H
ATOM   1051 2HD2 ASN A  69      16.388   5.551  25.285  1.00  0.61
H
ATOM   1052  N   ASP A  70      16.434   0.596  22.841  1.00  0.41
N
ATOM   1053  CA  ASP A  70      15.568   0.019  21.829  1.00  0.37
C
```

```
ATOM   1054  C   ASP A  70      14.512   1.084  21.537  1.00  0.48
C
ATOM   1055  O   ASP A  70      14.139   1.851  22.428  1.00  1.81
O
ATOM   1056  CB  ASP A  70      14.934  -1.303  22.298  1.00  0.55
C
ATOM   1057  CG  ASP A  70      15.918  -2.478  22.326  1.00  0.55
C
ATOM   1058  OD1 ASP A  70      16.872  -2.451  21.587  1.00  0.55
O
ATOM   1059  OD2 ASP A  70      15.722  -3.388  23.098  1.00  0.55
O
ATOM   1060  H   ASP A  70      16.391   0.252  23.790  1.00  0.49
H
ATOM   1061  HA  ASP A  70      16.139  -0.165  20.920  1.00  0.44
H
ATOM   1062 1HB  ASP A  70      14.517  -1.172  23.296  1.00  0.67
H
ATOM   1063 2HB  ASP A  70      14.110  -1.562  21.633  1.00  0.67
H
ATOM   1064  N   ARG A  71      14.068   1.172  20.295  1.00  0.15
N
ATOM   1065  CA  ARG A  71      13.102   2.194  19.925  1.00  0.16
C
ATOM   1066  C   ARG A  71      11.689   1.647  19.900  1.00  0.16
C
ATOM   1067  O   ARG A  71      11.471   0.486  19.561  1.00  0.29
O
ATOM   1068  CB  ARG A  71      13.435   2.773  18.559  1.00  0.24
C
ATOM   1069  CG  ARG A  71      14.410   3.950  18.525  1.00  0.24
C
ATOM   1070  CD  ARG A  71      15.779   3.589  18.972  1.00  0.24
C
ATOM   1071  NE  ARG A  71      16.751   4.623  18.622  1.00  0.24
N
ATOM   1072  CZ  ARG A  71      18.029   4.655  19.053  1.00  0.24
C
ATOM   1073  NH1 ARG A  71      18.473   3.759  19.887  1.00  0.24
N
ATOM   1074  NH2 ARG A  71      18.882   5.578  18.656  1.00  0.24
N
ATOM   1075  H   ARG A  71      14.385   0.505  19.607  1.00  0.18
H
ATOM   1076  HA  ARG A  71      13.150   2.997  20.661  1.00  0.19
H
ATOM   1077 1HB  ARG A  71      13.865   1.987  17.950  1.00  0.29
H
ATOM   1078 2HB  ARG A  71      12.516   3.097  18.071  1.00  0.29
H
ATOM   1079 1HG  ARG A  71      14.484   4.326  17.506  1.00  0.29
H
ATOM   1080 2HG  ARG A  71      14.039   4.743  19.175  1.00  0.29
H
ATOM   1081 1HD  ARG A  71      15.786   3.470  20.054  1.00  0.29
H
ATOM   1082 2HD  ARG A  71      16.086   2.656  18.501  1.00  0.29
H
```

```
ATOM   1083  HE  ARG A  71      16.457   5.341  17.974  1.00  0.29
H
ATOM   1084 1HH1 ARG A  71      17.877   3.034  20.246  1.00  0.29
H
ATOM   1085 2HH1 ARG A  71      19.434   3.800  20.186  1.00  0.29
H
ATOM   1086 1HH2 ARG A  71      18.599   6.298  18.006  1.00  0.29
H
ATOM   1087 2HH2 ARG A  71      19.829   5.528  19.017  1.00  0.29
H
ATOM   1088  N   PHE A  72      10.730   2.496  20.243  1.00  0.18
N
ATOM   1089  CA  PHE A  72       9.327   2.120  20.190  1.00  0.20
C
ATOM   1090  C   PHE A  72       8.490   3.238  19.632  1.00  0.16
C
ATOM   1091  O   PHE A  72       8.416   4.323  20.212  1.00  0.18
O
ATOM   1092  CB  PHE A  72       8.754   1.751  21.552  1.00  0.30
C
ATOM   1093  CG  PHE A  72       7.299   1.369  21.468  1.00  0.30
C
ATOM   1094  CD1 PHE A  72       6.919   0.100  21.063  1.00  0.30
C
ATOM   1095  CD2 PHE A  72       6.306   2.289  21.774  1.00  0.30
C
ATOM   1096  CE1 PHE A  72       5.582  -0.243  20.968  1.00  0.30
C
ATOM   1097  CE2 PHE A  72       4.971   1.948  21.684  1.00  0.30
C
ATOM   1098  CZ  PHE A  72       4.610   0.681  21.280  1.00  0.30
C
ATOM   1099  H   PHE A  72      10.977   3.432  20.531  1.00  0.22
H
ATOM   1100  HA  PHE A  72       9.229   1.258  19.538  1.00  0.24
H
ATOM   1101 1HB  PHE A  72       9.297   0.907  21.959  1.00  0.36
H
ATOM   1102 2HB  PHE A  72       8.856   2.586  22.244  1.00  0.36
H
ATOM   1103  HD1 PHE A  72       7.686  -0.630  20.818  1.00  0.36
H
ATOM   1104  HD2 PHE A  72       6.590   3.294  22.089  1.00  0.36
H
ATOM   1105  HE1 PHE A  72       5.298  -1.245  20.648  1.00  0.36
H
ATOM   1106  HE2 PHE A  72       4.203   2.678  21.929  1.00  0.36
H
ATOM   1107  HZ  PHE A  72       3.558   0.413  21.210  1.00  0.36
H
ATOM   1108  N   ILE A  73       7.836   2.965  18.518  1.00  0.17
N
ATOM   1109  CA  ILE A  73       6.950   3.943  17.940  1.00  0.16
C
ATOM   1110  C   ILE A  73       5.523   3.497  17.990  1.00  0.19
C
ATOM   1111  O   ILE A  73       5.129   2.553  17.307  1.00  0.34
O
```

```
ATOM   1112  CB  ILE A  73       7.281   4.266  16.477  1.00  0.24
C
ATOM   1113  CG1 ILE A  73       8.672   4.853  16.344  1.00  0.24
C
ATOM   1114  CG2 ILE A  73       6.247   5.214  15.894  1.00  0.24
C
ATOM   1115  CD1 ILE A  73       9.039   5.055  14.907  1.00  0.24
C
ATOM   1116  H   ILE A  73       7.951   2.063  18.079  1.00  0.20
H
ATOM   1117  HA  ILE A  73       7.034   4.865  18.513  1.00  0.19
H
ATOM   1118  HB  ILE A  73       7.273   3.341  15.902  1.00  0.29
H
ATOM   1119 1HG1 ILE A  73       8.717   5.812  16.858  1.00  0.29
H
ATOM   1120 2HG1 ILE A  73       9.400   4.182  16.800  1.00  0.29
H
ATOM   1121 1HG2 ILE A  73       6.487   5.414  14.851  1.00  0.29
H
ATOM   1122 2HG2 ILE A  73       5.260   4.760  15.959  1.00  0.29
H
ATOM   1123 3HG2 ILE A  73       6.255   6.150  16.454  1.00  0.29
H
ATOM   1124 1HD1 ILE A  73      10.034   5.476  14.825  1.00  0.29
H
ATOM   1125 2HD1 ILE A  73       9.008   4.100  14.399  1.00  0.29
H
ATOM   1126 3HD1 ILE A  73       8.324   5.735  14.445  1.00  0.29
H
ATOM   1127  N   LEU A  74       4.738   4.219  18.761  1.00  0.21
N
ATOM   1128  CA  LEU A  74       3.318   4.001  18.745  1.00  0.36
C
ATOM   1129  C   LEU A  74       2.829   4.659  17.480  1.00  0.29
C
ATOM   1130  O   LEU A  74       2.783   5.885  17.418  1.00  0.60
O
ATOM   1131  CB  LEU A  74       2.656   4.632  19.969  1.00  0.54
C
ATOM   1132  CG  LEU A  74       1.140   4.491  20.034  1.00  0.54
C
ATOM   1133  CD1 LEU A  74       0.768   3.024  20.126  1.00  0.54
C
ATOM   1134  CD2 LEU A  74       0.612   5.276  21.220  1.00  0.54
C
ATOM   1135  H   LEU A  74       5.130   4.956  19.333  1.00  0.25
H
ATOM   1136  HA  LEU A  74       3.105   2.934  18.709  1.00  0.43
H
ATOM   1137 1HB  LEU A  74       3.070   4.179  20.866  1.00  0.65
H
ATOM   1138 2HB  LEU A  74       2.890   5.697  19.983  1.00  0.65
H
ATOM   1139  HG  LEU A  74       0.708   4.888  19.120  1.00  0.65
H
ATOM   1140 1HD1 LEU A  74      -0.316   2.926  20.156  1.00  0.65
H
```

```
ATOM   1141 2HD1 LEU A  74       1.157   2.494  19.256  1.00  0.65
H
ATOM   1142 3HD1 LEU A  74       1.197   2.599  21.033  1.00  0.65
H
ATOM   1143 1HD2 LEU A  74      -0.471   5.188  21.255  1.00  0.65
H
ATOM   1144 2HD2 LEU A  74       1.041   4.877  22.138  1.00  0.65
H
ATOM   1145 3HD2 LEU A  74       0.887   6.325  21.114  1.00  0.65
H
ATOM   1146  N   SER A  75       2.514   3.857  16.467  1.00  0.35
N
ATOM   1147  CA  SER A  75       2.064   4.392  15.191  1.00  0.29
C
ATOM   1148  C   SER A  75       0.652   4.891  15.372  1.00  0.33
C
ATOM   1149  O   SER A  75       0.316   6.000  14.962  1.00  0.35
O
ATOM   1150  CB  SER A  75       2.160   3.357  14.103  1.00  0.43
C
ATOM   1151  OG  SER A  75       1.791   3.899  12.867  1.00  0.43
O
ATOM   1152  H   SER A  75       2.559   2.856  16.591  1.00  0.42
H
ATOM   1153  HA  SER A  75       2.694   5.238  14.920  1.00  0.35
H
ATOM   1154 1HB  SER A  75       3.181   2.989  14.049  1.00  0.52
H
ATOM   1155 2HB  SER A  75       1.515   2.515  14.340  1.00  0.52
H
ATOM   1156  HG  SER A  75       0.837   4.017  12.895  1.00  0.52
H
ATOM   1157  N   ARG A  76      -0.177   4.091  16.037  1.00  0.58
N
ATOM   1158  CA  ARG A  76      -1.515   4.565  16.368  1.00  0.92
C
ATOM   1159  C   ARG A  76      -1.453   5.482  17.583  1.00  0.59
C
ATOM   1160  O   ARG A  76      -1.849   5.093  18.686  1.00  1.13
O
ATOM   1161  CB  ARG A  76      -2.457   3.436  16.674  1.00  1.38
C
ATOM   1162  CG  ARG A  76      -3.878   3.891  16.964  1.00  1.38
C
ATOM   1163  CD  ARG A  76      -4.718   2.738  17.315  1.00  1.38
C
ATOM   1164  NE  ARG A  76      -4.240   2.128  18.539  1.00  1.38
N
ATOM   1165  CZ  ARG A  76      -4.436   0.844  18.876  1.00  1.38
C
ATOM   1166  NH1 ARG A  76      -5.108   0.044  18.093  1.00  1.38
N
ATOM   1167  NH2 ARG A  76      -3.954   0.353  19.998  1.00  1.38
N
ATOM   1168  H   ARG A  76       0.137   3.166  16.313  1.00  0.70
H
ATOM   1169  HA  ARG A  76      -1.905   5.128  15.522  1.00  1.10
H
```

```
ATOM   1170 1HB  ARG A  76      -2.490   2.747  15.831  1.00  1.66
H
ATOM   1171 2HB  ARG A  76      -2.099   2.881  17.541  1.00  1.66
H
ATOM   1172 1HG  ARG A  76      -3.879   4.591  17.798  1.00  1.66
H
ATOM   1173 2HG  ARG A  76      -4.298   4.371  16.080  1.00  1.66
H
ATOM   1174 1HD  ARG A  76      -5.748   3.059  17.469  1.00  1.66
H
ATOM   1175 2HD  ARG A  76      -4.678   1.993  16.522  1.00  1.66
H
ATOM   1176  HE  ARG A  76      -3.707   2.704  19.177  1.00  1.66
H
ATOM   1177 1HH1 ARG A  76      -5.498   0.378  17.224  1.00  1.66
H
ATOM   1178 2HH1 ARG A  76      -5.226  -0.917  18.390  1.00  1.66
H
ATOM   1179 1HH2 ARG A  76      -3.429   0.934  20.636  1.00  1.66
H
ATOM   1180 2HH2 ARG A  76      -4.125  -0.624  20.198  1.00  1.66
H
ATOM   1181  N   GLY A  77      -0.974   6.710  17.348  1.00  0.30
N
ATOM   1182  CA  GLY A  77      -0.742   7.752  18.355  1.00  0.39
C
ATOM   1183  C   GLY A  77      -1.928   8.063  19.252  1.00  0.39
C
ATOM   1184  O   GLY A  77      -1.765   8.618  20.337  1.00  0.41
O
ATOM   1185  H   GLY A  77      -0.708   6.915  16.392  1.00  0.36
H
ATOM   1186 1HA  GLY A  77       0.103   7.457  18.975  1.00  0.47
H
ATOM   1187 2HA  GLY A  77      -0.438   8.666  17.844  1.00  0.47
H
ATOM   1188  N   HIS A  78      -3.119   7.690  18.820  1.00  0.44
N
ATOM   1189  CA  HIS A  78      -4.325   7.948  19.558  1.00  0.54
C
ATOM   1190  C   HIS A  78      -4.344   7.253  20.925  1.00  0.42
C
ATOM   1191  O   HIS A  78      -5.060   7.696  21.823  1.00  0.54
O
ATOM   1192  CB  HIS A  78      -5.506   7.462  18.759  1.00  0.81
C
ATOM   1193  CG  HIS A  78      -5.736   8.149  17.441  1.00  0.81
C
ATOM   1194  ND1 HIS A  78      -6.056   9.491  17.319  1.00  0.81
N
ATOM   1195  CD2 HIS A  78      -5.719   7.649  16.185  1.00  0.81
C
ATOM   1196  CE1 HIS A  78      -6.225   9.777  16.034  1.00  0.81
C
ATOM   1197  NE2 HIS A  78      -6.023   8.680  15.333  1.00  0.81
N
ATOM   1198  H   HIS A  78      -3.200   7.234  17.924  1.00  0.53
H
```

```
ATOM   1199  HA  HIS A  78      -4.427   9.021  19.721  1.00  0.65
H
ATOM   1200 1HB  HIS A  78      -5.404   6.392  18.573  1.00  0.97
H
ATOM   1201 2HB  HIS A  78      -6.385   7.600  19.371  1.00  0.97
H
ATOM   1202  HD2 HIS A  78      -5.508   6.617  15.904  1.00  0.97
H
ATOM   1203  HE1 HIS A  78      -6.484  10.753  15.624  1.00  0.97
H
ATOM   1204  HE2 HIS A  78      -6.083   8.605  14.327  1.00  0.97
H
ATOM   1205  N   ALA A  79      -3.545   6.183  21.074  1.00  0.38
N
ATOM   1206  CA  ALA A  79      -3.422   5.434  22.324  1.00  0.35
C
ATOM   1207  C   ALA A  79      -2.342   6.024  23.258  1.00  0.32
C
ATOM   1208  O   ALA A  79      -1.931   5.371  24.218  1.00  0.37
O
ATOM   1209  CB  ALA A  79      -3.115   3.971  22.021  1.00  0.52
C
ATOM   1210  H   ALA A  79      -2.987   5.873  20.286  1.00  0.46
H
ATOM   1211  HA  ALA A  79      -4.377   5.493  22.844  1.00  0.42
H
ATOM   1212 1HB  ALA A  79      -3.061   3.412  22.955  1.00  0.63
H
ATOM   1213 2HB  ALA A  79      -3.905   3.559  21.396  1.00  0.63
H
ATOM   1214 3HB  ALA A  79      -2.164   3.896  21.500  1.00  0.63
H
ATOM   1215  N   ALA A  80      -1.909   7.263  22.987  1.00  0.32
N
ATOM   1216  CA  ALA A  80      -0.909   7.971  23.789  1.00  0.33
C
ATOM   1217  C   ALA A  80      -1.077   7.852  25.319  1.00  0.36
C
ATOM   1218  O   ALA A  80      -0.063   7.825  26.006  1.00  0.50
O
ATOM   1219  CB  ALA A  80      -0.892   9.440  23.403  1.00  0.49
C
ATOM   1220  H   ALA A  80      -2.251   7.751  22.172  1.00  0.38
H
ATOM   1221  HA  ALA A  80       0.061   7.545  23.537  1.00  0.40
H
ATOM   1222 1HB  ALA A  80      -0.099   9.945  23.947  1.00  0.59
H
ATOM   1223 2HB  ALA A  80      -0.706   9.523  22.341  1.00  0.59
H
ATOM   1224 3HB  ALA A  80      -1.839   9.916  23.631  1.00  0.59
H
ATOM   1225  N   PRO A  81      -2.289   7.812  25.902  1.00  0.39
N
ATOM   1226  CA  PRO A  81      -2.494   7.649  27.338  1.00  0.46
C
ATOM   1227  C   PRO A  81      -1.838   6.392  27.908  1.00  0.43
C
```

```
ATOM   1228  O   PRO A  81       -1.341   6.422  29.035  1.00  0.46
O
ATOM   1229  CB  PRO A  81       -4.011   7.629  27.463  1.00  0.69
C
ATOM   1230  CG  PRO A  81       -4.464   8.537  26.371  1.00  0.69
C
ATOM   1231  CD  PRO A  81       -3.514   8.329  25.245  1.00  0.69
C
ATOM   1232  HA  PRO A  81       -2.092   8.539  27.845  1.00  0.55
H
ATOM   1233 1HB  PRO A  81       -4.387   6.602  27.353  1.00  0.83
H
ATOM   1234 2HB  PRO A  81       -4.311   7.977  28.462  1.00  0.83
H
ATOM   1235 1HG  PRO A  81       -5.507   8.316  26.100  1.00  0.83
H
ATOM   1236 2HG  PRO A  81       -4.433   9.573  26.710  1.00  0.83
H
ATOM   1237 1HD  PRO A  81       -3.951   7.593  24.562  1.00  0.83
H
ATOM   1238 2HD  PRO A  81       -3.327   9.288  24.753  1.00  0.83
H
ATOM   1239  N   ILE A  82       -1.836   5.289  27.150  1.00  0.41
N
ATOM   1240  CA  ILE A  82       -1.234   4.063  27.664  1.00  0.43
C
ATOM   1241  C   ILE A  82        0.260   4.143  27.516  1.00  0.39
C
ATOM   1242  O   ILE A  82        1.012   3.572  28.306  1.00  0.43
O
ATOM   1243  CB  ILE A  82       -1.767   2.805  26.961  1.00  0.65
C
ATOM   1244  CG1 ILE A  82       -1.545   1.622  27.877  1.00  0.65
C
ATOM   1245  CG2 ILE A  82       -1.071   2.536  25.632  1.00  0.65
C
ATOM   1246  CD1 ILE A  82       -2.292   0.394  27.455  1.00  0.65
C
ATOM   1247  H   ILE A  82       -2.224   5.306  26.216  1.00  0.49
H
ATOM   1248  HA  ILE A  82       -1.468   3.978  28.724  1.00  0.52
H
ATOM   1249  HB  ILE A  82       -2.834   2.913  26.798  1.00  0.77
H
ATOM   1250 1HG1 ILE A  82       -0.482   1.390  27.913  1.00  0.77
H
ATOM   1251 2HG1 ILE A  82       -1.871   1.897  28.881  1.00  0.77
H
ATOM   1252 1HG2 ILE A  82       -1.482   1.629  25.190  1.00  0.77
H
ATOM   1253 2HG2 ILE A  82       -1.223   3.363  24.954  1.00  0.77
H
ATOM   1254 3HG2 ILE A  82       -0.003   2.400  25.800  1.00  0.77
H
ATOM   1255 1HD1 ILE A  82       -2.091  -0.401  28.169  1.00  0.77
H
ATOM   1256 2HD1 ILE A  82       -3.362   0.608  27.439  1.00  0.77
H
```

```
ATOM   1257 3HD1 ILE A  82      -1.966   0.089  26.461  1.00  0.77
H
ATOM   1258  N   LEU A  83       0.676   4.883  26.507  1.00  0.37
N
ATOM   1259  CA  LEU A  83       2.067   5.111  26.254  1.00  0.50
C
ATOM   1260  C   LEU A  83       2.648   5.895  27.449  1.00  0.51
C
ATOM   1261  O   LEU A  83       3.693   5.536  27.999  1.00  0.69
O
ATOM   1262  CB  LEU A  83       2.176   5.842  24.916  1.00  0.75
C
ATOM   1263  CG  LEU A  83       3.551   6.104  24.418  1.00  0.75
C
ATOM   1264  CD1 LEU A  83       4.260   4.772  24.194  1.00  0.75
C
ATOM   1265  CD2 LEU A  83       3.486   6.931  23.141  1.00  0.75
C
ATOM   1266  H   LEU A  83      -0.013   5.287  25.876  1.00  0.44
H
ATOM   1267  HA  LEU A  83       2.579   4.151  26.181  1.00  0.60
H
ATOM   1268 1HB  LEU A  83       1.654   5.256  24.162  1.00  0.90
H
ATOM   1269 2HB  LEU A  83       1.691   6.805  24.997  1.00  0.90
H
ATOM   1270  HG  LEU A  83       4.073   6.652  25.182  1.00  0.90
H
ATOM   1271 1HD1 LEU A  83       5.278   4.951  23.850  1.00  0.90
H
ATOM   1272 2HD1 LEU A  83       4.286   4.213  25.128  1.00  0.90
H
ATOM   1273 3HD1 LEU A  83       3.720   4.200  23.441  1.00  0.90
H
ATOM   1274 1HD2 LEU A  83       4.497   7.137  22.789  1.00  0.90
H
ATOM   1275 2HD2 LEU A  83       2.943   6.380  22.378  1.00  0.90
H
ATOM   1276 3HD2 LEU A  83       2.973   7.871  23.346  1.00  0.90
H
ATOM   1277  N   TYR A  84       1.914   6.925  27.875  1.00  0.45
N
ATOM   1278  CA  TYR A  84       2.285   7.750  29.012  1.00  0.54
C
ATOM   1279  C   TYR A  84       2.273   6.934  30.301  1.00  0.53
C
ATOM   1280  O   TYR A  84       3.159   7.081  31.145  1.00  0.58
O
ATOM   1281  CB  TYR A  84       1.296   8.905  29.138  1.00  0.81
C
ATOM   1282  CG  TYR A  84       1.276   9.807  27.935  1.00  0.81
C
ATOM   1283  CD1 TYR A  84       2.321   9.770  27.033  1.00  0.81
C
ATOM   1284  CD2 TYR A  84       0.213  10.665  27.728  1.00  0.81
C
ATOM   1285  CE1 TYR A  84       2.315  10.595  25.928  1.00  0.81
C
```

```
ATOM   1286  CE2 TYR A  84       0.205  11.494  26.625  1.00  0.81
C
ATOM   1287  CZ  TYR A  84       1.252  11.465  25.730  1.00  0.81
C
ATOM   1288  OH  TYR A  84       1.248  12.294  24.635  1.00  0.81
O
ATOM   1289  H   TYR A  84       1.074   7.163  27.366  1.00  0.54
H
ATOM   1290  HA  TYR A  84       3.292   8.135  28.859  1.00  0.65
H
ATOM   1291 1HB  TYR A  84       0.289   8.511  29.286  1.00  0.97
H
ATOM   1292 2HB  TYR A  84       1.546   9.506  30.012  1.00  0.97
H
ATOM   1293  HD1 TYR A  84       3.146   9.085  27.200  1.00  0.97
H
ATOM   1294  HD2 TYR A  84      -0.612  10.689  28.438  1.00  0.97
H
ATOM   1295  HE1 TYR A  84       3.140  10.565  25.218  1.00  0.97
H
ATOM   1296  HE2 TYR A  84      -0.630  12.176  26.463  1.00  0.97
H
ATOM   1297  HH  TYR A  84       0.432  12.807  24.628  1.00  0.97
H
ATOM   1298  N   ALA A  85       1.277   6.053  30.425  1.00  0.46
N
ATOM   1299  CA  ALA A  85       1.144   5.186  31.582  1.00  0.49
C
ATOM   1300  C   ALA A  85       2.356   4.286  31.726  1.00  0.50
C
ATOM   1301  O   ALA A  85       2.851   4.099  32.834  1.00  0.60
O
ATOM   1302  CB  ALA A  85      -0.124   4.363  31.467  1.00  0.73
C
ATOM   1303  H   ALA A  85       0.563   6.001  29.710  1.00  0.55
H
ATOM   1304  HA  ALA A  85       1.082   5.813  32.472  1.00  0.59
H
ATOM   1305 1HB  ALA A  85      -0.228   3.734  32.348  1.00  0.88
H
ATOM   1306 2HB  ALA A  85      -0.983   5.029  31.390  1.00  0.88
H
ATOM   1307 3HB  ALA A  85      -0.072   3.736  30.580  1.00  0.88
H
ATOM   1308  N   ALA A  86       2.847   3.748  30.607  1.00  0.44
N
ATOM   1309  CA  ALA A  86       4.035   2.908  30.632  1.00  0.46
C
ATOM   1310  C   ALA A  86       5.226   3.698  31.152  1.00  0.45
C
ATOM   1311  O   ALA A  86       5.986   3.197  31.975  1.00  0.49
O
ATOM   1312  CB  ALA A  86       4.331   2.356  29.246  1.00  0.69
C
ATOM   1313  H   ALA A  86       2.376   3.907  29.724  1.00  0.53
H
ATOM   1314  HA  ALA A  86       3.853   2.079  31.315  1.00  0.55
H
```

```
ATOM   1315 1HB  ALA A  86        5.211   1.714  29.289  1.00  0.83
H
ATOM   1316 2HB  ALA A  86        3.475   1.778  28.900  1.00  0.83
H
ATOM   1317 3HB  ALA A  86        4.516   3.178  28.557  1.00  0.83
H
ATOM   1318  N   TRP A  87        5.355   4.949  30.700  1.00  0.47
N
ATOM   1319  CA  TRP A  87        6.447   5.834  31.104  1.00  0.59
C
ATOM   1320  C   TRP A  87        6.415   6.152  32.604  1.00  0.93
C
ATOM   1321  O   TRP A  87        7.461   6.371  33.230  1.00  1.52
O
ATOM   1322  CB  TRP A  87        6.378   7.084  30.264  1.00  0.89
C
ATOM   1323  CG  TRP A  87        6.548   6.720  28.835  1.00  0.89
C
ATOM   1324  CD1 TRP A  87        6.976   5.511  28.373  1.00  0.89
C
ATOM   1325  CD2 TRP A  87        6.296   7.527  27.670  1.00  0.89
C
ATOM   1326  NE1 TRP A  87        7.012   5.513  27.007  1.00  0.89
N
ATOM   1327  CE2 TRP A  87        6.605   6.739  26.558  1.00  0.89
C
ATOM   1328  CE3 TRP A  87        5.849   8.838  27.481  1.00  0.89
C
ATOM   1329  CZ2 TRP A  87        6.485   7.218  25.271  1.00  0.89
C
ATOM   1330  CZ3 TRP A  87        5.717   9.311  26.183  1.00  0.89
C
ATOM   1331  CH2 TRP A  87        6.025   8.519  25.111  1.00  0.89
C
ATOM   1332  H   TRP A  87        4.690   5.287  30.009  1.00  0.56
H
ATOM   1333  HA  TRP A  87        7.386   5.333  30.878  1.00  0.71
H
ATOM   1334 1HB  TRP A  87        5.424   7.590  30.398  1.00  1.06
H
ATOM   1335 2HB  TRP A  87        7.172   7.770  30.547  1.00  1.06
H
ATOM   1336  HD1 TRP A  87        7.250   4.665  29.001  1.00  1.06
H
ATOM   1337  HE1 TRP A  87        7.297   4.741  26.423  1.00  1.06
H
ATOM   1338  HE3 TRP A  87        5.605   9.471  28.334  1.00  1.06
H
ATOM   1339  HZ2 TRP A  87        6.725   6.608  24.399  1.00  1.06
H
ATOM   1340  HZ3 TRP A  87        5.363  10.328  26.036  1.00  1.06
H
ATOM   1341  HH2 TRP A  87        5.910   8.911  24.108  1.00  1.06
H
ATOM   1342  N   VAL A  88        5.218   6.123  33.179  1.00  0.68
N
ATOM   1343  CA  VAL A  88        5.081   6.206  34.618  1.00  0.72
C
```

```
ATOM   1344  C   VAL A  88       5.542   4.930  35.278  1.00  0.68
C
ATOM   1345  O   VAL A  88       6.321   4.959  36.228  1.00  0.75
O
ATOM   1346  CB  VAL A  88       3.633   6.453  35.031  1.00  1.08
C
ATOM   1347  CG1 VAL A  88       3.505   6.304  36.533  1.00  1.08
C
ATOM   1348  CG2 VAL A  88       3.225   7.839  34.614  1.00  1.08
C
ATOM   1349  H   VAL A  88       4.390   6.033  32.603  1.00  0.82
H
ATOM   1350  HA  VAL A  88       5.697   7.025  34.972  1.00  0.86
H
ATOM   1351  HB  VAL A  88       2.985   5.715  34.567  1.00  1.30
H
ATOM   1352 1HG1 VAL A  88       2.469   6.466  36.824  1.00  1.30
H
ATOM   1353 2HG1 VAL A  88       3.806   5.299  36.826  1.00  1.30
H
ATOM   1354 3HG1 VAL A  88       4.144   7.034  37.029  1.00  1.30
H
ATOM   1355 1HG2 VAL A  88       2.208   8.009  34.924  1.00  1.30
H
ATOM   1356 2HG2 VAL A  88       3.872   8.567  35.094  1.00  1.30
H
ATOM   1357 3HG2 VAL A  88       3.305   7.938  33.532  1.00  1.30
H
ATOM   1358  N   GLU A  89       5.062   3.806  34.761  1.00  0.62
N
ATOM   1359  CA  GLU A  89       5.356   2.498  35.320  1.00  0.60
C
ATOM   1360  C   GLU A  89       6.853   2.141  35.252  1.00  0.52
C
ATOM   1361  O   GLU A  89       7.348   1.425  36.124  1.00  0.57
O
ATOM   1362  CB  GLU A  89       4.494   1.452  34.608  1.00  0.90
C
ATOM   1363  CG  GLU A  89       2.994   1.574  34.890  1.00  0.90
C
ATOM   1364  CD  GLU A  89       2.597   1.277  36.312  1.00  0.90
C
ATOM   1365  OE1 GLU A  89       2.856   0.194  36.769  1.00  0.90
O
ATOM   1366  OE2 GLU A  89       2.005   2.119  36.950  1.00  0.90
O
ATOM   1367  H   GLU A  89       4.436   3.859  33.968  1.00  0.74
H
ATOM   1368  HA  GLU A  89       5.068   2.510  36.371  1.00  0.72
H
ATOM   1369 1HB  GLU A  89       4.630   1.542  33.531  1.00  1.08
H
ATOM   1370 2HB  GLU A  89       4.813   0.451  34.899  1.00  1.08
H
ATOM   1371 1HG  GLU A  89       2.679   2.588  34.658  1.00  1.08
H
ATOM   1372 2HG  GLU A  89       2.458   0.900  34.222  1.00  1.08
H
```

```
ATOM   1373  N   VAL A  90       7.591   2.671  34.258  1.00  0.58
N
ATOM   1374  CA  VAL A  90       9.042   2.434  34.200  1.00  0.71
C
ATOM   1375  C   VAL A  90       9.798   3.297  35.222  1.00  0.71
C
ATOM   1376  O   VAL A  90      10.992   3.092  35.431  1.00  1.62
O
ATOM   1377  CB  VAL A  90       9.650   2.725  32.801  1.00  1.06
C
ATOM   1378  CG1 VAL A  90       8.995   1.869  31.732  1.00  1.06
C
ATOM   1379  CG2 VAL A  90       9.520   4.185  32.469  1.00  1.06
C
ATOM   1380  H   VAL A  90       7.143   3.219  33.536  1.00  0.70
H
ATOM   1381  HA  VAL A  90       9.224   1.385  34.436  1.00  0.85
H
ATOM   1382  HB  VAL A  90      10.707   2.468  32.822  1.00  1.28
H
ATOM   1383 1HG1 VAL A  90       9.458   2.083  30.769  1.00  1.28
H
ATOM   1384 2HG1 VAL A  90       9.128   0.817  31.970  1.00  1.28
H
ATOM   1385 3HG1 VAL A  90       7.938   2.090  31.675  1.00  1.28
H
ATOM   1386 1HG2 VAL A  90       9.970   4.383  31.498  1.00  1.28
H
ATOM   1387 2HG2 VAL A  90       8.468   4.423  32.440  1.00  1.28
H
ATOM   1388 3HG2 VAL A  90      10.017   4.784  33.231  1.00  1.28
H
ATOM   1389  N   GLY A  91       9.113   4.251  35.862  1.00  0.50
N
ATOM   1390  CA  GLY A  91       9.738   5.088  36.871  1.00  0.64
C
ATOM   1391  C   GLY A  91      10.338   6.397  36.360  1.00  0.63
C
ATOM   1392  O   GLY A  91      11.319   6.877  36.930  1.00  1.22
O
ATOM   1393  H   GLY A  91       8.133   4.397  35.683  1.00  0.60
H
ATOM   1394 1HA  GLY A  91       8.996   5.317  37.638  1.00  0.77
H
ATOM   1395 2HA  GLY A  91      10.518   4.513  37.369  1.00  0.77
H
ATOM   1396  N   ASP A  92       9.762   7.000  35.320  1.00  0.76
N
ATOM   1397  CA  ASP A  92      10.307   8.279  34.871  1.00  1.18
C
ATOM   1398  C   ASP A  92       9.272   9.383  34.997  1.00  0.84
C
ATOM   1399  O   ASP A  92       9.589  10.493  35.422  1.00  0.79
O
ATOM   1400  CB  ASP A  92      10.810   8.222  33.442  1.00  1.77
C
ATOM   1401  CG  ASP A  92      11.614   9.473  33.099  1.00  1.77
C
```

ATOM 1402 OD1 ASP A 92 12.642 9.671 33.702 1.00 1.77
O
ATOM 1403 OD2 ASP A 92 11.185 10.234 32.265 1.00 1.77
O
ATOM 1404 H ASP A 92 8.962 6.602 34.842 1.00 0.91
H
ATOM 1405 HA ASP A 92 11.149 8.542 35.511 1.00 1.42
H
ATOM 1406 1HB ASP A 92 11.439 7.342 33.305 1.00 2.12
H
ATOM 1407 2HB ASP A 92 9.965 8.141 32.760 1.00 2.12
H
ATOM 1408 N ILE A 93 8.036 9.081 34.621 1.00 0.88
N
ATOM 1409 CA ILE A 93 6.962 10.056 34.719 1.00 0.93
C
ATOM 1410 C ILE A 93 6.222 9.873 36.042 1.00 0.96
C
ATOM 1411 O ILE A 93 5.962 8.748 36.460 1.00 0.90
O
ATOM 1412 CB ILE A 93 5.999 9.922 33.523 1.00 1.40
C
ATOM 1413 CG1 ILE A 93 6.782 10.062 32.213 1.00 1.40
C
ATOM 1414 CG2 ILE A 93 4.888 10.965 33.597 1.00 1.40
C
ATOM 1415 CD1 ILE A 93 7.514 11.375 32.068 1.00 1.40
C
ATOM 1416 H ILE A 93 7.829 8.158 34.260 1.00 1.06
H
ATOM 1417 HA ILE A 93 7.393 11.056 34.702 1.00 1.12
H
ATOM 1418 HB ILE A 93 5.561 8.928 33.525 1.00 1.67
H
ATOM 1419 1HG1 ILE A 93 7.514 9.257 32.154 1.00 1.67
H
ATOM 1420 2HG1 ILE A 93 6.093 9.961 31.376 1.00 1.67
H
ATOM 1421 1HG2 ILE A 93 4.220 10.845 32.745 1.00 1.67
H
ATOM 1422 2HG2 ILE A 93 4.324 10.834 34.519 1.00 1.67
H
ATOM 1423 3HG2 ILE A 93 5.325 11.964 33.578 1.00 1.67
H
ATOM 1424 1HD1 ILE A 93 8.044 11.393 31.116 1.00 1.67
H
ATOM 1425 2HD1 ILE A 93 6.798 12.196 32.101 1.00 1.67
H
ATOM 1426 3HD1 ILE A 93 8.229 11.484 32.883 1.00 1.67
H
ATOM 1427 N SER A 94 5.926 10.967 36.734 1.00 1.18
N
ATOM 1428 CA SER A 94 5.189 10.848 37.986 1.00 1.38
C
ATOM 1429 C SER A 94 3.769 10.388 37.703 1.00 1.30
C
ATOM 1430 O SER A 94 3.121 10.899 36.795 1.00 1.33
O

```
ATOM   1431  CB  SER A  94       5.157  12.164  38.732  1.00  2.07
C
ATOM   1432  OG  SER A  94       4.375  12.059  39.890  1.00  2.07
O
ATOM   1433  H   SER A  94       6.178  11.881  36.380  1.00  1.42
H
ATOM   1434  HA  SER A  94       5.677  10.099  38.611  1.00  1.66
H
ATOM   1435 1HB  SER A  94       6.172  12.460  38.999  1.00  2.48
H
ATOM   1436 2HB  SER A  94       4.751  12.940  38.084  1.00  2.48
H
ATOM   1437  HG  SER A  94       4.309  12.949  40.247  1.00  2.48
H
ATOM   1438  N   GLU A  95       3.282   9.434  38.489  1.00  1.37
N
ATOM   1439  CA  GLU A  95       1.935   8.885  38.316  1.00  1.63
C
ATOM   1440  C   GLU A  95       0.858   9.953  38.288  1.00  1.64
C
ATOM   1441  O   GLU A  95      -0.069   9.884  37.480  1.00  1.87
O
ATOM   1442  CB  GLU A  95       1.635   7.875  39.419  1.00  2.44
C
ATOM   1443  CG  GLU A  95       0.292   7.172  39.283  1.00  2.44
C
ATOM   1444  CD  GLU A  95       0.076   6.134  40.350  1.00  2.44
C
ATOM   1445  OE1 GLU A  95       0.946   5.964  41.169  1.00  2.44
O
ATOM   1446  OE2 GLU A  95      -0.958   5.508  40.346  1.00  2.44
O
ATOM   1447  H   GLU A  95       3.868   9.067  39.226  1.00  1.64
H
ATOM   1448  HA  GLU A  95       1.898   8.375  37.358  1.00  1.96
H
ATOM   1449 1HB  GLU A  95       2.413   7.111  39.433  1.00  2.93
H
ATOM   1450 2HB  GLU A  95       1.653   8.376  40.386  1.00  2.93
H
ATOM   1451 1HG  GLU A  95      -0.503   7.914  39.346  1.00  2.93
H
ATOM   1452 2HG  GLU A  95       0.236   6.701  38.302  1.00  2.93
H
ATOM   1453  N   SER A  96       0.980  10.947  39.167  1.00  1.53
N
ATOM   1454  CA  SER A  96       0.006  12.027  39.253  1.00  1.78
C
ATOM   1455  C   SER A  96      -0.099  12.848  37.966  1.00  1.75
C
ATOM   1456  O   SER A  96      -1.140  13.450  37.702  1.00  2.49
O
ATOM   1457  CB  SER A  96       0.359  12.947  40.404  1.00  2.67
C
ATOM   1458  OG  SER A  96       1.556  13.632  40.159  1.00  2.67
O
ATOM   1459  H   SER A  96       1.768  10.950  39.799  1.00  1.84
H
```

```
ATOM   1460  HA  SER A  96      -0.972  11.584  39.449  1.00  2.14
H
ATOM   1461 1HB  SER A  96      -0.448  13.663  40.557  1.00  3.20
H
ATOM   1462 2HB  SER A  96       0.453  12.363  41.319  1.00  3.20
H
ATOM   1463  HG  SER A  96       1.428  14.109  39.334  1.00  3.20
H
ATOM   1464  N   ASP A  97       0.958  12.848  37.147  1.00  1.41
N
ATOM   1465  CA  ASP A  97       0.960  13.603  35.904  1.00  1.47
C
ATOM   1466  C   ASP A  97      -0.012  13.023  34.890  1.00  1.43
C
ATOM   1467  O   ASP A  97      -0.451  13.731  33.987  1.00  1.24
O
ATOM   1468  CB  ASP A  97       2.361  13.645  35.292  1.00  2.21
C
ATOM   1469  CG  ASP A  97       3.328  14.498  36.097  1.00  2.21
C
ATOM   1470  OD1 ASP A  97       2.875  15.270  36.910  1.00  2.21
O
ATOM   1471  OD2 ASP A  97       4.512  14.369  35.894  1.00  2.21
O
ATOM   1472  H   ASP A  97       1.782  12.309  37.371  1.00  1.69
H
ATOM   1473  HA  ASP A  97       0.651  14.624  36.124  1.00  1.76
H
ATOM   1474 1HB  ASP A  97       2.760  12.636  35.210  1.00  2.65
H
ATOM   1475 2HB  ASP A  97       2.301  14.052  34.283  1.00  2.65
H
ATOM   1476  N   LEU A  98      -0.382  11.749  35.050  1.00  2.03
N
ATOM   1477  CA  LEU A  98      -1.305  11.103  34.128  1.00  2.12
C
ATOM   1478  C   LEU A  98      -2.680  11.719  34.156  1.00  2.70
C
ATOM   1479  O   LEU A  98      -3.423  11.618  33.183  1.00  4.40
O
ATOM   1480  CB  LEU A  98      -1.450   9.623  34.456  1.00  3.18
C
ATOM   1481  CG  LEU A  98      -0.214   8.784  34.210  1.00  3.18
C
ATOM   1482  CD1 LEU A  98      -0.464   7.375  34.717  1.00  3.18
C
ATOM   1483  CD2 LEU A  98       0.109   8.785  32.725  1.00  3.18
C
ATOM   1484  H   LEU A  98      -0.029  11.202  35.825  1.00  2.44
H
ATOM   1485  HA  LEU A  98      -0.905  11.204  33.119  1.00  2.54
H
ATOM   1486 1HB  LEU A  98      -1.718   9.522  35.506  1.00  3.82
H
ATOM   1487 2HB  LEU A  98      -2.259   9.210  33.854  1.00  3.82
H
ATOM   1488  HG  LEU A  98       0.619   9.209  34.765  1.00  3.82
H
```

```
ATOM   1489 1HD1 LEU A  98       0.419   6.760  34.555  1.00  3.82
H
ATOM   1490 2HD1 LEU A  98      -0.694   7.406  35.783  1.00  3.82
H
ATOM   1491 3HD1 LEU A  98      -1.306   6.948  34.178  1.00  3.82
H
ATOM   1492 1HD2 LEU A  98       1.001   8.187  32.550  1.00  3.82
H
ATOM   1493 2HD2 LEU A  98      -0.728   8.362  32.170  1.00  3.82
H
ATOM   1494 3HD2 LEU A  98       0.285   9.808  32.392  1.00  3.82
H
ATOM   1495  N   LEU A  99      -3.035  12.365  35.263  1.00  2.64
N
ATOM   1496  CA  LEU A  99      -4.347  12.962  35.373  1.00  3.10
C
ATOM   1497  C   LEU A  99      -4.386  14.354  34.752  1.00  2.87
C
ATOM   1498  O   LEU A  99      -5.438  14.995  34.733  1.00  3.58
O
ATOM   1499  CB  LEU A  99      -4.768  13.020  36.839  1.00  4.65
C
ATOM   1500  CG  LEU A  99      -4.937  11.653  37.510  1.00  4.65
C
ATOM   1501  CD1 LEU A  99      -5.256  11.851  38.984  1.00  4.65
C
ATOM   1502  CD2 LEU A  99      -6.040  10.879  36.798  1.00  4.65
C
ATOM   1503  H   LEU A  99      -2.394  12.462  36.044  1.00  3.17
H
ATOM   1504  HA  LEU A  99      -5.056  12.333  34.838  1.00  3.72
H
ATOM   1505 1HB  LEU A  99      -4.016  13.577  37.397  1.00  5.58
H
ATOM   1506 2HB  LEU A  99      -5.718  13.550  36.912  1.00  5.58
H
ATOM   1507  HG  LEU A  99      -4.003  11.095  37.442  1.00  5.58
H
ATOM   1508 1HD1 LEU A  99      -5.367  10.880  39.467  1.00  5.58
H
ATOM   1509 2HD1 LEU A  99      -4.446  12.402  39.461  1.00  5.58
H
ATOM   1510 3HD1 LEU A  99      -6.185  12.412  39.083  1.00  5.58
H
ATOM   1511 1HD2 LEU A  99      -6.159   9.903  37.268  1.00  5.58
H
ATOM   1512 2HD2 LEU A  99      -6.977  11.433  36.867  1.00  5.58
H
ATOM   1513 3HD2 LEU A  99      -5.774  10.747  35.749  1.00  5.58
H
ATOM   1514  N   ASN A 100      -3.242  14.819  34.240  1.00  2.31
N
ATOM   1515  CA  ASN A 100      -3.151  16.113  33.593  1.00  2.33
C
ATOM   1516  C   ASN A 100      -3.156  15.971  32.063  1.00  1.83
C
ATOM   1517  O   ASN A 100      -2.741  16.883  31.344  1.00  2.27
O
```

```
ATOM   1518  CB  ASN A 100      -1.922  16.848  34.075  1.00  3.50
C
ATOM   1519  CG  ASN A 100      -1.999  17.205  35.540  1.00  3.50
C
ATOM   1520  OD1 ASN A 100      -3.045  17.611  36.069  1.00  3.50
O
ATOM   1521  ND2 ASN A 100      -0.888  17.060  36.216  1.00  3.50
N
ATOM   1522  H   ASN A 100      -2.393  14.275  34.280  1.00  2.77
H
ATOM   1523  HA  ASN A 100      -4.030  16.690  33.863  1.00  2.80
H
ATOM   1524 1HB  ASN A 100      -1.037  16.235  33.908  1.00  4.19
H
ATOM   1525 2HB  ASN A 100      -1.802  17.765  33.502  1.00  4.19
H
ATOM   1526 1HD2 ASN A 100      -0.860  17.277  37.192  1.00  4.19
H
ATOM   1527 2HD2 ASN A 100      -0.062  16.730  35.757  1.00  4.19
H
ATOM   1528  N   LEU A 101      -3.616  14.820  31.586  1.00  1.90
N
ATOM   1529  CA  LEU A 101      -3.700  14.514  30.174  1.00  1.56
C
ATOM   1530  C   LEU A 101      -4.535  15.557  29.426  1.00  2.19
C
ATOM   1531  O   LEU A 101      -5.671  15.857  29.803  1.00  3.03
O
ATOM   1532  CB  LEU A 101      -4.294  13.104  30.040  1.00  2.34
C
ATOM   1533  CG  LEU A 101      -4.502  12.541  28.639  1.00  2.34
C
ATOM   1534  CD1 LEU A 101      -3.173  12.315  27.945  1.00  2.34
C
ATOM   1535  CD2 LEU A 101      -5.277  11.250  28.758  1.00  2.34
C
ATOM   1536  H   LEU A 101      -3.935  14.111  32.229  1.00  2.28
H
ATOM   1537  HA  LEU A 101      -2.693  14.516  29.758  1.00  1.87
H
ATOM   1538 1HB  LEU A 101      -3.633  12.415  30.565  1.00  2.81
H
ATOM   1539 2HB  LEU A 101      -5.257  13.088  30.548  1.00  2.81
H
ATOM   1540  HG  LEU A 101      -5.082  13.246  28.058  1.00  2.81
H
ATOM   1541 1HD1 LEU A 101      -3.345  11.915  26.945  1.00  2.81
H
ATOM   1542 2HD1 LEU A 101      -2.640  13.258  27.870  1.00  2.81
H
ATOM   1543 3HD1 LEU A 101      -2.583  11.607  28.523  1.00  2.81
H
ATOM   1544 1HD2 LEU A 101      -5.470  10.854  27.768  1.00  2.81
H
ATOM   1545 2HD2 LEU A 101      -4.699  10.530  29.334  1.00  2.81
H
ATOM   1546 3HD2 LEU A 101      -6.226  11.445  29.256  1.00  2.81
H
```

```
ATOM   1547  N   ARG A 102      -3.941  16.118  28.369  1.00  2.39
N
ATOM   1548  CA  ARG A 102      -4.552  17.137  27.503  1.00  3.37
C
ATOM   1549  C   ARG A 102      -4.776  18.489  28.162  1.00  3.65
C
ATOM   1550  O   ARG A 102      -5.498  19.323  27.615  1.00  9.35
O
ATOM   1551  CB  ARG A 102      -5.856  16.644  26.940  1.00  5.05
C
ATOM   1552  CG  ARG A 102      -5.638  15.429  26.109  1.00  5.05
C
ATOM   1553  CD  ARG A 102      -6.840  14.926  25.457  1.00  5.05
C
ATOM   1554  NE  ARG A 102      -6.512  13.652  24.899  1.00  5.05
N
ATOM   1555  CZ  ARG A 102      -6.730  12.480  25.506  1.00  5.05
C
ATOM   1556  NH1 ARG A 102      -7.371  12.477  26.642  1.00  5.05
N
ATOM   1557  NH2 ARG A 102      -6.309  11.346  24.973  1.00  5.05
N
ATOM   1558  H   ARG A 102      -3.006  15.809  28.134  1.00  2.87
H
ATOM   1559  HA  ARG A 102      -3.877  17.292  26.660  1.00  4.04
H
ATOM   1560 1HB  ARG A 102      -6.568  16.411  27.727  1.00  6.07
H
ATOM   1561 2HB  ARG A 102      -6.300  17.411  26.308  1.00  6.07
H
ATOM   1562 1HG  ARG A 102      -4.923  15.675  25.324  1.00  6.07
H
ATOM   1563 2HG  ARG A 102      -5.224  14.637  26.719  1.00  6.07
H
ATOM   1564 1HD  ARG A 102      -7.650  14.799  26.175  1.00  6.07
H
ATOM   1565 2HD  ARG A 102      -7.148  15.593  24.653  1.00  6.07
H
ATOM   1566  HE  ARG A 102      -5.999  13.650  24.044  1.00  6.07
H
ATOM   1567 1HH1 ARG A 102      -7.696  13.345  27.042  1.00  6.07
H
ATOM   1568 2HH1 ARG A 102      -7.560  11.607  27.117  1.00  6.07
H
ATOM   1569 1HH2 ARG A 102      -5.804  11.353  24.102  1.00  6.07
H
ATOM   1570 2HH2 ARG A 102      -6.482  10.473  25.449  1.00  6.07
H
ATOM   1571  N   LYS A 103      -4.171  18.722  29.323  1.00  2.35
N
ATOM   1572  CA  LYS A 103      -4.303  20.028  29.940  1.00  2.61
C
ATOM   1573  C   LYS A 103      -3.260  20.982  29.393  1.00  2.93
C
ATOM   1574  O   LYS A 103      -2.140  20.582  29.074  1.00  5.60
O
ATOM   1575  CB  LYS A 103      -4.161  19.956  31.456  1.00  3.92
C
```

```
ATOM   1576  CG  LYS A 103      -5.267  19.207  32.175  1.00  3.92
C
ATOM   1577  CD  LYS A 103      -5.050  19.285  33.673  1.00  3.92
C
ATOM   1578  CE  LYS A 103      -6.086  18.491  34.446  1.00  3.92
C
ATOM   1579  NZ  LYS A 103      -5.778  18.493  35.898  1.00  3.92
N
ATOM   1580  H   LYS A 103      -3.610  18.015  29.782  1.00  2.82
H
ATOM   1581  HA  LYS A 103      -5.287  20.431  29.701  1.00  3.13
H
ATOM   1582 1HB  LYS A 103      -3.219  19.469  31.708  1.00  4.70
H
ATOM   1583 2HB  LYS A 103      -4.127  20.964  31.867  1.00  4.70
H
ATOM   1584 1HG  LYS A 103      -6.233  19.649  31.928  1.00  4.70
H
ATOM   1585 2HG  LYS A 103      -5.271  18.163  31.863  1.00  4.70
H
ATOM   1586 1HD  LYS A 103      -4.056  18.909  33.918  1.00  4.70
H
ATOM   1587 2HD  LYS A 103      -5.106  20.327  33.988  1.00  4.70
H
ATOM   1588 1HE  LYS A 103      -7.073  18.923  34.290  1.00  4.70
H
ATOM   1589 2HE  LYS A 103      -6.091  17.462  34.087  1.00  4.70
H
ATOM   1590 1HZ  LYS A 103      -6.467  17.949  36.397  1.00  4.70
H
ATOM   1591 2HZ  LYS A 103      -4.857  18.087  36.028  1.00  4.70
H
ATOM   1592 3HZ  LYS A 103      -5.779  19.441  36.245  1.00  4.70
H
ATOM   1593  N   LEU A 104      -3.640  22.250  29.312  1.00  3.54
N
ATOM   1594  CA  LEU A 104      -2.788  23.328  28.821  1.00  5.78
C
ATOM   1595  C   LEU A 104      -1.425  23.397  29.505  1.00  7.11
C
ATOM   1596  O   LEU A 104      -0.424  23.737  28.874  1.00 11.19
O
ATOM   1597  CB  LEU A 104      -3.500  24.672  29.007  1.00  8.67
C
ATOM   1598  CG  LEU A 104      -2.755  25.893  28.462  1.00  8.67
C
ATOM   1599  CD1 LEU A 104      -2.576  25.735  26.957  1.00  8.67
C
ATOM   1600  CD2 LEU A 104      -3.539  27.154  28.792  1.00  8.67
C
ATOM   1601  H   LEU A 104      -4.580  22.485  29.598  1.00  4.25
H
ATOM   1602  HA  LEU A 104      -2.624  23.166  27.755  1.00  6.94
H
ATOM   1603 1HB  LEU A 104      -4.467  24.626  28.509  1.00 10.40
H
ATOM   1604 2HB  LEU A 104      -3.671  24.834  30.071  1.00 10.40
H
```

```
ATOM   1605  HG  LEU A 104      -1.765  25.952  28.917  1.00 10.40
H
ATOM   1606 1HD1 LEU A 104      -2.038  26.595  26.564  1.00 10.40
H
ATOM   1607 2HD1 LEU A 104      -2.010  24.826  26.751  1.00 10.40
H
ATOM   1608 3HD1 LEU A 104      -3.554  25.668  26.482  1.00 10.40
H
ATOM   1609 1HD2 LEU A 104      -3.011  28.025  28.406  1.00 10.40
H
ATOM   1610 2HD2 LEU A 104      -4.527  27.095  28.334  1.00 10.40
H
ATOM   1611 3HD2 LEU A 104      -3.647  27.242  29.873  1.00 10.40
H
ATOM   1612  N   HIS A 105      -1.387  23.096  30.799  1.00  6.58
N
ATOM   1613  CA  HIS A 105      -0.151  23.203  31.556  1.00  9.72
C
ATOM   1614  C   HIS A 105       0.593  21.875  31.714  1.00  6.52
C
ATOM   1615  O   HIS A 105       1.443  21.741  32.596  1.00  6.71
O
ATOM   1616  CB  HIS A 105      -0.460  23.799  32.924  1.00 14.58
C
ATOM   1617  CG  HIS A 105      -1.017  25.184  32.822  1.00 14.58
C
ATOM   1618  ND1 HIS A 105      -0.250  26.270  32.458  1.00 14.58
N
ATOM   1619  CD2 HIS A 105      -2.270  25.658  33.022  1.00 14.58
C
ATOM   1620  CE1 HIS A 105      -1.007  27.354  32.442  1.00 14.58
C
ATOM   1621  NE2 HIS A 105      -2.235  27.009  32.782  1.00 14.58
N
ATOM   1622  H   HIS A 105      -2.231  22.810  31.273  1.00  7.90
H
ATOM   1623  HA  HIS A 105       0.521  23.889  31.040  1.00 11.66
H
ATOM   1624 1HB  HIS A 105      -1.182  23.171  33.446  1.00 17.50
H
ATOM   1625 2HB  HIS A 105       0.447  23.833  33.525  1.00 17.50
H
ATOM   1626  HD2 HIS A 105      -3.143  25.076  33.318  1.00 17.50
H
ATOM   1627  HE1 HIS A 105      -0.673  28.361  32.192  1.00 17.50
H
ATOM   1628  HE2 HIS A 105      -3.026  27.633  32.854  1.00 17.50
H
ATOM   1629  N   SER A 106       0.299  20.911  30.845  1.00  5.11
N
ATOM   1630  CA  SER A 106       0.973  19.622  30.854  1.00  4.10
C
ATOM   1631  C   SER A 106       1.639  19.345  29.523  1.00  3.74
C
ATOM   1632  O   SER A 106       1.285  19.934  28.501  1.00 10.51
O
ATOM   1633  CB  SER A 106       0.012  18.501  31.155  1.00  6.15
C
```

ATOM 1634 OG SER A 106 0.669 17.260 31.105 1.00 6.15
O
ATOM 1635 H SER A 106 -0.425 21.051 30.151 1.00 6.13
H
ATOM 1636 HA SER A 106 1.745 19.639 31.624 1.00 4.92
H
ATOM 1637 1HB SER A 106 -0.417 18.652 32.143 1.00 7.38
H
ATOM 1638 2HB SER A 106 -0.805 18.517 30.435 1.00 7.38
H
ATOM 1639 HG SER A 106 0.007 16.607 31.350 1.00 7.38
H
ATOM 1640 N ASP A 107 2.614 18.452 29.543 1.00 1.22
N
ATOM 1641 CA ASP A 107 3.307 18.041 28.333 1.00 0.87
C
ATOM 1642 C ASP A 107 2.656 16.783 27.744 1.00 0.70
C
ATOM 1643 O ASP A 107 2.930 16.403 26.604 1.00 0.78
O
ATOM 1644 CB ASP A 107 4.788 17.787 28.627 1.00 1.30
C
ATOM 1645 CG ASP A 107 5.564 19.041 29.022 1.00 1.30
C
ATOM 1646 OD1 ASP A 107 5.535 20.002 28.286 1.00 1.30
O
ATOM 1647 OD2 ASP A 107 6.176 19.026 30.065 1.00 1.30
O
ATOM 1648 H ASP A 107 2.867 18.027 30.426 1.00 1.46
H
ATOM 1649 HA ASP A 107 3.227 18.842 27.600 1.00 1.04
H
ATOM 1650 1HB ASP A 107 4.879 17.055 29.429 1.00 1.57
H
ATOM 1651 2HB ASP A 107 5.260 17.363 27.741 1.00 1.57
H
ATOM 1652 N LEU A 108 1.782 16.134 28.523 1.00 1.00
N
ATOM 1653 CA LEU A 108 1.149 14.895 28.077 1.00 1.28
C
ATOM 1654 C LEU A 108 -0.062 15.187 27.199 1.00 1.18
C
ATOM 1655 O LEU A 108 -1.206 15.134 27.649 1.00 1.89
O
ATOM 1656 CB LEU A 108 0.739 14.034 29.275 1.00 1.92
C
ATOM 1657 CG LEU A 108 1.879 13.598 30.209 1.00 1.92
C
ATOM 1658 CD1 LEU A 108 1.306 12.711 31.306 1.00 1.92
C
ATOM 1659 CD2 LEU A 108 2.963 12.886 29.416 1.00 1.92
C
ATOM 1660 H LEU A 108 1.549 16.495 29.441 1.00 1.20
H
ATOM 1661 HA LEU A 108 1.872 14.335 27.484 1.00 1.54
H
ATOM 1662 1HB LEU A 108 0.029 14.603 29.874 1.00 2.30
H

```
ATOM   1663 2HB  LEU A 108       0.236  13.142  28.917  1.00  2.30
H
ATOM   1664  HG  LEU A 108       2.309  14.479  30.687  1.00  2.30
H
ATOM   1665 1HD1 LEU A 108       2.101  12.414  31.989  1.00  2.30
H
ATOM   1666 2HD1 LEU A 108       0.544  13.262  31.850  1.00  2.30
H
ATOM   1667 3HD1 LEU A 108       0.862  11.822  30.858  1.00  2.30
H
ATOM   1668 1HD2 LEU A 108       3.771  12.590  30.086  1.00  2.30
H
ATOM   1669 2HD2 LEU A 108       2.548  12.005  28.940  1.00  2.30
H
ATOM   1670 3HD2 LEU A 108       3.354  13.559  28.653  1.00  2.30
H
ATOM   1671  N   GLU A 109       0.236  15.507  25.946  1.00  0.72
N
ATOM   1672  CA  GLU A 109      -0.727  15.855  24.907  1.00  0.84
C
ATOM   1673  C   GLU A 109      -1.521  14.677  24.320  1.00  0.81
C
ATOM   1674  O   GLU A 109      -1.108  13.522  24.411  1.00  0.85
O
ATOM   1675  CB  GLU A 109       0.015  16.587  23.788  1.00  1.26
C
ATOM   1676  CG  GLU A 109       0.497  17.991  24.181  1.00  1.26
C
ATOM   1677  CD  GLU A 109       1.079  18.795  23.039  1.00  1.26
C
ATOM   1678  OE1 GLU A 109       0.378  19.629  22.502  1.00  1.26
O
ATOM   1679  OE2 GLU A 109       2.224  18.592  22.695  1.00  1.26
O
ATOM   1680  H   GLU A 109       1.222  15.530  25.713  1.00  0.86
H
ATOM   1681  HA  GLU A 109      -1.447  16.547  25.345  1.00  1.01
H
ATOM   1682 1HB  GLU A 109       0.902  16.011  23.523  1.00  1.51
H
ATOM   1683 2HB  GLU A 109      -0.603  16.631  22.900  1.00  1.51
H
ATOM   1684 1HG  GLU A 109      -0.344  18.541  24.602  1.00  1.51
H
ATOM   1685 2HG  GLU A 109       1.250  17.888  24.961  1.00  1.51
H
ATOM   1686  N   ARG A 110      -2.667  14.997  23.694  1.00  1.02
N
ATOM   1687  CA  ARG A 110      -3.549  14.025  23.009  1.00  1.08
C
ATOM   1688  C   ARG A 110      -2.820  12.970  22.190  1.00  0.86
C
ATOM   1689  O   ARG A 110      -3.195  11.798  22.217  1.00  1.14
O
ATOM   1690  CB  ARG A 110      -4.483  14.745  22.052  1.00  1.62
C
ATOM   1691  CG  ARG A 110      -5.340  13.881  21.126  1.00  1.62
C
```

ATOM 1692 CD ARG A 110 -6.484 13.208 21.754 1.00 1.62
C
ATOM 1693 NE ARG A 110 -7.192 12.361 20.827 1.00 1.62
N
ATOM 1694 CZ ARG A 110 -6.932 11.052 20.675 1.00 1.62
C
ATOM 1695 NH1 ARG A 110 -6.012 10.486 21.426 1.00 1.62
N
ATOM 1696 NH2 ARG A 110 -7.601 10.349 19.786 1.00 1.62
N
ATOM 1697 H ARG A 110 -2.939 15.968 23.683 1.00 1.22
H
ATOM 1698 HA ARG A 110 -4.132 13.513 23.769 1.00 1.30
H
ATOM 1699 1HB ARG A 110 -5.160 15.374 22.625 1.00 1.94
H
ATOM 1700 2HB ARG A 110 -3.904 15.390 21.415 1.00 1.94
H
ATOM 1701 1HG ARG A 110 -5.707 14.478 20.298 1.00 1.94
H
ATOM 1702 2HG ARG A 110 -4.702 13.090 20.734 1.00 1.94
H
ATOM 1703 1HD ARG A 110 -6.112 12.569 22.545 1.00 1.94
H
ATOM 1704 2HD ARG A 110 -7.183 13.941 22.156 1.00 1.94
H
ATOM 1705 HE ARG A 110 -7.902 12.785 20.243 1.00 1.94
H
ATOM 1706 1HH1 ARG A 110 -5.502 11.043 22.093 1.00 1.94
H
ATOM 1707 2HH1 ARG A 110 -5.807 9.497 21.352 1.00 1.94
H
ATOM 1708 1HH2 ARG A 110 -8.304 10.796 19.215 1.00 1.94
H
ATOM 1709 2HH2 ARG A 110 -7.400 9.368 19.664 1.00 1.94
H
ATOM 1710 N HIS A 111 -1.785 13.386 21.463 1.00 0.78
N
ATOM 1711 CA HIS A 111 -0.985 12.459 20.679 1.00 0.74
C
ATOM 1712 C HIS A 111 0.460 12.712 21.048 1.00 1.21
C
ATOM 1713 O HIS A 111 0.783 13.825 21.457 1.00 6.58
O
ATOM 1714 CB HIS A 111 -1.149 12.664 19.166 1.00 1.11
C
ATOM 1715 CG HIS A 111 -2.544 12.563 18.671 1.00 1.11
C
ATOM 1716 ND1 HIS A 111 -3.324 13.669 18.395 1.00 1.11
N
ATOM 1717 CD2 HIS A 111 -3.312 11.483 18.424 1.00 1.11
C
ATOM 1718 CE1 HIS A 111 -4.515 13.257 17.975 1.00 1.11
C
ATOM 1719 NE2 HIS A 111 -4.528 11.935 17.998 1.00 1.11
N
ATOM 1720 H HIS A 111 -1.535 14.364 21.471 1.00 0.94
H

```
ATOM   1721  HA  HIS A 111      -1.255  11.439  20.931  1.00  0.89
H
ATOM   1722 1HB  HIS A 111      -0.749  13.632  18.883  1.00  1.33
H
ATOM   1723 2HB  HIS A 111      -0.566  11.919  18.631  1.00  1.33
H
ATOM   1724  HD2 HIS A 111      -3.021  10.445  18.544  1.00  1.33
H
ATOM   1725  HE1 HIS A 111      -5.345  13.899  17.680  1.00  1.33
H
ATOM   1726  HE2 HIS A 111      -5.283  11.309  17.753  1.00  1.33
H
ATOM   1727  N   PRO A 112       1.347  11.718  20.921  1.00  0.68
N
ATOM   1728  CA  PRO A 112       2.759  11.770  21.244  1.00  0.56
C
ATOM   1729  C   PRO A 112       3.424  12.894  20.471  1.00  0.90
C
ATOM   1730  O   PRO A 112       3.328  12.954  19.246  1.00  3.49
O
ATOM   1731  CB  PRO A 112       3.291  10.409  20.764  1.00  0.84
C
ATOM   1732  CG  PRO A 112       2.098   9.530  20.763  1.00  0.84
C
ATOM   1733  CD  PRO A 112       1.011  10.441  20.266  1.00  0.84
C
ATOM   1734  HA  PRO A 112       2.890  11.901  22.328  1.00  0.67
H
ATOM   1735 1HB  PRO A 112       3.747  10.510  19.768  1.00  1.01
H
ATOM   1736 2HB  PRO A 112       4.078  10.054  21.443  1.00  1.01
H
ATOM   1737 1HG  PRO A 112       2.261   8.659  20.111  1.00  1.01
H
ATOM   1738 2HG  PRO A 112       1.901   9.144  21.775  1.00  1.01
H
ATOM   1739 1HD  PRO A 112       1.071  10.551  19.174  1.00  1.01
H
ATOM   1740 2HD  PRO A 112       0.040  10.068  20.583  1.00  1.01
H
ATOM   1741  N   THR A 113       4.108  13.770  21.190  1.00  0.48
N
ATOM   1742  CA  THR A 113       4.854  14.860  20.584  1.00  0.51
C
ATOM   1743  C   THR A 113       6.265  14.877  21.150  1.00  0.51
C
ATOM   1744  O   THR A 113       6.449  14.533  22.318  1.00  0.55
O
ATOM   1745  CB  THR A 113       4.146  16.215  20.836  1.00  0.77
C
ATOM   1746  OG1 THR A 113       3.974  16.416  22.244  1.00  0.77
O
ATOM   1747  CG2 THR A 113       2.812  16.269  20.134  1.00  0.77
C
ATOM   1748  H   THR A 113       4.123  13.669  22.195  1.00  0.58
H
ATOM   1749  HA  THR A 113       4.906  14.686  19.510  1.00  0.61
H
```

ATOM 1750 HB THR A 113 4.758 17.027 20.456 1.00 0.92
H
ATOM 1751 HG1 THR A 113 3.370 17.157 22.398 1.00 0.92
H
ATOM 1752 1HG2 THR A 113 2.358 17.238 20.328 1.00 0.92
H
ATOM 1753 2HG2 THR A 113 2.960 16.140 19.062 1.00 0.92
H
ATOM 1754 3HG2 THR A 113 2.165 15.483 20.507 1.00 0.92
H
ATOM 1755 N PRO A 114 7.250 15.352 20.367 1.00 0.56
N
ATOM 1756 CA PRO A 114 8.697 15.422 20.617 1.00 0.63
C
ATOM 1757 C PRO A 114 9.090 16.236 21.847 1.00 0.50
C
ATOM 1758 O PRO A 114 10.249 16.203 22.262 1.00 0.71
O
ATOM 1759 CB PRO A 114 9.242 16.084 19.345 1.00 0.95
C
ATOM 1760 CG PRO A 114 8.081 16.819 18.786 1.00 0.95
C
ATOM 1761 CD PRO A 114 6.929 15.908 19.038 1.00 0.95
C
ATOM 1762 HA PRO A 114 9.086 14.397 20.707 1.00 0.76
H
ATOM 1763 1HB PRO A 114 10.084 16.745 19.596 1.00 1.13
H
ATOM 1764 2HB PRO A 114 9.630 15.317 18.659 1.00 1.13
H
ATOM 1765 1HG PRO A 114 7.969 17.786 19.297 1.00 1.13
H
ATOM 1766 2HG PRO A 114 8.229 17.041 17.728 1.00 1.13
H
ATOM 1767 1HD PRO A 114 6.004 16.490 19.036 1.00 1.13
H
ATOM 1768 2HD PRO A 114 6.902 15.101 18.291 1.00 1.13
H
ATOM 1769 N ARG A 115 8.135 16.957 22.439 1.00 0.59
N
ATOM 1770 CA ARG A 115 8.384 17.684 23.672 1.00 0.67
C
ATOM 1771 C ARG A 115 8.626 16.706 24.817 1.00 0.55
C
ATOM 1772 O ARG A 115 9.260 17.054 25.815 1.00 0.57
O
ATOM 1773 CB ARG A 115 7.195 18.556 24.028 1.00 1.01
C
ATOM 1774 CG ARG A 115 5.975 17.769 24.487 1.00 1.01
C
ATOM 1775 CD ARG A 115 4.748 18.597 24.483 1.00 1.01
C
ATOM 1776 NE ARG A 115 4.759 19.643 25.487 1.00 1.01
N
ATOM 1777 CZ ARG A 115 4.010 20.756 25.426 1.00 1.01
C
ATOM 1778 NH1 ARG A 115 3.220 20.968 24.399 1.00 1.01
N

```
ATOM   1779  NH2 ARG A 115       4.081  21.630  26.411  1.00  1.01
N
ATOM   1780  H   ARG A 115       7.209  16.993  22.044  1.00  0.71
H
ATOM   1781  HA  ARG A 115       9.268  18.309  23.543  1.00  0.80
H
ATOM   1782 1HB  ARG A 115       7.469  19.245  24.825  1.00  1.21
H
ATOM   1783 2HB  ARG A 115       6.903  19.148  23.162  1.00  1.21
H
ATOM   1784 1HG  ARG A 115       5.820  16.919  23.824  1.00  1.21
H
ATOM   1785 2HG  ARG A 115       6.137  17.409  25.505  1.00  1.21
H
ATOM   1786 1HD  ARG A 115       4.638  19.067  23.506  1.00  1.21
H
ATOM   1787 2HD  ARG A 115       3.886  17.957  24.671  1.00  1.21
H
ATOM   1788  HE  ARG A 115       5.338  19.529  26.311  1.00  1.21
H
ATOM   1789 1HH1 ARG A 115       3.157  20.281  23.656  1.00  1.21
H
ATOM   1790 2HH1 ARG A 115       2.666  21.814  24.361  1.00  1.21
H
ATOM   1791 1HH2 ARG A 115       4.689  21.425  27.199  1.00  1.21
H
ATOM   1792 2HH2 ARG A 115       3.525  22.473  26.387  1.00  1.21
H
ATOM   1793  N   LEU A 116       8.134  15.475  24.661  1.00  0.55
N
ATOM   1794  CA  LEU A 116       8.347  14.450  25.653  1.00  0.45
C
ATOM   1795  C   LEU A 116       9.620  13.682  25.312  1.00  0.46
C
ATOM   1796  O   LEU A 116       9.817  13.323  24.153  1.00  0.48
O
ATOM   1797  CB  LEU A 116       7.149  13.504  25.692  1.00  0.68
C
ATOM   1798  CG  LEU A 116       5.835  14.153  26.124  1.00  0.68
C
ATOM   1799  CD1 LEU A 116       4.697  13.165  25.982  1.00  0.68
C
ATOM   1800  CD2 LEU A 116       5.973  14.598  27.567  1.00  0.68
C
ATOM   1801  H   LEU A 116       7.610  15.232  23.831  1.00  0.66
H
ATOM   1802  HA  LEU A 116       8.429  14.931  26.619  1.00  0.54
H
ATOM   1803 1HB  LEU A 116       7.002  13.097  24.696  1.00  0.81
H
ATOM   1804 2HB  LEU A 116       7.364  12.685  26.378  1.00  0.81
H
ATOM   1805  HG  LEU A 116       5.621  15.012  25.490  1.00  0.81
H
ATOM   1806 1HD1 LEU A 116       3.764  13.638  26.292  1.00  0.81
H
ATOM   1807 2HD1 LEU A 116       4.614  12.850  24.941  1.00  0.81
H
```

```
ATOM   1808 3HD1 LEU A 116       4.889  12.300  26.612  1.00  0.81
H
ATOM   1809 1HD2 LEU A 116       5.043  15.062  27.893  1.00  0.81
H
ATOM   1810 2HD2 LEU A 116       6.184  13.732  28.196  1.00  0.81
H
ATOM   1811 3HD2 LEU A 116       6.789  15.316  27.653  1.00  0.81
H
ATOM   1812  N   PRO A 117      10.475  13.400  26.305  1.00  0.45
N
ATOM   1813  CA  PRO A 117      11.746  12.674  26.242  1.00  0.48
C
ATOM   1814  C   PRO A 117      11.639  11.317  25.566  1.00  0.39
C
ATOM   1815  O   PRO A 117      12.579  10.854  24.924  1.00  0.50
O
ATOM   1816  CB  PRO A 117      12.123  12.518  27.716  1.00  0.72
C
ATOM   1817  CG  PRO A 117      11.536  13.710  28.376  1.00  0.72
C
ATOM   1818  CD  PRO A 117      10.241  13.949  27.659  1.00  0.72
C
ATOM   1819  HA  PRO A 117      12.484  13.300  25.721  1.00  0.58
H
ATOM   1820 1HB  PRO A 117      11.719  11.576  28.113  1.00  0.86
H
ATOM   1821 2HB  PRO A 117      13.217  12.468  27.822  1.00  0.86
H
ATOM   1822 1HG  PRO A 117      11.396  13.518  29.449  1.00  0.86
H
ATOM   1823 2HG  PRO A 117      12.226  14.563  28.293  1.00  0.86
H
ATOM   1824 1HD  PRO A 117       9.409  13.423  28.150  1.00  0.86
H
ATOM   1825 2HD  PRO A 117      10.078  15.035  27.611  1.00  0.86
H
ATOM   1826  N   PHE A 118      10.491  10.678  25.711  1.00  0.41
N
ATOM   1827  CA  PHE A 118      10.292   9.367  25.140  1.00  0.42
C
ATOM   1828  C   PHE A 118       9.659   9.391  23.749  1.00  0.50
C
ATOM   1829  O   PHE A 118       9.375   8.330  23.192  1.00  0.94
O
ATOM   1830  CB  PHE A 118       9.340   8.598  26.003  1.00  0.63
C
ATOM   1831  CG  PHE A 118       9.734   8.180  27.380  1.00  0.63
C
ATOM   1832  CD1 PHE A 118       9.588   9.042  28.452  1.00  0.63
C
ATOM   1833  CD2 PHE A 118      10.164   6.883  27.618  1.00  0.63
C
ATOM   1834  CE1 PHE A 118       9.889   8.622  29.730  1.00  0.63
C
ATOM   1835  CE2 PHE A 118      10.465   6.460  28.897  1.00  0.63
C
ATOM   1836  CZ  PHE A 118      10.331   7.334  29.951  1.00  0.63
C
```

```
ATOM   1837  H   PHE A 118       9.745  11.101  26.242  1.00  0.49
H
ATOM   1838  HA  PHE A 118      11.252   8.859  25.077  1.00  0.50
H
ATOM   1839 1HB  PHE A 118       8.445   9.199  26.102  1.00  0.76
H
ATOM   1840 2HB  PHE A 118       9.075   7.697  25.461  1.00  0.76
H
ATOM   1841  HD1 PHE A 118       9.225  10.056  28.278  1.00  0.76
H
ATOM   1842  HD2 PHE A 118      10.256   6.192  26.780  1.00  0.76
H
ATOM   1843  HE1 PHE A 118       9.770   9.308  30.564  1.00  0.76
H
ATOM   1844  HE2 PHE A 118      10.805   5.440  29.071  1.00  0.76
H
ATOM   1845  HZ  PHE A 118      10.567   6.999  30.958  1.00  0.76
H
ATOM   1846  N   VAL A 119       9.432  10.578  23.182  1.00  0.35
N
ATOM   1847  CA  VAL A 119       8.840  10.653  21.852  1.00  0.34
C
ATOM   1848  C   VAL A 119       9.831  11.134  20.822  1.00  0.26
C
ATOM   1849  O   VAL A 119      10.380  12.231  20.921  1.00  0.34
O
ATOM   1850  CB  VAL A 119       7.631  11.592  21.794  1.00  0.51
C
ATOM   1851  CG1 VAL A 119       7.112  11.626  20.360  1.00  0.51
C
ATOM   1852  CG2 VAL A 119       6.544  11.120  22.730  1.00  0.51
C
ATOM   1853  H   VAL A 119       9.671  11.439  23.653  1.00  0.42
H
ATOM   1854  HA  VAL A 119       8.508   9.655  21.568  1.00  0.41
H
ATOM   1855  HB  VAL A 119       7.945  12.597  22.069  1.00  0.61
H
ATOM   1856 1HG1 VAL A 119       6.258  12.292  20.289  1.00  0.61
H
ATOM   1857 2HG1 VAL A 119       7.899  11.979  19.694  1.00  0.61
H
ATOM   1858 3HG1 VAL A 119       6.807  10.623  20.062  1.00  0.61
H
ATOM   1859 1HG2 VAL A 119       5.693  11.799  22.673  1.00  0.61
H
ATOM   1860 2HG2 VAL A 119       6.231  10.120  22.435  1.00  0.61
H
ATOM   1861 3HG2 VAL A 119       6.924  11.099  23.750  1.00  0.61
H
ATOM   1862  N   ASP A 120      10.033  10.306  19.823  1.00  0.28
N
ATOM   1863  CA  ASP A 120      10.917  10.598  18.720  1.00  0.35
C
ATOM   1864  C   ASP A 120      10.221  11.191  17.507  1.00  0.40
C
ATOM   1865  O   ASP A 120      10.809  11.978  16.763  1.00  1.90
O
```

ATOM 1866 CB ASP A 120 11.623 9.328 18.342 1.00 0.52
C
ATOM 1867 CG ASP A 120 12.617 8.906 19.406 1.00 0.52
C
ATOM 1868 OD1 ASP A 120 13.440 9.725 19.739 1.00 0.52
O
ATOM 1869 OD2 ASP A 120 12.567 7.799 19.885 1.00 0.52
O
ATOM 1870 H ASP A 120 9.546 9.420 19.829 1.00 0.34
H
ATOM 1871 HA ASP A 120 11.660 11.317 19.065 1.00 0.42
H
ATOM 1872 1HB ASP A 120 10.889 8.531 18.225 1.00 0.63
H
ATOM 1873 2HB ASP A 120 12.105 9.459 17.387 1.00 0.63
H
ATOM 1874 N VAL A 121 8.973 10.801 17.310 1.00 0.26
N
ATOM 1875 CA VAL A 121 8.190 11.244 16.173 1.00 0.17
C
ATOM 1876 C VAL A 121 6.731 11.370 16.555 1.00 0.15
C
ATOM 1877 O VAL A 121 6.212 10.553 17.316 1.00 0.15
O
ATOM 1878 CB VAL A 121 8.338 10.233 15.020 1.00 0.26
C
ATOM 1879 CG1 VAL A 121 7.803 8.878 15.469 1.00 0.26
C
ATOM 1880 CG2 VAL A 121 7.592 10.719 13.783 1.00 0.26
C
ATOM 1881 H VAL A 121 8.550 10.161 17.965 1.00 0.31
H
ATOM 1882 HA VAL A 121 8.559 12.218 15.848 1.00 0.20
H
ATOM 1883 HB VAL A 121 9.395 10.115 14.783 1.00 0.31
H
ATOM 1884 1HG1 VAL A 121 7.919 8.152 14.666 1.00 0.31
H
ATOM 1885 2HG1 VAL A 121 8.357 8.542 16.345 1.00 0.31
H
ATOM 1886 3HG1 VAL A 121 6.750 8.972 15.722 1.00 0.31
H
ATOM 1887 1HG2 VAL A 121 7.718 9.995 12.978 1.00 0.31
H
ATOM 1888 2HG2 VAL A 121 6.533 10.825 14.006 1.00 0.31
H
ATOM 1889 3HG2 VAL A 121 7.996 11.682 13.469 1.00 0.31
H
ATOM 1890 N ALA A 122 6.066 12.390 16.033 1.00 0.20
N
ATOM 1891 CA ALA A 122 4.657 12.563 16.307 1.00 0.22
C
ATOM 1892 C ALA A 122 3.866 11.744 15.303 1.00 0.21
C
ATOM 1893 O ALA A 122 4.090 11.843 14.096 1.00 0.24
O
ATOM 1894 CB ALA A 122 4.278 14.030 16.249 1.00 0.33
C

ATOM	1895	H	ALA	A	122	6.528	13.035	15.413	1.00	0.24	H
ATOM	1896	HA	ALA	A	122	4.447	12.174	17.300	1.00	0.26	H
ATOM	1897	1HB	ALA	A	122	3.215	14.140	16.466	1.00	0.40	H
ATOM	1898	2HB	ALA	A	122	4.857	14.584	16.987	1.00	0.40	H
ATOM	1899	3HB	ALA	A	122	4.488	14.420	15.255	1.00	0.40	H
ATOM	1900	N	THR	A	123	2.949	10.923	15.806	1.00	0.21	N
ATOM	1901	CA	THR	A	123	2.158	10.044	14.950	1.00	0.22	C
ATOM	1902	C	THR	A	123	0.659	10.193	15.148	1.00	0.24	C
ATOM	1903	O	THR	A	123	-0.052	9.196	15.253	1.00	0.30	O
ATOM	1904	CB	THR	A	123	2.510	8.579	15.200	1.00	0.33	C
ATOM	1905	OG1	THR	A	123	2.301	8.298	16.585	1.00	0.33	O
ATOM	1906	CG2	THR	A	123	3.953	8.278	14.825	1.00	0.33	C
ATOM	1907	H	THR	A	123	2.809	10.896	16.806	1.00	0.25	H
ATOM	1908	HA	THR	A	123	2.385	10.283	13.911	1.00	0.26	H
ATOM	1909	HB	THR	A	123	1.850	7.948	14.607	1.00	0.40	H
ATOM	1910	HG1	THR	A	123	2.449	7.365	16.748	1.00	0.40	H
ATOM	1911	1HG2	THR	A	123	4.166	7.227	15.019	1.00	0.40	H
ATOM	1912	2HG2	THR	A	123	4.105	8.491	13.767	1.00	0.40	H
ATOM	1913	3HG2	THR	A	123	4.619	8.899	15.421	1.00	0.40	H
ATOM	1914	N	GLY	A	124	0.171	11.422	15.206	1.00	0.25	N
ATOM	1915	CA	GLY	A	124	-1.266	11.627	15.325	1.00	0.29	C
ATOM	1916	C	GLY	A	124	-1.958	11.197	14.036	1.00	0.31	C
ATOM	1917	O	GLY	A	124	-3.045	10.616	14.060	1.00	0.61	O
ATOM	1918	H	GLY	A	124	0.792	12.215	15.147	1.00	0.30	H
ATOM	1919	1HA	GLY	A	124	-1.648	11.039	16.159	1.00	0.35	H
ATOM	1920	2HA	GLY	A	124	-1.477	12.670	15.539	1.00	0.35	H
ATOM	1921	N	SER	A	125	-1.302	11.466	12.907	1.00	0.27	N
ATOM	1922	CA	SER	A	125	-1.810	11.047	11.618	1.00	0.28	C
ATOM	1923	C	SER	A	125	-1.421	9.585	11.424	1.00	0.37	C

ATOM 1924 O SER A 125 -0.273 9.199 11.647 1.00 1.04
O
ATOM 1925 CB SER A 125 -1.258 11.936 10.527 1.00 0.42
C
ATOM 1926 OG SER A 125 -1.704 11.529 9.267 1.00 0.42
O
ATOM 1927 H SER A 125 -0.425 11.963 12.948 1.00 0.32
H
ATOM 1928 HA SER A 125 -2.898 11.123 11.619 1.00 0.34
H
ATOM 1929 1HB SER A 125 -1.567 12.964 10.709 1.00 0.50
H
ATOM 1930 2HB SER A 125 -0.170 11.912 10.553 1.00 0.50
H
ATOM 1931 HG SER A 125 -1.500 12.257 8.670 1.00 0.50
H
ATOM 1932 N LEU A 126 -2.396 8.774 11.048 1.00 0.28
N
ATOM 1933 CA LEU A 126 -2.212 7.334 10.943 1.00 0.27
C
ATOM 1934 C LEU A 126 -1.466 6.856 9.698 1.00 0.27
C
ATOM 1935 O LEU A 126 -1.503 7.488 8.637 1.00 0.55
O
ATOM 1936 CB LEU A 126 -3.586 6.666 11.021 1.00 0.41
C
ATOM 1937 CG LEU A 126 -4.301 6.866 12.360 1.00 0.41
C
ATOM 1938 CD1 LEU A 126 -5.681 6.239 12.305 1.00 0.41
C
ATOM 1939 CD2 LEU A 126 -3.459 6.239 13.460 1.00 0.41
C
ATOM 1940 H LEU A 126 -3.307 9.166 10.857 1.00 0.34
H
ATOM 1941 HA LEU A 126 -1.637 7.014 11.812 1.00 0.32
H
ATOM 1942 1HB LEU A 126 -4.221 7.079 10.239 1.00 0.49
H
ATOM 1943 2HB LEU A 126 -3.481 5.601 10.852 1.00 0.49
H
ATOM 1944 HG LEU A 126 -4.419 7.932 12.557 1.00 0.49
H
ATOM 1945 1HD1 LEU A 126 -6.187 6.387 13.261 1.00 0.49
H
ATOM 1946 2HD1 LEU A 126 -6.263 6.711 11.512 1.00 0.49
H
ATOM 1947 3HD1 LEU A 126 -5.589 5.172 12.106 1.00 0.49
H
ATOM 1948 1HD2 LEU A 126 -3.946 6.376 14.421 1.00 0.49
H
ATOM 1949 2HD2 LEU A 126 -3.344 5.173 13.263 1.00 0.49
H
ATOM 1950 3HD2 LEU A 126 -2.476 6.710 13.480 1.00 0.49
H
ATOM 1951 N GLY A 127 -0.774 5.724 9.867 1.00 0.19
N
ATOM 1952 CA GLY A 127 -0.017 5.065 8.810 1.00 0.32
C

```
ATOM   1953  C   GLY A 127       1.408   5.589   8.606  1.00  0.77
C
ATOM   1954  O   GLY A 127       2.062   5.227   7.627  1.00  3.84
O
ATOM   1955  H   GLY A 127      -0.789   5.274  10.776  1.00  0.23
H
ATOM   1956 1HA  GLY A 127       0.029   4.002   9.038  1.00  0.38
H
ATOM   1957 2HA  GLY A 127      -0.567   5.155   7.875  1.00  0.38
H
ATOM   1958  N   GLN A 128       1.903   6.447   9.497  1.00  0.65
N
ATOM   1959  CA  GLN A 128       3.239   7.011   9.283  1.00  0.80
C
ATOM   1960  C   GLN A 128       4.328   6.486  10.219  1.00  0.37
C
ATOM   1961  O   GLN A 128       5.518   6.593   9.901  1.00  1.08
O
ATOM   1962  CB  GLN A 128       3.196   8.528   9.425  1.00  1.20
C
ATOM   1963  CG  GLN A 128       2.363   9.247   8.387  1.00  1.20
C
ATOM   1964  CD  GLN A 128       2.451  10.740   8.581  1.00  1.20
C
ATOM   1965  OE1 GLN A 128       3.245  11.198   9.407  1.00  1.20
O
ATOM   1966  NE2 GLN A 128       1.665  11.500   7.834  1.00  1.20
N
ATOM   1967  H   GLN A 128       1.357   6.724  10.301  1.00  0.78
H
ATOM   1968  HA  GLN A 128       3.543   6.773   8.264  1.00  0.96
H
ATOM   1969 1HB  GLN A 128       2.799   8.787  10.406  1.00  1.44
H
ATOM   1970 2HB  GLN A 128       4.210   8.923   9.370  1.00  1.44
H
ATOM   1971 1HG  GLN A 128       2.734   9.001   7.393  1.00  1.44
H
ATOM   1972 2HG  GLN A 128       1.321   8.945   8.488  1.00  1.44
H
ATOM   1973 1HE2 GLN A 128       1.692  12.498   7.928  1.00  1.44
H
ATOM   1974 2HE2 GLN A 128       1.041  11.083   7.175  1.00  1.44
H
ATOM   1975  N   GLY A 129       3.924   5.935  11.362  1.00  0.46
N
ATOM   1976  CA  GLY A 129       4.868   5.462  12.368  1.00  0.25
C
ATOM   1977  C   GLY A 129       5.815   4.387  11.859  1.00  0.25
C
ATOM   1978  O   GLY A 129       7.005   4.419  12.170  1.00  0.40
O
ATOM   1979  H   GLY A 129       2.937   5.856  11.554  1.00  0.55
H
ATOM   1980 1HA  GLY A 129       5.450   6.309  12.733  1.00  0.30
H
ATOM   1981 2HA  GLY A 129       4.314   5.077  13.222  1.00  0.30
H
```

```
ATOM   1982  N   LEU A 130       5.285   3.441  11.089  1.00  0.18
N
ATOM   1983  CA  LEU A 130       6.085   2.341  10.565  1.00  0.19
C
ATOM   1984  C   LEU A 130       7.194   2.818   9.636  1.00  0.17
C
ATOM   1985  O   LEU A 130       8.298   2.280   9.674  1.00  0.35
O
ATOM   1986  CB  LEU A 130       5.208   1.337   9.839  1.00  0.29
C
ATOM   1987  CG  LEU A 130       5.962   0.111   9.331  1.00  0.29
C
ATOM   1988  CD1 LEU A 130       6.595  -0.625  10.508  1.00  0.29
C
ATOM   1989  CD2 LEU A 130       5.004  -0.781   8.588  1.00  0.29
C
ATOM   1990  H   LEU A 130       4.299   3.476  10.870  1.00  0.22
H
ATOM   1991  HA  LEU A 130       6.551   1.833  11.408  1.00  0.23
H
ATOM   1992 1HB  LEU A 130       4.426   0.998  10.515  1.00  0.34
H
ATOM   1993 2HB  LEU A 130       4.741   1.825   8.983  1.00  0.34
H
ATOM   1994  HG  LEU A 130       6.764   0.423   8.666  1.00  0.34
H
ATOM   1995 1HD1 LEU A 130       7.138  -1.496  10.142  1.00  0.34
H
ATOM   1996 2HD1 LEU A 130       7.284   0.042  11.024  1.00  0.34
H
ATOM   1997 3HD1 LEU A 130       5.814  -0.947  11.197  1.00  0.34
H
ATOM   1998 1HD2 LEU A 130       5.551  -1.648   8.228  1.00  0.34
H
ATOM   1999 2HD2 LEU A 130       4.209  -1.102   9.259  1.00  0.34
H
ATOM   2000 3HD2 LEU A 130       4.569  -0.243   7.747  1.00  0.34
H
ATOM   2001  N   GLY A 131       6.898   3.802   8.787  1.00  0.13
N
ATOM   2002  CA  GLY A 131       7.907   4.355   7.887  1.00  0.14
C
ATOM   2003  C   GLY A 131       9.055   4.957   8.695  1.00  0.16
C
ATOM   2004  O   GLY A 131      10.228   4.756   8.369  1.00  0.21
O
ATOM   2005  H   GLY A 131       5.966   4.192   8.777  1.00  0.16
H
ATOM   2006 1HA  GLY A 131       8.285   3.571   7.231  1.00  0.17
H
ATOM   2007 2HA  GLY A 131       7.456   5.118   7.255  1.00  0.17
H
ATOM   2008  N   THR A 132       8.702   5.677   9.766  1.00  0.17
N
ATOM   2009  CA  THR A 132       9.700   6.256  10.655  1.00  0.17
C
ATOM   2010  C   THR A 132      10.513   5.146  11.320  1.00  0.13
C
```

```
ATOM   2011  O   THR A 132      11.736   5.256  11.436  1.00  0.19
O
ATOM   2012  CB  THR A 132       9.055   7.138  11.738  1.00  0.26
C
ATOM   2013  OG1 THR A 132       8.337   8.216  11.124  1.00  0.26
O
ATOM   2014  CG2 THR A 132      10.138   7.713  12.633  1.00  0.26
C
ATOM   2015  H   THR A 132       7.716   5.833   9.961  1.00  0.20
H
ATOM   2016  HA  THR A 132      10.381   6.869  10.069  1.00  0.20
H
ATOM   2017  HB  THR A 132       8.365   6.545  12.335  1.00  0.31
H
ATOM   2018  HG1 THR A 132       7.610   7.859  10.606  1.00  0.31
H
ATOM   2019 1HG2 THR A 132       9.688   8.339  13.397  1.00  0.31
H
ATOM   2020 2HG2 THR A 132      10.691   6.903  13.106  1.00  0.31
H
ATOM   2021 3HG2 THR A 132      10.815   8.314  12.032  1.00  0.31
H
ATOM   2022  N   ALA A 133       9.822   4.077  11.738  1.00  0.15
N
ATOM   2023  CA  ALA A 133      10.449   2.912  12.356  1.00  0.16
C
ATOM   2024  C   ALA A 133      11.453   2.281  11.412  1.00  0.16
C
ATOM   2025  O   ALA A 133      12.530   1.874  11.842  1.00  0.18
O
ATOM   2026  CB  ALA A 133       9.400   1.881  12.754  1.00  0.24
C
ATOM   2027  H   ALA A 133       8.817   4.075  11.637  1.00  0.18
H
ATOM   2028  HA  ALA A 133      10.981   3.245  13.246  1.00  0.19
H
ATOM   2029 1HB  ALA A 133       9.887   1.034  13.232  1.00  0.29
H
ATOM   2030 2HB  ALA A 133       8.687   2.322  13.447  1.00  0.29
H
ATOM   2031 3HB  ALA A 133       8.871   1.536  11.871  1.00  0.29
H
ATOM   2032  N   CYS A 134      11.103   2.217  10.125  1.00  0.15
N
ATOM   2033  CA  CYS A 134      11.994   1.684   9.110  1.00  0.15
C
ATOM   2034  C   CYS A 134      13.253   2.530   9.001  1.00  0.14
C
ATOM   2035  O   CYS A 134      14.347   1.985   8.877  1.00  0.14
O
ATOM   2036  CB  CYS A 134      11.301   1.627   7.750  1.00  0.22
C
ATOM   2037  SG  CYS A 134      10.000   0.381   7.618  1.00  0.22
S
ATOM   2038  H   CYS A 134      10.186   2.534   9.845  1.00  0.18
H
ATOM   2039  HA  CYS A 134      12.278   0.672   9.395  1.00  0.18
H
```

```
ATOM   2040 1HB  CYS A 134      10.862   2.594   7.519  1.00  0.27
H
ATOM   2041 2HB  CYS A 134      12.043   1.419   6.988  1.00  0.27
H
ATOM   2042  HG  CYS A 134       9.658   0.669   6.367  1.00  0.27
H
ATOM   2043  N   GLY A 135      13.104   3.858   9.073  1.00  0.13
N
ATOM   2044  CA  GLY A 135      14.257   4.756   9.055  1.00  0.13
C
ATOM   2045  C   GLY A 135      15.174   4.491  10.249  1.00  0.14
C
ATOM   2046  O   GLY A 135      16.397   4.422  10.097  1.00  0.16
O
ATOM   2047  H   GLY A 135      12.169   4.249   9.127  1.00  0.16
H
ATOM   2048 1HA  GLY A 135      14.816   4.611   8.133  1.00  0.16
H
ATOM   2049 2HA  GLY A 135      13.916   5.790   9.071  1.00  0.16
H
ATOM   2050  N   MET A 136      14.566   4.327  11.431  1.00  0.13
N
ATOM   2051  CA  MET A 136      15.297   4.016  12.654  1.00  0.15
C
ATOM   2052  C   MET A 136      16.060   2.711  12.543  1.00  0.15
C
ATOM   2053  O   MET A 136      17.243   2.646  12.889  1.00  0.16
O
ATOM   2054  CB  MET A 136      14.341   3.918  13.842  1.00  0.22
C
ATOM   2055  CG  MET A 136      13.702   5.213  14.298  1.00  0.22
C
ATOM   2056  SD  MET A 136      12.545   4.931  15.643  1.00  0.22
S
ATOM   2057  CE  MET A 136      12.150   6.610  16.100  1.00  0.22
C
ATOM   2058  H   MET A 136      13.559   4.428  11.479  1.00  0.16
H
ATOM   2059  HA  MET A 136      16.021   4.807  12.832  1.00  0.18
H
ATOM   2060 1HB  MET A 136      13.534   3.229  13.603  1.00  0.27
H
ATOM   2061 2HB  MET A 136      14.873   3.502  14.697  1.00  0.27
H
ATOM   2062 1HG  MET A 136      14.466   5.905  14.645  1.00  0.27
H
ATOM   2063 2HG  MET A 136      13.168   5.676  13.470  1.00  0.27
H
ATOM   2064 1HE  MET A 136      11.434   6.603  16.923  1.00  0.27
H
ATOM   2065 2HE  MET A 136      13.059   7.115  16.415  1.00  0.27
H
ATOM   2066 3HE  MET A 136      11.717   7.131  15.245  1.00  0.27
H
ATOM   2067  N   ALA A 137      15.359   1.675  12.084  1.00  0.17
N
ATOM   2068  CA  ALA A 137      15.902   0.337  11.931  1.00  0.18
C
```

ATOM 2069 C ALA A 137 17.017 0.313 10.906 1.00 0.19
C
ATOM 2070 O ALA A 137 18.047 -0.323 11.133 1.00 0.21
O
ATOM 2071 CB ALA A 137 14.792 -0.620 11.541 1.00 0.27
C
ATOM 2072 H ALA A 137 14.390 1.818 11.836 1.00 0.20
H
ATOM 2073 HA ALA A 137 16.320 0.026 12.889 1.00 0.22
H
ATOM 2074 1HB ALA A 137 15.189 -1.629 11.446 1.00 0.32
H
ATOM 2075 2HB ALA A 137 14.016 -0.606 12.305 1.00 0.32
H
ATOM 2076 3HB ALA A 137 14.369 -0.305 10.592 1.00 0.32
H
ATOM 2077 N TYR A 138 16.813 1.022 9.792 1.00 0.20
N
ATOM 2078 CA TYR A 138 17.801 1.133 8.733 1.00 0.21
C
ATOM 2079 C TYR A 138 19.087 1.701 9.295 1.00 0.23
C
ATOM 2080 O TYR A 138 20.164 1.169 9.045 1.00 0.26
O
ATOM 2081 CB TYR A 138 17.300 2.033 7.599 1.00 0.32
C
ATOM 2082 CG TYR A 138 18.290 2.150 6.460 1.00 0.32
C
ATOM 2083 CD1 TYR A 138 18.150 1.346 5.337 1.00 0.32
C
ATOM 2084 CD2 TYR A 138 19.361 3.028 6.559 1.00 0.32
C
ATOM 2085 CE1 TYR A 138 19.073 1.431 4.315 1.00 0.32
C
ATOM 2086 CE2 TYR A 138 20.287 3.105 5.537 1.00 0.32
C
ATOM 2087 CZ TYR A 138 20.144 2.309 4.420 1.00 0.32
C
ATOM 2088 OH TYR A 138 21.066 2.382 3.402 1.00 0.32
O
ATOM 2089 H TYR A 138 15.933 1.498 9.663 1.00 0.24
H
ATOM 2090 HA TYR A 138 18.013 0.141 8.339 1.00 0.25
H
ATOM 2091 1HB TYR A 138 16.366 1.636 7.201 1.00 0.38
H
ATOM 2092 2HB TYR A 138 17.097 3.032 7.981 1.00 0.38
H
ATOM 2093 HD1 TYR A 138 17.315 0.650 5.263 1.00 0.38
H
ATOM 2094 HD2 TYR A 138 19.478 3.652 7.444 1.00 0.38
H
ATOM 2095 HE1 TYR A 138 18.969 0.807 3.432 1.00 0.38
H
ATOM 2096 HE2 TYR A 138 21.130 3.791 5.615 1.00 0.38
H
ATOM 2097 HH TYR A 138 20.799 1.801 2.686 1.00 0.38
H

ATOM	2098	N	THR	A	139	18.971	2.793	10.050	1.00	0.24	N
ATOM	2099	CA	THR	A	139	20.126	3.436	10.654	1.00	0.27	C
ATOM	2100	C	THR	A	139	20.828	2.491	11.636	1.00	0.24	C
ATOM	2101	O	THR	A	139	22.043	2.288	11.563	1.00	0.27	O
ATOM	2102	CB	THR	A	139	19.689	4.729	11.374	1.00	0.41	C
ATOM	2103	OG1	THR	A	139	19.114	5.633	10.422	1.00	0.41	O
ATOM	2104	CG2	THR	A	139	20.864	5.409	12.056	1.00	0.41	C
ATOM	2105	H	THR	A	139	18.056	3.203	10.200	1.00	0.29	H
ATOM	2106	HA	THR	A	139	20.831	3.696	9.865	1.00	0.32	H
ATOM	2107	HB	THR	A	139	18.938	4.484	12.125	1.00	0.49	H
ATOM	2108	HG1	THR	A	139	18.295	5.255	10.081	1.00	0.49	H
ATOM	2109	1HG2	THR	A	139	20.517	6.312	12.557	1.00	0.49	H
ATOM	2110	2HG2	THR	A	139	21.298	4.732	12.791	1.00	0.49	H
ATOM	2111	3HG2	THR	A	139	21.614	5.676	11.319	1.00	0.49	H
ATOM	2112	N	GLY	A	140	20.056	1.859	12.515	1.00	0.23	N
ATOM	2113	CA	GLY	A	140	20.618	0.924	13.482	1.00	0.22	C
ATOM	2114	C	GLY	A	140	21.380	-0.212	12.807	1.00	0.23	C
ATOM	2115	O	GLY	A	140	22.413	-0.656	13.301	1.00	0.29	O
ATOM	2116	H	GLY	A	140	19.062	2.044	12.538	1.00	0.28	H
ATOM	2117	1HA	GLY	A	140	21.284	1.460	14.156	1.00	0.26	H
ATOM	2118	2HA	GLY	A	140	19.815	0.511	14.091	1.00	0.26	H
ATOM	2119	N	LYS	A	141	20.875	-0.684	11.675	1.00	0.27	N
ATOM	2120	CA	LYS	A	141	21.502	-1.779	10.951	1.00	0.32	C
ATOM	2121	C	LYS	A	141	22.676	-1.378	10.050	1.00	0.31	C
ATOM	2122	O	LYS	A	141	23.704	-2.057	10.030	1.00	0.36	O
ATOM	2123	CB	LYS	A	141	20.447	-2.484	10.099	1.00	0.48	C
ATOM	2124	CG	LYS	A	141	20.966	-3.683	9.328	1.00	0.48	C
ATOM	2125	CD	LYS	A	141	19.840	-4.408	8.609	1.00	0.48	C
ATOM	2126	CE	LYS	A	141	20.383	-5.584	7.818	1.00	0.48	C

```
ATOM   2127  NZ  LYS A 141      20.951  -6.621   8.725  1.00  0.48
N
ATOM   2128  H   LYS A 141      20.010  -0.302  11.315  1.00  0.32
H
ATOM   2129  HA  LYS A 141      21.883  -2.488  11.686  1.00  0.38
H
ATOM   2130 1HB  LYS A 141      19.632  -2.826  10.739  1.00  0.58
H
ATOM   2131 2HB  LYS A 141      20.024  -1.779   9.383  1.00  0.58
H
ATOM   2132 1HG  LYS A 141      21.700  -3.352   8.594  1.00  0.58
H
ATOM   2133 2HG  LYS A 141      21.450  -4.375  10.016  1.00  0.58
H
ATOM   2134 1HD  LYS A 141      19.122  -4.779   9.342  1.00  0.58
H
ATOM   2135 2HD  LYS A 141      19.326  -3.725   7.937  1.00  0.58
H
ATOM   2136 1HE  LYS A 141      19.578  -6.027   7.234  1.00  0.58
H
ATOM   2137 2HE  LYS A 141      21.163  -5.240   7.138  1.00  0.58
H
ATOM   2138 1HZ  LYS A 141      21.298  -7.400   8.184  1.00  0.58
H
ATOM   2139 2HZ  LYS A 141      21.705  -6.225   9.269  1.00  0.58
H
ATOM   2140 3HZ  LYS A 141      20.217  -6.937   9.346  1.00  0.58
H
ATOM   2141  N   TYR A 142      22.505  -0.306   9.282  1.00  0.33
N
ATOM   2142  CA  TYR A 142      23.478   0.085   8.268  1.00  0.41
C
ATOM   2143  C   TYR A 142      24.372   1.284   8.580  1.00  0.44
C
ATOM   2144  O   TYR A 142      25.460   1.395   8.013  1.00  0.66
O
ATOM   2145  CB  TYR A 142      22.730   0.407   6.971  1.00  0.61
C
ATOM   2146  CG  TYR A 142      22.008  -0.755   6.325  1.00  0.61
C
ATOM   2147  CD1 TYR A 142      20.635  -0.898   6.467  1.00  0.61
C
ATOM   2148  CD2 TYR A 142      22.719  -1.663   5.563  1.00  0.61
C
ATOM   2149  CE1 TYR A 142      19.976  -1.940   5.846  1.00  0.61
C
ATOM   2150  CE2 TYR A 142      22.063  -2.706   4.944  1.00  0.61
C
ATOM   2151  CZ  TYR A 142      20.699  -2.844   5.079  1.00  0.61
C
ATOM   2152  OH  TYR A 142      20.063  -3.884   4.445  1.00  0.61
O
ATOM   2153  H   TYR A 142      21.657   0.231   9.362  1.00  0.40
H
ATOM   2154  HA  TYR A 142      24.134  -0.769   8.095  1.00  0.49
H
ATOM   2155 1HB  TYR A 142      21.996   1.188   7.170  1.00  0.74
H
```

```
ATOM   2156 2HB  TYR A 142      23.435   0.808   6.244  1.00  0.74
H
ATOM   2157  HD1 TYR A 142      20.074  -0.182   7.061  1.00  0.74
H
ATOM   2158  HD2 TYR A 142      23.797  -1.551   5.447  1.00  0.74
H
ATOM   2159  HE1 TYR A 142      18.897  -2.047   5.953  1.00  0.74
H
ATOM   2160  HE2 TYR A 142      22.625  -3.418   4.339  1.00  0.74
H
ATOM   2161  HH  TYR A 142      19.143  -3.924   4.726  1.00  0.74
H
ATOM   2162  N   LEU A 143      23.923   2.198   9.429  1.00  0.40
N
ATOM   2163  CA  LEU A 143      24.690   3.410   9.691  1.00  0.49
C
ATOM   2164  C   LEU A 143      25.358   3.353  11.057  1.00  0.56
C
ATOM   2165  O   LEU A 143      26.584   3.421  11.161  1.00  1.04
O
ATOM   2166  CB  LEU A 143      23.765   4.626   9.585  1.00  0.73
C
ATOM   2167  CG  LEU A 143      23.125   4.853   8.200  1.00  0.73
C
ATOM   2168  CD1 LEU A 143      22.170   6.038   8.265  1.00  0.73
C
ATOM   2169  CD2 LEU A 143      24.217   5.091   7.166  1.00  0.73
C
ATOM   2170  H   LEU A 143      23.043   2.078   9.908  1.00  0.48
H
ATOM   2171  HA  LEU A 143      25.468   3.499   8.933  1.00  0.59
H
ATOM   2172 1HB  LEU A 143      22.962   4.502  10.303  1.00  0.88
H
ATOM   2173 2HB  LEU A 143      24.329   5.521   9.846  1.00  0.88
H
ATOM   2174  HG  LEU A 143      22.549   3.971   7.919  1.00  0.88
H
ATOM   2175 1HD1 LEU A 143      21.710   6.189   7.288  1.00  0.88
H
ATOM   2176 2HD1 LEU A 143      21.393   5.841   9.004  1.00  0.88
H
ATOM   2177 3HD1 LEU A 143      22.721   6.934   8.548  1.00  0.88
H
ATOM   2178 1HD2 LEU A 143      23.764   5.243   6.187  1.00  0.88
H
ATOM   2179 2HD2 LEU A 143      24.792   5.976   7.441  1.00  0.88
H
ATOM   2180 3HD2 LEU A 143      24.879   4.225   7.129  1.00  0.88
H
ATOM   2181  N   ASP A 144      24.547   3.241  12.103  1.00  0.62
N
ATOM   2182  CA  ASP A 144      25.062   3.094  13.453  1.00  1.04
C
ATOM   2183  C   ASP A 144      25.672   1.726  13.623  1.00  0.51
C
ATOM   2184  O   ASP A 144      26.730   1.584  14.237  1.00  0.48
O
```

ATOM 2185 CB ASP A 144 23.965 3.264 14.507 1.00 1.56
C
ATOM 2186 CG ASP A 144 23.484 4.688 14.675 1.00 1.56
C
ATOM 2187 OD1 ASP A 144 24.149 5.586 14.216 1.00 1.56
O
ATOM 2188 OD2 ASP A 144 22.448 4.866 15.269 1.00 1.56
O
ATOM 2189 H ASP A 144 23.553 3.208 11.948 1.00 0.74
H
ATOM 2190 HA ASP A 144 25.835 3.843 13.617 1.00 1.25
H
ATOM 2191 1HB ASP A 144 23.112 2.643 14.235 1.00 1.87
H
ATOM 2192 2HB ASP A 144 24.331 2.907 15.469 1.00 1.87
H
ATOM 2193 N LYS A 145 24.986 0.724 13.069 1.00 0.58
N
ATOM 2194 CA LYS A 145 25.425 -0.670 13.151 1.00 0.59
C
ATOM 2195 C LYS A 145 25.502 -1.076 14.620 1.00 0.62
C
ATOM 2196 O LYS A 145 26.532 -1.549 15.104 1.00 1.01
O
ATOM 2197 CB LYS A 145 26.767 -0.858 12.447 1.00 0.89
C
ATOM 2198 CG LYS A 145 26.741 -0.492 10.969 1.00 0.89
C
ATOM 2199 CD LYS A 145 28.082 -0.756 10.309 1.00 0.89
C
ATOM 2200 CE LYS A 145 28.063 -0.346 8.845 1.00 0.89
C
ATOM 2201 NZ LYS A 145 29.377 -0.582 8.186 1.00 0.89
N
ATOM 2202 H LYS A 145 24.115 0.951 12.587 1.00 0.70
H
ATOM 2203 HA LYS A 145 24.686 -1.306 12.662 1.00 0.71
H
ATOM 2204 1HB LYS A 145 27.536 -0.257 12.931 1.00 1.06
H
ATOM 2205 2HB LYS A 145 27.073 -1.901 12.525 1.00 1.06
H
ATOM 2206 1HG LYS A 145 25.977 -1.081 10.463 1.00 1.06
H
ATOM 2207 2HG LYS A 145 26.494 0.563 10.858 1.00 1.06
H
ATOM 2208 1HD LYS A 145 28.860 -0.194 10.827 1.00 1.06
H
ATOM 2209 2HD LYS A 145 28.317 -1.818 10.376 1.00 1.06
H
ATOM 2210 1HE LYS A 145 27.298 -0.918 8.322 1.00 1.06
H
ATOM 2211 2HE LYS A 145 27.821 0.714 8.774 1.00 1.06
H
ATOM 2212 1HZ LYS A 145 29.324 -0.296 7.219 1.00 1.06
H
ATOM 2213 2HZ LYS A 145 30.092 -0.044 8.657 1.00 1.06
H

```
ATOM   2214 3HZ  LYS A 145      29.608  -1.563   8.235  1.00  1.06
H
ATOM   2215  N   ALA A 146      24.390  -0.873  15.315  1.00  0.39
N
ATOM   2216  CA  ALA A 146      24.274  -1.120  16.742  1.00  0.54
C
ATOM   2217  C   ALA A 146      23.194  -2.154  17.011  1.00  0.90
C
ATOM   2218  O   ALA A 146      22.282  -2.333  16.206  1.00  3.54
O
ATOM   2219  CB  ALA A 146      23.978   0.178  17.461  1.00  0.81
C
ATOM   2220  H   ALA A 146      23.584  -0.521  14.816  1.00  0.47
H
ATOM   2221  HA  ALA A 146      25.222  -1.520  17.104  1.00  0.65
H
ATOM   2222 1HB  ALA A 146      23.905   0.001  18.532  1.00  0.97
H
ATOM   2223 2HB  ALA A 146      24.779   0.891  17.263  1.00  0.97
H
ATOM   2224 3HB  ALA A 146      23.040   0.576  17.092  1.00  0.97
H
ATOM   2225  N   SER A 147      23.296  -2.846  18.140  1.00  1.26
N
ATOM   2226  CA  SER A 147      22.355  -3.906  18.481  1.00  1.59
C
ATOM   2227  C   SER A 147      20.977  -3.469  18.995  1.00  2.85
C
ATOM   2228  O   SER A 147      20.247  -4.306  19.522  1.00 16.16
O
ATOM   2229  CB  SER A 147      22.980  -4.831  19.503  1.00  2.39
C
ATOM   2230  OG  SER A 147      23.238  -4.155  20.698  1.00  2.39
O
ATOM   2231  H   SER A 147      24.062  -2.664  18.780  1.00  1.51
H
ATOM   2232  HA  SER A 147      22.188  -4.487  17.572  1.00  1.91
H
ATOM   2233 1HB  SER A 147      22.308  -5.668  19.693  1.00  2.86
H
ATOM   2234 2HB  SER A 147      23.908  -5.241  19.106  1.00  2.86
H
ATOM   2235  HG  SER A 147      23.960  -3.547  20.495  1.00  2.86
H
ATOM   2236  N   TYR A 148      20.596  -2.196  18.866  1.00  0.99
N
ATOM   2237  CA  TYR A 148      19.255  -1.852  19.314  1.00  0.49
C
ATOM   2238  C   TYR A 148      18.218  -2.302  18.282  1.00  0.58
C
ATOM   2239  O   TYR A 148      18.502  -2.401  17.087  1.00  2.18
O
ATOM   2240  CB  TYR A 148      19.111  -0.351  19.626  1.00  0.73
C
ATOM   2241  CG  TYR A 148      19.419   0.638  18.516  1.00  0.73
C
ATOM   2242  CD1 TYR A 148      18.467   0.943  17.549  1.00  0.73
C
```

ATOM	2243	CD2	TYR	A	148	20.645	1.286	18.498	1.00	0.73	C
ATOM	2244	CE1	TYR	A	148	18.749	1.884	16.569	1.00	0.73	C
ATOM	2245	CE2	TYR	A	148	20.925	2.227	17.522	1.00	0.73	C
ATOM	2246	CZ	TYR	A	148	19.987	2.529	16.561	1.00	0.73	C
ATOM	2247	OH	TYR	A	148	20.270	3.475	15.594	1.00	0.73	O
ATOM	2248	H	TYR	A	148	21.198	-1.497	18.460	1.00	1.19	H
ATOM	2249	HA	TYR	A	148	19.050	-2.399	20.236	1.00	0.59	H
ATOM	2250	1HB	TYR	A	148	18.089	-0.164	19.958	1.00	0.88	H
ATOM	2251	2HB	TYR	A	148	19.760	-0.112	20.467	1.00	0.88	H
ATOM	2252	HD1	TYR	A	148	17.496	0.445	17.565	1.00	0.88	H
ATOM	2253	HD2	TYR	A	148	21.390	1.059	19.261	1.00	0.88	H
ATOM	2254	HE1	TYR	A	148	18.001	2.123	15.813	1.00	0.88	H
ATOM	2255	HE2	TYR	A	148	21.891	2.735	17.519	1.00	0.88	H
ATOM	2256	HH	TYR	A	148	21.174	3.800	15.693	1.00	0.88	H
ATOM	2257	N	ARG	A	149	17.016	-2.564	18.776	1.00	0.30	N
ATOM	2258	CA	ARG	A	149	15.893	-3.059	17.985	1.00	0.33	C
ATOM	2259	C	ARG	A	149	14.774	-2.036	17.947	1.00	0.36	C
ATOM	2260	O	ARG	A	149	14.628	-1.245	18.876	1.00	1.58	O
ATOM	2261	CB	ARG	A	149	15.398	-4.388	18.555	1.00	0.49	C
ATOM	2262	CG	ARG	A	149	14.261	-5.056	17.795	1.00	0.49	C
ATOM	2263	CD	ARG	A	149	13.979	-6.424	18.299	1.00	0.49	C
ATOM	2264	NE	ARG	A	149	15.080	-7.328	18.013	1.00	0.49	N
ATOM	2265	CZ	ARG	A	149	15.264	-7.928	16.822	1.00	0.49	C
ATOM	2266	NH1	ARG	A	149	14.410	-7.706	15.849	1.00	0.49	N
ATOM	2267	NH2	ARG	A	149	16.302	-8.721	16.626	1.00	0.49	N
ATOM	2268	H	ARG	A	149	16.882	-2.437	19.772	1.00	0.36	H
ATOM	2269	HA	ARG	A	149	16.237	-3.232	16.966	1.00	0.40	H
ATOM	2270	1HB	ARG	A	149	16.227	-5.094	18.590	1.00	0.59	H
ATOM	2271	2HB	ARG	A	149	15.060	-4.237	19.580	1.00	0.59	H

ATOM	2272	1HG	ARG	A	149	13.352	-4.462	17.885	1.00	0.59	H
ATOM	2273	2HG	ARG	A	149	14.536	-5.149	16.749	1.00	0.59	H
ATOM	2274	1HD	ARG	A	149	13.843	-6.392	19.379	1.00	0.59	H
ATOM	2275	2HD	ARG	A	149	13.080	-6.816	17.826	1.00	0.59	H
ATOM	2276	HE	ARG	A	149	15.752	-7.509	18.746	1.00	0.59	H
ATOM	2277	1HH1	ARG	A	149	13.623	-7.093	16.001	1.00	0.59	H
ATOM	2278	2HH1	ARG	A	149	14.563	-8.117	14.935	1.00	0.59	H
ATOM	2279	1HH2	ARG	A	149	16.962	-8.887	17.373	1.00	0.59	H
ATOM	2280	2HH2	ARG	A	149	16.437	-9.161	15.726	1.00	0.59	H
ATOM	2281	N	VAL	A	150	14.021	-2.022	16.853	1.00	0.19	N
ATOM	2282	CA	VAL	A	150	12.934	-1.070	16.697	1.00	0.15	C
ATOM	2283	C	VAL	A	150	11.577	-1.770	16.653	1.00	0.17	C
ATOM	2284	O	VAL	A	150	11.349	-2.648	15.821	1.00	0.23	O
ATOM	2285	CB	VAL	A	150	13.124	-0.251	15.405	1.00	0.22	C
ATOM	2286	CG1	VAL	A	150	11.984	0.746	15.249	1.00	0.22	C
ATOM	2287	CG2	VAL	A	150	14.473	0.446	15.429	1.00	0.22	C
ATOM	2288	H	VAL	A	150	14.197	-2.693	16.119	1.00	0.23	H
ATOM	2289	HA	VAL	A	150	12.945	-0.387	17.546	1.00	0.18	H
ATOM	2290	HB	VAL	A	150	13.084	-0.925	14.549	1.00	0.27	H
ATOM	2291	1HG1	VAL	A	150	12.115	1.305	14.324	1.00	0.27	H
ATOM	2292	2HG1	VAL	A	150	11.035	0.213	15.219	1.00	0.27	H
ATOM	2293	3HG1	VAL	A	150	11.986	1.436	16.093	1.00	0.27	H
ATOM	2294	1HG2	VAL	A	150	14.602	1.008	14.509	1.00	0.27	H
ATOM	2295	2HG2	VAL	A	150	14.524	1.125	16.274	1.00	0.27	H
ATOM	2296	3HG2	VAL	A	150	15.266	-0.297	15.515	1.00	0.27	H
ATOM	2297	N	PHE	A	151	10.683	-1.366	17.549	1.00	0.16	N
ATOM	2298	CA	PHE	A	151	9.331	-1.895	17.596	1.00	0.19	C
ATOM	2299	C	PHE	A	151	8.334	-0.826	17.182	1.00	0.24	C
ATOM	2300	O	PHE	A	151	8.505	0.344	17.519	1.00	0.35	O

```
ATOM   2301  CB  PHE A 151       8.995  -2.370  19.006  1.00  0.29
C
ATOM   2302  CG  PHE A 151       9.771  -3.568  19.445  1.00  0.29
C
ATOM   2303  CD1 PHE A 151      11.011  -3.425  20.045  1.00  0.29
C
ATOM   2304  CD2 PHE A 151       9.259  -4.841  19.265  1.00  0.29
C
ATOM   2305  CE1 PHE A 151      11.722  -4.534  20.456  1.00  0.29
C
ATOM   2306  CE2 PHE A 151       9.967  -5.951  19.671  1.00  0.29
C
ATOM   2307  CZ  PHE A 151      11.198  -5.795  20.269  1.00  0.29
C
ATOM   2308  H   PHE A 151      10.949  -0.659  18.217  1.00  0.19
H
ATOM   2309  HA  PHE A 151       9.258  -2.733  16.908  1.00  0.23
H
ATOM   2310 1HB  PHE A 151       9.199  -1.566  19.709  1.00  0.34
H
ATOM   2311 2HB  PHE A 151       7.934  -2.606  19.072  1.00  0.34
H
ATOM   2312  HD1 PHE A 151      11.421  -2.425  20.192  1.00  0.34
H
ATOM   2313  HD2 PHE A 151       8.282  -4.960  18.794  1.00  0.34
H
ATOM   2314  HE1 PHE A 151      12.696  -4.411  20.929  1.00  0.34
H
ATOM   2315  HE2 PHE A 151       9.556  -6.948  19.524  1.00  0.34
H
ATOM   2316  HZ  PHE A 151      11.760  -6.670  20.592  1.00  0.34
H
ATOM   2317  N   CYS A 152       7.289  -1.231  16.473  1.00  0.19
N
ATOM   2318  CA  CYS A 152       6.261  -0.294  16.039  1.00  0.20
C
ATOM   2319  C   CYS A 152       4.873  -0.878  16.240  1.00  0.27
C
ATOM   2320  O   CYS A 152       4.600  -1.984  15.787  1.00  0.47
O
ATOM   2321  CB  CYS A 152       6.454   0.062  14.569  1.00  0.30
C
ATOM   2322  SG  CYS A 152       5.244   1.239  13.937  1.00  0.30
S
ATOM   2323  H   CYS A 152       7.227  -2.206  16.206  1.00  0.23
H
ATOM   2324  HA  CYS A 152       6.349   0.614  16.631  1.00  0.24
H
ATOM   2325 1HB  CYS A 152       7.446   0.489  14.428  1.00  0.36
H
ATOM   2326 2HB  CYS A 152       6.396  -0.841  13.962  1.00  0.36
H
ATOM   2327  HG  CYS A 152       5.544   2.217  14.786  1.00  0.36
H
ATOM   2328  N   LEU A 153       3.999  -0.134  16.912  1.00  0.23
N
ATOM   2329  CA  LEU A 153       2.651  -0.628  17.181  1.00  0.27
C
```

```
ATOM   2330  C   LEU A 153       1.601   0.115  16.368  1.00  0.24
C
ATOM   2331  O   LEU A 153       1.399   1.321  16.533  1.00  0.25
O
ATOM   2332  CB  LEU A 153       2.350  -0.526  18.681  1.00  0.41
C
ATOM   2333  CG  LEU A 153       0.936  -0.928  19.125  1.00  0.41
C
ATOM   2334  CD1 LEU A 153       0.680  -2.384  18.779  1.00  0.41
C
ATOM   2335  CD2 LEU A 153       0.820  -0.731  20.628  1.00  0.41
C
ATOM   2336  H   LEU A 153       4.279   0.782  17.237  1.00  0.28
H
ATOM   2337  HA  LEU A 153       2.610  -1.679  16.900  1.00  0.32
H
ATOM   2338 1HB  LEU A 153       3.055  -1.161  19.215  1.00  0.49
H
ATOM   2339 2HB  LEU A 153       2.515   0.504  18.996  1.00  0.49
H
ATOM   2340  HG  LEU A 153       0.198  -0.314  18.609  1.00  0.49
H
ATOM   2341 1HD1 LEU A 153      -0.323  -2.664  19.101  1.00  0.49
H
ATOM   2342 2HD1 LEU A 153       0.767  -2.523  17.702  1.00  0.49
H
ATOM   2343 3HD1 LEU A 153       1.412  -3.008  19.287  1.00  0.49
H
ATOM   2344 1HD2 LEU A 153      -0.181  -1.013  20.950  1.00  0.49
H
ATOM   2345 2HD2 LEU A 153       1.555  -1.356  21.136  1.00  0.49
H
ATOM   2346 3HD2 LEU A 153       0.999   0.312  20.878  1.00  0.49
H
ATOM   2347  N   MET A 154       0.949  -0.632  15.479  1.00  0.24
N
ATOM   2348  CA  MET A 154      -0.053  -0.105  14.562  1.00  0.15
C
ATOM   2349  C   MET A 154      -1.439  -0.623  14.874  1.00  0.16
C
ATOM   2350  O   MET A 154      -1.594  -1.686  15.474  1.00  0.24
O
ATOM   2351  CB  MET A 154       0.276  -0.485  13.119  1.00  0.22
C
ATOM   2352  CG  MET A 154       1.571   0.071  12.555  1.00  0.22
C
ATOM   2353  SD  MET A 154       1.839  -0.467  10.859  1.00  0.22
S
ATOM   2354  CE  MET A 154       2.231  -2.186  11.139  1.00  0.22
C
ATOM   2355  H   MET A 154       1.167  -1.618  15.432  1.00  0.29
H
ATOM   2356  HA  MET A 154      -0.074   0.981  14.655  1.00  0.18
H
ATOM   2357 1HB  MET A 154       0.326  -1.570  13.039  1.00  0.27
H
ATOM   2358 2HB  MET A 154      -0.532  -0.152  12.467  1.00  0.27
H
```

```
ATOM   2359 1HG  MET A 154       1.534   1.157  12.564  1.00  0.27
H
ATOM   2360 2HG  MET A 154       2.414  -0.255  13.163  1.00  0.27
H
ATOM   2361 1HE  MET A 154       2.430  -2.676  10.188  1.00  0.27
H
ATOM   2362 2HE  MET A 154       3.112  -2.248  11.776  1.00  0.27
H
ATOM   2363 3HE  MET A 154       1.391  -2.676  11.633  1.00  0.27
H
ATOM   2364  N   GLY A 155      -2.445   0.132  14.455  1.00  0.20
N
ATOM   2365  CA  GLY A 155      -3.820  -0.275  14.646  1.00  0.21
C
ATOM   2366  C   GLY A 155      -4.288  -1.142  13.487  1.00  0.23
C
ATOM   2367  O   GLY A 155      -3.555  -1.364  12.522  1.00  0.38
O
ATOM   2368  H   GLY A 155      -2.246   1.005  13.981  1.00  0.24
H
ATOM   2369 1HA  GLY A 155      -3.912  -0.826  15.582  1.00  0.25
H
ATOM   2370 2HA  GLY A 155      -4.453   0.607  14.725  1.00  0.25
H
ATOM   2371  N   ASP A 156      -5.523  -1.607  13.597  1.00  0.25
N
ATOM   2372  CA  ASP A 156      -6.165  -2.449  12.601  1.00  0.29
C
ATOM   2373  C   ASP A 156      -6.538  -1.618  11.369  1.00  0.27
C
ATOM   2374  O   ASP A 156      -5.961  -1.792  10.292  1.00  0.28
O
ATOM   2375  CB  ASP A 156      -7.384  -3.139  13.228  1.00  0.43
C
ATOM   2376  CG  ASP A 156      -7.964  -4.220  12.352  1.00  0.43
C
ATOM   2377  OD1 ASP A 156      -7.400  -4.470  11.311  1.00  0.43
O
ATOM   2378  OD2 ASP A 156      -8.949  -4.808  12.727  1.00  0.43
O
ATOM   2379  H   ASP A 156      -6.049  -1.373  14.426  1.00  0.30
H
ATOM   2380  HA  ASP A 156      -5.457  -3.219  12.291  1.00  0.35
H
ATOM   2381 1HB  ASP A 156      -7.098  -3.580  14.181  1.00  0.52
H
ATOM   2382 2HB  ASP A 156      -8.161  -2.403  13.434  1.00  0.52
H
ATOM   2383  N   GLY A 157      -7.509  -0.711  11.540  1.00  0.25
N
ATOM   2384  CA  GLY A 157      -7.961   0.181  10.469  1.00  0.25
C
ATOM   2385  C   GLY A 157      -6.802   0.975   9.868  1.00  0.22
C
ATOM   2386  O   GLY A 157      -6.783   1.236   8.668  1.00  0.23
O
ATOM   2387  H   GLY A 157      -7.948  -0.633  12.447  1.00  0.30
H
```

```
ATOM   2388 1HA  GLY A 157      -8.444  -0.408   9.689  1.00  0.30
H
ATOM   2389 2HA  GLY A 157      -8.712   0.866  10.862  1.00  0.30
H
ATOM   2390  N   GLU A 158      -5.831   1.335  10.711  1.00  0.26
N
ATOM   2391  CA  GLU A 158      -4.623   2.046  10.307  1.00  0.27
C
ATOM   2392  C   GLU A 158      -3.898   1.408   9.116  1.00  0.30
C
ATOM   2393  O   GLU A 158      -3.311   2.117   8.295  1.00  0.31
O
ATOM   2394  CB  GLU A 158      -3.662   2.152  11.491  1.00  0.41
C
ATOM   2395  CG  GLU A 158      -2.388   2.892  11.156  1.00  0.41
C
ATOM   2396  CD  GLU A 158      -1.513   3.190  12.333  1.00  0.41
C
ATOM   2397  OE1 GLU A 158      -1.610   2.544  13.348  1.00  0.41
O
ATOM   2398  OE2 GLU A 158      -0.748   4.119  12.219  1.00  0.41
O
ATOM   2399  H   GLU A 158      -5.932   1.098  11.687  1.00  0.31
H
ATOM   2400  HA  GLU A 158      -4.912   3.055  10.014  1.00  0.32
H
ATOM   2401 1HB  GLU A 158      -4.151   2.672  12.316  1.00  0.49
H
ATOM   2402 2HB  GLU A 158      -3.393   1.155  11.837  1.00  0.49
H
ATOM   2403 1HG  GLU A 158      -1.817   2.278  10.458  1.00  0.49
H
ATOM   2404 2HG  GLU A 158      -2.639   3.813  10.643  1.00  0.49
H
ATOM   2405  N   SER A 159      -3.917   0.071   9.037  1.00  0.34
N
ATOM   2406  CA  SER A 159      -3.213  -0.663   7.989  1.00  0.45
C
ATOM   2407  C   SER A 159      -3.764  -0.417   6.584  1.00  0.54
C
ATOM   2408  O   SER A 159      -3.095  -0.733   5.603  1.00  1.05
O
ATOM   2409  CB  SER A 159      -3.252  -2.148   8.282  1.00  0.68
C
ATOM   2410  OG  SER A 159      -4.555  -2.651   8.165  1.00  0.68
O
ATOM   2411  H   SER A 159      -4.447  -0.466   9.713  1.00  0.41
H
ATOM   2412  HA  SER A 159      -2.171  -0.340   8.000  1.00  0.54
H
ATOM   2413 1HB  SER A 159      -2.595  -2.671   7.587  1.00  0.81
H
ATOM   2414 2HB  SER A 159      -2.877  -2.331   9.287  1.00  0.81
H
ATOM   2415  HG  SER A 159      -5.057  -2.253   8.885  1.00  0.81
H
ATOM   2416  N   SER A 160      -4.971   0.150   6.478  1.00  0.40
N
```

ATOM 2417 CA SER A 160 -5.565 0.458 5.181 1.00 0.48
C
ATOM 2418 C SER A 160 -4.844 1.584 4.450 1.00 0.46
C
ATOM 2419 O SER A 160 -5.038 1.773 3.248 1.00 0.62
O
ATOM 2420 CB SER A 160 -7.017 0.830 5.353 1.00 0.72
C
ATOM 2421 OG SER A 160 -7.148 2.023 6.074 1.00 0.72
O
ATOM 2422 H SER A 160 -5.499 0.392 7.307 1.00 0.48
H
ATOM 2423 HA SER A 160 -5.510 -0.438 4.564 1.00 0.58
H
ATOM 2424 1HB SER A 160 -7.485 0.938 4.376 1.00 0.86
H
ATOM 2425 2HB SER A 160 -7.535 0.027 5.878 1.00 0.86
H
ATOM 2426 HG SER A 160 -6.767 2.732 5.543 1.00 0.86
H
ATOM 2427 N GLU A 161 -4.024 2.338 5.169 1.00 0.45
N
ATOM 2428 CA GLU A 161 -3.282 3.415 4.562 1.00 0.69
C
ATOM 2429 C GLU A 161 -2.098 2.910 3.738 1.00 0.30
C
ATOM 2430 O GLU A 161 -1.294 2.112 4.221 1.00 0.28
O
ATOM 2431 CB GLU A 161 -2.817 4.360 5.635 1.00 1.03
C
ATOM 2432 CG GLU A 161 -2.096 5.544 5.083 1.00 1.03
C
ATOM 2433 CD GLU A 161 -2.993 6.434 4.260 1.00 1.03
C
ATOM 2434 OE1 GLU A 161 -4.186 6.255 4.289 1.00 1.03
O
ATOM 2435 OE2 GLU A 161 -2.473 7.334 3.642 1.00 1.03
O
ATOM 2436 H GLU A 161 -3.893 2.165 6.160 1.00 0.54
H
ATOM 2437 HA GLU A 161 -3.953 3.955 3.892 1.00 0.83
H
ATOM 2438 1HB GLU A 161 -3.672 4.717 6.209 1.00 1.24
H
ATOM 2439 2HB GLU A 161 -2.150 3.840 6.323 1.00 1.24
H
ATOM 2440 1HG GLU A 161 -1.693 6.095 5.925 1.00 1.24
H
ATOM 2441 2HG GLU A 161 -1.264 5.202 4.474 1.00 1.24
H
ATOM 2442 N GLY A 162 -1.993 3.405 2.500 1.00 0.24
N
ATOM 2443 CA GLY A 162 -0.961 3.007 1.542 1.00 0.19
C
ATOM 2444 C GLY A 162 0.465 3.074 2.064 1.00 0.12
C
ATOM 2445 O GLY A 162 1.273 2.188 1.775 1.00 0.27
O

```
ATOM   2446  H   GLY A 162      -2.682   4.078   2.177  1.00  0.29
H
ATOM   2447 1HA  GLY A 162      -1.167   2.009   1.191  1.00  0.23
H
ATOM   2448 2HA  GLY A 162      -1.043   3.654   0.671  1.00  0.23
H
ATOM   2449  N   SER A 163       0.762   4.110   2.840  1.00  0.20
N
ATOM   2450  CA  SER A 163       2.089   4.320   3.408  1.00  0.38
C
ATOM   2451  C   SER A 163       2.574   3.173   4.301  1.00  0.15
C
ATOM   2452  O   SER A 163       3.783   2.974   4.438  1.00  0.36
O
ATOM   2453  CB  SER A 163       2.086   5.624   4.171  1.00  0.57
C
ATOM   2454  OG  SER A 163       1.187   5.578   5.242  1.00  0.57
O
ATOM   2455  H   SER A 163       0.044   4.793   3.035  1.00  0.24
H
ATOM   2456  HA  SER A 163       2.792   4.415   2.580  1.00  0.46
H
ATOM   2457 1HB  SER A 163       3.088   5.841   4.540  1.00  0.68
H
ATOM   2458 2HB  SER A 163       1.804   6.428   3.495  1.00  0.68
H
ATOM   2459  HG  SER A 163       1.651   5.179   5.982  1.00  0.68
H
ATOM   2460  N   VAL A 164       1.644   2.406   4.880  1.00  0.12
N
ATOM   2461  CA  VAL A 164       2.000   1.265   5.715  1.00  0.19
C
ATOM   2462  C   VAL A 164       2.602   0.178   4.851  1.00  0.20
C
ATOM   2463  O   VAL A 164       3.631  -0.409   5.187  1.00  0.53
O
ATOM   2464  CB  VAL A 164       0.760   0.703   6.441  1.00  0.29
C
ATOM   2465  CG1 VAL A 164       1.122  -0.585   7.159  1.00  0.29
C
ATOM   2466  CG2 VAL A 164       0.225   1.733   7.419  1.00  0.29
C
ATOM   2467  H   VAL A 164       0.661   2.593   4.726  1.00  0.14
H
ATOM   2468  HA  VAL A 164       2.736   1.582   6.454  1.00  0.23
H
ATOM   2469  HB  VAL A 164      -0.008   0.465   5.705  1.00  0.34
H
ATOM   2470 1HG1 VAL A 164       0.239  -0.986   7.655  1.00  0.34
H
ATOM   2471 2HG1 VAL A 164       1.492  -1.307   6.434  1.00  0.34
H
ATOM   2472 3HG1 VAL A 164       1.895  -0.384   7.900  1.00  0.34
H
ATOM   2473 1HG2 VAL A 164      -0.657   1.336   7.921  1.00  0.34
H
ATOM   2474 2HG2 VAL A 164       0.991   1.964   8.159  1.00  0.34
H
```

ATOM 2475 3HG2 VAL A 164 -0.044 2.640 6.879 1.00 0.34
H
ATOM 2476 N TRP A 165 1.931 -0.078 3.737 1.00 0.25
N
ATOM 2477 CA TRP A 165 2.338 -1.073 2.768 1.00 0.26
C
ATOM 2478 C TRP A 165 3.660 -0.672 2.093 1.00 0.26
C
ATOM 2479 O TRP A 165 4.490 -1.534 1.795 1.00 0.40
O
ATOM 2480 CB TRP A 165 1.197 -1.253 1.762 1.00 0.39
C
ATOM 2481 CG TRP A 165 -0.019 -1.930 2.348 1.00 0.39
C
ATOM 2482 CD1 TRP A 165 -0.024 -3.000 3.183 1.00 0.39
C
ATOM 2483 CD2 TRP A 165 -1.408 -1.559 2.183 1.00 0.39
C
ATOM 2484 NE1 TRP A 165 -1.307 -3.333 3.531 1.00 0.39
N
ATOM 2485 CE2 TRP A 165 -2.168 -2.460 2.931 1.00 0.39
C
ATOM 2486 CE3 TRP A 165 -2.057 -0.554 1.478 1.00 0.39
C
ATOM 2487 CZ2 TRP A 165 -3.552 -2.387 2.989 1.00 0.39
C
ATOM 2488 CZ3 TRP A 165 -3.437 -0.473 1.538 1.00 0.39
C
ATOM 2489 CH2 TRP A 165 -4.169 -1.367 2.269 1.00 0.39
C
ATOM 2490 H TRP A 165 1.091 0.449 3.548 1.00 0.30
H
ATOM 2491 HA TRP A 165 2.491 -2.017 3.286 1.00 0.31
H
ATOM 2492 1HB TRP A 165 0.895 -0.283 1.367 1.00 0.47
H
ATOM 2493 2HB TRP A 165 1.547 -1.858 0.931 1.00 0.47
H
ATOM 2494 HD1 TRP A 165 0.857 -3.519 3.516 1.00 0.47
H
ATOM 2495 HE1 TRP A 165 -1.575 -4.095 4.138 1.00 0.47
H
ATOM 2496 HE3 TRP A 165 -1.484 0.149 0.886 1.00 0.47
H
ATOM 2497 HZ2 TRP A 165 -4.145 -3.089 3.573 1.00 0.47
H
ATOM 2498 HZ3 TRP A 165 -3.924 0.323 0.985 1.00 0.47
H
ATOM 2499 HH2 TRP A 165 -5.254 -1.273 2.291 1.00 0.47
H
ATOM 2500 N GLU A 166 3.873 0.642 1.896 1.00 0.19
N
ATOM 2501 CA GLU A 166 5.141 1.127 1.344 1.00 0.18
C
ATOM 2502 C GLU A 166 6.278 0.799 2.294 1.00 0.18
C
ATOM 2503 O GLU A 166 7.336 0.326 1.870 1.00 0.21
O

ATOM 2504 CB GLU A 166 5.120 2.643 1.110 1.00 0.27
C
ATOM 2505 CG GLU A 166 4.215 3.140 0.000 1.00 0.27
C
ATOM 2506 CD GLU A 166 4.272 4.643 -0.170 1.00 0.27
C
ATOM 2507 OE1 GLU A 166 5.014 5.282 0.545 1.00 0.27
O
ATOM 2508 OE2 GLU A 166 3.573 5.144 -1.024 1.00 0.27
O
ATOM 2509 H GLU A 166 3.139 1.305 2.118 1.00 0.23
H
ATOM 2510 HA GLU A 166 5.337 0.621 0.400 1.00 0.22
H
ATOM 2511 1HB GLU A 166 4.827 3.148 2.030 1.00 0.32
H
ATOM 2512 2HB GLU A 166 6.129 2.971 0.866 1.00 0.32
H
ATOM 2513 1HG GLU A 166 4.511 2.668 -0.937 1.00 0.32
H
ATOM 2514 2HG GLU A 166 3.192 2.840 0.219 1.00 0.32
H
ATOM 2515 N ALA A 167 6.041 1.038 3.584 1.00 0.17
N
ATOM 2516 CA ALA A 167 7.014 0.739 4.616 1.00 0.18
C
ATOM 2517 C ALA A 167 7.279 -0.763 4.706 1.00 0.18
C
ATOM 2518 O ALA A 167 8.424 -1.168 4.922 1.00 0.19
O
ATOM 2519 CB ALA A 167 6.540 1.285 5.944 1.00 0.27
C
ATOM 2520 H ALA A 167 5.162 1.461 3.861 1.00 0.20
H
ATOM 2521 HA ALA A 167 7.951 1.228 4.350 1.00 0.22
H
ATOM 2522 1HB ALA A 167 7.290 1.086 6.707 1.00 0.32
H
ATOM 2523 2HB ALA A 167 6.387 2.361 5.857 1.00 0.32
H
ATOM 2524 3HB ALA A 167 5.604 0.809 6.217 1.00 0.32
H
ATOM 2525 N PHE A 168 6.240 -1.594 4.515 1.00 0.19
N
ATOM 2526 CA PHE A 168 6.452 -3.038 4.539 1.00 0.22
C
ATOM 2527 C PHE A 168 7.402 -3.466 3.449 1.00 0.22
C
ATOM 2528 O PHE A 168 8.302 -4.278 3.690 1.00 0.27
O
ATOM 2529 CB PHE A 168 5.176 -3.817 4.251 1.00 0.33
C
ATOM 2530 CG PHE A 168 4.189 -3.872 5.348 1.00 0.33
C
ATOM 2531 CD1 PHE A 168 4.552 -3.647 6.653 1.00 0.33
C
ATOM 2532 CD2 PHE A 168 2.881 -4.167 5.060 1.00 0.33
C

ATOM 2533 CE1 PHE A 168 3.614 -3.700 7.656 1.00 0.33
C
ATOM 2534 CE2 PHE A 168 1.933 -4.215 6.051 1.00 0.33
C
ATOM 2535 CZ PHE A 168 2.305 -3.974 7.349 1.00 0.33
C
ATOM 2536 H PHE A 168 5.307 -1.223 4.380 1.00 0.23
H
ATOM 2537 HA PHE A 168 6.862 -3.320 5.505 1.00 0.26
H
ATOM 2538 1HB PHE A 168 4.682 -3.392 3.380 1.00 0.40
H
ATOM 2539 2HB PHE A 168 5.443 -4.840 3.999 1.00 0.40
H
ATOM 2540 HD1 PHE A 168 5.589 -3.418 6.884 1.00 0.40
H
ATOM 2541 HD2 PHE A 168 2.608 -4.354 4.025 1.00 0.40
H
ATOM 2542 HE1 PHE A 168 3.907 -3.515 8.688 1.00 0.40
H
ATOM 2543 HE2 PHE A 168 0.894 -4.440 5.810 1.00 0.40
H
ATOM 2544 HZ PHE A 168 1.565 -3.997 8.134 1.00 0.40
H
ATOM 2545 N ALA A 169 7.205 -2.903 2.260 1.00 0.19
N
ATOM 2546 CA ALA A 169 8.035 -3.218 1.117 1.00 0.20
C
ATOM 2547 C ALA A 169 9.476 -2.804 1.367 1.00 0.22
C
ATOM 2548 O ALA A 169 10.392 -3.572 1.072 1.00 0.35
O
ATOM 2549 CB ALA A 169 7.489 -2.533 -0.127 1.00 0.30
C
ATOM 2550 H ALA A 169 6.432 -2.257 2.137 1.00 0.23
H
ATOM 2551 HA ALA A 169 8.015 -4.297 0.968 1.00 0.24
H
ATOM 2552 1HB ALA A 169 8.105 -2.797 -0.988 1.00 0.36
H
ATOM 2553 2HB ALA A 169 6.464 -2.861 -0.301 1.00 0.36
H
ATOM 2554 3HB ALA A 169 7.505 -1.454 0.012 1.00 0.36
H
ATOM 2555 N PHE A 170 9.664 -1.602 1.922 1.00 0.17
N
ATOM 2556 CA PHE A 170 10.990 -1.063 2.227 1.00 0.18
C
ATOM 2557 C PHE A 170 11.762 -1.977 3.160 1.00 0.22
C
ATOM 2558 O PHE A 170 12.917 -2.310 2.892 1.00 0.25
O
ATOM 2559 CB PHE A 170 10.869 0.308 2.902 1.00 0.27
C
ATOM 2560 CG PHE A 170 12.184 0.971 3.266 1.00 0.27
C
ATOM 2561 CD1 PHE A 170 12.806 1.841 2.380 1.00 0.27
C

ATOM 2562 CD2 PHE A 170 12.805 0.730 4.484 1.00 0.27
C
ATOM 2563 CE1 PHE A 170 13.997 2.464 2.708 1.00 0.27
C
ATOM 2564 CE2 PHE A 170 14.001 1.346 4.813 1.00 0.27
C
ATOM 2565 CZ PHE A 170 14.597 2.217 3.926 1.00 0.27
C
ATOM 2566 H PHE A 170 8.857 -1.021 2.122 1.00 0.20
H
ATOM 2567 HA PHE A 170 11.547 -0.962 1.295 1.00 0.22
H
ATOM 2568 1HB PHE A 170 10.326 0.979 2.240 1.00 0.32
H
ATOM 2569 2HB PHE A 170 10.280 0.211 3.812 1.00 0.32
H
ATOM 2570 HD1 PHE A 170 12.337 2.042 1.416 1.00 0.32
H
ATOM 2571 HD2 PHE A 170 12.334 0.045 5.190 1.00 0.32
H
ATOM 2572 HE1 PHE A 170 14.463 3.148 2.001 1.00 0.32
H
ATOM 2573 HE2 PHE A 170 14.472 1.148 5.774 1.00 0.32
H
ATOM 2574 HZ PHE A 170 15.536 2.704 4.183 1.00 0.32
H
ATOM 2575 N ALA A 171 11.126 -2.355 4.266 1.00 0.22
N
ATOM 2576 CA ALA A 171 11.765 -3.186 5.272 1.00 0.26
C
ATOM 2577 C ALA A 171 12.202 -4.524 4.714 1.00 0.29
C
ATOM 2578 O ALA A 171 13.288 -5.008 5.030 1.00 0.33
O
ATOM 2579 CB ALA A 171 10.828 -3.395 6.431 1.00 0.39
C
ATOM 2580 H ALA A 171 10.176 -2.041 4.426 1.00 0.26
H
ATOM 2581 HA ALA A 171 12.654 -2.666 5.614 1.00 0.31
H
ATOM 2582 1HB ALA A 171 11.319 -3.989 7.199 1.00 0.47
H
ATOM 2583 2HB ALA A 171 10.540 -2.430 6.846 1.00 0.47
H
ATOM 2584 3HB ALA A 171 9.942 -3.917 6.075 1.00 0.47
H
ATOM 2585 N SER A 172 11.362 -5.144 3.892 1.00 0.31
N
ATOM 2586 CA SER A 172 11.755 -6.410 3.298 1.00 0.47
C
ATOM 2587 C SER A 172 12.871 -6.221 2.288 1.00 0.48
C
ATOM 2588 O SER A 172 13.823 -6.999 2.260 1.00 0.54
O
ATOM 2589 CB SER A 172 10.578 -7.063 2.641 1.00 0.70
C
ATOM 2590 OG SER A 172 9.639 -7.443 3.598 1.00 0.70
O

ATOM 2591 H SER A 172 10.453 -4.746 3.675 1.00 0.37
H
ATOM 2592 HA SER A 172 12.118 -7.065 4.090 1.00 0.56
H
ATOM 2593 1HB SER A 172 10.122 -6.367 1.937 1.00 0.85
H
ATOM 2594 2HB SER A 172 10.904 -7.936 2.077 1.00 0.85
H
ATOM 2595 HG SER A 172 8.871 -7.720 3.101 1.00 0.85
H
ATOM 2596 N HIS A 173 12.779 -5.154 1.493 1.00 0.48
N
ATOM 2597 CA HIS A 173 13.789 -4.837 0.489 1.00 0.52
C
ATOM 2598 C HIS A 173 15.164 -4.678 1.133 1.00 0.45
C
ATOM 2599 O HIS A 173 16.168 -5.149 0.594 1.00 0.55
O
ATOM 2600 CB HIS A 173 13.423 -3.557 -0.266 1.00 0.78
C
ATOM 2601 CG HIS A 173 14.300 -3.289 -1.450 1.00 0.78
C
ATOM 2602 ND1 HIS A 173 14.144 -3.956 -2.650 1.00 0.78
N
ATOM 2603 CD2 HIS A 173 15.345 -2.448 -1.623 1.00 0.78
C
ATOM 2604 CE1 HIS A 173 15.051 -3.527 -3.510 1.00 0.78
C
ATOM 2605 NE2 HIS A 173 15.791 -2.613 -2.912 1.00 0.78
N
ATOM 2606 H HIS A 173 11.974 -4.549 1.558 1.00 0.58
H
ATOM 2607 HA HIS A 173 13.853 -5.650 -0.233 1.00 0.62
H
ATOM 2608 1HB HIS A 173 12.391 -3.621 -0.613 1.00 0.94
H
ATOM 2609 2HB HIS A 173 13.488 -2.702 0.407 1.00 0.94
H
ATOM 2610 HD2 HIS A 173 15.755 -1.767 -0.882 1.00 0.94
H
ATOM 2611 HE1 HIS A 173 15.170 -3.872 -4.537 1.00 0.94
H
ATOM 2612 HE2 HIS A 173 16.565 -2.113 -3.327 1.00 0.94
H
ATOM 2613 N TYR A 174 15.205 -4.020 2.294 1.00 0.35
N
ATOM 2614 CA TYR A 174 16.456 -3.815 3.010 1.00 0.33
C
ATOM 2615 C TYR A 174 16.684 -4.784 4.168 1.00 0.32
C
ATOM 2616 O TYR A 174 17.565 -4.546 4.997 1.00 0.37
O
ATOM 2617 CB TYR A 174 16.531 -2.378 3.520 1.00 0.49
C
ATOM 2618 CG TYR A 174 16.752 -1.381 2.412 1.00 0.49
C
ATOM 2619 CD1 TYR A 174 15.714 -0.604 1.925 1.00 0.49
C

ATOM 2620 CD2 TYR A 174 18.022 -1.260 1.874 1.00 0.49
C
ATOM 2621 CE1 TYR A 174 15.954 0.298 0.903 1.00 0.49
C
ATOM 2622 CE2 TYR A 174 18.260 -0.360 0.858 1.00 0.49
C
ATOM 2623 CZ TYR A 174 17.233 0.415 0.371 1.00 0.49
C
ATOM 2624 OH TYR A 174 17.478 1.303 -0.647 1.00 0.49
O
ATOM 2625 H TYR A 174 14.353 -3.633 2.677 1.00 0.42
H
ATOM 2626 HA TYR A 174 17.272 -3.964 2.303 1.00 0.40
H
ATOM 2627 1HB TYR A 174 15.605 -2.120 4.035 1.00 0.59
H
ATOM 2628 2HB TYR A 174 17.346 -2.281 4.236 1.00 0.59
H
ATOM 2629 HD1 TYR A 174 14.713 -0.699 2.344 1.00 0.59
H
ATOM 2630 HD2 TYR A 174 18.835 -1.874 2.257 1.00 0.59
H
ATOM 2631 HE1 TYR A 174 15.143 0.911 0.512 1.00 0.59
H
ATOM 2632 HE2 TYR A 174 19.261 -0.267 0.436 1.00 0.59
H
ATOM 2633 HH TYR A 174 16.643 1.605 -1.019 1.00 0.59
H
ATOM 2634 N ASN A 175 15.924 -5.884 4.212 1.00 0.32
N
ATOM 2635 CA ASN A 175 16.106 -6.927 5.224 1.00 0.36
C
ATOM 2636 C ASN A 175 16.230 -6.409 6.651 1.00 0.29
C
ATOM 2637 O ASN A 175 17.149 -6.805 7.369 1.00 0.35
O
ATOM 2638 CB ASN A 175 17.314 -7.778 4.878 1.00 0.54
C
ATOM 2639 CG ASN A 175 17.113 -8.580 3.623 1.00 0.54
C
ATOM 2640 OD1 ASN A 175 16.057 -9.202 3.450 1.00 0.54
O
ATOM 2641 ND2 ASN A 175 18.091 -8.579 2.752 1.00 0.54
N
ATOM 2642 H ASN A 175 15.215 -6.043 3.511 1.00 0.38
H
ATOM 2643 HA ASN A 175 15.224 -7.569 5.200 1.00 0.43
H
ATOM 2644 1HB ASN A 175 18.187 -7.136 4.748 1.00 0.65
H
ATOM 2645 2HB ASN A 175 17.527 -8.460 5.701 1.00 0.65
H
ATOM 2646 1HD2 ASN A 175 18.006 -9.095 1.899 1.00 0.65
H
ATOM 2647 2HD2 ASN A 175 18.924 -8.058 2.938 1.00 0.65
H
ATOM 2648 N LEU A 176 15.320 -5.529 7.067 1.00 0.26
N

```
ATOM   2649  CA  LEU A 176      15.374  -4.959   8.414  1.00  0.26
C
ATOM   2650  C   LEU A 176      14.999  -5.973   9.502  1.00  0.28
C
ATOM   2651  O   LEU A 176      13.908  -5.926  10.072  1.00  0.29
O
ATOM   2652  CB  LEU A 176      14.481  -3.716   8.504  1.00  0.39
C
ATOM   2653  CG  LEU A 176      14.875  -2.578   7.546  1.00  0.39
C
ATOM   2654  CD1 LEU A 176      13.927  -1.401   7.728  1.00  0.39
C
ATOM   2655  CD2 LEU A 176      16.319  -2.171   7.807  1.00  0.39
C
ATOM   2656  H   LEU A 176      14.583  -5.244   6.435  1.00  0.31
H
ATOM   2657  HA  LEU A 176      16.400  -4.642   8.599  1.00  0.31
H
ATOM   2658 1HB  LEU A 176      13.455  -4.003   8.282  1.00  0.47
H
ATOM   2659 2HB  LEU A 176      14.522  -3.327   9.521  1.00  0.47
H
ATOM   2660  HG  LEU A 176      14.781  -2.925   6.516  1.00  0.47
H
ATOM   2661 1HD1 LEU A 176      14.200  -0.605   7.039  1.00  0.47
H
ATOM   2662 2HD1 LEU A 176      12.905  -1.716   7.530  1.00  0.47
H
ATOM   2663 3HD1 LEU A 176      13.996  -1.035   8.746  1.00  0.47
H
ATOM   2664 1HD2 LEU A 176      16.602  -1.372   7.121  1.00  0.47
H
ATOM   2665 2HD2 LEU A 176      16.419  -1.821   8.834  1.00  0.47
H
ATOM   2666 3HD2 LEU A 176      16.972  -3.031   7.652  1.00  0.47
H
ATOM   2667  N   ASP A 177      15.936  -6.874   9.795  1.00  0.33
N
ATOM   2668  CA  ASP A 177      15.780  -7.934  10.786  1.00  0.37
C
ATOM   2669  C   ASP A 177      15.634  -7.434  12.215  1.00  0.31
C
ATOM   2670  O   ASP A 177      15.160  -8.172  13.087  1.00  0.40
O
ATOM   2671  CB  ASP A 177      16.985  -8.864  10.723  1.00  0.55
C
ATOM   2672  CG  ASP A 177      18.319  -8.168  10.961  1.00  0.55
C
ATOM   2673  OD1 ASP A 177      18.643  -7.281  10.208  1.00  0.55
O
ATOM   2674  OD2 ASP A 177      19.002  -8.526  11.892  1.00  0.55
O
ATOM   2675  H   ASP A 177      16.796  -6.830   9.266  1.00  0.40
H
ATOM   2676  HA  ASP A 177      14.885  -8.503  10.533  1.00  0.44
H
ATOM   2677 1HB  ASP A 177      16.879  -9.624  11.493  1.00  0.67
H
```

```
ATOM   2678 2HB  ASP A 177      17.015  -9.366   9.757  1.00  0.67
H
ATOM   2679  N   ASN A 178      16.010  -6.177  12.440  1.00  0.43
N
ATOM   2680  CA  ASN A 178      15.878  -5.538  13.730  1.00  0.65
C
ATOM   2681  C   ASN A 178      14.577  -4.737  13.831  1.00  0.89
C
ATOM   2682  O   ASN A 178      14.419  -3.935  14.747  1.00  2.76
O
ATOM   2683  CB  ASN A 178      17.084  -4.662  14.007  1.00  0.98
C
ATOM   2684  CG  ASN A 178      17.204  -3.525  13.040  1.00  0.98
C
ATOM   2685  OD1 ASN A 178      16.583  -3.549  11.972  1.00  0.98
O
ATOM   2686  ND2 ASN A 178      17.988  -2.537  13.387  1.00  0.98
N
ATOM   2687  H   ASN A 178      16.410  -5.640  11.685  1.00  0.52
H
ATOM   2688  HA  ASN A 178      15.855  -6.309  14.488  1.00  0.78
H
ATOM   2689 1HB  ASN A 178      17.019  -4.259  15.019  1.00  1.17
H
ATOM   2690 2HB  ASN A 178      17.990  -5.265  13.954  1.00  1.17
H
ATOM   2691 1HD2 ASN A 178      18.107  -1.754  12.774  1.00  1.17
H
ATOM   2692 2HD2 ASN A 178      18.471  -2.565  14.263  1.00  1.17
H
ATOM   2693  N   LEU A 179      13.639  -4.965  12.907  1.00  0.22
N
ATOM   2694  CA  LEU A 179      12.366  -4.258  12.925  1.00  0.22
C
ATOM   2695  C   LEU A 179      11.201  -5.212  13.172  1.00  0.23
C
ATOM   2696  O   LEU A 179      11.011  -6.176  12.424  1.00  0.32
O
ATOM   2697  CB  LEU A 179      12.159  -3.522  11.596  1.00  0.33
C
ATOM   2698  CG  LEU A 179      10.840  -2.760  11.439  1.00  0.33
C
ATOM   2699  CD1 LEU A 179      10.784  -1.630  12.454  1.00  0.33
C
ATOM   2700  CD2 LEU A 179      10.750  -2.214  10.025  1.00  0.33
C
ATOM   2701  H   LEU A 179      13.803  -5.613  12.148  1.00  0.26
H
ATOM   2702  HA  LEU A 179      12.388  -3.527  13.731  1.00  0.26
H
ATOM   2703 1HB  LEU A 179      12.970  -2.810  11.466  1.00  0.40
H
ATOM   2704 2HB  LEU A 179      12.213  -4.249  10.786  1.00  0.40
H
ATOM   2705  HG  LEU A 179      10.001  -3.431  11.628  1.00  0.40
H
ATOM   2706 1HD1 LEU A 179       9.843  -1.092  12.344  1.00  0.40
H
```

```
ATOM   2707 2HD1 LEU A 179      10.849  -2.044  13.459  1.00  0.40
H
ATOM   2708 3HD1 LEU A 179      11.615  -0.948  12.286  1.00  0.40
H
ATOM   2709 1HD2 LEU A 179       9.812  -1.670   9.903  1.00  0.40
H
ATOM   2710 2HD2 LEU A 179      11.586  -1.541   9.841  1.00  0.40
H
ATOM   2711 3HD2 LEU A 179      10.784  -3.041   9.318  1.00  0.40
H
ATOM   2712  N   VAL A 180      10.438  -4.931  14.228  1.00  0.23
N
ATOM   2713  CA  VAL A 180       9.273  -5.725  14.589  1.00  0.28
C
ATOM   2714  C   VAL A 180       8.017  -4.865  14.615  1.00  0.23
C
ATOM   2715  O   VAL A 180       7.922  -3.917  15.395  1.00  0.28
O
ATOM   2716  CB  VAL A 180       9.446  -6.364  15.976  1.00  0.42
C
ATOM   2717  CG1 VAL A 180       8.204  -7.177  16.326  1.00  0.42
C
ATOM   2718  CG2 VAL A 180      10.695  -7.228  16.010  1.00  0.42
C
ATOM   2719  H   VAL A 180      10.673  -4.128  14.797  1.00  0.28
H
ATOM   2720  HA  VAL A 180       9.145  -6.514  13.848  1.00  0.34
H
ATOM   2721  HB  VAL A 180       9.539  -5.573  16.711  1.00  0.50
H
ATOM   2722 1HG1 VAL A 180       8.320  -7.607  17.320  1.00  0.50
H
ATOM   2723 2HG1 VAL A 180       7.328  -6.529  16.311  1.00  0.50
H
ATOM   2724 3HG1 VAL A 180       8.077  -7.977  15.597  1.00  0.50
H
ATOM   2725 1HG2 VAL A 180      10.810  -7.660  17.004  1.00  0.50
H
ATOM   2726 2HG2 VAL A 180      10.612  -8.028  15.277  1.00  0.50
H
ATOM   2727 3HG2 VAL A 180      11.565  -6.615  15.778  1.00  0.50
H
ATOM   2728  N   ALA A 181       7.056  -5.210  13.774  1.00  0.23
N
ATOM   2729  CA  ALA A 181       5.795  -4.495  13.733  1.00  0.28
C
ATOM   2730  C   ALA A 181       4.749  -5.297  14.470  1.00  0.20
C
ATOM   2731  O   ALA A 181       4.682  -6.515  14.322  1.00  0.27
O
ATOM   2732  CB  ALA A 181       5.357  -4.244  12.304  1.00  0.42
C
ATOM   2733  H   ALA A 181       7.195  -6.004  13.168  1.00  0.28
H
ATOM   2734  HA  ALA A 181       5.919  -3.541  14.244  1.00  0.34
H
ATOM   2735 1HB  ALA A 181       4.415  -3.706  12.309  1.00  0.50
H
```

```
ATOM   2736 2HB  ALA A 181       6.114  -3.652  11.789  1.00  0.50
H
ATOM   2737 3HB  ALA A 181       5.228  -5.196  11.790  1.00  0.50
H
ATOM   2738  N   VAL A 182       3.934  -4.615  15.258  1.00  0.20
N
ATOM   2739  CA  VAL A 182       2.882  -5.262  16.007  1.00  0.20
C
ATOM   2740  C   VAL A 182       1.548  -4.740  15.518  1.00  0.23
C
ATOM   2741  O   VAL A 182       1.343  -3.531  15.412  1.00  0.26
O
ATOM   2742  CB  VAL A 182       3.024  -5.002  17.506  1.00  0.30
C
ATOM   2743  CG1 VAL A 182       1.908  -5.724  18.250  1.00  0.30
C
ATOM   2744  CG2 VAL A 182       4.395  -5.443  17.968  1.00  0.30
C
ATOM   2745  H   VAL A 182       4.055  -3.619  15.346  1.00  0.24
H
ATOM   2746  HA  VAL A 182       2.929  -6.336  15.832  1.00  0.24
H
ATOM   2747  HB  VAL A 182       2.910  -3.936  17.699  1.00  0.36
H
ATOM   2748 1HG1 VAL A 182       1.997  -5.531  19.314  1.00  0.36
H
ATOM   2749 2HG1 VAL A 182       0.941  -5.366  17.897  1.00  0.36
H
ATOM   2750 3HG1 VAL A 182       1.986  -6.796  18.069  1.00  0.36
H
ATOM   2751 1HG2 VAL A 182       4.501  -5.250  19.034  1.00  0.36
H
ATOM   2752 2HG2 VAL A 182       4.509  -6.509  17.774  1.00  0.36
H
ATOM   2753 3HG2 VAL A 182       5.160  -4.891  17.421  1.00  0.36
H
ATOM   2754  N   PHE A 183       0.651  -5.655  15.196  1.00  0.29
N
ATOM   2755  CA  PHE A 183      -0.642  -5.292  14.652  1.00  0.43
C
ATOM   2756  C   PHE A 183      -1.724  -5.494  15.670  1.00  0.40
C
ATOM   2757  O   PHE A 183      -2.071  -6.626  16.007  1.00  0.56
O
ATOM   2758  CB  PHE A 183      -0.965  -6.190  13.484  1.00  0.65
C
ATOM   2759  CG  PHE A 183       0.011  -6.074  12.376  1.00  0.65
C
ATOM   2760  CD1 PHE A 183       1.298  -6.562  12.530  1.00  0.65
C
ATOM   2761  CD2 PHE A 183      -0.358  -5.517  11.173  1.00  0.65
C
ATOM   2762  CE1 PHE A 183       2.210  -6.471  11.507  1.00  0.65
C
ATOM   2763  CE2 PHE A 183       0.544  -5.440  10.145  1.00  0.65
C
ATOM   2764  CZ  PHE A 183       1.834  -5.912  10.312  1.00  0.65
C
```

ATOM 2765 H PHE A 183 0.880 -6.630 15.320 1.00 0.35
H
ATOM 2766 HA PHE A 183 -0.626 -4.246 14.344 1.00 0.52
H
ATOM 2767 1HB PHE A 183 -1.018 -7.213 13.802 1.00 0.77
H
ATOM 2768 2HB PHE A 183 -1.945 -5.925 13.094 1.00 0.77
H
ATOM 2769 HD1 PHE A 183 1.589 -7.011 13.477 1.00 0.77
H
ATOM 2770 HD2 PHE A 183 -1.376 -5.145 11.042 1.00 0.77
H
ATOM 2771 HE1 PHE A 183 3.221 -6.847 11.643 1.00 0.77
H
ATOM 2772 HE2 PHE A 183 0.233 -5.004 9.200 1.00 0.77
H
ATOM 2773 HZ PHE A 183 2.551 -5.847 9.495 1.00 0.77
H
ATOM 2774 N ASP A 184 -2.280 -4.405 16.162 1.00 0.37
N
ATOM 2775 CA ASP A 184 -3.340 -4.514 17.138 1.00 0.43
C
ATOM 2776 C ASP A 184 -4.658 -4.780 16.426 1.00 0.44
C
ATOM 2777 O ASP A 184 -5.467 -3.870 16.239 1.00 0.60
O
ATOM 2778 CB ASP A 184 -3.436 -3.234 17.976 1.00 0.65
C
ATOM 2779 CG ASP A 184 -4.415 -3.384 19.121 1.00 0.65
C
ATOM 2780 OD1 ASP A 184 -4.770 -4.498 19.397 1.00 0.65
O
ATOM 2781 OD2 ASP A 184 -4.820 -2.397 19.692 1.00 0.65
O
ATOM 2782 H ASP A 184 -1.973 -3.490 15.864 1.00 0.44
H
ATOM 2783 HA ASP A 184 -3.127 -5.354 17.798 1.00 0.52
H
ATOM 2784 1HB ASP A 184 -2.454 -2.988 18.380 1.00 0.77
H
ATOM 2785 2HB ASP A 184 -3.750 -2.404 17.344 1.00 0.77
H
ATOM 2786 N VAL A 185 -4.870 -6.038 16.015 1.00 0.32
N
ATOM 2787 CA VAL A 185 -6.089 -6.395 15.295 1.00 0.31
C
ATOM 2788 C VAL A 185 -7.248 -6.522 16.259 1.00 0.27
C
ATOM 2789 O VAL A 185 -7.647 -7.625 16.637 1.00 0.30
O
ATOM 2790 CB VAL A 185 -5.912 -7.703 14.501 1.00 0.46
C
ATOM 2791 CG1 VAL A 185 -7.195 -8.030 13.755 1.00 0.46
C
ATOM 2792 CG2 VAL A 185 -4.756 -7.546 13.524 1.00 0.46
C
ATOM 2793 H VAL A 185 -4.175 -6.752 16.216 1.00 0.38
H

ATOM 2794 HA VAL A 185 -6.315 -5.601 14.585 1.00 0.37
H
ATOM 2795 HB VAL A 185 -5.713 -8.525 15.186 1.00 0.56
H
ATOM 2796 1HG1 VAL A 185 -7.069 -8.960 13.201 1.00 0.56
H
ATOM 2797 2HG1 VAL A 185 -8.012 -8.141 14.467 1.00 0.56
H
ATOM 2798 3HG1 VAL A 185 -7.429 -7.223 13.060 1.00 0.56
H
ATOM 2799 1HG2 VAL A 185 -4.633 -8.470 12.961 1.00 0.56
H
ATOM 2800 2HG2 VAL A 185 -4.971 -6.728 12.837 1.00 0.56
H
ATOM 2801 3HG2 VAL A 185 -3.841 -7.329 14.074 1.00 0.56
H
ATOM 2802 N ASN A 186 -7.791 -5.362 16.618 1.00 0.32
N
ATOM 2803 CA ASN A 186 -8.889 -5.221 17.555 1.00 0.43
C
ATOM 2804 C ASN A 186 -10.260 -5.147 16.892 1.00 0.62
C
ATOM 2805 O ASN A 186 -11.226 -4.695 17.513 1.00 1.35
O
ATOM 2806 CB ASN A 186 -8.628 -4.017 18.440 1.00 0.65
C
ATOM 2807 CG ASN A 186 -8.605 -2.721 17.701 1.00 0.65
C
ATOM 2808 OD1 ASN A 186 -8.983 -2.643 16.526 1.00 0.65
O
ATOM 2809 ND2 ASN A 186 -8.146 -1.692 18.369 1.00 0.65
N
ATOM 2810 H ASN A 186 -7.372 -4.524 16.237 1.00 0.38
H
ATOM 2811 HA ASN A 186 -8.896 -6.099 18.188 1.00 0.52
H
ATOM 2812 1HB ASN A 186 -9.392 -3.961 19.210 1.00 0.77
H
ATOM 2813 2HB ASN A 186 -7.671 -4.135 18.943 1.00 0.77
H
ATOM 2814 1HD2 ASN A 186 -8.093 -0.794 17.935 1.00 0.77
H
ATOM 2815 2HD2 ASN A 186 -7.834 -1.812 19.318 1.00 0.77
H
ATOM 2816 N ARG A 187 -10.342 -5.631 15.640 1.00 0.43
N
ATOM 2817 CA ARG A 187 -11.557 -5.718 14.817 1.00 0.50
C
ATOM 2818 C ARG A 187 -12.131 -4.409 14.291 1.00 0.61
C
ATOM 2819 O ARG A 187 -12.423 -4.311 13.098 1.00 1.68
O
ATOM 2820 CB ARG A 187 -12.681 -6.379 15.594 1.00 0.75
C
ATOM 2821 CG ARG A 187 -13.937 -6.633 14.786 1.00 0.75
C
ATOM 2822 CD ARG A 187 -15.003 -7.231 15.621 1.00 0.75
C

```
ATOM   2823  NE  ARG A 187     -16.169  -7.586  14.837  1.00  0.75
N
ATOM   2824  CZ  ARG A 187     -17.326  -8.040  15.346  1.00  0.75
C
ATOM   2825  NH1 ARG A 187     -17.486  -8.171  16.646  1.00  0.75
N
ATOM   2826  NH2 ARG A 187     -18.296  -8.353  14.504  1.00  0.75
N
ATOM   2827  H   ARG A 187      -9.495  -5.967  15.207  1.00  0.52
H
ATOM   2828  HA  ARG A 187     -11.323  -6.347  13.961  1.00  0.60
H
ATOM   2829 1HB  ARG A 187     -12.339  -7.330  15.990  1.00  0.90
H
ATOM   2830 2HB  ARG A 187     -12.979  -5.761  16.438  1.00  0.90
H
ATOM   2831 1HG  ARG A 187     -14.307  -5.694  14.375  1.00  0.90
H
ATOM   2832 2HG  ARG A 187     -13.714  -7.324  13.973  1.00  0.90
H
ATOM   2833 1HD  ARG A 187     -14.634  -8.130  16.107  1.00  0.90
H
ATOM   2834 2HD  ARG A 187     -15.312  -6.514  16.382  1.00  0.90
H
ATOM   2835  HE  ARG A 187     -16.113  -7.513  13.826  1.00  0.90
H
ATOM   2836 1HH1 ARG A 187     -16.737  -7.924  17.287  1.00  0.90
H
ATOM   2837 2HH1 ARG A 187     -18.358  -8.514  17.018  1.00  0.90
H
ATOM   2838 1HH2 ARG A 187     -18.128  -8.246  13.506  1.00  0.90
H
ATOM   2839 2HH2 ARG A 187     -19.179  -8.699  14.846  1.00  0.90
H
ATOM   2840  N   LEU A 188     -12.350  -3.445  15.171  1.00  0.94
N
ATOM   2841  CA  LEU A 188     -13.020  -2.214  14.796  1.00  0.83
C
ATOM   2842  C   LEU A 188     -12.092  -1.029  14.516  1.00  0.77
C
ATOM   2843  O   LEU A 188     -10.989  -0.932  15.058  1.00  1.28
O
ATOM   2844  CB  LEU A 188     -13.985  -1.839  15.919  1.00  1.24
C
ATOM   2845  CG  LEU A 188     -15.022  -2.900  16.318  1.00  1.24
C
ATOM   2846  CD1 LEU A 188     -15.820  -2.386  17.502  1.00  1.24
C
ATOM   2847  CD2 LEU A 188     -15.929  -3.216  15.145  1.00  1.24
C
ATOM   2848  H   LEU A 188     -12.080  -3.591  16.132  1.00  1.13
H
ATOM   2849  HA  LEU A 188     -13.583  -2.411  13.897  1.00  1.00
H
ATOM   2850 1HB  LEU A 188     -13.397  -1.627  16.800  1.00  1.49
H
ATOM   2851 2HB  LEU A 188     -14.521  -0.934  15.634  1.00  1.49
H
```

```
ATOM   2852  HG  LEU A 188      -14.511  -3.811  16.630  1.00  1.49
H
ATOM   2853 1HD1 LEU A 188      -16.549  -3.139  17.803  1.00  1.49
H
ATOM   2854 2HD1 LEU A 188      -15.146  -2.183  18.335  1.00  1.49
H
ATOM   2855 3HD1 LEU A 188      -16.337  -1.470  17.221  1.00  1.49
H
ATOM   2856 1HD2 LEU A 188      -16.661  -3.968  15.444  1.00  1.49
H
ATOM   2857 2HD2 LEU A 188      -16.450  -2.312  14.828  1.00  1.49
H
ATOM   2858 3HD2 LEU A 188      -15.332  -3.603  14.323  1.00  1.49
H
ATOM   2859  N   GLY A 189      -12.576  -0.116  13.672  1.00  0.45
N
ATOM   2860  CA  GLY A 189      -11.886   1.124  13.322  1.00  0.37
C
ATOM   2861  C   GLY A 189      -12.546   2.299  14.052  1.00  0.31
C
ATOM   2862  O   GLY A 189      -12.836   2.200  15.246  1.00  0.63
O
ATOM   2863  H   GLY A 189      -13.485  -0.273  13.263  1.00  0.54
H
ATOM   2864 1HA  GLY A 189      -10.834   1.055  13.599  1.00  0.44
H
ATOM   2865 2HA  GLY A 189      -11.932   1.281  12.244  1.00  0.44
H
ATOM   2866  N   GLN A 190      -12.781   3.408  13.338  1.00  0.25
N
ATOM   2867  CA  GLN A 190      -13.429   4.568  13.949  1.00  0.39
C
ATOM   2868  C   GLN A 190      -14.948   4.532  13.912  1.00  0.61
C
ATOM   2869  O   GLN A 190      -15.600   4.466  14.956  1.00  2.37
O
ATOM   2870  CB  GLN A 190      -12.952   5.874  13.299  1.00  0.58
C
ATOM   2871  CG  GLN A 190      -13.652   7.122  13.822  1.00  0.58
C
ATOM   2872  CD  GLN A 190      -13.036   8.407  13.309  1.00  0.58
C
ATOM   2873  OE1 GLN A 190      -11.828   8.483  13.068  1.00  0.58
O
ATOM   2874  NE2 GLN A 190      -13.865   9.432  13.146  1.00  0.58
N
ATOM   2875  H   GLN A 190      -12.519   3.461  12.355  1.00  0.30
H
ATOM   2876  HA  GLN A 190      -13.137   4.589  14.989  1.00  0.47
H
ATOM   2877 1HB  GLN A 190      -11.880   5.991  13.461  1.00  0.70
H
ATOM   2878 2HB  GLN A 190      -13.118   5.837  12.228  1.00  0.70
H
ATOM   2879 1HG  GLN A 190      -14.686   7.100  13.481  1.00  0.70
H
ATOM   2880 2HG  GLN A 190      -13.629   7.136  14.904  1.00  0.70
H
```

ATOM 2881 1HE2 GLN A 190 -13.516 10.309 12.813 1.00 0.70
H
ATOM 2882 2HE2 GLN A 190 -14.843 9.332 13.349 1.00 0.70
H
ATOM 2883 N SER A 191 -15.514 4.624 12.713 1.00 0.31
N
ATOM 2884 CA SER A 191 -16.962 4.680 12.555 1.00 0.34
C
ATOM 2885 C SER A 191 -17.611 3.322 12.310 1.00 0.42
C
ATOM 2886 O SER A 191 -18.832 3.232 12.169 1.00 1.60
O
ATOM 2887 CB SER A 191 -17.311 5.601 11.412 1.00 0.51
C
ATOM 2888 OG SER A 191 -16.889 5.068 10.185 1.00 0.51
O
ATOM 2889 H SER A 191 -14.933 4.658 11.881 1.00 0.37
H
ATOM 2890 HA SER A 191 -17.385 5.093 13.471 1.00 0.41
H
ATOM 2891 1HB SER A 191 -18.389 5.757 11.393 1.00 0.61
H
ATOM 2892 2HB SER A 191 -16.840 6.570 11.571 1.00 0.61
H
ATOM 2893 HG SER A 191 -15.931 4.975 10.240 1.00 0.61
H
ATOM 2894 N GLY A 192 -16.809 2.272 12.259 1.00 0.35
N
ATOM 2895 CA GLY A 192 -17.325 0.937 11.998 1.00 0.37
C
ATOM 2896 C GLY A 192 -16.195 -0.060 12.135 1.00 0.40
C
ATOM 2897 O GLY A 192 -15.156 0.278 12.697 1.00 0.52
O
ATOM 2898 H GLY A 192 -15.817 2.393 12.389 1.00 0.42
H
ATOM 2899 1HA GLY A 192 -18.124 0.702 12.703 1.00 0.44
H
ATOM 2900 2HA GLY A 192 -17.743 0.907 10.995 1.00 0.44
H
ATOM 2901 N PRO A 193 -16.396 -1.289 11.671 1.00 0.43
N
ATOM 2902 CA PRO A 193 -15.458 -2.393 11.667 1.00 0.43
C
ATOM 2903 C PRO A 193 -14.391 -2.208 10.611 1.00 0.37
C
ATOM 2904 O PRO A 193 -14.657 -1.677 9.532 1.00 0.38
O
ATOM 2905 CB PRO A 193 -16.343 -3.610 11.379 1.00 0.65
C
ATOM 2906 CG PRO A 193 -17.501 -3.062 10.618 1.00 0.65
C
ATOM 2907 CD PRO A 193 -17.735 -1.697 11.212 1.00 0.65
C
ATOM 2908 HA PRO A 193 -15.007 -2.471 12.650 1.00 0.52
H
ATOM 2909 1HB PRO A 193 -15.776 -4.360 10.806 1.00 0.77
H

```
ATOM   2910 2HB  PRO A 193     -16.644  -4.090  12.322  1.00  0.77
H
ATOM   2911 1HG  PRO A 193     -17.264  -3.022   9.545  1.00  0.77
H
ATOM   2912 2HG  PRO A 193     -18.372  -3.725  10.726  1.00  0.77
H
ATOM   2913 1HD  PRO A 193     -18.100  -1.015  10.432  1.00  0.77
H
ATOM   2914 2HD  PRO A 193     -18.427  -1.752  12.065  1.00  0.77
H
ATOM   2915  N   ALA A 194     -13.182  -2.658  10.916  1.00  0.49
N
ATOM   2916  CA  ALA A 194     -12.112  -2.598   9.944  1.00  0.52
C
ATOM   2917  C   ALA A 194     -12.381  -3.684   8.905  1.00  0.53
C
ATOM   2918  O   ALA A 194     -12.851  -4.760   9.270  1.00  0.88
O
ATOM   2919  CB  ALA A 194     -10.762  -2.777  10.622  1.00  0.78
C
ATOM   2920  H   ALA A 194     -13.007  -3.075  11.818  1.00  0.59
H
ATOM   2921  HA  ALA A 194     -12.148  -1.625   9.459  1.00  0.62
H
ATOM   2922 1HB  ALA A 194      -9.960  -2.728   9.888  1.00  0.94
H
ATOM   2923 2HB  ALA A 194     -10.622  -1.988  11.361  1.00  0.94
H
ATOM   2924 3HB  ALA A 194     -10.734  -3.745  11.119  1.00  0.94
H
ATOM   2925  N   PRO A 195     -12.104  -3.419   7.621  1.00  0.54
N
ATOM   2926  CA  PRO A 195     -12.335  -4.276   6.446  1.00  0.53
C
ATOM   2927  C   PRO A 195     -12.213  -5.789   6.672  1.00  0.69
C
ATOM   2928  O   PRO A 195     -13.046  -6.555   6.187  1.00  1.29
O
ATOM   2929  CB  PRO A 195     -11.241  -3.827   5.470  1.00  0.80
C
ATOM   2930  CG  PRO A 195     -11.006  -2.410   5.817  1.00  0.80
C
ATOM   2931  CD  PRO A 195     -11.074  -2.399   7.314  1.00  0.80
C
ATOM   2932  HA  PRO A 195     -13.327  -4.041   6.034  1.00  0.64
H
ATOM   2933 1HB  PRO A 195     -10.344  -4.450   5.590  1.00  0.95
H
ATOM   2934 2HB  PRO A 195     -11.587  -3.959   4.433  1.00  0.95
H
ATOM   2935 1HG  PRO A 195     -10.032  -2.077   5.430  1.00  0.95
H
ATOM   2936 2HG  PRO A 195     -11.771  -1.774   5.351  1.00  0.95
H
ATOM   2937 1HD  PRO A 195     -10.106  -2.692   7.745  1.00  0.95
H
ATOM   2938 2HD  PRO A 195     -11.393  -1.404   7.654  1.00  0.95
H
```

ATOM 2939 N LEU A 196 -11.164 -6.223 7.367 1.00 0.48
N
ATOM 2940 CA LEU A 196 -10.915 -7.652 7.542 1.00 0.56
C
ATOM 2941 C LEU A 196 -11.309 -8.235 8.907 1.00 0.58
C
ATOM 2942 O LEU A 196 -11.085 -9.423 9.136 1.00 0.95
O
ATOM 2943 CB LEU A 196 -9.430 -7.915 7.324 1.00 0.84
C
ATOM 2944 CG LEU A 196 -8.875 -7.541 5.952 1.00 0.84
C
ATOM 2945 CD1 LEU A 196 -7.375 -7.797 5.949 1.00 0.84
C
ATOM 2946 CD2 LEU A 196 -9.589 -8.351 4.885 1.00 0.84
C
ATOM 2947 H LEU A 196 -10.515 -5.557 7.762 1.00 0.58
H
ATOM 2948 HA LEU A 196 -11.479 -8.184 6.776 1.00 0.67
H
ATOM 2949 1HB LEU A 196 -8.878 -7.336 8.054 1.00 1.01
H
ATOM 2950 2HB LEU A 196 -9.228 -8.971 7.492 1.00 1.01
H
ATOM 2951 HG LEU A 196 -9.035 -6.480 5.762 1.00 1.01
H
ATOM 2952 1HD1 LEU A 196 -6.959 -7.529 4.978 1.00 1.01
H
ATOM 2953 2HD1 LEU A 196 -6.904 -7.192 6.724 1.00 1.01
H
ATOM 2954 3HD1 LEU A 196 -7.187 -8.849 6.151 1.00 1.01
H
ATOM 2955 1HD2 LEU A 196 -9.195 -8.089 3.903 1.00 1.01
H
ATOM 2956 2HD2 LEU A 196 -9.429 -9.411 5.071 1.00 1.01
H
ATOM 2957 3HD2 LEU A 196 -10.657 -8.134 4.916 1.00 1.01
H
ATOM 2958 N GLU A 197 -11.887 -7.410 9.785 1.00 1.11
N
ATOM 2959 CA GLU A 197 -12.261 -7.779 11.164 1.00 2.07
C
ATOM 2960 C GLU A 197 -11.257 -8.724 11.846 1.00 0.94
C
ATOM 2961 O GLU A 197 -10.162 -8.296 12.204 1.00 2.62
O
ATOM 2962 CB GLU A 197 -13.668 -8.379 11.201 1.00 3.10
C
ATOM 2963 CG GLU A 197 -14.776 -7.373 10.933 1.00 3.10
C
ATOM 2964 CD GLU A 197 -16.147 -7.986 11.020 1.00 3.10
C
ATOM 2965 OE1 GLU A 197 -16.560 -8.618 10.077 1.00 3.10
O
ATOM 2966 OE2 GLU A 197 -16.771 -7.843 12.049 1.00 3.10
O
ATOM 2967 H GLU A 197 -12.075 -6.459 9.505 1.00 1.33
H

```
ATOM   2968  HA  GLU A 197     -12.290  -6.863  11.752  1.00  2.48
H
ATOM   2969 1HB  GLU A 197     -13.749  -9.171  10.458  1.00  3.73
H
ATOM   2970 2HB  GLU A 197     -13.856  -8.819  12.181  1.00  3.73
H
ATOM   2971 1HG  GLU A 197     -14.703  -6.564  11.659  1.00  3.73
H
ATOM   2972 2HG  GLU A 197     -14.634  -6.947   9.941  1.00  3.73
H
ATOM   2973  N   HIS A 198     -11.635  -9.989  12.061  1.00  1.43
N
ATOM   2974  CA  HIS A 198     -10.742 -10.948  12.721  1.00  1.07
C
ATOM   2975  C   HIS A 198     -10.108 -11.981  11.801  1.00  0.72
C
ATOM   2976  O   HIS A 198      -9.625 -13.006  12.286  1.00  0.81
O
ATOM   2977  CB  HIS A 198     -11.448 -11.713  13.844  1.00  1.60
C
ATOM   2978  CG  HIS A 198     -11.804 -10.908  15.048  1.00  1.60
C
ATOM   2979  ND1 HIS A 198     -10.849 -10.432  15.923  1.00  1.60
N
ATOM   2980  CD2 HIS A 198     -12.999 -10.526  15.549  1.00  1.60
C
ATOM   2981  CE1 HIS A 198     -11.445  -9.790  16.910  1.00  1.60
C
ATOM   2982  NE2 HIS A 198     -12.747  -9.836  16.708  1.00  1.60
N
ATOM   2983  H   HIS A 198     -12.544 -10.304  11.759  1.00  1.72
H
ATOM   2984  HA  HIS A 198      -9.924 -10.397  13.181  1.00  1.28
H
ATOM   2985 1HB  HIS A 198     -12.367 -12.153  13.454  1.00  1.93
H
ATOM   2986 2HB  HIS A 198     -10.812 -12.536  14.168  1.00  1.93
H
ATOM   2987  HD1 HIS A 198      -9.875 -10.338  15.718  1.00  1.93
H
ATOM   2988  HD2 HIS A 198     -14.026 -10.679  15.215  1.00  1.93
H
ATOM   2989  HE1 HIS A 198     -10.862  -9.340  17.710  1.00  1.93
H
ATOM   2990  N   GLY A 199     -10.120 -11.745  10.491  1.00  0.72
N
ATOM   2991  CA  GLY A 199      -9.537 -12.690   9.529  1.00  0.84
C
ATOM   2992  C   GLY A 199      -8.008 -12.720   9.601  1.00  0.74
C
ATOM   2993  O   GLY A 199      -7.336 -12.247   8.684  1.00  0.78
O
ATOM   2994  H   GLY A 199     -10.529 -10.891  10.140  1.00  0.86
H
ATOM   2995 1HA  GLY A 199      -9.932 -13.688   9.721  1.00  1.01
H
ATOM   2996 2HA  GLY A 199      -9.849 -12.415   8.522  1.00  1.01
H
```

```
ATOM   2997  N   ALA A 200      -7.485 -13.314  10.680  1.00  0.65
N
ATOM   2998  CA  ALA A 200      -6.055 -13.394  10.990  1.00  0.58
C
ATOM   2999  C   ALA A 200      -5.239 -13.972   9.853  1.00  0.55
C
ATOM   3000  O   ALA A 200      -4.133 -13.503   9.587  1.00  0.57
O
ATOM   3001  CB  ALA A 200      -5.851 -14.229  12.242  1.00  0.87
C
ATOM   3002  H   ALA A 200      -8.135 -13.694  11.352  1.00  0.78
H
ATOM   3003  HA  ALA A 200      -5.695 -12.387  11.186  1.00  0.70
H
ATOM   3004 1HB  ALA A 200      -4.793 -14.255  12.494  1.00  1.04
H
ATOM   3005 2HB  ALA A 200      -6.410 -13.788  13.068  1.00  1.04
H
ATOM   3006 3HB  ALA A 200      -6.206 -15.243  12.065  1.00  1.04
H
ATOM   3007  N   ASP A 201      -5.791 -14.975   9.179  1.00  0.54
N
ATOM   3008  CA  ASP A 201      -5.115 -15.628   8.071  1.00  0.55
C
ATOM   3009  C   ASP A 201      -4.911 -14.692   6.885  1.00  0.52
C
ATOM   3010  O   ASP A 201      -3.976 -14.877   6.108  1.00  0.54
O
ATOM   3011  CB  ASP A 201      -5.907 -16.853   7.624  1.00  0.83
C
ATOM   3012  CG  ASP A 201      -5.857 -17.991   8.634  1.00  0.83
C
ATOM   3013  OD1 ASP A 201      -5.031 -17.948   9.513  1.00  0.83
O
ATOM   3014  OD2 ASP A 201      -6.650 -18.893   8.517  1.00  0.83
O
ATOM   3015  H   ASP A 201      -6.705 -15.306   9.452  1.00  0.65
H
ATOM   3016  HA  ASP A 201      -4.134 -15.957   8.415  1.00  0.66
H
ATOM   3017 1HB  ASP A 201      -6.949 -16.576   7.462  1.00  0.99
H
ATOM   3018 2HB  ASP A 201      -5.513 -17.214   6.673  1.00  0.99
H
ATOM   3019  N   ILE A 202      -5.790 -13.702   6.734  1.00  0.56
N
ATOM   3020  CA  ILE A 202      -5.680 -12.755   5.643  1.00  0.55
C
ATOM   3021  C   ILE A 202      -4.559 -11.794   5.962  1.00  0.49
C
ATOM   3022  O   ILE A 202      -3.743 -11.478   5.095  1.00  0.48
O
ATOM   3023  CB  ILE A 202      -6.980 -11.983   5.410  1.00  0.83
C
ATOM   3024  CG1 ILE A 202      -8.080 -12.940   4.956  1.00  0.83
C
ATOM   3025  CG2 ILE A 202      -6.731 -10.936   4.335  1.00  0.83
C
```

```
ATOM   3026  CD1 ILE A 202      -9.458 -12.323   4.966  1.00  0.83
C
ATOM   3027  H   ILE A 202      -6.535 -13.572   7.403  1.00  0.67
H
ATOM   3028  HA  ILE A 202      -5.427 -13.292   4.730  1.00  0.66
H
ATOM   3029  HB  ILE A 202      -7.306 -11.499   6.329  1.00  0.99
H
ATOM   3030 1HG1 ILE A 202      -7.861 -13.281   3.946  1.00  0.99
H
ATOM   3031 2HG1 ILE A 202      -8.090 -13.808   5.616  1.00  0.99
H
ATOM   3032 1HG2 ILE A 202      -7.647 -10.379   4.149  1.00  0.99
H
ATOM   3033 2HG2 ILE A 202      -5.952 -10.251   4.667  1.00  0.99
H
ATOM   3034 3HG2 ILE A 202      -6.415 -11.427   3.415  1.00  0.99
H
ATOM   3035 1HD1 ILE A 202     -10.189 -13.059   4.635  1.00  0.99
H
ATOM   3036 2HD1 ILE A 202      -9.703 -11.996   5.978  1.00  0.99
H
ATOM   3037 3HD1 ILE A 202      -9.476 -11.468   4.292  1.00  0.99
H
ATOM   3038  N   TYR A 203      -4.516 -11.347   7.223  1.00  0.48
N
ATOM   3039  CA  TYR A 203      -3.453 -10.447   7.663  1.00  0.44
C
ATOM   3040  C   TYR A 203      -2.110 -11.141   7.485  1.00  0.41
C
ATOM   3041  O   TYR A 203      -1.138 -10.516   7.052  1.00  0.39
O
ATOM   3042  CB  TYR A 203      -3.636 -10.063   9.137  1.00  0.66
C
ATOM   3043  CG  TYR A 203      -4.805  -9.149   9.421  1.00  0.66
C
ATOM   3044  CD1 TYR A 203      -5.978  -9.693   9.901  1.00  0.66
C
ATOM   3045  CD2 TYR A 203      -4.708  -7.781   9.222  1.00  0.66
C
ATOM   3046  CE1 TYR A 203      -7.064  -8.894  10.175  1.00  0.66
C
ATOM   3047  CE2 TYR A 203      -5.795  -6.972   9.502  1.00  0.66
C
ATOM   3048  CZ  TYR A 203      -6.967  -7.529   9.973  1.00  0.66
C
ATOM   3049  OH  TYR A 203      -8.053  -6.736  10.243  1.00  0.66
O
ATOM   3050  H   TYR A 203      -5.241 -11.639   7.874  1.00  0.58
H
ATOM   3051  HA  TYR A 203      -3.464  -9.551   7.043  1.00  0.53
H
ATOM   3052 1HB  TYR A 203      -3.765 -10.968   9.732  1.00  0.79
H
ATOM   3053 2HB  TYR A 203      -2.732  -9.571   9.492  1.00  0.79
H
ATOM   3054  HD1 TYR A 203      -6.042 -10.761  10.059  1.00  0.79
H
```

ATOM	3055	HD2	TYR	A	203	-3.782	-7.345	8.848	1.00	0.79	H
ATOM	3056	HE1	TYR	A	203	-7.988	-9.332	10.548	1.00	0.79	H
ATOM	3057	HE2	TYR	A	203	-5.728	-5.894	9.348	1.00	0.79	H
ATOM	3058	HH	TYR	A	203	-7.759	-5.852	10.481	1.00	0.79	H
ATOM	3059	N	GLN	A	204	-2.087	-12.447	7.780	1.00	0.46	N
ATOM	3060	CA	GLN	A	204	-0.912	-13.276	7.592	1.00	0.55	C
ATOM	3061	C	GLN	A	204	-0.475	-13.313	6.150	1.00	0.52	C
ATOM	3062	O	GLN	A	204	0.692	-13.063	5.849	1.00	0.63	O
ATOM	3063	CB	GLN	A	204	-1.166	-14.708	8.067	1.00	0.83	C
ATOM	3064	CG	GLN	A	204	0.038	-15.613	7.908	1.00	0.83	C
ATOM	3065	CD	GLN	A	204	-0.227	-17.049	8.315	1.00	0.83	C
ATOM	3066	OE1	GLN	A	204	-1.332	-17.412	8.726	1.00	0.83	O
ATOM	3067	NE2	GLN	A	204	0.800	-17.883	8.195	1.00	0.83	N
ATOM	3068	H	GLN	A	204	-2.914	-12.881	8.167	1.00	0.55	H
ATOM	3069	HA	GLN	A	204	-0.104	-12.850	8.158	1.00	0.66	H
ATOM	3070	1HB	GLN	A	204	-1.457	-14.706	9.114	1.00	0.99	H
ATOM	3071	2HB	GLN	A	204	-1.985	-15.145	7.501	1.00	0.99	H
ATOM	3072	1HG	GLN	A	204	0.336	-15.612	6.859	1.00	0.99	H
ATOM	3073	2HG	GLN	A	204	0.852	-15.232	8.524	1.00	0.99	H
ATOM	3074	1HE2	GLN	A	204	0.697	-18.847	8.443	1.00	0.99	H
ATOM	3075	2HE2	GLN	A	204	1.681	-17.544	7.853	1.00	0.99	H
ATOM	3076	N	ASN	A	205	-1.419	-13.607	5.258	1.00	0.49	N
ATOM	3077	CA	ASN	A	205	-1.126	-13.689	3.840	1.00	0.48	C
ATOM	3078	C	ASN	A	205	-0.535	-12.401	3.310	1.00	0.43	C
ATOM	3079	O	ASN	A	205	0.425	-12.441	2.540	1.00	0.45	O
ATOM	3080	CB	ASN	A	205	-2.378	-14.041	3.057	1.00	0.72	C
ATOM	3081	CG	ASN	A	205	-2.789	-15.477	3.221	1.00	0.72	C
ATOM	3082	OD1	ASN	A	205	-1.989	-16.329	3.620	1.00	0.72	O
ATOM	3083	ND2	ASN	A	205	-4.030	-15.761	2.915	1.00	0.72	N

```
ATOM   3084  H   ASN A 205      -2.360 -13.814   5.571  1.00  0.59
H
ATOM   3085  HA  ASN A 205      -0.385 -14.474   3.686  1.00  0.58
H
ATOM   3086 1HB  ASN A 205      -3.200 -13.404   3.384  1.00  0.86
H
ATOM   3087 2HB  ASN A 205      -2.213 -13.844   1.998  1.00  0.86
H
ATOM   3088 1HD2 ASN A 205      -4.362 -16.701   3.005  1.00  0.86
H
ATOM   3089 2HD2 ASN A 205      -4.644 -15.041   2.595  1.00  0.86
H
ATOM   3090  N   CYS A 206      -1.091 -11.261   3.726  1.00  0.42
N
ATOM   3091  CA  CYS A 206      -0.598  -9.972   3.265  1.00  0.47
C
ATOM   3092  C   CYS A 206       0.816  -9.697   3.721  1.00  0.59
C
ATOM   3093  O   CYS A 206       1.676  -9.376   2.901  1.00  0.78
O
ATOM   3094  CB  CYS A 206      -1.501  -8.840   3.759  1.00  0.70
C
ATOM   3095  SG  CYS A 206      -3.128  -8.768   2.969  1.00  0.70
S
ATOM   3096  H   CYS A 206      -1.887 -11.292   4.352  1.00  0.50
H
ATOM   3097  HA  CYS A 206      -0.605  -9.967   2.179  1.00  0.56
H
ATOM   3098 1HB  CYS A 206      -1.657  -8.951   4.832  1.00  0.85
H
ATOM   3099 2HB  CYS A 206      -1.007  -7.883   3.598  1.00  0.85
H
ATOM   3100  HG  CYS A 206      -3.439 -10.044   3.165  1.00  0.85
H
ATOM   3101  N   CYS A 207       1.067  -9.838   5.013  1.00  0.61
N
ATOM   3102  CA  CYS A 207       2.387  -9.537   5.537  1.00  1.00
C
ATOM   3103  C   CYS A 207       3.458 -10.478   4.995  1.00  0.53
C
ATOM   3104  O   CYS A 207       4.558 -10.028   4.668  1.00  0.44
O
ATOM   3105  CB  CYS A 207       2.357  -9.577   7.052  1.00  1.50
C
ATOM   3106  SG  CYS A 207       1.442  -8.211   7.796  1.00  1.50
S
ATOM   3107  H   CYS A 207       0.333 -10.126   5.651  1.00  0.73
H
ATOM   3108  HA  CYS A 207       2.644  -8.522   5.236  1.00  1.20
H
ATOM   3109 1HB  CYS A 207       1.880 -10.503   7.371  1.00  1.80
H
ATOM   3110 2HB  CYS A 207       3.372  -9.568   7.448  1.00  1.80
H
ATOM   3111  HG  CYS A 207       0.290  -8.468   7.186  1.00  1.80
H
ATOM   3112  N   GLU A 208       3.130 -11.763   4.853  1.00  0.51
N
```

```
ATOM   3113  CA  GLU A 208       4.076 -12.710   4.285  1.00  0.53
C
ATOM   3114  C   GLU A 208       4.313 -12.409   2.811  1.00  0.44
C
ATOM   3115  O   GLU A 208       5.443 -12.512   2.336  1.00  0.54
O
ATOM   3116  CB  GLU A 208       3.595 -14.145   4.480  1.00  0.80
C
ATOM   3117  CG  GLU A 208       3.640 -14.610   5.930  1.00  0.80
C
ATOM   3118  CD  GLU A 208       3.164 -16.022   6.118  1.00  0.80
C
ATOM   3119  OE1 GLU A 208       2.772 -16.633   5.154  1.00  0.80
O
ATOM   3120  OE2 GLU A 208       3.189 -16.495   7.231  1.00  0.80
O
ATOM   3121  H   GLU A 208       2.220 -12.096   5.146  1.00  0.61
H
ATOM   3122  HA  GLU A 208       5.027 -12.598   4.806  1.00  0.64
H
ATOM   3123 1HB  GLU A 208       2.567 -14.237   4.130  1.00  0.95
H
ATOM   3124 2HB  GLU A 208       4.209 -14.823   3.886  1.00  0.95
H
ATOM   3125 1HG  GLU A 208       4.667 -14.534   6.290  1.00  0.95
H
ATOM   3126 2HG  GLU A 208       3.024 -13.942   6.528  1.00  0.95
H
ATOM   3127  N   ALA A 209       3.269 -11.981   2.095  1.00  0.40
N
ATOM   3128  CA  ALA A 209       3.411 -11.590   0.696  1.00  0.47
C
ATOM   3129  C   ALA A 209       4.389 -10.425   0.556  1.00  0.49
C
ATOM   3130  O   ALA A 209       5.185 -10.384  -0.384  1.00  0.74
O
ATOM   3131  CB  ALA A 209       2.070 -11.218   0.115  1.00  0.70
C
ATOM   3132  H   ALA A 209       2.347 -11.939   2.509  1.00  0.48
H
ATOM   3133  HA  ALA A 209       3.816 -12.439   0.144  1.00  0.56
H
ATOM   3134 1HB  ALA A 209       2.207 -10.956  -0.929  1.00  0.85
H
ATOM   3135 2HB  ALA A 209       1.389 -12.065   0.195  1.00  0.85
H
ATOM   3136 3HB  ALA A 209       1.657 -10.368   0.653  1.00  0.85
H
ATOM   3137  N   PHE A 210       4.359  -9.500   1.524  1.00  0.53
N
ATOM   3138  CA  PHE A 210       5.301  -8.383   1.553  1.00  1.02
C
ATOM   3139  C   PHE A 210       6.710  -8.747   2.039  1.00  0.59
C
ATOM   3140  O   PHE A 210       7.568  -7.868   2.065  1.00  1.07
O
ATOM   3141  CB  PHE A 210       4.784  -7.211   2.412  1.00  1.53
C
```

```
ATOM   3142  CG  PHE A 210       3.757  -6.321   1.755  1.00  1.53
C
ATOM   3143  CD1 PHE A 210       2.405  -6.419   2.043  1.00  1.53
C
ATOM   3144  CD2 PHE A 210       4.163  -5.354   0.847  1.00  1.53
C
ATOM   3145  CE1 PHE A 210       1.485  -5.590   1.430  1.00  1.53
C
ATOM   3146  CE2 PHE A 210       3.248  -4.520   0.240  1.00  1.53
C
ATOM   3147  CZ  PHE A 210       1.906  -4.640   0.526  1.00  1.53
C
ATOM   3148  H   PHE A 210       3.653  -9.563   2.247  1.00  0.64
H
ATOM   3149  HA  PHE A 210       5.396  -8.014   0.532  1.00  1.22
H
ATOM   3150 1HB  PHE A 210       4.346  -7.608   3.328  1.00  1.84
H
ATOM   3151 2HB  PHE A 210       5.628  -6.590   2.703  1.00  1.84
H
ATOM   3152  HD1 PHE A 210       2.069  -7.155   2.763  1.00  1.84
H
ATOM   3153  HD2 PHE A 210       5.224  -5.257   0.615  1.00  1.84
H
ATOM   3154  HE1 PHE A 210       0.426  -5.684   1.667  1.00  1.84
H
ATOM   3155  HE2 PHE A 210       3.590  -3.768  -0.466  1.00  1.84
H
ATOM   3156  HZ  PHE A 210       1.184  -3.982   0.044  1.00  1.84
H
ATOM   3157  N   GLY A 211       6.965 -10.014   2.398  1.00  0.47
N
ATOM   3158  CA  GLY A 211       8.292 -10.440   2.844  1.00  0.60
C
ATOM   3159  C   GLY A 211       8.514 -10.415   4.366  1.00  0.75
C
ATOM   3160  O   GLY A 211       9.660 -10.417   4.815  1.00  1.31
O
ATOM   3161  H   GLY A 211       6.247 -10.718   2.354  1.00  0.56
H
ATOM   3162 1HA  GLY A 211       8.472 -11.451   2.480  1.00  0.72
H
ATOM   3163 2HA  GLY A 211       9.041  -9.809   2.368  1.00  0.72
H
ATOM   3164  N   TRP A 212       7.436 -10.383   5.156  1.00  0.61
N
ATOM   3165  CA  TRP A 212       7.566 -10.335   6.618  1.00  0.63
C
ATOM   3166  C   TRP A 212       7.313 -11.676   7.314  1.00  0.86
C
ATOM   3167  O   TRP A 212       6.445 -12.459   6.916  1.00  1.44
O
ATOM   3168  CB  TRP A 212       6.592  -9.296   7.194  1.00  0.95
C
ATOM   3169  CG  TRP A 212       6.984  -7.878   6.917  1.00  0.95
C
ATOM   3170  CD1 TRP A 212       7.156  -7.295   5.704  1.00  0.95
C
```

```
ATOM   3171  CD2 TRP A 212       7.233  -6.844   7.896  1.00  0.95
C
ATOM   3172  NE1 TRP A 212       7.525  -5.985   5.860  1.00  0.95
N
ATOM   3173  CE2 TRP A 212       7.570  -5.691   7.196  1.00  0.95
C
ATOM   3174  CE3 TRP A 212       7.206  -6.808   9.285  1.00  0.95
C
ATOM   3175  CZ2 TRP A 212       7.880  -4.503   7.845  1.00  0.95
C
ATOM   3176  CZ3 TRP A 212       7.524  -5.626   9.937  1.00  0.95
C
ATOM   3177  CH2 TRP A 212       7.850  -4.499   9.236  1.00  0.95
C
ATOM   3178  H   TRP A 212       6.506 -10.383   4.758  1.00  0.73
H
ATOM   3179  HA  TRP A 212       8.582 -10.020   6.855  1.00  0.76
H
ATOM   3180 1HB  TRP A 212       5.595  -9.461   6.792  1.00  1.13
H
ATOM   3181 2HB  TRP A 212       6.530  -9.425   8.274  1.00  1.13
H
ATOM   3182  HD1 TRP A 212       7.036  -7.798   4.747  1.00  1.13
H
ATOM   3183  HE1 TRP A 212       7.729  -5.344   5.109  1.00  1.13
H
ATOM   3184  HE3 TRP A 212       6.944  -7.700   9.840  1.00  1.13
H
ATOM   3185  HZ2 TRP A 212       8.140  -3.596   7.302  1.00  1.13
H
ATOM   3186  HZ3 TRP A 212       7.505  -5.617  11.025  1.00  1.13
H
ATOM   3187  HH2 TRP A 212       8.092  -3.586   9.778  1.00  1.13
H
ATOM   3188  N   ASN A 213       8.063 -11.916   8.390  1.00  0.79
N
ATOM   3189  CA  ASN A 213       7.922 -13.127   9.193  1.00  1.42
C
ATOM   3190  C   ASN A 213       6.724 -12.977  10.089  1.00  0.94
C
ATOM   3191  O   ASN A 213       6.842 -12.510  11.220  1.00  1.58
O
ATOM   3192  CB  ASN A 213       9.151 -13.400  10.038  1.00  2.13
C
ATOM   3193  CG  ASN A 213      10.361 -13.779   9.247  1.00  2.13
C
ATOM   3194  OD1 ASN A 213      10.266 -14.438   8.205  1.00  2.13
O
ATOM   3195  ND2 ASN A 213      11.507 -13.386   9.737  1.00  2.13
N
ATOM   3196  H   ASN A 213       8.745 -11.230   8.672  1.00  0.95
H
ATOM   3197  HA  ASN A 213       7.742 -13.976   8.531  1.00  1.70
H
ATOM   3198 1HB  ASN A 213       9.389 -12.512  10.626  1.00  2.56
H
ATOM   3199 2HB  ASN A 213       8.934 -14.203  10.740  1.00  2.56
H
```

```
ATOM   3200 1HD2 ASN A 213      12.360 -13.614   9.263  1.00  2.56
H
ATOM   3201 2HD2 ASN A 213      11.534 -12.857  10.585  1.00  2.56
H
ATOM   3202  N   THR A 214       5.568 -13.349   9.574  1.00  0.80
N
ATOM   3203  CA  THR A 214       4.343 -13.176  10.324  1.00  0.60
C
ATOM   3204  C   THR A 214       4.035 -14.272  11.312  1.00  0.88
C
ATOM   3205  O   THR A 214       4.119 -15.462  11.004  1.00  2.62
O
ATOM   3206  CB  THR A 214       3.149 -13.035   9.397  1.00  0.90
C
ATOM   3207  OG1 THR A 214       3.368 -11.917   8.540  1.00  0.90
O
ATOM   3208  CG2 THR A 214       1.906 -12.779  10.241  1.00  0.90
C
ATOM   3209  H   THR A 214       5.551 -13.708   8.626  1.00  0.96
H
ATOM   3210  HA  THR A 214       4.437 -12.254  10.887  1.00  0.72
H
ATOM   3211  HB  THR A 214       3.019 -13.933   8.796  1.00  1.08
H
ATOM   3212  HG1 THR A 214       4.159 -12.070   8.017  1.00  1.08
H
ATOM   3213 1HG2 THR A 214       1.049 -12.637   9.608  1.00  1.08
H
ATOM   3214 2HG2 THR A 214       1.722 -13.626  10.899  1.00  1.08
H
ATOM   3215 3HG2 THR A 214       2.057 -11.881  10.836  1.00  1.08
H
ATOM   3216  N   TYR A 215       3.645 -13.848  12.504  1.00  0.68
N
ATOM   3217  CA  TYR A 215       3.209 -14.750  13.542  1.00  0.82
C
ATOM   3218  C   TYR A 215       1.823 -14.322  14.019  1.00  1.19
C
ATOM   3219  O   TYR A 215       1.558 -13.134  14.200  1.00  3.66
O
ATOM   3220  CB  TYR A 215       4.222 -14.758  14.692  1.00  1.23
C
ATOM   3221  CG  TYR A 215       5.590 -15.275  14.286  1.00  1.23
C
ATOM   3222  CD1 TYR A 215       6.578 -14.404  13.840  1.00  1.23
C
ATOM   3223  CD2 TYR A 215       5.850 -16.634  14.348  1.00  1.23
C
ATOM   3224  CE1 TYR A 215       7.821 -14.893  13.463  1.00  1.23
C
ATOM   3225  CE2 TYR A 215       7.087 -17.122  13.974  1.00  1.23
C
ATOM   3226  CZ  TYR A 215       8.072 -16.262  13.531  1.00  1.23
C
ATOM   3227  OH  TYR A 215       9.299 -16.778  13.165  1.00  1.23
O
ATOM   3228  H   TYR A 215       3.634 -12.852  12.676  1.00  0.82
H
```

```
ATOM   3229  HA  TYR A 215       3.130 -15.756  13.130  1.00  0.98
H
ATOM   3230 1HB  TYR A 215       4.340 -13.746  15.081  1.00  1.48
H
ATOM   3231 2HB  TYR A 215       3.849 -15.381  15.504  1.00  1.48
H
ATOM   3232  HD1 TYR A 215       6.376 -13.335  13.786  1.00  1.48
H
ATOM   3233  HD2 TYR A 215       5.077 -17.320  14.694  1.00  1.48
H
ATOM   3234  HE1 TYR A 215       8.593 -14.208  13.115  1.00  1.48
H
ATOM   3235  HE2 TYR A 215       7.285 -18.193  14.026  1.00  1.48
H
ATOM   3236  HH  TYR A 215       9.314 -17.720  13.353  1.00  1.48
H
ATOM   3237  N   LEU A 216       0.935 -15.292  14.217  1.00  0.57
N
ATOM   3238  CA  LEU A 216      -0.401 -14.978  14.709  1.00  0.70
C
ATOM   3239  C   LEU A 216      -0.423 -15.291  16.192  1.00  1.26
C
ATOM   3240  O   LEU A 216      -0.048 -16.389  16.604  1.00  3.68
O
ATOM   3241  CB  LEU A 216      -1.469 -15.790  13.959  1.00  1.05
C
ATOM   3242  CG  LEU A 216      -1.502 -15.604  12.431  1.00  1.05
C
ATOM   3243  CD1 LEU A 216      -2.604 -16.469  11.834  1.00  1.05
C
ATOM   3244  CD2 LEU A 216      -1.711 -14.139  12.112  1.00  1.05
C
ATOM   3245  H   LEU A 216       1.190 -16.254  14.038  1.00  0.68
H
ATOM   3246  HA  LEU A 216      -0.597 -13.916  14.570  1.00  0.84
H
ATOM   3247 1HB  LEU A 216      -1.307 -16.847  14.160  1.00  1.26
H
ATOM   3248 2HB  LEU A 216      -2.450 -15.514  14.346  1.00  1.26
H
ATOM   3249  HG  LEU A 216      -0.554 -15.933  12.003  1.00  1.26
H
ATOM   3250 1HD1 LEU A 216      -2.620 -16.344  10.751  1.00  1.26
H
ATOM   3251 2HD1 LEU A 216      -2.417 -17.514  12.075  1.00  1.26
H
ATOM   3252 3HD1 LEU A 216      -3.567 -16.169  12.245  1.00  1.26
H
ATOM   3253 1HD2 LEU A 216      -1.729 -13.991  11.035  1.00  1.26
H
ATOM   3254 2HD2 LEU A 216      -2.654 -13.800  12.539  1.00  1.26
H
ATOM   3255 3HD2 LEU A 216      -0.891 -13.573  12.543  1.00  1.26
H
ATOM   3256  N   VAL A 217      -0.874 -14.339  16.994  1.00  0.56
N
ATOM   3257  CA  VAL A 217      -0.814 -14.499  18.438  1.00  0.84
C
```

ATOM 3258 C VAL A 217 -2.016 -13.906 19.154 1.00 0.81
C
ATOM 3259 O VAL A 217 -2.579 -12.912 18.707 1.00 1.44
O
ATOM 3260 CB VAL A 217 0.487 -13.823 18.949 1.00 1.26
C
ATOM 3261 CG1 VAL A 217 0.441 -12.332 18.678 1.00 1.26
C
ATOM 3262 CG2 VAL A 217 0.688 -14.071 20.426 1.00 1.26
C
ATOM 3263 H VAL A 217 -1.196 -13.463 16.598 1.00 0.67
H
ATOM 3264 HA VAL A 217 -0.768 -15.566 18.661 1.00 1.01
H
ATOM 3265 HB VAL A 217 1.333 -14.234 18.398 1.00 1.51
H
ATOM 3266 1HG1 VAL A 217 1.368 -11.876 19.022 1.00 1.51
H
ATOM 3267 2HG1 VAL A 217 0.325 -12.161 17.610 1.00 1.51
H
ATOM 3268 3HG1 VAL A 217 -0.400 -11.890 19.213 1.00 1.51
H
ATOM 3269 1HG2 VAL A 217 1.618 -13.604 20.749 1.00 1.51
H
ATOM 3270 2HG2 VAL A 217 -0.139 -13.651 20.995 1.00 1.51
H
ATOM 3271 3HG2 VAL A 217 0.740 -15.143 20.591 1.00 1.51
H
ATOM 3272 N ASP A 218 -2.412 -14.531 20.260 1.00 0.59
N
ATOM 3273 CA ASP A 218 -3.465 -13.974 21.096 1.00 0.59
C
ATOM 3274 C ASP A 218 -2.900 -12.742 21.767 1.00 0.51
C
ATOM 3275 O ASP A 218 -2.013 -12.843 22.616 1.00 0.49
O
ATOM 3276 CB ASP A 218 -3.954 -14.978 22.140 1.00 0.89
C
ATOM 3277 CG ASP A 218 -5.114 -14.452 22.987 1.00 0.89
C
ATOM 3278 OD1 ASP A 218 -5.264 -13.256 23.108 1.00 0.89
O
ATOM 3279 OD2 ASP A 218 -5.847 -15.263 23.502 1.00 0.89
O
ATOM 3280 H ASP A 218 -1.942 -15.376 20.550 1.00 0.71
H
ATOM 3281 HA ASP A 218 -4.306 -13.677 20.466 1.00 0.71
H
ATOM 3282 1HB ASP A 218 -4.276 -15.892 21.640 1.00 1.06
H
ATOM 3283 2HB ASP A 218 -3.130 -15.242 22.802 1.00 1.06
H
ATOM 3284 N GLY A 219 -3.405 -11.574 21.389 1.00 0.49
N
ATOM 3285 CA GLY A 219 -2.853 -10.321 21.876 1.00 0.45
C
ATOM 3286 C GLY A 219 -3.166 -10.000 23.323 1.00 0.58
C

ATOM 3287 O GLY A 219 -2.666 -9.007 23.851 1.00 0.69
O
ATOM 3288 H GLY A 219 -4.181 -11.546 20.739 1.00 0.59
H
ATOM 3289 1HA GLY A 219 -1.772 -10.340 21.741 1.00 0.54
H
ATOM 3290 2HA GLY A 219 -3.223 -9.516 21.254 1.00 0.54
H
ATOM 3291 N HIS A 220 -3.972 -10.827 23.981 1.00 0.74
N
ATOM 3292 CA HIS A 220 -4.246 -10.590 25.378 1.00 1.07
C
ATOM 3293 C HIS A 220 -3.548 -11.624 26.252 1.00 0.61
C
ATOM 3294 O HIS A 220 -3.730 -11.643 27.471 1.00 0.61
O
ATOM 3295 CB HIS A 220 -5.744 -10.538 25.639 1.00 1.60
C
ATOM 3296 CG HIS A 220 -6.391 -9.426 24.879 1.00 1.60
C
ATOM 3297 ND1 HIS A 220 -5.985 -8.117 25.002 1.00 1.60
N
ATOM 3298 CD2 HIS A 220 -7.419 -9.420 24.002 1.00 1.60
C
ATOM 3299 CE1 HIS A 220 -6.722 -7.354 24.219 1.00 1.60
C
ATOM 3300 NE2 HIS A 220 -7.612 -8.116 23.613 1.00 1.60
N
ATOM 3301 H HIS A 220 -4.394 -11.630 23.525 1.00 0.89
H
ATOM 3302 HA HIS A 220 -3.842 -9.618 25.654 1.00 1.28
H
ATOM 3303 1HB HIS A 220 -6.205 -11.481 25.343 1.00 1.93
H
ATOM 3304 2HB HIS A 220 -5.932 -10.390 26.703 1.00 1.93
H
ATOM 3305 HD1 HIS A 220 -5.298 -7.758 25.641 1.00 1.93
H
ATOM 3306 HD2 HIS A 220 -8.057 -10.211 23.604 1.00 1.93
H
ATOM 3307 HE1 HIS A 220 -6.545 -6.282 24.179 1.00 1.93
H
ATOM 3308 N ASP A 221 -2.740 -12.481 25.629 1.00 0.65
N
ATOM 3309 CA ASP A 221 -1.972 -13.466 26.366 1.00 0.78
C
ATOM 3310 C ASP A 221 -0.521 -13.043 26.398 1.00 0.69
C
ATOM 3311 O ASP A 221 0.199 -13.224 25.415 1.00 0.66
O
ATOM 3312 CB ASP A 221 -2.096 -14.852 25.744 1.00 1.17
C
ATOM 3313 CG ASP A 221 -1.356 -15.905 26.554 1.00 1.17
C
ATOM 3314 OD1 ASP A 221 -0.686 -15.539 27.495 1.00 1.17
O
ATOM 3315 OD2 ASP A 221 -1.453 -17.062 26.223 1.00 1.17
O

```
ATOM   3316  H   ASP A 221      -2.624 -12.451  24.623  1.00  0.78
H
ATOM   3317  HA  ASP A 221      -2.342 -13.508  27.390  1.00  0.94
H
ATOM   3318 1HB  ASP A 221      -3.149 -15.132  25.680  1.00  1.40
H
ATOM   3319 2HB  ASP A 221      -1.697 -14.838  24.730  1.00  1.40
H
ATOM   3320  N   VAL A 222      -0.093 -12.487  27.527  1.00  0.77
N
ATOM   3321  CA  VAL A 222       1.273 -12.010  27.664  1.00  0.97
C
ATOM   3322  C   VAL A 222       2.321 -13.122  27.554  1.00  0.77
C
ATOM   3323  O   VAL A 222       3.481 -12.833  27.274  1.00  0.94
O
ATOM   3324  CB  VAL A 222       1.457 -11.279  29.001  1.00  1.46
C
ATOM   3325  CG1 VAL A 222       0.537 -10.074  29.085  1.00  1.46
C
ATOM   3326  CG2 VAL A 222       1.203 -12.230  30.153  1.00  1.46
C
ATOM   3327  H   VAL A 222      -0.735 -12.381  28.301  1.00  0.92
H
ATOM   3328  HA  VAL A 222       1.456 -11.293  26.864  1.00  1.16
H
ATOM   3329  HB  VAL A 222       2.473 -10.907  29.049  1.00  1.75
H
ATOM   3330 1HG1 VAL A 222       0.699  -9.553  30.028  1.00  1.75
H
ATOM   3331 2HG1 VAL A 222       0.754  -9.399  28.257  1.00  1.75
H
ATOM   3332 3HG1 VAL A 222      -0.501 -10.403  29.026  1.00  1.75
H
ATOM   3333 1HG2 VAL A 222       1.359 -11.705  31.095  1.00  1.75
H
ATOM   3334 2HG2 VAL A 222       0.177 -12.595  30.104  1.00  1.75
H
ATOM   3335 3HG2 VAL A 222       1.893 -13.070  30.090  1.00  1.75
H
ATOM   3336  N   GLU A 223       1.928 -14.387  27.765  1.00  0.83
N
ATOM   3337  CA  GLU A 223       2.879 -15.485  27.644  1.00  1.05
C
ATOM   3338  C   GLU A 223       3.214 -15.690  26.190  1.00  1.02
C
ATOM   3339  O   GLU A 223       4.379 -15.827  25.815  1.00  1.11
O
ATOM   3340  CB  GLU A 223       2.304 -16.795  28.193  1.00  1.58
C
ATOM   3341  CG  GLU A 223       2.094 -16.837  29.689  1.00  1.58
C
ATOM   3342  CD  GLU A 223       1.489 -18.136  30.157  1.00  1.58
C
ATOM   3343  OE1 GLU A 223       1.163 -18.952  29.328  1.00  1.58
O
ATOM   3344  OE2 GLU A 223       1.355 -18.313  31.346  1.00  1.58
O
```

```
ATOM   3345  H   GLU A 223       0.960 -14.602  27.980  1.00  1.00
H
ATOM   3346  HA  GLU A 223       3.792 -15.230  28.183  1.00  1.26
H
ATOM   3347 1HB  GLU A 223       1.344 -16.996  27.720  1.00  1.89
H
ATOM   3348 2HB  GLU A 223       2.972 -17.616  27.934  1.00  1.89
H
ATOM   3349 1HG  GLU A 223       3.059 -16.707  30.169  1.00  1.89
H
ATOM   3350 2HG  GLU A 223       1.450 -16.010  29.981  1.00  1.89
H
ATOM   3351  N   ALA A 224       2.163 -15.698  25.377  1.00  1.05
N
ATOM   3352  CA  ALA A 224       2.282 -15.879  23.950  1.00  1.17
C
ATOM   3353  C   ALA A 224       3.026 -14.707  23.334  1.00  0.85
C
ATOM   3354  O   ALA A 224       3.872 -14.901  22.461  1.00  0.90
O
ATOM   3355  CB  ALA A 224       0.903 -16.047  23.343  1.00  1.75
C
ATOM   3356  H   ALA A 224       1.240 -15.581  25.772  1.00  1.26
H
ATOM   3357  HA  ALA A 224       2.863 -16.784  23.768  1.00  1.40
H
ATOM   3358 1HB  ALA A 224       1.000 -16.222  22.276  1.00  2.11
H
ATOM   3359 2HB  ALA A 224       0.408 -16.902  23.803  1.00  2.11
H
ATOM   3360 3HB  ALA A 224       0.312 -15.150  23.519  1.00  2.11
H
ATOM   3361  N   LEU A 225       2.719 -13.496  23.810  1.00  0.67
N
ATOM   3362  CA  LEU A 225       3.391 -12.302  23.328  1.00  0.60
C
ATOM   3363  C   LEU A 225       4.869 -12.328  23.663  1.00  0.56
C
ATOM   3364  O   LEU A 225       5.693 -12.019  22.805  1.00  0.62
O
ATOM   3365  CB  LEU A 225       2.749 -11.060  23.935  1.00  0.90
C
ATOM   3366  CG  LEU A 225       1.341 -10.717  23.440  1.00  0.90
C
ATOM   3367  CD1 LEU A 225       0.778  -9.596  24.298  1.00  0.90
C
ATOM   3368  CD2 LEU A 225       1.411 -10.299  21.977  1.00  0.90
C
ATOM   3369  H   LEU A 225       1.995 -13.400  24.512  1.00  0.80
H
ATOM   3370  HA  LEU A 225       3.286 -12.260  22.245  1.00  0.72
H
ATOM   3371 1HB  LEU A 225       2.688 -11.205  25.009  1.00  1.08
H
ATOM   3372 2HB  LEU A 225       3.391 -10.201  23.738  1.00  1.08
H
ATOM   3373  HG  LEU A 225       0.691 -11.586  23.540  1.00  1.08
H
```

```
ATOM   3374 1HD1 LEU A 225      -0.224  -9.345  23.957  1.00  1.08
H
ATOM   3375 2HD1 LEU A 225       0.736  -9.918  25.338  1.00  1.08
H
ATOM   3376 3HD1 LEU A 225       1.419  -8.721  24.216  1.00  1.08
H
ATOM   3377 1HD2 LEU A 225       0.411 -10.052  21.620  1.00  1.08
H
ATOM   3378 2HD2 LEU A 225       2.057  -9.428  21.877  1.00  1.08
H
ATOM   3379 3HD2 LEU A 225       1.814 -11.120  21.383  1.00  1.08
H
ATOM   3380  N   CYS A 226       5.216 -12.752  24.882  1.00  0.57
N
ATOM   3381  CA  CYS A 226       6.618 -12.841  25.254  1.00  0.67
C
ATOM   3382  C   CYS A 226       7.357 -13.783  24.335  1.00  0.75
C
ATOM   3383  O   CYS A 226       8.412 -13.427  23.817  1.00  0.84
O
ATOM   3384  CB  CYS A 226       6.792 -13.322  26.696  1.00  1.01
C
ATOM   3385  SG  CYS A 226       6.345 -12.111  27.955  1.00  1.01
S
ATOM   3386  H   CYS A 226       4.511 -12.984  25.570  1.00  0.68
H
ATOM   3387  HA  CYS A 226       7.062 -11.850  25.165  1.00  0.80
H
ATOM   3388 1HB  CYS A 226       6.178 -14.209  26.854  1.00  1.21
H
ATOM   3389 2HB  CYS A 226       7.828 -13.610  26.862  1.00  1.21
H
ATOM   3390  HG  CYS A 226       7.302 -11.241  27.651  1.00  1.21
H
ATOM   3391  N   GLN A 227       6.797 -14.970  24.111  1.00  0.91
N
ATOM   3392  CA  GLN A 227       7.428 -15.956  23.243  1.00  1.27
C
ATOM   3393  C   GLN A 227       7.653 -15.406  21.841  1.00  1.23
C
ATOM   3394  O   GLN A 227       8.740 -15.553  21.267  1.00  1.92
O
ATOM   3395  CB  GLN A 227       6.556 -17.209  23.172  1.00  1.91
C
ATOM   3396  CG  GLN A 227       6.512 -18.010  24.460  1.00  1.91
C
ATOM   3397  CD  GLN A 227       5.522 -19.156  24.391  1.00  1.91
C
ATOM   3398  OE1 GLN A 227       4.630 -19.176  23.538  1.00  1.91
O
ATOM   3399  NE2 GLN A 227       5.671 -20.120  25.292  1.00  1.91
N
ATOM   3400  H   GLN A 227       5.923 -15.207  24.568  1.00  1.09
H
ATOM   3401  HA  GLN A 227       8.398 -16.220  23.666  1.00  1.52
H
ATOM   3402 1HB  GLN A 227       5.535 -16.928  22.918  1.00  2.29
H
```

```
ATOM   3403 2HB  GLN A 227       6.923 -17.864  22.382  1.00  2.29
H
ATOM   3404 1HG  GLN A 227       7.501 -18.426  24.647  1.00  2.29
H
ATOM   3405 2HG  GLN A 227       6.225 -17.354  25.279  1.00  2.29
H
ATOM   3406 1HE2 GLN A 227       5.047 -20.901  25.298  1.00  2.29
H
ATOM   3407 2HE2 GLN A 227       6.407 -20.062  25.967  1.00  2.29
H
ATOM   3408  N   ALA A 228       6.621 -14.752  21.307  1.00  1.01
N
ATOM   3409  CA  ALA A 228       6.665 -14.162  19.982  1.00  1.26
C
ATOM   3410  C   ALA A 228       7.757 -13.107  19.865  1.00  1.10
C
ATOM   3411  O   ALA A 228       8.456 -13.042  18.853  1.00  2.26
O
ATOM   3412  CB  ALA A 228       5.311 -13.548  19.652  1.00  1.89
C
ATOM   3413  H   ALA A 228       5.757 -14.678  21.829  1.00  1.21
H
ATOM   3414  HA  ALA A 228       6.883 -14.953  19.265  1.00  1.51
H
ATOM   3415 1HB  ALA A 228       5.339 -13.139  18.647  1.00  2.27
H
ATOM   3416 2HB  ALA A 228       4.541 -14.317  19.710  1.00  2.27
H
ATOM   3417 3HB  ALA A 228       5.086 -12.754  20.363  1.00  2.27
H
ATOM   3418  N   PHE A 229       7.910 -12.285  20.900  1.00  0.69
N
ATOM   3419  CA  PHE A 229       8.907 -11.230  20.864  1.00  0.80
C
ATOM   3420  C   PHE A 229      10.327 -11.676  21.241  1.00  1.50
C
ATOM   3421  O   PHE A 229      11.293 -11.061  20.789  1.00  4.90
O
ATOM   3422  CB  PHE A 229       8.498 -10.084  21.780  1.00  1.20
C
ATOM   3423  CG  PHE A 229       7.306  -9.300  21.314  1.00  1.20
C
ATOM   3424  CD1 PHE A 229       6.192  -9.166  22.124  1.00  1.20
C
ATOM   3425  CD2 PHE A 229       7.288  -8.705  20.067  1.00  1.20
C
ATOM   3426  CE1 PHE A 229       5.082  -8.458  21.712  1.00  1.20
C
ATOM   3427  CE2 PHE A 229       6.184  -7.994  19.650  1.00  1.20
C
ATOM   3428  CZ  PHE A 229       5.078  -7.870  20.471  1.00  1.20
C
ATOM   3429  H   PHE A 229       7.306 -12.367  21.708  1.00  0.83
H
ATOM   3430  HA  PHE A 229       8.943 -10.846  19.844  1.00  0.96
H
ATOM   3431 1HB  PHE A 229       8.280 -10.474  22.774  1.00  1.44
H
```

```
ATOM   3432 2HB  PHE A 229       9.331  -9.401  21.874  1.00  1.44
H
ATOM   3433  HD1 PHE A 229       6.202  -9.635  23.103  1.00  1.44
H
ATOM   3434  HD2 PHE A 229       8.156  -8.805  19.415  1.00  1.44
H
ATOM   3435  HE1 PHE A 229       4.215  -8.366  22.367  1.00  1.44
H
ATOM   3436  HE2 PHE A 229       6.188  -7.534  18.665  1.00  1.44
H
ATOM   3437  HZ  PHE A 229       4.205  -7.312  20.139  1.00  1.44
H
ATOM   3438  N   TRP A 230      10.465 -12.732  22.059  1.00  2.90
N
ATOM   3439  CA  TRP A 230      11.801 -13.188  22.473  1.00  5.13
C
ATOM   3440  C   TRP A 230      12.603 -13.802  21.354  1.00  6.38
C
ATOM   3441  O   TRP A 230      13.806 -13.575  21.243  1.00 29.64
O
ATOM   3442  CB  TRP A 230      11.751 -14.268  23.571  1.00  7.70
C
ATOM   3443  CG  TRP A 230      11.538 -13.826  24.993  1.00  7.70
C
ATOM   3444  CD1 TRP A 230      10.497 -14.167  25.799  1.00  7.70
C
ATOM   3445  CD2 TRP A 230      12.399 -12.984  25.796  1.00  7.70
C
ATOM   3446  NE1 TRP A 230      10.638 -13.590  27.031  1.00  7.70
N
ATOM   3447  CE2 TRP A 230      11.794 -12.864  27.052  1.00  7.70
C
ATOM   3448  CE3 TRP A 230      13.616 -12.333  25.558  1.00  7.70
C
ATOM   3449  CZ2 TRP A 230      12.356 -12.118  28.068  1.00  7.70
C
ATOM   3450  CZ3 TRP A 230      14.181 -11.585  26.581  1.00  7.70
C
ATOM   3451  CH2 TRP A 230      13.563 -11.481  27.801  1.00  7.70
C
ATOM   3452  H   TRP A 230       9.650 -13.204  22.421  1.00  3.48
H
ATOM   3453  HA  TRP A 230      12.349 -12.327  22.853  1.00  6.16
H
ATOM   3454 1HB  TRP A 230      10.958 -14.977  23.329  1.00  9.23
H
ATOM   3455 2HB  TRP A 230      12.686 -14.823  23.541  1.00  9.23
H
ATOM   3456  HD1 TRP A 230       9.671 -14.810  25.506  1.00  9.23
H
ATOM   3457  HE1 TRP A 230       9.998 -13.689  27.805  1.00  9.23
H
ATOM   3458  HE3 TRP A 230      14.106 -12.414  24.587  1.00  9.23
H
ATOM   3459  HZ2 TRP A 230      11.886 -12.023  29.047  1.00  9.23
H
ATOM   3460  HZ3 TRP A 230      15.128 -11.078  26.390  1.00  9.23
H
```

```
ATOM   3461  HH2 TRP A 230      14.027 -10.885  28.583  1.00  9.23
H
ATOM   3462  N   GLN A 231      11.961 -14.587  20.526  1.00 16.93
N
ATOM   3463  CA  GLN A 231      12.692 -15.213  19.453  1.00 28.43
C
ATOM   3464  C   GLN A 231      12.791 -14.282  18.292  1.00 16.96
C
ATOM   3465  O   GLN A 231      11.963 -13.385  18.138  1.00 51.15
O
ATOM   3466  CB  GLN A 231      12.019 -16.521  19.039  1.00 42.64
C
ATOM   3467  CG  GLN A 231      12.030 -17.596  20.113  1.00 42.64
C
ATOM   3468  CD  GLN A 231      13.438 -18.094  20.387  1.00 42.64
C
ATOM   3469  OE1 GLN A 231      14.163 -18.439  19.449  1.00 42.64
O
ATOM   3470  NE2 GLN A 231      13.837 -18.145  21.653  1.00 42.64
N
ATOM   3471  H   GLN A 231      10.968 -14.771  20.650  1.00 20.32
H
ATOM   3472  HA  GLN A 231      13.702 -15.434  19.799  1.00 34.12
H
ATOM   3473 1HB  GLN A 231      10.980 -16.325  18.771  1.00 51.17
H
ATOM   3474 2HB  GLN A 231      12.514 -16.924  18.155  1.00 51.17
H
ATOM   3475 1HG  GLN A 231      11.625 -17.181  21.036  1.00 51.17
H
ATOM   3476 2HG  GLN A 231      11.423 -18.439  19.782  1.00 51.17
H
ATOM   3477 1HE2 GLN A 231      14.756 -18.471  21.874  1.00 51.17
H
ATOM   3478 2HE2 GLN A 231      13.224 -17.864  22.396  1.00 51.17
H
ATOM   3479  N   ALA A 232      13.769 -14.511  17.443  1.00 23.33
N
ATOM   3480  CA  ALA A 232      13.838 -13.704  16.258  1.00 39.84
C
ATOM   3481  C   ALA A 232      13.130 -14.547  15.219  1.00 64.01
C
ATOM   3482  O   ALA A 232      11.921 -14.735  15.322  1.00102.78
O
ATOM   3483  CB  ALA A 232      15.277 -13.368  15.875  1.00 59.76
C
ATOM   3484  H   ALA A 232      14.442 -15.244  17.613  1.00 28.00
H
ATOM   3485  HA  ALA A 232      13.276 -12.783  16.402  1.00 47.81
H
ATOM   3486 1HB  ALA A 232      15.283 -12.786  14.954  1.00 71.71
H
ATOM   3487 2HB  ALA A 232      15.732 -12.786  16.676  1.00 71.71
H
ATOM   3488 3HB  ALA A 232      15.844 -14.285  15.731  1.00 71.71
H
ATOM   3489  N   SER A 233      13.828 -15.166  14.303  1.00115.90
N
```

```
ATOM   3490  CA  SER A 233      13.108 -16.056  13.416  1.00186.57
C
ATOM   3491  C   SER A 233      14.057 -17.078  12.868  1.00146.34
C
ATOM   3492  O   SER A 233      15.269 -16.923  12.991  1.00185.17
O
ATOM   3493  CB  SER A 233      12.430 -15.302  12.301  1.00279.86
C
ATOM   3494  OG  SER A 233      11.628 -16.166  11.541  1.00279.86
O
ATOM   3495  H   SER A 233      14.822 -15.027  14.202  1.00139.08
H
ATOM   3496  HA  SER A 233      12.342 -16.579  13.991  1.00223.88
H
ATOM   3497 1HB  SER A 233      11.815 -14.503  12.716  1.00335.83
H
ATOM   3498 2HB  SER A 233      13.178 -14.840  11.663  1.00335.83
H
ATOM   3499  HG  SER A 233      10.833 -16.317  12.066  1.00335.83
H
ATOM   3500  N   GLN A 234      13.513 -18.143  12.278  1.00134.90
N
ATOM   3501  CA  GLN A 234      14.365 -19.154  11.655  1.00176.88
C
ATOM   3502  C   GLN A 234      15.114 -18.474  10.530  1.00167.68
C
ATOM   3503  O   GLN A 234      16.317 -18.666  10.341  1.00198.90
O
ATOM   3504  CB  GLN A 234      13.542 -20.329  11.126  1.00265.32
C
ATOM   3505  CG  GLN A 234      14.377 -21.457  10.550  1.00265.32
C
ATOM   3506  CD  GLN A 234      15.253 -22.119  11.597  1.00265.32
C
ATOM   3507  OE1 GLN A 234      14.769 -22.555  12.646  1.00265.32
O
ATOM   3508  NE2 GLN A 234      16.549 -22.197  11.319  1.00265.32
N
ATOM   3509  H   GLN A 234      12.508 -18.232  12.233  1.00161.88
H
ATOM   3510  HA  GLN A 234      15.088 -19.518  12.384  1.00212.26
H
ATOM   3511 1HB  GLN A 234      12.932 -20.739  11.931  1.00318.38
H
ATOM   3512 2HB  GLN A 234      12.864 -19.980  10.347  1.00318.38
H
ATOM   3513 1HG  GLN A 234      13.712 -22.213  10.134  1.00318.38
H
ATOM   3514 2HG  GLN A 234      15.022 -21.055   9.768  1.00318.38
H
ATOM   3515 1HE2 GLN A 234      17.177 -22.623  11.972  1.00318.38
H
ATOM   3516 2HE2 GLN A 234      16.899 -21.829  10.457  1.00318.38
H
ATOM   3517  N   VAL A 235      14.385 -17.612   9.835  1.00176.02
N
ATOM   3518  CA  VAL A 235      14.923 -16.726   8.838  1.00198.64
C
```

```
ATOM   3519  C   VAL A 235      14.997 -15.368   9.490  1.00106.91
C
ATOM   3520  O   VAL A 235      14.286 -14.438   9.093  1.00158.29
O
ATOM   3521  CB  VAL A 235      14.045 -16.671   7.577  1.00297.96
C
ATOM   3522  CG1 VAL A 235      14.665 -15.717   6.563  1.00297.96
C
ATOM   3523  CG2 VAL A 235      13.906 -18.068   6.992  1.00297.96
C
ATOM   3524  H   VAL A 235      13.397 -17.548  10.034  1.00211.22
H
ATOM   3525  HA  VAL A 235      15.926 -17.051   8.562  1.00238.37
H
ATOM   3526  HB  VAL A 235      13.061 -16.281   7.841  1.00357.55
H
ATOM   3527 1HG1 VAL A 235      14.034 -15.669   5.676  1.00357.55
H
ATOM   3528 2HG1 VAL A 235      14.751 -14.723   7.002  1.00357.55
H
ATOM   3529 3HG1 VAL A 235      15.655 -16.077   6.285  1.00357.55
H
ATOM   3530 1HG2 VAL A 235      13.279 -18.028   6.103  1.00357.55
H
ATOM   3531 2HG2 VAL A 235      14.892 -18.449   6.725  1.00357.55
H
ATOM   3532 3HG2 VAL A 235      13.449 -18.728   7.730  1.00357.55
H
ATOM   3533  N   LYS A 236      15.852 -15.264  10.519  1.00 58.37
N
ATOM   3534  CA  LYS A 236      16.027 -14.050  11.313  1.00 70.49
C
ATOM   3535  C   LYS A 236      16.342 -12.832  10.472  1.00 33.24
C
ATOM   3536  O   LYS A 236      16.157 -11.713  10.933  1.00 80.30
O
ATOM   3537  CB  LYS A 236      17.140 -14.239  12.341  1.00105.73
C
ATOM   3538  CG  LYS A 236      18.531 -14.371  11.740  1.00105.73
C
ATOM   3539  CD  LYS A 236      19.577 -14.597  12.819  1.00105.73
C
ATOM   3540  CE  LYS A 236      20.975 -14.689  12.224  1.00105.73
C
ATOM   3541  NZ  LYS A 236      22.005 -14.939  13.270  1.00105.73
N
ATOM   3542  H   LYS A 236      16.400 -16.076  10.769  1.00 70.04
H
ATOM   3543  HA  LYS A 236      15.098 -13.850  11.844  1.00 84.59
H
ATOM   3544 1HB  LYS A 236      17.151 -13.391  13.025  1.00126.88
H
ATOM   3545 2HB  LYS A 236      16.947 -15.135  12.931  1.00126.88
H
ATOM   3546 1HG  LYS A 236      18.550 -15.211  11.043  1.00126.88
H
ATOM   3547 2HG  LYS A 236      18.778 -13.462  11.191  1.00126.88
H
```

```
ATOM   3548 1HD  LYS A 236      19.548 -13.772  13.532  1.00126.88
H
ATOM   3549 2HD  LYS A 236      19.357 -15.523  13.351  1.00126.88
H
ATOM   3550 1HE  LYS A 236      21.003 -15.501  11.500  1.00126.88
H
ATOM   3551 2HE  LYS A 236      21.209 -13.755  11.715  1.00126.88
H
ATOM   3552 1HZ  LYS A 236      22.916 -14.994  12.838  1.00126.88
H
ATOM   3553 2HZ  LYS A 236      21.995 -14.183  13.941  1.00126.88
H
ATOM   3554 3HZ  LYS A 236      21.803 -15.809  13.741  1.00126.88
H
ATOM   3555  N   ASN A 237      16.754 -13.042   9.233  1.00 13.24
N
ATOM   3556  CA  ASN A 237      17.046 -11.979   8.314  1.00 29.11
C
ATOM   3557  C   ASN A 237      15.838 -11.170   7.828  1.00 16.82
C
ATOM   3558  O   ASN A 237      16.032 -10.165   7.152  1.00 34.86
O
ATOM   3559  CB  ASN A 237      17.781 -12.546   7.119  1.00 43.66
C
ATOM   3560  CG  ASN A 237      19.184 -12.963   7.457  1.00 43.66
C
ATOM   3561  OD1 ASN A 237      19.791 -12.444   8.401  1.00 43.66
O
ATOM   3562  ND2 ASN A 237      19.712 -13.894   6.706  1.00 43.66
N
ATOM   3563  H   ASN A 237      16.906 -13.984   8.910  1.00 15.89
H
ATOM   3564  HA  ASN A 237      17.711 -11.279   8.821  1.00 34.93
H
ATOM   3565 1HB  ASN A 237      17.239 -13.409   6.731  1.00 52.40
H
ATOM   3566 2HB  ASN A 237      17.816 -11.799   6.326  1.00 52.40
H
ATOM   3567 1HD2 ASN A 237      20.643 -14.213   6.883  1.00 52.40
H
ATOM   3568 2HD2 ASN A 237      19.185 -14.287   5.952  1.00 52.40
H
ATOM   3569  N   LYS A 238      14.608 -11.551   8.156  1.00  6.51
N
ATOM   3570  CA  LYS A 238      13.490 -10.729   7.692  1.00  3.39
C
ATOM   3571  C   LYS A 238      12.790 -10.016   8.858  1.00  3.28
C
ATOM   3572  O   LYS A 238      12.869 -10.476   9.998  1.00 10.10
O
ATOM   3573  CB  LYS A 238      12.489 -11.583   6.906  1.00  5.08
C
ATOM   3574  CG  LYS A 238      13.069 -12.283   5.687  1.00  5.08
C
ATOM   3575  CD  LYS A 238      13.479 -11.294   4.608  1.00  5.08
C
ATOM   3576  CE  LYS A 238      13.990 -12.021   3.370  1.00  5.08
C
```

ATOM 3577 NZ LYS A 238 14.381 -11.077 2.286 1.00 5.08
N
ATOM 3578 H LYS A 238 14.446 -12.393 8.693 1.00 7.81
H
ATOM 3579 HA LYS A 238 13.890 -9.985 7.013 1.00 4.07
H
ATOM 3580 1HB LYS A 238 12.082 -12.354 7.546 1.00 6.10
H
ATOM 3581 2HB LYS A 238 11.656 -10.965 6.571 1.00 6.10
H
ATOM 3582 1HG LYS A 238 13.944 -12.860 5.983 1.00 6.10
H
ATOM 3583 2HG LYS A 238 12.328 -12.969 5.276 1.00 6.10
H
ATOM 3584 1HD LYS A 238 12.622 -10.678 4.332 1.00 6.10
H
ATOM 3585 2HD LYS A 238 14.266 -10.642 4.987 1.00 6.10
H
ATOM 3586 1HE LYS A 238 14.859 -12.616 3.646 1.00 6.10
H
ATOM 3587 2HE LYS A 238 13.214 -12.688 2.994 1.00 6.10
H
ATOM 3588 1HZ LYS A 238 14.723 -11.592 1.489 1.00 6.10
H
ATOM 3589 2HZ LYS A 238 13.577 -10.529 2.014 1.00 6.10
H
ATOM 3590 3HZ LYS A 238 15.106 -10.457 2.626 1.00 6.10
H
ATOM 3591 N PRO A 239 12.130 -8.875 8.588 1.00 0.78
N
ATOM 3592 CA PRO A 239 11.316 -8.081 9.501 1.00 0.45
C
ATOM 3593 C PRO A 239 10.204 -8.963 10.049 1.00 0.62
C
ATOM 3594 O PRO A 239 9.722 -9.846 9.342 1.00 1.70
O
ATOM 3595 CB PRO A 239 10.788 -6.954 8.621 1.00 0.68
C
ATOM 3596 CG PRO A 239 11.801 -6.802 7.548 1.00 0.68
C
ATOM 3597 CD PRO A 239 12.320 -8.185 7.294 1.00 0.68
C
ATOM 3598 HA PRO A 239 11.946 -7.692 10.314 1.00 0.54
H
ATOM 3599 1HB PRO A 239 9.816 -7.243 8.216 1.00 0.81
H
ATOM 3600 2HB PRO A 239 10.639 -6.041 9.216 1.00 0.81
H
ATOM 3601 1HG PRO A 239 11.323 -6.373 6.656 1.00 0.81
H
ATOM 3602 2HG PRO A 239 12.585 -6.100 7.859 1.00 0.81
H
ATOM 3603 1HD PRO A 239 11.740 -8.684 6.503 1.00 0.81
H
ATOM 3604 2HD PRO A 239 13.387 -8.113 7.043 1.00 0.81
H
ATOM 3605 N THR A 240 9.807 -8.728 11.296 1.00 0.54
N

```
ATOM   3606  CA  THR A 240       8.818  -9.581  11.955  1.00  0.78
C
ATOM   3607  C   THR A 240       7.471  -8.898  12.116  1.00  0.50
C
ATOM   3608  O   THR A 240       7.398  -7.724  12.472  1.00  0.75
O
ATOM   3609  CB  THR A 240       9.333 -10.042  13.328  1.00  1.17
C
ATOM   3610  OG1 THR A 240      10.537 -10.804  13.159  1.00  1.17
O
ATOM   3611  CG2 THR A 240       8.299 -10.898  14.027  1.00  1.17
C
ATOM   3612  H   THR A 240      10.217  -7.953  11.805  1.00  0.65
H
ATOM   3613  HA  THR A 240       8.667 -10.467  11.339  1.00  0.94
H
ATOM   3614  HB  THR A 240       9.549  -9.170  13.941  1.00  1.40
H
ATOM   3615  HG1 THR A 240      11.210 -10.253  12.752  1.00  1.40
H
ATOM   3616 1HG2 THR A 240       8.685 -11.214  14.996  1.00  1.40
H
ATOM   3617 2HG2 THR A 240       7.387 -10.322  14.172  1.00  1.40
H
ATOM   3618 3HG2 THR A 240       8.084 -11.775  13.420  1.00  1.40
H
ATOM   3619  N   ALA A 241       6.408  -9.638  11.819  1.00  0.48
N
ATOM   3620  CA  ALA A 241       5.048  -9.122  11.884  1.00  0.42
C
ATOM   3621  C   ALA A 241       4.238  -9.862  12.941  1.00  0.58
C
ATOM   3622  O   ALA A 241       3.761 -10.969  12.712  1.00  1.84
O
ATOM   3623  CB  ALA A 241       4.381  -9.242  10.521  1.00  0.63
C
ATOM   3624  H   ALA A 241       6.549 -10.602  11.545  1.00  0.58
H
ATOM   3625  HA  ALA A 241       5.093  -8.071  12.169  1.00  0.50
H
ATOM   3626 1HB  ALA A 241       3.371  -8.841  10.567  1.00  0.76
H
ATOM   3627 2HB  ALA A 241       4.956  -8.687   9.781  1.00  0.76
H
ATOM   3628 3HB  ALA A 241       4.336 -10.287  10.227  1.00  0.76
H
ATOM   3629  N   ILE A 242       4.081  -9.246  14.103  1.00  0.28
N
ATOM   3630  CA  ILE A 242       3.328  -9.862  15.180  1.00  0.33
C
ATOM   3631  C   ILE A 242       1.885  -9.433  15.095  1.00  0.33
C
ATOM   3632  O   ILE A 242       1.515  -8.362  15.573  1.00  0.69
O
ATOM   3633  CB  ILE A 242       3.927  -9.481  16.545  1.00  0.49
C
ATOM   3634  CG1 ILE A 242       5.403  -9.866  16.571  1.00  0.49
C
```

ATOM 3635 CG2 ILE A 242 3.170 -10.145 17.682 1.00 0.49
C
ATOM 3636 CD1 ILE A 242 5.631 -11.334 16.313 1.00 0.49
C
ATOM 3637 H ILE A 242 4.474 -8.327 14.239 1.00 0.34
H
ATOM 3638 HA ILE A 242 3.374 -10.945 15.068 1.00 0.40
H
ATOM 3639 HB ILE A 242 3.873 -8.401 16.668 1.00 0.59
H
ATOM 3640 1HG1 ILE A 242 5.942 -9.288 15.822 1.00 0.59
H
ATOM 3641 2HG1 ILE A 242 5.815 -9.632 17.548 1.00 0.59
H
ATOM 3642 1HG2 ILE A 242 3.610 -9.849 18.634 1.00 0.59
H
ATOM 3643 2HG2 ILE A 242 2.126 -9.835 17.654 1.00 0.59
H
ATOM 3644 3HG2 ILE A 242 3.228 -11.228 17.576 1.00 0.59
H
ATOM 3645 1HD1 ILE A 242 6.697 -11.553 16.358 1.00 0.59
H
ATOM 3646 2HD1 ILE A 242 5.108 -11.915 17.066 1.00 0.59
H
ATOM 3647 3HD1 ILE A 242 5.248 -11.594 15.327 1.00 0.59
H
ATOM 3648 N VAL A 243 1.067 -10.287 14.492 1.00 0.51
N
ATOM 3649 CA VAL A 243 -0.335 -9.977 14.322 1.00 0.39
C
ATOM 3650 C VAL A 243 -1.094 -10.461 15.524 1.00 0.41
C
ATOM 3651 O VAL A 243 -1.228 -11.666 15.745 1.00 0.82
O
ATOM 3652 CB VAL A 243 -0.881 -10.604 13.033 1.00 0.58
C
ATOM 3653 CG1 VAL A 243 -2.363 -10.310 12.885 1.00 0.58
C
ATOM 3654 CG2 VAL A 243 -0.117 -10.054 11.837 1.00 0.58
C
ATOM 3655 H VAL A 243 1.424 -11.172 14.154 1.00 0.61
H
ATOM 3656 HA VAL A 243 -0.439 -8.910 14.259 1.00 0.47
H
ATOM 3657 HB VAL A 243 -0.754 -11.677 13.090 1.00 0.70
H
ATOM 3658 1HG1 VAL A 243 -2.737 -10.774 11.972 1.00 0.70
H
ATOM 3659 2HG1 VAL A 243 -2.903 -10.711 13.743 1.00 0.70
H
ATOM 3660 3HG1 VAL A 243 -2.511 -9.233 12.831 1.00 0.70
H
ATOM 3661 1HG2 VAL A 243 -0.494 -10.511 10.922 1.00 0.70
H
ATOM 3662 2HG2 VAL A 243 -0.253 -8.975 11.785 1.00 0.70
H
ATOM 3663 3HG2 VAL A 243 0.943 -10.282 11.946 1.00 0.70
H

```
ATOM   3664  N   ALA A 244      -1.574  -9.510  16.312  1.00  0.38
N
ATOM   3665  CA  ALA A 244      -2.220  -9.838  17.561  1.00  0.46
C
ATOM   3666  C   ALA A 244      -3.732  -9.752  17.479  1.00  0.40
C
ATOM   3667  O   ALA A 244      -4.295  -8.732  17.080  1.00  0.37
O
ATOM   3668  CB  ALA A 244      -1.697  -8.909  18.643  1.00  0.69
C
ATOM   3669  H   ALA A 244      -1.475  -8.536  16.056  1.00  0.46
H
ATOM   3670  HA  ALA A 244      -1.956 -10.858  17.815  1.00  0.55
H
ATOM   3671 1HB  ALA A 244      -2.145  -9.169  19.593  1.00  0.83
H
ATOM   3672 2HB  ALA A 244      -0.615  -9.006  18.714  1.00  0.83
H
ATOM   3673 3HB  ALA A 244      -1.953  -7.880  18.392  1.00  0.83
H
ATOM   3674  N   LYS A 245      -4.398 -10.825  17.897  1.00  0.47
N
ATOM   3675  CA  LYS A 245      -5.851 -10.840  17.925  1.00  0.51
C
ATOM   3676  C   LYS A 245      -6.305 -10.255  19.240  1.00  0.54
C
ATOM   3677  O   LYS A 245      -6.008 -10.797  20.307  1.00  0.63
O
ATOM   3678  CB  LYS A 245      -6.403 -12.254  17.755  1.00  0.77
C
ATOM   3679  CG  LYS A 245      -7.928 -12.325  17.693  1.00  0.77
C
ATOM   3680  CD  LYS A 245      -8.404 -13.755  17.476  1.00  0.77
C
ATOM   3681  CE  LYS A 245      -9.924 -13.846  17.436  1.00  0.77
C
ATOM   3682  NZ  LYS A 245     -10.385 -15.249  17.232  1.00  0.77
N
ATOM   3683  H   LYS A 245      -3.879 -11.634  18.209  1.00  0.56
H
ATOM   3684  HA  LYS A 245      -6.231 -10.209  17.121  1.00  0.61
H
ATOM   3685 1HB  LYS A 245      -6.008 -12.691  16.837  1.00  0.92
H
ATOM   3686 2HB  LYS A 245      -6.071 -12.878  18.584  1.00  0.92
H
ATOM   3687 1HG  LYS A 245      -8.350 -11.948  18.626  1.00  0.92
H
ATOM   3688 2HG  LYS A 245      -8.287 -11.704  16.875  1.00  0.92
H
ATOM   3689 1HD  LYS A 245      -8.005 -14.131  16.533  1.00  0.92
H
ATOM   3690 2HD  LYS A 245      -8.035 -14.387  18.283  1.00  0.92
H
ATOM   3691 1HE  LYS A 245     -10.330 -13.473  18.375  1.00  0.92
H
ATOM   3692 2HE  LYS A 245     -10.301 -13.228  16.621  1.00  0.92
H
```

```
ATOM   3693 1HZ  LYS A 245     -11.395 -15.273  17.212  1.00  0.92
H
ATOM   3694 2HZ  LYS A 245     -10.022 -15.598  16.355  1.00  0.92
H
ATOM   3695 3HZ  LYS A 245     -10.050 -15.827  17.989  1.00  0.92
H
ATOM   3696  N   THR A 246      -6.985  -9.119  19.165  1.00  0.57
N
ATOM   3697  CA  THR A 246      -7.418  -8.406  20.354  1.00  0.65
C
ATOM   3698  C   THR A 246      -8.858  -7.943  20.268  1.00  0.58
C
ATOM   3699  O   THR A 246      -9.506  -8.074  19.233  1.00  0.99
O
ATOM   3700  CB  THR A 246      -6.529  -7.174  20.605  1.00  0.98
C
ATOM   3701  OG1 THR A 246      -6.707  -6.252  19.537  1.00  0.98
O
ATOM   3702  CG2 THR A 246      -5.063  -7.545  20.708  1.00  0.98
C
ATOM   3703  H   THR A 246      -7.201  -8.721  18.259  1.00  0.68
H
ATOM   3704  HA  THR A 246      -7.330  -9.080  21.203  1.00  0.78
H
ATOM   3705  HB  THR A 246      -6.833  -6.691  21.529  1.00  1.17
H
ATOM   3706  HG1 THR A 246      -6.048  -5.555  19.605  1.00  1.17
H
ATOM   3707 1HG2 THR A 246      -4.474  -6.646  20.888  1.00  1.17
H
ATOM   3708 2HG2 THR A 246      -4.929  -8.239  21.537  1.00  1.17
H
ATOM   3709 3HG2 THR A 246      -4.733  -8.012  19.783  1.00  1.17
H
ATOM   3710  N   PHE A 247      -9.354  -7.390  21.365  1.00  0.64
N
ATOM   3711  CA  PHE A 247     -10.700  -6.835  21.389  1.00  0.59
C
ATOM   3712  C   PHE A 247     -10.713  -5.390  21.877  1.00  0.62
C
ATOM   3713  O   PHE A 247     -10.330  -5.115  23.014  1.00  1.40
O
ATOM   3714  CB  PHE A 247     -11.615  -7.701  22.249  1.00  0.89
C
ATOM   3715  CG  PHE A 247     -11.773  -9.082  21.689  1.00  0.89
C
ATOM   3716  CD1 PHE A 247     -10.915 -10.104  22.069  1.00  0.89
C
ATOM   3717  CD2 PHE A 247     -12.766  -9.357  20.763  1.00  0.89
C
ATOM   3718  CE1 PHE A 247     -11.045 -11.371  21.534  1.00  0.89
C
ATOM   3719  CE2 PHE A 247     -12.901 -10.623  20.228  1.00  0.89
C
ATOM   3720  CZ  PHE A 247     -12.039 -11.630  20.613  1.00  0.89
C
ATOM   3721  H   PHE A 247      -8.776  -7.369  22.196  1.00  0.77
H
```

```
ATOM   3722  HA  PHE A 247     -11.091  -6.845  20.370  1.00  0.71
H
ATOM   3723 1HB  PHE A 247     -11.213  -7.782  23.258  1.00  1.06
H
ATOM   3724 2HB  PHE A 247     -12.600  -7.243  22.318  1.00  1.06
H
ATOM   3725  HD1 PHE A 247     -10.128  -9.895  22.794  1.00  1.06
H
ATOM   3726  HD2 PHE A 247     -13.442  -8.560  20.454  1.00  1.06
H
ATOM   3727  HE1 PHE A 247     -10.363 -12.164  21.839  1.00  1.06
H
ATOM   3728  HE2 PHE A 247     -13.686 -10.826  19.499  1.00  1.06
H
ATOM   3729  HZ  PHE A 247     -12.144 -12.628  20.190  1.00  1.06
H
ATOM   3730  N   LYS A 248     -11.147  -4.475  21.003  1.00  0.58
N
ATOM   3731  CA  LYS A 248     -11.253  -3.051  21.321  1.00  0.51
C
ATOM   3732  C   LYS A 248     -12.034  -2.846  22.619  1.00  0.48
C
ATOM   3733  O   LYS A 248     -13.146  -3.349  22.760  1.00  0.57
O
ATOM   3734  CB  LYS A 248     -11.925  -2.335  20.155  1.00  0.77
C
ATOM   3735  CG  LYS A 248     -11.962  -0.818  20.218  1.00  0.77
C
ATOM   3736  CD  LYS A 248     -12.620  -0.302  18.954  1.00  0.77
C
ATOM   3737  CE  LYS A 248     -12.638   1.206  18.837  1.00  0.77
C
ATOM   3738  NZ  LYS A 248     -11.289   1.732  18.517  1.00  0.77
N
ATOM   3739  H   LYS A 248     -11.398  -4.762  20.058  1.00  0.70
H
ATOM   3740  HA  LYS A 248     -10.259  -2.638  21.454  1.00  0.61
H
ATOM   3741 1HB  LYS A 248     -11.419  -2.609  19.228  1.00  0.92
H
ATOM   3742 2HB  LYS A 248     -12.956  -2.680  20.071  1.00  0.92
H
ATOM   3743 1HG  LYS A 248     -12.540  -0.494  21.082  1.00  0.92
H
ATOM   3744 2HG  LYS A 248     -10.950  -0.418  20.295  1.00  0.92
H
ATOM   3745 1HD  LYS A 248     -12.072  -0.697  18.098  1.00  0.92
H
ATOM   3746 2HD  LYS A 248     -13.644  -0.667  18.907  1.00  0.92
H
ATOM   3747 1HE  LYS A 248     -13.320   1.483  18.034  1.00  0.92
H
ATOM   3748 2HE  LYS A 248     -12.986   1.654  19.767  1.00  0.92
H
ATOM   3749 1HZ  LYS A 248     -11.338   2.733  18.400  1.00  0.92
H
ATOM   3750 2HZ  LYS A 248     -10.639   1.503  19.255  1.00  0.92
H
```

```
ATOM   3751 3HZ  LYS A 248     -10.973   1.323  17.650  1.00  0.92
H
ATOM   3752  N   GLY A 249     -11.459  -2.118  23.577  1.00  0.50
N
ATOM   3753  CA  GLY A 249     -12.122  -1.921  24.867  1.00  0.58
C
ATOM   3754  C   GLY A 249     -11.895  -3.079  25.849  1.00  0.66
C
ATOM   3755  O   GLY A 249     -12.557  -3.138  26.887  1.00  2.17
O
ATOM   3756  H   GLY A 249     -10.561  -1.675  23.418  1.00  0.60
H
ATOM   3757 1HA  GLY A 249     -11.766  -0.998  25.317  1.00  0.70
H
ATOM   3758 2HA  GLY A 249     -13.192  -1.794  24.702  1.00  0.70
H
ATOM   3759  N   ARG A 250     -10.958  -3.975  25.516  1.00  0.80
N
ATOM   3760  CA  ARG A 250     -10.625  -5.139  26.337  1.00  0.82
C
ATOM   3761  C   ARG A 250     -10.512  -4.889  27.813  1.00  0.83
C
ATOM   3762  O   ARG A 250      -9.656  -4.120  28.254  1.00  0.91
O
ATOM   3763  CB  ARG A 250      -9.310  -5.748  25.923  1.00  1.23
C
ATOM   3764  CG  ARG A 250      -8.855  -6.883  26.833  1.00  1.23
C
ATOM   3765  CD  ARG A 250      -9.670  -8.107  26.642  1.00  1.23
C
ATOM   3766  NE  ARG A 250      -9.113  -9.241  27.354  1.00  1.23
N
ATOM   3767  CZ  ARG A 250      -9.722 -10.434  27.495  1.00  1.23
C
ATOM   3768  NH1 ARG A 250     -10.918 -10.643  26.988  1.00  1.23
N
ATOM   3769  NH2 ARG A 250      -9.109 -11.404  28.154  1.00  1.23
N
ATOM   3770  H   ARG A 250     -10.471  -3.878  24.636  1.00  0.96
H
ATOM   3771  HA  ARG A 250     -11.397  -5.879  26.183  1.00  0.98
H
ATOM   3772 1HB  ARG A 250      -9.390  -6.156  24.918  1.00  1.48
H
ATOM   3773 2HB  ARG A 250      -8.531  -4.986  25.914  1.00  1.48
H
ATOM   3774 1HG  ARG A 250      -7.814  -7.125  26.649  1.00  1.48
H
ATOM   3775 2HG  ARG A 250      -8.962  -6.574  27.872  1.00  1.48
H
ATOM   3776 1HD  ARG A 250     -10.665  -7.928  27.034  1.00  1.48
H
ATOM   3777 2HD  ARG A 250      -9.731  -8.356  25.583  1.00  1.48
H
ATOM   3778  HE  ARG A 250      -8.194  -9.132  27.761  1.00  1.48
H
ATOM   3779 1HH1 ARG A 250     -11.408  -9.906  26.486  1.00  1.48
H
```

```
ATOM   3780 2HH1 ARG A 250     -11.366 -11.540  27.104  1.00  1.48
H
ATOM   3781 1HH2 ARG A 250      -8.189 -11.245  28.546  1.00  1.48
H
ATOM   3782 2HH2 ARG A 250      -9.558 -12.300  28.267  1.00  1.48
H
ATOM   3783  N   GLY A 251     -11.352  -5.598  28.568  1.00  0.91
N
ATOM   3784  CA  GLY A 251     -11.356  -5.534  30.020  1.00  0.98
C
ATOM   3785  C   GLY A 251     -12.424  -4.609  30.571  1.00  1.14
C
ATOM   3786  O   GLY A 251     -12.640  -4.572  31.783  1.00  2.09
O
ATOM   3787  H   GLY A 251     -12.020  -6.214  28.108  1.00  1.09
H
ATOM   3788 1HA  GLY A 251     -11.509  -6.537  30.418  1.00  1.18
H
ATOM   3789 2HA  GLY A 251     -10.381  -5.209  30.368  1.00  1.18
H
ATOM   3790  N   ILE A 252     -13.108  -3.878  29.697  1.00  0.95
N
ATOM   3791  CA  ILE A 252     -14.159  -2.990  30.153  1.00  1.02
C
ATOM   3792  C   ILE A 252     -15.564  -3.576  29.912  1.00  1.11
C
ATOM   3793  O   ILE A 252     -15.970  -3.757  28.759  1.00  1.03
O
ATOM   3794  CB  ILE A 252     -14.073  -1.637  29.464  1.00  1.53
C
ATOM   3795  CG1 ILE A 252     -12.732  -0.968  29.737  1.00  1.53
C
ATOM   3796  CG2 ILE A 252     -15.190  -0.777  29.985  1.00  1.53
C
ATOM   3797  CD1 ILE A 252     -12.530   0.273  28.910  1.00  1.53
C
ATOM   3798  H   ILE A 252     -12.894  -3.913  28.709  1.00  1.14
H
ATOM   3799  HA  ILE A 252     -14.004  -2.811  31.209  1.00  1.22
H
ATOM   3800  HB  ILE A 252     -14.168  -1.761  28.387  1.00  1.84
H
ATOM   3801 1HG1 ILE A 252     -12.675  -0.697  30.790  1.00  1.84
H
ATOM   3802 2HG1 ILE A 252     -11.926  -1.667  29.517  1.00  1.84
H
ATOM   3803 1HG2 ILE A 252     -15.156   0.203  29.509  1.00  1.84
H
ATOM   3804 2HG2 ILE A 252     -16.145  -1.255  29.778  1.00  1.84
H
ATOM   3805 3HG2 ILE A 252     -15.067  -0.670  31.058  1.00  1.84
H
ATOM   3806 1HD1 ILE A 252     -11.563   0.715  29.148  1.00  1.84
H
ATOM   3807 2HD1 ILE A 252     -12.562   0.014  27.851  1.00  1.84
H
ATOM   3808 3HD1 ILE A 252     -13.319   0.990  29.132  1.00  1.84
H
```

ATOM 3809 N PRO A 253 -16.295 -3.913 30.991 1.00 1.59
N
ATOM 3810 CA PRO A 253 -17.662 -4.434 31.033 1.00 1.93
C
ATOM 3811 C PRO A 253 -18.582 -3.742 30.040 1.00 1.63
C
ATOM 3812 O PRO A 253 -18.786 -2.530 30.114 1.00 1.66
O
ATOM 3813 CB PRO A 253 -18.110 -4.124 32.464 1.00 2.90
C
ATOM 3814 CG PRO A 253 -16.856 -4.152 33.251 1.00 2.90
C
ATOM 3815 CD PRO A 253 -15.842 -3.527 32.345 1.00 2.90
C
ATOM 3816 HA PRO A 253 -17.642 -5.519 30.853 1.00 2.32
H
ATOM 3817 1HB PRO A 253 -18.613 -3.145 32.498 1.00 3.47
H
ATOM 3818 2HB PRO A 253 -18.842 -4.873 32.799 1.00 3.47
H
ATOM 3819 1HG PRO A 253 -16.987 -3.589 34.185 1.00 3.47
H
ATOM 3820 2HG PRO A 253 -16.603 -5.185 33.531 1.00 3.47
H
ATOM 3821 1HD PRO A 253 -15.835 -2.435 32.449 1.00 3.47
H
ATOM 3822 2HD PRO A 253 -14.860 -3.968 32.564 1.00 3.47
H
ATOM 3823 N ASN A 254 -19.163 -4.533 29.139 1.00 1.64
N
ATOM 3824 CA ASN A 254 -20.114 -4.084 28.113 1.00 1.64
C
ATOM 3825 C ASN A 254 -19.554 -3.096 27.071 1.00 1.81
C
ATOM 3826 O ASN A 254 -20.320 -2.546 26.277 1.00 3.21
O
ATOM 3827 CB ASN A 254 -21.341 -3.476 28.772 1.00 2.46
C
ATOM 3828 CG ASN A 254 -22.085 -4.468 29.622 1.00 2.46
C
ATOM 3829 OD1 ASN A 254 -22.220 -5.641 29.257 1.00 2.46
O
ATOM 3830 ND2 ASN A 254 -22.570 -4.018 30.750 1.00 2.46
N
ATOM 3831 H ASN A 254 -18.932 -5.516 29.163 1.00 1.97
H
ATOM 3832 HA ASN A 254 -20.436 -4.966 27.557 1.00 1.97
H
ATOM 3833 1HB ASN A 254 -21.057 -2.624 29.389 1.00 2.95
H
ATOM 3834 2HB ASN A 254 -22.016 -3.106 28.001 1.00 2.95
H
ATOM 3835 1HD2 ASN A 254 -23.076 -4.631 31.358 1.00 2.95
H
ATOM 3836 2HD2 ASN A 254 -22.437 -3.060 31.006 1.00 2.95
H
ATOM 3837 N ILE A 255 -18.239 -2.888 27.051 1.00 0.94
N

```
ATOM   3838  CA  ILE A 255     -17.608  -2.001  26.082  1.00  0.94
C
ATOM   3839  C   ILE A 255     -16.758  -2.819  25.127  1.00  1.12
C
ATOM   3840  O   ILE A 255     -16.760  -2.572  23.921  1.00  1.62
O
ATOM   3841  CB  ILE A 255     -16.752  -0.932  26.766  1.00  1.41
C
ATOM   3842  CG1 ILE A 255     -17.613  -0.093  27.717  1.00  1.41
C
ATOM   3843  CG2 ILE A 255     -16.081  -0.050  25.731  1.00  1.41
C
ATOM   3844  CD1 ILE A 255     -18.745   0.630  27.047  1.00  1.41
C
ATOM   3845  H   ILE A 255     -17.641  -3.350  27.721  1.00  1.13
H
ATOM   3846  HA  ILE A 255     -18.385  -1.501  25.504  1.00  1.13
H
ATOM   3847  HB  ILE A 255     -15.989  -1.423  27.362  1.00  1.69
H
ATOM   3848 1HG1 ILE A 255     -18.029  -0.745  28.483  1.00  1.69
H
ATOM   3849 2HG1 ILE A 255     -16.983   0.651  28.205  1.00  1.69
H
ATOM   3850 1HG2 ILE A 255     -15.464   0.694  26.233  1.00  1.69
H
ATOM   3851 2HG2 ILE A 255     -15.457  -0.662  25.082  1.00  1.69
H
ATOM   3852 3HG2 ILE A 255     -16.842   0.452  25.135  1.00  1.69
H
ATOM   3853 1HD1 ILE A 255     -19.305   1.199  27.789  1.00  1.69
H
ATOM   3854 2HD1 ILE A 255     -18.340   1.308  26.297  1.00  1.69
H
ATOM   3855 3HD1 ILE A 255     -19.407  -0.089  26.567  1.00  1.69
H
ATOM   3856  N   GLU A 256     -16.030  -3.787  25.686  1.00  1.03
N
ATOM   3857  CA  GLU A 256     -15.188  -4.687  24.910  1.00  1.21
C
ATOM   3858  C   GLU A 256     -15.921  -5.299  23.719  1.00  1.11
C
ATOM   3859  O   GLU A 256     -16.984  -5.902  23.875  1.00  1.49
O
ATOM   3860  CB  GLU A 256     -14.643  -5.797  25.806  1.00  1.81
C
ATOM   3861  CG  GLU A 256     -13.768  -6.807  25.083  1.00  1.81
C
ATOM   3862  CD  GLU A 256     -13.158  -7.821  26.010  1.00  1.81
C
ATOM   3863  OE1 GLU A 256     -13.139  -7.583  27.196  1.00  1.81
O
ATOM   3864  OE2 GLU A 256     -12.687  -8.829  25.534  1.00  1.81
O
ATOM   3865  H   GLU A 256     -16.051  -3.895  26.693  1.00  1.24
H
ATOM   3866  HA  GLU A 256     -14.348  -4.115  24.535  1.00  1.45
H
```

```
ATOM   3867 1HB  GLU A 256     -14.055  -5.356  26.611  1.00  2.18
H
ATOM   3868 2HB  GLU A 256     -15.474  -6.336  26.264  1.00  2.18
H
ATOM   3869 1HG  GLU A 256     -14.371  -7.328  24.339  1.00  2.18
H
ATOM   3870 2HG  GLU A 256     -12.975  -6.274  24.559  1.00  2.18
H
ATOM   3871  N   ASP A 257     -15.315  -5.155  22.529  1.00  0.89
N
ATOM   3872  CA  ASP A 257     -15.855  -5.640  21.252  1.00  0.99
C
ATOM   3873  C   ASP A 257     -17.098  -4.893  20.727  1.00  2.40
C
ATOM   3874  O   ASP A 257     -17.625  -5.260  19.677  1.00 11.32
O
ATOM   3875  CB  ASP A 257     -16.177  -7.140  21.347  1.00  1.48
C
ATOM   3876  CG  ASP A 257     -16.334  -7.824  19.986  1.00  1.48
C
ATOM   3877  OD1 ASP A 257     -15.781  -7.357  19.019  1.00  1.48
O
ATOM   3878  OD2 ASP A 257     -17.020  -8.817  19.928  1.00  1.48
O
ATOM   3879  H   ASP A 257     -14.443  -4.647  22.509  1.00  1.07
H
ATOM   3880  HA  ASP A 257     -15.069  -5.521  20.505  1.00  1.19
H
ATOM   3881 1HB  ASP A 257     -15.389  -7.647  21.904  1.00  1.78
H
ATOM   3882 2HB  ASP A 257     -17.107  -7.279  21.900  1.00  1.78
H
ATOM   3883  N   ALA A 258     -17.577  -3.873  21.441  1.00  1.19
N
ATOM   3884  CA  ALA A 258     -18.753  -3.127  21.003  1.00  1.12
C
ATOM   3885  C   ALA A 258     -18.366  -1.907  20.169  1.00  0.93
C
ATOM   3886  O   ALA A 258     -17.299  -1.321  20.356  1.00  1.17
O
ATOM   3887  CB  ALA A 258     -19.580  -2.701  22.207  1.00  1.68
C
ATOM   3888  H   ALA A 258     -17.128  -3.585  22.299  1.00  1.43
H
ATOM   3889  HA  ALA A 258     -19.355  -3.785  20.377  1.00  1.34
H
ATOM   3890 1HB  ALA A 258     -20.470  -2.173  21.869  1.00  2.02
H
ATOM   3891 2HB  ALA A 258     -19.874  -3.584  22.776  1.00  2.02
H
ATOM   3892 3HB  ALA A 258     -18.988  -2.044  22.842  1.00  2.02
H
ATOM   3893  N   GLU A 259     -19.256  -1.528  19.258  1.00  0.96
N
ATOM   3894  CA  GLU A 259     -19.058  -0.372  18.388  1.00  0.78
C
ATOM   3895  C   GLU A 259     -19.545   0.916  19.045  1.00  0.88
C
```

ATOM 3896 O GLU A 259 -20.212 0.882 20.078 1.00 2.45
O
ATOM 3897 CB GLU A 259 -19.787 -0.577 17.054 1.00 1.17
C
ATOM 3898 CG GLU A 259 -19.251 -1.720 16.205 1.00 1.17
C
ATOM 3899 CD GLU A 259 -19.987 -1.891 14.905 1.00 1.17
C
ATOM 3900 OE1 GLU A 259 -20.977 -1.229 14.711 1.00 1.17
O
ATOM 3901 OE2 GLU A 259 -19.558 -2.688 14.104 1.00 1.17
O
ATOM 3902 H GLU A 259 -20.109 -2.062 19.161 1.00 1.15
H
ATOM 3903 HA GLU A 259 -17.992 -0.270 18.190 1.00 0.94
H
ATOM 3904 1HB GLU A 259 -20.843 -0.772 17.245 1.00 1.40
H
ATOM 3905 2HB GLU A 259 -19.727 0.334 16.459 1.00 1.40
H
ATOM 3906 1HG GLU A 259 -18.204 -1.520 15.985 1.00 1.40
H
ATOM 3907 2HG GLU A 259 -19.306 -2.645 16.776 1.00 1.40
H
ATOM 3908 N ASN A 260 -19.207 2.042 18.417 1.00 0.78
N
ATOM 3909 CA ASN A 260 -19.575 3.392 18.852 1.00 0.79
C
ATOM 3910 C ASN A 260 -18.945 3.821 20.174 1.00 2.37
C
ATOM 3911 O ASN A 260 -19.534 4.612 20.909 1.00 14.62
O
ATOM 3912 CB ASN A 260 -21.087 3.529 18.941 1.00 1.19
C
ATOM 3913 CG ASN A 260 -21.760 3.351 17.611 1.00 1.19
C
ATOM 3914 OD1 ASN A 260 -21.277 3.852 16.588 1.00 1.19
O
ATOM 3915 ND2 ASN A 260 -22.865 2.649 17.603 1.00 1.19
N
ATOM 3916 H ASN A 260 -18.658 1.959 17.573 1.00 0.94
H
ATOM 3917 HA ASN A 260 -19.220 4.093 18.095 1.00 0.95
H
ATOM 3918 1HB ASN A 260 -21.497 2.805 19.645 1.00 1.42
H
ATOM 3919 2HB ASN A 260 -21.335 4.519 19.321 1.00 1.42
H
ATOM 3920 1HD2 ASN A 260 -23.355 2.498 16.743 1.00 1.42
H
ATOM 3921 2HD2 ASN A 260 -23.219 2.264 18.454 1.00 1.42
H
ATOM 3922 N TRP A 261 -17.734 3.342 20.453 1.00 1.23
N
ATOM 3923 CA TRP A 261 -17.011 3.757 21.650 1.00 1.54
C
ATOM 3924 C TRP A 261 -15.653 4.369 21.311 1.00 3.33
C

ATOM 3925 O TRP A 261 -14.933 4.824 22.197 1.00 23.85
O
ATOM 3926 CB TRP A 261 -16.840 2.579 22.604 1.00 2.31
C
ATOM 3927 CG TRP A 261 -18.156 2.093 23.110 1.00 2.31
C
ATOM 3928 CD1 TRP A 261 -18.760 0.907 22.832 1.00 2.31
C
ATOM 3929 CD2 TRP A 261 -19.065 2.815 23.968 1.00 2.31
C
ATOM 3930 NE1 TRP A 261 -19.979 0.838 23.459 1.00 2.31
N
ATOM 3931 CE2 TRP A 261 -20.183 1.999 24.157 1.00 2.31
C
ATOM 3932 CE3 TRP A 261 -19.022 4.074 24.578 1.00 2.31
C
ATOM 3933 CZ2 TRP A 261 -21.257 2.397 24.935 1.00 2.31
C
ATOM 3934 CZ3 TRP A 261 -20.098 4.473 25.360 1.00 2.31
C
ATOM 3935 CH2 TRP A 261 -21.186 3.655 25.534 1.00 2.31
C
ATOM 3936 H TRP A 261 -17.306 2.678 19.824 1.00 1.48
H
ATOM 3937 HA TRP A 261 -17.601 4.516 22.161 1.00 1.85
H
ATOM 3938 1HB TRP A 261 -16.336 1.758 22.095 1.00 2.77
H
ATOM 3939 2HB TRP A 261 -16.226 2.875 23.454 1.00 2.77
H
ATOM 3940 HD1 TRP A 261 -18.338 0.130 22.201 1.00 2.77
H
ATOM 3941 HE1 TRP A 261 -20.622 0.062 23.410 1.00 2.77
H
ATOM 3942 HE3 TRP A 261 -18.160 4.727 24.442 1.00 2.77
H
ATOM 3943 HZ2 TRP A 261 -22.131 1.762 25.086 1.00 2.77
H
ATOM 3944 HZ3 TRP A 261 -20.057 5.455 25.832 1.00 2.77
H
ATOM 3945 HH2 TRP A 261 -22.015 4.000 26.153 1.00 2.77
H
ATOM 3946 N HIS A 262 -15.304 4.371 20.027 1.00 1.85
N
ATOM 3947 CA HIS A 262 -14.043 4.930 19.571 1.00 1.04
C
ATOM 3948 C HIS A 262 -13.949 6.412 19.888 1.00 1.72
C
ATOM 3949 O HIS A 262 -14.722 7.212 19.365 1.00 8.29
O
ATOM 3950 CB HIS A 262 -13.911 4.748 18.066 1.00 1.56
C
ATOM 3951 CG HIS A 262 -12.583 5.122 17.498 1.00 1.56
C
ATOM 3952 ND1 HIS A 262 -11.513 4.254 17.465 1.00 1.56
N
ATOM 3953 CD2 HIS A 262 -12.153 6.271 16.934 1.00 1.56
C

ATOM	3954	CE1	HIS	A	262	-10.493	4.845	16.869	1.00	1.56	C
ATOM	3955	NE2	HIS	A	262	-10.858	6.067	16.537	1.00	1.56	N
ATOM	3956	H	HIS	A	262	-15.929	3.984	19.337	1.00	2.22	H
ATOM	3957	HA	HIS	A	262	-13.214	4.423	20.063	1.00	1.25	H
ATOM	3958	1HB	HIS	A	262	-14.107	3.707	17.809	1.00	1.87	H
ATOM	3959	2HB	HIS	A	262	-14.669	5.350	17.565	1.00	1.87	H
ATOM	3960	HD2	HIS	A	262	-12.735	7.181	16.799	1.00	1.87	H
ATOM	3961	HE1	HIS	A	262	-9.514	4.401	16.688	1.00	1.87	H
ATOM	3962	HE2	HIS	A	262	-10.280	6.751	16.070	1.00	1.87	H
ATOM	3963	N	GLY	A	263	-12.975	6.783	20.707	1.00	1.30	N
ATOM	3964	CA	GLY	A	263	-12.768	8.184	21.060	1.00	1.05	C
ATOM	3965	C	GLY	A	263	-13.677	8.667	22.171	1.00	1.07	C
ATOM	3966	O	GLY	A	263	-13.671	9.854	22.498	1.00	3.35	O
ATOM	3967	H	GLY	A	263	-12.365	6.080	21.099	1.00	1.56	H
ATOM	3968	1HA	GLY	A	263	-11.738	8.336	21.370	1.00	1.26	H
ATOM	3969	2HA	GLY	A	263	-12.923	8.800	20.175	1.00	1.26	H
ATOM	3970	N	LYS	A	264	-14.448	7.766	22.769	1.00	1.12	N
ATOM	3971	CA	LYS	A	264	-15.332	8.199	23.824	1.00	1.41	C
ATOM	3972	C	LYS	A	264	-14.767	7.838	25.190	1.00	1.64	C
ATOM	3973	O	LYS	A	264	-14.324	6.707	25.398	1.00	4.00	O
ATOM	3974	CB	LYS	A	264	-16.710	7.577	23.653	1.00	2.11	C
ATOM	3975	CG	LYS	A	264	-17.437	8.034	22.403	1.00	2.11	C
ATOM	3976	CD	LYS	A	264	-18.811	7.409	22.313	1.00	2.11	C
ATOM	3977	CE	LYS	A	264	-19.526	7.829	21.037	1.00	2.11	C
ATOM	3978	NZ	LYS	A	264	-20.817	7.114	20.874	1.00	2.11	N
ATOM	3979	H	LYS	A	264	-14.443	6.791	22.495	1.00	1.34	H
ATOM	3980	HA	LYS	A	264	-15.436	9.274	23.734	1.00	1.69	H
ATOM	3981	1HB	LYS	A	264	-16.618	6.491	23.610	1.00	2.54	H
ATOM	3982	2HB	LYS	A	264	-17.332	7.821	24.514	1.00	2.54	H

```
ATOM   3983 1HG  LYS A 264     -17.539   9.121  22.416  1.00  2.54
H
ATOM   3984 2HG  LYS A 264     -16.860   7.752  21.522  1.00  2.54
H
ATOM   3985 1HD  LYS A 264     -18.721   6.327  22.344  1.00  2.54
H
ATOM   3986 2HD  LYS A 264     -19.409   7.727  23.167  1.00  2.54
H
ATOM   3987 1HE  LYS A 264     -19.717   8.901  21.069  1.00  2.54
H
ATOM   3988 2HE  LYS A 264     -18.890   7.609  20.179  1.00  2.54
H
ATOM   3989 1HZ  LYS A 264     -21.268   7.408  20.019  1.00  2.54
H
ATOM   3990 2HZ  LYS A 264     -20.617   6.121  20.836  1.00  2.54
H
ATOM   3991 3HZ  LYS A 264     -21.421   7.312  21.660  1.00  2.54
H
ATOM   3992  N   PRO A 265     -14.768   8.793  26.124  1.00  1.04
N
ATOM   3993  CA  PRO A 265     -14.371   8.694  27.511  1.00  1.03
C
ATOM   3994  C   PRO A 265     -15.476   8.037  28.303  1.00  0.72
C
ATOM   3995  O   PRO A 265     -16.644   8.109  27.922  1.00  1.00
O
ATOM   3996  CB  PRO A 265     -14.171  10.152  27.933  1.00  1.54
C
ATOM   3997  CG  PRO A 265     -15.145  10.901  27.097  1.00  1.54
C
ATOM   3998  CD  PRO A 265     -15.155  10.177  25.780  1.00  1.54
C
ATOM   3999  HA  PRO A 265     -13.442   8.114  27.583  1.00  1.24
H
ATOM   4000 1HB  PRO A 265     -14.356  10.264  29.010  1.00  1.85
H
ATOM   4001 2HB  PRO A 265     -13.134  10.465  27.748  1.00  1.85
H
ATOM   4002 1HG  PRO A 265     -16.132  10.906  27.581  1.00  1.85
H
ATOM   4003 2HG  PRO A 265     -14.839  11.949  26.994  1.00  1.85
H
ATOM   4004 1HD  PRO A 265     -16.174  10.196  25.366  1.00  1.85
H
ATOM   4005 2HD  PRO A 265     -14.421  10.617  25.087  1.00  1.85
H
ATOM   4006  N   VAL A 266     -15.121   7.414  29.411  1.00  1.27
N
ATOM   4007  CA  VAL A 266     -16.123   6.818  30.275  1.00  1.24
C
ATOM   4008  C   VAL A 266     -16.489   7.779  31.410  1.00  1.24
C
ATOM   4009  O   VAL A 266     -15.591   8.304  32.070  1.00  1.49
O
ATOM   4010  CB  VAL A 266     -15.623   5.497  30.853  1.00  1.86
C
ATOM   4011  CG1 VAL A 266     -16.639   4.960  31.824  1.00  1.86
C
```

```
ATOM   4012  CG2 VAL A 266     -15.423   4.502  29.728  1.00  1.86
C
ATOM   4013  H   VAL A 266     -14.147   7.367  29.669  1.00  1.52
H
ATOM   4014  HA  VAL A 266     -16.996   6.595  29.669  1.00  1.49
H
ATOM   4015  HB  VAL A 266     -14.688   5.662  31.387  1.00  2.23
H
ATOM   4016 1HG1 VAL A 266     -16.277   4.029  32.234  1.00  2.23
H
ATOM   4017 2HG1 VAL A 266     -16.786   5.678  32.628  1.00  2.23
H
ATOM   4018 3HG1 VAL A 266     -17.584   4.790  31.309  1.00  2.23
H
ATOM   4019 1HG2 VAL A 266     -15.067   3.556  30.135  1.00  2.23
H
ATOM   4020 2HG2 VAL A 266     -16.370   4.343  29.212  1.00  2.23
H
ATOM   4021 3HG2 VAL A 266     -14.692   4.898  29.026  1.00  2.23
H
ATOM   4022  N   PRO A 267     -17.788   8.047  31.630  1.00  1.36
N
ATOM   4023  CA  PRO A 267     -18.359   8.888  32.680  1.00  1.55
C
ATOM   4024  C   PRO A 267     -17.855   8.451  34.041  1.00  2.54
C
ATOM   4025  O   PRO A 267     -17.901   7.266  34.354  1.00 10.63
O
ATOM   4026  CB  PRO A 267     -19.864   8.666  32.526  1.00  2.33
C
ATOM   4027  CG  PRO A 267     -20.039   8.386  31.073  1.00  2.33
C
ATOM   4028  CD  PRO A 267     -18.828   7.587  30.684  1.00  2.33
C
ATOM   4029  HA  PRO A 267     -18.092   9.938  32.487  1.00  1.86
H
ATOM   4030 1HB  PRO A 267     -20.191   7.833  33.165  1.00  2.79
H
ATOM   4031 2HB  PRO A 267     -20.412   9.560  32.857  1.00  2.79
H
ATOM   4032 1HG  PRO A 267     -20.978   7.842  30.902  1.00  2.79
H
ATOM   4033 2HG  PRO A 267     -20.114   9.331  30.515  1.00  2.79
H
ATOM   4034 1HD  PRO A 267     -19.007   6.510  30.806  1.00  2.79
H
ATOM   4035 2HD  PRO A 267     -18.546   7.846  29.653  1.00  2.79
H
ATOM   4036  N   LYS A 268     -17.412   9.422  34.839  1.00  2.14
N
ATOM   4037  CA  LYS A 268     -16.828   9.227  36.173  1.00  1.97
C
ATOM   4038  C   LYS A 268     -17.351   8.036  36.968  1.00  1.70
C
ATOM   4039  O   LYS A 268     -16.564   7.194  37.394  1.00  2.32
O
ATOM   4040  CB  LYS A 268     -17.014  10.486  37.011  1.00  2.96
C
```

ATOM 4041 CG LYS A 268 -16.354 10.405 38.382 1.00 2.96
C
ATOM 4042 CD LYS A 268 -14.841 10.290 38.235 1.00 2.96
C
ATOM 4043 CE LYS A 268 -14.117 10.549 39.548 1.00 2.96
C
ATOM 4044 NZ LYS A 268 -14.445 9.531 40.578 1.00 2.96
N
ATOM 4045 H LYS A 268 -17.451 10.367 34.484 1.00 2.57
H
ATOM 4046 HA LYS A 268 -15.759 9.070 36.042 1.00 2.36
H
ATOM 4047 1HB LYS A 268 -16.592 11.343 36.484 1.00 3.55
H
ATOM 4048 2HB LYS A 268 -18.077 10.680 37.158 1.00 3.55
H
ATOM 4049 1HG LYS A 268 -16.591 11.300 38.958 1.00 3.55
H
ATOM 4050 2HG LYS A 268 -16.727 9.534 38.920 1.00 3.55
H
ATOM 4051 1HD LYS A 268 -14.588 9.286 37.890 1.00 3.55
H
ATOM 4052 2HD LYS A 268 -14.492 11.007 37.493 1.00 3.55
H
ATOM 4053 1HE LYS A 268 -13.043 10.536 39.372 1.00 3.55
H
ATOM 4054 2HE LYS A 268 -14.399 11.532 39.924 1.00 3.55
H
ATOM 4055 1HZ LYS A 268 -13.947 9.735 41.432 1.00 3.55
H
ATOM 4056 2HZ LYS A 268 -15.439 9.535 40.762 1.00 3.55
H
ATOM 4057 3HZ LYS A 268 -14.180 8.610 40.249 1.00 3.55
H
ATOM 4058 N GLU A 269 -18.661 7.980 37.191 1.00 1.69
N
ATOM 4059 CA GLU A 269 -19.254 6.911 37.990 1.00 2.29
C
ATOM 4060 C GLU A 269 -19.036 5.534 37.377 1.00 2.69
C
ATOM 4061 O GLU A 269 -18.812 4.553 38.092 1.00 7.02
O
ATOM 4062 CB GLU A 269 -20.750 7.162 38.163 1.00 3.44
C
ATOM 4063 CG GLU A 269 -21.085 8.350 39.052 1.00 3.44
C
ATOM 4064 CD GLU A 269 -22.562 8.613 39.144 1.00 3.44
C
ATOM 4065 OE1 GLU A 269 -23.303 7.999 38.413 1.00 3.44
O
ATOM 4066 OE2 GLU A 269 -22.952 9.429 39.945 1.00 3.44
O
ATOM 4067 H GLU A 269 -19.259 8.699 36.810 1.00 2.03
H
ATOM 4068 HA GLU A 269 -18.783 6.920 38.973 1.00 2.75
H
ATOM 4069 1HB GLU A 269 -21.207 7.334 37.188 1.00 4.12
H

ATOM 4070 2HB GLU A 269 -21.221 6.278 38.594 1.00 4.12
H
ATOM 4071 1HG GLU A 269 -20.697 8.162 40.053 1.00 4.12
H
ATOM 4072 2HG GLU A 269 -20.587 9.236 38.659 1.00 4.12
H
ATOM 4073 N ARG A 270 -19.110 5.465 36.055 1.00 1.27
N
ATOM 4074 CA ARG A 270 -18.925 4.217 35.351 1.00 1.31
C
ATOM 4075 C ARG A 270 -17.464 3.840 35.393 1.00 1.45
C
ATOM 4076 O ARG A 270 -17.127 2.676 35.596 1.00 1.64
O
ATOM 4077 CB ARG A 270 -19.389 4.346 33.912 1.00 1.97
C
ATOM 4078 CG ARG A 270 -20.885 4.499 33.730 1.00 1.97
C
ATOM 4079 CD ARG A 270 -21.237 4.709 32.305 1.00 1.97
C
ATOM 4080 NE ARG A 270 -22.668 4.870 32.115 1.00 1.97
N
ATOM 4081 CZ ARG A 270 -23.248 5.232 30.955 1.00 1.97
C
ATOM 4082 NH1 ARG A 270 -22.509 5.469 29.894 1.00 1.97
N
ATOM 4083 NH2 ARG A 270 -24.563 5.352 30.883 1.00 1.97
N
ATOM 4084 H ARG A 270 -19.267 6.304 35.516 1.00 1.52
H
ATOM 4085 HA ARG A 270 -19.507 3.439 35.846 1.00 1.57
H
ATOM 4086 1HB ARG A 270 -18.926 5.218 33.458 1.00 2.36
H
ATOM 4087 2HB ARG A 270 -19.073 3.472 33.344 1.00 2.36
H
ATOM 4088 1HG ARG A 270 -21.390 3.598 34.080 1.00 2.36
H
ATOM 4089 2HG ARG A 270 -21.237 5.358 34.302 1.00 2.36
H
ATOM 4090 1HD ARG A 270 -20.742 5.608 31.940 1.00 2.36
H
ATOM 4091 2HD ARG A 270 -20.909 3.851 31.719 1.00 2.36
H
ATOM 4092 HE ARG A 270 -23.270 4.697 32.909 1.00 2.36
H
ATOM 4093 1HH1 ARG A 270 -21.505 5.377 29.948 1.00 2.36
H
ATOM 4094 2HH1 ARG A 270 -22.946 5.739 29.025 1.00 2.36
H
ATOM 4095 1HH2 ARG A 270 -25.133 5.169 31.698 1.00 2.36
H
ATOM 4096 2HH2 ARG A 270 -24.999 5.622 30.014 1.00 2.36
H
ATOM 4097 N ALA A 271 -16.606 4.848 35.237 1.00 1.41
N
ATOM 4098 CA ALA A 271 -15.166 4.673 35.257 1.00 1.49
C

ATOM 4099 C ALA A 271 -14.724 4.117 36.595 1.00 1.58
C
ATOM 4100 O ALA A 271 -13.891 3.214 36.641 1.00 1.73
O
ATOM 4101 CB ALA A 271 -14.476 5.998 34.967 1.00 2.23
C
ATOM 4102 H ALA A 271 -16.975 5.774 35.078 1.00 1.69
H
ATOM 4103 HA ALA A 271 -14.897 3.953 34.484 1.00 1.79
H
ATOM 4104 1HB ALA A 271 -13.397 5.855 34.966 1.00 2.68
H
ATOM 4105 2HB ALA A 271 -14.795 6.368 33.992 1.00 2.68
H
ATOM 4106 3HB ALA A 271 -14.742 6.723 35.732 1.00 2.68
H
ATOM 4107 N ASP A 272 -15.304 4.635 37.680 1.00 1.54
N
ATOM 4108 CA ASP A 272 -14.999 4.157 39.019 1.00 1.65
C
ATOM 4109 C ASP A 272 -15.306 2.674 39.154 1.00 1.63
C
ATOM 4110 O ASP A 272 -14.468 1.907 39.628 1.00 1.71
O
ATOM 4111 CB ASP A 272 -15.809 4.926 40.068 1.00 2.47
C
ATOM 4112 CG ASP A 272 -15.363 6.369 40.285 1.00 2.47
C
ATOM 4113 OD1 ASP A 272 -14.283 6.734 39.876 1.00 2.47
O
ATOM 4114 OD2 ASP A 272 -16.115 7.109 40.873 1.00 2.47
O
ATOM 4115 H ASP A 272 -15.964 5.393 37.575 1.00 1.85
H
ATOM 4116 HA ASP A 272 -13.937 4.307 39.209 1.00 1.98
H
ATOM 4117 1HB ASP A 272 -16.858 4.935 39.775 1.00 2.97
H
ATOM 4118 2HB ASP A 272 -15.746 4.403 41.023 1.00 2.97
H
ATOM 4119 N ALA A 273 -16.507 2.276 38.724 1.00 1.61
N
ATOM 4120 CA ALA A 273 -16.928 0.881 38.803 1.00 1.72
C
ATOM 4121 C ALA A 273 -16.015 -0.033 37.995 1.00 1.49
C
ATOM 4122 O ALA A 273 -15.615 -1.094 38.476 1.00 1.51
O
ATOM 4123 CB ALA A 273 -18.361 0.745 38.313 1.00 2.58
C
ATOM 4124 H ALA A 273 -17.160 2.964 38.354 1.00 1.93
H
ATOM 4125 HA ALA A 273 -16.877 0.571 39.846 1.00 2.06
H
ATOM 4126 1HB ALA A 273 -18.680 -0.294 38.403 1.00 3.10
H
ATOM 4127 2HB ALA A 273 -19.012 1.378 38.916 1.00 3.10
H

```
ATOM   4128 3HB  ALA A 273     -18.421   1.052  37.271  1.00  3.10
H
ATOM   4129  N   ILE A 274     -15.682   0.398  36.780  1.00  1.29
N
ATOM   4130  CA  ILE A 274     -14.829  -0.359  35.877  1.00  1.12
C
ATOM   4131  C   ILE A 274     -13.450  -0.559  36.459  1.00  1.03
C
ATOM   4132  O   ILE A 274     -12.948  -1.682  36.476  1.00  1.02
O
ATOM   4133  CB  ILE A 274     -14.726   0.365  34.538  1.00  1.68
C
ATOM   4134  CG1 ILE A 274     -16.080   0.328  33.841  1.00  1.68
C
ATOM   4135  CG2 ILE A 274     -13.664  -0.290  33.671  1.00  1.68
C
ATOM   4136  CD1 ILE A 274     -16.173   1.307  32.710  1.00  1.68
C
ATOM   4137  H   ILE A 274     -16.055   1.281  36.452  1.00  1.55
H
ATOM   4138  HA  ILE A 274     -15.279  -1.337  35.713  1.00  1.34
H
ATOM   4139  HB  ILE A 274     -14.468   1.410  34.704  1.00  2.02
H
ATOM   4140 1HG1 ILE A 274     -16.245  -0.669  33.443  1.00  2.02
H
ATOM   4141 2HG1 ILE A 274     -16.870   0.539  34.558  1.00  2.02
H
ATOM   4142 1HG2 ILE A 274     -13.596   0.235  32.720  1.00  2.02
H
ATOM   4143 2HG2 ILE A 274     -12.702  -0.242  34.181  1.00  2.02
H
ATOM   4144 3HG2 ILE A 274     -13.928  -1.332  33.494  1.00  2.02
H
ATOM   4145 1HD1 ILE A 274     -17.153   1.238  32.239  1.00  2.02
H
ATOM   4146 2HD1 ILE A 274     -16.032   2.302  33.121  1.00  2.02
H
ATOM   4147 3HD1 ILE A 274     -15.398   1.111  31.978  1.00  2.02
H
ATOM   4148  N   VAL A 275     -12.853   0.525  36.953  1.00  1.02
N
ATOM   4149  CA  VAL A 275     -11.544   0.464  37.578  1.00  0.98
C
ATOM   4150  C   VAL A 275     -11.523  -0.479  38.756  1.00  1.10
C
ATOM   4151  O   VAL A 275     -10.590  -1.265  38.877  1.00  1.09
O
ATOM   4152  CB  VAL A 275     -11.076   1.858  38.016  1.00  1.47
C
ATOM   4153  CG1 VAL A 275      -9.850   1.733  38.900  1.00  1.47
C
ATOM   4154  CG2 VAL A 275     -10.716   2.677  36.781  1.00  1.47
C
ATOM   4155  H   VAL A 275     -13.317   1.421  36.894  1.00  1.22
H
ATOM   4156  HA  VAL A 275     -10.834   0.096  36.840  1.00  1.18
H
```

```
ATOM   4157  HB  VAL A 275     -11.865   2.352  38.583  1.00  1.76
H
ATOM   4158 1HG1 VAL A 275      -9.520   2.725  39.203  1.00  1.76
H
ATOM   4159 2HG1 VAL A 275     -10.100   1.147  39.785  1.00  1.76
H
ATOM   4160 3HG1 VAL A 275      -9.053   1.236  38.353  1.00  1.76
H
ATOM   4161 1HG2 VAL A 275     -10.379   3.667  37.087  1.00  1.76
H
ATOM   4162 2HG2 VAL A 275      -9.918   2.174  36.235  1.00  1.76
H
ATOM   4163 3HG2 VAL A 275     -11.587   2.773  36.138  1.00  1.76
H
ATOM   4164  N   LYS A 276     -12.543  -0.422  39.614  1.00  1.24
N
ATOM   4165  CA  LYS A 276     -12.608  -1.325  40.758  1.00  1.40
C
ATOM   4166  C   LYS A 276     -12.628  -2.785  40.311  1.00  1.38
C
ATOM   4167  O   LYS A 276     -11.949  -3.630  40.904  1.00  1.52
O
ATOM   4168  CB  LYS A 276     -13.843  -1.020  41.600  1.00  2.10
C
ATOM   4169  CG  LYS A 276     -13.771   0.289  42.374  1.00  2.10
C
ATOM   4170  CD  LYS A 276     -15.057   0.543  43.143  1.00  2.10
C
ATOM   4171  CE  LYS A 276     -15.002   1.861  43.900  1.00  2.10
C
ATOM   4172  NZ  LYS A 276     -16.267   2.126  44.642  1.00  2.10
N
ATOM   4173  H   LYS A 276     -13.280   0.262  39.484  1.00  1.49
H
ATOM   4174  HA  LYS A 276     -11.719  -1.173  41.370  1.00  1.68
H
ATOM   4175 1HB  LYS A 276     -14.722  -0.978  40.957  1.00  2.52
H
ATOM   4176 2HB  LYS A 276     -14.001  -1.824  42.318  1.00  2.52
H
ATOM   4177 1HG  LYS A 276     -12.942   0.239  43.082  1.00  2.52
H
ATOM   4178 2HG  LYS A 276     -13.586   1.115  41.693  1.00  2.52
H
ATOM   4179 1HD  LYS A 276     -15.898   0.568  42.447  1.00  2.52
H
ATOM   4180 2HD  LYS A 276     -15.222  -0.267  43.853  1.00  2.52
H
ATOM   4181 1HE  LYS A 276     -14.177   1.832  44.610  1.00  2.52
H
ATOM   4182 2HE  LYS A 276     -14.831   2.674  43.195  1.00  2.52
H
ATOM   4183 1HZ  LYS A 276     -16.193   3.007  45.130  1.00  2.52
H
ATOM   4184 2HZ  LYS A 276     -17.037   2.167  43.989  1.00  2.52
H
ATOM   4185 3HZ  LYS A 276     -16.429   1.385  45.307  1.00  2.52
H
```

ATOM	4186	N	LEU	A	277	-13.378	-3.074	39.248	1.00	1.27	N
ATOM	4187	CA	LEU	A	277	-13.436	-4.424	38.708	1.00	1.32	C
ATOM	4188	C	LEU	A	277	-12.082	-4.854	38.148	1.00	1.38	C
ATOM	4189	O	LEU	A	277	-11.672	-6.002	38.323	1.00	1.65	O
ATOM	4190	CB	LEU	A	277	-14.515	-4.497	37.625	1.00	1.98	C
ATOM	4191	CG	LEU	A	277	-15.954	-4.363	38.140	1.00	1.98	C
ATOM	4192	CD1	LEU	A	277	-16.905	-4.246	36.962	1.00	1.98	C
ATOM	4193	CD2	LEU	A	277	-16.291	-5.574	38.994	1.00	1.98	C
ATOM	4194	H	LEU	A	277	-13.941	-2.349	38.814	1.00	1.52	H
ATOM	4195	HA	LEU	A	277	-13.706	-5.104	39.515	1.00	1.58	H
ATOM	4196	1HB	LEU	A	277	-14.346	-3.701	36.903	1.00	2.38	H
ATOM	4197	2HB	LEU	A	277	-14.432	-5.454	37.111	1.00	2.38	H
ATOM	4198	HG	LEU	A	277	-16.047	-3.461	38.743	1.00	2.38	H
ATOM	4199	1HD1	LEU	A	277	-17.927	-4.145	37.327	1.00	2.38	H
ATOM	4200	2HD1	LEU	A	277	-16.642	-3.368	36.372	1.00	2.38	H
ATOM	4201	3HD1	LEU	A	277	-16.827	-5.138	36.342	1.00	2.38	H
ATOM	4202	1HD2	LEU	A	277	-17.312	-5.482	39.367	1.00	2.38	H
ATOM	4203	2HD2	LEU	A	277	-16.204	-6.479	38.393	1.00	2.38	H
ATOM	4204	3HD2	LEU	A	277	-15.601	-5.629	39.837	1.00	2.38	H
ATOM	4205	N	ILE	A	278	-11.364	-3.921	37.527	1.00	1.16	N
ATOM	4206	CA	ILE	A	278	-10.035	-4.207	37.008	1.00	1.12	C
ATOM	4207	C	ILE	A	278	-9.079	-4.485	38.154	1.00	1.39	C
ATOM	4208	O	ILE	A	278	-8.301	-5.435	38.107	1.00	1.63	O
ATOM	4209	CB	ILE	A	278	-9.489	-3.044	36.197	1.00	1.68	C
ATOM	4210	CG1	ILE	A	278	-10.314	-2.837	34.946	1.00	1.68	C
ATOM	4211	CG2	ILE	A	278	-8.061	-3.383	35.798	1.00	1.68	C
ATOM	4212	CD1	ILE	A	278	-10.028	-1.514	34.304	1.00	1.68	C
ATOM	4213	H	ILE	A	278	-11.759	-3.000	37.385	1.00	1.39	H
ATOM	4214	HA	ILE	A	278	-10.086	-5.092	36.375	1.00	1.34	H

ATOM 4215 HB ILE A 278 -9.510 -2.124 36.779 1.00 2.02
H
ATOM 4216 1HG1 ILE A 278 -10.086 -3.628 34.232 1.00 2.02
H
ATOM 4217 2HG1 ILE A 278 -11.372 -2.894 35.189 1.00 2.02
H
ATOM 4218 1HG2 ILE A 278 -7.647 -2.575 35.200 1.00 2.02
H
ATOM 4219 2HG2 ILE A 278 -7.456 -3.524 36.691 1.00 2.02
H
ATOM 4220 3HG2 ILE A 278 -8.060 -4.300 35.210 1.00 2.02
H
ATOM 4221 1HD1 ILE A 278 -10.635 -1.411 33.406 1.00 2.02
H
ATOM 4222 2HD1 ILE A 278 -10.270 -0.713 34.999 1.00 2.02
H
ATOM 4223 3HD1 ILE A 278 -8.973 -1.458 34.041 1.00 2.02
H
ATOM 4224 N GLU A 279 -9.154 -3.649 39.194 1.00 1.41
N
ATOM 4225 CA GLU A 279 -8.314 -3.777 40.378 1.00 1.61
C
ATOM 4226 C GLU A 279 -8.481 -5.126 41.051 1.00 1.76
C
ATOM 4227 O GLU A 279 -7.505 -5.698 41.534 1.00 1.70
O
ATOM 4228 CB GLU A 279 -8.618 -2.667 41.389 1.00 2.42
C
ATOM 4229 CG GLU A 279 -8.137 -1.277 40.996 1.00 2.42
C
ATOM 4230 CD GLU A 279 -8.555 -0.218 41.981 1.00 2.42
C
ATOM 4231 OE1 GLU A 279 -9.386 -0.502 42.810 1.00 2.42
O
ATOM 4232 OE2 GLU A 279 -8.037 0.873 41.912 1.00 2.42
O
ATOM 4233 H GLU A 279 -9.808 -2.884 39.160 1.00 1.69
H
ATOM 4234 HA GLU A 279 -7.277 -3.685 40.071 1.00 1.93
H
ATOM 4235 1HB GLU A 279 -9.695 -2.609 41.550 1.00 2.90
H
ATOM 4236 2HB GLU A 279 -8.160 -2.915 42.346 1.00 2.90
H
ATOM 4237 1HG GLU A 279 -7.049 -1.289 40.936 1.00 2.90
H
ATOM 4238 2HG GLU A 279 -8.521 -1.026 40.012 1.00 2.90
H
ATOM 4239 N SER A 280 -9.702 -5.677 41.035 1.00 2.13
N
ATOM 4240 CA SER A 280 -9.938 -6.995 41.629 1.00 2.22
C
ATOM 4241 C SER A 280 -9.173 -8.111 40.907 1.00 2.22
C
ATOM 4242 O SER A 280 -9.004 -9.199 41.457 1.00 4.00
O
ATOM 4243 CB SER A 280 -11.417 -7.354 41.609 1.00 3.33
C

```
ATOM   4244  OG  SER A 280     -11.862  -7.660  40.311  1.00  3.33
O
ATOM   4245  H   SER A 280     -10.484  -5.157  40.650  1.00  2.56
H
ATOM   4246  HA  SER A 280      -9.600  -6.967  42.666  1.00  2.66
H
ATOM   4247 1HB  SER A 280     -11.591  -8.205  42.265  1.00  4.00
H
ATOM   4248 2HB  SER A 280     -11.995  -6.517  42.002  1.00  4.00
H
ATOM   4249  HG  SER A 280     -11.713  -6.876  39.776  1.00  4.00
H
ATOM   4250  N   GLN A 281      -8.723  -7.849  39.679  1.00  1.79
N
ATOM   4251  CA  GLN A 281      -7.996  -8.824  38.892  1.00  1.71
C
ATOM   4252  C   GLN A 281      -6.480  -8.634  39.007  1.00  1.68
C
ATOM   4253  O   GLN A 281      -5.713  -9.386  38.403  1.00  4.55
O
ATOM   4254  CB  GLN A 281      -8.436  -8.727  37.434  1.00  2.56
C
ATOM   4255  CG  GLN A 281      -9.926  -8.953  37.260  1.00  2.56
C
ATOM   4256  CD  GLN A 281     -10.366 -10.318  37.749  1.00  2.56
C
ATOM   4257  OE1 GLN A 281      -9.923 -11.350  37.235  1.00  2.56
O
ATOM   4258  NE2 GLN A 281     -11.231 -10.332  38.758  1.00  2.56
N
ATOM   4259  H   GLN A 281      -8.869  -6.945  39.259  1.00  2.15
H
ATOM   4260  HA  GLN A 281      -8.239  -9.818  39.264  1.00  2.05
H
ATOM   4261 1HB  GLN A 281      -8.189  -7.742  37.037  1.00  3.08
H
ATOM   4262 2HB  GLN A 281      -7.905  -9.469  36.838  1.00  3.08
H
ATOM   4263 1HG  GLN A 281     -10.465  -8.198  37.831  1.00  3.08
H
ATOM   4264 2HG  GLN A 281     -10.176  -8.871  36.202  1.00  3.08
H
ATOM   4265 1HE2 GLN A 281     -11.553 -11.203  39.130  1.00  3.08
H
ATOM   4266 2HE2 GLN A 281     -11.551  -9.470  39.158  1.00  3.08
H
ATOM   4267  N   ILE A 282      -6.048  -7.629  39.775  1.00  1.51
N
ATOM   4268  CA  ILE A 282      -4.628  -7.380  39.979  1.00  1.14
C
ATOM   4269  C   ILE A 282      -4.136  -8.119  41.214  1.00  1.38
C
ATOM   4270  O   ILE A 282      -4.585  -7.852  42.330  1.00  1.74
O
ATOM   4271  CB  ILE A 282      -4.338  -5.868  40.110  1.00  1.71
C
ATOM   4272  CG1 ILE A 282      -4.735  -5.156  38.818  1.00  1.71
C
```

```
ATOM   4273  CG2 ILE A 282      -2.881  -5.619  40.439  1.00  1.71
C
ATOM   4274  CD1 ILE A 282      -4.656  -3.649  38.893  1.00  1.71
C
ATOM   4275  H   ILE A 282      -6.700  -7.028  40.256  1.00  1.81
H
ATOM   4276  HA  ILE A 282      -4.082  -7.756  39.116  1.00  1.37
H
ATOM   4277  HB  ILE A 282      -4.955  -5.458  40.908  1.00  2.05
H
ATOM   4278 1HG1 ILE A 282      -4.078  -5.496  38.022  1.00  2.05
H
ATOM   4279 2HG1 ILE A 282      -5.758  -5.432  38.562  1.00  2.05
H
ATOM   4280 1HG2 ILE A 282      -2.706  -4.548  40.540  1.00  2.05
H
ATOM   4281 2HG2 ILE A 282      -2.626  -6.117  41.373  1.00  2.05
H
ATOM   4282 3HG2 ILE A 282      -2.260  -6.012  39.639  1.00  2.05
H
ATOM   4283 1HD1 ILE A 282      -4.951  -3.223  37.936  1.00  2.05
H
ATOM   4284 2HD1 ILE A 282      -5.318  -3.282  39.675  1.00  2.05
H
ATOM   4285 3HD1 ILE A 282      -3.633  -3.354  39.122  1.00  2.05
H
ATOM   4286  N   GLN A 283      -3.226  -9.068  40.999  1.00  1.52
N
ATOM   4287  CA  GLN A 283      -2.681  -9.886  42.080  1.00  2.06
C
ATOM   4288  C   GLN A 283      -1.735  -9.136  43.016  1.00  1.27
C
ATOM   4289  O   GLN A 283      -1.827  -9.281  44.236  1.00  2.09
O
ATOM   4290  CB  GLN A 283      -1.945 -11.092  41.498  1.00  3.09
C
ATOM   4291  CG  GLN A 283      -1.419 -12.065  42.535  1.00  3.09
C
ATOM   4292  CD  GLN A 283      -2.533 -12.710  43.337  1.00  3.09
C
ATOM   4293  OE1 GLN A 283      -3.492 -13.246  42.774  1.00  3.09
O
ATOM   4294  NE2 GLN A 283      -2.412 -12.663  44.659  1.00  3.09
N
ATOM   4295  H   GLN A 283      -2.902  -9.224  40.051  1.00  1.82
H
ATOM   4296  HA  GLN A 283      -3.516 -10.250  42.679  1.00  2.47
H
ATOM   4297 1HB  GLN A 283      -2.614 -11.637  40.831  1.00  3.71
H
ATOM   4298 2HB  GLN A 283      -1.100 -10.749  40.901  1.00  3.71
H
ATOM   4299 1HG  GLN A 283      -0.861 -12.853  42.030  1.00  3.71
H
ATOM   4300 2HG  GLN A 283      -0.767 -11.529  43.224  1.00  3.71
H
ATOM   4301 1HE2 GLN A 283      -3.117 -13.073  45.241  1.00  3.71
H
```

```
ATOM   4302 2HE2 GLN A 283      -1.618 -12.220  45.074  1.00  3.71
H
ATOM   4303  N   THR A 284      -0.848  -8.316  42.460  1.00  0.79
N
ATOM   4304  CA  THR A 284       0.096  -7.591  43.306  1.00  1.17
C
ATOM   4305  C   THR A 284       0.365  -6.174  42.875  1.00  0.86
C
ATOM   4306  O   THR A 284       0.055  -5.765  41.757  1.00  3.08
O
ATOM   4307  CB  THR A 284       1.450  -8.299  43.385  1.00  1.75
C
ATOM   4308  OG1 THR A 284       2.260  -7.631  44.366  1.00  1.75
O
ATOM   4309  CG2 THR A 284       2.155  -8.254  42.041  1.00  1.75
C
ATOM   4310  H   THR A 284      -0.799  -8.231  41.450  1.00  0.95
H
ATOM   4311  HA  THR A 284      -0.322  -7.551  44.312  1.00  1.40
H
ATOM   4312  HB  THR A 284       1.303  -9.336  43.688  1.00  2.11
H
ATOM   4313  HG1 THR A 284       3.052  -8.149  44.529  1.00  2.11
H
ATOM   4314 1HG2 THR A 284       3.120  -8.754  42.121  1.00  2.11
H
ATOM   4315 2HG2 THR A 284       1.546  -8.760  41.294  1.00  2.11
H
ATOM   4316 3HG2 THR A 284       2.307  -7.218  41.741  1.00  2.11
H
ATOM   4317  N   ASN A 285       0.957  -5.423  43.798  1.00  1.16
N
ATOM   4318  CA  ASN A 285       1.309  -4.033  43.565  1.00  1.72
C
ATOM   4319  C   ASN A 285       2.780  -3.882  43.185  1.00  2.02
C
ATOM   4320  O   ASN A 285       3.247  -2.771  42.935  1.00  5.90
O
ATOM   4321  CB  ASN A 285       0.990  -3.199  44.790  1.00  2.58
C
ATOM   4322  CG  ASN A 285      -0.488  -3.094  45.049  1.00  2.58
C
ATOM   4323  OD1 ASN A 285      -1.289  -3.003  44.112  1.00  2.58
O
ATOM   4324  ND2 ASN A 285      -0.864  -3.105  46.302  1.00  2.58
N
ATOM   4325  H   ASN A 285       1.179  -5.846  44.690  1.00  1.39
H
ATOM   4326  HA  ASN A 285       0.719  -3.662  42.727  1.00  2.06
H
ATOM   4327 1HB  ASN A 285       1.469  -3.639  45.666  1.00  3.10
H
ATOM   4328 2HB  ASN A 285       1.397  -2.196  44.664  1.00  3.10
H
ATOM   4329 1HD2 ASN A 285      -1.834  -3.037  46.534  1.00  3.10
H
ATOM   4330 2HD2 ASN A 285      -0.181  -3.179  47.028  1.00  3.10
H
```

```
ATOM   4331  N   GLU A 286       3.505  -5.002  43.138  1.00  2.27
N
ATOM   4332  CA  GLU A 286       4.903  -4.986  42.727  1.00  3.70
C
ATOM   4333  C   GLU A 286       5.023  -4.586  41.270  1.00  1.36
C
ATOM   4334  O   GLU A 286       4.336  -5.136  40.410  1.00  2.76
O
ATOM   4335  CB  GLU A 286       5.552  -6.357  42.921  1.00  5.55
C
ATOM   4336  CG  GLU A 286       7.037  -6.389  42.572  1.00  5.55
C
ATOM   4337  CD  GLU A 286       7.667  -7.742  42.759  1.00  5.55
C
ATOM   4338  OE1 GLU A 286       6.979  -8.654  43.152  1.00  5.55
O
ATOM   4339  OE2 GLU A 286       8.839  -7.875  42.498  1.00  5.55
O
ATOM   4340  H   GLU A 286       3.083  -5.888  43.386  1.00  2.72
H
ATOM   4341  HA  GLU A 286       5.436  -4.251  43.330  1.00  4.44
H
ATOM   4342 1HB  GLU A 286       5.442  -6.669  43.959  1.00  6.66
H
ATOM   4343 2HB  GLU A 286       5.044  -7.093  42.299  1.00  6.66
H
ATOM   4344 1HG  GLU A 286       7.162  -6.087  41.533  1.00  6.66
H
ATOM   4345 2HG  GLU A 286       7.559  -5.664  43.196  1.00  6.66
H
ATOM   4346  N   ASN A 287       5.913  -3.647  40.989  1.00  1.83
N
ATOM   4347  CA  ASN A 287       6.110  -3.186  39.626  1.00  2.91
C
ATOM   4348  C   ASN A 287       7.362  -3.787  39.004  1.00  1.80
C
ATOM   4349  O   ASN A 287       8.357  -4.033  39.688  1.00  2.33
O
ATOM   4350  CB  ASN A 287       6.160  -1.673  39.588  1.00  4.37
C
ATOM   4351  CG  ASN A 287       4.843  -1.054  39.973  1.00  4.37
C
ATOM   4352  OD1 ASN A 287       3.775  -1.554  39.601  1.00  4.37
O
ATOM   4353  ND2 ASN A 287       4.897   0.025  40.714  1.00  4.37
N
ATOM   4354  H   ASN A 287       6.455  -3.236  41.736  1.00  2.20
H
ATOM   4355  HA  ASN A 287       5.262  -3.516  39.025  1.00  3.49
H
ATOM   4356 1HB  ASN A 287       6.932  -1.314  40.271  1.00  5.24
H
ATOM   4357 2HB  ASN A 287       6.428  -1.340  38.585  1.00  5.24
H
ATOM   4358 1HD2 ASN A 287       4.053   0.478  40.999  1.00  5.24
H
ATOM   4359 2HD2 ASN A 287       5.782   0.396  40.991  1.00  5.24
H
```

```
ATOM   4360  N   LEU A 288       7.295  -4.021  37.700  1.00  1.26
N
ATOM   4361  CA  LEU A 288       8.402  -4.571  36.932  1.00  0.94
C
ATOM   4362  C   LEU A 288       9.465  -3.514  36.675  1.00  0.67
C
ATOM   4363  O   LEU A 288       9.162  -2.327  36.563  1.00  0.87
O
ATOM   4364  CB  LEU A 288       7.871  -5.136  35.611  1.00  1.41
C
ATOM   4365  CG  LEU A 288       6.914  -6.318  35.764  1.00  1.41
C
ATOM   4366  CD1 LEU A 288       6.296  -6.688  34.424  1.00  1.41
C
ATOM   4367  CD2 LEU A 288       7.685  -7.501  36.333  1.00  1.41
C
ATOM   4368  H   LEU A 288       6.436  -3.799  37.215  1.00  1.51
H
ATOM   4369  HA  LEU A 288       8.853  -5.381  37.504  1.00  1.13
H
ATOM   4370 1HB  LEU A 288       7.335  -4.349  35.088  1.00  1.69
H
ATOM   4371 2HB  LEU A 288       8.712  -5.457  34.998  1.00  1.69
H
ATOM   4372  HG  LEU A 288       6.111  -6.037  36.440  1.00  1.69
H
ATOM   4373 1HD1 LEU A 288       5.616  -7.528  34.559  1.00  1.69
H
ATOM   4374 2HD1 LEU A 288       5.745  -5.835  34.028  1.00  1.69
H
ATOM   4375 3HD1 LEU A 288       7.083  -6.969  33.728  1.00  1.69
H
ATOM   4376 1HD2 LEU A 288       7.014  -8.350  36.458  1.00  1.69
H
ATOM   4377 2HD2 LEU A 288       8.488  -7.773  35.648  1.00  1.69
H
ATOM   4378 3HD2 LEU A 288       8.110  -7.228  37.299  1.00  1.69
H
ATOM   4379  N   ILE A 289      10.713  -3.959  36.594  1.00  0.68
N
ATOM   4380  CA  ILE A 289      11.851  -3.074  36.390  1.00  0.65
C
ATOM   4381  C   ILE A 289      12.488  -3.291  35.011  1.00  0.62
C
ATOM   4382  O   ILE A 289      12.994  -4.380  34.739  1.00  1.10
O
ATOM   4383  CB  ILE A 289      12.900  -3.323  37.488  1.00  0.98
C
ATOM   4384  CG1 ILE A 289      12.287  -3.056  38.866  1.00  0.98
C
ATOM   4385  CG2 ILE A 289      14.116  -2.451  37.269  1.00  0.98
C
ATOM   4386  CD1 ILE A 289      13.176  -3.472  40.013  1.00  0.98
C
ATOM   4387  H   ILE A 289      10.881  -4.951  36.687  1.00  0.82
H
ATOM   4388  HA  ILE A 289      11.509  -2.047  36.479  1.00  0.78
H
```

ATOM   4389 HB ILE A 289   13.202 -4.369 37.462 1.00 1.17
H
ATOM   4390 1HG1 ILE A 289   12.077 -1.991 38.961 1.00 1.17
H
ATOM   4391 2HG1 ILE A 289   11.347 -3.600 38.951 1.00 1.17
H
ATOM   4392 1HG2 ILE A 289   14.852 -2.649 38.048 1.00 1.17
H
ATOM   4393 2HG2 ILE A 289   14.552 -2.665 36.294 1.00 1.17
H
ATOM   4394 3HG2 ILE A 289   13.813 -1.407 37.314 1.00 1.17
H
ATOM   4395 1HD1 ILE A 289   12.678 -3.253 40.957 1.00 1.17
H
ATOM   4396 2HD1 ILE A 289   13.377 -4.542 39.949 1.00 1.17
H
ATOM   4397 3HD1 ILE A 289   14.116 -2.923 39.965 1.00 1.17
H
ATOM   4398 N   PRO A 290   12.446 -2.289 34.126 1.00 0.59
N
ATOM   4399 CA PRO A 290   13.032 -2.288 32.789 1.00 0.81
C
ATOM   4400 C   PRO A 290   14.541 -2.459 32.838 1.00 0.35
C
ATOM   4401 O   PRO A 290   15.227 -1.846 33.660 1.00 1.40
O
ATOM   4402 CB PRO A 290   12.619 -0.940 32.219 1.00 1.22
C
ATOM   4403 CG PRO A 290   11.349 -0.616 32.921 1.00 1.22
C
ATOM   4404 CD PRO A 290   11.485 -1.188 34.299 1.00 1.22
C
ATOM   4405 HA PRO A 290   12.579 -3.104 32.206 1.00 0.97
H
ATOM   4406 1HB PRO A 290   13.413 -0.202 32.390 1.00 1.46
H
ATOM   4407 2HB PRO A 290   12.493 -1.024 31.132 1.00 1.46
H
ATOM   4408 1HG PRO A 290   11.214   0.469 32.942 1.00 1.46
H
ATOM   4409 2HG PRO A 290   10.497 -1.033 32.367 1.00 1.46
H
ATOM   4410 1HD PRO A 290   11.884 -0.438 34.996 1.00 1.46
H
ATOM   4411 2HD PRO A 290   10.516 -1.582 34.634 1.00 1.46
H
ATOM   4412 N   LYS A 291   15.053 -3.265 31.918 1.00 0.76
N
ATOM   4413 CA LYS A 291   16.477 -3.551 31.844 1.00 0.78
C
ATOM   4414 C   LYS A 291   17.177 -2.656 30.836 1.00 0.48
C
ATOM   4415 O   LYS A 291   16.579 -2.265 29.835 1.00 0.70
O
ATOM   4416 CB LYS A 291   16.687 -5.016 31.480 1.00 1.17
C
ATOM   4417 CG LYS A 291   16.047 -5.996 32.452 1.00 1.17
C

ATOM 4418 CD LYS A 291 16.686 -5.907 33.827 1.00 1.17
C
ATOM 4419 CE LYS A 291 16.132 -6.971 34.763 1.00 1.17
C
ATOM 4420 NZ LYS A 291 16.755 -6.903 36.112 1.00 1.17
N
ATOM 4421 H LYS A 291 14.429 -3.716 31.263 1.00 0.91
H
ATOM 4422 HA LYS A 291 16.920 -3.365 32.822 1.00 0.94
H
ATOM 4423 1HB LYS A 291 16.280 -5.210 30.486 1.00 1.40
H
ATOM 4424 2HB LYS A 291 17.755 -5.231 31.447 1.00 1.40
H
ATOM 4425 1HG LYS A 291 14.983 -5.775 32.542 1.00 1.40
H
ATOM 4426 2HG LYS A 291 16.155 -7.013 32.076 1.00 1.40
H
ATOM 4427 1HD LYS A 291 17.765 -6.037 33.739 1.00 1.40
H
ATOM 4428 2HD LYS A 291 16.490 -4.923 34.256 1.00 1.40
H
ATOM 4429 1HE LYS A 291 15.056 -6.833 34.863 1.00 1.40
H
ATOM 4430 2HE LYS A 291 16.322 -7.957 34.336 1.00 1.40
H
ATOM 4431 1HZ LYS A 291 16.362 -7.622 36.702 1.00 1.40
H
ATOM 4432 2HZ LYS A 291 17.752 -7.041 36.031 1.00 1.40
H
ATOM 4433 3HZ LYS A 291 16.573 -5.997 36.520 1.00 1.40
H
ATOM 4434 N SER A 292 18.444 -2.350 31.119 1.00 0.71
N
ATOM 4435 CA SER A 292 19.274 -1.505 30.270 1.00 0.94
C
ATOM 4436 C SER A 292 19.761 -2.249 29.014 1.00 0.84
C
ATOM 4437 O SER A 292 19.854 -3.481 29.012 1.00 3.54
O
ATOM 4438 CB SER A 292 20.468 -1.008 31.074 1.00 1.41
C
ATOM 4439 OG SER A 292 21.322 -2.069 31.408 1.00 1.41
O
ATOM 4440 H SER A 292 18.850 -2.719 31.968 1.00 0.85
H
ATOM 4441 HA SER A 292 18.672 -0.649 29.972 1.00 1.13
H
ATOM 4442 1HB SER A 292 21.022 -0.263 30.508 1.00 1.69
H
ATOM 4443 2HB SER A 292 20.114 -0.526 31.983 1.00 1.69
H
ATOM 4444 HG SER A 292 22.065 -1.675 31.872 1.00 1.69
H
ATOM 4445 N PRO A 293 20.093 -1.505 27.952 1.00 0.94
N
ATOM 4446 CA PRO A 293 20.594 -1.934 26.654 1.00 0.69
C

ATOM 4447 C PRO A 293 22.107 -2.063 26.624 1.00 0.95
C
ATOM 4448 O PRO A 293 22.796 -1.692 27.574 1.00 2.14
O
ATOM 4449 CB PRO A 293 20.111 -0.829 25.728 1.00 1.03
C
ATOM 4450 CG PRO A 293 20.200 0.394 26.564 1.00 1.03
C
ATOM 4451 CD PRO A 293 19.783 -0.061 27.935 1.00 1.03
C
ATOM 4452 HA PRO A 293 20.132 -2.893 26.389 1.00 0.83
H
ATOM 4453 1HB PRO A 293 20.751 -0.786 24.837 1.00 1.24
H
ATOM 4454 2HB PRO A 293 19.088 -1.041 25.382 1.00 1.24
H
ATOM 4455 1HG PRO A 293 21.225 0.794 26.544 1.00 1.24
H
ATOM 4456 2HG PRO A 293 19.547 1.177 26.158 1.00 1.24
H
ATOM 4457 1HD PRO A 293 20.368 0.472 28.693 1.00 1.24
H
ATOM 4458 2HD PRO A 293 18.713 0.086 28.072 1.00 1.24
H
ATOM 4459 N VAL A 294 22.613 -2.579 25.510 1.00 1.14
N
ATOM 4460 CA VAL A 294 24.046 -2.720 25.300 1.00 1.83
C
ATOM 4461 C VAL A 294 24.620 -1.445 24.701 1.00 1.22
C
ATOM 4462 O VAL A 294 24.193 -0.998 23.636 1.00 1.49
O
ATOM 4463 CB VAL A 294 24.345 -3.905 24.364 1.00 2.75
C
ATOM 4464 CG1 VAL A 294 25.842 -3.995 24.101 1.00 2.75
C
ATOM 4465 CG2 VAL A 294 23.826 -5.189 24.992 1.00 2.75
C
ATOM 4466 H VAL A 294 21.984 -2.874 24.777 1.00 1.37
H
ATOM 4467 HA VAL A 294 24.524 -2.904 26.263 1.00 2.20
H
ATOM 4468 HB VAL A 294 23.851 -3.740 23.406 1.00 3.29
H
ATOM 4469 1HG1 VAL A 294 26.044 -4.828 23.427 1.00 3.29
H
ATOM 4470 2HG1 VAL A 294 26.188 -3.068 23.644 1.00 3.29
H
ATOM 4471 3HG1 VAL A 294 26.369 -4.155 25.042 1.00 3.29
H
ATOM 4472 1HG2 VAL A 294 24.028 -6.028 24.326 1.00 3.29
H
ATOM 4473 2HG2 VAL A 294 24.326 -5.355 25.947 1.00 3.29
H
ATOM 4474 3HG2 VAL A 294 22.751 -5.107 25.154 1.00 3.29
H
ATOM 4475 N GLU A 295 25.578 -0.854 25.403 1.00 1.52
N

```
ATOM   4476  CA  GLU A 295      26.200   0.391  24.971  1.00  1.39
C
ATOM   4477  C   GLU A 295      27.121   0.252  23.755  1.00  1.34
C
ATOM   4478  O   GLU A 295      28.343   0.225  23.904  1.00  2.56
O
ATOM   4479  CB  GLU A 295      26.951   1.026  26.139  1.00  2.08
C
ATOM   4480  CG  GLU A 295      26.027   1.502  27.256  1.00  2.08
C
ATOM   4481  CD  GLU A 295      26.754   2.161  28.395  1.00  2.08
C
ATOM   4482  OE1 GLU A 295      27.952   2.028  28.464  1.00  2.08
O
ATOM   4483  OE2 GLU A 295      26.111   2.813  29.189  1.00  2.08
O
ATOM   4484  H   GLU A 295      25.884  -1.278  26.268  1.00  1.82
H
ATOM   4485  HA  GLU A 295      25.399   1.060  24.707  1.00  1.67
H
ATOM   4486 1HB  GLU A 295      27.652   0.306  26.560  1.00  2.50
H
ATOM   4487 2HB  GLU A 295      27.525   1.882  25.784  1.00  2.50
H
ATOM   4488 1HG  GLU A 295      25.313   2.211  26.836  1.00  2.50
H
ATOM   4489 2HG  GLU A 295      25.468   0.648  27.635  1.00  2.50
H
ATOM   4490  N   ASP A 296      26.540   0.170  22.556  1.00  1.05
N
ATOM   4491  CA  ASP A 296      27.355   0.009  21.351  1.00  1.04
C
ATOM   4492  C   ASP A 296      27.080   0.986  20.189  1.00  0.66
C
ATOM   4493  O   ASP A 296      27.637   0.806  19.105  1.00  1.33
O
ATOM   4494  CB  ASP A 296      27.200  -1.418  20.838  1.00  1.56
C
ATOM   4495  CG  ASP A 296      25.772  -1.716  20.434  1.00  1.56
C
ATOM   4496  OD1 ASP A 296      24.947  -0.850  20.575  1.00  1.56
O
ATOM   4497  OD2 ASP A 296      25.506  -2.801  19.978  1.00  1.56
O
ATOM   4498  H   ASP A 296      25.528   0.189  22.506  1.00  1.26
H
ATOM   4499  HA  ASP A 296      28.396   0.145  21.643  1.00  1.25
H
ATOM   4500 1HB  ASP A 296      27.852  -1.573  19.977  1.00  1.87
H
ATOM   4501 2HB  ASP A 296      27.502  -2.122  21.613  1.00  1.87
H
ATOM   4502  N   SER A 297      26.260   2.018  20.396  1.00  0.76
N
ATOM   4503  CA  SER A 297      26.025   2.985  19.324  1.00  0.74
C
ATOM   4504  C   SER A 297      27.216   3.936  19.260  1.00  1.22
C
```

ATOM	4505	O	SER	A	297	27.769	4.291	20.301	1.00	3.68	O
ATOM	4506	CB	SER	A	297	24.755	3.781	19.568	1.00	1.11	C
ATOM	4507	OG	SER	A	297	23.637	2.940	19.609	1.00	1.11	O
ATOM	4508	H	SER	A	297	25.788	2.140	21.280	1.00	0.91	H
ATOM	4509	HA	SER	A	297	25.935	2.441	18.386	1.00	0.89	H
ATOM	4510	1HB	SER	A	297	24.839	4.325	20.508	1.00	1.33	H
ATOM	4511	2HB	SER	A	297	24.631	4.516	18.774	1.00	1.33	H
ATOM	4512	HG	SER	A	297	23.695	2.497	20.463	1.00	1.33	H
ATOM	4513	N	PRO	A	298	27.614	4.371	18.064	1.00	0.44	N
ATOM	4514	CA	PRO	A	298	28.726	5.264	17.770	1.00	0.55	C
ATOM	4515	C	PRO	A	298	28.421	6.703	18.151	1.00	0.48	C
ATOM	4516	O	PRO	A	298	27.266	7.130	18.133	1.00	0.49	O
ATOM	4517	CB	PRO	A	298	28.898	5.122	16.255	1.00	0.83	C
ATOM	4518	CG	PRO	A	298	27.542	4.764	15.755	1.00	0.83	C
ATOM	4519	CD	PRO	A	298	26.953	3.895	16.834	1.00	0.83	C
ATOM	4520	HA	PRO	A	298	29.622	4.908	18.299	1.00	0.66	H
ATOM	4521	1HB	PRO	A	298	29.273	6.065	15.830	1.00	0.99	H
ATOM	4522	2HB	PRO	A	298	29.650	4.350	16.034	1.00	0.99	H
ATOM	4523	1HG	PRO	A	298	26.950	5.673	15.572	1.00	0.99	H
ATOM	4524	2HG	PRO	A	298	27.627	4.241	14.792	1.00	0.99	H
ATOM	4525	1HD	PRO	A	298	25.867	4.055	16.896	1.00	0.99	H
ATOM	4526	2HD	PRO	A	298	27.201	2.838	16.655	1.00	0.99	H
ATOM	4527	N	GLN	A	299	29.466	7.454	18.478	1.00	0.56	N
ATOM	4528	CA	GLN	A	299	29.305	8.862	18.801	1.00	0.57	C
ATOM	4529	C	GLN	A	299	29.161	9.679	17.532	1.00	0.47	C
ATOM	4530	O	GLN	A	299	30.033	9.653	16.662	1.00	0.45	O
ATOM	4531	CB	GLN	A	299	30.482	9.392	19.617	1.00	0.85	C
ATOM	4532	CG	GLN	A	299	30.365	10.874	19.938	1.00	0.85	C
ATOM	4533	CD	GLN	A	299	29.221	11.175	20.891	1.00	0.85	C

ATOM 4534 OE1 GLN A 299 29.190 10.663 22.014 1.00 0.85
O
ATOM 4535 NE2 GLN A 299 28.273 11.999 20.452 1.00 0.85
N
ATOM 4536 H GLN A 299 30.389 7.043 18.490 1.00 0.67
H
ATOM 4537 HA GLN A 299 28.394 8.983 19.387 1.00 0.68
H
ATOM 4538 1HB GLN A 299 30.553 8.845 20.556 1.00 1.03
H
ATOM 4539 2HB GLN A 299 31.410 9.232 19.070 1.00 1.03
H
ATOM 4540 1HG GLN A 299 31.292 11.209 20.401 1.00 1.03
H
ATOM 4541 2HG GLN A 299 30.191 11.424 19.012 1.00 1.03
H
ATOM 4542 1HE2 GLN A 299 27.498 12.226 21.043 1.00 1.03
H
ATOM 4543 2HE2 GLN A 299 28.326 12.390 19.530 1.00 1.03
H
ATOM 4544 N ILE A 300 28.058 10.400 17.435 1.00 0.52
N
ATOM 4545 CA ILE A 300 27.777 11.230 16.278 1.00 0.53
C
ATOM 4546 C ILE A 300 28.216 12.671 16.538 1.00 0.75
C
ATOM 4547 O ILE A 300 27.949 13.223 17.607 1.00 0.86
O
ATOM 4548 CB ILE A 300 26.275 11.176 15.933 1.00 0.80
C
ATOM 4549 CG1 ILE A 300 25.836 9.717 15.735 1.00 0.80
C
ATOM 4550 CG2 ILE A 300 25.994 11.983 14.676 1.00 0.80
C
ATOM 4551 CD1 ILE A 300 26.576 8.992 14.635 1.00 0.80
C
ATOM 4552 H ILE A 300 27.386 10.370 18.189 1.00 0.62
H
ATOM 4553 HA ILE A 300 28.341 10.847 15.428 1.00 0.64
H
ATOM 4554 HB ILE A 300 25.695 11.583 16.761 1.00 0.95
H
ATOM 4555 1HG1 ILE A 300 25.992 9.173 16.667 1.00 0.95
H
ATOM 4556 2HG1 ILE A 300 24.774 9.694 15.502 1.00 0.95
H
ATOM 4557 1HG2 ILE A 300 24.930 11.936 14.447 1.00 0.95
H
ATOM 4558 2HG2 ILE A 300 26.284 13.020 14.836 1.00 0.95
H
ATOM 4559 3HG2 ILE A 300 26.561 11.569 13.843 1.00 0.95
H
ATOM 4560 1HD1 ILE A 300 26.207 7.968 14.563 1.00 0.95
H
ATOM 4561 2HD1 ILE A 300 26.411 9.505 13.687 1.00 0.95
H
ATOM 4562 3HD1 ILE A 300 27.642 8.977 14.860 1.00 0.95
H

ATOM 4563 N SER A 301 28.931 13.257 15.577 1.00 1.10
N
ATOM 4564 CA SER A 301 29.394 14.638 15.698 1.00 1.81
C
ATOM 4565 C SER A 301 28.240 15.619 15.661 1.00 2.32
C
ATOM 4566 O SER A 301 27.272 15.427 14.928 1.00 5.83
O
ATOM 4567 CB SER A 301 30.357 15.003 14.589 1.00 2.71
C
ATOM 4568 OG SER A 301 30.726 16.356 14.691 1.00 2.71
O
ATOM 4569 H SER A 301 29.139 12.740 14.734 1.00 1.32
H
ATOM 4570 HA SER A 301 29.904 14.746 16.657 1.00 2.17
H
ATOM 4571 1HB SER A 301 31.244 14.372 14.648 1.00 3.26
H
ATOM 4572 2HB SER A 301 29.886 14.821 13.624 1.00 3.26
H
ATOM 4573 HG SER A 301 31.264 16.418 15.486 1.00 3.26
H
ATOM 4574 N ILE A 302 28.357 16.682 16.442 1.00 2.15
N
ATOM 4575 CA ILE A 302 27.344 17.727 16.482 1.00 2.45
C
ATOM 4576 C ILE A 302 27.886 19.037 15.914 1.00 3.10
C
ATOM 4577 O ILE A 302 27.366 20.110 16.212 1.00 11.20
O
ATOM 4578 CB ILE A 302 26.855 17.955 17.924 1.00 3.68
C
ATOM 4579 CG1 ILE A 302 28.033 18.373 18.809 1.00 3.68
C
ATOM 4580 CG2 ILE A 302 26.204 16.690 18.463 1.00 3.68
C
ATOM 4581 CD1 ILE A 302 27.618 18.857 20.179 1.00 3.68
C
ATOM 4582 H ILE A 302 29.176 16.772 17.026 1.00 2.58
H
ATOM 4583 HA ILE A 302 26.496 17.415 15.876 1.00 2.94
H
ATOM 4584 HB ILE A 302 26.130 18.767 17.938 1.00 4.41
H
ATOM 4585 1HG1 ILE A 302 28.704 17.523 18.933 1.00 4.41
H
ATOM 4586 2HG1 ILE A 302 28.583 19.175 18.315 1.00 4.41
H
ATOM 4587 1HG2 ILE A 302 25.856 16.866 19.480 1.00 4.41
H
ATOM 4588 2HG2 ILE A 302 25.357 16.420 17.833 1.00 4.41
H
ATOM 4589 3HG2 ILE A 302 26.930 15.876 18.464 1.00 4.41
H
ATOM 4590 1HD1 ILE A 302 28.503 19.137 20.751 1.00 4.41
H
ATOM 4591 2HD1 ILE A 302 26.963 19.724 20.075 1.00 4.41
H

```
ATOM   4592 3HD1 ILE A 302      27.086  18.061  20.700  1.00  4.41
H
ATOM   4593  N   THR A 303      28.948  18.944  15.112  1.00  2.57
N
ATOM   4594  CA  THR A 303      29.591  20.124  14.543  1.00  3.07
C
ATOM   4595  C   THR A 303      28.961  20.591  13.230  1.00  2.91
C
ATOM   4596  O   THR A 303      28.132  19.898  12.636  1.00  3.37
O
ATOM   4597  CB  THR A 303      31.092  19.851  14.326  1.00  4.60
C
ATOM   4598  OG1 THR A 303      31.257  18.783  13.380  1.00  4.60
O
ATOM   4599  CG2 THR A 303      31.752  19.470  15.642  1.00  4.60
C
ATOM   4600  H   THR A 303      29.338  18.034  14.895  1.00  3.08
H
ATOM   4601  HA  THR A 303      29.497  20.939  15.262  1.00  3.68
H
ATOM   4602  HB  THR A 303      31.570  20.747  13.931  1.00  5.53
H
ATOM   4603  HG1 THR A 303      30.972  17.951  13.777  1.00  5.53
H
ATOM   4604 1HG2 THR A 303      32.812  19.282  15.476  1.00  5.53
H
ATOM   4605 2HG2 THR A 303      31.636  20.285  16.357  1.00  5.53
H
ATOM   4606 3HG2 THR A 303      31.283  18.572  16.039  1.00  5.53
H
ATOM   4607  N   ASP A 304      29.360  21.782  12.789  1.00  2.70
N
ATOM   4608  CA  ASP A 304      28.868  22.349  11.538  1.00  2.98
C
ATOM   4609  C   ASP A 304      29.500  21.658  10.336  1.00  2.86
C
ATOM   4610  O   ASP A 304      30.705  21.396  10.326  1.00  7.16
O
ATOM   4611  CB  ASP A 304      29.163  23.850  11.475  1.00  4.47
C
ATOM   4612  CG  ASP A 304      28.340  24.665  12.464  1.00  4.47
C
ATOM   4613  OD1 ASP A 304      27.370  24.153  12.970  1.00  4.47
O
ATOM   4614  OD2 ASP A 304      28.695  25.792  12.709  1.00  4.47
O
ATOM   4615  H   ASP A 304      30.033  22.305  13.331  1.00  3.24
H
ATOM   4616  HA  ASP A 304      27.788  22.202  11.490  1.00  3.58
H
ATOM   4617 1HB  ASP A 304      30.220  24.020  11.680  1.00  5.36
H
ATOM   4618 2HB  ASP A 304      28.962  24.218  10.469  1.00  5.36
H
ATOM   4619  N   ILE A 305      28.697  21.384   9.313  1.00  1.26
N
ATOM   4620  CA  ILE A 305      29.224  20.746   8.117  1.00  1.97
C
```

ATOM 4621 C ILE A 305 29.643 21.822 7.138 1.00 4.76
C
ATOM 4622 O ILE A 305 28.827 22.644 6.723 1.00 19.68
O
ATOM 4623 CB ILE A 305 28.193 19.827 7.429 1.00 2.96
C
ATOM 4624 CG1 ILE A 305 27.726 18.720 8.367 1.00 2.96
C
ATOM 4625 CG2 ILE A 305 28.846 19.200 6.207 1.00 2.96
C
ATOM 4626 CD1 ILE A 305 26.553 17.929 7.829 1.00 2.96
C
ATOM 4627 H ILE A 305 27.715 21.625 9.369 1.00 1.51
H
ATOM 4628 HA ILE A 305 30.099 20.156 8.383 1.00 2.36
H
ATOM 4629 HB ILE A 305 27.321 20.406 7.126 1.00 3.55
H
ATOM 4630 1HG1 ILE A 305 28.552 18.036 8.547 1.00 3.55
H
ATOM 4631 2HG1 ILE A 305 27.432 19.163 9.320 1.00 3.55
H
ATOM 4632 1HG2 ILE A 305 28.131 18.550 5.704 1.00 3.55
H
ATOM 4633 2HG2 ILE A 305 29.168 19.985 5.526 1.00 3.55
H
ATOM 4634 3HG2 ILE A 305 29.711 18.616 6.518 1.00 3.55
H
ATOM 4635 1HD1 ILE A 305 26.272 17.160 8.548 1.00 3.55
H
ATOM 4636 2HD1 ILE A 305 25.708 18.599 7.666 1.00 3.55
H
ATOM 4637 3HD1 ILE A 305 26.833 17.462 6.888 1.00 3.55
H
ATOM 4638 N LYS A 306 30.913 21.839 6.779 1.00 1.46
N
ATOM 4639 CA LYS A 306 31.383 22.849 5.854 1.00 2.63
C
ATOM 4640 C LYS A 306 31.852 22.215 4.566 1.00 1.90
C
ATOM 4641 O LYS A 306 32.405 21.120 4.578 1.00 2.98
O
ATOM 4642 CB LYS A 306 32.498 23.666 6.494 1.00 3.94
C
ATOM 4643 CG LYS A 306 32.051 24.463 7.713 1.00 3.94
C
ATOM 4644 CD LYS A 306 33.196 25.270 8.300 1.00 3.94
C
ATOM 4645 CE LYS A 306 32.753 26.041 9.535 1.00 3.94
C
ATOM 4646 NZ LYS A 306 33.872 26.826 10.126 1.00 3.94
N
ATOM 4647 H LYS A 306 31.555 21.149 7.146 1.00 1.75
H
ATOM 4648 HA LYS A 306 30.554 23.505 5.620 1.00 3.16
H
ATOM 4649 1HB LYS A 306 33.306 23.003 6.804 1.00 4.73
H

```
ATOM   4650 2HB  LYS A 306      32.907  24.365   5.764  1.00  4.73
H
ATOM   4651 1HG  LYS A 306      31.246  25.142   7.429  1.00  4.73
H
ATOM   4652 2HG  LYS A 306      31.673  23.781   8.474  1.00  4.73
H
ATOM   4653 1HD  LYS A 306      34.011  24.598   8.573  1.00  4.73
H
ATOM   4654 2HD  LYS A 306      33.564  25.975   7.555  1.00  4.73
H
ATOM   4655 1HE  LYS A 306      31.949  26.722   9.263  1.00  4.73
H
ATOM   4656 2HE  LYS A 306      32.382  25.339  10.282  1.00  4.73
H
ATOM   4657 1HZ  LYS A 306      33.541  27.322  10.941  1.00  4.73
H
ATOM   4658 2HZ  LYS A 306      34.619  26.199  10.393  1.00  4.73
H
ATOM   4659 3HZ  LYS A 306      34.216  27.488   9.446  1.00  4.73
H
ATOM   4660  N   MET A 307      31.634  22.903   3.455  1.00  1.63
N
ATOM   4661  CA  MET A 307      32.036  22.391   2.158  1.00  1.66
C
ATOM   4662  C   MET A 307      33.563  22.370   2.093  1.00  1.45
C
ATOM   4663  O   MET A 307      34.220  23.256   2.642  1.00  3.02
O
ATOM   4664  CB  MET A 307      31.423  23.241   1.048  1.00  2.49
C
ATOM   4665  CG  MET A 307      31.548  22.643  -0.341  1.00  2.49
C
ATOM   4666  SD  MET A 307      30.615  21.107  -0.495  1.00  2.49
S
ATOM   4667  CE  MET A 307      28.917  21.682  -0.523  1.00  2.49
C
ATOM   4668  H   MET A 307      31.178  23.801   3.510  1.00  1.96
H
ATOM   4669  HA  MET A 307      31.681  21.365   2.056  1.00  1.99
H
ATOM   4670 1HB  MET A 307      30.365  23.397   1.251  1.00  2.99
H
ATOM   4671 2HB  MET A 307      31.902  24.221   1.033  1.00  2.99
H
ATOM   4672 1HG  MET A 307      31.173  23.350  -1.080  1.00  2.99
H
ATOM   4673 2HG  MET A 307      32.592  22.436  -0.566  1.00  2.99
H
ATOM   4674 1HE  MET A 307      28.244  20.829  -0.616  1.00  2.99
H
ATOM   4675 2HE  MET A 307      28.699  22.218   0.402  1.00  2.99
H
ATOM   4676 3HE  MET A 307      28.773  22.351  -1.372  1.00  2.99
H
ATOM   4677  N   THR A 308      34.130  21.358   1.433  1.00  1.35
N
ATOM   4678  CA  THR A 308      35.588  21.200   1.376  1.00  1.59
C
```

ATOM 4679 C THR A 308 36.286 22.327 0.608 1.00 1.80
C
ATOM 4680 O THR A 308 37.465 22.601 0.835 1.00 4.40
O
ATOM 4681 CB THR A 308 35.969 19.860 0.713 1.00 2.39
C
ATOM 4682 OG1 THR A 308 35.476 19.835 -0.634 1.00 2.39
O
ATOM 4683 CG2 THR A 308 35.370 18.690 1.480 1.00 2.39
C
ATOM 4684 H THR A 308 33.541 20.669 0.984 1.00 1.62
H
ATOM 4685 HA THR A 308 35.970 21.204 2.397 1.00 1.91
H
ATOM 4686 HB THR A 308 37.054 19.762 0.696 1.00 2.86
H
ATOM 4687 HG1 THR A 308 35.878 20.552 -1.134 1.00 2.86
H
ATOM 4688 1HG2 THR A 308 35.651 17.755 0.997 1.00 2.86
H
ATOM 4689 2HG2 THR A 308 35.744 18.697 2.504 1.00 2.86
H
ATOM 4690 3HG2 THR A 308 34.286 18.781 1.489 1.00 2.86
H
ATOM 4691 N SER A 309 35.557 22.981 -0.286 1.00 0.74
N
ATOM 4692 CA SER A 309 36.093 24.080 -1.071 1.00 0.95
C
ATOM 4693 C SER A 309 34.936 24.956 -1.536 1.00 1.60
C
ATOM 4694 O SER A 309 33.779 24.579 -1.365 1.00 5.27
O
ATOM 4695 CB SER A 309 36.860 23.530 -2.267 1.00 1.42
C
ATOM 4696 OG SER A 309 35.987 22.937 -3.189 1.00 1.42
O
ATOM 4697 H SER A 309 34.597 22.709 -0.431 1.00 0.89
H
ATOM 4698 HA SER A 309 36.763 24.670 -0.444 1.00 1.14
H
ATOM 4699 1HB SER A 309 37.424 24.319 -2.758 1.00 1.71
H
ATOM 4700 2HB SER A 309 37.578 22.789 -1.920 1.00 1.71
H
ATOM 4701 HG SER A 309 35.437 23.653 -3.524 1.00 1.71
H
ATOM 4702 N PRO A 310 35.216 26.141 -2.076 1.00 0.91
N
ATOM 4703 CA PRO A 310 34.267 27.045 -2.703 1.00 0.93
C
ATOM 4704 C PRO A 310 33.865 26.455 -4.055 1.00 1.15
C
ATOM 4705 O PRO A 310 34.578 25.601 -4.581 1.00 2.62
O
ATOM 4706 CB PRO A 310 35.035 28.371 -2.803 1.00 1.40
C
ATOM 4707 CG PRO A 310 36.467 27.976 -2.761 1.00 1.40
C

```
ATOM   4708  CD  PRO A 310      36.483  26.834  -1.776  1.00  1.40
C
ATOM   4709  HA  PRO A 310      33.403  27.143  -2.037  1.00  1.12
H
ATOM   4710 1HB  PRO A 310      34.764  28.893  -3.733  1.00  1.67
H
ATOM   4711 2HB  PRO A 310      34.749  29.031  -1.970  1.00  1.67
H
ATOM   4712 1HG  PRO A 310      36.812  27.682  -3.763  1.00  1.67
H
ATOM   4713 2HG  PRO A 310      37.090  28.823  -2.442  1.00  1.67
H
ATOM   4714 1HD  PRO A 310      37.352  26.192  -1.962  1.00  1.67
H
ATOM   4715 2HD  PRO A 310      36.470  27.210  -0.742  1.00  1.67
H
ATOM   4716  N   PRO A 311      32.722  26.875  -4.609  1.00  1.04
N
ATOM   4717  CA  PRO A 311      32.146  26.472  -5.887  1.00  1.27
C
ATOM   4718  C   PRO A 311      33.150  26.589  -7.022  1.00  2.14
C
ATOM   4719  O   PRO A 311      33.888  27.564  -7.124  1.00  9.80
O
ATOM   4720  CB  PRO A 311      30.995  27.455  -6.067  1.00  1.91
C
ATOM   4721  CG  PRO A 311      30.578  27.794  -4.682  1.00  1.91
C
ATOM   4722  CD  PRO A 311      31.865  27.856  -3.913  1.00  1.91
C
ATOM   4723  HA  PRO A 311      31.782  25.441  -5.810  1.00  1.52
H
ATOM   4724 1HB  PRO A 311      31.356  28.330  -6.620  1.00  2.29
H
ATOM   4725 2HB  PRO A 311      30.192  26.996  -6.659  1.00  2.29
H
ATOM   4726 1HG  PRO A 311      30.030  28.748  -4.668  1.00  2.29
H
ATOM   4727 2HG  PRO A 311      29.890  27.026  -4.296  1.00  2.29
H
ATOM   4728 1HD  PRO A 311      32.314  28.856  -3.968  1.00  2.29
H
ATOM   4729 2HD  PRO A 311      31.676  27.544  -2.877  1.00  2.29
H
ATOM   4730  N   ALA A 312      33.168  25.587  -7.887  1.00  1.35
N
ATOM   4731  CA  ALA A 312      34.099  25.561  -9.006  1.00  1.38
C
ATOM   4732  C   ALA A 312      33.413  25.864 -10.331  1.00  1.35
C
ATOM   4733  O   ALA A 312      33.552  25.099 -11.284  1.00  3.68
O
ATOM   4734  CB  ALA A 312      34.782  24.205  -9.080  1.00  2.07
C
ATOM   4735  H   ALA A 312      32.527  24.817  -7.760  1.00  1.62
H
ATOM   4736  HA  ALA A 312      34.854  26.329  -8.837  1.00  1.66
H
```

```
ATOM   4737 1HB  ALA A 312      35.501  24.205  -9.900  1.00  2.48
H
ATOM   4738 2HB  ALA A 312      35.300  24.009  -8.143  1.00  2.48
H
ATOM   4739 3HB  ALA A 312      34.036  23.432  -9.253  1.00  2.48
H
ATOM   4740  N   TYR A 313      32.679  26.971 -10.401  1.00  0.73
N
ATOM   4741  CA  TYR A 313      32.003  27.319 -11.647  1.00  0.68
C
ATOM   4742  C   TYR A 313      32.402  28.712 -12.070  1.00  0.79
C
ATOM   4743  O   TYR A 313      32.654  29.572 -11.226  1.00  1.25
O
ATOM   4744  CB  TYR A 313      30.492  27.267 -11.490  1.00  1.02
C
ATOM   4745  CG  TYR A 313      29.969  25.939 -11.037  1.00  1.02
C
ATOM   4746  CD1 TYR A 313      29.850  25.707  -9.680  1.00  1.02
C
ATOM   4747  CD2 TYR A 313      29.607  24.960 -11.953  1.00  1.02
C
ATOM   4748  CE1 TYR A 313      29.373  24.504  -9.235  1.00  1.02
C
ATOM   4749  CE2 TYR A 313      29.126  23.745 -11.500  1.00  1.02
C
ATOM   4750  CZ  TYR A 313      29.011  23.520 -10.143  1.00  1.02
C
ATOM   4751  OH  TYR A 313      28.538  22.321  -9.670  1.00  1.02
O
ATOM   4752  H   TYR A 313      32.592  27.573  -9.596  1.00  0.88
H
ATOM   4753  HA  TYR A 313      32.312  26.626 -12.430  1.00  0.82
H
ATOM   4754 1HB  TYR A 313      30.166  28.023 -10.780  1.00  1.22
H
ATOM   4755 2HB  TYR A 313      30.030  27.499 -12.447  1.00  1.22
H
ATOM   4756  HD1 TYR A 313      30.135  26.479  -8.966  1.00  1.22
H
ATOM   4757  HD2 TYR A 313      29.703  25.148 -13.022  1.00  1.22
H
ATOM   4758  HE1 TYR A 313      29.278  24.327  -8.171  1.00  1.22
H
ATOM   4759  HE2 TYR A 313      28.841  22.970 -12.211  1.00  1.22
H
ATOM   4760  HH  TYR A 313      28.508  22.352  -8.708  1.00  1.22
H
ATOM   4761  N   LYS A 314      32.455  28.948 -13.374  1.00  0.68
N
ATOM   4762  CA  LYS A 314      32.821  30.266 -13.855  1.00  0.78
C
ATOM   4763  C   LYS A 314      31.597  30.988 -14.372  1.00  0.63
C
ATOM   4764  O   LYS A 314      30.699  30.370 -14.940  1.00  0.59
O
ATOM   4765  CB  LYS A 314      33.887  30.172 -14.946  1.00  1.17
C
```

ATOM	4766	CG	LYS	A	314	35.218	29.606	-14.472	1.00	1.17	C
ATOM	4767	CD	LYS	A	314	36.238	29.574	-15.600	1.00	1.17	C
ATOM	4768	CE	LYS	A	314	37.567	29.000	-15.132	1.00	1.17	C
ATOM	4769	NZ	LYS	A	314	38.566	28.949	-16.233	1.00	1.17	N
ATOM	4770	H	LYS	A	314	32.242	28.221	-14.053	1.00	0.82	H
ATOM	4771	HA	LYS	A	314	33.226	30.844	-13.024	1.00	0.94	H
ATOM	4772	1HB	LYS	A	314	33.527	29.544	-15.759	1.00	1.40	H
ATOM	4773	2HB	LYS	A	314	34.074	31.164	-15.359	1.00	1.40	H
ATOM	4774	1HG	LYS	A	314	35.607	30.221	-13.659	1.00	1.40	H
ATOM	4775	2HG	LYS	A	314	35.070	28.593	-14.099	1.00	1.40	H
ATOM	4776	1HD	LYS	A	314	35.855	28.963	-16.418	1.00	1.40	H
ATOM	4777	2HD	LYS	A	314	36.401	30.585	-15.972	1.00	1.40	H
ATOM	4778	1HE	LYS	A	314	37.961	29.618	-14.326	1.00	1.40	H
ATOM	4779	2HE	LYS	A	314	37.408	27.990	-14.755	1.00	1.40	H
ATOM	4780	1HZ	LYS	A	314	39.432	28.563	-15.885	1.00	1.40	H
ATOM	4781	2HZ	LYS	A	314	38.215	28.369	-16.981	1.00	1.40	H
ATOM	4782	3HZ	LYS	A	314	38.731	29.883	-16.582	1.00	1.40	H
ATOM	4783	N	VAL	A	315	31.572	32.298	-14.172	1.00	0.62	N
ATOM	4784	CA	VAL	A	315	30.451	33.106	-14.616	1.00	0.56	C
ATOM	4785	C	VAL	A	315	30.334	33.021	-16.125	1.00	0.50	C
ATOM	4786	O	VAL	A	315	31.313	33.222	-16.847	1.00	0.51	O
ATOM	4787	CB	VAL	A	315	30.631	34.568	-14.157	1.00	0.84	C
ATOM	4788	CG1	VAL	A	315	29.541	35.454	-14.736	1.00	0.84	C
ATOM	4789	CG2	VAL	A	315	30.594	34.618	-12.636	1.00	0.84	C
ATOM	4790	H	VAL	A	315	32.344	32.744	-13.698	1.00	0.74	H
ATOM	4791	HA	VAL	A	315	29.539	32.714	-14.170	1.00	0.67	H
ATOM	4792	HB	VAL	A	315	31.590	34.940	-14.517	1.00	1.01	H
ATOM	4793	1HG1	VAL	A	315	29.696	36.482	-14.406	1.00	1.01	H
ATOM	4794	2HG1	VAL	A	315	29.578	35.416	-15.825	1.00	1.01	H

ATOM 4795 3HG1 VAL A 315 28.569 35.110 -14.393 1.00 1.01
H
ATOM 4796 1HG2 VAL A 315 30.733 35.646 -12.302 1.00 1.01
H
ATOM 4797 2HG2 VAL A 315 29.630 34.251 -12.284 1.00 1.01
H
ATOM 4798 3HG2 VAL A 315 31.390 33.994 -12.230 1.00 1.01
H
ATOM 4799 N GLY A 316 29.134 32.696 -16.592 1.00 0.48
N
ATOM 4800 CA GLY A 316 28.872 32.511 -18.010 1.00 0.52
C
ATOM 4801 C GLY A 316 28.677 31.032 -18.372 1.00 0.54
C
ATOM 4802 O GLY A 316 28.074 30.724 -19.401 1.00 0.63
O
ATOM 4803 H GLY A 316 28.373 32.589 -15.935 1.00 0.58
H
ATOM 4804 1HA GLY A 316 27.983 33.076 -18.290 1.00 0.62
H
ATOM 4805 2HA GLY A 316 29.701 32.920 -18.588 1.00 0.62
H
ATOM 4806 N ASP A 317 29.176 30.122 -17.527 1.00 0.55
N
ATOM 4807 CA ASP A 317 29.007 28.687 -17.753 1.00 0.69
C
ATOM 4808 C ASP A 317 27.543 28.315 -17.622 1.00 0.52
C
ATOM 4809 O ASP A 317 26.860 28.820 -16.741 1.00 0.52
O
ATOM 4810 CB ASP A 317 29.810 27.865 -16.730 1.00 1.03
C
ATOM 4811 CG ASP A 317 31.319 27.901 -16.930 1.00 1.03
C
ATOM 4812 OD1 ASP A 317 31.756 28.277 -17.992 1.00 1.03
O
ATOM 4813 OD2 ASP A 317 32.024 27.558 -16.006 1.00 1.03
O
ATOM 4814 H ASP A 317 29.677 30.414 -16.696 1.00 0.66
H
ATOM 4815 HA ASP A 317 29.342 28.443 -18.762 1.00 0.83
H
ATOM 4816 1HB ASP A 317 29.588 28.228 -15.727 1.00 1.24
H
ATOM 4817 2HB ASP A 317 29.486 26.825 -16.774 1.00 1.24
H
ATOM 4818 N LYS A 318 27.040 27.428 -18.472 1.00 0.51
N
ATOM 4819 CA LYS A 318 25.639 27.053 -18.323 1.00 0.53
C
ATOM 4820 C LYS A 318 25.480 25.649 -17.770 1.00 0.56
C
ATOM 4821 O LYS A 318 26.132 24.708 -18.222 1.00 1.12
O
ATOM 4822 CB LYS A 318 24.896 27.185 -19.645 1.00 0.80
C
ATOM 4823 CG LYS A 318 24.777 28.618 -20.140 1.00 0.80
C

ATOM	4824	CD	LYS	A	318	23.958	28.698	-21.415	1.00	0.80
C										
ATOM	4825	CE	LYS	A	318	23.841	30.132	-21.906	1.00	0.80
C										
ATOM	4826	NZ	LYS	A	318	23.049	30.223	-23.163	1.00	0.80
N										
ATOM	4827	H	LYS	A	318	27.612	27.023	-19.200	1.00	0.61
H										
ATOM	4828	HA	LYS	A	318	25.182	27.737	-17.617	1.00	0.64
H										
ATOM	4829	1HB	LYS	A	318	25.408	26.605	-20.414	1.00	0.95
H										
ATOM	4830	2HB	LYS	A	318	23.889	26.779	-19.544	1.00	0.95
H										
ATOM	4831	1HG	LYS	A	318	24.297	29.228	-19.377	1.00	0.95
H										
ATOM	4832	2HG	LYS	A	318	25.772	29.022	-20.331	1.00	0.95
H										
ATOM	4833	1HD	LYS	A	318	24.432	28.093	-22.192	1.00	0.95
H										
ATOM	4834	2HD	LYS	A	318	22.959	28.303	-21.232	1.00	0.95
H										
ATOM	4835	1HE	LYS	A	318	23.357	30.735	-21.139	1.00	0.95
H										
ATOM	4836	2HE	LYS	A	318	24.839	30.532	-22.089	1.00	0.95
H										
ATOM	4837	1HZ	LYS	A	318	22.993	31.188	-23.458	1.00	0.95
H										
ATOM	4838	2HZ	LYS	A	318	23.498	29.676	-23.885	1.00	0.95
H										
ATOM	4839	3HZ	LYS	A	318	22.118	29.866	-23.001	1.00	0.95
H										
ATOM	4840	N	ILE	A	319	24.608	25.525	-16.775	1.00	0.52
N										
ATOM	4841	CA	ILE	A	319	24.330	24.240	-16.153	1.00	0.81
C										
ATOM	4842	C	ILE	A	319	23.011	24.283	-15.391	1.00	0.48
C										
ATOM	4843	O	ILE	A	319	22.619	25.323	-14.861	1.00	0.56
O										
ATOM	4844	CB	ILE	A	319	25.494	23.811	-15.243	1.00	1.22
C										
ATOM	4845	CG1	ILE	A	319	25.284	22.371	-14.759	1.00	1.22
C										
ATOM	4846	CG2	ILE	A	319	25.624	24.776	-14.083	1.00	1.22
C										
ATOM	4847	CD1	ILE	A	319	26.525	21.739	-14.168	1.00	1.22
C										
ATOM	4848	H	ILE	A	319	24.118	26.352	-16.456	1.00	0.62
H										
ATOM	4849	HA	ILE	A	319	24.237	23.493	-16.941	1.00	0.97
H										
ATOM	4850	HB	ILE	A	319	26.420	23.823	-15.816	1.00	1.46
H										
ATOM	4851	1HG1	ILE	A	319	24.504	22.365	-13.999	1.00	1.46
H										
ATOM	4852	2HG1	ILE	A	319	24.952	21.757	-15.596	1.00	1.46
H										

```
ATOM   4853 1HG2 ILE A 319      26.460  24.476 -13.453  1.00  1.46
H
ATOM   4854 2HG2 ILE A 319      25.799  25.782 -14.463  1.00  1.46
H
ATOM   4855 3HG2 ILE A 319      24.704  24.765 -13.497  1.00  1.46
H
ATOM   4856 1HD1 ILE A 319      26.298  20.724 -13.843  1.00  1.46
H
ATOM   4857 2HD1 ILE A 319      27.311  21.712 -14.923  1.00  1.46
H
ATOM   4858 3HD1 ILE A 319      26.864  22.325 -13.317  1.00  1.46
H
ATOM   4859  N   ALA A 320      22.296  23.165 -15.392  1.00  0.48
N
ATOM   4860  CA  ALA A 320      21.021  23.080 -14.705  1.00  0.38
C
ATOM   4861  C   ALA A 320      21.254  23.105 -13.222  1.00  0.37
C
ATOM   4862  O   ALA A 320      22.243  22.546 -12.738  1.00  0.46
O
ATOM   4863  CB  ALA A 320      20.270  21.821 -15.107  1.00  0.57
C
ATOM   4864  H   ALA A 320      22.658  22.347 -15.864  1.00  0.58
H
ATOM   4865  HA  ALA A 320      20.426  23.954 -14.968  1.00  0.46
H
ATOM   4866 1HB  ALA A 320      19.312  21.787 -14.587  1.00  0.68
H
ATOM   4867 2HB  ALA A 320      20.097  21.829 -16.183  1.00  0.68
H
ATOM   4868 3HB  ALA A 320      20.858  20.947 -14.841  1.00  0.68
H
ATOM   4869  N   THR A 321      20.339  23.726 -12.488  1.00  0.33
N
ATOM   4870  CA  THR A 321      20.543  23.795 -11.065  1.00  0.45
C
ATOM   4871  C   THR A 321      20.404  22.390 -10.479  1.00  0.67
C
ATOM   4872  O   THR A 321      21.164  22.048  -9.576  1.00  1.94
O
ATOM   4873  CB  THR A 321      19.555  24.780 -10.413  1.00  0.68
C
ATOM   4874  OG1 THR A 321      18.227  24.333 -10.649  1.00  0.68
O
ATOM   4875  CG2 THR A 321      19.729  26.180 -10.983  1.00  0.68
C
ATOM   4876  H   THR A 321      19.534  24.160 -12.920  1.00  0.40
H
ATOM   4877  HA  THR A 321      21.555  24.147 -10.870  1.00  0.54
H
ATOM   4878  HB  THR A 321      19.720  24.805  -9.342  1.00  0.81
H
ATOM   4879  HG1 THR A 321      18.083  23.535 -10.133  1.00  0.81
H
ATOM   4880 1HG2 THR A 321      19.019  26.857 -10.508  1.00  0.81
H
ATOM   4881 2HG2 THR A 321      20.744  26.528 -10.792  1.00  0.81
H
```

```
ATOM   4882 3HG2 THR A 321      19.549  26.162 -12.057  1.00  0.81
H
ATOM   4883  N   GLN A 322      19.546  21.543 -11.090  1.00  0.32
N
ATOM   4884  CA  GLN A 322      19.411  20.128 -10.693  1.00  0.34
C
ATOM   4885  C   GLN A 322      20.755  19.422 -10.680  1.00  0.34
C
ATOM   4886  O   GLN A 322      21.101  18.736  -9.712  1.00  0.41
O
ATOM   4887  CB  GLN A 322      18.480  19.364 -11.664  1.00  0.51
C
ATOM   4888  CG  GLN A 322      17.005  19.670 -11.558  1.00  0.51
C
ATOM   4889  CD  GLN A 322      16.178  19.089 -12.680  1.00  0.51
C
ATOM   4890  OE1 GLN A 322      14.962  19.296 -12.740  1.00  0.51
O
ATOM   4891  NE2 GLN A 322      16.826  18.369 -13.586  1.00  0.51
N
ATOM   4892  H   GLN A 322      18.937  21.894 -11.824  1.00  0.38
H
ATOM   4893  HA  GLN A 322      18.996  20.082  -9.688  1.00  0.41
H
ATOM   4894 1HB  GLN A 322      18.788  19.555 -12.693  1.00  0.61
H
ATOM   4895 2HB  GLN A 322      18.581  18.297 -11.485  1.00  0.61
H
ATOM   4896 1HG  GLN A 322      16.648  19.226 -10.633  1.00  0.61
H
ATOM   4897 2HG  GLN A 322      16.852  20.739 -11.543  1.00  0.61
H
ATOM   4898 1HE2 GLN A 322      16.325  17.979 -14.363  1.00  0.61
H
ATOM   4899 2HE2 GLN A 322      17.811  18.229 -13.503  1.00  0.61
H
ATOM   4900  N   LYS A 323      21.505  19.601 -11.765  1.00  0.36
N
ATOM   4901  CA  LYS A 323      22.802  18.971 -11.923  1.00  0.42
C
ATOM   4902  C   LYS A 323      23.770  19.411 -10.852  1.00  0.40
C
ATOM   4903  O   LYS A 323      24.502  18.586 -10.295  1.00  0.36
O
ATOM   4904  CB  LYS A 323      23.380  19.299 -13.301  1.00  0.63
C
ATOM   4905  CG  LYS A 323      22.663  18.625 -14.458  1.00  0.63
C
ATOM   4906  CD  LYS A 323      23.263  19.001 -15.807  1.00  0.63
C
ATOM   4907  CE  LYS A 323      22.524  18.296 -16.939  1.00  0.63
C
ATOM   4908  NZ  LYS A 323      23.096  18.605 -18.283  1.00  0.63
N
ATOM   4909  H   LYS A 323      21.158  20.187 -12.511  1.00  0.43
H
ATOM   4910  HA  LYS A 323      22.677  17.893 -11.838  1.00  0.50
H
```

```
ATOM   4911 1HB  LYS A 323      23.341  20.376 -13.467  1.00  0.76
H
ATOM   4912 2HB  LYS A 323      24.428  19.001 -13.337  1.00  0.76
H
ATOM   4913 1HG  LYS A 323      22.734  17.544 -14.336  1.00  0.76
H
ATOM   4914 2HG  LYS A 323      21.611  18.905 -14.446  1.00  0.76
H
ATOM   4915 1HD  LYS A 323      23.194  20.080 -15.952  1.00  0.76
H
ATOM   4916 2HD  LYS A 323      24.313  18.712 -15.834  1.00  0.76
H
ATOM   4917 1HE  LYS A 323      22.578  17.220 -16.775  1.00  0.76
H
ATOM   4918 2HE  LYS A 323      21.477  18.599 -16.925  1.00  0.76
H
ATOM   4919 1HZ  LYS A 323      22.575  18.106 -18.989  1.00  0.76
H
ATOM   4920 2HZ  LYS A 323      23.047  19.598 -18.485  1.00  0.76
H
ATOM   4921 3HZ  LYS A 323      24.062  18.313 -18.313  1.00  0.76
H
ATOM   4922  N   THR A 324      23.756  20.703 -10.536  1.00  0.52
N
ATOM   4923  CA  THR A 324      24.688  21.199  -9.548  1.00  0.54
C
ATOM   4924  C   THR A 324      24.295  20.827  -8.136  1.00  0.68
C
ATOM   4925  O   THR A 324      25.169  20.732  -7.282  1.00  1.45
O
ATOM   4926  CB  THR A 324      24.836  22.702  -9.655  1.00  0.81
C
ATOM   4927  OG1 THR A 324      23.587  23.329  -9.370  1.00  0.81
O
ATOM   4928  CG2 THR A 324      25.244  23.035 -11.059  1.00  0.81
C
ATOM   4929  H   THR A 324      23.133  21.342 -11.022  1.00  0.62
H
ATOM   4930  HA  THR A 324      25.661  20.754  -9.748  1.00  0.65
H
ATOM   4931  HB  THR A 324      25.590  23.063  -8.956  1.00  0.97
H
ATOM   4932  HG1 THR A 324      22.880  22.834  -9.791  1.00  0.97
H
ATOM   4933 1HG2 THR A 324      25.339  24.107 -11.168  1.00  0.97
H
ATOM   4934 2HG2 THR A 324      26.198  22.560 -11.278  1.00  0.97
H
ATOM   4935 3HG2 THR A 324      24.489  22.671 -11.751  1.00  0.97
H
ATOM   4936  N   TYR A 325      23.013  20.559  -7.875  1.00  0.57
N
ATOM   4937  CA  TYR A 325      22.666  20.106  -6.533  1.00  0.66
C
ATOM   4938  C   TYR A 325      23.182  18.691  -6.335  1.00  0.47
C
ATOM   4939  O   TYR A 325      23.630  18.350  -5.244  1.00  0.51
O
```

ATOM 4940 CB TYR A 325 21.172 20.142 -6.272 1.00 0.99
C
ATOM 4941 CG TYR A 325 20.570 21.516 -6.100 1.00 0.99
C
ATOM 4942 CD1 TYR A 325 19.761 21.984 -7.082 1.00 0.99
C
ATOM 4943 CD2 TYR A 325 20.792 22.306 -4.986 1.00 0.99
C
ATOM 4944 CE1 TYR A 325 19.161 23.205 -6.964 1.00 0.99
C
ATOM 4945 CE2 TYR A 325 20.165 23.528 -4.866 1.00 0.99
C
ATOM 4946 CZ TYR A 325 19.321 23.958 -5.862 1.00 0.99
C
ATOM 4947 OH TYR A 325 18.599 25.124 -5.801 1.00 0.99
O
ATOM 4948 H TYR A 325 22.295 20.687 -8.579 1.00 0.68
H
ATOM 4949 HA TYR A 325 23.164 20.750 -5.807 1.00 0.79
H
ATOM 4950 1HB TYR A 325 20.656 19.653 -7.099 1.00 1.19
H
ATOM 4951 2HB TYR A 325 20.954 19.567 -5.373 1.00 1.19
H
ATOM 4952 HD1 TYR A 325 19.582 21.373 -7.963 1.00 1.19
H
ATOM 4953 HD2 TYR A 325 21.438 21.970 -4.196 1.00 1.19
H
ATOM 4954 HE1 TYR A 325 18.512 23.581 -7.745 1.00 1.19
H
ATOM 4955 HE2 TYR A 325 20.322 24.138 -3.976 1.00 1.19
H
ATOM 4956 HH TYR A 325 17.692 24.969 -6.118 1.00 1.19
H
ATOM 4957 N GLY A 326 23.160 17.880 -7.396 1.00 0.45
N
ATOM 4958 CA GLY A 326 23.717 16.534 -7.313 1.00 0.43
C
ATOM 4959 C GLY A 326 25.211 16.603 -6.996 1.00 0.39
C
ATOM 4960 O GLY A 326 25.703 15.892 -6.113 1.00 0.39
O
ATOM 4961 H GLY A 326 22.738 18.194 -8.267 1.00 0.54
H
ATOM 4962 1HA GLY A 326 23.197 15.966 -6.542 1.00 0.52
H
ATOM 4963 2HA GLY A 326 23.563 16.020 -8.260 1.00 0.52
H
ATOM 4964 N LEU A 327 25.913 17.489 -7.707 1.00 0.42
N
ATOM 4965 CA LEU A 327 27.339 17.698 -7.502 1.00 0.50
C
ATOM 4966 C LEU A 327 27.631 18.231 -6.100 1.00 0.46
C
ATOM 4967 O LEU A 327 28.571 17.780 -5.445 1.00 0.53
O
ATOM 4968 CB LEU A 327 27.856 18.688 -8.554 1.00 0.75
C

```
ATOM   4969  CG  LEU A 327      27.885  18.168  -9.998  1.00  0.75
C
ATOM   4970  CD1 LEU A 327      28.179  19.318 -10.952  1.00  0.75
C
ATOM   4971  CD2 LEU A 327      28.952  17.091 -10.117  1.00  0.75
C
ATOM   4972  H   LEU A 327      25.443  18.019  -8.434  1.00  0.50
H
ATOM   4973  HA  LEU A 327      27.852  16.745  -7.624  1.00  0.60
H
ATOM   4974 1HB  LEU A 327      27.228  19.576  -8.534  1.00  0.90
H
ATOM   4975 2HB  LEU A 327      28.871  18.980  -8.287  1.00  0.90
H
ATOM   4976  HG  LEU A 327      26.912  17.749 -10.258  1.00  0.90
H
ATOM   4977 1HD1 LEU A 327      28.196  18.945 -11.976  1.00  0.90
H
ATOM   4978 2HD1 LEU A 327      27.406  20.080 -10.857  1.00  0.90
H
ATOM   4979 3HD1 LEU A 327      29.148  19.753 -10.709  1.00  0.90
H
ATOM   4980 1HD2 LEU A 327      28.976  16.714 -11.140  1.00  0.90
H
ATOM   4981 2HD2 LEU A 327      29.924  17.515  -9.864  1.00  0.90
H
ATOM   4982 3HD2 LEU A 327      28.725  16.275  -9.435  1.00  0.90
H
ATOM   4983  N   ALA A 328      26.793  19.164  -5.645  1.00  0.51
N
ATOM   4984  CA  ALA A 328      26.907  19.776  -4.332  1.00  0.61
C
ATOM   4985  C   ALA A 328      26.753  18.761  -3.222  1.00  0.48
C
ATOM   4986  O   ALA A 328      27.527  18.781  -2.265  1.00  0.52
O
ATOM   4987  CB  ALA A 328      25.860  20.846  -4.177  1.00  0.92
C
ATOM   4988  H   ALA A 328      26.055  19.491  -6.246  1.00  0.61
H
ATOM   4989  HA  ALA A 328      27.898  20.224  -4.250  1.00  0.73
H
ATOM   4990 1HB  ALA A 328      25.955  21.325  -3.203  1.00  1.10
H
ATOM   4991 2HB  ALA A 328      25.969  21.586  -4.962  1.00  1.10
H
ATOM   4992 3HB  ALA A 328      24.889  20.380  -4.269  1.00  1.10
H
ATOM   4993  N   LEU A 329      25.759  17.872  -3.354  1.00  0.58
N
ATOM   4994  CA  LEU A 329      25.536  16.829  -2.362  1.00  0.71
C
ATOM   4995  C   LEU A 329      26.725  15.915  -2.245  1.00  0.65
C
ATOM   4996  O   LEU A 329      27.130  15.576  -1.134  1.00  0.84
O
ATOM   4997  CB  LEU A 329      24.318  15.959  -2.717  1.00  1.06
C
```

```
ATOM   4998  CG  LEU A 329      22.937  16.591  -2.557  1.00  1.06
C
ATOM   4999  CD1 LEU A 329      21.887  15.663  -3.149  1.00  1.06
C
ATOM   5000  CD2 LEU A 329      22.678  16.829  -1.078  1.00  1.06
C
ATOM   5001  H   LEU A 329      25.140  17.927  -4.151  1.00  0.70
H
ATOM   5002  HA  LEU A 329      25.366  17.301  -1.395  1.00  0.85
H
ATOM   5003 1HB  LEU A 329      24.411  15.651  -3.757  1.00  1.28
H
ATOM   5004 2HB  LEU A 329      24.341  15.064  -2.095  1.00  1.28
H
ATOM   5005  HG  LEU A 329      22.898  17.533  -3.094  1.00  1.28
H
ATOM   5006 1HD1 LEU A 329      20.899  16.111  -3.039  1.00  1.28
H
ATOM   5007 2HD1 LEU A 329      22.097  15.506  -4.207  1.00  1.28
H
ATOM   5008 3HD1 LEU A 329      21.910  14.707  -2.629  1.00  1.28
H
ATOM   5009 1HD2 LEU A 329      21.694  17.282  -0.954  1.00  1.28
H
ATOM   5010 2HD2 LEU A 329      22.711  15.880  -0.544  1.00  1.28
H
ATOM   5011 3HD2 LEU A 329      23.438  17.499  -0.677  1.00  1.28
H
ATOM   5012  N   ALA A 330      27.293  15.522  -3.388  1.00  0.58
N
ATOM   5013  CA  ALA A 330      28.448  14.637  -3.385  1.00  0.68
C
ATOM   5014  C   ALA A 330      29.622  15.283  -2.671  1.00  0.85
C
ATOM   5015  O   ALA A 330      30.304  14.629  -1.875  1.00  1.22
O
ATOM   5016  CB  ALA A 330      28.826  14.266  -4.801  1.00  1.02
C
ATOM   5017  H   ALA A 330      26.903  15.821  -4.277  1.00  0.70
H
ATOM   5018  HA  ALA A 330      28.184  13.731  -2.842  1.00  0.82
H
ATOM   5019 1HB  ALA A 330      29.675  13.582  -4.786  1.00  1.22
H
ATOM   5020 2HB  ALA A 330      27.976  13.781  -5.277  1.00  1.22
H
ATOM   5021 3HB  ALA A 330      29.092  15.163  -5.357  1.00  1.22
H
ATOM   5022  N   LYS A 331      29.843  16.572  -2.932  1.00  0.71
N
ATOM   5023  CA  LYS A 331      30.923  17.289  -2.277  1.00  0.81
C
ATOM   5024  C   LYS A 331      30.689  17.372  -0.776  1.00  0.86
C
ATOM   5025  O   LYS A 331      31.615  17.172   0.014  1.00  1.27
O
ATOM   5026  CB  LYS A 331      31.053  18.691  -2.848  1.00  1.22
C
```

ATOM	5027	CG	LYS	A	331	31.578	18.761	-4.266	1.00	1.22	C
ATOM	5028	CD	LYS	A	331	31.649	20.204	-4.728	1.00	1.22	C
ATOM	5029	CE	LYS	A	331	32.134	20.306	-6.161	1.00	1.22	C
ATOM	5030	NZ	LYS	A	331	32.221	21.720	-6.600	1.00	1.22	N
ATOM	5031	H	LYS	A	331	29.268	17.058	-3.613	1.00	0.85	H
ATOM	5032	HA	LYS	A	331	31.854	16.748	-2.445	1.00	0.97	H
ATOM	5033	1HB	LYS	A	331	30.084	19.189	-2.826	1.00	1.46	H
ATOM	5034	2HB	LYS	A	331	31.735	19.269	-2.225	1.00	1.46	H
ATOM	5035	1HG	LYS	A	331	32.577	18.324	-4.307	1.00	1.46	H
ATOM	5036	2HG	LYS	A	331	30.927	18.200	-4.933	1.00	1.46	H
ATOM	5037	1HD	LYS	A	331	30.660	20.659	-4.656	1.00	1.46	H
ATOM	5038	2HD	LYS	A	331	32.332	20.758	-4.084	1.00	1.46	H
ATOM	5039	1HE	LYS	A	331	33.118	19.847	-6.246	1.00	1.46	H
ATOM	5040	2HE	LYS	A	331	31.439	19.777	-6.813	1.00	1.46	H
ATOM	5041	1HZ	LYS	A	331	32.539	21.759	-7.557	1.00	1.46	H
ATOM	5042	2HZ	LYS	A	331	31.306	22.150	-6.527	1.00	1.46	H
ATOM	5043	3HZ	LYS	A	331	32.870	22.217	-6.007	1.00	1.46	H
ATOM	5044	N	LEU	A	332	29.445	17.653	-0.391	1.00	0.69	N
ATOM	5045	CA	LEU	A	332	29.068	17.744	1.008	1.00	0.94	C
ATOM	5046	C	LEU	A	332	29.353	16.407	1.707	1.00	0.86	C
ATOM	5047	O	LEU	A	332	29.829	16.376	2.843	1.00	1.35	O
ATOM	5048	CB	LEU	A	332	27.585	18.072	1.139	1.00	1.41	C
ATOM	5049	CG	LEU	A	332	27.139	18.433	2.547	1.00	1.41	C
ATOM	5050	CD1	LEU	A	332	27.805	19.746	2.937	1.00	1.41	C
ATOM	5051	CD2	LEU	A	332	25.636	18.503	2.613	1.00	1.41	C
ATOM	5052	H	LEU	A	332	28.735	17.826	-1.090	1.00	0.83	H
ATOM	5053	HA	LEU	A	332	29.658	18.526	1.484	1.00	1.13	H
ATOM	5054	1HB	LEU	A	332	27.353	18.913	0.487	1.00	1.69	H
ATOM	5055	2HB	LEU	A	332	27.002	17.212	0.814	1.00	1.69	H

ATOM	5056	HG	LEU	A	332	27.496	17.682	3.240	1.00	1.69	H
ATOM	5057	1HD1	LEU	A	332	27.509	20.016	3.949	1.00	1.69	H
ATOM	5058	2HD1	LEU	A	332	28.887	19.638	2.888	1.00	1.69	H
ATOM	5059	3HD1	LEU	A	332	27.485	20.528	2.252	1.00	1.69	H
ATOM	5060	1HD2	LEU	A	332	25.329	18.754	3.626	1.00	1.69	H
ATOM	5061	2HD2	LEU	A	332	25.289	19.265	1.929	1.00	1.69	H
ATOM	5062	3HD2	LEU	A	332	25.212	17.539	2.333	1.00	1.69	H
ATOM	5063	N	GLY	A	333	29.054	15.306	1.005	1.00	0.76	N
ATOM	5064	CA	GLY	A	333	29.292	13.953	1.495	1.00	0.96	C
ATOM	5065	C	GLY	A	333	30.765	13.723	1.803	1.00	0.90	C
ATOM	5066	O	GLY	A	333	31.099	13.078	2.797	1.00	2.06	O
ATOM	5067	H	GLY	A	333	28.629	15.409	0.092	1.00	0.91	H
ATOM	5068	1HA	GLY	A	333	28.699	13.782	2.393	1.00	1.15	H
ATOM	5069	2HA	GLY	A	333	28.961	13.235	0.748	1.00	1.15	H
ATOM	5070	N	ARG	A	334	31.648	14.268	0.962	1.00	0.81	N
ATOM	5071	CA	ARG	A	334	33.083	14.162	1.214	1.00	0.75	C
ATOM	5072	C	ARG	A	334	33.470	14.914	2.485	1.00	0.89	C
ATOM	5073	O	ARG	A	334	34.353	14.481	3.227	1.00	2.38	O
ATOM	5074	CB	ARG	A	334	33.889	14.724	0.053	1.00	1.12	C
ATOM	5075	CG	ARG	A	334	33.853	13.907	-1.221	1.00	1.12	C
ATOM	5076	CD	ARG	A	334	34.630	14.558	-2.309	1.00	1.12	C
ATOM	5077	NE	ARG	A	334	34.608	13.762	-3.520	1.00	1.12	N
ATOM	5078	CZ	ARG	A	334	35.036	14.161	-4.734	1.00	1.12	C
ATOM	5079	NH1	ARG	A	334	35.544	15.365	-4.895	1.00	1.12	N
ATOM	5080	NH2	ARG	A	334	34.938	13.336	-5.766	1.00	1.12	N
ATOM	5081	H	ARG	A	334	31.317	14.742	0.128	1.00	0.97	H
ATOM	5082	HA	ARG	A	334	33.336	13.109	1.340	1.00	0.90	H
ATOM	5083	1HB	ARG	A	334	33.537	15.723	-0.190	1.00	1.35	H
ATOM	5084	2HB	ARG	A	334	34.934	14.813	0.351	1.00	1.35	H

```
ATOM   5085 1HG  ARG A 334      34.279  12.920  -1.037  1.00  1.35
H
ATOM   5086 2HG  ARG A 334      32.820  13.798  -1.555  1.00  1.35
H
ATOM   5087 1HD  ARG A 334      34.202  15.534  -2.532  1.00  1.35
H
ATOM   5088 2HD  ARG A 334      35.667  14.677  -1.998  1.00  1.35
H
ATOM   5089  HE  ARG A 334      34.242  12.821  -3.444  1.00  1.35
H
ATOM   5090 1HH1 ARG A 334      35.615  15.992  -4.105  1.00  1.35
H
ATOM   5091 2HH1 ARG A 334      35.862  15.661  -5.806  1.00  1.35
H
ATOM   5092 1HH2 ARG A 334      34.524  12.414  -5.643  1.00  1.35
H
ATOM   5093 2HH2 ARG A 334      35.254  13.627  -6.678  1.00  1.35
H
ATOM   5094  N   ALA A 335      32.803  16.042   2.728  1.00  1.29
N
ATOM   5095  CA  ALA A 335      33.048  16.848   3.916  1.00  1.17
C
ATOM   5096  C   ALA A 335      32.625  16.158   5.215  1.00  1.05
C
ATOM   5097  O   ALA A 335      33.333  16.250   6.222  1.00  1.11
O
ATOM   5098  CB  ALA A 335      32.325  18.174   3.802  1.00  1.75
C
ATOM   5099  H   ALA A 335      32.118  16.362   2.050  1.00  1.55
H
ATOM   5100  HA  ALA A 335      34.121  17.035   3.974  1.00  1.40
H
ATOM   5101 1HB  ALA A 335      32.557  18.772   4.682  1.00  2.11
H
ATOM   5102 2HB  ALA A 335      32.650  18.697   2.906  1.00  2.11
H
ATOM   5103 3HB  ALA A 335      31.252  18.011   3.754  1.00  2.11
H
ATOM   5104  N   ASN A 336      31.480  15.469   5.216  1.00  1.01
N
ATOM   5105  CA  ASN A 336      31.060  14.864   6.474  1.00  1.06
C
ATOM   5106  C   ASN A 336      30.180  13.625   6.331  1.00  0.92
C
ATOM   5107  O   ASN A 336      29.207  13.600   5.577  1.00  1.20
O
ATOM   5108  CB  ASN A 336      30.368  15.899   7.323  1.00  1.59
C
ATOM   5109  CG  ASN A 336      30.197  15.453   8.739  1.00  1.59
C
ATOM   5110  OD1 ASN A 336      29.299  14.681   9.091  1.00  1.59
O
ATOM   5111  ND2 ASN A 336      31.083  15.921   9.580  1.00  1.59
N
ATOM   5112  H   ASN A 336      30.910  15.411   4.380  1.00  1.21
H
ATOM   5113  HA  ASN A 336      31.959  14.544   7.003  1.00  1.27
H
```

```
ATOM   5114 1HB  ASN A 336      30.947  16.823   7.314  1.00  1.91
H
ATOM   5115 2HB  ASN A 336      29.388  16.123   6.902  1.00  1.91
H
ATOM   5116 1HD2 ASN A 336      31.048  15.659  10.546  1.00  1.91
H
ATOM   5117 2HD2 ASN A 336      31.803  16.535   9.258  1.00  1.91
H
ATOM   5118  N   GLU A 337      30.540  12.611   7.119  1.00  0.77
N
ATOM   5119  CA  GLU A 337      29.929  11.285   7.149  1.00  0.75
C
ATOM   5120  C   GLU A 337      28.451  11.234   7.541  1.00  0.52
C
ATOM   5121  O   GLU A 337      27.778  10.235   7.273  1.00  0.70
O
ATOM   5122  CB  GLU A 337      30.720  10.412   8.117  1.00  1.12
C
ATOM   5123  CG  GLU A 337      32.124  10.067   7.641  1.00  1.12
C
ATOM   5124  CD  GLU A 337      32.856   9.164   8.591  1.00  1.12
C
ATOM   5125  OE1 GLU A 337      32.314   8.863   9.629  1.00  1.12
O
ATOM   5126  OE2 GLU A 337      33.953   8.767   8.277  1.00  1.12
O
ATOM   5127  H   GLU A 337      31.336  12.761   7.724  1.00  0.92
H
ATOM   5128  HA  GLU A 337      30.018  10.861   6.149  1.00  0.90
H
ATOM   5129 1HB  GLU A 337      30.820  10.936   9.068  1.00  1.35
H
ATOM   5130 2HB  GLU A 337      30.178   9.492   8.315  1.00  1.35
H
ATOM   5131 1HG  GLU A 337      32.059   9.579   6.668  1.00  1.35
H
ATOM   5132 2HG  GLU A 337      32.689  10.989   7.517  1.00  1.35
H
ATOM   5133  N   ARG A 338      27.940  12.293   8.168  1.00  0.49
N
ATOM   5134  CA  ARG A 338      26.536  12.337   8.552  1.00  0.50
C
ATOM   5135  C   ARG A 338      25.601  12.568   7.370  1.00  0.53
C
ATOM   5136  O   ARG A 338      24.393  12.412   7.508  1.00  0.66
O
ATOM   5137  CB  ARG A 338      26.296  13.424   9.589  1.00  0.75
C
ATOM   5138  CG  ARG A 338      26.910  13.158  10.954  1.00  0.75
C
ATOM   5139  CD  ARG A 338      26.816  14.351  11.832  1.00  0.75
C
ATOM   5140  NE  ARG A 338      27.688  15.409  11.356  1.00  0.75
N
ATOM   5141  CZ  ARG A 338      27.725  16.673  11.825  1.00  0.75
C
ATOM   5142  NH1 ARG A 338      26.933  17.077  12.789  1.00  0.75
N
```

```
ATOM   5143  NH2 ARG A 338      28.572  17.541  11.317  1.00  0.75
N
ATOM   5144  H   ARG A 338      28.521  13.094   8.385  1.00  0.59
H
ATOM   5145  HA  ARG A 338      26.281  11.377   9.004  1.00  0.60
H
ATOM   5146 1HB  ARG A 338      26.698  14.368   9.225  1.00  0.90
H
ATOM   5147 2HB  ARG A 338      25.226  13.556   9.734  1.00  0.90
H
ATOM   5148 1HG  ARG A 338      26.385  12.334  11.437  1.00  0.90
H
ATOM   5149 2HG  ARG A 338      27.963  12.902  10.837  1.00  0.90
H
ATOM   5150 1HD  ARG A 338      25.793  14.726  11.842  1.00  0.90
H
ATOM   5151 2HD  ARG A 338      27.119  14.085  12.843  1.00  0.90
H
ATOM   5152  HE  ARG A 338      28.329  15.168  10.607  1.00  0.90
H
ATOM   5153 1HH1 ARG A 338      26.275  16.437  13.215  1.00  0.90
H
ATOM   5154 2HH1 ARG A 338      27.008  18.037  13.104  1.00  0.90
H
ATOM   5155 1HH2 ARG A 338      29.199  17.269  10.575  1.00  0.90
H
ATOM   5156 2HH2 ARG A 338      28.585  18.484  11.680  1.00  0.90
H
ATOM   5157  N   VAL A 339      26.135  12.953   6.216  1.00  0.57
N
ATOM   5158  CA  VAL A 339      25.285  13.227   5.066  1.00  0.84
C
ATOM   5159  C   VAL A 339      24.815  11.974   4.337  1.00  0.60
C
ATOM   5160  O   VAL A 339      25.627  11.231   3.784  1.00  0.90
O
ATOM   5161  CB  VAL A 339      26.053  14.117   4.080  1.00  1.26
C
ATOM   5162  CG1 VAL A 339      25.234  14.370   2.819  1.00  1.26
C
ATOM   5163  CG2 VAL A 339      26.404  15.415   4.776  1.00  1.26
C
ATOM   5164  H   VAL A 339      27.138  13.072   6.115  1.00  0.68
H
ATOM   5165  HA  VAL A 339      24.407  13.771   5.417  1.00  1.01
H
ATOM   5166  HB  VAL A 339      26.968  13.607   3.781  1.00  1.51
H
ATOM   5167 1HG1 VAL A 339      25.804  14.994   2.132  1.00  1.51
H
ATOM   5168 2HG1 VAL A 339      25.003  13.419   2.338  1.00  1.51
H
ATOM   5169 3HG1 VAL A 339      24.309  14.873   3.085  1.00  1.51
H
ATOM   5170 1HG2 VAL A 339      26.971  16.033   4.095  1.00  1.51
H
ATOM   5171 2HG2 VAL A 339      25.491  15.933   5.073  1.00  1.51
H
```

```
ATOM   5172 3HG2 VAL A 339      27.007  15.203   5.659  1.00  1.51
H
ATOM   5173  N   ILE A 340      23.499  11.767   4.301  1.00  0.33
N
ATOM   5174  CA  ILE A 340      22.929  10.656   3.540  1.00  0.21
C
ATOM   5175  C   ILE A 340      21.899  11.214   2.570  1.00  0.35
C
ATOM   5176  O   ILE A 340      21.305  12.262   2.826  1.00  1.22
O
ATOM   5177  CB  ILE A 340      22.252   9.590   4.431  1.00  0.32
C
ATOM   5178  CG1 ILE A 340      21.076  10.208   5.184  1.00  0.32
C
ATOM   5179  CG2 ILE A 340      23.253   8.980   5.404  1.00  0.32
C
ATOM   5180  CD1 ILE A 340      20.219   9.193   5.899  1.00  0.32
C
ATOM   5181  H   ILE A 340      22.885  12.400   4.797  1.00  0.40
H
ATOM   5182  HA  ILE A 340      23.719  10.176   2.964  1.00  0.25
H
ATOM   5183  HB  ILE A 340      21.849   8.799   3.799  1.00  0.38
H
ATOM   5184 1HG1 ILE A 340      21.456  10.920   5.916  1.00  0.38
H
ATOM   5185 2HG1 ILE A 340      20.448  10.743   4.473  1.00  0.38
H
ATOM   5186 1HG2 ILE A 340      22.755   8.225   6.011  1.00  0.38
H
ATOM   5187 2HG2 ILE A 340      24.066   8.519   4.850  1.00  0.38
H
ATOM   5188 3HG2 ILE A 340      23.653   9.760   6.052  1.00  0.38
H
ATOM   5189 1HD1 ILE A 340      19.402   9.703   6.410  1.00  0.38
H
ATOM   5190 2HD1 ILE A 340      19.812   8.487   5.176  1.00  0.38
H
ATOM   5191 3HD1 ILE A 340      20.825   8.658   6.628  1.00  0.38
H
ATOM   5192  N   VAL A 341      21.692  10.510   1.466  1.00  0.26
N
ATOM   5193  CA  VAL A 341      20.775  10.975   0.431  1.00  0.30
C
ATOM   5194  C   VAL A 341      19.771   9.896   0.052  1.00  0.30
C
ATOM   5195  O   VAL A 341      20.133   8.730  -0.070  1.00  0.46
O
ATOM   5196  CB  VAL A 341      21.561  11.449  -0.818  1.00  0.45
C
ATOM   5197  CG1 VAL A 341      20.609  11.874  -1.922  1.00  0.45
C
ATOM   5198  CG2 VAL A 341      22.460  12.624  -0.454  1.00  0.45
C
ATOM   5199  H   VAL A 341      22.195   9.638   1.345  1.00  0.31
H
ATOM   5200  HA  VAL A 341      20.229  11.829   0.824  1.00  0.36
H
```

```
ATOM   5201  HB  VAL A 341      22.162  10.625  -1.193  1.00  0.54
H
ATOM   5202 1HG1 VAL A 341      21.186  12.194  -2.788  1.00  0.54
H
ATOM   5203 2HG1 VAL A 341      19.971  11.037  -2.201  1.00  0.54
H
ATOM   5204 3HG1 VAL A 341      19.993  12.701  -1.574  1.00  0.54
H
ATOM   5205 1HG2 VAL A 341      23.010  12.945  -1.337  1.00  0.54
H
ATOM   5206 2HG2 VAL A 341      21.855  13.450  -0.091  1.00  0.54
H
ATOM   5207 3HG2 VAL A 341      23.167  12.325   0.319  1.00  0.54
H
ATOM   5208  N   LEU A 342      18.509  10.294  -0.106  1.00  0.22
N
ATOM   5209  CA  LEU A 342      17.433   9.364  -0.430  1.00  0.20
C
ATOM   5210  C   LEU A 342      16.777   9.754  -1.755  1.00  0.21
C
ATOM   5211  O   LEU A 342      16.668  10.940  -2.055  1.00  0.31
O
ATOM   5212  CB  LEU A 342      16.354   9.401   0.669  1.00  0.30
C
ATOM   5213  CG  LEU A 342      16.792   9.051   2.098  1.00  0.30
C
ATOM   5214  CD1 LEU A 342      17.353  10.295   2.777  1.00  0.30
C
ATOM   5215  CD2 LEU A 342      15.597   8.509   2.868  1.00  0.30
C
ATOM   5216  H   LEU A 342      18.299  11.276   0.004  1.00  0.26
H
ATOM   5217  HA  LEU A 342      17.844   8.361  -0.515  1.00  0.24
H
ATOM   5218 1HB  LEU A 342      15.927  10.400   0.697  1.00  0.36
H
ATOM   5219 2HB  LEU A 342      15.563   8.706   0.393  1.00  0.36
H
ATOM   5220  HG  LEU A 342      17.581   8.308   2.068  1.00  0.36
H
ATOM   5221 1HD1 LEU A 342      17.669  10.043   3.789  1.00  0.36
H
ATOM   5222 2HD1 LEU A 342      18.206  10.672   2.217  1.00  0.36
H
ATOM   5223 3HD1 LEU A 342      16.582  11.063   2.820  1.00  0.36
H
ATOM   5224 1HD2 LEU A 342      15.903   8.257   3.884  1.00  0.36
H
ATOM   5225 2HD2 LEU A 342      14.810   9.264   2.902  1.00  0.36
H
ATOM   5226 3HD2 LEU A 342      15.219   7.616   2.372  1.00  0.36
H
ATOM   5227  N   SER A 343      16.311   8.775  -2.534  1.00  0.20
N
ATOM   5228  CA  SER A 343      15.618   9.113  -3.789  1.00  0.26
C
ATOM   5229  C   SER A 343      14.525   8.143  -4.177  1.00  0.30
C
```

```
ATOM   5230  O   SER A 343      14.698   6.926  -4.137  1.00  0.31
O
ATOM   5231  CB  SER A 343      16.564   9.171  -4.969  1.00  0.39
C
ATOM   5232  OG  SER A 343      15.854   9.481  -6.147  1.00  0.39
O
ATOM   5233  H   SER A 343      16.455   7.810  -2.256  1.00  0.24
H
ATOM   5234  HA  SER A 343      15.163  10.095  -3.664  1.00  0.31
H
ATOM   5235 1HB  SER A 343      17.336   9.918  -4.790  1.00  0.47
H
ATOM   5236 2HB  SER A 343      17.052   8.206  -5.081  1.00  0.47
H
ATOM   5237  HG  SER A 343      15.572  10.395  -6.044  1.00  0.47
H
ATOM   5238  N   GLY A 344      13.403   8.701  -4.629  1.00  0.38
N
ATOM   5239  CA  GLY A 344      12.248   7.895  -5.050  1.00  0.38
C
ATOM   5240  C   GLY A 344      12.340   7.423  -6.511  1.00  0.32
C
ATOM   5241  O   GLY A 344      11.457   7.721  -7.315  1.00  0.37
O
ATOM   5242  H   GLY A 344      13.353   9.718  -4.651  1.00  0.46
H
ATOM   5243 1HA  GLY A 344      12.167   7.031  -4.394  1.00  0.46
H
ATOM   5244 2HA  GLY A 344      11.338   8.478  -4.914  1.00  0.46
H
ATOM   5245  N   ASP A 345      13.412   6.694  -6.836  1.00  0.27
N
ATOM   5246  CA  ASP A 345      13.695   6.223  -8.201  1.00  0.25
C
ATOM   5247  C   ASP A 345      13.785   7.377  -9.202  1.00  0.34
C
ATOM   5248  O   ASP A 345      13.327   7.249 -10.338  1.00  0.53
O
ATOM   5249  CB  ASP A 345      12.619   5.215  -8.670  1.00  0.38
C
ATOM   5250  CG  ASP A 345      13.006   4.430  -9.930  1.00  0.38
C
ATOM   5251  OD1 ASP A 345      14.170   4.392 -10.253  1.00  0.38
O
ATOM   5252  OD2 ASP A 345      12.135   3.868 -10.560  1.00  0.38
O
ATOM   5253  H   ASP A 345      14.061   6.473  -6.093  1.00  0.32
H
ATOM   5254  HA  ASP A 345      14.658   5.712  -8.192  1.00  0.30
H
ATOM   5255 1HB  ASP A 345      12.417   4.506  -7.873  1.00  0.45
H
ATOM   5256 2HB  ASP A 345      11.689   5.748  -8.871  1.00  0.45
H
ATOM   5257  N   THR A 346      14.394   8.496  -8.784  1.00  0.40
N
ATOM   5258  CA  THR A 346      14.547   9.667  -9.647  1.00  0.43
C
```

```
ATOM   5259  C   THR A 346      15.986  10.165  -9.680  1.00  0.33
C
ATOM   5260  O   THR A 346      16.235  11.303 -10.072  1.00  0.34
O
ATOM   5261  CB  THR A 346      13.647  10.832  -9.180  1.00  0.65
C
ATOM   5262  OG1 THR A 346      13.975  11.155  -7.823  1.00  0.65
O
ATOM   5263  CG2 THR A 346      12.170  10.495  -9.285  1.00  0.65
C
ATOM   5264  H   THR A 346      14.771   8.559  -7.837  1.00  0.48
H
ATOM   5265  HA  THR A 346      14.262   9.391 -10.662  1.00  0.52
H
ATOM   5266  HB  THR A 346      13.848  11.702  -9.803  1.00  0.77
H
ATOM   5267  HG1 THR A 346      13.831  10.381  -7.272  1.00  0.77
H
ATOM   5268 1HG2 THR A 346      11.579  11.347  -8.951  1.00  0.77
H
ATOM   5269 2HG2 THR A 346      11.923  10.263 -10.320  1.00  0.77
H
ATOM   5270 3HG2 THR A 346      11.947   9.635  -8.660  1.00  0.77
H
ATOM   5271  N   MET A 347      16.922   9.331  -9.231  1.00  0.31
N
ATOM   5272  CA  MET A 347      18.329   9.716  -9.096  1.00  0.30
C
ATOM   5273  C   MET A 347      19.008  10.200 -10.382  1.00  0.31
C
ATOM   5274  O   MET A 347      19.975  10.961 -10.322  1.00  0.32
O
ATOM   5275  CB  MET A 347      19.104   8.537  -8.520  1.00  0.45
C
ATOM   5276  CG  MET A 347      19.230   7.372  -9.478  1.00  0.45
C
ATOM   5277  SD  MET A 347      20.180   6.004  -8.811  1.00  0.45
S
ATOM   5278  CE  MET A 347      19.011   5.333  -7.638  1.00  0.45
C
ATOM   5279  H   MET A 347      16.653   8.399  -8.950  1.00  0.37
H
ATOM   5280  HA  MET A 347      18.376  10.535  -8.384  1.00  0.36
H
ATOM   5281 1HB  MET A 347      20.101   8.853  -8.230  1.00  0.54
H
ATOM   5282 2HB  MET A 347      18.601   8.178  -7.622  1.00  0.54
H
ATOM   5283 1HG  MET A 347      18.231   7.015  -9.711  1.00  0.54
H
ATOM   5284 2HG  MET A 347      19.704   7.697 -10.403  1.00  0.54
H
ATOM   5285 1HE  MET A 347      19.454   4.474  -7.134  1.00  0.54
H
ATOM   5286 2HE  MET A 347      18.753   6.097  -6.905  1.00  0.54
H
ATOM   5287 3HE  MET A 347      18.109   5.018  -8.163  1.00  0.54
H
```

ATOM 5288 N ASN A 348 18.506 9.767 -11.539 1.00 0.33
N
ATOM 5289 CA ASN A 348 19.077 10.163 -12.823 1.00 0.38
C
ATOM 5290 C ASN A 348 18.405 11.400 -13.411 1.00 0.38
C
ATOM 5291 O ASN A 348 18.713 11.809 -14.531 1.00 0.45
O
ATOM 5292 CB ASN A 348 18.960 9.011 -13.799 1.00 0.57
C
ATOM 5293 CG ASN A 348 17.520 8.688 -14.132 1.00 0.57
C
ATOM 5294 OD1 ASN A 348 16.588 9.420 -13.763 1.00 0.57
O
ATOM 5295 ND2 ASN A 348 17.324 7.598 -14.831 1.00 0.57
N
ATOM 5296 H ASN A 348 17.717 9.139 -11.534 1.00 0.40
H
ATOM 5297 HA ASN A 348 20.132 10.399 -12.675 1.00 0.46
H
ATOM 5298 1HB ASN A 348 19.489 9.259 -14.720 1.00 0.68
H
ATOM 5299 2HB ASN A 348 19.436 8.126 -13.376 1.00 0.68
H
ATOM 5300 1HD2 ASN A 348 16.396 7.327 -15.091 1.00 0.68
H
ATOM 5301 2HD2 ASN A 348 18.104 7.037 -15.110 1.00 0.68
H
ATOM 5302 N SER A 349 17.499 11.996 -12.646 1.00 0.33
N
ATOM 5303 CA SER A 349 16.742 13.157 -13.072 1.00 0.31
C
ATOM 5304 C SER A 349 16.991 14.299 -12.096 1.00 0.30
C
ATOM 5305 O SER A 349 17.108 15.460 -12.488 1.00 0.49
O
ATOM 5306 CB SER A 349 15.274 12.781 -13.137 1.00 0.46
C
ATOM 5307 OG SER A 349 15.050 11.786 -14.101 1.00 0.46
O
ATOM 5308 H SER A 349 17.284 11.618 -11.737 1.00 0.40
H
ATOM 5309 HA SER A 349 17.083 13.460 -14.063 1.00 0.37
H
ATOM 5310 1HB SER A 349 14.947 12.425 -12.160 1.00 0.56
H
ATOM 5311 2HB SER A 349 14.680 13.652 -13.382 1.00 0.56
H
ATOM 5312 HG SER A 349 15.639 11.052 -13.887 1.00 0.56
H
ATOM 5313 N THR A 350 17.118 13.934 -10.819 1.00 0.27
N
ATOM 5314 CA THR A 350 17.466 14.840 -9.735 1.00 0.26
C
ATOM 5315 C THR A 350 18.966 14.986 -9.640 1.00 0.24
C
ATOM 5316 O THR A 350 19.470 15.902 -8.991 1.00 0.29
O

ATOM   5317 CB THR A 350   16.993 14.286 -8.383 1.00 0.39
C
ATOM   5318 OG1 THR A 350   17.648 13.019 -8.167 1.00 0.39
O
ATOM   5319 CG2 THR A 350   15.481 14.105 -8.362 1.00 0.39
C
ATOM   5320 H   THR A 350   16.960 12.968 -10.579 1.00 0.32
H
ATOM   5321 HA THR A 350   17.022 15.818 -9.922 1.00 0.31
H
ATOM   5322 HB THR A 350   17.282 14.975 -7.589 1.00 0.47
H
ATOM   5323 HG1 THR A 350   17.279 12.372 -8.779 1.00 0.47
H
ATOM   5324 1HG2 THR A 350   15.176 13.703 -7.395 1.00 0.47
H
ATOM   5325 2HG2 THR A 350   14.997 15.067 -8.524 1.00 0.47
H
ATOM   5326 3HG2 THR A 350   15.185 13.414 -9.150 1.00 0.47
H
ATOM   5327 N   PHE A 351   19.671 14.043 -10.266 1.00 0.23
N
ATOM   5328 CA PHE A 351   21.119 13.949 -10.246 1.00 0.24
C
ATOM   5329 C   PHE A 351   21.680 13.583 -8.875 1.00 0.32
C
ATOM   5330 O   PHE A 351   22.885 13.681 -8.649 1.00 0.38
O
ATOM   5331 CB PHE A 351   21.759 15.190 -10.856 1.00 0.36
C
ATOM   5332 CG PHE A 351   21.351 15.338 -12.291 1.00 0.36
C
ATOM   5333 CD1 PHE A 351   20.380 16.246 -12.666 1.00 0.36
C
ATOM   5334 CD2 PHE A 351   21.927 14.546 -13.270 1.00 0.36
C
ATOM   5335 CE1 PHE A 351   19.989 16.363 -13.984 1.00 0.36
C
ATOM   5336 CE2 PHE A 351   21.540 14.659 -14.590 1.00 0.36
C
ATOM   5337 CZ PHE A 351   20.567 15.569 -14.946 1.00 0.36
C
ATOM   5338 H   PHE A 351   19.180 13.331 -10.783 1.00 0.28
H
ATOM   5339 HA PHE A 351   21.393 13.131 -10.914 1.00 0.29
H
ATOM   5340 1HB PHE A 351   21.466 16.087 -10.315 1.00 0.43
H
ATOM   5341 2HB PHE A 351   22.842 15.111 -10.813 1.00 0.43
H
ATOM   5342 HD1 PHE A 351   19.924 16.863 -11.899 1.00 0.43
H
ATOM   5343 HD2 PHE A 351   22.692 13.822 -12.987 1.00 0.43
H
ATOM   5344 HE1 PHE A 351   19.220 17.080 -14.265 1.00 0.43
H
ATOM   5345 HE2 PHE A 351   22.000 14.027 -15.350 1.00 0.43
H

```
ATOM   5346  HZ  PHE A 351      20.258  15.657 -15.987  1.00  0.43
H
ATOM   5347  N   SER A 352      20.816  13.066  -7.982  1.00  0.35
N
ATOM   5348  CA  SER A 352      21.271  12.561  -6.692  1.00  0.45
C
ATOM   5349  C   SER A 352      22.112  11.282  -6.870  1.00  0.36
C
ATOM   5350  O   SER A 352      22.864  10.899  -5.972  1.00  0.47
O
ATOM   5351  CB  SER A 352      20.086  12.364  -5.772  1.00  0.68
C
ATOM   5352  OG  SER A 352      19.181  11.436  -6.291  1.00  0.68
O
ATOM   5353  H   SER A 352      19.820  13.016  -8.184  1.00  0.42
H
ATOM   5354  HA  SER A 352      21.914  13.318  -6.240  1.00  0.54
H
ATOM   5355 1HB  SER A 352      20.444  12.016  -4.806  1.00  0.81
H
ATOM   5356 2HB  SER A 352      19.583  13.316  -5.610  1.00  0.81
H
ATOM   5357  HG  SER A 352      18.644  11.942  -6.916  1.00  0.81
H
ATOM   5358  N   GLU A 353      22.030  10.681  -8.067  1.00  0.36
N
ATOM   5359  CA  GLU A 353      22.873   9.570  -8.494  1.00  0.41
C
ATOM   5360  C   GLU A 353      24.358   9.903  -8.375  1.00  0.44
C
ATOM   5361  O   GLU A 353      25.175   9.004  -8.190  1.00  0.49
O
ATOM   5362  CB  GLU A 353      22.569   9.186  -9.936  1.00  0.61
C
ATOM   5363  CG  GLU A 353      23.317   7.967 -10.434  1.00  0.61
C
ATOM   5364  CD  GLU A 353      22.915   7.573 -11.829  1.00  0.61
C
ATOM   5365  OE1 GLU A 353      22.021   8.180 -12.367  1.00  0.61
O
ATOM   5366  OE2 GLU A 353      23.511   6.665 -12.361  1.00  0.61
O
ATOM   5367  H   GLU A 353      21.345  11.006  -8.738  1.00  0.43
H
ATOM   5368  HA  GLU A 353      22.661   8.715  -7.853  1.00  0.49
H
ATOM   5369 1HB  GLU A 353      21.510   8.981 -10.043  1.00  0.74
H
ATOM   5370 2HB  GLU A 353      22.809  10.021 -10.595  1.00  0.74
H
ATOM   5371 1HG  GLU A 353      24.385   8.183 -10.420  1.00  0.74
H
ATOM   5372 2HG  GLU A 353      23.131   7.135  -9.757  1.00  0.74
H
ATOM   5373  N   ILE A 354      24.714  11.184  -8.529  1.00  0.43
N
ATOM   5374  CA  ILE A 354      26.098  11.614  -8.414  1.00  0.47
C
```

```
ATOM   5375  C   ILE A 354      26.609  11.272  -7.015  1.00  0.46
C
ATOM   5376  O   ILE A 354      27.694  10.701  -6.865  1.00  0.58
O
ATOM   5377  CB  ILE A 354      26.217  13.126  -8.685  1.00  0.70
C
ATOM   5378  CG1 ILE A 354      25.892  13.422 -10.154  1.00  0.70
C
ATOM   5379  CG2 ILE A 354      27.612  13.613  -8.348  1.00  0.70
C
ATOM   5380  CD1 ILE A 354      25.700  14.892 -10.451  1.00  0.70
C
ATOM   5381  H   ILE A 354      24.010  11.894  -8.695  1.00  0.52
H
ATOM   5382  HA  ILE A 354      26.699  11.074  -9.146  1.00  0.56
H
ATOM   5383  HB  ILE A 354      25.491  13.660  -8.073  1.00  0.85
H
ATOM   5384 1HG1 ILE A 354      26.704  13.048 -10.777  1.00  0.85
H
ATOM   5385 2HG1 ILE A 354      24.979  12.895 -10.429  1.00  0.85
H
ATOM   5386 1HG2 ILE A 354      27.674  14.682  -8.531  1.00  0.85
H
ATOM   5387 2HG2 ILE A 354      27.820  13.408  -7.304  1.00  0.85
H
ATOM   5388 3HG2 ILE A 354      28.340  13.093  -8.971  1.00  0.85
H
ATOM   5389 1HD1 ILE A 354      25.475  15.023 -11.510  1.00  0.85
H
ATOM   5390 2HD1 ILE A 354      24.876  15.279  -9.853  1.00  0.85
H
ATOM   5391 3HD1 ILE A 354      26.611  15.431 -10.206  1.00  0.85
H
ATOM   5392  N   PHE A 355      25.804  11.612  -6.003  1.00  0.40
N
ATOM   5393  CA  PHE A 355      26.097  11.266  -4.618  1.00  0.43
C
ATOM   5394  C   PHE A 355      26.164   9.758  -4.466  1.00  0.44
C
ATOM   5395  O   PHE A 355      27.072   9.245  -3.826  1.00  0.50
O
ATOM   5396  CB  PHE A 355      25.044  11.792  -3.652  1.00  0.65
C
ATOM   5397  CG  PHE A 355      25.336  11.421  -2.222  1.00  0.65
C
ATOM   5398  CD1 PHE A 355      26.043  12.273  -1.390  1.00  0.65
C
ATOM   5399  CD2 PHE A 355      24.924  10.201  -1.710  1.00  0.65
C
ATOM   5400  CE1 PHE A 355      26.312  11.927  -0.078  1.00  0.65
C
ATOM   5401  CE2 PHE A 355      25.191   9.851  -0.406  1.00  0.65
C
ATOM   5402  CZ  PHE A 355      25.883  10.715   0.416  1.00  0.65
C
ATOM   5403  H   PHE A 355      24.946  12.106  -6.205  1.00  0.48
H
```

```
ATOM   5404  HA  PHE A 355      27.067  11.686  -4.348  1.00  0.52
H
ATOM   5405 1HB  PHE A 355      24.991  12.878  -3.721  1.00  0.77
H
ATOM   5406 2HB  PHE A 355      24.065  11.396  -3.913  1.00  0.77
H
ATOM   5407  HD1 PHE A 355      26.380  13.228  -1.779  1.00  0.77
H
ATOM   5408  HD2 PHE A 355      24.381   9.514  -2.356  1.00  0.77
H
ATOM   5409  HE1 PHE A 355      26.862  12.612   0.565  1.00  0.77
H
ATOM   5410  HE2 PHE A 355      24.856   8.892  -0.026  1.00  0.77
H
ATOM   5411  HZ  PHE A 355      26.093  10.439   1.447  1.00  0.77
H
ATOM   5412  N   ARG A 356      25.179   9.052  -5.031  1.00  0.42
N
ATOM   5413  CA  ARG A 356      25.135   7.589  -4.980  1.00  0.49
C
ATOM   5414  C   ARG A 356      26.441   6.960  -5.453  1.00  0.57
C
ATOM   5415  O   ARG A 356      26.950   6.033  -4.832  1.00  0.70
O
ATOM   5416  CB  ARG A 356      24.004   7.059  -5.840  1.00  0.73
C
ATOM   5417  CG  ARG A 356      23.810   5.555  -5.822  1.00  0.73
C
ATOM   5418  CD  ARG A 356      22.719   5.174  -6.748  1.00  0.73
C
ATOM   5419  NE  ARG A 356      22.428   3.749  -6.741  1.00  0.73
N
ATOM   5420  CZ  ARG A 356      23.094   2.814  -7.445  1.00  0.73
C
ATOM   5421  NH1 ARG A 356      24.121   3.143  -8.194  1.00  0.73
N
ATOM   5422  NH2 ARG A 356      22.690   1.560  -7.369  1.00  0.73
N
ATOM   5423  H   ARG A 356      24.438   9.548  -5.516  1.00  0.50
H
ATOM   5424  HA  ARG A 356      24.960   7.287  -3.948  1.00  0.59
H
ATOM   5425 1HB  ARG A 356      23.066   7.519  -5.538  1.00  0.88
H
ATOM   5426 2HB  ARG A 356      24.177   7.330  -6.876  1.00  0.88
H
ATOM   5427 1HG  ARG A 356      24.727   5.061  -6.144  1.00  0.88
H
ATOM   5428 2HG  ARG A 356      23.549   5.223  -4.817  1.00  0.88
H
ATOM   5429 1HD  ARG A 356      21.812   5.706  -6.466  1.00  0.88
H
ATOM   5430 2HD  ARG A 356      22.998   5.455  -7.764  1.00  0.88
H
ATOM   5431  HE  ARG A 356      21.638   3.420  -6.192  1.00  0.88
H
ATOM   5432 1HH1 ARG A 356      24.421   4.107  -8.248  1.00  0.88
H
```

```
ATOM   5433 2HH1 ARG A 356      24.610   2.432  -8.718  1.00  0.88
H
ATOM   5434 1HH2 ARG A 356      21.880   1.343  -6.792  1.00  0.88
H
ATOM   5435 2HH2 ARG A 356      23.164   0.835  -7.885  1.00  0.88
H
ATOM   5436  N   LYS A 357      26.977   7.455  -6.564  1.00  0.54
N
ATOM   5437  CA  LYS A 357      28.235   6.946  -7.092  1.00  0.64
C
ATOM   5438  C   LYS A 357      29.415   7.266  -6.175  1.00  0.65
C
ATOM   5439  O   LYS A 357      30.303   6.434  -5.987  1.00  0.72
O
ATOM   5440  CB  LYS A 357      28.482   7.521  -8.484  1.00  0.96
C
ATOM   5441  CG  LYS A 357      27.550   6.975  -9.556  1.00  0.96
C
ATOM   5442  CD  LYS A 357      27.827   7.607 -10.911  1.00  0.96
C
ATOM   5443  CE  LYS A 357      26.911   7.032 -11.979  1.00  0.96
C
ATOM   5444  NZ  LYS A 357      27.127   7.668 -13.307  1.00  0.96
N
ATOM   5445  H   LYS A 357      26.500   8.195  -7.058  1.00  0.65
H
ATOM   5446  HA  LYS A 357      28.160   5.862  -7.172  1.00  0.77
H
ATOM   5447 1HB  LYS A 357      28.363   8.605  -8.458  1.00  1.15
H
ATOM   5448 2HB  LYS A 357      29.507   7.310  -8.792  1.00  1.15
H
ATOM   5449 1HG  LYS A 357      27.683   5.895  -9.636  1.00  1.15
H
ATOM   5450 2HG  LYS A 357      26.516   7.174  -9.278  1.00  1.15
H
ATOM   5451 1HD  LYS A 357      27.670   8.685 -10.850  1.00  1.15
H
ATOM   5452 2HD  LYS A 357      28.864   7.424 -11.195  1.00  1.15
H
ATOM   5453 1HE  LYS A 357      27.093   5.962 -12.068  1.00  1.15
H
ATOM   5454 2HE  LYS A 357      25.878   7.187 -11.680  1.00  1.15
H
ATOM   5455 1HZ  LYS A 357      26.494   7.256 -13.980  1.00  1.15
H
ATOM   5456 2HZ  LYS A 357      26.945   8.659 -13.237  1.00  1.15
H
ATOM   5457 3HZ  LYS A 357      28.080   7.520 -13.604  1.00  1.15
H
ATOM   5458  N   GLU A 358      29.408   8.464  -5.593  1.00  0.64
N
ATOM   5459  CA  GLU A 358      30.469   8.902  -4.694  1.00  0.73
C
ATOM   5460  C   GLU A 358      30.451   8.176  -3.340  1.00  0.79
C
ATOM   5461  O   GLU A 358      31.492   7.735  -2.849  1.00  1.07
O
```

```
ATOM   5462  CB  GLU A 358      30.337  10.407  -4.465  1.00  1.09
C
ATOM   5463  CG  GLU A 358      31.410  11.008  -3.582  1.00  1.09
C
ATOM   5464  CD  GLU A 358      32.777  11.006  -4.191  1.00  1.09
C
ATOM   5465  OE1 GLU A 358      32.904  10.988  -5.391  1.00  1.09
O
ATOM   5466  OE2 GLU A 358      33.722  11.069  -3.441  1.00  1.09
O
ATOM   5467  H   GLU A 358      28.660   9.116  -5.806  1.00  0.77
H
ATOM   5468  HA  GLU A 358      31.427   8.699  -5.174  1.00  0.88
H
ATOM   5469 1HB  GLU A 358      30.366  10.923  -5.424  1.00  1.31
H
ATOM   5470 2HB  GLU A 358      29.371  10.621  -4.008  1.00  1.31
H
ATOM   5471 1HG  GLU A 358      31.134  12.031  -3.330  1.00  1.31
H
ATOM   5472 2HG  GLU A 358      31.452  10.433  -2.663  1.00  1.31
H
ATOM   5473  N   HIS A 359      29.262   8.062  -2.754  1.00  0.66
N
ATOM   5474  CA  HIS A 359      29.034   7.418  -1.463  1.00  0.75
C
ATOM   5475  C   HIS A 359      27.795   6.538  -1.455  1.00  0.71
C
ATOM   5476  O   HIS A 359      26.818   6.877  -0.787  1.00  0.78
O
ATOM   5477  CB  HIS A 359      28.834   8.447  -0.347  1.00  1.12
C
ATOM   5478  CG  HIS A 359      30.024   9.285  -0.061  1.00  1.12
C
ATOM   5479  ND1 HIS A 359      31.148   8.771   0.545  1.00  1.12
N
ATOM   5480  CD2 HIS A 359      30.271  10.598  -0.269  1.00  1.12
C
ATOM   5481  CE1 HIS A 359      32.047   9.731   0.683  1.00  1.12
C
ATOM   5482  NE2 HIS A 359      31.539  10.849   0.196  1.00  1.12
N
ATOM   5483  H   HIS A 359      28.466   8.447  -3.223  1.00  0.79
H
ATOM   5484  HA  HIS A 359      29.894   6.808  -1.194  1.00  0.90
H
ATOM   5485 1HB  HIS A 359      28.016   9.111  -0.620  1.00  1.35
H
ATOM   5486 2HB  HIS A 359      28.551   7.934   0.572  1.00  1.35
H
ATOM   5487  HD1 HIS A 359      31.219   7.844   0.930  1.00  1.35
H
ATOM   5488  HD2 HIS A 359      29.680  11.402  -0.708  1.00  1.35
H
ATOM   5489  HE1 HIS A 359      33.015   9.525   1.139  1.00  1.35
H
ATOM   5490  N   PRO A 360      27.809   5.400  -2.156  1.00  0.70
N
```

```
ATOM   5491  CA  PRO A 360      26.718   4.437  -2.278  1.00  0.81
C
ATOM   5492  C   PRO A 360      26.324   3.863  -0.922  1.00  1.02
C
ATOM   5493  O   PRO A 360      25.159   3.532  -0.695  1.00  2.07
O
ATOM   5494  CB  PRO A 360      27.305   3.345  -3.182  1.00  1.22
C
ATOM   5495  CG  PRO A 360      28.787   3.461  -3.019  1.00  1.22
C
ATOM   5496  CD  PRO A 360      29.048   4.923  -2.799  1.00  1.22
C
ATOM   5497  HA  PRO A 360      25.857   4.925  -2.757  1.00  0.97
H
ATOM   5498 1HB  PRO A 360      26.923   2.360  -2.876  1.00  1.46
H
ATOM   5499 2HB  PRO A 360      26.980   3.504  -4.220  1.00  1.46
H
ATOM   5500 1HG  PRO A 360      29.122   2.844  -2.172  1.00  1.46
H
ATOM   5501 2HG  PRO A 360      29.296   3.074  -3.913  1.00  1.46
H
ATOM   5502 1HD  PRO A 360      29.909   5.046  -2.129  1.00  1.46
H
ATOM   5503 2HD  PRO A 360      29.200   5.438  -3.757  1.00  1.46
H
ATOM   5504  N   GLU A 361      27.277   3.826   0.009  1.00  0.92
N
ATOM   5505  CA  GLU A 361      27.039   3.325   1.355  1.00  1.82
C
ATOM   5506  C   GLU A 361      26.151   4.266   2.191  1.00  2.00
C
ATOM   5507  O   GLU A 361      25.694   3.892   3.272  1.00  3.78
O
ATOM   5508  CB  GLU A 361      28.375   3.110   2.064  1.00  2.73
C
ATOM   5509  CG  GLU A 361      29.076   4.397   2.462  1.00  2.73
C
ATOM   5510  CD  GLU A 361      29.911   5.036   1.390  1.00  2.73
C
ATOM   5511  OE1 GLU A 361      29.986   4.508   0.308  1.00  2.73
O
ATOM   5512  OE2 GLU A 361      30.451   6.087   1.664  1.00  2.73
O
ATOM   5513  H   GLU A 361      28.210   4.124  -0.232  1.00  1.10
H
ATOM   5514  HA  GLU A 361      26.529   2.364   1.275  1.00  2.18
H
ATOM   5515 1HB  GLU A 361      28.220   2.519   2.967  1.00  3.28
H
ATOM   5516 2HB  GLU A 361      29.047   2.546   1.416  1.00  3.28
H
ATOM   5517 1HG  GLU A 361      28.320   5.103   2.771  1.00  3.28
H
ATOM   5518 2HG  GLU A 361      29.707   4.189   3.325  1.00  3.28
H
ATOM   5519  N   ARG A 362      25.918   5.483   1.695  1.00  1.12
N
```

```
ATOM   5520  CA  ARG A 362      25.072   6.462   2.357  1.00  1.32
C
ATOM   5521  C   ARG A 362      23.885   6.871   1.490  1.00  2.33
C
ATOM   5522  O   ARG A 362      23.244   7.887   1.764  1.00 10.36
O
ATOM   5523  CB  ARG A 362      25.896   7.689   2.741  1.00  1.98
C
ATOM   5524  CG  ARG A 362      26.872   7.444   3.882  1.00  1.98
C
ATOM   5525  CD  ARG A 362      27.882   8.521   4.055  1.00  1.98
C
ATOM   5526  NE  ARG A 362      28.647   8.307   5.275  1.00  1.98
N
ATOM   5527  CZ  ARG A 362      29.721   7.496   5.408  1.00  1.98
C
ATOM   5528  NH1 ARG A 362      30.223   6.841   4.385  1.00  1.98
N
ATOM   5529  NH2 ARG A 362      30.284   7.352   6.591  1.00  1.98
N
ATOM   5530  H   ARG A 362      26.328   5.761   0.816  1.00  1.34
H
ATOM   5531  HA  ARG A 362      24.685   6.015   3.273  1.00  1.58
H
ATOM   5532 1HB  ARG A 362      26.472   8.023   1.880  1.00  2.38
H
ATOM   5533 2HB  ARG A 362      25.237   8.504   3.032  1.00  2.38
H
ATOM   5534 1HG  ARG A 362      26.308   7.374   4.812  1.00  2.38
H
ATOM   5535 2HG  ARG A 362      27.400   6.512   3.725  1.00  2.38
H
ATOM   5536 1HD  ARG A 362      28.572   8.522   3.211  1.00  2.38
H
ATOM   5537 2HD  ARG A 362      27.387   9.489   4.125  1.00  2.38
H
ATOM   5538  HE  ARG A 362      28.324   8.796   6.105  1.00  2.38
H
ATOM   5539 1HH1 ARG A 362      29.832   6.922   3.455  1.00  2.38
H
ATOM   5540 2HH1 ARG A 362      31.026   6.242   4.518  1.00  2.38
H
ATOM   5541 1HH2 ARG A 362      29.912   7.840   7.393  1.00  2.38
H
ATOM   5542 2HH2 ARG A 362      31.088   6.749   6.698  1.00  2.38
H
ATOM   5543  N   PHE A 363      23.597   6.098   0.441  1.00  0.50
N
ATOM   5544  CA  PHE A 363      22.489   6.449  -0.432  1.00  0.41
C
ATOM   5545  C   PHE A 363      21.356   5.433  -0.290  1.00  0.35
C
ATOM   5546  O   PHE A 363      21.589   4.223  -0.339  1.00  0.59
O
ATOM   5547  CB  PHE A 363      22.954   6.514  -1.880  1.00  0.61
C
ATOM   5548  CG  PHE A 363      21.892   6.979  -2.830  1.00  0.61
C
```

ATOM	5549	CD1	PHE	A	363	21.743	8.330	-3.101	1.00	0.61	C
ATOM	5550	CD2	PHE	A	363	21.035	6.083	-3.446	1.00	0.61	C
ATOM	5551	CE1	PHE	A	363	20.779	8.778	-3.977	1.00	0.61	C
ATOM	5552	CE2	PHE	A	363	20.063	6.532	-4.317	1.00	0.61	C
ATOM	5553	CZ	PHE	A	363	19.941	7.877	-4.587	1.00	0.61	C
ATOM	5554	H	PHE	A	363	24.134	5.262	0.235	1.00	0.60	H
ATOM	5555	HA	PHE	A	363	22.115	7.429	-0.142	1.00	0.49	H
ATOM	5556	1HB	PHE	A	363	23.803	7.192	-1.957	1.00	0.74	H
ATOM	5557	2HB	PHE	A	363	23.292	5.529	-2.198	1.00	0.74	H
ATOM	5558	HD1	PHE	A	363	22.405	9.043	-2.620	1.00	0.74	H
ATOM	5559	HD2	PHE	A	363	21.133	5.018	-3.237	1.00	0.74	H
ATOM	5560	HE1	PHE	A	363	20.681	9.842	-4.185	1.00	0.74	H
ATOM	5561	HE2	PHE	A	363	19.395	5.823	-4.797	1.00	0.74	H
ATOM	5562	HZ	PHE	A	363	19.181	8.228	-5.280	1.00	0.74	H
ATOM	5563	N	ILE	A	364	20.134	5.931	-0.107	1.00	0.22	N
ATOM	5564	CA	ILE	A	364	18.971	5.076	0.079	1.00	0.21	C
ATOM	5565	C	ILE	A	364	18.029	5.127	-1.115	1.00	0.20	C
ATOM	5566	O	ILE	A	364	17.428	6.162	-1.413	1.00	0.29	O
ATOM	5567	CB	ILE	A	364	18.181	5.474	1.339	1.00	0.32	C
ATOM	5568	CG1	ILE	A	364	19.069	5.382	2.580	1.00	0.32	C
ATOM	5569	CG2	ILE	A	364	16.967	4.569	1.496	1.00	0.32	C
ATOM	5570	CD1	ILE	A	364	18.431	5.938	3.836	1.00	0.32	C
ATOM	5571	H	ILE	A	364	20.013	6.931	-0.088	1.00	0.26	H
ATOM	5572	HA	ILE	A	364	19.315	4.049	0.197	1.00	0.25	H
ATOM	5573	HB	ILE	A	364	17.849	6.501	1.242	1.00	0.38	H
ATOM	5574	1HG1	ILE	A	364	19.322	4.339	2.751	1.00	0.38	H
ATOM	5575	2HG1	ILE	A	364	19.993	5.932	2.397	1.00	0.38	H
ATOM	5576	1HG2	ILE	A	364	16.404	4.863	2.382	1.00	0.38	H
ATOM	5577	2HG2	ILE	A	364	16.332	4.656	0.617	1.00	0.38	H

ATOM 5578 3HG2 ILE A 364 17.297 3.534 1.603 1.00 0.38
H
ATOM 5579 1HD1 ILE A 364 19.123 5.834 4.671 1.00 0.38
H
ATOM 5580 2HD1 ILE A 364 18.194 6.990 3.690 1.00 0.38
H
ATOM 5581 3HD1 ILE A 364 17.517 5.386 4.052 1.00 0.38
H
ATOM 5582 N GLU A 365 17.882 3.988 -1.771 1.00 0.28
N
ATOM 5583 CA GLU A 365 16.972 3.851 -2.894 1.00 0.25
C
ATOM 5584 C GLU A 365 15.591 3.567 -2.326 1.00 0.29
C
ATOM 5585 O GLU A 365 15.356 2.493 -1.772 1.00 0.58
O
ATOM 5586 CB GLU A 365 17.441 2.722 -3.815 1.00 0.38
C
ATOM 5587 CG GLU A 365 18.795 2.986 -4.457 1.00 0.38
C
ATOM 5588 CD GLU A 365 19.308 1.848 -5.290 1.00 0.38
C
ATOM 5589 OE1 GLU A 365 18.646 0.842 -5.377 1.00 0.38
O
ATOM 5590 OE2 GLU A 365 20.378 1.987 -5.839 1.00 0.38
O
ATOM 5591 H GLU A 365 18.415 3.180 -1.475 1.00 0.34
H
ATOM 5592 HA GLU A 365 16.940 4.789 -3.450 1.00 0.30
H
ATOM 5593 1HB GLU A 365 17.511 1.792 -3.251 1.00 0.45
H
ATOM 5594 2HB GLU A 365 16.711 2.571 -4.611 1.00 0.45
H
ATOM 5595 1HG GLU A 365 18.702 3.863 -5.093 1.00 0.45
H
ATOM 5596 2HG GLU A 365 19.518 3.213 -3.674 1.00 0.45
H
ATOM 5597 N CYS A 366 14.702 4.554 -2.388 1.00 0.18
N
ATOM 5598 CA CYS A 366 13.382 4.397 -1.795 1.00 0.18
C
ATOM 5599 C CYS A 366 12.341 3.872 -2.766 1.00 0.20
C
ATOM 5600 O CYS A 366 11.290 3.396 -2.333 1.00 0.64
O
ATOM 5601 CB CYS A 366 12.906 5.725 -1.221 1.00 0.27
C
ATOM 5602 SG CYS A 366 13.920 6.312 0.150 1.00 0.27
S
ATOM 5603 H CYS A 366 14.931 5.417 -2.864 1.00 0.22
H
ATOM 5604 HA CYS A 366 13.465 3.686 -0.972 1.00 0.22
H
ATOM 5605 1HB CYS A 366 12.914 6.486 -2.002 1.00 0.32
H
ATOM 5606 2HB CYS A 366 11.880 5.624 -0.868 1.00 0.32
H

ATOM 5607 HG CYS A 366 15.035 6.454 -0.558 1.00 0.32
H
ATOM 5608 N ILE A 367 12.651 3.936 -4.069 1.00 0.25
N
ATOM 5609 CA ILE A 367 11.760 3.476 -5.135 1.00 0.31
C
ATOM 5610 C ILE A 367 10.545 4.435 -5.166 1.00 0.32
C
ATOM 5611 O ILE A 367 10.462 5.347 -4.342 1.00 1.53
O
ATOM 5612 CB ILE A 367 11.402 1.958 -4.898 1.00 0.46
C
ATOM 5613 CG1 ILE A 367 12.710 1.155 -4.827 1.00 0.46
C
ATOM 5614 CG2 ILE A 367 10.545 1.337 -5.997 1.00 0.46
C
ATOM 5615 CD1 ILE A 367 13.538 1.238 -6.087 1.00 0.46
C
ATOM 5616 H ILE A 367 13.540 4.333 -4.335 1.00 0.30
H
ATOM 5617 HA ILE A 367 12.281 3.549 -6.084 1.00 0.37
H
ATOM 5618 HB ILE A 367 10.882 1.835 -3.952 1.00 0.56
H
ATOM 5619 1HG1 ILE A 367 13.314 1.521 -3.997 1.00 0.56
H
ATOM 5620 2HG1 ILE A 367 12.474 0.109 -4.640 1.00 0.56
H
ATOM 5621 1HG2 ILE A 367 10.378 0.288 -5.764 1.00 0.56
H
ATOM 5622 2HG2 ILE A 367 9.583 1.828 -6.062 1.00 0.56
H
ATOM 5623 3HG2 ILE A 367 11.062 1.416 -6.954 1.00 0.56
H
ATOM 5624 1HD1 ILE A 367 14.446 0.645 -5.968 1.00 0.56
H
ATOM 5625 2HD1 ILE A 367 12.961 0.851 -6.929 1.00 0.56
H
ATOM 5626 3HD1 ILE A 367 13.807 2.276 -6.280 1.00 0.56
H
ATOM 5627 N ILE A 368 9.624 4.264 -6.113 1.00 0.39
N
ATOM 5628 CA ILE A 368 8.445 5.127 -6.218 1.00 0.24
C
ATOM 5629 C ILE A 368 7.512 4.983 -5.003 1.00 0.17
C
ATOM 5630 O ILE A 368 6.514 4.263 -5.045 1.00 0.35
O
ATOM 5631 CB ILE A 368 7.674 4.785 -7.507 1.00 0.36
C
ATOM 5632 CG1 ILE A 368 8.567 5.059 -8.718 1.00 0.36
C
ATOM 5633 CG2 ILE A 368 6.378 5.584 -7.587 1.00 0.36
C
ATOM 5634 CD1 ILE A 368 8.009 4.507 -10.004 1.00 0.36
C
ATOM 5635 H ILE A 368 9.738 3.536 -6.796 1.00 0.47
H

```
ATOM   5636  HA  ILE A 368       8.776   6.160  -6.276  1.00  0.29
H
ATOM   5637  HB  ILE A 368       7.438   3.726  -7.520  1.00  0.43
H
ATOM   5638 1HG1 ILE A 368       8.694   6.135  -8.830  1.00  0.43
H
ATOM   5639 2HG1 ILE A 368       9.549   4.615  -8.549  1.00  0.43
H
ATOM   5640 1HG2 ILE A 368       5.845   5.322  -8.501  1.00  0.43
H
ATOM   5641 2HG2 ILE A 368       5.752   5.353  -6.725  1.00  0.43
H
ATOM   5642 3HG2 ILE A 368       6.607   6.650  -7.593  1.00  0.43
H
ATOM   5643 1HD1 ILE A 368       8.688   4.739 -10.825  1.00  0.43
H
ATOM   5644 2HD1 ILE A 368       7.900   3.426  -9.917  1.00  0.43
H
ATOM   5645 3HD1 ILE A 368       7.038   4.956 -10.193  1.00  0.43
H
ATOM   5646  N   ALA A 369       7.901   5.634  -3.910  1.00  0.30
N
ATOM   5647  CA  ALA A 369       7.180   5.643  -2.643  1.00  0.26
C
ATOM   5648  C   ALA A 369       7.500   6.935  -1.897  1.00  0.31
C
ATOM   5649  O   ALA A 369       8.249   6.906  -0.916  1.00  1.26
O
ATOM   5650  CB  ALA A 369       7.555   4.421  -1.817  1.00  0.39
C
ATOM   5651  H   ALA A 369       8.759   6.160  -3.973  1.00  0.36
H
ATOM   5652  HA  ALA A 369       6.108   5.627  -2.852  1.00  0.31
H
ATOM   5653 1HB  ALA A 369       7.008   4.433  -0.875  1.00  0.47
H
ATOM   5654 2HB  ALA A 369       7.304   3.517  -2.370  1.00  0.47
H
ATOM   5655 3HB  ALA A 369       8.625   4.436  -1.613  1.00  0.47
H
ATOM   5656  N   GLU A 370       6.965   8.052  -2.381  1.00  0.27
N
ATOM   5657  CA  GLU A 370       7.258   9.384  -1.847  1.00  0.22
C
ATOM   5658  C   GLU A 370       7.008   9.522  -0.348  1.00  0.22
C
ATOM   5659  O   GLU A 370       7.828  10.097   0.375  1.00  0.22
O
ATOM   5660  CB  GLU A 370       6.411  10.423  -2.572  1.00  0.33
C
ATOM   5661  CG  GLU A 370       6.803  11.851  -2.278  1.00  0.33
C
ATOM   5662  CD  GLU A 370       8.088  12.205  -2.946  1.00  0.33
C
ATOM   5663  OE1 GLU A 370       8.458  11.517  -3.869  1.00  0.33
O
ATOM   5664  OE2 GLU A 370       8.702  13.160  -2.545  1.00  0.33
O
```

```
ATOM   5665  H   GLU A 370       6.339   7.977  -3.172  1.00  0.32
H
ATOM   5666  HA  GLU A 370       8.312   9.597  -2.033  1.00  0.26
H
ATOM   5667 1HB  GLU A 370       6.486  10.267  -3.648  1.00  0.40
H
ATOM   5668 2HB  GLU A 370       5.363  10.296  -2.297  1.00  0.40
H
ATOM   5669 1HG  GLU A 370       6.018  12.526  -2.606  1.00  0.40
H
ATOM   5670 2HG  GLU A 370       6.924  11.969  -1.204  1.00  0.40
H
ATOM   5671  N   GLN A 371       5.860   9.008   0.099  1.00  0.27
N
ATOM   5672  CA  GLN A 371       5.442   9.083   1.492  1.00  0.32
C
ATOM   5673  C   GLN A 371       6.445   8.413   2.389  1.00  0.22
C
ATOM   5674  O   GLN A 371       6.944   9.014   3.347  1.00  0.28
O
ATOM   5675  CB  GLN A 371       4.096   8.400   1.667  1.00  0.48
C
ATOM   5676  CG  GLN A 371       2.929   9.097   1.012  1.00  0.48
C
ATOM   5677  CD  GLN A 371       1.659   8.297   1.192  1.00  0.48
C
ATOM   5678  OE1 GLN A 371       0.960   8.450   2.199  1.00  0.48
O
ATOM   5679  NE2 GLN A 371       1.369   7.422   0.235  1.00  0.48
N
ATOM   5680  H   GLN A 371       5.248   8.546  -0.561  1.00  0.32
H
ATOM   5681  HA  GLN A 371       5.363  10.123   1.789  1.00  0.38
H
ATOM   5682 1HB  GLN A 371       4.144   7.399   1.244  1.00  0.58
H
ATOM   5683 2HB  GLN A 371       3.881   8.289   2.728  1.00  0.58
H
ATOM   5684 1HG  GLN A 371       2.792  10.078   1.465  1.00  0.58
H
ATOM   5685 2HG  GLN A 371       3.126   9.198  -0.055  1.00  0.58
H
ATOM   5686 1HE2 GLN A 371       0.546   6.855   0.304  1.00  0.58
H
ATOM   5687 2HE2 GLN A 371       1.976   7.321  -0.556  1.00  0.58
H
ATOM   5688  N   ASN A 372       6.749   7.164   2.044  1.00  0.16
N
ATOM   5689  CA  ASN A 372       7.731   6.381   2.754  1.00  0.16
C
ATOM   5690  C   ASN A 372       9.091   7.051   2.773  1.00  0.14
C
ATOM   5691  O   ASN A 372       9.732   7.073   3.816  1.00  0.17
O
ATOM   5692  CB  ASN A 372       7.835   5.002   2.163  1.00  0.24
C
ATOM   5693  CG  ASN A 372       8.758   4.121   2.939  1.00  0.24
C
```

ATOM 5694 OD1 ASN A 372 8.517 3.811 4.112 1.00 0.24
O
ATOM 5695 ND2 ASN A 372 9.824 3.718 2.307 1.00 0.24
N
ATOM 5696 H ASN A 372 6.255 6.722 1.271 1.00 0.19
H
ATOM 5697 HA ASN A 372 7.408 6.285 3.791 1.00 0.19
H
ATOM 5698 1HB ASN A 372 6.848 4.548 2.151 1.00 0.29
H
ATOM 5699 2HB ASN A 372 8.182 5.067 1.131 1.00 0.29
H
ATOM 5700 1HD2 ASN A 372 10.487 3.131 2.773 1.00 0.29
H
ATOM 5701 2HD2 ASN A 372 9.980 3.997 1.361 1.00 0.29
H
ATOM 5702 N MET A 373 9.535 7.592 1.631 1.00 0.15
N
ATOM 5703 CA MET A 373 10.834 8.260 1.555 1.00 0.16
C
ATOM 5704 C MET A 373 10.977 9.332 2.621 1.00 0.15
C
ATOM 5705 O MET A 373 11.983 9.367 3.337 1.00 0.17
O
ATOM 5706 CB MET A 373 11.055 8.876 0.172 1.00 0.24
C
ATOM 5707 CG MET A 373 12.382 9.616 0.030 1.00 0.24
C
ATOM 5708 SD MET A 373 12.598 10.392 -1.584 1.00 0.24
S
ATOM 5709 CE MET A 373 11.439 11.748 -1.464 1.00 0.24
C
ATOM 5710 H MET A 373 8.969 7.530 0.794 1.00 0.18
H
ATOM 5711 HA MET A 373 11.613 7.518 1.731 1.00 0.19
H
ATOM 5712 1HB MET A 373 11.021 8.093 -0.585 1.00 0.29
H
ATOM 5713 2HB MET A 373 10.253 9.580 -0.050 1.00 0.29
H
ATOM 5714 1HG MET A 373 12.439 10.393 0.792 1.00 0.29
H
ATOM 5715 2HG MET A 373 13.209 8.928 0.192 1.00 0.29
H
ATOM 5716 1HE MET A 373 11.454 12.324 -2.389 1.00 0.29
H
ATOM 5717 2HE MET A 373 10.435 11.355 -1.300 1.00 0.29
H
ATOM 5718 3HE MET A 373 11.714 12.394 -0.630 1.00 0.29
H
ATOM 5719 N VAL A 374 9.970 10.201 2.725 1.00 0.14
N
ATOM 5720 CA VAL A 374 9.984 11.253 3.731 1.00 0.16
C
ATOM 5721 C VAL A 374 10.006 10.666 5.135 1.00 0.16
C
ATOM 5722 O VAL A 374 10.786 11.113 5.974 1.00 0.16
O

ATOM 5723 CB VAL A 374 8.763 12.180 3.575 1.00 0.24
C
ATOM 5724 CG1 VAL A 374 8.684 13.136 4.760 1.00 0.24
C
ATOM 5725 CG2 VAL A 374 8.880 12.955 2.268 1.00 0.24
C
ATOM 5726 H VAL A 374 9.182 10.130 2.087 1.00 0.17
H
ATOM 5727 HA VAL A 374 10.885 11.850 3.590 1.00 0.19
H
ATOM 5728 HB VAL A 374 7.853 11.581 3.568 1.00 0.29
H
ATOM 5729 1HG1 VAL A 374 7.812 13.781 4.649 1.00 0.29
H
ATOM 5730 2HG1 VAL A 374 8.595 12.565 5.684 1.00 0.29
H
ATOM 5731 3HG1 VAL A 374 9.586 13.748 4.796 1.00 0.29
H
ATOM 5732 1HG2 VAL A 374 8.015 13.607 2.153 1.00 0.29
H
ATOM 5733 2HG2 VAL A 374 9.788 13.556 2.281 1.00 0.29
H
ATOM 5734 3HG2 VAL A 374 8.921 12.256 1.433 1.00 0.29
H
ATOM 5735 N SER A 375 9.158 9.661 5.379 1.00 0.18
N
ATOM 5736 CA SER A 375 9.075 9.005 6.681 1.00 0.20
C
ATOM 5737 C SER A 375 10.413 8.389 7.102 1.00 0.19
C
ATOM 5738 O SER A 375 10.818 8.510 8.259 1.00 0.27
O
ATOM 5739 CB SER A 375 8.006 7.931 6.643 1.00 0.30
C
ATOM 5740 OG SER A 375 6.737 8.488 6.436 1.00 0.30
O
ATOM 5741 H SER A 375 8.534 9.347 4.643 1.00 0.22
H
ATOM 5742 HA SER A 375 8.796 9.749 7.424 1.00 0.24
H
ATOM 5743 1HB SER A 375 8.228 7.216 5.853 1.00 0.36
H
ATOM 5744 2HB SER A 375 8.012 7.391 7.585 1.00 0.36
H
ATOM 5745 HG SER A 375 6.756 8.861 5.550 1.00 0.36
H
ATOM 5746 N VAL A 376 11.099 7.749 6.152 1.00 0.20
N
ATOM 5747 CA VAL A 376 12.409 7.158 6.396 1.00 0.17
C
ATOM 5748 C VAL A 376 13.413 8.233 6.754 1.00 0.15
C
ATOM 5749 O VAL A 376 14.189 8.050 7.689 1.00 0.17
O
ATOM 5750 CB VAL A 376 12.891 6.348 5.170 1.00 0.26
C
ATOM 5751 CG1 VAL A 376 14.348 5.933 5.337 1.00 0.26
C

ATOM 5752 CG2 VAL A 376 12.033 5.095 5.034 1.00 0.26
C
ATOM 5753 H VAL A 376 10.698 7.664 5.231 1.00 0.24
H
ATOM 5754 HA VAL A 376 12.323 6.472 7.238 1.00 0.20
H
ATOM 5755 HB VAL A 376 12.808 6.964 4.274 1.00 0.31
H
ATOM 5756 1HG1 VAL A 376 14.665 5.365 4.462 1.00 0.31
H
ATOM 5757 2HG1 VAL A 376 14.969 6.821 5.438 1.00 0.31
H
ATOM 5758 3HG1 VAL A 376 14.449 5.315 6.223 1.00 0.31
H
ATOM 5759 1HG2 VAL A 376 12.363 4.522 4.169 1.00 0.31
H
ATOM 5760 2HG2 VAL A 376 12.136 4.487 5.933 1.00 0.31
H
ATOM 5761 3HG2 VAL A 376 10.989 5.372 4.907 1.00 0.31
H
ATOM 5762 N ALA A 377 13.389 9.354 6.027 1.00 0.13
N
ATOM 5763 CA ALA A 377 14.275 10.475 6.322 1.00 0.12
C
ATOM 5764 C ALA A 377 14.028 11.006 7.740 1.00 0.14
C
ATOM 5765 O ALA A 377 14.981 11.321 8.453 1.00 0.21
O
ATOM 5766 CB ALA A 377 14.083 11.586 5.296 1.00 0.18
C
ATOM 5767 H ALA A 377 12.750 9.432 5.244 1.00 0.16
H
ATOM 5768 HA ALA A 377 15.304 10.118 6.271 1.00 0.14
H
ATOM 5769 1HB ALA A 377 14.769 12.406 5.512 1.00 0.22
H
ATOM 5770 2HB ALA A 377 14.287 11.196 4.299 1.00 0.22
H
ATOM 5771 3HB ALA A 377 13.059 11.951 5.339 1.00 0.22
H
ATOM 5772 N LEU A 378 12.753 11.078 8.146 1.00 0.13
N
ATOM 5773 CA LEU A 378 12.384 11.539 9.487 1.00 0.17
C
ATOM 5774 C LEU A 378 12.944 10.596 10.562 1.00 0.20
C
ATOM 5775 O LEU A 378 13.459 11.045 11.596 1.00 0.40
O
ATOM 5776 CB LEU A 378 10.845 11.601 9.615 1.00 0.26
C
ATOM 5777 CG LEU A 378 10.120 12.665 8.769 1.00 0.26
C
ATOM 5778 CD1 LEU A 378 8.613 12.460 8.858 1.00 0.26
C
ATOM 5779 CD2 LEU A 378 10.485 14.033 9.266 1.00 0.26
C
ATOM 5780 H LEU A 378 12.014 10.829 7.501 1.00 0.16
H

ATOM	5781	HA	LEU	A	378	12.800	12.533	9.641	1.00	0.20
H										
ATOM	5782	1HB	LEU	A	378	10.431	10.634	9.345	1.00	0.31
H										
ATOM	5783	2HB	LEU	A	378	10.598	11.794	10.658	1.00	0.31
H										
ATOM	5784	HG	LEU	A	378	10.420	12.571	7.730	1.00	0.31
H										
ATOM	5785	1HD1	LEU	A	378	8.107	13.212	8.254	1.00	0.31
H										
ATOM	5786	2HD1	LEU	A	378	8.354	11.469	8.492	1.00	0.31
H										
ATOM	5787	3HD1	LEU	A	378	8.296	12.555	9.896	1.00	0.31
H										
ATOM	5788	1HD2	LEU	A	378	9.977	14.786	8.663	1.00	0.31
H										
ATOM	5789	2HD2	LEU	A	378	10.174	14.126	10.305	1.00	0.31
H										
ATOM	5790	3HD2	LEU	A	378	11.562	14.172	9.191	1.00	0.31
H										
ATOM	5791	N	GLY	A	379	12.871	9.289	10.286	1.00	0.15
N										
ATOM	5792	CA	GLY	A	379	13.413	8.275	11.177	1.00	0.17
C										
ATOM	5793	C	GLY	A	379	14.923	8.387	11.260	1.00	0.23
C										
ATOM	5794	O	GLY	A	379	15.488	8.389	12.349	1.00	0.61
O										
ATOM	5795	H	GLY	A	379	12.404	8.990	9.438	1.00	0.18
H										
ATOM	5796	1HA	GLY	A	379	12.983	8.390	12.170	1.00	0.20
H										
ATOM	5797	2HA	GLY	A	379	13.136	7.287	10.813	1.00	0.20
H										
ATOM	5798	N	CYS	A	380	15.572	8.527	10.114	1.00	0.23
N										
ATOM	5799	CA	CYS	A	380	17.022	8.658	10.049	1.00	0.26
C										
ATOM	5800	C	CYS	A	380	17.531	9.879	10.827	1.00	0.29
C										
ATOM	5801	O	CYS	A	380	18.509	9.787	11.571	1.00	0.37
O										
ATOM	5802	CB	CYS	A	380	17.457	8.746	8.587	1.00	0.39
C										
ATOM	5803	SG	CYS	A	380	17.277	7.193	7.672	1.00	0.39
S										
ATOM	5804	H	CYS	A	380	15.054	8.520	9.248	1.00	0.28
H										
ATOM	5805	HA	CYS	A	380	17.464	7.763	10.486	1.00	0.31
H										
ATOM	5806	1HB	CYS	A	380	16.863	9.504	8.078	1.00	0.47
H										
ATOM	5807	2HB	CYS	A	380	18.495	9.050	8.524	1.00	0.47
H										
ATOM	5808	HG	CYS	A	380	17.666	7.675	6.498	1.00	0.47
H										
ATOM	5809	N	ALA	A	381	16.838	11.011	10.690	1.00	0.31
N										

```
ATOM   5810  CA  ALA A 381      17.201  12.251  11.376  1.00  0.43
C
ATOM   5811  C   ALA A 381      16.941  12.206  12.888  1.00  0.34
C
ATOM   5812  O   ALA A 381      17.389  13.093  13.623  1.00  1.11
O
ATOM   5813  CB  ALA A 381      16.436  13.417  10.766  1.00  0.65
C
ATOM   5814  H   ALA A 381      16.046  11.030  10.060  1.00  0.37
H
ATOM   5815  HA  ALA A 381      18.268  12.411  11.230  1.00  0.52
H
ATOM   5816 1HB  ALA A 381      16.739  14.346  11.249  1.00  0.77
H
ATOM   5817 2HB  ALA A 381      16.654  13.475   9.699  1.00  0.77
H
ATOM   5818 3HB  ALA A 381      15.367  13.263  10.910  1.00  0.77
H
ATOM   5819  N   THR A 382      16.197  11.205  13.343  1.00  0.74
N
ATOM   5820  CA  THR A 382      15.856  11.079  14.736  1.00  0.63
C
ATOM   5821  C   THR A 382      17.109  10.851  15.586  1.00  0.52
C
ATOM   5822  O   THR A 382      18.030  10.130  15.188  1.00  0.67
O
ATOM   5823  CB  THR A 382      14.830   9.966  14.913  1.00  0.95
C
ATOM   5824  OG1 THR A 382      13.631  10.276  14.172  1.00  0.95
O
ATOM   5825  CG2 THR A 382      14.494   9.817  16.359  1.00  0.95
C
ATOM   5826  H   THR A 382      15.861  10.482  12.724  1.00  0.89
H
ATOM   5827  HA  THR A 382      15.395  12.013  15.062  1.00  0.76
H
ATOM   5828  HB  THR A 382      15.236   9.030  14.557  1.00  1.13
H
ATOM   5829  HG1 THR A 382      13.829  10.454  13.239  1.00  1.13
H
ATOM   5830 1HG2 THR A 382      13.778   9.013  16.466  1.00  1.13
H
ATOM   5831 2HG2 THR A 382      15.385   9.573  16.910  1.00  1.13
H
ATOM   5832 3HG2 THR A 382      14.070  10.743  16.740  1.00  1.13
H
ATOM   5833  N   ARG A 383      17.148  11.545  16.729  1.00  0.65
N
ATOM   5834  CA  ARG A 383      18.260  11.569  17.690  1.00  0.72
C
ATOM   5835  C   ARG A 383      19.497  12.276  17.131  1.00  0.51
C
ATOM   5836  O   ARG A 383      20.553  12.255  17.763  1.00  0.69
O
ATOM   5837  CB  ARG A 383      18.698  10.161  18.119  1.00  1.08
C
ATOM   5838  CG  ARG A 383      17.643   9.271  18.747  1.00  1.08
C
```

ATOM 5839 CD ARG A 383 17.107 9.785 20.028 1.00 1.08
C
ATOM 5840 NE ARG A 383 16.000 8.956 20.468 1.00 1.08
N
ATOM 5841 CZ ARG A 383 16.112 7.812 21.165 1.00 1.08
C
ATOM 5842 NH1 ARG A 383 17.299 7.381 21.526 1.00 1.08
N
ATOM 5843 NH2 ARG A 383 15.022 7.129 21.474 1.00 1.08
N
ATOM 5844 H ARG A 383 16.334 12.101 16.957 1.00 0.78
H
ATOM 5845 HA ARG A 383 17.930 12.108 18.578 1.00 0.86
H
ATOM 5846 1HB ARG A 383 19.089 9.624 17.264 1.00 1.30
H
ATOM 5847 2HB ARG A 383 19.511 10.248 18.841 1.00 1.30
H
ATOM 5848 1HG ARG A 383 16.823 9.124 18.064 1.00 1.30
H
ATOM 5849 2HG ARG A 383 18.096 8.303 18.959 1.00 1.30
H
ATOM 5850 1HD ARG A 383 17.882 9.764 20.794 1.00 1.30
H
ATOM 5851 2HD ARG A 383 16.744 10.804 19.898 1.00 1.30
H
ATOM 5852 HE ARG A 383 15.063 9.252 20.218 1.00 1.30
H
ATOM 5853 1HH1 ARG A 383 18.122 7.915 21.289 1.00 1.30
H
ATOM 5854 2HH1 ARG A 383 17.392 6.523 22.054 1.00 1.30
H
ATOM 5855 1HH2 ARG A 383 14.111 7.462 21.166 1.00 1.30
H
ATOM 5856 2HH2 ARG A 383 15.094 6.271 21.998 1.00 1.30
H
ATOM 5857 N GLY A 384 19.383 12.897 15.949 1.00 0.40
N
ATOM 5858 CA GLY A 384 20.517 13.578 15.353 1.00 0.41
C
ATOM 5859 C GLY A 384 21.527 12.594 14.769 1.00 0.45
C
ATOM 5860 O GLY A 384 22.682 12.958 14.556 1.00 0.58
O
ATOM 5861 H GLY A 384 18.511 12.914 15.437 1.00 0.48
H
ATOM 5862 1HA GLY A 384 20.163 14.247 14.569 1.00 0.49
H
ATOM 5863 2HA GLY A 384 21.003 14.198 16.106 1.00 0.49
H
ATOM 5864 N ARG A 385 21.112 11.349 14.515 1.00 0.44
N
ATOM 5865 CA ARG A 385 22.063 10.346 14.039 1.00 0.55
C
ATOM 5866 C ARG A 385 22.595 10.595 12.629 1.00 0.47
C
ATOM 5867 O ARG A 385 23.714 10.190 12.315 1.00 0.57
O

```
ATOM   5868  CB  ARG A 385      21.419   8.979  14.087  1.00  0.83
C
ATOM   5869  CG  ARG A 385      21.176   8.458  15.490  1.00  0.83
C
ATOM   5870  CD  ARG A 385      20.352   7.230  15.464  1.00  0.83
C
ATOM   5871  NE  ARG A 385      19.032   7.525  14.956  1.00  0.83
N
ATOM   5872  CZ  ARG A 385      18.086   6.620  14.636  1.00  0.83
C
ATOM   5873  NH1 ARG A 385      18.267   5.323  14.798  1.00  0.83
N
ATOM   5874  NH2 ARG A 385      16.949   7.058  14.140  1.00  0.83
N
ATOM   5875  H   ARG A 385      20.147  11.074  14.685  1.00  0.53
H
ATOM   5876  HA  ARG A 385      22.909  10.345  14.722  1.00  0.66
H
ATOM   5877 1HB  ARG A 385      20.459   9.007  13.573  1.00  0.99
H
ATOM   5878 2HB  ARG A 385      22.051   8.257  13.569  1.00  0.99
H
ATOM   5879 1HG  ARG A 385      22.131   8.219  15.960  1.00  0.99
H
ATOM   5880 2HG  ARG A 385      20.666   9.220  16.073  1.00  0.99
H
ATOM   5881 1HD  ARG A 385      20.811   6.500  14.804  1.00  0.99
H
ATOM   5882 2HD  ARG A 385      20.258   6.817  16.467  1.00  0.99
H
ATOM   5883  HE  ARG A 385      18.805   8.502  14.800  1.00  0.99
H
ATOM   5884 1HH1 ARG A 385      19.128   4.946  15.169  1.00  0.99
H
ATOM   5885 2HH1 ARG A 385      17.533   4.682  14.538  1.00  0.99
H
ATOM   5886 1HH2 ARG A 385      16.811   8.050  13.994  1.00  0.99
H
ATOM   5887 2HH2 ARG A 385      16.223   6.412  13.882  1.00  0.99
H
ATOM   5888  N   THR A 386      21.824  11.280  11.789  1.00  0.36
N
ATOM   5889  CA  THR A 386      22.288  11.583  10.438  1.00  0.26
C
ATOM   5890  C   THR A 386      21.480  12.686   9.762  1.00  0.25
C
ATOM   5891  O   THR A 386      20.320  12.927  10.094  1.00  0.39
O
ATOM   5892  CB  THR A 386      22.295  10.321   9.561  1.00  0.39
C
ATOM   5893  OG1 THR A 386      22.782  10.651   8.259  1.00  0.39
O
ATOM   5894  CG2 THR A 386      20.920   9.726   9.452  1.00  0.39
C
ATOM   5895  H   THR A 386      20.910  11.591  12.083  1.00  0.43
H
ATOM   5896  HA  THR A 386      23.317  11.933  10.509  1.00  0.31
H
```

ATOM 5897 HB THR A 386 22.962 9.583 10.005 1.00 0.47
H
ATOM 5898 HG1 THR A 386 23.621 11.109 8.333 1.00 0.47
H
ATOM 5899 1HG2 THR A 386 20.959 8.832 8.833 1.00 0.47
H
ATOM 5900 2HG2 THR A 386 20.569 9.461 10.447 1.00 0.47
H
ATOM 5901 3HG2 THR A 386 20.242 10.451 9.005 1.00 0.47
H
ATOM 5902 N ILE A 387 22.131 13.366 8.822 1.00 0.24
N
ATOM 5903 CA ILE A 387 21.546 14.473 8.071 1.00 0.25
C
ATOM 5904 C ILE A 387 21.001 13.959 6.746 1.00 0.23
C
ATOM 5905 O ILE A 387 21.765 13.621 5.844 1.00 0.25
O
ATOM 5906 CB ILE A 387 22.600 15.567 7.807 1.00 0.38
C
ATOM 5907 CG1 ILE A 387 23.248 15.999 9.125 1.00 0.38
C
ATOM 5908 CG2 ILE A 387 21.961 16.759 7.117 1.00 0.38
C
ATOM 5909 CD1 ILE A 387 22.266 16.531 10.139 1.00 0.38
C
ATOM 5910 H ILE A 387 23.072 13.088 8.601 1.00 0.29
H
ATOM 5911 HA ILE A 387 20.724 14.899 8.647 1.00 0.30
H
ATOM 5912 HB ILE A 387 23.387 15.162 7.172 1.00 0.45
H
ATOM 5913 1HG1 ILE A 387 23.763 15.146 9.563 1.00 0.45
H
ATOM 5914 2HG1 ILE A 387 23.984 16.777 8.921 1.00 0.45
H
ATOM 5915 1HG2 ILE A 387 22.718 17.519 6.926 1.00 0.45
H
ATOM 5916 2HG2 ILE A 387 21.521 16.436 6.176 1.00 0.45
H
ATOM 5917 3HG2 ILE A 387 21.183 17.174 7.757 1.00 0.45
H
ATOM 5918 1HD1 ILE A 387 22.798 16.817 11.047 1.00 0.45
H
ATOM 5919 2HD1 ILE A 387 21.754 17.401 9.728 1.00 0.45
H
ATOM 5920 3HD1 ILE A 387 21.533 15.759 10.377 1.00 0.45
H
ATOM 5921 N ALA A 388 19.680 13.903 6.629 1.00 0.24
N
ATOM 5922 CA ALA A 388 19.063 13.335 5.439 1.00 0.25
C
ATOM 5923 C ALA A 388 18.653 14.367 4.395 1.00 0.25
C
ATOM 5924 O ALA A 388 18.002 15.361 4.712 1.00 0.29
O
ATOM 5925 CB ALA A 388 17.843 12.523 5.854 1.00 0.38
C

ATOM 5926 H ALA A 388 19.097 14.224 7.390 1.00 0.29
H
ATOM 5927 HA ALA A 388 19.789 12.675 4.974 1.00 0.30
H
ATOM 5928 1HB ALA A 388 17.399 12.058 4.978 1.00 0.45
H
ATOM 5929 2HB ALA A 388 18.145 11.753 6.563 1.00 0.45
H
ATOM 5930 3HB ALA A 388 17.112 13.181 6.323 1.00 0.45
H
ATOM 5931 N PHE A 389 19.002 14.067 3.141 1.00 0.40
N
ATOM 5932 CA PHE A 389 18.634 14.847 1.960 1.00 0.28
C
ATOM 5933 C PHE A 389 17.818 13.980 1.012 1.00 0.23
C
ATOM 5934 O PHE A 389 18.382 13.175 0.276 1.00 0.31
O
ATOM 5935 CB PHE A 389 19.871 15.333 1.214 1.00 0.42
C
ATOM 5936 CG PHE A 389 20.696 16.332 1.955 1.00 0.42
C
ATOM 5937 CD1 PHE A 389 21.644 15.929 2.880 1.00 0.42
C
ATOM 5938 CD2 PHE A 389 20.534 17.686 1.705 1.00 0.42
C
ATOM 5939 CE1 PHE A 389 22.405 16.865 3.546 1.00 0.42
C
ATOM 5940 CE2 PHE A 389 21.293 18.621 2.370 1.00 0.42
C
ATOM 5941 CZ PHE A 389 22.228 18.207 3.292 1.00 0.42
C
ATOM 5942 H PHE A 389 19.578 13.249 2.992 1.00 0.48
H
ATOM 5943 HA PHE A 389 18.036 15.701 2.269 1.00 0.34
H
ATOM 5944 1HB PHE A 389 20.501 14.478 0.994 1.00 0.50
H
ATOM 5945 2HB PHE A 389 19.573 15.774 0.264 1.00 0.50
H
ATOM 5946 HD1 PHE A 389 21.783 14.866 3.077 1.00 0.50
H
ATOM 5947 HD2 PHE A 389 19.793 18.006 0.971 1.00 0.50
H
ATOM 5948 HE1 PHE A 389 23.148 16.542 4.275 1.00 0.50
H
ATOM 5949 HE2 PHE A 389 21.157 19.683 2.169 1.00 0.50
H
ATOM 5950 HZ PHE A 389 22.827 18.940 3.820 1.00 0.50
H
ATOM 5951 N ALA A 390 16.500 14.129 1.034 1.00 0.21
N
ATOM 5952 CA ALA A 390 15.654 13.322 0.159 1.00 0.19
C
ATOM 5953 C ALA A 390 15.317 14.098 -1.109 1.00 0.16
C
ATOM 5954 O ALA A 390 15.005 15.286 -1.044 1.00 0.29
O

```
ATOM   5955  CB  ALA A 390      14.391  12.895   0.885  1.00  0.29
C
ATOM   5956  H   ALA A 390      16.089  14.823   1.644  1.00  0.25
H
ATOM   5957  HA  ALA A 390      16.211  12.432  -0.128  1.00  0.23
H
ATOM   5958 1HB  ALA A 390      13.789  12.274   0.226  1.00  0.34
H
ATOM   5959 2HB  ALA A 390      14.657  12.325   1.776  1.00  0.34
H
ATOM   5960 3HB  ALA A 390      13.823  13.772   1.174  1.00  0.34
H
ATOM   5961  N   GLY A 391      15.385  13.418  -2.253  1.00  0.14
N
ATOM   5962  CA  GLY A 391      15.132  14.050  -3.539  1.00  0.14
C
ATOM   5963  C   GLY A 391      14.090  13.341  -4.405  1.00  0.14
C
ATOM   5964  O   GLY A 391      13.992  12.108  -4.435  1.00  0.25
O
ATOM   5965  H   GLY A 391      15.652  12.448  -2.229  1.00  0.17
H
ATOM   5966 1HA  GLY A 391      14.812  15.075  -3.366  1.00  0.17
H
ATOM   5967 2HA  GLY A 391      16.070  14.113  -4.090  1.00  0.17
H
ATOM   5968  N   ALA A 392      13.323  14.166  -5.107  1.00  0.16
N
ATOM   5969  CA  ALA A 392      12.280  13.747  -6.038  1.00  0.14
C
ATOM   5970  C   ALA A 392      11.900  14.953  -6.863  1.00  0.16
C
ATOM   5971  O   ALA A 392      12.487  16.016  -6.681  1.00  0.20
O
ATOM   5972  CB  ALA A 392      11.073  13.179  -5.305  1.00  0.21
C
ATOM   5973  H   ALA A 392      13.491  15.162  -4.984  1.00  0.19
H
ATOM   5974  HA  ALA A 392      12.687  12.989  -6.705  1.00  0.17
H
ATOM   5975 1HB  ALA A 392      10.313  12.879  -6.026  1.00  0.25
H
ATOM   5976 2HB  ALA A 392      11.376  12.311  -4.720  1.00  0.25
H
ATOM   5977 3HB  ALA A 392      10.658  13.934  -4.641  1.00  0.25
H
ATOM   5978  N   PHE A 393      10.947  14.811  -7.768  1.00  0.25
N
ATOM   5979  CA  PHE A 393      10.476  16.000  -8.447  1.00  0.28
C
ATOM   5980  C   PHE A 393       9.707  16.780  -7.393  1.00  0.30
C
ATOM   5981  O   PHE A 393       9.052  16.184  -6.537  1.00  0.40
O
ATOM   5982  CB  PHE A 393       9.517  15.684  -9.589  1.00  0.42
C
ATOM   5983  CG  PHE A 393      10.124  14.955 -10.745  1.00  0.42
C
```

```
ATOM   5984  CD1 PHE A 393      11.286  14.215 -10.589  1.00  0.42
C
ATOM   5985  CD2 PHE A 393       9.520  14.993 -11.991  1.00  0.42
C
ATOM   5986  CE1 PHE A 393      11.829  13.524 -11.653  1.00  0.42
C
ATOM   5987  CE2 PHE A 393      10.062  14.305 -13.057  1.00  0.42
C
ATOM   5988  CZ  PHE A 393      11.217  13.567 -12.886  1.00  0.42
C
ATOM   5989  H   PHE A 393      10.522  13.912  -7.942  1.00  0.30
H
ATOM   5990  HA  PHE A 393      11.316  16.591  -8.801  1.00  0.34
H
ATOM   5991 1HB  PHE A 393       8.688  15.097  -9.216  1.00  0.50
H
ATOM   5992 2HB  PHE A 393       9.101  16.616  -9.968  1.00  0.50
H
ATOM   5993  HD1 PHE A 393      11.769  14.176  -9.615  1.00  0.50
H
ATOM   5994  HD2 PHE A 393       8.603  15.570 -12.124  1.00  0.50
H
ATOM   5995  HE1 PHE A 393      12.739  12.944 -11.516  1.00  0.50
H
ATOM   5996  HE2 PHE A 393       9.577  14.339 -14.032  1.00  0.50
H
ATOM   5997  HZ  PHE A 393      11.642  13.021 -13.728  1.00  0.50
H
ATOM   5998  N   ALA A 394       9.760  18.097  -7.461  1.00  0.24
N
ATOM   5999  CA  ALA A 394       9.040  18.947  -6.523  1.00  0.24
C
ATOM   6000  C   ALA A 394       7.545  18.653  -6.593  1.00  0.24
C
ATOM   6001  O   ALA A 394       6.845  18.729  -5.584  1.00  0.72
O
ATOM   6002  CB  ALA A 394       9.333  20.399  -6.809  1.00  0.36
C
ATOM   6003  H   ALA A 394      10.314  18.521  -8.185  1.00  0.29
H
ATOM   6004  HA  ALA A 394       9.378  18.726  -5.519  1.00  0.29
H
ATOM   6005 1HB  ALA A 394       8.797  21.030  -6.100  1.00  0.43
H
ATOM   6006 2HB  ALA A 394      10.398  20.588  -6.719  1.00  0.43
H
ATOM   6007 3HB  ALA A 394       9.024  20.636  -7.816  1.00  0.43
H
ATOM   6008  N   ALA A 395       7.063  18.299  -7.786  1.00  0.16
N
ATOM   6009  CA  ALA A 395       5.679  17.918  -7.983  1.00  0.17
C
ATOM   6010  C   ALA A 395       5.370  16.644  -7.201  1.00  0.24
C
ATOM   6011  O   ALA A 395       4.305  16.526  -6.593  1.00  1.04
O
ATOM   6012  CB  ALA A 395       5.392  17.718  -9.466  1.00  0.26
C
```

```
ATOM   6013  H   ALA A 395       7.685  18.282  -8.581  1.00  0.19
H
ATOM   6014  HA  ALA A 395       5.043  18.716  -7.598  1.00  0.20
H
ATOM   6015 1HB  ALA A 395       4.346  17.449  -9.602  1.00  0.31
H
ATOM   6016 2HB  ALA A 395       5.603  18.642 -10.003  1.00  0.31
H
ATOM   6017 3HB  ALA A 395       6.024  16.921  -9.854  1.00  0.31
H
ATOM   6018  N   PHE A 396       6.324  15.704  -7.196  1.00  0.22
N
ATOM   6019  CA  PHE A 396       6.155  14.428  -6.506  1.00  0.22
C
ATOM   6020  C   PHE A 396       6.012  14.623  -5.010  1.00  0.18
C
ATOM   6021  O   PHE A 396       5.238  13.910  -4.376  1.00  0.53
O
ATOM   6022  CB  PHE A 396       7.337  13.486  -6.757  1.00  0.33
C
ATOM   6023  CG  PHE A 396       7.437  12.979  -8.164  1.00  0.33
C
ATOM   6024  CD1 PHE A 396       6.466  13.295  -9.104  1.00  0.33
C
ATOM   6025  CD2 PHE A 396       8.505  12.183  -8.553  1.00  0.33
C
ATOM   6026  CE1 PHE A 396       6.566  12.835 -10.402  1.00  0.33
C
ATOM   6027  CE2 PHE A 396       8.603  11.719  -9.849  1.00  0.33
C
ATOM   6028  CZ  PHE A 396       7.634  12.046 -10.773  1.00  0.33
C
ATOM   6029  H   PHE A 396       7.183  15.870  -7.699  1.00  0.26
H
ATOM   6030  HA  PHE A 396       5.246  13.953  -6.878  1.00  0.26
H
ATOM   6031 1HB  PHE A 396       8.270  13.981  -6.507  1.00  0.40
H
ATOM   6032 2HB  PHE A 396       7.250  12.623  -6.097  1.00  0.40
H
ATOM   6033  HD1 PHE A 396       5.621  13.919  -8.810  1.00  0.40
H
ATOM   6034  HD2 PHE A 396       9.271  11.926  -7.822  1.00  0.40
H
ATOM   6035  HE1 PHE A 396       5.799  13.092 -11.133  1.00  0.40
H
ATOM   6036  HE2 PHE A 396       9.445  11.095 -10.144  1.00  0.40
H
ATOM   6037  HZ  PHE A 396       7.713  11.680 -11.796  1.00  0.40
H
ATOM   6038  N   PHE A 397       6.708  15.623  -4.454  1.00  0.45
N
ATOM   6039  CA  PHE A 397       6.622  15.921  -3.018  1.00  0.28
C
ATOM   6040  C   PHE A 397       5.214  16.209  -2.500  1.00  0.29
C
ATOM   6041  O   PHE A 397       4.964  16.058  -1.304  1.00  0.42
O
```

ATOM 6042 CB PHE A 397 7.572 17.053 -2.615 1.00 0.42
C
ATOM 6043 CG PHE A 397 8.983 16.589 -2.402 1.00 0.42
C
ATOM 6044 CD1 PHE A 397 9.964 16.774 -3.358 1.00 0.42
C
ATOM 6045 CD2 PHE A 397 9.326 15.947 -1.223 1.00 0.42
C
ATOM 6046 CE1 PHE A 397 11.252 16.331 -3.145 1.00 0.42
C
ATOM 6047 CE2 PHE A 397 10.611 15.497 -1.014 1.00 0.42
C
ATOM 6048 CZ PHE A 397 11.579 15.691 -1.974 1.00 0.42
C
ATOM 6049 H PHE A 397 7.345 16.154 -5.039 1.00 0.54
H
ATOM 6050 HA PHE A 397 6.973 15.035 -2.490 1.00 0.34
H
ATOM 6051 1HB PHE A 397 7.580 17.821 -3.385 1.00 0.50
H
ATOM 6052 2HB PHE A 397 7.223 17.514 -1.692 1.00 0.50
H
ATOM 6053 HD1 PHE A 397 9.708 17.266 -4.286 1.00 0.50
H
ATOM 6054 HD2 PHE A 397 8.563 15.789 -0.461 1.00 0.50
H
ATOM 6055 HE1 PHE A 397 12.006 16.480 -3.913 1.00 0.50
H
ATOM 6056 HE2 PHE A 397 10.856 14.981 -0.090 1.00 0.50
H
ATOM 6057 HZ PHE A 397 12.594 15.333 -1.811 1.00 0.50
H
ATOM 6058 N THR A 398 4.275 16.568 -3.383 1.00 0.28
N
ATOM 6059 CA THR A 398 2.907 16.816 -2.950 1.00 0.42
C
ATOM 6060 C THR A 398 2.249 15.515 -2.471 1.00 0.39
C
ATOM 6061 O THR A 398 1.346 15.533 -1.633 1.00 0.57
O
ATOM 6062 CB THR A 398 2.085 17.441 -4.095 1.00 0.63
C
ATOM 6063 OG1 THR A 398 2.075 16.556 -5.217 1.00 0.63
O
ATOM 6064 CG2 THR A 398 2.683 18.774 -4.522 1.00 0.63
C
ATOM 6065 H THR A 398 4.491 16.669 -4.369 1.00 0.34
H
ATOM 6066 HA THR A 398 2.926 17.510 -2.114 1.00 0.50
H
ATOM 6067 HB THR A 398 1.061 17.591 -3.762 1.00 0.76
H
ATOM 6068 HG1 THR A 398 2.960 16.490 -5.587 1.00 0.76
H
ATOM 6069 1HG2 THR A 398 2.088 19.196 -5.331 1.00 0.76
H
ATOM 6070 2HG2 THR A 398 2.688 19.461 -3.677 1.00 0.76
H

```
ATOM   6071 3HG2 THR A 398       3.705  18.618  -4.867  1.00  0.76
H
ATOM   6072  N   ARG A 399       2.763  14.380  -2.950  1.00  0.32
N
ATOM   6073  CA  ARG A 399       2.279  13.054  -2.594  1.00  0.35
C
ATOM   6074  C   ARG A 399       2.539  12.756  -1.111  1.00  0.36
C
ATOM   6075  O   ARG A 399       1.856  11.918  -0.518  1.00  0.79
O
ATOM   6076  CB  ARG A 399       2.955  11.987  -3.460  1.00  0.52
C
ATOM   6077  CG  ARG A 399       2.398  10.570  -3.334  1.00  0.52
C
ATOM   6078  CD  ARG A 399       1.024  10.468  -3.918  1.00  0.52
C
ATOM   6079  NE  ARG A 399       1.031  10.629  -5.376  1.00  0.52
N
ATOM   6080  CZ  ARG A 399       1.103   9.637  -6.285  1.00  0.52
C
ATOM   6081  NH1 ARG A 399       1.129   8.388  -5.950  1.00  0.52
N
ATOM   6082  NH2 ARG A 399       1.148   9.919  -7.565  1.00  0.52
N
ATOM   6083  H   ARG A 399       3.524  14.422  -3.610  1.00  0.38
H
ATOM   6084  HA  ARG A 399       1.204  13.018  -2.773  1.00  0.42
H
ATOM   6085 1HB  ARG A 399       2.880  12.274  -4.507  1.00  0.63
H
ATOM   6086 2HB  ARG A 399       4.013  11.943  -3.214  1.00  0.63
H
ATOM   6087 1HG  ARG A 399       3.048   9.873  -3.862  1.00  0.63
H
ATOM   6088 2HG  ARG A 399       2.345  10.291  -2.281  1.00  0.63
H
ATOM   6089 1HD  ARG A 399       0.609   9.486  -3.688  1.00  0.63
H
ATOM   6090 2HD  ARG A 399       0.387  11.241  -3.491  1.00  0.63
H
ATOM   6091  HE  ARG A 399       1.021  11.561  -5.763  1.00  0.63
H
ATOM   6092 1HH1 ARG A 399       1.123   8.107  -4.976  1.00  0.63
H
ATOM   6093 2HH1 ARG A 399       1.183   7.700  -6.694  1.00  0.63
H
ATOM   6094 1HH2 ARG A 399       1.127  10.885  -7.854  1.00  0.63
H
ATOM   6095 2HH2 ARG A 399       1.218   9.163  -8.237  1.00  0.63
H
ATOM   6096  N   ALA A 400       3.541  13.417  -0.515  1.00  0.25
N
ATOM   6097  CA  ALA A 400       3.870  13.174   0.879  1.00  0.25
C
ATOM   6098  C   ALA A 400       3.688  14.416   1.724  1.00  0.21
C
ATOM   6099  O   ALA A 400       4.342  14.553   2.755  1.00  0.36
O
```

```
ATOM   6100  CB  ALA A 400       5.300  12.700   1.000  1.00  0.38
C
ATOM   6101  H   ALA A 400       4.076  14.116  -1.014  1.00  0.30
H
ATOM   6102  HA  ALA A 400       3.199  12.403   1.257  1.00  0.30
H
ATOM   6103 1HB  ALA A 400       5.531  12.495   2.044  1.00  0.45
H
ATOM   6104 2HB  ALA A 400       5.426  11.795   0.412  1.00  0.45
H
ATOM   6105 3HB  ALA A 400       5.971  13.472   0.626  1.00  0.45
H
ATOM   6106  N   PHE A 401       2.767  15.287   1.322  1.00  0.27
N
ATOM   6107  CA  PHE A 401       2.527  16.530   2.036  1.00  0.38
C
ATOM   6108  C   PHE A 401       2.153  16.333   3.505  1.00  0.36
C
ATOM   6109  O   PHE A 401       2.617  17.084   4.362  1.00  0.45
O
ATOM   6110  CB  PHE A 401       1.502  17.363   1.282  1.00  0.57
C
ATOM   6111  CG  PHE A 401       1.199  18.675   1.919  1.00  0.57
C
ATOM   6112  CD1 PHE A 401       2.085  19.240   2.824  1.00  0.57
C
ATOM   6113  CD2 PHE A 401       0.046  19.364   1.590  1.00  0.57
C
ATOM   6114  CE1 PHE A 401       1.808  20.462   3.397  1.00  0.57
C
ATOM   6115  CE2 PHE A 401      -0.229  20.587   2.160  1.00  0.57
C
ATOM   6116  CZ  PHE A 401       0.652  21.134   3.064  1.00  0.57
C
ATOM   6117  H   PHE A 401       2.241  15.123   0.468  1.00  0.32
H
ATOM   6118  HA  PHE A 401       3.452  17.097   2.021  1.00  0.46
H
ATOM   6119 1HB  PHE A 401       1.863  17.551   0.271  1.00  0.68
H
ATOM   6120 2HB  PHE A 401       0.575  16.807   1.195  1.00  0.68
H
ATOM   6121  HD1 PHE A 401       3.001  18.710   3.080  1.00  0.68
H
ATOM   6122  HD2 PHE A 401      -0.647  18.925   0.871  1.00  0.68
H
ATOM   6123  HE1 PHE A 401       2.504  20.898   4.111  1.00  0.68
H
ATOM   6124  HE2 PHE A 401      -1.140  21.122   1.898  1.00  0.68
H
ATOM   6125  HZ  PHE A 401       0.438  22.100   3.514  1.00  0.68
H
ATOM   6126  N   ASP A 402       1.320  15.343   3.802  1.00  0.30
N
ATOM   6127  CA  ASP A 402       0.947  15.088   5.187  1.00  0.32
C
ATOM   6128  C   ASP A 402       2.126  14.551   6.006  1.00  0.27
C
```

ATOM 6129 O ASP A 402 2.207 14.809 7.204 1.00 0.52
O
ATOM 6130 CB ASP A 402 -0.237 14.145 5.259 1.00 0.48
C
ATOM 6131 CG ASP A 402 -0.842 14.034 6.662 1.00 0.48
C
ATOM 6132 OD1 ASP A 402 -1.123 15.035 7.281 1.00 0.48
O
ATOM 6133 OD2 ASP A 402 -1.056 12.922 7.079 1.00 0.48
O
ATOM 6134 H ASP A 402 0.949 14.759 3.066 1.00 0.36
H
ATOM 6135 HA ASP A 402 0.639 16.033 5.632 1.00 0.38
H
ATOM 6136 1HB ASP A 402 -1.009 14.484 4.572 1.00 0.58
H
ATOM 6137 2HB ASP A 402 0.081 13.158 4.931 1.00 0.58
H
ATOM 6138 N GLN A 403 3.042 13.819 5.367 1.00 0.20
N
ATOM 6139 CA GLN A 403 4.226 13.340 6.074 1.00 0.25
C
ATOM 6140 C GLN A 403 5.142 14.507 6.371 1.00 0.35
C
ATOM 6141 O GLN A 403 5.770 14.555 7.430 1.00 0.45
O
ATOM 6142 CB GLN A 403 4.956 12.267 5.264 1.00 0.38
C
ATOM 6143 CG GLN A 403 4.256 10.922 5.247 1.00 0.38
C
ATOM 6144 CD GLN A 403 3.037 10.860 4.352 1.00 0.38
C
ATOM 6145 OE1 GLN A 403 2.668 11.818 3.667 1.00 0.38
O
ATOM 6146 NE2 GLN A 403 2.404 9.693 4.354 1.00 0.38
N
ATOM 6147 H GLN A 403 2.931 13.608 4.385 1.00 0.24
H
ATOM 6148 HA GLN A 403 3.917 12.905 7.024 1.00 0.30
H
ATOM 6149 1HB GLN A 403 5.071 12.601 4.233 1.00 0.45
H
ATOM 6150 2HB GLN A 403 5.955 12.119 5.674 1.00 0.45
H
ATOM 6151 1HG GLN A 403 4.965 10.174 4.894 1.00 0.45
H
ATOM 6152 2HG GLN A 403 3.947 10.682 6.259 1.00 0.45
H
ATOM 6153 1HE2 GLN A 403 1.609 9.539 3.760 1.00 0.45
H
ATOM 6154 2HE2 GLN A 403 2.744 8.944 4.921 1.00 0.45
H
ATOM 6155 N LEU A 404 5.175 15.467 5.451 1.00 0.41
N
ATOM 6156 CA LEU A 404 5.959 16.670 5.627 1.00 0.47
C
ATOM 6157 C LEU A 404 5.387 17.496 6.779 1.00 0.26
C

ATOM 6158 O LEU A 404 6.128 18.003 7.625 1.00 0.26
O
ATOM 6159 CB LEU A 404 5.930 17.481 4.330 1.00 0.70
C
ATOM 6160 CG LEU A 404 6.604 16.811 3.136 1.00 0.70
C
ATOM 6161 CD1 LEU A 404 6.372 17.639 1.884 1.00 0.70
C
ATOM 6162 CD2 LEU A 404 8.055 16.648 3.440 1.00 0.70
C
ATOM 6163 H LEU A 404 4.658 15.347 4.589 1.00 0.49
H
ATOM 6164 HA LEU A 404 6.985 16.391 5.865 1.00 0.56
H
ATOM 6165 1HB LEU A 404 4.899 17.693 4.060 1.00 0.85
H
ATOM 6166 2HB LEU A 404 6.441 18.425 4.504 1.00 0.85
H
ATOM 6167 HG LEU A 404 6.176 15.831 2.973 1.00 0.85
H
ATOM 6168 1HD1 LEU A 404 6.854 17.158 1.033 1.00 0.85
H
ATOM 6169 2HD1 LEU A 404 5.309 17.730 1.688 1.00 0.85
H
ATOM 6170 3HD1 LEU A 404 6.793 18.628 2.032 1.00 0.85
H
ATOM 6171 1HD2 LEU A 404 8.554 16.162 2.601 1.00 0.85
H
ATOM 6172 2HD2 LEU A 404 8.484 17.631 3.607 1.00 0.85
H
ATOM 6173 3HD2 LEU A 404 8.175 16.041 4.336 1.00 0.85
H
ATOM 6174 N ARG A 405 4.056 17.582 6.819 1.00 0.24
N
ATOM 6175 CA ARG A 405 3.337 18.283 7.868 1.00 0.22
C
ATOM 6176 C ARG A 405 3.660 17.694 9.230 1.00 0.21
C
ATOM 6177 O ARG A 405 3.976 18.428 10.169 1.00 0.24
O
ATOM 6178 CB ARG A 405 1.843 18.214 7.606 1.00 0.33
C
ATOM 6179 CG ARG A 405 0.965 18.984 8.574 1.00 0.33
C
ATOM 6180 CD ARG A 405 -0.462 18.884 8.173 1.00 0.33
C
ATOM 6181 NE ARG A 405 -1.021 17.580 8.468 1.00 0.33
N
ATOM 6182 CZ ARG A 405 -1.535 17.240 9.664 1.00 0.33
C
ATOM 6183 NH1 ARG A 405 -1.584 18.127 10.633 1.00 0.33
N
ATOM 6184 NH2 ARG A 405 -1.998 16.019 9.849 1.00 0.33
N
ATOM 6185 H ARG A 405 3.515 17.162 6.075 1.00 0.29
H
ATOM 6186 HA ARG A 405 3.645 19.328 7.859 1.00 0.26
H

```
ATOM   6187 1HB  ARG A 405       1.636  18.596   6.607  1.00  0.40
H
ATOM   6188 2HB  ARG A 405       1.514  17.177   7.630  1.00  0.40
H
ATOM   6189 1HG  ARG A 405       1.068  18.553   9.570  1.00  0.40
H
ATOM   6190 2HG  ARG A 405       1.264  20.029   8.603  1.00  0.40
H
ATOM   6191 1HD  ARG A 405      -1.043  19.633   8.709  1.00  0.40
H
ATOM   6192 2HD  ARG A 405      -0.549  19.057   7.101  1.00  0.40
H
ATOM   6193  HE  ARG A 405      -0.998  16.861   7.751  1.00  0.40
H
ATOM   6194 1HH1 ARG A 405      -1.233  19.062  10.478  1.00  0.40
H
ATOM   6195 2HH1 ARG A 405      -1.978  17.880  11.529  1.00  0.40
H
ATOM   6196 1HH2 ARG A 405      -1.939  15.360   9.078  1.00  0.40
H
ATOM   6197 2HH2 ARG A 405      -2.407  15.764  10.738  1.00  0.40
H
ATOM   6198  N   MET A 406       3.603  16.364   9.321  1.00  0.20
N
ATOM   6199  CA  MET A 406       3.909  15.675  10.561  1.00  0.20
C
ATOM   6200  C   MET A 406       5.385  15.788  10.917  1.00  0.18
C
ATOM   6201  O   MET A 406       5.729  15.770  12.096  1.00  0.19
O
ATOM   6202  CB  MET A 406       3.478  14.222  10.481  1.00  0.30
C
ATOM   6203  CG  MET A 406       1.966  14.014  10.426  1.00  0.30
C
ATOM   6204  SD  MET A 406       1.073  14.775  11.792  1.00  0.30
S
ATOM   6205  CE  MET A 406       1.717  13.881  13.194  1.00  0.30
C
ATOM   6206  H   MET A 406       3.317  15.816   8.518  1.00  0.24
H
ATOM   6207  HA  MET A 406       3.346  16.148  11.360  1.00  0.24
H
ATOM   6208 1HB  MET A 406       3.909  13.765   9.591  1.00  0.36
H
ATOM   6209 2HB  MET A 406       3.860  13.679  11.345  1.00  0.36
H
ATOM   6210 1HG  MET A 406       1.576  14.423   9.498  1.00  0.36
H
ATOM   6211 2HG  MET A 406       1.755  12.945  10.439  1.00  0.36
H
ATOM   6212 1HE  MET A 406       1.250  14.249  14.108  1.00  0.36
H
ATOM   6213 2HE  MET A 406       1.506  12.818  13.076  1.00  0.36
H
ATOM   6214 3HE  MET A 406       2.796  14.028  13.254  1.00  0.36
H
ATOM   6215  N   GLY A 407       6.254  15.918   9.908  1.00  0.17
N
```

ATOM 6216 CA GLY A 407 7.680 16.127 10.141 1.00 0.17
C
ATOM 6217 C GLY A 407 7.907 17.438 10.889 1.00 0.18
C
ATOM 6218 O GLY A 407 8.708 17.498 11.825 1.00 0.19
O
ATOM 6219 H GLY A 407 5.929 15.862 8.953 1.00 0.20
H
ATOM 6220 1HA GLY A 407 8.084 15.294 10.715 1.00 0.20
H
ATOM 6221 2HA GLY A 407 8.205 16.150 9.187 1.00 0.20
H
ATOM 6222 N ALA A 408 7.178 18.487 10.485 1.00 0.19
N
ATOM 6223 CA ALA A 408 7.242 19.779 11.172 1.00 0.21
C
ATOM 6224 C ALA A 408 6.733 19.635 12.600 1.00 0.22
C
ATOM 6225 O ALA A 408 7.361 20.109 13.546 1.00 0.23
O
ATOM 6226 CB ALA A 408 6.431 20.825 10.428 1.00 0.32
C
ATOM 6227 H ALA A 408 6.572 18.382 9.677 1.00 0.23
H
ATOM 6228 HA ALA A 408 8.285 20.095 11.212 1.00 0.25
H
ATOM 6229 1HB ALA A 408 6.508 21.782 10.944 1.00 0.38
H
ATOM 6230 2HB ALA A 408 6.826 20.923 9.420 1.00 0.38
H
ATOM 6231 3HB ALA A 408 5.388 20.520 10.383 1.00 0.38
H
ATOM 6232 N ILE A 409 5.621 18.923 12.752 1.00 0.23
N
ATOM 6233 CA ILE A 409 5.028 18.636 14.054 1.00 0.24
C
ATOM 6234 C ILE A 409 6.006 17.862 14.944 1.00 0.26
C
ATOM 6235 O ILE A 409 6.066 18.075 16.156 1.00 0.31
O
ATOM 6236 CB ILE A 409 3.720 17.863 13.874 1.00 0.36
C
ATOM 6237 CG1 ILE A 409 2.690 18.783 13.214 1.00 0.36
C
ATOM 6238 CG2 ILE A 409 3.221 17.371 15.218 1.00 0.36
C
ATOM 6239 CD1 ILE A 409 1.463 18.070 12.707 1.00 0.36
C
ATOM 6240 H ILE A 409 5.145 18.578 11.925 1.00 0.28
H
ATOM 6241 HA ILE A 409 4.801 19.582 14.544 1.00 0.29
H
ATOM 6242 HB ILE A 409 3.881 17.014 13.217 1.00 0.43
H
ATOM 6243 1HG1 ILE A 409 2.380 19.532 13.940 1.00 0.43
H
ATOM 6244 2HG1 ILE A 409 3.160 19.297 12.377 1.00 0.43
H

```
ATOM   6245 1HG2 ILE A 409       2.290  16.822  15.083  1.00  0.43
H
ATOM   6246 2HG2 ILE A 409       3.970  16.719  15.661  1.00  0.43
H
ATOM   6247 3HG2 ILE A 409       3.052  18.222  15.876  1.00  0.43
H
ATOM   6248 1HD1 ILE A 409       0.783  18.792  12.256  1.00  0.43
H
ATOM   6249 2HD1 ILE A 409       1.756  17.342  11.958  1.00  0.43
H
ATOM   6250 3HD1 ILE A 409       0.963  17.566  13.532  1.00  0.43
H
ATOM   6251  N   SER A 410       6.782  16.972  14.321  1.00  0.23
N
ATOM   6252  CA  SER A 410       7.773  16.146  14.994  1.00  0.23
C
ATOM   6253  C   SER A 410       9.092  16.878  15.259  1.00  0.25
C
ATOM   6254  O   SER A 410      10.018  16.279  15.806  1.00  0.33
O
ATOM   6255  CB  SER A 410       8.082  14.933  14.145  1.00  0.35
C
ATOM   6256  OG  SER A 410       6.943  14.147  13.957  1.00  0.35
O
ATOM   6257  H   SER A 410       6.661  16.834  13.330  1.00  0.28
H
ATOM   6258  HA  SER A 410       7.358  15.825  15.950  1.00  0.28
H
ATOM   6259 1HB  SER A 410       8.470  15.252  13.178  1.00  0.41
H
ATOM   6260 2HB  SER A 410       8.858  14.343  14.629  1.00  0.41
H
ATOM   6261  HG  SER A 410       6.369  14.654  13.371  1.00  0.41
H
ATOM   6262  N   GLN A 411       9.186  18.159  14.871  1.00  0.20
N
ATOM   6263  CA  GLN A 411      10.391  18.959  15.066  1.00  0.19
C
ATOM   6264  C   GLN A 411      11.601  18.320  14.401  1.00  0.18
C
ATOM   6265  O   GLN A 411      12.715  18.390  14.924  1.00  0.19
O
ATOM   6266  CB  GLN A 411      10.659  19.174  16.559  1.00  0.29
C
ATOM   6267  CG  GLN A 411       9.549  19.913  17.287  1.00  0.29
C
ATOM   6268  CD  GLN A 411       9.887  20.171  18.744  1.00  0.29
C
ATOM   6269  OE1 GLN A 411      11.048  20.075  19.151  1.00  0.29
O
ATOM   6270  NE2 GLN A 411       8.876  20.504  19.537  1.00  0.29
N
ATOM   6271  H   GLN A 411       8.406  18.622  14.429  1.00  0.24
H
ATOM   6272  HA  GLN A 411      10.233  19.932  14.602  1.00  0.23
H
ATOM   6273 1HB  GLN A 411      10.809  18.215  17.053  1.00  0.34
H
```

```
ATOM   6274 2HB  GLN A 411      11.577  19.748  16.681  1.00  0.34
H
ATOM   6275 1HG  GLN A 411       9.389  20.875  16.799  1.00  0.34
H
ATOM   6276 2HG  GLN A 411       8.638  19.319  17.245  1.00  0.34
H
ATOM   6277 1HE2 GLN A 411       9.040  20.689  20.507  1.00  0.34
H
ATOM   6278 2HE2 GLN A 411       7.950  20.573  19.165  1.00  0.34
H
ATOM   6279  N   ALA A 412      11.374  17.680  13.257  1.00  0.18
N
ATOM   6280  CA  ALA A 412      12.431  16.999  12.537  1.00  0.19
C
ATOM   6281  C   ALA A 412      13.310  17.972  11.786  1.00  0.20
C
ATOM   6282  O   ALA A 412      12.906  19.095  11.481  1.00  0.24
O
ATOM   6283  CB  ALA A 412      11.847  15.997  11.579  1.00  0.29
C
ATOM   6284  H   ALA A 412      10.441  17.662  12.866  1.00  0.22
H
ATOM   6285  HA  ALA A 412      13.052  16.478  13.264  1.00  0.23
H
ATOM   6286 1HB  ALA A 412      12.652  15.470  11.065  1.00  0.34
H
ATOM   6287 2HB  ALA A 412      11.240  15.279  12.131  1.00  0.34
H
ATOM   6288 3HB  ALA A 412      11.226  16.514  10.850  1.00  0.34
H
ATOM   6289  N   ASN A 413      14.515  17.520  11.487  1.00  0.26
N
ATOM   6290  CA  ASN A 413      15.485  18.320  10.773  1.00  0.31
C
ATOM   6291  C   ASN A 413      15.868  17.622   9.474  1.00  0.49
C
ATOM   6292  O   ASN A 413      16.931  17.006   9.392  1.00  1.69
O
ATOM   6293  CB  ASN A 413      16.702  18.561  11.640  1.00  0.46
C
ATOM   6294  CG  ASN A 413      16.359  19.248  12.936  1.00  0.46
C
ATOM   6295  OD1 ASN A 413      15.857  20.377  12.971  1.00  0.46
O
ATOM   6296  ND2 ASN A 413      16.625  18.571  14.024  1.00  0.46
N
ATOM   6297  H   ASN A 413      14.772  16.586  11.771  1.00  0.31
H
ATOM   6298  HA  ASN A 413      15.035  19.275  10.521  1.00  0.37
H
ATOM   6299 1HB  ASN A 413      17.184  17.608  11.864  1.00  0.56
H
ATOM   6300 2HB  ASN A 413      17.422  19.173  11.096  1.00  0.56
H
ATOM   6301 1HD2 ASN A 413      16.422  18.965  14.921  1.00  0.56
H
ATOM   6302 2HD2 ASN A 413      17.029  17.659  13.959  1.00  0.56
H
```

ATOM	6303	N	ILE	A	414	14.993	17.685	8.468	1.00	0.58	N
ATOM	6304	CA	ILE	A	414	15.290	17.009	7.206	1.00	0.80	C
ATOM	6305	C	ILE	A	414	15.376	17.985	6.044	1.00	0.52	C
ATOM	6306	O	ILE	A	414	14.720	19.030	6.039	1.00	0.99	O
ATOM	6307	CB	ILE	A	414	14.232	15.927	6.881	1.00	1.20	C
ATOM	6308	CG1	ILE	A	414	12.845	16.559	6.720	1.00	1.20	C
ATOM	6309	CG2	ILE	A	414	14.207	14.866	7.974	1.00	1.20	C
ATOM	6310	CD1	ILE	A	414	11.809	15.590	6.189	1.00	1.20	C
ATOM	6311	H	ILE	A	414	14.133	18.204	8.576	1.00	0.70	H
ATOM	6312	HA	ILE	A	414	16.256	16.515	7.299	1.00	0.96	H
ATOM	6313	HB	ILE	A	414	14.485	15.454	5.933	1.00	1.44	H
ATOM	6314	1HG1	ILE	A	414	12.507	16.931	7.687	1.00	1.44	H
ATOM	6315	2HG1	ILE	A	414	12.911	17.402	6.032	1.00	1.44	H
ATOM	6316	1HG2	ILE	A	414	13.471	14.103	7.723	1.00	1.44	H
ATOM	6317	2HG2	ILE	A	414	15.191	14.407	8.057	1.00	1.44	H
ATOM	6318	3HG2	ILE	A	414	13.941	15.328	8.925	1.00	1.44	H
ATOM	6319	1HD1	ILE	A	414	10.847	16.091	6.097	1.00	1.44	H
ATOM	6320	2HD1	ILE	A	414	12.123	15.224	5.214	1.00	1.44	H
ATOM	6321	3HD1	ILE	A	414	11.717	14.750	6.873	1.00	1.44	H
ATOM	6322	N	ASN	A	415	16.186	17.614	5.059	1.00	0.31	N
ATOM	6323	CA	ASN	A	415	16.403	18.417	3.877	1.00	0.31	C
ATOM	6324	C	ASN	A	415	15.739	17.749	2.688	1.00	0.24	C
ATOM	6325	O	ASN	A	415	15.830	16.532	2.522	1.00	0.50	O
ATOM	6326	CB	ASN	A	415	17.884	18.595	3.661	1.00	0.46	C
ATOM	6327	CG	ASN	A	415	18.540	19.304	4.813	1.00	0.46	C
ATOM	6328	OD1	ASN	A	415	18.149	20.408	5.194	1.00	0.46	O
ATOM	6329	ND2	ASN	A	415	19.529	18.679	5.392	1.00	0.46	N
ATOM	6330	H	ASN	A	415	16.692	16.742	5.129	1.00	0.37	H
ATOM	6331	HA	ASN	A	415	15.936	19.392	4.018	1.00	0.37	H

```
ATOM   6332 1HB  ASN A 415      18.350  17.618   3.540  1.00  0.56
H
ATOM   6333 2HB  ASN A 415      18.057  19.162   2.747  1.00  0.56
H
ATOM   6334 1HD2 ASN A 415      19.998  19.101   6.169  1.00  0.56
H
ATOM   6335 2HD2 ASN A 415      19.816  17.781   5.059  1.00  0.56
H
ATOM   6336  N   LEU A 416      15.046  18.540   1.888  1.00  0.27
N
ATOM   6337  CA  LEU A 416      14.321  18.027   0.749  1.00  0.18
C
ATOM   6338  C   LEU A 416      14.679  18.818  -0.500  1.00  0.28
C
ATOM   6339  O   LEU A 416      14.673  20.043  -0.465  1.00  0.60
O
ATOM   6340  CB  LEU A 416      12.845  18.160   1.058  1.00  0.27
C
ATOM   6341  CG  LEU A 416      12.413  17.483   2.357  1.00  0.27
C
ATOM   6342  CD1 LEU A 416      11.013  17.893   2.651  1.00  0.27
C
ATOM   6343  CD2 LEU A 416      12.532  15.978   2.241  1.00  0.27
C
ATOM   6344  H   LEU A 416      15.008  19.525   2.087  1.00  0.32
H
ATOM   6345  HA  LEU A 416      14.581  16.983   0.602  1.00  0.22
H
ATOM   6346 1HB  LEU A 416      12.603  19.215   1.150  1.00  0.32
H
ATOM   6347 2HB  LEU A 416      12.265  17.734   0.240  1.00  0.32
H
ATOM   6348  HG  LEU A 416      13.037  17.834   3.178  1.00  0.32
H
ATOM   6349 1HD1 LEU A 416      10.703  17.437   3.589  1.00  0.32
H
ATOM   6350 2HD1 LEU A 416      10.968  18.978   2.740  1.00  0.32
H
ATOM   6351 3HD1 LEU A 416      10.360  17.564   1.844  1.00  0.32
H
ATOM   6352 1HD2 LEU A 416      12.216  15.514   3.172  1.00  0.32
H
ATOM   6353 2HD2 LEU A 416      11.902  15.628   1.437  1.00  0.32
H
ATOM   6354 3HD2 LEU A 416      13.565  15.710   2.035  1.00  0.32
H
ATOM   6355  N   ILE A 417      14.963  18.137  -1.600  1.00  0.22
N
ATOM   6356  CA  ILE A 417      15.284  18.850  -2.832  1.00  0.23
C
ATOM   6357  C   ILE A 417      14.317  18.408  -3.914  1.00  0.14
C
ATOM   6358  O   ILE A 417      14.260  17.224  -4.256  1.00  0.26
O
ATOM   6359  CB  ILE A 417      16.748  18.617  -3.283  1.00  0.35
C
ATOM   6360  CG1 ILE A 417      17.729  19.066  -2.187  1.00  0.35
C
```

```
ATOM   6361  CG2 ILE A 417      17.017  19.404  -4.563  1.00  0.35
C
ATOM   6362  CD1 ILE A 417      19.169  18.703  -2.468  1.00  0.35
C
ATOM   6363  H   ILE A 417      14.958  17.126  -1.580  1.00  0.26
H
ATOM   6364  HA  ILE A 417      15.147  19.917  -2.669  1.00  0.28
H
ATOM   6365  HB  ILE A 417      16.912  17.556  -3.463  1.00  0.41
H
ATOM   6366 1HG1 ILE A 417      17.666  20.144  -2.073  1.00  0.41
H
ATOM   6367 2HG1 ILE A 417      17.445  18.605  -1.242  1.00  0.41
H
ATOM   6368 1HG2 ILE A 417      18.045  19.236  -4.884  1.00  0.41
H
ATOM   6369 2HG2 ILE A 417      16.335  19.071  -5.345  1.00  0.41
H
ATOM   6370 3HG2 ILE A 417      16.864  20.467  -4.375  1.00  0.41
H
ATOM   6371 1HD1 ILE A 417      19.800  19.052  -1.652  1.00  0.41
H
ATOM   6372 2HD1 ILE A 417      19.260  17.621  -2.560  1.00  0.41
H
ATOM   6373 3HD1 ILE A 417      19.487  19.174  -3.397  1.00  0.41
H
ATOM   6374  N   GLY A 418      13.547  19.361  -4.428  1.00  0.13
N
ATOM   6375  CA  GLY A 418      12.527  19.058  -5.420  1.00  0.17
C
ATOM   6376  C   GLY A 418      12.902  19.579  -6.791  1.00  0.33
C
ATOM   6377  O   GLY A 418      13.125  20.779  -6.966  1.00  1.13
O
ATOM   6378  H   GLY A 418      13.685  20.316  -4.124  1.00  0.16
H
ATOM   6379 1HA  GLY A 418      12.371  17.985  -5.462  1.00  0.20
H
ATOM   6380 2HA  GLY A 418      11.583  19.500  -5.110  1.00  0.20
H
ATOM   6381  N   SER A 419      12.932  18.661  -7.759  1.00  0.19
N
ATOM   6382  CA  SER A 419      13.325  18.955  -9.129  1.00  0.22
C
ATOM   6383  C   SER A 419      12.190  19.194 -10.082  1.00  0.21
C
ATOM   6384  O   SER A 419      11.026  19.017  -9.739  1.00  0.35
O
ATOM   6385  CB  SER A 419      14.200  17.827  -9.648  1.00  0.33
C
ATOM   6386  OG  SER A 419      13.461  16.663  -9.873  1.00  0.33
O
ATOM   6387  H   SER A 419      12.704  17.707  -7.513  1.00  0.23
H
ATOM   6388  HA  SER A 419      13.875  19.871  -9.124  1.00  0.26
H
ATOM   6389 1HB  SER A 419      14.687  18.127 -10.567  1.00  0.40
H
```

```
ATOM   6390 2HB  SER A 419      14.981  17.624  -8.917  1.00  0.40
H
ATOM   6391  HG  SER A 419      13.112  16.750 -10.766  1.00  0.40
H
ATOM   6392  N   HIS A 420      12.532  19.649 -11.286  1.00  0.20
N
ATOM   6393  CA  HIS A 420      11.529  19.904 -12.307  1.00  0.28
C
ATOM   6394  C   HIS A 420      10.490  20.928 -11.874  1.00  0.27
C
ATOM   6395  O   HIS A 420       9.286  20.725 -12.064  1.00  0.34
O
ATOM   6396  CB  HIS A 420      10.866  18.583 -12.711  1.00  0.42
C
ATOM   6397  CG  HIS A 420      11.858  17.673 -13.337  1.00  0.42
C
ATOM   6398  ND1 HIS A 420      12.750  16.930 -12.601  1.00  0.42
N
ATOM   6399  CD2 HIS A 420      12.114  17.396 -14.635  1.00  0.42
C
ATOM   6400  CE1 HIS A 420      13.525  16.245 -13.420  1.00  0.42
C
ATOM   6401  NE2 HIS A 420      13.156  16.505 -14.660  1.00  0.42
N
ATOM   6402  H   HIS A 420      13.510  19.775 -11.528  1.00  0.24
H
ATOM   6403  HA  HIS A 420      12.024  20.303 -13.191  1.00  0.34
H
ATOM   6404 1HB  HIS A 420      10.435  18.084 -11.844  1.00  0.50
H
ATOM   6405 2HB  HIS A 420      10.062  18.764 -13.420  1.00  0.50
H
ATOM   6406  HD2 HIS A 420      11.597  17.810 -15.501  1.00  0.50
H
ATOM   6407  HE1 HIS A 420      14.331  15.585 -13.117  1.00  0.50
H
ATOM   6408  HE2 HIS A 420      13.573  16.121 -15.496  1.00  0.50
H
ATOM   6409  N   CYS A 421      10.959  22.038 -11.317  1.00  0.23
N
ATOM   6410  CA  CYS A 421      10.045  23.080 -10.905  1.00  0.33
C
ATOM   6411  C   CYS A 421       9.606  23.927 -12.083  1.00  0.32
C
ATOM   6412  O   CYS A 421      10.432  24.366 -12.885  1.00  0.98
O
ATOM   6413  CB  CYS A 421      10.698  23.992  -9.863  1.00  0.49
C
ATOM   6414  SG  CYS A 421      11.097  23.183  -8.301  1.00  0.49
S
ATOM   6415  H   CYS A 421      11.954  22.160 -11.157  1.00  0.28
H
ATOM   6416  HA  CYS A 421       9.169  22.615 -10.468  1.00  0.40
H
ATOM   6417 1HB  CYS A 421      11.622  24.402 -10.272  1.00  0.59
H
ATOM   6418 2HB  CYS A 421      10.037  24.831  -9.647  1.00  0.59
H
```

```
ATOM   6419  HG  CYS A 421       9.836  22.968  -7.947  1.00  0.59
H
ATOM   6420  N   GLY A 422       8.304  24.168 -12.179  1.00  0.20
N
ATOM   6421  CA  GLY A 422       7.780  25.065 -13.192  1.00  0.22
C
ATOM   6422  C   GLY A 422       7.509  24.499 -14.588  1.00  0.30
C
ATOM   6423  O   GLY A 422       7.879  23.369 -14.917  1.00  0.42
O
ATOM   6424  H   GLY A 422       7.668  23.779 -11.497  1.00  0.24
H
ATOM   6425 1HA  GLY A 422       6.878  25.523 -12.811  1.00  0.26
H
ATOM   6426 2HA  GLY A 422       8.498  25.870 -13.289  1.00  0.26
H
ATOM   6427  N   VAL A 423       6.894  25.347 -15.428  1.00  0.41
N
ATOM   6428  CA  VAL A 423       6.567  25.009 -16.808  1.00  0.74
C
ATOM   6429  C   VAL A 423       7.831  24.922 -17.647  1.00  0.51
C
ATOM   6430  O   VAL A 423       7.836  24.257 -18.683  1.00  0.63
O
ATOM   6431  CB  VAL A 423       5.605  26.041 -17.443  1.00  1.11
C
ATOM   6432  CG1 VAL A 423       4.351  26.147 -16.591  1.00  1.11
C
ATOM   6433  CG2 VAL A 423       6.294  27.383 -17.626  1.00  1.11
C
ATOM   6434  H   VAL A 423       6.640  26.262 -15.080  1.00  0.49
H
ATOM   6435  HA  VAL A 423       6.086  24.043 -16.812  1.00  0.89
H
ATOM   6436  HB  VAL A 423       5.293  25.670 -18.419  1.00  1.33
H
ATOM   6437 1HG1 VAL A 423       3.651  26.844 -17.047  1.00  1.33
H
ATOM   6438 2HG1 VAL A 423       3.886  25.168 -16.509  1.00  1.33
H
ATOM   6439 3HG1 VAL A 423       4.620  26.506 -15.598  1.00  1.33
H
ATOM   6440 1HG2 VAL A 423       5.606  28.088 -18.087  1.00  1.33
H
ATOM   6441 2HG2 VAL A 423       6.605  27.768 -16.661  1.00  1.33
H
ATOM   6442 3HG2 VAL A 423       7.166  27.264 -18.266  1.00  1.33
H
ATOM   6443  N   SER A 424       8.909  25.573 -17.182  1.00  0.33
N
ATOM   6444  CA  SER A 424      10.225  25.533 -17.831  1.00  0.28
C
ATOM   6445  C   SER A 424      10.765  24.114 -18.136  1.00  0.29
C
ATOM   6446  O   SER A 424      11.668  23.960 -18.959  1.00  0.34
O
ATOM   6447  CB  SER A 424      11.230  26.273 -16.980  1.00  0.42
C
```

```
ATOM   6448  OG  SER A 424      11.463  25.602 -15.772  1.00  0.42
O
ATOM   6449  H   SER A 424       8.824  26.141 -16.340  1.00  0.40
H
ATOM   6450  HA  SER A 424      10.133  26.061 -18.782  1.00  0.34
H
ATOM   6451 1HB  SER A 424      12.162  26.378 -17.530  1.00  0.50
H
ATOM   6452 2HB  SER A 424      10.856  27.274 -16.774  1.00  0.50
H
ATOM   6453  HG  SER A 424      10.661  25.713 -15.251  1.00  0.50
H
ATOM   6454  N   THR A 425      10.194  23.069 -17.512  1.00  0.32
N
ATOM   6455  CA  THR A 425      10.610  21.696 -17.802  1.00  0.37
C
ATOM   6456  C   THR A 425      10.254  21.280 -19.236  1.00  0.35
C
ATOM   6457  O   THR A 425      10.865  20.365 -19.791  1.00  0.38
O
ATOM   6458  CB  THR A 425       9.953  20.724 -16.829  1.00  0.55
C
ATOM   6459  OG1 THR A 425       8.533  20.805 -16.971  1.00  0.55
O
ATOM   6460  CG2 THR A 425      10.328  21.090 -15.415  1.00  0.55
C
ATOM   6461  H   THR A 425       9.469  23.213 -16.814  1.00  0.38
H
ATOM   6462  HA  THR A 425      11.691  21.631 -17.688  1.00  0.44
H
ATOM   6463  HB  THR A 425      10.277  19.705 -17.045  1.00  0.67
H
ATOM   6464  HG1 THR A 425       8.236  21.664 -16.659  1.00  0.67
H
ATOM   6465 1HG2 THR A 425       9.841  20.403 -14.728  1.00  0.67
H
ATOM   6466 2HG2 THR A 425      11.408  21.028 -15.295  1.00  0.67
H
ATOM   6467 3HG2 THR A 425       9.998  22.106 -15.202  1.00  0.67
H
ATOM   6468  N   GLY A 426       9.287  21.968 -19.840  1.00  0.44
N
ATOM   6469  CA  GLY A 426       8.926  21.731 -21.227  1.00  0.40
C
ATOM   6470  C   GLY A 426       8.018  20.535 -21.502  1.00  1.12
C
ATOM   6471  O   GLY A 426       6.955  20.377 -20.899  1.00  2.41
O
ATOM   6472  H   GLY A 426       8.802  22.699 -19.340  1.00  0.53
H
ATOM   6473 1HA  GLY A 426       8.454  22.628 -21.621  1.00  0.48
H
ATOM   6474 2HA  GLY A 426       9.844  21.609 -21.801  1.00  0.48
H
ATOM   6475  N   GLU A 427       8.465  19.731 -22.468  1.00  5.01
N
ATOM   6476  CA  GLU A 427       7.752  18.606 -23.082  1.00 10.12
C
```

```
ATOM   6477  C   GLU A 427       7.112  17.551 -22.189  1.00  6.93
C
ATOM   6478  O   GLU A 427       6.292  16.780 -22.678  1.00 11.00
O
ATOM   6479  CB  GLU A 427       8.705  17.862 -24.005  1.00 15.18
C
ATOM   6480  CG  GLU A 427       9.135  18.611 -25.252  1.00 15.18
C
ATOM   6481  CD  GLU A 427      10.093  17.782 -26.048  1.00 15.18
C
ATOM   6482  OE1 GLU A 427      10.385  16.701 -25.597  1.00 15.18
O
ATOM   6483  OE2 GLU A 427      10.553  18.218 -27.076  1.00 15.18
O
ATOM   6484  H   GLU A 427       9.375  19.949 -22.850  1.00  6.01
H
ATOM   6485  HA  GLU A 427       6.955  19.033 -23.693  1.00 12.14
H
ATOM   6486 1HB  GLU A 427       9.607  17.601 -23.452  1.00 18.22
H
ATOM   6487 2HB  GLU A 427       8.240  16.929 -24.324  1.00 18.22
H
ATOM   6488 1HG  GLU A 427       8.260  18.837 -25.861  1.00 18.22
H
ATOM   6489 2HG  GLU A 427       9.607  19.550 -24.966  1.00 18.22
H
ATOM   6490  N   ASP A 428       7.478  17.465 -20.919  1.00  3.28
N
ATOM   6491  CA  ASP A 428       6.833  16.479 -20.062  1.00  2.23
C
ATOM   6492  C   ASP A 428       5.394  16.869 -19.722  1.00  2.08
C
ATOM   6493  O   ASP A 428       4.523  16.008 -19.593  1.00  4.32
O
ATOM   6494  CB  ASP A 428       7.636  16.256 -18.788  1.00  3.34
C
ATOM   6495  CG  ASP A 428       8.919  15.478 -19.047  1.00  3.34
C
ATOM   6496  OD1 ASP A 428       9.016  14.860 -20.082  1.00  3.34
O
ATOM   6497  OD2 ASP A 428       9.786  15.503 -18.208  1.00  3.34
O
ATOM   6498  H   ASP A 428       8.177  18.085 -20.537  1.00  3.94
H
ATOM   6499  HA  ASP A 428       6.811  15.539 -20.603  1.00  2.68
H
ATOM   6500 1HB  ASP A 428       7.892  17.218 -18.343  1.00  4.01
H
ATOM   6501 2HB  ASP A 428       7.031  15.709 -18.069  1.00  4.01
H
ATOM   6502  N   GLY A 429       5.157  18.173 -19.583  1.00  1.50
N
ATOM   6503  CA  GLY A 429       3.844  18.709 -19.249  1.00  2.03
C
ATOM   6504  C   GLY A 429       3.529  18.747 -17.778  1.00  1.13
C
ATOM   6505  O   GLY A 429       4.308  18.269 -16.953  1.00  0.73
O
```

```
ATOM   6506  H   GLY A 429       5.910  18.829 -19.749  1.00  1.80
H
ATOM   6507 1HA  GLY A 429       3.757  19.714 -19.663  1.00  2.44
H
ATOM   6508 2HA  GLY A 429       3.094  18.135 -19.711  1.00  2.44
H
ATOM   6509  N   VAL A 430       2.358  19.313 -17.478  1.00  1.07
N
ATOM   6510  CA  VAL A 430       1.895  19.594 -16.119  1.00  0.75
C
ATOM   6511  C   VAL A 430       2.000  18.497 -15.072  1.00  0.51
C
ATOM   6512  O   VAL A 430       2.211  18.803 -13.898  1.00  0.44
O
ATOM   6513  CB  VAL A 430       0.436  20.050 -16.156  1.00  1.12
C
ATOM   6514  CG1 VAL A 430      -0.486  18.931 -16.601  1.00  1.12
C
ATOM   6515  CG2 VAL A 430       0.080  20.534 -14.762  1.00  1.12
C
ATOM   6516  H   VAL A 430       1.776  19.622 -18.242  1.00  1.28
H
ATOM   6517  HA  VAL A 430       2.478  20.438 -15.763  1.00  0.90
H
ATOM   6518  HB  VAL A 430       0.322  20.858 -16.874  1.00  1.35
H
ATOM   6519 1HG1 VAL A 430      -1.511  19.293 -16.619  1.00  1.35
H
ATOM   6520 2HG1 VAL A 430      -0.202  18.602 -17.600  1.00  1.35
H
ATOM   6521 3HG1 VAL A 430      -0.418  18.093 -15.911  1.00  1.35
H
ATOM   6522 1HG2 VAL A 430      -0.949  20.881 -14.736  1.00  1.35
H
ATOM   6523 2HG2 VAL A 430       0.203  19.716 -14.058  1.00  1.35
H
ATOM   6524 3HG2 VAL A 430       0.744  21.350 -14.480  1.00  1.35
H
ATOM   6525  N   SER A 431       1.911  17.229 -15.453  1.00  0.52
N
ATOM   6526  CA  SER A 431       1.993  16.182 -14.442  1.00  0.36
C
ATOM   6527  C   SER A 431       3.369  16.135 -13.779  1.00  0.36
C
ATOM   6528  O   SER A 431       3.507  15.632 -12.660  1.00  0.61
O
ATOM   6529  CB  SER A 431       1.699  14.830 -15.046  1.00  0.54
C
ATOM   6530  OG  SER A 431       2.745  14.420 -15.886  1.00  0.54
O
ATOM   6531  H   SER A 431       1.761  16.991 -16.423  1.00  0.62
H
ATOM   6532  HA  SER A 431       1.248  16.390 -13.673  1.00  0.43
H
ATOM   6533 1HB  SER A 431       1.562  14.101 -14.249  1.00  0.65
H
ATOM   6534 2HB  SER A 431       0.771  14.876 -15.612  1.00  0.65
H
```

ATOM 6535 HG SER A 431 2.757 15.040 -16.619 1.00 0.65
H
ATOM 6536 N GLN A 432 4.386 16.669 -14.465 1.00 0.38
N
ATOM 6537 CA GLN A 432 5.742 16.673 -13.948 1.00 0.66
C
ATOM 6538 C GLN A 432 6.203 18.103 -13.619 1.00 0.47
C
ATOM 6539 O GLN A 432 7.399 18.339 -13.448 1.00 0.74
O
ATOM 6540 CB GLN A 432 6.688 16.072 -14.993 1.00 0.99
C
ATOM 6541 CG GLN A 432 6.273 14.697 -15.499 1.00 0.99
C
ATOM 6542 CD GLN A 432 6.193 13.618 -14.445 1.00 0.99
C
ATOM 6543 OE1 GLN A 432 7.169 13.290 -13.769 1.00 0.99
O
ATOM 6544 NE2 GLN A 432 5.000 13.052 -14.304 1.00 0.99
N
ATOM 6545 H GLN A 432 4.225 17.087 -15.370 1.00 0.46
H
ATOM 6546 HA GLN A 432 5.776 16.079 -13.037 1.00 0.79
H
ATOM 6547 1HB GLN A 432 6.749 16.741 -15.853 1.00 1.19
H
ATOM 6548 2HB GLN A 432 7.689 15.988 -14.573 1.00 1.19
H
ATOM 6549 1HG GLN A 432 5.291 14.785 -15.959 1.00 1.19
H
ATOM 6550 2HG GLN A 432 6.999 14.377 -16.244 1.00 1.19
H
ATOM 6551 1HE2 GLN A 432 4.862 12.327 -13.628 1.00 1.19
H
ATOM 6552 2HE2 GLN A 432 4.235 13.366 -14.873 1.00 1.19
H
ATOM 6553 N MET A 433 5.262 19.055 -13.555 1.00 0.47
N
ATOM 6554 CA MET A 433 5.591 20.460 -13.308 1.00 0.49
C
ATOM 6555 C MET A 433 5.172 20.919 -11.937 1.00 0.53
C
ATOM 6556 O MET A 433 3.984 20.917 -11.611 1.00 1.13
O
ATOM 6557 CB MET A 433 4.908 21.357 -14.337 1.00 0.73
C
ATOM 6558 CG MET A 433 5.395 21.194 -15.757 1.00 0.73
C
ATOM 6559 SD MET A 433 4.407 22.131 -16.932 1.00 0.73
S
ATOM 6560 CE MET A 433 5.333 21.878 -18.438 1.00 0.73
C
ATOM 6561 H MET A 433 4.288 18.818 -13.680 1.00 0.56
H
ATOM 6562 HA MET A 433 6.671 20.584 -13.385 1.00 0.59
H
ATOM 6563 1HB MET A 433 3.837 21.164 -14.322 1.00 0.88
H

```
ATOM   6564 2HB  MET A 433       5.054  22.400 -14.057  1.00  0.88
H
ATOM   6565 1HG  MET A 433       6.422  21.542 -15.819  1.00  0.88
H
ATOM   6566 2HG  MET A 433       5.381  20.146 -16.037  1.00  0.88
H
ATOM   6567 1HE  MET A 433       4.829  22.382 -19.261  1.00  0.88
H
ATOM   6568 2HE  MET A 433       6.338  22.283 -18.320  1.00  0.88
H
ATOM   6569 3HE  MET A 433       5.404  20.815 -18.652  1.00  0.88
H
ATOM   6570  N   ALA A 434       6.133  21.357 -11.139  1.00  0.24
N
ATOM   6571  CA  ALA A 434       5.767  21.845  -9.827  1.00  0.27
C
ATOM   6572  C   ALA A 434       5.414  23.305  -9.883  1.00  0.42
C
ATOM   6573  O   ALA A 434       6.259  24.153 -10.166  1.00  1.76
O
ATOM   6574  CB  ALA A 434       6.871  21.645  -8.850  1.00  0.41
C
ATOM   6575  H   ALA A 434       7.108  21.329 -11.435  1.00  0.29
H
ATOM   6576  HA  ALA A 434       4.890  21.293  -9.492  1.00  0.32
H
ATOM   6577 1HB  ALA A 434       6.556  21.993  -7.868  1.00  0.49
H
ATOM   6578 2HB  ALA A 434       7.109  20.590  -8.804  1.00  0.49
H
ATOM   6579 3HB  ALA A 434       7.746  22.200  -9.161  1.00  0.49
H
ATOM   6580  N   LEU A 435       4.158  23.595  -9.626  1.00  0.61
N
ATOM   6581  CA  LEU A 435       3.676  24.958  -9.625  1.00  0.46
C
ATOM   6582  C   LEU A 435       2.911  25.233  -8.337  1.00  0.85
C
ATOM   6583  O   LEU A 435       2.302  26.288  -8.177  1.00  2.89
O
ATOM   6584  CB  LEU A 435       2.800  25.180 -10.859  1.00  0.69
C
ATOM   6585  CG  LEU A 435       3.504  25.078 -12.219  1.00  0.69
C
ATOM   6586  CD1 LEU A 435       2.489  25.056 -13.326  1.00  0.69
C
ATOM   6587  CD2 LEU A 435       4.391  26.280 -12.408  1.00  0.69
C
ATOM   6588  H   LEU A 435       3.515  22.843  -9.426  1.00  0.73
H
ATOM   6589  HA  LEU A 435       4.528  25.632  -9.672  1.00  0.55
H
ATOM   6590 1HB  LEU A 435       2.021  24.424 -10.854  1.00  0.83
H
ATOM   6591 2HB  LEU A 435       2.335  26.162 -10.796  1.00  0.83
H
ATOM   6592  HG  LEU A 435       4.092  24.162 -12.268  1.00  0.83
H
```

```
ATOM   6593 1HD1 LEU A 435       3.008  24.992 -14.280  1.00  0.83
H
ATOM   6594 2HD1 LEU A 435       1.834  24.194 -13.208  1.00  0.83
H
ATOM   6595 3HD1 LEU A 435       1.902  25.970 -13.293  1.00  0.83
H
ATOM   6596 1HD2 LEU A 435       4.887  26.218 -13.376  1.00  0.83
H
ATOM   6597 2HD2 LEU A 435       3.787  27.187 -12.368  1.00  0.83
H
ATOM   6598 3HD2 LEU A 435       5.141  26.306 -11.617  1.00  0.83
H
ATOM   6599  N   GLU A 436       2.953  24.270  -7.413  1.00  0.46
N
ATOM   6600  CA  GLU A 436       2.294  24.388  -6.120  1.00  0.58
C
ATOM   6601  C   GLU A 436       3.250  24.164  -4.950  1.00  0.41
C
ATOM   6602  O   GLU A 436       2.841  24.287  -3.794  1.00  0.80
O
ATOM   6603  CB  GLU A 436       1.157  23.377  -6.028  1.00  0.87
C
ATOM   6604  CG  GLU A 436       0.091  23.580  -7.075  1.00  0.87
C
ATOM   6605  CD  GLU A 436      -1.038  22.613  -7.006  1.00  0.87
C
ATOM   6606  OE1 GLU A 436      -1.840  22.722  -6.111  1.00  0.87
O
ATOM   6607  OE2 GLU A 436      -1.109  21.767  -7.860  1.00  0.87
O
ATOM   6608  H   GLU A 436       3.462  23.422  -7.604  1.00  0.55
H
ATOM   6609  HA  GLU A 436       1.883  25.394  -6.034  1.00  0.70
H
ATOM   6610 1HB  GLU A 436       1.551  22.365  -6.131  1.00  1.04
H
ATOM   6611 2HB  GLU A 436       0.687  23.452  -5.049  1.00  1.04
H
ATOM   6612 1HG  GLU A 436      -0.306  24.584  -6.956  1.00  1.04
H
ATOM   6613 2HG  GLU A 436       0.551  23.514  -8.059  1.00  1.04
H
ATOM   6614  N   ASP A 437       4.502  23.800  -5.251  1.00  0.46
N
ATOM   6615  CA  ASP A 437       5.458  23.415  -4.213  1.00  0.38
C
ATOM   6616  C   ASP A 437       5.820  24.546  -3.271  1.00  0.48
C
ATOM   6617  O   ASP A 437       5.957  24.325  -2.073  1.00  2.61
O
ATOM   6618  CB  ASP A 437       6.749  22.872  -4.841  1.00  0.57
C
ATOM   6619  CG  ASP A 437       7.459  23.882  -5.745  1.00  0.57
C
ATOM   6620  OD1 ASP A 437       6.827  24.799  -6.215  1.00  0.57
O
ATOM   6621  OD2 ASP A 437       8.648  23.757  -5.916  1.00  0.57
O
```

ATOM	6622	H	ASP	A	437	4.793	23.755	-6.214	1.00	0.55	H
ATOM	6623	HA	ASP	A	437	5.005	22.619	-3.620	1.00	0.46	H
ATOM	6624	1HB	ASP	A	437	7.437	22.571	-4.051	1.00	0.68	H
ATOM	6625	2HB	ASP	A	437	6.519	21.983	-5.428	1.00	0.68	H
ATOM	6626	N	LEU	A	438	5.951	25.755	-3.789	1.00	0.63	N
ATOM	6627	CA	LEU	A	438	6.255	26.880	-2.921	1.00	0.32	C
ATOM	6628	C	LEU	A	438	5.099	27.149	-1.985	1.00	0.32	C
ATOM	6629	O	LEU	A	438	5.304	27.317	-0.786	1.00	0.43	O
ATOM	6630	CB	LEU	A	438	6.595	28.120	-3.718	1.00	0.48	C
ATOM	6631	CG	LEU	A	438	6.894	29.345	-2.891	1.00	0.48	C
ATOM	6632	CD1	LEU	A	438	8.058	29.059	-1.972	1.00	0.48	C
ATOM	6633	CD2	LEU	A	438	7.227	30.462	-3.833	1.00	0.48	C
ATOM	6634	H	LEU	A	438	5.868	25.863	-4.794	1.00	0.76	H
ATOM	6635	HA	LEU	A	438	7.124	26.621	-2.318	1.00	0.38	H
ATOM	6636	1HB	LEU	A	438	7.458	27.912	-4.344	1.00	0.58	H
ATOM	6637	2HB	LEU	A	438	5.759	28.379	-4.350	1.00	0.58	H
ATOM	6638	HG	LEU	A	438	6.023	29.607	-2.290	1.00	0.58	H
ATOM	6639	1HD1	LEU	A	438	8.280	29.939	-1.376	1.00	0.58	H
ATOM	6640	2HD1	LEU	A	438	7.804	28.230	-1.311	1.00	0.58	H
ATOM	6641	3HD1	LEU	A	438	8.929	28.792	-2.569	1.00	0.58	H
ATOM	6642	1HD2	LEU	A	438	7.431	31.370	-3.275	1.00	0.58	H
ATOM	6643	2HD2	LEU	A	438	8.101	30.188	-4.421	1.00	0.58	H
ATOM	6644	3HD2	LEU	A	438	6.396	30.635	-4.505	1.00	0.58	H
ATOM	6645	N	ALA	A	439	3.881	27.174	-2.523	1.00	0.42	N
ATOM	6646	CA	ALA	A	439	2.705	27.349	-1.666	1.00	0.55	C
ATOM	6647	C	ALA	A	439	2.687	26.308	-0.540	1.00	0.68	C
ATOM	6648	O	ALA	A	439	2.477	26.644	0.631	1.00	1.10	O
ATOM	6649	CB	ALA	A	439	1.429	27.238	-2.487	1.00	0.83	C
ATOM	6650	H	ALA	A	439	3.780	27.076	-3.536	1.00	0.50	H

```
ATOM   6651  HA  ALA A 439       2.757  28.336  -1.210  1.00  0.66
H
ATOM   6652 1HB  ALA A 439       0.565  27.390  -1.840  1.00  0.99
H
ATOM   6653 2HB  ALA A 439       1.433  27.997  -3.268  1.00  0.99
H
ATOM   6654 3HB  ALA A 439       1.371  26.252  -2.942  1.00  0.99
H
ATOM   6655  N   MET A 440       2.912  25.049  -0.915  1.00  0.52
N
ATOM   6656  CA  MET A 440       2.915  23.929   0.010  1.00  0.51
C
ATOM   6657  C   MET A 440       3.998  24.022   1.072  1.00  0.38
C
ATOM   6658  O   MET A 440       3.719  23.883   2.263  1.00  0.73
O
ATOM   6659  CB  MET A 440       3.051  22.630  -0.770  1.00  0.77
C
ATOM   6660  CG  MET A 440       3.054  21.391   0.097  1.00  0.77
C
ATOM   6661  SD  MET A 440       3.149  19.870  -0.849  1.00  0.77
S
ATOM   6662  CE  MET A 440       4.839  19.893  -1.390  1.00  0.77
C
ATOM   6663  H   MET A 440       3.071  24.851  -1.896  1.00  0.62
H
ATOM   6664  HA  MET A 440       1.956  23.920   0.527  1.00  0.61
H
ATOM   6665 1HB  MET A 440       2.233  22.552  -1.482  1.00  0.92
H
ATOM   6666 2HB  MET A 440       3.980  22.644  -1.341  1.00  0.92
H
ATOM   6667 1HG  MET A 440       3.899  21.426   0.784  1.00  0.92
H
ATOM   6668 2HG  MET A 440       2.140  21.374   0.682  1.00  0.92
H
ATOM   6669 1HE  MET A 440       5.041  19.014  -2.000  1.00  0.92
H
ATOM   6670 2HE  MET A 440       5.022  20.793  -1.979  1.00  0.92
H
ATOM   6671 3HE  MET A 440       5.487  19.887  -0.517  1.00  0.92
H
ATOM   6672  N   PHE A 441       5.235  24.240   0.653  1.00  0.30
N
ATOM   6673  CA  PHE A 441       6.320  24.304   1.609  1.00  0.30
C
ATOM   6674  C   PHE A 441       6.299  25.549   2.481  1.00  0.28
C
ATOM   6675  O   PHE A 441       6.625  25.463   3.661  1.00  0.32
O
ATOM   6676  CB  PHE A 441       7.662  24.090   0.923  1.00  0.45
C
ATOM   6677  CG  PHE A 441       7.946  22.635   0.656  1.00  0.45
C
ATOM   6678  CD1 PHE A 441       7.765  22.063  -0.593  1.00  0.45
C
ATOM   6679  CD2 PHE A 441       8.412  21.828   1.679  1.00  0.45
C
```

```
ATOM   6680  CE1 PHE A 441       8.049  20.729  -0.812  1.00  0.45
C
ATOM   6681  CE2 PHE A 441       8.693  20.498   1.463  1.00  0.45
C
ATOM   6682  CZ  PHE A 441       8.513  19.943   0.215  1.00  0.45
C
ATOM   6683  H   PHE A 441       5.429  24.349  -0.329  1.00  0.36
H
ATOM   6684  HA  PHE A 441       6.199  23.458   2.280  1.00  0.36
H
ATOM   6685 1HB  PHE A 441       7.681  24.624  -0.026  1.00  0.54
H
ATOM   6686 2HB  PHE A 441       8.461  24.486   1.546  1.00  0.54
H
ATOM   6687  HD1 PHE A 441       7.406  22.674  -1.415  1.00  0.54
H
ATOM   6688  HD2 PHE A 441       8.560  22.260   2.669  1.00  0.54
H
ATOM   6689  HE1 PHE A 441       7.906  20.299  -1.803  1.00  0.54
H
ATOM   6690  HE2 PHE A 441       9.056  19.884   2.282  1.00  0.54
H
ATOM   6691  HZ  PHE A 441       8.738  18.892   0.045  1.00  0.54
H
ATOM   6692  N   ARG A 442       5.842  26.692   1.958  1.00  0.24
N
ATOM   6693  CA  ARG A 442       5.738  27.866   2.825  1.00  0.30
C
ATOM   6694  C   ARG A 442       4.720  27.639   3.923  1.00  0.37
C
ATOM   6695  O   ARG A 442       4.916  28.096   5.050  1.00  1.19
O
ATOM   6696  CB  ARG A 442       5.326  29.125   2.084  1.00  0.45
C
ATOM   6697  CG  ARG A 442       6.364  29.738   1.184  1.00  0.45
C
ATOM   6698  CD  ARG A 442       5.852  30.985   0.562  1.00  0.45
C
ATOM   6699  NE  ARG A 442       5.648  32.056   1.541  1.00  0.45
N
ATOM   6700  CZ  ARG A 442       6.620  32.884   1.966  1.00  0.45
C
ATOM   6701  NH1 ARG A 442       7.833  32.704   1.527  1.00  0.45
N
ATOM   6702  NH2 ARG A 442       6.354  33.860   2.801  1.00  0.45
N
ATOM   6703  H   ARG A 442       5.585  26.761   0.984  1.00  0.29
H
ATOM   6704  HA  ARG A 442       6.710  28.041   3.285  1.00  0.36
H
ATOM   6705 1HB  ARG A 442       4.450  28.914   1.473  1.00  0.54
H
ATOM   6706 2HB  ARG A 442       5.043  29.889   2.807  1.00  0.54
H
ATOM   6707 1HG  ARG A 442       7.258  29.978   1.758  1.00  0.54
H
ATOM   6708 2HG  ARG A 442       6.616  29.032   0.400  1.00  0.54
H
```

```
ATOM   6709 1HD  ARG A 442       6.567  31.335  -0.183  1.00  0.54
H
ATOM   6710 2HD  ARG A 442       4.898  30.781   0.074  1.00  0.54
H
ATOM   6711  HE  ARG A 442       4.717  32.215   1.923  1.00  0.54
H
ATOM   6712 1HH1 ARG A 442       8.013  31.943   0.889  1.00  0.54
H
ATOM   6713 2HH1 ARG A 442       8.591  33.304   1.813  1.00  0.54
H
ATOM   6714 1HH2 ARG A 442       5.399  33.972   3.131  1.00  0.54
H
ATOM   6715 2HH2 ARG A 442       7.080  34.480   3.124  1.00  0.54
H
ATOM   6716  N   SER A 443       3.641  26.912   3.620  1.00  0.41
N
ATOM   6717  CA  SER A 443       2.629  26.632   4.631  1.00  0.44
C
ATOM   6718  C   SER A 443       3.128  25.713   5.758  1.00  0.39
C
ATOM   6719  O   SER A 443       2.482  25.627   6.799  1.00  0.71
O
ATOM   6720  CB  SER A 443       1.393  26.035   3.981  1.00  0.66
C
ATOM   6721  OG  SER A 443       1.629  24.743   3.492  1.00  0.66
O
ATOM   6722  H   SER A 443       3.492  26.574   2.673  1.00  0.49
H
ATOM   6723  HA  SER A 443       2.340  27.578   5.085  1.00  0.53
H
ATOM   6724 1HB  SER A 443       0.574  26.014   4.698  1.00  0.79
H
ATOM   6725 2HB  SER A 443       1.085  26.678   3.158  1.00  0.79
H
ATOM   6726  HG  SER A 443       1.731  24.171   4.262  1.00  0.79
H
ATOM   6727  N   ILE A 444       4.266  25.035   5.569  1.00  0.27
N
ATOM   6728  CA  ILE A 444       4.814  24.153   6.595  1.00  0.25
C
ATOM   6729  C   ILE A 444       5.604  24.962   7.638  1.00  0.39
C
ATOM   6730  O   ILE A 444       6.477  25.746   7.266  1.00  0.58
O
ATOM   6731  CB  ILE A 444       5.719  23.073   5.956  1.00  0.38
C
ATOM   6732  CG1 ILE A 444       4.883  22.191   5.026  1.00  0.38
C
ATOM   6733  CG2 ILE A 444       6.398  22.231   7.021  1.00  0.38
C
ATOM   6734  CD1 ILE A 444       5.698  21.252   4.170  1.00  0.38
C
ATOM   6735  H   ILE A 444       4.796  25.136   4.714  1.00  0.32
H
ATOM   6736  HA  ILE A 444       3.986  23.640   7.071  1.00  0.30
H
ATOM   6737  HB  ILE A 444       6.480  23.558   5.347  1.00  0.45
H
```

ATOM 6738 1HG1 ILE A 444 4.195 21.597 5.628 1.00 0.45
H
ATOM 6739 2HG1 ILE A 444 4.295 22.830 4.372 1.00 0.45
H
ATOM 6740 1HG2 ILE A 444 7.034 21.485 6.547 1.00 0.45
H
ATOM 6741 2HG2 ILE A 444 7.005 22.872 7.659 1.00 0.45
H
ATOM 6742 3HG2 ILE A 444 5.640 21.732 7.624 1.00 0.45
H
ATOM 6743 1HD1 ILE A 444 5.032 20.664 3.539 1.00 0.45
H
ATOM 6744 2HD1 ILE A 444 6.375 21.829 3.547 1.00 0.45
H
ATOM 6745 3HD1 ILE A 444 6.273 20.588 4.812 1.00 0.45
H
ATOM 6746 N PRO A 445 5.290 24.825 8.935 1.00 0.46
N
ATOM 6747 CA PRO A 445 5.956 25.449 10.072 1.00 0.61
C
ATOM 6748 C PRO A 445 7.412 25.062 10.125 1.00 0.41
C
ATOM 6749 O PRO A 445 7.765 23.913 9.862 1.00 0.49
O
ATOM 6750 CB PRO A 445 5.209 24.904 11.276 1.00 0.92
C
ATOM 6751 CG PRO A 445 3.864 24.587 10.758 1.00 0.92
C
ATOM 6752 CD PRO A 445 4.080 24.099 9.357 1.00 0.92
C
ATOM 6753 HA PRO A 445 5.846 26.542 10.000 1.00 0.73
H
ATOM 6754 1HB PRO A 445 5.733 24.022 11.670 1.00 1.10
H
ATOM 6755 2HB PRO A 445 5.190 25.655 12.079 1.00 1.10
H
ATOM 6756 1HG PRO A 445 3.366 23.848 11.401 1.00 1.10
H
ATOM 6757 2HG PRO A 445 3.257 25.501 10.786 1.00 1.10
H
ATOM 6758 1HD PRO A 445 4.258 23.014 9.335 1.00 1.10
H
ATOM 6759 2HD PRO A 445 3.220 24.389 8.741 1.00 1.10
H
ATOM 6760 N ASN A 446 8.250 26.019 10.503 1.00 0.39
N
ATOM 6761 CA ASN A 446 9.689 25.816 10.599 1.00 0.54
C
ATOM 6762 C ASN A 446 10.270 25.301 9.287 1.00 0.67
C
ATOM 6763 O ASN A 446 11.145 24.433 9.296 1.00 2.31
O
ATOM 6764 CB ASN A 446 10.023 24.872 11.740 1.00 0.81
C
ATOM 6765 CG ASN A 446 9.637 25.438 13.078 1.00 0.81
C
ATOM 6766 OD1 ASN A 446 9.786 26.641 13.322 1.00 0.81
O

ATOM 6767 ND2 ASN A 446 9.147 24.596 13.952 1.00 0.81
N
ATOM 6768 H ASN A 446 7.875 26.933 10.719 1.00 0.47
H
ATOM 6769 HA ASN A 446 10.159 26.780 10.801 1.00 0.65
H
ATOM 6770 1HB ASN A 446 9.509 23.920 11.599 1.00 0.97
H
ATOM 6771 2HB ASN A 446 11.093 24.669 11.742 1.00 0.97
H
ATOM 6772 1HD2 ASN A 446 8.876 24.917 14.859 1.00 0.97
H
ATOM 6773 2HD2 ASN A 446 9.045 23.631 13.712 1.00 0.97
H
ATOM 6774 N CYS A 447 9.795 25.852 8.170 1.00 0.78
N
ATOM 6775 CA CYS A 447 10.284 25.451 6.867 1.00 0.63
C
ATOM 6776 C CYS A 447 11.026 26.584 6.173 1.00 0.55
C
ATOM 6777 O CYS A 447 10.490 27.678 6.002 1.00 1.27
O
ATOM 6778 CB CYS A 447 9.150 24.981 5.961 1.00 0.95
C
ATOM 6779 SG CYS A 447 9.697 24.431 4.326 1.00 0.95
S
ATOM 6780 H CYS A 447 9.078 26.560 8.227 1.00 0.94
H
ATOM 6781 HA CYS A 447 10.963 24.622 7.012 1.00 0.76
H
ATOM 6782 1HB CYS A 447 8.618 24.158 6.438 1.00 1.13
H
ATOM 6783 2HB CYS A 447 8.440 25.794 5.819 1.00 1.13
H
ATOM 6784 HG CYS A 447 10.474 23.449 4.765 1.00 1.13
H
ATOM 6785 N THR A 448 12.269 26.316 5.799 1.00 0.41
N
ATOM 6786 CA THR A 448 13.080 27.267 5.059 1.00 0.33
C
ATOM 6787 C THR A 448 13.028 26.886 3.594 1.00 0.28
C
ATOM 6788 O THR A 448 13.341 25.751 3.241 1.00 0.39
O
ATOM 6789 CB THR A 448 14.548 27.292 5.547 1.00 0.49
C
ATOM 6790 OG1 THR A 448 14.594 27.703 6.920 1.00 0.49
O
ATOM 6791 CG2 THR A 448 15.384 28.248 4.702 1.00 0.49
C
ATOM 6792 H THR A 448 12.639 25.398 5.991 1.00 0.49
H
ATOM 6793 HA THR A 448 12.654 28.259 5.177 1.00 0.40
H
ATOM 6794 HB THR A 448 14.968 26.290 5.468 1.00 0.59
H
ATOM 6795 HG1 THR A 448 14.113 27.071 7.459 1.00 0.59
H

```
ATOM   6796 1HG2 THR A 448      16.412  28.243   5.060  1.00  0.59
H
ATOM   6797 2HG2 THR A 448      15.360  27.928   3.661  1.00  0.59
H
ATOM   6798 3HG2 THR A 448      14.976  29.255   4.781  1.00  0.59
H
ATOM   6799  N   VAL A 449      12.613  27.813   2.744  1.00  0.27
N
ATOM   6800  CA  VAL A 449      12.536  27.509   1.329  1.00  0.25
C
ATOM   6801  C   VAL A 449      13.582  28.268   0.553  1.00  0.23
C
ATOM   6802  O   VAL A 449      13.597  29.499   0.541  1.00  0.26
O
ATOM   6803  CB  VAL A 449      11.151  27.816   0.745  1.00  0.38
C
ATOM   6804  CG1 VAL A 449      11.173  27.490  -0.743  1.00  0.38
C
ATOM   6805  CG2 VAL A 449      10.089  26.994   1.465  1.00  0.38
C
ATOM   6806  H   VAL A 449      12.377  28.732   3.088  1.00  0.32
H
ATOM   6807  HA  VAL A 449      12.718  26.443   1.198  1.00  0.30
H
ATOM   6808  HB  VAL A 449      10.931  28.878   0.855  1.00  0.45
H
ATOM   6809 1HG1 VAL A 449      10.203  27.701  -1.177  1.00  0.45
H
ATOM   6810 2HG1 VAL A 449      11.933  28.092  -1.239  1.00  0.45
H
ATOM   6811 3HG1 VAL A 449      11.402  26.433  -0.877  1.00  0.45
H
ATOM   6812 1HG2 VAL A 449       9.109  27.213   1.042  1.00  0.45
H
ATOM   6813 2HG2 VAL A 449      10.309  25.933   1.346  1.00  0.45
H
ATOM   6814 3HG2 VAL A 449      10.089  27.246   2.526  1.00  0.45
H
ATOM   6815  N   PHE A 450      14.445  27.510  -0.108  1.00  0.31
N
ATOM   6816  CA  PHE A 450      15.510  28.040  -0.929  1.00  0.67
C
ATOM   6817  C   PHE A 450      15.131  27.886  -2.385  1.00  1.10
C
ATOM   6818  O   PHE A 450      14.596  26.854  -2.784  1.00  6.59
O
ATOM   6819  CB  PHE A 450      16.773  27.187  -0.773  1.00  1.01
C
ATOM   6820  CG  PHE A 450      17.464  27.163   0.554  1.00  1.01
C
ATOM   6821  CD1 PHE A 450      17.053  26.284   1.547  1.00  1.01
C
ATOM   6822  CD2 PHE A 450      18.563  27.972   0.792  1.00  1.01
C
ATOM   6823  CE1 PHE A 450      17.724  26.221   2.749  1.00  1.01
C
ATOM   6824  CE2 PHE A 450      19.235  27.909   1.994  1.00  1.01
C
```

```
ATOM   6825  CZ  PHE A 450      18.815  27.030   2.970  1.00  1.01
C
ATOM   6826  H   PHE A 450      14.348  26.508  -0.043  1.00  0.37
H
ATOM   6827  HA  PHE A 450      15.686  29.087  -0.690  1.00  0.80
H
ATOM   6828 1HB  PHE A 450      16.519  26.163  -1.025  1.00  1.21
H
ATOM   6829 2HB  PHE A 450      17.505  27.515  -1.506  1.00  1.21
H
ATOM   6830  HD1 PHE A 450      16.196  25.635   1.361  1.00  1.21
H
ATOM   6831  HD2 PHE A 450      18.899  28.656   0.012  1.00  1.21
H
ATOM   6832  HE1 PHE A 450      17.396  25.527   3.522  1.00  1.21
H
ATOM   6833  HE2 PHE A 450      20.101  28.547   2.169  1.00  1.21
H
ATOM   6834  HZ  PHE A 450      19.346  26.972   3.916  1.00  1.21
H
ATOM   6835  N   TYR A 451      15.482  28.868  -3.190  1.00  0.43
N
ATOM   6836  CA  TYR A 451      15.301  28.765  -4.626  1.00  0.28
C
ATOM   6837  C   TYR A 451      16.438  29.451  -5.359  1.00  0.39
C
ATOM   6838  O   TYR A 451      16.298  30.596  -5.790  1.00  0.84
O
ATOM   6839  CB  TYR A 451      13.980  29.363  -5.075  1.00  0.42
C
ATOM   6840  CG  TYR A 451      13.657  29.055  -6.525  1.00  0.42
C
ATOM   6841  CD1 TYR A 451      14.468  28.189  -7.247  1.00  0.42
C
ATOM   6842  CD2 TYR A 451      12.551  29.625  -7.133  1.00  0.42
C
ATOM   6843  CE1 TYR A 451      14.169  27.893  -8.562  1.00  0.42
C
ATOM   6844  CE2 TYR A 451      12.251  29.330  -8.448  1.00  0.42
C
ATOM   6845  CZ  TYR A 451      13.053  28.466  -9.161  1.00  0.42
C
ATOM   6846  OH  TYR A 451      12.750  28.173 -10.471  1.00  0.42
O
ATOM   6847  H   TYR A 451      15.891  29.707  -2.808  1.00  0.52
H
ATOM   6848  HA  TYR A 451      15.316  27.711  -4.904  1.00  0.34
H
ATOM   6849 1HB  TYR A 451      13.176  28.970  -4.453  1.00  0.50
H
ATOM   6850 2HB  TYR A 451      13.999  30.445  -4.949  1.00  0.50
H
ATOM   6851  HD1 TYR A 451      15.340  27.736  -6.777  1.00  0.50
H
ATOM   6852  HD2 TYR A 451      11.912  30.305  -6.575  1.00  0.50
H
ATOM   6853  HE1 TYR A 451      14.804  27.210  -9.126  1.00  0.50
H
```

```
ATOM   6854  HE2 TYR A 451      11.378  29.779  -8.921  1.00  0.50
H
ATOM   6855  HH  TYR A 451      11.909  28.577 -10.703  1.00  0.50
H
ATOM   6856  N   PRO A 452      17.584  28.771  -5.461  1.00  0.79
N
ATOM   6857  CA  PRO A 452      18.767  29.167  -6.202  1.00  0.97
C
ATOM   6858  C   PRO A 452      18.508  29.365  -7.673  1.00  0.65
C
ATOM   6859  O   PRO A 452      17.571  28.812  -8.251  1.00  2.78
O
ATOM   6860  CB  PRO A 452      19.738  28.050  -5.927  1.00  1.46
C
ATOM   6861  CG  PRO A 452      19.411  27.635  -4.546  1.00  1.46
C
ATOM   6862  CD  PRO A 452      17.944  27.824  -4.396  1.00  1.46
C
ATOM   6863  HA  PRO A 452      19.147  30.100  -5.769  1.00  1.16
H
ATOM   6864 1HB  PRO A 452      19.581  27.249  -6.658  1.00  1.75
H
ATOM   6865 2HB  PRO A 452      20.764  28.401  -6.050  1.00  1.75
H
ATOM   6866 1HG  PRO A 452      19.738  26.604  -4.362  1.00  1.75
H
ATOM   6867 2HG  PRO A 452      19.974  28.269  -3.847  1.00  1.75
H
ATOM   6868 1HD  PRO A 452      17.392  26.880  -4.506  1.00  1.75
H
ATOM   6869 2HD  PRO A 452      17.792  28.284  -3.415  1.00  1.75
H
ATOM   6870  N   SER A 453      19.328  30.219  -8.252  1.00  0.76
N
ATOM   6871  CA  SER A 453      19.183  30.620  -9.636  1.00  0.40
C
ATOM   6872  C   SER A 453      20.276  30.054 -10.514  1.00  0.40
C
ATOM   6873  O   SER A 453      20.070  29.836 -11.707  1.00  0.64
O
ATOM   6874  CB  SER A 453      19.150  32.114  -9.715  1.00  0.60
C
ATOM   6875  OG  SER A 453      18.020  32.602  -9.047  1.00  0.60
O
ATOM   6876  H   SER A 453      20.113  30.567  -7.710  1.00  0.91
H
ATOM   6877  HA  SER A 453      18.233  30.238  -9.999  1.00  0.48
H
ATOM   6878 1HB  SER A 453      20.050  32.515  -9.247  1.00  0.72
H
ATOM   6879 2HB  SER A 453      19.143  32.440 -10.753  1.00  0.72
H
ATOM   6880  HG  SER A 453      18.346  33.342  -8.520  1.00  0.72
H
ATOM   6881  N   ASP A 454      21.444  29.812  -9.926  1.00  0.43
N
ATOM   6882  CA  ASP A 454      22.540  29.226 -10.680  1.00  0.46
C
```

```
ATOM   6883  C   ASP A 454      23.369  28.325  -9.792  1.00  0.82
C
ATOM   6884  O   ASP A 454      23.176  28.304  -8.579  1.00  3.74
O
ATOM   6885  CB  ASP A 454      23.478  30.288 -11.242  1.00  0.69
C
ATOM   6886  CG  ASP A 454      24.243  31.014 -10.143  1.00  0.69
C
ATOM   6887  OD1 ASP A 454      24.122  30.614  -8.995  1.00  0.69
O
ATOM   6888  OD2 ASP A 454      24.969  31.934 -10.459  1.00  0.69
O
ATOM   6889  H   ASP A 454      21.570  30.016  -8.948  1.00  0.52
H
ATOM   6890  HA  ASP A 454      22.131  28.631 -11.498  1.00  0.55
H
ATOM   6891 1HB  ASP A 454      24.188  29.823 -11.923  1.00  0.83
H
ATOM   6892 2HB  ASP A 454      22.904  31.019 -11.814  1.00  0.83
H
ATOM   6893  N   ALA A 455      24.343  27.669 -10.411  1.00  0.31
N
ATOM   6894  CA  ALA A 455      25.274  26.724  -9.794  1.00  0.40
C
ATOM   6895  C   ALA A 455      25.872  27.156  -8.460  1.00  0.41
C
ATOM   6896  O   ALA A 455      26.033  26.336  -7.552  1.00  0.74
O
ATOM   6897  CB  ALA A 455      26.413  26.486 -10.752  1.00  0.60
C
ATOM   6898  H   ALA A 455      24.421  27.797 -11.410  1.00  0.37
H
ATOM   6899  HA  ALA A 455      24.733  25.793  -9.633  1.00  0.48
H
ATOM   6900 1HB  ALA A 455      27.079  25.738 -10.332  1.00  0.72
H
ATOM   6901 2HB  ALA A 455      26.020  26.132 -11.700  1.00  0.72
H
ATOM   6902 3HB  ALA A 455      26.959  27.414 -10.909  1.00  0.72
H
ATOM   6903  N   ILE A 456      26.215  28.429  -8.351  1.00  0.32
N
ATOM   6904  CA  ILE A 456      26.891  28.938  -7.175  1.00  0.36
C
ATOM   6905  C   ILE A 456      25.916  28.980  -6.025  1.00  0.40
C
ATOM   6906  O   ILE A 456      26.182  28.447  -4.943  1.00  0.49
O
ATOM   6907  CB  ILE A 456      27.424  30.353  -7.452  1.00  0.54
C
ATOM   6908  CG1 ILE A 456      28.389  30.315  -8.626  1.00  0.54
C
ATOM   6909  CG2 ILE A 456      28.110  30.934  -6.227  1.00  0.54
C
ATOM   6910  CD1 ILE A 456      29.583  29.443  -8.431  1.00  0.54
C
ATOM   6911  H   ILE A 456      26.026  29.062  -9.117  1.00  0.38
H
```

```
ATOM   6912  HA  ILE A 456      27.716  28.276  -6.919  1.00  0.43
H
ATOM   6913  HB  ILE A 456      26.592  30.995  -7.738  1.00  0.65
H
ATOM   6914 1HG1 ILE A 456      27.854  29.968  -9.505  1.00  0.65
H
ATOM   6915 2HG1 ILE A 456      28.740  31.321  -8.812  1.00  0.65
H
ATOM   6916 1HG2 ILE A 456      28.470  31.938  -6.452  1.00  0.65
H
ATOM   6917 2HG2 ILE A 456      27.399  30.980  -5.407  1.00  0.65
H
ATOM   6918 3HG2 ILE A 456      28.952  30.303  -5.945  1.00  0.65
H
ATOM   6919 1HD1 ILE A 456      30.217  29.490  -9.315  1.00  0.65
H
ATOM   6920 2HD1 ILE A 456      30.142  29.794  -7.569  1.00  0.65
H
ATOM   6921 3HD1 ILE A 456      29.259  28.418  -8.265  1.00  0.65
H
ATOM   6922  N   SER A 457      24.763  29.583  -6.290  1.00  0.38
N
ATOM   6923  CA  SER A 457      23.711  29.678  -5.306  1.00  0.41
C
ATOM   6924  C   SER A 457      23.177  28.295  -4.896  1.00  0.34
C
ATOM   6925  O   SER A 457      22.748  28.123  -3.755  1.00  0.35
O
ATOM   6926  CB  SER A 457      22.617  30.597  -5.858  1.00  0.61
C
ATOM   6927  OG  SER A 457      22.074  30.098  -7.054  1.00  0.61
O
ATOM   6928  H   SER A 457      24.612  29.993  -7.209  1.00  0.46
H
ATOM   6929  HA  SER A 457      24.125  30.152  -4.415  1.00  0.49
H
ATOM   6930 1HB  SER A 457      21.827  30.706  -5.116  1.00  0.74
H
ATOM   6931 2HB  SER A 457      23.034  31.587  -6.037  1.00  0.74
H
ATOM   6932  HG  SER A 457      22.809  30.087  -7.689  1.00  0.74
H
ATOM   6933  N   THR A 458      23.268  27.295  -5.791  1.00  0.35
N
ATOM   6934  CA  THR A 458      22.794  25.951  -5.448  1.00  0.43
C
ATOM   6935  C   THR A 458      23.721  25.234  -4.489  1.00  0.37
C
ATOM   6936  O   THR A 458      23.252  24.560  -3.565  1.00  0.42
O
ATOM   6937  CB  THR A 458      22.699  25.047  -6.680  1.00  0.65
C
ATOM   6938  OG1 THR A 458      23.988  24.933  -7.272  1.00  0.65
O
ATOM   6939  CG2 THR A 458      21.732  25.569  -7.706  1.00  0.65
C
ATOM   6940  H   THR A 458      23.610  27.481  -6.726  1.00  0.42
H
```

```
ATOM   6941  HA  THR A 458      21.816  26.034  -4.981  1.00  0.52
H
ATOM   6942  HB  THR A 458      22.383  24.057  -6.372  1.00  0.77
H
ATOM   6943  HG1 THR A 458      23.913  24.381  -8.052  1.00  0.77
H
ATOM   6944 1HG2 THR A 458      21.714  24.887  -8.553  1.00  0.77
H
ATOM   6945 2HG2 THR A 458      20.741  25.643  -7.278  1.00  0.77
H
ATOM   6946 3HG2 THR A 458      22.035  26.541  -8.037  1.00  0.77
H
ATOM   6947  N   GLU A 459      25.037  25.421  -4.658  1.00  0.42
N
ATOM   6948  CA  GLU A 459      25.954  24.773  -3.727  1.00  0.48
C
ATOM   6949  C   GLU A 459      25.859  25.415  -2.366  1.00  0.64
C
ATOM   6950  O   GLU A 459      25.933  24.721  -1.347  1.00  1.04
O
ATOM   6951  CB  GLU A 459      27.421  24.815  -4.185  1.00  0.72
C
ATOM   6952  CG  GLU A 459      27.754  23.947  -5.387  1.00  0.72
C
ATOM   6953  CD  GLU A 459      29.228  23.715  -5.533  1.00  0.72
C
ATOM   6954  OE1 GLU A 459      29.978  24.341  -4.830  1.00  0.72
O
ATOM   6955  OE2 GLU A 459      29.607  22.880  -6.325  1.00  0.72
O
ATOM   6956  H   GLU A 459      25.386  25.959  -5.445  1.00  0.50
H
ATOM   6957  HA  GLU A 459      25.662  23.730  -3.628  1.00  0.58
H
ATOM   6958 1HB  GLU A 459      27.690  25.840  -4.436  1.00  0.86
H
ATOM   6959 2HB  GLU A 459      28.064  24.501  -3.363  1.00  0.86
H
ATOM   6960 1HG  GLU A 459      27.266  22.991  -5.287  1.00  0.86
H
ATOM   6961 2HG  GLU A 459      27.371  24.429  -6.286  1.00  0.86
H
ATOM   6962  N   HIS A 460      25.653  26.729  -2.343  1.00  0.55
N
ATOM   6963  CA  HIS A 460      25.514  27.416  -1.077  1.00  0.62
C
ATOM   6964  C   HIS A 460      24.197  27.072  -0.395  1.00  0.56
C
ATOM   6965  O   HIS A 460      24.154  26.995   0.830  1.00  0.82
O
ATOM   6966  CB  HIS A 460      25.679  28.923  -1.245  1.00  0.93
C
ATOM   6967  CG  HIS A 460      27.106  29.334  -1.472  1.00  0.93
C
ATOM   6968  ND1 HIS A 460      28.056  29.295  -0.472  1.00  0.93
N
ATOM   6969  CD2 HIS A 460      27.743  29.784  -2.575  1.00  0.93
C
```

```
ATOM   6970  CE1 HIS A 460      29.213  29.713  -0.956  1.00  0.93
C
ATOM   6971  NE2 HIS A 460      29.049  30.016  -2.229  1.00  0.93
N
ATOM   6972  H   HIS A 460      25.619  27.261  -3.207  1.00  0.66
H
ATOM   6973  HA  HIS A 460      26.310  27.081  -0.414  1.00  0.74
H
ATOM   6974 1HB  HIS A 460      25.084  29.267  -2.092  1.00  1.12
H
ATOM   6975 2HB  HIS A 460      25.312  29.431  -0.355  1.00  1.12
H
ATOM   6976  HD1 HIS A 460      27.872  29.186   0.505  1.00  1.12
H
ATOM   6977  HD2 HIS A 460      27.409  29.978  -3.589  1.00  1.12
H
ATOM   6978  HE1 HIS A 460      30.097  29.761  -0.320  1.00  1.12
H
ATOM   6979  N   ALA A 461      23.140  26.824  -1.175  1.00  0.53
N
ATOM   6980  CA  ALA A 461      21.856  26.431  -0.601  1.00  0.59
C
ATOM   6981  C   ALA A 461      21.956  25.114   0.146  1.00  0.49
C
ATOM   6982  O   ALA A 461      21.469  25.021   1.270  1.00  0.53
O
ATOM   6983  CB  ALA A 461      20.804  26.321  -1.675  1.00  0.89
C
ATOM   6984  H   ALA A 461      23.209  26.932  -2.179  1.00  0.64
H
ATOM   6985  HA  ALA A 461      21.557  27.199   0.112  1.00  0.71
H
ATOM   6986 1HB  ALA A 461      19.848  26.058  -1.227  1.00  1.06
H
ATOM   6987 2HB  ALA A 461      20.722  27.282  -2.174  1.00  1.06
H
ATOM   6988 3HB  ALA A 461      21.093  25.557  -2.393  1.00  1.06
H
ATOM   6989  N   ILE A 462      22.582  24.097  -0.459  1.00  0.65
N
ATOM   6990  CA  ILE A 462      22.759  22.827   0.256  1.00  0.74
C
ATOM   6991  C   ILE A 462      23.657  22.955   1.466  1.00  0.40
C
ATOM   6992  O   ILE A 462      23.365  22.364   2.505  1.00  0.48
O
ATOM   6993  CB  ILE A 462      23.237  21.694  -0.642  1.00  1.11
C
ATOM   6994  CG1 ILE A 462      22.110  21.276  -1.568  1.00  1.11
C
ATOM   6995  CG2 ILE A 462      23.728  20.527   0.174  1.00  1.11
C
ATOM   6996  CD1 ILE A 462      22.559  20.320  -2.623  1.00  1.11
C
ATOM   6997  H   ILE A 462      22.939  24.208  -1.404  1.00  0.78
H
ATOM   6998  HA  ILE A 462      21.779  22.524   0.621  1.00  0.89
H
```

```
ATOM   6999  HB  ILE A 462      24.052  22.056  -1.269  1.00  1.33
H
ATOM   7000 1HG1 ILE A 462      21.334  20.794  -0.977  1.00  1.33
H
ATOM   7001 2HG1 ILE A 462      21.681  22.157  -2.044  1.00  1.33
H
ATOM   7002 1HG2 ILE A 462      24.074  19.735  -0.490  1.00  1.33
H
ATOM   7003 2HG2 ILE A 462      24.549  20.858   0.807  1.00  1.33
H
ATOM   7004 3HG2 ILE A 462      22.917  20.154   0.795  1.00  1.33
H
ATOM   7005 1HD1 ILE A 462      21.714  20.035  -3.248  1.00  1.33
H
ATOM   7006 2HD1 ILE A 462      23.306  20.812  -3.228  1.00  1.33
H
ATOM   7007 3HD1 ILE A 462      22.980  19.438  -2.153  1.00  1.33
H
ATOM   7008  N   TYR A 463      24.734  23.721   1.338  1.00  0.46
N
ATOM   7009  CA  TYR A 463      25.617  24.030   2.457  1.00  1.04
C
ATOM   7010  C   TYR A 463      24.834  24.597   3.644  1.00  0.47
C
ATOM   7011  O   TYR A 463      24.978  24.137   4.779  1.00  1.05
O
ATOM   7012  CB  TYR A 463      26.673  25.034   1.968  1.00  1.56
C
ATOM   7013  CG  TYR A 463      27.539  25.691   3.018  1.00  1.56
C
ATOM   7014  CD1 TYR A 463      28.696  25.094   3.493  1.00  1.56
C
ATOM   7015  CD2 TYR A 463      27.157  26.938   3.495  1.00  1.56
C
ATOM   7016  CE1 TYR A 463      29.464  25.757   4.437  1.00  1.56
C
ATOM   7017  CE2 TYR A 463      27.919  27.589   4.440  1.00  1.56
C
ATOM   7018  CZ  TYR A 463      29.072  27.002   4.911  1.00  1.56
C
ATOM   7019  OH  TYR A 463      29.841  27.652   5.848  1.00  1.56
O
ATOM   7020  H   TYR A 463      24.952  24.136   0.439  1.00  0.55
H
ATOM   7021  HA  TYR A 463      26.107  23.112   2.781  1.00  1.25
H
ATOM   7022 1HB  TYR A 463      27.336  24.526   1.265  1.00  1.87
H
ATOM   7023 2HB  TYR A 463      26.177  25.823   1.412  1.00  1.87
H
ATOM   7024  HD1 TYR A 463      29.004  24.118   3.118  1.00  1.87
H
ATOM   7025  HD2 TYR A 463      26.250  27.404   3.116  1.00  1.87
H
ATOM   7026  HE1 TYR A 463      30.380  25.310   4.811  1.00  1.87
H
ATOM   7027  HE2 TYR A 463      27.614  28.569   4.809  1.00  1.87
H
```

```
ATOM   7028  HH  TYR A 463      29.453  28.508   6.042  1.00  1.87
H
ATOM   7029  N   LEU A 464      24.010  25.608   3.371  1.00  0.52
N
ATOM   7030  CA  LEU A 464      23.206  26.237   4.403  1.00  0.57
C
ATOM   7031  C   LEU A 464      22.130  25.303   4.938  1.00  0.47
C
ATOM   7032  O   LEU A 464      21.931  25.227   6.150  1.00  0.63
O
ATOM   7033  CB  LEU A 464      22.564  27.512   3.842  1.00  0.85
C
ATOM   7034  CG  LEU A 464      23.537  28.662   3.548  1.00  0.85
C
ATOM   7035  CD1 LEU A 464      22.812  29.767   2.806  1.00  0.85
C
ATOM   7036  CD2 LEU A 464      24.111  29.181   4.856  1.00  0.85
C
ATOM   7037  H   LEU A 464      23.947  25.959   2.425  1.00  0.62
H
ATOM   7038  HA  LEU A 464      23.862  26.511   5.227  1.00  0.68
H
ATOM   7039 1HB  LEU A 464      22.053  27.263   2.913  1.00  1.03
H
ATOM   7040 2HB  LEU A 464      21.825  27.875   4.556  1.00  1.03
H
ATOM   7041  HG  LEU A 464      24.345  28.303   2.913  1.00  1.03
H
ATOM   7042 1HD1 LEU A 464      23.509  30.576   2.591  1.00  1.03
H
ATOM   7043 2HD1 LEU A 464      22.415  29.371   1.873  1.00  1.03
H
ATOM   7044 3HD1 LEU A 464      21.997  30.144   3.416  1.00  1.03
H
ATOM   7045 1HD2 LEU A 464      24.806  29.995   4.650  1.00  1.03
H
ATOM   7046 2HD2 LEU A 464      23.303  29.545   5.489  1.00  1.03
H
ATOM   7047 3HD2 LEU A 464      24.636  28.375   5.367  1.00  1.03
H
ATOM   7048  N   ALA A 465      21.477  24.550   4.048  1.00  0.34
N
ATOM   7049  CA  ALA A 465      20.430  23.608   4.439  1.00  0.32
C
ATOM   7050  C   ALA A 465      20.967  22.555   5.393  1.00  0.29
C
ATOM   7051  O   ALA A 465      20.325  22.222   6.388  1.00  0.36
O
ATOM   7052  CB  ALA A 465      19.837  22.939   3.212  1.00  0.48
C
ATOM   7053  H   ALA A 465      21.682  24.648   3.066  1.00  0.41
H
ATOM   7054  HA  ALA A 465      19.648  24.164   4.956  1.00  0.38
H
ATOM   7055 1HB  ALA A 465      19.042  22.260   3.517  1.00  0.58
H
ATOM   7056 2HB  ALA A 465      19.432  23.702   2.548  1.00  0.58
H
```

```
ATOM   7057 3HB  ALA A 465      20.614  22.380   2.692  1.00  0.58
H
ATOM   7058  N   ALA A 466      22.177  22.071   5.118  1.00  0.33
N
ATOM   7059  CA  ALA A 466      22.847  21.077   5.948  1.00  0.45
C
ATOM   7060  C   ALA A 466      22.988  21.515   7.403  1.00  0.63
C
ATOM   7061  O   ALA A 466      23.039  20.675   8.301  1.00  2.72
O
ATOM   7062  CB  ALA A 466      24.232  20.784   5.390  1.00  0.68
C
ATOM   7063  H   ALA A 466      22.649  22.377   4.279  1.00  0.40
H
ATOM   7064  HA  ALA A 466      22.250  20.166   5.926  1.00  0.54
H
ATOM   7065 1HB  ALA A 466      24.714  20.013   5.993  1.00  0.81
H
ATOM   7066 2HB  ALA A 466      24.147  20.444   4.361  1.00  0.81
H
ATOM   7067 3HB  ALA A 466      24.834  21.692   5.419  1.00  0.81
H
ATOM   7068  N   ASN A 467      23.091  22.824   7.638  1.00  0.81
N
ATOM   7069  CA  ASN A 467      23.288  23.334   8.979  1.00  0.92
C
ATOM   7070  C   ASN A 467      22.076  24.099   9.496  1.00  1.30
C
ATOM   7071  O   ASN A 467      22.179  24.826  10.485  1.00  7.26
O
ATOM   7072  CB  ASN A 467      24.517  24.208   9.005  1.00  1.38
C
ATOM   7073  CG  ASN A 467      25.761  23.418   8.730  1.00  1.38
C
ATOM   7074  OD1 ASN A 467      26.103  22.491   9.474  1.00  1.38
O
ATOM   7075  ND2 ASN A 467      26.439  23.764   7.668  1.00  1.38
N
ATOM   7076  H   ASN A 467      23.012  23.495   6.885  1.00  0.97
H
ATOM   7077  HA  ASN A 467      23.444  22.489   9.651  1.00  1.10
H
ATOM   7078 1HB  ASN A 467      24.424  24.994   8.255  1.00  1.66
H
ATOM   7079 2HB  ASN A 467      24.609  24.688   9.979  1.00  1.66
H
ATOM   7080 1HD2 ASN A 467      27.278  23.276   7.419  1.00  1.66
H
ATOM   7081 2HD2 ASN A 467      26.118  24.517   7.094  1.00  1.66
H
ATOM   7082  N   THR A 468      20.930  23.943   8.837  1.00  0.89
N
ATOM   7083  CA  THR A 468      19.727  24.652   9.242  1.00  0.60
C
ATOM   7084  C   THR A 468      18.700  23.688   9.810  1.00  0.82
C
ATOM   7085  O   THR A 468      18.444  22.626   9.244  1.00  3.32
O
```

ATOM 7086 CB THR A 468 19.138 25.435 8.058 1.00 0.90
C
ATOM 7087 OG1 THR A 468 20.101 26.394 7.611 1.00 0.90
O
ATOM 7088 CG2 THR A 468 17.860 26.157 8.455 1.00 0.90
C
ATOM 7089 H THR A 468 20.872 23.326 8.036 1.00 1.07
H
ATOM 7090 HA THR A 468 19.990 25.364 10.025 1.00 0.72
H
ATOM 7091 HB THR A 468 18.927 24.746 7.242 1.00 1.08
H
ATOM 7092 HG1 THR A 468 20.874 25.934 7.268 1.00 1.08
H
ATOM 7093 1HG2 THR A 468 17.471 26.705 7.597 1.00 1.08
H
ATOM 7094 2HG2 THR A 468 17.119 25.431 8.786 1.00 1.08
H
ATOM 7095 3HG2 THR A 468 18.072 26.854 9.264 1.00 1.08
H
ATOM 7096 N LYS A 469 18.129 24.059 10.948 1.00 0.44
N
ATOM 7097 CA LYS A 469 17.140 23.230 11.623 1.00 0.35
C
ATOM 7098 C LYS A 469 15.756 23.374 11.004 1.00 0.33
C
ATOM 7099 O LYS A 469 15.438 24.394 10.391 1.00 0.39
O
ATOM 7100 CB LYS A 469 17.087 23.578 13.108 1.00 0.52
C
ATOM 7101 CG LYS A 469 18.358 23.238 13.874 1.00 0.52
C
ATOM 7102 CD LYS A 469 18.223 23.581 15.349 1.00 0.52
C
ATOM 7103 CE LYS A 469 19.493 23.243 16.115 1.00 0.52
C
ATOM 7104 NZ LYS A 469 19.378 23.586 17.558 1.00 0.52
N
ATOM 7105 H LYS A 469 18.386 24.945 11.359 1.00 0.53
H
ATOM 7106 HA LYS A 469 17.441 22.187 11.522 1.00 0.42
H
ATOM 7107 1HB LYS A 469 16.897 24.644 13.227 1.00 0.63
H
ATOM 7108 2HB LYS A 469 16.261 23.042 13.578 1.00 0.63
H
ATOM 7109 1HG LYS A 469 18.567 22.171 13.774 1.00 0.63
H
ATOM 7110 2HG LYS A 469 19.195 23.794 13.454 1.00 0.63
H
ATOM 7111 1HD LYS A 469 18.015 24.645 15.457 1.00 0.63
H
ATOM 7112 2HD LYS A 469 17.392 23.020 15.778 1.00 0.63
H
ATOM 7113 1HE LYS A 469 19.695 22.177 16.020 1.00 0.63
H
ATOM 7114 2HE LYS A 469 20.329 23.797 15.686 1.00 0.63
H

ATOM 7115 1HZ LYS A 469 20.238 23.349 18.031 1.00 0.63
H
ATOM 7116 2HZ LYS A 469 19.204 24.577 17.655 1.00 0.63
H
ATOM 7117 3HZ LYS A 469 18.614 23.068 17.968 1.00 0.63
H
ATOM 7118 N GLY A 470 14.934 22.348 11.185 1.00 0.32
N
ATOM 7119 CA GLY A 470 13.581 22.335 10.649 1.00 0.37
C
ATOM 7120 C GLY A 470 13.552 21.681 9.280 1.00 0.45
C
ATOM 7121 O GLY A 470 14.396 20.847 8.959 1.00 1.16
O
ATOM 7122 H GLY A 470 15.262 21.547 11.712 1.00 0.38
H
ATOM 7123 1HA GLY A 470 12.925 21.793 11.331 1.00 0.44
H
ATOM 7124 2HA GLY A 470 13.205 23.354 10.580 1.00 0.44
H
ATOM 7125 N MET A 471 12.578 22.051 8.469 1.00 0.49
N
ATOM 7126 CA MET A 471 12.485 21.469 7.147 1.00 0.69
C
ATOM 7127 C MET A 471 13.078 22.409 6.138 1.00 1.03
C
ATOM 7128 O MET A 471 12.653 23.552 6.026 1.00 3.12
O
ATOM 7129 CB MET A 471 11.049 21.154 6.794 1.00 1.03
C
ATOM 7130 CG MET A 471 10.885 20.524 5.425 1.00 1.03
C
ATOM 7131 SD MET A 471 9.181 20.093 5.097 1.00 1.03
S
ATOM 7132 CE MET A 471 9.051 18.763 6.280 1.00 1.03
C
ATOM 7133 H MET A 471 11.916 22.756 8.763 1.00 0.59
H
ATOM 7134 HA MET A 471 13.064 20.547 7.127 1.00 0.83
H
ATOM 7135 1HB MET A 471 10.635 20.468 7.532 1.00 1.24
H
ATOM 7136 2HB MET A 471 10.453 22.066 6.823 1.00 1.24
H
ATOM 7137 1HG MET A 471 11.222 21.217 4.655 1.00 1.24
H
ATOM 7138 2HG MET A 471 11.491 19.619 5.360 1.00 1.24
H
ATOM 7139 1HE MET A 471 8.055 18.341 6.236 1.00 1.24
H
ATOM 7140 2HE MET A 471 9.783 17.992 6.041 1.00 1.24
H
ATOM 7141 3HE MET A 471 9.241 19.147 7.283 1.00 1.24
H
ATOM 7142 N CYS A 472 14.060 21.942 5.397 1.00 0.44
N
ATOM 7143 CA CYS A 472 14.659 22.808 4.402 1.00 0.49
C

ATOM	7144	C	CYS	A	472	14.333	22.285	3.037	1.00	0.54	C
ATOM	7145	O	CYS	A	472	14.719	21.175	2.692	1.00	2.33	O
ATOM	7146	CB	CYS	A	472	16.164	22.884	4.560	1.00	0.73	C
ATOM	7147	SG	CYS	A	472	16.708	23.526	6.156	1.00	0.73	S
ATOM	7148	H	CYS	A	472	14.387	20.989	5.520	1.00	0.53	H
ATOM	7149	HA	CYS	A	472	14.245	23.809	4.505	1.00	0.59	H
ATOM	7150	1HB	CYS	A	472	16.584	21.893	4.427	1.00	0.88	H
ATOM	7151	2HB	CYS	A	472	16.579	23.520	3.780	1.00	0.88	H
ATOM	7152	HG	CYS	A	472	16.297	22.484	6.873	1.00	0.88	H
ATOM	7153	N	PHE	A	473	13.619	23.082	2.266	1.00	0.30	N
ATOM	7154	CA	PHE	A	473	13.244	22.679	0.932	1.00	0.26	C
ATOM	7155	C	PHE	A	473	13.978	23.492	-0.087	1.00	0.40	C
ATOM	7156	O	PHE	A	473	13.979	24.718	-0.029	1.00	0.55	O
ATOM	7157	CB	PHE	A	473	11.755	22.793	0.701	1.00	0.39	C
ATOM	7158	CG	PHE	A	473	11.365	22.390	-0.691	1.00	0.39	C
ATOM	7159	CD1	PHE	A	473	11.568	21.089	-1.123	1.00	0.39	C
ATOM	7160	CD2	PHE	A	473	10.775	23.292	-1.561	1.00	0.39	C
ATOM	7161	CE1	PHE	A	473	11.197	20.691	-2.390	1.00	0.39	C
ATOM	7162	CE2	PHE	A	473	10.394	22.891	-2.826	1.00	0.39	C
ATOM	7163	CZ	PHE	A	473	10.608	21.595	-3.243	1.00	0.39	C
ATOM	7164	H	PHE	A	473	13.347	23.990	2.610	1.00	0.36	H
ATOM	7165	HA	PHE	A	473	13.519	21.636	0.800	1.00	0.31	H
ATOM	7166	1HB	PHE	A	473	11.228	22.154	1.406	1.00	0.47	H
ATOM	7167	2HB	PHE	A	473	11.428	23.818	0.870	1.00	0.47	H
ATOM	7168	HD1	PHE	A	473	12.025	20.375	-0.446	1.00	0.47	H
ATOM	7169	HD2	PHE	A	473	10.600	24.316	-1.235	1.00	0.47	H
ATOM	7170	HE1	PHE	A	473	11.365	19.663	-2.709	1.00	0.47	H
ATOM	7171	HE2	PHE	A	473	9.916	23.599	-3.500	1.00	0.47	H
ATOM	7172	HZ	PHE	A	473	10.306	21.287	-4.242	1.00	0.47	H

```
ATOM   7173  N   ILE A 474      14.608  22.809  -1.019  1.00  0.41
N
ATOM   7174  CA  ILE A 474      15.351  23.497  -2.027  1.00  0.59
C
ATOM   7175  C   ILE A 474      14.752  23.219  -3.398  1.00  0.60
C
ATOM   7176  O   ILE A 474      14.597  22.067  -3.805  1.00  1.35
O
ATOM   7177  CB  ILE A 474      16.809  23.080  -1.994  1.00  0.89
C
ATOM   7178  CG1 ILE A 474      17.444  23.314  -0.626  1.00  0.89
C
ATOM   7179  CG2 ILE A 474      17.531  23.937  -2.963  1.00  0.89
C
ATOM   7180  CD1 ILE A 474      18.832  22.728  -0.518  1.00  0.89
C
ATOM   7181  H   ILE A 474      14.579  21.800  -1.010  1.00  0.49
H
ATOM   7182  HA  ILE A 474      15.292  24.567  -1.840  1.00  0.71
H
ATOM   7183  HB  ILE A 474      16.919  22.033  -2.257  1.00  1.06
H
ATOM   7184 1HG1 ILE A 474      17.513  24.381  -0.442  1.00  1.06
H
ATOM   7185 2HG1 ILE A 474      16.821  22.867   0.147  1.00  1.06
H
ATOM   7186 1HG2 ILE A 474      18.573  23.654  -2.921  1.00  1.06
H
ATOM   7187 2HG2 ILE A 474      17.134  23.785  -3.964  1.00  1.06
H
ATOM   7188 3HG2 ILE A 474      17.426  24.984  -2.684  1.00  1.06
H
ATOM   7189 1HD1 ILE A 474      19.236  22.929   0.470  1.00  1.06
H
ATOM   7190 2HD1 ILE A 474      18.786  21.651  -0.679  1.00  1.06
H
ATOM   7191 3HD1 ILE A 474      19.476  23.180  -1.270  1.00  1.06
H
ATOM   7192  N   ARG A 475      14.389  24.286  -4.092  1.00  0.71
N
ATOM   7193  CA  ARG A 475      13.787  24.172  -5.406  1.00  0.79
C
ATOM   7194  C   ARG A 475      14.871  24.099  -6.475  1.00  1.33
C
ATOM   7195  O   ARG A 475      15.877  24.823  -6.405  1.00  6.32
O
ATOM   7196  CB  ARG A 475      12.888  25.375  -5.662  1.00  1.19
C
ATOM   7197  CG  ARG A 475      11.789  25.549  -4.633  1.00  1.19
C
ATOM   7198  CD  ARG A 475      10.917  26.727  -4.871  1.00  1.19
C
ATOM   7199  NE  ARG A 475       9.886  26.470  -5.863  1.00  1.19
N
ATOM   7200  CZ  ARG A 475       9.193  27.429  -6.509  1.00  1.19
C
ATOM   7201  NH1 ARG A 475       9.475  28.695  -6.312  1.00  1.19
N
```

ATOM 7202 NH2 ARG A 475 8.215 27.095 -7.325 1.00 1.19
N
ATOM 7203 H ARG A 475 14.530 25.203 -3.696 1.00 0.85
H
ATOM 7204 HA ARG A 475 13.188 23.261 -5.444 1.00 0.95
H
ATOM 7205 1HB ARG A 475 13.487 26.278 -5.640 1.00 1.42
H
ATOM 7206 2HB ARG A 475 12.428 25.299 -6.647 1.00 1.42
H
ATOM 7207 1HG ARG A 475 11.153 24.673 -4.690 1.00 1.42
H
ATOM 7208 2HG ARG A 475 12.222 25.628 -3.634 1.00 1.42
H
ATOM 7209 1HD ARG A 475 10.428 27.005 -3.939 1.00 1.42
H
ATOM 7210 2HD ARG A 475 11.522 27.559 -5.221 1.00 1.42
H
ATOM 7211 HE ARG A 475 9.618 25.503 -6.031 1.00 1.42
H
ATOM 7212 1HH1 ARG A 475 10.217 28.953 -5.678 1.00 1.42
H
ATOM 7213 2HH1 ARG A 475 8.934 29.418 -6.775 1.00 1.42
H
ATOM 7214 1HH2 ARG A 475 7.958 26.119 -7.428 1.00 1.42
H
ATOM 7215 2HH2 ARG A 475 7.691 27.812 -7.801 1.00 1.42
H
ATOM 7216 N THR A 476 14.649 23.259 -7.484 1.00 0.55
N
ATOM 7217 CA THR A 476 15.608 23.172 -8.566 1.00 0.37
C
ATOM 7218 C THR A 476 14.951 23.013 -9.927 1.00 0.61
C
ATOM 7219 O THR A 476 13.836 22.496 -10.043 1.00 2.33
O
ATOM 7220 CB THR A 476 16.609 22.048 -8.317 1.00 0.55
C
ATOM 7221 OG1 THR A 476 17.608 22.115 -9.329 1.00 0.55
O
ATOM 7222 CG2 THR A 476 16.085 20.678 -8.308 1.00 0.55
C
ATOM 7223 H THR A 476 13.835 22.654 -7.483 1.00 0.66
H
ATOM 7224 HA THR A 476 16.170 24.105 -8.589 1.00 0.44
H
ATOM 7225 HB THR A 476 17.018 22.191 -7.337 1.00 0.67
H
ATOM 7226 HG1 THR A 476 17.223 21.838 -10.159 1.00 0.67
H
ATOM 7227 1HG2 THR A 476 16.897 19.979 -8.110 1.00 0.67
H
ATOM 7228 2HG2 THR A 476 15.335 20.582 -7.537 1.00 0.67
H
ATOM 7229 3HG2 THR A 476 15.663 20.478 -9.283 1.00 0.67
H
ATOM 7230 N SER A 477 15.655 23.505 -10.950 1.00 0.19
N

ATOM 7231 CA SER A 477 15.164 23.547 -12.310 1.00 0.17
C
ATOM 7232 C SER A 477 15.904 22.659 -13.308 1.00 0.19
C
ATOM 7233 O SER A 477 17.105 22.386 -13.195 1.00 0.25
O
ATOM 7234 CB SER A 477 15.257 24.977 -12.797 1.00 0.26
C
ATOM 7235 OG SER A 477 16.599 25.393 -12.863 1.00 0.26
O
ATOM 7236 H SER A 477 16.554 23.903 -10.774 1.00 0.23
H
ATOM 7237 HA SER A 477 14.117 23.242 -12.298 1.00 0.20
H
ATOM 7238 1HB SER A 477 14.792 25.070 -13.778 1.00 0.31
H
ATOM 7239 2HB SER A 477 14.707 25.622 -12.113 1.00 0.31
H
ATOM 7240 HG SER A 477 17.037 25.067 -12.065 1.00 0.31
H
ATOM 7241 N GLN A 478 15.139 22.247 -14.314 1.00 0.20
N
ATOM 7242 CA GLN A 478 15.575 21.413 -15.424 1.00 0.23
C
ATOM 7243 C GLN A 478 16.444 22.078 -16.501 1.00 0.29
C
ATOM 7244 O GLN A 478 17.377 21.435 -16.981 1.00 0.42
O
ATOM 7245 CB GLN A 478 14.348 20.770 -16.089 1.00 0.35
C
ATOM 7246 CG GLN A 478 14.685 19.870 -17.267 1.00 0.35
C
ATOM 7247 CD GLN A 478 15.561 18.694 -16.882 1.00 0.35
C
ATOM 7248 OE1 GLN A 478 15.260 17.948 -15.947 1.00 0.35
O
ATOM 7249 NE2 GLN A 478 16.659 18.519 -17.609 1.00 0.35
N
ATOM 7250 H GLN A 478 14.171 22.533 -14.305 1.00 0.24
H
ATOM 7251 HA GLN A 478 16.172 20.608 -15.003 1.00 0.28
H
ATOM 7252 1HB GLN A 478 13.821 20.163 -15.353 1.00 0.41
H
ATOM 7253 2HB GLN A 478 13.655 21.536 -16.432 1.00 0.41
H
ATOM 7254 1HG GLN A 478 13.759 19.480 -17.687 1.00 0.41
H
ATOM 7255 2HG GLN A 478 15.215 20.458 -18.017 1.00 0.41
H
ATOM 7256 1HE2 GLN A 478 17.278 17.759 -17.407 1.00 0.41
H
ATOM 7257 2HE2 GLN A 478 16.868 19.146 -18.359 1.00 0.41
H
ATOM 7258 N PRO A 479 16.175 23.325 -16.927 1.00 0.36
N
ATOM 7259 CA PRO A 479 16.947 24.011 -17.956 1.00 0.57
C

ATOM 7260 C PRO A 479 18.301 24.469 -17.453 1.00 0.35
C
ATOM 7261 O PRO A 479 18.460 24.832 -16.288 1.00 0.45
O
ATOM 7262 CB PRO A 479 16.040 25.178 -18.375 1.00 0.85
C
ATOM 7263 CG PRO A 479 15.123 25.379 -17.230 1.00 0.85
C
ATOM 7264 CD PRO A 479 14.855 23.977 -16.754 1.00 0.85
C
ATOM 7265 HA PRO A 479 17.081 23.322 -18.802 1.00 0.68
H
ATOM 7266 1HB PRO A 479 16.649 26.068 -18.590 1.00 1.03
H
ATOM 7267 2HB PRO A 479 15.508 24.922 -19.303 1.00 1.03
H
ATOM 7268 1HG PRO A 479 15.599 26.007 -16.463 1.00 1.03
H
ATOM 7269 2HG PRO A 479 14.217 25.903 -17.557 1.00 1.03
H
ATOM 7270 1HD PRO A 479 14.542 24.039 -15.711 1.00 1.03
H
ATOM 7271 2HD PRO A 479 14.099 23.490 -17.387 1.00 1.03
H
ATOM 7272 N GLU A 480 19.274 24.466 -18.359 1.00 0.33
N
ATOM 7273 CA GLU A 480 20.612 24.910 -18.025 1.00 0.41
C
ATOM 7274 C GLU A 480 20.664 26.416 -18.104 1.00 0.65
C
ATOM 7275 O GLU A 480 20.256 27.003 -19.108 1.00 3.84
O
ATOM 7276 CB GLU A 480 21.644 24.284 -18.971 1.00 0.61
C
ATOM 7277 CG GLU A 480 21.765 22.767 -18.860 1.00 0.61
C
ATOM 7278 CD GLU A 480 22.801 22.188 -19.778 1.00 0.61
C
ATOM 7279 OE1 GLU A 480 23.226 22.880 -20.672 1.00 0.61
O
ATOM 7280 OE2 GLU A 480 23.179 21.054 -19.585 1.00 0.61
O
ATOM 7281 H GLU A 480 19.077 24.150 -19.298 1.00 0.40
H
ATOM 7282 HA GLU A 480 20.832 24.615 -17.007 1.00 0.49
H
ATOM 7283 1HB GLU A 480 21.383 24.522 -20.002 1.00 0.74
H
ATOM 7284 2HB GLU A 480 22.626 24.714 -18.773 1.00 0.74
H
ATOM 7285 1HG GLU A 480 22.014 22.498 -17.840 1.00 0.74
H
ATOM 7286 2HG GLU A 480 20.797 22.325 -19.094 1.00 0.74
H
ATOM 7287 N THR A 481 21.161 27.047 -17.047 1.00 0.85
N
ATOM 7288 CA THR A 481 21.207 28.498 -17.011 1.00 0.59
C

```
ATOM   7289  C   THR A 481      22.614  28.978 -16.733  1.00  0.59
C
ATOM   7290  O   THR A 481      23.439  28.241 -16.195  1.00  0.79
O
ATOM   7291  CB  THR A 481      20.251  29.063 -15.939  1.00  0.89
C
ATOM   7292  OG1 THR A 481      20.676  28.621 -14.643  1.00  0.89
O
ATOM   7293  CG2 THR A 481      18.815  28.614 -16.184  1.00  0.89
C
ATOM   7294  H   THR A 481      21.508  26.514 -16.255  1.00  1.02
H
ATOM   7295  HA  THR A 481      20.904  28.885 -17.985  1.00  0.71
H
ATOM   7296  HB  THR A 481      20.287  30.148 -15.971  1.00  1.06
H
ATOM   7297  HG1 THR A 481      20.121  29.025 -13.969  1.00  1.06
H
ATOM   7298 1HG2 THR A 481      18.168  29.036 -15.415  1.00  1.06
H
ATOM   7299 2HG2 THR A 481      18.487  28.960 -17.164  1.00  1.06
H
ATOM   7300 3HG2 THR A 481      18.760  27.527 -16.146  1.00  1.06
H
ATOM   7301  N   ALA A 482      22.895  30.214 -17.115  1.00  0.52
N
ATOM   7302  CA  ALA A 482      24.235  30.752 -16.954  1.00  0.56
C
ATOM   7303  C   ALA A 482      24.566  31.078 -15.507  1.00  0.54
C
ATOM   7304  O   ALA A 482      23.738  31.612 -14.765  1.00  0.62
O
ATOM   7305  CB  ALA A 482      24.398  31.998 -17.807  1.00  0.84
C
ATOM   7306  H   ALA A 482      22.175  30.784 -17.538  1.00  0.62
H
ATOM   7307  HA  ALA A 482      24.936  29.999 -17.299  1.00  0.67
H
ATOM   7308 1HB  ALA A 482      25.418  32.372 -17.712  1.00  1.01
H
ATOM   7309 2HB  ALA A 482      24.198  31.753 -18.851  1.00  1.01
H
ATOM   7310 3HB  ALA A 482      23.699  32.763 -17.473  1.00  1.01
H
ATOM   7311  N   VAL A 483      25.810  30.812 -15.129  1.00  0.48
N
ATOM   7312  CA  VAL A 483      26.297  31.181 -13.814  1.00  0.48
C
ATOM   7313  C   VAL A 483      26.445  32.678 -13.772  1.00  0.49
C
ATOM   7314  O   VAL A 483      27.064  33.265 -14.657  1.00  0.50
O
ATOM   7315  CB  VAL A 483      27.644  30.504 -13.508  1.00  0.72
C
ATOM   7316  CG1 VAL A 483      28.200  31.018 -12.191  1.00  0.72
C
ATOM   7317  CG2 VAL A 483      27.445  29.000 -13.427  1.00  0.72
C
```

ATOM 7318 H VAL A 483 26.429 30.336 -15.768 1.00 0.58
H
ATOM 7319 HA VAL A 483 25.571 30.876 -13.067 1.00 0.58
H
ATOM 7320 HB VAL A 483 28.357 30.743 -14.294 1.00 0.86
H
ATOM 7321 1HG1 VAL A 483 29.154 30.534 -11.981 1.00 0.86
H
ATOM 7322 2HG1 VAL A 483 28.347 32.097 -12.251 1.00 0.86
H
ATOM 7323 3HG1 VAL A 483 27.493 30.793 -11.395 1.00 0.86
H
ATOM 7324 1HG2 VAL A 483 28.395 28.519 -13.215 1.00 0.86
H
ATOM 7325 2HG2 VAL A 483 26.736 28.775 -12.632 1.00 0.86
H
ATOM 7326 3HG2 VAL A 483 27.054 28.629 -14.371 1.00 0.86
H
ATOM 7327 N ILE A 484 25.863 33.308 -12.759 1.00 0.50
N
ATOM 7328 CA ILE A 484 25.938 34.758 -12.668 1.00 0.52
C
ATOM 7329 C ILE A 484 26.632 35.220 -11.399 1.00 0.53
C
ATOM 7330 O ILE A 484 27.088 36.362 -11.328 1.00 0.57
O
ATOM 7331 CB ILE A 484 24.532 35.400 -12.739 1.00 0.78
C
ATOM 7332 CG1 ILE A 484 23.676 34.943 -11.557 1.00 0.78
C
ATOM 7333 CG2 ILE A 484 23.849 35.060 -14.057 1.00 0.78
C
ATOM 7334 CD1 ILE A 484 22.382 35.704 -11.422 1.00 0.78
C
ATOM 7335 H ILE A 484 25.373 32.776 -12.048 1.00 0.60
H
ATOM 7336 HA ILE A 484 26.514 35.125 -13.515 1.00 0.62
H
ATOM 7337 HB ILE A 484 24.633 36.482 -12.664 1.00 0.94
H
ATOM 7338 1HG1 ILE A 484 23.448 33.883 -11.669 1.00 0.94
H
ATOM 7339 2HG1 ILE A 484 24.240 35.077 -10.637 1.00 0.94
H
ATOM 7340 1HG2 ILE A 484 22.870 35.536 -14.093 1.00 0.94
H
ATOM 7341 2HG2 ILE A 484 24.459 35.418 -14.886 1.00 0.94
H
ATOM 7342 3HG2 ILE A 484 23.729 33.980 -14.135 1.00 0.94
H
ATOM 7343 1HD1 ILE A 484 21.830 35.330 -10.560 1.00 0.94
H
ATOM 7344 2HD1 ILE A 484 22.597 36.763 -11.283 1.00 0.94
H
ATOM 7345 3HD1 ILE A 484 21.784 35.569 -12.322 1.00 0.94
H
ATOM 7346 N TYR A 485 26.749 34.339 -10.408 1.00 0.53
N

```
ATOM   7347  CA  TYR A 485      27.435  34.732  -9.192  1.00  0.63
C
ATOM   7348  C   TYR A 485      28.879  34.255  -9.194  1.00  0.68
C
ATOM   7349  O   TYR A 485      29.230  33.275  -9.846  1.00  0.66
O
ATOM   7350  CB  TYR A 485      26.718  34.193  -7.953  1.00  0.95
C
ATOM   7351  CG  TYR A 485      25.328  34.753  -7.758  1.00  0.95
C
ATOM   7352  CD1 TYR A 485      24.238  33.935  -7.988  1.00  0.95
C
ATOM   7353  CD2 TYR A 485      25.132  36.069  -7.370  1.00  0.95
C
ATOM   7354  CE1 TYR A 485      22.956  34.409  -7.834  1.00  0.95
C
ATOM   7355  CE2 TYR A 485      23.844  36.552  -7.217  1.00  0.95
C
ATOM   7356  CZ  TYR A 485      22.762  35.723  -7.450  1.00  0.95
C
ATOM   7357  OH  TYR A 485      21.486  36.199  -7.304  1.00  0.95
O
ATOM   7358  H   TYR A 485      26.333  33.414 -10.478  1.00  0.64
H
ATOM   7359  HA  TYR A 485      27.448  35.820  -9.136  1.00  0.76
H
ATOM   7360 1HB  TYR A 485      26.637  33.110  -8.030  1.00  1.13
H
ATOM   7361 2HB  TYR A 485      27.304  34.416  -7.063  1.00  1.13
H
ATOM   7362  HD1 TYR A 485      24.400  32.908  -8.287  1.00  1.13
H
ATOM   7363  HD2 TYR A 485      25.986  36.722  -7.190  1.00  1.13
H
ATOM   7364  HE1 TYR A 485      22.103  33.755  -8.017  1.00  1.13
H
ATOM   7365  HE2 TYR A 485      23.683  37.587  -6.915  1.00  1.13
H
ATOM   7366  HH  TYR A 485      20.847  35.494  -7.477  1.00  1.13
H
ATOM   7367  N   THR A 486      29.713  34.974  -8.462  1.00  0.88
N
ATOM   7368  CA  THR A 486      31.108  34.616  -8.278  1.00  1.15
C
ATOM   7369  C   THR A 486      31.145  33.605  -7.127  1.00  1.50
C
ATOM   7370  O   THR A 486      30.413  33.785  -6.163  1.00  5.08
O
ATOM   7371  CB  THR A 486      31.959  35.871  -7.946  1.00  1.72
C
ATOM   7372  OG1 THR A 486      31.868  36.806  -9.030  1.00  1.72
O
ATOM   7373  CG2 THR A 486      33.424  35.525  -7.723  1.00  1.72
C
ATOM   7374  H   THR A 486      29.356  35.789  -7.984  1.00  1.06
H
ATOM   7375  HA  THR A 486      31.483  34.163  -9.191  1.00  1.38
H
```

```
ATOM   7376  HB  THR A 486      31.563  36.339  -7.045  1.00  2.07
H
ATOM   7377  HG1 THR A 486      32.387  37.586  -8.824  1.00  2.07
H
ATOM   7378 1HG2 THR A 486      33.979  36.434  -7.494  1.00  2.07
H
ATOM   7379 2HG2 THR A 486      33.517  34.830  -6.898  1.00  2.07
H
ATOM   7380 3HG2 THR A 486      33.831  35.072  -8.626  1.00  2.07
H
ATOM   7381  N   PRO A 487      31.933  32.525  -7.210  1.00  1.11
N
ATOM   7382  CA  PRO A 487      32.112  31.466  -6.205  1.00  1.90
C
ATOM   7383  C   PRO A 487      32.405  31.963  -4.789  1.00  4.99
C
ATOM   7384  O   PRO A 487      32.120  31.266  -3.818  1.00 30.39
O
ATOM   7385  CB  PRO A 487      33.281  30.677  -6.764  1.00  2.85
C
ATOM   7386  CG  PRO A 487      33.135  30.798  -8.244  1.00  2.85
C
ATOM   7387  CD  PRO A 487      32.601  32.183  -8.480  1.00  2.85
C
ATOM   7388  HA  PRO A 487      31.209  30.844  -6.192  1.00  2.28
H
ATOM   7389 1HB  PRO A 487      34.229  31.090  -6.394  1.00  3.42
H
ATOM   7390 2HB  PRO A 487      33.222  29.644  -6.403  1.00  3.42
H
ATOM   7391 1HG  PRO A 487      34.110  30.639  -8.730  1.00  3.42
H
ATOM   7392 2HG  PRO A 487      32.466  30.016  -8.623  1.00  3.42
H
ATOM   7393 1HD  PRO A 487      33.420  32.880  -8.689  1.00  3.42
H
ATOM   7394 2HD  PRO A 487      31.865  32.156  -9.300  1.00  3.42
H
ATOM   7395  N   GLN A 488      32.961  33.157  -4.662  1.00  2.46
N
ATOM   7396  CA  GLN A 488      33.269  33.738  -3.365  1.00  1.81
C
ATOM   7397  C   GLN A 488      32.084  34.512  -2.766  1.00  1.83
C
ATOM   7398  O   GLN A 488      32.174  35.002  -1.640  1.00  2.44
O
ATOM   7399  CB  GLN A 488      34.472  34.671  -3.491  1.00  2.71
C
ATOM   7400  CG  GLN A 488      35.744  33.992  -3.969  1.00  2.71
C
ATOM   7401  CD  GLN A 488      36.209  32.898  -3.031  1.00  2.71
C
ATOM   7402  OE1 GLN A 488      36.319  33.104  -1.818  1.00  2.71
O
ATOM   7403  NE2 GLN A 488      36.491  31.726  -3.586  1.00  2.71
N
ATOM   7404  H   GLN A 488      33.190  33.682  -5.488  1.00  2.95
H
```

```
ATOM   7405  HA  GLN A 488      33.522  32.928  -2.679  1.00  2.17
H
ATOM   7406 1HB  GLN A 488      34.238  35.476  -4.187  1.00  3.26
H
ATOM   7407 2HB  GLN A 488      34.681  35.128  -2.523  1.00  3.26
H
ATOM   7408 1HG  GLN A 488      35.559  33.549  -4.947  1.00  3.26
H
ATOM   7409 2HG  GLN A 488      36.535  34.738  -4.043  1.00  3.26
H
ATOM   7410 1HE2 GLN A 488      36.806  30.965  -3.018  1.00  3.26
H
ATOM   7411 2HE2 GLN A 488      36.391  31.603  -4.574  1.00  3.26
H
ATOM   7412  N   GLU A 489      30.991  34.643  -3.526  1.00  1.52
N
ATOM   7413  CA  GLU A 489      29.802  35.350  -3.064  1.00  1.75
C
ATOM   7414  C   GLU A 489      29.235  34.681  -1.823  1.00  1.35
C
ATOM   7415  O   GLU A 489      28.988  33.475  -1.812  1.00  1.58
O
ATOM   7416  CB  GLU A 489      28.733  35.403  -4.163  1.00  2.62
C
ATOM   7417  CG  GLU A 489      27.493  36.220  -3.808  1.00  2.62
C
ATOM   7418  CD  GLU A 489      27.786  37.697  -3.702  1.00  2.62
C
ATOM   7419  OE1 GLU A 489      28.625  38.164  -4.434  1.00  2.62
O
ATOM   7420  OE2 GLU A 489      27.169  38.364  -2.900  1.00  2.62
O
ATOM   7421  H   GLU A 489      30.962  34.233  -4.444  1.00  1.82
H
ATOM   7422  HA  GLU A 489      30.084  36.370  -2.802  1.00  2.10
H
ATOM   7423 1HB  GLU A 489      29.166  35.834  -5.066  1.00  3.15
H
ATOM   7424 2HB  GLU A 489      28.413  34.390  -4.407  1.00  3.15
H
ATOM   7425 1HG  GLU A 489      26.735  36.063  -4.575  1.00  3.15
H
ATOM   7426 2HG  GLU A 489      27.094  35.860  -2.861  1.00  3.15
H
ATOM   7427  N   ASN A 490      29.034  35.466  -0.774  1.00  1.59
N
ATOM   7428  CA  ASN A 490      28.516  34.928   0.472  1.00  1.96
C
ATOM   7429  C   ASN A 490      26.994  34.924   0.500  1.00  1.28
C
ATOM   7430  O   ASN A 490      26.357  35.972   0.384  1.00  2.46
O
ATOM   7431  CB  ASN A 490      29.074  35.702   1.648  1.00  2.94
C
ATOM   7432  CG  ASN A 490      30.559  35.516   1.800  1.00  2.94
C
ATOM   7433  OD1 ASN A 490      31.065  34.390   1.742  1.00  2.94
O
```

```
ATOM   7434  ND2 ASN A 490      31.268  36.600   1.993  1.00  2.94
N
ATOM   7435  H   ASN A 490      29.250  36.451  -0.842  1.00  1.91
H
ATOM   7436  HA  ASN A 490      28.841  33.889   0.558  1.00  2.35
H
ATOM   7437 1HB  ASN A 490      28.864  36.765   1.517  1.00  3.53
H
ATOM   7438 2HB  ASN A 490      28.583  35.380   2.566  1.00  3.53
H
ATOM   7439 1HD2 ASN A 490      32.260  36.536   2.099  1.00  3.53
H
ATOM   7440 2HD2 ASN A 490      30.817  37.492   2.032  1.00  3.53
H
ATOM   7441  N   PHE A 491      26.420  33.740   0.676  1.00  0.63
N
ATOM   7442  CA  PHE A 491      24.974  33.583   0.750  1.00  0.41
C
ATOM   7443  C   PHE A 491      24.513  33.319   2.168  1.00  0.45
C
ATOM   7444  O   PHE A 491      25.238  32.732   2.973  1.00  0.67
O
ATOM   7445  CB  PHE A 491      24.502  32.462  -0.163  1.00  0.61
C
ATOM   7446  CG  PHE A 491      24.616  32.789  -1.618  1.00  0.61
C
ATOM   7447  CD1 PHE A 491      25.805  32.593  -2.291  1.00  0.61
C
ATOM   7448  CD2 PHE A 491      23.530  33.287  -2.318  1.00  0.61
C
ATOM   7449  CE1 PHE A 491      25.913  32.881  -3.632  1.00  0.61
C
ATOM   7450  CE2 PHE A 491      23.632  33.577  -3.662  1.00  0.61
C
ATOM   7451  CZ  PHE A 491      24.827  33.370  -4.316  1.00  0.61
C
ATOM   7452  H   PHE A 491      27.006  32.920   0.760  1.00  0.76
H
ATOM   7453  HA  PHE A 491      24.511  34.511   0.413  1.00  0.49
H
ATOM   7454 1HB  PHE A 491      25.088  31.567   0.033  1.00  0.74
H
ATOM   7455 2HB  PHE A 491      23.463  32.229   0.054  1.00  0.74
H
ATOM   7456  HD1 PHE A 491      26.664  32.208  -1.745  1.00  0.74
H
ATOM   7457  HD2 PHE A 491      22.588  33.446  -1.794  1.00  0.74
H
ATOM   7458  HE1 PHE A 491      26.859  32.722  -4.148  1.00  0.74
H
ATOM   7459  HE2 PHE A 491      22.771  33.967  -4.207  1.00  0.74
H
ATOM   7460  HZ  PHE A 491      24.913  33.595  -5.370  1.00  0.74
H
ATOM   7461  N   GLU A 492      23.303  33.765   2.464  1.00  0.41
N
ATOM   7462  CA  GLU A 492      22.727  33.615   3.786  1.00  0.46
C
```

ATOM 7463 C GLU A 492 21.215 33.525 3.721 1.00 0.43
C
ATOM 7464 O GLU A 492 20.577 34.196 2.906 1.00 0.47
O
ATOM 7465 CB GLU A 492 23.145 34.780 4.685 1.00 0.69
C
ATOM 7466 CG GLU A 492 22.663 34.672 6.125 1.00 0.69
C
ATOM 7467 CD GLU A 492 23.185 35.778 6.999 1.00 0.69
C
ATOM 7468 OE1 GLU A 492 23.919 36.601 6.508 1.00 0.69
O
ATOM 7469 OE2 GLU A 492 22.856 35.796 8.162 1.00 0.69
O
ATOM 7470 H GLU A 492 22.765 34.232 1.748 1.00 0.49
H
ATOM 7471 HA GLU A 492 23.104 32.689 4.222 1.00 0.55
H
ATOM 7472 1HB GLU A 492 24.233 34.851 4.703 1.00 0.83
H
ATOM 7473 2HB GLU A 492 22.764 35.714 4.273 1.00 0.83
H
ATOM 7474 1HG GLU A 492 21.573 34.705 6.138 1.00 0.83
H
ATOM 7475 2HG GLU A 492 22.976 33.713 6.533 1.00 0.83
H
ATOM 7476 N ILE A 493 20.652 32.680 4.572 1.00 0.42
N
ATOM 7477 CA ILE A 493 19.215 32.493 4.632 1.00 0.45
C
ATOM 7478 C ILE A 493 18.547 33.778 5.076 1.00 0.42
C
ATOM 7479 O ILE A 493 18.925 34.358 6.095 1.00 0.46
O
ATOM 7480 CB ILE A 493 18.860 31.363 5.604 1.00 0.68
C
ATOM 7481 CG1 ILE A 493 19.422 30.050 5.081 1.00 0.68
C
ATOM 7482 CG2 ILE A 493 17.359 31.269 5.807 1.00 0.68
C
ATOM 7483 CD1 ILE A 493 19.333 28.936 6.084 1.00 0.68
C
ATOM 7484 H ILE A 493 21.242 32.157 5.206 1.00 0.50
H
ATOM 7485 HA ILE A 493 18.854 32.238 3.640 1.00 0.54
H
ATOM 7486 HB ILE A 493 19.336 31.556 6.564 1.00 0.81
H
ATOM 7487 1HG1 ILE A 493 18.878 29.760 4.185 1.00 0.81
H
ATOM 7488 2HG1 ILE A 493 20.468 30.191 4.820 1.00 0.81
H
ATOM 7489 1HG2 ILE A 493 17.137 30.466 6.510 1.00 0.81
H
ATOM 7490 2HG2 ILE A 493 16.988 32.211 6.207 1.00 0.81
H
ATOM 7491 3HG2 ILE A 493 16.870 31.061 4.857 1.00 0.81
H

ATOM 7492 1HD1 ILE A 493 19.756 28.027 5.661 1.00 0.81
H
ATOM 7493 2HD1 ILE A 493 19.892 29.209 6.980 1.00 0.81
H
ATOM 7494 3HD1 ILE A 493 18.290 28.764 6.346 1.00 0.81
H
ATOM 7495 N GLY A 494 17.562 34.237 4.306 1.00 0.43
N
ATOM 7496 CA GLY A 494 16.898 35.490 4.614 1.00 0.48
C
ATOM 7497 C GLY A 494 17.582 36.696 3.965 1.00 0.58
C
ATOM 7498 O GLY A 494 17.177 37.832 4.210 1.00 0.70
O
ATOM 7499 H GLY A 494 17.247 33.723 3.492 1.00 0.52
H
ATOM 7500 1HA GLY A 494 15.862 35.438 4.277 1.00 0.58
H
ATOM 7501 2HA GLY A 494 16.870 35.628 5.695 1.00 0.58
H
ATOM 7502 N GLN A 495 18.611 36.460 3.139 1.00 0.64
N
ATOM 7503 CA GLN A 495 19.297 37.556 2.455 1.00 0.87
C
ATOM 7504 C GLN A 495 19.164 37.452 0.933 1.00 0.94
C
ATOM 7505 O GLN A 495 19.557 36.450 0.332 1.00 1.76
O
ATOM 7506 CB GLN A 495 20.777 37.581 2.835 1.00 1.30
C
ATOM 7507 CG GLN A 495 21.582 38.645 2.109 1.00 1.30
C
ATOM 7508 CD GLN A 495 21.225 40.051 2.555 1.00 1.30
C
ATOM 7509 OE1 GLN A 495 21.392 40.403 3.727 1.00 1.30
O
ATOM 7510 NE2 GLN A 495 20.730 40.859 1.627 1.00 1.30
N
ATOM 7511 H GLN A 495 18.944 35.516 2.985 1.00 0.77
H
ATOM 7512 HA GLN A 495 18.841 38.496 2.765 1.00 1.04
H
ATOM 7513 1HB GLN A 495 20.876 37.754 3.907 1.00 1.57
H
ATOM 7514 2HB GLN A 495 21.227 36.613 2.617 1.00 1.57
H
ATOM 7515 1HG GLN A 495 22.640 38.481 2.306 1.00 1.57
H
ATOM 7516 2HG GLN A 495 21.384 38.568 1.039 1.00 1.57
H
ATOM 7517 1HE2 GLN A 495 20.473 41.797 1.864 1.00 1.57
H
ATOM 7518 2HE2 GLN A 495 20.601 40.531 0.689 1.00 1.57
H
ATOM 7519 N ALA A 496 18.613 38.502 0.323 1.00 0.69
N
ATOM 7520 CA ALA A 496 18.428 38.575 -1.125 1.00 0.66
C

ATOM 7521 C ALA A 496 19.606 39.259 -1.798 1.00 0.63
C
ATOM 7522 O ALA A 496 20.355 40.010 -1.169 1.00 0.72
O
ATOM 7523 CB ALA A 496 17.146 39.317 -1.469 1.00 0.99
C
ATOM 7524 H ALA A 496 18.312 39.292 0.886 1.00 0.83
H
ATOM 7525 HA ALA A 496 18.364 37.557 -1.511 1.00 0.79
H
ATOM 7526 1HB ALA A 496 17.016 39.338 -2.551 1.00 1.19
H
ATOM 7527 2HB ALA A 496 16.299 38.806 -1.013 1.00 1.19
H
ATOM 7528 3HB ALA A 496 17.204 40.336 -1.089 1.00 1.19
H
ATOM 7529 N LYS A 497 19.771 38.987 -3.086 1.00 0.64
N
ATOM 7530 CA LYS A 497 20.846 39.573 -3.866 1.00 0.79
C
ATOM 7531 C LYS A 497 20.362 40.598 -4.883 1.00 0.63
C
ATOM 7532 O LYS A 497 19.358 40.404 -5.569 1.00 0.77
O
ATOM 7533 CB LYS A 497 21.616 38.465 -4.589 1.00 1.19
C
ATOM 7534 CG LYS A 497 22.244 37.446 -3.654 1.00 1.19
C
ATOM 7535 CD LYS A 497 23.333 38.108 -2.831 1.00 1.19
C
ATOM 7536 CE LYS A 497 24.021 37.137 -1.893 1.00 1.19
C
ATOM 7537 NZ LYS A 497 25.090 37.818 -1.119 1.00 1.19
N
ATOM 7538 H LYS A 497 19.134 38.350 -3.540 1.00 0.77
H
ATOM 7539 HA LYS A 497 21.525 40.082 -3.182 1.00 0.95
H
ATOM 7540 1HB LYS A 497 20.945 37.932 -5.263 1.00 1.42
H
ATOM 7541 2HB LYS A 497 22.410 38.903 -5.193 1.00 1.42
H
ATOM 7542 1HG LYS A 497 21.485 37.036 -2.987 1.00 1.42
H
ATOM 7543 2HG LYS A 497 22.677 36.631 -4.234 1.00 1.42
H
ATOM 7544 1HD LYS A 497 24.081 38.538 -3.498 1.00 1.42
H
ATOM 7545 2HD LYS A 497 22.897 38.914 -2.240 1.00 1.42
H
ATOM 7546 1HE LYS A 497 23.287 36.729 -1.199 1.00 1.42
H
ATOM 7547 2HE LYS A 497 24.460 36.319 -2.463 1.00 1.42
H
ATOM 7548 1HZ LYS A 497 25.529 37.152 -0.500 1.00 1.42
H
ATOM 7549 2HZ LYS A 497 25.785 38.174 -1.762 1.00 1.42
H

ATOM	7550	3HZ	LYS	A	497	24.701	38.576	-0.580	1.00	1.42
H										
ATOM	7551	N	VAL	A	498	21.121	41.678	-5.019	1.00	1.00
N										
ATOM	7552	CA	VAL	A	498	20.832	42.660	-6.049	1.00	0.93
C										
ATOM	7553	C	VAL	A	498	21.682	42.270	-7.240	1.00	0.90
C										
ATOM	7554	O	VAL	A	498	22.901	42.444	-7.223	1.00	1.30
O										
ATOM	7555	CB	VAL	A	498	21.168	44.086	-5.593	1.00	1.40
C										
ATOM	7556	CG1	VAL	A	498	20.849	45.057	-6.715	1.00	1.40
C										
ATOM	7557	CG2	VAL	A	498	20.387	44.430	-4.333	1.00	1.40
C										
ATOM	7558	H	VAL	A	498	21.922	41.807	-4.417	1.00	1.20
H										
ATOM	7559	HA	VAL	A	498	19.781	42.604	-6.324	1.00	1.12
H										
ATOM	7560	HB	VAL	A	498	22.237	44.156	-5.389	1.00	1.67
H										
ATOM	7561	1HG1	VAL	A	498	21.103	46.070	-6.403	1.00	1.67
H										
ATOM	7562	2HG1	VAL	A	498	21.429	44.793	-7.599	1.00	1.67
H										
ATOM	7563	3HG1	VAL	A	498	19.787	45.002	-6.948	1.00	1.67
H										
ATOM	7564	1HG2	VAL	A	498	20.639	45.441	-4.015	1.00	1.67
H										
ATOM	7565	2HG2	VAL	A	498	19.320	44.371	-4.534	1.00	1.67
H										
ATOM	7566	3HG2	VAL	A	498	20.646	43.727	-3.541	1.00	1.67
H										
ATOM	7567	N	VAL	A	499	21.045	41.696	-8.248	1.00	0.69
N										
ATOM	7568	CA	VAL	A	499	21.755	41.162	-9.399	1.00	0.73
C										
ATOM	7569	C	VAL	A	499	22.127	42.274	-10.378	1.00	0.81
C										
ATOM	7570	O	VAL	A	499	23.267	42.347	-10.846	1.00	1.33
O										
ATOM	7571	CB	VAL	A	499	20.896	40.086	-10.089	1.00	1.09
C										
ATOM	7572	CG1	VAL	A	499	21.597	39.578	-11.329	1.00	1.09
C										
ATOM	7573	CG2	VAL	A	499	20.618	38.953	-9.116	1.00	1.09
C										
ATOM	7574	H	VAL	A	499	20.037	41.619	-8.218	1.00	0.83
H										
ATOM	7575	HA	VAL	A	499	22.674	40.690	-9.046	1.00	0.88
H										
ATOM	7576	HB	VAL	A	499	19.955	40.532	-10.405	1.00	1.31
H										
ATOM	7577	1HG1	VAL	A	499	20.974	38.828	-11.815	1.00	1.31
H										
ATOM	7578	2HG1	VAL	A	499	21.775	40.402	-12.012	1.00	1.31
H										

ATOM   7579 3HG1 VAL A 499   22.549 39.128 -11.048 1.00 1.31
H
ATOM   7580 1HG2 VAL A 499   19.997 38.202 -9.602 1.00 1.31
H
ATOM   7581 2HG2 VAL A 499   21.558 38.500 -8.803 1.00 1.31
H
ATOM   7582 3HG2 VAL A 499   20.095 39.344 -8.243 1.00 1.31
H
ATOM   7583 N   ARG A 500   21.162 43.135 -10.687 1.00 0.54
N
ATOM   7584 CA ARG A 500   21.424 44.273 -11.571 1.00 0.55
C
ATOM   7585 C   ARG A 500   20.851 45.545 -10.998 1.00 0.63
C
ATOM   7586 O   ARG A 500   19.747 45.555 -10.463 1.00 1.10
O
ATOM   7587 CB ARG A 500   20.853 44.062 -12.965 1.00 0.83
C
ATOM   7588 CG ARG A 500   21.488 42.940 -13.760 1.00 0.83
C
ATOM   7589 CD ARG A 500   22.876 43.291 -14.152 1.00 0.83
C
ATOM   7590 NE ARG A 500   23.496 42.270 -14.979 1.00 0.83
N
ATOM   7591 CZ ARG A 500   24.178 41.204 -14.513 1.00 0.83
C
ATOM   7592 NH1 ARG A 500   24.332 41.010 -13.219 1.00 0.83
N
ATOM   7593 NH2 ARG A 500   24.706 40.343 -15.368 1.00 0.83
N
ATOM   7594 H   ARG A 500   20.242 43.003 -10.279 1.00 0.65
H
ATOM   7595 HA ARG A 500   22.504 44.397 -11.662 1.00 0.66
H
ATOM   7596 1HB ARG A 500   19.788 43.859 -12.896 1.00 0.99
H
ATOM   7597 2HB ARG A 500   20.970 44.977 -13.545 1.00 0.99
H
ATOM   7598 1HG ARG A 500   21.511 42.031 -13.169 1.00 0.99
H
ATOM   7599 2HG ARG A 500   20.911 42.769 -14.669 1.00 0.99
H
ATOM   7600 1HD ARG A 500   22.867 44.224 -14.714 1.00 0.99
H
ATOM   7601 2HD ARG A 500   23.482 43.416 -13.255 1.00 0.99
H
ATOM   7602 HE ARG A 500   23.412 42.369 -15.982 1.00 0.99
H
ATOM   7603 1HH1 ARG A 500   23.936 41.654 -12.543 1.00 0.99
H
ATOM   7604 2HH1 ARG A 500   24.849 40.207 -12.890 1.00 0.99
H
ATOM   7605 1HH2 ARG A 500   24.594 40.487 -16.362 1.00 0.99
H
ATOM   7606 2HH2 ARG A 500   25.220 39.545 -15.027 1.00 0.99
H
ATOM   7607 N   HIS A 501   21.598 46.632 -11.130 1.00 0.70
N

```
ATOM   7608  CA  HIS A 501      21.146  47.902 -10.593  1.00  0.78
C
ATOM   7609  C   HIS A 501      21.818  49.114 -11.220  1.00  1.73
C
ATOM   7610  O   HIS A 501      22.858  49.001 -11.872  1.00 10.52
O
ATOM   7611  CB  HIS A 501      21.385  47.922  -9.087  1.00  1.17
C
ATOM   7612  CG  HIS A 501      22.822  47.760  -8.715  1.00  1.17
C
ATOM   7613  ND1 HIS A 501      23.440  46.527  -8.674  1.00  1.17
N
ATOM   7614  CD2 HIS A 501      23.766  48.666  -8.368  1.00  1.17
C
ATOM   7615  CE1 HIS A 501      24.702  46.683  -8.316  1.00  1.17
C
ATOM   7616  NE2 HIS A 501      24.924  47.971  -8.124  1.00  1.17
N
ATOM   7617  H   HIS A 501      22.494  46.576 -11.592  1.00  0.84
H
ATOM   7618  HA  HIS A 501      20.076  47.998 -10.766  1.00  0.94
H
ATOM   7619 1HB  HIS A 501      21.027  48.863  -8.669  1.00  1.40
H
ATOM   7620 2HB  HIS A 501      20.820  47.121  -8.622  1.00  1.40
H
ATOM   7621  HD2 HIS A 501      23.630  49.746  -8.295  1.00  1.40
H
ATOM   7622  HE1 HIS A 501      25.434  45.884  -8.198  1.00  1.40
H
ATOM   7623  HE2 HIS A 501      25.803  48.383  -7.844  1.00  1.40
H
ATOM   7624  N   GLY A 502      21.234  50.277 -10.964  1.00  1.06
N
ATOM   7625  CA  GLY A 502      21.758  51.559 -11.415  1.00  1.48
C
ATOM   7626  C   GLY A 502      20.933  52.711 -10.836  1.00  1.47
C
ATOM   7627  O   GLY A 502      19.846  52.511 -10.292  1.00  1.36
O
ATOM   7628  H   GLY A 502      20.363  50.259 -10.453  1.00  1.27
H
ATOM   7629 1HA  GLY A 502      22.799  51.657 -11.108  1.00  1.78
H
ATOM   7630 2HA  GLY A 502      21.735  51.600 -12.504  1.00  1.78
H
ATOM   7631  N   VAL A 503      21.447  53.929 -10.971  1.00  1.63
N
ATOM   7632  CA  VAL A 503      20.794  55.108 -10.403  1.00  1.63
C
ATOM   7633  C   VAL A 503      19.483  55.477 -11.091  1.00  1.54
C
ATOM   7634  O   VAL A 503      18.647  56.166 -10.507  1.00  1.62
O
ATOM   7635  CB  VAL A 503      21.754  56.315 -10.450  1.00  2.44
C
ATOM   7636  CG1 VAL A 503      23.005  56.011  -9.639  1.00  2.44
C
```

```
ATOM   7637  CG2 VAL A 503      22.120  56.630 -11.893  1.00  2.44
C
ATOM   7638  H   VAL A 503      22.331  54.039 -11.450  1.00  1.96
H
ATOM   7639  HA  VAL A 503      20.576  54.894  -9.356  1.00  1.96
H
ATOM   7640  HB  VAL A 503      21.266  57.178  -9.996  1.00  2.93
H
ATOM   7641 1HG1 VAL A 503      23.672  56.873  -9.663  1.00  2.93
H
ATOM   7642 2HG1 VAL A 503      22.726  55.797  -8.607  1.00  2.93
H
ATOM   7643 3HG1 VAL A 503      23.514  55.147 -10.065  1.00  2.93
H
ATOM   7644 1HG2 VAL A 503      22.795  57.487 -11.915  1.00  2.93
H
ATOM   7645 2HG2 VAL A 503      22.615  55.769 -12.341  1.00  2.93
H
ATOM   7646 3HG2 VAL A 503      21.220  56.868 -12.457  1.00  2.93
H
ATOM   7647  N   ASN A 504      19.309  55.023 -12.329  1.00  1.49
N
ATOM   7648  CA  ASN A 504      18.110  55.315 -13.094  1.00  1.43
C
ATOM   7649  C   ASN A 504      17.090  54.176 -13.103  1.00  1.28
C
ATOM   7650  O   ASN A 504      16.183  54.187 -13.936  1.00  1.28
O
ATOM   7651  CB  ASN A 504      18.480  55.679 -14.519  1.00  2.15
C
ATOM   7652  CG  ASN A 504      19.199  56.996 -14.612  1.00  2.15
C
ATOM   7653  OD1 ASN A 504      18.843  57.962 -13.928  1.00  2.15
O
ATOM   7654  ND2 ASN A 504      20.203  57.054 -15.451  1.00  2.15
N
ATOM   7655  H   ASN A 504      20.031  54.463 -12.759  1.00  1.79
H
ATOM   7656  HA  ASN A 504      17.619  56.176 -12.637  1.00  1.72
H
ATOM   7657 1HB  ASN A 504      19.118  54.900 -14.939  1.00  2.57
H
ATOM   7658 2HB  ASN A 504      17.578  55.727 -15.129  1.00  2.57
H
ATOM   7659 1HD2 ASN A 504      20.718  57.907 -15.555  1.00  2.57
H
ATOM   7660 2HD2 ASN A 504      20.455  56.249 -15.986  1.00  2.57
H
ATOM   7661  N   ASP A 505      17.228  53.197 -12.202  1.00  1.21
N
ATOM   7662  CA  ASP A 505      16.280  52.085 -12.191  1.00  1.11
C
ATOM   7663  C   ASP A 505      14.855  52.575 -11.981  1.00  1.06
C
ATOM   7664  O   ASP A 505      14.576  53.359 -11.073  1.00  1.07
O
ATOM   7665  CB  ASP A 505      16.654  51.090 -11.108  1.00  1.67
C
```

ATOM 7666 CG ASP A 505 17.919 50.339 -11.429 1.00 1.67
C
ATOM 7667 OD1 ASP A 505 18.262 50.232 -12.584 1.00 1.67
O
ATOM 7668 OD2 ASP A 505 18.574 49.915 -10.505 1.00 1.67
O
ATOM 7669 H ASP A 505 17.990 53.199 -11.532 1.00 1.45
H
ATOM 7670 HA ASP A 505 16.328 51.584 -13.155 1.00 1.33
H
ATOM 7671 1HB ASP A 505 16.780 51.609 -10.159 1.00 2.00
H
ATOM 7672 2HB ASP A 505 15.848 50.367 -10.989 1.00 2.00
H
ATOM 7673 N LYS A 506 13.956 52.088 -12.825 1.00 1.09
N
ATOM 7674 CA LYS A 506 12.569 52.519 -12.812 1.00 1.10
C
ATOM 7675 C LYS A 506 11.624 51.549 -12.126 1.00 1.03
C
ATOM 7676 O LYS A 506 10.504 51.919 -11.772 1.00 1.41
O
ATOM 7677 CB LYS A 506 12.105 52.737 -14.239 1.00 1.65
C
ATOM 7678 CG LYS A 506 12.897 53.780 -15.012 1.00 1.65
C
ATOM 7679 CD LYS A 506 12.755 55.155 -14.378 1.00 1.65
C
ATOM 7680 CE LYS A 506 13.475 56.219 -15.193 1.00 1.65
C
ATOM 7681 NZ LYS A 506 13.349 57.568 -14.577 1.00 1.65
N
ATOM 7682 H LYS A 506 14.259 51.435 -13.534 1.00 1.31
H
ATOM 7683 HA LYS A 506 12.515 53.467 -12.278 1.00 1.32
H
ATOM 7684 1HB LYS A 506 12.154 51.797 -14.790 1.00 1.98
H
ATOM 7685 2HB LYS A 506 11.065 53.055 -14.218 1.00 1.98
H
ATOM 7686 1HG LYS A 506 13.950 53.501 -15.026 1.00 1.98
H
ATOM 7687 2HG LYS A 506 12.536 53.825 -16.039 1.00 1.98
H
ATOM 7688 1HD LYS A 506 11.699 55.417 -14.306 1.00 1.98
H
ATOM 7689 2HD LYS A 506 13.176 55.136 -13.373 1.00 1.98
H
ATOM 7690 1HE LYS A 506 14.530 55.960 -15.264 1.00 1.98
H
ATOM 7691 2HE LYS A 506 13.053 56.248 -16.197 1.00 1.98
H
ATOM 7692 1HZ LYS A 506 13.841 58.244 -15.145 1.00 1.98
H
ATOM 7693 2HZ LYS A 506 12.373 57.823 -14.518 1.00 1.98
H
ATOM 7694 3HZ LYS A 506 13.751 57.552 -13.650 1.00 1.98
H

ATOM 7695 N VAL A 507 12.048 50.300 -11.978 1.00 1.01
N
ATOM 7696 CA VAL A 507 11.211 49.304 -11.329 1.00 1.04
C
ATOM 7697 C VAL A 507 12.048 48.144 -10.803 1.00 0.92
C
ATOM 7698 O VAL A 507 13.040 47.758 -11.425 1.00 0.98
O
ATOM 7699 CB VAL A 507 10.149 48.797 -12.317 1.00 1.56
C
ATOM 7700 CG1 VAL A 507 10.819 48.090 -13.472 1.00 1.56
C
ATOM 7701 CG2 VAL A 507 9.170 47.875 -11.620 1.00 1.56
C
ATOM 7702 H VAL A 507 12.967 50.040 -12.303 1.00 1.21
H
ATOM 7703 HA VAL A 507 10.702 49.774 -10.487 1.00 1.25
H
ATOM 7704 HB VAL A 507 9.611 49.655 -12.722 1.00 1.87
H
ATOM 7705 1HG1 VAL A 507 10.056 47.764 -14.172 1.00 1.87
H
ATOM 7706 2HG1 VAL A 507 11.504 48.774 -13.971 1.00 1.87
H
ATOM 7707 3HG1 VAL A 507 11.370 47.227 -13.102 1.00 1.87
H
ATOM 7708 1HG2 VAL A 507 8.419 47.555 -12.336 1.00 1.87
H
ATOM 7709 2HG2 VAL A 507 9.688 47.002 -11.227 1.00 1.87
H
ATOM 7710 3HG2 VAL A 507 8.686 48.408 -10.806 1.00 1.87
H
ATOM 7711 N THR A 508 11.634 47.589 -9.667 1.00 0.90
N
ATOM 7712 CA THR A 508 12.284 46.427 -9.088 1.00 0.82
C
ATOM 7713 C THR A 508 11.620 45.165 -9.567 1.00 0.78
C
ATOM 7714 O THR A 508 10.470 44.909 -9.236 1.00 0.88
O
ATOM 7715 CB THR A 508 12.233 46.440 -7.545 1.00 1.23
C
ATOM 7716 OG1 THR A 508 12.949 47.566 -7.019 1.00 1.23
O
ATOM 7717 CG2 THR A 508 12.818 45.157 -6.996 1.00 1.23
C
ATOM 7718 H THR A 508 10.826 47.970 -9.196 1.00 1.08
H
ATOM 7719 HA THR A 508 13.318 46.400 -9.411 1.00 0.98
H
ATOM 7720 HB THR A 508 11.194 46.516 -7.229 1.00 1.48
H
ATOM 7721 HG1 THR A 508 12.515 48.387 -7.280 1.00 1.48
H
ATOM 7722 1HG2 THR A 508 12.762 45.166 -5.907 1.00 1.48
H
ATOM 7723 2HG2 THR A 508 12.257 44.306 -7.381 1.00 1.48
H

```
ATOM   7724 3HG2 THR A 508      13.856  45.082  -7.308  1.00  1.48
H
ATOM   7725  N   VAL A 509      12.353  44.362 -10.318  1.00  0.70
N
ATOM   7726  CA  VAL A 509      11.831  43.100 -10.795  1.00  0.62
C
ATOM   7727  C   VAL A 509      12.440  41.952 -10.008  1.00  0.56
C
ATOM   7728  O   VAL A 509      13.655  41.755 -10.004  1.00  0.58
O
ATOM   7729  CB  VAL A 509      12.077  42.919 -12.303  1.00  0.93
C
ATOM   7730  CG1 VAL A 509      11.559  41.559 -12.756  1.00  0.93
C
ATOM   7731  CG2 VAL A 509      11.356  44.027 -13.059  1.00  0.93
C
ATOM   7732  H   VAL A 509      13.292  44.631 -10.553  1.00  0.84
H
ATOM   7733  HA  VAL A 509      10.759  43.101 -10.640  1.00  0.74
H
ATOM   7734  HB  VAL A 509      13.148  42.962 -12.504  1.00  1.12
H
ATOM   7735 1HG1 VAL A 509      11.738  41.438 -13.824  1.00  1.12
H
ATOM   7736 2HG1 VAL A 509      12.076  40.771 -12.209  1.00  1.12
H
ATOM   7737 3HG1 VAL A 509      10.489  41.495 -12.558  1.00  1.12
H
ATOM   7738 1HG2 VAL A 509      11.529  43.911 -14.128  1.00  1.12
H
ATOM   7739 2HG2 VAL A 509      10.289  43.971 -12.857  1.00  1.12
H
ATOM   7740 3HG2 VAL A 509      11.734  44.995 -12.732  1.00  1.12
H
ATOM   7741  N   ILE A 510      11.573  41.214  -9.332  1.00  0.53
N
ATOM   7742  CA  ILE A 510      11.970  40.094  -8.504  1.00  0.49
C
ATOM   7743  C   ILE A 510      11.733  38.790  -9.232  1.00  0.43
C
ATOM   7744  O   ILE A 510      10.609  38.491  -9.628  1.00  0.43
O
ATOM   7745  CB  ILE A 510      11.193  40.094  -7.174  1.00  0.73
C
ATOM   7746  CG1 ILE A 510      11.483  41.378  -6.392  1.00  0.73
C
ATOM   7747  CG2 ILE A 510      11.558  38.873  -6.355  1.00  0.73
C
ATOM   7748  CD1 ILE A 510      10.597  41.562  -5.184  1.00  0.73
C
ATOM   7749  H   ILE A 510      10.592  41.438  -9.413  1.00  0.64
H
ATOM   7750  HA  ILE A 510      13.034  40.178  -8.285  1.00  0.59
H
ATOM   7751  HB  ILE A 510      10.124  40.078  -7.382  1.00  0.88
H
ATOM   7752 1HG1 ILE A 510      12.517  41.365  -6.054  1.00  0.88
H
```

```
ATOM   7753 2HG1 ILE A 510      11.345  42.234  -7.052  1.00  0.88
H
ATOM   7754 1HG2 ILE A 510      10.994  38.876  -5.423  1.00  0.88
H
ATOM   7755 2HG2 ILE A 510      11.322  37.971  -6.919  1.00  0.88
H
ATOM   7756 3HG2 ILE A 510      12.625  38.894  -6.136  1.00  0.88
H
ATOM   7757 1HD1 ILE A 510      10.858  42.492  -4.683  1.00  0.88
H
ATOM   7758 2HD1 ILE A 510       9.554  41.599  -5.502  1.00  0.88
H
ATOM   7759 3HD1 ILE A 510      10.738  40.727  -4.499  1.00  0.88
H
ATOM   7760  N   GLY A 511      12.789  38.015  -9.414  1.00  0.40
N
ATOM   7761  CA  GLY A 511      12.651  36.733 -10.092  1.00  0.36
C
ATOM   7762  C   GLY A 511      13.493  35.676  -9.409  1.00  0.31
C
ATOM   7763  O   GLY A 511      14.249  35.972  -8.488  1.00  0.32
O
ATOM   7764  H   GLY A 511      13.694  38.316  -9.065  1.00  0.48
H
ATOM   7765 1HA  GLY A 511      11.604  36.427 -10.091  1.00  0.43
H
ATOM   7766 2HA  GLY A 511      12.956  36.835 -11.132  1.00  0.43
H
ATOM   7767  N   ALA A 512      13.371  34.438  -9.859  1.00  0.29
N
ATOM   7768  CA  ALA A 512      14.159  33.357  -9.283  1.00  0.28
C
ATOM   7769  C   ALA A 512      14.226  32.196 -10.253  1.00  0.26
C
ATOM   7770  O   ALA A 512      13.250  31.904 -10.958  1.00  0.24
O
ATOM   7771  CB  ALA A 512      13.569  32.915  -7.951  1.00  0.42
C
ATOM   7772  H   ALA A 512      12.729  34.243 -10.617  1.00  0.35
H
ATOM   7773  HA  ALA A 512      15.173  33.721  -9.119  1.00  0.34
H
ATOM   7774 1HB  ALA A 512      14.183  32.120  -7.528  1.00  0.50
H
ATOM   7775 2HB  ALA A 512      13.549  33.760  -7.263  1.00  0.50
H
ATOM   7776 3HB  ALA A 512      12.555  32.548  -8.105  1.00  0.50
H
ATOM   7777  N   GLY A 513      15.373  31.523 -10.274  1.00  0.35
N
ATOM   7778  CA  GLY A 513      15.544  30.402 -11.177  1.00  0.29
C
ATOM   7779  C   GLY A 513      15.483  30.919 -12.596  1.00  0.29
C
ATOM   7780  O   GLY A 513      16.061  31.964 -12.907  1.00  0.34
O
ATOM   7781  H   GLY A 513      16.153  31.815  -9.686  1.00  0.42
H
```

ATOM 7782 1HA GLY A 513 16.497 29.909 -10.996 1.00 0.35
H
ATOM 7783 2HA GLY A 513 14.758 29.670 -11.015 1.00 0.35
H
ATOM 7784 N VAL A 514 14.744 30.211 -13.444 1.00 0.27
N
ATOM 7785 CA VAL A 514 14.593 30.609 -14.836 1.00 0.28
C
ATOM 7786 C VAL A 514 13.991 32.004 -14.998 1.00 0.30
C
ATOM 7787 O VAL A 514 14.261 32.664 -15.997 1.00 0.33
O
ATOM 7788 CB VAL A 514 13.724 29.587 -15.604 1.00 0.42
C
ATOM 7789 CG1 VAL A 514 12.266 29.679 -15.171 1.00 0.42
C
ATOM 7790 CG2 VAL A 514 13.851 29.840 -17.099 1.00 0.42
C
ATOM 7791 H VAL A 514 14.288 29.368 -13.120 1.00 0.32
H
ATOM 7792 HA VAL A 514 15.585 30.614 -15.291 1.00 0.34
H
ATOM 7793 HB VAL A 514 14.073 28.580 -15.371 1.00 0.50
H
ATOM 7794 1HG1 VAL A 514 11.679 28.939 -15.712 1.00 0.50
H
ATOM 7795 2HG1 VAL A 514 12.191 29.487 -14.101 1.00 0.50
H
ATOM 7796 3HG1 VAL A 514 11.880 30.672 -15.393 1.00 0.50
H
ATOM 7797 1HG2 VAL A 514 13.254 29.109 -17.643 1.00 0.50
H
ATOM 7798 2HG2 VAL A 514 13.496 30.845 -17.331 1.00 0.50
H
ATOM 7799 3HG2 VAL A 514 14.896 29.748 -17.397 1.00 0.50
H
ATOM 7800 N THR A 515 13.199 32.464 -14.021 1.00 0.28
N
ATOM 7801 CA THR A 515 12.569 33.770 -14.140 1.00 0.31
C
ATOM 7802 C THR A 515 13.511 34.891 -13.753 1.00 0.33
C
ATOM 7803 O THR A 515 13.267 36.048 -14.102 1.00 0.38
O
ATOM 7804 CB THR A 515 11.267 33.835 -13.324 1.00 0.46
C
ATOM 7805 OG1 THR A 515 11.523 33.516 -11.948 1.00 0.46
O
ATOM 7806 CG2 THR A 515 10.237 32.880 -13.902 1.00 0.46
C
ATOM 7807 H THR A 515 13.033 31.915 -13.184 1.00 0.34
H
ATOM 7808 HA THR A 515 12.299 33.917 -15.186 1.00 0.37
H
ATOM 7809 HB THR A 515 10.871 34.845 -13.379 1.00 0.56
H
ATOM 7810 HG1 THR A 515 11.933 32.646 -11.887 1.00 0.56
H

```
ATOM   7811 1HG2 THR A 515       9.317  32.956 -13.331  1.00  0.56
H
ATOM   7812 2HG2 THR A 515      10.040  33.145 -14.942  1.00  0.56
H
ATOM   7813 3HG2 THR A 515      10.614  31.860 -13.853  1.00  0.56
H
ATOM   7814  N   LEU A 516      14.606  34.556 -13.070  1.00  0.32
N
ATOM   7815  CA  LEU A 516      15.624  35.545 -12.792  1.00  0.34
C
ATOM   7816  C   LEU A 516      16.298  35.844 -14.097  1.00  0.38
C
ATOM   7817  O   LEU A 516      16.485  37.000 -14.466  1.00  0.46
O
ATOM   7818  CB  LEU A 516      16.683  35.058 -11.812  1.00  0.51
C
ATOM   7819  CG  LEU A 516      17.771  36.099 -11.524  1.00  0.51
C
ATOM   7820  CD1 LEU A 516      17.135  37.312 -10.869  1.00  0.51
C
ATOM   7821  CD2 LEU A 516      18.843  35.496 -10.653  1.00  0.51
C
ATOM   7822  H   LEU A 516      14.778  33.601 -12.789  1.00  0.38
H
ATOM   7823  HA  LEU A 516      15.158  36.454 -12.412  1.00  0.41
H
ATOM   7824 1HB  LEU A 516      16.204  34.801 -10.869  1.00  0.61
H
ATOM   7825 2HB  LEU A 516      17.164  34.167 -12.214  1.00  0.61
H
ATOM   7826  HG  LEU A 516      18.219  36.425 -12.463  1.00  0.61
H
ATOM   7827 1HD1 LEU A 516      17.902  38.060 -10.676  1.00  0.61
H
ATOM   7828 2HD1 LEU A 516      16.376  37.729 -11.531  1.00  0.61
H
ATOM   7829 3HD1 LEU A 516      16.673  37.016  -9.929  1.00  0.61
H
ATOM   7830 1HD2 LEU A 516      19.614  36.239 -10.455  1.00  0.61
H
ATOM   7831 2HD2 LEU A 516      18.399  35.172  -9.715  1.00  0.61
H
ATOM   7832 3HD2 LEU A 516      19.284  34.642 -11.163  1.00  0.61
H
ATOM   7833  N   HIS A 517      16.659  34.770 -14.795  1.00  0.37
N
ATOM   7834  CA  HIS A 517      17.335  34.883 -16.071  1.00  0.39
C
ATOM   7835  C   HIS A 517      16.449  35.521 -17.130  1.00  0.40
C
ATOM   7836  O   HIS A 517      16.949  36.289 -17.953  1.00  0.41
O
ATOM   7837  CB  HIS A 517      17.832  33.512 -16.513  1.00  0.58
C
ATOM   7838  CG  HIS A 517      19.032  33.068 -15.732  1.00  0.58
C
ATOM   7839  ND1 HIS A 517      18.951  32.618 -14.430  1.00  0.58
N
```

ATOM 7840 CD2 HIS A 517 20.341 33.002 -16.072 1.00 0.58
C
ATOM 7841 CE1 HIS A 517 20.161 32.301 -14.002 1.00 0.58
C
ATOM 7842 NE2 HIS A 517 21.020 32.517 -14.982 1.00 0.58
N
ATOM 7843 H HIS A 517 16.468 33.848 -14.409 1.00 0.44
H
ATOM 7844 HA HIS A 517 18.207 35.523 -15.956 1.00 0.47
H
ATOM 7845 1HB HIS A 517 17.040 32.772 -16.386 1.00 0.70
H
ATOM 7846 2HB HIS A 517 18.096 33.538 -17.570 1.00 0.70
H
ATOM 7847 HD2 HIS A 517 20.774 33.276 -17.035 1.00 0.70
H
ATOM 7848 HE1 HIS A 517 20.408 31.918 -13.014 1.00 0.70
H
ATOM 7849 HE2 HIS A 517 22.017 32.348 -14.938 1.00 0.70
H
ATOM 7850 N GLU A 518 15.136 35.263 -17.088 1.00 0.40
N
ATOM 7851 CA GLU A 518 14.247 35.931 -18.030 1.00 0.43
C
ATOM 7852 C GLU A 518 14.193 37.428 -17.719 1.00 0.45
C
ATOM 7853 O GLU A 518 14.164 38.242 -18.641 1.00 0.49
O
ATOM 7854 CB GLU A 518 12.828 35.337 -17.991 1.00 0.65
C
ATOM 7855 CG GLU A 518 12.686 33.919 -18.553 1.00 0.65
C
ATOM 7856 CD GLU A 518 13.045 33.783 -20.010 1.00 0.65
C
ATOM 7857 OE1 GLU A 518 12.557 34.540 -20.820 1.00 0.65
O
ATOM 7858 OE2 GLU A 518 13.816 32.905 -20.320 1.00 0.65
O
ATOM 7859 H GLU A 518 14.760 34.592 -16.430 1.00 0.48
H
ATOM 7860 HA GLU A 518 14.648 35.805 -19.036 1.00 0.52
H
ATOM 7861 1HB GLU A 518 12.480 35.314 -16.958 1.00 0.77
H
ATOM 7862 2HB GLU A 518 12.152 35.984 -18.542 1.00 0.77
H
ATOM 7863 1HG GLU A 518 13.330 33.252 -17.993 1.00 0.77
H
ATOM 7864 2HG GLU A 518 11.659 33.587 -18.405 1.00 0.77
H
ATOM 7865 N ALA A 519 14.221 37.789 -16.426 1.00 0.44
N
ATOM 7866 CA ALA A 519 14.244 39.194 -16.016 1.00 0.46
C
ATOM 7867 C ALA A 519 15.537 39.871 -16.463 1.00 0.46
C
ATOM 7868 O ALA A 519 15.515 41.029 -16.880 1.00 0.51
O

```
ATOM   7869  CB  ALA A 519      14.081  39.316 -14.507  1.00  0.69
C
ATOM   7870  H   ALA A 519      14.201  37.081 -15.701  1.00  0.53
H
ATOM   7871  HA  ALA A 519      13.414  39.703 -16.504  1.00  0.55
H
ATOM   7872 1HB  ALA A 519      14.073  40.369 -14.227  1.00  0.83
H
ATOM   7873 2HB  ALA A 519      13.141  38.854 -14.205  1.00  0.83
H
ATOM   7874 3HB  ALA A 519      14.906  38.817 -14.005  1.00  0.83
H
ATOM   7875  N   LEU A 520      16.654  39.148 -16.383  1.00  0.44
N
ATOM   7876  CA  LEU A 520      17.941  39.671 -16.821  1.00  0.48
C
ATOM   7877  C   LEU A 520      17.958  39.931 -18.321  1.00  0.56
C
ATOM   7878  O   LEU A 520      18.467  40.961 -18.772  1.00  0.63
O
ATOM   7879  CB  LEU A 520      19.044  38.684 -16.432  1.00  0.72
C
ATOM   7880  CG  LEU A 520      19.300  38.614 -14.929  1.00  0.72
C
ATOM   7881  CD1 LEU A 520      20.254  37.470 -14.596  1.00  0.72
C
ATOM   7882  CD2 LEU A 520      19.864  39.949 -14.507  1.00  0.72
C
ATOM   7883  H   LEU A 520      16.614  38.215 -15.993  1.00  0.53
H
ATOM   7884  HA  LEU A 520      18.121  40.614 -16.309  1.00  0.58
H
ATOM   7885 1HB  LEU A 520      18.771  37.689 -16.776  1.00  0.86
H
ATOM   7886 2HB  LEU A 520      19.973  38.979 -16.919  1.00  0.86
H
ATOM   7887  HG  LEU A 520      18.364  38.439 -14.404  1.00  0.86
H
ATOM   7888 1HD1 LEU A 520      20.425  37.441 -13.520  1.00  0.86
H
ATOM   7889 2HD1 LEU A 520      19.818  36.525 -14.916  1.00  0.86
H
ATOM   7890 3HD1 LEU A 520      21.201  37.626 -15.110  1.00  0.86
H
ATOM   7891 1HD2 LEU A 520      20.034  39.949 -13.438  1.00  0.86
H
ATOM   7892 2HD2 LEU A 520      20.804  40.131 -15.029  1.00  0.86
H
ATOM   7893 3HD2 LEU A 520      19.153  40.736 -14.756  1.00  0.86
H
ATOM   7894  N   GLU A 521      17.349  39.020 -19.080  1.00  0.57
N
ATOM   7895  CA  GLU A 521      17.237  39.166 -20.520  1.00  0.62
C
ATOM   7896  C   GLU A 521      16.348  40.358 -20.836  1.00  0.67
C
ATOM   7897  O   GLU A 521      16.671  41.167 -21.710  1.00  0.86
O
```

ATOM 7898 CB GLU A 521 16.660 37.884 -21.133 1.00 0.93
C
ATOM 7899 CG GLU A 521 16.553 37.863 -22.655 1.00 0.93
C
ATOM 7900 CD GLU A 521 17.876 37.827 -23.366 1.00 0.93
C
ATOM 7901 OE1 GLU A 521 18.838 37.408 -22.769 1.00 0.93
O
ATOM 7902 OE2 GLU A 521 17.926 38.211 -24.515 1.00 0.93
O
ATOM 7903 H GLU A 521 16.974 38.185 -18.649 1.00 0.68
H
ATOM 7904 HA GLU A 521 18.229 39.350 -20.936 1.00 0.74
H
ATOM 7905 1HB GLU A 521 17.276 37.036 -20.836 1.00 1.12
H
ATOM 7906 2HB GLU A 521 15.662 37.712 -20.732 1.00 1.12
H
ATOM 7907 1HG GLU A 521 15.974 36.988 -22.949 1.00 1.12
H
ATOM 7908 2HG GLU A 521 16.002 38.743 -22.972 1.00 1.12
H
ATOM 7909 N ALA A 522 15.244 40.474 -20.093 1.00 0.54
N
ATOM 7910 CA ALA A 522 14.305 41.569 -20.249 1.00 0.56
C
ATOM 7911 C ALA A 522 14.990 42.898 -20.034 1.00 0.61
C
ATOM 7912 O ALA A 522 14.800 43.811 -20.829 1.00 0.68
O
ATOM 7913 CB ALA A 522 13.148 41.413 -19.276 1.00 0.84
C
ATOM 7914 H ALA A 522 15.032 39.764 -19.406 1.00 0.65
H
ATOM 7915 HA ALA A 522 13.923 41.551 -21.269 1.00 0.67
H
ATOM 7916 1HB ALA A 522 12.439 42.228 -19.414 1.00 1.01
H
ATOM 7917 2HB ALA A 522 12.647 40.463 -19.458 1.00 1.01
H
ATOM 7918 3HB ALA A 522 13.521 41.433 -18.255 1.00 1.01
H
ATOM 7919 N ALA A 523 15.804 42.991 -18.979 1.00 0.61
N
ATOM 7920 CA ALA A 523 16.531 44.213 -18.661 1.00 0.70
C
ATOM 7921 C ALA A 523 17.444 44.623 -19.809 1.00 0.73
C
ATOM 7922 O ALA A 523 17.518 45.806 -20.144 1.00 0.90
O
ATOM 7923 CB ALA A 523 17.333 44.025 -17.382 1.00 1.05
C
ATOM 7924 H ALA A 523 15.903 42.199 -18.359 1.00 0.73
H
ATOM 7925 HA ALA A 523 15.803 45.010 -18.508 1.00 0.84
H
ATOM 7926 1HB ALA A 523 17.850 44.952 -17.137 1.00 1.26
H

```
ATOM   7927 2HB  ALA A 523      16.659  43.757 -16.568  1.00  1.26
H
ATOM   7928 3HB  ALA A 523      18.062  43.230 -17.524  1.00  1.26
H
ATOM   7929  N   ASP A 524      18.122  43.651 -20.422  1.00  0.74
N
ATOM   7930  CA  ASP A 524      18.983  43.955 -21.556  1.00  0.90
C
ATOM   7931  C   ASP A 524      18.194  44.533 -22.733  1.00  0.92
C
ATOM   7932  O   ASP A 524      18.627  45.505 -23.355  1.00  1.08
O
ATOM   7933  CB  ASP A 524      19.758  42.712 -21.990  1.00  1.35
C
ATOM   7934  CG  ASP A 524      20.860  42.338 -21.001  1.00  1.35
C
ATOM   7935  OD1 ASP A 524      21.182  43.150 -20.164  1.00  1.35
O
ATOM   7936  OD2 ASP A 524      21.380  41.254 -21.103  1.00  1.35
O
ATOM   7937  H   ASP A 524      18.056  42.696 -20.084  1.00  0.89
H
ATOM   7938  HA  ASP A 524      19.708  44.706 -21.239  1.00  1.08
H
ATOM   7939 1HB  ASP A 524      19.075  41.868 -22.087  1.00  1.62
H
ATOM   7940 2HB  ASP A 524      20.208  42.884 -22.968  1.00  1.62
H
ATOM   7941  N   HIS A 525      17.014  43.984 -23.008  1.00  0.93
N
ATOM   7942  CA  HIS A 525      16.215  44.516 -24.106  1.00  0.99
C
ATOM   7943  C   HIS A 525      15.594  45.857 -23.735  1.00  0.99
C
ATOM   7944  O   HIS A 525      15.510  46.755 -24.566  1.00  1.30
O
ATOM   7945  CB  HIS A 525      15.146  43.519 -24.504  1.00  1.48
C
ATOM   7946  CG  HIS A 525      15.736  42.306 -25.133  1.00  1.48
C
ATOM   7947  ND1 HIS A 525      16.373  42.332 -26.356  1.00  1.48
N
ATOM   7948  CD2 HIS A 525      15.820  41.035 -24.692  1.00  1.48
C
ATOM   7949  CE1 HIS A 525      16.825  41.121 -26.637  1.00  1.48
C
ATOM   7950  NE2 HIS A 525      16.508  40.319 -25.638  1.00  1.48
N
ATOM   7951  H   HIS A 525      16.692  43.176 -22.481  1.00  1.12
H
ATOM   7952  HA  HIS A 525      16.856  44.681 -24.972  1.00  1.19
H
ATOM   7953 1HB  HIS A 525      14.592  43.215 -23.618  1.00  1.78
H
ATOM   7954 2HB  HIS A 525      14.445  43.975 -25.203  1.00  1.78
H
ATOM   7955  HD2 HIS A 525      15.429  40.653 -23.750  1.00  1.78
H
```

```
ATOM   7956  HE1 HIS A 525      17.377  40.837 -27.532  1.00  1.78
H
ATOM   7957  HE2 HIS A 525      16.738  39.335 -25.556  1.00  1.78
H
ATOM   7958  N   LEU A 526      15.229  46.022 -22.469  1.00  0.91
N
ATOM   7959  CA  LEU A 526      14.696  47.281 -21.973  1.00  1.03
C
ATOM   7960  C   LEU A 526      15.714  48.405 -22.075  1.00  1.20
C
ATOM   7961  O   LEU A 526      15.346  49.534 -22.404  1.00  1.51
O
ATOM   7962  CB  LEU A 526      14.216  47.083 -20.534  1.00  1.54
C
ATOM   7963  CG  LEU A 526      12.928  46.269 -20.423  1.00  1.54
C
ATOM   7964  CD1 LEU A 526      12.677  45.868 -18.995  1.00  1.54
C
ATOM   7965  CD2 LEU A 526      11.775  47.130 -20.901  1.00  1.54
C
ATOM   7966  H   LEU A 526      15.305  45.251 -21.825  1.00  1.09
H
ATOM   7967  HA  LEU A 526      13.836  47.550 -22.584  1.00  1.24
H
ATOM   7968 1HB  LEU A 526      14.989  46.559 -19.978  1.00  1.85
H
ATOM   7969 2HB  LEU A 526      14.051  48.048 -20.066  1.00  1.85
H
ATOM   7970  HG  LEU A 526      13.008  45.377 -21.032  1.00  1.85
H
ATOM   7971 1HD1 LEU A 526      11.752  45.296 -18.932  1.00  1.85
H
ATOM   7972 2HD1 LEU A 526      13.505  45.258 -18.634  1.00  1.85
H
ATOM   7973 3HD1 LEU A 526      12.590  46.767 -18.393  1.00  1.85
H
ATOM   7974 1HD2 LEU A 526      10.846  46.569 -20.824  1.00  1.85
H
ATOM   7975 2HD2 LEU A 526      11.708  48.022 -20.279  1.00  1.85
H
ATOM   7976 3HD2 LEU A 526      11.941  47.423 -21.938  1.00  1.85
H
ATOM   7977  N   SER A 527      17.001  48.091 -21.879  1.00  1.27
N
ATOM   7978  CA  SER A 527      18.047  49.100 -22.000  1.00  1.82
C
ATOM   7979  C   SER A 527      18.139  49.654 -23.424  1.00  2.39
C
ATOM   7980  O   SER A 527      18.602  50.777 -23.618  1.00  1.90
O
ATOM   7981  CB  SER A 527      19.404  48.540 -21.612  1.00  2.73
C
ATOM   7982  OG  SER A 527      19.907  47.665 -22.587  1.00  2.73
O
ATOM   7983  H   SER A 527      17.253  47.153 -21.594  1.00  1.52
H
ATOM   7984  HA  SER A 527      17.806  49.924 -21.328  1.00  2.18
H
```

```
ATOM   7985 1HB  SER A 527      20.105  49.361 -21.463  1.00  3.28
H
ATOM   7986 2HB  SER A 527      19.316  48.013 -20.663  1.00  3.28
H
ATOM   7987  HG  SER A 527      19.281  46.940 -22.666  1.00  3.28
H
ATOM   7988  N   GLN A 528      17.645  48.908 -24.422  1.00  4.05
N
ATOM   7989  CA  GLN A 528      17.661  49.373 -25.803  1.00  4.63
C
ATOM   7990  C   GLN A 528      16.655  50.509 -25.995  1.00  4.61
C
ATOM   7991  O   GLN A 528      16.752  51.281 -26.948  1.00  5.05
O
ATOM   7992  CB  GLN A 528      17.330  48.224 -26.761  1.00  6.95
C
ATOM   7993  CG  GLN A 528      18.371  47.116 -26.799  1.00  6.95
C
ATOM   7994  CD  GLN A 528      17.970  45.975 -27.717  1.00  6.95
C
ATOM   7995  OE1 GLN A 528      16.801  45.839 -28.089  1.00  6.95
O
ATOM   7996  NE2 GLN A 528      18.939  45.146 -28.092  1.00  6.95
N
ATOM   7997  H   GLN A 528      17.234  48.002 -24.242  1.00  4.86
H
ATOM   7998  HA  GLN A 528      18.655  49.753 -26.033  1.00  5.56
H
ATOM   7999 1HB  GLN A 528      16.370  47.786 -26.501  1.00  8.33
H
ATOM   8000 2HB  GLN A 528      17.239  48.619 -27.773  1.00  8.33
H
ATOM   8001 1HG  GLN A 528      19.314  47.529 -27.155  1.00  8.33
H
ATOM   8002 2HG  GLN A 528      18.496  46.716 -25.792  1.00  8.33
H
ATOM   8003 1HE2 GLN A 528      18.733  44.375 -28.695  1.00  8.33
H
ATOM   8004 2HE2 GLN A 528      19.874  45.291 -27.767  1.00  8.33
H
ATOM   8005  N   GLN A 529      15.689  50.601 -25.078  1.00  5.54
N
ATOM   8006  CA  GLN A 529      14.676  51.634 -25.091  1.00  5.84
C
ATOM   8007  C   GLN A 529      14.993  52.728 -24.074  1.00  5.77
C
ATOM   8008  O   GLN A 529      14.187  53.637 -23.875  1.00 11.84
O
ATOM   8009  CB  GLN A 529      13.308  51.027 -24.782  1.00  8.76
C
ATOM   8010  CG  GLN A 529      12.856  49.975 -25.779  1.00  8.76
C
ATOM   8011  CD  GLN A 529      12.705  50.538 -27.179  1.00  8.76
C
ATOM   8012  OE1 GLN A 529      12.048  51.564 -27.379  1.00  8.76
O
ATOM   8013  NE2 GLN A 529      13.306  49.870 -28.156  1.00  8.76
N
```

```
ATOM   8014  H   GLN A 529      15.650  49.944 -24.315  1.00  6.65
H
ATOM   8015  HA  GLN A 529      14.651  52.086 -26.082  1.00  7.01
H
ATOM   8016 1HB  GLN A 529      13.329  50.565 -23.794  1.00 10.51
H
ATOM   8017 2HB  GLN A 529      12.556  51.815 -24.760  1.00 10.51
H
ATOM   8018 1HG  GLN A 529      13.596  49.175 -25.810  1.00 10.51
H
ATOM   8019 2HG  GLN A 529      11.891  49.579 -25.462  1.00 10.51
H
ATOM   8020 1HE2 GLN A 529      13.239  50.196 -29.101  1.00 10.51
H
ATOM   8021 2HE2 GLN A 529      13.827  49.042 -27.950  1.00 10.51
H
ATOM   8022  N   GLY A 530      16.158  52.644 -23.420  1.00  3.79
N
ATOM   8023  CA  GLY A 530      16.520  53.611 -22.394  1.00  3.35
C
ATOM   8024  C   GLY A 530      15.869  53.302 -21.041  1.00  2.16
C
ATOM   8025  O   GLY A 530      15.752  54.188 -20.193  1.00  2.59
O
ATOM   8026  H   GLY A 530      16.823  51.912 -23.623  1.00  4.55
H
ATOM   8027 1HA  GLY A 530      17.605  53.626 -22.281  1.00  4.02
H
ATOM   8028 2HA  GLY A 530      16.225  54.608 -22.719  1.00  4.02
H
ATOM   8029  N   ILE A 531      15.427  52.058 -20.847  1.00  1.47
N
ATOM   8030  CA  ILE A 531      14.759  51.664 -19.610  1.00  1.09
C
ATOM   8031  C   ILE A 531      15.670  50.847 -18.701  1.00  0.94
C
ATOM   8032  O   ILE A 531      16.108  49.755 -19.065  1.00  1.27
O
ATOM   8033  CB  ILE A 531      13.502  50.845 -19.927  1.00  1.64
C
ATOM   8034  CG1 ILE A 531      12.536  51.672 -20.772  1.00  1.64
C
ATOM   8035  CG2 ILE A 531      12.846  50.377 -18.636  1.00  1.64
C
ATOM   8036  CD1 ILE A 531      11.407  50.856 -21.353  1.00  1.64
C
ATOM   8037  H   ILE A 531      15.548  51.352 -21.561  1.00  1.76
H
ATOM   8038  HA  ILE A 531      14.461  52.566 -19.076  1.00  1.31
H
ATOM   8039  HB  ILE A 531      13.779  49.985 -20.525  1.00  1.96
H
ATOM   8040 1HG1 ILE A 531      12.110  52.462 -20.155  1.00  1.96
H
ATOM   8041 2HG1 ILE A 531      13.083  52.135 -21.594  1.00  1.96
H
ATOM   8042 1HG2 ILE A 531      11.962  49.787 -18.874  1.00  1.96
H
```

ATOM	8043	2HG2	ILE	A	531	13.548	49.768	-18.070	1.00	1.96	H
ATOM	8044	3HG2	ILE	A	531	12.555	51.242	-18.041	1.00	1.96	H
ATOM	8045	1HD1	ILE	A	531	10.757	51.503	-21.941	1.00	1.96	H
ATOM	8046	2HD1	ILE	A	531	11.816	50.074	-21.992	1.00	1.96	H
ATOM	8047	3HD1	ILE	A	531	10.834	50.401	-20.546	1.00	1.96	H
ATOM	8048	N	SER	A	532	15.954	51.378	-17.515	1.00	1.10	N
ATOM	8049	CA	SER	A	532	16.805	50.680	-16.559	1.00	0.99	C
ATOM	8050	C	SER	A	532	15.948	49.965	-15.515	1.00	0.89	C
ATOM	8051	O	SER	A	532	14.951	50.509	-15.041	1.00	1.30	O
ATOM	8052	CB	SER	A	532	17.759	51.658	-15.910	1.00	1.48	C
ATOM	8053	OG	SER	A	532	18.603	51.019	-14.992	1.00	1.48	O
ATOM	8054	H	SER	A	532	15.570	52.280	-17.271	1.00	1.32	H
ATOM	8055	HA	SER	A	532	17.389	49.930	-17.094	1.00	1.19	H
ATOM	8056	1HB	SER	A	532	18.361	52.136	-16.683	1.00	1.78	H
ATOM	8057	2HB	SER	A	532	17.194	52.438	-15.409	1.00	1.78	H
ATOM	8058	HG	SER	A	532	18.075	50.799	-14.216	1.00	1.78	H
ATOM	8059	N	VAL	A	533	16.332	48.731	-15.192	1.00	0.92	N
ATOM	8060	CA	VAL	A	533	15.595	47.876	-14.262	1.00	1.53	C
ATOM	8061	C	VAL	A	533	16.460	47.258	-13.172	1.00	1.13	C
ATOM	8062	O	VAL	A	533	17.575	46.803	-13.425	1.00	1.42	O
ATOM	8063	CB	VAL	A	533	14.885	46.742	-15.031	1.00	2.29	C
ATOM	8064	CG1	VAL	A	533	14.209	45.782	-14.071	1.00	2.29	C
ATOM	8065	CG2	VAL	A	533	13.841	47.338	-15.948	1.00	2.29	C
ATOM	8066	H	VAL	A	533	17.172	48.365	-15.619	1.00	1.10	H
ATOM	8067	HA	VAL	A	533	14.830	48.487	-13.781	1.00	1.84	H
ATOM	8068	HB	VAL	A	533	15.617	46.178	-15.611	1.00	2.75	H
ATOM	8069	1HG1	VAL	A	533	13.715	44.994	-14.640	1.00	2.75	H
ATOM	8070	2HG1	VAL	A	533	14.950	45.335	-13.412	1.00	2.75	H
ATOM	8071	3HG1	VAL	A	533	13.470	46.319	-13.479	1.00	2.75	H

```
ATOM   8072 1HG2 VAL A 533      13.343  46.528 -16.471  1.00  2.75
H
ATOM   8073 2HG2 VAL A 533      13.111  47.891 -15.359  1.00  2.75
H
ATOM   8074 3HG2 VAL A 533      14.309  48.006 -16.669  1.00  2.75
H
ATOM   8075  N   ARG A 534      15.926  47.257 -11.958  1.00  0.90
N
ATOM   8076  CA  ARG A 534      16.562  46.655 -10.800  1.00  0.89
C
ATOM   8077  C   ARG A 534      16.215  45.172 -10.746  1.00  0.72
C
ATOM   8078  O   ARG A 534      15.042  44.820 -10.686  1.00  0.91
O
ATOM   8079  CB  ARG A 534      16.082  47.366  -9.553  1.00  1.33
C
ATOM   8080  CG  ARG A 534      16.560  46.843  -8.221  1.00  1.33
C
ATOM   8081  CD  ARG A 534      17.987  47.107  -8.028  1.00  1.33
C
ATOM   8082  NE  ARG A 534      18.257  48.518  -8.182  1.00  1.33
N
ATOM   8083  CZ  ARG A 534      18.241  49.448  -7.216  1.00  1.33
C
ATOM   8084  NH1 ARG A 534      18.008  49.130  -5.963  1.00  1.33
N
ATOM   8085  NH2 ARG A 534      18.471  50.694  -7.577  1.00  1.33
N
ATOM   8086  H   ARG A 534      15.004  47.652 -11.833  1.00  1.08
H
ATOM   8087  HA  ARG A 534      17.642  46.770 -10.888  1.00  1.07
H
ATOM   8088 1HB  ARG A 534      16.408  48.403  -9.605  1.00  1.60
H
ATOM   8089 2HB  ARG A 534      15.000  47.383  -9.537  1.00  1.60
H
ATOM   8090 1HG  ARG A 534      16.024  47.383  -7.451  1.00  1.60
H
ATOM   8091 2HG  ARG A 534      16.373  45.773  -8.134  1.00  1.60
H
ATOM   8092 1HD  ARG A 534      18.286  46.807  -7.024  1.00  1.60
H
ATOM   8093 2HD  ARG A 534      18.568  46.562  -8.761  1.00  1.60
H
ATOM   8094  HE  ARG A 534      18.428  48.857  -9.120  1.00  1.60
H
ATOM   8095 1HH1 ARG A 534      17.829  48.167  -5.713  1.00  1.60
H
ATOM   8096 2HH1 ARG A 534      18.003  49.847  -5.253  1.00  1.60
H
ATOM   8097 1HH2 ARG A 534      18.631  50.879  -8.563  1.00  1.60
H
ATOM   8098 2HH2 ARG A 534      18.471  51.438  -6.895  1.00  1.60
H
ATOM   8099  N   VAL A 535      17.208  44.293 -10.799  1.00  0.67
N
ATOM   8100  CA  VAL A 535      16.912  42.861 -10.789  1.00  0.57
C
```

ATOM 8101 C VAL A 535 17.313 42.225 -9.470 1.00 0.54
C
ATOM 8102 O VAL A 535 18.484 42.264 -9.089 1.00 0.54
O
ATOM 8103 CB VAL A 535 17.609 42.142 -11.956 1.00 0.85
C
ATOM 8104 CG1 VAL A 535 17.299 40.659 -11.888 1.00 0.85
C
ATOM 8105 CG2 VAL A 535 17.130 42.727 -13.280 1.00 0.85
C
ATOM 8106 H VAL A 535 18.163 44.616 -10.831 1.00 0.80
H
ATOM 8107 HA VAL A 535 15.837 42.733 -10.917 1.00 0.68
H
ATOM 8108 HB VAL A 535 18.687 42.261 -11.865 1.00 1.03
H
ATOM 8109 1HG1 VAL A 535 17.792 40.138 -12.704 1.00 1.03
H
ATOM 8110 2HG1 VAL A 535 17.650 40.261 -10.938 1.00 1.03
H
ATOM 8111 3HG1 VAL A 535 16.222 40.512 -11.967 1.00 1.03
H
ATOM 8112 1HG2 VAL A 535 17.624 42.217 -14.105 1.00 1.03
H
ATOM 8113 2HG2 VAL A 535 16.051 42.595 -13.365 1.00 1.03
H
ATOM 8114 3HG2 VAL A 535 17.370 43.790 -13.316 1.00 1.03
H
ATOM 8115 N ILE A 536 16.318 41.661 -8.781 1.00 0.54
N
ATOM 8116 CA ILE A 536 16.477 41.046 -7.466 1.00 0.51
C
ATOM 8117 C ILE A 536 16.311 39.531 -7.466 1.00 0.46
C
ATOM 8118 O ILE A 536 15.391 38.989 -8.082 1.00 0.48
O
ATOM 8119 CB ILE A 536 15.467 41.648 -6.470 1.00 0.77
C
ATOM 8120 CG1 ILE A 536 15.656 43.163 -6.396 1.00 0.77
C
ATOM 8121 CG2 ILE A 536 15.602 41.012 -5.087 1.00 0.77
C
ATOM 8122 CD1 ILE A 536 17.024 43.591 -5.936 1.00 0.77
C
ATOM 8123 H ILE A 536 15.392 41.671 -9.189 1.00 0.65
H
ATOM 8124 HA ILE A 536 17.481 41.272 -7.111 1.00 0.61
H
ATOM 8125 HB ILE A 536 14.462 41.472 -6.844 1.00 0.92
H
ATOM 8126 1HG1 ILE A 536 15.476 43.590 -7.382 1.00 0.92
H
ATOM 8127 2HG1 ILE A 536 14.922 43.575 -5.703 1.00 0.92
H
ATOM 8128 1HG2 ILE A 536 14.868 41.453 -4.414 1.00 0.92
H
ATOM 8129 2HG2 ILE A 536 15.428 39.941 -5.155 1.00 0.92
H

```
ATOM   8130 3HG2 ILE A 536      16.605  41.195  -4.699  1.00  0.92
H
ATOM   8131 1HD1 ILE A 536      17.073  44.679  -5.907  1.00  0.92
H
ATOM   8132 2HD1 ILE A 536      17.213  43.191  -4.938  1.00  0.92
H
ATOM   8133 3HD1 ILE A 536      17.771  43.210  -6.628  1.00  0.92
H
ATOM   8134  N   ASP A 537      17.207  38.865  -6.746  1.00  0.44
N
ATOM   8135  CA  ASP A 537      17.173  37.428  -6.546  1.00  0.44
C
ATOM   8136  C   ASP A 537      17.039  37.103  -5.069  1.00  0.35
C
ATOM   8137  O   ASP A 537      18.035  37.109  -4.349  1.00  0.56
O
ATOM   8138  CB  ASP A 537      18.424  36.762  -7.055  1.00  0.66
C
ATOM   8139  CG  ASP A 537      18.375  35.253  -6.866  1.00  0.66
C
ATOM   8140  OD1 ASP A 537      17.511  34.767  -6.166  1.00  0.66
O
ATOM   8141  OD2 ASP A 537      19.233  34.596  -7.411  1.00  0.66
O
ATOM   8142  H   ASP A 537      17.944  39.386  -6.292  1.00  0.53
H
ATOM   8143  HA  ASP A 537      16.329  37.017  -7.095  1.00  0.53
H
ATOM   8144 1HB  ASP A 537      18.550  36.983  -8.115  1.00  0.79
H
ATOM   8145 2HB  ASP A 537      19.289  37.161  -6.528  1.00  0.79
H
ATOM   8146  N   PRO A 538      15.831  36.818  -4.584  1.00  0.49
N
ATOM   8147  CA  PRO A 538      15.468  36.491  -3.213  1.00  0.34
C
ATOM   8148  C   PRO A 538      16.367  35.454  -2.539  1.00  1.79
C
ATOM   8149  O   PRO A 538      16.532  35.498  -1.323  1.00 20.83
O
ATOM   8150  CB  PRO A 538      14.052  35.919  -3.355  1.00  0.51
C
ATOM   8151  CG  PRO A 538      13.506  36.595  -4.552  1.00  0.51
C
ATOM   8152  CD  PRO A 538      14.676  36.673  -5.490  1.00  0.51
C
ATOM   8153  HA  PRO A 538      15.444  37.415  -2.622  1.00  0.41
H
ATOM   8154 1HB  PRO A 538      14.098  34.826  -3.466  1.00  0.61
H
ATOM   8155 2HB  PRO A 538      13.470  36.125  -2.445  1.00  0.61
H
ATOM   8156 1HG  PRO A 538      12.665  36.020  -4.968  1.00  0.61
H
ATOM   8157 2HG  PRO A 538      13.114  37.584  -4.276  1.00  0.61
H
ATOM   8158 1HD  PRO A 538      14.771  35.745  -6.073  1.00  0.61
H
```

ATOM 8159 2HD PRO A 538 14.578 37.557 -6.135 1.00 0.61
H
ATOM 8160 N PHE A 539 16.926 34.509 -3.307 1.00 2.28
N
ATOM 8161 CA PHE A 539 17.698 33.382 -2.765 1.00 0.73
C
ATOM 8162 C PHE A 539 16.785 32.482 -1.928 1.00 0.52
C
ATOM 8163 O PHE A 539 16.411 31.401 -2.383 1.00 0.78
O
ATOM 8164 CB PHE A 539 18.964 33.811 -2.015 1.00 1.09
C
ATOM 8165 CG PHE A 539 19.760 32.629 -1.525 1.00 1.09
C
ATOM 8166 CD1 PHE A 539 20.272 31.700 -2.424 1.00 1.09
C
ATOM 8167 CD2 PHE A 539 20.015 32.450 -0.176 1.00 1.09
C
ATOM 8168 CE1 PHE A 539 21.007 30.614 -1.983 1.00 1.09
C
ATOM 8169 CE2 PHE A 539 20.746 31.367 0.267 1.00 1.09
C
ATOM 8170 CZ PHE A 539 21.243 30.446 -0.636 1.00 1.09
C
ATOM 8171 H PHE A 539 16.819 34.561 -4.318 1.00 2.74
H
ATOM 8172 HA PHE A 539 18.047 32.783 -3.608 1.00 0.88
H
ATOM 8173 1HB PHE A 539 19.597 34.406 -2.672 1.00 1.31
H
ATOM 8174 2HB PHE A 539 18.705 34.427 -1.157 1.00 1.31
H
ATOM 8175 HD1 PHE A 539 20.084 31.836 -3.490 1.00 1.31
H
ATOM 8176 HD2 PHE A 539 19.625 33.176 0.540 1.00 1.31
H
ATOM 8177 HE1 PHE A 539 21.397 29.895 -2.700 1.00 1.31
H
ATOM 8178 HE2 PHE A 539 20.929 31.242 1.333 1.00 1.31
H
ATOM 8179 HZ PHE A 539 21.818 29.590 -0.287 1.00 1.31
H
ATOM 8180 N THR A 540 16.417 32.916 -0.723 1.00 0.38
N
ATOM 8181 CA THR A 540 15.414 32.179 0.020 1.00 0.33
C
ATOM 8182 C THR A 540 14.102 32.911 -0.155 1.00 0.33
C
ATOM 8183 O THR A 540 14.048 34.133 -0.279 1.00 0.38
O
ATOM 8184 CB THR A 540 15.729 32.014 1.528 1.00 0.49
C
ATOM 8185 OG1 THR A 540 15.821 33.297 2.166 1.00 0.49
O
ATOM 8186 CG2 THR A 540 17.019 31.247 1.714 1.00 0.49
C
ATOM 8187 H THR A 540 16.776 33.793 -0.375 1.00 0.46
H

```
ATOM   8188  HA  THR A 540      15.300  31.187  -0.414  1.00  0.40
H
ATOM   8189  HB  THR A 540      14.923  31.450   2.001  1.00  0.59
H
ATOM   8190  HG1 THR A 540      14.934  33.615   2.392  1.00  0.59
H
ATOM   8191 1HG2 THR A 540      17.219  31.121   2.775  1.00  0.59
H
ATOM   8192 2HG2 THR A 540      16.928  30.269   1.246  1.00  0.59
H
ATOM   8193 3HG2 THR A 540      17.833  31.797   1.256  1.00  0.59
H
ATOM   8194  N   ILE A 541      13.044  32.155  -0.153  1.00  0.31
N
ATOM   8195  CA  ILE A 541      11.728  32.689  -0.341  1.00  0.32
C
ATOM   8196  C   ILE A 541      11.094  32.754   1.030  1.00  0.37
C
ATOM   8197  O   ILE A 541      10.362  33.687   1.361  1.00  0.46
O
ATOM   8198  CB  ILE A 541      10.930  31.831  -1.308  1.00  0.48
C
ATOM   8199  CG1 ILE A 541      11.633  31.797  -2.665  1.00  0.48
C
ATOM   8200  CG2 ILE A 541       9.574  32.463  -1.494  1.00  0.48
C
ATOM   8201  CD1 ILE A 541      11.053  30.769  -3.597  1.00  0.48
C
ATOM   8202  H   ILE A 541      13.164  31.163  -0.026  1.00  0.37
H
ATOM   8203  HA  ILE A 541      11.798  33.700  -0.741  1.00  0.38
H
ATOM   8204  HB  ILE A 541      10.834  30.813  -0.937  1.00  0.58
H
ATOM   8205 1HG1 ILE A 541      11.546  32.777  -3.135  1.00  0.58
H
ATOM   8206 2HG1 ILE A 541      12.690  31.577  -2.523  1.00  0.58
H
ATOM   8207 1HG2 ILE A 541       9.011  31.874  -2.206  1.00  0.58
H
ATOM   8208 2HG2 ILE A 541       9.055  32.502  -0.561  1.00  0.58
H
ATOM   8209 3HG2 ILE A 541       9.697  33.474  -1.878  1.00  0.58
H
ATOM   8210 1HD1 ILE A 541      11.585  30.803  -4.547  1.00  0.58
H
ATOM   8211 2HD1 ILE A 541      11.154  29.776  -3.160  1.00  0.58
H
ATOM   8212 3HD1 ILE A 541      10.006  30.992  -3.765  1.00  0.58
H
ATOM   8213  N   LYS A 542      11.391  31.728   1.818  1.00  0.36
N
ATOM   8214  CA  LYS A 542      10.972  31.654   3.204  1.00  0.36
C
ATOM   8215  C   LYS A 542      12.203  31.394   4.069  1.00  0.28
C
ATOM   8216  O   LYS A 542      12.761  30.302   4.026  1.00  0.32
O
```

ATOM   8217 CB LYS A 542   9.939 30.544   3.400 1.00 0.54
C
ATOM   8218 CG LYS A 542   9.470 30.361   4.836 1.00 0.54
C
ATOM   8219 CD LYS A 542   8.622 31.529   5.317 1.00 0.54
C
ATOM   8220 CE LYS A 542   8.085 31.267   6.721 1.00 0.54
C
ATOM   8221 NZ LYS A 542   7.293 32.414   7.256 1.00 0.54
N
ATOM   8222 H   LYS A 542   11.976 30.980   1.455 1.00 0.43
H
ATOM   8223 HA LYS A 542   10.531 32.606   3.496 1.00 0.43
H
ATOM   8224 1HB LYS A 542   9.062 30.743   2.789 1.00 0.65
H
ATOM   8225 2HB LYS A 542   10.357 29.594   3.067 1.00 0.65
H
ATOM   8226 1HG LYS A 542   8.882 29.445   4.912 1.00 0.65
H
ATOM   8227 2HG LYS A 542   10.337 30.266   5.488 1.00 0.65
H
ATOM   8228 1HD LYS A 542   9.228 32.436   5.331 1.00 0.65
H
ATOM   8229 2HD LYS A 542   7.782 31.682   4.637 1.00 0.65
H
ATOM   8230 1HE LYS A 542   7.450 30.382   6.700 1.00 0.65
H
ATOM   8231 2HE LYS A 542   8.924 31.079   7.391 1.00 0.65
H
ATOM   8232 1HZ LYS A 542   6.964 32.192   8.184 1.00 0.65
H
ATOM   8233 2HZ LYS A 542   7.875 33.238   7.294 1.00 0.65
H
ATOM   8234 3HZ LYS A 542   6.498 32.596   6.657 1.00 0.65
H
ATOM   8235 N   PRO A 543   12.660 32.374   4.845 1.00 0.26
N
ATOM   8236 CA PRO A 543   12.211 33.751   5.030 1.00 0.30
C
ATOM   8237 C   PRO A 543   12.548 34.632   3.833 1.00 0.33
C
ATOM   8238 O   PRO A 543   13.512 34.387   3.100 1.00 0.38
O
ATOM   8239 CB PRO A 543   12.968 34.200   6.282 1.00 0.45
C
ATOM   8240 CG PRO A 543   14.203 33.371   6.293 1.00 0.45
C
ATOM   8241 CD PRO A 543   13.765 32.040   5.753 1.00 0.45
C
ATOM   8242 HA PRO A 543   11.126 33.753   5.216 1.00 0.36
H
ATOM   8243 1HB PRO A 543   13.183 35.278   6.225 1.00 0.54
H
ATOM   8244 2HB PRO A 543   12.346 34.046   7.176 1.00 0.54
H
ATOM   8245 1HG PRO A 543   14.983 33.837   5.678 1.00 0.54
H

```
ATOM   8246 2HG  PRO A 543      14.606  33.300   7.315  1.00  0.54
H
ATOM   8247 1HD  PRO A 543      14.577  31.564   5.183  1.00  0.54
H
ATOM   8248 2HD  PRO A 543      13.403  31.389   6.561  1.00  0.54
H
ATOM   8249  N   LEU A 544      11.741  35.675   3.653  1.00  0.45
N
ATOM   8250  CA  LEU A 544      11.909  36.606   2.550  1.00  0.68
C
ATOM   8251  C   LEU A 544      12.578  37.896   3.011  1.00  1.03
C
ATOM   8252  O   LEU A 544      12.133  38.527   3.973  1.00  1.89
O
ATOM   8253  CB  LEU A 544      10.543  36.940   1.941  1.00  1.02
C
ATOM   8254  CG  LEU A 544      10.560  37.887   0.745  1.00  1.02
C
ATOM   8255  CD1 LEU A 544      11.242  37.179  -0.425  1.00  1.02
C
ATOM   8256  CD2 LEU A 544       9.135  38.287   0.405  1.00  1.02
C
ATOM   8257  H   LEU A 544      10.978  35.818   4.299  1.00  0.54
H
ATOM   8258  HA  LEU A 544      12.537  36.140   1.791  1.00  0.82
H
ATOM   8259 1HB  LEU A 544      10.066  36.020   1.620  1.00  1.22
H
ATOM   8260 2HB  LEU A 544       9.921  37.395   2.712  1.00  1.22
H
ATOM   8261  HG  LEU A 544      11.139  38.777   0.988  1.00  1.22
H
ATOM   8262 1HD1 LEU A 544      11.271  37.840  -1.289  1.00  1.22
H
ATOM   8263 2HD1 LEU A 544      12.260  36.908  -0.142  1.00  1.22
H
ATOM   8264 3HD1 LEU A 544      10.685  36.278  -0.678  1.00  1.22
H
ATOM   8265 1HD2 LEU A 544       9.143  38.965  -0.447  1.00  1.22
H
ATOM   8266 2HD2 LEU A 544       8.555  37.399   0.158  1.00  1.22
H
ATOM   8267 3HD2 LEU A 544       8.684  38.789   1.261  1.00  1.22
H
ATOM   8268  N   ASP A 545      13.645  38.290   2.323  1.00  0.73
N
ATOM   8269  CA  ASP A 545      14.374  39.514   2.644  1.00  0.86
C
ATOM   8270  C   ASP A 545      13.625  40.800   2.314  1.00  0.95
C
ATOM   8271  O   ASP A 545      13.968  41.489   1.349  1.00  0.97
O
ATOM   8272  CB  ASP A 545      15.693  39.537   1.911  1.00  1.29
C
ATOM   8273  CG  ASP A 545      16.526  40.740   2.283  1.00  1.29
C
ATOM   8274  OD1 ASP A 545      16.029  41.593   2.982  1.00  1.29
O
```

ATOM 8275 OD2 ASP A 545 17.653 40.810 1.853 1.00 1.29
O
ATOM 8276 H ASP A 545 13.961 37.724 1.547 1.00 0.88
H
ATOM 8277 HA ASP A 545 14.576 39.510 3.716 1.00 1.03
H
ATOM 8278 1HB ASP A 545 16.254 38.627 2.118 1.00 1.55
H
ATOM 8279 2HB ASP A 545 15.499 39.568 0.842 1.00 1.55
H
ATOM 8280 N ALA A 546 12.652 41.142 3.151 1.00 1.00
N
ATOM 8281 CA ALA A 546 11.848 42.342 2.971 1.00 1.06
C
ATOM 8282 C ALA A 546 12.673 43.624 3.075 1.00 1.03
C
ATOM 8283 O ALA A 546 12.323 44.625 2.460 1.00 1.06
O
ATOM 8284 CB ALA A 546 10.722 42.372 3.991 1.00 1.59
C
ATOM 8285 H ALA A 546 12.446 40.513 3.916 1.00 1.20
H
ATOM 8286 HA ALA A 546 11.415 42.306 1.972 1.00 1.27
H
ATOM 8287 1HB ALA A 546 10.102 43.252 3.824 1.00 1.91
H
ATOM 8288 2HB ALA A 546 10.113 41.473 3.884 1.00 1.91
H
ATOM 8289 3HB ALA A 546 11.141 42.408 4.995 1.00 1.91
H
ATOM 8290 N ALA A 547 13.757 43.608 3.856 1.00 1.05
N
ATOM 8291 CA ALA A 547 14.589 44.800 4.015 1.00 1.09
C
ATOM 8292 C ALA A 547 15.177 45.244 2.676 1.00 1.02
C
ATOM 8293 O ALA A 547 15.071 46.420 2.309 1.00 1.10
O
ATOM 8294 CB ALA A 547 15.703 44.531 5.013 1.00 1.64
C
ATOM 8295 H ALA A 547 14.012 42.761 4.344 1.00 1.26
H
ATOM 8296 HA ALA A 547 13.959 45.606 4.390 1.00 1.31
H
ATOM 8297 1HB ALA A 547 16.300 45.434 5.143 1.00 1.96
H
ATOM 8298 2HB ALA A 547 15.270 44.243 5.971 1.00 1.96
H
ATOM 8299 3HB ALA A 547 16.335 43.726 4.644 1.00 1.96
H
ATOM 8300 N THR A 548 15.779 44.298 1.946 1.00 0.91
N
ATOM 8301 CA THR A 548 16.329 44.596 0.627 1.00 0.85
C
ATOM 8302 C THR A 548 15.232 44.916 -0.364 1.00 0.80
C
ATOM 8303 O THR A 548 15.380 45.848 -1.152 1.00 0.82
O

```
ATOM   8304  CB  THR A 548      17.194  43.448   0.058  1.00  1.27
C
ATOM   8305  OG1 THR A 548      18.339  43.232   0.897  1.00  1.27
O
ATOM   8306  CG2 THR A 548      17.654  43.778  -1.357  1.00  1.27
C
ATOM   8307  H   THR A 548      15.856  43.354   2.314  1.00  1.09
H
ATOM   8308  HA  THR A 548      16.964  45.477   0.714  1.00  1.02
H
ATOM   8309  HB  THR A 548      16.605  42.532   0.027  1.00  1.53
H
ATOM   8310  HG1 THR A 548      18.160  42.492   1.486  1.00  1.53
H
ATOM   8311 1HG2 THR A 548      18.255  42.955  -1.746  1.00  1.53
H
ATOM   8312 2HG2 THR A 548      16.784  43.925  -1.998  1.00  1.53
H
ATOM   8313 3HG2 THR A 548      18.251  44.688  -1.343  1.00  1.53
H
ATOM   8314  N   ILE A 549      14.137  44.149  -0.338  1.00  0.79
N
ATOM   8315  CA  ILE A 549      13.041  44.381  -1.276  1.00  0.82
C
ATOM   8316  C   ILE A 549      12.452  45.772  -1.150  1.00  0.84
C
ATOM   8317  O   ILE A 549      12.246  46.442  -2.159  1.00  0.87
O
ATOM   8318  CB  ILE A 549      11.916  43.352  -1.097  1.00  1.23
C
ATOM   8319  CG1 ILE A 549      12.402  41.973  -1.538  1.00  1.23
C
ATOM   8320  CG2 ILE A 549      10.679  43.773  -1.883  1.00  1.23
C
ATOM   8321  CD1 ILE A 549      11.472  40.859  -1.138  1.00  1.23
C
ATOM   8322  H   ILE A 549      14.063  43.384   0.327  1.00  0.95
H
ATOM   8323  HA  ILE A 549      13.434  44.275  -2.287  1.00  0.98
H
ATOM   8324  HB  ILE A 549      11.659  43.281  -0.041  1.00  1.48
H
ATOM   8325 1HG1 ILE A 549      12.505  41.963  -2.622  1.00  1.48
H
ATOM   8326 2HG1 ILE A 549      13.381  41.780  -1.099  1.00  1.48
H
ATOM   8327 1HG2 ILE A 549       9.888  43.038  -1.738  1.00  1.48
H
ATOM   8328 2HG2 ILE A 549      10.339  44.747  -1.534  1.00  1.48
H
ATOM   8329 3HG2 ILE A 549      10.927  43.835  -2.943  1.00  1.48
H
ATOM   8330 1HD1 ILE A 549      11.880  39.911  -1.486  1.00  1.48
H
ATOM   8331 2HD1 ILE A 549      11.375  40.839  -0.052  1.00  1.48
H
ATOM   8332 3HD1 ILE A 549      10.494  41.022  -1.587  1.00  1.48
H
```

```
ATOM   8333  N   ILE A 550      12.175  46.199   0.076  1.00  0.87
N
ATOM   8334  CA  ILE A 550      11.617  47.519   0.317  1.00  0.95
C
ATOM   8335  C   ILE A 550      12.599  48.625  -0.040  1.00  1.00
C
ATOM   8336  O   ILE A 550      12.209  49.611  -0.663  1.00  1.04
O
ATOM   8337  CB  ILE A 550      11.153  47.661   1.770  1.00  1.42
C
ATOM   8338  CG1 ILE A 550       9.952  46.742   2.011  1.00  1.42
C
ATOM   8339  CG2 ILE A 550      10.790  49.105   2.060  1.00  1.42
C
ATOM   8340  CD1 ILE A 550       9.575  46.585   3.464  1.00  1.42
C
ATOM   8341  H   ILE A 550      12.347  45.596   0.867  1.00  1.04
H
ATOM   8342  HA  ILE A 550      10.741  47.636  -0.319  1.00  1.14
H
ATOM   8343  HB  ILE A 550      11.950  47.343   2.443  1.00  1.71
H
ATOM   8344 1HG1 ILE A 550       9.092  47.141   1.475  1.00  1.71
H
ATOM   8345 2HG1 ILE A 550      10.177  45.755   1.607  1.00  1.71
H
ATOM   8346 1HG2 ILE A 550      10.463  49.196   3.096  1.00  1.71
H
ATOM   8347 2HG2 ILE A 550      11.662  49.737   1.898  1.00  1.71
H
ATOM   8348 3HG2 ILE A 550       9.985  49.418   1.396  1.00  1.71
H
ATOM   8349 1HD1 ILE A 550       8.716  45.919   3.549  1.00  1.71
H
ATOM   8350 2HD1 ILE A 550      10.418  46.162   4.014  1.00  1.71
H
ATOM   8351 3HD1 ILE A 550       9.321  47.558   3.882  1.00  1.71
H
ATOM   8352  N   SER A 551      13.869  48.471   0.353  1.00  1.06
N
ATOM   8353  CA  SER A 551      14.883  49.467   0.024  1.00  1.21
C
ATOM   8354  C   SER A 551      14.991  49.641  -1.489  1.00  1.19
C
ATOM   8355  O   SER A 551      15.043  50.765  -1.996  1.00  1.39
O
ATOM   8356  CB  SER A 551      16.226  49.040   0.588  1.00  1.81
C
ATOM   8357  OG  SER A 551      16.198  49.005   1.988  1.00  1.81
O
ATOM   8358  H   SER A 551      14.150  47.657   0.890  1.00  1.27
H
ATOM   8359  HA  SER A 551      14.593  50.422   0.466  1.00  1.45
H
ATOM   8360 1HB  SER A 551      16.489  48.056   0.202  1.00  2.18
H
ATOM   8361 2HB  SER A 551      16.993  49.738   0.257  1.00  2.18
H
```

```
ATOM   8362  HG  SER A 551      15.650  48.245   2.221  1.00  2.18
H
ATOM   8363  N   SER A 552      14.999  48.507  -2.192  1.00  1.01
N
ATOM   8364  CA  SER A 552      15.056  48.443  -3.641  1.00  1.05
C
ATOM   8365  C   SER A 552      13.844  49.106  -4.260  1.00  0.98
C
ATOM   8366  O   SER A 552      13.967  49.905  -5.192  1.00  1.16
O
ATOM   8367  CB  SER A 552      15.130  46.991  -4.065  1.00  1.58
C
ATOM   8368  OG  SER A 552      15.188  46.865  -5.452  1.00  1.58
O
ATOM   8369  H   SER A 552      14.975  47.628  -1.696  1.00  1.21
H
ATOM   8370  HA  SER A 552      15.951  48.966  -3.978  1.00  1.26
H
ATOM   8371 1HB  SER A 552      16.014  46.533  -3.621  1.00  1.89
H
ATOM   8372 2HB  SER A 552      14.262  46.454  -3.685  1.00  1.89
H
ATOM   8373  HG  SER A 552      14.344  47.170  -5.807  1.00  1.89
H
ATOM   8374  N   ALA A 553      12.674  48.771  -3.727  1.00  0.92
N
ATOM   8375  CA  ALA A 553      11.421  49.308  -4.201  1.00  0.95
C
ATOM   8376  C   ALA A 553      11.415  50.819  -4.136  1.00  1.07
C
ATOM   8377  O   ALA A 553      11.095  51.464  -5.128  1.00  1.29
O
ATOM   8378  CB  ALA A 553      10.280  48.743  -3.389  1.00  1.42
C
ATOM   8379  H   ALA A 553      12.644  48.097  -2.976  1.00  1.10
H
ATOM   8380  HA  ALA A 553      11.298  49.011  -5.243  1.00  1.14
H
ATOM   8381 1HB  ALA A 553       9.337  49.125  -3.777  1.00  1.71
H
ATOM   8382 2HB  ALA A 553      10.289  47.656  -3.461  1.00  1.71
H
ATOM   8383 3HB  ALA A 553      10.391  49.033  -2.347  1.00  1.71
H
ATOM   8384  N   LYS A 554      11.827  51.380  -2.999  1.00  1.15
N
ATOM   8385  CA  LYS A 554      11.878  52.829  -2.824  1.00  1.44
C
ATOM   8386  C   LYS A 554      12.857  53.479  -3.799  1.00  1.30
C
ATOM   8387  O   LYS A 554      12.562  54.527  -4.380  1.00  1.59
O
ATOM   8388  CB  LYS A 554      12.259  53.161  -1.385  1.00  2.16
C
ATOM   8389  CG  LYS A 554      11.170  52.851  -0.367  1.00  2.16
C
ATOM   8390  CD  LYS A 554      11.624  53.188   1.045  1.00  2.16
C
```

ATOM 8391 CE LYS A 554 10.556 52.835 2.070 1.00 2.16
C
ATOM 8392 NZ LYS A 554 9.370 53.727 1.987 1.00 2.16
N
ATOM 8393 H LYS A 554 12.083 50.787 -2.220 1.00 1.38
H
ATOM 8394 HA LYS A 554 10.887 53.236 -3.024 1.00 1.73
H
ATOM 8395 1HB LYS A 554 13.150 52.599 -1.104 1.00 2.59
H
ATOM 8396 2HB LYS A 554 12.501 54.221 -1.305 1.00 2.59
H
ATOM 8397 1HG LYS A 554 10.277 53.431 -0.600 1.00 2.59
H
ATOM 8398 2HG LYS A 554 10.916 51.793 -0.415 1.00 2.59
H
ATOM 8399 1HD LYS A 554 12.535 52.636 1.277 1.00 2.59
H
ATOM 8400 2HD LYS A 554 11.839 54.255 1.113 1.00 2.59
H
ATOM 8401 1HE LYS A 554 10.229 51.814 1.895 1.00 2.59
H
ATOM 8402 2HE LYS A 554 10.980 52.901 3.072 1.00 2.59
H
ATOM 8403 1HZ LYS A 554 8.698 53.423 2.684 1.00 2.59
H
ATOM 8404 2HZ LYS A 554 9.644 54.683 2.168 1.00 2.59
H
ATOM 8405 3HZ LYS A 554 8.956 53.661 1.069 1.00 2.59
H
ATOM 8406 N ALA A 555 14.001 52.825 -4.023 1.00 1.32
N
ATOM 8407 CA ALA A 555 14.985 53.308 -4.987 1.00 1.38
C
ATOM 8408 C ALA A 555 14.400 53.335 -6.400 1.00 1.50
C
ATOM 8409 O ALA A 555 14.794 54.158 -7.227 1.00 4.41
O
ATOM 8410 CB ALA A 555 16.231 52.436 -4.950 1.00 2.07
C
ATOM 8411 H ALA A 555 14.212 51.982 -3.499 1.00 1.58
H
ATOM 8412 HA ALA A 555 15.257 54.328 -4.714 1.00 1.66
H
ATOM 8413 1HB ALA A 555 16.966 52.824 -5.654 1.00 2.48
H
ATOM 8414 2HB ALA A 555 16.651 52.447 -3.944 1.00 2.48
H
ATOM 8415 3HB ALA A 555 15.970 51.416 -5.223 1.00 2.48
H
ATOM 8416 N THR A 556 13.440 52.446 -6.659 1.00 1.28
N
ATOM 8417 CA THR A 556 12.779 52.364 -7.949 1.00 1.13
C
ATOM 8418 C THR A 556 11.363 52.953 -7.950 1.00 1.52
C
ATOM 8419 O THR A 556 10.477 52.441 -8.633 1.00 3.27
O

```
ATOM   8420  CB  THR A 556      12.722  50.910  -8.409  1.00  1.69
C
ATOM   8421  OG1 THR A 556      12.040  50.119  -7.433  1.00  1.69
O
ATOM   8422  CG2 THR A 556      14.125  50.376  -8.585  1.00  1.69
C
ATOM   8423  H   THR A 556      13.188  51.769  -5.949  1.00  1.54
H
ATOM   8424  HA  THR A 556      13.373  52.926  -8.670  1.00  1.36
H
ATOM   8425  HB  THR A 556      12.188  50.856  -9.351  1.00  2.03
H
ATOM   8426  HG1 THR A 556      12.418  50.305  -6.567  1.00  2.03
H
ATOM   8427 1HG2 THR A 556      14.080  49.342  -8.918  1.00  2.03
H
ATOM   8428 2HG2 THR A 556      14.639  50.981  -9.325  1.00  2.03
H
ATOM   8429 3HG2 THR A 556      14.659  50.430  -7.637  1.00  2.03
H
ATOM   8430  N   GLY A 557      11.138  54.011  -7.164  1.00  1.91
N
ATOM   8431  CA  GLY A 557       9.863  54.733  -7.206  1.00  2.10
C
ATOM   8432  C   GLY A 557       8.717  54.025  -6.497  1.00  1.71
C
ATOM   8433  O   GLY A 557       7.551  54.367  -6.699  1.00  2.37
O
ATOM   8434  H   GLY A 557      11.865  54.347  -6.540  1.00  2.29
H
ATOM   8435 1HA  GLY A 557      10.002  55.719  -6.760  1.00  2.52
H
ATOM   8436 2HA  GLY A 557       9.588  54.903  -8.246  1.00  2.52
H
ATOM   8437  N   GLY A 558       9.047  53.027  -5.690  1.00  1.44
N
ATOM   8438  CA  GLY A 558       8.064  52.228  -4.982  1.00  1.52
C
ATOM   8439  C   GLY A 558       7.451  51.156  -5.884  1.00  2.06
C
ATOM   8440  O   GLY A 558       6.442  50.549  -5.538  1.00  7.51
O
ATOM   8441  H   GLY A 558      10.016  52.802  -5.552  1.00  1.73
H
ATOM   8442 1HA  GLY A 558       8.539  51.756  -4.124  1.00  1.82
H
ATOM   8443 2HA  GLY A 558       7.283  52.879  -4.599  1.00  1.82
H
ATOM   8444  N   ARG A 559       8.041  50.920  -7.047  1.00  1.33
N
ATOM   8445  CA  ARG A 559       7.447  49.989  -7.990  1.00  1.35
C
ATOM   8446  C   ARG A 559       8.137  48.626  -8.011  1.00  1.16
C
ATOM   8447  O   ARG A 559       9.338  48.540  -8.262  1.00  1.45
O
ATOM   8448  CB  ARG A 559       7.492  50.647  -9.356  1.00  2.03
C
```

```
ATOM   8449  CG  ARG A 559       6.656  51.924  -9.435  1.00  2.03
C
ATOM   8450  CD  ARG A 559       6.872  52.656 -10.704  1.00  2.03
C
ATOM   8451  NE  ARG A 559       6.089  53.884 -10.791  1.00  2.03
N
ATOM   8452  CZ  ARG A 559       4.827  53.958 -11.269  1.00  2.03
C
ATOM   8453  NH1 ARG A 559       4.207  52.871 -11.666  1.00  2.03
N
ATOM   8454  NH2 ARG A 559       4.207  55.122 -11.352  1.00  2.03
N
ATOM   8455  H   ARG A 559       8.884  51.413  -7.327  1.00  1.60
H
ATOM   8456  HA  ARG A 559       6.404  49.842  -7.712  1.00  1.62
H
ATOM   8457 1HB  ARG A 559       8.519  50.904  -9.611  1.00  2.43
H
ATOM   8458 2HB  ARG A 559       7.122  49.956 -10.113  1.00  2.43
H
ATOM   8459 1HG  ARG A 559       5.597  51.669  -9.361  1.00  2.43
H
ATOM   8460 2HG  ARG A 559       6.927  52.586  -8.612  1.00  2.43
H
ATOM   8461 1HD  ARG A 559       7.925  52.921 -10.785  1.00  2.43
H
ATOM   8462 2HD  ARG A 559       6.600  52.027 -11.538  1.00  2.43
H
ATOM   8463  HE  ARG A 559       6.532  54.744 -10.499  1.00  2.43
H
ATOM   8464 1HH1 ARG A 559       4.667  51.976 -11.615  1.00  2.43
H
ATOM   8465 2HH1 ARG A 559       3.263  52.935 -12.038  1.00  2.43
H
ATOM   8466 1HH2 ARG A 559       4.672  55.968 -11.057  1.00  2.43
H
ATOM   8467 2HH2 ARG A 559       3.269  55.162 -11.742  1.00  2.43
H
ATOM   8468  N   VAL A 560       7.358  47.568  -7.754  1.00  0.99
N
ATOM   8469  CA  VAL A 560       7.847  46.188  -7.744  1.00  0.88
C
ATOM   8470  C   VAL A 560       7.049  45.270  -8.671  1.00  0.81
C
ATOM   8471  O   VAL A 560       5.825  45.294  -8.655  1.00  0.85
O
ATOM   8472  CB  VAL A 560       7.767  45.596  -6.322  1.00  1.32
C
ATOM   8473  CG1 VAL A 560       8.218  44.142  -6.324  1.00  1.32
C
ATOM   8474  CG2 VAL A 560       8.620  46.405  -5.374  1.00  1.32
C
ATOM   8475  H   VAL A 560       6.385  47.721  -7.528  1.00  1.19
H
ATOM   8476  HA  VAL A 560       8.886  46.196  -8.064  1.00  1.06
H
ATOM   8477  HB  VAL A 560       6.733  45.623  -5.992  1.00  1.58
H
```

```
ATOM   8478 1HG1 VAL A 560       8.139  43.736  -5.315  1.00  1.58
H
ATOM   8479 2HG1 VAL A 560       7.586  43.561  -6.994  1.00  1.58
H
ATOM   8480 3HG1 VAL A 560       9.254  44.081  -6.658  1.00  1.58
H
ATOM   8481 1HG2 VAL A 560       8.543  45.988  -4.371  1.00  1.58
H
ATOM   8482 2HG2 VAL A 560       9.658  46.375  -5.699  1.00  1.58
H
ATOM   8483 3HG2 VAL A 560       8.274  47.438  -5.361  1.00  1.58
H
ATOM   8484  N   ILE A 561       7.748  44.469  -9.467  1.00  0.75
N
ATOM   8485  CA  ILE A 561       7.131  43.460 -10.324  1.00  0.68
C
ATOM   8486  C   ILE A 561       7.727  42.110  -9.978  1.00  0.63
C
ATOM   8487  O   ILE A 561       8.922  41.903 -10.159  1.00  0.62
O
ATOM   8488  CB  ILE A 561       7.371  43.719 -11.826  1.00  1.02
C
ATOM   8489  CG1 ILE A 561       6.814  45.071 -12.263  1.00  1.02
C
ATOM   8490  CG2 ILE A 561       6.740  42.600 -12.646  1.00  1.02
C
ATOM   8491  CD1 ILE A 561       7.224  45.446 -13.665  1.00  1.02
C
ATOM   8492  H   ILE A 561       8.751  44.534  -9.449  1.00  0.90
H
ATOM   8493  HA  ILE A 561       6.060  43.429 -10.130  1.00  0.82
H
ATOM   8494  HB  ILE A 561       8.440  43.737 -12.014  1.00  1.22
H
ATOM   8495 1HG1 ILE A 561       5.728  45.043 -12.226  1.00  1.22
H
ATOM   8496 2HG1 ILE A 561       7.160  45.841 -11.578  1.00  1.22
H
ATOM   8497 1HG2 ILE A 561       6.933  42.772 -13.704  1.00  1.22
H
ATOM   8498 2HG2 ILE A 561       7.170  41.644 -12.350  1.00  1.22
H
ATOM   8499 3HG2 ILE A 561       5.664  42.582 -12.472  1.00  1.22
H
ATOM   8500 1HD1 ILE A 561       6.797  46.414 -13.923  1.00  1.22
H
ATOM   8501 2HD1 ILE A 561       8.310  45.501 -13.723  1.00  1.22
H
ATOM   8502 3HD1 ILE A 561       6.862  44.692 -14.362  1.00  1.22
H
ATOM   8503  N   THR A 562       6.916  41.184  -9.500  1.00  0.65
N
ATOM   8504  CA  THR A 562       7.454  39.874  -9.175  1.00  0.58
C
ATOM   8505  C   THR A 562       7.003  38.851 -10.201  1.00  0.57
C
ATOM   8506  O   THR A 562       5.810  38.741 -10.484  1.00  0.64
O
```

```
ATOM   8507  CB  THR A 562       7.033  39.430  -7.769  1.00  0.87
C
ATOM   8508  OG1 THR A 562       7.522  40.368  -6.803  1.00  0.87
O
ATOM   8509  CG2 THR A 562       7.614  38.059  -7.470  1.00  0.87
C
ATOM   8510  H   THR A 562       5.933  41.382  -9.361  1.00  0.78
H
ATOM   8511  HA  THR A 562       8.542  39.924  -9.204  1.00  0.70
H
ATOM   8512  HB  THR A 562       5.946  39.387  -7.708  1.00  1.04
H
ATOM   8513  HG1 THR A 562       7.235  40.100  -5.927  1.00  1.04
H
ATOM   8514 1HG2 THR A 562       7.312  37.755  -6.473  1.00  1.04
H
ATOM   8515 2HG2 THR A 562       7.244  37.336  -8.197  1.00  1.04
H
ATOM   8516 3HG2 THR A 562       8.701  38.103  -7.526  1.00  1.04
H
ATOM   8517  N   VAL A 563       7.962  38.101 -10.739  1.00  0.50
N
ATOM   8518  CA  VAL A 563       7.682  37.080 -11.739  1.00  0.47
C
ATOM   8519  C   VAL A 563       8.153  35.715 -11.261  1.00  0.42
C
ATOM   8520  O   VAL A 563       9.253  35.581 -10.724  1.00  0.39
O
ATOM   8521  CB  VAL A 563       8.367  37.439 -13.077  1.00  0.70
C
ATOM   8522  CG1 VAL A 563       9.867  37.597 -12.868  1.00  0.70
C
ATOM   8523  CG2 VAL A 563       8.087  36.350 -14.111  1.00  0.70
C
ATOM   8524  H   VAL A 563       8.915  38.250 -10.439  1.00  0.60
H
ATOM   8525  HA  VAL A 563       6.609  37.045 -11.897  1.00  0.56
H
ATOM   8526  HB  VAL A 563       7.980  38.391 -13.432  1.00  0.85
H
ATOM   8527 1HG1 VAL A 563      10.344  37.863 -13.810  1.00  0.85
H
ATOM   8528 2HG1 VAL A 563      10.048  38.386 -12.138  1.00  0.85
H
ATOM   8529 3HG1 VAL A 563      10.286  36.663 -12.501  1.00  0.85
H
ATOM   8530 1HG2 VAL A 563       8.563  36.615 -15.055  1.00  0.85
H
ATOM   8531 2HG2 VAL A 563       8.487  35.400 -13.759  1.00  0.85
H
ATOM   8532 3HG2 VAL A 563       7.012  36.257 -14.262  1.00  0.85
H
ATOM   8533  N   GLU A 564       7.319  34.699 -11.457  1.00  0.41
N
ATOM   8534  CA  GLU A 564       7.672  33.366 -10.993  1.00  0.34
C
ATOM   8535  C   GLU A 564       7.022  32.227 -11.755  1.00  0.34
C
```

```
ATOM   8536  O   GLU A 564       5.836  32.263 -12.083  1.00  0.65
O
ATOM   8537  CB  GLU A 564       7.298  33.201  -9.534  1.00  0.51
C
ATOM   8538  CG  GLU A 564       7.728  31.869  -8.947  1.00  0.51
C
ATOM   8539  CD  GLU A 564       7.303  31.733  -7.543  1.00  0.51
C
ATOM   8540  OE1 GLU A 564       6.535  32.559  -7.119  1.00  0.51
O
ATOM   8541  OE2 GLU A 564       7.725  30.815  -6.881  1.00  0.51
O
ATOM   8542  H   GLU A 564       6.430  34.872 -11.907  1.00  0.49
H
ATOM   8543  HA  GLU A 564       8.753  33.256 -11.089  1.00  0.41
H
ATOM   8544 1HB  GLU A 564       7.761  33.995  -8.947  1.00  0.61
H
ATOM   8545 2HB  GLU A 564       6.219  33.293  -9.418  1.00  0.61
H
ATOM   8546 1HG  GLU A 564       7.281  31.059  -9.515  1.00  0.61
H
ATOM   8547 2HG  GLU A 564       8.812  31.777  -9.014  1.00  0.61
H
ATOM   8548  N   ASP A 565       7.799  31.174 -11.979  1.00  0.40
N
ATOM   8549  CA  ASP A 565       7.298  29.980 -12.632  1.00  0.41
C
ATOM   8550  C   ASP A 565       6.651  29.069 -11.557  1.00  0.49
C
ATOM   8551  O   ASP A 565       7.216  28.059 -11.135  1.00  1.97
O
ATOM   8552  CB  ASP A 565       8.458  29.315 -13.380  1.00  0.61
C
ATOM   8553  CG  ASP A 565       8.043  28.196 -14.290  1.00  0.61
C
ATOM   8554  OD1 ASP A 565       6.881  27.873 -14.302  1.00  0.61
O
ATOM   8555  OD2 ASP A 565       8.886  27.657 -14.977  1.00  0.61
O
ATOM   8556  H   ASP A 565       8.766  31.206 -11.686  1.00  0.48
H
ATOM   8557  HA  ASP A 565       6.529  30.263 -13.349  1.00  0.49
H
ATOM   8558 1HB  ASP A 565       8.979  30.066 -13.975  1.00  0.74
H
ATOM   8559 2HB  ASP A 565       9.174  28.924 -12.657  1.00  0.74
H
ATOM   8560  N   HIS A 566       5.454  29.488 -11.132  1.00  0.39
N
ATOM   8561  CA  HIS A 566       4.626  28.893 -10.066  1.00  0.28
C
ATOM   8562  C   HIS A 566       3.230  29.462 -10.222  1.00  0.32
C
ATOM   8563  O   HIS A 566       3.077  30.523 -10.822  1.00  0.45
O
ATOM   8564  CB  HIS A 566       5.179  29.252  -8.665  1.00  0.42
C
```

ATOM 8565 CG HIS A 566 4.521 28.602 -7.454 1.00 0.42
C
ATOM 8566 ND1 HIS A 566 3.389 29.118 -6.856 1.00 0.42
N
ATOM 8567 CD2 HIS A 566 4.853 27.509 -6.726 1.00 0.42
C
ATOM 8568 CE1 HIS A 566 3.059 28.373 -5.815 1.00 0.42
C
ATOM 8569 NE2 HIS A 566 3.932 27.391 -5.702 1.00 0.42
N
ATOM 8570 H HIS A 566 5.108 30.329 -11.577 1.00 0.47
H
ATOM 8571 HA HIS A 566 4.580 27.814 -10.161 1.00 0.34
H
ATOM 8572 1HB HIS A 566 6.241 29.014 -8.635 1.00 0.50
H
ATOM 8573 2HB HIS A 566 5.092 30.329 -8.534 1.00 0.50
H
ATOM 8574 HD1 HIS A 566 2.724 29.712 -7.307 1.00 0.50
H
ATOM 8575 HD2 HIS A 566 5.663 26.791 -6.810 1.00 0.50
H
ATOM 8576 HE1 HIS A 566 2.187 28.626 -5.215 1.00 0.50
H
ATOM 8577 N TYR A 567 2.214 28.757 -9.736 1.00 0.55
N
ATOM 8578 CA TYR A 567 0.873 29.307 -9.812 1.00 0.96
C
ATOM 8579 C TYR A 567 0.787 30.562 -8.957 1.00 1.37
C
ATOM 8580 O TYR A 567 1.433 30.676 -7.916 1.00 2.32
O
ATOM 8581 CB TYR A 567 -0.193 28.319 -9.332 1.00 1.44
C
ATOM 8582 CG TYR A 567 -0.405 27.132 -10.232 1.00 1.44
C
ATOM 8583 CD1 TYR A 567 -0.453 25.870 -9.684 1.00 1.44
C
ATOM 8584 CD2 TYR A 567 -0.529 27.294 -11.594 1.00 1.44
C
ATOM 8585 CE1 TYR A 567 -0.626 24.761 -10.483 1.00 1.44
C
ATOM 8586 CE2 TYR A 567 -0.695 26.188 -12.397 1.00 1.44
C
ATOM 8587 CZ TYR A 567 -0.741 24.925 -11.853 1.00 1.44
C
ATOM 8588 OH TYR A 567 -0.905 23.826 -12.664 1.00 1.44
O
ATOM 8589 H TYR A 567 2.366 27.868 -9.278 1.00 0.66
H
ATOM 8590 HA TYR A 567 0.664 29.584 -10.842 1.00 1.15
H
ATOM 8591 1HB TYR A 567 0.084 27.945 -8.344 1.00 1.73
H
ATOM 8592 2HB TYR A 567 -1.146 28.836 -9.227 1.00 1.73
H
ATOM 8593 HD1 TYR A 567 -0.351 25.763 -8.611 1.00 1.73
H

```
ATOM   8594  HD2 TYR A 567      -0.485  28.290 -12.030  1.00  1.73
H
ATOM   8595  HE1 TYR A 567      -0.663  23.766 -10.039  1.00  1.73
H
ATOM   8596  HE2 TYR A 567      -0.781  26.311 -13.467  1.00  1.73
H
ATOM   8597  HH  TYR A 567      -0.946  23.034 -12.122  1.00  1.73
H
ATOM   8598  N   ARG A 568      -0.043  31.496  -9.389  1.00  1.45
N
ATOM   8599  CA  ARG A 568      -0.253  32.760  -8.676  1.00  1.72
C
ATOM   8600  C   ARG A 568      -0.570  32.637  -7.175  1.00  3.14
C
ATOM   8601  O   ARG A 568      -0.287  33.551  -6.407  1.00 15.42
O
ATOM   8602  CB  ARG A 568      -1.395  33.530  -9.332  1.00  2.58
C
ATOM   8603  CG  ARG A 568      -1.069  34.110 -10.693  1.00  2.58
C
ATOM   8604  CD  ARG A 568      -2.184  34.874 -11.284  1.00  2.58
C
ATOM   8605  NE  ARG A 568      -1.831  35.352 -12.608  1.00  2.58
N
ATOM   8606  CZ  ARG A 568      -1.080  36.447 -12.845  1.00  2.58
C
ATOM   8607  NH1 ARG A 568      -0.607  37.165 -11.850  1.00  2.58
N
ATOM   8608  NH2 ARG A 568      -0.808  36.801 -14.090  1.00  2.58
N
ATOM   8609  H   ARG A 568      -0.515  31.334 -10.272  1.00  1.74
H
ATOM   8610  HA  ARG A 568       0.657  33.351  -8.777  1.00  2.06
H
ATOM   8611 1HB  ARG A 568      -2.258  32.876  -9.449  1.00  3.10
H
ATOM   8612 2HB  ARG A 568      -1.695  34.356  -8.687  1.00  3.10
H
ATOM   8613 1HG  ARG A 568      -0.254  34.819 -10.577  1.00  3.10
H
ATOM   8614 2HG  ARG A 568      -0.776  33.316 -11.381  1.00  3.10
H
ATOM   8615 1HD  ARG A 568      -3.064  34.237 -11.371  1.00  3.10
H
ATOM   8616 2HD  ARG A 568      -2.413  35.734 -10.656  1.00  3.10
H
ATOM   8617  HE  ARG A 568      -2.167  34.830 -13.405  1.00  3.10
H
ATOM   8618 1HH1 ARG A 568      -0.796  36.911 -10.884  1.00  3.10
H
ATOM   8619 2HH1 ARG A 568      -0.035  37.977 -12.044  1.00  3.10
H
ATOM   8620 1HH2 ARG A 568      -1.164  36.253 -14.861  1.00  3.10
H
ATOM   8621 2HH2 ARG A 568      -0.246  37.622 -14.269  1.00  3.10
H
ATOM   8622  N   GLU A 569      -1.160  31.524  -6.764  1.00  1.20
N
```

ATOM 8623 CA GLU A 569 -1.610 31.349 -5.397 1.00 1.14
C
ATOM 8624 C GLU A 569 -0.538 30.712 -4.483 1.00 1.01
C
ATOM 8625 O GLU A 569 -0.072 29.604 -4.743 1.00 1.33
O
ATOM 8626 CB GLU A 569 -2.856 30.483 -5.463 1.00 1.71
C
ATOM 8627 CG GLU A 569 -3.940 31.013 -6.409 1.00 1.71
C
ATOM 8628 CD GLU A 569 -4.604 32.283 -5.982 1.00 1.71
C
ATOM 8629 OE1 GLU A 569 -4.905 32.423 -4.824 1.00 1.71
O
ATOM 8630 OE2 GLU A 569 -4.812 33.127 -6.822 1.00 1.71
O
ATOM 8631 H GLU A 569 -1.343 30.787 -7.429 1.00 1.44
H
ATOM 8632 HA GLU A 569 -1.869 32.327 -4.992 1.00 1.37
H
ATOM 8633 1HB GLU A 569 -2.585 29.483 -5.794 1.00 2.05
H
ATOM 8634 2HB GLU A 569 -3.289 30.398 -4.480 1.00 2.05
H
ATOM 8635 1HG GLU A 569 -3.484 31.181 -7.385 1.00 2.05
H
ATOM 8636 2HG GLU A 569 -4.702 30.251 -6.534 1.00 2.05
H
ATOM 8637 N GLY A 570 -0.161 31.421 -3.412 1.00 0.90
N
ATOM 8638 CA GLY A 570 0.836 30.948 -2.439 1.00 0.82
C
ATOM 8639 C GLY A 570 2.313 31.071 -2.882 1.00 0.70
C
ATOM 8640 O GLY A 570 3.194 30.488 -2.246 1.00 1.03
O
ATOM 8641 H GLY A 570 -0.596 32.317 -3.254 1.00 1.08
H
ATOM 8642 1HA GLY A 570 0.700 31.498 -1.511 1.00 0.98
H
ATOM 8643 2HA GLY A 570 0.621 29.909 -2.201 1.00 0.98
H
ATOM 8644 N GLY A 571 2.589 31.814 -3.957 1.00 0.58
N
ATOM 8645 CA GLY A 571 3.962 31.908 -4.473 1.00 0.47
C
ATOM 8646 C GLY A 571 4.785 33.090 -3.928 1.00 0.47
C
ATOM 8647 O GLY A 571 4.443 33.674 -2.899 1.00 0.67
O
ATOM 8648 H GLY A 571 1.845 32.301 -4.436 1.00 0.70
H
ATOM 8649 1HA GLY A 571 4.467 30.976 -4.239 1.00 0.56
H
ATOM 8650 2HA GLY A 571 3.925 31.971 -5.559 1.00 0.56
H
ATOM 8651 N ILE A 572 5.881 33.426 -4.635 1.00 0.44
N

ATOM 8652 CA ILE A 572 6.778 34.520 -4.244 1.00 0.58
C
ATOM 8653 C ILE A 572 6.061 35.839 -4.313 1.00 0.46
C
ATOM 8654 O ILE A 572 6.222 36.669 -3.428 1.00 0.64
O
ATOM 8655 CB ILE A 572 8.043 34.668 -5.132 1.00 0.87
C
ATOM 8656 CG1 ILE A 572 9.004 33.498 -4.997 1.00 0.87
C
ATOM 8657 CG2 ILE A 572 8.782 35.930 -4.716 1.00 0.87
C
ATOM 8658 CD1 ILE A 572 10.100 33.503 -6.042 1.00 0.87
C
ATOM 8659 H ILE A 572 6.097 32.907 -5.476 1.00 0.53
H
ATOM 8660 HA ILE A 572 7.098 34.357 -3.216 1.00 0.70
H
ATOM 8661 HB ILE A 572 7.749 34.741 -6.179 1.00 1.04
H
ATOM 8662 1HG1 ILE A 572 9.472 33.535 -4.019 1.00 1.04
H
ATOM 8663 2HG1 ILE A 572 8.451 32.568 -5.083 1.00 1.04
H
ATOM 8664 1HG2 ILE A 572 9.662 36.062 -5.344 1.00 1.04
H
ATOM 8665 2HG2 ILE A 572 8.127 36.791 -4.824 1.00 1.04
H
ATOM 8666 3HG2 ILE A 572 9.089 35.843 -3.673 1.00 1.04
H
ATOM 8667 1HD1 ILE A 572 10.754 32.645 -5.890 1.00 1.04
H
ATOM 8668 2HD1 ILE A 572 9.655 33.448 -7.036 1.00 1.04
H
ATOM 8669 3HD1 ILE A 572 10.679 34.421 -5.955 1.00 1.04
H
ATOM 8670 N GLY A 573 5.290 36.048 -5.380 1.00 0.51
N
ATOM 8671 CA GLY A 573 4.549 37.293 -5.556 1.00 0.61
C
ATOM 8672 C GLY A 573 3.645 37.554 -4.366 1.00 0.66
C
ATOM 8673 O GLY A 573 3.533 38.686 -3.900 1.00 0.76
O
ATOM 8674 H GLY A 573 5.214 35.325 -6.083 1.00 0.61
H
ATOM 8675 1HA GLY A 573 5.245 38.122 -5.678 1.00 0.73
H
ATOM 8676 2HA GLY A 573 3.953 37.235 -6.465 1.00 0.73
H
ATOM 8677 N GLU A 574 3.021 36.491 -3.879 1.00 0.68
N
ATOM 8678 CA GLU A 574 2.129 36.546 -2.746 1.00 0.84
C
ATOM 8679 C GLU A 574 2.906 36.855 -1.473 1.00 0.78
C
ATOM 8680 O GLU A 574 2.475 37.669 -0.652 1.00 1.03
O

ATOM 8681 CB GLU A 574 1.433 35.212 -2.635 1.00 1.26
C
ATOM 8682 CG GLU A 574 0.524 34.887 -3.793 1.00 1.26
C
ATOM 8683 CD GLU A 574 -0.734 35.679 -3.833 1.00 1.26
C
ATOM 8684 OE1 GLU A 574 -1.434 35.708 -2.844 1.00 1.26
O
ATOM 8685 OE2 GLU A 574 -1.004 36.261 -4.857 1.00 1.26
O
ATOM 8686 H GLU A 574 3.168 35.596 -4.323 1.00 0.82
H
ATOM 8687 HA GLU A 574 1.393 37.335 -2.910 1.00 1.01
H
ATOM 8688 1HB GLU A 574 2.161 34.409 -2.526 1.00 1.51
H
ATOM 8689 2HB GLU A 574 0.815 35.218 -1.750 1.00 1.51
H
ATOM 8690 1HG GLU A 574 1.074 35.077 -4.714 1.00 1.51
H
ATOM 8691 2HG GLU A 574 0.286 33.838 -3.772 1.00 1.51
H
ATOM 8692 N ALA A 575 4.083 36.231 -1.345 1.00 0.62
N
ATOM 8693 CA ALA A 575 4.978 36.456 -0.222 1.00 0.66
C
ATOM 8694 C ALA A 575 5.420 37.910 -0.164 1.00 0.76
C
ATOM 8695 O ALA A 575 5.415 38.516 0.908 1.00 0.97
O
ATOM 8696 CB ALA A 575 6.194 35.548 -0.326 1.00 0.99
C
ATOM 8697 H ALA A 575 4.357 35.551 -2.044 1.00 0.74
H
ATOM 8698 HA ALA A 575 4.436 36.228 0.697 1.00 0.79
H
ATOM 8699 1HB ALA A 575 6.843 35.709 0.534 1.00 1.19
H
ATOM 8700 2HB ALA A 575 5.867 34.509 -0.346 1.00 1.19
H
ATOM 8701 3HB ALA A 575 6.746 35.771 -1.235 1.00 1.19
H
ATOM 8702 N VAL A 576 5.784 38.459 -1.329 1.00 0.72
N
ATOM 8703 CA VAL A 576 6.215 39.846 -1.436 1.00 0.88
C
ATOM 8704 C VAL A 576 5.091 40.790 -1.090 1.00 1.11
C
ATOM 8705 O VAL A 576 5.304 41.724 -0.321 1.00 1.33
O
ATOM 8706 CB VAL A 576 6.730 40.176 -2.860 1.00 1.32
C
ATOM 8707 CG1 VAL A 576 6.966 41.676 -2.987 1.00 1.32
C
ATOM 8708 CG2 VAL A 576 8.034 39.434 -3.133 1.00 1.32
C
ATOM 8709 H VAL A 576 5.774 37.893 -2.161 1.00 0.86
H

ATOM	8710	HA	VAL	A	576	7.030	40.009	-0.734	1.00	1.06	H
ATOM	8711	HB	VAL	A	576	5.977	39.884	-3.590	1.00	1.58	H
ATOM	8712	1HG1	VAL	A	576	7.317	41.907	-3.992	1.00	1.58	H
ATOM	8713	2HG1	VAL	A	576	6.031	42.207	-2.800	1.00	1.58	H
ATOM	8714	3HG1	VAL	A	576	7.713	41.990	-2.260	1.00	1.58	H
ATOM	8715	1HG2	VAL	A	576	8.383	39.669	-4.137	1.00	1.58	H
ATOM	8716	2HG2	VAL	A	576	8.785	39.741	-2.407	1.00	1.58	H
ATOM	8717	3HG2	VAL	A	576	7.874	38.360	-3.049	1.00	1.58	H
ATOM	8718	N	CYS	A	577	3.893	40.537	-1.634	1.00	1.24	N
ATOM	8719	CA	CYS	A	577	2.739	41.385	-1.358	1.00	1.84	C
ATOM	8720	C	CYS	A	577	2.458	41.457	0.127	1.00	2.04	C
ATOM	8721	O	CYS	A	577	2.263	42.543	0.666	1.00	3.34	O
ATOM	8722	CB	CYS	A	577	1.483	40.862	-2.064	1.00	2.76	C
ATOM	8723	SG	CYS	A	577	1.488	41.046	-3.860	1.00	2.76	S
ATOM	8724	H	CYS	A	577	3.780	39.757	-2.269	1.00	1.49	H
ATOM	8725	HA	CYS	A	577	2.952	42.389	-1.723	1.00	2.21	H
ATOM	8726	1HB	CYS	A	577	1.355	39.803	-1.836	1.00	3.31	H
ATOM	8727	2HB	CYS	A	577	0.608	41.385	-1.676	1.00	3.31	H
ATOM	8728	HG	CYS	A	577	1.513	42.374	-3.860	1.00	3.31	H
ATOM	8729	N	ALA	A	578	2.451	40.304	0.791	1.00	1.58	N
ATOM	8730	CA	ALA	A	578	2.211	40.256	2.222	1.00	1.96	C
ATOM	8731	C	ALA	A	578	3.301	40.999	2.990	1.00	2.05	C
ATOM	8732	O	ALA	A	578	3.010	41.730	3.939	1.00	3.13	O
ATOM	8733	CB	ALA	A	578	2.131	38.812	2.685	1.00	2.94	C
ATOM	8734	H	ALA	A	578	2.604	39.434	0.293	1.00	1.90	H
ATOM	8735	HA	ALA	A	578	1.262	40.751	2.424	1.00	2.35	H
ATOM	8736	1HB	ALA	A	578	1.925	38.783	3.755	1.00	3.53	H
ATOM	8737	2HB	ALA	A	578	1.332	38.302	2.147	1.00	3.53	H
ATOM	8738	3HB	ALA	A	578	3.078	38.312	2.484	1.00	3.53	H

ATOM 8739 N ALA A 579 4.554 40.817 2.573 1.00 1.47
N
ATOM 8740 CA ALA A 579 5.690 41.453 3.224 1.00 1.47
C
ATOM 8741 C ALA A 579 5.654 42.978 3.152 1.00 1.51
C
ATOM 8742 O ALA A 579 5.991 43.647 4.131 1.00 1.68
O
ATOM 8743 CB ALA A 579 6.987 40.954 2.601 1.00 2.21
C
ATOM 8744 H ALA A 579 4.741 40.194 1.797 1.00 1.76
H
ATOM 8745 HA ALA A 579 5.671 41.169 4.276 1.00 1.76
H
ATOM 8746 1HB ALA A 579 7.835 41.400 3.122 1.00 2.65
H
ATOM 8747 2HB ALA A 579 7.037 39.869 2.690 1.00 2.65
H
ATOM 8748 3HB ALA A 579 7.018 41.234 1.550 1.00 2.65
H
ATOM 8749 N VAL A 580 5.256 43.536 2.005 1.00 1.46
N
ATOM 8750 CA VAL A 580 5.293 44.990 1.868 1.00 1.69
C
ATOM 8751 C VAL A 580 3.942 45.679 2.033 1.00 2.43
C
ATOM 8752 O VAL A 580 3.903 46.908 2.116 1.00 5.83
O
ATOM 8753 CB VAL A 580 5.842 45.394 0.492 1.00 2.54
C
ATOM 8754 CG1 VAL A 580 7.223 44.792 0.277 1.00 2.54
C
ATOM 8755 CG2 VAL A 580 4.865 44.991 -0.591 1.00 2.54
C
ATOM 8756 H VAL A 580 4.986 42.955 1.221 1.00 1.75
H
ATOM 8757 HA VAL A 580 5.967 45.380 2.631 1.00 2.03
H
ATOM 8758 HB VAL A 580 5.966 46.476 0.472 1.00 3.04
H
ATOM 8759 1HG1 VAL A 580 7.615 45.112 -0.688 1.00 3.04
H
ATOM 8760 2HG1 VAL A 580 7.890 45.123 1.069 1.00 3.04
H
ATOM 8761 3HG1 VAL A 580 7.150 43.704 0.296 1.00 3.04
H
ATOM 8762 1HG2 VAL A 580 5.259 45.308 -1.552 1.00 3.04
H
ATOM 8763 2HG2 VAL A 580 4.729 43.917 -0.589 1.00 3.04
H
ATOM 8764 3HG2 VAL A 580 3.905 45.475 -0.415 1.00 3.04
H
ATOM 8765 N SER A 581 2.843 44.921 2.063 1.00 1.97
N
ATOM 8766 CA SER A 581 1.534 45.549 2.206 1.00 2.55
C
ATOM 8767 C SER A 581 1.455 46.325 3.499 1.00 2.91
C

```
ATOM   8768  O   SER A 581       1.623  45.748   4.569  1.00 31.80
O
ATOM   8769  CB  SER A 581       0.424  44.520   2.176  1.00  3.82
C
ATOM   8770  OG  SER A 581      -0.817  45.124   2.413  1.00  3.82
O
ATOM   8771  H   SER A 581       2.896  43.917   1.965  1.00  2.36
H
ATOM   8772  HA  SER A 581       1.391  46.244   1.378  1.00  3.06
H
ATOM   8773 1HB  SER A 581       0.408  44.030   1.204  1.00  4.59
H
ATOM   8774 2HB  SER A 581       0.613  43.755   2.929  1.00  4.59
H
ATOM   8775  HG  SER A 581      -0.953  45.738   1.689  1.00  4.59
H
ATOM   8776  N   ARG A 582       1.244  47.635   3.355  1.00 11.10
N
ATOM   8777  CA  ARG A 582       1.091  48.660   4.402  1.00 11.60
C
ATOM   8778  C   ARG A 582       2.127  49.760   4.211  1.00  4.92
C
ATOM   8779  O   ARG A 582       1.997  50.841   4.784  1.00  7.03
O
ATOM   8780  CB  ARG A 582       1.154  48.130   5.834  1.00 17.40
C
ATOM   8781  CG  ARG A 582      -0.093  47.376   6.287  1.00 17.40
C
ATOM   8782  CD  ARG A 582       0.099  46.685   7.573  1.00 17.40
C
ATOM   8783  NE  ARG A 582       1.056  45.614   7.439  1.00 17.40
N
ATOM   8784  CZ  ARG A 582       1.405  44.754   8.417  1.00 17.40
C
ATOM   8785  NH1 ARG A 582       0.843  44.822   9.608  1.00 17.40
N
ATOM   8786  NH2 ARG A 582       2.319  43.831   8.176  1.00 17.40
N
ATOM   8787  H   ARG A 582       1.147  47.961   2.405  1.00 13.32
H
ATOM   8788  HA  ARG A 582       0.108  49.109   4.283  1.00 13.92
H
ATOM   8789 1HB  ARG A 582       2.029  47.499   5.976  1.00 20.88
H
ATOM   8790 2HB  ARG A 582       1.267  48.975   6.511  1.00 20.88
H
ATOM   8791 1HG  ARG A 582      -0.898  48.090   6.430  1.00 20.88
H
ATOM   8792 2HG  ARG A 582      -0.396  46.642   5.544  1.00 20.88
H
ATOM   8793 1HD  ARG A 582       0.464  47.385   8.324  1.00 20.88
H
ATOM   8794 2HD  ARG A 582      -0.847  46.257   7.896  1.00 20.88
H
ATOM   8795  HE  ARG A 582       1.499  45.524   6.531  1.00 20.88
H
ATOM   8796 1HH1 ARG A 582       0.123  45.508   9.805  1.00 20.88
H
```

```
ATOM   8797 2HH1 ARG A 582       1.115  44.170  10.328  1.00 20.88
H
ATOM   8798 1HH2 ARG A 582       2.751  43.775   7.263  1.00 20.88
H
ATOM   8799 2HH2 ARG A 582       2.586  43.181   8.901  1.00 20.88
H
ATOM   8800  N   GLU A 583       3.150  49.493   3.396  1.00  1.91
N
ATOM   8801  CA  GLU A 583       4.183  50.494   3.148  1.00  3.73
C
ATOM   8802  C   GLU A 583       3.687  51.441   2.030  1.00  8.37
C
ATOM   8803  O   GLU A 583       3.601  51.025   0.875  1.00 13.03
O
ATOM   8804  CB  GLU A 583       5.508  49.819   2.768  1.00  5.59
C
ATOM   8805  CG  GLU A 583       6.698  50.767   2.627  1.00  5.59
C
ATOM   8806  CD  GLU A 583       7.105  51.395   3.934  1.00  5.59
C
ATOM   8807  OE1 GLU A 583       6.851  50.806   4.957  1.00  5.59
O
ATOM   8808  OE2 GLU A 583       7.681  52.462   3.911  1.00  5.59
O
ATOM   8809  H   GLU A 583       3.232  48.590   2.948  1.00  2.29
H
ATOM   8810  HA  GLU A 583       4.346  51.043   4.066  1.00  4.48
H
ATOM   8811 1HB  GLU A 583       5.763  49.077   3.525  1.00  6.71
H
ATOM   8812 2HB  GLU A 583       5.384  49.290   1.824  1.00  6.71
H
ATOM   8813 1HG  GLU A 583       7.543  50.214   2.222  1.00  6.71
H
ATOM   8814 2HG  GLU A 583       6.438  51.555   1.923  1.00  6.71
H
ATOM   8815  N   PRO A 584       3.344  52.695   2.384  1.00 13.55
N
ATOM   8816  CA  PRO A 584       2.691  53.752   1.570  1.00 25.25
C
ATOM   8817  C   PRO A 584       2.888  53.766   0.042  1.00 22.39
C
ATOM   8818  O   PRO A 584       1.923  53.605  -0.719  1.00 77.79
O
ATOM   8819  CB  PRO A 584       3.294  55.048   2.140  1.00 37.88
C
ATOM   8820  CG  PRO A 584       3.602  54.726   3.542  1.00 37.88
C
ATOM   8821  CD  PRO A 584       4.114  53.326   3.484  1.00 37.88
C
ATOM   8822  HA  PRO A 584       1.615  53.727   1.791  1.00 30.30
H
ATOM   8823 1HB  PRO A 584       4.185  55.351   1.573  1.00 45.45
H
ATOM   8824 2HB  PRO A 584       2.567  55.867   2.046  1.00 45.45
H
ATOM   8825 1HG  PRO A 584       4.345  55.434   3.942  1.00 45.45
H
```

```
ATOM   8826 2HG  PRO A 584       2.700  54.823   4.164  1.00 45.45
H
ATOM   8827 1HD  PRO A 584       5.188  53.314   3.255  1.00 45.45
H
ATOM   8828 2HD  PRO A 584       3.888  52.848   4.446  1.00 45.45
H
ATOM   8829  N   ASP A 585       4.090  53.999  -0.419  1.00  4.05
N
ATOM   8830  CA  ASP A 585       4.297  54.153  -1.849  1.00  4.33
C
ATOM   8831  C   ASP A 585       4.653  52.869  -2.571  1.00  2.30
C
ATOM   8832  O   ASP A 585       5.058  52.925  -3.729  1.00  3.84
O
ATOM   8833  CB  ASP A 585       5.396  55.183  -2.100  1.00  6.50
C
ATOM   8834  CG  ASP A 585       4.993  56.586  -1.666  1.00  6.50
C
ATOM   8835  OD1 ASP A 585       3.819  56.874  -1.666  1.00  6.50
O
ATOM   8836  OD2 ASP A 585       5.863  57.354  -1.334  1.00  6.50
O
ATOM   8837  H   ASP A 585       4.869  54.098   0.214  1.00  4.86
H
ATOM   8838  HA  ASP A 585       3.371  54.531  -2.283  1.00  5.20
H
ATOM   8839 1HB  ASP A 585       6.297  54.894  -1.561  1.00  7.79
H
ATOM   8840 2HB  ASP A 585       5.640  55.203  -3.162  1.00  7.79
H
ATOM   8841  N   ILE A 586       4.527  51.720  -1.908  1.00  1.77
N
ATOM   8842  CA  ILE A 586       4.931  50.472  -2.533  1.00  1.54
C
ATOM   8843  C   ILE A 586       3.791  49.777  -3.266  1.00  1.30
C
ATOM   8844  O   ILE A 586       2.776  49.418  -2.668  1.00  2.09
O
ATOM   8845  CB  ILE A 586       5.511  49.500  -1.496  1.00  2.31
C
ATOM   8846  CG1 ILE A 586       6.635  50.182  -0.722  1.00  2.31
C
ATOM   8847  CG2 ILE A 586       6.028  48.249  -2.193  1.00  2.31
C
ATOM   8848  CD1 ILE A 586       7.762  50.682  -1.577  1.00  2.31
C
ATOM   8849  H   ILE A 586       4.165  51.695  -0.963  1.00  2.12
H
ATOM   8850  HA  ILE A 586       5.712  50.695  -3.256  1.00  1.85
H
ATOM   8851  HB  ILE A 586       4.737  49.225  -0.780  1.00  2.77
H
ATOM   8852 1HG1 ILE A 586       6.220  51.028  -0.178  1.00  2.77
H
ATOM   8853 2HG1 ILE A 586       7.044  49.474  -0.001  1.00  2.77
H
ATOM   8854 1HG2 ILE A 586       6.437  47.562  -1.454  1.00  2.77
H
```

```
ATOM   8855 2HG2 ILE A 586       5.212  47.765  -2.726  1.00  2.77
H
ATOM   8856 3HG2 ILE A 586       6.810  48.524  -2.903  1.00  2.77
H
ATOM   8857 1HD1 ILE A 586       8.521  51.149  -0.950  1.00  2.77
H
ATOM   8858 2HD1 ILE A 586       8.194  49.842  -2.108  1.00  2.77
H
ATOM   8859 3HD1 ILE A 586       7.379  51.409  -2.289  1.00  2.77
H
ATOM   8860  N   LEU A 587       3.986  49.574  -4.563  1.00  1.72
N
ATOM   8861  CA  LEU A 587       3.032  48.882  -5.423  1.00  2.15
C
ATOM   8862  C   LEU A 587       3.655  47.590  -5.945  1.00  1.80
C
ATOM   8863  O   LEU A 587       4.801  47.599  -6.390  1.00  2.81
O
ATOM   8864  CB  LEU A 587       2.646  49.774  -6.613  1.00  3.22
C
ATOM   8865  CG  LEU A 587       1.689  50.938  -6.326  1.00  3.22
C
ATOM   8866  CD1 LEU A 587       2.480  52.141  -5.831  1.00  3.22
C
ATOM   8867  CD2 LEU A 587       0.918  51.270  -7.593  1.00  3.22
C
ATOM   8868  H   LEU A 587       4.844  49.914  -4.967  1.00  2.06
H
ATOM   8869  HA  LEU A 587       2.143  48.639  -4.843  1.00  2.58
H
ATOM   8870 1HB  LEU A 587       3.562  50.210  -7.011  1.00  3.87
H
ATOM   8871 2HB  LEU A 587       2.208  49.160  -7.392  1.00  3.87
H
ATOM   8872  HG  LEU A 587       0.992  50.655  -5.540  1.00  3.87
H
ATOM   8873 1HD1 LEU A 587       1.796  52.964  -5.621  1.00  3.87
H
ATOM   8874 2HD1 LEU A 587       3.016  51.880  -4.922  1.00  3.87
H
ATOM   8875 3HD1 LEU A 587       3.192  52.448  -6.596  1.00  3.87
H
ATOM   8876 1HD2 LEU A 587       0.229  52.092  -7.395  1.00  3.87
H
ATOM   8877 2HD2 LEU A 587       1.614  51.561  -8.380  1.00  3.87
H
ATOM   8878 3HD2 LEU A 587       0.353  50.395  -7.915  1.00  3.87
H
ATOM   8879  N   VAL A 588       2.904  46.483  -5.913  1.00  1.49
N
ATOM   8880  CA  VAL A 588       3.445  45.203  -6.381  1.00  1.30
C
ATOM   8881  C   VAL A 588       2.604  44.547  -7.481  1.00  1.39
C
ATOM   8882  O   VAL A 588       1.422  44.259  -7.292  1.00  2.04
O
ATOM   8883  CB  VAL A 588       3.580  44.198  -5.219  1.00  1.95
C
```

ATOM 8884 CG1 VAL A 588 4.127 42.872 -5.737 1.00 1.95
C
ATOM 8885 CG2 VAL A 588 4.494 44.752 -4.144 1.00 1.95
C
ATOM 8886 H VAL A 588 1.962 46.523 -5.547 1.00 1.79
H
ATOM 8887 HA VAL A 588 4.441 45.388 -6.775 1.00 1.56
H
ATOM 8888 HB VAL A 588 2.596 44.008 -4.798 1.00 2.34
H
ATOM 8889 1HG1 VAL A 588 4.202 42.163 -4.914 1.00 2.34
H
ATOM 8890 2HG1 VAL A 588 3.456 42.474 -6.498 1.00 2.34
H
ATOM 8891 3HG1 VAL A 588 5.114 43.032 -6.171 1.00 2.34
H
ATOM 8892 1HG2 VAL A 588 4.569 44.030 -3.334 1.00 2.34
H
ATOM 8893 2HG2 VAL A 588 5.483 44.930 -4.558 1.00 2.34
H
ATOM 8894 3HG2 VAL A 588 4.088 45.687 -3.761 1.00 2.34
H
ATOM 8895 N HIS A 589 3.246 44.289 -8.618 1.00 1.07
N
ATOM 8896 CA HIS A 589 2.648 43.617 -9.762 1.00 1.02
C
ATOM 8897 C HIS A 589 3.017 42.134 -9.718 1.00 1.06
C
ATOM 8898 O HIS A 589 4.021 41.765 -9.108 1.00 1.53
O
ATOM 8899 CB HIS A 589 3.144 44.231 -11.070 1.00 1.53
C
ATOM 8900 CG HIS A 589 2.785 45.675 -11.257 1.00 1.53
C
ATOM 8901 ND1 HIS A 589 1.518 46.094 -11.621 1.00 1.53
N
ATOM 8902 CD2 HIS A 589 3.531 46.797 -11.130 1.00 1.53
C
ATOM 8903 CE1 HIS A 589 1.510 47.414 -11.720 1.00 1.53
C
ATOM 8904 NE2 HIS A 589 2.715 47.862 -11.424 1.00 1.53
N
ATOM 8905 H HIS A 589 4.209 44.570 -8.689 1.00 1.28
H
ATOM 8906 HA HIS A 589 1.562 43.700 -9.724 1.00 1.22
H
ATOM 8907 1HB HIS A 589 4.227 44.143 -11.112 1.00 1.84
H
ATOM 8908 2HB HIS A 589 2.742 43.669 -11.912 1.00 1.84
H
ATOM 8909 HD2 HIS A 589 4.584 46.846 -10.850 1.00 1.84
H
ATOM 8910 HE1 HIS A 589 0.653 48.029 -11.995 1.00 1.84
H
ATOM 8911 HE2 HIS A 589 2.998 48.832 -11.416 1.00 1.84
H
ATOM 8912 N GLN A 590 2.216 41.285 -10.363 1.00 1.08
N

```
ATOM   8913  CA  GLN A 590       2.470  39.842 -10.324  1.00  1.07
C
ATOM   8914  C   GLN A 590       2.376  39.145 -11.683  1.00  0.94
C
ATOM   8915  O   GLN A 590       1.335  39.186 -12.345  1.00  1.11
O
ATOM   8916  CB  GLN A 590       1.472  39.170  -9.381  1.00  1.60
C
ATOM   8917  CG  GLN A 590       1.674  37.675  -9.187  1.00  1.60
C
ATOM   8918  CD  GLN A 590       0.575  37.079  -8.328  1.00  1.60
C
ATOM   8919  OE1 GLN A 590      -0.526  36.824  -8.828  1.00  1.60
O
ATOM   8920  NE2 GLN A 590       0.859  36.876  -7.045  1.00  1.60
N
ATOM   8921  H   GLN A 590       1.412  41.637 -10.863  1.00  1.30
H
ATOM   8922  HA  GLN A 590       3.478  39.687  -9.941  1.00  1.28
H
ATOM   8923 1HB  GLN A 590       1.533  39.643  -8.399  1.00  1.93
H
ATOM   8924 2HB  GLN A 590       0.460  39.327  -9.753  1.00  1.93
H
ATOM   8925 1HG  GLN A 590       1.664  37.180 -10.159  1.00  1.93
H
ATOM   8926 2HG  GLN A 590       2.630  37.506  -8.691  1.00  1.93
H
ATOM   8927 1HE2 GLN A 590       0.165  36.503  -6.418  1.00  1.93
H
ATOM   8928 2HE2 GLN A 590       1.762  37.110  -6.690  1.00  1.93
H
ATOM   8929  N   LEU A 591       3.455  38.459 -12.056  1.00  0.77
N
ATOM   8930  CA  LEU A 591       3.523  37.649 -13.269  1.00  0.71
C
ATOM   8931  C   LEU A 591       3.711  36.182 -12.886  1.00  0.70
C
ATOM   8932  O   LEU A 591       4.816  35.764 -12.547  1.00  1.26
O
ATOM   8933  CB  LEU A 591       4.693  38.086 -14.165  1.00  1.06
C
ATOM   8934  CG  LEU A 591       4.694  39.537 -14.660  1.00  1.06
C
ATOM   8935  CD1 LEU A 591       5.992  39.799 -15.414  1.00  1.06
C
ATOM   8936  CD2 LEU A 591       3.492  39.765 -15.562  1.00  1.06
C
ATOM   8937  H   LEU A 591       4.273  38.493 -11.468  1.00  0.92
H
ATOM   8938  HA  LEU A 591       2.589  37.752 -13.820  1.00  0.85
H
ATOM   8939 1HB  LEU A 591       5.614  37.939 -13.612  1.00  1.28
H
ATOM   8940 2HB  LEU A 591       4.716  37.440 -15.042  1.00  1.28
H
ATOM   8941  HG  LEU A 591       4.648  40.217 -13.810  1.00  1.28
H
```

```
ATOM   8942 1HD1 LEU A 591       6.006  40.829 -15.767  1.00  1.28
H
ATOM   8943 2HD1 LEU A 591       6.839  39.633 -14.750  1.00  1.28
H
ATOM   8944 3HD1 LEU A 591       6.059  39.123 -16.266  1.00  1.28
H
ATOM   8945 1HD2 LEU A 591       3.494  40.795 -15.918  1.00  1.28
H
ATOM   8946 2HD2 LEU A 591       3.545  39.086 -16.415  1.00  1.28
H
ATOM   8947 3HD2 LEU A 591       2.576  39.575 -15.002  1.00  1.28
H
ATOM   8948  N   ALA A 592       2.642  35.400 -12.925  1.00  0.47
N
ATOM   8949  CA  ALA A 592       2.752  33.995 -12.545  1.00  0.42
C
ATOM   8950  C   ALA A 592       1.755  33.151 -13.342  1.00  0.39
C
ATOM   8951  O   ALA A 592       0.980  33.688 -14.136  1.00  0.47
O
ATOM   8952  CB  ALA A 592       2.563  33.848 -11.047  1.00  0.63
C
ATOM   8953  H   ALA A 592       1.751  35.776 -13.217  1.00  0.56
H
ATOM   8954  HA  ALA A 592       3.752  33.646 -12.800  1.00  0.50
H
ATOM   8955 1HB  ALA A 592       2.673  32.809 -10.758  1.00  0.76
H
ATOM   8956 2HB  ALA A 592       3.316  34.444 -10.531  1.00  0.76
H
ATOM   8957 3HB  ALA A 592       1.586  34.197 -10.765  1.00  0.76
H
ATOM   8958  N   VAL A 593       1.823  31.830 -13.177  1.00  0.36
N
ATOM   8959  CA  VAL A 593       1.015  30.922 -13.986  1.00  0.41
C
ATOM   8960  C   VAL A 593      -0.456  30.949 -13.544  1.00  0.46
C
ATOM   8961  O   VAL A 593      -0.781  30.849 -12.357  1.00  0.51
O
ATOM   8962  CB  VAL A 593       1.593  29.488 -13.914  1.00  0.61
C
ATOM   8963  CG1 VAL A 593       0.724  28.529 -14.712  1.00  0.61
C
ATOM   8964  CG2 VAL A 593       3.026  29.485 -14.439  1.00  0.61
C
ATOM   8965  H   VAL A 593       2.441  31.440 -12.477  1.00  0.43
H
ATOM   8966  HA  VAL A 593       1.069  31.252 -15.024  1.00  0.49
H
ATOM   8967  HB  VAL A 593       1.587  29.153 -12.878  1.00  0.74
H
ATOM   8968 1HG1 VAL A 593       1.132  27.521 -14.641  1.00  0.74
H
ATOM   8969 2HG1 VAL A 593      -0.289  28.536 -14.313  1.00  0.74
H
ATOM   8970 3HG1 VAL A 593       0.706  28.838 -15.757  1.00  0.74
H
```

```
ATOM   8971 1HG2 VAL A 593       3.434  28.477 -14.374  1.00  0.74
H
ATOM   8972 2HG2 VAL A 593       3.034  29.812 -15.478  1.00  0.74
H
ATOM   8973 3HG2 VAL A 593       3.634  30.162 -13.839  1.00  0.74
H
ATOM   8974  N   SER A 594      -1.341  31.148 -14.523  1.00  0.55
N
ATOM   8975  CA  SER A 594      -2.779  31.289 -14.280  1.00  0.73
C
ATOM   8976  C   SER A 594      -3.602  29.992 -14.270  1.00  0.96
C
ATOM   8977  O   SER A 594      -4.745  30.001 -13.812  1.00  2.87
O
ATOM   8978  CB  SER A 594      -3.368  32.213 -15.324  1.00  1.09
C
ATOM   8979  OG  SER A 594      -3.310  31.630 -16.596  1.00  1.09
O
ATOM   8980  H   SER A 594      -1.003  31.217 -15.471  1.00  0.66
H
ATOM   8981  HA  SER A 594      -2.899  31.759 -13.303  1.00  0.88
H
ATOM   8982 1HB  SER A 594      -4.404  32.436 -15.070  1.00  1.31
H
ATOM   8983 2HB  SER A 594      -2.820  33.155 -15.327  1.00  1.31
H
ATOM   8984  HG  SER A 594      -3.819  30.817 -16.536  1.00  1.31
H
ATOM   8985  N   GLY A 595      -3.054  28.888 -14.771  1.00  0.79
N
ATOM   8986  CA  GLY A 595      -3.826  27.641 -14.809  1.00  0.83
C
ATOM   8987  C   GLY A 595      -2.994  26.462 -15.296  1.00  1.17
C
ATOM   8988  O   GLY A 595      -1.786  26.589 -15.466  1.00  5.02
O
ATOM   8989  H   GLY A 595      -2.113  28.909 -15.134  1.00  0.95
H
ATOM   8990 1HA  GLY A 595      -4.212  27.427 -13.813  1.00  1.00
H
ATOM   8991 2HA  GLY A 595      -4.688  27.772 -15.463  1.00  1.00
H
ATOM   8992  N   VAL A 596      -3.648  25.317 -15.526  1.00  0.79
N
ATOM   8993  CA  VAL A 596      -2.946  24.092 -15.929  1.00  0.64
C
ATOM   8994  C   VAL A 596      -2.180  24.266 -17.242  1.00  0.79
C
ATOM   8995  O   VAL A 596      -2.769  24.662 -18.249  1.00  1.41
O
ATOM   8996  CB  VAL A 596      -3.949  22.921 -16.104  1.00  0.96
C
ATOM   8997  CG1 VAL A 596      -3.244  21.698 -16.670  1.00  0.96
C
ATOM   8998  CG2 VAL A 596      -4.581  22.571 -14.770  1.00  0.96
C
ATOM   8999  H   VAL A 596      -4.648  25.287 -15.393  1.00  0.95
H
```

ATOM 9000 HA VAL A 596 -2.267 23.831 -15.124 1.00 0.77
H
ATOM 9001 HB VAL A 596 -4.722 23.216 -16.814 1.00 1.15
H
ATOM 9002 1HG1 VAL A 596 -3.965 20.890 -16.800 1.00 1.15
H
ATOM 9003 2HG1 VAL A 596 -2.800 21.945 -17.633 1.00 1.15
H
ATOM 9004 3HG1 VAL A 596 -2.466 21.380 -15.984 1.00 1.15
H
ATOM 9005 1HG2 VAL A 596 -5.289 21.753 -14.906 1.00 1.15
H
ATOM 9006 2HG2 VAL A 596 -3.807 22.268 -14.066 1.00 1.15
H
ATOM 9007 3HG2 VAL A 596 -5.104 23.444 -14.387 1.00 1.15
H
ATOM 9008 N PRO A 597 -0.869 23.978 -17.250 1.00 0.79
N
ATOM 9009 CA PRO A 597 0.023 24.058 -18.390 1.00 0.93
C
ATOM 9010 C PRO A 597 -0.035 22.854 -19.311 1.00 0.88
C
ATOM 9011 O PRO A 597 -0.514 21.782 -18.944 1.00 0.82
O
ATOM 9012 CB PRO A 597 1.399 24.237 -17.752 1.00 1.40
C
ATOM 9013 CG PRO A 597 1.295 23.565 -16.454 1.00 1.40
C
ATOM 9014 CD PRO A 597 -0.099 23.899 -15.992 1.00 1.40
C
ATOM 9015 HA PRO A 597 -0.268 24.947 -18.949 1.00 1.12
H
ATOM 9016 1HB PRO A 597 2.176 23.802 -18.399 1.00 1.67
H
ATOM 9017 2HB PRO A 597 1.629 25.307 -17.649 1.00 1.67
H
ATOM 9018 1HG PRO A 597 1.477 22.494 -16.574 1.00 1.67
H
ATOM 9019 2HG PRO A 597 2.075 23.938 -15.784 1.00 1.67
H
ATOM 9020 1HD PRO A 597 -0.480 23.095 -15.355 1.00 1.67
H
ATOM 9021 2HD PRO A 597 -0.109 24.868 -15.475 1.00 1.67
H
ATOM 9022 N GLN A 598 0.460 23.063 -20.525 1.00 1.19
N
ATOM 9023 CA GLN A 598 0.477 22.061 -21.579 1.00 1.18
C
ATOM 9024 C GLN A 598 1.907 21.711 -21.960 1.00 1.28
C
ATOM 9025 O GLN A 598 2.849 22.358 -21.504 1.00 1.85
O
ATOM 9026 CB GLN A 598 -0.260 22.596 -22.810 1.00 1.77
C
ATOM 9027 CG GLN A 598 -1.719 22.944 -22.570 1.00 1.77
C
ATOM 9028 CD GLN A 598 -2.374 23.548 -23.799 1.00 1.77
C

ATOM 9029 OE1 GLN A 598 -2.481 22.909 -24.850 1.00 1.77
O
ATOM 9030 NE2 GLN A 598 -2.824 24.792 -23.673 1.00 1.77
N
ATOM 9031 H GLN A 598 0.840 23.974 -20.733 1.00 1.43
H
ATOM 9032 HA GLN A 598 -0.017 21.158 -21.219 1.00 1.42
H
ATOM 9033 1HB GLN A 598 0.243 23.491 -23.174 1.00 2.12
H
ATOM 9034 2HB GLN A 598 -0.222 21.853 -23.607 1.00 2.12
H
ATOM 9035 1HG GLN A 598 -2.259 22.036 -22.304 1.00 2.12
H
ATOM 9036 2HG GLN A 598 -1.782 23.667 -21.758 1.00 2.12
H
ATOM 9037 1HE2 GLN A 598 -3.268 25.243 -24.448 1.00 2.12
H
ATOM 9038 2HE2 GLN A 598 -2.721 25.277 -22.804 1.00 2.12
H
ATOM 9039 N ARG A 599 2.069 20.702 -22.815 1.00 1.66
N
ATOM 9040 CA ARG A 599 3.406 20.343 -23.292 1.00 2.25
C
ATOM 9041 C ARG A 599 3.891 21.340 -24.347 1.00 2.51
C
ATOM 9042 O ARG A 599 3.097 21.886 -25.113 1.00 5.24
O
ATOM 9043 CB ARG A 599 3.418 18.944 -23.891 1.00 3.38
C
ATOM 9044 CG ARG A 599 3.488 17.845 -22.859 1.00 3.38
C
ATOM 9045 CD ARG A 599 2.214 17.778 -22.137 1.00 3.38
C
ATOM 9046 NE ARG A 599 2.127 16.700 -21.163 1.00 3.38
N
ATOM 9047 CZ ARG A 599 1.078 16.526 -20.346 1.00 3.38
C
ATOM 9048 NH1 ARG A 599 0.042 17.325 -20.466 1.00 3.38
N
ATOM 9049 NH2 ARG A 599 1.092 15.583 -19.418 1.00 3.38
N
ATOM 9050 H ARG A 599 1.252 20.170 -23.124 1.00 1.99
H
ATOM 9051 HA ARG A 599 4.094 20.366 -22.447 1.00 2.70
H
ATOM 9052 1HB ARG A 599 2.516 18.790 -24.482 1.00 4.05
H
ATOM 9053 2HB ARG A 599 4.274 18.835 -24.557 1.00 4.05
H
ATOM 9054 1HG ARG A 599 3.694 16.890 -23.335 1.00 4.05
H
ATOM 9055 2HG ARG A 599 4.271 18.079 -22.143 1.00 4.05
H
ATOM 9056 1HD ARG A 599 2.054 18.718 -21.612 1.00 4.05
H
ATOM 9057 2HD ARG A 599 1.435 17.652 -22.860 1.00 4.05
H

```
ATOM   9058  HE  ARG A 599       2.940  16.129 -20.991  1.00  4.05
H
ATOM   9059 1HH1 ARG A 599       0.026  18.045 -21.185  1.00  4.05
H
ATOM   9060 2HH1 ARG A 599      -0.753  17.220 -19.855  1.00  4.05
H
ATOM   9061 1HH2 ARG A 599       1.890  14.956 -19.310  1.00  4.05
H
ATOM   9062 2HH2 ARG A 599       0.295  15.471 -18.810  1.00  4.05
H
ATOM   9063  N   GLY A 600       5.197  21.574 -24.378  1.00  1.71
N
ATOM   9064  CA  GLY A 600       5.797  22.495 -25.343  1.00  2.41
C
ATOM   9065  C   GLY A 600       7.267  22.692 -25.021  1.00  1.91
C
ATOM   9066  O   GLY A 600       7.813  22.002 -24.165  1.00  3.03
O
ATOM   9067  H   GLY A 600       5.797  21.099 -23.718  1.00  2.05
H
ATOM   9068 1HA  GLY A 600       5.687  22.096 -26.352  1.00  2.89
H
ATOM   9069 2HA  GLY A 600       5.277  23.452 -25.311  1.00  2.89
H
ATOM   9070  N   LYS A 601       7.919  23.631 -25.692  1.00  1.14
N
ATOM   9071  CA  LYS A 601       9.319  23.883 -25.382  1.00  0.83
C
ATOM   9072  C   LYS A 601       9.396  24.879 -24.242  1.00  0.68
C
ATOM   9073  O   LYS A 601       8.534  25.749 -24.146  1.00  1.41
O
ATOM   9074  CB  LYS A 601      10.065  24.435 -26.596  1.00  1.24
C
ATOM   9075  CG  LYS A 601      10.034  23.549 -27.834  1.00  1.24
C
ATOM   9076  CD  LYS A 601      10.766  22.234 -27.621  1.00  1.24
C
ATOM   9077  CE  LYS A 601      10.830  21.444 -28.920  1.00  1.24
C
ATOM   9078  NZ  LYS A 601      11.551  20.156 -28.761  1.00  1.24
N
ATOM   9079  H   LYS A 601       7.451  24.176 -26.413  1.00  1.37
H
ATOM   9080  HA  LYS A 601       9.789  22.953 -25.061  1.00  1.00
H
ATOM   9081 1HB  LYS A 601       9.644  25.400 -26.871  1.00  1.49
H
ATOM   9082 2HB  LYS A 601      11.110  24.598 -26.335  1.00  1.49
H
ATOM   9083 1HG  LYS A 601       8.997  23.338 -28.099  1.00  1.49
H
ATOM   9084 2HG  LYS A 601      10.497  24.078 -28.666  1.00  1.49
H
ATOM   9085 1HD  LYS A 601      11.779  22.430 -27.269  1.00  1.49
H
ATOM   9086 2HD  LYS A 601      10.243  21.641 -26.870  1.00  1.49
H
```

```
ATOM   9087 1HE  LYS A 601       9.818  21.241 -29.266  1.00  1.49
H
ATOM   9088 2HE  LYS A 601      11.344  22.042 -29.673  1.00  1.49
H
ATOM   9089 1HZ  LYS A 601      11.572  19.669 -29.644  1.00  1.49
H
ATOM   9090 2HZ  LYS A 601      12.496  20.331 -28.452  1.00  1.49
H
ATOM   9091 3HZ  LYS A 601      11.082  19.576 -28.078  1.00  1.49
H
ATOM   9092  N   THR A 602      10.417  24.742 -23.388  1.00  0.63
N
ATOM   9093  CA  THR A 602      10.679  25.663 -22.270  1.00  0.45
C
ATOM   9094  C   THR A 602      10.234  27.093 -22.549  1.00  0.49
C
ATOM   9095  O   THR A 602       9.333  27.612 -21.888  1.00  0.63
O
ATOM   9096  CB  THR A 602      12.183  25.678 -21.903  1.00  0.68
C
ATOM   9097  OG1 THR A 602      12.580  24.379 -21.450  1.00  0.68
O
ATOM   9098  CG2 THR A 602      12.479  26.709 -20.819  1.00  0.68
C
ATOM   9099  H   THR A 602      11.060  23.976 -23.533  1.00  0.76
H
ATOM   9100  HA  THR A 602      10.123  25.307 -21.403  1.00  0.54
H
ATOM   9101  HB  THR A 602      12.761  25.926 -22.793  1.00  0.81
H
ATOM   9102  HG1 THR A 602      12.232  24.235 -20.562  1.00  0.81
H
ATOM   9103 1HG2 THR A 602      13.544  26.696 -20.591  1.00  0.81
H
ATOM   9104 2HG2 THR A 602      12.195  27.701 -21.165  1.00  0.81
H
ATOM   9105 3HG2 THR A 602      11.916  26.463 -19.923  1.00  0.81
H
ATOM   9106  N   SER A 603      10.897  27.720 -23.525  1.00  0.48
N
ATOM   9107  CA  SER A 603      10.643  29.101 -23.913  1.00  0.54
C
ATOM   9108  C   SER A 603       9.216  29.338 -24.381  1.00  0.58
C
ATOM   9109  O   SER A 603       8.626  30.368 -24.054  1.00  0.62
O
ATOM   9110  CB  SER A 603      11.603  29.513 -25.011  1.00  0.81
C
ATOM   9111  OG  SER A 603      12.924  29.546 -24.541  1.00  0.81
O
ATOM   9112  H   SER A 603      11.621  27.213 -24.015  1.00  0.58
H
ATOM   9113  HA  SER A 603      10.821  29.735 -23.043  1.00  0.65
H
ATOM   9114 1HB  SER A 603      11.528  28.812 -25.841  1.00  0.97
H
ATOM   9115 2HB  SER A 603      11.324  30.497 -25.387  1.00  0.97
H
```

ATOM 9116 HG SER A 603 12.944 30.219 -23.855 1.00 0.97
H
ATOM 9117 N GLU A 604 8.666 28.388 -25.141 1.00 0.66
N
ATOM 9118 CA GLU A 604 7.303 28.507 -25.648 1.00 0.75
C
ATOM 9119 C GLU A 604 6.308 28.590 -24.514 1.00 0.73
C
ATOM 9120 O GLU A 604 5.363 29.377 -24.568 1.00 0.86
O
ATOM 9121 CB GLU A 604 6.931 27.310 -26.534 1.00 1.12
C
ATOM 9122 CG GLU A 604 7.635 27.246 -27.877 1.00 1.12
C
ATOM 9123 CD GLU A 604 7.294 25.997 -28.644 1.00 1.12
C
ATOM 9124 OE1 GLU A 604 6.883 25.035 -28.036 1.00 1.12
O
ATOM 9125 OE2 GLU A 604 7.444 26.007 -29.844 1.00 1.12
O
ATOM 9126 H GLU A 604 9.202 27.564 -25.373 1.00 0.79
H
ATOM 9127 HA GLU A 604 7.230 29.423 -26.236 1.00 0.90
H
ATOM 9128 1HB GLU A 604 7.142 26.383 -26.006 1.00 1.35
H
ATOM 9129 2HB GLU A 604 5.860 27.332 -26.731 1.00 1.35
H
ATOM 9130 1HG GLU A 604 7.346 28.114 -28.469 1.00 1.35
H
ATOM 9131 2HG GLU A 604 8.711 27.290 -27.717 1.00 1.35
H
ATOM 9132 N LEU A 605 6.528 27.775 -23.488 1.00 0.65
N
ATOM 9133 CA LEU A 605 5.633 27.740 -22.354 1.00 0.73
C
ATOM 9134 C LEU A 605 5.820 28.964 -21.475 1.00 0.72
C
ATOM 9135 O LEU A 605 4.845 29.521 -20.978 1.00 0.83
O
ATOM 9136 CB LEU A 605 5.872 26.466 -21.556 1.00 1.09
C
ATOM 9137 CG LEU A 605 5.530 25.176 -22.306 1.00 1.09
C
ATOM 9138 CD1 LEU A 605 5.898 23.997 -21.435 1.00 1.09
C
ATOM 9139 CD2 LEU A 605 4.056 25.158 -22.682 1.00 1.09
C
ATOM 9140 H LEU A 605 7.322 27.148 -23.517 1.00 0.78
H
ATOM 9141 HA LEU A 605 4.609 27.732 -22.723 1.00 0.88
H
ATOM 9142 1HB LEU A 605 6.923 26.420 -21.276 1.00 1.31
H
ATOM 9143 2HB LEU A 605 5.272 26.499 -20.647 1.00 1.31
H
ATOM 9144 HG LEU A 605 6.130 25.119 -23.216 1.00 1.31
H

```
ATOM   9145 1HD1 LEU A 605       5.682  23.067 -21.963  1.00  1.31
H
ATOM   9146 2HD1 LEU A 605       6.959  24.043 -21.197  1.00  1.31
H
ATOM   9147 3HD1 LEU A 605       5.317  24.039 -20.517  1.00  1.31
H
ATOM   9148 1HD2 LEU A 605       3.831  24.238 -23.223  1.00  1.31
H
ATOM   9149 2HD2 LEU A 605       3.448  25.202 -21.781  1.00  1.31
H
ATOM   9150 3HD2 LEU A 605       3.831  26.016 -23.316  1.00  1.31
H
ATOM   9151  N   LEU A 606       7.058  29.432 -21.330  1.00  0.65
N
ATOM   9152  CA  LEU A 606       7.289  30.633 -20.541  1.00  0.69
C
ATOM   9153  C   LEU A 606       6.541  31.810 -21.184  1.00  0.81
C
ATOM   9154  O   LEU A 606       5.942  32.636 -20.489  1.00  1.07
O
ATOM   9155  CB  LEU A 606       8.793  30.925 -20.445  1.00  1.03
C
ATOM   9156  CG  LEU A 606       9.634  29.939 -19.615  1.00  1.03
C
ATOM   9157  CD1 LEU A 606      11.107  30.255 -19.809  1.00  1.03
C
ATOM   9158  CD2 LEU A 606       9.259  30.051 -18.148  1.00  1.03
C
ATOM   9159  H   LEU A 606       7.844  28.942 -21.738  1.00  0.78
H
ATOM   9160  HA  LEU A 606       6.894  30.478 -19.537  1.00  0.83
H
ATOM   9161 1HB  LEU A 606       9.210  30.952 -21.451  1.00  1.24
H
ATOM   9162 2HB  LEU A 606       8.923  31.905 -19.992  1.00  1.24
H
ATOM   9163  HG  LEU A 606       9.452  28.923 -19.953  1.00  1.24
H
ATOM   9164 1HD1 LEU A 606      11.709  29.556 -19.230  1.00  1.24
H
ATOM   9165 2HD1 LEU A 606      11.364  30.165 -20.864  1.00  1.24
H
ATOM   9166 3HD1 LEU A 606      11.310  31.272 -19.473  1.00  1.24
H
ATOM   9167 1HD2 LEU A 606       9.858  29.352 -17.564  1.00  1.24
H
ATOM   9168 2HD2 LEU A 606       9.445  31.068 -17.801  1.00  1.24
H
ATOM   9169 3HD2 LEU A 606       8.204  29.813 -18.028  1.00  1.24
H
ATOM   9170  N   ASP A 607       6.540  31.860 -22.524  1.00  0.77
N
ATOM   9171  CA  ASP A 607       5.764  32.870 -23.231  1.00  0.93
C
ATOM   9172  C   ASP A 607       4.267  32.655 -23.006  1.00  1.24
C
ATOM   9173  O   ASP A 607       3.542  33.592 -22.670  1.00  2.37
O
```

```
ATOM   9174  CB  ASP A 607       6.039  32.849 -24.744  1.00  1.40
C
ATOM   9175  CG  ASP A 607       7.394  33.402 -25.181  1.00  1.40
C
ATOM   9176  OD1 ASP A 607       8.081  34.013 -24.400  1.00  1.40
O
ATOM   9177  OD2 ASP A 607       7.713  33.247 -26.336  1.00  1.40
O
ATOM   9178  H   ASP A 607       7.082  31.191 -23.058  1.00  0.92
H
ATOM   9179  HA  ASP A 607       6.033  33.850 -22.841  1.00  1.12
H
ATOM   9180 1HB  ASP A 607       5.965  31.822 -25.101  1.00  1.67
H
ATOM   9181 2HB  ASP A 607       5.261  33.420 -25.252  1.00  1.67
H
ATOM   9182  N   MET A 608       3.819  31.408 -23.168  1.00  0.87
N
ATOM   9183  CA  MET A 608       2.417  31.033 -23.013  1.00  1.04
C
ATOM   9184  C   MET A 608       1.800  31.473 -21.697  1.00  1.32
C
ATOM   9185  O   MET A 608       0.652  31.922 -21.668  1.00  2.33
O
ATOM   9186  CB  MET A 608       2.261  29.520 -23.141  1.00  1.56
C
ATOM   9187  CG  MET A 608       0.817  29.045 -23.141  1.00  1.56
C
ATOM   9188  SD  MET A 608       0.669  27.253 -23.277  1.00  1.56
S
ATOM   9189  CE  MET A 608       1.364  26.988 -24.898  1.00  1.56
C
ATOM   9190  H   MET A 608       4.467  30.684 -23.446  1.00  1.04
H
ATOM   9191  HA  MET A 608       1.851  31.509 -23.812  1.00  1.25
H
ATOM   9192 1HB  MET A 608       2.730  29.181 -24.063  1.00  1.87
H
ATOM   9193 2HB  MET A 608       2.771  29.027 -22.312  1.00  1.87
H
ATOM   9194 1HG  MET A 608       0.332  29.361 -22.219  1.00  1.87
H
ATOM   9195 2HG  MET A 608       0.286  29.498 -23.979  1.00  1.87
H
ATOM   9196 1HE  MET A 608       1.345  25.924 -25.131  1.00  1.87
H
ATOM   9197 2HE  MET A 608       0.779  27.534 -25.639  1.00  1.87
H
ATOM   9198 3HE  MET A 608       2.395  27.345 -24.914  1.00  1.87
H
ATOM   9199  N   PHE A 609       2.550  31.342 -20.604  1.00  1.44
N
ATOM   9200  CA  PHE A 609       1.993  31.660 -19.298  1.00  2.63
C
ATOM   9201  C   PHE A 609       2.361  33.038 -18.770  1.00  2.51
C
ATOM   9202  O   PHE A 609       2.128  33.325 -17.596  1.00  6.05
O
```

```
ATOM   9203  CB  PHE A 609       2.384  30.577 -18.307  1.00  3.94
C
ATOM   9204  CG  PHE A 609       1.890  29.271 -18.821  1.00  3.94
C
ATOM   9205  CD1 PHE A 609       2.790  28.314 -19.229  1.00  3.94
C
ATOM   9206  CD2 PHE A 609       0.536  29.029 -18.974  1.00  3.94
C
ATOM   9207  CE1 PHE A 609       2.367  27.138 -19.789  1.00  3.94
C
ATOM   9208  CE2 PHE A 609       0.104  27.848 -19.536  1.00  3.94
C
ATOM   9209  CZ  PHE A 609       1.027  26.911 -19.951  1.00  3.94
C
ATOM   9210  H   PHE A 609       3.486  30.966 -20.678  1.00  1.73
H
ATOM   9211  HA  PHE A 609       0.906  31.631 -19.391  1.00  3.16
H
ATOM   9212 1HB  PHE A 609       3.466  30.528 -18.195  1.00  4.73
H
ATOM   9213 2HB  PHE A 609       1.939  30.766 -17.331  1.00  4.73
H
ATOM   9214  HD1 PHE A 609       3.852  28.510 -19.114  1.00  4.73
H
ATOM   9215  HD2 PHE A 609      -0.184  29.788 -18.666  1.00  4.73
H
ATOM   9216  HE1 PHE A 609       3.095  26.398 -20.111  1.00  4.73
H
ATOM   9217  HE2 PHE A 609      -0.962  27.661 -19.667  1.00  4.73
H
ATOM   9218  HZ  PHE A 609       0.690  25.986 -20.409  1.00  4.73
H
ATOM   9219  N   GLY A 610       2.905  33.907 -19.622  1.00  0.82
N
ATOM   9220  CA  GLY A 610       3.160  35.276 -19.195  1.00  0.83
C
ATOM   9221  C   GLY A 610       4.345  35.459 -18.247  1.00  0.78
C
ATOM   9222  O   GLY A 610       4.350  36.405 -17.456  1.00  0.74
O
ATOM   9223  H   GLY A 610       3.107  33.649 -20.582  1.00  0.98
H
ATOM   9224 1HA  GLY A 610       3.296  35.894 -20.073  1.00  1.00
H
ATOM   9225 2HA  GLY A 610       2.263  35.653 -18.705  1.00  1.00
H
ATOM   9226  N   ILE A 611       5.329  34.561 -18.287  1.00  0.89
N
ATOM   9227  CA  ILE A 611       6.447  34.649 -17.352  1.00  1.09
C
ATOM   9228  C   ILE A 611       7.813  34.602 -18.029  1.00  1.66
C
ATOM   9229  O   ILE A 611       8.774  34.080 -17.461  1.00  6.60
O
ATOM   9230  CB  ILE A 611       6.341  33.516 -16.312  1.00  1.64
C
ATOM   9231  CG1 ILE A 611       6.274  32.172 -17.033  1.00  1.64
C
```

ATOM 9232 CG2 ILE A 611 5.135 33.698 -15.403 1.00 1.64
C
ATOM 9233 CD1 ILE A 611 6.381 30.994 -16.107 1.00 1.64
C
ATOM 9234 H ILE A 611 5.318 33.808 -18.965 1.00 1.07
H
ATOM 9235 HA ILE A 611 6.371 35.599 -16.824 1.00 1.31
H
ATOM 9236 HB ILE A 611 7.243 33.511 -15.703 1.00 1.96
H
ATOM 9237 1HG1 ILE A 611 5.329 32.105 -17.571 1.00 1.96
H
ATOM 9238 2HG1 ILE A 611 7.085 32.117 -17.760 1.00 1.96
H
ATOM 9239 1HG2 ILE A 611 5.103 32.888 -14.675 1.00 1.96
H
ATOM 9240 2HG2 ILE A 611 5.218 34.651 -14.883 1.00 1.96
H
ATOM 9241 3HG2 ILE A 611 4.221 33.686 -15.995 1.00 1.96
H
ATOM 9242 1HD1 ILE A 611 6.325 30.073 -16.683 1.00 1.96
H
ATOM 9243 2HD1 ILE A 611 7.334 31.033 -15.577 1.00 1.96
H
ATOM 9244 3HD1 ILE A 611 5.565 31.020 -15.387 1.00 1.96
H
ATOM 9245 N SER A 612 7.905 35.171 -19.227 1.00 0.60
N
ATOM 9246 CA SER A 612 9.173 35.219 -19.942 1.00 0.61
C
ATOM 9247 C SER A 612 9.612 36.644 -20.173 1.00 0.62
C
ATOM 9248 O SER A 612 8.843 37.573 -19.934 1.00 0.73
O
ATOM 9249 CB SER A 612 9.064 34.562 -21.289 1.00 0.92
C
ATOM 9250 OG SER A 612 8.288 35.344 -22.151 1.00 0.92
O
ATOM 9251 H SER A 612 7.083 35.579 -19.649 1.00 0.72
H
ATOM 9252 HA SER A 612 9.933 34.710 -19.349 1.00 0.73
H
ATOM 9253 1HB SER A 612 10.056 34.414 -21.715 1.00 1.10
H
ATOM 9254 2HB SER A 612 8.608 33.587 -21.183 1.00 1.10
H
ATOM 9255 HG SER A 612 8.178 34.822 -22.952 1.00 1.10
H
ATOM 9256 N THR A 613 10.833 36.774 -20.689 1.00 0.61
N
ATOM 9257 CA THR A 613 11.472 38.023 -21.091 1.00 0.67
C
ATOM 9258 C THR A 613 10.514 39.112 -21.539 1.00 0.76
C
ATOM 9259 O THR A 613 10.447 40.172 -20.922 1.00 0.79
O
ATOM 9260 CB THR A 613 12.453 37.756 -22.254 1.00 1.01
C

```
ATOM   9261  OG1 THR A 613      13.486  36.863 -21.825  1.00  1.01
O
ATOM   9262  CG2 THR A 613      13.070  39.048 -22.755  1.00  1.01
C
ATOM   9263  H   THR A 613      11.374  35.925 -20.808  1.00  0.73
H
ATOM   9264  HA  THR A 613      12.034  38.401 -20.238  1.00  0.80
H
ATOM   9265  HB  THR A 613      11.910  37.285 -23.073  1.00  1.21
H
ATOM   9266  HG1 THR A 613      13.096  36.038 -21.518  1.00  1.21
H
ATOM   9267 1HG2 THR A 613      13.749  38.829 -23.578  1.00  1.21
H
ATOM   9268 2HG2 THR A 613      12.288  39.719 -23.099  1.00  1.21
H
ATOM   9269 3HG2 THR A 613      13.621  39.516 -21.947  1.00  1.21
H
ATOM   9270  N   ARG A 614       9.798  38.848 -22.633  1.00  0.80
N
ATOM   9271  CA  ARG A 614       8.921  39.830 -23.263  1.00  0.90
C
ATOM   9272  C   ARG A 614       7.687  40.195 -22.458  1.00  0.91
C
ATOM   9273  O   ARG A 614       7.103  41.258 -22.674  1.00  1.02
O
ATOM   9274  CB  ARG A 614       8.502  39.330 -24.635  1.00  1.35
C
ATOM   9275  CG  ARG A 614       9.648  39.269 -25.637  1.00  1.35
C
ATOM   9276  CD  ARG A 614       9.222  38.783 -26.979  1.00  1.35
C
ATOM   9277  NE  ARG A 614       8.861  37.369 -26.998  1.00  1.35
N
ATOM   9278  CZ  ARG A 614       8.388  36.734 -28.090  1.00  1.35
C
ATOM   9279  NH1 ARG A 614       8.227  37.406 -29.209  1.00  1.35
N
ATOM   9280  NH2 ARG A 614       8.085  35.447 -28.057  1.00  1.35
N
ATOM   9281  H   ARG A 614       9.888  37.937 -23.063  1.00  0.96
H
ATOM   9282  HA  ARG A 614       9.494  40.738 -23.409  1.00  1.08
H
ATOM   9283 1HB  ARG A 614       8.080  38.329 -24.549  1.00  1.62
H
ATOM   9284 2HB  ARG A 614       7.731  39.980 -25.046  1.00  1.62
H
ATOM   9285 1HG  ARG A 614      10.072  40.266 -25.758  1.00  1.62
H
ATOM   9286 2HG  ARG A 614      10.417  38.593 -25.262  1.00  1.62
H
ATOM   9287 1HD  ARG A 614       8.356  39.356 -27.307  1.00  1.62
H
ATOM   9288 2HD  ARG A 614      10.037  38.932 -27.686  1.00  1.62
H
ATOM   9289  HE  ARG A 614       8.977  36.830 -26.148  1.00  1.62
H
```

```
ATOM   9290 1HH1 ARG A 614       8.457  38.389 -29.245  1.00  1.62
H
ATOM   9291 2HH1 ARG A 614       7.875  36.939 -30.031  1.00  1.62
H
ATOM   9292 1HH2 ARG A 614       8.199  34.899 -27.209  1.00  1.62
H
ATOM   9293 2HH2 ARG A 614       7.734  34.991 -28.884  1.00  1.62
H
ATOM   9294  N   HIS A 615       7.295  39.343 -21.524  1.00  0.97
N
ATOM   9295  CA  HIS A 615       6.150  39.638 -20.691  1.00  0.92
C
ATOM   9296  C   HIS A 615       6.601  40.488 -19.534  1.00  0.87
C
ATOM   9297  O   HIS A 615       5.860  41.356 -19.069  1.00  0.93
O
ATOM   9298  CB  HIS A 615       5.494  38.356 -20.226  1.00  1.38
C
ATOM   9299  CG  HIS A 615       4.845  37.650 -21.363  1.00  1.38
C
ATOM   9300  ND1 HIS A 615       3.720  38.142 -21.992  1.00  1.38
N
ATOM   9301  CD2 HIS A 615       5.158  36.499 -21.998  1.00  1.38
C
ATOM   9302  CE1 HIS A 615       3.369  37.319 -22.963  1.00  1.38
C
ATOM   9303  NE2 HIS A 615       4.224  36.317 -22.987  1.00  1.38
N
ATOM   9304  H   HIS A 615       7.806  38.488 -21.361  1.00  1.16
H
ATOM   9305  HA  HIS A 615       5.414  40.206 -21.258  1.00  1.10
H
ATOM   9306 1HB  HIS A 615       6.237  37.694 -19.779  1.00  1.66
H
ATOM   9307 2HB  HIS A 615       4.739  38.574 -19.471  1.00  1.66
H
ATOM   9308  HD2 HIS A 615       5.991  35.841 -21.766  1.00  1.66
H
ATOM   9309  HE1 HIS A 615       2.517  37.447 -23.631  1.00  1.66
H
ATOM   9310  HE2 HIS A 615       4.200  35.535 -23.627  1.00  1.66
H
ATOM   9311  N   ILE A 616       7.842  40.265 -19.108  1.00  0.77
N
ATOM   9312  CA  ILE A 616       8.458  41.077 -18.082  1.00  0.74
C
ATOM   9313  C   ILE A 616       8.650  42.474 -18.654  1.00  0.81
C
ATOM   9314  O   ILE A 616       8.306  43.456 -18.001  1.00  0.88
O
ATOM   9315  CB  ILE A 616       9.786  40.463 -17.614  1.00  1.11
C
ATOM   9316  CG1 ILE A 616       9.496  39.139 -16.897  1.00  1.11
C
ATOM   9317  CG2 ILE A 616      10.512  41.429 -16.686  1.00  1.11
C
ATOM   9318  CD1 ILE A 616      10.721  38.299 -16.649  1.00  1.11
C
```

```
ATOM   9319  H   ILE A 616       8.373  39.502 -19.510  1.00  0.92
H
ATOM   9320  HA  ILE A 616       7.788  41.144 -17.229  1.00  0.89
H
ATOM   9321  HB  ILE A 616      10.416  40.243 -18.472  1.00  1.33
H
ATOM   9322 1HG1 ILE A 616       9.026  39.351 -15.939  1.00  1.33
H
ATOM   9323 2HG1 ILE A 616       8.799  38.557 -17.500  1.00  1.33
H
ATOM   9324 1HG2 ILE A 616      11.450  40.982 -16.358  1.00  1.33
H
ATOM   9325 2HG2 ILE A 616      10.719  42.357 -17.217  1.00  1.33
H
ATOM   9326 3HG2 ILE A 616       9.887  41.638 -15.818  1.00  1.33
H
ATOM   9327 1HD1 ILE A 616      10.435  37.378 -16.140  1.00  1.33
H
ATOM   9328 2HD1 ILE A 616      11.195  38.055 -17.600  1.00  1.33
H
ATOM   9329 3HD1 ILE A 616      11.417  38.854 -16.026  1.00  1.33
H
ATOM   9330  N   ILE A 617       9.151  42.546 -19.897  1.00  0.82
N
ATOM   9331  CA  ILE A 617       9.318  43.806 -20.618  1.00  0.90
C
ATOM   9332  C   ILE A 617       8.014  44.569 -20.704  1.00  0.99
C
ATOM   9333  O   ILE A 617       7.971  45.762 -20.392  1.00  1.01
O
ATOM   9334  CB  ILE A 617       9.839  43.560 -22.058  1.00  1.35
C
ATOM   9335  CG1 ILE A 617      11.284  43.070 -22.023  1.00  1.35
C
ATOM   9336  CG2 ILE A 617       9.676  44.787 -22.943  1.00  1.35
C
ATOM   9337  CD1 ILE A 617      11.795  42.551 -23.343  1.00  1.35
C
ATOM   9338  H   ILE A 617       9.435  41.692 -20.358  1.00  0.98
H
ATOM   9339  HA  ILE A 617      10.041  44.420 -20.083  1.00  1.08
H
ATOM   9340  HB  ILE A 617       9.253  42.754 -22.495  1.00  1.62
H
ATOM   9341 1HG1 ILE A 617      11.924  43.885 -21.711  1.00  1.62
H
ATOM   9342 2HG1 ILE A 617      11.364  42.277 -21.289  1.00  1.62
H
ATOM   9343 1HG2 ILE A 617      10.020  44.553 -23.949  1.00  1.62
H
ATOM   9344 2HG2 ILE A 617       8.626  45.075 -22.979  1.00  1.62
H
ATOM   9345 3HG2 ILE A 617      10.259  45.611 -22.551  1.00  1.62
H
ATOM   9346 1HD1 ILE A 617      12.824  42.219 -23.224  1.00  1.62
H
ATOM   9347 2HD1 ILE A 617      11.190  41.713 -23.673  1.00  1.62
H
```

```
ATOM   9348 3HD1 ILE A 617      11.750  43.343 -24.087  1.00  1.62
H
ATOM   9349  N   ALA A 618       6.952  43.873 -21.116  1.00  1.05
N
ATOM   9350  CA  ALA A 618       5.638  44.477 -21.223  1.00  1.14
C
ATOM   9351  C   ALA A 618       5.187  45.043 -19.889  1.00  1.19
C
ATOM   9352  O   ALA A 618       4.766  46.198 -19.828  1.00  1.45
O
ATOM   9353  CB  ALA A 618       4.630  43.455 -21.722  1.00  1.71
C
ATOM   9354  H   ALA A 618       7.057  42.903 -21.389  1.00  1.26
H
ATOM   9355  HA  ALA A 618       5.698  45.299 -21.938  1.00  1.37
H
ATOM   9356 1HB  ALA A 618       3.653  43.926 -21.823  1.00  2.05
H
ATOM   9357 2HB  ALA A 618       4.950  43.073 -22.692  1.00  2.05
H
ATOM   9358 3HB  ALA A 618       4.565  42.631 -21.013  1.00  2.05
H
ATOM   9359  N   ALA A 619       5.312  44.244 -18.824  1.00  1.05
N
ATOM   9360  CA  ALA A 619       4.914  44.659 -17.484  1.00  1.08
C
ATOM   9361  C   ALA A 619       5.657  45.911 -17.048  1.00  1.06
C
ATOM   9362  O   ALA A 619       5.054  46.810 -16.459  1.00  1.10
O
ATOM   9363  CB  ALA A 619       5.162  43.532 -16.493  1.00  1.62
C
ATOM   9364  H   ALA A 619       5.666  43.303 -18.945  1.00  1.26
H
ATOM   9365  HA  ALA A 619       3.850  44.887 -17.502  1.00  1.30
H
ATOM   9366 1HB  ALA A 619       4.835  43.842 -15.500  1.00  1.94
H
ATOM   9367 2HB  ALA A 619       4.602  42.649 -16.801  1.00  1.94
H
ATOM   9368 3HB  ALA A 619       6.224  43.296 -16.467  1.00  1.94
H
ATOM   9369  N   VAL A 620       6.958  45.970 -17.346  1.00  1.02
N
ATOM   9370  CA  VAL A 620       7.766  47.134 -17.013  1.00  1.01
C
ATOM   9371  C   VAL A 620       7.263  48.386 -17.698  1.00  1.09
C
ATOM   9372  O   VAL A 620       7.149  49.428 -17.056  1.00  1.22
O
ATOM   9373  CB  VAL A 620       9.246  46.914 -17.386  1.00  1.52
C
ATOM   9374  CG1 VAL A 620      10.007  48.226 -17.251  1.00  1.52
C
ATOM   9375  CG2 VAL A 620       9.853  45.861 -16.466  1.00  1.52
C
ATOM   9376  H   VAL A 620       7.403  45.185 -17.805  1.00  1.22
H
```

```
ATOM   9377  HA  VAL A 620       7.709  47.289 -15.936  1.00  1.21
H
ATOM   9378  HB  VAL A 620       9.318  46.586 -18.422  1.00  1.82
H
ATOM   9379 1HG1 VAL A 620      11.051  48.072 -17.519  1.00  1.82
H
ATOM   9380 2HG1 VAL A 620       9.569  48.971 -17.915  1.00  1.82
H
ATOM   9381 3HG1 VAL A 620       9.945  48.579 -16.223  1.00  1.82
H
ATOM   9382 1HG2 VAL A 620      10.899  45.704 -16.726  1.00  1.82
H
ATOM   9383 2HG2 VAL A 620       9.786  46.194 -15.435  1.00  1.82
H
ATOM   9384 3HG2 VAL A 620       9.314  44.926 -16.566  1.00  1.82
H
ATOM   9385  N   THR A 621       6.959  48.299 -18.995  1.00  1.16
N
ATOM   9386  CA  THR A 621       6.501  49.492 -19.694  1.00  1.38
C
ATOM   9387  C   THR A 621       5.087  49.884 -19.283  1.00  1.64
C
ATOM   9388  O   THR A 621       4.773  51.072 -19.240  1.00  2.03
O
ATOM   9389  CB  THR A 621       6.555  49.298 -21.217  1.00  2.07
C
ATOM   9390  OG1 THR A 621       5.694  48.218 -21.597  1.00  2.07
O
ATOM   9391  CG2 THR A 621       7.979  48.985 -21.650  1.00  2.07
C
ATOM   9392  H   THR A 621       7.075  47.422 -19.493  1.00  1.39
H
ATOM   9393  HA  THR A 621       7.161  50.314 -19.434  1.00  1.66
H
ATOM   9394  HB  THR A 621       6.220  50.208 -21.715  1.00  2.48
H
ATOM   9395  HG1 THR A 621       5.875  47.453 -21.042  1.00  2.48
H
ATOM   9396 1HG2 THR A 621       8.010  48.849 -22.731  1.00  2.48
H
ATOM   9397 2HG2 THR A 621       8.631  49.811 -21.369  1.00  2.48
H
ATOM   9398 3HG2 THR A 621       8.319  48.075 -21.162  1.00  2.48
H
ATOM   9399  N   LEU A 622       4.262  48.906 -18.909  1.00  1.91
N
ATOM   9400  CA  LEU A 622       2.919  49.198 -18.418  1.00  2.50
C
ATOM   9401  C   LEU A 622       3.005  49.928 -17.089  1.00  2.54
C
ATOM   9402  O   LEU A 622       2.234  50.850 -16.821  1.00  2.72
O
ATOM   9403  CB  LEU A 622       2.118  47.901 -18.259  1.00  3.75
C
ATOM   9404  CG  LEU A 622       1.727  47.193 -19.563  1.00  3.75
C
ATOM   9405  CD1 LEU A 622       1.141  45.825 -19.237  1.00  3.75
C
```

```
ATOM   9406  CD2 LEU A 622       0.716  48.048 -20.310  1.00  3.75
C
ATOM   9407  H   LEU A 622       4.553  47.941 -18.986  1.00  2.29
H
ATOM   9408  HA  LEU A 622       2.414  49.841 -19.137  1.00  3.00
H
ATOM   9409 1HB  LEU A 622       2.706  47.201 -17.668  1.00  4.50
H
ATOM   9410 2HB  LEU A 622       1.201  48.123 -17.715  1.00  4.50
H
ATOM   9411  HG  LEU A 622       2.605  47.051 -20.188  1.00  4.50
H
ATOM   9412 1HD1 LEU A 622       0.865  45.319 -20.162  1.00  4.50
H
ATOM   9413 2HD1 LEU A 622       1.884  45.229 -18.707  1.00  4.50
H
ATOM   9414 3HD1 LEU A 622       0.258  45.947 -18.611  1.00  4.50
H
ATOM   9415 1HD2 LEU A 622       0.434  47.550 -21.238  1.00  4.50
H
ATOM   9416 2HD2 LEU A 622      -0.170  48.189 -19.690  1.00  4.50
H
ATOM   9417 3HD2 LEU A 622       1.157  49.017 -20.537  1.00  4.50
H
ATOM   9418  N   THR A 623       3.985  49.523 -16.283  1.00  2.85
N
ATOM   9419  CA  THR A 623       4.283  50.136 -15.007  1.00  3.45
C
ATOM   9420  C   THR A 623       4.757  51.579 -15.222  1.00  3.57
C
ATOM   9421  O   THR A 623       4.281  52.502 -14.562  1.00  5.75
O
ATOM   9422  CB  THR A 623       5.346  49.284 -14.274  1.00  5.18
C
ATOM   9423  OG1 THR A 623       4.817  47.972 -14.048  1.00  5.18
O
ATOM   9424  CG2 THR A 623       5.742  49.890 -12.957  1.00  5.18
C
ATOM   9425  H   THR A 623       4.549  48.730 -16.559  1.00  3.42
H
ATOM   9426  HA  THR A 623       3.374  50.159 -14.406  1.00  4.14
H
ATOM   9427  HB  THR A 623       6.232  49.201 -14.896  1.00  6.21
H
ATOM   9428  HG1 THR A 623       4.692  47.530 -14.895  1.00  6.21
H
ATOM   9429 1HG2 THR A 623       6.491  49.262 -12.477  1.00  6.21
H
ATOM   9430 2HG2 THR A 623       6.156  50.878 -13.143  1.00  6.21
H
ATOM   9431 3HG2 THR A 623       4.867  49.970 -12.314  1.00  6.21
H
ATOM   9432  N   LEU A 624       5.652  51.776 -16.195  1.00  2.41
N
ATOM   9433  CA  LEU A 624       6.153  53.107 -16.548  1.00  3.33
C
ATOM   9434  C   LEU A 624       5.048  54.043 -17.013  1.00  2.55
C
```

```
ATOM   9435  O   LEU A 624       5.055  55.234 -16.696  1.00  2.98
O
ATOM   9436  CB  LEU A 624       7.187  53.007 -17.679  1.00  5.00
C
ATOM   9437  CG  LEU A 624       8.535  52.385 -17.329  1.00  5.00
C
ATOM   9438  CD1 LEU A 624       9.346  52.186 -18.597  1.00  5.00
C
ATOM   9439  CD2 LEU A 624       9.262  53.310 -16.385  1.00  5.00
C
ATOM   9440  H   LEU A 624       6.021  50.976 -16.692  1.00  2.89
H
ATOM   9441  HA  LEU A 624       6.625  53.541 -15.668  1.00  4.00
H
ATOM   9442 1HB  LEU A 624       6.758  52.419 -18.488  1.00  5.99
H
ATOM   9443 2HB  LEU A 624       7.379  54.011 -18.056  1.00  5.99
H
ATOM   9444  HG  LEU A 624       8.391  51.418 -16.853  1.00  5.99
H
ATOM   9445 1HD1 LEU A 624      10.309  51.749 -18.342  1.00  5.99
H
ATOM   9446 2HD1 LEU A 624       8.816  51.521 -19.276  1.00  5.99
H
ATOM   9447 3HD1 LEU A 624       9.501  53.148 -19.082  1.00  5.99
H
ATOM   9448 1HD2 LEU A 624      10.225  52.869 -16.146  1.00  5.99
H
ATOM   9449 2HD2 LEU A 624       9.412  54.277 -16.864  1.00  5.99
H
ATOM   9450 3HD2 LEU A 624       8.678  53.441 -15.475  1.00  5.99
H
ATOM   9451  N   MET A 625       4.089  53.495 -17.755  1.00  3.20
N
ATOM   9452  CA  MET A 625       2.965  54.258 -18.274  1.00  6.42
C
ATOM   9453  C   MET A 625       1.816  54.444 -17.278  1.00 15.15
C
ATOM   9454  O   MET A 625       0.763  54.952 -17.653  1.00 45.26
O
ATOM   9455  CB  MET A 625       2.437  53.585 -19.539  1.00  9.63
C
ATOM   9456  CG  MET A 625       3.380  53.656 -20.733  1.00  9.63
C
ATOM   9457  SD  MET A 625       2.634  53.019 -22.247  1.00  9.63
S
ATOM   9458  CE  MET A 625       2.636  51.261 -21.910  1.00  9.63
C
ATOM   9459  H   MET A 625       4.155  52.516 -18.002  1.00  3.84
H
ATOM   9460  HA  MET A 625       3.327  55.251 -18.537  1.00  7.70
H
ATOM   9461 1HB  MET A 625       2.239  52.534 -19.334  1.00 11.56
H
ATOM   9462 2HB  MET A 625       1.493  54.046 -19.831  1.00 11.56
H
ATOM   9463 1HG  MET A 625       3.668  54.693 -20.903  1.00 11.56
H
```

```
ATOM   9464 2HG  MET A 625       4.283  53.085 -20.527  1.00 11.56
H
ATOM   9465 1HE  MET A 625       2.201  50.727 -22.756  1.00 11.56
H
ATOM   9466 2HE  MET A 625       3.660  50.921 -21.752  1.00 11.56
H
ATOM   9467 3HE  MET A 625       2.045  51.063 -21.015  1.00 11.56
H
ATOM   9468  N   LYS A 626       2.000  54.035 -16.021  1.00 31.37
N
ATOM   9469  CA  LYS A 626       0.960  54.163 -15.002  1.00 51.67
C
ATOM   9470  C   LYS A 626       1.570  54.154 -13.598  1.00142.48
C
ATOM   9471  O   LYS A 626       1.939  55.203 -13.066  1.00255.29
O
ATOM   9472  OXT LYS A 626       1.696  53.093 -12.986  1.00  0.00
O
ATOM   9473  CB  LYS A 626      -0.062  53.034 -15.141  1.00 77.50
C
ATOM   9474  CG  LYS A 626      -1.245  53.131 -14.188  1.00 77.50
C
ATOM   9475  CD  LYS A 626      -2.262  52.032 -14.457  1.00 77.50
C
ATOM   9476  CE  LYS A 626      -3.439  52.118 -13.498  1.00 77.50
C
ATOM   9477  NZ  LYS A 626      -4.441  51.047 -13.754  1.00 77.50
N
ATOM   9478  H   LYS A 626       2.872  53.614 -15.733  1.00 37.64
H
ATOM   9479  HA  LYS A 626       0.451  55.117 -15.141  1.00 62.00
H
ATOM   9480 1HB  LYS A 626      -0.453  53.023 -16.159  1.00 93.01
H
ATOM   9481 2HB  LYS A 626       0.431  52.076 -14.969  1.00 93.01
H
ATOM   9482 1HG  LYS A 626      -0.894  53.045 -13.159  1.00 93.01
H
ATOM   9483 2HG  LYS A 626      -1.729  54.100 -14.306  1.00 93.01
H
ATOM   9484 1HD  LYS A 626      -2.630  52.122 -15.480  1.00 93.01
H
ATOM   9485 2HD  LYS A 626      -1.784  51.060 -14.346  1.00 93.01
H
ATOM   9486 1HE  LYS A 626      -3.076  52.023 -12.476  1.00 93.01
H
ATOM   9487 2HE  LYS A 626      -3.924  53.088 -13.610  1.00 93.01
H
ATOM   9488 1HZ  LYS A 626      -5.206  51.137 -13.099  1.00 93.01
H
ATOM   9489 2HZ  LYS A 626      -4.795  51.134 -14.696  1.00 93.01
H
ATOM   9490 3HZ  LYS A 626      -4.006  50.143 -13.640  1.00 93.01
H
TER    9491      LYS A 626
ATOM   9492  N   MET B   1     -21.327  -7.144 -29.787  1.00221.72
N
```

```
ATOM   9493  CA  MET B   1     -20.449  -8.272 -29.472  1.00178.33
C
ATOM   9494  C   MET B   1     -21.126  -9.324 -28.583  1.00 88.59
C
ATOM   9495  O   MET B   1     -21.903  -8.987 -27.691  1.00142.35
O
ATOM   9496  CB  MET B   1     -19.168  -7.770 -28.819  1.00267.50
C
ATOM   9497  CG  MET B   1     -18.178  -8.871 -28.462  1.00267.50
C
ATOM   9498  SD  MET B   1     -17.547  -9.745 -29.907  1.00267.50
S
ATOM   9499  CE  MET B   1     -17.050 -11.288 -29.147  1.00267.50
C
ATOM   9500 1H   MET B   1     -20.870  -6.273 -29.527  1.00266.06
H
ATOM   9501 2H   MET B   1     -21.523  -7.134 -30.778  1.00266.06
H
ATOM   9502 3H   MET B   1     -22.193  -7.234 -29.275  1.00266.06
H
ATOM   9503  HA  MET B   1     -20.182  -8.763 -30.409  1.00214.00
H
ATOM   9504 1HB  MET B   1     -18.670  -7.067 -29.482  1.00320.99
H
ATOM   9505 2HB  MET B   1     -19.421  -7.234 -27.908  1.00320.99
H
ATOM   9506 1HG  MET B   1     -17.332  -8.431 -27.936  1.00320.99
H
ATOM   9507 2HG  MET B   1     -18.648  -9.591 -27.797  1.00320.99
H
ATOM   9508 1HE  MET B   1     -16.630 -11.950 -29.905  1.00320.99
H
ATOM   9509 2HE  MET B   1     -16.302 -11.088 -28.381  1.00320.99
H
ATOM   9510 3HE  MET B   1     -17.917 -11.765 -28.688  1.00320.99
H
ATOM   9511  N   MET B   2     -20.832 -10.604 -28.852  1.00 34.52
N
ATOM   9512  CA  MET B   2     -21.376 -11.737 -28.113  1.00 18.91
C
ATOM   9513  C   MET B   2     -20.792 -11.878 -26.712  1.00  9.21
C
ATOM   9514  O   MET B   2     -19.708 -11.379 -26.415  1.00  9.07
O
ATOM   9515  CB  MET B   2     -21.128 -13.027 -28.890  1.00 28.37
C
ATOM   9516  CG  MET B   2     -21.822 -13.093 -30.241  1.00 28.37
C
ATOM   9517  SD  MET B   2     -23.616 -13.027 -30.092  1.00 28.37
S
ATOM   9518  CE  MET B   2     -23.944 -14.628 -29.358  1.00 28.37
C
ATOM   9519  H   MET B   2     -20.189 -10.795 -29.607  1.00 41.42
H
ATOM   9520  HA  MET B   2     -22.451 -11.590 -28.007  1.00 22.69
H
ATOM   9521 1HB  MET B   2     -20.059 -13.149 -29.060  1.00 34.04
H
```

```
ATOM   9522 2HB  MET B   2      -21.465 -13.880 -28.301  1.00 34.04
H
ATOM   9523 1HG  MET B   2      -21.495 -12.259 -30.861  1.00 34.04
H
ATOM   9524 2HG  MET B   2      -21.551 -14.020 -30.745  1.00 34.04
H
ATOM   9525 1HE  MET B   2      -25.017 -14.743 -29.201  1.00 34.04
H
ATOM   9526 2HE  MET B   2      -23.588 -15.414 -30.025  1.00 34.04
H
ATOM   9527 3HE  MET B   2      -23.427 -14.702 -28.401  1.00 34.04
H
ATOM   9528  N   ALA B   3      -21.529 -12.575 -25.851  1.00  7.91
N
ATOM   9529  CA  ALA B   3      -21.096 -12.838 -24.483  1.00  7.72
C
ATOM   9530  C   ALA B   3      -20.252 -14.118 -24.379  1.00  8.07
C
ATOM   9531  O   ALA B   3      -19.913 -14.548 -23.277  1.00 16.06
O
ATOM   9532  CB  ALA B   3      -22.306 -12.944 -23.569  1.00 11.58
C
ATOM   9533  H   ALA B   3      -22.419 -12.942 -26.157  1.00  9.49
H
ATOM   9534  HA  ALA B   3      -20.477 -12.002 -24.158  1.00  9.26
H
ATOM   9535 1HB  ALA B   3      -21.975 -13.108 -22.544  1.00 13.90
H
ATOM   9536 2HB  ALA B   3      -22.883 -12.021 -23.621  1.00 13.90
H
ATOM   9537 3HB  ALA B   3      -22.929 -13.779 -23.886  1.00 13.90
H
ATOM   9538  N   ASN B   4      -19.934 -14.733 -25.513  1.00  6.91
N
ATOM   9539  CA  ASN B   4      -19.175 -15.970 -25.519  1.00  9.81
C
ATOM   9540  C   ASN B   4      -17.680 -15.766 -25.757  1.00  7.13
C
ATOM   9541  O   ASN B   4      -17.251 -14.823 -26.429  1.00  6.89
O
ATOM   9542  CB  ASN B   4      -19.741 -16.909 -26.566  1.00 14.71
C
ATOM   9543  CG  ASN B   4      -21.140 -17.354 -26.243  1.00 14.71
C
ATOM   9544  OD1 ASN B   4      -21.473 -17.613 -25.081  1.00 14.71
O
ATOM   9545  ND2 ASN B   4      -21.968 -17.452 -27.252  1.00 14.71
N
ATOM   9546  H   ASN B   4      -20.225 -14.349 -26.398  1.00  8.29
H
ATOM   9547  HA  ASN B   4      -19.282 -16.439 -24.540  1.00 11.77
H
ATOM   9548 1HB  ASN B   4      -19.745 -16.412 -27.537  1.00 17.66
H
ATOM   9549 2HB  ASN B   4      -19.104 -17.789 -26.651  1.00 17.66
H
ATOM   9550 1HD2 ASN B   4      -22.912 -17.747 -27.098  1.00 17.66
H
```

ATOM 9551 2HD2 ASN B 4 -21.657 -17.234 -28.177 1.00 17.66
H
ATOM 9552 N ASP B 5 -16.890 -16.685 -25.214 1.00 7.53
N
ATOM 9553 CA ASP B 5 -15.449 -16.677 -25.401 1.00 8.13
C
ATOM 9554 C ASP B 5 -15.096 -17.018 -26.827 1.00 5.50
C
ATOM 9555 O ASP B 5 -14.959 -18.192 -27.175 1.00 8.84
O
ATOM 9556 CB ASP B 5 -14.795 -17.685 -24.451 1.00 12.20
C
ATOM 9557 CG ASP B 5 -13.280 -17.777 -24.577 1.00 12.20
C
ATOM 9558 OD1 ASP B 5 -12.763 -17.398 -25.600 1.00 12.20
O
ATOM 9559 OD2 ASP B 5 -12.653 -18.220 -23.642 1.00 12.20
O
ATOM 9560 H ASP B 5 -17.306 -17.423 -24.661 1.00 9.04
H
ATOM 9561 HA ASP B 5 -15.072 -15.678 -25.179 1.00 9.76
H
ATOM 9562 1HB ASP B 5 -15.039 -17.415 -23.423 1.00 14.63
H
ATOM 9563 2HB ASP B 5 -15.215 -18.674 -24.634 1.00 14.63
H
ATOM 9564 N ALA B 6 -14.899 -15.994 -27.648 1.00 4.74
N
ATOM 9565 CA ALA B 6 -14.592 -16.202 -29.059 1.00 3.31
C
ATOM 9566 C ALA B 6 -13.137 -16.602 -29.289 1.00 4.20
C
ATOM 9567 O ALA B 6 -12.343 -15.804 -29.792 1.00 9.79
O
ATOM 9568 CB ALA B 6 -14.919 -14.949 -29.852 1.00 4.96
C
ATOM 9569 H ALA B 6 -15.042 -15.059 -27.285 1.00 5.69
H
ATOM 9570 HA ALA B 6 -15.220 -17.020 -29.419 1.00 3.97
H
ATOM 9571 1HB ALA B 6 -14.726 -15.126 -30.910 1.00 5.96
H
ATOM 9572 2HB ALA B 6 -15.969 -14.694 -29.713 1.00 5.96
H
ATOM 9573 3HB ALA B 6 -14.299 -14.126 -29.506 1.00 5.96
H
ATOM 9574 N LYS B 7 -12.818 -17.848 -28.928 1.00 2.99
N
ATOM 9575 CA LYS B 7 -11.511 -18.478 -29.116 1.00 3.80
C
ATOM 9576 C LYS B 7 -10.868 -18.104 -30.453 1.00 3.39
C
ATOM 9577 O LYS B 7 -11.341 -18.550 -31.500 1.00 4.84
O
ATOM 9578 CB LYS B 7 -11.635 -19.995 -29.032 1.00 5.70
C
ATOM 9579 CG LYS B 7 -10.303 -20.726 -29.139 1.00 5.70
C

ATOM 9580 CD LYS B 7 -10.479 -22.228 -28.983 1.00 5.70
C
ATOM 9581 CE LYS B 7 -9.145 -22.953 -29.083 1.00 5.70
C
ATOM 9582 NZ LYS B 7 -9.302 -24.425 -28.926 1.00 5.70
N
ATOM 9583 H LYS B 7 -13.550 -18.395 -28.495 1.00 3.59
H
ATOM 9584 HA LYS B 7 -10.865 -18.161 -28.302 1.00 4.56
H
ATOM 9585 1HB LYS B 7 -12.097 -20.269 -28.083 1.00 6.84
H
ATOM 9586 2HB LYS B 7 -12.283 -20.354 -29.831 1.00 6.84
H
ATOM 9587 1HG LYS B 7 -9.852 -20.520 -30.111 1.00 6.84
H
ATOM 9588 2HG LYS B 7 -9.627 -20.365 -28.363 1.00 6.84
H
ATOM 9589 1HD LYS B 7 -10.929 -22.444 -28.013 1.00 6.84
H
ATOM 9590 2HD LYS B 7 -11.143 -22.599 -29.763 1.00 6.84
H
ATOM 9591 1HE LYS B 7 -8.697 -22.746 -30.054 1.00 6.84
H
ATOM 9592 2HE LYS B 7 -8.478 -22.585 -28.303 1.00 6.84
H
ATOM 9593 1HZ LYS B 7 -8.396 -24.870 -28.999 1.00 6.84
H
ATOM 9594 2HZ LYS B 7 -9.705 -24.627 -28.022 1.00 6.84
H
ATOM 9595 3HZ LYS B 7 -9.908 -24.779 -29.652 1.00 6.84
H
ATOM 9596 N PRO B 8 -9.802 -17.296 -30.442 1.00 3.55
N
ATOM 9597 CA PRO B 8 -9.036 -16.833 -31.593 1.00 4.67
C
ATOM 9598 C PRO B 8 -8.483 -17.991 -32.413 1.00 2.39
C
ATOM 9599 O PRO B 8 -7.984 -18.974 -31.864 1.00 4.16
O
ATOM 9600 CB PRO B 8 -7.900 -16.025 -30.948 1.00 7.00
C
ATOM 9601 CG PRO B 8 -8.468 -15.543 -29.660 1.00 7.00
C
ATOM 9602 CD PRO B 8 -9.336 -16.672 -29.188 1.00 7.00
C
ATOM 9603 HA PRO B 8 -9.674 -16.190 -32.214 1.00 5.60
H
ATOM 9604 1HB PRO B 8 -7.020 -16.667 -30.806 1.00 8.41
H
ATOM 9605 2HB PRO B 8 -7.592 -15.206 -31.610 1.00 8.41
H
ATOM 9606 1HG PRO B 8 -7.657 -15.308 -28.955 1.00 8.41
H
ATOM 9607 2HG PRO B 8 -9.033 -14.613 -29.818 1.00 8.41
H
ATOM 9608 1HD PRO B 8 -8.753 -17.395 -28.598 1.00 8.41
H

ATOM 9609 2HD PRO B 8 -10.187 -16.269 -28.618 1.00 8.41
H
ATOM 9610 N ASP B 9 -8.536 -17.854 -33.735 1.00 3.06
N
ATOM 9611 CA ASP B 9 -7.946 -18.863 -34.598 1.00 3.91
C
ATOM 9612 C ASP B 9 -6.529 -18.432 -34.941 1.00 3.22
C
ATOM 9613 O ASP B 9 -6.082 -17.385 -34.480 1.00 4.51
O
ATOM 9614 CB ASP B 9 -8.799 -19.094 -35.856 1.00 5.87
C
ATOM 9615 CG ASP B 9 -8.873 -17.901 -36.796 1.00 5.87
C
ATOM 9616 OD1 ASP B 9 -8.082 -16.999 -36.649 1.00 5.87
O
ATOM 9617 OD2 ASP B 9 -9.726 -17.900 -37.652 1.00 5.87
O
ATOM 9618 H ASP B 9 -8.977 -17.040 -34.137 1.00 3.67
H
ATOM 9619 HA ASP B 9 -7.892 -19.806 -34.052 1.00 4.69
H
ATOM 9620 1HB ASP B 9 -8.405 -19.946 -36.410 1.00 7.04
H
ATOM 9621 2HB ASP B 9 -9.814 -19.352 -35.553 1.00 7.04
H
ATOM 9622 N VAL B 10 -5.841 -19.220 -35.761 1.00 3.37
N
ATOM 9623 CA VAL B 10 -4.450 -18.947 -36.112 1.00 3.22
C
ATOM 9624 C VAL B 10 -4.248 -17.577 -36.740 1.00 1.85
C
ATOM 9625 O VAL B 10 -3.324 -16.855 -36.356 1.00 1.61
O
ATOM 9626 CB VAL B 10 -3.927 -20.020 -37.081 1.00 4.83
C
ATOM 9627 CG1 VAL B 10 -2.560 -19.618 -37.614 1.00 4.83
C
ATOM 9628 CG2 VAL B 10 -3.853 -21.358 -36.362 1.00 4.83
C
ATOM 9629 H VAL B 10 -6.282 -20.052 -36.127 1.00 4.04
H
ATOM 9630 HA VAL B 10 -3.859 -18.995 -35.196 1.00 3.86
H
ATOM 9631 HB VAL B 10 -4.605 -20.098 -37.932 1.00 5.80
H
ATOM 9632 1HG1 VAL B 10 -2.202 -20.379 -38.309 1.00 5.80
H
ATOM 9633 2HG1 VAL B 10 -2.635 -18.662 -38.132 1.00 5.80
H
ATOM 9634 3HG1 VAL B 10 -1.859 -19.528 -36.784 1.00 5.80
H
ATOM 9635 1HG2 VAL B 10 -3.492 -22.121 -37.050 1.00 5.80
H
ATOM 9636 2HG2 VAL B 10 -3.170 -21.278 -35.516 1.00 5.80
H
ATOM 9637 3HG2 VAL B 10 -4.845 -21.633 -36.003 1.00 5.80
H

```
ATOM   9638  N   LYS B  11      -5.091 -17.237 -37.716  1.00  1.74
N
ATOM   9639  CA  LYS B  11      -4.997 -15.942 -38.376  1.00  1.50
C
ATOM   9640  C   LYS B  11      -5.199 -14.812 -37.400  1.00  1.15
C
ATOM   9641  O   LYS B  11      -4.419 -13.865 -37.385  1.00  1.95
O
ATOM   9642  CB  LYS B  11      -6.014 -15.792 -39.501  1.00  2.25
C
ATOM   9643  CG  LYS B  11      -5.912 -14.426 -40.172  1.00  2.25
C
ATOM   9644  CD  LYS B  11      -6.900 -14.239 -41.311  1.00  2.25
C
ATOM   9645  CE  LYS B  11      -6.788 -12.821 -41.862  1.00  2.25
C
ATOM   9646  NZ  LYS B  11      -7.743 -12.553 -42.970  1.00  2.25
N
ATOM   9647  H   LYS B  11      -5.820 -17.879 -37.992  1.00  2.09
H
ATOM   9648  HA  LYS B  11      -3.997 -15.843 -38.798  1.00  1.80
H
ATOM   9649 1HB  LYS B  11      -5.851 -16.561 -40.256  1.00  2.70
H
ATOM   9650 2HB  LYS B  11      -7.024 -15.915 -39.111  1.00  2.70
H
ATOM   9651 1HG  LYS B  11      -6.092 -13.651 -39.427  1.00  2.70
H
ATOM   9652 2HG  LYS B  11      -4.904 -14.295 -40.562  1.00  2.70
H
ATOM   9653 1HD  LYS B  11      -6.689 -14.953 -42.108  1.00  2.70
H
ATOM   9654 2HD  LYS B  11      -7.916 -14.405 -40.952  1.00  2.70
H
ATOM   9655 1HE  LYS B  11      -6.982 -12.115 -41.056  1.00  2.70
H
ATOM   9656 2HE  LYS B  11      -5.774 -12.663 -42.229  1.00  2.70
H
ATOM   9657 1HZ  LYS B  11      -7.610 -11.594 -43.275  1.00  2.70
H
ATOM   9658 2HZ  LYS B  11      -7.568 -13.184 -43.740  1.00  2.70
H
ATOM   9659 3HZ  LYS B  11      -8.690 -12.674 -42.642  1.00  2.70
H
ATOM   9660  N   THR B  12      -6.260 -14.917 -36.597  1.00  1.14
N
ATOM   9661  CA  THR B  12      -6.607 -13.930 -35.587  1.00  2.63
C
ATOM   9662  C   THR B  12      -5.421 -13.623 -34.699  1.00  1.55
C
ATOM   9663  O   THR B  12      -5.097 -12.459 -34.464  1.00  1.47
O
ATOM   9664  CB  THR B  12      -7.771 -14.443 -34.726  1.00  3.94
C
ATOM   9665  OG1 THR B  12      -8.915 -14.673 -35.557  1.00  3.94
O
ATOM   9666  CG2 THR B  12      -8.120 -13.440 -33.662  1.00  3.94
C
```

```
ATOM   9667  H   THR B  12      -6.861 -15.729 -36.689  1.00  1.37
H
ATOM   9668  HA  THR B  12      -6.910 -13.010 -36.086  1.00  3.16
H
ATOM   9669  HB  THR B  12      -7.486 -15.378 -34.252  1.00  4.73
H
ATOM   9670  HG1 THR B  12      -8.715 -15.397 -36.164  1.00  4.73
H
ATOM   9671 1HG2 THR B  12      -8.946 -13.816 -33.061  1.00  4.73
H
ATOM   9672 2HG2 THR B  12      -7.254 -13.265 -33.023  1.00  4.73
H
ATOM   9673 3HG2 THR B  12      -8.411 -12.513 -34.146  1.00  4.73
H
ATOM   9674  N   VAL B  13      -4.769 -14.682 -34.227  1.00  1.60
N
ATOM   9675  CA  VAL B  13      -3.586 -14.573 -33.398  1.00  1.55
C
ATOM   9676  C   VAL B  13      -2.444 -13.876 -34.115  1.00  1.18
C
ATOM   9677  O   VAL B  13      -1.790 -13.012 -33.533  1.00  1.08
O
ATOM   9678  CB  VAL B  13      -3.140 -15.966 -32.950  1.00  2.33
C
ATOM   9679  CG1 VAL B  13      -1.781 -15.890 -32.286  1.00  2.33
C
ATOM   9680  CG2 VAL B  13      -4.171 -16.522 -31.982  1.00  2.33
C
ATOM   9681  H   VAL B  13      -5.115 -15.608 -34.449  1.00  1.92
H
ATOM   9682  HA  VAL B  13      -3.843 -13.993 -32.511  1.00  1.86
H
ATOM   9683  HB  VAL B  13      -3.055 -16.618 -33.819  1.00  2.79
H
ATOM   9684 1HG1 VAL B  13      -1.472 -16.888 -31.976  1.00  2.79
H
ATOM   9685 2HG1 VAL B  13      -1.051 -15.488 -32.989  1.00  2.79
H
ATOM   9686 3HG1 VAL B  13      -1.841 -15.244 -31.414  1.00  2.79
H
ATOM   9687 1HG2 VAL B  13      -3.866 -17.519 -31.664  1.00  2.79
H
ATOM   9688 2HG2 VAL B  13      -4.241 -15.869 -31.112  1.00  2.79
H
ATOM   9689 3HG2 VAL B  13      -5.141 -16.578 -32.467  1.00  2.79
H
ATOM   9690  N   GLN B  14      -2.197 -14.246 -35.370  1.00  1.17
N
ATOM   9691  CA  GLN B  14      -1.125 -13.612 -36.120  1.00  1.09
C
ATOM   9692  C   GLN B  14      -1.371 -12.139 -36.395  1.00  0.94
C
ATOM   9693  O   GLN B  14      -0.442 -11.345 -36.293  1.00  0.88
O
ATOM   9694  CB  GLN B  14      -0.895 -14.321 -37.449  1.00  1.64
C
ATOM   9695  CG  GLN B  14       0.265 -13.737 -38.245  1.00  1.64
C
```

ATOM 9696 CD GLN B 14 1.596 -13.877 -37.526 1.00 1.64
C
ATOM 9697 OE1 GLN B 14 1.949 -14.972 -37.076 1.00 1.64
O
ATOM 9698 NE2 GLN B 14 2.345 -12.783 -37.412 1.00 1.64
N
ATOM 9699 H GLN B 14 -2.744 -14.980 -35.804 1.00 1.40
H
ATOM 9700 HA GLN B 14 -0.211 -13.690 -35.533 1.00 1.31
H
ATOM 9701 1HB GLN B 14 -0.692 -15.378 -37.272 1.00 1.96
H
ATOM 9702 2HB GLN B 14 -1.795 -14.256 -38.061 1.00 1.96
H
ATOM 9703 1HG GLN B 14 0.336 -14.259 -39.199 1.00 1.96
H
ATOM 9704 2HG GLN B 14 0.078 -12.675 -38.414 1.00 1.96
H
ATOM 9705 1HE2 GLN B 14 3.230 -12.829 -36.949 1.00 1.96
H
ATOM 9706 2HE2 GLN B 14 2.035 -11.904 -37.787 1.00 1.96
H
ATOM 9707 N VAL B 15 -2.612 -11.770 -36.720 1.00 0.97
N
ATOM 9708 CA VAL B 15 -2.938 -10.377 -37.011 1.00 0.94
C
ATOM 9709 C VAL B 15 -2.829 -9.563 -35.731 1.00 0.86
C
ATOM 9710 O VAL B 15 -2.409 -8.407 -35.744 1.00 1.04
O
ATOM 9711 CB VAL B 15 -4.337 -10.231 -37.620 1.00 1.41
C
ATOM 9712 CG1 VAL B 15 -4.652 -8.756 -37.778 1.00 1.41
C
ATOM 9713 CG2 VAL B 15 -4.400 -10.935 -38.964 1.00 1.41
C
ATOM 9714 H VAL B 15 -3.336 -12.469 -36.794 1.00 1.16
H
ATOM 9715 HA VAL B 15 -2.213 -9.994 -37.730 1.00 1.13
H
ATOM 9716 HB VAL B 15 -5.073 -10.663 -36.942 1.00 1.69
H
ATOM 9717 1HG1 VAL B 15 -5.652 -8.636 -38.196 1.00 1.69
H
ATOM 9718 2HG1 VAL B 15 -4.600 -8.276 -36.804 1.00 1.69
H
ATOM 9719 3HG1 VAL B 15 -3.924 -8.298 -38.449 1.00 1.69
H
ATOM 9720 1HG2 VAL B 15 -5.399 -10.823 -39.388 1.00 1.69
H
ATOM 9721 2HG2 VAL B 15 -3.671 -10.490 -39.637 1.00 1.69
H
ATOM 9722 3HG2 VAL B 15 -4.176 -11.991 -38.840 1.00 1.69
H
ATOM 9723 N LEU B 16 -3.225 -10.178 -34.632 1.00 0.92
N
ATOM 9724 CA LEU B 16 -3.101 -9.625 -33.305 1.00 0.87
C

```
ATOM   9725  C   LEU B  16      -1.622  -9.346 -32.971  1.00  0.83
C
ATOM   9726  O   LEU B  16      -1.286  -8.274 -32.459  1.00  1.01
O
ATOM   9727  CB  LEU B  16      -3.788 -10.607 -32.373  1.00  1.30
C
ATOM   9728  CG  LEU B  16      -3.806 -10.327 -30.910  1.00  1.30
C
ATOM   9729  CD1 LEU B  16      -4.433  -8.978 -30.672  1.00  1.30
C
ATOM   9730  CD2 LEU B  16      -4.625 -11.442 -30.272  1.00  1.30
C
ATOM   9731  H   LEU B  16      -3.630 -11.104 -34.702  1.00  1.10
H
ATOM   9732  HA  LEU B  16      -3.642  -8.680 -33.272  1.00  1.04
H
ATOM   9733 1HB  LEU B  16      -4.827 -10.690 -32.689  1.00  1.57
H
ATOM   9734 2HB  LEU B  16      -3.327 -11.580 -32.500  1.00  1.57
H
ATOM   9735  HG  LEU B  16      -2.797 -10.315 -30.507  1.00  1.57
H
ATOM   9736 1HD1 LEU B  16      -4.457  -8.791 -29.607  1.00  1.57
H
ATOM   9737 2HD1 LEU B  16      -3.835  -8.216 -31.166  1.00  1.57
H
ATOM   9738 3HD1 LEU B  16      -5.439  -8.949 -31.071  1.00  1.57
H
ATOM   9739 1HD2 LEU B  16      -4.687 -11.296 -29.202  1.00  1.57
H
ATOM   9740 2HD2 LEU B  16      -5.628 -11.446 -30.693  1.00  1.57
H
ATOM   9741 3HD2 LEU B  16      -4.148 -12.401 -30.477  1.00  1.57
H
ATOM   9742  N   ARG B  17      -0.743 -10.296 -33.303  1.00  0.71
N
ATOM   9743  CA  ARG B  17       0.696 -10.110 -33.141  1.00  0.77
C
ATOM   9744  C   ARG B  17       1.219  -9.021 -34.086  1.00  0.72
C
ATOM   9745  O   ARG B  17       2.071  -8.222 -33.699  1.00  0.81
O
ATOM   9746  CB  ARG B  17       1.433 -11.411 -33.412  1.00  1.16
C
ATOM   9747  CG  ARG B  17       1.246 -12.482 -32.357  1.00  1.16
C
ATOM   9748  CD  ARG B  17       1.880 -13.764 -32.751  1.00  1.16
C
ATOM   9749  NE  ARG B  17       1.704 -14.786 -31.732  1.00  1.16
N
ATOM   9750  CZ  ARG B  17       1.914 -16.103 -31.923  1.00  1.16
C
ATOM   9751  NH1 ARG B  17       2.309 -16.539 -33.100  1.00  1.16
N
ATOM   9752  NH2 ARG B  17       1.721 -16.954 -30.930  1.00  1.16
N
ATOM   9753  H   ARG B  17      -1.079 -11.176 -33.672  1.00  0.85
H
```

ATOM 9754 HA ARG B 17 0.894 -9.804 -32.113 1.00 0.92
H
ATOM 9755 1HB ARG B 17 1.106 -11.827 -34.363 1.00 1.39
H
ATOM 9756 2HB ARG B 17 2.503 -11.213 -33.494 1.00 1.39
H
ATOM 9757 1HG ARG B 17 1.692 -12.152 -31.419 1.00 1.39
H
ATOM 9758 2HG ARG B 17 0.184 -12.658 -32.210 1.00 1.39
H
ATOM 9759 1HD ARG B 17 1.433 -14.123 -33.677 1.00 1.39
H
ATOM 9760 2HD ARG B 17 2.948 -13.611 -32.899 1.00 1.39
H
ATOM 9761 HE ARG B 17 1.400 -14.488 -30.815 1.00 1.39
H
ATOM 9762 1HH1 ARG B 17 2.453 -15.887 -33.859 1.00 1.39
H
ATOM 9763 2HH1 ARG B 17 2.463 -17.526 -33.245 1.00 1.39
H
ATOM 9764 1HH2 ARG B 17 1.413 -16.622 -30.025 1.00 1.39
H
ATOM 9765 2HH2 ARG B 17 1.873 -17.942 -31.073 1.00 1.39
H
ATOM 9766 N ASP B 18 0.678 -8.978 -35.310 1.00 0.66
N
ATOM 9767 CA ASP B 18 1.039 -7.958 -36.287 1.00 0.67
C
ATOM 9768 C ASP B 18 0.657 -6.581 -35.777 1.00 0.61
C
ATOM 9769 O ASP B 18 1.402 -5.623 -35.971 1.00 0.63
O
ATOM 9770 CB ASP B 18 0.351 -8.205 -37.632 1.00 1.01
C
ATOM 9771 CG ASP B 18 0.895 -9.396 -38.406 1.00 1.01
C
ATOM 9772 OD1 ASP B 18 1.938 -9.908 -38.069 1.00 1.01
O
ATOM 9773 OD2 ASP B 18 0.271 -9.766 -39.369 1.00 1.01
O
ATOM 9774 H ASP B 18 0.007 -9.681 -35.580 1.00 0.79
H
ATOM 9775 HA ASP B 18 2.119 -7.983 -36.435 1.00 0.80
H
ATOM 9776 1HB ASP B 18 -0.712 -8.363 -37.471 1.00 1.21
H
ATOM 9777 2HB ASP B 18 0.447 -7.315 -38.253 1.00 1.21
H
ATOM 9778 N THR B 19 -0.498 -6.498 -35.112 1.00 0.62
N
ATOM 9779 CA THR B 19 -0.980 -5.262 -34.529 1.00 0.69
C
ATOM 9780 C THR B 19 -0.013 -4.786 -33.465 1.00 0.59
C
ATOM 9781 O THR B 19 0.351 -3.613 -33.449 1.00 0.61
O
ATOM 9782 CB THR B 19 -2.383 -5.443 -33.920 1.00 1.03
C

```
ATOM   9783  OG1 THR B  19      -3.304  -5.830 -34.949  1.00  1.03
O
ATOM   9784  CG2 THR B  19      -2.853  -4.148 -33.279  1.00  1.03
C
ATOM   9785  H   THR B  19      -1.073  -7.323 -35.023  1.00  0.74
H
ATOM   9786  HA  THR B  19      -1.034  -4.501 -35.308  1.00  0.83
H
ATOM   9787  HB  THR B  19      -2.353  -6.223 -33.161  1.00  1.24
H
ATOM   9788  HG1 THR B  19      -3.024  -6.672 -35.326  1.00  1.24
H
ATOM   9789 1HG2 THR B  19      -3.842  -4.296 -32.855  1.00  1.24
H
ATOM   9790 2HG2 THR B  19      -2.158  -3.858 -32.490  1.00  1.24
H
ATOM   9791 3HG2 THR B  19      -2.894  -3.361 -34.032  1.00  1.24
H
ATOM   9792  N   ALA B  20       0.430  -5.709 -32.604  1.00  0.59
N
ATOM   9793  CA  ALA B  20       1.412  -5.391 -31.570  1.00  0.56
C
ATOM   9794  C   ALA B  20       2.695  -4.860 -32.192  1.00  0.58
C
ATOM   9795  O   ALA B  20       3.281  -3.901 -31.686  1.00  0.60
O
ATOM   9796  CB  ALA B  20       1.703  -6.622 -30.724  1.00  0.84
C
ATOM   9797  H   ALA B  20       0.061  -6.652 -32.655  1.00  0.71
H
ATOM   9798  HA  ALA B  20       0.998  -4.610 -30.936  1.00  0.67
H
ATOM   9799 1HB  ALA B  20       2.418  -6.367 -29.943  1.00  1.01
H
ATOM   9800 2HB  ALA B  20       0.778  -6.975 -30.268  1.00  1.01
H
ATOM   9801 3HB  ALA B  20       2.119  -7.408 -31.350  1.00  1.01
H
ATOM   9802  N   ASN B  21       3.110  -5.471 -33.303  1.00  0.62
N
ATOM   9803  CA  ASN B  21       4.303  -5.047 -34.015  1.00  0.68
C
ATOM   9804  C   ASN B  21       4.135  -3.639 -34.570  1.00  0.70
C
ATOM   9805  O   ASN B  21       5.050  -2.819 -34.472  1.00  0.81
O
ATOM   9806  CB  ASN B  21       4.623  -6.032 -35.123  1.00  1.02
C
ATOM   9807  CG  ASN B  21       5.146  -7.360 -34.635  1.00  1.02
C
ATOM   9808  OD1 ASN B  21       5.726  -7.486 -33.548  1.00  1.02
O
ATOM   9809  ND2 ASN B  21       4.942  -8.376 -35.434  1.00  1.02
N
ATOM   9810  H   ASN B  21       2.597  -6.272 -33.650  1.00  0.74
H
ATOM   9811  HA  ASN B  21       5.127  -5.016 -33.315  1.00  0.82
H
```

```
ATOM   9812 1HB  ASN B  21       3.725  -6.216 -35.709  1.00  1.22
H
ATOM   9813 2HB  ASN B  21       5.359  -5.593 -35.793  1.00  1.22
H
ATOM   9814 1HD2 ASN B  21       5.259  -9.289 -35.177  1.00  1.22
H
ATOM   9815 2HD2 ASN B  21       4.469  -8.243 -36.305  1.00  1.22
H
ATOM   9816  N   ARG B  22       2.955  -3.354 -35.117  1.00  0.64
N
ATOM   9817  CA  ARG B  22       2.643  -2.035 -35.642  1.00  0.68
C
ATOM   9818  C   ARG B  22       2.617  -0.989 -34.538  1.00  0.74
C
ATOM   9819  O   ARG B  22       3.082   0.132 -34.742  1.00  0.94
O
ATOM   9820  CB  ARG B  22       1.311  -2.066 -36.375  1.00  1.02
C
ATOM   9821  CG  ARG B  22       1.347  -2.779 -37.719  1.00  1.02
C
ATOM   9822  CD  ARG B  22      -0.008  -2.924 -38.314  1.00  1.02
C
ATOM   9823  NE  ARG B  22      -0.655  -1.635 -38.492  1.00  1.02
N
ATOM   9824  CZ  ARG B  22      -0.441  -0.784 -39.512  1.00  1.02
C
ATOM   9825  NH1 ARG B  22       0.419  -1.088 -40.462  1.00  1.02
N
ATOM   9826  NH2 ARG B  22      -1.097   0.363 -39.545  1.00  1.02
N
ATOM   9827  H   ARG B  22       2.255  -4.081 -35.190  1.00  0.77
H
ATOM   9828  HA  ARG B  22       3.419  -1.760 -36.359  1.00  0.82
H
ATOM   9829 1HB  ARG B  22       0.564  -2.562 -35.758  1.00  1.22
H
ATOM   9830 2HB  ARG B  22       0.966  -1.047 -36.550  1.00  1.22
H
ATOM   9831 1HG  ARG B  22       1.963  -2.207 -38.414  1.00  1.22
H
ATOM   9832 2HG  ARG B  22       1.774  -3.774 -37.594  1.00  1.22
H
ATOM   9833 1HD  ARG B  22       0.069  -3.404 -39.288  1.00  1.22
H
ATOM   9834 2HD  ARG B  22      -0.632  -3.530 -37.657  1.00  1.22
H
ATOM   9835  HE  ARG B  22      -1.342  -1.364 -37.795  1.00  1.22
H
ATOM   9836 1HH1 ARG B  22       0.917  -1.967 -40.430  1.00  1.22
H
ATOM   9837 2HH1 ARG B  22       0.578  -0.444 -41.223  1.00  1.22
H
ATOM   9838 1HH2 ARG B  22      -1.742   0.592 -38.793  1.00  1.22
H
ATOM   9839 2HH2 ARG B  22      -0.945   1.017 -40.298  1.00  1.22
H
ATOM   9840  N   LEU B  23       2.104  -1.367 -33.363  1.00  0.67
N
```

ATOM 9841 CA LEU B 23 2.081 -0.469 -32.220 1.00 0.67
C
ATOM 9842 C LEU B 23 3.499 -0.122 -31.780 1.00 0.69
C
ATOM 9843 O LEU B 23 3.781 1.033 -31.450 1.00 0.72
O
ATOM 9844 CB LEU B 23 1.336 -1.114 -31.049 1.00 1.01
C
ATOM 9845 CG LEU B 23 -0.179 -1.302 -31.189 1.00 1.01
C
ATOM 9846 CD1 LEU B 23 -0.674 -2.109 -30.003 1.00 1.01
C
ATOM 9847 CD2 LEU B 23 -0.865 0.049 -31.241 1.00 1.01
C
ATOM 9848 H LEU B 23 1.708 -2.291 -33.265 1.00 0.80
H
ATOM 9849 HA LEU B 23 1.571 0.450 -32.507 1.00 0.80
H
ATOM 9850 1HB LEU B 23 1.767 -2.096 -30.865 1.00 1.21
H
ATOM 9851 2HB LEU B 23 1.504 -0.505 -30.173 1.00 1.21
H
ATOM 9852 HG LEU B 23 -0.402 -1.852 -32.097 1.00 1.21
H
ATOM 9853 1HD1 LEU B 23 -1.750 -2.263 -30.088 1.00 1.21
H
ATOM 9854 2HD1 LEU B 23 -0.169 -3.071 -29.988 1.00 1.21
H
ATOM 9855 3HD1 LEU B 23 -0.454 -1.571 -29.082 1.00 1.21
H
ATOM 9856 1HD2 LEU B 23 -1.941 -0.095 -31.334 1.00 1.21
H
ATOM 9857 2HD2 LEU B 23 -0.650 0.603 -30.327 1.00 1.21
H
ATOM 9858 3HD2 LEU B 23 -0.497 0.608 -32.101 1.00 1.21
H
ATOM 9859 N ARG B 24 4.389 -1.127 -31.801 1.00 0.69
N
ATOM 9860 CA ARG B 24 5.798 -0.932 -31.473 1.00 0.68
C
ATOM 9861 C ARG B 24 6.421 0.083 -32.422 1.00 0.68
C
ATOM 9862 O ARG B 24 7.073 1.028 -31.980 1.00 0.63
O
ATOM 9863 CB ARG B 24 6.568 -2.251 -31.551 1.00 1.02
C
ATOM 9864 CG ARG B 24 8.023 -2.176 -31.105 1.00 1.02
C
ATOM 9865 CD ARG B 24 8.724 -3.487 -31.228 1.00 1.02
C
ATOM 9866 NE ARG B 24 8.179 -4.512 -30.364 1.00 1.02
N
ATOM 9867 CZ ARG B 24 7.425 -5.538 -30.798 1.00 1.02
C
ATOM 9868 NH1 ARG B 24 7.135 -5.615 -32.074 1.00 1.02
N
ATOM 9869 NH2 ARG B 24 6.985 -6.456 -29.955 1.00 1.02
N

```
ATOM   9870  H   ARG B  24       4.076  -2.060 -32.042  1.00  0.83
H
ATOM   9871  HA  ARG B  24       5.869  -0.548 -30.456  1.00  0.82
H
ATOM   9872 1HB  ARG B  24       6.072  -2.998 -30.933  1.00  1.22
H
ATOM   9873 2HB  ARG B  24       6.564  -2.621 -32.575  1.00  1.22
H
ATOM   9874 1HG  ARG B  24       8.554  -1.441 -31.711  1.00  1.22
H
ATOM   9875 2HG  ARG B  24       8.054  -1.873 -30.062  1.00  1.22
H
ATOM   9876 1HD  ARG B  24       8.636  -3.844 -32.251  1.00  1.22
H
ATOM   9877 2HD  ARG B  24       9.777  -3.359 -30.979  1.00  1.22
H
ATOM   9878  HE  ARG B  24       8.435  -4.489 -29.386  1.00  1.22
H
ATOM   9879 1HH1 ARG B  24       7.478  -4.908 -32.709  1.00  1.22
H
ATOM   9880 2HH1 ARG B  24       6.566  -6.373 -32.432  1.00  1.22
H
ATOM   9881 1HH2 ARG B  24       7.209  -6.390 -28.973  1.00  1.22
H
ATOM   9882 2HH2 ARG B  24       6.415  -7.222 -30.288  1.00  1.22
H
ATOM   9883  N   ILE B  25       6.183  -0.106 -33.725  1.00  0.76
N
ATOM   9884  CA  ILE B  25       6.695   0.790 -34.757  1.00  0.80
C
ATOM   9885  C   ILE B  25       6.198   2.207 -34.596  1.00  0.82
C
ATOM   9886  O   ILE B  25       6.980   3.151 -34.705  1.00  0.85
O
ATOM   9887  CB  ILE B  25       6.304   0.302 -36.152  1.00  1.20
C
ATOM   9888  CG1 ILE B  25       7.055  -0.979 -36.475  1.00  1.20
C
ATOM   9889  CG2 ILE B  25       6.571   1.387 -37.177  1.00  1.20
C
ATOM   9890  CD1 ILE B  25       6.533  -1.686 -37.700  1.00  1.20
C
ATOM   9891  H   ILE B  25       5.646  -0.916 -34.012  1.00  0.91
H
ATOM   9892  HA  ILE B  25       7.780   0.803 -34.691  1.00  0.96
H
ATOM   9893  HB  ILE B  25       5.243   0.059 -36.164  1.00  1.44
H
ATOM   9894 1HG1 ILE B  25       8.107  -0.748 -36.628  1.00  1.44
H
ATOM   9895 2HG1 ILE B  25       6.973  -1.648 -35.625  1.00  1.44
H
ATOM   9896 1HG2 ILE B  25       6.279   1.033 -38.165  1.00  1.44
H
ATOM   9897 2HG2 ILE B  25       5.995   2.276 -36.926  1.00  1.44
H
ATOM   9898 3HG2 ILE B  25       7.631   1.633 -37.178  1.00  1.44
H
```

```
ATOM   9899 1HD1 ILE B  25       7.110  -2.593 -37.873  1.00  1.44
H
ATOM   9900 2HD1 ILE B  25       5.485  -1.945 -37.550  1.00  1.44
H
ATOM   9901 3HD1 ILE B  25       6.626  -1.029 -38.564  1.00  1.44
H
ATOM   9902  N   HIS B  26       4.903   2.356 -34.341  1.00  0.86
N
ATOM   9903  CA  HIS B  26       4.320   3.667 -34.141  1.00  0.96
C
ATOM   9904  C   HIS B  26       4.918   4.369 -32.946  1.00  0.97
C
ATOM   9905  O   HIS B  26       5.196   5.567 -33.005  1.00  1.12
O
ATOM   9906  CB  HIS B  26       2.813   3.556 -33.956  1.00  1.44
C
ATOM   9907  CG  HIS B  26       2.056   3.344 -35.221  1.00  1.44
C
ATOM   9908  ND1 HIS B  26       0.761   2.875 -35.244  1.00  1.44
N
ATOM   9909  CD2 HIS B  26       2.391   3.590 -36.511  1.00  1.44
C
ATOM   9910  CE1 HIS B  26       0.334   2.837 -36.493  1.00  1.44
C
ATOM   9911  NE2 HIS B  26       1.302   3.270 -37.280  1.00  1.44
N
ATOM   9912  H   HIS B  26       4.303   1.542 -34.290  1.00  1.03
H
ATOM   9913  HA  HIS B  26       4.510   4.286 -35.018  1.00  1.15
H
ATOM   9914 1HB  HIS B  26       2.594   2.721 -33.289  1.00  1.73
H
ATOM   9915 2HB  HIS B  26       2.439   4.459 -33.477  1.00  1.73
H
ATOM   9916  HD2 HIS B  26       3.341   3.985 -36.867  1.00  1.73
H
ATOM   9917  HE1 HIS B  26      -0.650   2.519 -36.821  1.00  1.73
H
ATOM   9918  HE2 HIS B  26       1.241   3.366 -38.289  1.00  1.73
H
ATOM   9919  N   SER B  27       5.155   3.617 -31.871  1.00  0.89
N
ATOM   9920  CA  SER B  27       5.766   4.176 -30.682  1.00  0.92
C
ATOM   9921  C   SER B  27       7.170   4.677 -30.997  1.00  1.23
C
ATOM   9922  O   SER B  27       7.550   5.763 -30.559  1.00  1.91
O
ATOM   9923  CB  SER B  27       5.816   3.132 -29.583  1.00  1.38
C
ATOM   9924  OG  SER B  27       4.525   2.765 -29.184  1.00  1.38
O
ATOM   9925  H   SER B  27       4.886   2.642 -31.864  1.00  1.07
H
ATOM   9926  HA  SER B  27       5.165   5.021 -30.343  1.00  1.10
H
ATOM   9927 1HB  SER B  27       6.356   2.253 -29.932  1.00  1.66
H
```

```
ATOM   9928 2HB  SER B  27       6.362   3.533 -28.730  1.00  1.66
H
ATOM   9929  HG  SER B  27       4.141   2.303 -29.937  1.00  1.66
H
ATOM   9930  N   ILE B  28       7.919   3.892 -31.785  1.00  0.90
N
ATOM   9931  CA  ILE B  28       9.285   4.244 -32.175  1.00  0.96
C
ATOM   9932  C   ILE B  28       9.334   5.501 -32.999  1.00  0.98
C
ATOM   9933  O   ILE B  28      10.103   6.413 -32.697  1.00  1.08
O
ATOM   9934  CB  ILE B  28       9.945   3.134 -33.021  1.00  1.44
C
ATOM   9935  CG1 ILE B  28      10.180   1.881 -32.202  1.00  1.44
C
ATOM   9936  CG2 ILE B  28      11.270   3.636 -33.572  1.00  1.44
C
ATOM   9937  CD1 ILE B  28      10.542   0.690 -33.028  1.00  1.44
C
ATOM   9938  H   ILE B  28       7.545   3.004 -32.097  1.00  1.08
H
ATOM   9939  HA  ILE B  28       9.876   4.405 -31.277  1.00  1.15
H
ATOM   9940  HB  ILE B  28       9.292   2.865 -33.848  1.00  1.73
H
ATOM   9941 1HG1 ILE B  28      10.992   2.063 -31.511  1.00  1.73
H
ATOM   9942 2HG1 ILE B  28       9.287   1.650 -31.631  1.00  1.73
H
ATOM   9943 1HG2 ILE B  28      11.731   2.857 -34.177  1.00  1.73
H
ATOM   9944 2HG2 ILE B  28      11.100   4.519 -34.186  1.00  1.73
H
ATOM   9945 3HG2 ILE B  28      11.933   3.892 -32.745  1.00  1.73
H
ATOM   9946 1HD1 ILE B  28      10.704  -0.168 -32.376  1.00  1.73
H
ATOM   9947 2HD1 ILE B  28       9.731   0.473 -33.723  1.00  1.73
H
ATOM   9948 3HD1 ILE B  28      11.452   0.900 -33.588  1.00  1.73
H
ATOM   9949  N   ARG B  29       8.524   5.533 -34.052  1.00  1.00
N
ATOM   9950  CA  ARG B  29       8.515   6.650 -34.976  1.00  1.09
C
ATOM   9951  C   ARG B  29       8.063   7.933 -34.317  1.00  1.17
C
ATOM   9952  O   ARG B  29       8.659   8.985 -34.544  1.00  1.31
O
ATOM   9953  CB  ARG B  29       7.611   6.348 -36.160  1.00  1.64
C
ATOM   9954  CG  ARG B  29       8.133   5.295 -37.130  1.00  1.64
C
ATOM   9955  CD  ARG B  29       7.160   5.044 -38.228  1.00  1.64
C
ATOM   9956  NE  ARG B  29       7.653   4.072 -39.195  1.00  1.64
N
```

ATOM 9957 CZ ARG B 29 6.914 3.544 -40.194 1.00 1.64
C
ATOM 9958 NH1 ARG B 29 5.659 3.907 -40.343 1.00 1.64
N
ATOM 9959 NH2 ARG B 29 7.446 2.661 -41.023 1.00 1.64
N
ATOM 9960 H ARG B 29 7.913 4.749 -34.231 1.00 1.20
H
ATOM 9961 HA ARG B 29 9.531 6.793 -35.346 1.00 1.31
H
ATOM 9962 1HB ARG B 29 6.641 6.007 -35.800 1.00 1.96
H
ATOM 9963 2HB ARG B 29 7.445 7.261 -36.732 1.00 1.96
H
ATOM 9964 1HG ARG B 29 9.069 5.638 -37.571 1.00 1.96
H
ATOM 9965 2HG ARG B 29 8.302 4.359 -36.598 1.00 1.96
H
ATOM 9966 1HD ARG B 29 6.231 4.662 -37.808 1.00 1.96
H
ATOM 9967 2HD ARG B 29 6.962 5.976 -38.756 1.00 1.96
H
ATOM 9968 HE ARG B 29 8.614 3.768 -39.110 1.00 1.96
H
ATOM 9969 1HH1 ARG B 29 5.247 4.580 -39.712 1.00 1.96
H
ATOM 9970 2HH1 ARG B 29 5.106 3.511 -41.090 1.00 1.96
H
ATOM 9971 1HH2 ARG B 29 8.409 2.373 -40.914 1.00 1.96
H
ATOM 9972 2HH2 ARG B 29 6.892 2.260 -41.766 1.00 1.96
H
ATOM 9973 N ALA B 30 7.022 7.847 -33.495 1.00 1.12
N
ATOM 9974 CA ALA B 30 6.505 9.011 -32.804 1.00 1.24
C
ATOM 9975 C ALA B 30 7.521 9.548 -31.799 1.00 1.34
C
ATOM 9976 O ALA B 30 7.752 10.756 -31.738 1.00 1.47
O
ATOM 9977 CB ALA B 30 5.201 8.656 -32.120 1.00 1.86
C
ATOM 9978 H ALA B 30 6.557 6.961 -33.350 1.00 1.34
H
ATOM 9979 HA ALA B 30 6.318 9.791 -33.544 1.00 1.49
H
ATOM 9980 1HB ALA B 30 4.794 9.540 -31.637 1.00 2.23
H
ATOM 9981 2HB ALA B 30 4.491 8.295 -32.864 1.00 2.23
H
ATOM 9982 3HB ALA B 30 5.374 7.877 -31.380 1.00 2.23
H
ATOM 9983 N THR B 31 8.140 8.639 -31.037 1.00 1.42
N
ATOM 9984 CA THR B 31 9.147 8.979 -30.035 1.00 2.04
C
ATOM 9985 C THR B 31 10.357 9.650 -30.675 1.00 1.57
C

ATOM 9986 O THR B 31 10.854 10.670 -30.188 1.00 2.16
O
ATOM 9987 CB THR B 31 9.591 7.698 -29.287 1.00 3.06
C
ATOM 9988 OG1 THR B 31 8.471 7.124 -28.603 1.00 3.06
O
ATOM 9989 CG2 THR B 31 10.686 7.997 -28.292 1.00 3.06
C
ATOM 9990 H THR B 31 7.898 7.660 -31.132 1.00 1.70
H
ATOM 9991 HA THR B 31 8.708 9.674 -29.319 1.00 2.45
H
ATOM 9992 HB THR B 31 9.964 6.977 -30.013 1.00 3.67
H
ATOM 9993 HG1 THR B 31 7.860 6.759 -29.251 1.00 3.67
H
ATOM 9994 1HG2 THR B 31 10.985 7.079 -27.790 1.00 3.67
H
ATOM 9995 2HG2 THR B 31 11.536 8.418 -28.822 1.00 3.67
H
ATOM 9996 3HG2 THR B 31 10.324 8.708 -27.554 1.00 3.67
H
ATOM 9997 N CYS B 32 10.821 9.062 -31.777 1.00 1.11
N
ATOM 9998 CA CYS B 32 11.943 9.566 -32.553 1.00 1.20
C
ATOM 9999 C CYS B 32 11.660 10.949 -33.110 1.00 1.28
C
ATOM 10000 O CYS B 32 12.497 11.847 -33.008 1.00 2.03
O
ATOM 10001 CB CYS B 32 12.267 8.623 -33.707 1.00 1.80
C
ATOM 10002 SG CYS B 32 13.664 9.164 -34.714 1.00 1.80
S
ATOM 10003 H CYS B 32 10.379 8.215 -32.104 1.00 1.33
H
ATOM 10004 HA CYS B 32 12.815 9.626 -31.902 1.00 1.44
H
ATOM 10005 1HB CYS B 32 12.496 7.634 -33.314 1.00 2.16
H
ATOM 10006 2HB CYS B 32 11.397 8.524 -34.358 1.00 2.16
H
ATOM 10007 HG CYS B 32 14.531 9.245 -33.710 1.00 2.16
H
ATOM 10008 N ALA B 33 10.486 11.108 -33.726 1.00 1.26
N
ATOM 10009 CA ALA B 33 10.073 12.386 -34.290 1.00 1.51
C
ATOM 10010 C ALA B 33 10.033 13.466 -33.217 1.00 1.61
C
ATOM 10011 O ALA B 33 10.462 14.599 -33.451 1.00 1.95
O
ATOM 10012 CB ALA B 33 8.706 12.252 -34.952 1.00 2.27
C
ATOM 10013 H ALA B 33 9.851 10.326 -33.808 1.00 1.51
H
ATOM 10014 HA ALA B 33 10.807 12.680 -35.040 1.00 1.81
H

ATOM 10015 1HB ALA B 33   8.418 13.207 -35.391 1.00 2.72
H
ATOM 10016 2HB ALA B 33   8.755 11.493 -35.734 1.00 2.72
H
ATOM 10017 3HB ALA B 33   7.968 11.958 -34.207 1.00 2.72
H
ATOM 10018 N   SER B 34   9.530 13.108 -32.036 1.00 1.47
N
ATOM 10019 CA SER B 34   9.458 14.018 -30.907 1.00 2.07
C
ATOM 10020 C   SER B 34   10.840 14.346 -30.353 1.00 1.72
C
ATOM 10021 O   SER B 34   11.110 15.482 -29.961 1.00 2.09
O
ATOM 10022 CB SER B 34   8.638 13.389 -29.806 1.00 3.10
C
ATOM 10023 OG SER B 34   7.319 13.181 -30.216 1.00 3.10
O
ATOM 10024 H   SER B 34   9.163 12.173 -31.915 1.00 1.76
H
ATOM 10025 HA SER B 34   8.984 14.945 -31.235 1.00 2.48
H
ATOM 10026 1HB SER B 34   9.084 12.439 -29.520 1.00 3.73
H
ATOM 10027 2HB SER B 34   8.650 14.036 -28.929 1.00 3.73
H
ATOM 10028 HG SER B 34   6.915 14.044 -30.332 1.00 3.73
H
ATOM 10029 N   GLY B 35   11.714 13.338 -30.327 1.00 1.67
N
ATOM 10030 CA GLY B 35   13.038 13.483 -29.749 1.00 1.64
C
ATOM 10031 C   GLY B 35   12.991 13.195 -28.248 1.00 1.15
C
ATOM 10032 O   GLY B 35   13.882 13.611 -27.504 1.00 0.94
O
ATOM 10033 H   GLY B 35   11.443 12.430 -30.675 1.00 2.00
H
ATOM 10034 1HA GLY B 35   13.729 12.796 -30.238 1.00 1.97
H
ATOM 10035 2HA GLY B 35   13.407 14.494 -29.922 1.00 1.97
H
ATOM 10036 N   SER B 36   11.924 12.520 -27.802 1.00 2.30
N
ATOM 10037 CA SER B 36   11.738 12.197 -26.386 1.00 2.39
C
ATOM 10038 C   SER B 36   10.577 11.227 -26.200 1.00 3.33
C
ATOM 10039 O   SER B 36   9.750 11.078 -27.095 1.00 11.86
O
ATOM 10040 CB SER B 36   11.478 13.463 -25.592 1.00 3.58
C
ATOM 10041 OG SER B 36   10.246 14.027 -25.945 1.00 3.58
O
ATOM 10042 H   SER B 36   11.248 12.173 -28.472 1.00 2.76
H
ATOM 10043 HA SER B 36   12.647 11.723 -26.014 1.00 2.87
H

```
ATOM  10044 1HB  SER B  36      11.489  13.241 -24.526  1.00  4.30
H
ATOM  10045 2HB  SER B  36      12.272  14.184 -25.780  1.00  4.30
H
ATOM  10046  HG  SER B  36      10.322  14.968 -25.747  1.00  4.30
H
ATOM  10047  N   GLY B  37      10.489  10.603 -25.026  1.00  1.40
N
ATOM  10048  CA  GLY B  37       9.392   9.676 -24.729  1.00  1.13
C
ATOM  10049  C   GLY B  37       9.864   8.259 -24.398  1.00  1.15
C
ATOM  10050  O   GLY B  37      10.915   7.813 -24.857  1.00  3.78
O
ATOM  10051  H   GLY B  37      11.198  10.768 -24.326  1.00  1.68
H
ATOM  10052 1HA  GLY B  37       8.813  10.067 -23.893  1.00  1.36
H
ATOM  10053 2HA  GLY B  37       8.721   9.634 -25.584  1.00  1.36
H
ATOM  10054  N   GLN B  38       9.058   7.535 -23.612  1.00  1.41
N
ATOM  10055  CA  GLN B  38       9.408   6.178 -23.171  1.00  1.37
C
ATOM  10056  C   GLN B  38       9.068   5.045 -24.124  1.00  1.61
C
ATOM  10057  O   GLN B  38       8.245   4.185 -23.798  1.00  5.39
O
ATOM  10058  CB  GLN B  38       8.754   5.829 -21.844  1.00  2.06
C
ATOM  10059  CG  GLN B  38       9.229   6.606 -20.651  1.00  2.06
C
ATOM  10060  CD  GLN B  38       8.575   6.123 -19.390  1.00  2.06
C
ATOM  10061  OE1 GLN B  38       7.915   5.077 -19.364  1.00  2.06
O
ATOM  10062  NE2 GLN B  38       8.733   6.896 -18.327  1.00  2.06
N
ATOM  10063  H   GLN B  38       8.195   7.945 -23.288  1.00  1.69
H
ATOM  10064  HA  GLN B  38      10.487   6.154 -23.023  1.00  1.64
H
ATOM  10065 1HB  GLN B  38       7.674   5.963 -21.926  1.00  2.47
H
ATOM  10066 2HB  GLN B  38       8.937   4.777 -21.632  1.00  2.47
H
ATOM  10067 1HG  GLN B  38      10.297   6.456 -20.539  1.00  2.47
H
ATOM  10068 2HG  GLN B  38       9.005   7.663 -20.788  1.00  2.47
H
ATOM  10069 1HE2 GLN B  38       8.322   6.636 -17.451  1.00  2.47
H
ATOM  10070 2HE2 GLN B  38       9.270   7.736 -18.392  1.00  2.47
H
ATOM  10071  N   LEU B  39       9.728   5.030 -25.265  1.00  0.43
N
ATOM  10072  CA  LEU B  39       9.594   3.952 -26.231  1.00  0.40
C
```

ATOM 10073 C LEU B 39 9.577 2.540 -25.650 1.00 0.36
C
ATOM 10074 O LEU B 39 8.740 1.720 -26.030 1.00 0.36
O
ATOM 10075 CB LEU B 39 10.724 4.029 -27.230 1.00 0.60
C
ATOM 10076 CG LEU B 39 10.786 2.837 -28.155 1.00 0.60
C
ATOM 10077 CD1 LEU B 39 9.472 2.715 -28.881 1.00 0.60
C
ATOM 10078 CD2 LEU B 39 11.941 3.038 -29.108 1.00 0.60
C
ATOM 10079 H LEU B 39 10.368 5.798 -25.445 1.00 0.52
H
ATOM 10080 HA LEU B 39 8.656 4.103 -26.764 1.00 0.48
H
ATOM 10081 1HB LEU B 39 10.598 4.922 -27.838 1.00 0.72
H
ATOM 10082 2HB LEU B 39 11.672 4.099 -26.695 1.00 0.72
H
ATOM 10083 HG LEU B 39 10.943 1.925 -27.579 1.00 0.72
H
ATOM 10084 1HD1 LEU B 39 9.502 1.855 -29.545 1.00 0.72
H
ATOM 10085 2HD1 LEU B 39 8.669 2.581 -28.157 1.00 0.72
H
ATOM 10086 3HD1 LEU B 39 9.295 3.620 -29.450 1.00 0.72
H
ATOM 10087 1HD2 LEU B 39 12.016 2.189 -29.781 1.00 0.72
H
ATOM 10088 2HD2 LEU B 39 11.786 3.951 -29.684 1.00 0.72
H
ATOM 10089 3HD2 LEU B 39 12.857 3.121 -28.529 1.00 0.72
H
ATOM 10090 N THR B 40 10.519 2.256 -24.758 1.00 0.36
N
ATOM 10091 CA THR B 40 10.672 0.925 -24.195 1.00 0.39
C
ATOM 10092 C THR B 40 9.527 0.506 -23.269 1.00 0.42
C
ATOM 10093 O THR B 40 9.288 -0.694 -23.079 1.00 0.56
O
ATOM 10094 CB THR B 40 12.002 0.834 -23.431 1.00 0.58
C
ATOM 10095 OG1 THR B 40 12.011 1.789 -22.362 1.00 0.58
O
ATOM 10096 CG2 THR B 40 13.161 1.122 -24.367 1.00 0.58
C
ATOM 10097 H THR B 40 11.165 2.982 -24.478 1.00 0.43
H
ATOM 10098 HA THR B 40 10.705 0.215 -25.021 1.00 0.47
H
ATOM 10099 HB THR B 40 12.118 -0.164 -23.019 1.00 0.70
H
ATOM 10100 HG1 THR B 40 12.865 1.766 -21.922 1.00 0.70
H
ATOM 10101 1HG2 THR B 40 14.099 1.053 -23.817 1.00 0.70
H

```
ATOM  10102 2HG2 THR B  40      13.162   0.395 -25.178  1.00  0.70
H
ATOM  10103 3HG2 THR B  40      13.056   2.125 -24.780  1.00  0.70
H
ATOM  10104  N   SER B  41       8.812   1.477 -22.689  1.00  0.40
N
ATOM  10105  CA  SER B  41       7.708   1.114 -21.814  1.00  0.54
C
ATOM  10106  C   SER B  41       6.477   0.871 -22.660  1.00  1.01
C
ATOM  10107  O   SER B  41       5.596   0.098 -22.281  1.00  3.82
O
ATOM  10108  CB  SER B  41       7.451   2.174 -20.761  1.00  0.81
C
ATOM  10109  OG  SER B  41       6.968   3.361 -21.314  1.00  0.81
O
ATOM  10110  H   SER B  41       9.002   2.453 -22.873  1.00  0.48
H
ATOM  10111  HA  SER B  41       7.958   0.183 -21.303  1.00  0.65
H
ATOM  10112 1HB  SER B  41       6.741   1.796 -20.026  1.00  0.97
H
ATOM  10113 2HB  SER B  41       8.383   2.376 -20.233  1.00  0.97
H
ATOM  10114  HG  SER B  41       7.191   4.044 -20.669  1.00  0.97
H
ATOM  10115  N   CYS B  42       6.450   1.481 -23.844  1.00  0.44
N
ATOM  10116  CA  CYS B  42       5.396   1.197 -24.803  1.00  0.43
C
ATOM  10117  C   CYS B  42       5.570  -0.230 -25.320  1.00  0.59
C
ATOM  10118  O   CYS B  42       4.621  -1.014 -25.341  1.00  0.98
O
ATOM  10119  CB  CYS B  42       5.441   2.178 -25.974  1.00  0.65
C
ATOM  10120  SG  CYS B  42       4.969   3.869 -25.554  1.00  0.65
S
ATOM  10121  H   CYS B  42       7.168   2.161 -24.076  1.00  0.53
H
ATOM  10122  HA  CYS B  42       4.430   1.281 -24.305  1.00  0.52
H
ATOM  10123 1HB  CYS B  42       6.447   2.209 -26.383  1.00  0.77
H
ATOM  10124 2HB  CYS B  42       4.785   1.827 -26.767  1.00  0.77
H
ATOM  10125  HG  CYS B  42       5.886   4.037 -24.609  1.00  0.77
H
ATOM  10126  N   CYS B  43       6.808  -0.567 -25.697  1.00  0.40
N
ATOM  10127  CA  CYS B  43       7.150  -1.879 -26.243  1.00  0.45
C
ATOM  10128  C   CYS B  43       6.757  -3.046 -25.352  1.00  0.41
C
ATOM  10129  O   CYS B  43       6.157  -4.012 -25.826  1.00  0.44
O
ATOM  10130  CB  CYS B  43       8.649  -1.954 -26.496  1.00  0.68
C
```

ATOM 10131 SG CYS B 43 9.205 -0.938 -27.870 1.00 0.68
S
ATOM 10132 H CYS B 43 7.539 0.132 -25.642 1.00 0.48
H
ATOM 10133 HA CYS B 43 6.634 -1.989 -27.197 1.00 0.54
H
ATOM 10134 1HB CYS B 43 9.177 -1.623 -25.602 1.00 0.81
H
ATOM 10135 2HB CYS B 43 8.943 -2.983 -26.689 1.00 0.81
H
ATOM 10136 HG CYS B 43 8.355 0.060 -27.659 1.00 0.81
H
ATOM 10137 N SER B 44 7.037 -2.951 -24.053 1.00 0.46
N
ATOM 10138 CA SER B 44 6.689 -4.046 -23.147 1.00 0.52
C
ATOM 10139 C SER B 44 5.180 -4.285 -22.966 1.00 0.56
C
ATOM 10140 O SER B 44 4.788 -5.305 -22.400 1.00 0.78
O
ATOM 10141 CB SER B 44 7.343 -3.829 -21.799 1.00 0.78
C
ATOM 10142 OG SER B 44 6.860 -2.687 -21.145 1.00 0.78
O
ATOM 10143 H SER B 44 7.535 -2.141 -23.695 1.00 0.55
H
ATOM 10144 HA SER B 44 7.115 -4.956 -23.566 1.00 0.62
H
ATOM 10145 1HB SER B 44 7.182 -4.706 -21.171 1.00 0.94
H
ATOM 10146 2HB SER B 44 8.408 -3.737 -21.956 1.00 0.94
H
ATOM 10147 HG SER B 44 5.994 -2.917 -20.794 1.00 0.94
H
ATOM 10148 N ALA B 45 4.332 -3.372 -23.444 1.00 0.64
N
ATOM 10149 CA ALA B 45 2.892 -3.549 -23.323 1.00 0.73
C
ATOM 10150 C ALA B 45 2.209 -3.759 -24.678 1.00 0.50
C
ATOM 10151 O ALA B 45 0.983 -3.800 -24.732 1.00 6.34
O
ATOM 10152 CB ALA B 45 2.282 -2.344 -22.636 1.00 1.09
C
ATOM 10153 H ALA B 45 4.668 -2.541 -23.913 1.00 0.77
H
ATOM 10154 HA ALA B 45 2.711 -4.435 -22.717 1.00 0.88
H
ATOM 10155 1HB ALA B 45 1.210 -2.498 -22.521 1.00 1.31
H
ATOM 10156 2HB ALA B 45 2.739 -2.217 -21.654 1.00 1.31
H
ATOM 10157 3HB ALA B 45 2.460 -1.453 -23.237 1.00 1.31
H
ATOM 10158 N ALA B 46 2.985 -3.870 -25.763 1.00 1.48
N
ATOM 10159 CA ALA B 46 2.420 -3.958 -27.117 1.00 0.36
C

```
ATOM  10160  C   ALA B  46       1.366  -5.047 -27.290  1.00  0.40
C
ATOM  10161  O   ALA B  46       0.295  -4.786 -27.846  1.00  1.59
O
ATOM  10162  CB  ALA B  46       3.542  -4.181 -28.118  1.00  0.54
C
ATOM  10163  H   ALA B  46       3.995  -3.870 -25.671  1.00  1.78
H
ATOM  10164  HA  ALA B  46       1.935  -3.016 -27.331  1.00  0.43
H
ATOM  10165 1HB  ALA B  46       3.133  -4.197 -29.127  1.00  0.65
H
ATOM  10166 2HB  ALA B  46       4.269  -3.372 -28.034  1.00  0.65
H
ATOM  10167 3HB  ALA B  46       4.032  -5.131 -27.910  1.00  0.65
H
ATOM  10168  N   GLU B  47       1.664  -6.255 -26.830  1.00  0.76
N
ATOM  10169  CA  GLU B  47       0.724  -7.356 -26.962  1.00  0.58
C
ATOM  10170  C   GLU B  47      -0.505  -7.159 -26.091  1.00  0.40
C
ATOM  10171  O   GLU B  47      -1.613  -7.472 -26.516  1.00  0.32
O
ATOM  10172  CB  GLU B  47       1.415  -8.675 -26.646  1.00  0.87
C
ATOM  10173  CG  GLU B  47       2.445  -9.076 -27.692  1.00  0.87
C
ATOM  10174  CD  GLU B  47       3.765  -8.391 -27.506  1.00  0.87
C
ATOM  10175  OE1 GLU B  47       3.972  -7.841 -26.452  1.00  0.87
O
ATOM  10176  OE2 GLU B  47       4.566  -8.410 -28.411  1.00  0.87
O
ATOM  10177  H   GLU B  47       2.556  -6.415 -26.384  1.00  0.91
H
ATOM  10178  HA  GLU B  47       0.391  -7.395 -27.998  1.00  0.70
H
ATOM  10179 1HB  GLU B  47       1.928  -8.593 -25.689  1.00  1.04
H
ATOM  10180 2HB  GLU B  47       0.680  -9.473 -26.557  1.00  1.04
H
ATOM  10181 1HG  GLU B  47       2.596 -10.153 -27.646  1.00  1.04
H
ATOM  10182 2HG  GLU B  47       2.054  -8.836 -28.679  1.00  1.04
H
ATOM  10183  N   VAL B  48      -0.313  -6.622 -24.886  1.00  0.44
N
ATOM  10184  CA  VAL B  48      -1.425  -6.361 -23.976  1.00  0.35
C
ATOM  10185  C   VAL B  48      -2.421  -5.394 -24.587  1.00  0.28
C
ATOM  10186  O   VAL B  48      -3.631  -5.633 -24.551  1.00  0.23
O
ATOM  10187  CB  VAL B  48      -0.913  -5.781 -22.647  1.00  0.52
C
ATOM  10188  CG1 VAL B  48      -2.090  -5.308 -21.809  1.00  0.52
C
```

ATOM 10189 CG2 VAL B 48   -0.108 -6.840 -21.906 1.00 0.52
C
ATOM 10190 H   VAL B 48   0.625 -6.388 -24.590 1.00 0.53
H
ATOM 10191 HA VAL B 48   -1.935 -7.302 -23.773 1.00 0.42
H
ATOM 10192 HB VAL B 48   -0.284 -4.916 -22.850 1.00 0.63
H
ATOM 10193 1HG1 VAL B 48   -1.721 -4.884 -20.877 1.00 0.63
H
ATOM 10194 2HG1 VAL B 48   -2.646 -4.548 -22.357 1.00 0.63
H
ATOM 10195 3HG1 VAL B 48   -2.745 -6.152 -21.591 1.00 0.63
H
ATOM 10196 1HG2 VAL B 48   0.261 -6.427 -20.968 1.00 0.63
H
ATOM 10197 2HG2 VAL B 48   -0.744 -7.701 -21.699 1.00 0.63
H
ATOM 10198 3HG2 VAL B 48   0.736 -7.151 -22.522 1.00 0.63
H
ATOM 10199 N   VAL B 49   -1.897 -4.307 -25.151 1.00 0.35
N
ATOM 10200 CA VAL B 49   -2.707 -3.289 -25.797 1.00 0.28
C
ATOM 10201 C   VAL B 49   -3.430 -3.864 -27.002 1.00 0.32
C
ATOM 10202 O   VAL B 49   -4.623 -3.634 -27.184 1.00 0.36
O
ATOM 10203 CB VAL B 49   -1.821 -2.095 -26.220 1.00 0.42
C
ATOM 10204 CG1 VAL B 49   -2.609 -1.114 -27.075 1.00 0.42
C
ATOM 10205 CG2 VAL B 49   -1.297 -1.394 -24.976 1.00 0.42
C
ATOM 10206 H   VAL B 49   -0.894 -4.179 -25.124 1.00 0.42
H
ATOM 10207 HA VAL B 49   -3.450 -2.933 -25.082 1.00 0.34
H
ATOM 10208 HB VAL B 49   -0.987 -2.463 -26.817 1.00 0.50
H
ATOM 10209 1HG1 VAL B 49   -1.963 -0.287 -27.369 1.00 0.50
H
ATOM 10210 2HG1 VAL B 49   -2.978 -1.621 -27.967 1.00 0.50
H
ATOM 10211 3HG1 VAL B 49   -3.449 -0.728 -26.501 1.00 0.50
H
ATOM 10212 1HG2 VAL B 49   -0.661 -0.561 -25.271 1.00 0.50
H
ATOM 10213 2HG2 VAL B 49   -2.136 -1.021 -24.388 1.00 0.50
H
ATOM 10214 3HG2 VAL B 49   -0.720 -2.095 -24.376 1.00 0.50
H
ATOM 10215 N   SER B 50   -2.714 -4.630 -27.821 1.00 0.34
N
ATOM 10216 CA SER B 50   -3.309 -5.240 -28.998 1.00 0.49
C
ATOM 10217 C   SER B 50   -4.495 -6.126 -28.643 1.00 0.48
C

```
ATOM  10218  O   SER B  50      -5.547  -6.054 -29.279  1.00  0.68
O
ATOM  10219  CB  SER B  50      -2.272  -6.071 -29.710  1.00  0.73
C
ATOM  10220  OG  SER B  50      -1.235  -5.267 -30.173  1.00  0.73
O
ATOM  10221  H   SER B  50      -1.727  -4.784 -27.644  1.00  0.41
H
ATOM  10222  HA  SER B  50      -3.655  -4.446 -29.662  1.00  0.59
H
ATOM  10223 1HB  SER B  50      -1.879  -6.834 -29.039  1.00  0.88
H
ATOM  10224 2HB  SER B  50      -2.734  -6.578 -30.553  1.00  0.88
H
ATOM  10225  HG  SER B  50      -0.761  -4.975 -29.387  1.00  0.88
H
ATOM  10226  N   VAL B  51      -4.329  -6.952 -27.611  1.00  0.42
N
ATOM  10227  CA  VAL B  51      -5.379  -7.868 -27.191  1.00  0.49
C
ATOM  10228  C   VAL B  51      -6.585  -7.154 -26.640  1.00  0.40
C
ATOM  10229  O   VAL B  51      -7.714  -7.434 -27.053  1.00  0.51
O
ATOM  10230  CB  VAL B  51      -4.847  -8.846 -26.127  1.00  0.73
C
ATOM  10231  CG1 VAL B  51      -5.991  -9.660 -25.550  1.00  0.73
C
ATOM  10232  CG2 VAL B  51      -3.835  -9.778 -26.759  1.00  0.73
C
ATOM  10233  H   VAL B  51      -3.442  -6.972 -27.123  1.00  0.50
H
ATOM  10234  HA  VAL B  51      -5.699  -8.445 -28.055  1.00  0.59
H
ATOM  10235  HB  VAL B  51      -4.382  -8.283 -25.319  1.00  0.88
H
ATOM  10236 1HG1 VAL B  51      -5.600 -10.348 -24.802  1.00  0.88
H
ATOM  10237 2HG1 VAL B  51      -6.720  -8.995 -25.088  1.00  0.88
H
ATOM  10238 3HG1 VAL B  51      -6.469 -10.227 -26.347  1.00  0.88
H
ATOM  10239 1HG2 VAL B  51      -3.454 -10.465 -26.012  1.00  0.88
H
ATOM  10240 2HG2 VAL B  51      -4.321 -10.339 -27.546  1.00  0.88
H
ATOM  10241 3HG2 VAL B  51      -3.011  -9.208 -27.179  1.00  0.88
H
ATOM  10242  N   LEU B  52      -6.358  -6.227 -25.719  1.00  0.30
N
ATOM  10243  CA  LEU B  52      -7.473  -5.531 -25.122  1.00  0.27
C
ATOM  10244  C   LEU B  52      -8.228  -4.663 -26.108  1.00  0.23
C
ATOM  10245  O   LEU B  52      -9.455  -4.675 -26.136  1.00  0.23
O
ATOM  10246  CB  LEU B  52      -6.992  -4.678 -23.946  1.00  0.41
C
```

```
ATOM  10247  CG  LEU B  52      -6.543  -5.482 -22.723  1.00  0.41
C
ATOM  10248  CD1 LEU B  52      -5.952  -4.552 -21.675  1.00  0.41
C
ATOM  10249  CD2 LEU B  52      -7.743  -6.230 -22.184  1.00  0.41
C
ATOM  10250  H   LEU B  52      -5.414  -6.017 -25.414  1.00  0.36
H
ATOM  10251  HA  LEU B  52      -8.157  -6.274 -24.734  1.00  0.32
H
ATOM  10252 1HB  LEU B  52      -6.150  -4.069 -24.274  1.00  0.49
H
ATOM  10253 2HB  LEU B  52      -7.800  -4.015 -23.637  1.00  0.49
H
ATOM  10254  HG  LEU B  52      -5.770  -6.194 -23.013  1.00  0.49
H
ATOM  10255 1HD1 LEU B  52      -5.635  -5.134 -20.810  1.00  0.49
H
ATOM  10256 2HD1 LEU B  52      -5.092  -4.030 -22.096  1.00  0.49
H
ATOM  10257 3HD1 LEU B  52      -6.704  -3.826 -21.368  1.00  0.49
H
ATOM  10258 1HD2 LEU B  52      -7.447  -6.814 -21.316  1.00  0.49
H
ATOM  10259 2HD2 LEU B  52      -8.515  -5.518 -21.899  1.00  0.49
H
ATOM  10260 3HD2 LEU B  52      -8.129  -6.896 -22.954  1.00  0.49
H
ATOM  10261  N   PHE B  53      -7.523  -3.949 -26.965  1.00  0.23
N
ATOM  10262  CA  PHE B  53      -8.215  -3.083 -27.899  1.00  0.22
C
ATOM  10263  C   PHE B  53      -8.821  -3.787 -29.107  1.00  0.26
C
ATOM  10264  O   PHE B  53      -9.781  -3.278 -29.687  1.00  0.25
O
ATOM  10265  CB  PHE B  53      -7.307  -1.925 -28.323  1.00  0.33
C
ATOM  10266  CG  PHE B  53      -7.195  -0.845 -27.274  1.00  0.33
C
ATOM  10267  CD1 PHE B  53      -6.249  -0.906 -26.259  1.00  0.33
C
ATOM  10268  CD2 PHE B  53      -8.042   0.252 -27.309  1.00  0.33
C
ATOM  10269  CE1 PHE B  53      -6.155   0.093 -25.309  1.00  0.33
C
ATOM  10270  CE2 PHE B  53      -7.953   1.253 -26.363  1.00  0.33
C
ATOM  10271  CZ  PHE B  53      -7.007   1.174 -25.362  1.00  0.33
C
ATOM  10272  H   PHE B  53      -6.511  -3.964 -26.954  1.00  0.28
H
ATOM  10273  HA  PHE B  53      -9.044  -2.629 -27.355  1.00  0.26
H
ATOM  10274 1HB  PHE B  53      -6.307  -2.301 -28.536  1.00  0.40
H
ATOM  10275 2HB  PHE B  53      -7.689  -1.474 -29.238  1.00  0.40
H
```

ATOM 10276 HD1 PHE B 53 -5.573 -1.755 -26.209 1.00 0.40
H
ATOM 10277 HD2 PHE B 53 -8.787 0.322 -28.099 1.00 0.40
H
ATOM 10278 HE1 PHE B 53 -5.407 0.026 -24.520 1.00 0.40
H
ATOM 10279 HE2 PHE B 53 -8.628 2.107 -26.409 1.00 0.40
H
ATOM 10280 HZ PHE B 53 -6.936 1.964 -24.617 1.00 0.40
H
ATOM 10281 N PHE B 54 -8.286 -4.939 -29.511 1.00 0.33
N
ATOM 10282 CA PHE B 54 -8.831 -5.555 -30.709 1.00 0.43
C
ATOM 10283 C PHE B 54 -9.571 -6.878 -30.550 1.00 0.95
C
ATOM 10284 O PHE B 54 -10.209 -7.325 -31.503 1.00 3.13
O
ATOM 10285 CB PHE B 54 -7.753 -5.605 -31.761 1.00 0.65
C
ATOM 10286 CG PHE B 54 -7.344 -4.205 -32.101 1.00 0.65
C
ATOM 10287 CD1 PHE B 54 -6.218 -3.633 -31.531 1.00 0.65
C
ATOM 10288 CD2 PHE B 54 -8.117 -3.445 -32.960 1.00 0.65
C
ATOM 10289 CE1 PHE B 54 -5.878 -2.324 -31.804 1.00 0.65
C
ATOM 10290 CE2 PHE B 54 -7.778 -2.142 -33.240 1.00 0.65
C
ATOM 10291 CZ PHE B 54 -6.664 -1.582 -32.655 1.00 0.65
C
ATOM 10292 H PHE B 54 -7.490 -5.359 -29.046 1.00 0.40
H
ATOM 10293 HA PHE B 54 -9.568 -4.858 -31.108 1.00 0.52
H
ATOM 10294 1HB PHE B 54 -6.883 -6.156 -31.408 1.00 0.77
H
ATOM 10295 2HB PHE B 54 -8.134 -6.088 -32.655 1.00 0.77
H
ATOM 10296 HD1 PHE B 54 -5.607 -4.220 -30.850 1.00 0.77
H
ATOM 10297 HD2 PHE B 54 -9.008 -3.885 -33.407 1.00 0.77
H
ATOM 10298 HE1 PHE B 54 -4.997 -1.880 -31.344 1.00 0.77
H
ATOM 10299 HE2 PHE B 54 -8.396 -1.549 -33.913 1.00 0.77
H
ATOM 10300 HZ PHE B 54 -6.408 -0.549 -32.865 1.00 0.77
H
ATOM 10301 N HIS B 55 -9.565 -7.474 -29.356 1.00 0.67
N
ATOM 10302 CA HIS B 55 -10.332 -8.706 -29.156 1.00 1.58
C
ATOM 10303 C HIS B 55 -11.076 -8.804 -27.831 1.00 0.59
C
ATOM 10304 O HIS B 55 -12.141 -9.418 -27.784 1.00 2.15
O

```
ATOM  10305  CB  HIS B  55      -9.527  -9.977 -29.449  1.00  2.37
C
ATOM  10306  CG  HIS B  55      -9.237 -10.110 -30.906  1.00  2.37
C
ATOM  10307  ND1 HIS B  55     -10.241 -10.275 -31.838  1.00  2.37
N
ATOM  10308  CD2 HIS B  55      -8.081 -10.090 -31.598  1.00  2.37
C
ATOM  10309  CE1 HIS B  55      -9.707 -10.333 -33.044  1.00  2.37
C
ATOM  10310  NE2 HIS B  55      -8.399 -10.223 -32.924  1.00  2.37
N
ATOM  10311  H   HIS B  55      -9.014  -7.109 -28.593  1.00  0.80
H
ATOM  10312  HA  HIS B  55     -11.119  -8.700 -29.908  1.00  1.90
H
ATOM  10313 1HB  HIS B  55      -8.581  -9.952 -28.906  1.00  2.84
H
ATOM  10314 2HB  HIS B  55     -10.081 -10.854 -29.120  1.00  2.84
H
ATOM  10315  HD1 HIS B  55     -11.212 -10.102 -31.677  1.00  2.84
H
ATOM  10316  HD2 HIS B  55      -7.050  -9.985 -31.296  1.00  2.84
H
ATOM  10317  HE1 HIS B  55     -10.338 -10.458 -33.924  1.00  2.84
H
ATOM  10318  N   THR B  56     -10.557  -8.212 -26.761  1.00  0.70
N
ATOM  10319  CA  THR B  56     -11.270  -8.314 -25.489  1.00  0.57
C
ATOM  10320  C   THR B  56     -12.296  -7.208 -25.235  1.00  0.33
C
ATOM  10321  O   THR B  56     -13.459  -7.502 -24.952  1.00  0.54
O
ATOM  10322  CB  THR B  56     -10.298  -8.312 -24.312  1.00  0.85
C
ATOM  10323  OG1 THR B  56      -9.424  -9.444 -24.404  1.00  0.85
O
ATOM  10324  CG2 THR B  56     -11.063  -8.349 -23.003  1.00  0.85
C
ATOM  10325  H   THR B  56      -9.673  -7.725 -26.817  1.00  0.84
H
ATOM  10326  HA  THR B  56     -11.805  -9.264 -25.482  1.00  0.68
H
ATOM  10327  HB  THR B  56      -9.707  -7.406 -24.345  1.00  1.03
H
ATOM  10328  HG1 THR B  56      -9.944 -10.251 -24.366  1.00  1.03
H
ATOM  10329 1HG2 THR B  56     -10.363  -8.328 -22.172  1.00  1.03
H
ATOM  10330 2HG2 THR B  56     -11.719  -7.481 -22.942  1.00  1.03
H
ATOM  10331 3HG2 THR B  56     -11.661  -9.259 -22.956  1.00  1.03
H
ATOM  10332  N   MET B  57     -11.873  -5.948 -25.304  1.00  0.32
N
ATOM  10333  CA  MET B  57     -12.766  -4.830 -24.995  1.00  0.35
C
```

ATOM 10334 C   MET B 57   -13.804 -4.523 -26.060 1.00 0.42
C
ATOM 10335 O   MET B 57   -13.560 -4.670 -27.258 1.00 0.72
O
ATOM 10336 CB MET B 57   -11.965 -3.558 -24.764 1.00 0.52
C
ATOM 10337 CG MET B 57   -11.091 -3.542 -23.530 1.00 0.52
C
ATOM 10338 SD MET B 57   -10.148 -2.016 -23.426 1.00 0.52
S
ATOM 10339 CE MET B 57   -11.509 -0.871 -23.223 1.00 0.52
C
ATOM 10340 H   MET B 57   -10.916 -5.742 -25.554 1.00 0.38
H
ATOM 10341 HA MET B 57   -13.304 -5.076 -24.080 1.00 0.42
H
ATOM 10342 1HB MET B 57   -11.341 -3.353 -25.630 1.00 0.63
H
ATOM 10343 2HB MET B 57   -12.658 -2.722 -24.668 1.00 0.63
H
ATOM 10344 1HG MET B 57   -11.721 -3.621 -22.647 1.00 0.63
H
ATOM 10345 2HG MET B 57   -10.399 -4.384 -23.545 1.00 0.63
H
ATOM 10346 1HE MET B 57   -11.128   0.146 -23.151 1.00 0.63
H
ATOM 10347 2HE MET B 57   -12.177 -0.945 -24.083 1.00 0.63
H
ATOM 10348 3HE MET B 57   -12.059 -1.119 -22.315 1.00 0.63
H
ATOM 10349 N   LYS B 58   -14.951 -4.042 -25.594 1.00 0.37
N
ATOM 10350 CA LYS B 58   -16.039 -3.591 -26.447 1.00 0.54
C
ATOM 10351 C   LYS B 58   -16.138 -2.083 -26.315 1.00 0.43
C
ATOM 10352 O   LYS B 58   -16.097 -1.568 -25.202 1.00 0.55
O
ATOM 10353 CB LYS B 58   -17.356 -4.206 -26.018 1.00 0.81
C
ATOM 10354 CG LYS B 58   -17.411 -5.714 -26.048 1.00 0.81
C
ATOM 10355 CD LYS B 58   -18.733 -6.194 -25.476 1.00 0.81
C
ATOM 10356 CE LYS B 58   -18.872 -5.781 -24.017 1.00 0.81
C
ATOM 10357 NZ LYS B 58   -17.813 -6.376 -23.160 1.00 0.81
N
ATOM 10358 H   LYS B 58   -15.078 -3.982 -24.591 1.00 0.44
H
ATOM 10359 HA LYS B 58   -15.820 -3.845 -27.484 1.00 0.65
H
ATOM 10360 1HB LYS B 58   -17.594 -3.881 -25.004 1.00 0.97
H
ATOM 10361 2HB LYS B 58   -18.140 -3.842 -26.673 1.00 0.97
H
ATOM 10362 1HG LYS B 58   -17.309 -6.066 -27.074 1.00 0.97
H

ATOM 10363 2HG LYS B 58 -16.593 -6.123 -25.455 1.00 0.97
H
ATOM 10364 1HD LYS B 58 -19.558 -5.772 -26.050 1.00 0.97
H
ATOM 10365 2HD LYS B 58 -18.785 -7.281 -25.541 1.00 0.97
H
ATOM 10366 1HE LYS B 58 -18.814 -4.695 -23.944 1.00 0.97
H
ATOM 10367 2HE LYS B 58 -19.845 -6.105 -23.646 1.00 0.97
H
ATOM 10368 1HZ LYS B 58 -17.945 -6.073 -22.205 1.00 0.97
H
ATOM 10369 2HZ LYS B 58 -17.860 -7.383 -23.205 1.00 0.97
H
ATOM 10370 3HZ LYS B 58 -16.906 -6.063 -23.483 1.00 0.97
H
ATOM 10371 N TYR B 59 -16.289 -1.382 -27.425 1.00 0.40
N
ATOM 10372 CA TYR B 59 -16.369 0.076 -27.395 1.00 0.33
C
ATOM 10373 C TYR B 59 -16.559 0.644 -28.790 1.00 0.35
C
ATOM 10374 O TYR B 59 -16.073 0.089 -29.774 1.00 0.44
O
ATOM 10375 CB TYR B 59 -15.098 0.692 -26.782 1.00 0.49
C
ATOM 10376 CG TYR B 59 -13.840 0.319 -27.529 1.00 0.49
C
ATOM 10377 CD1 TYR B 59 -13.430 1.087 -28.610 1.00 0.49
C
ATOM 10378 CD2 TYR B 59 -13.096 -0.785 -27.134 1.00 0.49
C
ATOM 10379 CE1 TYR B 59 -12.287 0.743 -29.298 1.00 0.49
C
ATOM 10380 CE2 TYR B 59 -11.956 -1.130 -27.822 1.00 0.49
C
ATOM 10381 CZ TYR B 59 -11.557 -0.371 -28.899 1.00 0.49
C
ATOM 10382 OH TYR B 59 -10.432 -0.718 -29.590 1.00 0.49
O
ATOM 10383 H TYR B 59 -16.351 -1.872 -28.307 1.00 0.48
H
ATOM 10384 HA TYR B 59 -17.229 0.362 -26.793 1.00 0.40
H
ATOM 10385 1HB TYR B 59 -15.185 1.779 -26.798 1.00 0.59
H
ATOM 10386 2HB TYR B 59 -14.982 0.395 -25.743 1.00 0.59
H
ATOM 10387 HD1 TYR B 59 -14.013 1.955 -28.916 1.00 0.59
H
ATOM 10388 HD2 TYR B 59 -13.416 -1.383 -26.283 1.00 0.59
H
ATOM 10389 HE1 TYR B 59 -11.963 1.337 -30.152 1.00 0.59
H
ATOM 10390 HE2 TYR B 59 -11.373 -2.000 -27.520 1.00 0.59
H
ATOM 10391 HH TYR B 59 -10.272 -1.660 -29.491 1.00 0.59
H

ATOM 10392 N   LYS B 60   -17.246   1.776 -28.866 1.00 0.43
N
ATOM 10393 CA LYS B 60   -17.490   2.415 -30.143 1.00 0.91
C
ATOM 10394 C   LYS B 60   -16.251   3.171 -30.602 1.00 1.97
C
ATOM 10395 O   LYS B 60   -15.824   4.137 -29.975 1.00 15.61
O
ATOM 10396 CB LYS B 60   -18.713   3.323 -30.040 1.00 1.36
C
ATOM 10397 CG LYS B 60   -19.994   2.533 -29.785 1.00 1.36
C
ATOM 10398 CD LYS B 60   -21.236   3.407 -29.750 1.00 1.36
C
ATOM 10399 CE LYS B 60   -22.482   2.558 -29.502 1.00 1.36
C
ATOM 10400 NZ LYS B 60   -23.730   3.367 -29.523 1.00 1.36
N
ATOM 10401 H   LYS B 60   -17.629   2.183 -28.024 1.00 0.52
H
ATOM 10402 HA LYS B 60   -17.699   1.641 -30.883 1.00 1.09
H
ATOM 10403 1HB LYS B 60   -18.580   4.036 -29.225 1.00 1.64
H
ATOM 10404 2HB LYS B 60   -18.836   3.889 -30.963 1.00 1.64
H
ATOM 10405 1HG LYS B 60   -20.115   1.784 -30.567 1.00 1.64
H
ATOM 10406 2HG LYS B 60   -19.907   2.019 -28.828 1.00 1.64
H
ATOM 10407 1HD LYS B 60   -21.142   4.146 -28.953 1.00 1.64
H
ATOM 10408 2HD LYS B 60   -21.344   3.931 -30.699 1.00 1.64
H
ATOM 10409 1HE LYS B 60   -22.548   1.791 -30.273 1.00 1.64
H
ATOM 10410 2HE LYS B 60   -22.395   2.071 -28.532 1.00 1.64
H
ATOM 10411 1HZ LYS B 60   -24.535   2.770 -29.358 1.00 1.64
H
ATOM 10412 2HZ LYS B 60   -23.684   4.072 -28.802 1.00 1.64
H
ATOM 10413 3HZ LYS B 60   -23.828   3.813 -30.423 1.00 1.64
H
ATOM 10414 N   GLN B 61   -15.702   2.722 -31.724 1.00 2.29
N
ATOM 10415 CA GLN B 61   -14.463   3.224 -32.315 1.00 1.17
C
ATOM 10416 C   GLN B 61   -14.410   4.734 -32.448 1.00 1.62
C
ATOM 10417 O   GLN B 61   -13.440   5.374 -32.039 1.00 3.58
O
ATOM 10418 CB GLN B 61   -14.315   2.638 -33.717 1.00 1.75
C
ATOM 10419 CG GLN B 61   -14.061   1.152 -33.781 1.00 1.75
C
ATOM 10420 CD GLN B 61   -14.182   0.659 -35.207 1.00 1.75
C

```
ATOM  10421  OE1 GLN B  61      -13.949   1.417 -36.154  1.00  1.75
O
ATOM  10422  NE2 GLN B  61      -14.571  -0.595 -35.385  1.00  1.75
N
ATOM  10423  H   GLN B  61      -16.153   1.942 -32.182  1.00  2.75
H
ATOM  10424  HA  GLN B  61      -13.628   2.906 -31.690  1.00  1.40
H
ATOM  10425 1HB  GLN B  61      -15.221   2.840 -34.288  1.00  2.11
H
ATOM  10426 2HB  GLN B  61      -13.492   3.136 -34.228  1.00  2.11
H
ATOM  10427 1HG  GLN B  61      -13.052   0.946 -33.426  1.00  2.11
H
ATOM  10428 2HG  GLN B  61      -14.794   0.630 -33.168  1.00  2.11
H
ATOM  10429 1HE2 GLN B  61      -14.680  -0.947 -36.313  1.00  2.11
H
ATOM  10430 2HE2 GLN B  61      -14.764  -1.194 -34.605  1.00  2.11
H
ATOM  10431  N   THR B  62      -15.468   5.300 -33.025  1.00  0.88
N
ATOM  10432  CA  THR B  62      -15.534   6.729 -33.288  1.00  0.94
C
ATOM  10433  C   THR B  62      -16.345   7.486 -32.250  1.00  0.99
C
ATOM  10434  O   THR B  62      -16.803   8.599 -32.509  1.00  4.47
O
ATOM  10435  CB  THR B  62      -16.114   6.995 -34.687  1.00  1.41
C
ATOM  10436  OG1 THR B  62      -17.423   6.418 -34.785  1.00  1.41
O
ATOM  10437  CG2 THR B  62      -15.207   6.389 -35.748  1.00  1.41
C
ATOM  10438  H   THR B  62      -16.239   4.717 -33.317  1.00  1.06
H
ATOM  10439  HA  THR B  62      -14.519   7.123 -33.265  1.00  1.13
H
ATOM  10440  HB  THR B  62      -16.188   8.070 -34.849  1.00  1.69
H
ATOM  10441  HG1 THR B  62      -17.994   6.805 -34.117  1.00  1.69
H
ATOM  10442 1HG2 THR B  62      -15.622   6.585 -36.736  1.00  1.69
H
ATOM  10443 2HG2 THR B  62      -14.215   6.836 -35.674  1.00  1.69
H
ATOM  10444 3HG2 THR B  62      -15.132   5.313 -35.593  1.00  1.69
H
ATOM  10445  N   ASP B  63      -16.501   6.902 -31.071  1.00  0.74
N
ATOM  10446  CA  ASP B  63      -17.204   7.561 -29.992  1.00  0.76
C
ATOM  10447  C   ASP B  63      -16.755   6.978 -28.664  1.00  0.55
C
ATOM  10448  O   ASP B  63      -17.541   6.349 -27.956  1.00  0.64
O
ATOM  10449  CB  ASP B  63      -18.713   7.415 -30.147  1.00  1.14
C
```

ATOM 10450 CG ASP B 63 -19.469 8.263 -29.138 1.00 1.14
C
ATOM 10451 OD1 ASP B 63 -18.830 8.879 -28.318 1.00 1.14
O
ATOM 10452 OD2 ASP B 63 -20.676 8.285 -29.190 1.00 1.14
O
ATOM 10453 H ASP B 63 -16.121 5.982 -30.891 1.00 0.89
H
ATOM 10454 HA ASP B 63 -16.971 8.623 -30.014 1.00 0.91
H
ATOM 10455 1HB ASP B 63 -19.009 7.717 -31.152 1.00 1.37
H
ATOM 10456 2HB ASP B 63 -18.998 6.370 -30.017 1.00 1.37
H
ATOM 10457 N PRO B 64 -15.491 7.218 -28.293 1.00 0.47
N
ATOM 10458 CA PRO B 64 -14.786 6.748 -27.105 1.00 0.36
C
ATOM 10459 C PRO B 64 -15.325 7.338 -25.817 1.00 0.89
C
ATOM 10460 O PRO B 64 -15.045 6.828 -24.733 1.00 3.31
O
ATOM 10461 CB PRO B 64 -13.376 7.222 -27.331 1.00 0.54
C
ATOM 10462 CG PRO B 64 -13.503 8.425 -28.189 1.00 0.54
C
ATOM 10463 CD PRO B 64 -14.646 8.126 -29.094 1.00 0.54
C
ATOM 10464 HA PRO B 64 -14.828 5.651 -27.076 1.00 0.43
H
ATOM 10465 1HB PRO B 64 -12.887 7.416 -26.370 1.00 0.65
H
ATOM 10466 2HB PRO B 64 -12.832 6.413 -27.821 1.00 0.65
H
ATOM 10467 1HG PRO B 64 -13.690 9.309 -27.564 1.00 0.65
H
ATOM 10468 2HG PRO B 64 -12.568 8.611 -28.736 1.00 0.65
H
ATOM 10469 1HD PRO B 64 -15.176 9.059 -29.328 1.00 0.65
H
ATOM 10470 2HD PRO B 64 -14.281 7.618 -29.999 1.00 0.65
H
ATOM 10471 N GLU B 65 -16.106 8.402 -25.942 1.00 0.72
N
ATOM 10472 CA GLU B 65 -16.731 9.052 -24.814 1.00 1.12
C
ATOM 10473 C GLU B 65 -18.089 8.430 -24.489 1.00 1.10
C
ATOM 10474 O GLU B 65 -18.725 8.823 -23.509 1.00 1.56
O
ATOM 10475 CB GLU B 65 -16.916 10.542 -25.101 1.00 1.68
C
ATOM 10476 CG GLU B 65 -15.625 11.330 -25.275 1.00 1.68
C
ATOM 10477 CD GLU B 65 -15.867 12.787 -25.564 1.00 1.68
C
ATOM 10478 OE1 GLU B 65 -17.009 13.175 -25.644 1.00 1.68
O

ATOM 10479 OE2 GLU B 65 -14.913 13.513 -25.707 1.00 1.68
O
ATOM 10480 H GLU B 65 -16.283 8.783 -26.860 1.00 0.86
H
ATOM 10481 HA GLU B 65 -16.085 8.934 -23.944 1.00 1.34
H
ATOM 10482 1HB GLU B 65 -17.502 10.663 -26.013 1.00 2.02
H
ATOM 10483 2HB GLU B 65 -17.478 11.002 -24.289 1.00 2.02
H
ATOM 10484 1HG GLU B 65 -15.032 11.244 -24.364 1.00 2.02
H
ATOM 10485 2HG GLU B 65 -15.053 10.893 -26.090 1.00 2.02
H
ATOM 10486 N HIS B 66 -18.547 7.481 -25.313 1.00 0.85
N
ATOM 10487 CA HIS B 66 -19.871 6.914 -25.116 1.00 0.86
C
ATOM 10488 C HIS B 66 -19.966 6.140 -23.790 1.00 0.64
C
ATOM 10489 O HIS B 66 -19.106 5.306 -23.497 1.00 0.67
O
ATOM 10490 CB HIS B 66 -20.235 5.976 -26.266 1.00 1.29
C
ATOM 10491 CG HIS B 66 -21.694 5.702 -26.332 1.00 1.29
C
ATOM 10492 ND1 HIS B 66 -22.349 4.874 -25.449 1.00 1.29
N
ATOM 10493 CD2 HIS B 66 -22.636 6.192 -27.168 1.00 1.29
C
ATOM 10494 CE1 HIS B 66 -23.639 4.867 -25.746 1.00 1.29
C
ATOM 10495 NE2 HIS B 66 -23.837 5.656 -26.783 1.00 1.29
N
ATOM 10496 H HIS B 66 -18.008 7.163 -26.107 1.00 1.02
H
ATOM 10497 HA HIS B 66 -20.591 7.723 -25.127 1.00 1.03
H
ATOM 10498 1HB HIS B 66 -19.924 6.411 -27.212 1.00 1.55
H
ATOM 10499 2HB HIS B 66 -19.714 5.028 -26.150 1.00 1.55
H
ATOM 10500 HD2 HIS B 66 -22.473 6.890 -27.989 1.00 1.55
H
ATOM 10501 HE1 HIS B 66 -24.418 4.317 -25.230 1.00 1.55
H
ATOM 10502 HE2 HIS B 66 -24.728 5.844 -27.220 1.00 1.55
H
ATOM 10503 N PRO B 67 -21.024 6.391 -22.998 1.00 0.73
N
ATOM 10504 CA PRO B 67 -21.365 5.802 -21.702 1.00 0.67
C
ATOM 10505 C PRO B 67 -21.359 4.278 -21.652 1.00 0.53
C
ATOM 10506 O PRO B 67 -21.089 3.717 -20.590 1.00 0.55
O
ATOM 10507 CB PRO B 67 -22.772 6.346 -21.442 1.00 1.01
C

```
ATOM  10508  CG  PRO B  67     -22.769   7.684 -22.087  1.00  1.01
C
ATOM  10509  CD  PRO B  67     -21.922   7.529 -23.308  1.00  1.01
C
ATOM  10510  HA  PRO B  67     -20.670   6.198 -20.950  1.00  0.80
H
ATOM  10511 1HB  PRO B  67     -23.525   5.669 -21.871  1.00  1.21
H
ATOM  10512 2HB  PRO B  67     -22.964   6.397 -20.359  1.00  1.21
H
ATOM  10513 1HG  PRO B  67     -23.799   7.988 -22.327  1.00  1.21
H
ATOM  10514 2HG  PRO B  67     -22.372   8.438 -21.391  1.00  1.21
H
ATOM  10515 1HD  PRO B  67     -22.548   7.303 -24.182  1.00  1.21
H
ATOM  10516 2HD  PRO B  67     -21.335   8.449 -23.447  1.00  1.21
H
ATOM  10517  N   ASP B  68     -21.642   3.611 -22.770  1.00  0.75
N
ATOM  10518  CA  ASP B  68     -21.678   2.151 -22.789  1.00  0.99
C
ATOM  10519  C   ASP B  68     -20.363   1.460 -23.114  1.00  1.13
C
ATOM  10520  O   ASP B  68     -20.317   0.232 -23.154  1.00  2.62
O
ATOM  10521  CB  ASP B  68     -22.722   1.682 -23.788  1.00  1.48
C
ATOM  10522  CG  ASP B  68     -24.113   1.958 -23.293  1.00  1.48
C
ATOM  10523  OD1 ASP B  68     -24.352   1.664 -22.152  1.00  1.48
O
ATOM  10524  OD2 ASP B  68     -24.925   2.456 -24.030  1.00  1.48
O
ATOM  10525  H   ASP B  68     -21.851   4.115 -23.626  1.00  0.90
H
ATOM  10526  HA  ASP B  68     -21.986   1.817 -21.798  1.00  1.19
H
ATOM  10527 1HB  ASP B  68     -22.575   2.186 -24.742  1.00  1.78
H
ATOM  10528 2HB  ASP B  68     -22.614   0.609 -23.958  1.00  1.78
H
ATOM  10529  N   ASN B  69     -19.300   2.218 -23.330  1.00  0.46
N
ATOM  10530  CA  ASN B  69     -18.021   1.616 -23.687  1.00  0.34
C
ATOM  10531  C   ASN B  69     -17.291   0.955 -22.518  1.00  0.47
C
ATOM  10532  O   ASN B  69     -17.403   1.371 -21.369  1.00  0.79
O
ATOM  10533  CB  ASN B  69     -17.105   2.657 -24.298  1.00  0.51
C
ATOM  10534  CG  ASN B  69     -17.558   3.157 -25.636  1.00  0.51
C
ATOM  10535  OD1 ASN B  69     -18.315   2.498 -26.355  1.00  0.51
O
ATOM  10536  ND2 ASN B  69     -17.088   4.318 -25.999  1.00  0.51
N
```

ATOM 10537 H ASN B 69 -19.363 3.228 -23.278 1.00 0.55
H
ATOM 10538 HA ASN B 69 -18.208 0.836 -24.427 1.00 0.41
H
ATOM 10539 1HB ASN B 69 -17.031 3.508 -23.620 1.00 0.61
H
ATOM 10540 2HB ASN B 69 -16.102 2.240 -24.401 1.00 0.61
H
ATOM 10541 1HD2 ASN B 69 -17.343 4.715 -26.884 1.00 0.61
H
ATOM 10542 2HD2 ASN B 69 -16.471 4.818 -25.391 1.00 0.61
H
ATOM 10543 N ASP B 70 -16.505 -0.069 -22.811 1.00 0.41
N
ATOM 10544 CA ASP B 70 -15.636 -0.616 -21.783 1.00 0.38
C
ATOM 10545 C ASP B 70 -14.584 0.459 -21.521 1.00 0.48
C
ATOM 10546 O ASP B 70 -14.213 1.202 -22.432 1.00 1.81
O
ATOM 10547 CB ASP B 70 -15.000 -1.950 -22.217 1.00 0.57
C
ATOM 10548 CG ASP B 70 -15.982 -3.126 -22.213 1.00 0.57
C
ATOM 10549 OD1 ASP B 70 -16.936 -3.080 -21.476 1.00 0.57
O
ATOM 10550 OD2 ASP B 70 -15.782 -4.057 -22.959 1.00 0.57
O
ATOM 10551 H ASP B 70 -16.461 -0.438 -23.750 1.00 0.49
H
ATOM 10552 HA ASP B 70 -16.207 -0.777 -20.869 1.00 0.46
H
ATOM 10553 1HB ASP B 70 -14.583 -1.844 -23.217 1.00 0.68
H
ATOM 10554 2HB ASP B 70 -14.174 -2.188 -21.544 1.00 0.68
H
ATOM 10555 N ARG B 71 -14.140 0.583 -20.282 1.00 0.15
N
ATOM 10556 CA ARG B 71 -13.177 1.617 -19.939 1.00 0.16
C
ATOM 10557 C ARG B 71 -11.762 1.074 -19.899 1.00 0.16
C
ATOM 10558 O ARG B 71 -11.542 -0.078 -19.530 1.00 0.29
O
ATOM 10559 CB ARG B 71 -13.512 2.230 -18.589 1.00 0.24
C
ATOM 10560 CG ARG B 71 -14.489 3.406 -18.586 1.00 0.24
C
ATOM 10561 CD ARG B 71 -15.858 3.030 -19.023 1.00 0.24
C
ATOM 10562 NE ARG B 71 -16.831 4.071 -18.700 1.00 0.24
N
ATOM 10563 CZ ARG B 71 -18.109 4.089 -19.135 1.00 0.24
C
ATOM 10564 NH1 ARG B 71 -18.551 3.169 -19.943 1.00 0.24
N
ATOM 10565 NH2 ARG B 71 -18.964 5.020 -18.762 1.00 0.24
N

```
ATOM  10566  H   ARG B  71     -14.456  -0.066 -19.576  1.00  0.18
H
ATOM  10567  HA  ARG B  71     -13.227   2.400 -20.696  1.00  0.19
H
ATOM  10568 1HB  ARG B  71     -13.939   1.460 -17.958  1.00  0.29
H
ATOM  10569 2HB  ARG B  71     -12.594   2.570 -18.110  1.00  0.29
H
ATOM  10570 1HG  ARG B  71     -14.565   3.809 -17.577  1.00  0.29
H
ATOM  10571 2HG  ARG B  71     -14.120   4.182 -19.256  1.00  0.29
H
ATOM  10572 1HD  ARG B  71     -15.863   2.883 -20.102  1.00  0.29
H
ATOM  10573 2HD  ARG B  71     -16.163   2.111 -18.527  1.00  0.29
H
ATOM  10574  HE  ARG B  71     -16.538   4.807 -18.072  1.00  0.29
H
ATOM  10575 1HH1 ARG B  71     -17.954   2.437 -20.283  1.00  0.29
H
ATOM  10576 2HH1 ARG B  71     -19.511   3.201 -20.244  1.00  0.29
H
ATOM  10577 1HH2 ARG B  71     -18.683   5.758 -18.132  1.00  0.29
H
ATOM  10578 2HH2 ARG B  71     -19.912   4.959 -19.122  1.00  0.29
H
ATOM  10579  N   PHE B  72     -10.806   1.916 -20.261  1.00  0.17
N
ATOM  10580  CA  PHE B  72      -9.403   1.547 -20.198  1.00  0.20
C
ATOM  10581  C   PHE B  72      -8.568   2.681 -19.671  1.00  0.16
C
ATOM  10582  O   PHE B  72      -8.493   3.747 -20.284  1.00  0.18
O
ATOM  10583  CB  PHE B  72      -8.829   1.141 -21.549  1.00  0.30
C
ATOM  10584  CG  PHE B  72      -7.373   0.764 -21.456  1.00  0.30
C
ATOM  10585  CD1 PHE B  72      -6.989  -0.491 -21.012  1.00  0.30
C
ATOM  10586  CD2 PHE B  72      -6.382   1.676 -21.793  1.00  0.30
C
ATOM  10587  CE1 PHE B  72      -5.652  -0.829 -20.910  1.00  0.30
C
ATOM  10588  CE2 PHE B  72      -5.046   1.340 -21.695  1.00  0.30
C
ATOM  10589  CZ  PHE B  72      -4.682   0.086 -21.253  1.00  0.30
C
ATOM  10590  H   PHE B  72     -11.056   2.844 -20.573  1.00  0.20
H
ATOM  10591  HA  PHE B  72      -9.301   0.703 -19.521  1.00  0.24
H
ATOM  10592 1HB  PHE B  72      -9.371   0.284 -21.932  1.00  0.36
H
ATOM  10593 2HB  PHE B  72      -8.933   1.955 -22.264  1.00  0.36
H
ATOM  10594  HD1 PHE B  72      -7.754  -1.214 -20.743  1.00  0.36
H
```

```
ATOM  10595  HD2 PHE B  72      -6.669   2.670 -22.138  1.00  0.36
H
ATOM  10596  HE1 PHE B  72      -5.366  -1.819 -20.560  1.00  0.36
H
ATOM  10597  HE2 PHE B  72      -4.280   2.062 -21.965  1.00  0.36
H
ATOM  10598  HZ  PHE B  72      -3.629  -0.179 -21.177  1.00  0.36
H
ATOM  10599  N   ILE B  73      -7.917   2.444 -18.546  1.00  0.17
N
ATOM  10600  CA  ILE B  73      -7.030   3.440 -17.999  1.00  0.16
C
ATOM  10601  C   ILE B  73      -5.604   2.992 -18.039  1.00  0.19
C
ATOM  10602  O   ILE B  73      -5.208   2.067 -17.330  1.00  0.35
O
ATOM  10603  CB  ILE B  73      -7.356   3.812 -16.545  1.00  0.24
C
ATOM  10604  CG1 ILE B  73      -8.745   4.408 -16.420  1.00  0.24
C
ATOM  10605  CG2 ILE B  73      -6.317   4.776 -15.998  1.00  0.24
C
ATOM  10606  CD1 ILE B  73      -9.099   4.666 -14.987  1.00  0.24
C
ATOM  10607  H   ILE B  73      -8.032   1.556 -18.080  1.00  0.20
H
ATOM  10608  HA  ILE B  73      -7.114   4.343 -18.602  1.00  0.19
H
ATOM  10609  HB  ILE B  73      -7.347   2.907 -15.940  1.00  0.29
H
ATOM  10610 1HG1 ILE B  73      -8.792   5.347 -16.969  1.00  0.29
H
ATOM  10611 2HG1 ILE B  73      -9.479   3.722 -16.844  1.00  0.29
H
ATOM  10612 1HG2 ILE B  73      -6.551   5.009 -14.960  1.00  0.29
H
ATOM  10613 2HG2 ILE B  73      -5.331   4.317 -16.052  1.00  0.29
H
ATOM  10614 3HG2 ILE B  73      -6.324   5.693 -16.587  1.00  0.29
H
ATOM  10615 1HD1 ILE B  73     -10.094   5.091 -14.912  1.00  0.29
H
ATOM  10616 2HD1 ILE B  73      -9.065   3.731 -14.443  1.00  0.29
H
ATOM  10617 3HD1 ILE B  73      -8.380   5.361 -14.555  1.00  0.29
H
ATOM  10618  N   LEU B  74      -4.819   3.691 -18.832  1.00  0.21
N
ATOM  10619  CA  LEU B  74      -3.400   3.475 -18.811  1.00  0.36
C
ATOM  10620  C   LEU B  74      -2.909   4.172 -17.568  1.00  0.29
C
ATOM  10621  O   LEU B  74      -2.866   5.400 -17.542  1.00  0.60
O
ATOM  10622  CB  LEU B  74      -2.741   4.066 -20.057  1.00  0.54
C
ATOM  10623  CG  LEU B  74      -1.226   3.922 -20.124  1.00  0.54
C
```

ATOM 10624 CD1 LEU B 74   -0.854   2.454 -20.166 1.00 0.54
C
ATOM 10625 CD2 LEU B 74   -0.703   4.661 -21.342 1.00 0.54
C
ATOM 10626 H   LEU B 74   -5.211   4.411 -19.423 1.00 0.25
H
ATOM 10627 HA LEU B 74   -3.186   2.410 -18.741 1.00 0.43
H
ATOM 10628 1HB LEU B 74   -3.158   3.585 -20.938 1.00 0.65
H
ATOM 10629 2HB LEU B 74   -2.976   5.129 -20.105 1.00 0.65
H
ATOM 10630 HG LEU B 74   -0.790   4.353 -19.227 1.00 0.65
H
ATOM 10631 1HD1 LEU B 74   0.231   2.355 -20.197 1.00 0.65
H
ATOM 10632 2HD1 LEU B 74   -1.238   1.956 -19.275 1.00 0.65
H
ATOM 10633 3HD1 LEU B 74   -1.287   1.996 -21.054 1.00 0.65
H
ATOM 10634 1HD2 LEU B 74   0.380   4.572 -21.379 1.00 0.65
H
ATOM 10635 2HD2 LEU B 74   -1.137   4.228 -22.241 1.00 0.65
H
ATOM 10636 3HD2 LEU B 74   -0.978   5.714 -21.275 1.00 0.65
H
ATOM 10637 N   SER B 75   -2.593   3.403 -16.530 1.00 0.35
N
ATOM 10638 CA SER B 75   -2.145   3.976 -15.271 1.00 0.29
C
ATOM 10639 C   SER B 75   -0.734   4.472 -15.463 1.00 0.33
C
ATOM 10640 O   SER B 75   -0.401   5.595 -15.088 1.00 0.35
O
ATOM 10641 CB SER B 75   -2.243   2.975 -14.152 1.00 0.43
C
ATOM 10642 OG SER B 75   -1.874   3.553 -12.932 1.00 0.43
O
ATOM 10643 H   SER B 75   -2.635   2.397 -16.625 1.00 0.42
H
ATOM 10644 HA SER B 75   -2.778   4.829 -15.025 1.00 0.35
H
ATOM 10645 1HB SER B 75   -3.264   2.608 -14.086 1.00 0.52
H
ATOM 10646 2HB SER B 75   -1.599   2.124 -14.362 1.00 0.52
H
ATOM 10647 HG SER B 75   -0.920   3.665 -12.961 1.00 0.52
H
ATOM 10648 N   ARG B 76   0.099   3.653 -16.098 1.00 0.58
N
ATOM 10649 CA ARG B 76   1.435   4.124 -16.439 1.00 0.92
C
ATOM 10650 C   ARG B 76   1.356   4.990 -17.688 1.00 0.59
C
ATOM 10651 O   ARG B 76   1.734   4.557 -18.782 1.00 1.13
O
ATOM 10652 CB ARG B 76   2.386   2.992 -16.702 1.00 1.38
C

ATOM 10653 CG ARG B 76 3.806 3.442 -16.991 1.00 1.38
C
ATOM 10654 CD ARG B 76 4.647 2.279 -17.307 1.00 1.38
C
ATOM 10655 NE ARG B 76 4.182 1.644 -18.524 1.00 1.38
N
ATOM 10656 CZ ARG B 76 4.357 0.348 -18.820 1.00 1.38
C
ATOM 10657 NH1 ARG B 76 4.989 -0.446 -17.996 1.00 1.38
N
ATOM 10658 NH2 ARG B 76 3.895 -0.164 -19.940 1.00 1.38
N
ATOM 10659 H ARG B 76 -0.209 2.717 -16.344 1.00 0.70
H
ATOM 10660 HA ARG B 76 1.817 4.725 -15.618 1.00 1.10
H
ATOM 10661 1HB ARG B 76 2.414 2.329 -15.838 1.00 1.66
H
ATOM 10662 2HB ARG B 76 2.038 2.409 -17.554 1.00 1.66
H
ATOM 10663 1HG ARG B 76 3.812 4.121 -17.845 1.00 1.66
H
ATOM 10664 2HG ARG B 76 4.219 3.946 -16.117 1.00 1.66
H
ATOM 10665 1HD ARG B 76 5.680 2.594 -17.455 1.00 1.66
H
ATOM 10666 2HD ARG B 76 4.596 1.553 -16.498 1.00 1.66
H
ATOM 10667 HE ARG B 76 3.684 2.216 -19.193 1.00 1.66
H
ATOM 10668 1HH1 ARG B 76 5.358 -0.096 -17.125 1.00 1.66
H
ATOM 10669 2HH1 ARG B 76 5.093 -1.414 -18.266 1.00 1.66
H
ATOM 10670 1HH2 ARG B 76 3.405 0.410 -20.610 1.00 1.66
H
ATOM 10671 2HH2 ARG B 76 4.050 -1.151 -20.111 1.00 1.66
H
ATOM 10672 N GLY B 77 0.892 6.231 -17.500 1.00 0.30
N
ATOM 10673 CA GLY B 77 0.631 7.201 -18.567 1.00 0.39
C
ATOM 10674 C GLY B 77 1.830 7.552 -19.430 1.00 0.39
C
ATOM 10675 O GLY B 77 1.679 8.122 -20.508 1.00 0.41
O
ATOM 10676 H GLY B 77 0.650 6.489 -16.549 1.00 0.36
H
ATOM 10677 1HA GLY B 77 -0.164 6.814 -19.203 1.00 0.47
H
ATOM 10678 2HA GLY B 77 0.240 8.114 -18.117 1.00 0.47
H
ATOM 10679 N HIS B 78 3.015 7.175 -18.994 1.00 0.44
N
ATOM 10680 CA HIS B 78 4.215 7.464 -19.729 1.00 0.54
C
ATOM 10681 C HIS B 78 4.257 6.718 -21.071 1.00 0.42
C

```
ATOM  10682  O   HIS B  78       4.983   7.135 -21.974  1.00  0.54
O
ATOM  10683  CB  HIS B  78       5.410   7.056 -18.912  1.00  0.81
C
ATOM  10684  CG  HIS B  78       5.598   7.765 -17.599  1.00  0.81
C
ATOM  10685  ND1 HIS B  78       5.897   9.113 -17.487  1.00  0.81
N
ATOM  10686  CD2 HIS B  78       5.557   7.283 -16.338  1.00  0.81
C
ATOM  10687  CE1 HIS B  78       6.032   9.418 -16.203  1.00  0.81
C
ATOM  10688  NE2 HIS B  78       5.826   8.329 -15.492  1.00  0.81
N
ATOM  10689  H   HIS B  78       3.101   6.705 -18.106  1.00  0.53
H
ATOM  10690  HA  HIS B  78       4.271   8.532 -19.931  1.00  0.65
H
ATOM  10691 1HB  HIS B  78       5.377   5.983 -18.722  1.00  0.97
H
ATOM  10692 2HB  HIS B  78       6.282   7.254 -19.517  1.00  0.97
H
ATOM  10693  HD2 HIS B  78       5.355   6.252 -16.049  1.00  0.97
H
ATOM  10694  HE1 HIS B  78       6.269  10.403 -15.801  1.00  0.97
H
ATOM  10695  HE2 HIS B  78       5.865   8.266 -14.484  1.00  0.97
H
ATOM  10696  N   ALA B  79       3.463   5.638 -21.193  1.00  0.37
N
ATOM  10697  CA  ALA B  79       3.352   4.848 -22.420  1.00  0.35
C
ATOM  10698  C   ALA B  79       2.269   5.401 -23.374  1.00  0.32
C
ATOM  10699  O   ALA B  79       1.868   4.717 -24.317  1.00  0.37
O
ATOM  10700  CB  ALA B  79       3.058   3.394 -22.074  1.00  0.52
C
ATOM  10701  H   ALA B  79       2.900   5.348 -20.402  1.00  0.44
H
ATOM  10702  HA  ALA B  79       4.308   4.899 -22.938  1.00  0.42
H
ATOM  10703 1HB  ALA B  79       3.013   2.804 -22.988  1.00  0.63
H
ATOM  10704 2HB  ALA B  79       3.851   3.008 -21.433  1.00  0.63
H
ATOM  10705 3HB  ALA B  79       2.107   3.324 -21.551  1.00  0.63
H
ATOM  10706  N   ALA B  80       1.821   6.642 -23.138  1.00  0.32
N
ATOM  10707  CA  ALA B  80       0.818   7.318 -23.961  1.00  0.33
C
ATOM  10708  C   ALA B  80       0.985   7.159 -25.487  1.00  0.36
C
ATOM  10709  O   ALA B  80      -0.026   7.108 -26.174  1.00  0.50
O
ATOM  10710  CB  ALA B  80       0.788   8.798 -23.616  1.00  0.49
C
```

```
ATOM  10711  H   ALA B  80       2.154   7.155 -22.335  1.00  0.38
H
ATOM  10712  HA  ALA B  80      -0.150   6.893 -23.699  1.00  0.40
H
ATOM  10713 1HB  ALA B  80      -0.009   9.282 -24.173  1.00  0.59
H
ATOM  10714 2HB  ALA B  80       0.600   8.908 -22.556  1.00  0.59
H
ATOM  10715 3HB  ALA B  80       1.731   9.274 -23.855  1.00  0.59
H
ATOM  10716  N   PRO B  81       2.198   7.108 -26.068  1.00  0.39
N
ATOM  10717  CA  PRO B  81       2.403   6.907 -27.500  1.00  0.45
C
ATOM  10718  C   PRO B  81       1.755   5.631 -28.037  1.00  0.43
C
ATOM  10719  O   PRO B  81       1.261   5.626 -29.165  1.00  0.46
O
ATOM  10720  CB  PRO B  81       3.919   6.894 -27.625  1.00  0.68
C
ATOM  10721  CG  PRO B  81       4.366   7.841 -26.564  1.00  0.68
C
ATOM  10722  CD  PRO B  81       3.415   7.666 -25.432  1.00  0.68
C
ATOM  10723  HA  PRO B  81       1.995   7.779 -28.031  1.00  0.54
H
ATOM  10724 1HB  PRO B  81       4.304   5.874 -27.481  1.00  0.81
H
ATOM  10725 2HB  PRO B  81       4.218   7.213 -28.636  1.00  0.81
H
ATOM  10726 1HG  PRO B  81       5.410   7.635 -26.285  1.00  0.81
H
ATOM  10727 2HG  PRO B  81       4.329   8.865 -26.939  1.00  0.81
H
ATOM  10728 1HD  PRO B  81       3.858   6.966 -24.717  1.00  0.81
H
ATOM  10729 2HD  PRO B  81       3.215   8.642 -24.981  1.00  0.81
H
ATOM  10730  N   ILE B  82       1.757   4.550 -27.248  1.00  0.42
N
ATOM  10731  CA  ILE B  82       1.162   3.308 -27.728  1.00  0.43
C
ATOM  10732  C   ILE B  82      -0.332   3.386 -27.588  1.00  0.39
C
ATOM  10733  O   ILE B  82      -1.081   2.795 -28.367  1.00  0.43
O
ATOM  10734  CB  ILE B  82       1.700   2.074 -26.988  1.00  0.65
C
ATOM  10735  CG1 ILE B  82       1.484   0.863 -27.870  1.00  0.65
C
ATOM  10736  CG2 ILE B  82       1.002   1.840 -25.653  1.00  0.65
C
ATOM  10737  CD1 ILE B  82       2.237  -0.348 -27.410  1.00  0.65
C
ATOM  10738  H   ILE B  82       2.143   4.595 -26.314  1.00  0.50
H
ATOM  10739  HA  ILE B  82       1.398   3.194 -28.786  1.00  0.52
H
```

```
ATOM  10740  HB  ILE B  82       2.765   2.191 -26.827  1.00  0.77
H
ATOM  10741 1HG1 ILE B  82       0.422   0.624 -27.899  1.00  0.77
H
ATOM  10742 2HG1 ILE B  82       1.809   1.110 -28.880  1.00  0.77
H
ATOM  10743 1HG2 ILE B  82       1.417   0.950 -25.182  1.00  0.77
H
ATOM  10744 2HG2 ILE B  82       1.150   2.688 -25.000  1.00  0.77
H
ATOM  10745 3HG2 ILE B  82      -0.065   1.694 -25.818  1.00  0.77
H
ATOM  10746 1HD1 ILE B  82       2.041  -1.165 -28.100  1.00  0.77
H
ATOM  10747 2HD1 ILE B  82       3.305  -0.128 -27.400  1.00  0.77
H
ATOM  10748 3HD1 ILE B  82       1.913  -0.626 -26.408  1.00  0.77
H
ATOM  10749  N   LEU B  83      -0.753   4.151 -26.598  1.00  0.37
N
ATOM  10750  CA  LEU B  83      -2.146   4.382 -26.354  1.00  0.50
C
ATOM  10751  C   LEU B  83      -2.726   5.131 -27.570  1.00  0.52
C
ATOM  10752  O   LEU B  83      -3.772   4.758 -28.109  1.00  0.69
O
ATOM  10753  CB  LEU B  83      -2.259   5.148 -25.036  1.00  0.75
C
ATOM  10754  CG  LEU B  83      -3.637   5.419 -24.546  1.00  0.75
C
ATOM  10755  CD1 LEU B  83      -4.341   4.090 -24.288  1.00  0.75
C
ATOM  10756  CD2 LEU B  83      -3.576   6.279 -23.292  1.00  0.75
C
ATOM  10757  H   LEU B  83      -0.067   4.571 -25.975  1.00  0.44
H
ATOM  10758  HA  LEU B  83      -2.655   3.423 -26.255  1.00  0.60
H
ATOM  10759 1HB  LEU B  83      -1.736   4.585 -24.266  1.00  0.90
H
ATOM  10760 2HB  LEU B  83      -1.778   6.111 -25.143  1.00  0.90
H
ATOM  10761  HG  LEU B  83      -4.159   5.944 -25.325  1.00  0.90
H
ATOM  10762 1HD1 LEU B  83      -5.361   4.275 -23.952  1.00  0.90
H
ATOM  10763 2HD1 LEU B  83      -4.364   3.507 -25.208  1.00  0.90
H
ATOM  10764 3HD1 LEU B  83      -3.801   3.540 -23.521  1.00  0.90
H
ATOM  10765 1HD2 LEU B  83      -4.588   6.491 -22.946  1.00  0.90
H
ATOM  10766 2HD2 LEU B  83      -3.033   5.749 -22.513  1.00  0.90
H
ATOM  10767 3HD2 LEU B  83      -3.066   7.215 -23.520  1.00  0.90
H
ATOM  10768  N   TYR B  84      -1.991   6.148 -28.026  1.00  0.45
N
```

```
ATOM  10769  CA  TYR B  84      -2.362   6.940 -29.185  1.00  0.54
C
ATOM  10770  C   TYR B  84      -2.351   6.091 -30.453  1.00  0.52
C
ATOM  10771  O   TYR B  84      -3.241   6.214 -31.298  1.00  0.58
O
ATOM  10772  CB  TYR B  84      -1.373   8.091 -29.343  1.00  0.81
C
ATOM  10773  CG  TYR B  84      -1.358   9.030 -28.168  1.00  0.81
C
ATOM  10774  CD1 TYR B  84      -2.406   9.022 -27.272  1.00  0.81
C
ATOM  10775  CD2 TYR B  84      -0.292   9.891 -27.981  1.00  0.81
C
ATOM  10776  CE1 TYR B  84      -2.404   9.879 -26.191  1.00  0.81
C
ATOM  10777  CE2 TYR B  84      -0.288  10.753 -26.903  1.00  0.81
C
ATOM  10778  CZ  TYR B  84      -1.342  10.752 -26.014  1.00  0.81
C
ATOM  10779  OH  TYR B  84      -1.342  11.613 -24.944  1.00  0.81
O
ATOM  10780  H   TYR B  84      -1.150   6.400 -27.523  1.00  0.54
H
ATOM  10781  HA  TYR B  84      -3.369   7.329 -29.042  1.00  0.65
H
ATOM  10782 1HB  TYR B  84      -0.366   7.694 -29.478  1.00  0.97
H
ATOM  10783 2HB  TYR B  84      -1.622   8.667 -30.235  1.00  0.97
H
ATOM  10784  HD1 TYR B  84      -3.232   8.334 -27.423  1.00  0.97
H
ATOM  10785  HD2 TYR B  84       0.536   9.893 -28.687  1.00  0.97
H
ATOM  10786  HE1 TYR B  84      -3.233   9.873 -25.486  1.00  0.97
H
ATOM  10787  HE2 TYR B  84       0.547  11.437 -26.755  1.00  0.97
H
ATOM  10788  HH  TYR B  84      -0.527  12.126 -24.947  1.00  0.97
H
ATOM  10789  N   ALA B  85      -1.353   5.207 -30.561  1.00  0.46
N
ATOM  10790  CA  ALA B  85      -1.227   4.318 -31.704  1.00  0.49
C
ATOM  10791  C   ALA B  85      -2.437   3.408 -31.810  1.00  0.50
C
ATOM  10792  O   ALA B  85      -2.941   3.186 -32.908  1.00  0.60
O
ATOM  10793  CB  ALA B  85       0.049   3.502 -31.599  1.00  0.73
C
ATOM  10794  H   ALA B  85      -0.633   5.171 -29.848  1.00  0.55
H
ATOM  10795  HA  ALA B  85      -1.186   4.926 -32.607  1.00  0.59
H
ATOM  10796 1HB  ALA B  85       0.142   2.860 -32.475  1.00  0.88
H
ATOM  10797 2HB  ALA B  85       0.904   4.175 -31.549  1.00  0.88
H
```

ATOM 10798 3HB ALA B 85 0.018 2.890 -30.701 1.00 0.88
H
ATOM 10799 N ALA B 86 -2.920 2.902 -30.671 1.00 0.45
N
ATOM 10800 CA ALA B 86 -4.107 2.058 -30.665 1.00 0.46
C
ATOM 10801 C ALA B 86 -5.304 2.829 -31.203 1.00 0.45
C
ATOM 10802 O ALA B 86 -6.068 2.301 -32.006 1.00 0.49
O
ATOM 10803 CB ALA B 86 -4.395 1.547 -29.263 1.00 0.69
C
ATOM 10804 H ALA B 86 -2.445 3.088 -29.797 1.00 0.54
H
ATOM 10805 HA ALA B 86 -3.927 1.209 -31.324 1.00 0.55
H
ATOM 10806 1HB ALA B 86 -5.273 0.902 -29.281 1.00 0.83
H
ATOM 10807 2HB ALA B 86 -3.537 0.982 -28.903 1.00 0.83
H
ATOM 10808 3HB ALA B 86 -4.579 2.389 -28.597 1.00 0.83
H
ATOM 10809 N TRP B 87 -5.433 4.093 -30.789 1.00 0.47
N
ATOM 10810 CA TRP B 87 -6.529 4.964 -31.215 1.00 0.58
C
ATOM 10811 C TRP B 87 -6.499 5.240 -32.725 1.00 0.93
C
ATOM 10812 O TRP B 87 -7.546 5.441 -33.355 1.00 1.52
O
ATOM 10813 CB TRP B 87 -6.463 6.236 -30.412 1.00 0.87
C
ATOM 10814 CG TRP B 87 -6.633 5.912 -28.973 1.00 0.87
C
ATOM 10815 CD1 TRP B 87 -7.059 4.716 -28.478 1.00 0.87
C
ATOM 10816 CD2 TRP B 87 -6.381 6.754 -27.831 1.00 0.87
C
ATOM 10817 NE1 TRP B 87 -7.095 4.757 -27.112 1.00 0.87
N
ATOM 10818 CE2 TRP B 87 -6.688 5.996 -26.697 1.00 0.87
C
ATOM 10819 CE3 TRP B 87 -5.936 8.069 -27.679 1.00 0.87
C
ATOM 10820 CZ2 TRP B 87 -6.569 6.510 -25.424 1.00 0.87
C
ATOM 10821 CZ3 TRP B 87 -5.804 8.578 -26.395 1.00 0.87
C
ATOM 10822 CH2 TRP B 87 -6.112 7.817 -25.302 1.00 0.87
C
ATOM 10823 H TRP B 87 -4.767 4.454 -30.112 1.00 0.56
H
ATOM 10824 HA TRP B 87 -7.466 4.467 -30.975 1.00 0.70
H
ATOM 10825 1HB TRP B 87 -5.510 6.740 -30.560 1.00 1.04
H
ATOM 10826 2HB TRP B 87 -7.259 6.913 -30.714 1.00 1.04
H

ATOM 10827 HD1 TRP B 87 -7.332 3.853 -29.082 1.00 1.04
H
ATOM 10828 HE1 TRP B 87 -7.378 4.001 -26.507 1.00 1.04
H
ATOM 10829 HE3 TRP B 87 -5.693 8.678 -28.549 1.00 1.04
H
ATOM 10830 HZ2 TRP B 87 -6.808 5.925 -24.536 1.00 1.04
H
ATOM 10831 HZ3 TRP B 87 -5.452 9.600 -26.277 1.00 1.04
H
ATOM 10832 HH2 TRP B 87 -5.996 8.237 -24.309 1.00 1.04
H
ATOM 10833 N VAL B 88 -5.304 5.197 -33.300 1.00 0.68
N
ATOM 10834 CA VAL B 88 -5.169 5.237 -34.741 1.00 0.72
C
ATOM 10835 C VAL B 88 -5.635 3.944 -35.361 1.00 0.68
C
ATOM 10836 O VAL B 88 -6.416 3.945 -36.311 1.00 0.75
O
ATOM 10837 CB VAL B 88 -3.723 5.467 -35.166 1.00 1.08
C
ATOM 10838 CG1 VAL B 88 -3.603 5.276 -36.662 1.00 1.08
C
ATOM 10839 CG2 VAL B 88 -3.307 6.863 -34.790 1.00 1.08
C
ATOM 10840 H VAL B 88 -4.474 5.123 -32.723 1.00 0.82
H
ATOM 10841 HA VAL B 88 -5.784 6.046 -35.118 1.00 0.86
H
ATOM 10842 HB VAL B 88 -3.075 4.741 -34.685 1.00 1.30
H
ATOM 10843 1HG1 VAL B 88 -2.569 5.425 -36.963 1.00 1.30
H
ATOM 10844 2HG1 VAL B 88 -3.909 4.263 -36.923 1.00 1.30
H
ATOM 10845 3HG1 VAL B 88 -4.243 5.992 -37.176 1.00 1.30
H
ATOM 10846 1HG2 VAL B 88 -2.290 7.021 -35.109 1.00 1.30
H
ATOM 10847 2HG2 VAL B 88 -3.954 7.581 -35.288 1.00 1.30
H
ATOM 10848 3HG2 VAL B 88 -3.382 6.994 -33.711 1.00 1.30
H
ATOM 10849 N GLU B 89 -5.159 2.834 -34.809 1.00 0.62
N
ATOM 10850 CA GLU B 89 -5.457 1.506 -35.317 1.00 0.60
C
ATOM 10851 C GLU B 89 -6.950 1.156 -35.247 1.00 0.52
C
ATOM 10852 O GLU B 89 -7.443 0.413 -36.098 1.00 0.57
O
ATOM 10853 CB GLU B 89 -4.607 0.488 -34.558 1.00 0.90
C
ATOM 10854 CG GLU B 89 -3.117 0.602 -34.850 1.00 0.90
C
ATOM 10855 CD GLU B 89 -2.742 0.214 -36.251 1.00 0.90
C

ATOM 10856 OE1 GLU B 89   -2.729 -0.955 -36.540 1.00 0.90
O
ATOM 10857 OE2 GLU B 89   -2.500   1.083 -37.053 1.00 0.90
O
ATOM 10858 H   GLU B 89   -4.529   2.913 -34.020 1.00 0.74
H
ATOM 10859 HA GLU B 89   -5.161   1.475 -36.367 1.00 0.72
H
ATOM 10860 1HB GLU B 89   -4.742   0.625 -33.486 1.00 1.08
H
ATOM 10861 2HB GLU B 89   -4.926 -0.523 -34.810 1.00 1.08
H
ATOM 10862 1HG GLU B 89   -2.806   1.630 -34.690 1.00 1.08
H
ATOM 10863 2HG GLU B 89   -2.572 -0.026 -34.146 1.00 1.08
H
ATOM 10864 N   VAL B 90   -7.689   1.717 -34.272 1.00 0.58
N
ATOM 10865 CA VAL B 90   -9.139   1.482 -34.210 1.00 0.71
C
ATOM 10866 C   VAL B 90   -9.891   2.313 -35.261 1.00 0.71
C
ATOM 10867 O   VAL B 90   -11.084   2.103 -35.468 1.00 1.62
O
ATOM 10868 CB VAL B 90   -9.750   1.820 -32.821 1.00 1.06
C
ATOM 10869 CG1 VAL B 90   -9.095   0.999 -31.726 1.00 1.06
C
ATOM 10870 CG2 VAL B 90   -9.622   3.290 -32.536 1.00 1.06
C
ATOM 10871 H   VAL B 90   -7.242   2.288 -33.567 1.00 0.70
H
ATOM 10872 HA VAL B 90   -9.323   0.427 -34.412 1.00 0.85
H
ATOM 10873 HB VAL B 90   -10.806   1.559 -32.835 1.00 1.28
H
ATOM 10874 1HG1 VAL B 90   -9.559   1.243 -30.770 1.00 1.28
H
ATOM 10875 2HG1 VAL B 90   -9.225 -0.060 -31.931 1.00 1.28
H
ATOM 10876 3HG1 VAL B 90   -8.038   1.224 -31.675 1.00 1.28
H
ATOM 10877 1HG2 VAL B 90   -10.074   3.519 -31.573 1.00 1.28
H
ATOM 10878 2HG2 VAL B 90   -8.571   3.531 -32.513 1.00 1.28
H
ATOM 10879 3HG2 VAL B 90   -10.120   3.864 -33.318 1.00 1.28
H
ATOM 10880 N   GLY B 91   -9.203   3.247 -35.926 1.00 0.50
N
ATOM 10881 CA GLY B 91   -9.820   4.056 -36.962 1.00 0.64
C
ATOM 10882 C   GLY B 91   -10.424   5.377 -36.486 1.00 0.63
C
ATOM 10883 O   GLY B 91   -11.407   5.838 -37.067 1.00 1.22
O
ATOM 10884 H   GLY B 91   -8.223   3.397 -35.748 1.00 0.60
H

```
ATOM  10885 1HA  GLY B  91      -9.074   4.266 -37.729  1.00  0.77
H
ATOM  10886 2HA  GLY B  91     -10.597   3.468 -37.450  1.00  0.77
H
ATOM  10887  N   ASP B  92      -9.848   6.010 -35.465  1.00  0.76
N
ATOM  10888  CA  ASP B  92     -10.394   7.299 -35.050  1.00  1.17
C
ATOM  10889  C   ASP B  92      -9.361   8.400 -35.207  1.00  0.84
C
ATOM  10890  O   ASP B  92      -9.682   9.499 -35.663  1.00  0.79
O
ATOM  10891  CB  ASP B  92     -10.894   7.280 -33.618  1.00  1.75
C
ATOM  10892  CG  ASP B  92     -11.698   8.540 -33.307  1.00  1.75
C
ATOM  10893  OD1 ASP B  92     -12.731   8.716 -33.909  1.00  1.75
O
ATOM  10894  OD2 ASP B  92     -11.266   9.327 -32.501  1.00  1.75
O
ATOM  10895  H   ASP B  92      -9.046   5.625 -34.977  1.00  0.91
H
ATOM  10896  HA  ASP B  92     -11.239   7.542 -35.693  1.00  1.40
H
ATOM  10897 1HB  ASP B  92     -11.520   6.403 -33.455  1.00  2.11
H
ATOM  10898 2HB  ASP B  92     -10.046   7.220 -32.936  1.00  2.11
H
ATOM  10899  N   ILE B  93      -8.124   8.112 -34.827  1.00  0.88
N
ATOM  10900  CA  ILE B  93      -7.051   9.084 -34.954  1.00  0.93
C
ATOM  10901  C   ILE B  93      -6.311   8.864 -36.273  1.00  0.96
C
ATOM  10902  O   ILE B  93      -6.048   7.727 -36.656  1.00  0.90
O
ATOM  10903  CB  ILE B  93      -6.084   8.983 -33.756  1.00  1.40
C
ATOM  10904  CG1 ILE B  93      -6.866   9.159 -32.449  1.00  1.40
C
ATOM  10905  CG2 ILE B  93      -4.975  10.023 -33.861  1.00  1.40
C
ATOM  10906  CD1 ILE B  93      -7.597  10.474 -32.339  1.00  1.40
C
ATOM  10907  H   ILE B  93      -7.915   7.198 -34.441  1.00  1.06
H
ATOM  10908  HA  ILE B  93      -7.482  10.084 -34.965  1.00  1.12
H
ATOM  10909  HB  ILE B  93      -5.647   7.990 -33.734  1.00  1.67
H
ATOM  10910 1HG1 ILE B  93      -7.597   8.355 -32.367  1.00  1.67
H
ATOM  10911 2HG1 ILE B  93      -6.174   9.081 -31.611  1.00  1.67
H
ATOM  10912 1HG2 ILE B  93      -4.305   9.927 -33.007  1.00  1.67
H
ATOM  10913 2HG2 ILE B  93      -4.413   9.867 -34.780  1.00  1.67
H
```

```
ATOM  10914 3HG2 ILE B  93      -5.410  11.022 -33.868  1.00  1.67
H
ATOM  10915 1HD1 ILE B  93      -8.125  10.518 -31.386  1.00  1.67
H
ATOM  10916 2HD1 ILE B  93      -6.881  11.295 -32.396  1.00  1.67
H
ATOM  10917 3HD1 ILE B  93      -8.314  10.562 -33.153  1.00  1.67
H
ATOM  10918  N   SER B  94      -6.017   9.939 -37.000  1.00  1.18
N
ATOM  10919  CA  SER B  94      -5.276   9.782 -38.247  1.00  1.38
C
ATOM  10920  C   SER B  94      -3.858   9.333 -37.941  1.00  1.30
C
ATOM  10921  O   SER B  94      -3.218   9.876 -37.047  1.00  1.32
O
ATOM  10922  CB  SER B  94      -5.243  11.075 -39.033  1.00  2.07
C
ATOM  10923  OG  SER B  94      -4.456  10.932 -40.184  1.00  2.07
O
ATOM  10924  H   SER B  94      -6.273  10.862 -36.674  1.00  1.42
H
ATOM  10925  HA  SER B  94      -5.761   9.013 -38.850  1.00  1.66
H
ATOM  10926 1HB  SER B  94      -6.256  11.361 -39.312  1.00  2.48
H
ATOM  10927 2HB  SER B  94      -4.840  11.870 -38.409  1.00  2.48
H
ATOM  10928  HG  SER B  94      -4.385  11.812 -40.566  1.00  2.48
H
ATOM  10929  N   GLU B  95      -3.362   8.351 -38.687  1.00  1.38
N
ATOM  10930  CA  GLU B  95      -2.017   7.809 -38.465  1.00  1.63
C
ATOM  10931  C   GLU B  95      -0.944   8.884 -38.488  1.00  1.63
C
ATOM  10932  O   GLU B  95      -0.010   8.851 -37.687  1.00  1.87
O
ATOM  10933  CB  GLU B  95      -1.695   6.727 -39.496  1.00  2.44
C
ATOM  10934  CG  GLU B  95      -0.388   5.984 -39.238  1.00  2.44
C
ATOM  10935  CD  GLU B  95      -0.138   4.873 -40.228  1.00  2.44
C
ATOM  10936  OE1 GLU B  95      -0.790   4.861 -41.244  1.00  2.44
O
ATOM  10937  OE2 GLU B  95       0.675   4.017 -39.954  1.00  2.44
O
ATOM  10938  H   GLU B  95      -3.936   7.955 -39.419  1.00  1.66
H
ATOM  10939  HA  GLU B  95      -1.992   7.360 -37.475  1.00  1.96
H
ATOM  10940 1HB  GLU B  95      -2.502   5.994 -39.518  1.00  2.93
H
ATOM  10941 2HB  GLU B  95      -1.635   7.175 -40.488  1.00  2.93
H
ATOM  10942 1HG  GLU B  95       0.437   6.693 -39.290  1.00  2.93
H
```

```
ATOM  10943 2HG  GLU B  95      -0.412   5.568 -38.230  1.00  2.93
H
ATOM  10944  N   SER B  96      -1.077   9.847 -39.401  1.00  1.53
N
ATOM  10945  CA  SER B  96      -0.109  10.930 -39.530  1.00  1.78
C
ATOM  10946  C   SER B  96      -0.005  11.795 -38.270  1.00  1.75
C
ATOM  10947  O   SER B  96       1.035  12.408 -38.029  1.00  2.49
O
ATOM  10948  CB  SER B  96      -0.475  11.809 -40.709  1.00  2.67
C
ATOM  10949  OG  SER B  96      -1.677  12.495 -40.480  1.00  2.67
O
ATOM  10950  H   SER B  96      -1.868   9.824 -40.029  1.00  1.84
H
ATOM  10951  HA  SER B  96       0.870  10.488 -39.717  1.00  2.14
H
ATOM  10952 1HB  SER B  96       0.326  12.525 -40.890  1.00  3.20
H
ATOM  10953 2HB  SER B  96      -0.571  11.193 -41.603  1.00  3.20
H
ATOM  10954  HG  SER B  96      -1.547  12.999 -39.672  1.00  3.20
H
ATOM  10955  N   ASP B  97      -1.060  11.817 -37.450  1.00  1.42
N
ATOM  10956  CA  ASP B  97      -1.064  12.611 -36.230  1.00  1.47
C
ATOM  10957  C   ASP B  97      -0.090  12.065 -35.199  1.00  1.43
C
ATOM  10958  O   ASP B  97       0.345  12.801 -34.316  1.00  1.24
O
ATOM  10959  CB  ASP B  97      -2.465  12.666 -35.621  1.00  2.21
C
ATOM  10960  CG  ASP B  97      -3.434  13.491 -36.453  1.00  2.21
C
ATOM  10961  OD1 ASP B  97      -2.984  14.237 -37.290  1.00  2.21
O
ATOM  10962  OD2 ASP B  97      -4.618  13.362 -36.248  1.00  2.21
O
ATOM  10963  H   ASP B  97      -1.881  11.269 -37.656  1.00  1.70
H
ATOM  10964  HA  ASP B  97      -0.757  13.626 -36.482  1.00  1.76
H
ATOM  10965 1HB  ASP B  97      -2.860  11.659 -35.507  1.00  2.65
H
ATOM  10966 2HB  ASP B  97      -2.406  13.106 -34.625  1.00  2.65
H
ATOM  10967  N   LEU B  98       0.285  10.788 -35.322  1.00  2.03
N
ATOM  10968  CA  LEU B  98       1.208  10.171 -34.381  1.00  2.12
C
ATOM  10969  C   LEU B  98       2.582  10.789 -34.427  1.00  2.70
C
ATOM  10970  O   LEU B  98       3.326  10.717 -33.453  1.00  4.40
O
ATOM  10971  CB  LEU B  98       1.358   8.686 -34.665  1.00  3.18
C
```

```
ATOM  10972  CG  LEU B  98       0.125   7.850 -34.397  1.00  3.18
C
ATOM  10973  CD1 LEU B  98       0.383   6.430 -34.864  1.00  3.18
C
ATOM  10974  CD2 LEU B  98      -0.200   7.892 -32.915  1.00  3.18
C
ATOM  10975  H   LEU B  98      -0.064  10.217 -36.081  1.00  2.44
H
ATOM  10976  HA  LEU B  98       0.810  10.301 -33.375  1.00  2.54
H
ATOM  10977 1HB  LEU B  98       1.627   8.556 -35.712  1.00  3.82
H
ATOM  10978 2HB  LEU B  98       2.167   8.291 -34.051  1.00  3.82
H
ATOM  10979  HG  LEU B  98      -0.710   8.256 -34.966  1.00  3.82
H
ATOM  10980 1HD1 LEU B  98      -0.498   5.816 -34.686  1.00  3.82
H
ATOM  10981 2HD1 LEU B  98       0.614   6.433 -35.929  1.00  3.82
H
ATOM  10982 3HD1 LEU B  98       1.225   6.024 -34.310  1.00  3.82
H
ATOM  10983 1HD2 LEU B  98      -1.091   7.295 -32.723  1.00  3.82
H
ATOM  10984 2HD2 LEU B  98       0.639   7.487 -32.347  1.00  3.82
H
ATOM  10985 3HD2 LEU B  98      -0.381   8.923 -32.610  1.00  3.82
H
ATOM  10986  N   LEU B  99       2.935  11.403 -35.553  1.00  2.64
N
ATOM  10987  CA  LEU B  99       4.247  12.001 -35.680  1.00  3.10
C
ATOM  10988  C   LEU B  99       4.283  13.407 -35.096  1.00  2.87
C
ATOM  10989  O   LEU B  99       5.332  14.051 -35.094  1.00  3.58
O
ATOM  10990  CB  LEU B  99       4.666  12.021 -37.147  1.00  4.65
C
ATOM  10991  CG  LEU B  99       4.838  10.636 -37.782  1.00  4.65
C
ATOM  10992  CD1 LEU B  99       5.155  10.796 -39.261  1.00  4.65
C
ATOM  10993  CD2 LEU B  99       5.945   9.885 -37.052  1.00  4.65
C
ATOM  10994  H   LEU B  99       2.294  11.476 -36.337  1.00  3.17
H
ATOM  10995  HA  LEU B  99       4.958  11.387 -35.130  1.00  3.72
H
ATOM  10996 1HB  LEU B  99       3.912  12.559 -37.719  1.00  5.58
H
ATOM  10997 2HB  LEU B  99       5.614  12.551 -37.236  1.00  5.58
H
ATOM  10998  HG  LEU B  99       3.905  10.077 -37.697  1.00  5.58
H
ATOM  10999 1HD1 LEU B  99       5.267   9.814 -39.719  1.00  5.58
H
ATOM  11000 2HD1 LEU B  99       4.341  11.333 -39.751  1.00  5.58
H
```

```
ATOM  11001 3HD1 LEU B  99       6.081  11.358 -39.376  1.00  5.58
H
ATOM  11002 1HD2 LEU B  99       6.067   8.898 -37.497  1.00  5.58
H
ATOM  11003 2HD2 LEU B  99       6.880  10.440 -37.139  1.00  5.58
H
ATOM  11004 3HD2 LEU B  99       5.682   9.779 -36.000  1.00  5.58
H
ATOM  11005  N   ASN B 100       3.138  13.883 -34.594  1.00  2.31
N
ATOM  11006  CA  ASN B 100       3.044  15.192 -33.980  1.00  2.33
C
ATOM  11007  C   ASN B 100       3.048  15.090 -32.447  1.00  1.82
C
ATOM  11008  O   ASN B 100       2.627  16.018 -31.753  1.00  2.27
O
ATOM  11009  CB  ASN B 100       1.815  15.912 -34.483  1.00  3.50
C
ATOM  11010  CG  ASN B 100       1.890  16.230 -35.956  1.00  3.50
C
ATOM  11011  OD1 ASN B 100       2.936  16.622 -36.497  1.00  3.50
O
ATOM  11012  ND2 ASN B 100       0.778  16.065 -36.627  1.00  3.50
N
ATOM  11013  H   ASN B 100       2.289  13.335 -34.618  1.00  2.77
H
ATOM  11014  HA  ASN B 100       3.921  15.763 -34.266  1.00  2.80
H
ATOM  11015 1HB  ASN B 100       0.930  15.302 -34.298  1.00  4.19
H
ATOM  11016 2HB  ASN B 100       1.693  16.844 -33.933  1.00  4.19
H
ATOM  11017 1HD2 ASN B 100       0.751  16.256 -37.609  1.00  4.19
H
ATOM  11018 2HD2 ASN B 100      -0.047  15.748 -36.157  1.00  4.19
H
ATOM  11019  N   LEU B 101       3.516  13.955 -31.940  1.00  1.90
N
ATOM  11020  CA  LEU B 101       3.604  13.689 -30.519  1.00  1.56
C
ATOM  11021  C   LEU B 101       4.432  14.758 -29.801  1.00  2.19
C
ATOM  11022  O   LEU B 101       5.564  15.057 -30.187  1.00  3.03
O
ATOM  11023  CB  LEU B 101       4.208  12.287 -30.347  1.00  2.34
C
ATOM  11024  CG  LEU B 101       4.424  11.767 -28.930  1.00  2.34
C
ATOM  11025  CD1 LEU B 101       3.099  11.551 -28.225  1.00  2.34
C
ATOM  11026  CD2 LEU B 101       5.210  10.479 -29.012  1.00  2.34
C
ATOM  11027  H   LEU B 101       3.840  13.232 -32.564  1.00  2.28
H
ATOM  11028  HA  LEU B 101       2.597  13.693 -30.104  1.00  1.87
H
ATOM  11029 1HB  LEU B 101       3.550  11.579 -30.849  1.00  2.81
H
```

```
ATOM  11030 2HB  LEU B 101       5.169  12.262 -30.858  1.00  2.81
H
ATOM  11031  HG  LEU B 101       5.000  12.494 -28.373  1.00  2.81
H
ATOM  11032 1HD1 LEU B 101       3.279  11.183 -27.215  1.00  2.81
H
ATOM  11033 2HD1 LEU B 101       2.558  12.492 -28.176  1.00  2.81
H
ATOM  11034 3HD1 LEU B 101       2.512  10.821 -28.778  1.00  2.81
H
ATOM  11035 1HD2 LEU B 101       5.408  10.113 -28.011  1.00  2.81
H
ATOM  11036 2HD2 LEU B 101       4.637   9.737 -29.564  1.00  2.81
H
ATOM  11037 3HD2 LEU B 101       6.155  10.666 -29.518  1.00  2.81
H
ATOM  11038  N   ARG B 102       3.836  15.336 -28.756  1.00  2.39
N
ATOM  11039  CA  ARG B 102       4.432  16.385 -27.919  1.00  3.37
C
ATOM  11040  C   ARG B 102       4.664  17.713 -28.622  1.00  3.65
C
ATOM  11041  O   ARG B 102       5.394  18.561 -28.106  1.00  9.34
O
ATOM  11042  CB  ARG B 102       5.734  15.916 -27.331  1.00  5.05
C
ATOM  11043  CG  ARG B 102       5.521  14.724 -26.466  1.00  5.05
C
ATOM  11044  CD  ARG B 102       6.725  14.253 -25.793  1.00  5.05
C
ATOM  11045  NE  ARG B 102       6.408  12.992 -25.197  1.00  5.05
N
ATOM  11046  CZ  ARG B 102       6.638  11.806 -25.773  1.00  5.05
C
ATOM  11047  NH1 ARG B 102       7.277  11.778 -26.911  1.00  5.05
N
ATOM  11048  NH2 ARG B 102       6.228  10.683 -25.208  1.00  5.05
N
ATOM  11049  H   ARG B 102       2.904  15.022 -28.511  1.00  2.87
H
ATOM  11050  HA  ARG B 102       3.749  16.568 -27.090  1.00  4.04
H
ATOM  11051 1HB  ARG B 102       6.452  15.663 -28.107  1.00  6.07
H
ATOM  11052 2HB  ARG B 102       6.172  16.704 -26.719  1.00  6.07
H
ATOM  11053 1HG  ARG B 102       4.799  14.986 -25.693  1.00  6.07
H
ATOM  11054 2HG  ARG B 102       5.118  13.911 -27.055  1.00  6.07
H
ATOM  11055 1HD  ARG B 102       7.540  14.113 -26.503  1.00  6.07
H
ATOM  11056 2HD  ARG B 102       7.023  14.946 -25.007  1.00  6.07
H
ATOM  11057  HE  ARG B 102       5.896  13.009 -24.341  1.00  6.07
H
ATOM  11058 1HH1 ARG B 102       7.594  12.639 -27.334  1.00  6.07
H
```

```
ATOM  11059 2HH1 ARG B 102       7.476  10.898 -27.363  1.00  6.07
H
ATOM  11060 1HH2 ARG B 102       5.725  10.709 -24.336  1.00  6.07
H
ATOM  11061 2HH2 ARG B 102       6.409   9.798 -25.660  1.00  6.07
H
ATOM  11062  N   LYS B 103       4.051  17.916 -29.785  1.00  2.34
N
ATOM  11063  CA  LYS B 103       4.186  19.199 -30.443  1.00  2.61
C
ATOM  11064  C   LYS B 103       3.145  20.174 -29.930  1.00  2.93
C
ATOM  11065  O   LYS B 103       2.027  19.789 -29.588  1.00  5.60
O
ATOM  11066  CB  LYS B 103       4.047  19.081 -31.956  1.00  3.92
C
ATOM  11067  CG  LYS B 103       5.156  18.314 -32.650  1.00  3.92
C
ATOM  11068  CD  LYS B 103       4.941  18.354 -34.151  1.00  3.92
C
ATOM  11069  CE  LYS B 103       5.979  17.540 -34.901  1.00  3.92
C
ATOM  11070  NZ  LYS B 103       5.672  17.505 -36.354  1.00  3.92
N
ATOM  11071  H   LYS B 103       3.484  17.197 -30.219  1.00  2.81
H
ATOM  11072  HA  LYS B 103       5.170  19.607 -30.215  1.00  3.13
H
ATOM  11073 1HB  LYS B 103       3.106  18.586 -32.195  1.00  4.70
H
ATOM  11074 2HB  LYS B 103       4.012  20.078 -32.396  1.00  4.70
H
ATOM  11075 1HG  LYS B 103       6.121  18.761 -32.413  1.00  4.70
H
ATOM  11076 2HG  LYS B 103       5.158  17.278 -32.312  1.00  4.70
H
ATOM  11077 1HD  LYS B 103       3.949  17.973 -34.389  1.00  4.70
H
ATOM  11078 2HD  LYS B 103       5.000  19.387 -34.492  1.00  4.70
H
ATOM  11079 1HE  LYS B 103       6.967  17.975 -34.756  1.00  4.70
H
ATOM  11080 2HE  LYS B 103       5.984  16.521 -34.516  1.00  4.70
H
ATOM  11081 1HZ  LYS B 103       6.361  16.948 -36.838  1.00  4.70
H
ATOM  11082 2HZ  LYS B 103       4.750  17.095 -36.473  1.00  4.70
H
ATOM  11083 3HZ  LYS B 103       5.673  18.443 -36.725  1.00  4.70
H
ATOM  11084  N   LEU B 104       3.529  21.443 -29.909  1.00  3.54
N
ATOM  11085  CA  LEU B 104       2.691  22.548 -29.463  1.00  5.78
C
ATOM  11086  C   LEU B 104       1.316  22.589 -30.124  1.00  7.11
C
ATOM  11087  O   LEU B 104       0.324  22.948 -29.488  1.00 11.20
O
```

ATOM 11088 CB LEU B 104 3.410 23.870 -29.751 1.00 8.67
C
ATOM 11089 CG LEU B 104 2.682 25.129 -29.287 1.00 8.67
C
ATOM 11090 CD1 LEU B 104 2.530 25.076 -27.778 1.00 8.67
C
ATOM 11091 CD2 LEU B 104 3.466 26.358 -29.719 1.00 8.67
C
ATOM 11092 H LEU B 104 4.468 21.658 -30.211 1.00 4.25
H
ATOM 11093 HA LEU B 104 2.549 22.450 -28.387 1.00 6.94
H
ATOM 11094 1HB LEU B 104 4.382 23.852 -29.259 1.00 10.40
H
ATOM 11095 2HB LEU B 104 3.571 23.955 -30.825 1.00 10.40
H
ATOM 11096 HG LEU B 104 1.685 25.161 -29.728 1.00 10.40
H
ATOM 11097 1HD1 LEU B 104 2.003 25.967 -27.441 1.00 10.40
H
ATOM 11098 2HD1 LEU B 104 1.962 24.189 -27.499 1.00 10.40
H
ATOM 11099 3HD1 LEU B 104 3.516 25.038 -27.315 1.00 10.40
H
ATOM 11100 1HD2 LEU B 104 2.947 27.257 -29.386 1.00 10.40
H
ATOM 11101 2HD2 LEU B 104 4.461 26.328 -29.274 1.00 10.40
H
ATOM 11102 3HD2 LEU B 104 3.554 26.370 -30.805 1.00 10.40
H
ATOM 11103 N HIS B 105 1.261 22.245 -31.407 1.00 6.57
N
ATOM 11104 CA HIS B 105 0.018 22.327 -32.151 1.00 9.73
C
ATOM 11105 C HIS B 105 -0.723 20.993 -32.265 1.00 6.52
C
ATOM 11106 O HIS B 105 -1.578 20.830 -33.137 1.00 6.71
O
ATOM 11107 CB HIS B 105 0.308 22.884 -33.539 1.00 14.60
C
ATOM 11108 CG HIS B 105 0.863 24.273 -33.484 1.00 14.60
C
ATOM 11109 ND1 HIS B 105 0.099 25.366 -33.136 1.00 14.60
N
ATOM 11110 CD2 HIS B 105 2.109 24.744 -33.721 1.00 14.60
C
ATOM 11111 CE1 HIS B 105 0.852 26.452 -33.164 1.00 14.60
C
ATOM 11112 NE2 HIS B 105 2.075 26.102 -33.516 1.00 14.60
N
ATOM 11113 H HIS B 105 2.100 21.945 -31.881 1.00 7.88
H
ATOM 11114 HA HIS B 105 -0.652 23.024 -31.648 1.00 11.68
H
ATOM 11115 1HB HIS B 105 1.027 22.243 -34.052 1.00 17.51
H
ATOM 11116 2HB HIS B 105 -0.606 22.899 -34.130 1.00 17.51
H

```
ATOM  11117  HD2 HIS B 105       2.979  24.157 -34.016  1.00 17.51
H
ATOM  11118  HE1 HIS B 105       0.520  27.464 -32.935  1.00 17.51
H
ATOM  11119  HE2 HIS B 105       2.862  26.726 -33.619  1.00 17.51
H
ATOM  11120  N   SER B 106      -0.418  20.055 -31.370  1.00  5.11
N
ATOM  11121  CA  SER B 106      -1.089  18.764 -31.341  1.00  4.10
C
ATOM  11122  C   SER B 106      -1.754  18.525 -30.002  1.00  3.75
C
ATOM  11123  O   SER B 106      -1.400  19.142 -28.999  1.00 10.51
O
ATOM  11124  CB  SER B 106      -0.123  17.638 -31.608  1.00  6.15
C
ATOM  11125  OG  SER B 106      -0.774  16.396 -31.523  1.00  6.15
O
ATOM  11126  H   SER B 106       0.307  20.218 -30.684  1.00  6.13
H
ATOM  11127  HA  SER B 106      -1.860  18.756 -32.112  1.00  4.92
H
ATOM  11128 1HB  SER B 106       0.305  17.762 -32.601  1.00  7.38
H
ATOM  11129 2HB  SER B 106       0.695  17.678 -30.890  1.00  7.38
H
ATOM  11130  HG  SER B 106      -0.110  15.739 -31.747  1.00  7.38
H
ATOM  11131  N   ASP B 107      -2.728  17.628 -29.998  1.00  1.22
N
ATOM  11132  CA  ASP B 107      -3.418  17.250 -28.777  1.00  0.87
C
ATOM  11133  C   ASP B 107      -2.765  16.009 -28.155  1.00  0.70
C
ATOM  11134  O   ASP B 107      -3.039  15.660 -27.004  1.00  0.78
O
ATOM  11135  CB  ASP B 107      -4.900  16.985 -29.064  1.00  1.30
C
ATOM  11136  CG  ASP B 107      -5.678  18.227 -29.494  1.00  1.30
C
ATOM  11137  OD1 ASP B 107      -5.650  19.209 -28.785  1.00  1.30
O
ATOM  11138  OD2 ASP B 107      -6.290  18.182 -30.535  1.00  1.30
O
ATOM  11139  H   ASP B 107      -2.979  17.179 -30.868  1.00  1.46
H
ATOM  11140  HA  ASP B 107      -3.340  18.070 -28.066  1.00  1.04
H
ATOM  11141 1HB  ASP B 107      -4.989  16.231 -29.846  1.00  1.57
H
ATOM  11142 2HB  ASP B 107      -5.371  16.585 -28.167  1.00  1.57
H
ATOM  11143  N   LEU B 108      -1.890  15.342 -28.915  1.00  1.00
N
ATOM  11144  CA  LEU B 108      -1.255  14.116 -28.436  1.00  1.28
C
ATOM  11145  C   LEU B 108      -0.043  14.432 -27.567  1.00  1.18
C
```

ATOM 11146 O   LEU B 108   1.102 14.367 -28.015 1.00 1.89
O
ATOM 11147 CB LEU B 108   -0.843 13.224 -29.611 1.00 1.92
C
ATOM 11148 CG LEU B 108   -1.984 12.761 -30.531 1.00 1.92
C
ATOM 11149 CD1 LEU B 108   -1.411 11.846 -31.605 1.00 1.92
C
ATOM 11150 CD2 LEU B 108   -3.065 12.066 -29.718 1.00 1.92
C
ATOM 11151 H   LEU B 108   -1.658 15.678 -29.842 1.00 1.20
H
ATOM 11152 HA LEU B 108   -1.975 13.572 -27.827 1.00 1.54
H
ATOM 11153 1HB LEU B 108   -0.136 13.777 -30.225 1.00 2.30
H
ATOM 11154 2HB LEU B 108   -0.339 12.342 -29.230 1.00 2.30
H
ATOM 11155 HG LEU B 108   -2.417 13.628 -31.031 1.00 2.30
H
ATOM 11156 1HD1 LEU B 108   -2.207 11.530 -32.280 1.00 2.30
H
ATOM 11157 2HD1 LEU B 108   -0.652 12.386 -32.166 1.00 2.30
H
ATOM 11158 3HD1 LEU B 108   -0.964 10.971 -31.136 1.00 2.30
H
ATOM 11159 1HD2 LEU B 108   -3.873 11.750 -30.380 1.00 2.30
H
ATOM 11160 2HD2 LEU B 108   -2.646 11.199 -29.221 1.00 2.30
H
ATOM 11161 3HD2 LEU B 108   -3.456 12.758 -28.973 1.00 2.30
H
ATOM 11162 N   GLU B 109   -0.339 14.787 -26.324 1.00 0.72
N
ATOM 11163 CA GLU B 109   0.624 15.165 -25.294 1.00 0.84
C
ATOM 11164 C   GLU B 109   1.419 14.005 -24.671 1.00 0.81
C
ATOM 11165 O   GLU B 109   1.011 12.847 -24.736 1.00 0.85
O
ATOM 11166 CB GLU B 109   -0.121 15.928 -24.196 1.00 1.26
C
ATOM 11167 CG GLU B 109   -0.608 17.318 -24.631 1.00 1.26
C
ATOM 11168 CD GLU B 109   -1.193 18.155 -23.515 1.00 1.26
C
ATOM 11169 OE1 GLU B 109   -0.495 19.008 -23.005 1.00 1.26
O
ATOM 11170 OE2 GLU B 109   -2.337 17.958 -23.164 1.00 1.26
O
ATOM 11171 H   GLU B 109   -1.325 14.815 -26.091 1.00 0.86
H
ATOM 11172 HA GLU B 109   1.342 15.845 -25.751 1.00 1.01
H
ATOM 11173 1HB GLU B 109   -1.007 15.358 -23.916 1.00 1.51
H
ATOM 11174 2HB GLU B 109   0.495 15.999 -23.310 1.00 1.51
H

```
ATOM  11175 1HG  GLU B 109       0.232  17.859 -25.068  1.00  1.51
H
ATOM  11176 2HG  GLU B 109      -1.360  17.191 -25.409  1.00  1.51
H
ATOM  11177  N   ARG B 110       2.564  14.343 -24.050  1.00  1.02
N
ATOM  11178  CA  ARG B 110       3.448  13.392 -23.336  1.00  1.08
C
ATOM  11179  C   ARG B 110       2.716  12.359 -22.493  1.00  0.86
C
ATOM  11180  O   ARG B 110       3.092  11.187 -22.484  1.00  1.14
O
ATOM  11181  CB  ARG B 110       4.377  14.139 -22.393  1.00  1.62
C
ATOM  11182  CG  ARG B 110       5.247  13.308 -21.441  1.00  1.62
C
ATOM  11183  CD  ARG B 110       6.404  12.629 -22.043  1.00  1.62
C
ATOM  11184  NE  ARG B 110       7.130  11.828 -21.086  1.00  1.62
N
ATOM  11185  CZ  ARG B 110       6.899  10.519 -20.880  1.00  1.62
C
ATOM  11186  NH1 ARG B 110       5.986   9.911 -21.607  1.00  1.62
N
ATOM  11187  NH2 ARG B 110       7.588   9.864 -19.966  1.00  1.62
N
ATOM  11188  H   ARG B 110       2.833  15.315 -24.063  1.00  1.22
H
ATOM  11189  HA  ARG B 110       4.033  12.860 -24.080  1.00  1.30
H
ATOM  11190 1HB  ARG B 110       5.048  14.761 -22.980  1.00  1.94
H
ATOM  11191 2HB  ARG B 110       3.792  14.793 -21.771  1.00  1.94
H
ATOM  11192 1HG  ARG B 110       5.603  13.934 -20.629  1.00  1.94
H
ATOM  11193 2HG  ARG B 110       4.617  12.521 -21.028  1.00  1.94
H
ATOM  11194 1HD  ARG B 110       6.044  11.955 -22.811  1.00  1.94
H
ATOM  11195 2HD  ARG B 110       7.089  13.359 -22.473  1.00  1.94
H
ATOM  11196  HE  ARG B 110       7.836  12.288 -20.521  1.00  1.94
H
ATOM  11197 1HH1 ARG B 110       5.464  10.435 -22.291  1.00  1.94
H
ATOM  11198 2HH1 ARG B 110       5.796   8.922 -21.498  1.00  1.94
H
ATOM  11199 1HH2 ARG B 110       8.285  10.348 -19.418  1.00  1.94
H
ATOM  11200 2HH2 ARG B 110       7.412   8.885 -19.807  1.00  1.94
H
ATOM  11201  N   HIS B 111       1.678  12.795 -21.782  1.00  0.78
N
ATOM  11202  CA  HIS B 111       0.877  11.892 -20.972  1.00  0.74
C
ATOM  11203  C   HIS B 111      -0.569  12.128 -21.354  1.00  1.21
C
```

ATOM 11204 O HIS B 111 -0.892 13.228 -21.799 1.00 6.57
O
ATOM 11205 CB HIS B 111 1.029 12.148 -19.467 1.00 1.11
C
ATOM 11206 CG HIS B 111 2.421 12.080 -18.965 1.00 1.11
C
ATOM 11207 ND1 HIS B 111 3.187 13.204 -18.724 1.00 1.11
N
ATOM 11208 CD2 HIS B 111 3.201 11.020 -18.682 1.00 1.11
C
ATOM 11209 CE1 HIS B 111 4.381 12.822 -18.287 1.00 1.11
C
ATOM 11210 NE2 HIS B 111 4.410 11.501 -18.268 1.00 1.11
N
ATOM 11211 H HIS B 111 1.426 13.771 -21.819 1.00 0.94
H
ATOM 11212 HA HIS B 111 1.149 10.864 -21.188 1.00 0.89
H
ATOM 11213 1HB HIS B 111 0.616 13.120 -19.218 1.00 1.33
H
ATOM 11214 2HB HIS B 111 0.454 11.413 -18.910 1.00 1.33
H
ATOM 11215 HD2 HIS B 111 2.923 9.973 -18.768 1.00 1.33
H
ATOM 11216 HE1 HIS B 111 5.202 13.483 -18.012 1.00 1.33
H
ATOM 11217 HE2 HIS B 111 5.172 10.895 -17.999 1.00 1.33
H
ATOM 11218 N PRO B 112 -1.453 11.138 -21.201 1.00 0.68
N
ATOM 11219 CA PRO B 112 -2.864 11.177 -21.522 1.00 0.56
C
ATOM 11220 C PRO B 112 -3.528 12.322 -20.784 1.00 0.90
C
ATOM 11221 O PRO B 112 -3.429 12.417 -19.559 1.00 3.49
O
ATOM 11222 CB PRO B 112 -3.395 9.830 -21.006 1.00 0.84
C
ATOM 11223 CG PRO B 112 -2.200 8.952 -20.979 1.00 0.84
C
ATOM 11224 CD PRO B 112 -1.116 9.880 -20.508 1.00 0.84
C
ATOM 11225 HA PRO B 112 -2.995 11.279 -22.612 1.00 0.67
H
ATOM 11226 1HB PRO B 112 -3.852 9.958 -20.013 1.00 1.01
H
ATOM 11227 2HB PRO B 112 -4.181 9.452 -21.675 1.00 1.01
H
ATOM 11228 1HG PRO B 112 -2.364 8.101 -20.302 1.00 1.01
H
ATOM 11229 2HG PRO B 112 -2.003 8.538 -21.979 1.00 1.01
H
ATOM 11230 1HD PRO B 112 -1.175 10.020 -19.420 1.00 1.01
H
ATOM 11231 2HD PRO B 112 -0.147 9.501 -20.816 1.00 1.01
H
ATOM 11232 N THR B 113 -4.215 13.175 -21.524 1.00 0.48
N

ATOM 11233 CA THR B 113 -4.961 14.281 -20.948 1.00 0.51
C
ATOM 11234 C THR B 113 -6.372 14.278 -21.514 1.00 0.51
C
ATOM 11235 O THR B 113 -6.556 13.902 -22.671 1.00 0.55
O
ATOM 11236 CB THR B 113 -4.255 15.629 -21.239 1.00 0.77
C
ATOM 11237 OG1 THR B 113 -4.085 15.793 -22.652 1.00 0.77
O
ATOM 11238 CG2 THR B 113 -2.922 15.706 -20.538 1.00 0.77
C
ATOM 11239 H THR B 113 -4.234 13.044 -22.526 1.00 0.58
H
ATOM 11240 HA THR B 113 -5.012 14.136 -19.871 1.00 0.61
H
ATOM 11241 HB THR B 113 -4.870 16.451 -20.881 1.00 0.92
H
ATOM 11242 HG1 THR B 113 -3.481 16.529 -22.826 1.00 0.92
H
ATOM 11243 1HG2 THR B 113 -2.470 16.670 -20.760 1.00 0.92
H
ATOM 11244 2HG2 THR B 113 -3.069 15.606 -19.463 1.00 0.92
H
ATOM 11245 3HG2 THR B 113 -2.272 14.911 -20.891 1.00 0.92
H
ATOM 11246 N PRO B 114 -7.357 14.773 -20.744 1.00 0.56
N
ATOM 11247 CA PRO B 114 -8.804 14.833 -20.995 1.00 0.62
C
ATOM 11248 C PRO B 114 -9.201 15.611 -22.245 1.00 0.50
C
ATOM 11249 O PRO B 114 -10.358 15.562 -22.662 1.00 0.71
O
ATOM 11250 CB PRO B 114 -9.349 15.530 -19.741 1.00 0.93
C
ATOM 11251 CG PRO B 114 -8.191 16.282 -19.204 1.00 0.93
C
ATOM 11252 CD PRO B 114 -7.036 15.368 -19.431 1.00 0.93
C
ATOM 11253 HA PRO B 114 -9.192 13.805 -21.054 1.00 0.74
H
ATOM 11254 1HB PRO B 114 -10.194 16.181 -20.010 1.00 1.12
H
ATOM 11255 2HB PRO B 114 -9.735 14.781 -19.034 1.00 1.12
H
ATOM 11256 1HG PRO B 114 -8.080 17.234 -19.743 1.00 1.12
H
ATOM 11257 2HG PRO B 114 -8.337 16.535 -18.153 1.00 1.12
H
ATOM 11258 1HD PRO B 114 -6.113 15.951 -19.447 1.00 1.12
H
ATOM 11259 2HD PRO B 114 -7.006 14.581 -18.662 1.00 1.12
H
ATOM 11260 N ARG B 115 -8.247 16.318 -22.858 1.00 0.59
N
ATOM 11261 CA ARG B 115 -8.497 17.010 -24.112 1.00 0.67
C

ATOM 11262 C ARG B 115 -8.737 16.000 -25.230 1.00 0.54
C
ATOM 11263 O ARG B 115 -9.370 16.319 -26.236 1.00 0.57
O
ATOM 11264 CB ARG B 115 -7.311 17.875 -24.492 1.00 1.01
C
ATOM 11265 CG ARG B 115 -6.088 17.080 -24.928 1.00 1.01
C
ATOM 11266 CD ARG B 115 -4.864 17.910 -24.945 1.00 1.01
C
ATOM 11267 NE ARG B 115 -4.876 18.929 -25.977 1.00 1.01
N
ATOM 11268 CZ ARG B 115 -4.130 20.046 -25.944 1.00 1.01
C
ATOM 11269 NH1 ARG B 115 -3.342 20.289 -24.923 1.00 1.01
N
ATOM 11270 NH2 ARG B 115 -4.203 20.893 -26.953 1.00 1.01
N
ATOM 11271 H ARG B 115 -7.320 16.369 -22.464 1.00 0.71
H
ATOM 11272 HA ARG B 115 -9.383 17.635 -24.000 1.00 0.80
H
ATOM 11273 1HB ARG B 115 -7.586 18.542 -25.307 1.00 1.21
H
ATOM 11274 2HB ARG B 115 -7.020 18.493 -23.642 1.00 1.21
H
ATOM 11275 1HG ARG B 115 -5.932 16.248 -24.242 1.00 1.21
H
ATOM 11276 2HG ARG B 115 -6.249 16.693 -25.935 1.00 1.21
H
ATOM 11277 1HD ARG B 115 -4.755 18.407 -23.981 1.00 1.21
H
ATOM 11278 2HD ARG B 115 -3.999 17.269 -25.115 1.00 1.21
H
ATOM 11279 HE ARG B 115 -5.454 18.791 -26.799 1.00 1.21
H
ATOM 11280 1HH1 ARG B 115 -3.278 19.622 -24.161 1.00 1.21
H
ATOM 11281 2HH1 ARG B 115 -2.791 21.137 -24.907 1.00 1.21
H
ATOM 11282 1HH2 ARG B 115 -4.810 20.665 -27.735 1.00 1.21
H
ATOM 11283 2HH2 ARG B 115 -3.651 21.737 -26.950 1.00 1.21
H
ATOM 11284 N LEU B 116 -8.242 14.775 -25.039 1.00 0.55
N
ATOM 11285 CA LEU B 116 -8.452 13.722 -26.003 1.00 0.45
C
ATOM 11286 C LEU B 116 -9.721 12.961 -25.641 1.00 0.46
C
ATOM 11287 O LEU B 116 -9.918 12.633 -24.472 1.00 0.48
O
ATOM 11288 CB LEU B 116 -7.251 12.779 -26.014 1.00 0.68
C
ATOM 11289 CG LEU B 116 -5.939 13.420 -26.464 1.00 0.68
C
ATOM 11290 CD1 LEU B 116 -4.796 12.439 -26.292 1.00 0.68
C

```
ATOM  11291  CD2 LEU B 116      -6.076  13.823 -27.918  1.00  0.68
C
ATOM  11292  H   LEU B 116      -7.717  14.556 -24.202  1.00  0.66
H
ATOM  11293  HA  LEU B 116      -8.535  14.176 -26.982  1.00  0.54
H
ATOM  11294 1HB  LEU B 116      -7.104  12.401 -25.008  1.00  0.81
H
ATOM  11295 2HB  LEU B 116      -7.462  11.941 -26.677  1.00  0.81
H
ATOM  11296  HG  LEU B 116      -5.728  14.297 -25.852  1.00  0.81
H
ATOM  11297 1HD1 LEU B 116      -3.866  12.907 -26.614  1.00  0.81
H
ATOM  11298 2HD1 LEU B 116      -4.714  12.154 -25.243  1.00  0.81
H
ATOM  11299 3HD1 LEU B 116      -4.985  11.557 -26.898  1.00  0.81
H
ATOM  11300 1HD2 LEU B 116      -5.148  14.281 -28.255  1.00  0.81
H
ATOM  11301 2HD2 LEU B 116      -6.284  12.941 -28.522  1.00  0.81
H
ATOM  11302 3HD2 LEU B 116      -6.894  14.536 -28.024  1.00  0.81
H
ATOM  11303  N   PRO B 117     -10.577  12.650 -26.626  1.00  0.45
N
ATOM  11304  CA  PRO B 117     -11.846  11.922 -26.543  1.00  0.48
C
ATOM  11305  C   PRO B 117     -11.735  10.585 -25.828  1.00  0.39
C
ATOM  11306  O   PRO B 117     -12.675  10.137 -25.175  1.00  0.50
O
ATOM  11307  CB  PRO B 117     -12.223  11.724 -28.012  1.00  0.72
C
ATOM  11308  CG  PRO B 117     -11.639  12.900 -28.704  1.00  0.72
C
ATOM  11309  CD  PRO B 117     -10.344  13.162 -27.994  1.00  0.72
C
ATOM  11310  HA  PRO B 117     -12.585  12.560 -26.039  1.00  0.58
H
ATOM  11311 1HB  PRO B 117     -11.815  10.774 -28.383  1.00  0.86
H
ATOM  11312 2HB  PRO B 117     -13.317  11.670 -28.115  1.00  0.86
H
ATOM  11313 1HG  PRO B 117     -11.499  12.679 -29.771  1.00  0.86
H
ATOM  11314 2HG  PRO B 117     -12.331  13.754 -28.644  1.00  0.86
H
ATOM  11315 1HD  PRO B 117      -9.511  12.626 -28.470  1.00  0.86
H
ATOM  11316 2HD  PRO B 117     -10.184  14.250 -27.975  1.00  0.86
H
ATOM  11317  N   PHE B 118     -10.586   9.944 -25.956  1.00  0.41
N
ATOM  11318  CA  PHE B 118     -10.383   8.650 -25.351  1.00  0.42
C
ATOM  11319  C   PHE B 118      -9.752   8.714 -23.961  1.00  0.49
C
```

ATOM 11320 O   PHE B 118   -9.465   7.669 -23.374 1.00 0.95
O
ATOM 11321 CB PHE B 118   -9.428   7.860 -26.193 1.00 0.63
C
ATOM 11322 CG PHE B 118   -9.822   7.404 -27.558 1.00 0.63
C
ATOM 11323 CD1 PHE B 118   -9.676   8.237 -28.652 1.00 0.63
C
ATOM 11324 CD2 PHE B 118   -10.249   6.102 -27.760 1.00 0.63
C
ATOM 11325 CE1 PHE B 118   -9.976   7.783 -29.918 1.00 0.63
C
ATOM 11326 CE2 PHE B 118   -10.549   5.643 -29.027 1.00 0.63
C
ATOM 11327 CZ PHE B 118   -10.415   6.487 -30.105 1.00 0.63
C
ATOM 11328 H   PHE B 118   -9.841 10.356 -26.499 1.00 0.49
H
ATOM 11329 HA PHE B 118   -11.342   8.142 -25.273 1.00 0.50
H
ATOM 11330 1HB PHE B 118   -8.535   8.460 -26.307 1.00 0.76
H
ATOM 11331 2HB PHE B 118   -9.162   6.974 -25.625 1.00 0.76
H
ATOM 11332 HD1 PHE B 118   -9.315   9.256 -28.505 1.00 0.76
H
ATOM 11333 HD2 PHE B 118   -10.340   5.433 -26.904 1.00 0.76
H
ATOM 11334 HE1 PHE B 118   -9.858   8.447 -30.771 1.00 0.76
H
ATOM 11335 HE2 PHE B 118   -10.887   4.617 -29.174 1.00 0.76
H
ATOM 11336 HZ PHE B 118   -10.650   6.127 -31.103 1.00 0.76
H
ATOM 11337 N   VAL B 119   -9.529   9.916 -23.426 1.00 0.35
N
ATOM 11338 CA VAL B 119   -8.936 10.029 -22.098 1.00 0.34
C
ATOM 11339 C   VAL B 119   -9.928 10.536 -21.083 1.00 0.26
C
ATOM 11340 O   VAL B 119   -10.479 11.629 -21.211 1.00 0.34
O
ATOM 11341 CB VAL B 119   -7.730 10.972 -22.067 1.00 0.51
C
ATOM 11342 CG1 VAL B 119   -7.211 11.048 -20.635 1.00 0.51
C
ATOM 11343 CG2 VAL B 119   -6.641 10.478 -22.990 1.00 0.51
C
ATOM 11344 H   VAL B 119   -9.768 10.763 -23.921 1.00 0.42
H
ATOM 11345 HA VAL B 119   -8.601   9.040 -21.788 1.00 0.41
H
ATOM 11346 HB VAL B 119   -8.046 11.970 -22.370 1.00 0.61
H
ATOM 11347 1HG1 VAL B 119   -6.358 11.717 -20.582 1.00 0.61
H
ATOM 11348 2HG1 VAL B 119   -7.998 11.417 -19.979 1.00 0.61
H

ATOM 11349 3HG1 VAL B 119 -6.902 10.054 -20.309 1.00 0.61
H
ATOM 11350 1HG2 VAL B 119 -5.793 11.161 -22.951 1.00 0.61
H
ATOM 11351 2HG2 VAL B 119 -6.326 9.487 -22.668 1.00 0.61
H
ATOM 11352 3HG2 VAL B 119 -7.022 10.427 -24.010 1.00 0.61
H
ATOM 11353 N ASP B 120 -10.128 9.736 -20.062 1.00 0.28
N
ATOM 11354 CA ASP B 120 -11.013 10.055 -18.966 1.00 0.35
C
ATOM 11355 C ASP B 120 -10.319 10.684 -17.770 1.00 0.40
C
ATOM 11356 O ASP B 120 -10.909 11.489 -17.048 1.00 1.90
O
ATOM 11357 CB ASP B 120 -11.716 8.793 -18.554 1.00 0.52
C
ATOM 11358 CG ASP B 120 -12.708 8.340 -19.606 1.00 0.52
C
ATOM 11359 OD1 ASP B 120 -13.533 9.147 -19.960 1.00 0.52
O
ATOM 11360 OD2 ASP B 120 -12.654 7.221 -20.054 1.00 0.52
O
ATOM 11361 H ASP B 120 -9.639 8.852 -20.043 1.00 0.34
H
ATOM 11362 HA ASP B 120 -11.758 10.762 -19.331 1.00 0.42
H
ATOM 11363 1HB ASP B 120 -10.978 8.003 -18.415 1.00 0.63
H
ATOM 11364 2HB ASP B 120 -12.197 8.950 -17.602 1.00 0.63
H
ATOM 11365 N VAL B 121 -9.069 10.302 -17.562 1.00 0.26
N
ATOM 11366 CA VAL B 121 -8.288 10.778 -16.438 1.00 0.18
C
ATOM 11367 C VAL B 121 -6.828 10.895 -16.820 1.00 0.15
C
ATOM 11368 O VAL B 121 -6.307 10.060 -17.559 1.00 0.15
O
ATOM 11369 CB VAL B 121 -8.439 9.800 -15.257 1.00 0.27
C
ATOM 11370 CG1 VAL B 121 -7.910 8.431 -15.668 1.00 0.27
C
ATOM 11371 CG2 VAL B 121 -7.688 10.316 -14.034 1.00 0.27
C
ATOM 11372 H VAL B 121 -8.645 9.646 -18.200 1.00 0.31
H
ATOM 11373 HA VAL B 121 -8.658 11.759 -16.141 1.00 0.22
H
ATOM 11374 HB VAL B 121 -9.496 9.692 -15.016 1.00 0.32
H
ATOM 11375 1HG1 VAL B 121 -8.029 7.729 -14.844 1.00 0.32
H
ATOM 11376 2HG1 VAL B 121 -8.466 8.072 -16.533 1.00 0.32
H
ATOM 11377 3HG1 VAL B 121 -6.855 8.512 -15.924 1.00 0.32
H

```
ATOM  11378 1HG2 VAL B 121      -7.816   9.615 -13.209  1.00  0.32
H
ATOM  11379 2HG2 VAL B 121      -6.628  10.412 -14.262  1.00  0.32
H
ATOM  11380 3HG2 VAL B 121      -8.086  11.290 -13.746  1.00  0.32
H
ATOM  11381  N   ALA B 122      -6.167  11.933 -16.328  1.00  0.20
N
ATOM  11382  CA  ALA B 122      -4.758  12.104 -16.604  1.00  0.22
C
ATOM  11383  C   ALA B 122      -3.963  11.315 -15.579  1.00  0.21
C
ATOM  11384  O   ALA B 122      -4.186  11.448 -14.375  1.00  0.24
O
ATOM  11385  CB  ALA B 122      -4.384  13.574 -16.585  1.00  0.33
C
ATOM  11386  H   ALA B 122      -6.632  12.593 -15.726  1.00  0.24
H
ATOM  11387  HA  ALA B 122      -4.546  11.690 -17.587  1.00  0.26
H
ATOM  11388 1HB  ALA B 122      -3.323  13.682 -16.803  1.00  0.40
H
ATOM  11389 2HB  ALA B 122      -4.965  14.106 -17.337  1.00  0.40
H
ATOM  11390 3HB  ALA B 122      -4.597  13.988 -15.601  1.00  0.40
H
ATOM  11391  N   THR B 123      -3.047  10.481 -16.060  1.00  0.21
N
ATOM  11392  CA  THR B 123      -2.254   9.627 -15.182  1.00  0.22
C
ATOM  11393  C   THR B 123      -0.755   9.772 -15.386  1.00  0.24
C
ATOM  11394  O   THR B 123      -0.043   8.773 -15.468  1.00  0.30
O
ATOM  11395  CB  THR B 123      -2.605   8.155 -15.391  1.00  0.33
C
ATOM  11396  OG1 THR B 123      -2.399   7.836 -16.769  1.00  0.33
O
ATOM  11397  CG2 THR B 123      -4.047   7.864 -15.004  1.00  0.33
C
ATOM  11398  H   THR B 123      -2.907  10.426 -17.059  1.00  0.25
H
ATOM  11399  HA  THR B 123      -2.479   9.894 -14.150  1.00  0.26
H
ATOM  11400  HB  THR B 123      -1.943   7.541 -14.782  1.00  0.40
H
ATOM  11401  HG1 THR B 123      -2.544   6.899 -16.905  1.00  0.40
H
ATOM  11402 1HG2 THR B 123      -4.260   6.809 -15.169  1.00  0.40
H
ATOM  11403 2HG2 THR B 123      -4.197   8.107 -13.952  1.00  0.40
H
ATOM  11404 3HG2 THR B 123      -4.714   8.468 -15.615  1.00  0.40
H
ATOM  11405  N   GLY B 124      -0.267  10.999 -15.476  1.00  0.25
N
ATOM  11406  CA  GLY B 124       1.170  11.201 -15.603  1.00  0.29
C
```

ATOM 11407 C   GLY B 124   1.863 10.808 -14.301 1.00 0.31
C
ATOM 11408 O   GLY B 124   2.953 10.233 -14.309 1.00 0.61
O
ATOM 11409 H   GLY B 124   -0.888 11.793 -15.436 1.00 0.30
H
ATOM 11410 1HA GLY B 124   1.551 10.590 -16.419 1.00 0.35
H
ATOM 11411 2HA GLY B 124   1.381 12.238 -15.846 1.00 0.35
H
ATOM 11412 N   SER B 125   1.205 11.103 -13.182 1.00 0.27
N
ATOM 11413 CA SER B 125   1.710 10.724 -11.878 1.00 0.28
C
ATOM 11414 C   SER B 125   1.324   9.266 -11.641 1.00 0.37
C
ATOM 11415 O   SER B 125   0.175   8.874 -11.847 1.00 1.04
O
ATOM 11416 CB SER B 125   1.153 11.647 -10.818 1.00 0.42
C
ATOM 11417 OG SER B 125   1.596 11.285 -9.544 1.00 0.42
O
ATOM 11418 H   SER B 125   0.326 11.593 -13.239 1.00 0.32
H
ATOM 11419 HA SER B 125   2.798 10.802 -11.880 1.00 0.34
H
ATOM 11420 1HB SER B 125   1.460 12.671 -11.034 1.00 0.50
H
ATOM 11421 2HB SER B 125   0.066 11.619 -10.848 1.00 0.50
H
ATOM 11422 HG SER B 125   1.384 12.032 -8.971 1.00 0.50
H
ATOM 11423 N   LEU B 126   2.301   8.468 -11.246 1.00 0.28
N
ATOM 11424 CA LEU B 126   2.124   7.029 -11.097 1.00 0.27
C
ATOM 11425 C   LEU B 126   1.381   6.577 -9.841 1.00 0.27
C
ATOM 11426 O   LEU B 126   1.412   7.240 -8.797 1.00 0.55
O
ATOM 11427 CB LEU B 126   3.501   6.367 -11.156 1.00 0.41
C
ATOM 11428 CG LEU B 126   4.214   6.526 -12.502 1.00 0.41
C
ATOM 11429 CD1 LEU B 126   5.600   5.913 -12.428 1.00 0.41
C
ATOM 11430 CD2 LEU B 126   3.375   5.852 -13.575 1.00 0.41
C
ATOM 11431 H   LEU B 126   3.213   8.867 -11.071 1.00 0.34
H
ATOM 11432 HA LEU B 126   1.552   6.682 -11.957 1.00 0.32
H
ATOM 11433 1HB LEU B 126   4.135   6.809 -10.388 1.00 0.49
H
ATOM 11434 2HB LEU B 126   3.404   5.307 -10.951 1.00 0.49
H
ATOM 11435 HG LEU B 126   4.322   7.585 -12.737 1.00 0.49
H

```
ATOM  11436 1HD1 LEU B 126       6.102   6.032 -13.388  1.00  0.49
H
ATOM  11437 2HD1 LEU B 126       6.178   6.416 -11.654  1.00  0.49
H
ATOM  11438 3HD1 LEU B 126       5.516   4.853 -12.192  1.00  0.49
H
ATOM  11439 1HD2 LEU B 126       3.858   5.959 -14.543  1.00  0.49
H
ATOM  11440 2HD2 LEU B 126       3.271   4.794 -13.340  1.00  0.49
H
ATOM  11441 3HD2 LEU B 126       2.389   6.314 -13.611  1.00  0.49
H
ATOM  11442  N   GLY B 127       0.699   5.434  -9.980  1.00  0.19
N
ATOM  11443  CA  GLY B 127      -0.057   4.787  -8.913  1.00  0.32
C
ATOM  11444  C   GLY B 127      -1.481   5.317  -8.727  1.00  0.77
C
ATOM  11445  O   GLY B 127      -2.141   4.993  -7.738  1.00  3.84
O
ATOM  11446  H   GLY B 127       0.721   4.962 -10.879  1.00  0.23
H
ATOM  11447 1HA  GLY B 127      -0.103   3.721  -9.125  1.00  0.38
H
ATOM  11448 2HA  GLY B 127       0.491   4.894  -7.977  1.00  0.38
H
ATOM  11449  N   GLN B 128      -1.957   6.149  -9.646  1.00  0.65
N
ATOM  11450  CA  GLN B 128      -3.275   6.757  -9.469  1.00  0.80
C
ATOM  11451  C   GLN B 128      -4.393   6.172 -10.330  1.00  0.37
C
ATOM  11452  O   GLN B 128      -5.565   6.216  -9.938  1.00  1.08
O
ATOM  11453  CB  GLN B 128      -3.183   8.242  -9.746  1.00  1.20
C
ATOM  11454  CG  GLN B 128      -2.306   9.000  -8.774  1.00  1.20
C
ATOM  11455  CD  GLN B 128      -2.357  10.470  -9.064  1.00  1.20
C
ATOM  11456  OE1 GLN B 128      -2.952  10.854 -10.074  1.00  1.20
O
ATOM  11457  NE2 GLN B 128      -1.766  11.290  -8.204  1.00  1.20
N
ATOM  11458  H   GLN B 128      -1.403   6.385 -10.456  1.00  0.78
H
ATOM  11459  HA  GLN B 128      -3.564   6.624  -8.426  1.00  0.96
H
ATOM  11460 1HB  GLN B 128      -2.786   8.401 -10.749  1.00  1.44
H
ATOM  11461 2HB  GLN B 128      -4.180   8.679  -9.716  1.00  1.44
H
ATOM  11462 1HG  GLN B 128      -2.654   8.829  -7.757  1.00  1.44
H
ATOM  11463 2HG  GLN B 128      -1.276   8.663  -8.882  1.00  1.44
H
ATOM  11464 1HE2 GLN B 128      -1.782  12.278  -8.357  1.00  1.44
H
```

ATOM 11465 2HE2 GLN B 128   -1.302 10.919 -7.399 1.00 1.44
H
ATOM 11466 N   GLY B 129   -4.029   5.657 -11.503 1.00 0.46
N
ATOM 11467 CA GLY B 129   -4.990   5.145 -12.473 1.00 0.25
C
ATOM 11468 C   GLY B 129   -5.912   4.065 -11.929 1.00 0.25
C
ATOM 11469 O   GLY B 129   -7.102   4.068 -12.237 1.00 0.40
O
ATOM 11470 H   GLY B 129   -3.048   5.636 -11.745 1.00 0.55
H
ATOM 11471 1HA GLY B 129   -5.592   5.975 -12.843 1.00 0.30
H
ATOM 11472 2HA GLY B 129   -4.448   4.752 -13.332 1.00 0.30
H
ATOM 11473 N   LEU B 130   -5.366   3.145 -11.137 1.00 0.19
N
ATOM 11474 CA LEU B 130   -6.152   2.048 -10.589 1.00 0.19
C
ATOM 11475 C   LEU B 130   -7.273   2.523 -9.670 1.00 0.17
C
ATOM 11476 O   LEU B 130   -8.371   1.975 -9.712 1.00 0.35
O
ATOM 11477 CB LEU B 130   -5.263   1.073 -9.838 1.00 0.29
C
ATOM 11478 CG LEU B 130   -6.005 -0.144 -9.291 1.00 0.29
C
ATOM 11479 CD1 LEU B 130   -6.633 -0.920 -10.444 1.00 0.29
C
ATOM 11480 CD2 LEU B 130   -5.039 -1.003 -8.522 1.00 0.29
C
ATOM 11481 H   LEU B 130   -4.381   3.199 -10.921 1.00 0.23
H
ATOM 11482 HA LEU B 130   -6.608   1.514 -11.421 1.00 0.23
H
ATOM 11483 1HB LEU B 130   -4.483   0.719 -10.510 1.00 0.34
H
ATOM 11484 2HB LEU B 130   -4.794   1.590 -9.001 1.00 0.34
H
ATOM 11485 HG LEU B 130   -6.808   0.183 -8.636 1.00 0.34
H
ATOM 11486 1HD1 LEU B 130   -7.170 -1.784 -10.050 1.00 0.34
H
ATOM 11487 2HD1 LEU B 130   -7.329 -0.275 -10.980 1.00 0.34
H
ATOM 11488 3HD1 LEU B 130   -5.852 -1.258 -11.124 1.00 0.34
H
ATOM 11489 1HD2 LEU B 130   -5.579 -1.861 -8.133 1.00 0.34
H
ATOM 11490 2HD2 LEU B 130   -4.242 -1.338 -9.184 1.00 0.34
H
ATOM 11491 3HD2 LEU B 130   -4.609 -0.434 -7.699 1.00 0.34
H
ATOM 11492 N   GLY B 131   -6.996   3.513 -8.822 1.00 0.13
N
ATOM 11493 CA GLY B 131   -8.023   4.048 -7.930 1.00 0.14
C

ATOM 11494 C GLY B 131 -9.174 4.631 -8.744 1.00 0.16
C
ATOM 11495 O GLY B 131 -10.347 4.415 -8.430 1.00 0.21
O
ATOM 11496 H GLY B 131 -6.068 3.915 -8.807 1.00 0.16
H
ATOM 11497 1HA GLY B 131 -8.393 3.256 -7.278 1.00 0.17
H
ATOM 11498 2HA GLY B 131 -7.591 4.817 -7.291 1.00 0.17
H
ATOM 11499 N THR B 132 -8.823 5.365 -9.800 1.00 0.17
N
ATOM 11500 CA THR B 132 -9.814 5.931 -10.700 1.00 0.17
C
ATOM 11501 C THR B 132 -10.598 4.815 -11.395 1.00 0.13
C
ATOM 11502 O THR B 132 -11.817 4.918 -11.544 1.00 0.19
O
ATOM 11503 CB THR B 132 -9.148 6.875 -11.709 1.00 0.26
C
ATOM 11504 OG1 THR B 132 -8.543 7.971 -10.998 1.00 0.26
O
ATOM 11505 CG2 THR B 132 -10.170 7.414 -12.691 1.00 0.26
C
ATOM 11506 H THR B 132 -7.836 5.536 -9.983 1.00 0.20
H
ATOM 11507 HA THR B 132 -10.518 6.516 -10.113 1.00 0.20
H
ATOM 11508 HB THR B 132 -8.373 6.332 -12.248 1.00 0.31
H
ATOM 11509 HG1 THR B 132 -8.322 7.702 -10.094 1.00 0.31
H
ATOM 11510 1HG2 THR B 132 -9.681 8.081 -13.395 1.00 0.31
H
ATOM 11511 2HG2 THR B 132 -10.632 6.589 -13.231 1.00 0.31
H
ATOM 11512 3HG2 THR B 132 -10.930 7.964 -12.147 1.00 0.31
H
ATOM 11513 N ALA B 133 -9.895 3.745 -11.789 1.00 0.15
N
ATOM 11514 CA ALA B 133 -10.516 2.570 -12.397 1.00 0.16
C
ATOM 11515 C ALA B 133 -11.523 1.949 -11.444 1.00 0.16
C
ATOM 11516 O ALA B 133 -12.595 1.527 -11.871 1.00 0.18
O
ATOM 11517 CB ALA B 133 -9.464 1.534 -12.780 1.00 0.24
C
ATOM 11518 H ALA B 133 -8.891 3.745 -11.673 1.00 0.18
H
ATOM 11519 HA ALA B 133 -11.046 2.891 -13.294 1.00 0.19
H
ATOM 11520 1HB ALA B 133 -9.950 0.680 -13.248 1.00 0.29
H
ATOM 11521 2HB ALA B 133 -8.749 1.966 -13.477 1.00 0.29
H
ATOM 11522 3HB ALA B 133 -8.936 1.200 -11.891 1.00 0.29
H

ATOM 11523 N CYS B 134 -11.179 1.910 -10.153 1.00 0.15
N
ATOM 11524 CA CYS B 134 -12.074 1.387 -9.134 1.00 0.15
C
ATOM 11525 C CYS B 134 -13.332 2.238 -9.038 1.00 0.14
C
ATOM 11526 O CYS B 134 -14.425 1.698 -8.893 1.00 0.14
O
ATOM 11527 CB CYS B 134 -11.388 1.341 -7.770 1.00 0.22
C
ATOM 11528 SG CYS B 134 -10.082 0.101 -7.621 1.00 0.22
S
ATOM 11529 H CYS B 134 -10.265 2.237 -9.874 1.00 0.18
H
ATOM 11530 HA CYS B 134 -12.359 0.373 -9.412 1.00 0.18
H
ATOM 11531 1HB CYS B 134 -10.956 2.311 -7.539 1.00 0.27
H
ATOM 11532 2HB CYS B 134 -12.134 1.131 -7.012 1.00 0.27
H
ATOM 11533 HG CYS B 134 -9.749 0.402 -6.369 1.00 0.27
H
ATOM 11534 N GLY B 135 -13.183 3.563 -9.143 1.00 0.13
N
ATOM 11535 CA GLY B 135 -14.336 4.463 -9.144 1.00 0.13
C
ATOM 11536 C GLY B 135 -15.253 4.168 -10.330 1.00 0.14
C
ATOM 11537 O GLY B 135 -16.477 4.101 -10.174 1.00 0.16
O
ATOM 11538 H GLY B 135 -12.250 3.953 -9.212 1.00 0.16
H
ATOM 11539 1HA GLY B 135 -14.893 4.341 -8.217 1.00 0.16
H
ATOM 11540 2HA GLY B 135 -13.995 5.497 -9.185 1.00 0.16
H
ATOM 11541 N MET B 136 -14.647 3.976 -11.507 1.00 0.13
N
ATOM 11542 CA MET B 136 -15.377 3.633 -12.722 1.00 0.15
C
ATOM 11543 C MET B 136 -16.136 2.329 -12.577 1.00 0.15
C
ATOM 11544 O MET B 136 -17.318 2.251 -12.920 1.00 0.16
O
ATOM 11545 CB MET B 136 -14.418 3.509 -13.907 1.00 0.22
C
ATOM 11546 CG MET B 136 -13.783 4.794 -14.390 1.00 0.22
C
ATOM 11547 SD MET B 136 -12.619 4.491 -15.724 1.00 0.22
S
ATOM 11548 CE MET B 136 -12.221 6.164 -16.199 1.00 0.22
C
ATOM 11549 H MET B 136 -13.640 4.079 -11.559 1.00 0.16
H
ATOM 11550 HA MET B 136 -16.103 4.417 -12.920 1.00 0.18
H
ATOM 11551 1HB MET B 136 -13.612 2.828 -13.652 1.00 0.27
H

ATOM 11552 2HB MET B 136   -14.950   3.072 -14.753 1.00 0.27
H
ATOM 11553 1HG MET B 136   -14.548   5.475 -14.756 1.00 0.27
H
ATOM 11554 2HG MET B 136   -13.255   5.279 -13.571 1.00 0.27
H
ATOM 11555 1HE MET B 136   -11.501   6.148 -17.018 1.00 0.27
H
ATOM 11556 2HE MET B 136   -13.129   6.665 -16.524 1.00 0.27
H
ATOM 11557 3HE MET B 136   -11.793   6.695 -15.347 1.00 0.27
H
ATOM 11558 N   ALA B 137   -15.432   1.308 -12.090 1.00 0.17
N
ATOM 11559 CA ALA B 137   -15.972 -0.028 -11.900 1.00 0.18
C
ATOM 11560 C   ALA B 137   -17.088 -0.027 -10.875 1.00 0.19
C
ATOM 11561 O   ALA B 137   -18.115 -0.672 -11.085 1.00 0.21
O
ATOM 11562 CB ALA B 137   -14.859 -0.971 -11.482 1.00 0.27
C
ATOM 11563 H   ALA B 137   -14.463   1.459 -11.847 1.00 0.20
H
ATOM 11564 HA ALA B 137   -16.388 -0.365 -12.848 1.00 0.22
H
ATOM 11565 1HB ALA B 137   -15.254 -1.976 -11.361 1.00 0.32
H
ATOM 11566 2HB ALA B 137   -14.082 -0.975 -12.247 1.00 0.32
H
ATOM 11567 3HB ALA B 137   -14.437 -0.628 -10.544 1.00 0.32
H
ATOM 11568 N   TYR B 138   -16.885   0.711 -9.780 1.00 0.20
N
ATOM 11569 CA TYR B 138   -17.873   0.850 -8.725 1.00 0.21
C
ATOM 11570 C   TYR B 138   -19.162   1.398 -9.301 1.00 0.23
C
ATOM 11571 O   TYR B 138   -20.237   0.871 -9.037 1.00 0.26
O
ATOM 11572 CB TYR B 138   -17.375   1.780 -7.614 1.00 0.32
C
ATOM 11573 CG TYR B 138   -18.363   1.922 -6.479 1.00 0.32
C
ATOM 11574 CD1 TYR B 138   -18.217   1.150 -5.334 1.00 0.32
C
ATOM 11575 CD2 TYR B 138   -19.439   2.792 -6.598 1.00 0.32
C
ATOM 11576 CE1 TYR B 138   -19.138   1.259 -4.312 1.00 0.32
C
ATOM 11577 CE2 TYR B 138   -20.362   2.892 -5.577 1.00 0.32
C
ATOM 11578 CZ TYR B 138   -20.214   2.128 -4.438 1.00 0.32
C
ATOM 11579 OH TYR B 138   -21.134   2.225 -3.422 1.00 0.32
O
ATOM 11580 H   TYR B 138   -16.008   1.193 -9.665 1.00 0.24
H

```
ATOM  11581  HA  TYR B 138      -18.084  -0.133  -8.304  1.00  0.25
H
ATOM  11582 1HB  TYR B 138      -16.439   1.396  -7.208  1.00  0.38
H
ATOM  11583 2HB  TYR B 138      -17.176   2.769  -8.023  1.00  0.38
H
ATOM  11584  HD1 TYR B 138      -17.378   0.460  -5.243  1.00  0.38
H
ATOM  11585  HD2 TYR B 138      -19.560   3.392  -7.499  1.00  0.38
H
ATOM  11586  HE1 TYR B 138      -19.030   0.658  -3.413  1.00  0.38
H
ATOM  11587  HE2 TYR B 138      -21.210   3.572  -5.671  1.00  0.38
H
ATOM  11588  HH  TYR B 138      -20.861   1.666  -2.690  1.00  0.38
H
ATOM  11589  N   THR B 139      -19.048   2.470 -10.084  1.00  0.24
N
ATOM  11590  CA  THR B 139      -20.205   3.094 -10.706  1.00  0.27
C
ATOM  11591  C   THR B 139      -20.904   2.121 -11.663  1.00  0.24
C
ATOM  11592  O   THR B 139      -22.119   1.918 -11.584  1.00  0.27
O
ATOM  11593  CB  THR B 139      -19.770   4.368 -11.462  1.00  0.41
C
ATOM  11594  OG1 THR B 139      -19.196   5.298 -10.533  1.00  0.41
O
ATOM  11595  CG2 THR B 139      -20.946   5.026 -12.162  1.00  0.41
C
ATOM  11596  H   THR B 139      -18.134   2.878 -10.247  1.00  0.29
H
ATOM  11597  HA  THR B 139      -20.910   3.373  -9.924  1.00  0.32
H
ATOM  11598  HB  THR B 139      -19.019   4.104 -12.205  1.00  0.49
H
ATOM  11599  HG1 THR B 139      -18.378   4.932 -10.183  1.00  0.49
H
ATOM  11600 1HG2 THR B 139      -20.601   5.917 -12.686  1.00  0.49
H
ATOM  11601 2HG2 THR B 139      -21.379   4.329 -12.877  1.00  0.49
H
ATOM  11602 3HG2 THR B 139      -21.698   5.312 -11.432  1.00  0.49
H
ATOM  11603  N   GLY B 140      -20.131   1.467 -12.524  1.00  0.23
N
ATOM  11604  CA  GLY B 140      -20.691   0.504 -13.465  1.00  0.22
C
ATOM  11605  C   GLY B 140      -21.450  -0.614 -12.759  1.00  0.23
C
ATOM  11606  O   GLY B 140      -22.481  -1.074 -13.241  1.00  0.29
O
ATOM  11607  H   GLY B 140      -19.137   1.654 -12.553  1.00  0.28
H
ATOM  11608 1HA  GLY B 140      -21.357   1.020 -14.153  1.00  0.26
H
ATOM  11609 2HA  GLY B 140      -19.887   0.077 -14.062  1.00  0.26
H
```

ATOM 11610 N LYS B 141 -20.943 -1.054 -11.614 1.00 0.27
N
ATOM 11611 CA LYS B 141 -21.567 -2.130 -10.861 1.00 0.32
C
ATOM 11612 C LYS B 141 -22.742 -1.708 -9.972 1.00 0.31
C
ATOM 11613 O LYS B 141 -23.769 -2.388 -9.933 1.00 0.36
O
ATOM 11614 CB LYS B 141 -20.511 -2.809 -9.991 1.00 0.48
C
ATOM 11615 CG LYS B 141 -21.027 -3.988 -9.187 1.00 0.48
C
ATOM 11616 CD LYS B 141 -19.901 -4.690 -8.448 1.00 0.48
C
ATOM 11617 CE LYS B 141 -20.440 -5.846 -7.624 1.00 0.48
C
ATOM 11618 NZ LYS B 141 -21.007 -6.909 -8.503 1.00 0.48
N
ATOM 11619 H LYS B 141 -20.079 -0.660 -11.265 1.00 0.32
H
ATOM 11620 HA LYS B 141 -21.946 -2.861 -11.576 1.00 0.38
H
ATOM 11621 1HB LYS B 141 -19.696 -3.167 -10.621 1.00 0.58
H
ATOM 11622 2HB LYS B 141 -20.089 -2.084 -9.294 1.00 0.58
H
ATOM 11623 1HG LYS B 141 -21.763 -3.640 -8.461 1.00 0.58
H
ATOM 11624 2HG LYS B 141 -21.510 -4.701 -9.856 1.00 0.58
H
ATOM 11625 1HD LYS B 141 -19.181 -5.081 -9.171 1.00 0.58
H
ATOM 11626 2HD LYS B 141 -19.387 -3.988 -7.796 1.00 0.58
H
ATOM 11627 1HE LYS B 141 -19.634 -6.271 -7.029 1.00 0.58
H
ATOM 11628 2HE LYS B 141 -21.220 -5.485 -6.955 1.00 0.58
H
ATOM 11629 1HZ LYS B 141 -21.352 -7.673 -7.941 1.00 0.58
H
ATOM 11630 2HZ LYS B 141 -21.760 -6.529 -9.058 1.00 0.58
H
ATOM 11631 3HZ LYS B 141 -20.271 -7.240 -9.114 1.00 0.58
H
ATOM 11632 N TYR B 142 -22.574 -0.615 -9.233 1.00 0.33
N
ATOM 11633 CA TYR B 142 -23.548 -0.198 -8.230 1.00 0.41
C
ATOM 11634 C TYR B 142 -24.445 0.990 -8.575 1.00 0.44
C
ATOM 11635 O TYR B 142 -25.533 1.113 -8.011 1.00 0.66
O
ATOM 11636 CB TYR B 142 -22.802 0.160 -6.942 1.00 0.61
C
ATOM 11637 CG TYR B 142 -22.076 -0.982 -6.265 1.00 0.61
C
ATOM 11638 CD1 TYR B 142 -20.703 -1.125 -6.402 1.00 0.61
C

```
ATOM  11639  CD2 TYR B 142     -22.785  -1.871  -5.478  1.00  0.61
C
ATOM  11640  CE1 TYR B 142     -20.042  -2.148  -5.753  1.00  0.61
C
ATOM  11641  CE2 TYR B 142     -22.128  -2.894  -4.831  1.00  0.61
C
ATOM  11642  CZ  TYR B 142     -20.763  -3.034  -4.963  1.00  0.61
C
ATOM  11643  OH  TYR B 142     -20.123  -4.054  -4.301  1.00  0.61
O
ATOM  11644  H   TYR B 142     -21.729  -0.078  -9.328  1.00  0.40
H
ATOM  11645  HA  TYR B 142     -24.202  -1.049  -8.033  1.00  0.49
H
ATOM  11646 1HB  TYR B 142     -22.069   0.937  -7.163  1.00  0.74
H
ATOM  11647 2HB  TYR B 142     -23.507   0.580  -6.227  1.00  0.74
H
ATOM  11648  HD1 TYR B 142     -20.143  -0.425  -7.017  1.00  0.74
H
ATOM  11649  HD2 TYR B 142     -23.863  -1.758  -5.365  1.00  0.74
H
ATOM  11650  HE1 TYR B 142     -18.963  -2.256  -5.858  1.00  0.74
H
ATOM  11651  HE2 TYR B 142     -22.688  -3.592  -4.207  1.00  0.74
H
ATOM  11652  HH  TYR B 142     -19.203  -4.100  -4.580  1.00  0.74
H
ATOM  11653  N   LEU B 143     -23.998   1.880  -9.449  1.00  0.40
N
ATOM  11654  CA  LEU B 143     -24.768   3.084  -9.742  1.00  0.49
C
ATOM  11655  C   LEU B 143     -25.436   2.987 -11.107  1.00  0.56
C
ATOM  11656  O   LEU B 143     -26.662   3.049 -11.213  1.00  1.04
O
ATOM  11657  CB  LEU B 143     -23.847   4.305  -9.671  1.00  0.73
C
ATOM  11658  CG  LEU B 143     -23.208   4.572  -8.293  1.00  0.73
C
ATOM  11659  CD1 LEU B 143     -22.255   5.758  -8.391  1.00  0.73
C
ATOM  11660  CD2 LEU B 143     -24.300   4.835  -7.266  1.00  0.73
C
ATOM  11661  H   LEU B 143     -23.118   1.750  -9.923  1.00  0.48
H
ATOM  11662  HA  LEU B 143     -25.546   3.191  -8.988  1.00  0.59
H
ATOM  11663 1HB  LEU B 143     -23.043   4.162 -10.385  1.00  0.88
H
ATOM  11664 2HB  LEU B 143     -24.414   5.191  -9.957  1.00  0.88
H
ATOM  11665  HG  LEU B 143     -22.629   3.700  -7.987  1.00  0.88
H
ATOM  11666 1HD1 LEU B 143     -21.796   5.937  -7.419  1.00  0.88
H
ATOM  11667 2HD1 LEU B 143     -21.478   5.543  -9.124  1.00  0.88
H
```

```
ATOM  11668 3HD1 LEU B 143     -22.810   6.645  -8.698  1.00  0.88
H
ATOM  11669 1HD2 LEU B 143     -23.847   5.016  -6.291  1.00  0.88
H
ATOM  11670 2HD2 LEU B 143     -24.878   5.711  -7.565  1.00  0.88
H
ATOM  11671 3HD2 LEU B 143     -24.959   3.969  -7.204  1.00  0.88
H
ATOM  11672  N   ASP B 144     -24.625   2.848 -12.150  1.00  0.62
N
ATOM  11673  CA  ASP B 144     -25.140   2.664 -13.496  1.00  1.04
C
ATOM  11674  C   ASP B 144     -25.747   1.291 -13.627  1.00  0.51
C
ATOM  11675  O   ASP B 144     -26.804   1.128 -14.238  1.00  0.48
O
ATOM  11676  CB  ASP B 144     -24.043   2.807 -14.552  1.00  1.56
C
ATOM  11677  CG  ASP B 144     -23.566   4.226 -14.759  1.00  1.56
C
ATOM  11678  OD1 ASP B 144     -24.232   5.135 -14.325  1.00  1.56
O
ATOM  11679  OD2 ASP B 144     -22.530   4.391 -15.360  1.00  1.56
O
ATOM  11680  H   ASP B 144     -23.632   2.822 -11.994  1.00  0.74
H
ATOM  11681  HA  ASP B 144     -25.914   3.407 -13.679  1.00  1.25
H
ATOM  11682 1HB  ASP B 144     -23.188   2.196 -14.264  1.00  1.87
H
ATOM  11683 2HB  ASP B 144     -24.409   2.422 -15.505  1.00  1.87
H
ATOM  11684  N   LYS B 145     -25.058   0.305 -13.047  1.00  0.58
N
ATOM  11685  CA  LYS B 145     -25.493  -1.092 -13.091  1.00  0.59
C
ATOM  11686  C   LYS B 145     -25.569  -1.538 -14.549  1.00  0.61
C
ATOM  11687  O   LYS B 145     -26.598  -2.025 -15.020  1.00  1.01
O
ATOM  11688  CB  LYS B 145     -26.835  -1.263 -12.381  1.00  0.89
C
ATOM  11689  CG  LYS B 145     -26.809  -0.857 -10.914  1.00  0.89
C
ATOM  11690  CD  LYS B 145     -28.149  -1.106 -10.247  1.00  0.89
C
ATOM  11691  CE  LYS B 145     -28.132  -0.656  -8.794  1.00  0.89
C
ATOM  11692  NZ  LYS B 145     -29.445  -0.877  -8.130  1.00  0.89
N
ATOM  11693  H   LYS B 145     -24.189   0.547 -12.572  1.00  0.70
H
ATOM  11694  HA  LYS B 145     -24.753  -1.712 -12.585  1.00  0.71
H
ATOM  11695 1HB  LYS B 145     -27.605  -0.678 -12.882  1.00  1.06
H
ATOM  11696 2HB  LYS B 145     -27.138  -2.309 -12.431  1.00  1.06
H
```

```
ATOM  11697 1HG  LYS B 145     -26.044  -1.430 -10.392  1.00  1.06
H
ATOM  11698 2HG  LYS B 145     -26.564   0.201 -10.832  1.00  1.06
H
ATOM  11699 1HD  LYS B 145     -28.929  -0.560 -10.780  1.00  1.06
H
ATOM  11700 2HD  LYS B 145     -28.383  -2.170 -10.284  1.00  1.06
H
ATOM  11701 1HE  LYS B 145     -27.366  -1.211  -8.256  1.00  1.06
H
ATOM  11702 2HE  LYS B 145     -27.893   0.406  -8.752  1.00  1.06
H
ATOM  11703 1HZ  LYS B 145     -29.394  -0.565  -7.171  1.00  1.06
H
ATOM  11704 2HZ  LYS B 145     -30.161  -0.355  -8.616  1.00  1.06
H
ATOM  11705 3HZ  LYS B 145     -29.674  -1.861  -8.153  1.00  1.06
H
ATOM  11706  N   ALA B 146     -24.457  -1.351 -15.248  1.00  0.39
N
ATOM  11707  CA  ALA B 146     -24.340  -1.637 -16.668  1.00  0.54
C
ATOM  11708  C   ALA B 146     -23.257  -2.674 -16.909  1.00  0.90
C
ATOM  11709  O   ALA B 146     -22.339  -2.823 -16.104  1.00  3.54
O
ATOM  11710  CB  ALA B 146     -24.047  -0.358 -17.422  1.00  0.81
C
ATOM  11711  H   ALA B 146     -23.652  -0.985 -14.759  1.00  0.47
H
ATOM  11712  HA  ALA B 146     -25.285  -2.050 -17.018  1.00  0.65
H
ATOM  11713 1HB  ALA B 146     -23.973  -0.565 -18.488  1.00  0.97
H
ATOM  11714 2HB  ALA B 146     -24.850   0.357 -17.245  1.00  0.97
H
ATOM  11715 3HB  ALA B 146     -23.110   0.053 -17.063  1.00  0.97
H
ATOM  11716  N   SER B 147     -23.360  -3.401 -18.018  1.00  1.26
N
ATOM  11717  CA  SER B 147     -22.417  -4.467 -18.331  1.00  1.59
C
ATOM  11718  C   SER B 147     -21.038  -4.040 -18.854  1.00  2.85
C
ATOM  11719  O   SER B 147     -20.303  -4.891 -19.352  1.00 16.16
O
ATOM  11720  CB  SER B 147     -23.039  -5.416 -19.332  1.00  2.39
C
ATOM  11721  OG  SER B 147     -23.296  -4.768 -20.543  1.00  2.39
O
ATOM  11722  H   SER B 147     -24.131  -3.241 -18.658  1.00  1.51
H
ATOM  11723  HA  SER B 147     -22.251  -5.026 -17.407  1.00  1.91
H
ATOM  11724 1HB  SER B 147     -22.364  -6.255 -19.501  1.00  2.86
H
ATOM  11725 2HB  SER B 147     -23.967  -5.818 -18.928  1.00  2.86
H
```

```
ATOM  11726  HG  SER B 147     -24.025  -4.165 -20.359  1.00  2.86
H
ATOM  11727  N   TYR B 148     -20.662  -2.763 -18.764  1.00  0.99
N
ATOM  11728  CA  TYR B 148     -19.320  -2.427 -19.219  1.00  0.49
C
ATOM  11729  C   TYR B 148     -18.282  -2.845 -18.175  1.00  0.58
C
ATOM  11730  O   TYR B 148     -18.566  -2.911 -16.978  1.00  2.18
O
ATOM  11731  CB  TYR B 148     -19.178  -0.934 -19.571  1.00  0.73
C
ATOM  11732  CG  TYR B 148     -19.489   0.085 -18.487  1.00  0.73
C
ATOM  11733  CD1 TYR B 148     -18.538   0.418 -17.529  1.00  0.73
C
ATOM  11734  CD2 TYR B 148     -20.718   0.729 -18.488  1.00  0.73
C
ATOM  11735  CE1 TYR B 148     -18.823   1.386 -16.575  1.00  0.73
C
ATOM  11736  CE2 TYR B 148     -21.000   1.696 -17.538  1.00  0.73
C
ATOM  11737  CZ  TYR B 148     -20.063   2.028 -16.586  1.00  0.73
C
ATOM  11738  OH  TYR B 148     -20.349   2.998 -15.645  1.00  0.73
O
ATOM  11739  H   TYR B 148     -21.268  -2.053 -18.383  1.00  1.19
H
ATOM  11740  HA  TYR B 148     -19.113  -2.998 -20.125  1.00  0.59
H
ATOM  11741 1HB  TYR B 148     -18.157  -0.754 -19.907  1.00  0.88
H
ATOM  11742 2HB  TYR B 148     -19.827  -0.718 -20.420  1.00  0.88
H
ATOM  11743  HD1 TYR B 148     -17.566  -0.076 -17.531  1.00  0.88
H
ATOM  11744  HD2 TYR B 148     -21.461   0.479 -19.244  1.00  0.88
H
ATOM  11745  HE1 TYR B 148     -18.077   1.647 -15.827  1.00  0.88
H
ATOM  11746  HE2 TYR B 148     -21.967   2.201 -17.549  1.00  0.88
H
ATOM  11747  HH  TYR B 148     -21.255   3.317 -15.752  1.00  0.88
H
ATOM  11748  N   ARG B 149     -17.080  -3.119 -18.661  1.00  0.30
N
ATOM  11749  CA  ARG B 149     -15.954  -3.587 -17.859  1.00  0.33
C
ATOM  11750  C   ARG B 149     -14.837  -2.563 -17.848  1.00  0.36
C
ATOM  11751  O   ARG B 149     -14.692  -1.797 -18.798  1.00  1.58
O
ATOM  11752  CB  ARG B 149     -15.456  -4.931 -18.391  1.00  0.49
C
ATOM  11753  CG  ARG B 149     -14.317  -5.575 -17.614  1.00  0.49
C
ATOM  11754  CD  ARG B 149     -14.031  -6.955 -18.081  1.00  0.49
C
```

ATOM 11755 NE ARG B 149   -15.129 -7.854 -17.768 1.00 0.49
N
ATOM 11756 CZ ARG B 149   -15.313 -8.419 -16.561 1.00 0.49
C
ATOM 11757 NH1 ARG B 149   -14.459 -8.168 -15.595 1.00 0.49
N
ATOM 11758 NH2 ARG B 149   -16.348 -9.211 -16.341 1.00 0.49
N
ATOM 11759 H   ARG B 149   -16.946 -3.019 -19.660 1.00 0.36
H
ATOM 11760 HA ARG B 149   -16.298 -3.734 -16.834 1.00 0.40
H
ATOM 11761 1HB ARG B 149   -16.283 -5.640 -18.406 1.00 0.59
H
ATOM 11762 2HB ARG B 149   -15.119 -4.805 -19.420 1.00 0.59
H
ATOM 11763 1HG ARG B 149   -13.409 -4.981 -17.721 1.00 0.59
H
ATOM 11764 2HG ARG B 149   -14.591 -5.640 -16.565 1.00 0.59
H
ATOM 11765 1HD ARG B 149   -13.895 -6.954 -19.160 1.00 0.59
H
ATOM 11766 2HD ARG B 149   -13.131 -7.331 -17.597 1.00 0.59
H
ATOM 11767 HE ARG B 149   -15.801 -8.057 -18.496 1.00 0.59
H
ATOM 11768 1HH1 ARG B 149   -13.674 -7.556 -15.764 1.00 0.59
H
ATOM 11769 2HH1 ARG B 149   -14.610 -8.553 -14.671 1.00 0.59
H
ATOM 11770 1HH2 ARG B 149   -17.007 -9.400 -17.084 1.00 0.59
H
ATOM 11771 2HH2 ARG B 149   -16.481 -9.625 -15.430 1.00 0.59
H
ATOM 11772 N   VAL B 150   -14.085 -2.516 -16.755 1.00 0.19
N
ATOM 11773 CA VAL B 150   -13.000 -1.558 -16.625 1.00 0.15
C
ATOM 11774 C   VAL B 150   -11.641 -2.252 -16.562 1.00 0.17
C
ATOM 11775 O   VAL B 150   -11.412 -3.106 -15.705 1.00 0.23
O
ATOM 11776 CB VAL B 150   -13.192 -0.704 -15.356 1.00 0.22
C
ATOM 11777 CG1 VAL B 150   -12.054   0.300 -15.230 1.00 0.22
C
ATOM 11778 CG2 VAL B 150   -14.543 -0.011 -15.400 1.00 0.22
C
ATOM 11779 H   VAL B 150   -14.259 -3.166 -16.002 1.00 0.23
H
ATOM 11780 HA VAL B 150   -13.013 -0.899 -17.493 1.00 0.18
H
ATOM 11781 HB VAL B 150   -13.149 -1.354 -14.482 1.00 0.27
H
ATOM 11782 1HG1 VAL B 150   -12.186   0.885 -14.320 1.00 0.27
H
ATOM 11783 2HG1 VAL B 150   -11.104 -0.230 -15.185 1.00 0.27
H

ATOM 11784 3HG1 VAL B 150   -12.059   0.966 -16.093 1.00 0.27
H
ATOM 11785 1HG2 VAL B 150   -14.674   0.576 -14.495 1.00 0.27
H
ATOM 11786 2HG2 VAL B 150   -14.598   0.643 -16.262 1.00 0.27
H
ATOM 11787 3HG2 VAL B 150   -15.334 -0.759 -15.463 1.00 0.27
H
ATOM 11788 N   PHE B 151   -10.750 -1.873 -17.468 1.00 0.16
N
ATOM 11789 CA PHE B 151   -9.396 -2.399 -17.500 1.00 0.19
C
ATOM 11790 C   PHE B 151   -8.402 -1.316 -17.115 1.00 0.24
C
ATOM 11791 O   PHE B 151   -8.577 -0.156 -17.482 1.00 0.35
O
ATOM 11792 CB PHE B 151   -9.058 -2.912 -18.896 1.00 0.29
C
ATOM 11793 CG PHE B 151   -9.828 -4.127 -19.301 1.00 0.29
C
ATOM 11794 CD1 PHE B 151   -11.068 -4.008 -19.904 1.00 0.29
C
ATOM 11795 CD2 PHE B 151   -9.307 -5.391 -19.085 1.00 0.29
C
ATOM 11796 CE1 PHE B 151   -11.772 -5.131 -20.283 1.00 0.29
C
ATOM 11797 CE2 PHE B 151   -10.010 -6.516 -19.460 1.00 0.29
C
ATOM 11798 CZ PHE B 151   -11.243 -6.383 -20.060 1.00 0.29
C
ATOM 11799 H   PHE B 151   -11.016 -1.185 -18.156 1.00 0.19
H
ATOM 11800 HA PHE B 151   -9.321 -3.218 -16.789 1.00 0.23
H
ATOM 11801 1HB PHE B 151   -9.265 -2.129 -19.622 1.00 0.34
H
ATOM 11802 2HB PHE B 151   -7.995 -3.146 -18.956 1.00 0.34
H
ATOM 11803 HD1 PHE B 151   -11.483 -3.014 -20.079 1.00 0.34
H
ATOM 11804 HD2 PHE B 151   -8.331 -5.492 -18.611 1.00 0.34
H
ATOM 11805 HE1 PHE B 151   -12.748 -5.027 -20.758 1.00 0.34
H
ATOM 11806 HE2 PHE B 151   -9.594 -7.507 -19.284 1.00 0.34
H
ATOM 11807 HZ PHE B 151   -11.800 -7.270 -20.358 1.00 0.34
H
ATOM 11808 N   CYS B 152   -7.356 -1.699 -16.395 1.00 0.19
N
ATOM 11809 CA CYS B 152   -6.330 -0.748 -15.987 1.00 0.20
C
ATOM 11810 C   CYS B 152   -4.941 -1.334 -16.173 1.00 0.27
C
ATOM 11811 O   CYS B 152   -4.666 -2.428 -15.691 1.00 0.47
O
ATOM 11812 CB CYS B 152   -6.523 -0.353 -14.526 1.00 0.30
C

```
ATOM  11813  SG  CYS B 152      -5.315   0.844 -13.928  1.00  0.30
S
ATOM  11814  H   CYS B 152      -7.291  -2.667 -16.103  1.00  0.23
H
ATOM  11815  HA  CYS B 152      -6.421   0.143 -16.604  1.00  0.24
H
ATOM  11816 1HB  CYS B 152      -7.516   0.075 -14.398  1.00  0.36
H
ATOM  11817 2HB  CYS B 152      -6.462  -1.239 -13.896  1.00  0.36
H
ATOM  11818  HG  CYS B 152      -5.620   1.798 -14.802  1.00  0.36
H
ATOM  11819  N   LEU B 153      -4.068  -0.607 -16.864  1.00  0.23
N
ATOM  11820  CA  LEU B 153      -2.720  -1.105 -17.120  1.00  0.27
C
ATOM  11821  C   LEU B 153      -1.671  -0.337 -16.328  1.00  0.24
C
ATOM  11822  O   LEU B 153      -1.474   0.865 -16.525  1.00  0.25
O
ATOM  11823  CB  LEU B 153      -2.420  -1.045 -18.623  1.00  0.41
C
ATOM  11824  CG  LEU B 153      -1.007  -1.457 -19.056  1.00  0.41
C
ATOM  11825  CD1 LEU B 153      -0.745  -2.901 -18.667  1.00  0.41
C
ATOM  11826  CD2 LEU B 153      -0.892  -1.305 -20.565  1.00  0.41
C
ATOM  11827  H   LEU B 153      -4.351   0.300 -17.214  1.00  0.28
H
ATOM  11828  HA  LEU B 153      -2.676  -2.149 -16.810  1.00  0.32
H
ATOM  11829 1HB  LEU B 153      -3.125  -1.697 -19.138  1.00  0.49
H
ATOM  11830 2HB  LEU B 153      -2.587  -0.025 -18.968  1.00  0.49
H
ATOM  11831  HG  LEU B 153      -0.269  -0.826 -18.560  1.00  0.49
H
ATOM  11832 1HD1 LEU B 153       0.257  -3.188 -18.983  1.00  0.49
H
ATOM  11833 2HD1 LEU B 153      -0.831  -3.008 -17.587  1.00  0.49
H
ATOM  11834 3HD1 LEU B 153      -1.477  -3.542 -19.156  1.00  0.49
H
ATOM  11835 1HD2 LEU B 153       0.108  -1.595 -20.878  1.00  0.49
H
ATOM  11836 2HD2 LEU B 153      -1.626  -1.948 -21.051  1.00  0.49
H
ATOM  11837 3HD2 LEU B 153      -1.075  -0.271 -20.846  1.00  0.49
H
ATOM  11838  N   MET B 154      -1.016  -1.059 -15.421  1.00  0.24
N
ATOM  11839  CA  MET B 154      -0.016  -0.504 -14.519  1.00  0.15
C
ATOM  11840  C   MET B 154       1.370  -1.028 -14.815  1.00  0.16
C
ATOM  11841  O   MET B 154       1.528  -2.107 -15.386  1.00  0.24
O
```

ATOM 11842 CB MET B 154   -0.345 -0.845 -13.065 1.00 0.22
C
ATOM 11843 CG MET B 154   -1.640 -0.277 -12.517 1.00 0.22
C
ATOM 11844 SD MET B 154   -1.906 -0.768 -10.808 1.00 0.22
S
ATOM 11845 CE MET B 154   -2.295 -2.495 -11.042 1.00 0.22
C
ATOM 11846 H   MET B 154   -1.231 -2.044 -15.346 1.00 0.29
H
ATOM 11847 HA MET B 154   0.002   0.577 -14.641 1.00 0.18
H
ATOM 11848 1HB MET B 154   -0.392 -1.928 -12.955 1.00 0.27
H
ATOM 11849 2HB MET B 154   0.464 -0.492 -12.423 1.00 0.27
H
ATOM 11850 1HG MET B 154   -1.606   0.809 -12.557 1.00 0.27
H
ATOM 11851 2HG MET B 154   -2.484 -0.621 -13.116 1.00 0.27
H
ATOM 11852 1HE MET B 154   -2.492 -2.960 -10.077 1.00 0.27
H
ATOM 11853 2HE MET B 154   -3.175 -2.576 -11.675 1.00 0.27
H
ATOM 11854 3HE MET B 154   -1.454 -2.996 -11.522 1.00 0.27
H
ATOM 11855 N   GLY B 155   2.374 -0.261 -14.416 1.00 0.20
N
ATOM 11856 CA GLY B 155   3.750 -0.669 -14.594 1.00 0.21
C
ATOM 11857 C   GLY B 155   4.220 -1.503 -13.412 1.00 0.23
C
ATOM 11858 O   GLY B 155   3.488 -1.699 -12.440 1.00 0.38
O
ATOM 11859 H   GLY B 155   2.174   0.624 -13.966 1.00 0.24
H
ATOM 11860 1HA GLY B 155   3.844 -1.248 -15.513 1.00 0.25
H
ATOM 11861 2HA GLY B 155   4.382   0.211 -14.697 1.00 0.25
H
ATOM 11862 N   ASP B 156   5.456 -1.969 -13.508 1.00 0.25
N
ATOM 11863 CA ASP B 156   6.100 -2.781 -12.491 1.00 0.29
C
ATOM 11864 C   ASP B 156   6.472 -1.915 -11.282 1.00 0.27
C
ATOM 11865 O   ASP B 156   5.895 -2.060 -10.201 1.00 0.28
O
ATOM 11866 CB ASP B 156   7.320 -3.484 -13.098 1.00 0.43
C
ATOM 11867 CG ASP B 156   7.904 -4.539 -12.193 1.00 0.43
C
ATOM 11868 OD1 ASP B 156   7.340 -4.760 -11.145 1.00 0.43
O
ATOM 11869 OD2 ASP B 156   8.890 -5.135 -12.553 1.00 0.43
O
ATOM 11870 H   ASP B 156   5.982 -1.756 -14.345 1.00 0.30
H

ATOM 11871 HA ASP B 156 5.393 -3.543 -12.158 1.00 0.35
H
ATOM 11872 1HB ASP B 156 7.035 -3.952 -14.040 1.00 0.52
H
ATOM 11873 2HB ASP B 156 8.096 -2.752 -13.325 1.00 0.52
H
ATOM 11874 N GLY B 157 7.441 -1.010 -11.478 1.00 0.25
N
ATOM 11875 CA GLY B 157 7.890 -0.087 -10.433 1.00 0.25
C
ATOM 11876 C GLY B 157 6.729 0.721 -9.855 1.00 0.22
C
ATOM 11877 O GLY B 157 6.709 1.015 -8.662 1.00 0.23
O
ATOM 11878 H GLY B 157 7.879 -0.957 -12.386 1.00 0.30
H
ATOM 11879 1HA GLY B 157 8.375 -0.652 -9.637 1.00 0.30
H
ATOM 11880 2HA GLY B 157 8.638 0.589 -10.845 1.00 0.30
H
ATOM 11881 N GLU B 158 5.757 1.054 -10.708 1.00 0.26
N
ATOM 11882 CA GLU B 158 4.547 1.773 -10.325 1.00 0.27
C
ATOM 11883 C GLU B 158 3.825 1.168 -9.115 1.00 0.30
C
ATOM 11884 O GLU B 158 3.236 1.898 -8.315 1.00 0.31
O
ATOM 11885 CB GLU B 158 3.586 1.842 -11.511 1.00 0.41
C
ATOM 11886 CG GLU B 158 2.310 2.586 -11.195 1.00 0.41
C
ATOM 11887 CD GLU B 158 1.432 2.846 -12.379 1.00 0.41
C
ATOM 11888 OE1 GLU B 158 1.528 2.171 -13.375 1.00 0.41
O
ATOM 11889 OE2 GLU B 158 0.666 3.777 -12.290 1.00 0.41
O
ATOM 11890 H GLU B 158 5.859 0.790 -11.677 1.00 0.31
H
ATOM 11891 HA GLU B 158 4.834 2.792 -10.061 1.00 0.32
H
ATOM 11892 1HB GLU B 158 4.072 2.340 -12.350 1.00 0.49
H
ATOM 11893 2HB GLU B 158 3.320 0.834 -11.829 1.00 0.49
H
ATOM 11894 1HG GLU B 158 1.743 1.991 -10.479 1.00 0.49
H
ATOM 11895 2HG GLU B 158 2.559 3.523 -10.712 1.00 0.49
H
ATOM 11896 N SER B 159 3.846 -0.167 -9.000 1.00 0.34
N
ATOM 11897 CA SER B 159 3.143 -0.872 -7.930 1.00 0.45
C
ATOM 11898 C SER B 159 3.694 -0.586 -6.534 1.00 0.55
C
ATOM 11899 O SER B 159 3.025 -0.875 -5.543 1.00 1.05
O

ATOM 11900 CB SER B 159 3.186 -2.364 -8.182 1.00 0.68
C
ATOM 11901 OG SER B 159 4.489 -2.862 -8.051 1.00 0.68
O
ATOM 11902 H SER B 159 4.376 -0.722 -9.660 1.00 0.41
H
ATOM 11903 HA SER B 159 2.101 -0.552 -7.950 1.00 0.54
H
ATOM 11904 1HB SER B 159 2.530 -2.870 -7.473 1.00 0.81
H
ATOM 11905 2HB SER B 159 2.812 -2.577 -9.181 1.00 0.81
H
ATOM 11906 HG SER B 159 4.992 -2.481 -8.782 1.00 0.81
H
ATOM 11907 N SER B 160 4.900 -0.013 -6.444 1.00 0.40
N
ATOM 11908 CA SER B 160 5.494 0.332 -5.156 1.00 0.47
C
ATOM 11909 C SER B 160 4.770 1.475 -4.454 1.00 0.46
C
ATOM 11910 O SER B 160 4.964 1.695 -3.259 1.00 0.62
O
ATOM 11911 CB SER B 160 6.944 0.705 -5.340 1.00 0.70
C
ATOM 11912 OG SER B 160 7.068 1.880 -6.091 1.00 0.70
O
ATOM 11913 H SER B 160 5.427 0.208 -7.279 1.00 0.48
H
ATOM 11914 HA SER B 160 5.443 -0.547 -4.515 1.00 0.56
H
ATOM 11915 1HB SER B 160 7.413 0.840 -4.366 1.00 0.85
H
ATOM 11916 2HB SER B 160 7.463 -0.108 -5.845 1.00 0.85
H
ATOM 11917 HG SER B 160 6.685 2.601 -5.578 1.00 0.85
H
ATOM 11918 N GLU B 161 3.947 2.209 -5.193 1.00 0.45
N
ATOM 11919 CA GLU B 161 3.204 3.298 -4.609 1.00 0.70
C
ATOM 11920 C GLU B 161 2.019 2.811 -3.776 1.00 0.30
C
ATOM 11921 O GLU B 161 1.220 1.999 -4.240 1.00 0.27
O
ATOM 11922 CB GLU B 161 2.737 4.221 -5.701 1.00 1.05
C
ATOM 11923 CG GLU B 161 2.001 5.407 -5.172 1.00 1.05
C
ATOM 11924 CD GLU B 161 2.880 6.321 -4.356 1.00 1.05
C
ATOM 11925 OE1 GLU B 161 4.075 6.154 -4.367 1.00 1.05
O
ATOM 11926 OE2 GLU B 161 2.343 7.226 -3.759 1.00 1.05
O
ATOM 11927 H GLU B 161 3.817 2.011 -6.178 1.00 0.54
H
ATOM 11928 HA GLU B 161 3.873 3.853 -3.951 1.00 0.84
H

```
ATOM  11929 1HB  GLU B 161       3.593   4.578  -6.273  1.00  1.26
H
ATOM  11930 2HB  GLU B 161       2.080   3.684  -6.385  1.00  1.26
H
ATOM  11931 1HG  GLU B 161       1.597   5.940  -6.024  1.00  1.26
H
ATOM  11932 2HG  GLU B 161       1.168   5.066  -4.564  1.00  1.26
H
ATOM  11933  N   GLY B 162       1.909   3.338  -2.551  1.00  0.24
N
ATOM  11934  CA  GLY B 162       0.876   2.962  -1.587  1.00  0.19
C
ATOM  11935  C   GLY B 162      -0.549   3.013  -2.114  1.00  0.12
C
ATOM  11936  O   GLY B 162      -1.355   2.133  -1.806  1.00  0.27
O
ATOM  11937  H   GLY B 162       2.595   4.021  -2.243  1.00  0.29
H
ATOM  11938 1HA  GLY B 162       1.083   1.974  -1.209  1.00  0.23
H
ATOM  11939 2HA  GLY B 162       0.955   3.631  -0.731  1.00  0.23
H
ATOM  11940  N   SER B 163      -0.845   4.029  -2.918  1.00  0.19
N
ATOM  11941  CA  SER B 163      -2.169   4.221  -3.496  1.00  0.39
C
ATOM  11942  C   SER B 163      -2.653   3.047  -4.354  1.00  0.15
C
ATOM  11943  O   SER B 163      -3.861   2.842  -4.485  1.00  0.36
O
ATOM  11944  CB  SER B 163      -2.162   5.499  -4.305  1.00  0.58
C
ATOM  11945  OG  SER B 163      -1.261   5.413  -5.371  1.00  0.58
O
ATOM  11946  H   SER B 163      -0.127   4.709  -3.125  1.00  0.23
H
ATOM  11947  HA  SER B 163      -2.876   4.345  -2.675  1.00  0.47
H
ATOM  11948 1HB  SER B 163      -3.163   5.706  -4.683  1.00  0.70
H
ATOM  11949 2HB  SER B 163      -1.882   6.327  -3.659  1.00  0.70
H
ATOM  11950  HG  SER B 163      -1.723   4.989  -6.097  1.00  0.70
H
ATOM  11951  N   VAL B 164      -1.721   2.266  -4.909  1.00  0.12
N
ATOM  11952  CA  VAL B 164      -2.073   1.098  -5.710  1.00  0.19
C
ATOM  11953  C   VAL B 164      -2.673   0.037  -4.816  1.00  0.19
C
ATOM  11954  O   VAL B 164      -3.701  -0.561  -5.135  1.00  0.54
O
ATOM  11955  CB  VAL B 164      -0.830   0.519  -6.416  1.00  0.29
C
ATOM  11956  CG1 VAL B 164      -1.186  -0.790  -7.098  1.00  0.29
C
ATOM  11957  CG2 VAL B 164      -0.295   1.521  -7.422  1.00  0.29
C
```

```
ATOM  11958  H   VAL B 164      -0.739   2.459  -4.760  1.00  0.14
H
ATOM  11959  HA  VAL B 164      -2.808   1.392  -6.460  1.00  0.23
H
ATOM  11960  HB  VAL B 164      -0.063   0.304  -5.673  1.00  0.34
H
ATOM  11961 1HG1 VAL B 164      -0.300  -1.203  -7.580  1.00  0.34
H
ATOM  11962 2HG1 VAL B 164      -1.555  -1.492  -6.354  1.00  0.34
H
ATOM  11963 3HG1 VAL B 164      -1.957  -0.612  -7.847  1.00  0.34
H
ATOM  11964 1HG2 VAL B 164       0.589   1.112  -7.911  1.00  0.34
H
ATOM  11965 2HG2 VAL B 164      -1.060   1.730  -8.171  1.00  0.34
H
ATOM  11966 3HG2 VAL B 164      -0.030   2.445  -6.907  1.00  0.34
H
ATOM  11967  N   TRP B 165      -2.002  -0.187  -3.696  1.00  0.25
N
ATOM  11968  CA  TRP B 165      -2.407  -1.154  -2.699  1.00  0.26
C
ATOM  11969  C   TRP B 165      -3.730  -0.736  -2.035  1.00  0.26
C
ATOM  11970  O   TRP B 165      -4.559  -1.590  -1.713  1.00  0.40
O
ATOM  11971  CB  TRP B 165      -1.265  -1.303  -1.690  1.00  0.39
C
ATOM  11972  CG  TRP B 165      -0.049  -1.993  -2.257  1.00  0.39
C
ATOM  11973  CD1 TRP B 165      -0.042  -3.085  -3.064  1.00  0.39
C
ATOM  11974  CD2 TRP B 165       1.341  -1.616  -2.104  1.00  0.39
C
ATOM  11975  NE1 TRP B 165       1.241  -3.425  -3.405  1.00  0.39
N
ATOM  11976  CE2 TRP B 165       2.102  -2.535  -2.829  1.00  0.39
C
ATOM  11977  CE3 TRP B 165       1.990  -0.592  -1.425  1.00  0.39
C
ATOM  11978  CZ2 TRP B 165       3.484  -2.463  -2.890  1.00  0.39
C
ATOM  11979  CZ3 TRP B 165       3.369  -0.512  -1.490  1.00  0.39
C
ATOM  11980  CH2 TRP B 165       4.101  -1.424  -2.198  1.00  0.39
C
ATOM  11981  H   TRP B 165      -1.162   0.347  -3.523  1.00  0.30
H
ATOM  11982  HA  TRP B 165      -2.559  -2.113  -3.189  1.00  0.31
H
ATOM  11983 1HB  TRP B 165      -0.965  -0.322  -1.322  1.00  0.47
H
ATOM  11984 2HB  TRP B 165      -1.612  -1.886  -0.842  1.00  0.47
H
ATOM  11985  HD1 TRP B 165      -0.924  -3.615  -3.383  1.00  0.47
H
ATOM  11986  HE1 TRP B 165       1.510  -4.203  -3.991  1.00  0.47
H
```

ATOM 11987 HE3 TRP B 165 1.416 0.125 -0.853 1.00 0.47
H
ATOM 11988 HZ2 TRP B 165 4.080 -3.180 -3.455 1.00 0.47
H
ATOM 11989 HZ3 TRP B 165 3.856 0.300 -0.959 1.00 0.47
H
ATOM 11990 HH2 TRP B 165 5.187 -1.329 -2.224 1.00 0.47
H
ATOM 11991 N GLU B 166 -3.944 0.584 -1.877 1.00 0.19
N
ATOM 11992 CA GLU B 166 -5.212 1.084 -1.338 1.00 0.18
C
ATOM 11993 C GLU B 166 -6.350 0.725 -2.278 1.00 0.18
C
ATOM 11994 O GLU B 166 -7.405 0.260 -1.838 1.00 0.21
O
ATOM 11995 CB GLU B 166 -5.193 2.608 -1.152 1.00 0.27
C
ATOM 11996 CG GLU B 166 -4.288 3.143 -0.058 1.00 0.27
C
ATOM 11997 CD GLU B 166 -4.339 4.653 0.072 1.00 0.27
C
ATOM 11998 OE1 GLU B 166 -5.050 5.285 -0.681 1.00 0.27
O
ATOM 11999 OE2 GLU B 166 -3.667 5.163 0.940 1.00 0.27
O
ATOM 12000 H GLU B 166 -3.212 1.240 -2.119 1.00 0.23
H
ATOM 12001 HA GLU B 166 -5.406 0.608 -0.380 1.00 0.22
H
ATOM 12002 1HB GLU B 166 -4.901 3.086 -2.087 1.00 0.32
H
ATOM 12003 2HB GLU B 166 -6.202 2.941 -0.916 1.00 0.32
H
ATOM 12004 1HG GLU B 166 -4.583 2.698 0.892 1.00 0.32
H
ATOM 12005 2HG GLU B 166 -3.265 2.835 -0.269 1.00 0.32
H
ATOM 12006 N ALA B 167 -6.114 0.926 -3.574 1.00 0.17
N
ATOM 12007 CA ALA B 167 -7.087 0.593 -4.597 1.00 0.18
C
ATOM 12008 C ALA B 167 -7.348 -0.911 -4.645 1.00 0.18
C
ATOM 12009 O ALA B 167 -8.491 -1.325 -4.849 1.00 0.19
O
ATOM 12010 CB ALA B 167 -6.615 1.103 -5.939 1.00 0.27
C
ATOM 12011 H ALA B 167 -5.237 1.345 -3.865 1.00 0.20
H
ATOM 12012 HA ALA B 167 -8.025 1.087 -4.345 1.00 0.22
H
ATOM 12013 1HB ALA B 167 -7.365 0.878 -6.696 1.00 0.32
H
ATOM 12014 2HB ALA B 167 -6.465 2.181 -5.884 1.00 0.32
H
ATOM 12015 3HB ALA B 167 -5.678 0.621 -6.198 1.00 0.32
H

```
ATOM  12016  N   PHE B 168      -6.307  -1.733  -4.431  1.00  0.19
N
ATOM  12017  CA  PHE B 168      -6.515  -3.178  -4.415  1.00  0.22
C
ATOM  12018  C   PHE B 168      -7.464  -3.579  -3.315  1.00  0.22
C
ATOM  12019  O   PHE B 168      -8.362  -4.399  -3.534  1.00  0.27
O
ATOM  12020  CB  PHE B 168      -5.236  -3.945  -4.107  1.00  0.33
C
ATOM  12021  CG  PHE B 168      -4.251  -4.027  -5.204  1.00  0.33
C
ATOM  12022  CD1 PHE B 168      -4.617  -3.834  -6.514  1.00  0.33
C
ATOM  12023  CD2 PHE B 168      -2.942  -4.313  -4.910  1.00  0.33
C
ATOM  12024  CE1 PHE B 168      -3.680  -3.912  -7.516  1.00  0.33
C
ATOM  12025  CE2 PHE B 168      -1.996  -4.387  -5.901  1.00  0.33
C
ATOM  12026  CZ  PHE B 168      -2.370  -4.177  -7.205  1.00  0.33
C
ATOM  12027  H   PHE B 168      -5.374  -1.356  -4.306  1.00  0.23
H
ATOM  12028  HA  PHE B 168      -6.924  -3.486  -5.374  1.00  0.26
H
ATOM  12029 1HB  PHE B 168      -4.743  -3.496  -3.249  1.00  0.40
H
ATOM  12030 2HB  PHE B 168      -5.499  -4.962  -3.827  1.00  0.40
H
ATOM  12031  HD1 PHE B 168      -5.654  -3.612  -6.749  1.00  0.40
H
ATOM  12032  HD2 PHE B 168      -2.667  -4.476  -3.871  1.00  0.40
H
ATOM  12033  HE1 PHE B 168      -3.974  -3.753  -8.552  1.00  0.40
H
ATOM  12034  HE2 PHE B 168      -0.957  -4.604  -5.656  1.00  0.40
H
ATOM  12035  HZ  PHE B 168      -1.631  -4.220  -7.990  1.00  0.40
H
ATOM  12036  N   ALA B 169      -7.268  -2.983  -2.142  1.00  0.19
N
ATOM  12037  CA  ALA B 169      -8.097  -3.268  -0.990  1.00  0.20
C
ATOM  12038  C   ALA B 169      -9.540  -2.864  -1.252  1.00  0.22
C
ATOM  12039  O   ALA B 169     -10.455  -3.625  -0.937  1.00  0.35
O
ATOM  12040  CB  ALA B 169      -7.553  -2.548   0.235  1.00  0.30
C
ATOM  12041  H   ALA B 169      -6.497  -2.331  -2.037  1.00  0.23
H
ATOM  12042  HA  ALA B 169      -8.076  -4.342  -0.813  1.00  0.24
H
ATOM  12043 1HB  ALA B 169      -8.169  -2.789   1.102  1.00  0.36
H
ATOM  12044 2HB  ALA B 169      -6.527  -2.869   0.417  1.00  0.36
H
```

```
ATOM  12045 3HB  ALA B 169      -7.571  -1.473   0.066  1.00  0.36
H
ATOM  12046  N   PHE B 170      -9.730  -1.677  -1.841  1.00  0.17
N
ATOM  12047  CA  PHE B 170     -11.055  -1.149  -2.161  1.00  0.18
C
ATOM  12048  C   PHE B 170     -11.826  -2.090  -3.068  1.00  0.22
C
ATOM  12049  O   PHE B 170     -12.981  -2.417  -2.791  1.00  0.25
O
ATOM  12050  CB  PHE B 170     -10.934   0.202  -2.873  1.00  0.27
C
ATOM  12051  CG  PHE B 170     -12.249   0.857  -3.256  1.00  0.27
C
ATOM  12052  CD1 PHE B 170     -12.867   1.757  -2.396  1.00  0.27
C
ATOM  12053  CD2 PHE B 170     -12.871   0.578  -4.464  1.00  0.27
C
ATOM  12054  CE1 PHE B 170     -14.058   2.370  -2.743  1.00  0.27
C
ATOM  12055  CE2 PHE B 170     -14.067   1.186  -4.811  1.00  0.27
C
ATOM  12056  CZ  PHE B 170     -14.660   2.085  -3.951  1.00  0.27
C
ATOM  12057  H   PHE B 170      -8.923  -1.102  -2.055  1.00  0.20
H
ATOM  12058  HA  PHE B 170     -11.613  -1.022  -1.232  1.00  0.22
H
ATOM  12059 1HB  PHE B 170     -10.390   0.891  -2.230  1.00  0.32
H
ATOM  12060 2HB  PHE B 170     -10.344   0.080  -3.780  1.00  0.32
H
ATOM  12061  HD1 PHE B 170     -12.395   1.985  -1.441  1.00  0.32
H
ATOM  12062  HD2 PHE B 170     -12.403  -0.129  -5.150  1.00  0.32
H
ATOM  12063  HE1 PHE B 170     -14.520   3.077  -2.057  1.00  0.32
H
ATOM  12064  HE2 PHE B 170     -14.540   0.958  -5.766  1.00  0.32
H
ATOM  12065  HZ  PHE B 170     -15.598   2.566  -4.223  1.00  0.32
H
ATOM  12066  N   ALA B 171     -11.189  -2.498  -4.164  1.00  0.22
N
ATOM  12067  CA  ALA B 171     -11.828  -3.358  -5.145  1.00  0.26
C
ATOM  12068  C   ALA B 171     -12.261  -4.681  -4.552  1.00  0.29
C
ATOM  12069  O   ALA B 171     -13.347  -5.176  -4.853  1.00  0.33
O
ATOM  12070  CB  ALA B 171     -10.889  -3.598  -6.298  1.00  0.39
C
ATOM  12071  H   ALA B 171     -10.240  -2.188  -4.331  1.00  0.26
H
ATOM  12072  HA  ALA B 171     -12.717  -2.850  -5.502  1.00  0.31
H
ATOM  12073 1HB  ALA B 171     -11.380  -4.214  -7.050  1.00  0.47
H
```

```
ATOM  12074 2HB  ALA B 171     -10.604  -2.645  -6.741  1.00  0.47
H
ATOM  12075 3HB  ALA B 171     -10.003  -4.109  -5.929  1.00  0.47
H
ATOM  12076  N   SER B 172     -11.420  -5.276  -3.712  1.00  0.31
N
ATOM  12077  CA  SER B 172     -11.809  -6.526  -3.083  1.00  0.47
C
ATOM  12078  C   SER B 172     -12.926  -6.311  -2.079  1.00  0.48
C
ATOM  12079  O   SER B 172     -13.877  -7.091  -2.030  1.00  0.54
O
ATOM  12080  CB  SER B 172     -10.632  -7.159  -2.410  1.00  0.70
C
ATOM  12081  OG  SER B 172      -9.692  -7.563  -3.356  1.00  0.70
O
ATOM  12082  H   SER B 172     -10.511  -4.871  -3.507  1.00  0.37
H
ATOM  12083  HA  SER B 172     -12.172  -7.203  -3.858  1.00  0.56
H
ATOM  12084 1HB  SER B 172     -10.176  -6.443  -1.725  1.00  0.85
H
ATOM  12085 2HB  SER B 172     -10.956  -8.016  -1.822  1.00  0.85
H
ATOM  12086  HG  SER B 172      -8.923  -7.825  -2.851  1.00  0.85
H
ATOM  12087  N   HIS B 173     -12.837  -5.223  -1.314  1.00  0.48
N
ATOM  12088  CA  HIS B 173     -13.846  -4.880  -0.319  1.00  0.52
C
ATOM  12089  C   HIS B 173     -15.222  -4.743  -0.969  1.00  0.45
C
ATOM  12090  O   HIS B 173     -16.225  -5.200  -0.416  1.00  0.55
O
ATOM  12091  CB  HIS B 173     -13.484  -3.577   0.399  1.00  0.78
C
ATOM  12092  CG  HIS B 173     -14.361  -3.278   1.574  1.00  0.78
C
ATOM  12093  ND1 HIS B 173     -14.207  -3.911   2.792  1.00  0.78
N
ATOM  12094  CD2 HIS B 173     -15.406  -2.431   1.722  1.00  0.78
C
ATOM  12095  CE1 HIS B 173     -15.114  -3.458   3.638  1.00  0.78
C
ATOM  12096  NE2 HIS B 173     -15.853  -2.561   3.014  1.00  0.78
N
ATOM  12097  H   HIS B 173     -12.032  -4.618  -1.396  1.00  0.58
H
ATOM  12098  HA  HIS B 173     -13.908  -5.673   0.425  1.00  0.62
H
ATOM  12099 1HB  HIS B 173     -12.452  -3.631   0.748  1.00  0.94
H
ATOM  12100 2HB  HIS B 173     -13.550  -2.744  -0.299  1.00  0.94
H
ATOM  12101  HD2 HIS B 173     -15.816  -1.772   0.962  1.00  0.94
H
ATOM  12102  HE1 HIS B 173     -15.233  -3.774   4.675  1.00  0.94
H
```

ATOM 12103 HE2 HIS B 173   -16.628 -2.050   3.416 1.00 0.94
H
ATOM 12104 N   TYR B 174   -15.266 -4.118 -2.147 1.00 0.35
N
ATOM 12105 CA TYR B 174   -16.516 -3.936 -2.868 1.00 0.33
C
ATOM 12106 C   TYR B 174   -16.743 -4.938 -3.998 1.00 0.32
C
ATOM 12107 O   TYR B 174   -17.624 -4.724 -4.833 1.00 0.37
O
ATOM 12108 CB TYR B 174   -16.597 -2.513 -3.420 1.00 0.49
C
ATOM 12109 CG TYR B 174   -16.817 -1.487 -2.340 1.00 0.49
C
ATOM 12110 CD1 TYR B 174   -15.784 -0.687 -1.882 1.00 0.49
C
ATOM 12111 CD2 TYR B 174   -18.085 -1.360 -1.797 1.00 0.49
C
ATOM 12112 CE1 TYR B 174   -16.026   0.241 -0.884 1.00 0.49
C
ATOM 12113 CE2 TYR B 174   -18.326 -0.434 -0.805 1.00 0.49
C
ATOM 12114 CZ TYR B 174   -17.302   0.364 -0.349 1.00 0.49
C
ATOM 12115 OH TYR B 174   -17.550   1.278   0.645 1.00 0.49
O
ATOM 12116 H   TYR B 174   -14.414 -3.740 -2.541 1.00 0.42
H
ATOM 12117 HA TYR B 174   -17.333 -4.067 -2.157 1.00 0.40
H
ATOM 12118 1HB TYR B 174   -15.670 -2.268 -3.941 1.00 0.59
H
ATOM 12119 2HB TYR B 174   -17.412 -2.439 -4.136 1.00 0.59
H
ATOM 12120 HD1 TYR B 174   -14.785 -0.787 -2.303 1.00 0.59
H
ATOM 12121 HD2 TYR B 174   -18.896 -1.994 -2.158 1.00 0.59
H
ATOM 12122 HE1 TYR B 174   -15.217   0.872 -0.517 1.00 0.59
H
ATOM 12123 HE2 TYR B 174   -19.325 -0.337 -0.381 1.00 0.59
H
ATOM 12124 HH TYR B 174   -16.717   1.592   1.009 1.00 0.59
H
ATOM 12125 N   ASN B 175   -15.980 -6.036 -4.013 1.00 0.33
N
ATOM 12126 CA ASN B 175   -16.158 -7.107 -4.995 1.00 0.36
C
ATOM 12127 C   ASN B 175   -16.284 -6.628 -6.436 1.00 0.29
C
ATOM 12128 O   ASN B 175   -17.203 -7.046 -7.143 1.00 0.35
O
ATOM 12129 CB ASN B 175   -17.364 -7.951 -4.626 1.00 0.54
C
ATOM 12130 CG ASN B 175   -17.160 -8.720 -3.349 1.00 0.54
C
ATOM 12131 OD1 ASN B 175   -16.103 -9.334 -3.162 1.00 0.54
O

```
ATOM  12132  ND2 ASN B 175     -18.137  -8.696  -2.478  1.00  0.54
N
ATOM  12133  H   ASN B 175     -15.270  -6.174  -3.307  1.00  0.40
H
ATOM  12134  HA  ASN B 175     -15.275  -7.745  -4.955  1.00  0.43
H
ATOM  12135 1HB  ASN B 175     -18.239  -7.310  -4.513  1.00  0.65
H
ATOM  12136 2HB  ASN B 175     -17.575  -8.656  -5.430  1.00  0.65
H
ATOM  12137 1HD2 ASN B 175     -18.050  -9.189  -1.612  1.00  0.65
H
ATOM  12138 2HD2 ASN B 175     -18.971  -8.183  -2.678  1.00  0.65
H
ATOM  12139  N   LEU B 176     -15.376  -5.758  -6.876  1.00  0.26
N
ATOM  12140  CA  LEU B 176     -15.432  -5.226  -8.238  1.00  0.26
C
ATOM  12141  C   LEU B 176     -15.053  -6.267  -9.298  1.00  0.28
C
ATOM  12142  O   LEU B 176     -13.964  -6.232  -9.869  1.00  0.28
O
ATOM  12143  CB  LEU B 176     -14.543  -3.983  -8.362  1.00  0.39
C
ATOM  12144  CG  LEU B 176     -14.940  -2.819  -7.435  1.00  0.39
C
ATOM  12145  CD1 LEU B 176     -13.994  -1.646  -7.649  1.00  0.39
C
ATOM  12146  CD2 LEU B 176     -16.385  -2.425  -7.707  1.00  0.39
C
ATOM  12147  H   LEU B 176     -14.640  -5.453  -6.252  1.00  0.31
H
ATOM  12148  HA  LEU B 176     -16.458  -4.916  -8.431  1.00  0.31
H
ATOM  12149 1HB  LEU B 176     -13.516  -4.260  -8.132  1.00  0.47
H
ATOM  12150 2HB  LEU B 176     -14.584  -3.622  -9.389  1.00  0.47
H
ATOM  12151  HG  LEU B 176     -14.845  -3.138  -6.397  1.00  0.47
H
ATOM  12152 1HD1 LEU B 176     -14.271  -0.832  -6.982  1.00  0.47
H
ATOM  12153 2HD1 LEU B 176     -12.972  -1.952  -7.443  1.00  0.47
H
ATOM  12154 3HD1 LEU B 176     -14.065  -1.308  -8.677  1.00  0.47
H
ATOM  12155 1HD2 LEU B 176     -16.670  -1.608  -7.044  1.00  0.47
H
ATOM  12156 2HD2 LEU B 176     -16.485  -2.102  -8.744  1.00  0.47
H
ATOM  12157 3HD2 LEU B 176     -17.035  -3.281  -7.530  1.00  0.47
H
ATOM  12158  N   ASP B 177     -15.989  -7.178  -9.566  1.00  0.33
N
ATOM  12159  CA  ASP B 177     -15.830  -8.265 -10.528  1.00  0.37
C
ATOM  12160  C   ASP B 177     -15.686  -7.804 -11.970  1.00  0.31
C
```

ATOM 12161 O   ASP B 177   -15.209 -8.563 -12.821 1.00 0.40
O
ATOM 12162 CB ASP B 177   -17.033 -9.196 -10.439 1.00 0.55
C
ATOM 12163 CG ASP B 177   -18.368 -8.510 -10.697 1.00 0.55
C
ATOM 12164 OD1 ASP B 177   -18.696 -7.606 -9.965 1.00 0.55
O
ATOM 12165 OD2 ASP B 177   -19.047 -8.889 -11.621 1.00 0.55
O
ATOM 12166 H   ASP B 177   -16.849 -7.122 -9.039 1.00 0.40
H
ATOM 12167 HA ASP B 177   -14.934 -8.825 -10.260 1.00 0.44
H
ATOM 12168 1HB ASP B 177   -16.924 -9.977 -11.187 1.00 0.67
H
ATOM 12169 2HB ASP B 177   -17.061 -9.669 -9.460 1.00 0.67
H
ATOM 12170 N   ASN B 178   -16.064 -6.554 -12.228 1.00 0.43
N
ATOM 12171 CA ASN B 178   -15.934 -5.950 -13.536 1.00 0.65
C
ATOM 12172 C   ASN B 178   -14.635 -5.149 -13.660 1.00 0.89
C
ATOM 12173 O   ASN B 178   -14.480 -4.371 -14.597 1.00 2.76
O
ATOM 12174 CB ASN B 178   -17.142 -5.085 -13.836 1.00 0.98
C
ATOM 12175 CG ASN B 178   -17.265 -3.922 -12.900 1.00 0.98
C
ATOM 12176 OD1 ASN B 178   -16.643 -3.915 -11.833 1.00 0.98
O
ATOM 12177 ND2 ASN B 178   -18.052 -2.946 -13.273 1.00 0.98
N
ATOM 12178 H   ASN B 178   -16.466 -5.998 -11.488 1.00 0.52
H
ATOM 12179 HA ASN B 178   -15.910 -6.742 -14.273 1.00 0.78
H
ATOM 12180 1HB ASN B 178   -17.079 -4.710 -14.859 1.00 1.17
H
ATOM 12181 2HB ASN B 178   -18.048 -5.689 -13.767 1.00 1.17
H
ATOM 12182 1HD2 ASN B 178   -18.173 -2.147 -12.682 1.00 1.17
H
ATOM 12183 2HD2 ASN B 178   -18.535 -3.000 -14.148 1.00 1.17
H
ATOM 12184 N   LEU B 179   -13.697 -5.350 -12.730 1.00 0.23
N
ATOM 12185 CA LEU B 179   -12.425 -4.640 -12.769 1.00 0.22
C
ATOM 12186 C   LEU B 179   -11.259 -5.597 -12.990 1.00 0.23
C
ATOM 12187 O   LEU B 179   -11.066 -6.539 -12.216 1.00 0.32
O
ATOM 12188 CB LEU B 179   -12.220 -3.866 -11.461 1.00 0.33
C
ATOM 12189 CG LEU B 179   -10.903 -3.094 -11.327 1.00 0.33
C

```
ATOM  12190  CD1 LEU B 179     -10.854  -1.990 -12.370  1.00  0.33
C
ATOM  12191  CD2 LEU B 179     -10.812  -2.514  -9.928  1.00  0.33
C
ATOM  12192  H   LEU B 179     -13.859  -5.976 -11.953  1.00  0.28
H
ATOM  12193  HA  LEU B 179     -12.449  -3.931 -13.595  1.00  0.26
H
ATOM  12194 1HB  LEU B 179     -13.034  -3.155 -11.350  1.00  0.40
H
ATOM  12195 2HB  LEU B 179     -12.270  -4.571 -10.632  1.00  0.40
H
ATOM  12196  HG  LEU B 179     -10.062  -3.766 -11.502  1.00  0.40
H
ATOM  12197 1HD1 LEU B 179      -9.916  -1.444 -12.275  1.00  0.40
H
ATOM  12198 2HD1 LEU B 179     -10.920  -2.428 -13.365  1.00  0.40
H
ATOM  12199 3HD1 LEU B 179     -11.689  -1.307 -12.216  1.00  0.40
H
ATOM  12200 1HD2 LEU B 179      -9.877  -1.964  -9.822  1.00  0.40
H
ATOM  12201 2HD2 LEU B 179     -11.652  -1.841  -9.757  1.00  0.40
H
ATOM  12202 3HD2 LEU B 179     -10.841  -3.325  -9.200  1.00  0.40
H
ATOM  12203  N   VAL B 180     -10.495  -5.343 -14.052  1.00  0.23
N
ATOM  12204  CA  VAL B 180      -9.327  -6.145 -14.390  1.00  0.28
C
ATOM  12205  C   VAL B 180      -8.074  -5.283 -14.440  1.00  0.23
C
ATOM  12206  O   VAL B 180      -7.982  -4.357 -15.246  1.00  0.28
O
ATOM  12207  CB  VAL B 180      -9.499  -6.824 -15.757  1.00  0.42
C
ATOM  12208  CG1 VAL B 180      -8.255  -7.644 -16.083  1.00  0.42
C
ATOM  12209  CG2 VAL B 180     -10.747  -7.690 -15.768  1.00  0.42
C
ATOM  12210  H   VAL B 180     -10.732  -4.556 -14.642  1.00  0.28
H
ATOM  12211  HA  VAL B 180      -9.199  -6.912 -13.627  1.00  0.34
H
ATOM  12212  HB  VAL B 180      -9.593  -6.054 -16.515  1.00  0.50
H
ATOM  12213 1HG1 VAL B 180      -8.369  -8.103 -17.065  1.00  0.50
H
ATOM  12214 2HG1 VAL B 180      -7.380  -6.994 -16.086  1.00  0.50
H
ATOM  12215 3HG1 VAL B 180      -8.127  -8.423 -15.332  1.00  0.50
H
ATOM  12216 1HG2 VAL B 180     -10.860  -8.149 -16.750  1.00  0.50
H
ATOM  12217 2HG2 VAL B 180     -10.663  -8.467 -15.014  1.00  0.50
H
ATOM  12218 3HG2 VAL B 180     -11.618  -7.072 -15.556  1.00  0.50
H
```

ATOM 12219 N ALA B 181 -7.112 -5.602 -13.590 1.00 0.23
N
ATOM 12220 CA ALA B 181 -5.853 -4.884 -13.569 1.00 0.28
C
ATOM 12221 C ALA B 181 -4.806 -5.704 -14.284 1.00 0.20
C
ATOM 12222 O ALA B 181 -4.738 -6.918 -14.104 1.00 0.27
O
ATOM 12223 CB ALA B 181 -5.415 -4.595 -12.146 1.00 0.42
C
ATOM 12224 H ALA B 181 -7.251 -6.380 -12.961 1.00 0.28
H
ATOM 12225 HA ALA B 181 -5.978 -3.945 -14.104 1.00 0.34
H
ATOM 12226 1HB ALA B 181 -4.473 -4.056 -12.167 1.00 0.50
H
ATOM 12227 2HB ALA B 181 -6.172 -3.990 -11.648 1.00 0.50
H
ATOM 12228 3HB ALA B 181 -5.284 -5.532 -11.608 1.00 0.50
H
ATOM 12229 N VAL B 182 -3.992 -5.041 -15.090 1.00 0.20
N
ATOM 12230 CA VAL B 182 -2.939 -5.708 -15.822 1.00 0.20
C
ATOM 12231 C VAL B 182 -1.605 -5.172 -15.346 1.00 0.23
C
ATOM 12232 O VAL B 182 -1.401 -3.959 -15.273 1.00 0.26
O
ATOM 12233 CB VAL B 182 -3.082 -5.488 -17.327 1.00 0.30
C
ATOM 12234 CG1 VAL B 182 -1.964 -6.227 -18.052 1.00 0.30
C
ATOM 12235 CG2 VAL B 182 -4.451 -5.945 -17.775 1.00 0.30
C
ATOM 12236 H VAL B 182 -4.114 -4.050 -15.205 1.00 0.24
H
ATOM 12237 HA VAL B 182 -2.985 -6.777 -15.617 1.00 0.24
H
ATOM 12238 HB VAL B 182 -2.970 -4.427 -17.549 1.00 0.36
H
ATOM 12239 1HG1 VAL B 182 -2.054 -6.064 -19.121 1.00 0.36
H
ATOM 12240 2HG1 VAL B 182 -0.998 -5.858 -17.708 1.00 0.36
H
ATOM 12241 3HG1 VAL B 182 -2.040 -7.295 -17.842 1.00 0.36
H
ATOM 12242 1HG2 VAL B 182 -4.558 -5.781 -18.847 1.00 0.36
H
ATOM 12243 2HG2 VAL B 182 -4.562 -7.005 -17.553 1.00 0.36
H
ATOM 12244 3HG2 VAL B 182 -5.218 -5.380 -17.244 1.00 0.36
H
ATOM 12245 N PHE B 183 -0.707 -6.077 -14.999 1.00 0.29
N
ATOM 12246 CA PHE B 183 0.584 -5.697 -14.463 1.00 0.43
C
ATOM 12247 C PHE B 183 1.667 -5.924 -15.475 1.00 0.40
C

ATOM 12248 O PHE B 183 2.022 -7.063 -15.778 1.00 0.56
O
ATOM 12249 CB PHE B 183 0.908 -6.559 -13.269 1.00 0.65
C
ATOM 12250 CG PHE B 183 -0.069 -6.413 -12.166 1.00 0.65
C
ATOM 12251 CD1 PHE B 183 -1.356 -6.906 -12.311 1.00 0.65
C
ATOM 12252 CD2 PHE B 183 0.298 -5.826 -10.978 1.00 0.65
C
ATOM 12253 CE1 PHE B 183 -2.270 -6.787 -11.294 1.00 0.65
C
ATOM 12254 CE2 PHE B 183 -0.608 -5.720 -9.955 1.00 0.65
C
ATOM 12255 CZ PHE B 183 -1.898 -6.196 -10.112 1.00 0.65
C
ATOM 12256 H PHE B 183 -0.936 -7.056 -15.097 1.00 0.35
H
ATOM 12257 HA PHE B 183 0.565 -4.642 -14.183 1.00 0.52
H
ATOM 12258 1HB PHE B 183 0.968 -7.591 -13.556 1.00 0.77
H
ATOM 12259 2HB PHE B 183 1.888 -6.279 -12.885 1.00 0.77
H
ATOM 12260 HD1 PHE B 183 -1.644 -7.380 -13.247 1.00 0.77
H
ATOM 12261 HD2 PHE B 183 1.314 -5.451 -10.856 1.00 0.77
H
ATOM 12262 HE1 PHE B 183 -3.281 -7.166 -11.422 1.00 0.77
H
ATOM 12263 HE2 PHE B 183 -0.298 -5.259 -9.022 1.00 0.77
H
ATOM 12264 HZ PHE B 183 -2.617 -6.110 -9.300 1.00 0.77
H
ATOM 12265 N ASP B 184 2.218 -4.847 -15.998 1.00 0.37
N
ATOM 12266 CA ASP B 184 3.276 -4.978 -16.970 1.00 0.43
C
ATOM 12267 C ASP B 184 4.596 -5.222 -16.253 1.00 0.44
C
ATOM 12268 O ASP B 184 5.402 -4.303 -16.091 1.00 0.60
O
ATOM 12269 CB ASP B 184 3.363 -3.723 -17.841 1.00 0.65
C
ATOM 12270 CG ASP B 184 4.336 -3.892 -18.986 1.00 0.65
C
ATOM 12271 OD1 ASP B 184 4.695 -5.009 -19.246 1.00 0.65
O
ATOM 12272 OD2 ASP B 184 4.737 -2.914 -19.574 1.00 0.65
O
ATOM 12273 H ASP B 184 1.906 -3.925 -15.726 1.00 0.44
H
ATOM 12274 HA ASP B 184 3.066 -5.837 -17.609 1.00 0.52
H
ATOM 12275 1HB ASP B 184 2.379 -3.490 -18.246 1.00 0.77
H
ATOM 12276 2HB ASP B 184 3.675 -2.876 -17.230 1.00 0.77
H

ATOM 12277 N VAL B 185 4.813 -6.466 -15.808 1.00 0.32
N
ATOM 12278 CA VAL B 185 6.034 -6.802 -15.082 1.00 0.31
C
ATOM 12279 C VAL B 185 7.192 -6.952 -16.040 1.00 0.27
C
ATOM 12280 O VAL B 185 7.596 -8.064 -16.386 1.00 0.30
O
ATOM 12281 CB VAL B 185 5.860 -8.086 -14.251 1.00 0.46
C
ATOM 12282 CG1 VAL B 185 7.146 -8.391 -13.500 1.00 0.46
C
ATOM 12283 CG2 VAL B 185 4.709 -7.900 -13.274 1.00 0.46
C
ATOM 12284 H VAL B 185 4.120 -7.188 -15.991 1.00 0.38
H
ATOM 12285 HA VAL B 185 6.258 -5.987 -14.393 1.00 0.37
H
ATOM 12286 HB VAL B 185 5.656 -8.926 -14.911 1.00 0.56
H
ATOM 12287 1HG1 VAL B 185 7.021 -9.304 -12.919 1.00 0.56
H
ATOM 12288 2HG1 VAL B 185 7.960 -8.524 -14.211 1.00 0.56
H
ATOM 12289 3HG1 VAL B 185 7.381 -7.564 -12.829 1.00 0.56
H
ATOM 12290 1HG2 VAL B 185 4.588 -8.806 -12.683 1.00 0.56
H
ATOM 12291 2HG2 VAL B 185 4.930 -7.061 -12.611 1.00 0.56
H
ATOM 12292 3HG2 VAL B 185 3.792 -7.697 -13.826 1.00 0.56
H
ATOM 12293 N ASN B 186 7.728 -5.801 -16.433 1.00 0.32
N
ATOM 12294 CA ASN B 186 8.825 -5.676 -17.373 1.00 0.43
C
ATOM 12295 C ASN B 186 10.196 -5.574 -16.712 1.00 0.62
C
ATOM 12296 O ASN B 186 11.160 -5.135 -17.347 1.00 1.36
O
ATOM 12297 CB ASN B 186 8.546 -4.502 -18.292 1.00 0.65
C
ATOM 12298 CG ASN B 186 8.481 -3.187 -17.590 1.00 0.65
C
ATOM 12299 OD1 ASN B 186 8.892 -3.051 -16.432 1.00 0.65
O
ATOM 12300 ND2 ASN B 186 7.946 -2.207 -18.274 1.00 0.65
N
ATOM 12301 H ASN B 186 7.303 -4.955 -16.076 1.00 0.38
H
ATOM 12302 HA ASN B 186 8.839 -6.571 -17.980 1.00 0.52
H
ATOM 12303 1HB ASN B 186 9.316 -4.447 -19.055 1.00 0.77
H
ATOM 12304 2HB ASN B 186 7.598 -4.657 -18.803 1.00 0.77
H
ATOM 12305 1HD2 ASN B 186 7.858 -1.300 -17.865 1.00 0.77
H

ATOM 12306 2HD2 ASN B 186   7.615 -2.369 -19.209 1.00 0.77
H
ATOM 12307 N   ARG B 187   10.279 -6.021 -15.447 1.00 0.43
N
ATOM 12308 CA ARG B 187   11.499 -6.083 -14.626 1.00 0.49
C
ATOM 12309 C   ARG B 187   12.065 -4.754 -14.140 1.00 0.61
C
ATOM 12310 O   ARG B 187   12.358 -4.617 -12.953 1.00 1.68
O
ATOM 12311 CB ARG B 187   12.622 -6.766 -15.384 1.00 0.73
C
ATOM 12312 CG ARG B 187   13.877 -7.001 -14.568 1.00 0.73
C
ATOM 12313 CD ARG B 187   14.938 -7.628 -15.387 1.00 0.73
C
ATOM 12314 NE ARG B 187   16.095 -7.984 -14.592 1.00 0.73
N
ATOM 12315 CZ ARG B 187   17.251 -8.458 -15.085 1.00 0.73
C
ATOM 12316 NH1 ARG B 187   17.416 -8.612 -16.383 1.00 0.73
N
ATOM 12317 NH2 ARG B 187   18.213 -8.769 -14.235 1.00 0.73
N
ATOM 12318 H   ARG B 187   9.436 -6.348 -15.004 1.00 0.52
H
ATOM 12319 HA ARG B 187   11.266 -6.685 -13.748 1.00 0.59
H
ATOM 12320 1HB ARG B 187   12.278 -7.725 -15.754 1.00 0.88
H
ATOM 12321 2HB ARG B 187   12.919 -6.170 -16.243 1.00 0.88
H
ATOM 12322 1HG ARG B 187   14.252 -6.052 -14.187 1.00 0.88
H
ATOM 12323 2HG ARG B 187   13.651 -7.666 -13.734 1.00 0.88
H
ATOM 12324 1HD ARG B 187   14.561 -8.531 -15.858 1.00 0.88
H
ATOM 12325 2HD ARG B 187   15.261 -6.929 -16.159 1.00 0.88
H
ATOM 12326 HE ARG B 187   16.034 -7.895 -13.581 1.00 0.88
H
ATOM 12327 1HH1 ARG B 187   16.672 -8.368 -17.032 1.00 0.88
H
ATOM 12328 2HH1 ARG B 187   18.286 -8.970 -16.746 1.00 0.88
H
ATOM 12329 1HH2 ARG B 187   18.040 -8.644 -13.240 1.00 0.88
H
ATOM 12330 2HH2 ARG B 187   19.095 -9.129 -14.567 1.00 0.88
H
ATOM 12331 N   LEU B 188   12.277 -3.819 -15.056 1.00 0.94
N
ATOM 12332 CA LEU B 188   12.933 -2.568 -14.732 1.00 0.83
C
ATOM 12333 C   LEU B 188   12.000 -1.397 -14.434 1.00 0.77
C
ATOM 12334 O   LEU B 188   10.876 -1.325 -14.932 1.00 1.28
O

```
ATOM  12335  CB  LEU B 188      13.840  -2.190 -15.903  1.00  1.24
C
ATOM  12336  CG  LEU B 188      14.884  -3.244 -16.299  1.00  1.24
C
ATOM  12337  CD1 LEU B 188      15.653  -2.749 -17.509  1.00  1.24
C
ATOM  12338  CD2 LEU B 188      15.808  -3.524 -15.126  1.00  1.24
C
ATOM  12339  H   LEU B 188      12.007  -4.003 -16.009  1.00  1.13
H
ATOM  12340  HA  LEU B 188      13.541  -2.737 -13.858  1.00  1.00
H
ATOM  12341 1HB  LEU B 188      13.220  -1.999 -16.776  1.00  1.49
H
ATOM  12342 2HB  LEU B 188      14.373  -1.274 -15.650  1.00  1.49
H
ATOM  12343  HG  LEU B 188      14.379  -4.168 -16.583  1.00  1.49
H
ATOM  12344 1HD1 LEU B 188      16.386  -3.499 -17.808  1.00  1.49
H
ATOM  12345 2HD1 LEU B 188      14.962  -2.571 -18.333  1.00  1.49
H
ATOM  12346 3HD1 LEU B 188      16.165  -1.821 -17.258  1.00  1.49
H
ATOM  12347 1HD2 LEU B 188      16.545  -4.274 -15.413  1.00  1.49
H
ATOM  12348 2HD2 LEU B 188      16.319  -2.607 -14.835  1.00  1.49
H
ATOM  12349 3HD2 LEU B 188      15.222  -3.897 -14.288  1.00  1.49
H
ATOM  12350  N   GLY B 189      12.506  -0.461 -13.635  1.00  0.45
N
ATOM  12351  CA  GLY B 189      11.819   0.782 -13.297  1.00  0.37
C
ATOM  12352  C   GLY B 189      12.471   1.933 -14.070  1.00  0.31
C
ATOM  12353  O   GLY B 189      12.730   1.803 -15.272  1.00  0.63
O
ATOM  12354  H   GLY B 189      13.429  -0.598 -13.252  1.00  0.54
H
ATOM  12355 1HA  GLY B 189      10.762   0.707 -13.552  1.00  0.44
H
ATOM  12356 2HA  GLY B 189      11.889   0.965 -12.225  1.00  0.44
H
ATOM  12357  N   GLN B 190      12.715   3.061 -13.389  1.00  0.25
N
ATOM  12358  CA  GLN B 190      13.355   4.210 -14.030  1.00  0.39
C
ATOM  12359  C   GLN B 190      14.871   4.183 -13.993  1.00  0.61
C
ATOM  12360  O   GLN B 190      15.522   4.093 -15.035  1.00  2.37
O
ATOM  12361  CB  GLN B 190      12.866   5.527 -13.416  1.00  0.58
C
ATOM  12362  CG  GLN B 190      13.552   6.765 -13.975  1.00  0.58
C
ATOM  12363  CD  GLN B 190      12.916   8.058 -13.510  1.00  0.58
C
```

ATOM 12364 OE1 GLN B 190   11.721   8.105 -13.205 1.00 0.58
O
ATOM 12365 NE2 GLN B 190   13.708   9.124 -13.459 1.00 0.58
N
ATOM 12366 H   GLN B 190   12.468   3.136 -12.403 1.00 0.30
H
ATOM 12367 HA GLN B 190   13.066   4.205 -15.070 1.00 0.47
H
ATOM 12368 1HB GLN B 190   11.793   5.631 -13.580 1.00 0.70
H
ATOM 12369 2HB GLN B 190   13.031   5.522 -12.345 1.00 0.70
H
ATOM 12370 1HG GLN B 190   14.585   6.768 -13.632 1.00 0.70
H
ATOM 12371 2HG GLN B 190   13.531   6.736 -15.058 1.00 0.70
H
ATOM 12372 1HE2 GLN B 190   13.340 10.007 -13.164 1.00 0.70
H
ATOM 12373 2HE2 GLN B 190   14.677   9.054 -13.710 1.00 0.70
H
ATOM 12374 N   SER B 191   15.436   4.309 -12.796 1.00 0.31
N
ATOM 12375 CA SER B 191   16.884   4.374 -12.639 1.00 0.34
C
ATOM 12376 C   SER B 191   17.537   3.024 -12.360 1.00 0.42
C
ATOM 12377 O   SER B 191   18.757   2.941 -12.218 1.00 1.60
O
ATOM 12378 CB SER B 191   17.230   5.323 -11.521 1.00 0.51
C
ATOM 12379 OG SER B 191   16.812   4.820 -10.281 1.00 0.51
O
ATOM 12380 H   SER B 191   14.855   4.363 -11.965 1.00 0.37
H
ATOM 12381 HA SER B 191   17.305   4.764 -13.567 1.00 0.41
H
ATOM 12382 1HB SER B 191   18.308   5.483 -11.507 1.00 0.61
H
ATOM 12383 2HB SER B 191   16.757   6.286 -11.703 1.00 0.61
H
ATOM 12384 HG SER B 191   15.855   4.725 -10.330 1.00 0.61
H
ATOM 12385 N   GLY B 192   16.737   1.973 -12.283 1.00 0.35
N
ATOM 12386 CA GLY B 192   17.254   0.647 -11.989 1.00 0.37
C
ATOM 12387 C   GLY B 192   16.124 -0.353 -12.105 1.00 0.40
C
ATOM 12388 O   GLY B 192   15.086 -0.028 -12.677 1.00 0.52
O
ATOM 12389 H   GLY B 192   15.744   2.090 -12.415 1.00 0.42
H
ATOM 12390 1HA GLY B 192   18.055   0.396 -12.685 1.00 0.44
H
ATOM 12391 2HA GLY B 192   17.670   0.640 -10.983 1.00 0.44
H
ATOM 12392 N   PRO B 193   16.325 -1.573 -11.615 1.00 0.43
N

```
ATOM  12393  CA  PRO B 193      15.387  -2.676 -11.585  1.00  0.43
C
ATOM  12394  C   PRO B 193      14.327  -2.467 -10.525  1.00  0.37
C
ATOM  12395  O   PRO B 193      14.600  -1.912  -9.460  1.00  0.37
O
ATOM  12396  CB  PRO B 193      16.273  -3.888 -11.281  1.00  0.65
C
ATOM  12397  CG  PRO B 193      17.434  -3.326 -10.534  1.00  0.65
C
ATOM  12398  CD  PRO B 193      17.665  -1.971 -11.151  1.00  0.65
C
ATOM  12399  HA  PRO B 193      14.931  -2.774 -12.565  1.00  0.52
H
ATOM  12400 1HB  PRO B 193      15.709  -4.628 -10.695  1.00  0.77
H
ATOM  12401 2HB  PRO B 193      16.571  -4.382 -12.218  1.00  0.77
H
ATOM  12402 1HG  PRO B 193      17.202  -3.269  -9.460  1.00  0.77
H
ATOM  12403 2HG  PRO B 193      18.305  -3.991 -10.635  1.00  0.77
H
ATOM  12404 1HD  PRO B 193      18.030  -1.274 -10.383  1.00  0.77
H
ATOM  12405 2HD  PRO B 193      18.357  -2.039 -12.002  1.00  0.77
H
ATOM  12406  N   ALA B 194      13.116  -2.922 -10.811  1.00  0.49
N
ATOM  12407  CA  ALA B 194      12.050  -2.838  -9.835  1.00  0.52
C
ATOM  12408  C   ALA B 194      12.322  -3.896  -8.767  1.00  0.53
C
ATOM  12409  O   ALA B 194      12.792  -4.982  -9.104  1.00  0.88
O
ATOM  12410  CB  ALA B 194      10.697  -3.036 -10.504  1.00  0.78
C
ATOM  12411  H   ALA B 194      12.934  -3.360 -11.702  1.00  0.59
H
ATOM  12412  HA  ALA B 194      12.086  -1.853  -9.376  1.00  0.62
H
ATOM  12413 1HB  ALA B 194       9.898  -2.969  -9.769  1.00  0.94
H
ATOM  12414 2HB  ALA B 194      10.553  -2.268 -11.263  1.00  0.94
H
ATOM  12415 3HB  ALA B 194      10.668  -4.018 -10.976  1.00  0.94
H
ATOM  12416  N   PRO B 195      12.046  -3.596  -7.491  1.00  0.54
N
ATOM  12417  CA  PRO B 195      12.279  -4.422  -6.293  1.00  0.53
C
ATOM  12418  C   PRO B 195      12.157  -5.940  -6.476  1.00  0.69
C
ATOM  12419  O   PRO B 195      12.992  -6.692  -5.971  1.00  1.29
O
ATOM  12420  CB  PRO B 195      11.186  -3.947  -5.328  1.00  0.80
C
ATOM  12421  CG  PRO B 195      10.949  -2.540  -5.713  1.00  0.80
C
```

ATOM 12422 CD PRO B 195 11.016 -2.570 -7.211 1.00 0.80
C
ATOM 12423 HA PRO B 195 13.271 -4.174 -5.889 1.00 0.64
H
ATOM 12424 1HB PRO B 195 10.289 -4.574 -5.430 1.00 0.95
H
ATOM 12425 2HB PRO B 195 11.533 -4.049 -4.289 1.00 0.95
H
ATOM 12426 1HG PRO B 195 9.975 -2.197 -5.334 1.00 0.95
H
ATOM 12427 2HG PRO B 195 11.715 -1.891 -5.265 1.00 0.95
H
ATOM 12428 1HD PRO B 195 10.047 -2.875 -7.631 1.00 0.95
H
ATOM 12429 2HD PRO B 195 11.332 -1.584 -7.577 1.00 0.95
H
ATOM 12430 N LEU B 196 11.108 -6.395 -7.158 1.00 0.48
N
ATOM 12431 CA LEU B 196 10.861 -7.828 -7.293 1.00 0.56
C
ATOM 12432 C LEU B 196 11.256 -8.447 -8.641 1.00 0.58
C
ATOM 12433 O LEU B 196 11.035 -9.643 -8.837 1.00 0.95
O
ATOM 12434 CB LEU B 196 9.375 -8.086 -7.069 1.00 0.84
C
ATOM 12435 CG LEU B 196 8.818 -7.675 -5.709 1.00 0.84
C
ATOM 12436 CD1 LEU B 196 7.320 -7.934 -5.700 1.00 0.84
C
ATOM 12437 CD2 LEU B 196 9.534 -8.451 -4.619 1.00 0.84
C
ATOM 12438 H LEU B 196 10.459 -5.741 -7.572 1.00 0.58
H
ATOM 12439 HA LEU B 196 11.424 -8.337 -6.514 1.00 0.67
H
ATOM 12440 1HB LEU B 196 8.823 -7.530 -7.817 1.00 1.01
H
ATOM 12441 2HB LEU B 196 9.176 -9.148 -7.208 1.00 1.01
H
ATOM 12442 HG LEU B 196 8.977 -6.608 -5.552 1.00 1.01
H
ATOM 12443 1HD1 LEU B 196 6.903 -7.639 -4.737 1.00 1.01
H
ATOM 12444 2HD1 LEU B 196 6.847 -7.352 -6.492 1.00 1.01
H
ATOM 12445 3HD1 LEU B 196 7.134 -8.992 -5.871 1.00 1.01
H
ATOM 12446 1HD2 LEU B 196 9.138 -8.163 -3.645 1.00 1.01
H
ATOM 12447 2HD2 LEU B 196 9.377 -9.517 -4.774 1.00 1.01
H
ATOM 12448 3HD2 LEU B 196 10.601 -8.234 -4.657 1.00 1.01
H
ATOM 12449 N GLU B 197 11.833 -7.646 -9.542 1.00 1.11
N
ATOM 12450 CA GLU B 197 12.212 -8.051 -10.907 1.00 2.07
C

```
ATOM  12451  C   GLU B 197      11.210  -9.016 -11.564  1.00  0.94
C
ATOM  12452  O   GLU B 197      10.113  -8.602 -11.933  1.00  2.62
O
ATOM  12453  CB  GLU B 197      13.621  -8.645 -10.926  1.00  3.10
C
ATOM  12454  CG  GLU B 197      14.722  -7.628 -10.666  1.00  3.10
C
ATOM  12455  CD  GLU B 197      16.099  -8.228 -10.741  1.00  3.10
C
ATOM  12456  OE1 GLU B 197      16.671  -8.214 -11.808  1.00  3.10
O
ATOM  12457  OE2 GLU B 197      16.577  -8.707  -9.739  1.00  3.10
O
ATOM  12458  H   GLU B 197      12.018  -6.688  -9.285  1.00  1.33
H
ATOM  12459  HA  GLU B 197      12.238  -7.149 -11.521  1.00  2.48
H
ATOM  12460 1HB  GLU B 197      13.701  -9.424 -10.167  1.00  3.73
H
ATOM  12461 2HB  GLU B 197      13.818  -9.101 -11.896  1.00  3.73
H
ATOM  12462 1HG  GLU B 197      14.645  -6.827 -11.401  1.00  3.73
H
ATOM  12463 2HG  GLU B 197      14.573  -7.192  -9.680  1.00  3.73
H
ATOM  12464  N   HIS B 198      11.591 -10.286 -11.748  1.00  1.43
N
ATOM  12465  CA  HIS B 198      10.701 -11.265 -12.379  1.00  1.07
C
ATOM  12466  C   HIS B 198      10.068 -12.275 -11.432  1.00  0.72
C
ATOM  12467  O   HIS B 198       9.586 -13.313 -11.890  1.00  0.81
O
ATOM  12468  CB  HIS B 198      11.407 -12.059 -13.481  1.00  1.60
C
ATOM  12469  CG  HIS B 198      11.757 -11.286 -14.706  1.00  1.60
C
ATOM  12470  ND1 HIS B 198      10.799 -10.834 -15.590  1.00  1.60
N
ATOM  12471  CD2 HIS B 198      12.950 -10.917 -15.221  1.00  1.60
C
ATOM  12472  CE1 HIS B 198      11.390 -10.218 -16.596  1.00  1.60
C
ATOM  12473  NE2 HIS B 198      12.694 -10.257 -16.397  1.00  1.60
N
ATOM  12474  H   HIS B 198      12.503 -10.589 -11.438  1.00  1.72
H
ATOM  12475  HA  HIS B 198       9.880 -10.728 -12.854  1.00  1.28
H
ATOM  12476 1HB  HIS B 198      12.328 -12.484 -13.080  1.00  1.93
H
ATOM  12477 2HB  HIS B 198      10.773 -12.892 -13.782  1.00  1.93
H
ATOM  12478  HD1 HIS B 198       9.825 -10.736 -15.384  1.00  1.93
H
ATOM  12479  HD2 HIS B 198      13.978 -11.059 -14.887  1.00  1.93
H
```

```
ATOM  12480  HE1 HIS B 198      10.804  -9.789 -17.405  1.00  1.93
H
ATOM  12481  N   GLY B 199      10.077 -12.004 -10.129  1.00  0.72
N
ATOM  12482  CA  GLY B 199       9.495 -12.925  -9.144  1.00  0.83
C
ATOM  12483  C   GLY B 199       7.965 -12.962  -9.215  1.00  0.73
C
ATOM  12484  O   GLY B 199       7.293 -12.464  -8.311  1.00  0.78
O
ATOM  12485  H   GLY B 199      10.483 -11.141  -9.801  1.00  0.86
H
ATOM  12486 1HA  GLY B 199       9.892 -13.927  -9.309  1.00  1.00
H
ATOM  12487 2HA  GLY B 199       9.805 -12.624  -8.144  1.00  1.00
H
ATOM  12488  N   ALA B 200       7.442 -13.585 -10.278  1.00  0.65
N
ATOM  12489  CA  ALA B 200       6.011 -13.676 -10.583  1.00  0.58
C
ATOM  12490  C   ALA B 200       5.199 -14.224  -9.429  1.00  0.55
C
ATOM  12491  O   ALA B 200       4.093 -13.751  -9.174  1.00  0.57
O
ATOM  12492  CB  ALA B 200       5.802 -14.547 -11.809  1.00  0.87
C
ATOM  12493  H   ALA B 200       8.093 -13.981 -10.940  1.00  0.78
H
ATOM  12494  HA  ALA B 200       5.649 -12.676 -10.806  1.00  0.70
H
ATOM  12495 1HB  ALA B 200       4.742 -14.582 -12.056  1.00  1.04
H
ATOM  12496 2HB  ALA B 200       6.356 -14.130 -12.650  1.00  1.04
H
ATOM  12497 3HB  ALA B 200       6.158 -15.556 -11.605  1.00  1.04
H
ATOM  12498  N   ASP B 201       5.754 -15.207  -8.728  1.00  0.54
N
ATOM  12499  CA  ASP B 201       5.082 -15.830  -7.601  1.00  0.55
C
ATOM  12500  C   ASP B 201       4.878 -14.864  -6.441  1.00  0.52
C
ATOM  12501  O   ASP B 201       3.944 -15.029  -5.658  1.00  0.55
O
ATOM  12502  CB  ASP B 201       5.878 -17.041  -7.122  1.00  0.83
C
ATOM  12503  CG  ASP B 201       5.830 -18.206  -8.101  1.00  0.83
C
ATOM  12504  OD1 ASP B 201       5.003 -18.187  -8.982  1.00  0.83
O
ATOM  12505  OD2 ASP B 201       6.625 -19.103  -7.961  1.00  0.83
O
ATOM  12506  H   ASP B 201       6.669 -15.543  -8.993  1.00  0.65
H
ATOM  12507  HA  ASP B 201       4.102 -16.172  -7.935  1.00  0.66
H
ATOM  12508 1HB  ASP B 201       6.920 -16.757  -6.969  1.00  0.99
H
```

ATOM 12509 2HB ASP B 201   5.486 -17.377 -6.162 1.00 0.99
H
ATOM 12510 N   ILE B 202   5.754 -13.866 -6.319 1.00 0.56
N
ATOM 12511 CA ILE B 202   5.641 -12.889 -5.256 1.00 0.55
C
ATOM 12512 C   ILE B 202   4.517 -11.941 -5.601 1.00 0.49
C
ATOM 12513 O   ILE B 202   3.700 -11.603 -4.744 1.00 0.48
O
ATOM 12514 CB ILE B 202   6.938 -12.106 -5.046 1.00 0.83
C
ATOM 12515 CG1 ILE B 202   8.043 -13.047 -4.566 1.00 0.83
C
ATOM 12516 CG2 ILE B 202   6.687 -11.031 -4.001 1.00 0.83
C
ATOM 12517 CD1 ILE B 202   9.418 -12.425 -4.595 1.00 0.83
C
ATOM 12518 H   ILE B 202   6.497 -13.752 -6.993 1.00 0.67
H
ATOM 12519 HA ILE B 202   5.390 -13.401 -4.328 1.00 0.66
H
ATOM 12520 HB ILE B 202   7.262 -11.648 -5.978 1.00 0.99
H
ATOM 12521 1HG1 ILE B 202   7.825 -13.360 -3.546 1.00 0.99
H
ATOM 12522 2HG1 ILE B 202   8.055 -13.933 -5.202 1.00 0.99
H
ATOM 12523 1HG2 ILE B 202   7.600 -10.466 -3.831 1.00 0.99
H
ATOM 12524 2HG2 ILE B 202   5.905 -10.358 -4.352 1.00 0.99
H
ATOM 12525 3HG2 ILE B 202   6.372 -11.497 -3.067 1.00 0.99
H
ATOM 12526 1HD1 ILE B 202   10.153 -13.150 -4.245 1.00 0.99
H
ATOM 12527 2HD1 ILE B 202   9.662 -12.125 -5.615 1.00 0.99
H
ATOM 12528 3HD1 ILE B 202   9.434 -11.552 -3.945 1.00 0.99
H
ATOM 12529 N   TYR B 203   4.471 -11.531 -6.875 1.00 0.48
N
ATOM 12530 CA TYR B 203   3.405 -10.649 -7.341 1.00 0.44
C
ATOM 12531 C   TYR B 203   2.064 -11.342 -7.143 1.00 0.42
C
ATOM 12532 O   TYR B 203   1.089 -10.709 -6.729 1.00 0.39
O
ATOM 12533 CB TYR B 203   3.587 -10.306 -8.825 1.00 0.66
C
ATOM 12534 CG TYR B 203   4.752 -9.396 -9.134 1.00 0.66
C
ATOM 12535 CD1 TYR B 203   5.928 -9.949 -9.599 1.00 0.66
C
ATOM 12536 CD2 TYR B 203   4.652 -8.023 -8.973 1.00 0.66
C
ATOM 12537 CE1 TYR B 203   7.010 -9.156 -9.893 1.00 0.66
C

ATOM 12538 CE2 TYR B 203   5.736 -7.219 -9.274 1.00 0.66
C
ATOM 12539 CZ TYR B 203   6.910 -7.786 -9.730 1.00 0.66
C
ATOM 12540 OH TYR B 203   7.995 -6.998 -10.021 1.00 0.66
O
ATOM 12541 H   TYR B 203   5.196 -11.840 -7.518 1.00 0.58
H
ATOM 12542 HA TYR B 203   3.411 -9.734 -6.746 1.00 0.53
H
ATOM 12543 1HB TYR B 203   3.719 -11.226 -9.394 1.00 0.79
H
ATOM 12544 2HB TYR B 203   2.680 -9.827 -9.194 1.00 0.79
H
ATOM 12545 HD1 TYR B 203   5.994 -11.021 -9.726 1.00 0.79
H
ATOM 12546 HD2 TYR B 203   3.724 -7.579 -8.612 1.00 0.79
H
ATOM 12547 HE1 TYR B 203   7.937 -9.602 -10.254 1.00 0.79
H
ATOM 12548 HE2 TYR B 203   5.666 -6.137 -9.150 1.00 0.79
H
ATOM 12549 HH TYR B 203   7.699 -6.121 -10.280 1.00 0.79
H
ATOM 12550 N   GLN B 204   2.045 -12.657 -7.399 1.00 0.46
N
ATOM 12551 CA GLN B 204   0.874 -13.487 -7.184 1.00 0.55
C
ATOM 12552 C   GLN B 204   0.435 -13.476 -5.744 1.00 0.52
C
ATOM 12553 O   GLN B 204   -0.731 -13.220 -5.451 1.00 0.63
O
ATOM 12554 CB GLN B 204   1.135 -14.934 -7.606 1.00 0.83
C
ATOM 12555 CG GLN B 204   -0.067 -15.837 -7.413 1.00 0.83
C
ATOM 12556 CD GLN B 204   0.205 -17.290 -7.752 1.00 0.83
C
ATOM 12557 OE1 GLN B 204   1.320 -17.673 -8.116 1.00 0.83
O
ATOM 12558 NE2 GLN B 204   -0.828 -18.116 -7.625 1.00 0.83
N
ATOM 12559 H   GLN B 204   2.875 -13.099 -7.772 1.00 0.55
H
ATOM 12560 HA GLN B 204   0.064 -13.085 -7.768 1.00 0.66
H
ATOM 12561 1HB GLN B 204   1.428 -14.971 -8.653 1.00 0.99
H
ATOM 12562 2HB GLN B 204   1.955 -15.346 -7.024 1.00 0.99
H
ATOM 12563 1HG GLN B 204   -0.372 -15.788 -6.369 1.00 0.99
H
ATOM 12564 2HG GLN B 204   -0.877 -15.487 -8.053 1.00 0.99
H
ATOM 12565 1HE2 GLN B 204   -0.721 -19.090 -7.828 1.00 0.99
H
ATOM 12566 2HE2 GLN B 204   -1.717 -17.760 -7.322 1.00 0.99
H

ATOM 12567 N ASN B 205 1.380 -13.743 -4.844 1.00 0.49
N
ATOM 12568 CA ASN B 205 1.088 -13.784 -3.424 1.00 0.48
C
ATOM 12569 C ASN B 205 0.494 -12.483 -2.930 1.00 0.43
C
ATOM 12570 O ASN B 205 -0.465 -12.503 -2.161 1.00 0.45
O
ATOM 12571 CB ASN B 205 2.342 -14.110 -2.632 1.00 0.72
C
ATOM 12572 CG ASN B 205 2.757 -15.548 -2.754 1.00 0.72
C
ATOM 12573 OD1 ASN B 205 1.956 -16.414 -3.125 1.00 0.72
O
ATOM 12574 ND2 ASN B 205 3.997 -15.821 -2.442 1.00 0.72
N
ATOM 12575 H ASN B 205 2.321 -13.956 -5.151 1.00 0.59
H
ATOM 12576 HA ASN B 205 0.350 -14.566 -3.246 1.00 0.58
H
ATOM 12577 1HB ASN B 205 3.163 -13.480 -2.978 1.00 0.86
H
ATOM 12578 2HB ASN B 205 2.177 -13.881 -1.580 1.00 0.86
H
ATOM 12579 1HD2 ASN B 205 4.333 -16.762 -2.504 1.00 0.86
H
ATOM 12580 2HD2 ASN B 205 4.610 -15.090 -2.145 1.00 0.86
H
ATOM 12581 N CYS B 206 1.049 -11.354 -3.379 1.00 0.42
N
ATOM 12582 CA CYS B 206 0.552 -10.054 -2.955 1.00 0.47
C
ATOM 12583 C CYS B 206 -0.863 -9.796 -3.418 1.00 0.59
C
ATOM 12584 O CYS B 206 -1.724 -9.455 -2.608 1.00 0.78
O
ATOM 12585 CB CYS B 206 1.451 -8.935 -3.480 1.00 0.70
C
ATOM 12586 SG CYS B 206 3.078 -8.836 -2.693 1.00 0.70
S
ATOM 12587 H CYS B 206 1.845 -11.401 -4.004 1.00 0.50
H
ATOM 12588 HA CYS B 206 0.558 -10.018 -1.869 1.00 0.56
H
ATOM 12589 1HB CYS B 206 1.608 -9.075 -4.550 1.00 0.85
H
ATOM 12590 2HB CYS B 206 0.955 -7.973 -3.347 1.00 0.85
H
ATOM 12591 HG CYS B 206 3.393 -10.116 -2.853 1.00 0.85
H
ATOM 12592 N CYS B 207 -1.114 -9.974 -4.706 1.00 0.61
N
ATOM 12593 CA CYS B 207 -2.434 -9.693 -5.238 1.00 1.00
C
ATOM 12594 C CYS B 207 -3.503 -10.619 -4.670 1.00 0.53
C
ATOM 12595 O CYS B 207 -4.605 -10.162 -4.356 1.00 0.44
O

ATOM 12596 CB CYS B 207 -2.404 -9.776 -6.753 1.00 1.50
C
ATOM 12597 SG CYS B 207 -1.493 -8.431 -7.536 1.00 1.50
S
ATOM 12598 H CYS B 207 -0.379 -10.279 -5.336 1.00 0.73
H
ATOM 12599 HA CYS B 207 -2.694 -8.669 -4.968 1.00 1.20
H
ATOM 12600 1HB CYS B 207 -1.925 -10.711 -7.044 1.00 1.80
H
ATOM 12601 2HB CYS B 207 -3.419 -9.780 -7.148 1.00 1.80
H
ATOM 12602 HG CYS B 207 -0.339 -8.667 -6.919 1.00 1.80
H
ATOM 12603 N GLU B 208 -3.172 -11.900 -4.493 1.00 0.51
N
ATOM 12604 CA GLU B 208 -4.118 -12.831 -3.899 1.00 0.53
C
ATOM 12605 C GLU B 208 -4.354 -12.492 -2.434 1.00 0.44
C
ATOM 12606 O GLU B 208 -5.484 -12.584 -1.955 1.00 0.55
O
ATOM 12607 CB GLU B 208 -3.636 -14.270 -4.057 1.00 0.80
C
ATOM 12608 CG GLU B 208 -3.682 -14.773 -5.494 1.00 0.80
C
ATOM 12609 CD GLU B 208 -3.207 -16.190 -5.644 1.00 0.80
C
ATOM 12610 OE1 GLU B 208 -2.820 -16.776 -4.662 1.00 0.80
O
ATOM 12611 OE2 GLU B 208 -3.227 -16.690 -6.745 1.00 0.80
O
ATOM 12612 H GLU B 208 -2.262 -12.238 -4.776 1.00 0.61
H
ATOM 12613 HA GLU B 208 -5.069 -12.734 -4.422 1.00 0.64
H
ATOM 12614 1HB GLU B 208 -2.607 -14.352 -3.704 1.00 0.95
H
ATOM 12615 2HB GLU B 208 -4.249 -14.933 -3.445 1.00 0.95
H
ATOM 12616 1HG GLU B 208 -4.707 -14.705 -5.856 1.00 0.95
H
ATOM 12617 2HG GLU B 208 -3.064 -14.122 -6.110 1.00 0.95
H
ATOM 12618 N ALA B 209 -3.310 -12.042 -1.731 1.00 0.40
N
ATOM 12619 CA ALA B 209 -3.453 -11.613 -0.342 1.00 0.47
C
ATOM 12620 C ALA B 209 -4.434 -10.447 -0.234 1.00 0.49
C
ATOM 12621 O ALA B 209 -5.230 -10.383 0.705 1.00 0.74
O
ATOM 12622 CB ALA B 209 -2.114 -11.223 0.228 1.00 0.70
C
ATOM 12623 H ALA B 209 -2.389 -12.008 -2.145 1.00 0.48
H
ATOM 12624 HA ALA B 209 -3.857 -12.448 0.232 1.00 0.56
H

ATOM 12625 1HB ALA B 209   -2.250 -10.932   1.266 1.00 0.85
H
ATOM 12626 2HB ALA B 209   -1.430 -12.070   0.172 1.00 0.85
H
ATOM 12627 3HB ALA B 209   -1.703 -10.387 -0.333 1.00 0.85
H
ATOM 12628 N   PHE B 210   -4.407 -9.548 -1.226 1.00 0.53
N
ATOM 12629 CA PHE B 210   -5.351 -8.436 -1.286 1.00 1.02
C
ATOM 12630 C   PHE B 210   -6.759 -8.815 -1.762 1.00 0.59
C
ATOM 12631 O   PHE B 210   -7.620 -7.941 -1.812 1.00 1.07
O
ATOM 12632 CB PHE B 210   -4.837 -7.286 -2.176 1.00 1.53
C
ATOM 12633 CG PHE B 210   -3.813 -6.376 -1.544 1.00 1.53
C
ATOM 12634 CD1 PHE B 210   -2.461 -6.478 -1.829 1.00 1.53
C
ATOM 12635 CD2 PHE B 210   -4.222 -5.386 -0.662 1.00 1.53
C
ATOM 12636 CE1 PHE B 210   -1.543 -5.629 -1.238 1.00 1.53
C
ATOM 12637 CE2 PHE B 210   -3.309 -4.533 -0.078 1.00 1.53
C
ATOM 12638 CZ PHE B 210   -1.967 -4.657 -0.362 1.00 1.53
C
ATOM 12639 H   PHE B 210   -3.700 -9.630 -1.947 1.00 0.64
H
ATOM 12640 HA PHE B 210   -5.448 -8.038 -0.276 1.00 1.22
H
ATOM 12641 1HB PHE B 210   -4.398 -7.706 -3.081 1.00 1.84
H
ATOM 12642 2HB PHE B 210   -5.682 -6.675 -2.485 1.00 1.84
H
ATOM 12643 HD1 PHE B 210   -2.122 -7.233 -2.528 1.00 1.84
H
ATOM 12644 HD2 PHE B 210   -5.283 -5.285 -0.433 1.00 1.84
H
ATOM 12645 HE1 PHE B 210   -0.483 -5.727 -1.472 1.00 1.84
H
ATOM 12646 HE2 PHE B 210   -3.653 -3.764   0.607 1.00 1.84
H
ATOM 12647 HZ PHE B 210   -1.247 -3.985   0.102 1.00 1.84
H
ATOM 12648 N   GLY B 211   -7.011 -10.093 -2.087 1.00 0.47
N
ATOM 12649 CA GLY B 211   -8.337 -10.534 -2.523 1.00 0.60
C
ATOM 12650 C   GLY B 211   -8.558 -10.552 -4.045 1.00 0.75
C
ATOM 12651 O   GLY B 211   -9.705 -10.569 -4.493 1.00 1.31
O
ATOM 12652 H   GLY B 211   -6.291 -10.793 -2.024 1.00 0.56
H
ATOM 12653 1HA GLY B 211   -8.514 -11.536 -2.129 1.00 0.72
H

```
ATOM  12654 2HA  GLY B 211      -9.087  -9.892  -2.063  1.00  0.72
H
ATOM  12655  N   TRP B 212      -7.481 -10.541  -4.836  1.00  0.61
N
ATOM  12656  CA  TRP B 212      -7.612 -10.534  -6.298  1.00  0.63
C
ATOM  12657  C   TRP B 212      -7.355 -11.893  -6.958  1.00  0.86
C
ATOM  12658  O   TRP B 212      -6.485 -12.662  -6.540  1.00  1.44
O
ATOM  12659  CB  TRP B 212      -6.640  -9.509  -6.903  1.00  0.95
C
ATOM  12660  CG  TRP B 212      -7.035  -8.085  -6.664  1.00  0.95
C
ATOM  12661  CD1 TRP B 212      -7.206  -7.469  -5.466  1.00  0.95
C
ATOM  12662  CD2 TRP B 212      -7.286  -7.077  -7.670  1.00  0.95
C
ATOM  12663  NE1 TRP B 212      -7.578  -6.164  -5.657  1.00  0.95
N
ATOM  12664  CE2 TRP B 212      -7.625  -5.906  -7.000  1.00  0.95
C
ATOM  12665  CE3 TRP B 212      -7.261  -7.077  -9.059  1.00  0.95
C
ATOM  12666  CZ2 TRP B 212      -7.937  -4.736  -7.680  1.00  0.95
C
ATOM  12667  CZ3 TRP B 212      -7.582  -5.913  -9.743  1.00  0.95
C
ATOM  12668  CH2 TRP B 212      -7.910  -4.769  -9.070  1.00  0.95
C
ATOM  12669  H   TRP B 212      -6.551 -10.529  -4.438  1.00  0.73
H
ATOM  12670  HA  TRP B 212      -8.629 -10.228  -6.544  1.00  0.76
H
ATOM  12671 1HB  TRP B 212      -5.643  -9.661  -6.497  1.00  1.13
H
ATOM  12672 2HB  TRP B 212      -6.579  -9.665  -7.979  1.00  1.13
H
ATOM  12673  HD1 TRP B 212      -7.085  -7.946  -4.497  1.00  1.13
H
ATOM  12674  HE1 TRP B 212      -7.782  -5.504  -4.921  1.00  1.13
H
ATOM  12675  HE3 TRP B 212      -6.999  -7.983  -9.591  1.00  1.13
H
ATOM  12676  HZ2 TRP B 212      -8.199  -3.815  -7.161  1.00  1.13
H
ATOM  12677  HZ3 TRP B 212      -7.565  -5.933 -10.830  1.00  1.13
H
ATOM  12678  HH2 TRP B 212      -8.153  -3.870  -9.636  1.00  1.13
H
ATOM  12679  N   ASN B 213      -8.105 -12.164  -8.029  1.00  0.79
N
ATOM  12680  CA  ASN B 213      -7.961 -13.395  -8.801  1.00  1.42
C
ATOM  12681  C   ASN B 213      -6.763 -13.266  -9.701  1.00  0.94
C
ATOM  12682  O   ASN B 213      -6.882 -12.833 -10.845  1.00  1.58
O
```

ATOM 12683 CB ASN B 213   -9.190 -13.691 -9.644 1.00 2.13
C
ATOM 12684 CG ASN B 213   -10.400 -14.056 -8.848 1.00 2.13
C
ATOM 12685 OD1 ASN B 213   -10.308 -14.694 -7.791 1.00 2.13
O
ATOM 12686 ND2 ASN B 213   -11.546 -13.676 -9.349 1.00 2.13
N
ATOM 12687 H   ASN B 213   -8.789 -11.487 -8.329 1.00 0.95
H
ATOM 12688 HA ASN B 213   -7.780 -14.226 -8.118 1.00 1.70
H
ATOM 12689 1HB ASN B 213   -9.428 -12.817 -10.251 1.00 2.56
H
ATOM 12690 2HB ASN B 213   -8.966 -14.509 -10.327 1.00 2.56
H
ATOM 12691 1HD2 ASN B 213   -12.400 -13.897 -8.873 1.00 2.56
H
ATOM 12692 2HD2 ASN B 213   -11.571 -13.165 -10.208 1.00 2.56
H
ATOM 12693 N   THR B 214   -5.605 -13.623 -9.177 1.00 0.80
N
ATOM 12694 CA THR B 214   -4.383 -13.465 -9.934 1.00 0.60
C
ATOM 12695 C   THR B 214   -4.070 -14.584 -10.899 1.00 0.87
C
ATOM 12696 O   THR B 214   -4.142 -15.766 -10.563 1.00 2.63
O
ATOM 12697 CB THR B 214   -3.187 -13.299 -9.012 1.00 0.90
C
ATOM 12698 OG1 THR B 214   -3.405 -12.161 -8.182 1.00 0.90
O
ATOM 12699 CG2 THR B 214   -1.944 -13.064 -9.861 1.00 0.90
C
ATOM 12700 H   THR B 214   -5.586 -13.956 -8.219 1.00 0.96
H
ATOM 12701 HA THR B 214   -4.479 -12.556 -10.517 1.00 0.72
H
ATOM 12702 HB THR B 214   -3.056 -14.183 -8.389 1.00 1.08
H
ATOM 12703 HG1 THR B 214   -4.196 -12.301 -7.653 1.00 1.08
H
ATOM 12704 1HG2 THR B 214   -1.086 -12.907 -9.230 1.00 1.08
H
ATOM 12705 2HG2 THR B 214   -1.761 -13.926 -10.498 1.00 1.08
H
ATOM 12706 3HG2 THR B 214   -2.095 -12.181 -10.478 1.00 1.08
H
ATOM 12707 N   TYR B 215   -3.692 -14.181 -12.103 1.00 0.68
N
ATOM 12708 CA TYR B 215   -3.245 -15.095 -13.129 1.00 0.82
C
ATOM 12709 C   TYR B 215   -1.848 -14.681 -13.581 1.00 1.19
C
ATOM 12710 O   TYR B 215   -1.556 -13.493 -13.715 1.00 3.66
O
ATOM 12711 CB TYR B 215   -4.230 -15.105 -14.299 1.00 1.23
C

```
ATOM  12712  CG  TYR B 215      -5.601 -15.637 -13.927  1.00  1.23
C
ATOM  12713  CD1 TYR B 215      -6.599 -14.786 -13.467  1.00  1.23
C
ATOM  12714  CD2 TYR B 215      -5.853 -16.994 -14.041  1.00  1.23
C
ATOM  12715  CE1 TYR B 215      -7.844 -15.295 -13.125  1.00  1.23
C
ATOM  12716  CE2 TYR B 215      -7.091 -17.501 -13.703  1.00  1.23
C
ATOM  12717  CZ  TYR B 215      -8.086 -16.662 -13.247  1.00  1.23
C
ATOM  12718  OH  TYR B 215      -9.316 -17.197 -12.921  1.00  1.23
O
ATOM  12719  H   TYR B 215      -3.695 -13.189 -12.295  1.00  0.82
H
ATOM  12720  HA  TYR B 215      -3.181 -16.099 -12.707  1.00  0.98
H
ATOM  12721 1HB  TYR B 215      -4.349 -14.094 -14.684  1.00  1.48
H
ATOM  12722 2HB  TYR B 215      -3.833 -15.720 -15.106  1.00  1.48
H
ATOM  12723  HD1 TYR B 215      -6.404 -13.717 -13.374  1.00  1.48
H
ATOM  12724  HD2 TYR B 215      -5.071 -17.664 -14.398  1.00  1.48
H
ATOM  12725  HE1 TYR B 215      -8.626 -14.626 -12.767  1.00  1.48
H
ATOM  12726  HE2 TYR B 215      -7.282 -18.570 -13.796  1.00  1.48
H
ATOM  12727  HH  TYR B 215      -9.327 -18.127 -13.161  1.00  1.48
H
ATOM  12728  N   LEU B 216      -0.986 -15.661 -13.818  1.00  0.57
N
ATOM  12729  CA  LEU B 216       0.370 -15.377 -14.271  1.00  0.70
C
ATOM  12730  C   LEU B 216       0.482 -15.775 -15.730  1.00  1.26
C
ATOM  12731  O   LEU B 216       0.323 -16.949 -16.068  1.00  3.67
O
ATOM  12732  CB  LEU B 216       1.408 -16.142 -13.430  1.00  1.05
C
ATOM  12733  CG  LEU B 216       1.383 -15.889 -11.912  1.00  1.05
C
ATOM  12734  CD1 LEU B 216       2.456 -16.732 -11.236  1.00  1.05
C
ATOM  12735  CD2 LEU B 216       1.593 -14.416 -11.646  1.00  1.05
C
ATOM  12736  H   LEU B 216      -1.275 -16.621 -13.687  1.00  0.68
H
ATOM  12737  HA  LEU B 216       0.560 -14.307 -14.185  1.00  0.84
H
ATOM  12738 1HB  LEU B 216       1.260 -17.209 -13.588  1.00  1.26
H
ATOM  12739 2HB  LEU B 216       2.404 -15.881 -13.790  1.00  1.26
H
ATOM  12740  HG  LEU B 216       0.417 -16.194 -11.507  1.00  1.26
H
```

```
ATOM  12741 1HD1 LEU B 216       2.432 -16.560 -10.161  1.00  1.26
H
ATOM  12742 2HD1 LEU B 216       2.273 -17.787 -11.439  1.00  1.26
H
ATOM  12743 3HD1 LEU B 216       3.435 -16.454 -11.624  1.00  1.26
H
ATOM  12744 1HD2 LEU B 216       1.569 -14.223 -10.576  1.00  1.26
H
ATOM  12745 2HD2 LEU B 216       2.556 -14.102 -12.050  1.00  1.26
H
ATOM  12746 3HD2 LEU B 216       0.793 -13.866 -12.131  1.00  1.26
H
ATOM  12747  N   VAL B 217       0.723 -14.800 -16.600  1.00  0.56
N
ATOM  12748  CA  VAL B 217       0.790 -15.081 -18.030  1.00  0.84
C
ATOM  12749  C   VAL B 217       2.020 -14.503 -18.698  1.00  0.81
C
ATOM  12750  O   VAL B 217       2.637 -13.570 -18.187  1.00  1.44
O
ATOM  12751  CB  VAL B 217      -0.447 -14.505 -18.757  1.00  1.26
C
ATOM  12752  CG1 VAL B 217      -1.729 -15.119 -18.223  1.00  1.26
C
ATOM  12753  CG2 VAL B 217      -0.465 -12.992 -18.588  1.00  1.26
C
ATOM  12754  H   VAL B 217       0.855 -13.854 -16.265  1.00  0.67
H
ATOM  12755  HA  VAL B 217       0.801 -16.163 -18.165  1.00  1.01
H
ATOM  12756  HB  VAL B 217      -0.383 -14.752 -19.817  1.00  1.51
H
ATOM  12757 1HG1 VAL B 217      -2.575 -14.705 -18.765  1.00  1.51
H
ATOM  12758 2HG1 VAL B 217      -1.701 -16.200 -18.363  1.00  1.51
H
ATOM  12759 3HG1 VAL B 217      -1.834 -14.892 -17.164  1.00  1.51
H
ATOM  12760 1HG2 VAL B 217      -1.324 -12.579 -19.114  1.00  1.51
H
ATOM  12761 2HG2 VAL B 217      -0.534 -12.745 -17.528  1.00  1.51
H
ATOM  12762 3HG2 VAL B 217       0.451 -12.569 -19.000  1.00  1.51
H
ATOM  12763  N   ASP B 218       2.343 -15.049 -19.865  1.00  0.59
N
ATOM  12764  CA  ASP B 218       3.408 -14.517 -20.693  1.00  0.59
C
ATOM  12765  C   ASP B 218       2.850 -13.303 -21.389  1.00  0.51
C
ATOM  12766  O   ASP B 218       1.962 -13.422 -22.234  1.00  0.49
O
ATOM  12767  CB  ASP B 218       3.907 -15.542 -21.711  1.00  0.89
C
ATOM  12768  CG  ASP B 218       5.075 -15.033 -22.559  1.00  0.89
C
ATOM  12769  OD1 ASP B 218       5.240 -13.840 -22.682  1.00  0.89
O
```

```
ATOM  12770  OD2 ASP B 218       5.795 -15.855 -23.075  1.00  0.89
O
ATOM  12771  H   ASP B 218       1.809 -15.839 -20.200  1.00  0.71
H
ATOM  12772  HA  ASP B 218       4.240 -14.208 -20.059  1.00  0.71
H
ATOM  12773 1HB  ASP B 218       4.226 -16.445 -21.189  1.00  1.06
H
ATOM  12774 2HB  ASP B 218       3.090 -15.822 -22.375  1.00  1.06
H
ATOM  12775  N   GLY B 219       3.353 -12.130 -21.034  1.00  0.50
N
ATOM  12776  CA  GLY B 219       2.802 -10.891 -21.554  1.00  0.45
C
ATOM  12777  C   GLY B 219       3.116 -10.614 -23.010  1.00  0.58
C
ATOM  12778  O   GLY B 219       2.609  -9.643 -23.570  1.00  0.69
O
ATOM  12779  H   GLY B 219       4.128 -12.086 -20.383  1.00  0.60
H
ATOM  12780 1HA  GLY B 219       1.721 -10.903 -21.420  1.00  0.54
H
ATOM  12781 2HA  GLY B 219       3.174 -10.068 -20.956  1.00  0.54
H
ATOM  12782  N   HIS B 220       3.930 -11.455 -23.640  1.00  0.75
N
ATOM  12783  CA  HIS B 220       4.206 -11.259 -25.043  1.00  1.07
C
ATOM  12784  C   HIS B 220       3.512 -12.323 -25.887  1.00  0.61
C
ATOM  12785  O   HIS B 220       3.697 -12.379 -27.104  1.00  0.61
O
ATOM  12786  CB  HIS B 220       5.706 -11.211 -25.302  1.00  1.60
C
ATOM  12787  CG  HIS B 220       6.346 -10.076 -24.571  1.00  1.60
C
ATOM  12788  ND1 HIS B 220       5.934  -8.771 -24.729  1.00  1.60
N
ATOM  12789  CD2 HIS B 220       7.376 -10.041 -23.694  1.00  1.60
C
ATOM  12790  CE1 HIS B 220       6.668  -7.985 -23.968  1.00  1.60
C
ATOM  12791  NE2 HIS B 220       7.562  -8.725 -23.340  1.00  1.60
N
ATOM  12792  H   HIS B 220       4.358 -12.238 -23.157  1.00  0.90
H
ATOM  12793  HA  HIS B 220       3.800 -10.298 -25.349  1.00  1.28
H
ATOM  12794 1HB  HIS B 220       6.169 -12.143 -24.977  1.00  1.93
H
ATOM  12795 2HB  HIS B 220       5.896 -11.093 -26.368  1.00  1.93
H
ATOM  12796  HD1 HIS B 220       5.247  -8.434 -25.378  1.00  1.93
H
ATOM  12797  HD2 HIS B 220       8.016 -10.817 -23.275  1.00  1.93
H
ATOM  12798  HE1 HIS B 220       6.485  -6.913 -23.956  1.00  1.93
H
```

ATOM 12799 N ASP B 221 2.704 -13.161 -25.239 1.00 0.65
N
ATOM 12800 CA ASP B 221 1.940 -14.169 -25.946 1.00 0.78
C
ATOM 12801 C ASP B 221 0.487 -13.751 -25.990 1.00 0.69
C
ATOM 12802 O ASP B 221 -0.231 -13.898 -25.000 1.00 0.66
O
ATOM 12803 CB ASP B 221 2.067 -15.536 -25.283 1.00 1.17
C
ATOM 12804 CG ASP B 221 1.334 -16.615 -26.064 1.00 1.17
C
ATOM 12805 OD1 ASP B 221 0.673 -16.279 -27.021 1.00 1.17
O
ATOM 12806 OD2 ASP B 221 1.426 -17.761 -25.694 1.00 1.17
O
ATOM 12807 H ASP B 221 2.585 -13.100 -24.235 1.00 0.78
H
ATOM 12808 HA ASP B 221 2.310 -14.241 -26.968 1.00 0.94
H
ATOM 12809 1HB ASP B 221 3.121 -15.810 -25.208 1.00 1.40
H
ATOM 12810 2HB ASP B 221 1.665 -15.494 -24.271 1.00 1.40
H
ATOM 12811 N VAL B 222 0.056 -13.237 -27.137 1.00 0.77
N
ATOM 12812 CA VAL B 222 -1.313 -12.770 -27.287 1.00 0.97
C
ATOM 12813 C VAL B 222 -2.354 -13.884 -27.136 1.00 0.77
C
ATOM 12814 O VAL B 222 -3.517 -13.593 -26.866 1.00 0.94
O
ATOM 12815 CB VAL B 222 -1.502 -12.085 -28.647 1.00 1.46
C
ATOM 12816 CG1 VAL B 222 -0.585 -10.880 -28.773 1.00 1.46
C
ATOM 12817 CG2 VAL B 222 -1.245 -13.075 -29.767 1.00 1.46
C
ATOM 12818 H VAL B 222 0.696 -13.156 -27.915 1.00 0.92
H
ATOM 12819 HA VAL B 222 -1.498 -12.027 -26.513 1.00 1.16
H
ATOM 12820 HB VAL B 222 -2.518 -11.720 -28.706 1.00 1.75
H
ATOM 12821 1HG1 VAL B 222 -0.749 -10.392 -29.734 1.00 1.75
H
ATOM 12822 2HG1 VAL B 222 -0.804 -10.179 -27.969 1.00 1.75
H
ATOM 12823 3HG1 VAL B 222 0.453 -11.204 -28.705 1.00 1.75
H
ATOM 12824 1HG2 VAL B 222 -1.406 -12.583 -30.725 1.00 1.75
H
ATOM 12825 2HG2 VAL B 222 -0.218 -13.434 -29.706 1.00 1.75
H
ATOM 12826 3HG2 VAL B 222 -1.932 -13.914 -29.673 1.00 1.75
H
ATOM 12827 N GLU B 223 -1.951 -15.154 -27.296 1.00 0.83
N

ATOM 12828 CA GLU B 223 -2.892 -16.255 -27.123 1.00 1.05
C
ATOM 12829 C GLU B 223 -3.211 -16.407 -25.654 1.00 1.02
C
ATOM 12830 O GLU B 223 -4.375 -16.550 -25.271 1.00 1.11
O
ATOM 12831 CB GLU B 223 -2.313 -17.575 -27.640 1.00 1.58
C
ATOM 12832 CG GLU B 223 -2.126 -17.659 -29.138 1.00 1.58
C
ATOM 12833 CD GLU B 223 -1.520 -18.966 -29.580 1.00 1.58
C
ATOM 12834 OE1 GLU B 223 -1.182 -19.759 -28.734 1.00 1.58
O
ATOM 12835 OE2 GLU B 223 -1.397 -19.171 -30.765 1.00 1.58
O
ATOM 12836 H GLU B 223 -0.982 -15.370 -27.505 1.00 1.00
H
ATOM 12837 HA GLU B 223 -3.812 -16.025 -27.661 1.00 1.26
H
ATOM 12838 1HB GLU B 223 -1.345 -17.756 -27.174 1.00 1.89
H
ATOM 12839 2HB GLU B 223 -2.970 -18.394 -27.345 1.00 1.89
H
ATOM 12840 1HG GLU B 223 -3.099 -17.546 -29.609 1.00 1.89
H
ATOM 12841 2HG GLU B 223 -1.491 -16.836 -29.462 1.00 1.89
H
ATOM 12842 N ALA B 224 -2.157 -16.355 -24.838 1.00 1.05
N
ATOM 12843 CA ALA B 224 -2.271 -16.461 -23.393 1.00 1.17
C
ATOM 12844 C ALA B 224 -3.060 -15.295 -22.851 1.00 0.85
C
ATOM 12845 O ALA B 224 -3.921 -15.475 -21.987 1.00 0.90
O
ATOM 12846 CB ALA B 224 -0.891 -16.504 -22.748 1.00 1.75
C
ATOM 12847 H ALA B 224 -1.235 -16.242 -25.239 1.00 1.26
H
ATOM 12848 HA ALA B 224 -2.809 -17.379 -23.158 1.00 1.40
H
ATOM 12849 1HB ALA B 224 -0.998 -16.601 -21.668 1.00 2.11
H
ATOM 12850 2HB ALA B 224 -0.336 -17.360 -23.135 1.00 2.11
H
ATOM 12851 3HB ALA B 224 -0.350 -15.589 -22.980 1.00 2.11
H
ATOM 12852 N LEU B 225 -2.768 -14.102 -23.371 1.00 0.67
N
ATOM 12853 CA LEU B 225 -3.458 -12.900 -22.951 1.00 0.60
C
ATOM 12854 C LEU B 225 -4.932 -12.960 -23.288 1.00 0.56
C
ATOM 12855 O LEU B 225 -5.761 -12.633 -22.444 1.00 0.62
O
ATOM 12856 CB LEU B 225 -2.824 -11.683 -23.615 1.00 0.90
C

ATOM 12857 CG LEU B 225 -1.421 -11.308 -23.129 1.00 0.90
C
ATOM 12858 CD1 LEU B 225 -0.853 -10.232 -24.039 1.00 0.90
C
ATOM 12859 CD2 LEU B 225 -1.505 -10.812 -21.692 1.00 0.90
C
ATOM 12860 H LEU B 225 -2.034 -14.027 -24.066 1.00 0.80
H
ATOM 12861 HA LEU B 225 -3.355 -12.805 -21.869 1.00 0.72
H
ATOM 12862 1HB LEU B 225 -2.754 -11.884 -24.681 1.00 1.08
H
ATOM 12863 2HB LEU B 225 -3.473 -10.822 -23.466 1.00 1.08
H
ATOM 12864 HG LEU B 225 -0.767 -12.179 -23.176 1.00 1.08
H
ATOM 12865 1HD1 LEU B 225 0.144 -9.959 -23.701 1.00 1.08
H
ATOM 12866 2HD1 LEU B 225 -0.799 -10.607 -25.059 1.00 1.08
H
ATOM 12867 3HD1 LEU B 225 -1.499 -9.358 -24.009 1.00 1.08
H
ATOM 12868 1HD2 LEU B 225 -0.508 -10.543 -21.341 1.00 1.08
H
ATOM 12869 2HD2 LEU B 225 -2.154 -9.938 -21.645 1.00 1.08
H
ATOM 12870 3HD2 LEU B 225 -1.911 -11.601 -21.059 1.00 1.08
H
ATOM 12871 N CYS B 226 -5.268 -13.433 -24.490 1.00 0.57
N
ATOM 12872 CA CYS B 226 -6.667 -13.553 -24.863 1.00 0.67
C
ATOM 12873 C CYS B 226 -7.401 -14.470 -23.917 1.00 0.75
C
ATOM 12874 O CYS B 226 -8.460 -14.107 -23.414 1.00 0.84
O
ATOM 12875 CB CYS B 226 -6.828 -14.084 -26.289 1.00 1.01
C
ATOM 12876 SG CYS B 226 -6.388 -12.910 -27.585 1.00 1.01
S
ATOM 12877 H CYS B 226 -4.557 -13.683 -25.166 1.00 0.68
H
ATOM 12878 HA CYS B 226 -7.123 -12.563 -24.811 1.00 0.80
H
ATOM 12879 1HB CYS B 226 -6.203 -14.968 -26.415 1.00 1.21
H
ATOM 12880 2HB CYS B 226 -7.861 -14.389 -26.450 1.00 1.21
H
ATOM 12881 HG CYS B 226 -7.346 -12.032 -27.301 1.00 1.21
H
ATOM 12882 N GLN B 227 -6.832 -15.644 -23.653 1.00 0.91
N
ATOM 12883 CA GLN B 227 -7.461 -16.607 -22.759 1.00 1.27
C
ATOM 12884 C GLN B 227 -7.695 -16.018 -21.373 1.00 1.23
C
ATOM 12885 O GLN B 227 -8.780 -16.155 -20.797 1.00 1.92
O

```
ATOM  12886  CB  GLN B 227      -6.584 -17.853 -22.645  1.00  1.91
C
ATOM  12887  CG  GLN B 227      -6.531 -18.692 -23.909  1.00  1.91
C
ATOM  12888  CD  GLN B 227      -5.541 -19.835 -23.797  1.00  1.91
C
ATOM  12889  OE1 GLN B 227      -4.668 -19.839 -22.924  1.00  1.91
O
ATOM  12890  NE2 GLN B 227      -5.666 -20.813 -24.686  1.00  1.91
N
ATOM  12891  H   GLN B 227      -5.953 -15.890 -24.097  1.00  1.09
H
ATOM  12892  HA  GLN B 227      -8.428 -16.888 -23.176  1.00  1.52
H
ATOM  12893 1HB  GLN B 227      -5.565 -17.558 -22.397  1.00  2.29
H
ATOM  12894 2HB  GLN B 227      -6.950 -18.484 -21.836  1.00  2.29
H
ATOM  12895 1HG  GLN B 227      -7.519 -19.116 -24.089  1.00  2.29
H
ATOM  12896 2HG  GLN B 227      -6.239 -18.061 -24.746  1.00  2.29
H
ATOM  12897 1HE2 GLN B 227      -5.040 -21.593 -24.665  1.00  2.29
H
ATOM  12898 2HE2 GLN B 227      -6.386 -20.769 -25.379  1.00  2.29
H
ATOM  12899  N   ALA B 228      -6.669 -15.342 -20.855  1.00  1.01
N
ATOM  12900  CA  ALA B 228      -6.721 -14.715 -19.548  1.00  1.26
C
ATOM  12901  C   ALA B 228      -7.810 -13.660 -19.461  1.00  1.09
C
ATOM  12902  O   ALA B 228      -8.510 -13.568 -18.453  1.00  2.26
O
ATOM  12903  CB  ALA B 228      -5.377 -14.093 -19.230  1.00  1.89
C
ATOM  12904  H   ALA B 228      -5.805 -15.274 -21.378  1.00  1.21
H
ATOM  12905  HA  ALA B 228      -6.942 -15.489 -18.813  1.00  1.51
H
ATOM  12906 1HB  ALA B 228      -5.408 -13.659 -18.237  1.00  2.27
H
ATOM  12907 2HB  ALA B 228      -4.611 -14.866 -19.266  1.00  2.27
H
ATOM  12908 3HB  ALA B 228      -5.148 -13.319 -19.960  1.00  2.27
H
ATOM  12909  N   PHE B 229      -7.957 -12.864 -20.517  1.00  0.68
N
ATOM  12910  CA  PHE B 229      -8.951 -11.809 -20.507  1.00  0.80
C
ATOM  12911  C   PHE B 229     -10.367 -12.269 -20.871  1.00  1.50
C
ATOM  12912  O   PHE B 229     -11.336 -11.646 -20.434  1.00  4.90
O
ATOM  12913  CB  PHE B 229      -8.543 -10.687 -21.456  1.00  1.20
C
ATOM  12914  CG  PHE B 229      -7.350  -9.893 -21.010  1.00  1.20
C
```

ATOM 12915 CD1 PHE B 229   -6.244 -9.764 -21.833 1.00 1.20
C
ATOM 12916 CD2 PHE B 229   -7.324 -9.286 -19.770 1.00 1.20
C
ATOM 12917 CE1 PHE B 229   -5.134 -9.047 -21.439 1.00 1.20
C
ATOM 12918 CE2 PHE B 229   -6.218 -8.567 -19.370 1.00 1.20
C
ATOM 12919 CZ PHE B 229   -5.120 -8.447 -20.204 1.00 1.20
C
ATOM 12920 H   PHE B 229   -7.351 -12.966 -21.319 1.00 0.82
H
ATOM 12921 HA PHE B 229   -8.986 -11.397 -19.498 1.00 0.96
H
ATOM 12922 1HB PHE B 229   -8.326 -11.104 -22.438 1.00 1.44
H
ATOM 12923 2HB PHE B 229   -9.376 -10.007 -21.567 1.00 1.44
H
ATOM 12924 HD1 PHE B 229   -6.263 -10.243 -22.807 1.00 1.44
H
ATOM 12925 HD2 PHE B 229   -8.185 -9.382 -19.109 1.00 1.44
H
ATOM 12926 HE1 PHE B 229   -4.275 -8.959 -22.103 1.00 1.44
H
ATOM 12927 HE2 PHE B 229   -6.213 -8.095 -18.390 1.00 1.44
H
ATOM 12928 HZ PHE B 229   -4.246 -7.882 -19.886 1.00 1.44
H
ATOM 12929 N   TRP B 230   -10.502 -13.347 -21.657 1.00 2.90
N
ATOM 12930 CA TRP B 230   -11.835 -13.818 -22.054 1.00 5.13
C
ATOM 12931 C   TRP B 230   -12.638 -14.401 -20.916 1.00 6.38
C
ATOM 12932 O   TRP B 230   -13.842 -14.178 -20.822 1.00 29.64
O
ATOM 12933 CB TRP B 230   -11.786 -14.921 -23.129 1.00 7.70
C
ATOM 12934 CG TRP B 230   -11.610 -14.496 -24.565 1.00 7.70
C
ATOM 12935 CD1 TRP B 230   -10.576 -14.822 -25.388 1.00 7.70
C
ATOM 12936 CD2 TRP B 230   -12.510 -13.688 -25.362 1.00 7.70
C
ATOM 12937 NE1 TRP B 230   -10.759 -14.267 -26.626 1.00 7.70
N
ATOM 12938 CE2 TRP B 230   -11.935 -13.572 -26.631 1.00 7.70
C
ATOM 12939 CE3 TRP B 230   -13.738 -13.066 -25.106 1.00 7.70
C
ATOM 12940 CZ2 TRP B 230   -12.540 -12.854 -27.647 1.00 7.70
C
ATOM 12941 CZ3 TRP B 230   -14.346 -12.347 -26.128 1.00 7.70
C
ATOM 12942 CH2 TRP B 230   -13.758 -12.244 -27.364 1.00 7.70
C
ATOM 12943 H   TRP B 230   -9.686 -13.826 -22.010 1.00 3.48
H

```
ATOM  12944  HA  TRP B 230     -12.386 -12.967 -22.455  1.00  6.16
H
ATOM  12945 1HB  TRP B 230     -10.973 -15.606 -22.890  1.00  9.23
H
ATOM  12946 2HB  TRP B 230     -12.709 -15.494 -23.070  1.00  9.23
H
ATOM  12947  HD1 TRP B 230      -9.728 -15.439 -25.104  1.00  9.23
H
ATOM  12948  HE1 TRP B 230     -10.134 -14.361 -27.412  1.00  9.23
H
ATOM  12949  HE3 TRP B 230     -14.206 -13.145 -24.125  1.00  9.23
H
ATOM  12950  HZ2 TRP B 230     -12.092 -12.761 -28.637  1.00  9.23
H
ATOM  12951  HZ3 TRP B 230     -15.302 -11.864 -25.923  1.00  9.23
H
ATOM  12952  HH2 TRP B 230     -14.257 -11.671 -28.143  1.00  9.23
H
ATOM  12953  N   GLN B 231     -12.000 -15.155 -20.053  1.00 16.92
N
ATOM  12954  CA  GLN B 231     -12.761 -15.737 -18.972  1.00 28.44
C
ATOM  12955  C   GLN B 231     -12.838 -14.801 -17.807  1.00 16.96
C
ATOM  12956  O   GLN B 231     -11.931 -13.997 -17.593  1.00 51.14
O
ATOM  12957  CB  GLN B 231     -12.136 -17.063 -18.534  1.00 42.66
C
ATOM  12958  CG  GLN B 231     -12.174 -18.157 -19.588  1.00 42.66
C
ATOM  12959  CD  GLN B 231     -13.594 -18.612 -19.869  1.00 42.66
C
ATOM  12960  OE1 GLN B 231     -14.350 -18.894 -18.934  1.00 42.66
O
ATOM  12961  NE2 GLN B 231     -13.969 -18.697 -21.138  1.00 42.66
N
ATOM  12962  H   GLN B 231     -11.006 -15.345 -20.155  1.00 20.30
H
ATOM  12963  HA  GLN B 231     -13.774 -15.927 -19.321  1.00 34.13
H
ATOM  12964 1HB  GLN B 231     -11.094 -16.900 -18.260  1.00 51.19
H
ATOM  12965 2HB  GLN B 231     -12.652 -17.434 -17.649  1.00 51.19
H
ATOM  12966 1HG  GLN B 231     -11.745 -17.774 -20.515  1.00 51.19
H
ATOM  12967 2HG  GLN B 231     -11.600 -19.014 -19.235  1.00 51.19
H
ATOM  12968 1HE2 GLN B 231     -14.895 -18.998 -21.367  1.00 51.19
H
ATOM  12969 2HE2 GLN B 231     -13.330 -18.469 -21.879  1.00 51.19
H
ATOM  12970  N   ALA B 232     -13.914 -14.911 -17.045  1.00 23.34
N
ATOM  12971  CA  ALA B 232     -13.994 -14.167 -15.809  1.00 39.85
C
ATOM  12972  C   ALA B 232     -13.212 -15.018 -14.815  1.00 64.03
C
```

```
ATOM  12973  O   ALA B 232     -12.040 -15.313 -15.044  1.00102.79
O
ATOM  12974  CB  ALA B 232     -15.441 -13.937 -15.380  1.00 59.78
C
ATOM  12975  H   ALA B 232     -14.652 -15.549 -17.304  1.00 28.01
H
ATOM  12976  HA  ALA B 232     -13.492 -13.209 -15.924  1.00 47.82
H
ATOM  12977 1HB  ALA B 232     -15.459 -13.398 -14.433  1.00 71.73
H
ATOM  12978 2HB  ALA B 232     -15.951 -13.348 -16.142  1.00 71.73
H
ATOM  12979 3HB  ALA B 232     -15.945 -14.894 -15.266  1.00 71.73
H
ATOM  12980  N   SER B 233     -13.839 -15.519 -13.776  1.00115.90
N
ATOM  12981  CA  SER B 233     -13.104 -16.468 -12.964  1.00186.59
C
ATOM  12982  C   SER B 233     -14.050 -17.493 -12.408  1.00146.30
C
ATOM  12983  O   SER B 233     -15.256 -17.417 -12.641  1.00185.17
O
ATOM  12984  CB  SER B 233     -12.357 -15.793 -11.844  1.00279.88
C
ATOM  12985  OG  SER B 233     -11.513 -16.715 -11.207  1.00279.88
O
ATOM  12986  H   SER B 233     -14.791 -15.273 -13.554  1.00139.08
H
ATOM  12987  HA  SER B 233     -12.381 -16.985 -13.596  1.00223.91
H
ATOM  12988 1HB  SER B 233     -11.768 -14.964 -12.239  1.00335.86
H
ATOM  12989 2HB  SER B 233     -13.064 -15.383 -11.127  1.00335.86
H
ATOM  12990  HG  SER B 233     -10.740 -16.804 -11.778  1.00335.86
H
ATOM  12991  N   GLN B 234     -13.505 -18.453 -11.666  1.00134.92
N
ATOM  12992  CA  GLN B 234     -14.340 -19.473 -11.035  1.00176.88
C
ATOM  12993  C   GLN B 234     -15.126 -18.771  -9.964  1.00167.67
C
ATOM  12994  O   GLN B 234     -16.335 -18.959  -9.809  1.00198.91
O
ATOM  12995  CB  GLN B 234     -13.497 -20.603 -10.444  1.00265.32
C
ATOM  12996  CG  GLN B 234     -14.316 -21.738  -9.860  1.00265.32
C
ATOM  12997  CD  GLN B 234     -15.155 -22.442 -10.909  1.00265.32
C
ATOM  12998  OE1 GLN B 234     -14.642 -22.874 -11.945  1.00265.32
O
ATOM  12999  NE2 GLN B 234     -16.451 -22.564 -10.647  1.00265.32
N
ATOM  13000  H   GLN B 234     -12.503 -18.447 -11.520  1.00161.90
H
ATOM  13001  HA  GLN B 234     -15.033 -19.882 -11.768  1.00212.26
H
```

ATOM 13002 1HB GLN B 234 -12.850 -21.017 -11.218 1.00318.38
H
ATOM 13003 2HB GLN B 234 -12.855 -20.209 -9.656 1.00318.38
H
ATOM 13004 1HG GLN B 234 -13.640 -22.467 -9.413 1.00318.38
H
ATOM 13005 2HG GLN B 234 -14.985 -21.334 -9.099 1.00318.38
H
ATOM 13006 1HE2 GLN B 234 -17.054 -23.020 -11.302 1.00318.38
H
ATOM 13007 2HE2 GLN B 234 -16.825 -22.200 -9.794 1.00318.38
H
ATOM 13008 N VAL B 235 -14.417 -17.892 -9.278 1.00176.03
N
ATOM 13009 CA VAL B 235 -14.970 -16.979 -8.320 1.00198.62
C
ATOM 13010 C VAL B 235 -15.031 -15.649 -9.021 1.00106.92
C
ATOM 13011 O VAL B 235 -14.319 -14.709 -8.656 1.00158.30
O
ATOM 13012 CB VAL B 235 -14.112 -16.879 -7.050 1.00297.93
C
ATOM 13013 CG1 VAL B 235 -14.755 -15.899 -6.074 1.00297.93
C
ATOM 13014 CG2 VAL B 235 -13.976 -18.257 -6.418 1.00297.93
C
ATOM 13015 H VAL B 235 -13.423 -17.837 -9.453 1.00211.24
H
ATOM 13016 HA VAL B 235 -15.978 -17.294 -8.051 1.00238.34
H
ATOM 13017 HB VAL B 235 -13.127 -16.492 -7.309 1.00357.52
H
ATOM 13018 1HG1 VAL B 235 -14.137 -15.820 -5.179 1.00357.52
H
ATOM 13019 2HG1 VAL B 235 -14.839 -14.919 -6.544 1.00357.52
H
ATOM 13020 3HG1 VAL B 235 -15.746 -16.257 -5.800 1.00357.52
H
ATOM 13021 1HG2 VAL B 235 -13.361 -18.186 -5.521 1.00357.52
H
ATOM 13022 2HG2 VAL B 235 -14.963 -18.636 -6.155 1.00357.52
H
ATOM 13023 3HG2 VAL B 235 -13.503 -18.936 -7.128 1.00357.52
H
ATOM 13024 N LYS B 236 -15.877 -15.582 -10.058 1.00 58.36
N
ATOM 13025 CA LYS B 236 -16.045 -14.397 -10.893 1.00 70.52
C
ATOM 13026 C LYS B 236 -16.369 -13.152 -10.092 1.00 33.24
C
ATOM 13027 O LYS B 236 -16.184 -12.048 -10.585 1.00 80.30
O
ATOM 13028 CB LYS B 236 -17.145 -14.629 -11.924 1.00105.78
C
ATOM 13029 CG LYS B 236 -18.540 -14.742 -11.331 1.00105.78
C
ATOM 13030 CD LYS B 236 -19.578 -15.009 -12.409 1.00105.78
C

ATOM 13031 CE LYS B 236 -20.980 -15.082 -11.823 1.00105.78
C
ATOM 13032 NZ LYS B 236 -22.003 -15.373 -12.865 1.00105.78
N
ATOM 13033 H LYS B 236 -16.427 -16.401 -10.282 1.00 70.03
H
ATOM 13034 HA LYS B 236 -15.108 -14.215 -11.419 1.00 84.62
H
ATOM 13035 1HB LYS B 236 -17.151 -13.807 -12.642 1.00126.94
H
ATOM 13036 2HB LYS B 236 -16.942 -15.546 -12.477 1.00126.94
H
ATOM 13037 1HG LYS B 236 -18.564 -15.556 -10.606 1.00126.94
H
ATOM 13038 2HG LYS B 236 -18.796 -13.814 -10.818 1.00126.94
H
ATOM 13039 1HD LYS B 236 -19.546 -14.211 -13.153 1.00126.94
H
ATOM 13040 2HD LYS B 236 -19.352 -15.953 -12.905 1.00126.94
H
ATOM 13041 1HE LYS B 236 -21.013 -15.867 -11.068 1.00126.94
H
ATOM 13042 2HE LYS B 236 -21.220 -14.131 -11.349 1.00126.94
H
ATOM 13043 1HZ LYS B 236 -22.917 -15.413 -12.438 1.00126.94
H
ATOM 13044 2HZ LYS B 236 -21.989 -14.642 -13.563 1.00126.94
H
ATOM 13045 3HZ LYS B 236 -21.797 -16.259 -13.302 1.00126.94
H
ATOM 13046 N ASN B 237 -16.792 -13.327 -8.849 1.00 13.24
N
ATOM 13047 CA ASN B 237 -17.090 -12.239 -7.961 1.00 29.10
C
ATOM 13048 C ASN B 237 -15.883 -11.415 -7.491 1.00 16.82
C
ATOM 13049 O ASN B 237 -16.079 -10.395 -6.840 1.00 34.85
O
ATOM 13050 CB ASN B 237 -17.831 -12.771 -6.755 1.00 43.65
C
ATOM 13051 CG ASN B 237 -19.232 -13.199 -7.089 1.00 43.65
C
ATOM 13052 OD1 ASN B 237 -19.834 -12.707 -8.051 1.00 43.65
O
ATOM 13053 ND2 ASN B 237 -19.763 -14.108 -6.314 1.00 43.65
N
ATOM 13054 H ASN B 237 -16.948 -14.257 -8.500 1.00 15.89
H
ATOM 13055 HA ASN B 237 -17.751 -11.552 -8.491 1.00 34.92
H
ATOM 13056 1HB ASN B 237 -17.291 -13.623 -6.341 1.00 52.38
H
ATOM 13057 2HB ASN B 237 -17.871 -12.002 -5.982 1.00 52.38
H
ATOM 13058 1HD2 ASN B 237 -20.694 -14.431 -6.487 1.00 52.38
H
ATOM 13059 2HD2 ASN B 237 -19.241 -14.479 -5.547 1.00 52.38
H

```
ATOM  13060  N   LYS B 238     -14.652 -11.804  -7.805  1.00  6.50
N
ATOM  13061  CA  LYS B 238     -13.534 -10.970  -7.361  1.00  3.40
C
ATOM  13062  C   LYS B 238     -12.836 -10.286  -8.545  1.00  3.28
C
ATOM  13063  O   LYS B 238     -12.913 -10.775  -9.672  1.00 10.09
O
ATOM  13064  CB  LYS B 238     -12.532 -11.805  -6.557  1.00  5.10
C
ATOM  13065  CG  LYS B 238     -13.111 -12.475  -5.320  1.00  5.10
C
ATOM  13066  CD  LYS B 238     -13.521 -11.457  -4.267  1.00  5.10
C
ATOM  13067  CE  LYS B 238     -14.032 -12.152  -3.010  1.00  5.10
C
ATOM  13068  NZ  LYS B 238     -14.423 -11.179  -1.952  1.00  5.10
N
ATOM  13069  H   LYS B 238     -14.488 -12.661  -8.319  1.00  7.80
H
ATOM  13070  HA  LYS B 238     -13.935 -10.211  -6.701  1.00  4.08
H
ATOM  13071 1HB  LYS B 238     -12.126 -12.591  -7.178  1.00  6.12
H
ATOM  13072 2HB  LYS B 238     -11.700 -11.178  -6.237  1.00  6.12
H
ATOM  13073 1HG  LYS B 238     -13.986 -13.058  -5.602  1.00  6.12
H
ATOM  13074 2HG  LYS B 238     -12.370 -13.148  -4.893  1.00  6.12
H
ATOM  13075 1HD  LYS B 238     -12.664 -10.834  -4.008  1.00  6.12
H
ATOM  13076 2HD  LYS B 238     -14.309 -10.816  -4.663  1.00  6.12
H
ATOM  13077 1HE  LYS B 238     -14.901 -12.754  -3.271  1.00  6.12
H
ATOM  13078 2HE  LYS B 238     -13.255 -12.808  -2.618  1.00  6.12
H
ATOM  13079 1HZ  LYS B 238     -14.765 -11.674  -1.141  1.00  6.12
H
ATOM  13080 2HZ  LYS B 238     -13.620 -10.624  -1.695  1.00  6.12
H
ATOM  13081 3HZ  LYS B 238     -15.148 -10.568  -2.307  1.00  6.12
H
ATOM  13082  N   PRO B 239     -12.179  -9.137  -8.304  1.00  0.78
N
ATOM  13083  CA  PRO B 239     -11.366  -8.365  -9.237  1.00  0.45
C
ATOM  13084  C   PRO B 239     -10.254  -9.259  -9.761  1.00  0.62
C
ATOM  13085  O   PRO B 239      -9.768 -10.123  -9.031  1.00  1.70
O
ATOM  13086  CB  PRO B 239     -10.841  -7.215  -8.387  1.00  0.68
C
ATOM  13087  CG  PRO B 239     -11.855  -7.037  -7.319  1.00  0.68
C
ATOM  13088  CD  PRO B 239     -12.371  -8.414  -7.029  1.00  0.68
C
```

```
ATOM  13089  HA  PRO B 239     -11.997  -7.999 -10.060  1.00  0.54
H
ATOM  13090 1HB  PRO B 239      -9.869  -7.491  -7.975  1.00  0.81
H
ATOM  13091 2HB  PRO B 239     -10.695  -6.317  -9.005  1.00  0.81
H
ATOM  13092 1HG  PRO B 239     -11.380  -6.584  -6.439  1.00  0.81
H
ATOM  13093 2HG  PRO B 239     -12.641  -6.345  -7.648  1.00  0.81
H
ATOM  13094 1HD  PRO B 239     -11.790  -8.892  -6.226  1.00  0.81
H
ATOM  13095 2HD  PRO B 239     -13.438  -8.338  -6.781  1.00  0.81
H
ATOM  13096  N   THR B 240      -9.857  -9.057 -11.016  1.00  0.54
N
ATOM  13097  CA  THR B 240      -8.866  -9.925 -11.651  1.00  0.78
C
ATOM  13098  C   THR B 240      -7.524  -9.242 -11.834  1.00  0.50
C
ATOM  13099  O   THR B 240      -7.455  -8.077 -12.220  1.00  0.76
O
ATOM  13100  CB  THR B 240      -9.384 -10.426 -13.011  1.00  1.17
C
ATOM  13101  OG1 THR B 240     -10.585 -11.187 -12.818  1.00  1.17
O
ATOM  13102  CG2 THR B 240      -8.348 -11.298 -13.689  1.00  1.17
C
ATOM  13103  H   THR B 240     -10.269  -8.296 -11.545  1.00  0.65
H
ATOM  13104  HA  THR B 240      -8.711 -10.793 -11.011  1.00  0.94
H
ATOM  13105  HB  THR B 240      -9.604  -9.573 -13.646  1.00  1.40
H
ATOM  13106  HG1 THR B 240     -11.257 -10.627 -12.422  1.00  1.40
H
ATOM  13107 1HG2 THR B 240      -8.735 -11.641 -14.648  1.00  1.40
H
ATOM  13108 2HG2 THR B 240      -7.438 -10.723 -13.852  1.00  1.40
H
ATOM  13109 3HG2 THR B 240      -8.128 -12.157 -13.059  1.00  1.40
H
ATOM  13110  N   ALA B 241      -6.458  -9.970 -11.520  1.00  0.48
N
ATOM  13111  CA  ALA B 241      -5.100  -9.450 -11.601  1.00  0.42
C
ATOM  13112  C   ALA B 241      -4.287 -10.217 -12.635  1.00  0.58
C
ATOM  13113  O   ALA B 241      -3.802 -11.313 -12.368  1.00  1.84
O
ATOM  13114  CB  ALA B 241      -4.432  -9.529 -10.234  1.00  0.63
C
ATOM  13115  H   ALA B 241      -6.595 -10.927 -11.221  1.00  0.58
H
ATOM  13116  HA  ALA B 241      -5.149  -8.408 -11.917  1.00  0.50
H
ATOM  13117 1HB  ALA B 241      -3.423  -9.125 -10.293  1.00  0.76
H
```

```
ATOM  13118 2HB  ALA B 241      -5.009  -8.954  -9.512  1.00  0.76
H
ATOM  13119 3HB  ALA B 241      -4.383 -10.563  -9.911  1.00  0.76
H
ATOM  13120  N   ILE B 242      -4.137  -9.643 -13.821  1.00  0.28
N
ATOM  13121  CA  ILE B 242      -3.377 -10.304 -14.866  1.00  0.33
C
ATOM  13122  C   ILE B 242      -1.941  -9.857 -14.799  1.00  0.33
C
ATOM  13123  O   ILE B 242      -1.582  -8.801 -15.317  1.00  0.69
O
ATOM  13124  CB  ILE B 242      -3.963 -10.005 -16.258  1.00  0.49
C
ATOM  13125  CG1 ILE B 242      -5.438 -10.394 -16.280  1.00  0.49
C
ATOM  13126  CG2 ILE B 242      -3.188 -10.738 -17.342  1.00  0.49
C
ATOM  13127  CD1 ILE B 242      -5.670 -11.842 -15.938  1.00  0.49
C
ATOM  13128  H   ILE B 242      -4.535  -8.734 -13.994  1.00  0.34
H
ATOM  13129  HA  ILE B 242      -3.410 -11.381 -14.700  1.00  0.40
H
ATOM  13130  HB  ILE B 242      -3.909  -8.933 -16.445  1.00  0.59
H
ATOM  13131 1HG1 ILE B 242      -5.991  -9.773 -15.576  1.00  0.59
H
ATOM  13132 2HG1 ILE B 242      -5.836 -10.222 -17.276  1.00  0.59
H
ATOM  13133 1HG2 ILE B 242      -3.616 -10.503 -18.317  1.00  0.59
H
ATOM  13134 2HG2 ILE B 242      -2.145 -10.426 -17.320  1.00  0.59
H
ATOM  13135 3HG2 ILE B 242      -3.248 -11.813 -17.170  1.00  0.59
H
ATOM  13136 1HD1 ILE B 242      -6.735 -12.066 -15.987  1.00  0.59
H
ATOM  13137 2HD1 ILE B 242      -5.133 -12.467 -16.646  1.00  0.59
H
ATOM  13138 3HD1 ILE B 242      -5.302 -12.040 -14.933  1.00  0.59
H
ATOM  13139  N   VAL B 243      -1.117 -10.679 -14.166  1.00  0.51
N
ATOM  13140  CA  VAL B 243       0.282 -10.358 -14.022  1.00  0.39
C
ATOM  13141  C   VAL B 243       1.019 -10.880 -15.220  1.00  0.41
C
ATOM  13142  O   VAL B 243       1.101 -12.090 -15.434  1.00  0.81
O
ATOM  13143  CB  VAL B 243       0.848 -10.939 -12.722  1.00  0.58
C
ATOM  13144  CG1 VAL B 243       2.330 -10.637 -12.604  1.00  0.58
C
ATOM  13145  CG2 VAL B 243       0.096 -10.359 -11.532  1.00  0.58
C
ATOM  13146  H   VAL B 243      -1.465 -11.551 -13.788  1.00  0.61
H
```

```
ATOM  13147  HA  VAL B 243       0.383  -9.288 -13.999  1.00  0.47
H
ATOM  13148  HB  VAL B 243       0.725 -12.013 -12.746  1.00  0.70
H
ATOM  13149 1HG1 VAL B 243       2.719 -11.072 -11.683  1.00  0.70
H
ATOM  13150 2HG1 VAL B 243       2.858 -11.061 -13.457  1.00  0.70
H
ATOM  13151 3HG1 VAL B 243       2.474  -9.558 -12.585  1.00  0.70
H
ATOM  13152 1HG2 VAL B 243       0.488 -10.788 -10.610  1.00  0.70
H
ATOM  13153 2HG2 VAL B 243       0.227  -9.278 -11.513  1.00  0.70
H
ATOM  13154 3HG2 VAL B 243      -0.963 -10.595 -11.620  1.00  0.70
H
ATOM  13155  N   ALA B 244       1.538  -9.960 -16.016  1.00  0.38
N
ATOM  13156  CA  ALA B 244       2.175 -10.335 -17.256  1.00  0.46
C
ATOM  13157  C   ALA B 244       3.688 -10.232 -17.183  1.00  0.40
C
ATOM  13158  O   ALA B 244       4.244  -9.192 -16.828  1.00  0.38
O
ATOM  13159  CB  ALA B 244       1.637  -9.454 -18.369  1.00  0.69
C
ATOM  13160  H   ALA B 244       1.476  -8.979 -15.774  1.00  0.46
H
ATOM  13161  HA  ALA B 244       1.920 -11.370 -17.469  1.00  0.55
H
ATOM  13162 1HB  ALA B 244       2.078  -9.748 -19.310  1.00  0.83
H
ATOM  13163 2HB  ALA B 244       0.553  -9.565 -18.426  1.00  0.83
H
ATOM  13164 3HB  ALA B 244       1.883  -8.414 -18.161  1.00  0.83
H
ATOM  13165  N   LYS B 245       4.361 -11.318 -17.557  1.00  0.47
N
ATOM  13166  CA  LYS B 245       5.814 -11.321 -17.589  1.00  0.51
C
ATOM  13167  C   LYS B 245       6.261 -10.765 -18.920  1.00  0.54
C
ATOM  13168  O   LYS B 245       5.963 -11.333 -19.971  1.00  0.63
O
ATOM  13169  CB  LYS B 245       6.378 -12.726 -17.386  1.00  0.77
C
ATOM  13170  CG  LYS B 245       7.904 -12.785 -17.326  1.00  0.77
C
ATOM  13171  CD  LYS B 245       8.391 -14.204 -17.073  1.00  0.77
C
ATOM  13172  CE  LYS B 245       9.911 -14.283 -17.038  1.00  0.77
C
ATOM  13173  NZ  LYS B 245      10.382 -15.678 -16.799  1.00  0.77
N
ATOM  13174  H   LYS B 245       3.851 -12.148 -17.830  1.00  0.56
H
ATOM  13175  HA  LYS B 245       6.192 -10.668 -16.801  1.00  0.61
H
```

ATOM 13176 1HB LYS B 245   5.990 -13.143 -16.455 1.00 0.92
H
ATOM 13177 2HB LYS B 245   6.048 -13.373 -18.198 1.00 0.92
H
ATOM 13178 1HG LYS B 245   8.320 -12.428 -18.269 1.00 0.92
H
ATOM 13179 2HG LYS B 245   8.261 -12.138 -16.525 1.00 0.92
H
ATOM 13180 1HD LYS B 245   7.998 -14.557 -16.119 1.00 0.92
H
ATOM 13181 2HD LYS B 245   8.023 -14.860 -17.862 1.00 0.92
H
ATOM 13182 1HE LYS B 245   10.312 -13.933 -17.988 1.00 0.92
H
ATOM 13183 2HE LYS B 245   10.287 -13.642 -16.241 1.00 0.92
H
ATOM 13184 1HZ LYS B 245   11.392 -15.695 -16.782 1.00 0.92
H
ATOM 13185 2HZ LYS B 245   10.025 -16.005 -15.911 1.00 0.92
H
ATOM 13186 3HZ LYS B 245   10.048 -16.279 -17.539 1.00 0.92
H
ATOM 13187 N   THR B 246   6.938 -9.625 -18.873 1.00 0.57
N
ATOM 13188 CA THR B 246   7.369 -8.938 -20.077 1.00 0.65
C
ATOM 13189 C   THR B 246   8.806 -8.465 -19.996 1.00 0.58
C
ATOM 13190 O   THR B 246   9.453 -8.574 -18.958 1.00 1.00
O
ATOM 13191 CB THR B 246   6.472 -7.719 -20.360 1.00 0.98
C
ATOM 13192 OG1 THR B 246   6.635 -6.773 -19.310 1.00 0.98
O
ATOM 13193 CG2 THR B 246   5.010 -8.101 -20.464 1.00 0.98
C
ATOM 13194 H   THR B 246   7.153 -9.206 -17.976 1.00 0.68
H
ATOM 13195 HA THR B 246   7.289 -9.633 -20.909 1.00 0.78
H
ATOM 13196 HB THR B 246   6.779 -7.253 -21.291 1.00 1.17
H
ATOM 13197 HG1 THR B 246   5.973 -6.082 -19.399 1.00 1.17
H
ATOM 13198 1HG2 THR B 246   4.417 -7.209 -20.666 1.00 1.17
H
ATOM 13199 2HG2 THR B 246   4.886 -8.812 -21.280 1.00 1.17
H
ATOM 13200 3HG2 THR B 246   4.676 -8.550 -19.531 1.00 1.17
H
ATOM 13201 N   PHE B 247   9.303 -7.927 -21.101 1.00 0.64
N
ATOM 13202 CA PHE B 247   10.648 -7.362 -21.125 1.00 0.59
C
ATOM 13203 C   PHE B 247   10.653 -5.928 -21.652 1.00 0.62
C
ATOM 13204 O   PHE B 247   10.259 -5.689 -22.794 1.00 1.40
O

ATOM 13205 CB PHE B 247 11.575 -8.241 -21.961 1.00 0.89
C
ATOM 13206 CG PHE B 247 11.738 -9.610 -21.373 1.00 0.89
C
ATOM 13207 CD1 PHE B 247 10.890 -10.645 -21.739 1.00 0.89
C
ATOM 13208 CD2 PHE B 247 12.728 -9.860 -20.436 1.00 0.89
C
ATOM 13209 CE1 PHE B 247 11.026 -11.901 -21.179 1.00 0.89
C
ATOM 13210 CE2 PHE B 247 12.869 -11.115 -19.878 1.00 0.89
C
ATOM 13211 CZ PHE B 247 12.016 -12.136 -20.248 1.00 0.89
C
ATOM 13212 H PHE B 247 8.729 -7.927 -21.935 1.00 0.77
H
ATOM 13213 HA PHE B 247 11.030 -7.345 -20.104 1.00 0.71
H
ATOM 13214 1HB PHE B 247 11.180 -8.345 -22.971 1.00 1.06
H
ATOM 13215 2HB PHE B 247 12.558 -7.778 -22.033 1.00 1.06
H
ATOM 13216 HD1 PHE B 247 10.106 -10.456 -22.473 1.00 1.06
H
ATOM 13217 HD2 PHE B 247 13.397 -9.052 -20.139 1.00 1.06
H
ATOM 13218 HE1 PHE B 247 10.351 -12.705 -21.472 1.00 1.06
H
ATOM 13219 HE2 PHE B 247 13.651 -11.298 -19.141 1.00 1.06
H
ATOM 13220 HZ PHE B 247 12.126 -13.124 -19.805 1.00 1.06
H
ATOM 13221 N LYS B 248 11.101 -4.981 -20.814 1.00 0.58
N
ATOM 13222 CA LYS B 248 11.181 -3.572 -21.213 1.00 0.51
C
ATOM 13223 C LYS B 248 12.001 -3.415 -22.489 1.00 0.48
C
ATOM 13224 O LYS B 248 13.117 -3.918 -22.586 1.00 0.57
O
ATOM 13225 CB LYS B 248 11.757 -2.718 -20.069 1.00 0.77
C
ATOM 13226 CG LYS B 248 11.700 -1.220 -20.342 1.00 0.77
C
ATOM 13227 CD LYS B 248 12.051 -0.362 -19.128 1.00 0.77
C
ATOM 13228 CE LYS B 248 10.959 -0.470 -18.075 1.00 0.77
C
ATOM 13229 NZ LYS B 248 10.992 0.634 -17.073 1.00 0.77
N
ATOM 13230 H LYS B 248 11.365 -5.225 -19.861 1.00 0.70
H
ATOM 13231 HA LYS B 248 10.184 -3.207 -21.420 1.00 0.61
H
ATOM 13232 1HB LYS B 248 11.207 -2.916 -19.149 1.00 0.92
H
ATOM 13233 2HB LYS B 248 12.799 -2.990 -19.896 1.00 0.92
H

ATOM 13234 1HG LYS B 248   12.412 -0.987 -21.133 1.00 0.92
H
ATOM 13235 2HG LYS B 248   10.702 -0.951 -20.689 1.00 0.92
H
ATOM 13236 1HD LYS B 248   12.996 -0.700 -18.700 1.00 0.92
H
ATOM 13237 2HD LYS B 248   12.157   0.679 -19.428 1.00 0.92
H
ATOM 13238 1HE LYS B 248   9.993 -0.457 -18.576 1.00 0.92
H
ATOM 13239 2HE LYS B 248   11.070 -1.420 -17.554 1.00 0.92
H
ATOM 13240 1HZ LYS B 248   10.237   0.494 -16.417 1.00 0.92
H
ATOM 13241 2HZ LYS B 248   11.861   0.680 -16.550 1.00 0.92
H
ATOM 13242 3HZ LYS B 248   10.860   1.509 -17.553 1.00 0.92
H
ATOM 13243 N   GLY B 249   11.435 -2.731 -23.487 1.00 0.50
N
ATOM 13244 CA GLY B 249   12.101 -2.576 -24.778 1.00 0.58
C
ATOM 13245 C   GLY B 249   11.858 -3.758 -25.724 1.00 0.65
C
ATOM 13246 O   GLY B 249   12.510 -3.849 -26.766 1.00 2.18
O
ATOM 13247 H   GLY B 249   10.533 -2.290 -23.351 1.00 0.60
H
ATOM 13248 1HA GLY B 249   11.755 -1.660 -25.252 1.00 0.70
H
ATOM 13249 2HA GLY B 249   13.172 -2.457 -24.615 1.00 0.70
H
ATOM 13250 N   ARG B 250   10.921 -4.643 -25.360 1.00 0.81
N
ATOM 13251 CA ARG B 250   10.579 -5.827 -26.146 1.00 0.82
C
ATOM 13252 C   ARG B 250   10.460 -5.621 -27.629 1.00 0.83
C
ATOM 13253 O   ARG B 250   9.601 -4.865 -28.090 1.00 0.91
O
ATOM 13254 CB ARG B 250   9.265 -6.421 -25.707 1.00 1.23
C
ATOM 13255 CG ARG B 250   8.806 -7.579 -26.585 1.00 1.23
C
ATOM 13256 CD ARG B 250   9.620 -8.798 -26.365 1.00 1.23
C
ATOM 13257 NE ARG B 250   9.063 -9.950 -27.047 1.00 1.23
N
ATOM 13258 CZ ARG B 250   9.671 -11.147 -27.159 1.00 1.23
C
ATOM 13259 NH1 ARG B 250   10.868 -11.343 -26.647 1.00 1.23
N
ATOM 13260 NH2 ARG B 250   9.057 -12.132 -27.793 1.00 1.23
N
ATOM 13261 H   ARG B 250   10.440 -4.520 -24.478 1.00 0.97
H
ATOM 13262 HA ARG B 250   11.351 -6.565 -25.975 1.00 0.98
H

```
ATOM  13263 1HB  ARG B 250       9.351  -6.801 -24.693  1.00  1.48
H
ATOM  13264 2HB  ARG B 250       8.487  -5.658 -25.717  1.00  1.48
H
ATOM  13265 1HG  ARG B 250       7.766  -7.816 -26.391  1.00  1.48
H
ATOM  13266 2HG  ARG B 250       8.909  -7.298 -27.632  1.00  1.48
H
ATOM  13267 1HD  ARG B 250      10.615  -8.630 -26.763  1.00  1.48
H
ATOM  13268 2HD  ARG B 250       9.684  -9.021 -25.301  1.00  1.48
H
ATOM  13269  HE  ARG B 250       8.144  -9.850 -27.457  1.00  1.48
H
ATOM  13270 1HH1 ARG B 250      11.357 -10.595 -26.164  1.00  1.48
H
ATOM  13271 2HH1 ARG B 250      11.315 -12.243 -26.740  1.00  1.48
H
ATOM  13272 1HH2 ARG B 250       8.138 -11.983 -28.189  1.00  1.48
H
ATOM  13273 2HH2 ARG B 250       9.507 -13.032 -27.883  1.00  1.48
H
ATOM  13274  N   GLY B 251      11.298  -6.350 -28.366  1.00  0.91
N
ATOM  13275  CA  GLY B 251      11.299  -6.327 -29.819  1.00  0.98
C
ATOM  13276  C   GLY B 251      12.365  -5.416 -30.398  1.00  1.14
C
ATOM  13277  O   GLY B 251      12.578  -5.411 -31.611  1.00  2.09
O
ATOM  13278  H   GLY B 251      11.967  -6.953 -27.890  1.00  1.09
H
ATOM  13279 1HA  GLY B 251      11.453  -7.341 -30.189  1.00  1.18
H
ATOM  13280 2HA  GLY B 251      10.322  -6.014 -30.175  1.00  1.18
H
ATOM  13281  N   ILE B 252      13.049  -4.660 -29.546  1.00  0.95
N
ATOM  13282  CA  ILE B 252      14.097  -3.782 -30.026  1.00  1.02
C
ATOM  13283  C   ILE B 252      15.504  -4.356 -29.768  1.00  1.12
C
ATOM  13284  O   ILE B 252      15.909  -4.505 -28.610  1.00  1.03
O
ATOM  13285  CB  ILE B 252      14.007  -2.411 -29.373  1.00  1.53
C
ATOM  13286  CG1 ILE B 252      12.664  -1.755 -29.664  1.00  1.53
C
ATOM  13287  CG2 ILE B 252      15.120  -1.562 -29.918  1.00  1.53
C
ATOM  13288  CD1 ILE B 252      12.457  -0.493 -28.870  1.00  1.53
C
ATOM  13289  H   ILE B 252      12.836  -4.671 -28.556  1.00  1.14
H
ATOM  13290  HA  ILE B 252      13.941  -3.633 -31.086  1.00  1.22
H
ATOM  13291  HB  ILE B 252      14.103  -2.505 -28.294  1.00  1.84
H
```

ATOM 13292 1HG1 ILE B 252 12.606 -1.512 -30.724 1.00 1.84
H
ATOM 13293 2HG1 ILE B 252 11.860 -2.452 -29.426 1.00 1.84
H
ATOM 13294 1HG2 ILE B 252 15.085 -0.570 -29.469 1.00 1.84
H
ATOM 13295 2HG2 ILE B 252 16.077 -2.031 -29.699 1.00 1.84
H
ATOM 13296 3HG2 ILE B 252 14.996 -1.484 -30.994 1.00 1.84
H
ATOM 13297 1HD1 ILE B 252 11.489 -0.061 -29.119 1.00 1.84
H
ATOM 13298 2HD1 ILE B 252 12.490 -0.724 -27.805 1.00 1.84
H
ATOM 13299 3HD1 ILE B 252 13.243 0.219 -29.111 1.00 1.84
H
ATOM 13300 N PRO B 253 16.236 -4.721 -30.837 1.00 1.59
N
ATOM 13301 CA PRO B 253 17.604 -5.240 -30.864 1.00 1.93
C
ATOM 13302 C PRO B 253 18.522 -4.517 -29.890 1.00 1.63
C
ATOM 13303 O PRO B 253 18.723 -3.307 -29.999 1.00 1.66
O
ATOM 13304 CB PRO B 253 18.052 -4.968 -32.303 1.00 2.90
C
ATOM 13305 CG PRO B 253 16.798 -5.022 -33.089 1.00 2.90
C
ATOM 13306 CD PRO B 253 15.781 -4.375 -32.202 1.00 2.90
C
ATOM 13307 HA PRO B 253 17.587 -6.319 -30.655 1.00 2.32
H
ATOM 13308 1HB PRO B 253 18.551 -3.989 -32.363 1.00 3.47
H
ATOM 13309 2HB PRO B 253 18.786 -5.723 -32.617 1.00 3.47
H
ATOM 13310 1HG PRO B 253 16.929 -4.484 -34.037 1.00 3.47
H
ATOM 13311 2HG PRO B 253 16.548 -6.064 -33.340 1.00 3.47
H
ATOM 13312 1HD PRO B 253 15.772 -3.286 -32.335 1.00 3.47
H
ATOM 13313 2HD PRO B 253 14.801 -4.826 -32.408 1.00 3.47
H
ATOM 13314 N ASN B 254 19.104 -5.282 -28.968 1.00 1.64
N
ATOM 13315 CA ASN B 254 20.053 -4.802 -27.953 1.00 1.64
C
ATOM 13316 C ASN B 254 19.491 -3.788 -26.940 1.00 1.81
C
ATOM 13317 O ASN B 254 20.255 -3.213 -26.162 1.00 3.21
O
ATOM 13318 CB ASN B 254 21.279 -4.209 -28.630 1.00 2.46
C
ATOM 13319 CG ASN B 254 22.026 -5.220 -29.452 1.00 2.46
C
ATOM 13320 OD1 ASN B 254 22.166 -6.383 -29.055 1.00 2.46
O

ATOM 13321 ND2 ASN B 254 22.511 -4.800 -30.592 1.00 2.46
N
ATOM 13322 H ASN B 254 18.876 -6.266 -28.965 1.00 1.97
H
ATOM 13323 HA ASN B 254 20.379 -5.668 -27.374 1.00 1.97
H
ATOM 13324 1HB ASN B 254 20.993 -3.375 -29.269 1.00 2.95
H
ATOM 13325 2HB ASN B 254 21.953 -3.815 -27.869 1.00 2.95
H
ATOM 13326 1HD2 ASN B 254 23.019 -5.429 -31.183 1.00 2.95
H
ATOM 13327 2HD2 ASN B 254 22.375 -3.851 -30.873 1.00 2.95
H
ATOM 13328 N ILE B 255 18.174 -3.584 -26.926 1.00 0.94
N
ATOM 13329 CA ILE B 255 17.540 -2.673 -25.981 1.00 0.94
C
ATOM 13330 C ILE B 255 16.694 -3.467 -25.003 1.00 1.12
C
ATOM 13331 O ILE B 255 16.697 -3.186 -23.805 1.00 1.62
O
ATOM 13332 CB ILE B 255 16.680 -1.627 -26.695 1.00 1.41
C
ATOM 13333 CG1 ILE B 255 17.538 -0.811 -27.668 1.00 1.41
C
ATOM 13334 CG2 ILE B 255 16.005 -0.720 -25.685 1.00 1.41
C
ATOM 13335 CD1 ILE B 255 18.668 -0.064 -27.017 1.00 1.41
C
ATOM 13336 H ILE B 255 17.578 -4.066 -27.583 1.00 1.13
H
ATOM 13337 HA ILE B 255 18.315 -2.154 -25.418 1.00 1.13
H
ATOM 13338 HB ILE B 255 15.920 -2.136 -27.277 1.00 1.69
H
ATOM 13339 1HG1 ILE B 255 17.957 -1.482 -28.416 1.00 1.69
H
ATOM 13340 2HG1 ILE B 255 16.906 -0.082 -28.176 1.00 1.69
H
ATOM 13341 1HG2 ILE B 255 15.384 0.007 -26.208 1.00 1.69
H
ATOM 13342 2HG2 ILE B 255 15.382 -1.317 -25.019 1.00 1.69
H
ATOM 13343 3HG2 ILE B 255 16.762 -0.198 -25.102 1.00 1.69
H
ATOM 13344 1HD1 ILE B 255 19.224 0.487 -27.775 1.00 1.69
H
ATOM 13345 2HD1 ILE B 255 18.260 0.632 -26.287 1.00 1.69
H
ATOM 13346 3HD1 ILE B 255 19.332 -0.768 -26.519 1.00 1.69
H
ATOM 13347 N GLU B 256 15.967 -4.451 -25.535 1.00 1.03
N
ATOM 13348 CA GLU B 256 15.129 -5.332 -24.732 1.00 1.21
C
ATOM 13349 C GLU B 256 15.865 -5.911 -23.527 1.00 1.12
C

ATOM 13350 O GLU B 256 16.930 -6.514 -23.669 1.00 1.50
O
ATOM 13351 CB GLU B 256 14.585 -6.467 -25.597 1.00 1.81
C
ATOM 13352 CG GLU B 256 13.716 -7.461 -24.844 1.00 1.81
C
ATOM 13353 CD GLU B 256 13.108 -8.501 -25.742 1.00 1.81
C
ATOM 13354 OE1 GLU B 256 13.087 -8.296 -26.934 1.00 1.81
O
ATOM 13355 OE2 GLU B 256 12.638 -9.496 -25.239 1.00 1.81
O
ATOM 13356 H GLU B 256 15.988 -4.587 -26.538 1.00 1.24
H
ATOM 13357 HA GLU B 256 14.287 -4.752 -24.370 1.00 1.45
H
ATOM 13358 1HB GLU B 256 13.995 -6.051 -26.412 1.00 2.18
H
ATOM 13359 2HB GLU B 256 15.416 -7.016 -26.040 1.00 2.18
H
ATOM 13360 1HG GLU B 256 14.323 -7.959 -24.088 1.00 2.18
H
ATOM 13361 2HG GLU B 256 12.923 -6.917 -24.332 1.00 2.18
H
ATOM 13362 N ASP B 257 15.262 -5.735 -22.342 1.00 0.89
N
ATOM 13363 CA ASP B 257 15.803 -6.184 -21.052 1.00 0.99
C
ATOM 13364 C ASP B 257 17.044 -5.420 -20.551 1.00 2.41
C
ATOM 13365 O ASP B 257 17.574 -5.755 -19.491 1.00 11.33
O
ATOM 13366 CB ASP B 257 16.131 -7.686 -21.107 1.00 1.48
C
ATOM 13367 CG ASP B 257 16.289 -8.332 -19.729 1.00 1.48
C
ATOM 13368 OD1 ASP B 257 15.732 -7.842 -18.775 1.00 1.48
O
ATOM 13369 OD2 ASP B 257 16.978 -9.320 -19.643 1.00 1.48
O
ATOM 13370 H ASP B 257 14.389 -5.229 -22.332 1.00 1.07
H
ATOM 13371 HA ASP B 257 15.018 -6.048 -20.307 1.00 1.19
H
ATOM 13372 1HB ASP B 257 15.345 -8.210 -21.651 1.00 1.78
H
ATOM 13373 2HB ASP B 257 17.061 -7.835 -21.656 1.00 1.78
H
ATOM 13374 N ALA B 258 17.520 -4.418 -21.293 1.00 1.20
N
ATOM 13375 CA ALA B 258 18.693 -3.658 -20.877 1.00 1.12
C
ATOM 13376 C ALA B 258 18.302 -2.414 -20.082 1.00 0.92
C
ATOM 13377 O ALA B 258 17.236 -1.834 -20.289 1.00 1.18
O
ATOM 13378 CB ALA B 258 19.519 -3.265 -22.093 1.00 1.68
C

```
ATOM  13379  H   ALA B 258      17.069  -4.155 -22.157  1.00  1.44
H
ATOM  13380  HA  ALA B 258      19.296  -4.294 -20.230  1.00  1.34
H
ATOM  13381 1HB  ALA B 258      20.409  -2.725 -21.770  1.00  2.02
H
ATOM  13382 2HB  ALA B 258      19.817  -4.163 -22.635  1.00  2.02
H
ATOM  13383 3HB  ALA B 258      18.926  -2.630 -22.747  1.00  2.02
H
ATOM  13384  N   GLU B 259      19.186  -2.009 -19.176  1.00  0.96
N
ATOM  13385  CA  GLU B 259      18.981  -0.830 -18.341  1.00  0.78
C
ATOM  13386  C   GLU B 259      19.470   0.440 -19.028  1.00  0.88
C
ATOM  13387  O   GLU B 259      20.142   0.380 -20.057  1.00  2.45
O
ATOM  13388  CB  GLU B 259      19.696  -1.001 -16.996  1.00  1.17
C
ATOM  13389  CG  GLU B 259      19.146  -2.125 -16.131  1.00  1.17
C
ATOM  13390  CD  GLU B 259      19.860  -2.267 -14.814  1.00  1.17
C
ATOM  13391  OE1 GLU B 259      20.833  -1.583 -14.608  1.00  1.17
O
ATOM  13392  OE2 GLU B 259      19.431  -3.065 -14.014  1.00  1.17
O
ATOM  13393  H   GLU B 259      20.039  -2.539 -19.059  1.00  1.15
H
ATOM  13394  HA  GLU B 259      17.913  -0.723 -18.155  1.00  0.94
H
ATOM  13395 1HB  GLU B 259      20.754  -1.200 -17.169  1.00  1.40
H
ATOM  13396 2HB  GLU B 259      19.627  -0.076 -16.422  1.00  1.40
H
ATOM  13397 1HG  GLU B 259      18.096  -1.923 -15.937  1.00  1.40
H
ATOM  13398 2HG  GLU B 259      19.215  -3.062 -16.681  1.00  1.40
H
ATOM  13399  N   ASN B 260      19.129   1.581 -18.431  1.00  0.78
N
ATOM  13400  CA  ASN B 260      19.495   2.919 -18.903  1.00  0.79
C
ATOM  13401  C   ASN B 260      18.865   3.306 -20.238  1.00  2.37
C
ATOM  13402  O   ASN B 260      19.450   4.079 -20.995  1.00 14.62
O
ATOM  13403  CB  ASN B 260      21.008   3.058 -18.992  1.00  1.19
C
ATOM  13404  CG  ASN B 260      21.677   2.917 -17.656  1.00  1.19
C
ATOM  13405  OD1 ASN B 260      21.191   3.444 -16.649  1.00  1.19
O
ATOM  13406  ND2 ASN B 260      22.783   2.219 -17.626  1.00  1.19
N
ATOM  13407  H   ASN B 260      18.577   1.521 -17.588  1.00  0.94
H
```

ATOM 13408 HA ASN B 260   19.136   3.640 -18.167 1.00 0.95
H
ATOM 13409 1HB ASN B 260   21.420   2.317 -19.675 1.00 1.42
H
ATOM 13410 2HB ASN B 260   21.253   4.039 -19.398 1.00 1.42
H
ATOM 13411 1HD2 ASN B 260   23.271   2.091 -16.761 1.00 1.42
H
ATOM 13412 2HD2 ASN B 260   23.139   1.810 -18.466 1.00 1.42
H
ATOM 13413 N   TRP B 261   17.656   2.812 -20.502 1.00 1.23
N
ATOM 13414 CA TRP B 261   16.934   3.188 -21.714 1.00 1.54
C
ATOM 13415 C   TRP B 261   15.575   3.807 -21.392 1.00 3.34
C
ATOM 13416 O   TRP B 261   14.862   4.251 -22.291 1.00 23.85
O
ATOM 13417 CB TRP B 261   16.769   1.983 -22.632 1.00 2.31
C
ATOM 13418 CG TRP B 261   18.087   1.491 -23.126 1.00 2.31
C
ATOM 13419 CD1 TRP B 261   18.699   0.317 -22.817 1.00 2.31
C
ATOM 13420 CD2 TRP B 261   18.992   2.197 -24.004 1.00 2.31
C
ATOM 13421 NE1 TRP B 261   19.918   0.240 -23.443 1.00 2.31
N
ATOM 13422 CE2 TRP B 261   20.114   1.384 -24.171 1.00 2.31
C
ATOM 13423 CE3 TRP B 261   18.939   3.439 -24.647 1.00 2.31
C
ATOM 13424 CZ2 TRP B 261   21.185   1.767 -24.962 1.00 2.31
C
ATOM 13425 CZ3 TRP B 261   20.012   3.824 -25.439 1.00 2.31
C
ATOM 13426 CH2 TRP B 261   21.106   3.008 -25.594 1.00 2.31
C
ATOM 13427 H   TRP B 261   17.233   2.163 -19.856 1.00 1.48
H
ATOM 13428 HA TRP B 261   17.522   3.932 -22.245 1.00 1.85
H
ATOM 13429 1HB TRP B 261   16.271   1.173 -22.099 1.00 2.77
H
ATOM 13430 2HB TRP B 261   16.152   2.251 -23.490 1.00 2.77
H
ATOM 13431 HD1 TRP B 261   18.283 -0.445 -22.165 1.00 2.77
H
ATOM 13432 HE1 TRP B 261   20.566 -0.531 -23.374 1.00 2.77
H
ATOM 13433 HE3 TRP B 261   18.073   4.089 -24.527 1.00 2.77
H
ATOM 13434 HZ2 TRP B 261   22.063   1.134 -25.096 1.00 2.77
H
ATOM 13435 HZ3 TRP B 261   19.964   4.792 -25.939 1.00 2.77
H
ATOM 13436 HH2 TRP B 261   21.932   3.342 -26.222 1.00 2.77
H

```
ATOM  13437  N   HIS B 262      15.222   3.837 -20.111  1.00  1.85
N
ATOM  13438  CA  HIS B 262      13.971   4.435 -19.674  1.00  1.04
C
ATOM  13439  C   HIS B 262      13.875   5.906 -20.017  1.00  1.72
C
ATOM  13440  O   HIS B 262      14.653   6.721 -19.523  1.00  8.28
O
ATOM  13441  CB  HIS B 262      13.830   4.270 -18.167  1.00  1.56
C
ATOM  13442  CG  HIS B 262      12.626   4.937 -17.597  1.00  1.56
C
ATOM  13443  ND1 HIS B 262      12.578   6.297 -17.390  1.00  1.56
N
ATOM  13444  CD2 HIS B 262      11.439   4.449 -17.172  1.00  1.56
C
ATOM  13445  CE1 HIS B 262      11.422   6.615 -16.846  1.00  1.56
C
ATOM  13446  NE2 HIS B 262      10.711   5.512 -16.702  1.00  1.56
N
ATOM  13447  H   HIS B 262      15.836   3.452 -19.409  1.00  2.22
H
ATOM  13448  HA  HIS B 262      13.135   3.925 -20.152  1.00  1.25
H
ATOM  13449 1HB  HIS B 262      13.783   3.210 -17.919  1.00  1.87
H
ATOM  13450 2HB  HIS B 262      14.710   4.681 -17.673  1.00  1.87
H
ATOM  13451  HD1 HIS B 262      13.329   6.942 -17.530  1.00  1.87
H
ATOM  13452  HD2 HIS B 262      11.021   3.445 -17.131  1.00  1.87
H
ATOM  13453  HE1 HIS B 262      11.192   7.651 -16.597  1.00  1.87
H
ATOM  13454  N   GLY B 263      12.886   6.249 -20.831  1.00  1.30
N
ATOM  13455  CA  GLY B 263      12.653   7.631 -21.226  1.00  1.05
C
ATOM  13456  C   GLY B 263      13.560   8.091 -22.356  1.00  1.07
C
ATOM  13457  O   GLY B 263      13.550   9.270 -22.714  1.00  3.36
O
ATOM  13458  H   GLY B 263      12.278   5.527 -21.193  1.00  1.56
H
ATOM  13459 1HA  GLY B 263      11.618   7.752 -21.537  1.00  1.26
H
ATOM  13460 2HA  GLY B 263      12.798   8.278 -20.363  1.00  1.26
H
ATOM  13461  N   LYS B 264      14.338   7.178 -22.931  1.00  1.12
N
ATOM  13462  CA  LYS B 264      15.223   7.588 -23.996  1.00  1.41
C
ATOM  13463  C   LYS B 264      14.668   7.186 -25.357  1.00  1.64
C
ATOM  13464  O   LYS B 264      14.234   6.048 -25.536  1.00  4.00
O
ATOM  13465  CB  LYS B 264      16.604   6.977 -23.803  1.00  2.11
C
```

ATOM 13466 CG LYS B 264 17.324 7.469 -22.562 1.00 2.11
C
ATOM 13467 CD LYS B 264 18.700 6.849 -22.451 1.00 2.11
C
ATOM 13468 CE LYS B 264 19.409 7.302 -21.184 1.00 2.11
C
ATOM 13469 NZ LYS B 264 20.704 6.599 -21.003 1.00 2.11
N
ATOM 13470 H LYS B 264 14.337 6.211 -22.634 1.00 1.34
H
ATOM 13471 HA LYS B 264 15.322 8.665 -23.936 1.00 1.69
H
ATOM 13472 1HB LYS B 264 16.516 5.892 -23.733 1.00 2.54
H
ATOM 13473 2HB LYS B 264 17.228 7.202 -24.668 1.00 2.54
H
ATOM 13474 1HG LYS B 264 17.424 8.553 -22.603 1.00 2.54
H
ATOM 13475 2HG LYS B 264 16.745 7.207 -21.676 1.00 2.54
H
ATOM 13476 1HD LYS B 264 18.611 5.766 -22.454 1.00 2.54
H
ATOM 13477 2HD LYS B 264 19.299 7.146 -23.311 1.00 2.54
H
ATOM 13478 1HE LYS B 264 19.593 8.374 -21.239 1.00 2.54
H
ATOM 13479 2HE LYS B 264 18.773 7.097 -20.323 1.00 2.54
H
ATOM 13480 1HZ LYS B 264 21.151 6.913 -20.154 1.00 2.54
H
ATOM 13481 2HZ LYS B 264 20.510 5.605 -20.943 1.00 2.54
H
ATOM 13482 3HZ LYS B 264 21.308 6.782 -21.792 1.00 2.54
H
ATOM 13483 N PRO B 265 14.671 8.114 -26.316 1.00 1.04
N
ATOM 13484 CA PRO B 265 14.279 7.975 -27.702 1.00 1.03
C
ATOM 13485 C PRO B 265 15.388 7.297 -28.474 1.00 0.72
C
ATOM 13486 O PRO B 265 16.556 7.382 -28.094 1.00 1.00
O
ATOM 13487 CB PRO B 265 14.080 9.420 -28.165 1.00 1.54
C
ATOM 13488 CG PRO B 265 15.048 10.194 -27.346 1.00 1.54
C
ATOM 13489 CD PRO B 265 15.052 9.508 -26.010 1.00 1.54
C
ATOM 13490 HA PRO B 265 13.351 7.392 -27.761 1.00 1.24
H
ATOM 13491 1HB PRO B 265 14.270 9.503 -29.246 1.00 1.85
H
ATOM 13492 2HB PRO B 265 13.040 9.735 -27.995 1.00 1.85
H
ATOM 13493 1HG PRO B 265 16.037 10.187 -27.826 1.00 1.85
H
ATOM 13494 2HG PRO B 265 14.741 11.244 -27.276 1.00 1.85
H

```
ATOM  13495 1HD  PRO B 265      16.069   9.541 -25.590  1.00  1.85
H
ATOM  13496 2HD  PRO B 265      14.313   9.965 -25.334  1.00  1.85
H
ATOM  13497  N   VAL B 266      15.036   6.645 -29.565  1.00  1.27
N
ATOM  13498  CA  VAL B 266      16.039   6.028 -30.411  1.00  1.24
C
ATOM  13499  C   VAL B 266      16.402   6.957 -31.574  1.00  1.24
C
ATOM  13500  O   VAL B 266      15.501   7.460 -32.248  1.00  1.49
O
ATOM  13501  CB  VAL B 266      15.545   4.689 -30.952  1.00  1.86
C
ATOM  13502  CG1 VAL B 266      16.561   4.128 -31.908  1.00  1.86
C
ATOM  13503  CG2 VAL B 266      15.349   3.725 -29.799  1.00  1.86
C
ATOM  13504  H   VAL B 266      14.061   6.588 -29.823  1.00  1.52
H
ATOM  13505  HA  VAL B 266      16.912   5.826 -29.800  1.00  1.49
H
ATOM  13506  HB  VAL B 266      14.608   4.835 -31.489  1.00  2.23
H
ATOM  13507 1HG1 VAL B 266      16.203   3.185 -32.291  1.00  2.23
H
ATOM  13508 2HG1 VAL B 266      16.705   4.823 -32.733  1.00  2.23
H
ATOM  13509 3HG1 VAL B 266      17.507   3.977 -31.389  1.00  2.23
H
ATOM  13510 1HG2 VAL B 266      14.996   2.766 -30.178  1.00  2.23
H
ATOM  13511 2HG2 VAL B 266      16.297   3.584 -29.279  1.00  2.23
H
ATOM  13512 3HG2 VAL B 266      14.618   4.137 -29.107  1.00  2.23
H
ATOM  13513  N   PRO B 267      17.701   7.223 -31.802  1.00  1.36
N
ATOM  13514  CA  PRO B 267      18.267   8.037 -32.876  1.00  1.55
C
ATOM  13515  C   PRO B 267      17.765   7.559 -34.224  1.00  2.54
C
ATOM  13516  O   PRO B 267      17.816   6.367 -34.505  1.00 10.63
O
ATOM  13517  CB  PRO B 267      19.774   7.822 -32.716  1.00  2.33
C
ATOM  13518  CG  PRO B 267      19.950   7.585 -31.256  1.00  2.33
C
ATOM  13519  CD  PRO B 267      18.741   6.794 -30.844  1.00  2.33
C
ATOM  13520  HA  PRO B 267      17.998   9.090 -32.712  1.00  1.86
H
ATOM  13521 1HB  PRO B 267      20.104   6.975 -33.333  1.00  2.79
H
ATOM  13522 2HB  PRO B 267      20.319   8.710 -33.071  1.00  2.79
H
ATOM  13523 1HG  PRO B 267      20.892   7.046 -31.071  1.00  2.79
H
```

ATOM 13524 2HG PRO B 267 20.025 8.544 -30.725 1.00 2.79
H
ATOM 13525 1HD PRO B 267 18.923 5.714 -30.933 1.00 2.79
H
ATOM 13526 2HD PRO B 267 18.459 7.082 -29.820 1.00 2.79
H
ATOM 13527 N LYS B 268 17.320 8.507 -35.049 1.00 2.14
N
ATOM 13528 CA LYS B 268 16.736 8.273 -36.376 1.00 1.97
C
ATOM 13529 C LYS B 268 17.262 7.063 -37.139 1.00 1.70
C
ATOM 13530 O LYS B 268 16.478 6.205 -37.540 1.00 2.32
O
ATOM 13531 CB LYS B 268 16.920 9.509 -37.250 1.00 2.96
C
ATOM 13532 CG LYS B 268 16.259 9.390 -38.616 1.00 2.96
C
ATOM 13533 CD LYS B 268 14.746 9.274 -38.467 1.00 2.96
C
ATOM 13534 CE LYS B 268 14.022 9.496 -39.787 1.00 2.96
C
ATOM 13535 NZ LYS B 268 14.352 8.451 -40.789 1.00 2.96
N
ATOM 13536 H LYS B 268 17.354 9.461 -34.719 1.00 2.57
H
ATOM 13537 HA LYS B 268 15.667 8.117 -36.241 1.00 2.36
H
ATOM 13538 1HB LYS B 268 16.497 10.379 -36.746 1.00 3.55
H
ATOM 13539 2HB LYS B 268 17.982 9.700 -37.402 1.00 3.55
H
ATOM 13540 1HG LYS B 268 16.493 10.269 -39.217 1.00 3.55
H
ATOM 13541 2HG LYS B 268 16.635 8.506 -39.132 1.00 3.55
H
ATOM 13542 1HD LYS B 268 14.496 8.281 -38.094 1.00 3.55
H
ATOM 13543 2HD LYS B 268 14.396 10.011 -37.746 1.00 3.55
H
ATOM 13544 1HE LYS B 268 12.947 9.486 -39.610 1.00 3.55
H
ATOM 13545 2HE LYS B 268 14.302 10.469 -40.190 1.00 3.55
H
ATOM 13546 1HZ LYS B 268 13.854 8.630 -41.647 1.00 3.55
H
ATOM 13547 2HZ LYS B 268 15.347 8.453 -40.972 1.00 3.55
H
ATOM 13548 3HZ LYS B 268 14.089 7.539 -40.434 1.00 3.55
H
ATOM 13549 N GLU B 269 18.572 7.003 -37.360 1.00 1.69
N
ATOM 13550 CA GLU B 269 19.169 5.915 -38.129 1.00 2.29
C
ATOM 13551 C GLU B 269 18.955 4.554 -37.478 1.00 2.69
C
ATOM 13552 O GLU B 269 18.732 3.553 -38.168 1.00 7.02
O

ATOM 13553 CB GLU B 269   20.664   6.165 -38.309 1.00 3.44
C
ATOM 13554 CG GLU B 269   20.997   7.329 -39.230 1.00 3.44
C
ATOM 13555 CD GLU B 269   22.472   7.592 -39.328 1.00 3.44
C
ATOM 13556 OE1 GLU B 269   23.214   7.000 -38.581 1.00 3.44
O
ATOM 13557 OE2 GLU B 269   22.860   8.387 -40.153 1.00 3.44
O
ATOM 13558 H   GLU B 269   19.169   7.735 -37.001 1.00 2.03
H
ATOM 13559 HA GLU B 269   18.698   5.895 -39.113 1.00 2.75
H
ATOM 13560 1HB GLU B 269   21.120   6.365 -37.339 1.00 4.12
H
ATOM 13561 2HB GLU B 269   21.136   5.271 -38.715 1.00 4.12
H
ATOM 13562 1HG GLU B 269   20.609   7.113 -40.226 1.00 4.12
H
ATOM 13563 2HG GLU B 269   20.496   8.223 -38.862 1.00 4.12
H
ATOM 13564 N   ARG B 270   19.028   4.521 -36.155 1.00 1.27
N
ATOM 13565 CA ARG B 270   18.845   3.293 -35.418 1.00 1.31
C
ATOM 13566 C   ARG B 270   17.385   2.912 -35.448 1.00 1.45
C
ATOM 13567 O   ARG B 270   17.051   1.742 -35.620 1.00 1.64
O
ATOM 13568 CB ARG B 270   19.310   3.464 -33.982 1.00 1.97
C
ATOM 13569 CG ARG B 270   20.806   3.625 -33.805 1.00 1.97
C
ATOM 13570 CD ARG B 270   21.155   3.875 -32.386 1.00 1.97
C
ATOM 13571 NE ARG B 270   22.587   4.045 -32.200 1.00 1.97
N
ATOM 13572 CZ ARG B 270   23.166   4.441 -31.050 1.00 1.97
C
ATOM 13573 NH1 ARG B 270   22.426   4.704 -29.996 1.00 1.97
N
ATOM 13574 NH2 ARG B 270   24.481   4.565 -30.981 1.00 1.97
N
ATOM 13575 H   ARG B 270   19.183   5.376 -35.640 1.00 1.52
H
ATOM 13576 HA ARG B 270   19.429   2.503 -35.891 1.00 1.57
H
ATOM 13577 1HB ARG B 270   18.844   4.348 -33.554 1.00 2.36
H
ATOM 13578 2HB ARG B 270   18.994   2.606 -33.390 1.00 2.36
H
ATOM 13579 1HG ARG B 270   21.312   2.716 -34.130 1.00 2.36
H
ATOM 13580 2HG ARG B 270   21.154   4.469 -34.401 1.00 2.36
H
ATOM 13581 1HD ARG B 270   20.659   4.783 -32.045 1.00 2.36
H

```
ATOM  13582 2HD  ARG B 270      20.830   3.033 -31.777  1.00  2.36
H
ATOM  13583  HE  ARG B 270      23.190   3.852 -32.988  1.00  2.36
H
ATOM  13584 1HH1 ARG B 270      21.421   4.609 -30.047  1.00  2.36
H
ATOM  13585 2HH1 ARG B 270      22.862   4.999 -29.135  1.00  2.36
H
ATOM  13586 1HH2 ARG B 270      25.051   4.361 -31.791  1.00  2.36
H
ATOM  13587 2HH2 ARG B 270      24.915   4.861 -30.120  1.00  2.36
H
ATOM  13588  N   ALA B 271      16.526   3.922 -35.321  1.00  1.41
N
ATOM  13589  CA  ALA B 271      15.086   3.744 -35.336  1.00  1.50
C
ATOM  13590  C   ALA B 271      14.644   3.149 -36.657  1.00  1.58
C
ATOM  13591  O   ALA B 271      13.815   2.244 -36.678  1.00  1.73
O
ATOM  13592  CB  ALA B 271      14.394   5.074 -35.083  1.00  2.25
C
ATOM  13593  H   ALA B 271      16.892   4.853 -35.187  1.00  1.69
H
ATOM  13594  HA  ALA B 271      14.818   3.044 -34.544  1.00  1.80
H
ATOM  13595 1HB  ALA B 271      13.315   4.930 -35.078  1.00  2.70
H
ATOM  13596 2HB  ALA B 271      14.711   5.472 -34.118  1.00  2.70
H
ATOM  13597 3HB  ALA B 271      14.657   5.779 -35.868  1.00  2.70
H
ATOM  13598  N   ASP B 272      15.223   3.639 -37.756  1.00  1.54
N
ATOM  13599  CA  ASP B 272      14.920   3.122 -39.083  1.00  1.65
C
ATOM  13600  C   ASP B 272      15.231   1.638 -39.176  1.00  1.63
C
ATOM  13601  O   ASP B 272      14.394   0.857 -39.630  1.00  1.71
O
ATOM  13602  CB  ASP B 272      15.728   3.865 -40.152  1.00  2.47
C
ATOM  13603  CG  ASP B 272      15.277   5.301 -40.408  1.00  2.47
C
ATOM  13604  OD1 ASP B 272      14.198   5.674 -40.009  1.00  2.47
O
ATOM  13605  OD2 ASP B 272      16.028   6.026 -41.017  1.00  2.47
O
ATOM  13606  H   ASP B 272      15.880   4.401 -37.673  1.00  1.85
H
ATOM  13607  HA  ASP B 272      13.857   3.265 -39.277  1.00  1.98
H
ATOM  13608 1HB  ASP B 272      16.777   3.885 -39.858  1.00  2.97
H
ATOM  13609 2HB  ASP B 272      15.667   3.315 -41.092  1.00  2.97
H
ATOM  13610  N   ALA B 273      16.433   1.255 -38.734  1.00  1.62
N
```

ATOM 13611 CA ALA B 273 16.858 -0.141 -38.776 1.00 1.72
C
ATOM 13612 C ALA B 273 15.946 -1.033 -37.944 1.00 1.49
C
ATOM 13613 O ALA B 273 15.548 -2.109 -38.396 1.00 1.51
O
ATOM 13614 CB ALA B 273 18.290 -0.260 -38.283 1.00 2.58
C
ATOM 13615 H ALA B 273 17.084 1.954 -38.385 1.00 1.94
H
ATOM 13616 HA ALA B 273 16.807 -0.479 -39.810 1.00 2.06
H
ATOM 13617 1HB ALA B 273 18.612 -1.300 -38.344 1.00 3.10
H
ATOM 13618 2HB ALA B 273 18.940 0.358 -38.902 1.00 3.10
H
ATOM 13619 3HB ALA B 273 18.349 0.077 -37.249 1.00 3.10
H
ATOM 13620 N ILE B 274 15.613 -0.571 -36.740 1.00 1.29
N
ATOM 13621 CA ILE B 274 14.760 -1.305 -35.818 1.00 1.12
C
ATOM 13622 C ILE B 274 13.383 -1.524 -36.395 1.00 1.04
C
ATOM 13623 O ILE B 274 12.883 -2.649 -36.381 1.00 1.02
O
ATOM 13624 CB ILE B 274 14.656 -0.546 -34.499 1.00 1.68
C
ATOM 13625 CG1 ILE B 274 16.009 -0.559 -33.800 1.00 1.68
C
ATOM 13626 CG2 ILE B 274 13.594 -1.180 -33.616 1.00 1.68
C
ATOM 13627 CD1 ILE B 274 16.097 0.452 -32.697 1.00 1.68
C
ATOM 13628 H ILE B 274 15.983 0.321 -36.438 1.00 1.55
H
ATOM 13629 HA ILE B 274 15.213 -2.277 -35.627 1.00 1.34
H
ATOM 13630 HB ILE B 274 14.393 0.494 -34.694 1.00 2.02
H
ATOM 13631 1HG1 ILE B 274 16.176 -1.544 -33.375 1.00 2.02
H
ATOM 13632 2HG1 ILE B 274 16.799 -0.366 -34.523 1.00 2.02
H
ATOM 13633 1HG2 ILE B 274 13.524 -0.630 -32.678 1.00 2.02
H
ATOM 13634 2HG2 ILE B 274 12.634 -1.149 -34.126 1.00 2.02
H
ATOM 13635 3HG2 ILE B 274 13.862 -2.216 -33.410 1.00 2.02
H
ATOM 13636 1HD1 ILE B 274 17.078 0.398 -32.223 1.00 2.02
H
ATOM 13637 2HD1 ILE B 274 15.954 1.436 -33.135 1.00 2.02
H
ATOM 13638 3HD1 ILE B 274 15.323 0.274 -31.960 1.00 2.02
H
ATOM 13639 N VAL B 275 12.783 -0.456 -36.918 1.00 1.02
N

ATOM 13640 CA VAL B 275   11.475 -0.537 -37.541 1.00 0.98
C
ATOM 13641 C   VAL B 275   11.456 -1.512 -38.693 1.00 1.10
C
ATOM 13642 O   VAL B 275   10.525 -2.304 -38.792 1.00 1.10
O
ATOM 13643 CB VAL B 275   11.003   0.844 -38.019 1.00 1.47
C
ATOM 13644 CG1 VAL B 275   9.779   0.690 -38.900 1.00 1.47
C
ATOM 13645 CG2 VAL B 275   10.641   1.696 -36.808 1.00 1.47
C
ATOM 13646 H   VAL B 275   13.244   0.443 -36.883 1.00 1.22
H
ATOM 13647 HA VAL B 275   10.765 -0.885 -36.793 1.00 1.18
H
ATOM 13648 HB VAL B 275   11.792   1.323 -38.599 1.00 1.76
H
ATOM 13649 1HG1 VAL B 275   9.447   1.672 -39.231 1.00 1.76
H
ATOM 13650 2HG1 VAL B 275   10.031   0.081 -39.768 1.00 1.76
H
ATOM 13651 3HG1 VAL B 275   8.983   0.208 -38.339 1.00 1.76
H
ATOM 13652 1HG2 VAL B 275   10.302   2.676 -37.140 1.00 1.76
H
ATOM 13653 2HG2 VAL B 275   9.844   1.208 -36.248 1.00 1.76
H
ATOM 13654 3HG2 VAL B 275   11.511   1.812 -36.166 1.00 1.76
H
ATOM 13655 N   LYS B 276   12.476 -1.477 -39.552 1.00 1.24
N
ATOM 13656 CA LYS B 276   12.543 -2.411 -40.670 1.00 1.40
C
ATOM 13657 C   LYS B 276   12.565 -3.858 -40.184 1.00 1.38
C
ATOM 13658 O   LYS B 276   11.889 -4.719 -40.753 1.00 1.52
O
ATOM 13659 CB LYS B 276   13.778 -2.126 -41.520 1.00 2.10
C
ATOM 13660 CG LYS B 276   13.702 -0.839 -42.330 1.00 2.10
C
ATOM 13661 CD LYS B 276   14.988 -0.603 -43.105 1.00 2.10
C
ATOM 13662 CE LYS B 276   14.930   0.694 -43.899 1.00 2.10
C
ATOM 13663 NZ LYS B 276   16.194   0.942 -44.647 1.00 2.10
N
ATOM 13664 H   LYS B 276   13.211 -0.787 -39.441 1.00 1.49
H
ATOM 13665 HA LYS B 276   11.654 -2.277 -41.287 1.00 1.68
H
ATOM 13666 1HB LYS B 276   14.657 -2.064 -40.878 1.00 2.52
H
ATOM 13667 2HB LYS B 276   13.938 -2.949 -42.217 1.00 2.52
H
ATOM 13668 1HG LYS B 276   12.874 -0.909 -43.037 1.00 2.52
H

ATOM 13669 2HG LYS B 276   13.517   0.006 -41.672 1.00 2.52
H
ATOM 13670 1HD LYS B 276   15.828 -0.557 -42.411 1.00 2.52
H
ATOM 13671 2HD LYS B 276   15.155 -1.432 -43.794 1.00 2.52
H
ATOM 13672 1HE LYS B 276   14.105   0.643 -44.607 1.00 2.52
H
ATOM 13673 2HE LYS B 276   14.756   1.525 -43.215 1.00 2.52
H
ATOM 13674 1HZ LYS B 276   16.118   1.809 -45.160 1.00 2.52
H
ATOM 13675 2HZ LYS B 276   16.965   1.003 -43.996 1.00 2.52
H
ATOM 13676 3HZ LYS B 276   16.358   0.182 -45.292 1.00 2.52
H
ATOM 13677 N   LEU B 277   13.318 -4.115 -39.113 1.00 1.27
N
ATOM 13678 CA LEU B 277   13.378 -5.449 -38.536 1.00 1.32
C
ATOM 13679 C   LEU B 277   12.025 -5.867 -37.964 1.00 1.38
C
ATOM 13680 O   LEU B 277   11.617 -7.021 -38.107 1.00 1.66
O
ATOM 13681 CB LEU B 277   14.457 -5.490 -37.452 1.00 1.98
C
ATOM 13682 CG LEU B 277   15.894 -5.367 -37.970 1.00 1.98
C
ATOM 13683 CD1 LEU B 277   16.846 -5.216 -36.796 1.00 1.98
C
ATOM 13684 CD2 LEU B 277   16.235 -6.601 -38.791 1.00 1.98
C
ATOM 13685 H   LEU B 277   13.878 -3.377 -38.700 1.00 1.52
H
ATOM 13686 HA LEU B 277   13.648 -6.152 -39.323 1.00 1.58
H
ATOM 13687 1HB LEU B 277   14.286 -4.676 -36.752 1.00 2.38
H
ATOM 13688 2HB LEU B 277   14.376 -6.434 -36.912 1.00 2.38
H
ATOM 13689 HG LEU B 277   15.985 -4.483 -38.597 1.00 2.38
H
ATOM 13690 1HD1 LEU B 277   17.868 -5.123 -37.163 1.00 2.38
H
ATOM 13691 2HD1 LEU B 277   16.581 -4.324 -36.230 1.00 2.38
H
ATOM 13692 3HD1 LEU B 277   16.770 -6.091 -36.151 1.00 2.38
H
ATOM 13693 1HD2 LEU B 277   17.256 -6.518 -39.166 1.00 2.38
H
ATOM 13694 2HD2 LEU B 277   16.150 -7.490 -38.165 1.00 2.38
H
ATOM 13695 3HD2 LEU B 277   15.545 -6.681 -39.631 1.00 2.38
H
ATOM 13696 N   ILE B 278   11.305 -4.919 -37.369 1.00 1.16
N
ATOM 13697 CA ILE B 278   9.975 -5.192 -36.843 1.00 1.12
C

ATOM 13698 C   ILE B 278   9.020 -5.505 -37.982 1.00 1.40
C
ATOM 13699 O   ILE B 278   8.245 -6.455 -37.907 1.00 1.63
O
ATOM 13700 CB ILE B 278   9.426 -4.008 -36.067 1.00 1.68
C
ATOM 13701 CG1 ILE B 278   10.249 -3.763 -34.820 1.00 1.68
C
ATOM 13702 CG2 ILE B 278   7.999 -4.336 -35.659 1.00 1.68
C
ATOM 13703 CD1 ILE B 278   9.959 -2.424 -34.218 1.00 1.68
C
ATOM 13704 H   ILE B 278   11.698 -3.992 -37.253 1.00 1.39
H
ATOM 13705 HA ILE B 278   10.027 -6.058 -36.184 1.00 1.34
H
ATOM 13706 HB ILE B 278   9.446 -3.105 -36.674 1.00 2.02
H
ATOM 13707 1HG1 ILE B 278   10.023 -4.534 -34.086 1.00 2.02
H
ATOM 13708 2HG1 ILE B 278   11.308 -3.825 -35.062 1.00 2.02
H
ATOM 13709 1HG2 ILE B 278   7.582 -3.513 -35.086 1.00 2.02
H
ATOM 13710 2HG2 ILE B 278   7.394 -4.503 -36.549 1.00 2.02
H
ATOM 13711 3HG2 ILE B 278   7.997 -5.237 -35.046 1.00 2.02
H
ATOM 13712 1HD1 ILE B 278   10.566 -2.293 -33.321 1.00 2.02
H
ATOM 13713 2HD1 ILE B 278   10.199 -1.642 -34.934 1.00 2.02
H
ATOM 13714 3HD1 ILE B 278   8.905 -2.363 -33.957 1.00 2.02
H
ATOM 13715 N   GLU B 279   9.093 -4.697 -39.044 1.00 1.41
N
ATOM 13716 CA GLU B 279   8.254 -4.861 -40.224 1.00 1.61
C
ATOM 13717 C   GLU B 279   8.426 -6.228 -40.859 1.00 1.75
C
ATOM 13718 O   GLU B 279   7.451 -6.815 -41.326 1.00 1.70
O
ATOM 13719 CB GLU B 279   8.556 -3.778 -41.264 1.00 2.42
C
ATOM 13720 CG GLU B 279   8.071 -2.379 -40.909 1.00 2.42
C
ATOM 13721 CD GLU B 279   8.487 -1.346 -41.923 1.00 2.42
C
ATOM 13722 OE1 GLU B 279   9.318 -1.651 -42.745 1.00 2.42
O
ATOM 13723 OE2 GLU B 279   7.967 -0.254 -41.883 1.00 2.42
O
ATOM 13724 H   GLU B 279   9.746 -3.931 -39.031 1.00 1.69
H
ATOM 13725 HA GLU B 279   7.217 -4.764 -39.920 1.00 1.93
H
ATOM 13726 1HB GLU B 279   9.632 -3.722 -41.427 1.00 2.90
H

ATOM	13727	2HB	GLU	B	279	8.098	-4.054	-42.215	1.00	2.90	H
ATOM	13728	1HG	GLU	B	279	6.984	-2.392	-40.850	1.00	2.90	H
ATOM	13729	2HG	GLU	B	279	8.454	-2.101	-39.932	1.00	2.90	H
ATOM	13730	N	SER	B	280	9.647	-6.775	-40.828	1.00	2.13	N
ATOM	13731	CA	SER	B	280	9.888	-8.108	-41.385	1.00	2.22	C
ATOM	13732	C	SER	B	280	9.124	-9.205	-40.634	1.00	2.22	C
ATOM	13733	O	SER	B	280	8.957	-10.309	-41.153	1.00	3.99	O
ATOM	13734	CB	SER	B	280	11.367	-8.462	-41.354	1.00	3.33	C
ATOM	13735	OG	SER	B	280	11.811	-8.732	-40.049	1.00	3.33	O
ATOM	13736	H	SER	B	280	10.427	-6.243	-40.458	1.00	2.56	H
ATOM	13737	HA	SER	B	280	9.550	-8.109	-42.423	1.00	2.66	H
ATOM	13738	1HB	SER	B	280	11.543	-9.331	-41.987	1.00	4.00	H
ATOM	13739	2HB	SER	B	280	11.942	-7.636	-41.770	1.00	4.00	H
ATOM	13740	HG	SER	B	280	11.661	-7.934	-39.537	1.00	4.00	H
ATOM	13741	N	GLN	B	281	8.672	-8.910	-39.413	1.00	1.79	N
ATOM	13742	CA	GLN	B	281	7.947	-9.866	-38.600	1.00	1.72	C
ATOM	13743	C	GLN	B	281	6.431	-9.683	-38.721	1.00	1.68	C
ATOM	13744	O	GLN	B	281	5.666	-10.419	-38.097	1.00	4.55	O
ATOM	13745	CB	GLN	B	281	8.386	-9.727	-37.145	1.00	2.58	C
ATOM	13746	CG	GLN	B	281	9.877	-9.945	-36.965	1.00	2.58	C
ATOM	13747	CD	GLN	B	281	10.320	-11.322	-37.415	1.00	2.58	C
ATOM	13748	OE1	GLN	B	281	9.879	-12.340	-36.873	1.00	2.58	O
ATOM	13749	NE2	GLN	B	281	11.185	-11.362	-38.423	1.00	2.58	N
ATOM	13750	H	GLN	B	281	8.817	-7.996	-39.018	1.00	2.15	H
ATOM	13751	HA	GLN	B	281	8.193	-10.870	-38.945	1.00	2.06	H
ATOM	13752	1HB	GLN	B	281	8.137	-8.733	-36.775	1.00	3.10	H
ATOM	13753	2HB	GLN	B	281	7.857	-10.453	-36.528	1.00	3.10	H
ATOM	13754	1HG	GLN	B	281	10.414	-9.205	-37.555	1.00	3.10	H
ATOM	13755	2HG	GLN	B	281	10.127	-9.834	-35.909	1.00	3.10	H

```
ATOM  13756 1HE2 GLN B 281      11.510 -12.242 -38.770  1.00  3.10
H
ATOM  13757 2HE2 GLN B 281      11.504 -10.510 -38.847  1.00  3.10
H
ATOM  13758  N   ILE B 282       5.996  -8.700 -39.515  1.00  1.51
N
ATOM  13759  CA  ILE B 282       4.576  -8.459 -39.728  1.00  1.14
C
ATOM  13760  C   ILE B 282       4.087  -9.236 -40.942  1.00  1.38
C
ATOM  13761  O   ILE B 282       4.536  -9.000 -42.065  1.00  1.74
O
ATOM  13762  CB  ILE B 282       4.283  -6.953 -39.905  1.00  1.71
C
ATOM  13763  CG1 ILE B 282       4.676  -6.200 -38.635  1.00  1.71
C
ATOM  13764  CG2 ILE B 282       2.823  -6.718 -40.241  1.00  1.71
C
ATOM  13765  CD1 ILE B 282       4.591  -4.698 -38.757  1.00  1.71
C
ATOM  13766  H   ILE B 282       6.647  -8.110 -40.013  1.00  1.81
H
ATOM  13767  HA  ILE B 282       4.030  -8.811 -38.855  1.00  1.37
H
ATOM  13768  HB  ILE B 282       4.899  -6.565 -40.715  1.00  2.05
H
ATOM  13769 1HG1 ILE B 282       4.019  -6.518 -37.829  1.00  2.05
H
ATOM  13770 2HG1 ILE B 282       5.699  -6.465 -38.371  1.00  2.05
H
ATOM  13771 1HG2 ILE B 282       2.646  -5.652 -40.374  1.00  2.05
H
ATOM  13772 2HG2 ILE B 282       2.572  -7.245 -41.160  1.00  2.05
H
ATOM  13773 3HG2 ILE B 282       2.204  -7.089 -39.431  1.00  2.05
H
ATOM  13774 1HD1 ILE B 282       4.884  -4.240 -37.813  1.00  2.05
H
ATOM  13775 2HD1 ILE B 282       5.253  -4.353 -39.549  1.00  2.05
H
ATOM  13776 3HD1 ILE B 282       3.567  -4.412 -38.995  1.00  2.05
H
ATOM  13777  N   GLN B 283       3.180 -10.180 -40.700  1.00  1.52
N
ATOM  13778  CA  GLN B 283       2.636 -11.028 -41.758  1.00  2.06
C
ATOM  13779  C   GLN B 283       1.689 -10.307 -42.714  1.00  1.27
C
ATOM  13780  O   GLN B 283       1.779 -10.487 -43.930  1.00  2.09
O
ATOM  13781  CB  GLN B 283       1.902 -12.219 -41.142  1.00  3.09
C
ATOM  13782  CG  GLN B 283       1.379 -13.223 -42.152  1.00  3.09
C
ATOM  13783  CD  GLN B 283       2.495 -13.886 -42.936  1.00  3.09
C
ATOM  13784  OE1 GLN B 283       3.455 -14.404 -42.359  1.00  3.09
O
```

```
ATOM  13785  NE2 GLN B 283       2.375 -13.877 -44.259  1.00  3.09
N
ATOM  13786  H   GLN B 283       2.856 -10.309 -39.747  1.00  1.82
H
ATOM  13787  HA  GLN B 283       3.472 -11.406 -42.346  1.00  2.47
H
ATOM  13788 1HB  GLN B 283       2.573 -12.744 -40.461  1.00  3.71
H
ATOM  13789 2HB  GLN B 283       1.057 -11.861 -40.555  1.00  3.71
H
ATOM  13790 1HG  GLN B 283       0.823 -13.998 -41.625  1.00  3.71
H
ATOM  13791 2HG  GLN B 283       0.726 -12.706 -42.856  1.00  3.71
H
ATOM  13792 1HE2 GLN B 283       3.080 -14.301 -44.828  1.00  3.71
H
ATOM  13793 2HE2 GLN B 283       1.579 -13.447 -44.685  1.00  3.71
H
ATOM  13794  N   THR B 284       0.800  -9.474 -42.181  1.00  0.79
N
ATOM  13795  CA  THR B 284      -0.147  -8.776 -43.046  1.00  1.17
C
ATOM  13796  C   THR B 284      -0.423  -7.348 -42.648  1.00  0.86
C
ATOM  13797  O   THR B 284      -0.127  -6.917 -41.536  1.00  3.09
O
ATOM  13798  CB  THR B 284      -1.499  -9.493 -43.106  1.00  1.75
C
ATOM  13799  OG1 THR B 284      -2.315  -8.857 -44.100  1.00  1.75
O
ATOM  13800  CG2 THR B 284      -2.202  -9.420 -41.761  1.00  1.75
C
ATOM  13801  H   THR B 284       0.752  -9.359 -41.175  1.00  0.95
H
ATOM  13802  HA  THR B 284       0.270  -8.758 -44.052  1.00  1.40
H
ATOM  13803  HB  THR B 284      -1.346 -10.537 -43.382  1.00  2.11
H
ATOM  13804  HG1 THR B 284      -3.103  -9.386 -44.250  1.00  2.11
H
ATOM  13805 1HG2 THR B 284      -3.163  -9.928 -41.828  1.00  2.11
H
ATOM  13806 2HG2 THR B 284      -1.588  -9.904 -41.003  1.00  2.11
H
ATOM  13807 3HG2 THR B 284      -2.362  -8.378 -41.488  1.00  2.11
H
ATOM  13808  N   ASN B 285      -1.005  -6.618 -43.595  1.00  1.16
N
ATOM  13809  CA  ASN B 285      -1.362  -5.225 -43.392  1.00  1.72
C
ATOM  13810  C   ASN B 285      -2.836  -5.070 -43.028  1.00  2.02
C
ATOM  13811  O   ASN B 285      -3.311  -3.954 -42.811  1.00  5.90
O
ATOM  13812  CB  ASN B 285      -1.034  -4.413 -44.631  1.00  2.58
C
ATOM  13813  CG  ASN B 285       0.445  -4.309 -44.878  1.00  2.58
C
```

ATOM 13814 OD1 ASN B 285   1.238 -4.194 -43.937 1.00 2.58
O
ATOM 13815 ND2 ASN B 285   0.832 -4.346 -46.127 1.00 2.58
N
ATOM 13816 H   ASN B 285   -1.212 -7.056 -44.483 1.00 1.39
H
ATOM 13817 HA ASN B 285   -0.779 -4.834 -42.556 1.00 2.06
H
ATOM 13818 1HB ASN B 285   -1.505 -4.872 -45.502 1.00 3.10
H
ATOM 13819 2HB ASN B 285   -1.445 -3.409 -44.528 1.00 3.10
H
ATOM 13820 1HD2 ASN B 285   1.805 -4.280 -46.353 1.00 3.10
H
ATOM 13821 2HD2 ASN B 285   0.157 -4.439 -46.859 1.00 3.10
H
ATOM 13822 N   GLU B 286   -3.558 -6.192 -42.955 1.00 2.26
N
ATOM 13823 CA GLU B 286   -4.959 -6.173 -42.558 1.00 3.70
C
ATOM 13824 C   GLU B 286   -5.091 -5.748 -41.107 1.00 1.35
C
ATOM 13825 O   GLU B 286   -4.404 -6.274 -40.232 1.00 2.76
O
ATOM 13826 CB GLU B 286   -5.599 -7.551 -42.760 1.00 5.55
C
ATOM 13827 CG GLU B 286   -7.088 -7.611 -42.430 1.00 5.55
C
ATOM 13828 CD GLU B 286   -7.711 -8.957 -42.708 1.00 5.55
C
ATOM 13829 OE1 GLU B 286   -7.049 -9.805 -43.257 1.00 5.55
O
ATOM 13830 OE2 GLU B 286   -8.856 -9.143 -42.365 1.00 5.55
O
ATOM 13831 H   GLU B 286   -3.128 -7.083 -43.174 1.00 2.71
H
ATOM 13832 HA GLU B 286   -5.487 -5.447 -43.178 1.00 4.44
H
ATOM 13833 1HB GLU B 286   -5.474 -7.861 -43.797 1.00 6.66
H
ATOM 13834 2HB GLU B 286   -5.089 -8.284 -42.135 1.00 6.66
H
ATOM 13835 1HG GLU B 286   -7.223 -7.373 -41.375 1.00 6.66
H
ATOM 13836 2HG GLU B 286   -7.606 -6.850 -43.013 1.00 6.66
H
ATOM 13837 N   ASN B 287   -5.985 -4.803 -40.852 1.00 1.83
N
ATOM 13838 CA ASN B 287   -6.186 -4.297 -39.505 1.00 2.91
C
ATOM 13839 C   ASN B 287   -7.431 -4.886 -38.857 1.00 1.80
C
ATOM 13840 O   ASN B 287   -8.426 -5.160 -39.528 1.00 2.33
O
ATOM 13841 CB ASN B 287   -6.258 -2.784 -39.526 1.00 4.37
C
ATOM 13842 CG ASN B 287   -4.955 -2.157 -39.944 1.00 4.37
C

ATOM 13843 OD1 ASN B 287   -3.878 -2.583 -39.510 1.00 4.37
O
ATOM 13844 ND2 ASN B 287   -5.032 -1.155 -40.782 1.00 4.37
N
ATOM 13845 H   ASN B 287   -6.527 -4.416 -41.611 1.00 2.20
H
ATOM 13846 HA ASN B 287   -5.333 -4.594 -38.893 1.00 3.49
H
ATOM 13847 1HB ASN B 287   -7.040 -2.465 -40.217 1.00 5.24
H
ATOM 13848 2HB ASN B 287   -6.526 -2.416 -38.535 1.00 5.24
H
ATOM 13849 1HD2 ASN B 287   -4.198 -0.699 -41.094 1.00 5.24
H
ATOM 13850 2HD2 ASN B 287   -5.925 -0.843 -41.108 1.00 5.24
H
ATOM 13851 N   LEU B 288   -7.357 -5.073 -37.545 1.00 1.26
N
ATOM 13852 CA LEU B 288   -8.460 -5.602 -36.755 1.00 0.94
C
ATOM 13853 C   LEU B 288   -9.526 -4.541 -36.528 1.00 0.67
C
ATOM 13854 O   LEU B 288   -9.225 -3.350 -36.451 1.00 0.87
O
ATOM 13855 CB LEU B 288   -7.924 -6.124 -35.421 1.00 1.41
C
ATOM 13856 CG LEU B 288   -6.962 -7.308 -35.539 1.00 1.41
C
ATOM 13857 CD1 LEU B 288   -6.343 -7.637 -34.189 1.00 1.41
C
ATOM 13858 CD2 LEU B 288   -7.731 -8.508 -36.073 1.00 1.41
C
ATOM 13859 H   LEU B 288   -6.499 -4.830 -37.070 1.00 1.51
H
ATOM 13860 HA LEU B 288   -8.910 -6.430 -37.301 1.00 1.13
H
ATOM 13861 1HB LEU B 288   -7.390 -5.319 -34.921 1.00 1.69
H
ATOM 13862 2HB LEU B 288   -8.763 -6.431 -34.795 1.00 1.69
H
ATOM 13863 HG LEU B 288   -6.161 -7.043 -36.223 1.00 1.69
H
ATOM 13864 1HD1 LEU B 288   -5.659 -8.479 -34.301 1.00 1.69
H
ATOM 13865 2HD1 LEU B 288   -5.794 -6.771 -33.819 1.00 1.69
H
ATOM 13866 3HD1 LEU B 288   -7.127 -7.900 -33.484 1.00 1.69
H
ATOM 13867 1HD2 LEU B 288   -7.056 -9.359 -36.174 1.00 1.69
H
ATOM 13868 2HD2 LEU B 288   -8.531 -8.763 -35.379 1.00 1.69
H
ATOM 13869 3HD2 LEU B 288   -8.157 -8.265 -37.047 1.00 1.69
H
ATOM 13870 N   ILE B 289   -10.772 -4.986 -36.433 1.00 0.68
N
ATOM 13871 CA ILE B 289   -11.913 -4.099 -36.254 1.00 0.65
C

ATOM 13872 C ILE B 289 -12.549 -4.280 -34.870 1.00 0.63
C
ATOM 13873 O ILE B 289 -13.052 -5.362 -34.568 1.00 1.10
O
ATOM 13874 CB ILE B 289 -12.960 -4.379 -37.345 1.00 0.98
C
ATOM 13875 CG1 ILE B 289 -12.347 -4.147 -38.729 1.00 0.98
C
ATOM 13876 CG2 ILE B 289 -14.179 -3.504 -37.150 1.00 0.98
C
ATOM 13877 CD1 ILE B 289 -13.236 -4.596 -39.866 1.00 0.98
C
ATOM 13878 H ILE B 289 -10.938 -5.980 -36.498 1.00 0.82
H
ATOM 13879 HA ILE B 289 -11.573 -3.073 -36.370 1.00 0.78
H
ATOM 13880 HB ILE B 289 -13.260 -5.425 -37.292 1.00 1.17
H
ATOM 13881 1HG1 ILE B 289 -12.139 -3.084 -38.853 1.00 1.17
H
ATOM 13882 2HG1 ILE B 289 -11.406 -4.691 -38.800 1.00 1.17
H
ATOM 13883 1HG2 ILE B 289 -14.915 -3.724 -37.924 1.00 1.17
H
ATOM 13884 2HG2 ILE B 289 -14.614 -3.694 -36.169 1.00 1.17
H
ATOM 13885 3HG2 ILE B 289 -13.878 -2.460 -37.222 1.00 1.17
H
ATOM 13886 1HD1 ILE B 289 -12.737 -4.401 -40.815 1.00 1.17
H
ATOM 13887 2HD1 ILE B 289 -13.434 -5.664 -39.772 1.00 1.17
H
ATOM 13888 3HD1 ILE B 289 -14.176 -4.047 -39.832 1.00 1.17
H
ATOM 13889 N PRO B 290 -12.512 -3.254 -34.012 1.00 0.59
N
ATOM 13890 CA PRO B 290 -13.098 -3.219 -32.676 1.00 0.80
C
ATOM 13891 C PRO B 290 -14.607 -3.396 -32.720 1.00 0.35
C
ATOM 13892 O PRO B 290 -15.296 -2.807 -33.554 1.00 1.40
O
ATOM 13893 CB PRO B 290 -12.693 -1.854 -32.143 1.00 1.20
C
ATOM 13894 CG PRO B 290 -11.424 -1.542 -32.854 1.00 1.20
C
ATOM 13895 CD PRO B 290 -11.556 -2.153 -34.215 1.00 1.20
C
ATOM 13896 HA PRO B 290 -12.641 -4.016 -32.070 1.00 0.96
H
ATOM 13897 1HB PRO B 290 -13.490 -1.125 -32.334 1.00 1.44
H
ATOM 13898 2HB PRO B 290 -12.566 -1.908 -31.053 1.00 1.44
H
ATOM 13899 1HG PRO B 290 -11.294 -0.457 -32.905 1.00 1.44
H
ATOM 13900 2HG PRO B 290 -10.570 -1.939 -32.288 1.00 1.44
H

ATOM 13901 1HD PRO B 290 -11.960 -1.424 -34.932 1.00 1.44
H
ATOM 13902 2HD PRO B 290 -10.585 -2.550 -34.540 1.00 1.44
H
ATOM 13903 N LYS B 291 -15.117 -4.181 -31.779 1.00 0.76
N
ATOM 13904 CA LYS B 291 -16.540 -4.467 -31.693 1.00 0.78
C
ATOM 13905 C LYS B 291 -17.241 -3.541 -30.715 1.00 0.48
C
ATOM 13906 O LYS B 291 -16.642 -3.119 -29.726 1.00 0.70
O
ATOM 13907 CB LYS B 291 -16.755 -5.918 -31.278 1.00 1.17
C
ATOM 13908 CG LYS B 291 -16.122 -6.939 -32.214 1.00 1.17
C
ATOM 13909 CD LYS B 291 -16.759 -6.891 -33.592 1.00 1.17
C
ATOM 13910 CE LYS B 291 -16.214 -7.991 -34.490 1.00 1.17
C
ATOM 13911 NZ LYS B 291 -16.832 -7.960 -35.844 1.00 1.17
N
ATOM 13912 H LYS B 291 -14.491 -4.614 -31.114 1.00 0.91
H
ATOM 13913 HA LYS B 291 -16.985 -4.313 -32.677 1.00 0.94
H
ATOM 13914 1HB LYS B 291 -16.348 -6.078 -30.280 1.00 1.40
H
ATOM 13915 2HB LYS B 291 -17.824 -6.126 -31.237 1.00 1.40
H
ATOM 13916 1HG LYS B 291 -15.055 -6.732 -32.309 1.00 1.40
H
ATOM 13917 2HG LYS B 291 -16.244 -7.939 -31.801 1.00 1.40
H
ATOM 13918 1HD LYS B 291 -17.839 -7.009 -33.501 1.00 1.40
H
ATOM 13919 2HD LYS B 291 -16.554 -5.925 -34.055 1.00 1.40
H
ATOM 13920 1HE LYS B 291 -15.136 -7.868 -34.592 1.00 1.40
H
ATOM 13921 2HE LYS B 291 -16.415 -8.960 -34.033 1.00 1.40
H
ATOM 13922 1HZ LYS B 291 -16.443 -8.704 -36.408 1.00 1.40
H
ATOM 13923 2HZ LYS B 291 -17.831 -8.086 -35.762 1.00 1.40
H
ATOM 13924 3HZ LYS B 291 -16.639 -7.071 -36.280 1.00 1.40
H
ATOM 13925 N SER B 292 -18.509 -3.247 -31.005 1.00 0.71
N
ATOM 13926 CA SER B 292 -19.340 -2.378 -30.180 1.00 0.94
C
ATOM 13927 C SER B 292 -19.823 -3.088 -28.903 1.00 0.84
C
ATOM 13928 O SER B 292 -19.908 -4.321 -28.865 1.00 3.54
O
ATOM 13929 CB SER B 292 -20.535 -1.909 -30.997 1.00 1.41
C

```
ATOM  13930  OG  SER B 292     -21.387  -2.980 -31.299  1.00  1.41
O
ATOM  13931  H   SER B 292     -18.913  -3.643 -31.842  1.00  0.85
H
ATOM  13932  HA  SER B 292     -18.740  -1.514 -29.907  1.00  1.13
H
ATOM  13933 1HB  SER B 292     -21.090  -1.148 -30.452  1.00  1.69
H
ATOM  13934 2HB  SER B 292     -20.182  -1.452 -31.921  1.00  1.69
H
ATOM  13935  HG  SER B 292     -22.131  -2.600 -31.774  1.00  1.69
H
ATOM  13936  N   PRO B 293     -20.160  -2.317 -27.863  1.00  0.94
N
ATOM  13937  CA  PRO B 293     -20.659  -2.711 -26.554  1.00  0.69
C
ATOM  13938  C   PRO B 293     -22.171  -2.844 -26.520  1.00  0.95
C
ATOM  13939  O   PRO B 293     -22.862  -2.501 -27.481  1.00  2.14
O
ATOM  13940  CB  PRO B 293     -20.179  -1.580 -25.659  1.00  1.03
C
ATOM  13941  CG  PRO B 293     -20.271  -0.381 -26.527  1.00  1.03
C
ATOM  13942  CD  PRO B 293     -19.852  -0.872 -27.886  1.00  1.03
C
ATOM  13943  HA  PRO B 293     -20.195  -3.661 -26.263  1.00  0.83
H
ATOM  13944 1HB  PRO B 293     -20.819  -1.514 -24.768  1.00  1.24
H
ATOM  13945 2HB  PRO B 293     -19.155  -1.780 -25.308  1.00  1.24
H
ATOM  13946 1HG  PRO B 293     -21.297   0.016 -26.519  1.00  1.24
H
ATOM  13947 2HG  PRO B 293     -19.620   0.415 -26.143  1.00  1.24
H
ATOM  13948 1HD  PRO B 293     -20.439  -0.362 -28.658  1.00  1.24
H
ATOM  13949 2HD  PRO B 293     -18.783  -0.725 -28.027  1.00  1.24
H
ATOM  13950  N   VAL B 294     -22.677  -3.331 -25.393  1.00  1.15
N
ATOM  13951  CA  VAL B 294     -24.109  -3.470 -25.179  1.00  1.83
C
ATOM  13952  C   VAL B 294     -24.687  -2.181 -24.615  1.00  1.22
C
ATOM  13953  O   VAL B 294     -24.262  -1.704 -23.561  1.00  1.49
O
ATOM  13954  CB  VAL B 294     -24.405  -4.630 -24.212  1.00  2.75
C
ATOM  13955  CG1 VAL B 294     -25.902  -4.718 -23.948  1.00  2.75
C
ATOM  13956  CG2 VAL B 294     -23.883  -5.930 -24.806  1.00  2.75
C
ATOM  13957  H   VAL B 294     -22.046  -3.603 -24.652  1.00  1.38
H
ATOM  13958  HA  VAL B 294     -24.586  -3.680 -26.136  1.00  2.20
H
```

```
ATOM  13959  HB  VAL B 294     -23.911  -4.438 -23.259  1.00  3.29
H
ATOM  13960 1HG1 VAL B 294     -26.100  -5.534 -23.251  1.00  3.29
H
ATOM  13961 2HG1 VAL B 294     -26.250  -3.781 -23.515  1.00  3.29
H
ATOM  13962 3HG1 VAL B 294     -26.427  -4.905 -24.883  1.00  3.29
H
ATOM  13963 1HG2 VAL B 294     -24.082  -6.750 -24.117  1.00  3.29
H
ATOM  13964 2HG2 VAL B 294     -24.381  -6.122 -25.756  1.00  3.29
H
ATOM  13965 3HG2 VAL B 294     -22.808  -5.849 -24.971  1.00  3.29
H
ATOM  13966  N   GLU B 295     -25.647  -1.611 -25.333  1.00  1.52
N
ATOM  13967  CA  GLU B 295     -26.270  -0.356 -24.935  1.00  1.39
C
ATOM  13968  C   GLU B 295     -27.192  -0.463 -23.716  1.00  1.34
C
ATOM  13969  O   GLU B 295     -28.414  -0.499 -23.864  1.00  2.57
O
ATOM  13970  CB  GLU B 295     -27.023   0.244 -26.120  1.00  2.08
C
ATOM  13971  CG  GLU B 295     -26.100   0.692 -27.249  1.00  2.08
C
ATOM  13972  CD  GLU B 295     -26.828   1.317 -28.405  1.00  2.08
C
ATOM  13973  OE1 GLU B 295     -28.025   1.181 -28.471  1.00  2.08
O
ATOM  13974  OE2 GLU B 295     -26.187   1.950 -29.216  1.00  2.08
O
ATOM  13975  H   GLU B 295     -25.950  -2.059 -26.187  1.00  1.82
H
ATOM  13976  HA  GLU B 295     -25.472   0.321 -24.690  1.00  1.67
H
ATOM  13977 1HB  GLU B 295     -27.723  -0.488 -26.522  1.00  2.50
H
ATOM  13978 2HB  GLU B 295     -27.599   1.108 -25.789  1.00  2.50
H
ATOM  13979 1HG  GLU B 295     -25.388   1.414 -26.848  1.00  2.50
H
ATOM  13980 2HG  GLU B 295     -25.539  -0.170 -27.605  1.00  2.50
H
ATOM  13981  N   ASP B 296     -26.611  -0.511 -22.516  1.00  1.05
N
ATOM  13982  CA  ASP B 296     -27.425  -0.641 -21.307  1.00  1.04
C
ATOM  13983  C   ASP B 296     -27.153   0.368 -20.172  1.00  0.66
C
ATOM  13984  O   ASP B 296     -27.711   0.218 -19.084  1.00  1.33
O
ATOM  13985  CB  ASP B 296     -27.266  -2.052 -20.755  1.00  1.56
C
ATOM  13986  CG  ASP B 296     -25.837  -2.334 -20.343  1.00  1.56
C
ATOM  13987  OD1 ASP B 296     -25.014  -1.472 -20.508  1.00  1.56
O
```

```
ATOM  13988  OD2 ASP B 296     -25.568  -3.407 -19.857  1.00  1.56
O
ATOM  13989  H   ASP B 296     -25.598  -0.487 -22.466  1.00  1.26
H
ATOM  13990  HA  ASP B 296     -28.467  -0.516 -21.602  1.00  1.25
H
ATOM  13991 1HB  ASP B 296     -27.918  -2.186 -19.890  1.00  1.87
H
ATOM  13992 2HB  ASP B 296     -27.566  -2.778 -21.512  1.00  1.87
H
ATOM  13993  N   SER B 297     -26.336   1.395 -20.406  1.00  0.76
N
ATOM  13994  CA  SER B 297     -26.103   2.390 -19.361  1.00  0.74
C
ATOM  13995  C   SER B 297     -27.296   3.341 -19.324  1.00  1.22
C
ATOM  13996  O   SER B 297     -27.850   3.666 -20.373  1.00  3.68
O
ATOM  13997  CB  SER B 297     -24.835   3.182 -19.627  1.00  1.11
C
ATOM  13998  OG  SER B 297     -23.715   2.344 -19.645  1.00  1.11
O
ATOM  13999  H   SER B 297     -25.862   1.494 -21.293  1.00  0.91
H
ATOM  14000  HA  SER B 297     -26.012   1.873 -18.409  1.00  0.89
H
ATOM  14001 1HB  SER B 297     -24.919   3.700 -20.582  1.00  1.33
H
ATOM  14002 2HB  SER B 297     -24.712   3.939 -18.853  1.00  1.33
H
ATOM  14003  HG  SER B 297     -23.771   1.878 -20.488  1.00  1.33
H
ATOM  14004  N   PRO B 298     -27.695   3.806 -18.140  1.00  0.44
N
ATOM  14005  CA  PRO B 298     -28.809   4.706 -17.870  1.00  0.55
C
ATOM  14006  C   PRO B 298     -28.508   6.133 -18.290  1.00  0.48
C
ATOM  14007  O   PRO B 298     -27.353   6.564 -18.284  1.00  0.49
O
ATOM  14008  CB  PRO B 298     -28.980   4.604 -16.352  1.00  0.83
C
ATOM  14009  CG  PRO B 298     -27.623   4.264 -15.842  1.00  0.83
C
ATOM  14010  CD  PRO B 298     -27.032   3.367 -16.897  1.00  0.83
C
ATOM  14011  HA  PRO B 298     -29.704   4.332 -18.388  1.00  0.66
H
ATOM  14012 1HB  PRO B 298     -29.357   5.557 -15.952  1.00  0.99
H
ATOM  14013 2HB  PRO B 298     -29.730   3.836 -16.109  1.00  0.99
H
ATOM  14014 1HG  PRO B 298     -27.033   5.179 -15.684  1.00  0.99
H
ATOM  14015 2HG  PRO B 298     -27.707   3.766 -14.865  1.00  0.99
H
ATOM  14016 1HD  PRO B 298     -25.947   3.529 -16.964  1.00  0.99
H
```

```
ATOM  14017 2HD  PRO B 298     -27.277   2.314 -16.689  1.00  0.99
H
ATOM  14018  N   GLN B 299     -29.555   6.873 -18.638  1.00  0.56
N
ATOM  14019  CA  GLN B 299     -29.398   8.272 -18.999  1.00  0.57
C
ATOM  14020  C   GLN B 299     -29.256   9.123 -17.753  1.00  0.47
C
ATOM  14021  O   GLN B 299     -30.127   9.120 -16.883  1.00  0.45
O
ATOM  14022  CB  GLN B 299     -30.575   8.776 -19.829  1.00  0.85
C
ATOM  14023  CG  GLN B 299     -30.463  10.250 -20.190  1.00  0.85
C
ATOM  14024  CD  GLN B 299     -29.321  10.526 -21.152  1.00  0.85
C
ATOM  14025  OE1 GLN B 299     -29.288   9.984 -22.260  1.00  0.85
O
ATOM  14026  NE2 GLN B 299     -28.374  11.364 -20.736  1.00  0.85
N
ATOM  14027  H   GLN B 299     -30.477   6.460 -18.638  1.00  0.67
H
ATOM  14028  HA  GLN B 299     -28.487   8.379 -19.588  1.00  0.68
H
ATOM  14029 1HB  GLN B 299     -30.646   8.203 -20.752  1.00  1.03
H
ATOM  14030 2HB  GLN B 299     -31.505   8.629 -19.276  1.00  1.03
H
ATOM  14031 1HG  GLN B 299     -31.391  10.569 -20.662  1.00  1.03
H
ATOM  14032 2HG  GLN B 299     -30.290  10.825 -19.280  1.00  1.03
H
ATOM  14033 1HE2 GLN B 299     -27.600  11.577 -21.333  1.00  1.03
H
ATOM  14034 2HE2 GLN B 299     -28.426  11.781 -19.824  1.00  1.03
H
ATOM  14035  N   ILE B 300     -28.153   9.849 -17.676  1.00  0.52
N
ATOM  14036  CA  ILE B 300     -27.874  10.710 -16.542  1.00  0.53
C
ATOM  14037  C   ILE B 300     -28.317  12.143 -16.841  1.00  0.75
C
ATOM  14038  O   ILE B 300     -28.050  12.667 -17.924  1.00  0.86
O
ATOM  14039  CB  ILE B 300     -26.372  10.671 -16.198  1.00  0.80
C
ATOM  14040  CG1 ILE B 300     -25.929   9.219 -15.961  1.00  0.80
C
ATOM  14041  CG2 ILE B 300     -26.089  11.511 -14.964  1.00  0.80
C
ATOM  14042  CD1 ILE B 300     -26.665   8.522 -14.841  1.00  0.80
C
ATOM  14043  H   ILE B 300     -27.481   9.799 -18.429  1.00  0.62
H
ATOM  14044  HA  ILE B 300     -28.436  10.349 -15.681  1.00  0.64
H
ATOM  14045  HB  ILE B 300     -25.794  11.056 -17.036  1.00  0.95
H
```

```
ATOM  14046 1HG1 ILE B 300     -26.084   8.649 -16.877  1.00  0.95
H
ATOM  14047 2HG1 ILE B 300     -24.865   9.205 -15.729  1.00  0.95
H
ATOM  14048 1HG2 ILE B 300     -25.026  11.474 -14.735  1.00  0.95
H
ATOM  14049 2HG2 ILE B 300     -26.383  12.543 -15.150  1.00  0.95
H
ATOM  14050 3HG2 ILE B 300     -26.655  11.119 -14.119  1.00  0.95
H
ATOM  14051 1HD1 ILE B 300     -26.293   7.501 -14.742  1.00  0.95
H
ATOM  14052 2HD1 ILE B 300     -26.500   9.060 -13.907  1.00  0.95
H
ATOM  14053 3HD1 ILE B 300     -27.731   8.498 -15.065  1.00  0.95
H
ATOM  14054  N   SER B 301     -29.035  12.753 -15.898  1.00  1.11
N
ATOM  14055  CA  SER B 301     -29.502  14.129 -16.059  1.00  1.81
C
ATOM  14056  C   SER B 301     -28.350  15.113 -16.049  1.00  2.32
C
ATOM  14057  O   SER B 301     -27.379  14.941 -15.315  1.00  5.82
O
ATOM  14058  CB  SER B 301     -30.465  14.525 -14.960  1.00  2.71
C
ATOM  14059  OG  SER B 301     -30.837  15.873 -15.102  1.00  2.71
O
ATOM  14060  H   SER B 301     -29.245  12.259 -15.041  1.00  1.33
H
ATOM  14061  HA  SER B 301     -30.012  14.209 -17.020  1.00  2.17
H
ATOM  14062 1HB  SER B 301     -31.351  13.891 -15.000  1.00  3.26
H
ATOM  14063 2HB  SER B 301     -29.994  14.373 -13.991  1.00  3.26
H
ATOM  14064  HG  SER B 301     -31.376  15.909 -15.898  1.00  3.26
H
ATOM  14065  N   ILE B 302     -28.470  16.155 -16.858  1.00  2.15
N
ATOM  14066  CA  ILE B 302     -27.457  17.200 -16.927  1.00  2.46
C
ATOM  14067  C   ILE B 302     -28.003  18.524 -16.394  1.00  3.10
C
ATOM  14068  O   ILE B 302     -27.484  19.590 -16.719  1.00 11.19
O
ATOM  14069  CB  ILE B 302     -26.970  17.391 -18.374  1.00  3.69
C
ATOM  14070  CG1 ILE B 302     -28.148  17.782 -19.270  1.00  3.69
C
ATOM  14071  CG2 ILE B 302     -26.314  16.113 -18.879  1.00  3.69
C
ATOM  14072  CD1 ILE B 302     -27.731  18.230 -20.653  1.00  3.69
C
ATOM  14073  H   ILE B 302     -29.288  16.228 -17.445  1.00  2.58
H
ATOM  14074  HA  ILE B 302     -26.609  16.907 -16.311  1.00  2.95
H
```

```
ATOM  14075  HB  ILE B 302     -26.246  18.205 -18.409  1.00  4.43
H
ATOM  14076 1HG1 ILE B 302     -28.817  16.928 -19.371  1.00  4.43
H
ATOM  14077 2HG1 ILE B 302     -28.698  18.595 -18.798  1.00  4.43
H
ATOM  14078 1HG2 ILE B 302     -25.966  16.262 -19.901  1.00  4.43
H
ATOM  14079 2HG2 ILE B 302     -25.468  15.863 -18.241  1.00  4.43
H
ATOM  14080 3HG2 ILE B 302     -27.038  15.298 -18.858  1.00  4.43
H
ATOM  14081 1HD1 ILE B 302     -28.617  18.493 -21.232  1.00  4.43
H
ATOM  14082 2HD1 ILE B 302     -27.079  19.100 -20.572  1.00  4.43
H
ATOM  14083 3HD1 ILE B 302     -27.199  17.422 -21.153  1.00  4.43
H
ATOM  14084  N   THR B 303     -29.065  18.452 -15.592  1.00  2.57
N
ATOM  14085  CA  THR B 303     -29.711  19.646 -15.057  1.00  3.07
C
ATOM  14086  C   THR B 303     -29.086  20.151 -13.756  1.00  2.91
C
ATOM  14087  O   THR B 303     -28.269  19.470 -13.132  1.00  3.37
O
ATOM  14088  CB  THR B 303     -31.212  19.378 -14.836  1.00  4.60
C
ATOM  14089  OG1 THR B 303     -31.377  18.336 -13.862  1.00  4.60
O
ATOM  14090  CG2 THR B 303     -31.869  18.959 -16.143  1.00  4.60
C
ATOM  14091  H   THR B 303     -29.455  17.547 -15.350  1.00  3.08
H
ATOM  14092  HA  THR B 303     -29.616  20.440 -15.798  1.00  3.68
H
ATOM  14093  HB  THR B 303     -31.691  20.283 -14.467  1.00  5.53
H
ATOM  14094  HG1 THR B 303     -31.090  17.493 -14.235  1.00  5.53
H
ATOM  14095 1HG2 THR B 303     -32.930  18.775 -15.973  1.00  5.53
H
ATOM  14096 2HG2 THR B 303     -31.753  19.754 -16.879  1.00  5.53
H
ATOM  14097 3HG2 THR B 303     -31.398  18.050 -16.514  1.00  5.53
H
ATOM  14098  N   ASP B 304     -29.480  21.359 -13.358  1.00  2.70
N
ATOM  14099  CA  ASP B 304     -28.998  21.967 -12.122  1.00  2.98
C
ATOM  14100  C   ASP B 304     -29.635  21.307 -10.906  1.00  2.86
C
ATOM  14101  O   ASP B 304     -30.837  21.038 -10.896  1.00  7.16
O
ATOM  14102  CB  ASP B 304     -29.302  23.467 -12.105  1.00  4.47
C
ATOM  14103  CG  ASP B 304     -28.479  24.258 -13.111  1.00  4.47
C
```

ATOM 14104 OD1 ASP B 304   -27.503 23.737 -13.599 1.00 4.47
O
ATOM 14105 OD2 ASP B 304   -28.838 25.378 -13.387 1.00 4.47
O
ATOM 14106 H   ASP B 304   -30.144 21.869 -13.925 1.00 3.24
H
ATOM 14107 HA ASP B 304   -27.918 21.826 -12.064 1.00 3.58
H
ATOM 14108 1HB ASP B 304   -30.359 23.624 -12.321 1.00 5.36
H
ATOM 14109 2HB ASP B 304   -29.109 23.865 -11.109 1.00 5.36
H
ATOM 14110 N   ILE B 305   -28.839 21.069 -9.871 1.00 1.26
N
ATOM 14111 CA ILE B 305   -29.375 20.462 -8.664 1.00 1.97
C
ATOM 14112 C   ILE B 305   -29.757 21.559 -7.695 1.00 4.77
C
ATOM 14113 O   ILE B 305   -28.926 22.380 -7.310 1.00 19.68
O
ATOM 14114 CB ILE B 305   -28.375 19.512 -7.991 1.00 2.96
C
ATOM 14115 CG1 ILE B 305   -27.978 18.391 -8.944 1.00 2.96
C
ATOM 14116 CG2 ILE B 305   -29.035 18.913 -6.763 1.00 2.96
C
ATOM 14117 CD1 ILE B 305   -26.829 17.546 -8.444 1.00 2.96
C
ATOM 14118 H   ILE B 305   -27.858 21.315 -9.924 1.00 1.51
H
ATOM 14119 HA ILE B 305   -30.272 19.899 -8.919 1.00 2.36
H
ATOM 14120 HB ILE B 305   -27.472 20.050 -7.706 1.00 3.55
H
ATOM 14121 1HG1 ILE B 305   -28.837 17.744 -9.105 1.00 3.55
H
ATOM 14122 2HG1 ILE B 305   -27.690 18.826 -9.901 1.00 3.55
H
ATOM 14123 1HG2 ILE B 305   -28.342 18.225 -6.284 1.00 3.55
H
ATOM 14124 2HG2 ILE B 305   -29.306 19.706 -6.068 1.00 3.55
H
ATOM 14125 3HG2 ILE B 305   -29.932 18.371 -7.061 1.00 3.55
H
ATOM 14126 1HD1 ILE B 305   -26.601 16.769 -9.174 1.00 3.55
H
ATOM 14127 2HD1 ILE B 305   -25.950 18.176 -8.299 1.00 3.55
H
ATOM 14128 3HD1 ILE B 305   -27.108 17.087 -7.499 1.00 3.55
H
ATOM 14129 N   LYS B 306   -31.019 21.598 -7.314 1.00 1.46
N
ATOM 14130 CA LYS B 306   -31.471 22.642 -6.419 1.00 2.63
C
ATOM 14131 C   LYS B 306   -31.972 22.067 -5.116 1.00 1.90
C
ATOM 14132 O   LYS B 306   -32.482 20.950 -5.075 1.00 2.97
O

```
ATOM  14133  CB  LYS B 306     -32.560  23.467  -7.097  1.00  3.94
C
ATOM  14134  CG  LYS B 306     -32.079  24.213  -8.337  1.00  3.94
C
ATOM  14135  CD  LYS B 306     -33.189  25.047  -8.954  1.00  3.94
C
ATOM  14136  CE  LYS B 306     -32.704  25.770 -10.203  1.00  3.94
C
ATOM  14137  NZ  LYS B 306     -33.781  26.590 -10.823  1.00  3.94
N
ATOM  14138  H   LYS B 306     -31.671  20.904  -7.652  1.00  1.75
H
ATOM  14139  HA  LYS B 306     -30.630  23.296  -6.191  1.00  3.16
H
ATOM  14140 1HB  LYS B 306     -33.383  22.816  -7.395  1.00  4.73
H
ATOM  14141 2HB  LYS B 306     -32.959  24.200  -6.395  1.00  4.73
H
ATOM  14142 1HG  LYS B 306     -31.251  24.869  -8.068  1.00  4.73
H
ATOM  14143 2HG  LYS B 306     -31.726  23.496  -9.077  1.00  4.73
H
ATOM  14144 1HD  LYS B 306     -34.026  24.401  -9.220  1.00  4.73
H
ATOM  14145 2HD  LYS B 306     -33.536  25.786  -8.231  1.00  4.73
H
ATOM  14146 1HE  LYS B 306     -31.871  26.421  -9.940  1.00  4.73
H
ATOM  14147 2HE  LYS B 306     -32.358  25.035 -10.930  1.00  4.73
H
ATOM  14148 1HZ  LYS B 306     -33.421  27.051 -11.646  1.00  4.73
H
ATOM  14149 2HZ  LYS B 306     -34.554  25.992 -11.082  1.00  4.73
H
ATOM  14150 3HZ  LYS B 306     -34.100  27.283 -10.161  1.00  4.73
H
ATOM  14151  N   MET B 307     -31.828  22.847  -4.056  1.00  1.63
N
ATOM  14152  CA  MET B 307     -32.286  22.472  -2.731  1.00  1.66
C
ATOM  14153  C   MET B 307     -33.784  22.262  -2.729  1.00  1.45
C
ATOM  14154  O   MET B 307     -34.509  22.987  -3.409  1.00  3.02
O
ATOM  14155  CB  MET B 307     -31.919  23.582  -1.760  1.00  2.49
C
ATOM  14156  CG  MET B 307     -32.612  24.909  -2.031  1.00  2.49
C
ATOM  14157  SD  MET B 307     -32.049  26.224  -0.930  1.00  2.49
S
ATOM  14158  CE  MET B 307     -33.120  27.569  -1.430  1.00  2.49
C
ATOM  14159  H   MET B 307     -31.387  23.747  -4.176  1.00  1.96
H
ATOM  14160  HA  MET B 307     -31.806  21.539  -2.437  1.00  1.99
H
ATOM  14161 1HB  MET B 307     -32.138  23.277  -0.738  1.00  2.99
H
```

```
ATOM  14162 2HB  MET B 307     -30.854  23.775  -1.832  1.00  2.99
H
ATOM  14163 1HG  MET B 307     -32.422  25.215  -3.059  1.00  2.99
H
ATOM  14164 2HG  MET B 307     -33.688  24.789  -1.904  1.00  2.99
H
ATOM  14165 1HE  MET B 307     -32.892  28.455  -0.836  1.00  2.99
H
ATOM  14166 2HE  MET B 307     -32.961  27.787  -2.486  1.00  2.99
H
ATOM  14167 3HE  MET B 307     -34.160  27.284  -1.271  1.00  2.99
H
ATOM  14168  N   THR B 308     -34.266  21.309  -1.932  1.00  1.35
N
ATOM  14169  CA  THR B 308     -35.704  21.052  -1.869  1.00  1.60
C
ATOM  14170  C   THR B 308     -36.436  22.196  -1.166  1.00  1.80
C
ATOM  14171  O   THR B 308     -37.618  22.437  -1.413  1.00  4.40
O
ATOM  14172  CB  THR B 308     -36.004  19.736  -1.120  1.00  2.40
C
ATOM  14173  OG1 THR B 308     -35.528  19.836   0.228  1.00  2.40
O
ATOM  14174  CG2 THR B 308     -35.318  18.558  -1.800  1.00  2.40
C
ATOM  14175  H   THR B 308     -33.632  20.746  -1.382  1.00  1.62
H
ATOM  14176  HA  THR B 308     -36.089  20.973  -2.887  1.00  1.92
H
ATOM  14177  HB  THR B 308     -37.080  19.565  -1.105  1.00  2.88
H
ATOM  14178  HG1 THR B 308     -35.995  20.543   0.681  1.00  2.88
H
ATOM  14179 1HG2 THR B 308     -35.542  17.642  -1.256  1.00  2.88
H
ATOM  14180 2HG2 THR B 308     -35.679  18.470  -2.825  1.00  2.88
H
ATOM  14181 3HG2 THR B 308     -34.242  18.721  -1.807  1.00  2.88
H
ATOM  14182  N   SER B 309     -35.717  22.907  -0.308  1.00  0.74
N
ATOM  14183  CA  SER B 309     -36.253  24.039   0.426  1.00  0.95
C
ATOM  14184  C   SER B 309     -35.085  24.889   0.906  1.00  1.59
C
ATOM  14185  O   SER B 309     -33.940  24.447   0.824  1.00  5.27
O
ATOM  14186  CB  SER B 309     -37.076  23.545   1.609  1.00  1.42
C
ATOM  14187  OG  SER B 309     -36.252  22.960   2.579  1.00  1.42
O
ATOM  14188  H   SER B 309     -34.753  22.653  -0.151  1.00  0.89
H
ATOM  14189  HA  SER B 309     -36.881  24.630  -0.242  1.00  1.14
H
ATOM  14190 1HB  SER B 309     -37.636  24.364   2.053  1.00  1.71
H
```

ATOM 14191 2HB SER B 309   -37.802 22.811   1.258 1.00 1.71
H
ATOM 14192 HG SER B 309   -35.695 23.671   2.911 1.00 1.71
H
ATOM 14193 N   PRO B 310   -35.339 26.107   1.381 1.00 0.92
N
ATOM 14194 CA PRO B 310   -34.381 27.013   1.991 1.00 0.93
C
ATOM 14195 C   PRO B 310   -33.996 26.473   3.367 1.00 1.15
C
ATOM 14196 O   PRO B 310   -34.727 25.652   3.925 1.00 2.61
O
ATOM 14197 CB PRO B 310   -35.119 28.357   2.031 1.00 1.40
C
ATOM 14198 CG PRO B 310   -36.559 27.994   1.960 1.00 1.40
C
ATOM 14199 CD PRO B 310   -36.575 26.821   1.014 1.00 1.40
C
ATOM 14200 HA PRO B 310   -33.507 27.070   1.335 1.00 1.12
H
ATOM 14201 1HB PRO B 310   -34.865 28.899   2.954 1.00 1.67
H
ATOM 14202 2HB PRO B 310   -34.797 28.988   1.190 1.00 1.67
H
ATOM 14203 1HG PRO B 310   -36.940 27.741   2.960 1.00 1.67
H
ATOM 14204 2HG PRO B 310   -37.153 28.844   1.595 1.00 1.67
H
ATOM 14205 1HD PRO B 310   -37.467 26.209   1.192 1.00 1.67
H
ATOM 14206 2HD PRO B 310   -36.517 27.161 -0.031 1.00 1.67
H
ATOM 14207 N   PRO B 311   -32.847 26.896   3.906 1.00 1.04
N
ATOM 14208 CA PRO B 311   -32.272 26.536   5.197 1.00 1.27
C
ATOM 14209 C   PRO B 311   -33.277 26.685   6.328 1.00 2.14
C
ATOM 14210 O   PRO B 311   -34.011 27.666   6.404 1.00 9.80
O
ATOM 14211 CB PRO B 311   -31.126 27.529   5.346 1.00 1.91
C
ATOM 14212 CG PRO B 311   -30.698 27.812   3.952 1.00 1.91
C
ATOM 14213 CD PRO B 311   -31.980 27.844   3.173 1.00 1.91
C
ATOM 14214 HA PRO B 311   -31.902 25.504   5.151 1.00 1.52
H
ATOM 14215 1HB PRO B 311   -31.492 28.425   5.861 1.00 2.29
H
ATOM 14216 2HB PRO B 311   -30.325 27.095   5.963 1.00 2.29
H
ATOM 14217 1HG PRO B 311   -30.150 28.763   3.903 1.00 2.29
H
ATOM 14218 2HG PRO B 311   -30.010 27.028   3.600 1.00 2.29
H
ATOM 14219 1HD PRO B 311   -32.424 28.849   3.182 1.00 2.29
H

```
ATOM  14220 2HD  PRO B 311     -31.783  27.488   2.154  1.00  2.29
H
ATOM  14221  N   ALA B 312     -33.300  25.706   7.218  1.00  1.35
N
ATOM  14222  CA  ALA B 312     -34.234  25.717   8.336  1.00  1.38
C
ATOM  14223  C   ALA B 312     -33.548  26.057   9.653  1.00  1.35
C
ATOM  14224  O   ALA B 312     -33.684  25.316  10.626  1.00  3.68
O
ATOM  14225  CB  ALA B 312     -34.921  24.366   8.450  1.00  2.07
C
ATOM  14226  H   ALA B 312     -32.663  24.930   7.114  1.00  1.62
H
ATOM  14227  HA  ALA B 312     -34.985  26.482   8.143  1.00  1.66
H
ATOM  14228 1HB  ALA B 312     -35.642  24.394   9.268  1.00  2.48
H
ATOM  14229 2HB  ALA B 312     -35.439  24.142   7.518  1.00  2.48
H
ATOM  14230 3HB  ALA B 312     -34.179  23.595   8.648  1.00  2.48
H
ATOM  14231  N   TYR B 313     -32.816  27.165   9.693  1.00  0.73
N
ATOM  14232  CA  TYR B 313     -32.139  27.548  10.928  1.00  0.68
C
ATOM  14233  C   TYR B 313     -32.541  28.950  11.316  1.00  0.79
C
ATOM  14234  O   TYR B 313     -32.795  29.788  10.449  1.00  1.25
O
ATOM  14235  CB  TYR B 313     -30.628  27.494  10.768  1.00  1.02
C
ATOM  14236  CG  TYR B 313     -30.106  26.156  10.337  1.00  1.02
C
ATOM  14237  CD1 TYR B 313     -29.991  25.902   8.985  1.00  1.02
C
ATOM  14238  CD2 TYR B 313     -29.743  25.192  11.268  1.00  1.02
C
ATOM  14239  CE1 TYR B 313     -29.515  24.691   8.557  1.00  1.02
C
ATOM  14240  CE2 TYR B 313     -29.264  23.970  10.833  1.00  1.02
C
ATOM  14241  CZ  TYR B 313     -29.152  23.721   9.480  1.00  1.02
C
ATOM  14242  OH  TYR B 313     -28.681  22.514   9.027  1.00  1.02
O
ATOM  14243  H   TYR B 313     -32.732  27.748   8.872  1.00  0.88
H
ATOM  14244  HA  TYR B 313     -32.445  26.874  11.729  1.00  0.82
H
ATOM  14245 1HB  TYR B 313     -30.305  28.236  10.044  1.00  1.22
H
ATOM  14246 2HB  TYR B 313     -30.164  27.742  11.719  1.00  1.22
H
ATOM  14247  HD1 TYR B 313     -30.276  26.662   8.258  1.00  1.22
H
ATOM  14248  HD2 TYR B 313     -29.836  25.397  12.335  1.00  1.22
H
```

```
ATOM  14249  HE1 TYR B 313      -29.423  24.497   7.495  1.00  1.22
H
ATOM  14250  HE2 TYR B 313      -28.979  23.205  11.557  1.00  1.22
H
ATOM  14251  HH  TYR B 313      -28.663  22.524   8.065  1.00  1.22
H
ATOM  14252  N   LYS B 314      -32.594  29.222  12.613  1.00  0.68
N
ATOM  14253  CA  LYS B 314      -32.963  30.551  13.058  1.00  0.78
C
ATOM  14254  C   LYS B 314      -31.742  31.291  13.555  1.00  0.63
C
ATOM  14255  O   LYS B 314      -30.842  30.691  14.141  1.00  0.59
O
ATOM  14256  CB  LYS B 314      -34.028  30.483  14.152  1.00  1.17
C
ATOM  14257  CG  LYS B 314      -35.359  29.901  13.694  1.00  1.17
C
ATOM  14258  CD  LYS B 314      -36.378  29.896  14.824  1.00  1.17
C
ATOM  14259  CE  LYS B 314      -37.705  29.305  14.371  1.00  1.17
C
ATOM  14260  NZ  LYS B 314      -38.703  29.281  15.474  1.00  1.17
N
ATOM  14261  H   LYS B 314      -32.377  28.514  13.312  1.00  0.82
H
ATOM  14262  HA  LYS B 314      -33.369  31.106  12.213  1.00  0.94
H
ATOM  14263 1HB  LYS B 314      -33.666  29.878  14.982  1.00  1.40
H
ATOM  14264 2HB  LYS B 314      -34.218  31.485  14.539  1.00  1.40
H
ATOM  14265 1HG  LYS B 314      -35.749  30.493  12.865  1.00  1.40
H
ATOM  14266 2HG  LYS B 314      -35.207  28.879  13.349  1.00  1.40
H
ATOM  14267 1HD  LYS B 314      -35.992  29.307  15.658  1.00  1.40
H
ATOM  14268 2HD  LYS B 314      -36.544  30.916  15.167  1.00  1.40
H
ATOM  14269 1HE  LYS B 314      -38.100  29.899  13.549  1.00  1.40
H
ATOM  14270 2HE  LYS B 314      -37.542  28.285  14.021  1.00  1.40
H
ATOM  14271 1HZ  LYS B 314      -39.569  28.883  15.136  1.00  1.40
H
ATOM  14272 2HZ  LYS B 314      -38.351  28.720  16.237  1.00  1.40
H
ATOM  14273 3HZ  LYS B 314      -38.871  30.223  15.797  1.00  1.40
H
ATOM  14274  N   VAL B 315      -31.720  32.595  13.319  1.00  0.62
N
ATOM  14275  CA  VAL B 315      -30.602  33.419  13.740  1.00  0.56
C
ATOM  14276  C   VAL B 315      -30.486  33.377  15.252  1.00  0.50
C
ATOM  14277  O   VAL B 315      -31.465  33.595  15.968  1.00  0.51
O
```

ATOM 14278 CB VAL B 315   -30.786 34.866 13.241 1.00 0.84
C
ATOM 14279 CG1 VAL B 315   -29.700 35.772 13.796 1.00 0.84
C
ATOM 14280 CG2 VAL B 315   -30.749 34.874 11.719 1.00 0.84
C
ATOM 14281 H   VAL B 315   -32.494 33.024 12.833 1.00 0.74
H
ATOM 14282 HA VAL B 315   -29.688 33.017 13.306 1.00 0.67
H
ATOM 14283 HB VAL B 315   -31.747 35.246 13.591 1.00 1.01
H
ATOM 14284 1HG1 VAL B 315   -29.857 36.789 13.437 1.00 1.01
H
ATOM 14285 2HG1 VAL B 315   -29.736 35.764 14.885 1.00 1.01
H
ATOM 14286 3HG1 VAL B 315   -28.726 35.422 13.462 1.00 1.01
H
ATOM 14287 1HG2 VAL B 315   -30.892 35.892 11.358 1.00 1.01
H
ATOM 14288 2HG2 VAL B 315   -29.784 34.501 11.377 1.00 1.01
H
ATOM 14289 3HG2 VAL B 315   -31.543 34.237 11.331 1.00 1.01
H
ATOM 14290 N   GLY B 316   -29.285 33.068 15.727 1.00 0.48
N
ATOM 14291 CA GLY B 316   -29.023 32.923 17.151 1.00 0.52
C
ATOM 14292 C   GLY B 316   -28.823 31.455 17.553 1.00 0.54
C
ATOM 14293 O   GLY B 316   -28.218 31.178 18.591 1.00 0.63
O
ATOM 14294 H   GLY B 316   -28.523 32.945 15.074 1.00 0.58
H
ATOM 14295 1HA GLY B 316   -28.135 33.497 17.414 1.00 0.62
H
ATOM 14296 2HA GLY B 316   -29.852 33.344 17.716 1.00 0.62
H
ATOM 14297 N   ASP B 317   -29.319 30.520 16.733 1.00 0.55
N
ATOM 14298 CA ASP B 317   -29.144 29.092 17.000 1.00 0.70
C
ATOM 14299 C   ASP B 317   -27.679 28.721 16.881 1.00 0.52
C
ATOM 14300 O   ASP B 317   -26.998 29.201 15.983 1.00 0.52
O
ATOM 14301 CB ASP B 317   -29.944 28.239 16.000 1.00 1.05
C
ATOM 14302 CG ASP B 317   -31.454 28.275 16.197 1.00 1.05
C
ATOM 14303 OD1 ASP B 317   -31.892 28.679 17.249 1.00 1.05
O
ATOM 14304 OD2 ASP B 317   -32.155 27.905 15.281 1.00 1.05
O
ATOM 14305 H   ASP B 317   -29.820 30.788 15.895 1.00 0.66
H
ATOM 14306 HA ASP B 317   -29.480 28.876 18.015 1.00 0.84
H

ATOM	14307	1HB	ASP	B	317	-29.721	28.575	14.988	1.00	1.26	H
ATOM	14308	2HB	ASP	B	317	-29.616	27.203	16.074	1.00	1.26	H
ATOM	14309	N	LYS	B	318	-27.173	27.864	17.759	1.00	0.52	N
ATOM	14310	CA	LYS	B	318	-25.772	27.489	17.622	1.00	0.53	C
ATOM	14311	C	LYS	B	318	-25.611	26.073	17.101	1.00	0.56	C
ATOM	14312	O	LYS	B	318	-26.267	25.143	17.570	1.00	1.12	O
ATOM	14313	CB	LYS	B	318	-25.029	27.655	18.941	1.00	0.80	C
ATOM	14314	CG	LYS	B	318	-24.913	29.100	19.398	1.00	0.80	C
ATOM	14315	CD	LYS	B	318	-24.095	29.214	20.671	1.00	0.80	C
ATOM	14316	CE	LYS	B	318	-23.982	30.661	21.124	1.00	0.80	C
ATOM	14317	NZ	LYS	B	318	-23.190	30.786	22.379	1.00	0.80	N
ATOM	14318	H	LYS	B	318	-27.743	27.480	18.499	1.00	0.62	H
ATOM	14319	HA	LYS	B	318	-25.315	28.155	16.900	1.00	0.64	H
ATOM	14320	1HB	LYS	B	318	-25.542	27.094	19.723	1.00	0.95	H
ATOM	14321	2HB	LYS	B	318	-24.021	27.247	18.850	1.00	0.95	H
ATOM	14322	1HG	LYS	B	318	-24.433	29.690	18.619	1.00	0.95	H
ATOM	14323	2HG	LYS	B	318	-25.908	29.507	19.578	1.00	0.95	H
ATOM	14324	1HD	LYS	B	318	-24.569	28.630	21.461	1.00	0.95	H
ATOM	14325	2HD	LYS	B	318	-23.095	28.816	20.498	1.00	0.95	H
ATOM	14326	1HE	LYS	B	318	-23.497	31.244	20.341	1.00	0.95	H
ATOM	14327	2HE	LYS	B	318	-24.980	31.063	21.295	1.00	0.95	H
ATOM	14328	1HZ	LYS	B	318	-23.136	31.759	22.648	1.00	0.95	H
ATOM	14329	2HZ	LYS	B	318	-23.639	30.259	23.114	1.00	0.95	H
ATOM	14330	3HZ	LYS	B	318	-22.259	30.427	22.227	1.00	0.95	H
ATOM	14331	N	ILE	B	319	-24.735	25.926	16.115	1.00	0.52	N
ATOM	14332	CA	ILE	B	319	-24.468	24.630	15.516	1.00	0.81	C
ATOM	14333	C	ILE	B	319	-23.141	24.646	14.763	1.00	0.48	C
ATOM	14334	O	ILE	B	319	-22.744	25.669	14.204	1.00	0.56	O
ATOM	14335	CB	ILE	B	319	-25.632	24.216	14.601	1.00	1.22	C

ATOM 14336 CG1 ILE B 319   -25.459 22.770 14.128 1.00 1.22
C
ATOM 14337 CG2 ILE B 319   -25.727 25.171 13.431 1.00 1.22
C
ATOM 14338 CD1 ILE B 319   -26.721 22.184 13.532 1.00 1.22
C
ATOM 14339 H   ILE B 319   -24.239 26.745 15.781 1.00 0.62
H
ATOM 14340 HA ILE B 319   -24.393 23.893 16.314 1.00 0.97
H
ATOM 14341 HB ILE B 319   -26.563 24.258 15.166 1.00 1.46
H
ATOM 14342 1HG1 ILE B 319   -24.675 22.735 13.375 1.00 1.46
H
ATOM 14343 2HG1 ILE B 319   -25.153 22.148 14.970 1.00 1.46
H
ATOM 14344 1HG2 ILE B 319   -26.566 24.886 12.797 1.00 1.46
H
ATOM 14345 2HG2 ILE B 319   -25.880 26.185 13.800 1.00 1.46
H
ATOM 14346 3HG2 ILE B 319   -24.804 25.130 12.853 1.00 1.46
H
ATOM 14347 1HD1 ILE B 319   -26.532 21.161 13.208 1.00 1.46
H
ATOM 14348 2HD1 ILE B 319   -27.512 22.187 14.282 1.00 1.46
H
ATOM 14349 3HD1 ILE B 319   -27.033 22.781 12.679 1.00 1.46
H
ATOM 14350 N   ALA B 320   -22.429 23.527 14.803 1.00 0.48
N
ATOM 14351 CA ALA B 320   -21.151 23.421 14.120 1.00 0.38
C
ATOM 14352 C   ALA B 320   -21.380 23.408 12.635 1.00 0.37
C
ATOM 14353 O   ALA B 320   -22.361 22.826 12.165 1.00 0.46
O
ATOM 14354 CB ALA B 320   -20.405 22.170 14.557 1.00 0.57
C
ATOM 14355 H   ALA B 320   -22.793 22.724 15.298 1.00 0.58
H
ATOM 14356 HA ALA B 320   -20.554 24.300 14.364 1.00 0.46
H
ATOM 14357 1HB ALA B 320   -19.446 22.119 14.040 1.00 0.68
H
ATOM 14358 2HB ALA B 320   -20.236 22.204 15.633 1.00 0.68
H
ATOM 14359 3HB ALA B 320   -20.997 21.289 14.312 1.00 0.68
H
ATOM 14360 N   THR B 321   -20.467 24.018 11.886 1.00 0.34
N
ATOM 14361 CA THR B 321   -20.668 24.042 10.461 1.00 0.45
C
ATOM 14362 C   THR B 321   -20.529 22.629   9.906 1.00 0.67
C
ATOM 14363 O   THR B 321   -21.296 22.267   9.016 1.00 1.94
O
ATOM 14364 CB THR B 321   -19.689 25.017   9.775 1.00 0.68
C

ATOM 14365 OG1 THR B 321 -18.347 24.631 10.053 1.00 0.68
O
ATOM 14366 CG2 THR B 321 -19.918 26.446 10.248 1.00 0.68
C
ATOM 14367 H THR B 321 -19.665 24.471 12.306 1.00 0.41
H
ATOM 14368 HA THR B 321 -21.682 24.387 10.258 1.00 0.54
H
ATOM 14369 HB THR B 321 -19.837 24.967 8.702 1.00 0.81
H
ATOM 14370 HG1 THR B 321 -18.046 25.012 10.891 1.00 0.81
H
ATOM 14371 1HG2 THR B 321 -19.220 27.113 9.743 1.00 0.81
H
ATOM 14372 2HG2 THR B 321 -20.939 26.747 10.018 1.00 0.81
H
ATOM 14373 3HG2 THR B 321 -19.757 26.502 11.324 1.00 0.81
H
ATOM 14374 N GLN B 322 -19.669 21.798 10.528 1.00 0.32
N
ATOM 14375 CA GLN B 322 -19.535 20.380 10.158 1.00 0.34
C
ATOM 14376 C GLN B 322 -20.870 19.662 10.154 1.00 0.34
C
ATOM 14377 O GLN B 322 -21.206 18.961 9.194 1.00 0.41
O
ATOM 14378 CB GLN B 322 -18.599 19.647 11.136 1.00 0.51
C
ATOM 14379 CG GLN B 322 -17.130 19.965 11.002 1.00 0.51
C
ATOM 14380 CD GLN B 322 -16.296 19.424 12.144 1.00 0.51
C
ATOM 14381 OE1 GLN B 322 -15.082 19.644 12.207 1.00 0.51
O
ATOM 14382 NE2 GLN B 322 -16.941 18.719 13.066 1.00 0.51
N
ATOM 14383 H GLN B 322 -19.052 22.165 11.248 1.00 0.38
H
ATOM 14384 HA GLN B 322 -19.116 20.318 9.154 1.00 0.41
H
ATOM 14385 1HB GLN B 322 -18.896 19.869 12.161 1.00 0.61
H
ATOM 14386 2HB GLN B 322 -18.699 18.576 10.992 1.00 0.61
H
ATOM 14387 1HG GLN B 322 -16.784 19.472 10.099 1.00 0.61
H
ATOM 14388 2HG GLN B 322 -16.989 21.038 10.916 1.00 0.61
H
ATOM 14389 1HE2 GLN B 322 -16.439 18.351 13.853 1.00 0.61
H
ATOM 14390 2HE2 GLN B 322 -17.924 18.564 12.981 1.00 0.61
H
ATOM 14391 N LYS B 323 -21.624 19.833 11.235 1.00 0.36
N
ATOM 14392 CA LYS B 323 -22.909 19.175 11.366 1.00 0.42
C
ATOM 14393 C LYS B 323 -23.876 19.626 10.306 1.00 0.40
C

```
ATOM  14394  O   LYS B 323     -24.602  18.801   9.744  1.00  0.36
O
ATOM  14395  CB  LYS B 323     -23.494  19.386  12.753  1.00  0.63
C
ATOM  14396  CG  LYS B 323     -24.796  18.630  12.971  1.00  0.63
C
ATOM  14397  CD  LYS B 323     -25.260  18.718  14.414  1.00  0.63
C
ATOM  14398  CE  LYS B 323     -26.569  17.967  14.614  1.00  0.63
C
ATOM  14399  NZ  LYS B 323     -27.029  18.012  16.032  1.00  0.63
N
ATOM  14400  H   LYS B 323     -21.295  20.426  11.984  1.00  0.43
H
ATOM  14401  HA  LYS B 323     -22.766  18.112  11.222  1.00  0.50
H
ATOM  14402 1HB  LYS B 323     -22.780  19.058  13.508  1.00  0.76
H
ATOM  14403 2HB  LYS B 323     -23.684  20.447  12.914  1.00  0.76
H
ATOM  14404 1HG  LYS B 323     -25.569  19.047  12.324  1.00  0.76
H
ATOM  14405 2HG  LYS B 323     -24.655  17.582  12.708  1.00  0.76
H
ATOM  14406 1HD  LYS B 323     -24.501  18.286  15.067  1.00  0.76
H
ATOM  14407 2HD  LYS B 323     -25.402  19.762  14.692  1.00  0.76
H
ATOM  14408 1HE  LYS B 323     -27.337  18.413  13.981  1.00  0.76
H
ATOM  14409 2HE  LYS B 323     -26.433  16.928  14.321  1.00  0.76
H
ATOM  14410 1HZ  LYS B 323     -27.897  17.503  16.122  1.00  0.76
H
ATOM  14411 2HZ  LYS B 323     -26.329  17.589  16.627  1.00  0.76
H
ATOM  14412 3HZ  LYS B 323     -27.171  18.972  16.312  1.00  0.76
H
ATOM  14413  N   THR B 324     -23.881  20.922  10.010  1.00  0.52
N
ATOM  14414  CA  THR B 324     -24.806  21.409   9.008  1.00  0.54
C
ATOM  14415  C   THR B 324     -24.413  20.979   7.607  1.00  0.68
C
ATOM  14416  O   THR B 324     -25.286  20.862   6.756  1.00  1.45
O
ATOM  14417  CB  THR B 324     -24.923  22.917   9.062  1.00  0.81
C
ATOM  14418  OG1 THR B 324     -23.664  23.508   8.754  1.00  0.81
O
ATOM  14419  CG2 THR B 324     -25.323  23.307  10.451  1.00  0.81
C
ATOM  14420  H   THR B 324     -23.272  21.568  10.505  1.00  0.62
H
ATOM  14421  HA  THR B 324     -25.788  20.990   9.220  1.00  0.65
H
ATOM  14422  HB  THR B 324     -25.671  23.268   8.351  1.00  0.97
H
```

ATOM 14423 HG1 THR B 324 -22.963 23.004 9.178 1.00 0.97
H
ATOM 14424 1HG2 THR B 324 -25.394 24.382 10.524 1.00 0.97
H
ATOM 14425 2HG2 THR B 324 -26.286 22.857 10.683 1.00 0.97
H
ATOM 14426 3HG2 THR B 324 -24.576 22.951 11.156 1.00 0.97
H
ATOM 14427 N TYR B 325 -23.131 20.690 7.360 1.00 0.57
N
ATOM 14428 CA TYR B 325 -22.776 20.177 6.041 1.00 0.66
C
ATOM 14429 C TYR B 325 -23.302 18.770 5.873 1.00 0.47
C
ATOM 14430 O TYR B 325 -23.751 18.409 4.790 1.00 0.51
O
ATOM 14431 CB TYR B 325 -21.281 20.172 5.808 1.00 0.99
C
ATOM 14432 CG TYR B 325 -20.685 21.522 5.568 1.00 0.99
C
ATOM 14433 CD1 TYR B 325 -19.897 22.067 6.527 1.00 0.99
C
ATOM 14434 CD2 TYR B 325 -20.909 22.216 4.402 1.00 0.99
C
ATOM 14435 CE1 TYR B 325 -19.318 23.286 6.333 1.00 0.99
C
ATOM 14436 CE2 TYR B 325 -20.306 23.440 4.199 1.00 0.99
C
ATOM 14437 CZ TYR B 325 -19.480 23.958 5.176 1.00 0.99
C
ATOM 14438 OH TYR B 325 -18.779 25.132 5.042 1.00 0.99
O
ATOM 14439 H TYR B 325 -22.417 20.840 8.063 1.00 0.68
H
ATOM 14440 HA TYR B 325 -23.247 20.804 5.284 1.00 0.79
H
ATOM 14441 1HB TYR B 325 -20.783 19.732 6.673 1.00 1.19
H
ATOM 14442 2HB TYR B 325 -21.052 19.544 4.947 1.00 1.19
H
ATOM 14443 HD1 TYR B 325 -19.721 21.520 7.448 1.00 1.19
H
ATOM 14444 HD2 TYR B 325 -21.544 21.783 3.646 1.00 1.19
H
ATOM 14445 HE1 TYR B 325 -18.685 23.723 7.097 1.00 1.19
H
ATOM 14446 HE2 TYR B 325 -20.466 23.979 3.266 1.00 1.19
H
ATOM 14447 HH TYR B 325 -17.873 25.019 5.388 1.00 1.19
H
ATOM 14448 N GLY B 326 -23.284 17.985 6.952 1.00 0.45
N
ATOM 14449 CA GLY B 326 -23.848 16.642 6.899 1.00 0.43
C
ATOM 14450 C GLY B 326 -25.337 16.718 6.575 1.00 0.39
C
ATOM 14451 O GLY B 326 -25.836 15.986 5.713 1.00 0.38
O

ATOM 14452 H GLY B 326 -22.861 18.319 7.814 1.00 0.54
H
ATOM 14453 1HA GLY B 326 -23.330 16.053 6.141 1.00 0.52
H
ATOM 14454 2HA GLY B 326 -23.700 16.147 7.856 1.00 0.52
H
ATOM 14455 N LEU B 327 -26.027 17.631 7.259 1.00 0.42
N
ATOM 14456 CA LEU B 327 -27.450 17.855 7.052 1.00 0.50
C
ATOM 14457 C LEU B 327 -27.736 18.358 5.635 1.00 0.46
C
ATOM 14458 O LEU B 327 -28.686 17.904 4.993 1.00 0.53
O
ATOM 14459 CB LEU B 327 -27.943 18.876 8.090 1.00 0.75
C
ATOM 14460 CG LEU B 327 -27.957 18.383 9.548 1.00 0.75
C
ATOM 14461 CD1 LEU B 327 -28.223 19.552 10.488 1.00 0.75
C
ATOM 14462 CD2 LEU B 327 -29.036 17.323 9.702 1.00 0.75
C
ATOM 14463 H LEU B 327 -25.549 18.179 7.967 1.00 0.50
H
ATOM 14464 HA LEU B 327 -27.974 16.912 7.199 1.00 0.60
H
ATOM 14465 1HB LEU B 327 -27.307 19.757 8.042 1.00 0.90
H
ATOM 14466 2HB LEU B 327 -28.959 19.172 7.831 1.00 0.90
H
ATOM 14467 HG LEU B 327 -26.986 17.957 9.799 1.00 0.90
H
ATOM 14468 1HD1 LEU B 327 -28.228 19.196 11.518 1.00 0.90
H
ATOM 14469 2HD1 LEU B 327 -27.443 20.302 10.370 1.00 0.90
H
ATOM 14470 3HD1 LEU B 327 -29.189 19.996 10.252 1.00 0.90
H
ATOM 14471 1HD2 LEU B 327 -29.049 16.965 10.732 1.00 0.90
H
ATOM 14472 2HD2 LEU B 327 -30.006 17.755 9.456 1.00 0.90
H
ATOM 14473 3HD2 LEU B 327 -28.829 16.492 9.032 1.00 0.90
H
ATOM 14474 N ALA B 328 -26.882 19.265 5.151 1.00 0.51
N
ATOM 14475 CA ALA B 328 -26.986 19.848 3.821 1.00 0.61
C
ATOM 14476 C ALA B 328 -26.870 18.801 2.743 1.00 0.48
C
ATOM 14477 O ALA B 328 -27.658 18.799 1.797 1.00 0.52
O
ATOM 14478 CB ALA B 328 -25.900 20.886 3.617 1.00 0.92
C
ATOM 14479 H ALA B 328 -26.136 19.595 5.743 1.00 0.61
H
ATOM 14480 HA ALA B 328 -27.963 20.323 3.735 1.00 0.73
H

ATOM 14481 1HB ALA B 328 -26.004 21.335 2.629 1.00 1.10
H
ATOM 14482 2HB ALA B 328 -25.977 21.656 4.378 1.00 1.10
H
ATOM 14483 3HB ALA B 328 -24.928 20.406 3.697 1.00 1.10
H
ATOM 14484 N LEU B 329 -25.886 17.909 2.892 1.00 0.58
N
ATOM 14485 CA LEU B 329 -25.676 16.841 1.930 1.00 0.72
C
ATOM 14486 C LEU B 329 -26.851 15.900 1.870 1.00 0.65
C
ATOM 14487 O LEU B 329 -27.240 15.479 0.783 1.00 0.84
O
ATOM 14488 CB LEU B 329 -24.433 16.024 2.279 1.00 1.08
C
ATOM 14489 CG LEU B 329 -23.092 16.717 2.078 1.00 1.08
C
ATOM 14490 CD1 LEU B 329 -21.990 15.830 2.639 1.00 1.08
C
ATOM 14491 CD2 LEU B 329 -22.894 16.978 0.598 1.00 1.08
C
ATOM 14492 H LEU B 329 -25.259 17.983 3.681 1.00 0.70
H
ATOM 14493 HA LEU B 329 -25.540 17.286 0.946 1.00 0.86
H
ATOM 14494 1HB LEU B 329 -24.495 15.732 3.327 1.00 1.30
H
ATOM 14495 2HB LEU B 329 -24.430 15.119 1.673 1.00 1.30
H
ATOM 14496 HG LEU B 329 -23.076 17.663 2.614 1.00 1.30
H
ATOM 14497 1HD1 LEU B 329 -21.025 16.312 2.506 1.00 1.30
H
ATOM 14498 2HD1 LEU B 329 -22.166 15.663 3.702 1.00 1.30
H
ATOM 14499 3HD1 LEU B 329 -21.991 14.875 2.118 1.00 1.30
H
ATOM 14500 1HD2 LEU B 329 -21.937 17.475 0.450 1.00 1.30
H
ATOM 14501 2HD2 LEU B 329 -22.903 16.033 0.056 1.00 1.30
H
ATOM 14502 3HD2 LEU B 329 -23.697 17.617 0.229 1.00 1.30
H
ATOM 14503 N ALA B 330 -27.426 15.573 3.025 1.00 0.58
N
ATOM 14504 CA ALA B 330 -28.580 14.686 3.048 1.00 0.68
C
ATOM 14505 C ALA B 330 -29.751 15.307 2.295 1.00 0.85
C
ATOM 14506 O ALA B 330 -30.423 14.622 1.518 1.00 1.22
O
ATOM 14507 CB ALA B 330 -28.971 14.369 4.474 1.00 1.02
C
ATOM 14508 H ALA B 330 -27.050 15.922 3.902 1.00 0.70
H
ATOM 14509 HA ALA B 330 -28.310 13.761 2.542 1.00 0.82
H

ATOM 14510 1HB ALA B 330 -29.819 13.685 4.478 1.00 1.22
H
ATOM 14511 2HB ALA B 330 -28.124 13.904 4.973 1.00 1.22
H
ATOM 14512 3HB ALA B 330 -29.241 15.287 4.993 1.00 1.22
H
ATOM 14513 N LYS B 331 -29.977 16.608 2.508 1.00 0.71
N
ATOM 14514 CA LYS B 331 -31.050 17.315 1.815 1.00 0.81
C
ATOM 14515 C LYS B 331 -30.800 17.337 0.313 1.00 0.85
C
ATOM 14516 O LYS B 331 -31.715 17.103 -0.481 1.00 1.27
O
ATOM 14517 CB LYS B 331 -31.171 18.736 2.339 1.00 1.22
C
ATOM 14518 CG LYS B 331 -31.713 18.847 3.750 1.00 1.22
C
ATOM 14519 CD LYS B 331 -31.782 20.299 4.179 1.00 1.22
C
ATOM 14520 CE LYS B 331 -32.281 20.437 5.604 1.00 1.22
C
ATOM 14521 NZ LYS B 331 -32.362 21.862 6.011 1.00 1.22
N
ATOM 14522 H LYS B 331 -29.407 17.115 3.177 1.00 0.85
H
ATOM 14523 HA LYS B 331 -31.987 16.790 1.994 1.00 0.97
H
ATOM 14524 1HB LYS B 331 -30.191 19.213 2.324 1.00 1.46
H
ATOM 14525 2HB LYS B 331 -31.825 19.312 1.683 1.00 1.46
H
ATOM 14526 1HG LYS B 331 -32.714 18.416 3.793 1.00 1.46
H
ATOM 14527 2HG LYS B 331 -31.071 18.300 4.438 1.00 1.46
H
ATOM 14528 1HD LYS B 331 -30.790 20.749 4.108 1.00 1.46
H
ATOM 14529 2HD LYS B 331 -32.454 20.844 3.516 1.00 1.46
H
ATOM 14530 1HE LYS B 331 -33.268 19.986 5.691 1.00 1.46
H
ATOM 14531 2HE LYS B 331 -31.595 19.918 6.275 1.00 1.46
H
ATOM 14532 1HZ LYS B 331 -32.688 21.926 6.964 1.00 1.46
H
ATOM 14533 2HZ LYS B 331 -31.442 22.282 5.936 1.00 1.46
H
ATOM 14534 3HZ LYS B 331 -33.002 22.350 5.401 1.00 1.46
H
ATOM 14535 N LEU B 332 -29.548 17.584 -0.056 1.00 0.69
N
ATOM 14536 CA LEU B 332 -29.089 17.565 -1.434 1.00 0.94
C
ATOM 14537 C LEU B 332 -29.403 16.222 -2.094 1.00 0.86
C
ATOM 14538 O LEU B 332 -29.901 16.176 -3.221 1.00 1.35
O

```
ATOM  14539  CB  LEU B 332     -27.583  17.847  -1.458  1.00  1.41
C
ATOM  14540  CG  LEU B 332     -26.919  17.799  -2.814  1.00  1.41
C
ATOM  14541  CD1 LEU B 332     -27.490  18.865  -3.707  1.00  1.41
C
ATOM  14542  CD2 LEU B 332     -25.434  17.997  -2.657  1.00  1.41
C
ATOM  14543  H   LEU B 332     -28.864  17.800   0.655  1.00  0.83
H
ATOM  14544  HA  LEU B 332     -29.609  18.346  -1.985  1.00  1.13
H
ATOM  14545 1HB  LEU B 332     -27.412  18.837  -1.040  1.00  1.69
H
ATOM  14546 2HB  LEU B 332     -27.076  17.124  -0.831  1.00  1.69
H
ATOM  14547  HG  LEU B 332     -27.122  16.832  -3.257  1.00  1.69
H
ATOM  14548 1HD1 LEU B 332     -27.009  18.810  -4.683  1.00  1.69
H
ATOM  14549 2HD1 LEU B 332     -28.564  18.719  -3.817  1.00  1.69
H
ATOM  14550 3HD1 LEU B 332     -27.295  19.835  -3.265  1.00  1.69
H
ATOM  14551 1HD2 LEU B 332     -24.965  17.947  -3.638  1.00  1.69
H
ATOM  14552 2HD2 LEU B 332     -25.249  18.971  -2.218  1.00  1.69
H
ATOM  14553 3HD2 LEU B 332     -25.023  17.221  -2.012  1.00  1.69
H
ATOM  14554  N   GLY B 333     -29.108  15.137  -1.376  1.00  0.76
N
ATOM  14555  CA  GLY B 333     -29.378  13.782  -1.837  1.00  0.96
C
ATOM  14556  C   GLY B 333     -30.857  13.574  -2.143  1.00  0.90
C
ATOM  14557  O   GLY B 333     -31.199  12.916  -3.127  1.00  2.05
O
ATOM  14558  H   GLY B 333     -28.668  15.254  -0.473  1.00  0.91
H
ATOM  14559 1HA  GLY B 333     -28.787  13.578  -2.729  1.00  1.15
H
ATOM  14560 2HA  GLY B 333     -29.064  13.073  -1.074  1.00  1.15
H
ATOM  14561  N   ARG B 334     -31.736  14.146  -1.315  1.00  0.82
N
ATOM  14562  CA  ARG B 334     -33.174  14.047  -1.564  1.00  0.75
C
ATOM  14563  C   ARG B 334     -33.559  14.770  -2.852  1.00  0.90
C
ATOM  14564  O   ARG B 334     -34.447  14.323  -3.581  1.00  2.38
O
ATOM  14565  CB  ARG B 334     -33.973  14.646  -0.417  1.00  1.12
C
ATOM  14566  CG  ARG B 334     -33.946  13.861   0.877  1.00  1.12
C
ATOM  14567  CD  ARG B 334     -34.716  14.548   1.949  1.00  1.12
C
```

```
ATOM  14568  NE  ARG B 334     -34.697  13.784   3.181  1.00  1.12
N
ATOM  14569  CZ  ARG B 334     -35.119  14.217   4.385  1.00  1.12
C
ATOM  14570  NH1 ARG B 334     -35.619  15.428   4.513  1.00  1.12
N
ATOM  14571  NH2 ARG B 334     -35.024  13.419   5.438  1.00  1.12
N
ATOM  14572  H   ARG B 334     -31.401  14.633  -0.490  1.00  0.98
H
ATOM  14573  HA  ARG B 334     -33.436  12.994  -1.662  1.00  0.90
H
ATOM  14574 1HB  ARG B 334     -33.612  15.648  -0.198  1.00  1.35
H
ATOM  14575 2HB  ARG B 334     -35.018  14.738  -0.716  1.00  1.35
H
ATOM  14576 1HG  ARG B 334     -34.384  12.875   0.718  1.00  1.35
H
ATOM  14577 2HG  ARG B 334     -32.914  13.748   1.214  1.00  1.35
H
ATOM  14578 1HD  ARG B 334     -34.279  15.526   2.146  1.00  1.35
H
ATOM  14579 2HD  ARG B 334     -35.752  14.667   1.636  1.00  1.35
H
ATOM  14580  HE  ARG B 334     -34.337  12.838   3.128  1.00  1.35
H
ATOM  14581 1HH1 ARG B 334     -35.689  16.034   3.708  1.00  1.35
H
ATOM  14582 2HH1 ARG B 334     -35.934  15.750   5.417  1.00  1.35
H
ATOM  14583 1HH2 ARG B 334     -34.616  12.492   5.338  1.00  1.35
H
ATOM  14584 2HH2 ARG B 334     -35.336  13.736   6.342  1.00  1.35
H
ATOM  14585  N   ALA B 335     -32.887  15.888  -3.124  1.00  1.29
N
ATOM  14586  CA  ALA B 335     -33.131  16.668  -4.330  1.00  1.17
C
ATOM  14587  C   ALA B 335     -32.724  15.939  -5.611  1.00  1.05
C
ATOM  14588  O   ALA B 335     -33.445  16.002  -6.610  1.00  1.11
O
ATOM  14589  CB  ALA B 335     -32.388  17.988  -4.252  1.00  1.75
C
ATOM  14590  H   ALA B 335     -32.199  16.221  -2.457  1.00  1.55
H
ATOM  14591  HA  ALA B 335     -34.200  16.867  -4.385  1.00  1.40
H
ATOM  14592 1HB  ALA B 335     -32.616  18.571  -5.142  1.00  2.11
H
ATOM  14593 2HB  ALA B 335     -32.703  18.537  -3.365  1.00  2.11
H
ATOM  14594 3HB  ALA B 335     -31.318  17.811  -4.203  1.00  2.11
H
ATOM  14595  N   ASN B 336     -31.578  15.251  -5.605  1.00  1.00
N
ATOM  14596  CA  ASN B 336     -31.183  14.599  -6.848  1.00  1.06
C
```

ATOM 14597 C ASN B 336 -30.280 13.378 -6.692 1.00 0.92
C
ATOM 14598 O ASN B 336 -29.267 13.402 -5.988 1.00 1.21
O
ATOM 14599 CB ASN B 336 -30.529 15.607 -7.759 1.00 1.59
C
ATOM 14600 CG ASN B 336 -30.357 15.087 -9.147 1.00 1.59
C
ATOM 14601 OD1 ASN B 336 -29.411 14.357 -9.464 1.00 1.59
O
ATOM 14602 ND2 ASN B 336 -31.285 15.439 -9.999 1.00 1.59
N
ATOM 14603 H ASN B 336 -30.996 15.220 -4.775 1.00 1.20
H
ATOM 14604 HA ASN B 336 -32.091 14.247 -7.339 1.00 1.27
H
ATOM 14605 1HB ASN B 336 -31.137 16.512 -7.795 1.00 1.91
H
ATOM 14606 2HB ASN B 336 -29.553 15.882 -7.358 1.00 1.91
H
ATOM 14607 1HD2 ASN B 336 -31.248 15.115 -10.945 1.00 1.91
H
ATOM 14608 2HD2 ASN B 336 -32.041 16.022 -9.701 1.00 1.91
H
ATOM 14609 N GLU B 337 -30.679 12.320 -7.404 1.00 0.77
N
ATOM 14610 CA GLU B 337 -30.049 11.001 -7.443 1.00 0.75
C
ATOM 14611 C GLU B 337 -28.571 10.943 -7.840 1.00 0.53
C
ATOM 14612 O GLU B 337 -27.902 9.942 -7.567 1.00 0.70
O
ATOM 14613 CB GLU B 337 -30.835 10.133 -8.428 1.00 1.12
C
ATOM 14614 CG GLU B 337 -30.823 10.632 -9.871 1.00 1.12
C
ATOM 14615 CD GLU B 337 -31.886 11.658 -10.151 1.00 1.12
C
ATOM 14616 OE1 GLU B 337 -32.410 12.215 -9.218 1.00 1.12
O
ATOM 14617 OE2 GLU B 337 -32.175 11.884 -11.303 1.00 1.12
O
ATOM 14618 H GLU B 337 -31.518 12.437 -7.955 1.00 0.92
H
ATOM 14619 HA GLU B 337 -30.138 10.569 -6.446 1.00 0.90
H
ATOM 14620 1HB GLU B 337 -30.418 9.133 -8.445 1.00 1.35
H
ATOM 14621 2HB GLU B 337 -31.873 10.054 -8.103 1.00 1.35
H
ATOM 14622 1HG GLU B 337 -29.851 11.063 -10.098 1.00 1.35
H
ATOM 14623 2HG GLU B 337 -30.970 9.781 -10.534 1.00 1.35
H
ATOM 14624 N ARG B 338 -28.051 12.002 -8.457 1.00 0.49
N
ATOM 14625 CA ARG B 338 -26.657 12.027 -8.863 1.00 0.50
C

```
ATOM  14626  C   ARG B 338     -25.702  12.285  -7.715  1.00  0.53
C
ATOM  14627  O   ARG B 338     -24.499  12.108  -7.869  1.00  0.66
O
ATOM  14628  CB  ARG B 338     -26.433  13.077  -9.939  1.00  0.75
C
ATOM  14629  CG  ARG B 338     -27.066  12.763 -11.285  1.00  0.75
C
ATOM  14630  CD  ARG B 338     -26.994  13.923 -12.208  1.00  0.75
C
ATOM  14631  NE  ARG B 338     -27.884  14.983 -11.774  1.00  0.75
N
ATOM  14632  CZ  ARG B 338     -27.919  16.237 -12.270  1.00  0.75
C
ATOM  14633  NH1 ARG B 338     -27.101  16.629 -13.218  1.00  0.75
N
ATOM  14634  NH2 ARG B 338     -28.791  17.108 -11.810  1.00  0.75
N
ATOM  14635  H   ARG B 338     -28.627  12.809  -8.669  1.00  0.59
H
ATOM  14636  HA  ARG B 338     -26.415  11.054  -9.290  1.00  0.60
H
ATOM  14637 1HB  ARG B 338     -26.830  14.034  -9.603  1.00  0.90
H
ATOM  14638 2HB  ARG B 338     -25.364  13.206 -10.104  1.00  0.90
H
ATOM  14639 1HG  ARG B 338     -26.546  11.923 -11.746  1.00  0.90
H
ATOM  14640 2HG  ARG B 338     -28.117  12.505 -11.141  1.00  0.90
H
ATOM  14641 1HD  ARG B 338     -25.977  14.315 -12.232  1.00  0.90
H
ATOM  14642 2HD  ARG B 338     -27.292  13.613 -13.207  1.00  0.90
H
ATOM  14643  HE  ARG B 338     -28.545  14.751 -11.039  1.00  0.90
H
ATOM  14644 1HH1 ARG B 338     -26.424  15.988 -13.608  1.00  0.90
H
ATOM  14645 2HH1 ARG B 338     -27.174  17.583 -13.554  1.00  0.90
H
ATOM  14646 1HH2 ARG B 338     -29.439  16.845 -11.082  1.00  0.90
H
ATOM  14647 2HH2 ARG B 338     -28.800  18.042 -12.194  1.00  0.90
H
ATOM  14648  N   VAL B 339     -26.201  12.715  -6.569  1.00  0.57
N
ATOM  14649  CA  VAL B 339     -25.287  13.020  -5.485  1.00  0.84
C
ATOM  14650  C   VAL B 339     -24.894  11.815  -4.642  1.00  0.60
C
ATOM  14651  O   VAL B 339     -25.738  11.146  -4.049  1.00  0.90
O
ATOM  14652  CB  VAL B 339     -25.897  14.110  -4.616  1.00  1.26
C
ATOM  14653  CG1 VAL B 339     -25.005  14.395  -3.416  1.00  1.26
C
ATOM  14654  CG2 VAL B 339     -26.064  15.336  -5.501  1.00  1.26
C
```

```
ATOM  14655  H   VAL B 339     -27.200  12.849  -6.438  1.00  0.68
H
ATOM  14656  HA  VAL B 339     -24.380  13.429  -5.927  1.00  1.01
H
ATOM  14657  HB  VAL B 339     -26.868  13.783  -4.240  1.00  1.51
H
ATOM  14658 1HG1 VAL B 339     -25.453  15.176  -2.807  1.00  1.51
H
ATOM  14659 2HG1 VAL B 339     -24.897  13.490  -2.819  1.00  1.51
H
ATOM  14660 3HG1 VAL B 339     -24.025  14.719  -3.763  1.00  1.51
H
ATOM  14661 1HG2 VAL B 339     -26.509  16.150  -4.948  1.00  1.51
H
ATOM  14662 2HG2 VAL B 339     -25.089  15.646  -5.874  1.00  1.51
H
ATOM  14663 3HG2 VAL B 339     -26.710  15.085  -6.342  1.00  1.51
H
ATOM  14664  N   ILE B 340     -23.585  11.568  -4.586  1.00  0.33
N
ATOM  14665  CA  ILE B 340     -23.030  10.492  -3.772  1.00  0.21
C
ATOM  14666  C   ILE B 340     -22.001  11.085  -2.823  1.00  0.36
C
ATOM  14667  O   ILE B 340     -21.412  12.127  -3.111  1.00  1.22
O
ATOM  14668  CB  ILE B 340     -22.351   9.391  -4.621  1.00  0.32
C
ATOM  14669  CG1 ILE B 340     -21.169   9.981  -5.388  1.00  0.32
C
ATOM  14670  CG2 ILE B 340     -23.348   8.754  -5.581  1.00  0.32
C
ATOM  14671  CD1 ILE B 340     -20.310   8.942  -6.066  1.00  0.32
C
ATOM  14672  H   ILE B 340     -22.958  12.156  -5.118  1.00  0.40
H
ATOM  14673  HA  ILE B 340     -23.826  10.042  -3.183  1.00  0.25
H
ATOM  14674  HB  ILE B 340     -21.956   8.622  -3.960  1.00  0.38
H
ATOM  14675 1HG1 ILE B 340     -21.544  10.670  -6.145  1.00  0.38
H
ATOM  14676 2HG1 ILE B 340     -20.542  10.540  -4.693  1.00  0.38
H
ATOM  14677 1HG2 ILE B 340     -22.850   7.978  -6.159  1.00  0.38
H
ATOM  14678 2HG2 ILE B 340     -24.168   8.315  -5.019  1.00  0.38
H
ATOM  14679 3HG2 ILE B 340     -23.739   9.513  -6.257  1.00  0.38
H
ATOM  14680 1HD1 ILE B 340     -19.490   9.433  -6.589  1.00  0.38
H
ATOM  14681 2HD1 ILE B 340     -19.908   8.259  -5.318  1.00  0.38
H
ATOM  14682 3HD1 ILE B 340     -20.913   8.384  -6.781  1.00  0.38
H
ATOM  14683  N   VAL B 341     -21.790  10.415  -1.697  1.00  0.26
N
```

ATOM 14684 CA VAL B 341 -20.867 10.907 -0.678 1.00 0.30
C
ATOM 14685 C VAL B 341 -19.864 9.839 -0.278 1.00 0.30
C
ATOM 14686 O VAL B 341 -20.224 8.675 -0.127 1.00 0.46
O
ATOM 14687 CB VAL B 341 -21.641 11.415 0.566 1.00 0.45
C
ATOM 14688 CG1 VAL B 341 -20.678 11.857 1.659 1.00 0.45
C
ATOM 14689 CG2 VAL B 341 -22.525 12.595 0.175 1.00 0.45
C
ATOM 14690 H VAL B 341 -22.292 9.547 -1.549 1.00 0.31
H
ATOM 14691 HA VAL B 341 -20.323 11.748 -1.098 1.00 0.36
H
ATOM 14692 HB VAL B 341 -22.251 10.608 0.962 1.00 0.54
H
ATOM 14693 1HG1 VAL B 341 -21.245 12.203 2.523 1.00 0.54
H
ATOM 14694 2HG1 VAL B 341 -20.048 11.019 1.955 1.00 0.54
H
ATOM 14695 3HG1 VAL B 341 -20.053 12.668 1.290 1.00 0.54
H
ATOM 14696 1HG2 VAL B 341 -23.068 12.944 1.052 1.00 0.54
H
ATOM 14697 2HG2 VAL B 341 -21.905 13.402 -0.209 1.00 0.54
H
ATOM 14698 3HG2 VAL B 341 -23.237 12.288 -0.589 1.00 0.54
H
ATOM 14699 N LEU B 342 -18.602 10.241 -0.134 1.00 0.22
N
ATOM 14700 CA LEU B 342 -17.525 9.323 0.211 1.00 0.20
C
ATOM 14701 C LEU B 342 -16.870 9.753 1.523 1.00 0.21
C
ATOM 14702 O LEU B 342 -16.762 10.947 1.788 1.00 0.31
O
ATOM 14703 CB LEU B 342 -16.448 9.333 -0.890 1.00 0.30
C
ATOM 14704 CG LEU B 342 -16.887 8.942 -2.309 1.00 0.30
C
ATOM 14705 CD1 LEU B 342 -17.454 10.162 -3.023 1.00 0.30
C
ATOM 14706 CD2 LEU B 342 -15.690 8.381 -3.064 1.00 0.30
C
ATOM 14707 H LEU B 342 -18.393 11.223 -0.268 1.00 0.26
H
ATOM 14708 HA LEU B 342 -17.934 8.323 0.326 1.00 0.24
H
ATOM 14709 1HB LEU B 342 -16.026 10.331 -0.948 1.00 0.36
H
ATOM 14710 2HB LEU B 342 -15.655 8.648 -0.597 1.00 0.36
H
ATOM 14711 HG LEU B 342 -17.674 8.195 -2.256 1.00 0.36
H
ATOM 14712 1HD1 LEU B 342 -17.770 9.880 -4.027 1.00 0.36
H

ATOM 14713 2HD1 LEU B 342   -18.307 10.553 -2.474 1.00 0.36
H
ATOM 14714 3HD1 LEU B 342   -16.686 10.932 -3.088 1.00 0.36
H
ATOM 14715 1HD2 LEU B 342   -15.997   8.099 -4.072 1.00 0.36
H
ATOM 14716 2HD2 LEU B 342   -14.907   9.138 -3.121 1.00 0.36
H
ATOM 14717 3HD2 LEU B 342   -15.308   7.503 -2.543 1.00 0.36
H
ATOM 14718 N   SER B 343   -16.402   8.798   2.330 1.00 0.20
N
ATOM 14719 CA SER B 343   -15.712   9.174   3.576 1.00 0.26
C
ATOM 14720 C   SER B 343   -14.617   8.218   3.990 1.00 0.30
C
ATOM 14721 O   SER B 343   -14.789   6.998   3.981 1.00 0.31
O
ATOM 14722 CB SER B 343   -16.665   9.263   4.749 1.00 0.39
C
ATOM 14723 OG SER B 343   -15.964   9.614   5.921 1.00 0.39
O
ATOM 14724 H   SER B 343   -16.541   7.826   2.078 1.00 0.24
H
ATOM 14725 HA SER B 343   -15.260 10.153   3.424 1.00 0.31
H
ATOM 14726 1HB SER B 343   -17.440 10.001   4.542 1.00 0.47
H
ATOM 14727 2HB SER B 343   -17.149   8.299   4.888 1.00 0.47
H
ATOM 14728 HG SER B 343   -15.685 10.526   5.792 1.00 0.47
H
ATOM 14729 N   GLY B 344   -13.498   8.787   4.429 1.00 0.38
N
ATOM 14730 CA GLY B 344   -12.341   7.993   4.865 1.00 0.38
C
ATOM 14731 C   GLY B 344   -12.425   7.559   6.337 1.00 0.32
C
ATOM 14732 O   GLY B 344   -11.537   7.874   7.128 1.00 0.37
O
ATOM 14733 H   GLY B 344   -13.449   9.805   4.426 1.00 0.46
H
ATOM 14734 1HA GLY B 344   -12.263   7.113   4.231 1.00 0.46
H
ATOM 14735 2HA GLY B 344   -11.431   8.573   4.710 1.00 0.46
H
ATOM 14736 N   ASP B 345   -13.500   6.845   6.687 1.00 0.27
N
ATOM 14737 CA ASP B 345   -13.777   6.414   8.066 1.00 0.25
C
ATOM 14738 C   ASP B 345   -13.865   7.602   9.024 1.00 0.34
C
ATOM 14739 O   ASP B 345   -13.395   7.520 10.159 1.00 0.53
O
ATOM 14740 CB ASP B 345   -12.698   5.423   8.562 1.00 0.38
C
ATOM 14741 CG ASP B 345   -13.079   4.678   9.847 1.00 0.38
C

ATOM	14742	OD1	ASP	B	345	-14.241	4.647	10.175	1.00	0.38	O
ATOM	14743	OD2	ASP	B	345	-12.205	4.140	10.492	1.00	0.38	O
ATOM	14744	H	ASP	B	345	-14.154	6.606	5.953	1.00	0.32	H
ATOM	14745	HA	ASP	B	345	-14.740	5.902	8.076	1.00	0.30	H
ATOM	14746	1HB	ASP	B	345	-12.497	4.690	7.788	1.00	0.45	H
ATOM	14747	2HB	ASP	B	345	-11.768	5.964	8.744	1.00	0.45	H
ATOM	14748	N	THR	B	346	-14.489	8.700	8.569	1.00	0.40	N
ATOM	14749	CA	THR	B	346	-14.636	9.906	9.382	1.00	0.43	C
ATOM	14750	C	THR	B	346	-16.076	10.397	9.424	1.00	0.33	C
ATOM	14751	O	THR	B	346	-16.326	11.532	9.834	1.00	0.34	O
ATOM	14752	CB	THR	B	346	-13.756	11.055	8.839	1.00	0.65	C
ATOM	14753	OG1	THR	B	346	-14.127	11.315	7.479	1.00	0.65	O
ATOM	14754	CG2	THR	B	346	-12.274	10.735	8.915	1.00	0.65	C
ATOM	14755	H	THR	B	346	-14.877	8.723	7.626	1.00	0.48	H
ATOM	14756	HA	THR	B	346	-14.324	9.677	10.401	1.00	0.52	H
ATOM	14757	HB	THR	B	346	-13.946	11.950	9.429	1.00	0.77	H
ATOM	14758	HG1	THR	B	346	-14.011	10.513	6.962	1.00	0.77	H
ATOM	14759	1HG2	THR	B	346	-11.701	11.576	8.528	1.00	0.77	H
ATOM	14760	2HG2	THR	B	346	-11.995	10.553	9.954	1.00	0.77	H
ATOM	14761	3HG2	THR	B	346	-12.062	9.851	8.324	1.00	0.77	H
ATOM	14762	N	MET	B	347	-17.010	9.561	8.974	1.00	0.31	N
ATOM	14763	CA	MET	B	347	-18.420	9.932	8.839	1.00	0.30	C
ATOM	14764	C	MET	B	347	-19.100	10.433	10.118	1.00	0.31	C
ATOM	14765	O	MET	B	347	-20.073	11.183	10.042	1.00	0.32	O
ATOM	14766	CB	MET	B	347	-19.188	8.733	8.296	1.00	0.45	C
ATOM	14767	CG	MET	B	347	-19.304	7.593	9.288	1.00	0.45	C
ATOM	14768	SD	MET	B	347	-20.244	6.199	8.663	1.00	0.45	S
ATOM	14769	CE	MET	B	347	-19.066	5.495	7.517	1.00	0.45	C
ATOM	14770	H	MET	B	347	-16.736	8.633	8.685	1.00	0.37	H

```
ATOM  14771  HA  MET B 347     -18.478  10.737   8.110  1.00  0.36
H
ATOM  14772 1HB  MET B 347     -20.189   9.031   8.002  1.00  0.54
H
ATOM  14773 2HB  MET B 347     -18.684   8.351   7.408  1.00  0.54
H
ATOM  14774 1HG  MET B 347     -18.303   7.250   9.532  1.00  0.54
H
ATOM  14775 2HG  MET B 347     -19.781   7.941  10.202  1.00  0.54
H
ATOM  14776 1HE  MET B 347     -19.505   4.618   7.039  1.00  0.54
H
ATOM  14777 2HE  MET B 347     -18.809   6.235   6.759  1.00  0.54
H
ATOM  14778 3HE  MET B 347     -18.166   5.202   8.054  1.00  0.54
H
ATOM  14779  N   ASN B 348     -18.598  10.030  11.286  1.00  0.33
N
ATOM  14780  CA  ASN B 348     -19.187  10.447  12.552  1.00  0.38
C
ATOM  14781  C   ASN B 348     -18.514  11.688  13.138  1.00  0.38
C
ATOM  14782  O   ASN B 348     -18.818  12.097  14.260  1.00  0.45
O
ATOM  14783  CB  ASN B 348     -19.124   9.296  13.538  1.00  0.57
C
ATOM  14784  CG  ASN B 348     -17.708   8.925  13.889  1.00  0.57
C
ATOM  14785  OD1 ASN B 348     -16.759   9.468  13.307  1.00  0.57
O
ATOM  14786  ND2 ASN B 348     -17.548   8.021  14.821  1.00  0.57
N
ATOM  14787  H   ASN B 348     -17.802   9.411  11.300  1.00  0.40
H
ATOM  14788  HA  ASN B 348     -20.235  10.697  12.378  1.00  0.46
H
ATOM  14789 1HB  ASN B 348     -19.655   9.568  14.451  1.00  0.68
H
ATOM  14790 2HB  ASN B 348     -19.626   8.425  13.114  1.00  0.68
H
ATOM  14791 1HD2 ASN B 348     -16.630   7.738  15.097  1.00  0.68
H
ATOM  14792 2HD2 ASN B 348     -18.348   7.613  15.261  1.00  0.68
H
ATOM  14793  N   SER B 349     -17.624  12.301  12.365  1.00  0.33
N
ATOM  14794  CA  SER B 349     -16.914  13.494  12.793  1.00  0.31
C
ATOM  14795  C   SER B 349     -17.142  14.604  11.775  1.00  0.30
C
ATOM  14796  O   SER B 349     -17.261  15.776  12.131  1.00  0.49
O
ATOM  14797  CB  SER B 349     -15.447  13.178  12.939  1.00  0.46
C
ATOM  14798  OG  SER B 349     -14.891  12.846  11.701  1.00  0.46
O
ATOM  14799  H   SER B 349     -17.399  11.922  11.459  1.00  0.40
H
```

ATOM 14800 HA SER B 349 -17.310 13.818 13.756 1.00 0.37
H
ATOM 14801 1HB SER B 349 -14.922 14.019 13.379 1.00 0.56
H
ATOM 14802 2HB SER B 349 -15.336 12.336 13.622 1.00 0.56
H
ATOM 14803 HG SER B 349 -15.501 12.224 11.293 1.00 0.56
H
ATOM 14804 N THR B 350 -17.252 14.207 10.505 1.00 0.27
N
ATOM 14805 CA THR B 350 -17.573 15.114 9.414 1.00 0.26
C
ATOM 14806 C THR B 350 -19.075 15.227 9.271 1.00 0.24
C
ATOM 14807 O THR B 350 -19.571 16.099 8.557 1.00 0.29
O
ATOM 14808 CB THR B 350 -17.047 14.602 8.066 1.00 0.39
C
ATOM 14809 OG1 THR B 350 -17.713 13.365 7.757 1.00 0.39
O
ATOM 14810 CG2 THR B 350 -15.545 14.375 8.113 1.00 0.39
C
ATOM 14811 H THR B 350 -17.098 13.236 10.279 1.00 0.32
H
ATOM 14812 HA THR B 350 -17.162 16.100 9.628 1.00 0.31
H
ATOM 14813 HB THR B 350 -17.276 15.332 7.290 1.00 0.47
H
ATOM 14814 HG1 THR B 350 -17.369 12.675 8.333 1.00 0.47
H
ATOM 14815 1HG2 THR B 350 -15.201 14.006 7.146 1.00 0.47
H
ATOM 14816 2HG2 THR B 350 -15.043 15.312 8.340 1.00 0.47
H
ATOM 14817 3HG2 THR B 350 -15.309 13.643 8.883 1.00 0.47
H
ATOM 14818 N PHE B 351 -19.792 14.306 9.925 1.00 0.23
N
ATOM 14819 CA PHE B 351 -21.243 14.198 9.861 1.00 0.24
C
ATOM 14820 C PHE B 351 -21.758 13.730 8.503 1.00 0.32
C
ATOM 14821 O PHE B 351 -22.959 13.802 8.238 1.00 0.39
O
ATOM 14822 CB PHE B 351 -21.914 15.485 10.324 1.00 0.36
C
ATOM 14823 CG PHE B 351 -21.654 15.785 11.772 1.00 0.36
C
ATOM 14824 CD1 PHE B 351 -20.615 16.614 12.154 1.00 0.36
C
ATOM 14825 CD2 PHE B 351 -22.473 15.255 12.754 1.00 0.36
C
ATOM 14826 CE1 PHE B 351 -20.391 16.905 13.485 1.00 0.36
C
ATOM 14827 CE2 PHE B 351 -22.254 15.543 14.086 1.00 0.36
C
ATOM 14828 CZ PHE B 351 -21.211 16.370 14.451 1.00 0.36
C

```
ATOM  14829  H   PHE B 351     -19.313  13.629  10.497  1.00  0.28
H
ATOM  14830  HA  PHE B 351     -21.537  13.436  10.585  1.00  0.29
H
ATOM  14831 1HB  PHE B 351     -21.577  16.333   9.731  1.00  0.43
H
ATOM  14832 2HB  PHE B 351     -22.986  15.400  10.186  1.00  0.43
H
ATOM  14833  HD1 PHE B 351     -19.973  17.036  11.387  1.00  0.43
H
ATOM  14834  HD2 PHE B 351     -23.299  14.606  12.465  1.00  0.43
H
ATOM  14835  HE1 PHE B 351     -19.567  17.559  13.770  1.00  0.43
H
ATOM  14836  HE2 PHE B 351     -22.906  15.120  14.850  1.00  0.43
H
ATOM  14837  HZ  PHE B 351     -21.038  16.598  15.502  1.00  0.43
H
ATOM  14838  N   SER B 352     -20.859  13.160   7.679  1.00  0.35
N
ATOM  14839  CA  SER B 352     -21.224  12.562   6.394  1.00  0.45
C
ATOM  14840  C   SER B 352     -22.201  11.398   6.619  1.00  0.36
C
ATOM  14841  O   SER B 352     -23.012  11.075   5.749  1.00  0.47
O
ATOM  14842  CB  SER B 352     -19.982  12.071   5.679  1.00  0.68
C
ATOM  14843  OG  SER B 352     -19.120  13.133   5.377  1.00  0.68
O
ATOM  14844  H   SER B 352     -19.871  13.151   7.921  1.00  0.42
H
ATOM  14845  HA  SER B 352     -21.717  13.318   5.782  1.00  0.54
H
ATOM  14846 1HB  SER B 352     -19.464  11.330   6.286  1.00  0.81
H
ATOM  14847 2HB  SER B 352     -20.280  11.587   4.754  1.00  0.81
H
ATOM  14848  HG  SER B 352     -18.639  13.309   6.195  1.00  0.81
H
ATOM  14849  N   GLU B 353     -22.110  10.792   7.811  1.00  0.36
N
ATOM  14850  CA  GLU B 353     -22.990   9.733   8.292  1.00  0.41
C
ATOM  14851  C   GLU B 353     -24.469  10.076   8.149  1.00  0.44
C
ATOM  14852  O   GLU B 353     -25.289   9.177   7.982  1.00  0.49
O
ATOM  14853  CB  GLU B 353     -22.690   9.424   9.753  1.00  0.61
C
ATOM  14854  CG  GLU B 353     -23.477   8.265  10.329  1.00  0.61
C
ATOM  14855  CD  GLU B 353     -23.087   7.952  11.747  1.00  0.61
C
ATOM  14856  OE1 GLU B 353     -22.196   8.589  12.255  1.00  0.61
O
ATOM  14857  OE2 GLU B 353     -23.688   7.077  12.327  1.00  0.61
O
```

```
ATOM  14858  H   GLU B 353     -21.385  11.090   8.451  1.00  0.43
H
ATOM  14859  HA  GLU B 353     -22.792   8.838   7.704  1.00  0.49
H
ATOM  14860 1HB  GLU B 353     -21.637   9.187   9.867  1.00  0.74
H
ATOM  14861 2HB  GLU B 353     -22.894  10.304  10.362  1.00  0.74
H
ATOM  14862 1HG  GLU B 353     -24.537   8.513  10.298  1.00  0.74
H
ATOM  14863 2HG  GLU B 353     -23.319   7.384   9.707  1.00  0.74
H
ATOM  14864  N   ILE B 354     -24.818  11.360   8.272  1.00  0.43
N
ATOM  14865  CA  ILE B 354     -26.203  11.786   8.142  1.00  0.47
C
ATOM  14866  C   ILE B 354     -26.712  11.405   6.749  1.00  0.46
C
ATOM  14867  O   ILE B 354     -27.795  10.828   6.614  1.00  0.58
O
ATOM  14868  CB  ILE B 354     -26.325  13.306   8.375  1.00  0.70
C
ATOM  14869  CG1 ILE B 354     -26.006  13.640   9.838  1.00  0.70
C
ATOM  14870  CG2 ILE B 354     -27.721  13.780   8.023  1.00  0.70
C
ATOM  14871  CD1 ILE B 354     -25.823  15.118  10.102  1.00  0.70
C
ATOM  14872  H   ILE B 354     -24.112  12.071   8.421  1.00  0.52
H
ATOM  14873  HA  ILE B 354     -26.805  11.267   8.887  1.00  0.56
H
ATOM  14874  HB  ILE B 354     -25.599  13.826   7.751  1.00  0.85
H
ATOM  14875 1HG1 ILE B 354     -26.815  13.275  10.470  1.00  0.85
H
ATOM  14876 2HG1 ILE B 354     -25.089  13.126  10.126  1.00  0.85
H
ATOM  14877 1HG2 ILE B 354     -27.789  14.854   8.179  1.00  0.85
H
ATOM  14878 2HG2 ILE B 354     -27.925  13.549   6.984  1.00  0.85
H
ATOM  14879 3HG2 ILE B 354     -28.449  13.273   8.656  1.00  0.85
H
ATOM  14880 1HD1 ILE B 354     -25.600  15.277  11.157  1.00  0.85
H
ATOM  14881 2HD1 ILE B 354     -25.003  15.498   9.495  1.00  0.85
H
ATOM  14882 3HD1 ILE B 354     -26.738  15.645   9.842  1.00  0.85
H
ATOM  14883  N   PHE B 355     -25.908  11.717   5.728  1.00  0.40
N
ATOM  14884  CA  PHE B 355     -26.197  11.331   4.352  1.00  0.43
C
ATOM  14885  C   PHE B 355     -26.261   9.819   4.240  1.00  0.44
C
ATOM  14886  O   PHE B 355     -27.163   9.287   3.609  1.00  0.50
O
```

```
ATOM  14887  CB  PHE B 355     -25.143  11.833   3.374  1.00  0.65
C
ATOM  14888  CG  PHE B 355     -25.425  11.420   1.954  1.00  0.65
C
ATOM  14889  CD1 PHE B 355     -26.137  12.242   1.097  1.00  0.65
C
ATOM  14890  CD2 PHE B 355     -24.997  10.193   1.478  1.00  0.65
C
ATOM  14891  CE1 PHE B 355     -26.393  11.859  -0.208  1.00  0.65
C
ATOM  14892  CE2 PHE B 355     -25.253   9.805   0.181  1.00  0.65
C
ATOM  14893  CZ  PHE B 355     -25.948  10.640  -0.667  1.00  0.65
C
ATOM  14894  H   PHE B 355     -25.049  12.217   5.916  1.00  0.48
H
ATOM  14895  HA  PHE B 355     -27.168  11.741   4.069  1.00  0.52
H
ATOM  14896 1HB  PHE B 355     -25.096  12.920   3.413  1.00  0.77
H
ATOM  14897 2HB  PHE B 355     -24.165  11.450   3.651  1.00  0.77
H
ATOM  14898  HD1 PHE B 355     -26.487  13.203   1.460  1.00  0.77
H
ATOM  14899  HD2 PHE B 355     -24.452   9.529   2.145  1.00  0.77
H
ATOM  14900  HE1 PHE B 355     -26.946  12.522  -0.871  1.00  0.77
H
ATOM  14901  HE2 PHE B 355     -24.906   8.839  -0.171  1.00  0.77
H
ATOM  14902  HZ  PHE B 355     -26.149  10.335  -1.692  1.00  0.77
H
ATOM  14903  N   ARG B 356     -25.275   9.132   4.828  1.00  0.42
N
ATOM  14904  CA  ARG B 356     -25.224   7.668   4.815  1.00  0.49
C
ATOM  14905  C   ARG B 356     -26.529   7.046   5.300  1.00  0.57
C
ATOM  14906  O   ARG B 356     -27.032   6.100   4.703  1.00  0.70
O
ATOM  14907  CB  ARG B 356     -24.093   7.166   5.692  1.00  0.73
C
ATOM  14908  CG  ARG B 356     -23.896   5.663   5.721  1.00  0.73
C
ATOM  14909  CD  ARG B 356     -22.802   5.312   6.656  1.00  0.73
C
ATOM  14910  NE  ARG B 356     -22.506   3.889   6.690  1.00  0.73
N
ATOM  14911  CZ  ARG B 356     -23.166   2.975   7.425  1.00  0.73
C
ATOM  14912  NH1 ARG B 356     -24.192   3.324   8.170  1.00  0.73
N
ATOM  14913  NH2 ARG B 356     -22.761   1.718   7.386  1.00  0.73
N
ATOM  14914  H   ARG B 356     -24.542   9.648   5.303  1.00  0.50
H
ATOM  14915  HA  ARG B 356     -25.044   7.340   3.792  1.00  0.59
H
```

ATOM 14916 1HB ARG B 356   -23.156   7.618   5.377 1.00 0.88
H
ATOM 14917 2HB ARG B 356   -24.269   7.468   6.720 1.00 0.88
H
ATOM 14918 1HG ARG B 356   -24.811   5.177   6.061 1.00 0.88
H
ATOM 14919 2HG ARG B 356   -23.636   5.300   4.726 1.00 0.88
H
ATOM 14920 1HD ARG B 356   -21.898   5.838   6.358 1.00 0.88
H
ATOM 14921 2HD ARG B 356   -23.080   5.622   7.662 1.00 0.88
H
ATOM 14922 HE ARG B 356   -21.719   3.545   6.148 1.00 0.88
H
ATOM 14923 1HH1 ARG B 356   -24.492   4.289   8.195 1.00 0.88
H
ATOM 14924 2HH1 ARG B 356   -24.677   2.629   8.717 1.00 0.88
H
ATOM 14925 1HH2 ARG B 356   -21.954   1.485   6.812 1.00 0.88
H
ATOM 14926 2HH2 ARG B 356   -23.232   1.009   7.925 1.00 0.88
H
ATOM 14927 N   LYS B 357   -27.071   7.566   6.397 1.00 0.54
N
ATOM 14928 CA LYS B 357   -28.328   7.067   6.933 1.00 0.64
C
ATOM 14929 C   LYS B 357   -29.506   7.360   6.007 1.00 0.66
C
ATOM 14930 O   LYS B 357   -30.392   6.521   5.838 1.00 0.72
O
ATOM 14931 CB LYS B 357   -28.584   7.675   8.311 1.00 0.96
C
ATOM 14932 CG LYS B 357   -27.658   7.156   9.400 1.00 0.96
C
ATOM 14933 CD LYS B 357   -27.947   7.818 10.737 1.00 0.96
C
ATOM 14934 CE LYS B 357   -27.043   7.262 11.825 1.00 0.96
C
ATOM 14935 NZ LYS B 357   -27.261   7.932 13.134 1.00 0.96
N
ATOM 14936 H   LYS B 357   -26.597   8.322   6.873 1.00 0.65
H
ATOM 14937 HA LYS B 357   -28.250   5.985   7.039 1.00 0.77
H
ATOM 14938 1HB LYS B 357   -28.467   8.757   8.259 1.00 1.15
H
ATOM 14939 2HB LYS B 357   -29.610   7.469   8.616 1.00 1.15
H
ATOM 14940 1HG LYS B 357   -27.788   6.078   9.503 1.00 1.15
H
ATOM 14941 2HG LYS B 357   -26.623   7.352   9.123 1.00 1.15
H
ATOM 14942 1HD LYS B 357   -27.784   8.893 10.655 1.00 1.15
H
ATOM 14943 2HD LYS B 357   -28.986   7.643 11.016 1.00 1.15
H
ATOM 14944 1HE LYS B 357   -27.235   6.196 11.938 1.00 1.15
H

```
ATOM  14945 2HE  LYS B 357     -26.008   7.400  11.527  1.00  1.15
H
ATOM  14946 1HZ  LYS B 357     -26.635   7.532  13.820  1.00  1.15
H
ATOM  14947 2HZ  LYS B 357     -27.069   8.919  13.043  1.00  1.15
H
ATOM  14948 3HZ  LYS B 357     -28.216   7.799  13.430  1.00  1.15
H
ATOM  14949  N   GLU B 358     -29.502   8.544   5.395  1.00  0.64
N
ATOM  14950  CA  GLU B 358     -30.562   8.954   4.484  1.00  0.73
C
ATOM  14951  C   GLU B 358     -30.541   8.193   3.151  1.00  0.79
C
ATOM  14952  O   GLU B 358     -31.579   7.736   2.669  1.00  1.08
O
ATOM  14953  CB  GLU B 358     -30.433  10.454   4.213  1.00  1.09
C
ATOM  14954  CG  GLU B 358     -31.506  11.029   3.315  1.00  1.09
C
ATOM  14955  CD  GLU B 358     -32.874  11.043   3.921  1.00  1.09
C
ATOM  14956  OE1 GLU B 358     -33.002  11.058   5.120  1.00  1.09
O
ATOM  14957  OE2 GLU B 358     -33.817  11.084   3.168  1.00  1.09
O
ATOM  14958  H   GLU B 358     -28.755   9.203   5.593  1.00  0.77
H
ATOM  14959  HA  GLU B 358     -31.521   8.763   4.968  1.00  0.88
H
ATOM  14960 1HB  GLU B 358     -30.463  10.995   5.160  1.00  1.31
H
ATOM  14961 2HB  GLU B 358     -29.468  10.657   3.752  1.00  1.31
H
ATOM  14962 1HG  GLU B 358     -31.230  12.045   3.034  1.00  1.31
H
ATOM  14963 2HG  GLU B 358     -31.546  10.429   2.412  1.00  1.31
H
ATOM  14964  N   HIS B 359     -29.350   8.064   2.570  1.00  0.66
N
ATOM  14965  CA  HIS B 359     -29.120   7.386   1.297  1.00  0.75
C
ATOM  14966  C   HIS B 359     -27.880   6.508   1.313  1.00  0.71
C
ATOM  14967  O   HIS B 359     -26.903   6.831   0.637  1.00  0.78
O
ATOM  14968  CB  HIS B 359     -28.921   8.385   0.154  1.00  1.12
C
ATOM  14969  CG  HIS B 359     -30.113   9.211  -0.153  1.00  1.12
C
ATOM  14970  ND1 HIS B 359     -31.237   8.679  -0.743  1.00  1.12
N
ATOM  14971  CD2 HIS B 359     -30.362  10.529   0.018  1.00  1.12
C
ATOM  14972  CE1 HIS B 359     -32.138   9.632  -0.906  1.00  1.12
C
ATOM  14973  NE2 HIS B 359     -31.632  10.763  -0.450  1.00  1.12
N
```

```
ATOM  14974  H   HIS B 359     -28.555   8.464   3.029  1.00  0.79
H
ATOM  14975  HA  HIS B 359     -29.978   6.767   1.045  1.00  0.90
H
ATOM  14976 1HB  HIS B 359     -28.102   9.056   0.407  1.00  1.35
H
ATOM  14977 2HB  HIS B 359     -28.638   7.848  -0.751  1.00  1.35
H
ATOM  14978  HD1 HIS B 359     -31.322   7.729  -1.066  1.00  1.35
H
ATOM  14979  HD2 HIS B 359     -29.772  11.347   0.434  1.00  1.35
H
ATOM  14980  HE1 HIS B 359     -33.106   9.411  -1.353  1.00  1.35
H
ATOM  14981  N   PRO B 360     -27.891   5.390   2.046  1.00  0.70
N
ATOM  14982  CA  PRO B 360     -26.799   4.432   2.191  1.00  0.81
C
ATOM  14983  C   PRO B 360     -26.406   3.823   0.850  1.00  1.02
C
ATOM  14984  O   PRO B 360     -25.241   3.491   0.628  1.00  2.07
O
ATOM  14985  CB  PRO B 360     -27.384   3.363   3.124  1.00  1.22
C
ATOM  14986  CG  PRO B 360     -28.865   3.475   2.964  1.00  1.22
C
ATOM  14987  CD  PRO B 360     -29.127   4.930   2.705  1.00  1.22
C
ATOM  14988  HA  PRO B 360     -25.937   4.933   2.657  1.00  0.97
H
ATOM  14989 1HB  PRO B 360     -27.001   2.370   2.844  1.00  1.46
H
ATOM  14990 2HB  PRO B 360     -27.055   3.549   4.158  1.00  1.46
H
ATOM  14991 1HG  PRO B 360     -29.204   2.836   2.135  1.00  1.46
H
ATOM  14992 2HG  PRO B 360     -29.372   3.111   3.870  1.00  1.46
H
ATOM  14993 1HD  PRO B 360     -29.992   5.036   2.038  1.00  1.46
H
ATOM  14994 2HD  PRO B 360     -29.275   5.471   3.651  1.00  1.46
H
ATOM  14995  N   GLU B 361     -27.364   3.756  -0.076  1.00  0.92
N
ATOM  14996  CA  GLU B 361     -27.127   3.228  -1.413  1.00  1.82
C
ATOM  14997  C   GLU B 361     -26.238   4.147  -2.272  1.00  2.00
C
ATOM  14998  O   GLU B 361     -25.777   3.745  -3.340  1.00  3.78
O
ATOM  14999  CB  GLU B 361     -28.467   3.003  -2.116  1.00  2.73
C
ATOM  15000  CG  GLU B 361     -29.181   4.283  -2.510  1.00  2.73
C
ATOM  15001  CD  GLU B 361     -30.024   4.905  -1.435  1.00  2.73
C
ATOM  15002  OE1 GLU B 361     -30.094   4.366  -0.359  1.00  2.73
O
```

ATOM 15003 OE2 GLU B 361 -30.576 5.953 -1.697 1.00 2.73
O
ATOM 15004 H GLU B 361 -28.296 4.055 0.163 1.00 1.10
H
ATOM 15005 HA GLU B 361 -26.621 2.267 -1.313 1.00 2.18
H
ATOM 15006 1HB GLU B 361 -28.310 2.412 -3.019 1.00 3.28
H
ATOM 15007 2HB GLU B 361 -29.130 2.433 -1.464 1.00 3.28
H
ATOM 15008 1HG GLU B 361 -28.433 5.001 -2.813 1.00 3.28
H
ATOM 15009 2HG GLU B 361 -29.809 4.074 -3.375 1.00 3.28
H
ATOM 15010 N ARG B 362 -26.007 5.378 -1.808 1.00 1.13
N
ATOM 15011 CA ARG B 362 -25.164 6.341 -2.494 1.00 1.32
C
ATOM 15012 C ARG B 362 -23.973 6.763 -1.640 1.00 2.32
C
ATOM 15013 O ARG B 362 -23.330 7.772 -1.934 1.00 10.36
O
ATOM 15014 CB ARG B 362 -25.985 7.563 -2.893 1.00 1.98
C
ATOM 15015 CG ARG B 362 -26.983 7.302 -4.010 1.00 1.98
C
ATOM 15016 CD ARG B 362 -27.968 8.397 -4.202 1.00 1.98
C
ATOM 15017 NE ARG B 362 -28.778 8.147 -5.387 1.00 1.98
N
ATOM 15018 CZ ARG B 362 -29.881 7.368 -5.442 1.00 1.98
C
ATOM 15019 NH1 ARG B 362 -30.372 6.781 -4.372 1.00 1.98
N
ATOM 15020 NH2 ARG B 362 -30.488 7.180 -6.597 1.00 1.98
N
ATOM 15021 H ARG B 362 -26.420 5.677 -0.937 1.00 1.36
H
ATOM 15022 HA ARG B 362 -24.783 5.876 -3.404 1.00 1.58
H
ATOM 15023 1HB ARG B 362 -26.544 7.922 -2.030 1.00 2.38
H
ATOM 15024 2HB ARG B 362 -25.325 8.368 -3.211 1.00 2.38
H
ATOM 15025 1HG ARG B 362 -26.435 7.188 -4.945 1.00 2.38
H
ATOM 15026 2HG ARG B 362 -27.529 6.390 -3.812 1.00 2.38
H
ATOM 15027 1HD ARG B 362 -28.632 8.457 -3.339 1.00 2.38
H
ATOM 15028 2HD ARG B 362 -27.451 9.346 -4.334 1.00 2.38
H
ATOM 15029 HE ARG B 362 -28.466 8.580 -6.251 1.00 2.38
H
ATOM 15030 1HH1 ARG B 362 -29.949 6.892 -3.459 1.00 2.38
H
ATOM 15031 2HH1 ARG B 362 -31.196 6.204 -4.450 1.00 2.38
H

ATOM 15032 1HH2 ARG B 362   -30.120   7.608 -7.432 1.00 2.38
H
ATOM 15033 2HH2 ARG B 362   -31.311   6.597 -6.646 1.00 2.38
H
ATOM 15034 N   PHE B 363   -23.686   6.009 -0.578 1.00 0.50
N
ATOM 15035 CA PHE B 363   -22.581   6.377   0.290 1.00 0.41
C
ATOM 15036 C   PHE B 363   -21.442   5.369   0.169 1.00 0.36
C
ATOM 15037 O   PHE B 363   -21.665   4.159   0.243 1.00 0.59
O
ATOM 15038 CB PHE B 363   -23.051   6.452   1.732 1.00 0.61
C
ATOM 15039 CG PHE B 363   -21.990   6.935   2.667 1.00 0.61
C
ATOM 15040 CD1 PHE B 363   -21.832   8.293   2.880 1.00 0.61
C
ATOM 15041 CD2 PHE B 363   -21.142   6.058   3.319 1.00 0.61
C
ATOM 15042 CE1 PHE B 363   -20.866   8.766   3.734 1.00 0.61
C
ATOM 15043 CE2 PHE B 363   -20.169   6.535   4.171 1.00 0.61
C
ATOM 15044 CZ PHE B 363   -20.035   7.888   4.383 1.00 0.61
C
ATOM 15045 H   PHE B 363   -24.224   5.178 -0.356 1.00 0.60
H
ATOM 15046 HA PHE B 363   -22.214   7.356 -0.010 1.00 0.49
H
ATOM 15047 1HB PHE B 363   -23.905   7.124   1.804 1.00 0.74
H
ATOM 15048 2HB PHE B 363   -23.377   5.467   2.064 1.00 0.74
H
ATOM 15049 HD1 PHE B 363   -22.486   8.990   2.366 1.00 0.74
H
ATOM 15050 HD2 PHE B 363   -21.250   4.986   3.154 1.00 0.74
H
ATOM 15051 HE1 PHE B 363   -20.763   9.834   3.891 1.00 0.74
H
ATOM 15052 HE2 PHE B 363   -19.506   5.844   4.682 1.00 0.74
H
ATOM 15053 HZ PHE B 363   -19.272   8.262   5.061 1.00 0.74
H
ATOM 15054 N   ILE B 364   -20.223   5.872 -0.019 1.00 0.22
N
ATOM 15055 CA ILE B 364   -19.055   5.019 -0.180 1.00 0.21
C
ATOM 15056 C   ILE B 364   -18.111   5.107   1.012 1.00 0.20
C
ATOM 15057 O   ILE B 364   -17.515   6.153   1.283 1.00 0.29
O
ATOM 15058 CB ILE B 364   -18.265   5.386 -1.449 1.00 0.32
C
ATOM 15059 CG1 ILE B 364   -19.153   5.256 -2.688 1.00 0.32
C
ATOM 15060 CG2 ILE B 364   -17.048   4.482 -1.582 1.00 0.32
C

ATOM 15061 CD1 ILE B 364 -18.516 5.778 -3.959 1.00 0.32
C
ATOM 15062 H ILE B 364 -20.108 6.872 -0.056 1.00 0.26
H
ATOM 15063 HA ILE B 364 -19.392 3.987 -0.271 1.00 0.25
H
ATOM 15064 HB ILE B 364 -17.938 6.418 -1.380 1.00 0.38
H
ATOM 15065 1HG1 ILE B 364 -19.401 4.208 -2.829 1.00 0.38
H
ATOM 15066 2HG1 ILE B 364 -20.079 5.809 -2.521 1.00 0.38
H
ATOM 15067 1HG2 ILE B 364 -16.486 4.754 -2.475 1.00 0.38
H
ATOM 15068 2HG2 ILE B 364 -16.413 4.596 -0.705 1.00 0.38
H
ATOM 15069 3HG2 ILE B 364 -17.373 3.444 -1.661 1.00 0.38
H
ATOM 15070 1HD1 ILE B 364 -19.208 5.649 -4.791 1.00 0.38
H
ATOM 15071 2HD1 ILE B 364 -18.282 6.836 -3.843 1.00 0.38
H
ATOM 15072 3HD1 ILE B 364 -17.600 5.224 -4.159 1.00 0.38
H
ATOM 15073 N GLU B 365 -17.961 3.986 1.699 1.00 0.28
N
ATOM 15074 CA GLU B 365 -17.051 3.881 2.824 1.00 0.25
C
ATOM 15075 C GLU B 365 -15.670 3.588 2.265 1.00 0.29
C
ATOM 15076 O GLU B 365 -15.432 2.499 1.738 1.00 0.58
O
ATOM 15077 CB GLU B 365 -17.515 2.772 3.774 1.00 0.38
C
ATOM 15078 CG GLU B 365 -18.867 3.049 4.413 1.00 0.38
C
ATOM 15079 CD GLU B 365 -19.383 1.930 5.273 1.00 0.38
C
ATOM 15080 OE1 GLU B 365 -18.725 0.923 5.377 1.00 0.38
O
ATOM 15081 OE2 GLU B 365 -20.448 2.087 5.824 1.00 0.38
O
ATOM 15082 H GLU B 365 -18.491 3.168 1.423 1.00 0.34
H
ATOM 15083 HA GLU B 365 -17.022 4.832 3.358 1.00 0.30
H
ATOM 15084 1HB GLU B 365 -17.584 1.828 3.233 1.00 0.45
H
ATOM 15085 2HB GLU B 365 -16.784 2.642 4.572 1.00 0.45
H
ATOM 15086 1HG GLU B 365 -18.771 3.936 5.030 1.00 0.45
H
ATOM 15087 2HG GLU B 365 -19.592 3.260 3.628 1.00 0.45
H
ATOM 15088 N CYS B 366 -14.785 4.577 2.299 1.00 0.18
N
ATOM 15089 CA CYS B 366 -13.464 4.407 1.709 1.00 0.17
C

```
ATOM  15090  C   CYS B 366     -12.421   3.916   2.696  1.00  0.20
C
ATOM  15091  O   CYS B 366     -11.368   3.430   2.277  1.00  0.64
O
ATOM  15092  CB  CYS B 366     -12.994   5.718   1.094  1.00  0.26
C
ATOM  15093  SG  CYS B 366     -14.012   6.255  -0.293  1.00  0.26
S
ATOM  15094  H   CYS B 366     -15.015   5.453   2.751  1.00  0.22
H
ATOM  15095  HA  CYS B 366     -13.545   3.671   0.910  1.00  0.20
H
ATOM  15096 1HB  CYS B 366     -13.005   6.503   1.850  1.00  0.31
H
ATOM  15097 2HB  CYS B 366     -11.968   5.610   0.743  1.00  0.31
H
ATOM  15098  HG  CYS B 366     -15.127   6.418   0.412  1.00  0.31
H
ATOM  15099  N   ILE B 367     -12.730   4.017   3.995  1.00  0.25
N
ATOM  15100  CA  ILE B 367     -11.839   3.591   5.075  1.00  0.31
C
ATOM  15101  C   ILE B 367     -10.625   4.552   5.078  1.00  0.32
C
ATOM  15102  O   ILE B 367     -10.543   5.440   4.228  1.00  1.53
O
ATOM  15103  CB  ILE B 367     -11.478   2.066   4.883  1.00  0.46
C
ATOM  15104  CG1 ILE B 367     -12.786   1.259   4.837  1.00  0.46
C
ATOM  15105  CG2 ILE B 367     -10.620   1.478   6.000  1.00  0.46
C
ATOM  15106  CD1 ILE B 367     -13.613   1.379   6.096  1.00  0.46
C
ATOM  15107  H   ILE B 367     -13.620   4.419   4.251  1.00  0.30
H
ATOM  15108  HA  ILE B 367     -12.360   3.691   6.021  1.00  0.37
H
ATOM  15109  HB  ILE B 367     -10.959   1.915   3.941  1.00  0.56
H
ATOM  15110 1HG1 ILE B 367     -13.389   1.599   3.998  1.00  0.56
H
ATOM  15111 2HG1 ILE B 367     -12.548   0.207   4.683  1.00  0.56
H
ATOM  15112 1HG2 ILE B 367     -10.451   0.423   5.798  1.00  0.56
H
ATOM  15113 2HG2 ILE B 367      -9.658   1.973   6.049  1.00  0.56
H
ATOM  15114 3HG2 ILE B 367     -11.136   1.586   6.954  1.00  0.56
H
ATOM  15115 1HD1 ILE B 367     -14.520   0.781   5.993  1.00  0.56
H
ATOM  15116 2HD1 ILE B 367     -13.036   1.017   6.948  1.00  0.56
H
ATOM  15117 3HD1 ILE B 367     -13.883   2.421   6.256  1.00  0.56
H
ATOM  15118  N   ILE B 368      -9.705   4.411   6.029  1.00  0.39
N
```

ATOM	15119	CA	ILE	B	368	-8.525	5.274	6.110	1.00	0.25	C
ATOM	15120	C	ILE	B	368	-7.593	5.097	4.900	1.00	0.17	C
ATOM	15121	O	ILE	B	368	-6.594	4.379	4.962	1.00	0.35	O
ATOM	15122	CB	ILE	B	368	-7.753	4.970	7.407	1.00	0.38	C
ATOM	15123	CG1	ILE	B	368	-8.646	5.278	8.611	1.00	0.38	C
ATOM	15124	CG2	ILE	B	368	-6.458	5.773	7.462	1.00	0.38	C
ATOM	15125	CD1	ILE	B	368	-8.086	4.763	9.911	1.00	0.38	C
ATOM	15126	H	ILE	B	368	-9.816	3.702	6.733	1.00	0.47	H
ATOM	15127	HA	ILE	B	368	-8.858	6.310	6.139	1.00	0.30	H
ATOM	15128	HB	ILE	B	368	-7.516	3.913	7.450	1.00	0.45	H
ATOM	15129	1HG1	ILE	B	368	-8.773	6.356	8.692	1.00	0.45	H
ATOM	15130	2HG1	ILE	B	368	-9.627	4.828	8.454	1.00	0.45	H
ATOM	15131	1HG2	ILE	B	368	-5.925	5.537	8.383	1.00	0.45	H
ATOM	15132	2HG2	ILE	B	368	-5.834	5.517	6.607	1.00	0.45	H
ATOM	15133	3HG2	ILE	B	368	-6.688	6.837	7.438	1.00	0.45	H
ATOM	15134	1HD1	ILE	B	368	-8.765	5.017	10.727	1.00	0.45	H
ATOM	15135	2HD1	ILE	B	368	-7.975	3.681	9.856	1.00	0.45	H
ATOM	15136	3HD1	ILE	B	368	-7.116	5.219	10.088	1.00	0.45	H
ATOM	15137	N	ALA	B	369	-7.981	5.716	3.785	1.00	0.30	N
ATOM	15138	CA	ALA	B	369	-7.254	5.694	2.523	1.00	0.26	C
ATOM	15139	C	ALA	B	369	-7.583	6.960	1.735	1.00	0.31	C
ATOM	15140	O	ALA	B	369	-8.329	6.904	0.761	1.00	1.26	O
ATOM	15141	CB	ALA	B	369	-7.626	4.448	1.726	1.00	0.39	C
ATOM	15142	H	ALA	B	369	-8.840	6.244	3.835	1.00	0.36	H
ATOM	15143	HA	ALA	B	369	-6.185	5.686	2.731	1.00	0.31	H
ATOM	15144	1HB	ALA	B	369	-7.076	4.437	0.787	1.00	0.47	H
ATOM	15145	2HB	ALA	B	369	-7.373	3.558	2.305	1.00	0.47	H
ATOM	15146	3HB	ALA	B	369	-8.695	4.453	1.518	1.00	0.47	H
ATOM	15147	N	GLU	B	370	-7.046	8.093	2.190	1.00	0.27	N

```
ATOM  15148  CA  GLU B 370      -7.352   9.408   1.622  1.00  0.22
C
ATOM  15149  C   GLU B 370      -7.109   9.516   0.116  1.00  0.22
C
ATOM  15150  O   GLU B 370      -7.942  10.060  -0.613  1.00  0.22
O
ATOM  15151  CB  GLU B 370      -6.508  10.470   2.316  1.00  0.33
C
ATOM  15152  CG  GLU B 370      -6.907  11.888   1.981  1.00  0.33
C
ATOM  15153  CD  GLU B 370      -8.193  12.254   2.643  1.00  0.33
C
ATOM  15154  OE1 GLU B 370      -8.559  11.591   3.583  1.00  0.33
O
ATOM  15155  OE2 GLU B 370      -8.811  13.197   2.218  1.00  0.33
O
ATOM  15156  H   GLU B 370      -6.419   8.042   2.980  1.00  0.32
H
ATOM  15157  HA  GLU B 370      -8.405   9.619   1.808  1.00  0.26
H
ATOM  15158 1HB  GLU B 370      -6.583  10.345   3.397  1.00  0.40
H
ATOM  15159 2HB  GLU B 370      -5.460  10.341   2.046  1.00  0.40
H
ATOM  15160 1HG  GLU B 370      -6.127  12.578   2.287  1.00  0.40
H
ATOM  15161 2HG  GLU B 370      -7.032  11.973   0.904  1.00  0.40
H
ATOM  15162  N   GLN B 371      -5.954   9.017  -0.327  1.00  0.27
N
ATOM  15163  CA  GLN B 371      -5.561   9.086  -1.729  1.00  0.32
C
ATOM  15164  C   GLN B 371      -6.535   8.338  -2.600  1.00  0.22
C
ATOM  15165  O   GLN B 371      -7.054   8.891  -3.573  1.00  0.28
O
ATOM  15166  CB  GLN B 371      -4.149   8.534  -1.895  1.00  0.48
C
ATOM  15167  CG  GLN B 371      -3.106   9.430  -1.265  1.00  0.48
C
ATOM  15168  CD  GLN B 371      -1.701   8.878  -1.318  1.00  0.48
C
ATOM  15169  OE1 GLN B 371      -1.455   7.732  -1.703  1.00  0.48
O
ATOM  15170  NE2 GLN B 371      -0.759   9.717  -0.917  1.00  0.48
N
ATOM  15171  H   GLN B 371      -5.324   8.579   0.331  1.00  0.32
H
ATOM  15172  HA  GLN B 371      -5.567  10.122  -2.048  1.00  0.38
H
ATOM  15173 1HB  GLN B 371      -4.078   7.549  -1.433  1.00  0.58
H
ATOM  15174 2HB  GLN B 371      -3.915   8.424  -2.954  1.00  0.58
H
ATOM  15175 1HG  GLN B 371      -3.108  10.375  -1.801  1.00  0.58
H
ATOM  15176 2HG  GLN B 371      -3.367   9.593  -0.220  1.00  0.58
H
```

```
ATOM  15177 1HE2 GLN B 371       0.201   9.439  -0.919  1.00  0.58
H
ATOM  15178 2HE2 GLN B 371      -1.020  10.631  -0.600  1.00  0.58
H
ATOM  15179  N   ASN B 372      -6.810   7.097  -2.211  1.00  0.16
N
ATOM  15180  CA  ASN B 372      -7.785   6.281  -2.901  1.00  0.16
C
ATOM  15181  C   ASN B 372      -9.154   6.939  -2.943  1.00  0.14
C
ATOM  15182  O   ASN B 372      -9.788   6.936  -3.990  1.00  0.17
O
ATOM  15183  CB  ASN B 372      -7.877   4.919  -2.272  1.00  0.24
C
ATOM  15184  CG  ASN B 372      -8.793   4.009  -3.023  1.00  0.24
C
ATOM  15185  OD1 ASN B 372      -8.550   3.673  -4.188  1.00  0.24
O
ATOM  15186  ND2 ASN B 372      -9.856   3.612  -2.381  1.00  0.24
N
ATOM  15187  H   ASN B 372      -6.306   6.685  -1.429  1.00  0.19
H
ATOM  15188  HA  ASN B 372      -7.457   6.159  -3.935  1.00  0.19
H
ATOM  15189 1HB  ASN B 372      -6.886   4.475  -2.250  1.00  0.29
H
ATOM  15190 2HB  ASN B 372      -8.224   5.008  -1.242  1.00  0.29
H
ATOM  15191 1HD2 ASN B 372     -10.511   3.007  -2.830  1.00  0.29
H
ATOM  15192 2HD2 ASN B 372     -10.013   3.914  -1.441  1.00  0.29
H
ATOM  15193  N   MET B 373      -9.606   7.500  -1.813  1.00  0.15
N
ATOM  15194  CA  MET B 373     -10.914   8.158  -1.755  1.00  0.15
C
ATOM  15195  C   MET B 373     -11.076   9.201  -2.845  1.00  0.15
C
ATOM  15196  O   MET B 373     -12.087   9.212  -3.550  1.00  0.17
O
ATOM  15197  CB  MET B 373     -11.138   8.808  -0.388  1.00  0.22
C
ATOM  15198  CG  MET B 373     -12.470   9.547  -0.265  1.00  0.22
C
ATOM  15199  SD  MET B 373     -12.687  10.364   1.328  1.00  0.22
S
ATOM  15200  CE  MET B 373     -11.539  11.726   1.175  1.00  0.22
C
ATOM  15201  H   MET B 373      -9.045   7.461  -0.973  1.00  0.18
H
ATOM  15202  HA  MET B 373     -11.683   7.402  -1.909  1.00  0.18
H
ATOM  15203 1HB  MET B 373     -11.100   8.047   0.389  1.00  0.27
H
ATOM  15204 2HB  MET B 373     -10.341   9.523  -0.184  1.00  0.27
H
ATOM  15205 1HG  MET B 373     -12.528  10.303  -1.047  1.00  0.27
H
```

```
ATOM  15206 2HG  MET B 373     -13.293   8.851  -0.409  1.00  0.27
H
ATOM  15207 1HE  MET B 373     -11.558  12.325   2.085  1.00  0.27
H
ATOM  15208 2HE  MET B 373     -10.532  11.337   1.020  1.00  0.27
H
ATOM  15209 3HE  MET B 373     -11.820  12.349   0.326  1.00  0.27
H
ATOM  15210  N   VAL B 374     -10.081  10.078  -2.978  1.00  0.14
N
ATOM  15211  CA  VAL B 374     -10.142  11.112  -3.996  1.00  0.16
C
ATOM  15212  C   VAL B 374     -10.133  10.509  -5.400  1.00  0.16
C
ATOM  15213  O   VAL B 374     -10.910  10.937  -6.249  1.00  0.16
O
ATOM  15214  CB  VAL B 374      -8.982  12.106  -3.846  1.00  0.24
C
ATOM  15215  CG1 VAL B 374      -8.975  13.061  -5.032  1.00  0.24
C
ATOM  15216  CG2 VAL B 374      -9.132  12.872  -2.537  1.00  0.24
C
ATOM  15217  H   VAL B 374      -9.279  10.029  -2.354  1.00  0.17
H
ATOM  15218  HA  VAL B 374     -11.075  11.662  -3.867  1.00  0.19
H
ATOM  15219  HB  VAL B 374      -8.039  11.562  -3.845  1.00  0.29
H
ATOM  15220 1HG1 VAL B 374      -8.142  13.756  -4.933  1.00  0.29
H
ATOM  15221 2HG1 VAL B 374      -8.866  12.494  -5.956  1.00  0.29
H
ATOM  15222 3HG1 VAL B 374      -9.911  13.618  -5.058  1.00  0.29
H
ATOM  15223 1HG2 VAL B 374      -8.304  13.573  -2.428  1.00  0.29
H
ATOM  15224 2HG2 VAL B 374     -10.074  13.420  -2.540  1.00  0.29
H
ATOM  15225 3HG2 VAL B 374      -9.124  12.171  -1.702  1.00  0.29
H
ATOM  15226  N   SER B 375      -9.262   9.518  -5.638  1.00  0.18
N
ATOM  15227  CA  SER B 375      -9.173   8.867  -6.949  1.00  0.20
C
ATOM  15228  C   SER B 375     -10.505   8.216  -7.332  1.00  0.19
C
ATOM  15229  O   SER B 375     -10.938   8.320  -8.478  1.00  0.27
O
ATOM  15230  CB  SER B 375      -8.054   7.846  -6.955  1.00  0.30
C
ATOM  15231  OG  SER B 375      -7.882   7.267  -8.226  1.00  0.30
O
ATOM  15232  H   SER B 375      -8.636   9.213  -4.901  1.00  0.22
H
ATOM  15233  HA  SER B 375      -8.944   9.627  -7.693  1.00  0.24
H
ATOM  15234 1HB  SER B 375      -7.128   8.318  -6.639  1.00  0.36
H
```

ATOM 15235 2HB SER B 375 -8.277 7.065 -6.229 1.00 0.36
H
ATOM 15236 HG SER B 375 -7.061 6.769 -8.176 1.00 0.36
H
ATOM 15237 N VAL B 376 -11.148 7.548 -6.368 1.00 0.20
N
ATOM 15238 CA VAL B 376 -12.457 6.939 -6.579 1.00 0.17
C
ATOM 15239 C VAL B 376 -13.482 7.997 -6.951 1.00 0.15
C
ATOM 15240 O VAL B 376 -14.264 7.786 -7.875 1.00 0.17
O
ATOM 15241 CB VAL B 376 -12.915 6.156 -5.327 1.00 0.26
C
ATOM 15242 CG1 VAL B 376 -14.373 5.734 -5.459 1.00 0.26
C
ATOM 15243 CG2 VAL B 376 -12.052 4.909 -5.176 1.00 0.26
C
ATOM 15244 H VAL B 376 -10.722 7.467 -5.457 1.00 0.24
H
ATOM 15245 HA VAL B 376 -12.376 6.233 -7.405 1.00 0.20
H
ATOM 15246 HB VAL B 376 -12.820 6.792 -4.447 1.00 0.31
H
ATOM 15247 1HG1 VAL B 376 -14.675 5.186 -4.567 1.00 0.31
H
ATOM 15248 2HG1 VAL B 376 -15.000 6.619 -5.571 1.00 0.31
H
ATOM 15249 3HG1 VAL B 376 -14.489 5.095 -6.330 1.00 0.31
H
ATOM 15250 1HG2 VAL B 376 -12.364 4.355 -4.292 1.00 0.31
H
ATOM 15251 2HG2 VAL B 376 -12.167 4.280 -6.060 1.00 0.31
H
ATOM 15252 3HG2 VAL B 376 -11.006 5.193 -5.074 1.00 0.31
H
ATOM 15253 N ALA B 377 -13.471 9.135 -6.246 1.00 0.13
N
ATOM 15254 CA ALA B 377 -14.375 10.239 -6.562 1.00 0.12
C
ATOM 15255 C ALA B 377 -14.142 10.744 -7.992 1.00 0.14
C
ATOM 15256 O ALA B 377 -15.101 11.033 -8.707 1.00 0.21
O
ATOM 15257 CB ALA B 377 -14.196 11.374 -5.561 1.00 0.18
C
ATOM 15258 H ALA B 377 -12.828 9.239 -5.471 1.00 0.16
H
ATOM 15259 HA ALA B 377 -15.398 9.867 -6.499 1.00 0.14
H
ATOM 15260 1HB ALA B 377 -14.894 12.178 -5.791 1.00 0.22
H
ATOM 15261 2HB ALA B 377 -14.390 11.002 -4.555 1.00 0.22
H
ATOM 15262 3HB ALA B 377 -13.178 11.754 -5.614 1.00 0.22
H
ATOM 15263 N LEU B 378 -12.871 10.826 -8.408 1.00 0.13
N

ATOM 15264 CA LEU B 378   -12.518 11.265 -9.759 1.00 0.17
C
ATOM 15265 C   LEU B 378   -13.058 10.284 -10.812 1.00 0.20
C
ATOM 15266 O   LEU B 378   -13.572 10.698 -11.860 1.00 0.40
O
ATOM 15267 CB LEU B 378   -10.985 11.372 -9.885 1.00 0.26
C
ATOM 15268 CG LEU B 378   -10.308 12.481 -9.058 1.00 0.26
C
ATOM 15269 CD1 LEU B 378   -8.802 12.325 -9.127 1.00 0.26
C
ATOM 15270 CD2 LEU B 378   -10.696 13.821 -9.600 1.00 0.26
C
ATOM 15271 H   LEU B 378   -12.126 10.601 -7.762 1.00 0.16
H
ATOM 15272 HA LEU B 378   -12.963 12.244 -9.935 1.00 0.20
H
ATOM 15273 1HB LEU B 378   -10.540 10.426 -9.591 1.00 0.31
H
ATOM 15274 2HB LEU B 378   -10.739 11.550 -10.932 1.00 0.31
H
ATOM 15275 HG LEU B 378   -10.619 12.407 -8.021 1.00 0.31
H
ATOM 15276 1HD1 LEU B 378   -8.327 13.111 -8.538 1.00 0.31
H
ATOM 15277 2HD1 LEU B 378   -8.516 11.355 -8.730 1.00 0.31
H
ATOM 15278 3HD1 LEU B 378   -8.477 12.403 -10.163 1.00 0.31
H
ATOM 15279 1HD2 LEU B 378   -10.217 14.604 -9.014 1.00 0.31
H
ATOM 15280 2HD2 LEU B 378   -10.367 13.887 -10.636 1.00 0.31
H
ATOM 15281 3HD2 LEU B 378   -11.777 13.935 -9.548 1.00 0.31
H
ATOM 15282 N   GLY B 379   -12.969   8.986 -10.501 1.00 0.15
N
ATOM 15283 CA GLY B 379   -13.501   7.943 -11.364 1.00 0.17
C
ATOM 15284 C   GLY B 379   -15.010   8.041 -11.452 1.00 0.23
C
ATOM 15285 O   GLY B 379   -15.575   8.010 -12.539 1.00 0.61
O
ATOM 15286 H   GLY B 379   -12.502   8.715 -9.644 1.00 0.18
H
ATOM 15287 1HA GLY B 379   -13.070   8.035 -12.361 1.00 0.20
H
ATOM 15288 2HA GLY B 379   -13.215   6.966 -10.975 1.00 0.20
H
ATOM 15289 N   CYS B 380   -15.660   8.207 -10.307 1.00 0.23
N
ATOM 15290 CA CYS B 380   -17.110   8.334 -10.246 1.00 0.26
C
ATOM 15291 C   CYS B 380   -17.624   9.532 -11.056 1.00 0.29
C
ATOM 15292 O   CYS B 380   -18.600   9.416 -11.800 1.00 0.37
O

ATOM 15293 CB CYS B 380 -17.543 8.458 -8.785 1.00 0.39
C
ATOM 15294 SG CYS B 380 -17.353 6.932 -7.830 1.00 0.39
S
ATOM 15295 H CYS B 380 -15.140 8.226 -9.443 1.00 0.28
H
ATOM 15296 HA CYS B 380 -17.549 7.424 -10.658 1.00 0.31
H
ATOM 15297 1HB CYS B 380 -16.953 9.234 -8.299 1.00 0.47
H
ATOM 15298 2HB CYS B 380 -18.582 8.759 -8.729 1.00 0.47
H
ATOM 15299 HG CYS B 380 -17.744 7.444 -6.668 1.00 0.47
H
ATOM 15300 N ALA B 381 -16.934 10.669 -10.949 1.00 0.30
N
ATOM 15301 CA ALA B 381 -17.303 11.890 -11.670 1.00 0.43
C
ATOM 15302 C ALA B 381 -17.043 11.807 -13.179 1.00 0.34
C
ATOM 15303 O ALA B 381 -17.492 12.672 -13.938 1.00 1.11
O
ATOM 15304 CB ALA B 381 -16.542 13.075 -11.090 1.00 0.65
C
ATOM 15305 H ALA B 381 -16.145 10.710 -10.318 1.00 0.36
H
ATOM 15306 HA ALA B 381 -18.370 12.051 -11.527 1.00 0.52
H
ATOM 15307 1HB ALA B 381 -16.848 13.989 -11.598 1.00 0.77
H
ATOM 15308 2HB ALA B 381 -16.760 13.161 -10.026 1.00 0.77
H
ATOM 15309 3HB ALA B 381 -15.473 12.921 -11.230 1.00 0.77
H
ATOM 15310 N THR B 382 -16.296 10.796 -13.608 1.00 0.74
N
ATOM 15311 CA THR B 382 -15.954 10.635 -14.997 1.00 0.63
C
ATOM 15312 C THR B 382 -17.206 10.380 -15.841 1.00 0.52
C
ATOM 15313 O THR B 382 -18.125 9.668 -15.424 1.00 0.67
O
ATOM 15314 CB THR B 382 -14.925 9.521 -15.147 1.00 0.95
C
ATOM 15315 OG1 THR B 382 -13.728 9.853 -14.414 1.00 0.95
O
ATOM 15316 CG2 THR B 382 -14.589 9.340 -16.589 1.00 0.95
C
ATOM 15317 H THR B 382 -15.957 10.090 -12.971 1.00 0.89
H
ATOM 15318 HA THR B 382 -15.497 11.561 -15.347 1.00 0.76
H
ATOM 15319 HB THR B 382 -15.329 8.593 -14.769 1.00 1.13
H
ATOM 15320 HG1 THR B 382 -13.926 10.058 -13.488 1.00 1.13
H
ATOM 15321 1HG2 THR B 382 -13.871 8.536 -16.679 1.00 1.13
H

ATOM 15322 2HG2 THR B 382   -15.481   9.080 -17.136 1.00 1.13
H
ATOM 15323 3HG2 THR B 382   -14.169 10.259 -16.992 1.00 1.13
H
ATOM 15324 N   ARG B 383   -17.248 11.042 -17.003 1.00 0.65
N
ATOM 15325 CA ARG B 383   -18.359 11.037 -17.964 1.00 0.72
C
ATOM 15326 C   ARG B 383   -19.598 11.756 -17.424 1.00 0.51
C
ATOM 15327 O   ARG B 383   -20.654 11.715 -18.056 1.00 0.69
O
ATOM 15328 CB ARG B 383   -18.794   9.617 -18.355 1.00 1.08
C
ATOM 15329 CG ARG B 383   -17.737   8.711 -18.956 1.00 1.08
C
ATOM 15330 CD ARG B 383   -17.200   9.191 -20.250 1.00 1.08
C
ATOM 15331 NE ARG B 383   -16.091   8.352 -20.668 1.00 1.08
N
ATOM 15332 CZ ARG B 383   -16.201   7.191 -21.333 1.00 1.08
C
ATOM 15333 NH1 ARG B 383   -17.387   6.746 -21.684 1.00 1.08
N
ATOM 15334 NH2 ARG B 383   -15.109   6.501 -21.623 1.00 1.08
N
ATOM 15335 H   ARG B 383   -16.435 11.594 -17.245 1.00 0.78
H
ATOM 15336 HA ARG B 383   -18.030 11.553 -18.867 1.00 0.86
H
ATOM 15337 1HB ARG B 383   -19.184   9.103 -17.485 1.00 1.30
H
ATOM 15338 2HB ARG B 383   -19.606   9.681 -19.079 1.00 1.30
H
ATOM 15339 1HG ARG B 383   -16.917   8.584 -18.268 1.00 1.30
H
ATOM 15340 2HG ARG B 383   -18.188   7.737 -19.143 1.00 1.30
H
ATOM 15341 1HD ARG B 383   -17.974   9.148 -21.017 1.00 1.30
H
ATOM 15342 2HD ARG B 383   -16.839 10.214 -20.148 1.00 1.30
H
ATOM 15343 HE ARG B 383   -15.155   8.658 -20.425 1.00 1.30
H
ATOM 15344 1HH1 ARG B 383   -18.211   7.286 -21.461 1.00 1.30
H
ATOM 15345 2HH1 ARG B 383   -17.478   5.875 -22.188 1.00 1.30
H
ATOM 15346 1HH2 ARG B 383   -14.200   6.844 -21.323 1.00 1.30
H
ATOM 15347 2HH2 ARG B 383   -15.179   5.630 -22.123 1.00 1.30
H
ATOM 15348 N   GLY B 384   -19.485 12.410 -16.260 1.00 0.40
N
ATOM 15349 CA GLY B 384   -20.620 13.103 -15.683 1.00 0.41
C
ATOM 15350 C   GLY B 384   -21.626 12.132 -15.070 1.00 0.45
C

```
ATOM  15351  O   GLY B 384     -22.783  12.499 -14.866  1.00  0.58
O
ATOM  15352  H   GLY B 384     -18.613  12.443 -15.748  1.00  0.48
H
ATOM  15353 1HA  GLY B 384     -20.268  13.795 -14.918  1.00  0.49
H
ATOM  15354 2HA  GLY B 384     -21.108  13.700 -16.452  1.00  0.49
H
ATOM  15355  N   ARG B 385     -21.207  10.896 -14.783  1.00  0.44
N
ATOM  15356  CA  ARG B 385     -22.155   9.903 -14.278  1.00  0.55
C
ATOM  15357  C   ARG B 385     -22.686  10.191 -12.875  1.00  0.47
C
ATOM  15358  O   ARG B 385     -23.806   9.798 -12.552  1.00  0.57
O
ATOM  15359  CB  ARG B 385     -21.508   8.538 -14.290  1.00  0.83
C
ATOM  15360  CG  ARG B 385     -21.267   7.978 -15.678  1.00  0.83
C
ATOM  15361  CD  ARG B 385     -20.440   6.754 -15.619  1.00  0.83
C
ATOM  15362  NE  ARG B 385     -19.120   7.065 -15.121  1.00  0.83
N
ATOM  15363  CZ  ARG B 385     -18.172   6.173 -14.776  1.00  0.83
C
ATOM  15364  NH1 ARG B 385     -18.350   4.873 -14.900  1.00  0.83
N
ATOM  15365  NH2 ARG B 385     -17.035   6.628 -14.291  1.00  0.83
N
ATOM  15366  H   ARG B 385     -20.242  10.619 -14.948  1.00  0.53
H
ATOM  15367  HA  ARG B 385     -23.003   9.881 -14.960  1.00  0.66
H
ATOM  15368 1HB  ARG B 385     -20.547   8.582 -13.779  1.00  0.99
H
ATOM  15369 2HB  ARG B 385     -22.137   7.828 -13.751  1.00  0.99
H
ATOM  15370 1HG  ARG B 385     -22.222   7.724 -16.138  1.00  0.99
H
ATOM  15371 2HG  ARG B 385     -20.760   8.724 -16.282  1.00  0.99
H
ATOM  15372 1HD  ARG B 385     -20.895   6.040 -14.939  1.00  0.99
H
ATOM  15373 2HD  ARG B 385     -20.345   6.313 -16.610  1.00  0.99
H
ATOM  15374  HE  ARG B 385     -18.895   8.048 -14.993  1.00  0.99
H
ATOM  15375 1HH1 ARG B 385     -19.210   4.482 -15.260  1.00  0.99
H
ATOM  15376 2HH1 ARG B 385     -17.615   4.240 -14.621  1.00  0.99
H
ATOM  15377 1HH2 ARG B 385     -16.900   7.624 -14.175  1.00  0.99
H
ATOM  15378 2HH2 ARG B 385     -16.309   5.991 -14.017  1.00  0.99
H
ATOM  15379  N   THR B 386     -21.912  10.895 -12.053  1.00  0.36
N
```

ATOM 15380 CA THR B 386 -22.377 11.236 -10.712 1.00 0.27
C
ATOM 15381 C THR B 386 -21.579 12.367 -10.068 1.00 0.25
C
ATOM 15382 O THR B 386 -20.421 12.610 -10.404 1.00 0.38
O
ATOM 15383 CB THR B 386 -22.375 10.000 -9.797 1.00 0.41
C
ATOM 15384 OG1 THR B 386 -22.873 10.362 -8.508 1.00 0.41
O
ATOM 15385 CG2 THR B 386 -20.995 9.426 -9.661 1.00 0.41
C
ATOM 15386 H THR B 386 -20.996 11.195 -12.355 1.00 0.43
H
ATOM 15387 HA THR B 386 -23.410 11.576 -10.795 1.00 0.32
H
ATOM 15388 HB THR B 386 -23.031 9.241 -10.222 1.00 0.49
H
ATOM 15389 HG1 THR B 386 -23.729 10.784 -8.599 1.00 0.49
H
ATOM 15390 1HG2 THR B 386 -21.028 8.551 -9.014 1.00 0.49
H
ATOM 15391 2HG2 THR B 386 -20.635 9.134 -10.644 1.00 0.49
H
ATOM 15392 3HG2 THR B 386 -20.328 10.172 -9.231 1.00 0.49
H
ATOM 15393 N ILE B 387 -22.238 13.063 -9.147 1.00 0.24
N
ATOM 15394 CA ILE B 387 -21.678 14.194 -8.412 1.00 0.25
C
ATOM 15395 C ILE B 387 -21.123 13.715 -7.081 1.00 0.23
C
ATOM 15396 O ILE B 387 -21.880 13.377 -6.174 1.00 0.25
O
ATOM 15397 CB ILE B 387 -22.759 15.266 -8.163 1.00 0.38
C
ATOM 15398 CG1 ILE B 387 -23.408 15.673 -9.487 1.00 0.38
C
ATOM 15399 CG2 ILE B 387 -22.163 16.473 -7.459 1.00 0.38
C
ATOM 15400 CD1 ILE B 387 -22.430 16.219 -10.499 1.00 0.38
C
ATOM 15401 H ILE B 387 -23.178 12.779 -8.926 1.00 0.29
H
ATOM 15402 HA ILE B 387 -20.866 14.630 -8.994 1.00 0.30
H
ATOM 15403 HB ILE B 387 -23.543 14.841 -7.536 1.00 0.45
H
ATOM 15404 1HG1 ILE B 387 -23.901 14.805 -9.922 1.00 0.45
H
ATOM 15405 2HG1 ILE B 387 -24.162 16.436 -9.294 1.00 0.45
H
ATOM 15406 1HG2 ILE B 387 -22.943 17.212 -7.280 1.00 0.45
H
ATOM 15407 2HG2 ILE B 387 -21.729 16.161 -6.511 1.00 0.45
H
ATOM 15408 3HG2 ILE B 387 -21.385 16.912 -8.087 1.00 0.45
H

```
ATOM  15409 1HD1 ILE B 387      -22.962  16.486 -11.412  1.00  0.45
H
ATOM  15410 2HD1 ILE B 387      -21.941  17.105 -10.092  1.00  0.45
H
ATOM  15411 3HD1 ILE B 387      -21.680  15.463 -10.725  1.00  0.45
H
ATOM  15412  N   ALA B 388      -19.802  13.689  -6.958  1.00  0.24
N
ATOM  15413  CA  ALA B 388      -19.190  13.153  -5.751  1.00  0.25
C
ATOM  15414  C   ALA B 388      -18.763  14.213  -4.743  1.00  0.25
C
ATOM  15415  O   ALA B 388      -18.083  15.178  -5.089  1.00  0.29
O
ATOM  15416  CB  ALA B 388      -17.981  12.313  -6.144  1.00  0.38
C
ATOM  15417  H   ALA B 388      -19.220  14.007  -7.720  1.00  0.29
H
ATOM  15418  HA  ALA B 388      -19.921  12.516  -5.262  1.00  0.30
H
ATOM  15419 1HB  ALA B 388      -17.539  11.871  -5.255  1.00  0.45
H
ATOM  15420 2HB  ALA B 388      -18.294  11.524  -6.827  1.00  0.45
H
ATOM  15421 3HB  ALA B 388      -17.244  12.946  -6.636  1.00  0.45
H
ATOM  15422  N   PHE B 389      -19.130  13.971  -3.481  1.00  0.40
N
ATOM  15423  CA  PHE B 389      -18.732  14.766  -2.317  1.00  0.28
C
ATOM  15424  C   PHE B 389      -17.925  13.909  -1.354  1.00  0.23
C
ATOM  15425  O   PHE B 389      -18.494  13.116  -0.608  1.00  0.31
O
ATOM  15426  CB  PHE B 389      -19.936  15.341  -1.569  1.00  0.42
C
ATOM  15427  CG  PHE B 389      -20.667  16.447  -2.264  1.00  0.42
C
ATOM  15428  CD1 PHE B 389      -21.590  16.199  -3.266  1.00  0.42
C
ATOM  15429  CD2 PHE B 389      -20.443  17.761  -1.871  1.00  0.42
C
ATOM  15430  CE1 PHE B 389      -22.259  17.248  -3.866  1.00  0.42
C
ATOM  15431  CE2 PHE B 389      -21.115  18.808  -2.465  1.00  0.42
C
ATOM  15432  CZ  PHE B 389      -22.022  18.549  -3.465  1.00  0.42
C
ATOM  15433  H   PHE B 389      -19.735  13.178  -3.310  1.00  0.48
H
ATOM  15434  HA  PHE B 389      -18.110  15.591  -2.656  1.00  0.34
H
ATOM  15435 1HB  PHE B 389      -20.645  14.538  -1.389  1.00  0.50
H
ATOM  15436 2HB  PHE B 389      -19.611  15.709  -0.598  1.00  0.50
H
ATOM  15437  HD1 PHE B 389      -21.781  15.172  -3.576  1.00  0.50
H
```

```
ATOM  15438  HD2 PHE B 389     -19.725  17.959  -1.076  1.00  0.50
H
ATOM  15439  HE1 PHE B 389     -22.983  17.048  -4.656  1.00  0.50
H
ATOM  15440  HE2 PHE B 389     -20.929  19.833  -2.146  1.00  0.50
H
ATOM  15441  HZ  PHE B 389     -22.551  19.372  -3.935  1.00  0.50
H
ATOM  15442  N   ALA B 390     -16.606  14.056  -1.371  1.00  0.21
N
ATOM  15443  CA  ALA B 390     -15.764  13.266  -0.478  1.00  0.19
C
ATOM  15444  C   ALA B 390     -15.417  14.081   0.763  1.00  0.16
C
ATOM  15445  O   ALA B 390     -15.095  15.264   0.660  1.00  0.29
O
ATOM  15446  CB  ALA B 390     -14.507  12.805  -1.195  1.00  0.29
C
ATOM  15447  H   ALA B 390     -16.193  14.739  -1.993  1.00  0.25
H
ATOM  15448  HA  ALA B 390     -16.328  12.392  -0.162  1.00  0.23
H
ATOM  15449 1HB  ALA B 390     -13.908  12.199  -0.519  1.00  0.34
H
ATOM  15450 2HB  ALA B 390     -14.783  12.212  -2.067  1.00  0.34
H
ATOM  15451 3HB  ALA B 390     -13.932  13.667  -1.512  1.00  0.34
H
ATOM  15452  N   GLY B 391     -15.487  13.439   1.929  1.00  0.14
N
ATOM  15453  CA  GLY B 391     -15.226  14.111   3.193  1.00  0.14
C
ATOM  15454  C   GLY B 391     -14.186  13.425   4.081  1.00  0.14
C
ATOM  15455  O   GLY B 391     -14.087  12.193   4.142  1.00  0.25
O
ATOM  15456  H   GLY B 391     -15.762  12.469   1.937  1.00  0.17
H
ATOM  15457 1HA  GLY B 391     -14.900  15.127   2.986  1.00  0.17
H
ATOM  15458 2HA  GLY B 391     -16.162  14.197   3.744  1.00  0.17
H
ATOM  15459  N   ALA B 392     -13.419  14.268   4.761  1.00  0.16
N
ATOM  15460  CA  ALA B 392     -12.377  13.878   5.707  1.00  0.14
C
ATOM  15461  C   ALA B 392     -11.997  15.111   6.496  1.00  0.16
C
ATOM  15462  O   ALA B 392     -12.586  16.167   6.284  1.00  0.20
O
ATOM  15463  CB  ALA B 392     -11.169  13.288   4.993  1.00  0.21
C
ATOM  15464  H   ALA B 392     -13.587  15.260   4.610  1.00  0.19
H
ATOM  15465  HA  ALA B 392     -12.787  13.141   6.397  1.00  0.17
H
ATOM  15466 1HB  ALA B 392     -10.411  13.009   5.724  1.00  0.25
H
```

```
ATOM  15467 2HB  ALA B 392     -11.474  12.403   4.434  1.00  0.25
H
ATOM  15468 3HB  ALA B 392     -10.754  14.021   4.306  1.00  0.25
H
ATOM  15469  N   PHE B 393     -11.039  14.998   7.403  1.00  0.25
N
ATOM  15470  CA  PHE B 393     -10.562  16.209   8.040  1.00  0.28
C
ATOM  15471  C   PHE B 393      -9.814  16.962   6.954  1.00  0.30
C
ATOM  15472  O   PHE B 393      -9.165  16.344   6.110  1.00  0.40
O
ATOM  15473  CB  PHE B 393      -9.564  15.941   9.160  1.00  0.42
C
ATOM  15474  CG  PHE B 393     -10.110  15.250  10.362  1.00  0.42
C
ATOM  15475  CD1 PHE B 393     -11.197  14.398  10.258  1.00  0.42
C
ATOM  15476  CD2 PHE B 393      -9.521  15.435  11.602  1.00  0.42
C
ATOM  15477  CE1 PHE B 393     -11.680  13.741  11.368  1.00  0.42
C
ATOM  15478  CE2 PHE B 393     -10.007  14.780  12.714  1.00  0.42
C
ATOM  15479  CZ  PHE B 393     -11.086  13.930  12.597  1.00  0.42
C
ATOM  15480  H   PHE B 393     -10.612  14.105   7.604  1.00  0.30
H
ATOM  15481  HA  PHE B 393     -11.398  16.808   8.394  1.00  0.34
H
ATOM  15482 1HB  PHE B 393      -8.742  15.352   8.776  1.00  0.50
H
ATOM  15483 2HB  PHE B 393      -9.145  16.891   9.488  1.00  0.50
H
ATOM  15484  HD1 PHE B 393     -11.666  14.242   9.287  1.00  0.50
H
ATOM  15485  HD2 PHE B 393      -8.663  16.102  11.692  1.00  0.50
H
ATOM  15486  HE1 PHE B 393     -12.529  13.068  11.272  1.00  0.50
H
ATOM  15487  HE2 PHE B 393      -9.537  14.930  13.686  1.00  0.50
H
ATOM  15488  HZ  PHE B 393     -11.468  13.409  13.474  1.00  0.50
H
ATOM  15489  N   ALA B 394      -9.868  18.279   6.990  1.00  0.24
N
ATOM  15490  CA  ALA B 394      -9.153  19.103   6.027  1.00  0.24
C
ATOM  15491  C   ALA B 394      -7.656  18.811   6.109  1.00  0.24
C
ATOM  15492  O   ALA B 394      -6.954  18.859   5.100  1.00  0.72
O
ATOM  15493  CB  ALA B 394      -9.446  20.564   6.269  1.00  0.36
C
ATOM  15494  H   ALA B 394     -10.419  18.723   7.707  1.00  0.29
H
ATOM  15495  HA  ALA B 394      -9.489  18.850   5.029  1.00  0.29
H
```

ATOM 15496 1HB ALA B 394 -8.911 21.172 5.540 1.00 0.43
H
ATOM 15497 2HB ALA B 394 -10.511 20.750 6.174 1.00 0.43
H
ATOM 15498 3HB ALA B 394 -9.138 20.831 7.269 1.00 0.43
H
ATOM 15499 N ALA B 395 -7.177 18.491 7.313 1.00 0.16
N
ATOM 15500 CA ALA B 395 -5.793 18.116 7.525 1.00 0.17
C
ATOM 15501 C ALA B 395 -5.482 16.821 6.781 1.00 0.24
C
ATOM 15502 O ALA B 395 -4.416 16.685 6.180 1.00 1.04
O
ATOM 15503 CB ALA B 395 -5.512 17.957 9.014 1.00 0.26
C
ATOM 15504 H ALA B 395 -7.800 18.496 8.107 1.00 0.19
H
ATOM 15505 HA ALA B 395 -5.156 18.902 7.120 1.00 0.20
H
ATOM 15506 1HB ALA B 395 -4.466 17.693 9.161 1.00 0.31
H
ATOM 15507 2HB ALA B 395 -5.724 18.896 9.524 1.00 0.31
H
ATOM 15508 3HB ALA B 395 -6.146 17.172 9.423 1.00 0.31
H
ATOM 15509 N PHE B 396 -6.436 15.880 6.799 1.00 0.22
N
ATOM 15510 CA PHE B 396 -6.265 14.586 6.146 1.00 0.21
C
ATOM 15511 C PHE B 396 -6.122 14.741 4.644 1.00 0.18
C
ATOM 15512 O PHE B 396 -5.352 14.008 4.029 1.00 0.53
O
ATOM 15513 CB PHE B 396 -7.442 13.646 6.423 1.00 0.32
C
ATOM 15514 CG PHE B 396 -7.533 13.168 7.841 1.00 0.32
C
ATOM 15515 CD1 PHE B 396 -6.569 13.522 8.775 1.00 0.32
C
ATOM 15516 CD2 PHE B 396 -8.586 12.362 8.246 1.00 0.32
C
ATOM 15517 CE1 PHE B 396 -6.661 13.086 10.083 1.00 0.32
C
ATOM 15518 CE2 PHE B 396 -8.678 11.922 9.552 1.00 0.32
C
ATOM 15519 CZ PHE B 396 -7.716 12.287 10.470 1.00 0.32
C
ATOM 15520 H PHE B 396 -7.295 16.059 7.297 1.00 0.26
H
ATOM 15521 HA PHE B 396 -5.354 14.124 6.529 1.00 0.25
H
ATOM 15522 1HB PHE B 396 -8.378 14.136 6.170 1.00 0.38
H
ATOM 15523 2HB PHE B 396 -7.358 12.770 5.780 1.00 0.38
H
ATOM 15524 HD1 PHE B 396 -5.735 14.154 8.469 1.00 0.38
H

ATOM 15525 HD2 PHE B 396   -9.348 12.075   7.521 1.00 0.38
H
ATOM 15526 HE1 PHE B 396   -5.902 13.374 10.808 1.00 0.38
H
ATOM 15527 HE2 PHE B 396   -9.509 11.290   9.859 1.00 0.38
H
ATOM 15528 HZ PHE B 396   -7.790 11.943 11.501 1.00 0.38
H
ATOM 15529 N   PHE B 397   -6.813 15.727   4.062 1.00 0.45
N
ATOM 15530 CA PHE B 397   -6.726 15.985   2.619 1.00 0.28
C
ATOM 15531 C   PHE B 397   -5.319 16.268   2.097 1.00 0.29
C
ATOM 15532 O   PHE B 397   -5.066 16.093   0.906 1.00 0.42
O
ATOM 15533 CB PHE B 397   -7.681 17.099   2.179 1.00 0.42
C
ATOM 15534 CG PHE B 397   -9.087 16.621   1.979 1.00 0.42
C
ATOM 15535 CD1 PHE B 397   -10.070 16.827   2.927 1.00 0.42
C
ATOM 15536 CD2 PHE B 397   -9.423 15.941   0.820 1.00 0.42
C
ATOM 15537 CE1 PHE B 397   -11.354 16.369   2.727 1.00 0.42
C
ATOM 15538 CE2 PHE B 397   -10.704 15.476   0.622 1.00 0.42
C
ATOM 15539 CZ PHE B 397   -11.676 15.691   1.576 1.00 0.42
C
ATOM 15540 H   PHE B 397   -7.449 16.278   4.631 1.00 0.54
H
ATOM 15541 HA PHE B 397   -7.069 15.082   2.115 1.00 0.34
H
ATOM 15542 1HB PHE B 397   -7.694 17.889   2.927 1.00 0.50
H
ATOM 15543 2HB PHE B 397   -7.332 17.535   1.244 1.00 0.50
H
ATOM 15544 HD1 PHE B 397   -9.820 17.349   3.842 1.00 0.50
H
ATOM 15545 HD2 PHE B 397   -8.657 15.765   0.065 1.00 0.50
H
ATOM 15546 HE1 PHE B 397   -12.109 16.536   3.490 1.00 0.50
H
ATOM 15547 HE2 PHE B 397   -10.943 14.930 -0.285 1.00 0.50
H
ATOM 15548 HZ PHE B 397   -12.687 15.321   1.421 1.00 0.50
H
ATOM 15549 N   THR B 398   -4.383 16.647   2.976 1.00 0.28
N
ATOM 15550 CA THR B 398   -3.012 16.888   2.545 1.00 0.42
C
ATOM 15551 C   THR B 398   -2.356 15.580   2.093 1.00 0.40
C
ATOM 15552 O   THR B 398   -1.454 15.578   1.255 1.00 0.57
O
ATOM 15553 CB THR B 398   -2.191 17.526   3.683 1.00 0.63
C

```
ATOM  15554  OG1 THR B 398      -2.182  16.652   4.815  1.00  0.63
O
ATOM  15555  CG2 THR B 398      -2.790  18.864   4.097  1.00  0.63
C
ATOM  15556  H   THR B 398      -4.602  16.768   3.960  1.00  0.34
H
ATOM  15557  HA  THR B 398      -3.026  17.567   1.698  1.00  0.50
H
ATOM  15558  HB  THR B 398      -1.168  17.673   3.351  1.00  0.76
H
ATOM  15559  HG1 THR B 398      -3.066  16.597   5.191  1.00  0.76
H
ATOM  15560 1HG2 THR B 398      -2.195  19.294   4.902  1.00  0.76
H
ATOM  15561 2HG2 THR B 398      -2.794  19.543   3.246  1.00  0.76
H
ATOM  15562 3HG2 THR B 398      -3.812  18.712   4.444  1.00  0.76
H
ATOM  15563  N   ARG B 399      -2.872  14.458   2.597  1.00  0.32
N
ATOM  15564  CA  ARG B 399      -2.389  13.128   2.268  1.00  0.35
C
ATOM  15565  C   ARG B 399      -2.638  12.816   0.788  1.00  0.36
C
ATOM  15566  O   ARG B 399      -1.927  12.003   0.200  1.00  0.79
O
ATOM  15567  CB  ARG B 399      -3.078  12.077   3.143  1.00  0.52
C
ATOM  15568  CG  ARG B 399      -2.524  10.659   3.050  1.00  0.52
C
ATOM  15569  CD  ARG B 399      -1.156  10.570   3.644  1.00  0.52
C
ATOM  15570  NE  ARG B 399      -1.166  10.765   5.098  1.00  0.52
N
ATOM  15571  CZ  ARG B 399      -1.209   9.796   6.032  1.00  0.52
C
ATOM  15572  NH1 ARG B 399      -1.191   8.537   5.732  1.00  0.52
N
ATOM  15573  NH2 ARG B 399      -1.269  10.109   7.304  1.00  0.52
N
ATOM  15574  H   ARG B 399      -3.634  14.514   3.256  1.00  0.38
H
ATOM  15575  HA  ARG B 399      -1.316  13.091   2.456  1.00  0.42
H
ATOM  15576 1HB  ARG B 399      -3.014  12.383   4.187  1.00  0.63
H
ATOM  15577 2HB  ARG B 399      -4.132  12.032   2.887  1.00  0.63
H
ATOM  15578 1HG  ARG B 399      -3.177   9.974   3.591  1.00  0.63
H
ATOM  15579 2HG  ARG B 399      -2.465  10.355   2.004  1.00  0.63
H
ATOM  15580 1HD  ARG B 399      -0.752   9.581   3.445  1.00  0.63
H
ATOM  15581 2HD  ARG B 399      -0.509  11.325   3.199  1.00  0.63
H
ATOM  15582  HE  ARG B 399      -1.187  11.707   5.460  1.00  0.63
H
```

```
ATOM  15583 1HH1 ARG B 399      -1.178   8.231   4.766  1.00  0.63
H
ATOM  15584 2HH1 ARG B 399      -1.228   7.870   6.495  1.00  0.63
H
ATOM  15585 1HH2 ARG B 399      -1.280  11.084   7.568  1.00  0.63
H
ATOM  15586 2HH2 ARG B 399      -1.318   9.372   7.997  1.00  0.63
H
ATOM  15587  N   ALA B 400      -3.655  13.445   0.187  1.00  0.25
N
ATOM  15588  CA  ALA B 400      -3.976  13.177  -1.203  1.00  0.25
C
ATOM  15589  C   ALA B 400      -3.792  14.397  -2.074  1.00  0.21
C
ATOM  15590  O   ALA B 400      -4.444  14.508  -3.109  1.00  0.36
O
ATOM  15591  CB  ALA B 400      -5.408  12.704  -1.319  1.00  0.38
C
ATOM  15592  H   ALA B 400      -4.210  14.135   0.678  1.00  0.30
H
ATOM  15593  HA  ALA B 400      -3.304  12.404  -1.564  1.00  0.30
H
ATOM  15594 1HB  ALA B 400      -5.639  12.476  -2.359  1.00  0.45
H
ATOM  15595 2HB  ALA B 400      -5.537  11.810  -0.713  1.00  0.45
H
ATOM  15596 3HB  ALA B 400      -6.078  13.483  -0.961  1.00  0.45
H
ATOM  15597  N   PHE B 401      -2.865  15.272  -1.697  1.00  0.27
N
ATOM  15598  CA  PHE B 401      -2.628  16.497  -2.442  1.00  0.38
C
ATOM  15599  C   PHE B 401      -2.253  16.264  -3.906  1.00  0.36
C
ATOM  15600  O   PHE B 401      -2.732  16.980  -4.785  1.00  0.45
O
ATOM  15601  CB  PHE B 401      -1.605  17.350  -1.709  1.00  0.57
C
ATOM  15602  CG  PHE B 401      -1.310  18.649  -2.376  1.00  0.57
C
ATOM  15603  CD1 PHE B 401      -2.200  19.189  -3.290  1.00  0.57
C
ATOM  15604  CD2 PHE B 401      -0.159  19.350  -2.065  1.00  0.57
C
ATOM  15605  CE1 PHE B 401      -1.932  20.400  -3.891  1.00  0.57
C
ATOM  15606  CE2 PHE B 401       0.108  20.563  -2.661  1.00  0.57
C
ATOM  15607  CZ  PHE B 401      -0.779  21.086  -3.575  1.00  0.57
C
ATOM  15608  H   PHE B 401      -2.339  15.128  -0.840  1.00  0.32
H
ATOM  15609  HA  PHE B 401      -3.554  17.063  -2.441  1.00  0.46
H
ATOM  15610 1HB  PHE B 401      -1.964  17.559  -0.702  1.00  0.68
H
ATOM  15611 2HB  PHE B 401      -0.674  16.801  -1.611  1.00  0.68
H
```

```
ATOM  15612  HD1 PHE B 401      -3.115  18.649  -3.533  1.00  0.68
H
ATOM  15613  HD2 PHE B 401       0.536  18.931  -1.339  1.00  0.68
H
ATOM  15614  HE1 PHE B 401      -2.632  20.818  -4.611  1.00  0.68
H
ATOM  15615  HE2 PHE B 401       1.016  21.107  -2.413  1.00  0.68
H
ATOM  15616  HZ  PHE B 401      -0.571  22.043  -4.045  1.00  0.68
H
ATOM  15617  N   ASP B 402      -1.407  15.278  -4.177  1.00  0.30
N
ATOM  15618  CA  ASP B 402      -1.041  14.989  -5.556  1.00  0.32
C
ATOM  15619  C   ASP B 402      -2.222  14.418  -6.341  1.00  0.27
C
ATOM  15620  O   ASP B 402      -2.331  14.645  -7.544  1.00  0.52
O
ATOM  15621  CB  ASP B 402       0.150  14.051  -5.612  1.00  0.48
C
ATOM  15622  CG  ASP B 402       0.732  13.883  -7.018  1.00  0.48
C
ATOM  15623  OD1 ASP B 402       1.026  14.859  -7.671  1.00  0.48
O
ATOM  15624  OD2 ASP B 402       0.912  12.754  -7.403  1.00  0.48
O
ATOM  15625  H   ASP B 402      -1.020  14.724  -3.426  1.00  0.36
H
ATOM  15626  HA  ASP B 402      -0.747  15.925  -6.031  1.00  0.38
H
ATOM  15627 1HB  ASP B 402       0.931  14.420  -4.952  1.00  0.58
H
ATOM  15628 2HB  ASP B 402      -0.156  13.075  -5.239  1.00  0.58
H
ATOM  15629  N   GLN B 403      -3.099  13.669  -5.667  1.00  0.21
N
ATOM  15630  CA  GLN B 403      -4.280  13.128  -6.324  1.00  0.25
C
ATOM  15631  C   GLN B 403      -5.192  14.284  -6.725  1.00  0.35
C
ATOM  15632  O   GLN B 403      -5.788  14.279  -7.804  1.00  0.45
O
ATOM  15633  CB  GLN B 403      -5.023  12.163  -5.404  1.00  0.38
C
ATOM  15634  CG  GLN B 403      -6.108  11.399  -6.114  1.00  0.38
C
ATOM  15635  CD  GLN B 403      -5.523  10.469  -7.145  1.00  0.38
C
ATOM  15636  OE1 GLN B 403      -4.824   9.518  -6.787  1.00  0.38
O
ATOM  15637  NE2 GLN B 403      -5.778  10.740  -8.417  1.00  0.38
N
ATOM  15638  H   GLN B 403      -2.955  13.488  -4.684  1.00  0.25
H
ATOM  15639  HA  GLN B 403      -3.977  12.601  -7.227  1.00  0.30
H
ATOM  15640 1HB  GLN B 403      -4.321  11.446  -4.978  1.00  0.45
H
```

```
ATOM  15641 2HB  GLN B 403      -5.477  12.712  -4.581  1.00  0.45
H
ATOM  15642 1HG  GLN B 403      -6.668  10.807  -5.391  1.00  0.45
H
ATOM  15643 2HG  GLN B 403      -6.764  12.102  -6.624  1.00  0.45
H
ATOM  15644 1HE2 GLN B 403      -5.404  10.158  -9.140  1.00  0.45
H
ATOM  15645 2HE2 GLN B 403      -6.339  11.530  -8.656  1.00  0.45
H
ATOM  15646  N   LEU B 404      -5.265  15.284  -5.845  1.00  0.41
N
ATOM  15647  CA  LEU B 404      -6.056  16.477  -6.068  1.00  0.47
C
ATOM  15648  C   LEU B 404      -5.489  17.280  -7.234  1.00  0.27
C
ATOM  15649  O   LEU B 404      -6.235  17.756  -8.095  1.00  0.26
O
ATOM  15650  CB  LEU B 404      -6.040  17.328  -4.794  1.00  0.70
C
ATOM  15651  CG  LEU B 404      -6.724  16.694  -3.588  1.00  0.70
C
ATOM  15652  CD1 LEU B 404      -6.488  17.549  -2.353  1.00  0.70
C
ATOM  15653  CD2 LEU B 404      -8.175  16.549  -3.890  1.00  0.70
C
ATOM  15654  H   LEU B 404      -4.767  15.199  -4.968  1.00  0.49
H
ATOM  15655  HA  LEU B 404      -7.079  16.183  -6.303  1.00  0.56
H
ATOM  15656 1HB  LEU B 404      -5.014  17.550  -4.521  1.00  0.85
H
ATOM  15657 2HB  LEU B 404      -6.551  18.265  -5.003  1.00  0.85
H
ATOM  15658  HG  LEU B 404      -6.314  15.711  -3.401  1.00  0.85
H
ATOM  15659 1HD1 LEU B 404      -6.982  17.096  -1.495  1.00  0.85
H
ATOM  15660 2HD1 LEU B 404      -5.425  17.631  -2.154  1.00  0.85
H
ATOM  15661 3HD1 LEU B 404      -6.894  18.540  -2.528  1.00  0.85
H
ATOM  15662 1HD2 LEU B 404      -8.682  16.089  -3.041  1.00  0.85
H
ATOM  15663 2HD2 LEU B 404      -8.590  17.536  -4.077  1.00  0.85
H
ATOM  15664 3HD2 LEU B 404      -8.305  15.927  -4.774  1.00  0.85
H
ATOM  15665  N   ARG B 405      -4.162  17.384  -7.269  1.00  0.24
N
ATOM  15666  CA  ARG B 405      -3.451  18.067  -8.334  1.00  0.22
C
ATOM  15667  C   ARG B 405      -3.771  17.446  -9.679  1.00  0.21
C
ATOM  15668  O   ARG B 405      -4.094  18.153 -10.636  1.00  0.24
O
ATOM  15669  CB  ARG B 405      -1.956  18.018  -8.074  1.00  0.33
C
```

ATOM 15670 CG ARG B 405   -1.086 18.769 -9.062 1.00 0.33
C
ATOM 15671 CD ARG B 405   0.344 18.688 -8.661 1.00 0.33
C
ATOM 15672 NE ARG B 405   0.908 17.379 -8.920 1.00 0.33
N
ATOM 15673 CZ ARG B 405   1.425 17.007 -10.106 1.00 0.33
C
ATOM 15674 NH1 ARG B 405   1.473 17.866 -11.098 1.00 0.33
N
ATOM 15675 NH2 ARG B 405   1.893 15.782 -10.255 1.00 0.33
N
ATOM 15676 H   ARG B 405   -3.618 16.991 -6.513 1.00 0.29
H
ATOM 15677 HA ARG B 405   -3.768 19.112 -8.348 1.00 0.26
H
ATOM 15678 1HB ARG B 405   -1.750 18.428 -7.085 1.00 0.40
H
ATOM 15679 2HB ARG B 405   -1.619 16.982 -8.071 1.00 0.40
H
ATOM 15680 1HG ARG B 405   -1.186 18.315 -10.047 1.00 0.40
H
ATOM 15681 2HG ARG B 405   -1.389 19.812 -9.114 1.00 0.40
H
ATOM 15682 1HD ARG B 405   0.920 19.424 -9.220 1.00 0.40
H
ATOM 15683 2HD ARG B 405   0.432 18.891 -7.595 1.00 0.40
H
ATOM 15684 HE ARG B 405   0.889 16.678 -8.183 1.00 0.40
H
ATOM 15685 1HH1 ARG B 405   1.119 18.804 -10.972 1.00 0.40
H
ATOM 15686 2HH1 ARG B 405   1.868 17.597 -11.987 1.00 0.40
H
ATOM 15687 1HH2 ARG B 405   1.834 15.145 -9.466 1.00 0.40
H
ATOM 15688 2HH2 ARG B 405   2.305 15.503 -11.136 1.00 0.40
H
ATOM 15689 N   MET B 406   -3.704 16.117 -9.735 1.00 0.20
N
ATOM 15690 CA MET B 406   -4.003 15.393 -10.953 1.00 0.20
C
ATOM 15691 C   MET B 406   -5.485 15.471 -11.308 1.00 0.18
C
ATOM 15692 O   MET B 406   -5.831 15.416 -12.487 1.00 0.19
O
ATOM 15693 CB MET B 406   -3.530 13.960 -10.829 1.00 0.30
C
ATOM 15694 CG MET B 406   -2.009 13.814 -10.784 1.00 0.30
C
ATOM 15695 SD MET B 406   -1.166 14.541 -12.197 1.00 0.30
S
ATOM 15696 CE MET B 406   -1.793 13.546 -13.539 1.00 0.30
C
ATOM 15697 H   MET B 406   -3.416 15.595 -8.917 1.00 0.24
H
ATOM 15698 HA MET B 406   -3.451 15.855 -11.765 1.00 0.24
H

ATOM 15699 1HB MET B 406   -3.935 13.518 -9.920 1.00 0.36
H
ATOM 15700 2HB MET B 406   -3.900 13.375 -11.671 1.00 0.36
H
ATOM 15701 1HG MET B 406   -1.629 14.288 -9.882 1.00 0.36
H
ATOM 15702 2HG MET B 406   -1.747 12.762 -10.746 1.00 0.36
H
ATOM 15703 1HE MET B 406   -1.353 13.881 -14.478 1.00 0.36
H
ATOM 15704 2HE MET B 406   -1.536 12.500 -13.367 1.00 0.36
H
ATOM 15705 3HE MET B 406   -2.877 13.647 -13.589 1.00 0.36
H
ATOM 15706 N   GLY B 407   -6.354 15.622 -10.303 1.00 0.17
N
ATOM 15707 CA GLY B 407   -7.782 15.813 -10.540 1.00 0.17
C
ATOM 15708 C   GLY B 407   -8.021 17.105 -11.324 1.00 0.18
C
ATOM 15709 O   GLY B 407   -8.826 17.133 -12.258 1.00 0.19
O
ATOM 15710 H   GLY B 407   -6.027 15.594 -9.346 1.00 0.20
H
ATOM 15711 1HA GLY B 407   -8.179 14.961 -11.093 1.00 0.20
H
ATOM 15712 2HA GLY B 407   -8.308 15.856 -9.586 1.00 0.20
H
ATOM 15713 N   ALA B 408   -7.297 18.168 -10.951 1.00 0.19
N
ATOM 15714 CA ALA B 408   -7.372 19.442 -11.671 1.00 0.21
C
ATOM 15715 C   ALA B 408   -6.863 19.265 -13.095 1.00 0.22
C
ATOM 15716 O   ALA B 408   -7.493 19.712 -14.052 1.00 0.23
O
ATOM 15717 CB ALA B 408   -6.570 20.514 -10.954 1.00 0.32
C
ATOM 15718 H   ALA B 408   -6.689 18.089 -10.143 1.00 0.23
H
ATOM 15719 HA ALA B 408   -8.418 19.748 -11.718 1.00 0.25
H
ATOM 15720 1HB ALA B 408   -6.655 21.455 -11.495 1.00 0.38
H
ATOM 15721 2HB ALA B 408   -6.966 20.635 -9.949 1.00 0.38
H
ATOM 15722 3HB ALA B 408   -5.524 20.218 -10.902 1.00 0.38
H
ATOM 15723 N   ILE B 409   -5.746 18.559 -13.228 1.00 0.23
N
ATOM 15724 CA ILE B 409   -5.152 18.239 -14.521 1.00 0.24
C
ATOM 15725 C   ILE B 409   -6.123 17.433 -15.390 1.00 0.25
C
ATOM 15726 O   ILE B 409   -6.184 17.613 -16.607 1.00 0.31
O
ATOM 15727 CB ILE B 409   -3.838 17.489 -14.315 1.00 0.36
C

ATOM 15728 CG1 ILE B 409   -2.826 18.448 -13.684 1.00 0.36
C
ATOM 15729 CG2 ILE B 409   -3.329 16.955 -15.639 1.00 0.36
C
ATOM 15730 CD1 ILE B 409   -1.591 17.776 -13.142 1.00 0.36
C
ATOM 15731 H   ILE B 409   -5.268 18.239 -12.392 1.00 0.28
H
ATOM 15732 HA ILE B 409   -4.932 19.173 -15.037 1.00 0.29
H
ATOM 15733 HB ILE B 409   -3.989 16.663 -13.628 1.00 0.43
H
ATOM 15734 1HG1 ILE B 409   -2.521 19.172 -14.436 1.00 0.43
H
ATOM 15735 2HG1 ILE B 409   -3.308 18.986 -12.869 1.00 0.43
H
ATOM 15736 1HG2 ILE B 409   -2.391 16.424 -15.482 1.00 0.43
H
ATOM 15737 2HG2 ILE B 409   -4.067 16.278 -16.058 1.00 0.43
H
ATOM 15738 3HG2 ILE B 409   -3.170 17.784 -16.326 1.00 0.43
H
ATOM 15739 1HD1 ILE B 409   -0.927 18.524 -12.715 1.00 0.43
H
ATOM 15740 2HD1 ILE B 409   -1.880 17.074 -12.366 1.00 0.43
H
ATOM 15741 3HD1 ILE B 409   -1.078 17.246 -13.943 1.00 0.43
H
ATOM 15742 N   SER B 410   -6.895 16.556 -14.742 1.00 0.23
N
ATOM 15743 CA SER B 410   -7.882 15.709 -15.392 1.00 0.23
C
ATOM 15744 C   SER B 410   -9.201 16.432 -15.681 1.00 0.25
C
ATOM 15745 O   SER B 410   -10.126 15.819 -16.216 1.00 0.33
O
ATOM 15746 CB SER B 410   -8.191 14.521 -14.508 1.00 0.35
C
ATOM 15747 OG SER B 410   -7.052 13.741 -14.298 1.00 0.35
O
ATOM 15748 H   SER B 410   -6.771 16.444 -13.747 1.00 0.28
H
ATOM 15749 HA SER B 410   -7.466 15.360 -16.338 1.00 0.28
H
ATOM 15750 1HB SER B 410   -8.580 14.868 -13.550 1.00 0.41
H
ATOM 15751 2HB SER B 410   -8.968 13.916 -14.975 1.00 0.41
H
ATOM 15752 HG SER B 410   -6.478 14.265 -13.727 1.00 0.41
H
ATOM 15753 N   GLN B 411   -9.298 17.722 -15.326 1.00 0.20
N
ATOM 15754 CA GLN B 411   -10.502 18.519 -15.547 1.00 0.19
C
ATOM 15755 C   GLN B 411   -11.716 17.898 -14.875 1.00 0.18
C
ATOM 15756 O   GLN B 411   -12.827 17.955 -15.405 1.00 0.19
O

ATOM 15757 CB GLN B 411   -10.763 18.697 -17.047 1.00 0.29
C
ATOM 15758 CG GLN B 411   -9.648 19.417 -17.787 1.00 0.29
C
ATOM 15759 CD GLN B 411   -9.980 19.640 -19.252 1.00 0.29
C
ATOM 15760 OE1 GLN B 411   -11.140 19.539 -19.660 1.00 0.29
O
ATOM 15761 NE2 GLN B 411   -8.963 19.949 -20.049 1.00 0.29
N
ATOM 15762 H   GLN B 411   -8.521 18.197 -14.893 1.00 0.24
H
ATOM 15763 HA GLN B 411   -10.344 19.503 -15.108 1.00 0.23
H
ATOM 15764 1HB GLN B 411   -10.910 17.726 -17.518 1.00 0.34
H
ATOM 15765 2HB GLN B 411   -11.679 19.268 -17.188 1.00 0.34
H
ATOM 15766 1HG GLN B 411   -9.490 20.390 -17.322 1.00 0.34
H
ATOM 15767 2HG GLN B 411   -8.737 18.822 -17.727 1.00 0.34
H
ATOM 15768 1HE2 GLN B 411   -9.123 20.110 -21.024 1.00 0.34
H
ATOM 15769 2HE2 GLN B 411   -8.039 20.024 -19.675 1.00 0.34
H
ATOM 15770 N   ALA B 412   -11.497 17.288 -13.713 1.00 0.18
N
ATOM 15771 CA ALA B 412   -12.563 16.626 -12.990 1.00 0.19
C
ATOM 15772 C   ALA B 412   -13.433 17.612 -12.250 1.00 0.20
C
ATOM 15773 O   ALA B 412   -13.024 18.739 -11.966 1.00 0.24
O
ATOM 15774 CB ALA B 412   -11.994 15.627 -12.028 1.00 0.29
C
ATOM 15775 H   ALA B 412   -10.566 17.276 -13.315 1.00 0.22
H
ATOM 15776 HA ALA B 412   -13.188 16.105 -13.715 1.00 0.23
H
ATOM 15777 1HB ALA B 412   -12.804 15.113 -11.512 1.00 0.34
H
ATOM 15778 2HB ALA B 412   -11.392 14.899 -12.573 1.00 0.34
H
ATOM 15779 3HB ALA B 412   -11.368 16.144 -11.302 1.00 0.34
H
ATOM 15780 N   ASN B 413   -14.638 17.168 -11.935 1.00 0.26
N
ATOM 15781 CA ASN B 413   -15.603 17.983 -11.236 1.00 0.31
C
ATOM 15782 C   ASN B 413   -15.983 17.320 -9.917 1.00 0.48
C
ATOM 15783 O   ASN B 413   -17.048 16.708 -9.817 1.00 1.69
O
ATOM 15784 CB ASN B 413   -16.824 18.202 -12.103 1.00 0.46
C
ATOM 15785 CG ASN B 413   -16.488 18.855 -13.417 1.00 0.46
C

ATOM 15786 OD1 ASN B 413 -15.982 19.980 -13.487 1.00 0.46
O
ATOM 15787 ND2 ASN B 413 -16.763 18.149 -14.485 1.00 0.46
N
ATOM 15788 H ASN B 413 -14.897 16.228 -12.201 1.00 0.31
H
ATOM 15789 HA ASN B 413 -15.153 18.944 -11.010 1.00 0.37
H
ATOM 15790 1HB ASN B 413 -17.309 17.245 -12.298 1.00 0.56
H
ATOM 15791 2HB ASN B 413 -17.540 18.830 -11.571 1.00 0.56
H
ATOM 15792 1HD2 ASN B 413 -16.565 18.518 -15.394 1.00 0.56
H
ATOM 15793 2HD2 ASN B 413 -17.170 17.241 -14.393 1.00 0.56
H
ATOM 15794 N ILE B 414 -15.109 17.407 -8.913 1.00 0.58
N
ATOM 15795 CA ILE B 414 -15.411 16.769 -7.633 1.00 0.80
C
ATOM 15796 C ILE B 414 -15.493 17.778 -6.500 1.00 0.52
C
ATOM 15797 O ILE B 414 -14.844 18.826 -6.530 1.00 0.99
O
ATOM 15798 CB ILE B 414 -14.366 15.691 -7.268 1.00 1.20
C
ATOM 15799 CG1 ILE B 414 -12.976 16.317 -7.117 1.00 1.20
C
ATOM 15800 CG2 ILE B 414 -14.348 14.596 -8.325 1.00 1.20
C
ATOM 15801 CD1 ILE B 414 -11.958 15.358 -6.542 1.00 1.20
C
ATOM 15802 H ILE B 414 -14.246 17.920 -9.035 1.00 0.70
H
ATOM 15803 HA ILE B 414 -16.381 16.280 -7.714 1.00 0.96
H
ATOM 15804 HB ILE B 414 -14.629 15.251 -6.306 1.00 1.44
H
ATOM 15805 1HG1 ILE B 414 -12.627 16.649 -8.093 1.00 1.44
H
ATOM 15806 2HG1 ILE B 414 -13.042 17.186 -6.463 1.00 1.44
H
ATOM 15807 1HG2 ILE B 414 -13.619 13.834 -8.046 1.00 1.44
H
ATOM 15808 2HG2 ILE B 414 -15.335 14.144 -8.398 1.00 1.44
H
ATOM 15809 3HG2 ILE B 414 -14.072 15.024 -9.289 1.00 1.44
H
ATOM 15810 1HD1 ILE B 414 -10.991 15.854 -6.459 1.00 1.44
H
ATOM 15811 2HD1 ILE B 414 -12.285 15.033 -5.558 1.00 1.44
H
ATOM 15812 3HD1 ILE B 414 -11.866 14.492 -7.191 1.00 1.44
H
ATOM 15813 N ASN B 415 -16.296 17.432 -5.502 1.00 0.31
N
ATOM 15814 CA ASN B 415 -16.509 18.270 -4.345 1.00 0.31
C

```
ATOM  15815  C   ASN B 415     -15.846  17.634  -3.137  1.00  0.25
C
ATOM  15816  O   ASN B 415     -15.936  16.423  -2.939  1.00  0.50
O
ATOM  15817  CB  ASN B 415     -17.988  18.458  -4.138  1.00  0.46
C
ATOM  15818  CG  ASN B 415     -18.642  19.122  -5.318  1.00  0.46
C
ATOM  15819  OD1 ASN B 415     -18.290  20.238  -5.704  1.00  0.46
O
ATOM  15820  ND2 ASN B 415     -19.584  18.446  -5.915  1.00  0.46
N
ATOM  15821  H   ASN B 415     -16.797  16.555  -5.542  1.00  0.37
H
ATOM  15822  HA  ASN B 415     -16.039  19.239  -4.515  1.00  0.37
H
ATOM  15823 1HB  ASN B 415     -18.457  17.488  -3.977  1.00  0.56
H
ATOM  15824 2HB  ASN B 415     -18.158  19.063  -3.249  1.00  0.56
H
ATOM  15825 1HD2 ASN B 415     -20.050  18.834  -6.710  1.00  0.56
H
ATOM  15826 2HD2 ASN B 415     -19.838  17.540  -5.577  1.00  0.56
H
ATOM  15827  N   LEU B 416     -15.154  18.446  -2.359  1.00  0.27
N
ATOM  15828  CA  LEU B 416     -14.427  17.963  -1.208  1.00  0.18
C
ATOM  15829  C   LEU B 416     -14.780  18.784   0.020  1.00  0.28
C
ATOM  15830  O   LEU B 416     -14.797  20.008  -0.049  1.00  0.60
O
ATOM  15831  CB  LEU B 416     -12.953  18.077  -1.527  1.00  0.27
C
ATOM  15832  CG  LEU B 416     -12.536  17.347  -2.801  1.00  0.27
C
ATOM  15833  CD1 LEU B 416     -11.141  17.738  -3.126  1.00  0.27
C
ATOM  15834  CD2 LEU B 416     -12.657  15.849  -2.618  1.00  0.27
C
ATOM  15835  H   LEU B 416     -15.118  19.427  -2.581  1.00  0.32
H
ATOM  15836  HA  LEU B 416     -14.691  16.923  -1.033  1.00  0.22
H
ATOM  15837 1HB  LEU B 416     -12.706  19.127  -1.661  1.00  0.32
H
ATOM  15838 2HB  LEU B 416     -12.372  17.680  -0.696  1.00  0.32
H
ATOM  15839  HG  LEU B 416     -13.167  17.664  -3.630  1.00  0.32
H
ATOM  15840 1HD1 LEU B 416     -10.841  17.241  -4.048  1.00  0.32
H
ATOM  15841 2HD1 LEU B 416     -11.094  18.818  -3.264  1.00  0.32
H
ATOM  15842 3HD1 LEU B 416     -10.479  17.443  -2.313  1.00  0.32
H
ATOM  15843 1HD2 LEU B 416     -12.352  15.342  -3.530  1.00  0.32
H
```

```
ATOM  15844 2HD2 LEU B 416      -12.020  15.532  -1.806  1.00  0.32
H
ATOM  15845 3HD2 LEU B 416      -13.689  15.594  -2.389  1.00  0.32
H
ATOM  15846  N   ILE B 417      -15.040  18.128   1.140  1.00  0.22
N
ATOM  15847  CA  ILE B 417      -15.378  18.859   2.354  1.00  0.23
C
ATOM  15848  C   ILE B 417      -14.405  18.469   3.444  1.00  0.14
C
ATOM  15849  O   ILE B 417      -14.334  17.299   3.826  1.00  0.26
O
ATOM  15850  CB  ILE B 417      -16.822  18.576   2.807  1.00  0.35
C
ATOM  15851  CG1 ILE B 417      -17.808  18.942   1.695  1.00  0.35
C
ATOM  15852  CG2 ILE B 417      -17.134  19.392   4.060  1.00  0.35
C
ATOM  15853  CD1 ILE B 417      -19.209  18.475   1.974  1.00  0.35
C
ATOM  15854  H   ILE B 417      -15.014  17.117   1.144  1.00  0.26
H
ATOM  15855  HA  ILE B 417      -15.277  19.926   2.168  1.00  0.28
H
ATOM  15856  HB  ILE B 417      -16.938  17.514   3.022  1.00  0.41
H
ATOM  15857 1HG1 ILE B 417      -17.820  20.024   1.565  1.00  0.41
H
ATOM  15858 2HG1 ILE B 417      -17.481  18.487   0.761  1.00  0.41
H
ATOM  15859 1HG2 ILE B 417      -18.155  19.188   4.381  1.00  0.41
H
ATOM  15860 2HG2 ILE B 417      -16.442  19.117   4.855  1.00  0.41
H
ATOM  15861 3HG2 ILE B 417      -17.028  20.454   3.838  1.00  0.41
H
ATOM  15862 1HD1 ILE B 417      -19.855  18.755   1.145  1.00  0.41
H
ATOM  15863 2HD1 ILE B 417      -19.210  17.392   2.084  1.00  0.41
H
ATOM  15864 3HD1 ILE B 417      -19.572  18.935   2.892  1.00  0.41
H
ATOM  15865  N   GLY B 418      -13.648  19.448   3.928  1.00  0.13
N
ATOM  15866  CA  GLY B 418      -12.628  19.184   4.929  1.00  0.17
C
ATOM  15867  C   GLY B 418      -13.011  19.740   6.282  1.00  0.33
C
ATOM  15868  O   GLY B 418      -13.244  20.943   6.423  1.00  1.13
O
ATOM  15869  H   GLY B 418      -13.797  20.391   3.594  1.00  0.16
H
ATOM  15870 1HA  GLY B 418      -12.460  18.114   5.002  1.00  0.20
H
ATOM  15871 2HA  GLY B 418      -11.688  19.628   4.606  1.00  0.20
H
ATOM  15872  N   SER B 419      -13.036  18.851   7.274  1.00  0.19
N
```

ATOM 15873 CA SER B 419 -13.431 19.180 8.634 1.00 0.22
C
ATOM 15874 C SER B 419 -12.300 19.447 9.581 1.00 0.21
C
ATOM 15875 O SER B 419 -11.133 19.259 9.249 1.00 0.35
O
ATOM 15876 CB SER B 419 -14.296 18.063 9.181 1.00 0.33
C
ATOM 15877 OG SER B 419 -13.545 16.910 9.423 1.00 0.33
O
ATOM 15878 H SER B 419 -12.801 17.892 7.056 1.00 0.23
H
ATOM 15879 HA SER B 419 -13.984 20.093 8.604 1.00 0.26
H
ATOM 15880 1HB SER B 419 -14.780 18.379 10.097 1.00 0.40
H
ATOM 15881 2HB SER B 419 -15.081 17.839 8.460 1.00 0.40
H
ATOM 15882 HG SER B 419 -13.190 17.015 10.313 1.00 0.40
H
ATOM 15883 N HIS B 420 -12.646 19.936 10.769 1.00 0.20
N
ATOM 15884 CA HIS B 420 -11.651 20.222 11.789 1.00 0.28
C
ATOM 15885 C HIS B 420 -10.611 21.233 11.330 1.00 0.27
C
ATOM 15886 O HIS B 420 -9.408 21.035 11.532 1.00 0.34
O
ATOM 15887 CB HIS B 420 -10.982 18.918 12.232 1.00 0.42
C
ATOM 15888 CG HIS B 420 -11.970 18.000 12.853 1.00 0.42
C
ATOM 15889 ND1 HIS B 420 -12.801 17.197 12.108 1.00 0.42
N
ATOM 15890 CD2 HIS B 420 -12.274 17.765 14.149 1.00 0.42
C
ATOM 15891 CE1 HIS B 420 -13.584 16.512 12.919 1.00 0.42
C
ATOM 15892 NE2 HIS B 420 -13.282 16.833 14.164 1.00 0.42
N
ATOM 15893 H HIS B 420 -13.625 20.071 11.001 1.00 0.24
H
ATOM 15894 HA HIS B 420 -12.151 20.646 12.658 1.00 0.34
H
ATOM 15895 1HB HIS B 420 -10.524 18.412 11.384 1.00 0.50
H
ATOM 15896 2HB HIS B 420 -10.194 19.122 12.955 1.00 0.50
H
ATOM 15897 HD2 HIS B 420 -11.812 18.231 15.019 1.00 0.50
H
ATOM 15898 HE1 HIS B 420 -14.347 15.806 12.604 1.00 0.50
H
ATOM 15899 HE2 HIS B 420 -13.720 16.466 14.996 1.00 0.50
H
ATOM 15900 N CYS B 421 -11.074 22.324 10.731 1.00 0.23
N
ATOM 15901 CA CYS B 421 -10.153 23.351 10.299 1.00 0.33
C

ATOM 15902 C CYS B 421 -9.728 24.232 11.457 1.00 0.32
C
ATOM 15903 O CYS B 421 -10.562 24.680 12.244 1.00 0.98
O
ATOM 15904 CB CYS B 421 -10.784 24.233 9.217 1.00 0.49
C
ATOM 15905 SG CYS B 421 -11.152 23.386 7.665 1.00 0.49
S
ATOM 15906 H CYS B 421 -12.068 22.445 10.561 1.00 0.28
H
ATOM 15907 HA CYS B 421 -9.271 22.869 9.892 1.00 0.40
H
ATOM 15908 1HB CYS B 421 -11.718 24.648 9.597 1.00 0.59
H
ATOM 15909 2HB CYS B 421 -10.122 25.069 8.995 1.00 0.59
H
ATOM 15910 HG CYS B 421 -9.887 23.254 7.282 1.00 0.59
H
ATOM 15911 N GLY B 422 -8.430 24.489 11.550 1.00 0.20
N
ATOM 15912 CA GLY B 422 -7.922 25.418 12.544 1.00 0.22
C
ATOM 15913 C GLY B 422 -7.638 24.884 13.951 1.00 0.30
C
ATOM 15914 O GLY B 422 -7.999 23.759 14.307 1.00 0.42
O
ATOM 15915 H GLY B 422 -7.787 24.093 10.880 1.00 0.24
H
ATOM 15916 1HA GLY B 422 -7.029 25.886 12.154 1.00 0.26
H
ATOM 15917 2HA GLY B 422 -8.655 26.211 12.628 1.00 0.26
H
ATOM 15918 N VAL B 423 -7.027 25.755 14.772 1.00 0.41
N
ATOM 15919 CA VAL B 423 -6.697 25.450 16.157 1.00 0.74
C
ATOM 15920 C VAL B 423 -7.961 25.377 16.997 1.00 0.51
C
ATOM 15921 O VAL B 423 -7.968 24.733 18.044 1.00 0.63
O
ATOM 15922 CB VAL B 423 -5.739 26.496 16.774 1.00 1.11
C
ATOM 15923 CG1 VAL B 423 -4.481 26.593 15.927 1.00 1.11
C
ATOM 15924 CG2 VAL B 423 -6.427 27.842 16.929 1.00 1.11
C
ATOM 15925 H VAL B 423 -6.778 26.665 14.404 1.00 0.49
H
ATOM 15926 HA VAL B 423 -6.214 24.484 16.183 1.00 0.89
H
ATOM 15927 HB VAL B 423 -5.429 26.145 17.759 1.00 1.33
H
ATOM 15928 1HG1 VAL B 423 -3.786 27.300 16.376 1.00 1.33
H
ATOM 15929 2HG1 VAL B 423 -4.012 25.613 15.865 1.00 1.33
H
ATOM 15930 3HG1 VAL B 423 -4.746 26.933 14.927 1.00 1.33
H

ATOM 15931 1HG2 VAL B 423   -5.740 28.555 17.379 1.00 1.33
H
ATOM 15932 2HG2 VAL B 423   -6.735 28.209 15.956 1.00 1.33
H
ATOM 15933 3HG2 VAL B 423   -7.303 27.735 17.568 1.00 1.33
H
ATOM 15934 N   SER B 424   -9.042 26.017 16.518 1.00 0.33
N
ATOM 15935 CA SER B 424   -10.356 25.989 17.170 1.00 0.28
C
ATOM 15936 C   SER B 424   -10.893 24.577 17.506 1.00 0.29
C
ATOM 15937 O   SER B 424   -11.798 24.441 18.332 1.00 0.34
O
ATOM 15938 CB SER B 424   -11.365 26.707 16.301 1.00 0.42
C
ATOM 15939 OG SER B 424   -11.597 26.008 15.111 1.00 0.42
O
ATOM 15940 H   SER B 424   -8.956 26.568 15.665 1.00 0.40
H
ATOM 15941 HA SER B 424   -10.266 26.539 18.106 1.00 0.34
H
ATOM 15942 1HB SER B 424   -12.296 26.824 16.851 1.00 0.50
H
ATOM 15943 2HB SER B 424   -10.992 27.703 16.072 1.00 0.50
H
ATOM 15944 HG SER B 424   -10.797 26.109 14.586 1.00 0.50
H
ATOM 15945 N   THR B 425   -10.321 23.518 16.910 1.00 0.32
N
ATOM 15946 CA THR B 425   -10.735 22.152 17.233 1.00 0.37
C
ATOM 15947 C   THR B 425   -10.384 21.772 18.678 1.00 0.36
C
ATOM 15948 O   THR B 425   -10.995 20.870 19.252 1.00 0.38
O
ATOM 15949 CB THR B 425   -10.074 21.158 16.286 1.00 0.55
C
ATOM 15950 OG1 THR B 425   -8.655 21.244 16.430 1.00 0.55
O
ATOM 15951 CG2 THR B 425   -10.445 21.493 14.862 1.00 0.55
C
ATOM 15952 H   THR B 425   -9.596 23.647 16.209 1.00 0.38
H
ATOM 15953 HA THR B 425   -11.816 22.083 17.116 1.00 0.44
H
ATOM 15954 HB THR B 425   -10.398 20.145 16.524 1.00 0.67
H
ATOM 15955 HG1 THR B 425   -8.357 22.096 16.100 1.00 0.67
H
ATOM 15956 1HG2 THR B 425   -9.955 20.790 14.194 1.00 0.67
H
ATOM 15957 2HG2 THR B 425   -11.526 21.427 14.741 1.00 0.67
H
ATOM 15958 3HG2 THR B 425   -10.116 22.503 14.629 1.00 0.67
H
ATOM 15959 N   GLY B 426   -9.420 22.476 19.268 1.00 0.44
N

```
ATOM  15960  CA  GLY B 426      -9.071  22.270  20.664  1.00  0.40
C
ATOM  15961  C   GLY B 426      -8.151  21.091  20.970  1.00  1.12
C
ATOM  15962  O   GLY B 426      -7.085  20.927  20.371  1.00  2.41
O
ATOM  15963  H   GLY B 426      -8.934  23.196  18.755  1.00  0.53
H
ATOM  15964 1HA  GLY B 426      -8.619  23.180  21.049  1.00  0.48
H
ATOM  15965 2HA  GLY B 426      -9.994  22.143  21.228  1.00  0.48
H
ATOM  15966  N   GLU B 427      -8.591  20.311  21.957  1.00  5.00
N
ATOM  15967  CA  GLU B 427      -7.872  19.208  22.602  1.00 10.12
C
ATOM  15968  C   GLU B 427      -7.226  18.133  21.738  1.00  6.93
C
ATOM  15969  O   GLU B 427      -6.401  17.381  22.248  1.00 11.00
O
ATOM  15970  CB  GLU B 427      -8.822  18.486  23.544  1.00 15.18
C
ATOM  15971  CG  GLU B 427      -9.259  19.268  24.768  1.00 15.18
C
ATOM  15972  CD  GLU B 427     -10.220  18.462  25.581  1.00 15.18
C
ATOM  15973  OE1 GLU B 427     -10.515  17.370  25.156  1.00 15.18
O
ATOM  15974  OE2 GLU B 427     -10.680  18.922  26.600  1.00 15.18
O
ATOM  15975  H   GLU B 427      -9.503  20.532  22.332  1.00  6.00
H
ATOM  15976  HA  GLU B 427      -7.078  19.657  23.200  1.00 12.14
H
ATOM  15977 1HB  GLU B 427      -9.722  18.203  22.997  1.00 18.22
H
ATOM  15978 2HB  GLU B 427      -8.353  17.566  23.891  1.00 18.22
H
ATOM  15979 1HG  GLU B 427      -8.387  19.511  25.374  1.00 18.22
H
ATOM  15980 2HG  GLU B 427      -9.730  20.198  24.453  1.00 18.22
H
ATOM  15981  N   ASP B 428      -7.591  18.010  20.470  1.00  3.28
N
ATOM  15982  CA  ASP B 428      -6.940  17.001  19.643  1.00  2.23
C
ATOM  15983  C   ASP B 428      -5.504  17.389  19.290  1.00  2.08
C
ATOM  15984  O   ASP B 428      -4.630  16.527  19.182  1.00  4.33
O
ATOM  15985  CB  ASP B 428      -7.744  16.734  18.379  1.00  3.34
C
ATOM  15986  CG  ASP B 428      -9.019  15.952  18.665  1.00  3.34
C
ATOM  15987  OD1 ASP B 428      -9.107  15.364  19.719  1.00  3.34
O
ATOM  15988  OD2 ASP B 428      -9.888  15.943  17.827  1.00  3.34
O
```

```
ATOM  15989  H   ASP B 428      -8.294  18.615  20.072  1.00  3.94
H
ATOM  15990  HA  ASP B 428      -6.912  16.078  20.212  1.00  2.68
H
ATOM  15991 1HB  ASP B 428      -8.010  17.679  17.905  1.00  4.01
H
ATOM  15992 2HB  ASP B 428      -7.135  16.170  17.675  1.00  4.01
H
ATOM  15993  N   GLY B 429      -5.272  18.688  19.115  1.00  1.50
N
ATOM  15994  CA  GLY B 429      -3.961  19.222  18.766  1.00  2.03
C
ATOM  15995  C   GLY B 429      -3.647  19.220  17.293  1.00  1.13
C
ATOM  15996  O   GLY B 429      -4.422  18.718  16.481  1.00  0.73
O
ATOM  15997  H   GLY B 429      -6.028  19.347  19.266  1.00  1.80
H
ATOM  15998 1HA  GLY B 429      -3.878  20.239  19.151  1.00  2.44
H
ATOM  15999 2HA  GLY B 429      -3.209  18.665  19.242  1.00  2.44
H
ATOM  16000  N   VAL B 430      -2.476  19.781  16.978  1.00  1.07
N
ATOM  16001  CA  VAL B 430      -2.014  20.029  15.613  1.00  0.75
C
ATOM  16002  C   VAL B 430      -2.111  18.902  14.598  1.00  0.51
C
ATOM  16003  O   VAL B 430      -2.324  19.175  13.415  1.00  0.44
O
ATOM  16004  CB  VAL B 430      -0.554  20.493  15.639  1.00  1.12
C
ATOM  16005  CG1 VAL B 430       0.371  19.389  16.118  1.00  1.12
C
ATOM  16006  CG2 VAL B 430      -0.197  20.937  14.232  1.00  1.12
C
ATOM  16007  H   VAL B 430      -1.896  20.114  17.735  1.00  1.28
H
ATOM  16008  HA  VAL B 430      -2.599  20.859  15.232  1.00  0.90
H
ATOM  16009  HB  VAL B 430      -0.448  21.321  16.334  1.00  1.35
H
ATOM  16010 1HG1 VAL B 430       1.395  19.757  16.127  1.00  1.35
H
ATOM  16011 2HG1 VAL B 430       0.086  19.088  17.126  1.00  1.35
H
ATOM  16012 3HG1 VAL B 430       0.308  18.534  15.452  1.00  1.35
H
ATOM  16013 1HG2 VAL B 430       0.829  21.289  14.199  1.00  1.35
H
ATOM  16014 2HG2 VAL B 430      -0.314  20.097  13.552  1.00  1.35
H
ATOM  16015 3HG2 VAL B 430      -0.866  21.741  13.926  1.00  1.35
H
ATOM  16016  N   SER B 431      -2.018  17.646  15.014  1.00  0.52
N
ATOM  16017  CA  SER B 431      -2.095  16.570  14.034  1.00  0.37
C
```

ATOM 16018 C SER B 431 -3.471 16.497 13.372 1.00 0.36
C
ATOM 16019 O SER B 431 -3.608 15.962 12.269 1.00 0.61
O
ATOM 16020 CB SER B 431 -1.794 15.240 14.679 1.00 0.55
C
ATOM 16021 OG SER B 431 -2.833 14.853 15.535 1.00 0.55
O
ATOM 16022 H SER B 431 -1.867 17.437 15.991 1.00 0.62
H
ATOM 16023 HA SER B 431 -1.351 16.759 13.259 1.00 0.44
H
ATOM 16024 1HB SER B 431 -1.657 14.486 13.905 1.00 0.67
H
ATOM 16025 2HB SER B 431 -0.863 15.308 15.239 1.00 0.67
H
ATOM 16026 HG SER B 431 -2.852 15.501 16.245 1.00 0.67
H
ATOM 16027 N GLN B 432 -4.491 17.046 14.042 1.00 0.38
N
ATOM 16028 CA GLN B 432 -5.847 17.031 13.526 1.00 0.65
C
ATOM 16029 C GLN B 432 -6.315 18.448 13.156 1.00 0.47
C
ATOM 16030 O GLN B 432 -7.513 18.673 12.978 1.00 0.74
O
ATOM 16031 CB GLN B 432 -6.787 16.453 14.588 1.00 0.98
C
ATOM 16032 CG GLN B 432 -6.361 15.096 15.134 1.00 0.98
C
ATOM 16033 CD GLN B 432 -6.265 13.982 14.117 1.00 0.98
C
ATOM 16034 OE1 GLN B 432 -7.234 13.620 13.449 1.00 0.98
O
ATOM 16035 NE2 GLN B 432 -5.066 13.425 14.000 1.00 0.98
N
ATOM 16036 H GLN B 432 -4.332 17.493 14.934 1.00 0.46
H
ATOM 16037 HA GLN B 432 -5.878 16.410 12.631 1.00 0.78
H
ATOM 16038 1HB GLN B 432 -6.854 17.145 15.427 1.00 1.17
H
ATOM 16039 2HB GLN B 432 -7.788 16.349 14.170 1.00 1.17
H
ATOM 16040 1HG GLN B 432 -5.381 15.209 15.596 1.00 1.17
H
ATOM 16041 2HG GLN B 432 -7.086 14.792 15.888 1.00 1.17
H
ATOM 16042 1HE2 GLN B 432 -4.917 12.678 13.351 1.00 1.17
H
ATOM 16043 2HE2 GLN B 432 -4.307 13.765 14.562 1.00 1.17
H
ATOM 16044 N MET B 433 -5.379 19.403 13.069 1.00 0.47
N
ATOM 16045 CA MET B 433 -5.713 20.801 12.783 1.00 0.48
C
ATOM 16046 C MET B 433 -5.297 21.221 11.400 1.00 0.53
C

ATOM 16047 O MET B 433 -4.109 21.211 11.072 1.00 1.13
O
ATOM 16048 CB MET B 433 -5.032 21.729 13.784 1.00 0.72
C
ATOM 16049 CG MET B 433 -5.519 21.607 15.209 1.00 0.72
C
ATOM 16050 SD MET B 433 -4.531 22.581 16.355 1.00 0.72
S
ATOM 16051 CE MET B 433 -5.452 22.366 17.870 1.00 0.72
C
ATOM 16052 H MET B 433 -4.403 19.175 13.202 1.00 0.56
H
ATOM 16053 HA MET B 433 -6.794 20.922 12.857 1.00 0.58
H
ATOM 16054 1HB MET B 433 -3.961 21.541 13.775 1.00 0.86
H
ATOM 16055 2HB MET B 433 -5.183 22.763 13.474 1.00 0.86
H
ATOM 16056 1HG MET B 433 -6.545 21.956 15.261 1.00 0.86
H
ATOM 16057 2HG MET B 433 -5.503 20.567 15.520 1.00 0.86
H
ATOM 16058 1HE MET B 433 -4.949 22.895 18.677 1.00 0.86
H
ATOM 16059 2HE MET B 433 -6.459 22.763 17.743 1.00 0.86
H
ATOM 16060 3HE MET B 433 -5.519 21.309 18.114 1.00 0.86
H
ATOM 16061 N ALA B 434 -6.258 21.634 10.592 1.00 0.24
N
ATOM 16062 CA ALA B 434 -5.892 22.085 9.267 1.00 0.27
C
ATOM 16063 C ALA B 434 -5.538 23.545 9.281 1.00 0.42
C
ATOM 16064 O ALA B 434 -6.382 24.401 9.533 1.00 1.76
O
ATOM 16065 CB ALA B 434 -6.993 21.852 8.297 1.00 0.41
C
ATOM 16066 H ALA B 434 -7.232 21.616 10.888 1.00 0.29
H
ATOM 16067 HA ALA B 434 -5.015 21.521 8.949 1.00 0.32
H
ATOM 16068 1HB ALA B 434 -6.677 22.171 7.304 1.00 0.49
H
ATOM 16069 2HB ALA B 434 -7.232 20.797 8.284 1.00 0.49
H
ATOM 16070 3HB ALA B 434 -7.870 22.417 8.589 1.00 0.49
H
ATOM 16071 N LEU B 435 -4.280 23.825 9.026 1.00 0.61
N
ATOM 16072 CA LEU B 435 -3.793 25.185 8.993 1.00 0.46
C
ATOM 16073 C LEU B 435 -3.019 25.426 7.705 1.00 0.84
C
ATOM 16074 O LEU B 435 -2.412 26.479 7.523 1.00 2.89
O
ATOM 16075 CB LEU B 435 -2.924 25.437 10.225 1.00 0.69
C

```
ATOM  16076  CG  LEU B 435      -3.634  25.367  11.584  1.00  0.69
C
ATOM  16077  CD1 LEU B 435      -2.622  25.375  12.694  1.00  0.69
C
ATOM  16078  CD2 LEU B 435      -4.524  26.569  11.742  1.00  0.69
C
ATOM  16079  H   LEU B 435      -3.636  23.066   8.851  1.00  0.73
H
ATOM  16080  HA  LEU B 435      -4.645  25.862   9.017  1.00  0.55
H
ATOM  16081 1HB  LEU B 435      -2.143  24.683  10.241  1.00  0.83
H
ATOM  16082 2HB  LEU B 435      -2.462  26.418  10.140  1.00  0.83
H
ATOM  16083  HG  LEU B 435      -4.219  24.451  11.654  1.00  0.83
H
ATOM  16084 1HD1 LEU B 435      -3.145  25.333  13.647  1.00  0.83
H
ATOM  16085 2HD1 LEU B 435      -1.964  24.512  12.599  1.00  0.83
H
ATOM  16086 3HD1 LEU B 435      -2.038  26.290  12.642  1.00  0.83
H
ATOM  16087 1HD2 LEU B 435      -5.023  26.528  12.711  1.00  0.83
H
ATOM  16088 2HD2 LEU B 435      -3.924  27.477  11.683  1.00  0.83
H
ATOM  16089 3HD2 LEU B 435      -5.273  26.573  10.950  1.00  0.83
H
ATOM  16090  N   GLU B 436      -3.057  24.444   6.802  1.00  0.46
N
ATOM  16091  CA  GLU B 436      -2.390  24.533   5.510  1.00  0.59
C
ATOM  16092  C   GLU B 436      -3.342  24.287   4.343  1.00  0.41
C
ATOM  16093  O   GLU B 436      -2.936  24.388   3.183  1.00  0.80
O
ATOM  16094  CB  GLU B 436      -1.256  23.517   5.441  1.00  0.89
C
ATOM  16095  CG  GLU B 436      -0.194  23.738   6.492  1.00  0.89
C
ATOM  16096  CD  GLU B 436       0.944  22.780   6.433  1.00  0.89
C
ATOM  16097  OE1 GLU B 436       1.730  22.873   5.524  1.00  0.89
O
ATOM  16098  OE2 GLU B 436       1.036  21.960   7.311  1.00  0.89
O
ATOM  16099  H   GLU B 436      -3.566  23.600   7.009  1.00  0.55
H
ATOM  16100  HA  GLU B 436      -1.975  25.536   5.404  1.00  0.71
H
ATOM  16101 1HB  GLU B 436      -1.654  22.509   5.562  1.00  1.06
H
ATOM  16102 2HB  GLU B 436      -0.780  23.572   4.464  1.00  1.06
H
ATOM  16103 1HG  GLU B 436       0.196  24.742   6.364  1.00  1.06
H
ATOM  16104 2HG  GLU B 436      -0.658  23.678   7.474  1.00  1.06
H
```

ATOM 16105 N ASP B 437 -4.595 23.932 4.652 1.00 0.46
N
ATOM 16106 CA ASP B 437 -5.541 23.519 3.620 1.00 0.38
C
ATOM 16107 C ASP B 437 -5.971 24.633 2.683 1.00 0.48
C
ATOM 16108 O ASP B 437 -6.356 24.365 1.548 1.00 2.61
O
ATOM 16109 CB ASP B 437 -6.801 22.918 4.266 1.00 0.57
C
ATOM 16110 CG ASP B 437 -7.582 23.903 5.150 1.00 0.57
C
ATOM 16111 OD1 ASP B 437 -7.001 24.567 5.977 1.00 0.57
O
ATOM 16112 OD2 ASP B 437 -8.777 23.970 4.987 1.00 0.57
O
ATOM 16113 H ASP B 437 -4.888 23.910 5.615 1.00 0.55
H
ATOM 16114 HA ASP B 437 -5.062 22.745 3.021 1.00 0.46
H
ATOM 16115 1HB ASP B 437 -7.468 22.556 3.484 1.00 0.68
H
ATOM 16116 2HB ASP B 437 -6.519 22.058 4.873 1.00 0.68
H
ATOM 16117 N LEU B 438 -5.893 25.880 3.119 1.00 0.63
N
ATOM 16118 CA LEU B 438 -6.264 26.952 2.229 1.00 0.32
C
ATOM 16119 C LEU B 438 -5.176 27.180 1.239 1.00 0.32
C
ATOM 16120 O LEU B 438 -5.448 27.316 0.052 1.00 0.43
O
ATOM 16121 CB LEU B 438 -6.592 28.216 2.979 1.00 0.48
C
ATOM 16122 CG LEU B 438 -6.911 29.389 2.091 1.00 0.48
C
ATOM 16123 CD1 LEU B 438 -8.032 29.019 1.148 1.00 0.48
C
ATOM 16124 CD2 LEU B 438 -7.323 30.518 2.989 1.00 0.48
C
ATOM 16125 H LEU B 438 -5.543 26.109 4.042 1.00 0.76
H
ATOM 16126 HA LEU B 438 -7.157 26.652 1.683 1.00 0.38
H
ATOM 16127 1HB LEU B 438 -7.446 28.035 3.628 1.00 0.58
H
ATOM 16128 2HB LEU B 438 -5.744 28.507 3.586 1.00 0.58
H
ATOM 16129 HG LEU B 438 -6.033 29.668 1.509 1.00 0.58
H
ATOM 16130 1HD1 LEU B 438 -8.270 29.864 0.509 1.00 0.58
H
ATOM 16131 2HD1 LEU B 438 -7.719 28.178 0.528 1.00 0.58
H
ATOM 16132 3HD1 LEU B 438 -8.909 28.738 1.728 1.00 0.58
H
ATOM 16133 1HD2 LEU B 438 -7.555 31.401 2.400 1.00 0.58
H

```
ATOM  16134 2HD2 LEU B 438      -8.199  30.218   3.561  1.00  0.58
H
ATOM  16135 3HD2 LEU B 438      -6.525  30.751   3.682  1.00  0.58
H
ATOM  16136  N   ALA B 439      -3.938  27.213   1.724  1.00  0.42
N
ATOM  16137  CA  ALA B 439      -2.784  27.364   0.854  1.00  0.55
C
ATOM  16138  C   ALA B 439      -2.776  26.265  -0.198  1.00  0.68
C
ATOM  16139  O   ALA B 439      -2.560  26.525  -1.384  1.00  1.10
O
ATOM  16140  CB  ALA B 439      -1.501  27.316   1.667  1.00  0.83
C
ATOM  16141  H   ALA B 439      -3.795  27.113   2.719  1.00  0.50
H
ATOM  16142  HA  ALA B 439      -2.857  28.326   0.349  1.00  0.66
H
ATOM  16143 1HB  ALA B 439      -0.645  27.444   1.003  1.00  0.99
H
ATOM  16144 2HB  ALA B 439      -1.508  28.113   2.409  1.00  0.99
H
ATOM  16145 3HB  ALA B 439      -1.426  26.354   2.172  1.00  0.99
H
ATOM  16146  N   MET B 440      -3.020  25.036   0.252  1.00  0.52
N
ATOM  16147  CA  MET B 440      -3.044  23.877  -0.616  1.00  0.51
C
ATOM  16148  C   MET B 440      -4.139  23.940  -1.670  1.00  0.38
C
ATOM  16149  O   MET B 440      -3.869  23.773  -2.858  1.00  0.73
O
ATOM  16150  CB  MET B 440      -3.185  22.618   0.223  1.00  0.77
C
ATOM  16151  CG  MET B 440      -3.208  21.344  -0.591  1.00  0.77
C
ATOM  16152  SD  MET B 440      -3.297  19.868   0.421  1.00  0.77
S
ATOM  16153  CE  MET B 440      -4.973  19.936   0.999  1.00  0.77
C
ATOM  16154  H   MET B 440      -3.179  24.891   1.243  1.00  0.62
H
ATOM  16155  HA  MET B 440      -2.091  23.833  -1.144  1.00  0.61
H
ATOM  16156 1HB  MET B 440      -2.358  22.561   0.927  1.00  0.92
H
ATOM  16157 2HB  MET B 440      -4.108  22.664   0.801  1.00  0.92
H
ATOM  16158 1HG  MET B 440      -4.063  21.354  -1.266  1.00  0.92
H
ATOM  16159 2HG  MET B 440      -2.302  21.296  -1.188  1.00  0.92
H
ATOM  16160 1HE  MET B 440      -5.172  19.087   1.650  1.00  0.92
H
ATOM  16161 2HE  MET B 440      -5.132  20.864   1.550  1.00  0.92
H
ATOM  16162 3HE  MET B 440      -5.638  19.900   0.139  1.00  0.92
H
```

ATOM 16163 N PHE B 441 -5.376 24.168 -1.249 1.00 0.30
N
ATOM 16164 CA PHE B 441 -6.454 24.201 -2.216 1.00 0.30
C
ATOM 16165 C PHE B 441 -6.445 25.424 -3.123 1.00 0.28
C
ATOM 16166 O PHE B 441 -6.767 25.303 -4.302 1.00 0.31
O
ATOM 16167 CB PHE B 441 -7.803 23.986 -1.544 1.00 0.45
C
ATOM 16168 CG PHE B 441 -8.091 22.532 -1.268 1.00 0.45
C
ATOM 16169 CD1 PHE B 441 -7.910 21.962 -0.017 1.00 0.45
C
ATOM 16170 CD2 PHE B 441 -8.565 21.724 -2.285 1.00 0.45
C
ATOM 16171 CE1 PHE B 441 -8.203 20.631 0.208 1.00 0.45
C
ATOM 16172 CE2 PHE B 441 -8.854 20.397 -2.063 1.00 0.45
C
ATOM 16173 CZ PHE B 441 -8.676 19.846 -0.813 1.00 0.45
C
ATOM 16174 H PHE B 441 -5.580 24.301 -0.267 1.00 0.36
H
ATOM 16175 HA PHE B 441 -6.317 23.340 -2.866 1.00 0.36
H
ATOM 16176 1HB PHE B 441 -7.836 24.528 -0.599 1.00 0.54
H
ATOM 16177 2HB PHE B 441 -8.596 24.376 -2.178 1.00 0.54
H
ATOM 16178 HD1 PHE B 441 -7.544 22.571 0.802 1.00 0.54
H
ATOM 16179 HD2 PHE B 441 -8.713 22.153 -3.277 1.00 0.54
H
ATOM 16180 HE1 PHE B 441 -8.061 20.203 1.200 1.00 0.54
H
ATOM 16181 HE2 PHE B 441 -9.224 19.782 -2.878 1.00 0.54
H
ATOM 16182 HZ PHE B 441 -8.908 18.796 -0.639 1.00 0.54
H
ATOM 16183 N ARG B 442 -6.009 26.586 -2.635 1.00 0.24
N
ATOM 16184 CA ARG B 442 -5.953 27.728 -3.540 1.00 0.30
C
ATOM 16185 C ARG B 442 -4.856 27.539 -4.591 1.00 0.37
C
ATOM 16186 O ARG B 442 -4.975 28.034 -5.712 1.00 1.19
O
ATOM 16187 CB ARG B 442 -5.705 29.035 -2.802 1.00 0.45
C
ATOM 16188 CG ARG B 442 -4.317 29.156 -2.216 1.00 0.45
C
ATOM 16189 CD ARG B 442 -4.101 30.411 -1.463 1.00 0.45
C
ATOM 16190 NE ARG B 442 -4.078 31.582 -2.320 1.00 0.45
N
ATOM 16191 CZ ARG B 442 -3.559 32.770 -1.966 1.00 0.45
C

ATOM 16192 NH1 ARG B 442   -3.050 32.919 -0.770 1.00 0.45
N
ATOM 16193 NH2 ARG B 442   -3.556 33.755 -2.845 1.00 0.45
N
ATOM 16194 H   ARG B 442   -5.743 26.692 -1.668 1.00 0.29
H
ATOM 16195 HA ARG B 442   -6.911 27.804 -4.053 1.00 0.36
H
ATOM 16196 1HB ARG B 442   -5.856 29.876 -3.479 1.00 0.54
H
ATOM 16197 2HB ARG B 442   -6.419 29.135 -1.985 1.00 0.54
H
ATOM 16198 1HG ARG B 442   -4.153 28.336 -1.534 1.00 0.54
H
ATOM 16199 2HG ARG B 442   -3.574 29.100 -3.005 1.00 0.54
H
ATOM 16200 1HD ARG B 442   -4.895 30.540 -0.728 1.00 0.54
H
ATOM 16201 2HD ARG B 442   -3.140 30.350 -0.956 1.00 0.54
H
ATOM 16202 HE ARG B 442   -4.458 31.516 -3.258 1.00 0.54
H
ATOM 16203 1HH1 ARG B 442   -3.083 32.142 -0.121 1.00 0.54
H
ATOM 16204 2HH1 ARG B 442   -2.645 33.802 -0.497 1.00 0.54
H
ATOM 16205 1HH2 ARG B 442   -3.966 33.589 -3.757 1.00 0.54
H
ATOM 16206 2HH2 ARG B 442   -3.127 34.645 -2.629 1.00 0.54
H
ATOM 16207 N   SER B 443   -3.823 26.745 -4.282 1.00 0.41
N
ATOM 16208 CA SER B 443   -2.788 26.480 -5.275 1.00 0.44
C
ATOM 16209 C   SER B 443   -3.264 25.538 -6.393 1.00 0.39
C
ATOM 16210 O   SER B 443   -2.599 25.427 -7.420 1.00 0.71
O
ATOM 16211 CB SER B 443   -1.549 25.912 -4.607 1.00 0.66
C
ATOM 16212 OG SER B 443   -1.768 24.621 -4.111 1.00 0.66
O
ATOM 16213 H   SER B 443   -3.716 26.360 -3.348 1.00 0.49
H
ATOM 16214 HA SER B 443   -2.514 27.425 -5.738 1.00 0.53
H
ATOM 16215 1HB SER B 443   -0.723 25.901 -5.315 1.00 0.79
H
ATOM 16216 2HB SER B 443   -1.262 26.568 -3.787 1.00 0.79
H
ATOM 16217 HG SER B 443   -1.865 24.042 -4.876 1.00 0.79
H
ATOM 16218 N   ILE B 444   -4.412 24.876 -6.209 1.00 0.27
N
ATOM 16219 CA ILE B 444   -4.948 23.967 -7.217 1.00 0.25
C
ATOM 16220 C   ILE B 444   -5.742 24.746 -8.280 1.00 0.39
C

```
ATOM  16221  O   ILE B 444      -6.610  25.547  -7.931  1.00  0.58
O
ATOM  16222  CB  ILE B 444      -5.846  22.893  -6.558  1.00  0.38
C
ATOM  16223  CG1 ILE B 444      -5.007  22.039  -5.605  1.00  0.38
C
ATOM  16224  CG2 ILE B 444      -6.514  22.022  -7.603  1.00  0.38
C
ATOM  16225  CD1 ILE B 444      -5.819  21.111  -4.734  1.00  0.38
C
ATOM  16226  H   ILE B 444      -4.955  25.002  -5.366  1.00  0.32
H
ATOM  16227  HA  ILE B 444      -4.114  23.450  -7.678  1.00  0.30
H
ATOM  16228  HB  ILE B 444      -6.614  23.384  -5.962  1.00  0.45
H
ATOM  16229 1HG1 ILE B 444      -4.309  21.440  -6.188  1.00  0.45
H
ATOM  16230 2HG1 ILE B 444      -4.429  22.698  -4.960  1.00  0.45
H
ATOM  16231 1HG2 ILE B 444      -7.145  21.281  -7.112  1.00  0.45
H
ATOM  16232 2HG2 ILE B 444      -7.126  22.642  -8.256  1.00  0.45
H
ATOM  16233 3HG2 ILE B 444      -5.751  21.516  -8.193  1.00  0.45
H
ATOM  16234 1HD1 ILE B 444      -5.154  20.543  -4.085  1.00  0.45
H
ATOM  16235 2HD1 ILE B 444      -6.506  21.695  -4.128  1.00  0.45
H
ATOM  16236 3HD1 ILE B 444      -6.384  20.428  -5.365  1.00  0.45
H
ATOM  16237  N   PRO B 445      -5.427  24.573  -9.573  1.00  0.47
N
ATOM  16238  CA  PRO B 445      -6.094  25.156 -10.731  1.00  0.61
C
ATOM  16239  C   PRO B 445      -7.548  24.759 -10.775  1.00  0.41
C
ATOM  16240  O   PRO B 445      -7.894  23.617 -10.470  1.00  0.49
O
ATOM  16241  CB  PRO B 445      -5.339  24.581 -11.916  1.00  0.92
C
ATOM  16242  CG  PRO B 445      -3.993  24.290 -11.386  1.00  0.92
C
ATOM  16243  CD  PRO B 445      -4.208  23.848  -9.970  1.00  0.92
C
ATOM  16244  HA  PRO B 445      -5.991  26.251 -10.694  1.00  0.73
H
ATOM  16245 1HB  PRO B 445      -5.857  23.682 -12.282  1.00  1.10
H
ATOM  16246 2HB  PRO B 445      -5.325  25.305 -12.742  1.00  1.10
H
ATOM  16247 1HG  PRO B 445      -3.489  23.533 -12.003  1.00  1.10
H
ATOM  16248 2HG  PRO B 445      -3.393  25.206 -11.443  1.00  1.10
H
ATOM  16249 1HD  PRO B 445      -4.373  22.761  -9.912  1.00  1.10
H
```

ATOM 16250 2HD PRO B 445   -3.351 24.167 -9.362 1.00 1.10
H
ATOM 16251 N   ASN B 446   -8.392 25.696 -11.188 1.00 0.39
N
ATOM 16252 CA ASN B 446   -9.830 25.479 -11.278 1.00 0.54
C
ATOM 16253 C   ASN B 446   -10.411 25.004 -9.952 1.00 0.67
C
ATOM 16254 O   ASN B 446   -11.277 24.129 -9.933 1.00 2.31
O
ATOM 16255 CB ASN B 446   -10.154 24.494 -12.388 1.00 0.81
C
ATOM 16256 CG ASN B 446   -9.773 25.019 -13.743 1.00 0.81
C
ATOM 16257 OD1 ASN B 446   -9.930 26.212 -14.026 1.00 0.81
O
ATOM 16258 ND2 ASN B 446   -9.274 24.153 -14.587 1.00 0.81
N
ATOM 16259 H   ASN B 446   -8.023 26.602 -11.440 1.00 0.47
H
ATOM 16260 HA ASN B 446   -10.307 26.431 -11.515 1.00 0.65
H
ATOM 16261 1HB ASN B 446   -9.634 23.552 -12.215 1.00 0.97
H
ATOM 16262 2HB ASN B 446   -11.224 24.283 -12.383 1.00 0.97
H
ATOM 16263 1HD2 ASN B 446   -9.004 24.446 -15.505 1.00 0.97
H
ATOM 16264 2HD2 ASN B 446   -9.165 23.197 -14.316 1.00 0.97
H
ATOM 16265 N   CYS B 447   -9.943 25.596 -8.853 1.00 0.78
N
ATOM 16266 CA CYS B 447   -10.430 25.231 -7.538 1.00 0.63
C
ATOM 16267 C   CYS B 447   -11.184 26.378 -6.882 1.00 0.55
C
ATOM 16268 O   CYS B 447   -10.668 27.487 -6.765 1.00 1.27
O
ATOM 16269 CB CYS B 447   -9.294 24.799 -6.617 1.00 0.95
C
ATOM 16270 SG CYS B 447   -9.839 24.298 -4.965 1.00 0.95
S
ATOM 16271 H   CYS B 447   -9.234 26.309 -8.935 1.00 0.94
H
ATOM 16272 HA CYS B 447   -11.101 24.392 -7.658 1.00 0.76
H
ATOM 16273 1HB CYS B 447   -8.755 23.966 -7.065 1.00 1.13
H
ATOM 16274 2HB CYS B 447   -8.591 25.622 -6.498 1.00 1.13
H
ATOM 16275 HG CYS B 447   -10.588 23.281 -5.371 1.00 1.13
H
ATOM 16276 N   THR B 448   -12.417 26.108 -6.481 1.00 0.41
N
ATOM 16277 CA THR B 448   -13.229 27.079 -5.771 1.00 0.33
C
ATOM 16278 C   THR B 448   -13.168 26.749 -4.294 1.00 0.28
C

ATOM 16279 O THR B 448 -13.475 25.626 -3.901 1.00 0.39
O
ATOM 16280 CB THR B 448 -14.698 27.085 -6.252 1.00 0.49
C
ATOM 16281 OG1 THR B 448 -14.753 27.446 -7.639 1.00 0.49
O
ATOM 16282 CG2 THR B 448 -15.528 28.072 -5.438 1.00 0.49
C
ATOM 16283 H THR B 448 -12.777 25.177 -6.628 1.00 0.49
H
ATOM 16284 HA THR B 448 -12.808 28.070 -5.923 1.00 0.40
H
ATOM 16285 HB THR B 448 -15.117 26.086 -6.133 1.00 0.59
H
ATOM 16286 HG1 THR B 448 -14.261 26.804 -8.156 1.00 0.59
H
ATOM 16287 1HG2 THR B 448 -16.559 28.056 -5.788 1.00 0.59
H
ATOM 16288 2HG2 THR B 448 -15.496 27.792 -4.386 1.00 0.59
H
ATOM 16289 3HG2 THR B 448 -15.120 29.074 -5.558 1.00 0.59
H
ATOM 16290 N VAL B 449 -12.750 27.705 -3.481 1.00 0.27
N
ATOM 16291 CA VAL B 449 -12.657 27.454 -2.057 1.00 0.25
C
ATOM 16292 C VAL B 449 -13.715 28.219 -1.302 1.00 0.23
C
ATOM 16293 O VAL B 449 -13.748 29.449 -1.328 1.00 0.26
O
ATOM 16294 CB VAL B 449 -11.277 27.818 -1.499 1.00 0.38
C
ATOM 16295 CG1 VAL B 449 -11.274 27.548 -0.001 1.00 0.38
C
ATOM 16296 CG2 VAL B 449 -10.200 27.002 -2.204 1.00 0.38
C
ATOM 16297 H VAL B 449 -12.519 28.613 -3.860 1.00 0.32
H
ATOM 16298 HA VAL B 449 -12.815 26.390 -1.886 1.00 0.30
H
ATOM 16299 HB VAL B 449 -11.089 28.882 -1.650 1.00 0.45
H
ATOM 16300 1HG1 VAL B 449 -10.307 27.805 0.413 1.00 0.45
H
ATOM 16301 2HG1 VAL B 449 -12.046 28.147 0.480 1.00 0.45
H
ATOM 16302 3HG1 VAL B 449 -11.471 26.491 0.176 1.00 0.45
H
ATOM 16303 1HG2 VAL B 449 -9.222 27.263 -1.801 1.00 0.45
H
ATOM 16304 2HG2 VAL B 449 -10.387 25.940 -2.045 1.00 0.45
H
ATOM 16305 3HG2 VAL B 449 -10.220 27.217 -3.272 1.00 0.45
H
ATOM 16306 N PHE B 450 -14.567 27.471 -0.617 1.00 0.31
N
ATOM 16307 CA PHE B 450 -15.635 28.021 0.186 1.00 0.67
C

```
ATOM  16308  C   PHE B 450     -15.258  27.910   1.648  1.00  1.10
C
ATOM  16309  O   PHE B 450     -14.723  26.890   2.077  1.00  6.58
O
ATOM  16310  CB  PHE B 450     -16.894  27.165   0.051  1.00  1.01
C
ATOM  16311  CG  PHE B 450     -17.580  27.098  -1.278  1.00  1.01
C
ATOM  16312  CD1 PHE B 450     -17.157  26.198  -2.246  1.00  1.01
C
ATOM  16313  CD2 PHE B 450     -18.689  27.886  -1.539  1.00  1.01
C
ATOM  16314  CE1 PHE B 450     -17.827  26.092  -3.446  1.00  1.01
C
ATOM  16315  CE2 PHE B 450     -19.360  27.780  -2.738  1.00  1.01
C
ATOM  16316  CZ  PHE B 450     -18.929  26.879  -3.688  1.00  1.01
C
ATOM  16317  H   PHE B 450     -14.461  26.469  -0.649  1.00  0.37
H
ATOM  16318  HA  PHE B 450     -15.813  29.062  -0.082  1.00  0.80
H
ATOM  16319 1HB  PHE B 450     -16.634  26.150   0.334  1.00  1.21
H
ATOM  16320 2HB  PHE B 450     -17.628  27.512   0.772  1.00  1.21
H
ATOM  16321  HD1 PHE B 450     -16.293  25.564  -2.043  1.00  1.21
H
ATOM  16322  HD2 PHE B 450     -19.036  28.586  -0.777  1.00  1.21
H
ATOM  16323  HE1 PHE B 450     -17.491  25.381  -4.198  1.00  1.21
H
ATOM  16324  HE2 PHE B 450     -20.233  28.401  -2.931  1.00  1.21
H
ATOM  16325  HZ  PHE B 450     -19.460  26.787  -4.632  1.00  1.21
H
ATOM  16326  N   TYR B 451     -15.614  28.912   2.422  1.00  0.43
N
ATOM  16327  CA  TYR B 451     -15.428  28.855   3.859  1.00  0.28
C
ATOM  16328  C   TYR B 451     -16.571  29.551   4.575  1.00  0.39
C
ATOM  16329  O   TYR B 451     -16.442  30.708   4.974  1.00  0.84
O
ATOM  16330  CB  TYR B 451     -14.109  29.485   4.279  1.00  0.42
C
ATOM  16331  CG  TYR B 451     -13.775  29.229   5.735  1.00  0.42
C
ATOM  16332  CD1 TYR B 451     -14.576  28.385   6.491  1.00  0.42
C
ATOM  16333  CD2 TYR B 451     -12.667  29.825   6.314  1.00  0.42
C
ATOM  16334  CE1 TYR B 451     -14.269  28.135   7.813  1.00  0.42
C
ATOM  16335  CE2 TYR B 451     -12.359  29.577   7.637  1.00  0.42
C
ATOM  16336  CZ  TYR B 451     -13.153  28.735   8.384  1.00  0.42
C
```

ATOM 16337 OH TYR B 451 -12.843 28.487 9.702 1.00 0.42
O
ATOM 16338 H TYR B 451 -16.025 29.738 2.017 1.00 0.52
H
ATOM 16339 HA TYR B 451 -15.429 27.811 4.171 1.00 0.34
H
ATOM 16340 1HB TYR B 451 -13.304 29.083 3.665 1.00 0.50
H
ATOM 16341 2HB TYR B 451 -14.145 30.562 4.119 1.00 0.50
H
ATOM 16342 HD1 TYR B 451 -15.450 27.913 6.041 1.00 0.50
H
ATOM 16343 HD2 TYR B 451 -12.036 30.490 5.727 1.00 0.50
H
ATOM 16344 HE1 TYR B 451 -14.898 27.470 8.403 1.00 0.50
H
ATOM 16345 HE2 TYR B 451 -11.486 30.047 8.090 1.00 0.50
H
ATOM 16346 HH TYR B 451 -12.012 28.915 9.920 1.00 0.50
H
ATOM 16347 N PRO B 452 -17.710 28.861 4.706 1.00 0.78
N
ATOM 16348 CA PRO B 452 -18.895 29.273 5.433 1.00 0.97
C
ATOM 16349 C PRO B 452 -18.641 29.513 6.900 1.00 0.65
C
ATOM 16350 O PRO B 452 -17.702 28.982 7.494 1.00 2.78
O
ATOM 16351 CB PRO B 452 -19.864 28.146 5.186 1.00 1.46
C
ATOM 16352 CG PRO B 452 -19.533 27.692 3.818 1.00 1.46
C
ATOM 16353 CD PRO B 452 -18.065 27.875 3.671 1.00 1.46
C
ATOM 16354 HA PRO B 452 -19.277 30.194 4.972 1.00 1.16
H
ATOM 16355 1HB PRO B 452 -19.699 27.367 5.936 1.00 1.75
H
ATOM 16356 2HB PRO B 452 -20.890 28.496 5.302 1.00 1.75
H
ATOM 16357 1HG PRO B 452 -19.862 26.658 3.657 1.00 1.75
H
ATOM 16358 2HG PRO B 452 -20.089 28.310 3.099 1.00 1.75
H
ATOM 16359 1HD PRO B 452 -17.515 26.935 3.822 1.00 1.75
H
ATOM 16360 2HD PRO B 452 -17.908 28.296 2.676 1.00 1.75
H
ATOM 16361 N SER B 453 -19.467 30.379 7.455 1.00 0.76
N
ATOM 16362 CA SER B 453 -19.324 30.819 8.828 1.00 0.40
C
ATOM 16363 C SER B 453 -20.417 30.275 9.722 1.00 0.40
C
ATOM 16364 O SER B 453 -20.208 30.092 10.921 1.00 0.64
O
ATOM 16365 CB SER B 453 -19.298 32.316 8.862 1.00 0.60
C

ATOM 16366 OG SER B 453 -18.170 32.789 8.182 1.00 0.60
O
ATOM 16367 H SER B 453 -20.254 30.707 6.904 1.00 0.91
H
ATOM 16368 HA SER B 453 -18.373 30.453 9.202 1.00 0.48
H
ATOM 16369 1HB SER B 453 -20.198 32.697 8.381 1.00 0.72
H
ATOM 16370 2HB SER B 453 -19.295 32.673 9.891 1.00 0.72
H
ATOM 16371 HG SER B 453 -18.498 33.515 7.637 1.00 0.72
H
ATOM 16372 N ASP B 454 -21.584 30.015 9.143 1.00 0.43
N
ATOM 16373 CA ASP B 454 -22.680 29.449 9.912 1.00 0.46
C
ATOM 16374 C ASP B 454 -23.504 28.520 9.051 1.00 0.82
C
ATOM 16375 O ASP B 454 -23.304 28.454 7.841 1.00 3.73
O
ATOM 16376 CB ASP B 454 -23.621 30.525 10.444 1.00 0.69
C
ATOM 16377 CG ASP B 454 -24.387 31.219 9.326 1.00 0.69
C
ATOM 16378 OD1 ASP B 454 -24.264 30.789 8.190 1.00 0.69
O
ATOM 16379 OD2 ASP B 454 -25.115 32.146 9.617 1.00 0.69
O
ATOM 16380 H ASP B 454 -21.710 30.191 8.158 1.00 0.52
H
ATOM 16381 HA ASP B 454 -22.271 28.880 10.748 1.00 0.55
H
ATOM 16382 1HB ASP B 454 -24.330 30.078 11.137 1.00 0.83
H
ATOM 16383 2HB ASP B 454 -23.049 31.271 10.996 1.00 0.83
H
ATOM 16384 N ALA B 455 -24.485 27.885 9.684 1.00 0.31
N
ATOM 16385 CA ALA B 455 -25.412 26.922 9.092 1.00 0.40
C
ATOM 16386 C ALA B 455 -26.005 27.315 7.746 1.00 0.41
C
ATOM 16387 O ALA B 455 -26.160 26.472 6.859 1.00 0.74
O
ATOM 16388 CB ALA B 455 -26.555 26.708 10.051 1.00 0.60
C
ATOM 16389 H ALA B 455 -24.571 28.049 10.679 1.00 0.37
H
ATOM 16390 HA ALA B 455 -24.867 25.988 8.959 1.00 0.48
H
ATOM 16391 1HB ALA B 455 -27.217 25.945 9.650 1.00 0.72
H
ATOM 16392 2HB ALA B 455 -26.165 26.381 11.010 1.00 0.72
H
ATOM 16393 3HB ALA B 455 -27.104 27.637 10.180 1.00 0.72
H
ATOM 16394 N ILE B 456 -26.356 28.584 7.605 1.00 0.32
N

ATOM 16395 CA ILE B 456 -27.031 29.060 6.416 1.00 0.36
C
ATOM 16396 C ILE B 456 -26.057 29.076 5.265 1.00 0.40
C
ATOM 16397 O ILE B 456 -26.324 28.521 4.195 1.00 0.49
O
ATOM 16398 CB ILE B 456 -27.569 30.479 6.656 1.00 0.54
C
ATOM 16399 CG1 ILE B 456 -28.536 30.470 7.829 1.00 0.54
C
ATOM 16400 CG2 ILE B 456 -28.256 31.027 5.416 1.00 0.54
C
ATOM 16401 CD1 ILE B 456 -29.728 29.590 7.654 1.00 0.54
C
ATOM 16402 H ILE B 456 -26.172 29.235 8.356 1.00 0.38
H
ATOM 16403 HA ILE B 456 -27.854 28.388 6.175 1.00 0.43
H
ATOM 16404 HB ILE B 456 -26.740 31.132 6.925 1.00 0.65
H
ATOM 16405 1HG1 ILE B 456 -28.001 30.146 8.717 1.00 0.65
H
ATOM 16406 2HG1 ILE B 456 -28.891 31.479 7.988 1.00 0.65
H
ATOM 16407 1HG2 ILE B 456 -28.618 32.035 5.614 1.00 0.65
H
ATOM 16408 2HG2 ILE B 456 -27.543 31.054 4.596 1.00 0.65
H
ATOM 16409 3HG2 ILE B 456 -29.096 30.385 5.149 1.00 0.65
H
ATOM 16410 1HD1 ILE B 456 -30.363 29.658 8.535 1.00 0.65
H
ATOM 16411 2HD1 ILE B 456 -30.285 29.917 6.782 1.00 0.65
H
ATOM 16412 3HD1 ILE B 456 -29.400 28.562 7.515 1.00 0.65
H
ATOM 16413 N SER B 457 -24.904 29.687 5.516 1.00 0.38
N
ATOM 16414 CA SER B 457 -23.851 29.763 4.532 1.00 0.41
C
ATOM 16415 C SER B 457 -23.306 28.375 4.162 1.00 0.34
C
ATOM 16416 O SER B 457 -22.876 28.175 3.025 1.00 0.35
O
ATOM 16417 CB SER B 457 -22.760 30.699 5.061 1.00 0.61
C
ATOM 16418 OG SER B 457 -22.215 30.231 6.267 1.00 0.61
O
ATOM 16419 H SER B 457 -24.753 30.118 6.427 1.00 0.46
H
ATOM 16420 HA SER B 457 -24.266 30.210 3.628 1.00 0.49
H
ATOM 16421 1HB SER B 457 -21.973 30.795 4.315 1.00 0.74
H
ATOM 16422 2HB SER B 457 -23.183 31.691 5.216 1.00 0.74
H
ATOM 16423 HG SER B 457 -22.948 30.233 6.903 1.00 0.74
H

ATOM 16424 N THR B 458 -23.387 27.400 5.085 1.00 0.35
N
ATOM 16425 CA THR B 458 -22.902 26.055 4.773 1.00 0.43
C
ATOM 16426 C THR B 458 -23.825 25.314 3.841 1.00 0.37
C
ATOM 16427 O THR B 458 -23.353 24.634 2.927 1.00 0.42
O
ATOM 16428 CB THR B 458 -22.789 25.177 6.021 1.00 0.65
C
ATOM 16429 OG1 THR B 458 -24.070 25.071 6.630 1.00 0.65
O
ATOM 16430 CG2 THR B 458 -21.811 25.719 7.023 1.00 0.65
C
ATOM 16431 H THR B 458 -23.732 27.607 6.015 1.00 0.42
H
ATOM 16432 HA THR B 458 -21.926 26.136 4.297 1.00 0.52
H
ATOM 16433 HB THR B 458 -22.471 24.181 5.727 1.00 0.77
H
ATOM 16434 HG1 THR B 458 -23.985 24.536 7.420 1.00 0.77
H
ATOM 16435 1HG2 THR B 458 -21.784 25.052 7.882 1.00 0.77
H
ATOM 16436 2HG2 THR B 458 -20.825 25.780 6.581 1.00 0.77
H
ATOM 16437 3HG2 THR B 458 -22.107 26.699 7.343 1.00 0.77
H
ATOM 16438 N GLU B 459 -25.141 25.484 4.017 1.00 0.42
N
ATOM 16439 CA GLU B 459 -26.038 24.807 3.096 1.00 0.48
C
ATOM 16440 C GLU B 459 -25.921 25.412 1.724 1.00 0.64
C
ATOM 16441 O GLU B 459 -25.892 24.685 0.733 1.00 1.04
O
ATOM 16442 CB GLU B 459 -27.510 24.844 3.529 1.00 0.72
C
ATOM 16443 CG GLU B 459 -27.856 23.988 4.736 1.00 0.72
C
ATOM 16444 CD GLU B 459 -29.334 23.774 4.883 1.00 0.72
C
ATOM 16445 OE1 GLU B 459 -30.075 24.384 4.155 1.00 0.72
O
ATOM 16446 OE2 GLU B 459 -29.724 22.970 5.700 1.00 0.72
O
ATOM 16447 H GLU B 459 -25.499 26.029 4.795 1.00 0.50
H
ATOM 16448 HA GLU B 459 -25.735 23.762 3.027 1.00 0.58
H
ATOM 16449 1HB GLU B 459 -27.787 25.871 3.767 1.00 0.86
H
ATOM 16450 2HB GLU B 459 -28.139 24.521 2.703 1.00 0.86
H
ATOM 16451 1HG GLU B 459 -27.378 23.023 4.641 1.00 0.86
H
ATOM 16452 2HG GLU B 459 -27.468 24.470 5.633 1.00 0.86
H

```
ATOM  16453  N   HIS B 460      -25.793  26.734   1.661  1.00  0.55
N
ATOM  16454  CA  HIS B 460      -25.666  27.377   0.370  1.00  0.62
C
ATOM  16455  C   HIS B 460      -24.364  27.004  -0.321  1.00  0.55
C
ATOM  16456  O   HIS B 460      -24.356  26.830  -1.537  1.00  0.82
O
ATOM  16457  CB  HIS B 460      -25.835  28.889   0.489  1.00  0.93
C
ATOM  16458  CG  HIS B 460      -27.264  29.297   0.704  1.00  0.93
C
ATOM  16459  ND1 HIS B 460      -28.217  29.213  -0.290  1.00  0.93
N
ATOM  16460  CD2 HIS B 460      -27.902  29.784   1.793  1.00  0.93
C
ATOM  16461  CE1 HIS B 460      -29.377  29.640   0.182  1.00  0.93
C
ATOM  16462  NE2 HIS B 460      -29.211  29.993   1.441  1.00  0.93
N
ATOM  16463  H   HIS B 460      -25.819  27.298   2.505  1.00  0.66
H
ATOM  16464  HA  HIS B 460      -26.471  27.020  -0.271  1.00  0.74
H
ATOM  16465 1HB  HIS B 460      -25.239  29.261   1.323  1.00  1.12
H
ATOM  16466 2HB  HIS B 460      -25.471  29.368  -0.419  1.00  1.12
H
ATOM  16467  HD1 HIS B 460      -28.032  29.081  -1.263  1.00  1.12
H
ATOM  16468  HD2 HIS B 460      -27.567  30.020   2.797  1.00  1.12
H
ATOM  16469  HE1 HIS B 460      -30.263  29.656  -0.453  1.00  1.12
H
ATOM  16470  N   ALA B 461      -23.282  26.828   0.446  1.00  0.53
N
ATOM  16471  CA  ALA B 461      -22.008  26.399  -0.127  1.00  0.59
C
ATOM  16472  C   ALA B 461      -22.139  25.040  -0.789  1.00  0.49
C
ATOM  16473  O   ALA B 461      -21.627  24.843  -1.887  1.00  0.53
O
ATOM  16474  CB  ALA B 461      -20.933  26.348   0.933  1.00  0.89
C
ATOM  16475  H   ALA B 461      -23.324  27.015   1.440  1.00  0.64
H
ATOM  16476  HA  ALA B 461      -21.720  27.121  -0.891  1.00  0.71
H
ATOM  16477 1HB  ALA B 461      -19.988  26.049   0.482  1.00  1.06
H
ATOM  16478 2HB  ALA B 461      -20.831  27.336   1.376  1.00  1.06
H
ATOM  16479 3HB  ALA B 461      -21.212  25.629   1.703  1.00  1.06
H
ATOM  16480  N   ILE B 462      -22.835  24.109  -0.124  1.00  0.65
N
ATOM  16481  CA  ILE B 462      -23.085  22.782  -0.688  1.00  0.74
C
```

```
ATOM  16482  C   ILE B 462     -23.849  22.811  -1.986  1.00  0.40
C
ATOM  16483  O   ILE B 462     -23.492  22.105  -2.933  1.00  0.48
O
ATOM  16484  CB  ILE B 462     -23.859  21.906   0.311  1.00  1.11
C
ATOM  16485  CG1 ILE B 462     -22.966  21.587   1.482  1.00  1.11
C
ATOM  16486  CG2 ILE B 462     -24.374  20.643  -0.344  1.00  1.11
C
ATOM  16487  CD1 ILE B 462     -21.775  20.768   1.077  1.00  1.11
C
ATOM  16488  H   ILE B 462     -23.201  24.324   0.797  1.00  0.78
H
ATOM  16489  HA  ILE B 462     -22.122  22.312  -0.878  1.00  0.89
H
ATOM  16490  HB  ILE B 462     -24.705  22.472   0.697  1.00  1.33
H
ATOM  16491 1HG1 ILE B 462     -22.616  22.517   1.926  1.00  1.33
H
ATOM  16492 2HG1 ILE B 462     -23.522  21.033   2.231  1.00  1.33
H
ATOM  16493 1HG2 ILE B 462     -24.925  20.053   0.389  1.00  1.33
H
ATOM  16494 2HG2 ILE B 462     -25.035  20.905  -1.168  1.00  1.33
H
ATOM  16495 3HG2 ILE B 462     -23.535  20.059  -0.723  1.00  1.33
H
ATOM  16496 1HD1 ILE B 462     -21.154  20.566   1.947  1.00  1.33
H
ATOM  16497 2HD1 ILE B 462     -22.113  19.827   0.646  1.00  1.33
H
ATOM  16498 3HD1 ILE B 462     -21.198  21.314   0.337  1.00  1.33
H
ATOM  16499  N   TYR B 463     -24.892  23.628  -2.041  1.00  0.46
N
ATOM  16500  CA  TYR B 463     -25.701  23.682  -3.241  1.00  1.04
C
ATOM  16501  C   TYR B 463     -24.956  24.376  -4.377  1.00  0.47
C
ATOM  16502  O   TYR B 463     -25.087  23.981  -5.536  1.00  1.05
O
ATOM  16503  CB  TYR B 463     -27.035  24.365  -2.935  1.00  1.56
C
ATOM  16504  CG  TYR B 463     -27.875  23.522  -1.998  1.00  1.56
C
ATOM  16505  CD1 TYR B 463     -28.252  24.015  -0.762  1.00  1.56
C
ATOM  16506  CD2 TYR B 463     -28.231  22.237  -2.362  1.00  1.56
C
ATOM  16507  CE1 TYR B 463     -28.972  23.226   0.115  1.00  1.56
C
ATOM  16508  CE2 TYR B 463     -28.956  21.446  -1.491  1.00  1.56
C
ATOM  16509  CZ  TYR B 463     -29.321  21.936  -0.255  1.00  1.56
C
ATOM  16510  OH  TYR B 463     -30.039  21.155   0.614  1.00  1.56
O
```

```
ATOM  16511  H   TYR B 463      -25.148  24.184  -1.230  1.00  0.55
H
ATOM  16512  HA  TYR B 463      -25.907  22.661  -3.562  1.00  1.25
H
ATOM  16513 1HB  TYR B 463      -26.861  25.335  -2.467  1.00  1.87
H
ATOM  16514 2HB  TYR B 463      -27.595  24.527  -3.854  1.00  1.87
H
ATOM  16515  HD1 TYR B 463      -27.972  25.028  -0.474  1.00  1.87
H
ATOM  16516  HD2 TYR B 463      -27.933  21.850  -3.335  1.00  1.87
H
ATOM  16517  HE1 TYR B 463      -29.263  23.615   1.089  1.00  1.87
H
ATOM  16518  HE2 TYR B 463      -29.234  20.435  -1.779  1.00  1.87
H
ATOM  16519  HH  TYR B 463      -30.181  21.641   1.431  1.00  1.87
H
ATOM  16520  N   LEU B 464      -24.141  25.380  -4.047  1.00  0.52
N
ATOM  16521  CA  LEU B 464      -23.337  26.041  -5.063  1.00  0.57
C
ATOM  16522  C   LEU B 464      -22.254  25.109  -5.585  1.00  0.47
C
ATOM  16523  O   LEU B 464      -22.055  25.011  -6.796  1.00  0.63
O
ATOM  16524  CB  LEU B 464      -22.705  27.317  -4.494  1.00  0.85
C
ATOM  16525  CG  LEU B 464      -23.677  28.473  -4.218  1.00  0.85
C
ATOM  16526  CD1 LEU B 464      -22.962  29.564  -3.447  1.00  0.85
C
ATOM  16527  CD2 LEU B 464      -24.194  29.016  -5.540  1.00  0.85
C
ATOM  16528  H   LEU B 464      -24.081  25.699  -3.090  1.00  0.62
H
ATOM  16529  HA  LEU B 464      -23.986  26.313  -5.893  1.00  0.68
H
ATOM  16530 1HB  LEU B 464      -22.213  27.071  -3.554  1.00  1.03
H
ATOM  16531 2HB  LEU B 464      -21.953  27.677  -5.193  1.00  1.03
H
ATOM  16532  HG  LEU B 464      -24.513  28.117  -3.618  1.00  1.03
H
ATOM  16533 1HD1 LEU B 464      -23.653  30.382  -3.248  1.00  1.03
H
ATOM  16534 2HD1 LEU B 464      -22.600  29.156  -2.505  1.00  1.03
H
ATOM  16535 3HD1 LEU B 464      -22.121  29.934  -4.030  1.00  1.03
H
ATOM  16536 1HD2 LEU B 464      -24.886  29.838  -5.351  1.00  1.03
H
ATOM  16537 2HD2 LEU B 464      -23.357  29.378  -6.138  1.00  1.03
H
ATOM  16538 3HD2 LEU B 464      -24.711  28.224  -6.082  1.00  1.03
H
ATOM  16539  N   ALA B 465      -21.602  24.376  -4.680  1.00  0.35
N
```

ATOM 16540 CA ALA B 465 -20.556 23.428 -5.050 1.00 0.32
C
ATOM 16541 C ALA B 465 -21.098 22.343 -5.962 1.00 0.29
C
ATOM 16542 O ALA B 465 -20.464 21.987 -6.955 1.00 0.36
O
ATOM 16543 CB ALA B 465 -19.949 22.805 -3.810 1.00 0.48
C
ATOM 16544 H ALA B 465 -21.809 24.495 -3.700 1.00 0.42
H
ATOM 16545 HA ALA B 465 -19.782 23.971 -5.593 1.00 0.38
H
ATOM 16546 1HB ALA B 465 -19.154 22.120 -4.098 1.00 0.58
H
ATOM 16547 2HB ALA B 465 -19.540 23.592 -3.177 1.00 0.58
H
ATOM 16548 3HB ALA B 465 -20.717 22.260 -3.263 1.00 0.58
H
ATOM 16549 N ALA B 466 -22.303 21.855 -5.661 1.00 0.33
N
ATOM 16550 CA ALA B 466 -22.960 20.830 -6.467 1.00 0.45
C
ATOM 16551 C ALA B 466 -23.118 21.240 -7.934 1.00 0.63
C
ATOM 16552 O ALA B 466 -23.179 20.378 -8.811 1.00 2.72
O
ATOM 16553 CB ALA B 466 -24.337 20.512 -5.888 1.00 0.68
C
ATOM 16554 H ALA B 466 -22.776 22.175 -4.824 1.00 0.40
H
ATOM 16555 HA ALA B 466 -22.342 19.934 -6.432 1.00 0.54
H
ATOM 16556 1HB ALA B 466 -24.803 19.720 -6.476 1.00 0.81
H
ATOM 16557 2HB ALA B 466 -24.239 20.185 -4.855 1.00 0.81
H
ATOM 16558 3HB ALA B 466 -24.960 21.403 -5.926 1.00 0.81
H
ATOM 16559 N ASN B 467 -23.214 22.543 -8.204 1.00 0.81
N
ATOM 16560 CA ASN B 467 -23.414 23.018 -9.559 1.00 0.92
C
ATOM 16561 C ASN B 467 -22.206 23.772 -10.103 1.00 1.29
C
ATOM 16562 O ASN B 467 -22.315 24.468 -11.113 1.00 7.26
O
ATOM 16563 CB ASN B 467 -24.644 23.890 -9.608 1.00 1.38
C
ATOM 16564 CG ASN B 467 -25.886 23.107 -9.317 1.00 1.38
C
ATOM 16565 OD1 ASN B 467 -26.247 22.182 -10.057 1.00 1.38
O
ATOM 16566 ND2 ASN B 467 -26.545 23.456 -8.244 1.00 1.38
N
ATOM 16567 H ASN B 467 -23.128 23.236 -7.471 1.00 0.97
H
ATOM 16568 HA ASN B 467 -23.571 22.155 -10.207 1.00 1.10
H

```
ATOM  16569 1HB  ASN B 467      -24.553  24.693  -8.876  1.00  1.66
H
ATOM  16570 2HB  ASN B 467      -24.735  24.346 -10.593  1.00  1.66
H
ATOM  16571 1HD2 ASN B 467      -27.383  22.973  -7.985  1.00  1.66
H
ATOM  16572 2HD2 ASN B 467      -26.209  24.205  -7.674  1.00  1.66
H
ATOM  16573  N   THR B 468      -21.058  23.638  -9.443  1.00  0.89
N
ATOM  16574  CA  THR B 468      -19.859  24.341  -9.873  1.00  0.60
C
ATOM  16575  C   THR B 468      -18.829  23.366 -10.416  1.00  0.82
C
ATOM  16576  O   THR B 468      -18.570  22.320  -9.821  1.00  3.31
O
ATOM  16577  CB  THR B 468      -19.270  25.161  -8.716  1.00  0.90
C
ATOM  16578  OG1 THR B 468      -20.236  26.129  -8.295  1.00  0.90
O
ATOM  16579  CG2 THR B 468      -17.996  25.876  -9.137  1.00  0.90
C
ATOM  16580  H   THR B 468      -20.996  23.046  -8.625  1.00  1.07
H
ATOM  16581  HA  THR B 468      -20.128  25.028 -10.676  1.00  0.72
H
ATOM  16582  HB  THR B 468      -19.054  24.497  -7.881  1.00  1.08
H
ATOM  16583  HG1 THR B 468      -21.007  25.678  -7.937  1.00  1.08
H
ATOM  16584 1HG2 THR B 468      -17.607  26.451  -8.296  1.00  1.08
H
ATOM  16585 2HG2 THR B 468      -17.252  25.144  -9.448  1.00  1.08
H
ATOM  16586 3HG2 THR B 468      -18.212  26.549  -9.966  1.00  1.08
H
ATOM  16587  N   LYS B 469      -18.260  23.707 -11.565  1.00  0.44
N
ATOM  16588  CA  LYS B 469      -17.270  22.863 -12.217  1.00  0.35
C
ATOM  16589  C   LYS B 469      -15.886  23.027 -11.605  1.00  0.33
C
ATOM  16590  O   LYS B 469      -15.571  24.063 -11.019  1.00  0.39
O
ATOM  16591  CB  LYS B 469      -17.218  23.171 -13.712  1.00  0.52
C
ATOM  16592  CG  LYS B 469      -18.488  22.807 -14.467  1.00  0.52
C
ATOM  16593  CD  LYS B 469      -18.355  23.112 -15.950  1.00  0.52
C
ATOM  16594  CE  LYS B 469      -19.626  22.751 -16.706  1.00  0.52
C
ATOM  16595  NZ  LYS B 469      -19.512  23.058 -18.158  1.00  0.52
N
ATOM  16596  H   LYS B 469      -18.521  24.580 -11.999  1.00  0.53
H
ATOM  16597  HA  LYS B 469      -17.568  21.822 -12.089  1.00  0.42
H
```

```
ATOM  16598 1HB  LYS B 469     -17.031  24.235 -13.859  1.00  0.63
H
ATOM  16599 2HB  LYS B 469     -16.391  22.626 -14.168  1.00  0.63
H
ATOM  16600 1HG  LYS B 469     -18.694  21.744 -14.339  1.00  0.63
H
ATOM  16601 2HG  LYS B 469     -19.327  23.372 -14.062  1.00  0.63
H
ATOM  16602 1HD  LYS B 469     -18.150  24.175 -16.087  1.00  0.63
H
ATOM  16603 2HD  LYS B 469     -17.524  22.544 -16.365  1.00  0.63
H
ATOM  16604 1HE  LYS B 469     -19.824  21.687 -16.585  1.00  0.63
H
ATOM  16605 2HE  LYS B 469     -20.462  23.314 -16.292  1.00  0.63
H
ATOM  16606 1HZ  LYS B 469     -20.372  22.805 -18.625  1.00  0.63
H
ATOM  16607 2HZ  LYS B 469     -19.340  24.045 -18.282  1.00  0.63
H
ATOM  16608 3HZ  LYS B 469     -18.748  22.531 -18.555  1.00  0.63
H
ATOM  16609  N   GLY B 470     -15.062  21.998 -11.757  1.00  0.32
N
ATOM  16610  CA  GLY B 470     -13.709  22.004 -11.222  1.00  0.36
C
ATOM  16611  C   GLY B 470     -13.677  21.385  -9.837  1.00  0.45
C
ATOM  16612  O   GLY B 470     -14.521  20.559  -9.493  1.00  1.16
O
ATOM  16613  H   GLY B 470     -15.387  21.182 -12.262  1.00  0.38
H
ATOM  16614 1HA  GLY B 470     -13.051  21.447 -11.890  1.00  0.43
H
ATOM  16615 2HA  GLY B 470     -13.336  23.026 -11.178  1.00  0.43
H
ATOM  16616  N   MET B 471     -12.703  21.777  -9.037  1.00  0.49
N
ATOM  16617  CA  MET B 471     -12.607  21.227  -7.703  1.00  0.69
C
ATOM  16618  C   MET B 471     -13.197  22.193  -6.716  1.00  1.02
C
ATOM  16619  O   MET B 471     -12.766  23.335  -6.627  1.00  3.12
O
ATOM  16620  CB  MET B 471     -11.169  20.914  -7.353  1.00  1.03
C
ATOM  16621  CG  MET B 471     -10.992  20.316  -5.973  1.00  1.03
C
ATOM  16622  SD  MET B 471      -9.287  19.876  -5.663  1.00  1.03
S
ATOM  16623  CE  MET B 471      -9.191  18.508  -6.806  1.00  1.03
C
ATOM  16624  H   MET B 471     -12.042  22.474  -9.349  1.00  0.59
H
ATOM  16625  HA  MET B 471     -13.188  20.307  -7.658  1.00  0.83
H
ATOM  16626 1HB  MET B 471     -10.763  20.211  -8.079  1.00  1.24
H
```

```
ATOM  16627 2HB  MET B 471     -10.572  21.825  -7.408  1.00  1.24
H
ATOM  16628 1HG  MET B 471     -11.311  21.031  -5.215  1.00  1.24
H
ATOM  16629 2HG  MET B 471     -11.604  19.419  -5.877  1.00  1.24
H
ATOM  16630 1HE  MET B 471      -8.200  18.075  -6.765  1.00  1.24
H
ATOM  16631 2HE  MET B 471      -9.929  17.755  -6.531  1.00  1.24
H
ATOM  16632 3HE  MET B 471      -9.394  18.863  -7.818  1.00  1.24
H
ATOM  16633  N   CYS B 472     -14.186  21.749  -5.968  1.00  0.44
N
ATOM  16634  CA  CYS B 472     -14.783  22.643  -4.998  1.00  0.49
C
ATOM  16635  C   CYS B 472     -14.460  22.156  -3.621  1.00  0.54
C
ATOM  16636  O   CYS B 472     -14.843  21.054  -3.246  1.00  2.33
O
ATOM  16637  CB  CYS B 472     -16.289  22.715  -5.159  1.00  0.73
C
ATOM  16638  SG  CYS B 472     -16.831  23.314  -6.771  1.00  0.73
S
ATOM  16639  H   CYS B 472     -14.516  20.795  -6.069  1.00  0.53
H
ATOM  16640  HA  CYS B 472     -14.369  23.640  -5.127  1.00  0.59
H
ATOM  16641 1HB  CYS B 472     -16.708  21.727  -5.000  1.00  0.88
H
ATOM  16642 2HB  CYS B 472     -16.703  23.371  -4.396  1.00  0.88
H
ATOM  16643  HG  CYS B 472     -16.415  22.256  -7.461  1.00  0.88
H
ATOM  16644  N   PHE B 473     -13.751  22.975  -2.870  1.00  0.30
N
ATOM  16645  CA  PHE B 473     -13.373  22.606  -1.529  1.00  0.26
C
ATOM  16646  C   PHE B 473     -14.100  23.448  -0.531  1.00  0.40
C
ATOM  16647  O   PHE B 473     -14.100  24.673  -0.622  1.00  0.55
O
ATOM  16648  CB  PHE B 473     -11.886  22.720  -1.312  1.00  0.39
C
ATOM  16649  CG  PHE B 473     -11.490  22.361   0.089  1.00  0.39
C
ATOM  16650  CD1 PHE B 473     -11.685  21.073   0.565  1.00  0.39
C
ATOM  16651  CD2 PHE B 473     -10.901  23.294   0.924  1.00  0.39
C
ATOM  16652  CE1 PHE B 473     -11.309  20.721   1.843  1.00  0.39
C
ATOM  16653  CE2 PHE B 473     -10.513  22.938   2.199  1.00  0.39
C
ATOM  16654  CZ  PHE B 473     -10.720  21.656   2.662  1.00  0.39
C
ATOM  16655  H   PHE B 473     -13.483  23.875  -3.237  1.00  0.36
H
```

ATOM 16656 HA PHE B 473 -13.653 21.569 -1.369 1.00 0.31
H
ATOM 16657 1HB PHE B 473 -11.367 22.054 -1.998 1.00 0.47
H
ATOM 16658 2HB PHE B 473 -11.553 23.736 -1.517 1.00 0.47
H
ATOM 16659 HD1 PHE B 473 -12.143 20.336 -0.085 1.00 0.47
H
ATOM 16660 HD2 PHE B 473 -10.732 24.308 0.562 1.00 0.47
H
ATOM 16661 HE1 PHE B 473 -11.470 19.704 2.198 1.00 0.47
H
ATOM 16662 HE2 PHE B 473 -10.039 23.675 2.844 1.00 0.47
H
ATOM 16663 HZ PHE B 473 -10.412 21.387 3.670 1.00 0.47
H
ATOM 16664 N ILE B 474 -14.725 22.791 0.424 1.00 0.41
N
ATOM 16665 CA ILE B 474 -15.466 23.511 1.410 1.00 0.58
C
ATOM 16666 C ILE B 474 -14.880 23.266 2.789 1.00 0.60
C
ATOM 16667 O ILE B 474 -14.720 22.124 3.225 1.00 1.35
O
ATOM 16668 CB ILE B 474 -16.926 23.111 1.371 1.00 0.87
C
ATOM 16669 CG1 ILE B 474 -17.532 23.328 -0.009 1.00 0.87
C
ATOM 16670 CG2 ILE B 474 -17.648 24.001 2.313 1.00 0.87
C
ATOM 16671 CD1 ILE B 474 -18.921 22.767 -0.129 1.00 0.87
C
ATOM 16672 H ILE B 474 -14.696 21.783 0.445 1.00 0.49
H
ATOM 16673 HA ILE B 474 -15.395 24.574 1.196 1.00 0.70
H
ATOM 16674 HB ILE B 474 -17.057 22.068 1.651 1.00 1.04
H
ATOM 16675 1HG1 ILE B 474 -17.581 24.389 -0.215 1.00 1.04
H
ATOM 16676 2HG1 ILE B 474 -16.905 22.854 -0.763 1.00 1.04
H
ATOM 16677 1HG2 ILE B 474 -18.695 23.739 2.266 1.00 1.04
H
ATOM 16678 2HG2 ILE B 474 -17.265 23.864 3.322 1.00 1.04
H
ATOM 16679 3HG2 ILE B 474 -17.520 25.040 2.013 1.00 1.04
H
ATOM 16680 1HD1 ILE B 474 -19.306 22.957 -1.127 1.00 1.04
H
ATOM 16681 2HD1 ILE B 474 -18.898 21.693 0.052 1.00 1.04
H
ATOM 16682 3HD1 ILE B 474 -19.570 23.246 0.603 1.00 1.04
H
ATOM 16683 N ARG B 475 -14.535 24.353 3.459 1.00 0.71
N
ATOM 16684 CA ARG B 475 -13.937 24.280 4.774 1.00 0.79
C

```
ATOM  16685  C   ARG B 475     -15.022  24.232   5.845  1.00  1.32
C
ATOM  16686  O   ARG B 475     -16.041  24.937   5.748  1.00  6.33
O
ATOM  16687  CB  ARG B 475     -13.039  25.490   4.990  1.00  1.19
C
ATOM  16688  CG  ARG B 475     -11.943  25.628   3.950  1.00  1.19
C
ATOM  16689  CD  ARG B 475     -11.053  26.796   4.163  1.00  1.19
C
ATOM  16690  NE  ARG B 475     -10.051  26.541   5.183  1.00  1.19
N
ATOM  16691  CZ  ARG B 475      -9.363  27.493   5.839  1.00  1.19
C
ATOM  16692  NH1 ARG B 475      -9.607  28.763   5.611  1.00  1.19
N
ATOM  16693  NH2 ARG B 475      -8.431  27.145   6.703  1.00  1.19
N
ATOM  16694  H   ARG B 475     -14.681  25.259   3.038  1.00  0.85
H
ATOM  16695  HA  ARG B 475     -13.336  23.372   4.839  1.00  0.95
H
ATOM  16696 1HB  ARG B 475     -13.639  26.393   4.942  1.00  1.42
H
ATOM  16697 2HB  ARG B 475     -12.575  25.444   5.976  1.00  1.42
H
ATOM  16698 1HG  ARG B 475     -11.321  24.742   4.018  1.00  1.42
H
ATOM  16699 2HG  ARG B 475     -12.382  25.697   2.954  1.00  1.42
H
ATOM  16700 1HD  ARG B 475     -10.538  27.033   3.233  1.00  1.42
H
ATOM  16701 2HD  ARG B 475     -11.646  27.652   4.477  1.00  1.42
H
ATOM  16702  HE  ARG B 475      -9.800  25.574   5.366  1.00  1.42
H
ATOM  16703 1HH1 ARG B 475     -10.316  29.026   4.942  1.00  1.42
H
ATOM  16704 2HH1 ARG B 475      -9.070  29.485   6.080  1.00  1.42
H
ATOM  16705 1HH2 ARG B 475      -8.206  26.163   6.836  1.00  1.42
H
ATOM  16706 2HH2 ARG B 475      -7.909  27.855   7.192  1.00  1.42
H
ATOM  16707  N   THR B 476     -14.788  23.429   6.883  1.00  0.55
N
ATOM  16708  CA  THR B 476     -15.739  23.359   7.974  1.00  0.37
C
ATOM  16709  C   THR B 476     -15.075  23.240   9.332  1.00  0.61
C
ATOM  16710  O   THR B 476     -13.933  22.789   9.451  1.00  2.32
O
ATOM  16711  CB  THR B 476     -16.736  22.233   7.764  1.00  0.55
C
ATOM  16712  OG1 THR B 476     -17.724  22.315   8.789  1.00  0.55
O
ATOM  16713  CG2 THR B 476     -16.209  20.862   7.756  1.00  0.55
C
```

ATOM 16714 H THR B 476 -13.964 22.840 6.899 1.00 0.66
H
ATOM 16715 HA THR B 476 -16.307 24.290 7.980 1.00 0.44
H
ATOM 16716 HB THR B 476 -17.159 22.366 6.789 1.00 0.67
H
ATOM 16717 HG1 THR B 476 -17.890 23.230 9.027 1.00 0.67
H
ATOM 16718 1HG2 THR B 476 -17.022 20.159 7.578 1.00 0.67
H
ATOM 16719 2HG2 THR B 476 -15.469 20.762 6.974 1.00 0.67
H
ATOM 16720 3HG2 THR B 476 -15.771 20.673 8.726 1.00 0.67
H
ATOM 16721 N SER B 477 -15.807 23.702 10.344 1.00 0.19
N
ATOM 16722 CA SER B 477 -15.323 23.806 11.705 1.00 0.17
C
ATOM 16723 C SER B 477 -16.051 22.952 12.730 1.00 0.19
C
ATOM 16724 O SER B 477 -17.254 22.679 12.632 1.00 0.25
O
ATOM 16725 CB SER B 477 -15.440 25.251 12.154 1.00 0.26
C
ATOM 16726 OG SER B 477 -16.791 25.651 12.192 1.00 0.26
O
ATOM 16727 H SER B 477 -16.731 24.020 10.149 1.00 0.23
H
ATOM 16728 HA SER B 477 -14.270 23.515 11.710 1.00 0.20
H
ATOM 16729 1HB SER B 477 -14.997 25.364 13.141 1.00 0.31
H
ATOM 16730 2HB SER B 477 -14.885 25.892 11.470 1.00 0.31
H
ATOM 16731 HG SER B 477 -16.799 26.508 12.626 1.00 0.31
H
ATOM 16732 N GLN B 478 -15.281 22.571 13.742 1.00 0.20
N
ATOM 16733 CA GLN B 478 -15.713 21.772 14.877 1.00 0.23
C
ATOM 16734 C GLN B 478 -16.578 22.472 15.934 1.00 0.29
C
ATOM 16735 O GLN B 478 -17.509 21.847 16.440 1.00 0.42
O
ATOM 16736 CB GLN B 478 -14.486 21.144 15.556 1.00 0.35
C
ATOM 16737 CG GLN B 478 -14.824 20.266 16.748 1.00 0.35
C
ATOM 16738 CD GLN B 478 -15.697 19.084 16.376 1.00 0.35
C
ATOM 16739 OE1 GLN B 478 -15.390 18.328 15.450 1.00 0.35
O
ATOM 16740 NE2 GLN B 478 -16.797 18.915 17.101 1.00 0.35
N
ATOM 16741 H GLN B 478 -14.311 22.854 13.717 1.00 0.24
H
ATOM 16742 HA GLN B 478 -16.314 20.955 14.482 1.00 0.28
H

ATOM 16743 1HB GLN B 478 -13.954 20.527 14.833 1.00 0.41
H
ATOM 16744 2HB GLN B 478 -13.797 21.919 15.888 1.00 0.41
H
ATOM 16745 1HG GLN B 478 -13.898 19.884 17.178 1.00 0.41
H
ATOM 16746 2HG GLN B 478 -15.357 20.865 17.487 1.00 0.41
H
ATOM 16747 1HE2 GLN B 478 -17.414 18.150 16.907 1.00 0.41
H
ATOM 16748 2HE2 GLN B 478 -17.009 19.550 17.844 1.00 0.41
H
ATOM 16749 N PRO B 479 -16.306 23.734 16.317 1.00 0.36
N
ATOM 16750 CA PRO B 479 -17.075 24.456 17.323 1.00 0.57
C
ATOM 16751 C PRO B 479 -18.431 24.898 16.812 1.00 0.35
C
ATOM 16752 O PRO B 479 -18.596 25.224 15.635 1.00 0.45
O
ATOM 16753 CB PRO B 479 -16.165 25.636 17.699 1.00 0.85
C
ATOM 16754 CG PRO B 479 -15.248 25.793 16.549 1.00 0.85
C
ATOM 16755 CD PRO B 479 -14.982 24.373 16.128 1.00 0.85
C
ATOM 16756 HA PRO B 479 -17.206 23.797 18.193 1.00 0.68
H
ATOM 16757 1HB PRO B 479 -16.772 26.535 17.881 1.00 1.03
H
ATOM 16758 2HB PRO B 479 -15.633 25.414 18.636 1.00 1.03
H
ATOM 16759 1HG PRO B 479 -15.722 26.391 15.758 1.00 1.03
H
ATOM 16760 2HG PRO B 479 -14.341 26.327 16.857 1.00 1.03
H
ATOM 16761 1HD PRO B 479 -14.659 24.391 15.087 1.00 1.03
H
ATOM 16762 2HD PRO B 479 -14.234 23.907 16.786 1.00 1.03
H
ATOM 16763 N GLU B 480 -19.398 24.926 17.723 1.00 0.33
N
ATOM 16764 CA GLU B 480 -20.741 25.358 17.393 1.00 0.41
C
ATOM 16765 C GLU B 480 -20.791 26.866 17.413 1.00 0.66
C
ATOM 16766 O GLU B 480 -20.382 27.489 18.393 1.00 3.84
O
ATOM 16767 CB GLU B 480 -21.755 24.782 18.385 1.00 0.61
C
ATOM 16768 CG GLU B 480 -21.873 23.264 18.356 1.00 0.61
C
ATOM 16769 CD GLU B 480 -22.862 22.729 19.355 1.00 0.61
C
ATOM 16770 OE1 GLU B 480 -23.388 23.504 20.116 1.00 0.61
O
ATOM 16771 OE2 GLU B 480 -23.086 21.541 19.360 1.00 0.61
O

```
ATOM  16772  H   GLU B 480     -19.192  24.641  18.670  1.00  0.40
H
ATOM  16773  HA  GLU B 480     -20.980  25.020  16.392  1.00  0.49
H
ATOM  16774 1HB  GLU B 480     -21.479  25.074  19.398  1.00  0.74
H
ATOM  16775 2HB  GLU B 480     -22.742  25.198  18.181  1.00  0.74
H
ATOM  16776 1HG  GLU B 480     -22.178  22.947  17.362  1.00  0.74
H
ATOM  16777 2HG  GLU B 480     -20.894  22.832  18.559  1.00  0.74
H
ATOM  16778  N   THR B 481     -21.293  27.457  16.336  1.00  0.85
N
ATOM  16779  CA  THR B 481     -21.343  28.907  16.254  1.00  0.59
C
ATOM  16780  C   THR B 481     -22.752  29.374  15.967  1.00  0.59
C
ATOM  16781  O   THR B 481     -23.577  28.619  15.454  1.00  0.79
O
ATOM  16782  CB  THR B 481     -20.391  29.440  15.161  1.00  0.89
C
ATOM  16783  OG1 THR B 481     -20.815  28.954  13.881  1.00  0.89
O
ATOM  16784  CG2 THR B 481     -18.953  29.005  15.418  1.00  0.89
C
ATOM  16785  H   THR B 481     -21.641  26.897  15.564  1.00  1.02
H
ATOM  16786  HA  THR B 481     -21.036  29.325  17.213  1.00  0.71
H
ATOM  16787  HB  THR B 481     -20.431  30.525  15.158  1.00  1.06
H
ATOM  16788  HG1 THR B 481     -20.286  29.365  13.190  1.00  1.06
H
ATOM  16789 1HG2 THR B 481     -18.309  29.404  14.635  1.00  1.06
H
ATOM  16790 2HG2 THR B 481     -18.624  29.385  16.386  1.00  1.06
H
ATOM  16791 3HG2 THR B 481     -18.893  27.918  15.415  1.00  1.06
H
ATOM  16792  N   ALA B 482     -23.037  30.618  16.314  1.00  0.52
N
ATOM  16793  CA  ALA B 482     -24.379  31.146  16.142  1.00  0.56
C
ATOM  16794  C   ALA B 482     -24.712  31.432  14.686  1.00  0.54
C
ATOM  16795  O   ALA B 482     -23.887  31.948  13.930  1.00  0.62
O
ATOM  16796  CB  ALA B 482     -24.547  32.413  16.961  1.00  0.84
C
ATOM  16797  H   ALA B 482     -22.318  31.203  16.717  1.00  0.62
H
ATOM  16798  HA  ALA B 482     -25.076  30.399  16.507  1.00  0.67
H
ATOM  16799 1HB  ALA B 482     -25.568  32.781  16.857  1.00  1.01
H
ATOM  16800 2HB  ALA B 482     -24.345  32.198  18.010  1.00  1.01
H
```

ATOM 16801 3HB ALA B 482 -23.852 33.173 16.606 1.00 1.01
H
ATOM 16802 N VAL B 483 -25.956 31.151 14.315 1.00 0.48
N
ATOM 16803 CA VAL B 483 -26.445 31.482 12.992 1.00 0.48
C
ATOM 16804 C VAL B 483 -26.595 32.977 12.908 1.00 0.49
C
ATOM 16805 O VAL B 483 -27.216 33.588 13.777 1.00 0.50
O
ATOM 16806 CB VAL B 483 -27.790 30.794 12.703 1.00 0.72
C
ATOM 16807 CG1 VAL B 483 -28.346 31.271 11.373 1.00 0.72
C
ATOM 16808 CG2 VAL B 483 -27.588 29.289 12.664 1.00 0.72
C
ATOM 16809 H VAL B 483 -26.573 30.691 14.969 1.00 0.58
H
ATOM 16810 HA VAL B 483 -25.718 31.158 12.252 1.00 0.58
H
ATOM 16811 HB VAL B 483 -28.503 31.053 13.483 1.00 0.86
H
ATOM 16812 1HG1 VAL B 483 -29.299 30.780 11.177 1.00 0.86
H
ATOM 16813 2HG1 VAL B 483 -28.495 32.351 11.403 1.00 0.86
H
ATOM 16814 3HG1 VAL B 483 -27.639 31.026 10.584 1.00 0.86
H
ATOM 16815 1HG2 VAL B 483 -28.539 28.801 12.466 1.00 0.86
H
ATOM 16816 2HG2 VAL B 483 -26.879 29.044 11.875 1.00 0.86
H
ATOM 16817 3HG2 VAL B 483 -27.197 28.944 13.619 1.00 0.86
H
ATOM 16818 N ILE B 484 -26.015 33.581 11.879 1.00 0.50
N
ATOM 16819 CA ILE B 484 -26.093 35.028 11.748 1.00 0.51
C
ATOM 16820 C ILE B 484 -26.788 35.454 10.467 1.00 0.53
C
ATOM 16821 O ILE B 484 -27.247 36.592 10.364 1.00 0.57
O
ATOM 16822 CB ILE B 484 -24.689 35.676 11.802 1.00 0.77
C
ATOM 16823 CG1 ILE B 484 -23.831 35.189 10.633 1.00 0.77
C
ATOM 16824 CG2 ILE B 484 -24.006 35.373 13.129 1.00 0.77
C
ATOM 16825 CD1 ILE B 484 -22.540 35.949 10.478 1.00 0.77
C
ATOM 16826 H ILE B 484 -25.523 33.031 11.182 1.00 0.60
H
ATOM 16827 HA ILE B 484 -26.671 35.417 12.584 1.00 0.61
H
ATOM 16828 HB ILE B 484 -24.793 36.754 11.697 1.00 0.92
H
ATOM 16829 1HG1 ILE B 484 -23.600 34.133 10.774 1.00 0.92
H

ATOM 16830 2HG1 ILE B 484   -24.396 35.296   9.709 1.00 0.92
H
ATOM 16831 1HG2 ILE B 484   -23.028 35.853 13.152 1.00 0.92
H
ATOM 16832 2HG2 ILE B 484   -24.616 35.752 13.947 1.00 0.92
H
ATOM 16833 3HG2 ILE B 484   -23.882 34.297 13.237 1.00 0.92
H
ATOM 16834 1HD1 ILE B 484   -21.986 35.553   9.626 1.00 0.92
H
ATOM 16835 2HD1 ILE B 484   -22.759 37.004 10.309 1.00 0.92
H
ATOM 16836 3HD1 ILE B 484   -21.941 35.842 11.381 1.00 0.92
H
ATOM 16837 N   TYR B 485   -26.902 34.547   9.500 1.00 0.53
N
ATOM 16838 CA TYR B 485   -27.591 34.904   8.273 1.00 0.63
C
ATOM 16839 C   TYR B 485   -29.033 34.424   8.289 1.00 0.68
C
ATOM 16840 O   TYR B 485   -29.380 33.461   8.969 1.00 0.66
O
ATOM 16841 CB TYR B 485   -26.871 34.333   7.050 1.00 0.95
C
ATOM 16842 CG TYR B 485   -25.482 34.891   6.841 1.00 0.95
C
ATOM 16843 CD1 TYR B 485   -24.390 34.083   7.093 1.00 0.95
C
ATOM 16844 CD2 TYR B 485   -25.289 36.197   6.417 1.00 0.95
C
ATOM 16845 CE1 TYR B 485   -23.109 34.556   6.927 1.00 0.95
C
ATOM 16846 CE2 TYR B 485   -24.002 36.679   6.250 1.00 0.95
C
ATOM 16847 CZ TYR B 485   -22.919 35.860   6.507 1.00 0.95
C
ATOM 16848 OH TYR B 485   -21.644 36.334   6.348 1.00 0.95
O
ATOM 16849 H   TYR B 485   -26.484 33.624   9.595 1.00 0.64
H
ATOM 16850 HA TYR B 485   -27.606 35.990   8.187 1.00 0.76
H
ATOM 16851 1HB TYR B 485   -26.788 33.253   7.157 1.00 1.13
H
ATOM 16852 2HB TYR B 485   -27.458 34.530   6.154 1.00 1.13
H
ATOM 16853 HD1 TYR B 485   -24.550 33.064   7.420 1.00 1.13
H
ATOM 16854 HD2 TYR B 485   -26.145 36.842   6.217 1.00 1.13
H
ATOM 16855 HE1 TYR B 485   -22.255 33.910   7.127 1.00 1.13
H
ATOM 16856 HE2 TYR B 485   -23.844 37.705   5.920 1.00 1.13
H
ATOM 16857 HH TYR B 485   -21.003 35.635   6.538 1.00 1.13
H
ATOM 16858 N   THR B 486   -29.868 35.119   7.539 1.00 0.88
N

ATOM 16859 CA THR B 486 -31.263 34.755 7.363 1.00 1.15
C
ATOM 16860 C THR B 486 -31.295 33.709 6.244 1.00 1.49
C
ATOM 16861 O THR B 486 -30.562 33.863 5.275 1.00 5.08
O
ATOM 16862 CB THR B 486 -32.116 35.998 6.998 1.00 1.72
C
ATOM 16863 OG1 THR B 486 -32.026 36.961 8.056 1.00 1.72
O
ATOM 16864 CG2 THR B 486 -33.581 35.644 6.785 1.00 1.72
C
ATOM 16865 H THR B 486 -29.514 35.922 7.038 1.00 1.06
H
ATOM 16866 HA THR B 486 -31.636 34.325 8.290 1.00 1.38
H
ATOM 16867 HB THR B 486 -31.721 36.442 6.083 1.00 2.07
H
ATOM 16868 HG1 THR B 486 -32.549 37.735 7.830 1.00 2.07
H
ATOM 16869 1HG2 THR B 486 -34.137 36.546 6.530 1.00 2.07
H
ATOM 16870 2HG2 THR B 486 -33.674 34.926 5.977 1.00 2.07
H
ATOM 16871 3HG2 THR B 486 -33.988 35.215 7.699 1.00 2.07
H
ATOM 16872 N PRO B 487 -32.086 32.636 6.352 1.00 1.11
N
ATOM 16873 CA PRO B 487 -32.262 31.550 5.379 1.00 1.90
C
ATOM 16874 C PRO B 487 -32.555 31.998 3.949 1.00 4.99
C
ATOM 16875 O PRO B 487 -32.274 31.276 2.999 1.00 30.38
O
ATOM 16876 CB PRO B 487 -33.424 30.767 5.959 1.00 2.85
C
ATOM 16877 CG PRO B 487 -33.287 30.936 7.434 1.00 2.85
C
ATOM 16878 CD PRO B 487 -32.749 32.323 7.633 1.00 2.85
C
ATOM 16879 HA PRO B 487 -31.356 30.926 5.383 1.00 2.28
H
ATOM 16880 1HB PRO B 487 -34.376 31.175 5.576 1.00 3.42
H
ATOM 16881 2HB PRO B 487 -33.369 29.729 5.625 1.00 3.42
H
ATOM 16882 1HG PRO B 487 -34.260 30.791 7.923 1.00 3.42
H
ATOM 16883 2HG PRO B 487 -32.617 30.161 7.837 1.00 3.42
H
ATOM 16884 1HD PRO B 487 -33.571 33.026 7.825 1.00 3.42
H
ATOM 16885 2HD PRO B 487 -32.014 32.321 8.452 1.00 3.42
H
ATOM 16886 N GLN B 488 -33.111 33.194 3.788 1.00 2.46
N
ATOM 16887 CA GLN B 488 -33.421 33.734 2.476 1.00 1.81
C

```
ATOM  16888  C   GLN B 488     -32.239  34.497   1.858  1.00  1.83
C
ATOM  16889  O   GLN B 488     -32.330  34.958   0.719  1.00  2.44
O
ATOM  16890  CB  GLN B 488     -34.627  34.669   2.577  1.00  2.71
C
ATOM  16891  CG  GLN B 488     -35.895  34.001   3.076  1.00  2.71
C
ATOM  16892  CD  GLN B 488     -36.359  32.879   2.169  1.00  2.71
C
ATOM  16893  OE1 GLN B 488     -36.469  33.052   0.953  1.00  2.71
O
ATOM  16894  NE2 GLN B 488     -36.637  31.723   2.758  1.00  2.71
N
ATOM  16895  H   GLN B 488     -33.335  33.743   4.599  1.00  2.95
H
ATOM  16896  HA  GLN B 488     -33.670  32.907   1.813  1.00  2.17
H
ATOM  16897 1HB  GLN B 488     -34.394  35.493   3.250  1.00  3.26
H
ATOM  16898 2HB  GLN B 488     -34.838  35.097   1.597  1.00  3.26
H
ATOM  16899 1HG  GLN B 488     -35.710  33.585   4.066  1.00  3.26
H
ATOM  16900 2HG  GLN B 488     -36.689  34.746   3.128  1.00  3.26
H
ATOM  16901 1HE2 GLN B 488     -36.950  30.945   2.211  1.00  3.26
H
ATOM  16902 2HE2 GLN B 488     -36.535  31.628   3.748  1.00  3.26
H
ATOM  16903  N   GLU B 489     -31.146  34.650   2.613  1.00  1.52
N
ATOM  16904  CA  GLU B 489     -29.959  35.348   2.132  1.00  1.75
C
ATOM  16905  C   GLU B 489     -29.390  34.647   0.910  1.00  1.35
C
ATOM  16906  O   GLU B 489     -29.139  33.442   0.932  1.00  1.58
O
ATOM  16907  CB  GLU B 489     -28.889  35.433   3.229  1.00  2.62
C
ATOM  16908  CG  GLU B 489     -27.651  36.243   2.852  1.00  2.62
C
ATOM  16909  CD  GLU B 489     -27.947  37.716   2.705  1.00  2.62
C
ATOM  16910  OE1 GLU B 489     -28.788  38.199   3.424  1.00  2.62
O
ATOM  16911  OE2 GLU B 489     -27.332  38.362   1.886  1.00  2.62
O
ATOM  16912  H   GLU B 489     -31.115  34.264   3.542  1.00  1.82
H
ATOM  16913  HA  GLU B 489     -30.243  36.360   1.842  1.00  2.10
H
ATOM  16914 1HB  GLU B 489     -29.323  35.888   4.120  1.00  3.15
H
ATOM  16915 2HB  GLU B 489     -28.567  34.428   3.500  1.00  3.15
H
ATOM  16916 1HG  GLU B 489     -26.893  36.109   3.623  1.00  3.15
H
```

ATOM 16917 2HG GLU B 489 -27.250 35.858 1.915 1.00 3.15
H
ATOM 16918 N ASN B 490 -29.191 35.404 -0.159 1.00 1.59
N
ATOM 16919 CA ASN B 490 -28.672 34.831 -1.390 1.00 1.96
C
ATOM 16920 C ASN B 490 -27.151 34.831 -1.419 1.00 1.28
C
ATOM 16921 O ASN B 490 -26.515 35.882 -1.332 1.00 2.46
O
ATOM 16922 CB ASN B 490 -29.232 35.573 -2.587 1.00 2.94
C
ATOM 16923 CG ASN B 490 -30.716 35.380 -2.736 1.00 2.94
C
ATOM 16924 OD1 ASN B 490 -31.219 34.255 -2.647 1.00 2.94
O
ATOM 16925 ND2 ASN B 490 -31.425 36.458 -2.957 1.00 2.94
N
ATOM 16926 H ASN B 490 -29.410 36.390 -0.120 1.00 1.91
H
ATOM 16927 HA ASN B 490 -28.995 33.791 -1.448 1.00 2.35
H
ATOM 16928 1HB ASN B 490 -29.024 36.640 -2.486 1.00 3.53
H
ATOM 16929 2HB ASN B 490 -28.738 35.227 -3.496 1.00 3.53
H
ATOM 16930 1HD2 ASN B 490 -32.418 36.389 -3.063 1.00 3.53
H
ATOM 16931 2HD2 ASN B 490 -30.977 37.348 -3.020 1.00 3.53
H
ATOM 16932 N PHE B 491 -26.574 33.643 -1.561 1.00 0.62
N
ATOM 16933 CA PHE B 491 -25.127 33.488 -1.631 1.00 0.41
C
ATOM 16934 C PHE B 491 -24.667 33.188 -3.041 1.00 0.45
C
ATOM 16935 O PHE B 491 -25.388 32.578 -3.830 1.00 0.67
O
ATOM 16936 CB PHE B 491 -24.650 32.394 -0.689 1.00 0.61
C
ATOM 16937 CG PHE B 491 -24.765 32.760 0.757 1.00 0.61
C
ATOM 16938 CD1 PHE B 491 -25.952 32.581 1.436 1.00 0.61
C
ATOM 16939 CD2 PHE B 491 -23.679 33.278 1.442 1.00 0.61
C
ATOM 16940 CE1 PHE B 491 -26.059 32.905 2.769 1.00 0.61
C
ATOM 16941 CE2 PHE B 491 -23.779 33.605 2.778 1.00 0.61
C
ATOM 16942 CZ PHE B 491 -24.973 33.415 3.437 1.00 0.61
C
ATOM 16943 H PHE B 491 -27.157 32.820 -1.622 1.00 0.74
H
ATOM 16944 HA PHE B 491 -24.666 34.426 -1.319 1.00 0.49
H
ATOM 16945 1HB PHE B 491 -25.234 31.492 -0.860 1.00 0.74
H

ATOM 16946 2HB PHE B 491   -23.611 32.158 -0.901 1.00 0.74
H
ATOM 16947 HD1 PHE B 491   -26.811 32.180   0.901 1.00 0.74
H
ATOM 16948 HD2 PHE B 491   -22.738 33.425   0.913 1.00 0.74
H
ATOM 16949 HE1 PHE B 491   -27.004 32.759   3.290 1.00 0.74
H
ATOM 16950 HE2 PHE B 491   -22.919 34.012   3.310 1.00 0.74
H
ATOM 16951 HZ PHE B 491   -25.059 33.668   4.485 1.00 0.74
H
ATOM 16952 N   GLU B 492   -23.458 33.632 -3.348 1.00 0.41
N
ATOM 16953 CA GLU B 492   -22.883 33.450 -4.665 1.00 0.46
C
ATOM 16954 C   GLU B 492   -21.370 33.363 -4.595 1.00 0.43
C
ATOM 16955 O   GLU B 492   -20.734 34.055 -3.798 1.00 0.47
O
ATOM 16956 CB GLU B 492   -23.300 34.592 -5.593 1.00 0.69
C
ATOM 16957 CG GLU B 492   -22.815 34.449 -7.030 1.00 0.69
C
ATOM 16958 CD GLU B 492   -23.337 35.531 -7.933 1.00 0.69
C
ATOM 16959 OE1 GLU B 492   -24.074 36.365 -7.464 1.00 0.69
O
ATOM 16960 OE2 GLU B 492   -23.004 35.523 -9.094 1.00 0.69
O
ATOM 16961 H   GLU B 492   -22.922 34.119 -2.645 1.00 0.49
H
ATOM 16962 HA GLU B 492   -23.256 32.513 -5.079 1.00 0.55
H
ATOM 16963 1HB GLU B 492   -24.388 34.662 -5.613 1.00 0.83
H
ATOM 16964 2HB GLU B 492   -22.920 35.536 -5.203 1.00 0.83
H
ATOM 16965 1HG GLU B 492   -21.726 34.482 -7.042 1.00 0.83
H
ATOM 16966 2HG GLU B 492   -23.126 33.478 -7.414 1.00 0.83
H
ATOM 16967 N   ILE B 493   -20.807 32.496 -5.421 1.00 0.42
N
ATOM 16968 CA ILE B 493   -19.371 32.304 -5.474 1.00 0.45
C
ATOM 16969 C   ILE B 493   -18.701 33.576 -5.957 1.00 0.42
C
ATOM 16970 O   ILE B 493   -19.078 34.125 -6.993 1.00 0.47
O
ATOM 16971 CB ILE B 493   -19.027 31.144 -6.411 1.00 0.68
C
ATOM 16972 CG1 ILE B 493   -19.600 29.854 -5.847 1.00 0.68
C
ATOM 16973 CG2 ILE B 493   -17.526 31.031 -6.610 1.00 0.68
C
ATOM 16974 CD1 ILE B 493   -19.527 28.710 -6.815 1.00 0.68
C

ATOM 16975 H ILE B 493 -21.396 31.959 -6.043 1.00 0.50
H
ATOM 16976 HA ILE B 493 -19.010 32.079 -4.474 1.00 0.54
H
ATOM 16977 HB ILE B 493 -19.501 31.312 -7.377 1.00 0.81
H
ATOM 16978 1HG1 ILE B 493 -19.054 29.586 -4.945 1.00 0.81
H
ATOM 16979 2HG1 ILE B 493 -20.645 30.014 -5.584 1.00 0.81
H
ATOM 16980 1HG2 ILE B 493 -17.312 30.204 -7.287 1.00 0.81
H
ATOM 16981 2HG2 ILE B 493 -17.147 31.957 -7.038 1.00 0.81
H
ATOM 16982 3HG2 ILE B 493 -17.039 30.848 -5.654 1.00 0.81
H
ATOM 16983 1HD1 ILE B 493 -19.958 27.818 -6.364 1.00 0.81
H
ATOM 16984 2HD1 ILE B 493 -20.087 28.962 -7.717 1.00 0.81
H
ATOM 16985 3HD1 ILE B 493 -18.489 28.519 -7.077 1.00 0.81
H
ATOM 16986 N GLY B 494 -17.719 34.058 -5.200 1.00 0.43
N
ATOM 16987 CA GLY B 494 -17.056 35.305 -5.544 1.00 0.48
C
ATOM 16988 C GLY B 494 -17.742 36.527 -4.930 1.00 0.58
C
ATOM 16989 O GLY B 494 -17.339 37.656 -5.208 1.00 0.70
O
ATOM 16990 H GLY B 494 -17.403 33.570 -4.370 1.00 0.52
H
ATOM 16991 1HA GLY B 494 -16.021 35.263 -5.205 1.00 0.58
H
ATOM 16992 2HA GLY B 494 -17.029 35.410 -6.627 1.00 0.58
H
ATOM 16993 N GLN B 495 -18.771 36.313 -4.099 1.00 0.64
N
ATOM 16994 CA GLN B 495 -19.457 37.428 -3.445 1.00 0.87
C
ATOM 16995 C GLN B 495 -19.325 37.366 -1.920 1.00 0.94
C
ATOM 16996 O GLN B 495 -19.716 36.380 -1.292 1.00 1.76
O
ATOM 16997 CB GLN B 495 -20.939 37.441 -3.825 1.00 1.30
C
ATOM 16998 CG GLN B 495 -21.744 38.523 -3.129 1.00 1.30
C
ATOM 16999 CD GLN B 495 -21.389 39.916 -3.614 1.00 1.30
C
ATOM 17000 OE1 GLN B 495 -21.555 40.237 -4.795 1.00 1.30
O
ATOM 17001 NE2 GLN B 495 -20.897 40.751 -2.707 1.00 1.30
N
ATOM 17002 H GLN B 495 -19.103 35.374 -3.916 1.00 0.77
H
ATOM 17003 HA GLN B 495 -19.004 38.360 -3.781 1.00 1.04
H

ATOM	17004	1HB	GLN	B	495	-21.037	37.584	-4.901	1.00	1.57	H
ATOM	17005	2HB	GLN	B	495	-21.387	36.478	-3.581	1.00	1.57	H
ATOM	17006	1HG	GLN	B	495	-22.803	38.353	-3.323	1.00	1.57	H
ATOM	17007	2HG	GLN	B	495	-21.548	38.475	-2.058	1.00	1.57	H
ATOM	17008	1HE2	GLN	B	495	-20.640	41.682	-2.969	1.00	1.57	H
ATOM	17009	2HE2	GLN	B	495	-20.768	40.449	-1.759	1.00	1.57	H
ATOM	17010	N	ALA	B	496	-18.776	38.433	-1.340	1.00	0.69	N
ATOM	17011	CA	ALA	B	496	-18.591	38.546	0.106	1.00	0.66	C
ATOM	17012	C	ALA	B	496	-19.771	39.245	0.760	1.00	0.63	C
ATOM	17013	O	ALA	B	496	-20.522	39.976	0.110	1.00	0.72	O
ATOM	17014	CB	ALA	B	496	-17.311	39.301	0.430	1.00	0.99	C
ATOM	17015	H	ALA	B	496	-18.478	39.209	-1.925	1.00	0.83	H
ATOM	17016	HA	ALA	B	496	-18.524	37.540	0.519	1.00	0.79	H
ATOM	17017	1HB	ALA	B	496	-17.182	39.353	1.510	1.00	1.19	H
ATOM	17018	2HB	ALA	B	496	-16.463	38.780	-0.012	1.00	1.19	H
ATOM	17019	3HB	ALA	B	496	-17.371	40.310	0.022	1.00	1.19	H
ATOM	17020	N	LYS	B	497	-19.936	39.009	2.055	1.00	0.64	N
ATOM	17021	CA	LYS	B	497	-21.012	39.614	2.818	1.00	0.79	C
ATOM	17022	C	LYS	B	497	-20.531	40.667	3.808	1.00	0.63	C
ATOM	17023	O	LYS	B	497	-19.527	40.495	4.497	1.00	0.76	O
ATOM	17024	CB	LYS	B	497	-21.780	38.523	3.571	1.00	1.19	C
ATOM	17025	CG	LYS	B	497	-22.406	37.477	2.664	1.00	1.19	C
ATOM	17026	CD	LYS	B	497	-23.495	38.114	1.823	1.00	1.19	C
ATOM	17027	CE	LYS	B	497	-24.181	37.115	0.912	1.00	1.19	C
ATOM	17028	NZ	LYS	B	497	-25.251	37.771	0.119	1.00	1.19	N
ATOM	17029	H	LYS	B	497	-19.297	38.387	2.527	1.00	0.77	H
ATOM	17030	HA	LYS	B	497	-21.693	40.101	2.121	1.00	0.95	H
ATOM	17031	1HB	LYS	B	497	-21.108	38.011	4.260	1.00	1.42	H
ATOM	17032	2HB	LYS	B	497	-22.576	38.976	4.162	1.00	1.42	H

ATOM 17033 1HG LYS B 497 -21.645 37.051 2.009 1.00 1.42
H
ATOM 17034 2HG LYS B 497 -22.836 36.677 3.267 1.00 1.42
H
ATOM 17035 1HD LYS B 497 -24.245 38.560 2.479 1.00 1.42
H
ATOM 17036 2HD LYS B 497 -23.062 38.904 1.210 1.00 1.42
H
ATOM 17037 1HE LYS B 497 -23.446 36.691 0.231 1.00 1.42
H
ATOM 17038 2HE LYS B 497 -24.619 36.312 1.505 1.00 1.42
H
ATOM 17039 1HZ LYS B 497 -25.689 37.089 -0.480 1.00 1.42
H
ATOM 17040 2HZ LYS B 497 -25.948 38.144 0.753 1.00 1.42
H
ATOM 17041 3HZ LYS B 497 -24.864 38.516 -0.440 1.00 1.42
H
ATOM 17042 N VAL B 498 -21.293 41.748 3.913 1.00 1.00
N
ATOM 17043 CA VAL B 498 -21.007 42.759 4.916 1.00 0.93
C
ATOM 17044 C VAL B 498 -21.856 42.400 6.117 1.00 0.91
C
ATOM 17045 O VAL B 498 -23.075 42.570 6.095 1.00 1.29
O
ATOM 17046 CB VAL B 498 -21.347 44.172 4.420 1.00 1.40
C
ATOM 17047 CG1 VAL B 498 -21.030 45.173 5.515 1.00 1.40
C
ATOM 17048 CG2 VAL B 498 -20.566 44.482 3.151 1.00 1.40
C
ATOM 17049 H VAL B 498 -22.093 41.858 3.306 1.00 1.20
H
ATOM 17050 HA VAL B 498 -19.956 42.713 5.191 1.00 1.12
H
ATOM 17051 HB VAL B 498 -22.415 44.234 4.215 1.00 1.67
H
ATOM 17052 1HG1 VAL B 498 -21.286 46.176 5.176 1.00 1.67
H
ATOM 17053 2HG1 VAL B 498 -21.609 44.933 6.406 1.00 1.67
H
ATOM 17054 3HG1 VAL B 498 -19.967 45.128 5.749 1.00 1.67
H
ATOM 17055 1HG2 VAL B 498 -20.820 45.484 2.806 1.00 1.67
H
ATOM 17056 2HG2 VAL B 498 -19.499 44.431 3.354 1.00 1.67
H
ATOM 17057 3HG2 VAL B 498 -20.823 43.757 2.379 1.00 1.67
H
ATOM 17058 N VAL B 499 -21.217 41.854 7.140 1.00 0.69
N
ATOM 17059 CA VAL B 499 -21.926 41.351 8.306 1.00 0.73
C
ATOM 17060 C VAL B 499 -22.302 42.489 9.253 1.00 0.81
C
ATOM 17061 O VAL B 499 -23.441 42.572 9.719 1.00 1.33
O

```
ATOM  17062  CB  VAL B 499     -21.066  40.297   9.024  1.00  1.09
C
ATOM  17063  CG1 VAL B 499     -21.765  39.821  10.277  1.00  1.09
C
ATOM  17064  CG2 VAL B 499     -20.783  39.138   8.083  1.00  1.09
C
ATOM  17065  H   VAL B 499     -20.209  41.780   7.112  1.00  0.83
H
ATOM  17066  HA  VAL B 499     -22.843  40.867   7.965  1.00  0.88
H
ATOM  17067  HB  VAL B 499     -20.124  40.753   9.328  1.00  1.31
H
ATOM  17068 1HG1 VAL B 499     -21.139  39.087  10.784  1.00  1.31
H
ATOM  17069 2HG1 VAL B 499     -21.946  40.663  10.938  1.00  1.31
H
ATOM  17070 3HG1 VAL B 499     -22.716  39.361  10.009  1.00  1.31
H
ATOM  17071 1HG2 VAL B 499     -20.162  38.403   8.590  1.00  1.31
H
ATOM  17072 2HG2 VAL B 499     -21.724  38.675   7.782  1.00  1.31
H
ATOM  17073 3HG2 VAL B 499     -20.262  39.506   7.200  1.00  1.31
H
ATOM  17074  N   ARG B 500     -21.339  43.361   9.539  1.00  0.54
N
ATOM  17075  CA  ARG B 500     -21.603  44.521  10.391  1.00  0.55
C
ATOM  17076  C   ARG B 500     -21.032  45.779   9.784  1.00  0.63
C
ATOM  17077  O   ARG B 500     -19.927  45.778   9.250  1.00  1.10
O
ATOM  17078  CB  ARG B 500     -21.032  44.350  11.791  1.00  0.83
C
ATOM  17079  CG  ARG B 500     -21.663  43.249  12.616  1.00  0.83
C
ATOM  17080  CD  ARG B 500     -23.053  43.607  12.996  1.00  0.83
C
ATOM  17081  NE  ARG B 500     -23.671  42.608  13.851  1.00  0.83
N
ATOM  17082  CZ  ARG B 500     -24.351  41.528  13.416  1.00  0.83
C
ATOM  17083  NH1 ARG B 500     -24.504  41.298  12.128  1.00  0.83
N
ATOM  17084  NH2 ARG B 500     -24.875  40.689  14.294  1.00  0.83
N
ATOM  17085  H   ARG B 500     -20.419  43.220   9.135  1.00  0.65
H
ATOM  17086  HA  ARG B 500     -22.683  44.645  10.478  1.00  0.66
H
ATOM  17087 1HB  ARG B 500     -19.966  44.147  11.727  1.00  0.99
H
ATOM  17088 2HB  ARG B 500     -21.150  45.281  12.346  1.00  0.99
H
ATOM  17089 1HG  ARG B 500     -21.684  42.325  12.050  1.00  0.99
H
ATOM  17090 2HG  ARG B 500     -21.087  43.105  13.530  1.00  0.99
H
```

```
ATOM  17091 1HD  ARG B 500     -23.047  44.555  13.534  1.00  0.99
H
ATOM  17092 2HD  ARG B 500     -23.660  43.706  12.097  1.00  0.99
H
ATOM  17093  HE  ARG B 500     -23.586  42.735  14.852  1.00  0.99
H
ATOM  17094 1HH1 ARG B 500     -24.109  41.924  11.434  1.00  0.99
H
ATOM  17095 2HH1 ARG B 500     -25.019  40.485  11.821  1.00  0.99
H
ATOM  17096 1HH2 ARG B 500     -24.764  40.860  15.284  1.00  0.99
H
ATOM  17097 2HH2 ARG B 500     -25.387  39.880  13.975  1.00  0.99
H
ATOM  17098  N   HIS B 501     -21.782  46.867   9.885  1.00  0.70
N
ATOM  17099  CA  HIS B 501     -21.333  48.123   9.314  1.00  0.78
C
ATOM  17100  C   HIS B 501     -22.008  49.350   9.907  1.00  1.73
C
ATOM  17101  O   HIS B 501     -23.047  49.252  10.562  1.00 10.52
O
ATOM  17102  CB  HIS B 501     -21.572  48.101   7.808  1.00  1.17
C
ATOM  17103  CG  HIS B 501     -23.008  47.926   7.440  1.00  1.17
C
ATOM  17104  ND1 HIS B 501     -23.623  46.691   7.433  1.00  1.17
N
ATOM  17105  CD2 HIS B 501     -23.953  48.820   7.069  1.00  1.17
C
ATOM  17106  CE1 HIS B 501     -24.885  46.834   7.070  1.00  1.17
C
ATOM  17107  NE2 HIS B 501     -25.111  48.116   6.843  1.00  1.17
N
ATOM  17108  H   HIS B 501     -22.678  46.822  10.348  1.00  0.84
H
ATOM  17109  HA  HIS B 501     -20.263  48.226   9.486  1.00  0.94
H
ATOM  17110 1HB  HIS B 501     -21.215  49.032   7.365  1.00  1.40
H
ATOM  17111 2HB  HIS B 501     -21.004  47.289   7.365  1.00  1.40
H
ATOM  17112  HD2 HIS B 501     -23.821  49.898   6.965  1.00  1.40
H
ATOM  17113  HE1 HIS B 501     -25.615  46.031   6.974  1.00  1.40
H
ATOM  17114  HE2 HIS B 501     -25.990  48.519   6.552  1.00  1.40
H
ATOM  17115  N   GLY B 502     -21.427  50.507   9.620  1.00  1.06
N
ATOM  17116  CA  GLY B 502     -21.954  51.800  10.035  1.00  1.48
C
ATOM  17117  C   GLY B 502     -21.132  52.937   9.425  1.00  1.47
C
ATOM  17118  O   GLY B 502     -20.044  52.725   8.887  1.00  1.36
O
ATOM  17119  H   GLY B 502     -20.556  50.477   9.110  1.00  1.27
H
```

ATOM 17120 1HA GLY B 502 -22.995 51.887 9.726 1.00 1.78
H
ATOM 17121 2HA GLY B 502 -21.932 51.871 11.123 1.00 1.78
H
ATOM 17122 N VAL B 503 -21.649 54.157 9.527 1.00 1.63
N
ATOM 17123 CA VAL B 503 -20.999 55.322 8.926 1.00 1.64
C
ATOM 17124 C VAL B 503 -19.689 55.713 9.605 1.00 1.54
C
ATOM 17125 O VAL B 503 -18.854 56.388 9.001 1.00 1.62
O
ATOM 17126 CB VAL B 503 -21.961 56.527 8.940 1.00 2.46
C
ATOM 17127 CG1 VAL B 503 -23.212 56.198 8.138 1.00 2.46
C
ATOM 17128 CG2 VAL B 503 -22.329 56.881 10.373 1.00 2.46
C
ATOM 17129 H VAL B 503 -22.533 54.279 10.003 1.00 1.96
H
ATOM 17130 HA VAL B 503 -20.781 55.080 7.886 1.00 1.97
H
ATOM 17131 HB VAL B 503 -21.476 57.379 8.463 1.00 2.95
H
ATOM 17132 1HG1 VAL B 503 -23.881 57.060 8.139 1.00 2.95
H
ATOM 17133 2HG1 VAL B 503 -22.933 55.957 7.113 1.00 2.95
H
ATOM 17134 3HG1 VAL B 503 -23.719 55.345 8.588 1.00 2.95
H
ATOM 17135 1HG2 VAL B 503 -23.005 57.736 10.373 1.00 2.95
H
ATOM 17136 2HG2 VAL B 503 -22.822 56.031 10.845 1.00 2.95
H
ATOM 17137 3HG2 VAL B 503 -21.430 57.136 10.932 1.00 2.95
H
ATOM 17138 N ASN B 504 -19.513 55.293 10.853 1.00 1.49
N
ATOM 17139 CA ASN B 504 -18.315 55.609 11.610 1.00 1.43
C
ATOM 17140 C ASN B 504 -17.293 54.474 11.650 1.00 1.28
C
ATOM 17141 O ASN B 504 -16.386 54.509 12.483 1.00 1.28
O
ATOM 17142 CB ASN B 504 -18.686 56.011 13.026 1.00 2.15
C
ATOM 17143 CG ASN B 504 -19.409 57.328 13.083 1.00 2.15
C
ATOM 17144 OD1 ASN B 504 -19.055 58.276 12.372 1.00 2.15
O
ATOM 17145 ND2 ASN B 504 -20.413 57.407 13.919 1.00 2.15
N
ATOM 17146 H ASN B 504 -20.234 54.743 11.299 1.00 1.79
H
ATOM 17147 HA ASN B 504 -17.827 56.458 11.130 1.00 1.72
H
ATOM 17148 1HB ASN B 504 -19.322 55.242 13.466 1.00 2.57
H

```
ATOM  17149 2HB  ASN B 504     -17.785  56.078  13.633  1.00  2.57
H
ATOM  17150 1HD2 ASN B 504     -20.930  58.260  14.001  1.00  2.57
H
ATOM  17151 2HD2 ASN B 504     -20.663  56.616  14.477  1.00  2.57
H
ATOM  17152  N   ASP B 505     -17.427  53.469  10.777  1.00  1.21
N
ATOM  17153  CA  ASP B 505     -16.478  52.361  10.795  1.00  1.11
C
ATOM  17154  C   ASP B 505     -15.054  52.847  10.573  1.00  1.06
C
ATOM  17155  O   ASP B 505     -14.775  53.607   9.644  1.00  1.07
O
ATOM  17156  CB  ASP B 505     -16.848  51.335   9.741  1.00  1.67
C
ATOM  17157  CG  ASP B 505     -18.113  50.591  10.081  1.00  1.67
C
ATOM  17158  OD1 ASP B 505     -18.454  50.513  11.238  1.00  1.67
O
ATOM  17159  OD2 ASP B 505     -18.765  50.140   9.169  1.00  1.67
O
ATOM  17160  H   ASP B 505     -18.191  53.451  10.106  1.00  1.45
H
ATOM  17161  HA  ASP B 505     -16.525  51.885  11.774  1.00  1.33
H
ATOM  17162 1HB  ASP B 505     -16.977  51.828   8.778  1.00  2.00
H
ATOM  17163 2HB  ASP B 505     -16.040  50.611   9.640  1.00  2.00
H
ATOM  17164  N   LYS B 506     -14.154  52.386  11.429  1.00  1.08
N
ATOM  17165  CA  LYS B 506     -12.768  52.820  11.406  1.00  1.09
C
ATOM  17166  C   LYS B 506     -11.819  51.834  10.746  1.00  1.03
C
ATOM  17167  O   LYS B 506     -10.701  52.197  10.383  1.00  1.42
O
ATOM  17168  CB  LYS B 506     -12.304  53.078  12.825  1.00  1.64
C
ATOM  17169  CG  LYS B 506     -13.099  54.141  13.569  1.00  1.64
C
ATOM  17170  CD  LYS B 506     -12.960  55.498  12.899  1.00  1.64
C
ATOM  17171  CE  LYS B 506     -13.683  56.581  13.684  1.00  1.64
C
ATOM  17172  NZ  LYS B 506     -13.562  57.913  13.031  1.00  1.64
N
ATOM  17173  H   LYS B 506     -14.455  51.752  12.156  1.00  1.30
H
ATOM  17174  HA  LYS B 506     -12.715  53.754  10.845  1.00  1.31
H
ATOM  17175 1HB  LYS B 506     -12.350  52.153  13.402  1.00  1.96
H
ATOM  17176 2HB  LYS B 506     -11.265  53.399  12.797  1.00  1.96
H
ATOM  17177 1HG  LYS B 506     -14.151  53.860  13.591  1.00  1.96
H
```

```
ATOM  17178 2HG  LYS B 506     -12.738  54.214  14.594  1.00  1.96
H
ATOM  17179 1HD  LYS B 506     -11.905  55.760  12.819  1.00  1.96
H
ATOM  17180 2HD  LYS B 506     -13.381  55.450  11.894  1.00  1.96
H
ATOM  17181 1HE  LYS B 506     -14.738  56.322  13.761  1.00  1.96
H
ATOM  17182 2HE  LYS B 506     -13.261  56.639  14.686  1.00  1.96
H
ATOM  17183 1HZ  LYS B 506     -14.055  58.603  13.580  1.00  1.96
H
ATOM  17184 2HZ  LYS B 506     -12.586  58.170  12.965  1.00  1.96
H
ATOM  17185 3HZ  LYS B 506     -13.963  57.872  12.104  1.00  1.96
H
ATOM  17186  N   VAL B 507     -12.241  50.581  10.632  1.00  1.00
N
ATOM  17187  CA  VAL B 507     -11.402  49.569  10.011  1.00  1.05
C
ATOM  17188  C   VAL B 507     -12.235  48.393   9.518  1.00  0.92
C
ATOM  17189  O   VAL B 507     -13.227  48.022  10.149  1.00  0.98
O
ATOM  17190  CB  VAL B 507     -10.338  49.092  11.013  1.00  1.58
C
ATOM  17191  CG1 VAL B 507     -11.009  48.415  12.186  1.00  1.58
C
ATOM  17192  CG2 VAL B 507      -9.357  48.151  10.342  1.00  1.58
C
ATOM  17193  H   VAL B 507     -13.158  50.327  10.963  1.00  1.20
H
ATOM  17194  HA  VAL B 507     -10.893  50.016   9.158  1.00  1.26
H
ATOM  17195  HB  VAL B 507      -9.802  49.961  11.394  1.00  1.89
H
ATOM  17196 1HG1 VAL B 507     -10.244  48.110  12.896  1.00  1.89
H
ATOM  17197 2HG1 VAL B 507     -11.694  49.112  12.667  1.00  1.89
H
ATOM  17198 3HG1 VAL B 507     -11.558  47.541  11.840  1.00  1.89
H
ATOM  17199 1HG2 VAL B 507      -8.607  47.852  11.069  1.00  1.89
H
ATOM  17200 2HG2 VAL B 507      -9.876  47.267   9.974  1.00  1.89
H
ATOM  17201 3HG2 VAL B 507      -8.873  48.661   9.514  1.00  1.89
H
ATOM  17202  N   THR B 508     -11.819  47.808   8.397  1.00  0.90
N
ATOM  17203  CA  THR B 508     -12.467  46.629   7.852  1.00  0.82
C
ATOM  17204  C   THR B 508     -11.801  45.382   8.365  1.00  0.78
C
ATOM  17205  O   THR B 508     -10.649  45.120   8.041  1.00  0.88
O
ATOM  17206  CB  THR B 508     -12.415  46.600   6.309  1.00  1.23
C
```

ATOM 17207 OG1 THR B 508 -13.132 47.709 5.751 1.00 1.23
O
ATOM 17208 CG2 THR B 508 -12.996 45.301 5.795 1.00 1.23
C
ATOM 17209 H THR B 508 -11.012 48.178 7.917 1.00 1.08
H
ATOM 17210 HA THR B 508 -13.501 46.608 8.174 1.00 0.98
H
ATOM 17211 HB THR B 508 -11.375 46.669 5.993 1.00 1.48
H
ATOM 17212 HG1 THR B 508 -12.699 48.537 5.989 1.00 1.48
H
ATOM 17213 1HG2 THR B 508 -12.940 45.279 4.707 1.00 1.48
H
ATOM 17214 2HG2 THR B 508 -12.435 44.461 6.206 1.00 1.48
H
ATOM 17215 3HG2 THR B 508 -14.034 45.231 6.109 1.00 1.48
H
ATOM 17216 N VAL B 509 -12.532 44.599 9.136 1.00 0.70
N
ATOM 17217 CA VAL B 509 -12.006 43.351 9.647 1.00 0.62
C
ATOM 17218 C VAL B 509 -12.613 42.180 8.893 1.00 0.57
C
ATOM 17219 O VAL B 509 -13.827 41.981 8.896 1.00 0.58
O
ATOM 17220 CB VAL B 509 -12.251 43.211 11.159 1.00 0.93
C
ATOM 17221 CG1 VAL B 509 -11.729 41.865 11.650 1.00 0.93
C
ATOM 17222 CG2 VAL B 509 -11.530 44.341 11.884 1.00 0.93
C
ATOM 17223 H VAL B 509 -13.472 44.871 9.365 1.00 0.84
H
ATOM 17224 HA VAL B 509 -10.935 43.351 9.492 1.00 0.74
H
ATOM 17225 HB VAL B 509 -13.321 43.257 11.362 1.00 1.12
H
ATOM 17226 1HG1 VAL B 509 -11.907 41.773 12.720 1.00 1.12
H
ATOM 17227 2HG1 VAL B 509 -12.246 41.061 11.126 1.00 1.12
H
ATOM 17228 3HG1 VAL B 509 -10.660 41.797 11.452 1.00 1.12
H
ATOM 17229 1HG2 VAL B 509 -11.700 44.253 12.957 1.00 1.12
H
ATOM 17230 2HG2 VAL B 509 -10.464 44.279 11.682 1.00 1.12
H
ATOM 17231 3HG2 VAL B 509 -11.909 45.299 11.532 1.00 1.12
H
ATOM 17232 N ILE B 510 -11.745 41.427 8.236 1.00 0.53
N
ATOM 17233 CA ILE B 510 -12.139 40.285 7.438 1.00 0.49
C
ATOM 17234 C ILE B 510 -11.898 39.003 8.203 1.00 0.43
C
ATOM 17235 O ILE B 510 -10.773 38.715 8.605 1.00 0.43
O

```
ATOM  17236  CB  ILE B 510     -11.362  40.251   6.109  1.00  0.73
C
ATOM  17237  CG1 ILE B 510     -11.654  41.512   5.291  1.00  0.73
C
ATOM  17238  CG2 ILE B 510     -11.724  39.006   5.324  1.00  0.73
C
ATOM  17239  CD1 ILE B 510     -10.769  41.664   4.080  1.00  0.73
C
ATOM  17240  H   ILE B 510     -10.764  41.656   8.310  1.00  0.64
H
ATOM  17241  HA  ILE B 510     -13.203  40.361   7.219  1.00  0.59
H
ATOM  17242  HB  ILE B 510     -10.293  40.242   6.318  1.00  0.88
H
ATOM  17243 1HG1 ILE B 510     -12.688  41.488   4.956  1.00  0.88
H
ATOM  17244 2HG1 ILE B 510     -11.517  42.386   5.928  1.00  0.88
H
ATOM  17245 1HG2 ILE B 510     -11.160  38.986   4.393  1.00  0.88
H
ATOM  17246 2HG2 ILE B 510     -11.486  38.122   5.913  1.00  0.88
H
ATOM  17247 3HG2 ILE B 510     -12.790  39.021   5.104  1.00  0.88
H
ATOM  17248 1HD1 ILE B 510     -11.031  42.580   3.552  1.00  0.88
H
ATOM  17249 2HD1 ILE B 510      -9.726  41.712   4.395  1.00  0.88
H
ATOM  17250 3HD1 ILE B 510     -10.908  40.810   3.418  1.00  0.88
H
ATOM  17251  N   GLY B 511     -12.954  38.231   8.408  1.00  0.40
N
ATOM  17252  CA  GLY B 511     -12.812  36.969   9.121  1.00  0.36
C
ATOM  17253  C   GLY B 511     -13.651  35.891   8.468  1.00  0.31
C
ATOM  17254  O   GLY B 511     -14.408  36.161   7.539  1.00  0.32
O
ATOM  17255  H   GLY B 511     -13.859  38.520   8.052  1.00  0.48
H
ATOM  17256 1HA  GLY B 511     -11.764  36.665   9.128  1.00  0.43
H
ATOM  17257 2HA  GLY B 511     -13.118  37.099  10.158  1.00  0.43
H
ATOM  17258  N   ALA B 512     -13.527  34.667   8.953  1.00  0.29
N
ATOM  17259  CA  ALA B 512     -14.313  33.571   8.406  1.00  0.28
C
ATOM  17260  C   ALA B 512     -14.376  32.434   9.405  1.00  0.26
C
ATOM  17261  O   ALA B 512     -13.399  32.164  10.116  1.00  0.24
O
ATOM  17262  CB  ALA B 512     -13.726  33.097   7.084  1.00  0.42
C
ATOM  17263  H   ALA B 512     -12.886  34.495   9.716  1.00  0.35
H
ATOM  17264  HA  ALA B 512     -15.329  33.927   8.234  1.00  0.34
H
```

ATOM 17265 1HB ALA B 512   -14.339 32.291   6.682 1.00 0.50
H
ATOM 17266 2HB ALA B 512   -13.709 33.926   6.375 1.00 0.50
H
ATOM 17267 3HB ALA B 512   -12.711 32.739   7.245 1.00 0.50
H
ATOM 17268 N   GLY B 513   -15.520 31.757   9.443 1.00 0.35
N
ATOM 17269 CA GLY B 513   -15.686 30.661 10.376 1.00 0.29
C
ATOM 17270 C   GLY B 513   -15.627 31.216 11.780 1.00 0.29
C
ATOM 17271 O   GLY B 513   -16.209 32.267 12.064 1.00 0.34
O
ATOM 17272 H   GLY B 513   -16.301 32.031   8.849 1.00 0.42
H
ATOM 17273 1HA GLY B 513   -16.637 30.160 10.209 1.00 0.35
H
ATOM 17274 2HA GLY B 513   -14.897 29.927 10.233 1.00 0.35
H
ATOM 17275 N   VAL B 514   -14.885 30.533 12.648 1.00 0.27
N
ATOM 17276 CA VAL B 514   -14.737 30.969 14.028 1.00 0.28
C
ATOM 17277 C   VAL B 514   -14.139 32.369 14.153 1.00 0.29
C
ATOM 17278 O   VAL B 514   -14.411 33.056 15.133 1.00 0.33
O
ATOM 17279 CB VAL B 514   -13.864 29.971 14.825 1.00 0.42
C
ATOM 17280 CG1 VAL B 514   -12.406 30.056 14.388 1.00 0.42
C
ATOM 17281 CG2 VAL B 514   -13.992 30.265 16.311 1.00 0.42
C
ATOM 17282 H   VAL B 514   -14.426 29.684 12.346 1.00 0.32
H
ATOM 17283 HA VAL B 514   -15.728 30.983 14.483 1.00 0.34
H
ATOM 17284 HB VAL B 514   -14.210 28.958 14.619 1.00 0.50
H
ATOM 17285 1HG1 VAL B 514   -11.816 29.333 14.949 1.00 0.50
H
ATOM 17286 2HG1 VAL B 514   -12.330 29.835 13.324 1.00 0.50
H
ATOM 17287 3HG1 VAL B 514   -12.023 31.056 14.583 1.00 0.50
H
ATOM 17288 1HG2 VAL B 514   -13.392 29.553 16.876 1.00 0.50
H
ATOM 17289 2HG2 VAL B 514   -13.639 31.278 16.515 1.00 0.50
H
ATOM 17290 3HG2 VAL B 514   -15.035 30.178 16.613 1.00 0.50
H
ATOM 17291 N   THR B 515   -13.348 32.805 13.163 1.00 0.28
N
ATOM 17292 CA THR B 515   -12.723 34.116 13.247 1.00 0.31
C
ATOM 17293 C   THR B 515   -13.669 35.224 12.830 1.00 0.33
C

```
ATOM  17294  O   THR B 515      -13.428  36.390  13.146  1.00  0.38
O
ATOM  17295  CB  THR B 515      -11.422  34.166  12.433  1.00  0.46
C
ATOM  17296  OG1 THR B 515      -11.673  33.808  11.064  1.00  0.46
O
ATOM  17297  CG2 THR B 515      -10.388  33.231  13.038  1.00  0.46
C
ATOM  17298  H   THR B 515      -13.180  32.236  12.342  1.00  0.34
H
ATOM  17299  HA  THR B 515      -12.456  34.293  14.289  1.00  0.37
H
ATOM  17300  HB  THR B 515      -11.029  35.179  12.460  1.00  0.56
H
ATOM  17301  HG1 THR B 515      -12.082  32.935  11.027  1.00  0.56
H
ATOM  17302 1HG2 THR B 515       -9.468  33.296  12.467  1.00  0.56
H
ATOM  17303 2HG2 THR B 515      -10.194  33.524  14.070  1.00  0.56
H
ATOM  17304 3HG2 THR B 515      -10.761  32.209  13.015  1.00  0.56
H
ATOM  17305  N   LEU B 516      -14.762  34.867  12.156  1.00  0.32
N
ATOM  17306  CA  LEU B 516      -15.783  35.844  11.849  1.00  0.34
C
ATOM  17307  C   LEU B 516      -16.457  36.177  13.146  1.00  0.38
C
ATOM  17308  O   LEU B 516      -16.647  37.343  13.483  1.00  0.46
O
ATOM  17309  CB  LEU B 516      -16.840  35.327  10.883  1.00  0.51
C
ATOM  17310  CG  LEU B 516      -17.931  36.356  10.567  1.00  0.51
C
ATOM  17311  CD1 LEU B 516      -17.299  37.553   9.879  1.00  0.51
C
ATOM  17312  CD2 LEU B 516      -19.000  35.726   9.712  1.00  0.51
C
ATOM  17313  H   LEU B 516      -14.930  33.903  11.902  1.00  0.38
H
ATOM  17314  HA  LEU B 516      -15.320  36.744  11.444  1.00  0.41
H
ATOM  17315 1HB  LEU B 516      -16.359  35.047   9.948  1.00  0.61
H
ATOM  17316 2HB  LEU B 516      -17.318  34.446  11.311  1.00  0.61
H
ATOM  17317  HG  LEU B 516      -18.380  36.707  11.497  1.00  0.61
H
ATOM  17318 1HD1 LEU B 516      -18.068  38.294   9.666  1.00  0.61
H
ATOM  17319 2HD1 LEU B 516      -16.542  37.991  10.530  1.00  0.61
H
ATOM  17320 3HD1 LEU B 516      -16.835  37.233   8.947  1.00  0.61
H
ATOM  17321 1HD2 LEU B 516      -19.774  36.460   9.492  1.00  0.61
H
ATOM  17322 2HD2 LEU B 516      -18.555  35.377   8.784  1.00  0.61
H
```

ATOM 17323 3HD2 LEU B 516 -19.438 34.884 10.245 1.00 0.61
H
ATOM 17324 N HIS B 517 -16.815 35.122 13.874 1.00 0.37
N
ATOM 17325 CA HIS B 517 -17.491 35.268 15.146 1.00 0.39
C
ATOM 17326 C HIS B 517 -16.606 35.937 16.188 1.00 0.40
C
ATOM 17327 O HIS B 517 -17.108 36.726 16.989 1.00 0.41
O
ATOM 17328 CB HIS B 517 -17.986 33.909 15.625 1.00 0.58
C
ATOM 17329 CG HIS B 517 -19.184 33.441 14.854 1.00 0.58
C
ATOM 17330 ND1 HIS B 517 -19.101 32.959 13.565 1.00 0.58
N
ATOM 17331 CD2 HIS B 517 -20.493 33.381 15.196 1.00 0.58
C
ATOM 17332 CE1 HIS B 517 -20.311 32.626 13.145 1.00 0.58
C
ATOM 17333 NE2 HIS B 517 -21.170 32.868 14.119 1.00 0.58
N
ATOM 17334 H HIS B 517 -16.621 34.190 13.513 1.00 0.44
H
ATOM 17335 HA HIS B 517 -18.365 35.904 15.013 1.00 0.47
H
ATOM 17336 1HB HIS B 517 -17.193 33.168 15.518 1.00 0.70
H
ATOM 17337 2HB HIS B 517 -18.250 33.962 16.680 1.00 0.70
H
ATOM 17338 HD2 HIS B 517 -20.928 33.681 16.150 1.00 0.70
H
ATOM 17339 HE1 HIS B 517 -20.556 32.216 12.167 1.00 0.70
H
ATOM 17340 HE2 HIS B 517 -22.168 32.695 14.078 1.00 0.70
H
ATOM 17341 N GLU B 518 -15.293 35.681 16.153 1.00 0.40
N
ATOM 17342 CA GLU B 518 -14.405 36.377 17.076 1.00 0.43
C
ATOM 17343 C GLU B 518 -14.356 37.865 16.724 1.00 0.45
C
ATOM 17344 O GLU B 518 -14.329 38.704 17.624 1.00 0.49
O
ATOM 17345 CB GLU B 518 -12.986 35.785 17.054 1.00 0.65
C
ATOM 17346 CG GLU B 518 -12.840 34.384 17.654 1.00 0.65
C
ATOM 17347 CD GLU B 518 -13.199 34.287 19.115 1.00 0.65
C
ATOM 17348 OE1 GLU B 518 -12.711 35.066 19.904 1.00 0.65
O
ATOM 17349 OE2 GLU B 518 -13.969 33.417 19.448 1.00 0.65
O
ATOM 17350 H GLU B 518 -14.916 34.994 15.513 1.00 0.48
H
ATOM 17351 HA GLU B 518 -14.806 36.278 18.086 1.00 0.52
H

```
ATOM  17352 1HB  GLU B 518     -12.637  35.736  16.023  1.00  0.77
H
ATOM  17353 2HB  GLU B 518     -12.311  36.450  17.588  1.00  0.77
H
ATOM  17354 1HG  GLU B 518     -13.482  33.700  17.112  1.00  0.77
H
ATOM  17355 2HG  GLU B 518     -11.812  34.052  17.516  1.00  0.77
H
ATOM  17356  N   ALA B 519     -14.385  38.190  15.422  1.00  0.44
N
ATOM  17357  CA  ALA B 519     -14.411  39.583  14.974  1.00  0.46
C
ATOM  17358  C   ALA B 519     -15.705  40.270  15.402  1.00  0.46
C
ATOM  17359  O   ALA B 519     -15.686  41.438  15.787  1.00  0.51
O
ATOM  17360  CB  ALA B 519     -14.248  39.665  13.462  1.00  0.69
C
ATOM  17361  H   ALA B 519     -14.363  37.463  14.717  1.00  0.53
H
ATOM  17362  HA  ALA B 519     -13.583  40.108  15.448  1.00  0.55
H
ATOM  17363 1HB  ALA B 519     -14.243  40.711  13.154  1.00  0.83
H
ATOM  17364 2HB  ALA B 519     -13.308  39.197  13.173  1.00  0.83
H
ATOM  17365 3HB  ALA B 519     -15.072  39.150  12.973  1.00  0.83
H
ATOM  17366  N   LEU B 520     -16.821  39.542  15.341  1.00  0.44
N
ATOM  17367  CA  LEU B 520     -18.110  40.074  15.765  1.00  0.48
C
ATOM  17368  C   LEU B 520     -18.127  40.374  17.258  1.00  0.56
C
ATOM  17369  O   LEU B 520     -18.638  41.415  17.679  1.00  0.63
O
ATOM  17370  CB  LEU B 520     -19.210  39.074  15.403  1.00  0.72
C
ATOM  17371  CG  LEU B 520     -19.466  38.961  13.903  1.00  0.72
C
ATOM  17372  CD1 LEU B 520     -20.416  37.808  13.601  1.00  0.72
C
ATOM  17373  CD2 LEU B 520     -20.033  40.285  13.445  1.00  0.72
C
ATOM  17374  H   LEU B 520     -16.778  38.599  14.977  1.00  0.53
H
ATOM  17375  HA  LEU B 520     -18.291  41.003  15.227  1.00  0.58
H
ATOM  17376 1HB  LEU B 520     -18.933  38.089  15.774  1.00  0.86
H
ATOM  17377 2HB  LEU B 520     -20.139  39.380  15.883  1.00  0.86
H
ATOM  17378  HG  LEU B 520     -18.529  38.775  13.383  1.00  0.86
H
ATOM  17379 1HD1 LEU B 520     -20.589  37.749  12.527  1.00  0.86
H
ATOM  17380 2HD1 LEU B 520     -19.978  36.873  13.947  1.00  0.86
H
```

ATOM 17381 3HD1 LEU B 520   -21.364 37.975 14.112 1.00 0.86
H
ATOM 17382 1HD2 LEU B 520   -20.204 40.255 12.377 1.00 0.86
H
ATOM 17383 2HD2 LEU B 520   -20.973 40.478 13.962 1.00 0.86
H
ATOM 17384 3HD2 LEU B 520   -19.325 41.080 13.673 1.00 0.86
H
ATOM 17385 N   GLU B 521   -17.515 39.486 18.041 1.00 0.57
N
ATOM 17386 CA GLU B 521   -17.405 39.671 19.476 1.00 0.62
C
ATOM 17387 C   GLU B 521   -16.517 40.874 19.761 1.00 0.67
C
ATOM 17388 O   GLU B 521   -16.843 41.706 20.611 1.00 0.86
O
ATOM 17389 CB GLU B 521   -16.823 38.409 20.124 1.00 0.93
C
ATOM 17390 CG GLU B 521   -16.716 38.430 21.646 1.00 0.93
C
ATOM 17391 CD GLU B 521   -18.040 38.410 22.358 1.00 0.93
C
ATOM 17392 OE1 GLU B 521   -19.001 37.972 21.773 1.00 0.93
O
ATOM 17393 OE2 GLU B 521   -18.090 38.824 23.496 1.00 0.93
O
ATOM 17394 H   GLU B 521   -17.138 38.640 17.633 1.00 0.68
H
ATOM 17395 HA GLU B 521   -18.396 39.865 19.887 1.00 0.74
H
ATOM 17396 1HB GLU B 521   -17.438 37.551 19.850 1.00 1.12
H
ATOM 17397 2HB GLU B 521   -15.825 38.228 19.727 1.00 1.12
H
ATOM 17398 1HG GLU B 521   -16.136 37.564 21.963 1.00 1.12
H
ATOM 17399 2HG GLU B 521   -16.169 39.320 21.939 1.00 1.12
H
ATOM 17400 N   ALA B 522   -15.414 40.973 19.014 1.00 0.54
N
ATOM 17401 CA ALA B 522   -14.478 42.073 19.140 1.00 0.56
C
ATOM 17402 C   ALA B 522   -15.165 43.394 18.888 1.00 0.61
C
ATOM 17403 O   ALA B 522   -14.978 44.329 19.659 1.00 0.69
O
ATOM 17404 CB ALA B 522   -13.320 41.894 18.172 1.00 0.84
C
ATOM 17405 H   ALA B 522   -15.200 40.245 18.348 1.00 0.65
H
ATOM 17406 HA ALA B 522   -14.095 42.084 20.159 1.00 0.67
H
ATOM 17407 1HB ALA B 522   -12.613 42.714 18.288 1.00 1.01
H
ATOM 17408 2HB ALA B 522   -12.817 40.950 18.380 1.00 1.01
H
ATOM 17409 3HB ALA B 522   -13.693 41.885 17.151 1.00 1.01
H

```
ATOM  17410  N   ALA B 523     -15.980  43.457  17.831  1.00  0.61
N
ATOM  17411  CA  ALA B 523     -16.709  44.667  17.479  1.00  0.70
C
ATOM  17412  C   ALA B 523     -17.624  45.107  18.616  1.00  0.73
C
ATOM  17413  O   ALA B 523     -17.700  46.299  18.919  1.00  0.90
O
ATOM  17414  CB  ALA B 523     -17.511  44.443  16.207  1.00  1.05
C
ATOM  17415  H   ALA B 523     -16.076  42.647  17.233  1.00  0.73
H
ATOM  17416  HA  ALA B 523     -15.984  45.462  17.306  1.00  0.84
H
ATOM  17417 1HB  ALA B 523     -18.031  45.362  15.936  1.00  1.26
H
ATOM  17418 2HB  ALA B 523     -16.837  44.155  15.400  1.00  1.26
H
ATOM  17419 3HB  ALA B 523     -18.238  43.650  16.371  1.00  1.26
H
ATOM  17420  N   ASP B 524     -18.300  44.151  19.255  1.00  0.74
N
ATOM  17421  CA  ASP B 524     -19.161  44.484  20.381  1.00  0.90
C
ATOM  17422  C   ASP B 524     -18.374  45.095  21.541  1.00  0.92
C
ATOM  17423  O   ASP B 524     -18.809  46.083  22.137  1.00  1.08
O
ATOM  17424  CB  ASP B 524     -19.933  43.250  20.848  1.00  1.35
C
ATOM  17425  CG  ASP B 524     -21.034  42.848  19.870  1.00  1.35
C
ATOM  17426  OD1 ASP B 524     -21.358  43.634  19.011  1.00  1.35
O
ATOM  17427  OD2 ASP B 524     -21.553  41.765  20.001  1.00  1.35
O
ATOM  17428  H   ASP B 524     -18.231  43.186  18.944  1.00  0.89
H
ATOM  17429  HA  ASP B 524     -19.887  45.224  20.044  1.00  1.08
H
ATOM  17430 1HB  ASP B 524     -19.248  42.411  20.968  1.00  1.62
H
ATOM  17431 2HB  ASP B 524     -20.383  43.448  21.822  1.00  1.62
H
ATOM  17432  N   HIS B 525     -17.192  44.556  21.832  1.00  0.93
N
ATOM  17433  CA  HIS B 525     -16.395  45.120  22.914  1.00  0.98
C
ATOM  17434  C   HIS B 525     -15.777  46.452  22.506  1.00  1.00
C
ATOM  17435  O   HIS B 525     -15.695  47.373  23.313  1.00  1.30
O
ATOM  17436  CB  HIS B 525     -15.323  44.137  23.340  1.00  1.47
C
ATOM  17437  CG  HIS B 525     -15.910  42.940  24.002  1.00  1.47
C
ATOM  17438  ND1 HIS B 525     -16.546  42.998  25.224  1.00  1.47
N
```

ATOM 17439 CD2 HIS B 525 -15.991 41.657 23.595 1.00 1.47
C
ATOM 17440 CE1 HIS B 525 -16.997 41.795 25.536 1.00 1.47
C
ATOM 17441 NE2 HIS B 525 -16.677 40.966 24.560 1.00 1.47
N
ATOM 17442 H HIS B 525 -16.868 43.736 21.327 1.00 1.12
H
ATOM 17443 HA HIS B 525 -17.036 45.307 23.775 1.00 1.18
H
ATOM 17444 1HB HIS B 525 -14.769 43.811 22.462 1.00 1.76
H
ATOM 17445 2HB HIS B 525 -14.623 44.614 24.026 1.00 1.76
H
ATOM 17446 HD2 HIS B 525 -15.599 41.251 22.665 1.00 1.76
H
ATOM 17447 HE1 HIS B 525 -17.547 41.534 26.440 1.00 1.76
H
ATOM 17448 HE2 HIS B 525 -16.904 39.979 24.506 1.00 1.76
H
ATOM 17449 N LEU B 526 -15.412 46.584 21.237 1.00 0.91
N
ATOM 17450 CA LEU B 526 -14.882 47.830 20.706 1.00 1.03
C
ATOM 17451 C LEU B 526 -15.903 48.954 20.778 1.00 1.20
C
ATOM 17452 O LEU B 526 -15.538 50.092 21.076 1.00 1.51
O
ATOM 17453 CB LEU B 526 -14.401 47.594 19.274 1.00 1.54
C
ATOM 17454 CG LEU B 526 -13.110 46.780 19.184 1.00 1.54
C
ATOM 17455 CD1 LEU B 526 -12.859 46.340 17.769 1.00 1.54
C
ATOM 17456 CD2 LEU B 526 -11.960 47.657 19.639 1.00 1.54
C
ATOM 17457 H LEU B 526 -15.486 45.795 20.614 1.00 1.09
H
ATOM 17458 HA LEU B 526 -14.023 48.116 21.310 1.00 1.24
H
ATOM 17459 1HB LEU B 526 -15.174 47.053 18.732 1.00 1.85
H
ATOM 17460 2HB LEU B 526 -14.238 48.545 18.779 1.00 1.85
H
ATOM 17461 HG LEU B 526 -13.189 45.905 19.817 1.00 1.85
H
ATOM 17462 1HD1 LEU B 526 -11.932 45.771 17.720 1.00 1.85
H
ATOM 17463 2HD1 LEU B 526 -13.685 45.719 17.423 1.00 1.85
H
ATOM 17464 3HD1 LEU B 526 -12.774 47.223 17.142 1.00 1.85
H
ATOM 17465 1HD2 LEU B 526 -11.029 47.095 19.576 1.00 1.85
H
ATOM 17466 2HD2 LEU B 526 -11.896 48.531 18.993 1.00 1.85
H
ATOM 17467 3HD2 LEU B 526 -12.127 47.977 20.668 1.00 1.85
H

ATOM 17468 N SER B 527 -17.190 48.631 20.590 1.00 1.27
N
ATOM 17469 CA SER B 527 -18.238 49.641 20.684 1.00 1.82
C
ATOM 17470 C SER B 527 -18.331 50.233 22.092 1.00 2.38
C
ATOM 17471 O SER B 527 -18.797 51.360 22.255 1.00 1.90
O
ATOM 17472 CB SER B 527 -19.594 49.067 20.311 1.00 2.73
C
ATOM 17473 OG SER B 527 -20.094 48.218 21.310 1.00 2.73
O
ATOM 17474 H SER B 527 -17.439 47.684 20.332 1.00 1.52
H
ATOM 17475 HA SER B 527 -17.998 50.447 19.988 1.00 2.18
H
ATOM 17476 1HB SER B 527 -20.296 49.882 20.140 1.00 3.28
H
ATOM 17477 2HB SER B 527 -19.504 48.514 19.376 1.00 3.28
H
ATOM 17478 HG SER B 527 -19.466 47.497 21.409 1.00 3.28
H
ATOM 17479 N GLN B 528 -17.835 49.515 23.109 1.00 4.05
N
ATOM 17480 CA GLN B 528 -17.853 50.018 24.478 1.00 4.63
C
ATOM 17481 C GLN B 528 -16.850 51.162 24.638 1.00 4.61
C
ATOM 17482 O GLN B 528 -16.948 51.960 25.570 1.00 5.05
O
ATOM 17483 CB GLN B 528 -17.519 48.897 25.467 1.00 6.95
C
ATOM 17484 CG GLN B 528 -18.557 47.788 25.534 1.00 6.95
C
ATOM 17485 CD GLN B 528 -18.152 46.673 26.484 1.00 6.95
C
ATOM 17486 OE1 GLN B 528 -16.983 46.551 26.859 1.00 6.95
O
ATOM 17487 NE2 GLN B 528 -19.120 45.852 26.880 1.00 6.95
N
ATOM 17488 H GLN B 528 -17.422 48.607 22.953 1.00 4.86
H
ATOM 17489 HA GLN B 528 -18.847 50.402 24.697 1.00 5.56
H
ATOM 17490 1HB GLN B 528 -16.558 48.455 25.219 1.00 8.33
H
ATOM 17491 2HB GLN B 528 -17.429 49.320 26.467 1.00 8.33
H
ATOM 17492 1HG GLN B 528 -19.501 48.208 25.880 1.00 8.33
H
ATOM 17493 2HG GLN B 528 -18.681 47.360 24.540 1.00 8.33
H
ATOM 17494 1HE2 GLN B 528 -18.912 45.099 27.506 1.00 8.33
H
ATOM 17495 2HE2 GLN B 528 -20.055 45.986 26.553 1.00 8.33
H
ATOM 17496 N GLN B 529 -15.884 51.231 23.719 1.00 5.54
N

```
ATOM  17497  CA  GLN B 529     -14.874  52.267  23.704  1.00  5.84
C
ATOM  17498  C   GLN B 529     -15.193  53.332  22.657  1.00  5.77
C
ATOM  17499  O   GLN B 529     -14.389  54.237  22.432  1.00 11.84
O
ATOM  17500  CB  GLN B 529     -13.503  51.654  23.411  1.00  8.76
C
ATOM  17501  CG  GLN B 529     -13.049  50.632  24.437  1.00  8.76
C
ATOM  17502  CD  GLN B 529     -12.899  51.233  25.821  1.00  8.76
C
ATOM  17503  OE1 GLN B 529     -12.246  52.266  25.993  1.00  8.76
O
ATOM  17504  NE2 GLN B 529     -13.499  50.591  26.816  1.00  8.76
N
ATOM  17505  H   GLN B 529     -15.843  50.553  22.975  1.00  6.65
H
ATOM  17506  HA  GLN B 529     -14.849  52.746  24.683  1.00  7.01
H
ATOM  17507 1HB  GLN B 529     -13.523  51.166  22.437  1.00 10.51
H
ATOM  17508 2HB  GLN B 529     -12.753  52.444  23.368  1.00 10.51
H
ATOM  17509 1HG  GLN B 529     -13.787  49.831  24.490  1.00 10.51
H
ATOM  17510 2HG  GLN B 529     -12.083  50.230  24.132  1.00 10.51
H
ATOM  17511 1HE2 GLN B 529     -13.433  50.943  27.751  1.00 10.51
H
ATOM  17512 2HE2 GLN B 529     -14.018  49.755  26.633  1.00 10.51
H
ATOM  17513  N   GLY B 530     -16.357  53.227  22.005  1.00  3.80
N
ATOM  17514  CA  GLY B 530     -16.721  54.165  20.953  1.00  3.35
C
ATOM  17515  C   GLY B 530     -16.070  53.820  19.610  1.00  2.16
C
ATOM  17516  O   GLY B 530     -15.955  54.682  18.738  1.00  2.58
O
ATOM  17517  H   GLY B 530     -17.020  52.499  22.229  1.00  4.56
H
ATOM  17518 1HA  GLY B 530     -17.806  54.174  20.840  1.00  4.02
H
ATOM  17519 2HA  GLY B 530     -16.429  55.170  21.251  1.00  4.02
H
ATOM  17520  N   ILE B 531     -15.625  52.572  19.449  1.00  1.47
N
ATOM  17521  CA  ILE B 531     -14.956  52.146  18.224  1.00  1.08
C
ATOM  17522  C   ILE B 531     -15.865  51.302  17.338  1.00  0.94
C
ATOM  17523  O   ILE B 531     -16.300  50.220  17.731  1.00  1.27
O
ATOM  17524  CB  ILE B 531     -13.697  51.339  18.563  1.00  1.62
C
ATOM  17525  CG1 ILE B 531     -12.733  52.191  19.386  1.00  1.62
C
```

ATOM 17526 CG2 ILE B 531 -13.040 50.837 17.286 1.00 1.62
C
ATOM 17527 CD1 ILE B 531 -11.602 51.395 19.988 1.00 1.62
C
ATOM 17528 H ILE B 531 -15.744 51.886 20.182 1.00 1.76
H
ATOM 17529 HA ILE B 531 -14.660 53.033 17.665 1.00 1.30
H
ATOM 17530 HB ILE B 531 -13.971 50.496 19.184 1.00 1.94
H
ATOM 17531 1HG1 ILE B 531 -12.309 52.965 18.747 1.00 1.94
H
ATOM 17532 2HG1 ILE B 531 -13.281 52.675 20.194 1.00 1.94
H
ATOM 17533 1HG2 ILE B 531 -12.154 50.255 17.540 1.00 1.94
H
ATOM 17534 2HG2 ILE B 531 -13.741 50.211 16.737 1.00 1.94
H
ATOM 17535 3HG2 ILE B 531 -12.751 51.687 16.667 1.00 1.94
H
ATOM 17536 1HD1 ILE B 531 -10.954 52.059 20.559 1.00 1.94
H
ATOM 17537 2HD1 ILE B 531 -12.010 50.630 20.649 1.00 1.94
H
ATOM 17538 3HD1 ILE B 531 -11.028 50.919 19.195 1.00 1.94
H
ATOM 17539 N SER B 532 -16.150 51.799 16.138 1.00 1.10
N
ATOM 17540 CA SER B 532 -16.999 51.073 15.202 1.00 0.99
C
ATOM 17541 C SER B 532 -16.140 50.331 14.177 1.00 0.89
C
ATOM 17542 O SER B 532 -15.144 50.866 13.688 1.00 1.30
O
ATOM 17543 CB SER B 532 -17.957 52.030 14.525 1.00 1.48
C
ATOM 17544 OG SER B 532 -18.797 51.365 13.625 1.00 1.48
O
ATOM 17545 H SER B 532 -15.769 52.696 15.869 1.00 1.32
H
ATOM 17546 HA SER B 532 -17.582 50.337 15.756 1.00 1.19
H
ATOM 17547 1HB SER B 532 -18.559 52.528 15.285 1.00 1.78
H
ATOM 17548 2HB SER B 532 -17.392 52.799 14.003 1.00 1.78
H
ATOM 17549 HG SER B 532 -18.270 51.125 12.855 1.00 1.78
H
ATOM 17550 N VAL B 533 -16.522 49.090 13.887 1.00 0.92
N
ATOM 17551 CA VAL B 533 -15.781 48.211 12.981 1.00 1.53
C
ATOM 17552 C VAL B 533 -16.645 47.561 11.909 1.00 1.13
C
ATOM 17553 O VAL B 533 -17.760 47.111 12.174 1.00 1.42
O
ATOM 17554 CB VAL B 533 -15.069 47.100 13.781 1.00 2.29
C

```
ATOM  17555  CG1 VAL B 533     -14.390  46.116  12.847  1.00  2.29
C
ATOM  17556  CG2 VAL B 533     -14.028  47.723  14.682  1.00  2.29
C
ATOM  17557  H   VAL B 533     -17.360  48.732  14.325  1.00  1.10
H
ATOM  17558  HA  VAL B 533     -15.019  48.810  12.484  1.00  1.84
H
ATOM  17559  HB  VAL B 533     -15.799  46.549  14.375  1.00  2.75
H
ATOM  17560 1HG1 VAL B 533     -13.893  45.345  13.437  1.00  2.75
H
ATOM  17561 2HG1 VAL B 533     -15.130  45.649  12.200  1.00  2.75
H
ATOM  17562 3HG1 VAL B 533     -13.652  46.639  12.241  1.00  2.75
H
ATOM  17563 1HG2 VAL B 533     -13.528  46.929  15.226  1.00  2.75
H
ATOM  17564 2HG2 VAL B 533     -13.299  48.262  14.078  1.00  2.75
H
ATOM  17565 3HG2 VAL B 533     -14.498  48.410  15.383  1.00  2.75
H
ATOM  17566  N   ARG B 534     -16.112  47.528  10.695  1.00  0.90
N
ATOM  17567  CA  ARG B 534     -16.747  46.893   9.554  1.00  0.88
C
ATOM  17568  C   ARG B 534     -16.395  45.410   9.541  1.00  0.72
C
ATOM  17569  O   ARG B 534     -15.223  45.060   9.491  1.00  0.91
O
ATOM  17570  CB  ARG B 534     -16.268  47.570   8.287  1.00  1.32
C
ATOM  17571  CG  ARG B 534     -16.746  47.009   6.970  1.00  1.32
C
ATOM  17572  CD  ARG B 534     -18.173  47.266   6.769  1.00  1.32
C
ATOM  17573  NE  ARG B 534     -18.443  48.681   6.883  1.00  1.32
N
ATOM  17574  CZ  ARG B 534     -18.428  49.584   5.892  1.00  1.32
C
ATOM  17575  NH1 ARG B 534     -18.193  49.230   4.649  1.00  1.32
N
ATOM  17576  NH2 ARG B 534     -18.660  50.839   6.219  1.00  1.32
N
ATOM  17577  H   ARG B 534     -15.190  47.923  10.559  1.00  1.08
H
ATOM  17578  HA  ARG B 534     -17.827  47.008   9.639  1.00  1.06
H
ATOM  17579 1HB  ARG B 534     -16.596  48.608   8.309  1.00  1.58
H
ATOM  17580 2HB  ARG B 534     -15.186  47.588   8.271  1.00  1.58
H
ATOM  17581 1HG  ARG B 534     -16.210  47.526   6.186  1.00  1.58
H
ATOM  17582 2HG  ARG B 534     -16.557  45.937   6.914  1.00  1.58
H
ATOM  17583 1HD  ARG B 534     -18.471  46.938   5.775  1.00  1.58
H
```

```
ATOM  17584 2HD  ARG B 534     -18.753  46.743   7.518  1.00  1.58
H
ATOM  17585  HE  ARG B 534     -18.617  49.047   7.812  1.00  1.58
H
ATOM  17586 1HH1 ARG B 534     -18.014  48.260   4.426  1.00  1.58
H
ATOM  17587 2HH1 ARG B 534     -18.189  49.927   3.918  1.00  1.58
H
ATOM  17588 1HH2 ARG B 534     -18.821  51.051   7.198  1.00  1.58
H
ATOM  17589 2HH2 ARG B 534     -18.660  51.563   5.516  1.00  1.58
H
ATOM  17590  N   VAL B 535     -17.387  44.531   9.619  1.00  0.67
N
ATOM  17591  CA  VAL B 535     -17.087  43.099   9.648  1.00  0.58
C
ATOM  17592  C   VAL B 535     -17.487  42.426   8.346  1.00  0.54
C
ATOM  17593  O   VAL B 535     -18.658  42.451   7.966  1.00  0.54
O
ATOM  17594  CB  VAL B 535     -17.784  42.411  10.834  1.00  0.87
C
ATOM  17595  CG1 VAL B 535     -17.470  40.929  10.807  1.00  0.87
C
ATOM  17596  CG2 VAL B 535     -17.306  43.034  12.142  1.00  0.87
C
ATOM  17597  H   VAL B 535     -18.344  44.852   9.642  1.00  0.80
H
ATOM  17598  HA  VAL B 535     -16.012  42.977   9.780  1.00  0.70
H
ATOM  17599  HB  VAL B 535     -18.862  42.525  10.740  1.00  1.04
H
ATOM  17600 1HG1 VAL B 535     -17.962  40.428  11.637  1.00  1.04
H
ATOM  17601 2HG1 VAL B 535     -17.820  40.503   9.868  1.00  1.04
H
ATOM  17602 3HG1 VAL B 535     -16.392  40.786  10.891  1.00  1.04
H
ATOM  17603 1HG2 VAL B 535     -17.800  42.545  12.981  1.00  1.04
H
ATOM  17604 2HG2 VAL B 535     -16.227  42.908  12.231  1.00  1.04
H
ATOM  17605 3HG2 VAL B 535     -17.549  44.097  12.149  1.00  1.04
H
ATOM  17606  N   ILE B 536     -16.490  41.846   7.674  1.00  0.54
N
ATOM  17607  CA  ILE B 536     -16.647  41.196   6.377  1.00  0.52
C
ATOM  17608  C   ILE B 536     -16.479  39.683   6.417  1.00  0.46
C
ATOM  17609  O   ILE B 536     -15.557  39.159   7.046  1.00  0.48
O
ATOM  17610  CB  ILE B 536     -15.639  41.773   5.364  1.00  0.78
C
ATOM  17611  CG1 ILE B 536     -15.831  43.285   5.250  1.00  0.78
C
ATOM  17612  CG2 ILE B 536     -15.773  41.100   3.999  1.00  0.78
C
```

ATOM 17613 CD1 ILE B 536   -17.201 43.697   4.777 1.00 0.78
C
ATOM 17614 H   ILE B 536   -15.565 41.870   8.082 1.00 0.65
H
ATOM 17615 HA ILE B 536   -17.652 41.410   6.014 1.00 0.62
H
ATOM 17616 HB ILE B 536   -14.634 41.610   5.744 1.00 0.94
H
ATOM 17617 1HG1 ILE B 536   -15.653 43.738   6.224 1.00 0.94
H
ATOM 17618 2HG1 ILE B 536   -15.099 43.680   4.547 1.00 0.94
H
ATOM 17619 1HG2 ILE B 536   -15.040 41.524   3.313 1.00 0.94
H
ATOM 17620 2HG2 ILE B 536   -15.597 40.031   4.097 1.00 0.94
H
ATOM 17621 3HG2 ILE B 536   -16.777 41.270   3.607 1.00 0.94
H
ATOM 17622 1HD1 ILE B 536   -17.253 44.784   4.720 1.00 0.94
H
ATOM 17623 2HD1 ILE B 536   -17.389 43.270   3.792 1.00 0.94
H
ATOM 17624 3HD1 ILE B 536   -17.947 43.333   5.480 1.00 0.94
H
ATOM 17625 N   ASP B 537   -17.373 38.995   5.716 1.00 0.44
N
ATOM 17626 CA ASP B 537   -17.336 37.553   5.556 1.00 0.44
C
ATOM 17627 C   ASP B 537   -17.197 37.189   4.088 1.00 0.35
C
ATOM 17628 O   ASP B 537   -18.192 37.171   3.368 1.00 0.55
O
ATOM 17629 CB ASP B 537   -18.584 36.898   6.081 1.00 0.66
C
ATOM 17630 CG ASP B 537   -18.528 35.384   5.935 1.00 0.66
C
ATOM 17631 OD1 ASP B 537   -17.660 34.882   5.254 1.00 0.66
O
ATOM 17632 OD2 ASP B 537   -19.386 34.741   6.496 1.00 0.66
O
ATOM 17633 H   ASP B 537   -18.111 39.501   5.249 1.00 0.53
H
ATOM 17634 HA ASP B 537   -16.489 37.160   6.118 1.00 0.53
H
ATOM 17635 1HB ASP B 537   -18.713 37.149   7.133 1.00 0.79
H
ATOM 17636 2HB ASP B 537   -19.449 37.277   5.540 1.00 0.79
H
ATOM 17637 N   PRO B 538   -15.987 36.895   3.614 1.00 0.49
N
ATOM 17638 CA PRO B 538   -15.622 36.533   2.253 1.00 0.34
C
ATOM 17639 C   PRO B 538   -16.517 35.475   1.606 1.00 1.79
C
ATOM 17640 O   PRO B 538   -16.677 35.486   0.389 1.00 20.83
O
ATOM 17641 CB PRO B 538   -14.204 35.971   2.409 1.00 0.51
C

```
ATOM  17642  CG  PRO B 538     -13.662  36.680   3.590  1.00  0.51
C
ATOM  17643  CD  PRO B 538     -14.833  36.777   4.525  1.00  0.51
C
ATOM  17644  HA  PRO B 538     -15.601  37.442   1.637  1.00  0.41
H
ATOM  17645 1HB  PRO B 538     -14.246  34.881   2.550  1.00  0.61
H
ATOM  17646 2HB  PRO B 538     -13.622  36.155   1.495  1.00  0.61
H
ATOM  17647 1HG  PRO B 538     -12.818  36.120   4.021  1.00  0.61
H
ATOM  17648 2HG  PRO B 538     -13.275  37.662   3.287  1.00  0.61
H
ATOM  17649 1HD  PRO B 538     -14.925  35.864   5.129  1.00  0.61
H
ATOM  17650 2HD  PRO B 538     -14.738  37.678   5.147  1.00  0.61
H
ATOM  17651  N   PHE B 539     -17.074  34.552   2.402  1.00  2.28
N
ATOM  17652  CA  PHE B 539     -17.845  33.412   1.886  1.00  0.73
C
ATOM  17653  C   PHE B 539     -16.927  32.490   1.078  1.00  0.53
C
ATOM  17654  O   PHE B 539     -16.558  31.421   1.560  1.00  0.78
O
ATOM  17655  CB  PHE B 539     -19.109  33.820   1.128  1.00  1.09
C
ATOM  17656  CG  PHE B 539     -19.902  32.624   0.677  1.00  1.09
C
ATOM  17657  CD1 PHE B 539     -20.410  31.728   1.612  1.00  1.09
C
ATOM  17658  CD2 PHE B 539     -20.155  32.392  -0.663  1.00  1.09
C
ATOM  17659  CE1 PHE B 539     -21.139  30.623   1.213  1.00  1.09
C
ATOM  17660  CE2 PHE B 539     -20.881  31.290  -1.065  1.00  1.09
C
ATOM  17661  CZ  PHE B 539     -21.374  30.402  -0.125  1.00  1.09
C
ATOM  17662  H   PHE B 539     -16.968  34.631   3.409  1.00  2.74
H
ATOM  17663  HA  PHE B 539     -18.192  32.836   2.745  1.00  0.88
H
ATOM  17664 1HB  PHE B 539     -19.742  34.433   1.768  1.00  1.31
H
ATOM  17665 2HB  PHE B 539     -18.853  34.410   0.251  1.00  1.31
H
ATOM  17666  HD1 PHE B 539     -20.223  31.903   2.671  1.00  1.31
H
ATOM  17667  HD2 PHE B 539     -19.768  33.092  -1.406  1.00  1.31
H
ATOM  17668  HE1 PHE B 539     -21.525  29.929   1.957  1.00  1.31
H
ATOM  17669  HE2 PHE B 539     -21.064  31.124  -2.125  1.00  1.31
H
ATOM  17670  HZ  PHE B 539     -21.947  29.532  -0.441  1.00  1.31
H
```

```
ATOM  17671  N   THR B 540     -16.552  32.893  -0.139  1.00  0.38
N
ATOM  17672  CA  THR B 540     -15.544  32.132  -0.856  1.00  0.33
C
ATOM  17673  C   THR B 540     -14.221  32.860  -0.712  1.00  0.33
C
ATOM  17674  O   THR B 540     -14.162  34.088  -0.637  1.00  0.38
O
ATOM  17675  CB  THR B 540     -15.868  31.919  -2.359  1.00  0.49
C
ATOM  17676  OG1 THR B 540     -15.970  33.183  -3.036  1.00  0.49
O
ATOM  17677  CG2 THR B 540     -17.156  31.140  -2.517  1.00  0.49
C
ATOM  17678  H   THR B 540     -16.910  33.762  -0.511  1.00  0.46
H
ATOM  17679  HA  THR B 540     -15.441  31.153  -0.393  1.00  0.40
H
ATOM  17680  HB  THR B 540     -15.061  31.348  -2.817  1.00  0.59
H
ATOM  17681  HG1 THR B 540     -15.089  33.501  -3.275  1.00  0.59
H
ATOM  17682 1HG2 THR B 540     -17.358  30.978  -3.572  1.00  0.59
H
ATOM  17683 2HG2 THR B 540     -17.054  30.177  -2.017  1.00  0.59
H
ATOM  17684 3HG2 THR B 540     -17.971  31.698  -2.072  1.00  0.59
H
ATOM  17685  N   ILE B 541     -13.164  32.092  -0.680  1.00  0.31
N
ATOM  17686  CA  ILE B 541     -11.829  32.615  -0.542  1.00  0.32
C
ATOM  17687  C   ILE B 541     -11.235  32.670  -1.916  1.00  0.37
C
ATOM  17688  O   ILE B 541     -10.498  33.591  -2.265  1.00  0.46
O
ATOM  17689  CB  ILE B 541     -10.999  31.748   0.370  1.00  0.48
C
ATOM  17690  CG1 ILE B 541     -11.638  31.744   1.760  1.00  0.48
C
ATOM  17691  CG2 ILE B 541      -9.603  32.334   0.409  1.00  0.48
C
ATOM  17692  CD1 ILE B 541     -11.075  30.701   2.683  1.00  0.48
C
ATOM  17693  H   ILE B 541     -13.298  31.097  -0.758  1.00  0.37
H
ATOM  17694  HA  ILE B 541     -11.875  33.624  -0.136  1.00  0.38
H
ATOM  17695  HB  ILE B 541     -10.968  30.722   0.007  1.00  0.58
H
ATOM  17696 1HG1 ILE B 541     -11.500  32.722   2.216  1.00  0.58
H
ATOM  17697 2HG1 ILE B 541     -12.708  31.563   1.656  1.00  0.58
H
ATOM  17698 1HG2 ILE B 541      -8.987  31.744   1.070  1.00  0.58
H
ATOM  17699 2HG2 ILE B 541      -9.176  32.327  -0.593  1.00  0.58
H
```

ATOM 17700 3HG2 ILE B 541   -9.649 33.359   0.776 1.00 0.58
H
ATOM 17701 1HD1 ILE B 541   -11.577 30.763   3.645 1.00 0.58
H
ATOM 17702 2HD1 ILE B 541   -11.224 29.711   2.253 1.00 0.58
H
ATOM 17703 3HD1 ILE B 541   -10.016 30.885   2.815 1.00 0.58
H
ATOM 17704 N   LYS B 542   -11.553 31.647 -2.691 1.00 0.36
N
ATOM 17705 CA LYS B 542   -11.140 31.592 -4.069 1.00 0.37
C
ATOM 17706 C   LYS B 542   -12.365 31.268 -4.915 1.00 0.28
C
ATOM 17707 O   LYS B 542   -12.897 30.166 -4.835 1.00 0.32
O
ATOM 17708 CB LYS B 542   -10.054 30.546 -4.275 1.00 0.55
C
ATOM 17709 CG LYS B 542   -9.527 30.500 -5.698 1.00 0.55
C
ATOM 17710 CD LYS B 542   -8.442 29.453 -5.861 1.00 0.55
C
ATOM 17711 CE LYS B 542   -7.949 29.415 -7.298 1.00 0.55
C
ATOM 17712 NZ LYS B 542   -7.013 28.294 -7.529 1.00 0.55
N
ATOM 17713 H   LYS B 542   -12.140 30.904 -2.321 1.00 0.43
H
ATOM 17714 HA LYS B 542   -10.751 32.563 -4.365 1.00 0.44
H
ATOM 17715 1HB LYS B 542   -9.214 30.753 -3.611 1.00 0.67
H
ATOM 17716 2HB LYS B 542   -10.439 29.558 -4.025 1.00 0.67
H
ATOM 17717 1HG LYS B 542   -10.346 30.269 -6.381 1.00 0.67
H
ATOM 17718 2HG LYS B 542   -9.120 31.474 -5.965 1.00 0.67
H
ATOM 17719 1HD LYS B 542   -7.603 29.702 -5.212 1.00 0.67
H
ATOM 17720 2HD LYS B 542   -8.818 28.473 -5.577 1.00 0.67
H
ATOM 17721 1HE LYS B 542   -8.796 29.318 -7.976 1.00 0.67
H
ATOM 17722 2HE LYS B 542   -7.432 30.350 -7.513 1.00 0.67
H
ATOM 17723 1HZ LYS B 542   -6.689 28.306 -8.484 1.00 0.67
H
ATOM 17724 2HZ LYS B 542   -6.225 28.372 -6.902 1.00 0.67
H
ATOM 17725 3HZ LYS B 542   -7.483 27.417 -7.347 1.00 0.67
H
ATOM 17726 N   PRO B 543   -12.835 32.216 -5.719 1.00 0.25
N
ATOM 17727 CA PRO B 543   -12.399 33.589 -5.943 1.00 0.30
C
ATOM 17728 C   PRO B 543   -12.727 34.498 -4.762 1.00 0.33
C

ATOM 17729 O   PRO B 543   -13.685 34.270 -4.017 1.00 0.38
O
ATOM 17730 CB PRO B 543   -13.178 34.003 -7.196 1.00 0.45
C
ATOM 17731 CG PRO B 543   -14.402 33.154 -7.177 1.00 0.45
C
ATOM 17732 CD PRO B 543   -13.942 31.842 -6.609 1.00 0.45
C
ATOM 17733 HA PRO B 543   -11.318 33.595 -6.146 1.00 0.36
H
ATOM 17734 1HB PRO B 543   -13.406 35.078 -7.159 1.00 0.54
H
ATOM 17735 2HB PRO B 543   -12.562 33.838 -8.093 1.00 0.54
H
ATOM 17736 1HG PRO B 543   -15.181 33.625 -6.566 1.00 0.54
H
ATOM 17737 2HG PRO B 543   -14.811 33.055 -8.193 1.00 0.54
H
ATOM 17738 1HD PRO B 543   -14.741 31.368 -6.023 1.00 0.54
H
ATOM 17739 2HD PRO B 543   -13.575 31.178 -7.405 1.00 0.54
H
ATOM 17740 N   LEU B 544   -11.916 35.540 -4.611 1.00 0.45
N
ATOM 17741 CA LEU B 544   -12.073 36.494 -3.527 1.00 0.68
C
ATOM 17742 C   LEU B 544   -12.743 37.773 -4.016 1.00 1.03
C
ATOM 17743 O   LEU B 544   -12.300 38.381 -4.992 1.00 1.89
O
ATOM 17744 CB LEU B 544   -10.703 36.839 -2.937 1.00 1.02
C
ATOM 17745 CG LEU B 544   -10.712 37.815 -1.766 1.00 1.02
C
ATOM 17746 CD1 LEU B 544   -11.394 37.145 -0.575 1.00 1.02
C
ATOM 17747 CD2 LEU B 544   -9.283 38.216 -1.441 1.00 1.02
C
ATOM 17748 H   LEU B 544   -11.157 35.668 -5.265 1.00 0.54
H
ATOM 17749 HA LEU B 544   -12.697 36.047 -2.754 1.00 0.82
H
ATOM 17750 1HB LEU B 544   -10.224 35.925 -2.599 1.00 1.22
H
ATOM 17751 2HB LEU B 544   -10.087 37.274 -3.724 1.00 1.22
H
ATOM 17752 HG LEU B 544   -11.285 38.703 -2.031 1.00 1.22
H
ATOM 17753 1HD1 LEU B 544   -11.414 37.829   0.271 1.00 1.22
H
ATOM 17754 2HD1 LEU B 544   -12.414 36.873 -0.846 1.00 1.22
H
ATOM 17755 3HD1 LEU B 544   -10.841 36.248 -0.299 1.00 1.22
H
ATOM 17756 1HD2 LEU B 544   -9.285 38.915 -0.607 1.00 1.22
H
ATOM 17757 2HD2 LEU B 544   -8.709 37.330 -1.171 1.00 1.22
H

ATOM 17758 3HD2 LEU B 544 -8.833 38.690 -2.311 1.00 1.22
H
ATOM 17759 N ASP B 545 -13.809 38.180 -3.336 1.00 0.73
N
ATOM 17760 CA ASP B 545 -14.542 39.394 -3.690 1.00 0.86
C
ATOM 17761 C ASP B 545 -13.796 40.690 -3.395 1.00 0.95
C
ATOM 17762 O ASP B 545 -14.141 41.405 -2.449 1.00 0.97
O
ATOM 17763 CB ASP B 545 -15.861 39.433 -2.957 1.00 1.29
C
ATOM 17764 CG ASP B 545 -16.695 40.624 -3.359 1.00 1.29
C
ATOM 17765 OD1 ASP B 545 -16.200 41.459 -4.083 1.00 1.29
O
ATOM 17766 OD2 ASP B 545 -17.822 40.703 -2.931 1.00 1.29
O
ATOM 17767 H ASP B 545 -14.124 37.633 -2.546 1.00 0.88
H
ATOM 17768 HA ASP B 545 -14.745 39.361 -4.760 1.00 1.03
H
ATOM 17769 1HB ASP B 545 -16.420 38.518 -3.139 1.00 1.55
H
ATOM 17770 2HB ASP B 545 -15.666 39.492 -1.888 1.00 1.55
H
ATOM 17771 N ALA B 546 -12.824 41.011 -4.241 1.00 1.00
N
ATOM 17772 CA ALA B 546 -12.021 42.217 -4.095 1.00 1.06
C
ATOM 17773 C ALA B 546 -12.851 43.493 -4.234 1.00 1.04
C
ATOM 17774 O ALA B 546 -12.502 44.512 -3.646 1.00 1.06
O
ATOM 17775 CB ALA B 546 -10.897 42.221 -5.115 1.00 1.59
C
ATOM 17776 H ALA B 546 -12.617 40.361 -4.989 1.00 1.20
H
ATOM 17777 HA ALA B 546 -11.588 42.208 -3.096 1.00 1.27
H
ATOM 17778 1HB ALA B 546 -10.278 43.107 -4.973 1.00 1.91
H
ATOM 17779 2HB ALA B 546 -10.287 41.327 -4.986 1.00 1.91
H
ATOM 17780 3HB ALA B 546 -11.316 42.230 -6.120 1.00 1.91
H
ATOM 17781 N ALA B 547 -13.934 43.453 -5.012 1.00 1.05
N
ATOM 17782 CA ALA B 547 -14.770 44.639 -5.203 1.00 1.09
C
ATOM 17783 C ALA B 547 -15.358 45.118 -3.878 1.00 1.02
C
ATOM 17784 O ALA B 547 -15.254 46.305 -3.543 1.00 1.10
O
ATOM 17785 CB ALA B 547 -15.883 44.340 -6.193 1.00 1.64
C
ATOM 17786 H ALA B 547 -14.189 42.593 -5.477 1.00 1.26
H

```
ATOM  17787  HA  ALA B 547     -14.142  45.436  -5.602  1.00  1.31
H
ATOM  17788 1HB  ALA B 547     -16.484  45.236  -6.348  1.00  1.96
H
ATOM  17789 2HB  ALA B 547     -15.450  44.025  -7.143  1.00  1.96
H
ATOM  17790 3HB  ALA B 547     -16.514  43.542  -5.802  1.00  1.96
H
ATOM  17791  N   THR B 548     -15.958  44.192  -3.121  1.00  0.91
N
ATOM  17792  CA  THR B 548     -16.506  44.523  -1.809  1.00  0.85
C
ATOM  17793  C   THR B 548     -15.407  44.873  -0.829  1.00  0.81
C
ATOM  17794  O   THR B 548     -15.557  45.826  -0.067  1.00  0.82
O
ATOM  17795  CB  THR B 548     -17.367  43.389  -1.208  1.00  1.27
C
ATOM  17796  OG1 THR B 548     -18.512  43.148  -2.039  1.00  1.27
O
ATOM  17797  CG2 THR B 548     -17.826  43.757   0.199  1.00  1.27
C
ATOM  17798  H   THR B 548     -16.032  43.238  -3.461  1.00  1.09
H
ATOM  17799  HA  THR B 548     -17.143  45.400  -1.919  1.00  1.02
H
ATOM  17800  HB  THR B 548     -16.775  42.476  -1.152  1.00  1.53
H
ATOM  17801  HG1 THR B 548     -18.333  42.392  -2.608  1.00  1.53
H
ATOM  17802 1HG2 THR B 548     -18.424  42.944   0.609  1.00  1.53
H
ATOM  17803 2HG2 THR B 548     -16.956  43.924   0.833  1.00  1.53
H
ATOM  17804 3HG2 THR B 548     -18.427  44.665   0.160  1.00  1.53
H
ATOM  17805  N   ILE B 549     -14.310  44.109  -0.838  1.00  0.79
N
ATOM  17806  CA  ILE B 549     -13.211  44.369   0.091  1.00  0.82
C
ATOM  17807  C   ILE B 549     -12.629  45.759  -0.073  1.00  0.84
C
ATOM  17808  O   ILE B 549     -12.423  46.456   0.918  1.00  0.87
O
ATOM  17809  CB  ILE B 549     -12.081  43.340  -0.068  1.00  1.23
C
ATOM  17810  CG1 ILE B 549     -12.557  41.969   0.408  1.00  1.23
C
ATOM  17811  CG2 ILE B 549     -10.845  43.788   0.704  1.00  1.23
C
ATOM  17812  CD1 ILE B 549     -11.622  40.852   0.029  1.00  1.23
C
ATOM  17813  H   ILE B 549     -14.236  43.326  -1.482  1.00  0.95
H
ATOM  17814  HA  ILE B 549     -13.600  44.287   1.105  1.00  0.98
H
ATOM  17815  HB  ILE B 549     -11.827  43.247  -1.123  1.00  1.48
H
```

ATOM 17816 1HG1 ILE B 549 -12.656 41.984 1.493 1.00 1.48
H
ATOM 17817 2HG1 ILE B 549 -13.537 41.761 -0.022 1.00 1.48
H
ATOM 17818 1HG2 ILE B 549 -10.050 43.054 0.573 1.00 1.48
H
ATOM 17819 2HG2 ILE B 549 -10.511 44.754 0.331 1.00 1.48
H
ATOM 17820 3HG2 ILE B 549 -11.089 43.873 1.763 1.00 1.48
H
ATOM 17821 1HD1 ILE B 549 -12.021 39.911 0.401 1.00 1.48
H
ATOM 17822 2HD1 ILE B 549 -11.530 40.807 -1.056 1.00 1.48
H
ATOM 17823 3HD1 ILE B 549 -10.643 41.032 0.471 1.00 1.48
H
ATOM 17824 N ILE B 550 -12.355 46.156 -1.310 1.00 0.87
N
ATOM 17825 CA ILE B 550 -11.800 47.472 -1.588 1.00 0.95
C
ATOM 17826 C ILE B 550 -12.787 48.583 -1.258 1.00 1.00
C
ATOM 17827 O ILE B 550 -12.399 49.589 -0.665 1.00 1.04
O
ATOM 17828 CB ILE B 550 -11.342 47.579 -3.047 1.00 1.42
C
ATOM 17829 CG1 ILE B 550 -10.139 46.655 -3.268 1.00 1.42
C
ATOM 17830 CG2 ILE B 550 -10.978 49.018 -3.372 1.00 1.42
C
ATOM 17831 CD1 ILE B 550 -9.767 46.461 -4.719 1.00 1.42
C
ATOM 17832 H ILE B 550 -12.526 45.532 -2.085 1.00 1.04
H
ATOM 17833 HA ILE B 550 -10.924 47.608 -0.956 1.00 1.14
H
ATOM 17834 HB ILE B 550 -12.140 47.246 -3.709 1.00 1.71
H
ATOM 17835 1HG1 ILE B 550 -9.277 47.070 -2.747 1.00 1.71
H
ATOM 17836 2HG1 ILE B 550 -10.361 45.679 -2.837 1.00 1.71
H
ATOM 17837 1HG2 ILE B 550 -10.654 49.084 -4.410 1.00 1.71
H
ATOM 17838 2HG2 ILE B 550 -11.850 49.654 -3.222 1.00 1.71
H
ATOM 17839 3HG2 ILE B 550 -10.171 49.346 -2.717 1.00 1.71
H
ATOM 17840 1HD1 ILE B 550 -8.906 45.795 -4.788 1.00 1.71
H
ATOM 17841 2HD1 ILE B 550 -10.610 46.021 -5.255 1.00 1.71
H
ATOM 17842 3HD1 ILE B 550 -9.516 47.423 -5.163 1.00 1.71
H
ATOM 17843 N SER B 551 -14.056 48.412 -1.645 1.00 1.06
N
ATOM 17844 CA SER B 551 -15.075 49.411 -1.341 1.00 1.21
C

ATOM 17845 C SER B 551 -15.179 49.624 0.167 1.00 1.19
C
ATOM 17846 O SER B 551 -15.234 50.763 0.643 1.00 1.40
O
ATOM 17847 CB SER B 551 -16.417 48.960 -1.888 1.00 1.81
C
ATOM 17848 OG SER B 551 -16.392 48.884 -3.288 1.00 1.81
O
ATOM 17849 H SER B 551 -14.333 47.581 -2.157 1.00 1.27
H
ATOM 17850 HA SER B 551 -14.791 50.355 -1.810 1.00 1.45
H
ATOM 17851 1HB SER B 551 -16.673 47.986 -1.473 1.00 2.18
H
ATOM 17852 2HB SER B 551 -17.187 49.663 -1.575 1.00 2.18
H
ATOM 17853 HG SER B 551 -15.842 48.120 -3.498 1.00 2.18
H
ATOM 17854 N SER B 552 -15.180 48.511 0.900 1.00 1.01
N
ATOM 17855 CA SER B 552 -15.231 48.487 2.350 1.00 1.04
C
ATOM 17856 C SER B 552 -14.017 49.170 2.948 1.00 0.98
C
ATOM 17857 O SER B 552 -14.142 49.994 3.857 1.00 1.16
O
ATOM 17858 CB SER B 552 -15.302 47.047 2.815 1.00 1.56
C
ATOM 17859 OG SER B 552 -15.358 46.959 4.205 1.00 1.56
O
ATOM 17860 H SER B 552 -15.152 47.618 0.429 1.00 1.21
H
ATOM 17861 HA SER B 552 -16.126 49.018 2.677 1.00 1.25
H
ATOM 17862 1HB SER B 552 -16.184 46.575 2.384 1.00 1.87
H
ATOM 17863 2HB SER B 552 -14.433 46.502 2.449 1.00 1.87
H
ATOM 17864 HG SER B 552 -14.515 47.278 4.551 1.00 1.87
H
ATOM 17865 N ALA B 553 -12.847 48.821 2.423 1.00 0.92
N
ATOM 17866 CA ALA B 553 -11.591 49.376 2.875 1.00 0.95
C
ATOM 17867 C ALA B 553 -11.598 50.883 2.784 1.00 1.07
C
ATOM 17868 O ALA B 553 -11.297 51.551 3.768 1.00 1.29
O
ATOM 17869 CB ALA B 553 -10.447 48.812 2.056 1.00 1.42
C
ATOM 17870 H ALA B 553 -12.817 48.126 1.691 1.00 1.10
H
ATOM 17871 HA ALA B 553 -11.453 49.096 3.919 1.00 1.14
H
ATOM 17872 1HB ALA B 553 -9.503 49.212 2.425 1.00 1.71
H
ATOM 17873 2HB ALA B 553 -10.440 47.726 2.144 1.00 1.71
H

```
ATOM  17874 3HB  ALA B 553     -10.572  49.087   1.011  1.00  1.71
H
ATOM  17875  N   LYS B 554     -11.992  51.417   1.630  1.00  1.15
N
ATOM  17876  CA  LYS B 554     -12.044  52.858   1.424  1.00  1.44
C
ATOM  17877  C   LYS B 554     -13.047  53.531   2.361  1.00  1.30
C
ATOM  17878  O   LYS B 554     -12.769  54.601   2.911  1.00  1.59
O
ATOM  17879  CB  LYS B 554     -12.360  53.145  -0.040  1.00  2.16
C
ATOM  17880  CG  LYS B 554     -11.200  52.801  -0.969  1.00  2.16
C
ATOM  17881  CD  LYS B 554     -11.525  53.057  -2.430  1.00  2.16
C
ATOM  17882  CE  LYS B 554     -10.326  52.735  -3.317  1.00  2.16
C
ATOM  17883  NZ  LYS B 554     -10.632  52.913  -4.763  1.00  2.16
N
ATOM  17884  H   LYS B 554     -12.235  50.805   0.862  1.00  1.38
H
ATOM  17885  HA  LYS B 554     -11.059  53.270   1.645  1.00  1.73
H
ATOM  17886 1HB  LYS B 554     -13.230  52.566  -0.351  1.00  2.59
H
ATOM  17887 2HB  LYS B 554     -12.599  54.201  -0.168  1.00  2.59
H
ATOM  17888 1HG  LYS B 554     -10.330  53.398  -0.693  1.00  2.59
H
ATOM  17889 2HG  LYS B 554     -10.945  51.749  -0.847  1.00  2.59
H
ATOM  17890 1HD  LYS B 554     -12.369  52.434  -2.732  1.00  2.59
H
ATOM  17891 2HD  LYS B 554     -11.798  54.103  -2.568  1.00  2.59
H
ATOM  17892 1HE  LYS B 554      -9.499  53.392  -3.050  1.00  2.59
H
ATOM  17893 2HE  LYS B 554     -10.020  51.705  -3.146  1.00  2.59
H
ATOM  17894 1HZ  LYS B 554      -9.811  52.688  -5.310  1.00  2.59
H
ATOM  17895 2HZ  LYS B 554     -11.389  52.297  -5.026  1.00  2.59
H
ATOM  17896 3HZ  LYS B 554     -10.901  53.870  -4.938  1.00  2.59
H
ATOM  17897  N   ALA B 555     -14.187  52.875   2.599  1.00  1.32
N
ATOM  17898  CA  ALA B 555     -15.180  53.382   3.543  1.00  1.38
C
ATOM  17899  C   ALA B 555     -14.600  53.452   4.960  1.00  1.50
C
ATOM  17900  O   ALA B 555     -15.003  54.297   5.761  1.00  4.41
O
ATOM  17901  CB  ALA B 555     -16.421  52.505   3.526  1.00  2.07
C
ATOM  17902  H   ALA B 555     -14.388  52.014   2.102  1.00  1.58
H
```

```
ATOM  17903  HA  ALA B 555     -15.452  54.393   3.239  1.00   1.66
H
ATOM  17904 1HB  ALA B 555     -17.161  52.912   4.215  1.00   2.48
H
ATOM  17905 2HB  ALA B 555     -16.836  52.484   2.518  1.00   2.48
H
ATOM  17906 3HB  ALA B 555     -16.158  51.495   3.831  1.00   2.48
H
ATOM  17907  N   THR B 556     -13.636  52.577   5.249  1.00   1.28
N
ATOM  17908  CA  THR B 556     -12.980  52.532   6.542  1.00   1.13
C
ATOM  17909  C   THR B 556     -11.566  53.124   6.531  1.00   1.52
C
ATOM  17910  O   THR B 556     -10.680  52.634   7.229  1.00   3.27
O
ATOM  17911  CB  THR B 556     -12.920  51.093   7.044  1.00   1.69
C
ATOM  17912  OG1 THR B 556     -12.233  50.275   6.093  1.00   1.69
O
ATOM  17913  CG2 THR B 556     -14.322  50.558   7.231  1.00   1.69
C
ATOM  17914  H   THR B 556     -13.377  51.882   4.559  1.00   1.54
H
ATOM  17915  HA  THR B 556     -13.578  53.113   7.245  1.00   1.36
H
ATOM  17916  HB  THR B 556     -12.389  51.065   7.989  1.00   2.03
H
ATOM  17917  HG1 THR B 556     -12.607  50.436   5.220  1.00   2.03
H
ATOM  17918 1HG2 THR B 556     -14.275  49.534   7.594  1.00   2.03
H
ATOM  17919 2HG2 THR B 556     -14.841  51.182   7.952  1.00   2.03
H
ATOM  17920 3HG2 THR B 556     -14.853  50.584   6.280  1.00   2.03
H
ATOM  17921  N   GLY B 557     -11.341  54.161   5.715  1.00   1.91
N
ATOM  17922  CA  GLY B 557     -10.066  54.887   5.739  1.00   2.10
C
ATOM  17923  C   GLY B 557      -8.921  54.160   5.049  1.00   1.71
C
ATOM  17924  O   GLY B 557      -7.755  54.510   5.240  1.00   2.38
O
ATOM  17925  H   GLY B 557     -12.068  54.478   5.083  1.00   2.29
H
ATOM  17926 1HA  GLY B 557     -10.208  55.860   5.267  1.00   2.52
H
ATOM  17927 2HA  GLY B 557      -9.794  55.084   6.775  1.00   2.52
H
ATOM  17928  N   GLY B 558      -9.250  53.140   4.270  1.00   1.44
N
ATOM  17929  CA  GLY B 558      -8.264  52.324   3.585  1.00   1.51
C
ATOM  17930  C   GLY B 558      -7.649  51.280   4.517  1.00   2.07
C
ATOM  17931  O   GLY B 558      -6.639  50.667   4.190  1.00   7.51
O
```

ATOM 17932 H GLY B 558 -10.219 52.909 4.139 1.00 1.73
H
ATOM 17933 1HA GLY B 558 -8.738 51.826 2.741 1.00 1.81
H
ATOM 17934 2HA GLY B 558 -7.484 52.965 3.183 1.00 1.81
H
ATOM 17935 N ARG B 559 -8.240 51.075 5.686 1.00 1.33
N
ATOM 17936 CA ARG B 559 -7.643 50.174 6.655 1.00 1.35
C
ATOM 17937 C ARG B 559 -8.329 48.808 6.714 1.00 1.17
C
ATOM 17938 O ARG B 559 -9.528 48.725 6.969 1.00 1.45
O
ATOM 17939 CB ARG B 559 -7.688 50.867 8.002 1.00 2.03
C
ATOM 17940 CG ARG B 559 -6.855 52.148 8.045 1.00 2.03
C
ATOM 17941 CD ARG B 559 -7.072 52.913 9.296 1.00 2.03
C
ATOM 17942 NE ARG B 559 -6.292 54.145 9.350 1.00 2.03
N
ATOM 17943 CZ ARG B 559 -5.030 54.234 9.824 1.00 2.03
C
ATOM 17944 NH1 ARG B 559 -4.406 53.160 10.248 1.00 2.03
N
ATOM 17945 NH2 ARG B 559 -4.414 55.401 9.876 1.00 2.03
N
ATOM 17946 H ARG B 559 -9.084 51.573 5.951 1.00 1.60
H
ATOM 17947 HA ARG B 559 -6.599 50.021 6.381 1.00 1.62
H
ATOM 17948 1HB ARG B 559 -8.715 51.128 8.250 1.00 2.43
H
ATOM 17949 2HB ARG B 559 -7.316 50.198 8.778 1.00 2.43
H
ATOM 17950 1HG ARG B 559 -5.797 51.895 7.978 1.00 2.43
H
ATOM 17951 2HG ARG B 559 -7.129 52.788 7.206 1.00 2.43
H
ATOM 17952 1HD ARG B 559 -8.126 53.176 9.371 1.00 2.43
H
ATOM 17953 2HD ARG B 559 -6.797 52.307 10.146 1.00 2.43
H
ATOM 17954 HE ARG B 559 -6.739 54.996 9.037 1.00 2.43
H
ATOM 17955 1HH1 ARG B 559 -4.864 52.263 10.221 1.00 2.43
H
ATOM 17956 2HH1 ARG B 559 -3.462 53.237 10.617 1.00 2.43
H
ATOM 17957 1HH2 ARG B 559 -4.881 56.238 9.559 1.00 2.43
H
ATOM 17958 2HH2 ARG B 559 -3.475 55.456 10.263 1.00 2.43
H
ATOM 17959 N VAL B 560 -7.547 47.746 6.484 1.00 0.99
N
ATOM 17960 CA VAL B 560 -8.032 46.365 6.513 1.00 0.88
C

ATOM 17961 C   VAL B 560   -7.228 45.473   7.461 1.00 0.81
C
ATOM 17962 O   VAL B 560   -6.004 45.498   7.440 1.00 0.85
O
ATOM 17963 CB VAL B 560   -7.959 45.737   5.106 1.00 1.32
C
ATOM 17964 CG1 VAL B 560   -8.403 44.280   5.149 1.00 1.32
C
ATOM 17965 CG2 VAL B 560   -8.823 46.516   4.142 1.00 1.32
C
ATOM 17966 H   VAL B 560   -6.575 47.895   6.252 1.00 1.19
H
ATOM 17967 HA VAL B 560   -9.069 46.379   6.837 1.00 1.06
H
ATOM 17968 HB VAL B 560   -6.927 45.760   4.769 1.00 1.58
H
ATOM 17969 1HG1 VAL B 560   -8.330 43.850   4.150 1.00 1.58
H
ATOM 17970 2HG1 VAL B 560   -7.764 43.719   5.829 1.00 1.58
H
ATOM 17971 3HG1 VAL B 560   -9.437 44.226   5.492 1.00 1.58
H
ATOM 17972 1HG2 VAL B 560   -8.752 46.073   3.150 1.00 1.58
H
ATOM 17973 2HG2 VAL B 560   -9.859 46.490   4.476 1.00 1.58
H
ATOM 17974 3HG2 VAL B 560   -8.483 47.550   4.100 1.00 1.58
H
ATOM 17975 N   ILE B 561   -7.924 44.691   8.279 1.00 0.75
N
ATOM 17976 CA ILE B 561   -7.303 43.707   9.161 1.00 0.67
C
ATOM 17977 C   ILE B 561   -7.899 42.347   8.853 1.00 0.63
C
ATOM 17978 O   ILE B 561   -9.094 42.145   9.042 1.00 0.62
O
ATOM 17979 CB ILE B 561   -7.537 44.007 10.655 1.00 1.01
C
ATOM 17980 CG1 ILE B 561   -6.978 45.370 11.051 1.00 1.01
C
ATOM 17981 CG2 ILE B 561   -6.902 42.911 11.503 1.00 1.01
C
ATOM 17982 CD1 ILE B 561   -7.382 45.784 12.445 1.00 1.01
C
ATOM 17983 H   ILE B 561   -8.927 44.755   8.262 1.00 0.90
H
ATOM 17984 HA ILE B 561   -6.233 43.671   8.963 1.00 0.80
H
ATOM 17985 HB ILE B 561   -8.606 44.029 10.847 1.00 1.21
H
ATOM 17986 1HG1 ILE B 561   -5.893 45.342 11.009 1.00 1.21
H
ATOM 17987 2HG1 ILE B 561   -7.329 46.122 10.348 1.00 1.21
H
ATOM 17988 1HG2 ILE B 561   -7.091 43.111 12.557 1.00 1.21
H
ATOM 17989 2HG2 ILE B 561   -7.335 41.947 11.236 1.00 1.21
H

ATOM 17990 3HG2 ILE B 561   -5.828 42.888 11.324 1.00 1.21
H
ATOM 17991 1HD1 ILE B 561   -6.955 46.760 12.675 1.00 1.21
H
ATOM 17992 2HD1 ILE B 561   -8.469 45.841 12.507 1.00 1.21
H
ATOM 17993 3HD1 ILE B 561   -7.017 45.051 13.161 1.00 1.21
H
ATOM 17994 N   THR B 562   -7.089 41.407   8.400 1.00 0.65
N
ATOM 17995 CA THR B 562   -7.627 40.089   8.113 1.00 0.58
C
ATOM 17996 C   THR B 562   -7.171 39.097   9.167 1.00 0.57
C
ATOM 17997 O   THR B 562   -5.978 38.998   9.453 1.00 0.64
O
ATOM 17998 CB THR B 562   -7.208 39.604   6.717 1.00 0.87
C
ATOM 17999 OG1 THR B 562   -7.700 40.515   5.726 1.00 0.87
O
ATOM 18000 CG2 THR B 562   -7.791 38.225   6.458 1.00 0.87
C
ATOM 18001 H   THR B 562   -6.106 41.603   8.256 1.00 0.78
H
ATOM 18002 HA THR B 562   -8.715 40.138   8.144 1.00 0.70
H
ATOM 18003 HB THR B 562   -6.121 39.560   6.655 1.00 1.04
H
ATOM 18004 HG1 THR B 562   -7.420 40.220   4.857 1.00 1.04
H
ATOM 18005 1HG2 THR B 562   -7.492 37.892   5.468 1.00 1.04
H
ATOM 18006 2HG2 THR B 562   -7.419 37.523   7.203 1.00 1.04
H
ATOM 18007 3HG2 THR B 562   -8.877 38.271   6.514 1.00 1.04
H
ATOM 18008 N   VAL B 563   -8.127 38.361   9.726 1.00 0.49
N
ATOM 18009 CA VAL B 563   -7.845 37.368 10.753 1.00 0.47
C
ATOM 18010 C   VAL B 563   -8.315 35.991 10.314 1.00 0.42
C
ATOM 18011 O   VAL B 563   -9.414 35.840   9.782 1.00 0.39
O
ATOM 18012 CB VAL B 563   -8.530 37.763 12.083 1.00 0.70
C
ATOM 18013 CG1 VAL B 563   -10.030 37.915 11.868 1.00 0.70
C
ATOM 18014 CG2 VAL B 563   -8.249 36.704 13.146 1.00 0.70
C
ATOM 18015 H   VAL B 563   -9.081 38.499   9.422 1.00 0.59
H
ATOM 18016 HA VAL B 563   -6.772 37.340 10.912 1.00 0.56
H
ATOM 18017 HB VAL B 563   -8.143 38.726 12.410 1.00 0.85
H
ATOM 18018 1HG1 VAL B 563   -10.507 38.206 12.804 1.00 0.85
H

ATOM 18019 2HG1 VAL B 563   -10.212 38.681 11.117 1.00 0.85
H
ATOM 18020 3HG1 VAL B 563   -10.448 36.970 11.528 1.00 0.85
H
ATOM 18021 1HG2 VAL B 563   -8.725 36.995 14.082 1.00 0.85
H
ATOM 18022 2HG2 VAL B 563   -8.648 35.744 12.821 1.00 0.85
H
ATOM 18023 3HG2 VAL B 563   -7.174 36.616 13.300 1.00 0.85
H
ATOM 18024 N   GLU B 564   -7.479 34.982 10.537 1.00 0.41
N
ATOM 18025 CA GLU B 564   -7.832 33.636 10.112 1.00 0.34
C
ATOM 18026 C   GLU B 564   -7.183 32.524 10.913 1.00 0.34
C
ATOM 18027 O   GLU B 564   -5.997 32.574 11.242 1.00 0.65
O
ATOM 18028 CB GLU B 564   -7.456 33.426   8.656 1.00 0.51
C
ATOM 18029 CG GLU B 564   -7.895 32.080   8.102 1.00 0.51
C
ATOM 18030 CD GLU B 564   -7.469 31.904   6.698 1.00 0.51
C
ATOM 18031 OE1 GLU B 564   -6.707 32.723   6.248 1.00 0.51
O
ATOM 18032 OE2 GLU B 564   -7.882 30.962   6.065 1.00 0.51
O
ATOM 18033 H   GLU B 564   -6.589 35.167 10.980 1.00 0.49
H
ATOM 18034 HA GLU B 564   -8.912 33.528 10.209 1.00 0.41
H
ATOM 18035 1HB GLU B 564   -7.910 34.207   8.046 1.00 0.61
H
ATOM 18036 2HB GLU B 564   -6.374 33.504   8.541 1.00 0.61
H
ATOM 18037 1HG GLU B 564   -7.457 31.284   8.695 1.00 0.61
H
ATOM 18038 2HG GLU B 564   -8.979 32.000   8.170 1.00 0.61
H
ATOM 18039 N   ASP B 565   -7.959 31.479 11.174 1.00 0.40
N
ATOM 18040 CA ASP B 565   -7.458 30.312 11.874 1.00 0.41
C
ATOM 18041 C   ASP B 565   -6.798 29.376 10.839 1.00 0.49
C
ATOM 18042 O   ASP B 565   -7.357 28.357 10.431 1.00 1.97
O
ATOM 18043 CB ASP B 565   -8.618 29.661 12.633 1.00 0.61
C
ATOM 18044 CG ASP B 565   -8.197 28.571 13.575 1.00 0.61
C
ATOM 18045 OD1 ASP B 565   -7.033 28.255 13.595 1.00 0.61
O
ATOM 18046 OD2 ASP B 565   -9.037 28.047 14.277 1.00 0.61
O
ATOM 18047 H   ASP B 565   -8.924 31.498 10.877 1.00 0.48
H

ATOM 18048 HA ASP B 565 -6.697 30.625 12.588 1.00 0.49
H
ATOM 18049 1HB ASP B 565 -9.147 30.425 13.204 1.00 0.74
H
ATOM 18050 2HB ASP B 565 -9.327 29.244 11.918 1.00 0.74
H
ATOM 18051 N HIS B 566 -5.596 29.783 10.424 1.00 0.39
N
ATOM 18052 CA HIS B 566 -4.782 29.152 9.380 1.00 0.28
C
ATOM 18053 C HIS B 566 -3.383 29.724 9.481 1.00 0.32
C
ATOM 18054 O HIS B 566 -3.216 30.809 10.033 1.00 0.45
O
ATOM 18055 CB HIS B 566 -5.373 29.458 7.997 1.00 0.42
C
ATOM 18056 CG HIS B 566 -4.795 28.693 6.839 1.00 0.42
C
ATOM 18057 ND1 HIS B 566 -3.680 29.112 6.140 1.00 0.42
N
ATOM 18058 CD2 HIS B 566 -5.186 27.535 6.263 1.00 0.42
C
ATOM 18059 CE1 HIS B 566 -3.424 28.246 5.174 1.00 0.42
C
ATOM 18060 NE2 HIS B 566 -4.329 27.280 5.226 1.00 0.42
N
ATOM 18061 H HIS B 566 -5.245 30.632 10.851 1.00 0.47
H
ATOM 18062 HA HIS B 566 -4.735 28.077 9.510 1.00 0.34
H
ATOM 18063 1HB HIS B 566 -6.446 29.272 8.018 1.00 0.50
H
ATOM 18064 2HB HIS B 566 -5.235 30.517 7.788 1.00 0.50
H
ATOM 18065 HD1 HIS B 566 -2.977 29.731 6.499 1.00 0.50
H
ATOM 18066 HD2 HIS B 566 -5.994 26.840 6.465 1.00 0.50
H
ATOM 18067 HE1 HIS B 566 -2.579 28.402 4.504 1.00 0.50
H
ATOM 18068 N TYR B 567 -2.375 29.002 8.998 1.00 0.56
N
ATOM 18069 CA TYR B 567 -1.031 29.553 9.032 1.00 0.96
C
ATOM 18070 C TYR B 567 -0.951 30.773 8.130 1.00 1.37
C
ATOM 18071 O TYR B 567 -1.604 30.842 7.085 1.00 2.33
O
ATOM 18072 CB TYR B 567 0.028 28.546 8.580 1.00 1.44
C
ATOM 18073 CG TYR B 567 0.254 27.397 9.525 1.00 1.44
C
ATOM 18074 CD1 TYR B 567 0.319 26.117 9.025 1.00 1.44
C
ATOM 18075 CD2 TYR B 567 0.372 27.611 10.880 1.00 1.44
C
ATOM 18076 CE1 TYR B 567 0.504 25.040 9.863 1.00 1.44
C

```
ATOM  18077  CE2 TYR B 567       0.550  26.537  11.723  1.00  1.44
C
ATOM  18078  CZ  TYR B 567       0.613  25.256  11.226  1.00  1.44
C
ATOM  18079  OH  TYR B 567       0.788  24.189  12.078  1.00  1.44
O
ATOM  18080  H   TYR B 567      -2.533  28.097   8.576  1.00  0.67
H
ATOM  18081  HA  TYR B 567      -0.810  29.870  10.049  1.00  1.15
H
ATOM  18082 1HB  TYR B 567      -0.261  28.133   7.613  1.00  1.73
H
ATOM  18083 2HB  TYR B 567       0.979  29.059   8.441  1.00  1.73
H
ATOM  18084  HD1 TYR B 567       0.222  25.971   7.956  1.00  1.73
H
ATOM  18085  HD2 TYR B 567       0.314  28.622  11.278  1.00  1.73
H
ATOM  18086  HE1 TYR B 567       0.554  24.030   9.457  1.00  1.73
H
ATOM  18087  HE2 TYR B 567       0.631  26.700  12.788  1.00  1.73
H
ATOM  18088  HH  TYR B 567       0.838  23.379  11.565  1.00  1.73
H
ATOM  18089  N   ARG B 568      -0.120  31.725   8.532  1.00  1.45
N
ATOM  18090  CA  ARG B 568       0.097  32.971   7.792  1.00  1.72
C
ATOM  18091  C   ARG B 568       0.421  32.811   6.295  1.00  3.14
C
ATOM  18092  O   ARG B 568       0.118  33.706   5.502  1.00 15.42
O
ATOM  18093  CB  ARG B 568       1.234  33.753   8.449  1.00  2.58
C
ATOM  18094  CG  ARG B 568       0.881  34.358   9.793  1.00  2.58
C
ATOM  18095  CD  ARG B 568       1.988  35.104  10.425  1.00  2.58
C
ATOM  18096  NE  ARG B 568       1.592  35.584  11.738  1.00  2.58
N
ATOM  18097  CZ  ARG B 568       0.847  36.689  11.948  1.00  2.58
C
ATOM  18098  NH1 ARG B 568       0.439  37.410  10.927  1.00  2.58
N
ATOM  18099  NH2 ARG B 568       0.521  37.046  13.178  1.00  2.58
N
ATOM  18100  H   ARG B 568       0.349  31.585   9.421  1.00  1.74
H
ATOM  18101  HA  ARG B 568      -0.814  33.562   7.877  1.00  2.06
H
ATOM  18102 1HB  ARG B 568       2.093  33.101   8.593  1.00  3.10
H
ATOM  18103 2HB  ARG B 568       1.544  34.567   7.791  1.00  3.10
H
ATOM  18104 1HG  ARG B 568       0.087  35.084   9.641  1.00  3.10
H
ATOM  18105 2HG  ARG B 568       0.543  33.579  10.478  1.00  3.10
H
```

```
ATOM  18106 1HD  ARG B 568       2.853  34.451  10.544  1.00  3.10
H
ATOM  18107 2HD  ARG B 568       2.257  35.962   9.809  1.00  3.10
H
ATOM  18108  HE  ARG B 568       1.882  35.050  12.547  1.00  3.10
H
ATOM  18109 1HH1 ARG B 568       0.687  37.140   9.985  1.00  3.10
H
ATOM  18110 2HH1 ARG B 568      -0.132  38.232  11.083  1.00  3.10
H
ATOM  18111 1HH2 ARG B 568       0.830  36.491  13.964  1.00  3.10
H
ATOM  18112 2HH2 ARG B 568      -0.035  37.875  13.332  1.00  3.10
H
ATOM  18113  N   GLU B 569       1.024  31.686   5.915  1.00  1.20
N
ATOM  18114  CA  GLU B 569       1.440  31.479   4.540  1.00  1.14
C
ATOM  18115  C   GLU B 569       0.366  30.816   3.684  1.00  1.01
C
ATOM  18116  O   GLU B 569      -0.083  29.704   3.971  1.00  1.33
O
ATOM  18117  CB  GLU B 569       2.694  30.627   4.527  1.00  1.71
C
ATOM  18118  CG  GLU B 569       3.828  31.201   5.361  1.00  1.71
C
ATOM  18119  CD  GLU B 569       4.323  32.515   4.867  1.00  1.71
C
ATOM  18120  OE1 GLU B 569       3.974  32.901   3.776  1.00  1.71
O
ATOM  18121  OE2 GLU B 569       5.103  33.131   5.557  1.00  1.71
O
ATOM  18122  H   GLU B 569       1.229  30.973   6.599  1.00  1.44
H
ATOM  18123  HA  GLU B 569       1.671  32.452   4.102  1.00  1.37
H
ATOM  18124 1HB  GLU B 569       2.465  29.637   4.910  1.00  2.05
H
ATOM  18125 2HB  GLU B 569       3.047  30.513   3.503  1.00  2.05
H
ATOM  18126 1HG  GLU B 569       3.481  31.320   6.385  1.00  2.05
H
ATOM  18127 2HG  GLU B 569       4.653  30.493   5.371  1.00  2.05
H
ATOM  18128  N   GLY B 570      -0.020  31.503   2.621  1.00  0.90
N
ATOM  18129  CA  GLY B 570      -0.999  31.017   1.655  1.00  0.81
C
ATOM  18130  C   GLY B 570      -2.459  31.160   2.094  1.00  0.69
C
ATOM  18131  O   GLY B 570      -3.347  30.651   1.414  1.00  1.03
O
ATOM  18132  H   GLY B 570       0.406  32.405   2.467  1.00  1.08
H
ATOM  18133 1HA  GLY B 570      -0.857  31.555   0.718  1.00  0.97
H
ATOM  18134 2HA  GLY B 570      -0.786  29.976   1.437  1.00  0.97
H
```

ATOM 18135 N   GLY B 571   -2.726 31.880   3.182 1.00 0.59
N
ATOM 18136 CA GLY B 571   -4.099 31.999   3.673 1.00 0.47
C
ATOM 18137 C   GLY B 571   -4.903 33.168   3.073 1.00 0.47
C
ATOM 18138 O   GLY B 571   -4.524 33.732   2.044 1.00 0.67
O
ATOM 18139 H   GLY B 571   -1.974 32.318   3.695 1.00 0.71
H
ATOM 18140 1HA GLY B 571   -4.605 31.064   3.466 1.00 0.56
H
ATOM 18141 2HA GLY B 571   -4.078 32.101   4.758 1.00 0.56
H
ATOM 18142 N   ILE B 572   -6.017 33.516   3.741 1.00 0.44
N
ATOM 18143 CA ILE B 572   -6.908 34.595   3.303 1.00 0.58
C
ATOM 18144 C   ILE B 572   -6.198 35.919   3.352 1.00 0.46
C
ATOM 18145 O   ILE B 572   -6.358 36.727   2.448 1.00 0.63
O
ATOM 18146 CB ILE B 572   -8.196 34.748   4.154 1.00 0.87
C
ATOM 18147 CG1 ILE B 572   -9.139 33.564   4.004 1.00 0.87
C
ATOM 18148 CG2 ILE B 572   -8.931 36.005   3.710 1.00 0.87
C
ATOM 18149 CD1 ILE B 572   -10.271 33.570   5.011 1.00 0.87
C
ATOM 18150 H   ILE B 572   -6.257 33.014   4.585 1.00 0.53
H
ATOM 18151 HA ILE B 572   -7.202 34.404   2.272 1.00 0.70
H
ATOM 18152 HB ILE B 572   -7.930 34.832   5.208 1.00 1.04
H
ATOM 18153 1HG1 ILE B 572   -9.566 33.574   3.005 1.00 1.04
H
ATOM 18154 2HG1 ILE B 572   -8.577 32.643   4.132 1.00 1.04
H
ATOM 18155 1HG2 ILE B 572   -9.829 36.134   4.312 1.00 1.04
H
ATOM 18156 2HG2 ILE B 572   -8.283 36.869   3.831 1.00 1.04
H
ATOM 18157 3HG2 ILE B 572   -9.209 35.910   2.660 1.00 1.04
H
ATOM 18158 1HD1 ILE B 572   -10.906 32.700   4.855 1.00 1.04
H
ATOM 18159 2HD1 ILE B 572   -9.858 33.541   6.020 1.00 1.04
H
ATOM 18160 3HD1 ILE B 572   -10.861 34.477   4.889 1.00 1.04
H
ATOM 18161 N   GLY B 573   -5.435 36.155   4.421 1.00 0.51
N
ATOM 18162 CA GLY B 573   -4.712 37.413   4.575 1.00 0.61
C
ATOM 18163 C   GLY B 573   -3.817 37.666   3.377 1.00 0.66
C

```
ATOM  18164  O   GLY B 573      -3.752  38.784   2.869  1.00  0.76
O
ATOM  18165  H   GLY B 573      -5.356  35.448   5.139  1.00  0.61
H
ATOM  18166 1HA  GLY B 573      -5.419  38.234   4.683  1.00  0.73
H
ATOM  18167 2HA  GLY B 573      -4.111  37.381   5.483  1.00  0.73
H
ATOM  18168  N   GLU B 574      -3.142  36.618   2.930  1.00  0.67
N
ATOM  18169  CA  GLU B 574      -2.272  36.686   1.778  1.00  0.84
C
ATOM  18170  C   GLU B 574      -3.077  36.929   0.503  1.00  0.78
C
ATOM  18171  O   GLU B 574      -2.688  37.753  -0.326  1.00  1.03
O
ATOM  18172  CB  GLU B 574      -1.454  35.419   1.706  1.00  1.26
C
ATOM  18173  CG  GLU B 574      -0.409  35.395   0.627  1.00  1.26
C
ATOM  18174  CD  GLU B 574       0.458  34.205   0.796  1.00  1.26
C
ATOM  18175  OE1 GLU B 574       0.913  33.998   1.894  1.00  1.26
O
ATOM  18176  OE2 GLU B 574       0.632  33.461  -0.137  1.00  1.26
O
ATOM  18177  H   GLU B 574      -3.239  35.734   3.408  1.00  0.80
H
ATOM  18178  HA  GLU B 574      -1.590  37.527   1.910  1.00  1.01
H
ATOM  18179 1HB  GLU B 574      -0.950  35.263   2.660  1.00  1.51
H
ATOM  18180 2HB  GLU B 574      -2.117  34.569   1.546  1.00  1.51
H
ATOM  18181 1HG  GLU B 574      -0.892  35.359  -0.348  1.00  1.51
H
ATOM  18182 2HG  GLU B 574       0.196  36.301   0.680  1.00  1.51
H
ATOM  18183  N   ALA B 575      -4.207  36.223   0.362  1.00  0.62
N
ATOM  18184  CA  ALA B 575      -5.097  36.416  -0.779  1.00  0.66
C
ATOM  18185  C   ALA B 575      -5.573  37.868  -0.859  1.00  0.76
C
ATOM  18186  O   ALA B 575      -5.593  38.452  -1.945  1.00  0.97
O
ATOM  18187  CB  ALA B 575      -6.294  35.476  -0.680  1.00  0.99
C
ATOM  18188  H   ALA B 575      -4.455  35.530   1.059  1.00  0.74
H
ATOM  18189  HA  ALA B 575      -4.540  36.192  -1.689  1.00  0.79
H
ATOM  18190 1HB  ALA B 575      -6.937  35.613  -1.550  1.00  1.19
H
ATOM  18191 2HB  ALA B 575      -5.942  34.445  -0.648  1.00  1.19
H
ATOM  18192 3HB  ALA B 575      -6.858  35.694   0.224  1.00  1.19
H
```

ATOM 18193 N VAL B 576 -5.937 38.444 0.295 1.00 0.72
N
ATOM 18194 CA VAL B 576 -6.384 39.831 0.369 1.00 0.88
C
ATOM 18195 C VAL B 576 -5.270 40.784 0.004 1.00 1.11
C
ATOM 18196 O VAL B 576 -5.493 41.698 -0.786 1.00 1.33
O
ATOM 18197 CB VAL B 576 -6.907 40.191 1.782 1.00 1.32
C
ATOM 18198 CG1 VAL B 576 -7.157 41.693 1.874 1.00 1.32
C
ATOM 18199 CG2 VAL B 576 -8.204 39.446 2.066 1.00 1.32
C
ATOM 18200 H VAL B 576 -5.911 37.900 1.141 1.00 0.86
H
ATOM 18201 HA VAL B 576 -7.199 39.967 -0.341 1.00 1.06
H
ATOM 18202 HB VAL B 576 -6.153 39.923 2.523 1.00 1.58
H
ATOM 18203 1HG1 VAL B 576 -7.514 41.942 2.873 1.00 1.58
H
ATOM 18204 2HG1 VAL B 576 -6.228 42.228 1.677 1.00 1.58
H
ATOM 18205 3HG1 VAL B 576 -7.906 41.982 1.137 1.00 1.58
H
ATOM 18206 1HG2 VAL B 576 -8.560 39.702 3.063 1.00 1.58
H
ATOM 18207 2HG2 VAL B 576 -8.954 39.733 1.331 1.00 1.58
H
ATOM 18208 3HG2 VAL B 576 -8.034 38.373 2.009 1.00 1.58
H
ATOM 18209 N CYS B 577 -4.072 40.562 0.560 1.00 1.24
N
ATOM 18210 CA CYS B 577 -2.927 41.417 0.277 1.00 1.84
C
ATOM 18211 C CYS B 577 -2.647 41.477 -1.208 1.00 2.04
C
ATOM 18212 O CYS B 577 -2.458 42.558 -1.759 1.00 3.33
O
ATOM 18213 CB CYS B 577 -1.667 40.911 0.989 1.00 2.76
C
ATOM 18214 SG CYS B 577 -1.672 41.115 2.783 1.00 2.76
S
ATOM 18215 H CYS B 577 -3.955 39.797 1.212 1.00 1.49
H
ATOM 18216 HA CYS B 577 -3.149 42.424 0.632 1.00 2.21
H
ATOM 18217 1HB CYS B 577 -1.535 39.850 0.775 1.00 3.31
H
ATOM 18218 2HB CYS B 577 -0.794 41.432 0.594 1.00 3.31
H
ATOM 18219 HG CYS B 577 -1.677 42.444 2.768 1.00 3.31
H
ATOM 18220 N ALA B 578 -2.634 40.319 -1.855 1.00 1.58
N
ATOM 18221 CA ALA B 578 -2.396 40.254 -3.284 1.00 1.96
C

```
ATOM  18222  C   ALA B 578      -3.489  40.954  -4.068  1.00  2.05
C
ATOM  18223  O   ALA B 578      -3.207  41.668  -5.032  1.00  3.13
O
ATOM  18224  CB  ALA B 578      -2.300  38.821  -3.715  1.00  2.94
C
ATOM  18225  H   ALA B 578      -2.782  39.457  -1.344  1.00  1.90
H
ATOM  18226  HA  ALA B 578      -1.454  40.758  -3.495  1.00  2.35
H
ATOM  18227 1HB  ALA B 578      -2.092  38.772  -4.784  1.00  3.53
H
ATOM  18228 2HB  ALA B 578      -1.494  38.335  -3.166  1.00  3.53
H
ATOM  18229 3HB  ALA B 578      -3.238  38.312  -3.504  1.00  3.53
H
ATOM  18230  N   ALA B 579      -4.739  40.749  -3.654  1.00  1.47
N
ATOM  18231  CA  ALA B 579      -5.882  41.351  -4.322  1.00  1.47
C
ATOM  18232  C   ALA B 579      -5.848  42.874  -4.295  1.00  1.51
C
ATOM  18233  O   ALA B 579      -6.188  43.514  -5.294  1.00  1.68
O
ATOM  18234  CB  ALA B 579      -7.172  40.863  -3.677  1.00  2.21
C
ATOM  18235  H   ALA B 579      -4.918  40.137  -2.867  1.00  1.76
H
ATOM  18236  HA  ALA B 579      -5.863  41.033  -5.364  1.00  1.76
H
ATOM  18237 1HB  ALA B 579      -8.026  41.285  -4.207  1.00  2.65
H
ATOM  18238 2HB  ALA B 579      -7.214  39.775  -3.730  1.00  2.65
H
ATOM  18239 3HB  ALA B 579      -7.200  41.175  -2.635  1.00  2.65
H
ATOM  18240  N   VAL B 580      -5.444  43.465  -3.168  1.00  1.46
N
ATOM  18241  CA  VAL B 580      -5.469  44.920  -3.075  1.00  1.69
C
ATOM  18242  C   VAL B 580      -4.112  45.591  -3.254  1.00  2.43
C
ATOM  18243  O   VAL B 580      -4.058  46.816  -3.348  1.00  5.83
O
ATOM  18244  CB  VAL B 580      -6.022  45.372  -1.714  1.00  2.54
C
ATOM  18245  CG1 VAL B 580      -7.411  44.794  -1.491  1.00  2.54
C
ATOM  18246  CG2 VAL B 580      -5.058  44.990  -0.614  1.00  2.54
C
ATOM  18247  H   VAL B 580      -5.173  42.905  -2.369  1.00  1.75
H
ATOM  18248  HA  VAL B 580      -6.137  45.293  -3.853  1.00  2.03
H
ATOM  18249  HB  VAL B 580      -6.131  46.456  -1.728  1.00  3.04
H
ATOM  18250 1HG1 VAL B 580      -7.804  45.147  -0.537  1.00  3.04
H
```

```
ATOM  18251 2HG1 VAL B 580      -8.068  45.109  -2.296  1.00  3.04
H
ATOM  18252 3HG1 VAL B 580      -7.350  43.705  -1.476  1.00  3.04
H
ATOM  18253 1HG2 VAL B 580      -5.454  45.341   0.336  1.00  3.04
H
ATOM  18254 2HG2 VAL B 580      -4.937  43.914  -0.583  1.00  3.04
H
ATOM  18255 3HG2 VAL B 580      -4.090  45.455  -0.797  1.00  3.04
H
ATOM  18256  N   SER B 581      -3.020  44.823  -3.281  1.00  1.97
N
ATOM  18257  CA  SER B 581      -1.711  45.445  -3.438  1.00  2.55
C
ATOM  18258  C   SER B 581      -1.630  46.194  -4.748  1.00  2.91
C
ATOM  18259  O   SER B 581      -1.801  45.595  -5.805  1.00 31.80
O
ATOM  18260  CB  SER B 581      -0.600  44.417  -3.385  1.00  3.82
C
ATOM  18261  OG  SER B 581       0.641  45.016  -3.634  1.00  3.82
O
ATOM  18262  H   SER B 581      -3.080  43.820  -3.176  1.00  2.36
H
ATOM  18263  HA  SER B 581      -1.566  46.159  -2.626  1.00  3.06
H
ATOM  18264 1HB  SER B 581      -0.584  43.950  -2.401  1.00  4.59
H
ATOM  18265 2HB  SER B 581      -0.788  43.635  -4.120  1.00  4.59
H
ATOM  18266  HG  SER B 581       0.776  45.646  -2.923  1.00  4.59
H
ATOM  18267  N   ARG B 582      -1.415  47.503  -4.634  1.00 11.09
N
ATOM  18268  CA  ARG B 582      -1.264  48.512  -5.696  1.00 11.60
C
ATOM  18269  C   ARG B 582      -2.304  49.602  -5.508  1.00  4.92
C
ATOM  18270  O   ARG B 582      -2.191  50.684  -6.086  1.00  7.03
O
ATOM  18271  CB  ARG B 582      -1.334  47.954  -7.121  1.00 17.40
C
ATOM  18272  CG  ARG B 582      -0.087  47.190  -7.560  1.00 17.40
C
ATOM  18273  CD  ARG B 582      -0.277  46.475  -8.833  1.00 17.40
C
ATOM  18274  NE  ARG B 582      -1.235  45.408  -8.683  1.00 17.40
N
ATOM  18275  CZ  ARG B 582      -1.580  44.528  -9.645  1.00 17.40
C
ATOM  18276  NH1 ARG B 582      -1.011  44.573 -10.834  1.00 17.40
N
ATOM  18277  NH2 ARG B 582      -2.496  43.609  -9.392  1.00 17.40
N
ATOM  18278  H   ARG B 582      -1.314  47.848  -3.687  1.00 13.31
H
ATOM  18279  HA  ARG B 582      -0.283  48.967  -5.591  1.00 13.92
H
```

```
ATOM  18280 1HB  ARG B 582      -2.209  47.321  -7.247  1.00 20.88
H
ATOM  18281 2HB  ARG B 582      -1.446  48.787  -7.814  1.00 20.88
H
ATOM  18282 1HG  ARG B 582       0.720  47.896  -7.714  1.00 20.88
H
ATOM  18283 2HG  ARG B 582       0.212  46.466  -6.804  1.00 20.88
H
ATOM  18284 1HD  ARG B 582      -0.641  47.161  -9.598  1.00 20.88
H
ATOM  18285 2HD  ARG B 582       0.669  46.040  -9.146  1.00 20.88
H
ATOM  18286  HE  ARG B 582      -1.683  45.335  -7.774  1.00 20.88
H
ATOM  18287 1HH1 ARG B 582      -0.288  45.254 -11.041  1.00 20.88
H
ATOM  18288 2HH1 ARG B 582      -1.279  43.906 -11.542  1.00 20.88
H
ATOM  18289 1HH2 ARG B 582      -2.933  43.572  -8.479  1.00 20.88
H
ATOM  18290 2HH2 ARG B 582      -2.758  42.946 -10.104  1.00 20.88
H
ATOM  18291  N   GLU B 583      -3.314  49.325  -4.680  1.00  1.92
N
ATOM  18292  CA  GLU B 583      -4.349  50.303  -4.392  1.00  3.73
C
ATOM  18293  C   GLU B 583      -3.849  51.333  -3.366  1.00  8.37
C
ATOM  18294  O   GLU B 583      -3.589  50.975  -2.220  1.00 13.02
O
ATOM  18295  CB  GLU B 583      -5.611  49.608  -3.863  1.00  5.59
C
ATOM  18296  CG  GLU B 583      -6.351  48.752  -4.877  1.00  5.59
C
ATOM  18297  CD  GLU B 583      -7.055  49.574  -5.925  1.00  5.59
C
ATOM  18298  OE1 GLU B 583      -7.493  50.658  -5.612  1.00  5.59
O
ATOM  18299  OE2 GLU B 583      -7.155  49.117  -7.038  1.00  5.59
O
ATOM  18300  H   GLU B 583      -3.380  48.419  -4.237  1.00  2.30
H
ATOM  18301  HA  GLU B 583      -4.594  50.814  -5.322  1.00  4.48
H
ATOM  18302 1HB  GLU B 583      -5.348  48.973  -3.016  1.00  6.71
H
ATOM  18303 2HB  GLU B 583      -6.311  50.359  -3.500  1.00  6.71
H
ATOM  18304 1HG  GLU B 583      -5.641  48.088  -5.367  1.00  6.71
H
ATOM  18305 2HG  GLU B 583      -7.081  48.137  -4.354  1.00  6.71
H
ATOM  18306  N   PRO B 584      -3.768  52.599  -3.796  1.00 13.55
N
ATOM  18307  CA  PRO B 584      -3.230  53.781  -3.063  1.00 25.25
C
ATOM  18308  C   PRO B 584      -3.208  53.708  -1.519  1.00 22.38
C
```

```
ATOM  18309  O   PRO B 584      -2.196  53.353  -0.899  1.00 77.80
O
ATOM  18310  CB  PRO B 584      -4.123  54.943  -3.535  1.00 37.88
C
ATOM  18311  CG  PRO B 584      -5.399  54.321  -3.922  1.00 37.88
C
ATOM  18312  CD  PRO B 584      -4.996  53.017  -4.519  1.00 37.88
C
ATOM  18313  HA  PRO B 584      -2.204  53.955  -3.421  1.00 30.30
H
ATOM  18314 1HB  PRO B 584      -4.228  55.700  -2.748  1.00 45.45
H
ATOM  18315 2HB  PRO B 584      -3.642  55.446  -4.387  1.00 45.45
H
ATOM  18316 1HG  PRO B 584      -6.063  54.205  -3.054  1.00 45.45
H
ATOM  18317 2HG  PRO B 584      -5.932  54.963  -4.640  1.00 45.45
H
ATOM  18318 1HD  PRO B 584      -5.804  52.293  -4.369  1.00 45.45
H
ATOM  18319 2HD  PRO B 584      -4.767  53.147  -5.587  1.00 45.45
H
ATOM  18320  N   ASP B 585      -4.264  54.164  -0.890  1.00  4.05
N
ATOM  18321  CA  ASP B 585      -4.304  54.236   0.553  1.00  4.32
C
ATOM  18322  C   ASP B 585      -4.770  52.956   1.233  1.00  2.30
C
ATOM  18323  O   ASP B 585      -5.232  53.018   2.369  1.00  3.84
O
ATOM  18324  CB  ASP B 585      -5.209  55.387   0.986  1.00  6.48
C
ATOM  18325  CG  ASP B 585      -4.647  56.752   0.613  1.00  6.48
C
ATOM  18326  OD1 ASP B 585      -3.451  56.869   0.493  1.00  6.48
O
ATOM  18327  OD2 ASP B 585      -5.420  57.666   0.455  1.00  6.48
O
ATOM  18328  H   ASP B 585      -5.078  54.456  -1.405  1.00  4.86
H
ATOM  18329  HA  ASP B 585      -3.295  54.450   0.905  1.00  5.18
H
ATOM  18330 1HB  ASP B 585      -6.190  55.275   0.521  1.00  7.78
H
ATOM  18331 2HB  ASP B 585      -5.354  55.350   2.067  1.00  7.78
H
ATOM  18332  N   ILE B 586      -4.676  51.804   0.563  1.00  1.77
N
ATOM  18333  CA  ILE B 586      -5.139  50.569   1.169  1.00  1.54
C
ATOM  18334  C   ILE B 586      -4.012  49.849   1.914  1.00  1.31
C
ATOM  18335  O   ILE B 586      -3.007  49.457   1.320  1.00  2.09
O
ATOM  18336  CB  ILE B 586      -5.734  49.627   0.108  1.00  2.31
C
ATOM  18337  CG1 ILE B 586      -6.822  50.357  -0.691  1.00  2.31
C
```

ATOM 18338 CG2 ILE B 586   -6.310 48.389   0.784 1.00 2.31
C
ATOM 18339 CD1 ILE B 586   -7.949 50.896   0.144 1.00 2.31
C
ATOM 18340 H   ILE B 586   -4.280 51.759 -0.368 1.00 2.12
H
ATOM 18341 HA ILE B 586   -5.922 50.808   1.884 1.00 1.85
H
ATOM 18342 HB ILE B 586   -4.955 49.328 -0.593 1.00 2.77
H
ATOM 18343 1HG1 ILE B 586   -6.366 51.186 -1.232 1.00 2.77
H
ATOM 18344 2HG1 ILE B 586   -7.245 49.665 -1.419 1.00 2.77
H
ATOM 18345 1HG2 ILE B 586   -6.727 47.723   0.030 1.00 2.77
H
ATOM 18346 2HG2 ILE B 586   -5.522 47.873   1.329 1.00 2.77
H
ATOM 18347 3HG2 ILE B 586   -7.095 48.687   1.478 1.00 2.77
H
ATOM 18348 1HD1 ILE B 586   -8.674 51.391 -0.500 1.00 2.77
H
ATOM 18349 2HD1 ILE B 586   -8.427 50.076   0.670 1.00 2.77
H
ATOM 18350 3HD1 ILE B 586   -7.554 51.611   0.863 1.00 2.77
H
ATOM 18351 N   LEU B 587   -4.197 49.682   3.221 1.00 1.72
N
ATOM 18352 CA LEU B 587   -3.241 49.002   4.091 1.00 2.14
C
ATOM 18353 C   LEU B 587   -3.857 47.723   4.654 1.00 1.80
C
ATOM 18354 O   LEU B 587   -5.003 47.740   5.101 1.00 2.81
O
ATOM 18355 CB LEU B 587   -2.844 49.917   5.261 1.00 3.21
C
ATOM 18356 CG LEU B 587   -1.874 51.066   4.950 1.00 3.21
C
ATOM 18357 CD1 LEU B 587   -2.648 52.262   4.417 1.00 3.21
C
ATOM 18358 CD2 LEU B 587   -1.111 51.425   6.215 1.00 3.21
C
ATOM 18359 H   LEU B 587   -5.046 50.041   3.624 1.00 2.06
H
ATOM 18360 HA LEU B 587   -2.356 48.742   3.513 1.00 2.57
H
ATOM 18361 1HB LEU B 587   -3.754 50.369   5.652 1.00 3.85
H
ATOM 18362 2HB LEU B 587   -2.410 49.315   6.051 1.00 3.85
H
ATOM 18363 HG LEU B 587   -1.173 50.756   4.178 1.00 3.85
H
ATOM 18364 1HD1 LEU B 587   -1.955 53.072   4.192 1.00 3.85
H
ATOM 18365 2HD1 LEU B 587   -3.177 51.981   3.510 1.00 3.85
H
ATOM 18366 3HD1 LEU B 587   -3.366 52.597   5.165 1.00 3.85
H

ATOM 18367 1HD2 LEU B 587   -0.414 52.235   6.002 1.00 3.85
H
ATOM 18368 2HD2 LEU B 587   -1.813 51.742   6.987 1.00 3.85
H
ATOM 18369 3HD2 LEU B 587   -0.557 50.554   6.565 1.00 3.85
H
ATOM 18370 N   VAL B 588   -3.100 46.619   4.656 1.00 1.49
N
ATOM 18371 CA VAL B 588   -3.631 45.350   5.165 1.00 1.30
C
ATOM 18372 C   VAL B 588   -2.781 44.725   6.278 1.00 1.39
C
ATOM 18373 O   VAL B 588   -1.597 44.440   6.090 1.00 2.04
O
ATOM 18374 CB VAL B 588   -3.768 44.312   4.030 1.00 1.95
C
ATOM 18375 CG1 VAL B 588   -4.301 42.997   4.588 1.00 1.95
C
ATOM 18376 CG2 VAL B 588   -4.692 44.830   2.947 1.00 1.95
C
ATOM 18377 H   VAL B 588   -2.159 46.654   4.288 1.00 1.79
H
ATOM 18378 HA VAL B 588   -4.625 45.541   5.559 1.00 1.56
H
ATOM 18379 HB VAL B 588   -2.783 44.117   3.608 1.00 2.34
H
ATOM 18380 1HG1 VAL B 588   -4.377 42.265   3.784 1.00 2.34
H
ATOM 18381 2HG1 VAL B 588   -3.622 42.623   5.354 1.00 2.34
H
ATOM 18382 3HG1 VAL B 588   -5.287 43.159   5.024 1.00 2.34
H
ATOM 18383 1HG2 VAL B 588   -4.766 44.085   2.157 1.00 2.34
H
ATOM 18384 2HG2 VAL B 588   -5.679 45.011   3.363 1.00 2.34
H
ATOM 18385 3HG2 VAL B 588   -4.293 45.756   2.537 1.00 2.34
H
ATOM 18386 N   HIS B 589   -3.415 44.490   7.423 1.00 1.07
N
ATOM 18387 CA HIS B 589   -2.805 43.842   8.580 1.00 1.02
C
ATOM 18388 C   HIS B 589   -3.187 42.361   8.581 1.00 1.06
C
ATOM 18389 O   HIS B 589   -4.213 41.991   8.008 1.00 1.53
O
ATOM 18390 CB HIS B 589   -3.287 44.492   9.879 1.00 1.53
C
ATOM 18391 CG HIS B 589   -2.929 45.940 10.029 1.00 1.53
C
ATOM 18392 ND1 HIS B 589   -1.664 46.371 10.379 1.00 1.53
N
ATOM 18393 CD2 HIS B 589   -3.679 47.057   9.875 1.00 1.53
C
ATOM 18394 CE1 HIS B 589   -1.658 47.694 10.446 1.00 1.53
C
ATOM 18395 NE2 HIS B 589   -2.862 48.131 10.141 1.00 1.53
N

ATOM 18396 H HIS B 589 -4.380 44.769 7.495 1.00 1.28
H
ATOM 18397 HA HIS B 589 -1.720 43.918 8.526 1.00 1.22
H
ATOM 18398 1HB HIS B 589 -4.369 44.403 9.936 1.00 1.84
H
ATOM 18399 2HB HIS B 589 -2.872 43.951 10.730 1.00 1.84
H
ATOM 18400 HD2 HIS B 589 -4.733 47.096 9.597 1.00 1.84
H
ATOM 18401 HE1 HIS B 589 -0.803 48.317 10.705 1.00 1.84
H
ATOM 18402 HE2 HIS B 589 -3.151 49.099 10.110 1.00 1.84
H
ATOM 18403 N GLN B 590 -2.379 41.514 9.224 1.00 1.08
N
ATOM 18404 CA GLN B 590 -2.683 40.078 9.251 1.00 1.07
C
ATOM 18405 C GLN B 590 -2.557 39.430 10.630 1.00 0.94
C
ATOM 18406 O GLN B 590 -1.504 39.492 11.271 1.00 1.11
O
ATOM 18407 CB GLN B 590 -1.764 39.305 8.294 1.00 1.60
C
ATOM 18408 CG GLN B 590 -2.067 37.808 8.237 1.00 1.60
C
ATOM 18409 CD GLN B 590 -1.108 37.026 7.352 1.00 1.60
C
ATOM 18410 OE1 GLN B 590 0.063 37.388 7.217 1.00 1.60
O
ATOM 18411 NE2 GLN B 590 -1.597 35.940 6.759 1.00 1.60
N
ATOM 18412 H GLN B 590 -1.550 41.862 9.686 1.00 1.30
H
ATOM 18413 HA GLN B 590 -3.710 39.946 8.919 1.00 1.28
H
ATOM 18414 1HB GLN B 590 -1.863 39.709 7.286 1.00 1.93
H
ATOM 18415 2HB GLN B 590 -0.726 39.434 8.598 1.00 1.93
H
ATOM 18416 1HG GLN B 590 -2.002 37.400 9.246 1.00 1.93
H
ATOM 18417 2HG GLN B 590 -3.075 37.672 7.848 1.00 1.93
H
ATOM 18418 1HE2 GLN B 590 -1.007 35.365 6.183 1.00 1.93
H
ATOM 18419 2HE2 GLN B 590 -2.551 35.679 6.906 1.00 1.93
H
ATOM 18420 N LEU B 591 -3.631 38.763 11.045 1.00 0.77
N
ATOM 18421 CA LEU B 591 -3.691 37.989 12.280 1.00 0.71
C
ATOM 18422 C LEU B 591 -3.875 36.512 11.936 1.00 0.70
C
ATOM 18423 O LEU B 591 -4.978 36.084 11.606 1.00 1.26
O
ATOM 18424 CB LEU B 591 -4.857 38.447 13.169 1.00 1.06
C

```
ATOM  18425  CG  LEU B 591      -4.865  39.911  13.618  1.00  1.06
C
ATOM  18426  CD1 LEU B 591      -6.158  40.188  14.374  1.00  1.06
C
ATOM  18427  CD2 LEU B 591      -3.658  40.176  14.503  1.00  1.06
C
ATOM  18428  H   LEU B 591      -4.459  38.784  10.467  1.00  0.92
H
ATOM  18429  HA  LEU B 591      -2.754  38.111  12.823  1.00  0.85
H
ATOM  18430 1HB  LEU B 591      -5.781  38.276  12.625  1.00  1.28
H
ATOM  18431 2HB  LEU B 591      -4.871  37.828  14.065  1.00  1.28
H
ATOM  18432  HG  LEU B 591      -4.831  40.566  12.747  1.00  1.28
H
ATOM  18433 1HD1 LEU B 591      -6.177  41.229  14.693  1.00  1.28
H
ATOM  18434 2HD1 LEU B 591      -7.010  39.995  13.723  1.00  1.28
H
ATOM  18435 3HD1 LEU B 591      -6.215  39.540  15.247  1.00  1.28
H
ATOM  18436 1HD2 LEU B 591      -3.664  41.217  14.825  1.00  1.28
H
ATOM  18437 2HD2 LEU B 591      -3.698  39.525  15.376  1.00  1.28
H
ATOM  18438 3HD2 LEU B 591      -2.746  39.975  13.941  1.00  1.28
H
ATOM  18439  N   ALA B 592      -2.806  35.734  11.996  1.00  0.47
N
ATOM  18440  CA  ALA B 592      -2.913  34.319  11.653  1.00  0.42
C
ATOM  18441  C   ALA B 592      -1.909  33.503  12.466  1.00  0.39
C
ATOM  18442  O   ALA B 592      -1.131  34.061  13.242  1.00  0.47
O
ATOM  18443  CB  ALA B 592      -2.733  34.136  10.157  1.00  0.63
C
ATOM  18444  H   ALA B 592      -1.916  36.118  12.281  1.00  0.56
H
ATOM  18445  HA  ALA B 592      -3.910  33.975  11.922  1.00  0.50
H
ATOM  18446 1HB  ALA B 592      -2.841  33.089   9.895  1.00  0.76
H
ATOM  18447 2HB  ALA B 592      -3.493  34.716   9.633  1.00  0.76
H
ATOM  18448 3HB  ALA B 592      -1.760  34.482   9.858  1.00  0.76
H
ATOM  18449  N   VAL B 593      -1.974  32.176  12.339  1.00  0.35
N
ATOM  18450  CA  VAL B 593      -1.162  31.293  13.171  1.00  0.41
C
ATOM  18451  C   VAL B 593       0.308  31.310  12.726  1.00  0.46
C
ATOM  18452  O   VAL B 593       0.631  31.176  11.541  1.00  0.51
O
ATOM  18453  CB  VAL B 593      -1.739  29.858  13.141  1.00  0.61
C
```

ATOM 18454 CG1 VAL B 593   -0.868 28.923 13.963 1.00 0.61
C
ATOM 18455 CG2 VAL B 593   -3.169 29.868 13.669 1.00 0.61
C
ATOM 18456 H   VAL B 593   -2.594 31.766 11.653 1.00 0.42
H
ATOM 18457 HA VAL B 593   -1.213 31.653 14.200 1.00 0.49
H
ATOM 18458 HB VAL B 593   -1.734 29.495 12.114 1.00 0.74
H
ATOM 18459 1HG1 VAL B 593   -1.275 27.913 13.921 1.00 0.74
H
ATOM 18460 2HG1 VAL B 593   0.144 28.921 13.562 1.00 0.74
H
ATOM 18461 3HG1 VAL B 593   -0.847 29.261 14.999 1.00 0.74
H
ATOM 18462 1HG2 VAL B 593   -3.579 28.859 13.633 1.00 0.74
H
ATOM 18463 2HG2 VAL B 593   -3.176 30.225 14.699 1.00 0.74
H
ATOM 18464 3HG2 VAL B 593   -3.779 30.528 13.052 1.00 0.74
H
ATOM 18465 N   SER B 594   1.194 31.540 13.695 1.00 0.55
N
ATOM 18466 CA SER B 594   2.632 31.676 13.449 1.00 0.73
C
ATOM 18467 C   SER B 594   3.457 30.381 13.475 1.00 0.96
C
ATOM 18468 O   SER B 594   4.601 30.379 13.018 1.00 2.87
O
ATOM 18469 CB SER B 594   3.219 32.630 14.466 1.00 1.09
C
ATOM 18470 OG SER B 594   3.162 32.083 15.755 1.00 1.09
O
ATOM 18471 H   SER B 594   0.856 31.636 14.643 1.00 0.66
H
ATOM 18472 HA SER B 594   2.751 32.119 12.459 1.00 0.88
H
ATOM 18473 1HB SER B 594   4.255 32.849 14.206 1.00 1.31
H
ATOM 18474 2HB SER B 594   2.671 33.570 14.444 1.00 1.31
H
ATOM 18475 HG SER B 594   3.673 31.269 15.718 1.00 1.31
H
ATOM 18476 N   GLY B 595   2.912 29.291 14.007 1.00 0.79
N
ATOM 18477 CA GLY B 595   3.685 28.046 14.080 1.00 0.83
C
ATOM 18478 C   GLY B 595   2.855 26.882 14.603 1.00 1.17
C
ATOM 18479 O   GLY B 595   1.647 27.013 14.772 1.00 5.02
O
ATOM 18480 H   GLY B 595   1.971 29.320 14.369 1.00 0.95
H
ATOM 18481 1HA GLY B 595   4.068 27.803 13.089 1.00 1.00
H
ATOM 18482 2HA GLY B 595   4.549 28.197 14.725 1.00 1.00
H

ATOM 18483 N VAL B 596 3.510 25.745 14.868 1.00 0.79
N
ATOM 18484 CA VAL B 596 2.808 24.534 15.310 1.00 0.64
C
ATOM 18485 C VAL B 596 2.049 24.750 16.622 1.00 0.79
C
ATOM 18486 O VAL B 596 2.647 25.167 17.615 1.00 1.41
O
ATOM 18487 CB VAL B 596 3.811 23.368 15.515 1.00 0.96
C
ATOM 18488 CG1 VAL B 596 3.109 22.164 16.119 1.00 0.96
C
ATOM 18489 CG2 VAL B 596 4.435 22.978 14.189 1.00 0.96
C
ATOM 18490 H VAL B 596 4.510 25.711 14.734 1.00 0.95
H
ATOM 18491 HA VAL B 596 2.124 24.252 14.517 1.00 0.77
H
ATOM 18492 HB VAL B 596 4.588 23.684 16.212 1.00 1.15
H
ATOM 18493 1HG1 VAL B 596 3.830 21.360 16.269 1.00 1.15
H
ATOM 18494 2HG1 VAL B 596 2.670 22.439 17.078 1.00 1.15
H
ATOM 18495 3HG1 VAL B 596 2.328 21.825 15.447 1.00 1.15
H
ATOM 18496 1HG2 VAL B 596 5.144 22.165 14.344 1.00 1.15
H
ATOM 18497 2HG2 VAL B 596 3.656 22.655 13.497 1.00 1.15
H
ATOM 18498 3HG2 VAL B 596 4.957 23.839 13.777 1.00 1.15
H
ATOM 18499 N PRO B 597 0.736 24.471 16.645 1.00 0.79
N
ATOM 18500 CA PRO B 597 -0.153 24.585 17.786 1.00 0.93
C
ATOM 18501 C PRO B 597 -0.092 23.399 18.730 1.00 0.88
C
ATOM 18502 O PRO B 597 0.387 22.320 18.383 1.00 0.82
O
ATOM 18503 CB PRO B 597 -1.531 24.745 17.150 1.00 1.40
C
ATOM 18504 CG PRO B 597 -1.430 24.028 15.874 1.00 1.40
C
ATOM 18505 CD PRO B 597 -0.037 24.340 15.394 1.00 1.40
C
ATOM 18506 HA PRO B 597 0.131 25.490 18.325 1.00 1.12
H
ATOM 18507 1HB PRO B 597 -2.304 24.332 17.813 1.00 1.67
H
ATOM 18508 2HB PRO B 597 -1.757 25.812 17.015 1.00 1.67
H
ATOM 18509 1HG PRO B 597 -1.613 22.961 16.033 1.00 1.67
H
ATOM 18510 2HG PRO B 597 -2.212 24.375 15.195 1.00 1.67
H
ATOM 18511 1HD PRO B 597 0.342 23.509 14.792 1.00 1.67
H

```
ATOM  18512 2HD  PRO B 597      -0.027  25.286  14.835  1.00  1.67
H
ATOM  18513  N   GLN B 598      -0.587  23.631  19.938  1.00  1.19
N
ATOM  18514  CA  GLN B 598      -0.600  22.650  21.012  1.00  1.18
C
ATOM  18515  C   GLN B 598      -2.029  22.304  21.400  1.00  1.28
C
ATOM  18516  O   GLN B 598      -2.972  22.935  20.924  1.00  1.85
O
ATOM  18517  CB  GLN B 598       0.138  23.214  22.231  1.00  1.77
C
ATOM  18518  CG  GLN B 598       1.595  23.561  21.979  1.00  1.77
C
ATOM  18519  CD  GLN B 598       2.255  24.192  23.192  1.00  1.77
C
ATOM  18520  OE1 GLN B 598       2.354  23.580  24.261  1.00  1.77
O
ATOM  18521  NE2 GLN B 598       2.719  25.426  23.033  1.00  1.77
N
ATOM  18522  H   GLN B 598      -0.968  24.546  20.129  1.00  1.43
H
ATOM  18523  HA  GLN B 598      -0.102  21.742  20.670  1.00  1.42
H
ATOM  18524 1HB  GLN B 598      -0.368  24.115  22.576  1.00  2.12
H
ATOM  18525 2HB  GLN B 598       0.104  22.488  23.043  1.00  2.12
H
ATOM  18526 1HG  GLN B 598       2.137  22.648  21.728  1.00  2.12
H
ATOM  18527 2HG  GLN B 598       1.654  24.268  21.152  1.00  2.12
H
ATOM  18528 1HE2 GLN B 598       3.167  25.894  23.795  1.00  2.12
H
ATOM  18529 2HE2 GLN B 598       2.623  25.888  22.151  1.00  2.12
H
ATOM  18530  N   ARG B 599      -2.187  21.316  22.278  1.00  1.66
N
ATOM  18531  CA  ARG B 599      -3.524  20.964  22.763  1.00  2.25
C
ATOM  18532  C   ARG B 599      -4.015  21.987  23.790  1.00  2.51
C
ATOM  18533  O   ARG B 599      -3.223  22.554  24.542  1.00  5.24
O
ATOM  18534  CB  ARG B 599      -3.533  19.581  23.398  1.00  3.38
C
ATOM  18535  CG  ARG B 599      -3.601  18.455  22.396  1.00  3.38
C
ATOM  18536  CD  ARG B 599      -2.328  18.369  21.673  1.00  3.38
C
ATOM  18537  NE  ARG B 599      -2.244  17.264  20.730  1.00  3.38
N
ATOM  18538  CZ  ARG B 599      -1.197  17.064  19.916  1.00  3.38
C
ATOM  18539  NH1 ARG B 599      -0.159  17.864  20.011  1.00  3.38
N
ATOM  18540  NH2 ARG B 599      -1.217  16.094  19.015  1.00  3.38
N
```

ATOM 18541 H ARG B 599 -1.370 20.793 22.600 1.00 1.99
H
ATOM 18542 HA ARG B 599 -4.211 20.963 21.916 1.00 2.70
H
ATOM 18543 1HB ARG B 599 -2.629 19.446 23.991 1.00 4.05
H
ATOM 18544 2HB ARG B 599 -4.387 19.487 24.067 1.00 4.05
H
ATOM 18545 1HG ARG B 599 -3.806 17.514 22.898 1.00 4.05
H
ATOM 18546 2HG ARG B 599 -4.385 18.668 21.674 1.00 4.05
H
ATOM 18547 1HD ARG B 599 -2.168 19.294 21.123 1.00 4.05
H
ATOM 18548 2HD ARG B 599 -1.548 18.263 22.399 1.00 4.05
H
ATOM 18549 HE ARG B 599 -3.059 16.689 20.575 1.00 4.05
H
ATOM 18550 1HH1 ARG B 599 -0.141 18.603 20.709 1.00 4.05
H
ATOM 18551 2HH1 ARG B 599 0.633 17.738 19.401 1.00 4.05
H
ATOM 18552 1HH2 ARG B 599 -2.016 15.466 18.927 1.00 4.05
H
ATOM 18553 2HH2 ARG B 599 -0.422 15.962 18.409 1.00 4.05
H
ATOM 18554 N GLY B 600 -5.321 22.221 23.813 1.00 1.71
N
ATOM 18555 CA GLY B 600 -5.923 23.165 24.752 1.00 2.41
C
ATOM 18556 C GLY B 600 -7.396 23.349 24.426 1.00 1.91
C
ATOM 18557 O GLY B 600 -7.939 22.633 23.590 1.00 3.03
O
ATOM 18558 H GLY B 600 -5.918 21.726 23.165 1.00 2.05
H
ATOM 18559 1HA GLY B 600 -5.812 22.794 25.771 1.00 2.89
H
ATOM 18560 2HA GLY B 600 -5.407 24.123 24.695 1.00 2.89
H
ATOM 18561 N LYS B 601 -8.049 24.305 25.071 1.00 1.14
N
ATOM 18562 CA LYS B 601 -9.451 24.544 24.755 1.00 0.83
C
ATOM 18563 C LYS B 601 -9.532 25.508 23.589 1.00 0.68
C
ATOM 18564 O LYS B 601 -8.676 26.379 23.471 1.00 1.41
O
ATOM 18565 CB LYS B 601 -10.196 25.126 25.956 1.00 1.24
C
ATOM 18566 CG LYS B 601 -10.161 24.276 27.218 1.00 1.24
C
ATOM 18567 CD LYS B 601 -10.888 22.953 27.042 1.00 1.24
C
ATOM 18568 CE LYS B 601 -10.948 22.198 28.363 1.00 1.24
C
ATOM 18569 NZ LYS B 601 -11.665 20.905 28.240 1.00 1.24
N

```
ATOM  18570  H   LYS B 601      -7.582  24.871  25.775  1.00  1.37
H
ATOM  18571  HA  LYS B 601      -9.918  23.604  24.461  1.00  1.00
H
ATOM  18572 1HB  LYS B 601      -9.778  26.100  26.202  1.00  1.49
H
ATOM  18573 2HB  LYS B 601     -11.243  25.280  25.691  1.00  1.49
H
ATOM  18574 1HG  LYS B 601      -9.122  24.076  27.486  1.00  1.49
H
ATOM  18575 2HG  LYS B 601     -10.624  24.826  28.035  1.00  1.49
H
ATOM  18576 1HD  LYS B 601     -11.903  23.135  26.687  1.00  1.49
H
ATOM  18577 2HD  LYS B 601     -10.366  22.341  26.306  1.00  1.49
H
ATOM  18578 1HE  LYS B 601      -9.933  22.007  28.710  1.00  1.49
H
ATOM  18579 2HE  LYS B 601     -11.461  22.816  29.100  1.00  1.49
H
ATOM  18580 1HZ  LYS B 601     -11.682  20.442  29.137  1.00  1.49
H
ATOM  18581 2HZ  LYS B 601     -12.612  21.069  27.930  1.00  1.49
H
ATOM  18582 3HZ  LYS B 601     -11.198  20.307  27.571  1.00  1.49
H
ATOM  18583  N   THR B 602     -10.552  25.344  22.739  1.00  0.63
N
ATOM  18584  CA  THR B 602     -10.818  26.234  21.596  1.00  0.45
C
ATOM  18585  C   THR B 602     -10.378  27.673  21.835  1.00  0.49
C
ATOM  18586  O   THR B 602      -9.476  28.176  21.162  1.00  0.63
O
ATOM  18587  CB  THR B 602     -12.321  26.234  21.229  1.00  0.68
C
ATOM  18588  OG1 THR B 602     -12.714  24.923  20.810  1.00  0.68
O
ATOM  18589  CG2 THR B 602     -12.621  27.235  20.117  1.00  0.68
C
ATOM  18590  H   THR B 602     -11.193  24.579  22.903  1.00  0.76
H
ATOM  18591  HA  THR B 602     -10.261  25.856  20.738  1.00  0.54
H
ATOM  18592  HB  THR B 602     -12.901  26.504  22.111  1.00  0.81
H
ATOM  18593  HG1 THR B 602     -12.367  24.756  19.926  1.00  0.81
H
ATOM  18594 1HG2 THR B 602     -13.686  27.213  19.889  1.00  0.81
H
ATOM  18595 2HG2 THR B 602     -12.340  28.237  20.437  1.00  0.81
H
ATOM  18596 3HG2 THR B 602     -12.057  26.967  19.228  1.00  0.81
H
ATOM  18597  N   SER B 603     -11.048  28.325  22.789  1.00  0.48
N
ATOM  18598  CA  SER B 603     -10.800  29.719  23.139  1.00  0.54
C
```

ATOM 18599 C SER B 603 -9.376 29.973 23.601 1.00 0.58
C
ATOM 18600 O SER B 603 -8.788 30.994 23.244 1.00 0.62
O
ATOM 18601 CB SER B 603 -11.763 30.156 24.224 1.00 0.81
C
ATOM 18602 OG SER B 603 -13.084 30.169 23.755 1.00 0.81
O
ATOM 18603 H SER B 603 -11.771 27.830 23.291 1.00 0.58
H
ATOM 18604 HA SER B 603 -10.979 30.327 22.251 1.00 0.65
H
ATOM 18605 1HB SER B 603 -11.685 29.479 25.075 1.00 0.97
H
ATOM 18606 2HB SER B 603 -11.490 31.152 24.572 1.00 0.97
H
ATOM 18607 HG SER B 603 -13.107 30.823 23.050 1.00 0.97
H
ATOM 18608 N GLU B 604 -8.824 29.045 24.388 1.00 0.66
N
ATOM 18609 CA GLU B 604 -7.463 29.185 24.886 1.00 0.75
C
ATOM 18610 C GLU B 604 -6.476 29.238 23.746 1.00 0.73
C
ATOM 18611 O GLU B 604 -5.554 30.050 23.761 1.00 0.86
O
ATOM 18612 CB GLU B 604 -7.080 28.015 25.805 1.00 1.12
C
ATOM 18613 CG GLU B 604 -7.776 27.986 27.152 1.00 1.12
C
ATOM 18614 CD GLU B 604 -7.424 26.761 27.953 1.00 1.12
C
ATOM 18615 OE1 GLU B 604 -7.016 25.782 27.369 1.00 1.12
O
ATOM 18616 OE2 GLU B 604 -7.562 26.806 29.153 1.00 1.12
O
ATOM 18617 H GLU B 604 -9.358 28.228 24.644 1.00 0.79
H
ATOM 18618 HA GLU B 604 -7.390 30.117 25.447 1.00 0.90
H
ATOM 18619 1HB GLU B 604 -7.288 27.072 25.304 1.00 1.35
H
ATOM 18620 2HB GLU B 604 -6.008 28.048 25.995 1.00 1.35
H
ATOM 18621 1HG GLU B 604 -7.489 28.872 27.717 1.00 1.35
H
ATOM 18622 2HG GLU B 604 -8.854 28.020 26.997 1.00 1.35
H
ATOM 18623 N LEU B 605 -6.683 28.379 22.753 1.00 0.65
N
ATOM 18624 CA LEU B 605 -5.786 28.306 21.622 1.00 0.73
C
ATOM 18625 C LEU B 605 -5.956 29.514 20.715 1.00 0.72
C
ATOM 18626 O LEU B 605 -4.973 30.054 20.214 1.00 0.83
O
ATOM 18627 CB LEU B 605 -6.053 27.022 20.847 1.00 1.09
C

```
ATOM  18628  CG  LEU B 605      -5.751  25.733  21.619  1.00  1.09
C
ATOM  18629  CD1 LEU B 605      -6.156  24.552  20.771  1.00  1.09
C
ATOM  18630  CD2 LEU B 605      -4.283  25.668  21.994  1.00  1.09
C
ATOM  18631  H   LEU B 605      -7.466  27.741  22.810  1.00  0.78
H
ATOM  18632  HA  LEU B 605      -4.763  28.289  21.991  1.00  0.88
H
ATOM  18633 1HB  LEU B 605      -7.103  26.997  20.557  1.00  1.31
H
ATOM  18634 2HB  LEU B 605      -5.445  27.021  19.943  1.00  1.31
H
ATOM  18635  HG  LEU B 605      -6.351  25.713  22.530  1.00  1.31
H
ATOM  18636 1HD1 LEU B 605      -5.970  23.627  21.315  1.00  1.31
H
ATOM  18637 2HD1 LEU B 605      -7.214  24.631  20.532  1.00  1.31
H
ATOM  18638 3HD1 LEU B 605      -5.573  24.560  19.853  1.00  1.31
H
ATOM  18639 1HD2 LEU B 605      -4.091  24.752  22.553  1.00  1.31
H
ATOM  18640 2HD2 LEU B 605      -3.674  25.674  21.095  1.00  1.31
H
ATOM  18641 3HD2 LEU B 605      -4.034  26.528  22.610  1.00  1.31
H
ATOM  18642  N   LEU B 606      -7.192  29.985  20.553  1.00  0.65
N
ATOM  18643  CA  LEU B 606      -7.421  31.168  19.737  1.00  0.69
C
ATOM  18644  C   LEU B 606      -6.675  32.359  20.354  1.00  0.81
C
ATOM  18645  O   LEU B 606      -6.079  33.171  19.639  1.00  1.07
O
ATOM  18646  CB  LEU B 606      -8.927  31.456  19.633  1.00  1.03
C
ATOM  18647  CG  LEU B 606      -9.769  30.450  18.830  1.00  1.03
C
ATOM  18648  CD1 LEU B 606     -11.242  30.774  19.016  1.00  1.03
C
ATOM  18649  CD2 LEU B 606      -9.395  30.520  17.361  1.00  1.03
C
ATOM  18650  H   LEU B 606      -7.981  29.506  20.971  1.00  0.78
H
ATOM  18651  HA  LEU B 606      -7.026  30.988  18.738  1.00  0.83
H
ATOM  18652 1HB  LEU B 606      -9.343  31.511  20.639  1.00  1.24
H
ATOM  18653 2HB  LEU B 606      -9.057  32.424  19.155  1.00  1.24
H
ATOM  18654  HG  LEU B 606      -9.589  29.443  19.196  1.00  1.24
H
ATOM  18655 1HD1 LEU B 606     -11.844  30.059  18.456  1.00  1.24
H
ATOM  18656 2HD1 LEU B 606     -11.499  30.713  20.073  1.00  1.24
H
```

```
ATOM  18657 3HD1 LEU B 606     -11.442  31.781  18.652  1.00  1.24
H
ATOM  18658 1HD2 LEU B 606      -9.995  29.806  16.797  1.00  1.24
H
ATOM  18659 2HD2 LEU B 606      -9.579  31.526  16.985  1.00  1.24
H
ATOM  18660 3HD2 LEU B 606      -8.340  30.275  17.248  1.00  1.24
H
ATOM  18661  N   ASP B 607      -6.672  32.437  21.692  1.00  0.77
N
ATOM  18662  CA  ASP B 607      -5.899  33.465  22.378  1.00  0.93
C
ATOM  18663  C   ASP B 607      -4.397  33.257  22.164  1.00  1.24
C
ATOM  18664  O   ASP B 607      -3.683  34.193  21.802  1.00  2.37
O
ATOM  18665  CB  ASP B 607      -6.181  33.482  23.888  1.00  1.40
C
ATOM  18666  CG  ASP B 607      -7.538  34.051  24.303  1.00  1.40
C
ATOM  18667  OD1 ASP B 607      -8.215  34.645  23.500  1.00  1.40
O
ATOM  18668  OD2 ASP B 607      -7.866  33.926  25.457  1.00  1.40
O
ATOM  18669  H   ASP B 607      -7.212  31.779  22.241  1.00  0.92
H
ATOM  18670  HA  ASP B 607      -6.172  34.434  21.965  1.00  1.12
H
ATOM  18671 1HB  ASP B 607      -6.112  32.464  24.273  1.00  1.67
H
ATOM  18672 2HB  ASP B 607      -5.404  34.062  24.387  1.00  1.67
H
ATOM  18673  N   MET B 608      -3.925  32.021  22.356  1.00  0.87
N
ATOM  18674  CA  MET B 608      -2.507  31.694  22.202  1.00  1.04
C
ATOM  18675  C   MET B 608      -1.923  32.050  20.854  1.00  1.32
C
ATOM  18676  O   MET B 608      -0.769  32.476  20.779  1.00  2.33
O
ATOM  18677  CB  MET B 608      -2.268  30.205  22.446  1.00  1.56
C
ATOM  18678  CG  MET B 608      -2.380  29.754  23.891  1.00  1.56
C
ATOM  18679  SD  MET B 608      -2.309  27.963  24.054  1.00  1.56
S
ATOM  18680  CE  MET B 608      -0.628  27.610  23.551  1.00  1.56
C
ATOM  18681  H   MET B 608      -4.557  31.289  22.650  1.00  1.04
H
ATOM  18682  HA  MET B 608      -1.953  32.258  22.952  1.00  1.25
H
ATOM  18683 1HB  MET B 608      -2.978  29.625  21.862  1.00  1.87
H
ATOM  18684 2HB  MET B 608      -1.269  29.940  22.096  1.00  1.87
H
ATOM  18685 1HG  MET B 608      -1.554  30.180  24.459  1.00  1.87
H
```

ATOM 18686 2HG MET B 608 -3.306 30.111 24.331 1.00 1.87
H
ATOM 18687 1HE MET B 608 -0.450 26.536 23.605 1.00 1.87
H
ATOM 18688 2HE MET B 608 -0.474 27.954 22.528 1.00 1.87
H
ATOM 18689 3HE MET B 608 0.066 28.127 24.215 1.00 1.87
H
ATOM 18690 N PHE B 609 -2.700 31.895 19.787 1.00 1.44
N
ATOM 18691 CA PHE B 609 -2.148 32.150 18.471 1.00 2.63
C
ATOM 18692 C PHE B 609 -2.524 33.506 17.884 1.00 2.51
C
ATOM 18693 O PHE B 609 -2.300 33.747 16.698 1.00 6.05
O
ATOM 18694 CB PHE B 609 -2.514 30.999 17.545 1.00 3.94
C
ATOM 18695 CG PHE B 609 -1.996 29.735 18.152 1.00 3.94
C
ATOM 18696 CD1 PHE B 609 -2.872 28.759 18.577 1.00 3.94
C
ATOM 18697 CD2 PHE B 609 -0.642 29.556 18.376 1.00 3.94
C
ATOM 18698 CE1 PHE B 609 -2.421 27.631 19.218 1.00 3.94
C
ATOM 18699 CE2 PHE B 609 -0.182 28.424 19.020 1.00 3.94
C
ATOM 18700 CZ PHE B 609 -1.078 27.468 19.448 1.00 3.94
C
ATOM 18701 H PHE B 609 -3.643 31.541 19.888 1.00 1.73
H
ATOM 18702 HA PHE B 609 -1.061 32.137 18.564 1.00 3.16
H
ATOM 18703 1HB PHE B 609 -3.595 30.922 17.429 1.00 4.73
H
ATOM 18704 2HB PHE B 609 -2.065 31.133 16.562 1.00 4.73
H
ATOM 18705 HD1 PHE B 609 -3.936 28.899 18.412 1.00 4.73
H
ATOM 18706 HD2 PHE B 609 0.061 30.326 18.059 1.00 4.73
H
ATOM 18707 HE1 PHE B 609 -3.132 26.878 19.546 1.00 4.73
H
ATOM 18708 HE2 PHE B 609 0.884 28.294 19.203 1.00 4.73
H
ATOM 18709 HZ PHE B 609 -0.719 26.580 19.965 1.00 4.73
H
ATOM 18710 N GLY B 610 -3.056 34.410 18.712 1.00 0.82
N
ATOM 18711 CA GLY B 610 -3.310 35.767 18.244 1.00 0.83
C
ATOM 18712 C GLY B 610 -4.500 35.928 17.299 1.00 0.78
C
ATOM 18713 O GLY B 610 -4.511 36.853 16.486 1.00 0.75
O
ATOM 18714 H GLY B 610 -3.249 34.186 19.682 1.00 0.98
H

```
ATOM  18715 1HA  GLY B 610      -3.438  36.413  19.104  1.00  1.00
H
ATOM  18716 2HA  GLY B 610      -2.416  36.126  17.737  1.00  1.00
H
ATOM  18717  N   ILE B 611      -5.484  35.032  17.368  1.00  0.89
N
ATOM  18718  CA  ILE B 611      -6.605  35.094  16.435  1.00  1.08
C
ATOM  18719  C   ILE B 611      -7.971  35.062  17.115  1.00  1.66
C
ATOM  18720  O   ILE B 611      -8.932  34.523  16.564  1.00  6.60
O
ATOM  18721  CB  ILE B 611      -6.500  33.933  15.428  1.00  1.62
C
ATOM  18722  CG1 ILE B 611      -6.426  32.612  16.189  1.00  1.62
C
ATOM  18723  CG2 ILE B 611      -5.298  34.092  14.509  1.00  1.62
C
ATOM  18724  CD1 ILE B 611      -6.530  31.406  15.299  1.00  1.62
C
ATOM  18725  H   ILE B 611      -5.470  34.297  18.065  1.00  1.07
H
ATOM  18726  HA  ILE B 611      -6.533  36.028  15.881  1.00  1.30
H
ATOM  18727  HB  ILE B 611      -7.405  33.907  14.822  1.00  1.94
H
ATOM  18728 1HG1 ILE B 611      -5.479  32.565  16.727  1.00  1.94
H
ATOM  18729 2HG1 ILE B 611      -7.235  32.577  16.919  1.00  1.94
H
ATOM  18730 1HG2 ILE B 611      -5.266  33.261  13.805  1.00  1.94
H
ATOM  18731 2HG2 ILE B 611      -5.386  35.028  13.961  1.00  1.94
H
ATOM  18732 3HG2 ILE B 611      -4.382  34.100  15.098  1.00  1.94
H
ATOM  18733 1HD1 ILE B 611      -6.470  30.503  15.903  1.00  1.94
H
ATOM  18734 2HD1 ILE B 611      -7.484  31.426  14.772  1.00  1.94
H
ATOM  18735 3HD1 ILE B 611      -5.715  31.414  14.578  1.00  1.94
H
ATOM  18736  N   SER B 612      -8.061  35.663  18.297  1.00  0.60
N
ATOM  18737  CA  SER B 612      -9.327  35.728  19.013  1.00  0.61
C
ATOM  18738  C   SER B 612      -9.771  37.158  19.201  1.00  0.62
C
ATOM  18739  O   SER B 612      -9.005  38.083  18.936  1.00  0.73
O
ATOM  18740  CB  SER B 612      -9.213  35.110  20.381  1.00  0.92
C
ATOM  18741  OG  SER B 612      -8.439  35.921  21.217  1.00  0.92
O
ATOM  18742  H   SER B 612      -7.239  36.084  18.707  1.00  0.72
H
ATOM  18743  HA  SER B 612     -10.086  35.200  18.438  1.00  0.73
H
```

```
ATOM  18744 1HB  SER B 612     -10.204  34.971  20.811  1.00  1.10
H
ATOM  18745 2HB  SER B 612      -8.753  34.136  20.301  1.00  1.10
H
ATOM  18746  HG  SER B 612      -8.323  35.422  22.032  1.00  1.10
H
ATOM  18747  N   THR B 613     -10.993  37.299  19.713  1.00  0.61
N
ATOM  18748  CA  THR B 613     -11.635  38.557  20.078  1.00  0.67
C
ATOM  18749  C   THR B 613     -10.680  39.660  20.497  1.00  0.76
C
ATOM  18750  O   THR B 613     -10.615  40.705  19.851  1.00  0.79
O
ATOM  18751  CB  THR B 613     -12.616  38.321  21.248  1.00  1.01
C
ATOM  18752  OG1 THR B 613     -13.647  37.413  20.844  1.00  1.01
O
ATOM  18753  CG2 THR B 613     -13.237  39.625  21.711  1.00  1.01
C
ATOM  18754  H   THR B 613     -11.532  36.452  19.857  1.00  0.73
H
ATOM  18755  HA  THR B 613     -12.198  38.910  19.215  1.00  0.80
H
ATOM  18756  HB  THR B 613     -12.073  37.875  22.082  1.00  1.21
H
ATOM  18757  HG1 THR B 613     -13.255  36.581  20.561  1.00  1.21
H
ATOM  18758 1HG2 THR B 613     -13.916  39.429  22.542  1.00  1.21
H
ATOM  18759 2HG2 THR B 613     -12.456  40.308  22.038  1.00  1.21
H
ATOM  18760 3HG2 THR B 613     -13.788  40.068  20.891  1.00  1.21
H
ATOM  18761  N   ARG B 614      -9.961  39.428  21.597  1.00  0.80
N
ATOM  18762  CA  ARG B 614      -9.087  40.429  22.200  1.00  0.90
C
ATOM  18763  C   ARG B 614      -7.854  40.775  21.383  1.00  0.91
C
ATOM  18764  O   ARG B 614      -7.274  41.846  21.569  1.00  1.02
O
ATOM  18765  CB  ARG B 614      -8.667  39.967  23.585  1.00  1.35
C
ATOM  18766  CG  ARG B 614      -9.812  39.932  24.588  1.00  1.35
C
ATOM  18767  CD  ARG B 614      -9.385  39.484  25.943  1.00  1.35
C
ATOM  18768  NE  ARG B 614      -9.022  38.072  26.000  1.00  1.35
N
ATOM  18769  CZ  ARG B 614      -8.546  37.466  27.108  1.00  1.35
C
ATOM  18770  NH1 ARG B 614      -8.386  38.170  28.208  1.00  1.35
N
ATOM  18771  NH2 ARG B 614      -8.241  36.180  27.109  1.00  1.35
N
ATOM  18772  H   ARG B 614     -10.049  38.529  22.052  1.00  0.96
H
```

```
ATOM  18773  HA  ARG B 614      -9.662  41.340  22.321  1.00  1.08
H
ATOM  18774 1HB  ARG B 614      -8.242  38.966  23.526  1.00  1.62
H
ATOM  18775 2HB  ARG B 614      -7.898  40.632  23.977  1.00  1.62
H
ATOM  18776 1HG  ARG B 614     -10.238  40.931  24.683  1.00  1.62
H
ATOM  18777 2HG  ARG B 614     -10.579  39.245  24.232  1.00  1.62
H
ATOM  18778 1HD  ARG B 614      -8.519  40.067  26.254  1.00  1.62
H
ATOM  18779 2HD  ARG B 614     -10.200  39.649  26.646  1.00  1.62
H
ATOM  18780  HE  ARG B 614      -9.137  37.509  25.165  1.00  1.62
H
ATOM  18781 1HH1 ARG B 614      -8.617  39.153  28.218  1.00  1.62
H
ATOM  18782 2HH1 ARG B 614      -8.032  37.726  29.042  1.00  1.62
H
ATOM  18783 1HH2 ARG B 614      -8.354  35.608  26.278  1.00  1.62
H
ATOM  18784 2HH2 ARG B 614      -7.888  35.748  27.949  1.00  1.62
H
ATOM  18785  N   HIS B 615      -7.460  39.897  20.475  1.00  0.97
N
ATOM  18786  CA  HIS B 615      -6.317  40.171  19.632  1.00  0.92
C
ATOM  18787  C   HIS B 615      -6.770  40.992  18.454  1.00  0.87
C
ATOM  18788  O   HIS B 615      -6.033  41.849  17.967  1.00  0.93
O
ATOM  18789  CB  HIS B 615      -5.660  38.878  19.197  1.00  1.38
C
ATOM  18790  CG  HIS B 615      -5.005  38.201  20.348  1.00  1.38
C
ATOM  18791  ND1 HIS B 615      -3.879  38.711  20.960  1.00  1.38
N
ATOM  18792  CD2 HIS B 615      -5.310  37.064  21.010  1.00  1.38
C
ATOM  18793  CE1 HIS B 615      -3.519  37.913  21.948  1.00  1.38
C
ATOM  18794  NE2 HIS B 615      -4.371  36.909  22.000  1.00  1.38
N
ATOM  18795  H   HIS B 615      -7.969  39.037  20.338  1.00  1.16
H
ATOM  18796  HA  HIS B 615      -5.580  40.755  20.183  1.00  1.10
H
ATOM  18797 1HB  HIS B 615      -6.405  38.205  18.771  1.00  1.66
H
ATOM  18798 2HB  HIS B 615      -4.910  39.077  18.432  1.00  1.66
H
ATOM  18799  HD2 HIS B 615      -6.142  36.397  20.797  1.00  1.66
H
ATOM  18800  HE1 HIS B 615      -2.665  38.060  22.608  1.00  1.66
H
ATOM  18801  HE2 HIS B 615      -4.342  36.141  22.656  1.00  1.66
H
```

```
ATOM  18802  N   ILE B 616      -8.011  40.753  18.035  1.00  0.77
N
ATOM  18803  CA  ILE B 616      -8.630  41.537  16.988  1.00  0.74
C
ATOM  18804  C   ILE B 616      -8.825  42.948  17.522  1.00  0.81
C
ATOM  18805  O   ILE B 616      -8.483  43.912  16.842  1.00  0.88
O
ATOM  18806  CB  ILE B 616      -9.957  40.907  16.537  1.00  1.11
C
ATOM  18807  CG1 ILE B 616      -9.664  39.565  15.857  1.00  1.11
C
ATOM  18808  CG2 ILE B 616     -10.685  41.846  15.585  1.00  1.11
C
ATOM  18809  CD1 ILE B 616     -10.886  38.716  15.631  1.00  1.11
C
ATOM  18810  H   ILE B 616      -8.540  40.000  18.458  1.00  0.92
H
ATOM  18811  HA  ILE B 616      -7.961  41.583  16.132  1.00  0.89
H
ATOM  18812  HB  ILE B 616     -10.586  40.708  17.401  1.00  1.33
H
ATOM  18813 1HG1 ILE B 616      -9.194  39.753  14.892  1.00  1.33
H
ATOM  18814 2HG1 ILE B 616      -8.965  39.001  16.474  1.00  1.33
H
ATOM  18815 1HG2 ILE B 616     -11.623  41.388  15.269  1.00  1.33
H
ATOM  18816 2HG2 ILE B 616     -10.894  42.787  16.090  1.00  1.33
H
ATOM  18817 3HG2 ILE B 616     -10.061  42.034  14.711  1.00  1.33
H
ATOM  18818 1HD1 ILE B 616     -10.597  37.782  15.147  1.00  1.33
H
ATOM  18819 2HD1 ILE B 616     -11.358  38.496  16.589  1.00  1.33
H
ATOM  18820 3HD1 ILE B 616     -11.585  39.252  14.993  1.00  1.33
H
ATOM  18821  N   ILE B 617      -9.326  43.053  18.762  1.00  0.82
N
ATOM  18822  CA  ILE B 617      -9.495  44.333  19.448  1.00  0.90
C
ATOM  18823  C   ILE B 617      -8.194  45.101  19.512  1.00  0.99
C
ATOM  18824  O   ILE B 617      -8.154  46.284  19.168  1.00  1.00
O
ATOM  18825  CB  ILE B 617     -10.016  44.124  20.895  1.00  1.35
C
ATOM  18826  CG1 ILE B 617     -11.460  43.630  20.873  1.00  1.35
C
ATOM  18827  CG2 ILE B 617      -9.856  45.376  21.744  1.00  1.35
C
ATOM  18828  CD1 ILE B 617     -11.968  43.146  22.206  1.00  1.35
C
ATOM  18829  H   ILE B 617      -9.608  42.211  19.246  1.00  0.98
H
ATOM  18830  HA  ILE B 617     -10.220  44.930  18.896  1.00  1.08
H
```

```
ATOM  18831  HB  ILE B 617      -9.428  43.332  21.353  1.00  1.62
H
ATOM  18832 1HG1 ILE B 617     -12.102  44.434  20.539  1.00  1.62
H
ATOM  18833 2HG1 ILE B 617     -11.538  42.817  20.161  1.00  1.62
H
ATOM  18834 1HG2 ILE B 617     -10.198  45.169  22.758  1.00  1.62
H
ATOM  18835 2HG2 ILE B 617      -8.806  45.667  21.773  1.00  1.62
H
ATOM  18836 3HG2 ILE B 617     -10.441  46.187  21.331  1.00  1.62
H
ATOM  18837 1HD1 ILE B 617     -12.997  42.808  22.097  1.00  1.62
H
ATOM  18838 2HD1 ILE B 617     -11.361  42.319  22.560  1.00  1.62
H
ATOM  18839 3HD1 ILE B 617     -11.925  43.959  22.929  1.00  1.62
H
ATOM  18840  N   ALA B 618      -7.130  44.419  19.944  1.00  1.05
N
ATOM  18841  CA  ALA B 618      -5.818  45.030  20.034  1.00  1.14
C
ATOM  18842  C   ALA B 618      -5.368  45.560  18.684  1.00  1.19
C
ATOM  18843  O   ALA B 618      -4.948  46.712  18.590  1.00  1.45
O
ATOM  18844  CB  ALA B 618      -4.807  44.024  20.561  1.00  1.71
C
ATOM  18845  H   ALA B 618      -7.233  43.456  20.242  1.00  1.26
H
ATOM  18846  HA  ALA B 618      -5.880  45.870  20.724  1.00  1.37
H
ATOM  18847 1HB  ALA B 618      -3.831  44.500  20.649  1.00  2.05
H
ATOM  18848 2HB  ALA B 618      -5.126  43.668  21.541  1.00  2.05
H
ATOM  18849 3HB  ALA B 618      -4.740  43.181  19.876  1.00  2.05
H
ATOM  18850  N   ALA B 619      -5.490  44.730  17.641  1.00  1.05
N
ATOM  18851  CA  ALA B 619      -5.094  45.108  16.291  1.00  1.08
C
ATOM  18852  C   ALA B 619      -5.839  46.346  15.820  1.00  1.07
C
ATOM  18853  O   ALA B 619      -5.238  47.231  15.207  1.00  1.10
O
ATOM  18854  CB  ALA B 619      -5.338  43.956  15.332  1.00  1.62
C
ATOM  18855  H   ALA B 619      -5.843  43.793  17.789  1.00  1.26
H
ATOM  18856  HA  ALA B 619      -4.029  45.341  16.304  1.00  1.30
H
ATOM  18857 1HB  ALA B 619      -5.012  44.237  14.331  1.00  1.94
H
ATOM  18858 2HB  ALA B 619      -4.777  43.083  15.664  1.00  1.94
H
ATOM  18859 3HB  ALA B 619      -6.400  43.716  15.312  1.00  1.94
H
```

```
ATOM  18860  N   VAL B 620      -7.141  46.411  16.116  1.00  1.02
N
ATOM  18861  CA  VAL B 620      -7.950  47.563  15.752  1.00  1.01
C
ATOM  18862  C   VAL B 620      -7.452  48.835  16.402  1.00  1.09
C
ATOM  18863  O   VAL B 620      -7.339  49.858  15.731  1.00  1.22
O
ATOM  18864  CB  VAL B 620      -9.430  47.349  16.129  1.00  1.52
C
ATOM  18865  CG1 VAL B 620     -10.195  48.656  15.958  1.00  1.52
C
ATOM  18866  CG2 VAL B 620     -10.035  46.270  15.238  1.00  1.52
C
ATOM  18867  H   VAL B 620      -7.583  45.637  16.597  1.00  1.22
H
ATOM  18868  HA  VAL B 620      -7.894  47.688  14.670  1.00  1.21
H
ATOM  18869  HB  VAL B 620      -9.501  47.050  17.175  1.00  1.82
H
ATOM  18870 1HG1 VAL B 620     -11.238  48.506  16.229  1.00  1.82
H
ATOM  18871 2HG1 VAL B 620      -9.759  49.419  16.603  1.00  1.82
H
ATOM  18872 3HG1 VAL B 620     -10.133  48.981  14.921  1.00  1.82
H
ATOM  18873 1HG2 VAL B 620     -11.079  46.118  15.501  1.00  1.82
H
ATOM  18874 2HG2 VAL B 620      -9.967  46.575  14.199  1.00  1.82
H
ATOM  18875 3HG2 VAL B 620      -9.493  45.339  15.364  1.00  1.82
H
ATOM  18876  N   THR B 621      -7.148  48.784  17.701  1.00  1.16
N
ATOM  18877  CA  THR B 621      -6.694  49.998  18.366  1.00  1.38
C
ATOM  18878  C   THR B 621      -5.281  50.382  17.945  1.00  1.63
C
ATOM  18879  O   THR B 621      -4.969  51.569  17.870  1.00  2.03
O
ATOM  18880  CB  THR B 621      -6.747  49.845  19.894  1.00  2.07
C
ATOM  18881  OG1 THR B 621      -5.883  48.778  20.304  1.00  2.07
O
ATOM  18882  CG2 THR B 621      -8.171  49.541  20.335  1.00  2.07
C
ATOM  18883  H   THR B 621      -7.261  47.921  18.224  1.00  1.39
H
ATOM  18884  HA  THR B 621      -7.355  50.811  18.083  1.00  1.66
H
ATOM  18885  HB  THR B 621      -6.415  50.769  20.367  1.00  2.48
H
ATOM  18886  HG1 THR B 621      -6.063  47.998  19.771  1.00  2.48
H
ATOM  18887 1HG2 THR B 621      -8.202  49.435  21.419  1.00  2.48
H
ATOM  18888 2HG2 THR B 621      -8.824  50.357  20.032  1.00  2.48
H
```

ATOM 18889 3HG2 THR B 621 -8.508 48.616 19.871 1.00 2.48
H
ATOM 18890 N LEU B 622 -4.452 49.395 17.598 1.00 1.92
N
ATOM 18891 CA LEU B 622 -3.109 49.677 17.101 1.00 2.50
C
ATOM 18892 C LEU B 622 -3.198 50.371 15.753 1.00 2.54
C
ATOM 18893 O LEU B 622 -2.429 51.288 15.460 1.00 2.72
O
ATOM 18894 CB LEU B 622 -2.305 48.378 16.978 1.00 3.75
C
ATOM 18895 CG LEU B 622 -1.913 47.707 18.301 1.00 3.75
C
ATOM 18896 CD1 LEU B 622 -1.324 46.333 18.012 1.00 3.75
C
ATOM 18897 CD2 LEU B 622 -0.905 48.585 19.024 1.00 3.75
C
ATOM 18898 H LEU B 622 -4.741 48.432 17.702 1.00 2.30
H
ATOM 18899 HA LEU B 622 -2.606 50.341 17.802 1.00 3.00
H
ATOM 18900 1HB LEU B 622 -2.891 47.661 16.406 1.00 4.50
H
ATOM 18901 2HB LEU B 622 -1.388 48.589 16.429 1.00 4.50
H
ATOM 18902 HG LEU B 622 -2.791 47.580 18.928 1.00 4.50
H
ATOM 18903 1HD1 LEU B 622 -1.046 45.853 18.951 1.00 4.50
H
ATOM 18904 2HD1 LEU B 622 -2.064 45.720 17.498 1.00 4.50
H
ATOM 18905 3HD1 LEU B 622 -0.441 46.440 17.384 1.00 4.50
H
ATOM 18906 1HD2 LEU B 622 -0.622 48.114 19.966 1.00 4.50
H
ATOM 18907 2HD2 LEU B 622 -0.019 48.712 18.400 1.00 4.50
H
ATOM 18908 3HD2 LEU B 622 -1.349 49.559 19.224 1.00 4.50
H
ATOM 18909 N THR B 623 -4.176 49.942 14.958 1.00 2.84
N
ATOM 18910 CA THR B 623 -4.474 50.518 13.664 1.00 3.45
C
ATOM 18911 C THR B 623 -4.953 51.965 13.840 1.00 3.57
C
ATOM 18912 O THR B 623 -4.479 52.871 13.154 1.00 5.76
O
ATOM 18913 CB THR B 623 -5.532 49.642 12.954 1.00 5.18
C
ATOM 18914 OG1 THR B 623 -4.998 48.327 12.764 1.00 5.18
O
ATOM 18915 CG2 THR B 623 -5.928 50.211 11.620 1.00 5.18
C
ATOM 18916 H THR B 623 -4.738 49.155 15.254 1.00 3.41
H
ATOM 18917 HA THR B 623 -3.564 50.528 13.065 1.00 4.14
H

```
ATOM  18918  HB  THR B 623      -6.419  49.573  13.576  1.00  6.21
H
ATOM  18919  HG1 THR B 623      -4.872  47.909  13.624  1.00  6.21
H
ATOM  18920 1HG2 THR B 623      -6.674  49.566  11.156  1.00  6.21
H
ATOM  18921 2HG2 THR B 623      -6.347  51.202  11.778  1.00  6.21
H
ATOM  18922 3HG2 THR B 623      -5.052  50.277  10.977  1.00  6.21
H
ATOM  18923  N   LEU B 624      -5.848  52.187  14.806  1.00  2.41
N
ATOM  18924  CA  LEU B 624      -6.353  53.525  15.123  1.00  3.34
C
ATOM  18925  C   LEU B 624      -5.251  54.477  15.563  1.00  2.55
C
ATOM  18926  O   LEU B 624      -5.261  55.658  15.213  1.00  2.98
O
ATOM  18927  CB  LEU B 624      -7.387  53.453  16.256  1.00  5.01
C
ATOM  18928  CG  LEU B 624      -8.733  52.818  15.923  1.00  5.01
C
ATOM  18929  CD1 LEU B 624      -9.544  52.652  17.197  1.00  5.01
C
ATOM  18930  CD2 LEU B 624      -9.463  53.714  14.955  1.00  5.01
C
ATOM  18931  H   LEU B 624      -6.215  51.399  15.326  1.00  2.89
H
ATOM  18932  HA  LEU B 624      -6.826  53.934  14.231  1.00  4.01
H
ATOM  18933 1HB  LEU B 624      -6.956  52.888  17.080  1.00  6.01
H
ATOM  18934 2HB  LEU B 624      -7.582  54.466  16.606  1.00  6.01
H
ATOM  18935  HG  LEU B 624      -8.588  51.839  15.475  1.00  6.01
H
ATOM  18936 1HD1 LEU B 624     -10.506  52.205  16.955  1.00  6.01
H
ATOM  18937 2HD1 LEU B 624      -9.013  52.008  17.893  1.00  6.01
H
ATOM  18938 3HD1 LEU B 624      -9.702  53.627  17.656  1.00  6.01
H
ATOM  18939 1HD2 LEU B 624     -10.424  53.266  14.729  1.00  6.01
H
ATOM  18940 2HD2 LEU B 624      -9.615  54.694  15.407  1.00  6.01
H
ATOM  18941 3HD2 LEU B 624      -8.879  53.822  14.041  1.00  6.01
H
ATOM  18942  N   MET B 625      -4.290  53.952  16.318  1.00  3.20
N
ATOM  18943  CA  MET B 625      -3.168  54.732  16.817  1.00  6.42
C
ATOM  18944  C   MET B 625      -2.020  54.892  15.816  1.00 15.15
C
ATOM  18945  O   MET B 625      -0.968  55.414  16.177  1.00 45.26
O
ATOM  18946  CB  MET B 625      -2.639  54.095  18.100  1.00  9.63
C
```

```
ATOM  18947  CG  MET B 625      -3.583  54.197  19.291  1.00  9.63
C
ATOM  18948  SD  MET B 625      -2.835  53.604  20.823  1.00  9.63
S
ATOM  18949  CE  MET B 625      -2.833  51.837  20.534  1.00  9.63
C
ATOM  18950  H   MET B 625      -4.354  52.980  16.593  1.00  3.84
H
ATOM  18951  HA  MET B 625      -3.534  55.731  17.053  1.00  7.70
H
ATOM  18952 1HB  MET B 625      -2.438  53.040  17.925  1.00 11.56
H
ATOM  18953 2HB  MET B 625      -1.696  54.566  18.379  1.00 11.56
H
ATOM  18954 1HG  MET B 625      -3.873  55.238  19.432  1.00 11.56
H
ATOM  18955 2HG  MET B 625      -4.484  53.618  19.101  1.00 11.56
H
ATOM  18956 1HE  MET B 625      -2.397  51.328  21.394  1.00 11.56
H
ATOM  18957 2HE  MET B 625      -3.856  51.490  20.386  1.00 11.56
H
ATOM  18958 3HE  MET B 625      -2.242  51.616  19.644  1.00 11.56
H
ATOM  18959  N   LYS B 626      -2.202  54.449  14.571  1.00 31.37
N
ATOM  18960  CA  LYS B 626      -1.162  54.551  13.549  1.00 51.69
C
ATOM  18961  C   LYS B 626      -1.773  54.502  12.145  1.00142.48
C
ATOM  18962  O   LYS B 626      -2.144  55.535  11.585  1.00255.32
O
ATOM  18963  OXT LYS B 626      -1.896  53.425  11.562  1.00  0.00
O
ATOM  18964  CB  LYS B 626      -0.138  53.429  13.719  1.00 77.53
C
ATOM  18965  CG  LYS B 626       1.045  53.503  12.763  1.00 77.53
C
ATOM  18966  CD  LYS B 626       2.064  52.415  13.064  1.00 77.53
C
ATOM  18967  CE  LYS B 626       3.242  52.477  12.102  1.00 77.53
C
ATOM  18968  NZ  LYS B 626       4.247  51.417  12.387  1.00 77.53
N
ATOM  18969  H   LYS B 626      -3.074  54.018  14.295  1.00 37.64
H
ATOM  18970  HA  LYS B 626      -0.656  55.511  13.661  1.00 62.03
H
ATOM  18971 1HB  LYS B 626       0.252  53.447  14.737  1.00 93.04
H
ATOM  18972 2HB  LYS B 626      -0.629  52.466  13.574  1.00 93.04
H
ATOM  18973 1HG  LYS B 626       0.694  53.388  11.737  1.00 93.04
H
ATOM  18974 2HG  LYS B 626       1.526  54.476  12.855  1.00 93.04
H
ATOM  18975 1HD  LYS B 626       2.432  52.533  14.084  1.00 93.04
H
```

```
ATOM  18976 2HD  LYS B 626       1.588  51.439  12.979  1.00 93.04
H
ATOM  18977 1HE  LYS B 626       2.878  52.353  11.083  1.00 93.04
H
ATOM  18978 2HE  LYS B 626       3.723  53.451  12.187  1.00 93.04
H
ATOM  18979 1HZ  LYS B 626       5.011  51.490  11.731  1.00 93.04
H
ATOM  18980 2HZ  LYS B 626       4.599  51.530  13.328  1.00 93.04
H
ATOM  18981 3HZ  LYS B 626       3.814  50.508  12.299  1.00 93.04
H
ENDMDL
END
```
